# Identification of molecular targets for the targeted treatment of gastric cancer using dasatinib

**DOI:** 10.18632/oncotarget.27462

**Published:** 2020-02-04

**Authors:** Raquel Carvalho Montenegro, Alison Howarth, Alessandro Ceroni, Vita Fedele, Batoul Farran, Felipe Pantoja Mesquita, Martin Frejno, Benedict-Tilman Berger, Stephanie Heinzlmeir, Heba Z. Sailem, Roberta Tesch, Daniel Ebner, Stefan Knapp, Rommel Burbano, Bernhard Kuster, Susanne Müller

**Affiliations:** ^1^Drug Research and Development Center (NPDM), Federal University of Ceará, Fortaleza, CE, Brazil; ^2^Novo Nordisk Research Centre Oxford (NNRCO), Discovery Technologies and Genomics, Oxford, UK; ^3^Winship Cancer Institute, Emory University, Atlanta, GA, USA; ^4^Chair of Proteomics and Bioanalytics, Technical University of Munich, Freising, Germany; ^5^Structural Genomics Consortium, Buchmann Institute for Life Sciences, Goethe-University Frankfurt, Frankfurt, Germany; ^6^Institute of Pharmaceutical Chemistry, Goethe-University Frankfurt, Frankfurt, Germany; ^7^German Cancer Consortium (DKTK), German Cancer Research Center (DKFZ), Heidelberg, Germany; ^8^Institute of Biomedical Engineering, Department of Engineering, University of Oxford, Oxford, UK; ^9^Big Data Institute, University of Oxford, Li Ka Shing Centre for Health Information and Discovery, Old Road Campus Research Building, Oxford, UK; ^10^Ophir Loyola Hospital, Belém, PA, Brazil; ^11^Bavarian Center for Biomolecular Mass Spectrometry (BayBioMS), Technische Universität München, Freising, Germany

**Keywords:** gastric cancer, dasatinib, biomarker, SRC-kinases, SIK2

## Abstract

Gastric cancer (GC) remains the third leading cause of cancer-related death despite several improvements in targeted therapy. There is therefore an urgent need to investigate new treatment strategies, including the identification of novel biomarkers for patient stratification. In this study, we evaluated the effect of FDA-approved kinase inhibitors on GC. Through a combination of cell growth, migration and invasion assays, we identified dasatinib as an efficient inhibitor of GC proliferation. Mass-spectrometry-based selectivity profiling and subsequent knockdown experiments identified members of the SRC family of kinases including *SRC*, *FRK*, *LYN* and *YES*, as well as other kinases such as *DDR1*, *ABL2*, *SIK2*, *RIPK2*, *EPHA2*, and *EPHB2* as dasatinib targets. The expression levels of the identified kinases were investigated on RNA and protein level in 200 classified tumor samples from patients, who had undergone gastrectomy, but had received no treatment. Levels of FRK, DDR1 and SRC expression on both mRNA and protein level were significantly higher in metastatic patient samples regardless of the tumor stage, while expression levels of SIK2 correlated with tumor size. Collectively, our data suggest dasatinib for treatment of GC based on its unique property, inhibiting a small number of key kinases (SRC, FRK, DDR1 and SIK2), highly expressed in GC patients.

## INTRODUCTION

Despite a decreased incidence rate during the past years, GC represents a major health problem, remaining the fifth most common type of cancer and the third leading cause of cancer-related death worldwide [1]. The standard treatment of localized or locally advanced GC currently consists of surgical resection and D2 lymphadenectomy in combination with adjuvant chemotherapies including fluoropyrimidines, anthracyclines, platinum agents, taxanes, and irinotecan [2]. However, the overall survival (OS) for patients with locally advanced and metastatic GC remains poor with a median OS of 8–11 months [3, 4] and only about 6 months after second-line therapy [5].

The emergence of targeted therapies has markedly improved treatment outcomes for different types of cancers, including GC [6]. Trastuzumab, a recombinant humanized monoclonal antibody against ERBB2/Her2, was the first targeted therapy approved for the treatment of GC in 2010. The incorporation of trastuzumab to standard chemotherapy improved median OS compared to chemotherapy alone [2, 7]. More recently, the VEGFR2 inhibitor ramucirumab was approved by the FDA as a second line treatment alone or in combination with paclitaxel for patients with local relapsed or metastatic GC resulting in improvement on median OS [8, 9].

Kinase inhibitors are one of the most successful classes of small molecule modulators for the treatment of cancer with currently more than 50 kinase inhibitors being approved for treatment [10]. In particular, targeting members of the tyrosine kinase subfamily has proven to be a successful strategy [11]. Kinases are key signalling molecules and many of them do not only play crucial roles in cell proliferation, but have also been associated with metastasis and invasion of different types of cancer [11–13]. For example, overexpression of the receptor tyrosine kinase epidermal growth factor receptor (EGFR) has been implicated in metastatic colorectal and biliary tract cancers [14]. Overexpression of another receptor tyrosine kinase subfamily, the Ephrin receptor kinase family, has been linked to metastasis of a variety of tumors by mediating aberrant cell-cell communication [15, 16]. Similarly, signaling of the nonreceptor tyrosine kinase SRC is involved in tumor metastasis and invasion of for example bone metastasis derived from breast and prostate cancer [17]. Currently, the broad spectrum kinase inhibitor dasatinib, potently targeting SRC, in combination with zoledronic acid is under evaluation in phase1/2 for treatment of breast cancer with bone metastasis [18]. More recently, the receptor tyrosine kinase discoidin domain receptor-1 (DDR1) has emerged as an important player in the metastatic phenotype of gastric cancer [19, 20].

The efficacy of kinase inhibitors for many different types of cancers has prompted the investigation of these compounds also in GC [6, 21, 22]. However, so far most clinical trials (clinicaltrial.org NCT00215995, NCT00595985, NCT00725712) with for instance the dual MET/VEGFR2 inhibitor GSK1363089, the multi-target kinase inhibitor sunitinib and the Epidermal Growth Factor Receptor EGFR1 and EGFR2 inhibitor lapatinib, respectively, failed to show beneficial effects [23–25]. A notable exception is the Vascular Endothelial Growth Factor Receptor-2 (VEGFR2) kinase inhibitor, apatinib, which is the only small molecule investigational kinase inhibitor which has demonstrated significant benefit on median OS and progression free survival as a third line therapy for GC in recent clinical trials [26, 27].

The lack of novel small molecule inhibitors for targeted treatment of GC points to an urgent need in exploring new drugs and drug candidates to manage this tumor type. Herein, we describe the efficay of FDA-approved kinase inhibitors on the proliferation and migration of GC cells. Dasatinib was identified as a potent inhibitor of migration and invasion of gastric cancer cells. Moreover, kinome-wide analysis identified five kinase targets responsible for the invasion of GC cells and their expression pattern in GC clinical samples characterizes them as possible biomarker for GC screening and personalised therapy.

## RESULTS

### Profiling of clinical kinase inhibitors in gastric cancer cell lines

In order to identify new potential therapeutic approaches for the treatment of gastric cancer, an initial screening of 14 clinical kinase inhibitors was performed. The antiproliferative effect of these inhibitors was assessed using three diverse GC cell lines, representing diffuse type (ACP-02), intestinal type (ACP-03) and peritoneal carcinomatosis (AGP-01). Most inhibitors did not show significant antiproliferative effects on any of the tested GC lines after 72 h incubation. Only treatment with the pan-kinase inhibitor staurosporine or the tyrosine kinase inhibitor dasatinib resulted in inhibition of cell growth in all cell lines with an *IC_50_* of <0.015 μM and 1 μM, respectively (Table 1). AGP-01 and ACP-03 cells were cell lines more sensitive to dasatinib treatment than ACP-02. Hence, AGP-01, a cell-line derived from ascites of a patient representing peritoneal carcinomatosis, the most aggressive form of gastric cancer, was chosen for further investigation.

**Table 1:** Antiproliferative effects of FDA approved kinase inhibitors on 2D gastric cancer cell lines

	**IC50 μmol/L (± SD)**		
Compounds	AGP-01	ACP-02	ACP-03
Pimecrolimus	>20	>20	>20
PX-866	>20	>20	>20
Tivozanib	>20	>20	>20
Sunitinib	1.53 (± 0.74)	2.66 (± 0.68)	1.97 (± 0.73)
Axitinib	>20	>20	>20
Vemurafenib	>20	>20	>20
Everolimus	>20	>20	>20
Saracatinib	2.38 (± 0.75)	5.94 (± 0.72)	2.80 (± 0.88)
Ruxolitinib	>20	>20	>20
Gefitinib	>20	>20	>20
Pazopanib	>20	>20	>20
Dasatinib	0.35 (± 0.80)	1.02 (± 0.78)	0.36 (± 0.86)
Vandetanib	>20	>20	>20
Staurosporin	6.13 × 10^-6^ (± 0.017)	0.04 (± 0.81)	0.01 (± 0.43)

*IC_50_* values were calculated using non-linear regression analysis from two independent experiments in triplicate. Staurosporine was used as the positive control. The drug with the most potent proliferation inhibition is highlighted. Numbers in brackets indicate the range of observed *IC_50_* values. *IC_50_* = concentration in μM that results in 50% inhibition of cell growth.

### Dasatinib inhibits migration and invasion of gastric cancer cells by altering actin remodelling

**Figure 1:**
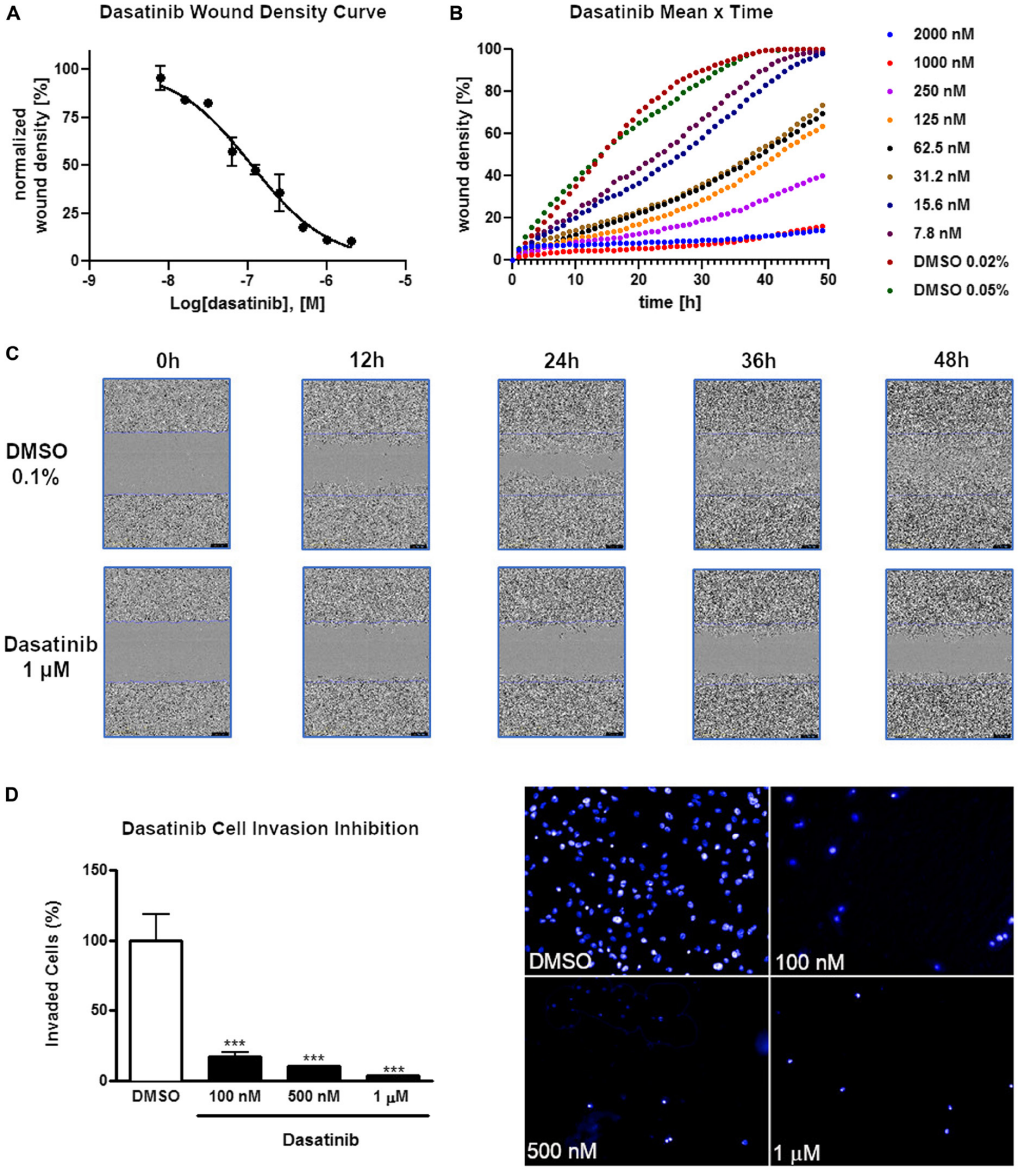
Inhibition of cell invasion and migration of AGP-01 cells by dasatinib. (**A**) Wound healing migration assay of cells exposed to dasatinib in concentration-dependent manner using an IncuCyte^®^ life cell imager after 24 h of treatment. (**B**) Wound density measured in a migration assay of GC cells in concentration- and time-dependent. (**C**) Representative images used for migration assay of AGP-01 cells exposed to dasatinib or DMSO at different time points. (**D**) Quantification of invasion inhibition of AGP-01 cells exposed to dasatinib at different concentrations for 8 h and representative images of the invasion assay. AGP-01 cells were stained with Hoechst 33342 after treatment. Quantitative data of invasion and migration are represented as mean ± SD of three independent experiments. ^***^ *P* < 0.0001, significant difference between control and treatment groups by analysis of variance and Tukey posttest.

Next, we explored, if treatment of GC cells with dasatinib would also influence cell migration and invasion. Treatment of AGP-01 cells with increasing concentrations of dasatinib significantly inhibited cell migration (Figure 1A–1C) and invasion after 24 h (Figure 1D). Significant effects on cell migration were already obvious after 4 h of exposure to dasatinib at all concentrations tested (*P* < 0.001). Interestingly, we also observed morphological changes of the gastric cancer cells exposed to different concentrations of dasatinib (100 nM, 500 nM, 1 μM) (Supplementary Figure 1A). Confocal imaging of these cells revealed a significant increase in cortical actin in the membrane region (Supplementary Figure 1B). We confirmed this observation by quantifying the amount of actin at the plasma membrane for individual cells (Supplementary Figure 1C–1D). This data suggests that the changes in the migration ability is linked to actin filaments dynamics. Taken together, the results demonstrate that dasatinib alters the metastatic phenotype of AGP-01 cells in a concentration-dependent manner.

### Kinase profile of AGP-01 cells reveals new potential targets

**Figure 2:**
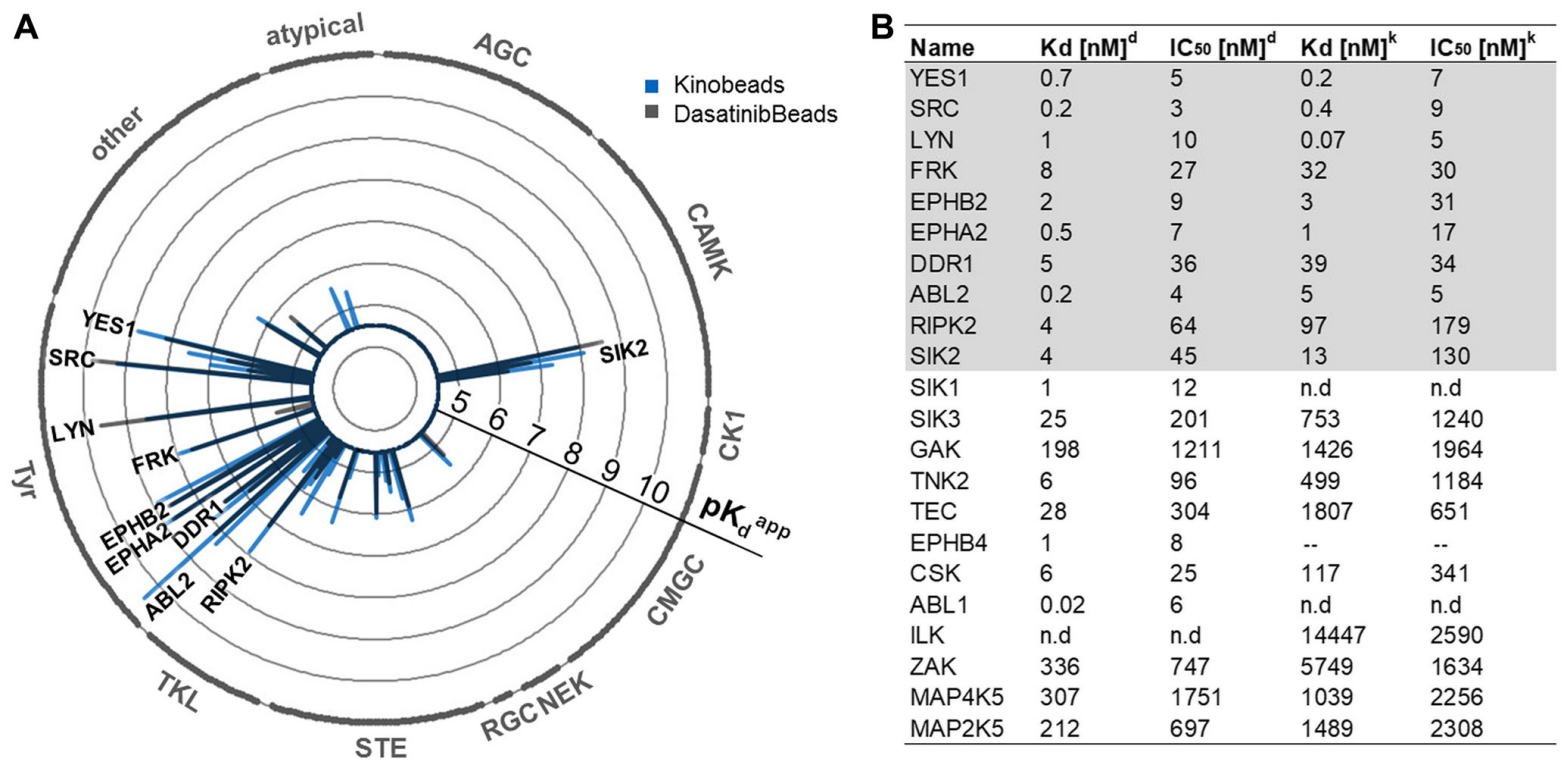
(**A**) Radarplot showing the kinome profile of GC AGP-01 cells using Kinobeads or Dasabeads, respectively. Kinases found to be potently bound in both types of experiments. Each spike is a protein target, and the length of the spike is indicative of apparent binding affinity depicted as pK_D_^app^. Kinase subfamilies are indicated around the circle. (**B**) Selectivity profiling of dasatinib using Dasabeads (d) or Kinobeads (k). Shown are the 22 most potently bound kinases.

Selectivity profiling of dasatinib was performed using two different types of affinity matrices containing either linkable dasatinib (Dasabeads) or a mixture of five immobilized broad-selectivity kinase inhibitors (Kinobeads γ) [28]. Dose-resolved competition experiments with dasatinib and subsequent quantitative mass spectrometric readout enabled the systematic determination of binding affinities of drug: protein interactions in native AGP-01 lysates (Supplementary Table 1 and Supplementary Figure 2A, 2B containing detailed information). Of the more than 200 protein kinases identified in the cell lysate, only 18 kinases presented apparent dissociation constants (K_d_^app^) of <100 nM (Figure 2) with 10 kinases common to both experiments (Figure 2). The comparison of both approaches highlighted kinases from the SRC family of kinases such as Rous Sarcoma oncogene (SRC) itself, Fyn Related Src family tyrosine Kinase (FRK), Lck/Yes-related novel protein tyrosine kinase (LYN) and Yamaguchi sarcoma oncogene (YES), as well as the receptor tyrosine kinase DDR1 and members of the Ephrin family (Ephrin Type-A Receptor 2 (EPHA2) and Ephrin Type-B Receptor 2 (EPHB2)), as potential dasatinib targets in AGP-01 cells (Supplementary Figure 2A–2B). In addition, the tyrosine kinase Abelson Tyrosine-Protein Kinase 2 (ABL2), the serine/threonine kinases Salt Inducible Kinase 2 (SIK2) and Receptor Interacting Serine/Threonine Kinase 2 (RIPK2) were identified as high affinity binders of dasatinib in AGP-01 cells.

### SRC family members and DDR1 drive the migration and invasion phenotype in APG-01 cells

**Figure 3:**
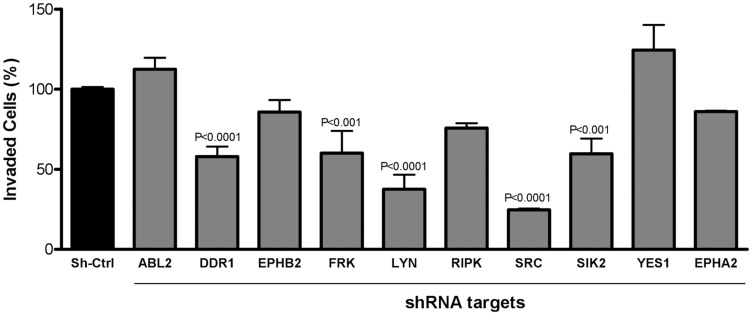
Inhibition of cell invasion 8 h after silencing target gene expression of kinases highlighted in the Kinobeads assay. Cell invasion was carried out with shCtrl cells (nonsilencing cells) and specific shRNA for the selected targets. The number of invading cells were counted automatically. Results are expressed as mean ± SD of three independent experiments after normalization by comparison with shCrtl. Significant differences: *P* < 0.001; *P* < 0.0001, significant difference between control and silenced cells by analysis of variance and Tukey posttest.

In order to identify, which of the kinases that were most potently inhibited by dasatinib in AGP-01 cells were responsible for its effect on invasion, we next validated the identified targets by knocking down the individual kinases using shRNAs and performing transwell assays. Of ten silenced kinases investigated, the most relevant kinases responsible for cell invasion were *DDR1*, *SIK2* and the SRC kinases *SRC*, *LYN* and *FRK*, whose knockdown significantly blocked the cell invasion capacity of AGP-01 cells compared to non-silenced cells or to cells treated with control shRNA (Figure 3). Unexpectedly, knockdown of *EPHA2* or *EPHB2*, both receptor tyrosine kinases linked to invasion of different types of cancer, had no effect on the metastatic ability of GC cells AGP-01. Similarly, knocking down of *ABL2*, *RIPK2* or the SRC family kinase *YES1* had no effect on the invasion capability of AGP-01 cells.

### Correlation between patient tumor samples and dasatinib targets

**Figure 4:**
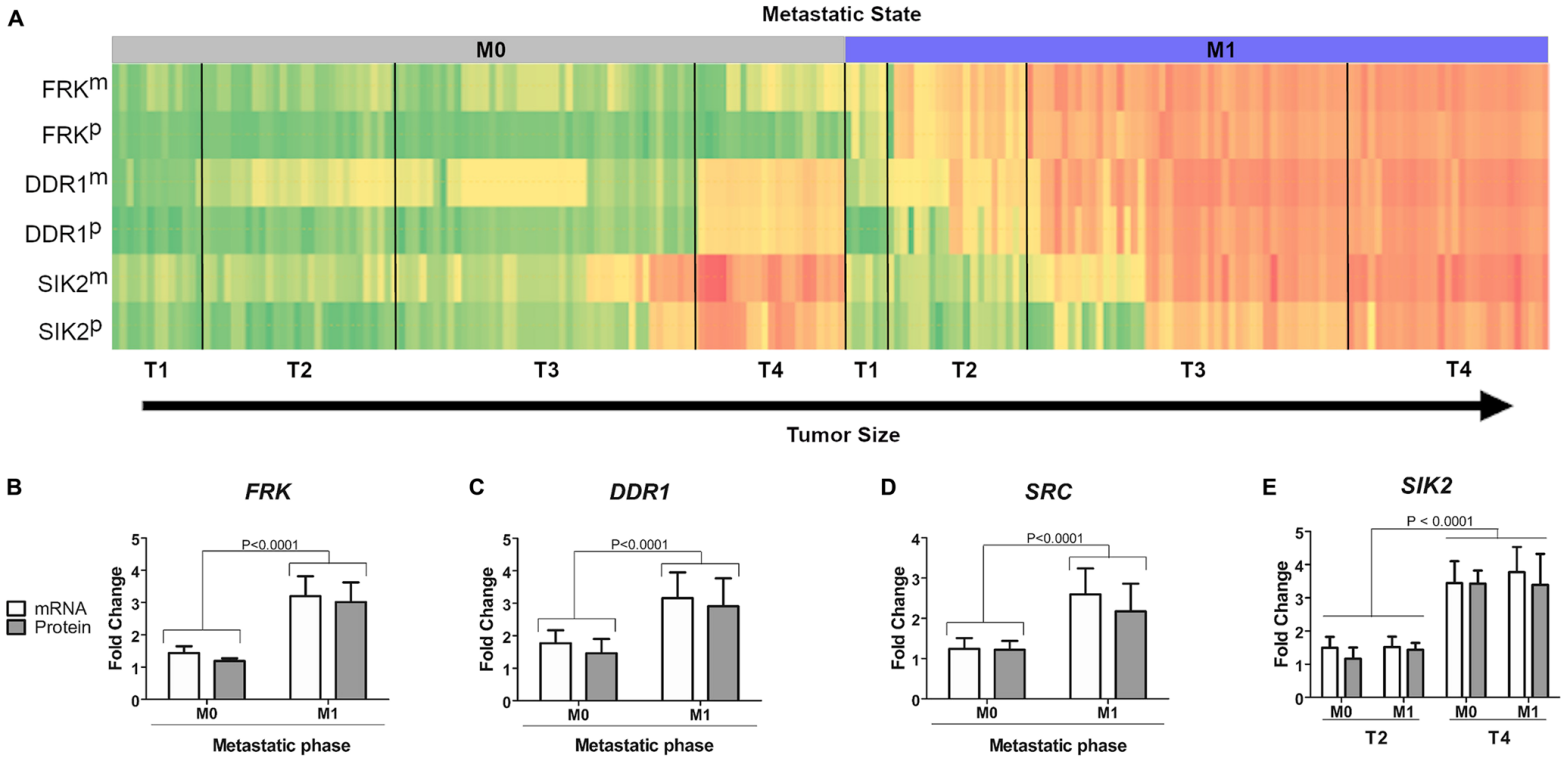
Correlation between kinase expression levels and tumor stage in patient samples. (**A**) Heatmap of expression levesl of different kinases in patient samples on mRNA level (m) or protein level (p). Samples were sorted according to tumor size (T1–T4) and metastatic stage (M0–M1). (**B–D**) Statistical analysis of mRNA and protein levels for each of the kinases FRK, DDR1, SRC and SIK2 was carried out using Mann-Whitney *U* test. Significant differences: *P* < 0.0001. (**E**) Analysis of SIK2 mRNA and protein expression, respectively in relationship to different tumor size (T2 or T4) and metastatic stage (M0 or M1).

In order to assess if the identified kinases in the invasion assay correlate with expression levels in tumors from GC patients, mRNA and protein expression levels for *FRK*, *DDR1*, *SIK2* and *SRC* were assessed in tumor samples from GC patients. We analysed 200 tumor samples from patients who had undergone gastrectomy, but had no chemotherapy or radiotherapy treatment prior to surgery. All samples were assessed for expression of FRK, DDR1 and SIK2, and wherever possible depending on the material available, also for SRC and LYN. The samples were classified according to tumor stage (T1-T4) and the presence (M1) or absence (M0) of metastatic tissue (Supplementary Table 2 and Figure 4A). In line with our findings in the metastatic cell line AGP-01, the median expression levels of both mRNA (measured by qPCR) and protein (assessed by Western blot) of FRK and DDR1 were higher in metastatic patient samples regardless of the tumor stage (*P* < 0.0001 Mann-Whitney *U* test) (Figure 4B–4C). This was also true for SRC and LYN (Figure 4D and Supplementary Figure 3), although less patient samples were tested due to limited availability of cancer tissue (Supplementary Figure 3). However, for SIK2, no clear correlation between mRNA and protein expression at later metastatic stages was seen (Figure 4E and Supplementary Figure 4). It is worth noting that larger tumors of stages T3M1, T4M0 and T4M1 had higher expression levels of SIK2 compared to early stages on both mRNA and protein level (Figure 4E, *P* < 0.001, Kruskal-Wallis test and Supplementary Figure 4). However, the statistically significant differences between T3M1 and T4M1 were only apparent at protein expression levels (*P* < 0.05) (Supplementary Figure 4). Thus, our results showed a statistically significant increase of *SIK2* mRNA and protein expression in advanced stages of gastric tumours compared to lower grade tumors.

Since we observed a strong correlation between the mRNA expression and protein levels of the different kinase targets in metastatic gastric samples (M1 stage), we performed the Spearman correlation test. There was a strong correlation between mRNA and protein expression for *SRC* (spearman r = 0.8967, *P* < 0.0001), *SIK2* (spearman r = 0.9220, *P* < 0.0001), *FRK* (spearman r = 0.8592, *P* < 0.0001) and *DDR1* (spearman r = 0.9502, *P* < 0.0001) suggesting that the identification of any one of these biomarkers could be performed on RNA or protein level, respectively (Supplementary Figure 5).

## DISCUSSION

In this study, we identified dasatinib as an efficient anti-proliferative inhibitor of GC cell growth, migration and invasion. Proteome wide analysis of dasatinib kinase targets in cells identified 10 high-affinity kinase targets (ABL2, DDR1, EPHB2, FRK, LYN, RIPK, SRC, SIK2, YES1 and EPHA2), in agreement with previous findings in other cancer types [29]. We further demonstrated by genetic knock down that five kinases (SRC, SIK2, LYN, FRK and DDR1) inhibited by dasatinib contribute to invasiveness of GC cells. In order to assess the relevance of these five kinases in gastric cancer tumors, we analysed the mRNA and protein levels in 200 biopsies from gastric cancer patients, revealing that transcript and protein levels of FRK, DDR1, SRC were elevated in metastatic tissues regardless of tumor stage. Contrary, transcript and protein levels of SIK2 correlated with tumor size, but not metastasis of GC. Thus, each of these individual targets could serve as diagnostic marker of metastasis, with SIK2 being a novel marker for tumor development.

Dasatinib, one of the most clinically investigated tyrosine kinase inhibitors, has been approved by the FDA for the first-line treatment of CML and it has been evaluated in additional cancer types such as advanced prostate and breast cancers [30, 31]. Originally developed as a dual BCR-ABL and SRC inhibitor, nearly 40 additional kinases have been identified as dasatinib targets operating at pivotal signalling nodes, including many SRC family kinases [29]. In a recent study, we identified 72 targets for dasatinib, of which 48 were targeted at submicromolar concentrations [32]. Our proliferation data already suggested that ABL was not a prominent target in GC cell lines as no proliferation inhibition was observed using the ABL inhibitors imatinib or nilotinib. On the other hand, the SRC family inhibitor saracatinib also did not show significant antiproliferativ effects, suggesting that SRC family members may only be involved in the invasion as opposed to proliferation of GC cells. Alternatively, the observed effect on invasion of GC cells in knockdown experiments could be at least partially attributed to roles of scaffolding functions of these kinases.

Preclinical and clinical studies have shown that dasatinib exerts various effects on cell proliferation, consistently inhibits cell adhesion, migration and invasion but infrequently induces apoptosis, thus acting as a cytostatic rather than cytotoxic agent [30]. Studies in non small cell lung cancer, NSCLC, and prostate cancer cell lines have revealed that dasatinib can inhibit cell migration and invasion by preventing SRC from relaying downstream signals to proteins such as focal adhesion kinase (FAK), paxillin and p130Cas, which mediate adhesion, and MAPK and p27, implicated in cell cycle regulation [33, 34]. Here, we extend this observation to gastric cells and show that dasatinib significantly increases actin accumulation in cell cortex which also implicate increased cell-cell adhesion [35].

Few studies have assessed the potential application of dasatinib in GC. A recent study showed the effect of dasatinib on proliferation of GC cells, promoting tumor necrosis factor-related apoptosis-inducing ligand (TRAIL)-mediated apoptosis via upregulation of CCAAT/enhancer-binding protein homologous protein (CHOP) -dependent expression of death receptor 5 [36]. While the study investigates the downstream targets involved in dasatinib-mediated apoptosis it does not identify the direct molecular targets inhibited by dasatinib. A previous report identified the dasatinib target discoidin-domain receptor 2 (DDR2) as a potential regulator of peritoneal dissemination, the most frequent and deadly form of metastasis in GC [37]. The therapeutic effects of dasatinib on peritoneal dissemination have been demonstrated in a xenograft mouse model suppressing peritoneal metastasis of GC both after oral and intraperitoneal administration. Together with our data these studies suggest dasatinib as a potential therapeutic avenue for the treatment of GC.

Upregulation of the transmembrane receptor tyrosine kinases DDR1, a close relative of DDR2, has been linked to poor prognosis in GC patients [38]. Xie et al. established a correlation between DDR1 upregulation and metastasis via the epithelial-mesenchymal transition in GC, demonstrating that DDR1 can enhance GC proliferation, invasion and micro vessel formation in a mouse model [39]. We did not identify DDR2, a close relative of DDR1 in our analysis as target for dasatinib. DDR2 has been identified in Singaporean and Japanese cohorts as being regulating metastasis. The present metatstatic cell line has been isolated from a Brazilian patient and shows different molecular make-up compared to several Asian GC cell lines [40]. It is thus possible that a diverse genetic and/or epigenetic make up of this cell line from a different ethnic origin may result in different expression pattern in GC.

Our results further corroborate previous findings from our group, reporting an association between SRC and GC invasiveness. The non-receptor tyrosine kinase SRC is one of the best studied kinases in solid cancers including GC [41]. Many human hematologic and epithelial malignancies harbor increased SRC activity, indicating its close association with oncogenesis [42]. SRC is implicated in cellular adhesion, invasion, proliferation and mutagenesis as well as the regulation of angiogenesis and bone remodelling leading to metastasis [41, 43], which together represent four of the six hallmarks of cancer as defined by Hanahan and Weinberg [44]. SRC can mediate a potential cross-talk between CXCR4 and EGFR and utilize the EGFR/AKT/ERK axis to promote cellular migration in GC [45]. Further, SRC upregulation was shown to mediate EGFR activation in GC cells through the RANKL/RANK pathway, thus abrogating sensitivity to the EGFR inhibitor cetuximab. These findings highlight an association between SRC overexpression and resistance to anti-EGFR therapies in GC, suggesting that combination treatment using anti-EGFR agents and SRC inhibitors such as dasatinib might be a promising therapeutic strategy in treatment-refractory GC patients.

The role of FRK and SIK2 in GC development remains unexplored in current literature. FRK belongs to the family of BRK family kinases (BFK), which is related to the SRC family kinases [46], but its functions remain largely unknown. FRK acts as an oncogene in hepatocellular carcinoma, contributing to enhanced proliferation and anchorage-dependent growth in liver cancer cells [46]. Studies in leukemia also indicate that an ETV6-FRK chimera could function as an oncogene contributing to leukemogenesis [47]. Additional research in GC is required to elucidate its downstream targets and functions as well as its role in metastasis.

SIK2 is a serine/threonine kinase belonging to the calcium calmodulin kinases (CAM) superfamily and the AMP-activated protein kinases (AMPK) subfamily and is overexpressed in many tumors [48]. SIK2 is implicated in a vast array of metabolic pathways such as the modulation of the insulin-signalling cascade of adipose tissue and hormonal signal transduction in adipocytes, cell-cycle progression and energy balance [49]. It is reactivated in refeeding from starvation and regulates homeostasis to enable adaptation to metabolic stresses by modulating CREB1-mediated gene transcription [50]. Interestingly, SIK2 is highly expressed in metastatic deposits in ovarian cancer but not in ovarian primary lesions promoting abdominal metastasis while SIK2 depletion was shown to prevent metastasis *in vivo* [51]. SIK2 also functions as a centrosome kinase that regulates centrosome splitting during mitosis in ovarian cancer cells, indicating that its depletion could inhibit cancer growth by targeting multiple phases of the cell-cycle such as G_1_ arrest [52]. In immune cells, SIK2 has been shown to regulate proinflammatory cytokines, while upregulating the anti-inflammatory cytokine IL-10. Targeting SIK2 may therefore also have a positive effect on local inflammation frequently associated with tumors [32, 53].

Our findings thus warrant further validation in the *in vivo* setting, which, if successful, could lead to clinical trials in gastric cancer patients. The cytotoxic effect of dasatinib, initially approved as an anti-leukemic agent in GC is intriguing as recent research suggests that gastric malignancies could arise from leukemia stem cells [54, 55]. For instance, strong preclinical and clinical evidence supports the existence of gastric cancer stem cells (gCSC) within the tumor and is emerging as a clinically relevant prognostic biomarker and in the management of GC [54, 55]. CSCs have been shown to be more resistant to treatment than more differentiated cancer cells, and, therefore, may lead to cancer recurrence after treatment [54, 56]. CSCs can be defined as small subpopulations of cells within a tumor that possess the capacity for self- renewal, are enhanced by chemotherapy and can generate heterogeneous lineages of cancer cells that can be enriched in the residual tumor [57]. The existence of gCSCs was demonstrated in human GCs based on tumorigenic functional assays demonstrating their ability to initiate tumors *in vitro* and *in vivo* [54, 58]. However, although dasatinib some years ago has been considered a promising agent for solid tumors [59], recent studies concentrate more on the use of dasatinib in combination with other agents (clinicaltrial.org), potentially due to the high doses required for the treatment of solid tumors with this broad-spectrum kinase inhibitor. Further studies will be necessary to establish the usefulness of dasatinib for GC and other solid tumors in a clinical setting.

In summary, our findings demonstrate that dasatinib could represent a potential targeted therapy for GC and provides mechanistic insight regarding the kinases FRK, DDR1, SRC and SIK2 targeted by this inhibitor. Moreover, this work is the first to elucidate the kinase targets of dasatinib in GC (SRC, ABL2, DDR1, EPHB2, FRK, LYN, RIPK, SRC, SIK2, YES1 and EPHA2). It is also the first to describe the involvement of FRK and SIK2 in GC invasiveness and to validate them through tissue studies as potential biomarkers of GC aggressiveness that can identify metastatic patients.

## MATERIALS AND METHODS

### Cell lines and cell culture

Our group established and cytogenetically characterized three new GC cell lines obtained from primary gastric adenocarcinoma (ACP-02, diffuse type and ACP-03, intestinal type) and peritoneal carcinomatosis (AGP-01, ascitic fluid) from a patient with a primary gastric tumor (Intestinal-type adenocarcinoma), each of which exhibited a composite karyotype with several clonal chromosome alterations similar to the primary tumor from the stomach as described previously [40].

All cell lines were cultured in Dulbecco’s Modified Eagle Medium, high Glucose (4.5 g/L), with Sodium Pyruvate and L-glutamin] (DMEM, Lonza, UK), containing 10% foetal bovine serum (FBS) (Invitrogen, USA), 1% antibiotic with 100 IU/ml Penicillin and 100 ug/ml Streptomycin (Invitrogen, USA). The culture medium was changed 2 to 3 times a week and cells were passaged using trypsin/EDTA (Invitrogen, USA).

### Tumor samples

Biomarker validations were performed on approximately 200 GC tissue samples obtained from patients who underwent gastrectomy in Northern Brazil. This study was approved by the ethics committee of João de Barros Barreto University Hospital (Protocol #316737). Written informed consent with approval of the ethics committee was obtained from all patients prior to specimen collection. All of the patients had negative histories of exposure to either chemotherapy or radiotherapy prior to surgery, and there was no co-occurrence of other diagnosed cancers. Each of the tumor tissue specimens were snap-frozen in liquid nitrogen and stored at –80°C until protein and nucleic acid purification. All of the samples were classified according to Laurén [60], and the tumors were staged according to the TNM staging criteria.

### Growth inhibition assay

All clinical kinase inhibitors (KI) (20–0.156 μM) were diluted in pure DMSO to obtain a stock solution of 50 mmol/L and stored at –20°C in aliquots. Resarzurin was used for growth inhibition assays. Briefly, cells (300/well) were plated in 384-well flat-bottomed plates and cultured for 24 h. Cells were exposed to serially diluted KI in DMEM with 1% FBS, for an additional 72 h. After the incubation time, resarzurin (10 ng/ml) was added for 2 h at 37°C before measurement of fluorescence at 579Ex/584Em on an Envision microplate spectrophotometer (PerkinElmer) and the concentration of KI resulting in 50% growth inhibition (*IC_50_*) was calculated for each cell line in GraphPad Prism 5.0. All the following *in vitro* experiments were performed on metastatic AGP-01 cells. For further validation of the biomarkers found in *in vitro* studies, expression analysis were performed on patient tumor samples from all GC histological types.

### Morphological analysis

In order to analyse the morphological changes, cells treated with dasatinib (1 μM, 500 nM and 100 nM) for 24 h were fixed in methanol (100%) and panoptic staining (Diff-Quik, Baxter Healthcare Co., Miami, Fla.) was performed. Cells were analyzed for morphological changes by optical microscope at 20 ×.

### Microfilament analysis

Cells exposed to dasatinib (1 μM, 500 nM and 100 nM) for 24 h were fixed in 4% PFA in PBS (20 min, RT), permeabilised with 0.5% Triton X-100 in PBS (20 min, RT) and blocked with 3% BSA. Between each step described above, cells were washed three times with PBS for 5 min at 37°C. Cortical actin was stained with 5 μg/mL Alexa 488-Phalloidin (Invitrogen; 1:200, 30 min, RT) and nuclei were stained with DAPI dye (Invitrogen; 100 ng/μl in PBS, 5 min, RT). Images were capture by a laser confocal microscope at 40× (Zeiss, Germany). Image analysis of actin fibers was performed using Acapella (Perkin Elmer). Nuclei were automatically segmented using DAPI channel. Cells were segmented using both DAPI and Actin channels. Dense actin was defined by thresholding to find pixels that were much brighter than their surrounding in the membrane region. The area of dense actin in the membrane region was calculated and normalized to the cell area. Similarly the actin intensity in the membrane region was measured and normalized to the cell area.

### Cell migration and invasion assay

Cell migration assays were performed using an IncuCyte^®^ life-cell analysis system (Essen Bioscience, USA). Briefly, 1.5–3.5 × 10^5^ AGP-01 cells per well were plated in 96-well flat-bottomed ImageLock plates (Essen Bioscience, USA) and cultured for 24 h. Subsequently, a scratch was made using a WoundMaker™ Tool (Essen Bioscience, USA), cells were washed with PBS and subsequently exposed to dasatinib (1 μM, 500 nM and 100 nM or as indicated) in DMEM supplemented with 10% FBS and PenStrep. Images were captured every 2 h for 48 h and evaluated using the InCucyte^®^ software where the wound width was calculated and expressed as percent relative to the control (0.1% DMSO). Cell invasion was assessed by using the cell invasion assay. A 24-well tissue culture plate (Corning) with cell culture inserts which contained an 8 μm pore size polycarbonate membrane was used. Cells (2.5 × 10^5^ cells/mL) were starved overnight (24 h) and then treated with dasatinib (1 μM, 500 nM and 100 nM) for 16 h at 37°C in a humidified incubator containing 5% CO_2_. Following this, AGP-01 cells (1,5 × 10^4^ cells/well) were plated into extra cellular matrix (ECM) coated inserts (Corning) in serum free DMEM containing Dasatinib (1 μM, 500 nM and 100 nM), and DMEM with 10% FBS was placed in the 24-well bottom plate as chemo attractant. After 8 h incubation, cells were removed from the inner surface of the insert using a cotton-tipped swab. Cells that had migrated through the ECM layer and clung to the bottom of the polycarbonate membrane were fixed and stained with Hoechst 33342 (1 μM). The number of migrating cells per insert was captured by the Operetta System (Perkin Elmer) and counted automatically.

### Compound synthesis

All compounds included in Kinobeads γ (KBγ) were synthesized according to the procedures described by Médard et al. [28] Linkable dasatinib was synthesized according to the procedures described in patent WO2013055780A1 by Johnson et al. [61].

### Generation of affinity matrices

Compounds were immobilized on sepharose beads through covalent linkage using their primary amino groups [62]. NHS-activated sepharose (GE Health-care, Freiburg, Germany) and the compounds were equilibrated in DMSO. Coupling densities of compounds included in KBγ were adjusted as previously described [28], linkable dasatinib was adjusted to a coupling density of 2 μmol per ml beads. Fifteen microliters of triethylamine was added to 1 ml beads to start the coupling reaction, and the mixture was incubated on an end-over-end shaker for 16–20 h in the dark. Free NHS groups on beads were blocked by adding 50 μl of amino ethanol and incubating on an end-over-end shaker for 16–20 h in the dark. Coupled beads were washed and stored in ethanol at 4°C in the dark. The coupling reaction was monitored by LC-MS.

### Generation of cell lysates for affinity enrichment

Lysates were prepared essentially as described [32]. Briefly, cells were lysed in lysis buffer containing 50 mM Tris/HCl, pH 7.5, 5% glycerol, 1.5 mM MgCl_2_, 150 mM NaCl, 1 mM Na_3_VO_4_, 0.8% NP40, 25 mM NaF, 1 mM DTT including protease inhibitors (SigmaFast, Sigma Aldrich, Munich, Germany) and phosphatase inhibitors. The lysate was ultracentrifuged for 20 min at 4°C and 145,000 ×g and the protein concentration was adjusted to 3 mg/mL using 1× compound pull down (CP) buffer (50 mM Tris/HCl, pH 7.5, 5% glycerol, 1.5 mM MgCl2, 150 mM NaCl, 20 mM NaF, 1 mM sodium ortho-vanadate, 1 mM DTT), protease inhibitor (SigmaFast, Sigma Aldrich, Munich, Germany) and phosphatase inhibitors.

### Protein enrichment using affinity matrices

For selectivity profiling experiments in 96-well filter plates, the cell lysates (3 mg of total proteins/well) were incubated for 45 min at 4°C in a end-over-end shaker with 0 nM (DMSO control), 3 nM, 10 nM, 30 nM, 100 nM, 300 nM, 1 μM, 3 μM, or 30 μM of the kinase inhibitor dissolved in DMSO [28, 32]. KBγ (35 μL settled beads resuspended with 50% glycerol, washed with 1× CP buffer and equilibrated with CP buffer containing 0.4% NP40) were incubated with the lysates at 4°C for 30 min. The DMSO control lysate was recovered and incubated similarly with KBγ as a pull down of pull down experiment in order to calculate the protein depletion factor [28, 32, 63]. The beads were then washed (3 mL of CP buffer containing 0.4% NP40, followed by 2 mL of CP buffer containing 0.2% NP40) and the bound proteins subsequently eluted by incubation for 30 min at 50°C with 40 μL of 2× NuPAGE LDS sample buffer (Invitrogen, Darmstadt, Germany) containing 50 mM DTT and centrifugation. The reduced eluates were alkylated with 55 mM chloroacetamide, and half of the eluate was desalted and concentrated by a short electrophoresis (about 0.5 cm) on a 4–12% NuPAGE gel (Invitrogen). In-gel trypsin digestion was performed according to standard procedures.

### LC-MS/MS analysis

The optimized conditions for the liquid chromatography tandem mass spectrometry measurements feature an Eksigent nanoLC-Ultra 1D+ (Eksigent, Dublin, CA) coupled to an Orbitrap Elite instrument (Thermo Scientific, Bremen, Germany). One half of the digested peptides was delivered to a trap column (75 μm × 2 cm, packed in-house with 5 μm C18 resin; Reprosil PUR AQ, Dr. Maisch, Ammerbruch-Entringen, Germany) at a flow rate of 5 μL/min in 100% solvent A (0.1% formic acid, FA, in HPLC grade water). After 10 min of loading and washing, peptides were transferred to an analytical column (75 μm × 45 cm, packed in-house with 3 μm C18 resin; Reprosil Gold, Dr. Maisch, Ammerbruch-Entringen, Germany) and separated at a flow rate of 300 nL/min using a 90 min gradient ranging from 4% to 32% solvent C in B (solvent B: 0.1% FA and 5% DMSO in HPLC grade water, solvent C: 0.1% FA and 5% DMSO in acetonitrile). The eluent was sprayed via stainless steel emitters (Thermo) at a spray voltage of 2.2 kV and a heated capillary temperature of 275°C. The Orbitrap Elite instrument was operated in data-dependent mode, automatically switching between MS and MS2. Full scan MS spectra (m/z 360–1300) were acquired in the Orbitrap at 30 000 (m/z 400) resolution using an automatic gain control (AGC) target value of 1e6 charges or maximum injection time of 100 ms. Tandem mass spectra of up to 15 precursors were generated in the multipole collision cell by using higher energy collisional dissociation (HCD; isolation width of 2 Th, maximum injection time of 100 ms, AGC value of 2e5) using 30% normalized collision energy (NCE) and analyzed in the Orbitrap (15,000 resolution). A previous experimentally-obtained inclusion list containing approximately 1,000 kinase peptide m/z and retention time values was enabled in the data acquisition regime. Dynamic exclusion was 20 s and singly-charged precursors were excluded [32].

### Peptide and protein identification and quantification

Peptide and protein identification plus quantification were performed [32] with MaxQuant [64] (version 1.4.0.5.) by searching the MS2 data against all canonical protein sequences as annotated in the UniProt reference database (human proteins only, 88,391 entries, downloaded 22.07.2013, internally annotated with PFAM domains) using the embedded search engine Andromeda [65]. Carbamidomethylated cysteine was a fixed modification; and phosphorylation of serine, threonine, and tyrosine, oxidation of methionine, and Nterminal protein acetylation were variable modifications. Trypsin/P was specified as the proteolytic enzyme and up to two missed cleavage sites were allowed. Precursor and fragment ion tolerances were 10 ppm and 20 ppm, respectively. Label-free quantification [66] and data matching between runs were enabled within MaxQuant.

Search results were filtered for a minimum peptide length of seven amino acids, 1% peptide and protein FDR plus common contaminants and reverse identifications. Each profile was analyzed separately.

### Data analysis

For the kinobeads competition binding assays, protein intensities were normalized to the respective DMSO control and IC50 and EC50 values were deduced by a four-parameter log-logistic regression using an internal pipeline that utilizes the ‘drc’ package in R [32, 67]. An apparent dissociation constant K_d_^app^ was calculated by multiplying the estimated EC50 with a protein-dependent correction factor (depletion factor) that was limited to a maximum value of 1. The correction factor (cf) for a protein is defined as the ratio of the amount of protein captured from two consecutive pulldowns of the same DMSO control lysate [28, 32, 63].

### Data deposition

Mass spectrometry data have been deposited at the ProteomeXchange Consortium (http://proteomecentral.proteomexchange.org) via the PRIDE partner repository with the dataset identifier PXD012953.

### Small hairpin RNA (shRNA) lentiviral vector

ShRNAs (Supplementary Table 3) lentiviral particles were produced in a 96 well format system using the protocol described [68]. Briefly, on Day 1, 17000 HEK293T cells were seeded in 96 well format plate to reach 40% confluency in 100 uL complete DMEM media containing 10%FBS, 1% L-Glutamine. On Day 2, media was replaced with 80 uL of complete media 1 hour prior transfection. The following DNA mix containing 53 ng of DECODE shRNA plasmid, 23.5 ng of psPAX2 and 9.6 ng of pMD2. G was produced and diluted in to a final volume of 10 uL of jetPRIME Buffer (Polyplus^®^). Concomitantly, the transfection mix containing 0,26 uL of jetPRIME was diluted in to 9.74 uL of jetPRIME Buffer, mixed to the DNA mix and incubated for 15 minutes at room temperature. The DNA-transfection reagent mix was added to the 96 well format plate. 18 hours post transfection, media was replaced and the plate was incubated for 48 hours. After 48 hours, the plate was centriguged for 5 minutes at 300xg to ensure cell debris are removed from supernatant before supernatants collection.

### Protein and mRNA purification

Total protein and mRNA were simultaneously isolated from gastric tissue samples using the AllPrep DNA/RNA/Protein Kit (Qiagen, Germany) according to the manufacturer’s instructions. The protein pellet was dissolved in a buffer containing 7 M urea, 2 M thiourea, 4% CHAPS, 50 mM DTT, 1% Protease Inhibitor Cocktail (Sigma-Aldrich, USA), and 0.5% each Phosphatase Inhibitor Cocktail 1 and 2 (Sigma-Aldrich, USA), as previously described [69]. Protein concentrations were determined by the Bradford method (Sigma-Aldrich, USA). RNA concentration and quality were determined using a NanoDrop spectrophotometer (Kisker, Germany) and 1% agarose gels, respectively. Samples were stored at –80°C until use.

### Protein expression analysis

Protein (25 μg) from each sample was separated by 12.5% homogeneous SDS-PAGE and electro-blotted onto a PVDF membrane (Hybond-P, GE Healthcare, USA) for expression. detection. The PVDF membrane was blocked with phosphate-buffered saline containing 0.1% Tween 20, and 5% low fat milk and incubated overnight at 4°C with the corresponding primary antibodies: anti-SRC (dilution 1:1000; clone 28, Life Technologies, USA), anti-LYN (dilution 1:1000; clone C13F9; Life Technologies, USA), anti-FRK (dilution 1:400; HPA001254, Santa Cruz Biotechnology, USA), anti-DDR1 (dilution 1:500), anti-SIK2 (dilution 1:500) and anti-ACTB (dilution 1:250; Ac-15, Life Technologies, USA). After several washing steps with TBS, a peroxidase-conjugated secondary antibody was added for 1 h at room temperature. Immunoreactive bands were visualized using the Western blotting Luminol reagent, and images were acquired using an ImageQuant 350 digital image system (GE Healthcare, Sweden). ACTB was used as a loading control.

### mRNA expression analysis

RNA was reverse-transcribed using the High-Capacity cDNA Archive kit according to the manufacturer’s instructions (Life Technologies, USA). Complementary DNA was then amplified by real-time reverse transcription quantitative PCR (RT-qPCR) using TaqMan probes purchased as Assays-on-demand Products for Gene Expression (Life Technologies, USA) and amplified in a 7500 Fast Real-Time PCR instrument (Life Technologies, USA). All qPCR reactions were performed in triplicates. Ct (threshold cycle number) and expression values with standard deviations were calculated. The GAPDH gene was selected as an internal control for RNA input and reverse-transcription efficiency. All RT-qPCRs were performed in triplicate for the target genes (SRC: Hs01082246_m1; LYN: Hs00176719_m1; FRK: Hs01547786_g1; *DDR1*: Hs01058424_g1; *SIK2*: Hs01568566_m1) and the internal control (GAPDH: NM_002046.3).

The relative quantification of gene expression was calculated according to Livak and Schmittgen [70]. The corresponding control sample was designated as a calibrator from each tumor.

### Statistical analysis

All experiments were repeated at least 3 times. Data are reported as means ± Standard Deviation (SD). Correlation coefficients (r) were calculated using the Pearson product–moment correlation, and statistical significance (*p*-value) was analysed using *F* test, *T* test or the corresponding nonparametric tests. The expression level of protein measured by western blot was analyzed by ImageJ software; *p*-values were calculated using Students *t*-test.

## SUPPLEMENTARY MATERIALS











## References

[1] Bray F , Ferlay J , Soerjomataram I , Siegel RL , Torre LA , Jemal A . Global cancer statistics 2018: GLOBOCAN estimates of incidence and mortality worldwide for 36 cancers in 185 countries. CA Cancer J Clin. 2018; 68:394–424. 10.3322/caac.21492. 30207593

[2] Jou E , Rajdev L . Current and emerging therapies in unresectable and recurrent gastric cancer. World J Gastroenterol. 2016; 22:4812–4823. 10.3748/wjg.v22.i20.4812. 27239108 PMC4873874

[3] Jacome AA , Coutinho AK , Lima EM , Andrade AC , Dos Santos JS . Personalized medicine in gastric cancer: Where are we and where are we going? World J Gastroenterol. 2016; 22:1160–1171. 10.3748/wjg.v22.i3.1160. 26811654 PMC4716027

[4] Lordick F , Kang YK , Chung HC , Salman P , Oh SC , Bodoky G , Kurteva G , Volovat C , Moiseyenko VM , Gorbunova V , Park JO , Sawaki A , Celik I , et al. Capecitabine and cisplatin with or without cetuximab for patients with previously untreated advanced gastric cancer (EXPAND): a randomised, open-label phase 3 trial. Lancet Oncol. 2013; 14:490–499. 10.1016/S1470-2045(13)70102-5. 23594786

[5] Janowitz T , Thuss-Patience P , Marshall A , Kang JH , Connell C , Cook N , Dunn J , Park SH , Ford H . Chemotherapy vs supportive care alone for relapsed gastric, gastroesophageal junction, and oesophageal adenocarcinoma: a meta-analysis of patient-level data. Br J Cancer. 2016; 114:381–387. 10.1038/bjc.2015.452. 26882063 PMC4815769

[6] Xu W , Yang Z , Lu N . Molecular targeted therapy for the treatment of gastric cancer. J Exp Clin Cancer Res. 2016; 35:1. 10.1186/s13046-015-0276-9. 26728266 PMC4700735

[7] Ang YL , Yong WP , Tan P . Translating gastric cancer genomics into targeted therapies. Crit Rev Oncol Hematol. 2016; 100:141–146. 10.1016/j.critrevonc.2016.02.007. 26947813

[8] Fuchs CS , Tabernero J , Tomasek J , Chau I , Melichar B , Safran H , Tehfe MA , Filip D , Topuzov E , Schlittler L , Udrea AA , Campbell W , Brincat S , et al. Biomarker analyses in REGARD gastric/GEJ carcinoma patients treated with VEGFR2-targeted antibody ramucirumab. Br J Cancer. 2016; 115:974–982. 10.1038/bjc.2016.293. 27623234 PMC5061911

[9] Hironaka S . Anti-angiogenic therapies for gastric cancer. Asia Pac J Clin Oncol. 2019; 15:208–217. 10.1111/ajco.13174. 31111678

[10] Chaikuad A , Koch P , Laufer SA , Knapp S . The Cysteinome of Protein Kinases as a Target in Drug Development. Angew Chem Int Ed Engl. 2018; 57:4372–4385. 10.1002/anie.201707875. 28994500

[11] Bhullar KS , Lagaron NO , McGowan EM , Parmar I , Jha A , Hubbard BP , Rupasinghe HPV . Kinase-targeted cancer therapies: progress, challenges and future directions. Mol Cancer. 2018; 17:48. 10.1186/s12943-018-0804-2. 29455673 PMC5817855

[12] Knapp S . New opportunities for kinase drug repurposing and target discovery. Br J Cancer. 2018; 118:936–937. 10.1038/s41416-018-0045-6. 29545596 PMC5931101

[13] Knapp S , Sundstrom M . Recently targeted kinases and their inhibitors-the path to clinical trials. Curr Opin Pharmacol. 2014; 17:58–63. 10.1016/j.coph.2014.07.015. 25113945

[14] Sasaki T , Hiroki K , Yamashita Y . The role of epidermal growth factor receptor in cancer metastasis and microenvironment. Biomed Res Int. 2013; 2013:546318. 10.1155/2013/546318. 23986907 PMC3748428

[15] Troster A , Heinzlmeir S , Berger BT , Gande SL , Saxena K , Sreeramulu S , Linhard V , Nasiri AH , Bolte M , Muller S , Kuster B , Medard G , Kudlinzki D , et al. NVP-BHG712: Effects of Regioisomers on the Affinity and Selectivity toward the EPHrin Family. ChemMedChem. 2018; 13:1629–1633. 10.1002/cmdc.201800398. 29928781

[16] Lodola A , Giorgio C , Incerti M , Zanotti I , Tognolini M . Targeting Eph/ephrin system in cancer therapy. Eur J Med Chem. 2017; 142:152–162. 10.1016/j.ejmech.2017.07.029. 28780190

[17] Patel A , Sabbineni H , Clarke A , Somanath PR . Novel roles of Src in cancer cell epithelial-to-mesenchymal transition, vascular permeability, microinvasion and metastasis. Life Sci. 2016; 157:52–61. 10.1016/j.lfs.2016.05.036. 27245276 PMC4956571

[18] https://clinicaltrials.gov/ct2/show/NCT00566618.

[19] Jin H , Ham IH , Oh HJ , Bae CA , Lee D , Kim YB , Son SY , Chwae YJ , Han SU , Brekken RA , Hur H . Inhibition of Discoidin Domain Receptor 1 Prevents Stroma-Induced Peritoneal Metastasis in Gastric Carcinoma. Mol Cancer Res. 2018; 16:1590–1600. 10.1158/1541-7786.MCR-17-0710. 29866925

[20] Yuge R , Kitadai Y , Takigawa H , Naito T , Oue N , Yasui W , Tanaka S , Chayama K . Silencing of Discoidin Domain Receptor-1 (DDR1) Concurrently Inhibits Multiple Steps of Metastasis Cascade in Gastric Cancer. Transl Oncol. 2018; 11:575–584. 10.1016/j.tranon.2018.02.003. 29547756 PMC5854925

[21] Farran B , Muller S , Montenegro RC . Gastric cancer management: Kinases as a target therapy. Clin Exp Pharmacol Physiol. 2017; 44:613–622. 10.1111/1440-1681.12743. 28271563

[22] Wu P , Nielsen TE , Clausen MH . FDA-approved small-molecule kinase inhibitors. Trends Pharmacol Sci. 2015; 36:422–439. 10.1016/j.tips.2015.04.005. 25975227

[23] Jhawer M , Kindler HL , Wainberg ZA , Ford JM , Kunz PL , Tang LA , McCallum S , Kallender H , Shah MA . Assessment of two dosing schedules of GSK1363089 (GSK089), a dual MET/VEGFR2 inhibitor, in metastatic gastric cancer (GC): Interim results of a multicenter phase II study. J Clin Oncol. 2009; 27.

[24] Moehler M , Gepfner-Tuma I , Maderer A , Thuss-Patience PC , Ruessel J , Hegewisch-Becker S , Wilke H , Al-Batran SE , Rafiyan MR , Weissinger F , Schmoll HJ , Kullmann F , von Weikersthal LF , et al. Sunitinib added to FOLFIRI versus FOLFIRI in patients with chemorefractory advanced adenocarcinoma of the stomach or lower esophagus: a randomized, placebo-controlled phase II AIO trial with serum biomarker program. BMC Cancer. 2016; 16:699. 10.1186/s12885-016-2736-9. 27582078 PMC5006426

[25] Satoh T , Xu RH , Chung HC , Sun GP , Doi T , Xu JM , Tsuji A , Omuro Y , Li J , Wang JW , Miwa H , Qin SK , Chung IJ , et al. Lapatinib plus paclitaxel versus paclitaxel alone in the second-line treatment of HER2-amplified advanced gastric cancer in Asian populations: TyTAN–a randomized, phase III study. J Clin Oncol. 2014; 32:2039–2049. 10.1200/JCO.2013.53.6136. 24868024

[26] Li J , Qin S , Xu J , Xiong J , Wu C , Bai Y , Liu W , Tong J , Liu Y , Xu R , Wang Z , Wang Q , Ouyang X , et al. Randomized, Double-Blind, Placebo-Controlled Phase III Trial of Apatinib in Patients With Chemotherapy-Refractory Advanced or Metastatic Adenocarcinoma of the Stomach or Gastroesophageal Junction. J Clin Oncol. 2016; 34:1448–1454. 10.1200/JCO.2015.63.5995. 26884585

[27] Roviello G , Ravelli A , Polom K , Petrioli R , Marano L , Marrelli D , Roviello F , Generali D . Apatinib: A novel receptor tyrosine kinase inhibitor for the treatment of gastric cancer. Cancer Lett. 2016; 372:187–191. 10.1016/j.canlet.2016.01.014. 26797419

[28] Medard G , Pachl F , Ruprecht B , Klaeger S , Heinzlmeir S , Helm D , Qiao H , Ku X , Wilhelm M , Kuehne T , Wu Z , Dittmann A , Hopf C , et al. Optimized chemical proteomics assay for kinase inhibitor profiling. J Proteome Res. 2015; 14:1574–1586. 10.1021/pr5012608. 25660469

[29] Li J , Rix U , Fang B , Bai Y , Edwards A , Colinge J , Bennett KL , Gao J , Song L , Eschrich S , Superti-Furga G , Koomen J , Haura EB . A chemical and phosphoproteomic characterization of dasatinib action in lung cancer. Nat Chem Biol. 2010; 6:291–299. 10.1038/nchembio.332. 20190765 PMC2842457

[30] Araujo J , Logothetis C . Dasatinib: a potent SRC inhibitor in clinical development for the treatment of solid tumors. Cancer Treat Rev. 2010; 36:492–500. 10.1016/j.ctrv.2010.02.015. 20226597 PMC3940067

[31] Shi H , Zhang CJ , Chen GY , Yao SQ . Cell-based proteome profiling of potential dasatinib targets by use of affinity-based probes. J Am Chem Soc. 2012; 134:3001–3014. 10.1021/ja208518u. 22242683

[32] Klaeger S , Heinzlmeir S , Wilhelm M , Polzer H , Vick B , Koenig PA , Reinecke M , Ruprecht B , Petzoldt S , Meng C , Zecha J , Reiter K , Qiao H , et al. The target landscape of clinical kinase drugs. Science. 2017; 358. 10.1126/science.aan4368. 29191878 PMC6542668

[33] Johnson FM , Saigal B , Talpaz M , Donato NJ . Dasatinib (BMS-354825) tyrosine kinase inhibitor suppresses invasion and induces cell cycle arrest and apoptosis of head and neck squamous cell carcinoma and non-small cell lung cancer cells. Clin Cancer Res. 2005; 11:6924–6932. 10.1158/1078-0432.CCR-05-0757. 16203784

[34] Nam S , Kim D , Cheng JQ , Zhang S , Lee JH , Buettner R , Mirosevich J , Lee FY , Jove R . Action of the Src family kinase inhibitor, dasatinib (BMS-354825), on human prostate cancer cells. Cancer Res. 2005; 65:9185–9189. 10.1158/0008-5472.CAN-05-1731. 16230377

[35] Fenton SE , Hutchens KA , Denning MF . Targeting Fyn in Ras-transformed cells induces F-actin to promote adherens junction-mediated cell-cell adhesion. Mol Carcinog. 2015; 54:1181–1193. 10.1002/mc.22190. 24976598

[36] Wang X , Xue Q , Wu L , Wang B , Liang H . Dasatinib promotes TRAIL-mediated apoptosis by upregulating CHOP-dependent death receptor 5 in gastric cancer. FEBS Open Bio. 2018; 8:732–742. 10.1002/2211-5463.12404. 29744288 PMC5929929

[37] Kurashige J , Hasegawa T , Niida A , Sugimachi K , Deng N , Mima K , Uchi R , Sawada G , Takahashi Y , Eguchi H , Inomata M , Kitano S , Fukagawa T , et al. Integrated Molecular Profiling of Human Gastric Cancer Identifies DDR2 as a Potential Regulator of Peritoneal Dissemination. Sci Rep. 2016; 6:22371. 10.1038/srep22371. 26934957 PMC4776110

[38] Hur H , Ham IH , Lee D , Jin H , Aguilera KY , Oh HJ , Han SU , Kwon JE , Kim YB , Ding K , Brekken RA . Discoidin domain receptor 1 activity drives an aggressive phenotype in gastric carcinoma. BMC Cancer. 2017; 17:87. 10.1186/s12885-017-3051-9. 28143619 PMC5286810

[39] Xie R , Wang X , Qi G , Wu Z , Wei R , Li P , Zhang D . DDR1 enhances invasion and metastasis of gastric cancer via epithelial-mesenchymal transition. Tumour Biol. 2016; 37:12049–12059. 10.1007/s13277-016-5070-6. 27179963

[40] Montenegro RC , Clark PG , Howarth A , Wan X , Ceroni A , Siejka P , Nunez-Alonso GA , Monteiro O , Rogers C , Gamble V , Burbano R , Brennan PE , Tallant C , et al. BET inhibition as a new strategy for the treatment of gastric cancer. Oncotarget. 2016; 7:43997–44012. 10.18632/oncotarget.9766. 27259267 PMC5190074

[41] Aleshin A , Finn RS . SRC: a century of science brought to the clinic. Neoplasia. 2010; 12:599–607. 10.1593/neo.10328. 20689754 PMC2915404

[42] Alvarez RH , Kantarjian HM , Cortes JE . The role of Src in solid and hematologic malignancies: development of new-generation Src inhibitors. Cancer. 2006; 107:1918–1929. 10.1002/cncr.22215. 16986126

[43] Steinberg M . Dasatinib: a tyrosine kinase inhibitor for the treatment of chronic myelogenous leukemia and philadelphia chromosome-positive acute lymphoblastic leukemia. Clin Ther. 2007; 29:2289–2308. 10.1016/j.clinthera.2007.11.005. 18158072

[44] Hanahan D , Weinberg RA . The hallmarks of cancer. Cell. 2000; 100:57–70. 10.1016/S0092-8674(00)81683-9. 10647931

[45] Cheng Y , Qu J , Che X , Xu L , Song N , Ma Y , Gong J , Qu X , Liu Y . CXCL12/SDF-1alpha induces migration via SRC-mediated CXCR4-EGFR cross-talk in gastric cancer cells. Oncol Lett. 2017; 14:2103–2110. 10.3892/ol.2017.6389. 28781651 PMC5530148

[46] Goel RK , Lukong KE . Understanding the cellular roles of Fyn-related kinase (FRK): implications in cancer biology. Cancer Metastasis Rev. 2016; 35:179–199. 10.1007/s10555-016-9623-3. 27067725

[47] Hosoya N , Qiao Y , Hangaishi A , Wang L , Nannya Y , Sanada M , Kurokawa M , Chiba S , Hirai H , Ogawa S . Identification of a SRC-like tyrosine kinase gene, FRK, fused with ETV6 in a patient with acute myelogenous leukemia carrying a t(6;12)(q21;p13) translocation. Genes Chromosomes Cancer. 2005; 42:269–279. 10.1002/gcc.20147. 15611931

[48] Chen F , Chen L , Qin Q , Sun X . Salt-Inducible Kinase 2: An Oncogenic Signal Transmitter and Potential Target for Cancer Therapy. Front Oncol. 2019; 9:18. 10.3389/fonc.2019.00018. 30723708 PMC6349817

[49] Du WQ , Zheng JN , Pei DS . The diverse oncogenic and tumor suppressor roles of salt-inducible kinase (SIK) in cancer. Expert Opin Ther Targets. 2016; 20:477–485. 10.1517/14728222.2016.1101452. 26549013

[50] Bon H , Wadhwa K , Schreiner A , Osborne M , Carroll T , Ramos-Montoya A , Ross-Adams H , Visser M , Hoffmann R , Ahmed AA , Neal DE , Mills IG . Salt-inducible kinase 2 regulates mitotic progression and transcription in prostate cancer. Mol Cancer Res. 2015; 13:620–635. 10.1158/1541-7786.MCR-13-0182-T. 25548099 PMC4383640

[51] Miranda F , Mannion D , Liu S , Zheng Y , Mangala LS , Redondo C , Herrero-Gonzalez S , Xu R , Taylor C , Chedom DF , Karaminejadranjbar M , Albukhari A , Jiang D , et al. Salt-Inducible Kinase 2 Couples Ovarian Cancer Cell Metabolism with Survival at the Adipocyte-Rich Metastatic Niche. Cancer Cell. 2016; 30:273–289. 10.1016/j.ccell.2016.06.020. 27478041

[52] Ahmed AA , Lu Z , Jennings NB , Etemadmoghadam D , Capalbo L , Jacamo RO , Barbosa-Morais N , Le XF . SIK2 is a centrosome kinase required for bipolar mitotic spindle formation that provides a potential target for therapy in ovarian cancer. Cancer Cell. 2010; 18:109–121. 10.1016/j.ccr.2010.06.018. 20708153 PMC3954541

[53] Lombardi MS , Gillieron C , Dietrich D , Gabay C . SIK inhibition in human myeloid cells modulates TLR and IL-1R signaling and induces an anti-inflammatory phenotype. J Leukoc Biol. 2016; 99:711–721. 10.1189/jlb.2A0715-307R. 26590148

[54] Chen XL , Chen XZ , Wang YG , He D , Lu ZH , Liu K , Zhang WH , Wang W , Li CC , Xue L , Zhao LY , Yang K , Liu JP , et al. Clinical significance of putative markers of cancer stem cells in gastric cancer: A retrospective cohort study. Oncotarget. 2016; 7:62049–62069. 10.18632/oncotarget.11384. 27557490 PMC5308710

[55] Nguyen PH , Giraud J , Chambonnier L , Dubus P , Wittkop L , Belleannee G , Collet D , Soubeyran I , Evrard S , Rousseau B , Senant-Dugot N , Megraud F , Mazurier F , et al. Characterization of Biomarkers of Tumorigenic and Chemoresistant Cancer Stem Cells in Human Gastric Carcinoma. Clin Cancer Res. 2017; 23:1586–1597. 10.1158/1078-0432.CCR-15-2157. 27620279

[56] Phi LTH , Sari IN , Yang YG , Lee SH , Jun N , Kim KS , Lee YK , Kwon HY . Cancer Stem Cells (CSCs) in Drug Resistance and their Therapeutic Implications in Cancer Treatment. Stem Cells Int. 2018; 2018:5416923. 10.1155/2018/5416923. 29681949 PMC5850899

[57] Ayob AZ , Ramasamy TS . Cancer stem cells as key drivers of tumour progression. J Biomed Sci. 2018; 25:20. 10.1186/s12929-018-0426-4. 29506506 PMC5838954

[58] Hayakawa Y , Fox JG , Wang TC . The Origins of Gastric Cancer From Gastric Stem Cells: Lessons From Mouse Models. Cell Mol Gastroenterol Hepatol. 2017; 3:331–338. 10.1016/j.jcmgh.2017.01.013. 28462375 PMC5404024

[59] Kim LC , Rix U , Haura EB . Dasatinib in solid tumors. Expert Opin Investig Drugs. 2010; 19:415–425. 10.1517/13543781003592097. 20113198

[60] Lauren P . The Two Histological Main Types of Gastric Carcinoma: Diffuse and So-Called Intestinal-Type Carcinoma. An Attempt at a Histo-Clinical Classification. Acta Pathol Microbiol Scand. 1965; 64:31–49. 10.1111/apm.1965.64.1.31. 14320675

[61] Johnson G , Duncan JS , Whittle MC , Jian J . Multiplexed kinase inhibitor beads and uses thereof, Patent WO2013055780A1. 2013.

[62] Bantscheff M , Eberhard D , Abraham Y , Bastuck S , Boesche M , Hobson S , Mathieson T , Perrin J , Raida M , Rau C , Reader V , Sweetman G , Bauer A , et al. Quantitative chemical proteomics reveals mechanisms of action of clinical ABL kinase inhibitors. Nat Biotechnol. 2007; 25:1035–1044. 10.1038/nbt1328. 17721511

[63] Daub H . Quantitative proteomics of kinase inhibitor targets and mechanisms. ACS Chem Biol. 2015; 10:201–212. 10.1021/cb5008794. 25474541

[64] Cox J , Mann M . MaxQuant enables high peptide identification rates, individualized p.p.b.-range mass accuracies and proteome-wide protein quantification. Nat Biotechnol. 2008; 26:1367–1372. 10.1038/nbt.1511. 19029910

[65] Cox J , Neuhauser N , Michalski A , Scheltema RA , Olsen JV , Mann M . Andromeda: a peptide search engine integrated into the MaxQuant environment. J Proteome Res. 2011; 10:1794–1805. 10.1021/pr101065j. 21254760

[66] Cox J , Hein MY , Luber CA , Paron I , Nagaraj N , Mann M . Accurate proteome-wide label-free quantification by delayed normalization and maximal peptide ratio extraction, termed MaxLFQ. Mol Cell Proteomics. 2014; 13:2513–2526. 10.1074/mcp.M113.031591. 24942700 PMC4159666

[67] Ritz C , Baty F , Streibig JC , Gerhard D . Dose-Response Analysis Using R. PLoS One. 2015; 10:e0146021. 10.1371/journal.pone.0146021. 26717316 PMC4696819

[68] Ceroni A , Higgins GS , Ebner DV . *In Vitro*-Pooled shRNA Screening to Identify Determinants of Radiosensitivity . Methods Mol Biol. 2016; 1470:103–119. 10.1007/978-1-4939-6337-9_9. 27581288

[69] Leal MF , Calcagno DQ , Demachki S , Assumpcao PP , Chammas R , Burbano RR , Smith Mde A . Clinical implication of 14-3-3 epsilon expression in gastric cancer. World J Gastroenterol. 2012; 18:1531–1537. 10.3748/wjg.v18.i13.1531. 22509086 PMC3319950

[70] Livak KJ , Schmittgen TD . Analysis of relative gene expression data using real-time quantitative PCR and the 2(-Delta Delta C(T)). Method. Methods. 2001; 25:402–408. 10.1006/meth.2001.1262. 11846609

