# Supplementary material for: Identification of molecular targets for the targeted treatment of gastric cancer using dasatinib
Source: Oncotarget. 2020 Feb 4;11(5):535–49. doi: 10.18632/oncotarget.27462 (PMC7007292; doi:10.18632/oncotarget.27462)
Supplement: Supplementary file 1 [file oncotarget-11-535-s001.pdf]

Identification of molecular targets for the targeted treatment of gastric cancer using dasatinib

SUPPLEMENTARY MATERIALS

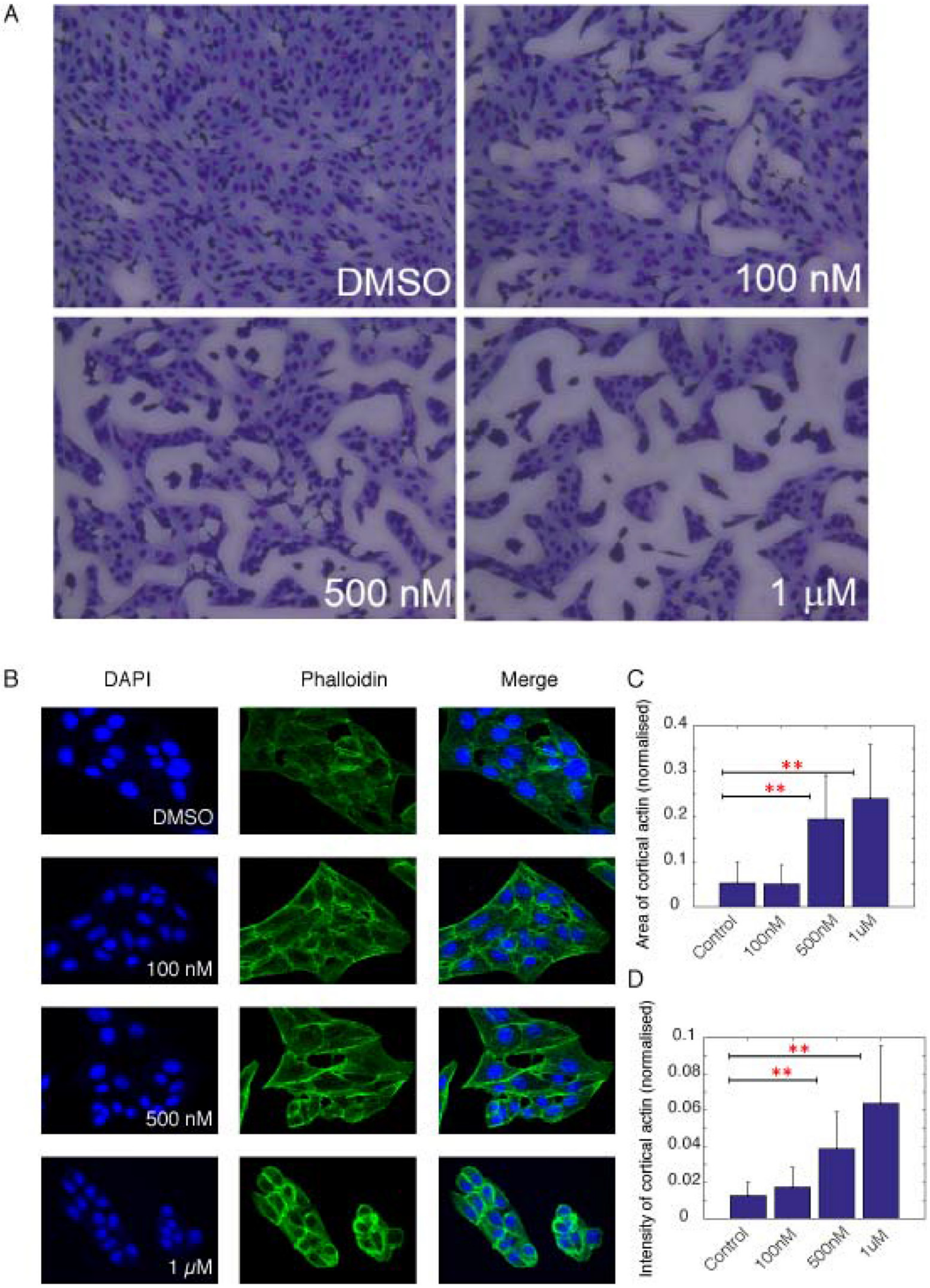

Supplementary Figure 1: Dasatinib effect on cell morphology and cortical actin. (**A**) Morphological effects of drug treatments. Optical microscope images of AGP-01 cells before and after dasatinib treatment. (**B**) Changes in actin cytoskeleton following dasatinib treatment in dose dependent fashion. (**C–D**) The area (C) and intensity (D) of cortical actin is significantly higher in cells treated with dasatinib. **indicate *p*-value < 0.01 using *t*-test. Error bars indicate 1 standard deviation based on measurements of single cells.

Supplementary Figure 2: (**A**) Binding curves for each kinase analysed in Kinobeads. (**B**) Binding curves for each kinase analysed in Dasabeads. See Supplementary Figure 2A and Supplementary Figure 2B

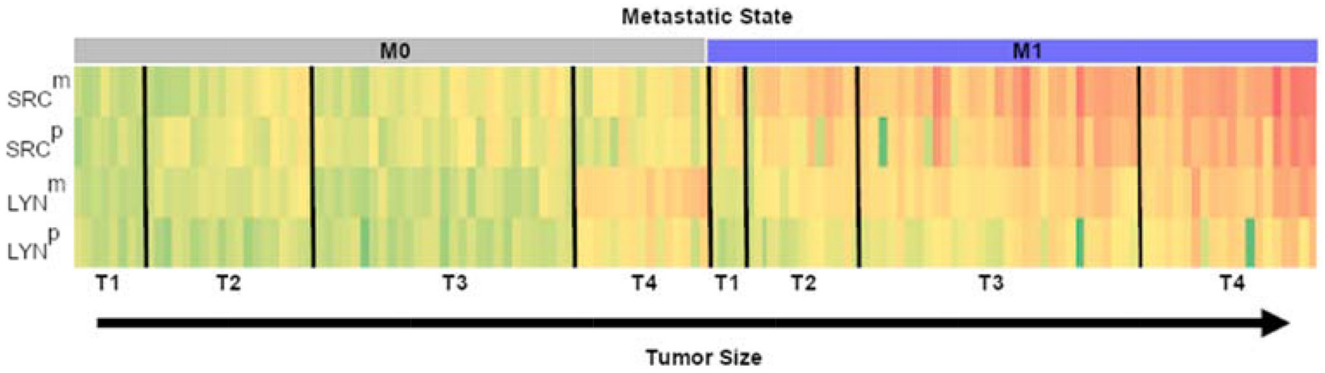

Supplementary Figure 3: Heatmap of mRNA and protein expression of SRC and LYN in patient samples ordered according to tumor size and stage.

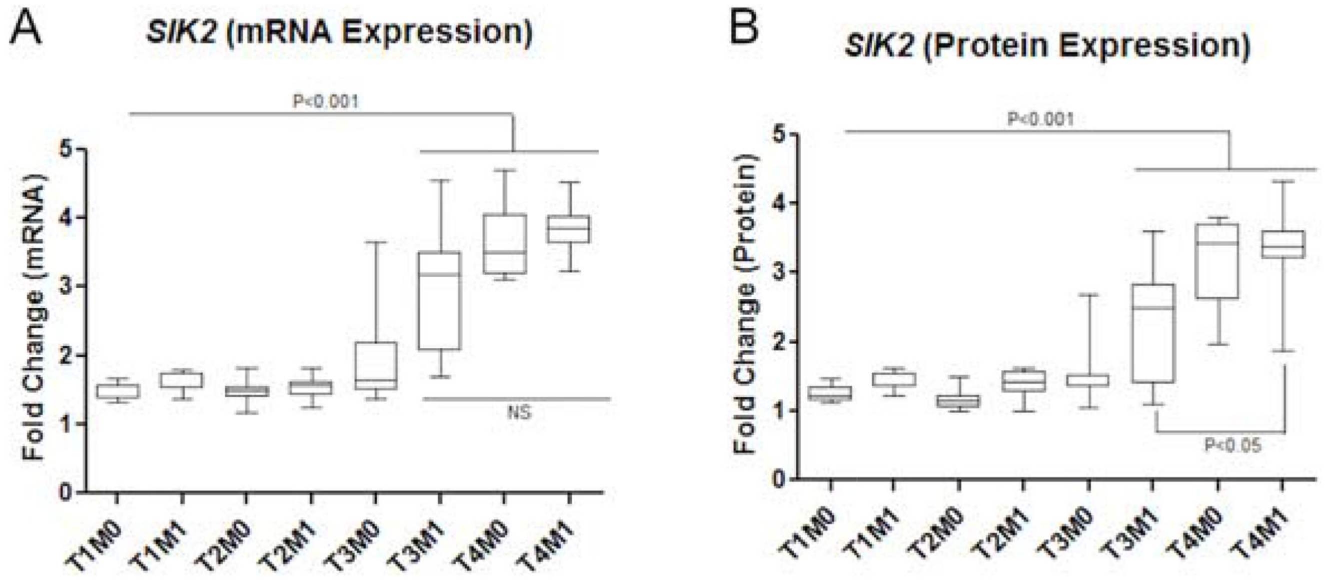

Supplementary Figure 4: Analysis of SIK2 mRNA (**A**) and protein expression (**B**), respectively in relation to tumor size (T1 to T4) and metastatic stage (M0 or M1).

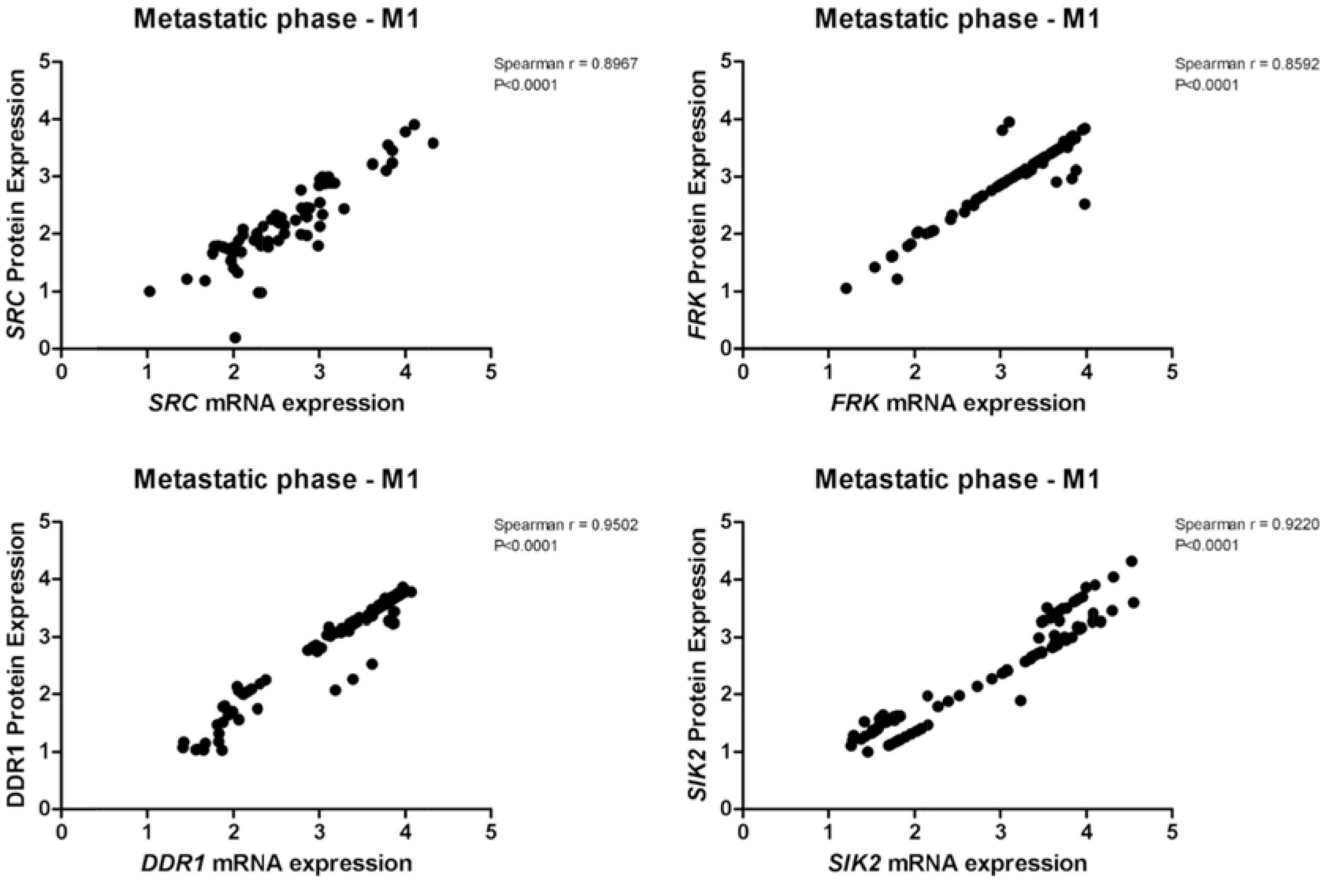

Supplementary Figure 5: Correlation between mRNA and protein expression in metastatic gastric samples for SRC, FRK, DDR1 and SIK2. Spearman coefficients and *P*-values are shown in the graphics, respectively. Significant correlation: $P < 0.05$.

Supplementary Table 1: Selectivity profiling of Dasatinib, using either Kinobeads gamma or Dasatinib beads. See Supplementary Table 1

Supplementary Table 2: Expression of different kinases in patient tumor samples. See Supplementary Table 2

Supplementary Table 3: List of shRNA used for silence target gene expression

Gene*	Full Hairpin Sequence	Sense Sequence	GenBank and Ref Seq Accessions	Entrez Gene ID
LYN	TGCTGTTGACAGTGAGCGA AAGAGTGGTTTTTCAAGGA TATAGTGAAGCCACAGATG TATATCCTTGAAAAACCACTC TTCTGCCTACTGCCTCGGA	AGAGTGGTTTTTCAAGGAT	BC075002, BC126458, BC075001, NM_001111097, NM_002350, BC059394, M16038, CB990993, M79321, BC068551, AK290494, AK298677, BC126456	4067
LYN	TGCTGTTGACAGTGAGCGAA AGGTGCTAAGTTCCCTATTATA GTGAAGCCACAGATGTATAATA GGGAACTTAGCACCTTCTGCCTA CTGCCTCGGA	AGGTGCTAAGTTCCCTATT	BC075002, BC126458, BC075001, NM_001111097, AK130135, NM_002350, BC059394, M16038, M79321, BC068551, AK290494, AK298677, BC126456	4067
LYN	TGCTGTTGACAGTGAGCGCCT CCGAGTCACTCATGTGCAATAGT GAAGCCACAGATGTATTGCACA TGAGTGACTCGGAGATGCCTACTGC CTCGGA	TCCGAGTCACTCATGTGCA	BC075002, BC126458, BC075001, NM_001111097, AK130135, NM_002350, BC059394, BC068551, AK290494, AK298677, BC126456	4067
YES1	TGCTGTTGACAGTGAGCGCAGGAA TATCATGGGACCAAATTAGTGAAGCC ACAGATGTAATTTGGTCCCATGATATTC CTTTGCCTACTGCCTCGGA	GGAATATCATGGGACCAAA	BC048960, NM_005433, M15990	7525
YES1	TGCTGTTGACAGTGAGCGAGGTG ACAATGTGAAACACTACTAGTGA AGCCACAGATGTAGTAGTGTTTC ACATTGTCACCCTGCCTACTGCCTC GGA	GTGACAATGTGAAACACTA	AK314063, BC031080, BC048960, NM_005433, M15990	7525
YES1	TGCTGTTGACAGTGAGCGCACAG TCAGTATGCAATCTTAATAGTGAAGC CACAGATGTATTAAGATTGCATACTG ACTGTTTGCCTACTGCCTCGGA	CAGTCAGTATGCAATCTTA	BC048960, NM_005433, M15990	7525
SRC	TGCTGTTGACAGTGAGCGATCGGCT CATTGAAGACAATGATAGTGAAGCCA CAGATGTATCATTGTCTTCAATGAGC CGAGTGCCTACTGCCTCGGA	CGGCTCATTGAAGACAATG	AK024281, NM_005417, AK091756, EU831426, BC011566, EU831519, BC051270, NM_198291	6714
SRC	TGCTGTTGACAGTGAGCGACGAGAAAG TGAGACCACGAAATAGTGAAGCCACAGA TGTATTTCGTGGTCTCACTTTCTCGCTGCC TACTGCCTCGGA	GAGAAAGTGAGACCACGAA	NM_005417, EU831426, BC011566, EU831519, BC051270, NM_198291	6714
SRC	TGCTGTTGACAGTGAGCGACTCCAGATTG TCAACAACACATAGTGAAGCCACAGATG TATGTGTTGTTGACAATCTGGAGCTGCCTA CTGCCTCGGA	TCCAGATTGTCAACAACAC	NM_005417, EU831426, BC011566, EU831519, BC051270, NM_198291	6714
EPHA2	TGCTGTTGACAGTGAGCGACAAGGAGACTT TCAACCTCTATAGTGAAGCCACAGATGTAT AGAGGTTGAAAGTCTCCTTGCTGCCTACTGCCT CGGA	AAGGAGACTTTCAACCTCT	CB991812, M59371, DQ895231, NM_004431, EU176326, EU826606, BC037166	1969
EPHA2	TGCTGTTGACAGTGAGCGATTGGTTG TGTTGAGGTTTTTATAGTGAAGCCAC AGATGTATAAAAACCTCAACACAAC CAAGTGCCTACTGCCTCGGA	TGGTTGTGTTGAGGTTTTT	M59371, BC008655, NM_004431, BC037166	1969
EPHA2	TGCTGTTGACAGTGAGCGCACCAGG CTGTGTTGAAGTTCATAGTGAAGCCA CAGATGTATGAACTTCAACACAGCCTGG TTTGCCTACTGCCTCGGA	CCAGGCTGTGTTGAAGTTC	M59371, DQ895231, NM_004431, AK296788, EU176326, BC037166	1969
EPHB2	TGCTGTTGACAGTGAGCGACCGAGAG GACCTCGTCTACAATAGTGAAGCCA CAGATGTATTGTAGACGAGGTCCTCTC GGCTGCCTACTGCCTCGGA	CGAGAGGACCTCGTCTACA	BC018763, L36643, BC007903, BC041017, NM_004442, L41939, D31661, NM_017449, AK299701, BC146296, AF025304, BC067861, AB210018	2048
EPHB2	TGCTGTTGACAGTGAGCGCCACGGAC AAGCTGCAACACTATAGTGAAGCCACA GATGTATAGTGTTGCAGCTTGTCCGTG TTGCCTACTGCCTCGGA	ACGGACAAGCTGCAACACT	BC018763, L36643, BC007903, BC041017, NM_004442, L41939, AK298898, D31661, NM_017449, D37827, BC146296, AF025304, AB210018	2048
EPHB2	TGCTGTTGACAGTGAGCGCACCCATCA AGCTCTACTGTAATAGTGAAGCCA CAGATGTATTACAGTAGAGCTTGATGG GTATGCCTACTGCCTCGGA	CCCATCAAGCTCTACTGTA	BC018763, L36643, BC007903, NM_004442, L41939, D31661, NM_017449, AK299701, BC146296, AF025304, BC067861, AB210018	2048

ABL2	TGCTGTTGACAGTGAGCGCGCCTACAATACCTTCTCAATTTAGTGAAGCCACAGATGTAAATTGAGAAGGTATTGTAGGCATGCCTACTGCCTCGGA	CCTACAATACCTTCTCAAT	BC065912, M35296, NM_001136001, NM_001100108, NM_001136000, NM_005158, AK311045, NM_007314, AK225255, BX538317, AK309549	27
ABL2	TGCTGTTGACAGTGAGCGAGCTTCTTTACACCACGCTTAATAGTGAAGCCACAGATGTATTAAGCGTGGTGTAAAGAAGCCTGCCTACTGCCTCGGA	CTTCTTTACACCACGCTTA	BC065912, M35296, NM_001100108, NM_005158, NM_007314	27
ABL2	TGCTGTTGACAGTGAGCGCCCACAGAGACCGGCTTCAATATAGTGAAGCCACAGATGTATATTGAAGCCGGTCTCTGTGGTTGCCTACTGCCTCGGA	CACAGAGACCGGCTTCAAT	M35296, NM_001136001, AK311045, NM_007314	27
SIK2	TGCTGTTGACAGTGAGCGCCCGAAGGATGTTGGTCCTAGATATAGTGAAGCCACAGATGTATCTAGGACCAACATCCTTCGGATGCCTACTGCCTCGGA	CGAAGGATGTTGGTCCTAG	BC117183, AK000396, AB018324, BC078150, AB096248, AL136764, NM_015191, AB084424, BC113459, AK291233	23235
SIK2	TGCTGTTGACAGTGAGCGACAGTTGTTGTATGAACAAATATAGTGAAGCCACAGATGTATATTTGTTCATACAACAACTGCTGCCTACTGCCTCGGA	AGTTGTTGTATGAACAAAT	BC117183, AB018324, BC078150, AB096248, AL136764, NM_015191, BC013612, BC113459, AK291233	23235
SIK2	TGCTGTTGACAGTGAGCGAAAGGAAGAGTGTATGTTCCTATAGTGAAGCCACAGATGTATAGGAACATACACTCTTCCTTCTGCCTACTGCCTCGGA	AGGAAGAGTGTATGTTCCT	AB018324, BC078150, AB096248, AL136764, NM_015191, BC013612, AK291233	23235
FRK	TGCTGTTGACAGTGAGCGCTGGAGCAGTTGTAAAACACTATAGTGAAGCCACAGATGTATAGTGTTTTACAACTGCTCCATTGCCTACTGCCTCGGA	GGAGCAGTTGTAAAACACT	U22322, NM_002031, AK302264, AK313314, U00803, DQ894643, BC012916	2444
FRK	TGCTGTTGACAGTGAGCGACCTAGACTTCTGAGTAATTAATAGTGAAGCCACAGATGTATTAATTACTCAGAAGTCTAGGCTGCCTACTGCCTCGGA	CTAGACTTCTGAGTAATTA	NM_002031, U00803	2444
FRK	TGCTGTTGACAGTGAGCGACCGAAGCCATTCGTAGTAATATAGTGAAGCCACAGATGTATATTACTACGAATGGCTTCGGGTGCCTACTGCCTCGGA	CGAAGCCATTCGTAGTAAT	U22322, NM_002031, AK302264, AK313314, U00803, DQ894643, BC012916	2444
DDR1	TGCTGTTGACAGTGAGCGACTGGTAGCTGTCAAGATCTTATAGTGAAGCCACAGATGTATAAGATCTTGACAGCTACCAGCTGCCTACTGCCTCGGA	TGGTAGCTGTCAAGATCTT	BI193181, L57508, BC008716, NM_013993, AB210021, NM_001954, L20817, BC013400, CR605641, Z29093, L11315, NM_013994, BC070070, AK130776, X74979, AK291621	780
DDR1	TGCTGTTGACAGTGAGCGATTGCAGGTGGATCTACAACGATAGTGAAGCCACAGATGTATCGTTGTAGATCCACCTGCAAGTGCCTACTGCCTCGGA	TGCAGGTGGATCTACAACG	L57508, BC008716, NM_013993, AB210021, NM_001954, EU826614, L20817, BC013400, Z29093, L11315, NM_013994, BC070070, X74979, AK291621, EU826613	780
DDR1	TGCTGTTGACAGTGAGCGAAAGCCAGTGACACTAAAACAATAGTGAAGCCACAGATGTATTGTTTTAGTGTCACTGGCTTCTGCCTACTGCCTCGGA	AGCCAGTGACACTAAAACA	L57508, BC008716, NM_013993, AB210021, NM_001954, L20817, BC013400, CR605641, Z29093, L11315, NM_013994, BC070070, X74979, AK291621	780
RIPK2	TGCTGTTGACAGTGAGCGACAGCTAAAGAAAACAAAGTTATAGTGAAGCCACAGATGTATAACTTTGTTTTCTTTAGCTGATGCCTACTGCCTCGGA	AGCTAAAGAAAACAAAGTT	CR596906, AY358813, AY358814, AF064824, AY524045, AF078530, AF027706, NM_003821, CR592019, AK075213, BC004553	8767
RIPK2	TGCTGTTGACAGTGAGCGAAAGCTCAAGACTGTTATTTTATAGTGAAGCCACAGATGTATAAAATAACAGTCTTGAGCTTTTGCCTACTGCCTCGGA	AGCTCAAGACTGTTATTTT	CR596906, AY358813, AY358814, AF064824, AY524045, AF078530, AF027706, NM_003821, CR592019, AK075213, BC004553	8767
RIPK2	TGCTGTTGACAGTGAGCGAAAGAAGAAATGTGTTTCATAATAGTGAAGCCACAGATGTATTATGAAACACATTTCTTCTTGTGCCTACTGCCTCGGA	AGAAGAAATGTGTTTCATA	AY358813, AY358814, AY524045, AF027706, NM_003821, AK075213, BC004553	8767
