# Supplementary material for: Identification of molecular targets for the targeted treatment of gastric cancer using dasatinib
Source: Oncotarget. 2020 Feb 4;11(5):535–49. doi: 10.18632/oncotarget.27462 (PMC7007292; doi:10.18632/oncotarget.27462)

## ABL1*

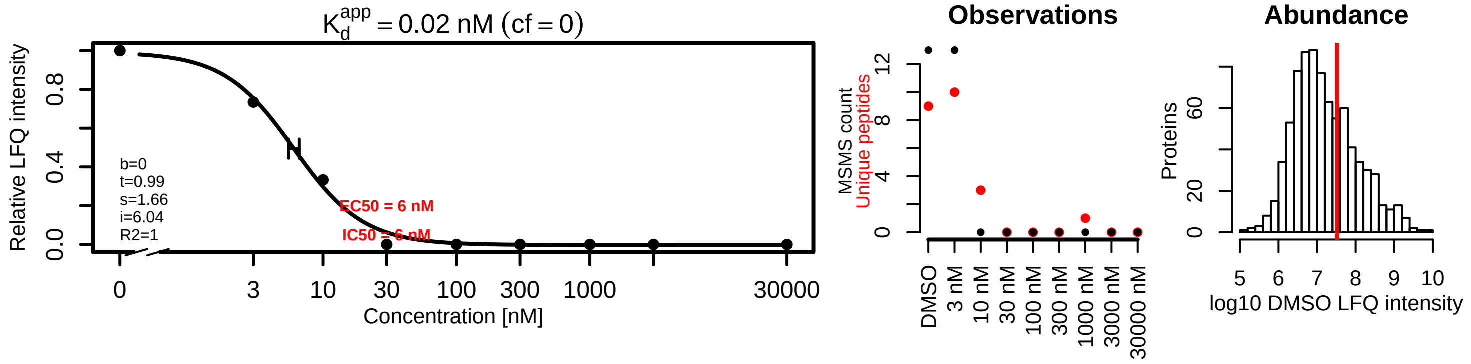


## SRC*

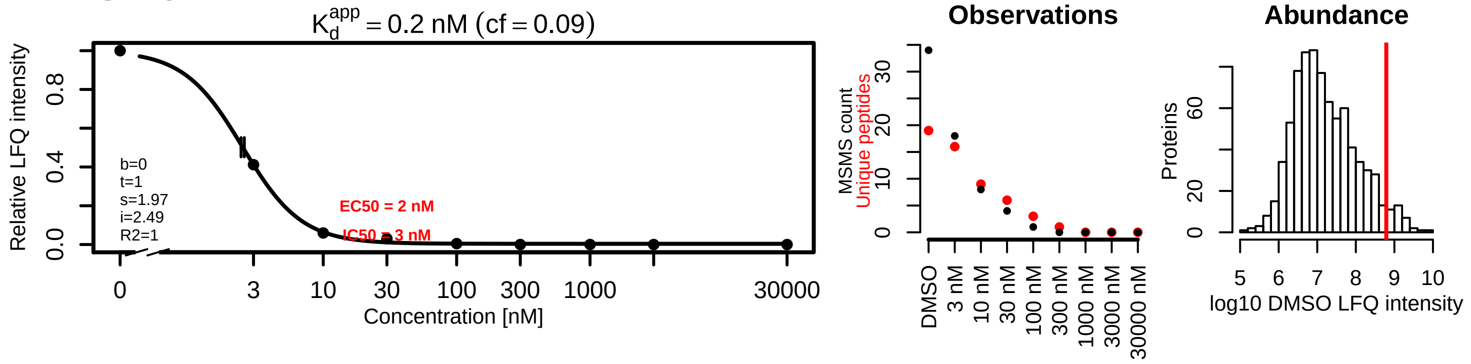


## ABL2*

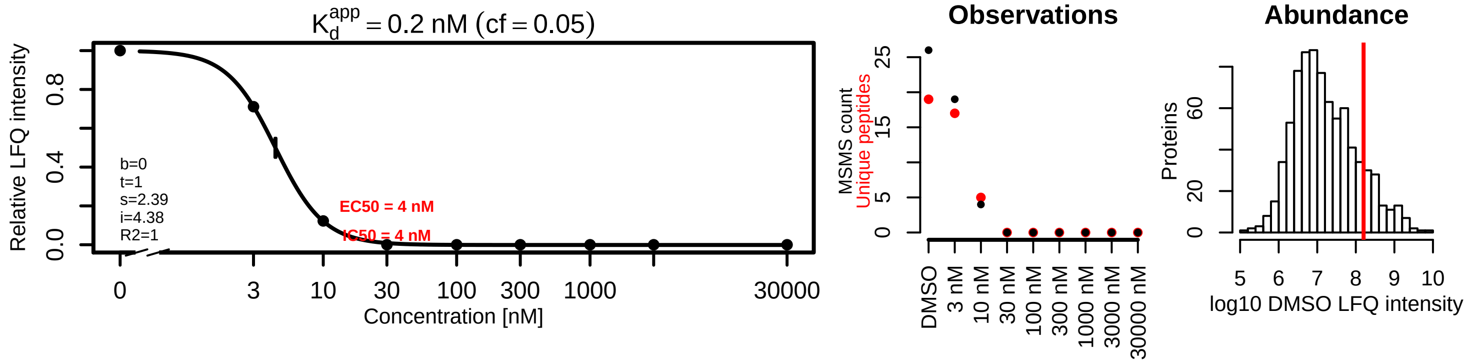


## EPHA2*

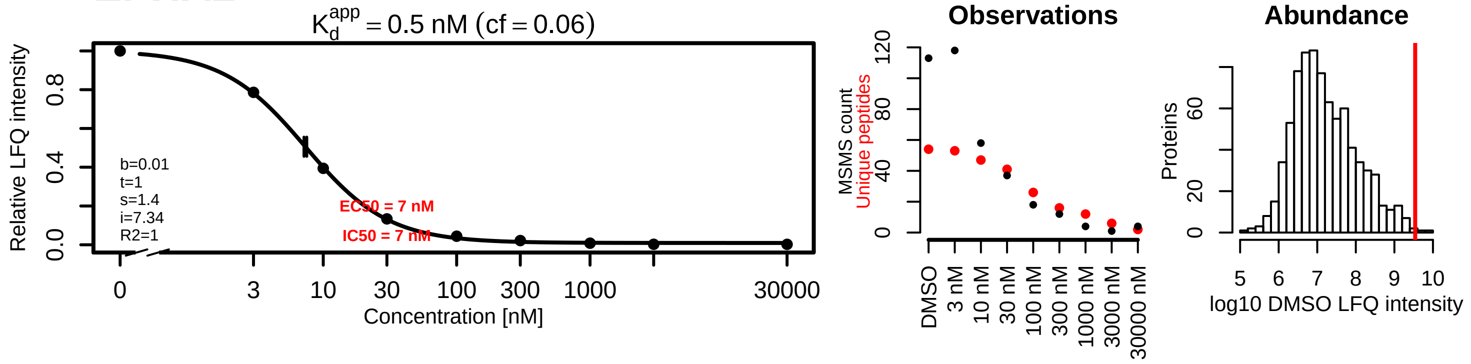


## YES1*

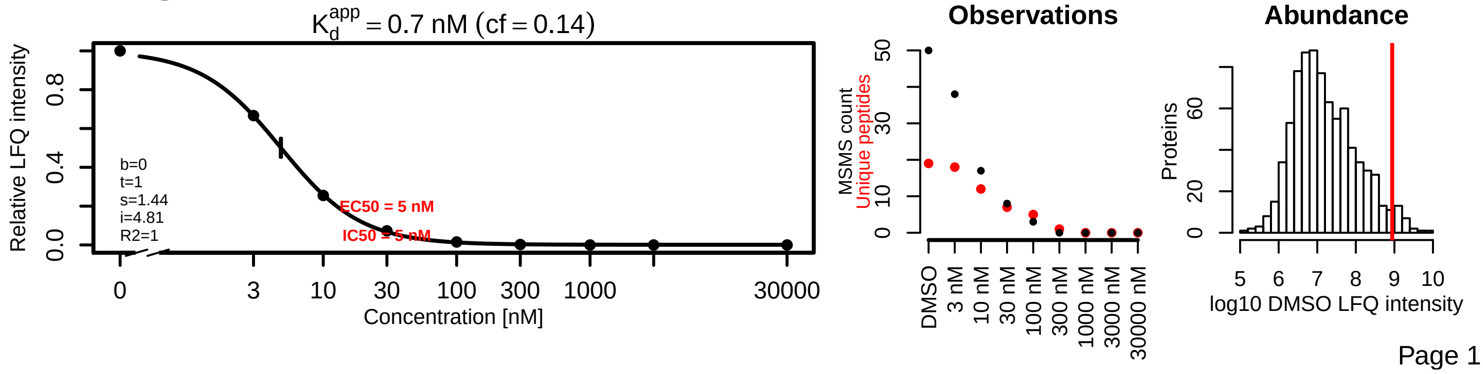

# EPHB4*

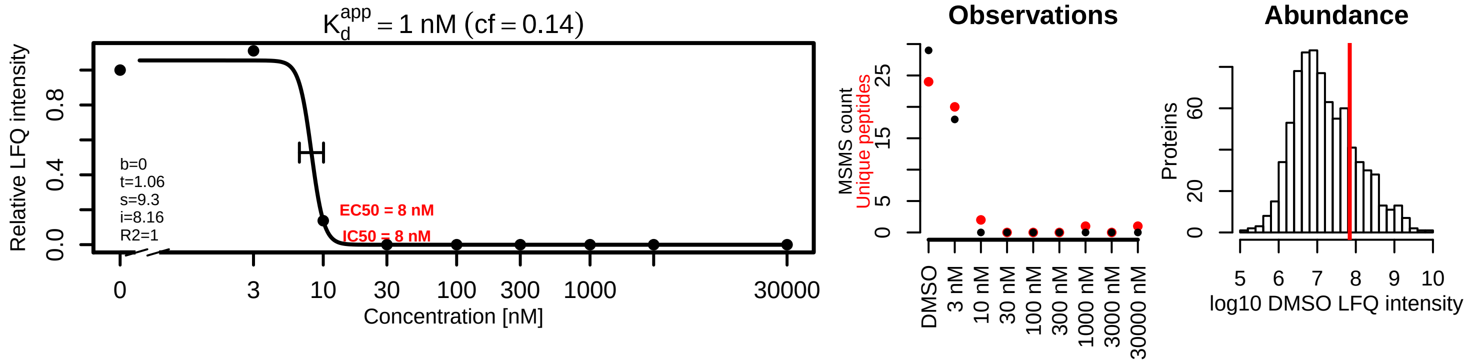


# LYN – P07948;E5RJ37*

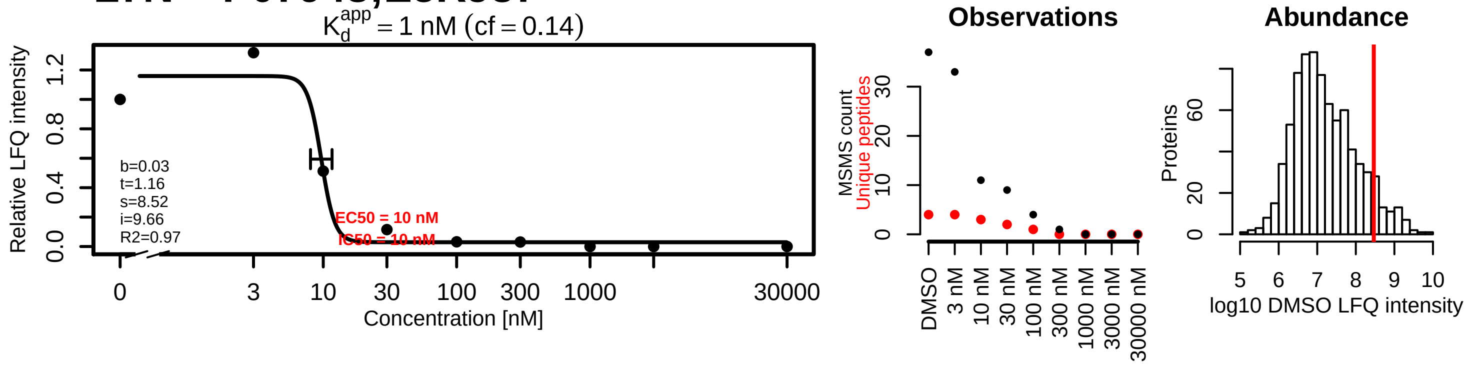


# SIK1*

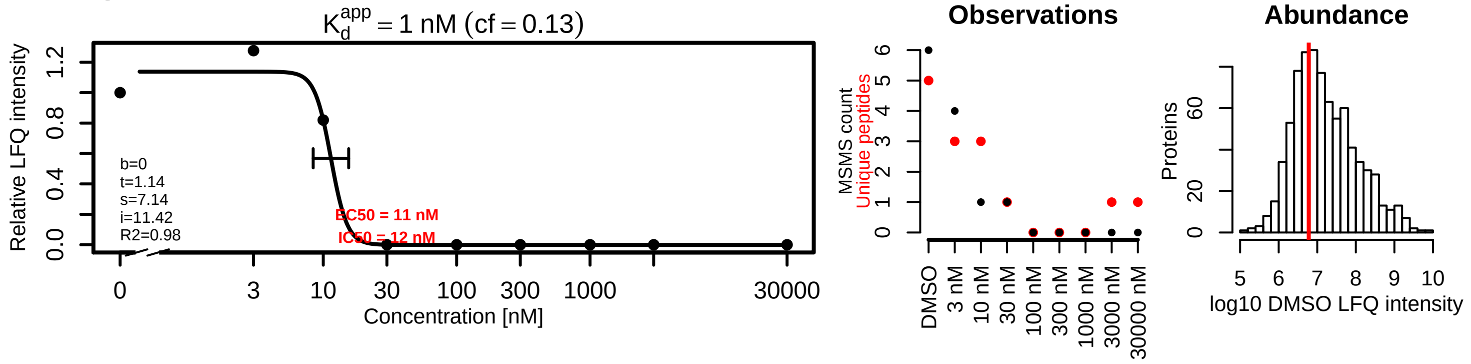


# EPHB2*

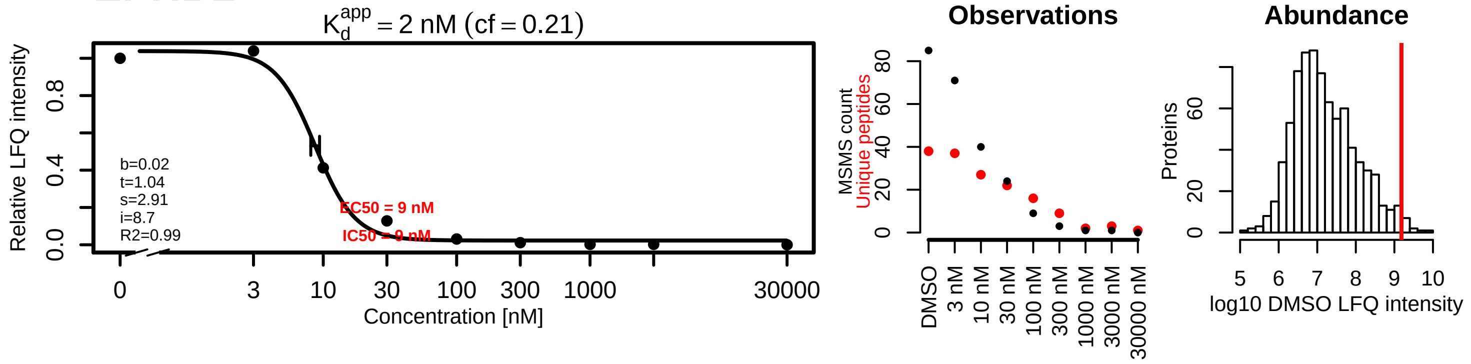


# SIK2*

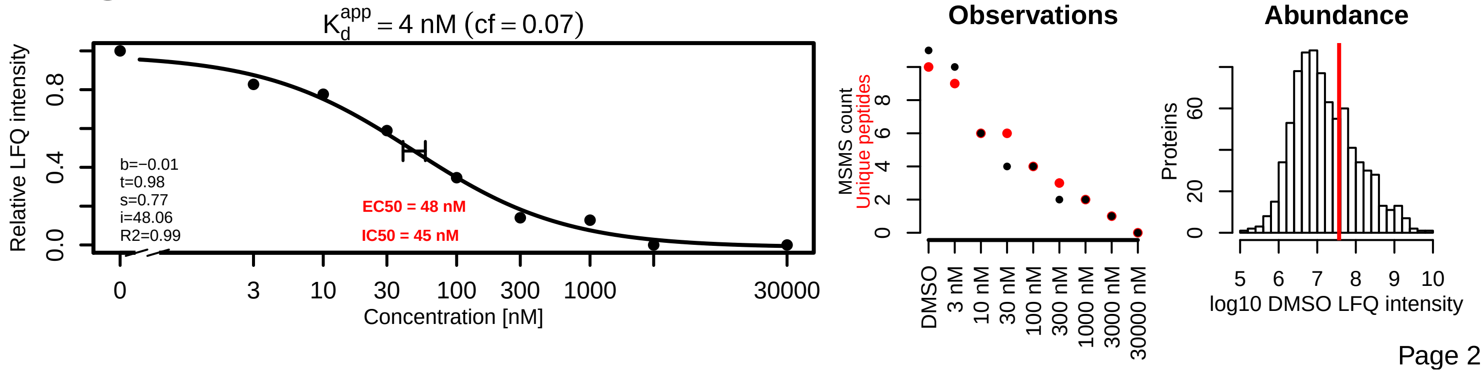

# RIPK2*

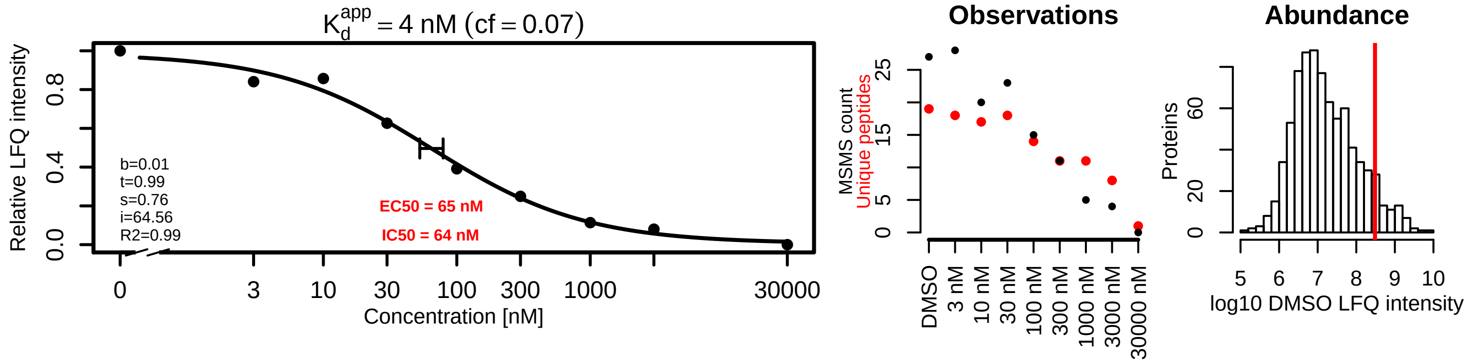


# DDR1*

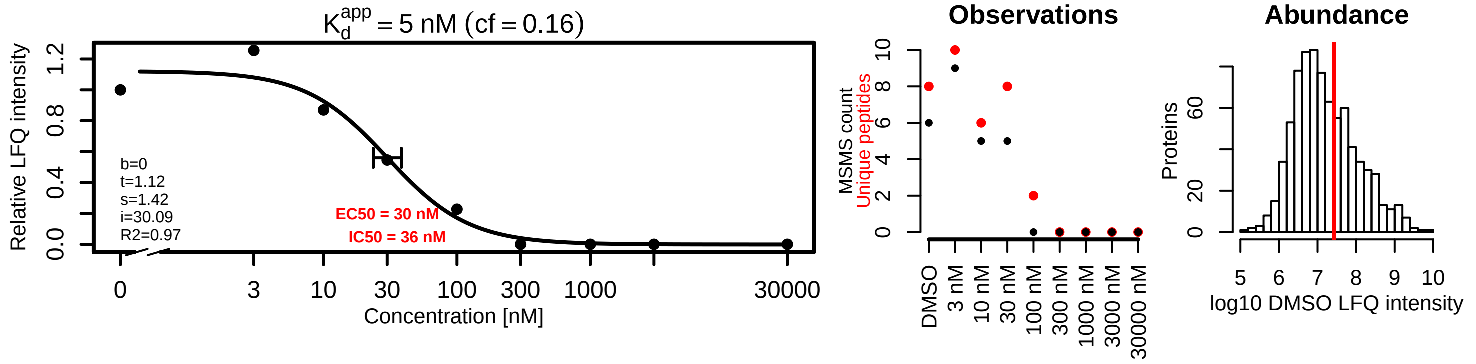


# CSK*

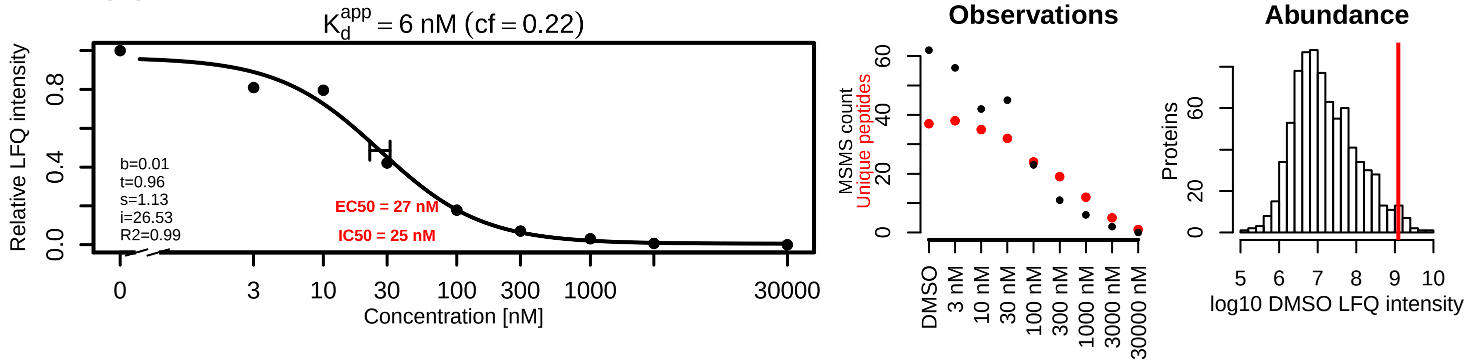


# TNK2*

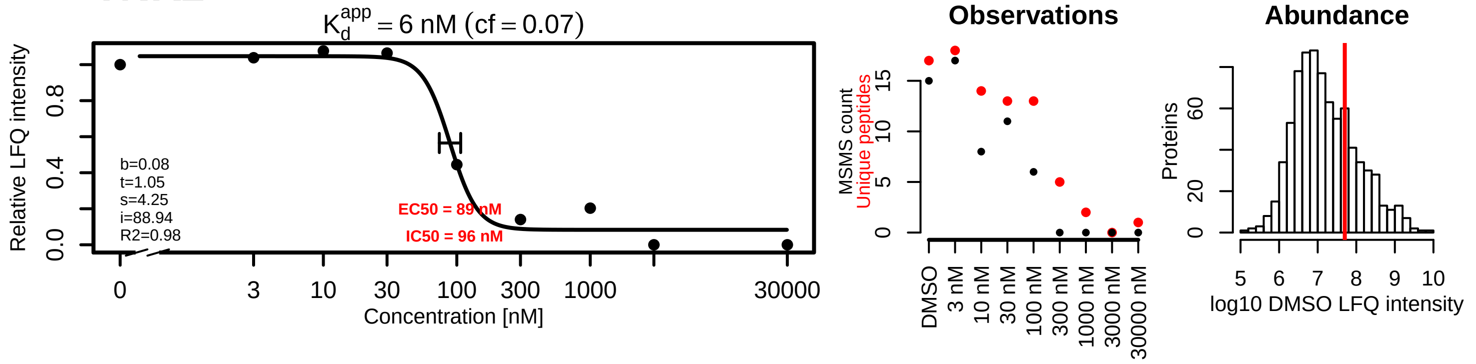


# FRK*

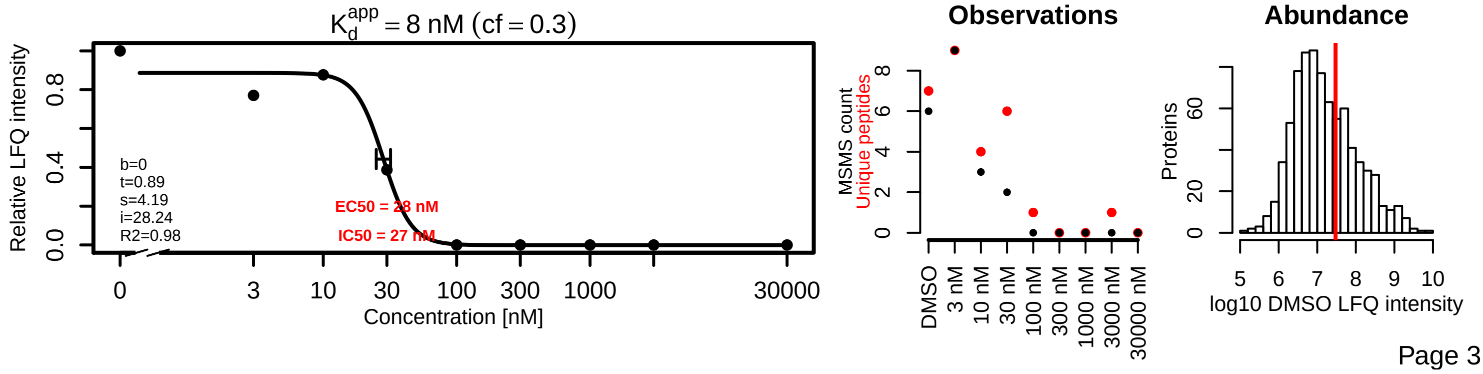

# SIK3;KIAA0999*

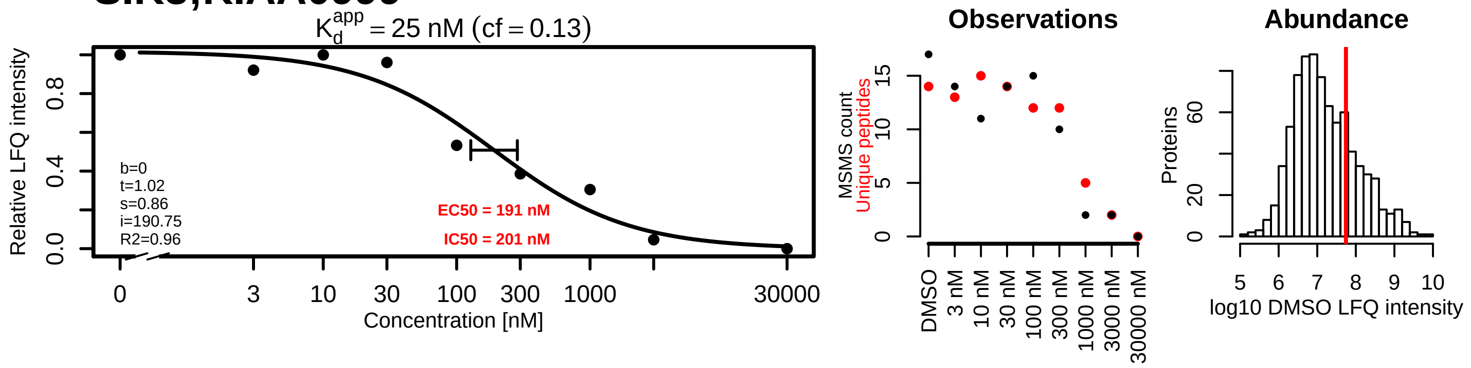


# TEC*

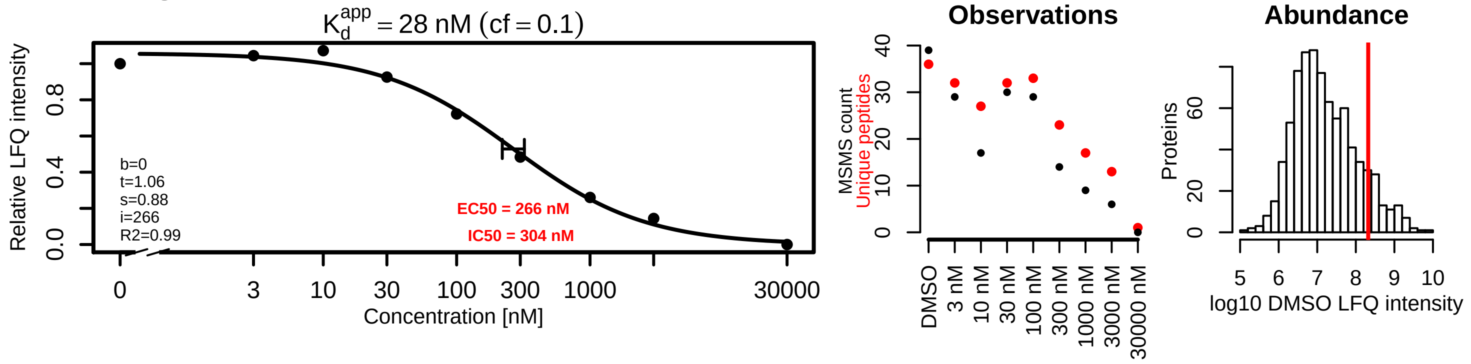


# RTL1*

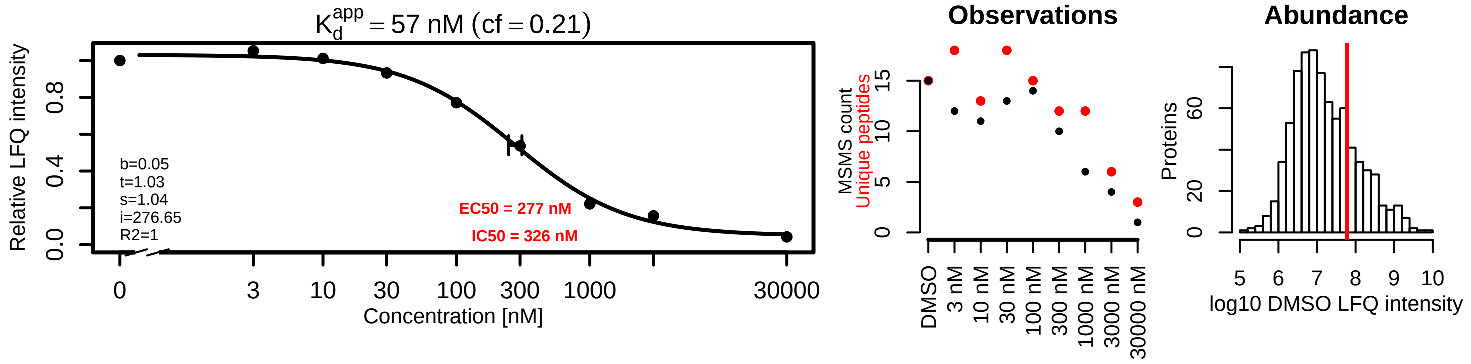


# MLTK*

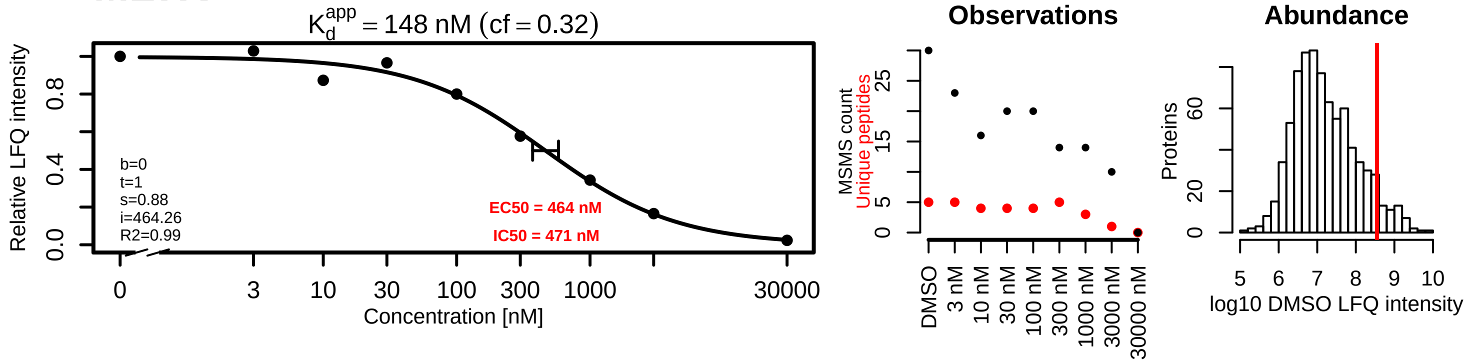


# GAK*

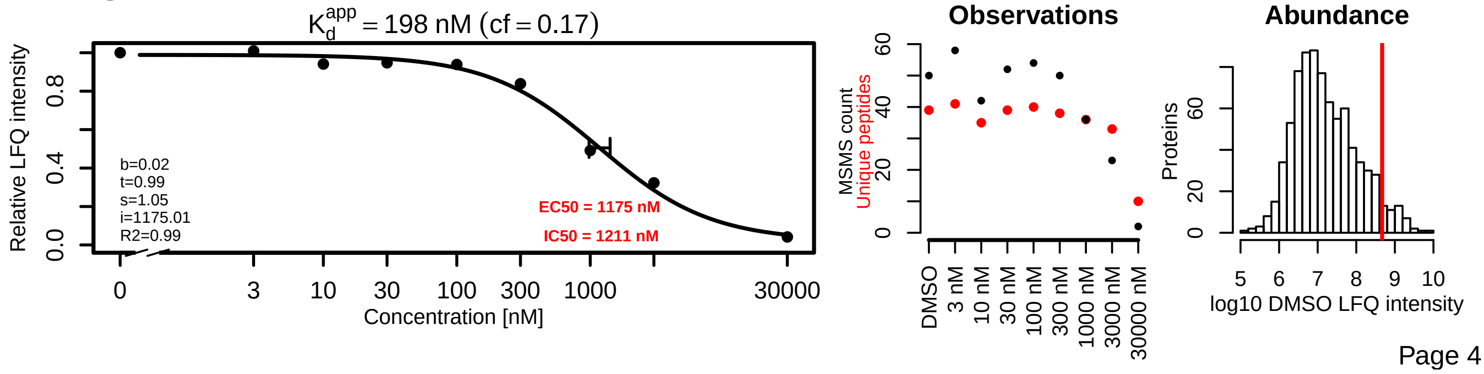

# MAP2K5*

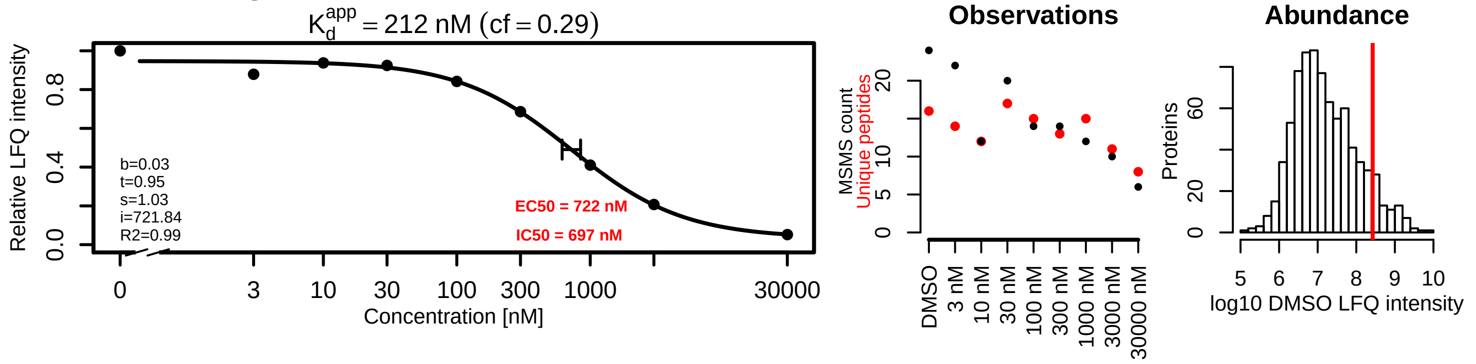


# MAP4K5*

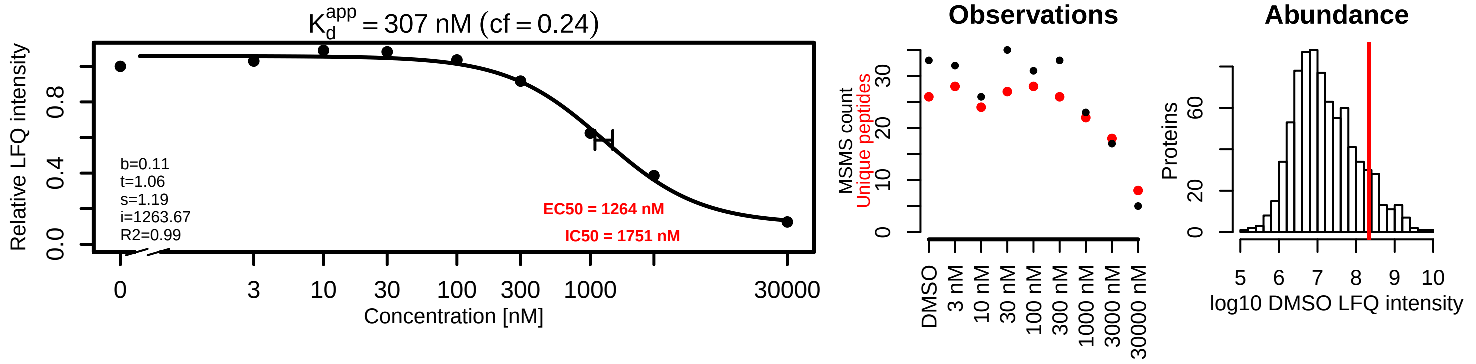


# ZAK*

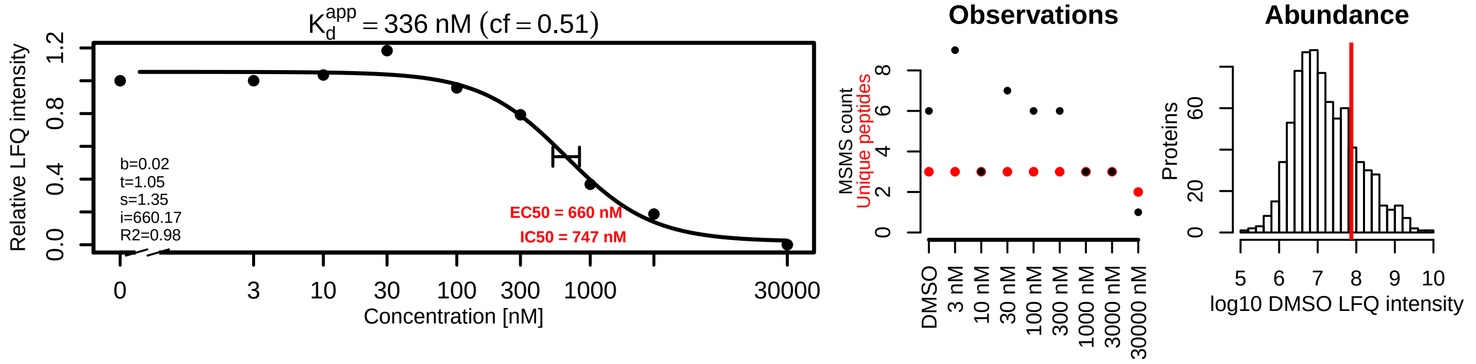


# LIMK2*

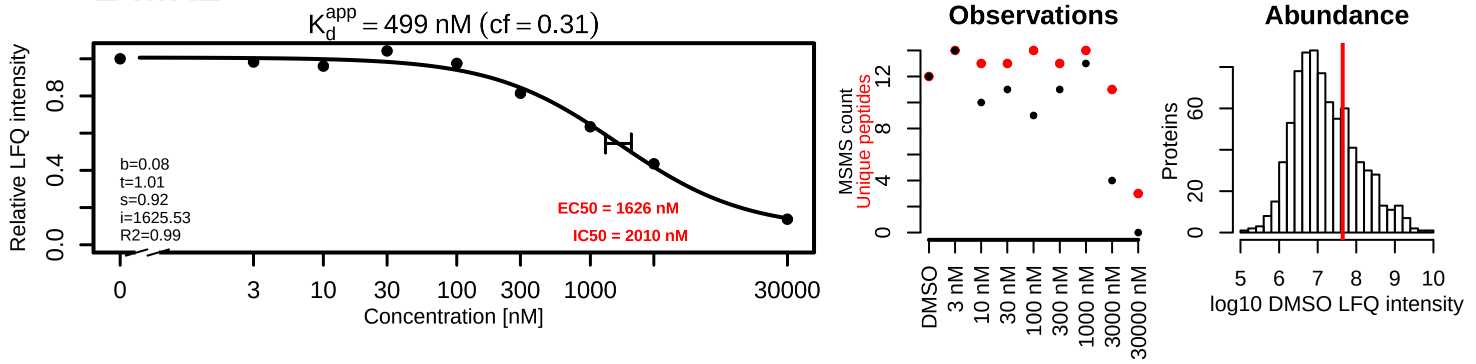


# TGFBR1*

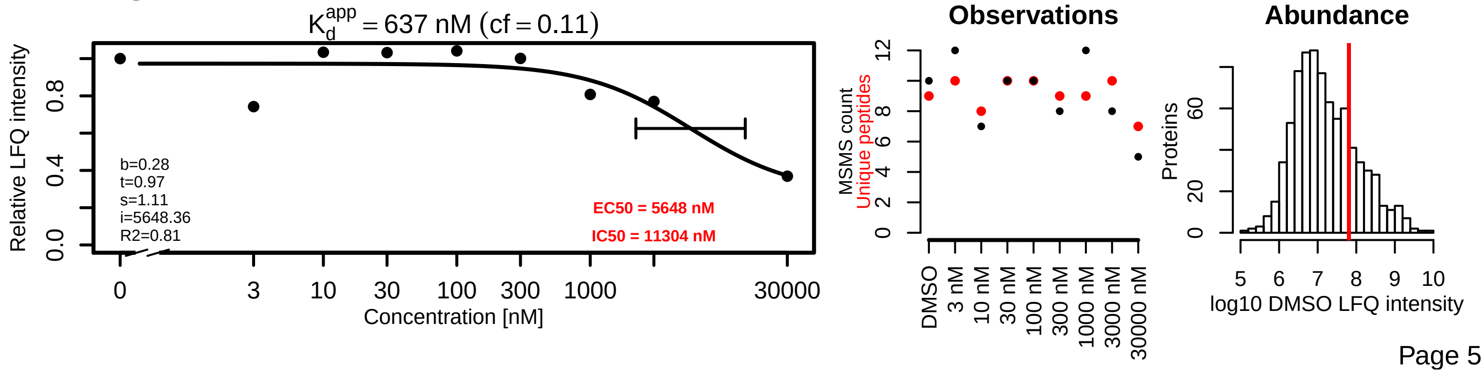

# LIMK1*

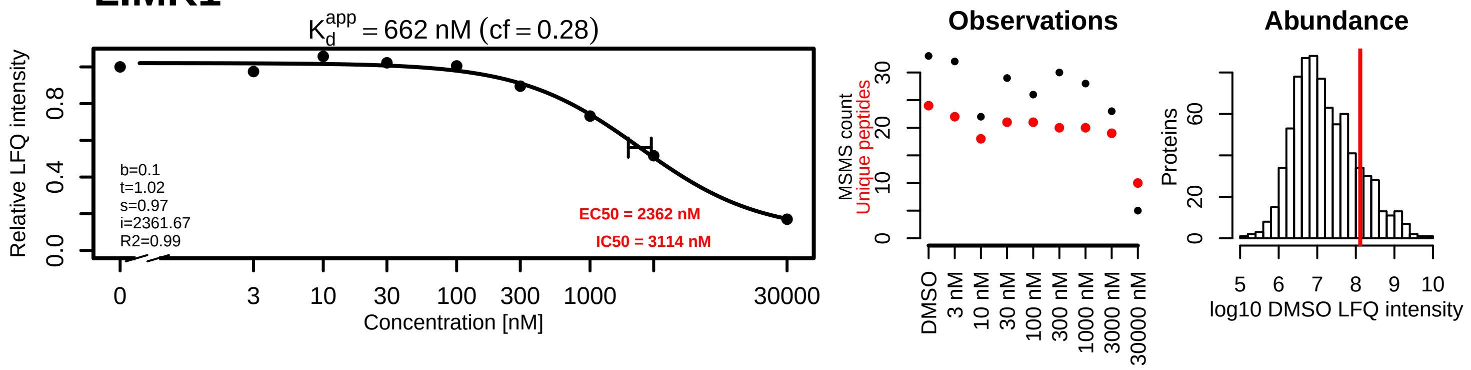


# MAP3K1*

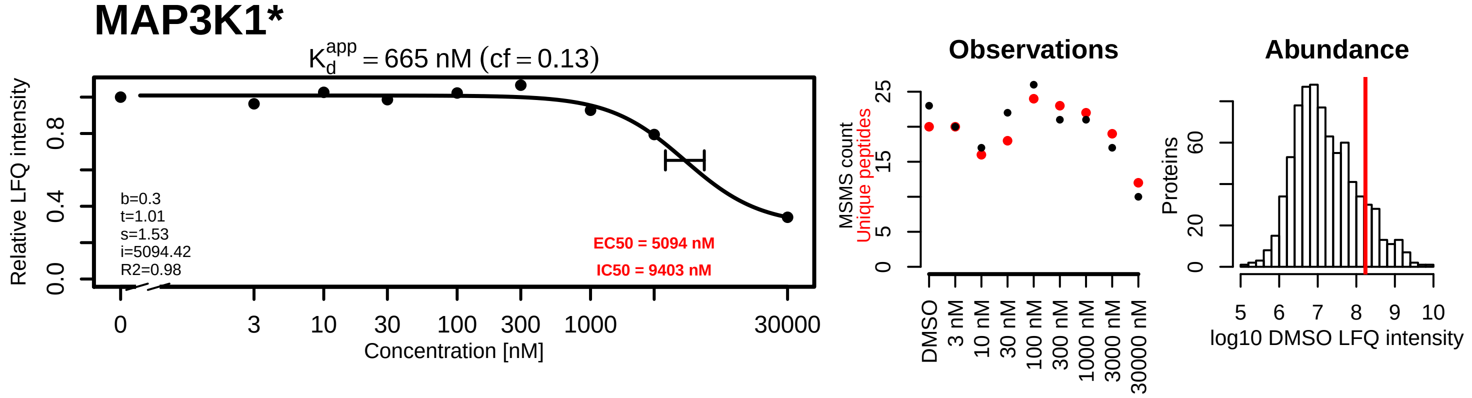


# ADCK3*

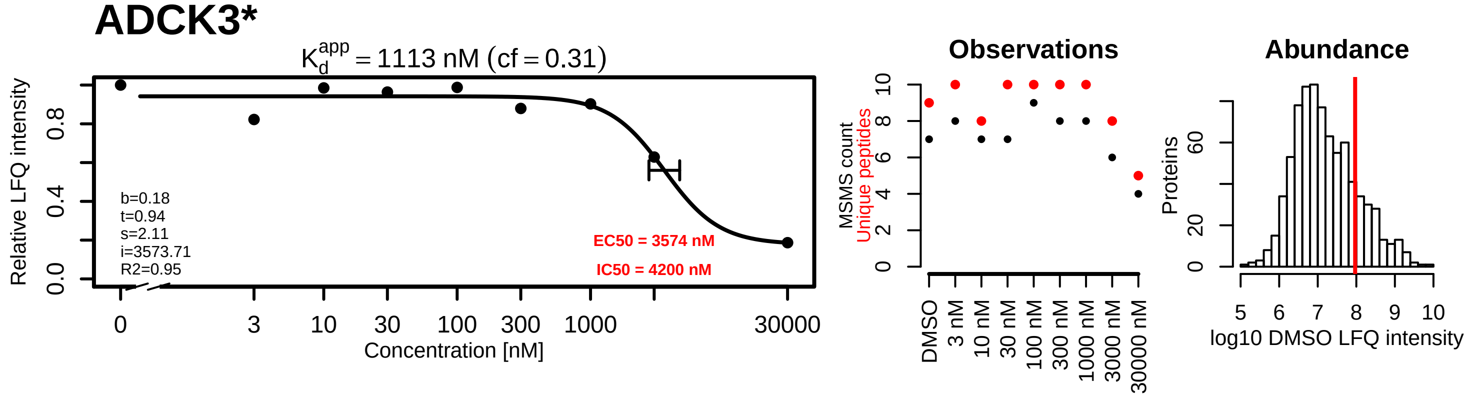


# MAP3K2*

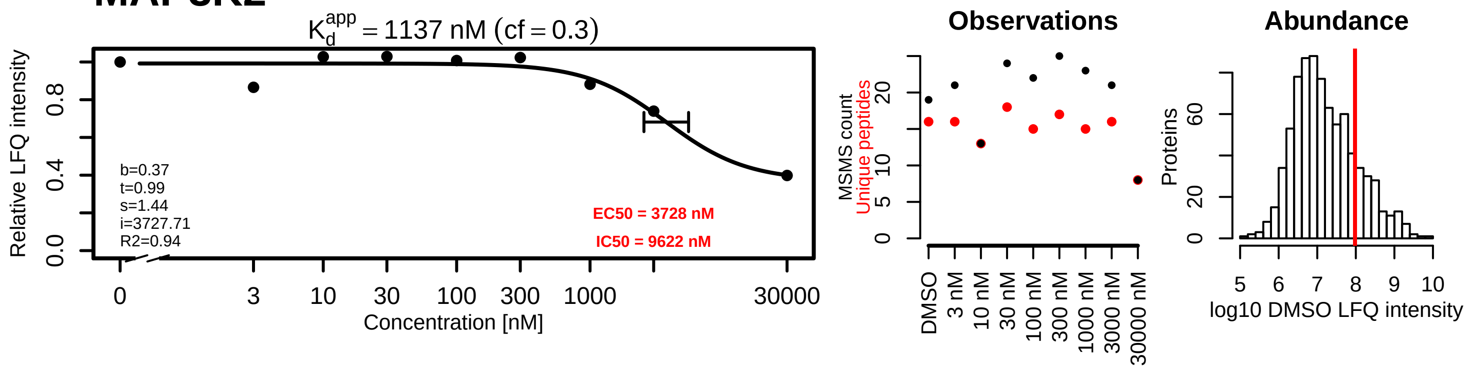


# MAPK14 – Q16539;Q16539−3;Q16539−4;B4E0K5;B5TY33;H7C4E2;E7

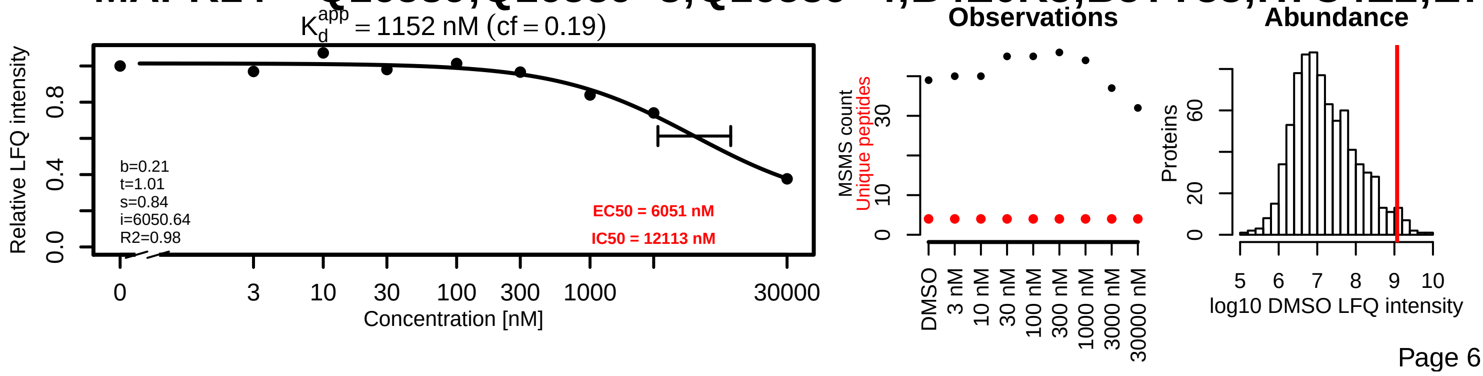

## YWHAZ*

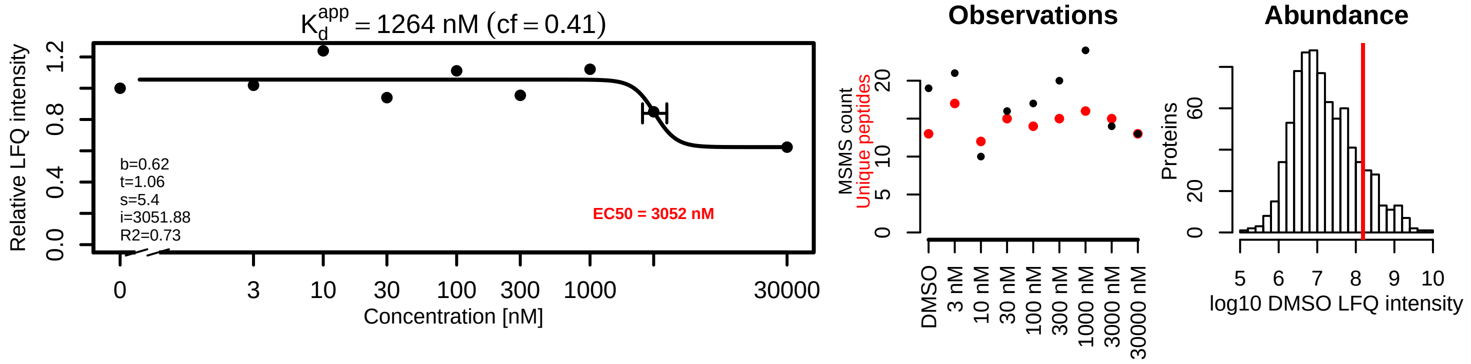


## YWHAE*

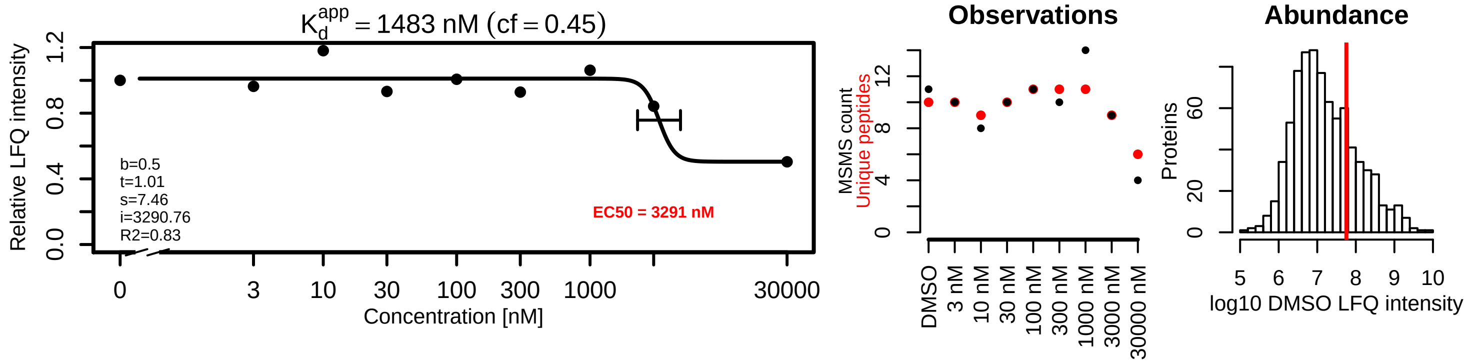


## MAP3K4*

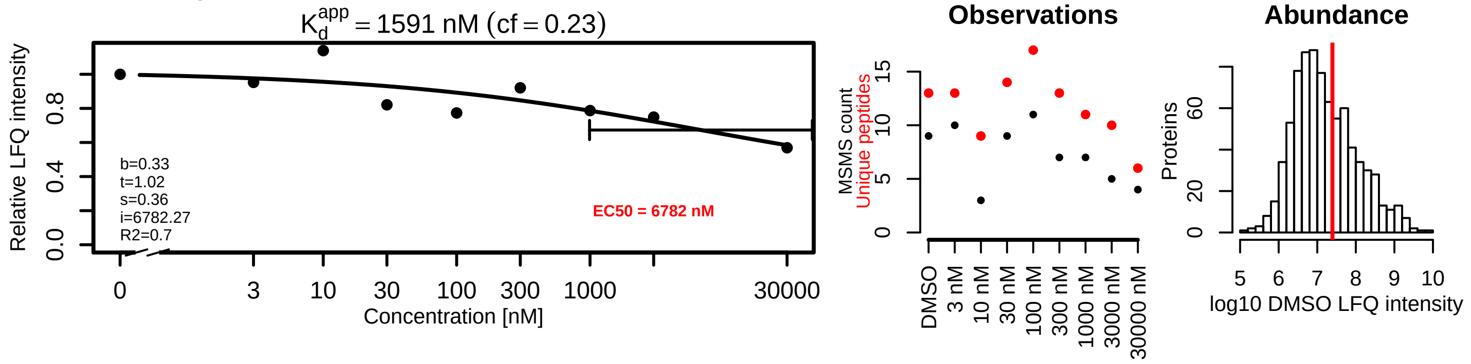


## EGFR*

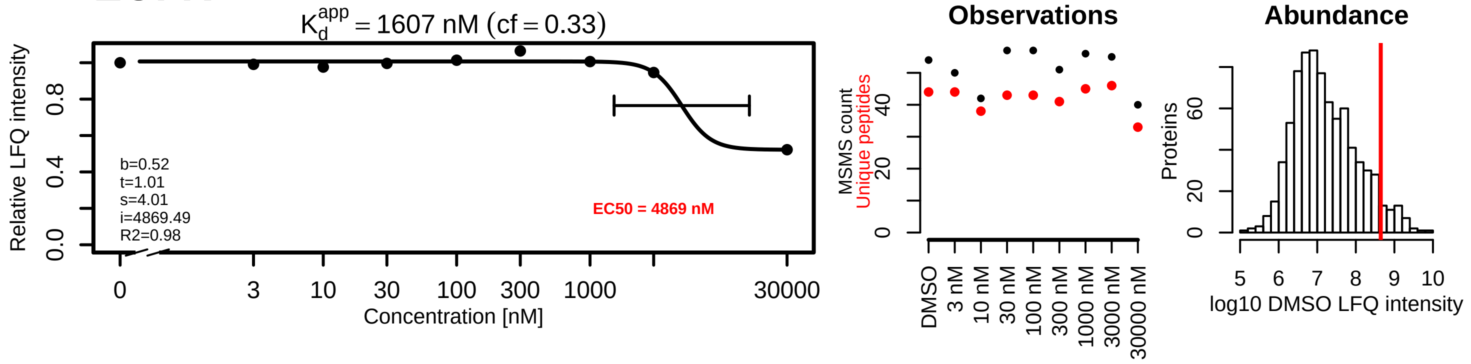


## YWHAQ*

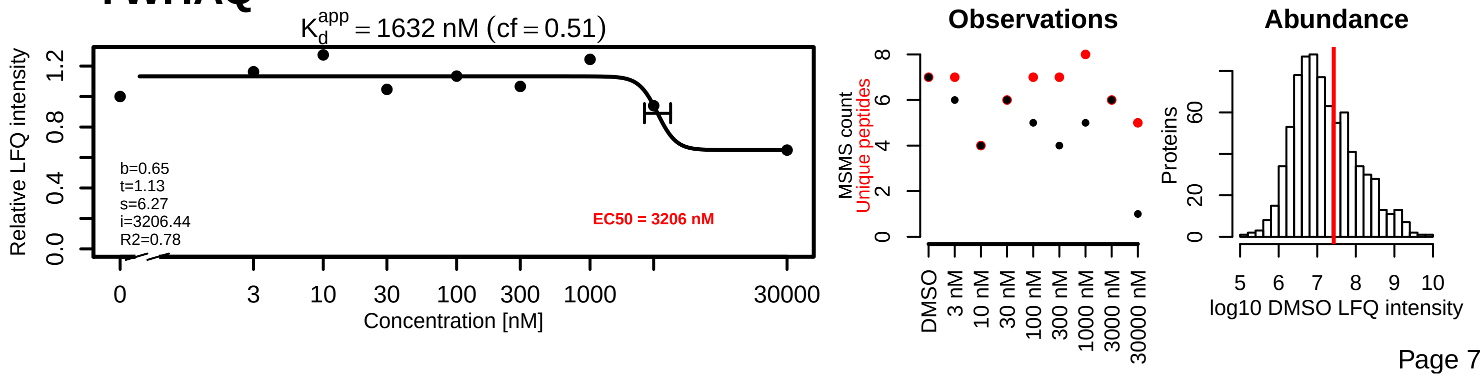

# YWHAH*

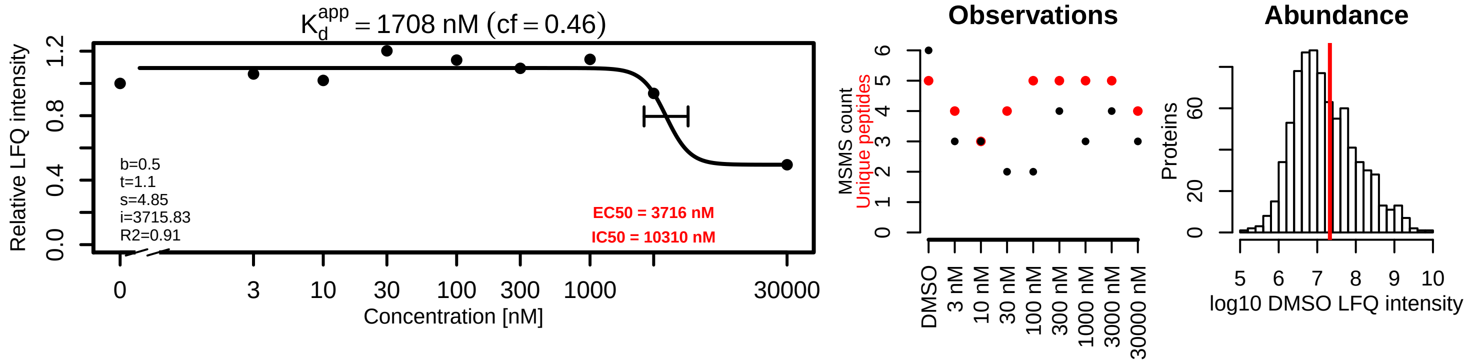


# YWHAG*

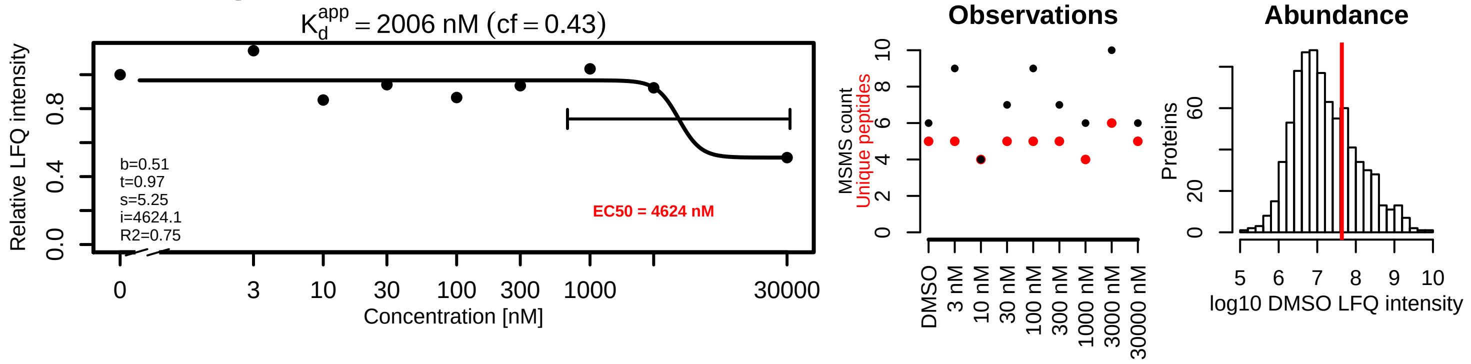


# YWHAB*

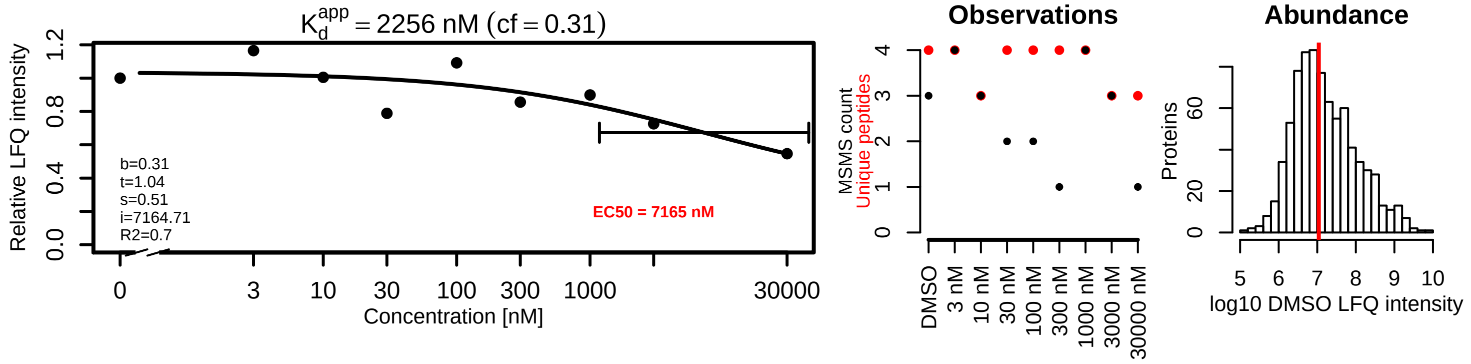


# PKMYT1*

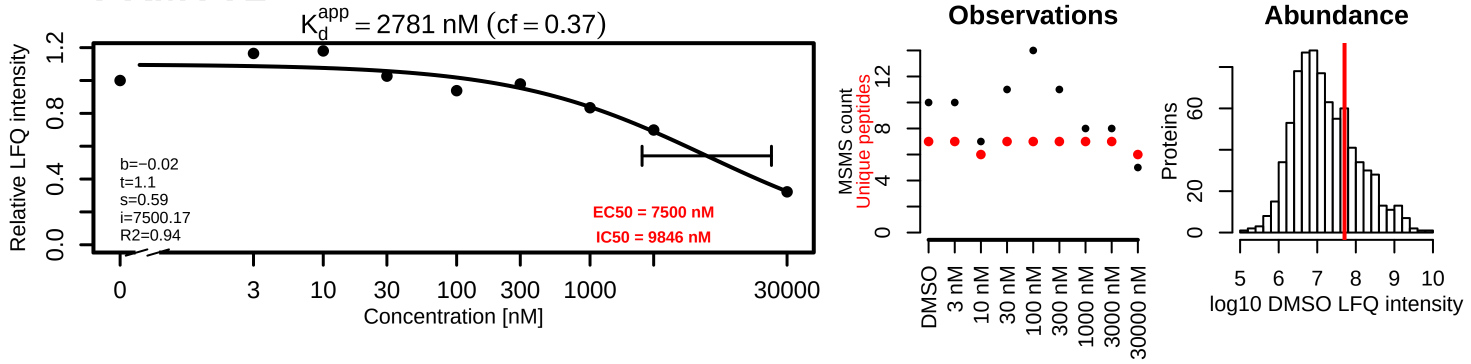


# TNIK*

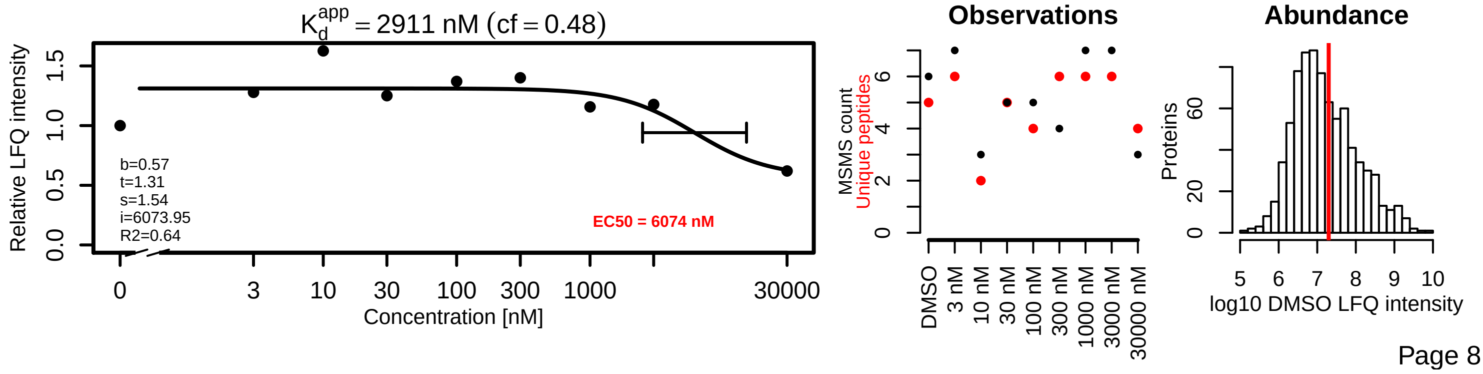

## BRAF – P15056;H7C5K3*

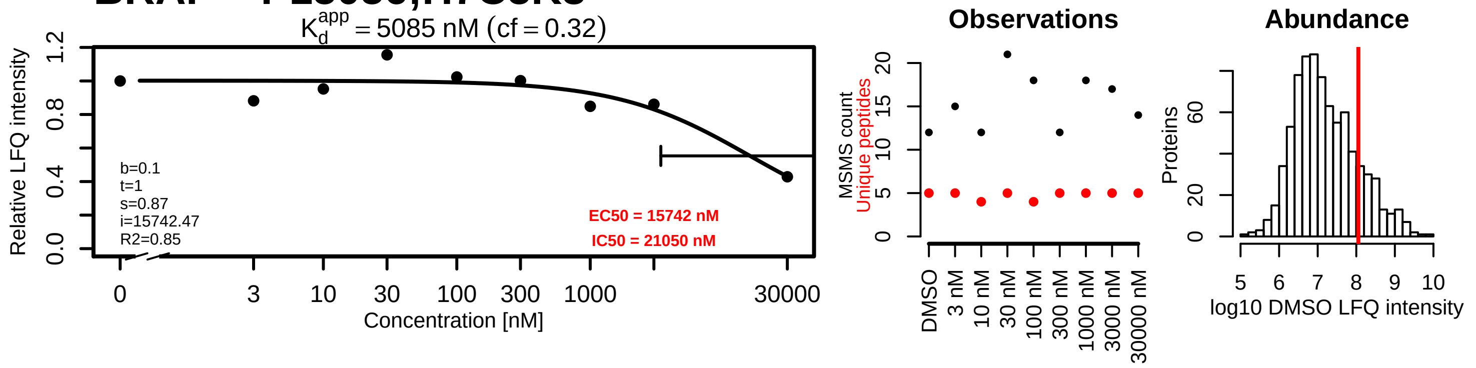


## TM9SF4

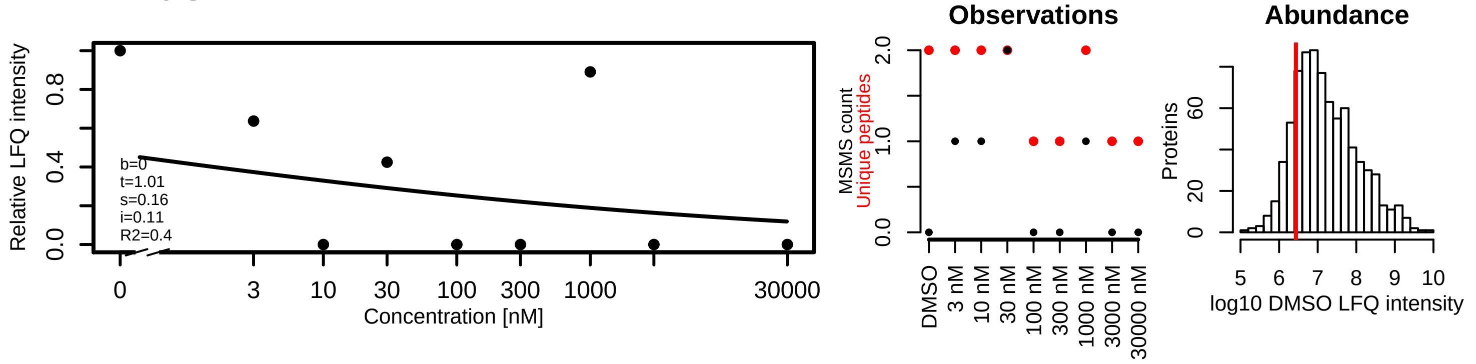


## ZW10

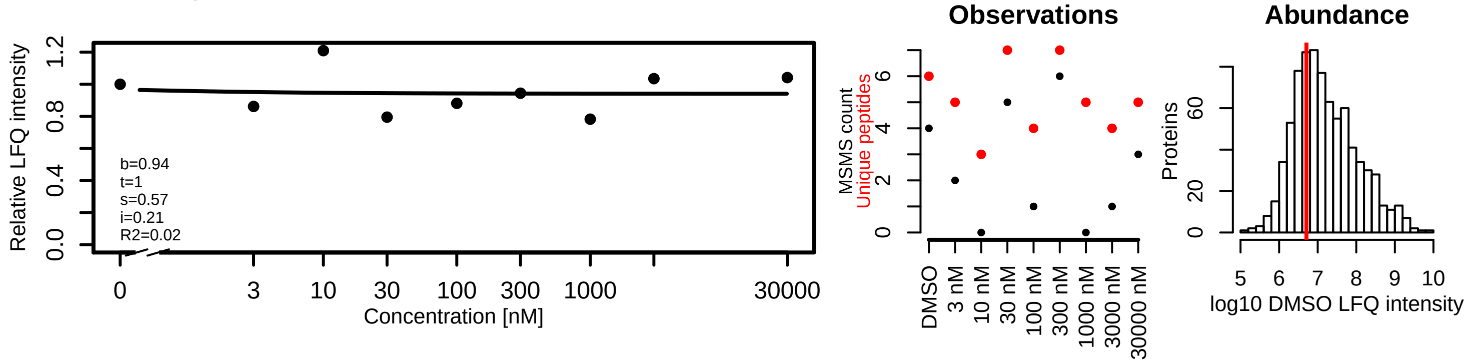


## TUBB6

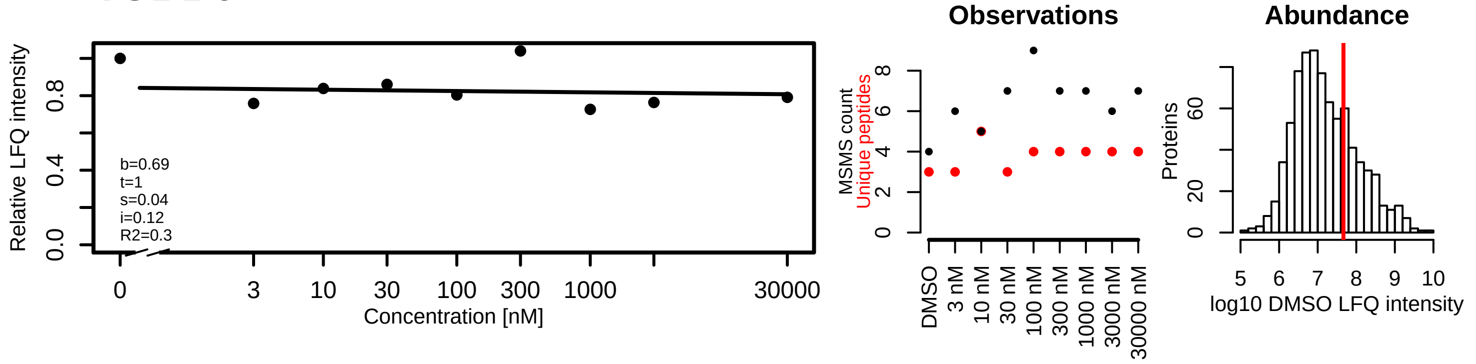


## MON2

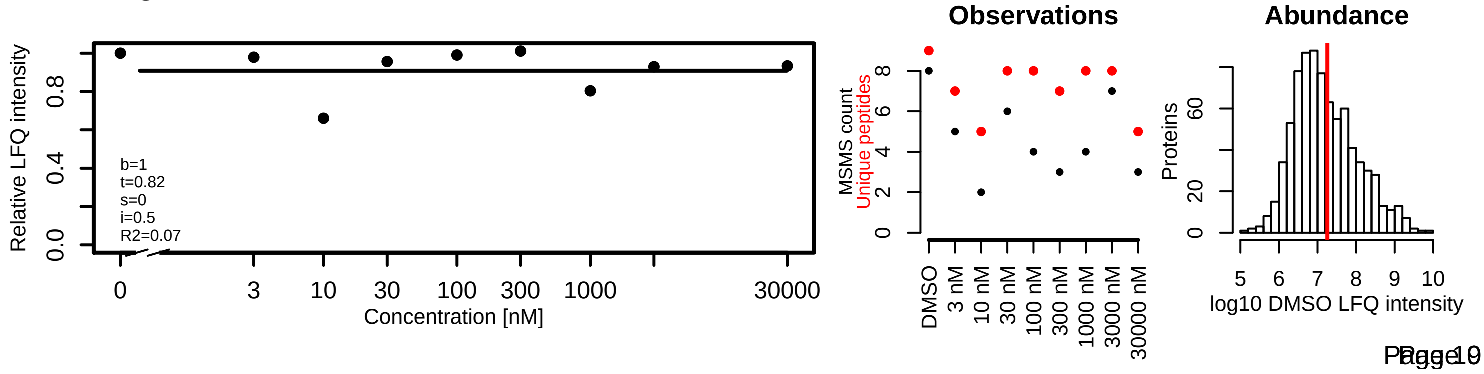

# KNTC1

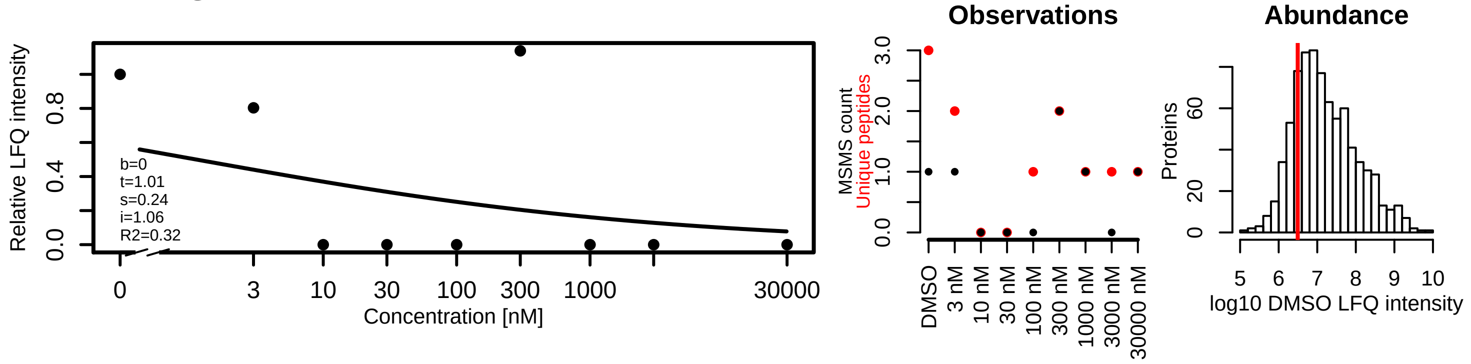


# SLC25A10

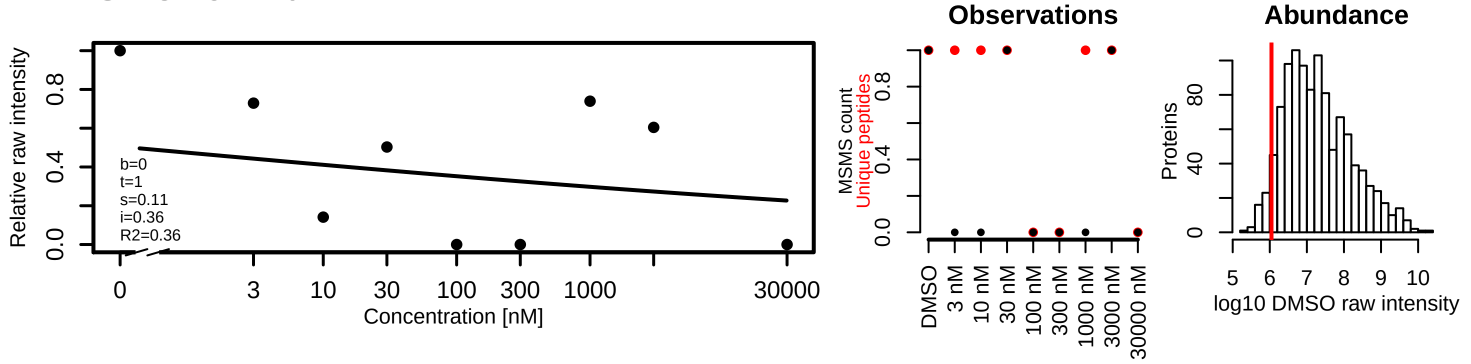


# CLK2

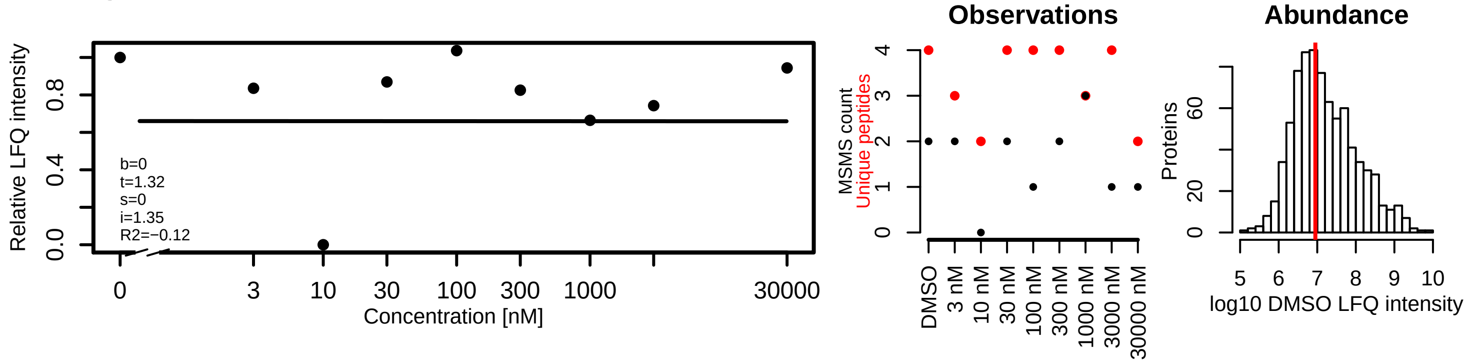


# C2orf18;SLC35F6

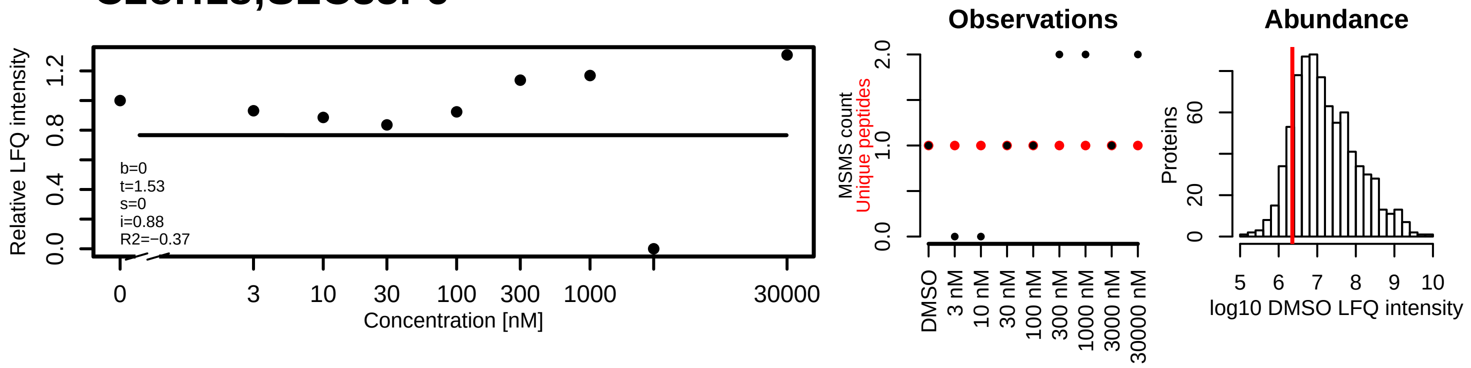


# ITGB3

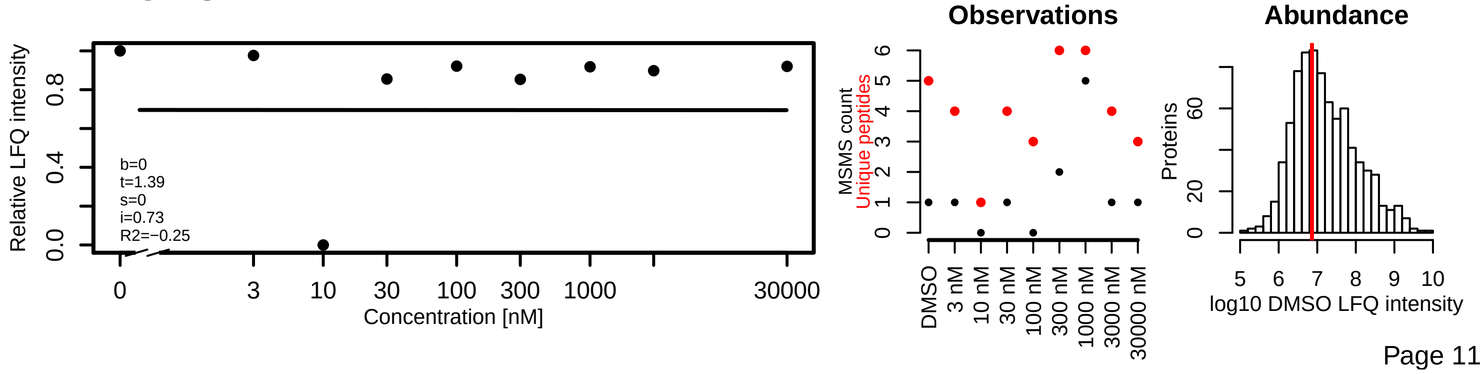

# PLK4

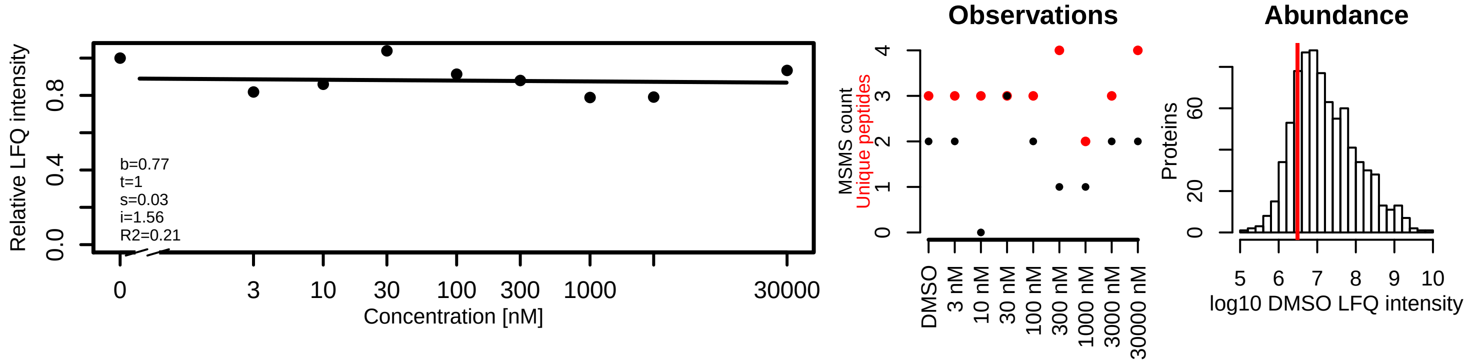


# SLC1A5

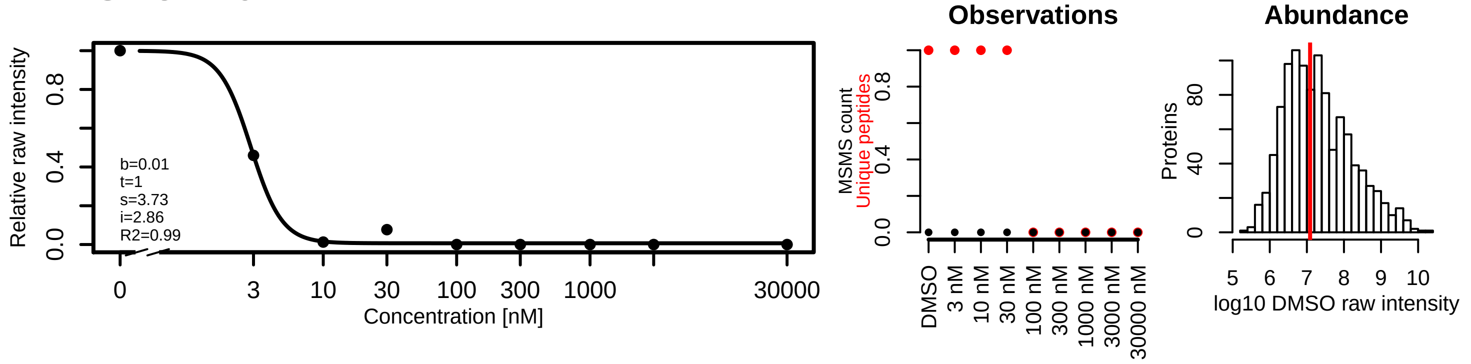


# MYLK

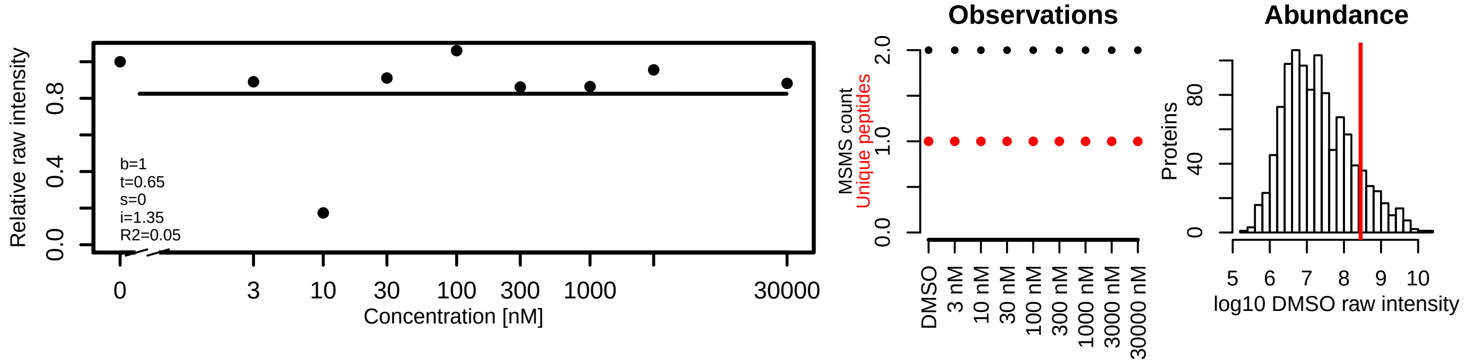


# C2orf47

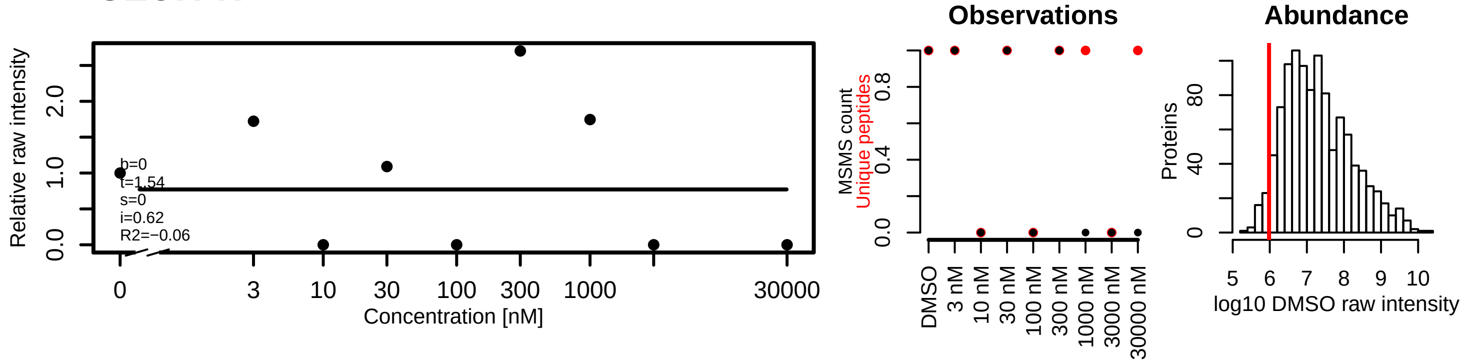


# LYN − P07948−2;P08631−3;P08631−2;A8K4G3;P08631−4;H0Y3C5;P08

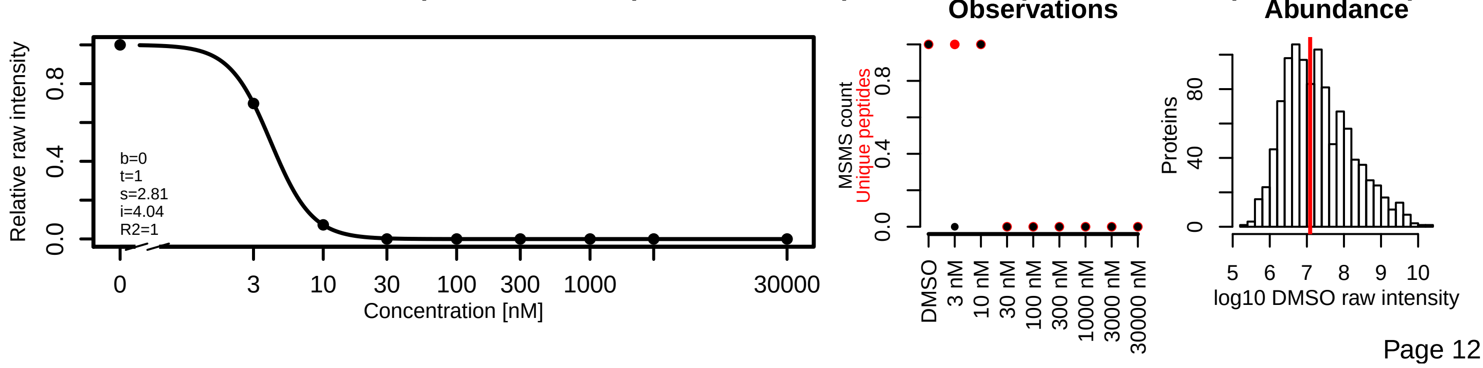

# PKN3

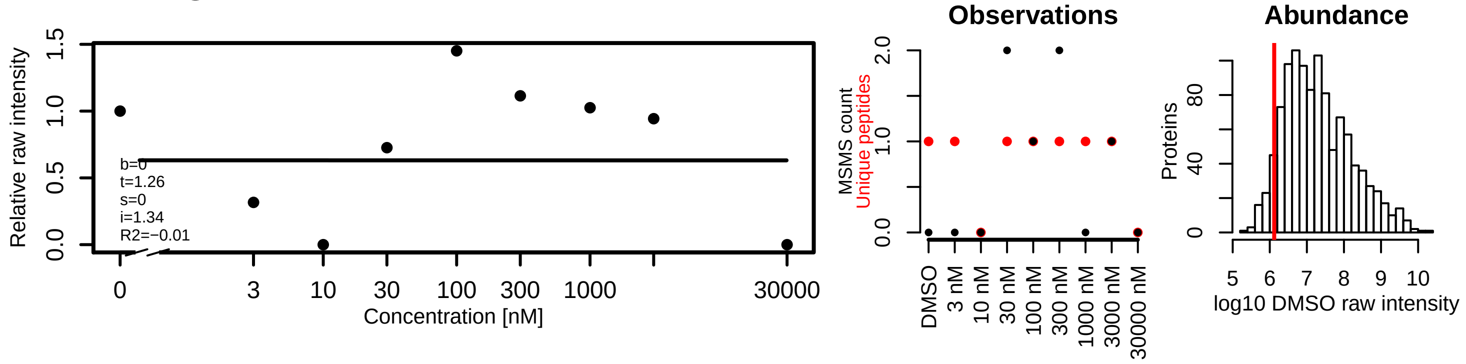


# CABLES2

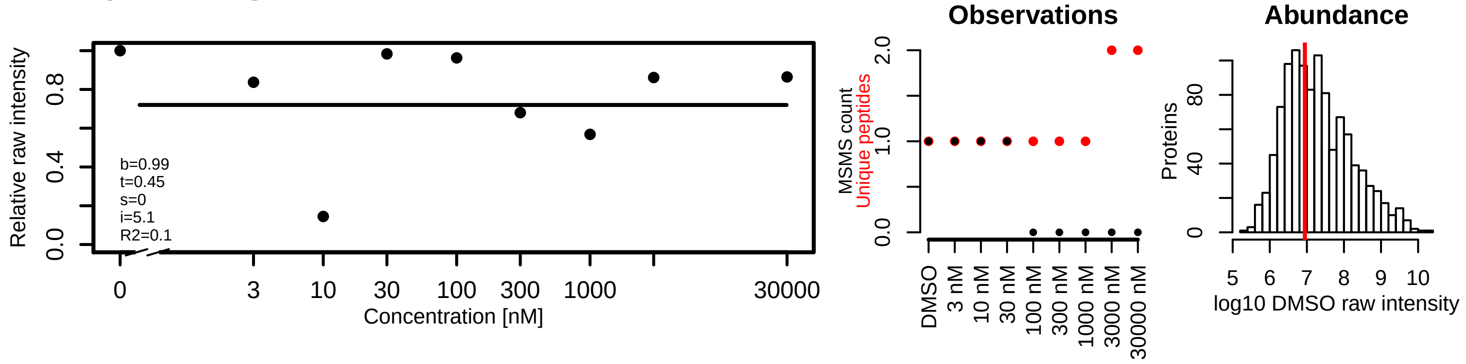


# ATP1B1

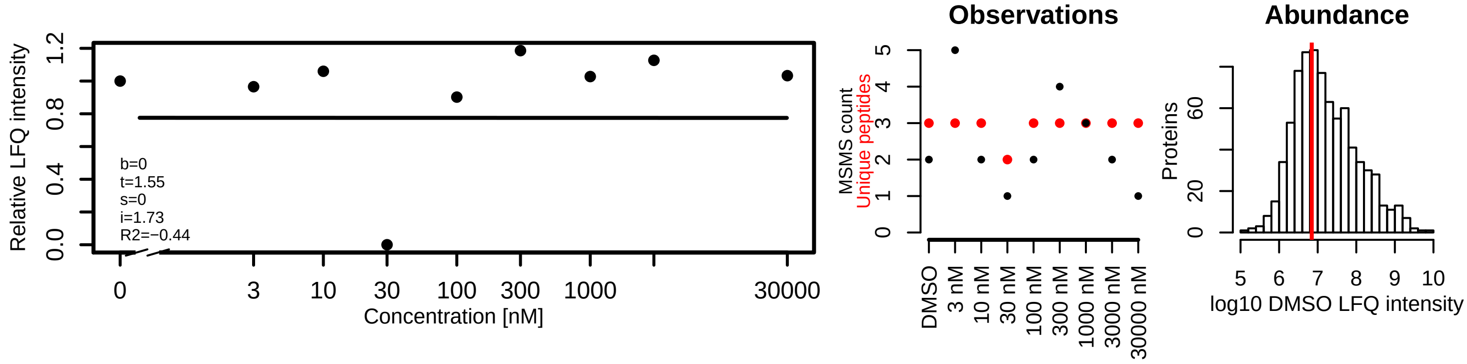


# RNF121

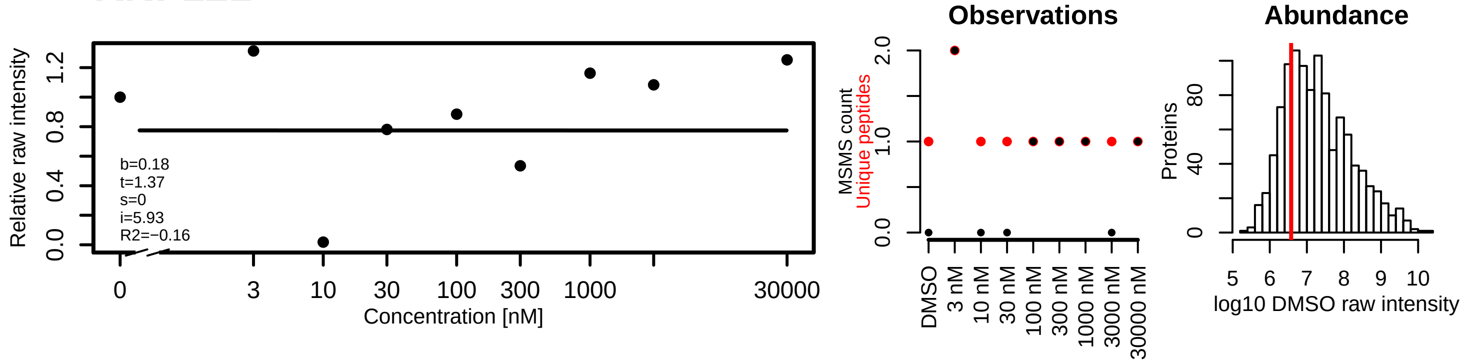


# LMNB1

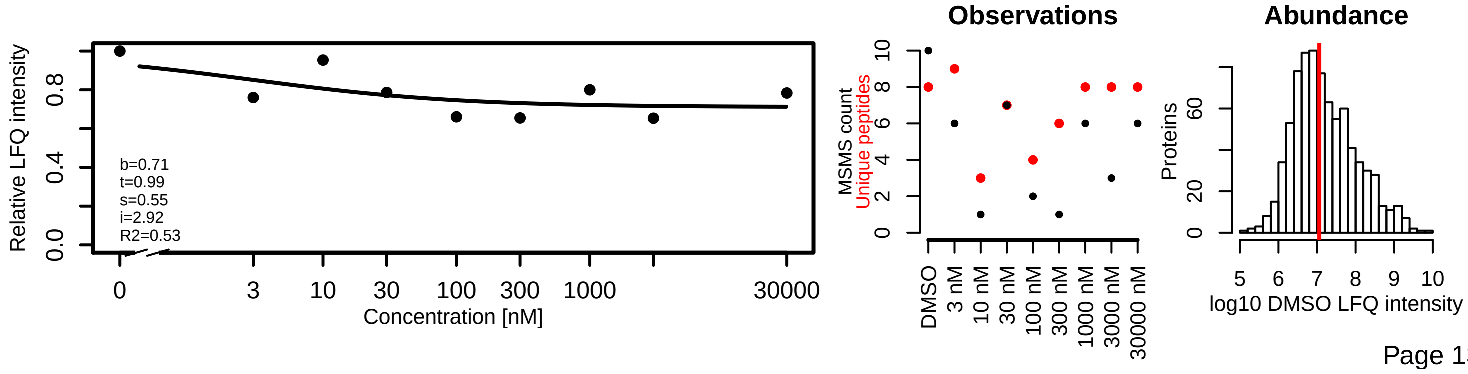

# ATP2B1;ATP2B2;ATP2B4

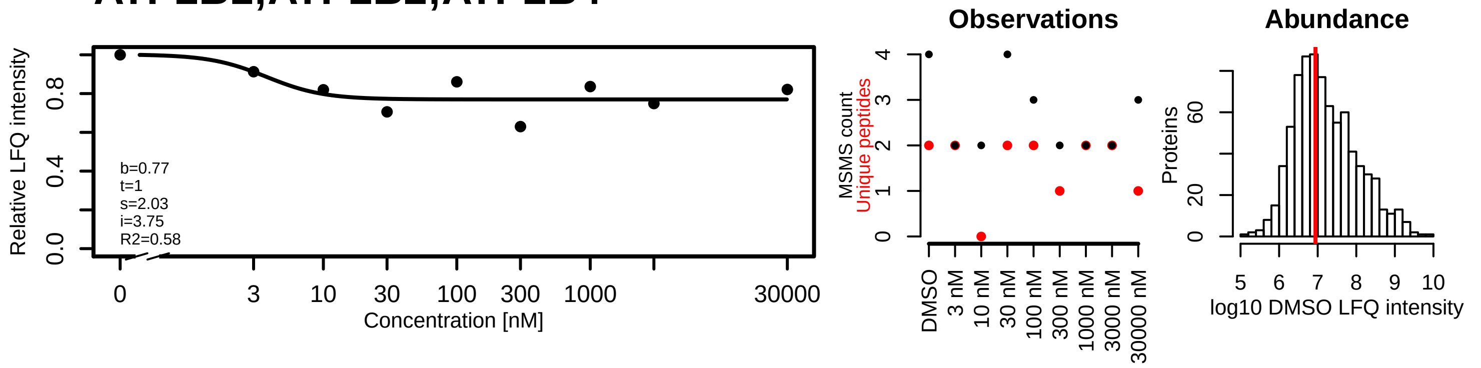


# SLC35B2

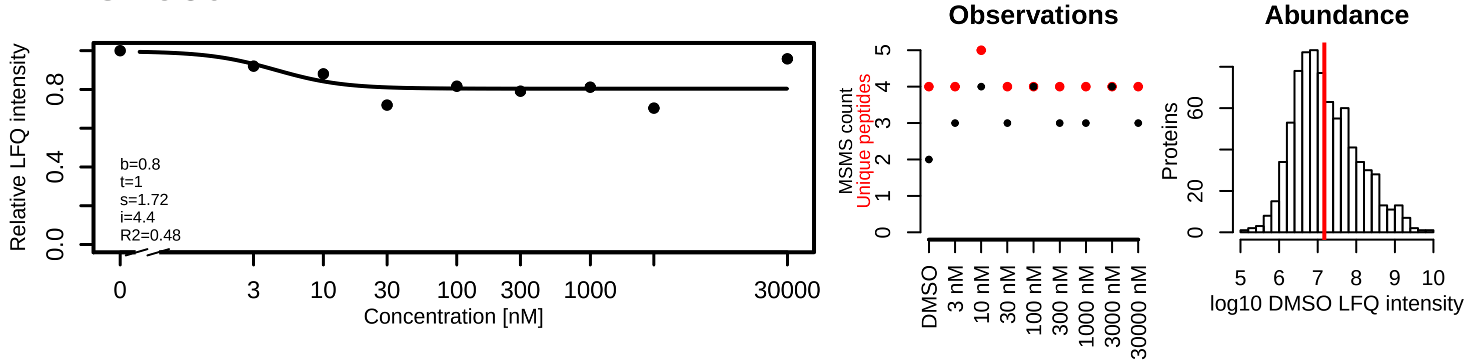


# ANKLE2

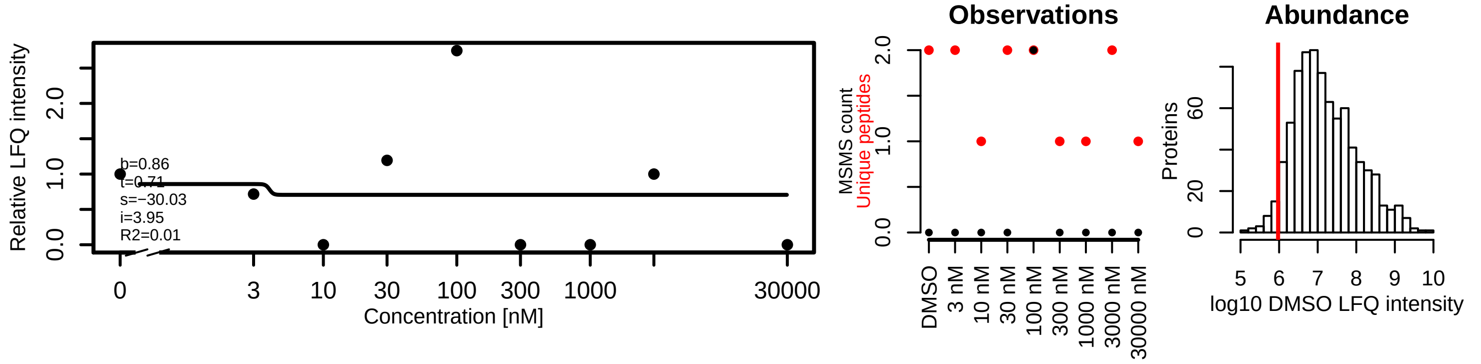


# TESK2

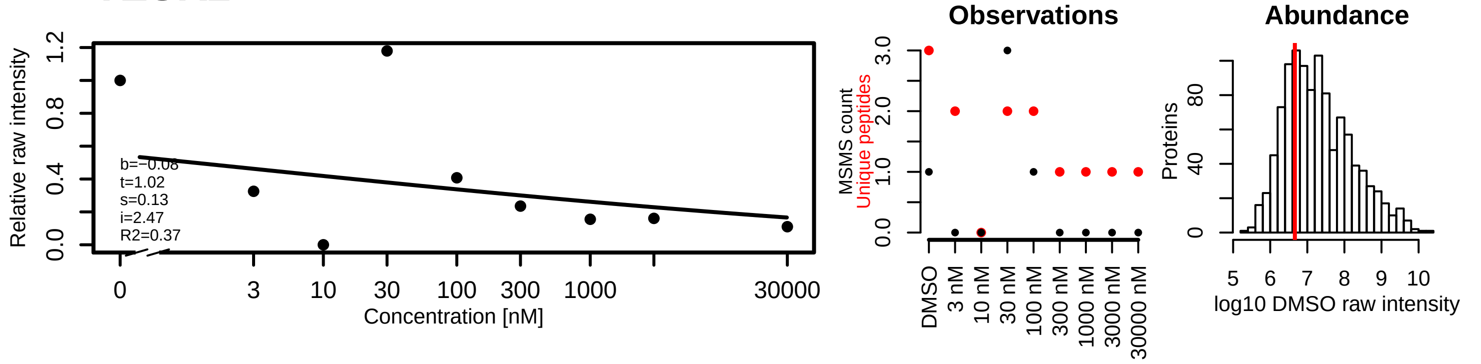


# LDHA

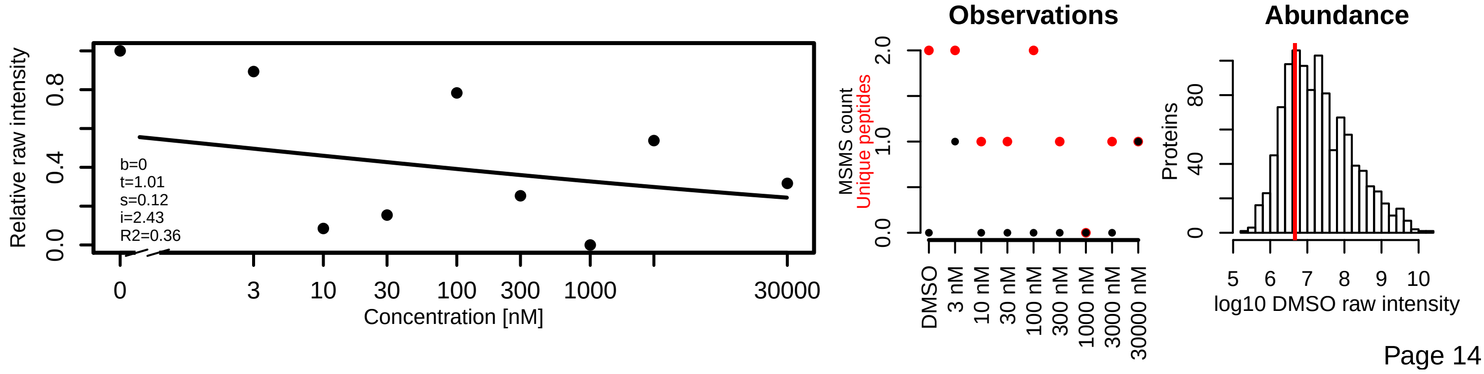

# UTP20

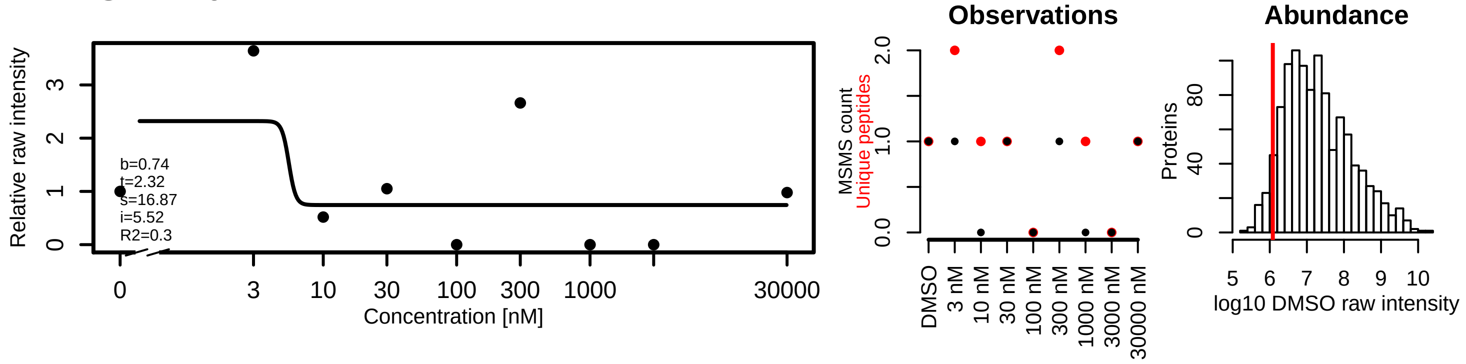


# FGFR1

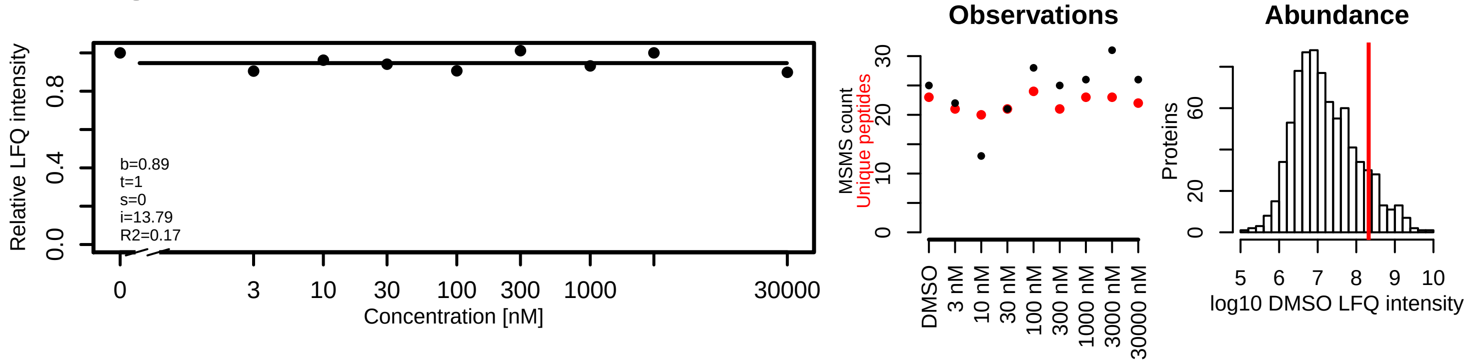


# MSH3

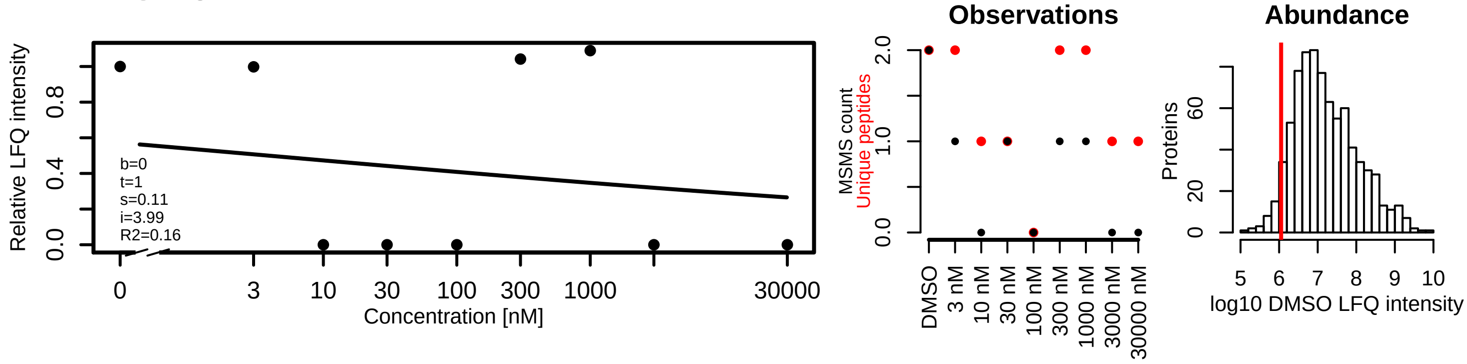


# TUBA4A

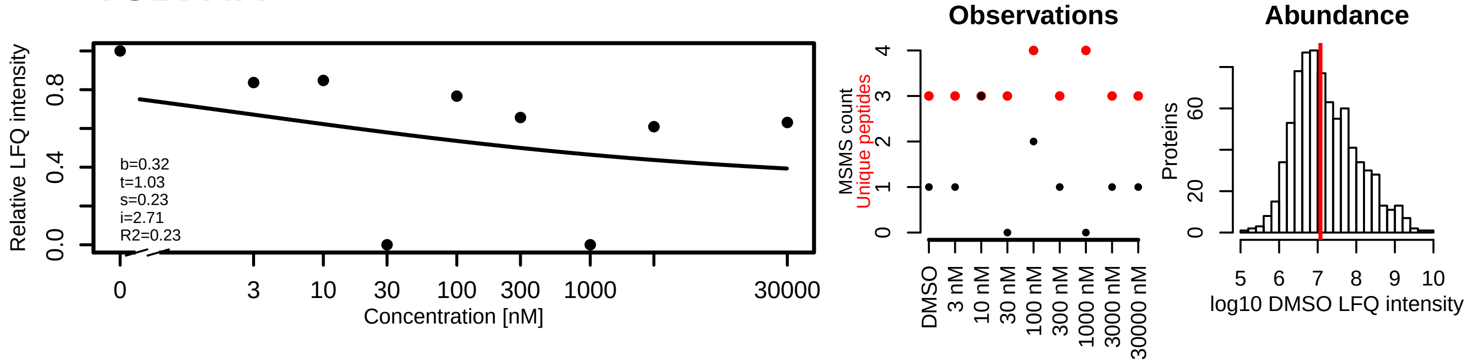


# PDE12

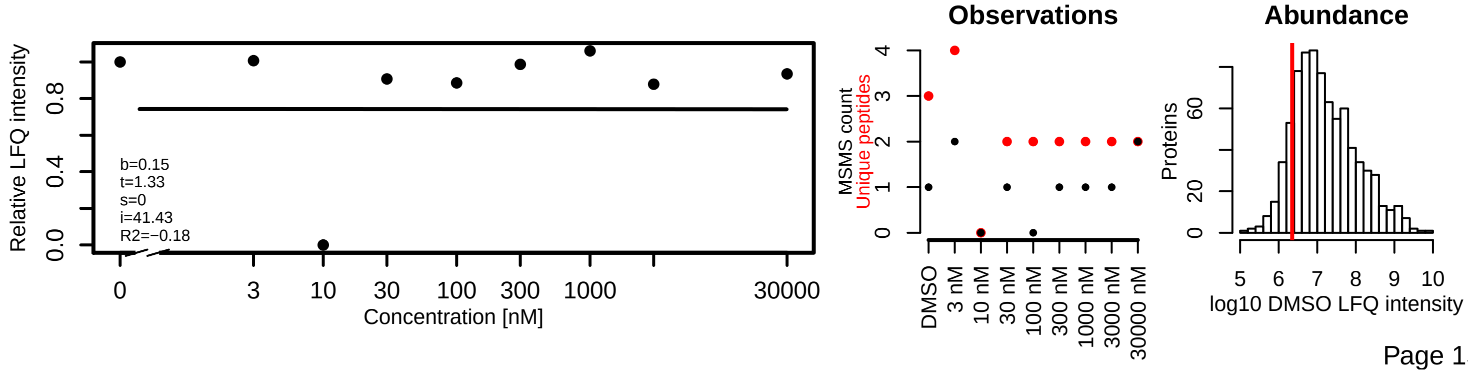

## HSPA1A

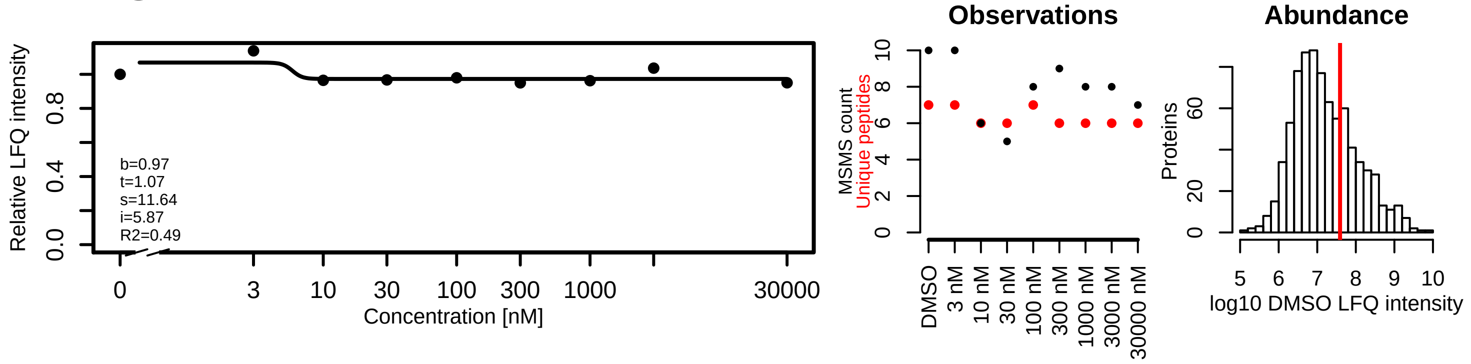


## PSMC4

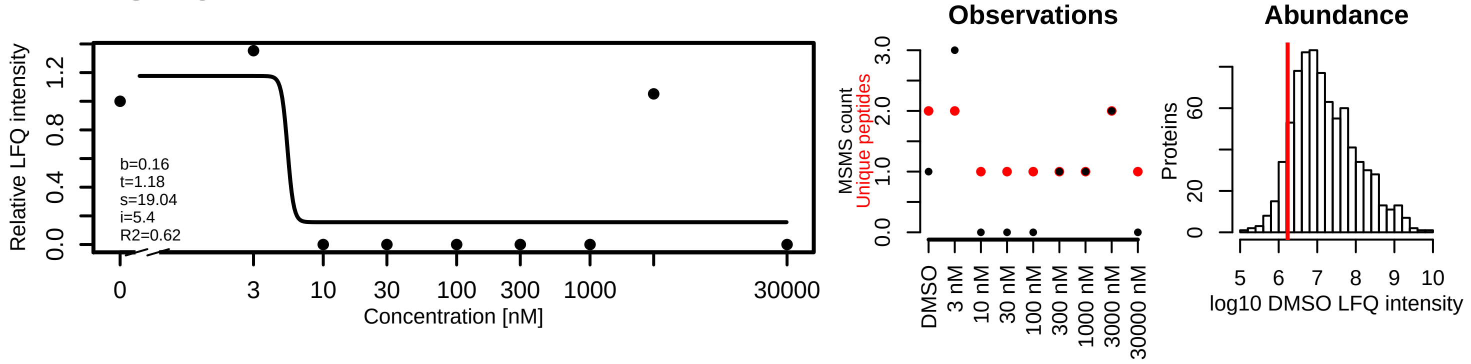


## SLC7A5

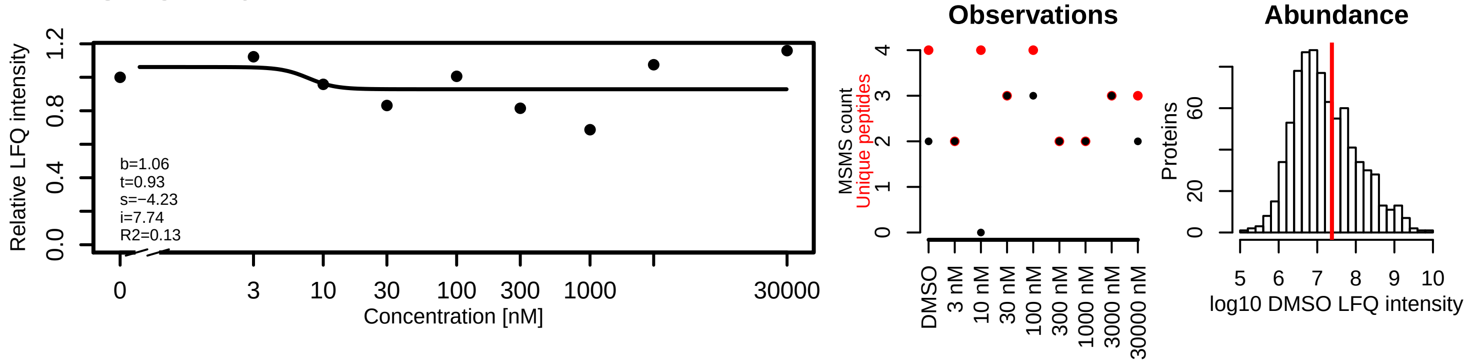


## PTK7

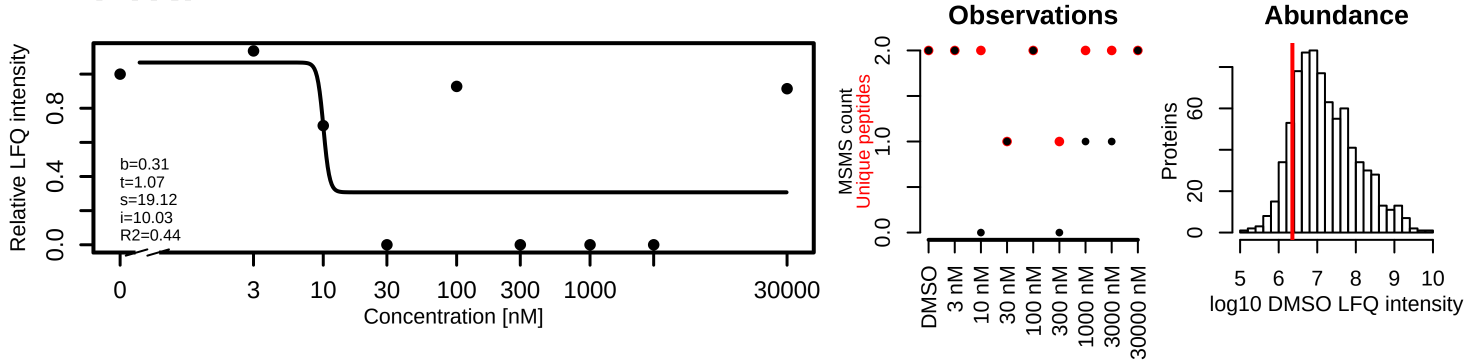


## PRKDC

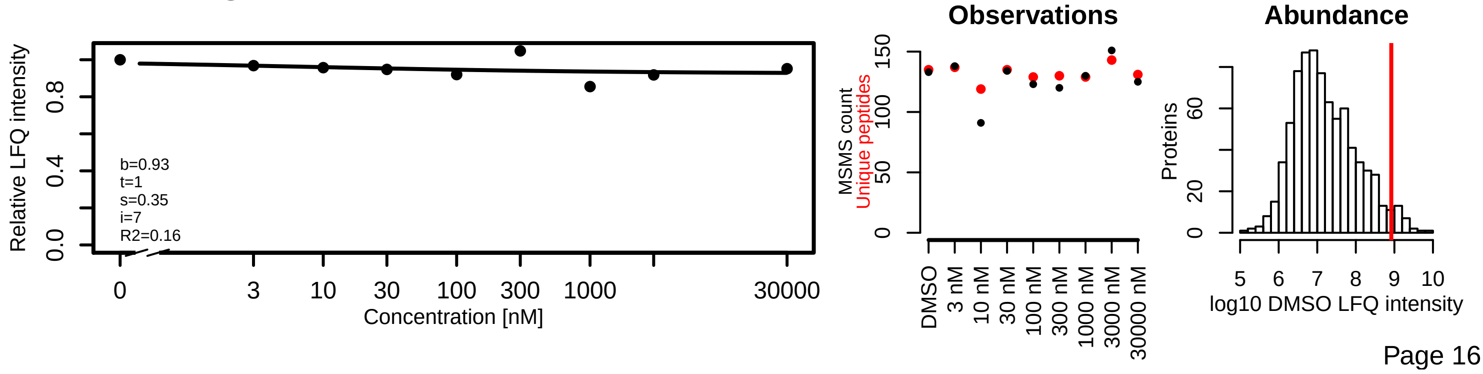

## COX6C

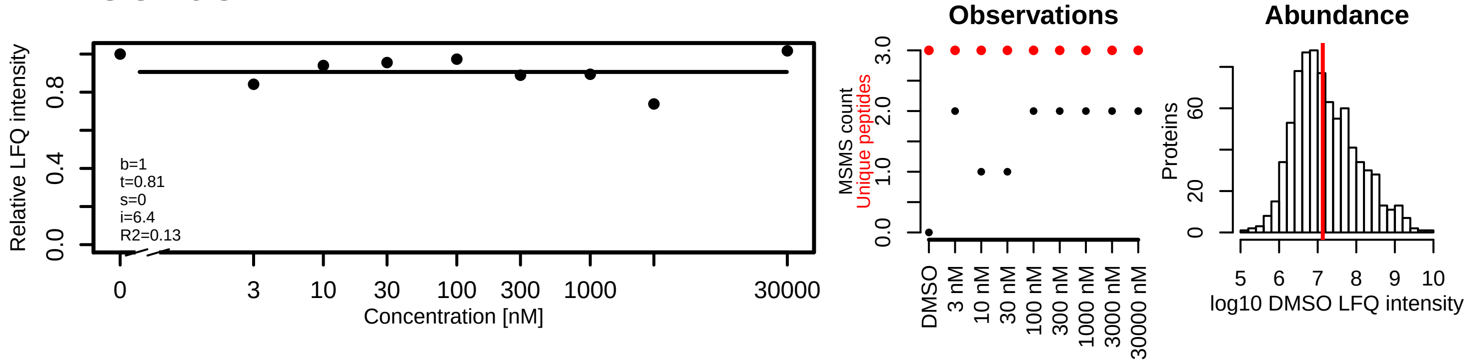


## ILF3

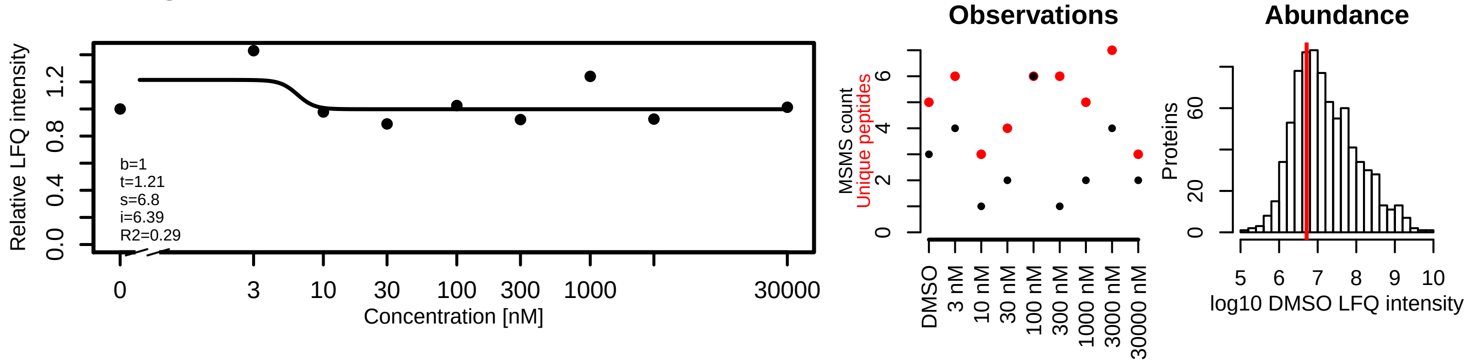


## VAC14

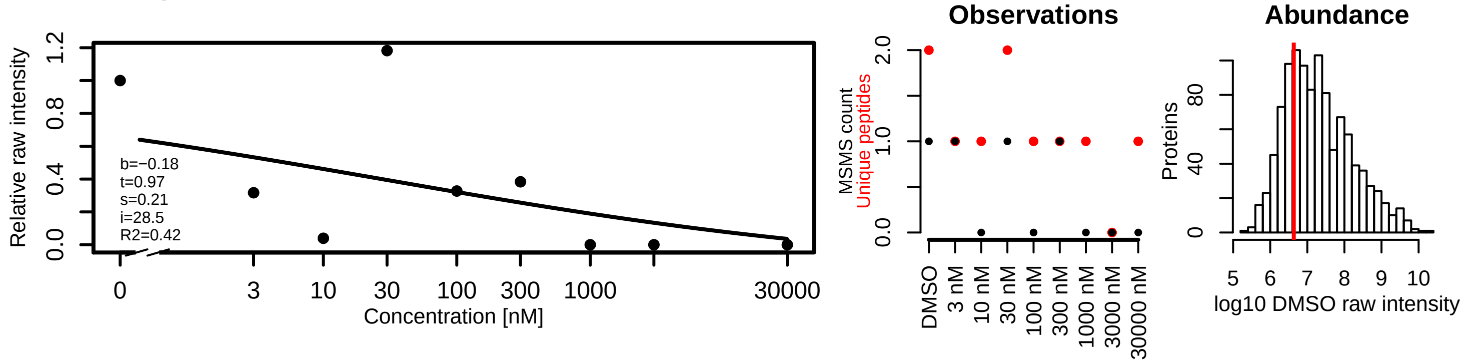


## MAP3K15

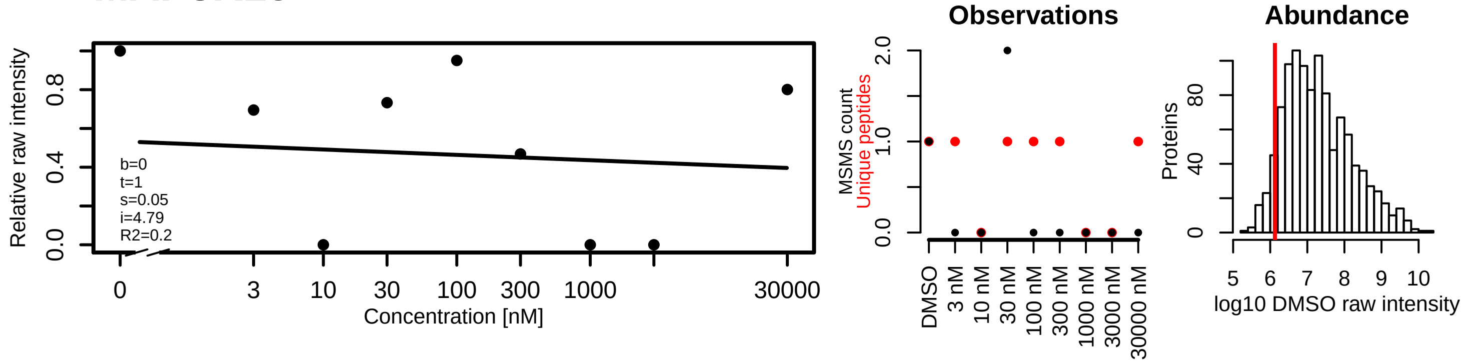


## SPTLC1

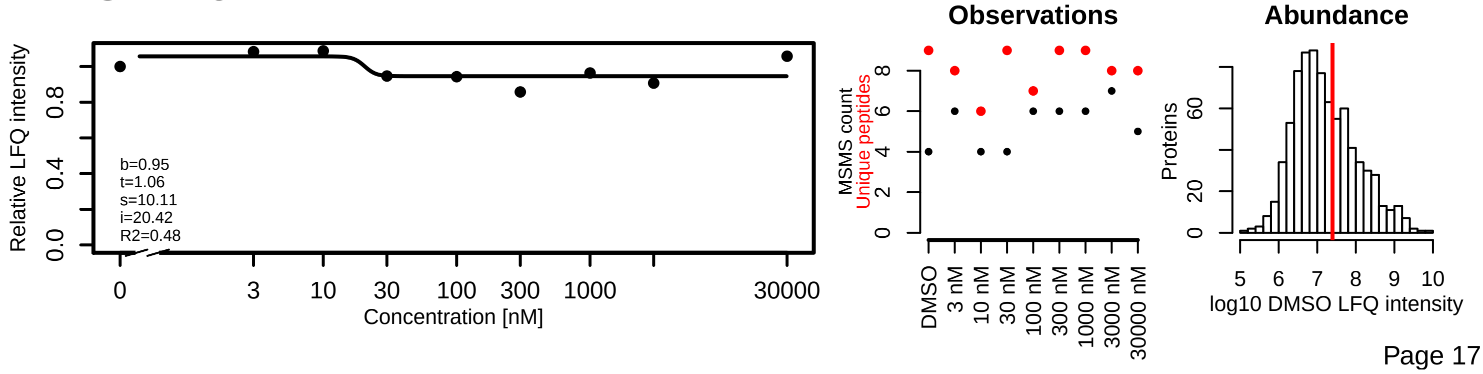

# ECHS1

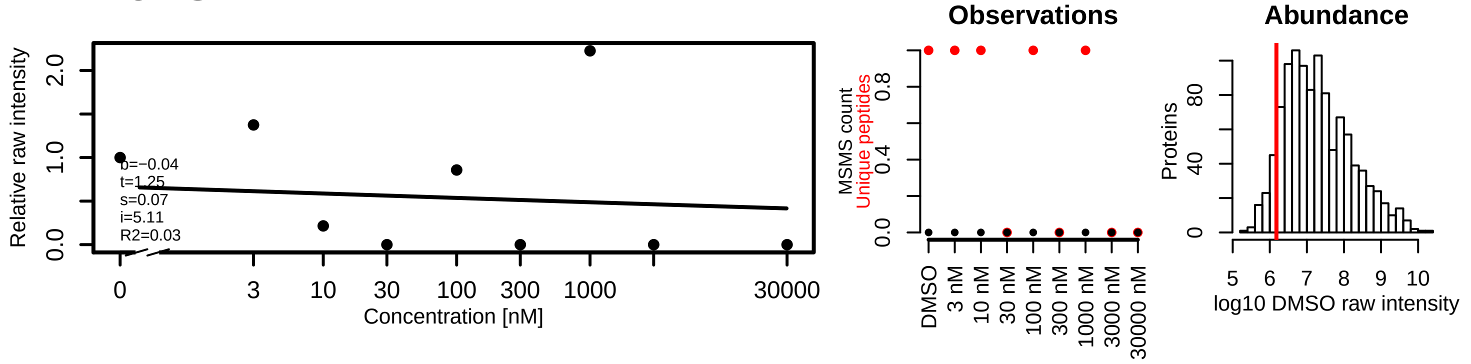


# CDC42

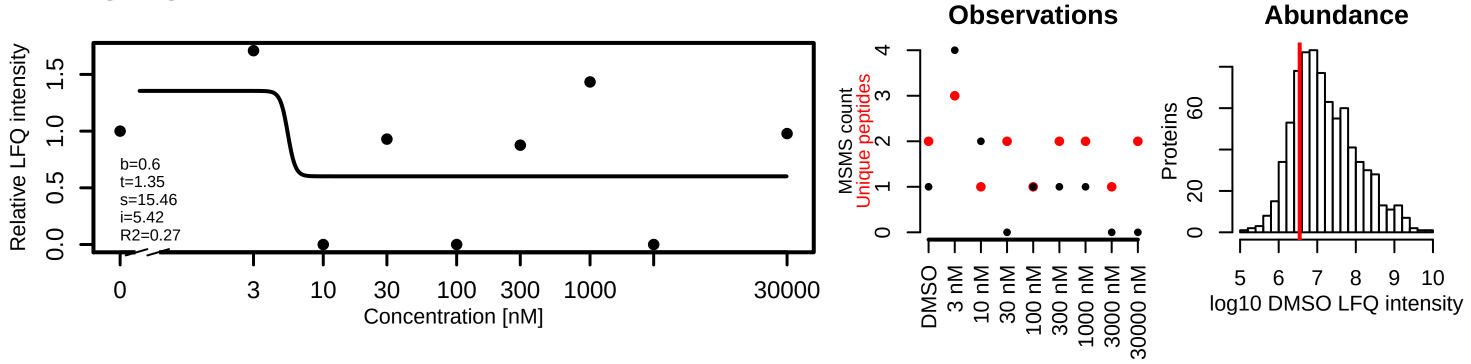


# ATAD3A

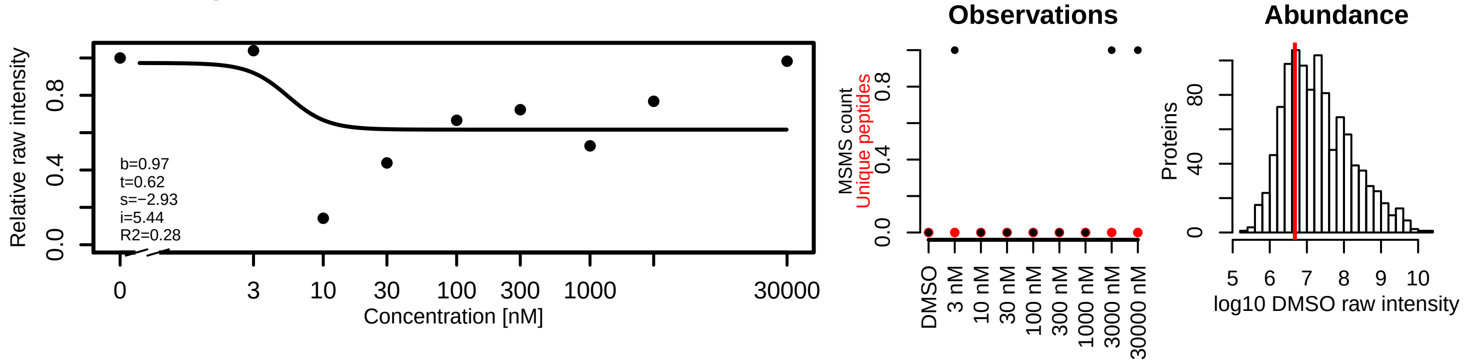


# TMEM245

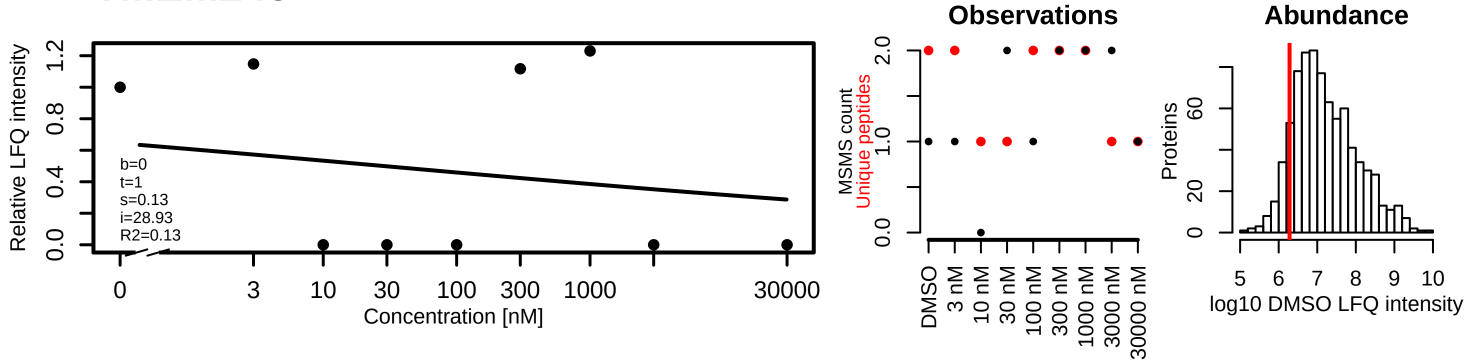


# C22orf28

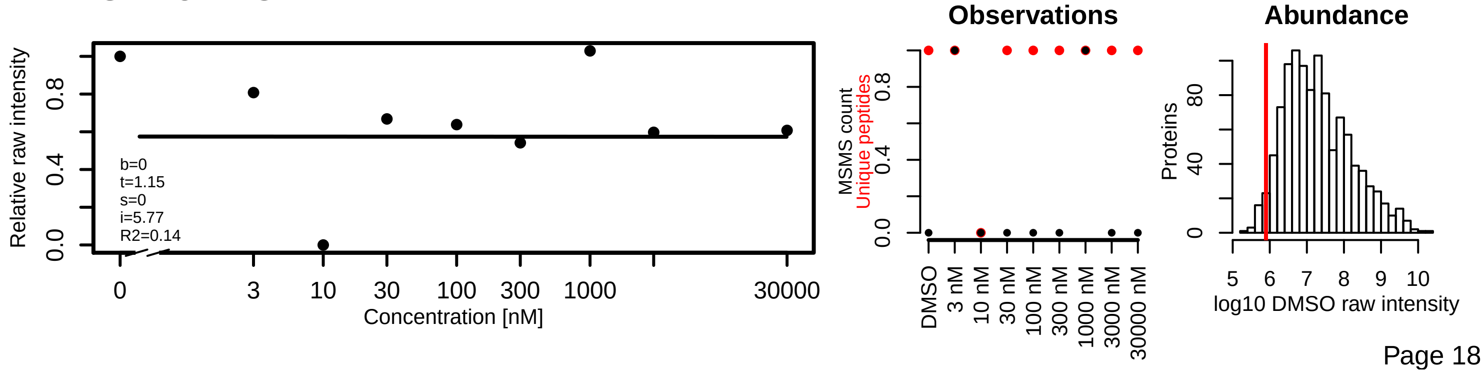

# DYM

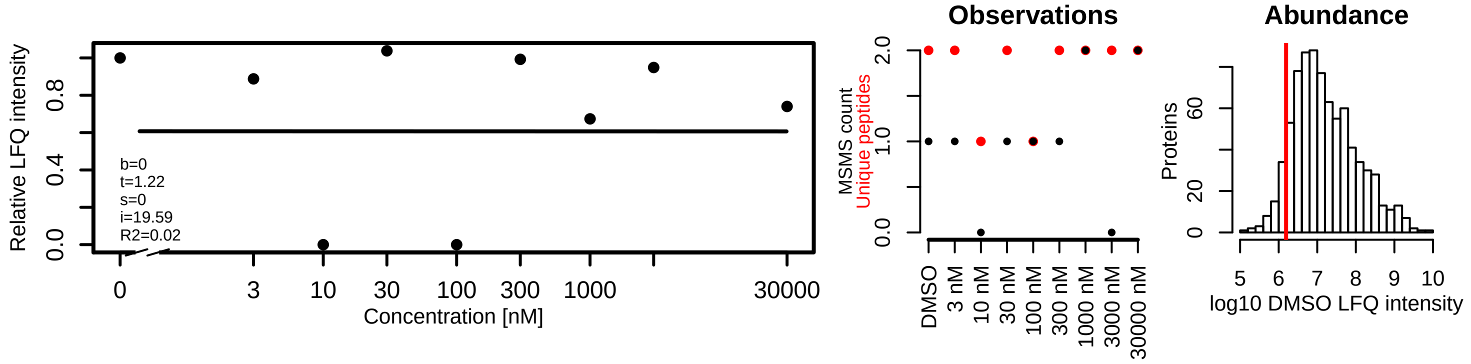


# APH1A

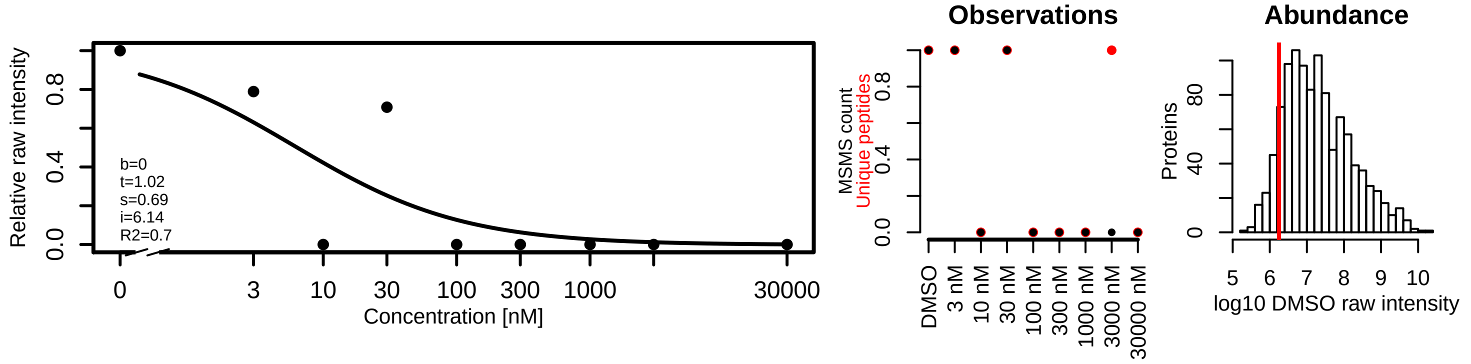


# DSP

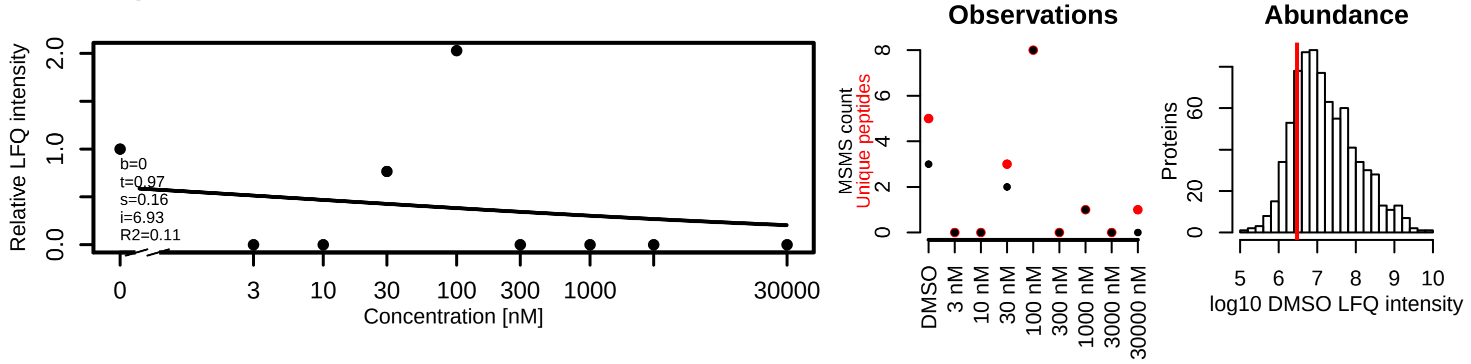


# TUBGCP2

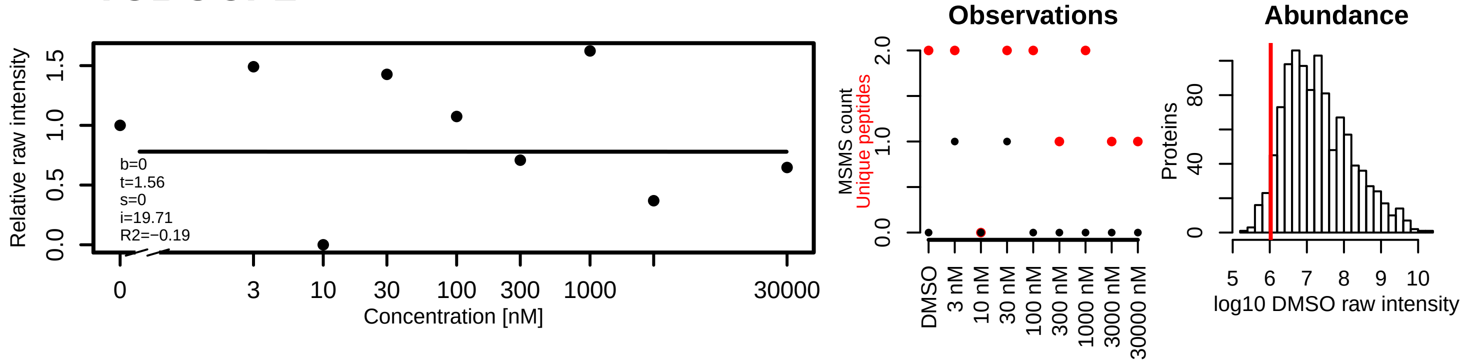


# EIF4G1

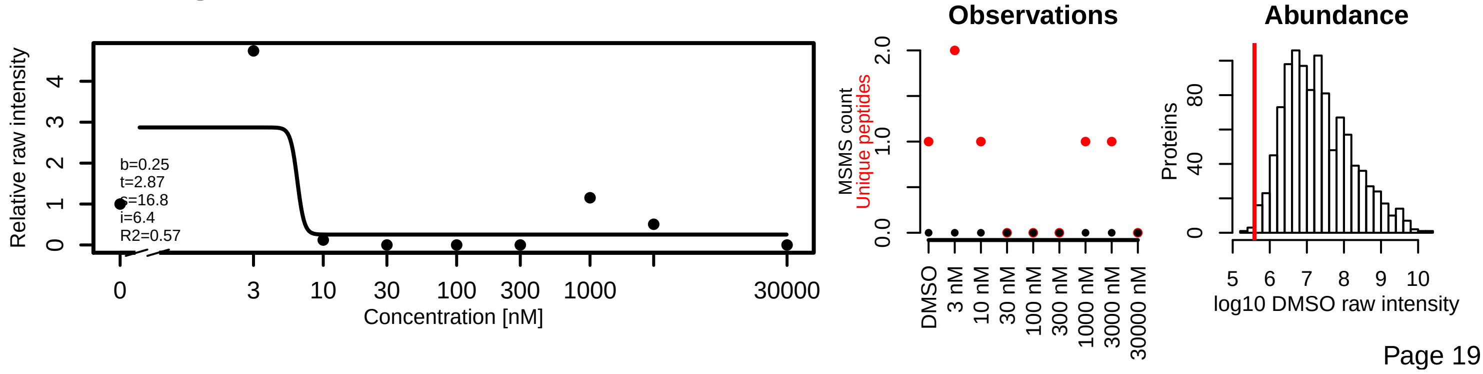

## RPS23

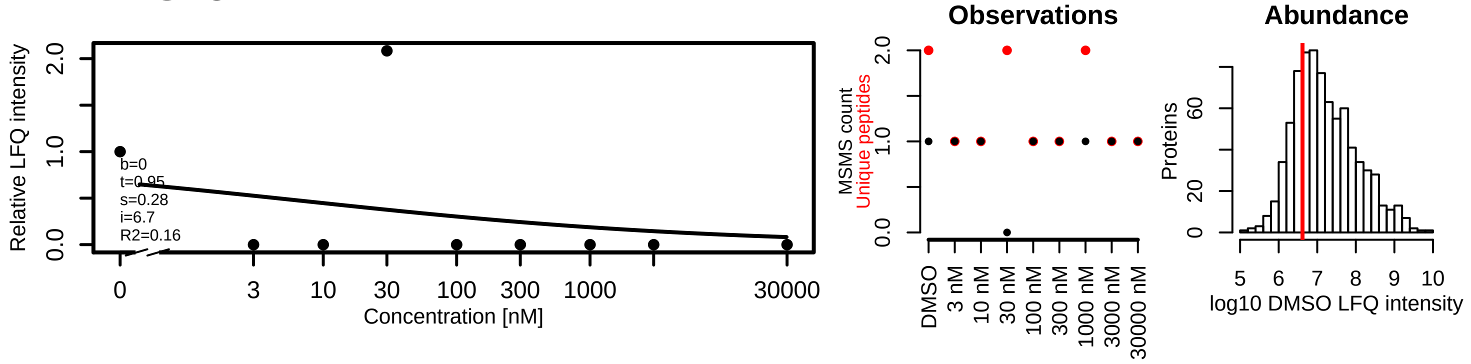


## SUCLG2

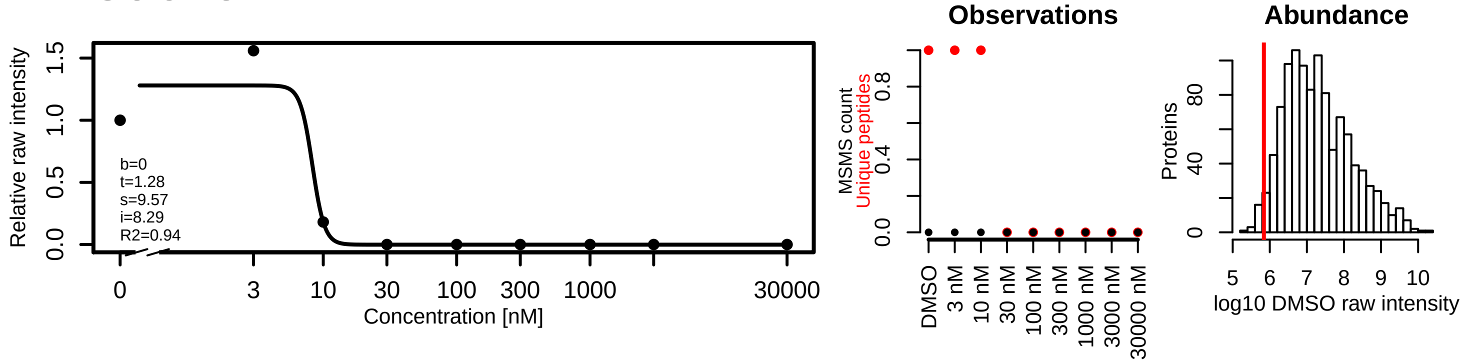


## NPEPPS;TBC1D3;NPEPPSL1

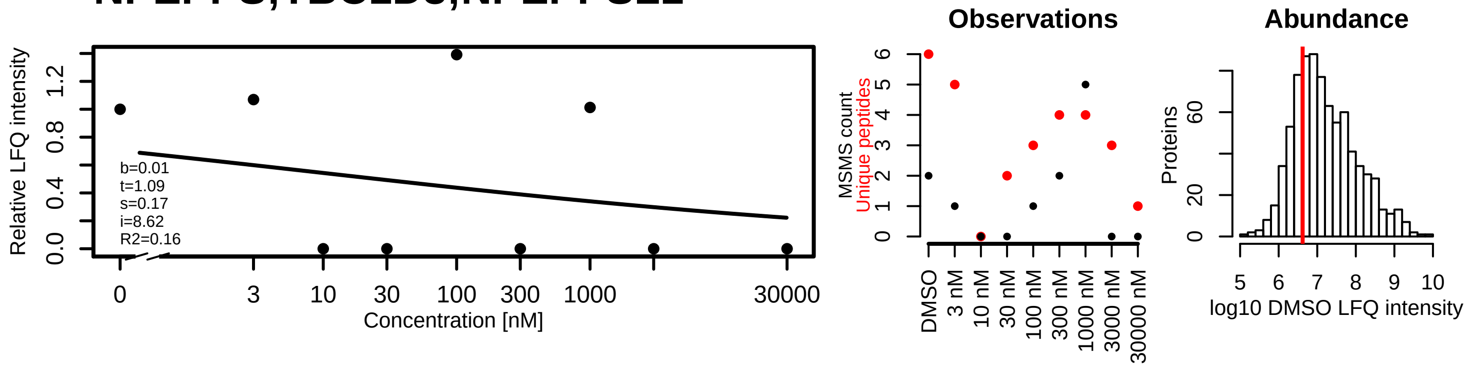


## PCBP2;PCBP3

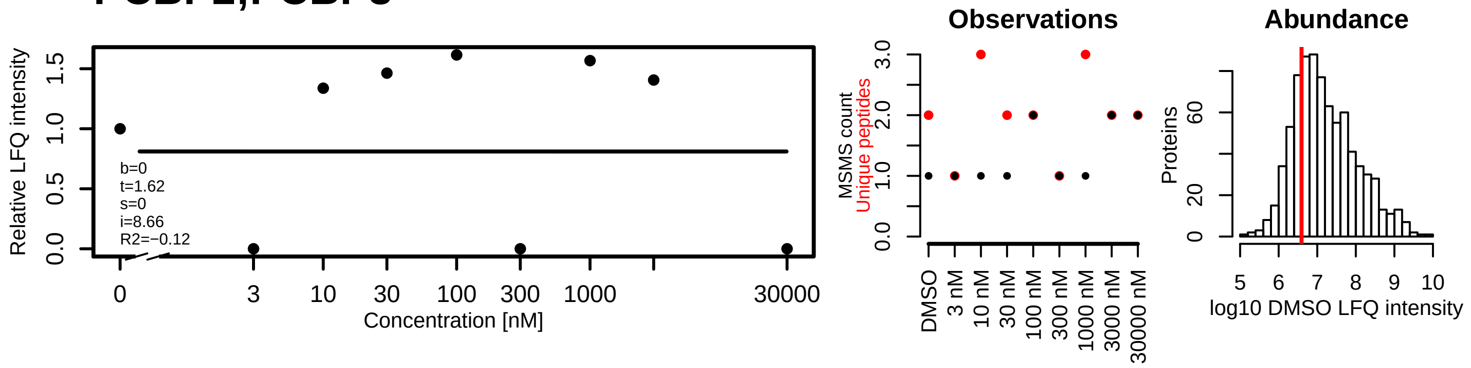


## TMEM120B

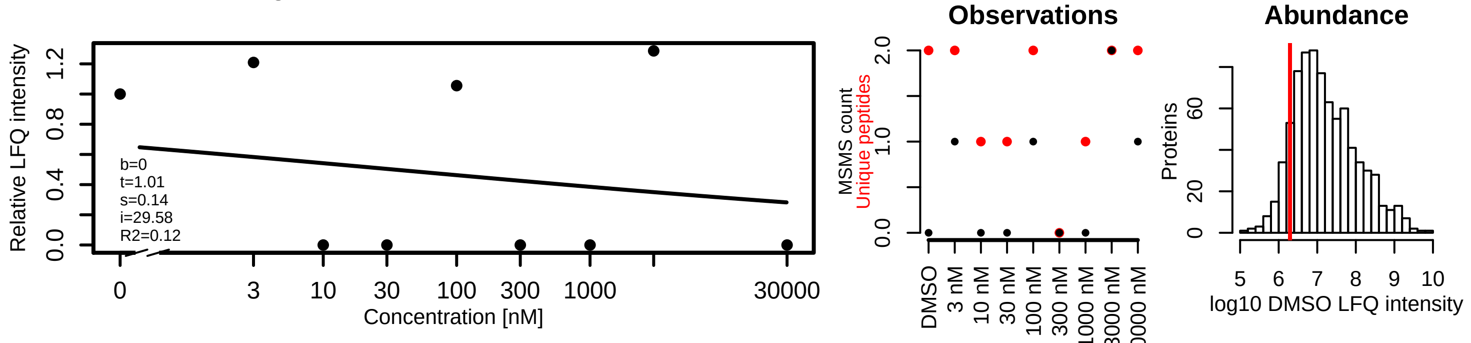

# HSPA8

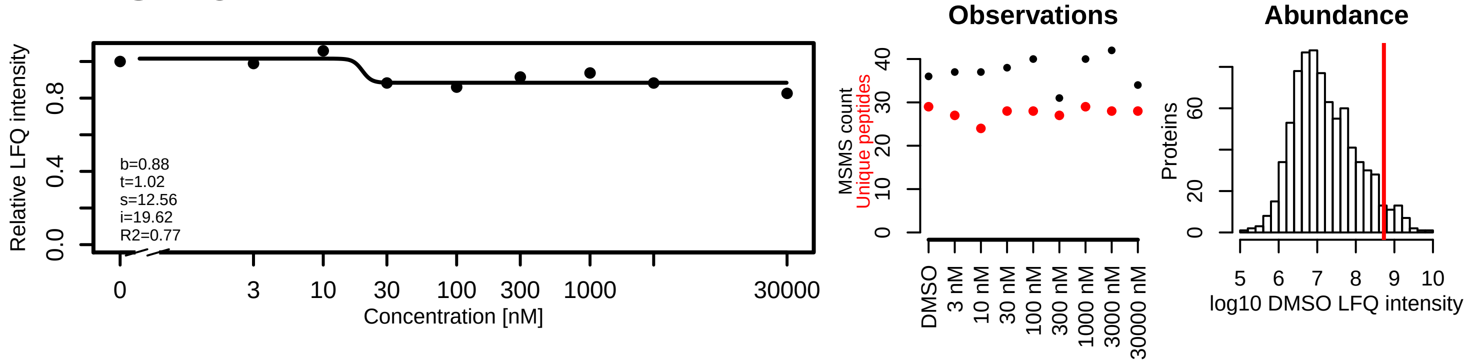


# PPM1A

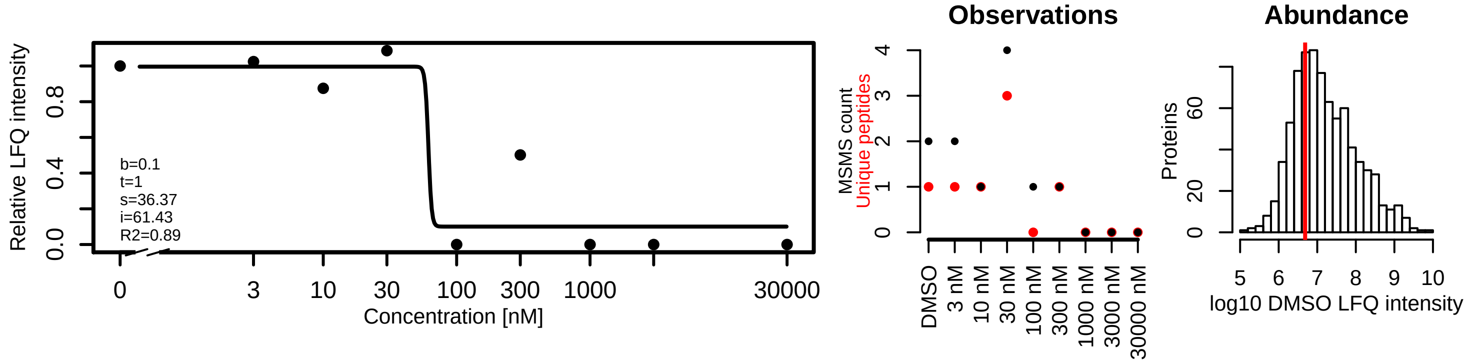


# MAP4K4

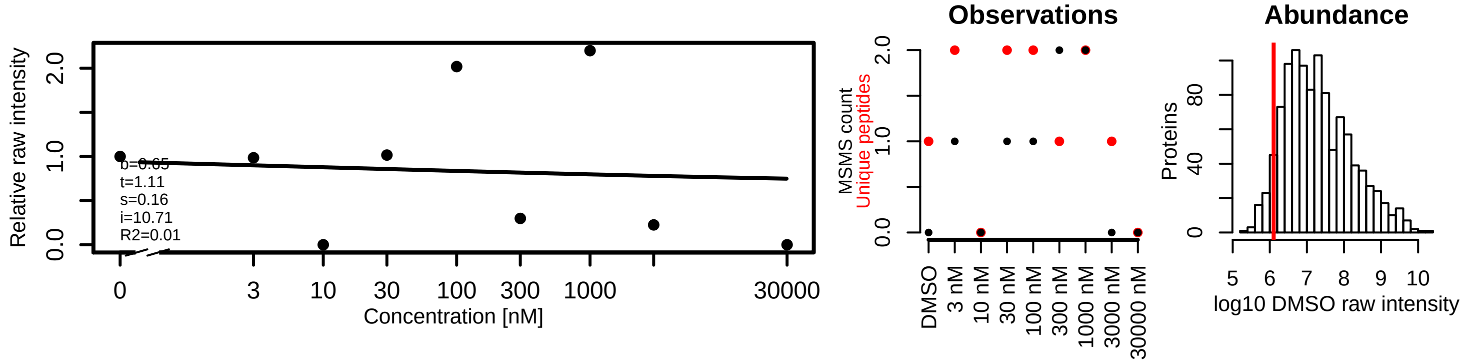


# SLC2A1

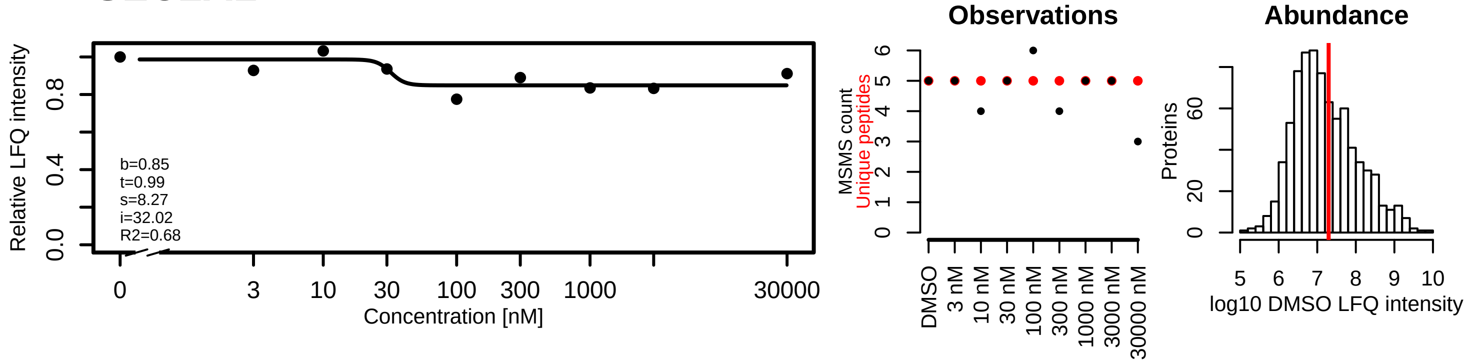


# COX4I1

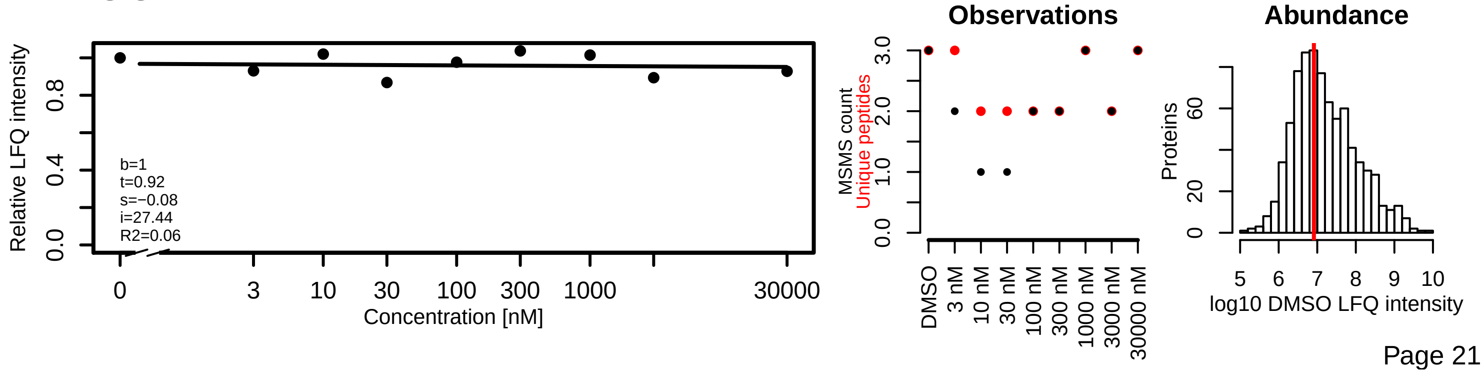

## THADA

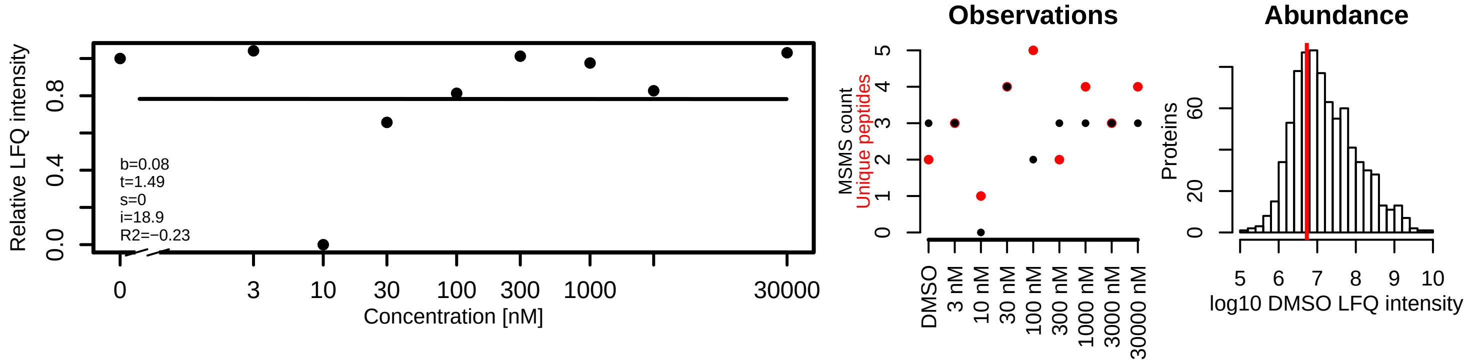


## RPS27

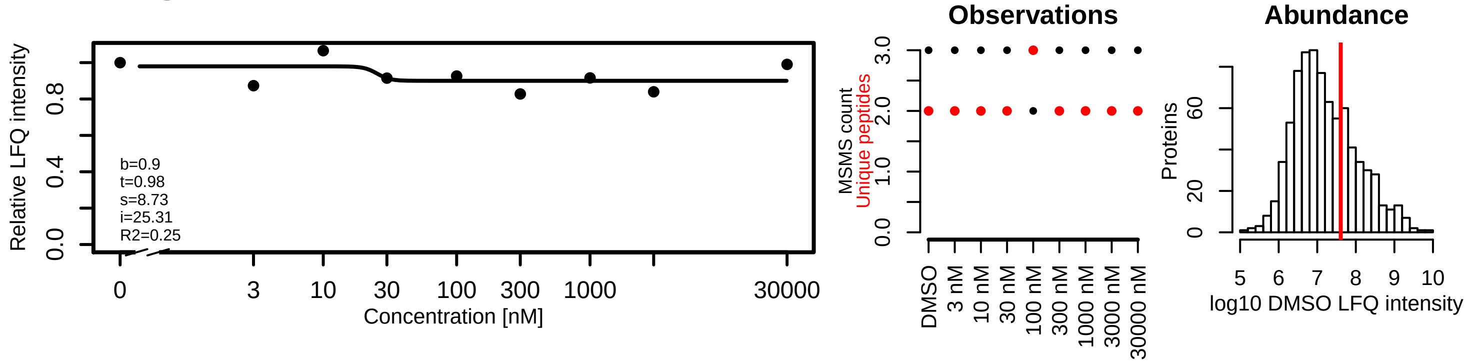


## TCEB1

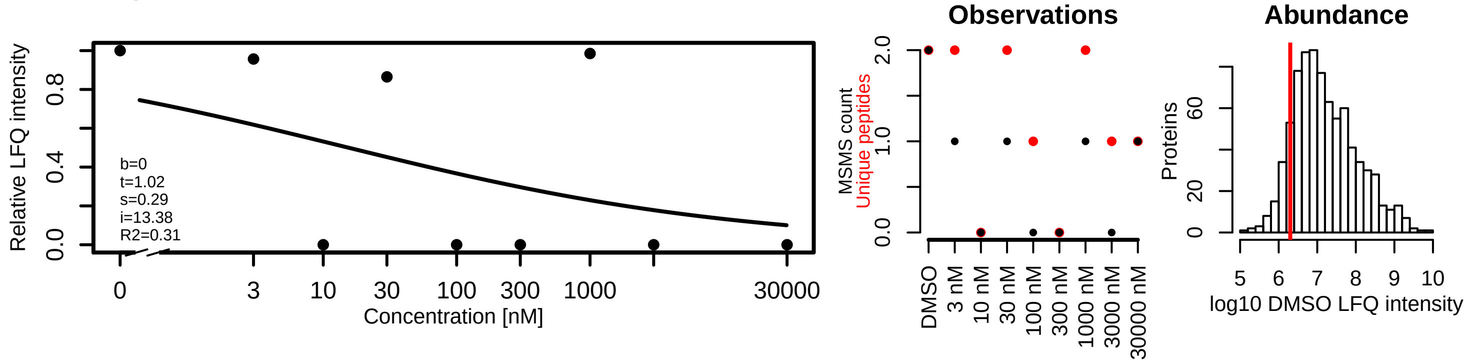


## MAPK14 – Q16539–2

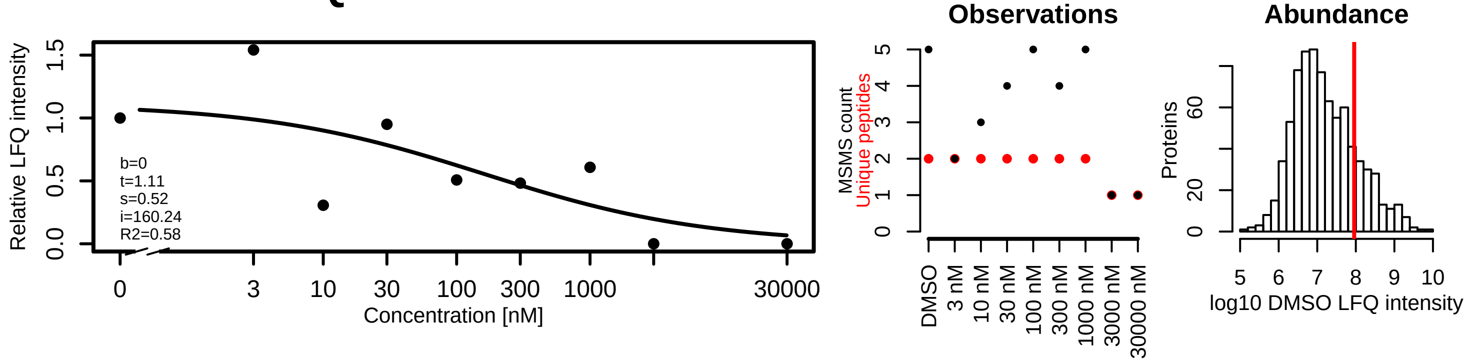


## RPS27A;UBB;UBC;UBA52

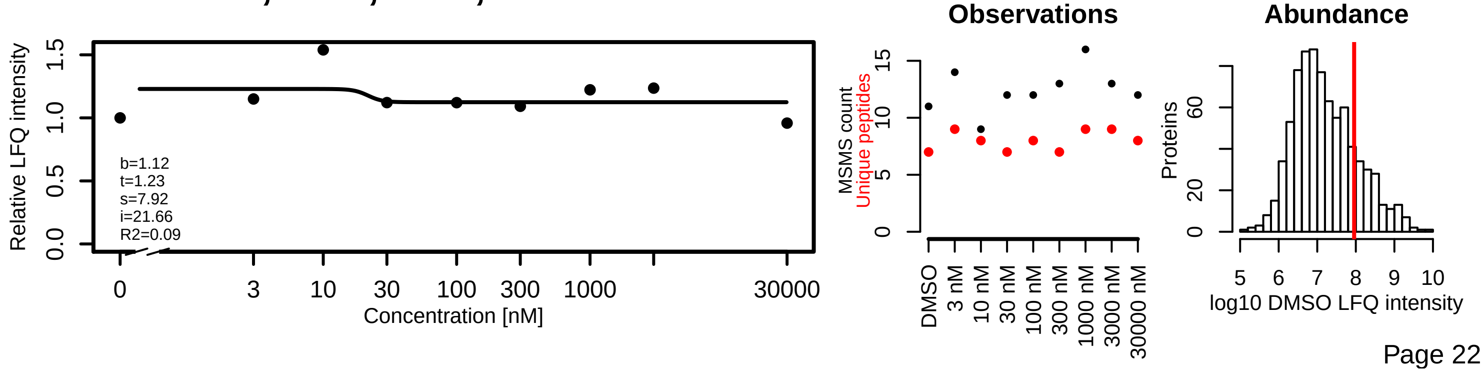

## PPM1B

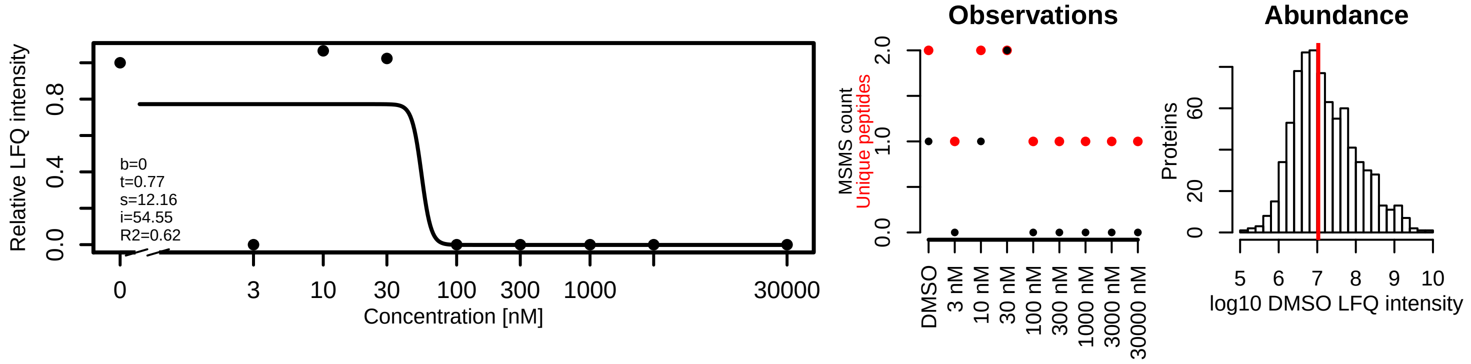


## CIT − H7BYJ3;O14578−3;F5H4K4

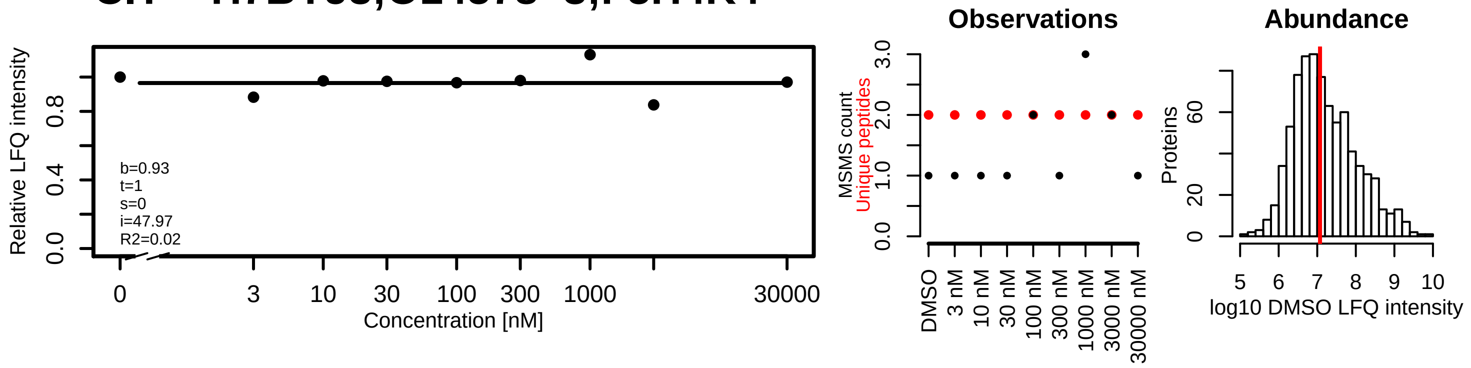


## HNRNPR

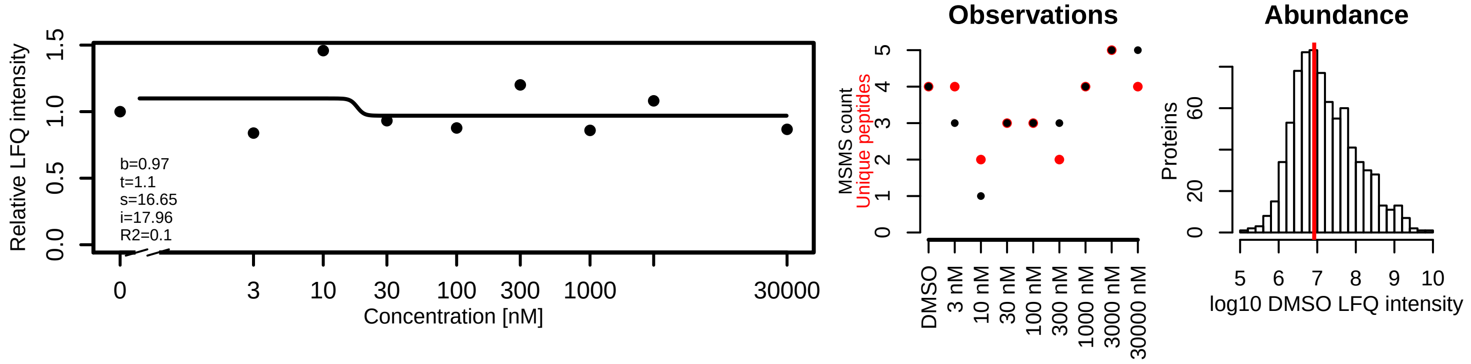


## MOB1A;MOB1B

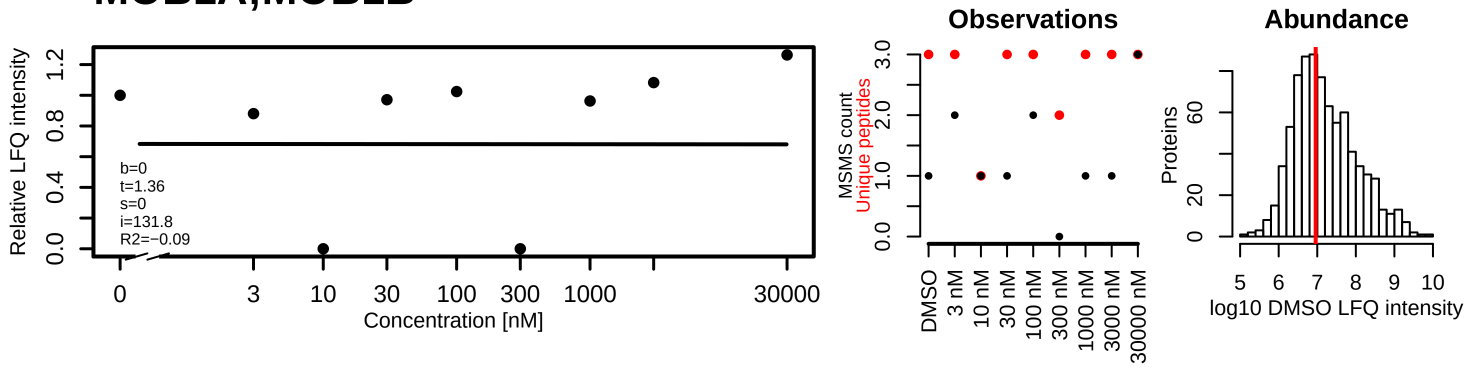


## ENDOD1

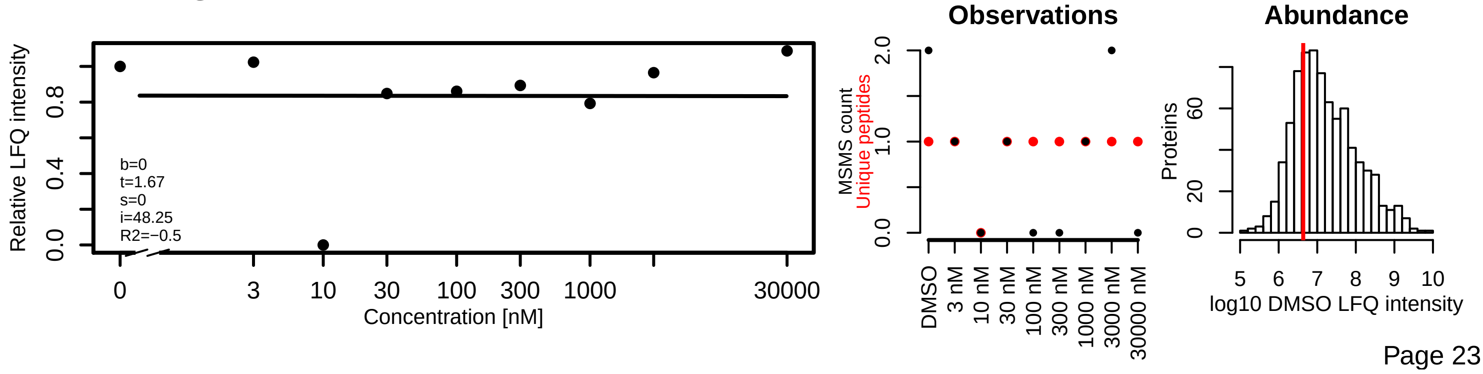

# NUP205

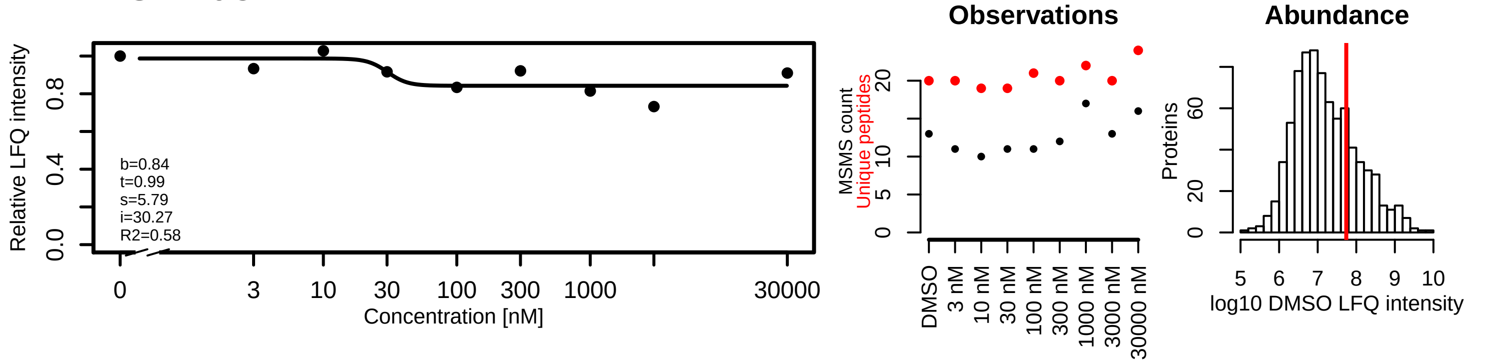


# HADHA

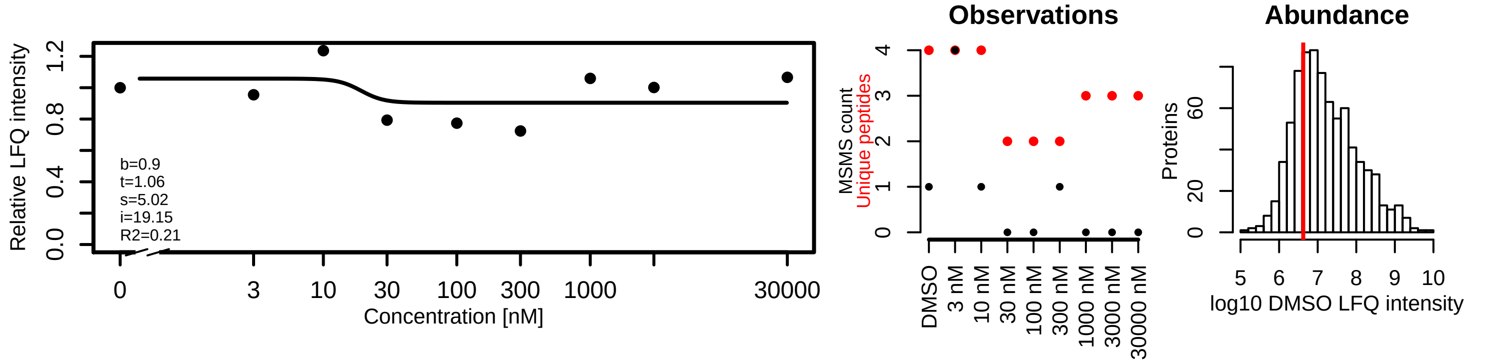


# STRADA

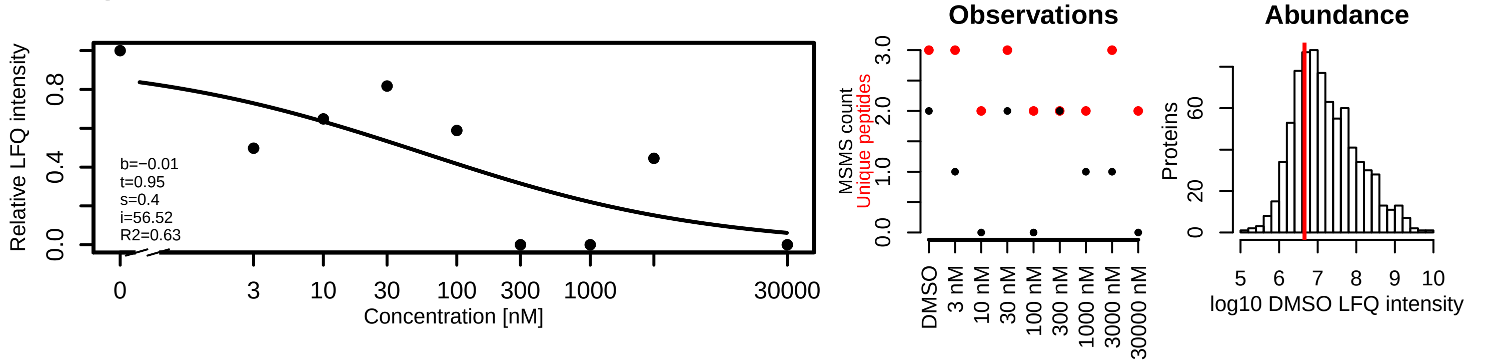


# PFKP

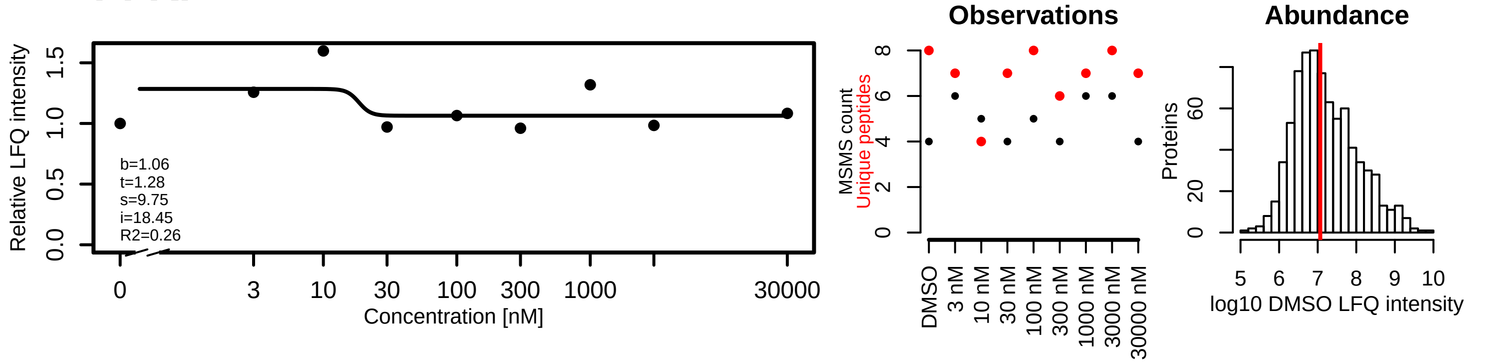


# EPRS

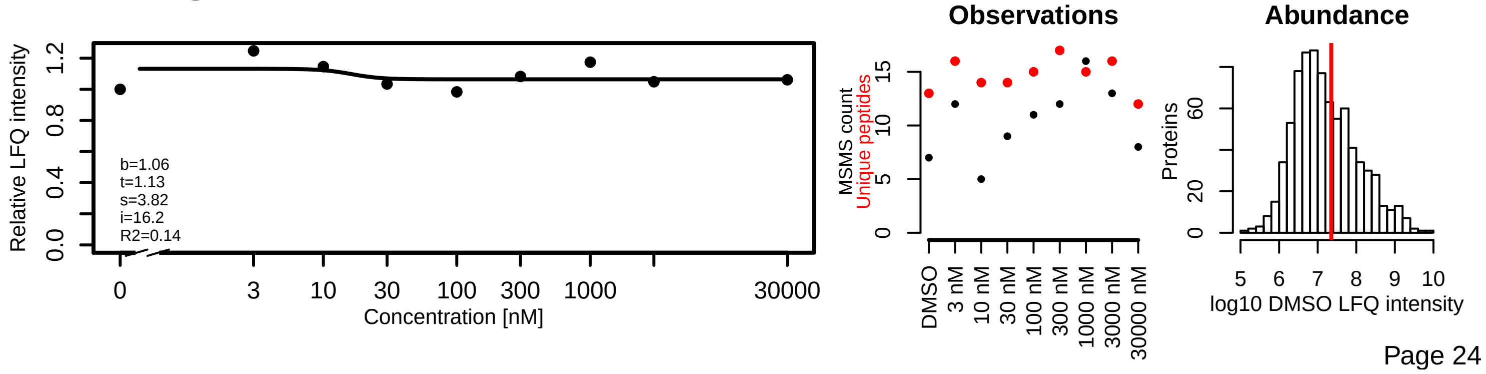

## LMNA

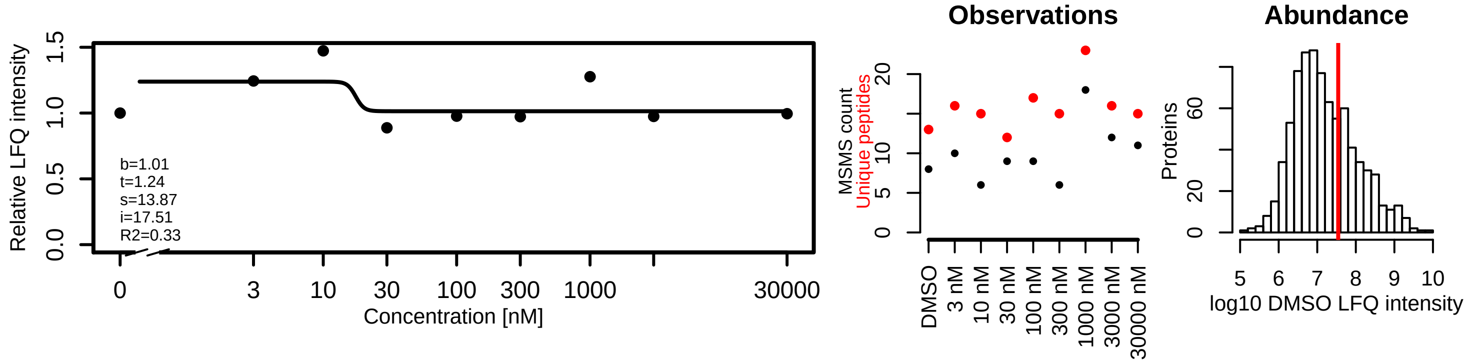


## RAC1;RAC2;RAC3

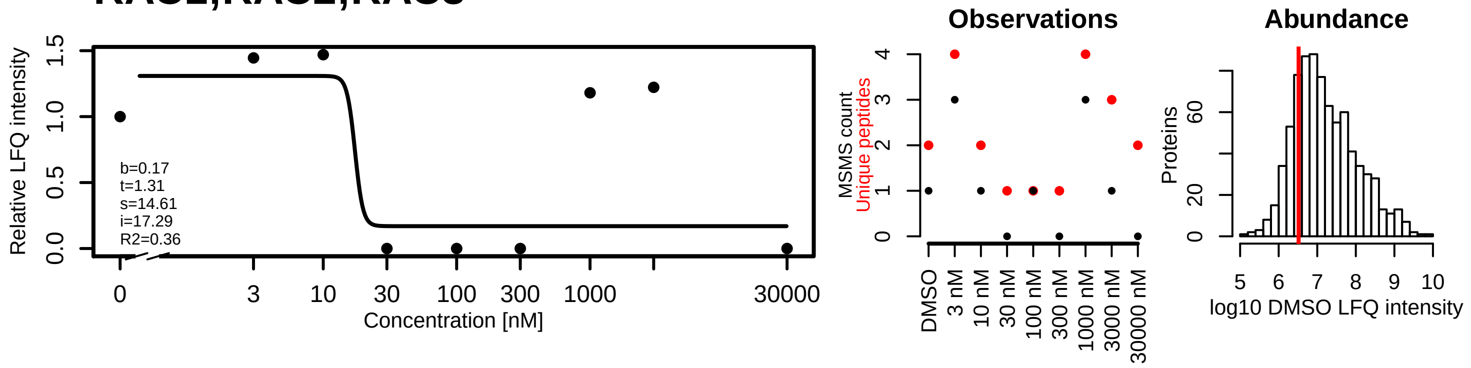


## USMG5

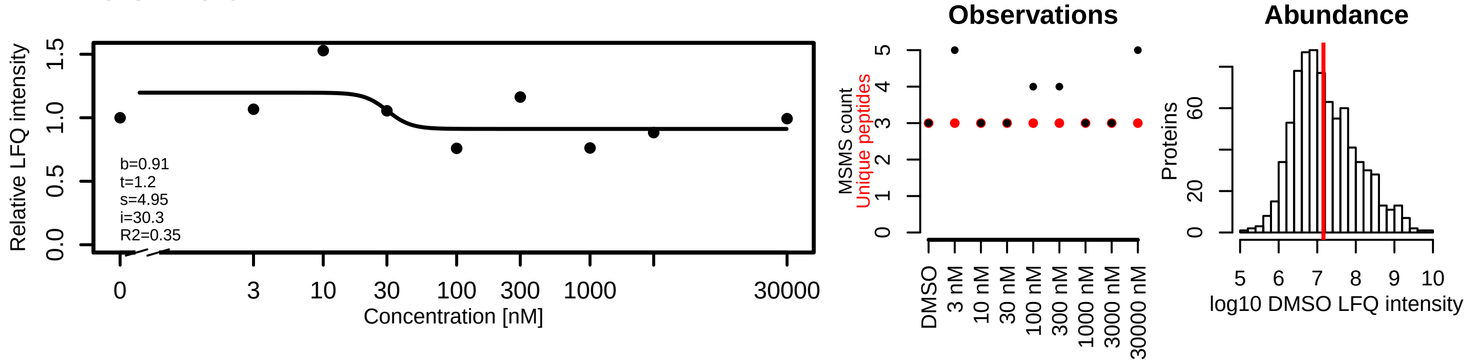


## GAPDH

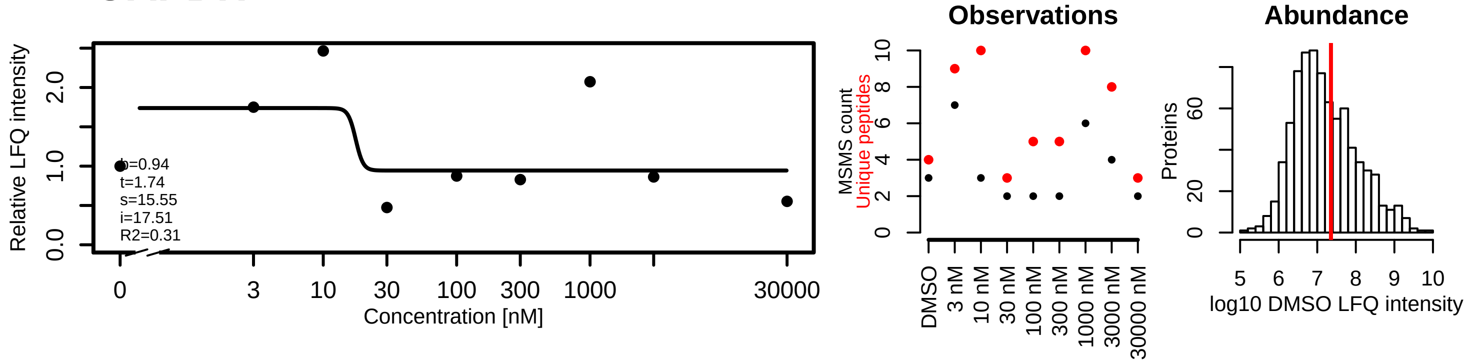


## HSPD1

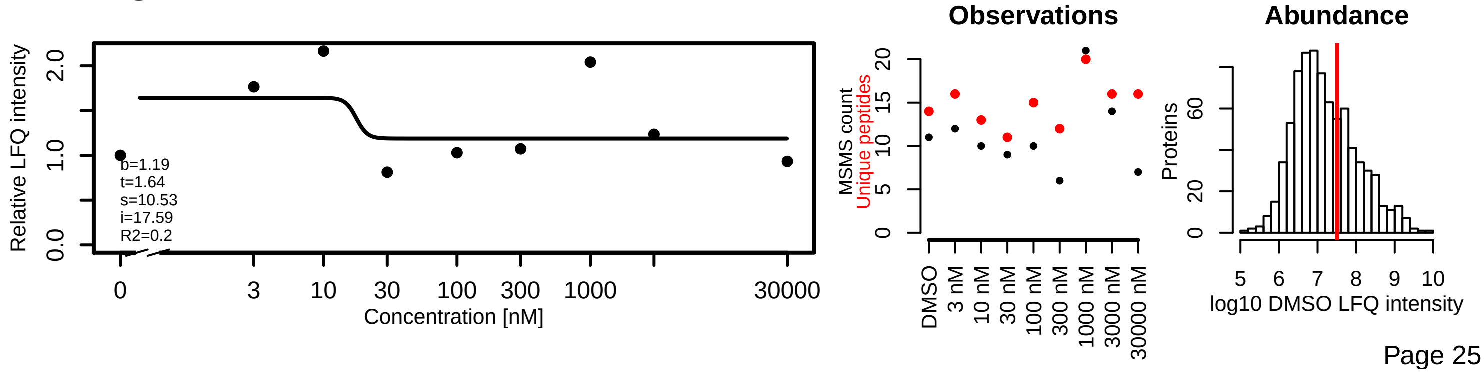

# LGALS1

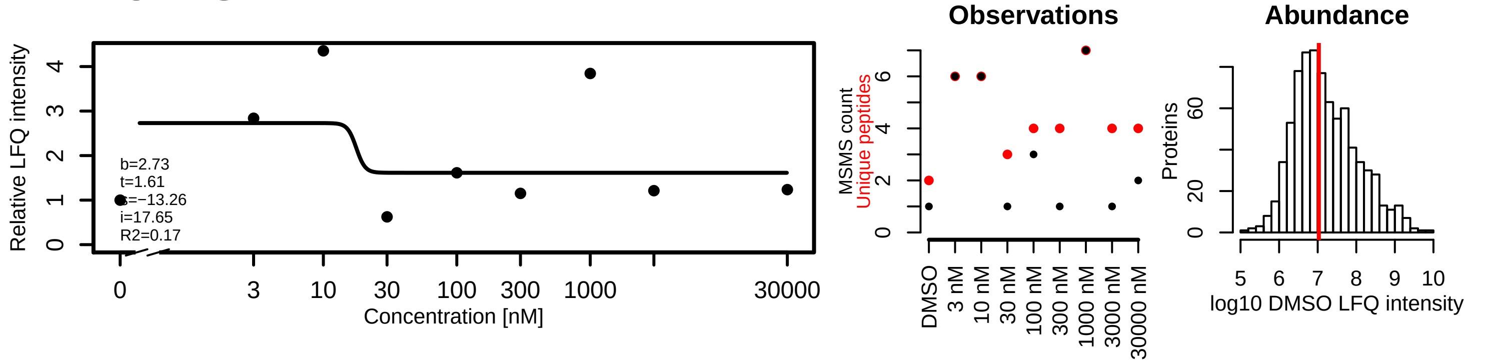


# ANXA2;ANXA2P2

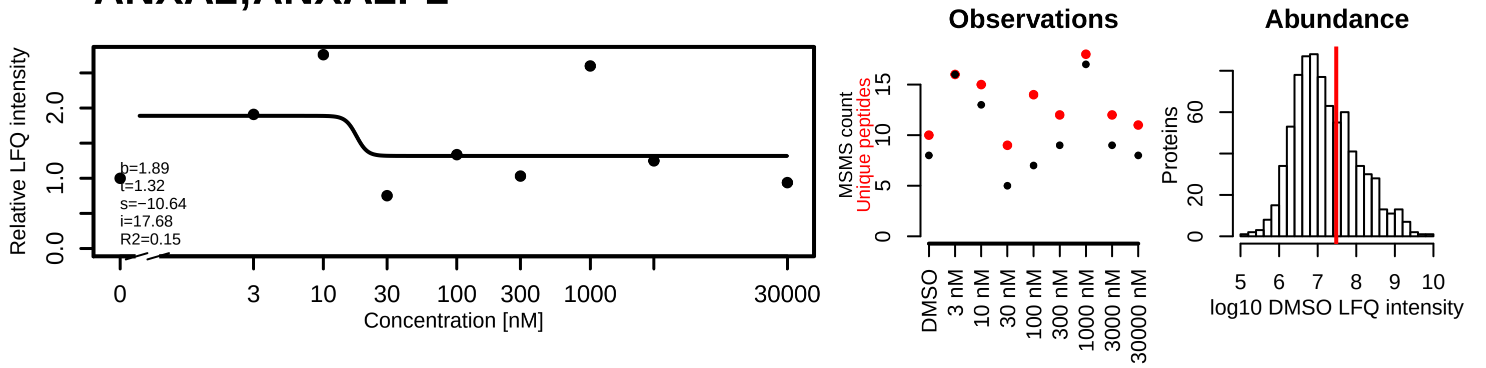


# EEF1A1P5;EEF1A1;EEF1A2

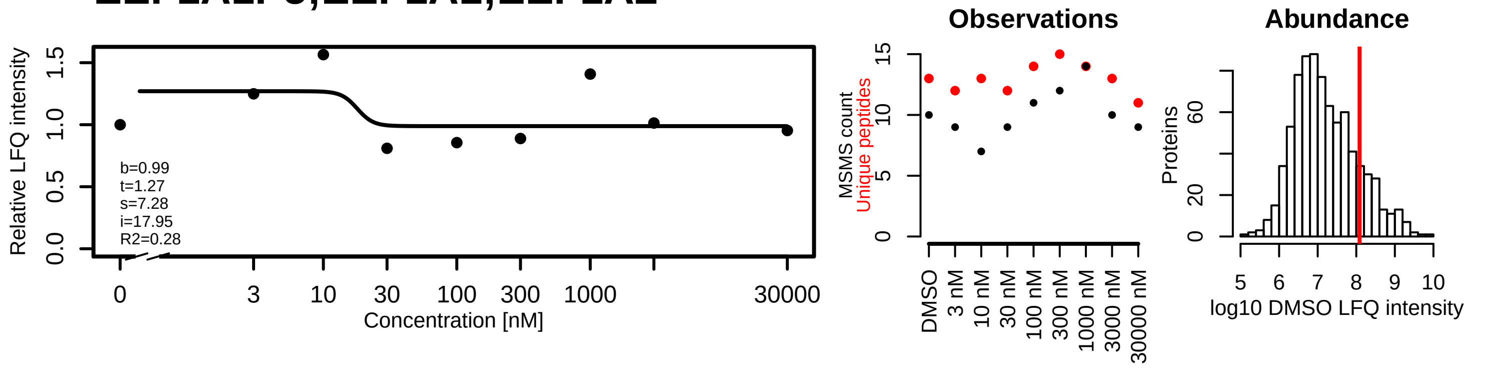


# VARS

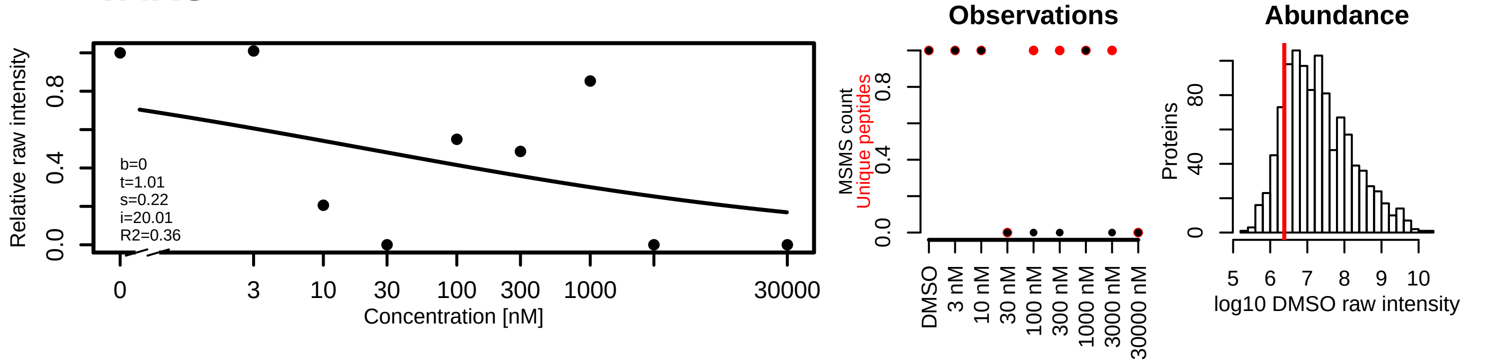


# TUBB

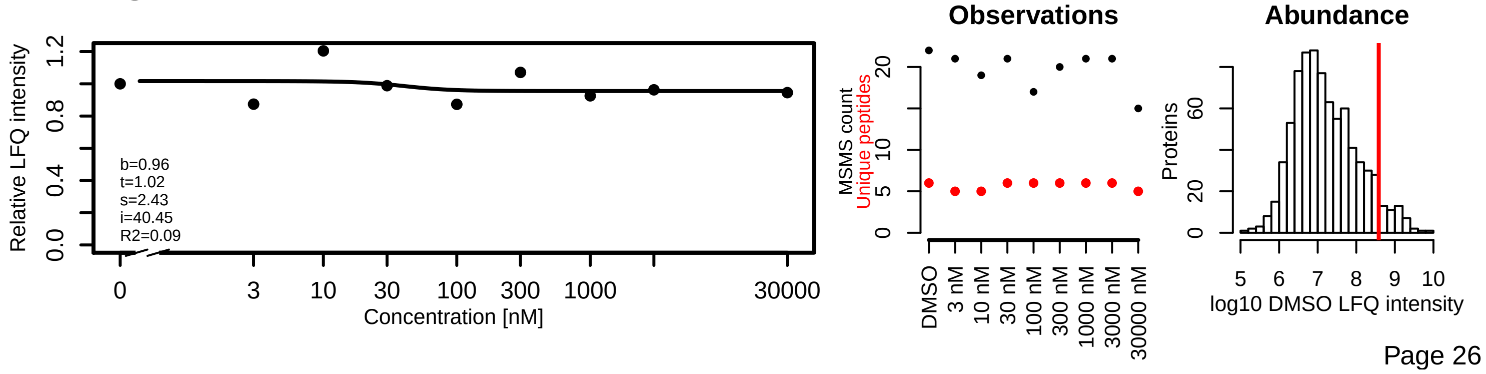

## ATP5L

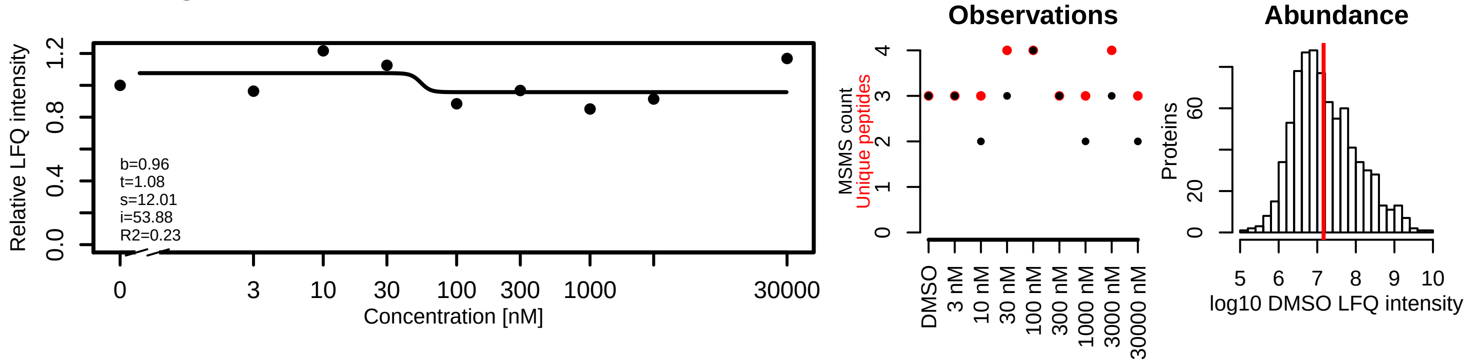


## AHNAK

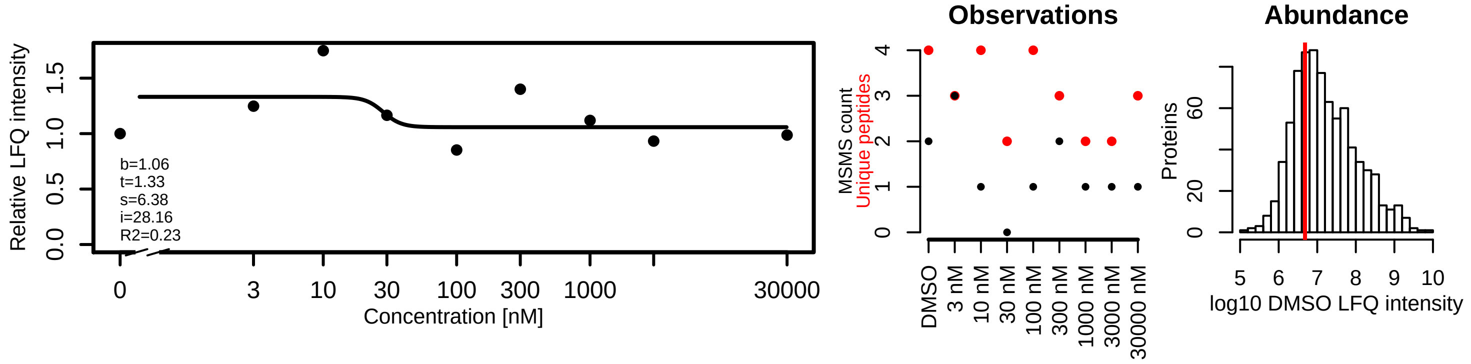


## TUBA1A;TUBA3C;TUBA3E

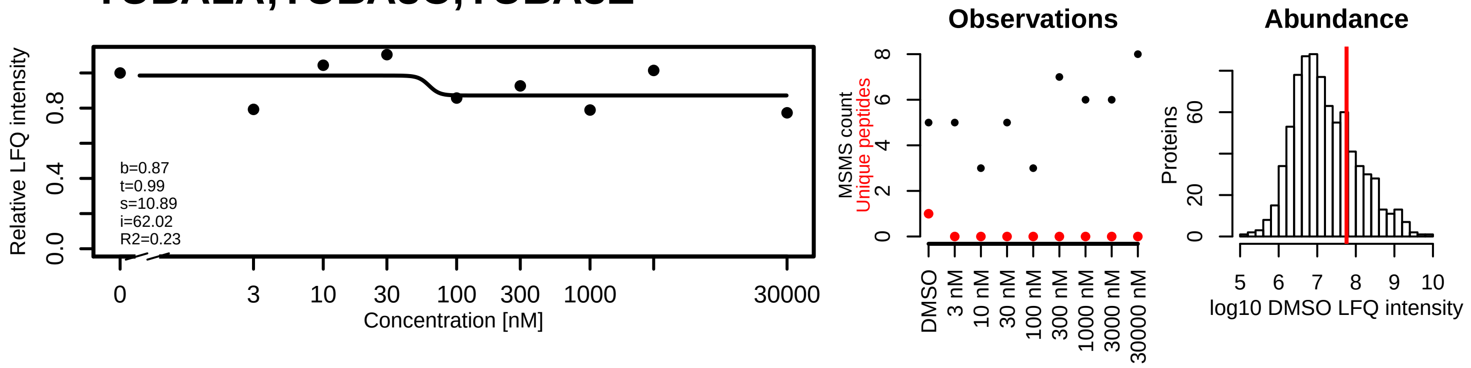


## RFT1

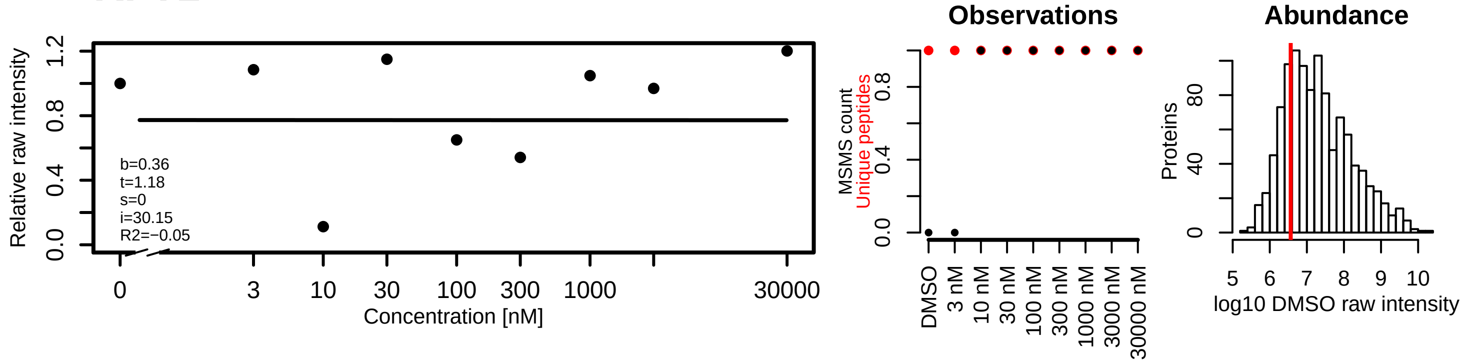


## TESK1

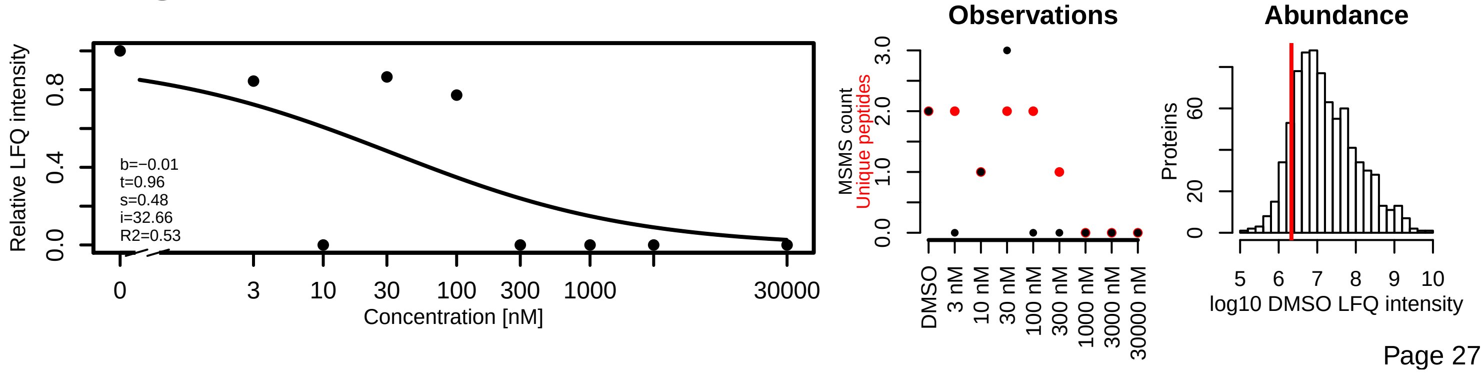

# LATS1

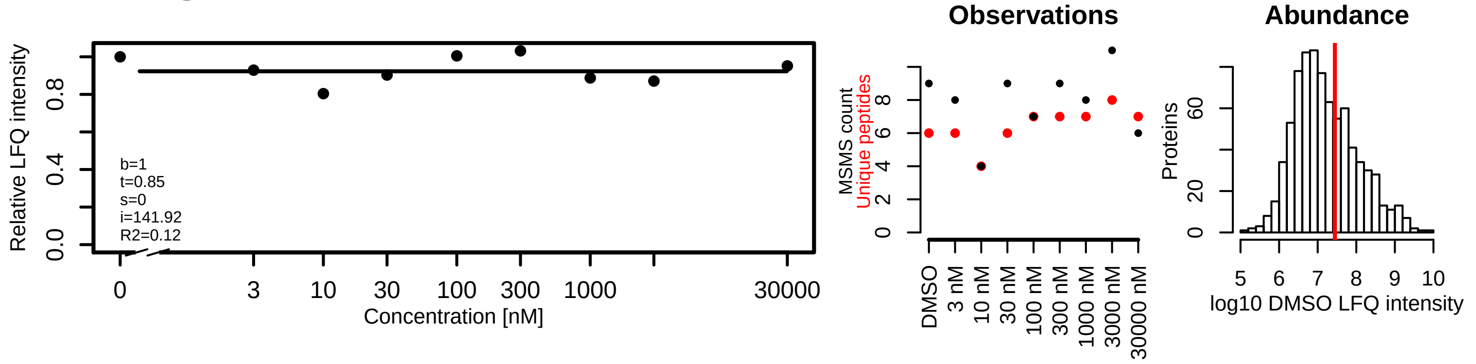


# DDX39B;DDX39A

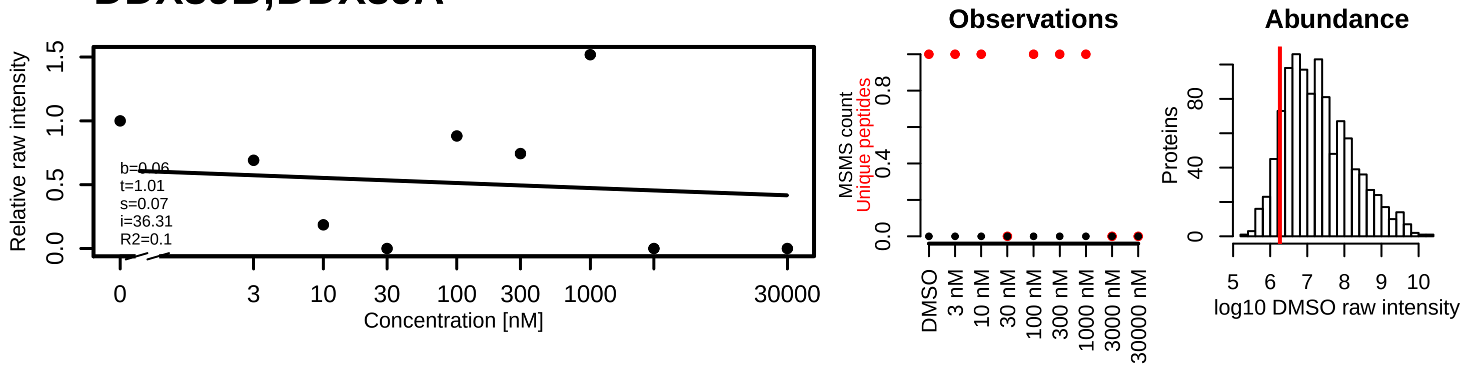


# PHKA2

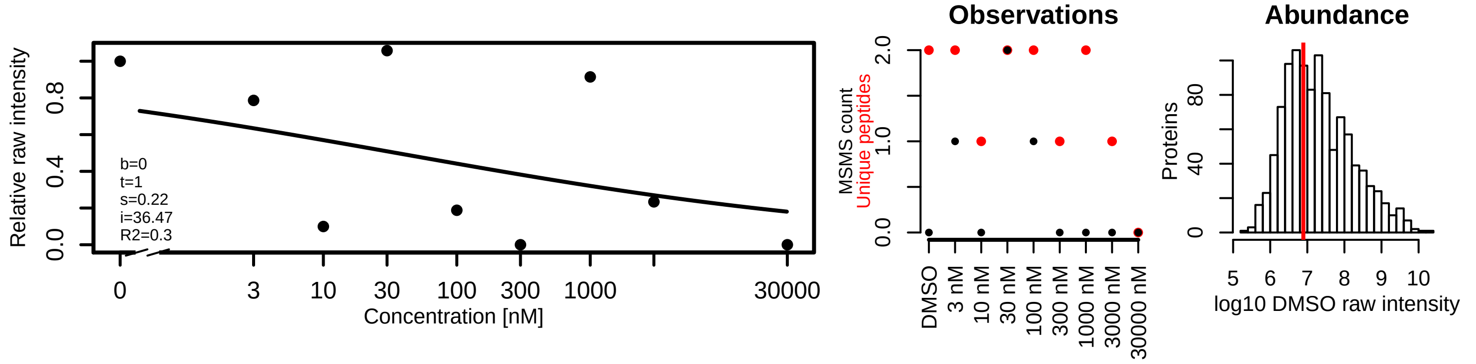


# SLC3A2

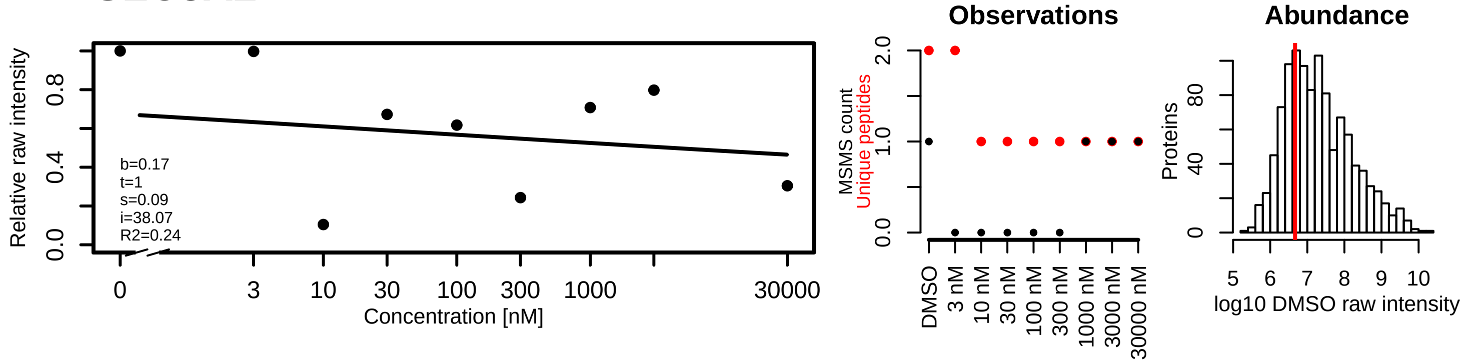


# CNIH4

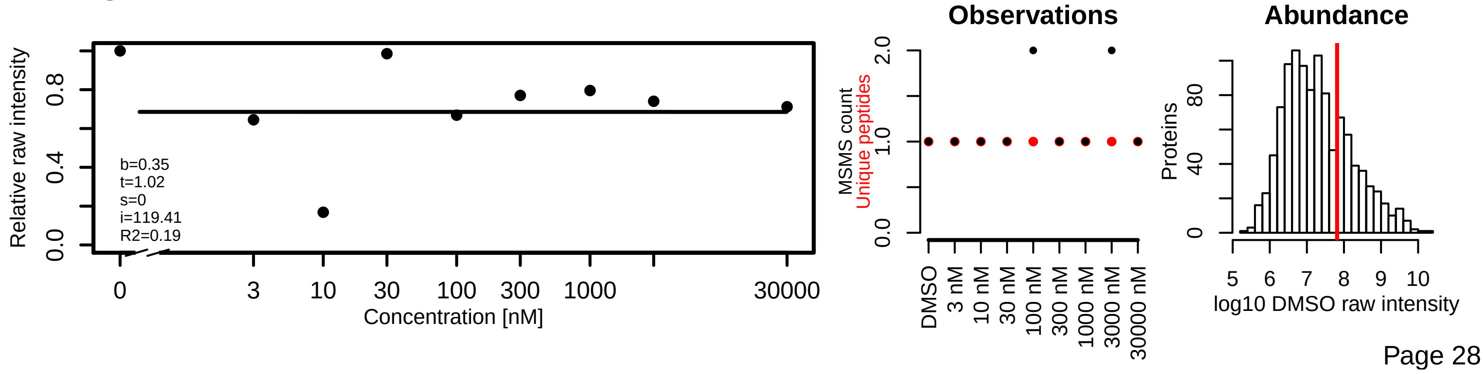

## SLC25A6

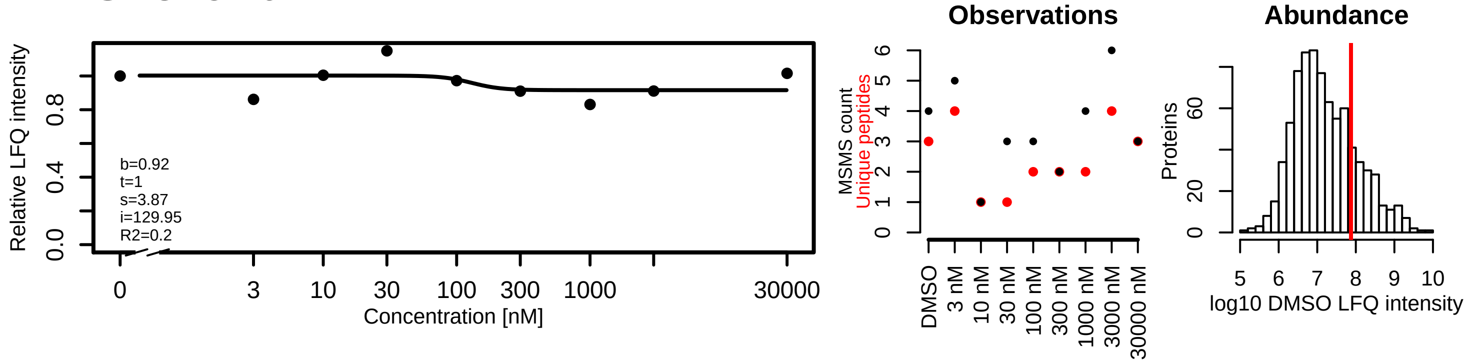


## TUBB4B

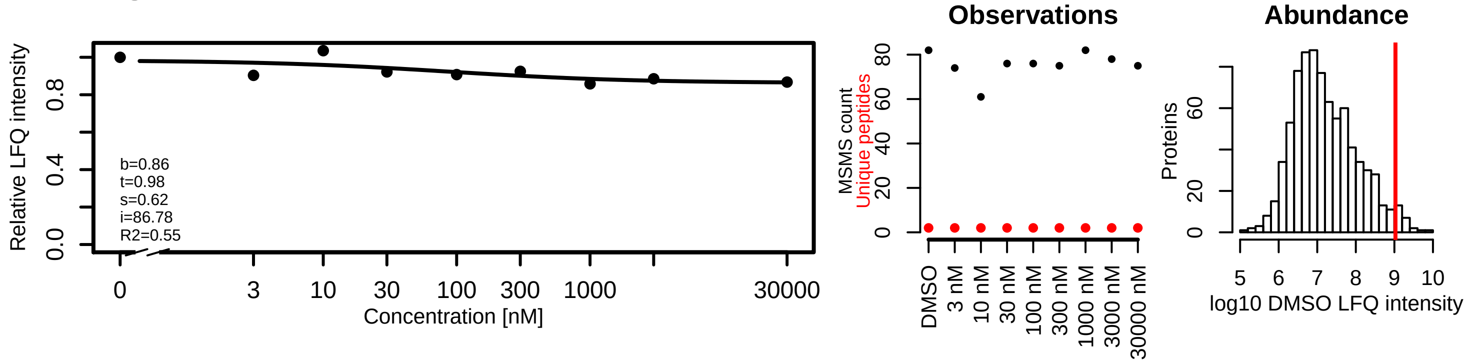


## CSNK1G2

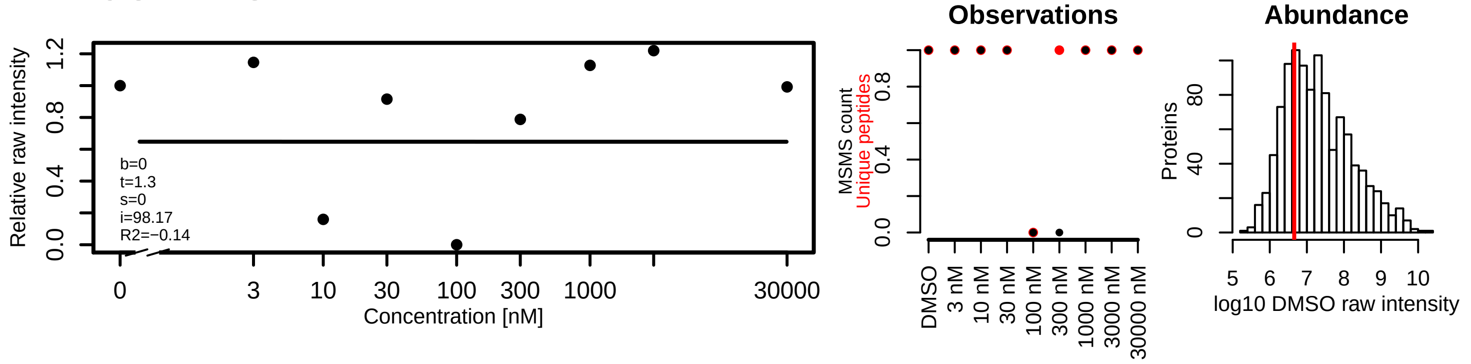


## SFPQ

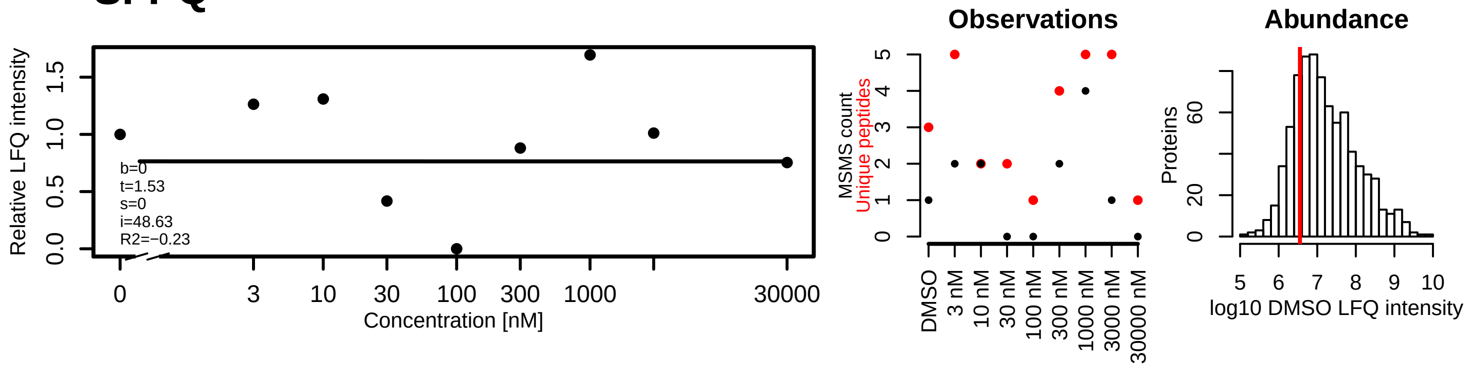


## HIST1H1B

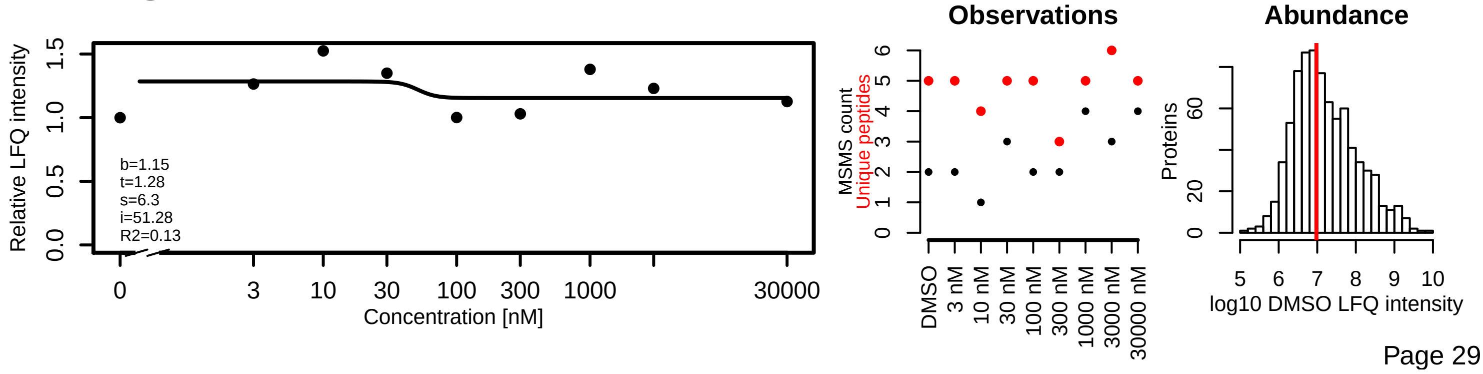

## PSMA1

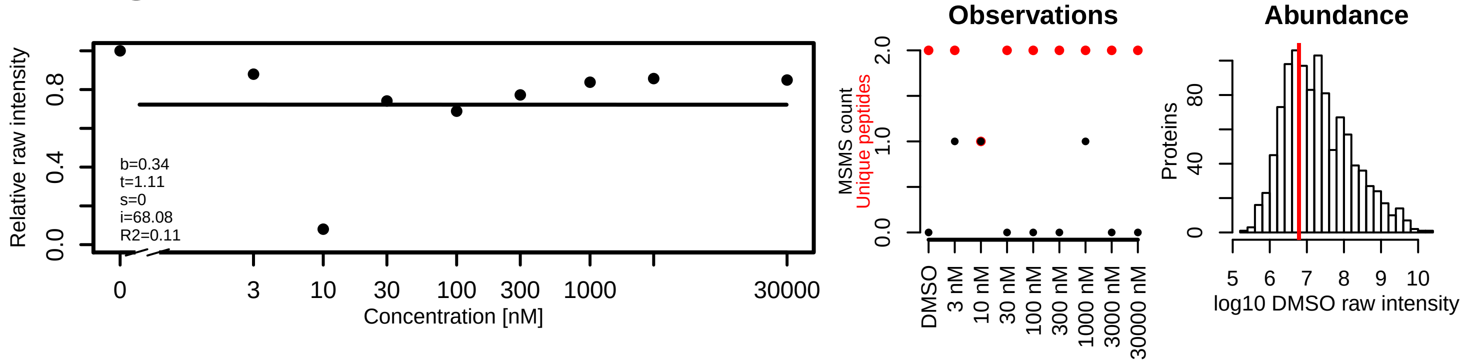


## AGK

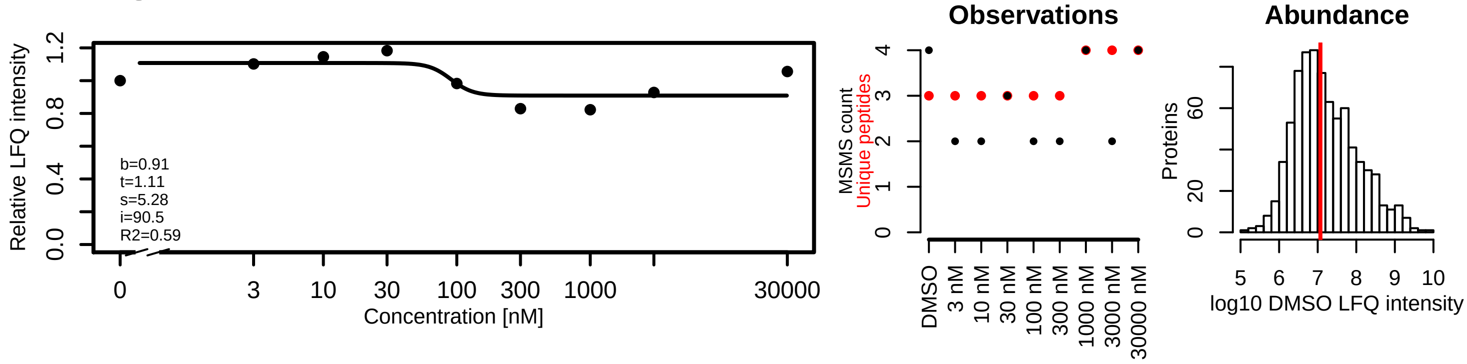


## PIGO

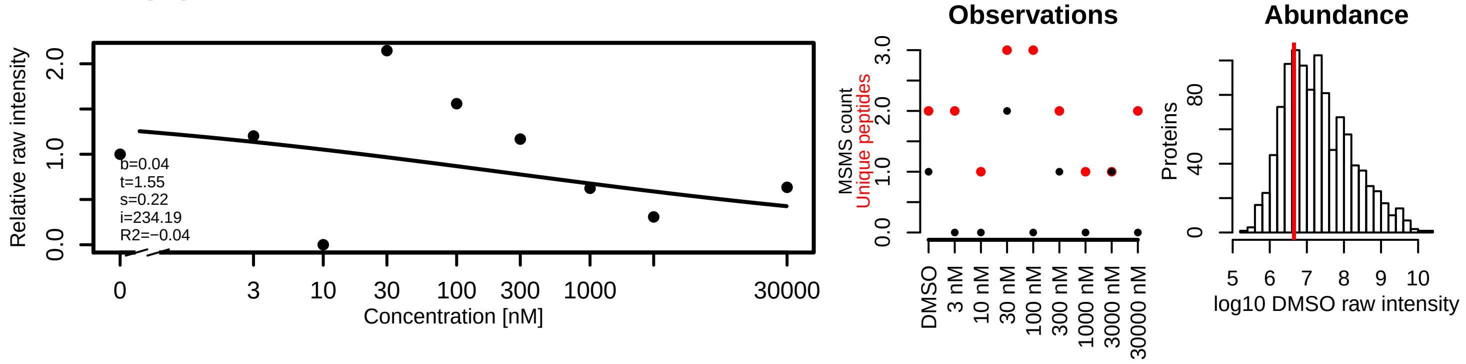


## SLC25A12

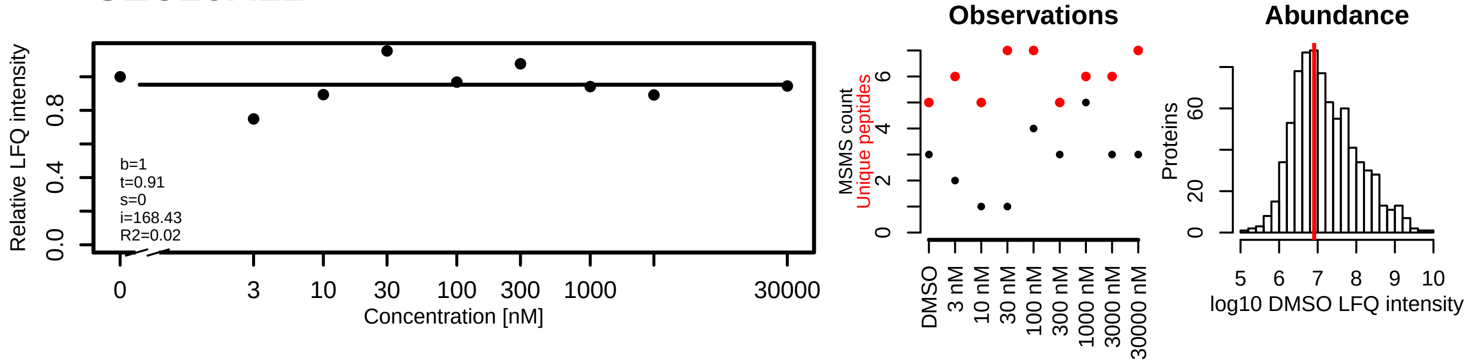


## PRKX;PRKY

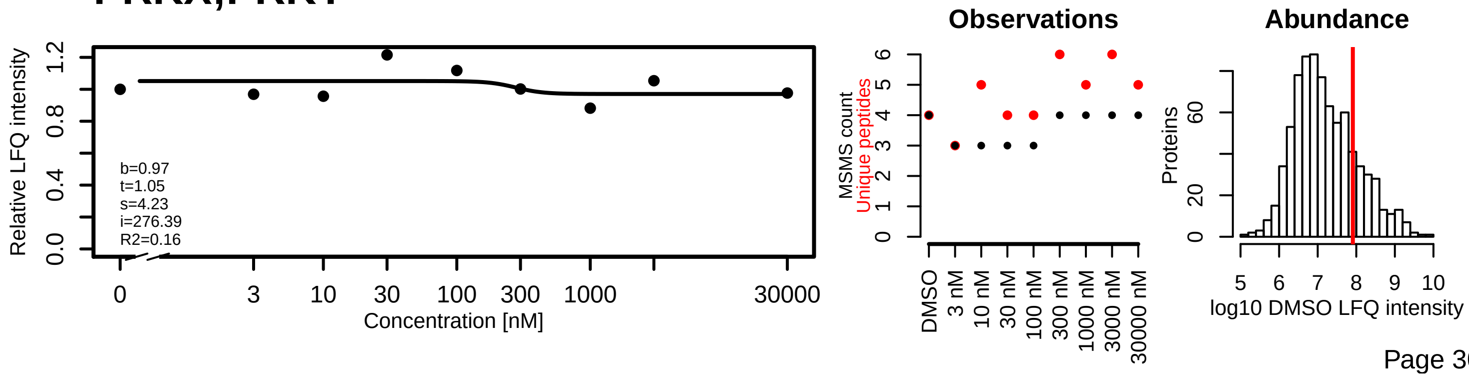

# STOM

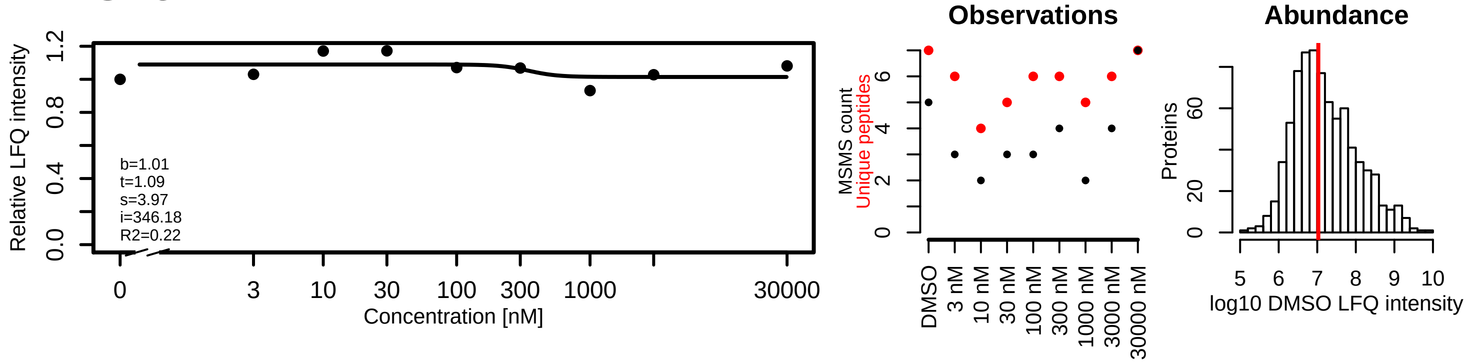


# MAPK7

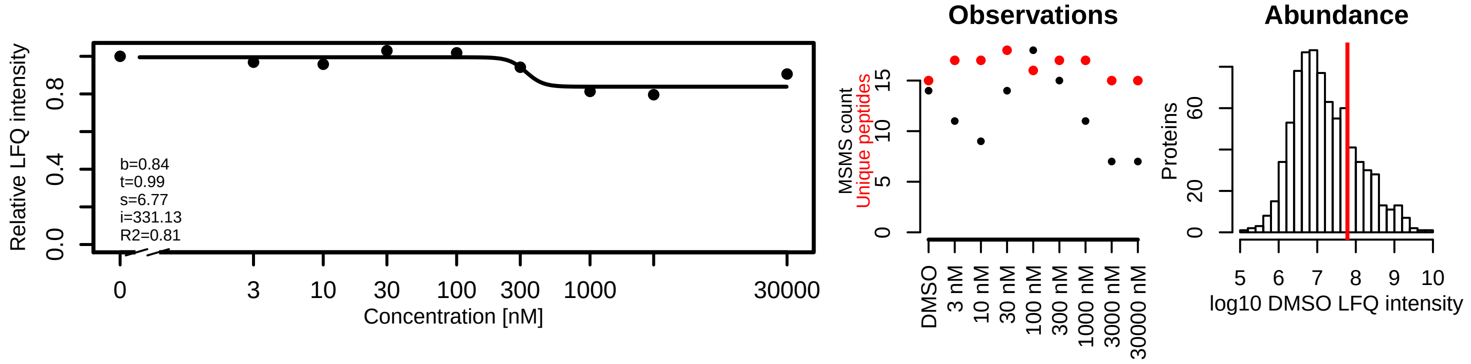


# CCDC127

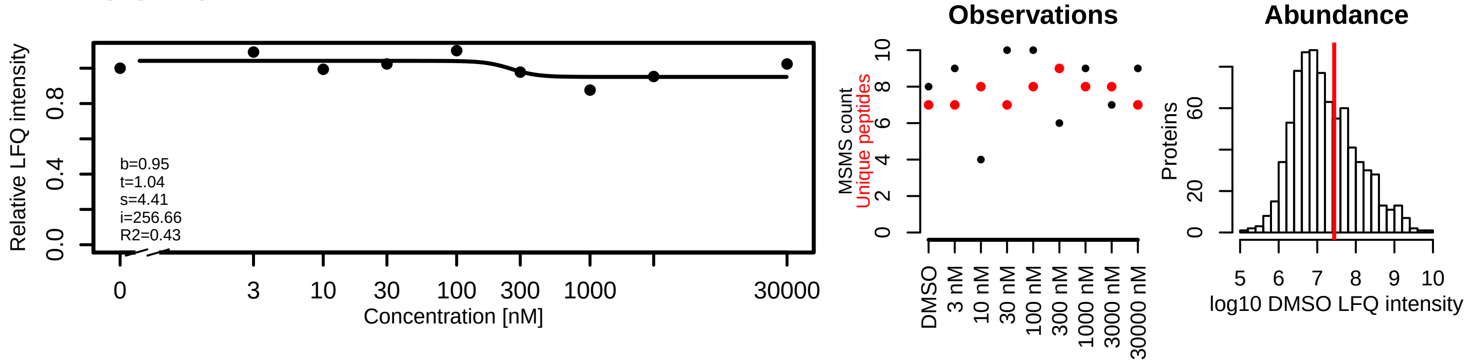


# HIST1H4A

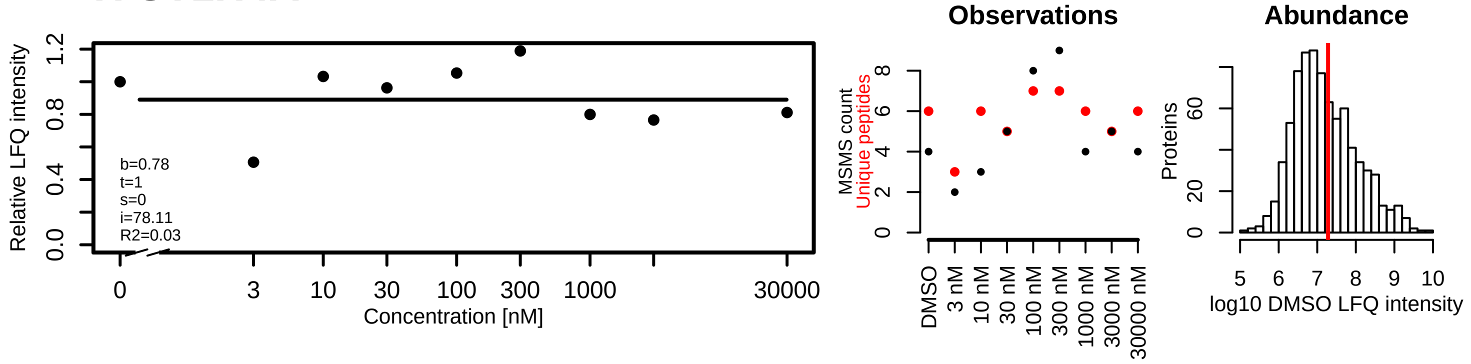


# ATP1A1;ATP1A3;ATP1A2

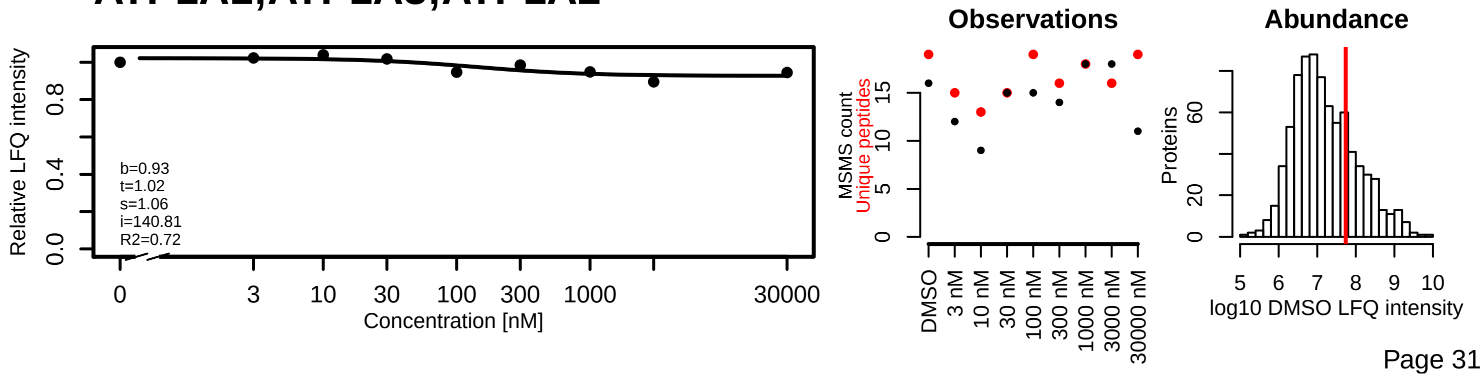

## RCN1

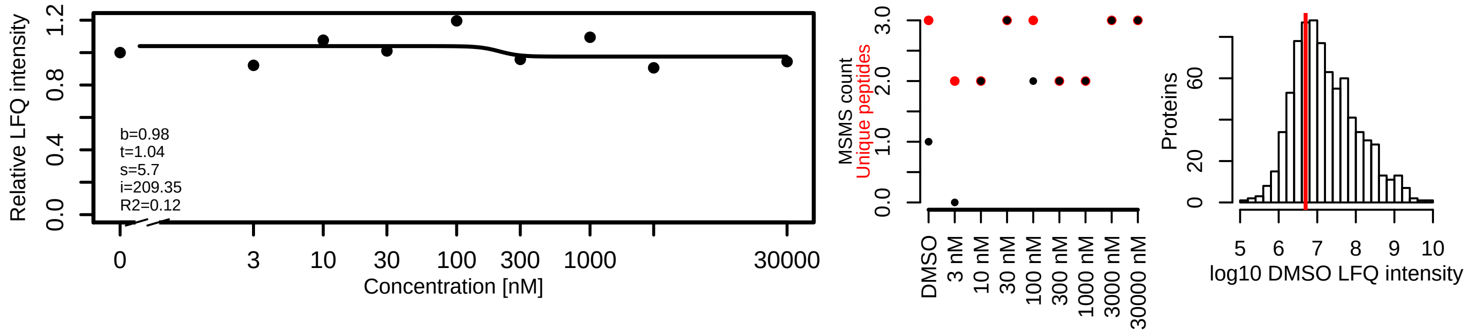


## JUP − P14923;C9JTX4;C9J826;C9JK18;C9JKY1

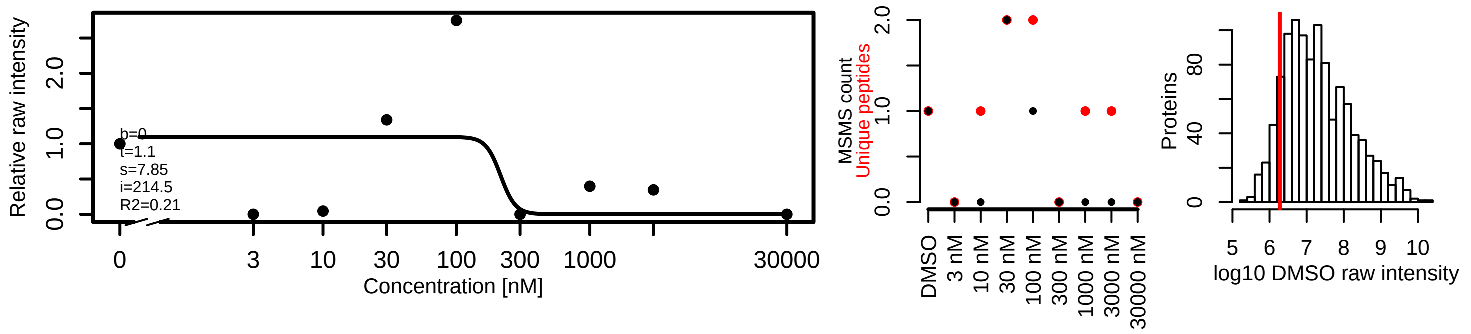


## PPP4R2

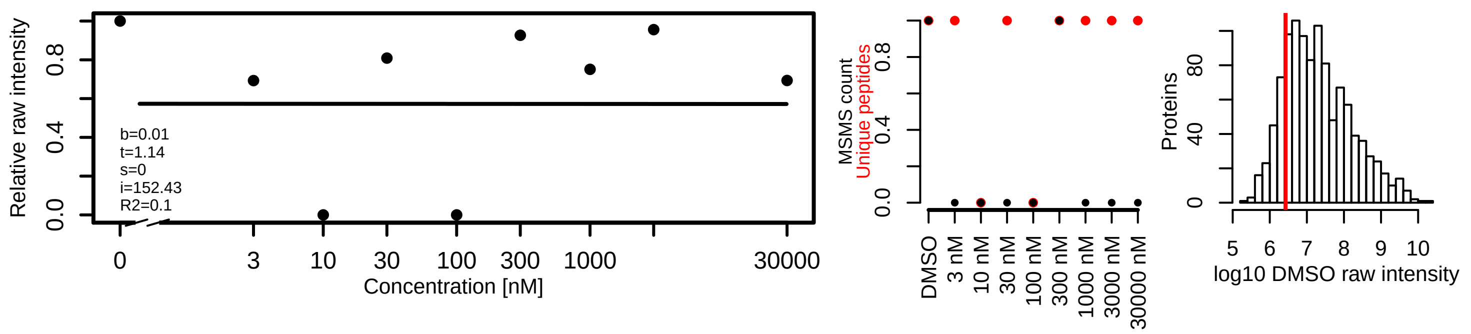


## XRCC5

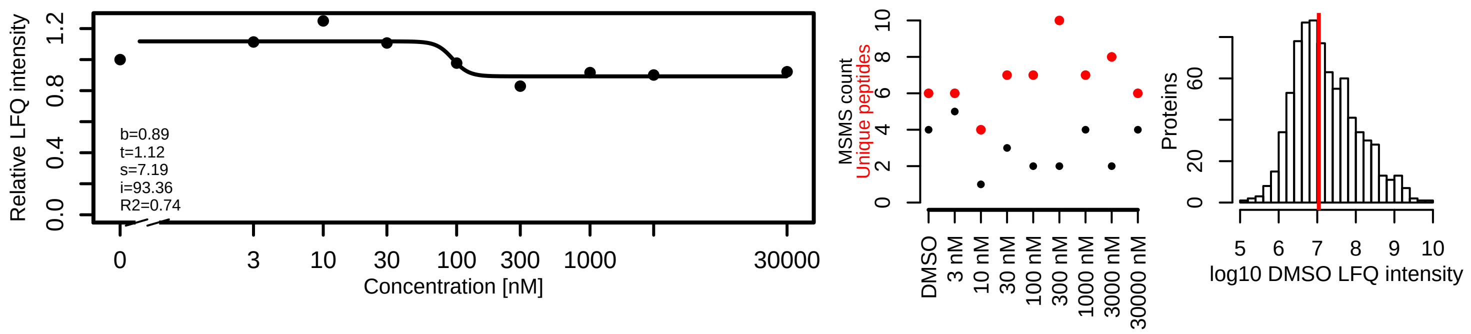


## IPO9

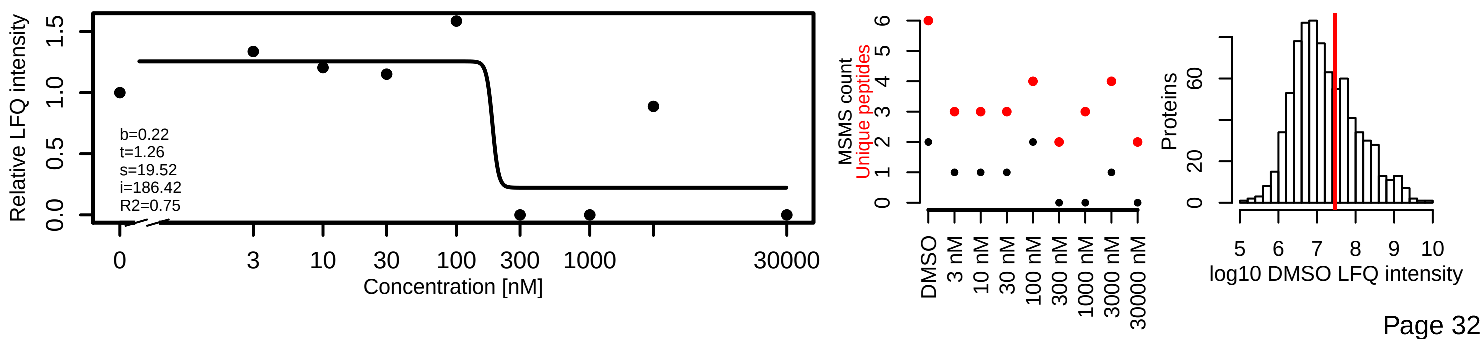

# PHKG2

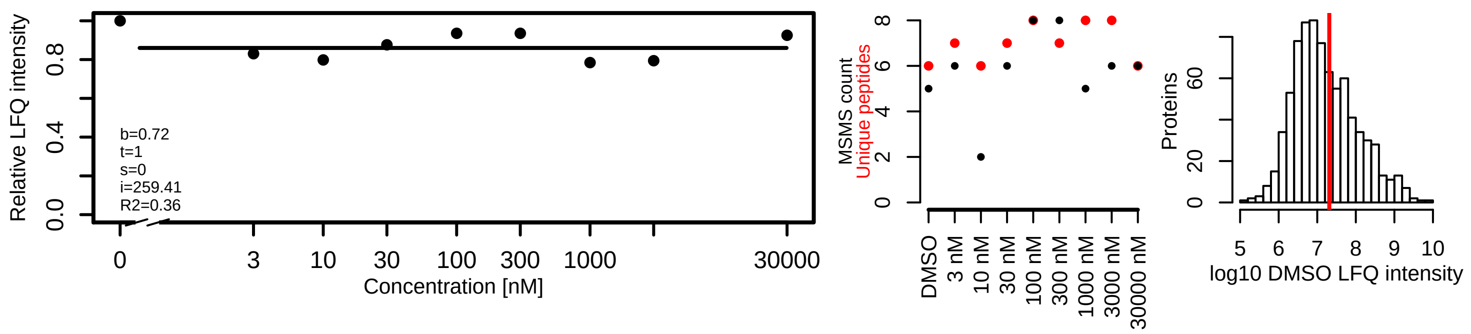


# XPO7

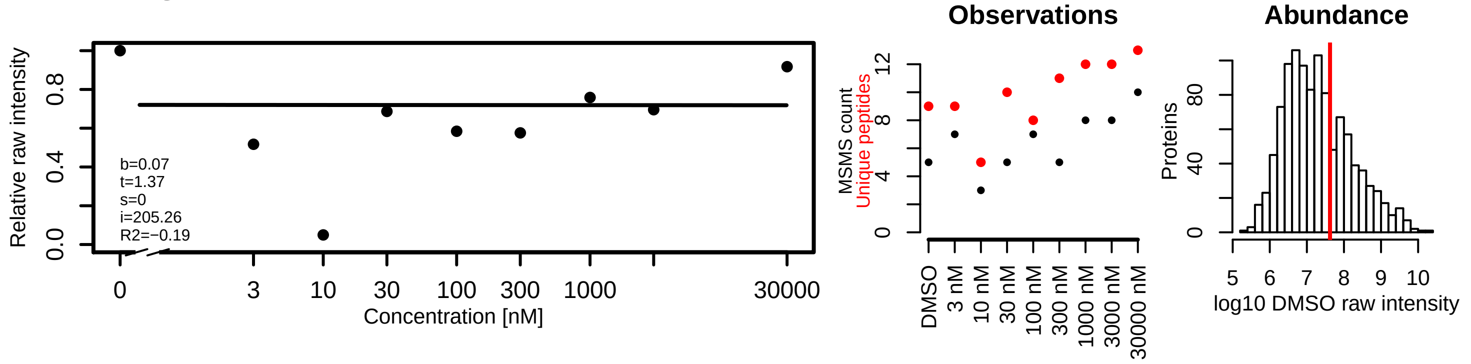


# PSMA3

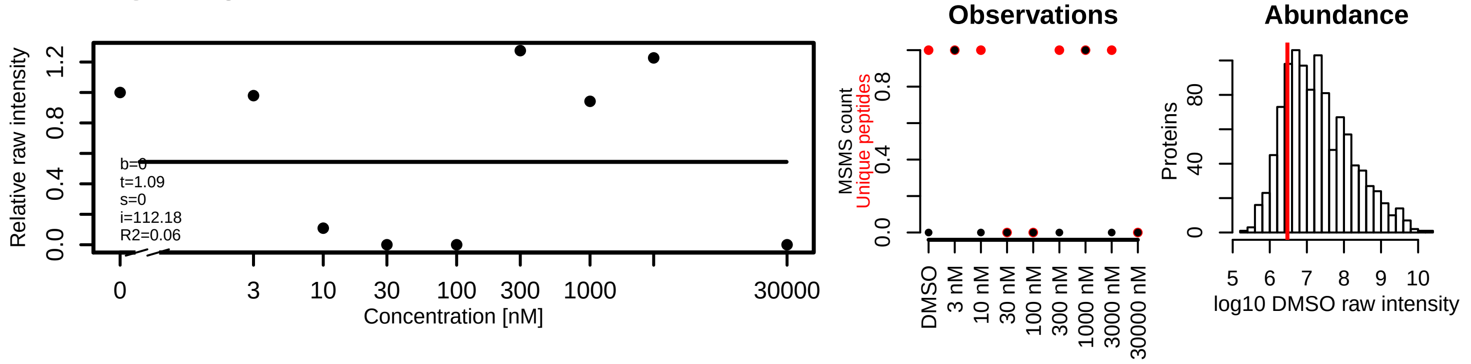


# SLC25A22;SLC25A18

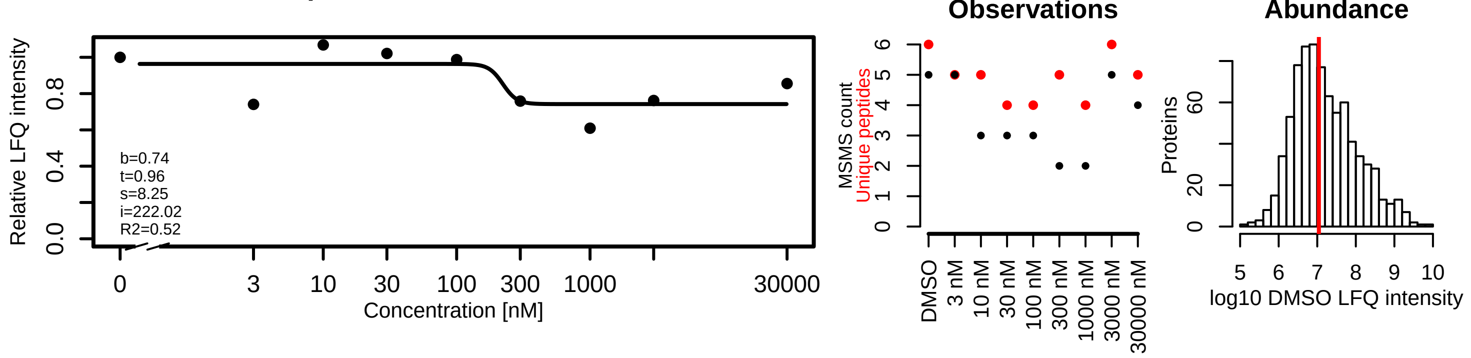


# KIF5A;KIF5C;KIF5B

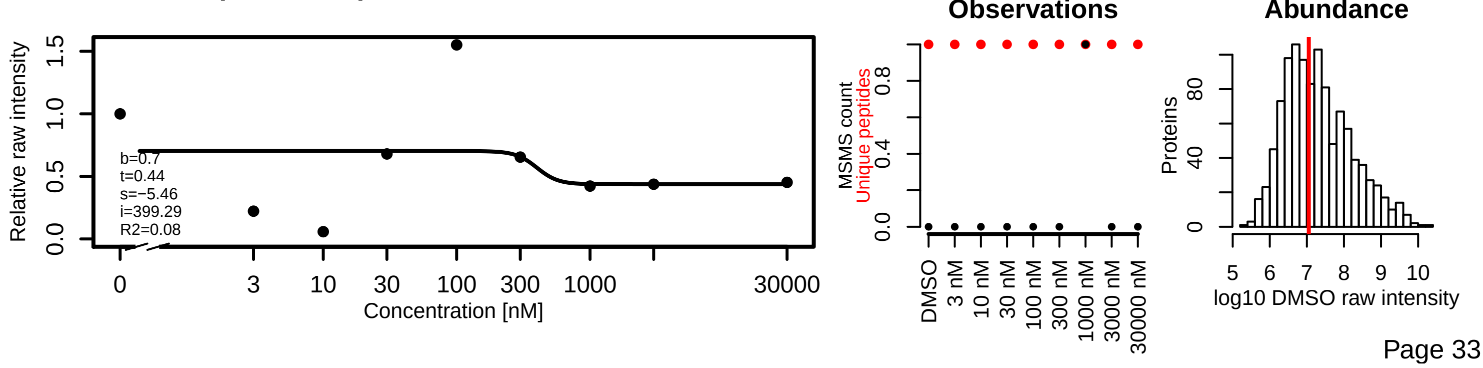

## RARS

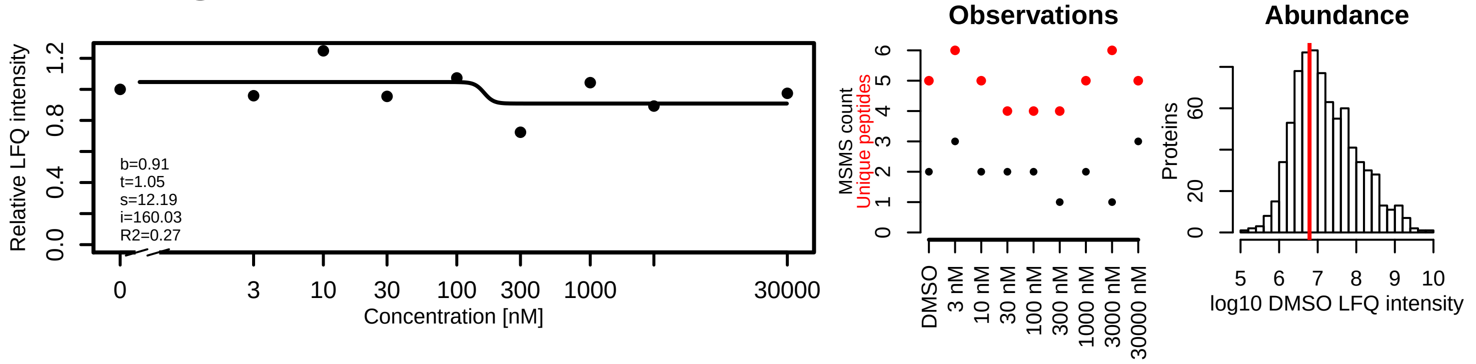


## RPN2

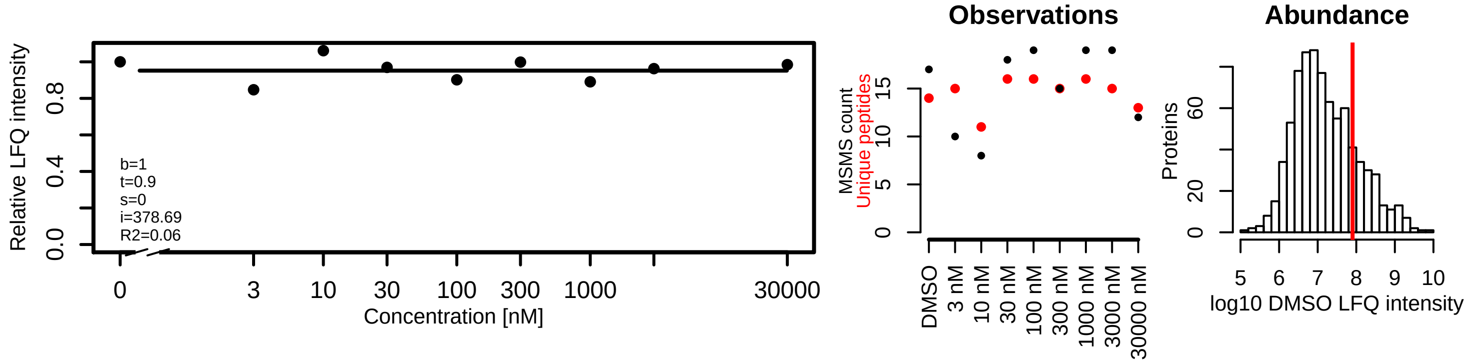


## CCT5

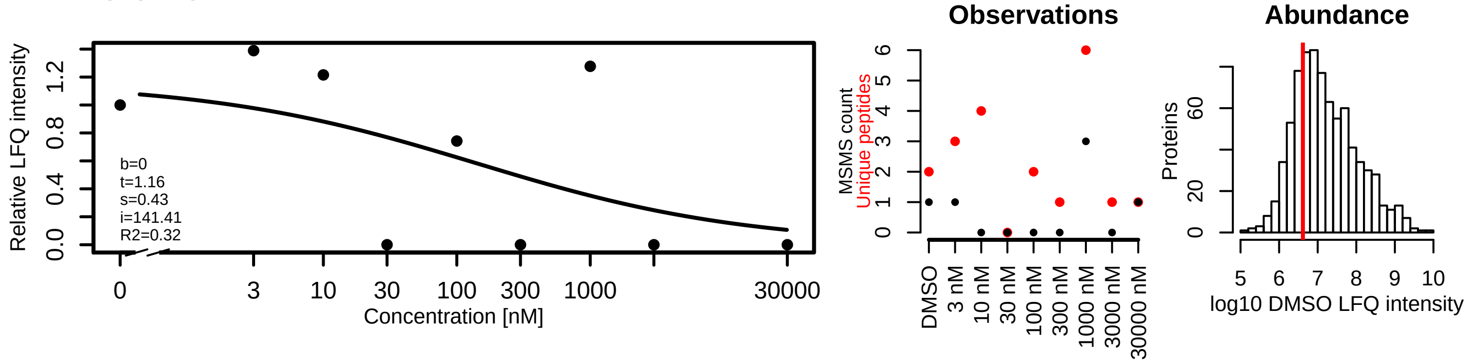


## DDOST

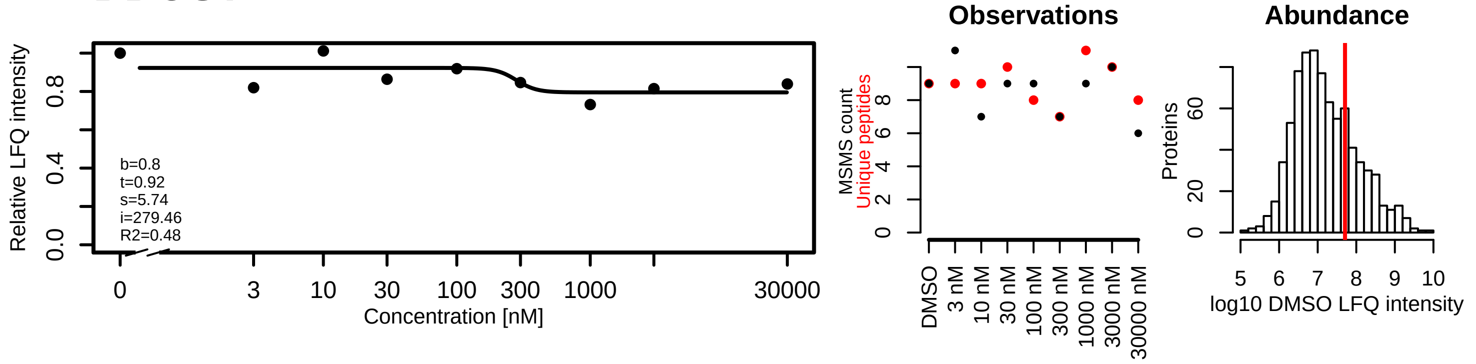


## RPL14

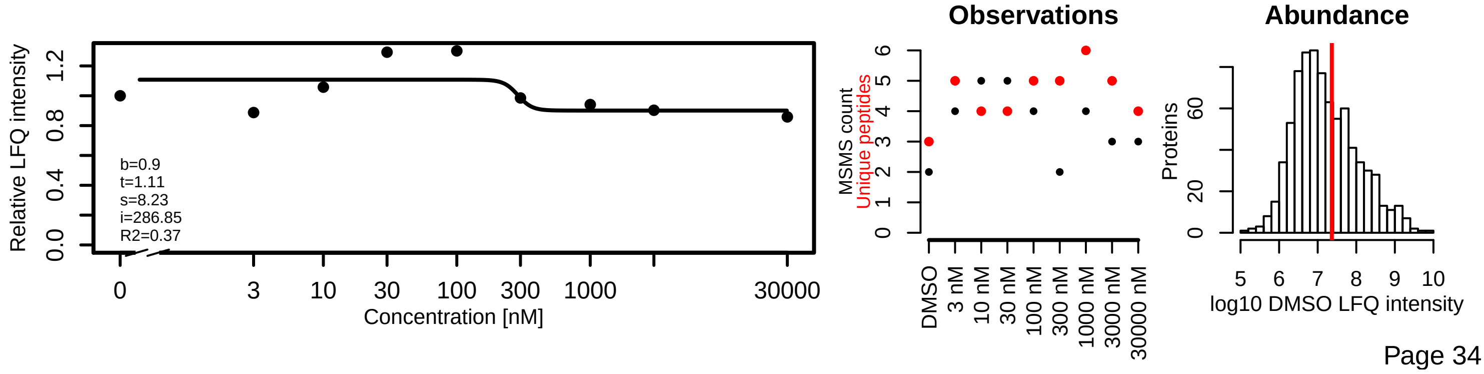

## TKT

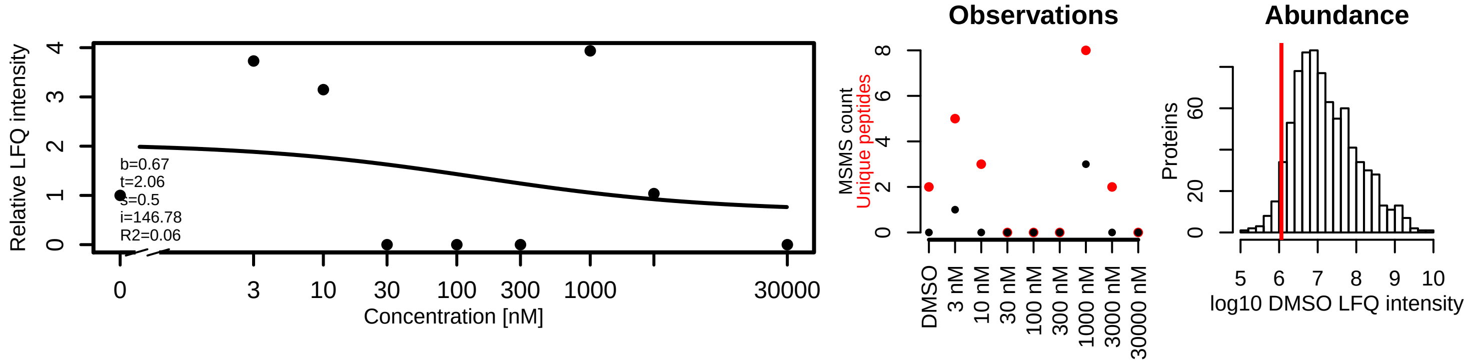


## SCAMP3

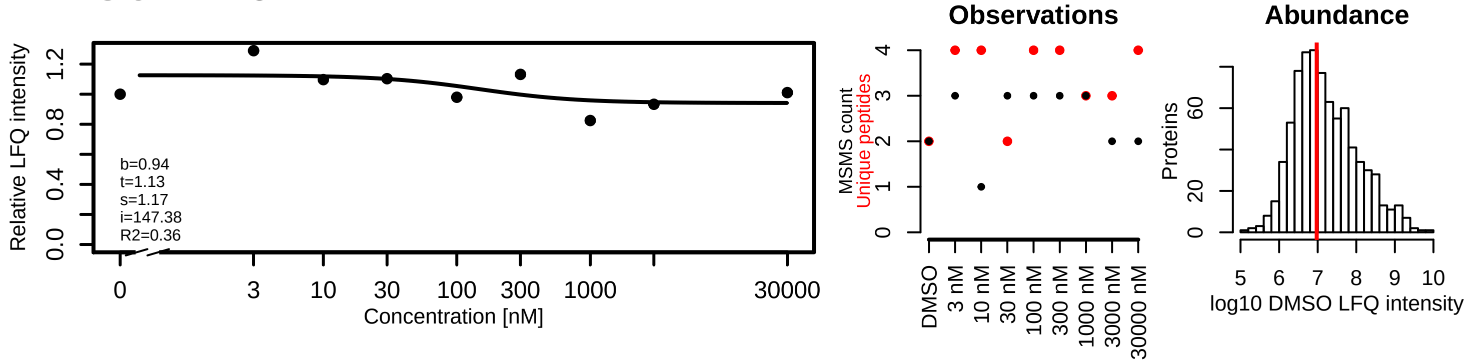


## NUP188

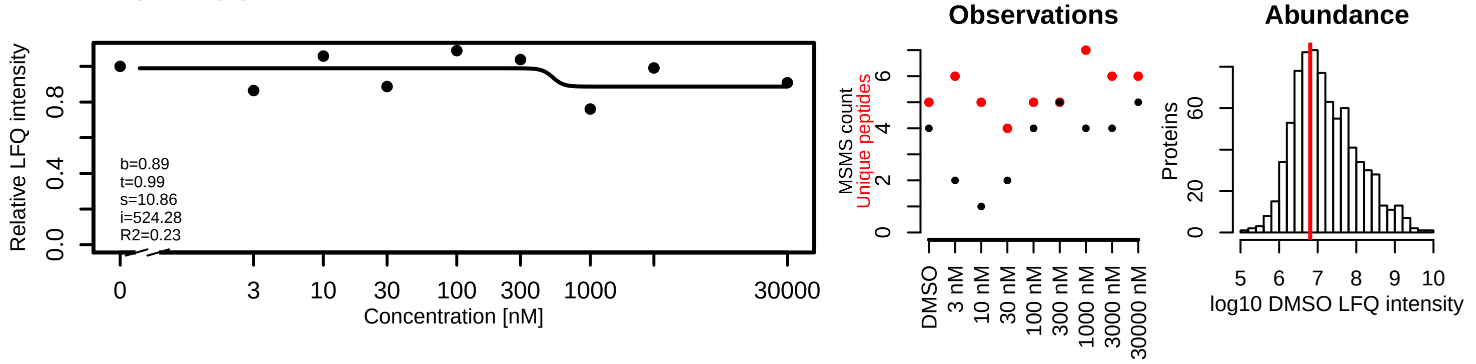


## CCT3

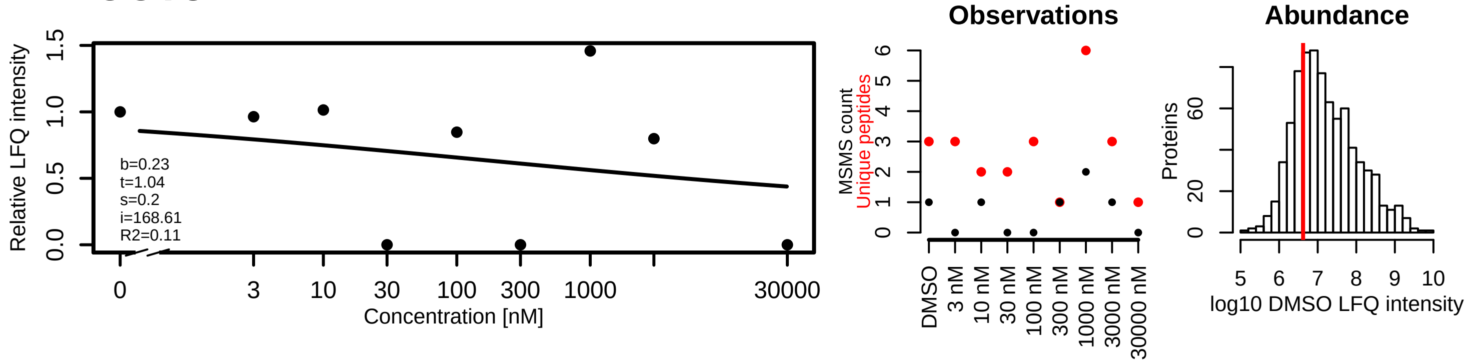


## HMOX2

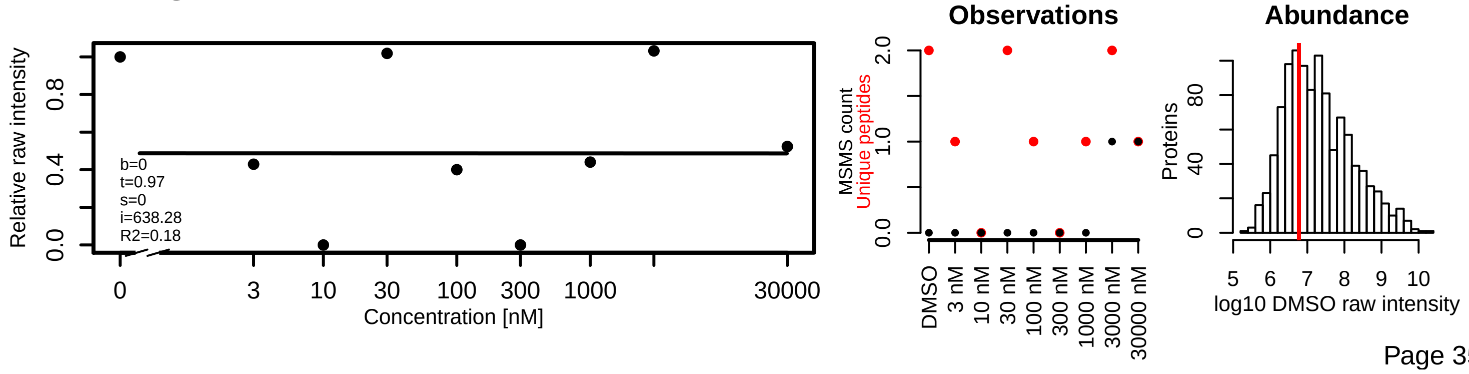

# SLC25A17

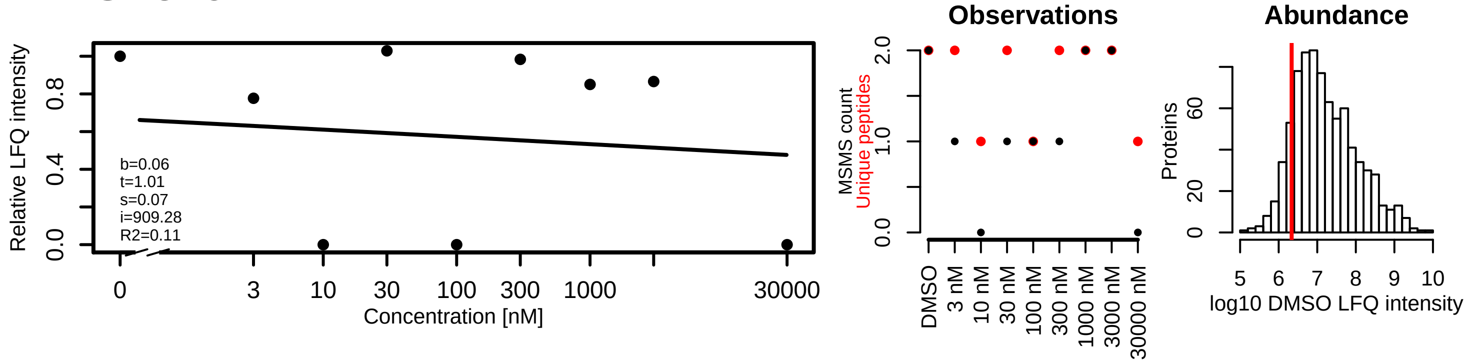


# AP2A1

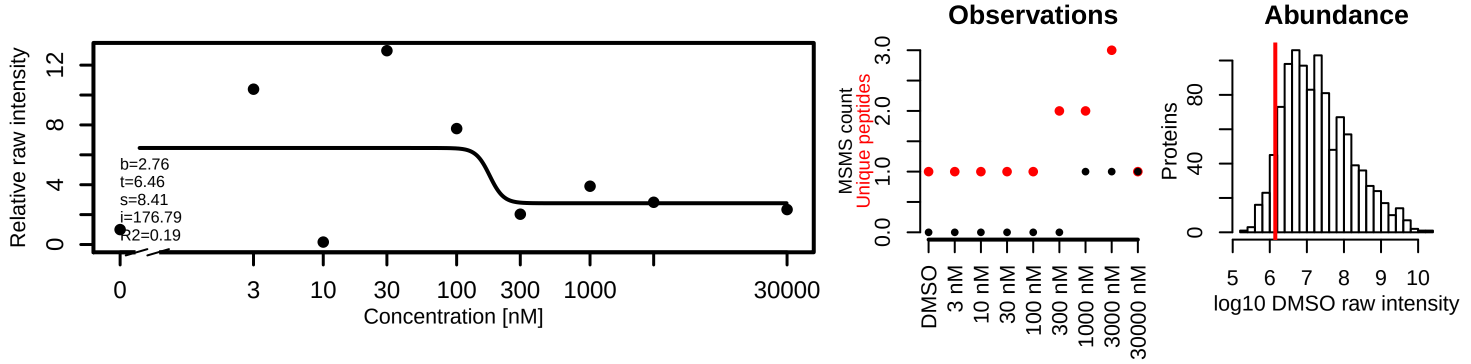


# ATP13A1

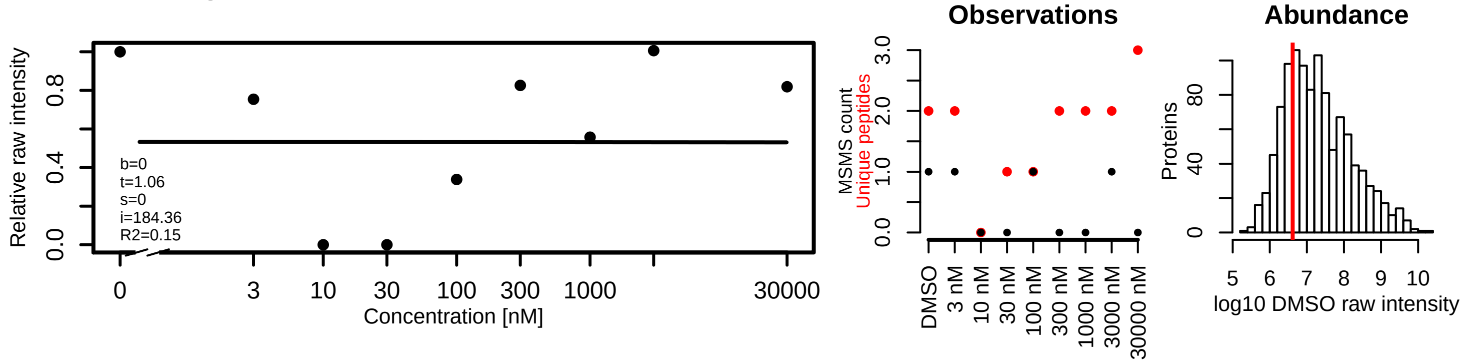


# RUVBL1

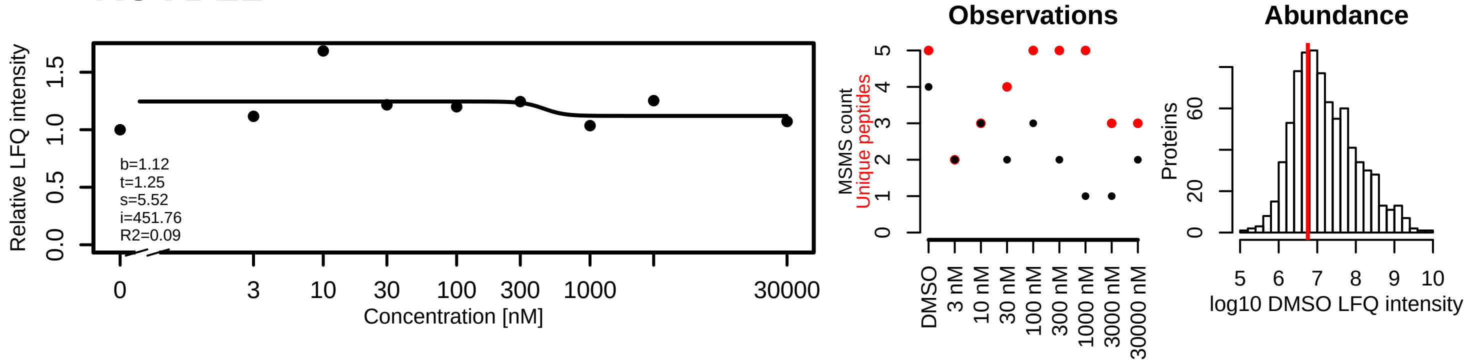


# DYNLRB2;DYNLRB1

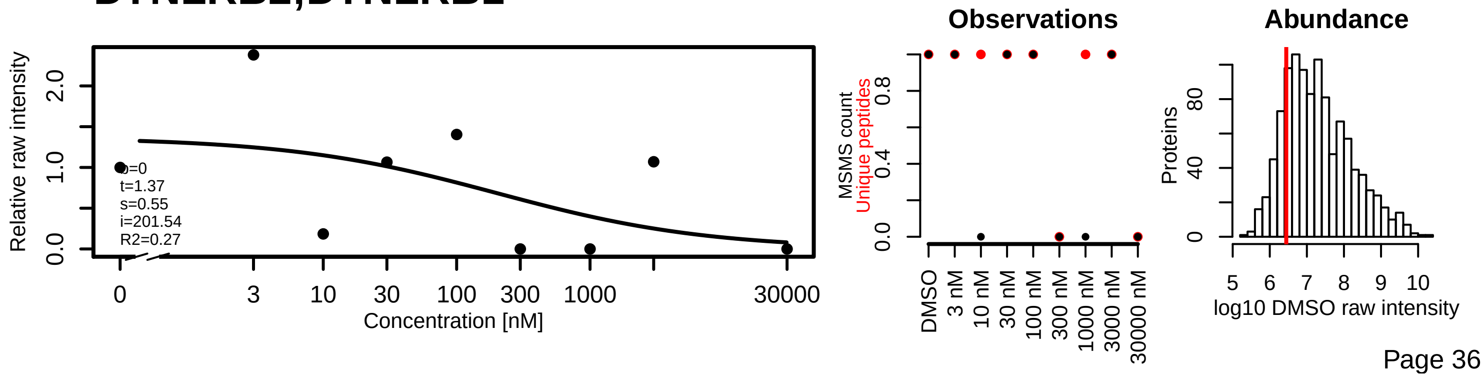

## HKDC1

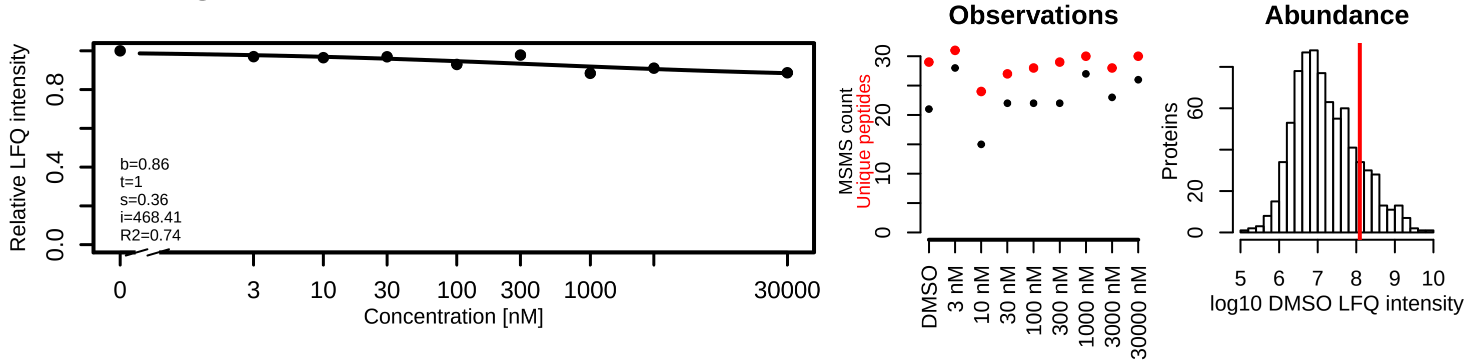


## TM9SF2

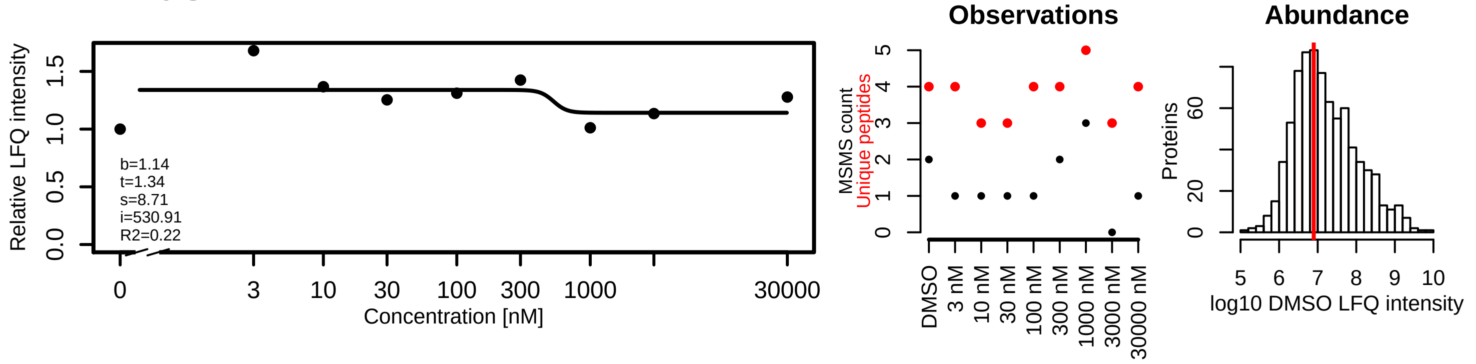


## PEX6

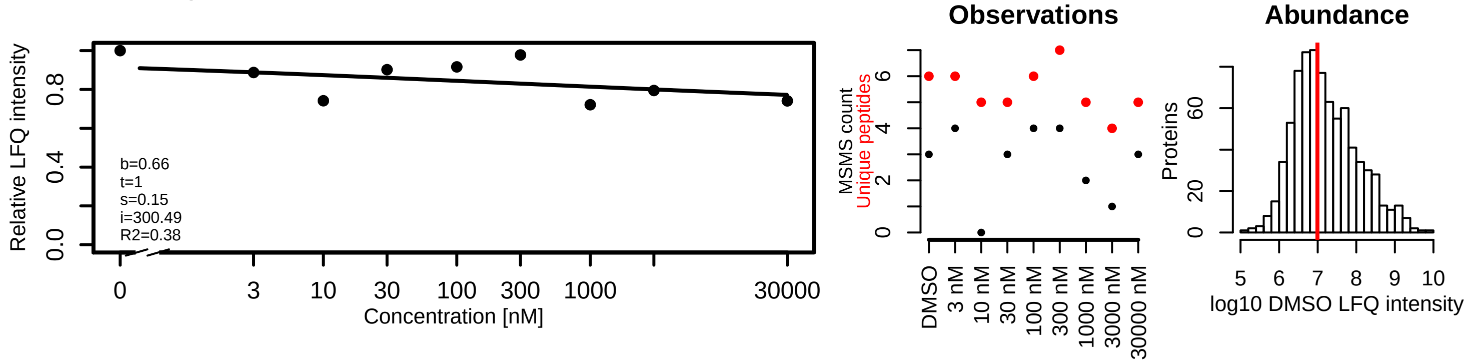


## RPS12

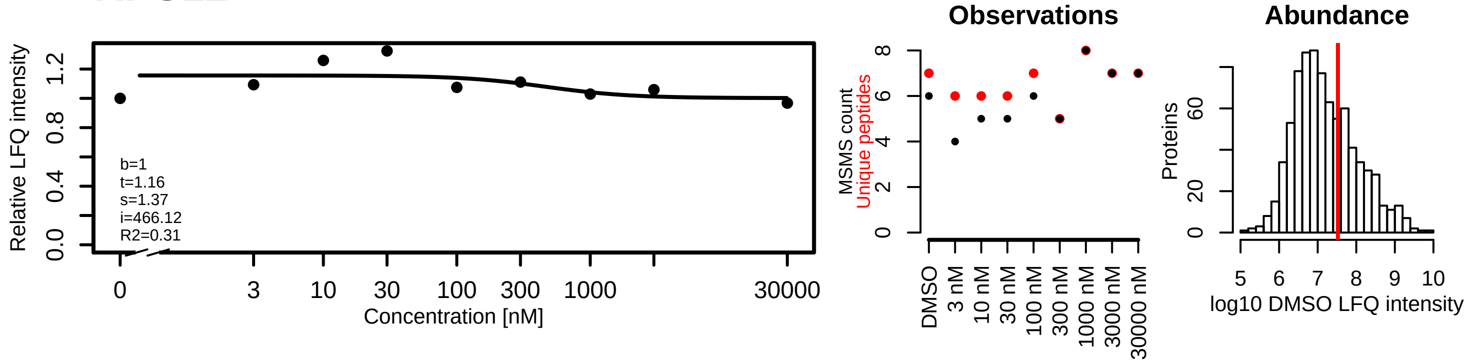


## ATP5O

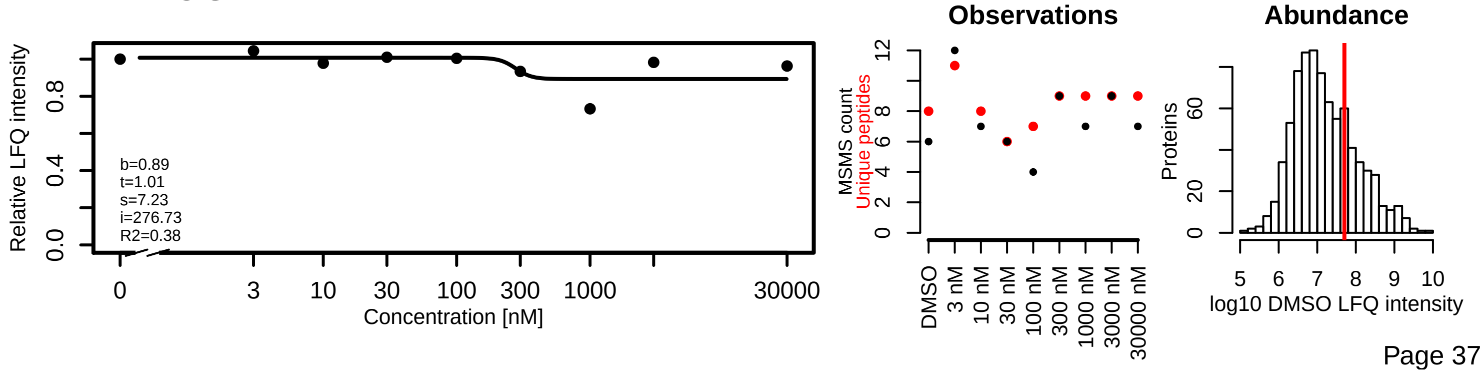

# OSBPL3

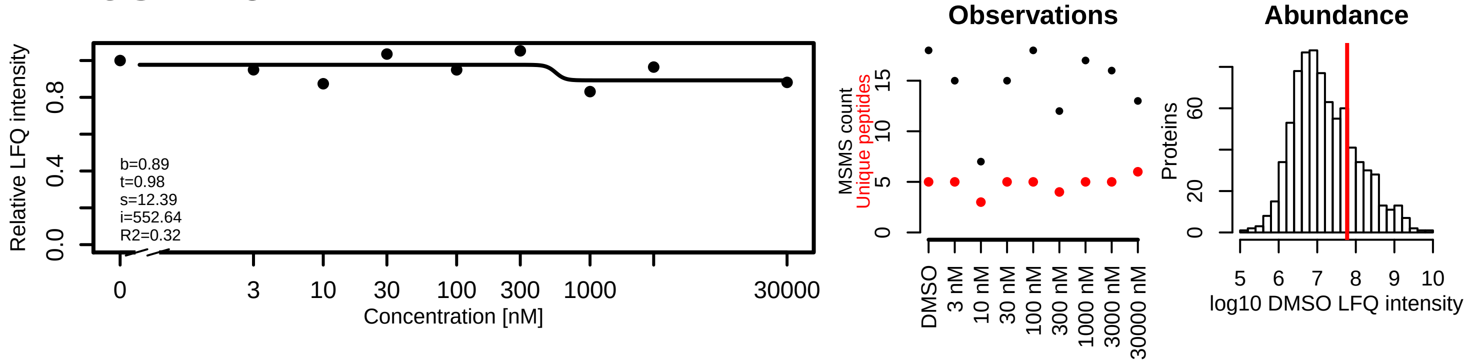


# SLC25A11

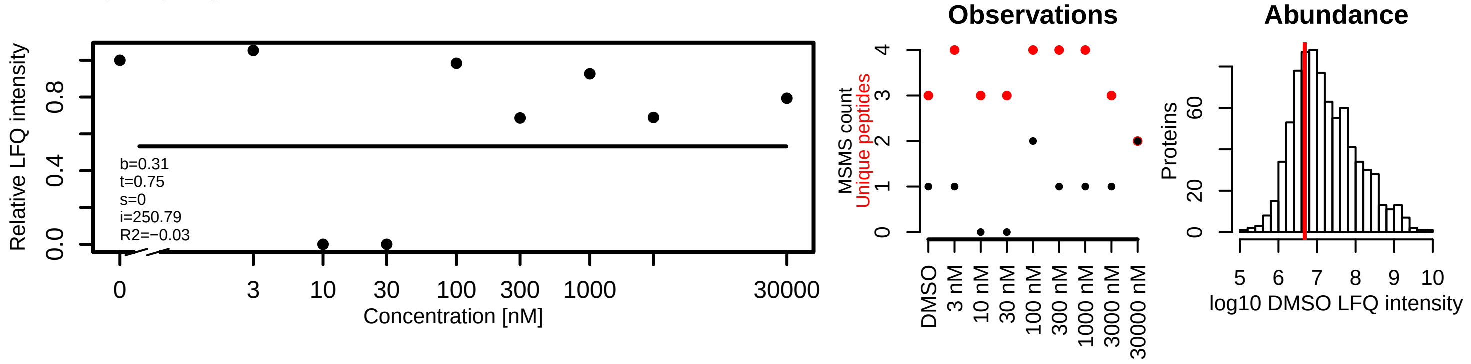


# CAD

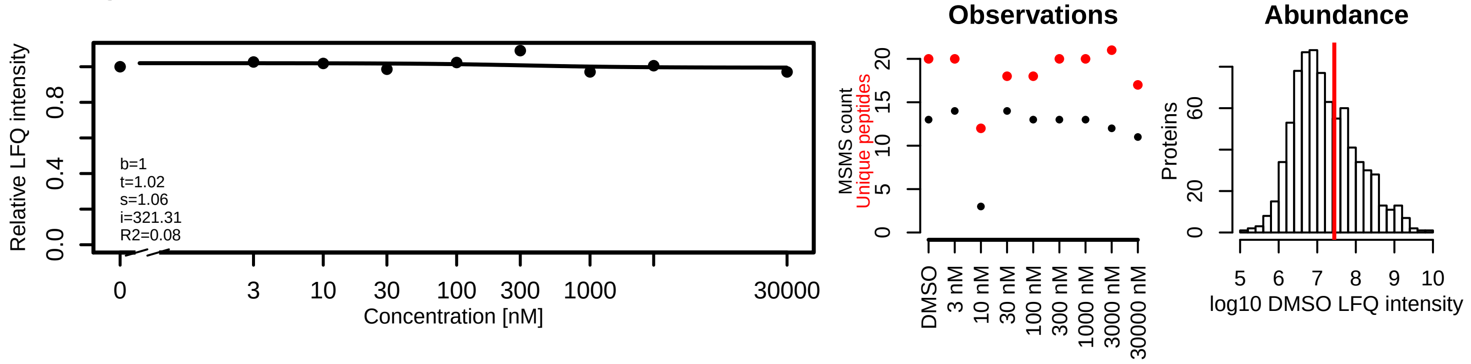


# CTSB

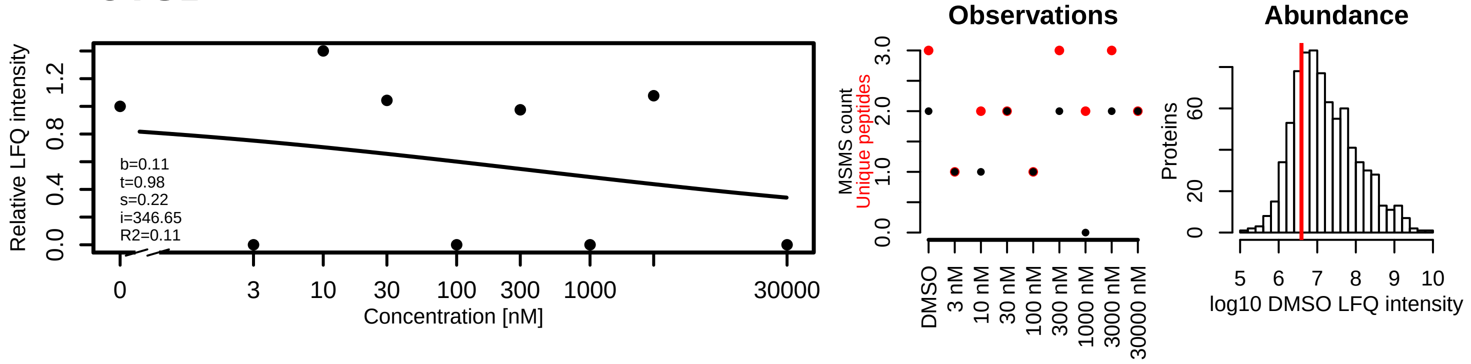


# RPS8

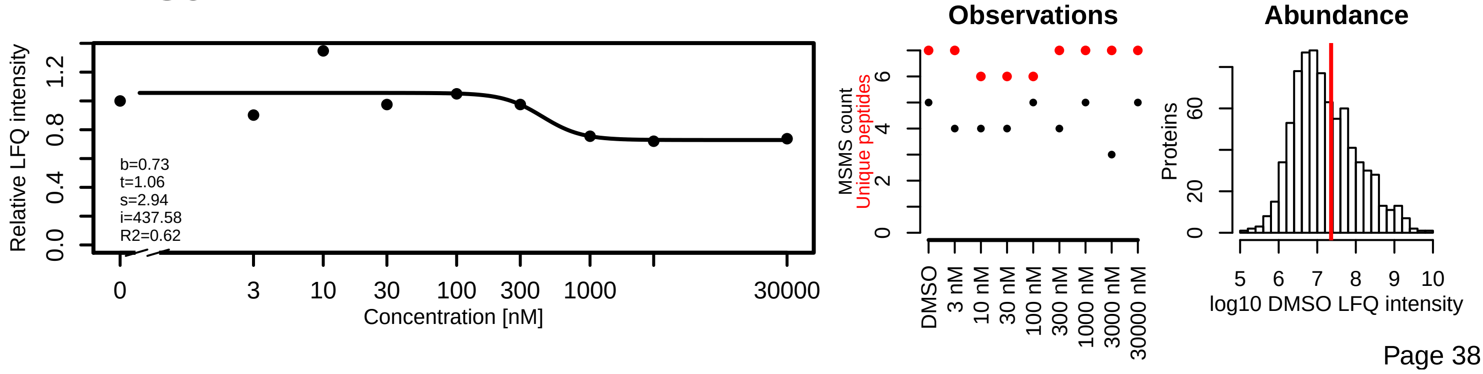

# CTNNB1

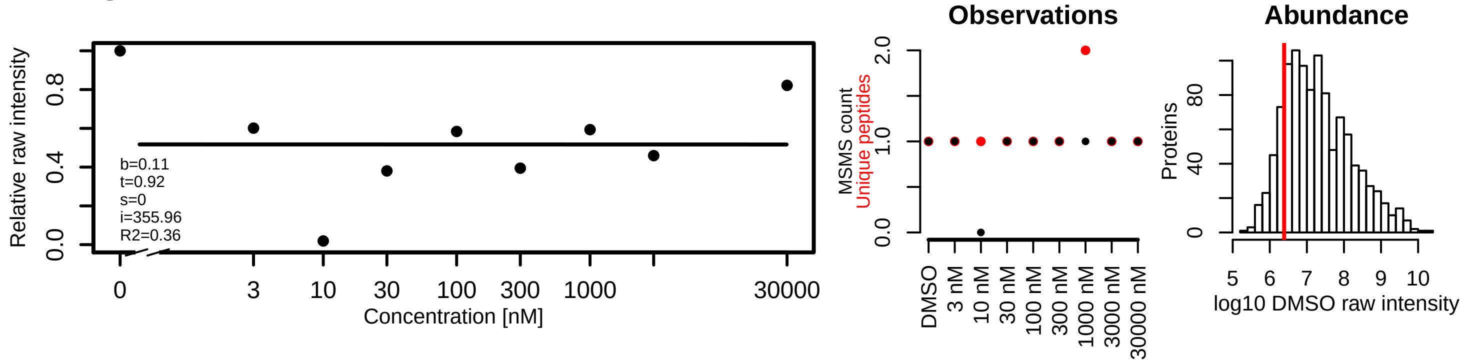


# AIFM1;PDCD8

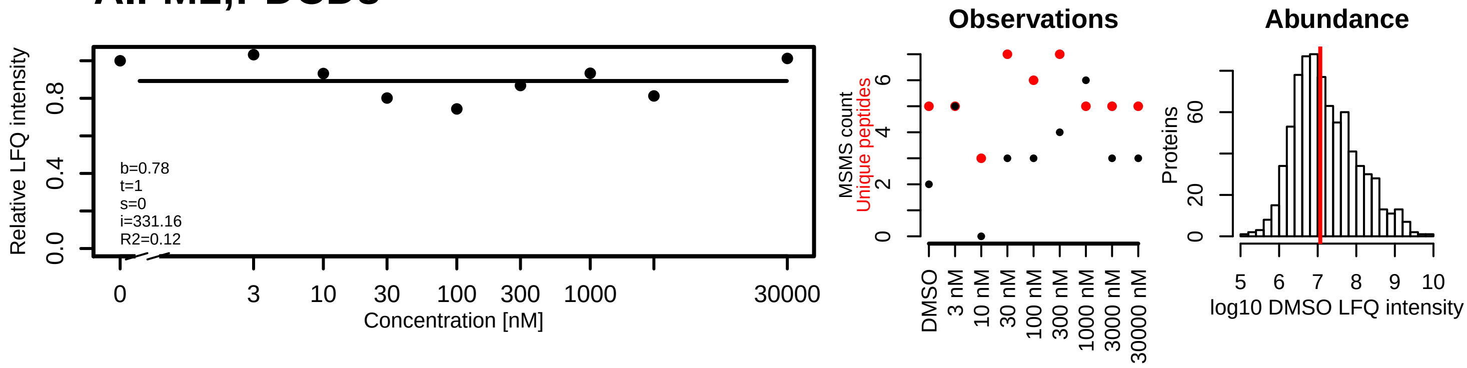


# ILF2

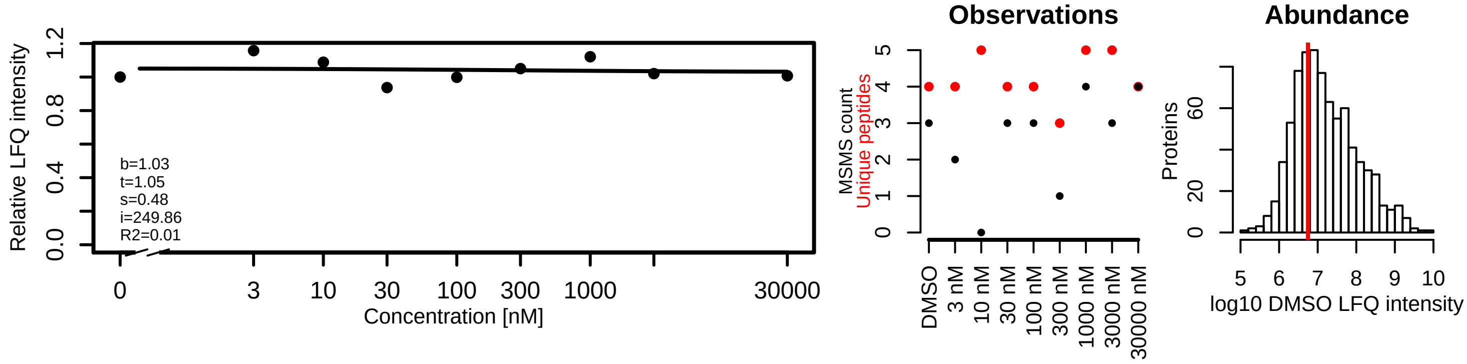


# STK38

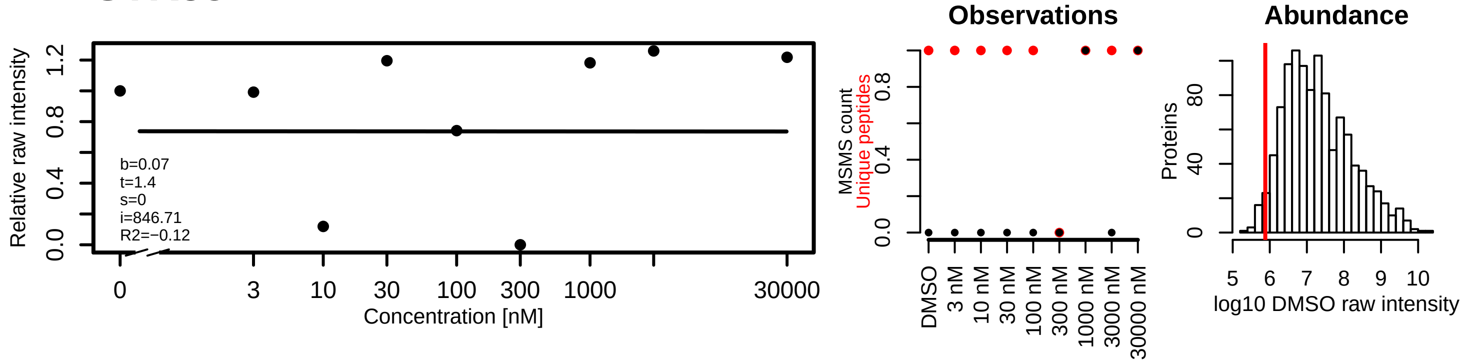


# FAM83H

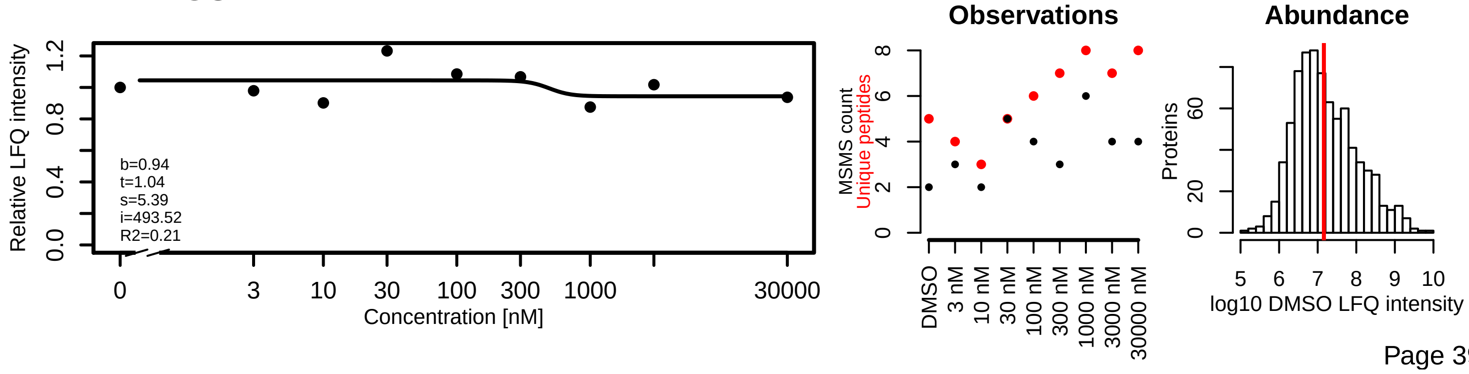

# ACVR1

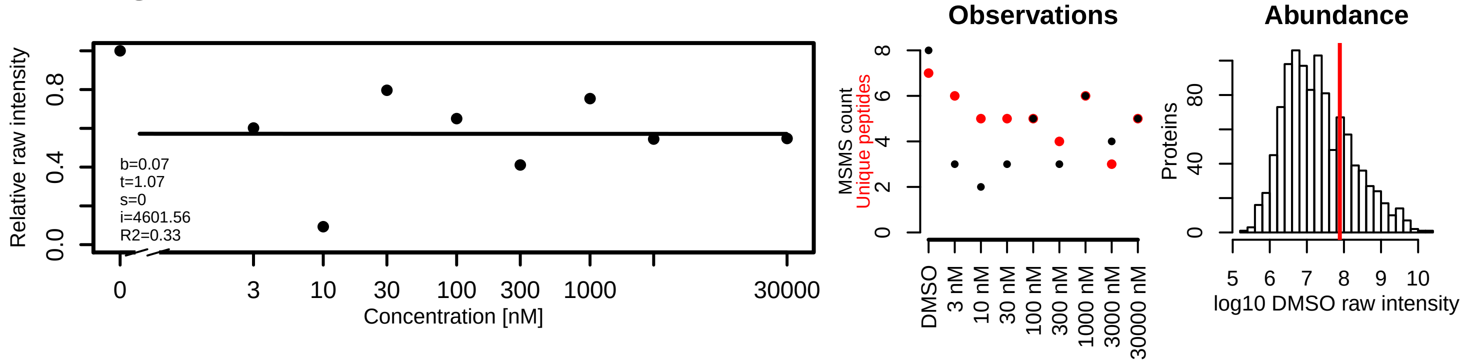


# PARVA;PARVB

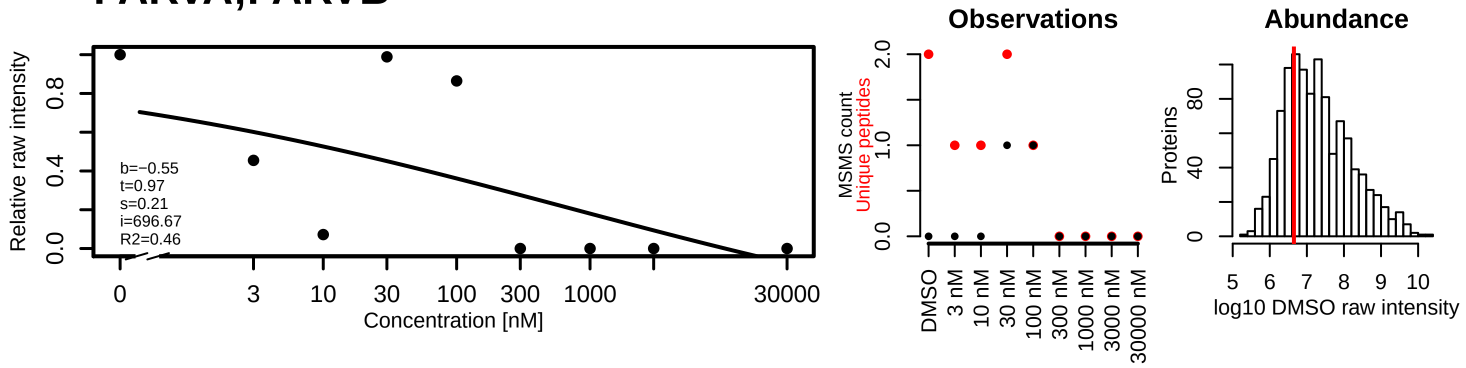


# SLC25A13

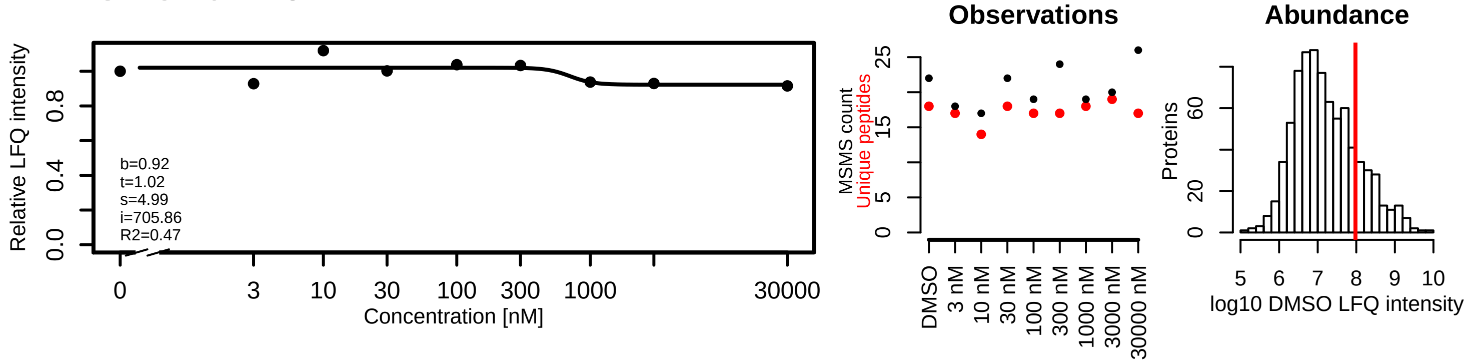


# MIF

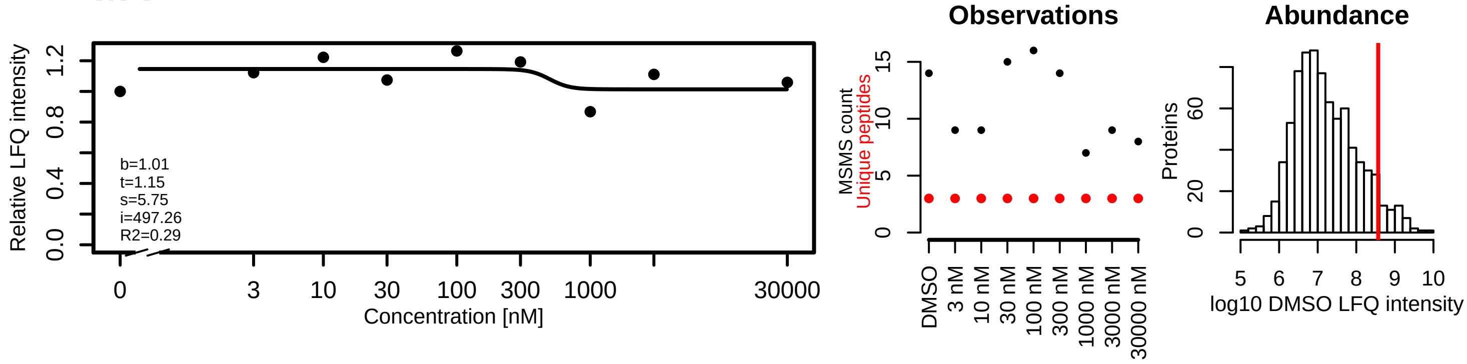


# NLK

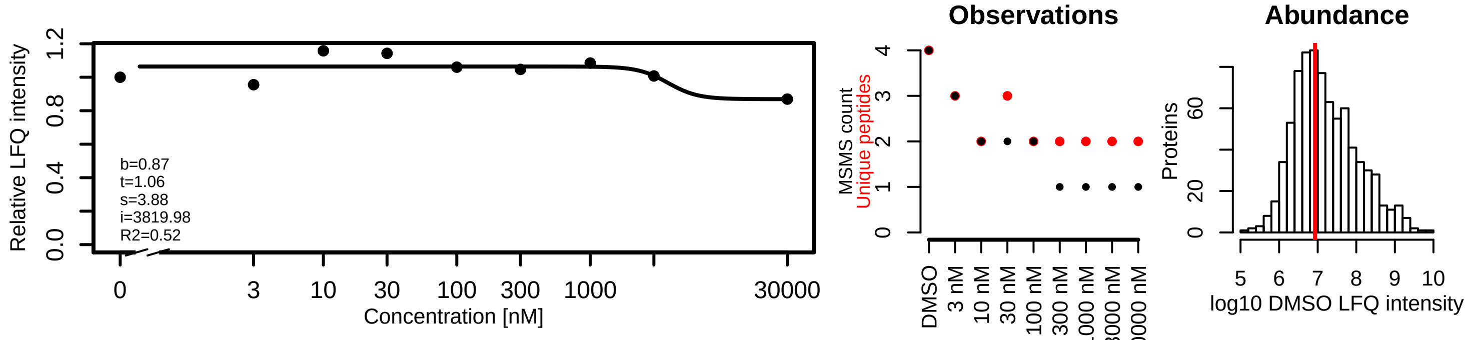

# DHX9

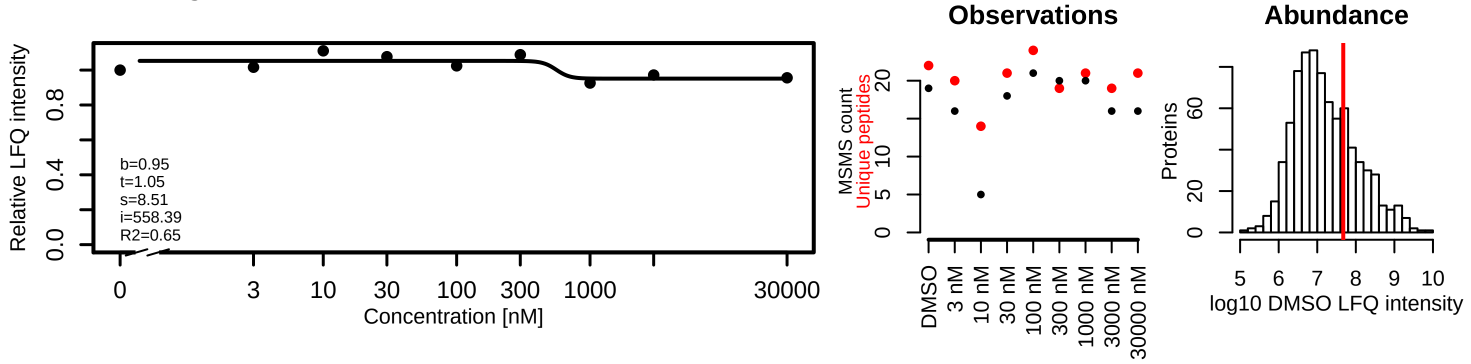


# RPL24

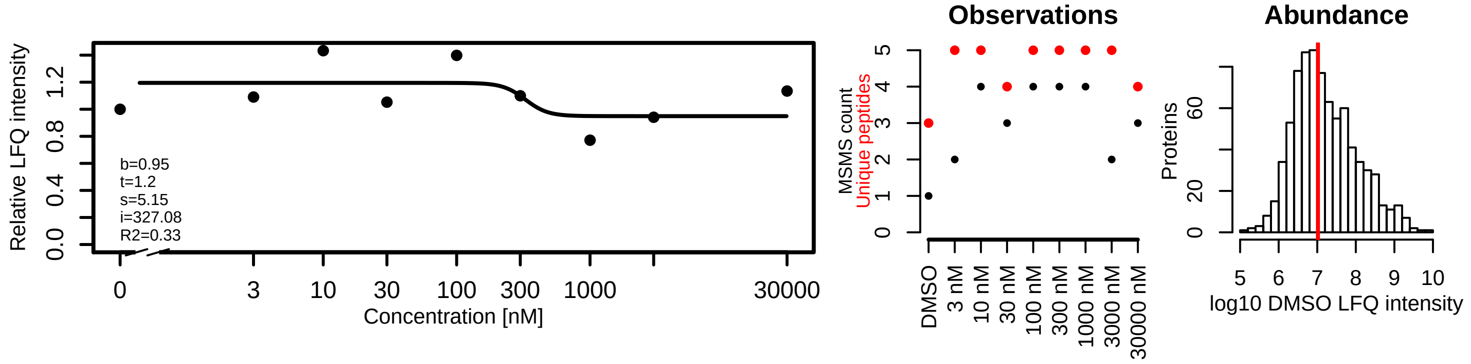


# TYRO3

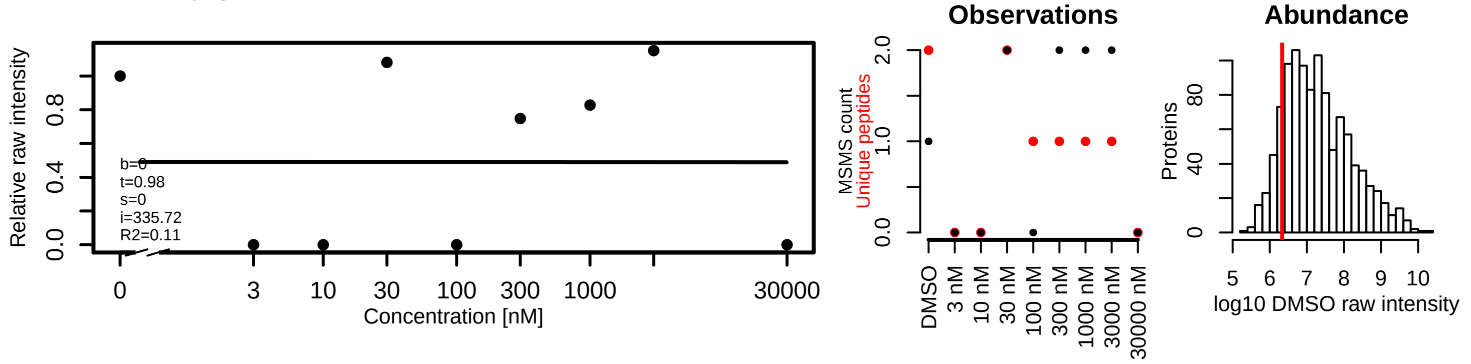


# XPO1

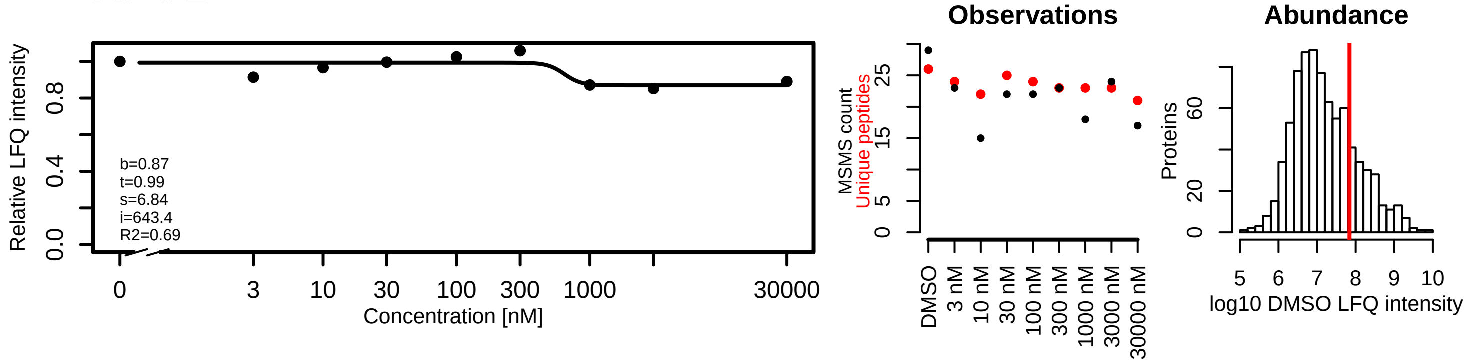


# ATP5B

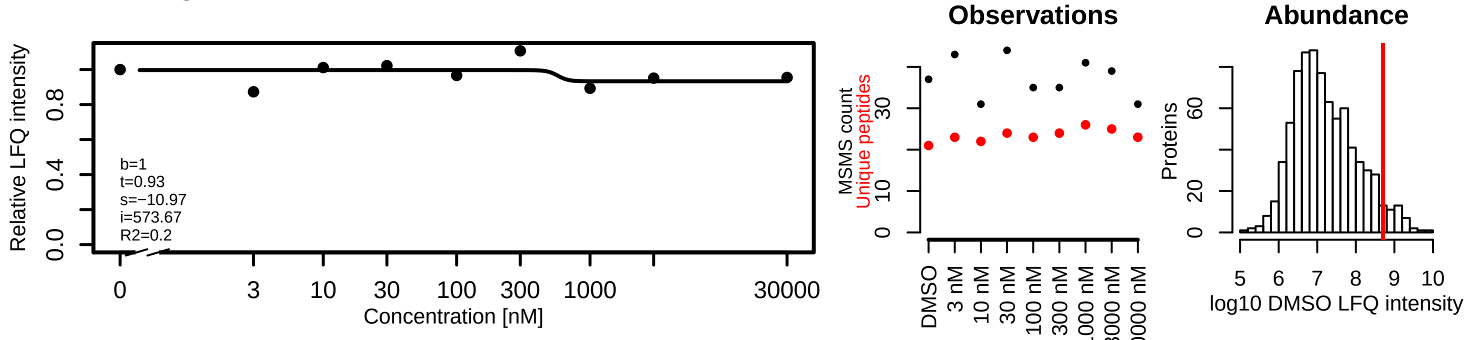

## GEMIN5

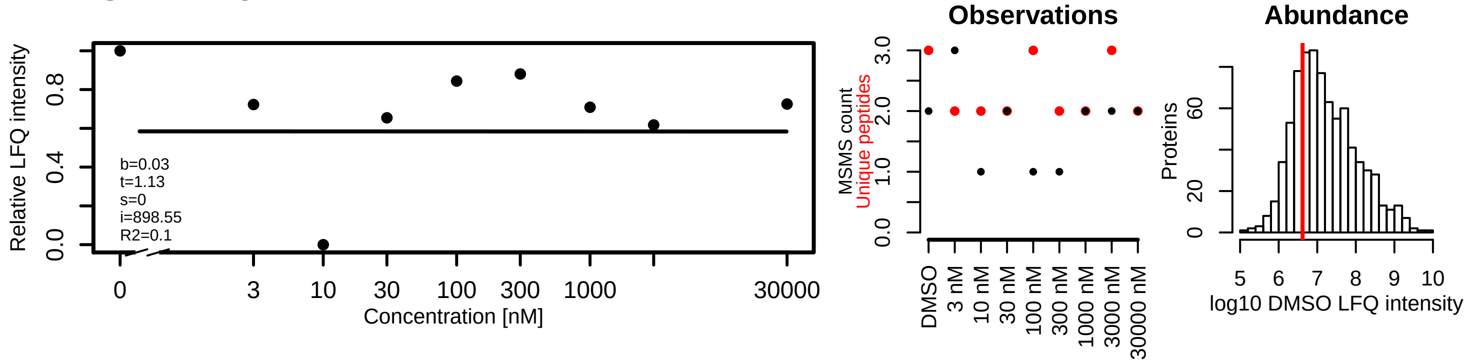


## PSMD2

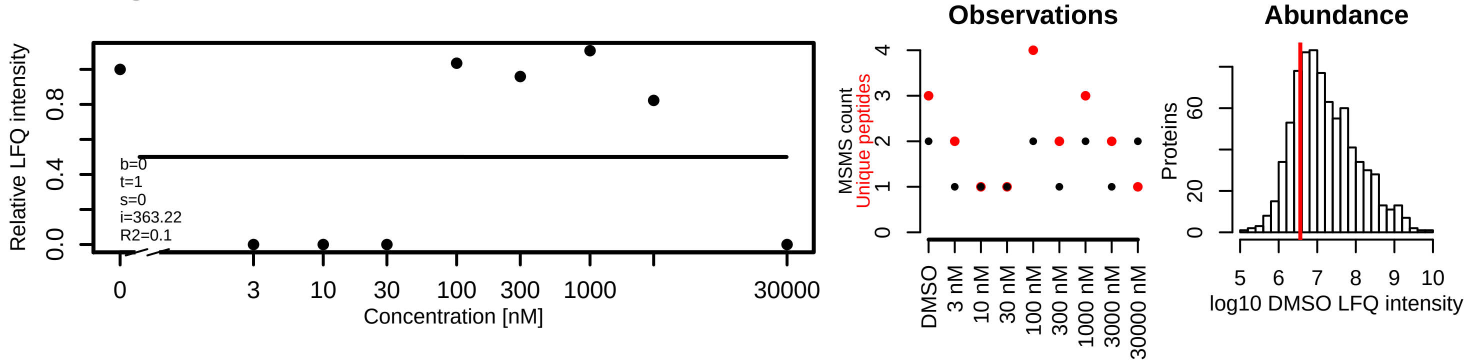


## PYGB

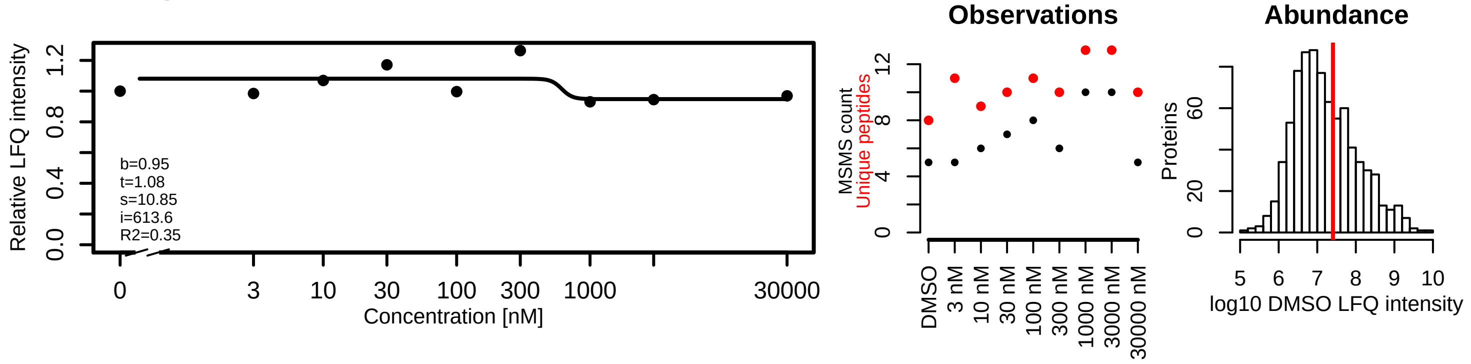


## SEC61A2

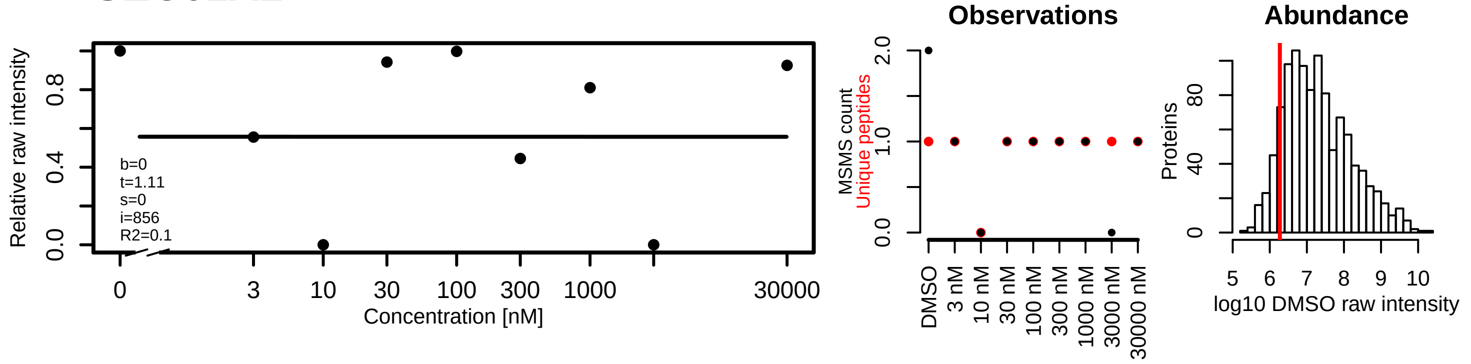


## GOLIM4

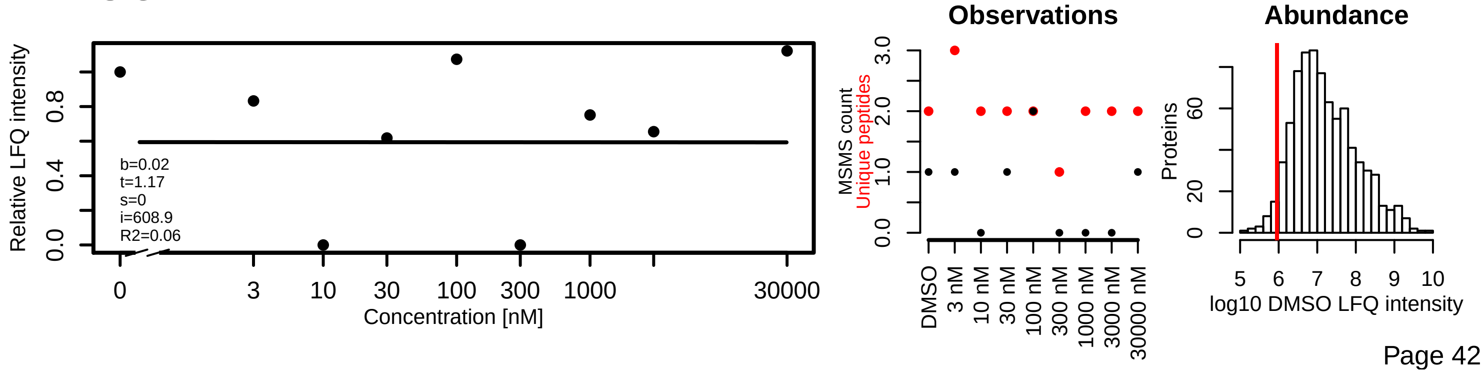

## HNRNPAB

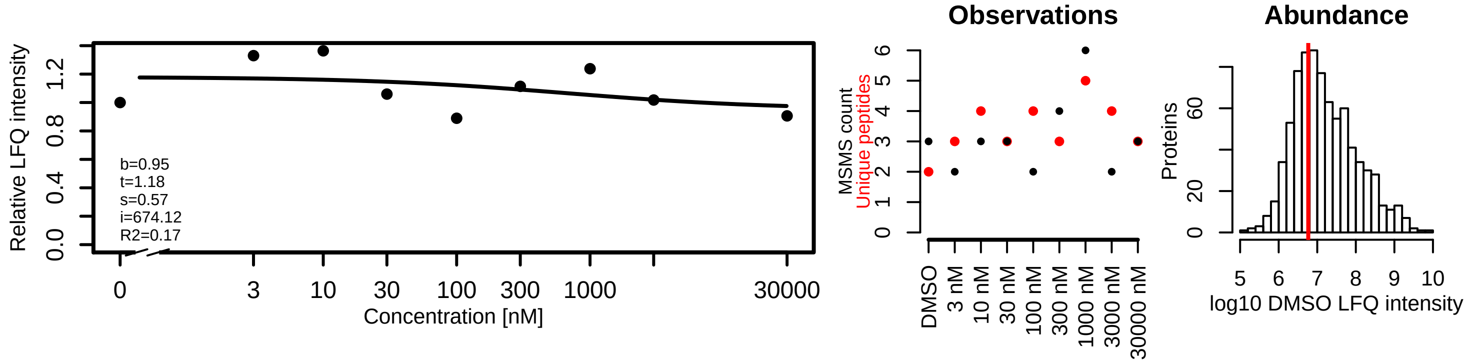


## ATP5C1

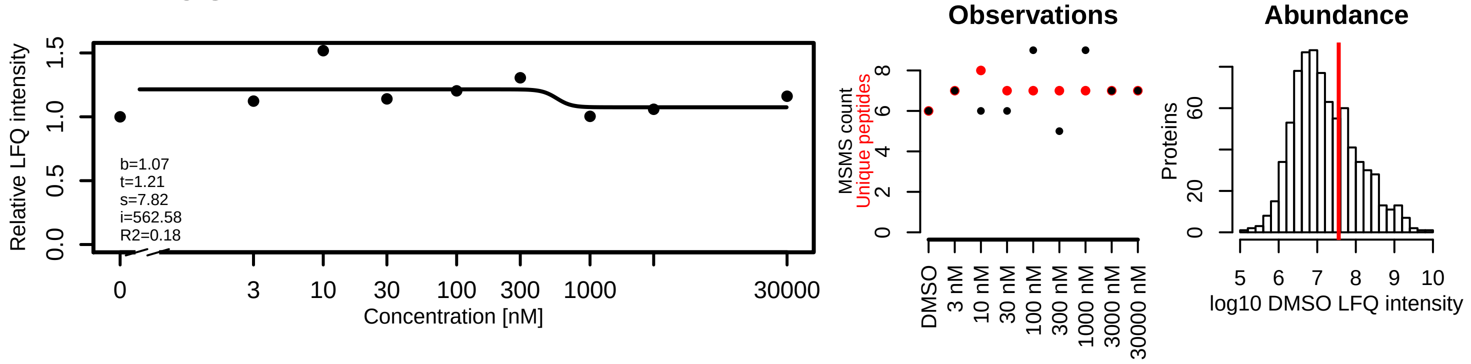


## URB1

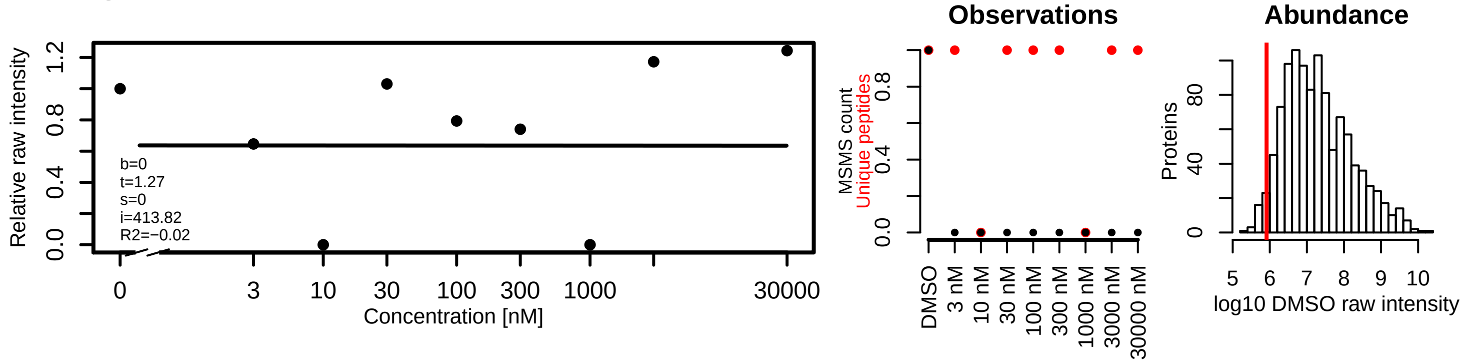


## RPL27

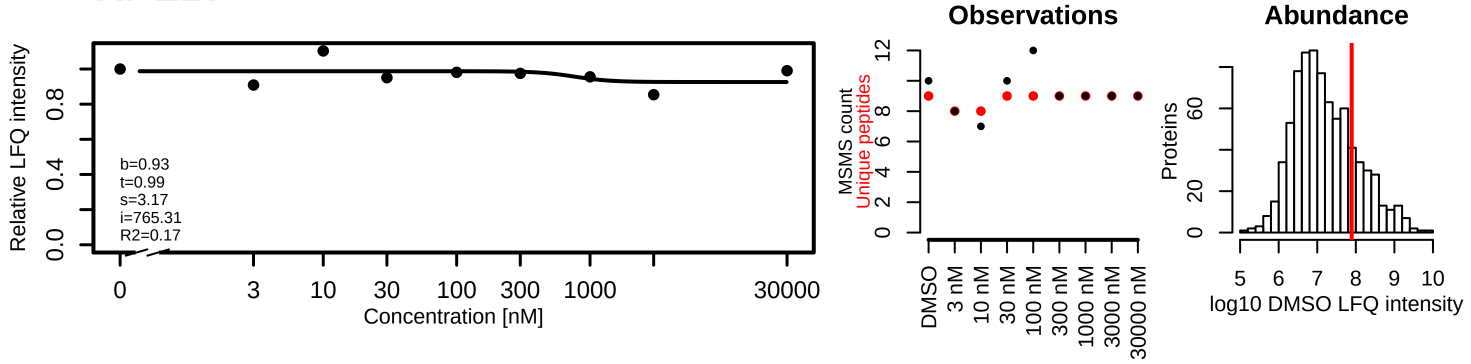


## RPS25

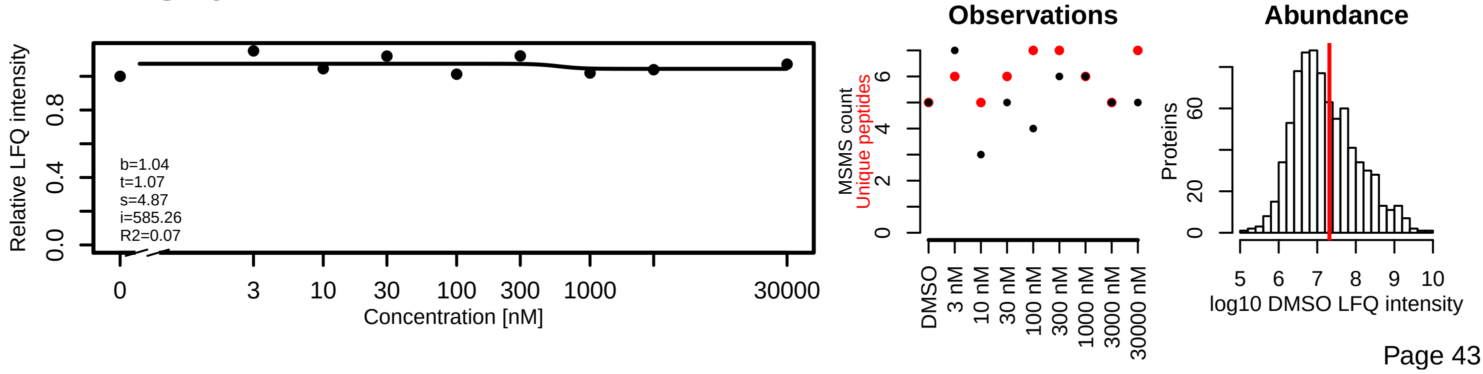

# TMCO1

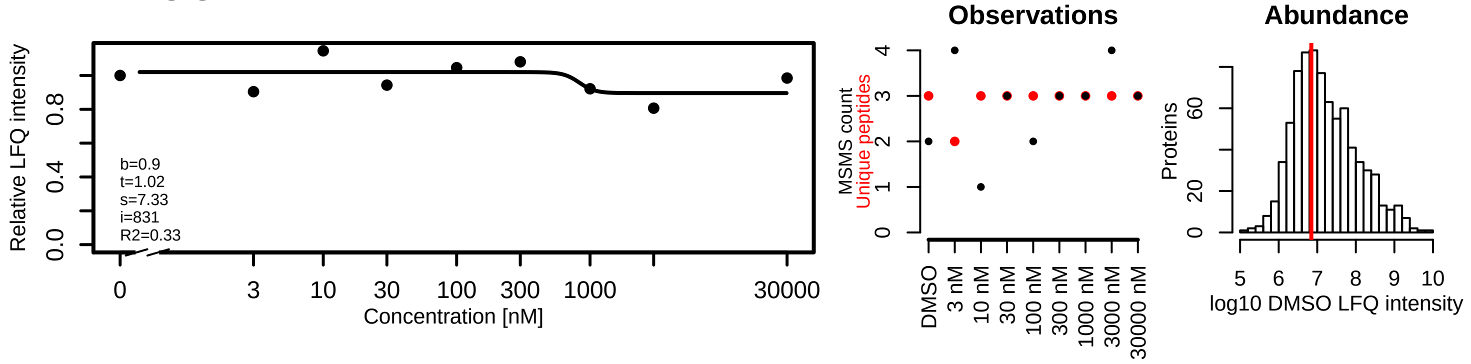


# RPL38

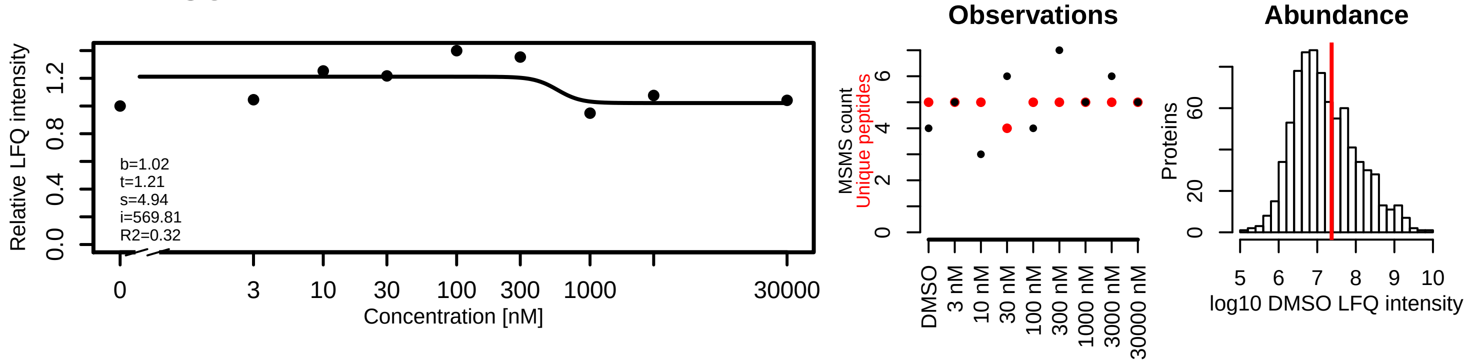


# RPL8

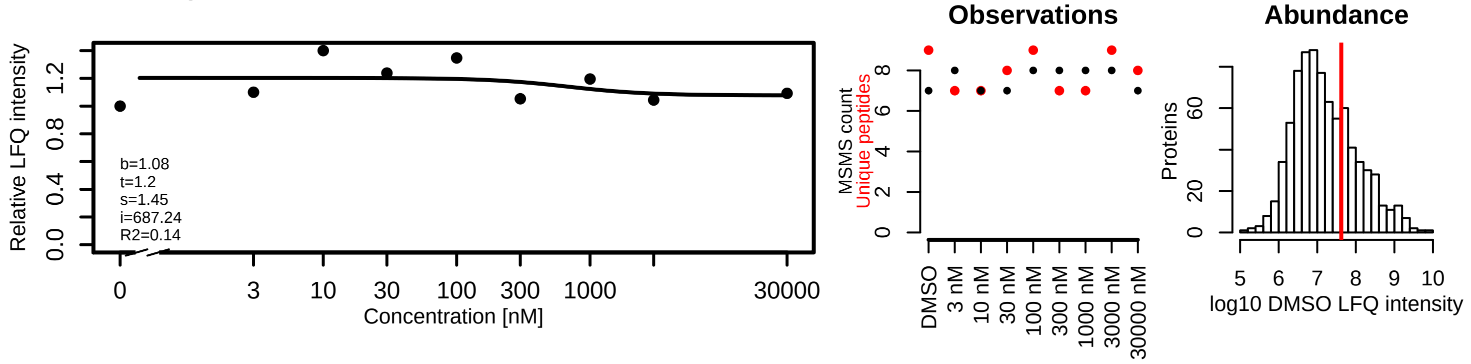


# TOP1

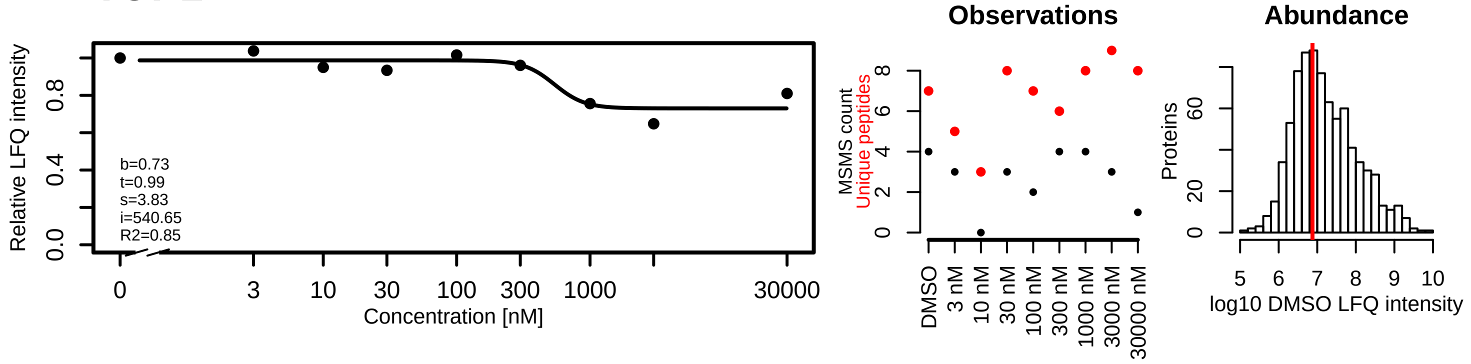


# ACVR2A

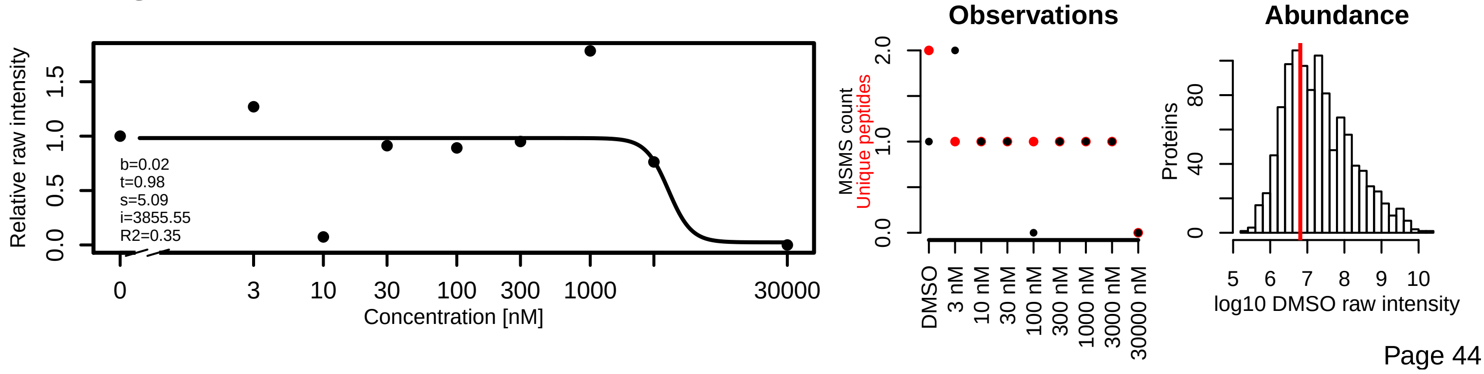

# IPO5

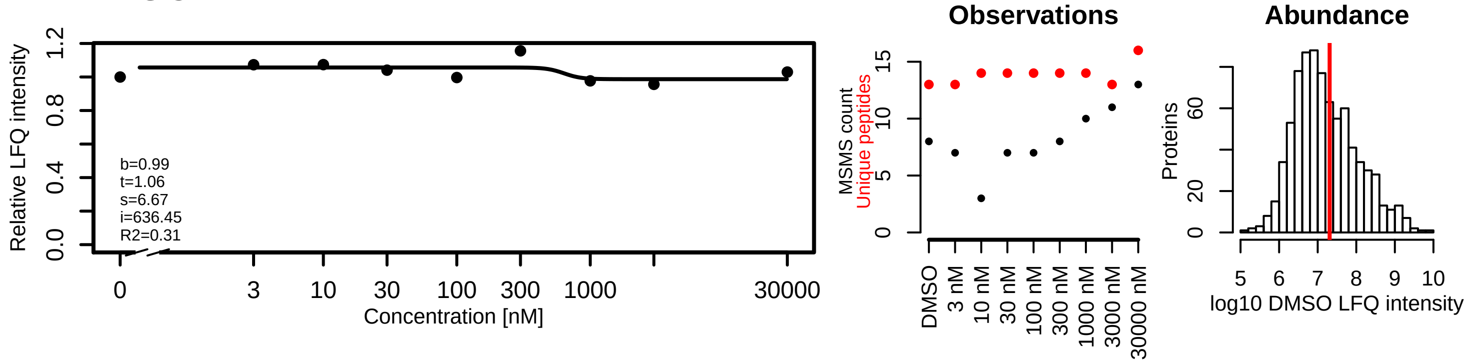


# WDR44

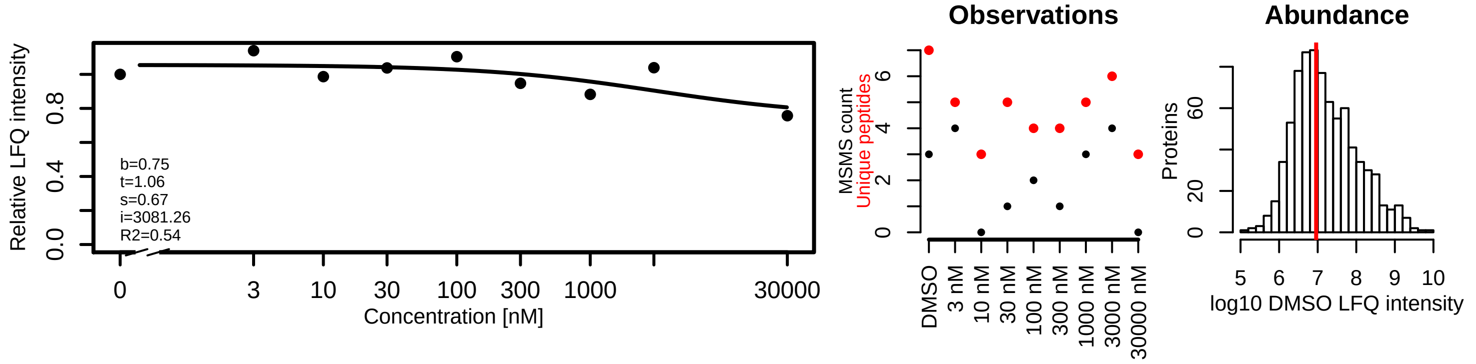


# ATP6V1H

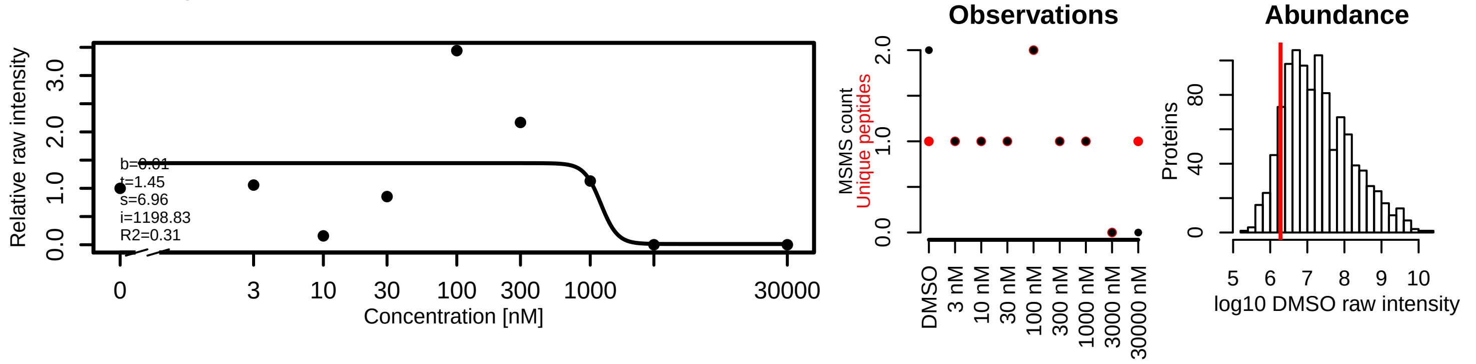


# FAM89A

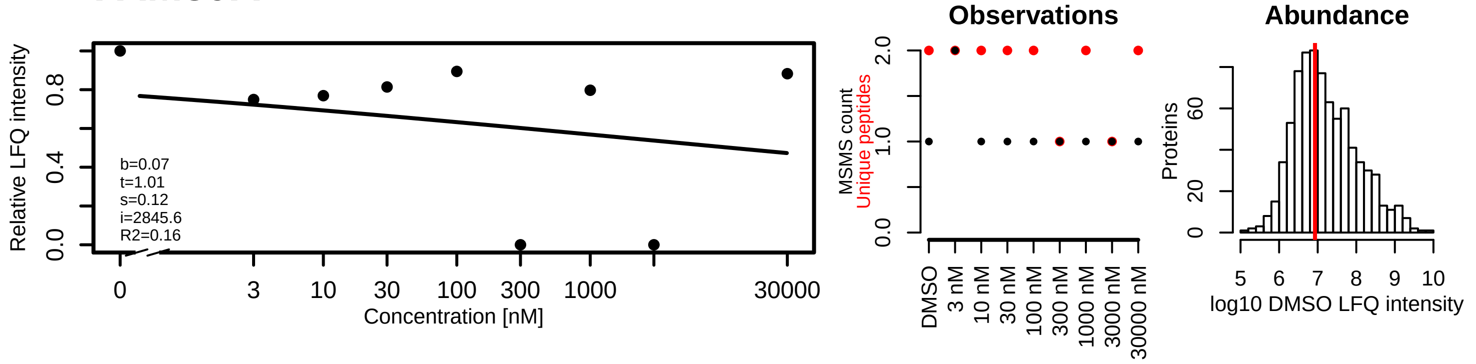


# ILK

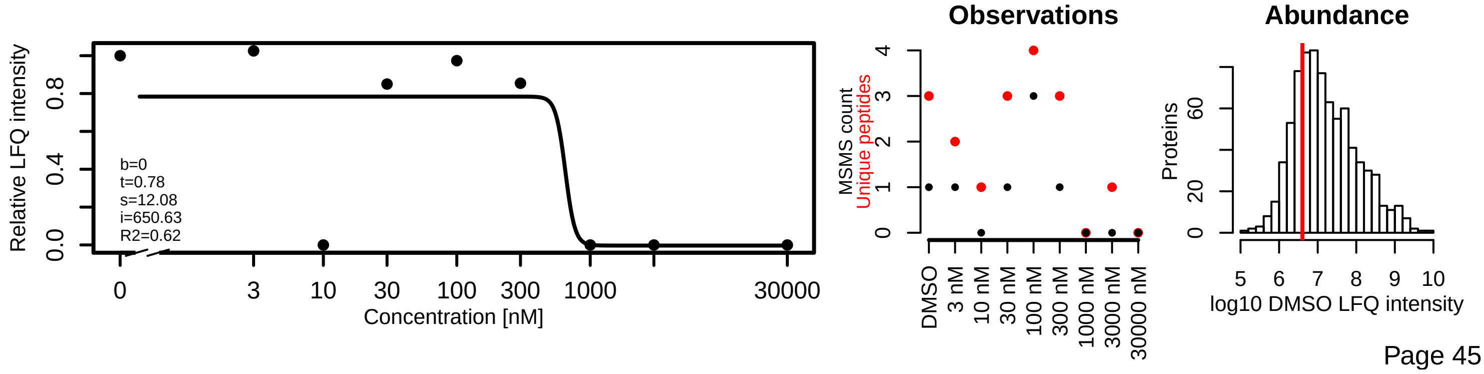

# IARS

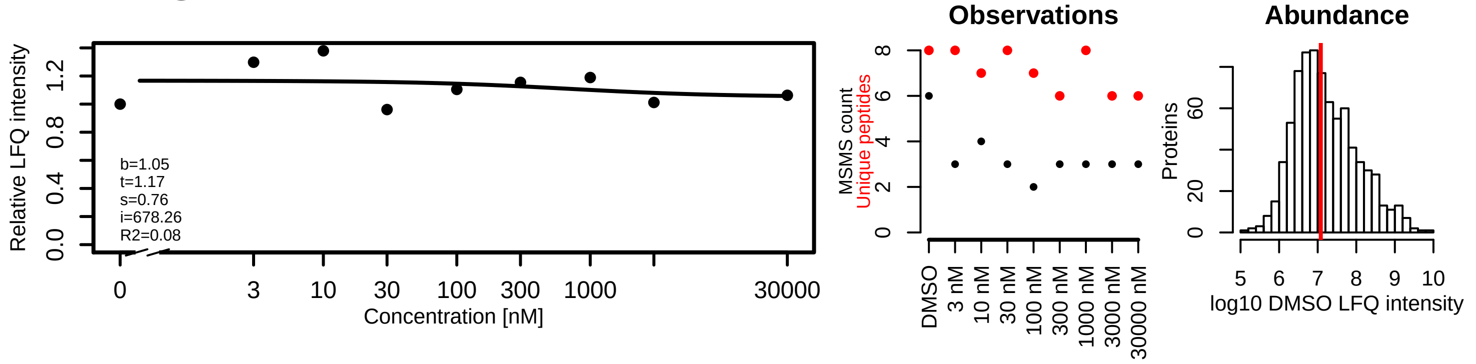


# RPLP2

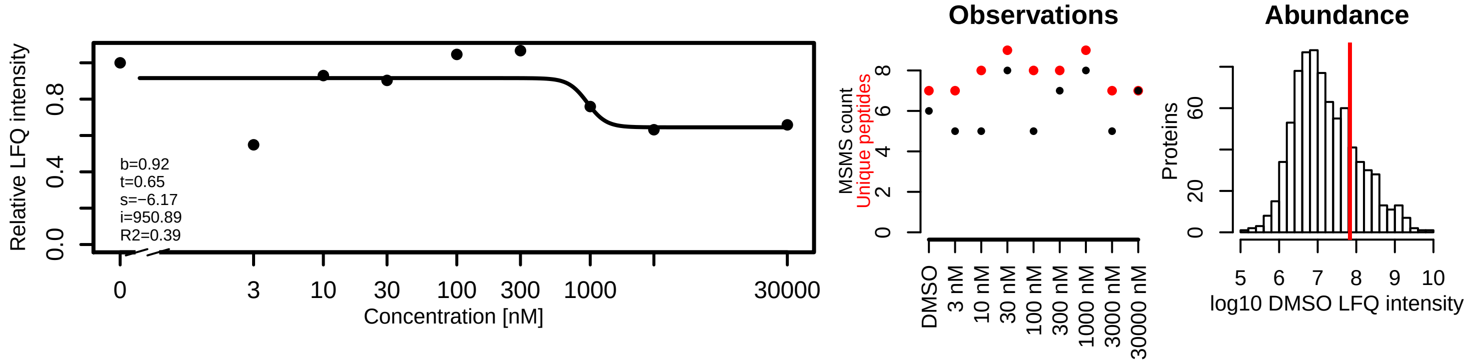


# CLTC

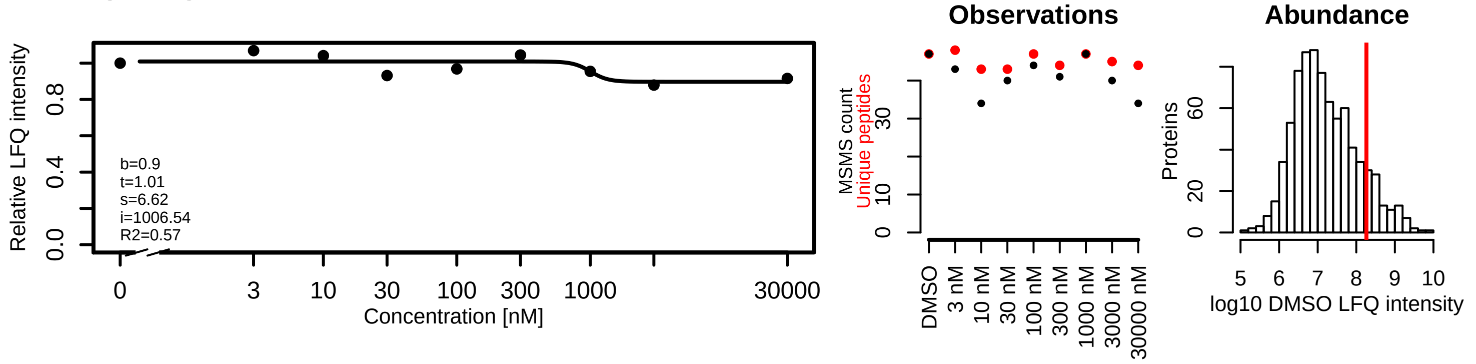


# MAPK11

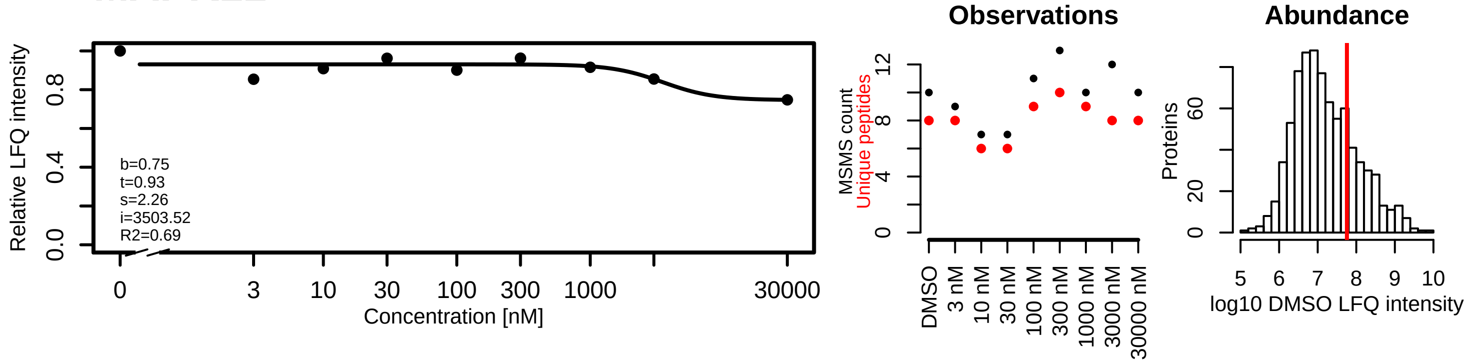


# FLNA

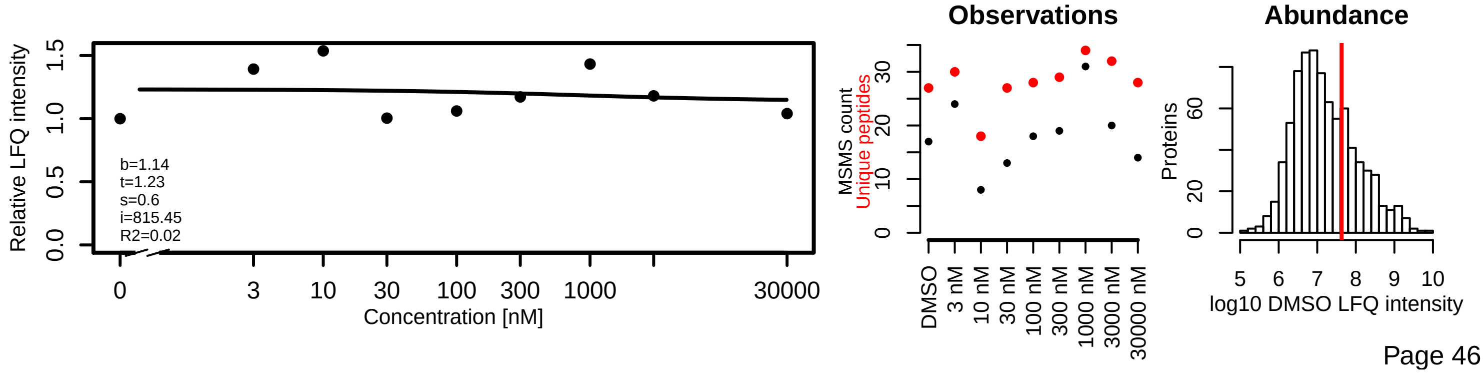

# ATG13

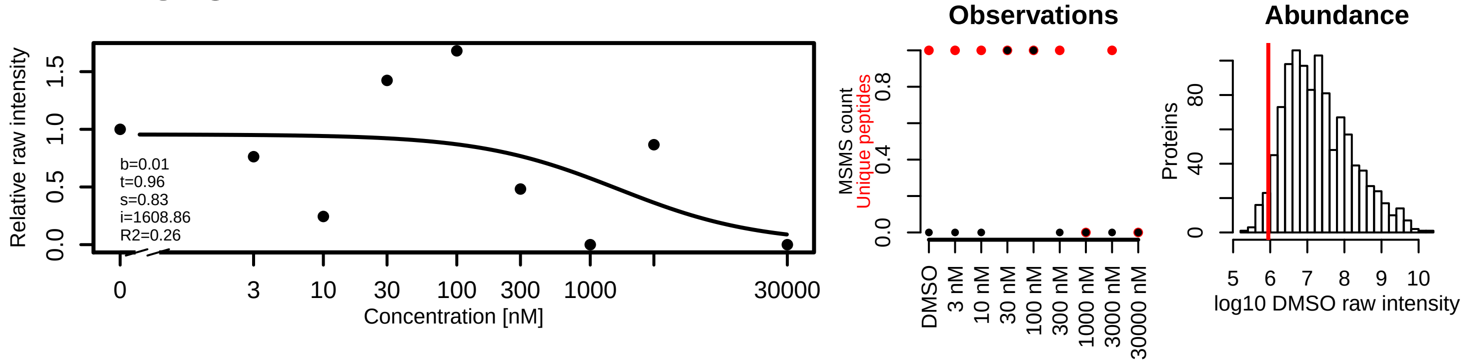


# MAP4K3

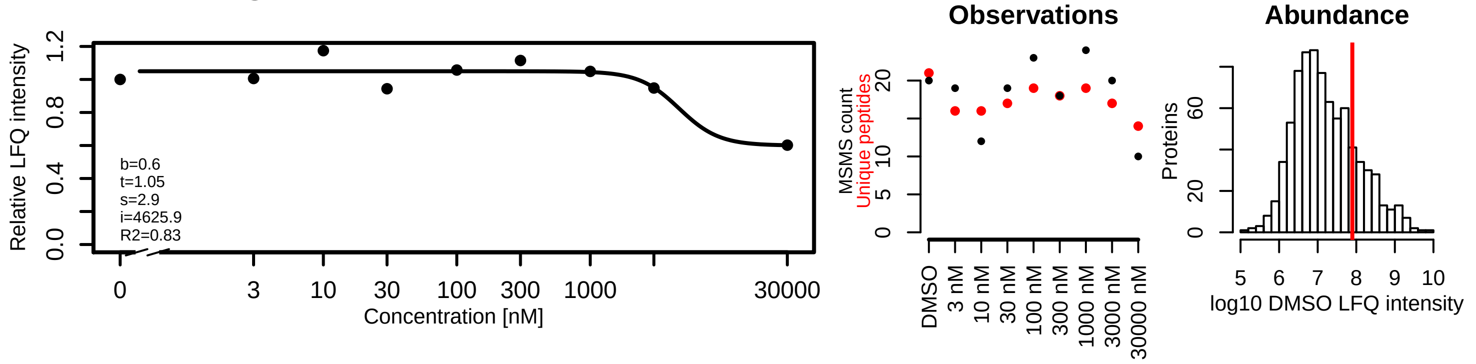


# HIGD1A

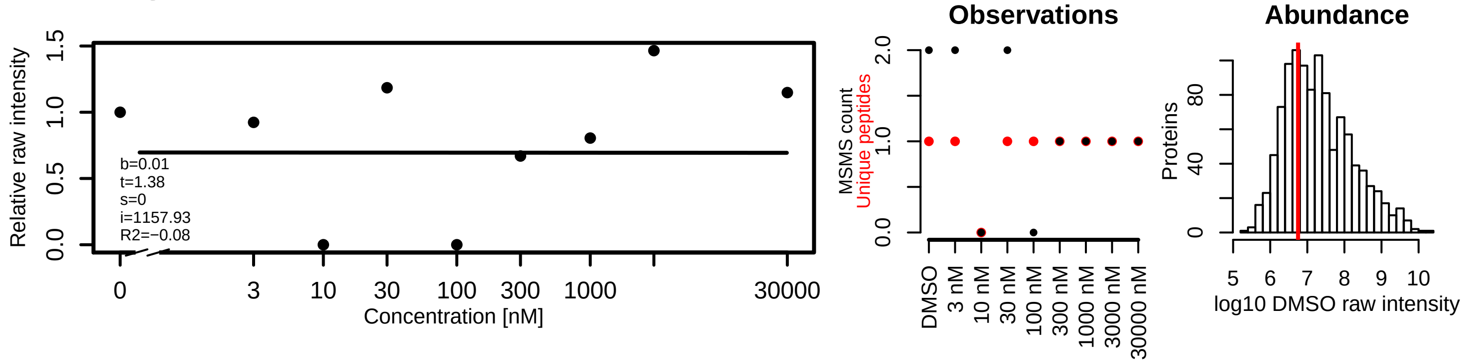


# DNAJA1

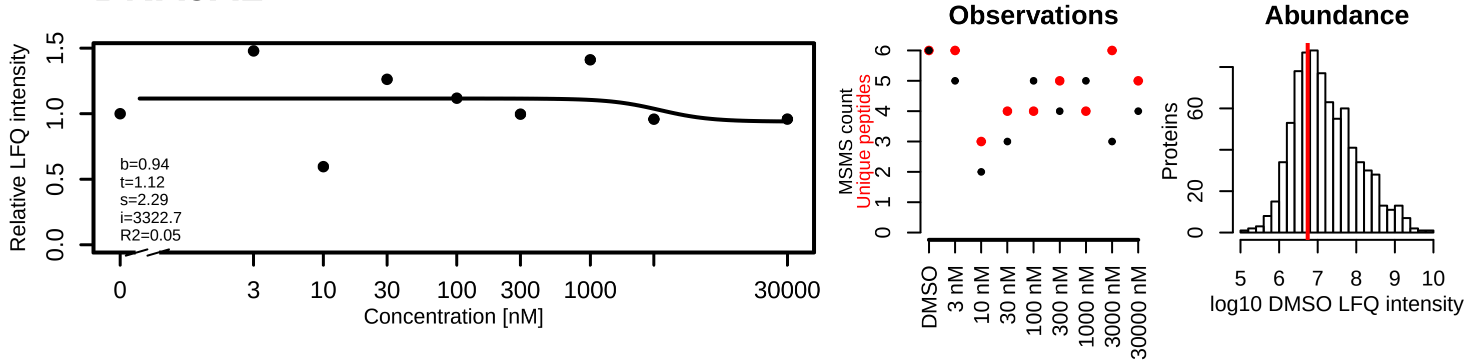


# MAP2K3 − P46734;P46734−3;J3QR49;J3KRV4;P52564−2;P52564;K7E

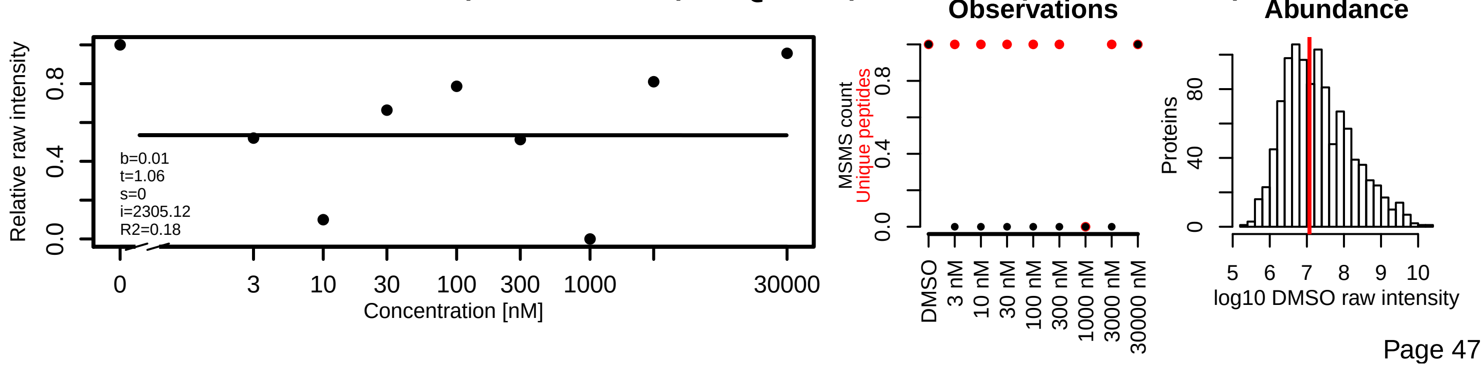

# COPB1

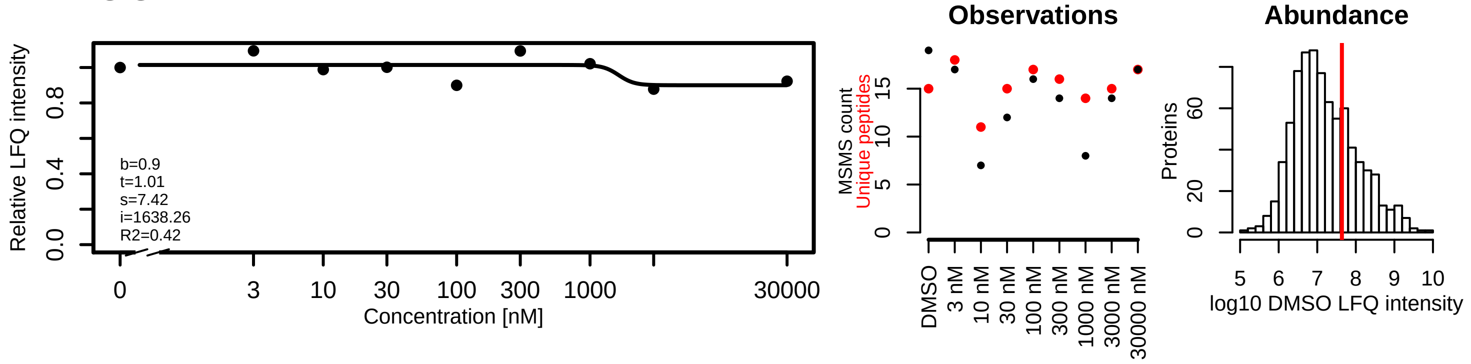


# WEE1

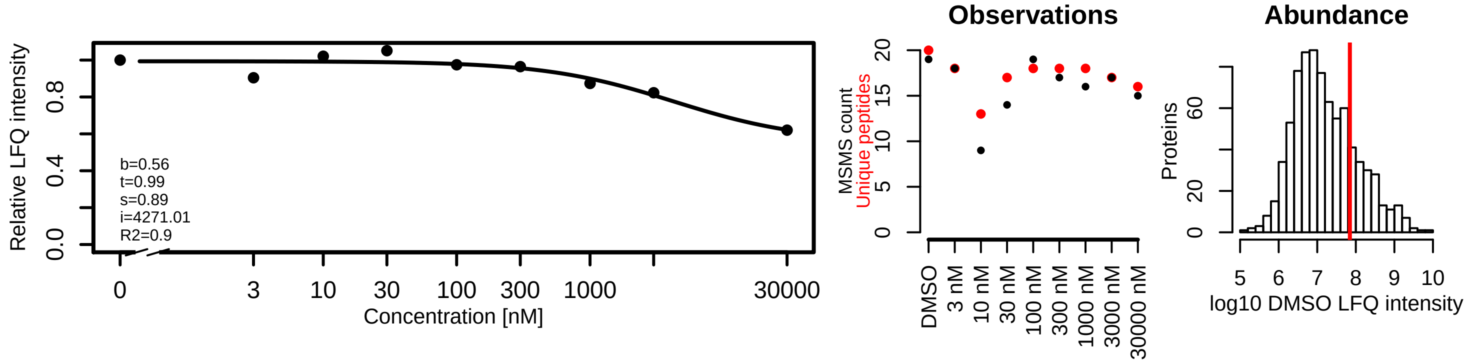


# SERPINH1

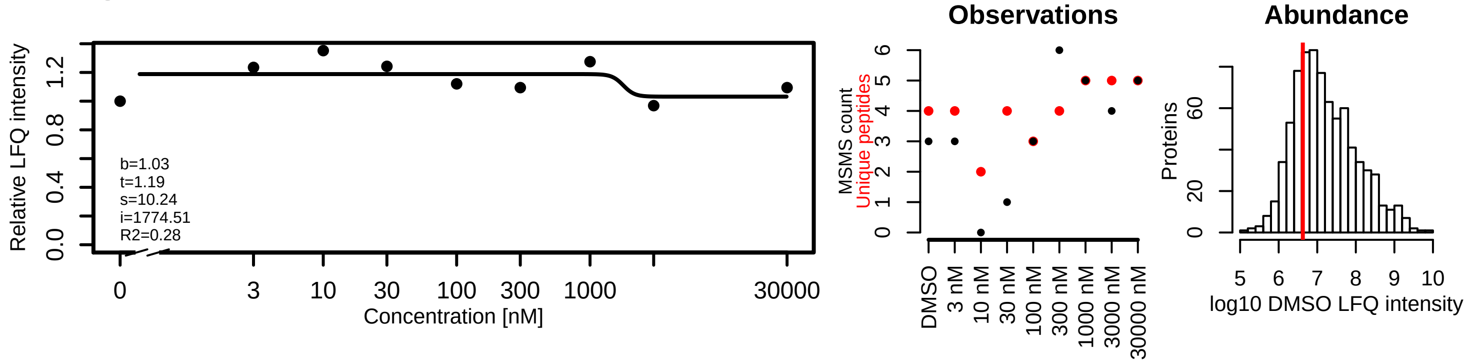


# CRELD1

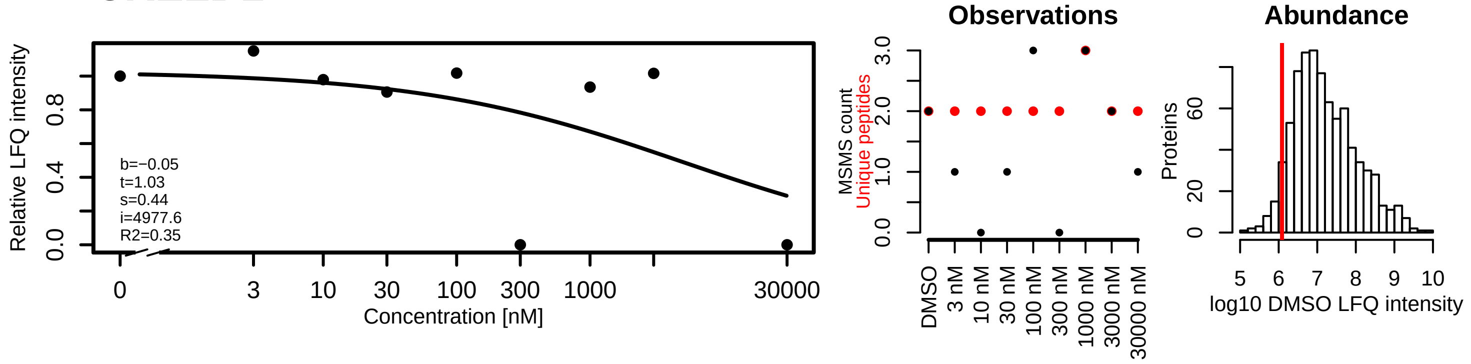


# YBX1;YBX3

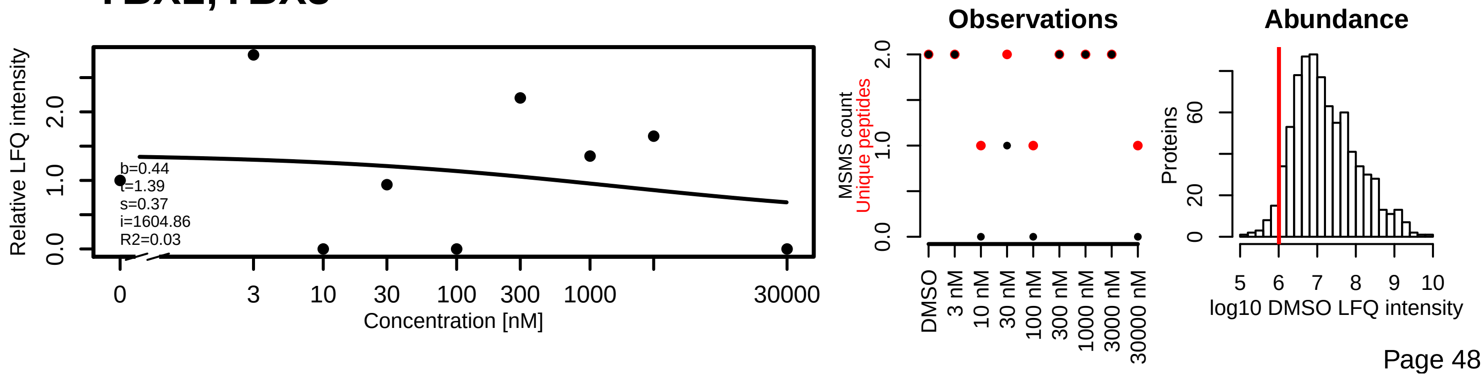

## TGFBR2

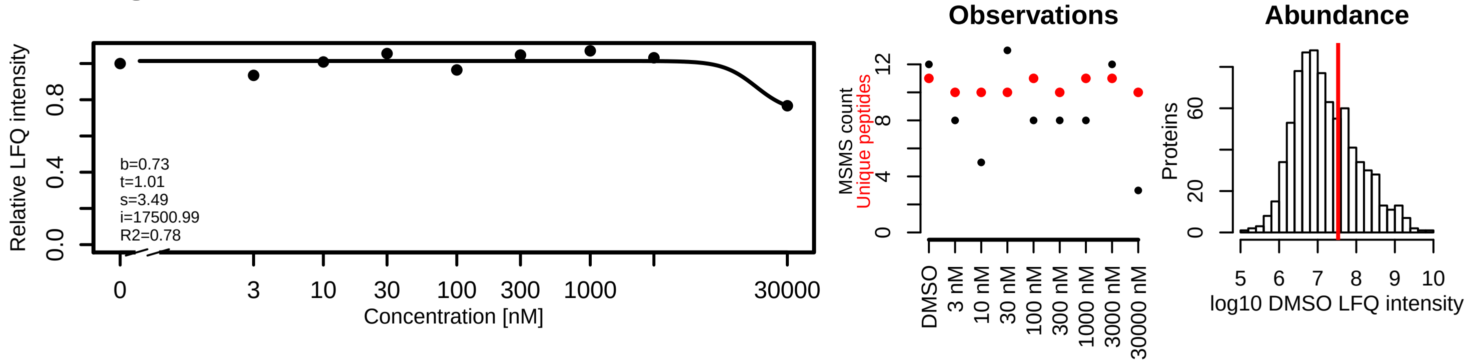


## TMED4

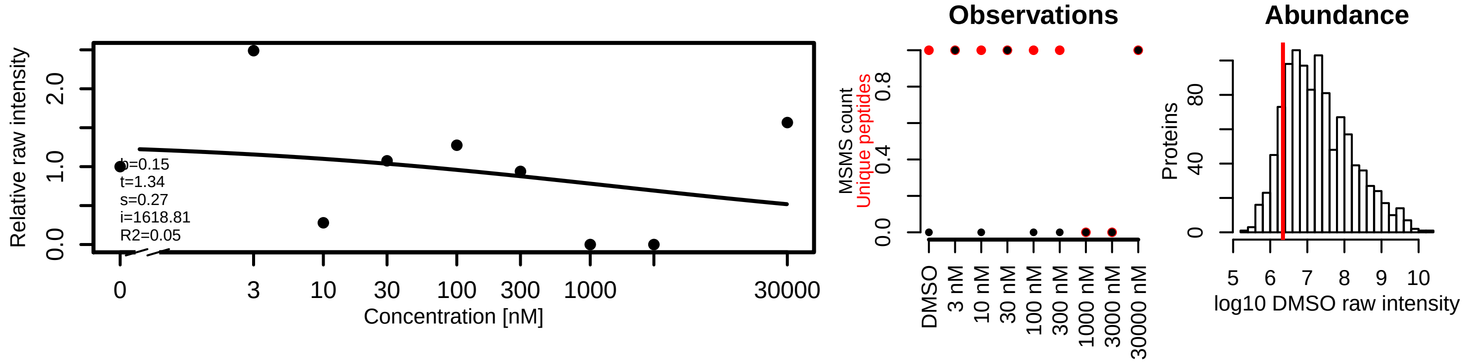


## HINT1

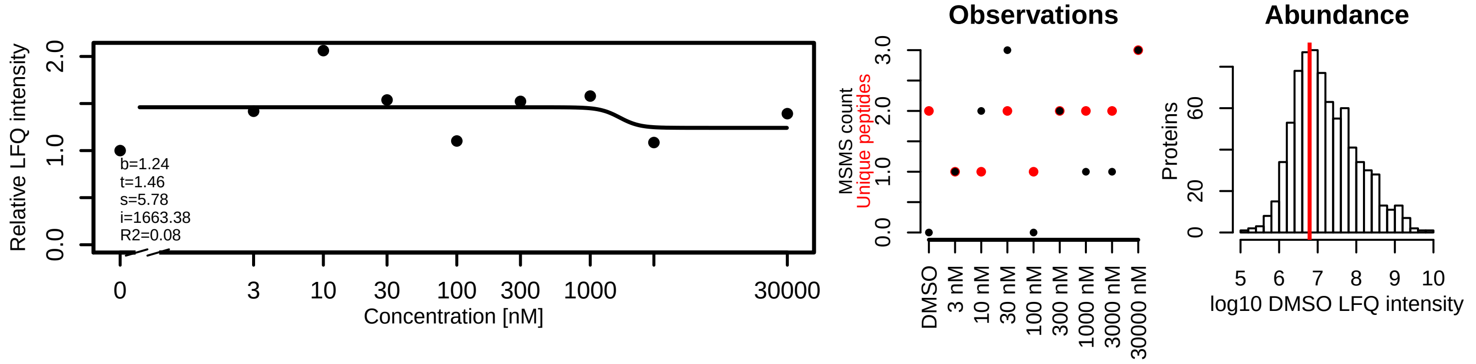


## LTN1

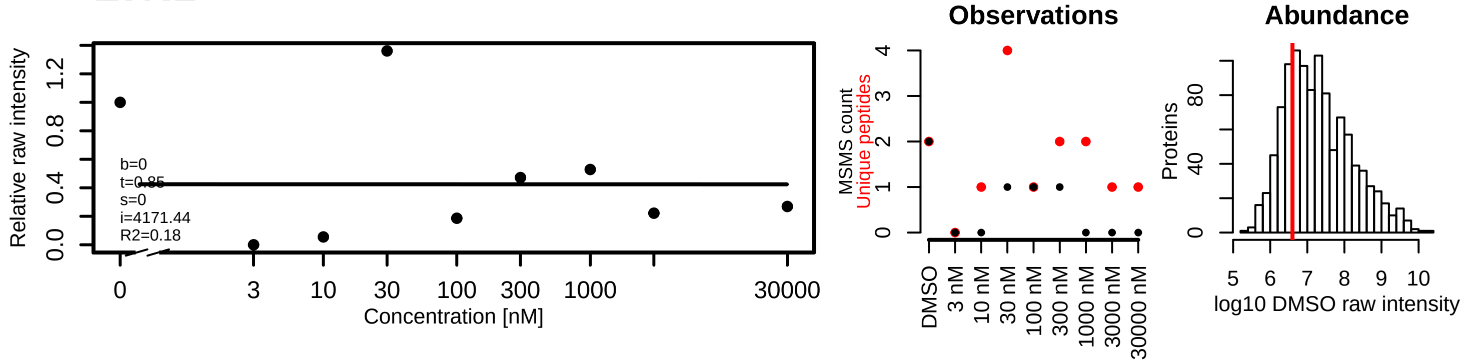


## HNRNPA0

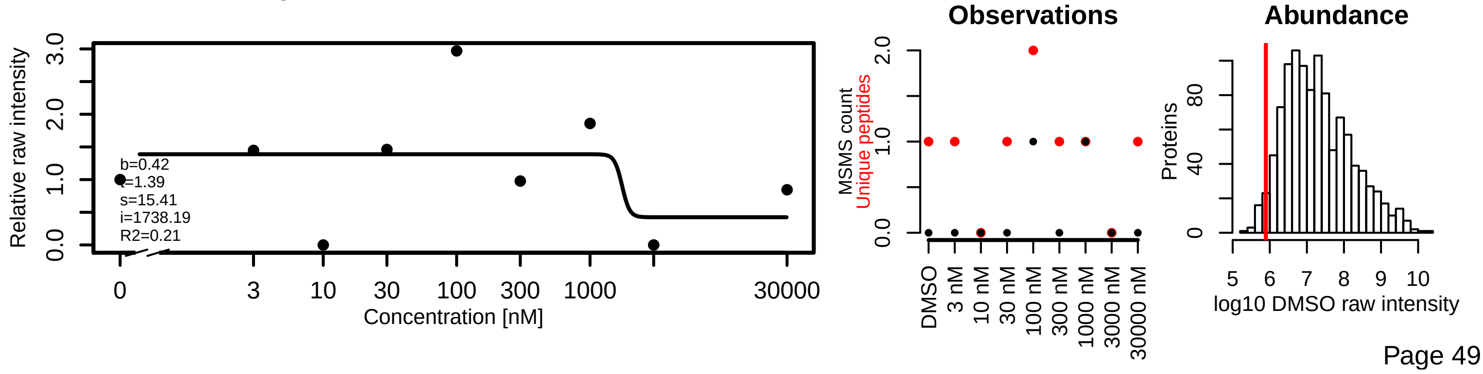

## TNPO1

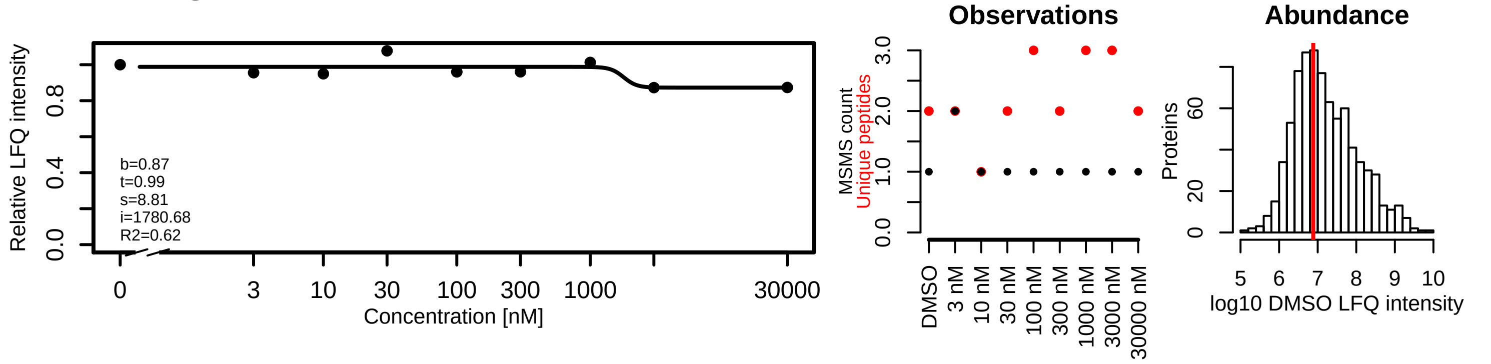


## CCT4

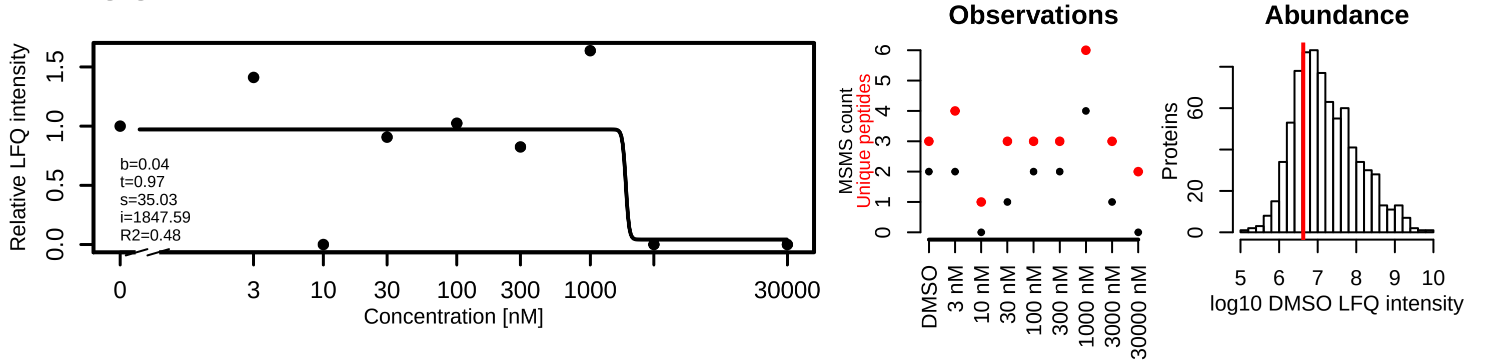


## CDK14

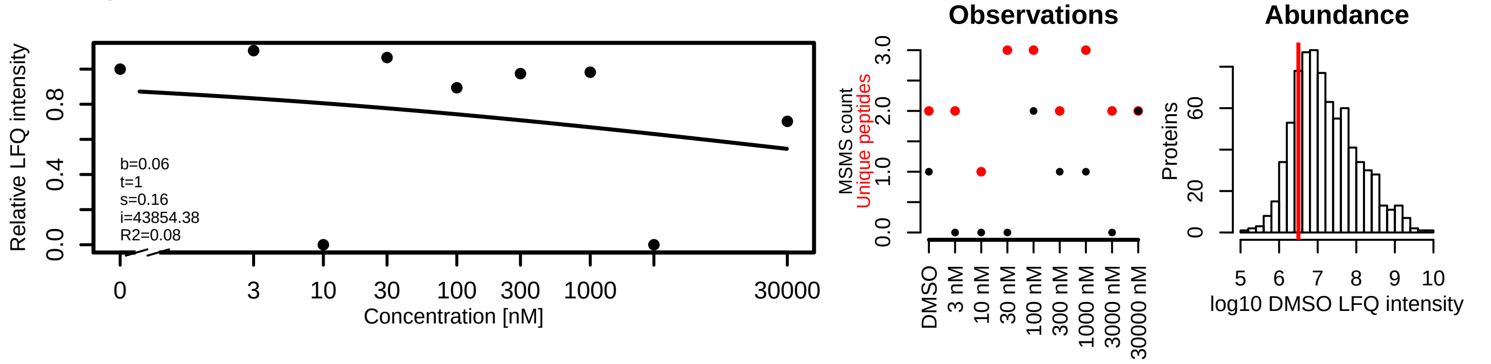


## CYFIP1;CYFIP2

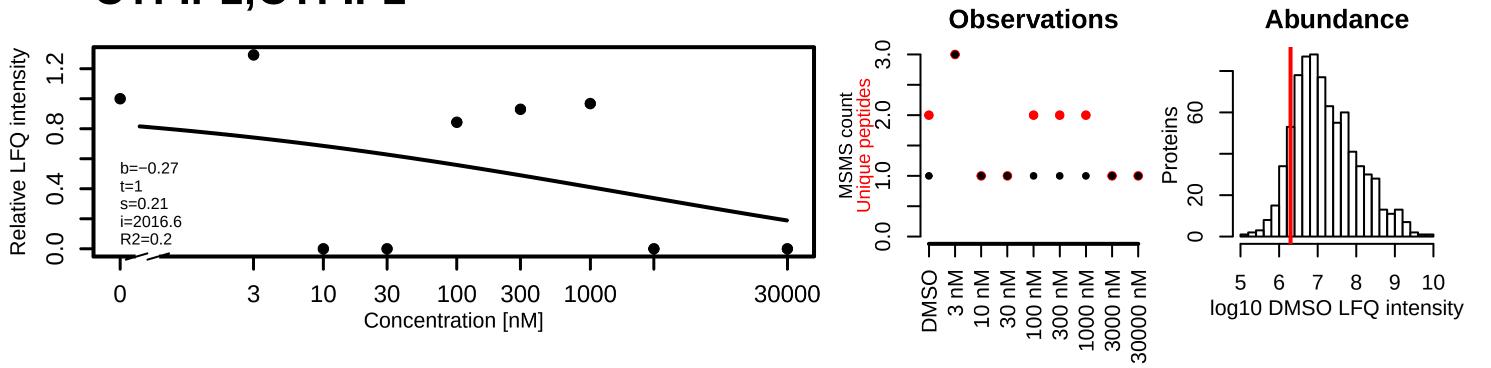


## PDIA3 − G5EA52;P30101

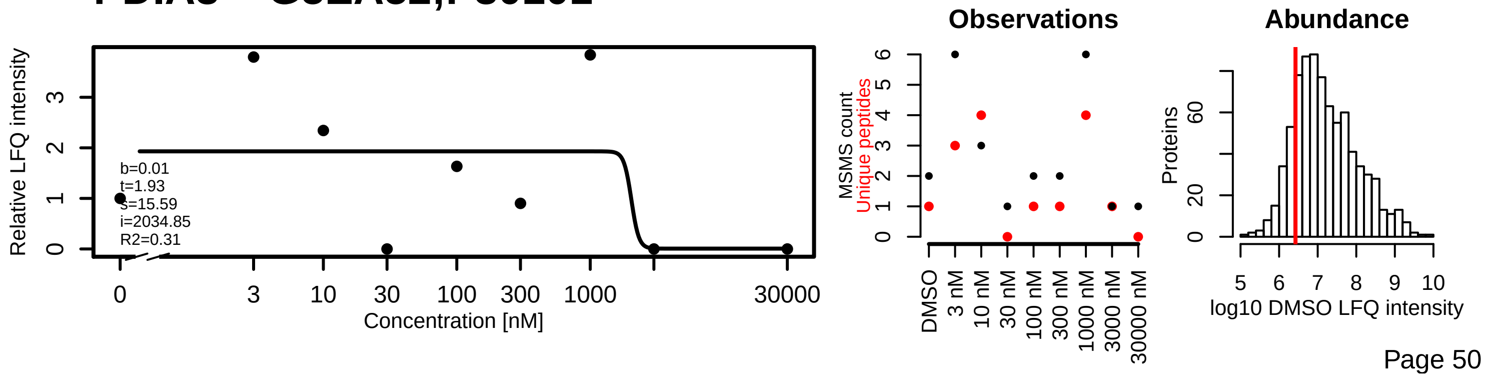

# MYL6

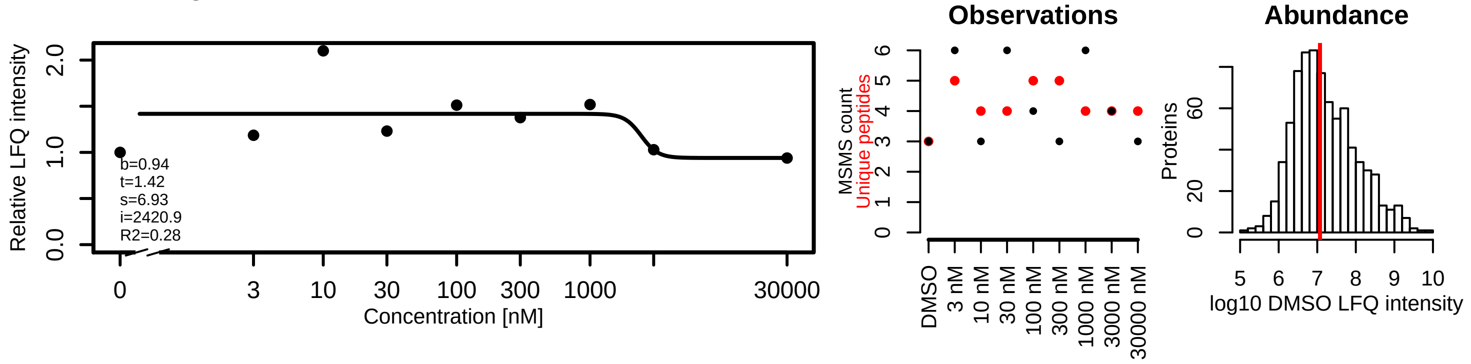


# ZDHHC6

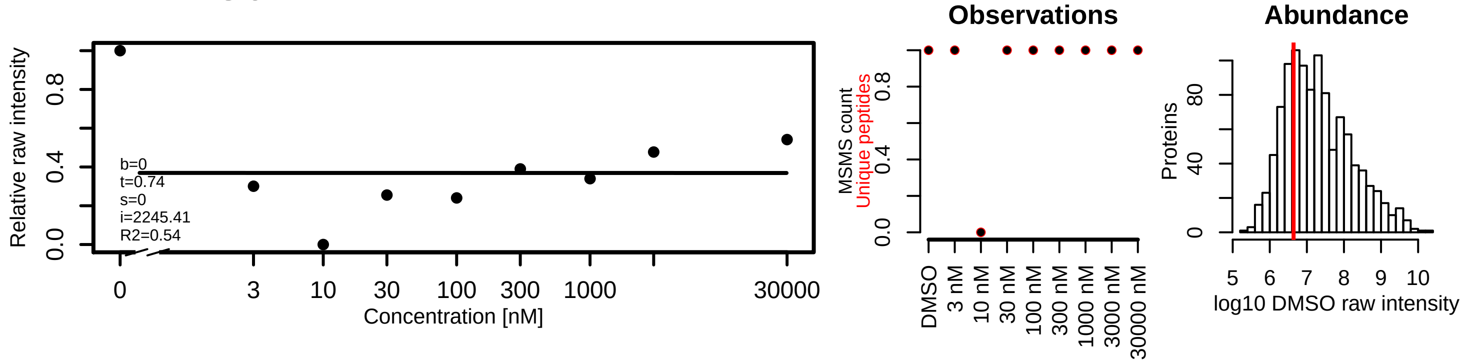


# RPL18A

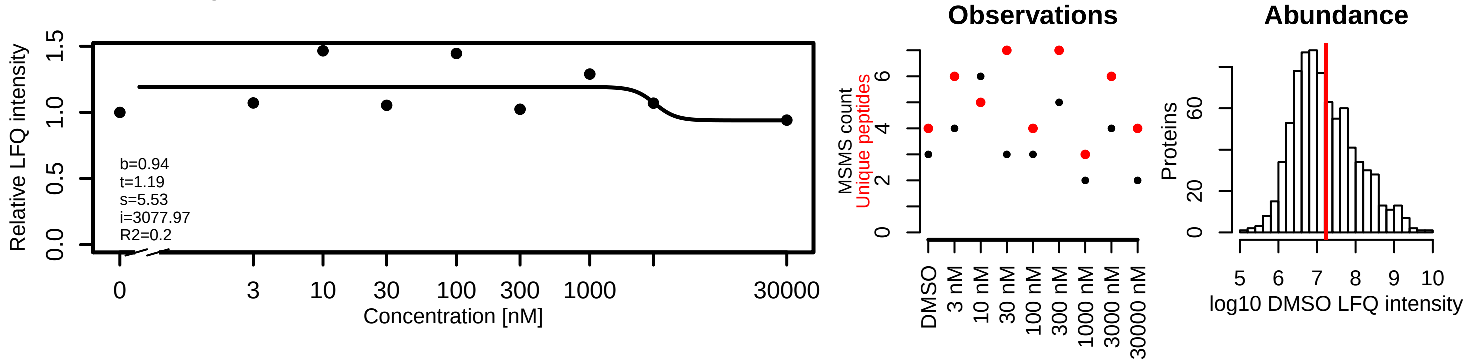


# HLA−F

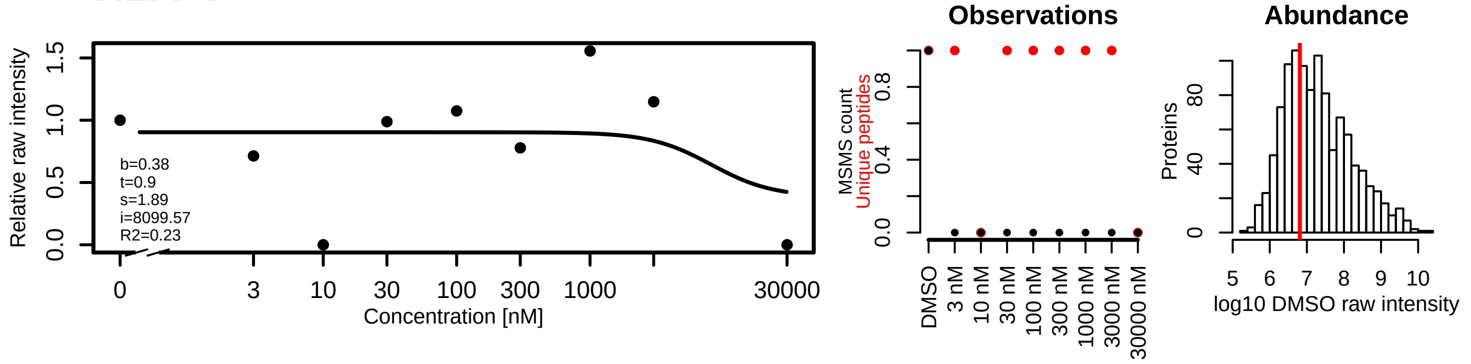


# SSR1

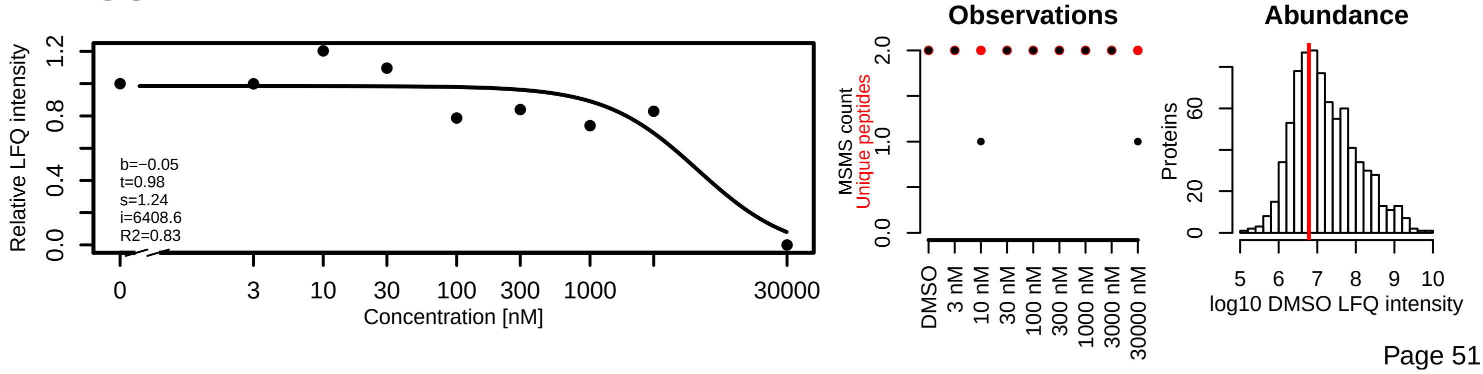

# TGM2

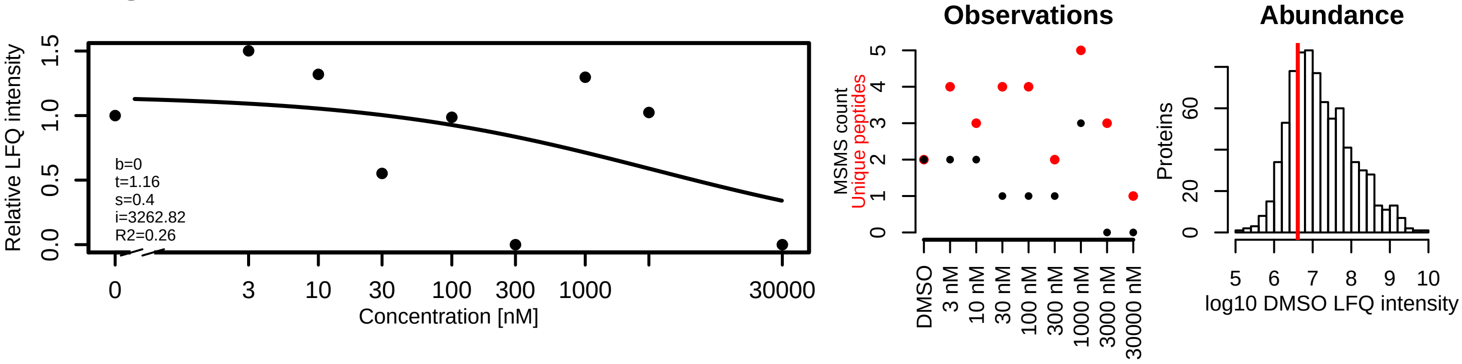


# MAP2K4

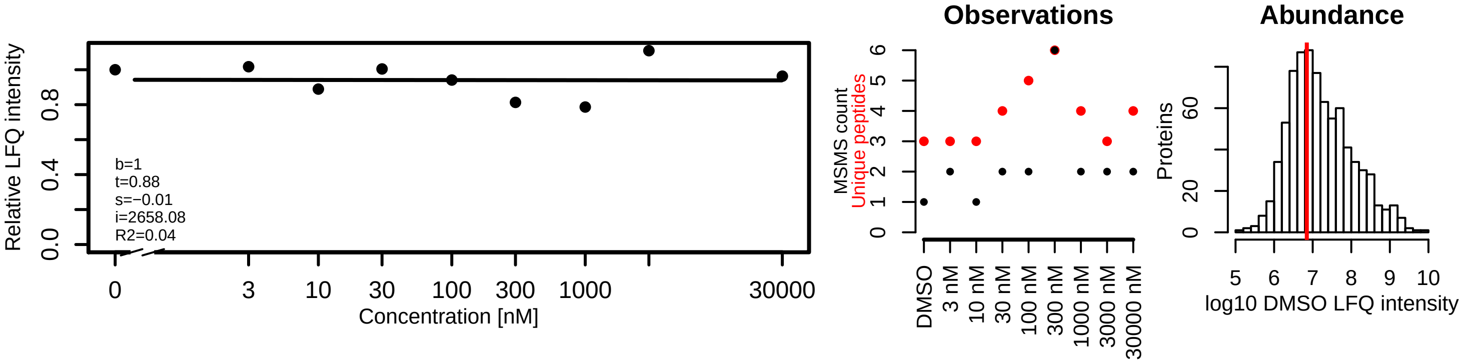


# VCP

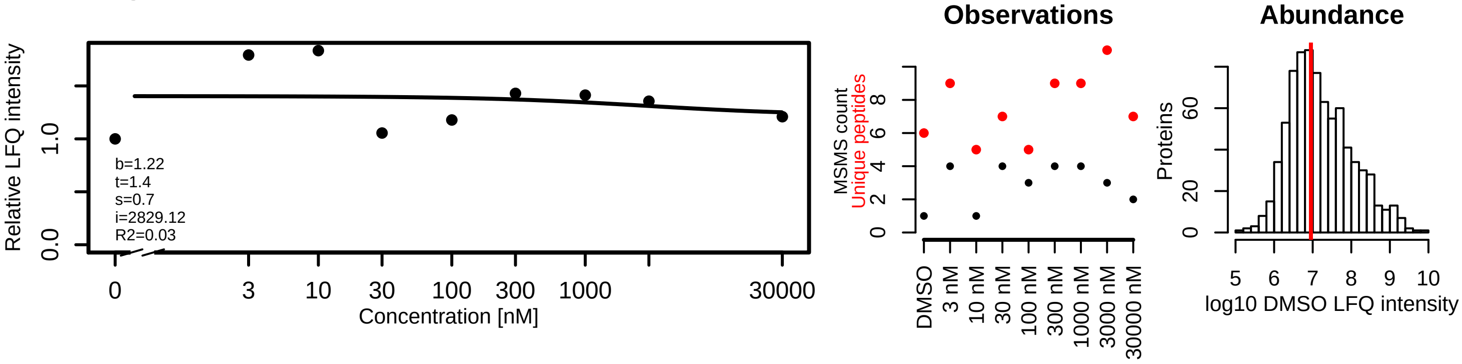


# NDUFS3

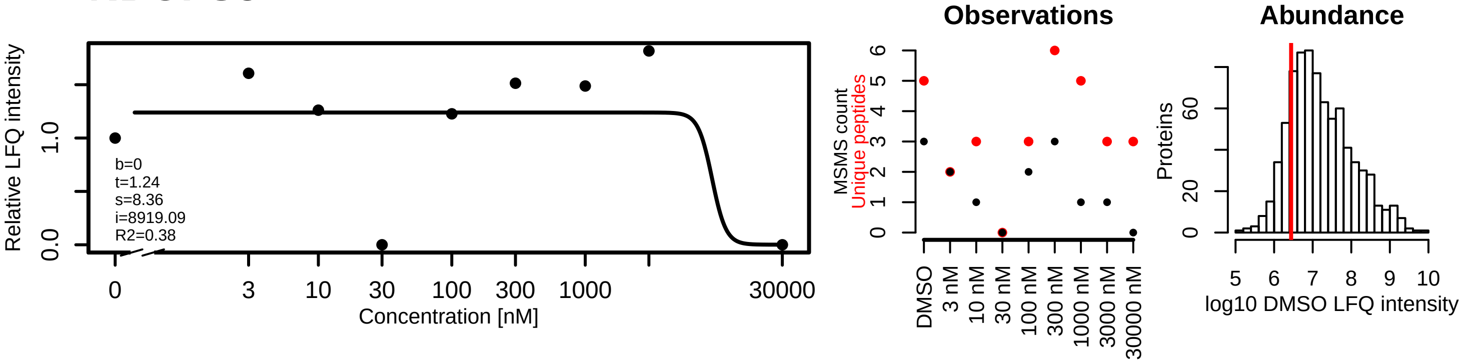


# MDH2

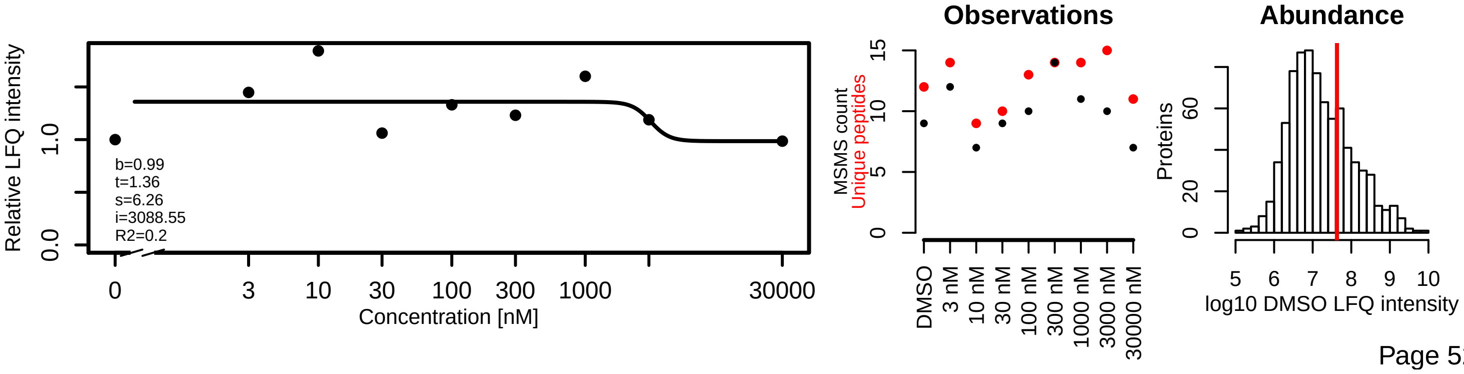

# RPLP1

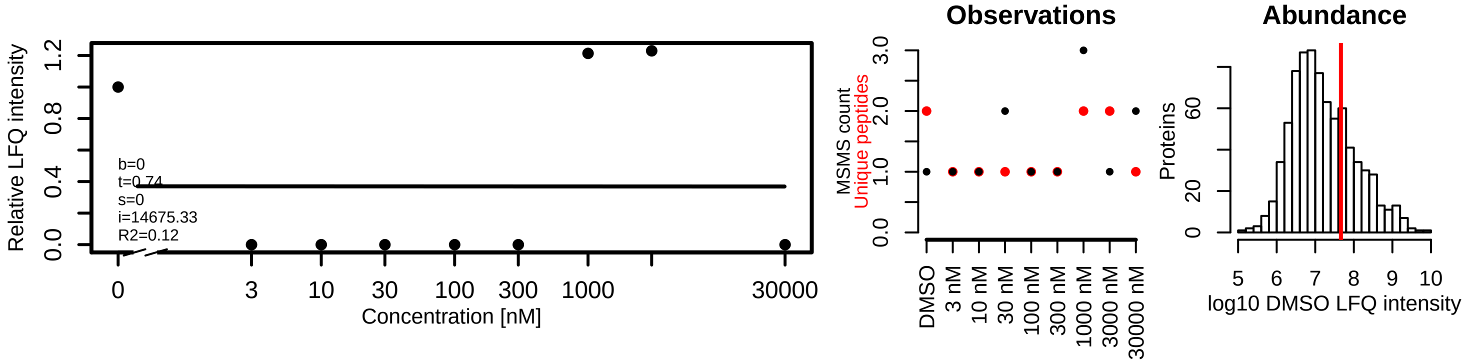


# SLC33A1

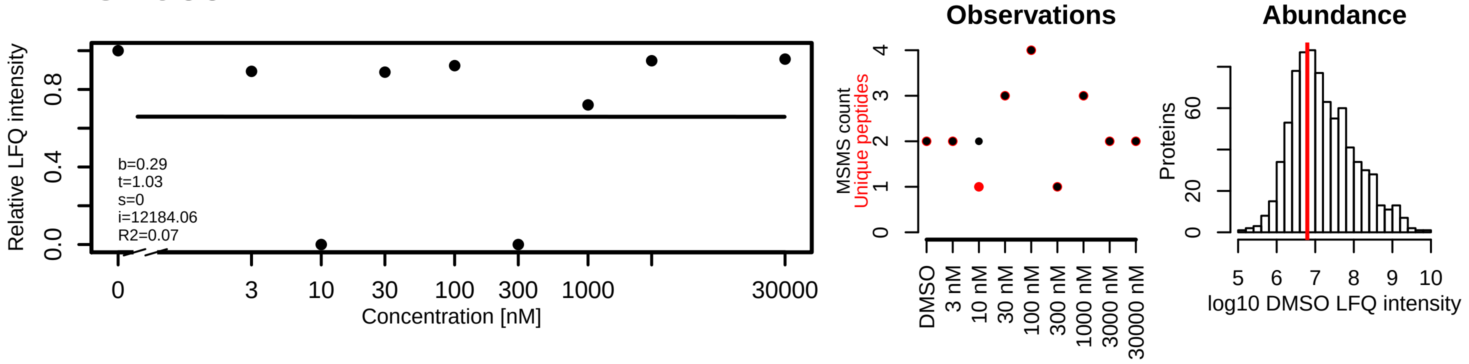


# DCTPP1

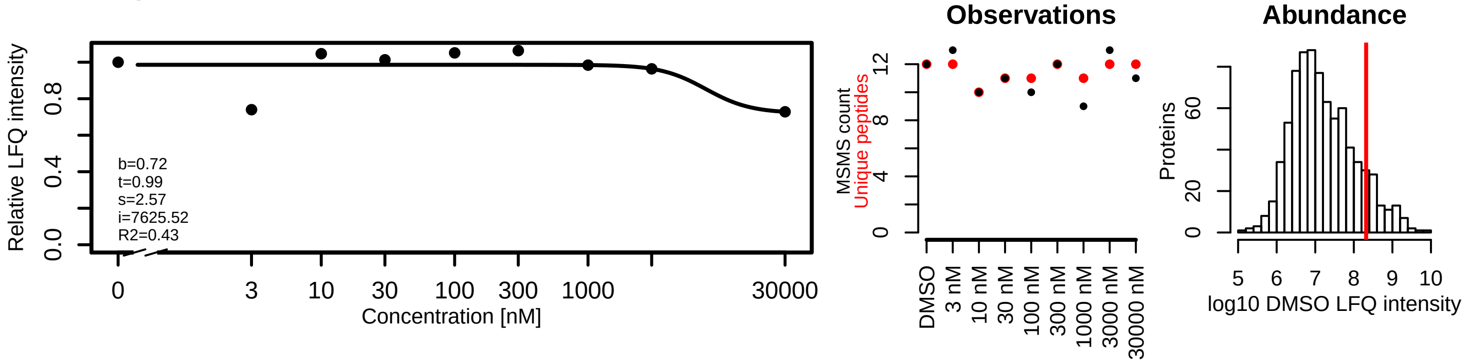


# HSPA5

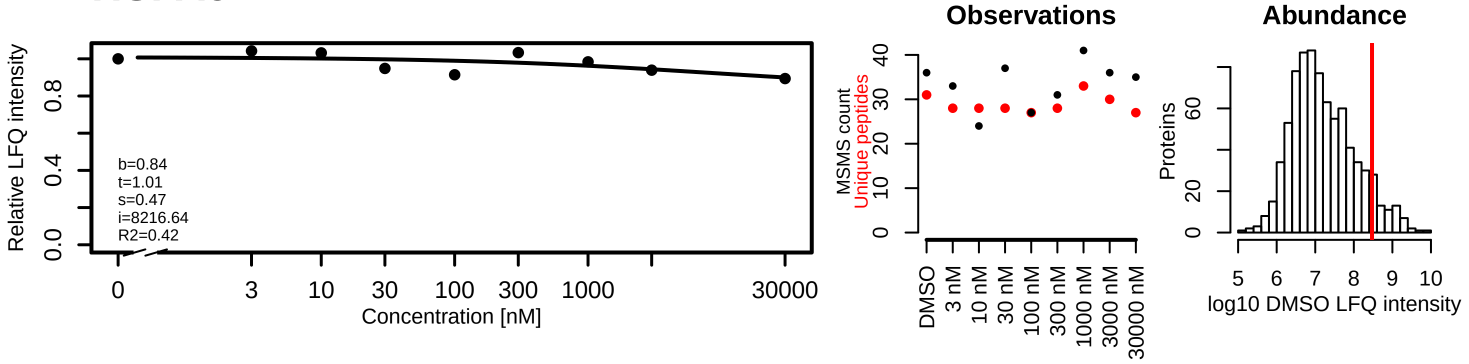


# CCT6A

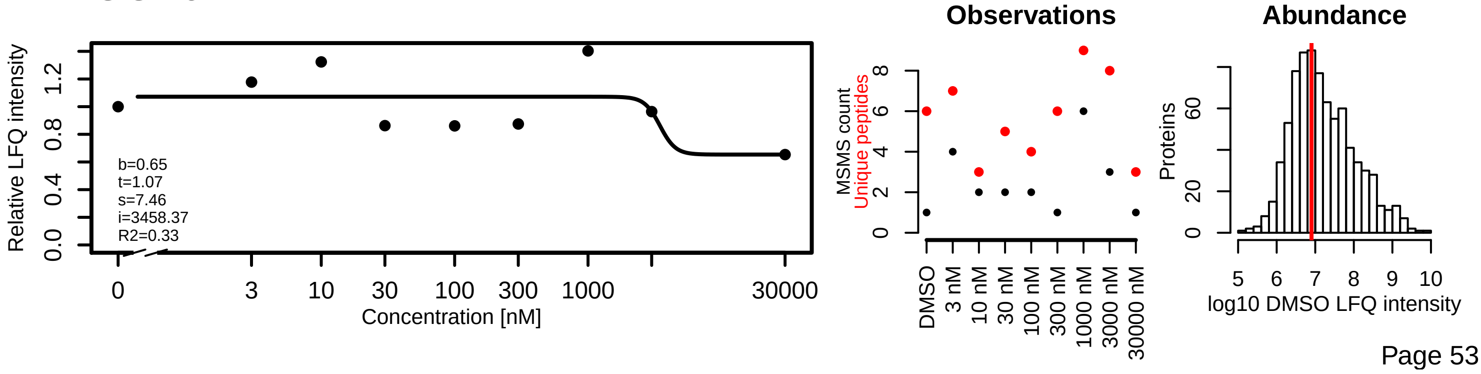

# MAP4K2

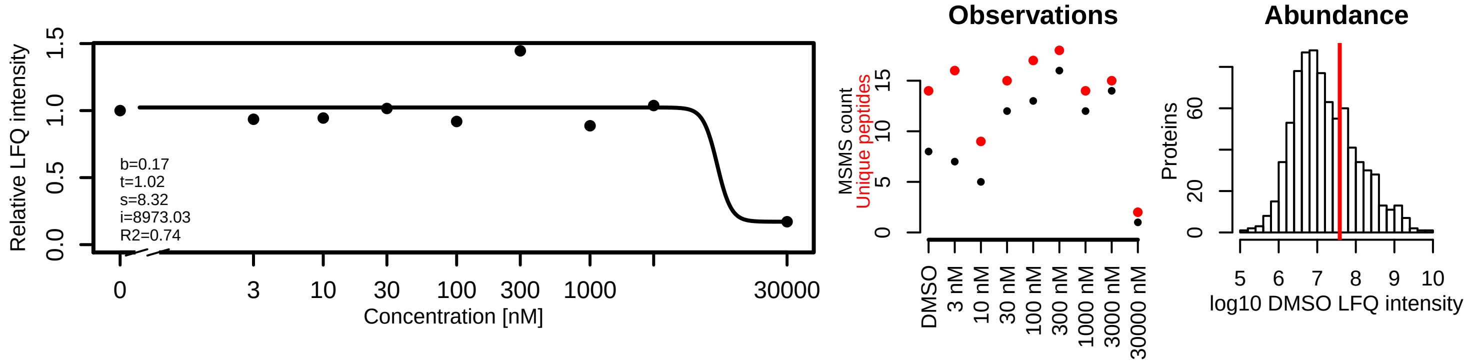


# STK35

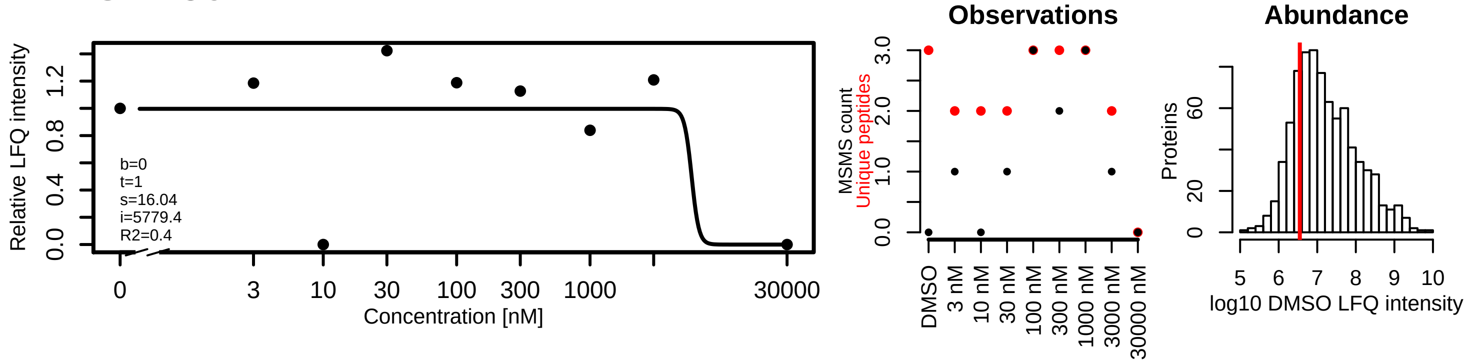


# RPL4

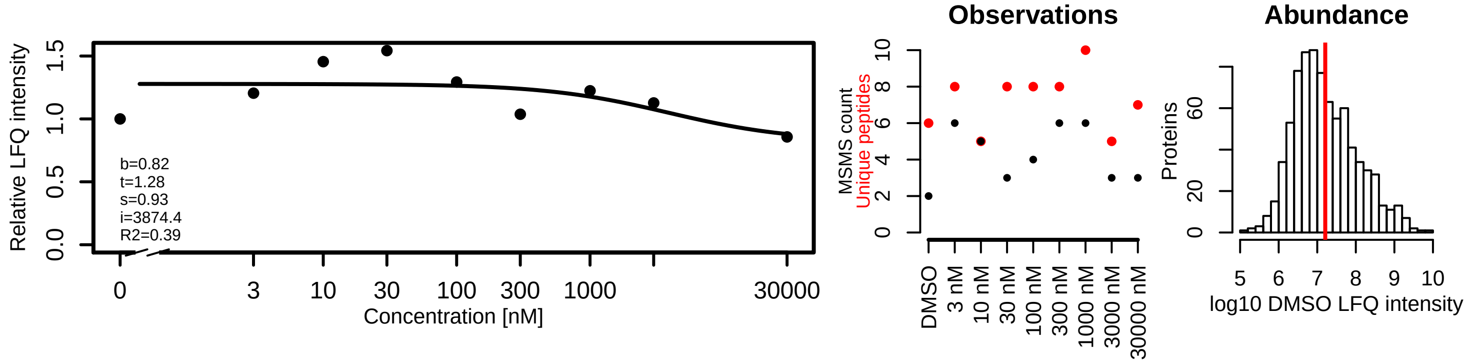


# VAPB

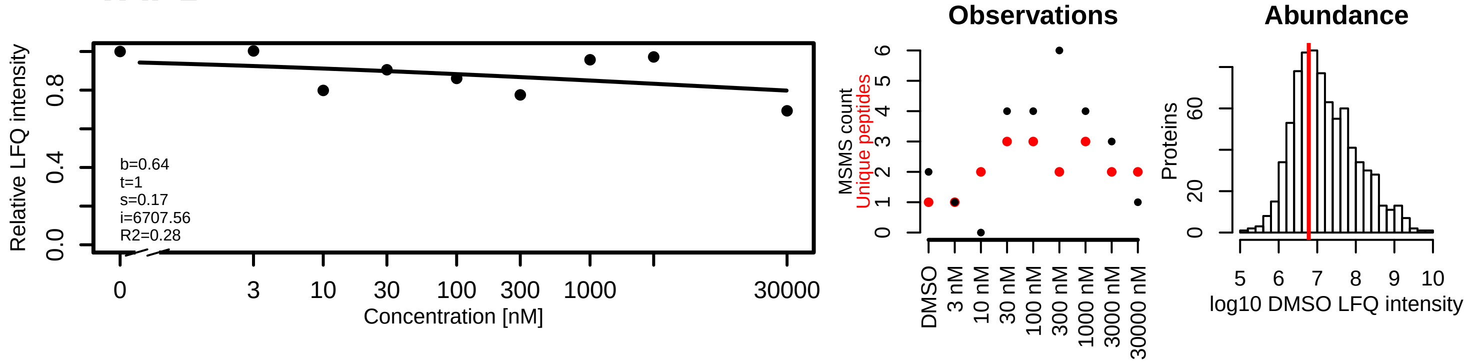


# DYNC1H1

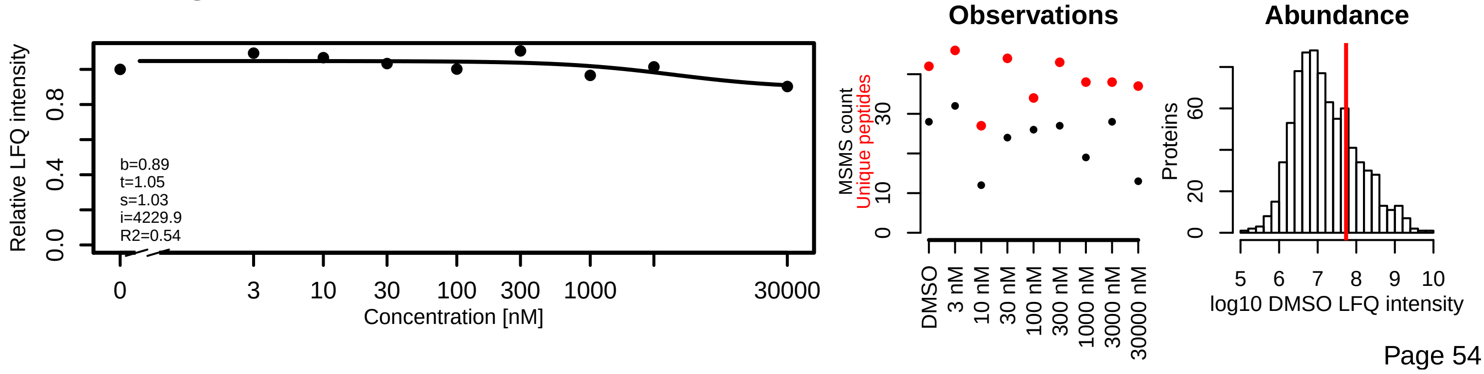

# GANAB

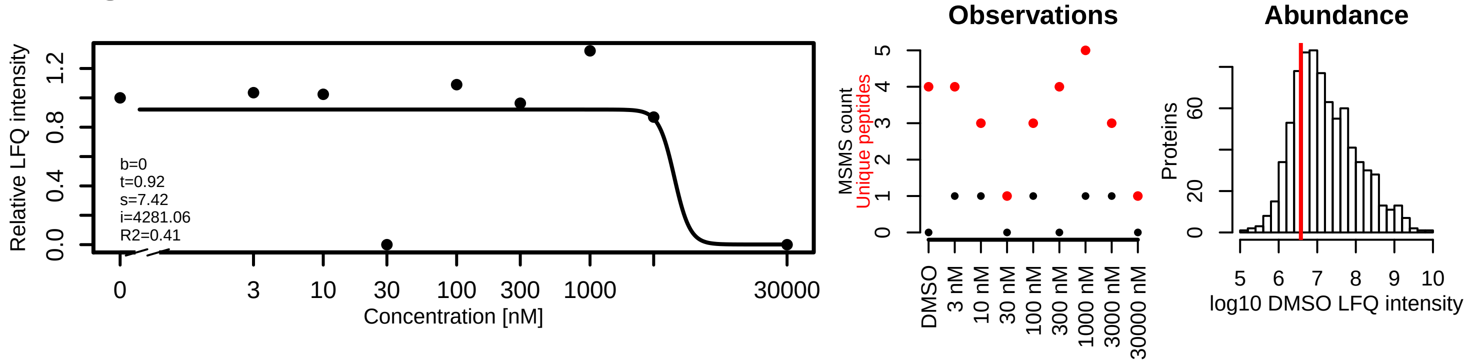


# RPL9

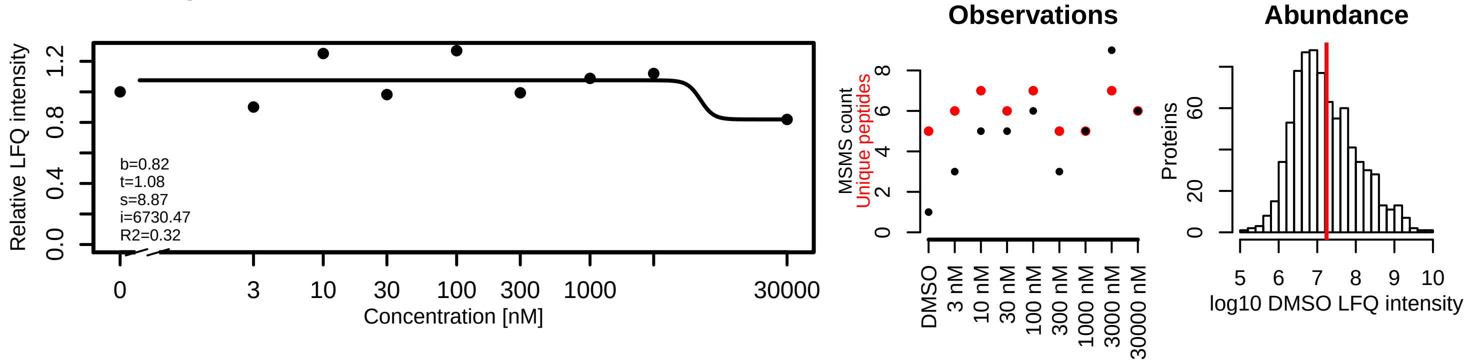


# PSMC5

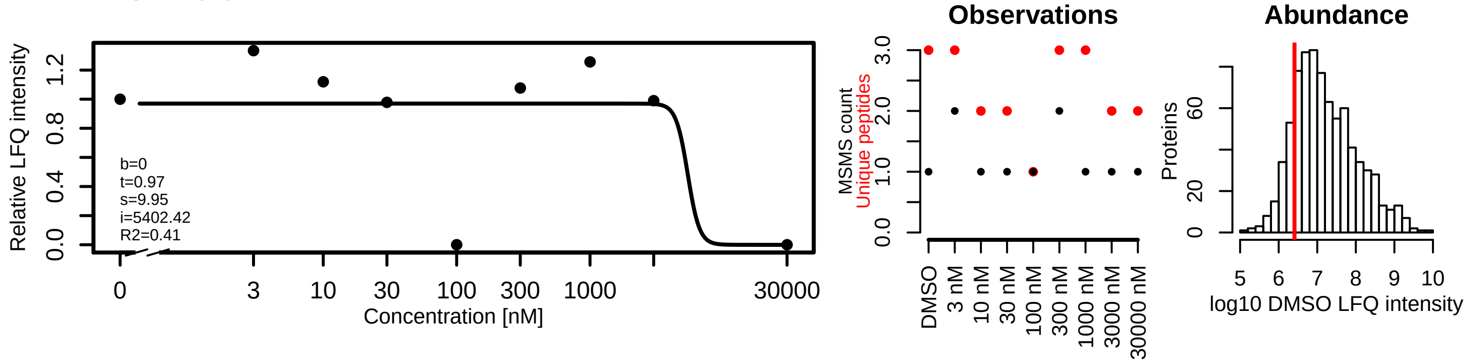


# SCAMP4

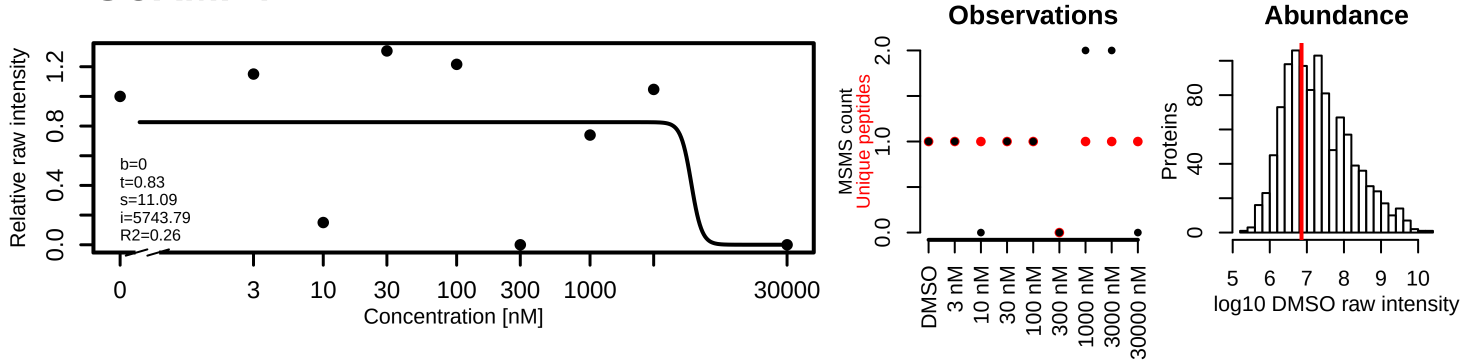


# BAX

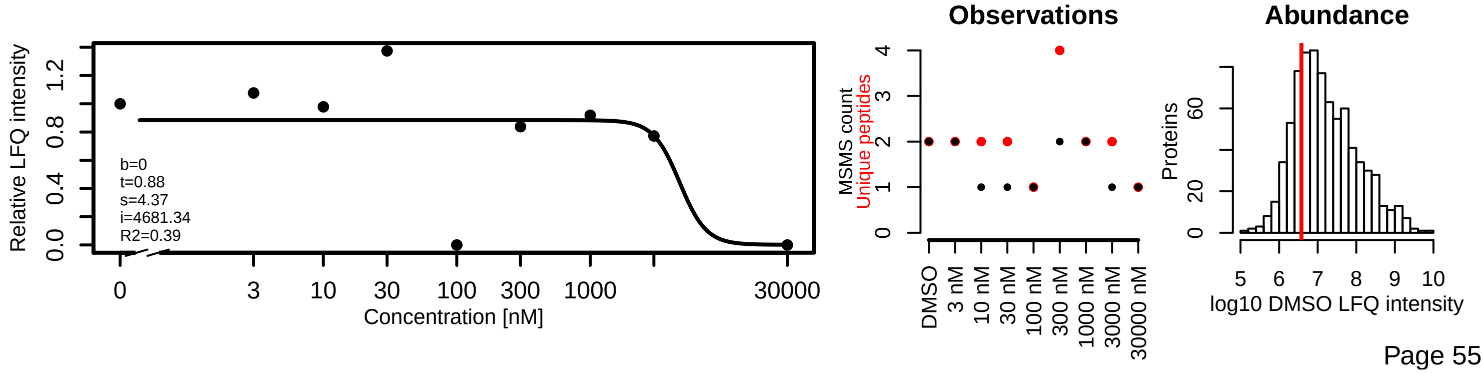

# NAP1L1

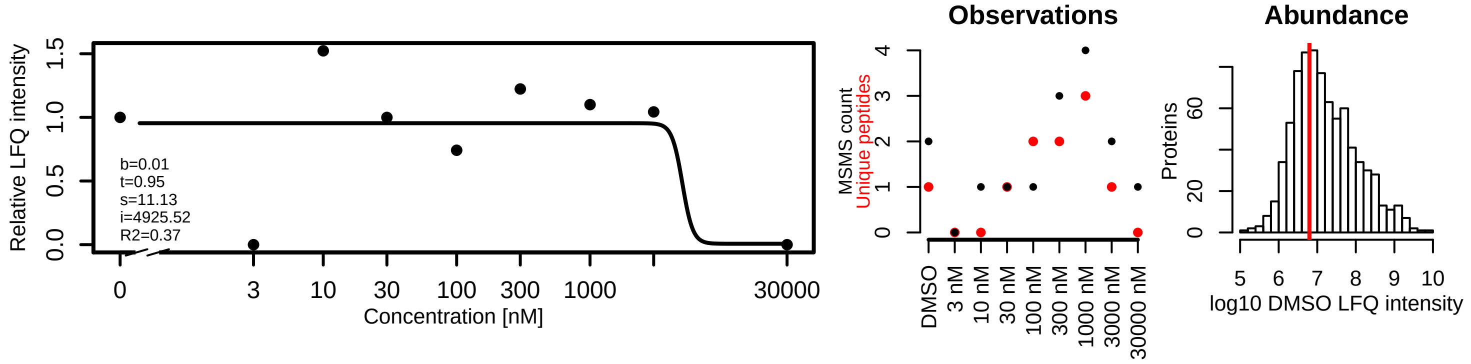


# LGMN

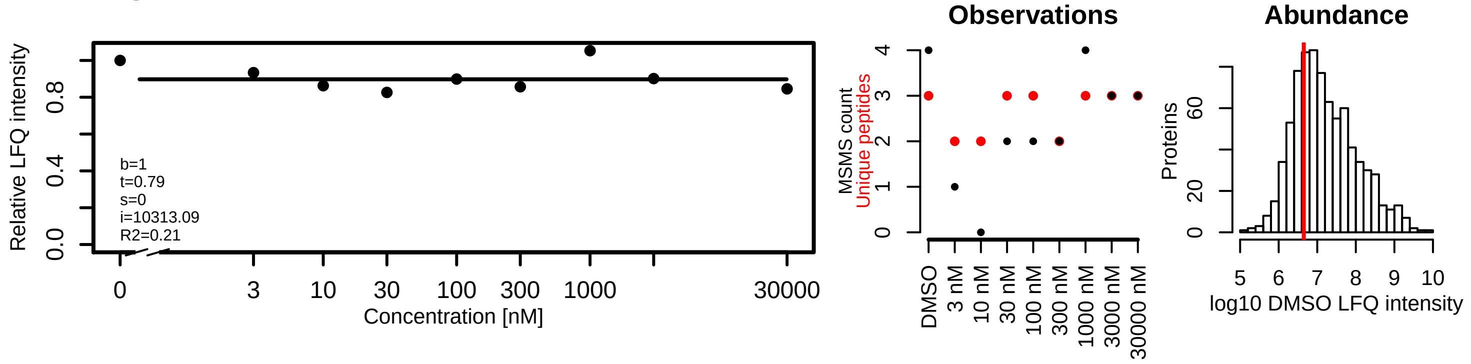


# RPL27A

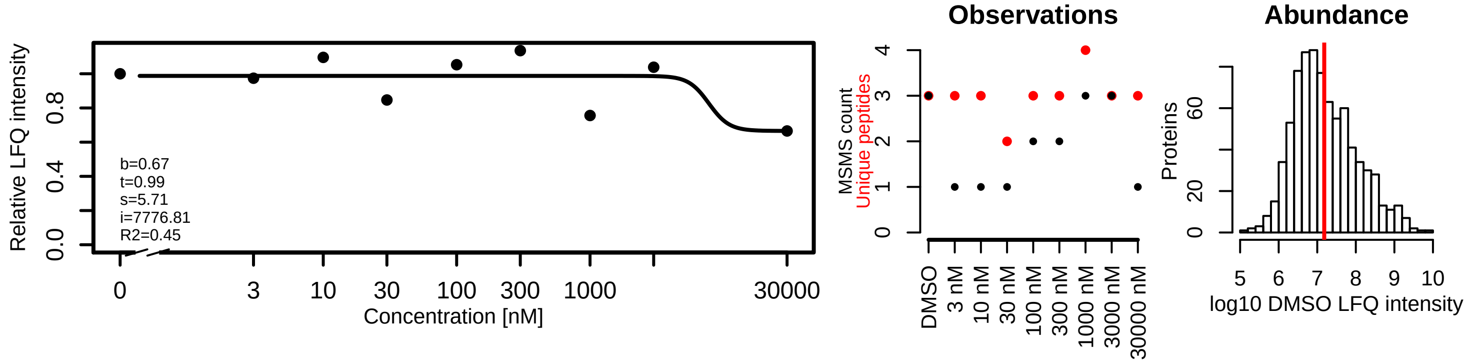


# EPT1

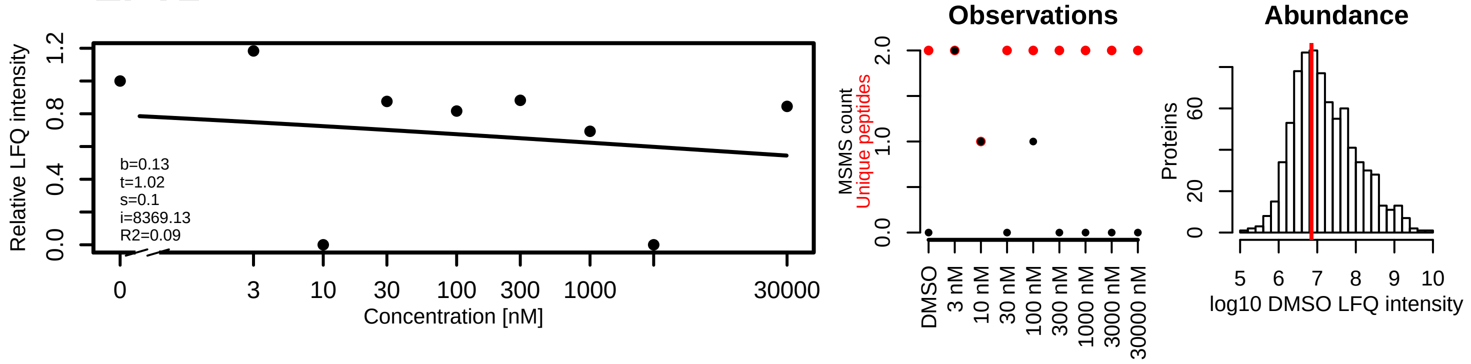


# PRPF19

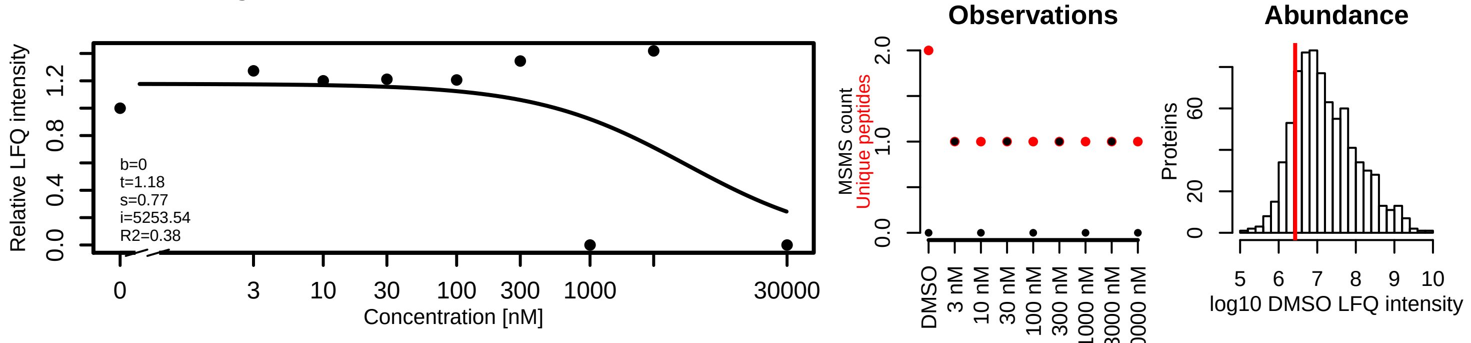

# MAP2K1

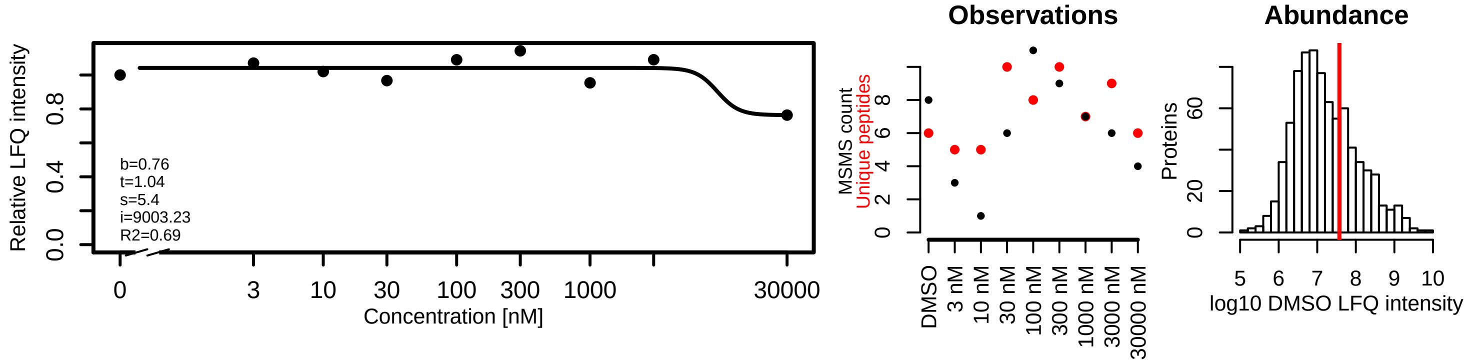


# HSPA9

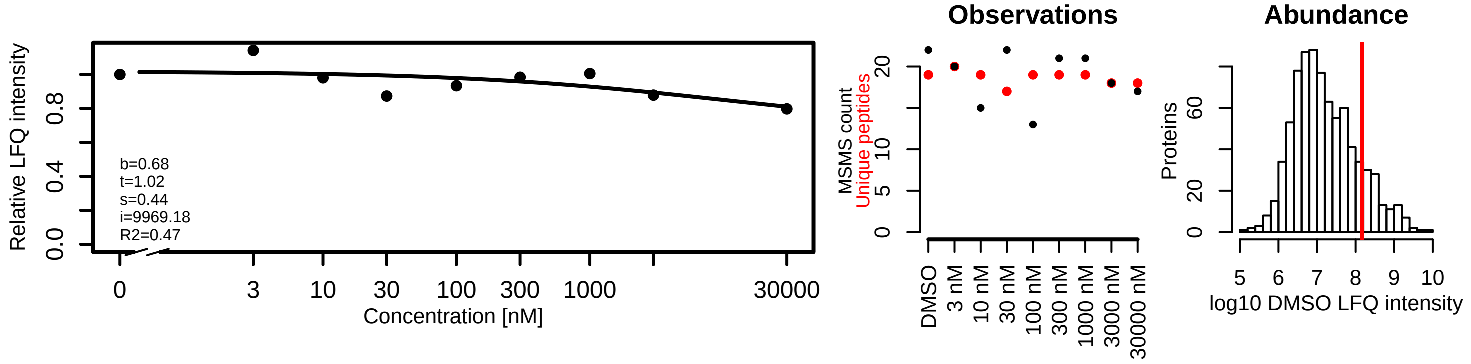


# TOMM22

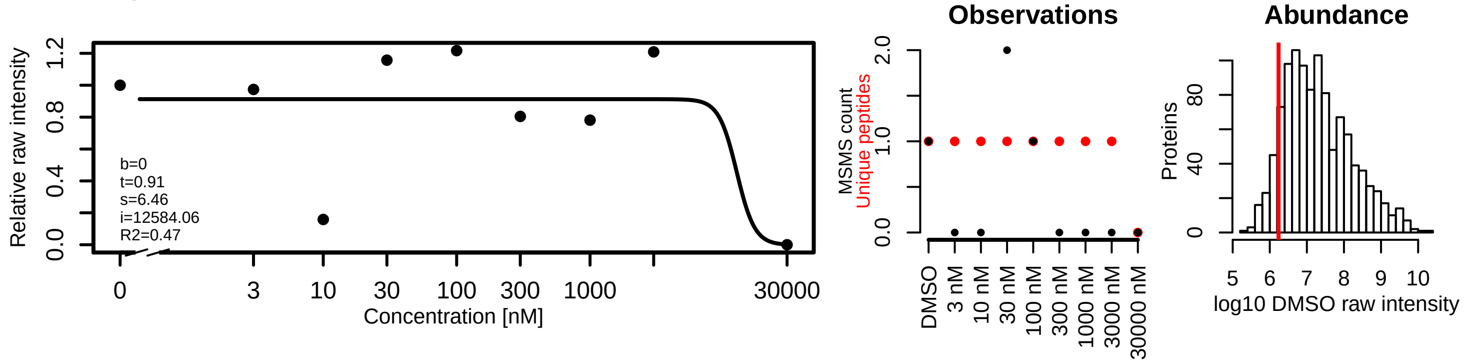


# SYNGR1

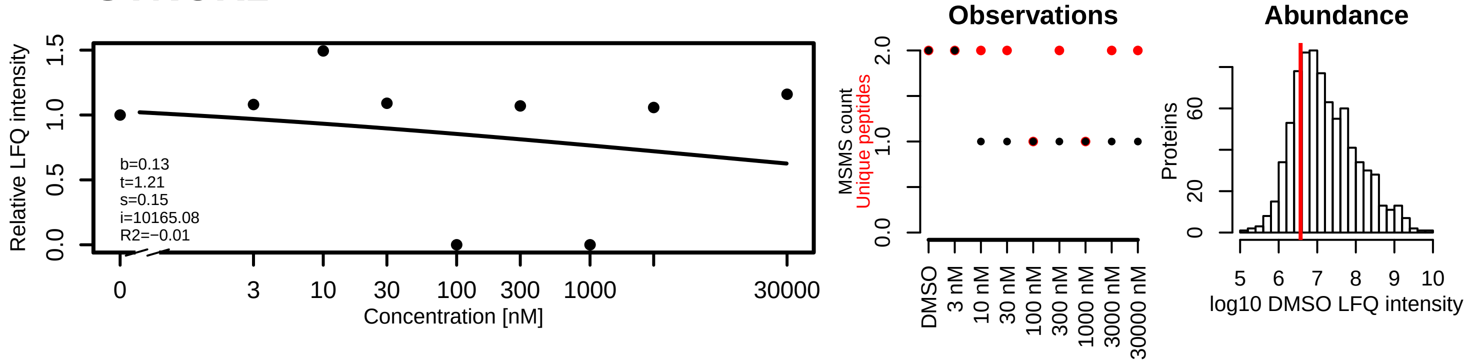


# ARL6IP5

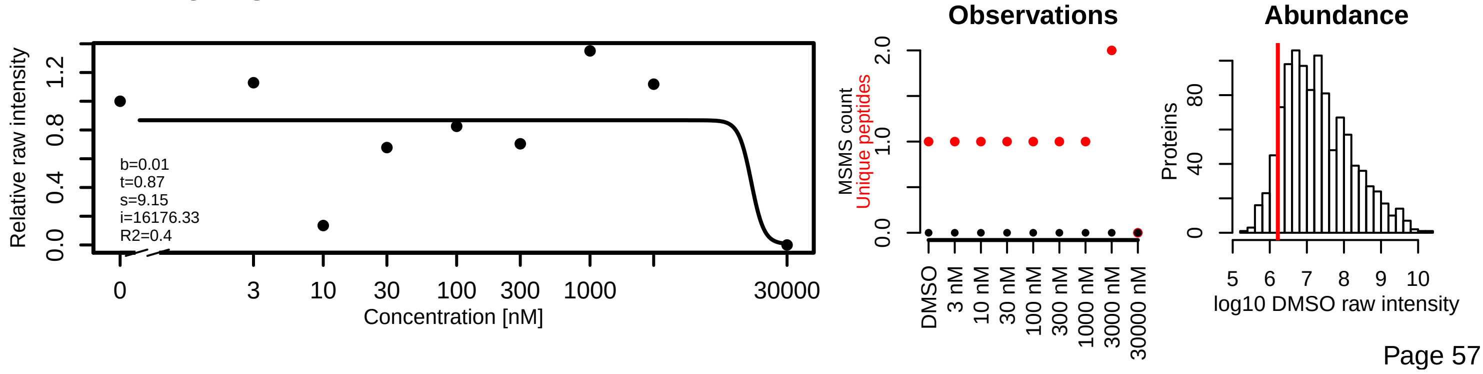

## DYNC1LI2

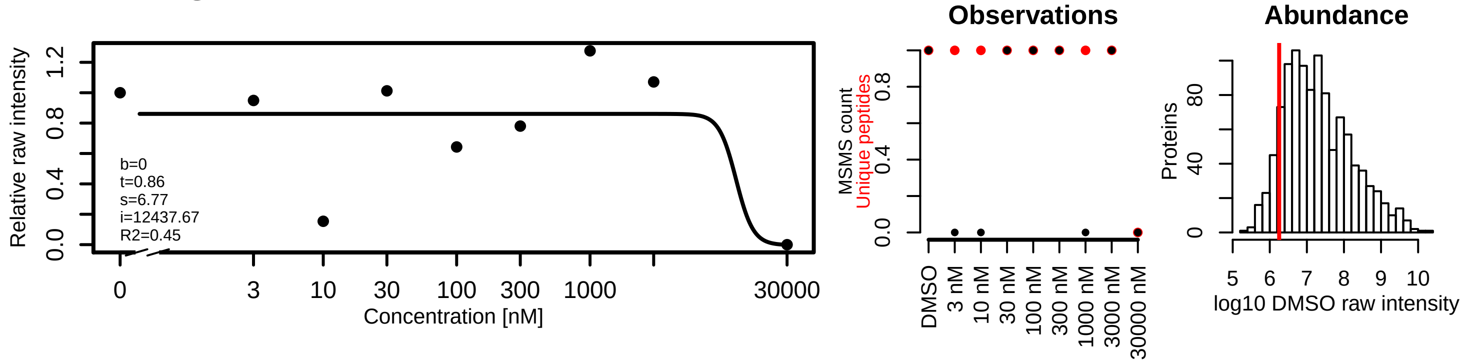


## CAND1

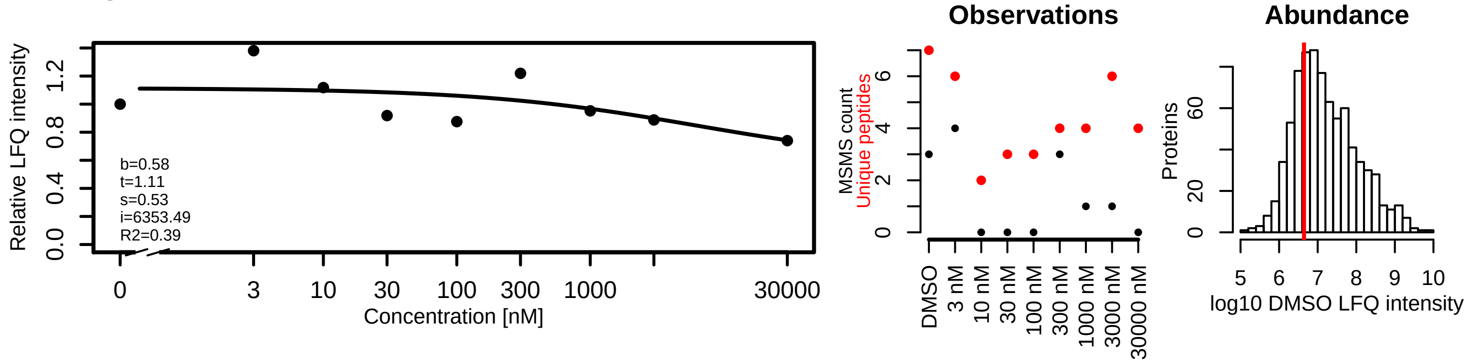


## RAB7A

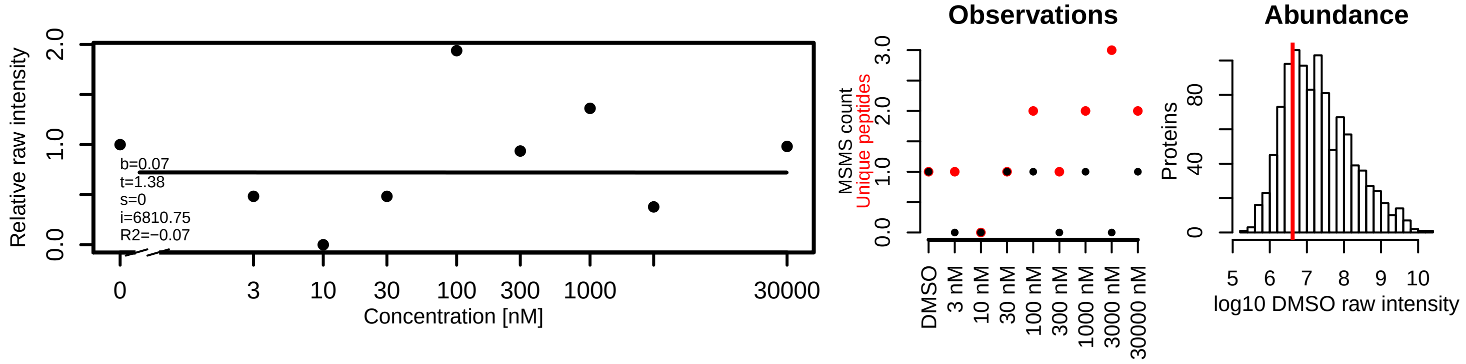


## CAPZB

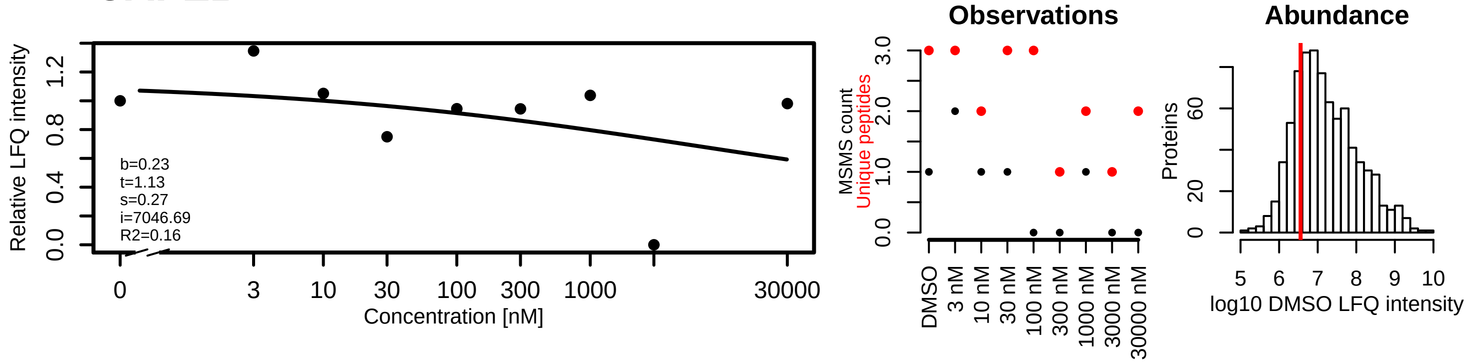


## RUVBL2

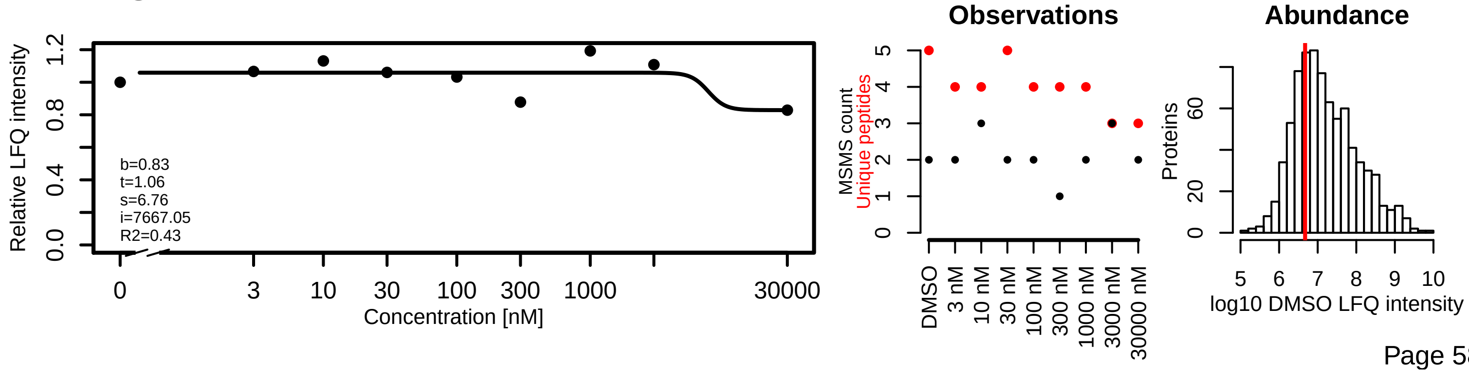

# RPS21

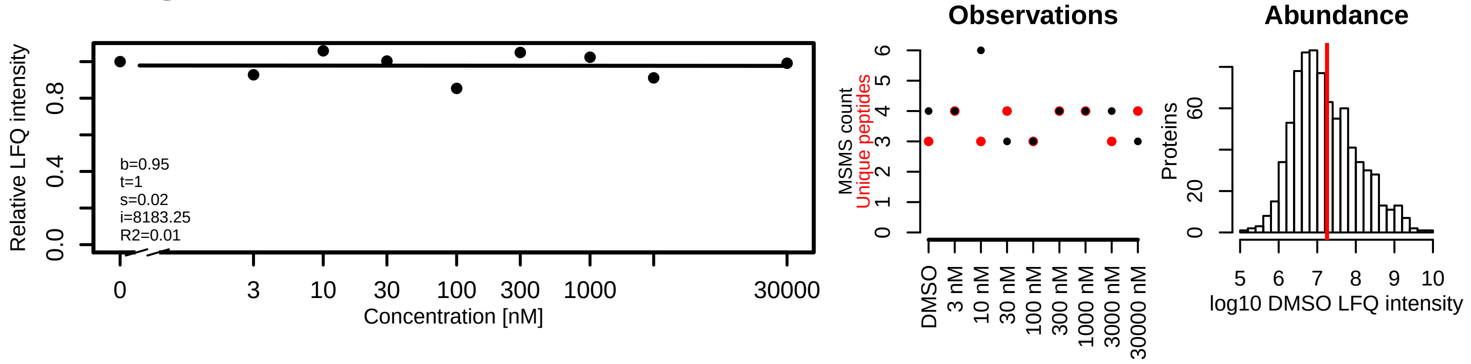


# PSMA2

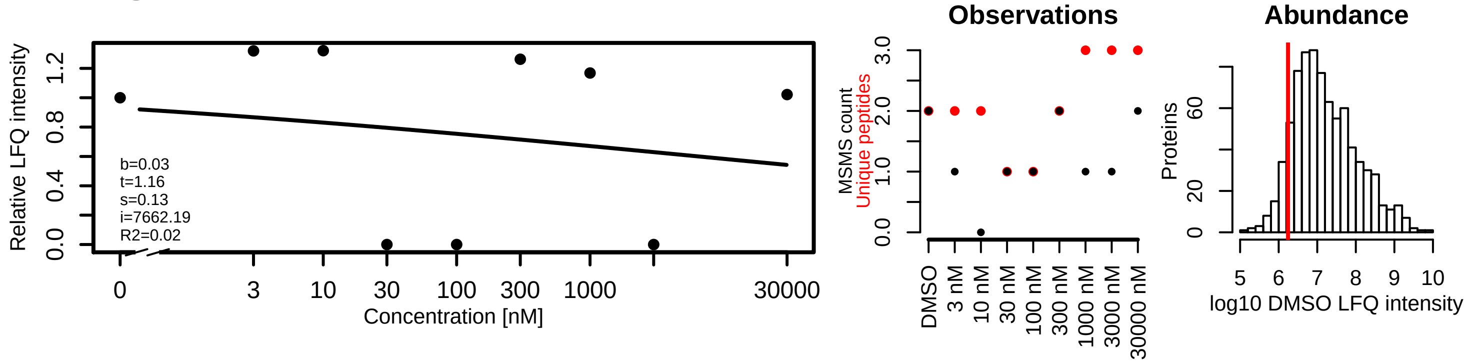


# RPL35

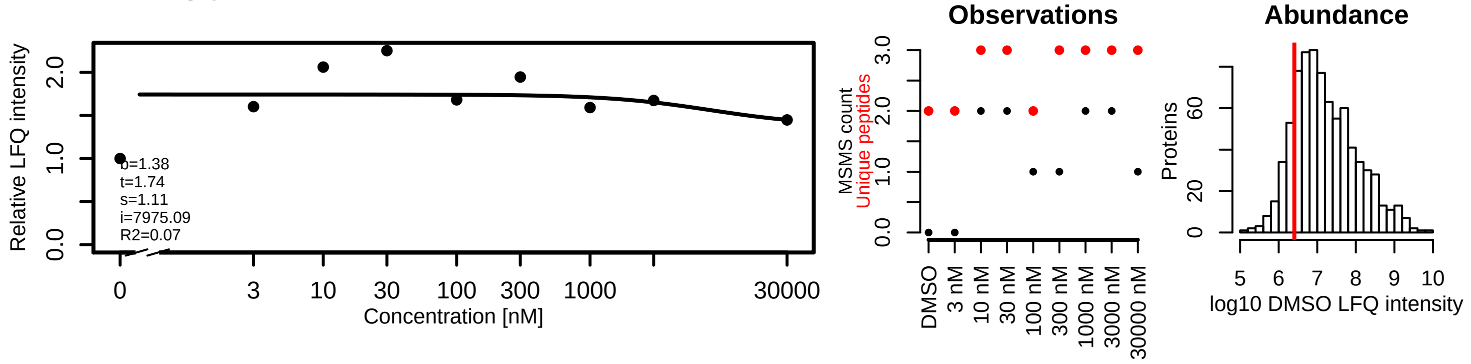


# SEC23A

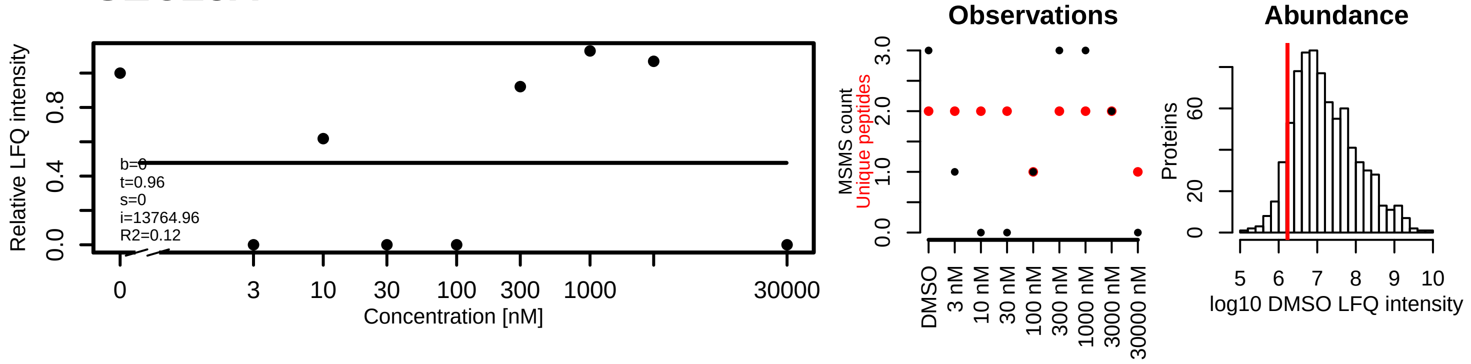


# DCK

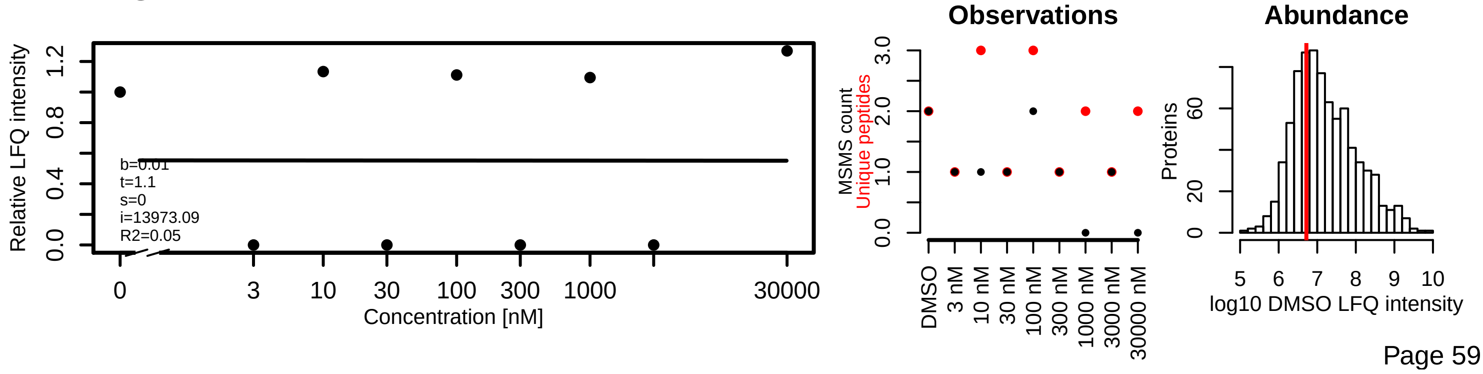

## CDS2

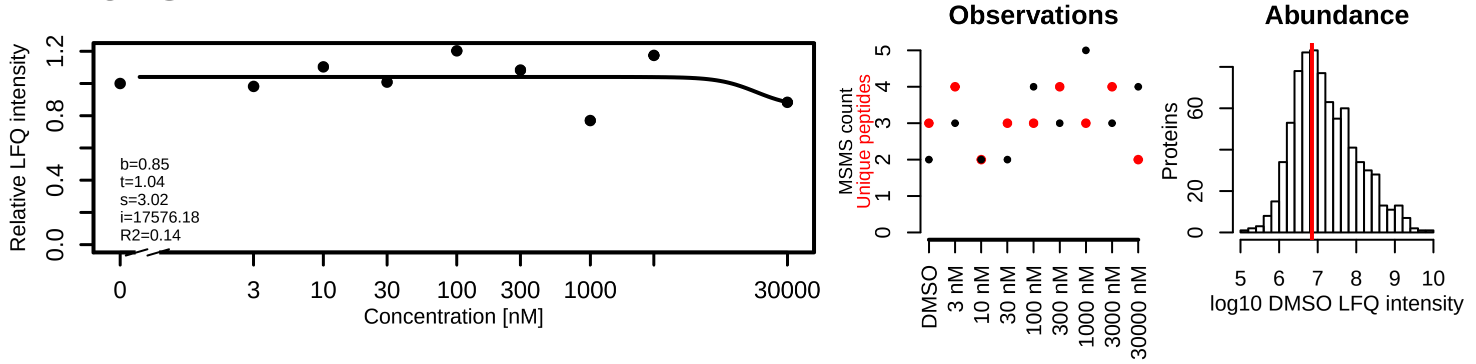


## LRP1

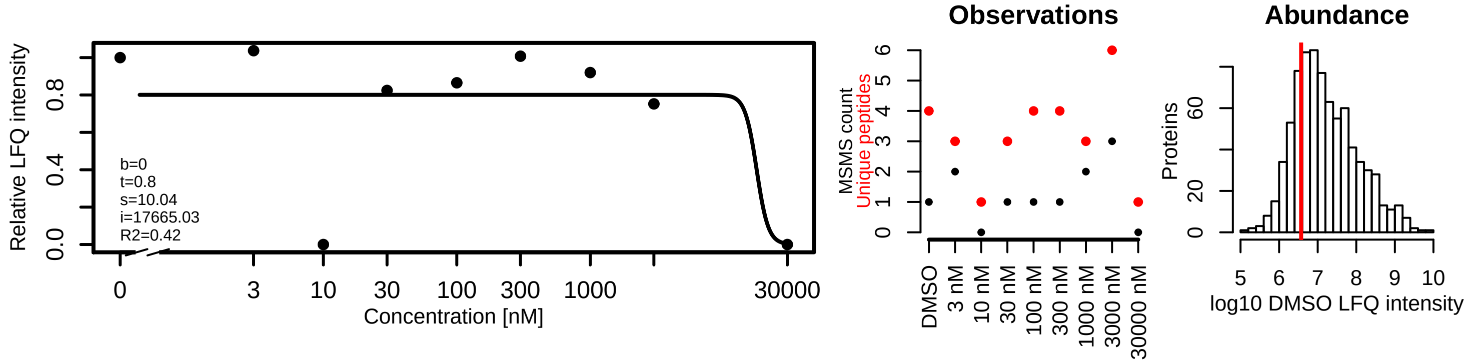


## RPL13A

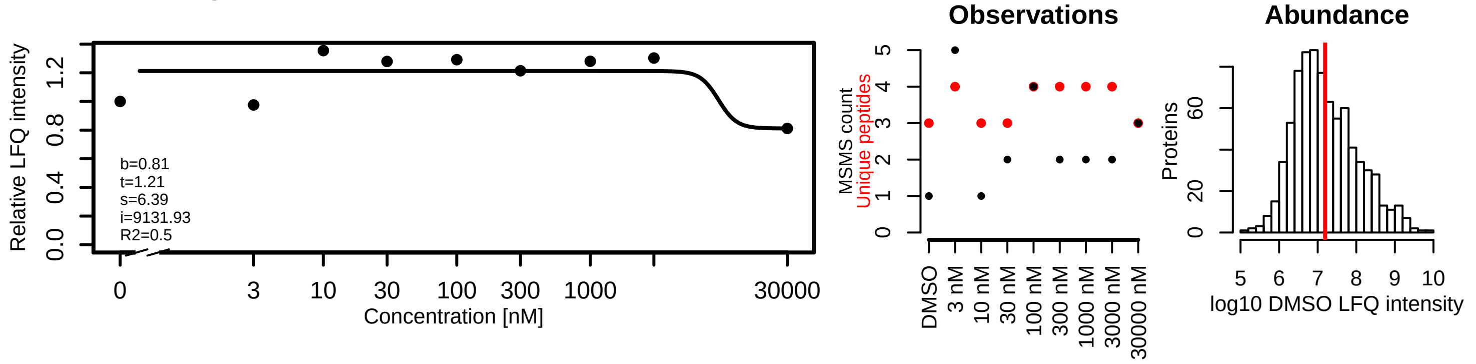


## SYK

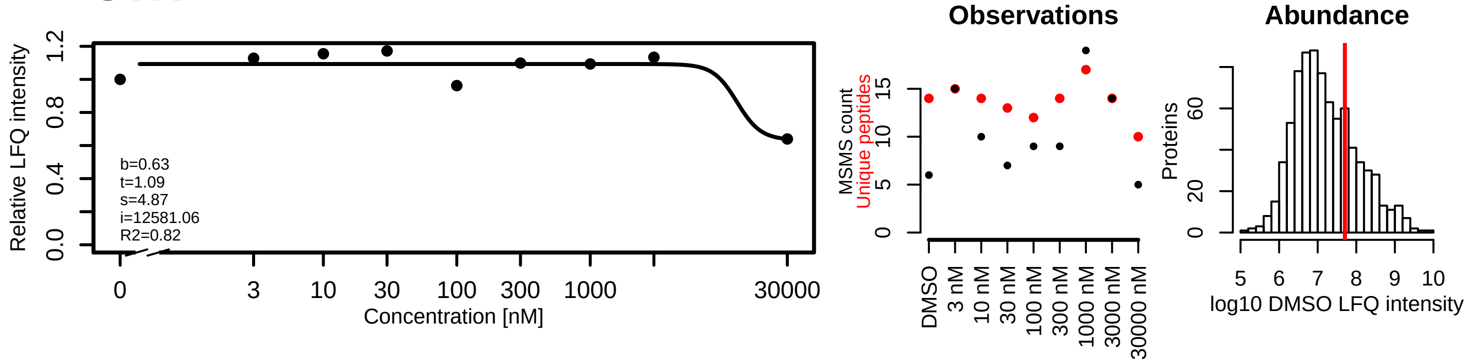


## COX6B1

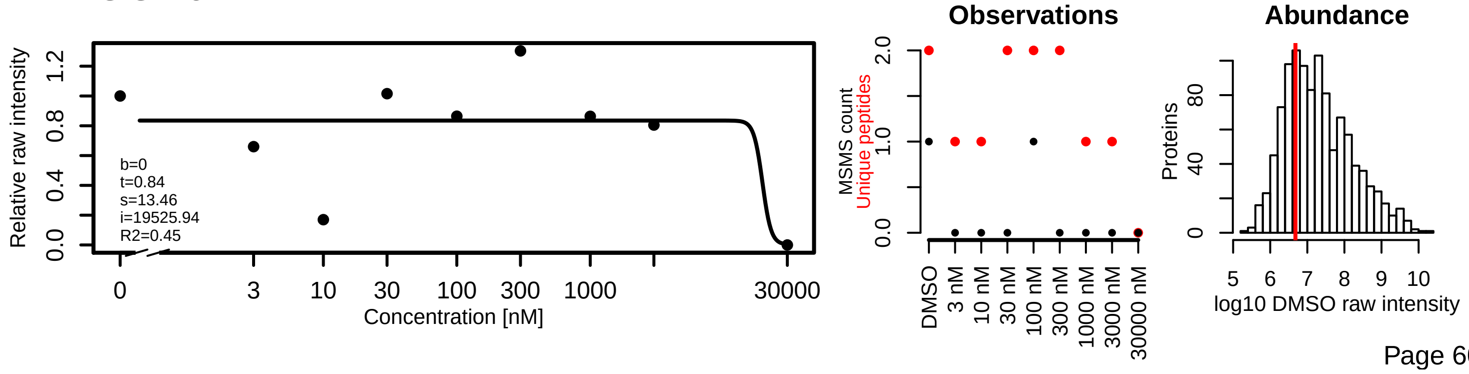

# NUP210

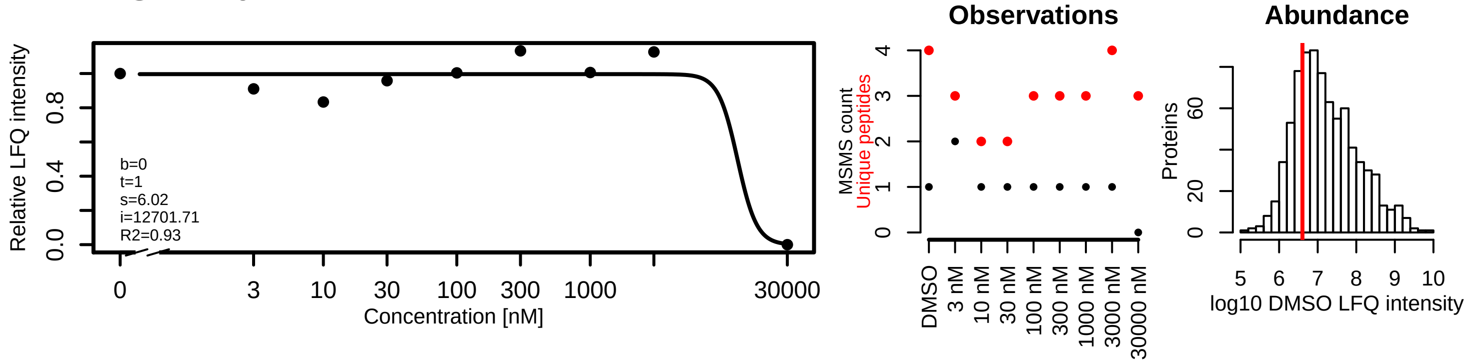


# MATR3

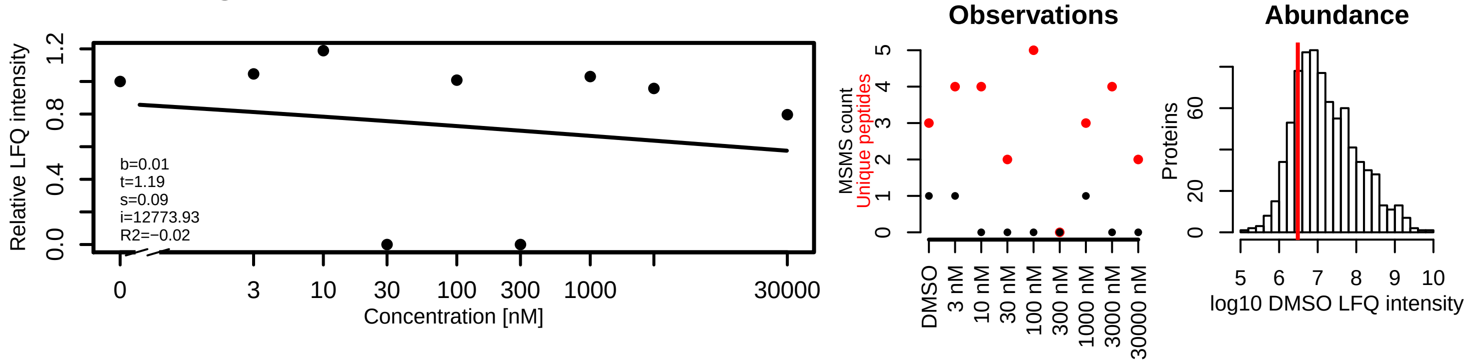


# COG5

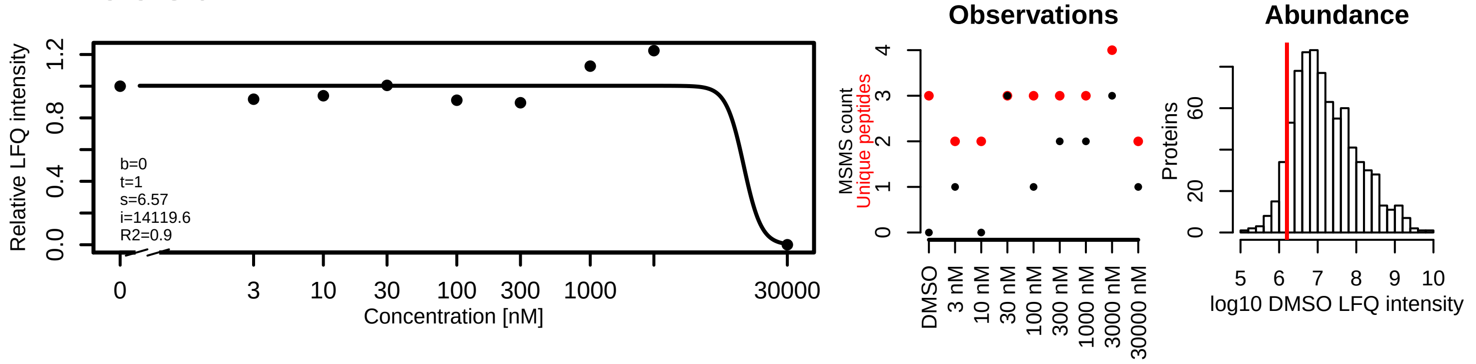


# IPO11

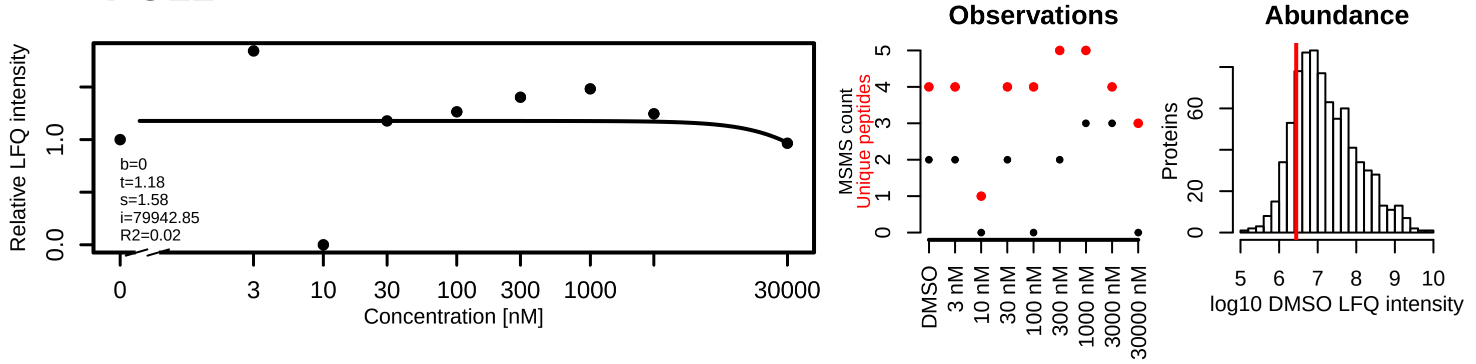


# SPTLC2

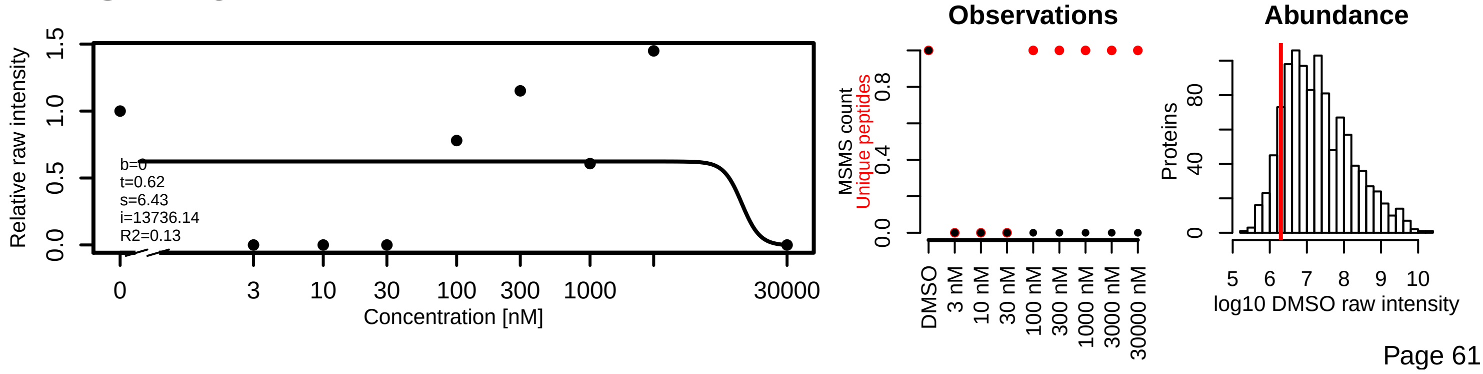

## NDUFV1

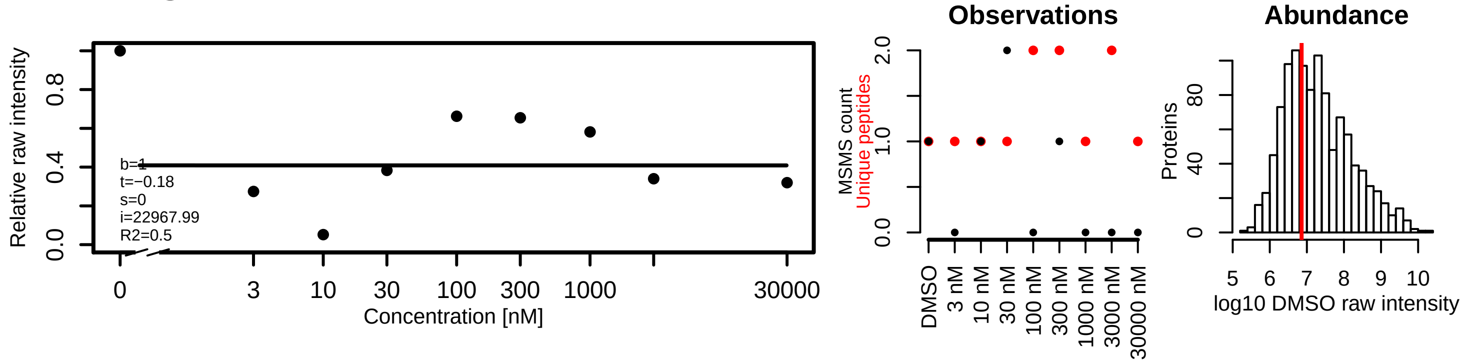


## EIF4A1;EIF4A2

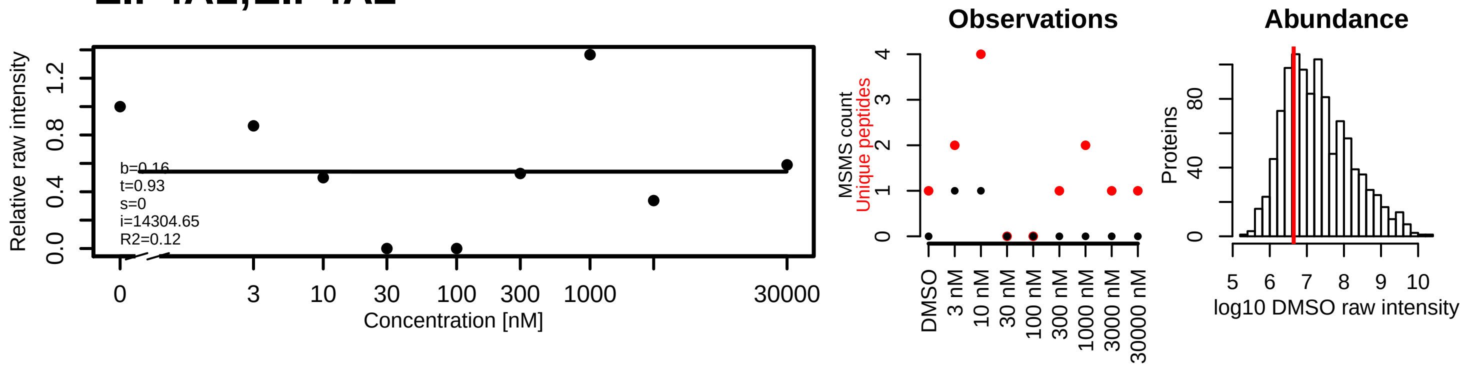


## EPDR1;UCC1

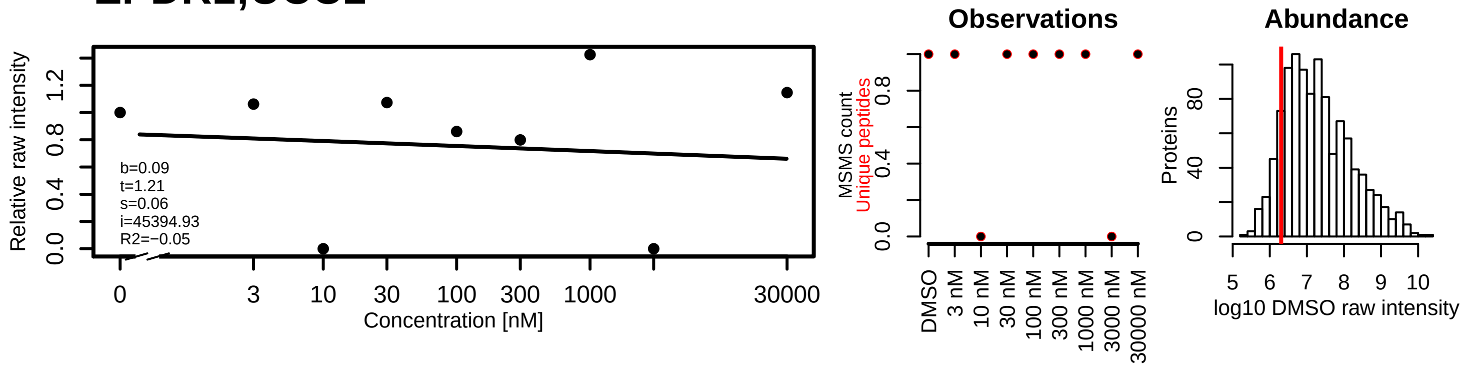


## DERL1

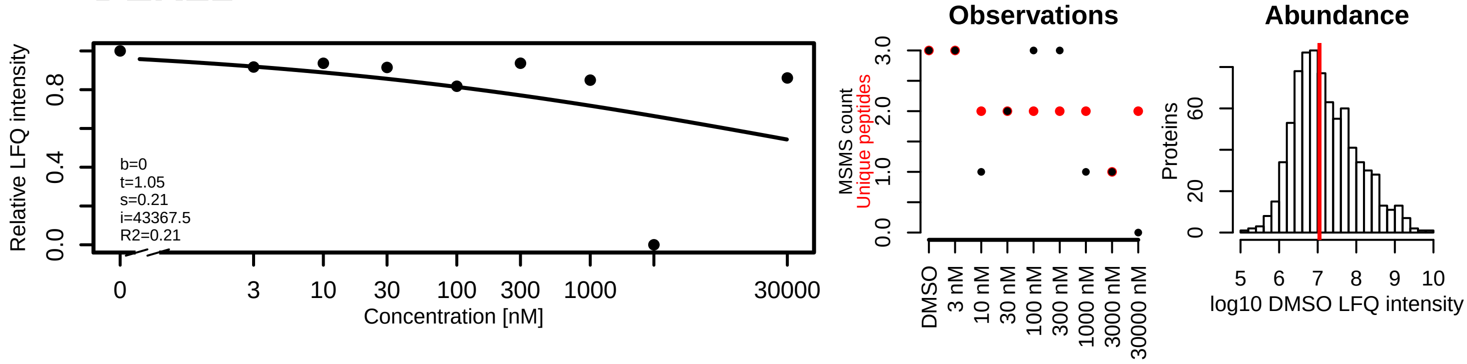


## TPM3;TPM4

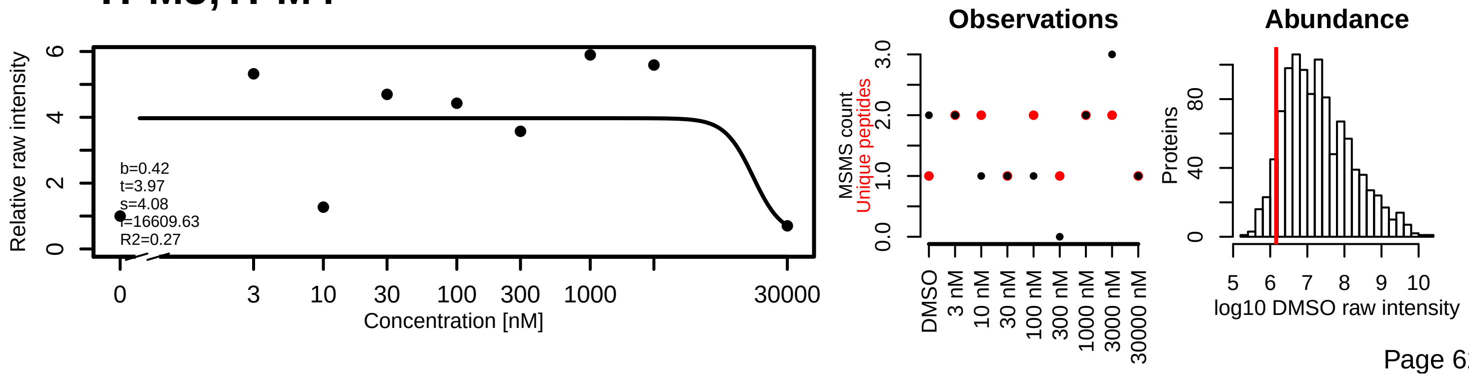

## LURAP1

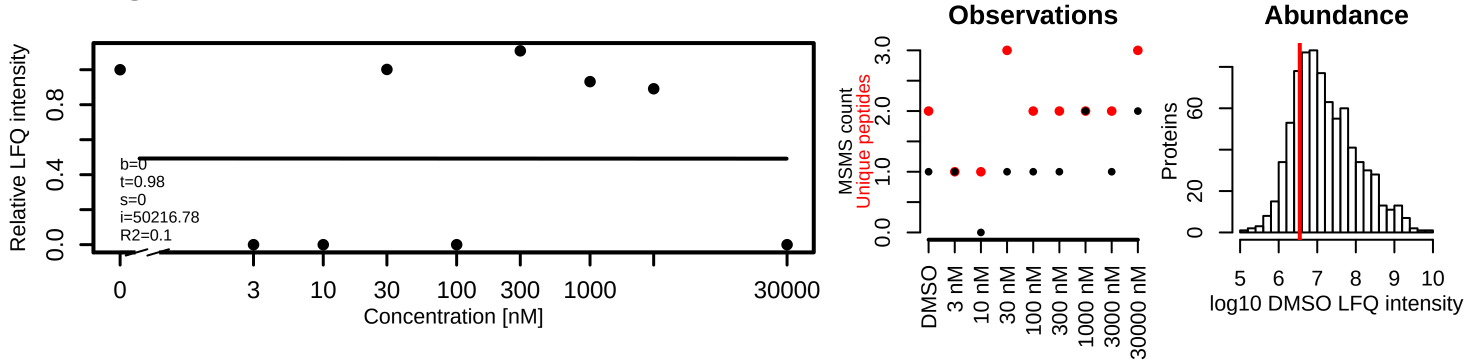


## SLC25A4

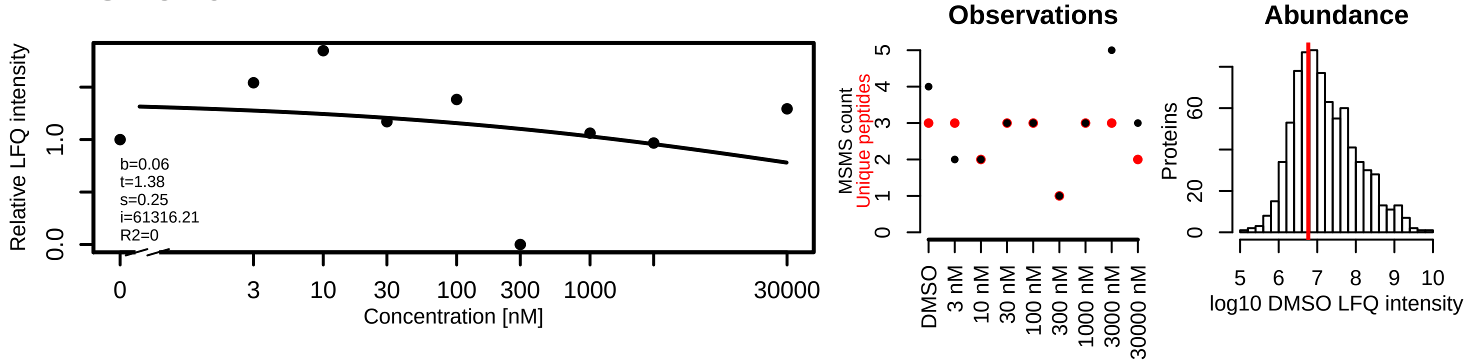


## LDHB

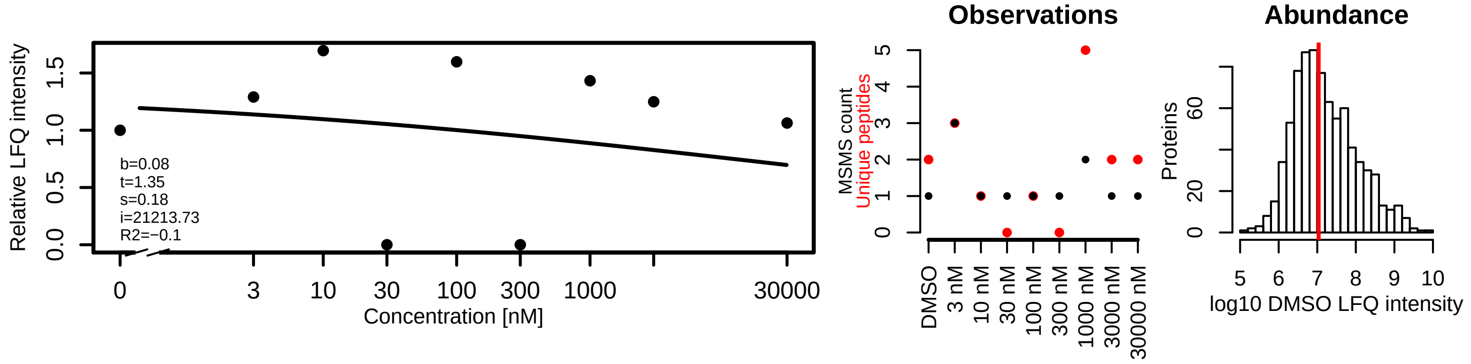


## TM9SF1

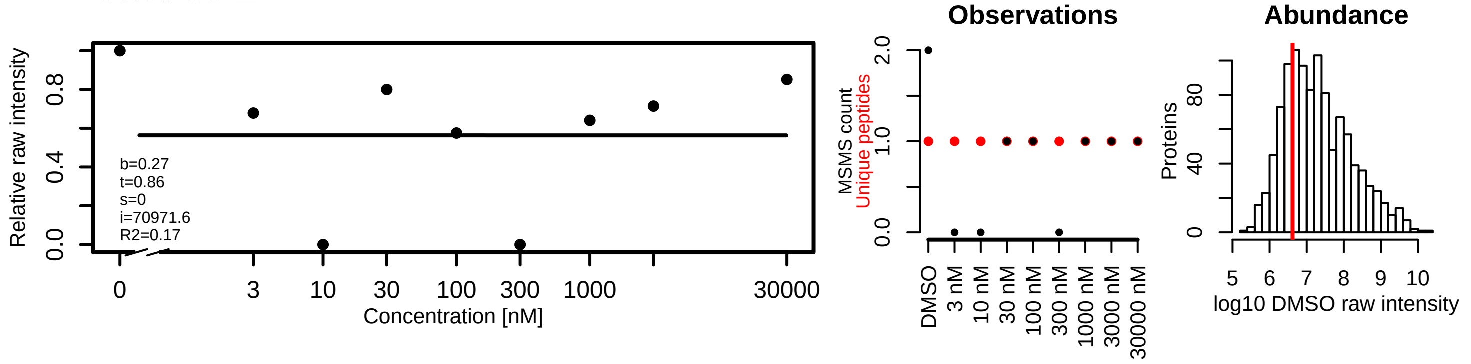


## TMEM209

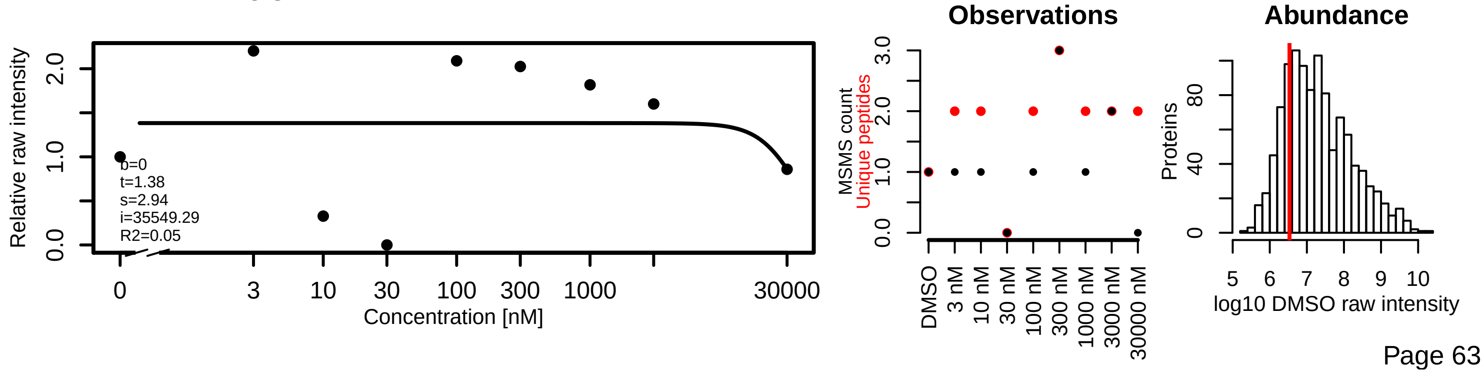

# PRSS3

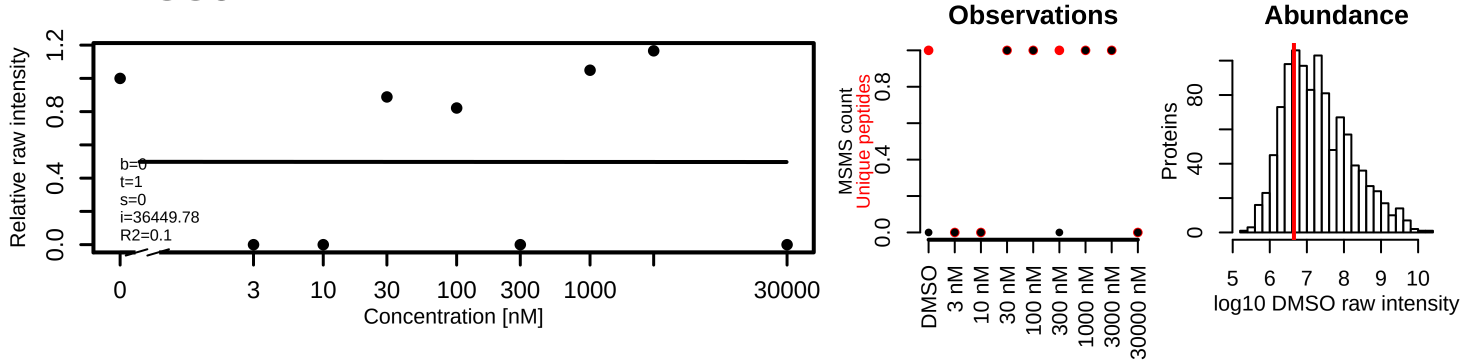


# TFRC

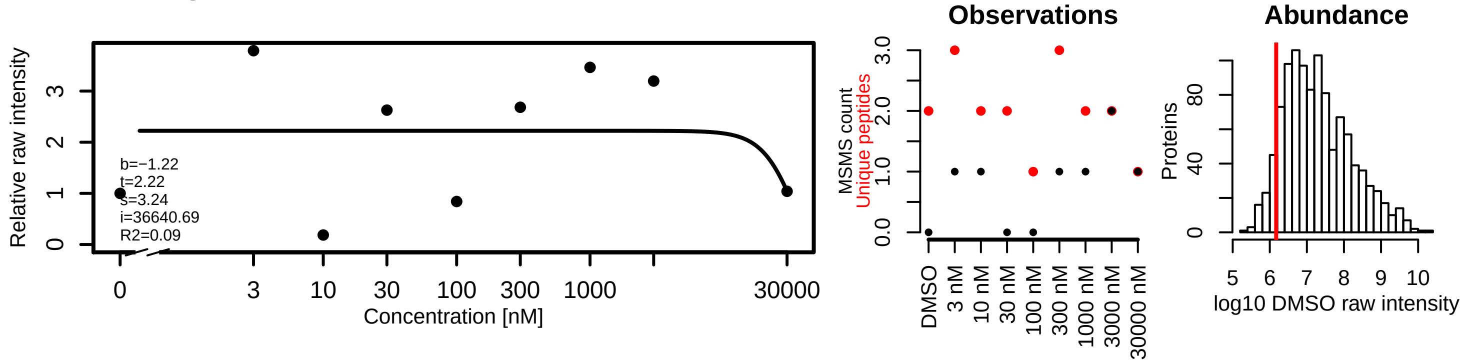


# TUBB2A;TUBB2B

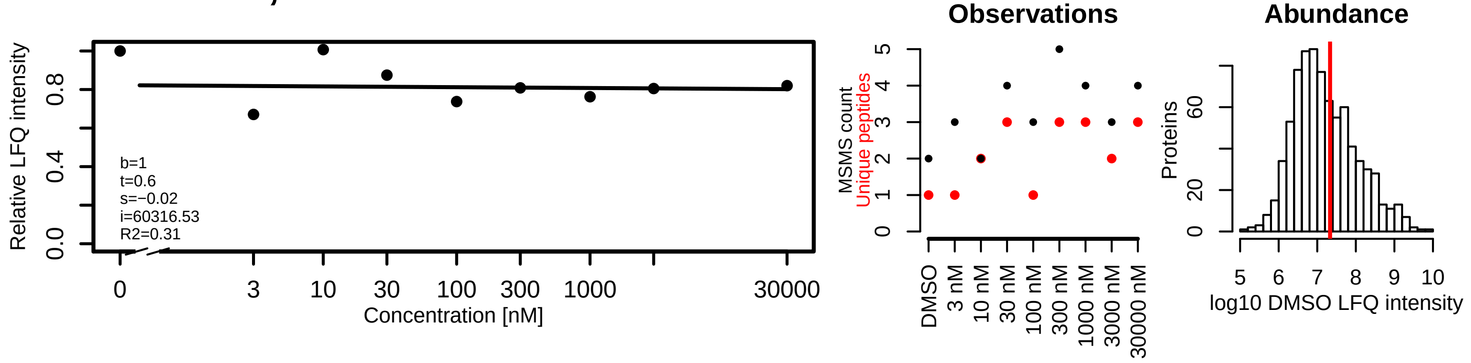


# MYOF

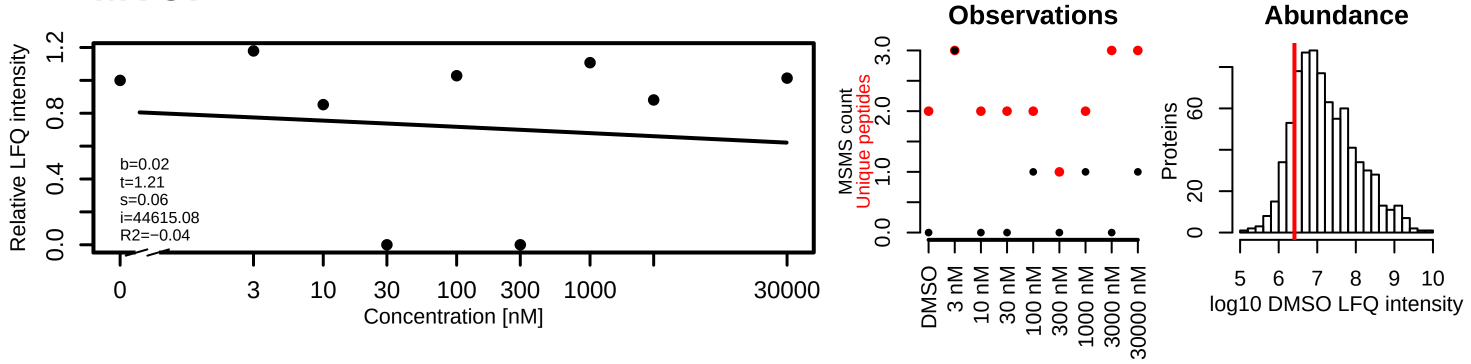


# NONO

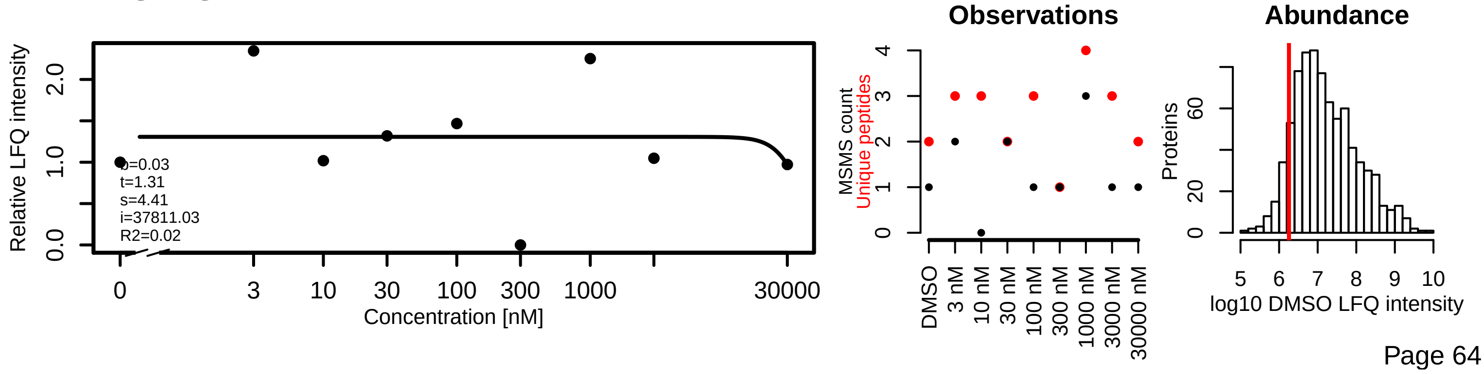

# UQCRC2

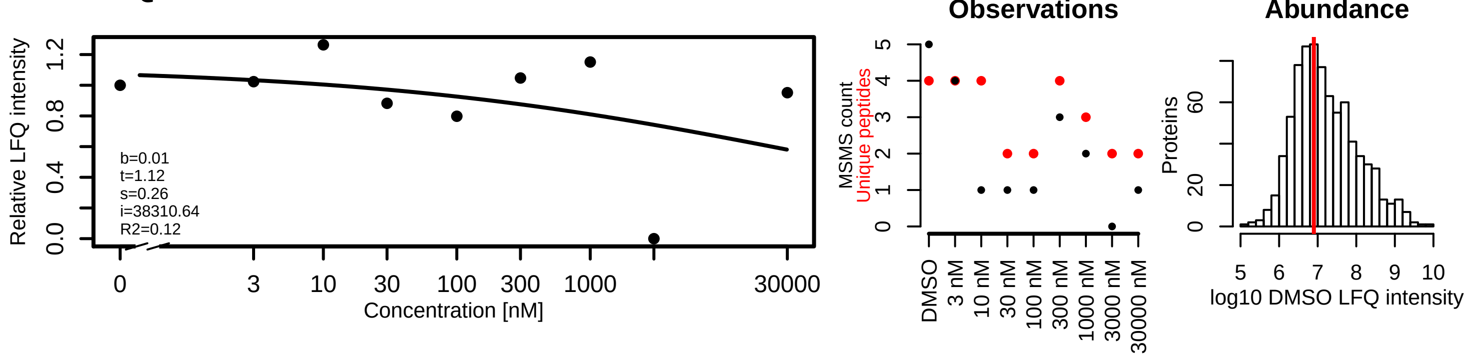


# TMED9

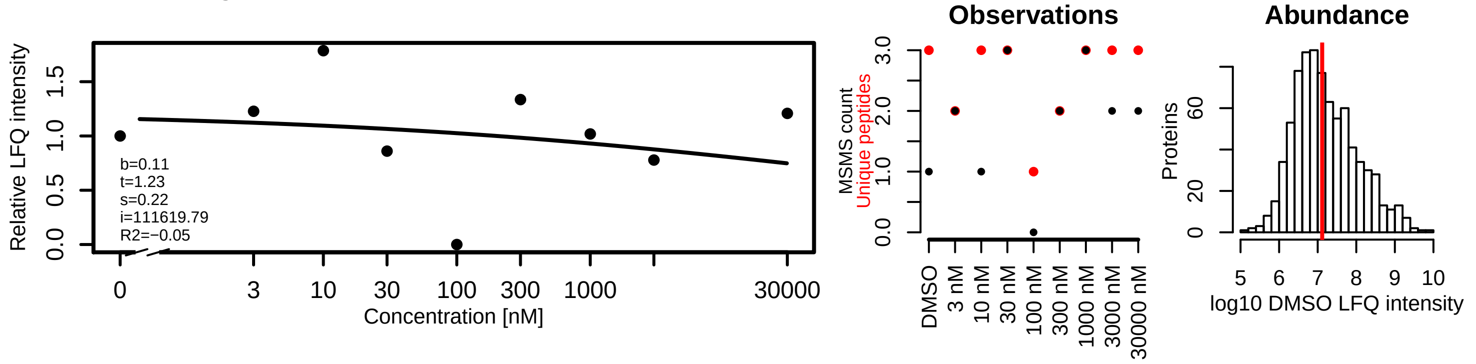


# SLC25A24

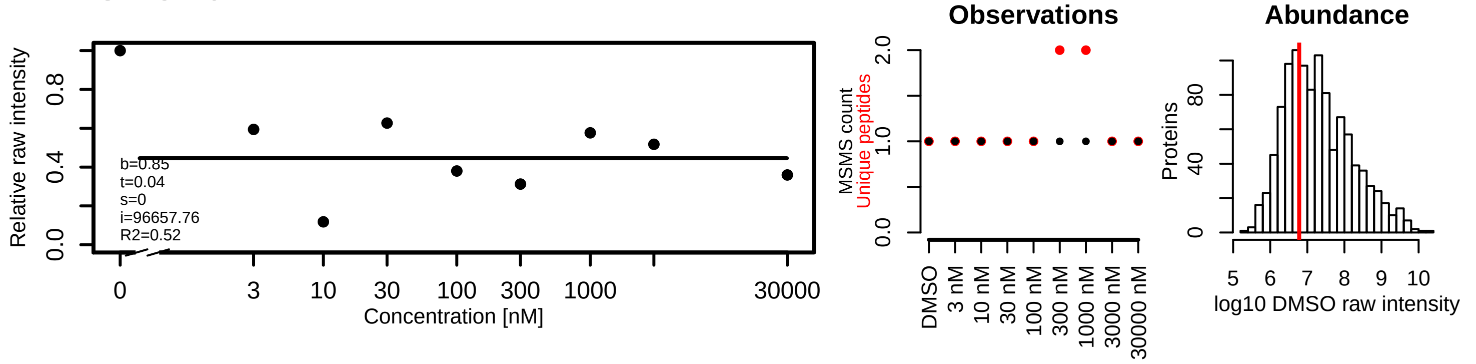


# PAICS

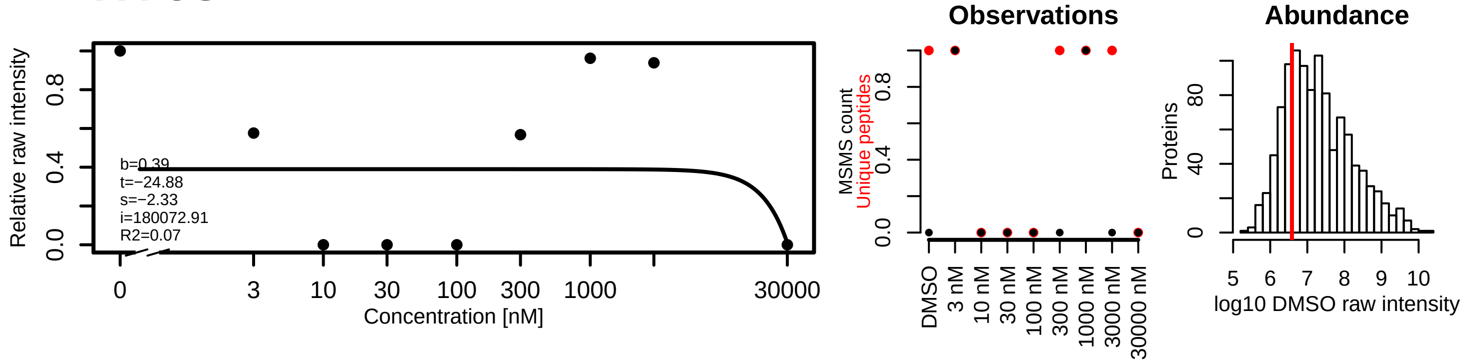


# KRT6B

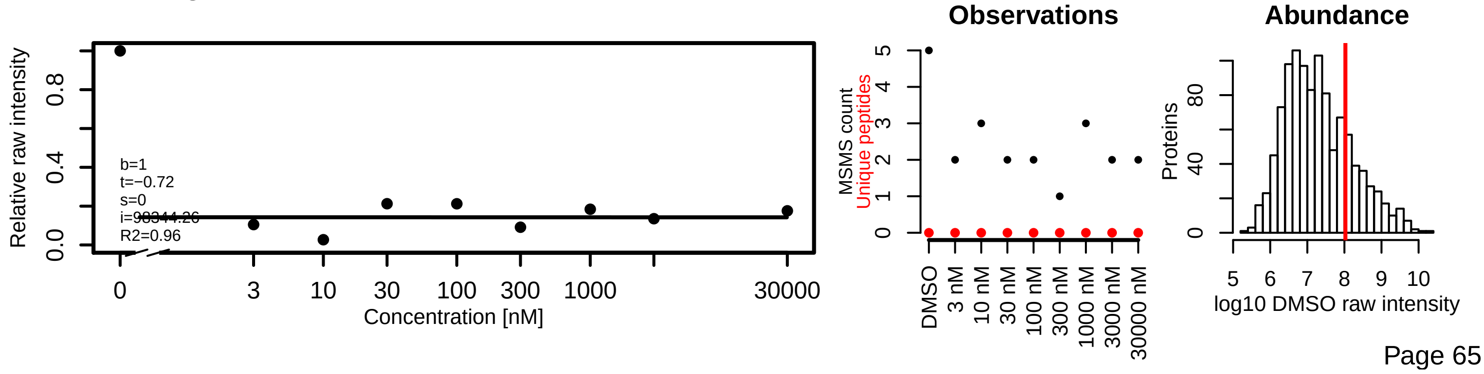

# CDK4

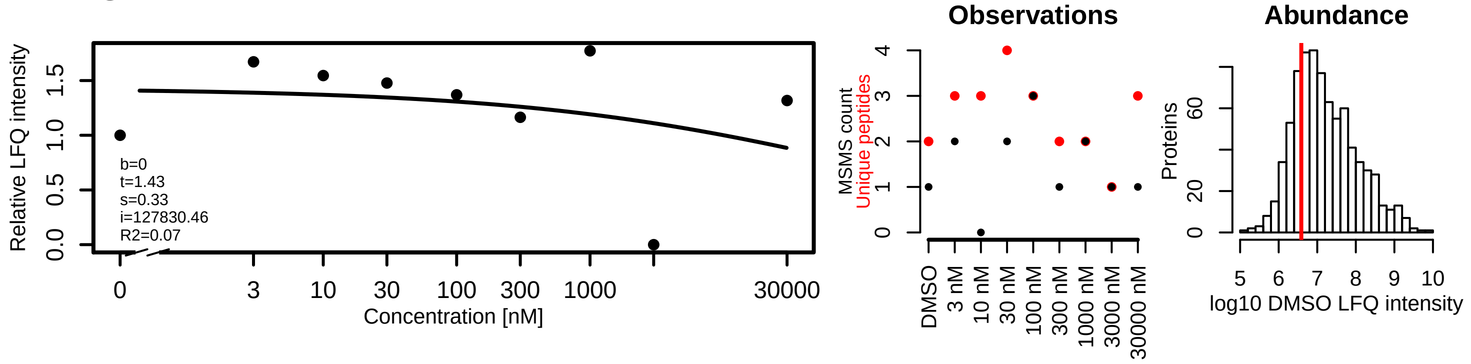


# NQO1

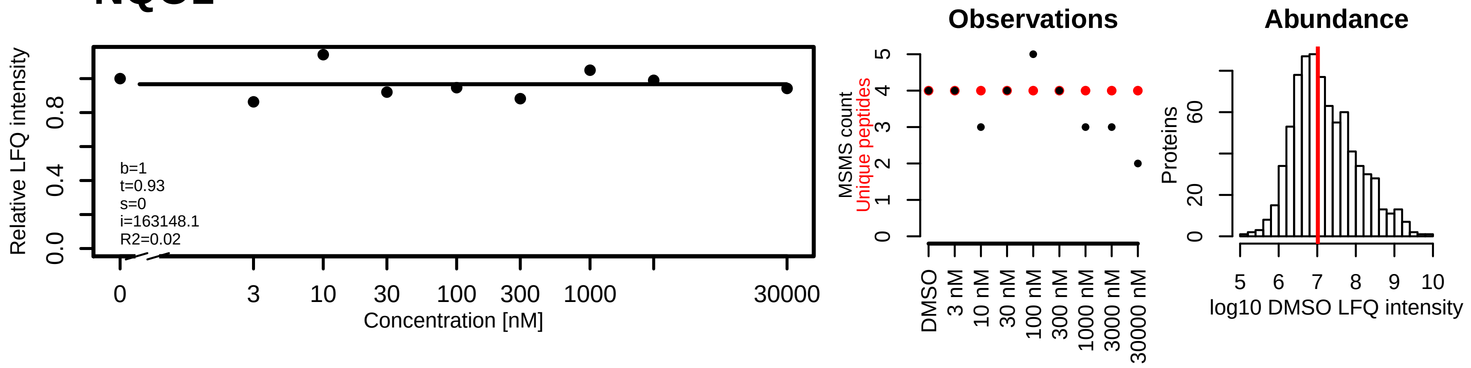


# DAD1

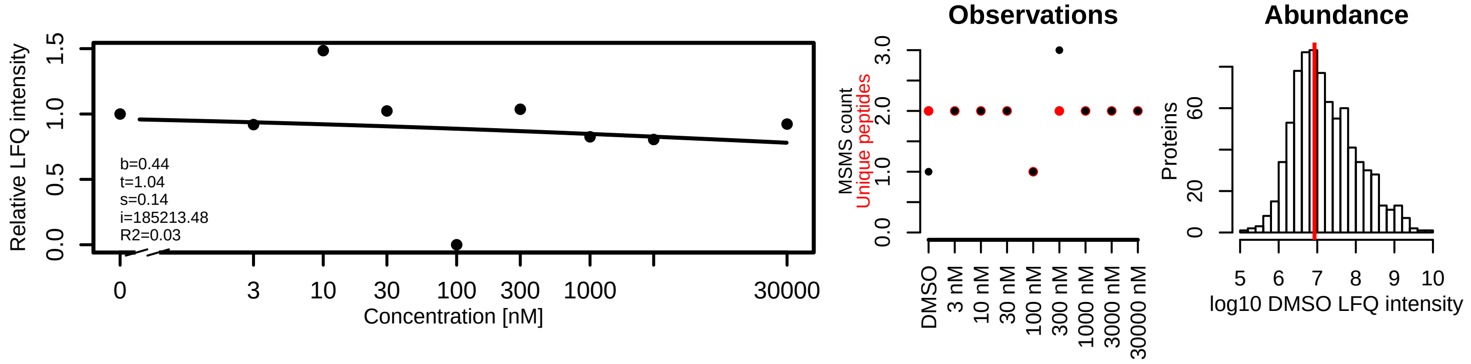


# TNPO3

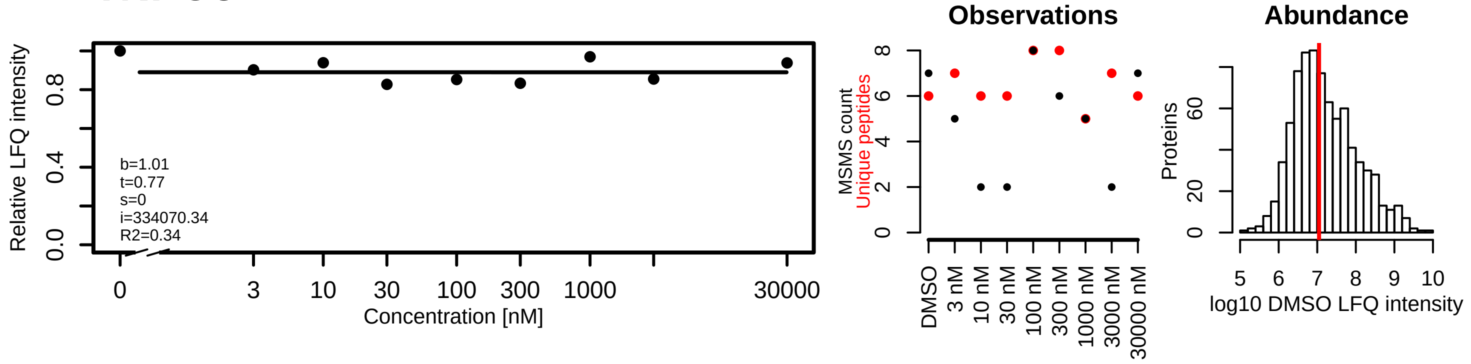


# IKBKE

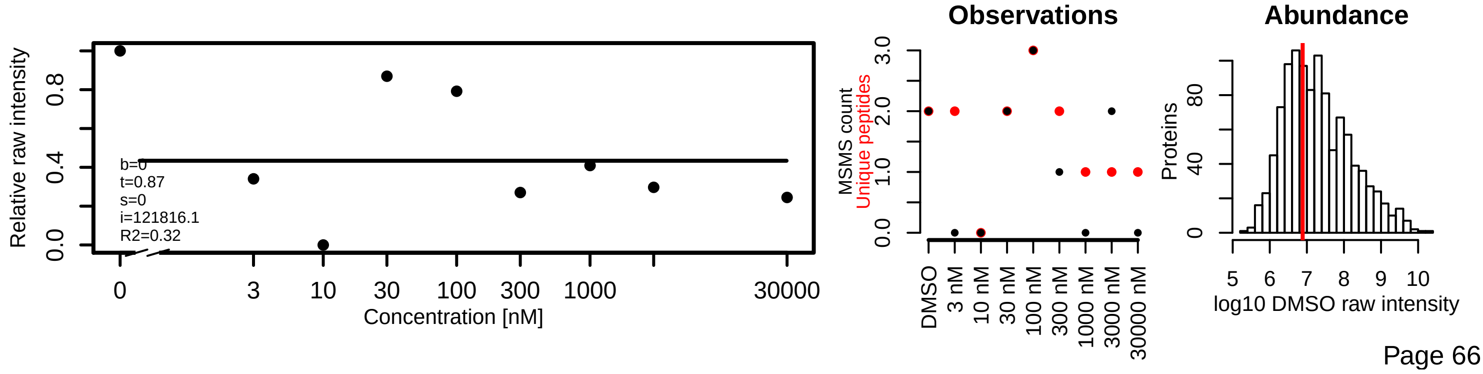

## ATM

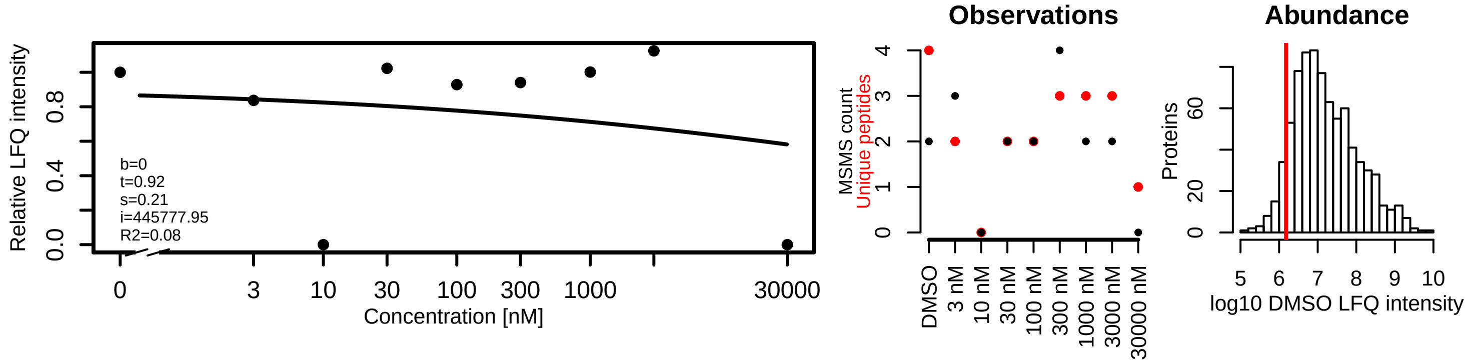


## CSRP1

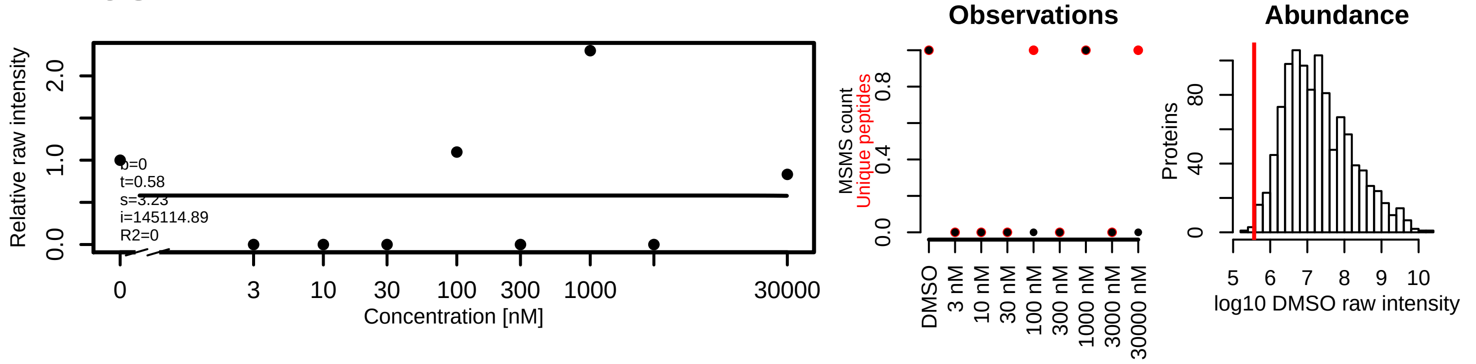


## SF3B3

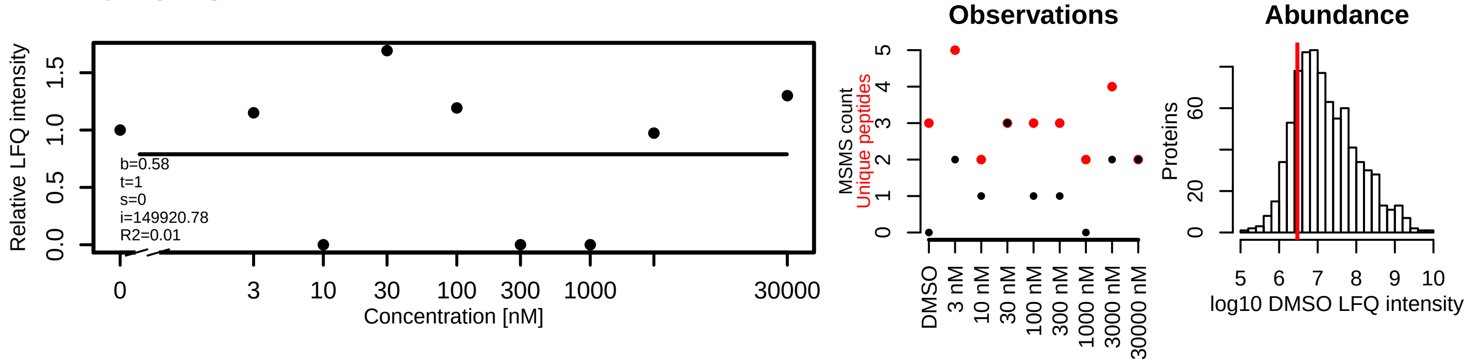


## DDX5

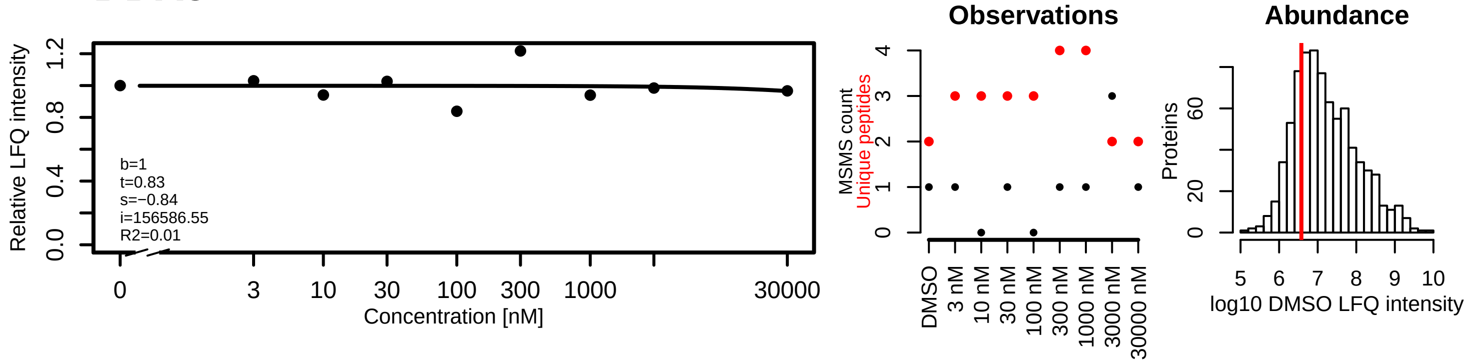


## EIF3A

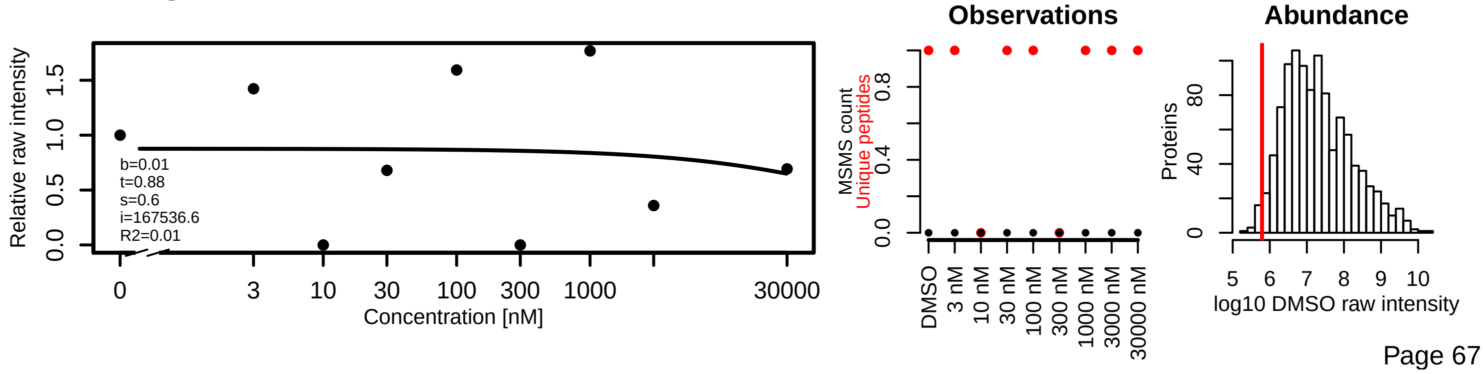

# RPL7

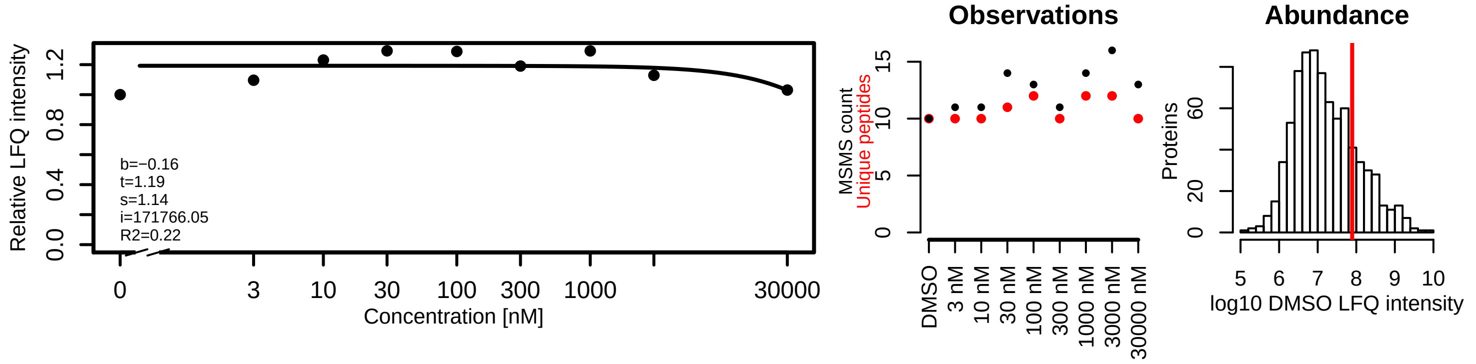


# PARD6B

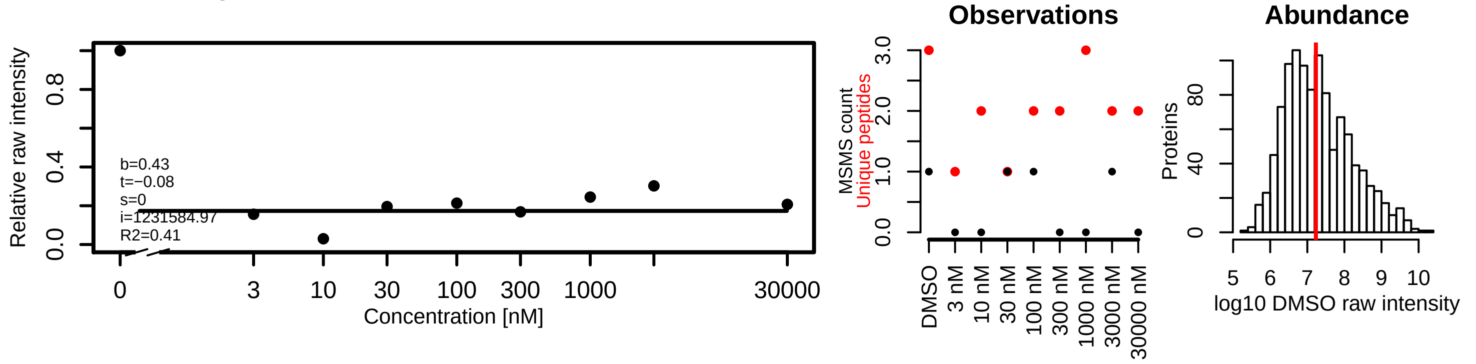


# CLTB

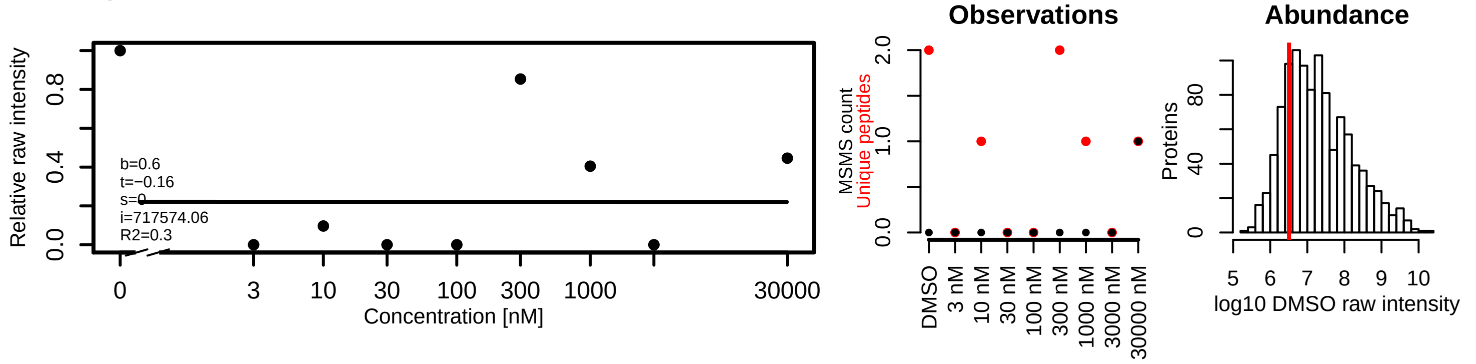


# SAV1

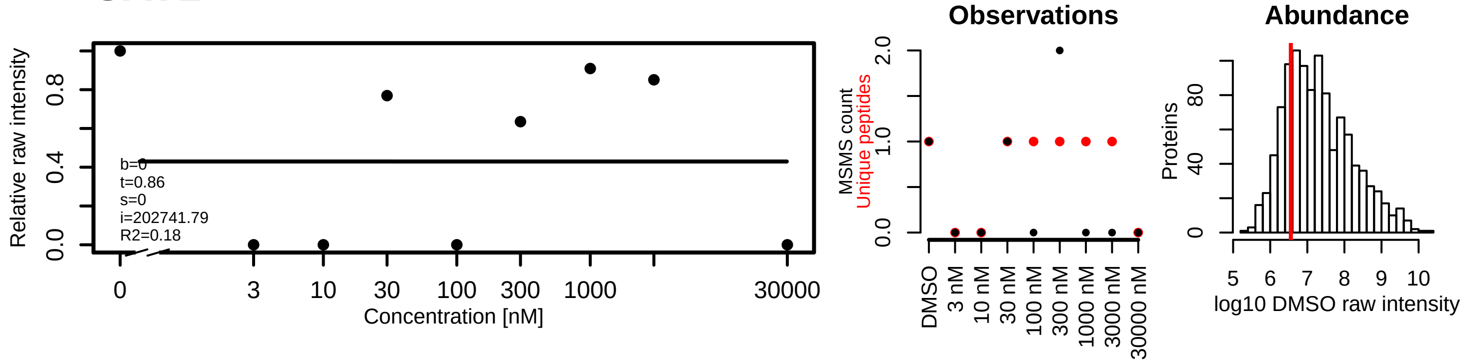


# PSMD1

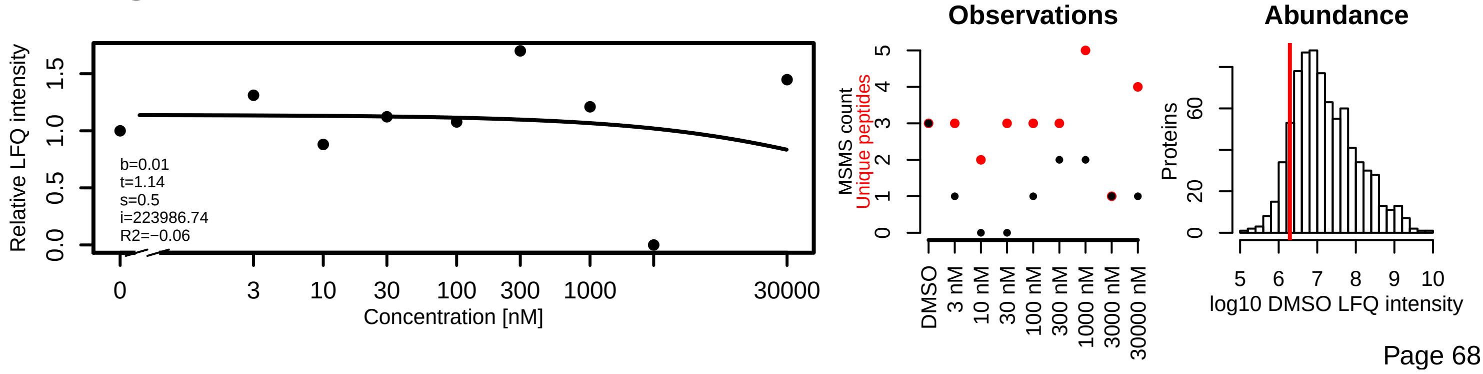

# ERH

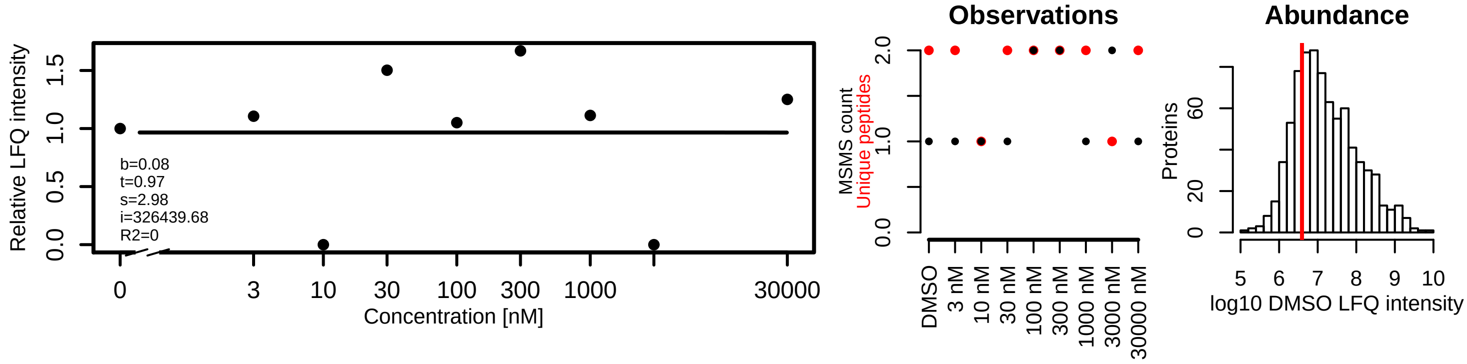


# OSTC

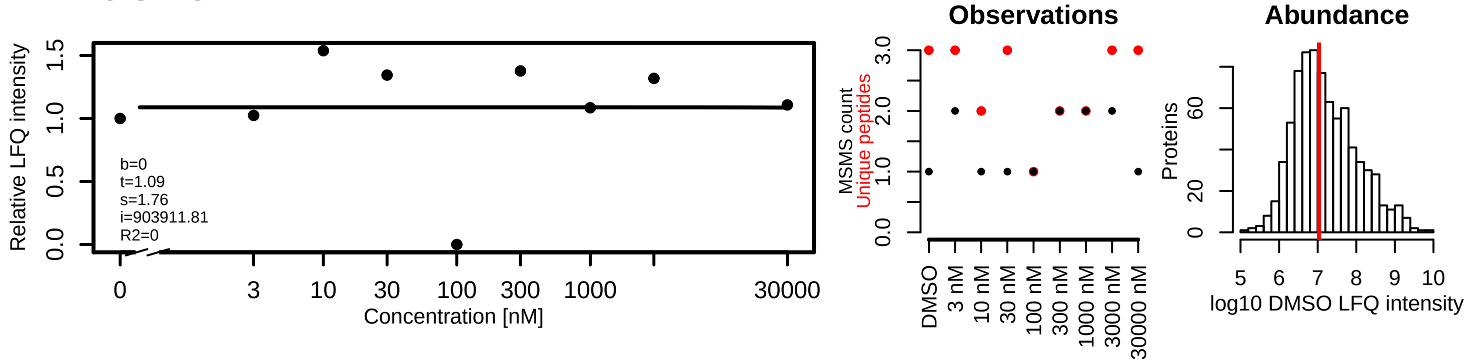


# CYC1

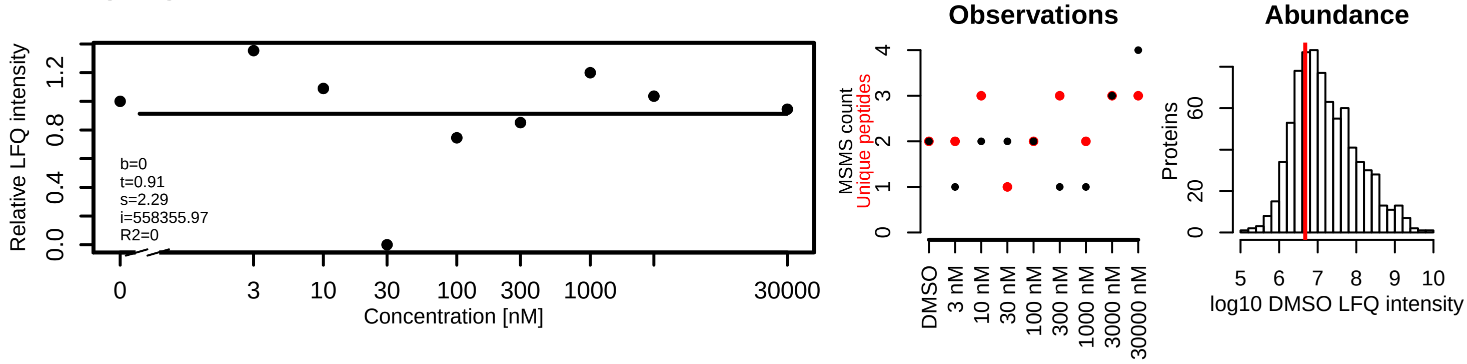


# SETDB1

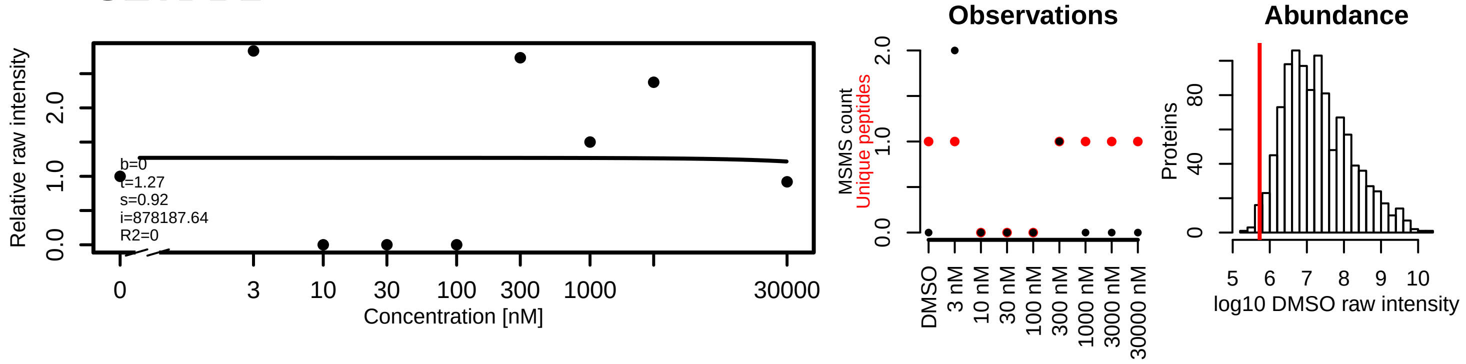


# NOMO1;NOMO2;NOMO3

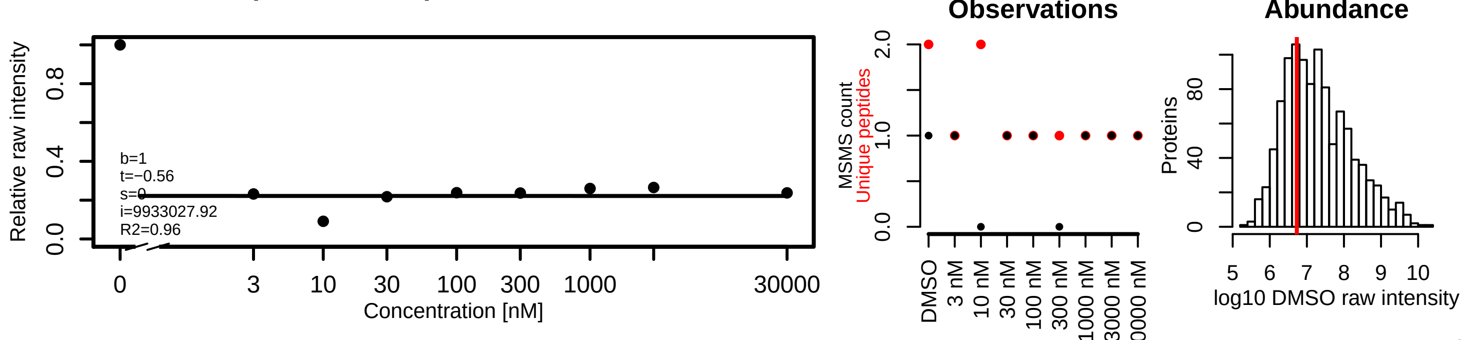

## B3GAT3

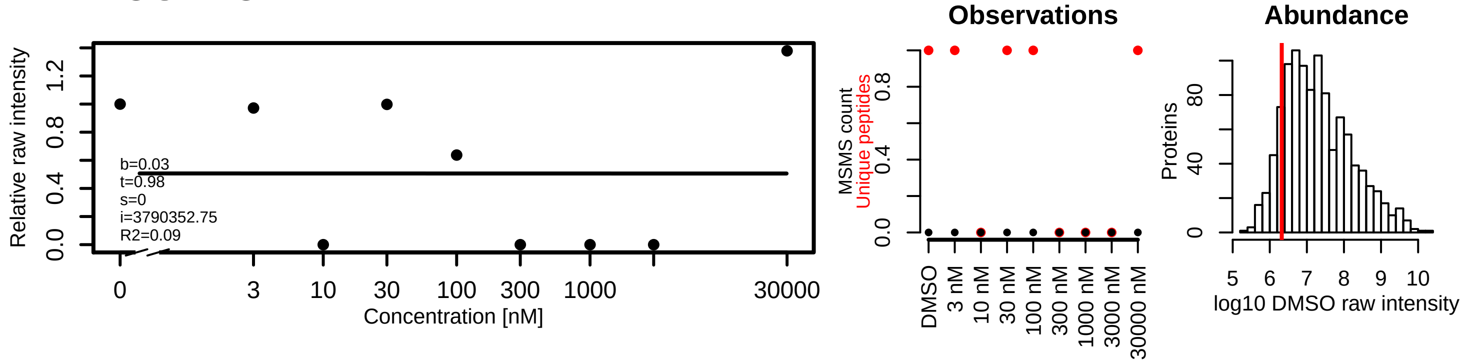


## LARS

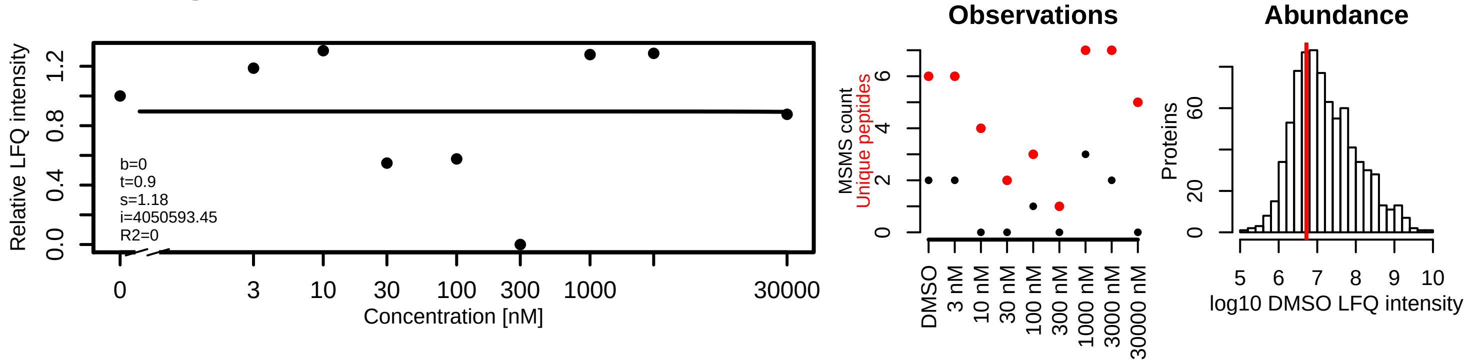


## CPT1A

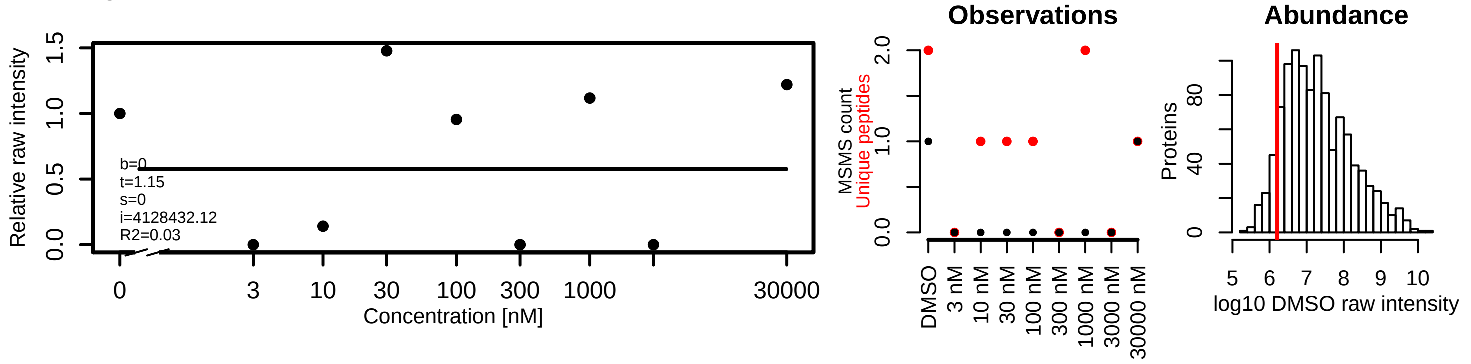


## STRADB

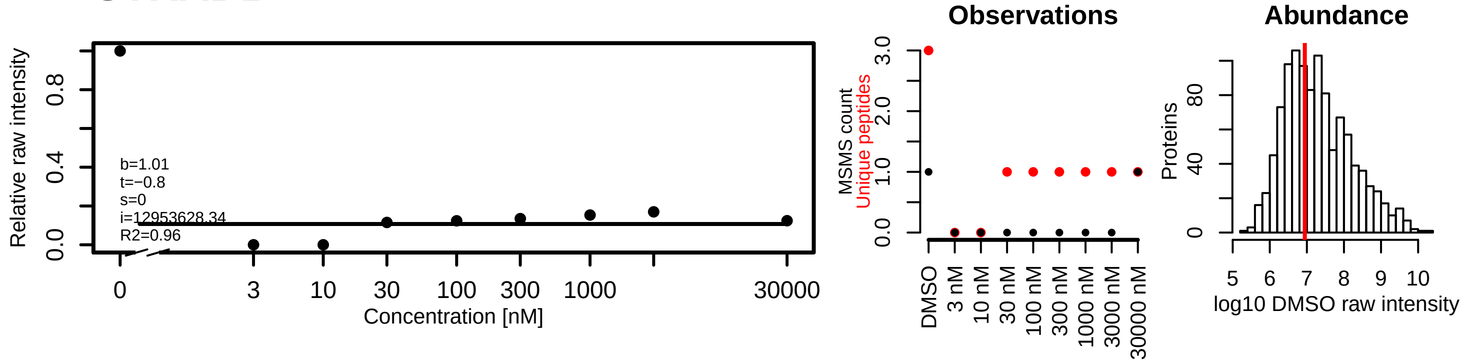


## MYEF2

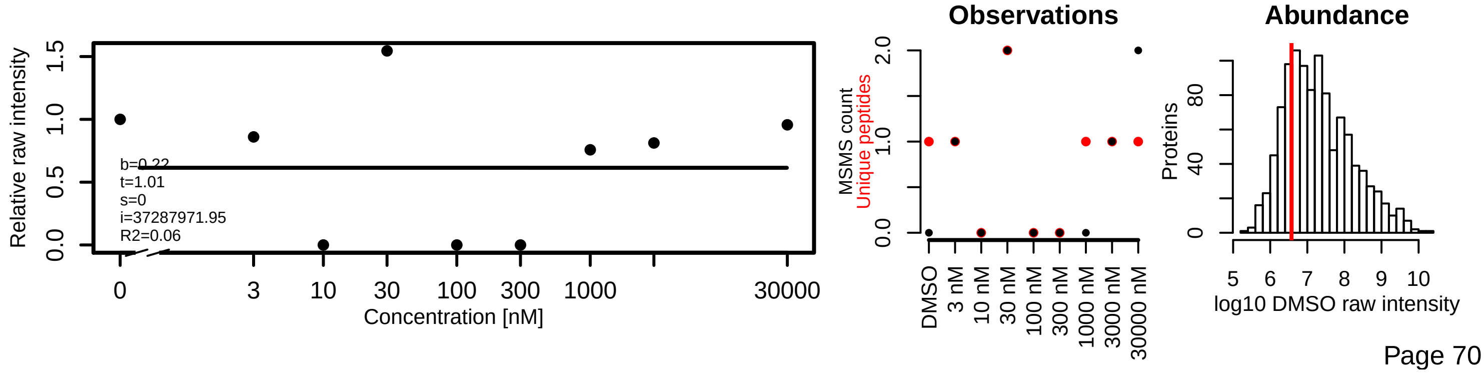

# PSMB1

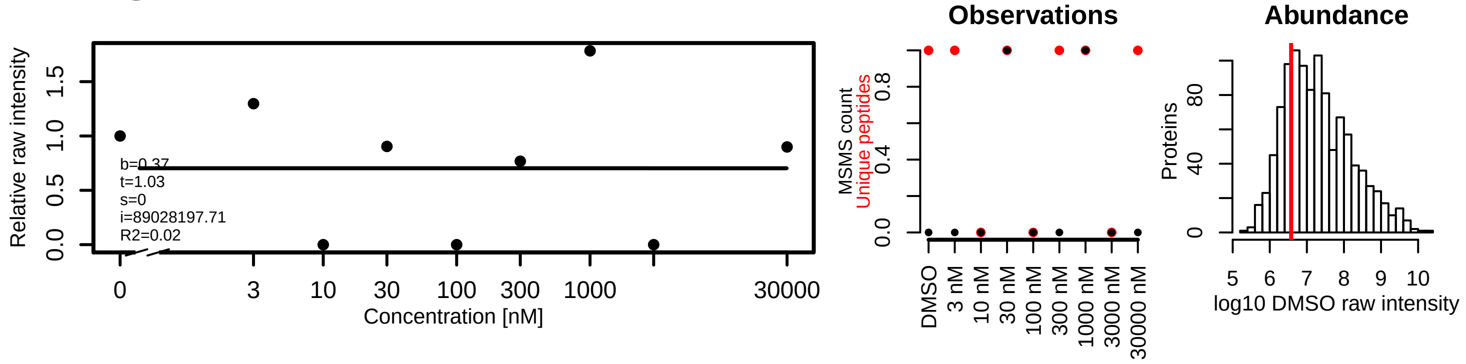


# RPS15A – H3BN98;I3L246;H3BV27;H3BT37;I3L303;H3BPJ2;F5GXP4;

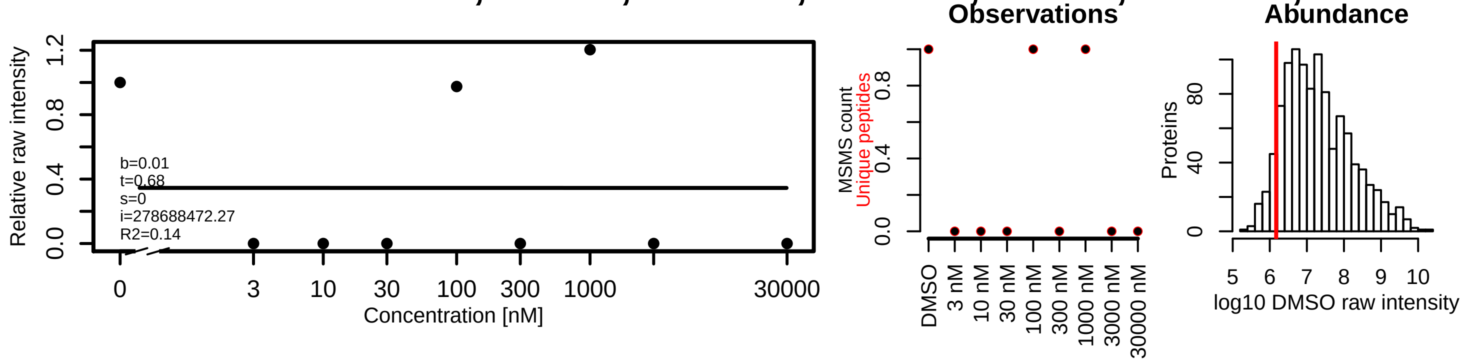


# RANBP1

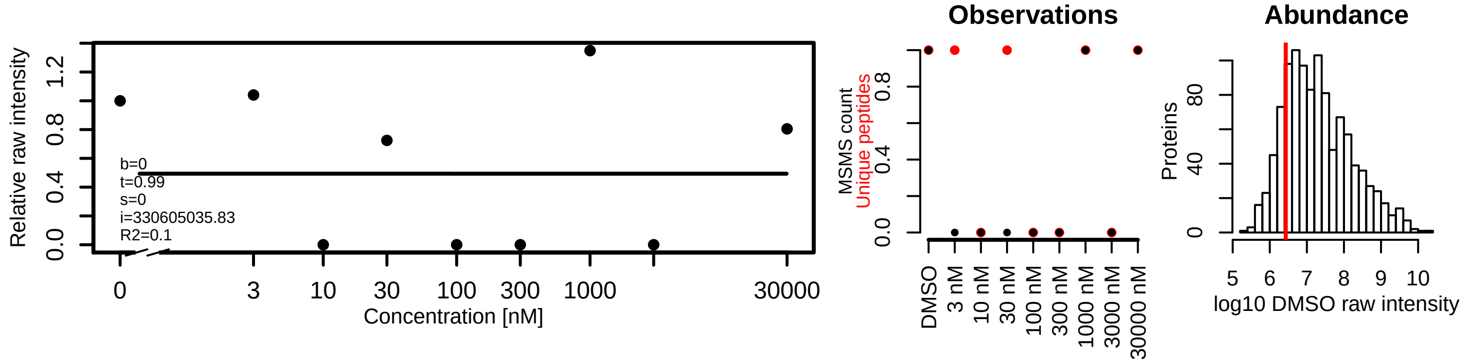


# RPL36A;RPL36AL

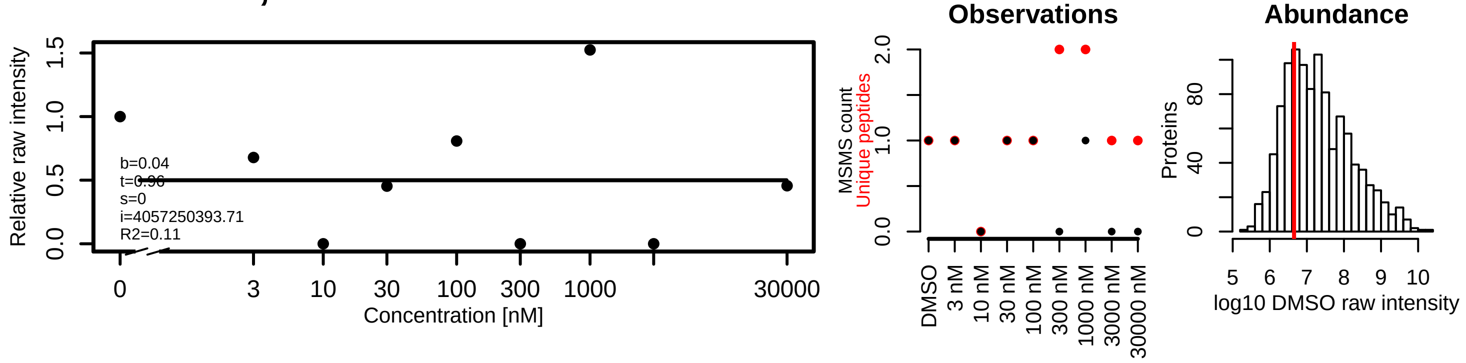


# NUP93

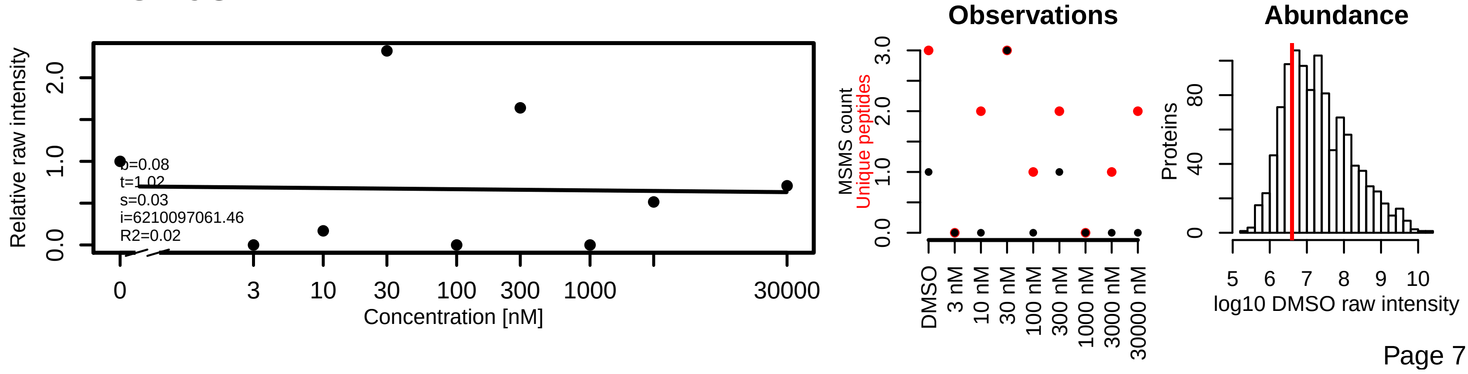

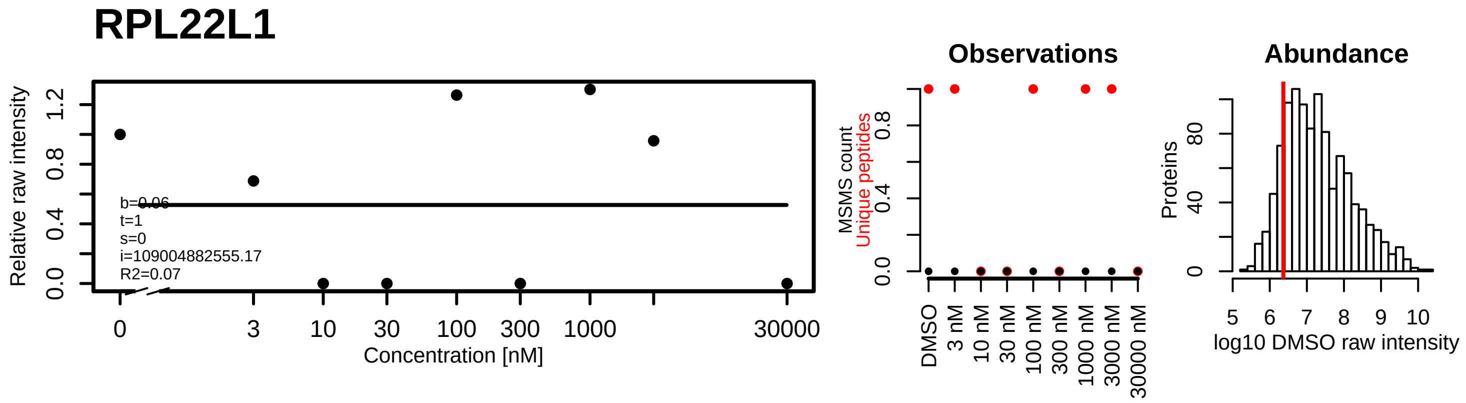
RPL22L1
Relative raw intensity
b=0.06
t=1
s=0
i=109004882555.17
R2=0.07
Concentration [nM]
Observations
MSMS count
Unique peptides
Abundance
Proteins
log10 DMSO raw intensity

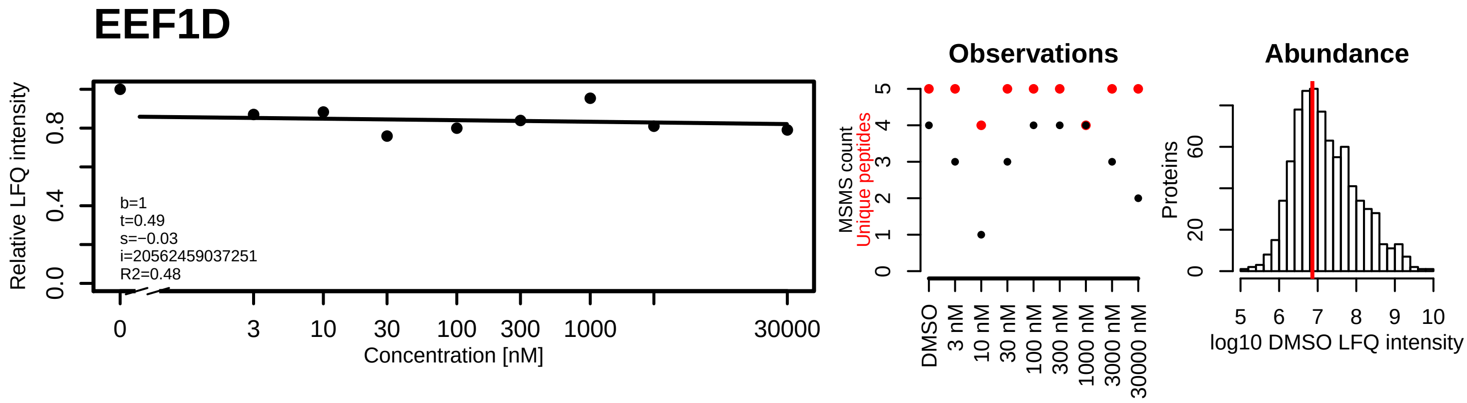
EEF1D
Relative LFQ intensity
b=1
t=0.49
s=−0.03
i=20562459037251
R2=0.48
Concentration [nM]
Observations
MSMS count
Unique peptides
Abundance
Proteins
log10 DMSO LFQ intensity

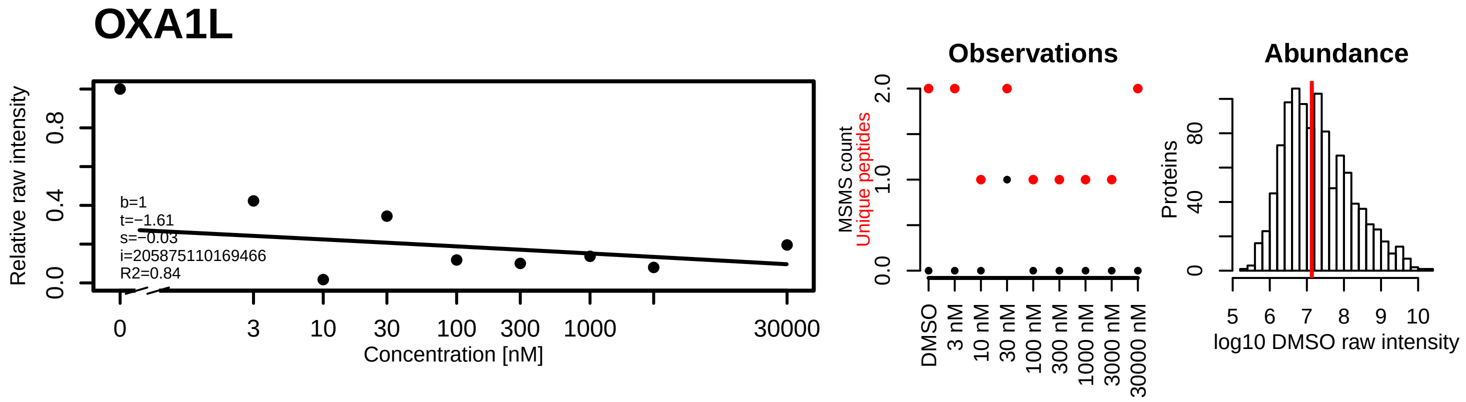
OXA1L
Relative raw intensity
b=1
t=−1.61
s=−0.03
i=205875110169466
R2=0.84
Concentration [nM]
Observations
MSMS count
Unique peptides
Abundance
Proteins
log10 DMSO raw intensity

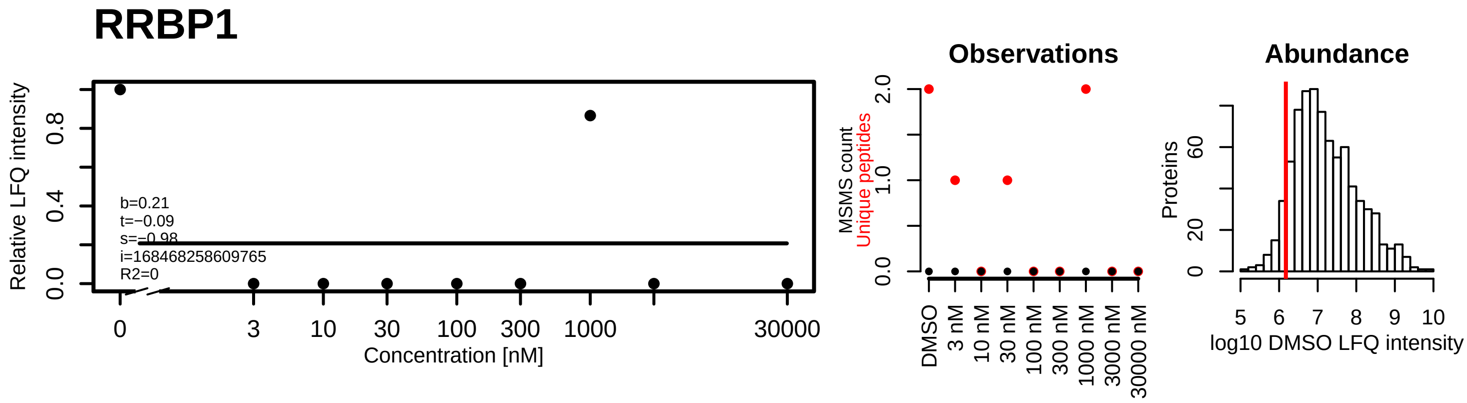
RRBP1
Relative LFQ intensity
b=0.21
t=−0.09
s=−0.98
i=168468258609765
R2=0
Concentration [nM]
Observations
MSMS count
Unique peptides
Abundance
Proteins
log10 DMSO LFQ intensity

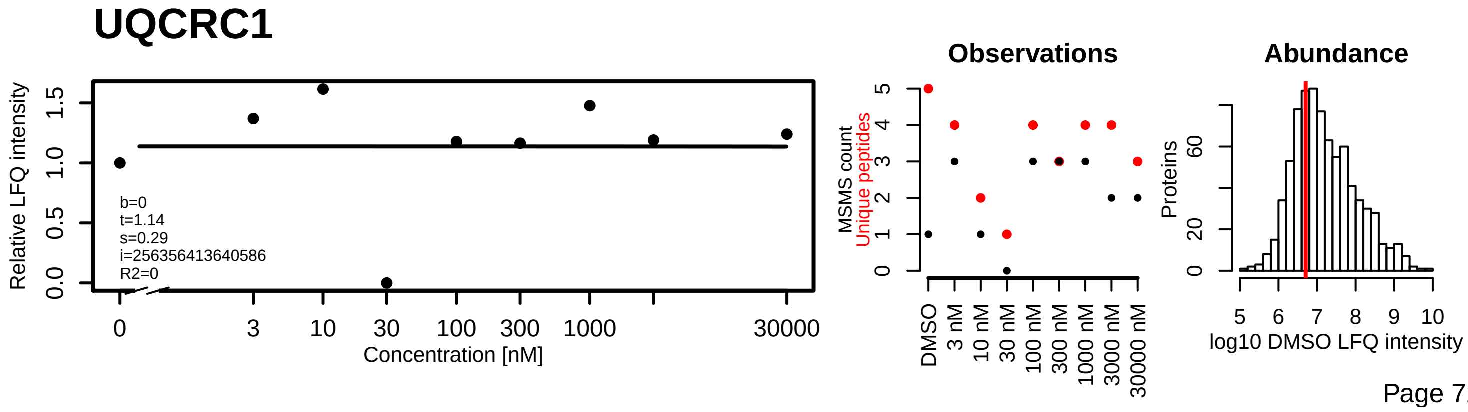
UQCRC1
Relative LFQ intensity
b=0
t=1.14
s=0.29
i=256356413640586
R2=0
Concentration [nM]
Observations
MSMS count
Unique peptides
Abundance
Proteins
log10 DMSO LFQ intensity

## JUP − F5GWP8

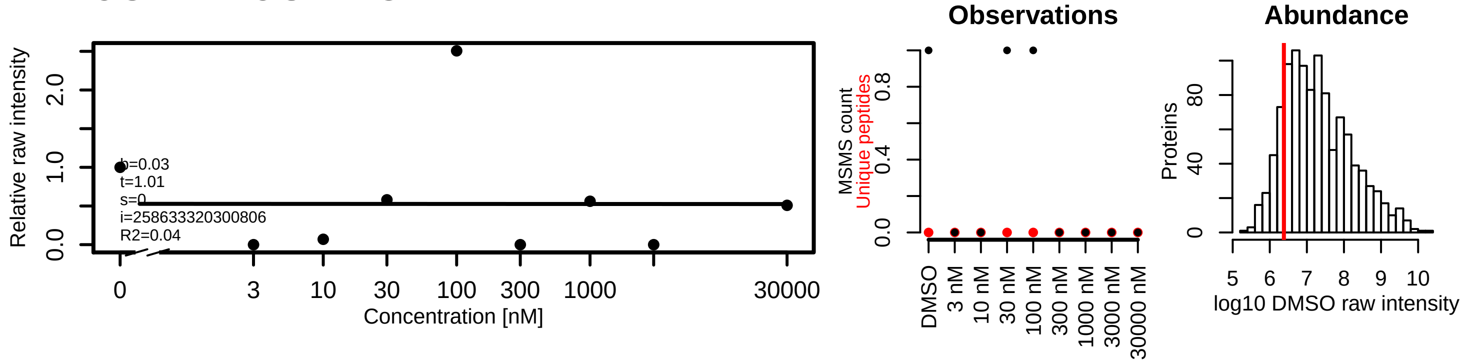


## EMD

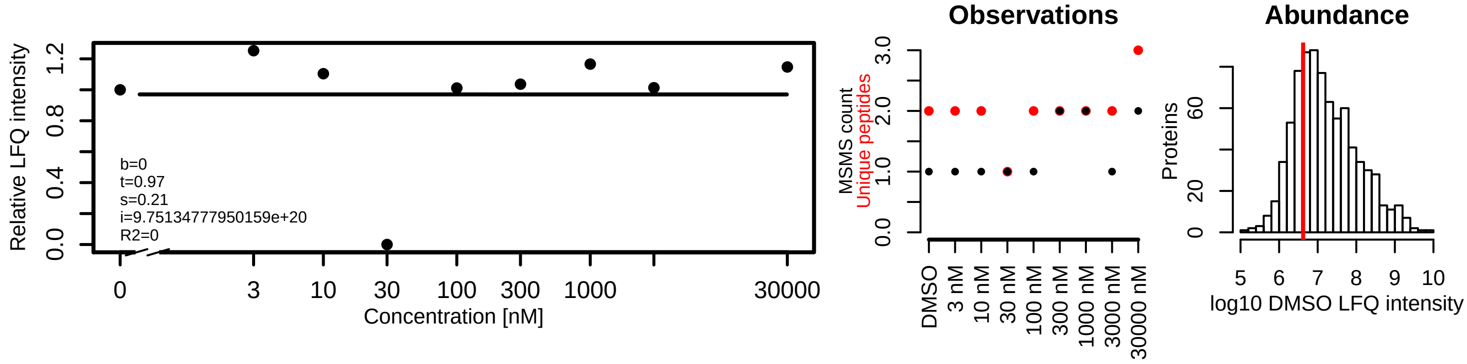


## EIF3I

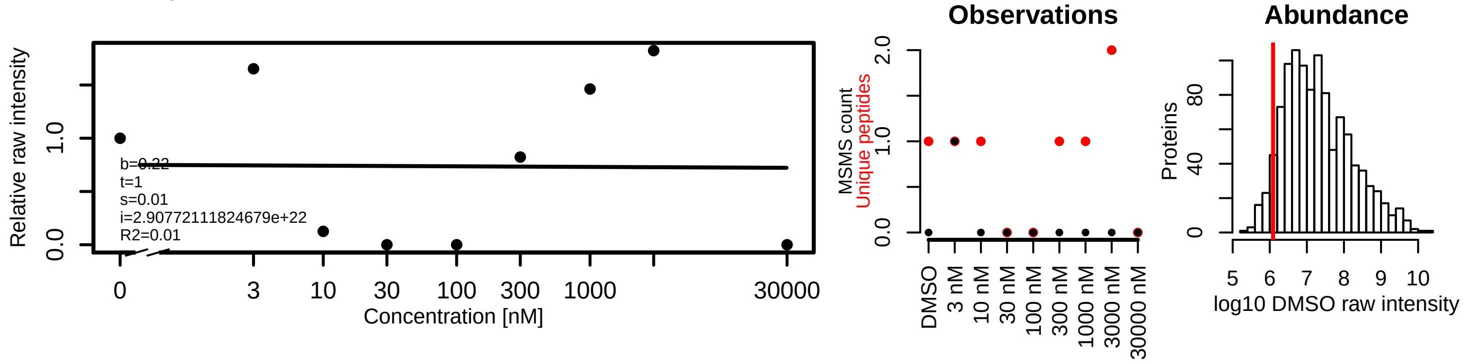


## DECR1

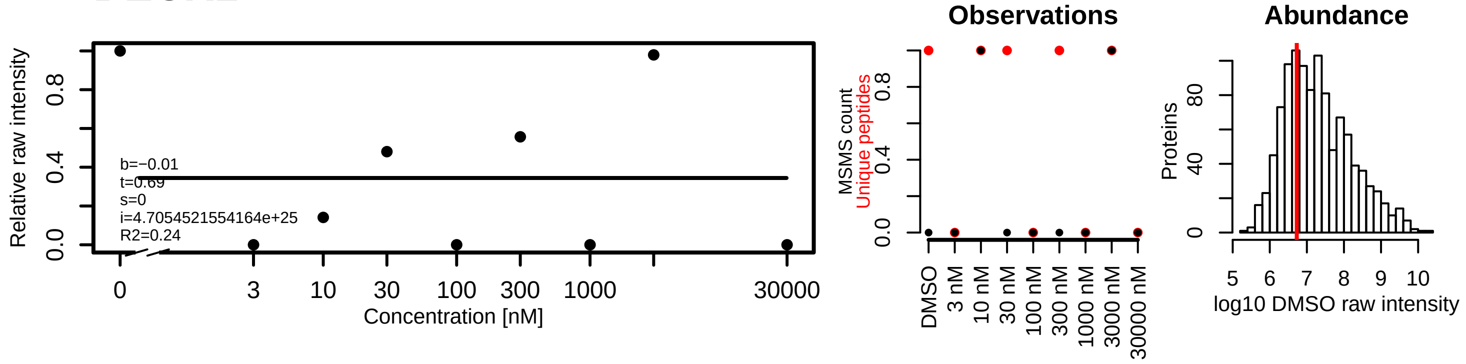


## TNPO2

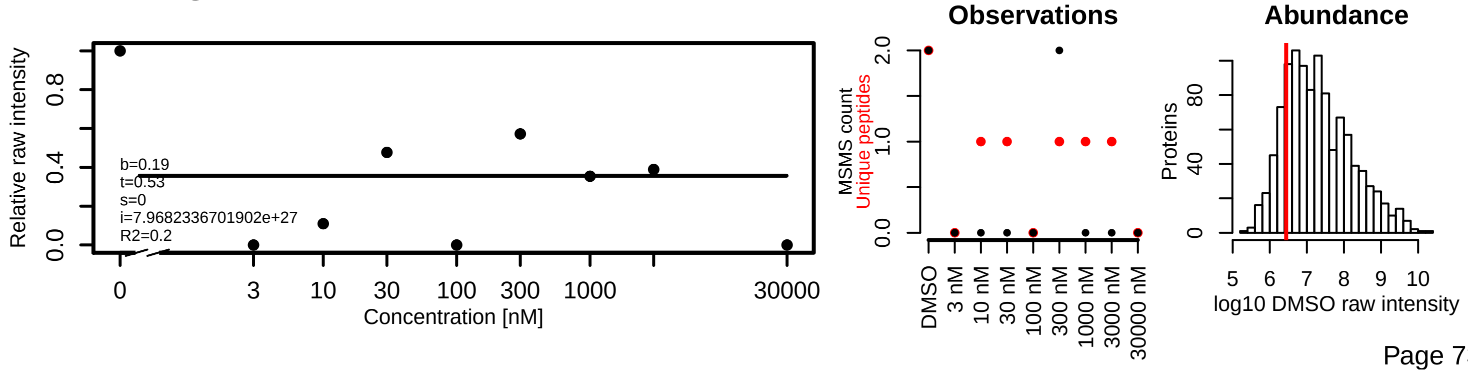

# STK33

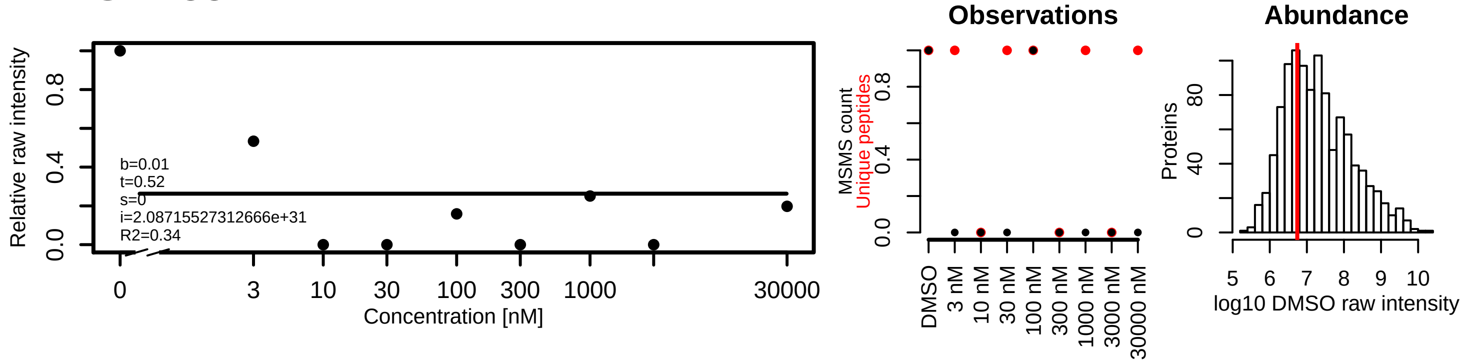


# NQO2

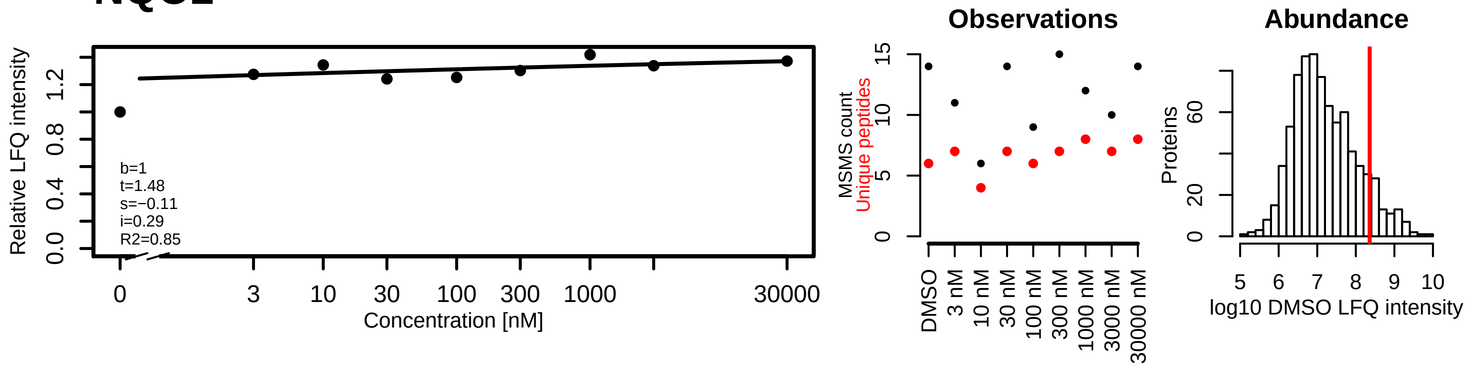


# RPL31

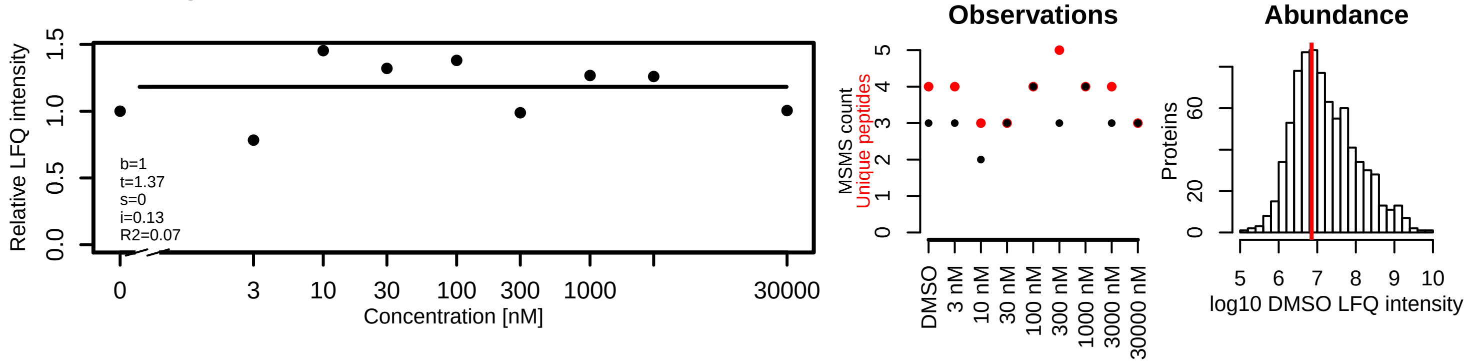


# PLEC

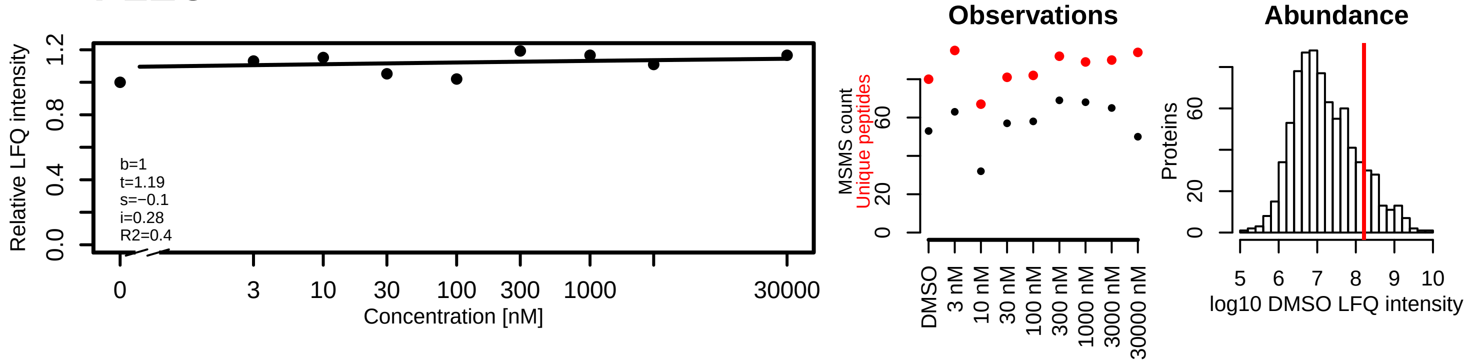


# TMEM48

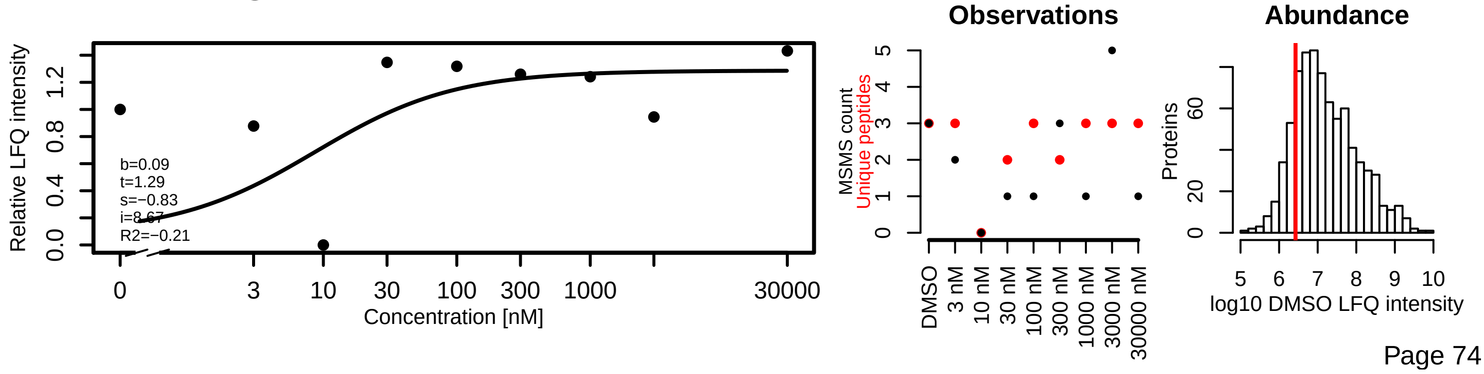

# STAU1

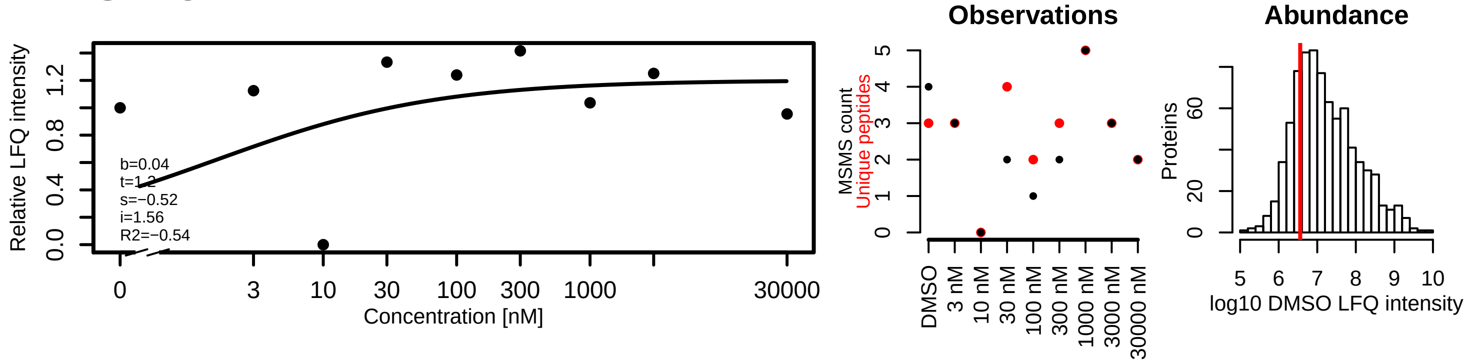


# AKR1B1

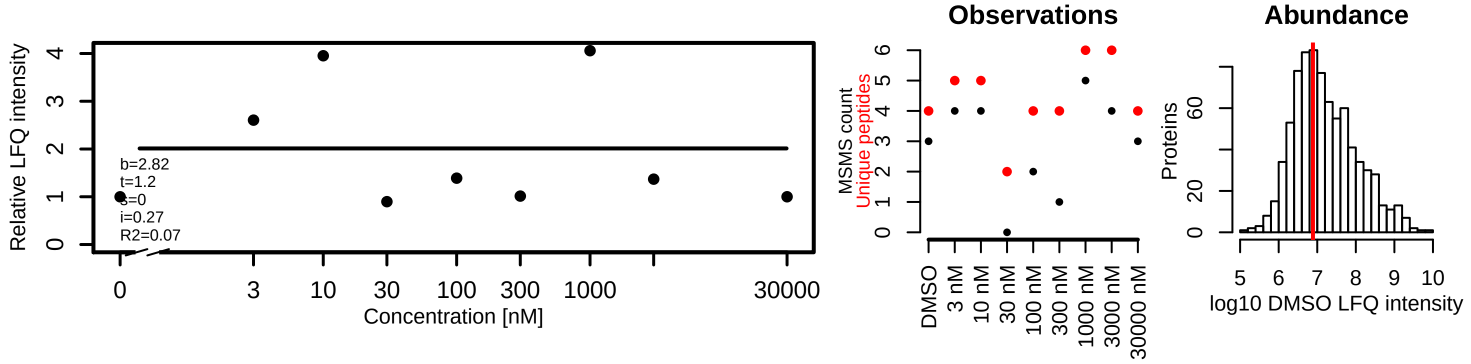


# RPL34

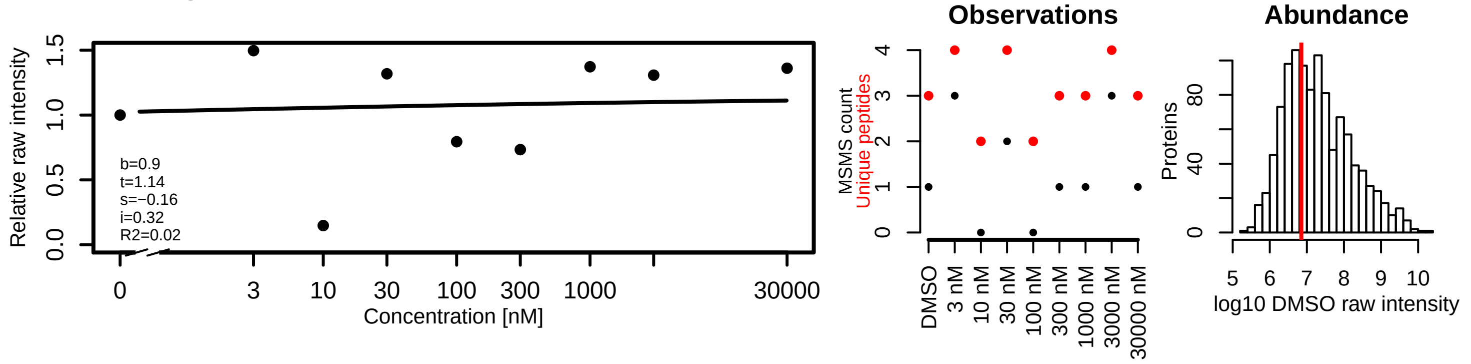


# ULK4

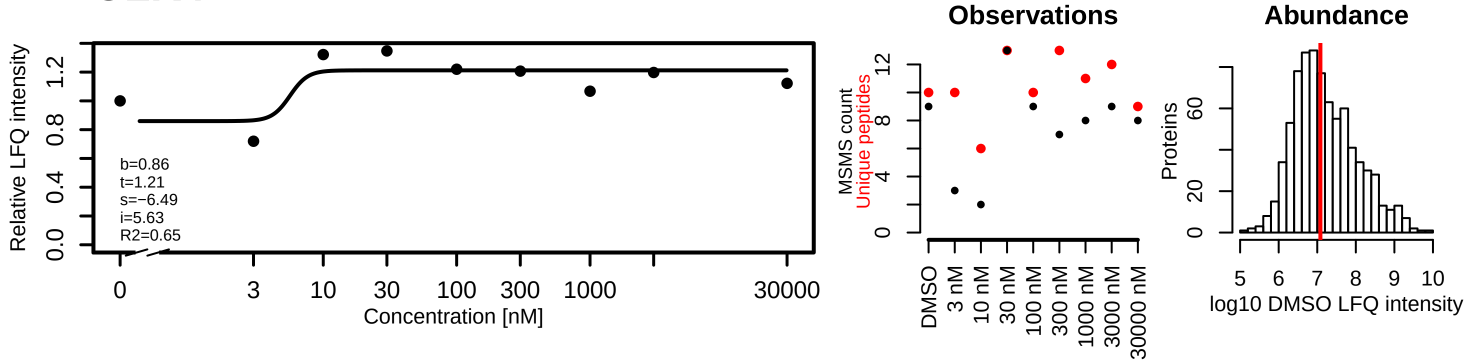


# PDHX

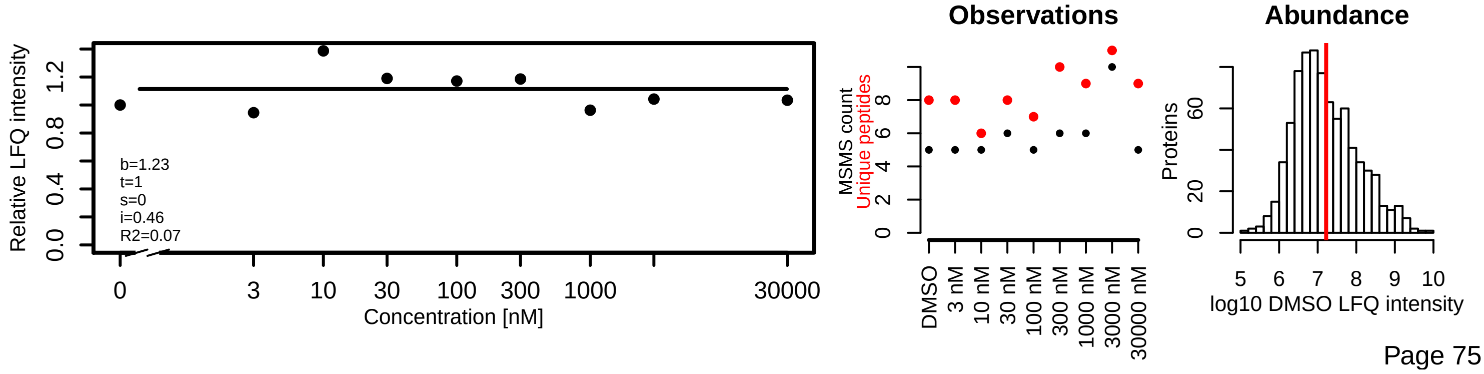

# RPS16

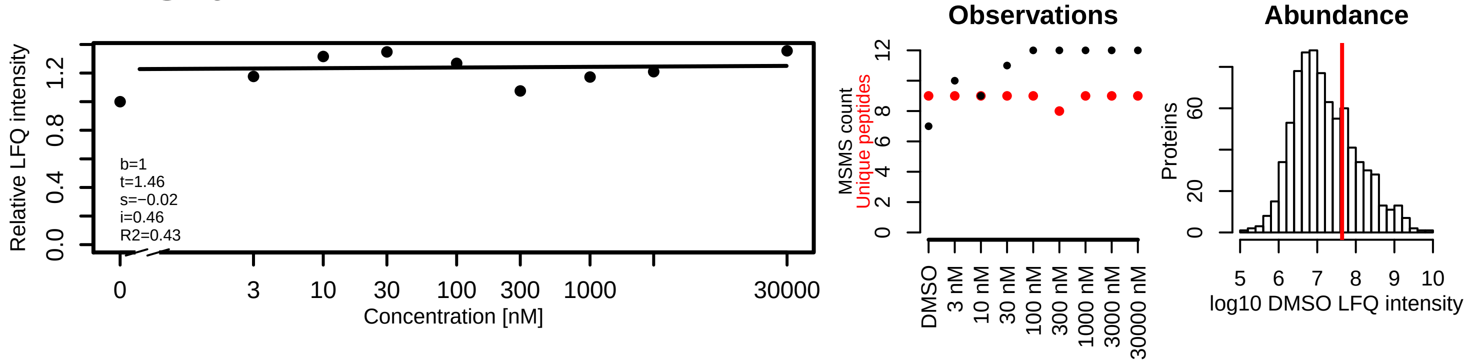


# RAN

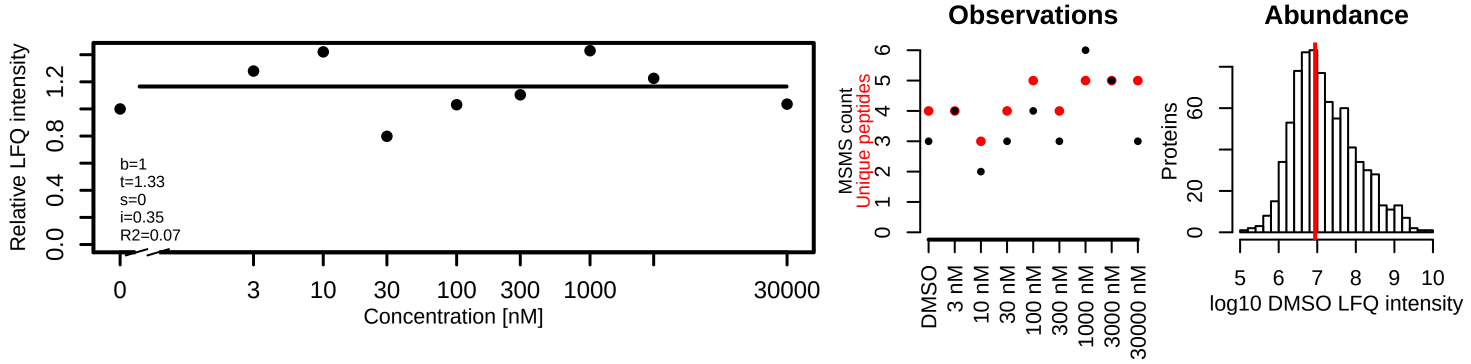


# DDX21

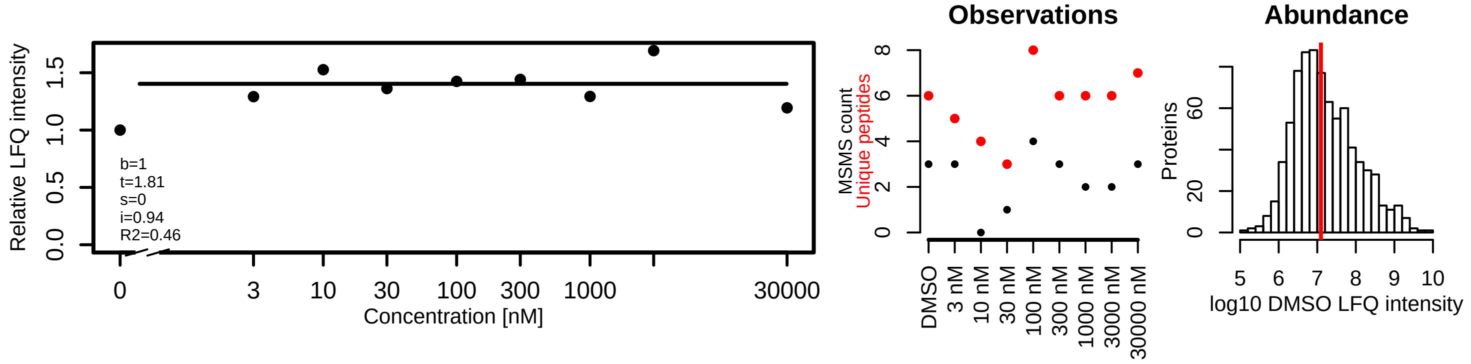


# ST7

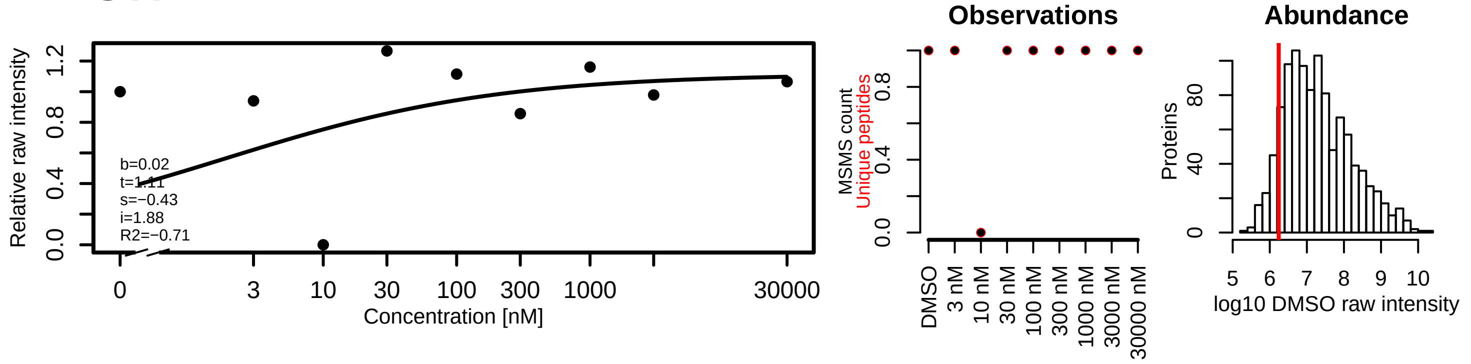


# MAP4

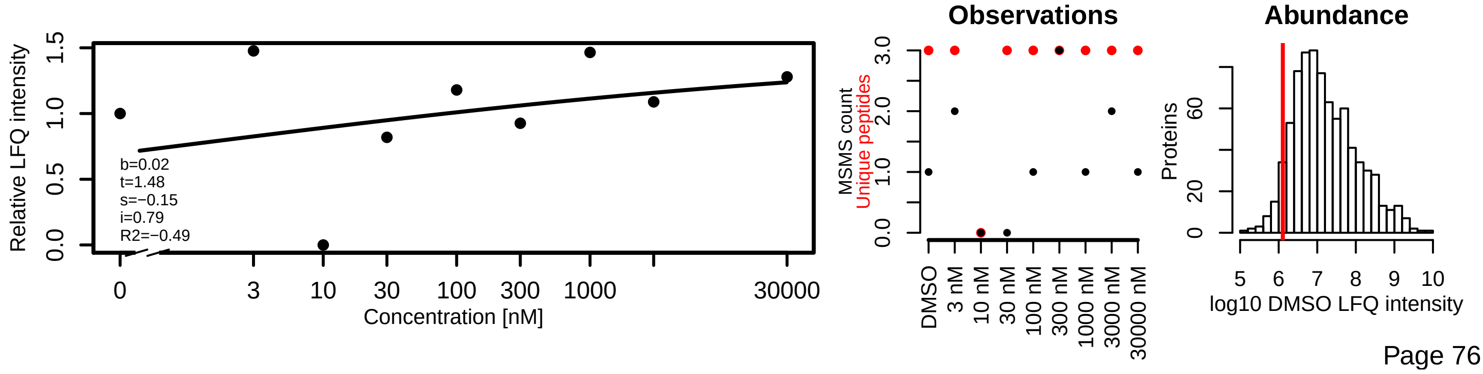

# ADCK4

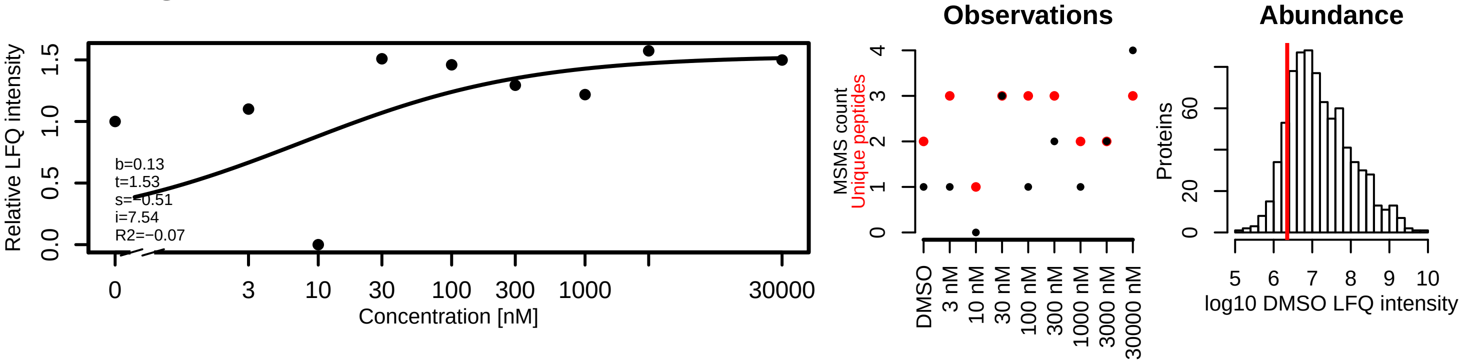


# DDX17

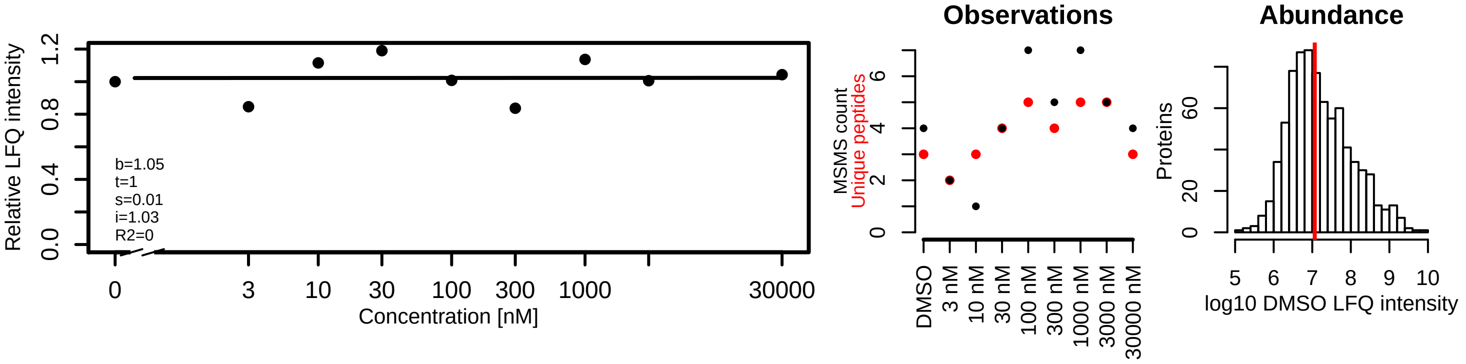


# RPS3

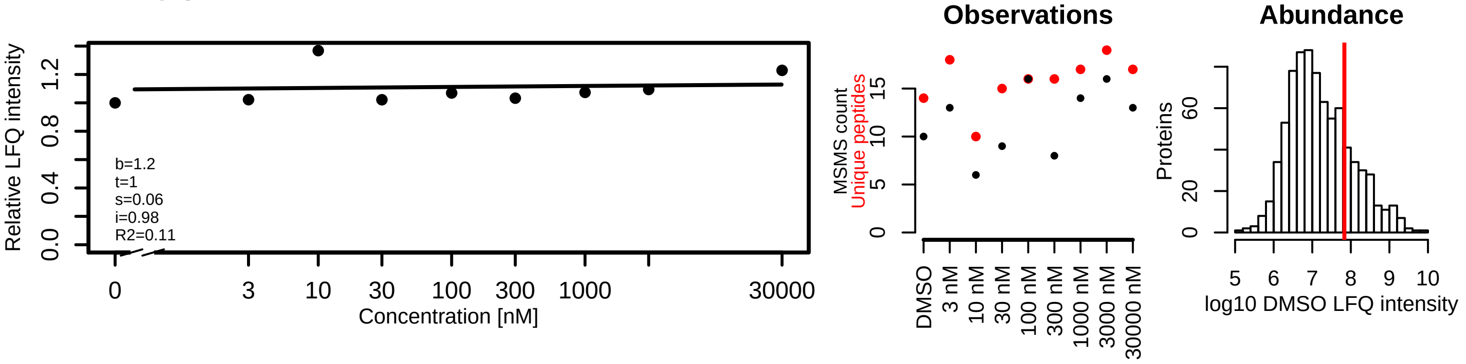


# IRAK3

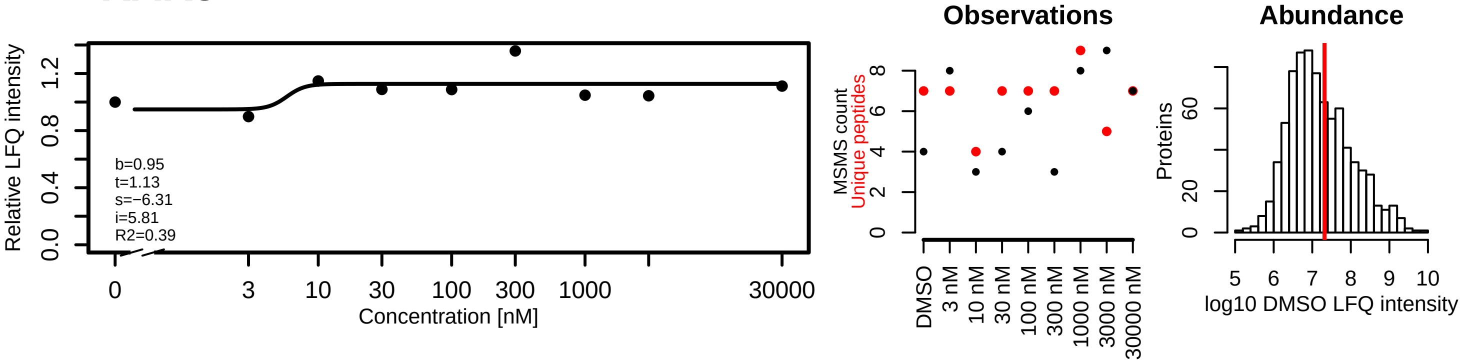


# CDIPT

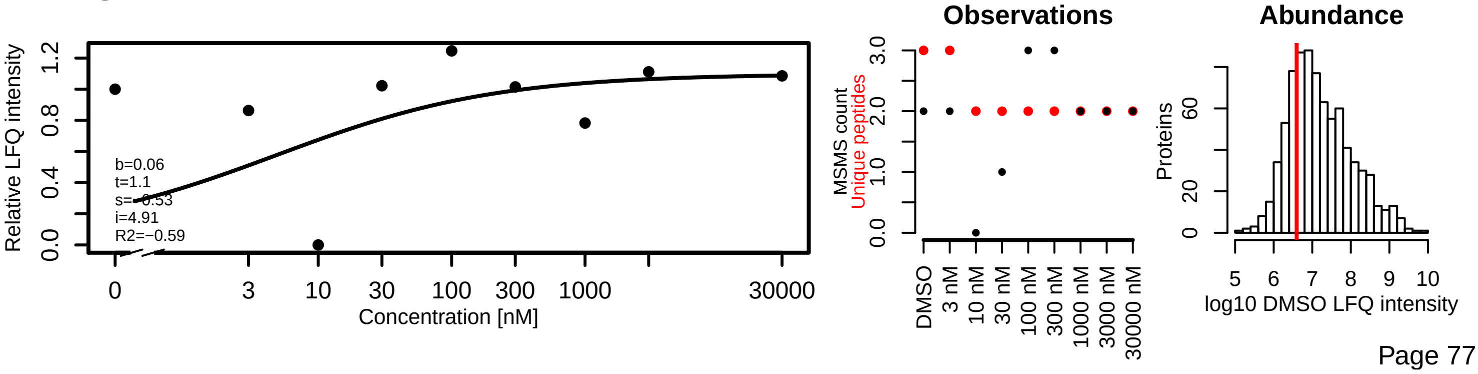

## RPS17L;RPS17

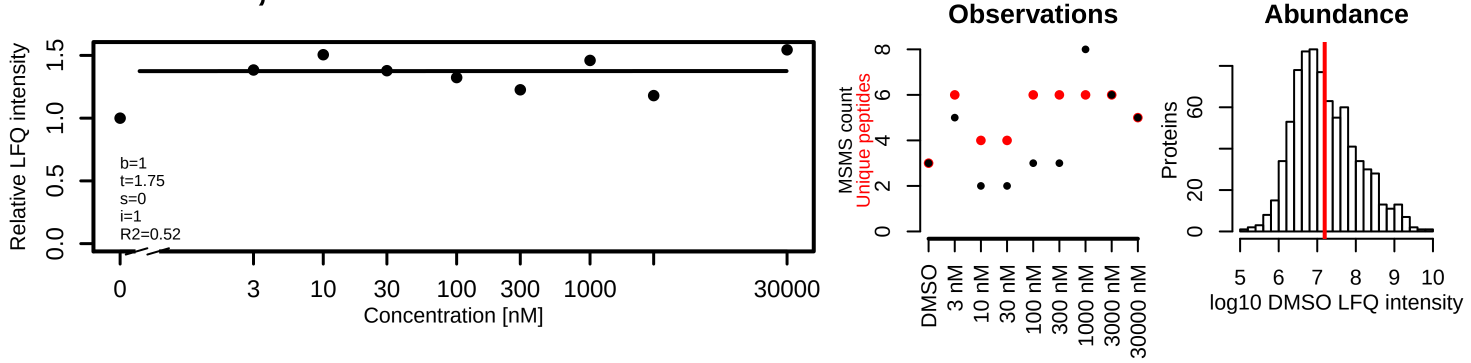


## NEK2

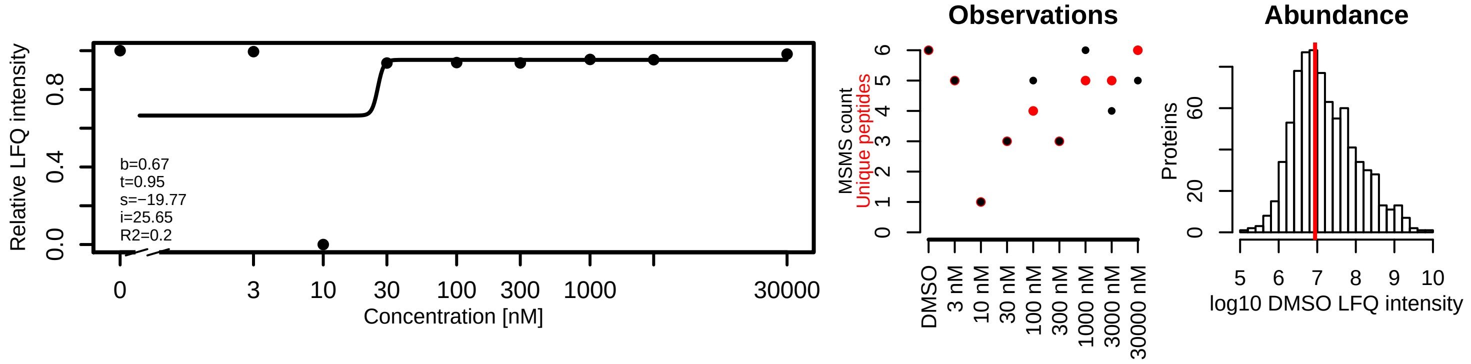


## TP53RK

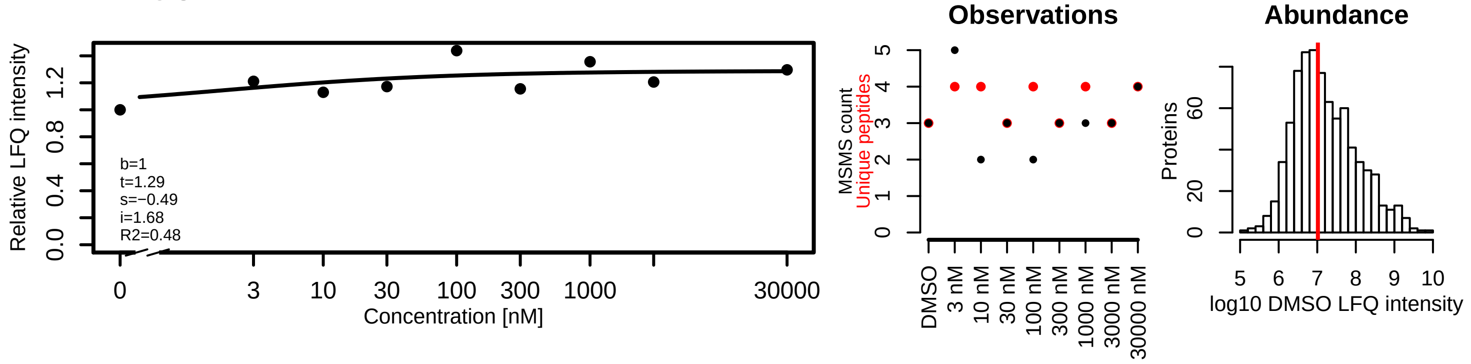


## KDELR1

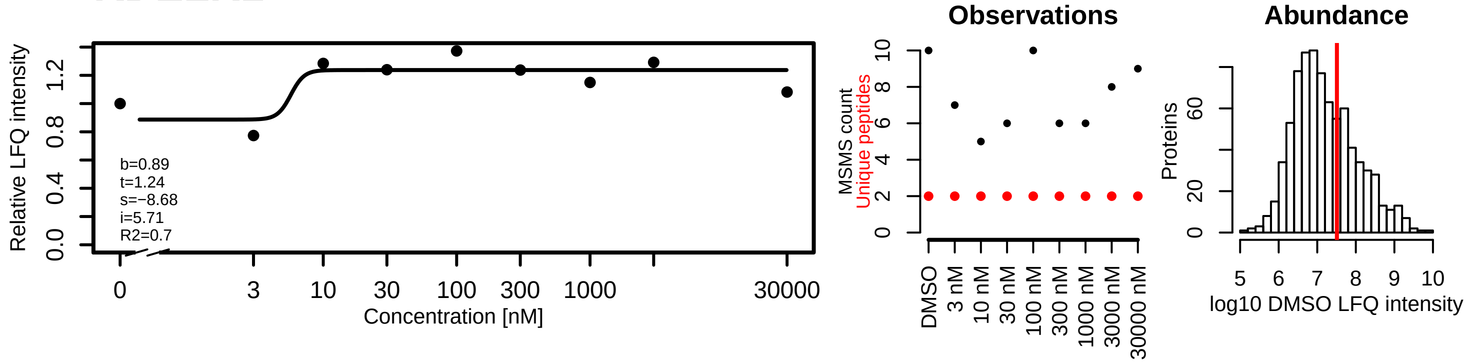


## SSR3

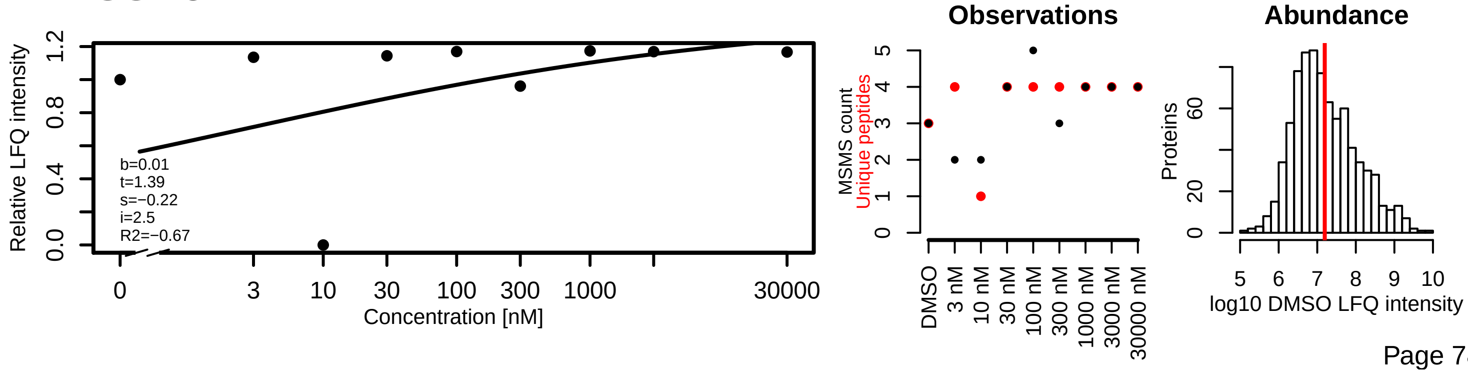

# RASSF2

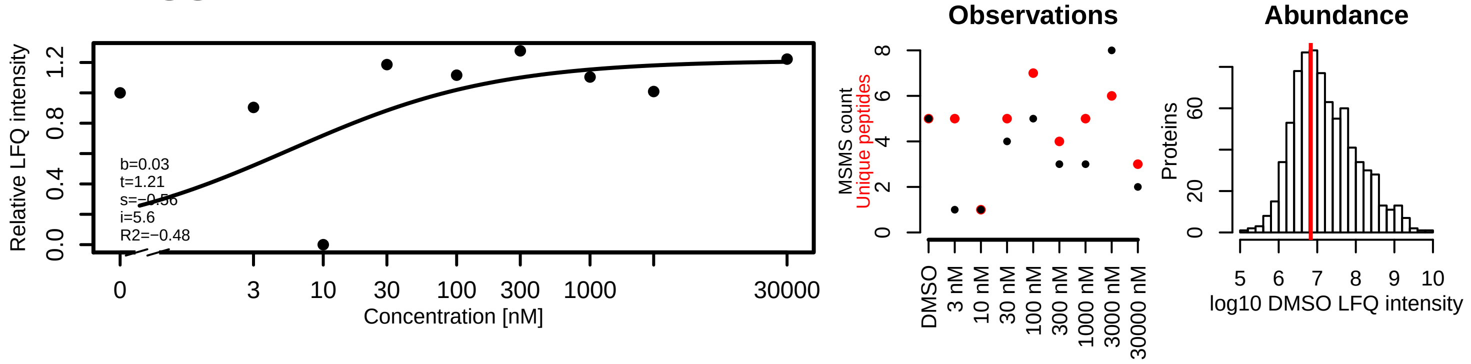


# ALG10;ALG10B

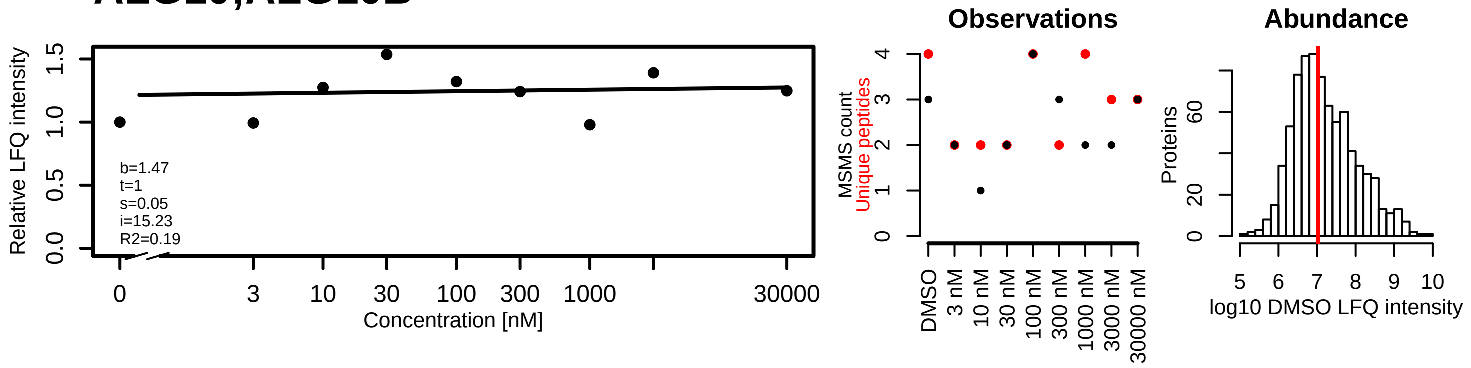


# CCNK

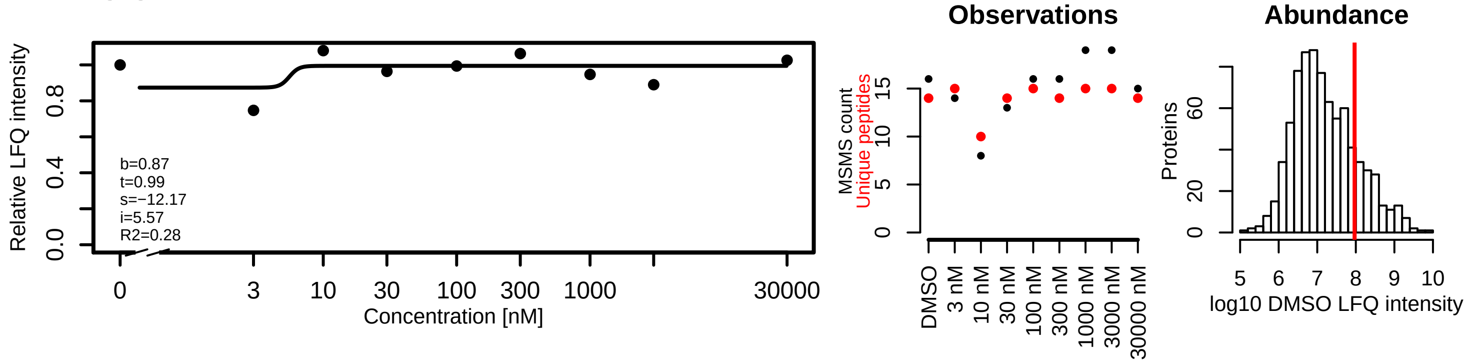


# AZI2

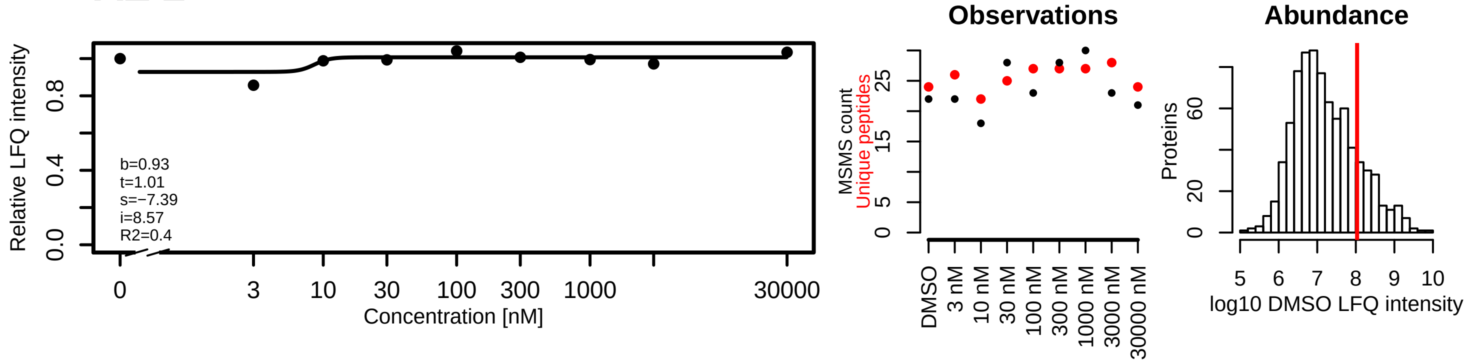


# PRKAB1

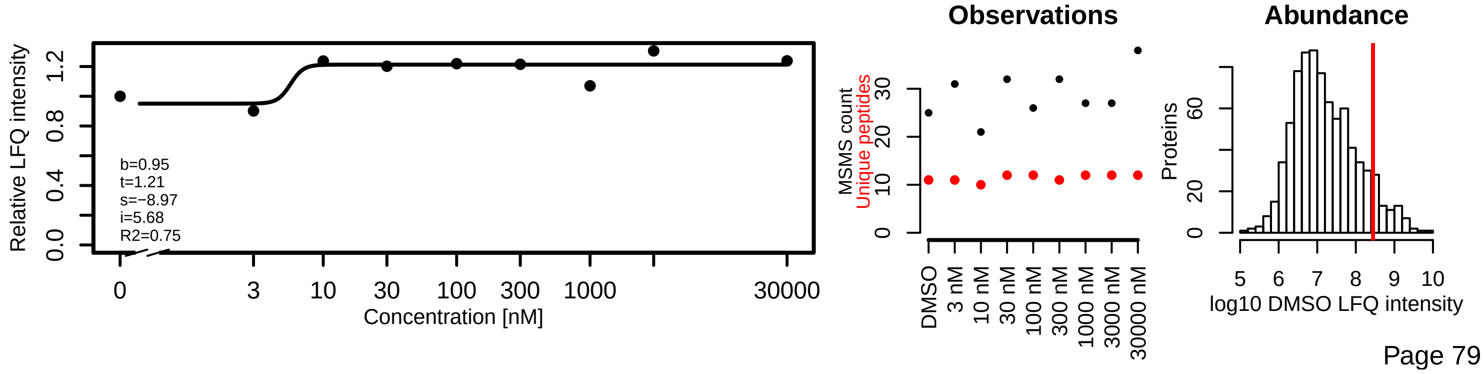

# STK4

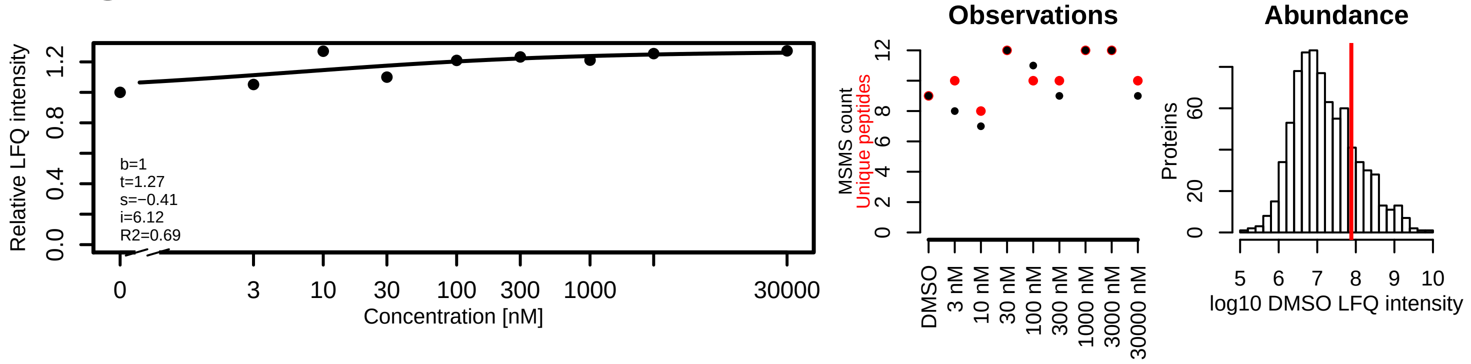


# PIM1

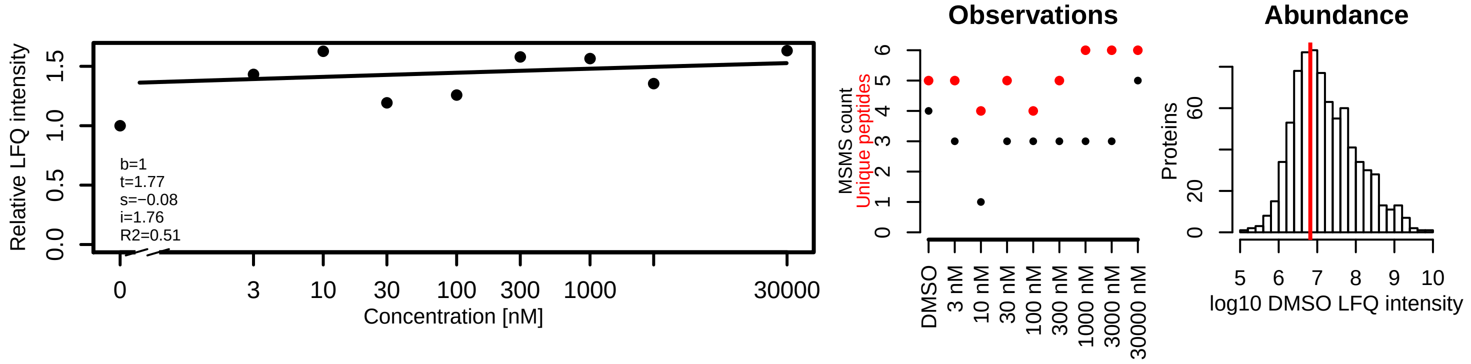


# NUAK1

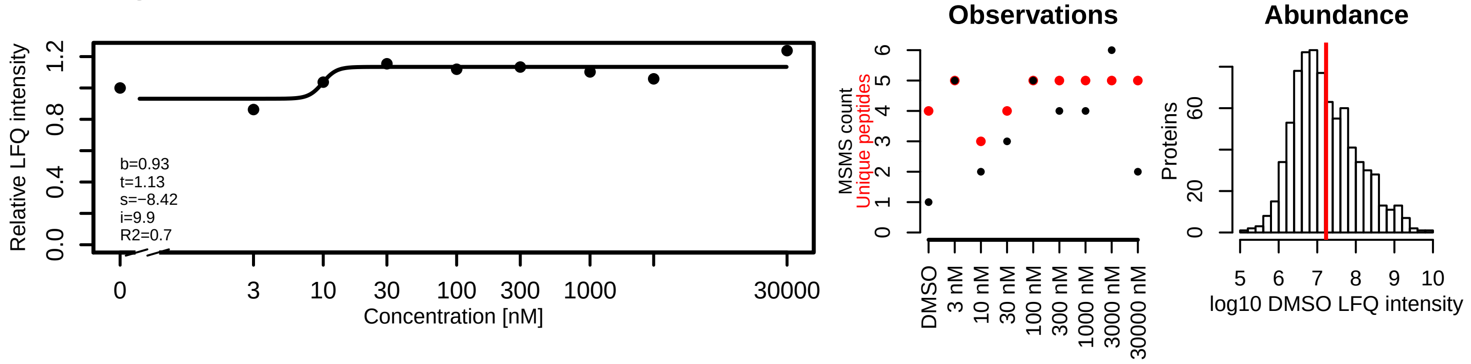


# SLC25A3

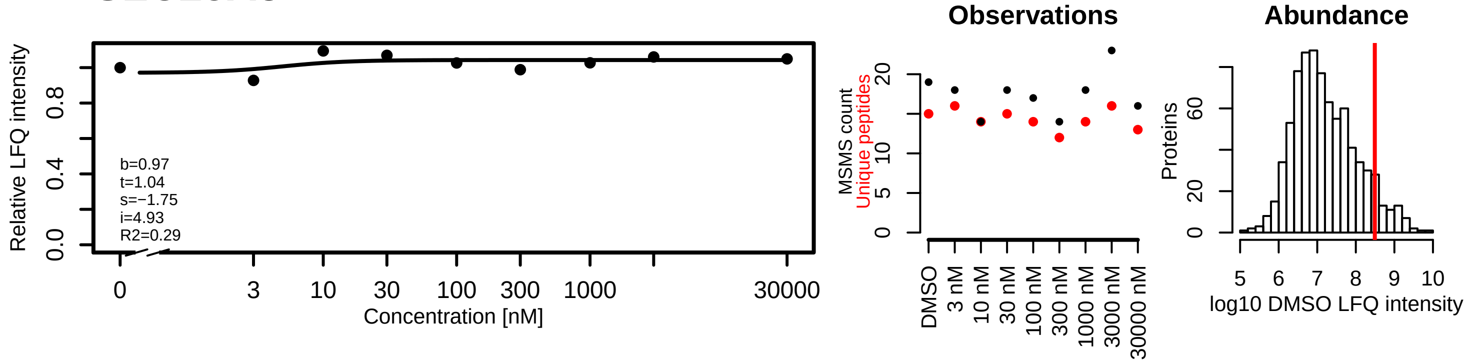


# ARAF

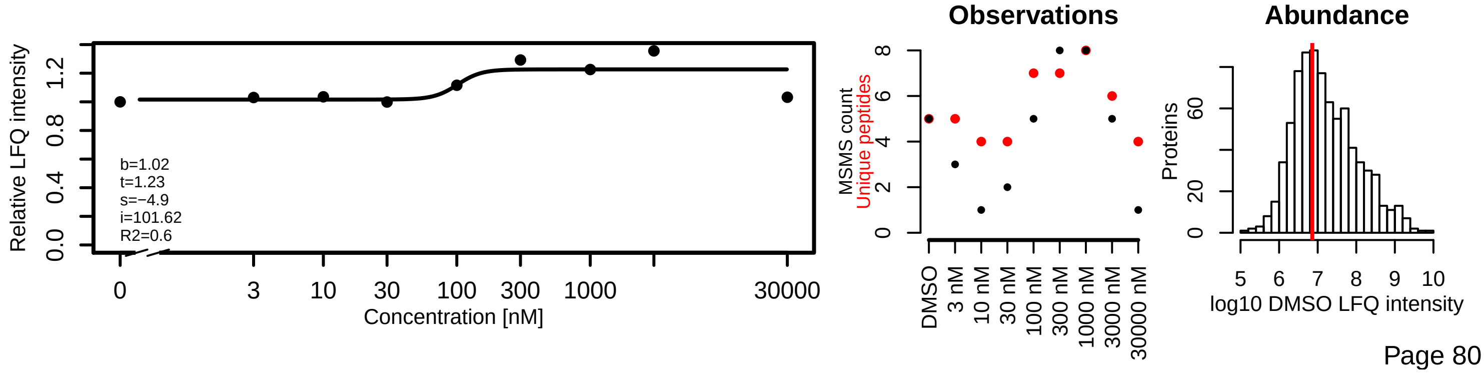

## RPL26;KRBA2;RPL26L1

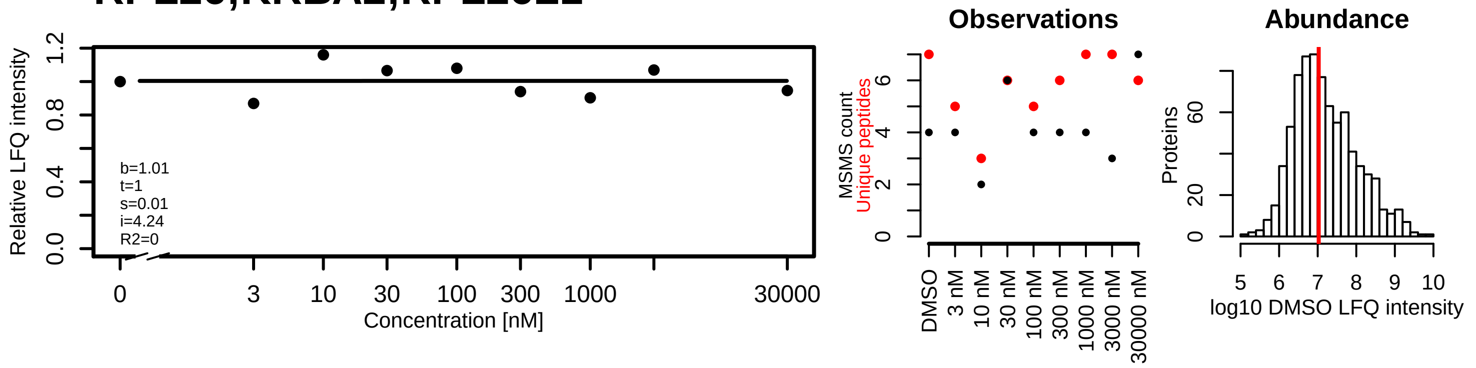


## IRAK4

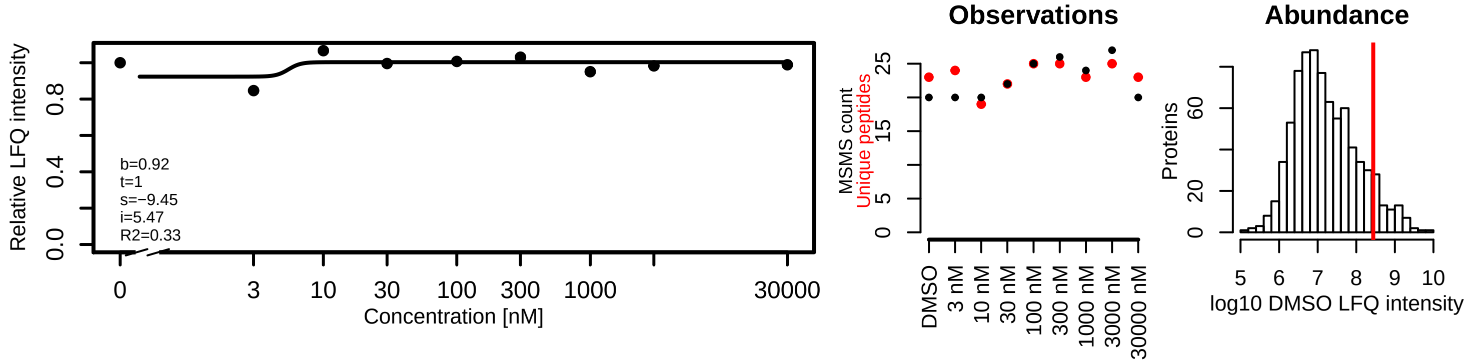


## NUAK2

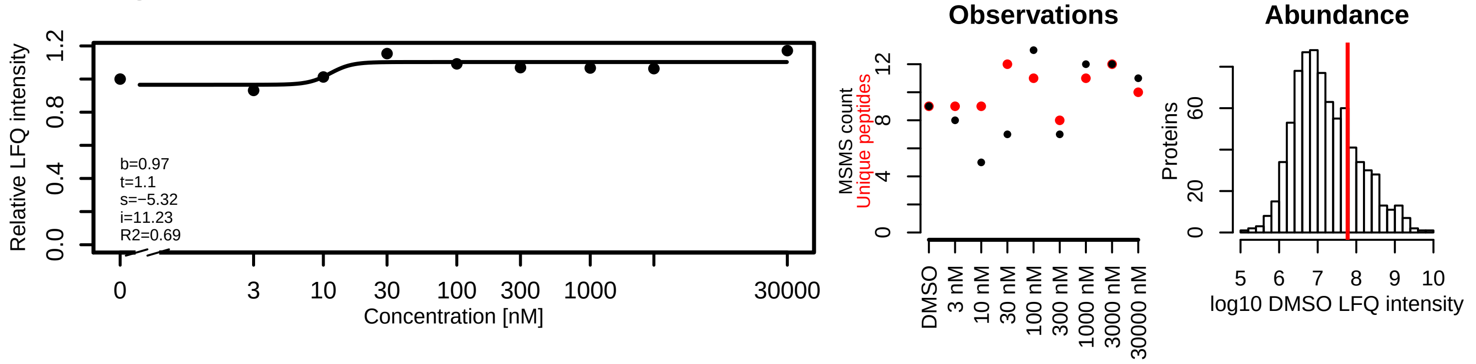


## BRI3BP

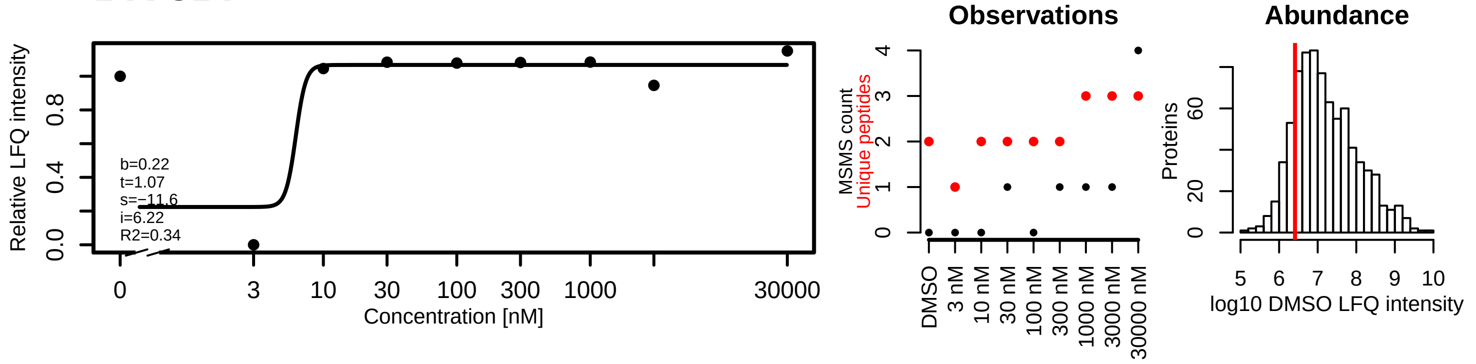


## JAK2

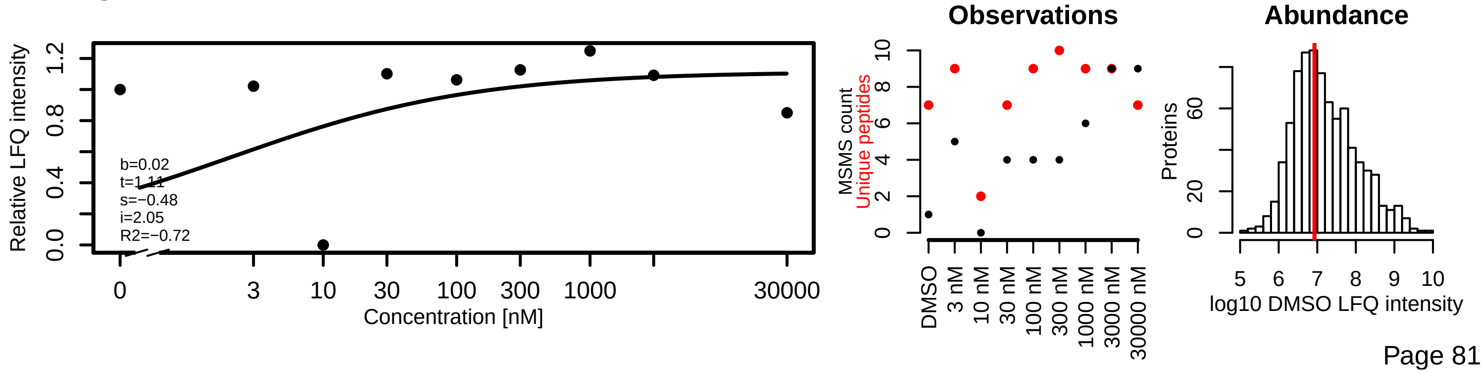

# DYRK1A

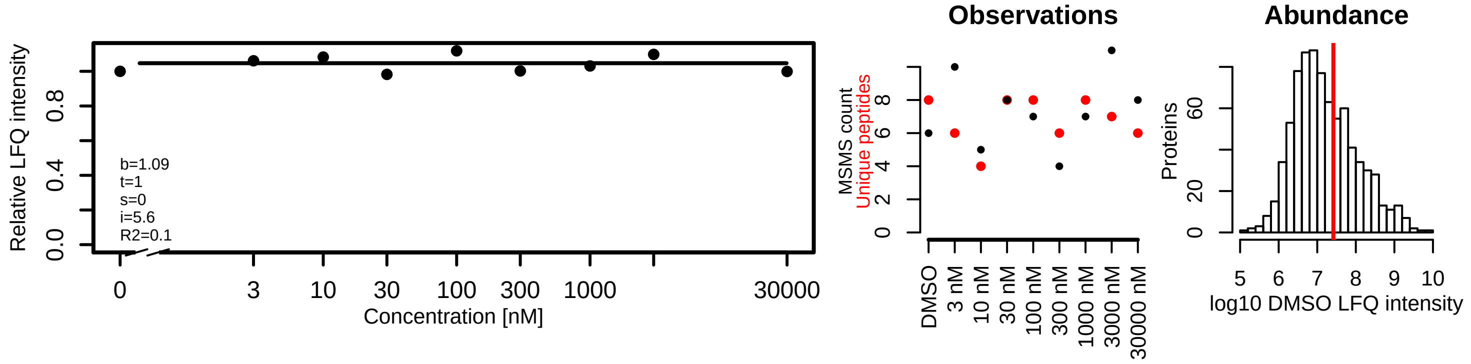


# ADCK1

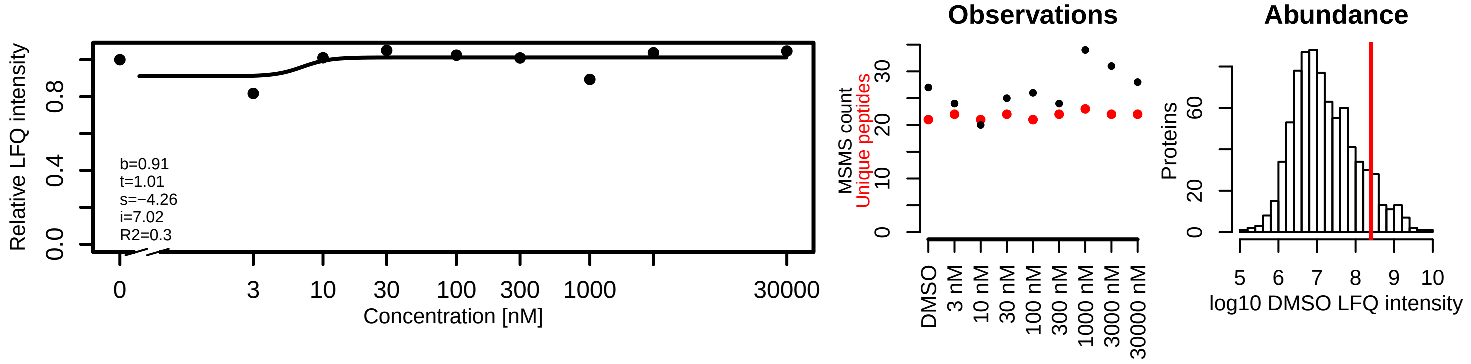


# MARK2 − E9PC69;Q7KZI7−11;Q7KZI7−8;Q7KZI7;Q7KZI7−14;Q7KZI7−

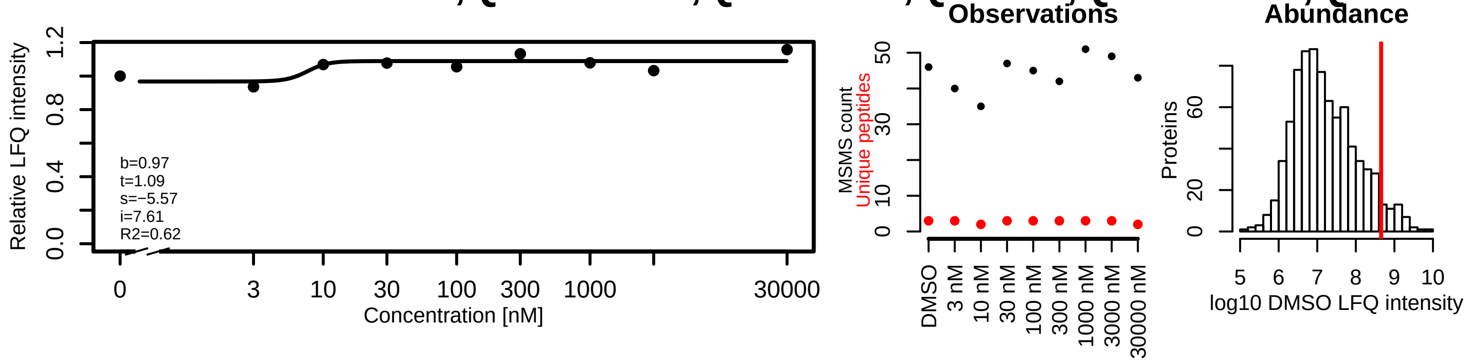


# ALDH3A2

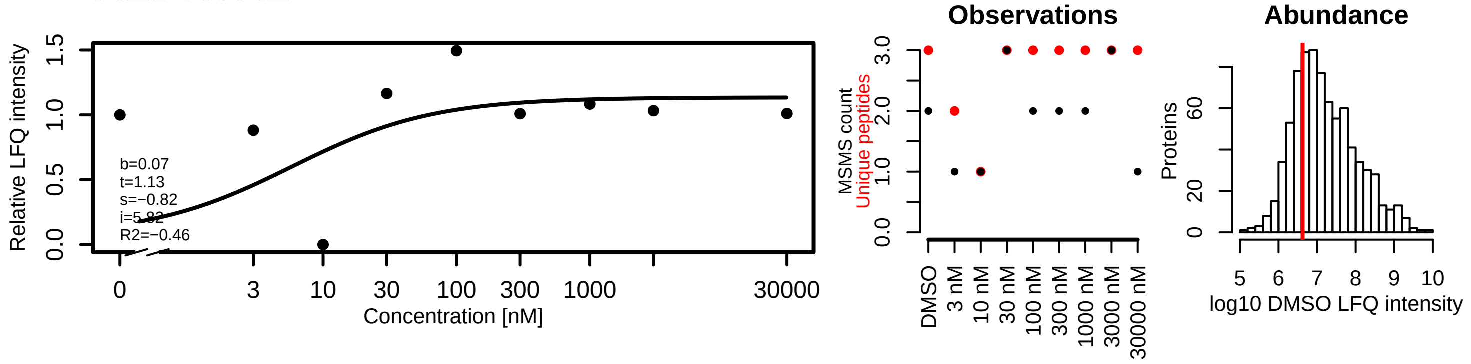


# CD9

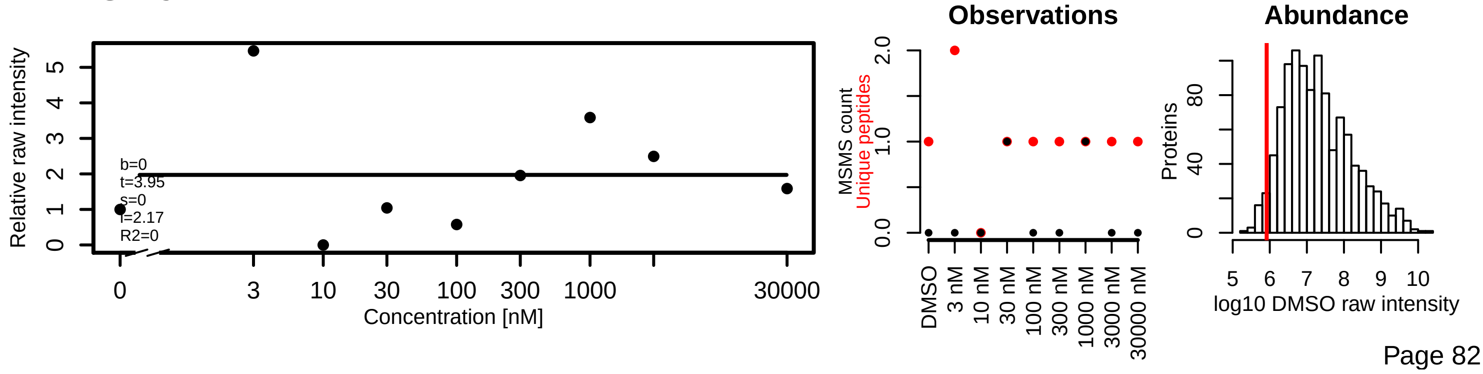

# RASSF3

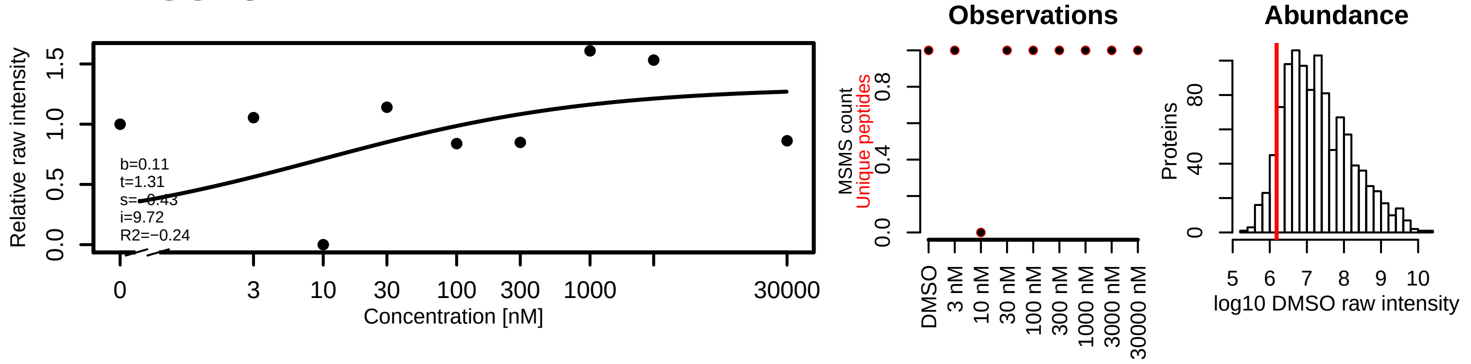


# SLMAP

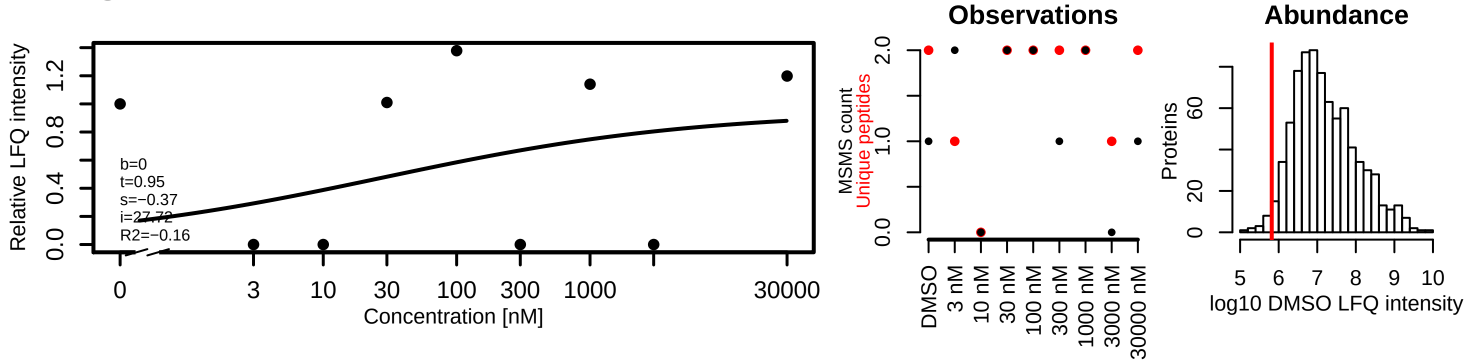


# SNRPD3

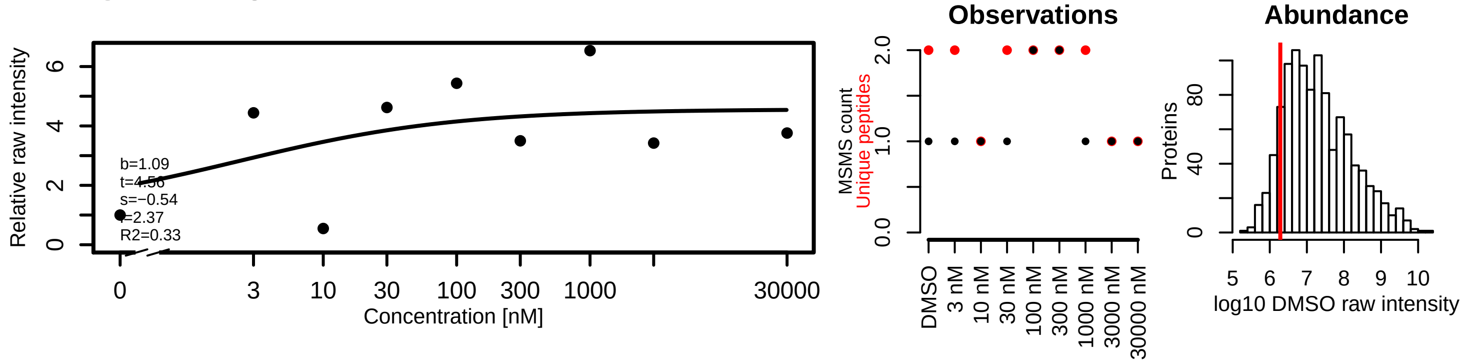


# ECM29;KIAA0368

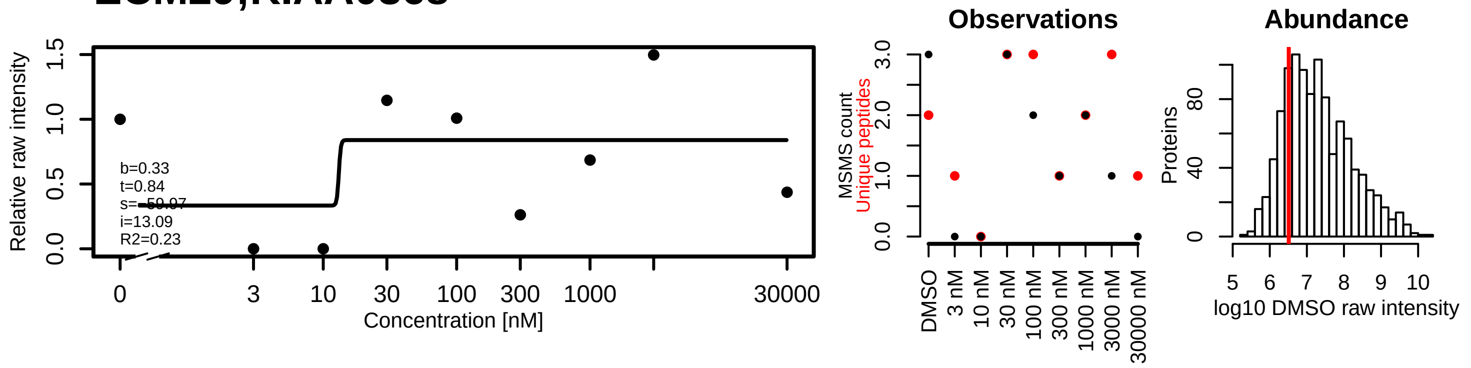


# CDK10

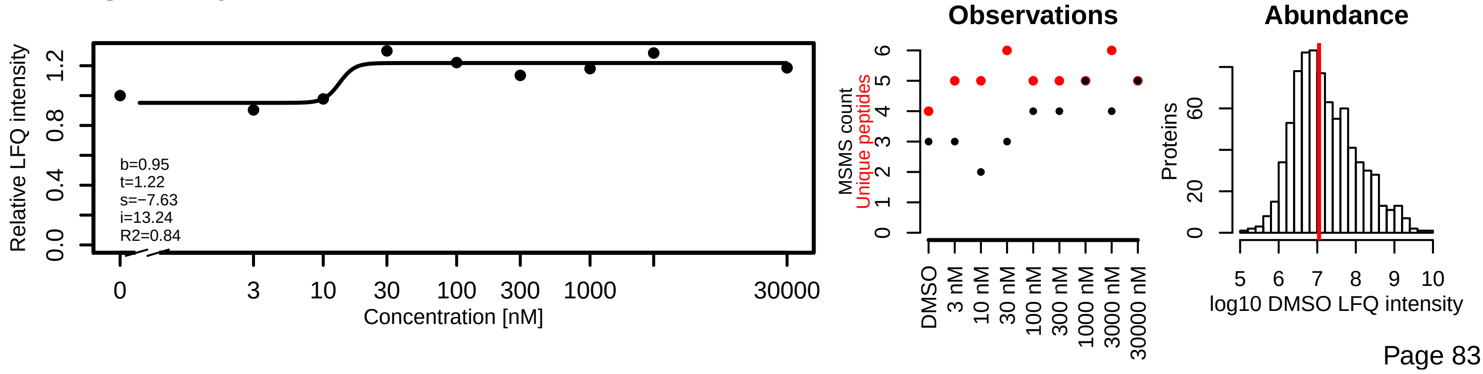

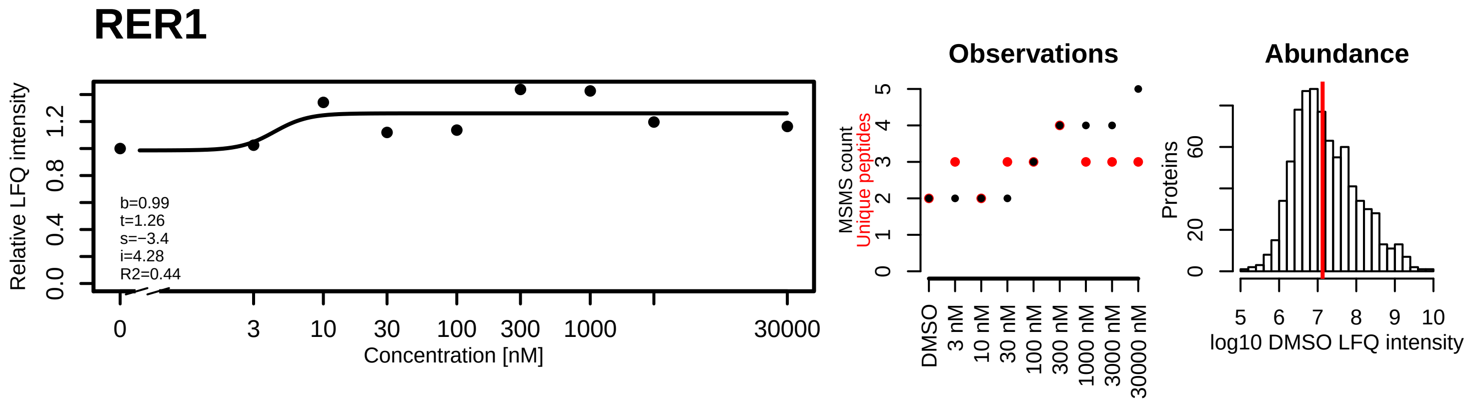
RER1
Relative LFQ intensity
Concentration [nM]
b=0.99
t=1.26
s=−3.4
i=4.28
R2=0.44
Observations
MSMS count
Unique peptides
Abundance
Proteins
log10 DMSO LFQ intensity

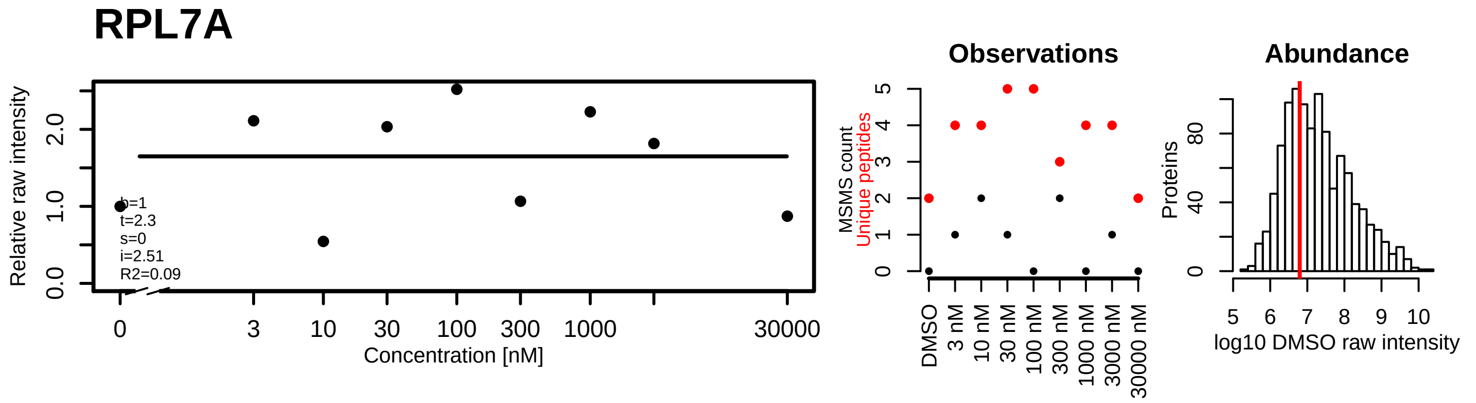
RPL7A
Relative raw intensity
Concentration [nM]
b=1
t=2.3
s=0
i=2.51
R2=0.09
Observations
MSMS count
Unique peptides
Abundance
Proteins
log10 DMSO raw intensity

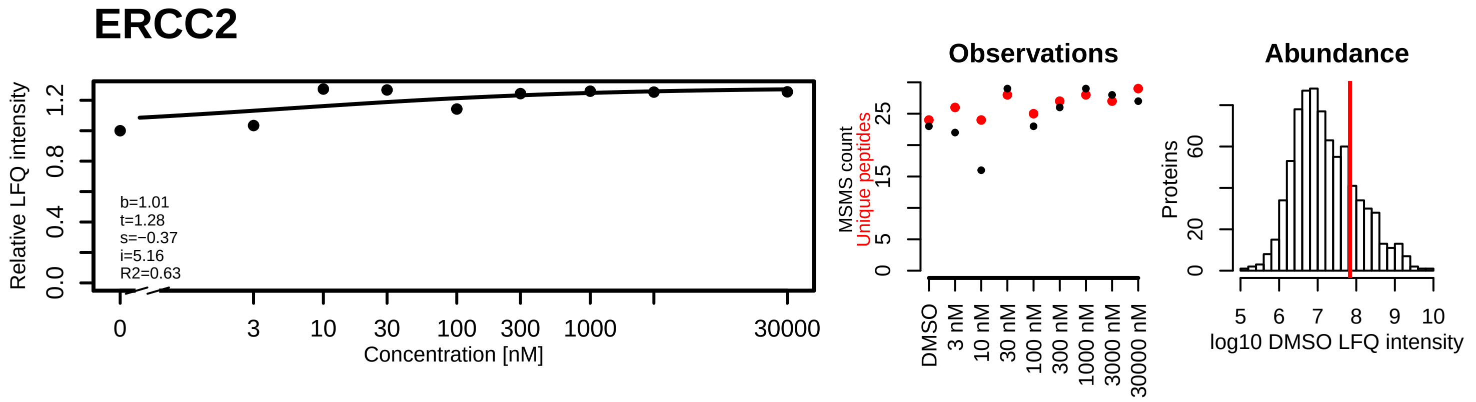
ERCC2
Relative LFQ intensity
Concentration [nM]
b=1.01
t=1.28
s=−0.37
i=5.16
R2=0.63
Observations
MSMS count
Unique peptides
Abundance
Proteins
log10 DMSO LFQ intensity

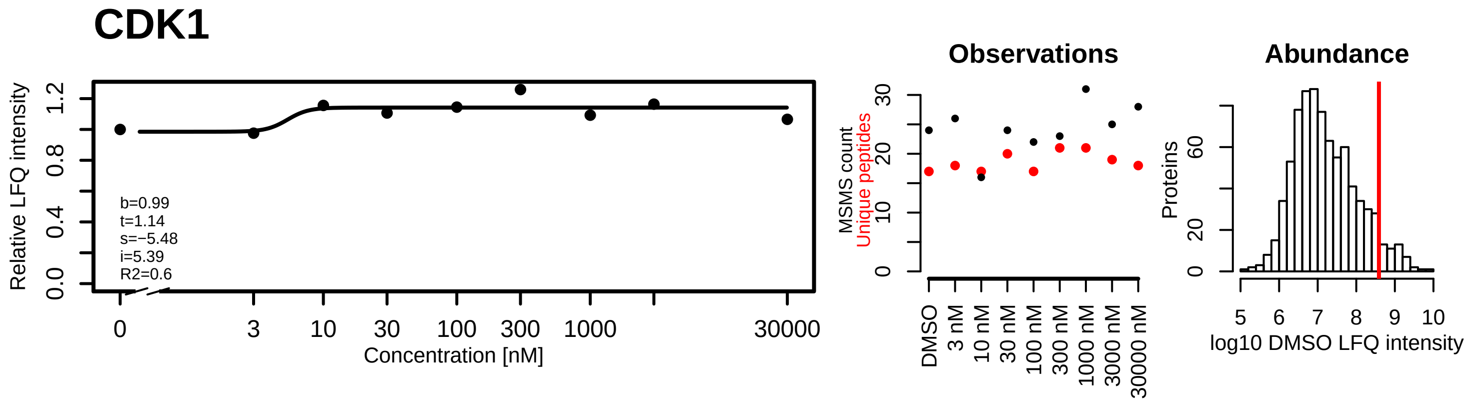
CDK1
Relative LFQ intensity
Concentration [nM]
b=0.99
t=1.14
s=−5.48
i=5.39
R2=0.6
Observations
MSMS count
Unique peptides
Abundance
Proteins
log10 DMSO LFQ intensity

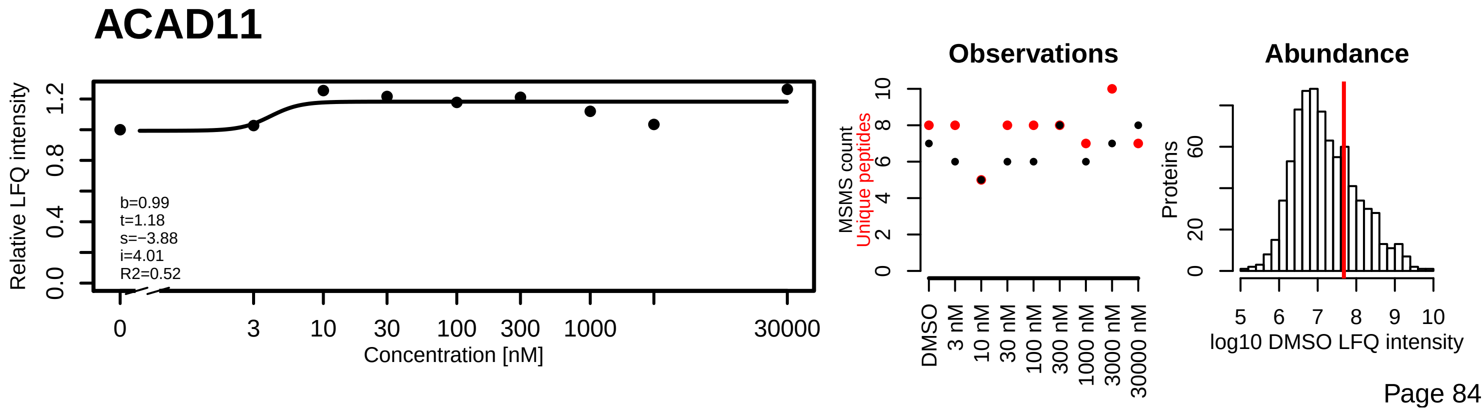
ACAD11
Relative LFQ intensity
Concentration [nM]
b=0.99
t=1.18
s=−3.88
i=4.01
R2=0.52
Observations
MSMS count
Unique peptides
Abundance
Proteins
log10 DMSO LFQ intensity

## SLC25A1

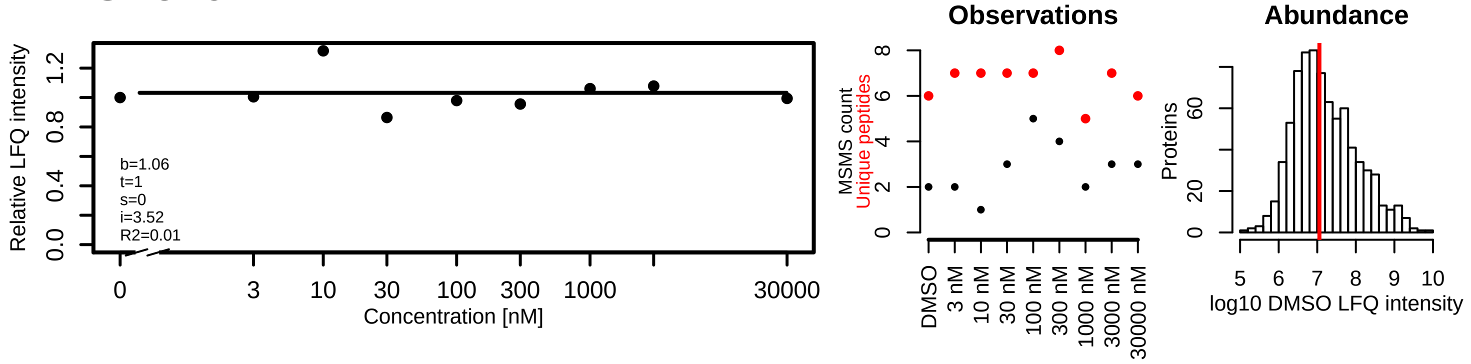


## CDK9

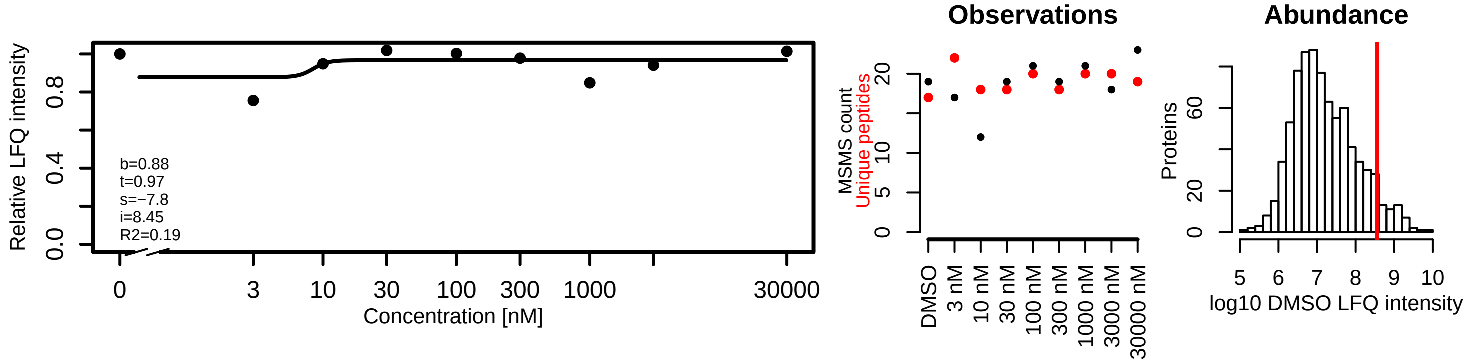


## HNRNPA1

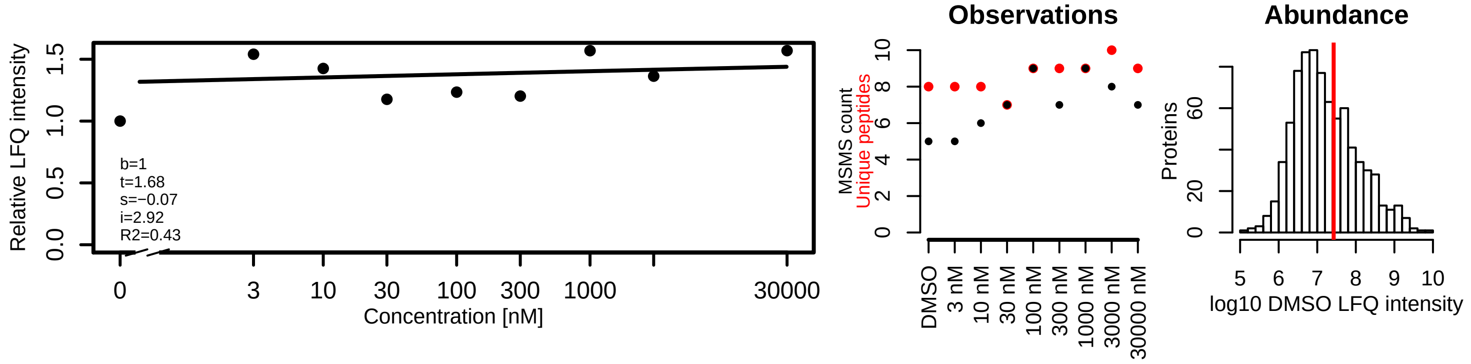


## MAP3K9;MLK4

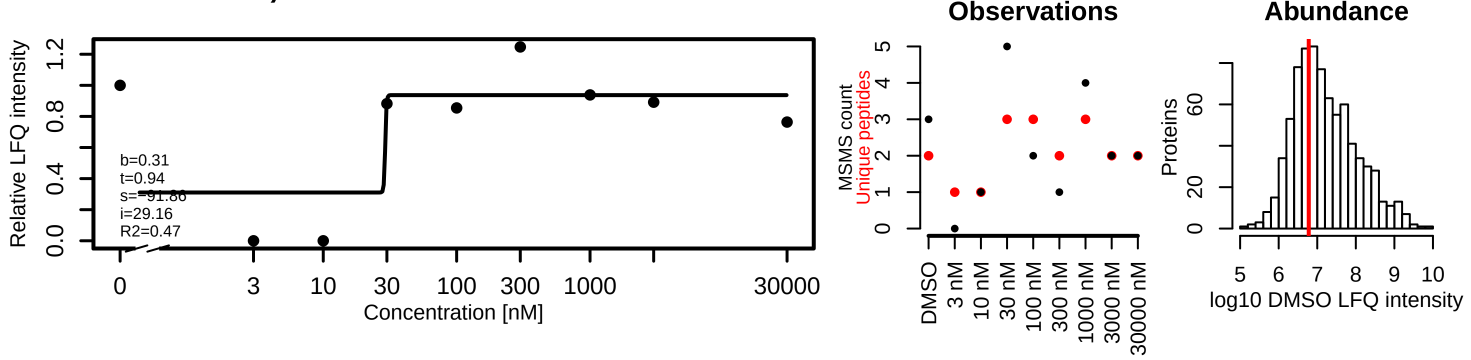


## RB1CC1

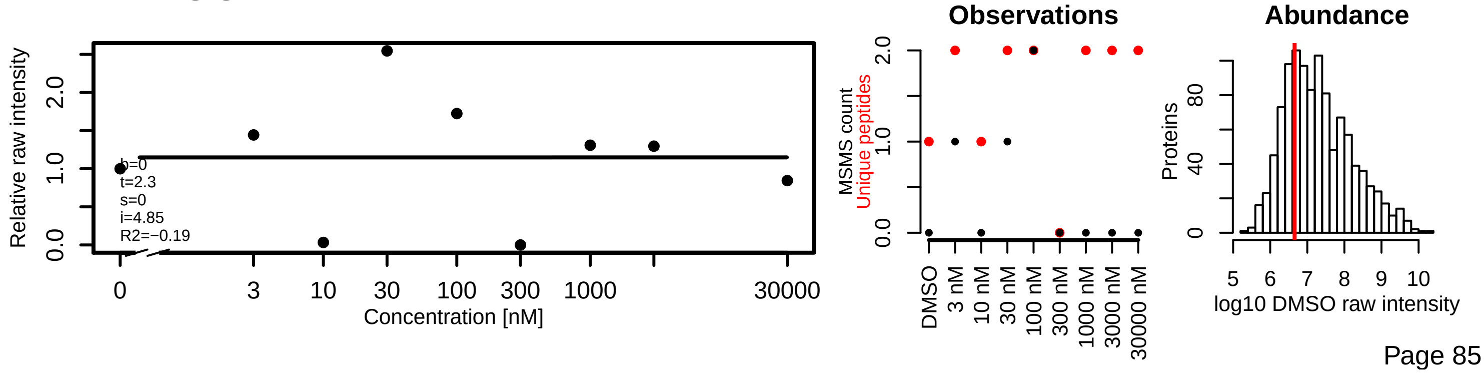

# HSP90AB1

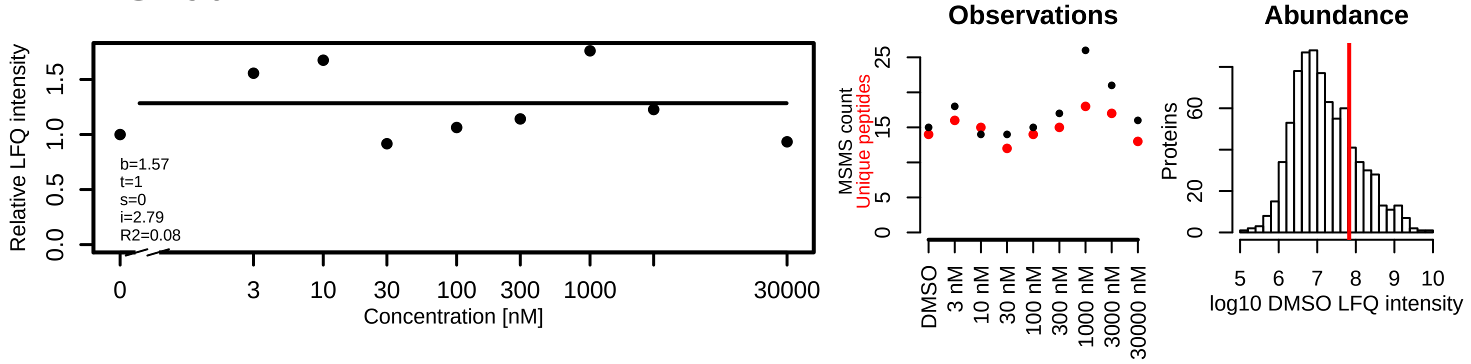


# CDC37

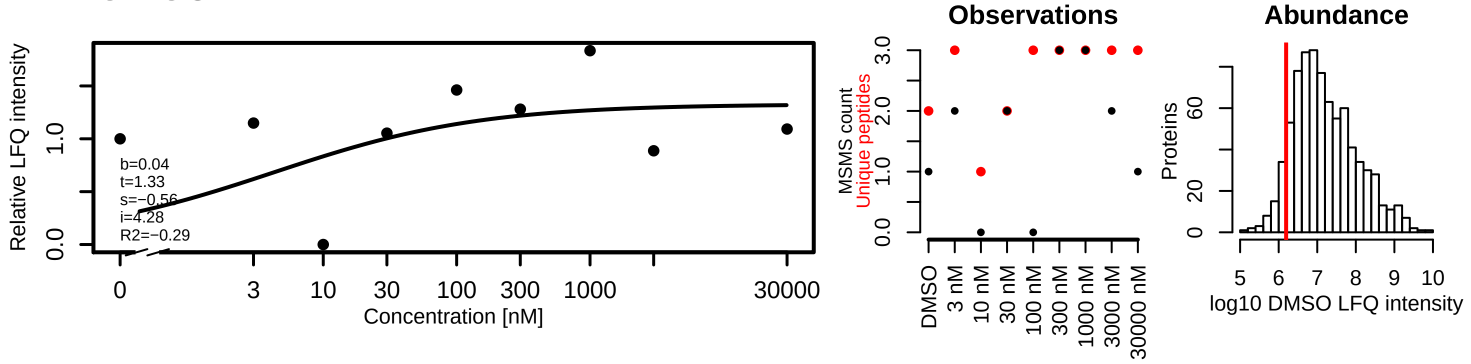


# SLC39A14

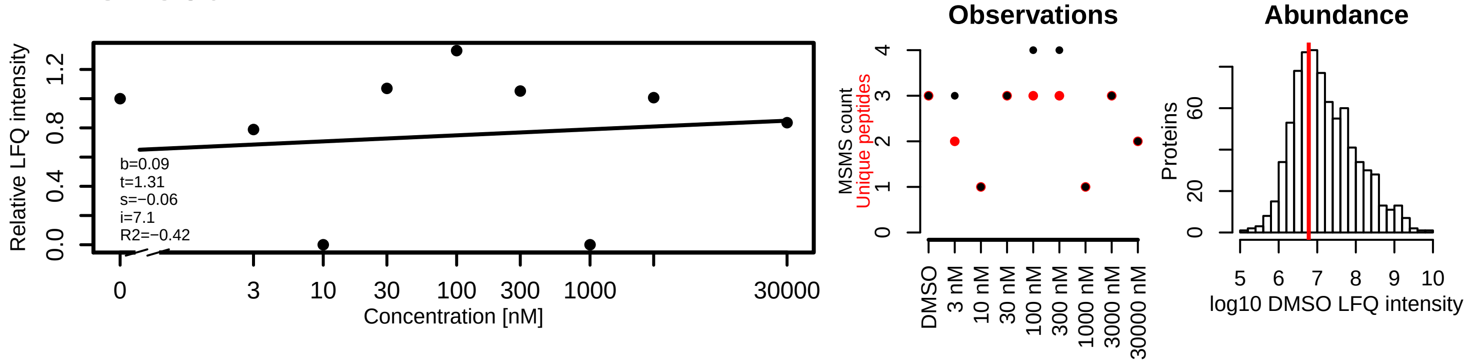


# FIBP

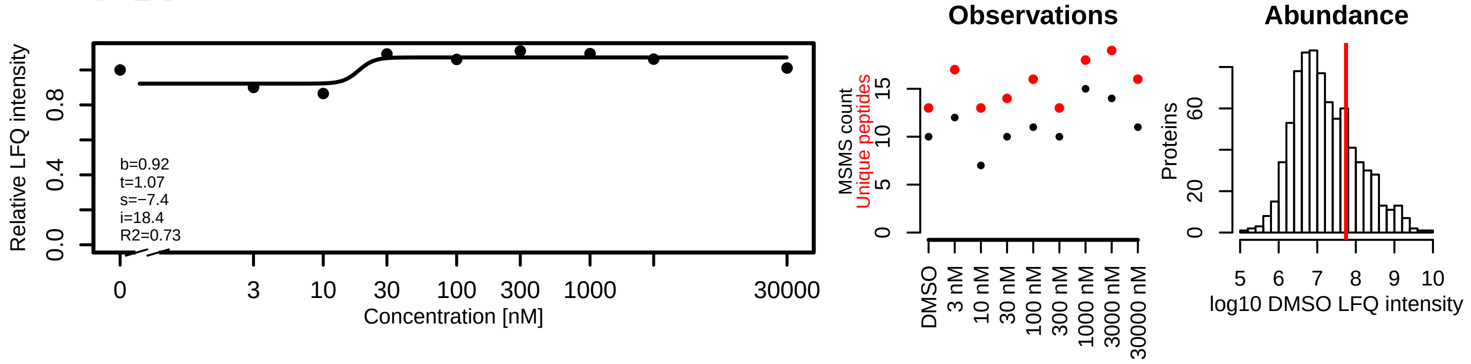


# PDHB

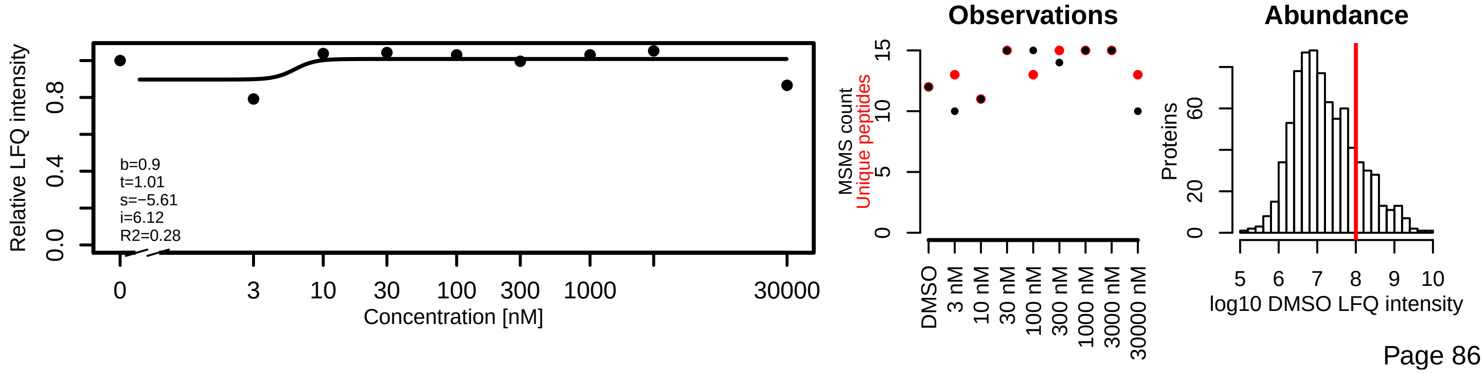

# SURF4

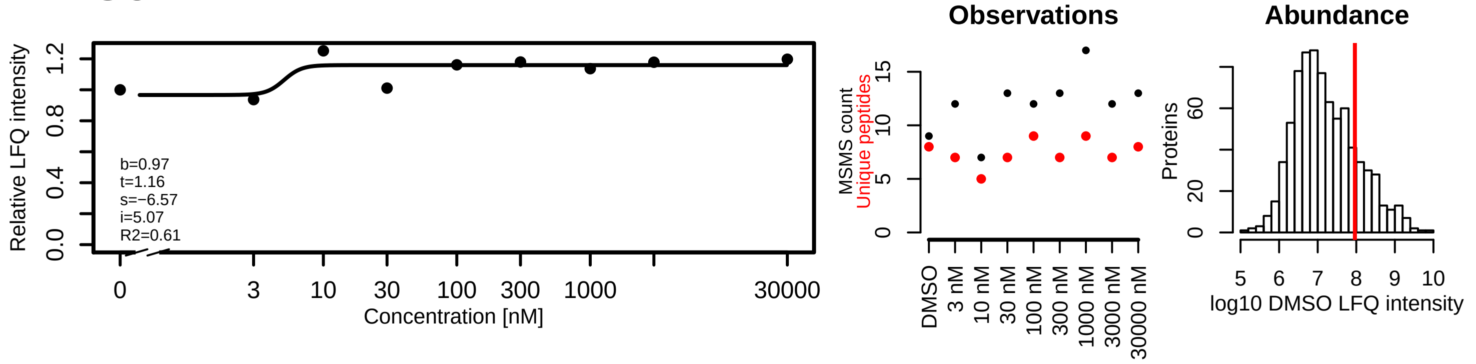


# OSGEP

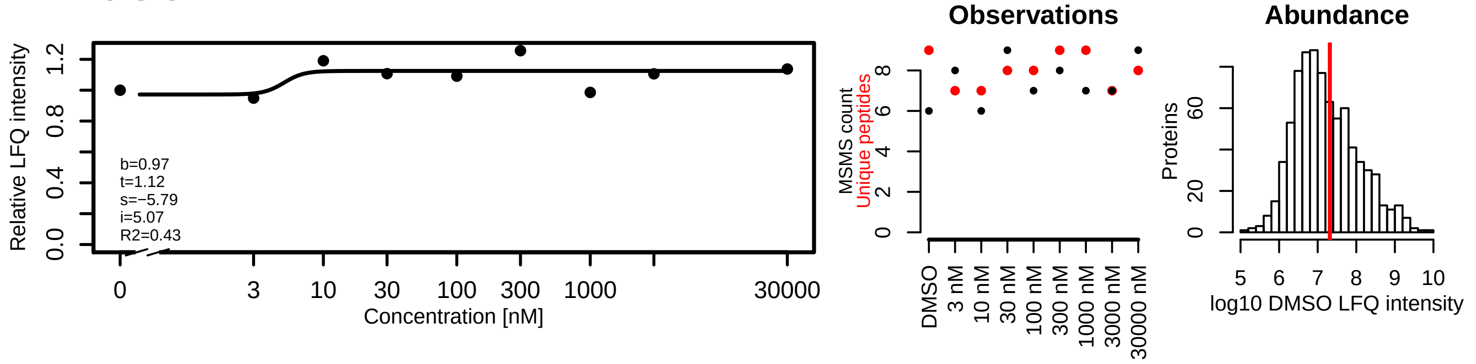


# PRKAB2

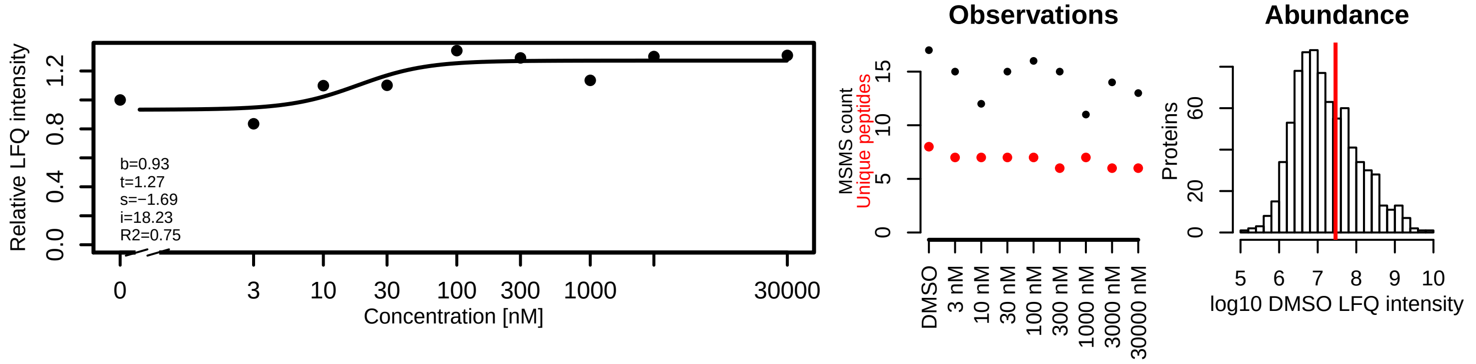


# RPS10

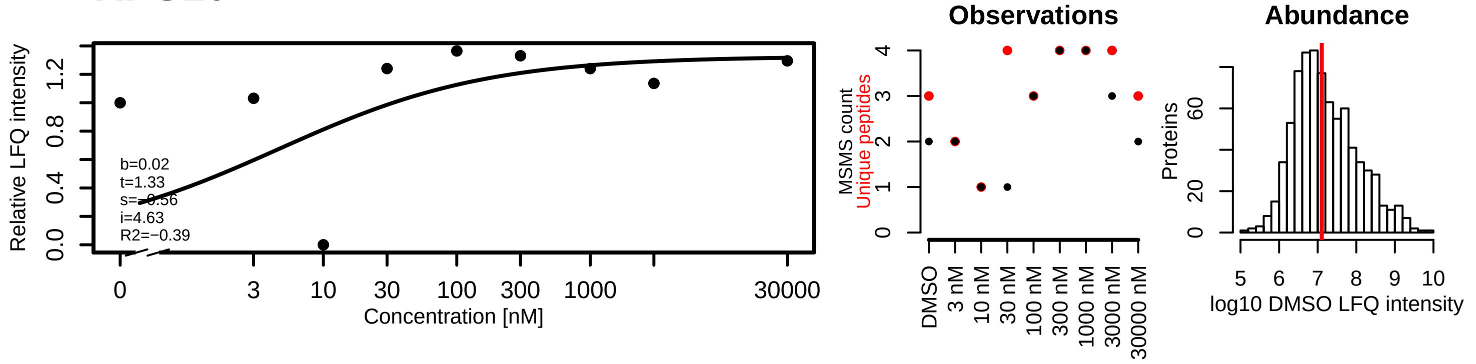


# ALDH1B1

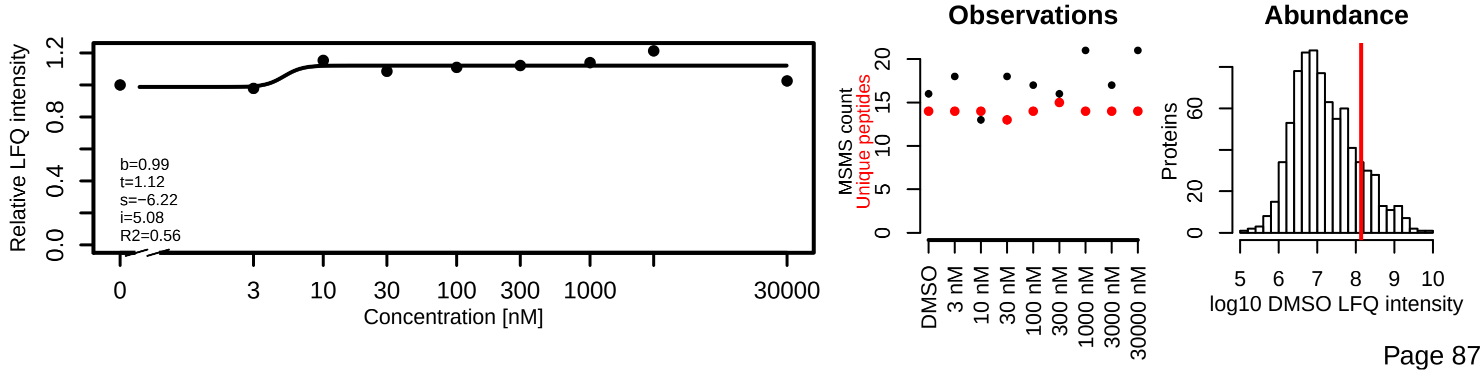

# ACVR1B

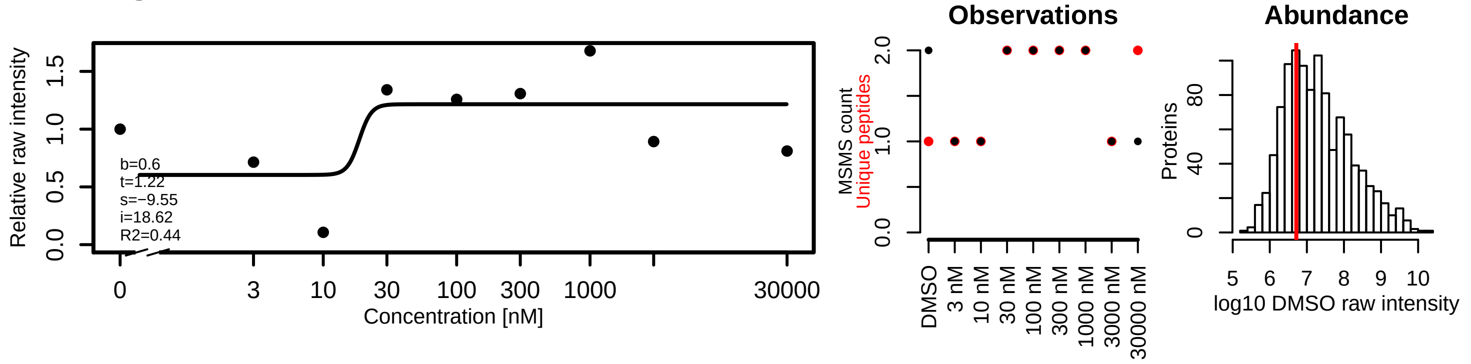


# MED23

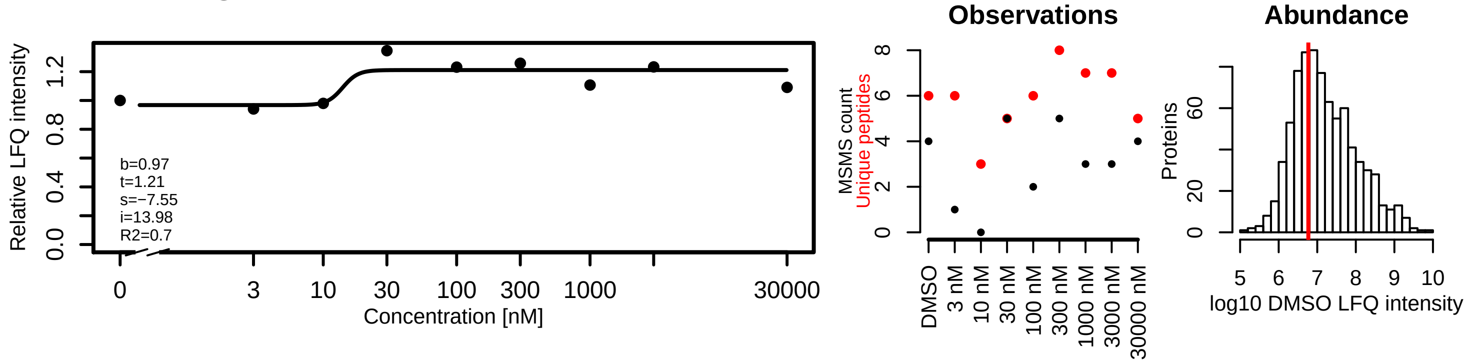


# TMEM33

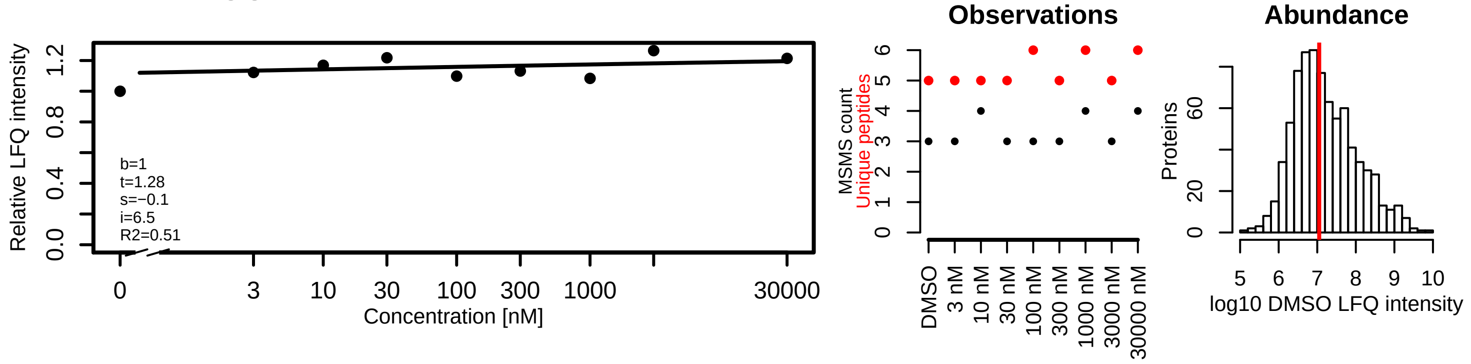


# RANBP2;RGPD8;RGPD5

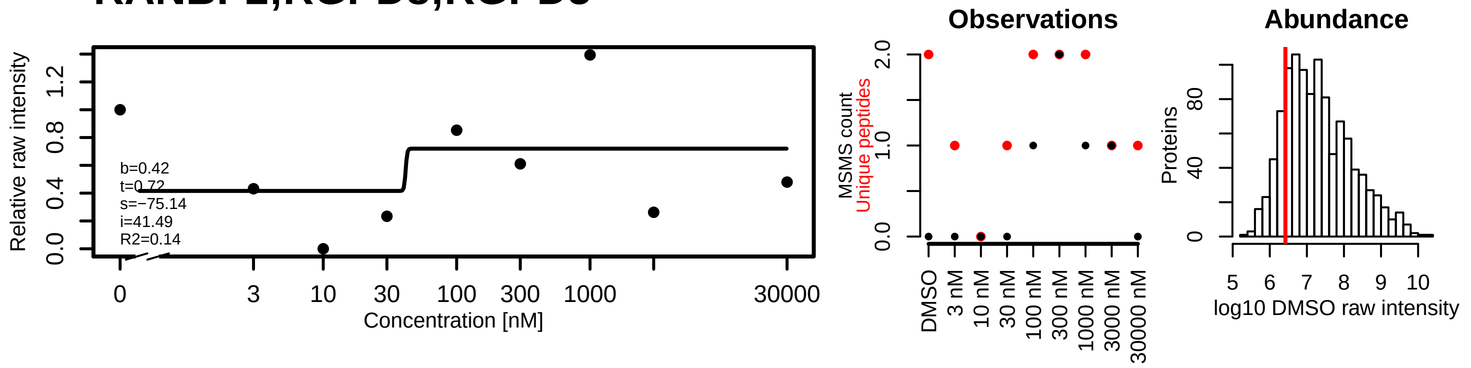


# RAB14

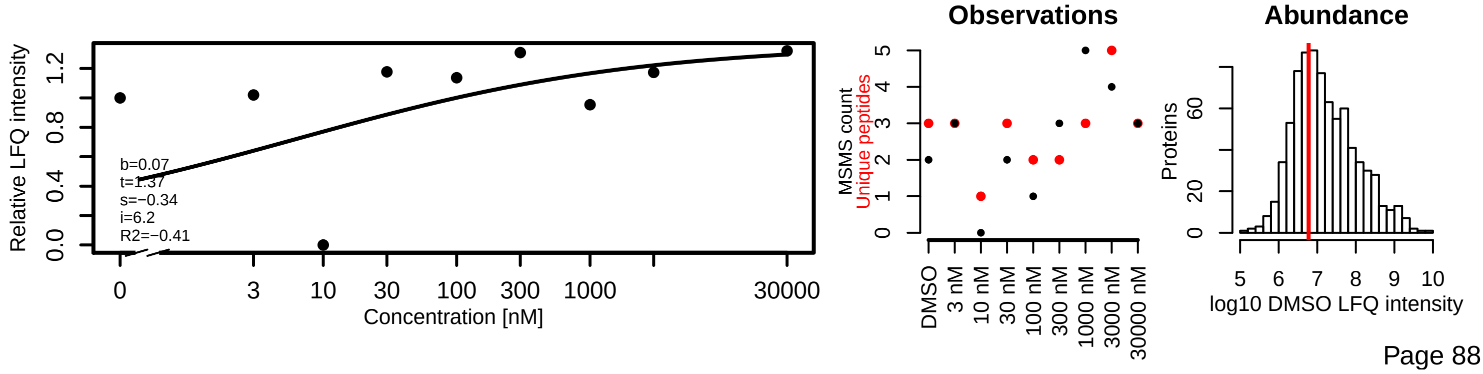

# MAP3K11

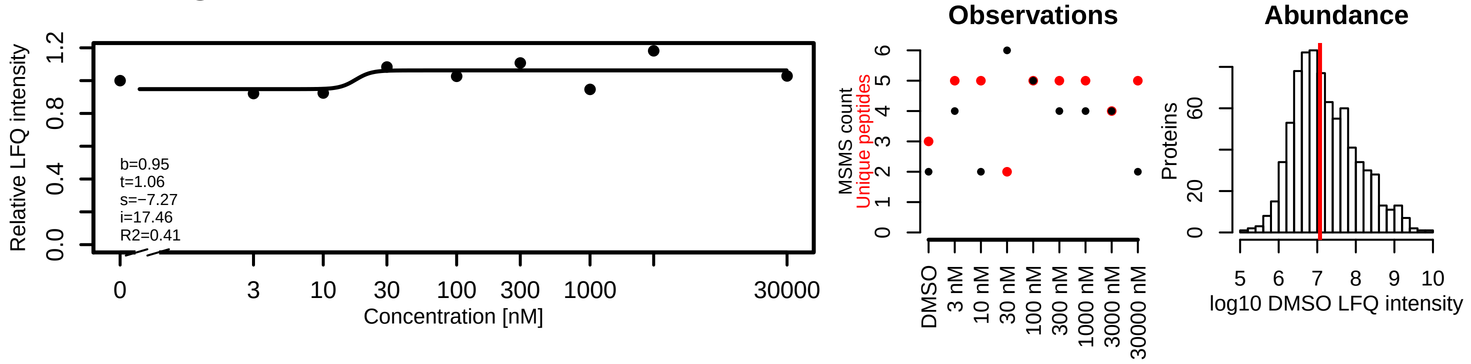


# MAPK1

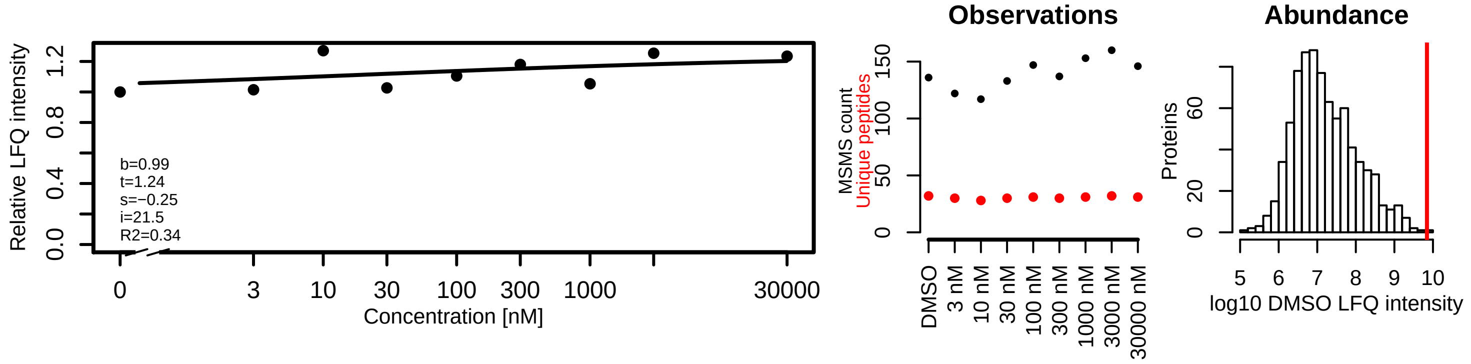


# MAP2K2

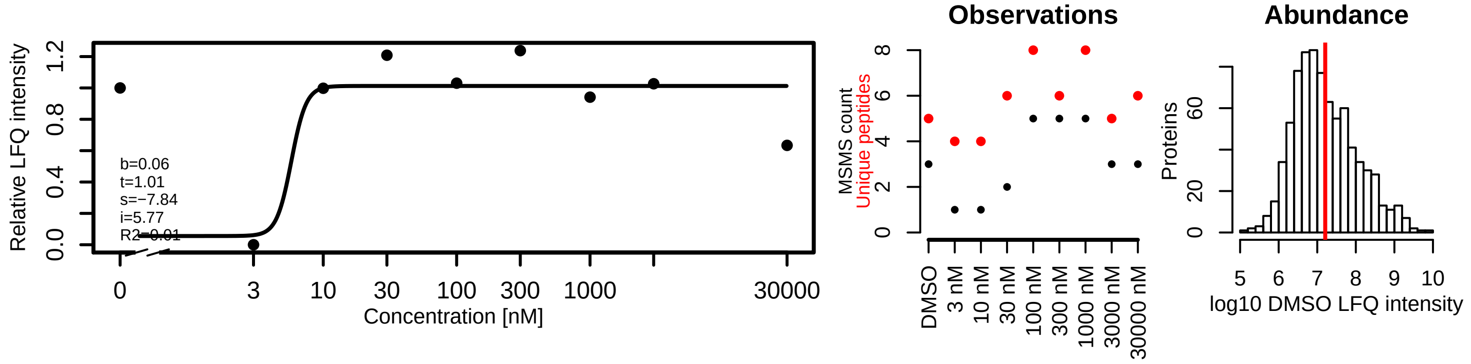


# RAB5A

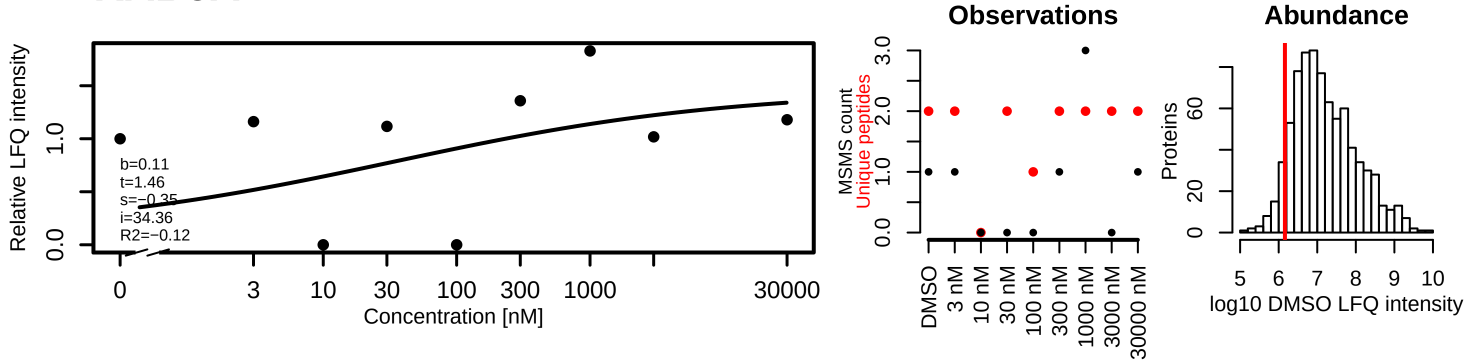


# GCDH

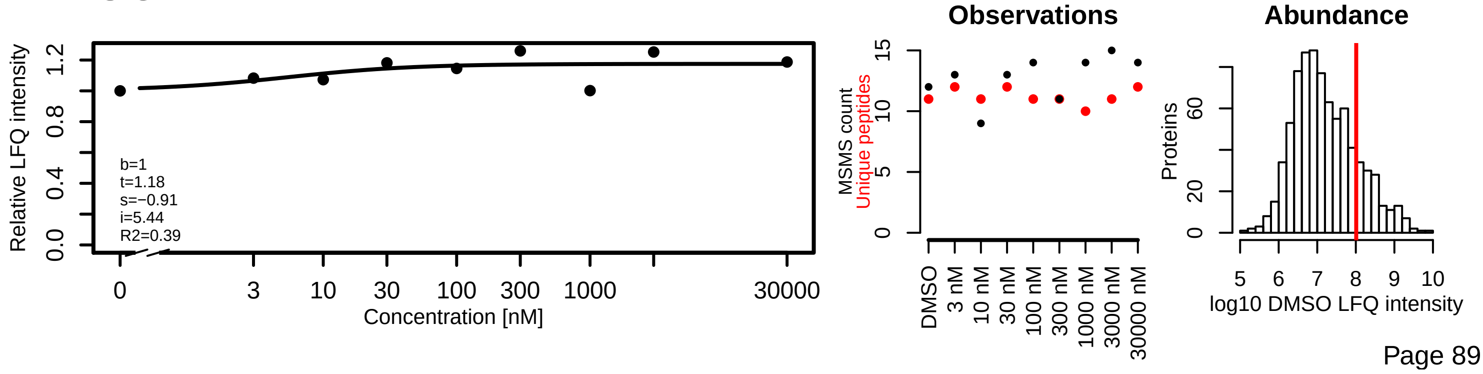

# RPL37A

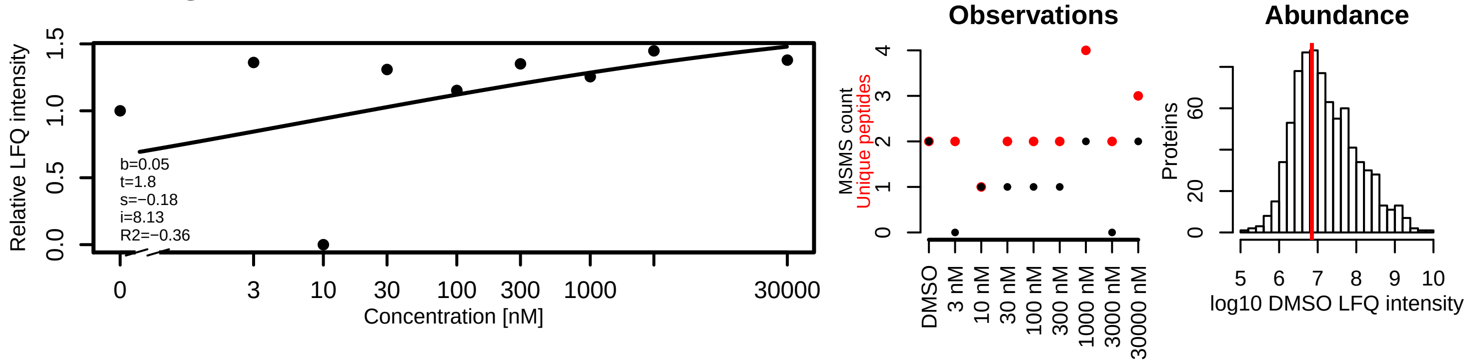


# EPHA7

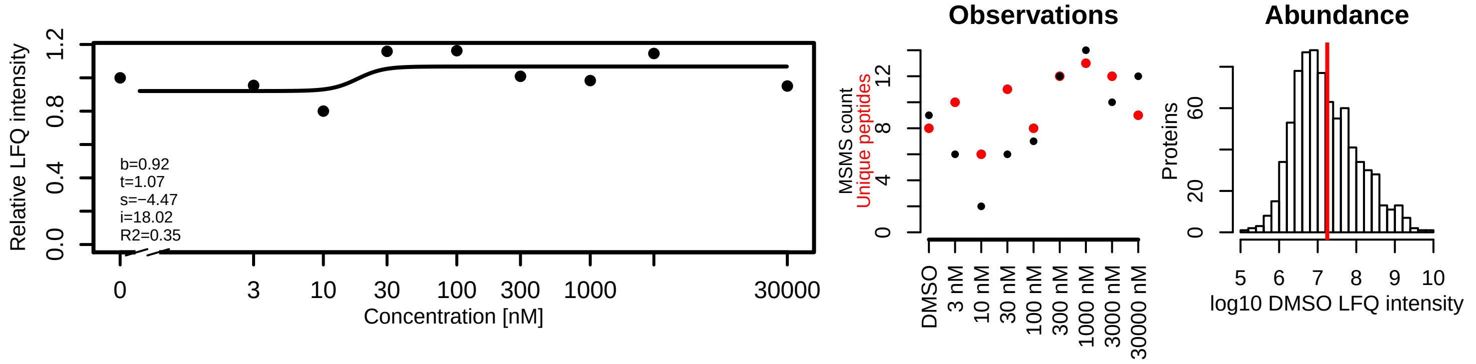


# MNAT1

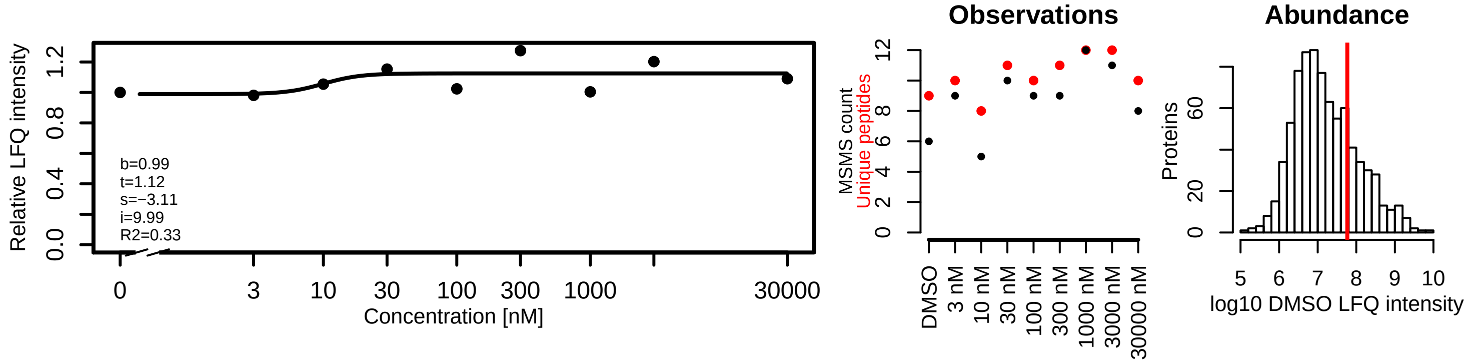


# DLAT

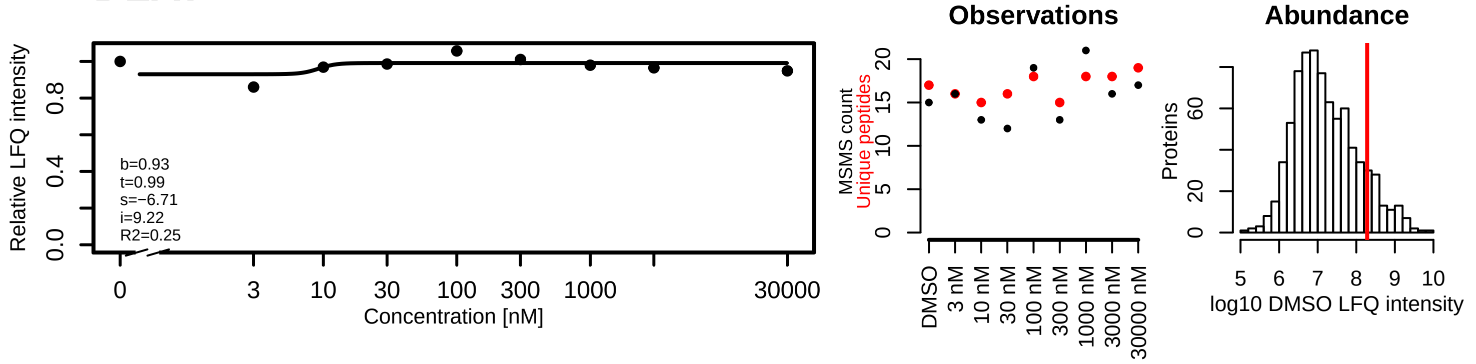


# PARP16

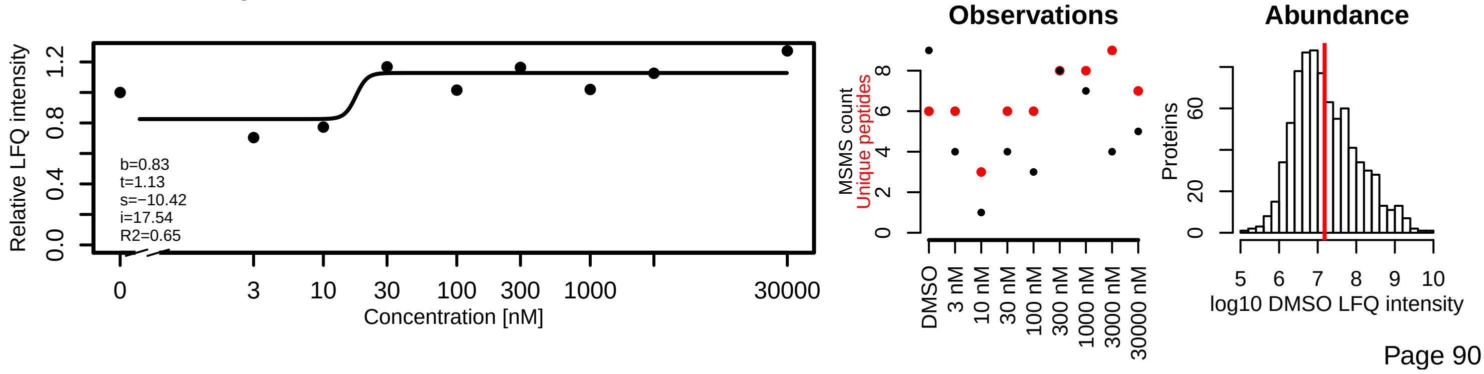

## Q6ZSR9;I3L097

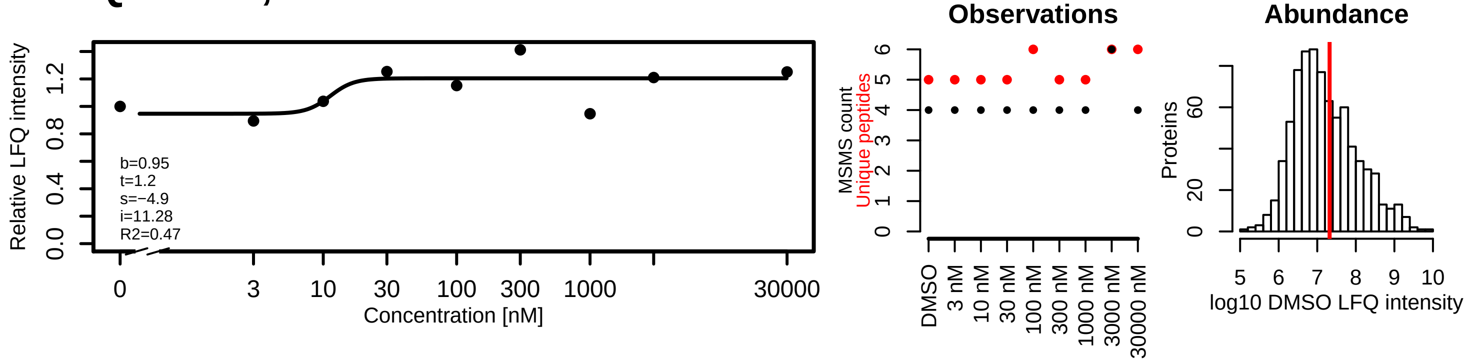


## CDC42BPA

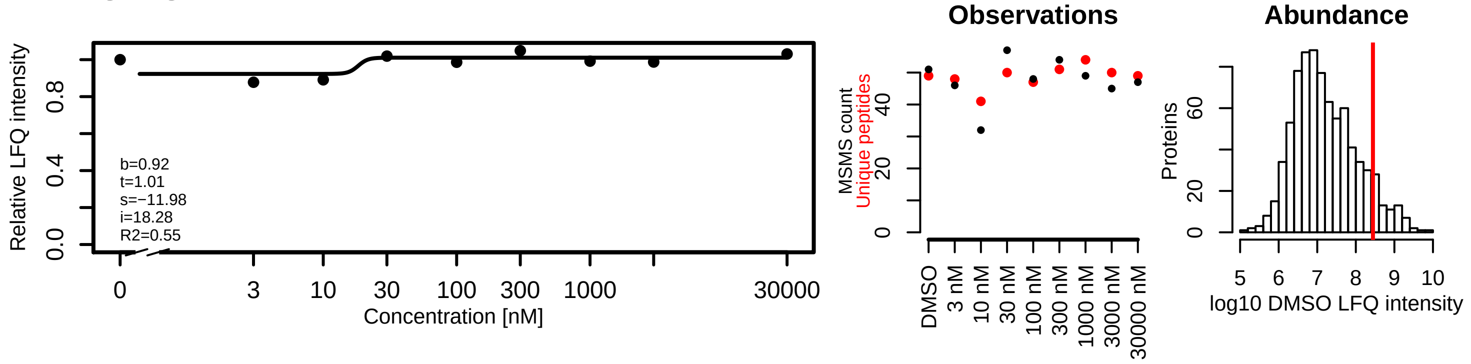


## RPLP0;RPLP0P6

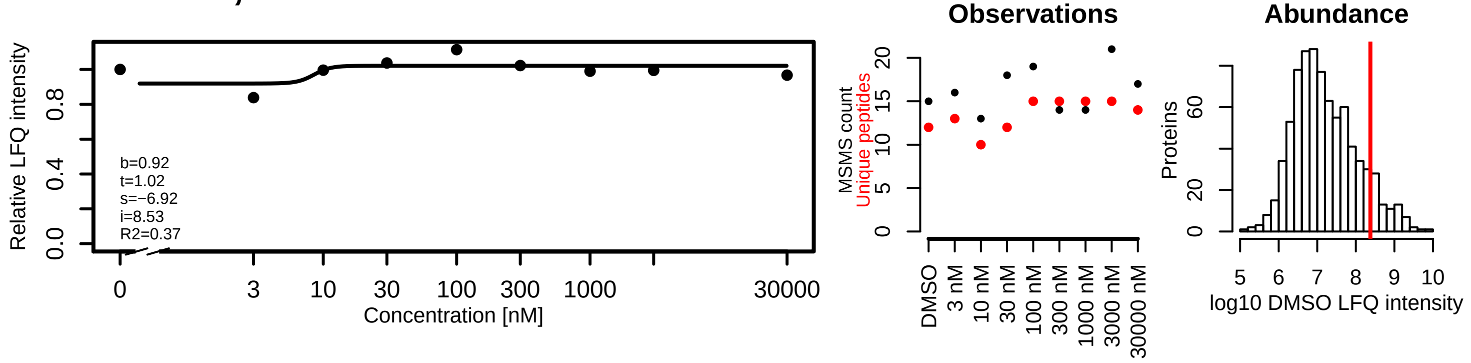


## HSD17B2

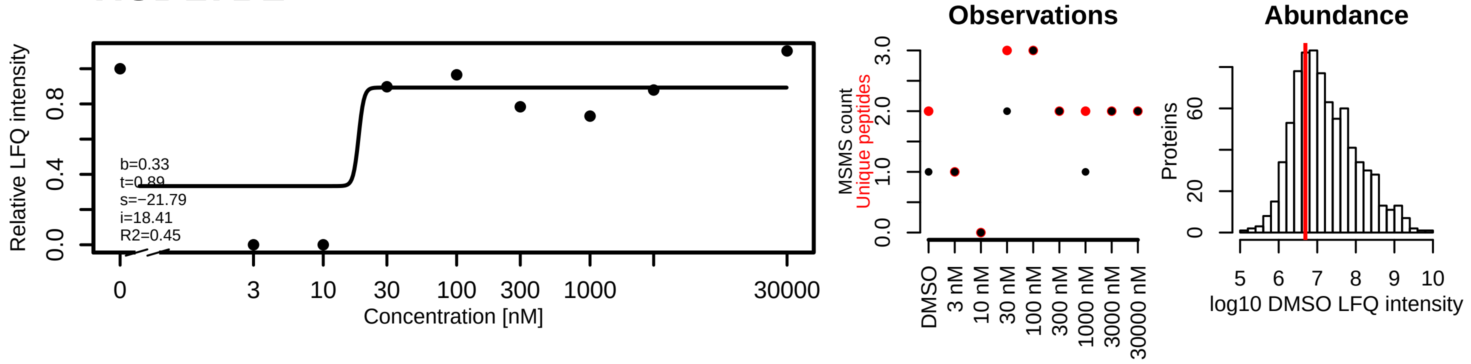


## AAK1

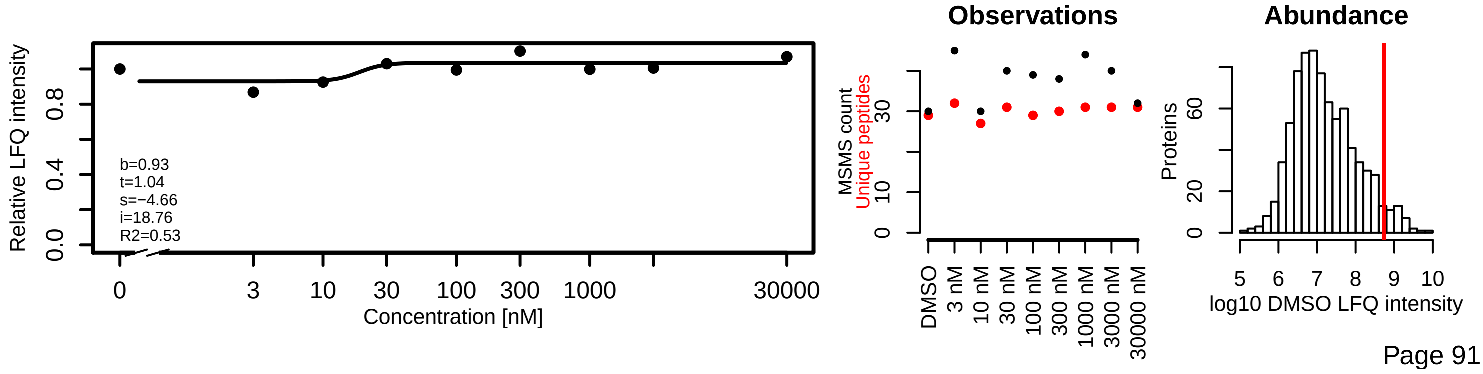

# SQRDL

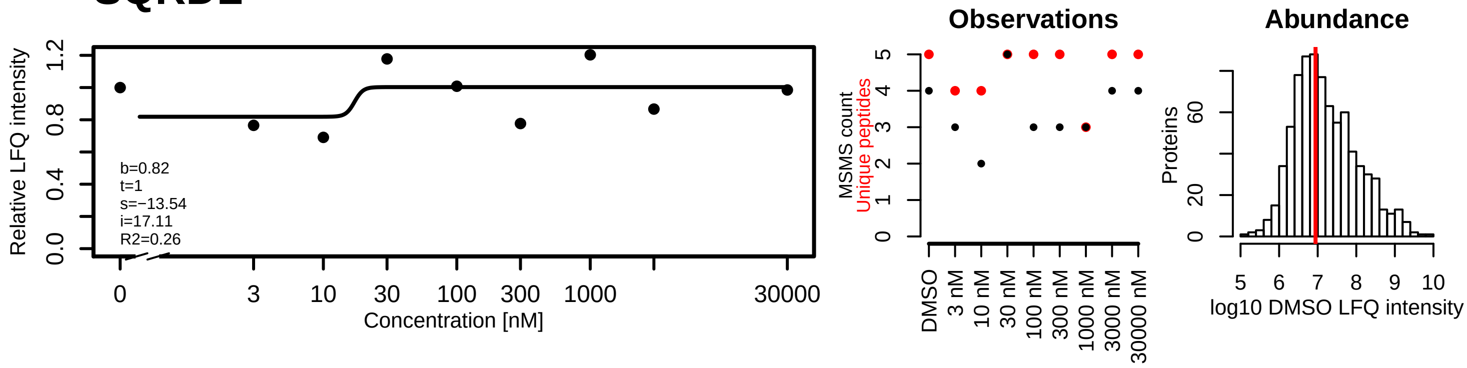


# P4HB

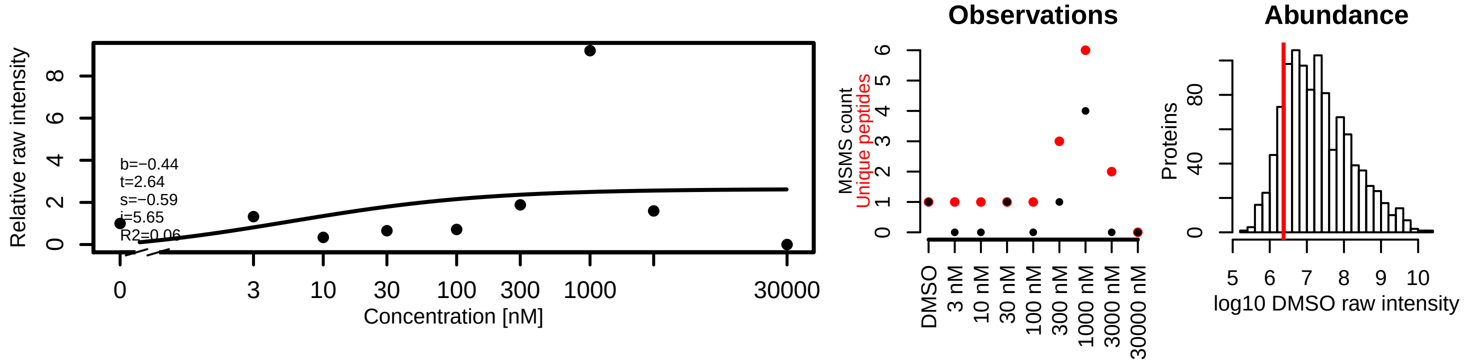


# CDKL5

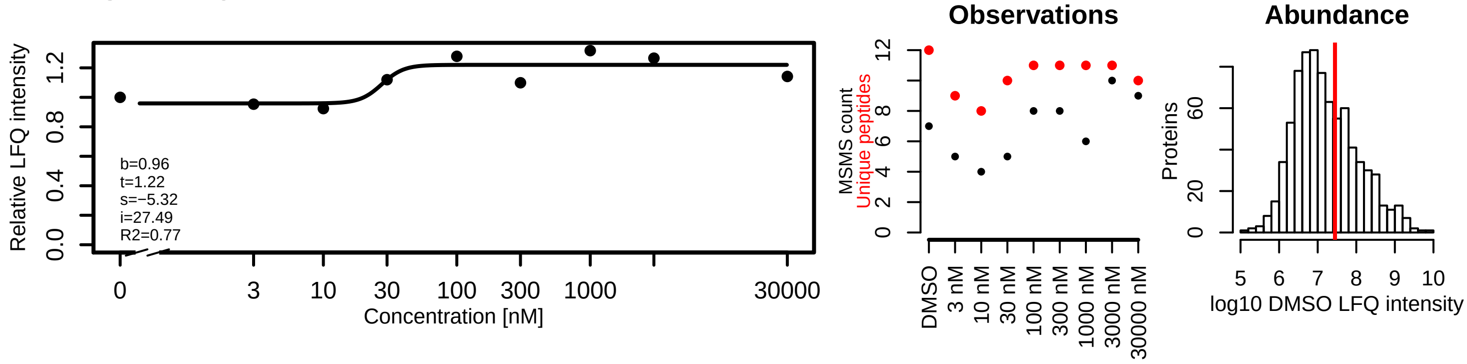


# PDPK1;PDPK2

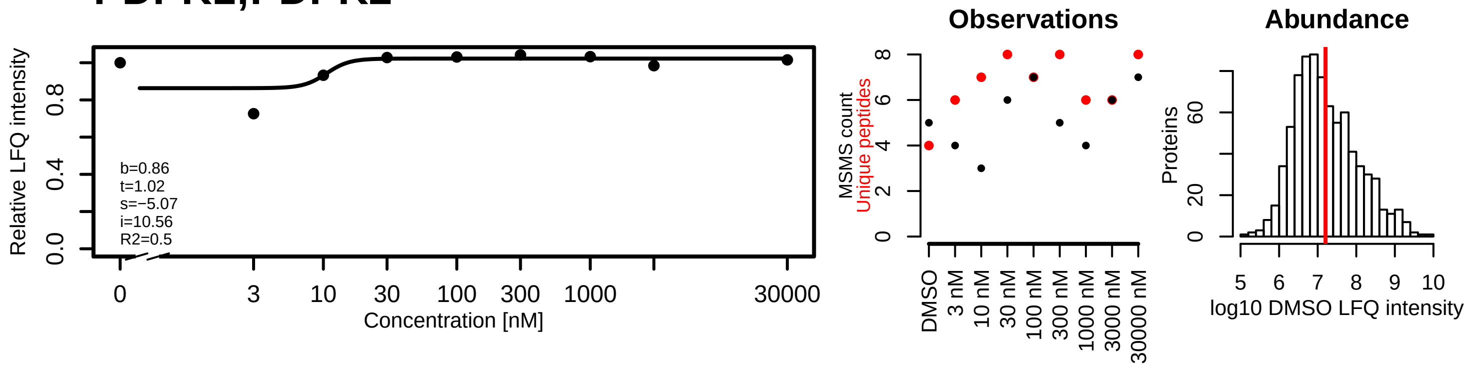


# DPY19L1

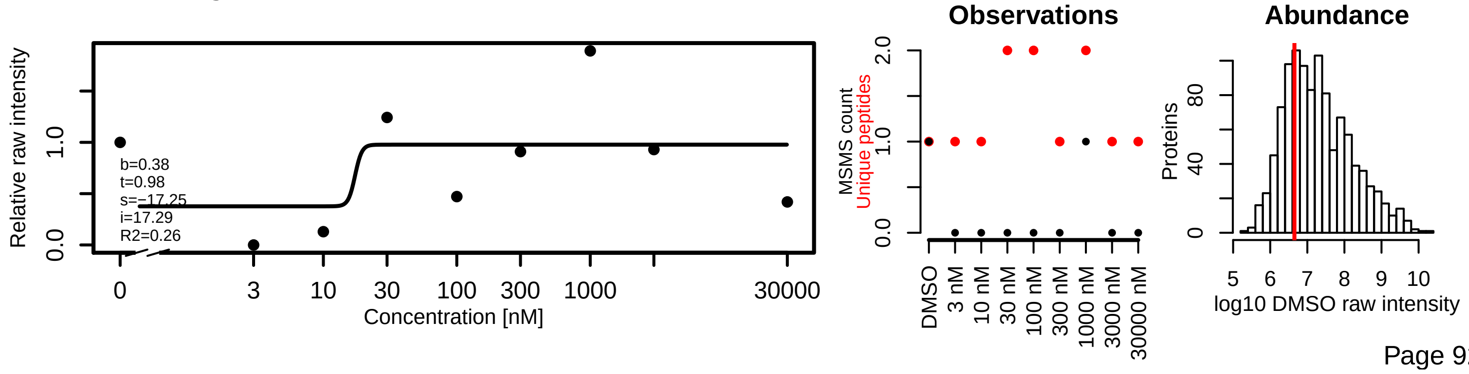

## TANK

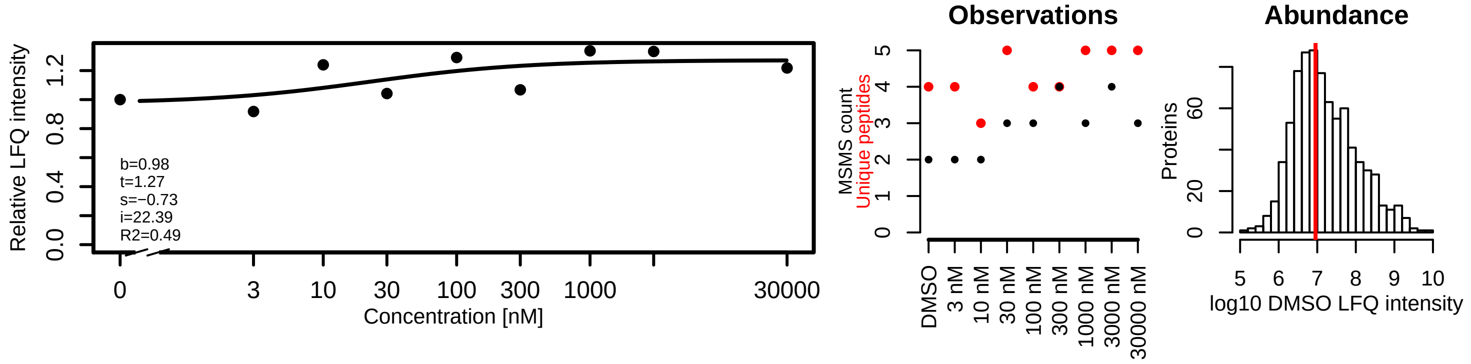


## DAPK3

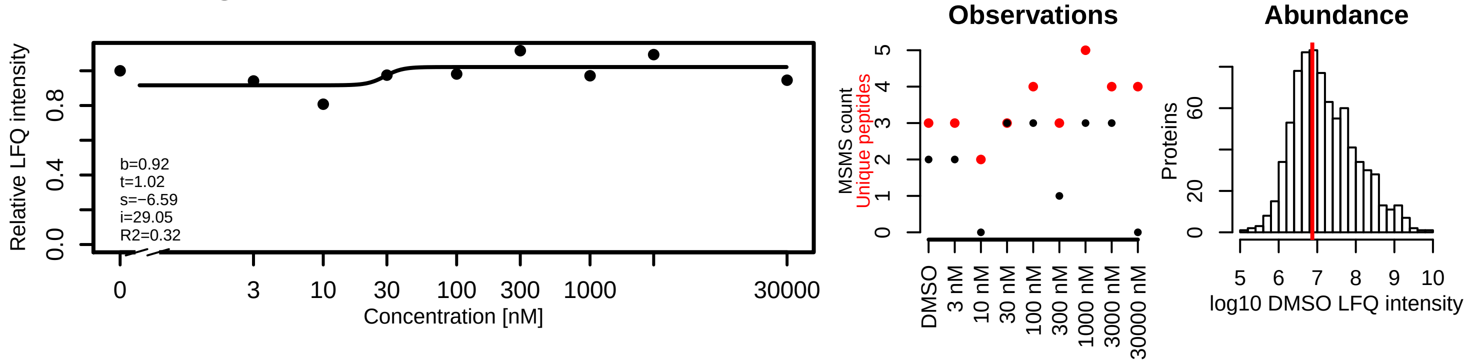


## H1F0

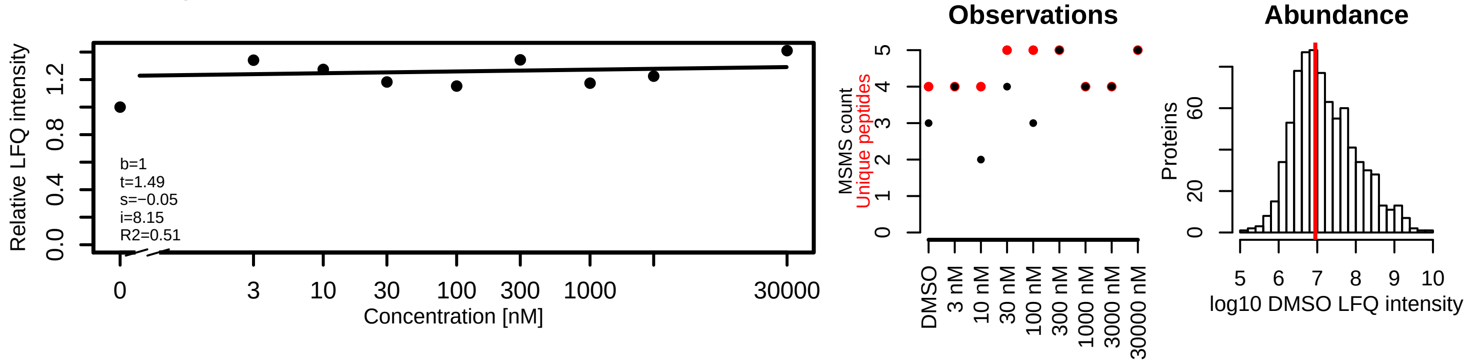


## SPTB

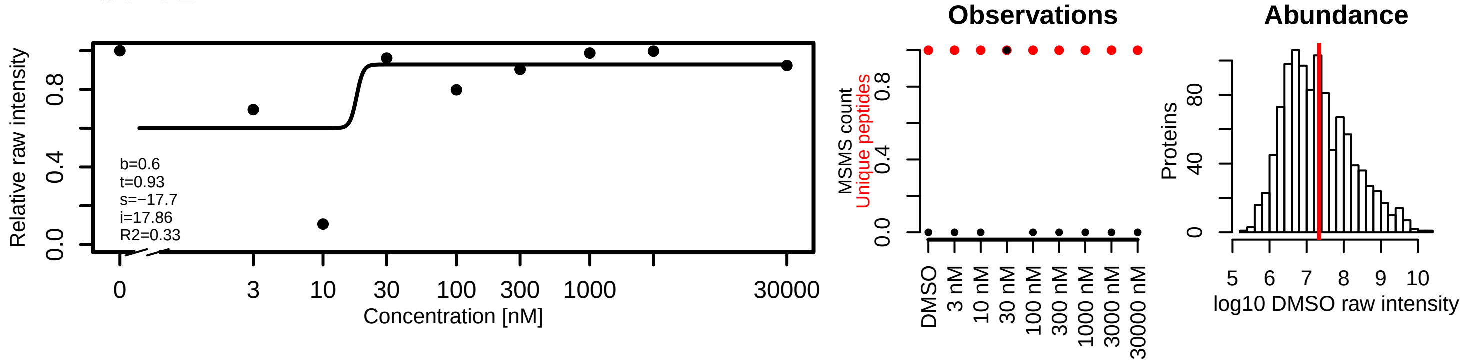


## CSNK1E

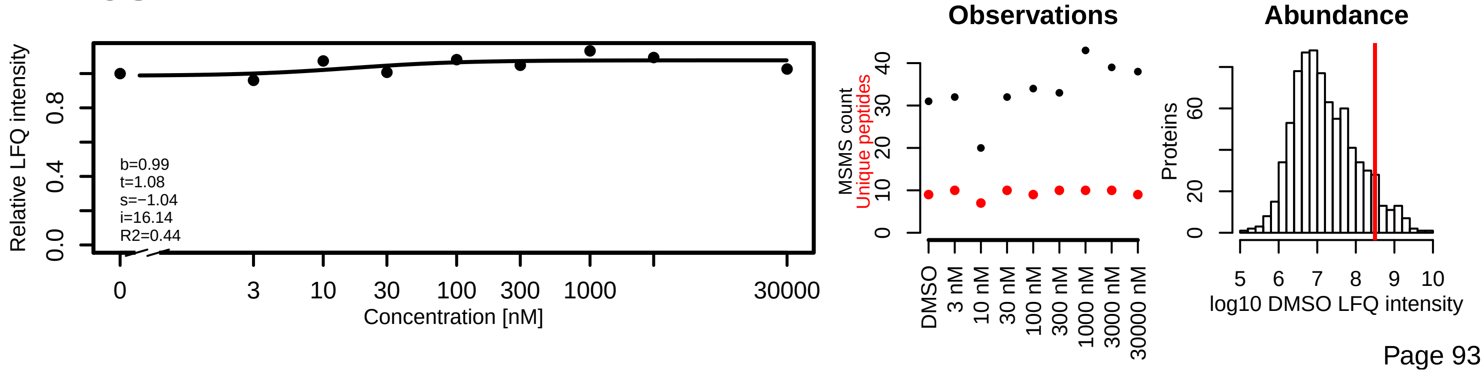

## CLK4

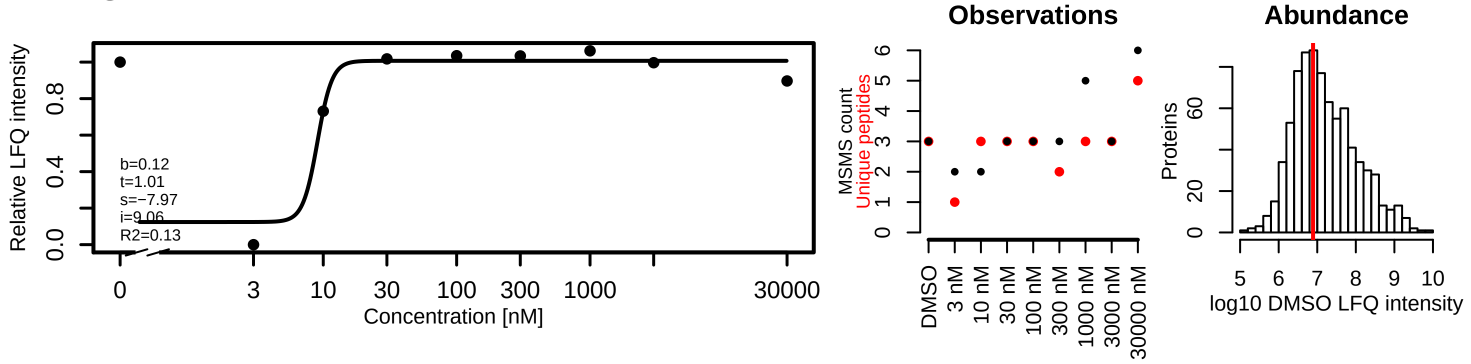


## GEMIN4

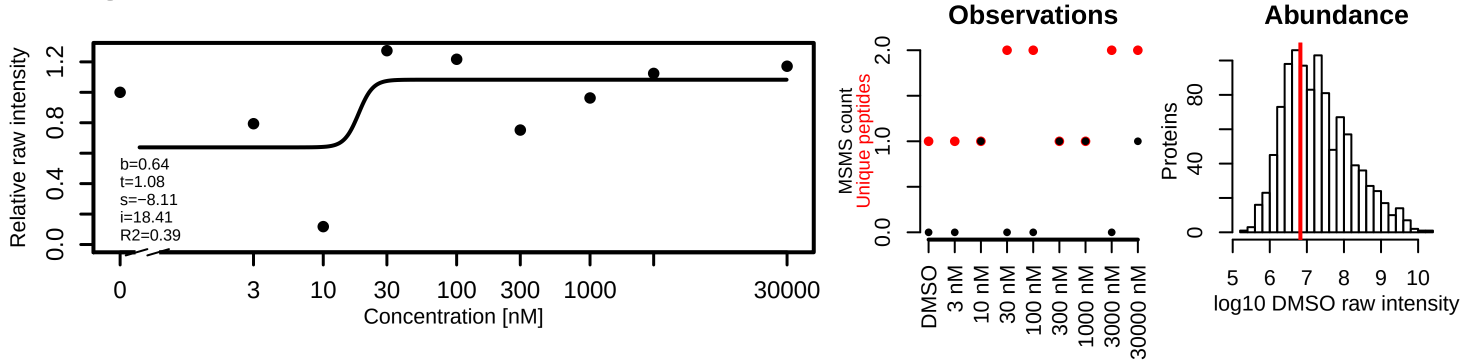


## TYK2

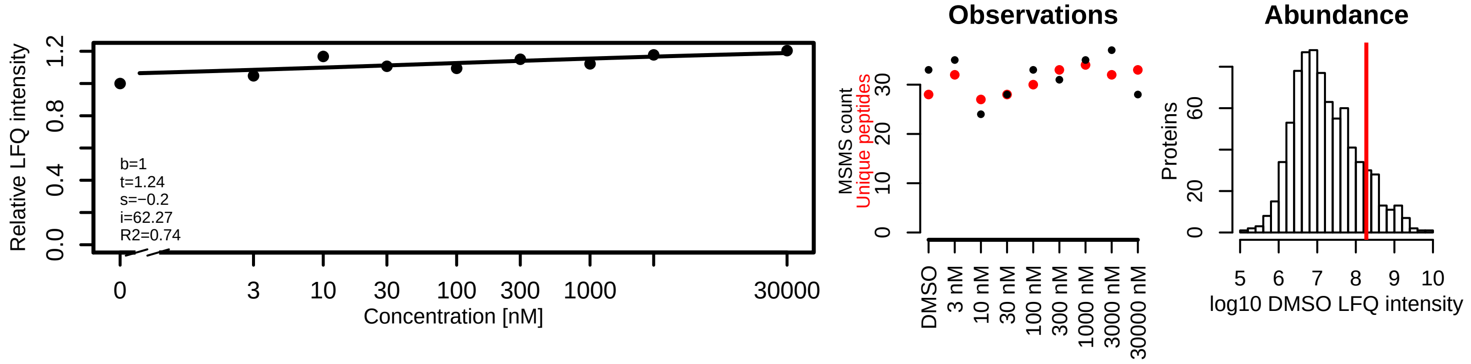


## TBK1

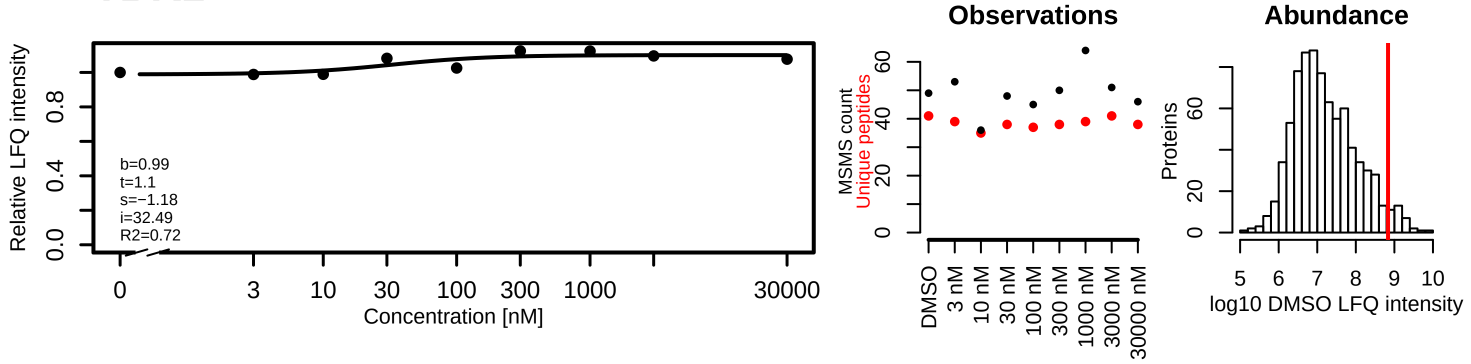


## DLD

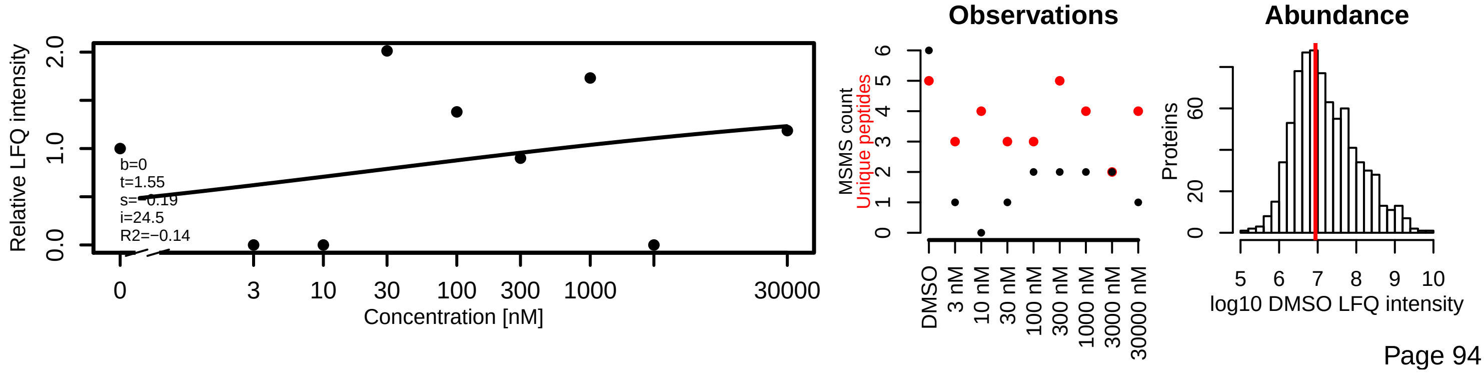

## VDAC1

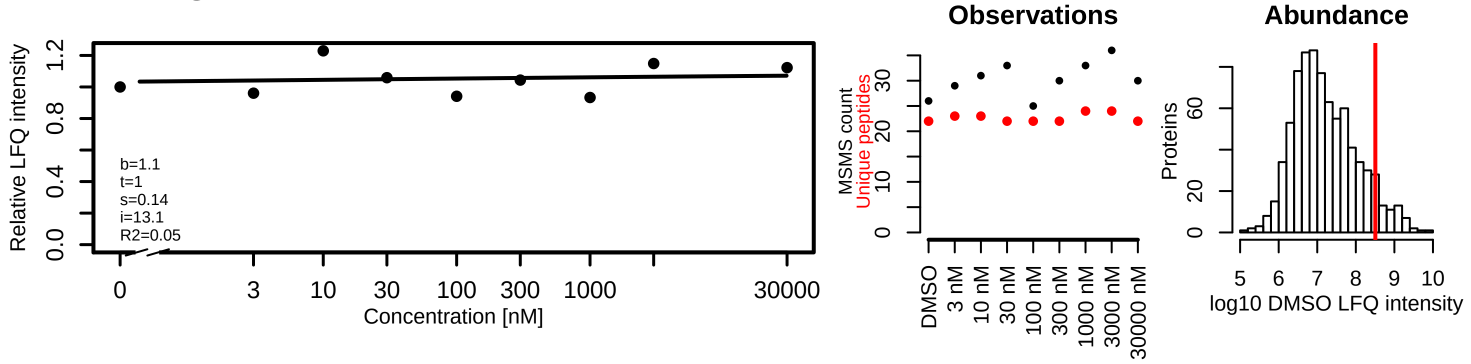


## AXL

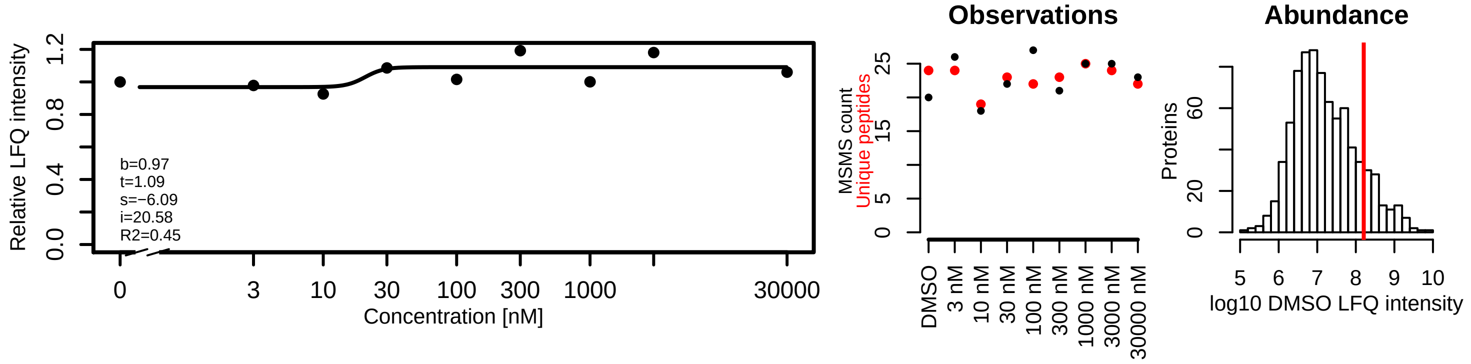


## MOV10

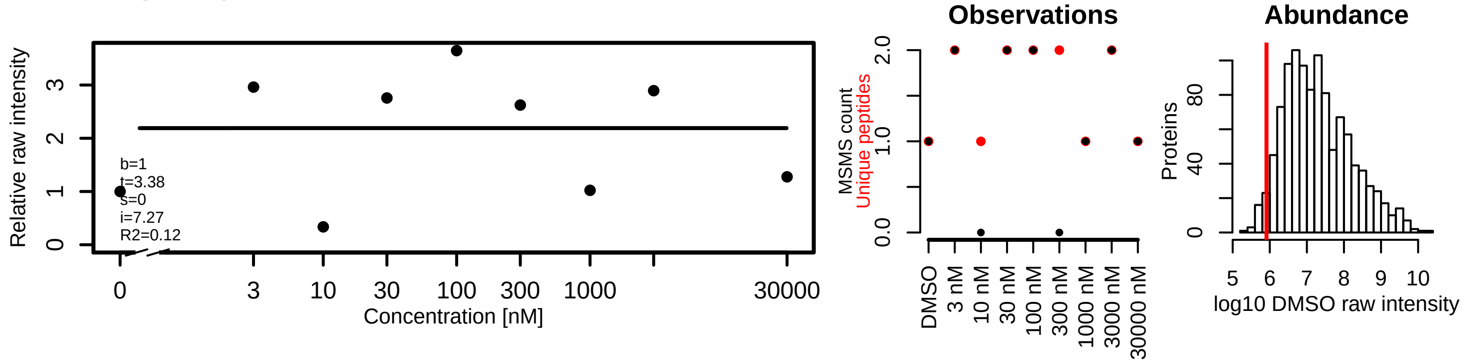


## GNB2L1

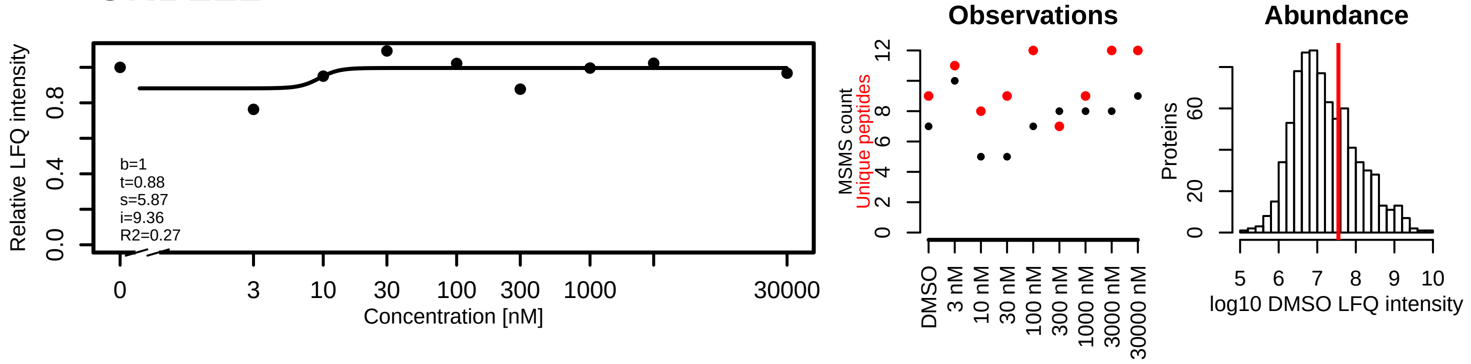


## PDHA1

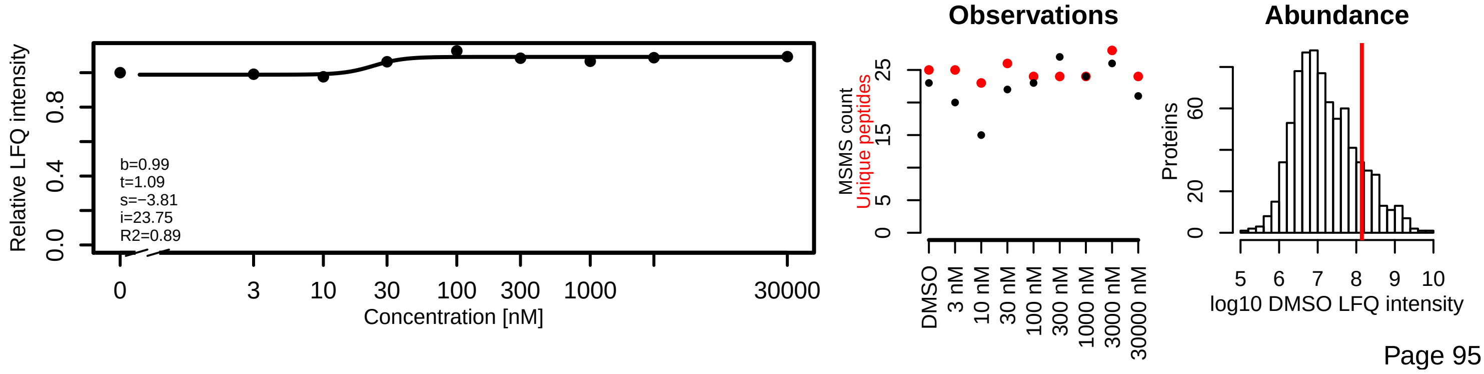

# LPCAT3

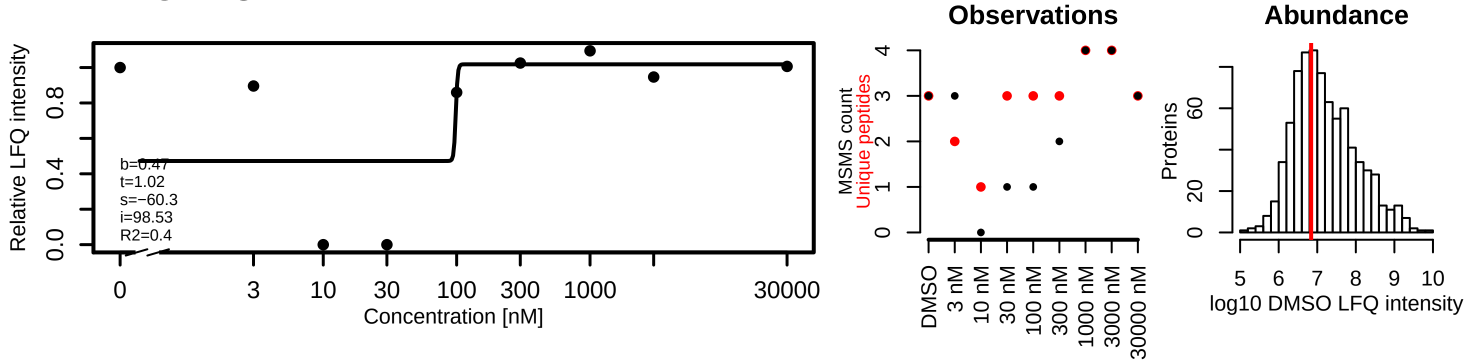


# GSKIP

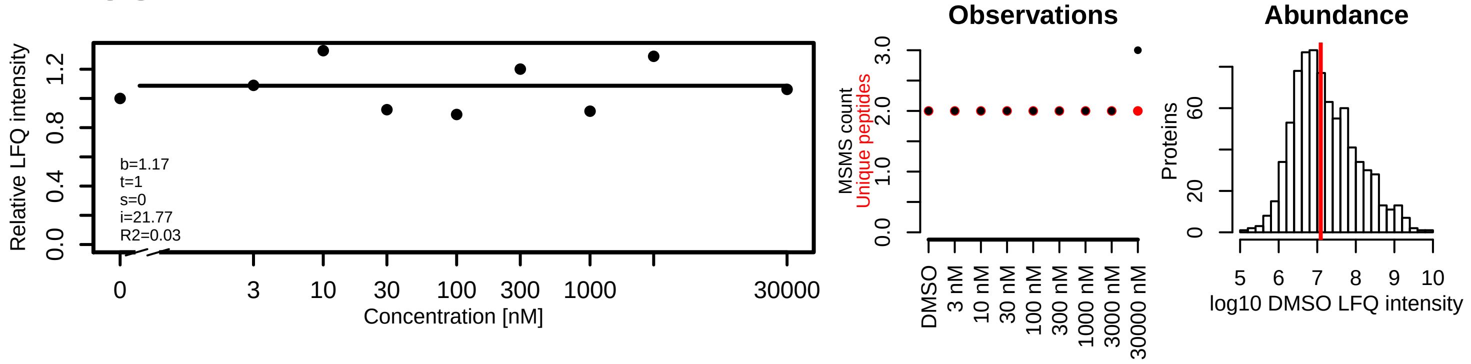


# SOAT1

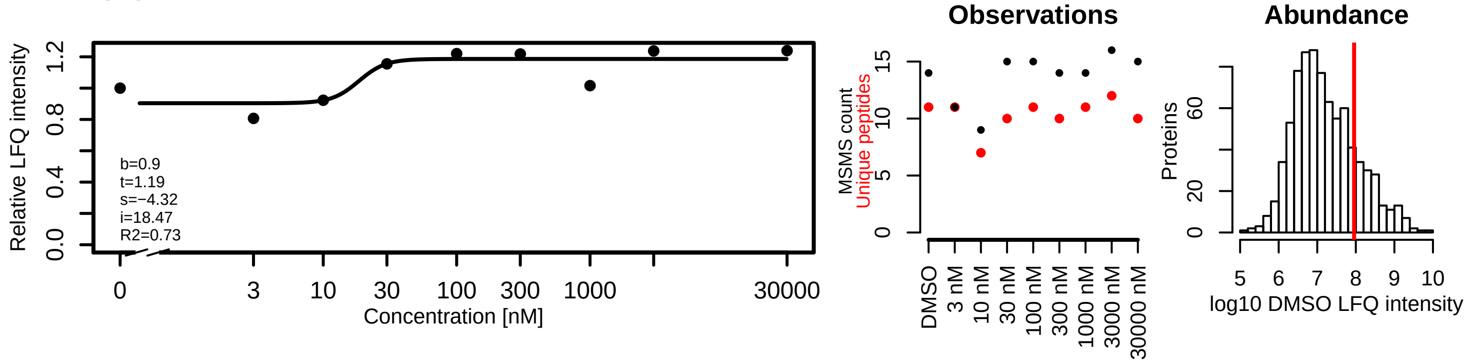


# ROCK1

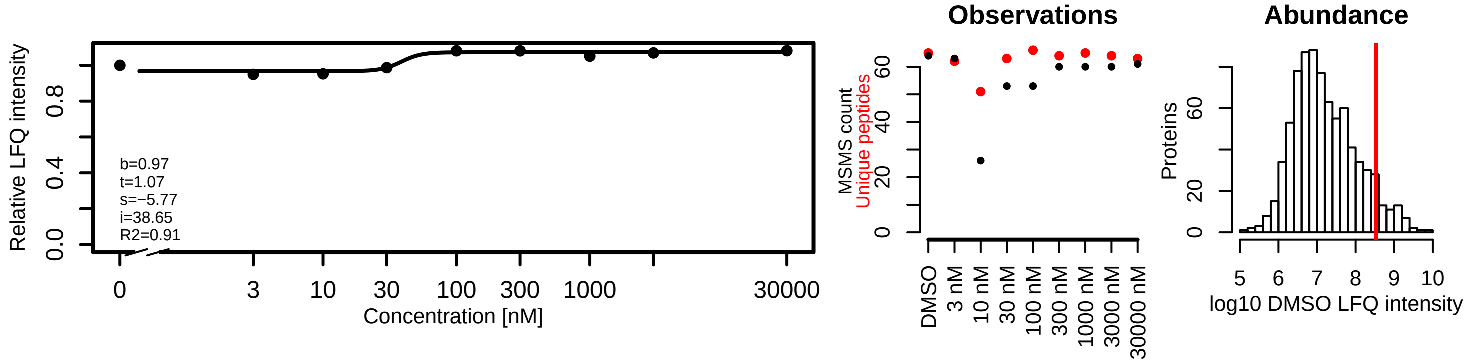


# CSNK1G1

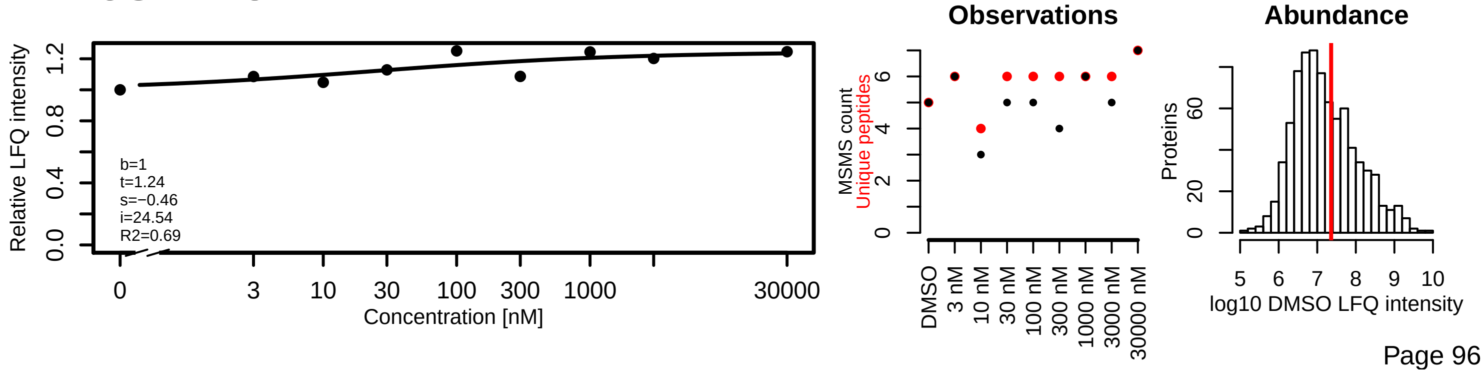

# PRKD2

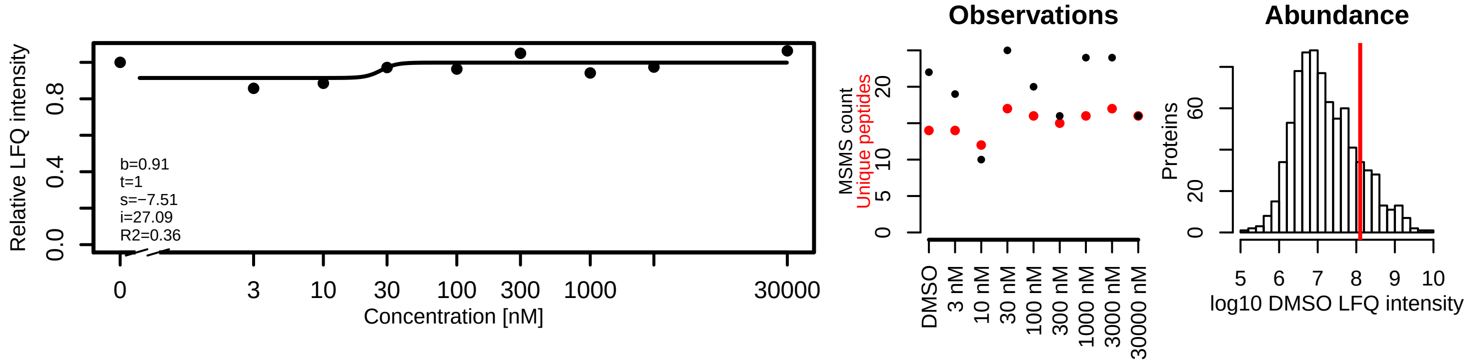


# PRKD3

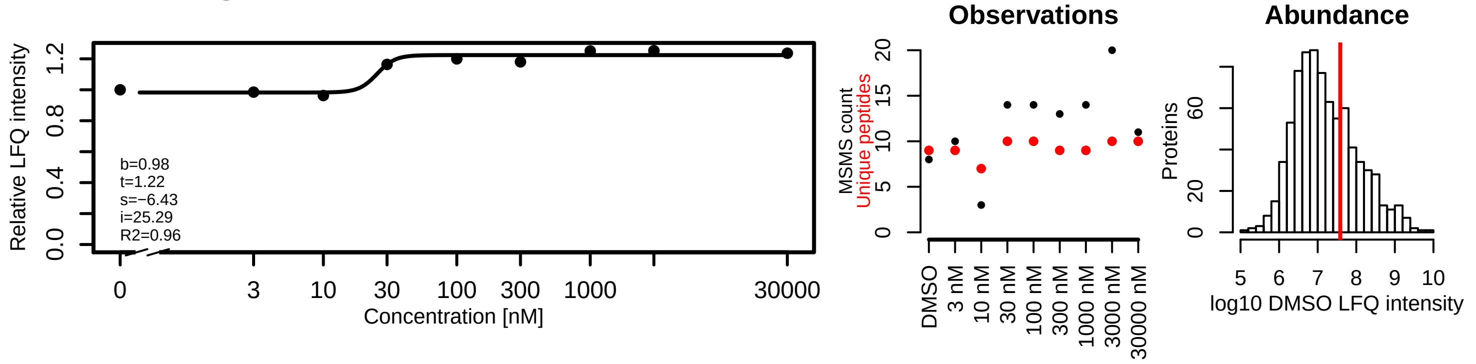


# CYBA

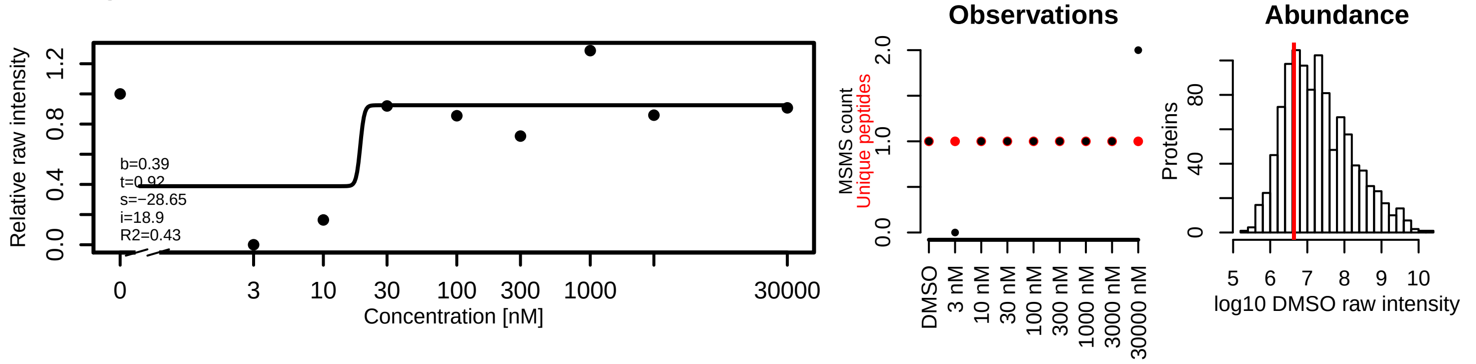


# CDK16

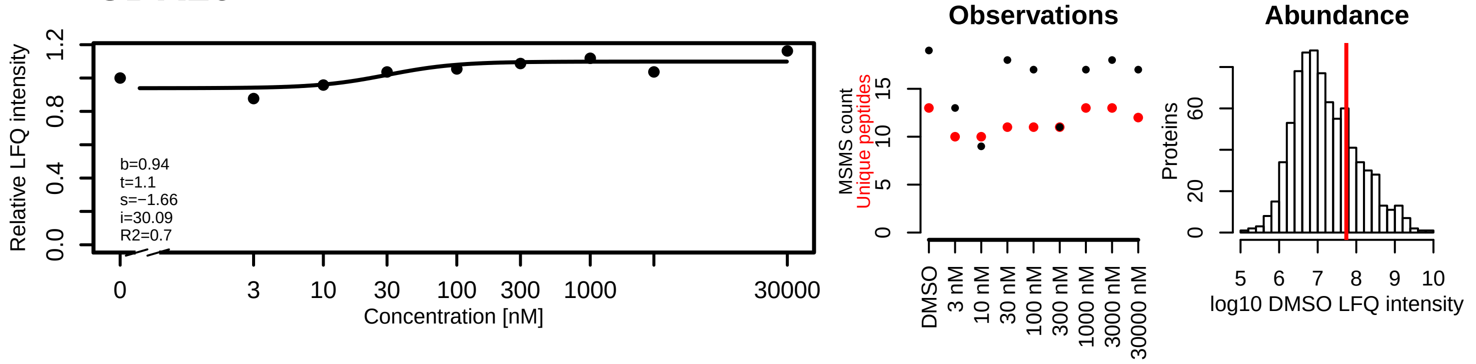


# ORMDL1;ORMDL2

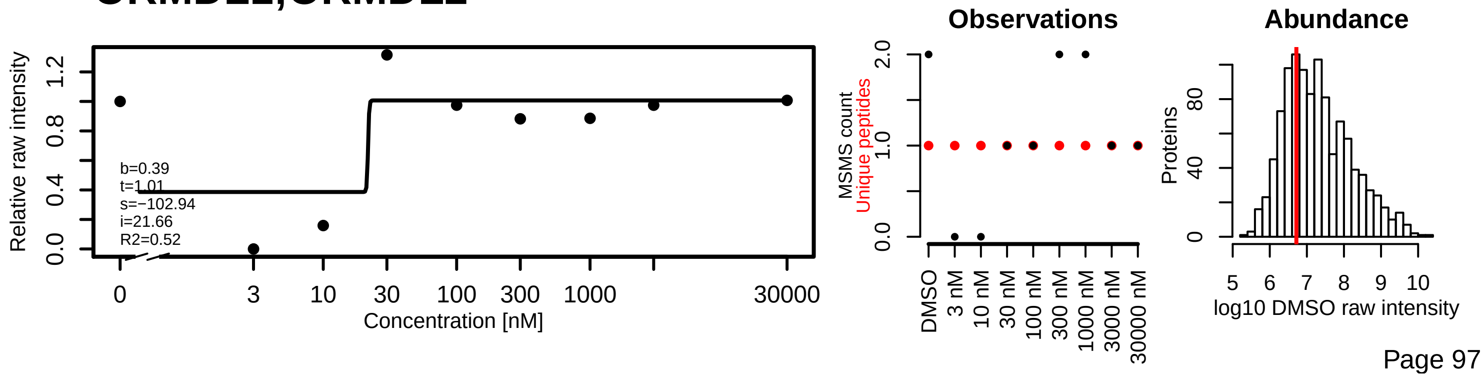

## SMPD4

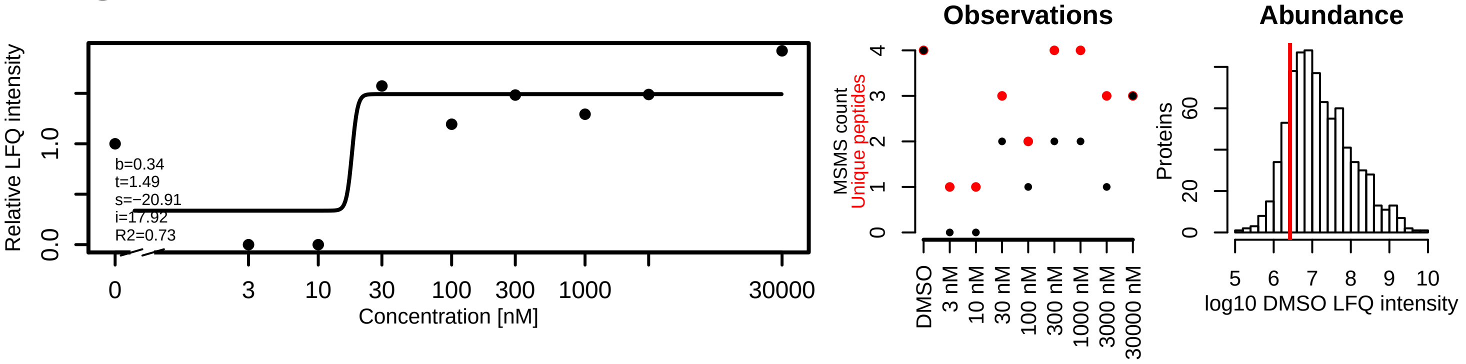


## RPL35A

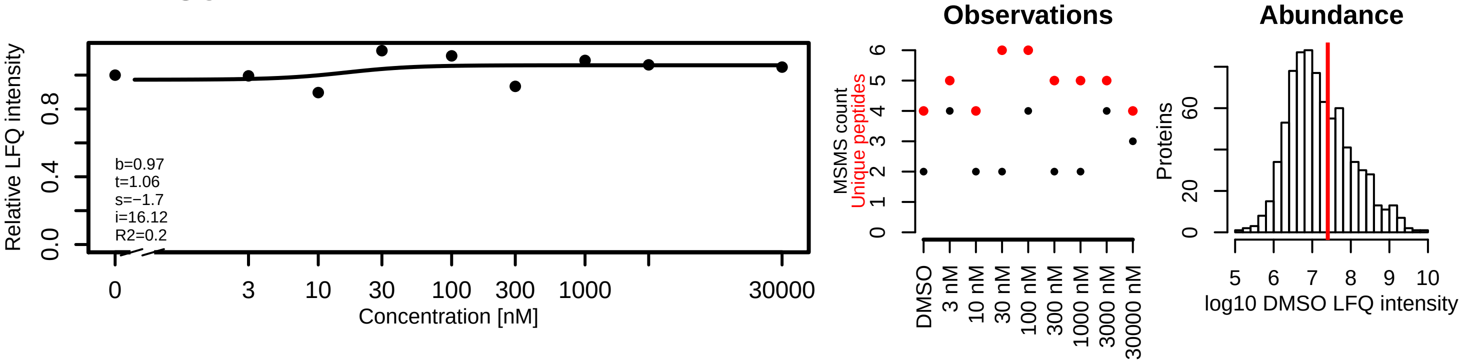


## FN1

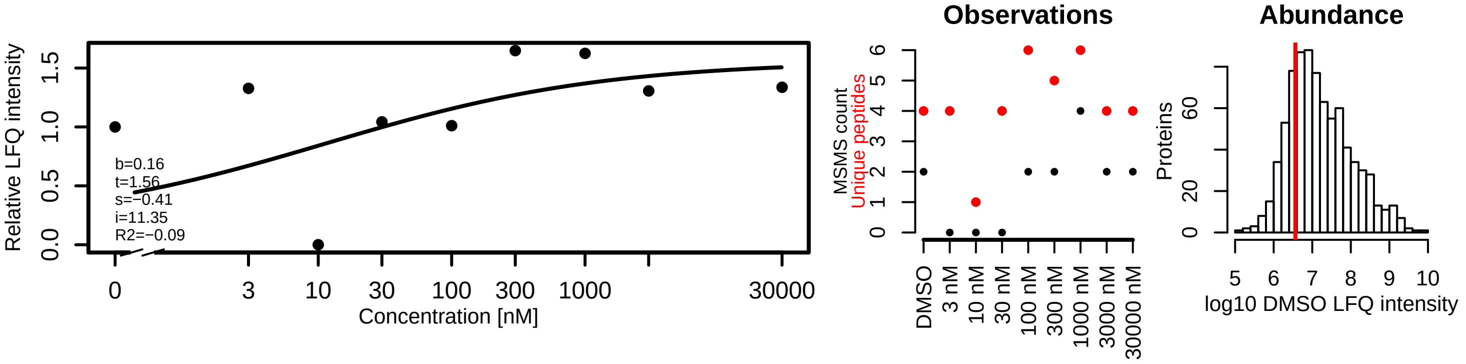


## PLK1

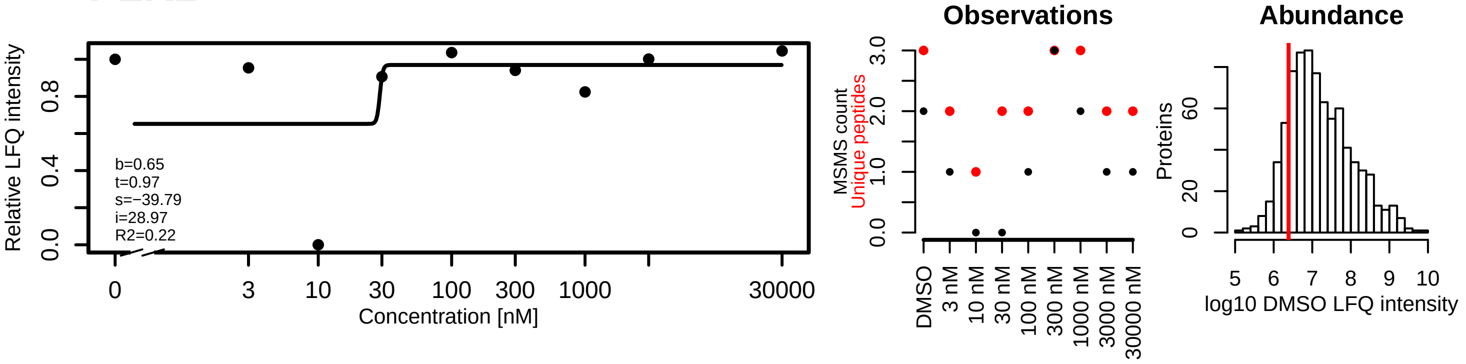


## NUP160

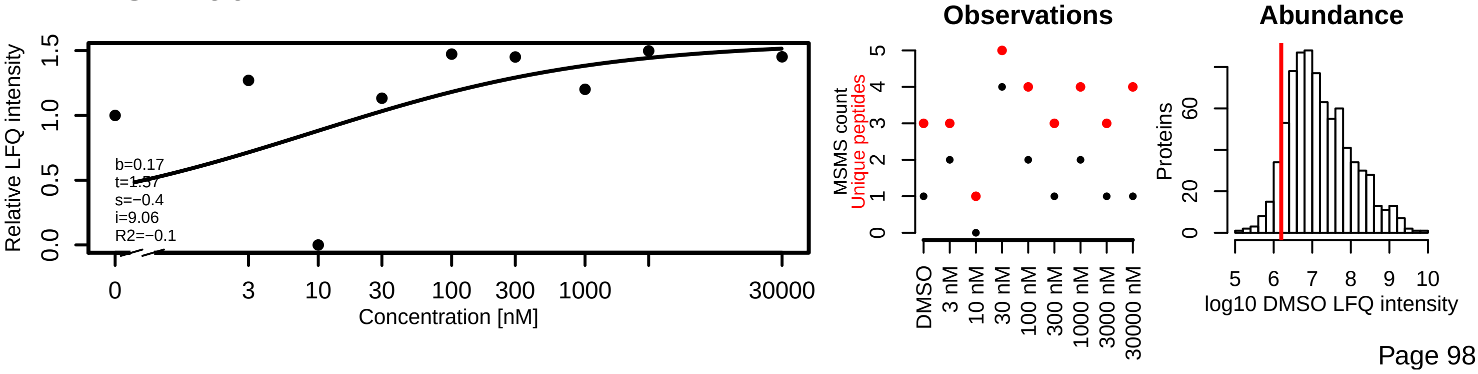

# MTX2

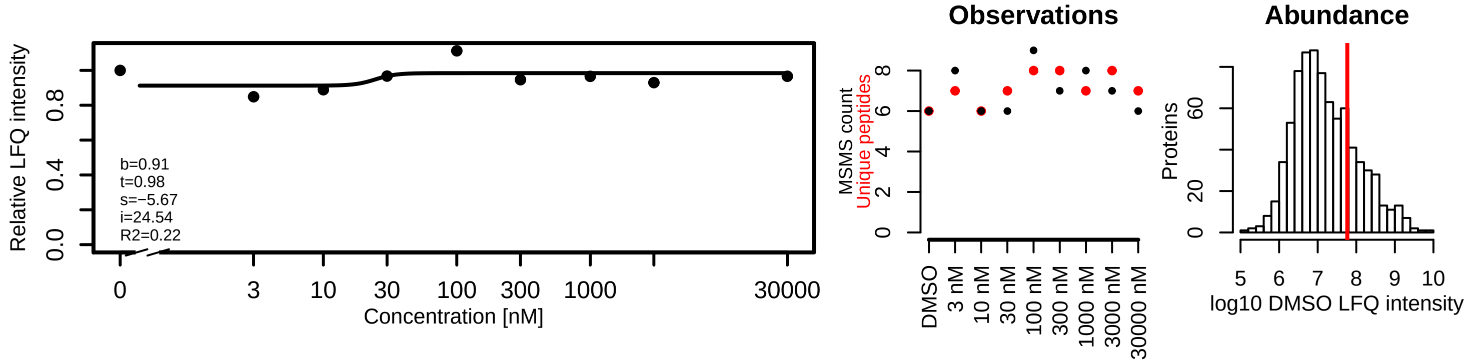


# DHCR7

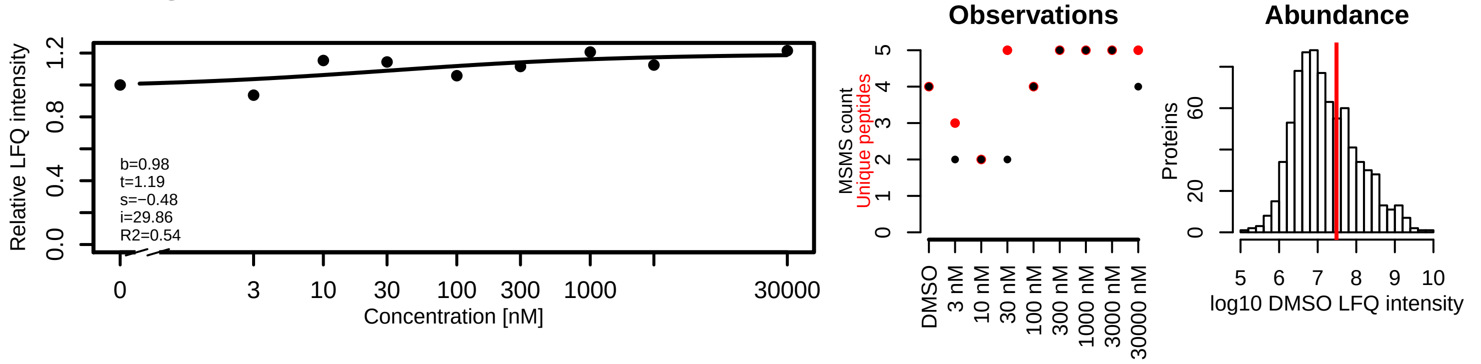


# SFN

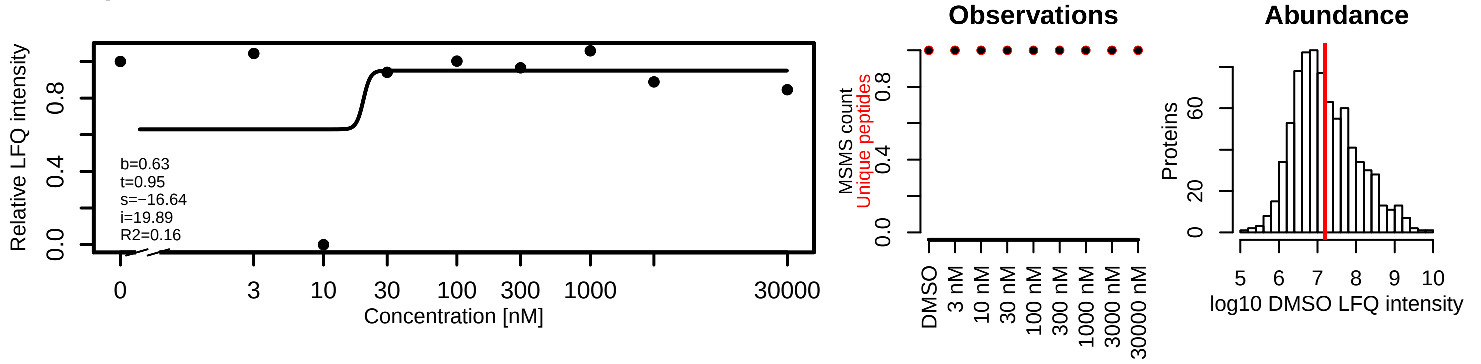


# ATP5F1

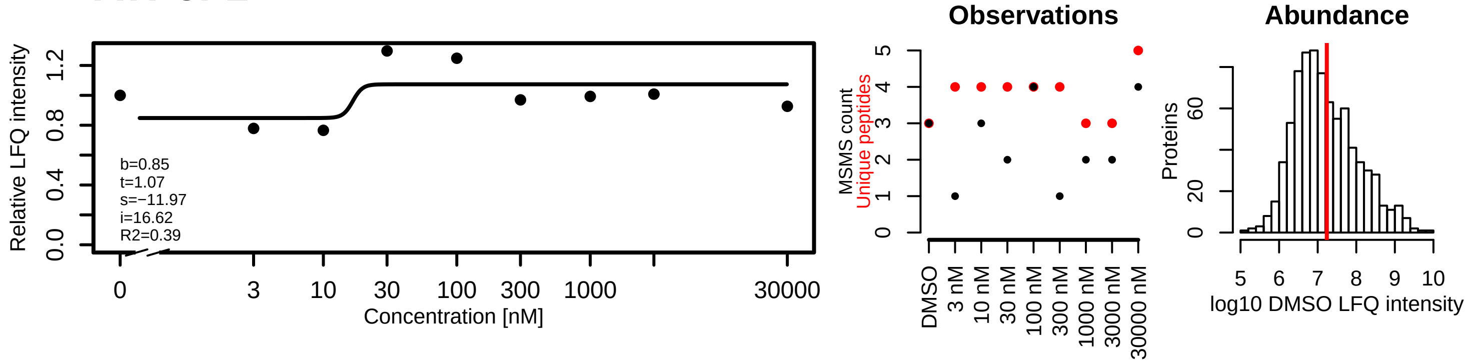


# PYGL

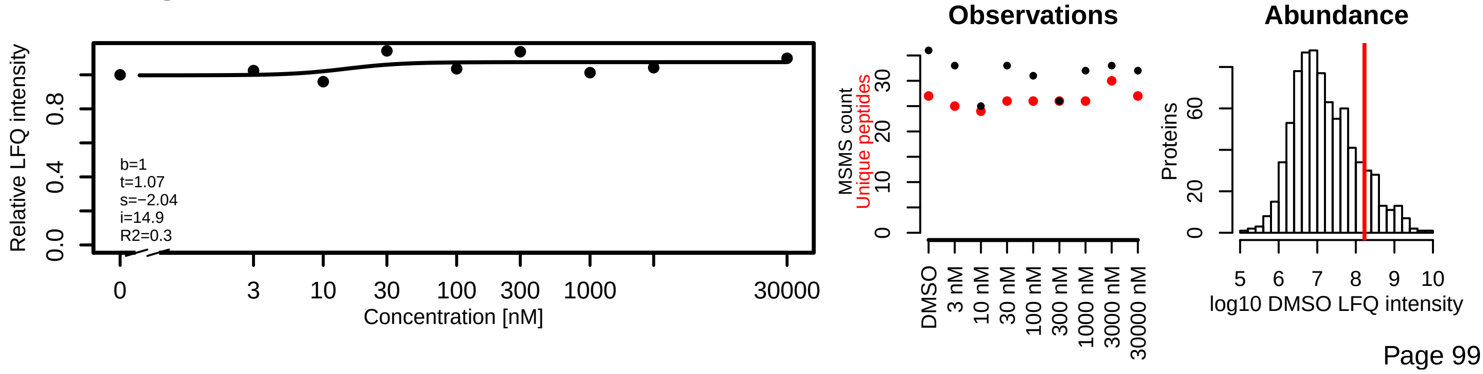

## TIMM23;TIMM23B

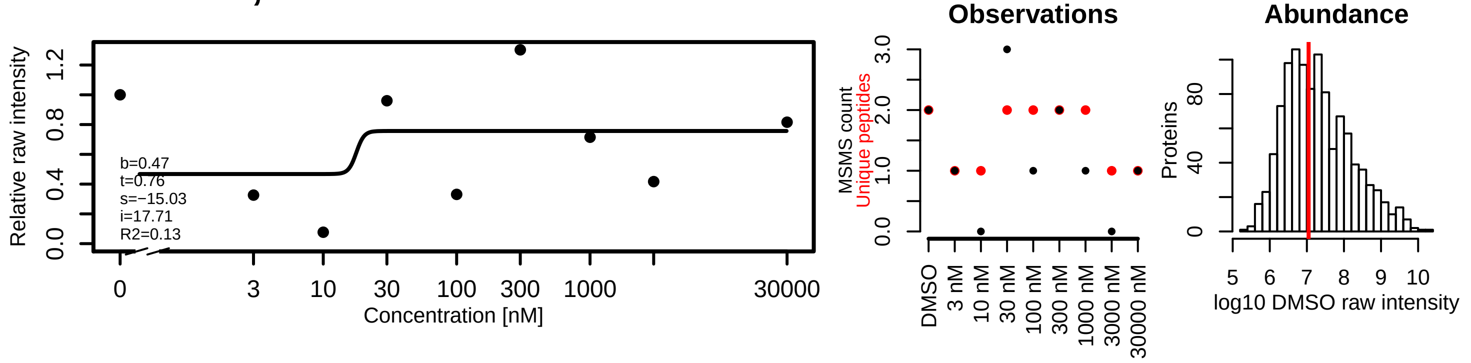


## MAGT1

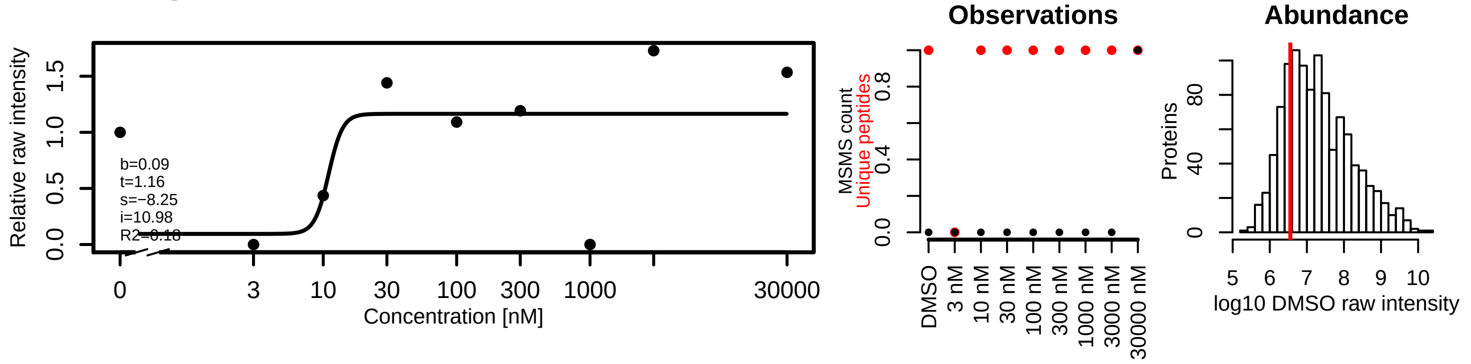


## NUMA1

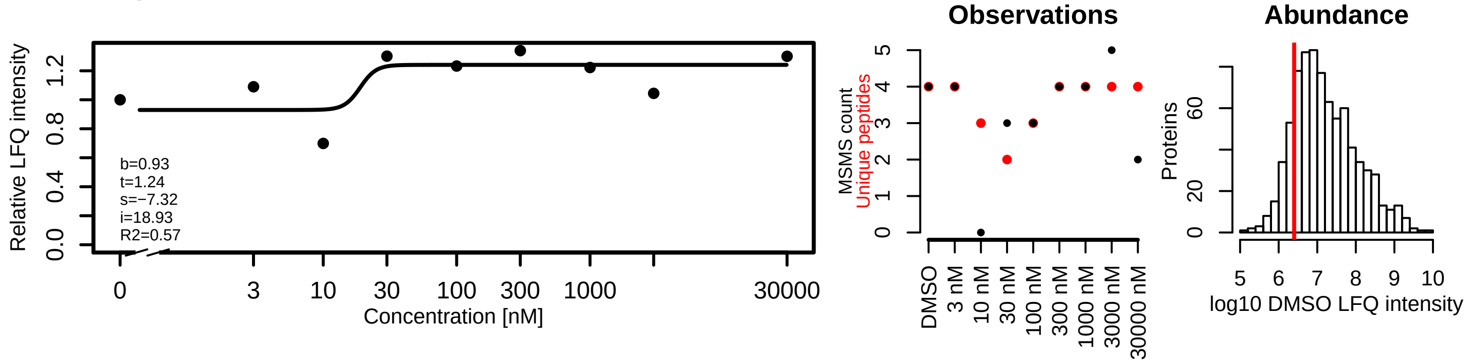


## TMED10

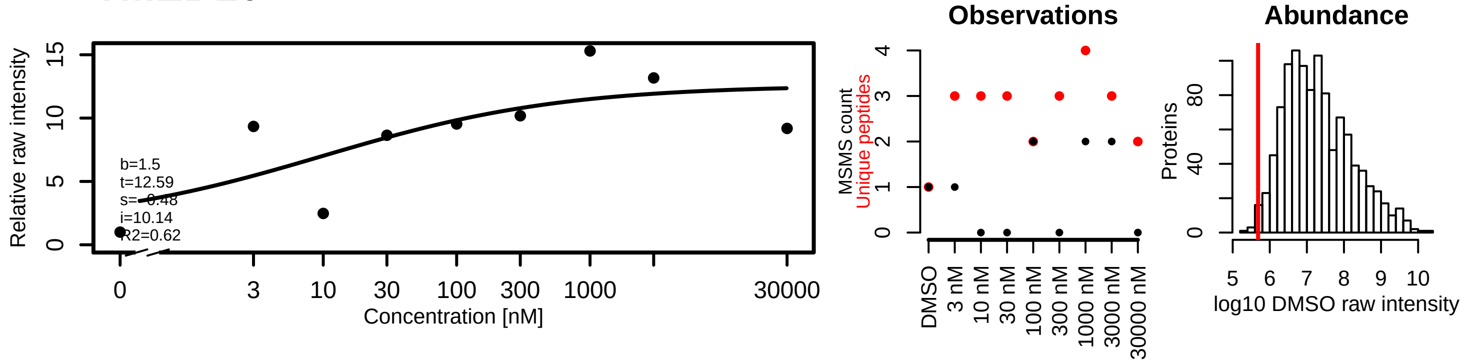


## DYNC1I2

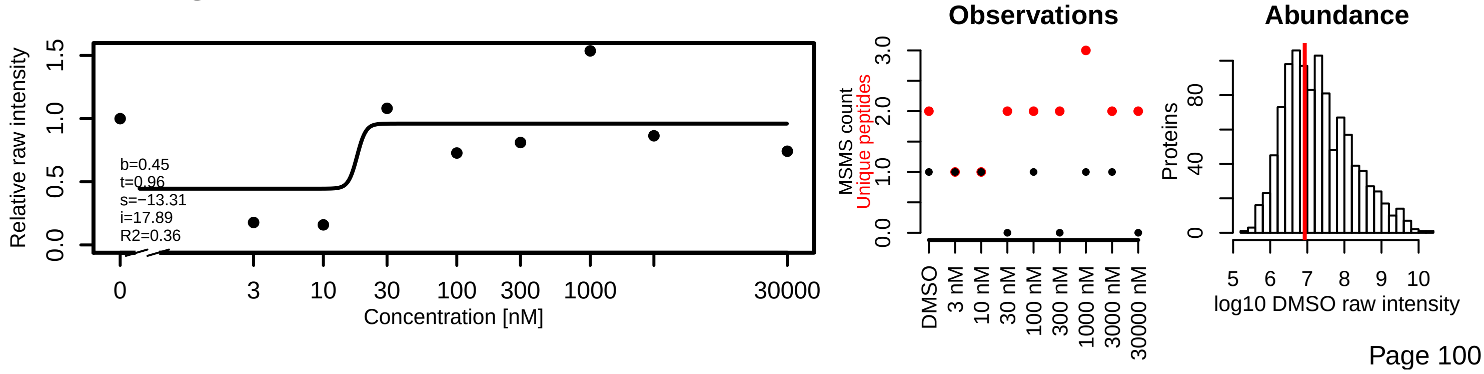

# INSR

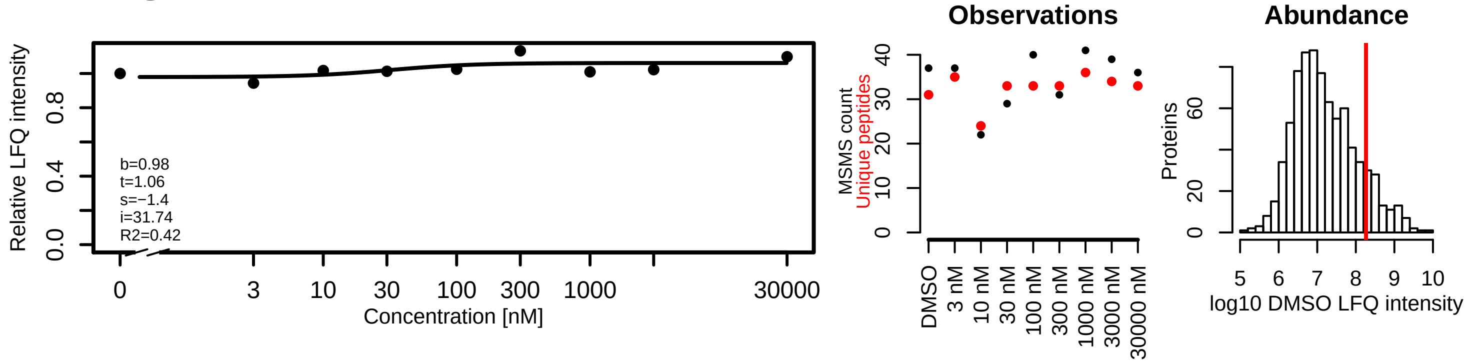


# BMPR1B

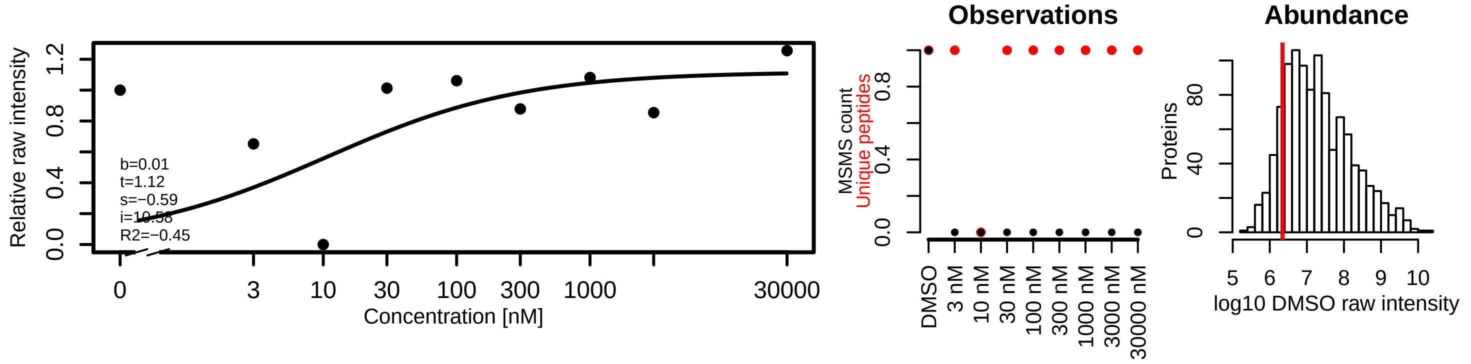


# PKN2

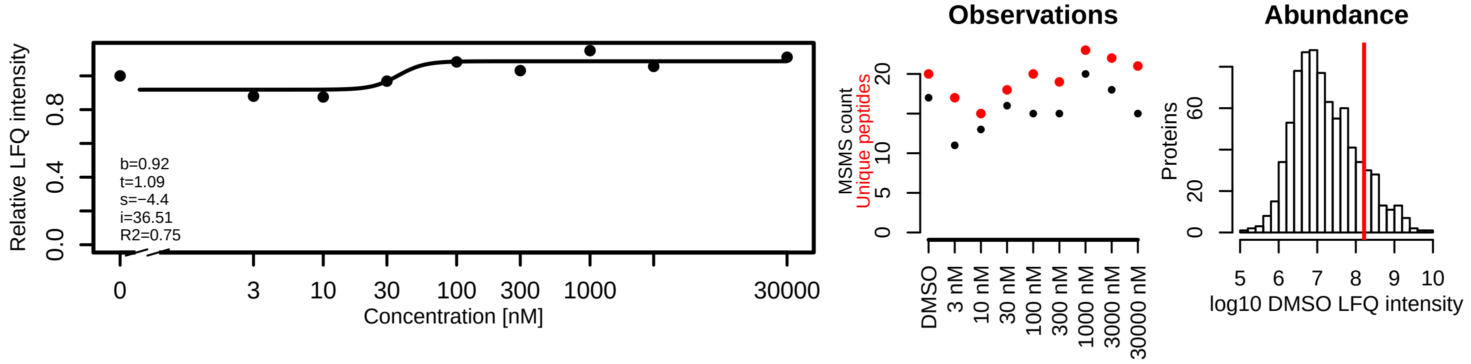


# PRKAR2A

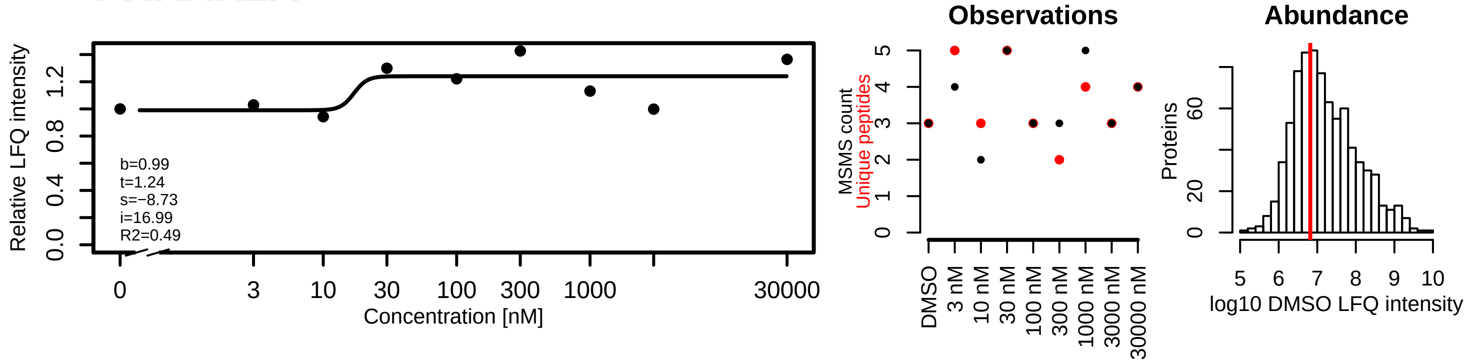


# RPL13

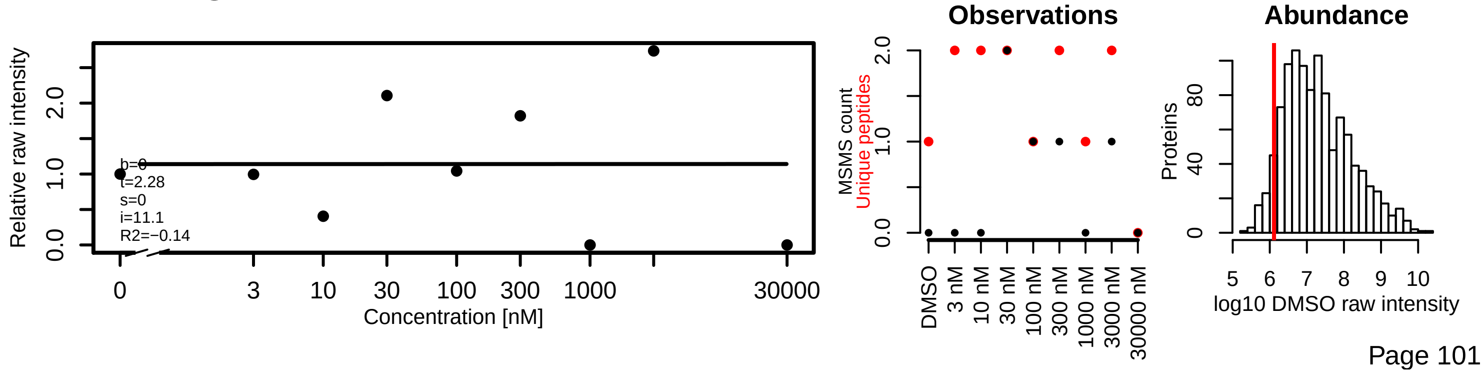

## CDR2

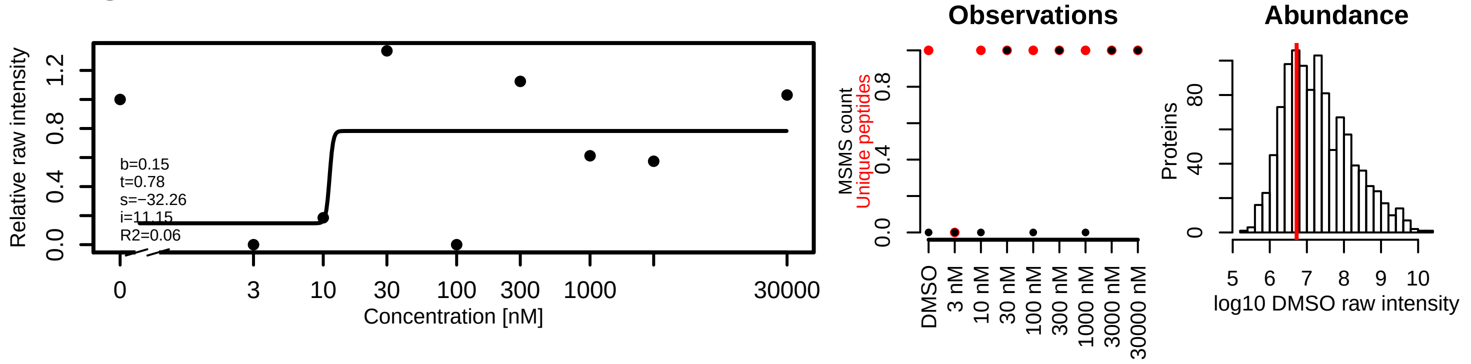


## STON2

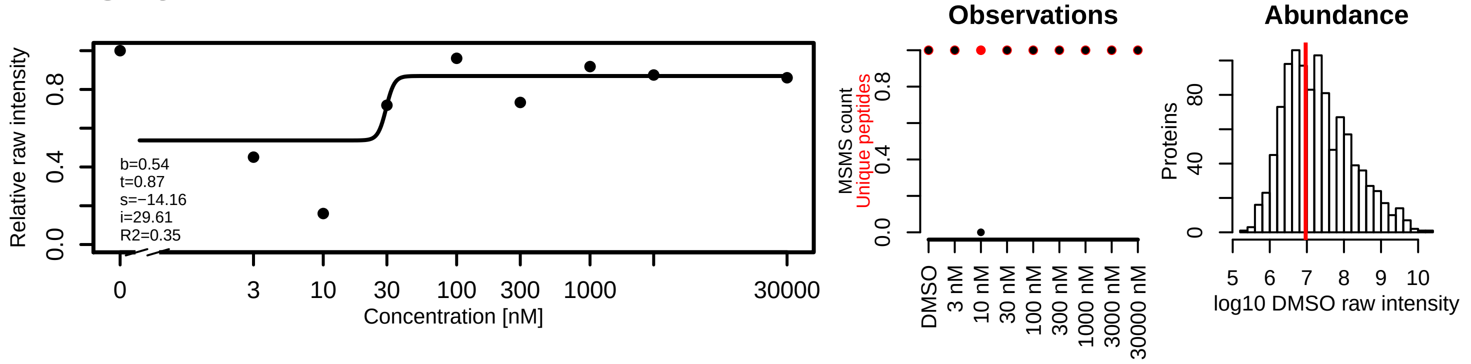


## FN3K

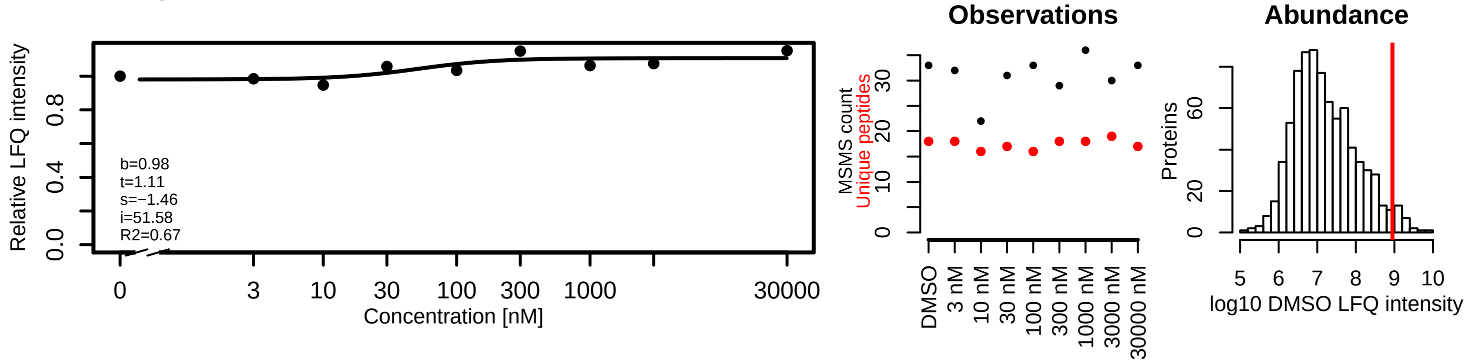


## PRDX1

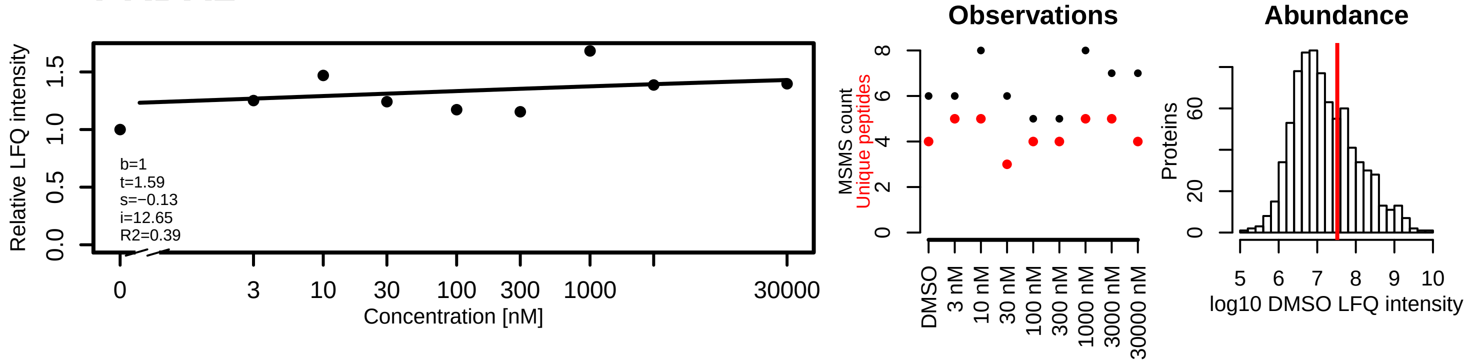


## RPL10A

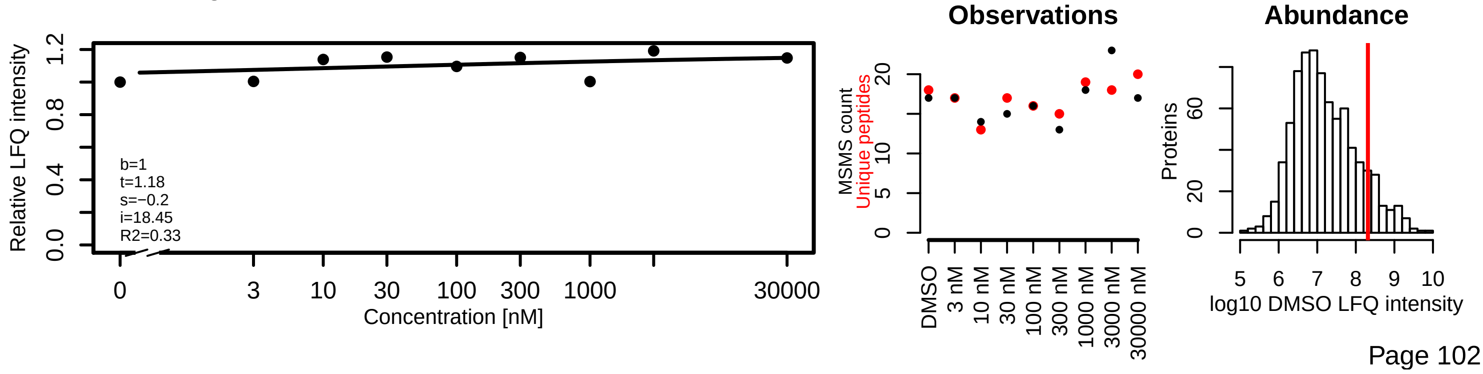

## RPL30

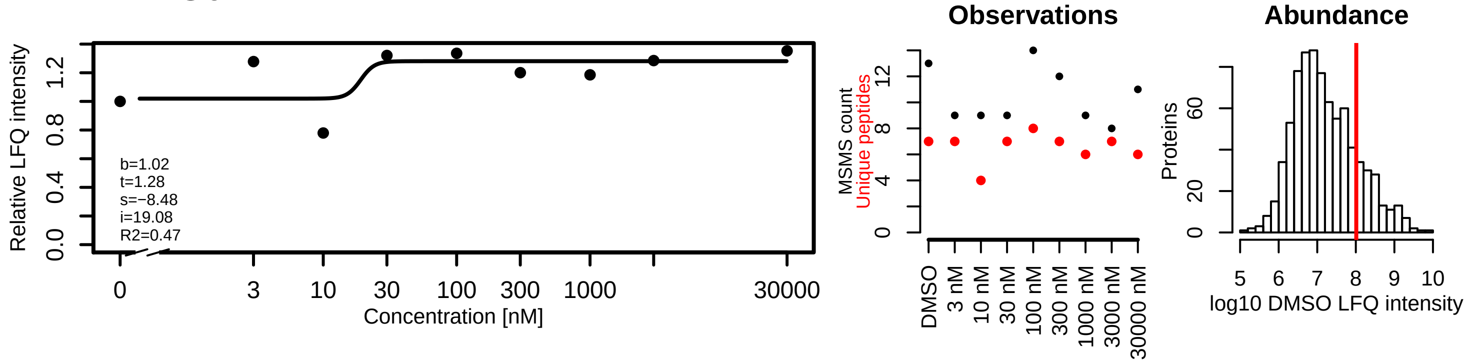


## FLOT2

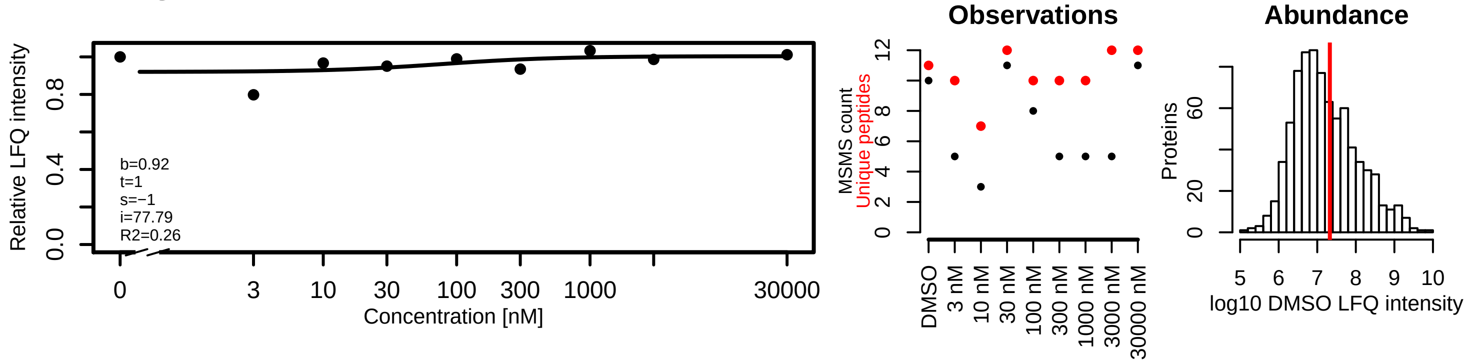


## JAK1

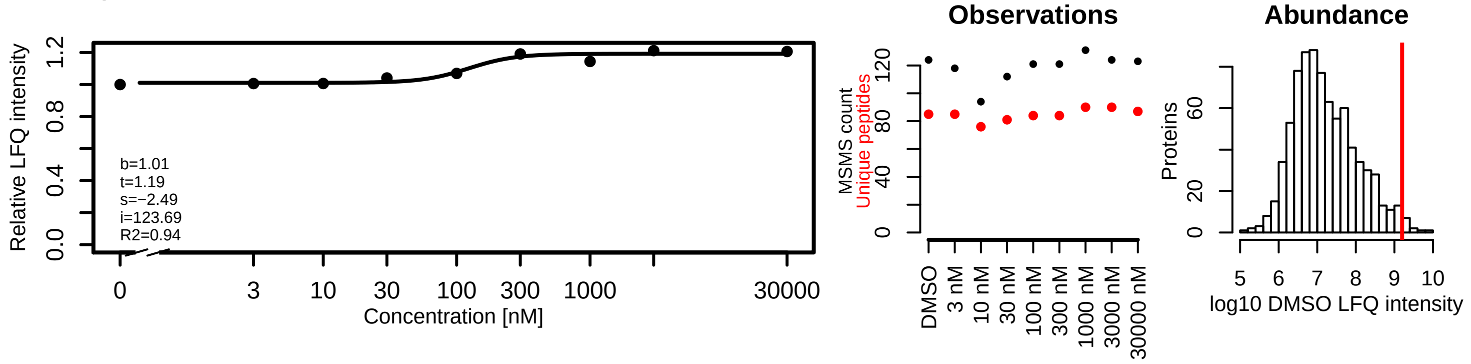


## STK10

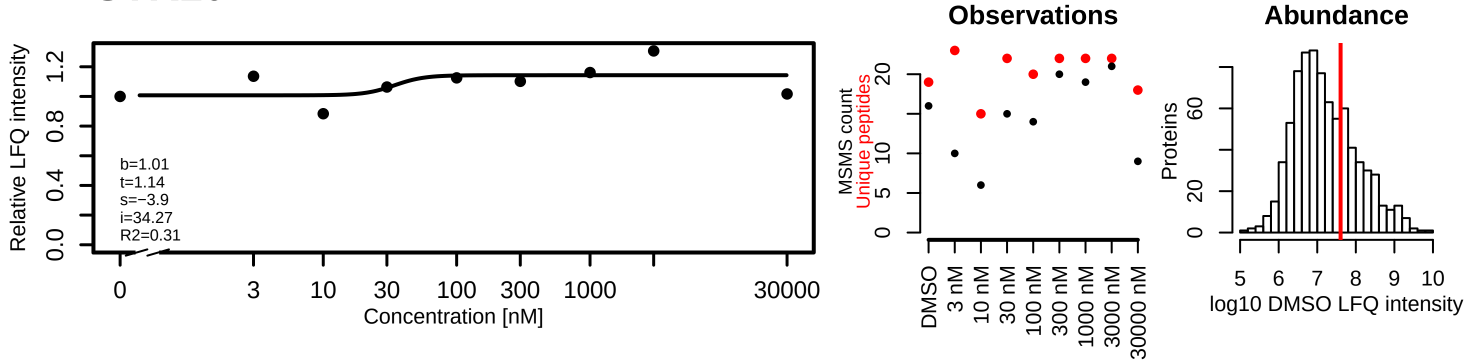


## MRPS28

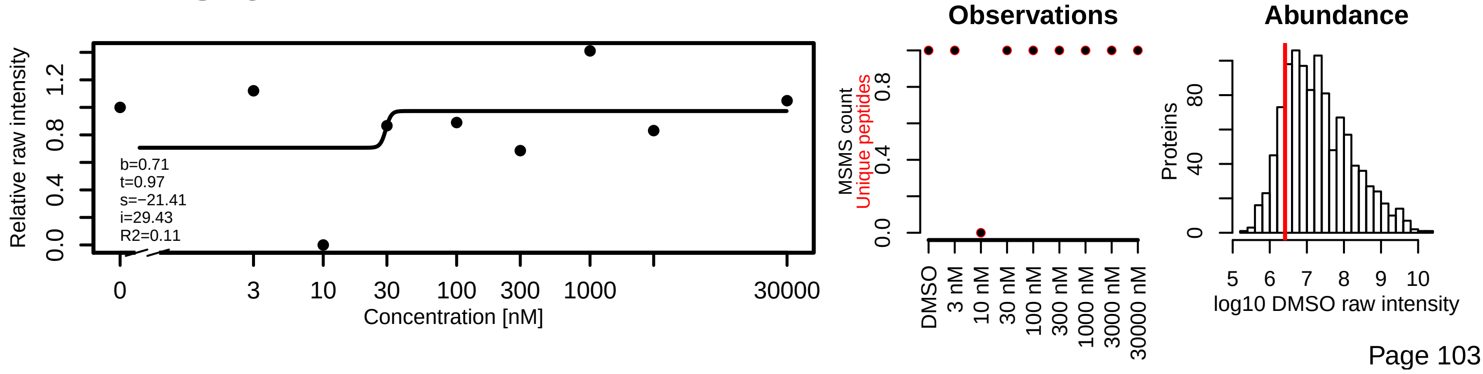

## MARS

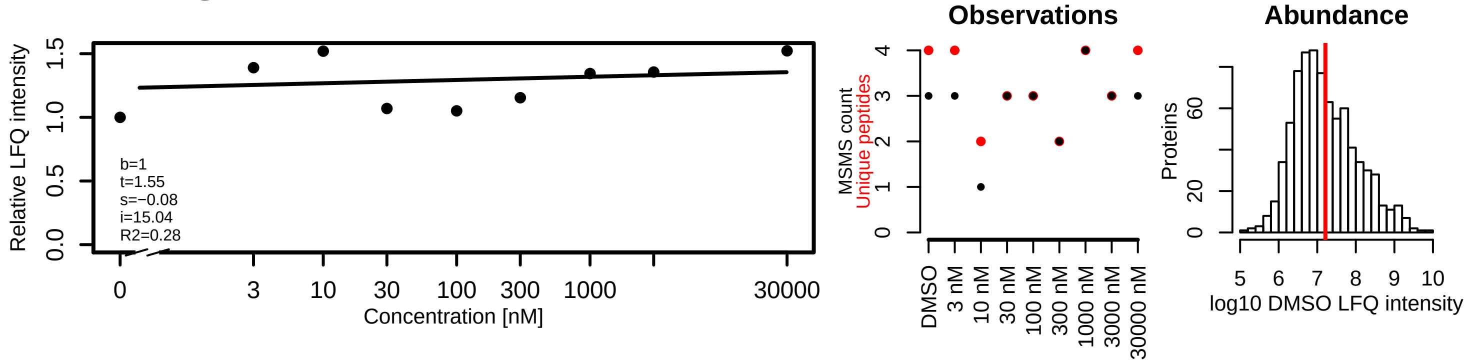


## CSNK1G3

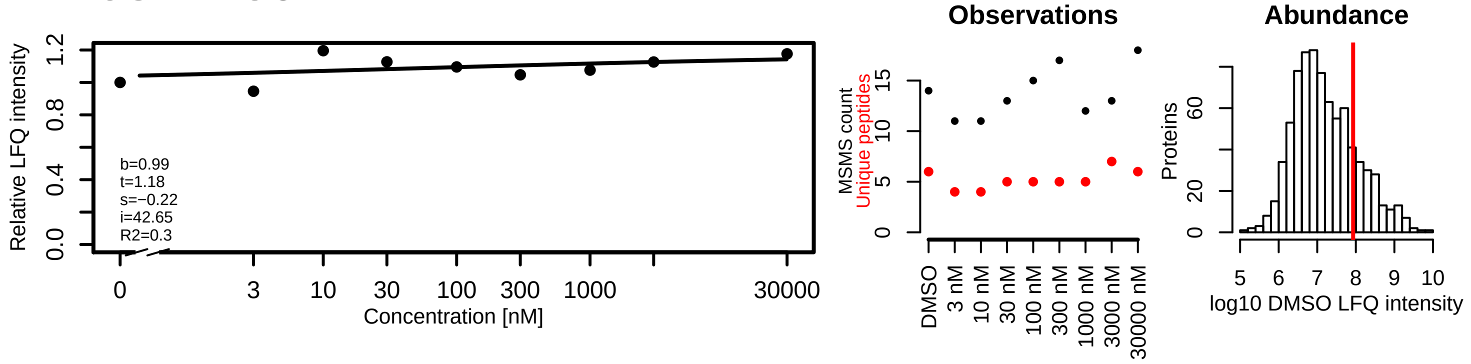


## TUBB4A

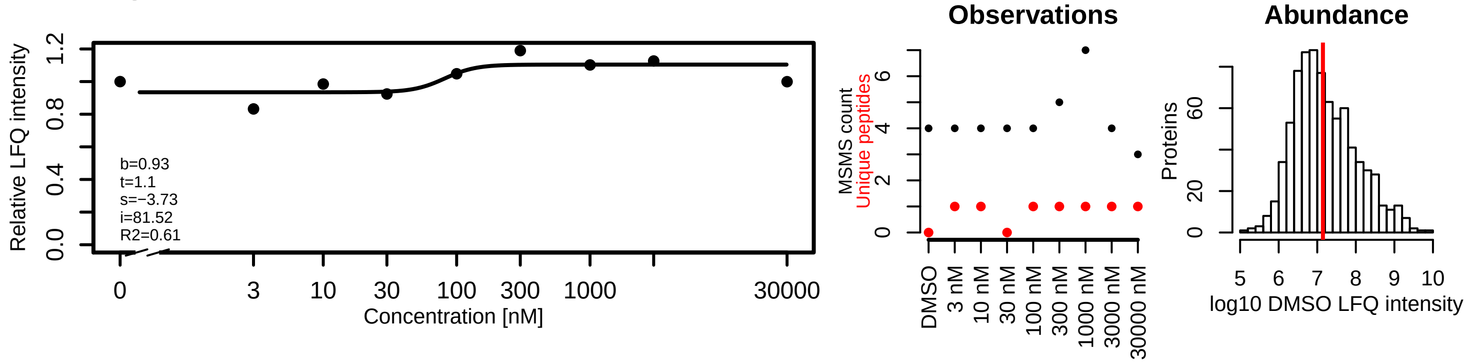


## LAMC3

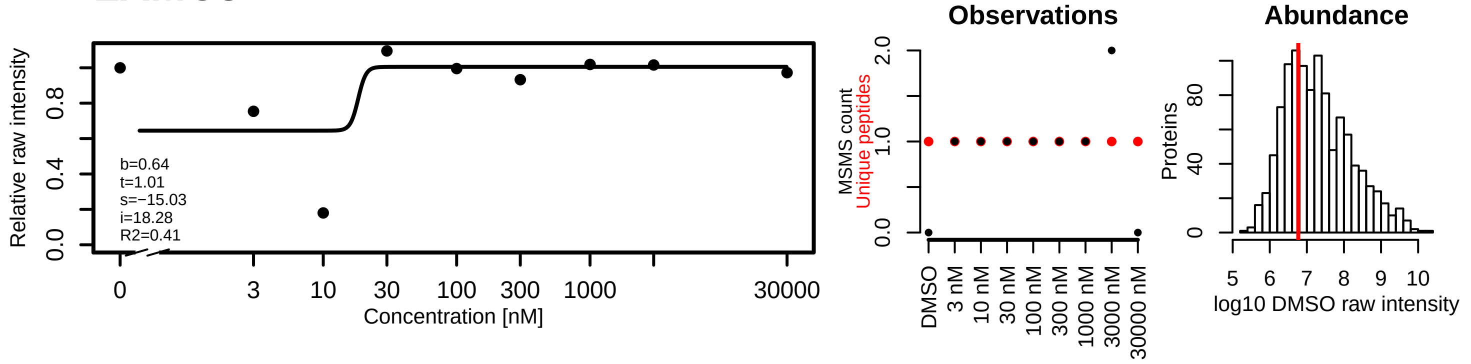


## RPS6KA3

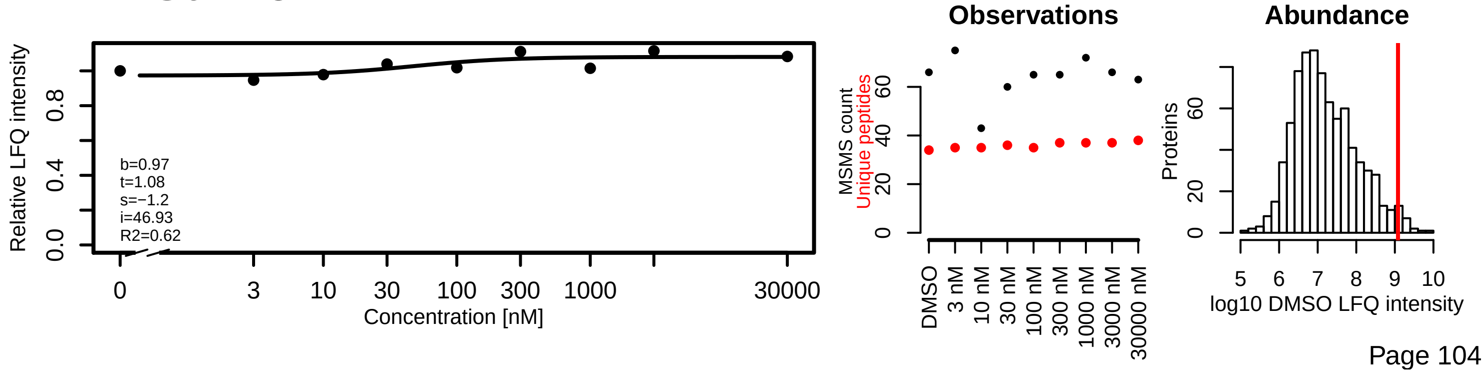

# PHKA1

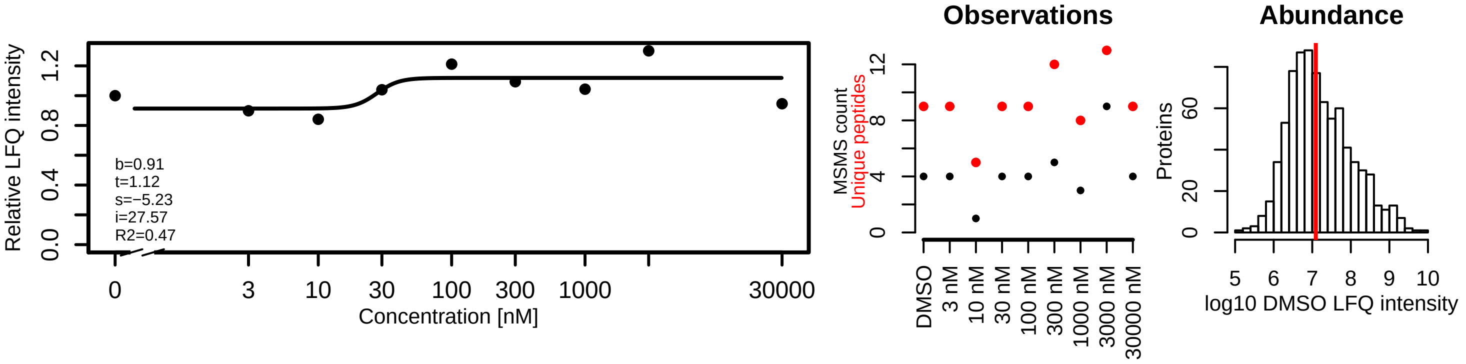


# ALDH2

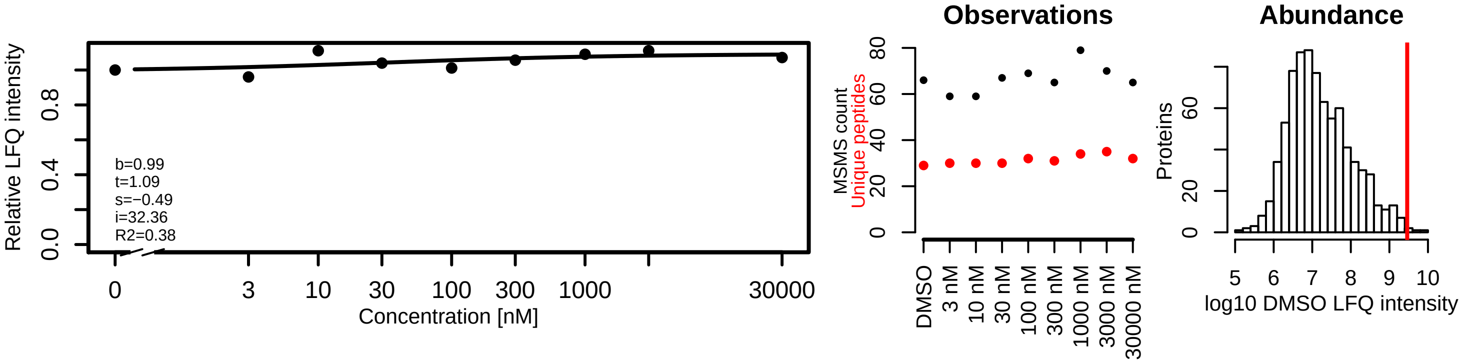


# DNAJA2

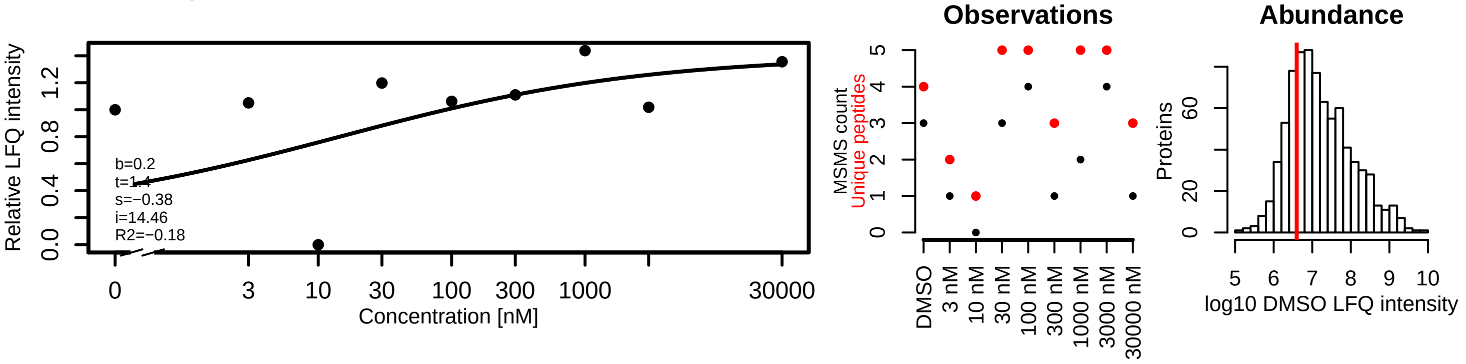


# SMEK1;SMEK2

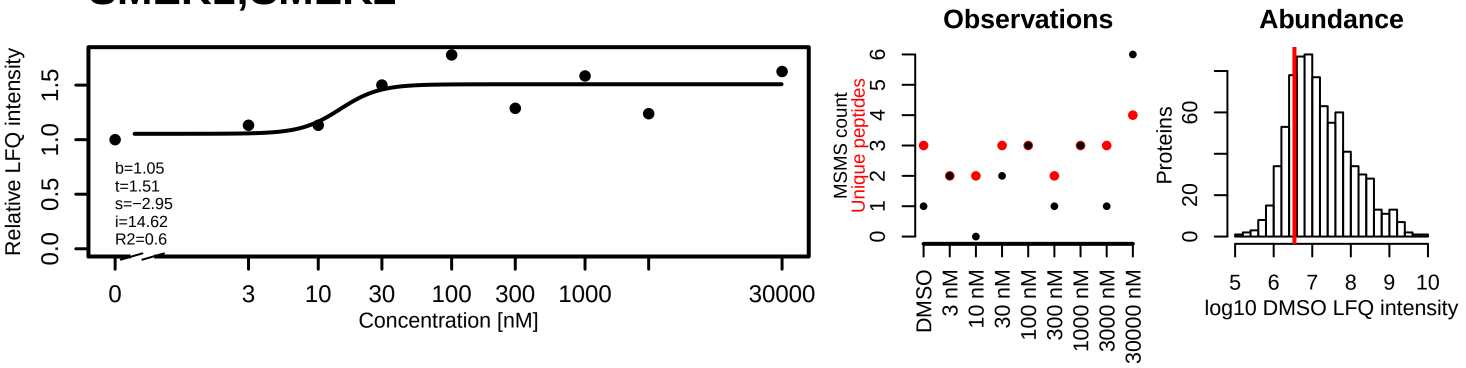


# SSRP1

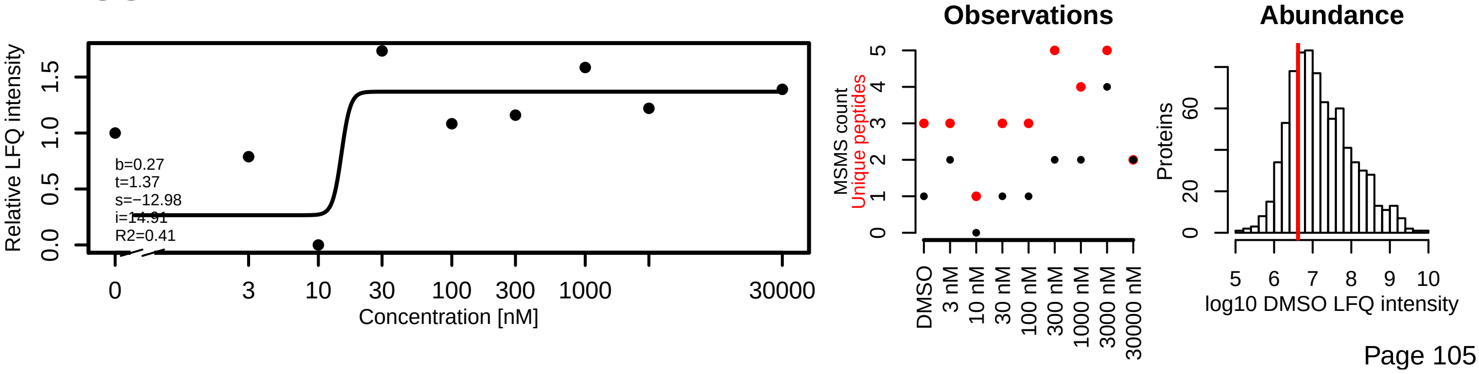

# FECH

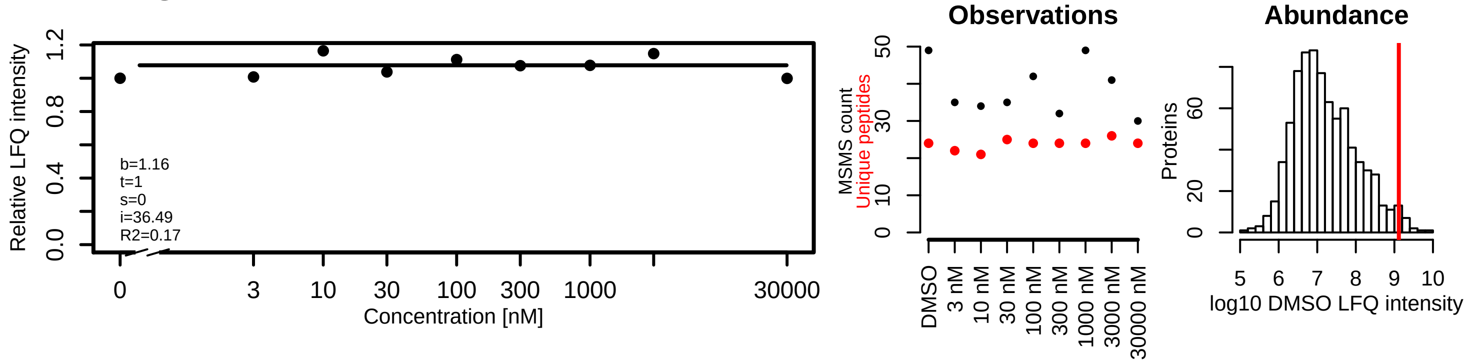


# MTOR

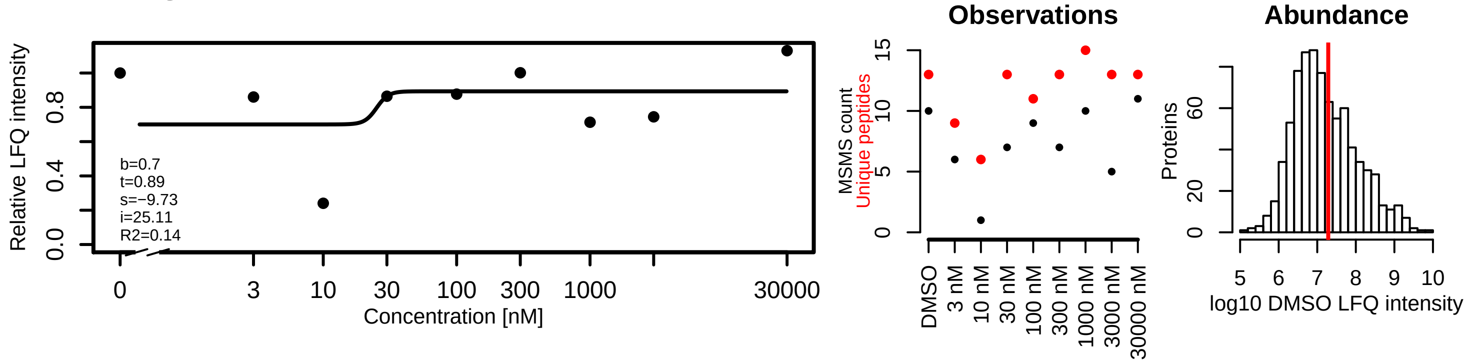


# TECR

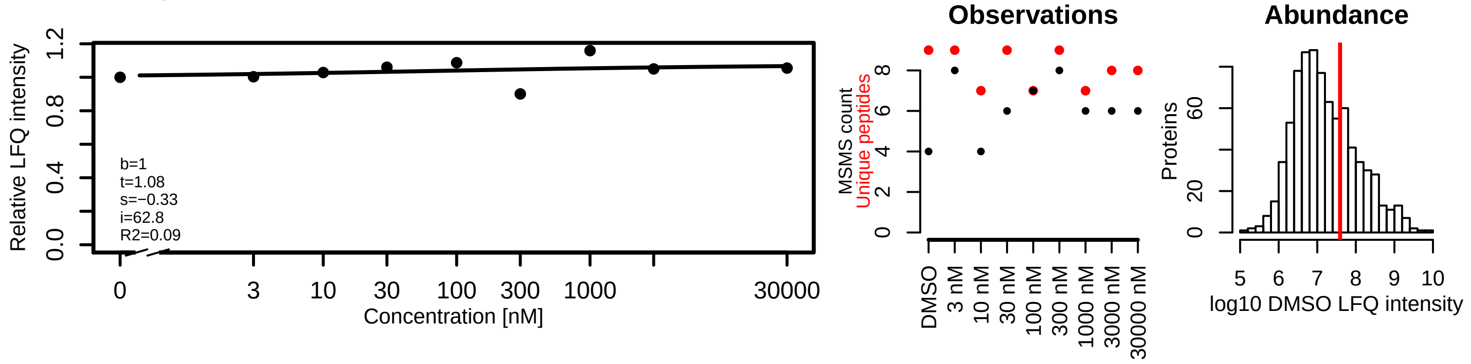


# NDUFA11

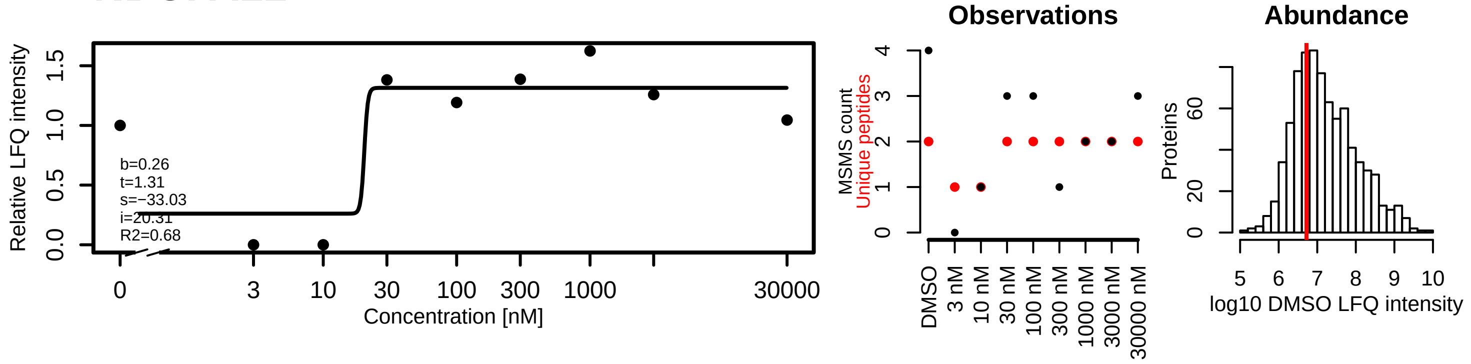


# FADS1

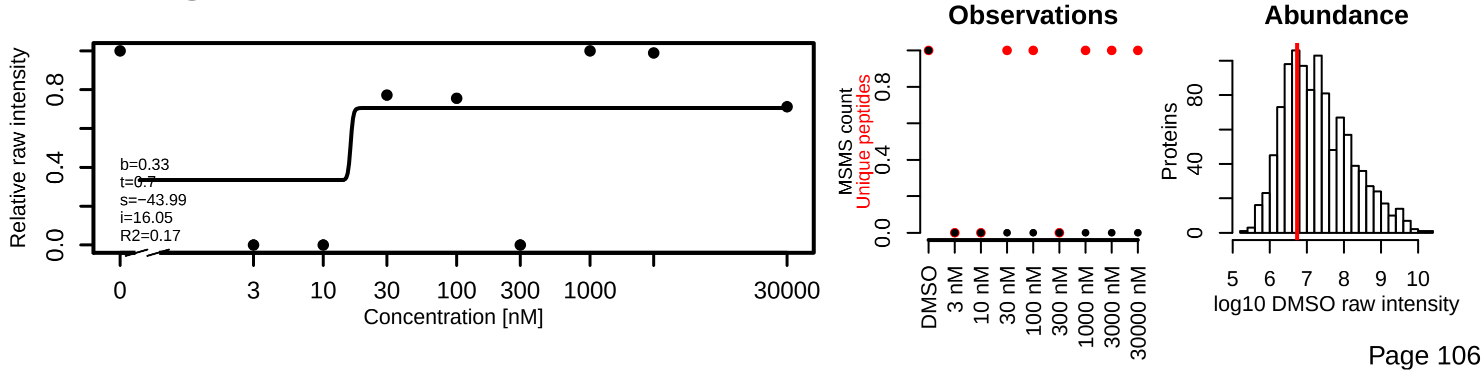

## RPL5

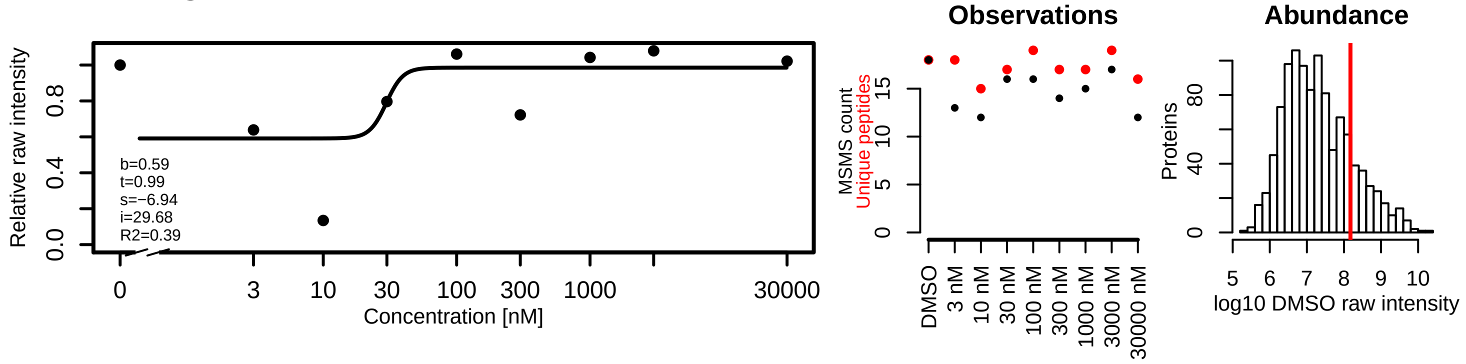


## RPS18

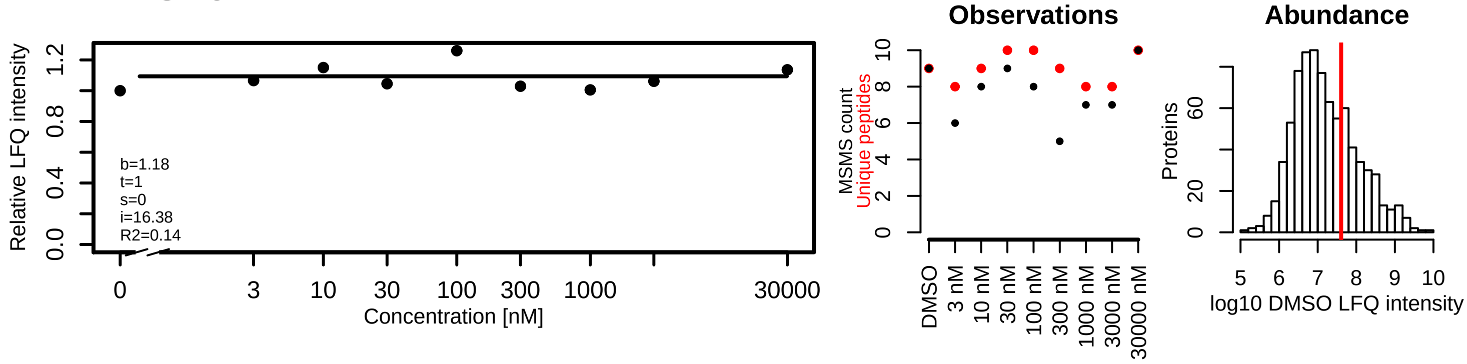


## STK17A

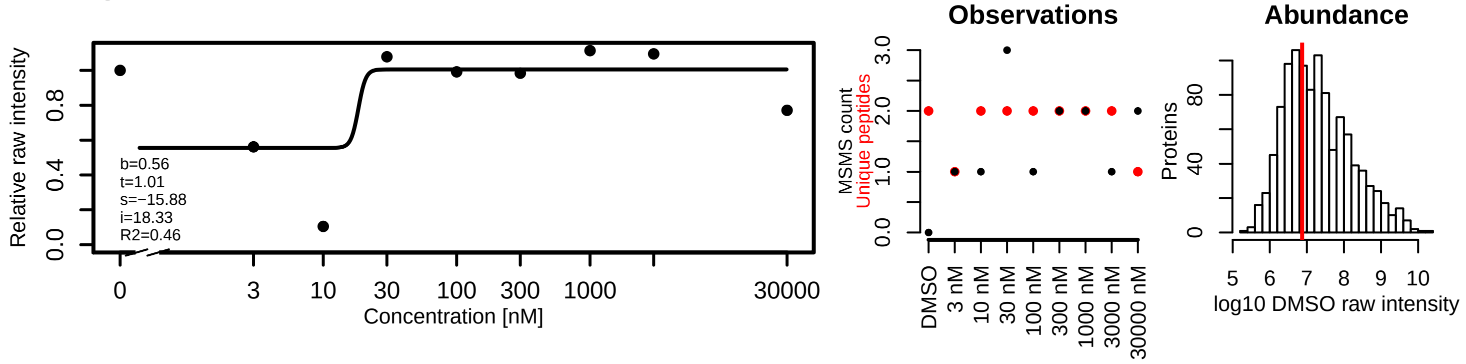


## NCBP2

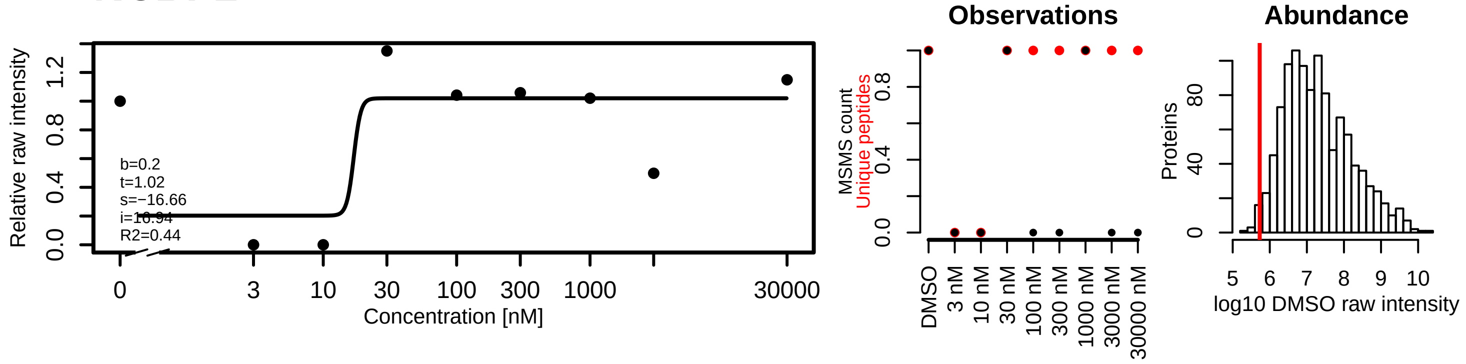


## RPS24

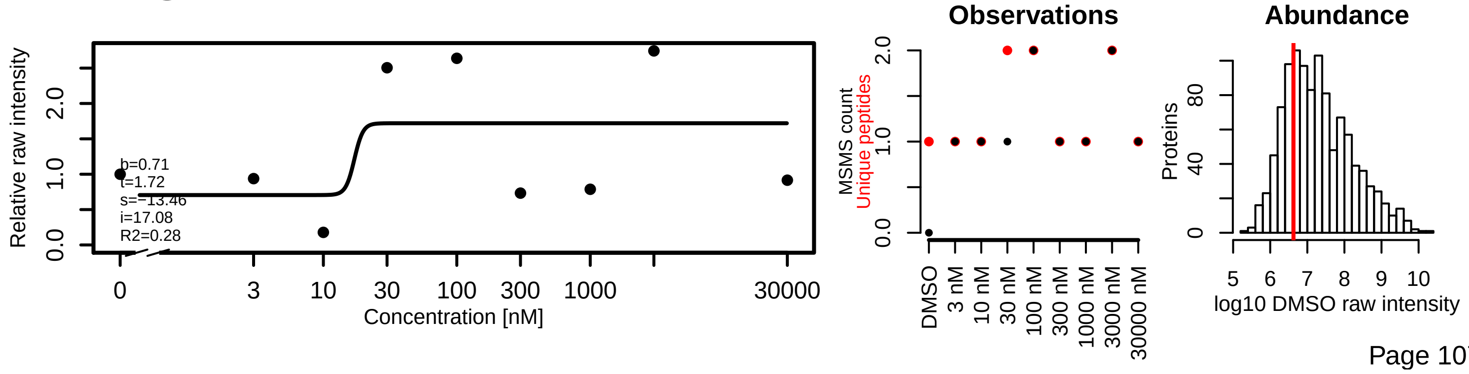

# SND1

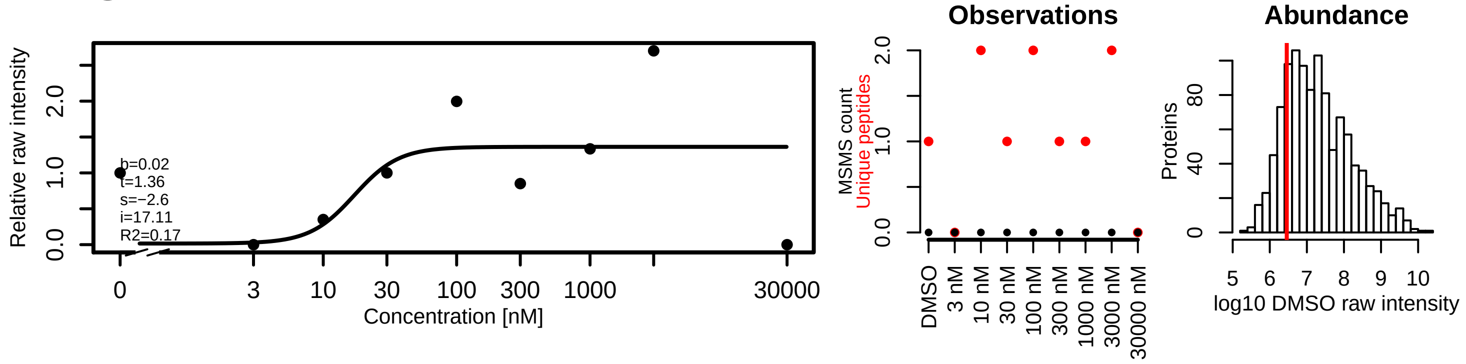


# ACADSB

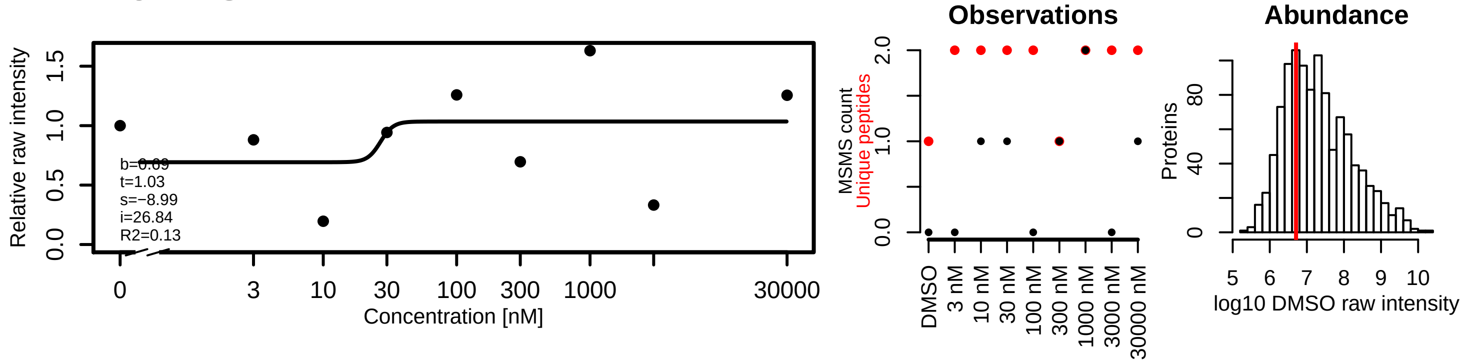


# GCN1L1

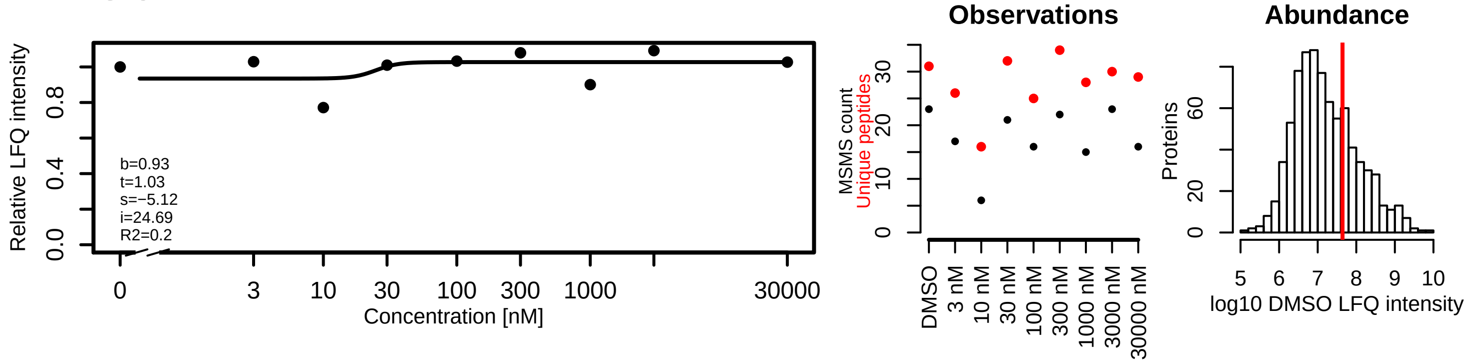


# PEX26

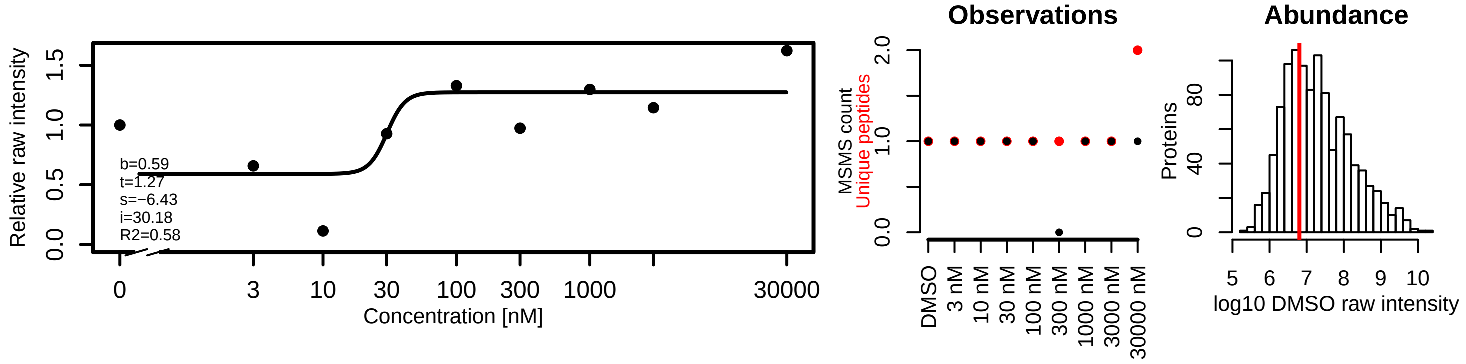


# CSNK2A2

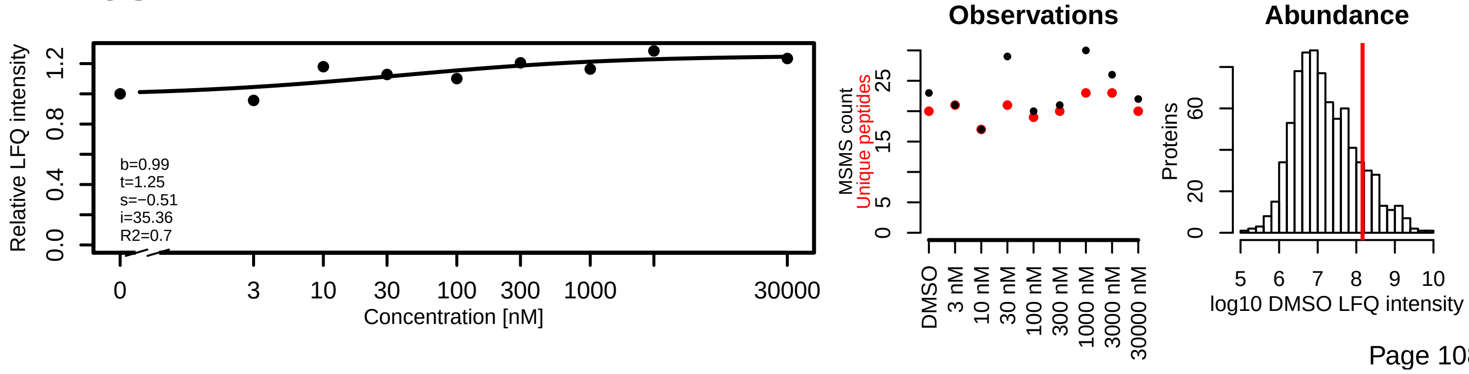

## HNRNPD

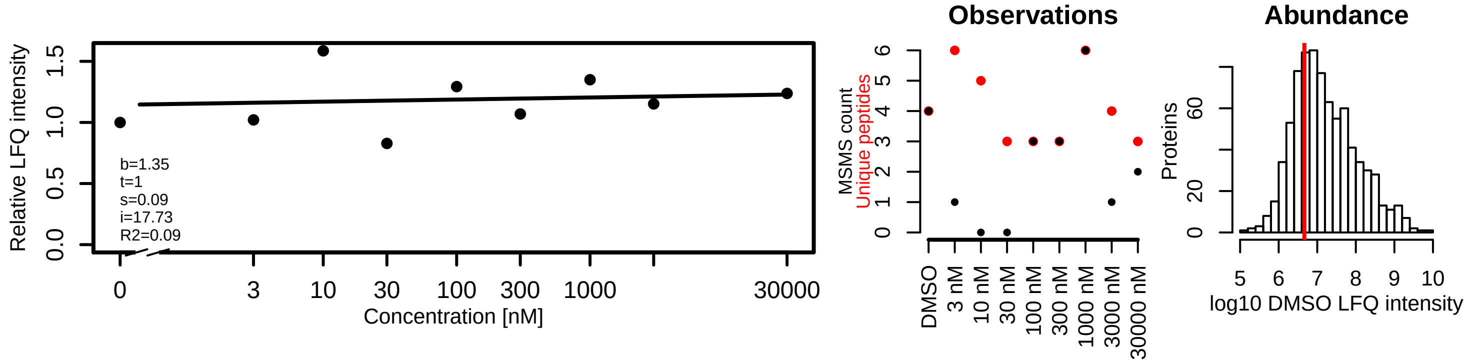


## ABCB6

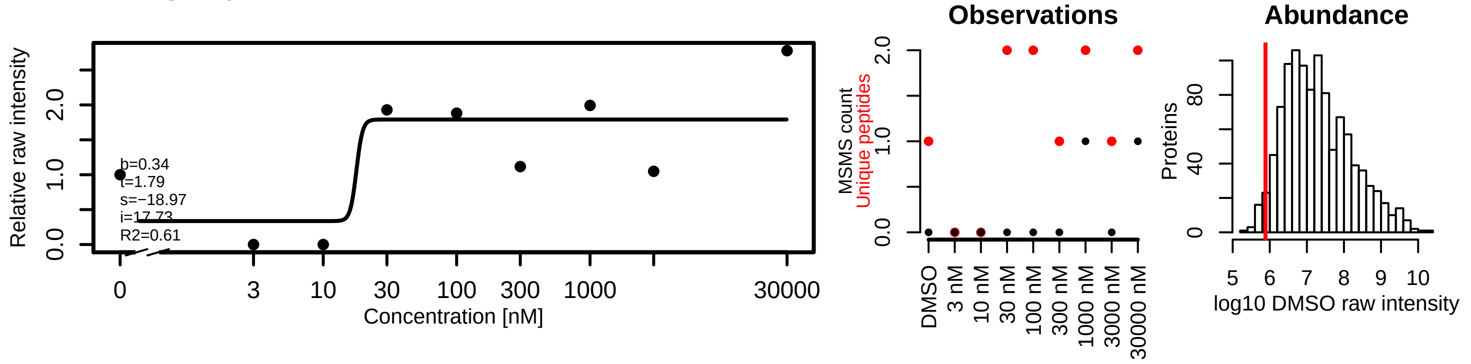


## MKRN3

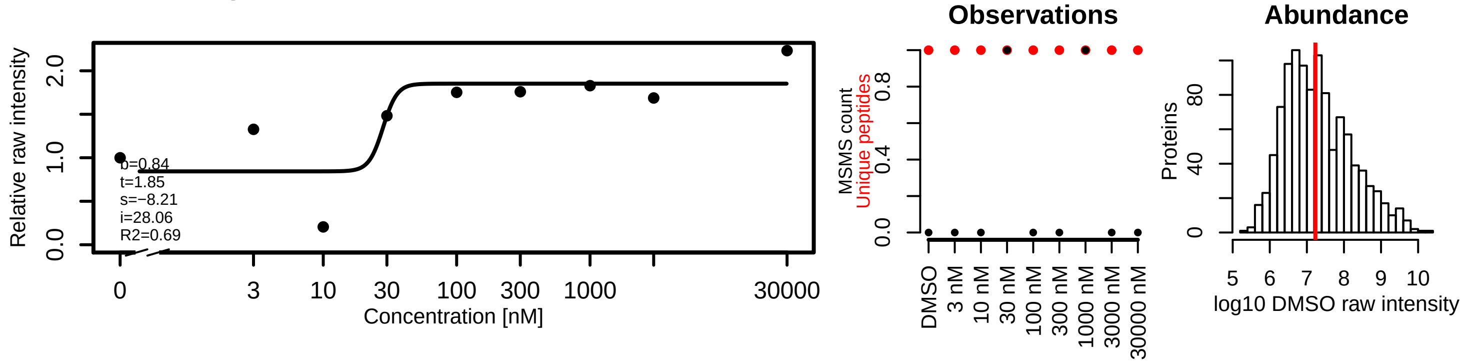


## MARK1

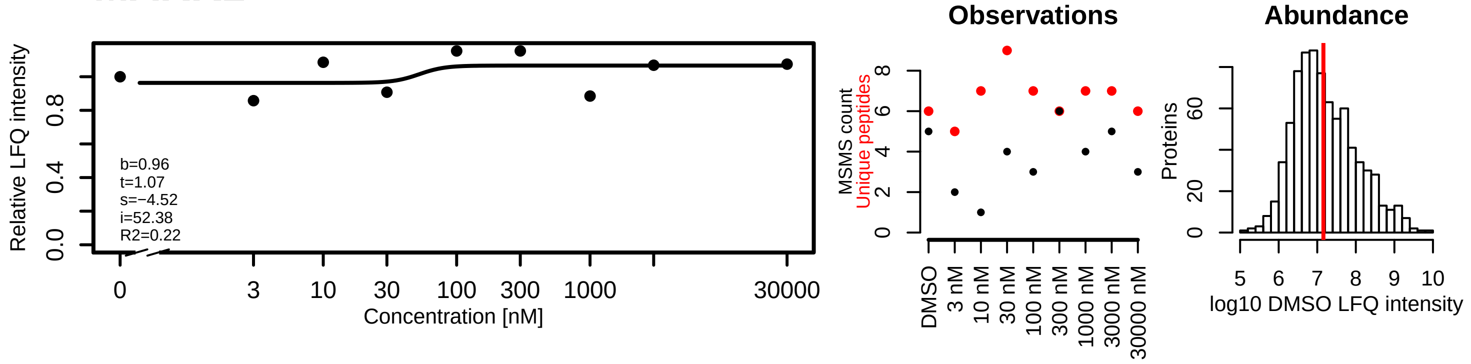


## PTK2 − Q8IYN9;Q05397;H0YBP1;Q05397−5;E7ESA6;J3QT16;E9PEI4;

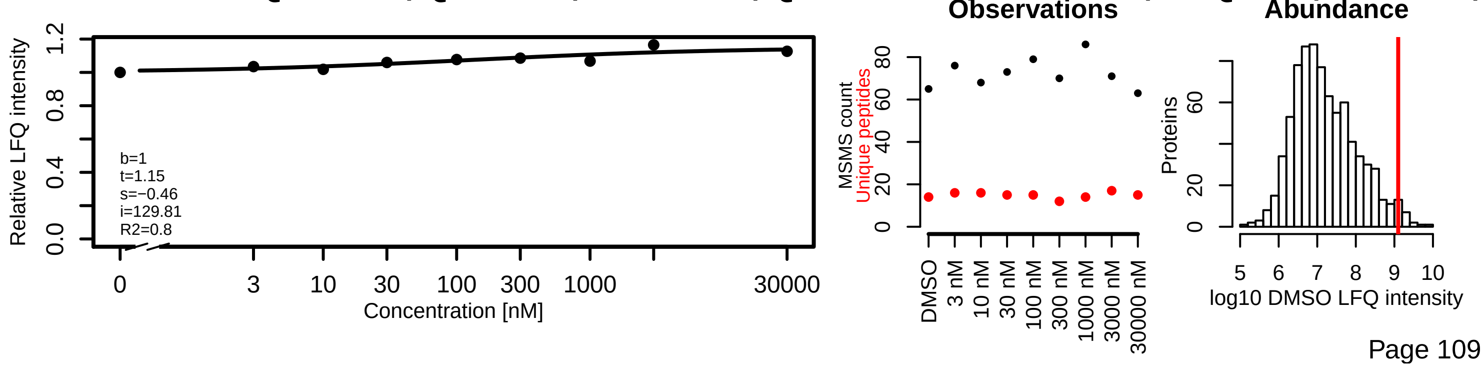

## COG7

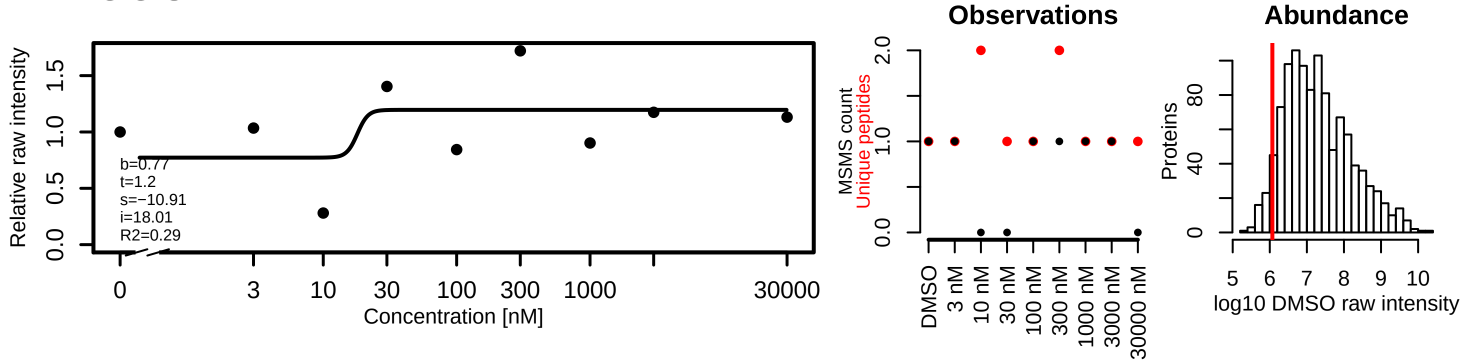


## AFG3L2

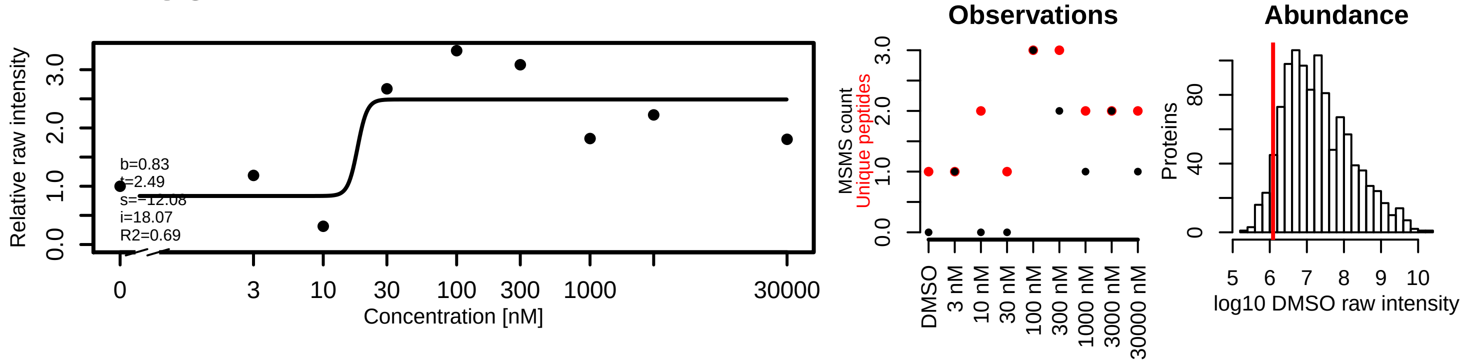


## COPB2

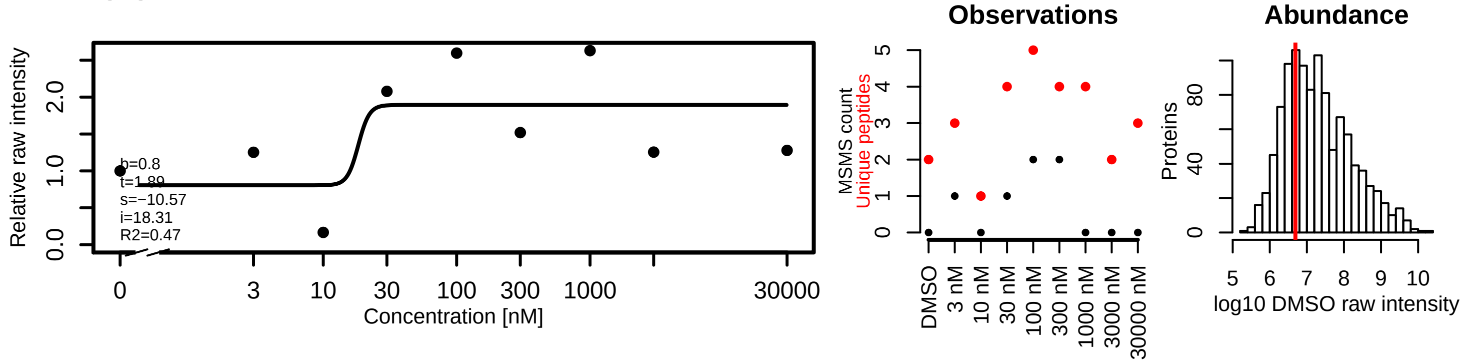


## SSBP1

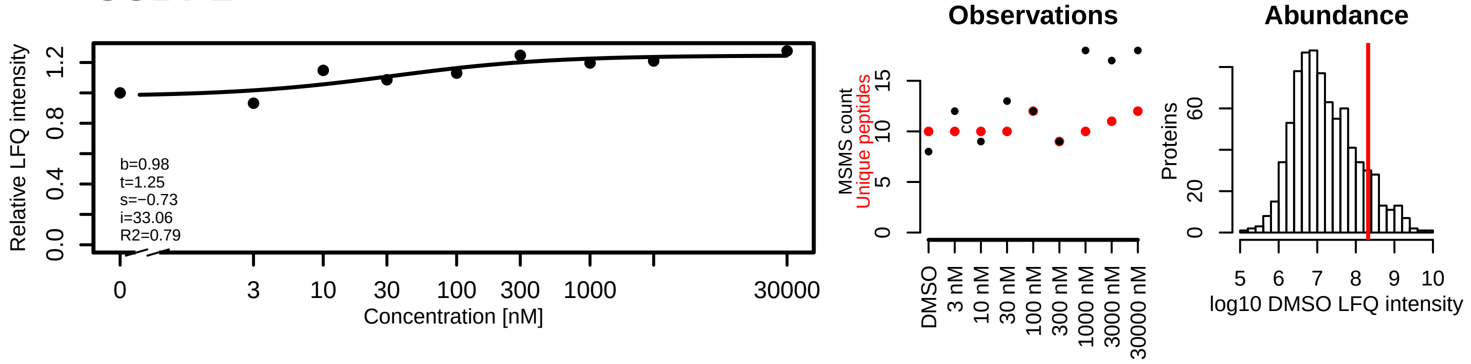


## FLNC

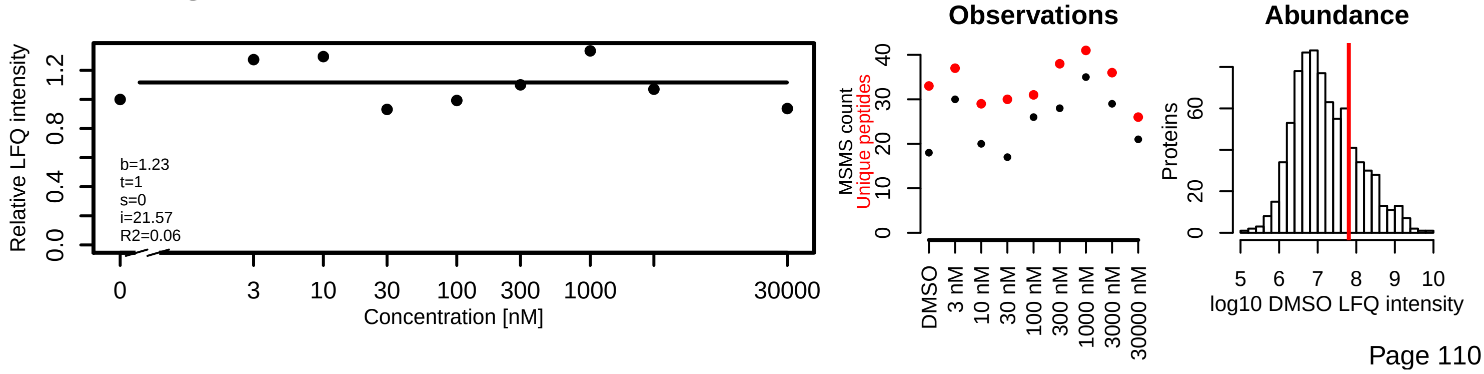

# CAMK2B − Q13554−4;Q13554−5;Q13554−8;Q13554−2;Q13554−7;Q13

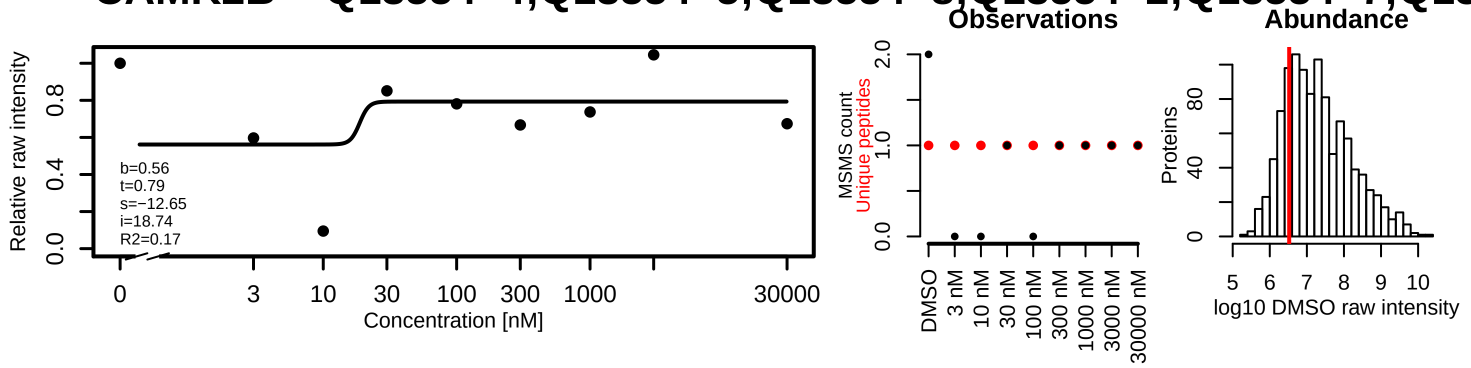


# MAPK3

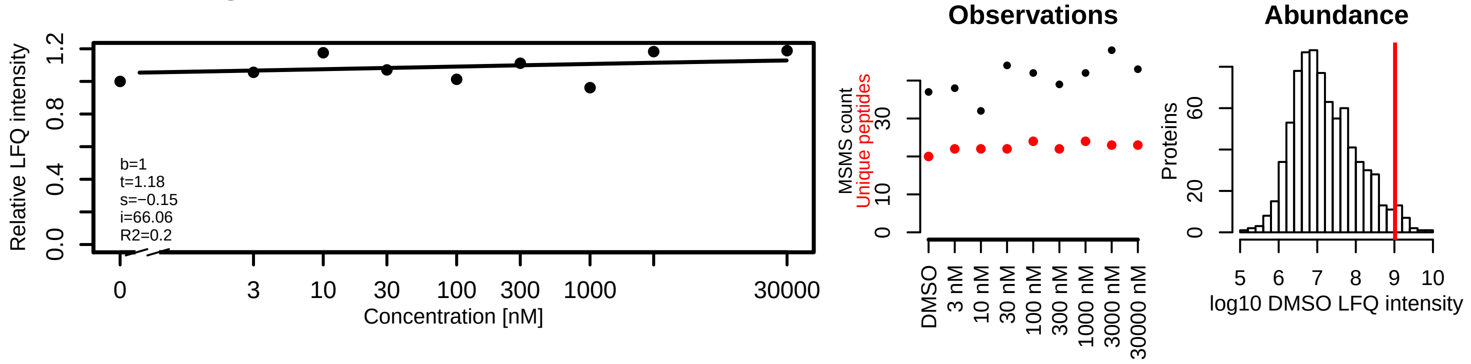


# ATP12A

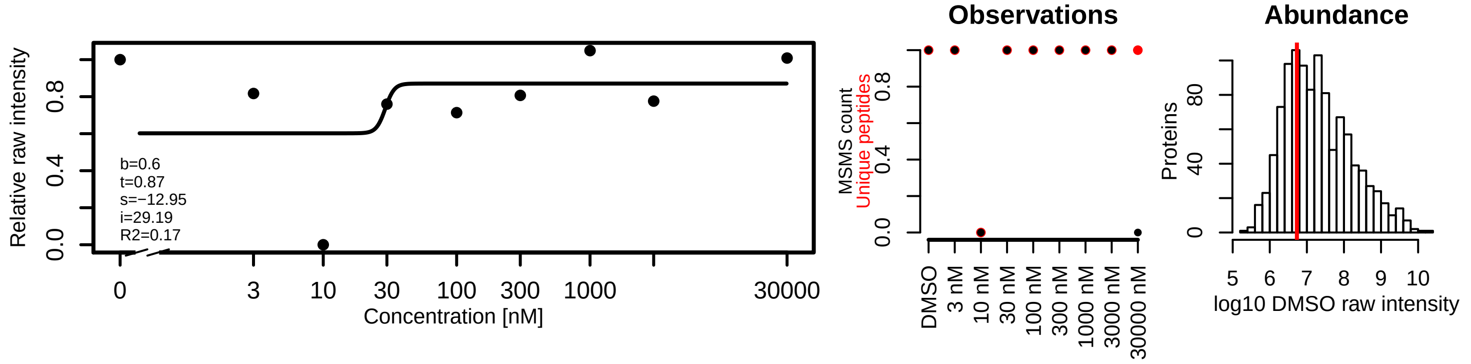


# CCNH

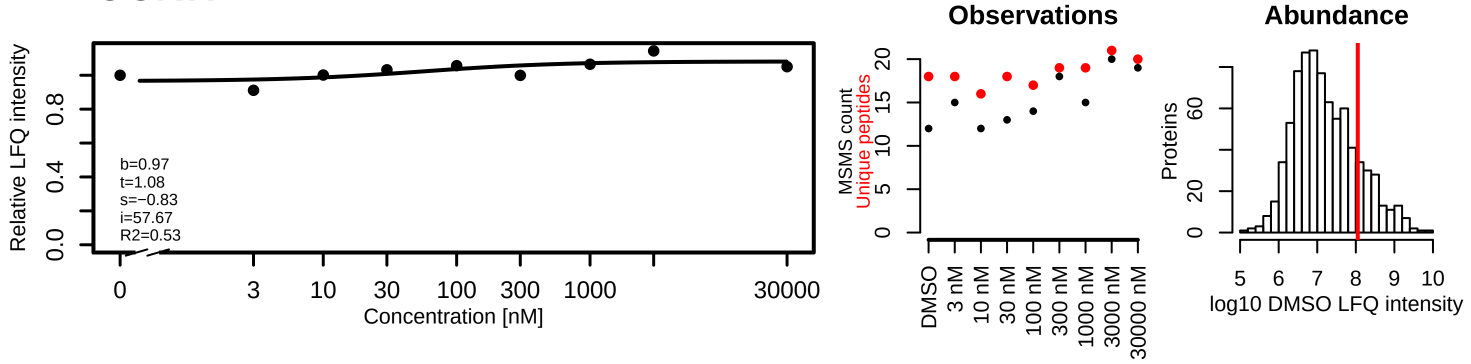


# RPL12

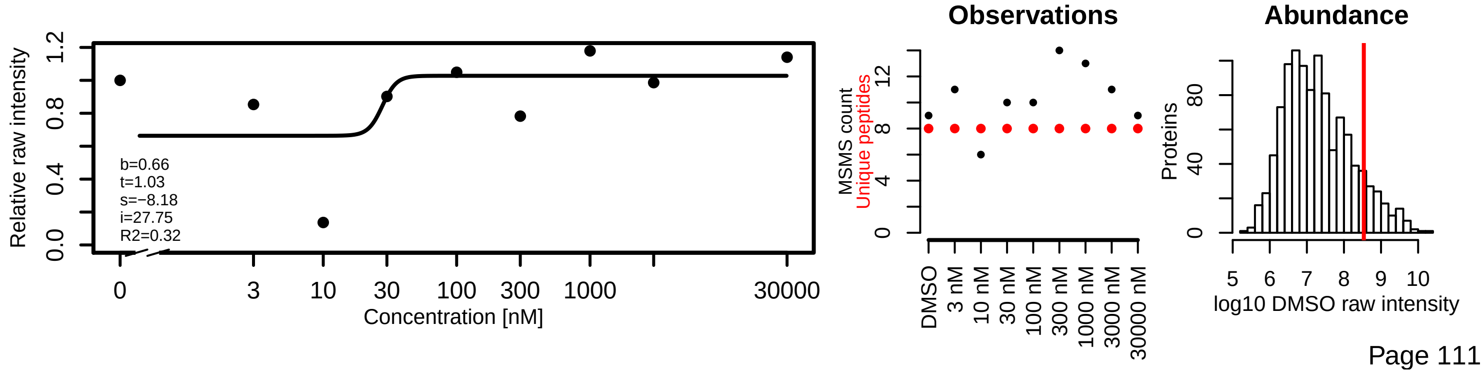

# SF3B1

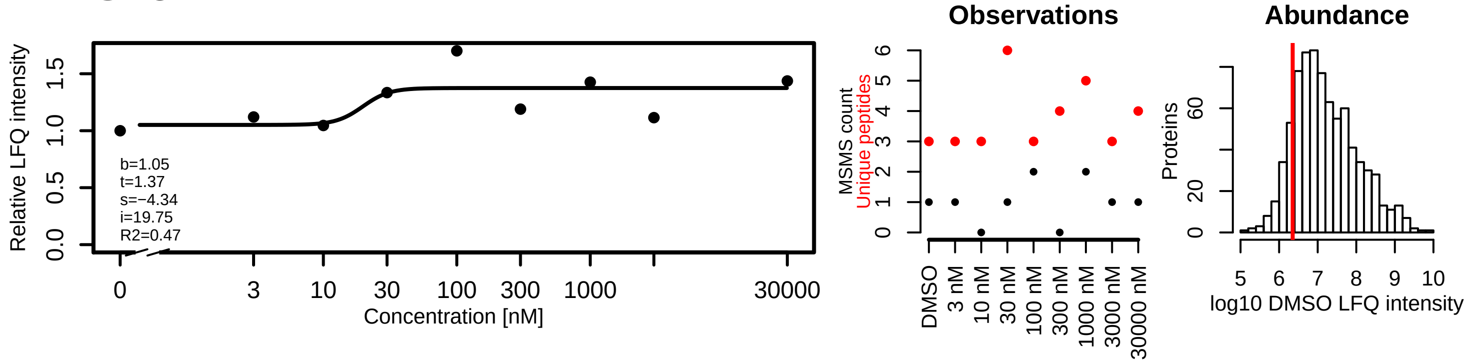


# PRKAG1

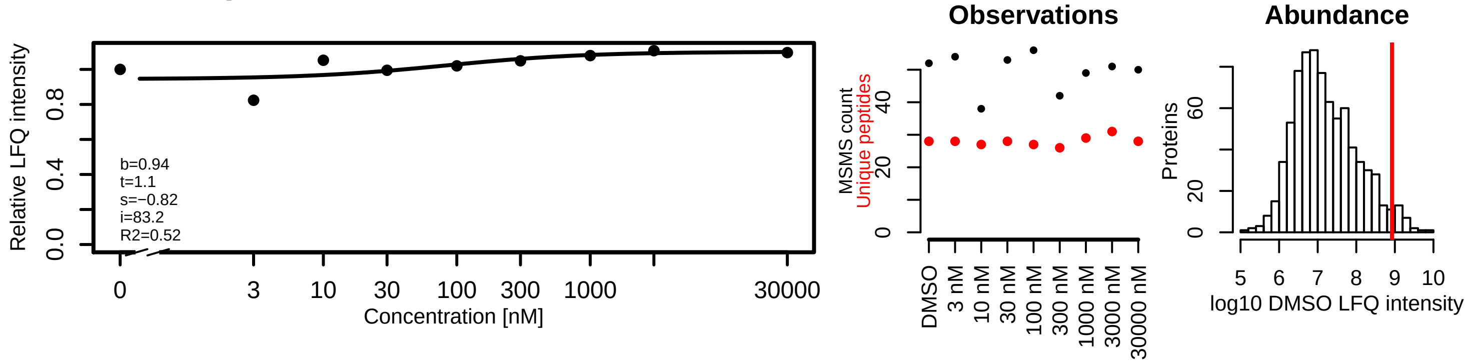


# MGST1

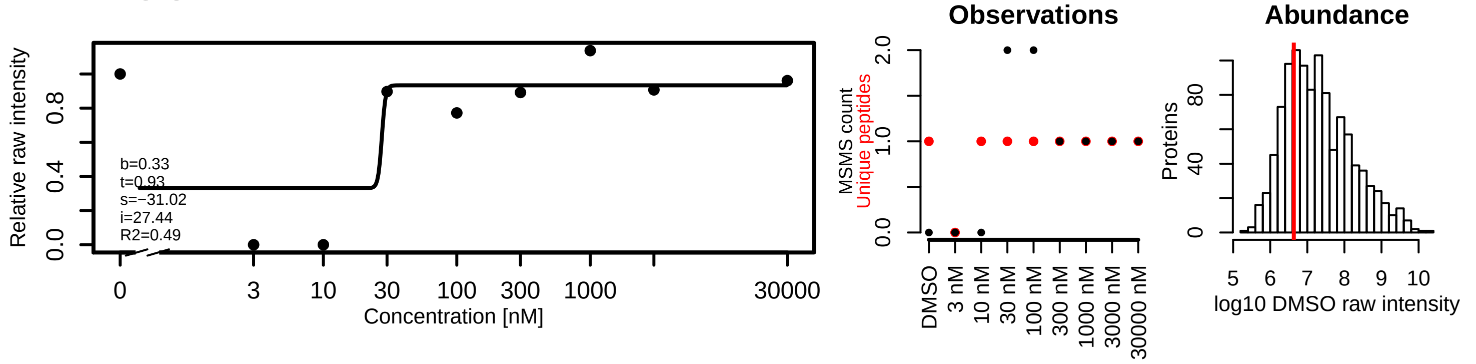


# DMPK

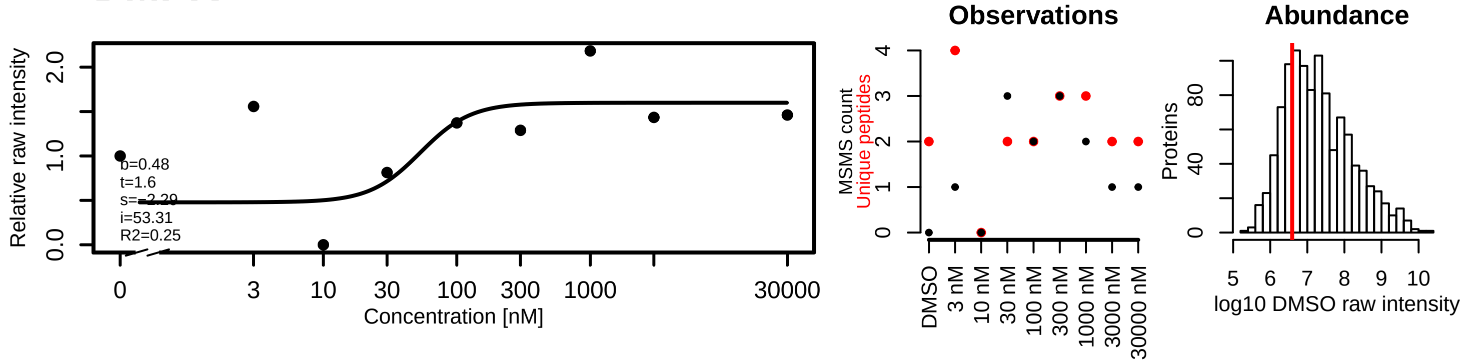


# STOML2

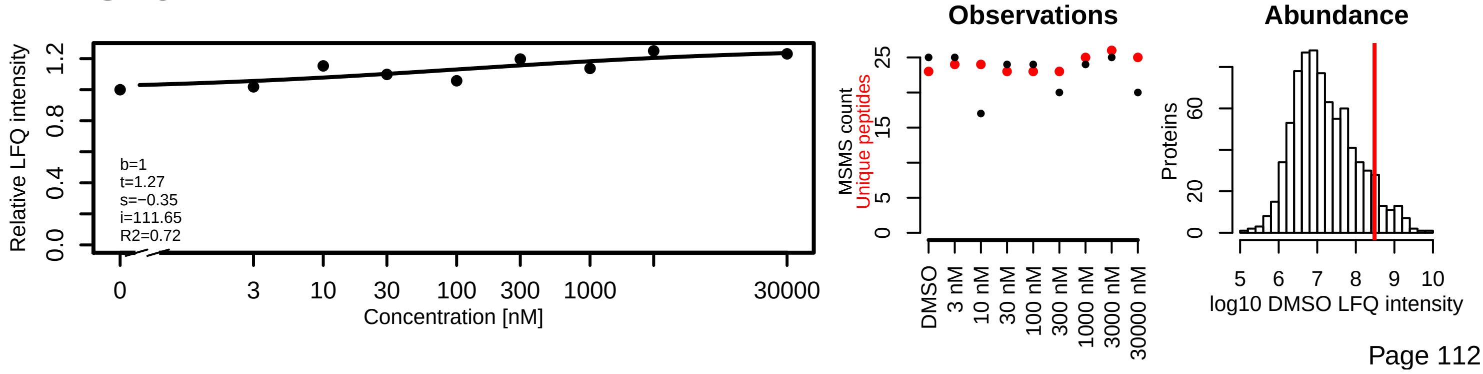

# PPP2R1A

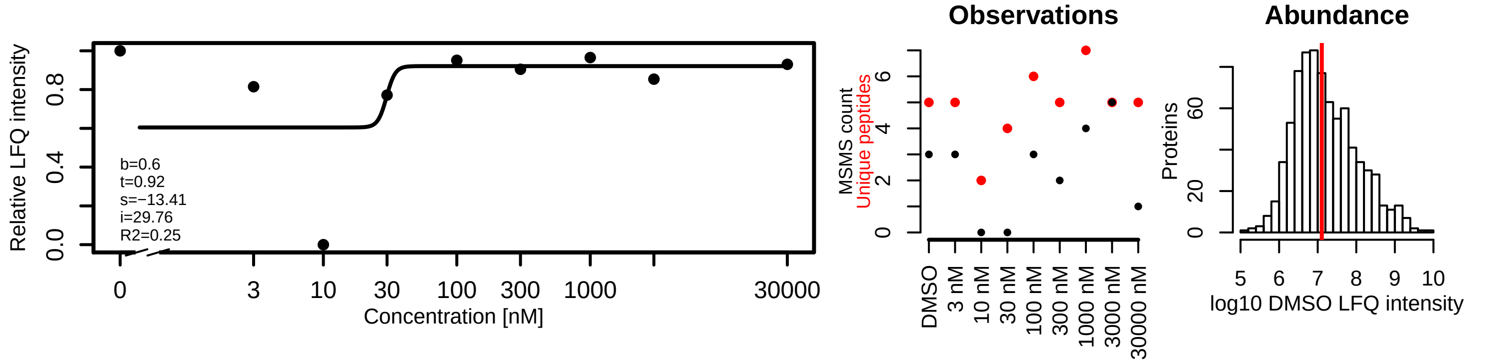


# CDC5L

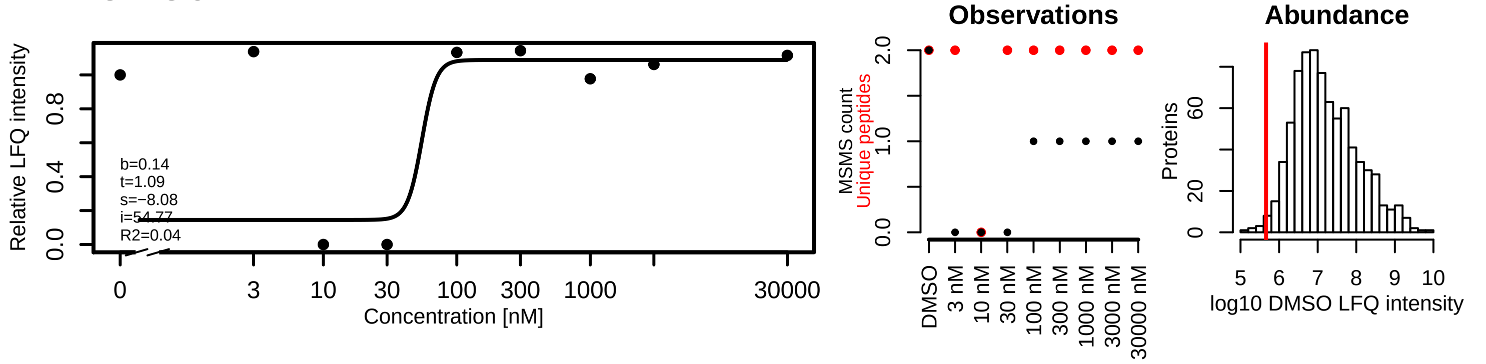


# RPL21

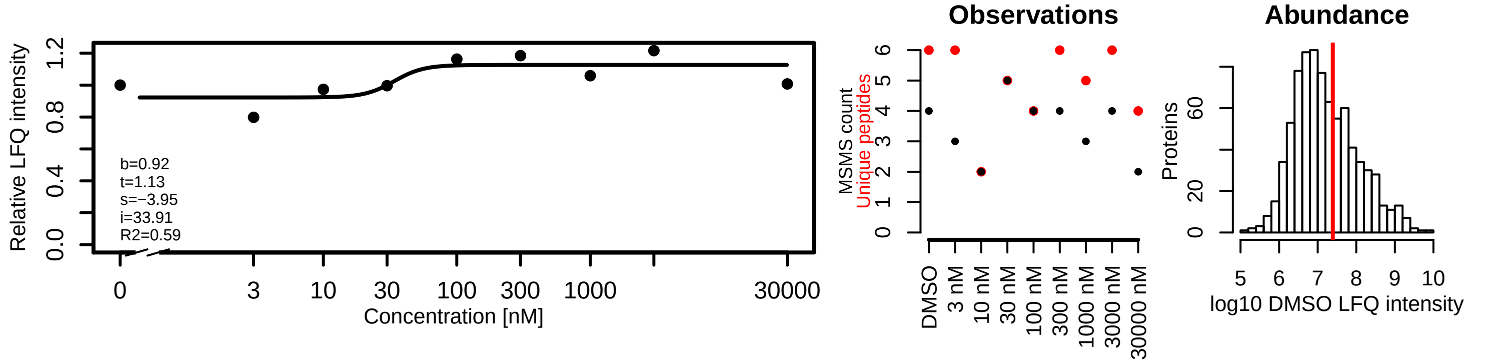


# SUN2

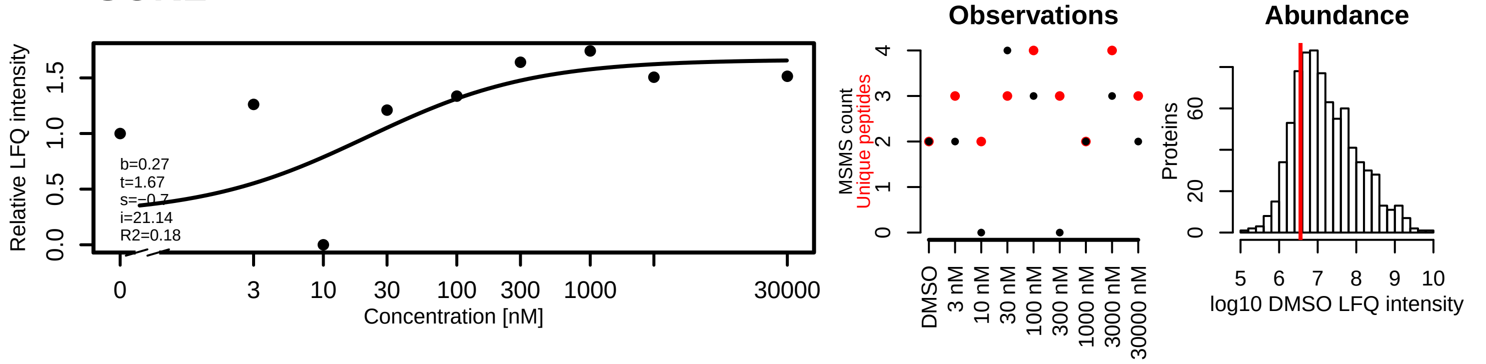


# RPS6KA6

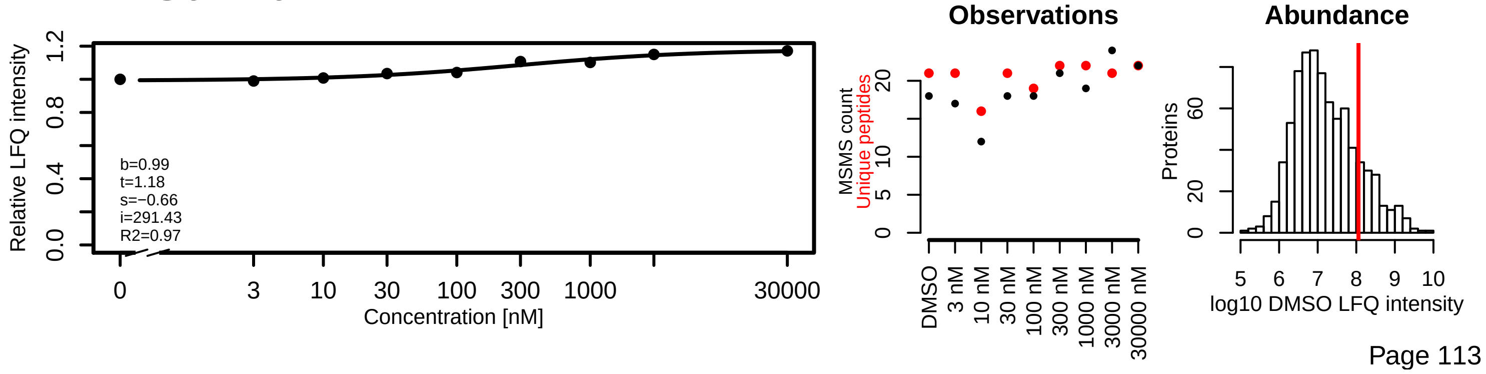

# CLTA

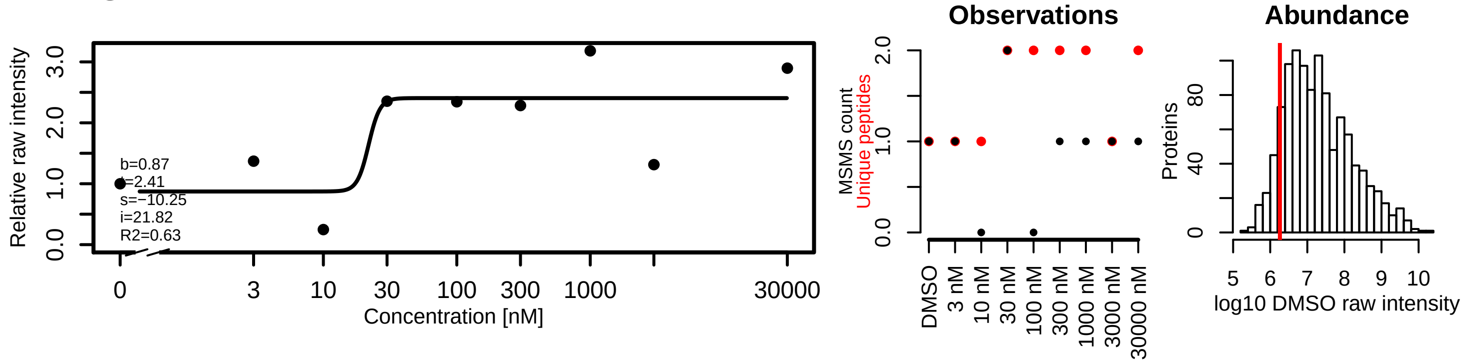


# SERBP1

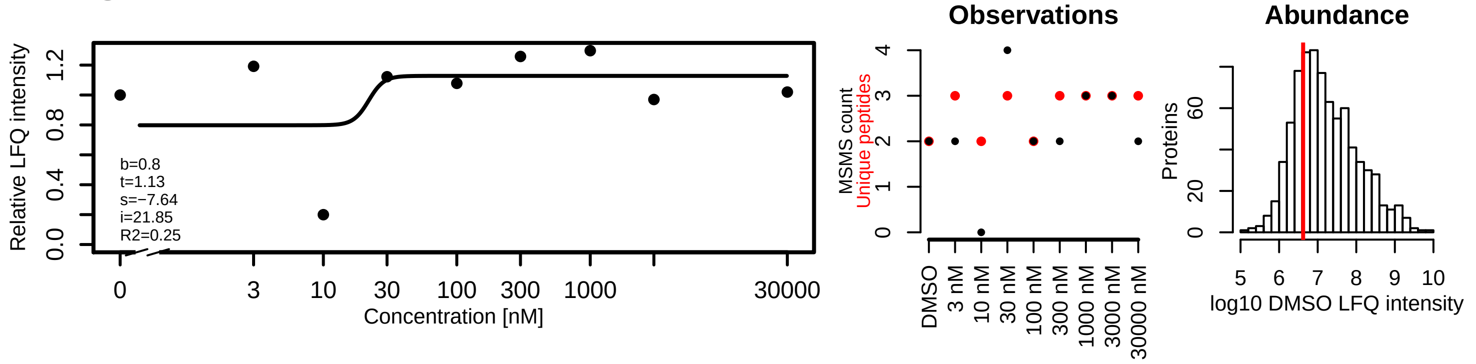


# MYO1B

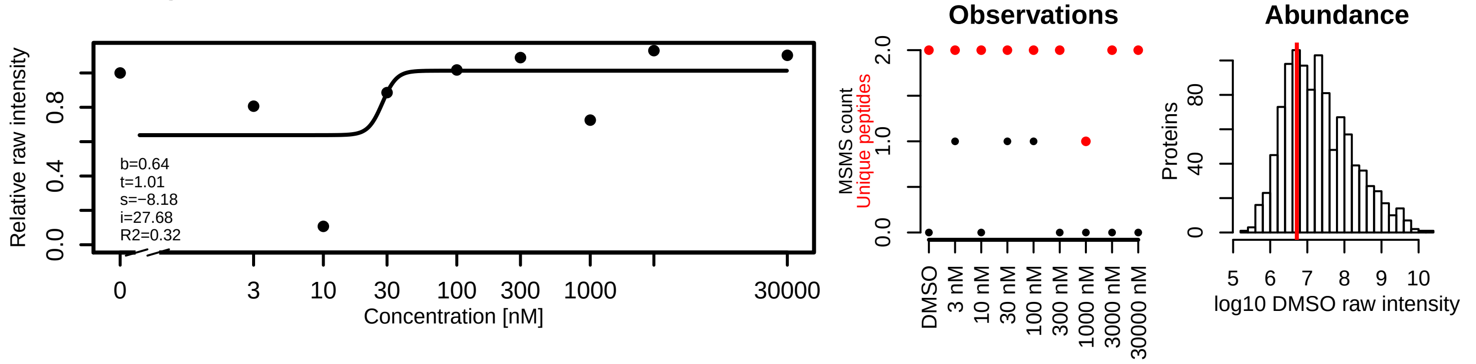


# COX5B

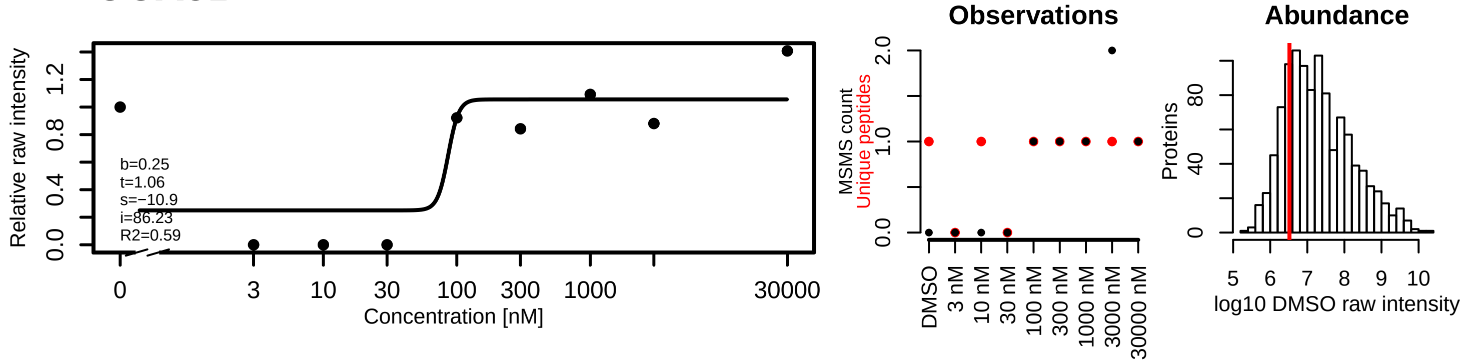


# CALM1;CALM2;CALM3;TNNC2;CALML3

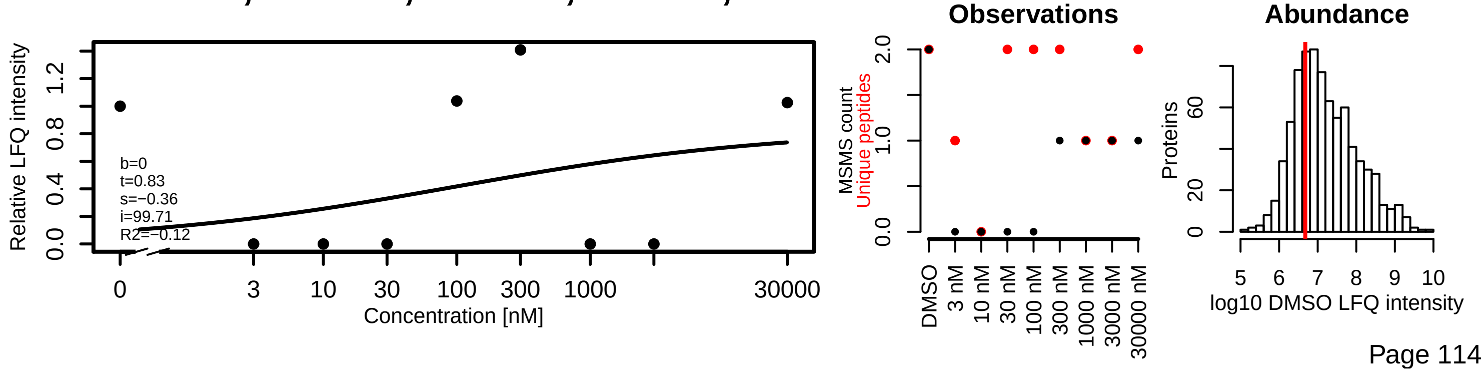

# XPOT

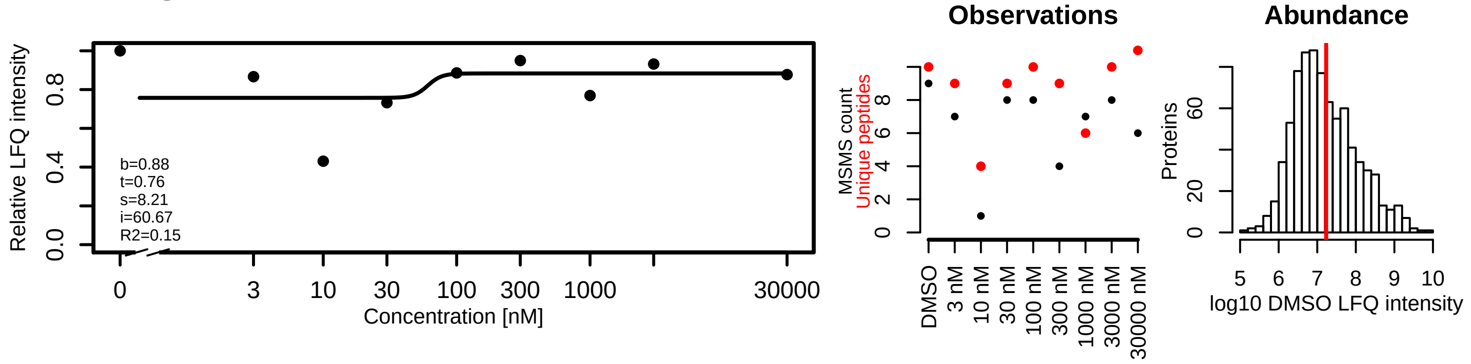


# HNRNPUL1

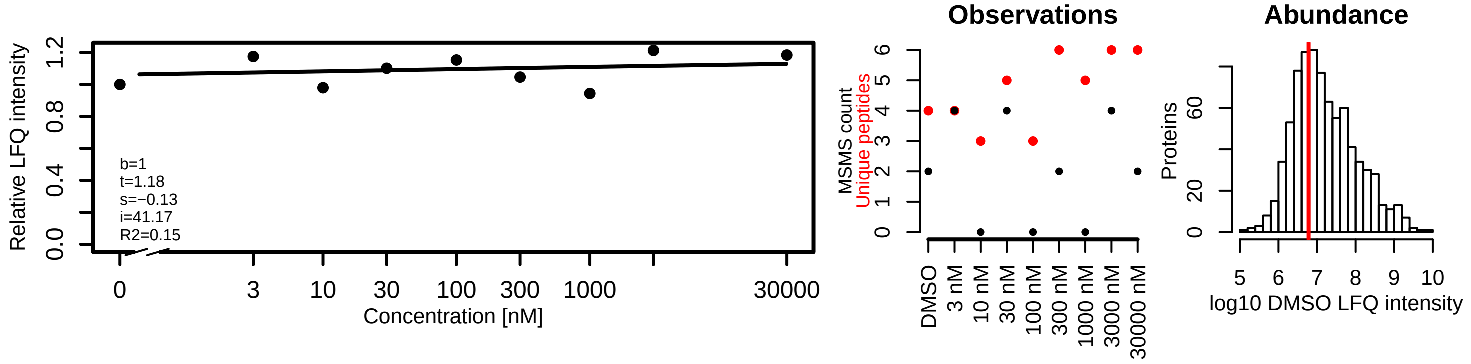


# PDCD6

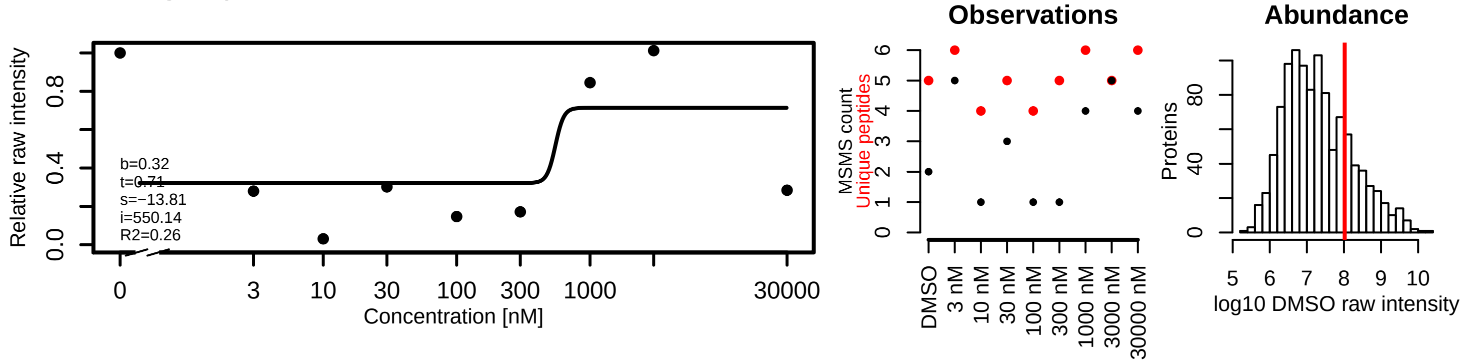


# EFHD2;EFHD1

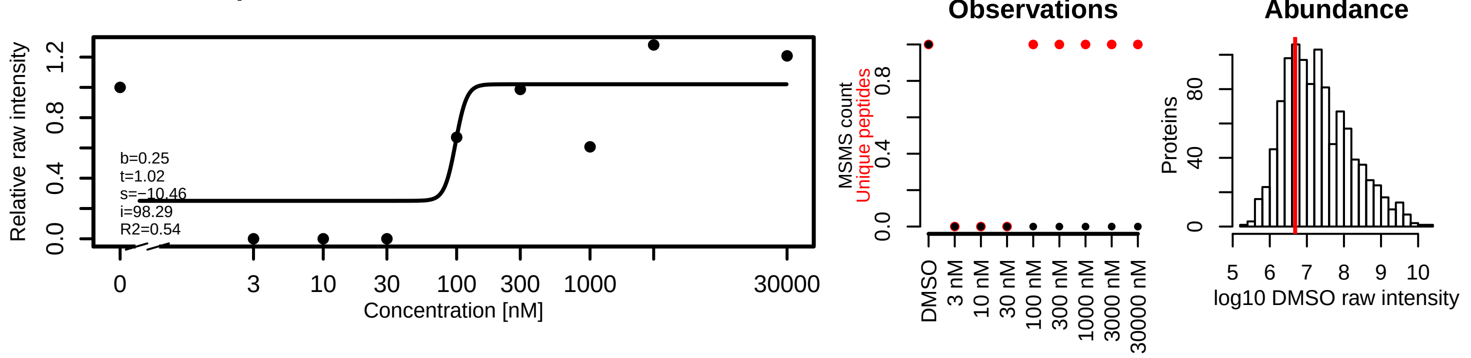


# SDHB

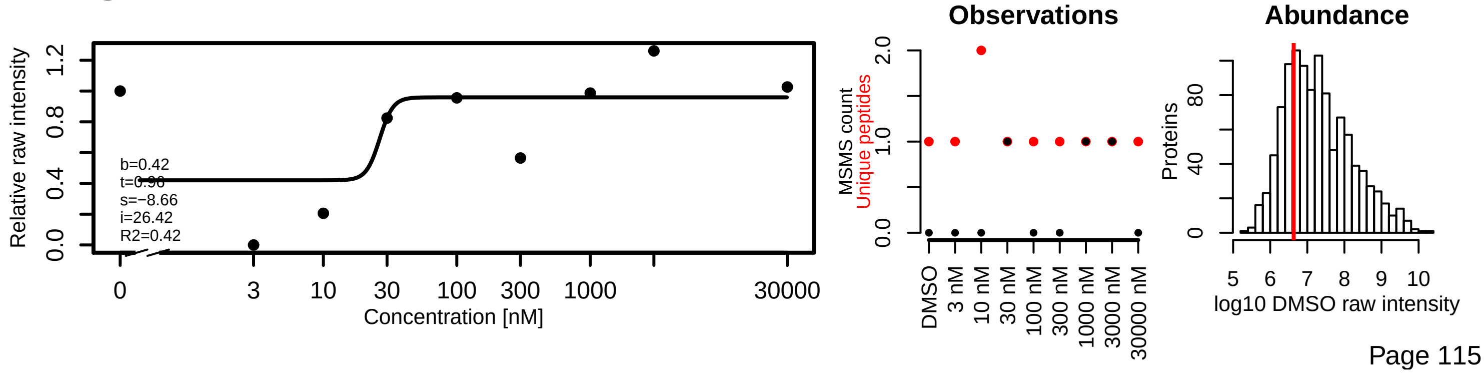

# TRAM1

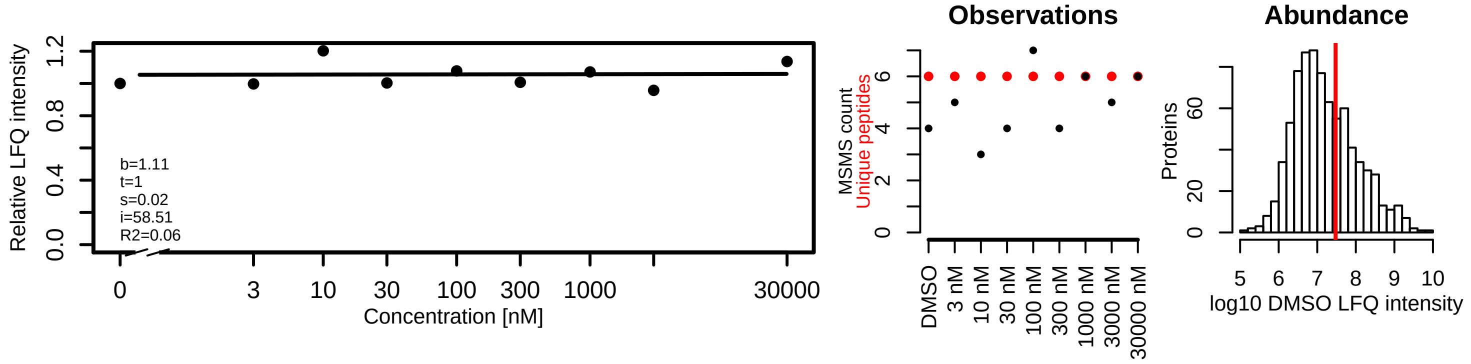


# PYCR1

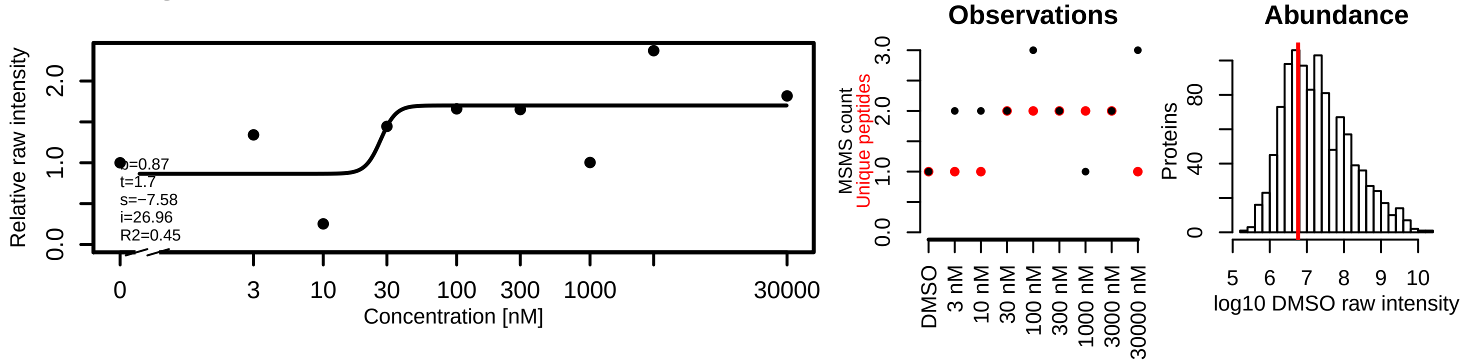


# LEPROT

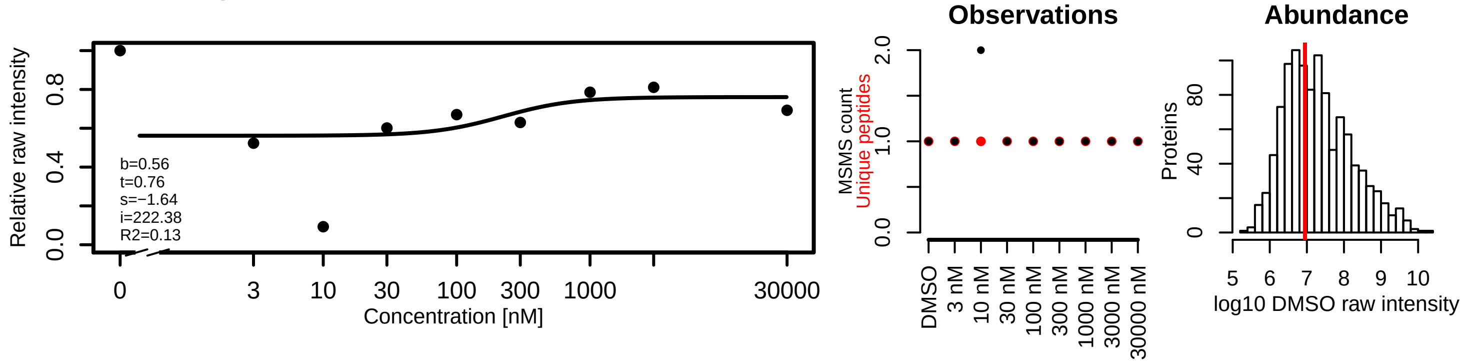


# RPL23A

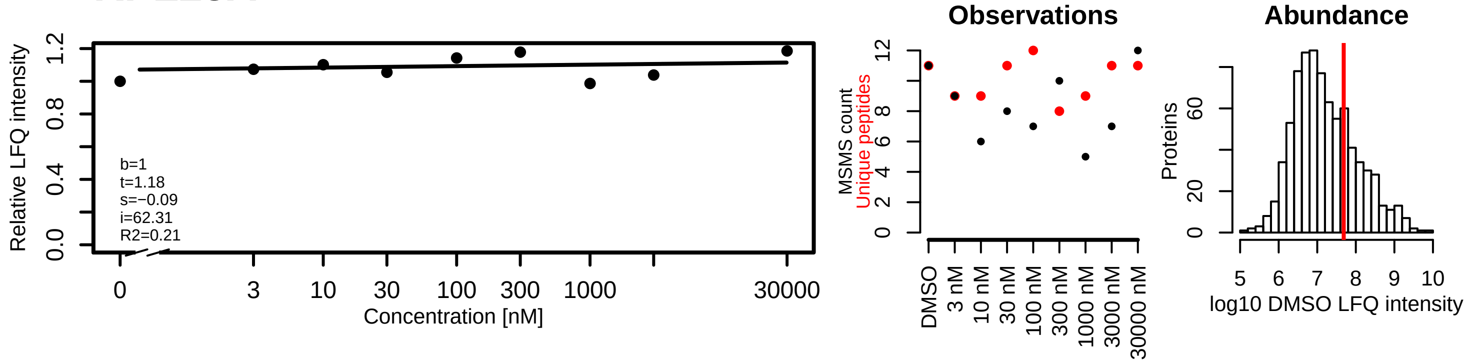


# TUBA1C;TUBA1B

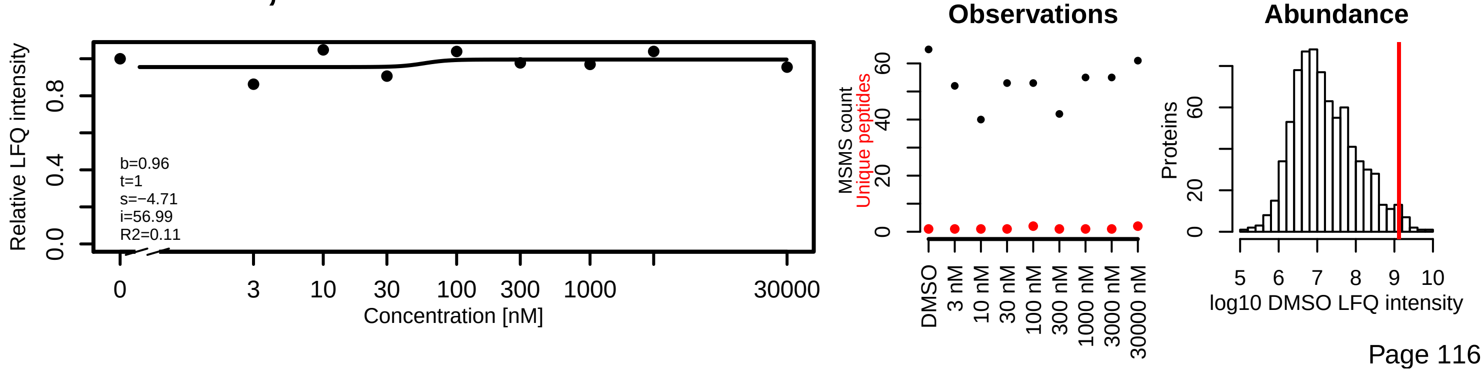

# KTN1

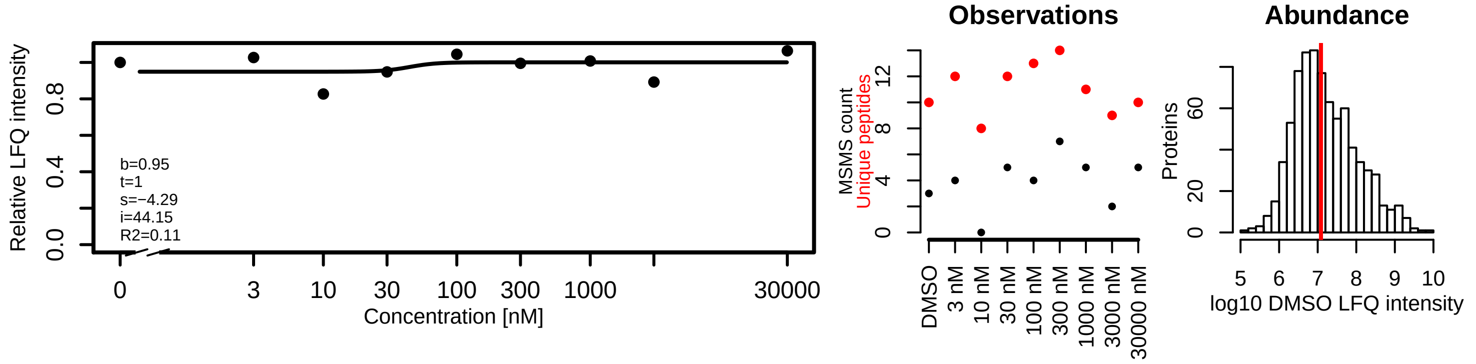


# EEF1G

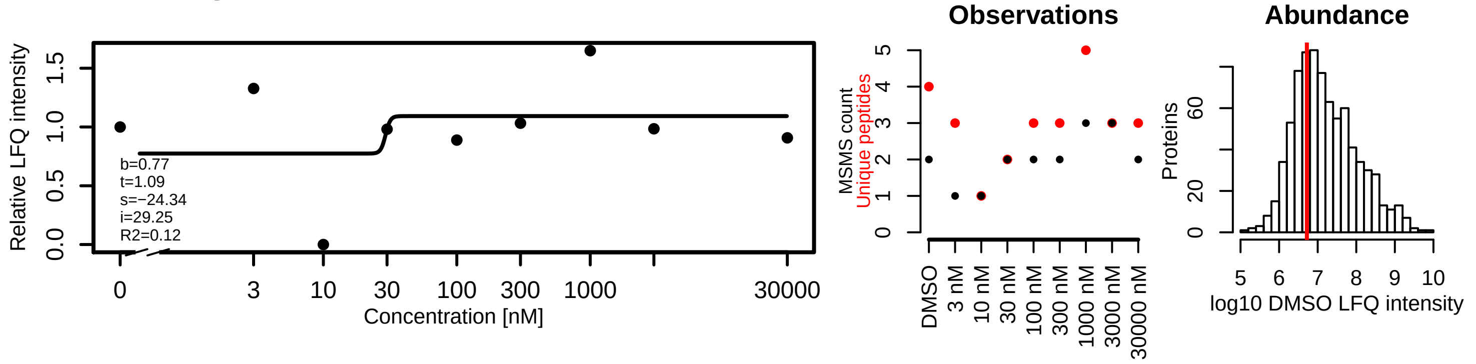


# NOP2

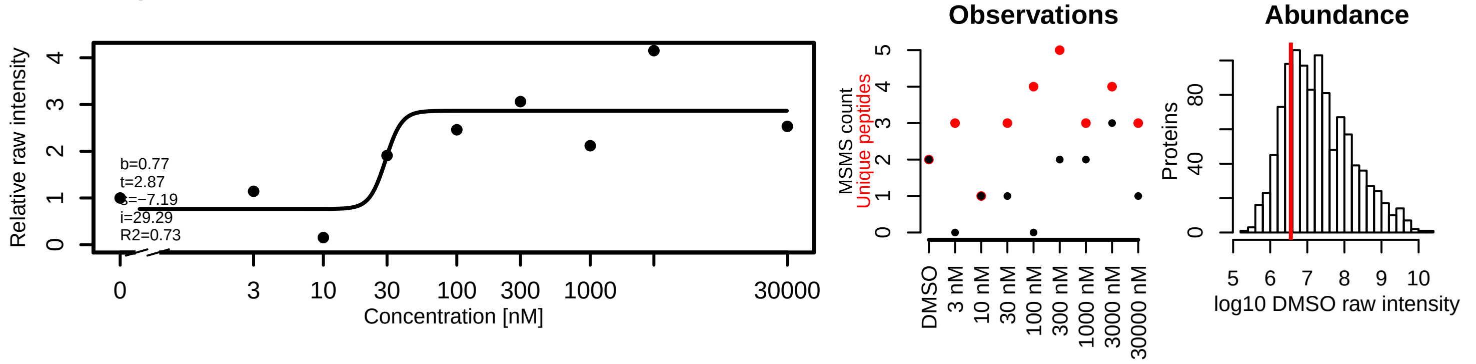


# RPL11

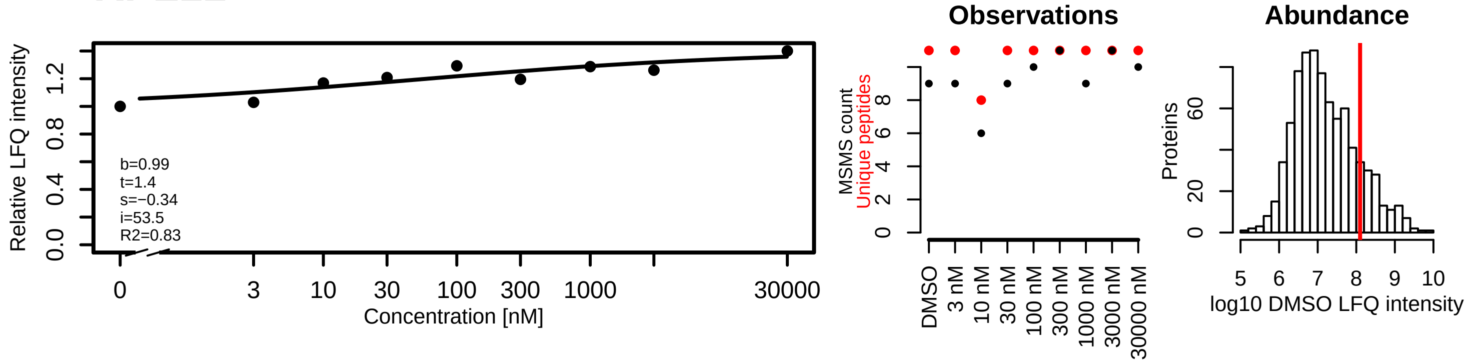


# RPS2

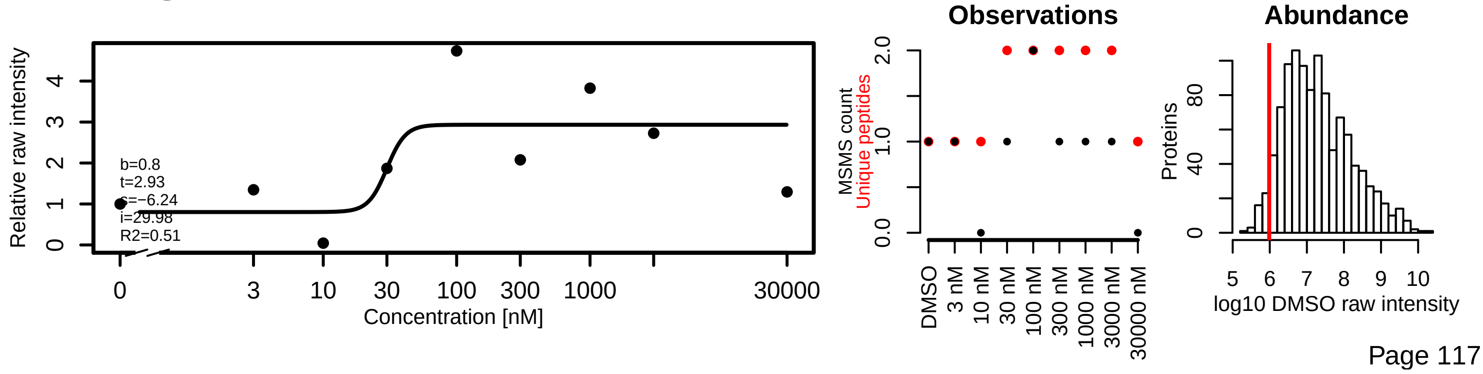

# ARL1

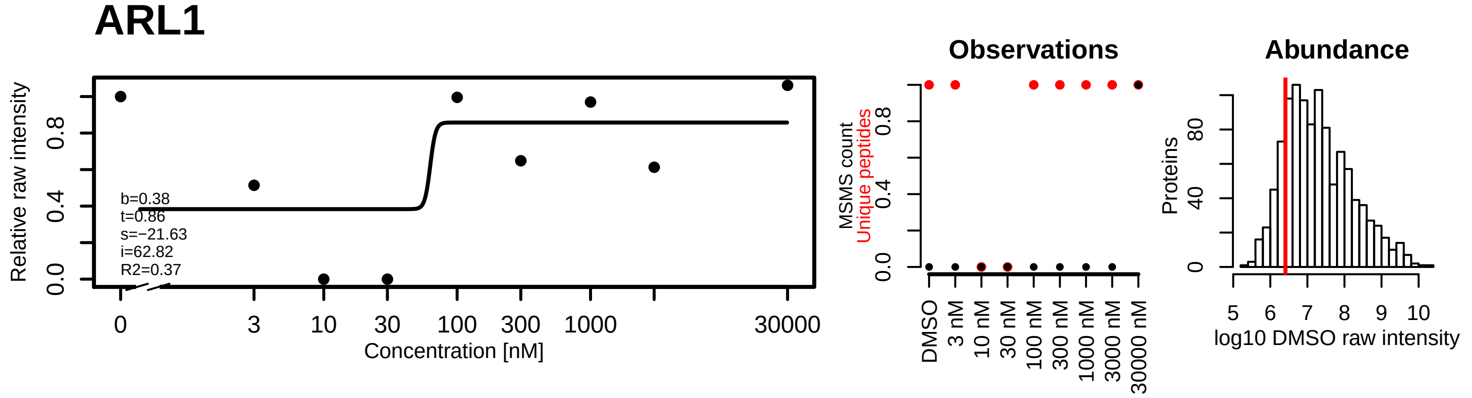


# RPS29

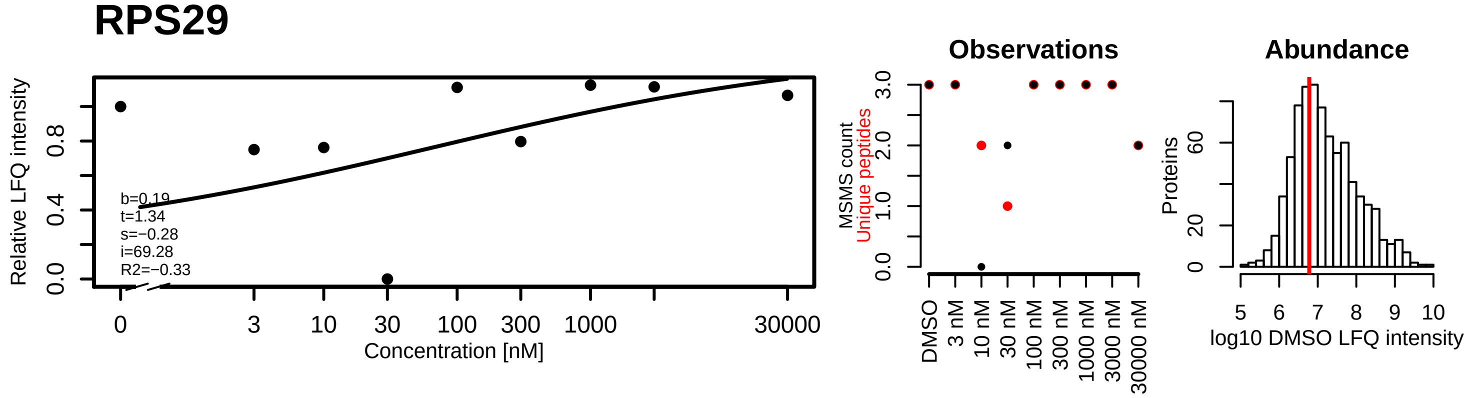


# ESYT2

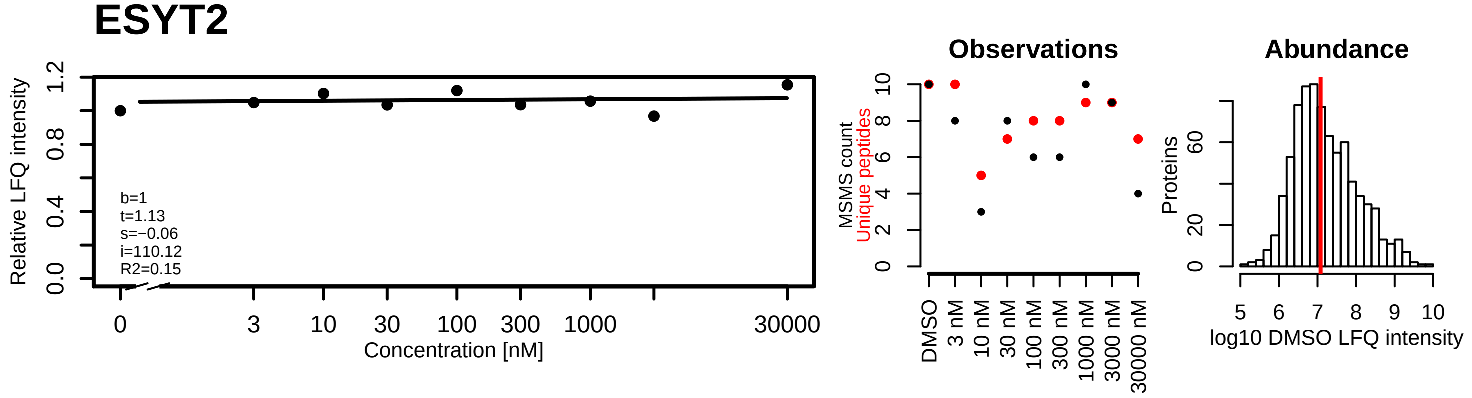


# CSNK1A1

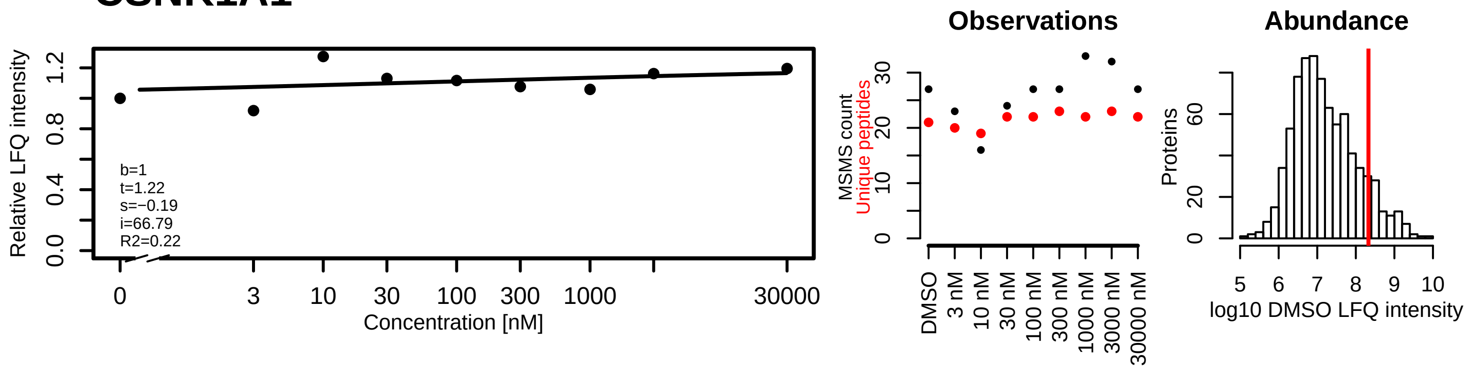


# KIAA0020

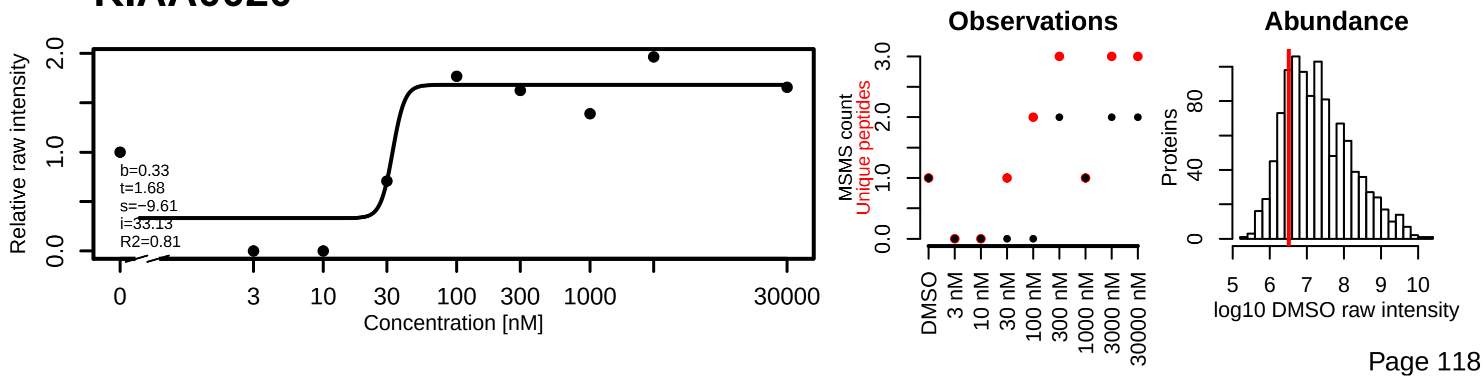

# CALR

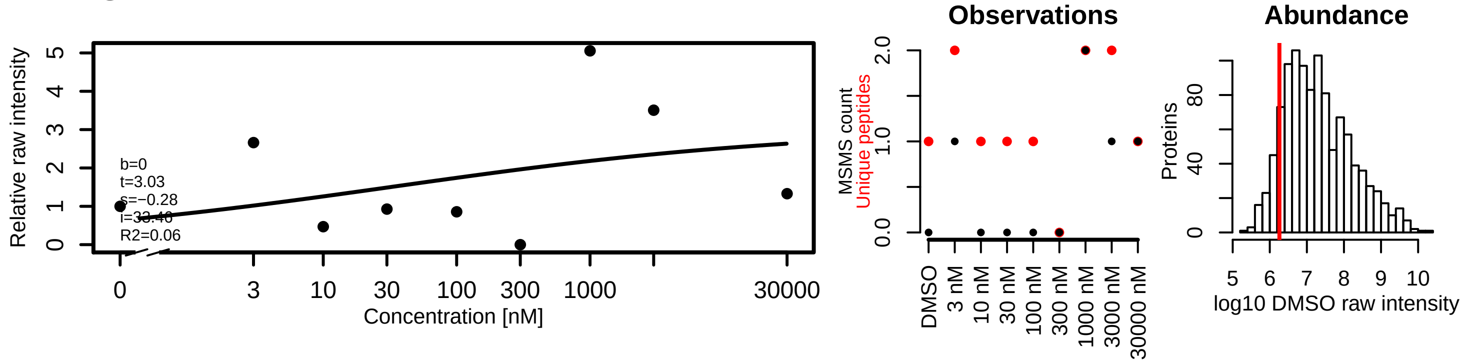


# SNRPE

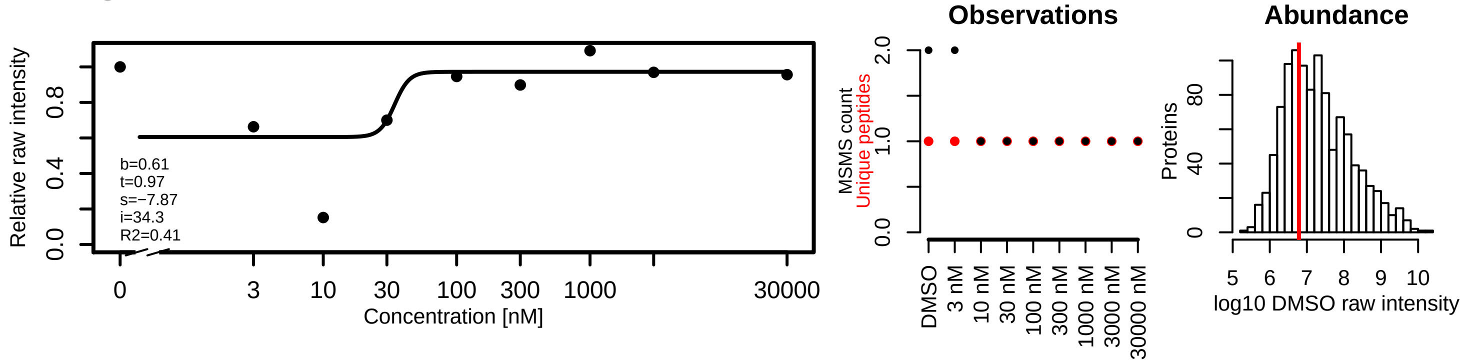


# PAK4

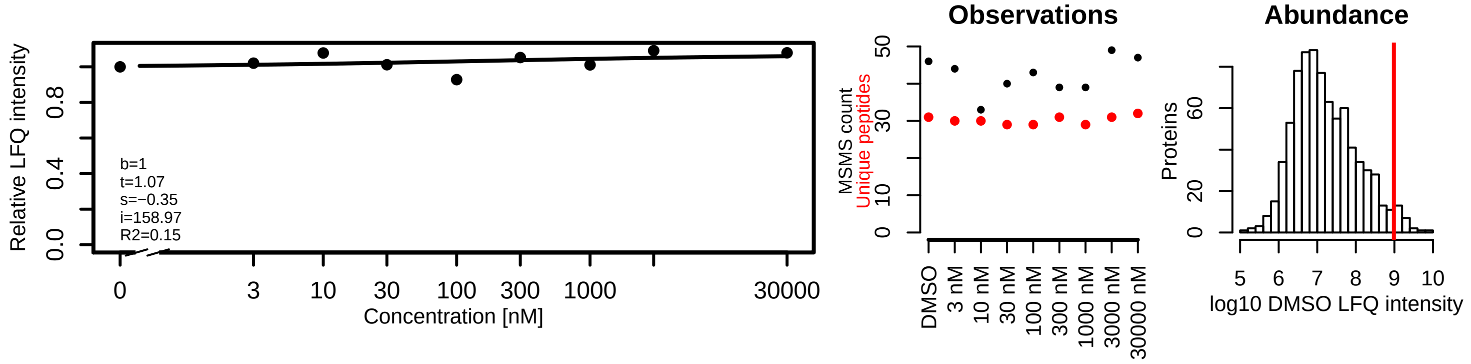


# CCNJL

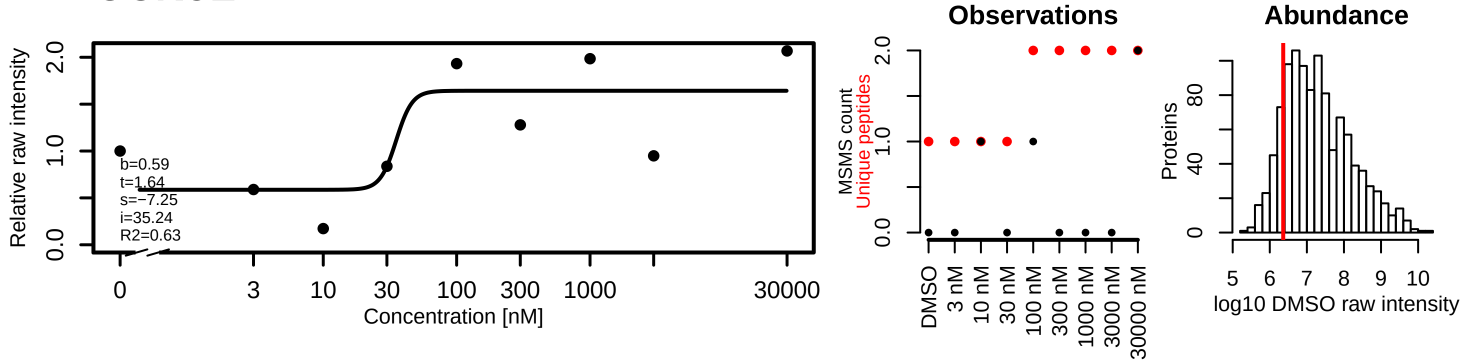


# PTMS

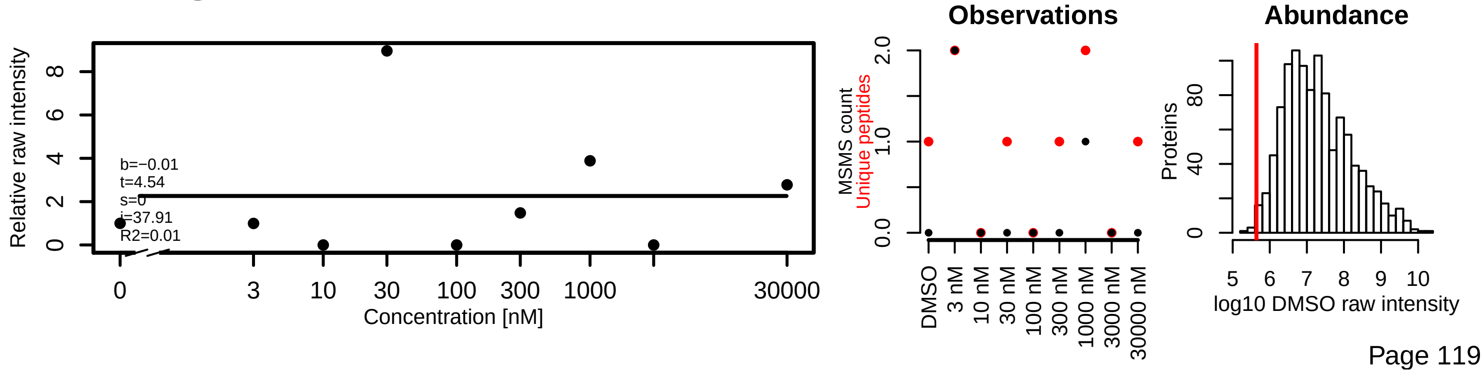

## CHPT1

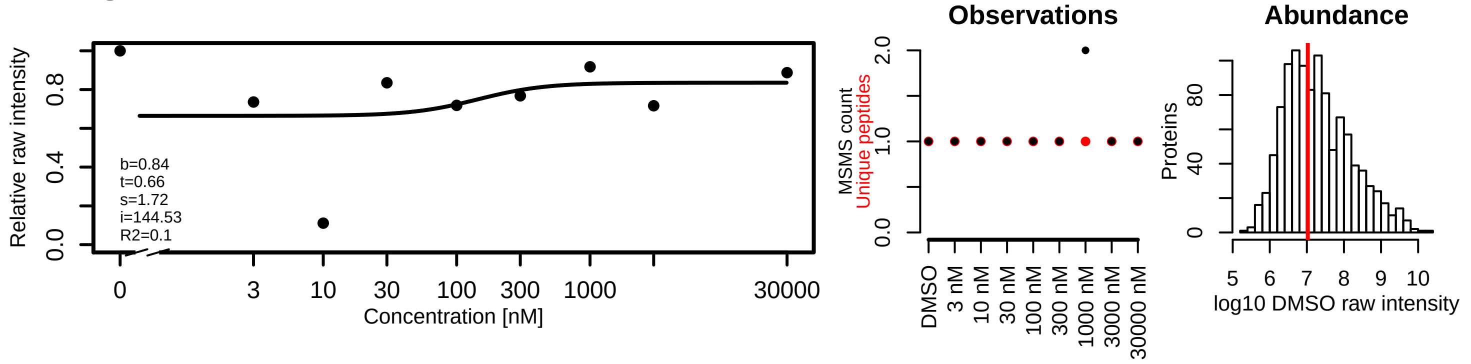


## GSK3A

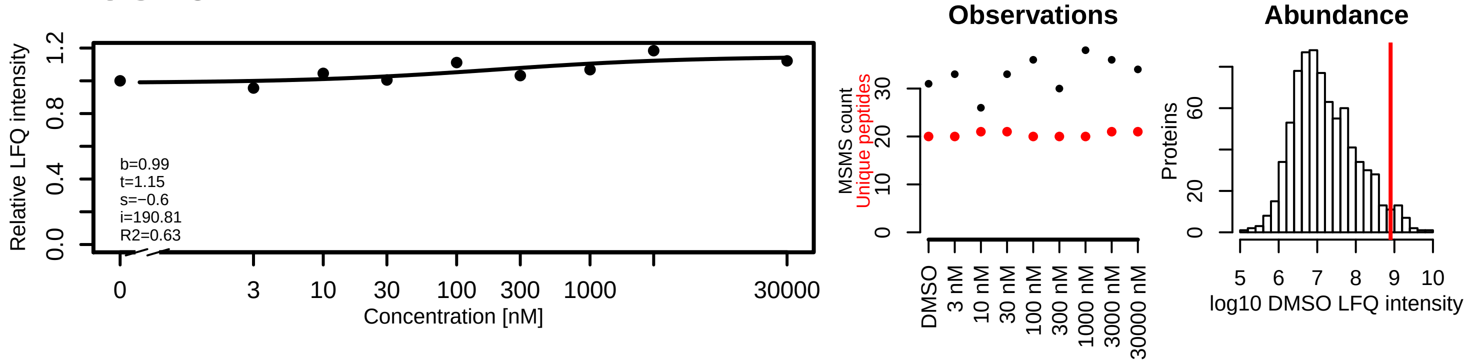


## SLC4A4

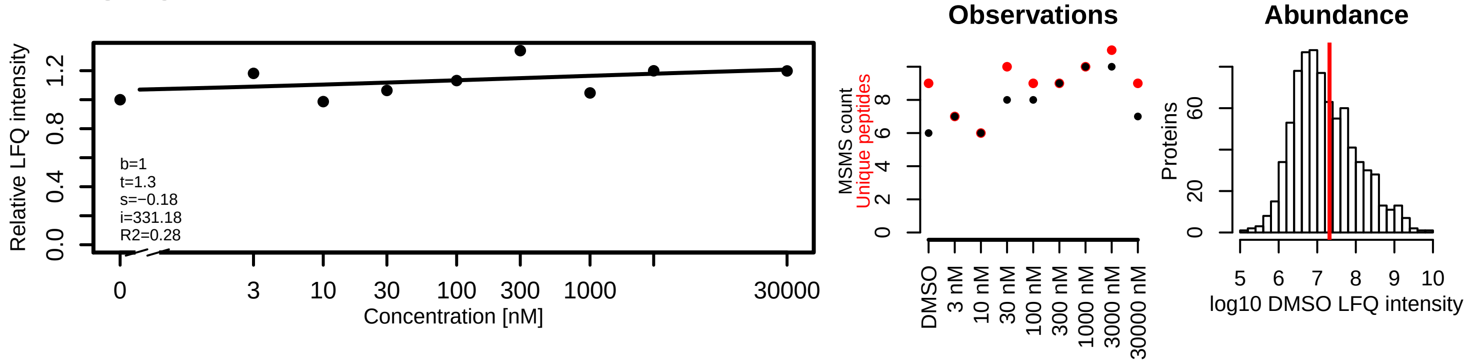


## GOLT1B

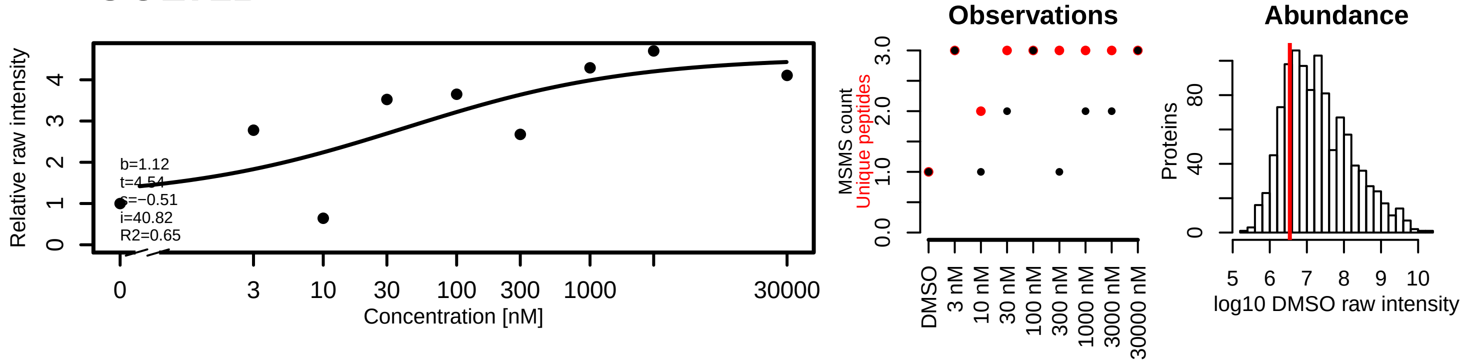


## IPO7

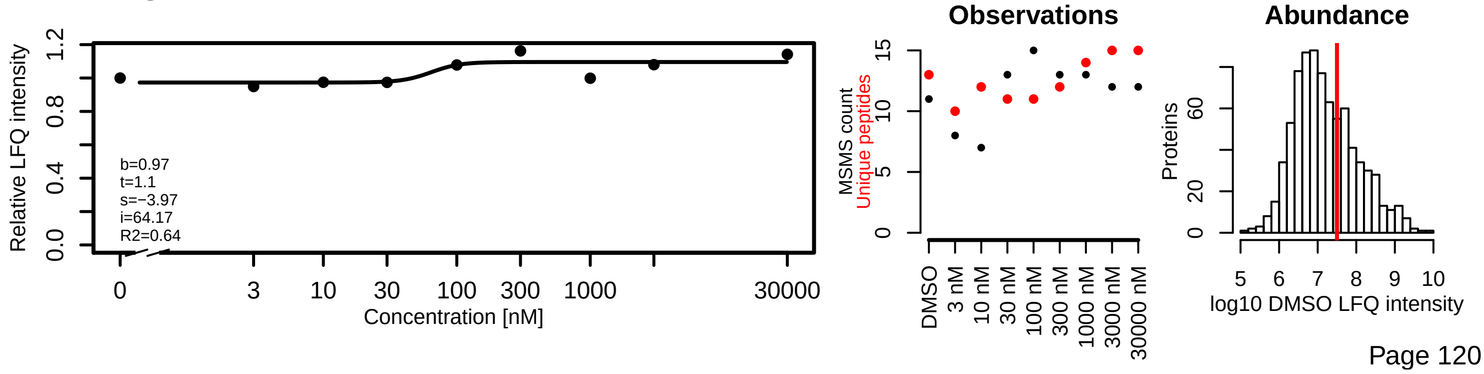

# HNRNPK

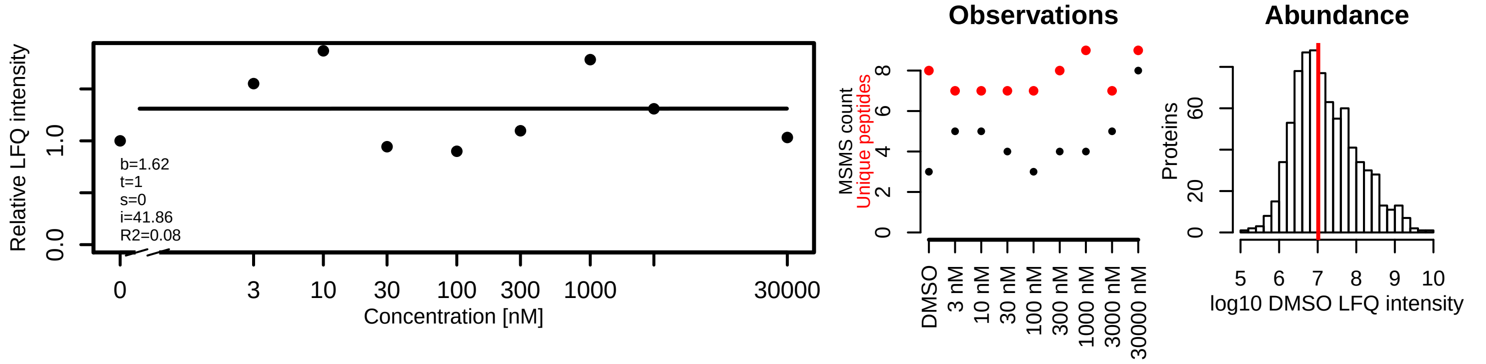


# RAB11A;RAB11B

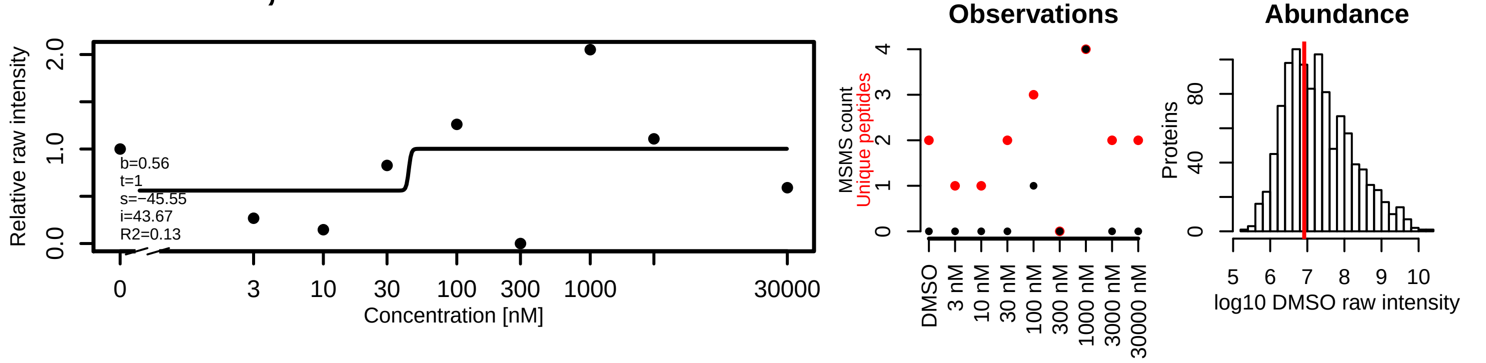


# ABCD3

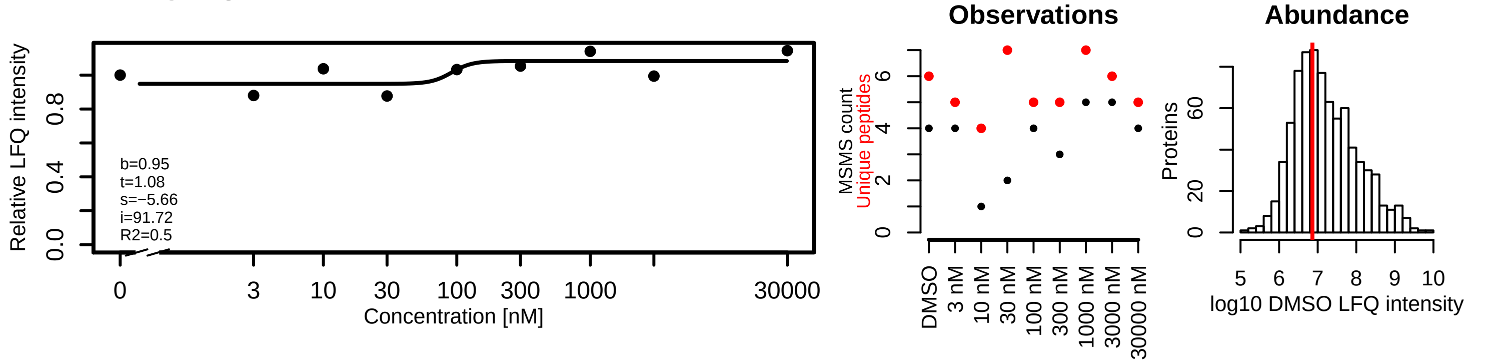


# IMMT

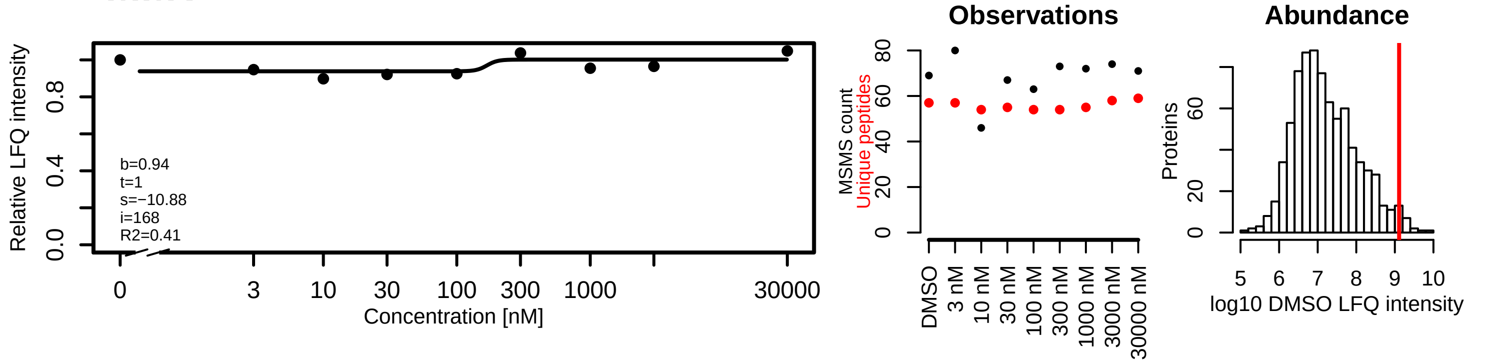


# RPSA;RPSAP58

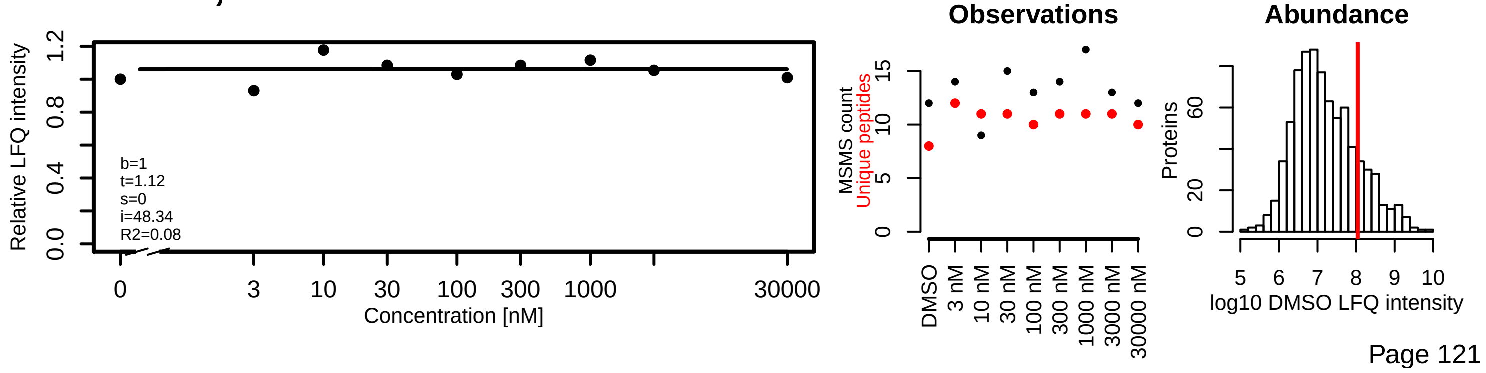

## SSR4

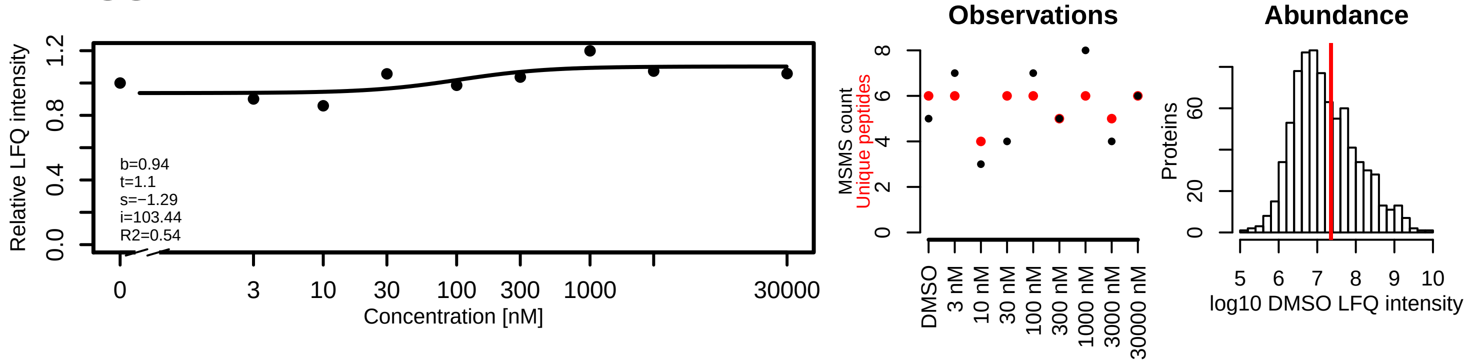


## BMPR1A

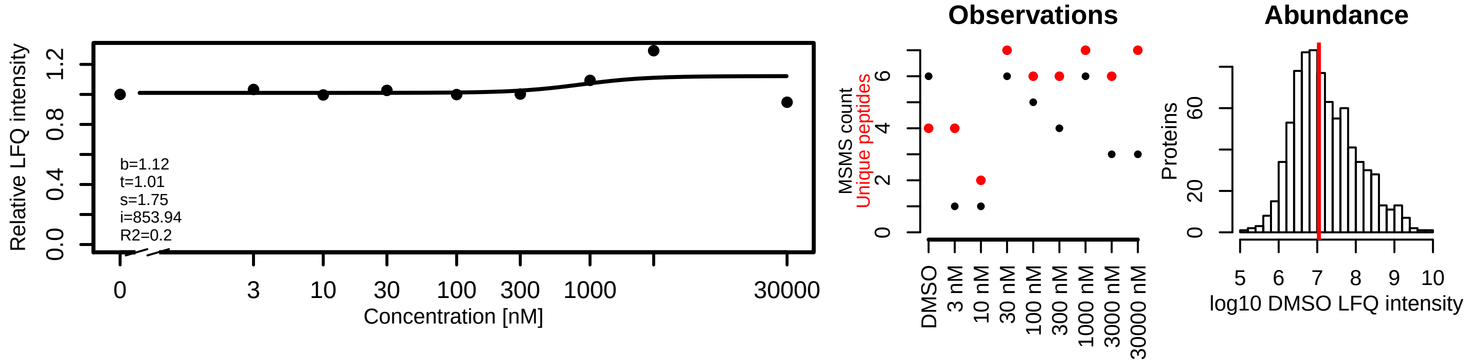


## RPS6KA5

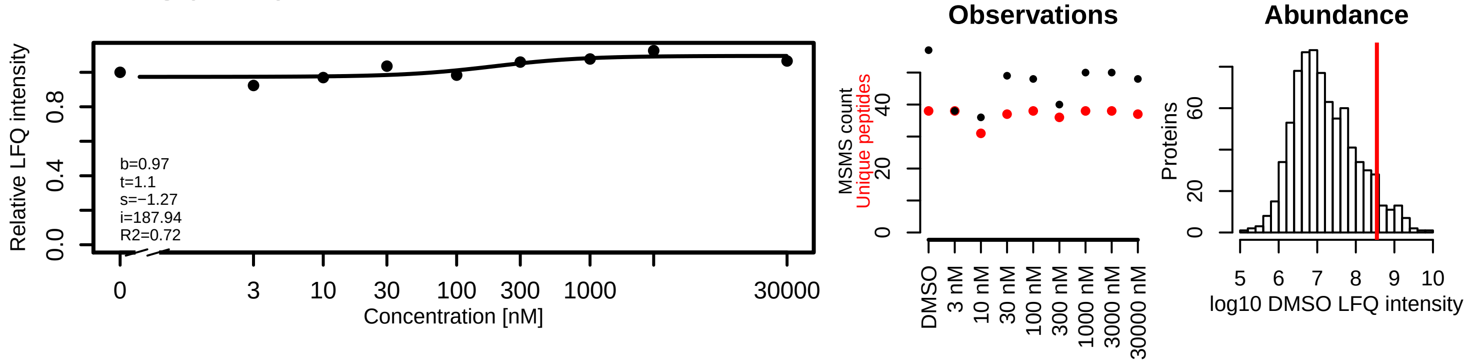


## HM13

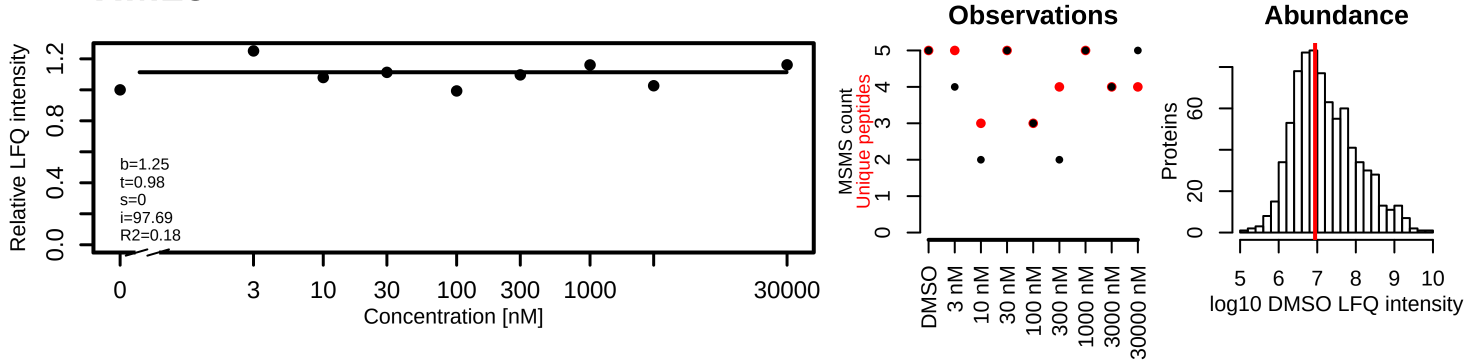


## RPS6KB1;RPS6KB2

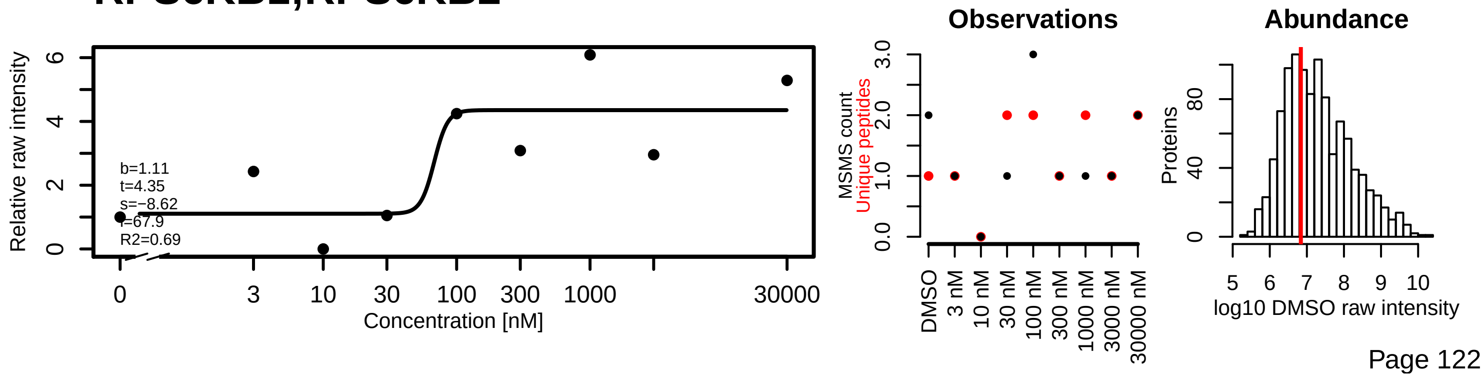

# PEX1

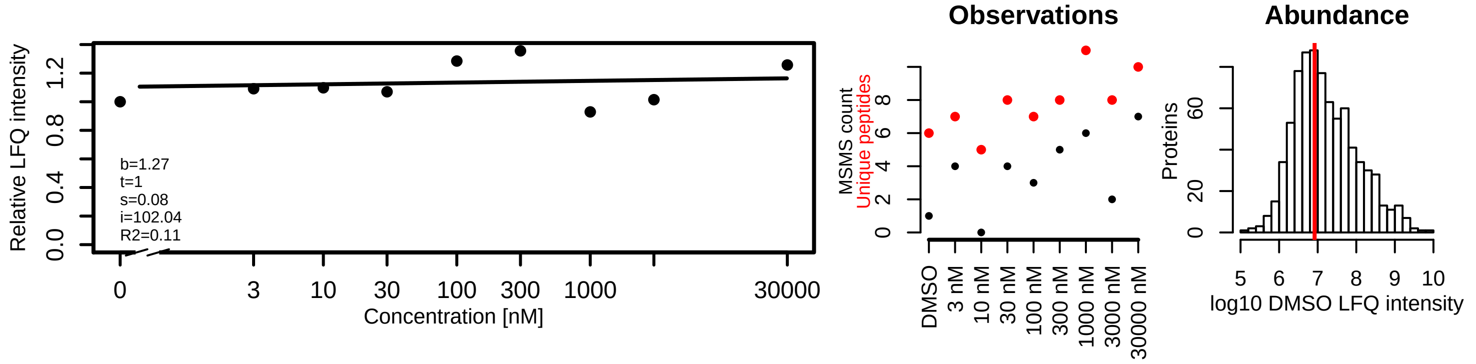


# GSTP1

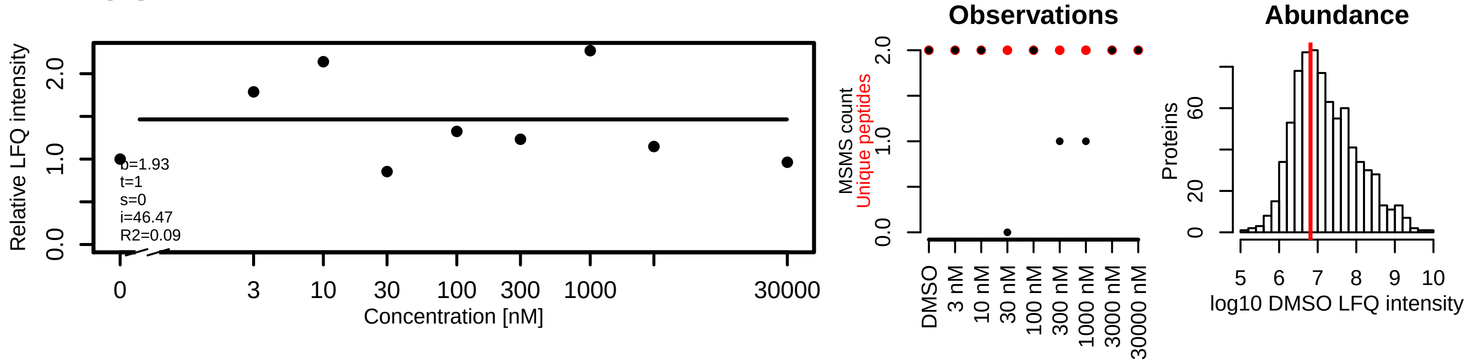


# HIPK2;HIPK1

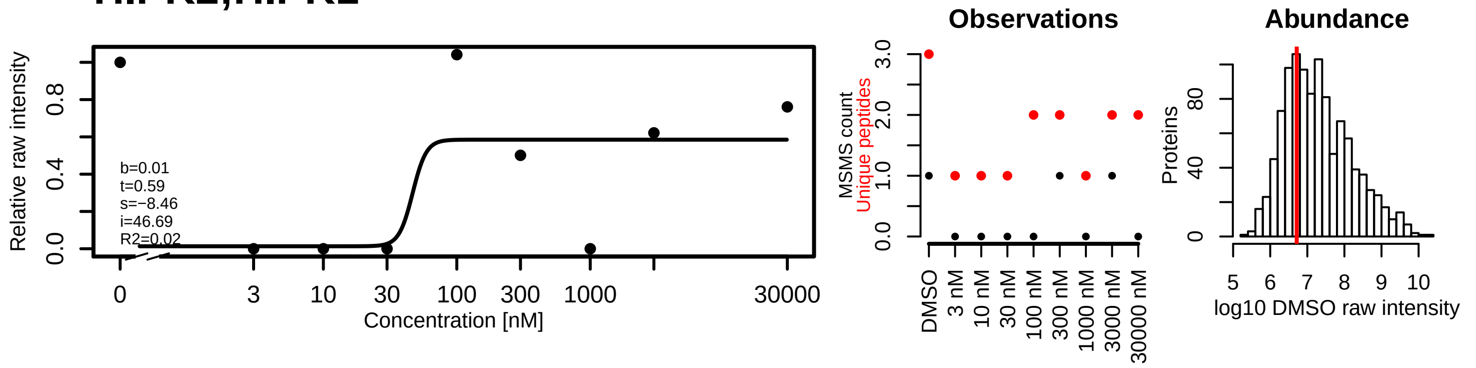


# EMC1

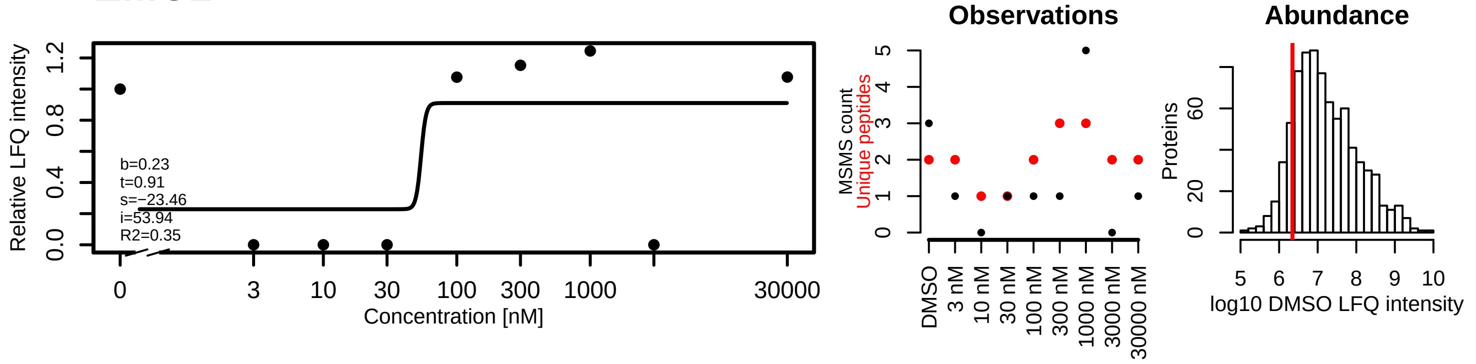


# RPL17

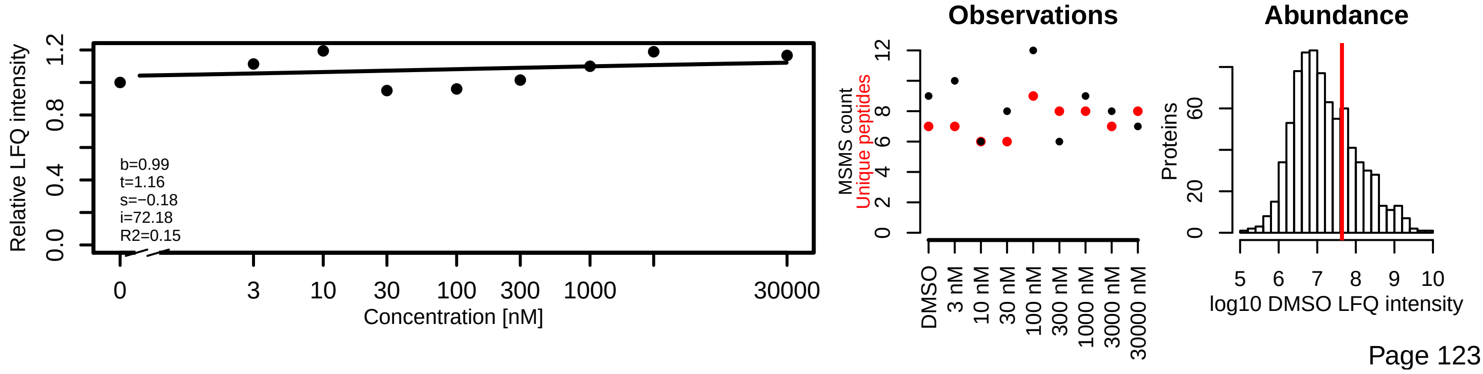

# AKT3

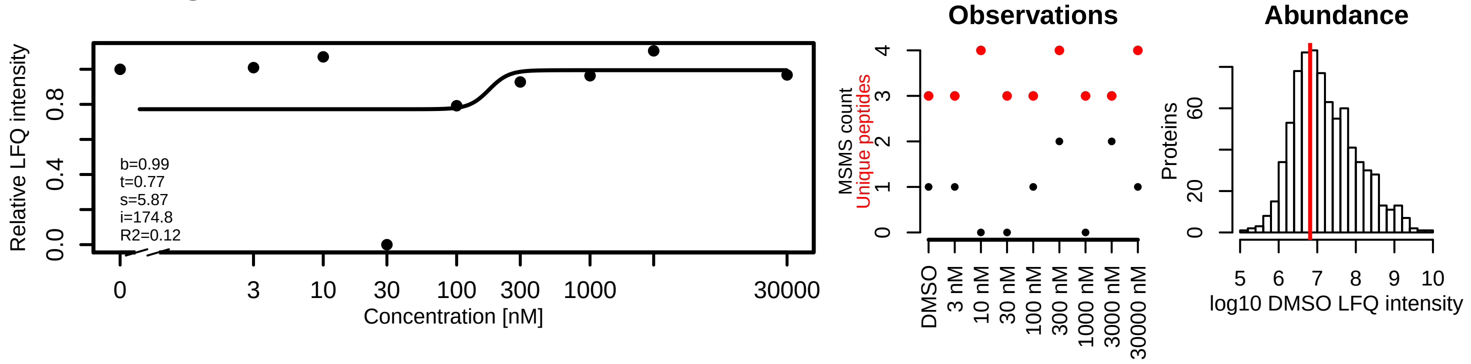


# CDK5

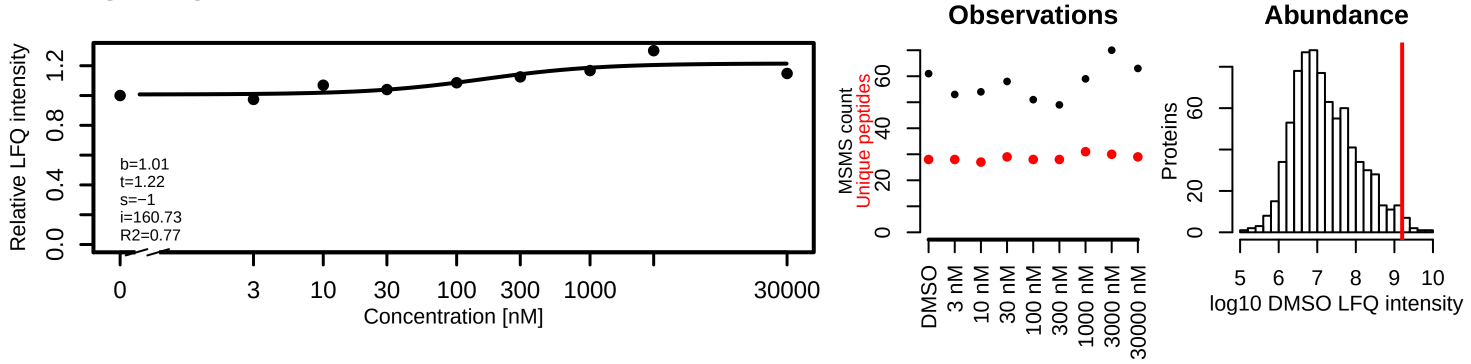


# ERLIN1

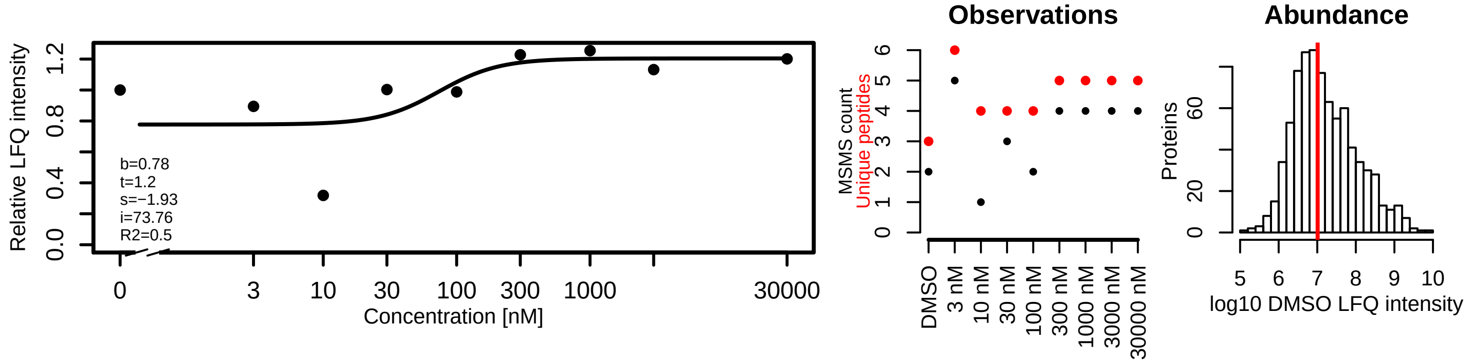


# PYCR2

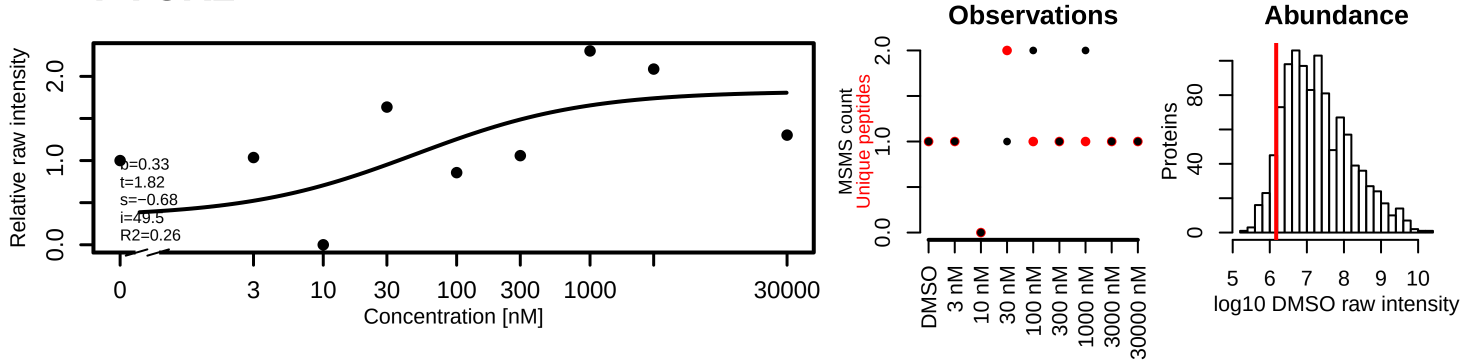


# GPR89B;GPR89A;GPR89C

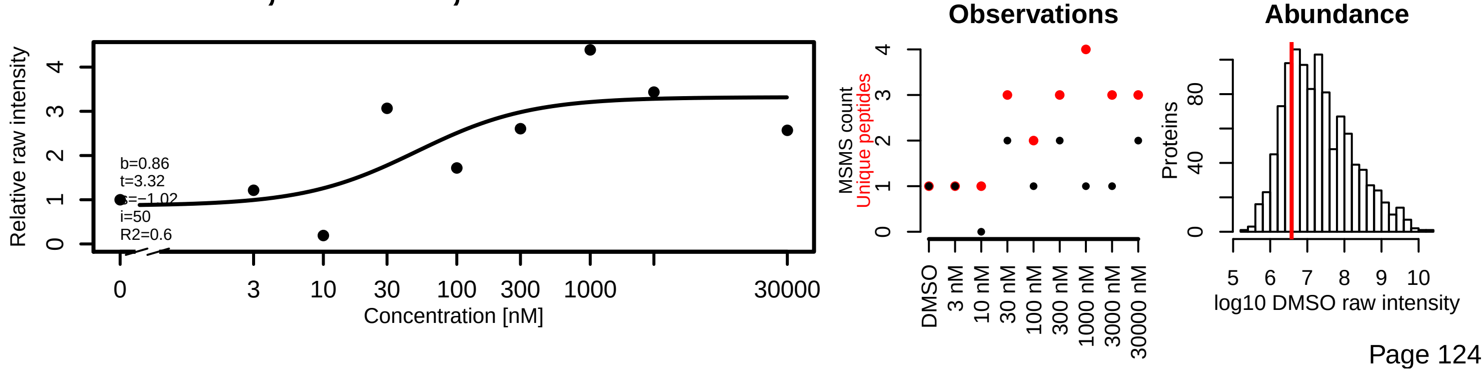

## ELOVL5

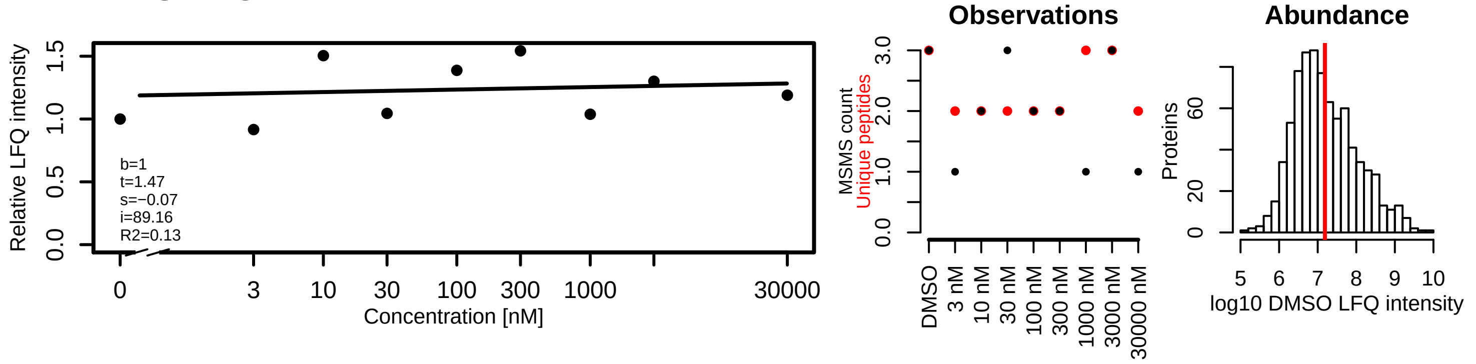


## SLC30A7

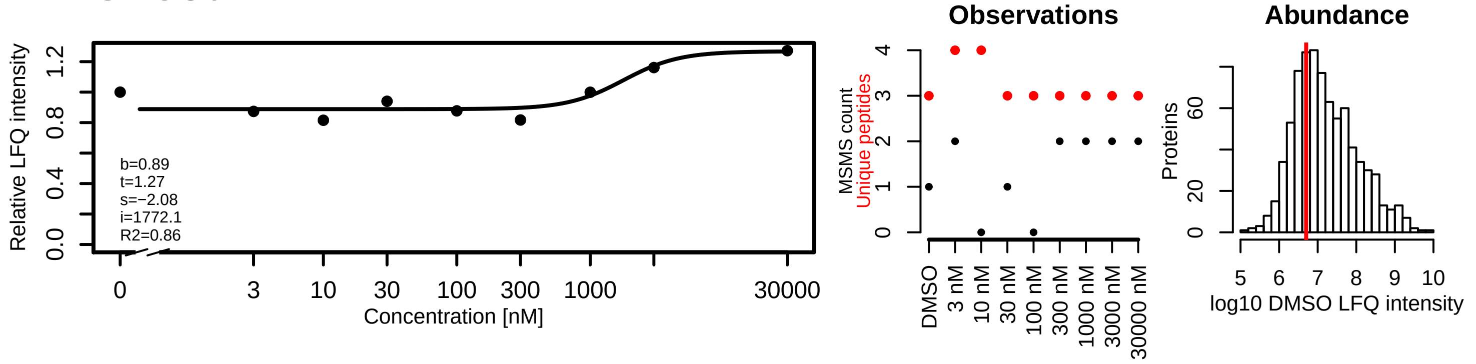


## CNN2

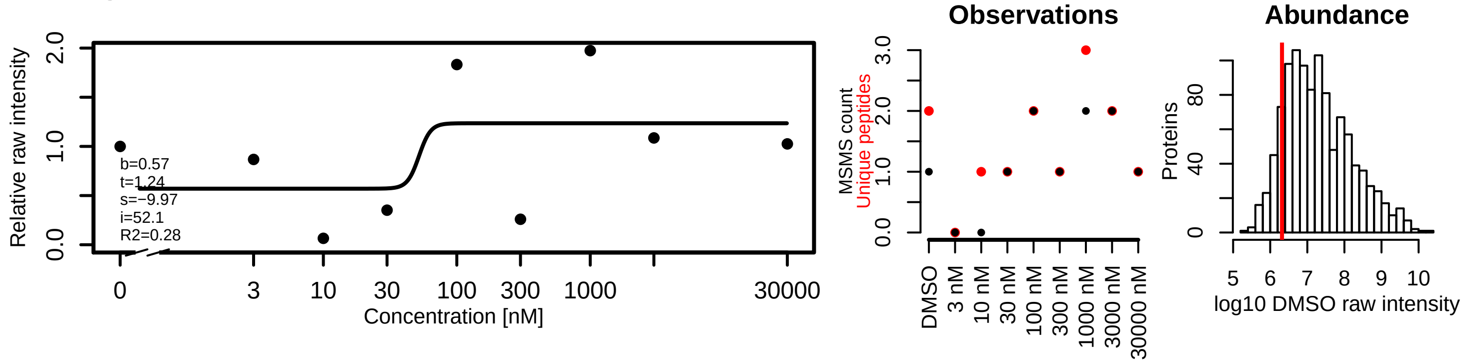


## SLK

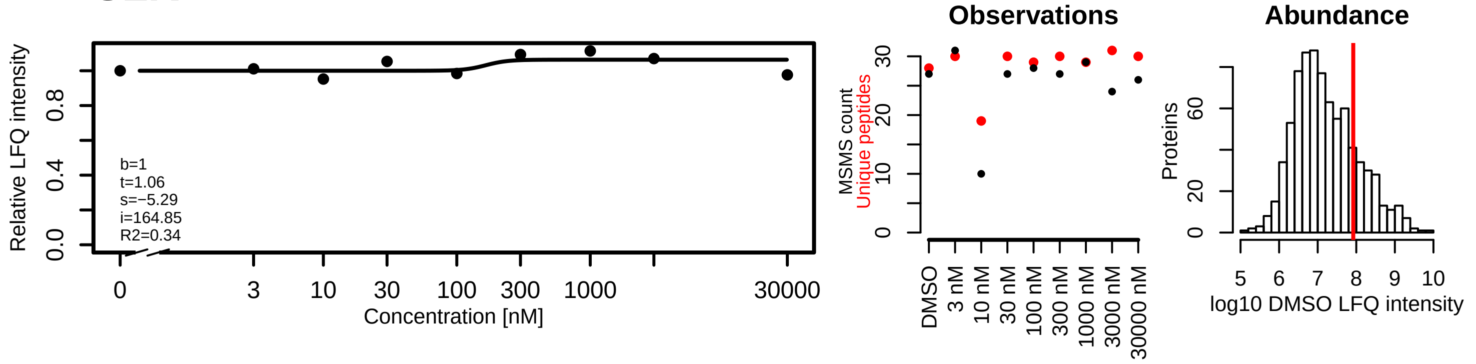


## PIP4K2C

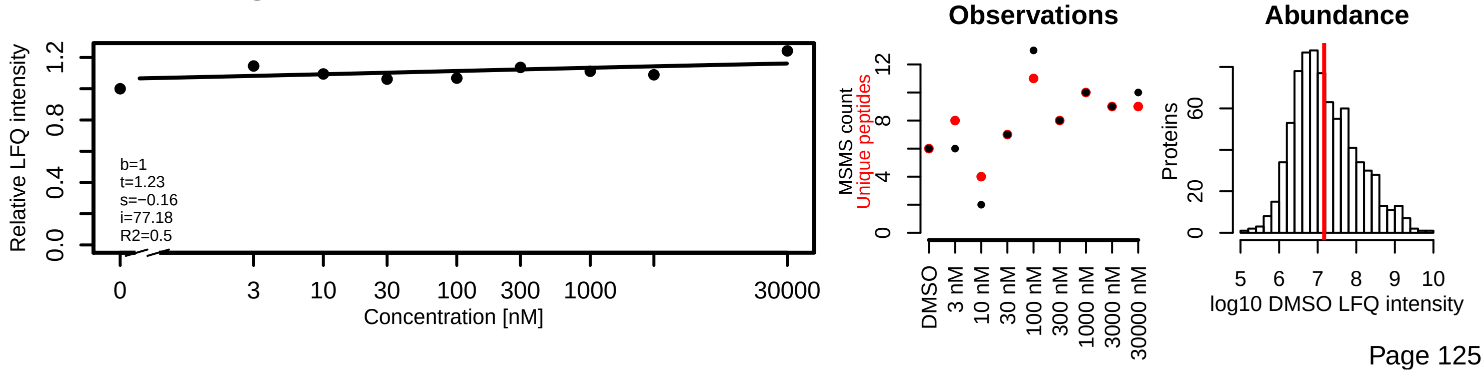

# CAMK2D – D6R938

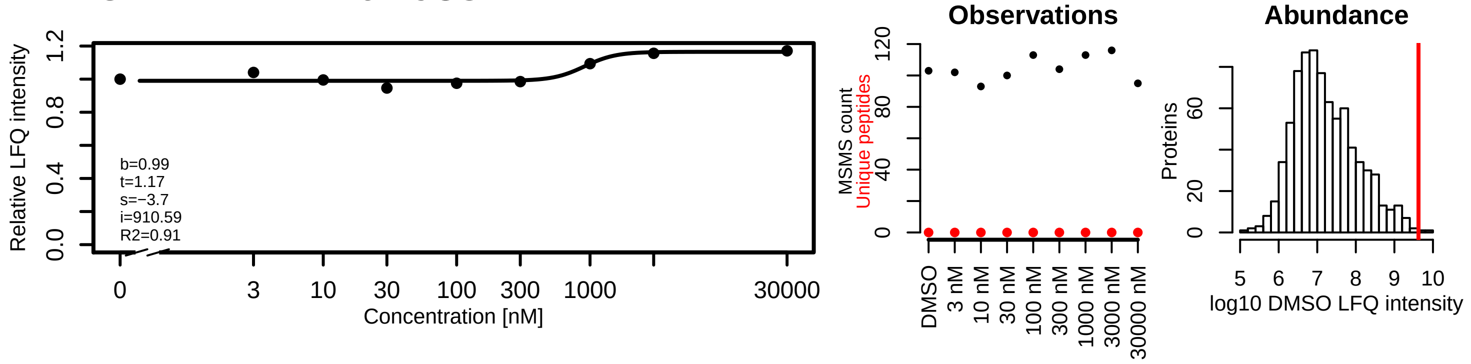


# CDK7

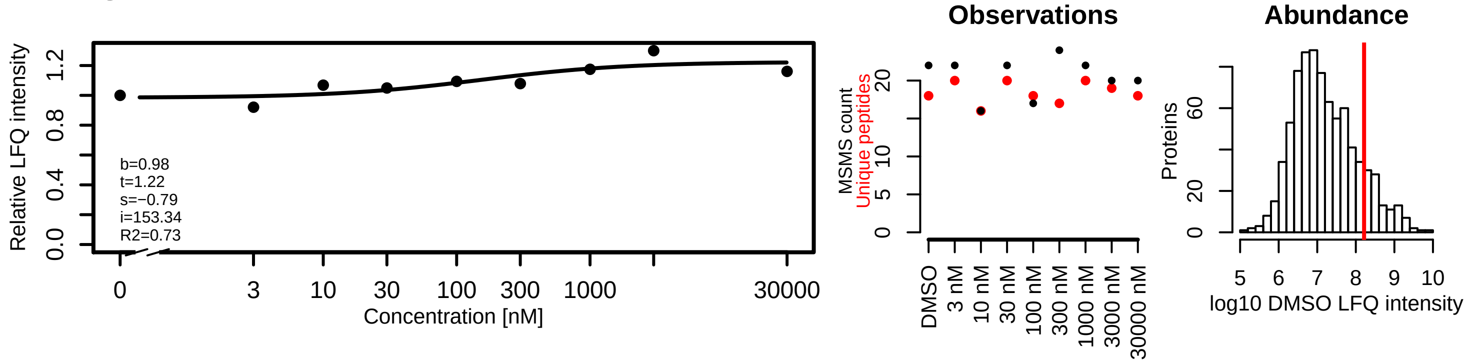


# DYNLL2

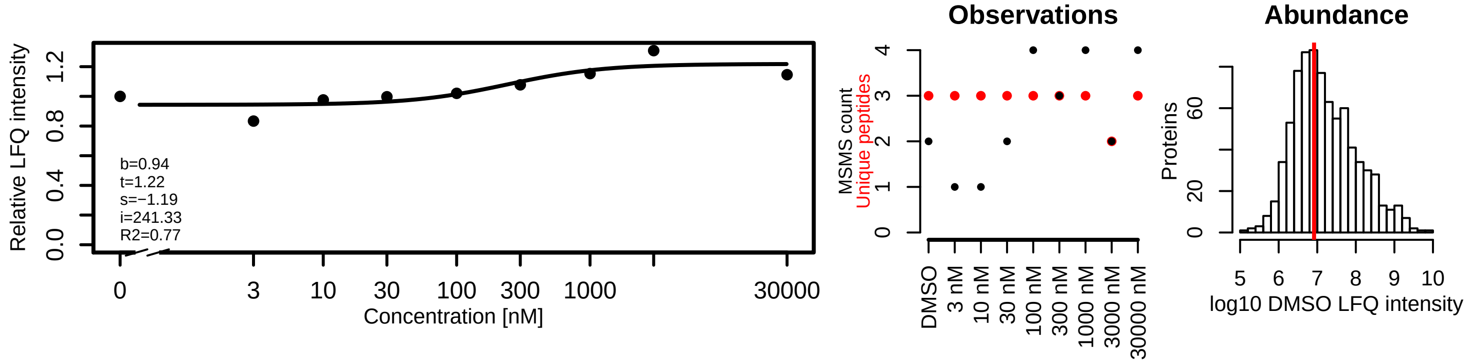


# HMGB1;HMGB1P1

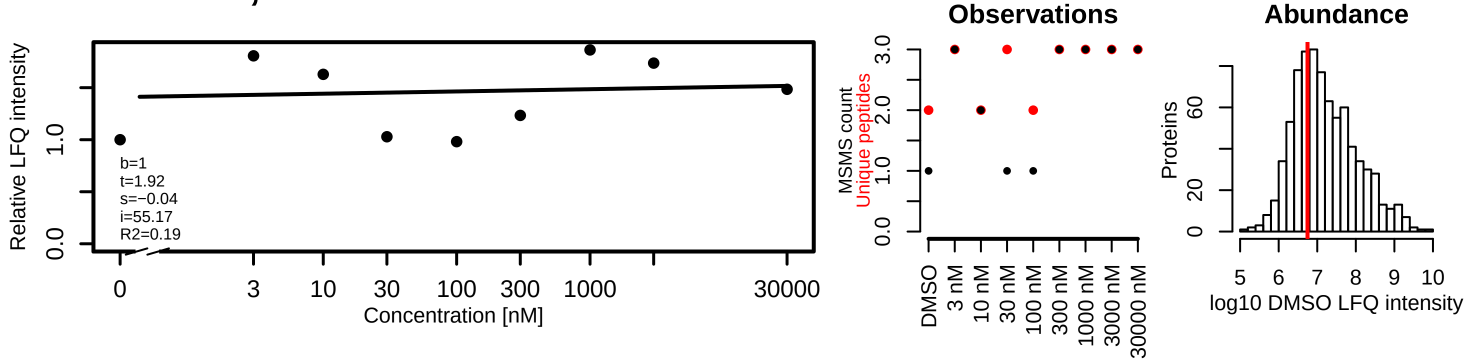


# HSP90B1

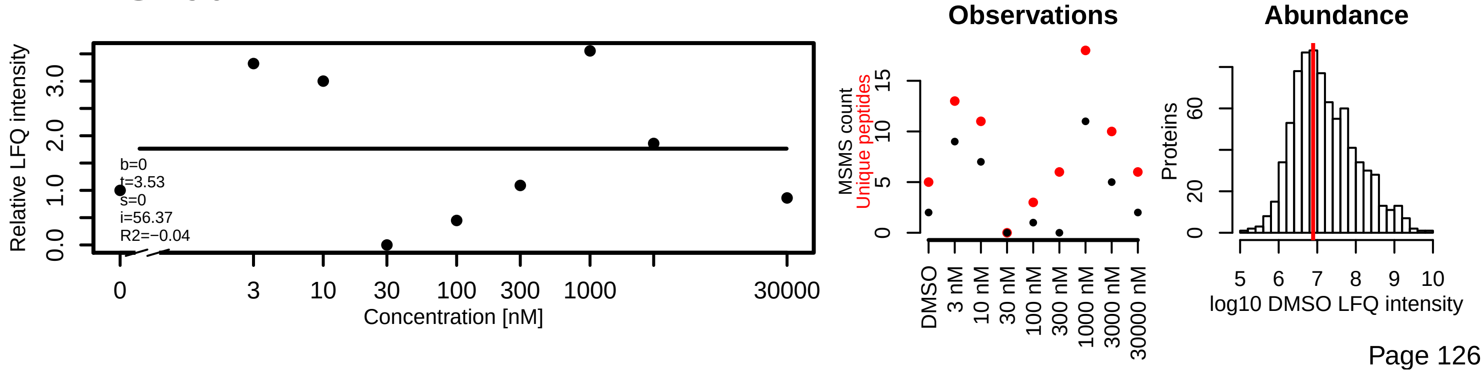

# AK2

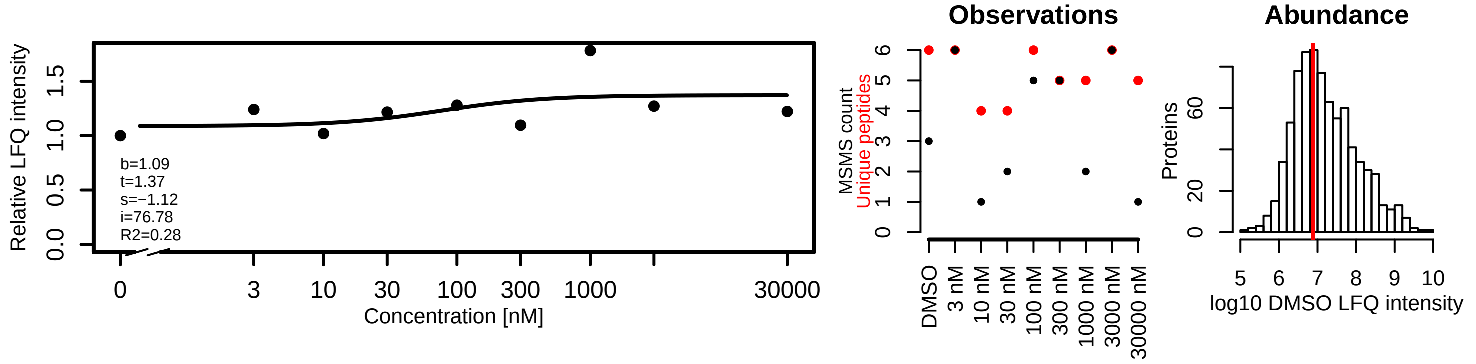


# ATP6V0D1

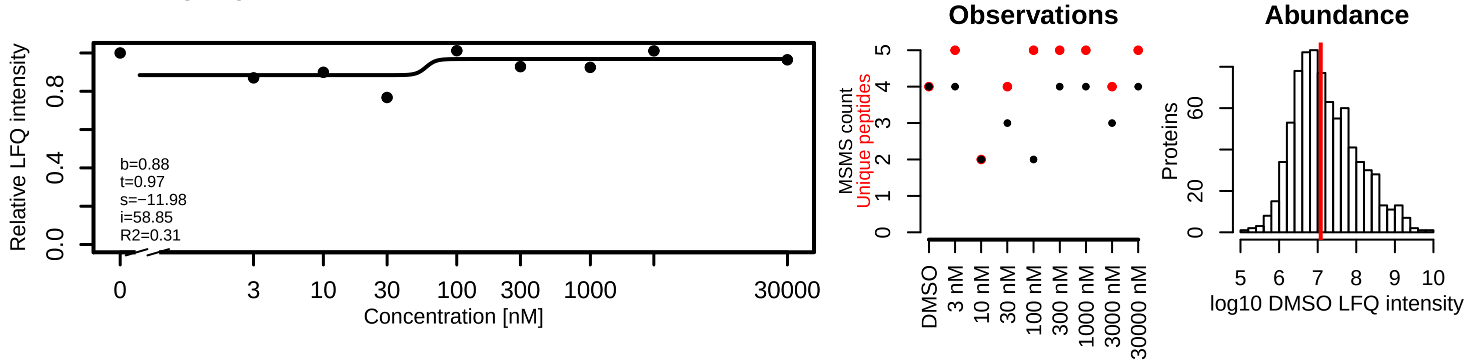


# COX5A

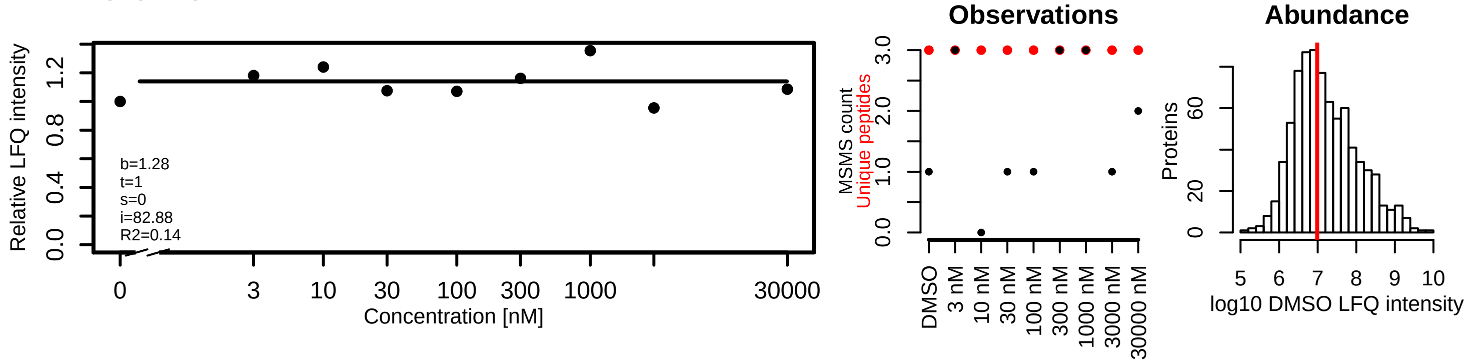


# UQCRFS1;UQCRFS1P1

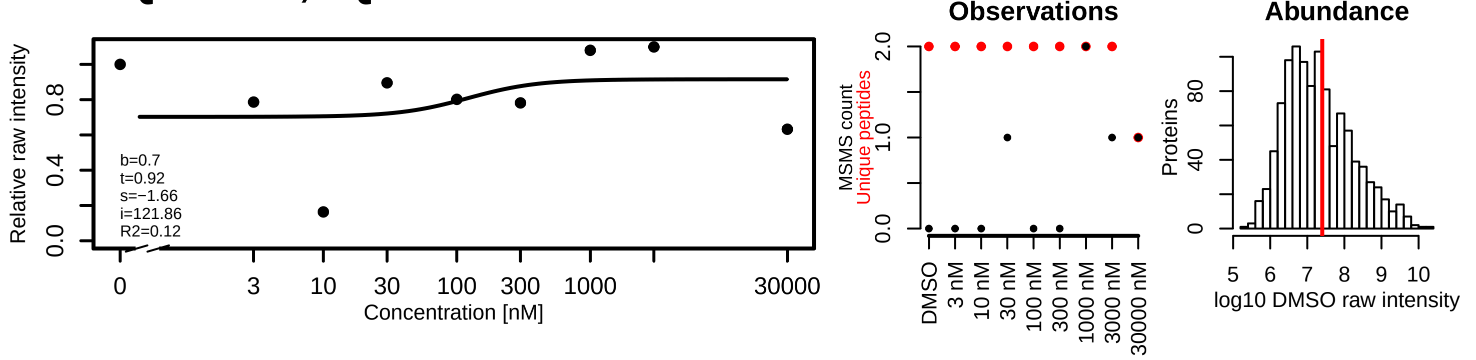


# SEC22B

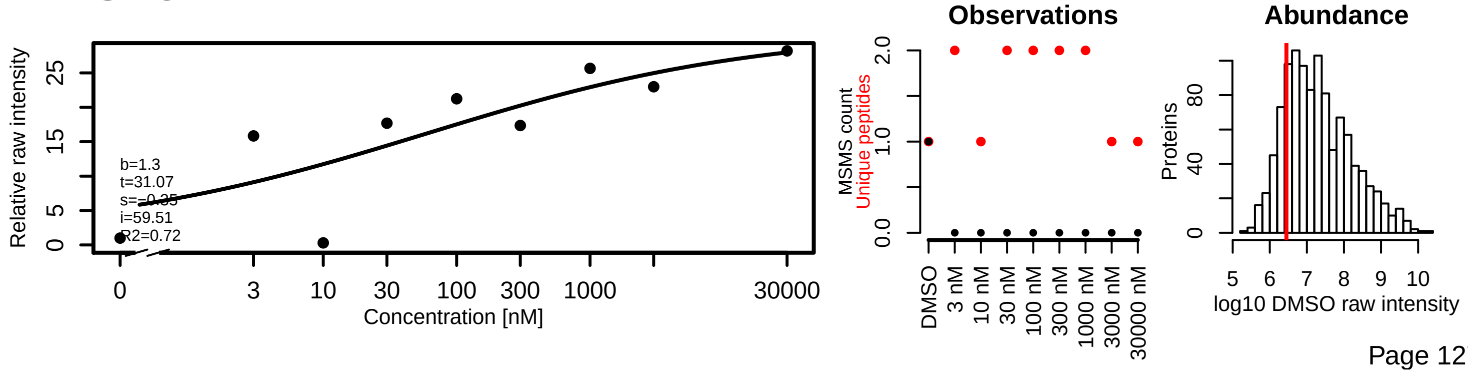

# ACAD10

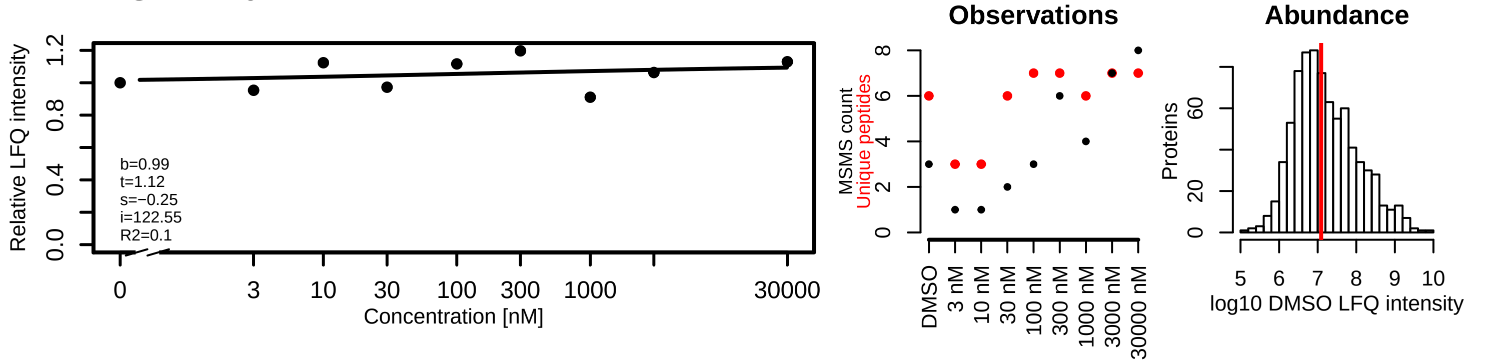


# CDK3

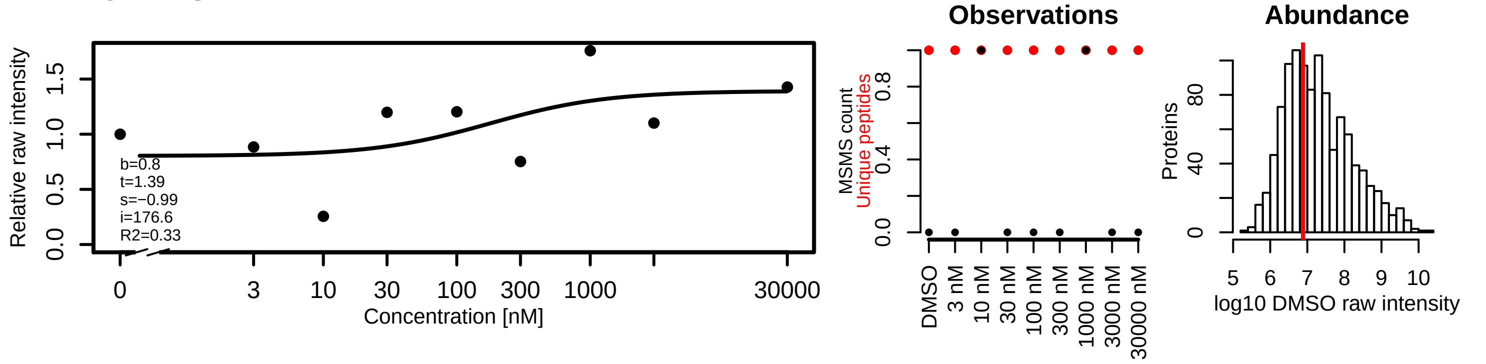


# CAMK2G − Q13555−7;Q13555−8;Q13555−3;Q13555−2;Q13555;H0Y6G[illegible]

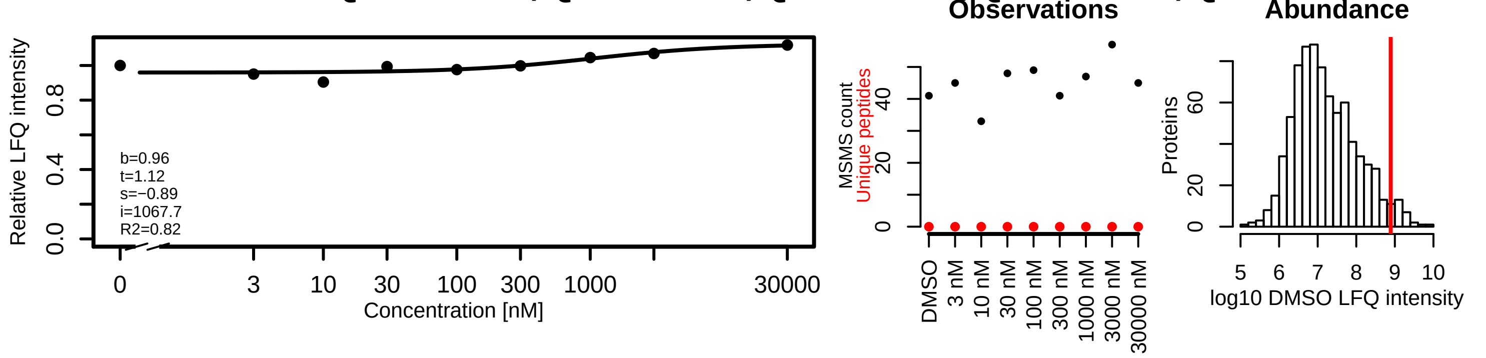


# RPS6KA1

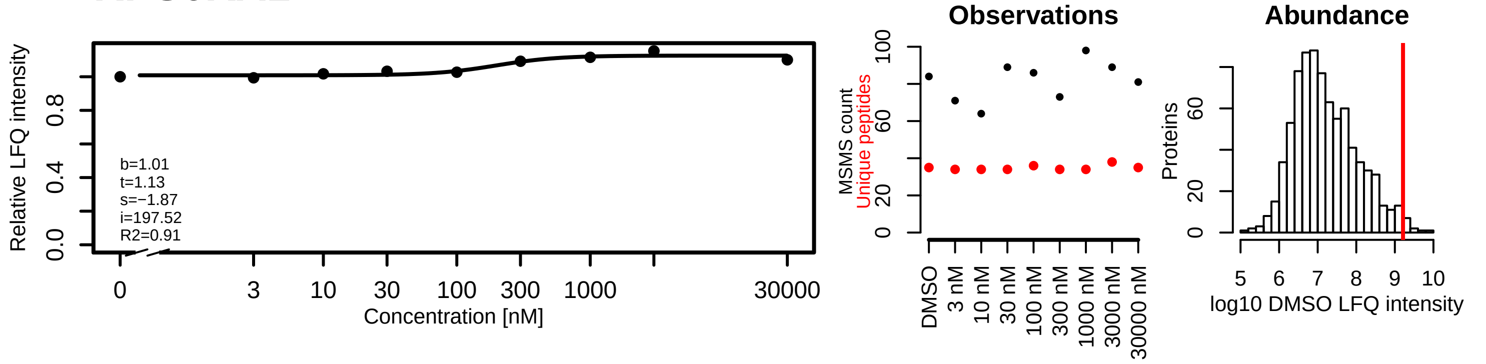


# ULK3

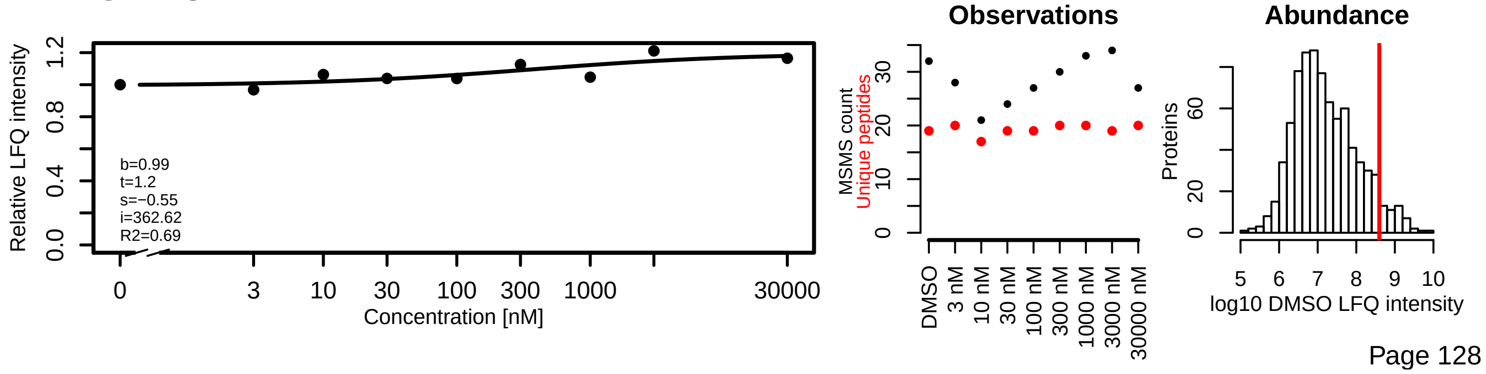

# SLC25A5

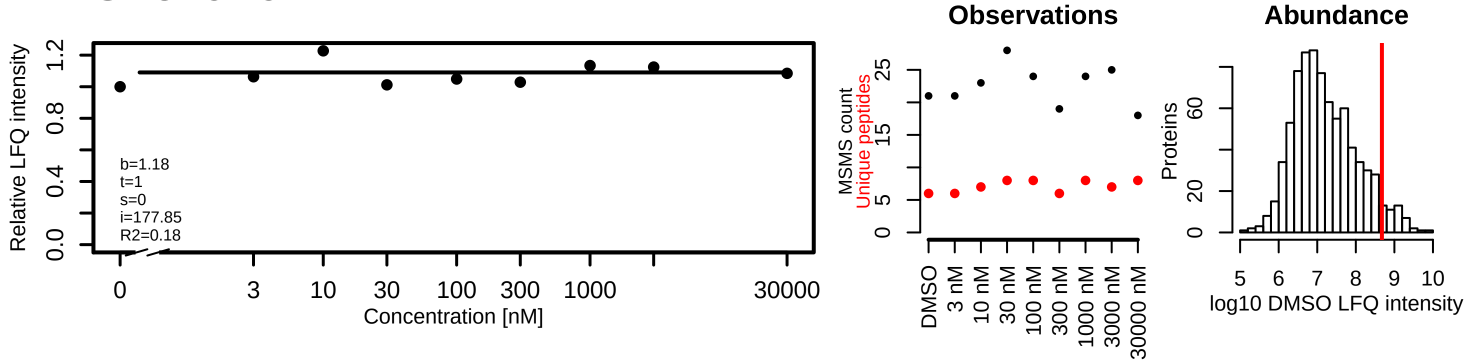


# NF1

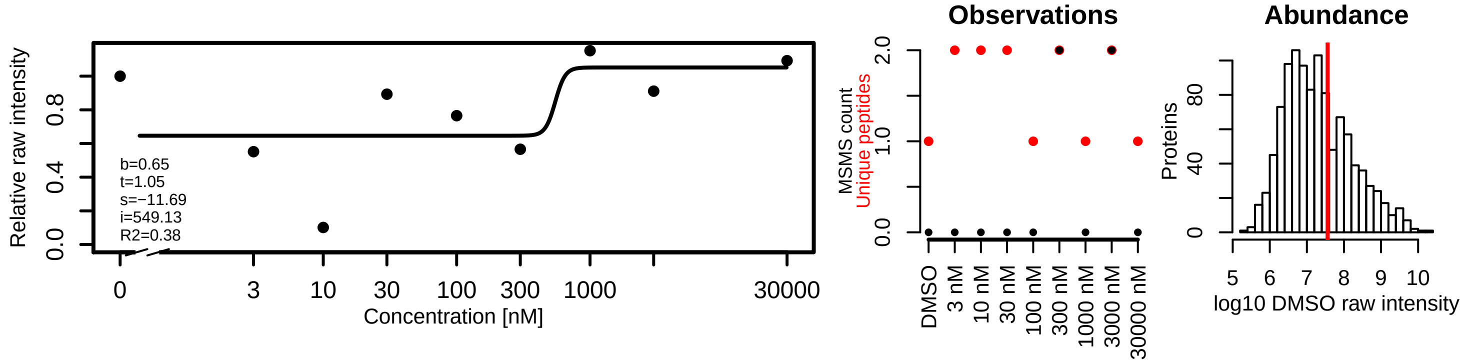


# OCIAD2

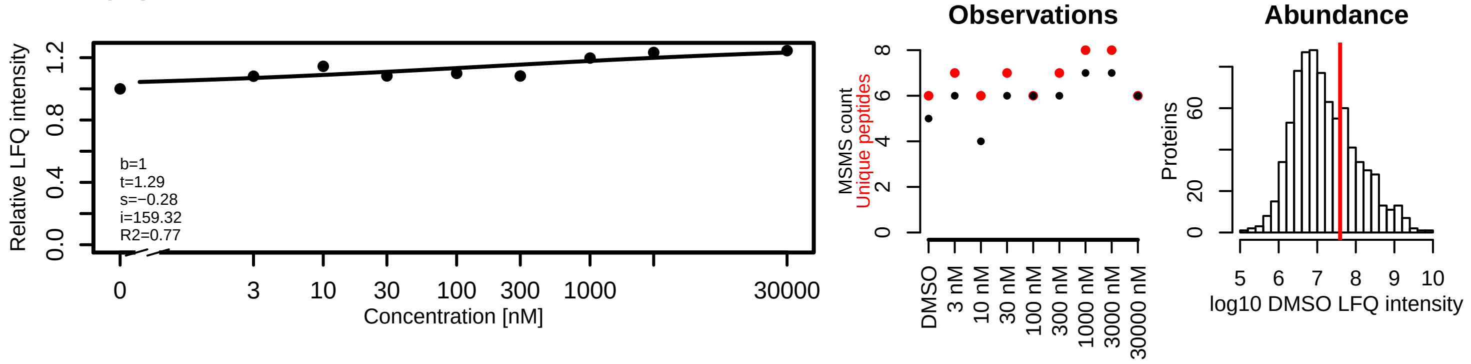


# TBKBP1

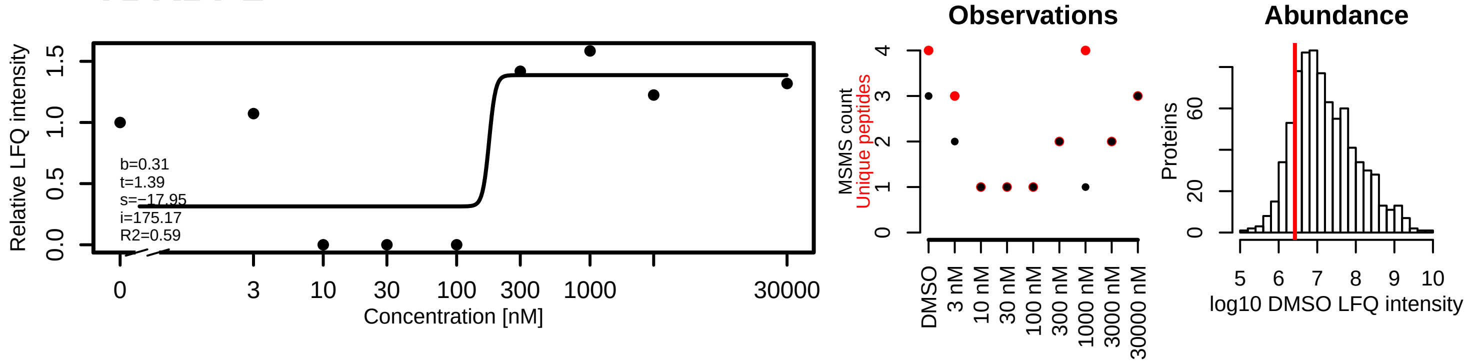


# SCN10A

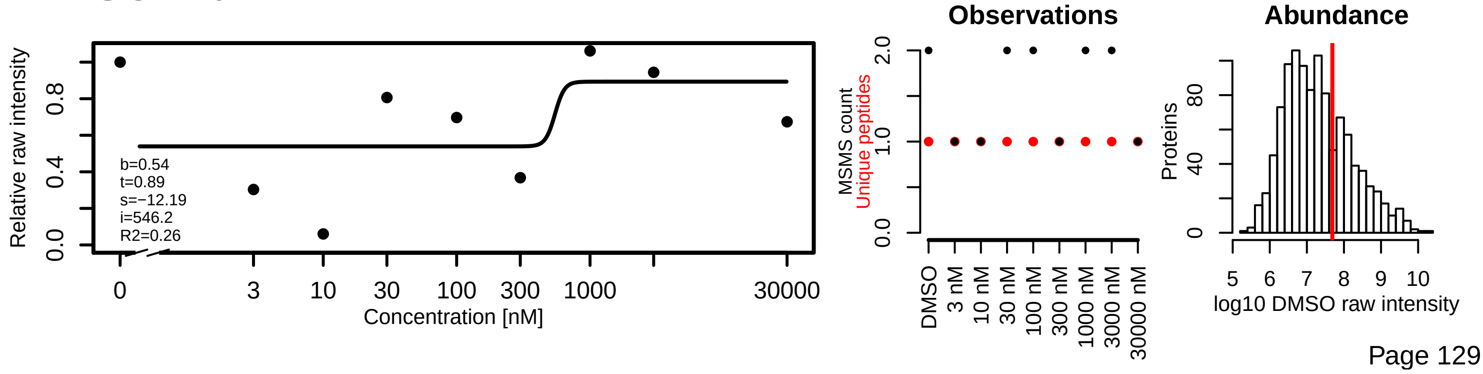

## CCDC88A;CCDC88B

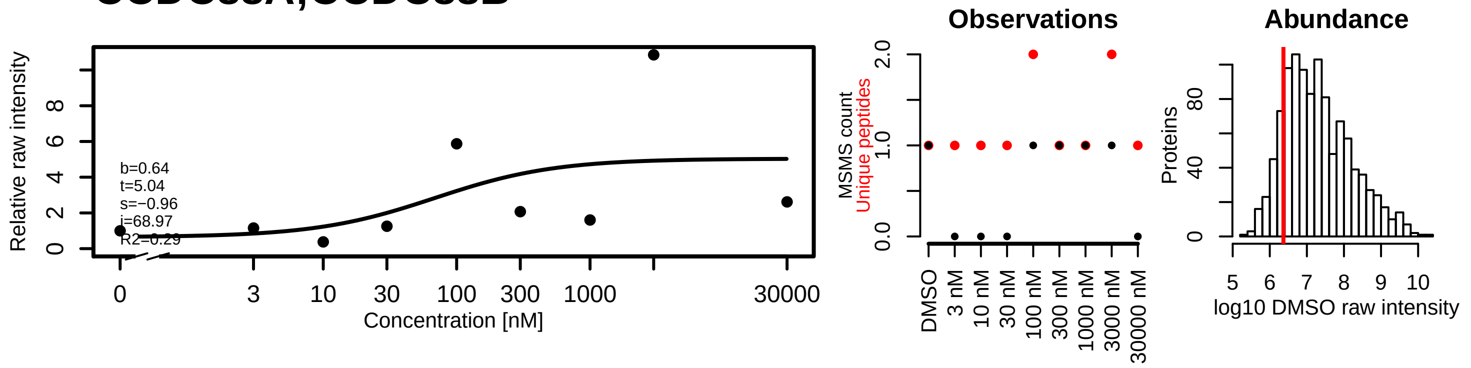


## PRKAG2

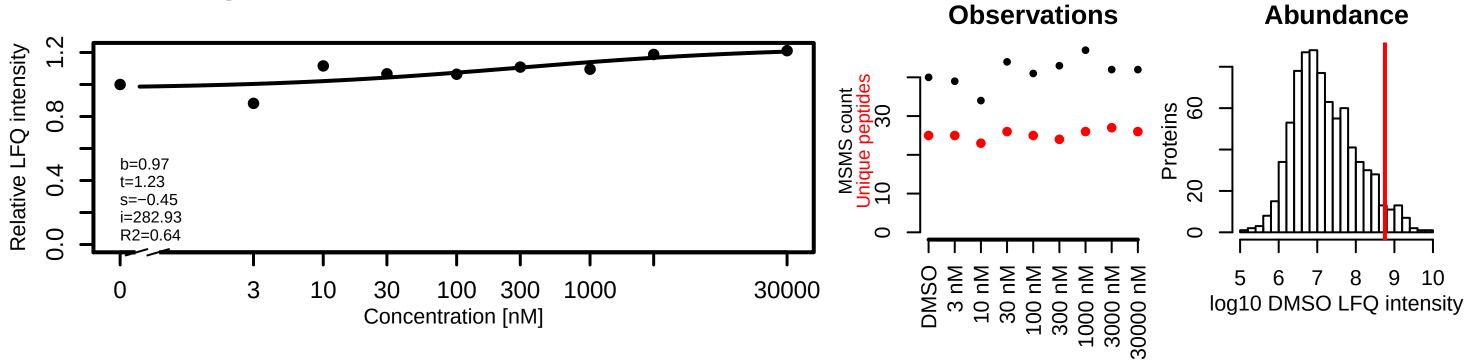


## RBMX;RBMXL1

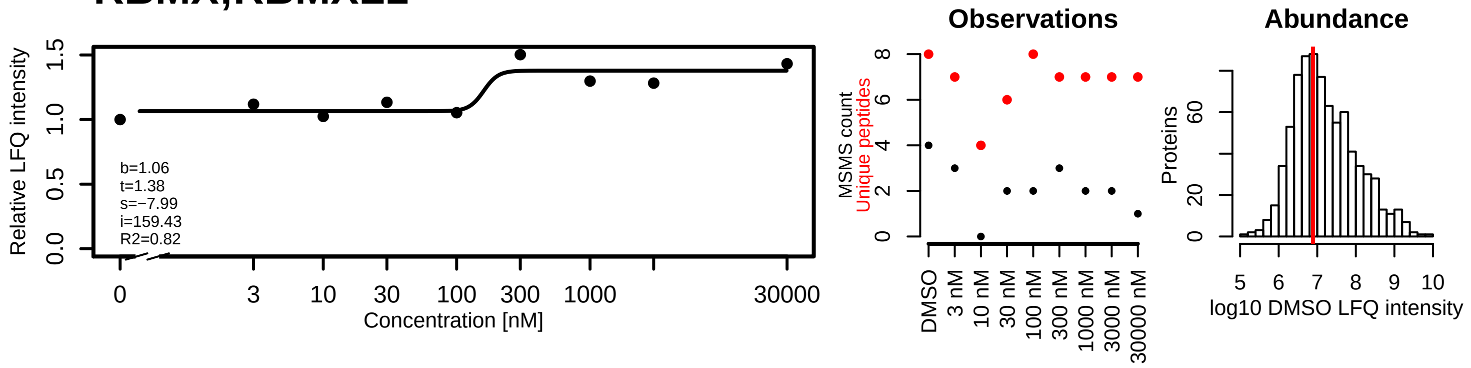


## ELOVL1

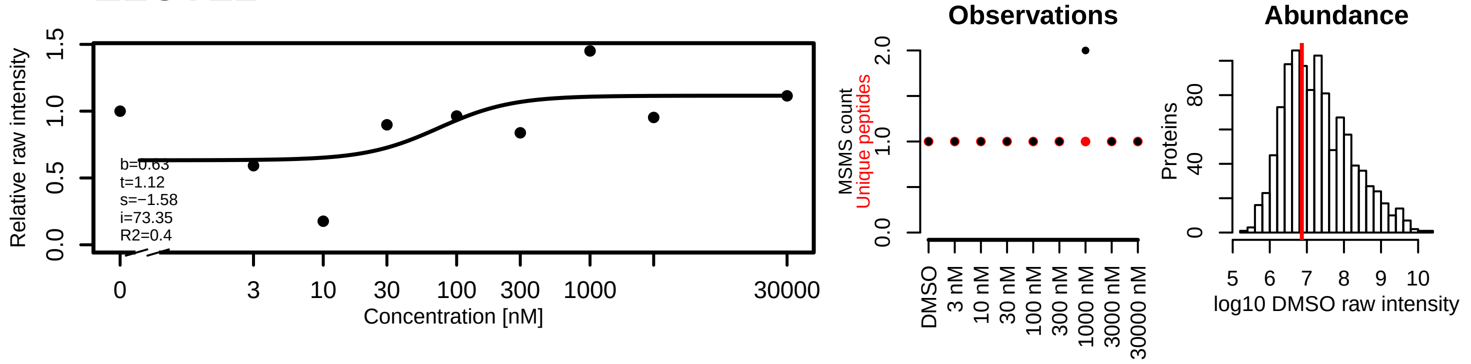


## LUZP1

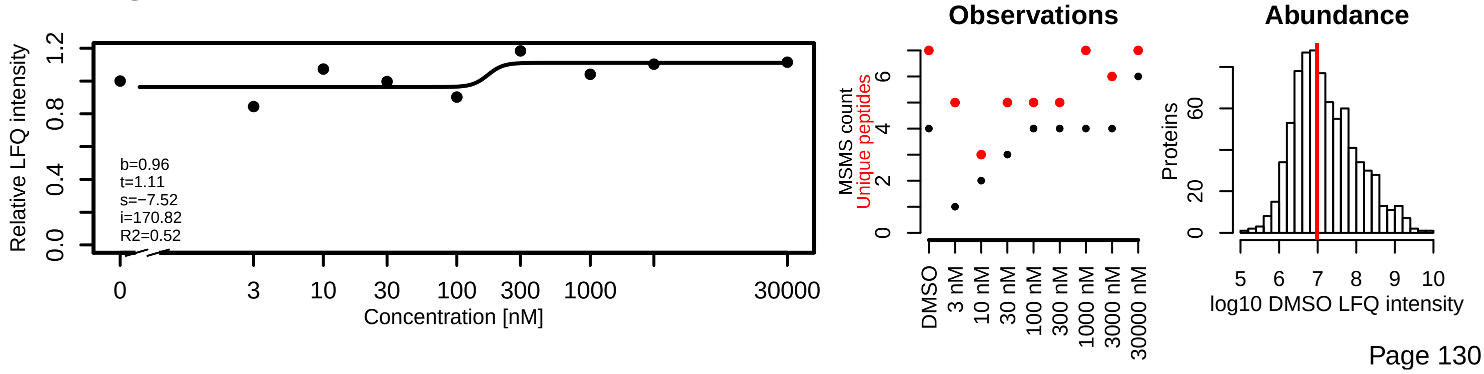

# STK16

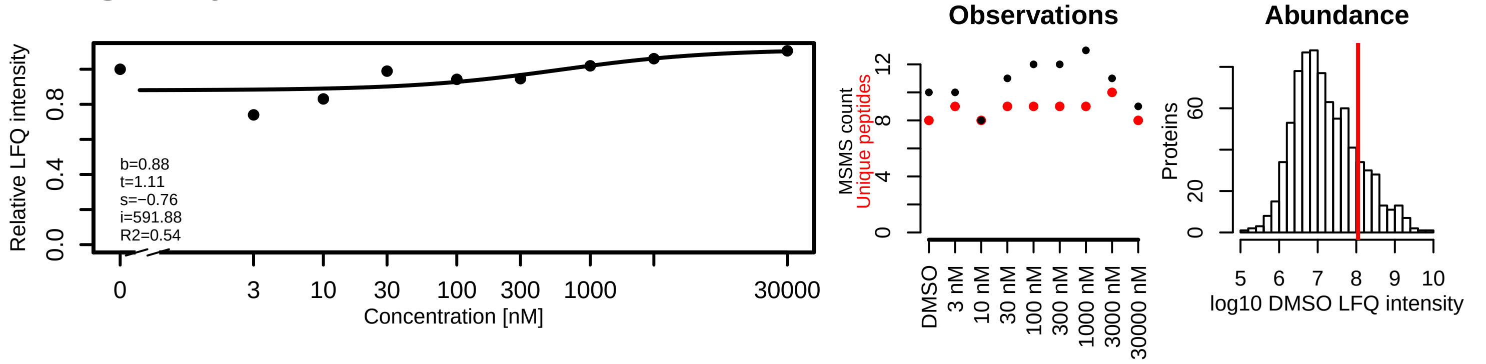


# ARF4;ARF3;ARF5;ARF1

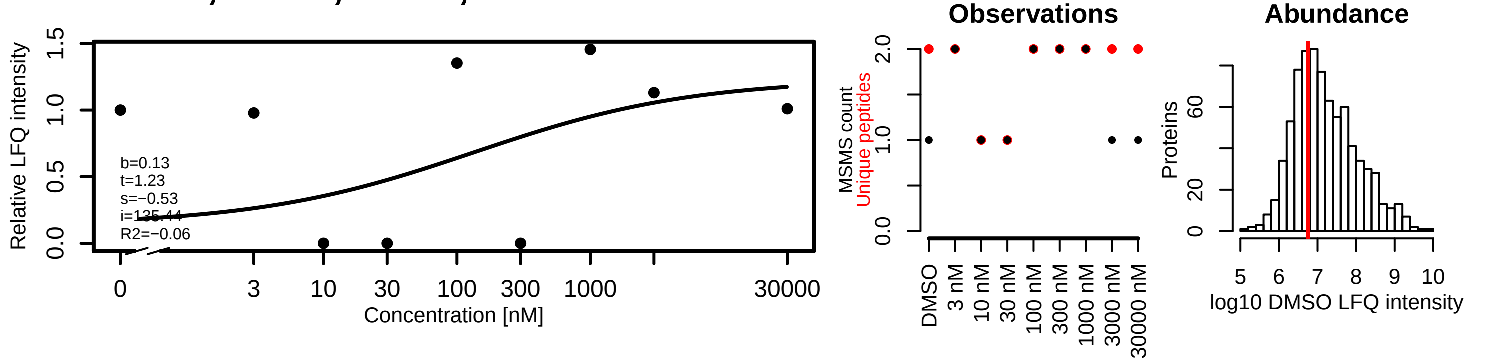


# C11orf58;SMAP

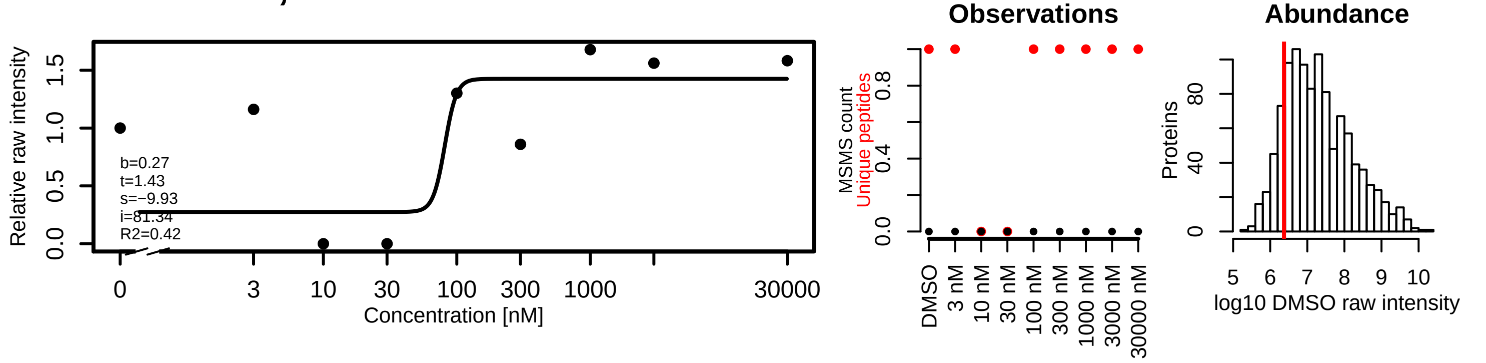


# CDK17

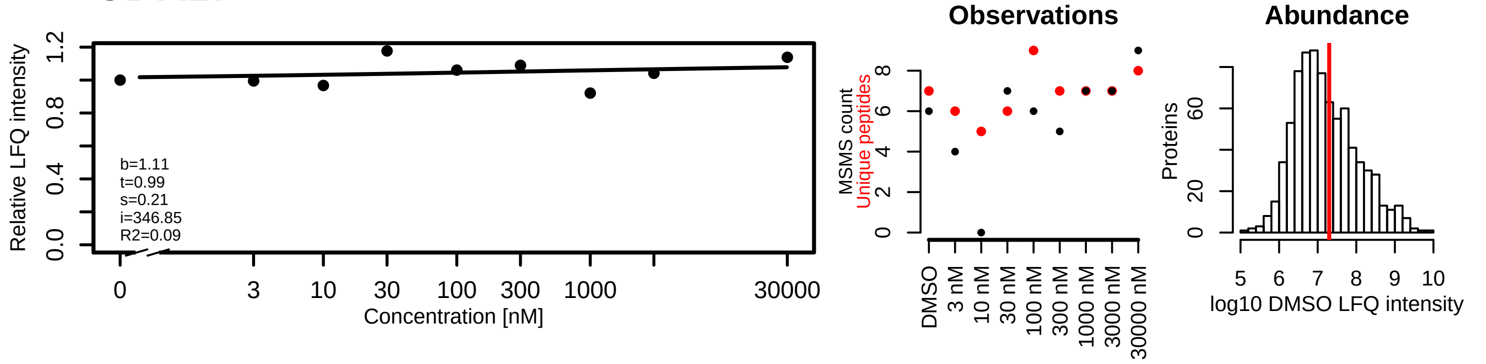


# AURKB − J9JID1;Q96GD4;J3KTD6;Q96GD4−2;Q96GD4−3;J3KRF8

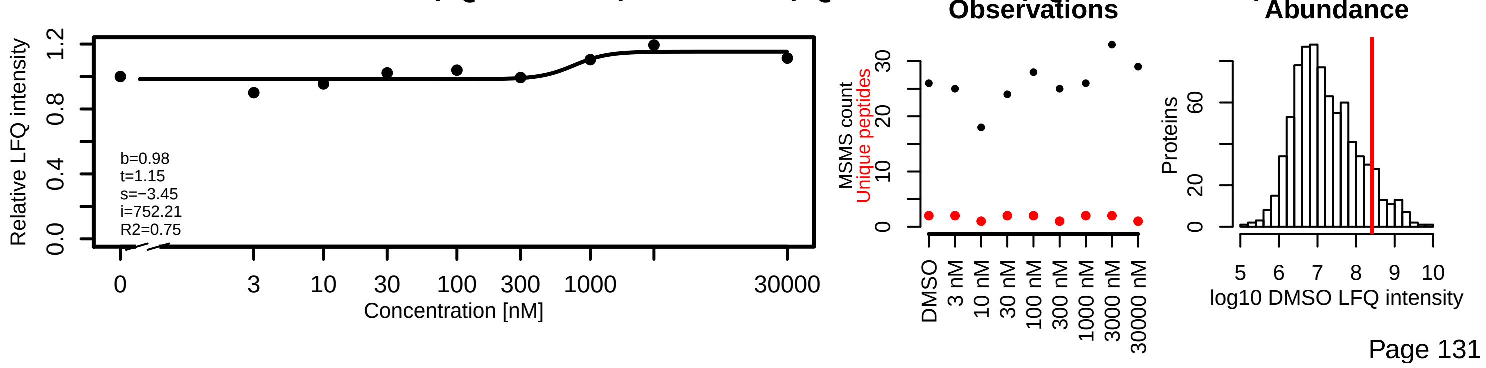

# LCK

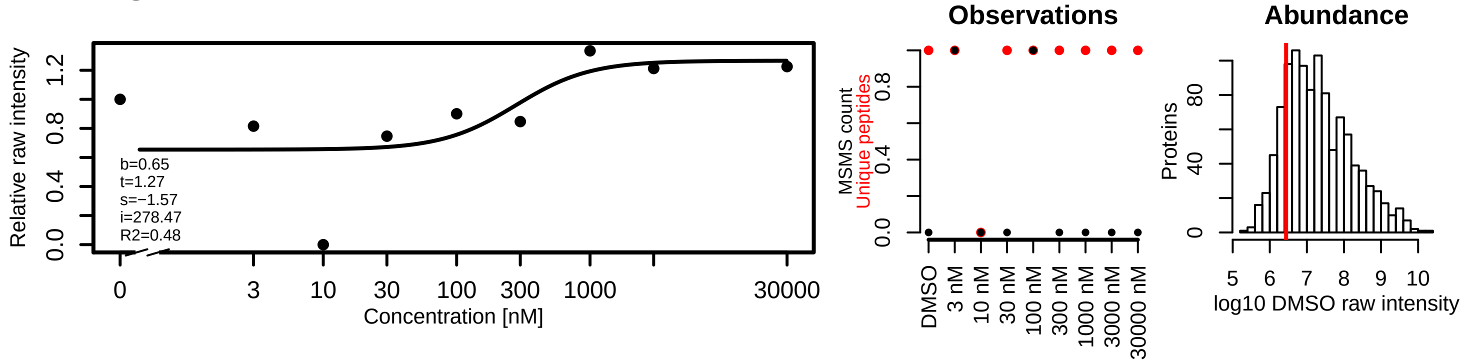


# HSD17B10

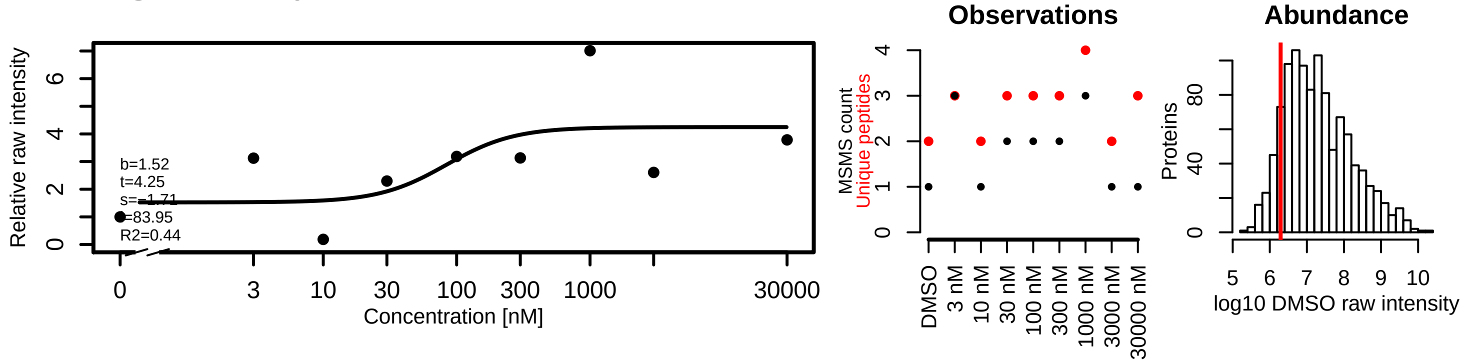


# C2CD5

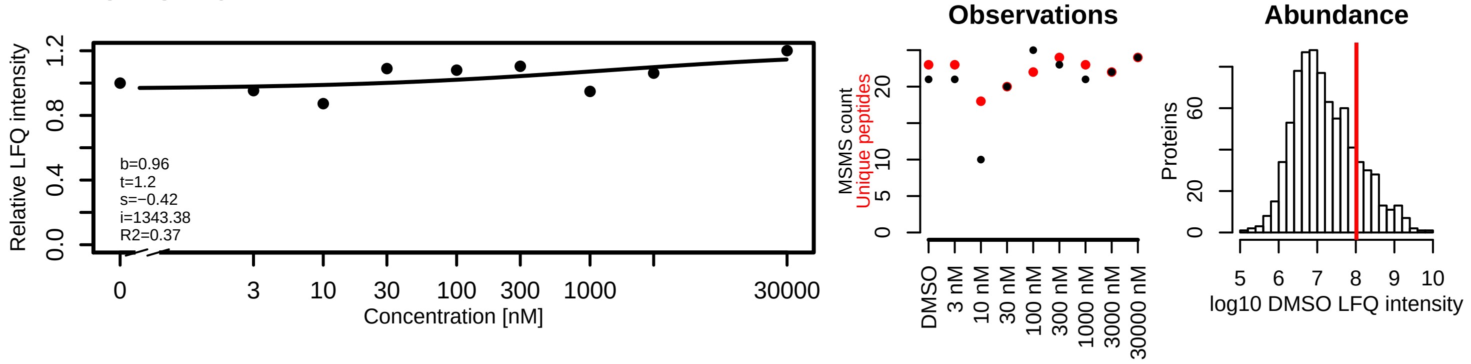


# HNRNPC

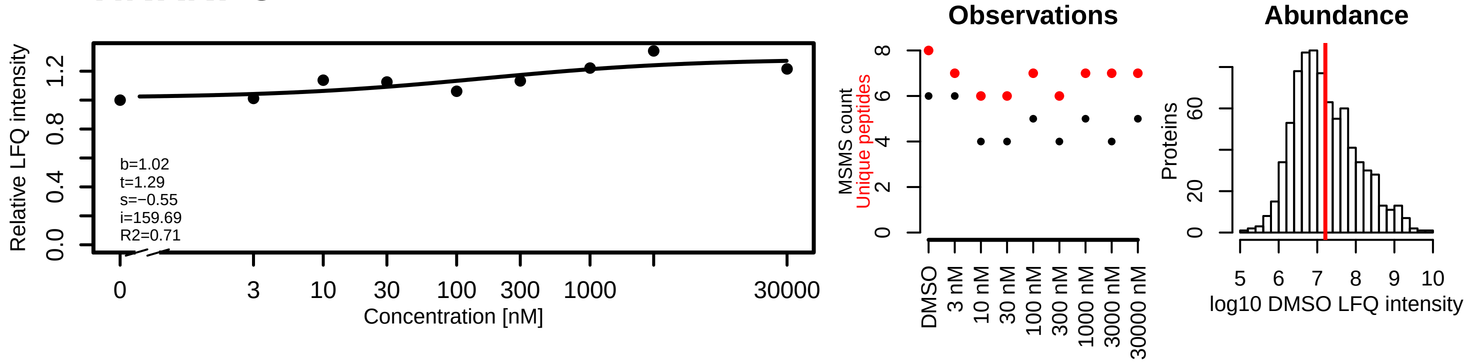


# H3F3B;H3F3A;HIST2H3A;HIST3H3;HIST1H3A;H3F3C

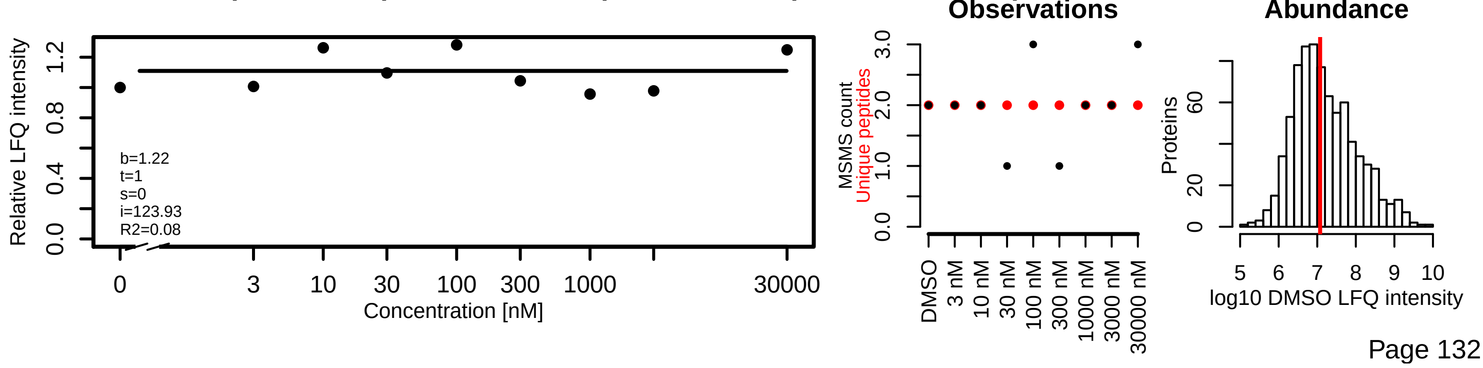

# TAOK1

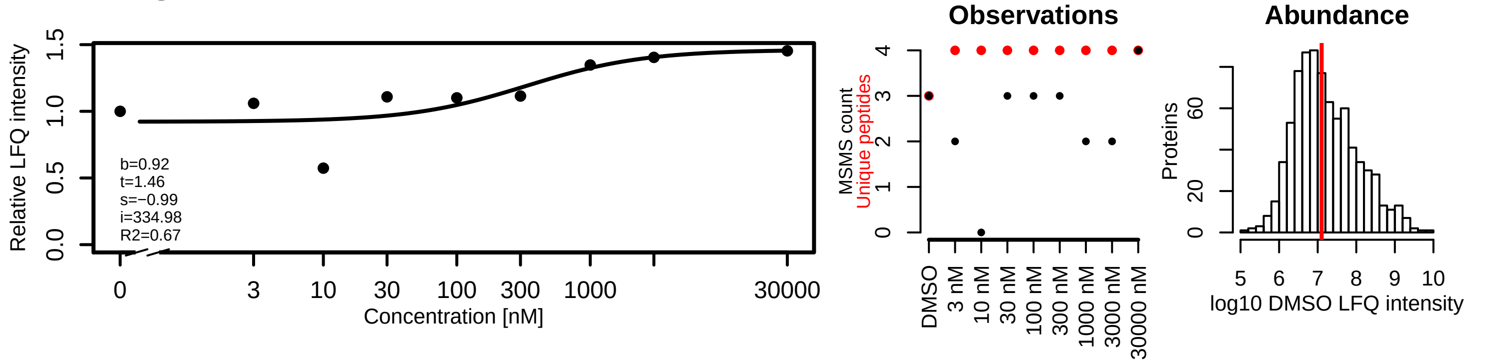


# NCL

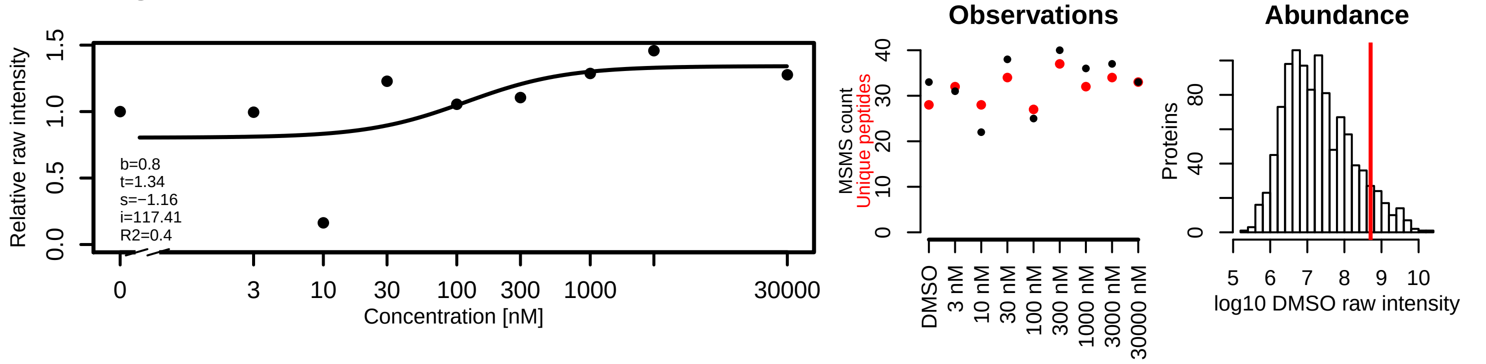


# SMN2;SMN1

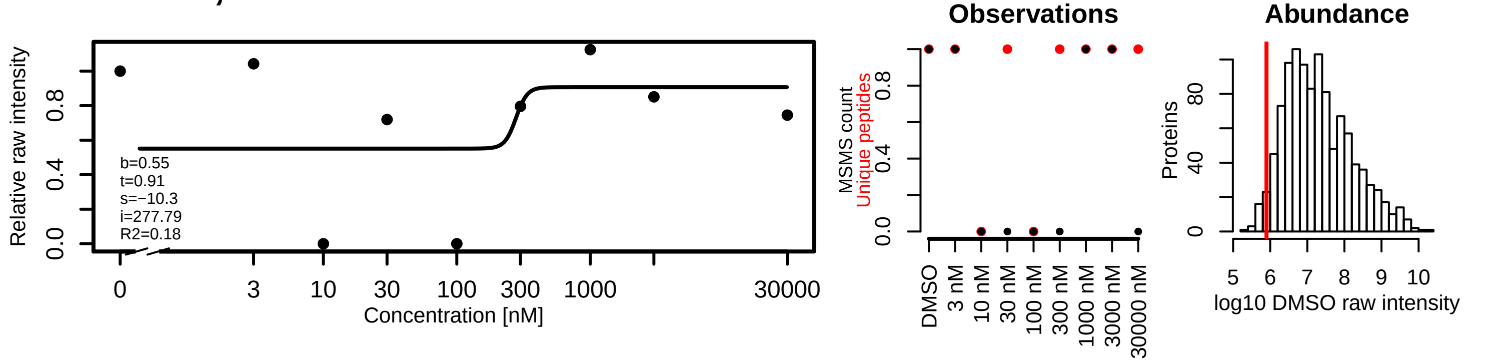


# AKT2

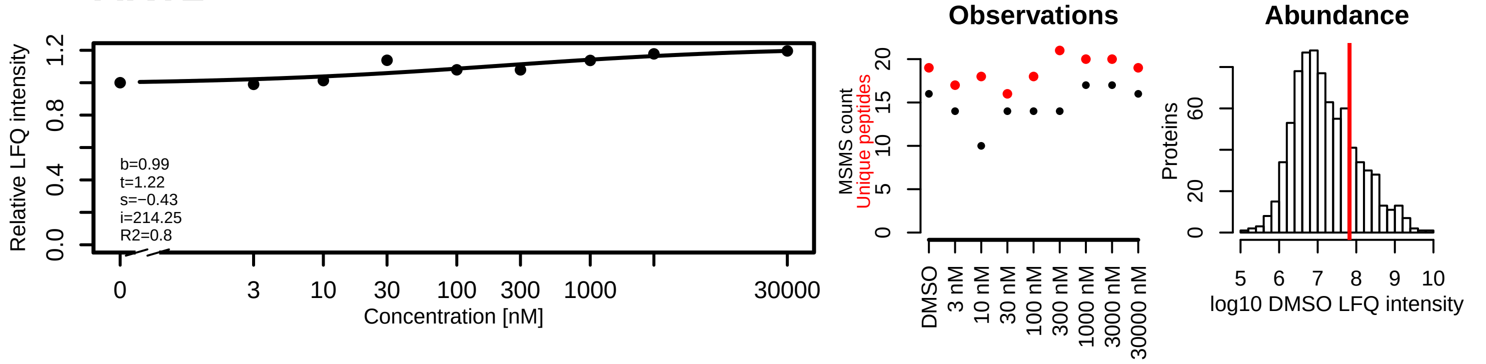


# DYNLL1

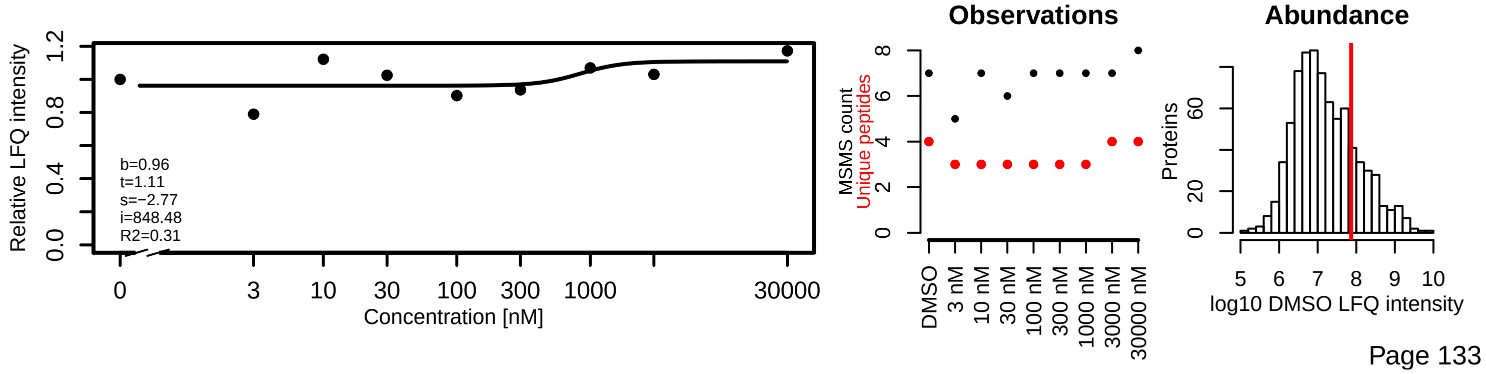

## BMP2K

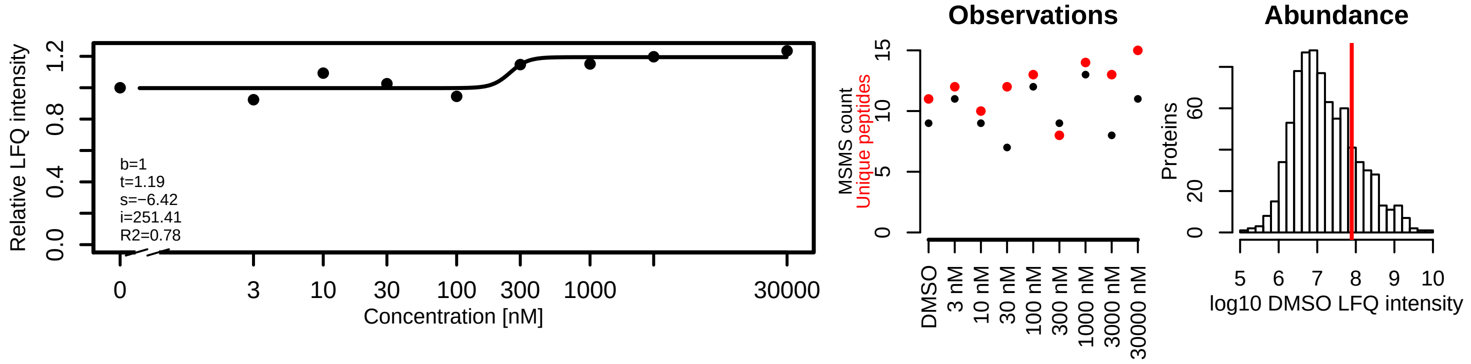


## MAPK9

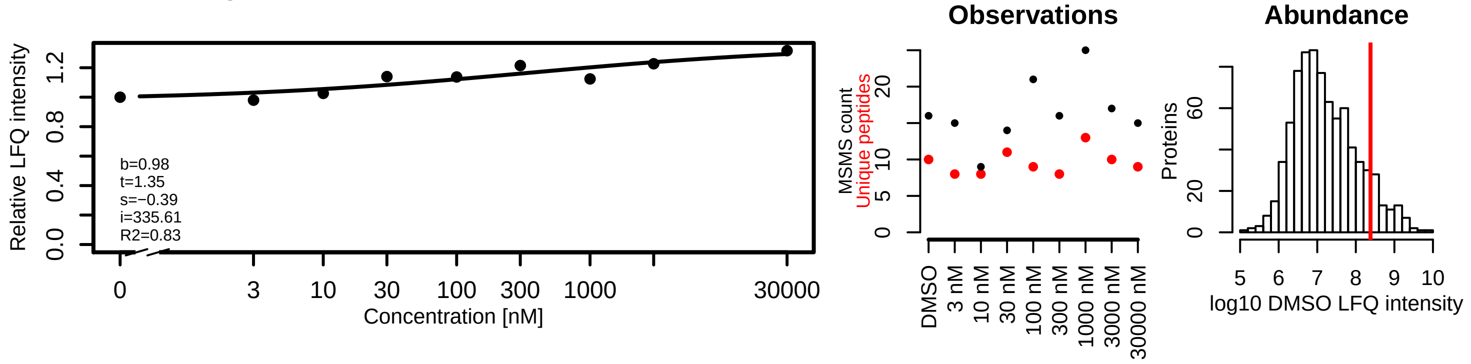


## RPS6KA4

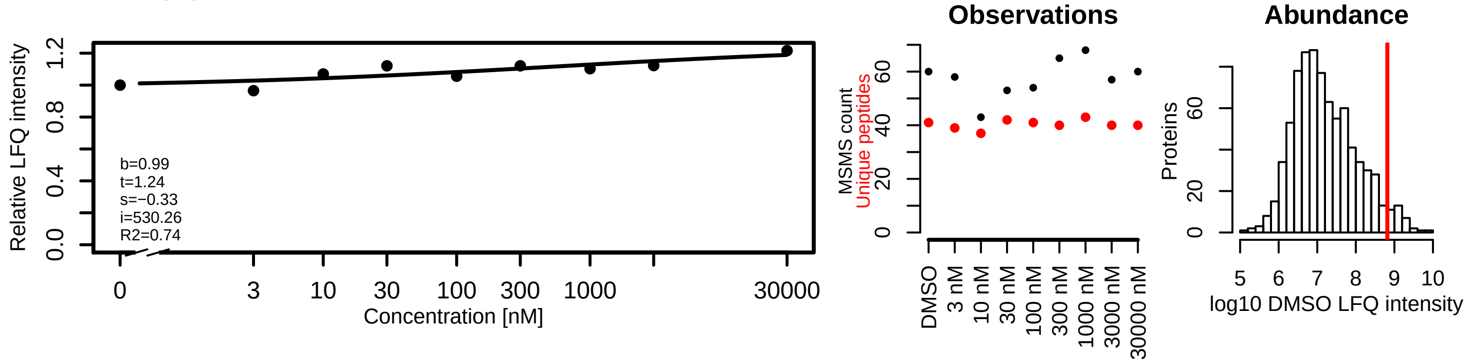


## PA2G4

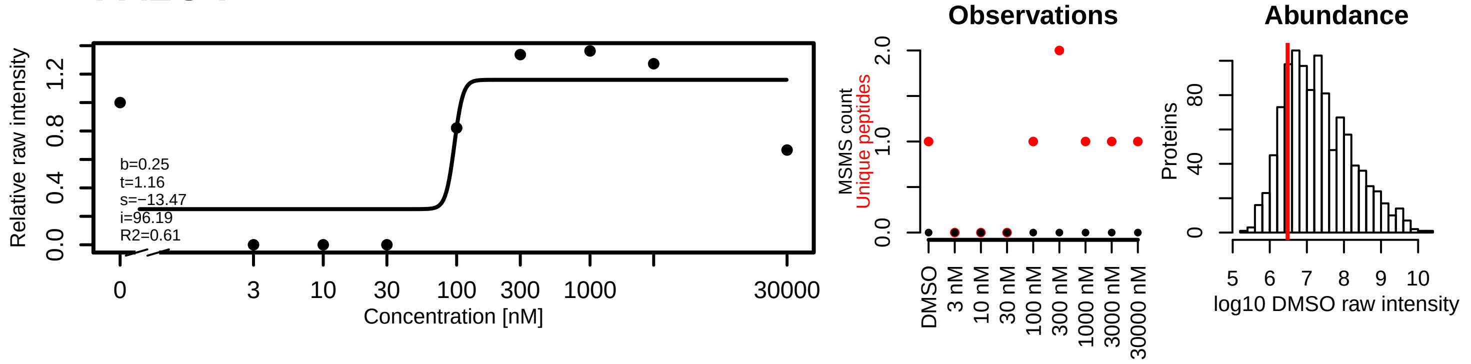


## CAMK2G − Q13555−6;B4DVQ3;D6RFJ0;D6RHX9

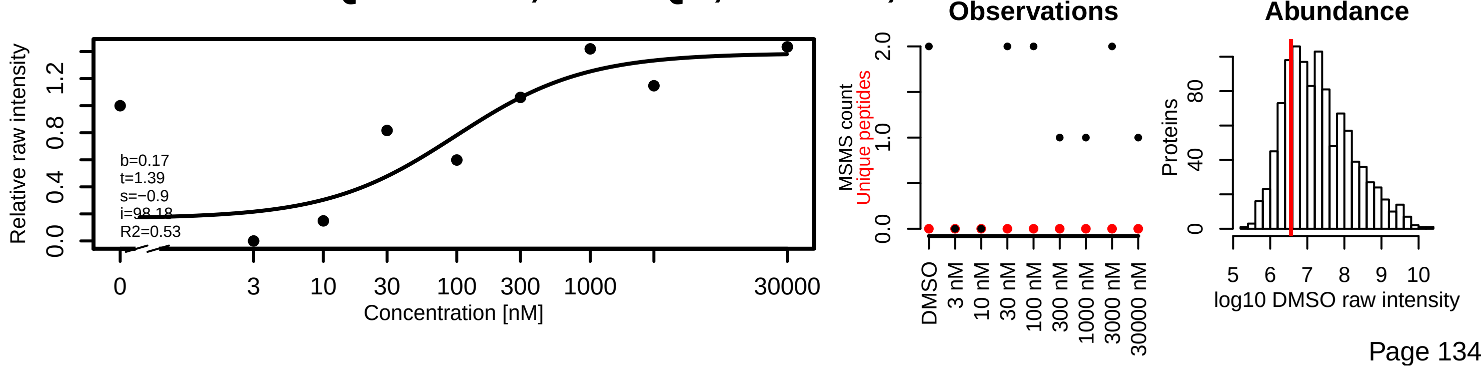

## KPNB1

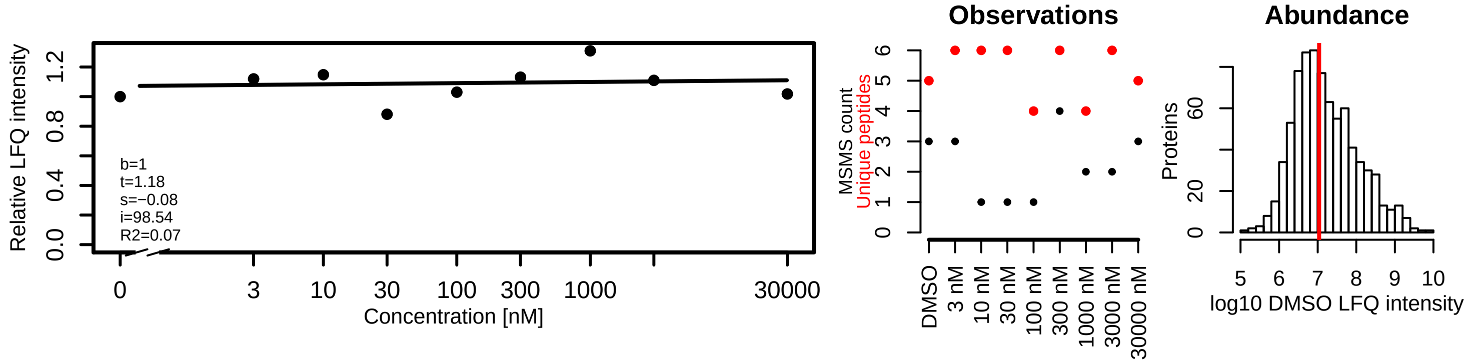


## CAV1 − C9JKI3;Q03135−2

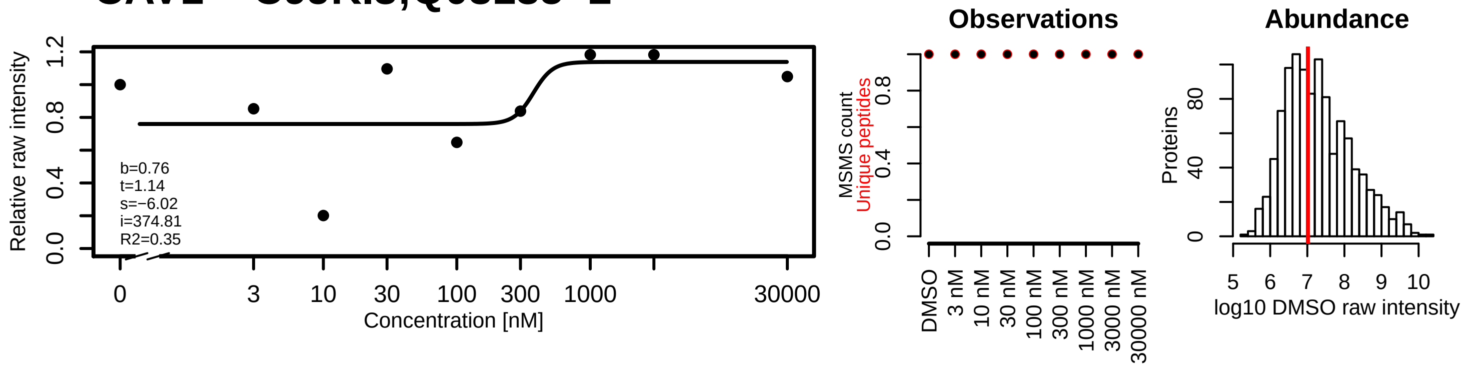


## NEK6;NEK7

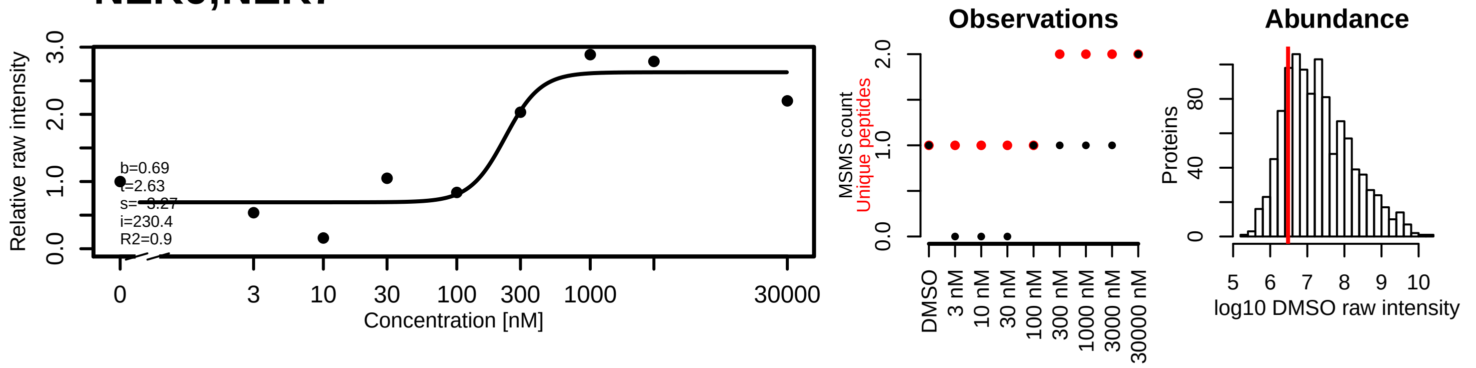


## NDUFS1

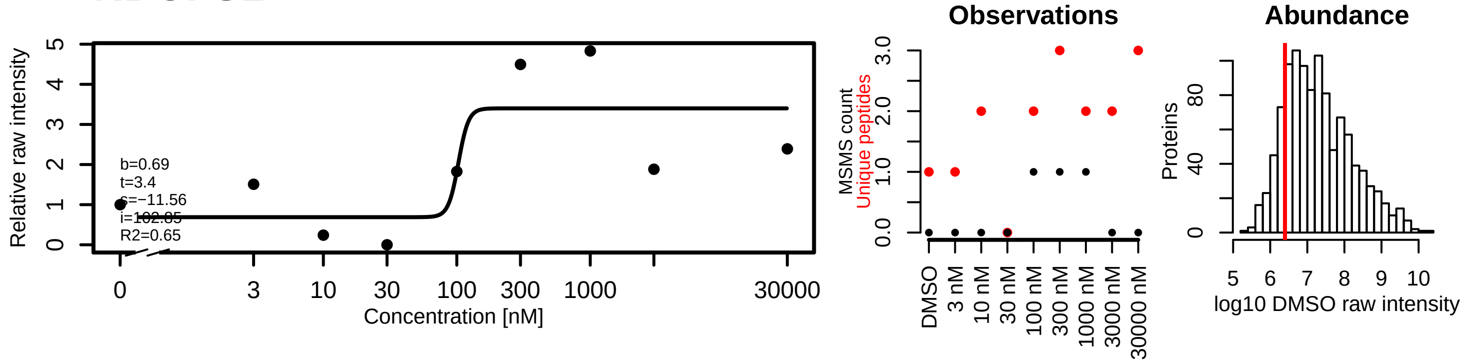


## SQSTM1

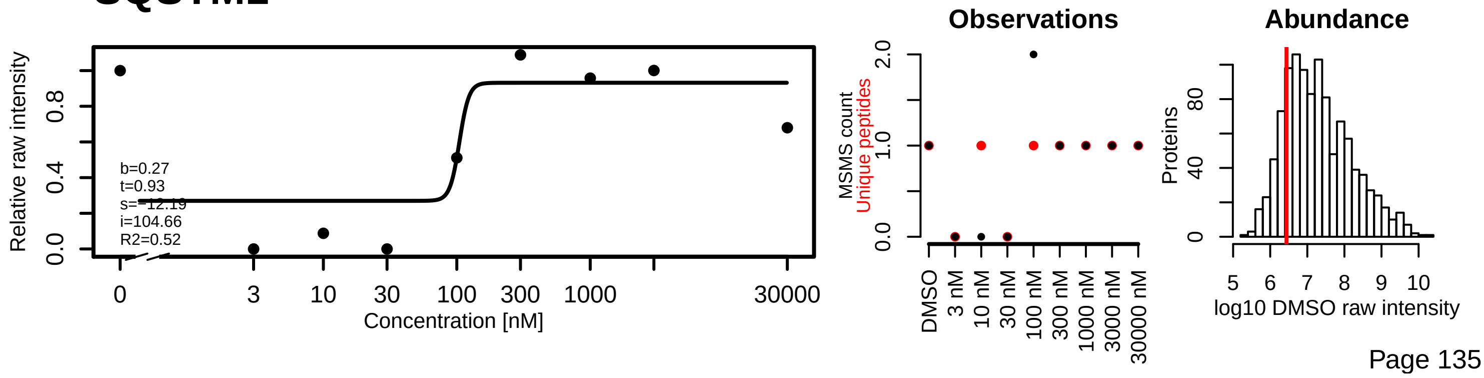

# SEC61B

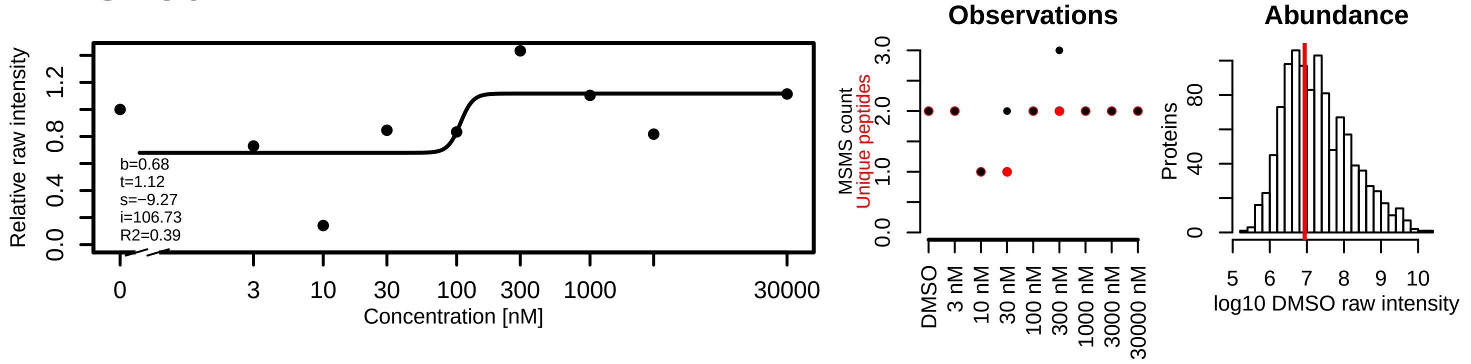


# BMPR2

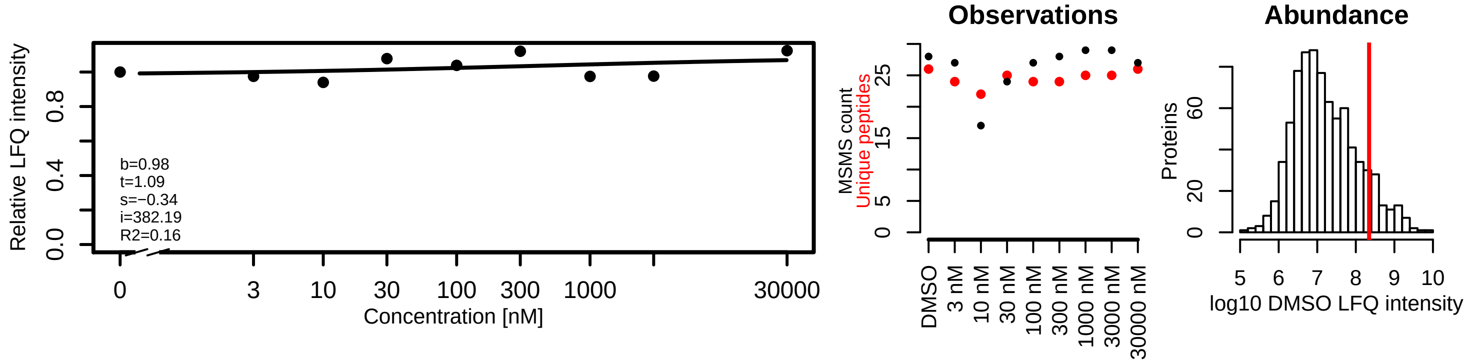


# STXBP1

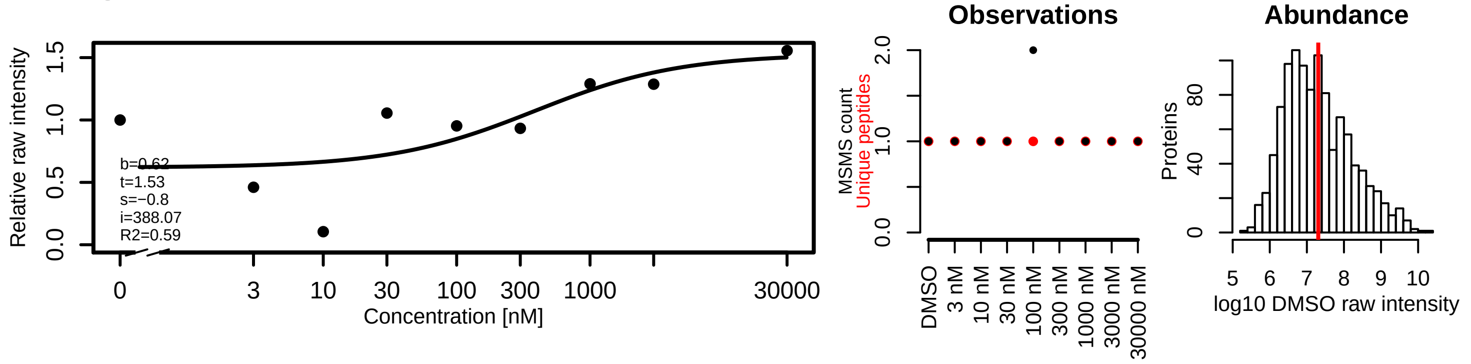


# QARS

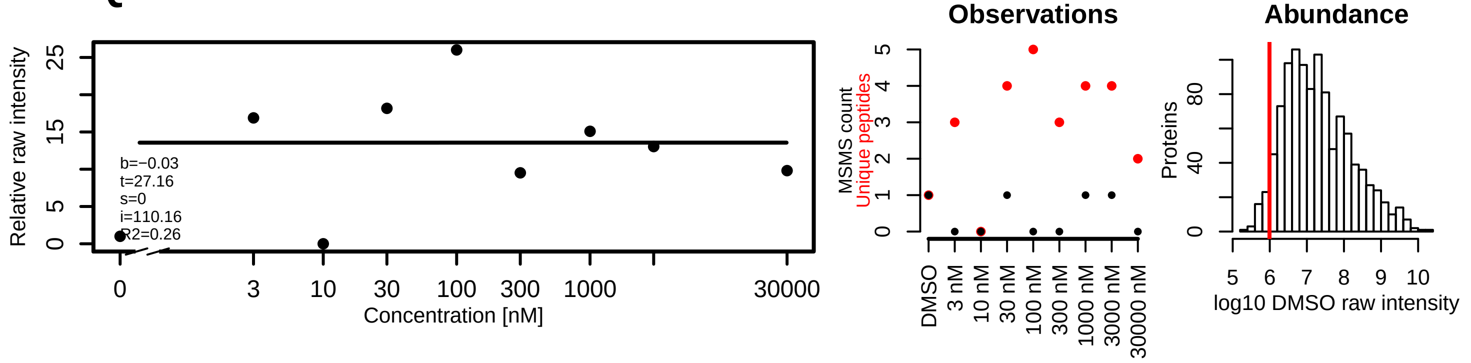


# EMC2

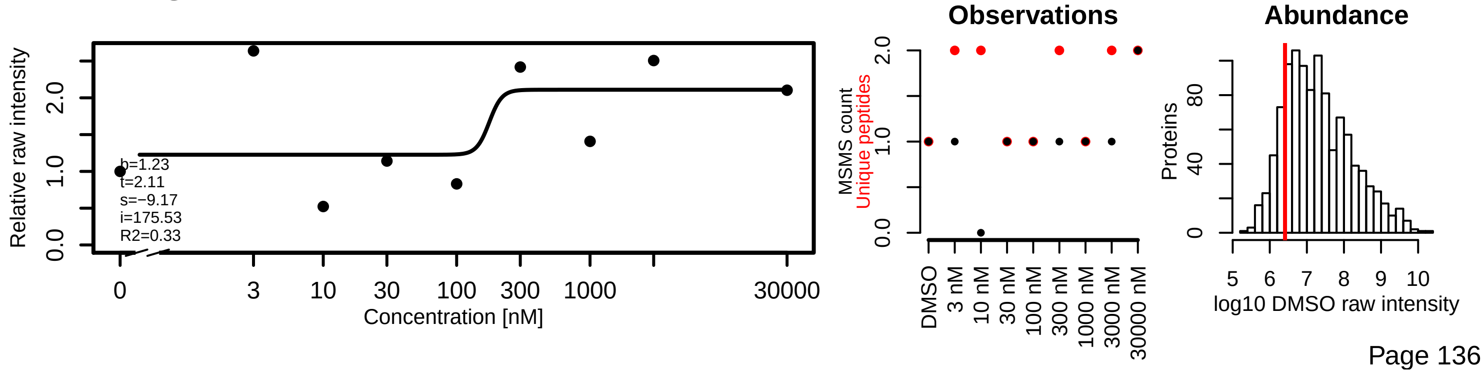

# MAPK8

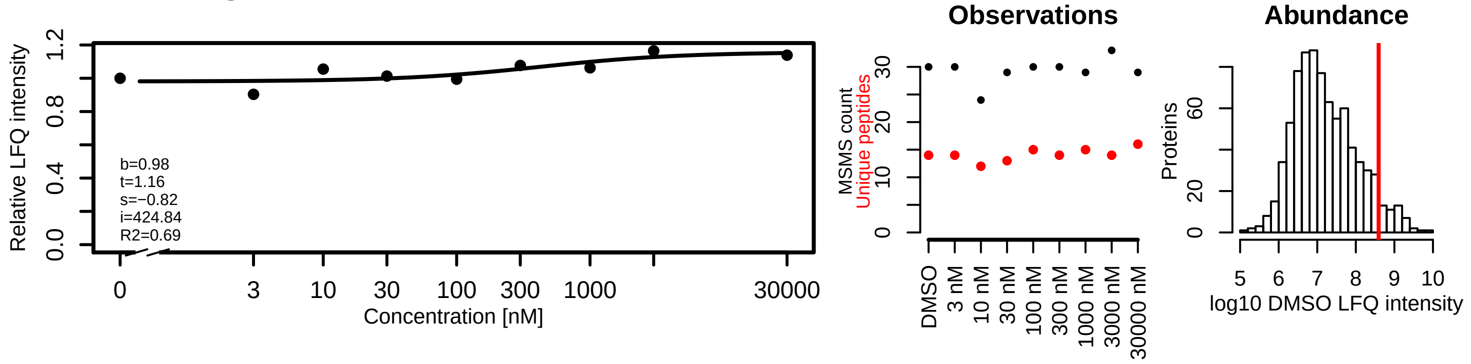


# PDXK

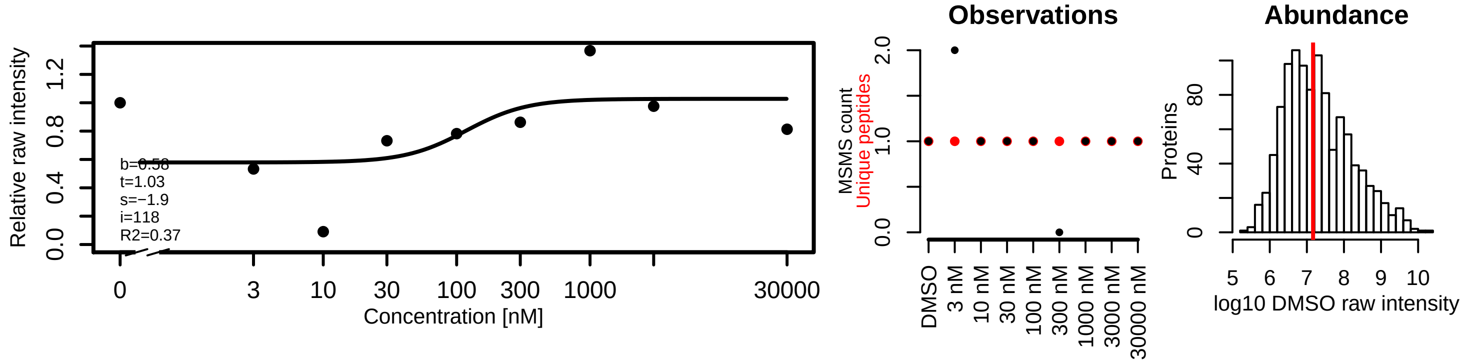


# BUB1

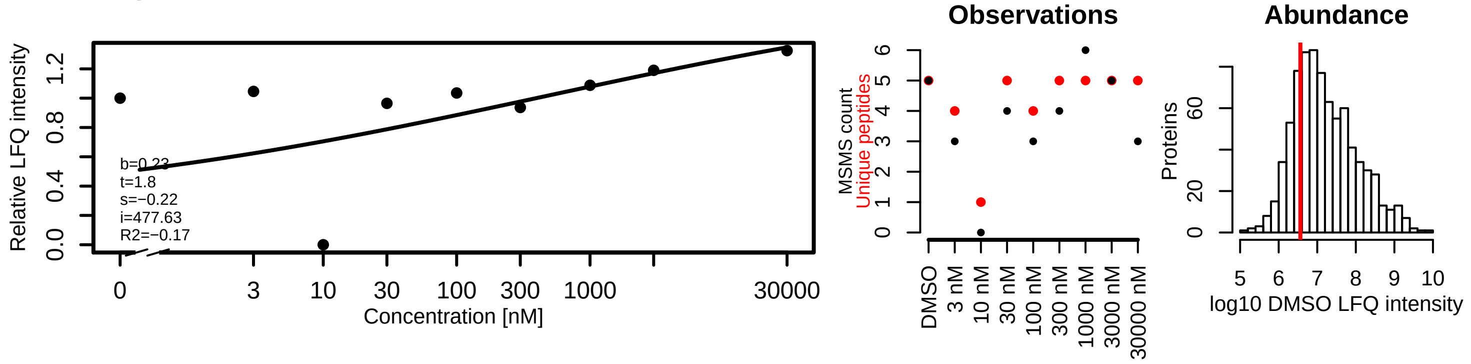


# THEM6

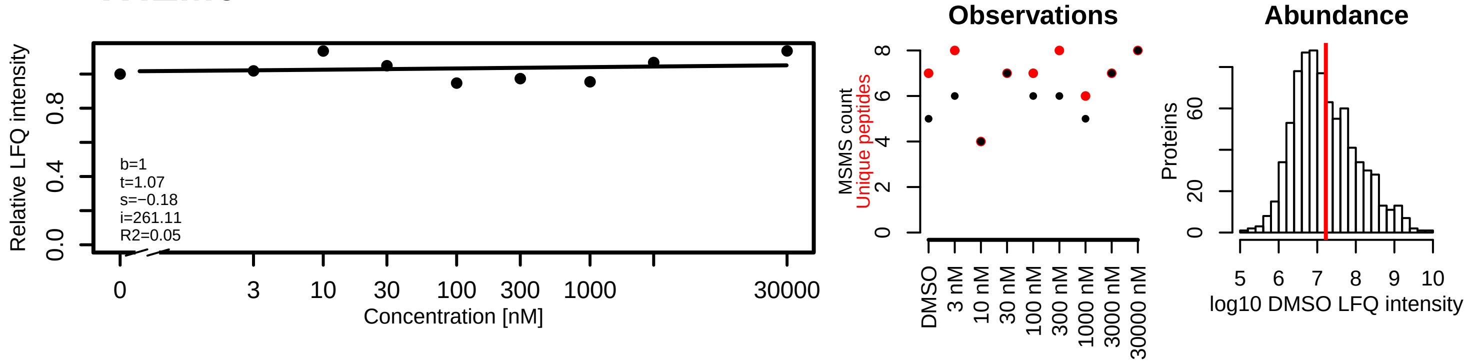


# SLC39A7

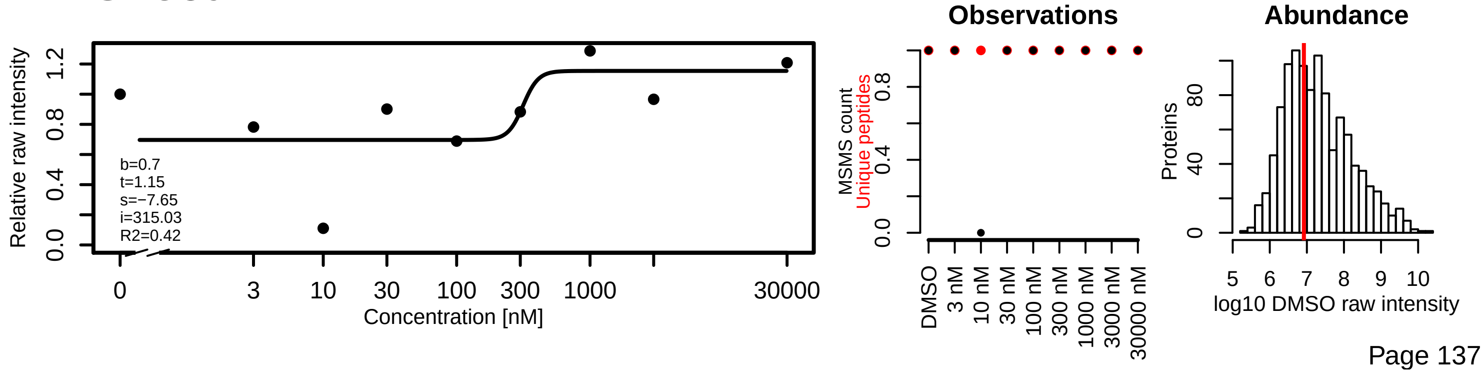

## HNRNPA3

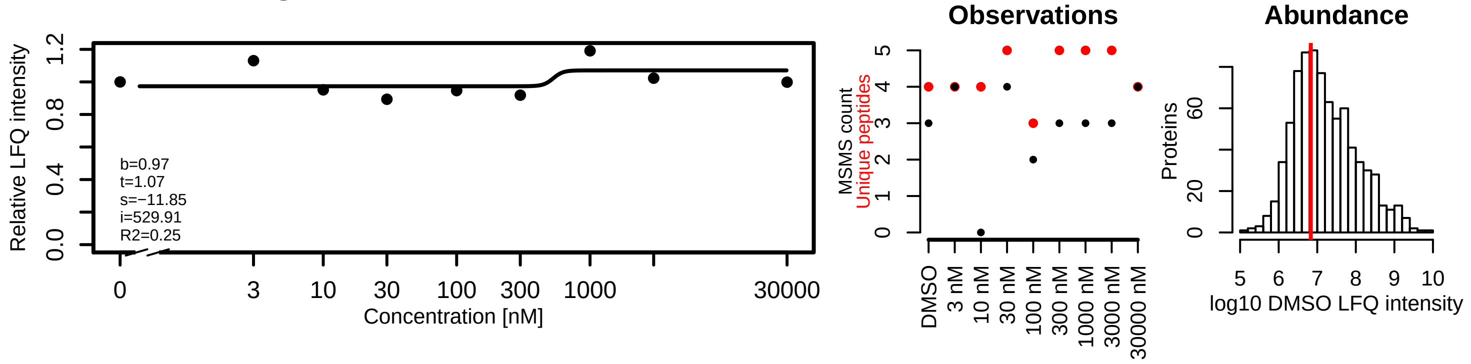


## HNRNPM

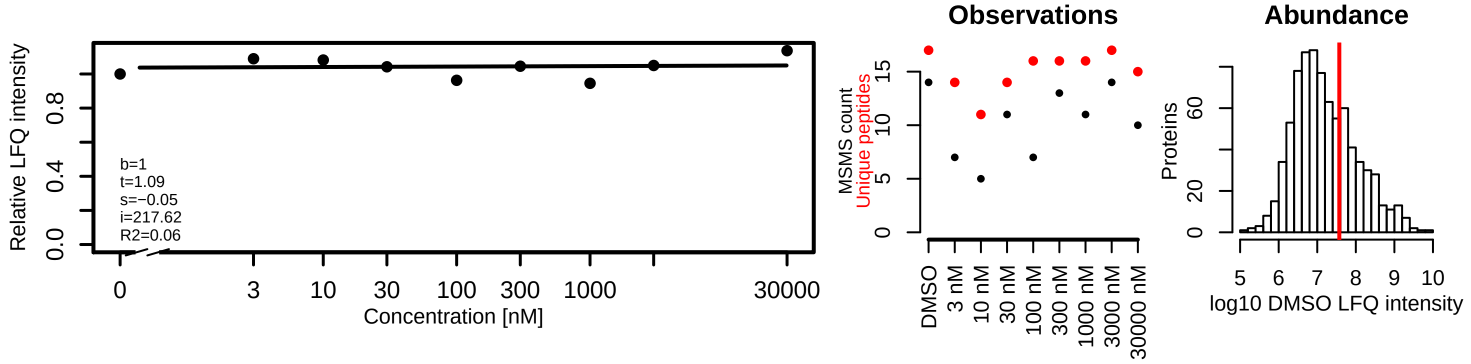


## PLK2

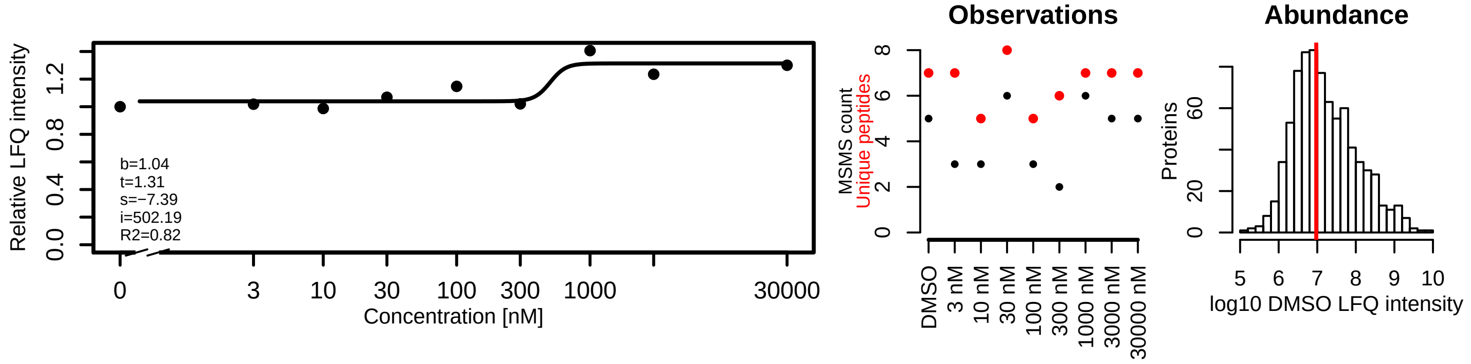


## MLEC

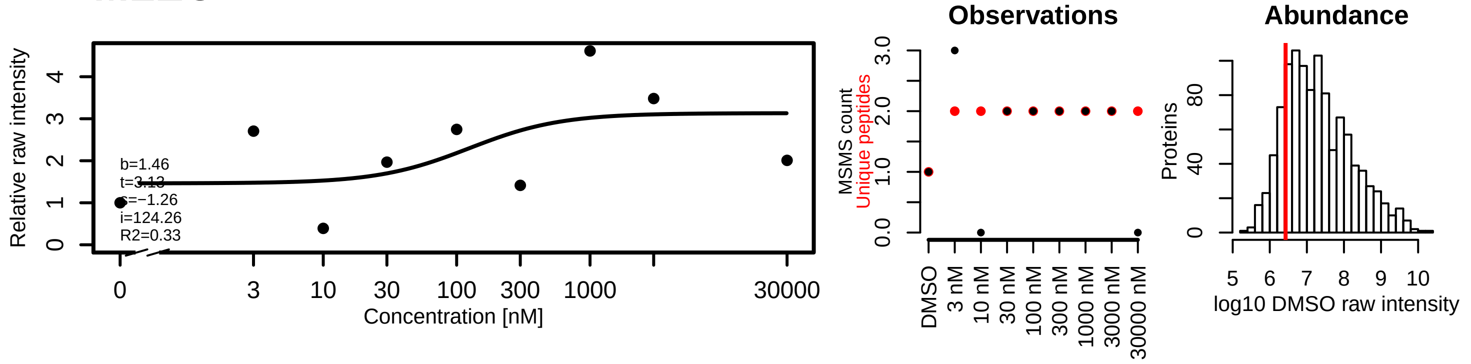


## COQ5

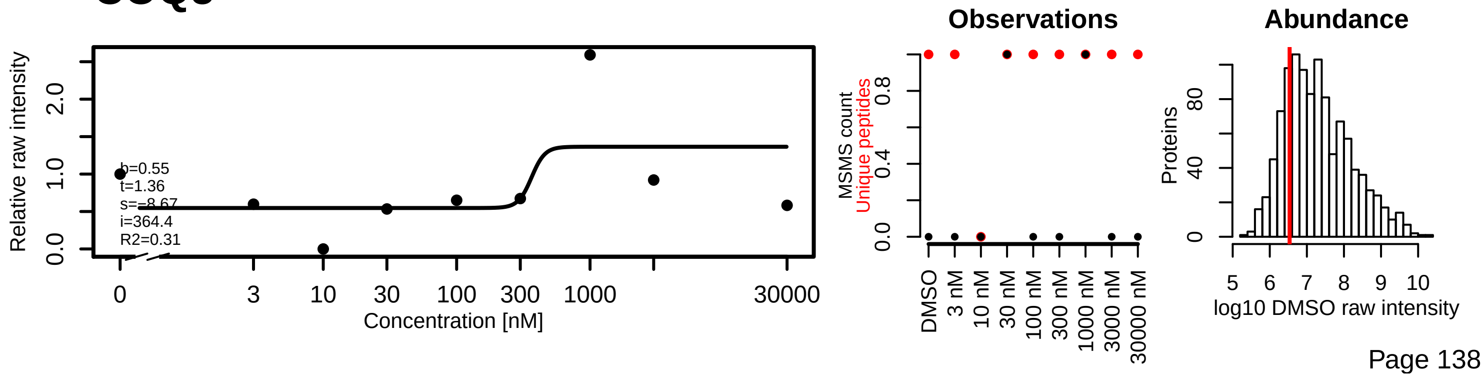

# RPA1

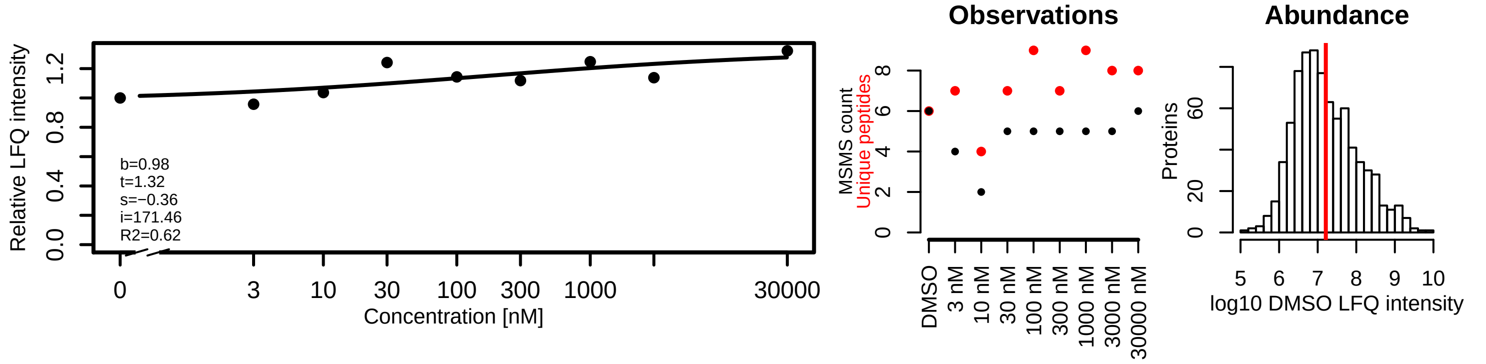


# KDELR2

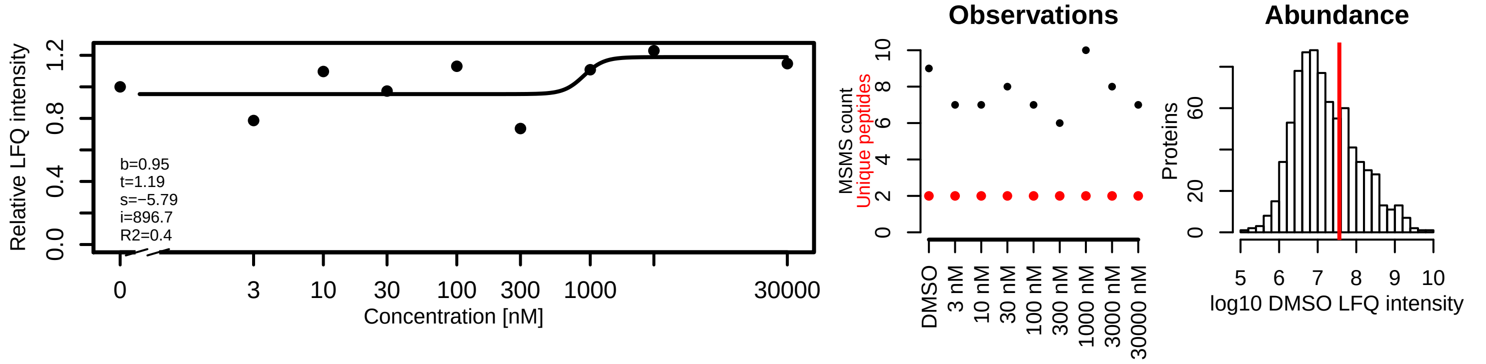


# RPS15

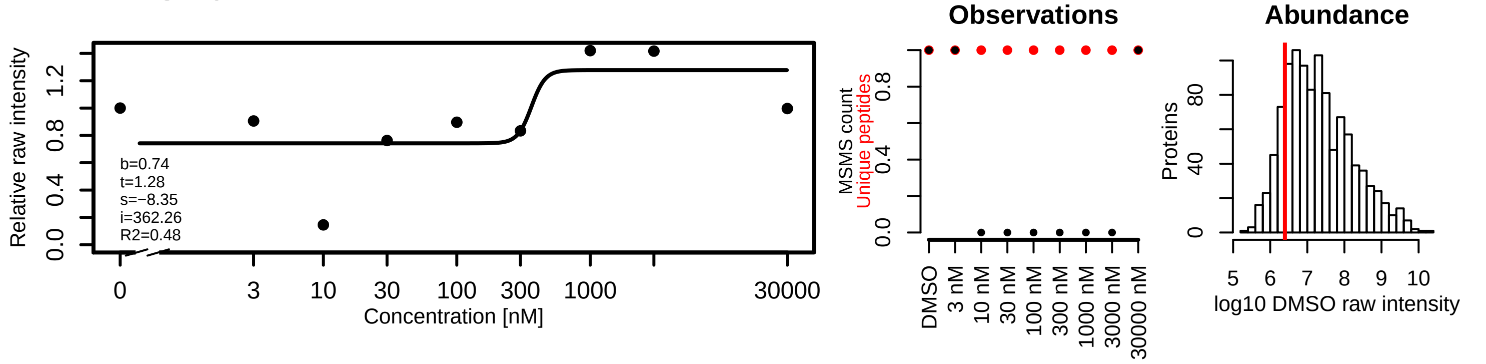


# LANCL2

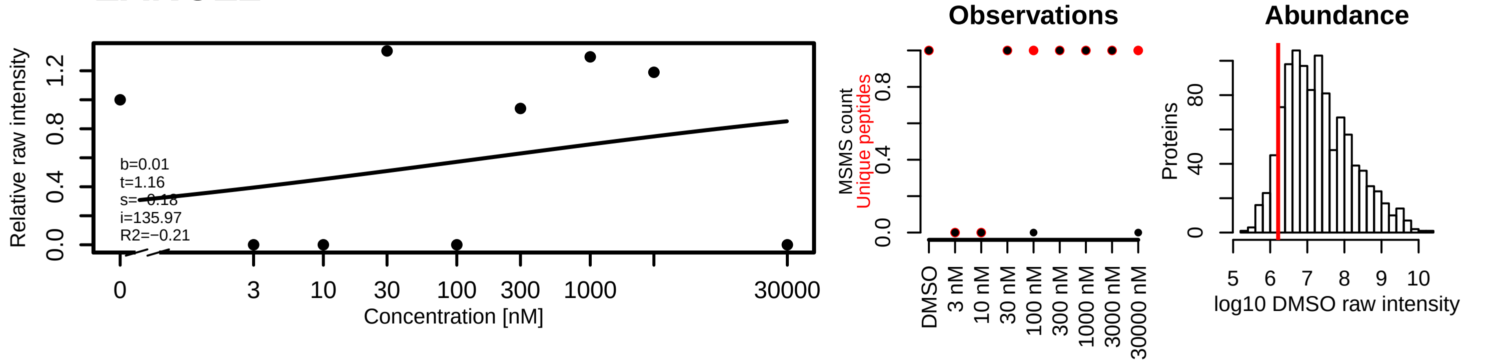


# ROCK2

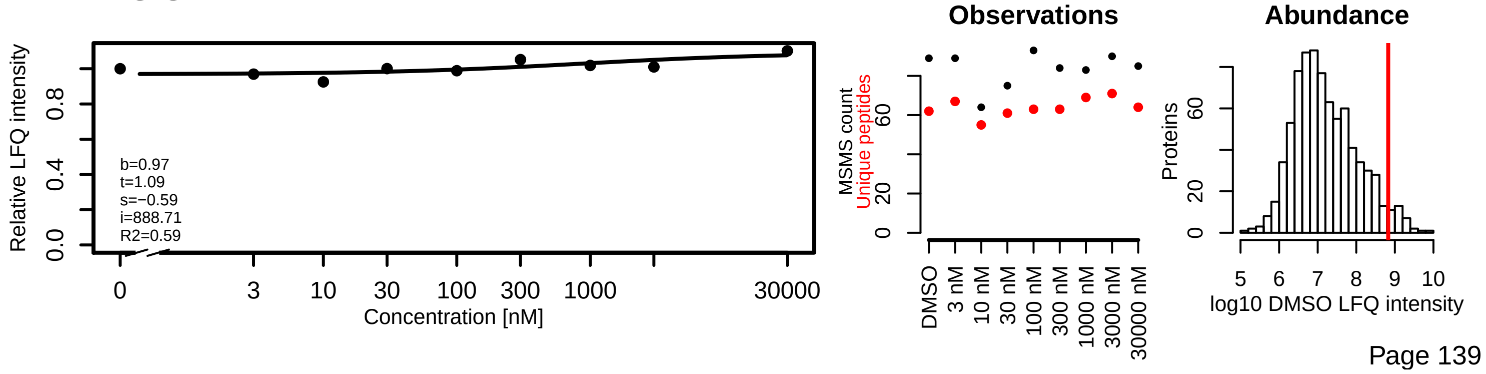

## AURKA

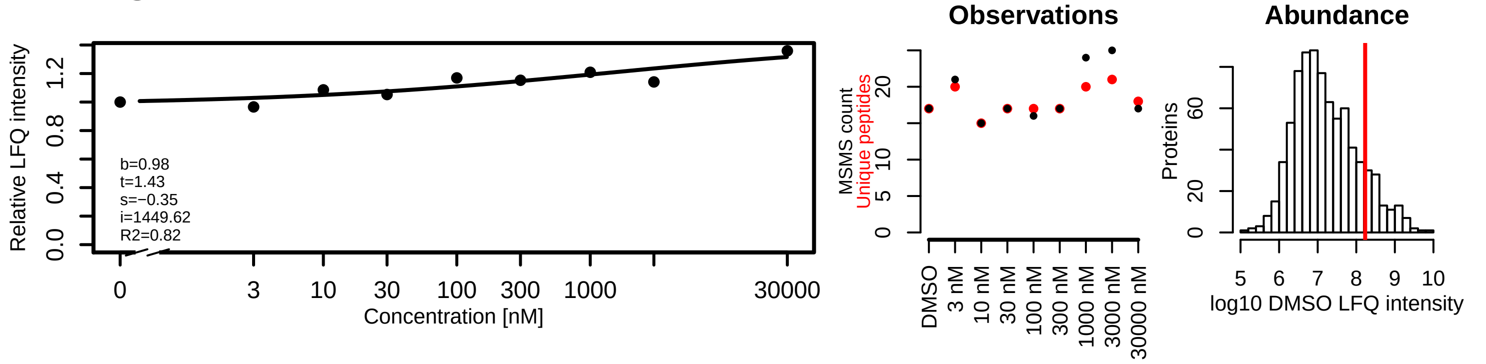


## MARK3 − P27448−3;P27448−4;P27448;P27448−2;J3KNR0;P27448−8;I

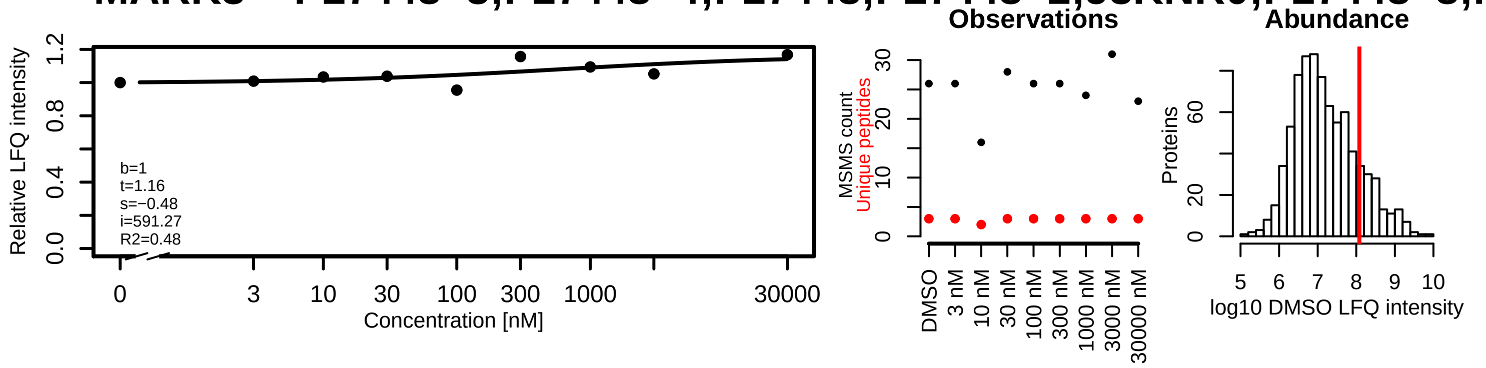


## RPS27L

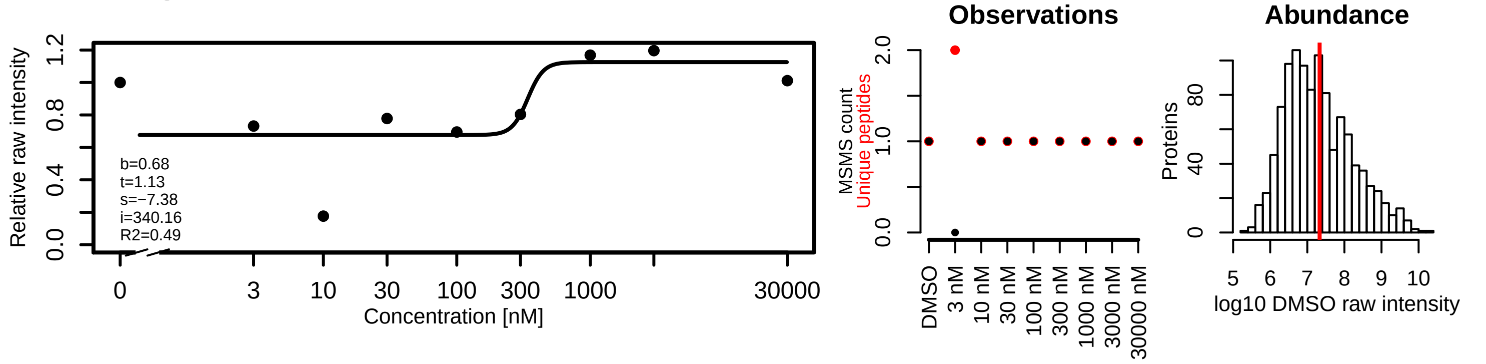


## EIF4B

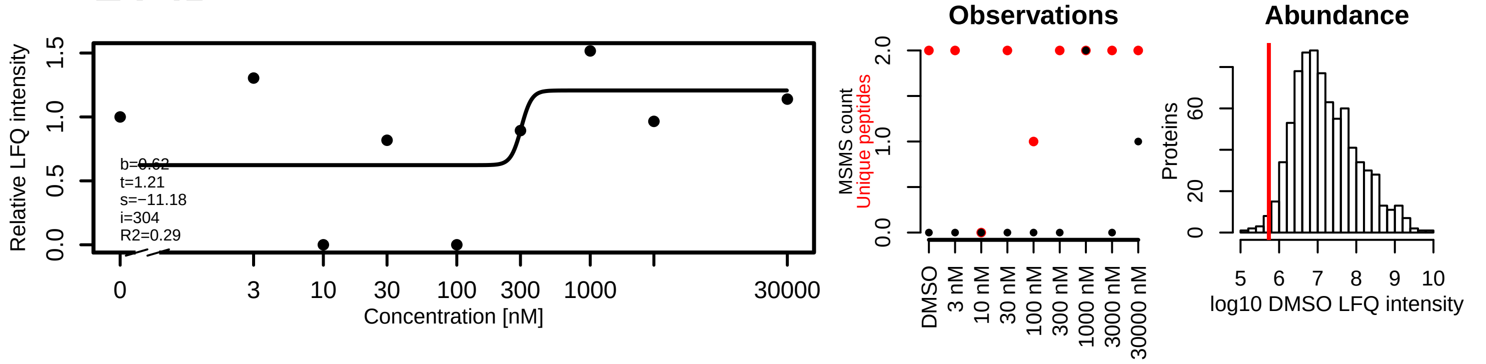


## TAOK3

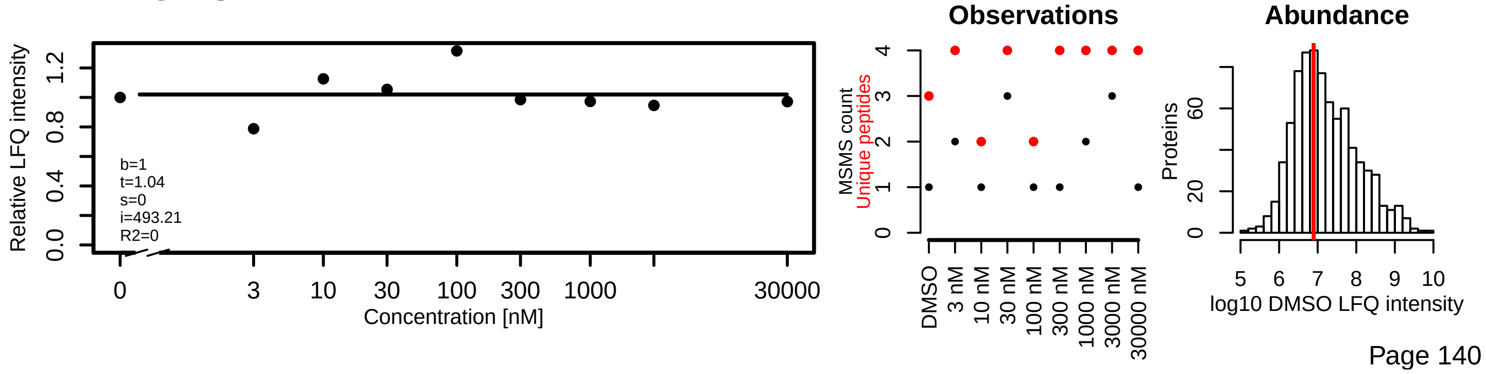

## VKORC1L1

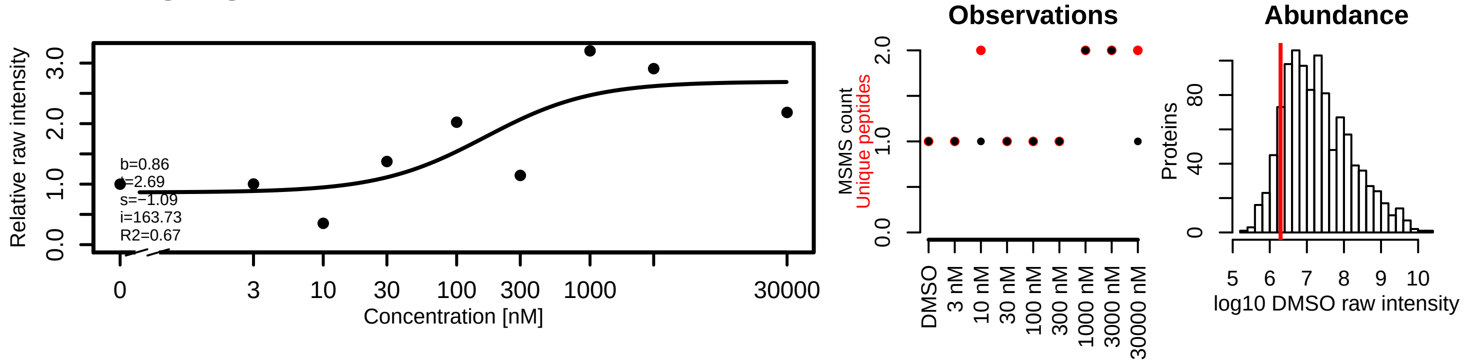


## GLUL

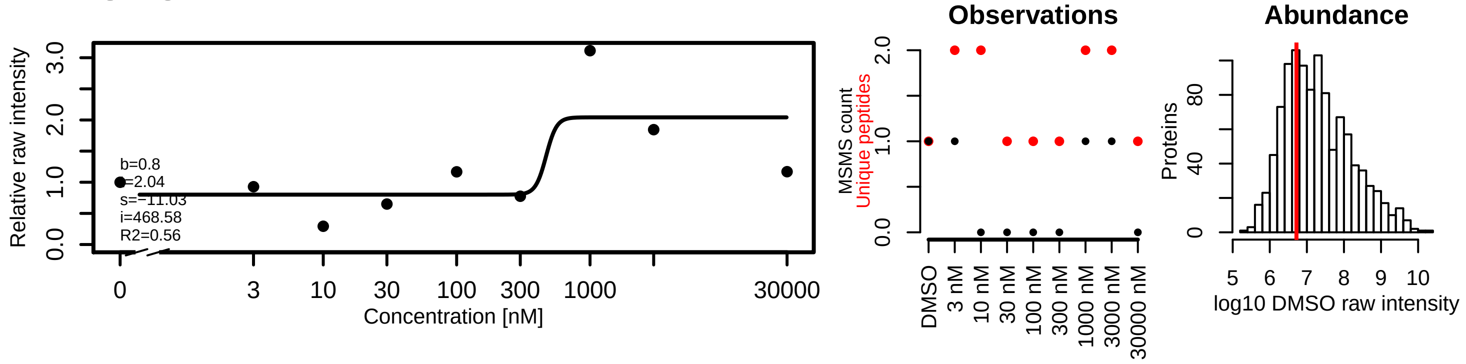


## GAPVD1

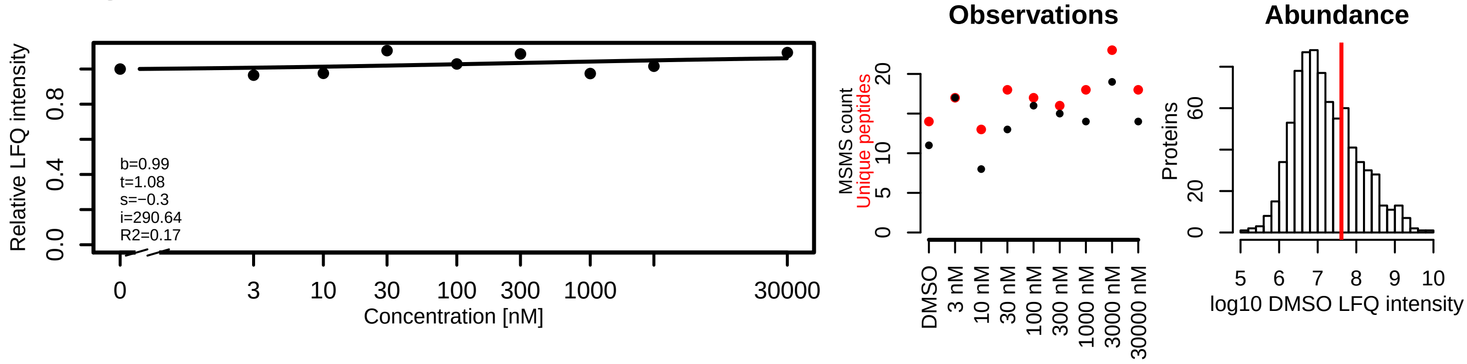


## MET

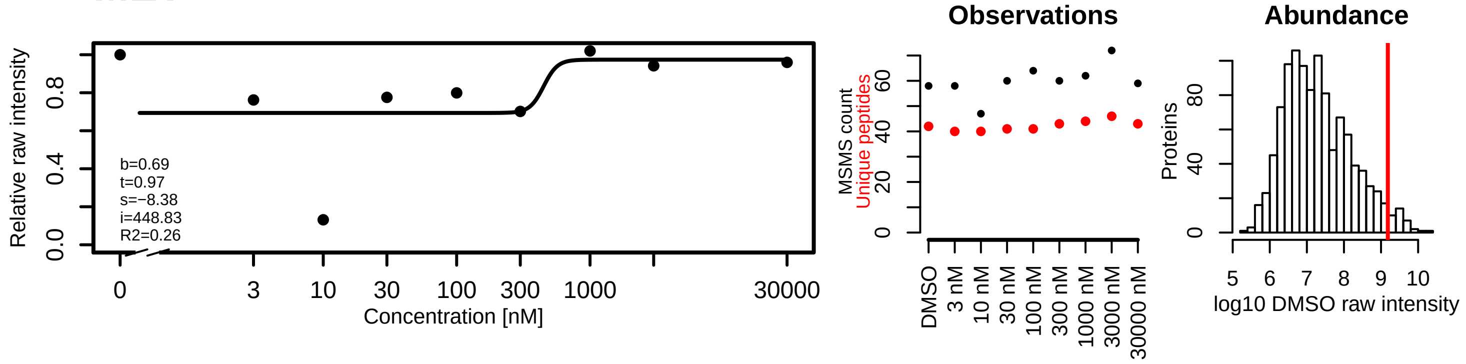


## CAMK2B − Q13554

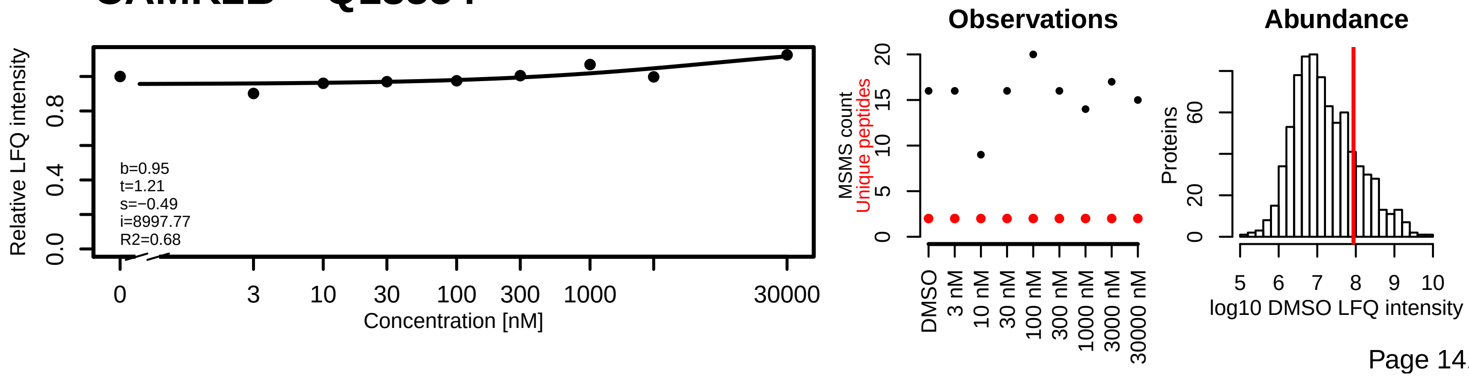

# NUP155

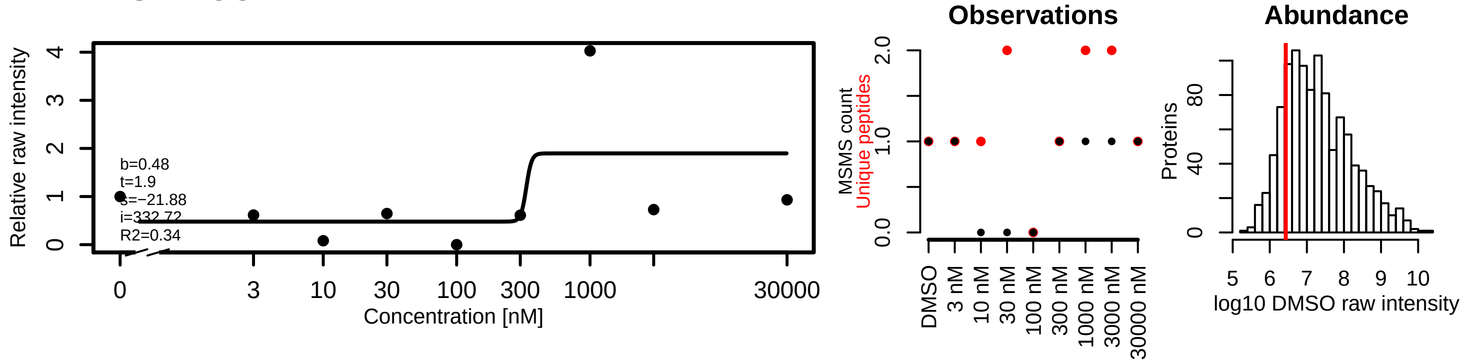


# SLC7A1

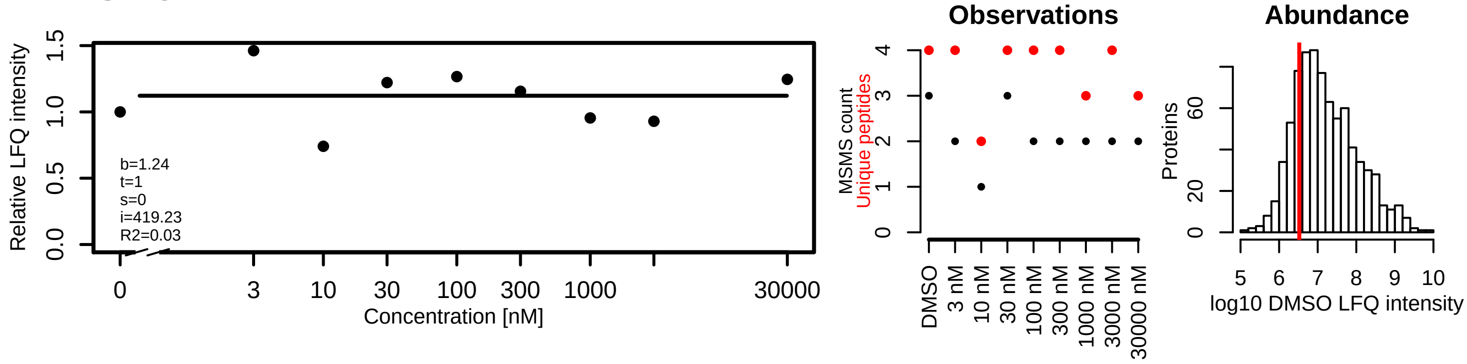


# FER

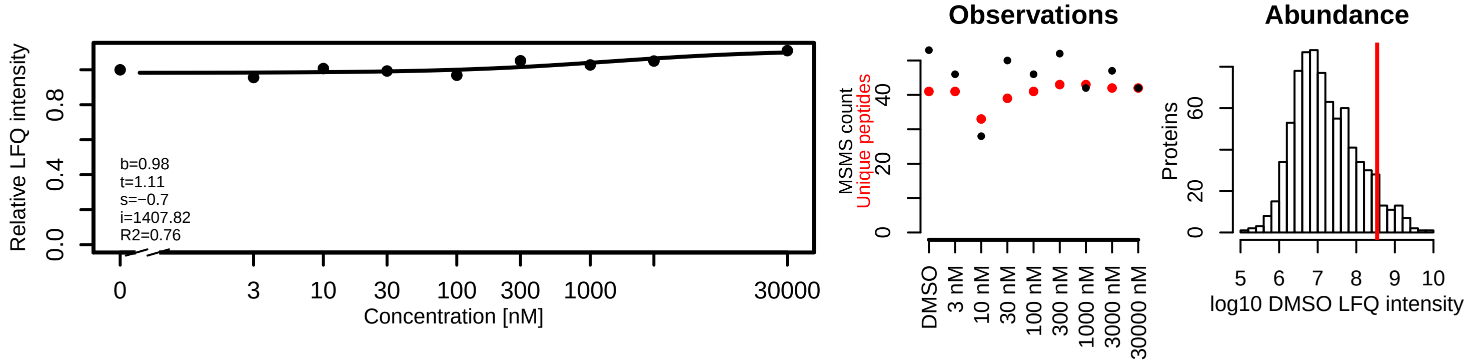


# HNRNPH3

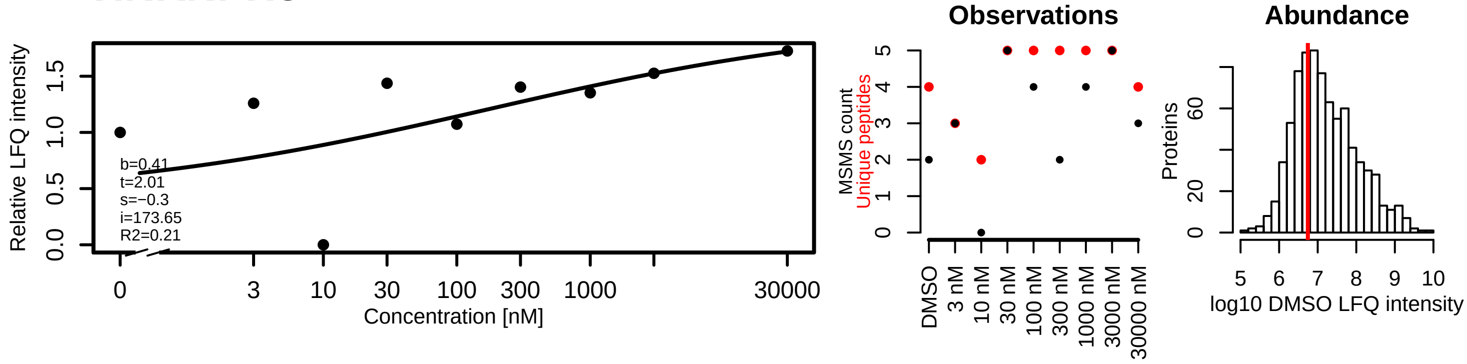


# AIFM2

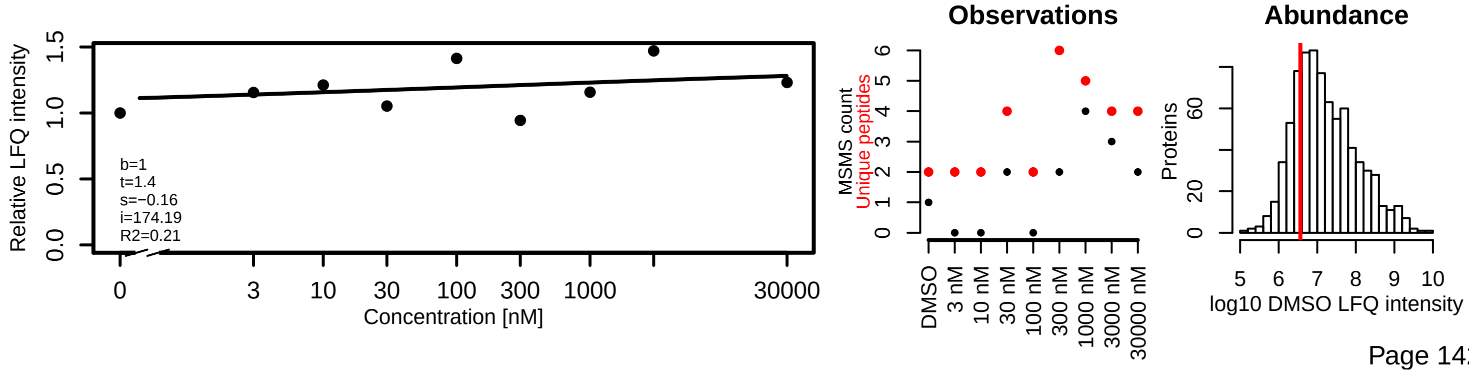

## PHGDH

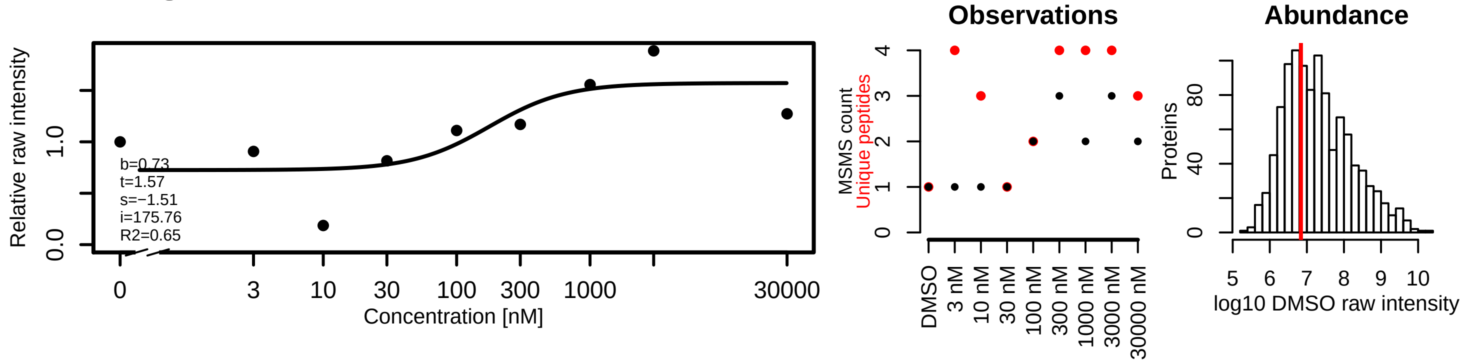


## RPA2

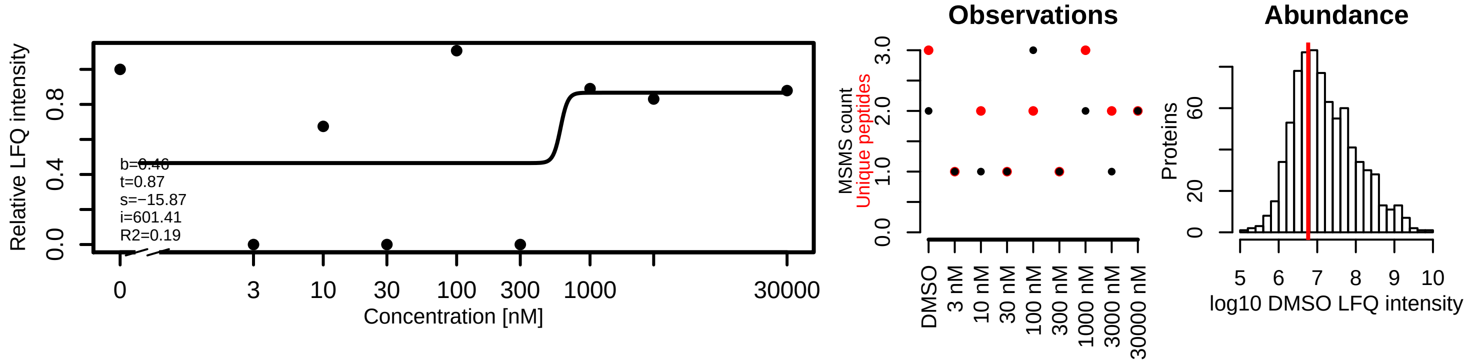


## CSNK1D

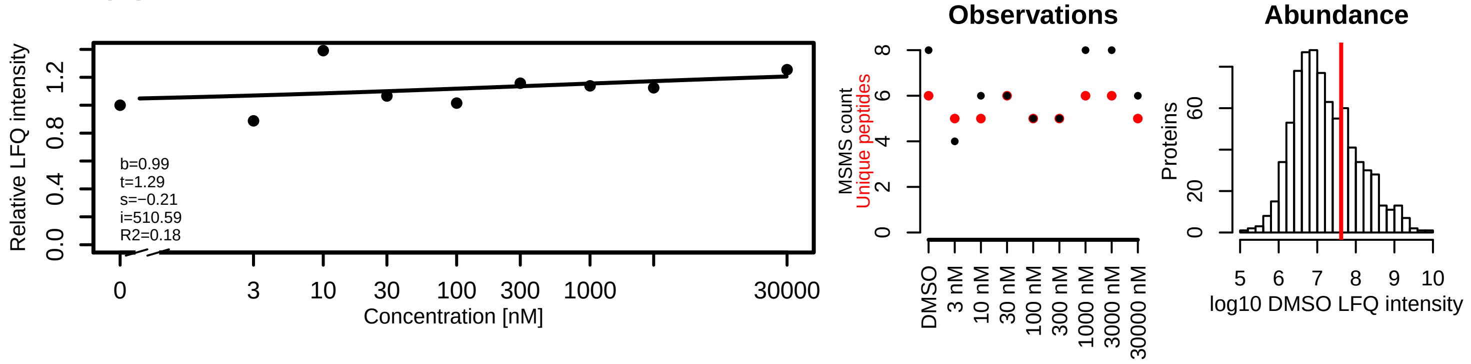


## SLC39A10

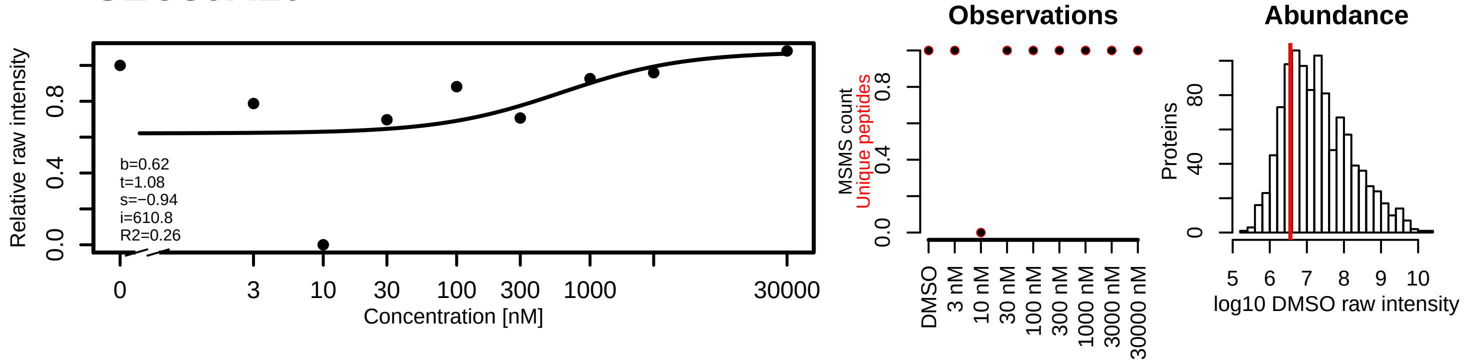


## CFL1

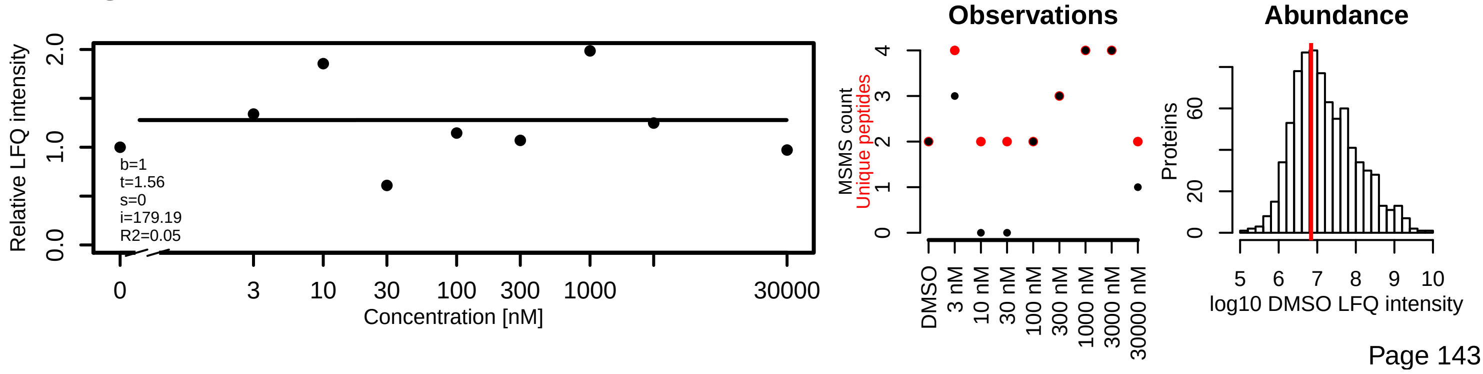

# CCT8

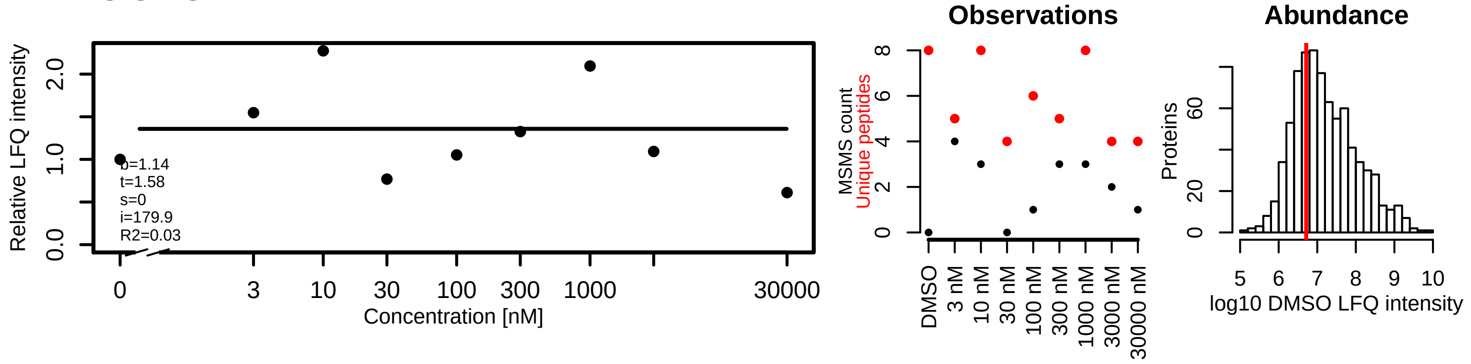


# YTHDF2

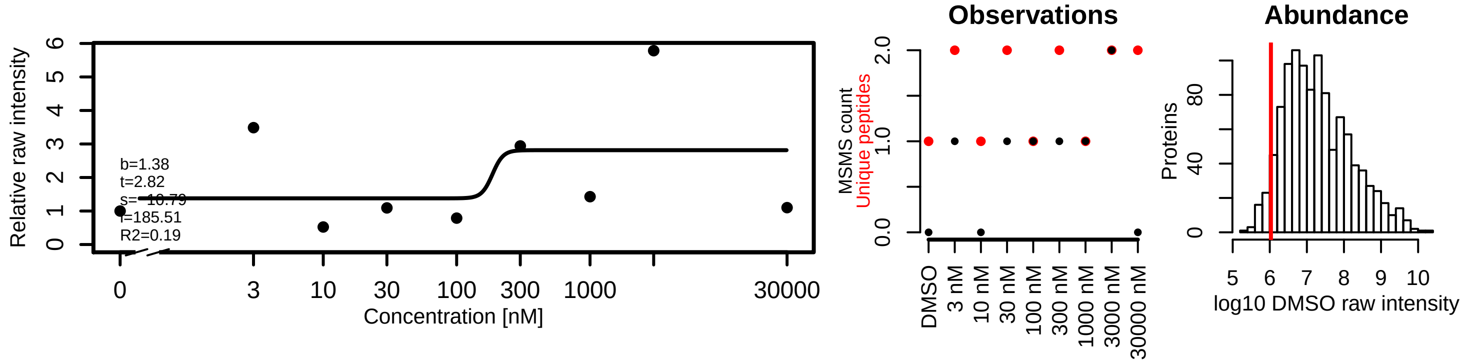


# HCCS

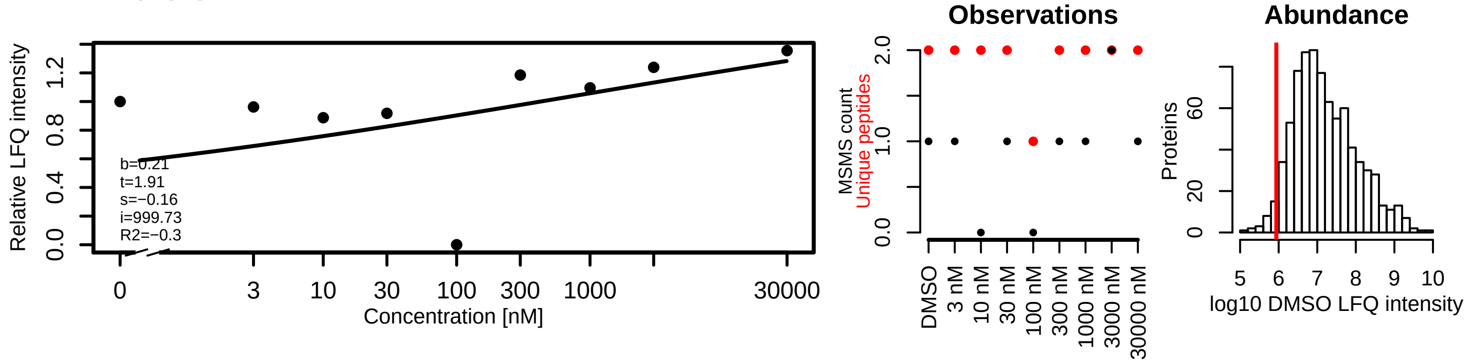


# SRSF9

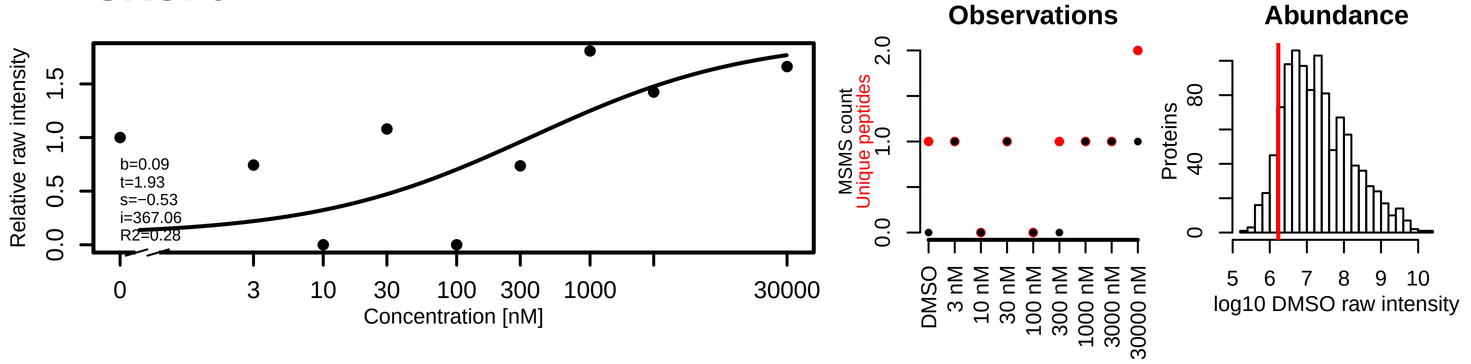


# PRKACA

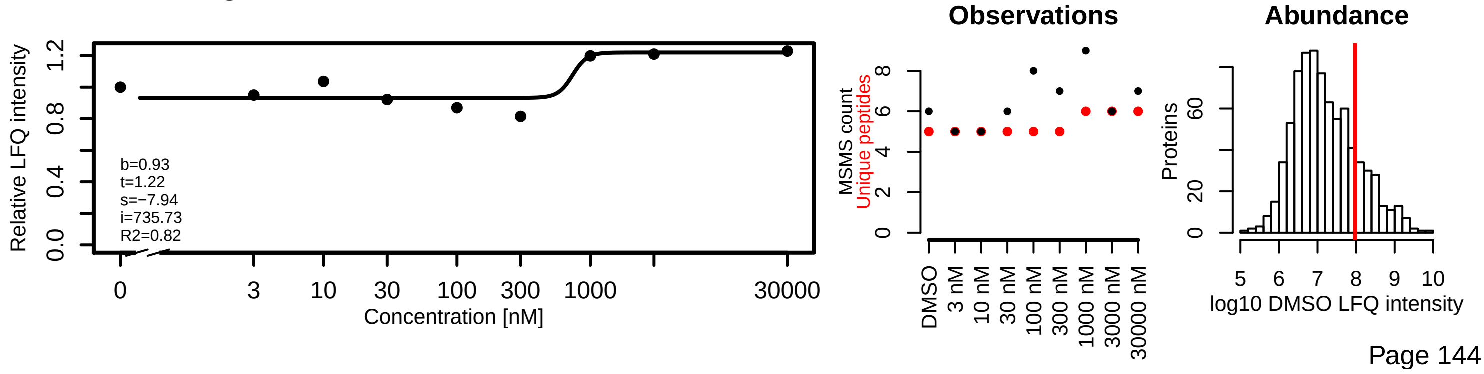

## CSNK2A1;CSNK2A3

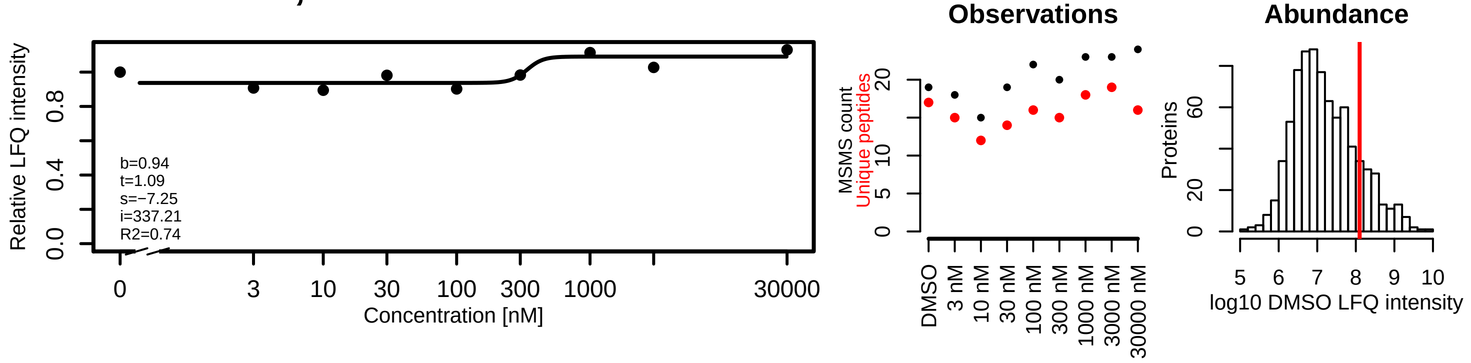


## PRKACB

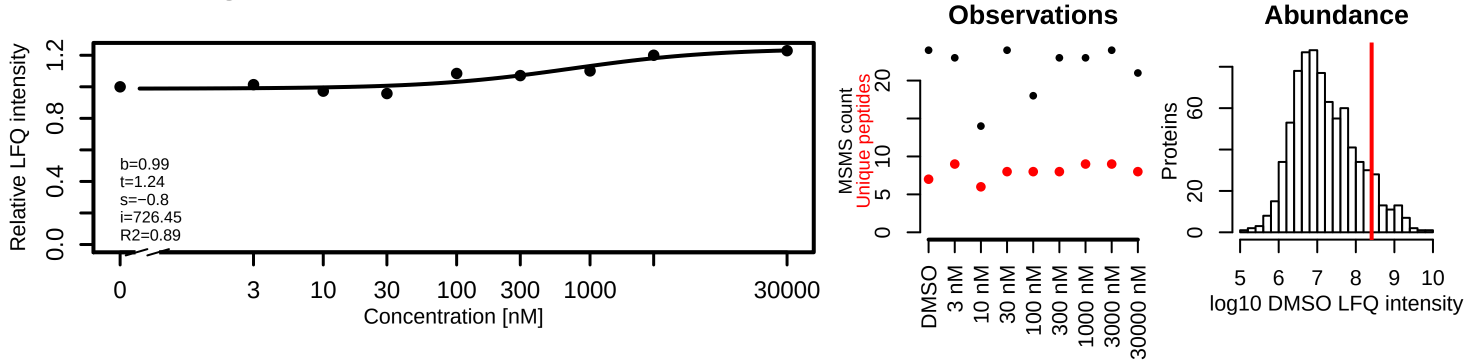


## ARFGEF1;ARFGEF2

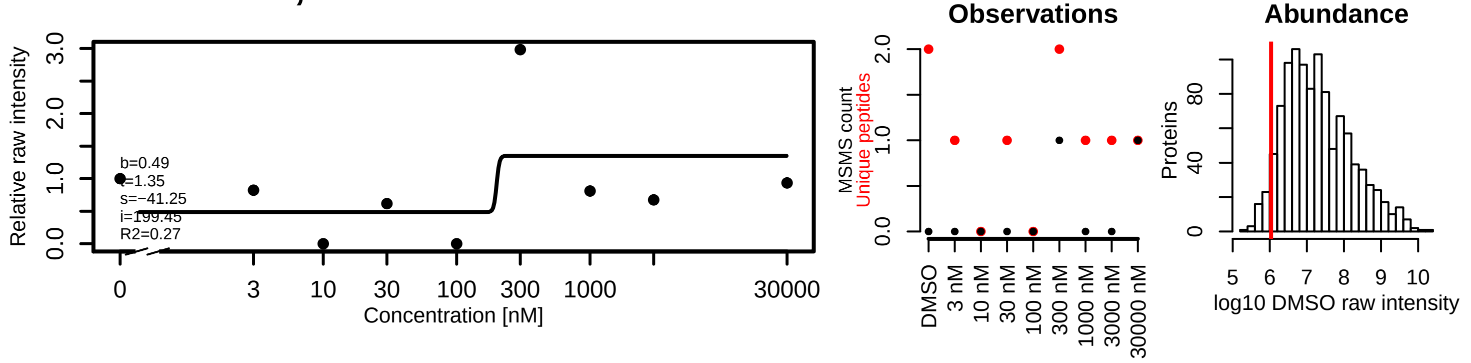


## EIF3J

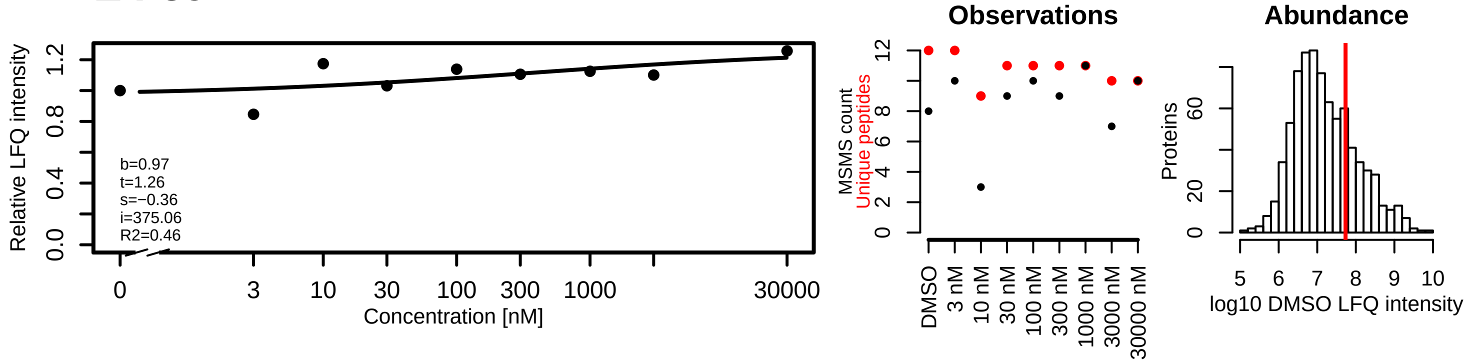


## SNRPF

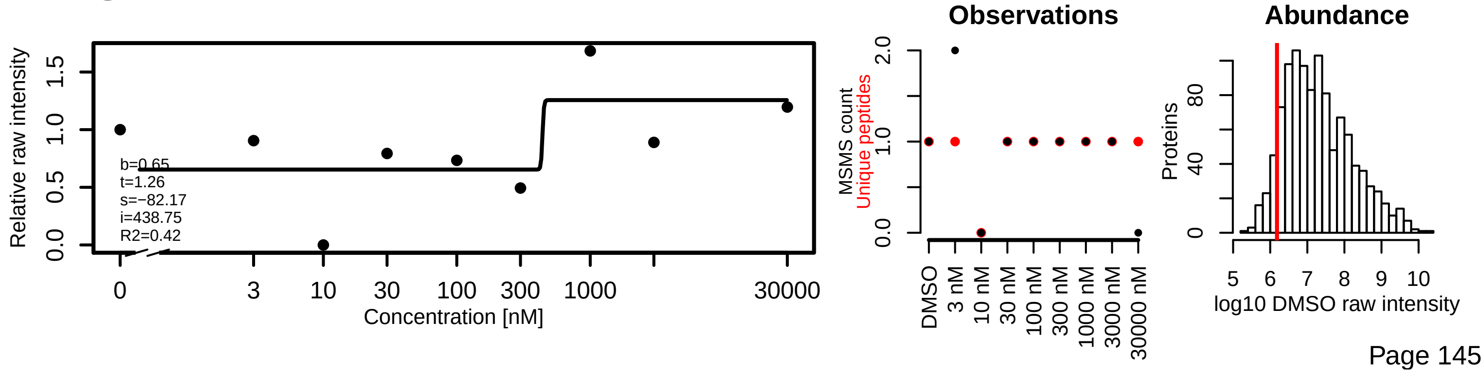

## TTC14

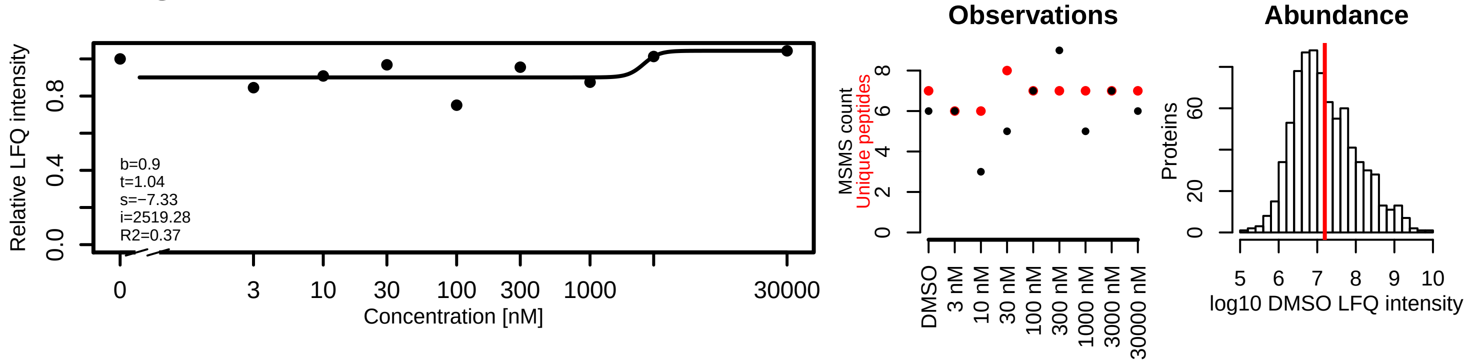


## MAP3K5

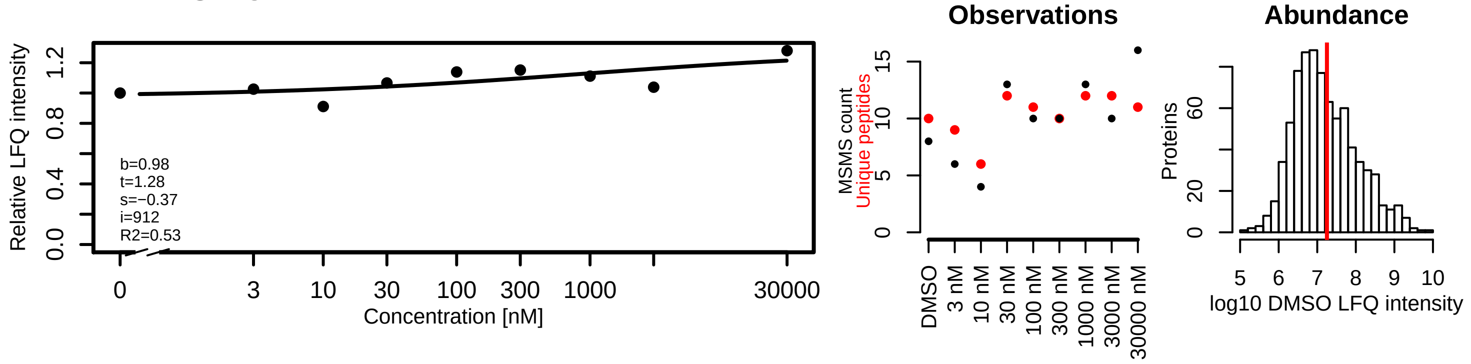


## EEA1

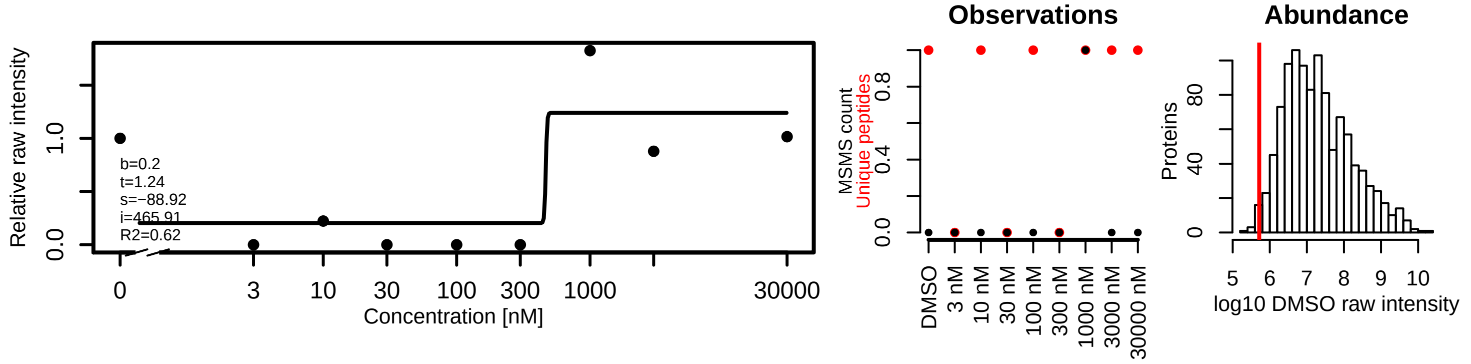


## COX7A2

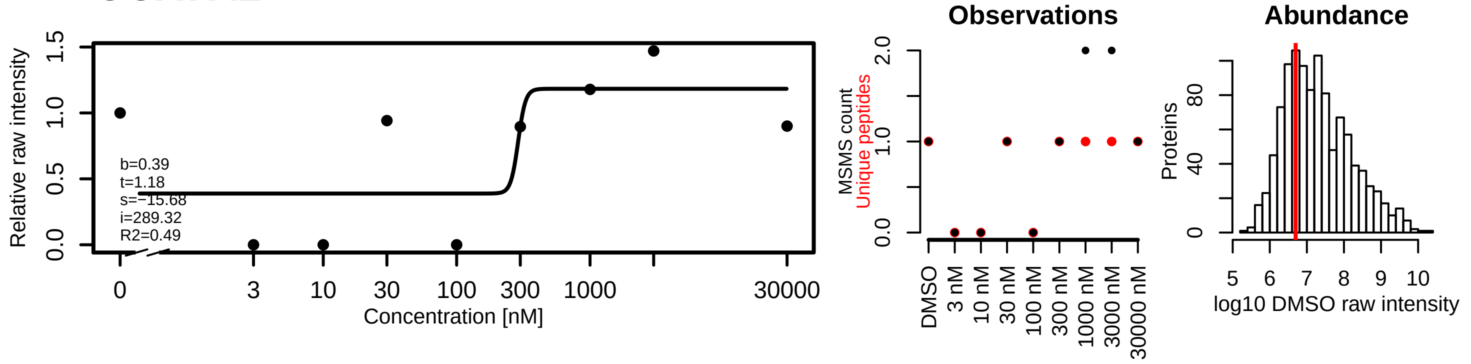


## WDR5

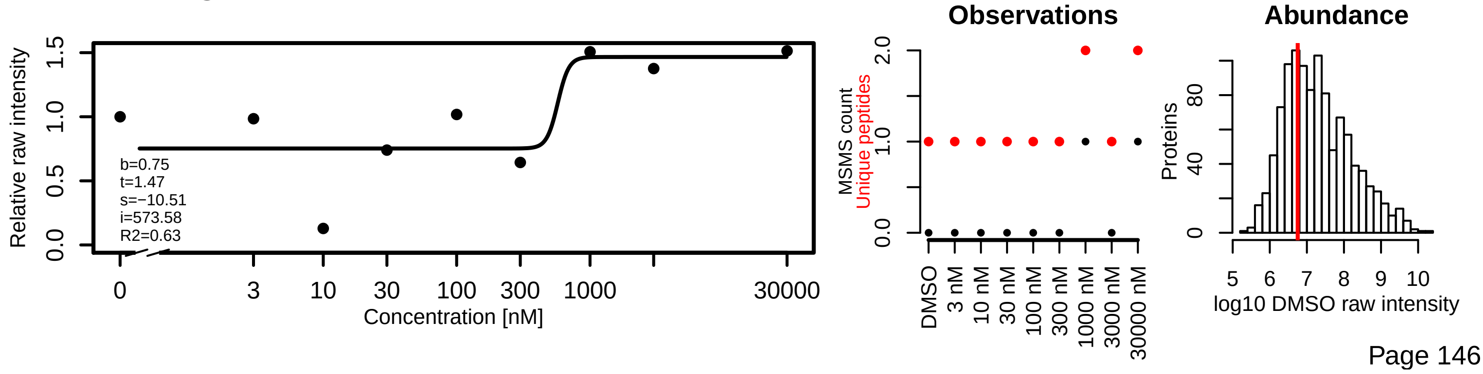

## MBOAT7

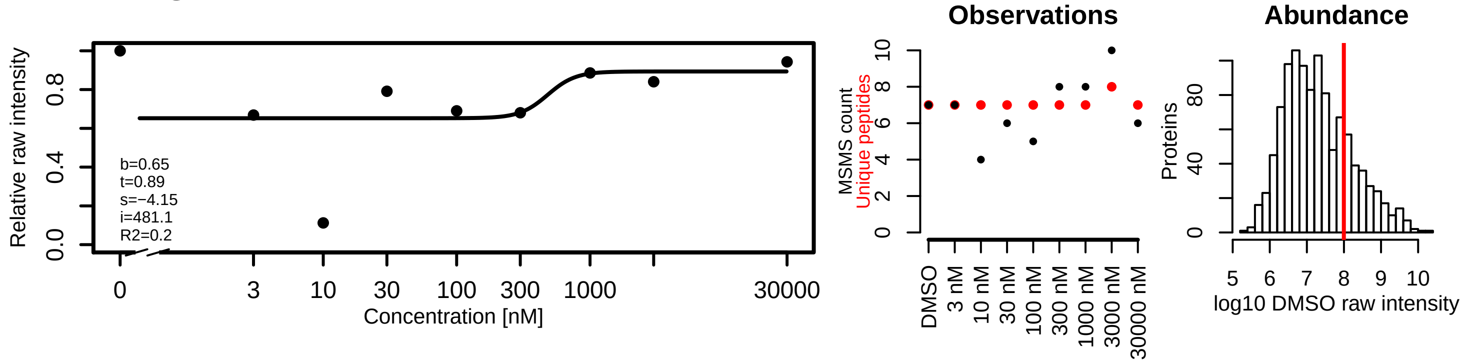


## VDAC3

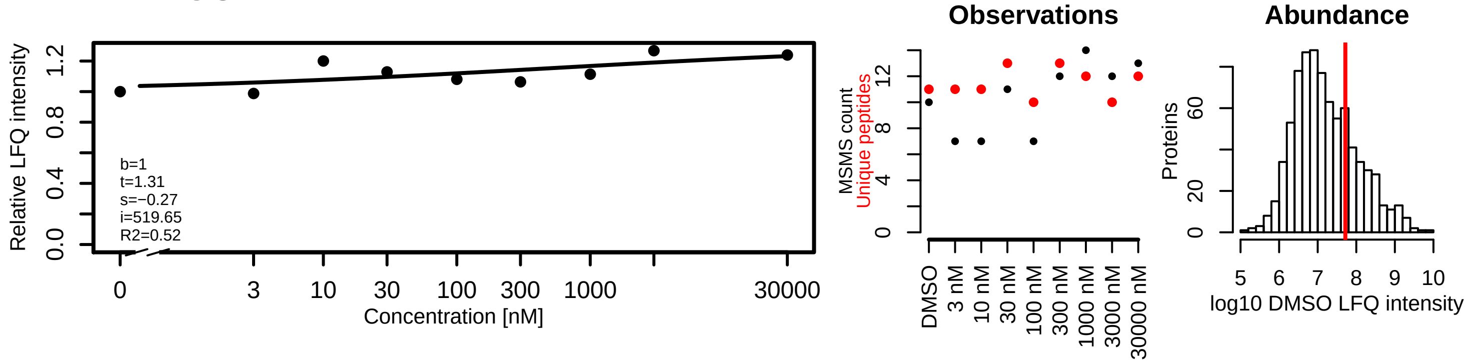


## RPN1

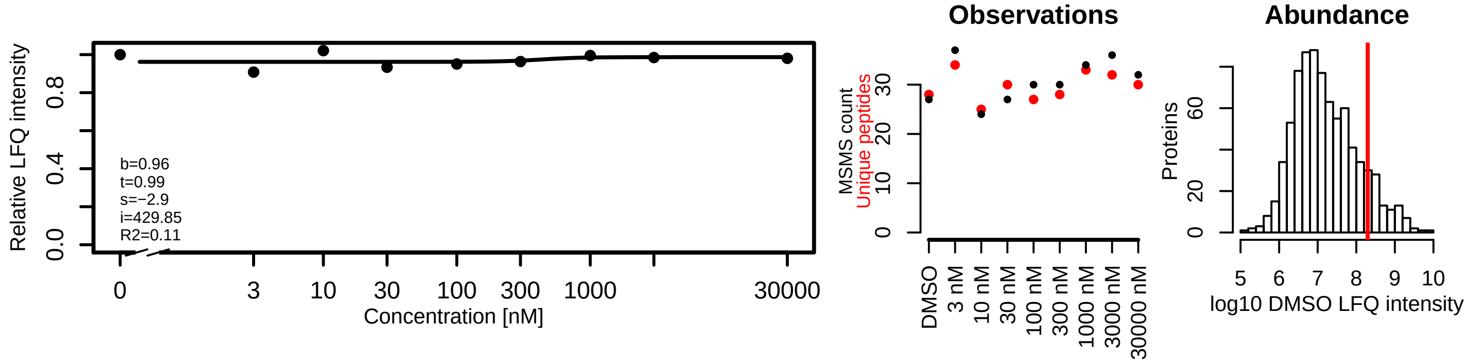


## FN3KRP

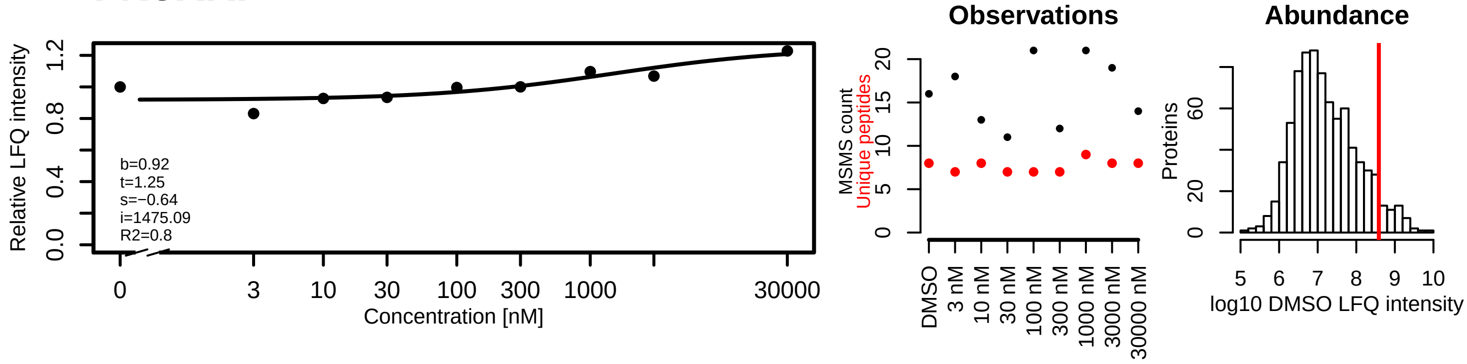


## CCNB2

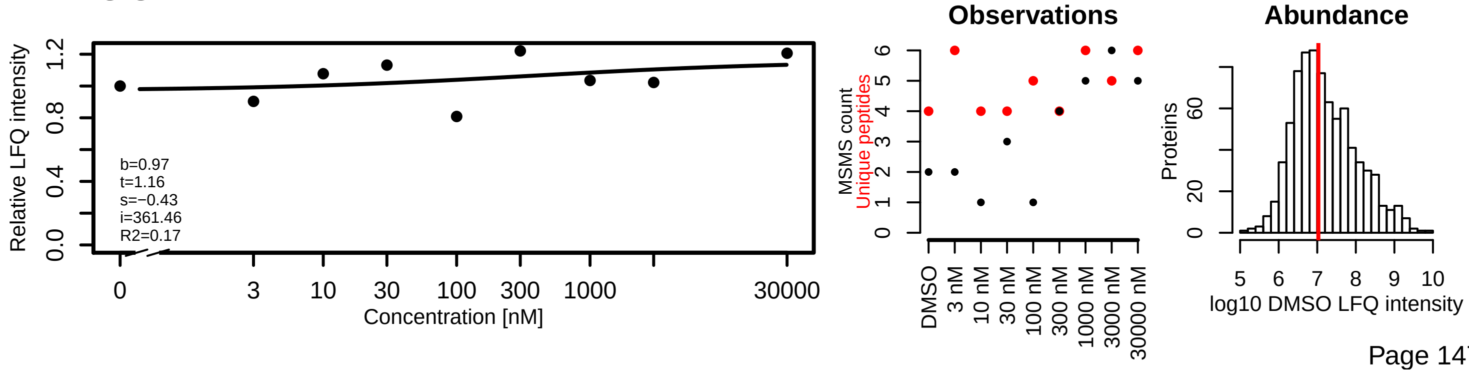

# ATP5A1

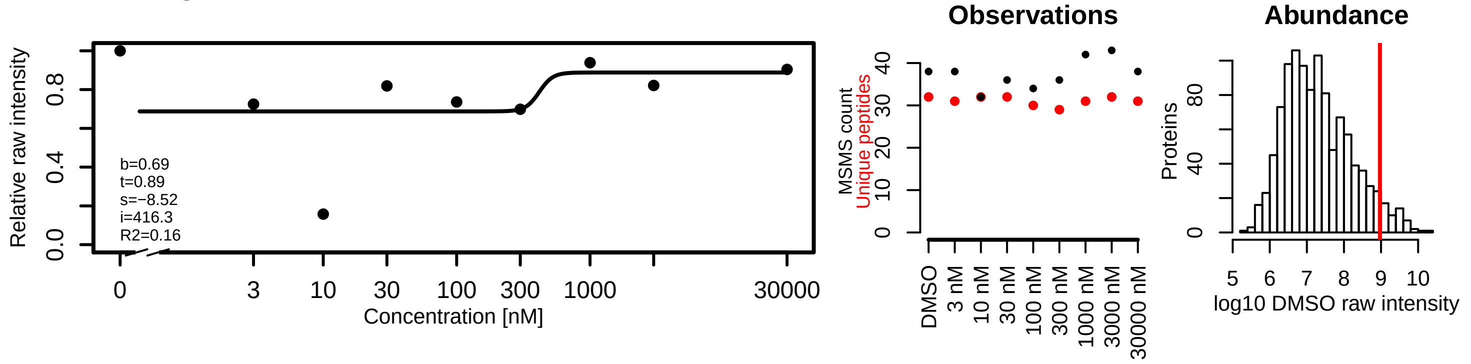


# PRKAA1

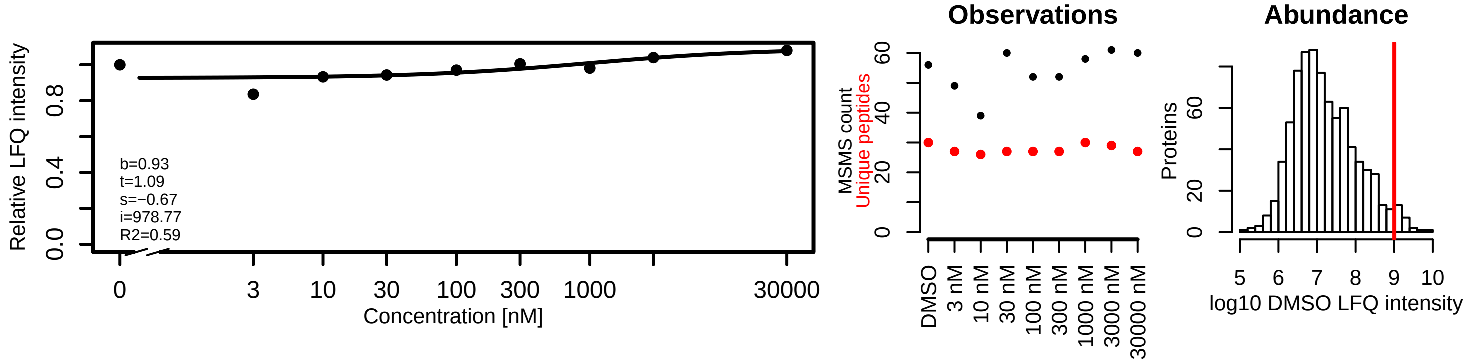


# CCNT2

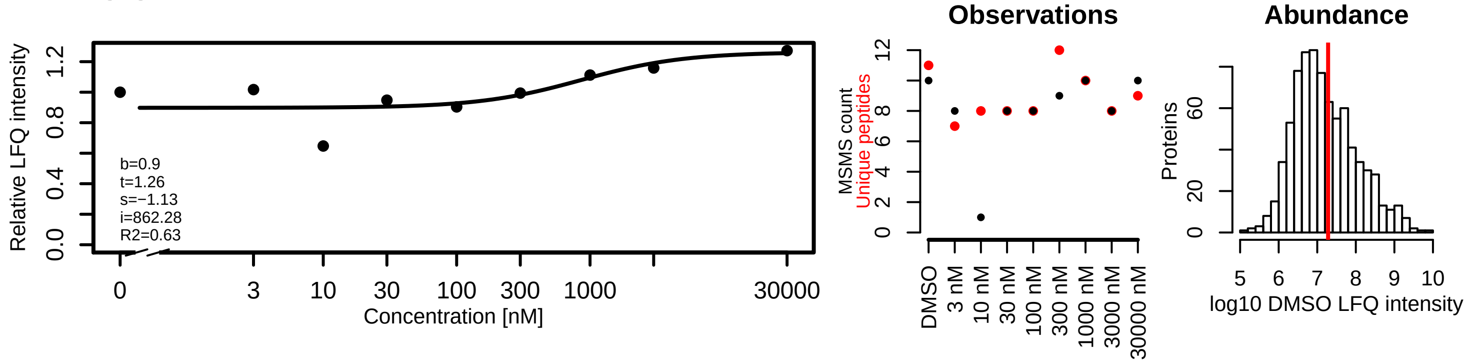


# CDK13

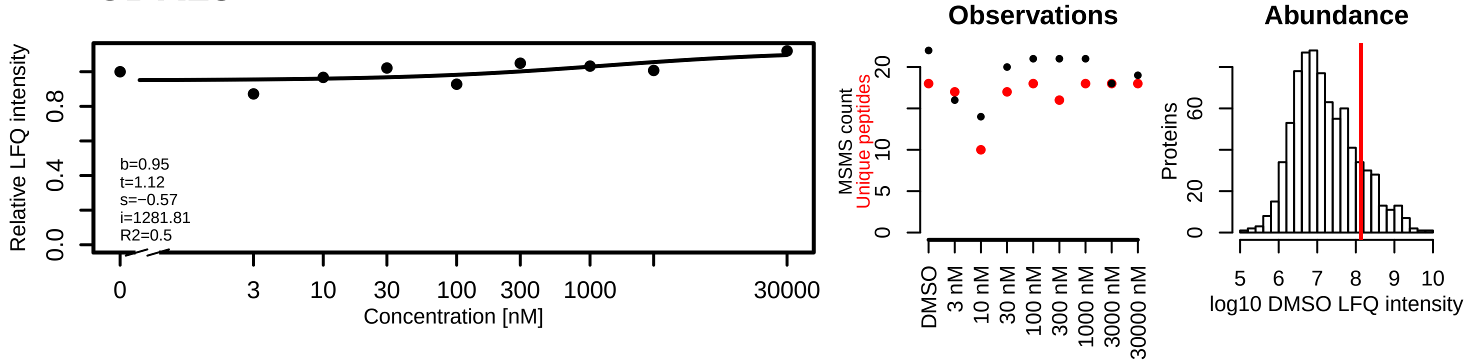


# PCBP1

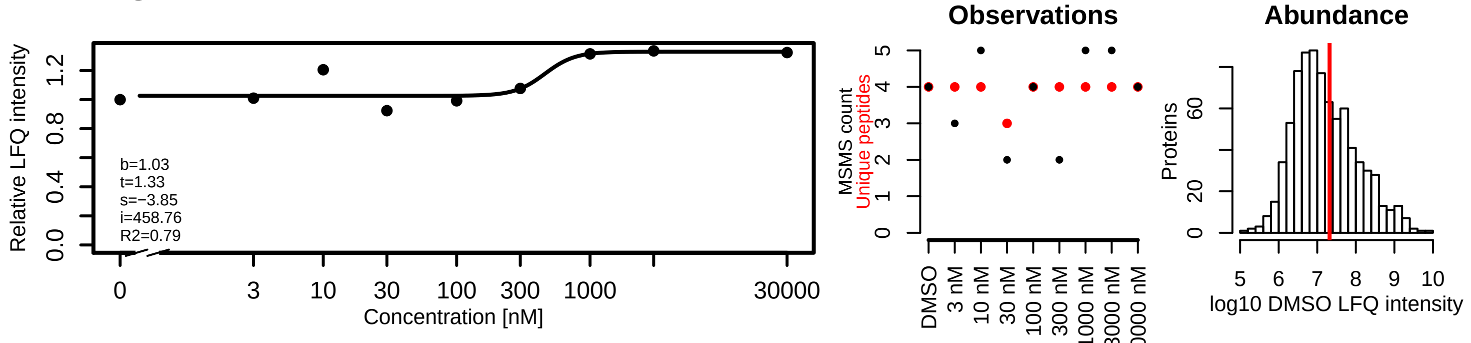

# PRKCA

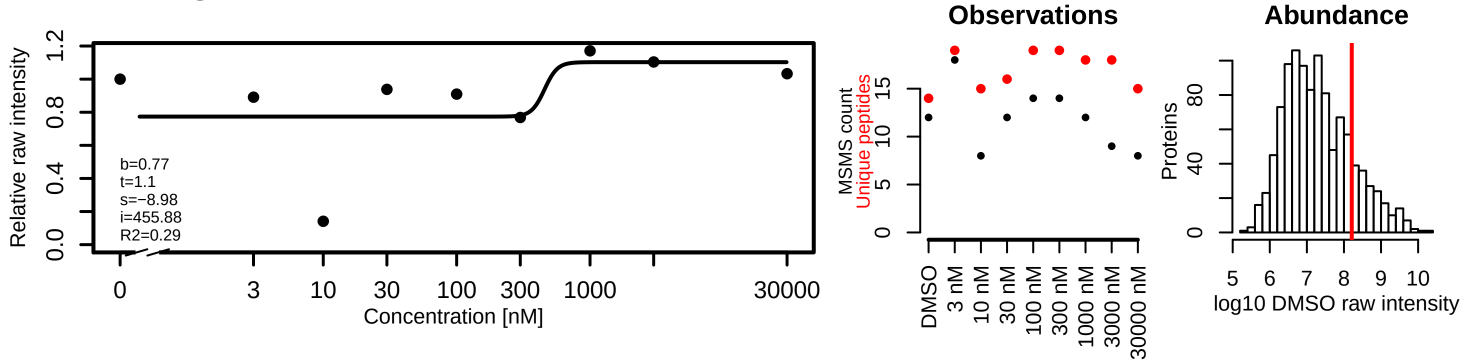


# YIPF5

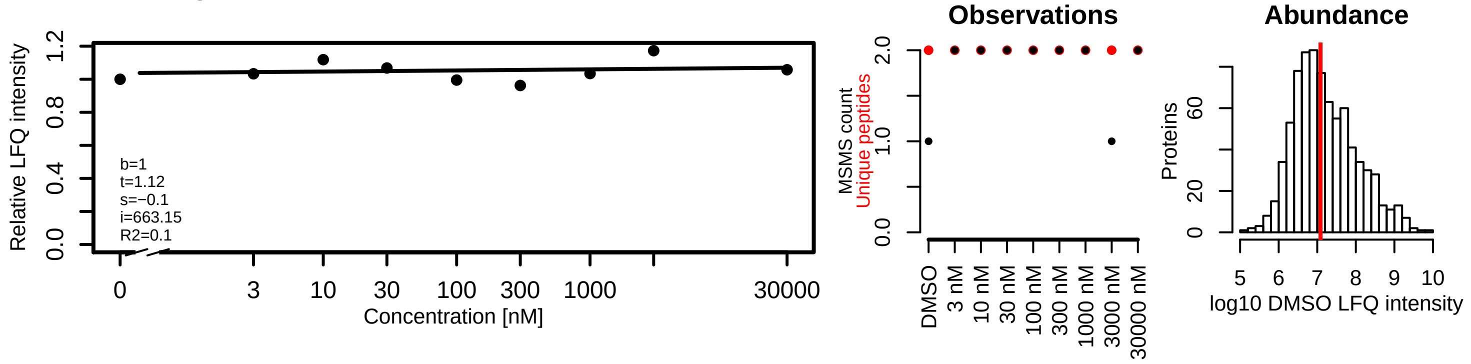


# CERS2

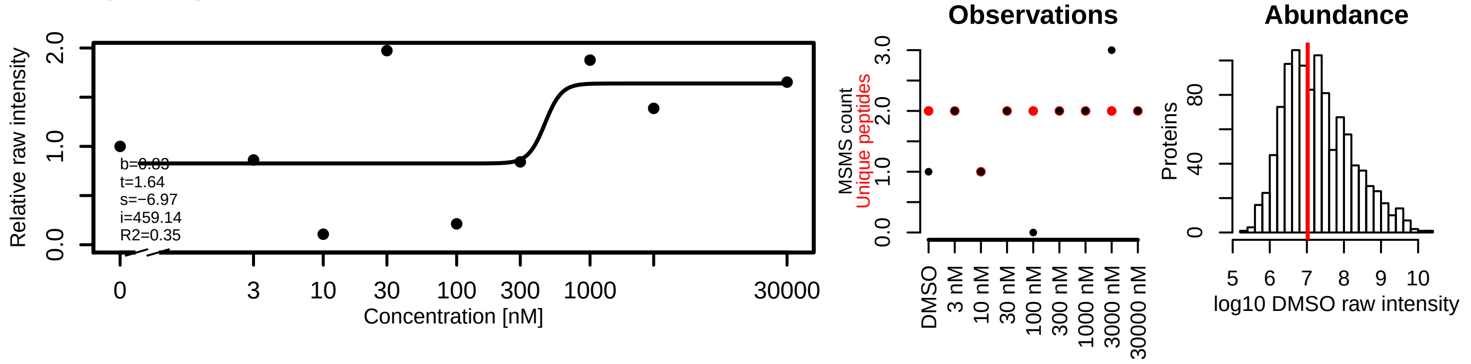


# CSNK2B

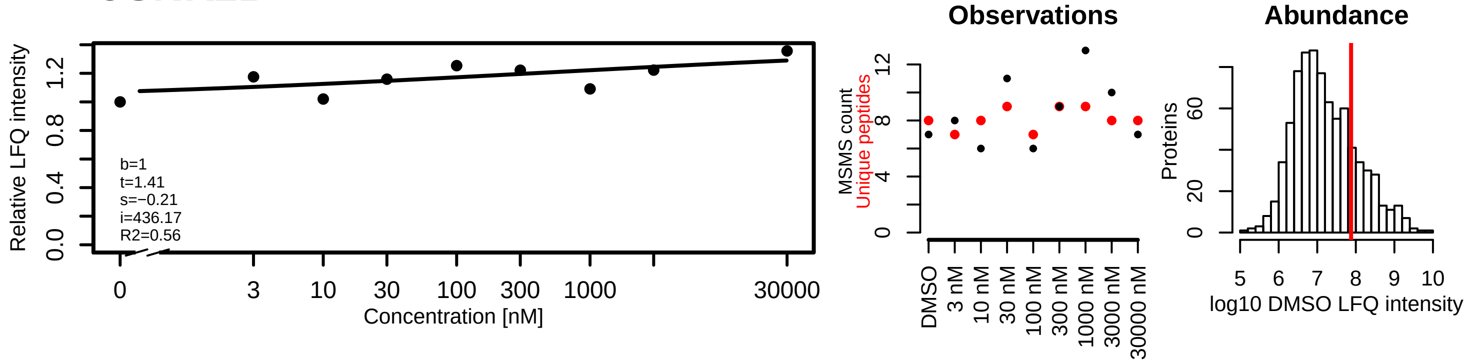


# STARD3NL

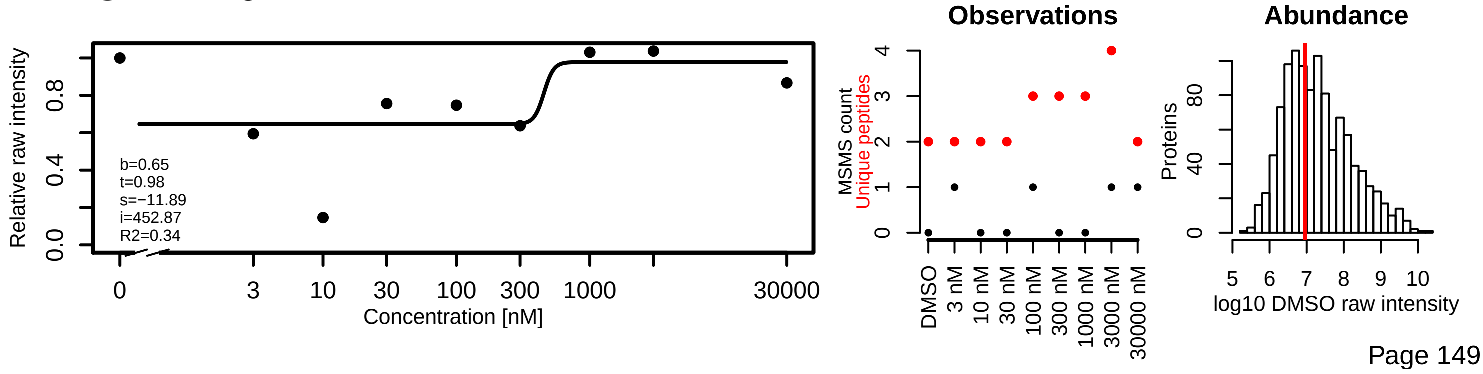

## RPS5

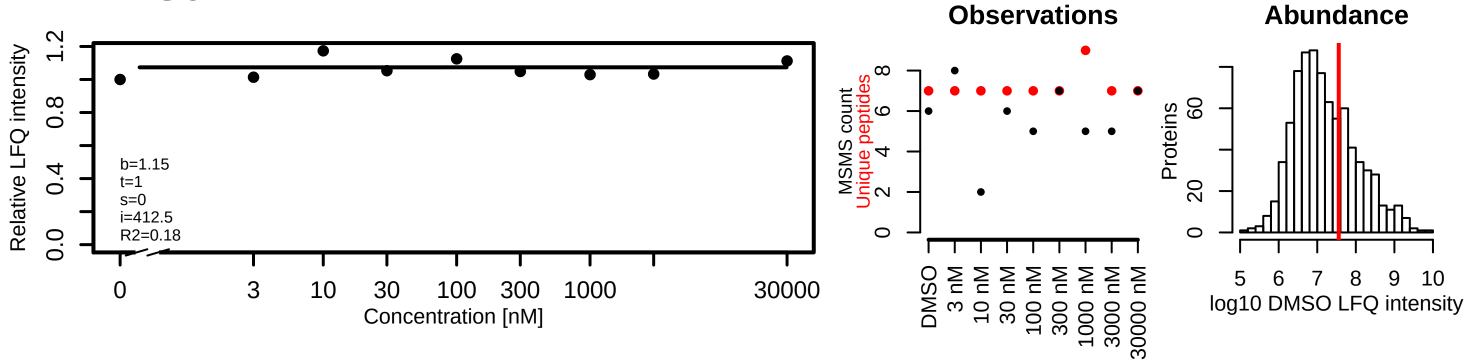


## DAZAP1

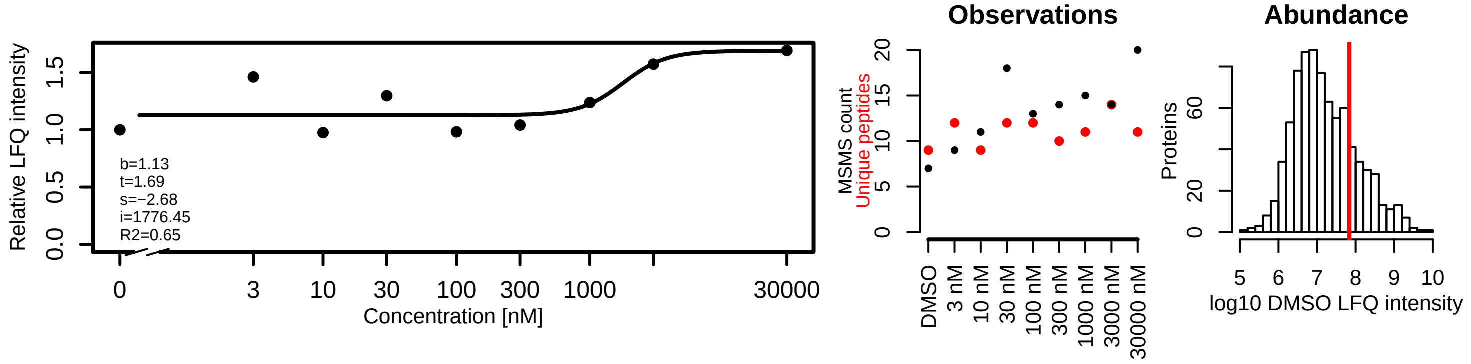


## GPNMB

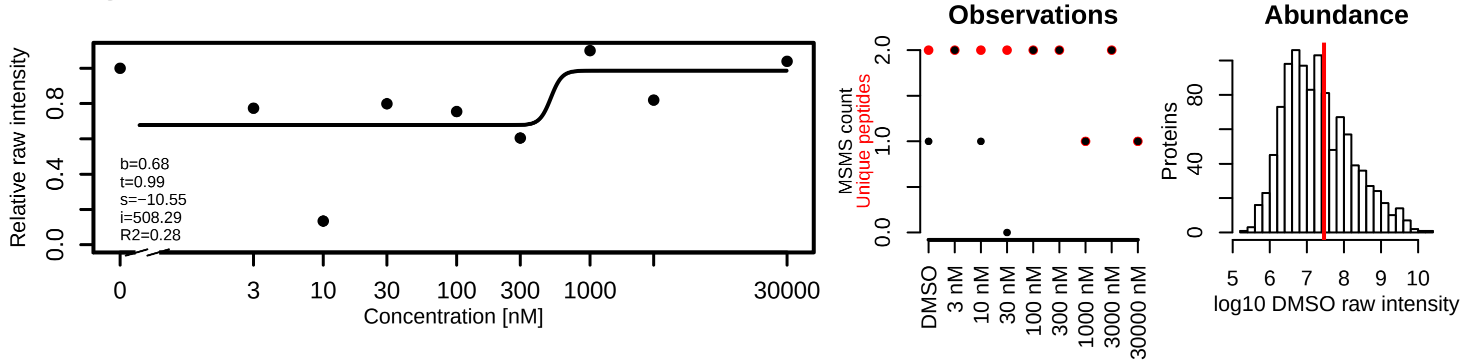


## SEPT7;DKFZp586I031

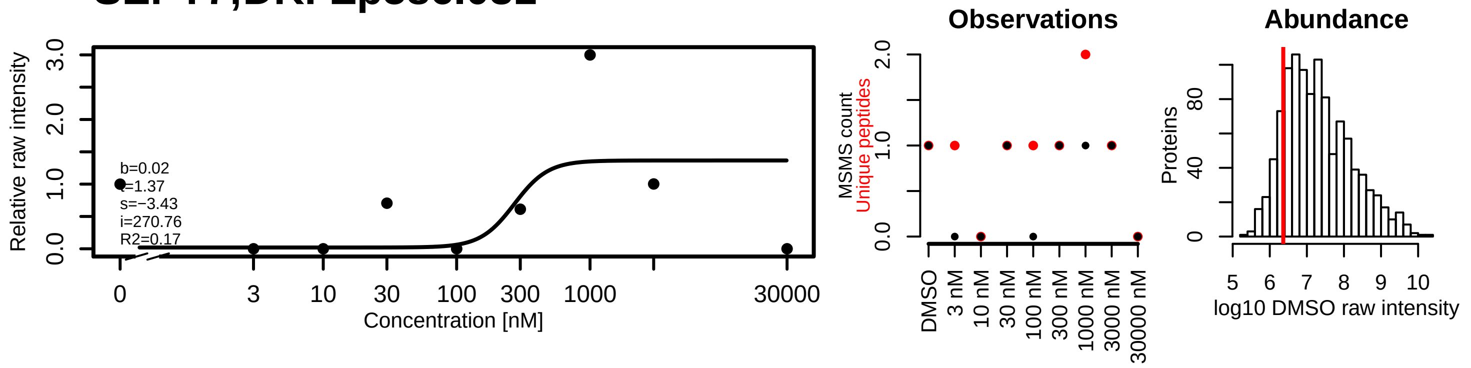


## NEK9

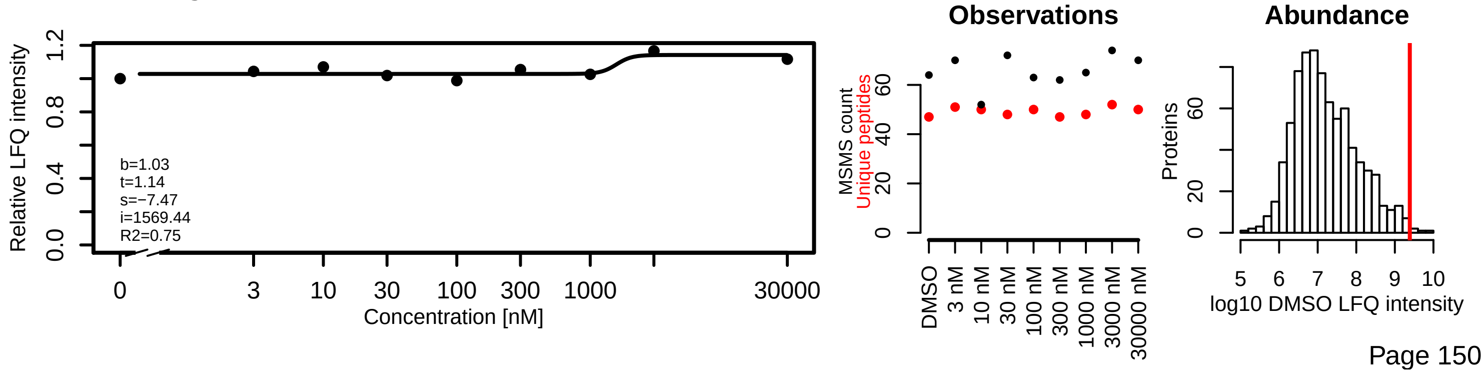

# HNRNPH1

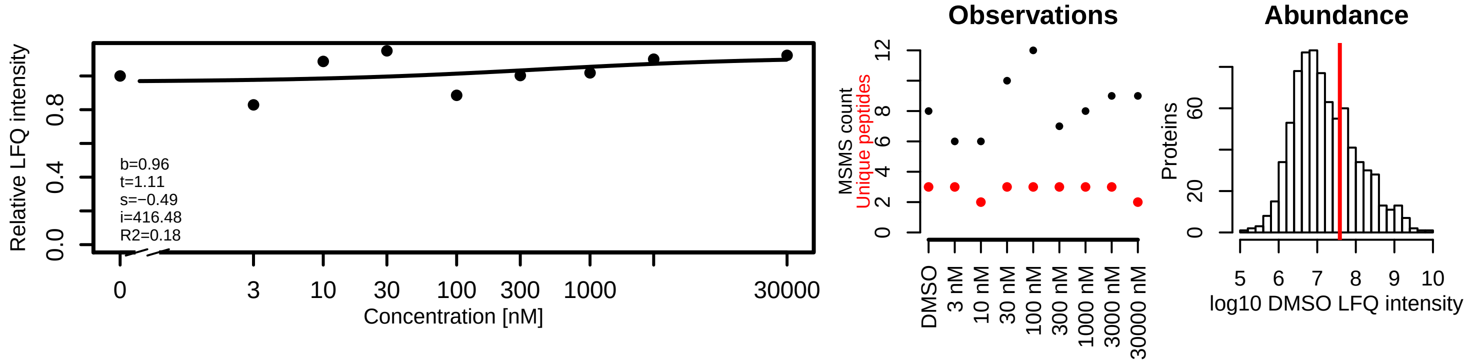


# HSPE1

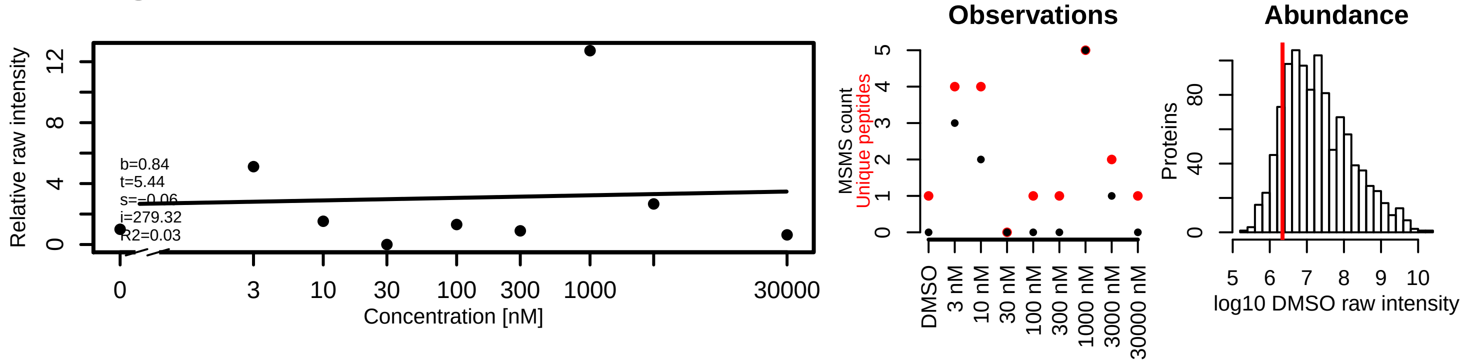


# ATPIF1

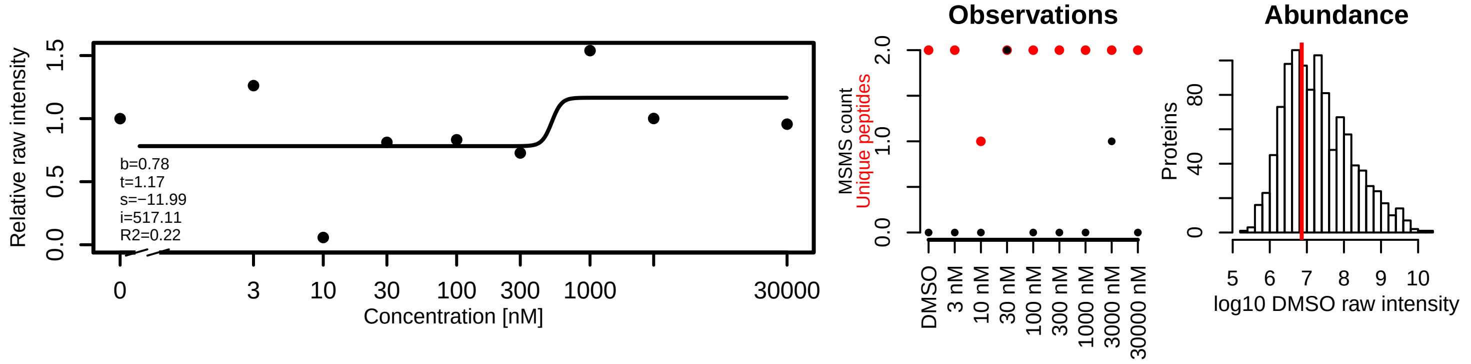


# RABL3

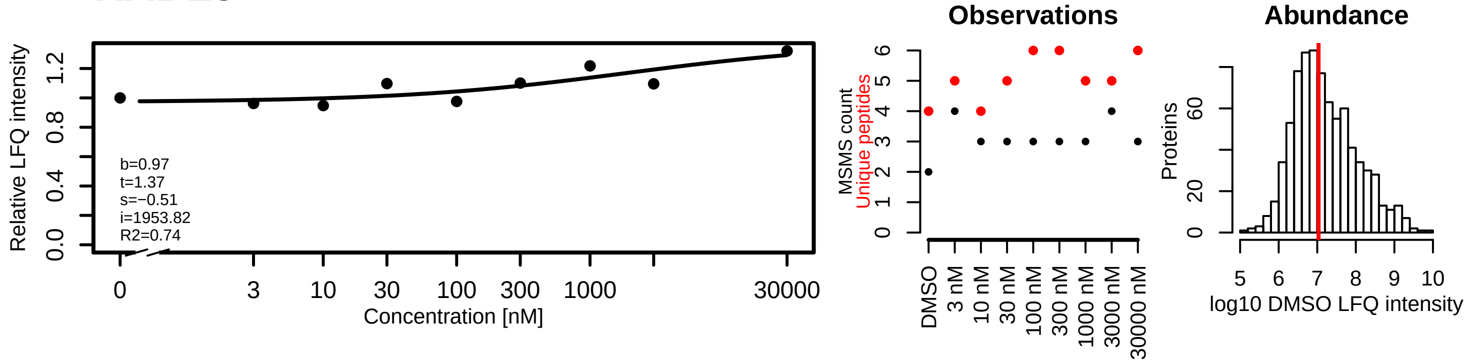


# SPTAN1

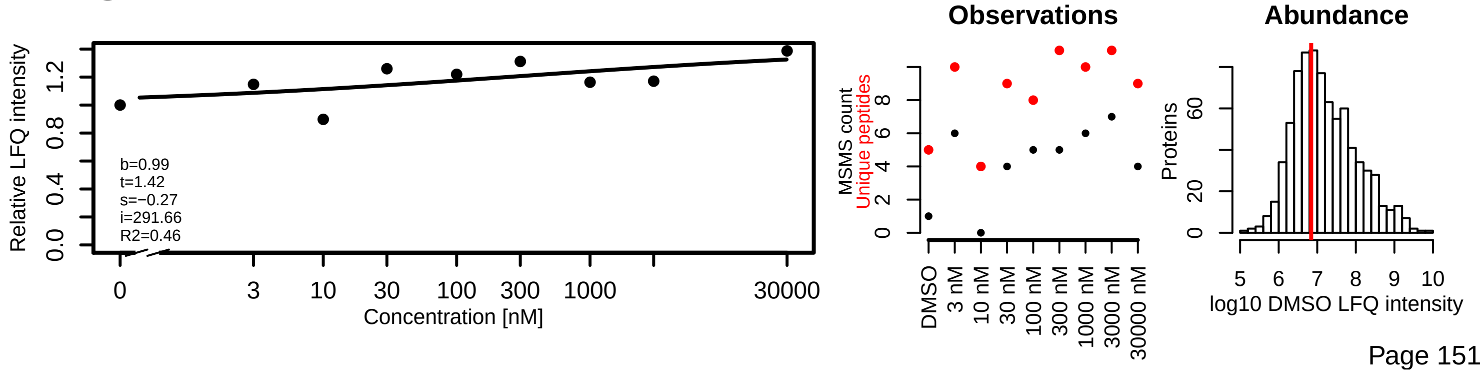

## LMBRD2

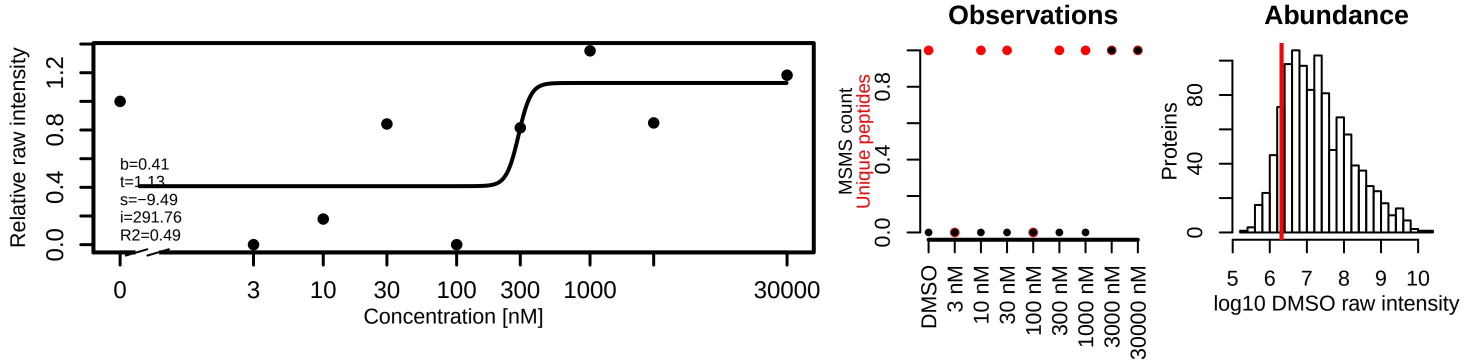


## KIAA1524

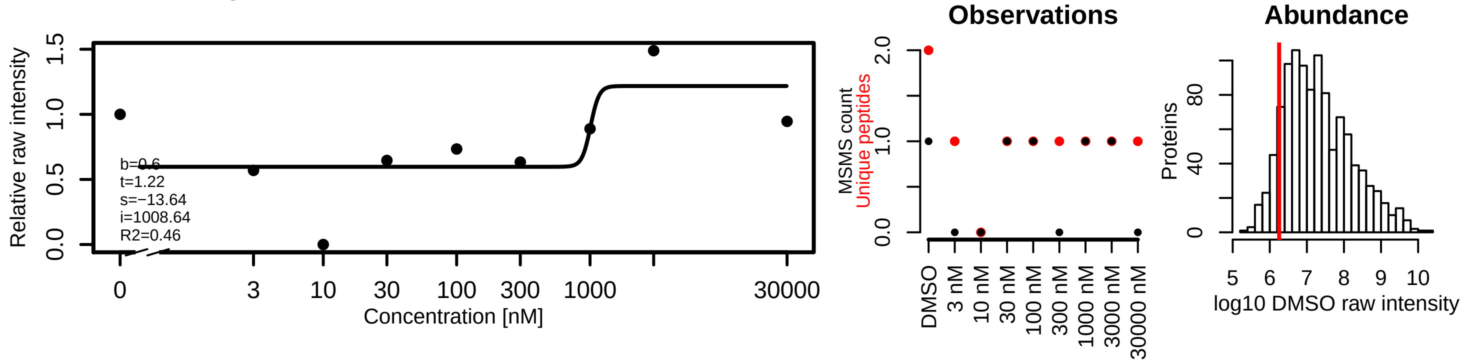


## CTSD

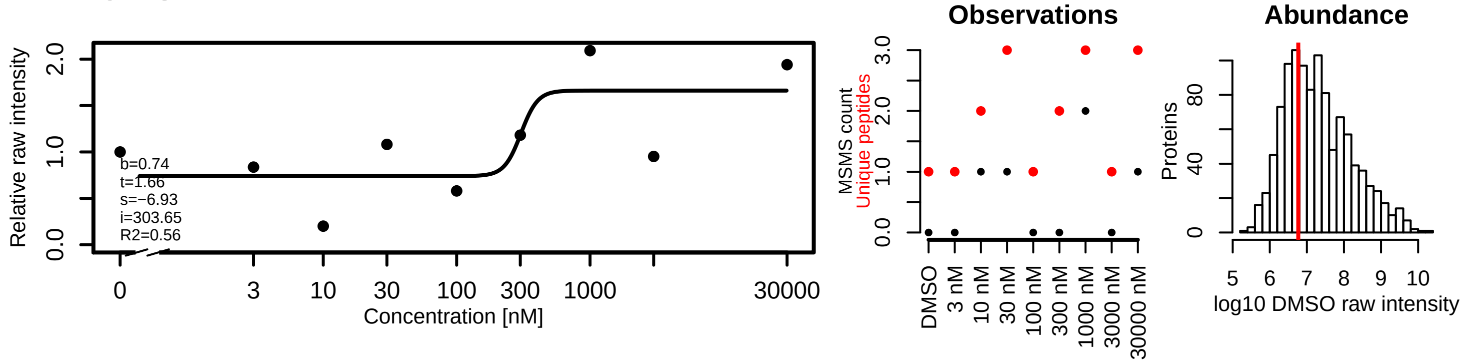


## CAPZA2

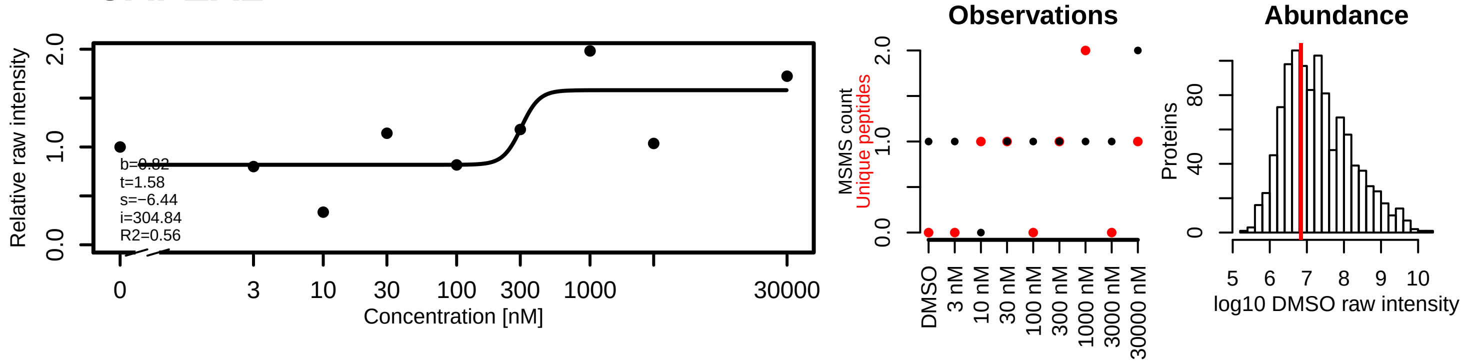


## CHCHD3

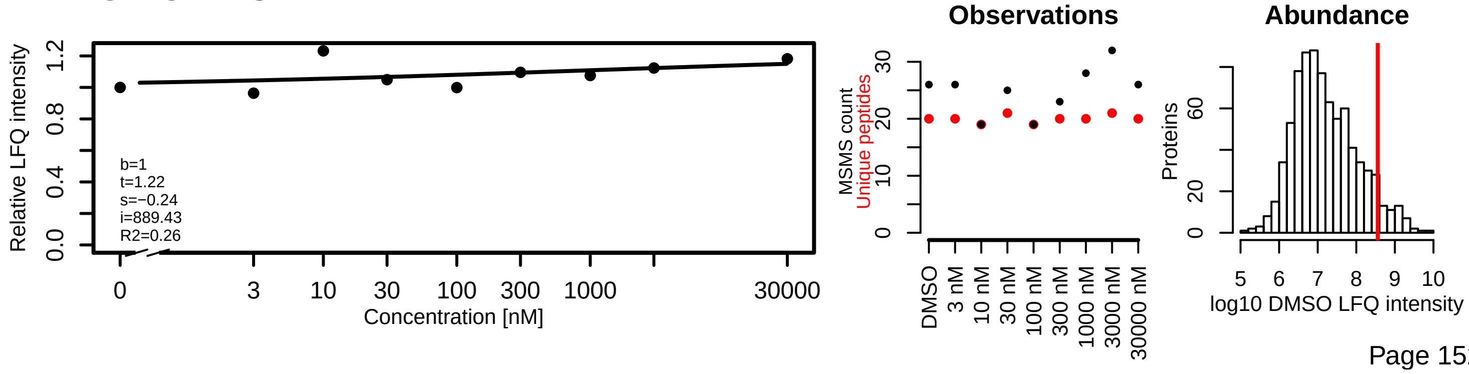

# AKT1

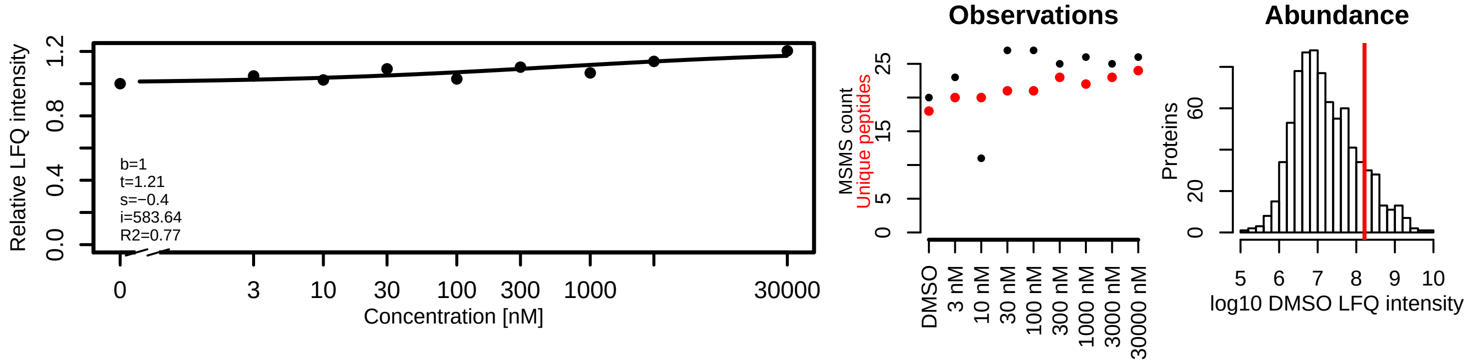


# KARS

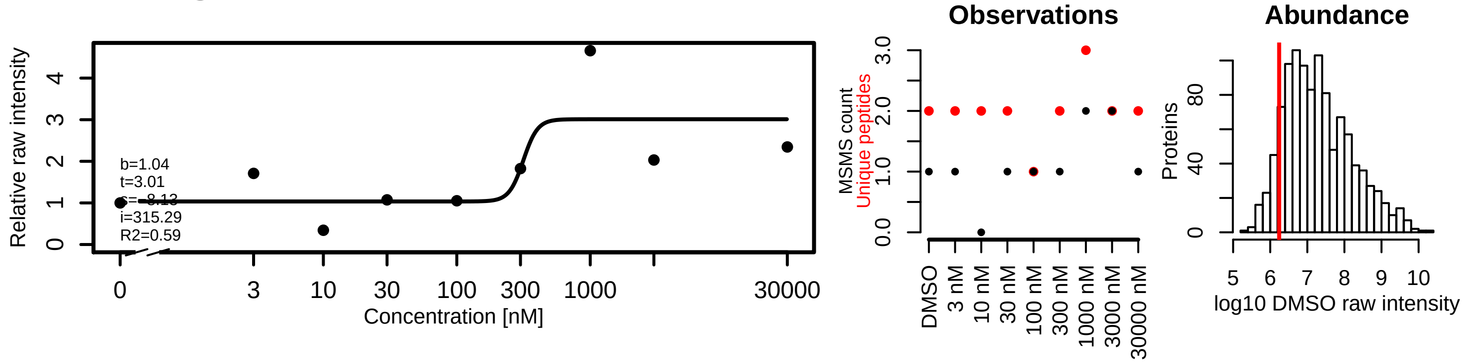


# HNRNPF

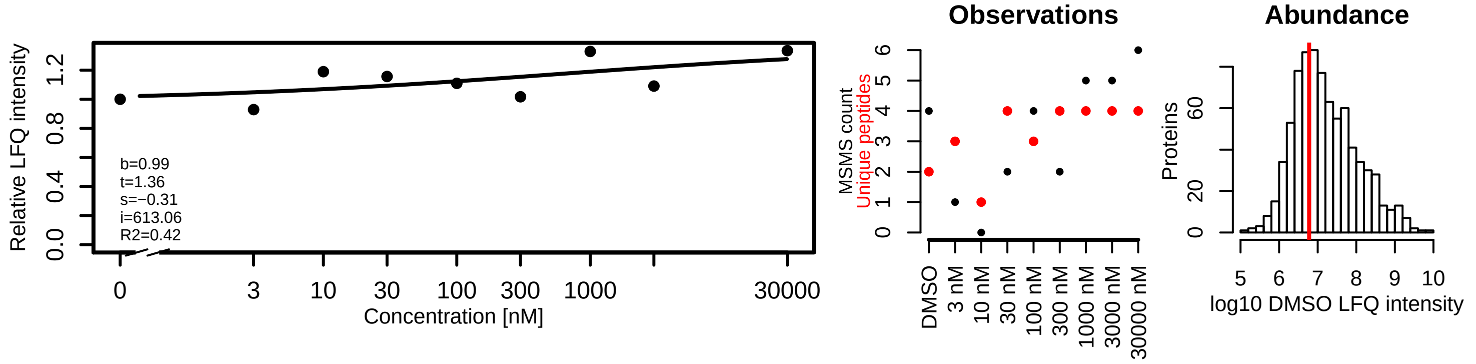


# SRP9

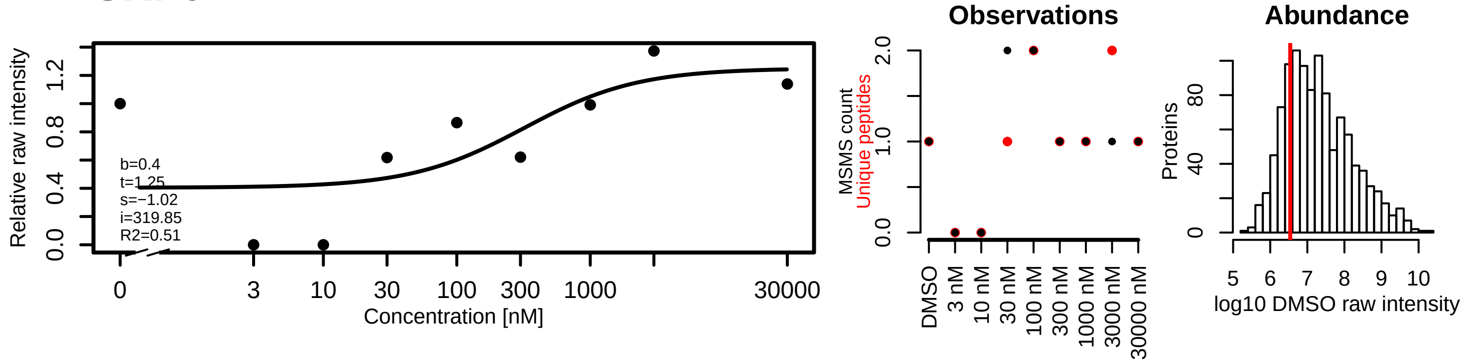


# MRPL46

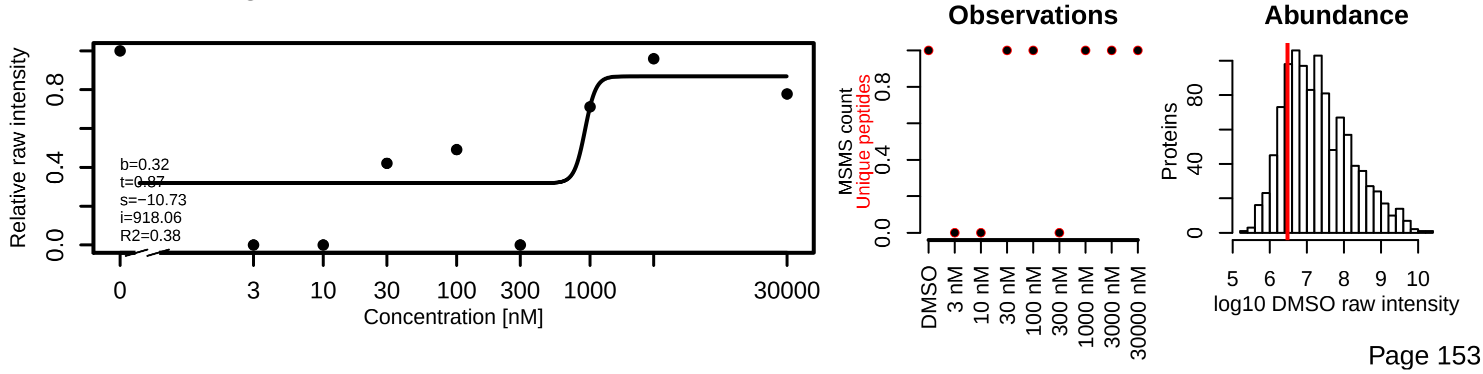

## CIT − O14578−4;O14578;O14578−2;H0YGG8

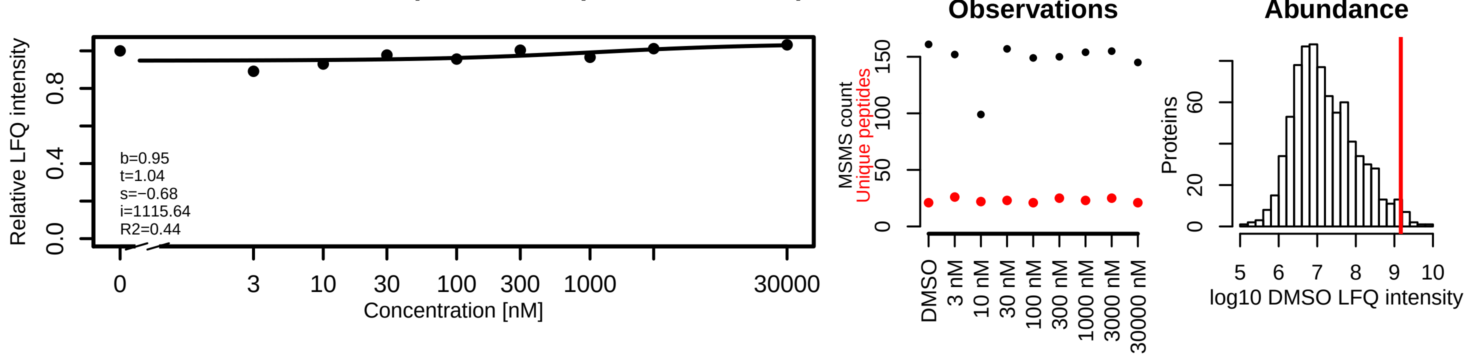


## GSK3B

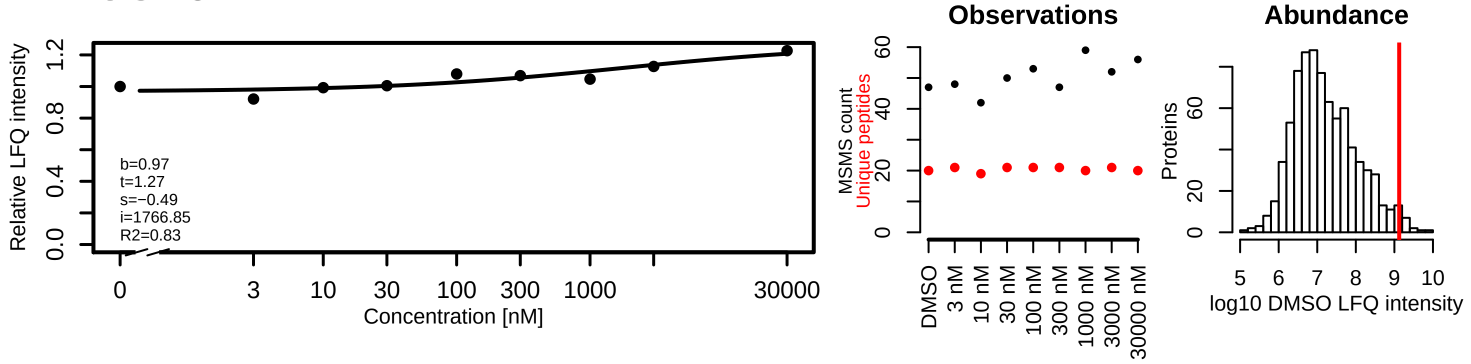


## EIF3L

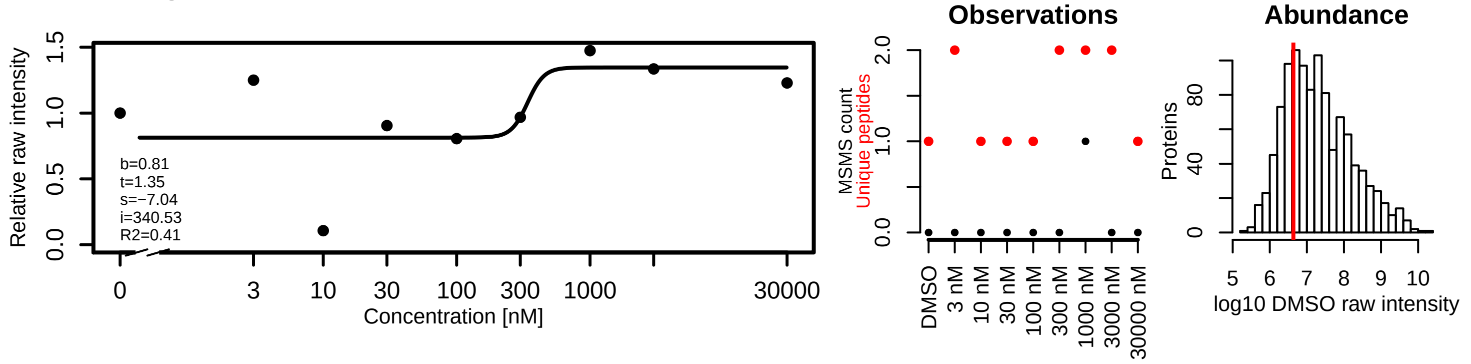


## CCT2

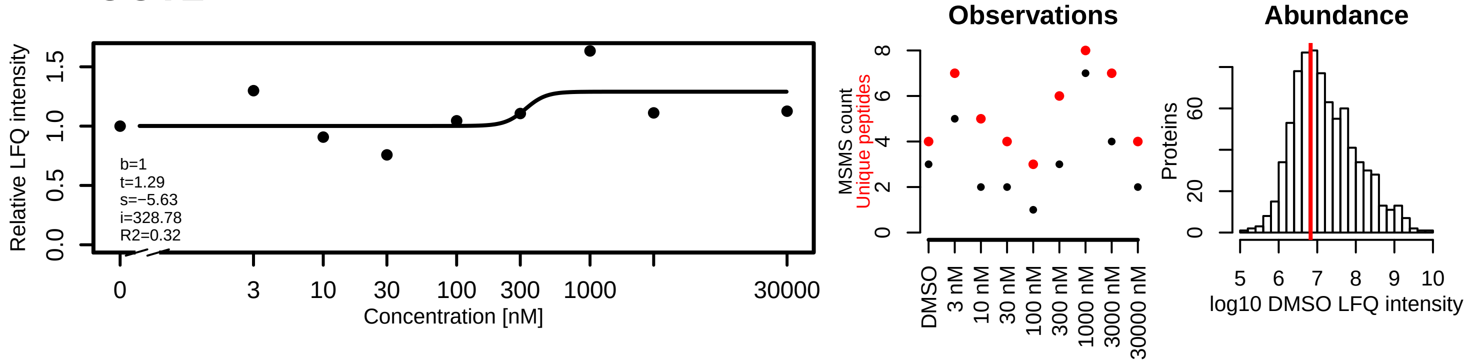


## ABCB1

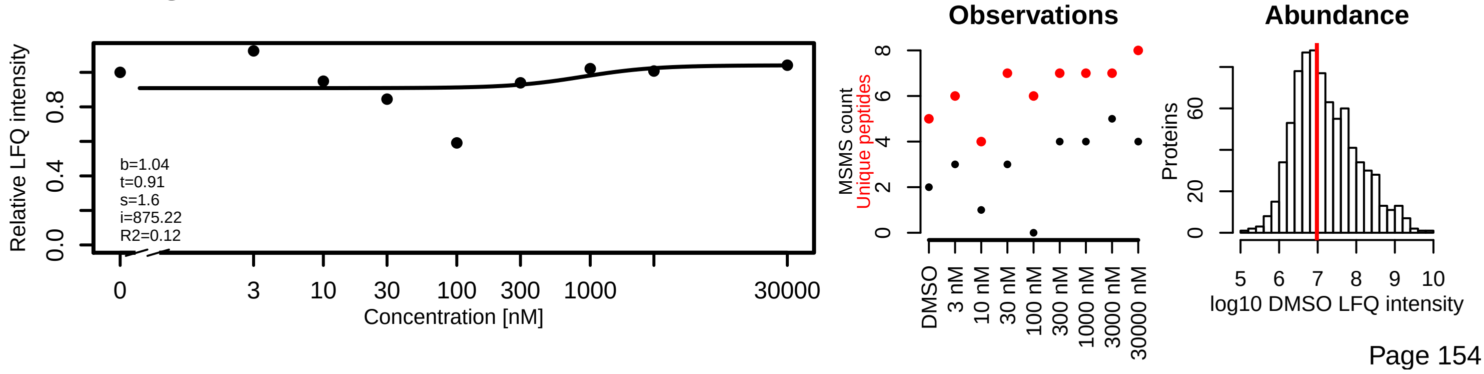

# RPL3

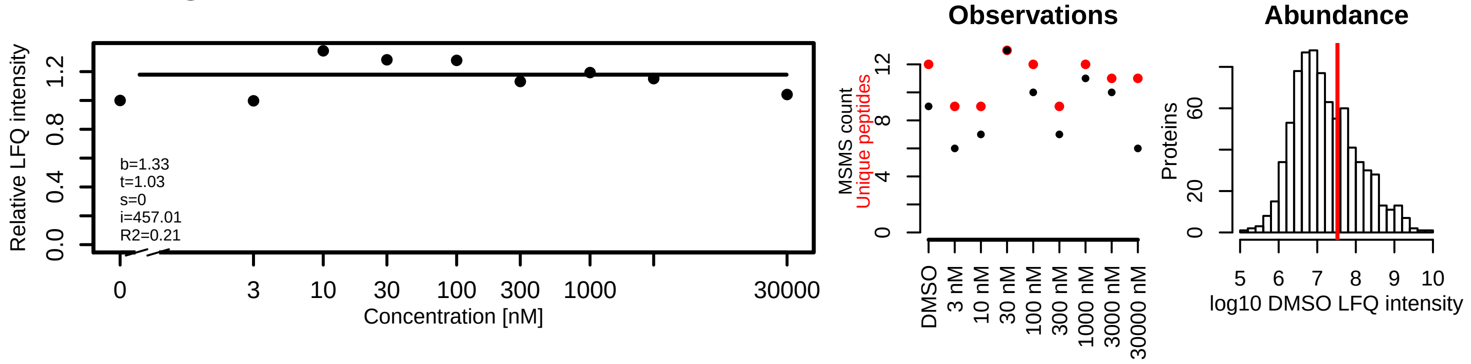


# CAMK2D − E9PF82;E9PBG7;Q9UQM7;Q9UQM7−2

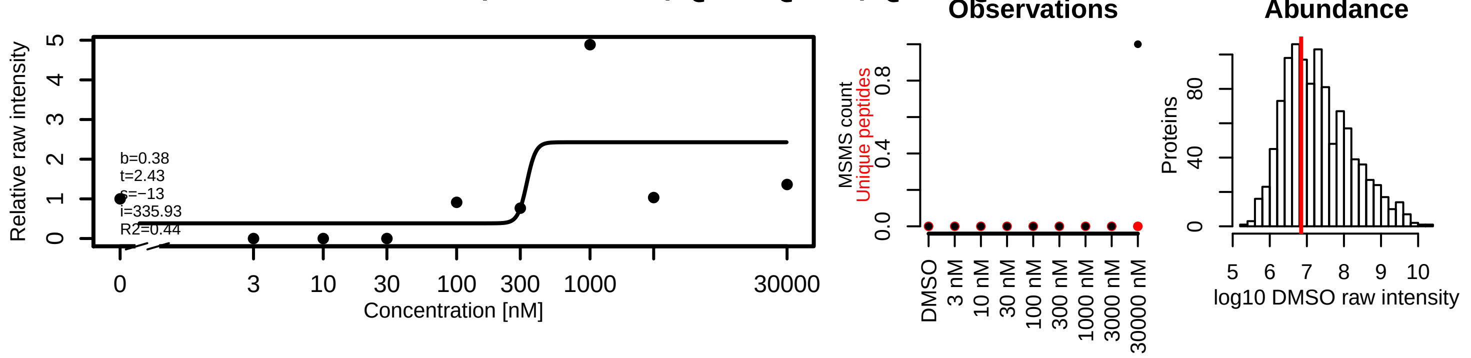


# TM9SF3

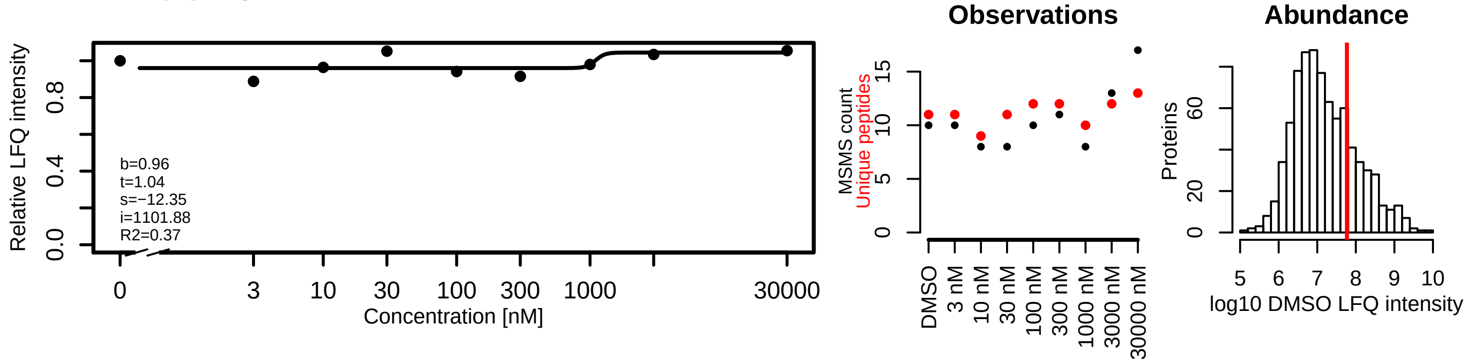


# HNRNPL

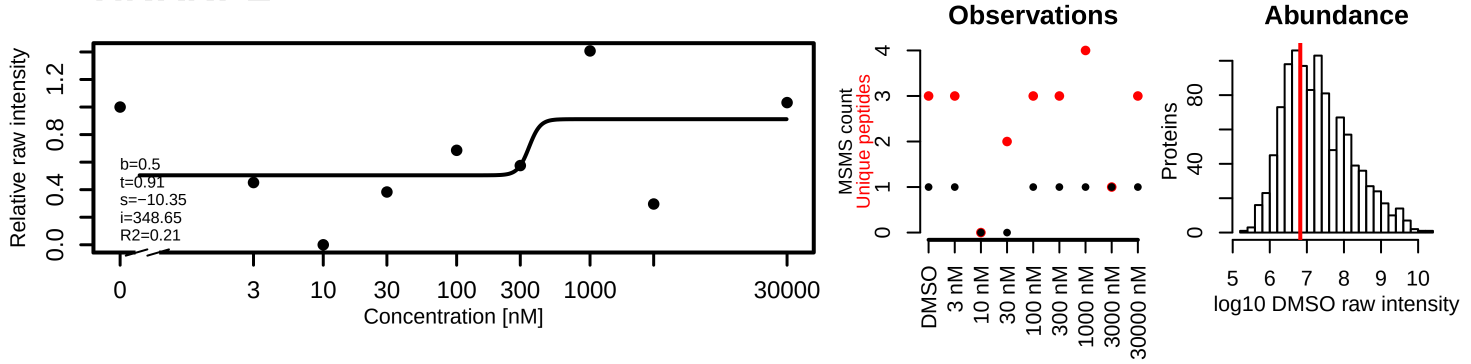


# GGCX

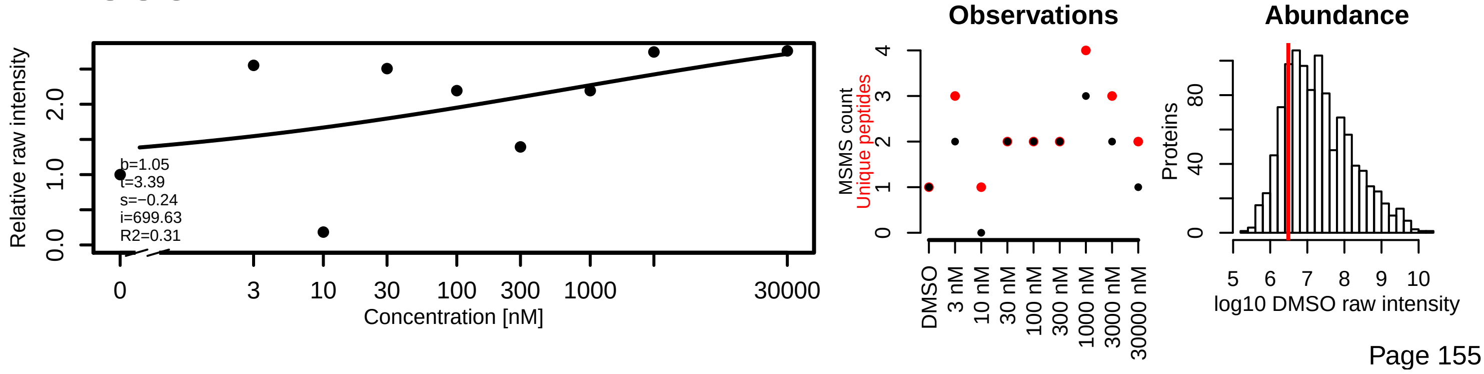

# LASP1

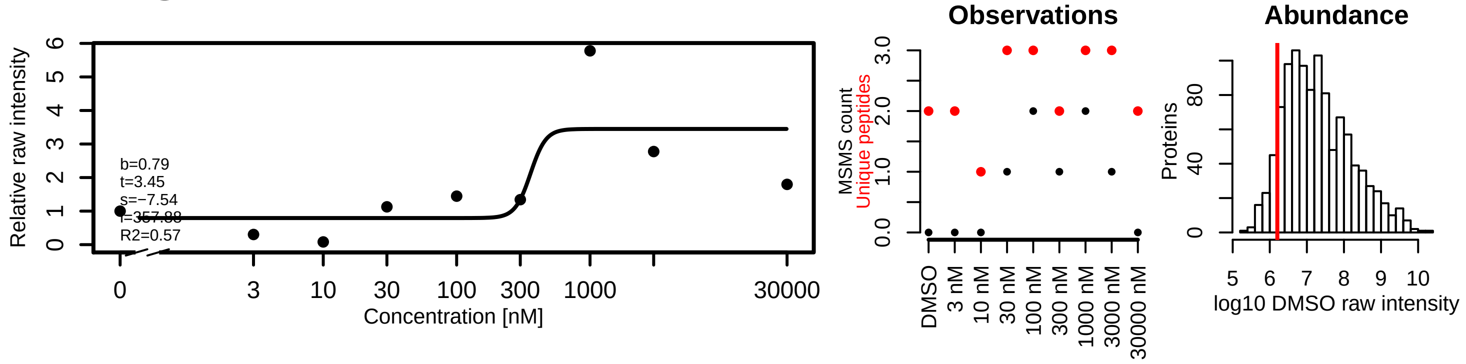


# ADRM1

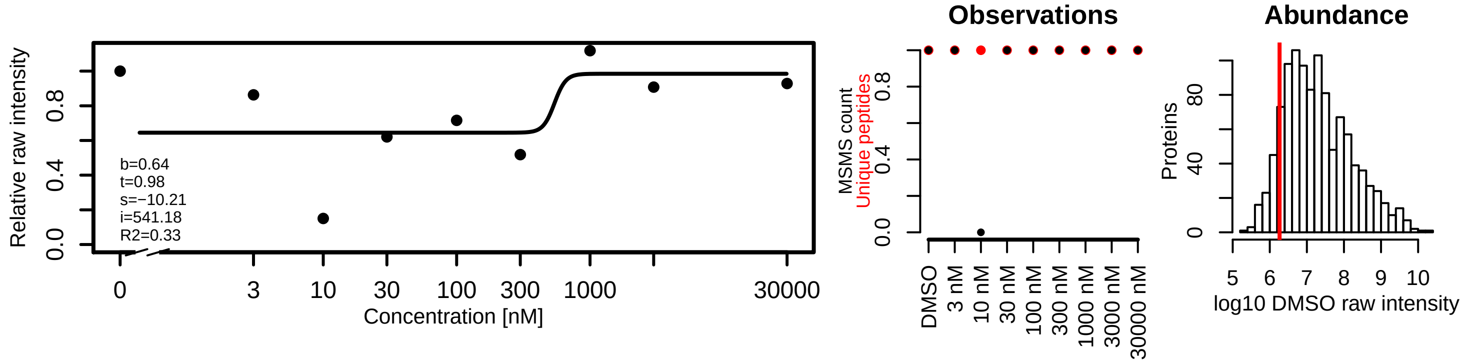


# YIF1A

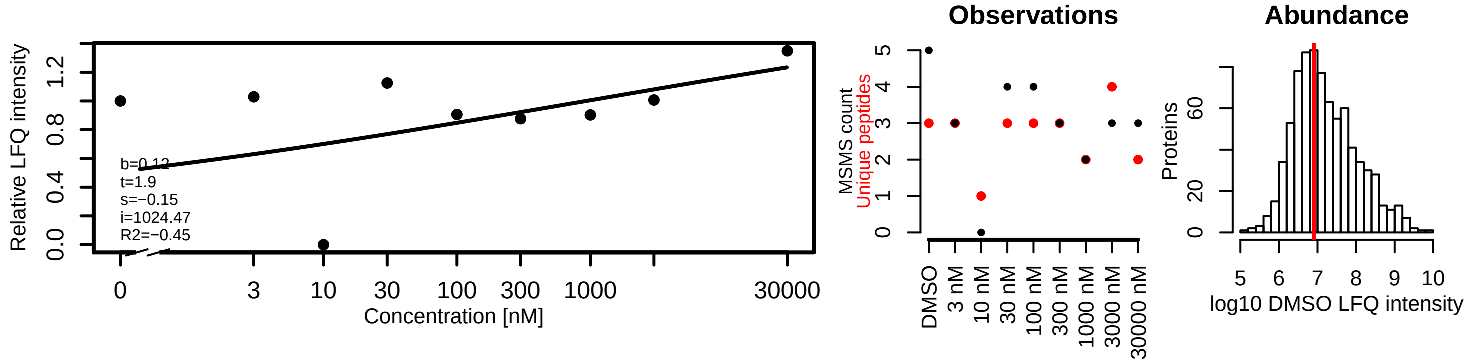


# INCENP

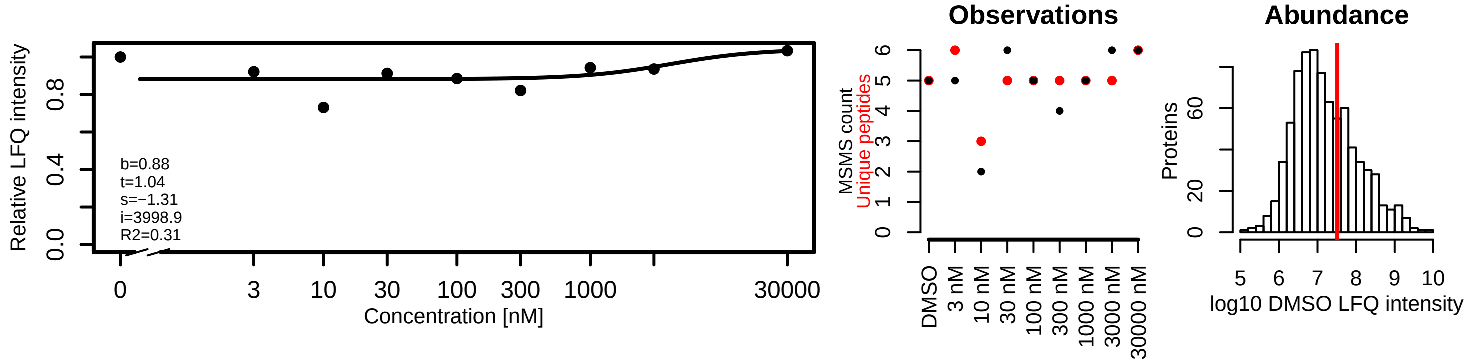


# DDX24

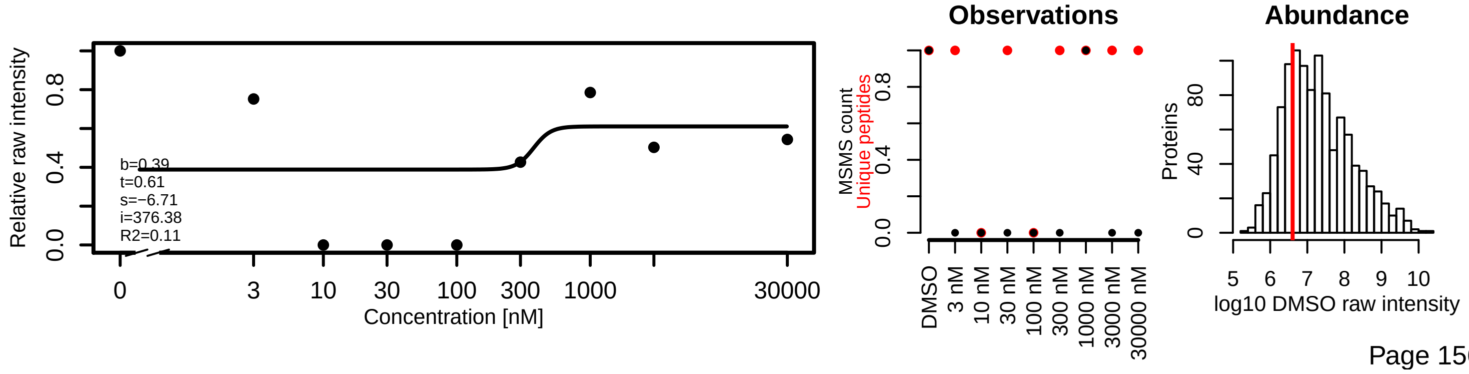

# AP2B1

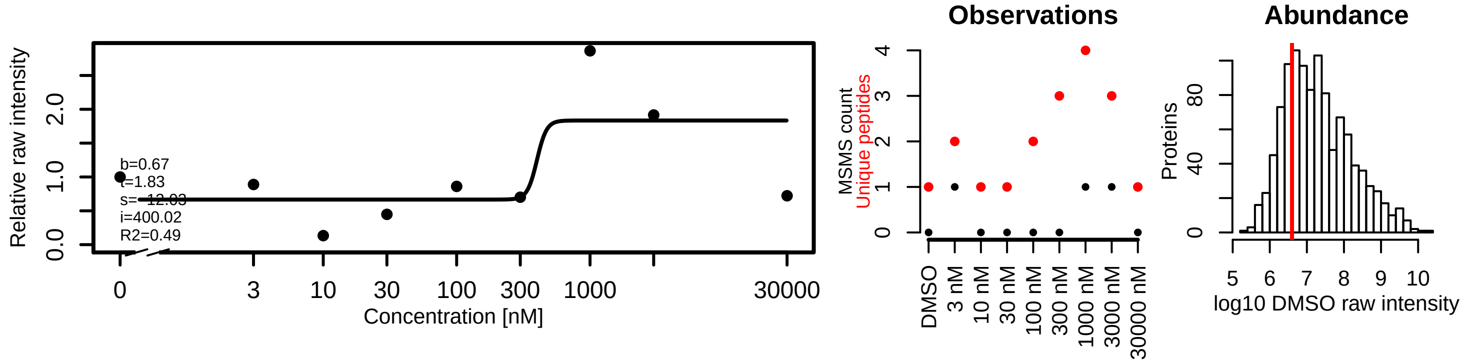


# RPS11

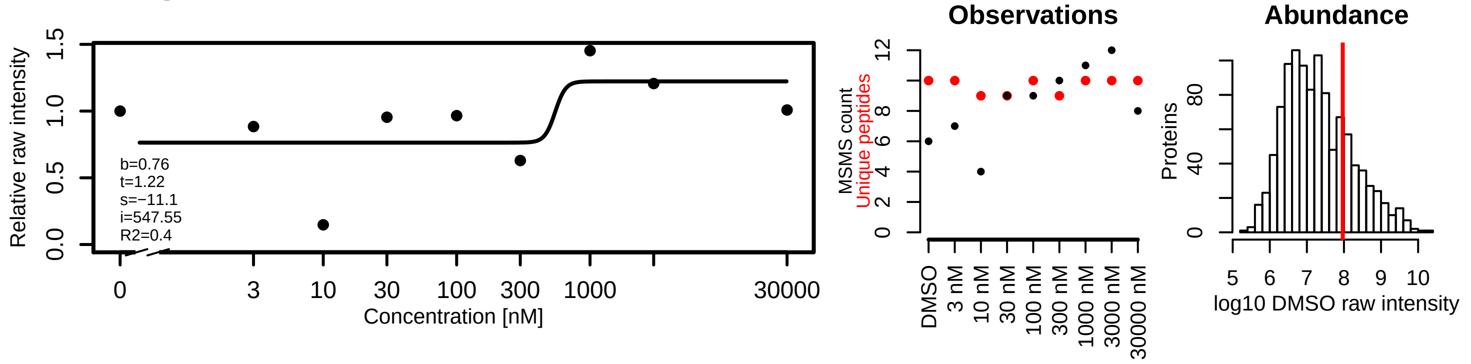


# AKAP8L

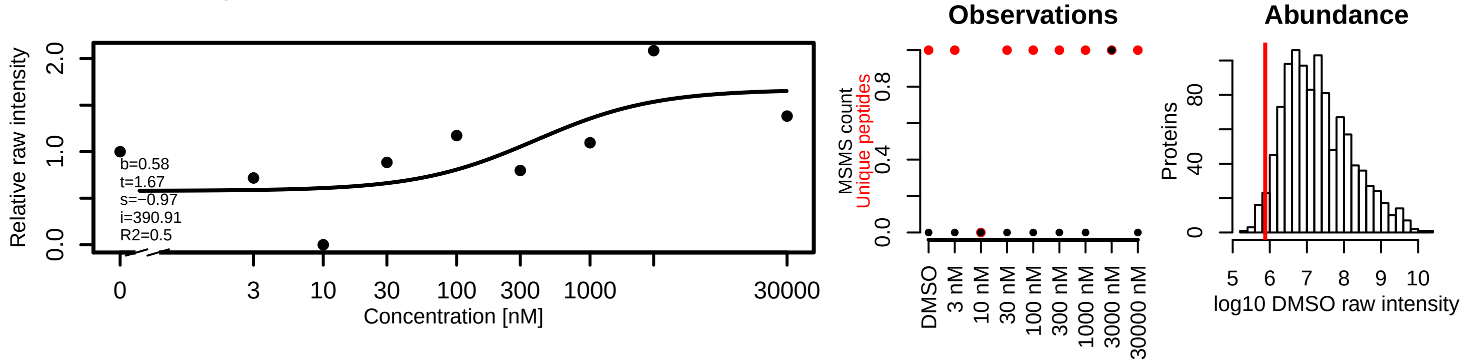


# IRAK1

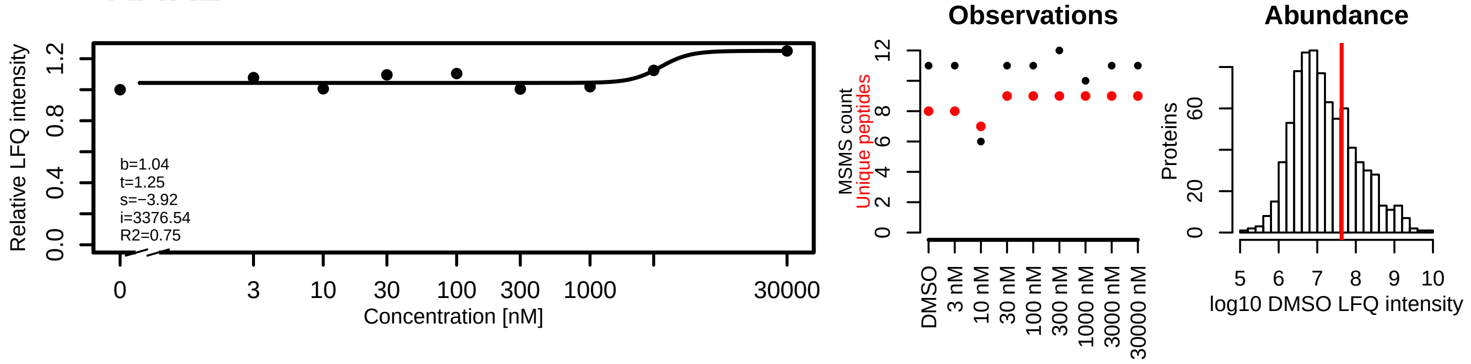


# ACOX3

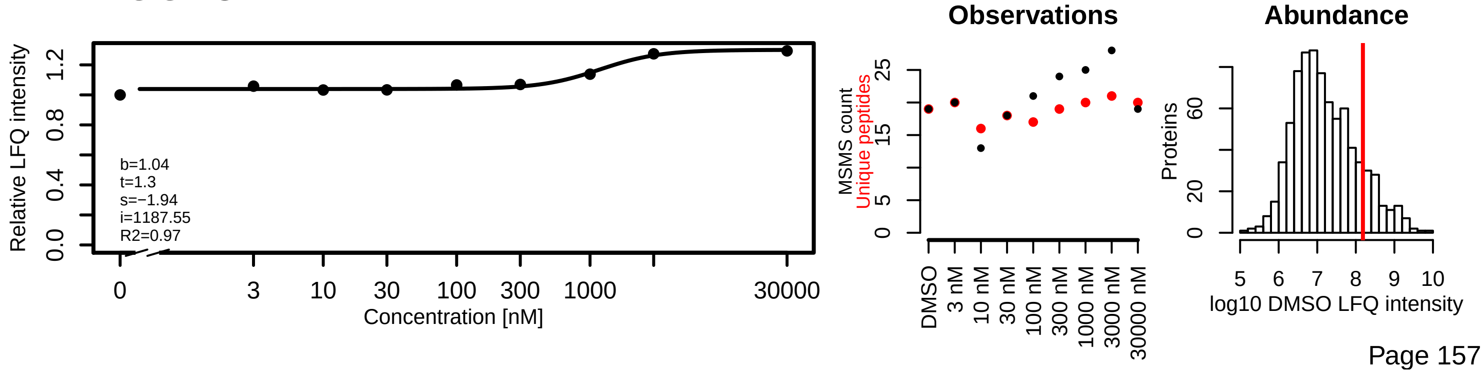

## SNRPD2

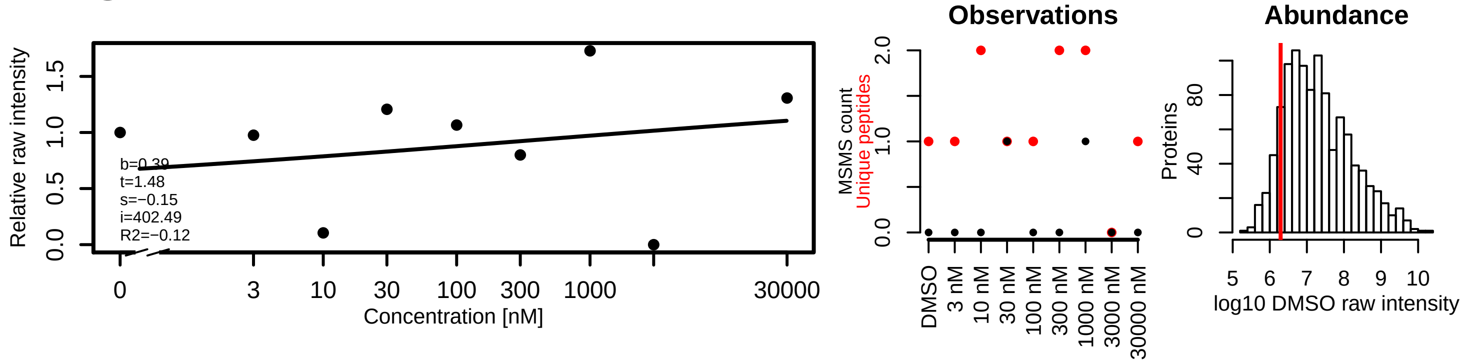


## HSPB1

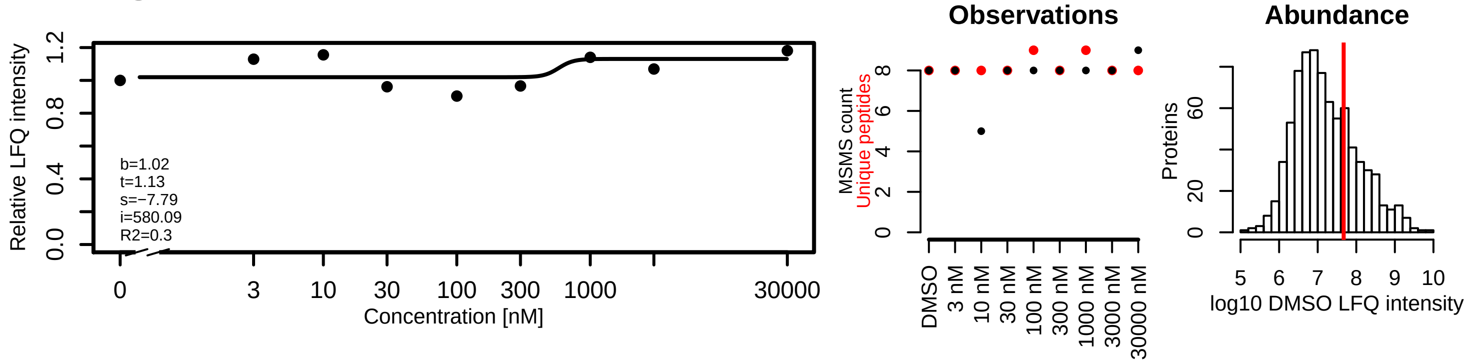


## AKAP9

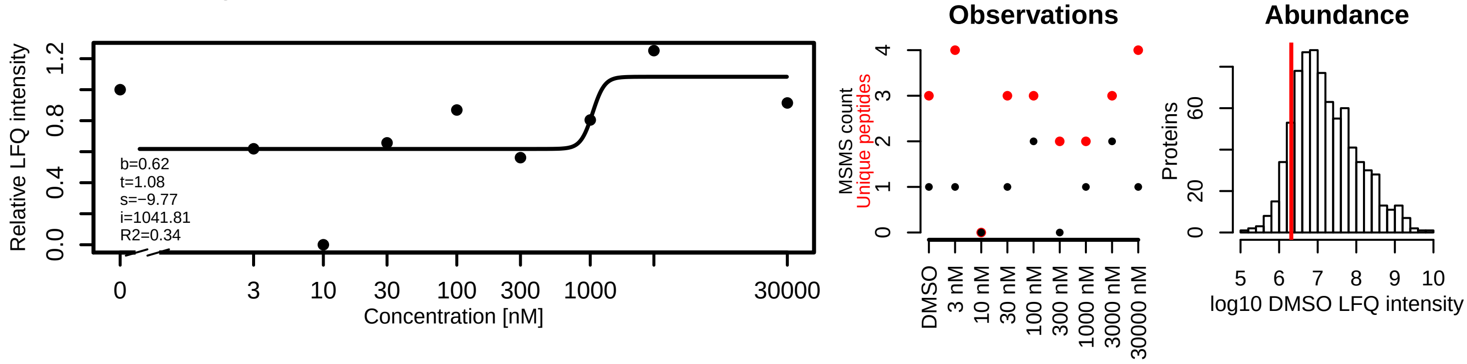


## HIST1H2BL;HIST1H2BM;HIST1H2BN;HIST1H2BH;HIST2H2BF;HIST1H

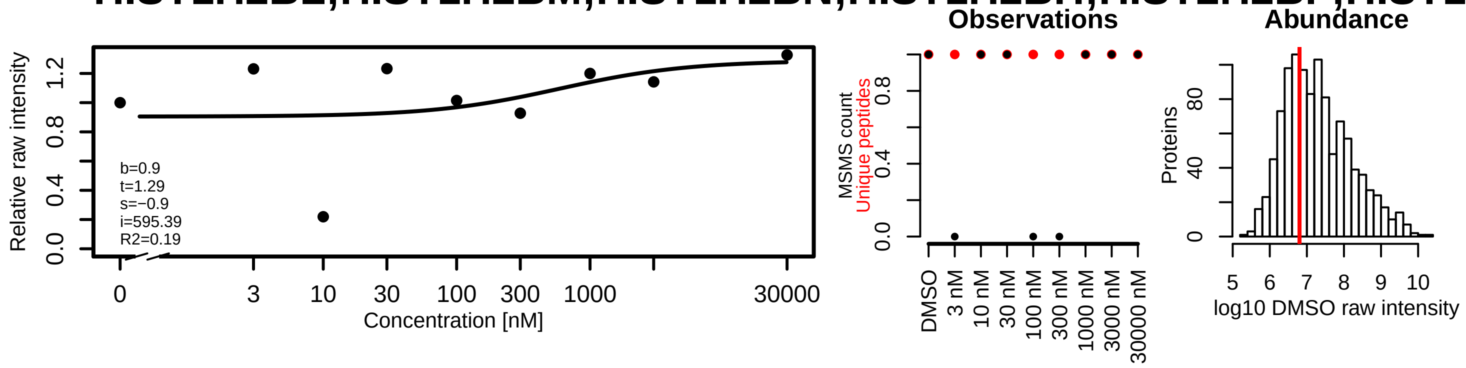


## HIST1H1E;HIST1H1C;HIST1H1D

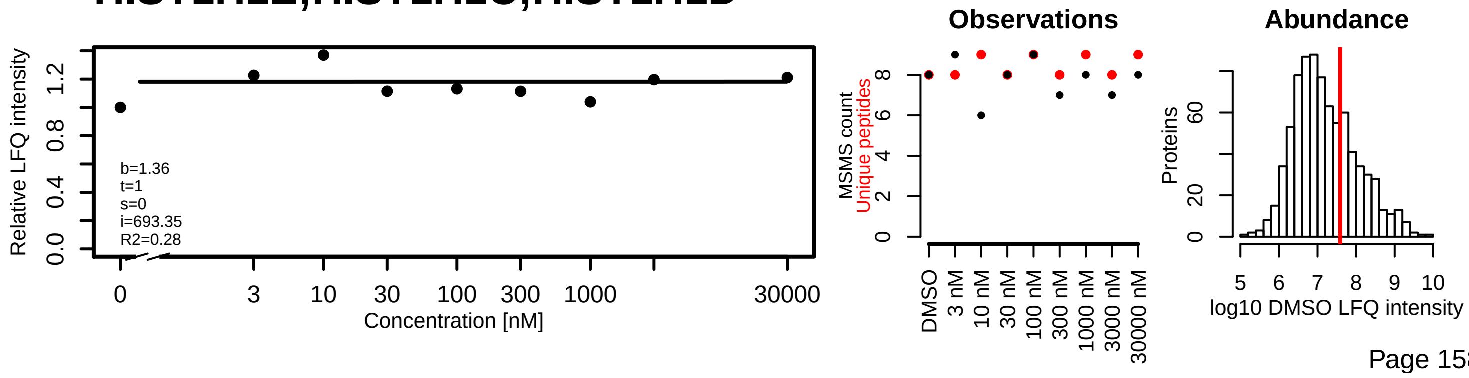

# GPC4;GPC6

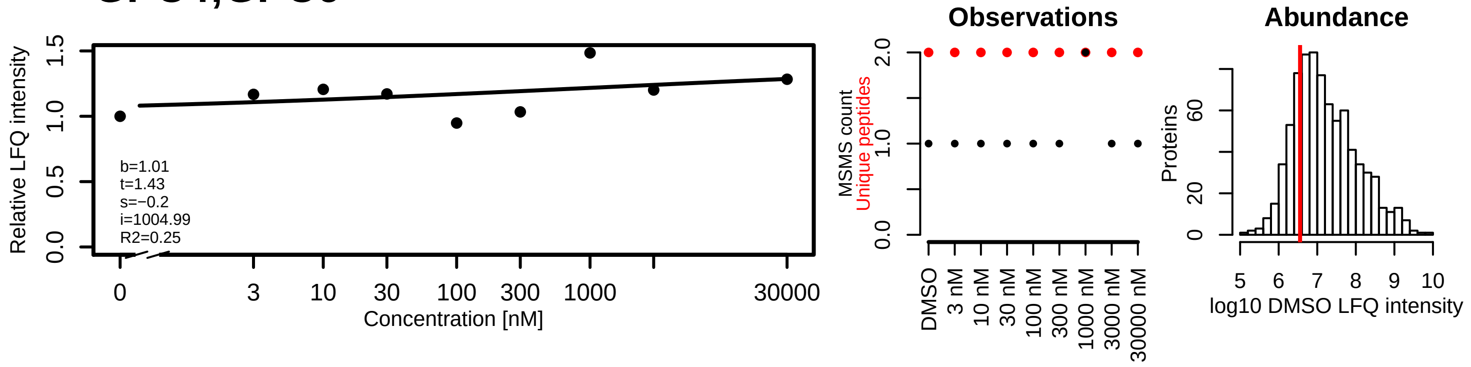


# CAB39

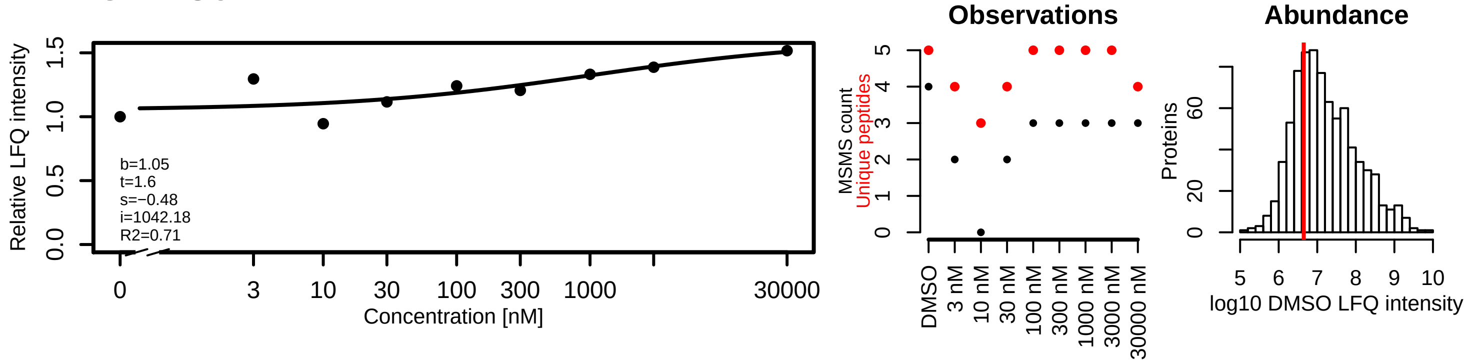


# CDK2

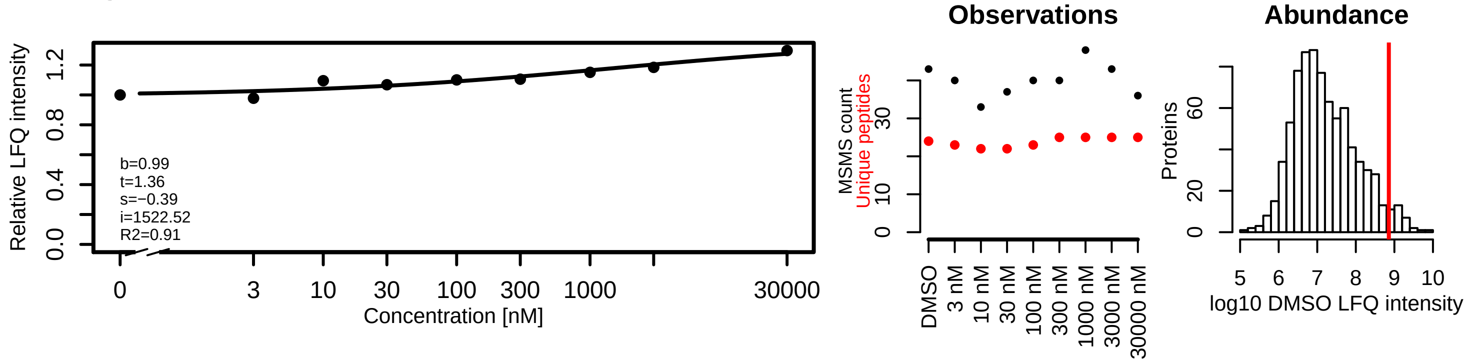


# PHKB

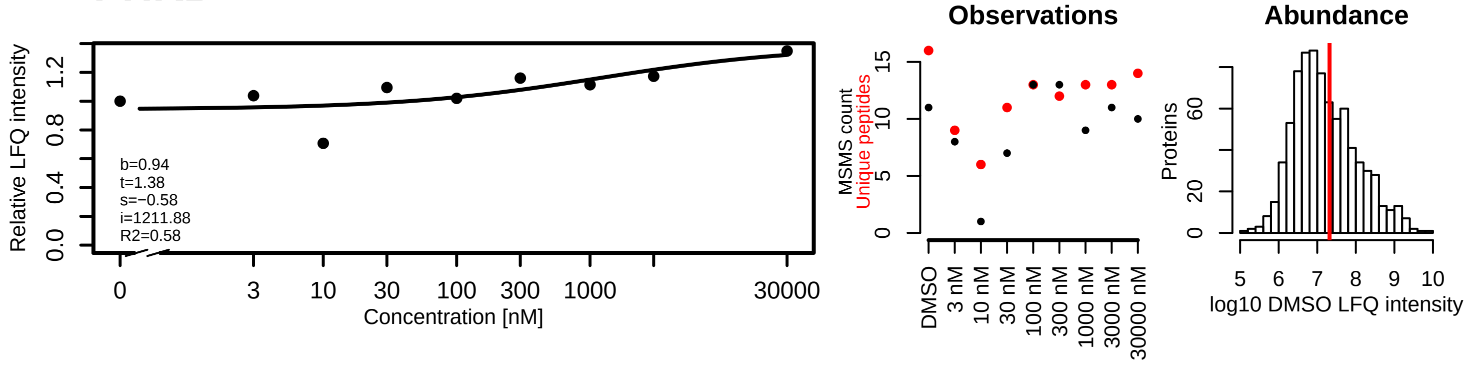


# UNC93B1

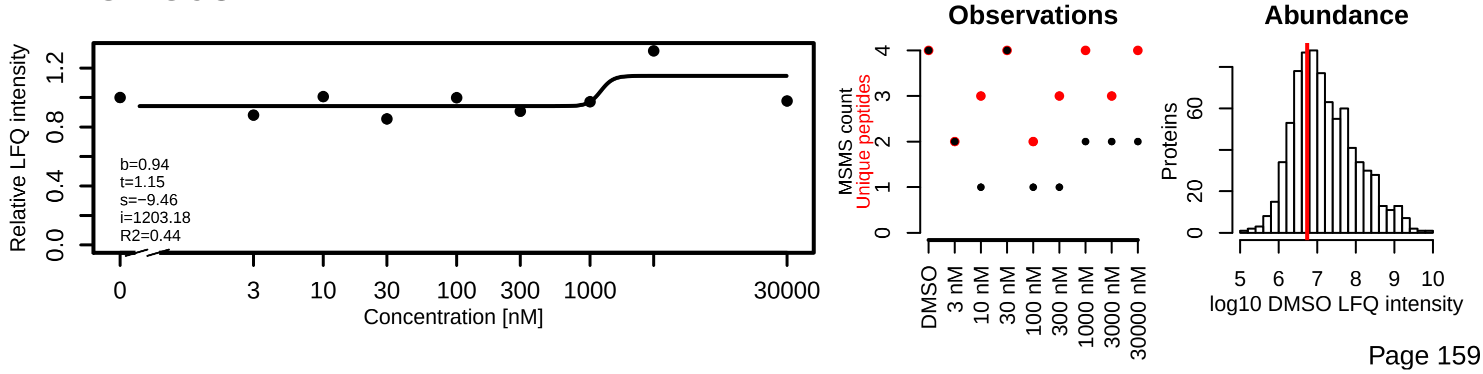

## RBMS1;RBMS3;RBMS2

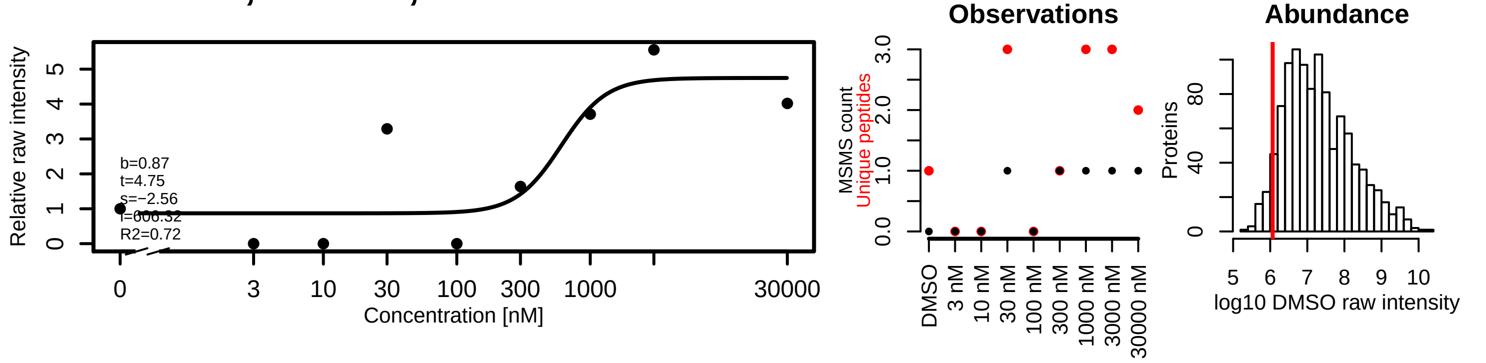


## AURKB − Q96GD4−4;J3KT86;J3QR41;J3KRJ2;Q9UQB9−2;Q9UQB9−

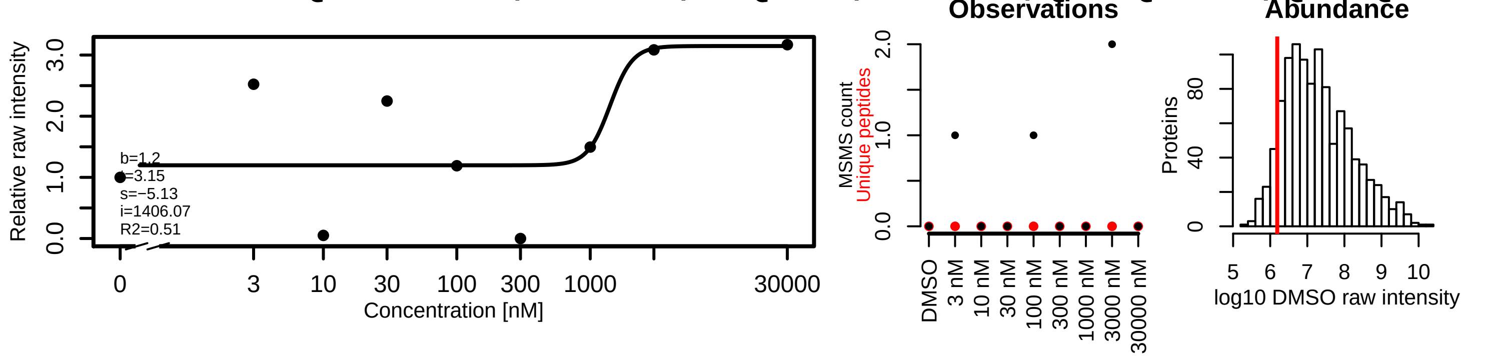


## FGF2

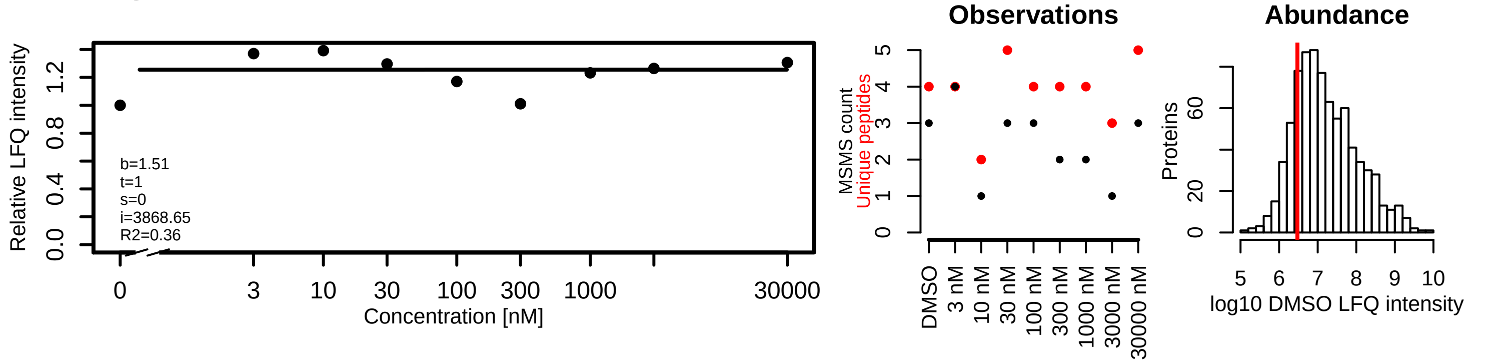


## RPL23

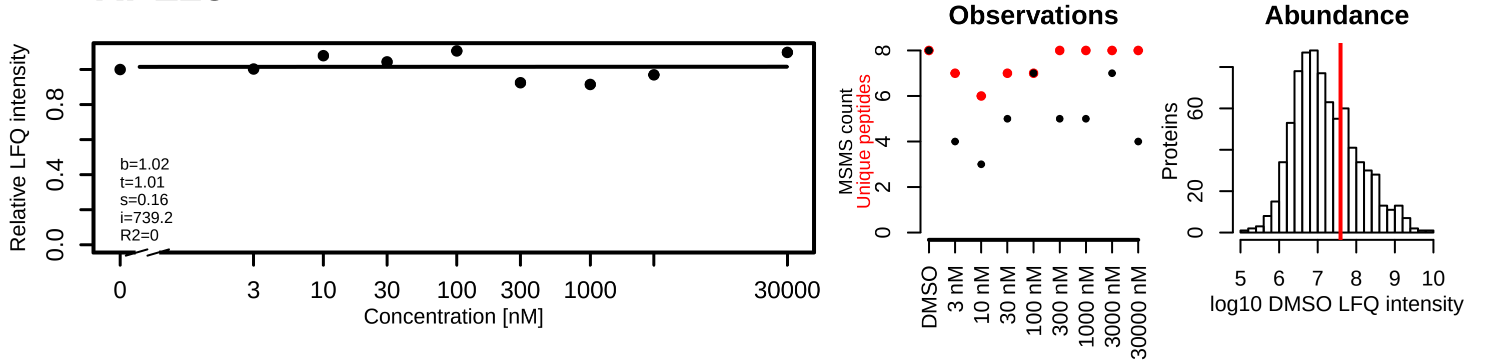


## PURB

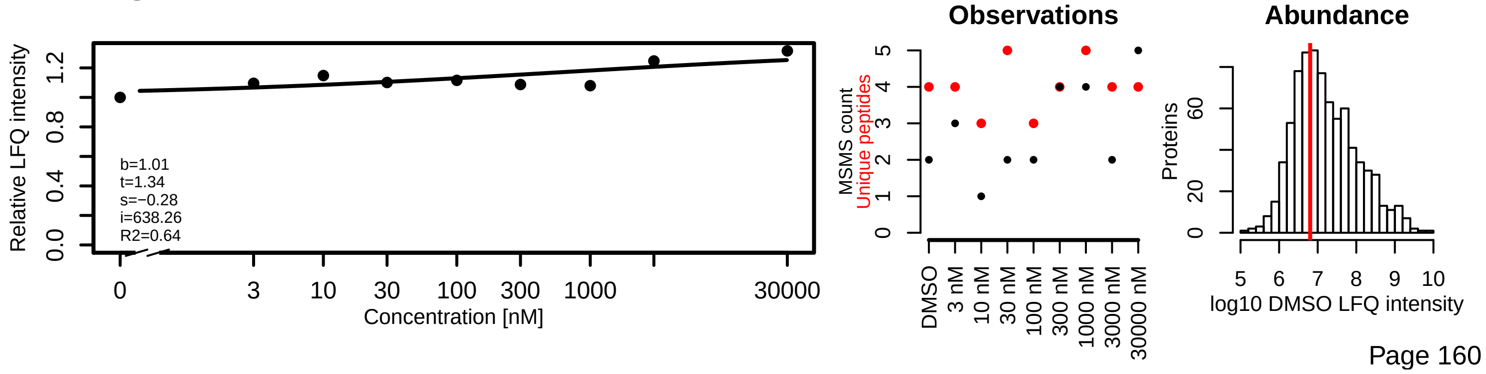

## ITGB1

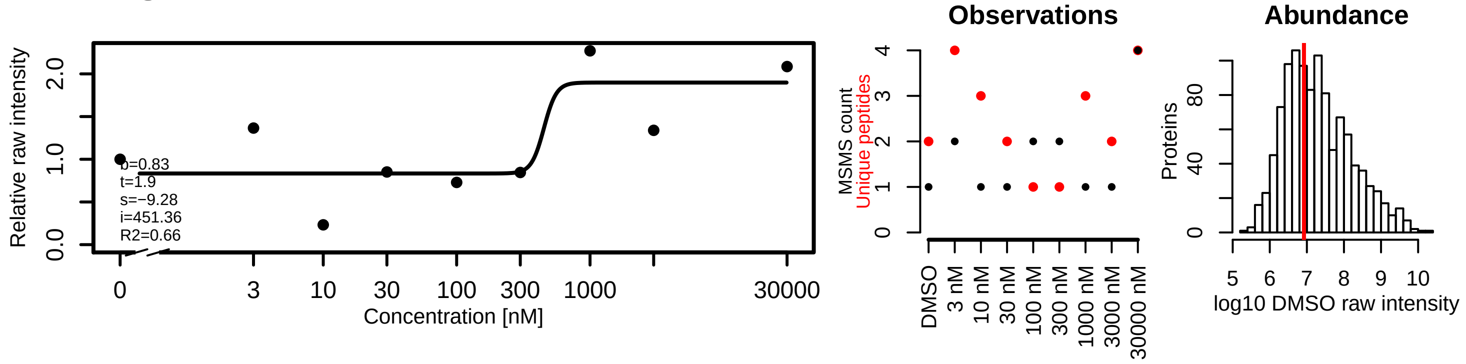


## ABCC1

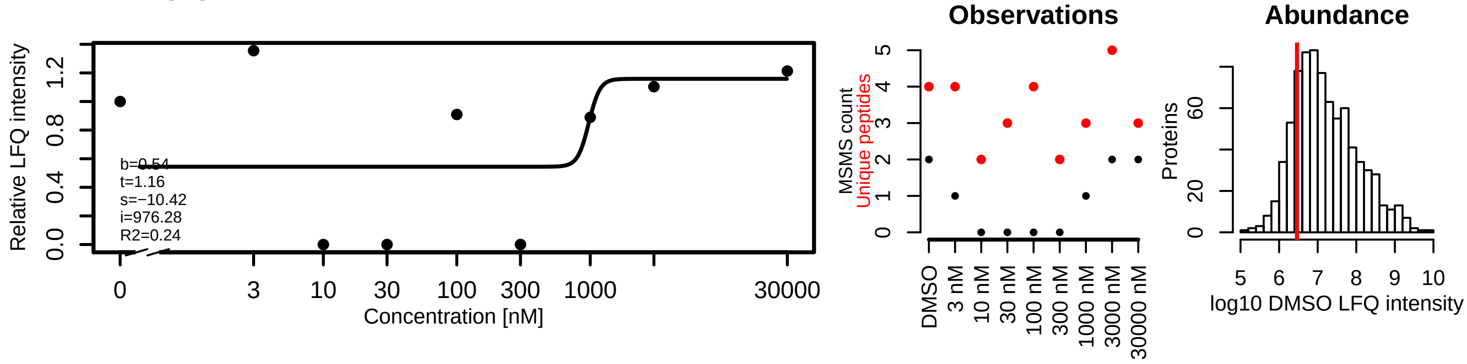


## SRSF3

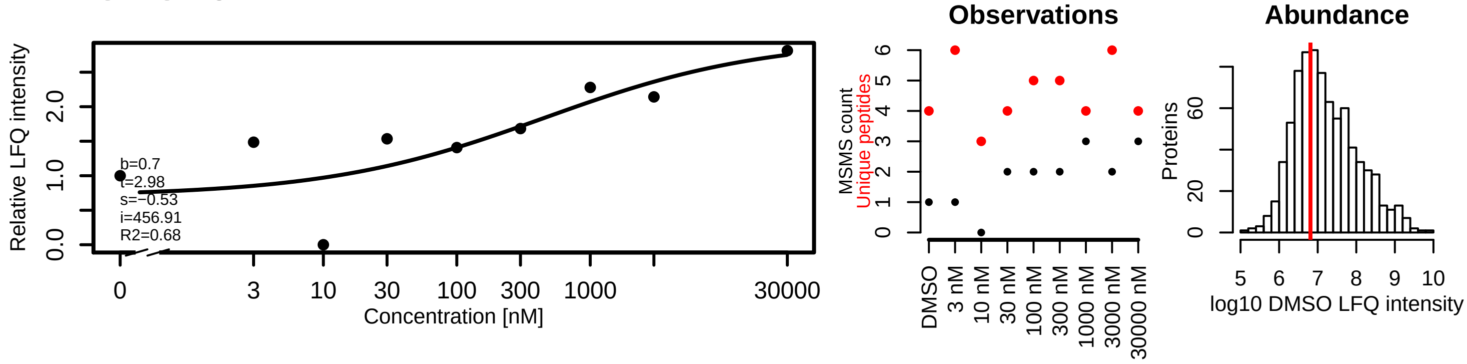


## AKR1B10

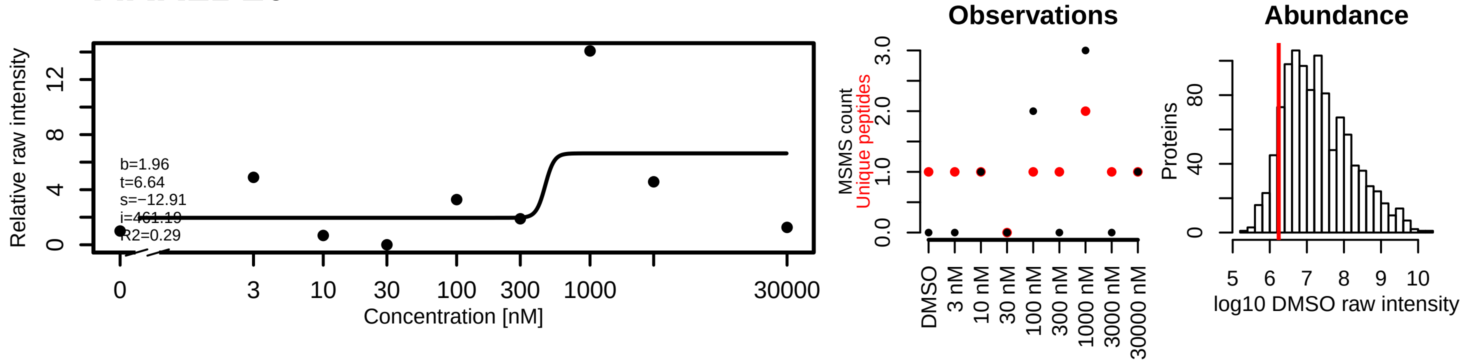


## TAOK2

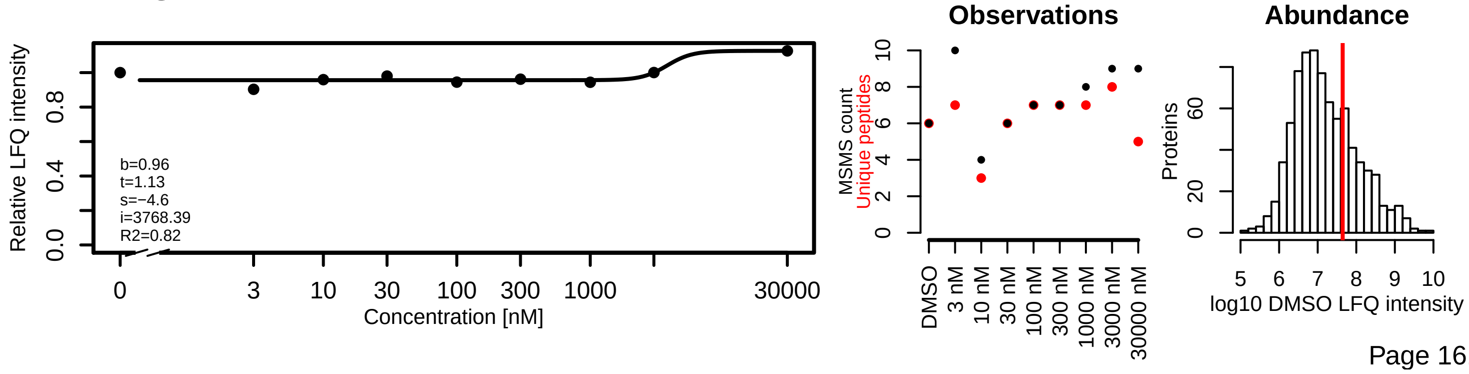

# LRRK2

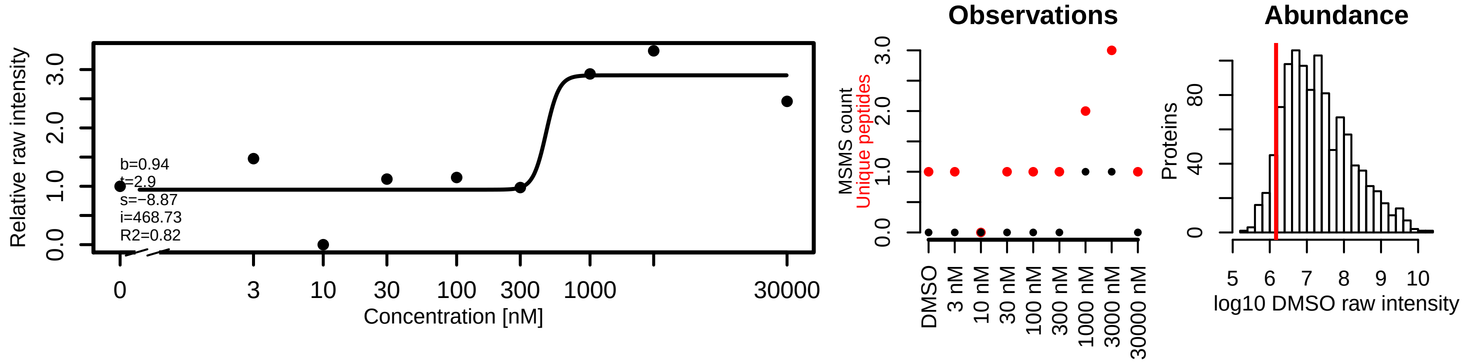


# KHDRBS1

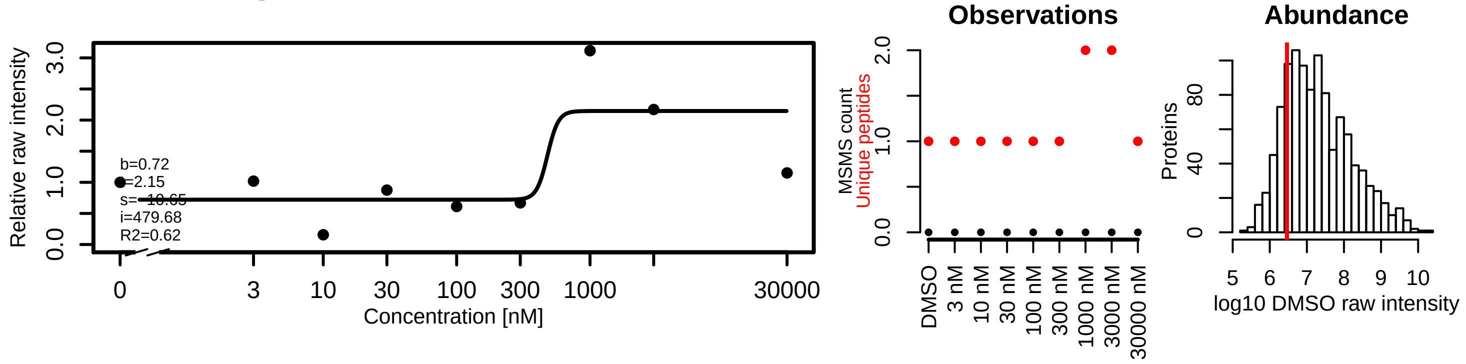


# ACTG1

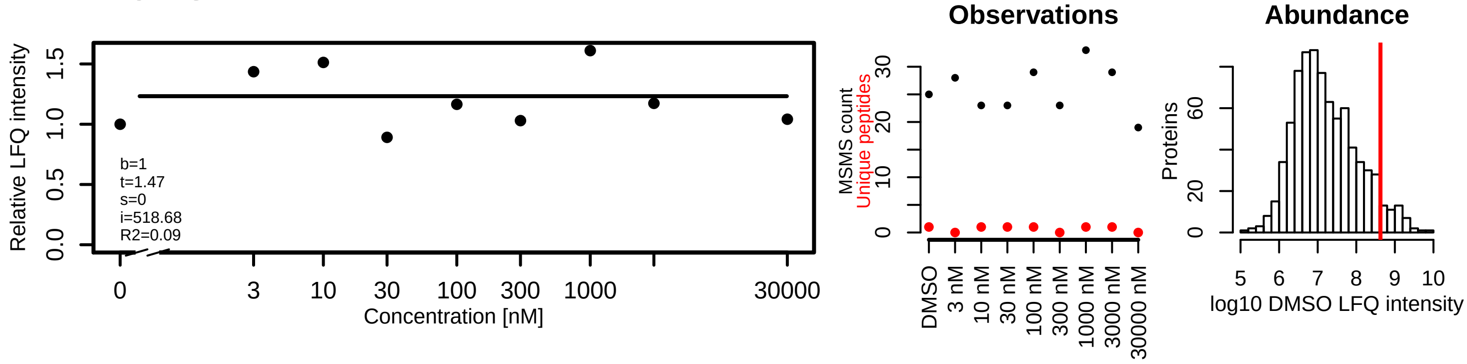


# TPT1;FKSG2

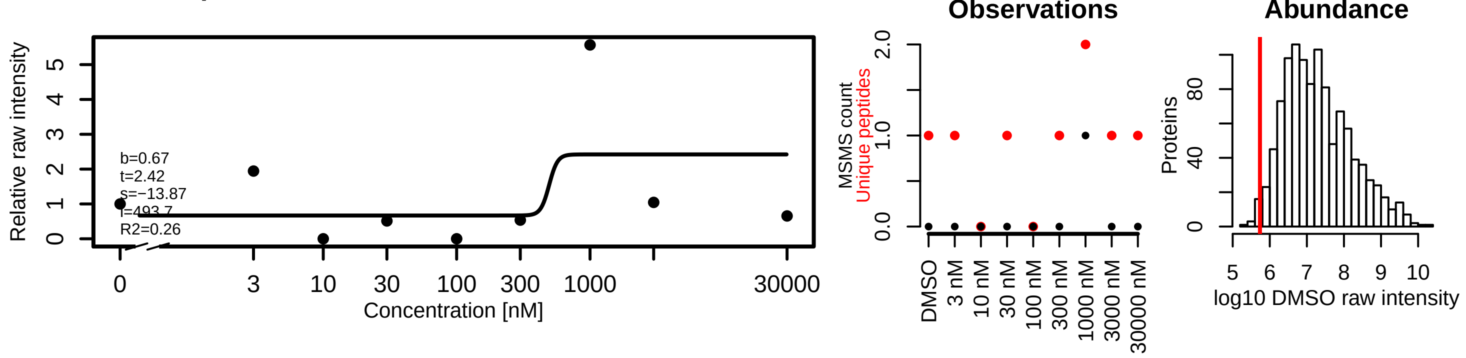


# CACYBP

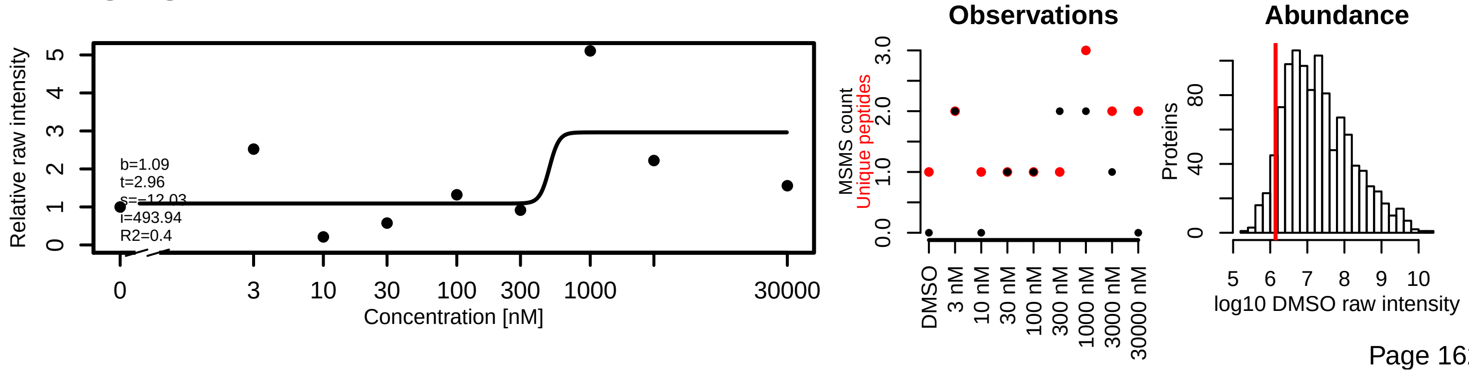

# FASN

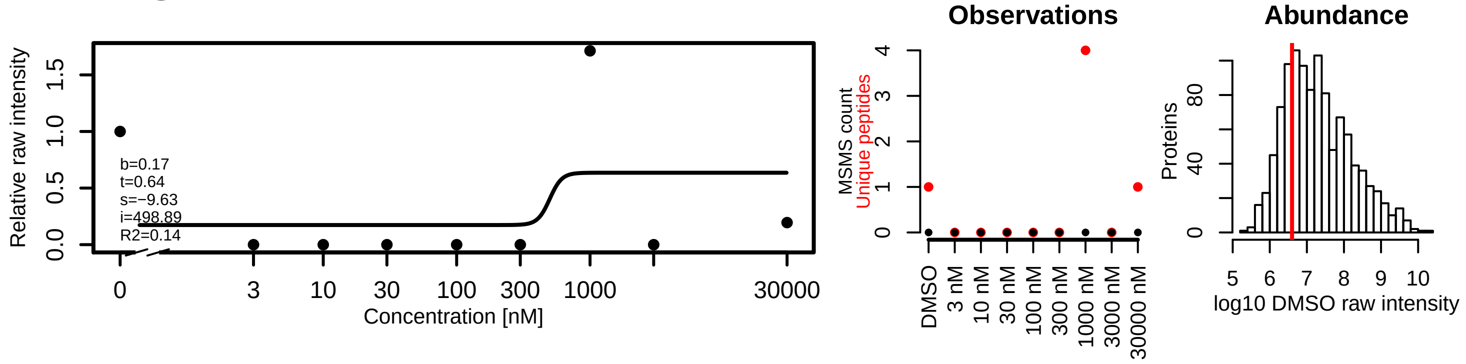


# ALDOA

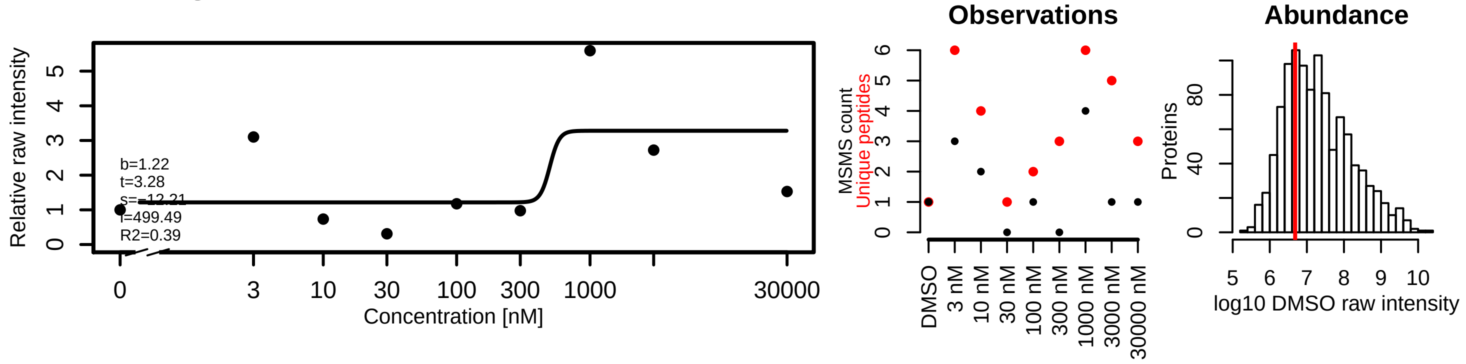


# CANX

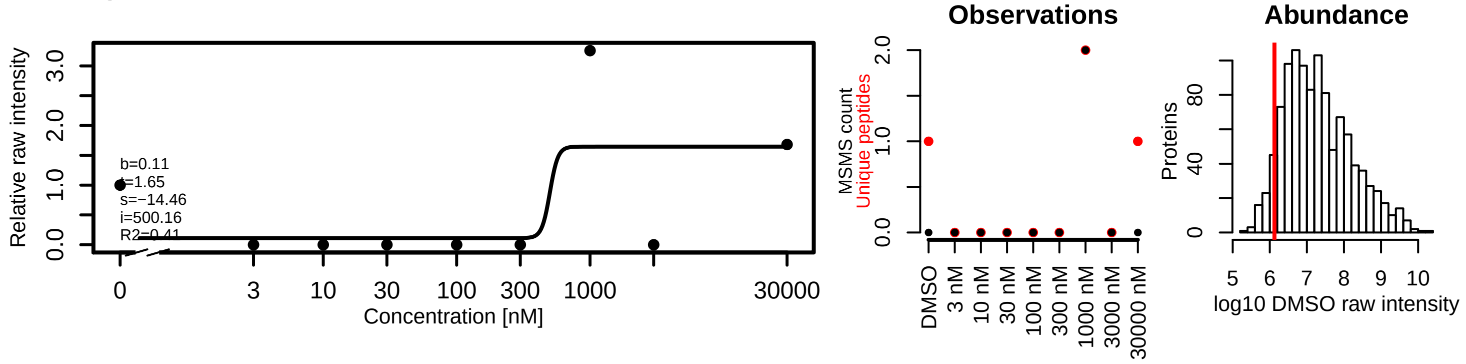


# PSMA5

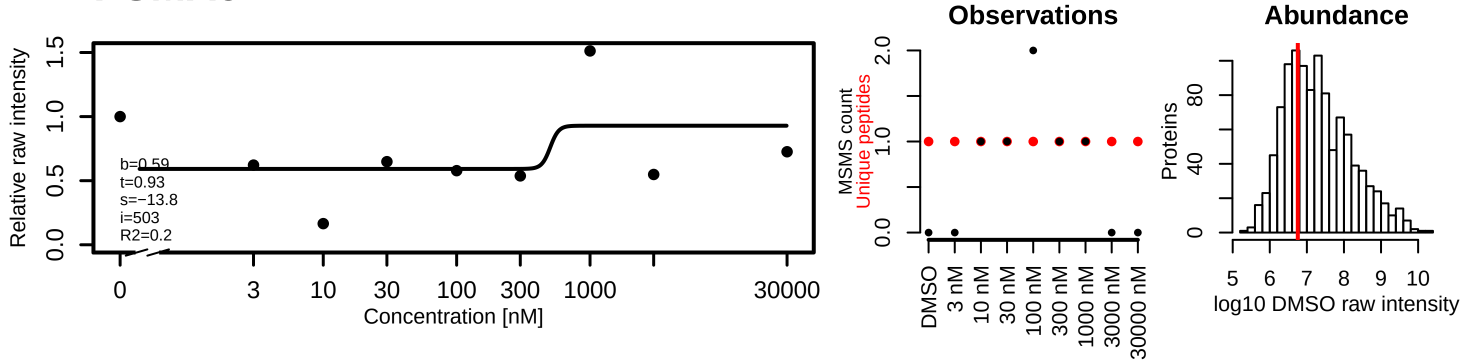


# CAMK2G − Q5SWX3;Q13555−5

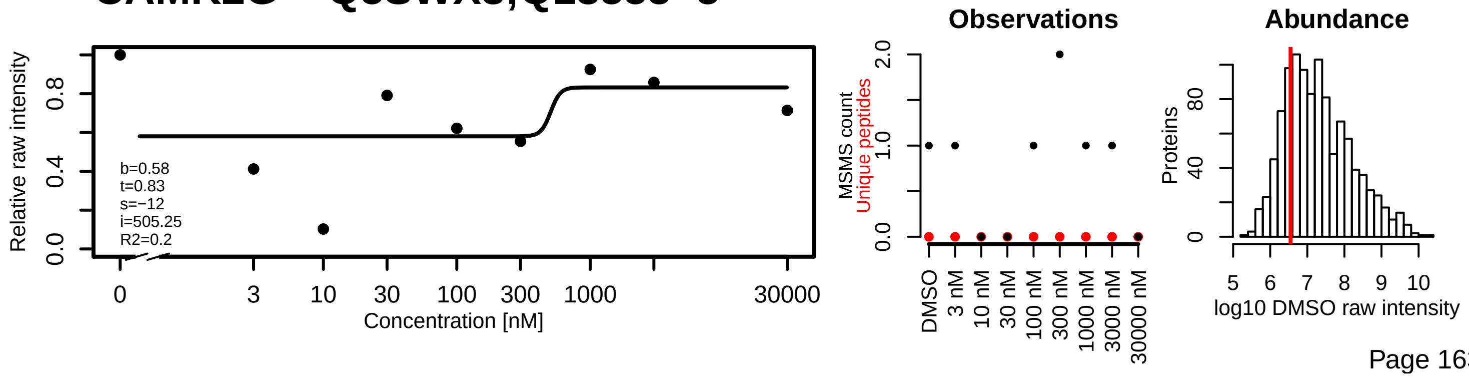

## NPM1

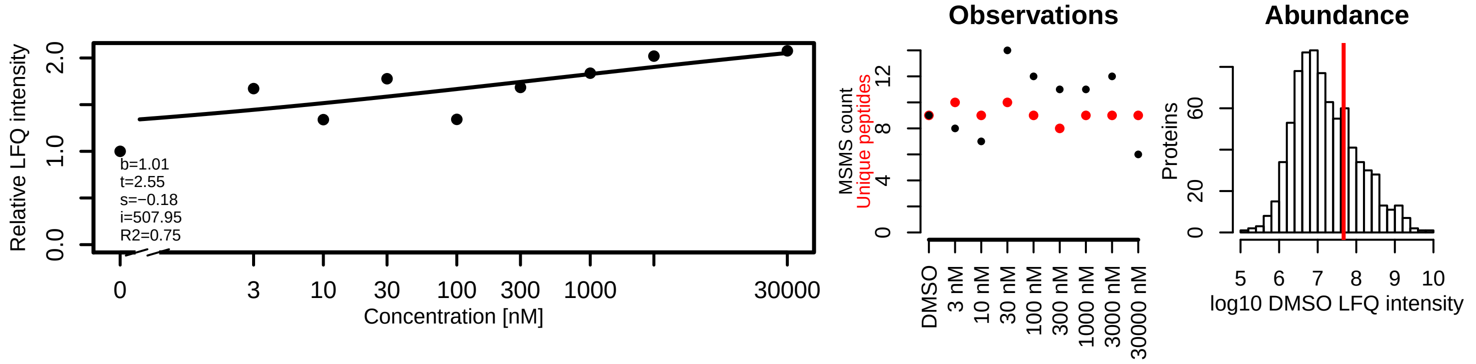


## CCT7

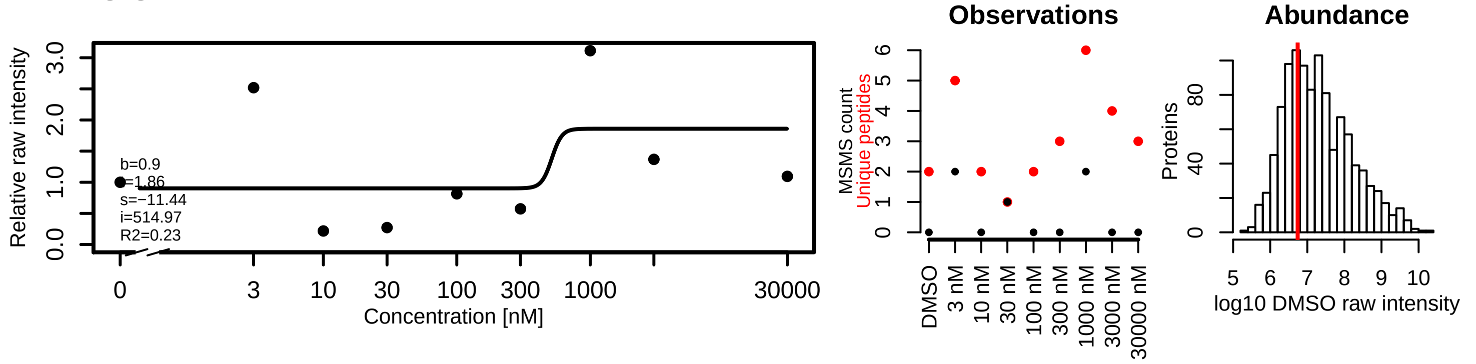


## MELK

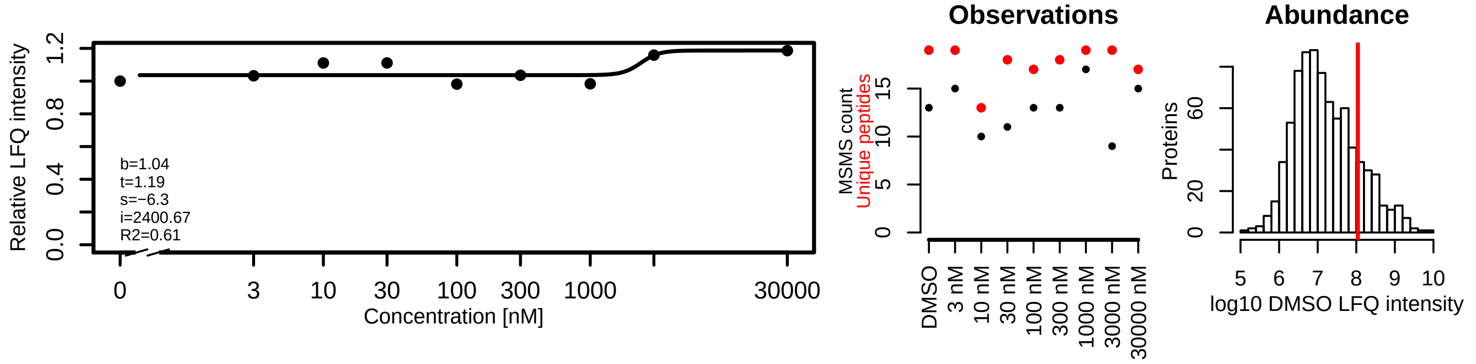


## ITPR3

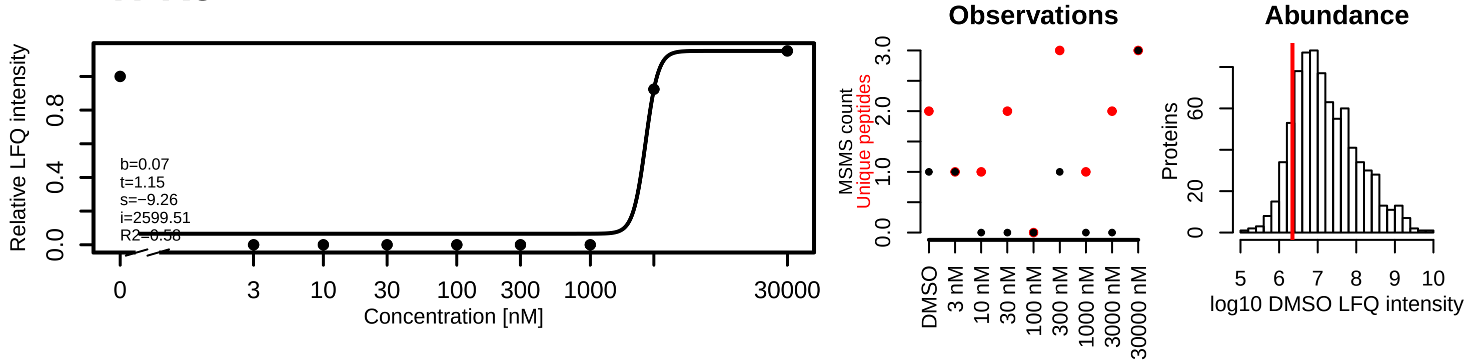


## LMAN1

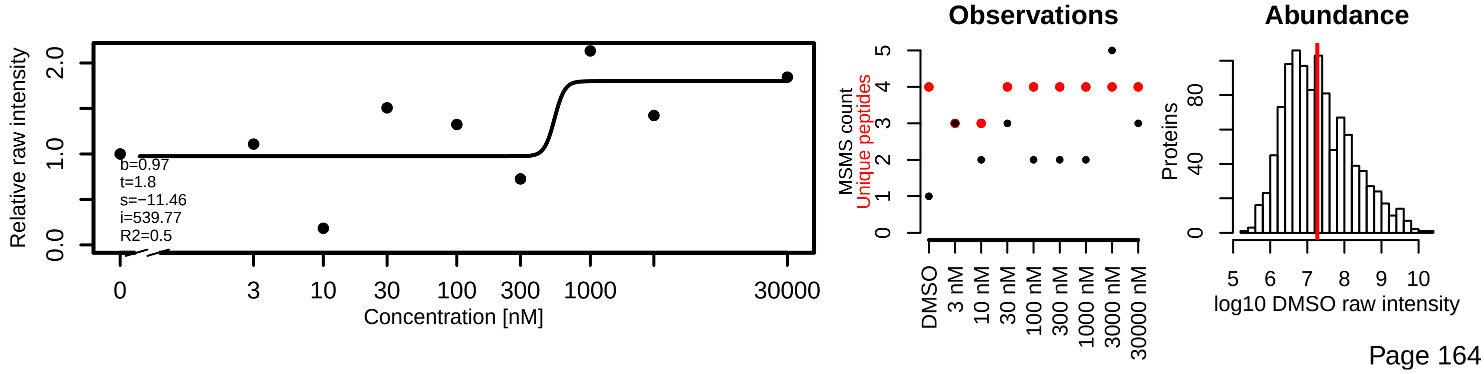

## PURA

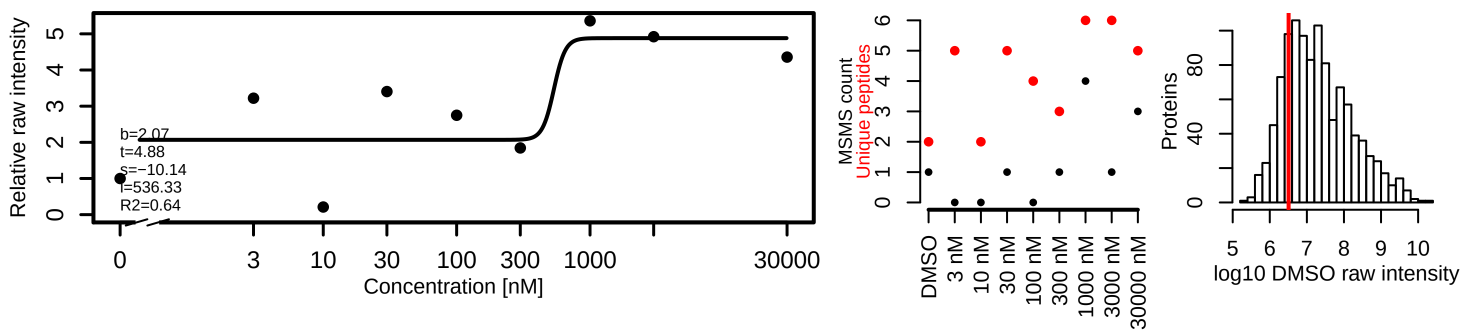


## MSN

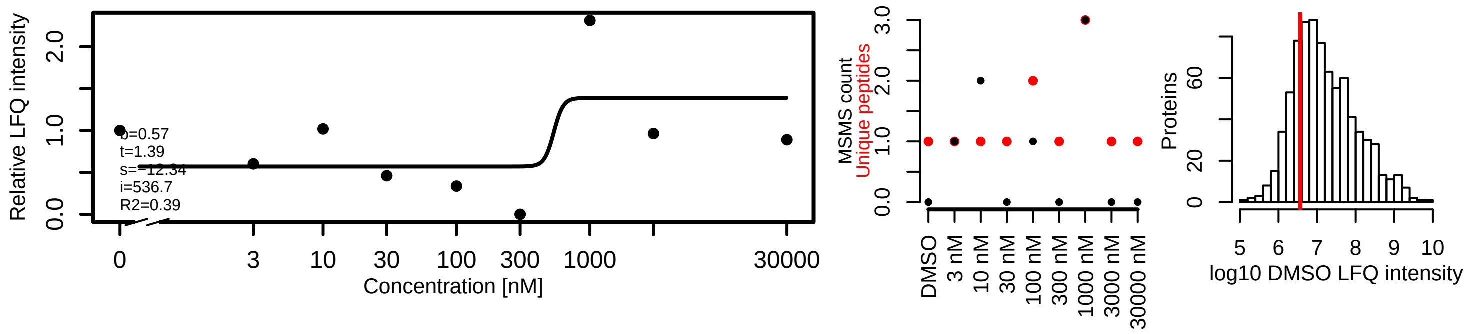


## RPS20

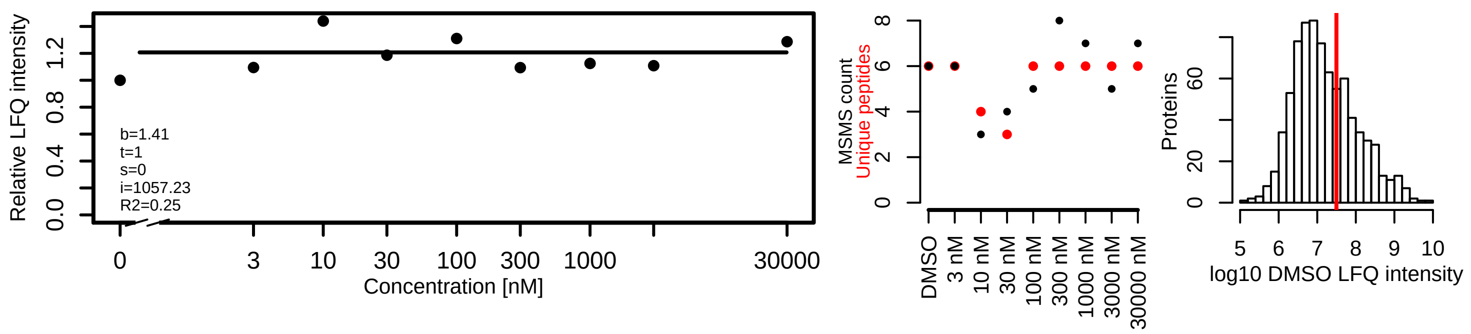


## S100A10

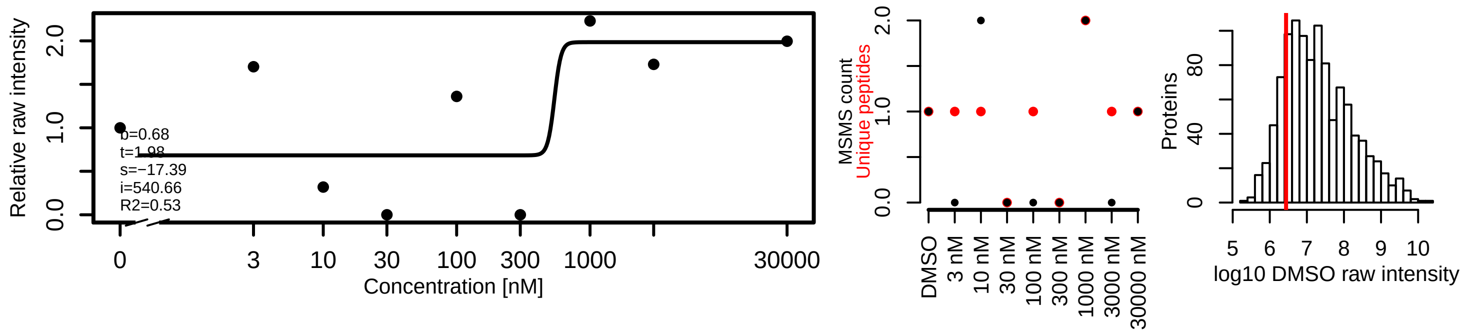


## RBFOX1;RBM9;RBFOX2;RBFOX3

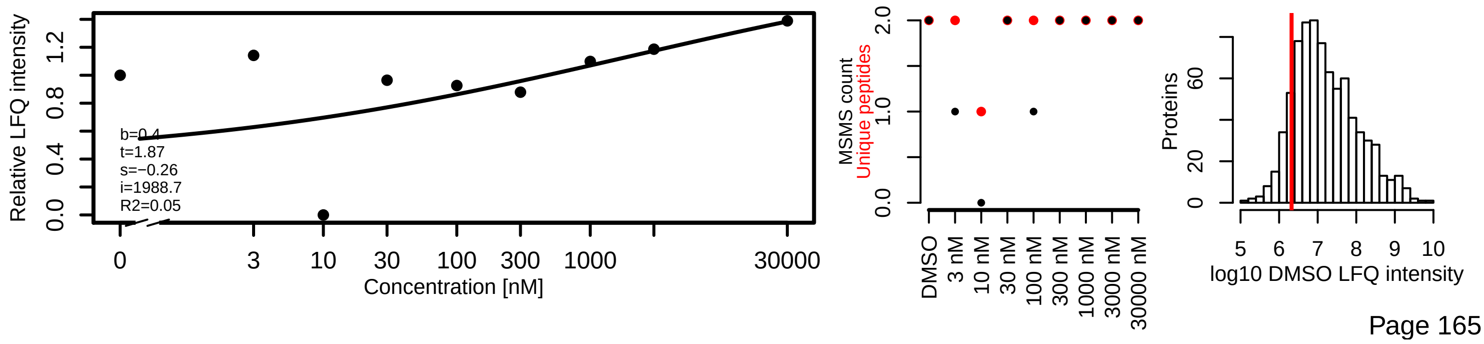

# ULK1

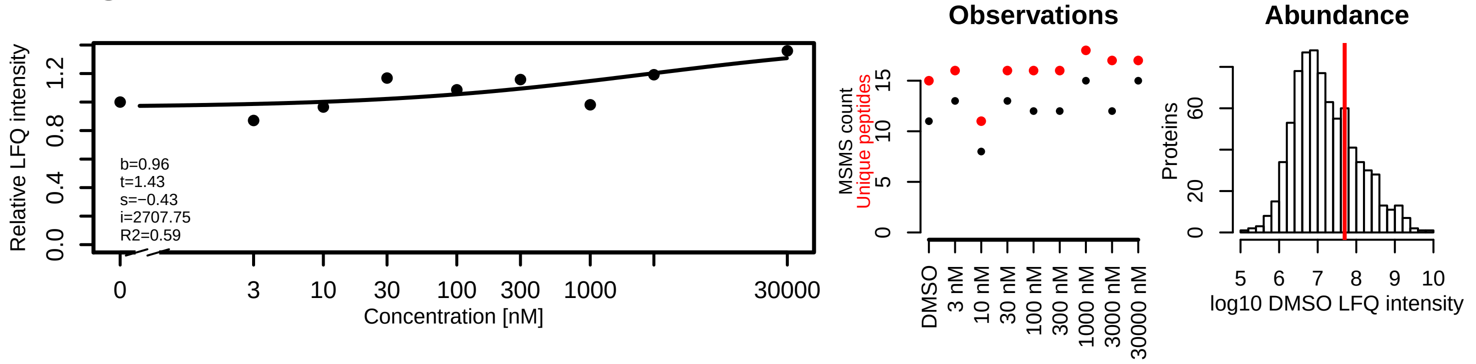


# MYH10

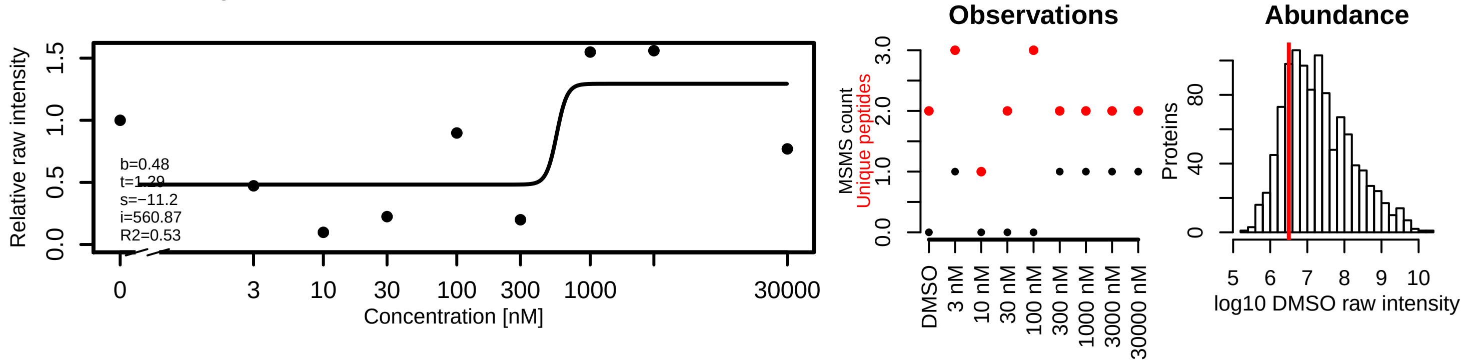


# GNB1

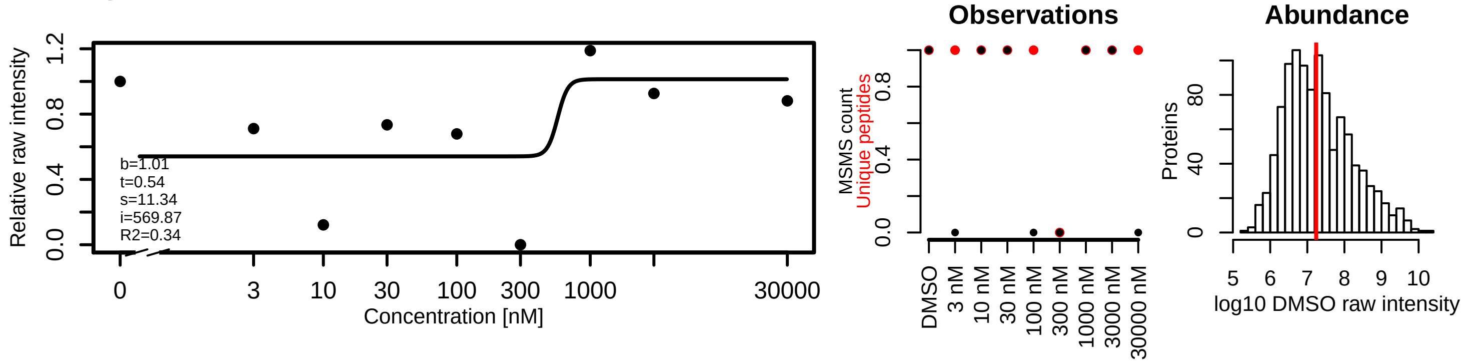


# MARK4

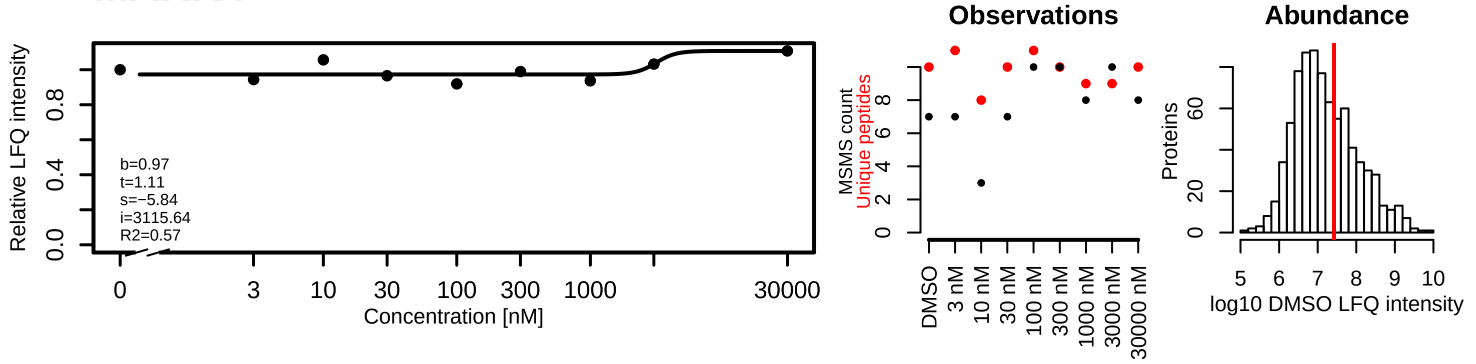


# SPTBN1

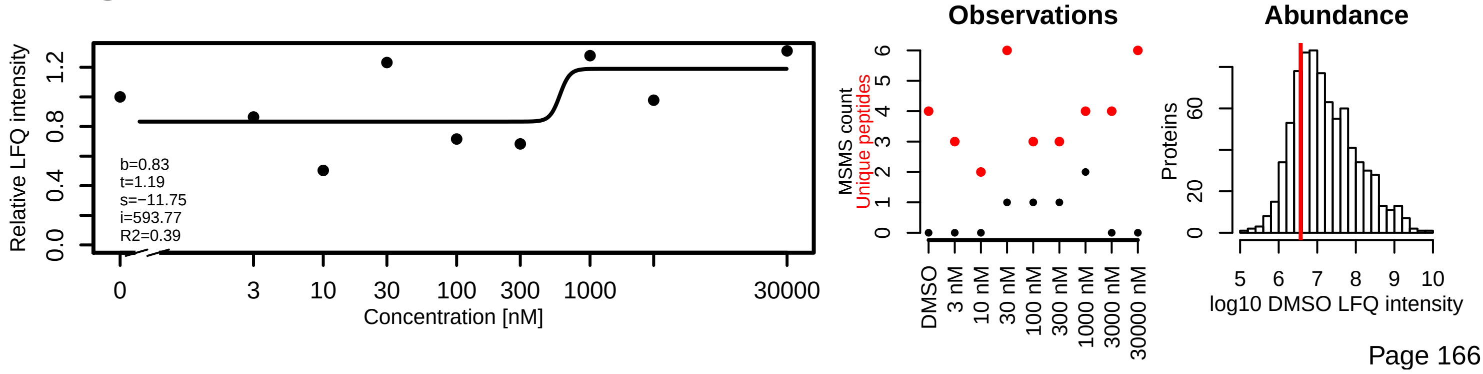

## HNRNPA2B1

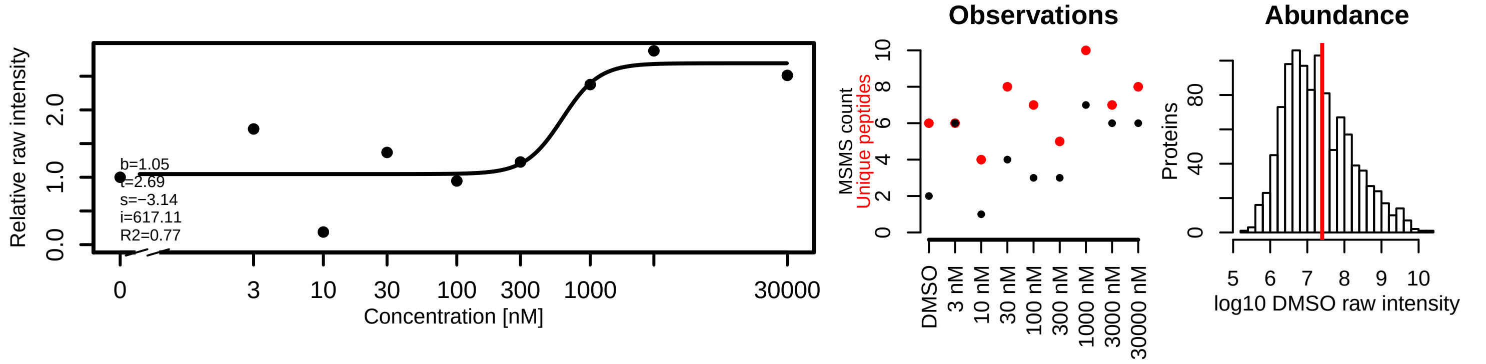


## BRSK1;BRSK2

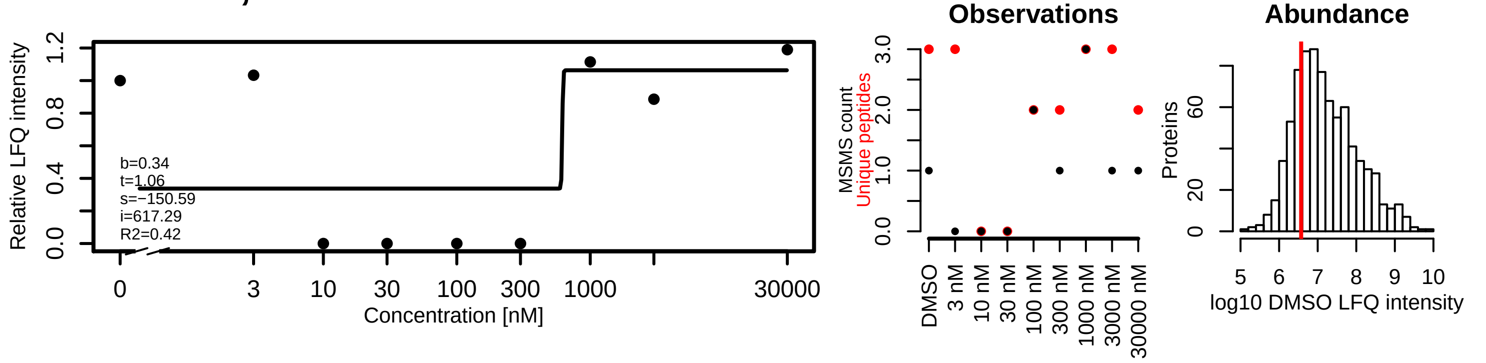


## DNAJC10

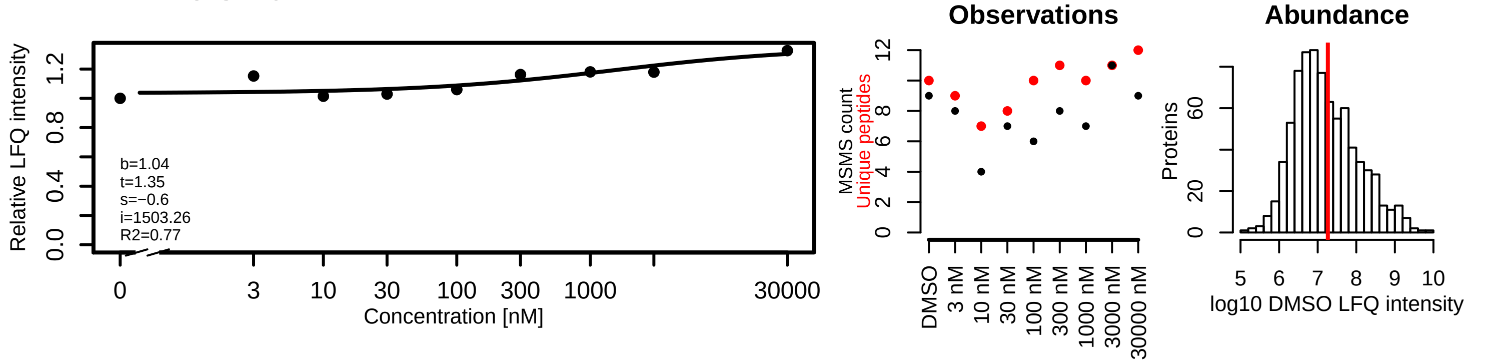


## EBP

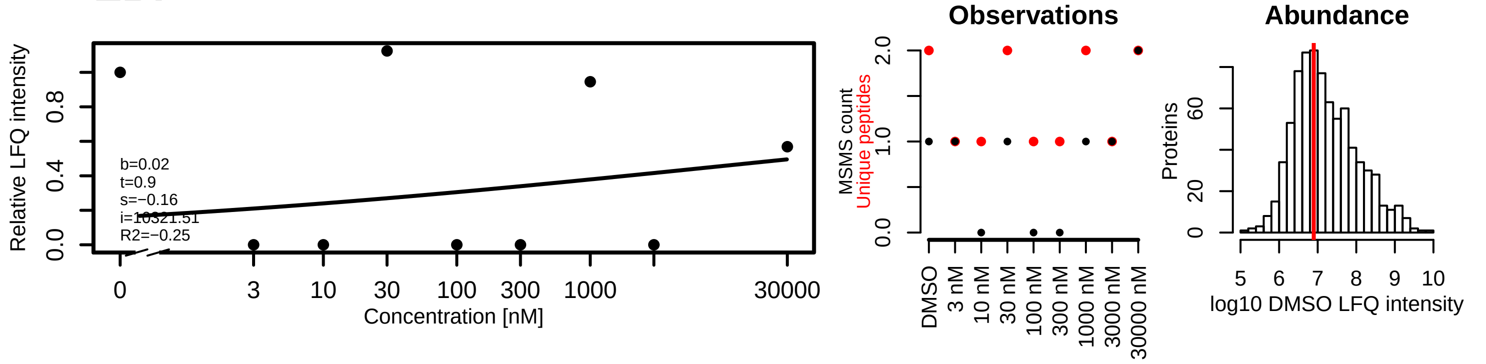


## PTPLB

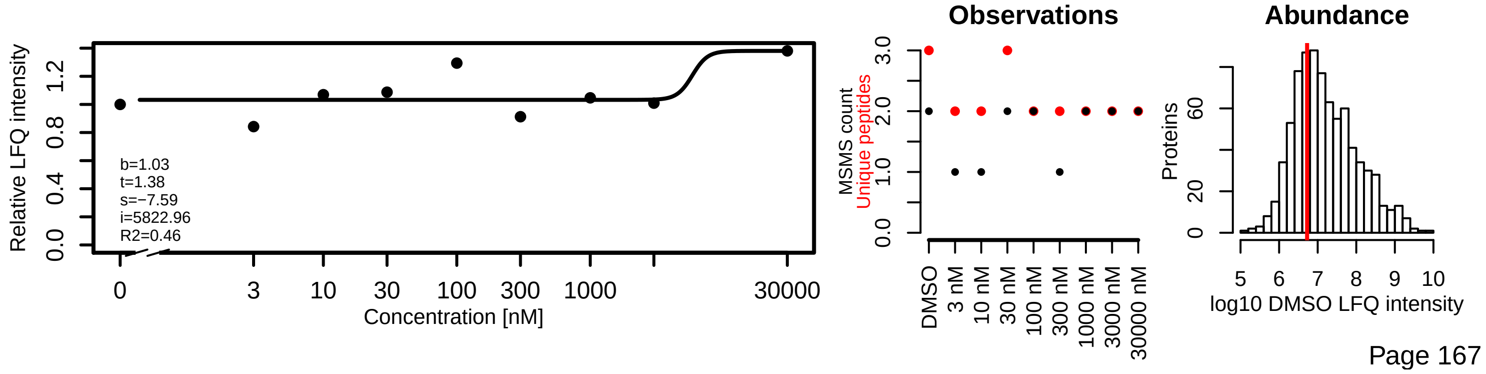

## TRA2B

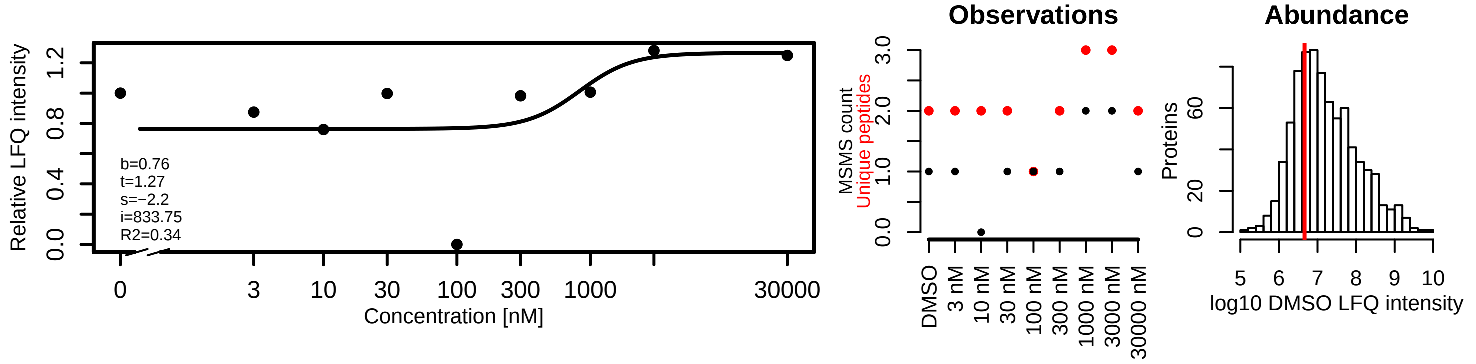


## CDK12

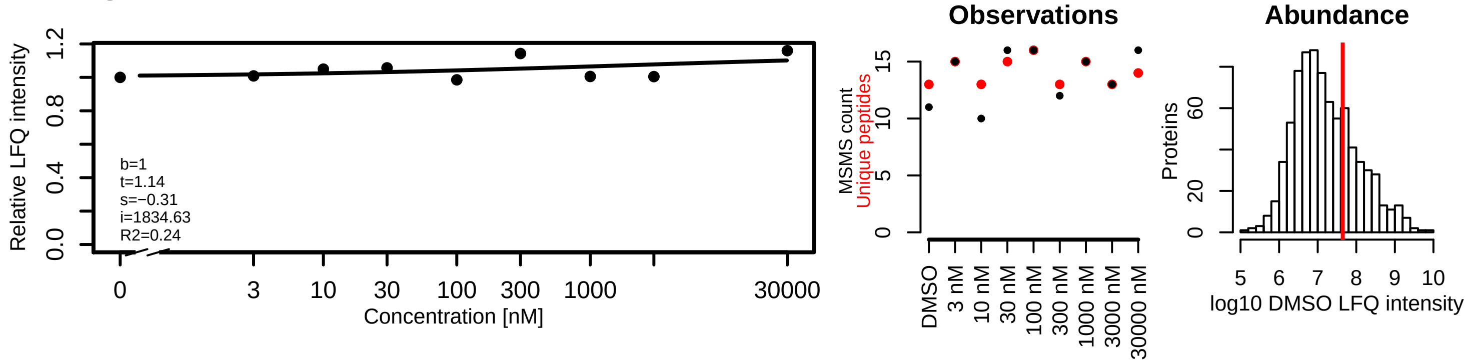


## S100A6

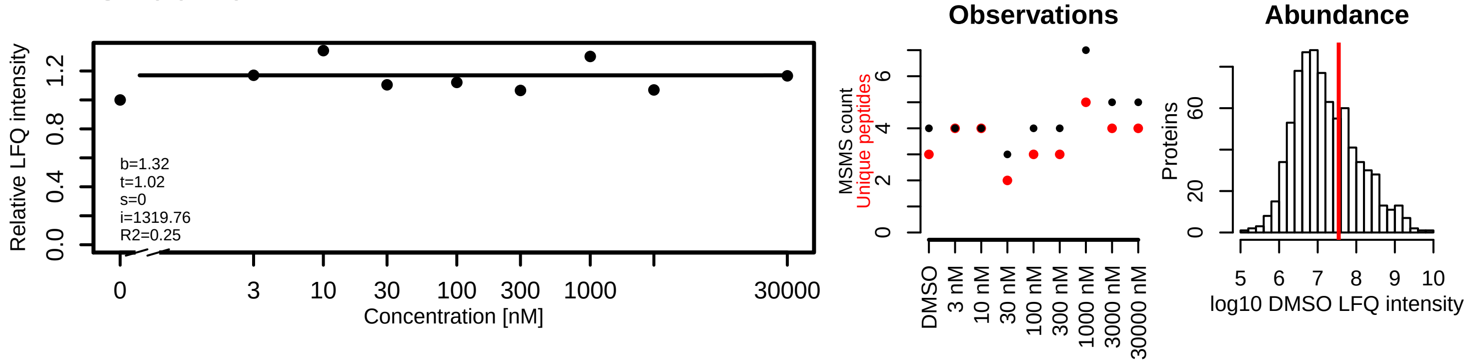


## PLGRKT

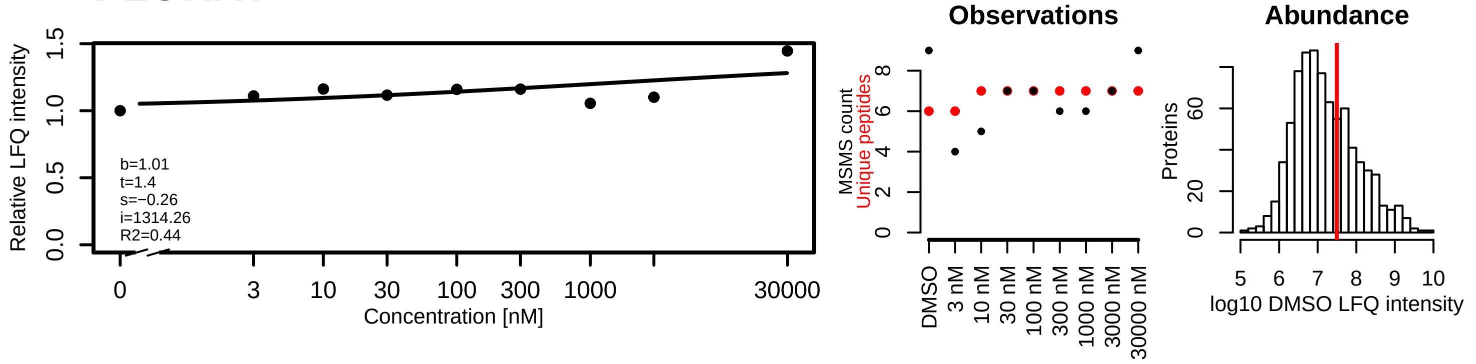


## PTDSS1

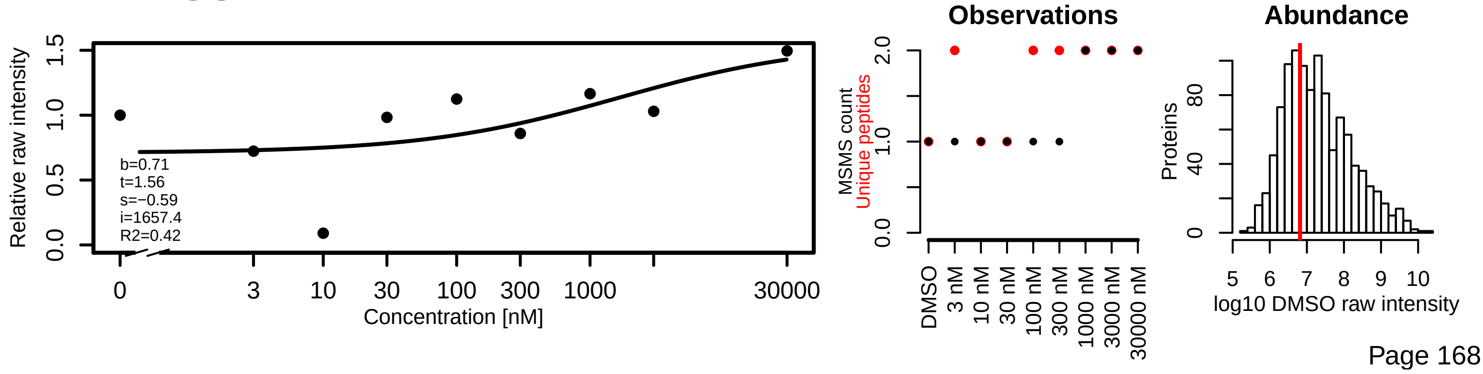

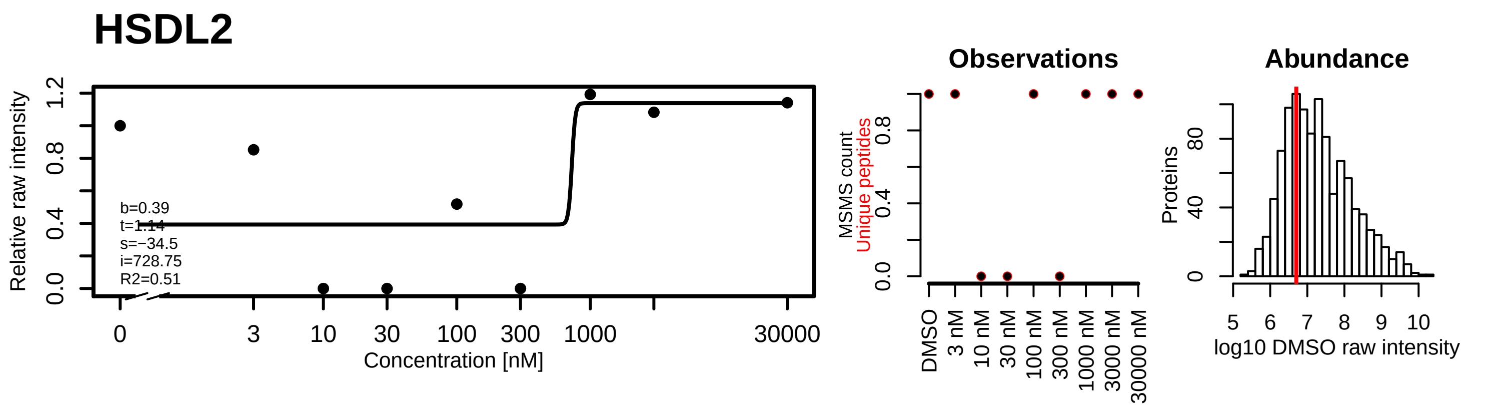
HSDL2
Relative raw intensity
b=0.39
t=1.14
s=−34.5
i=728.75
R2=0.51
Concentration [nM]
Observations
MSMS count
Unique peptides
Abundance
Proteins
log10 DMSO raw intensity

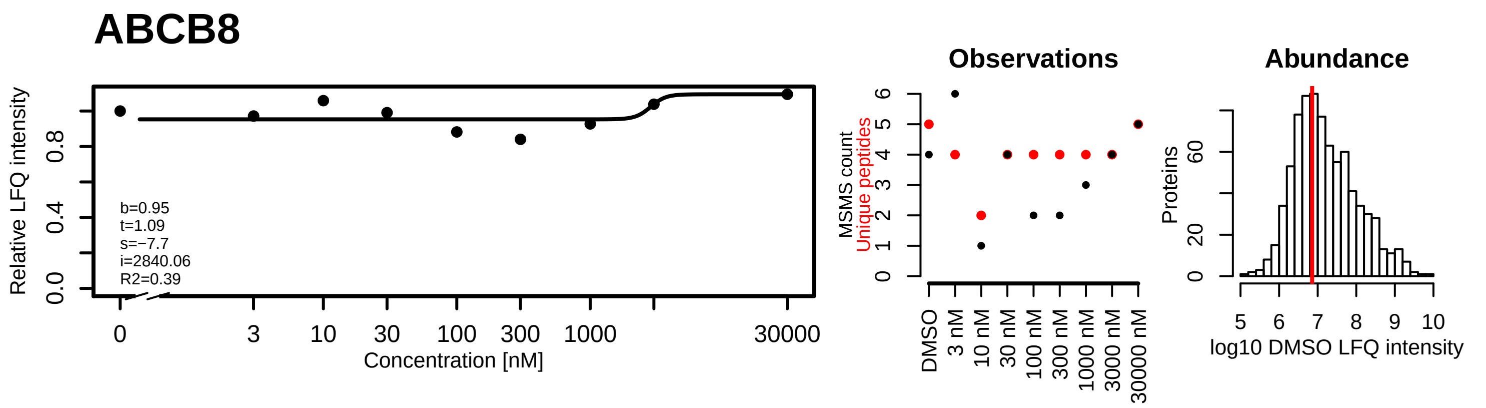
ABCB8
Relative LFQ intensity
b=0.95
t=1.09
s=−7.7
i=2840.06
R2=0.39
Concentration [nM]
Observations
MSMS count
Unique peptides
Abundance
Proteins
log10 DMSO LFQ intensity

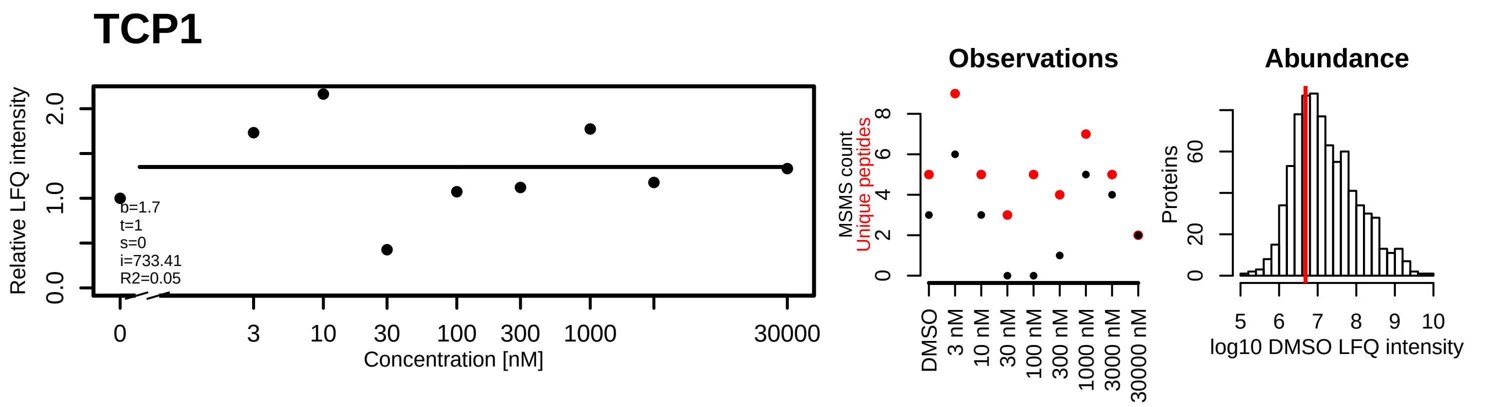
TCP1
Relative LFQ intensity
b=1.7
t=1
s=0
i=733.41
R2=0.05
Concentration [nM]
Observations
MSMS count
Unique peptides
Abundance
Proteins
log10 DMSO LFQ intensity

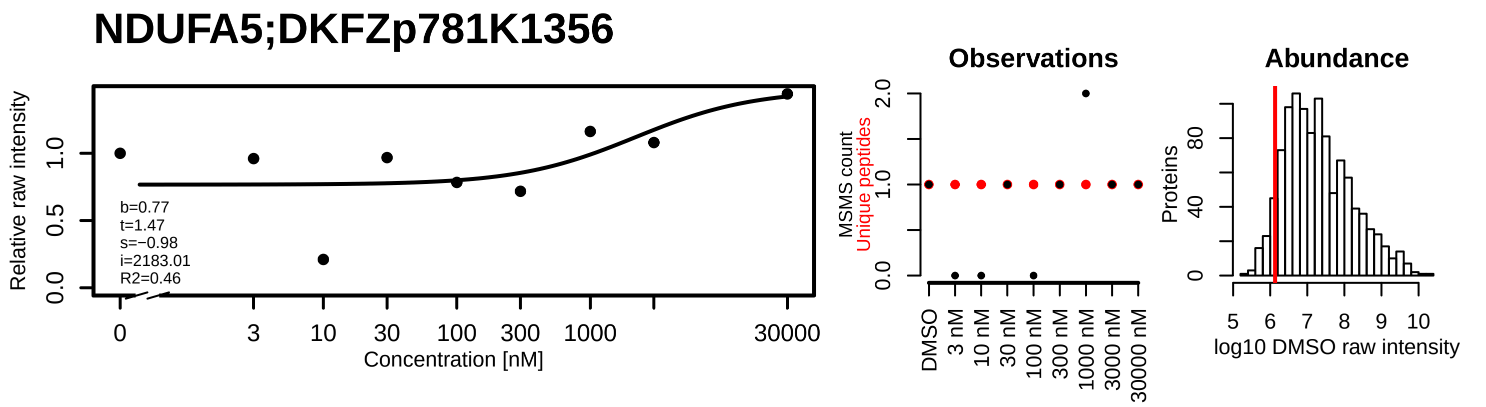
NDUFA5;DKFZp781K1356
Relative raw intensity
b=0.77
t=1.47
s=−0.98
i=2183.01
R2=0.46
Concentration [nM]
Observations
MSMS count
Unique peptides
Abundance
Proteins
log10 DMSO raw intensity

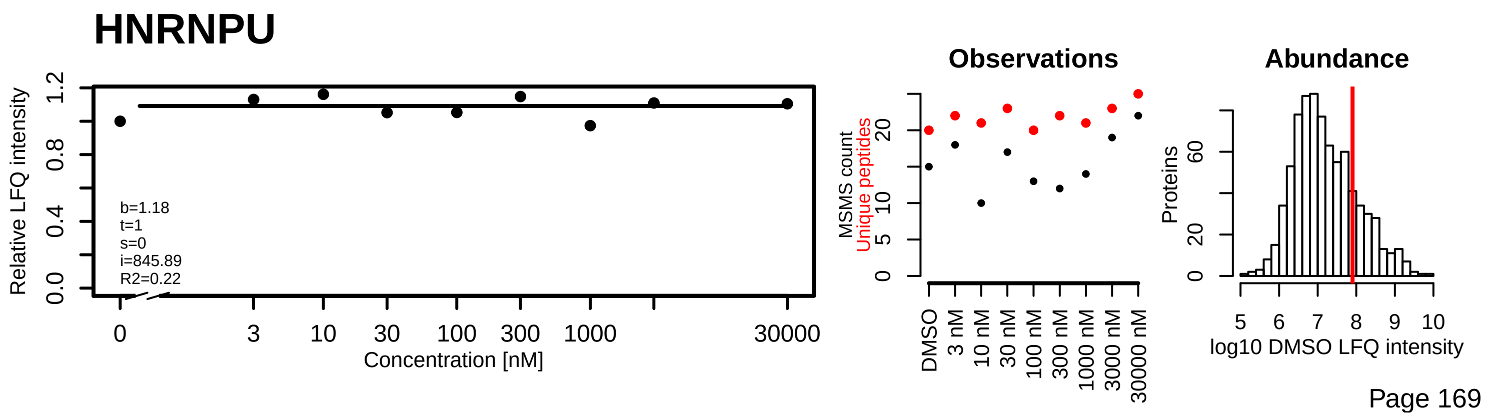
HNRNPU
Relative LFQ intensity
b=1.18
t=1
s=0
i=845.89
R2=0.22
Concentration [nM]
Observations
MSMS count
Unique peptides
Abundance
Proteins
log10 DMSO LFQ intensity

## KHSRP

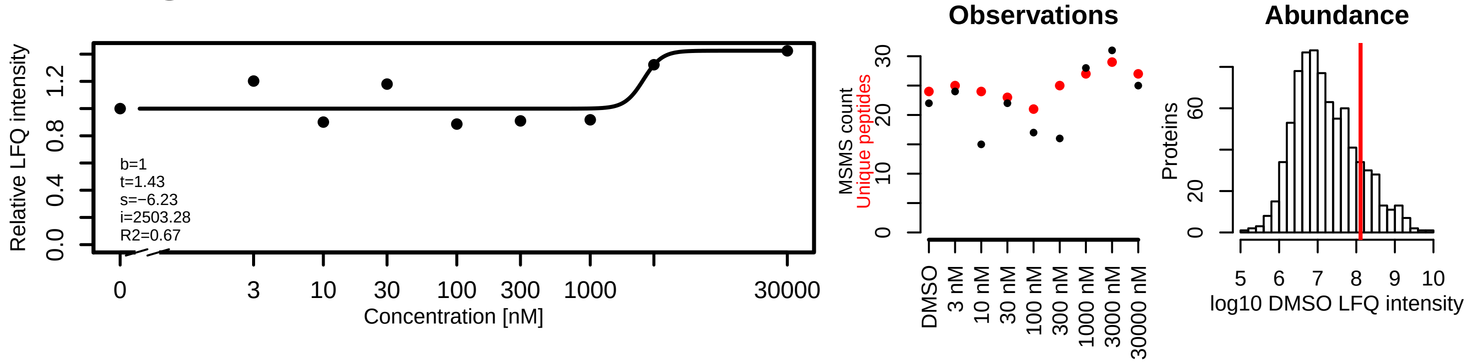


## RTN4

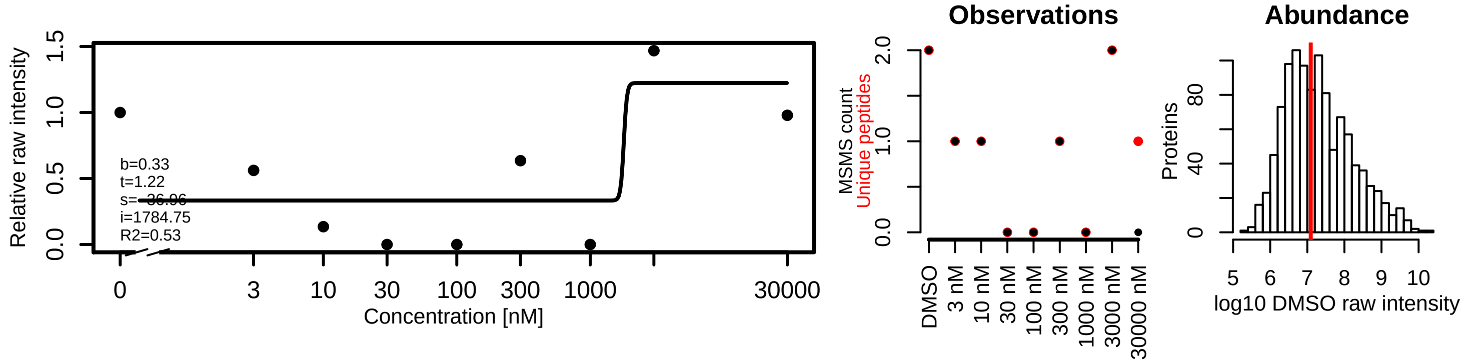


## CTSL1;CTSL3P;CTSK;CTSL2

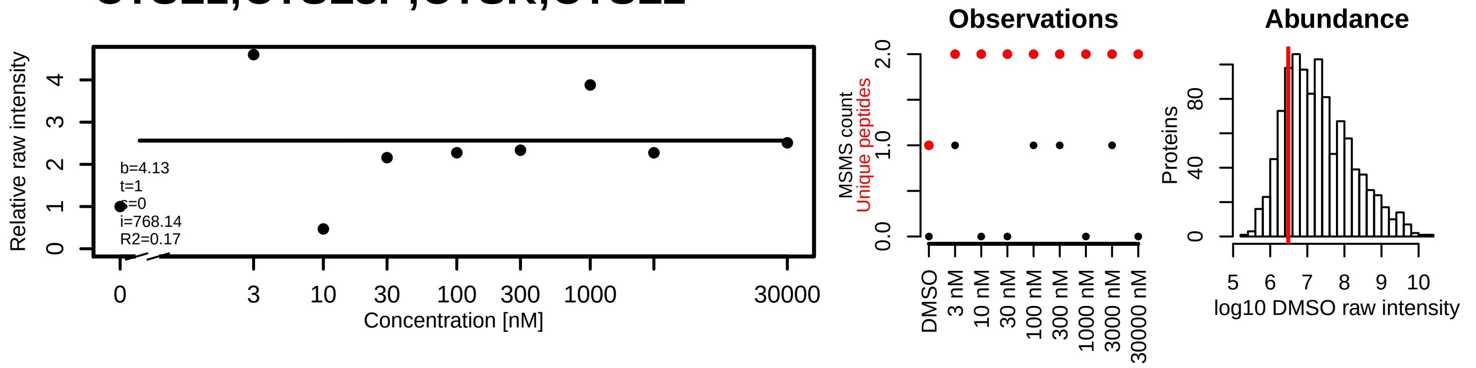


## ATP2A2

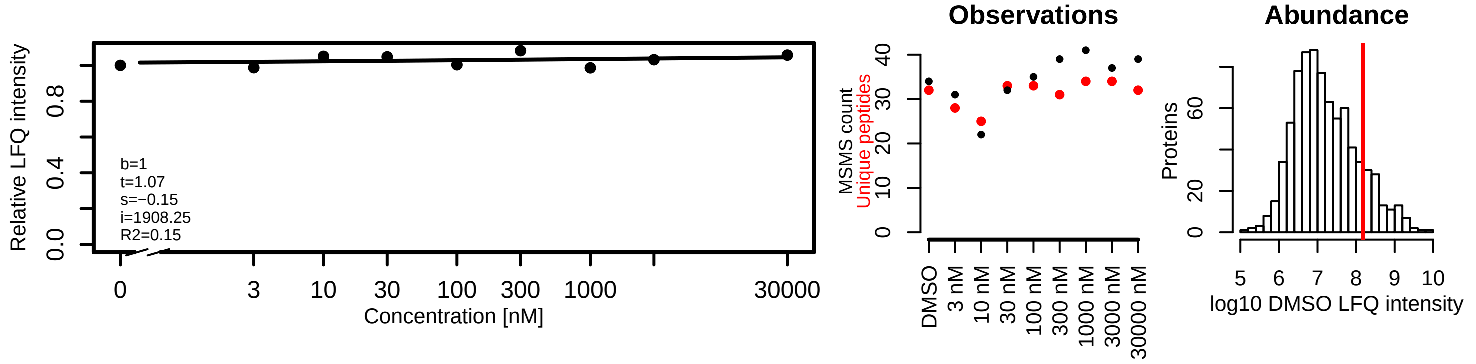


## RAB6B;RAB6A;RAB39A;RAB6C

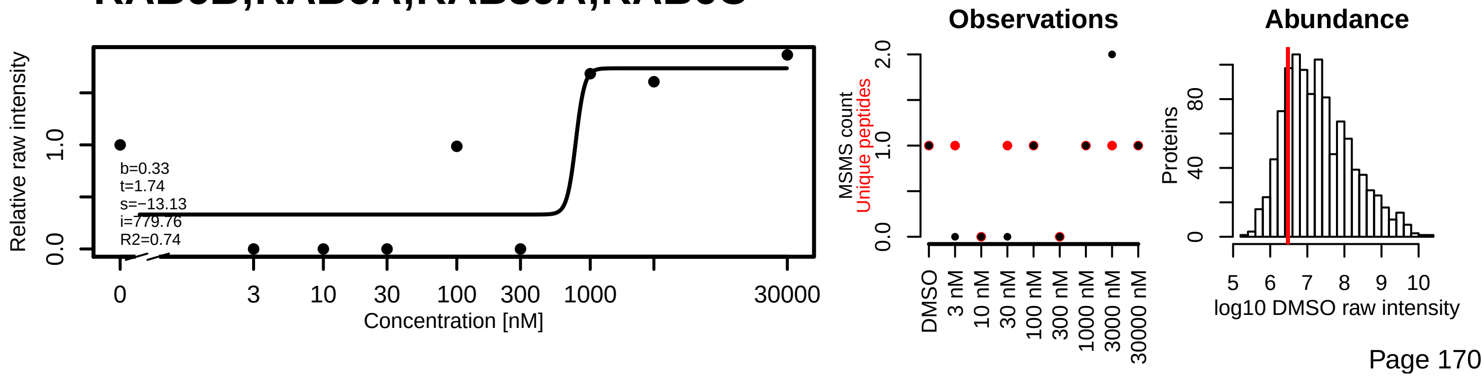

## SUPT16H

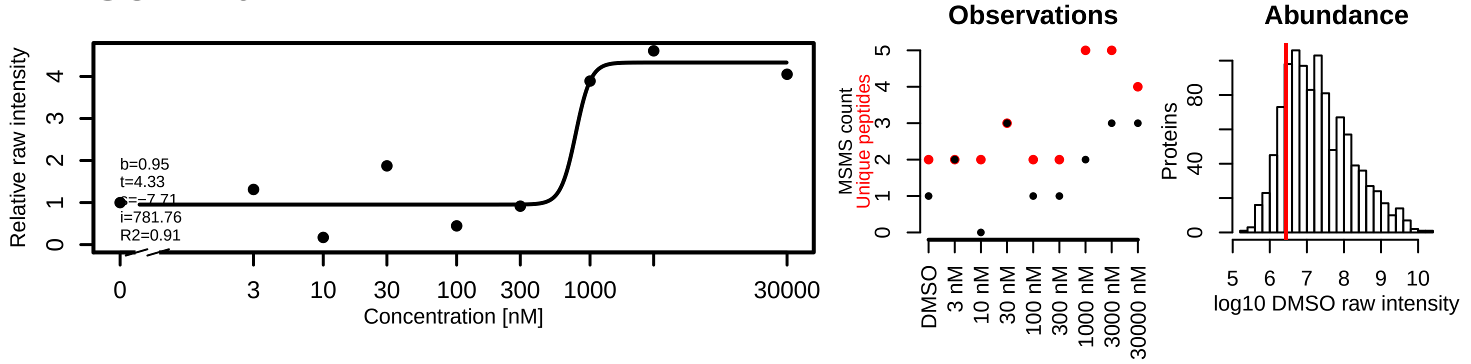


## TXN

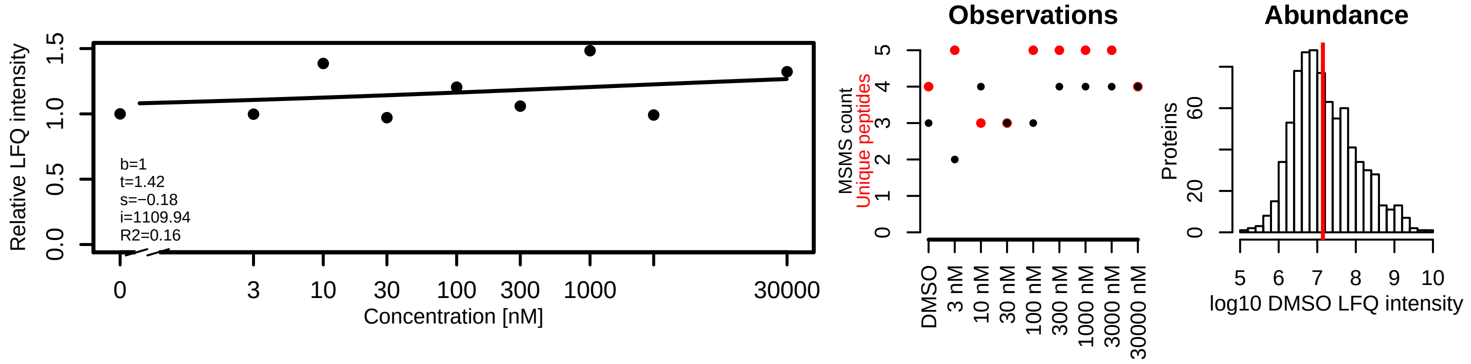


## PRKAA2

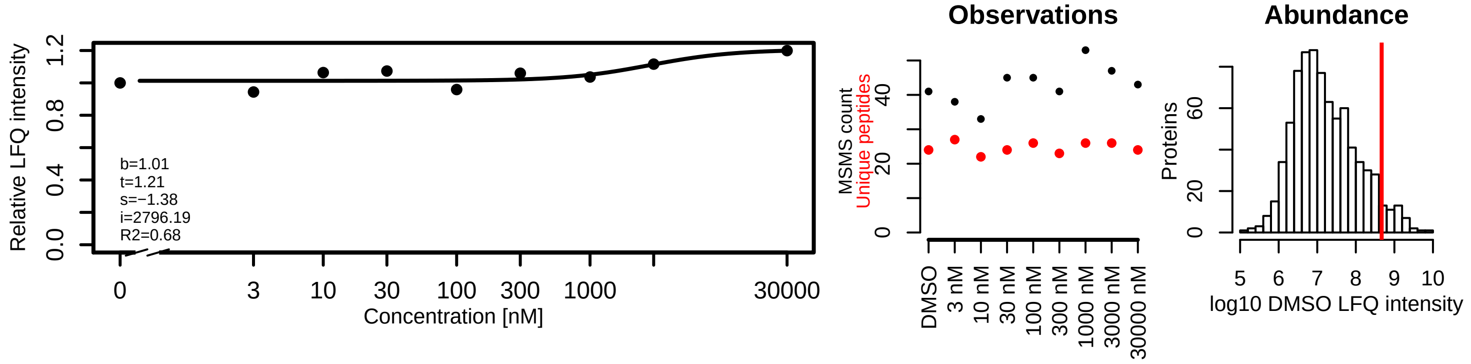


## MAPK15

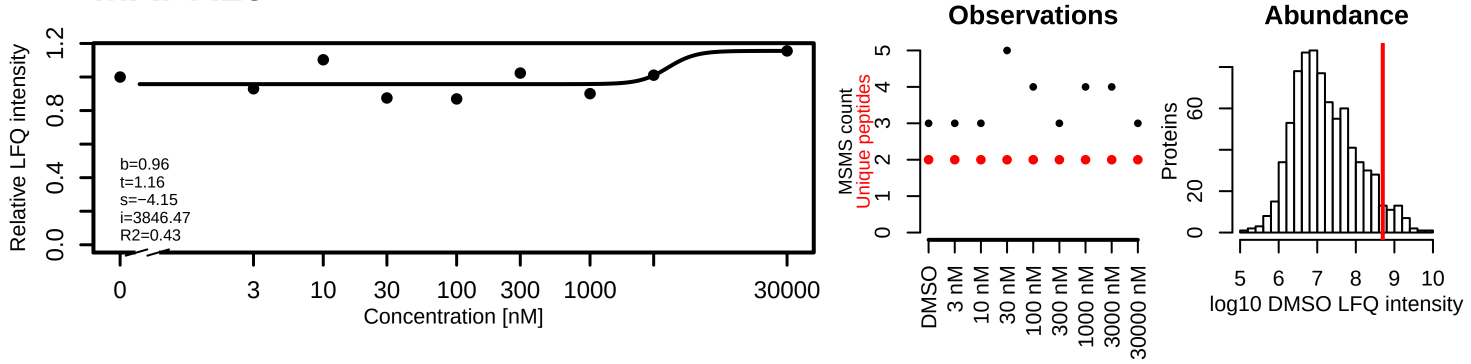


## KDELR3

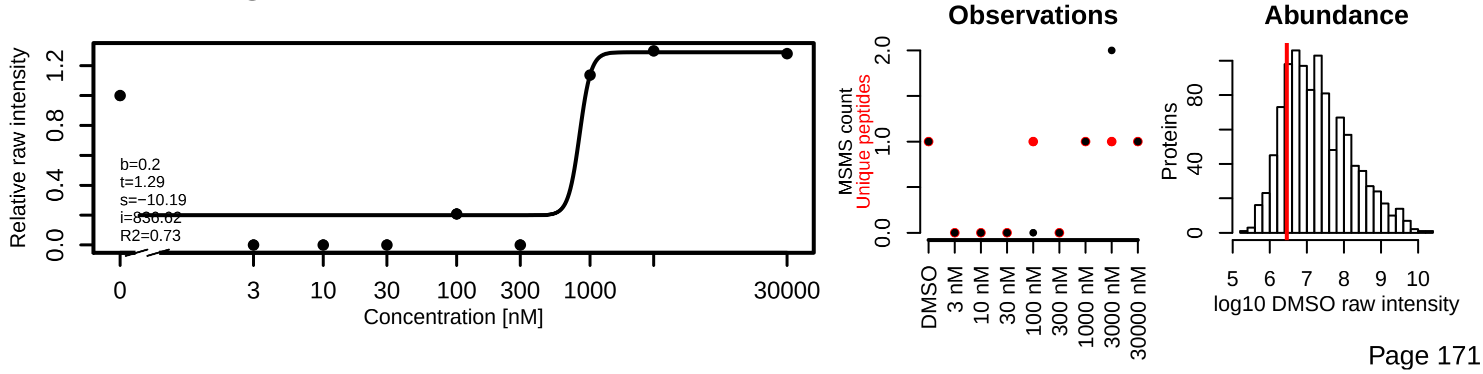

## SLC16A3

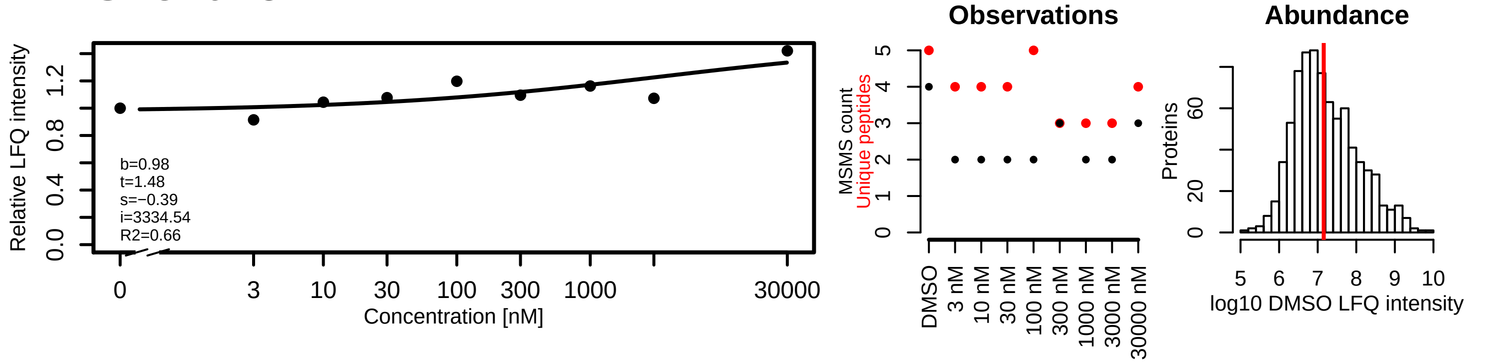


## PSMC6

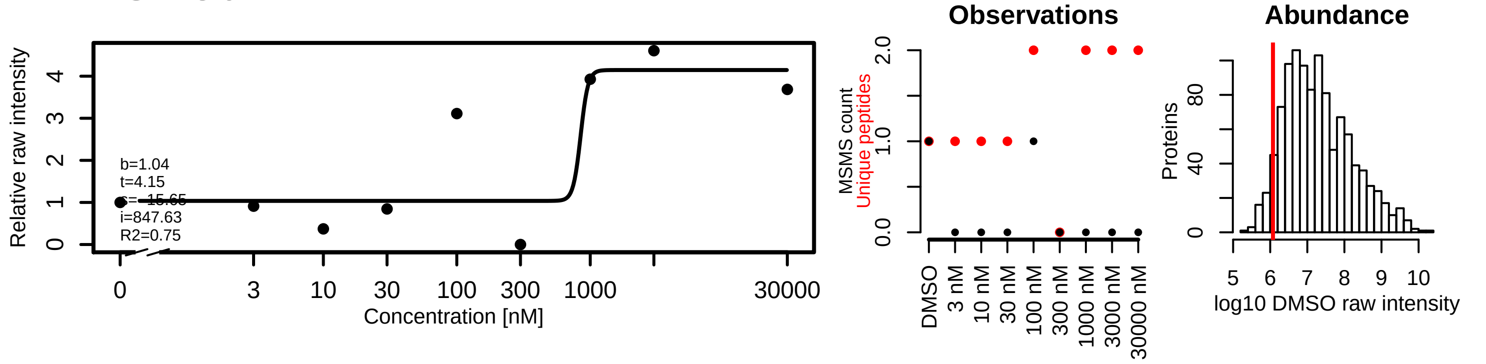


## NEK3

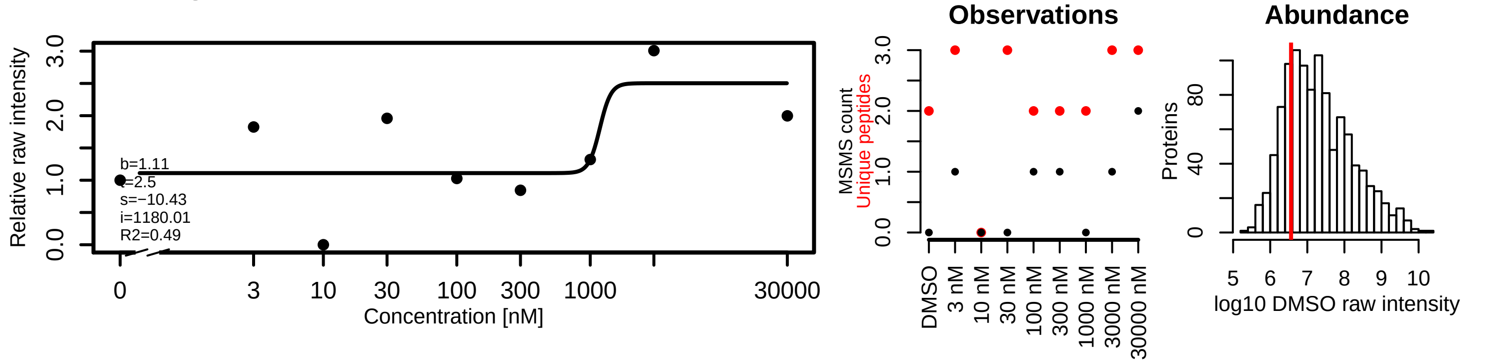


## FAM58BP

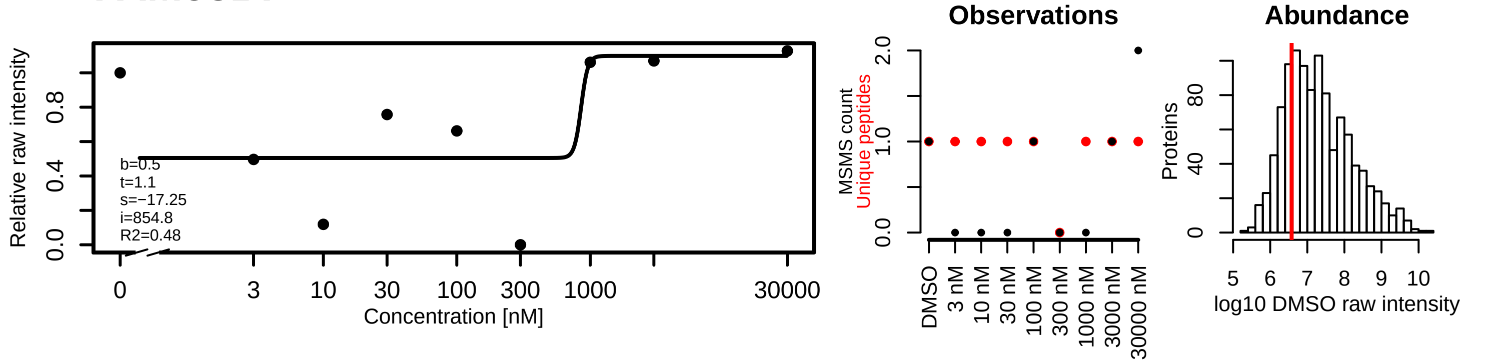


## PHB

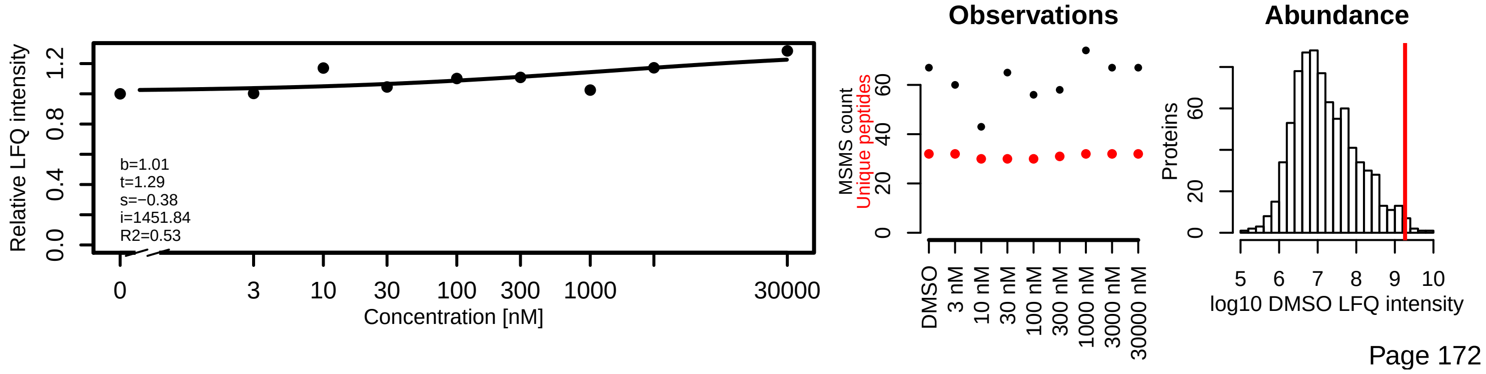

# ALDH1A2

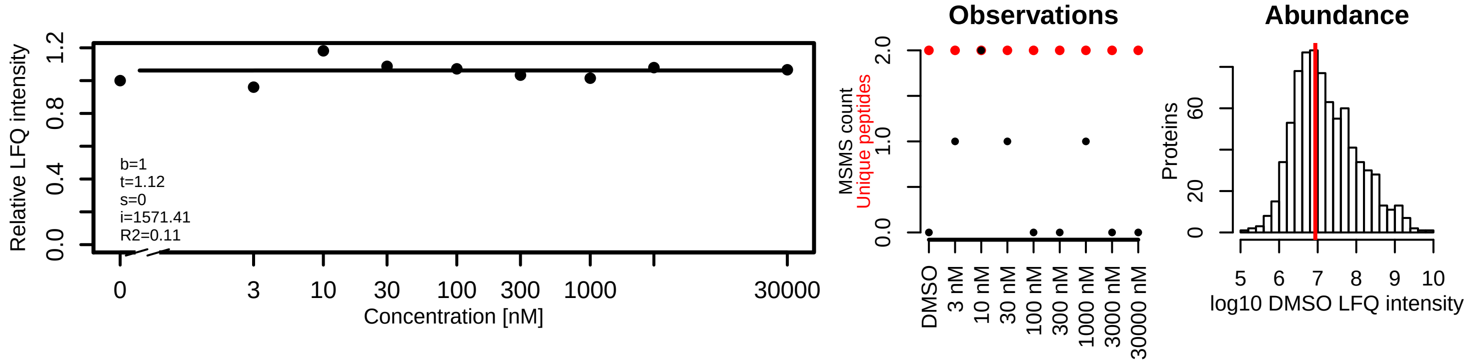


# LATS2

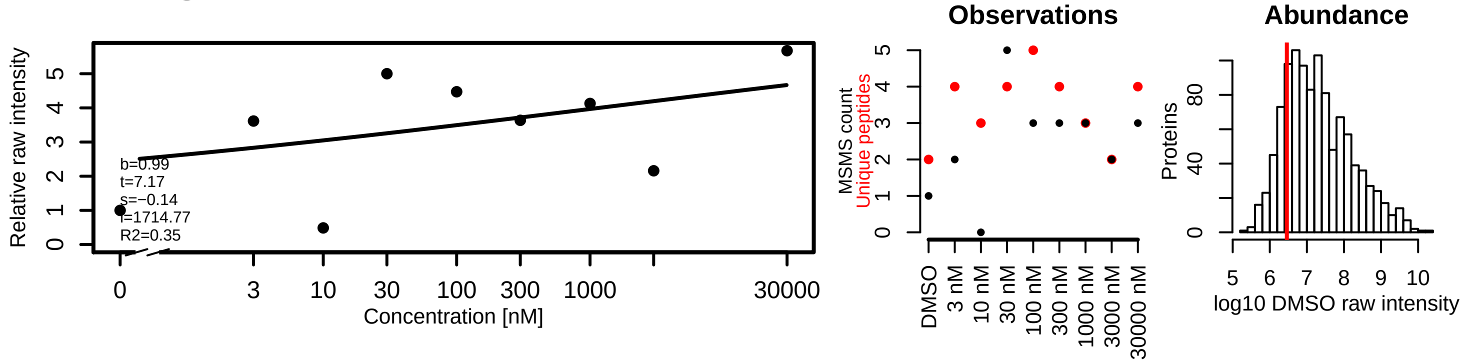


# BST2

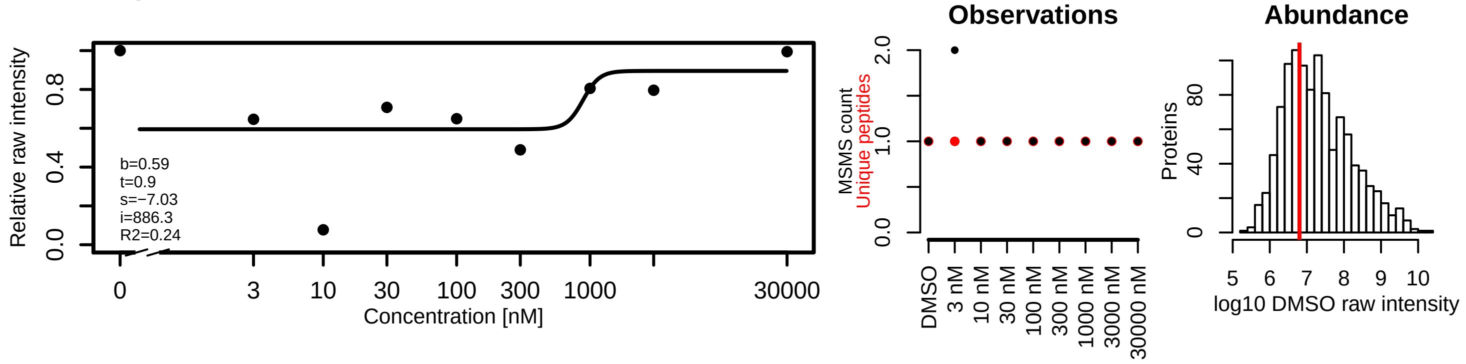


# VIM

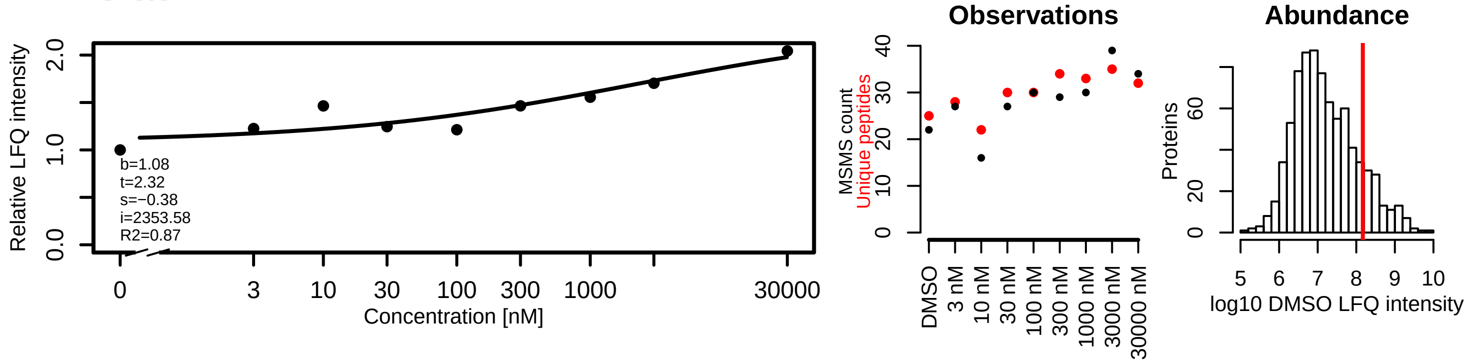


# RBM14 − Q96PK6;Q96PK6−2;Q96PK6−3;Q96PK6−4;F8WDX3;F2Z2W2

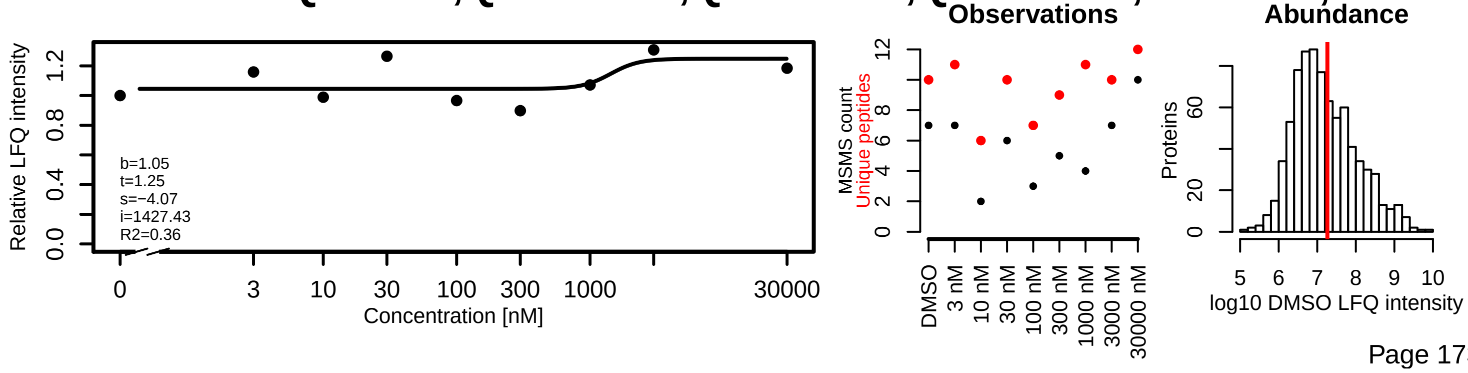

# TMEM43

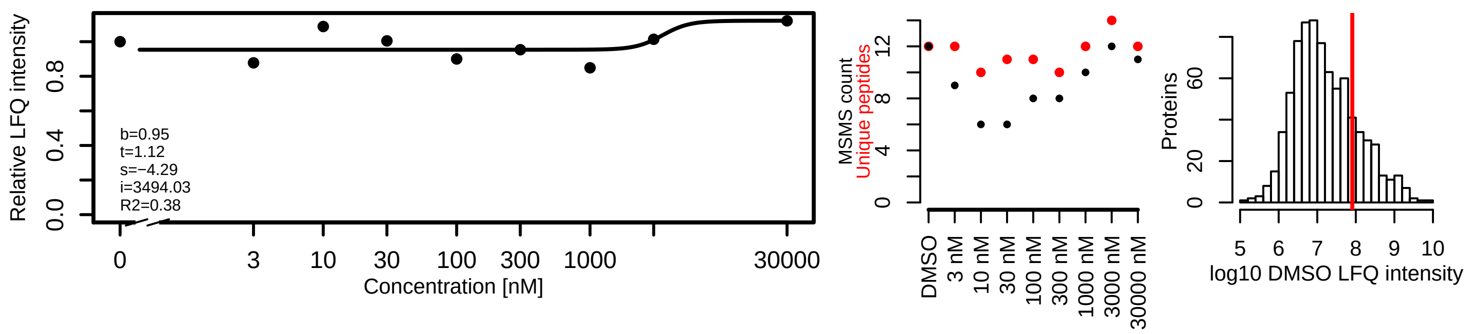


# ATP6V0A1

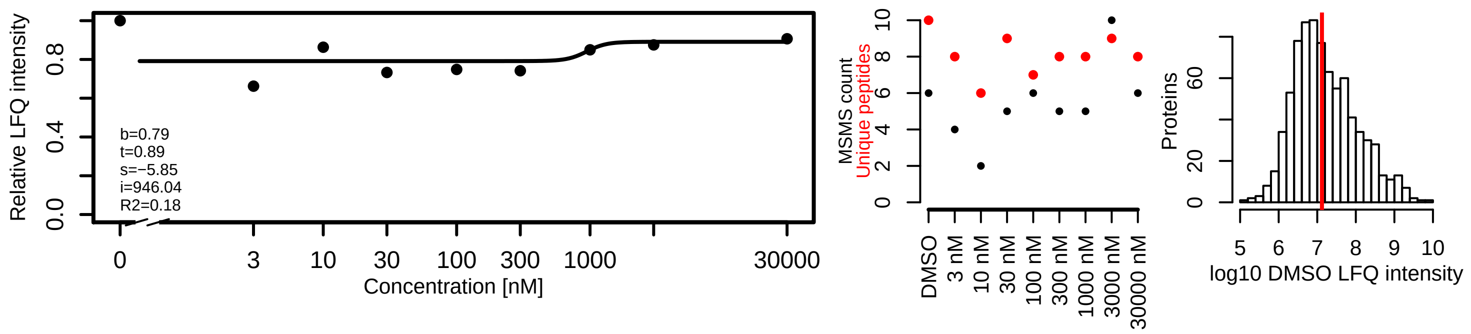


# BCAP31

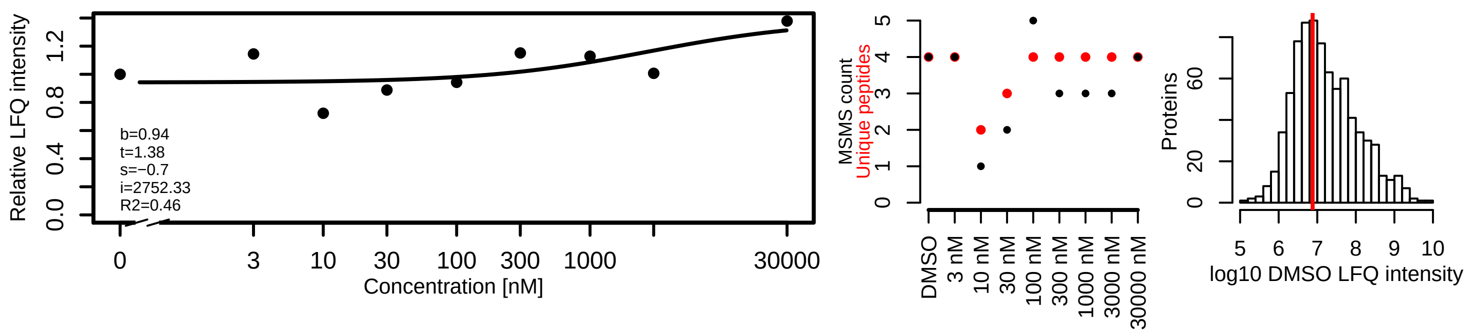


# PPM1G

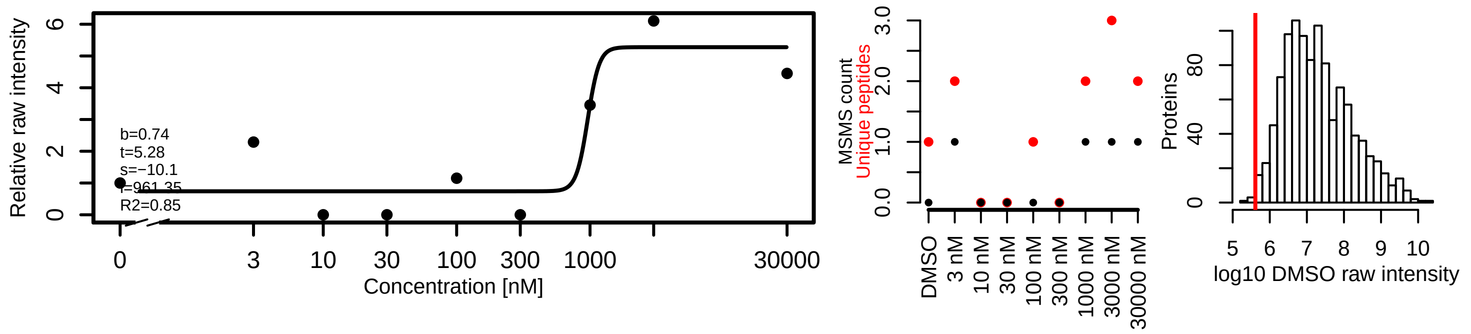


# ANP32B

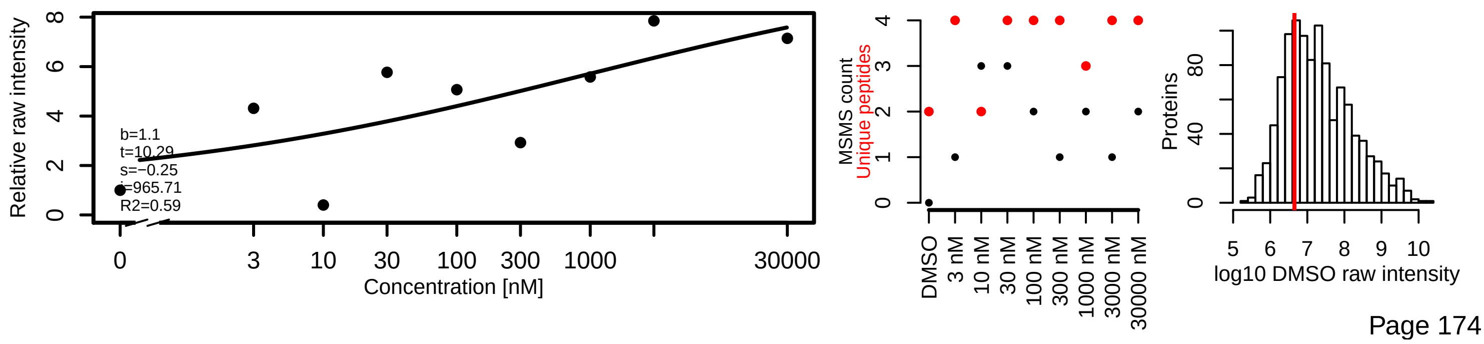

## STK3

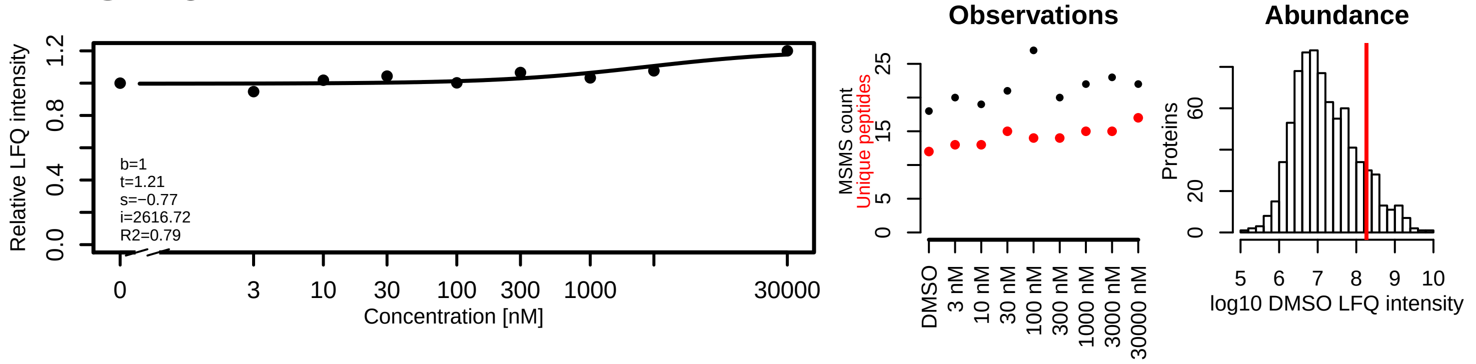


## ELAVL1

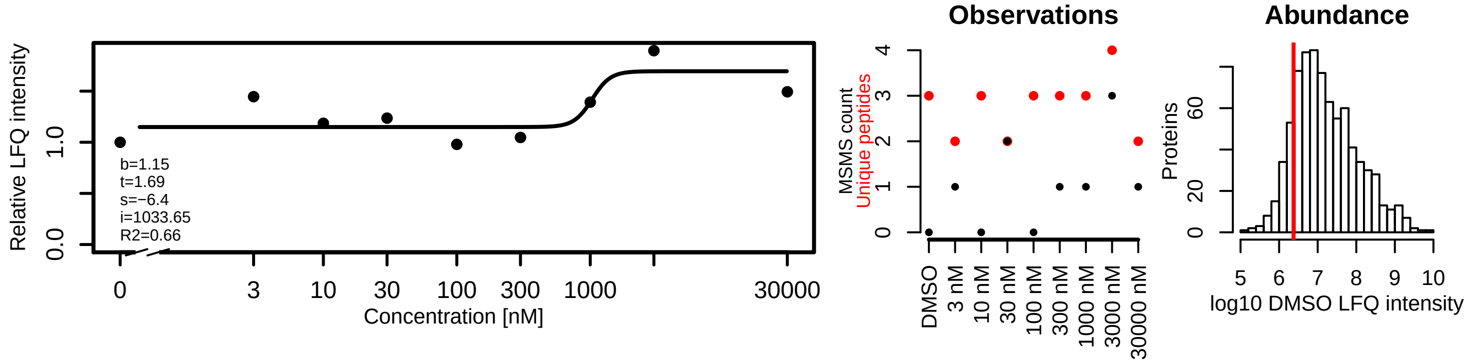


## FLOT1

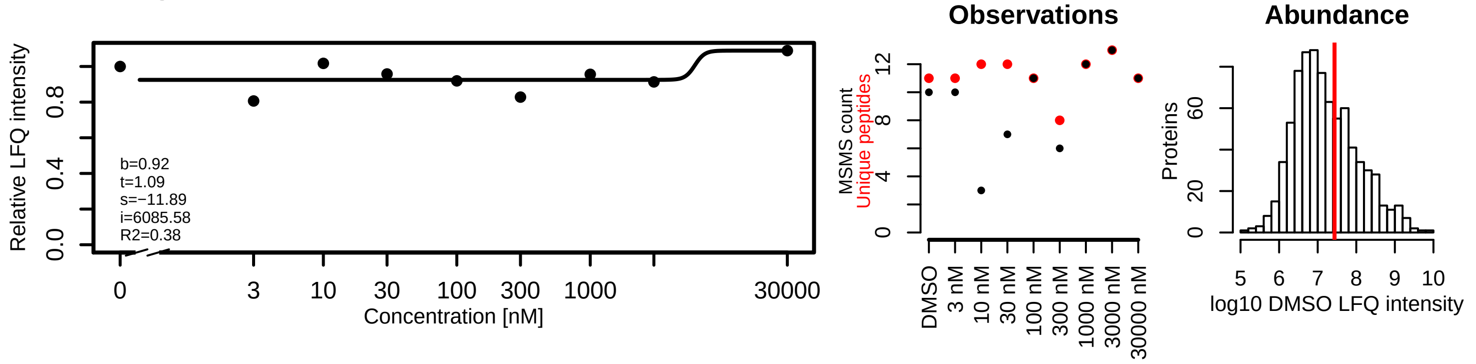


## PRKCI

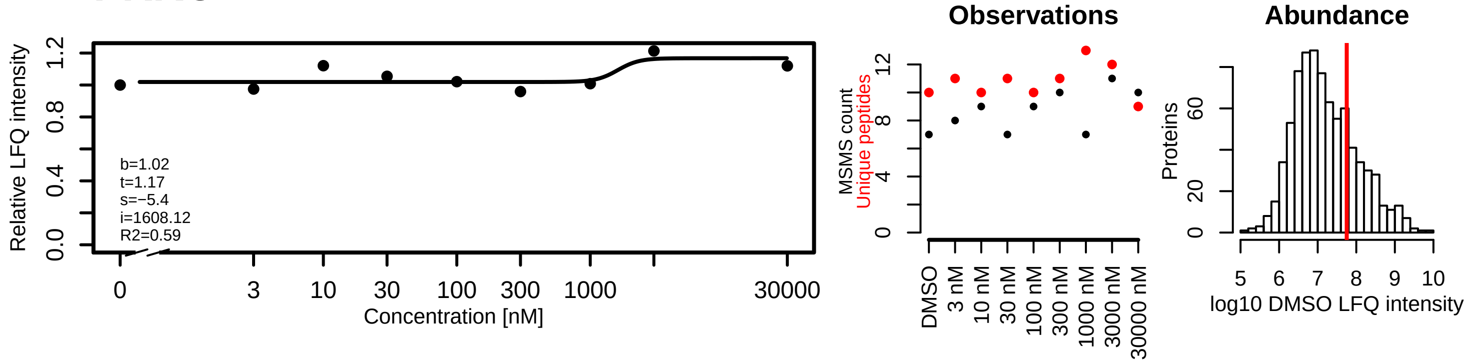


## AGPS

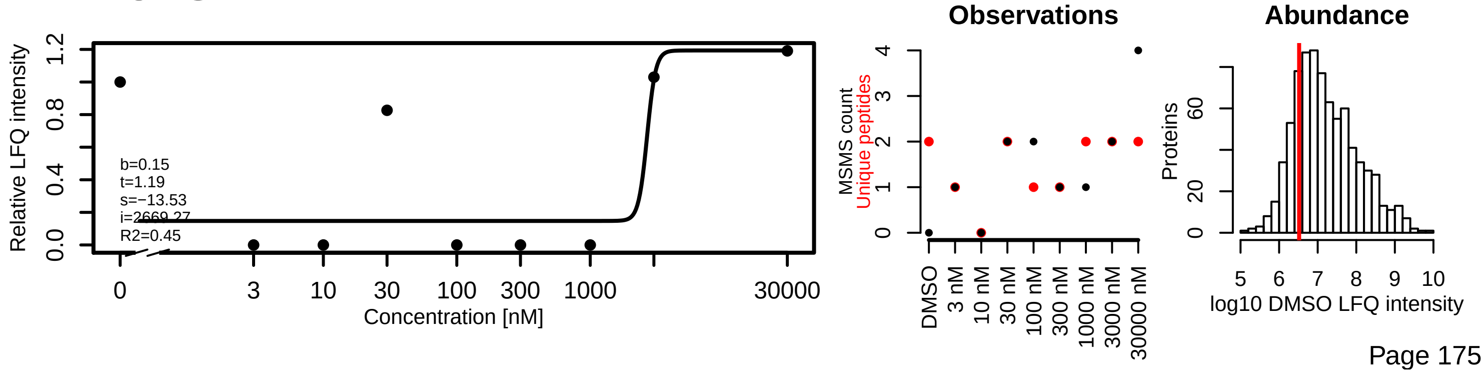

# SEC61A1

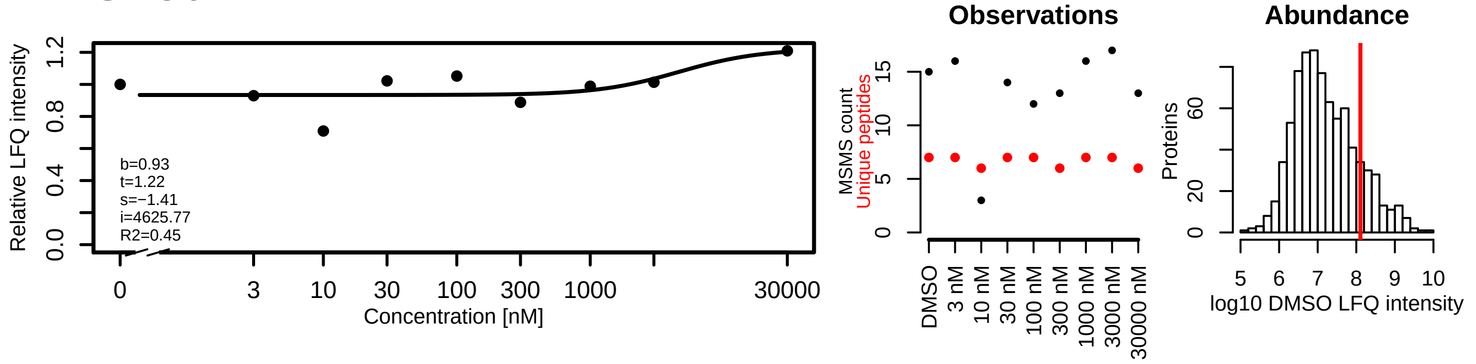


# UHMK1

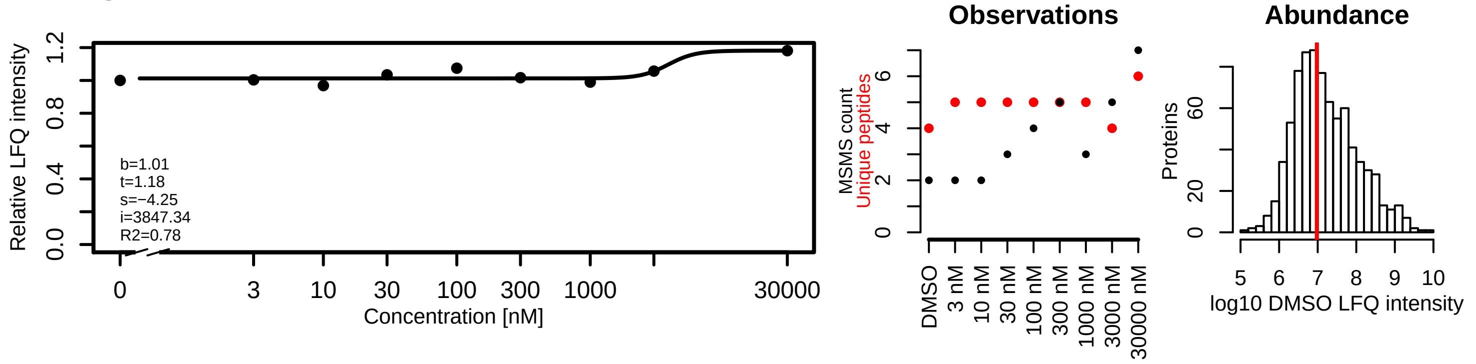


# RASSF1

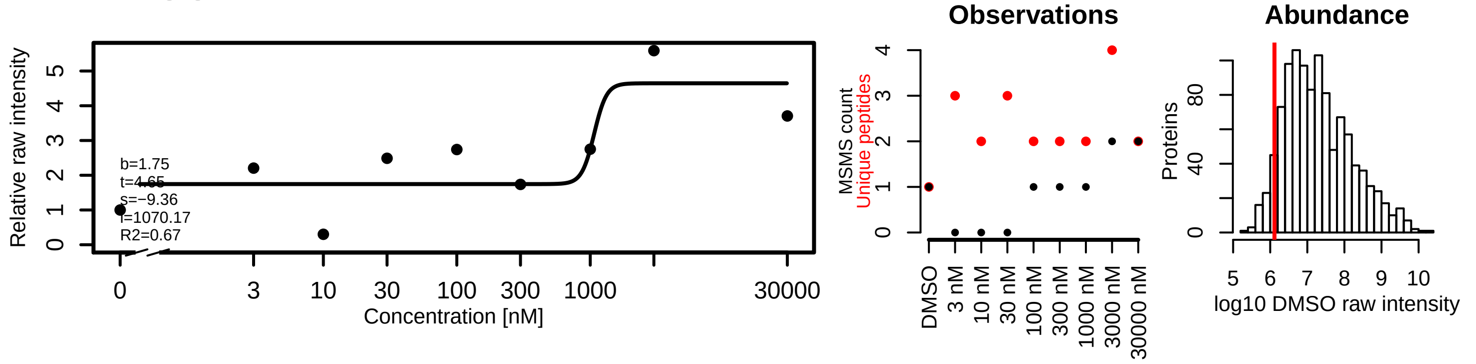


# PGAM5

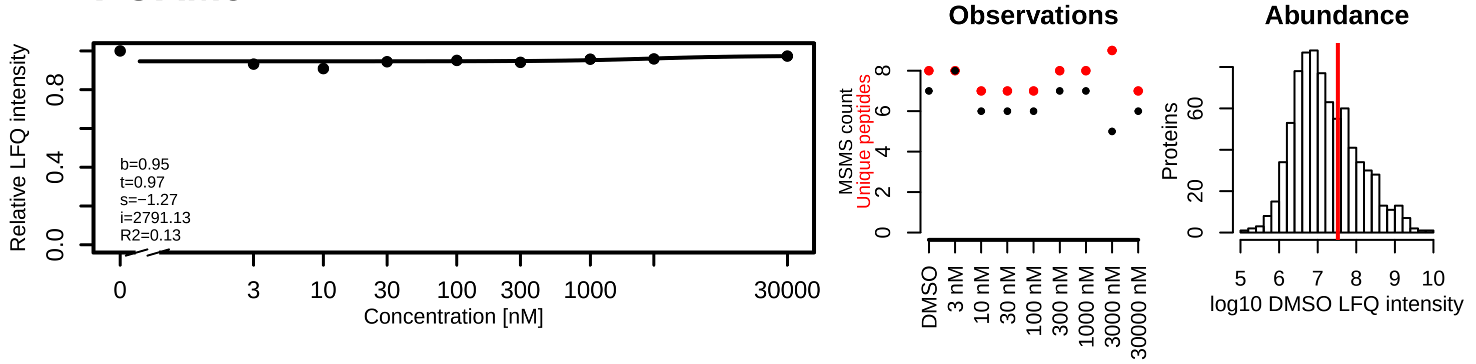


# CAMKK2

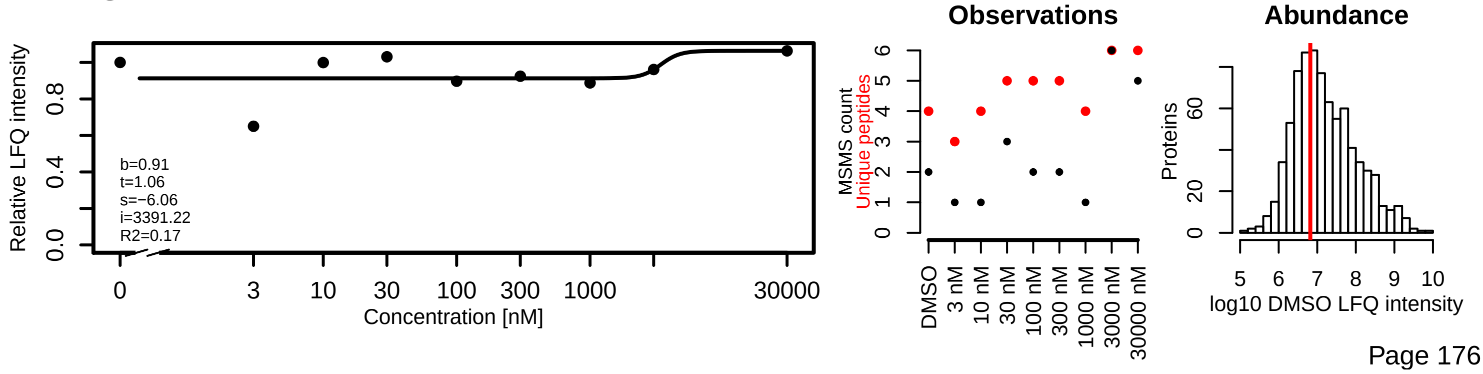

## TARDBP

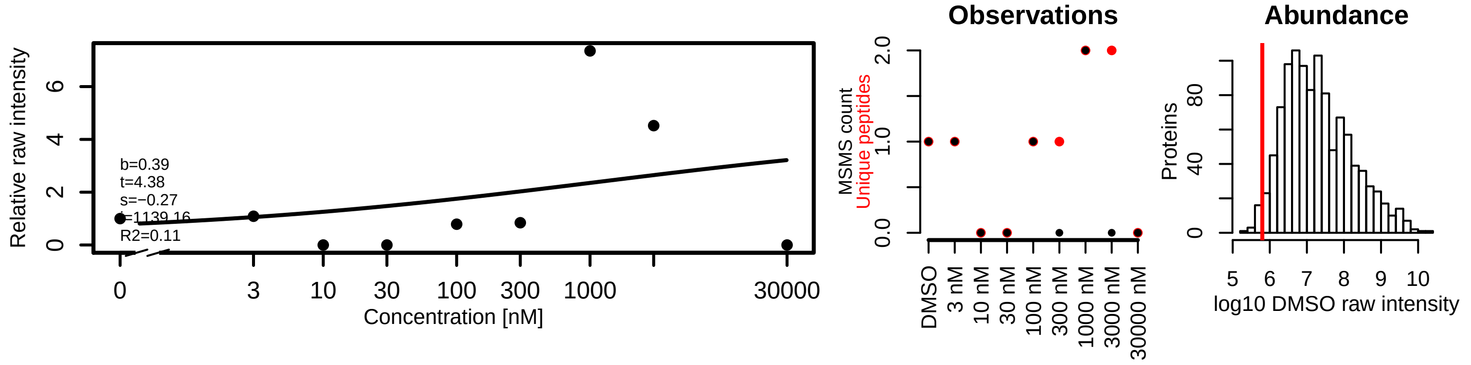


## ORMDL3

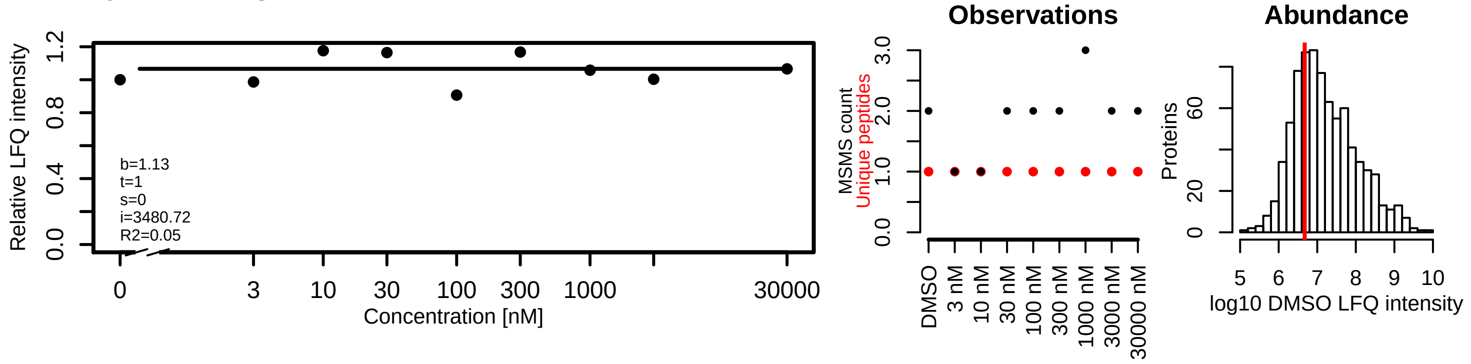


## PTPLAD1

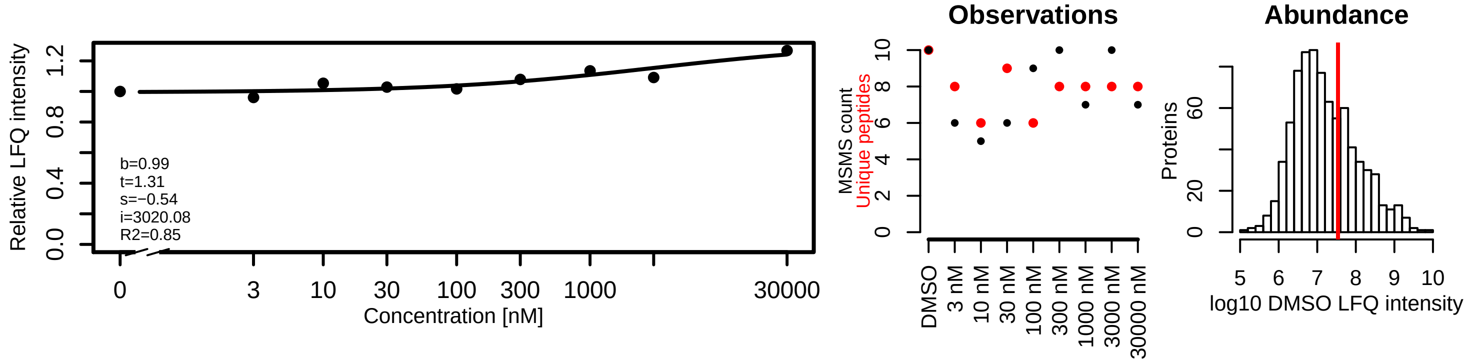


## RPS28

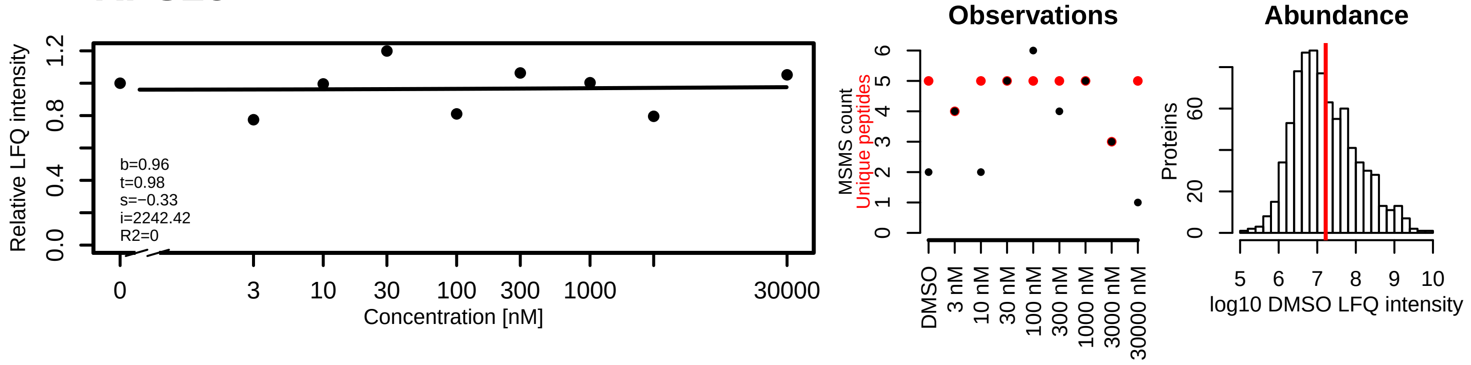


## OPA1

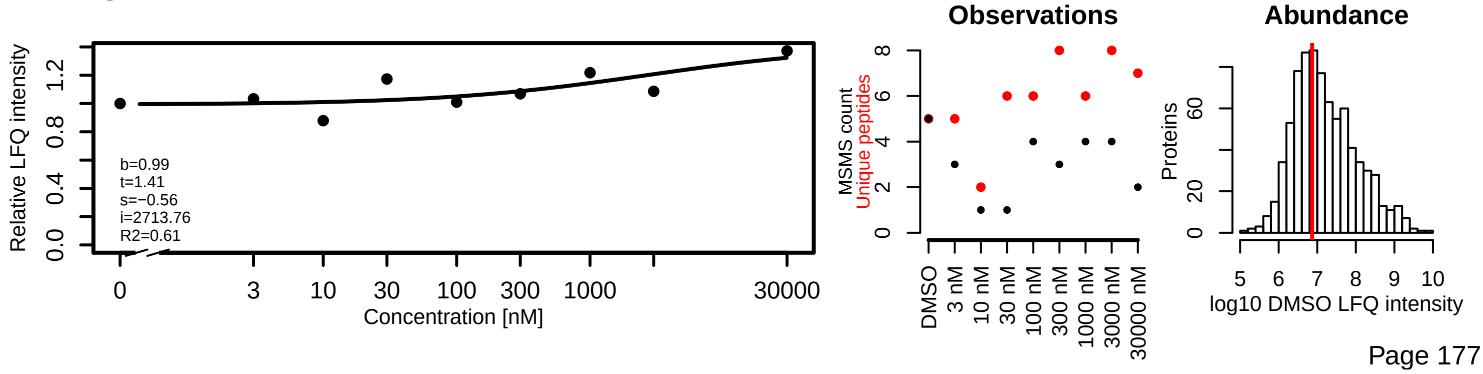

## CKAP4

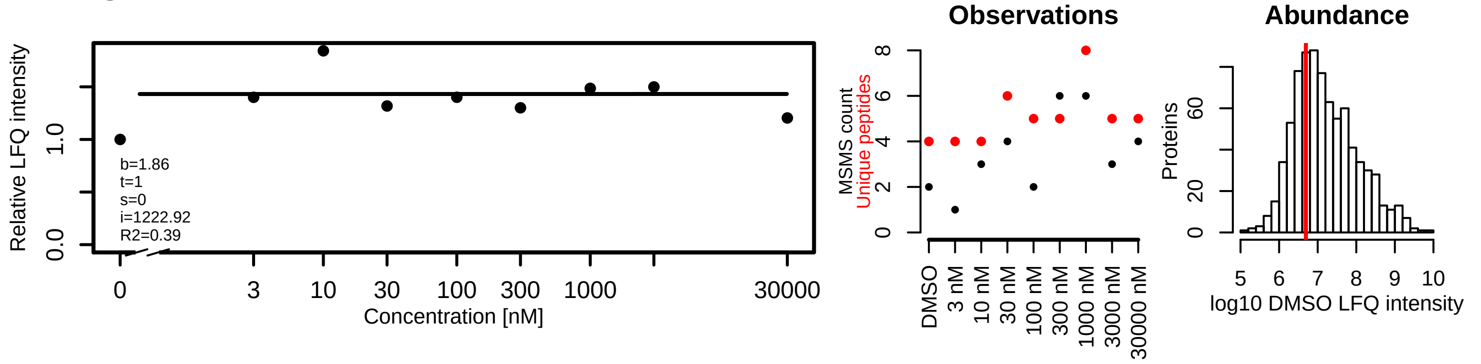


## MAP3K6

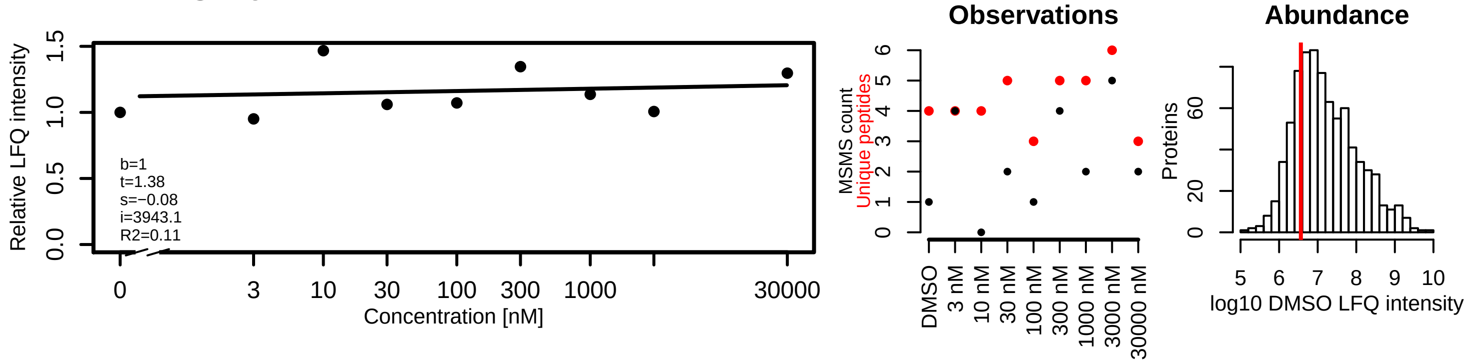


## RTN3

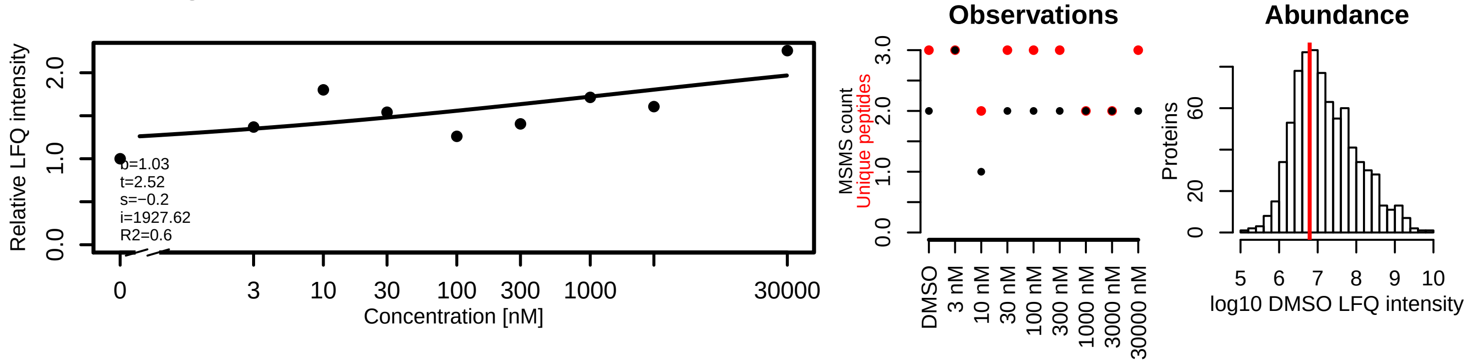


## LBR

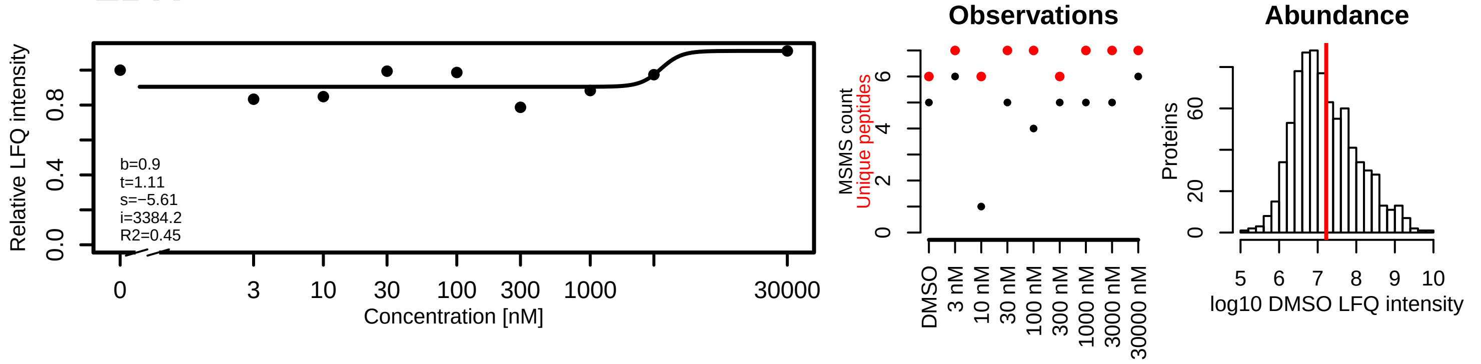


## RPS19

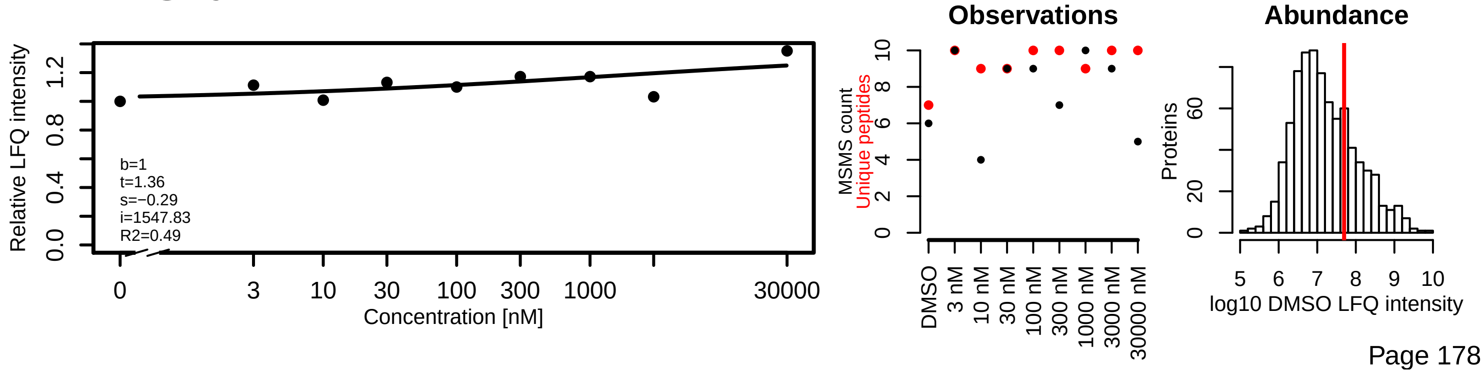

## ERLIN2

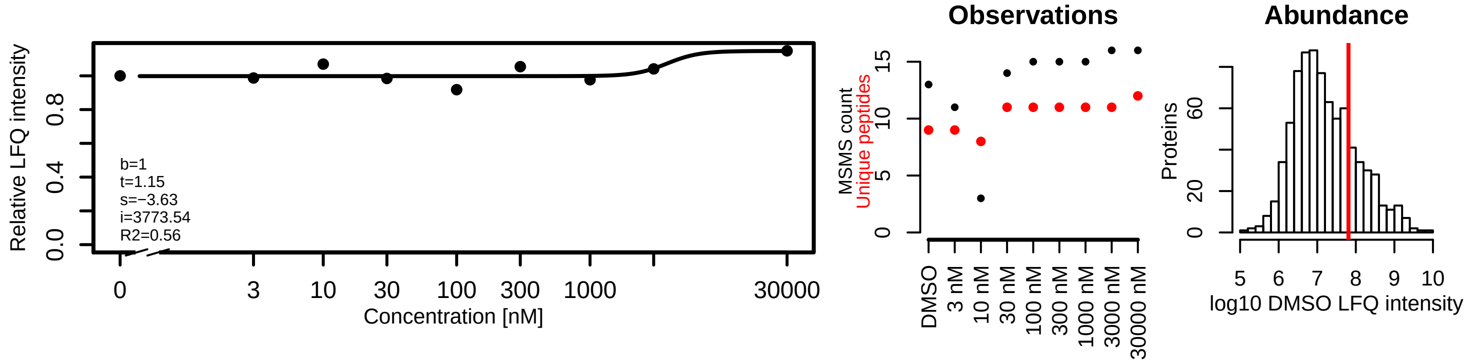


## WFS1

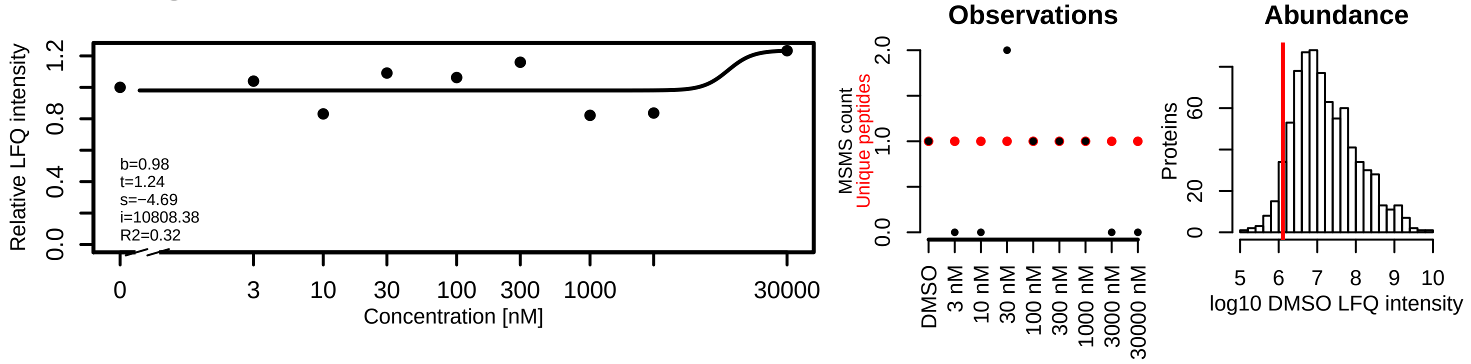


## CHEK1

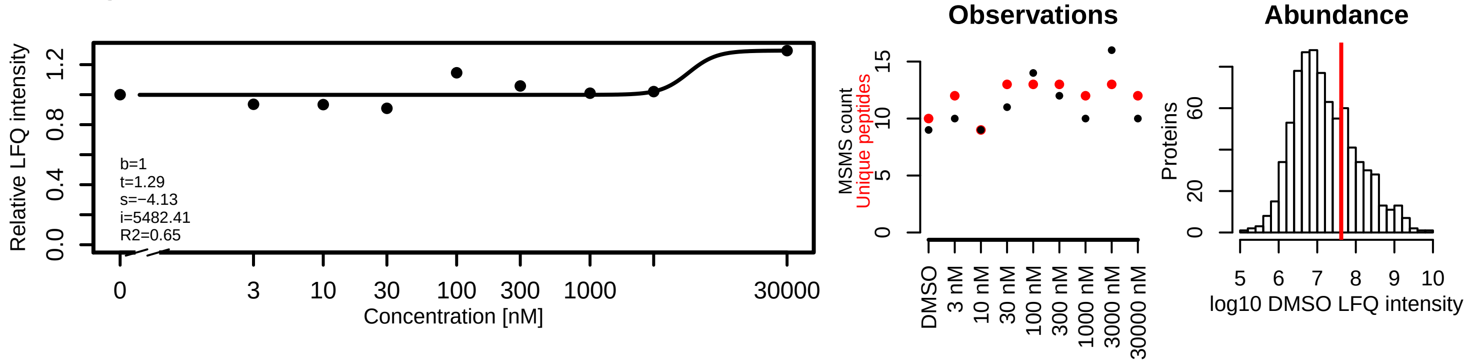


## PHB2

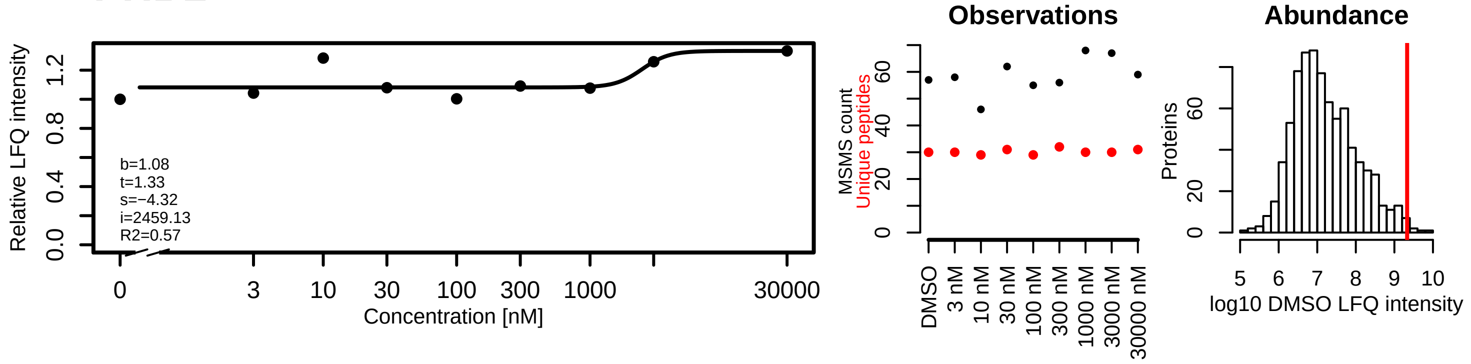


## SFXN2

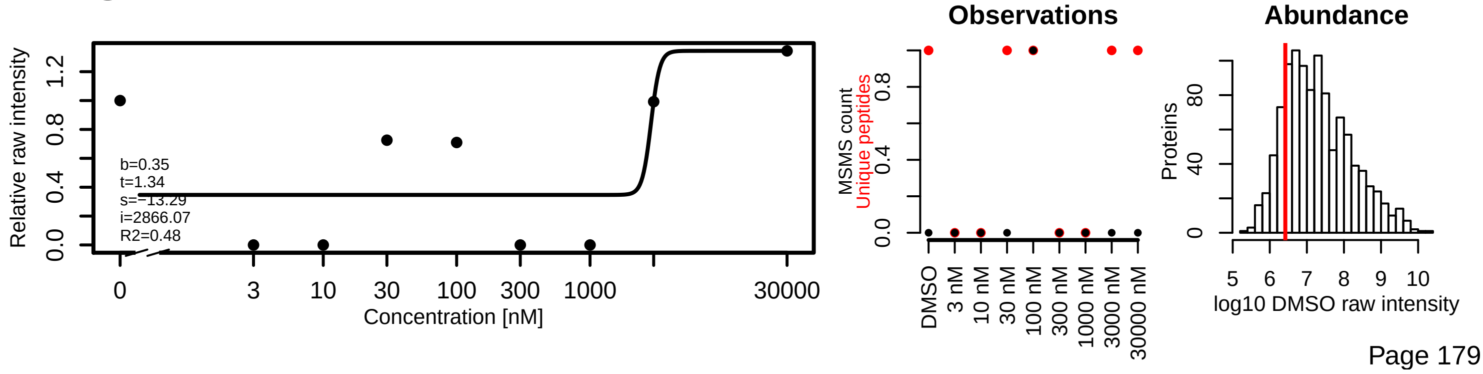

# VAPA

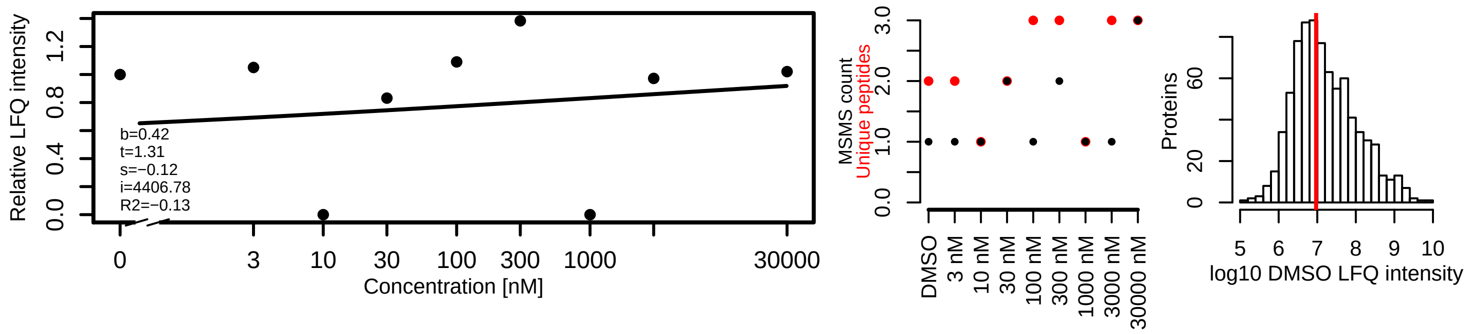


# MTCH2

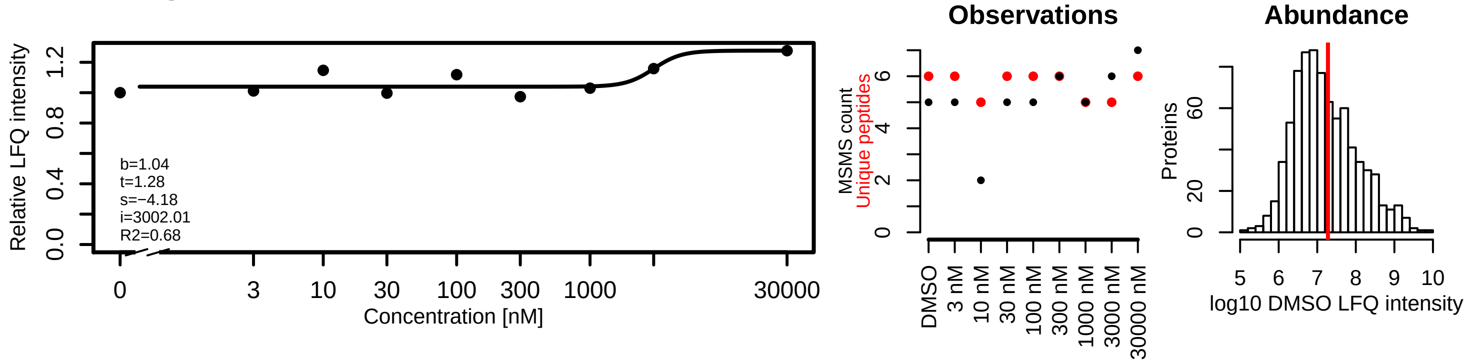


# STT3A

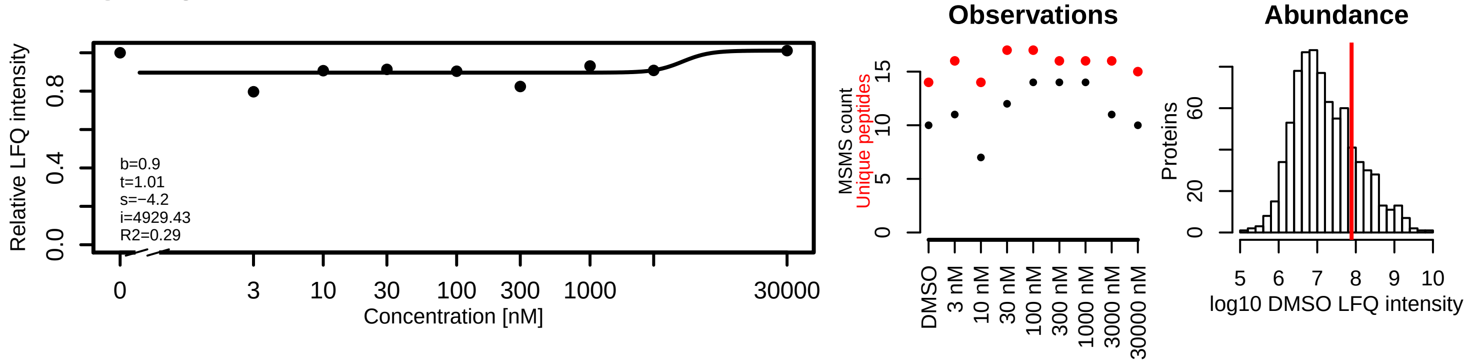


# SEC11A

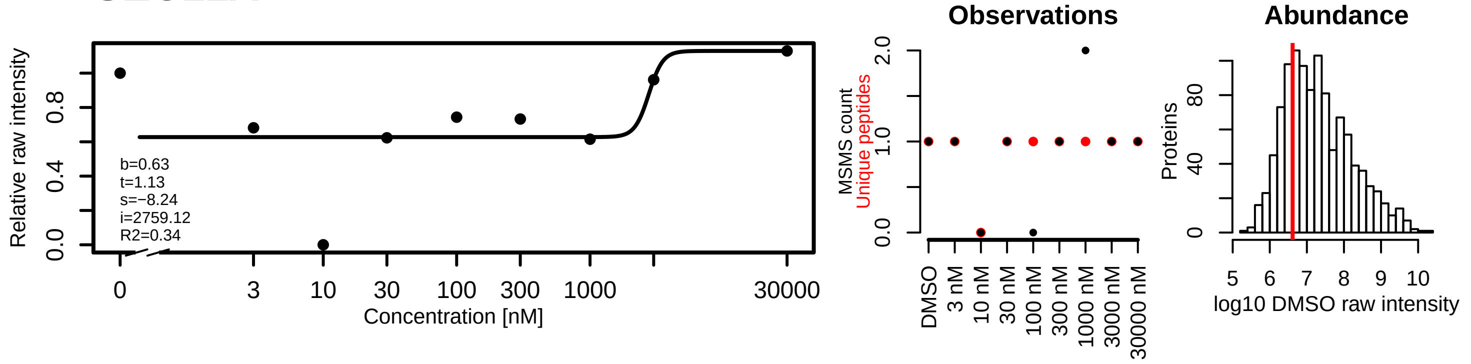


# PABPC1

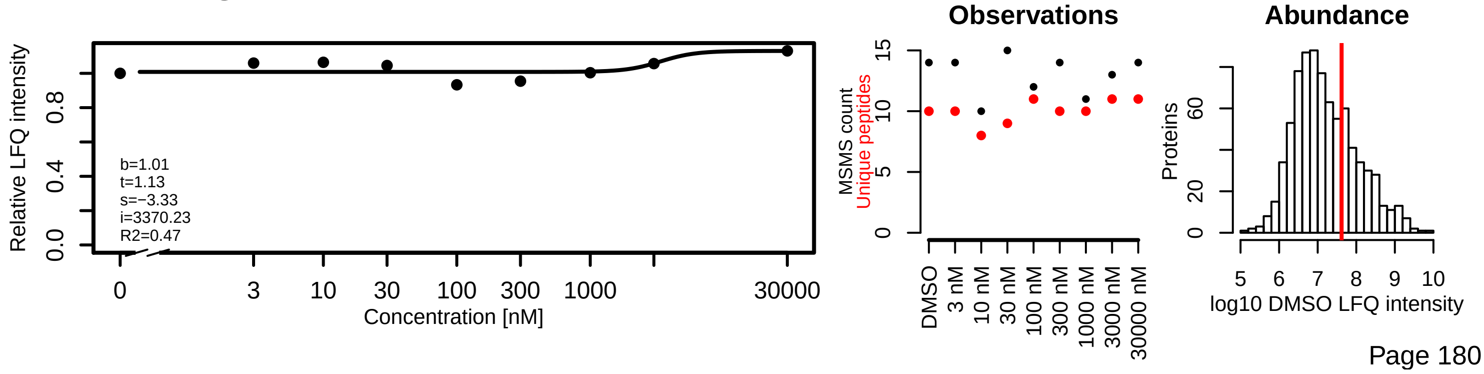

## ESYT1

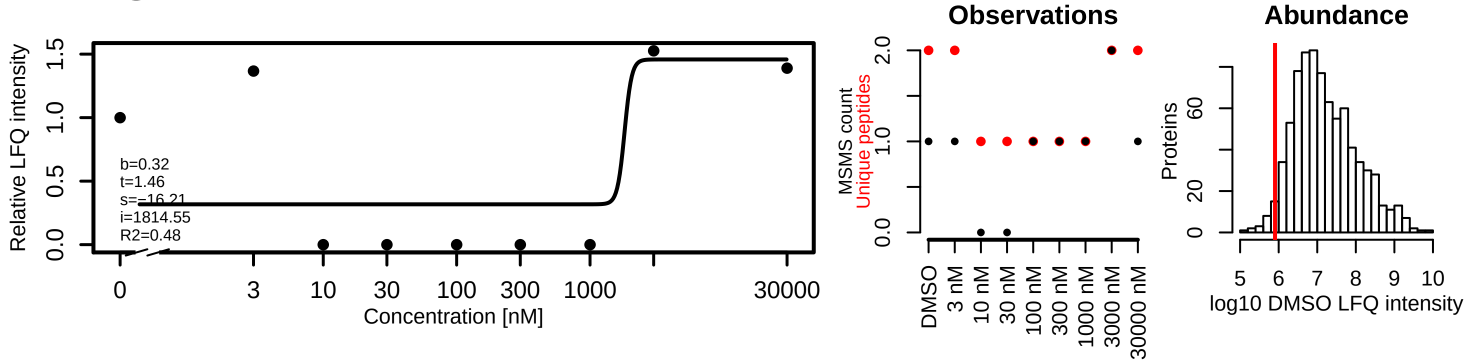


## MYADM

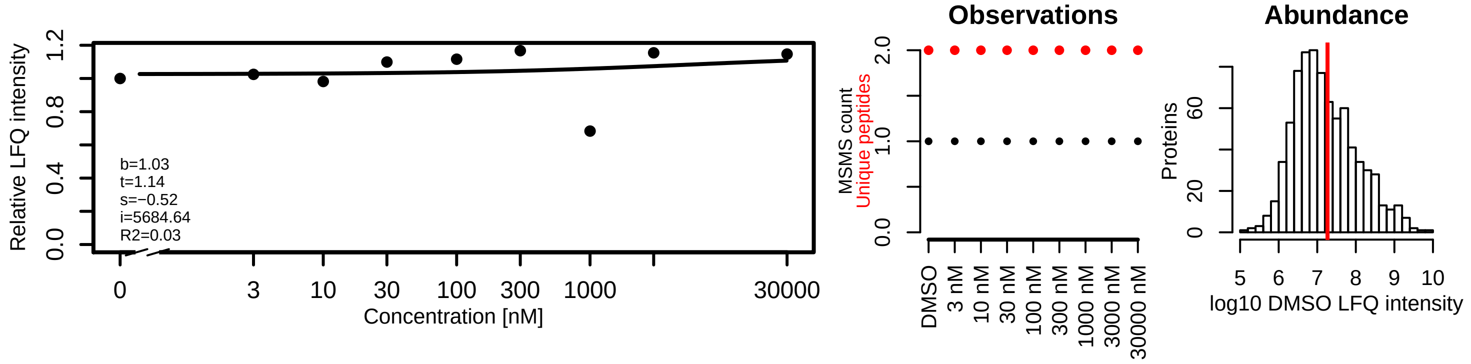


## SAMM50

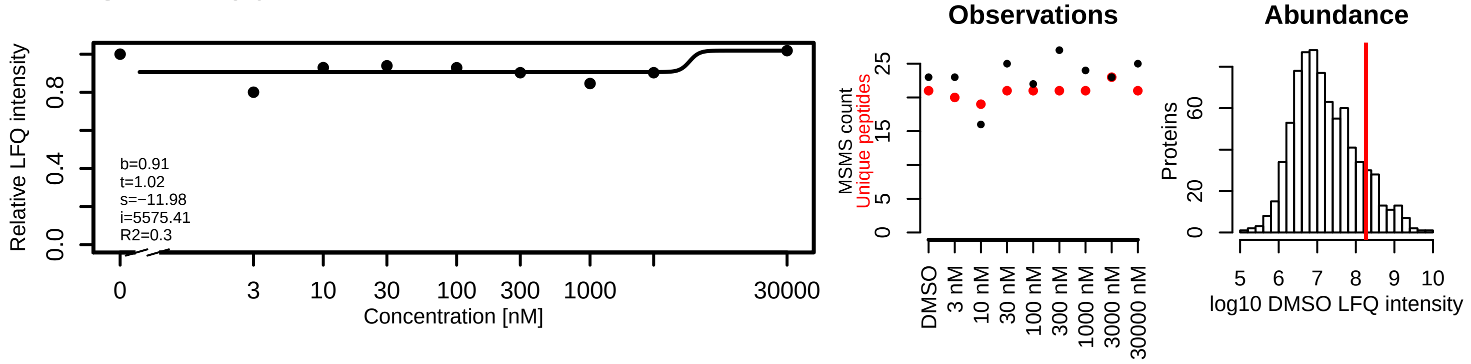


## SRSF6

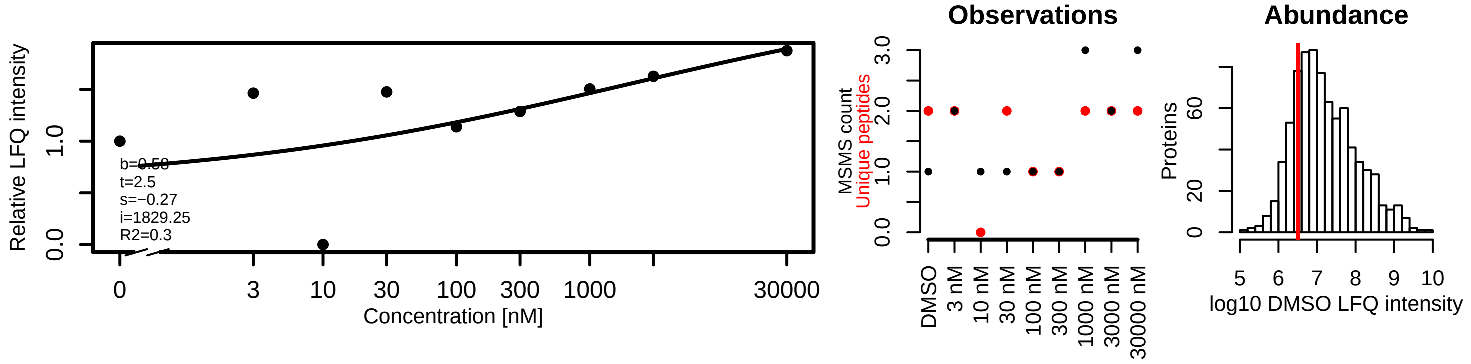


## URB2

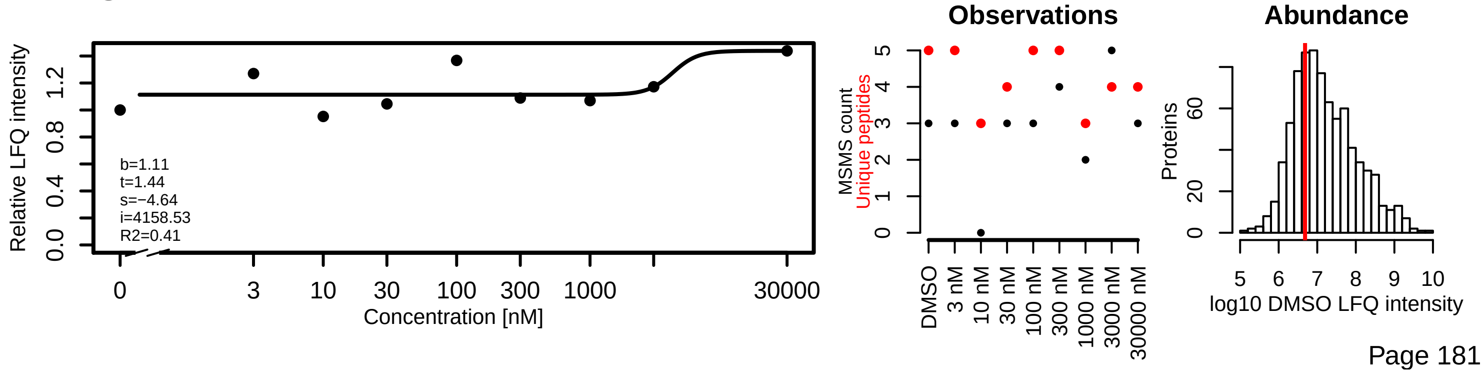

# HADHB

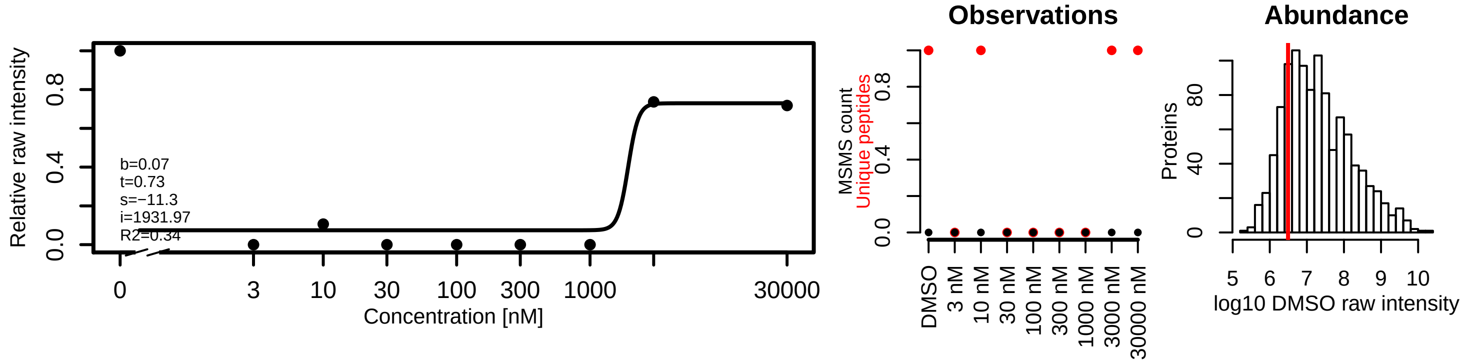


# HNRNPUL2;hCG_2044799

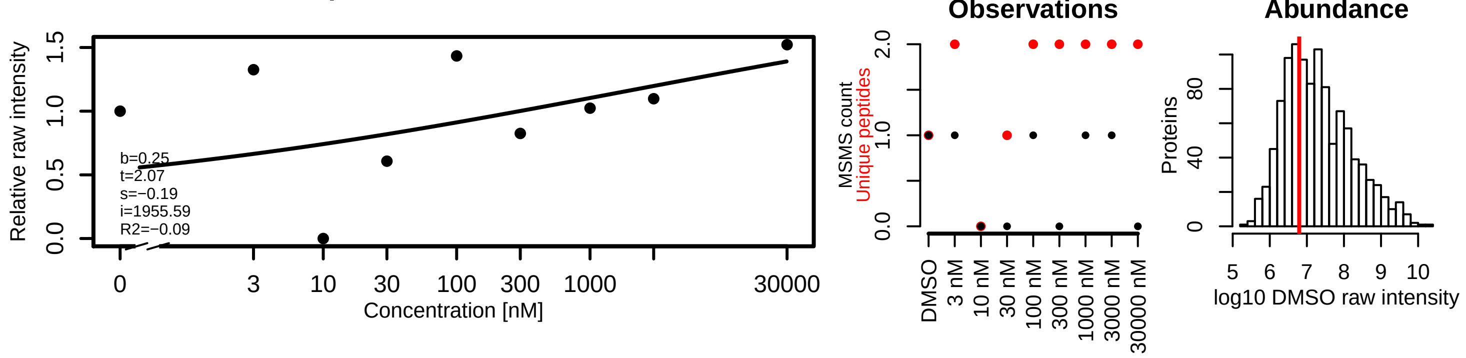


# TMEM120A

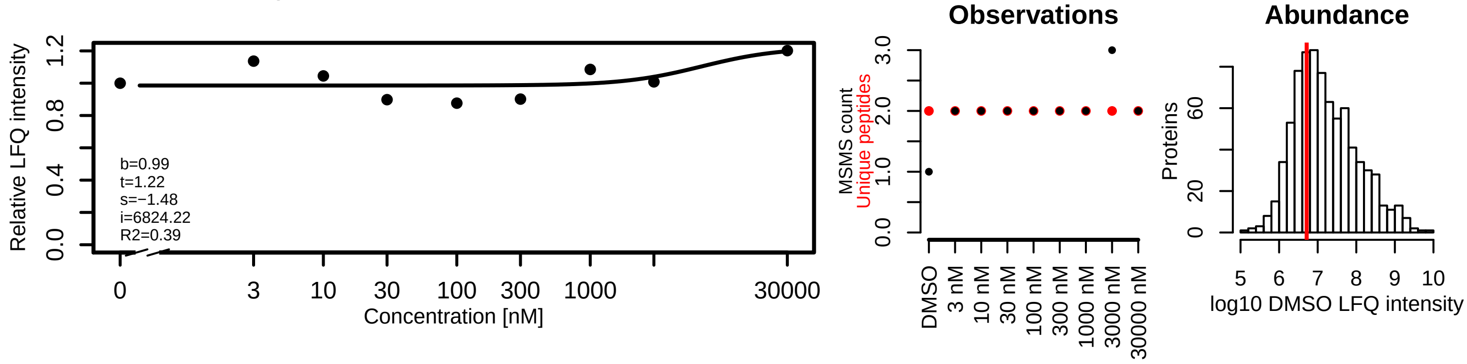


# HP1BP3

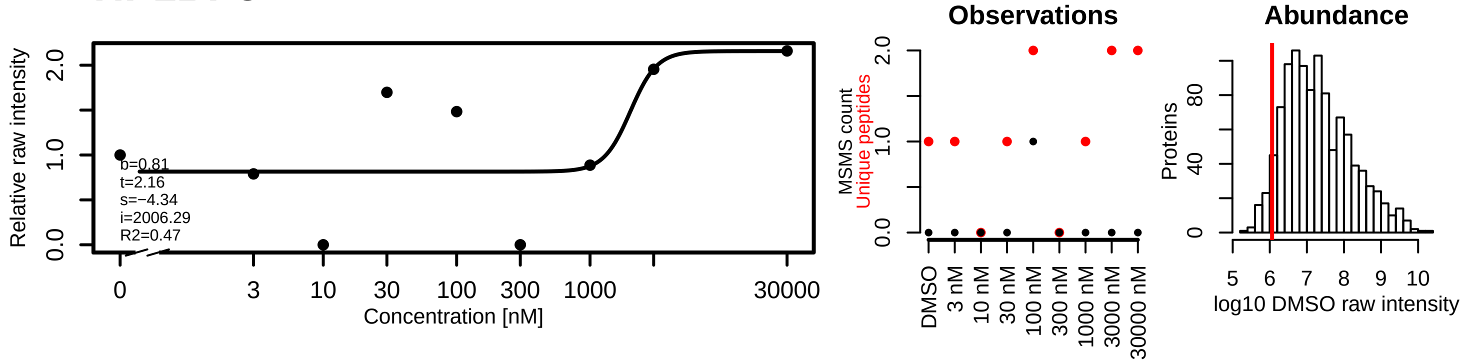


# XPO5

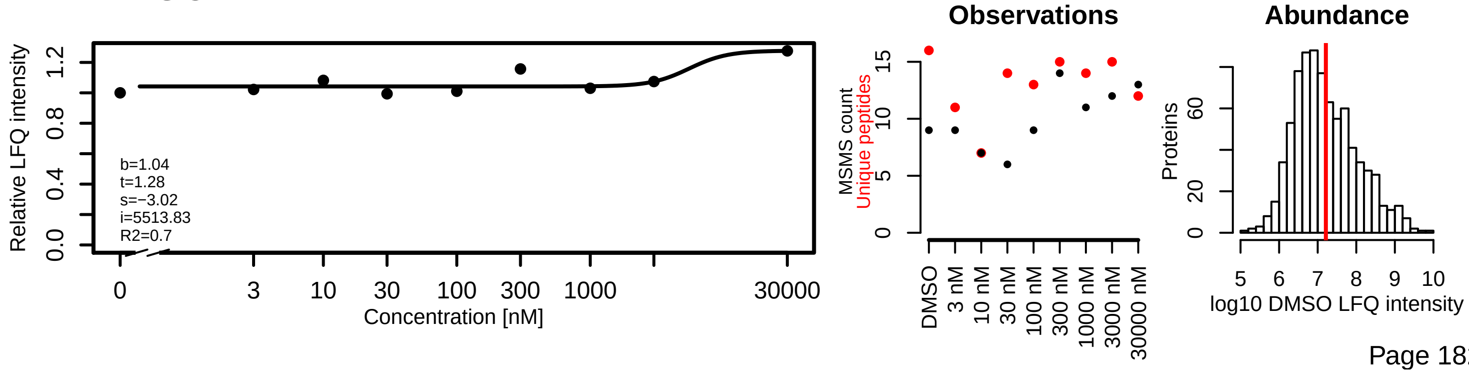

# RPS7

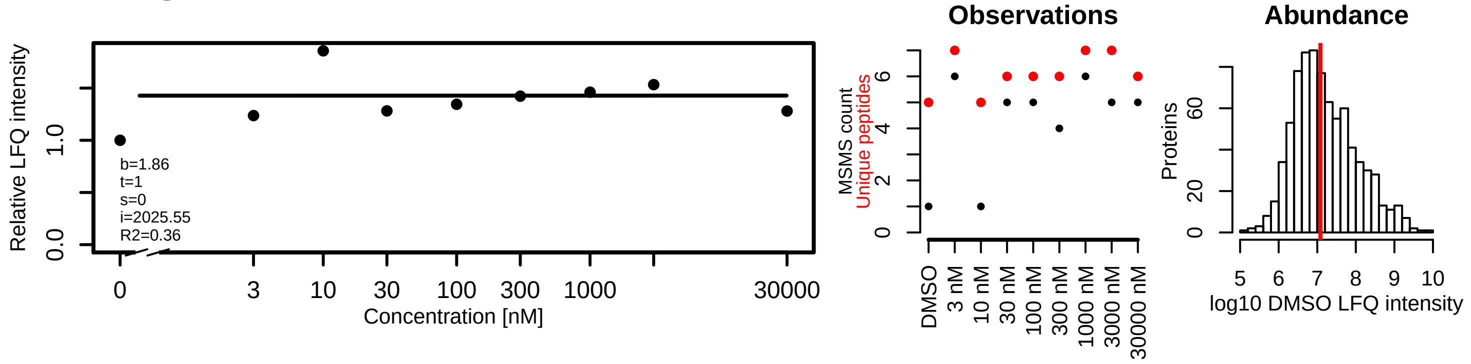


# PRDX4

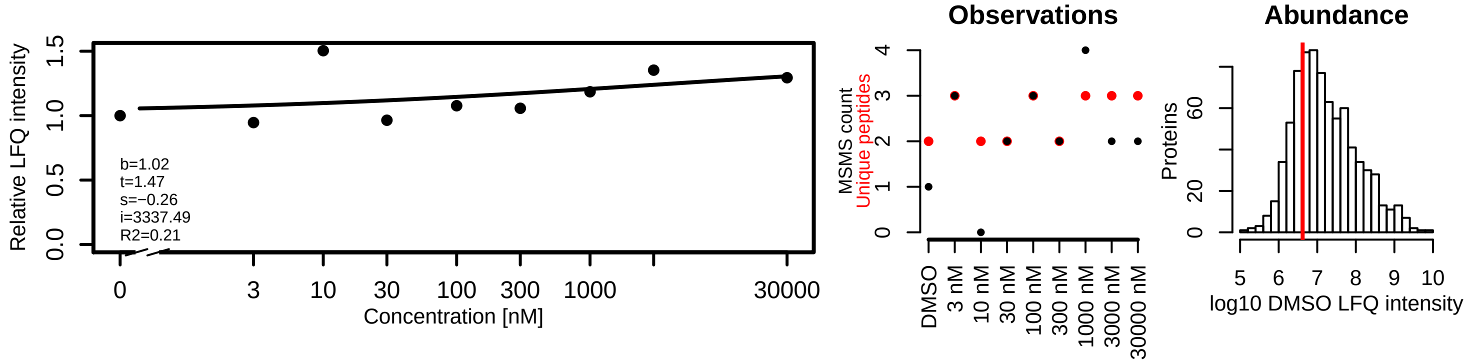


# CCNT1

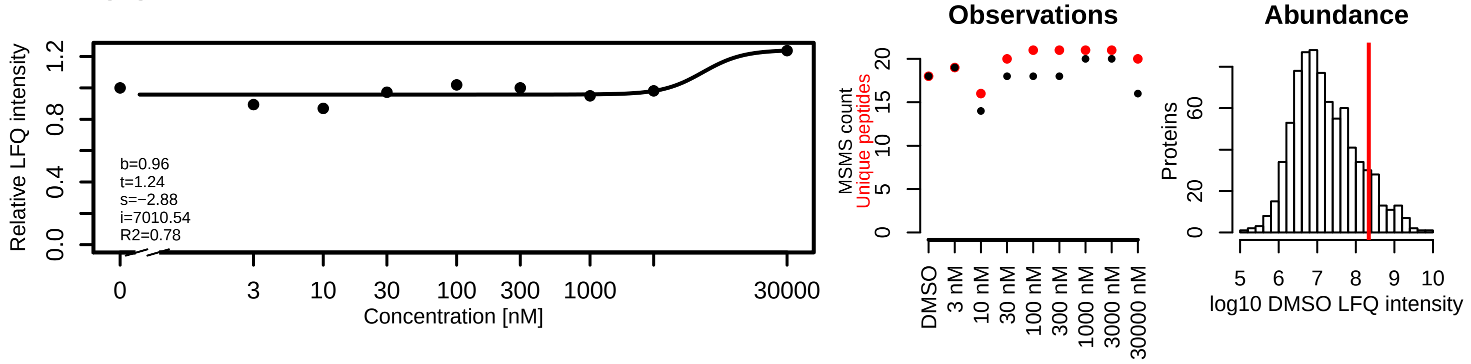


# DDX3X;DDX3Y

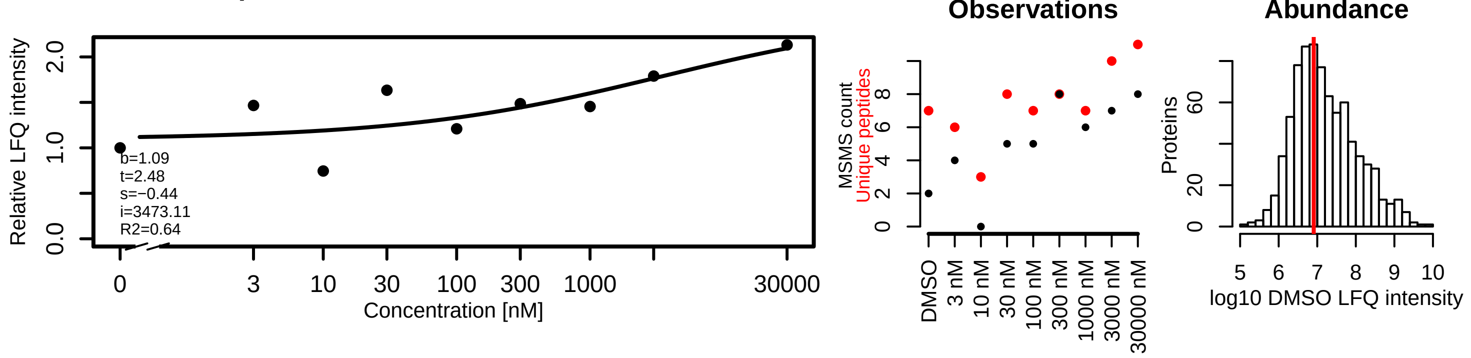


# CHP1

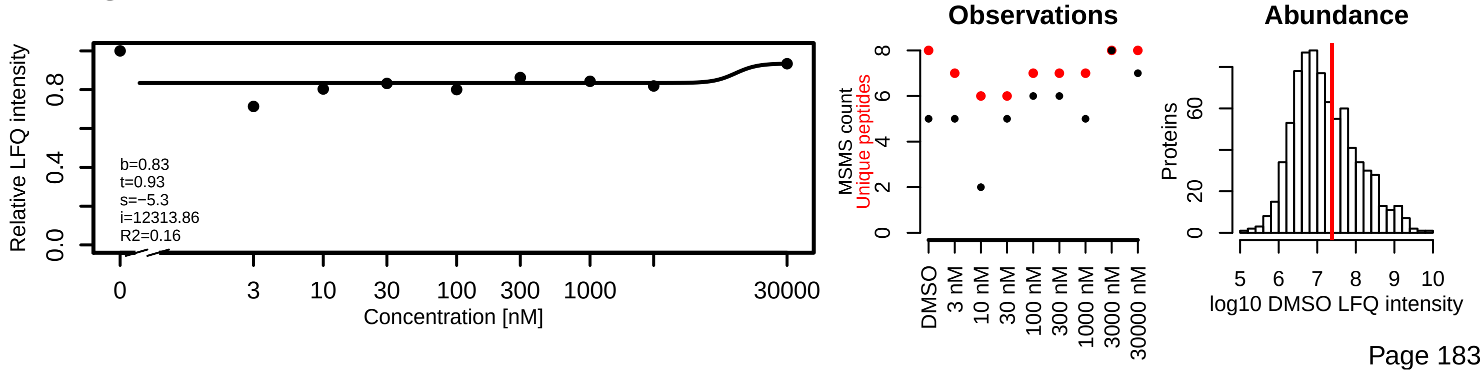

# NCKAP1

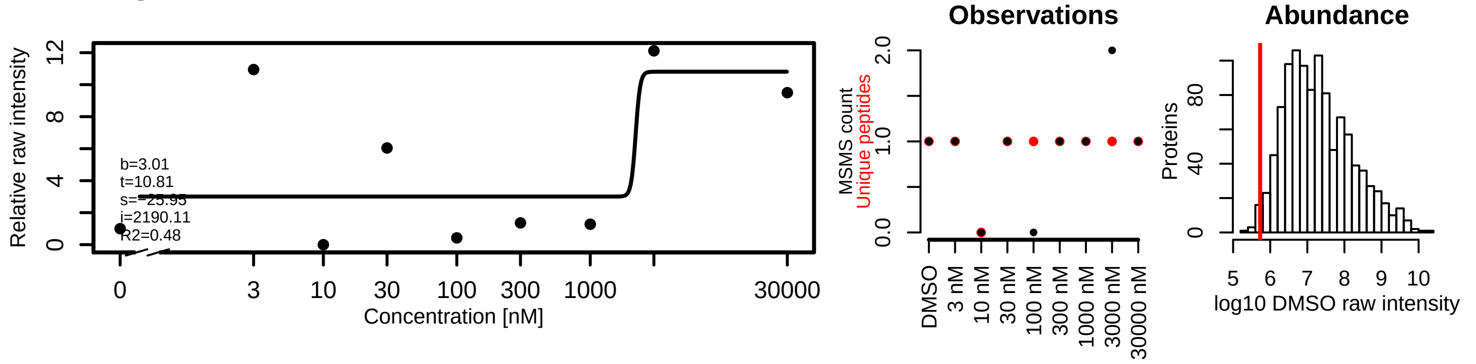


# MYH9

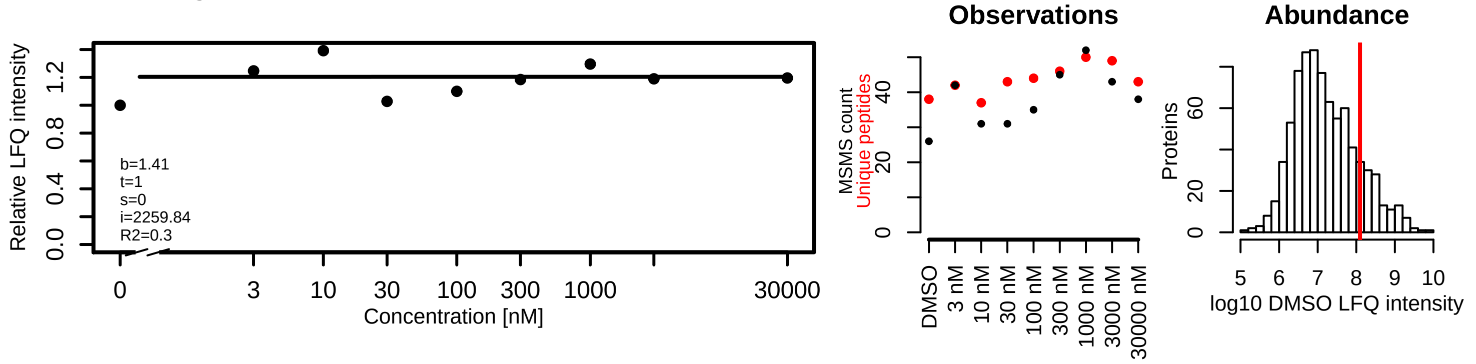


# EIF3C;EIF3CL

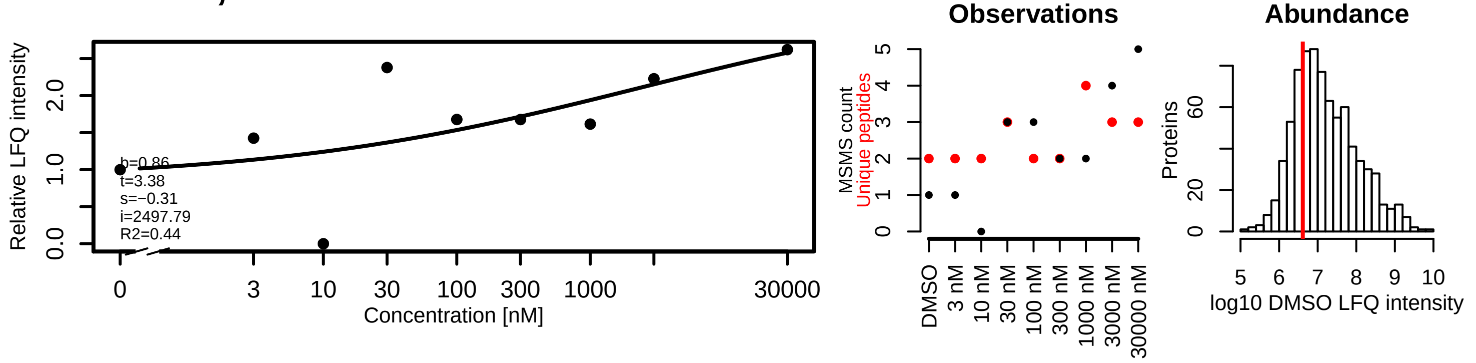


# VDAC2

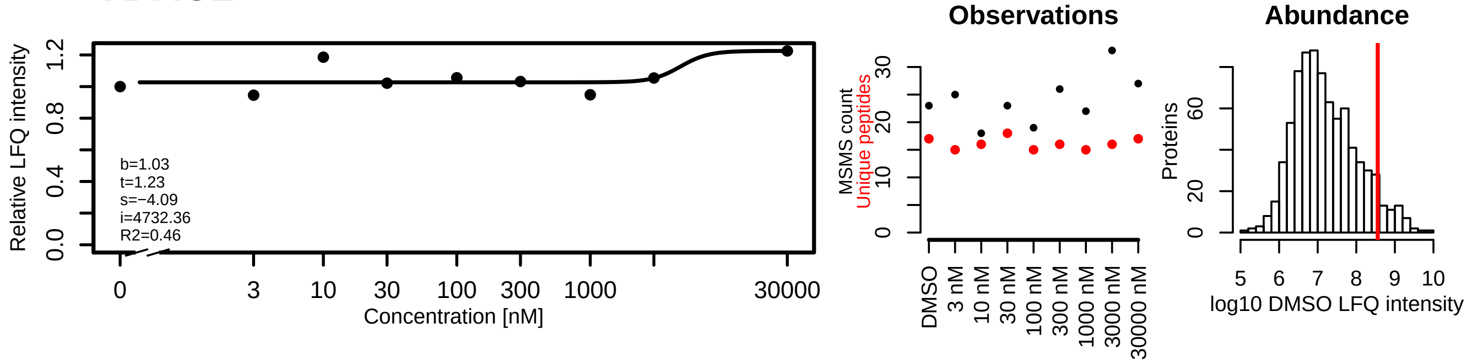


# TIAL1

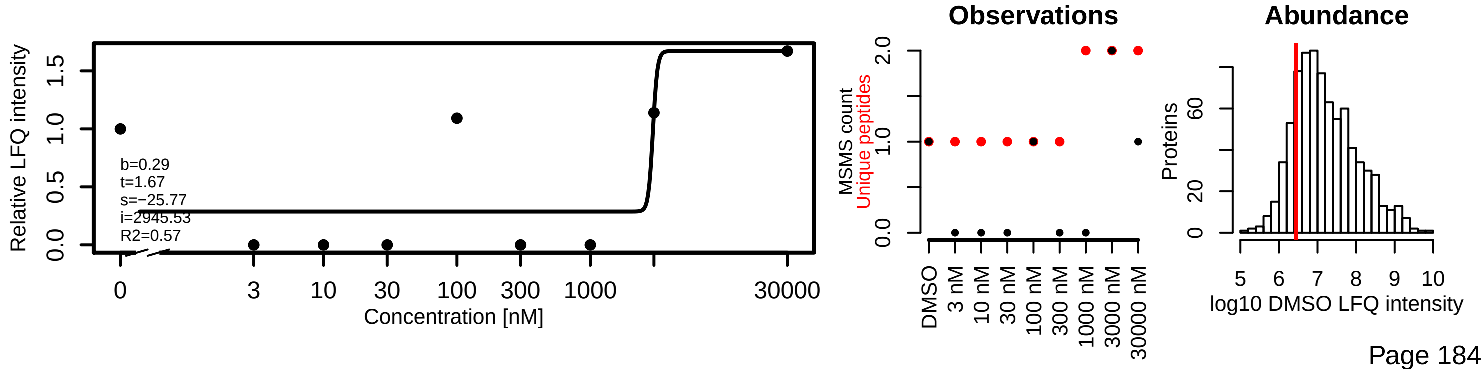

# OSBPL3;>sp|Q9H4L5−3|OSBL3_HUMAN;OSBPL3;>sp|Q9H4L5−8|OS

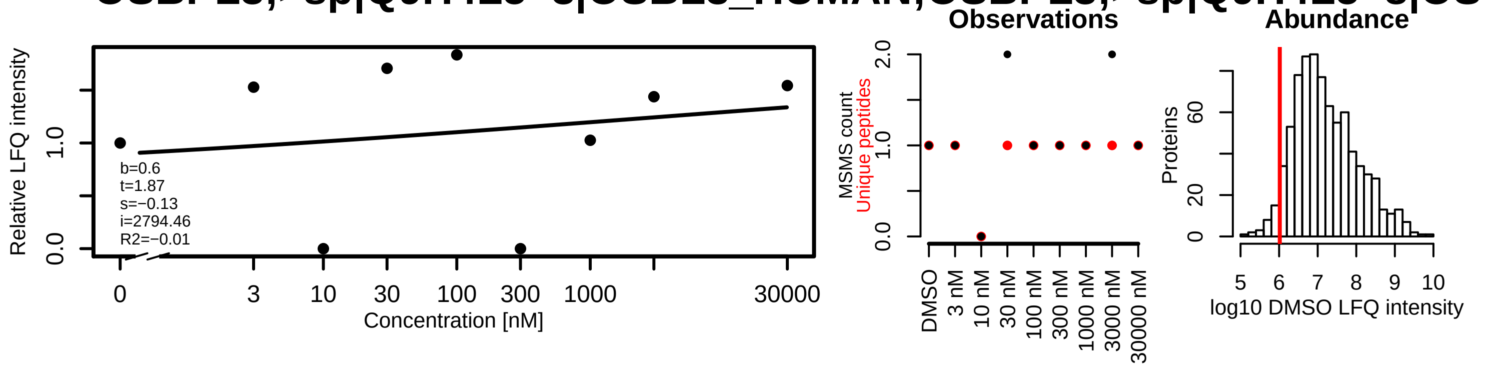


# DCAF7

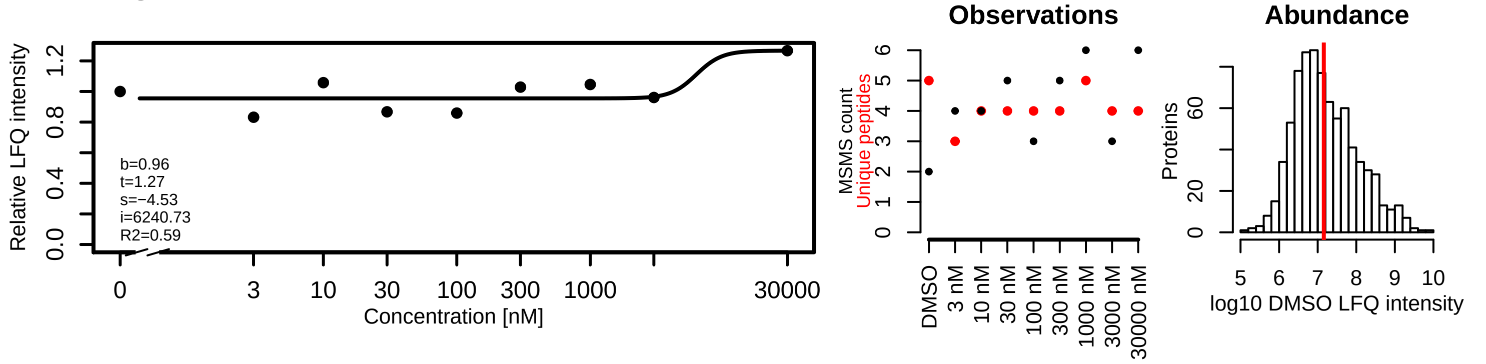


# NOP56

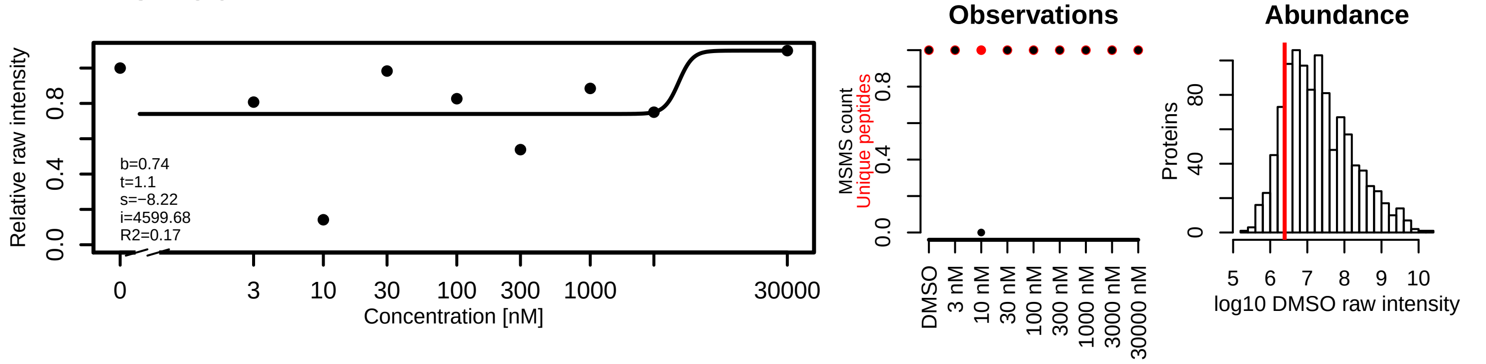


# TIMMDC1

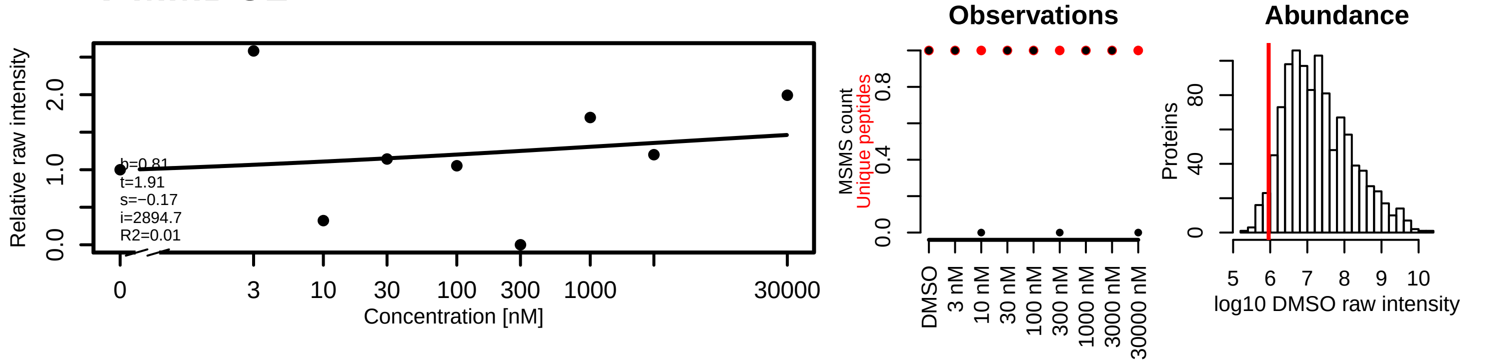


# PSMD3

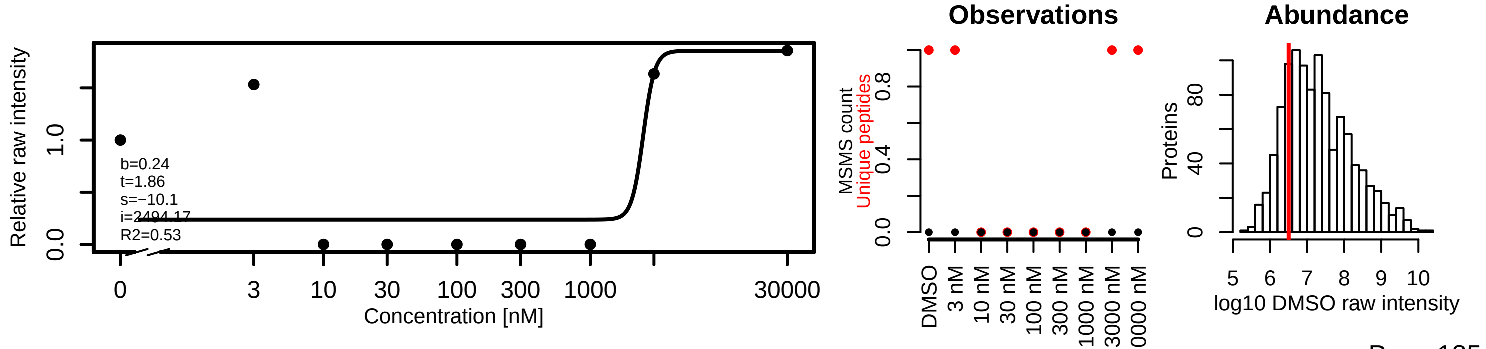

# OGDH

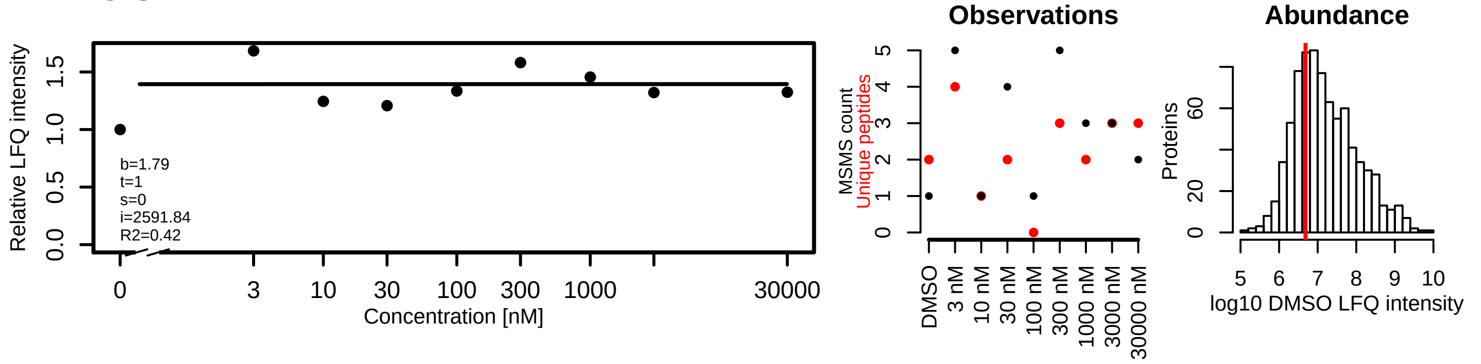


# DHRS3

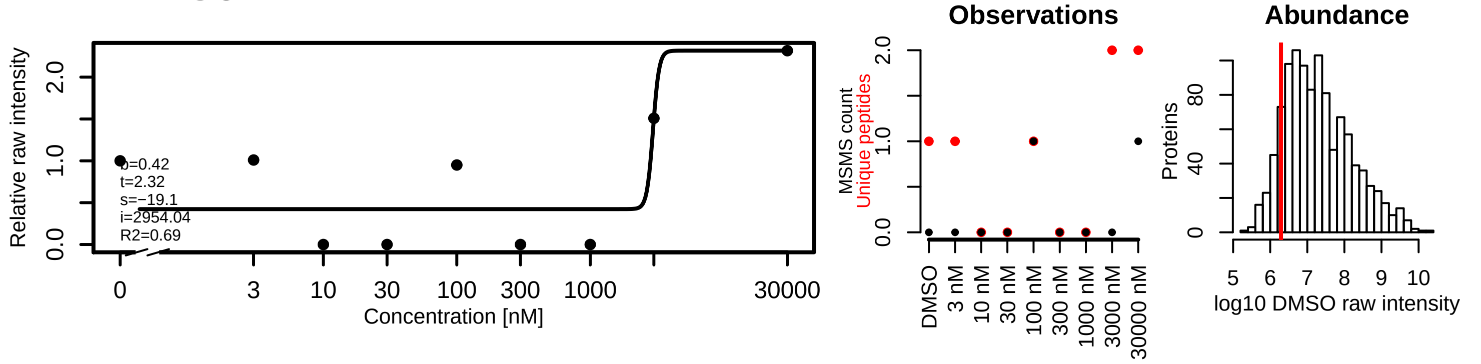


# MYBBP1A

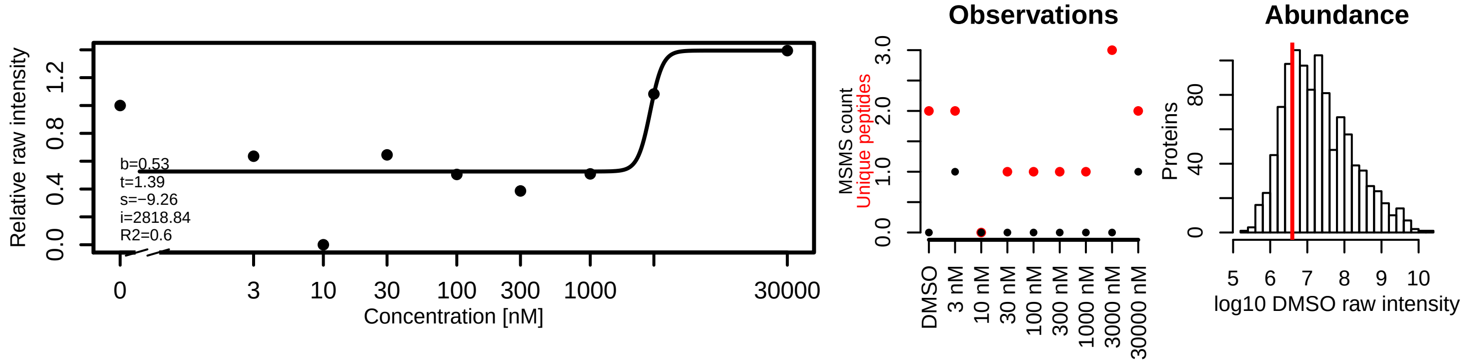


# ATP5EP2;ATP5E

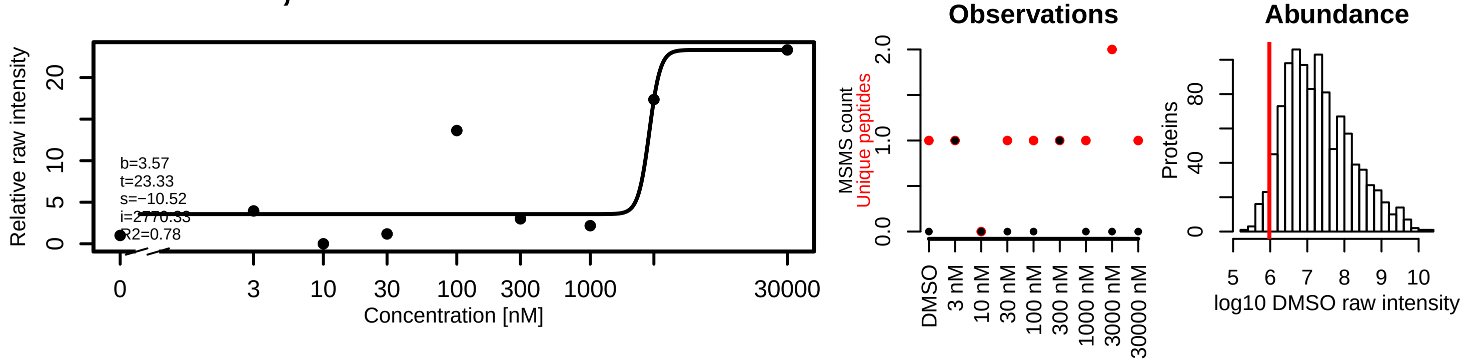


# MARK2 – Q7KZI7−15;Q7KZI7−16;Q7KZI7−5;Q7KZI7−4;Q7KZI7−9;Q7K

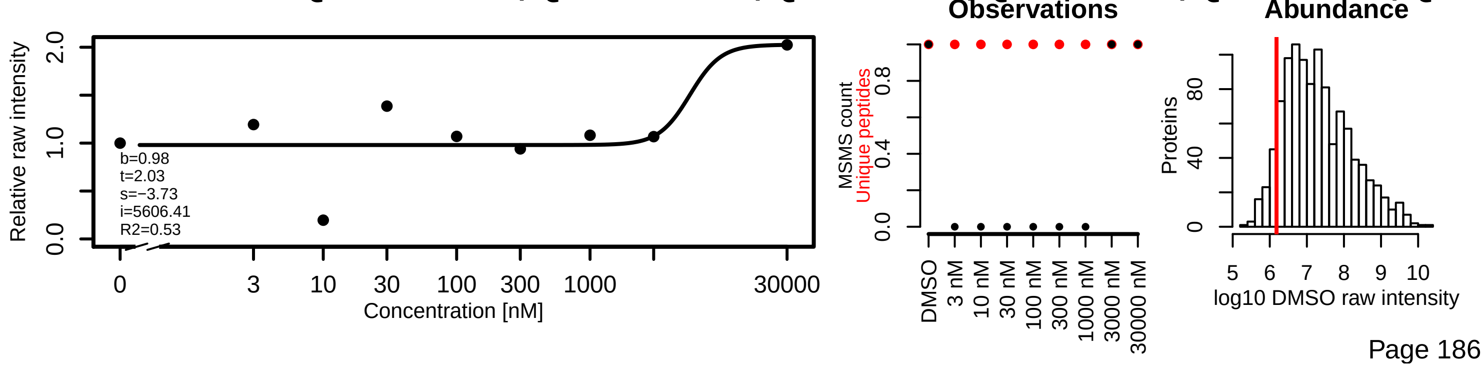

# RPS3A

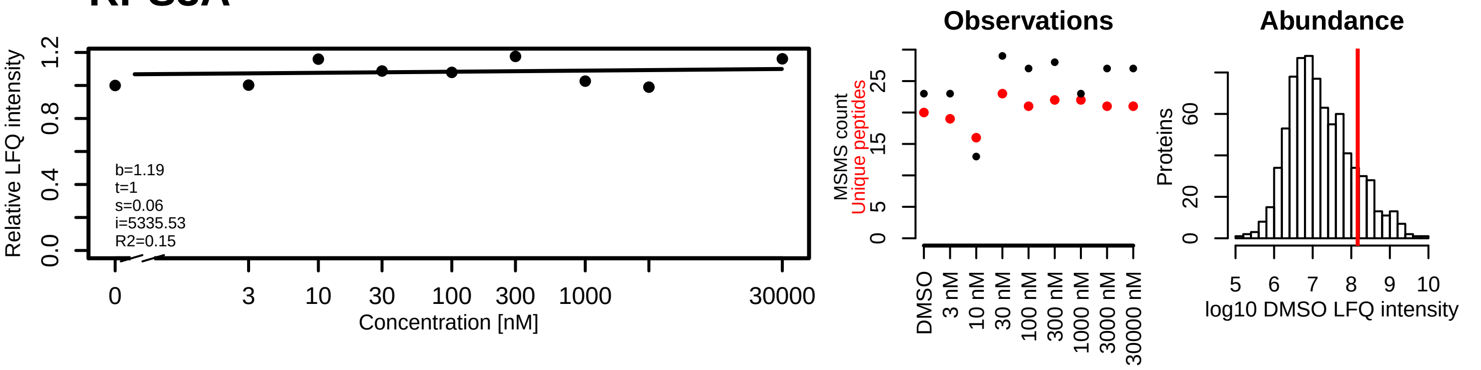


# XPO6

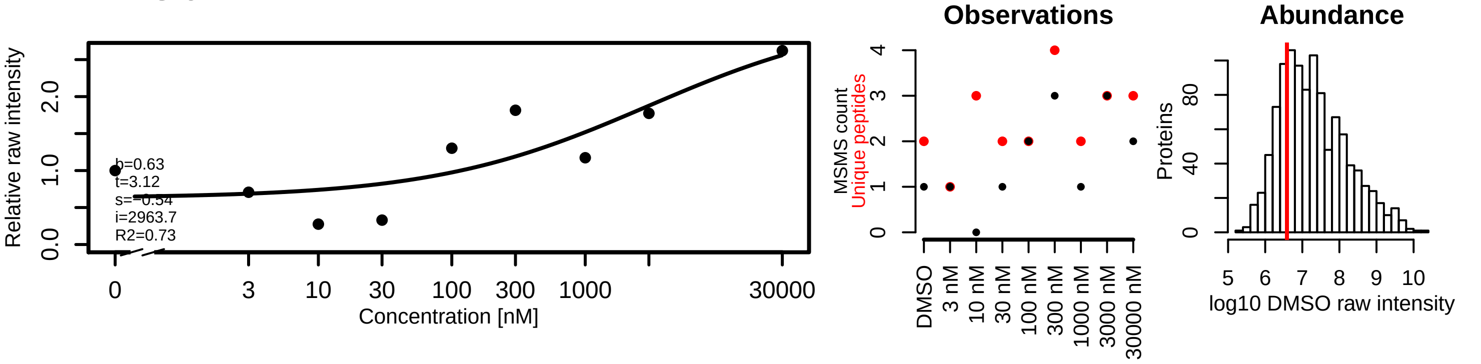


# CAV1 − Q03135;E9PCT5;P56539

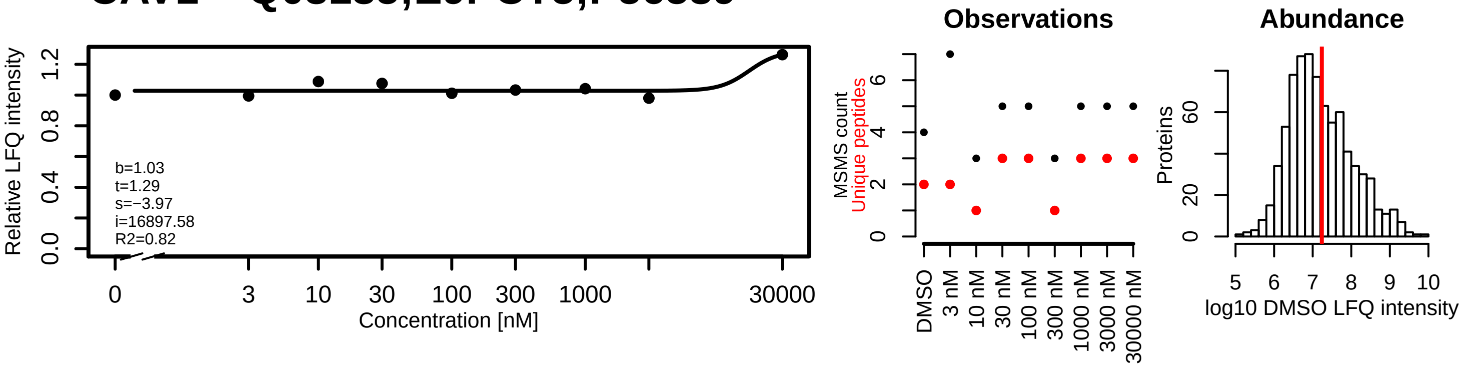


# SRSF2;SFRS2

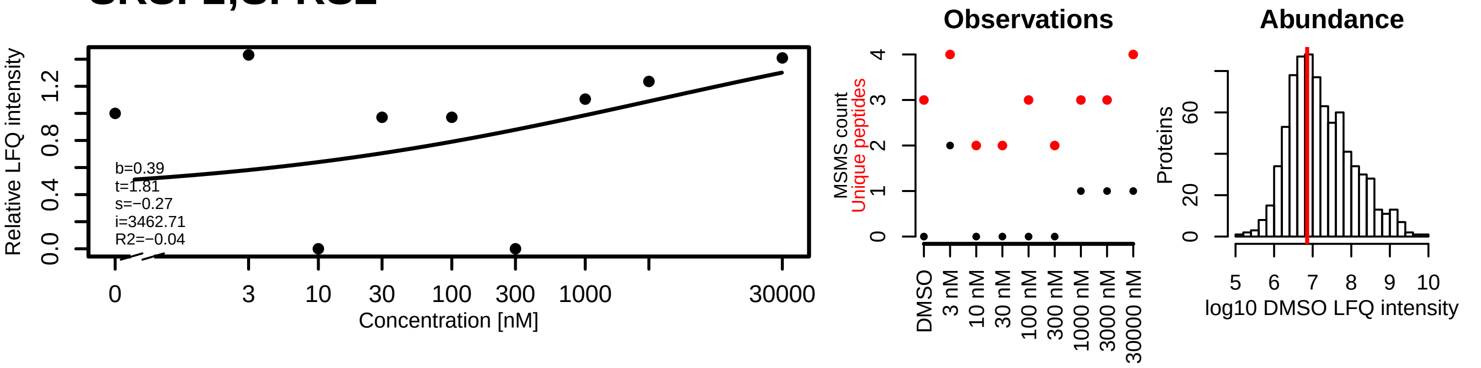


# CDC42BPB

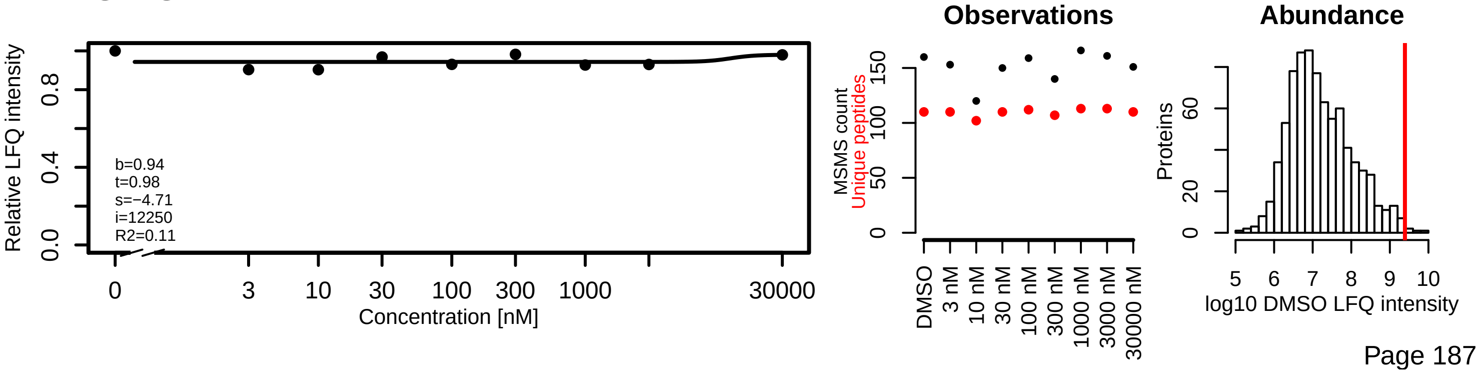

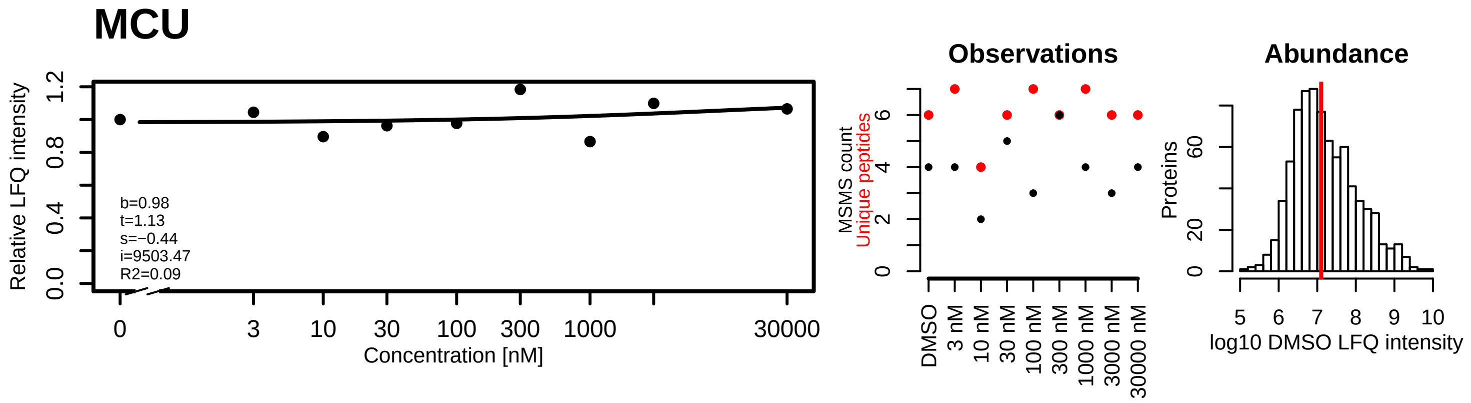
MCU
b=0.98
t=1.13
s=−0.44
i=9503.47
R2=0.09
Relative LFQ intensity
Concentration [nM]
Observations
MSMS count
Unique peptides
Abundance
Proteins
log10 DMSO LFQ intensity

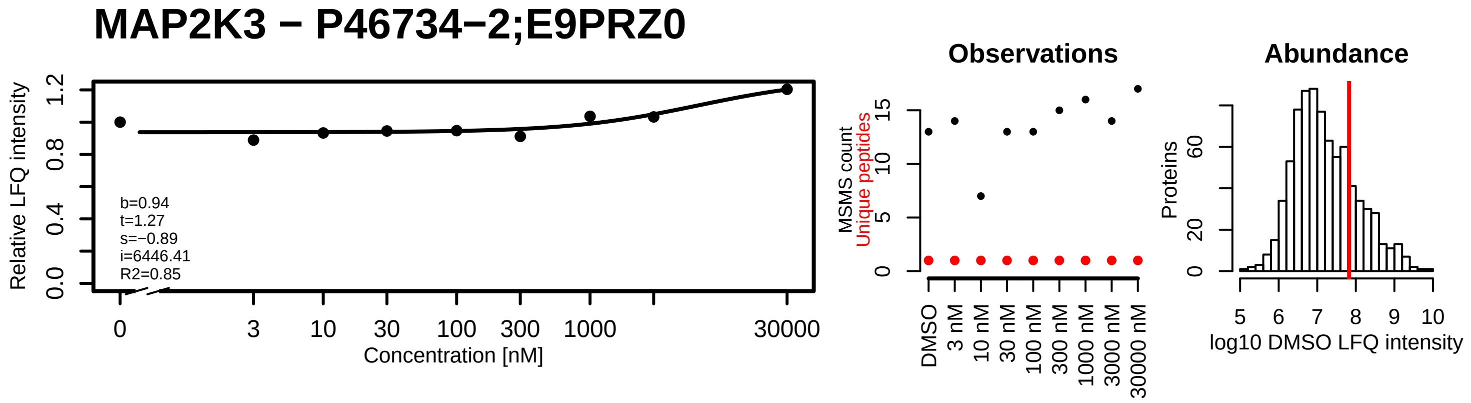
MAP2K3 − P46734−2;E9PRZ0
b=0.94
t=1.27
s=−0.89
i=6446.41
R2=0.85
Relative LFQ intensity
Concentration [nM]
Observations
MSMS count
Unique peptides
Abundance
Proteins
log10 DMSO LFQ intensity

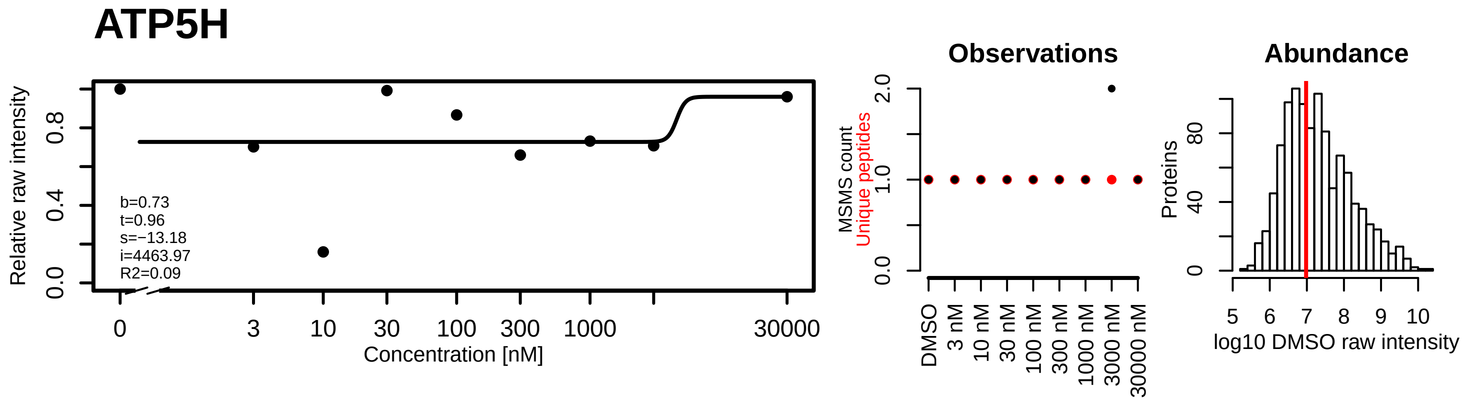
ATP5H
b=0.73
t=0.96
s=−13.18
i=4463.97
R2=0.09
Relative raw intensity
Concentration [nM]
Observations
MSMS count
Unique peptides
Abundance
Proteins
log10 DMSO raw intensity

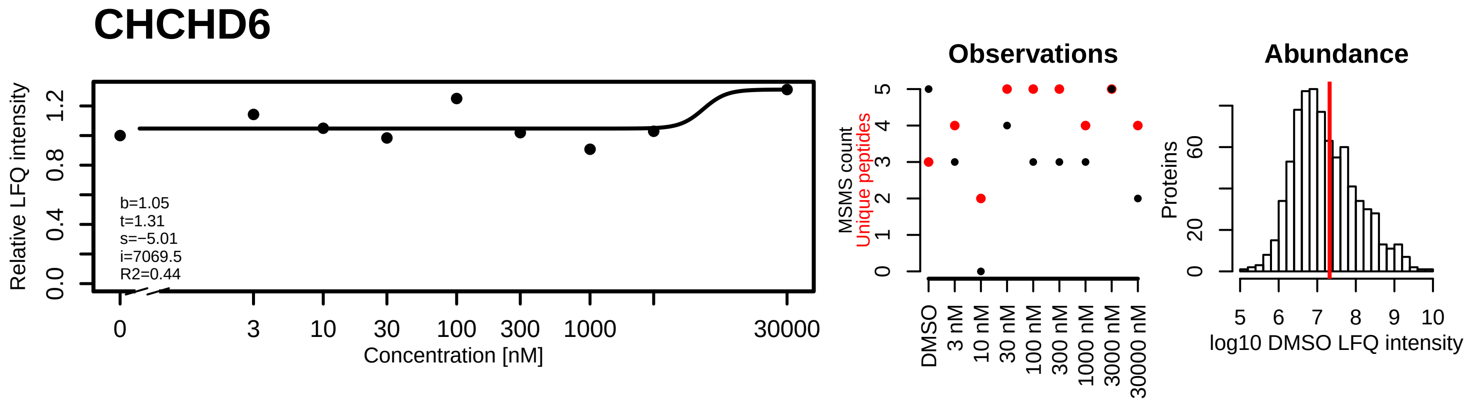
CHCHD6
b=1.05
t=1.31
s=−5.01
i=7069.5
R2=0.44
Relative LFQ intensity
Concentration [nM]
Observations
MSMS count
Unique peptides
Abundance
Proteins
log10 DMSO LFQ intensity

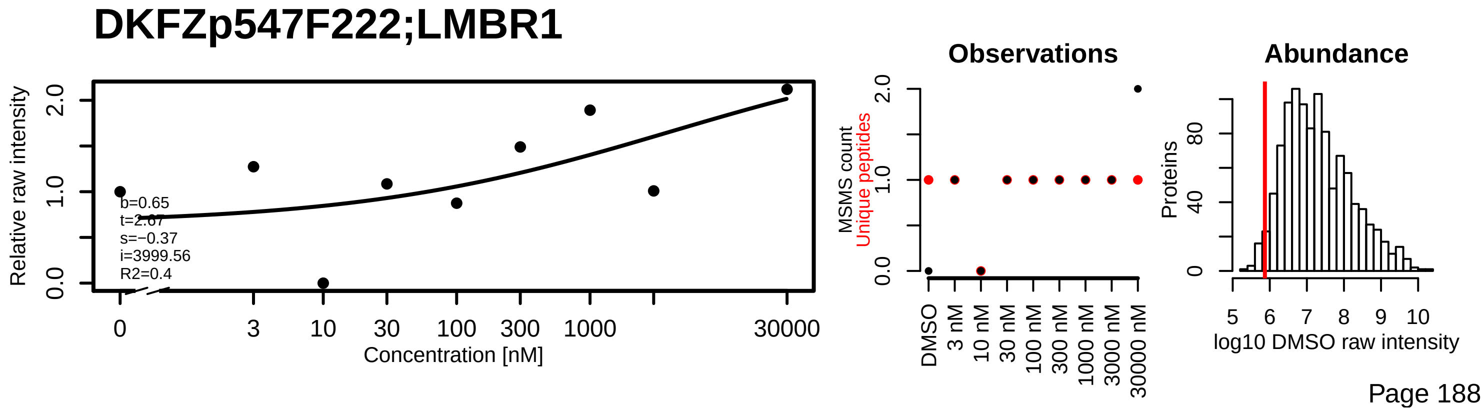
DKFZp547F222;LMBR1
b=0.65
t=2.07
s=−0.37
i=3999.56
R2=0.4
Relative raw intensity
Concentration [nM]
Observations
MSMS count
Unique peptides
Abundance
Proteins
log10 DMSO raw intensity

## TPR

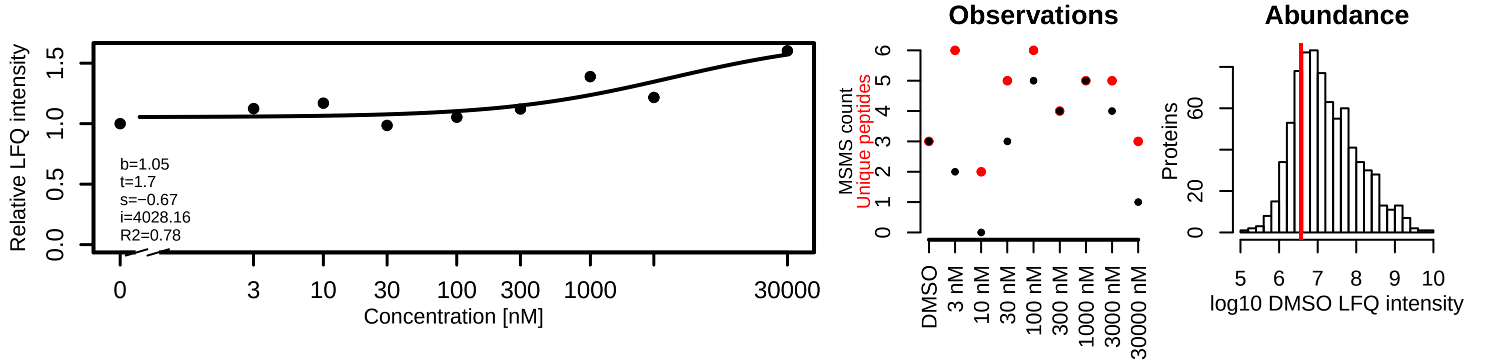


## MGST3

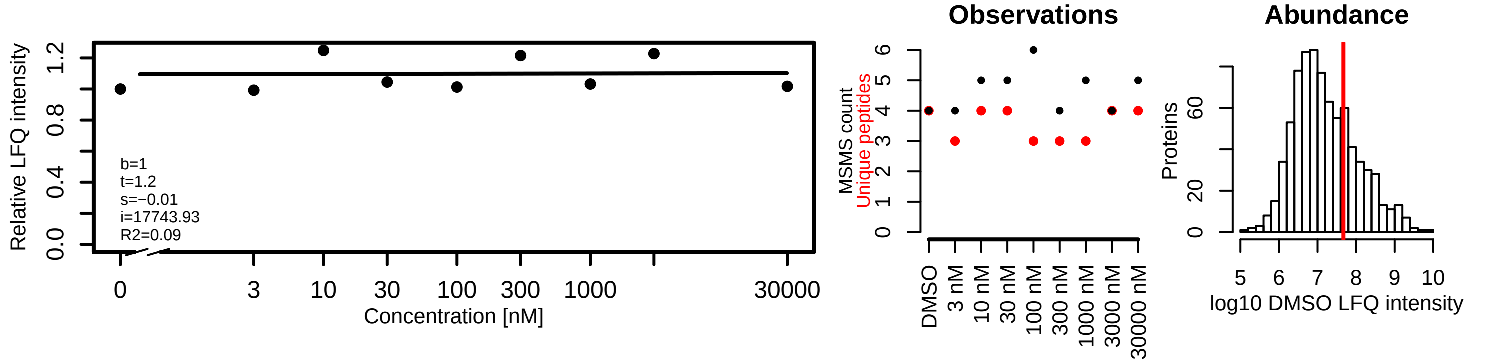


## ACLY

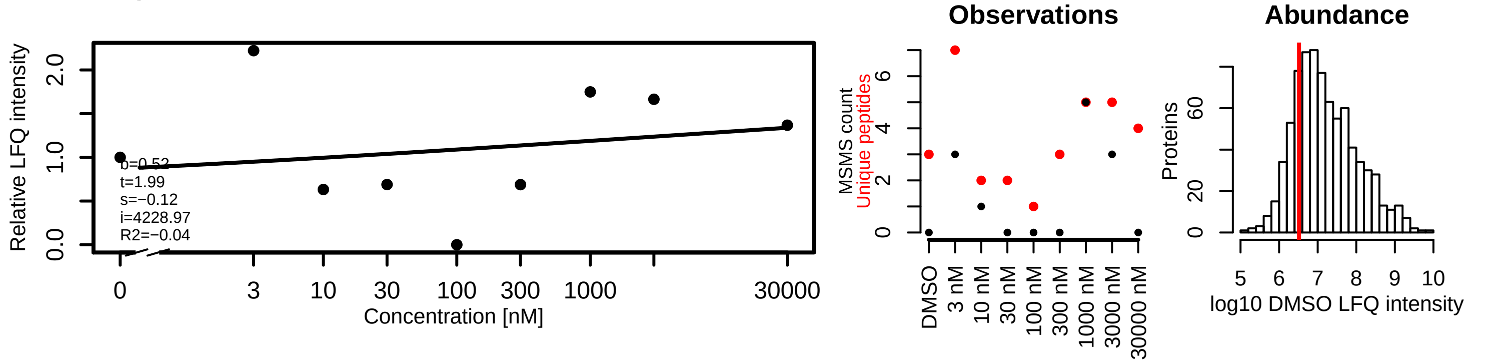


## GPR180

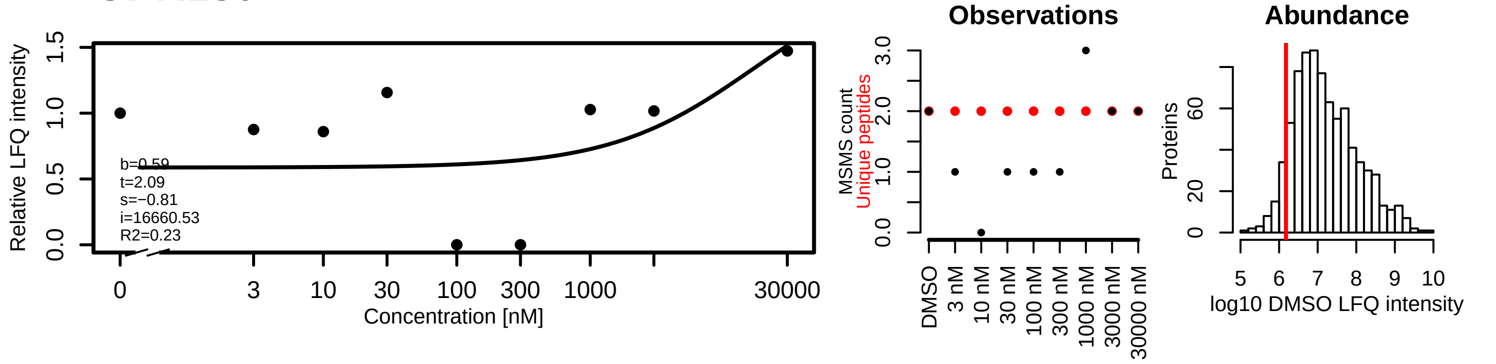


## STT3B

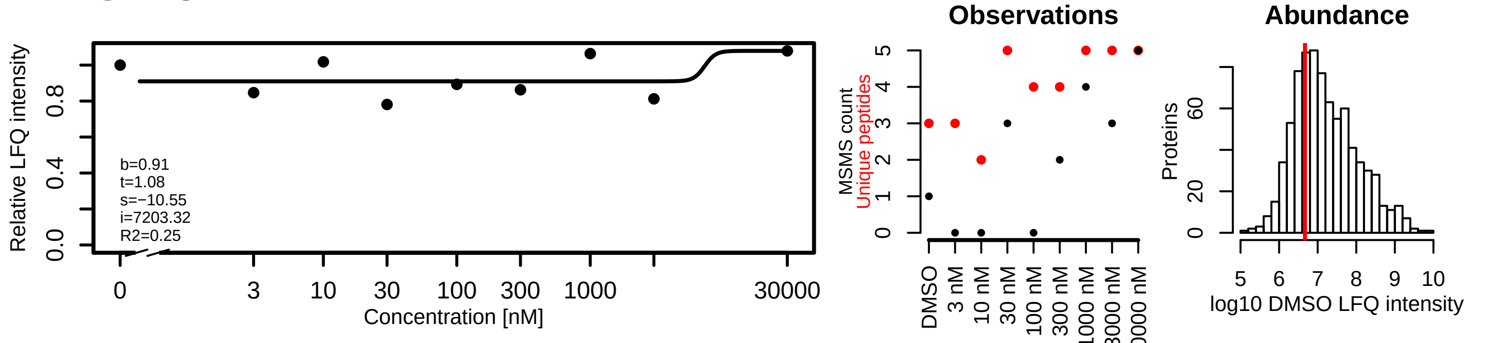

# NEK1

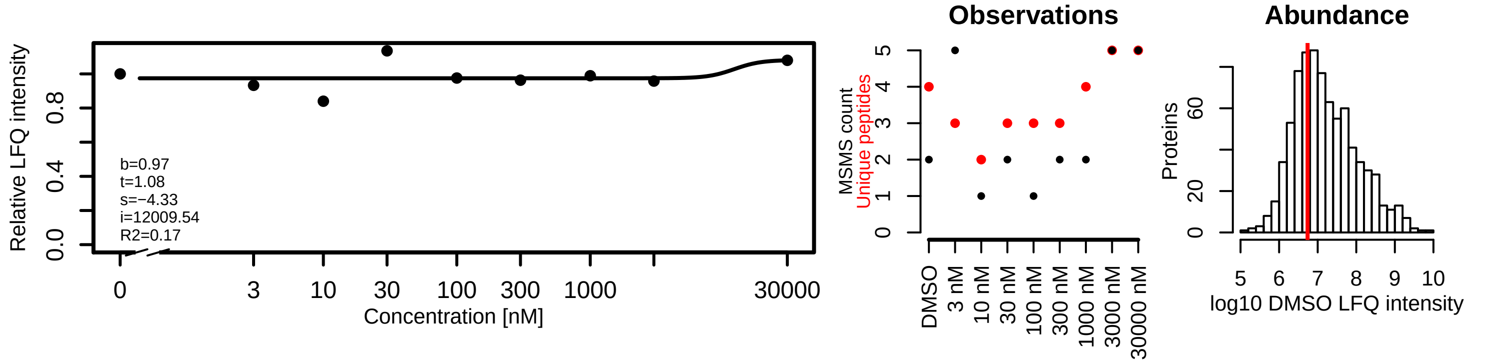


# TIA1

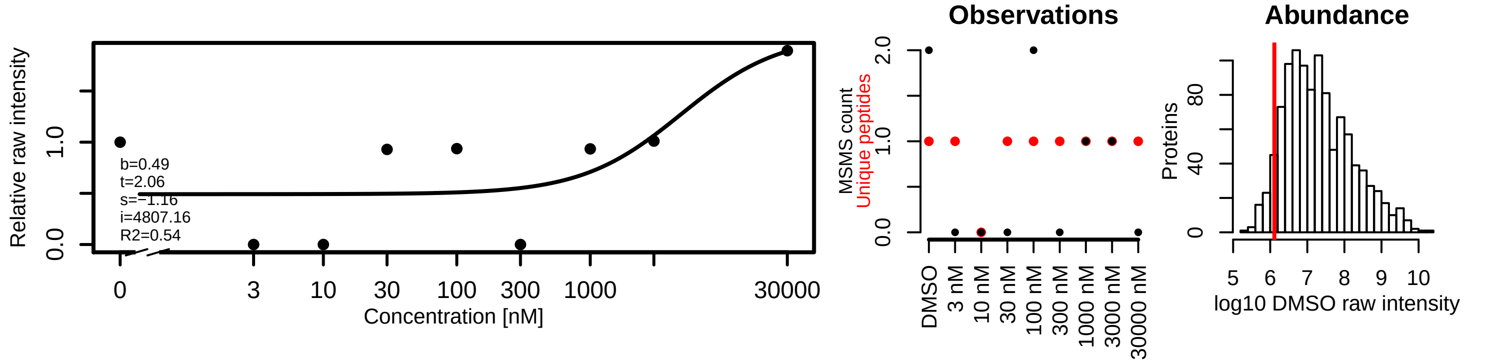


# SLC25A40

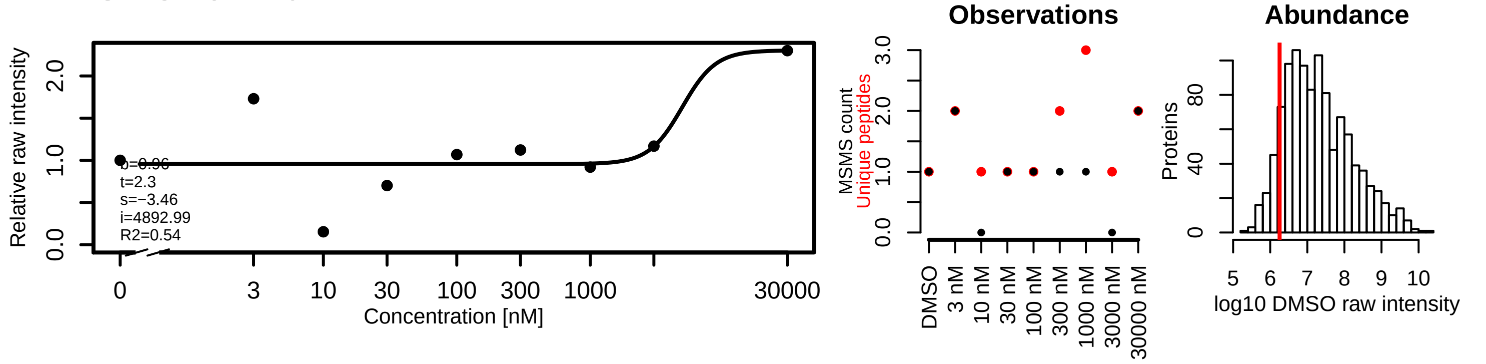


# CSE1L

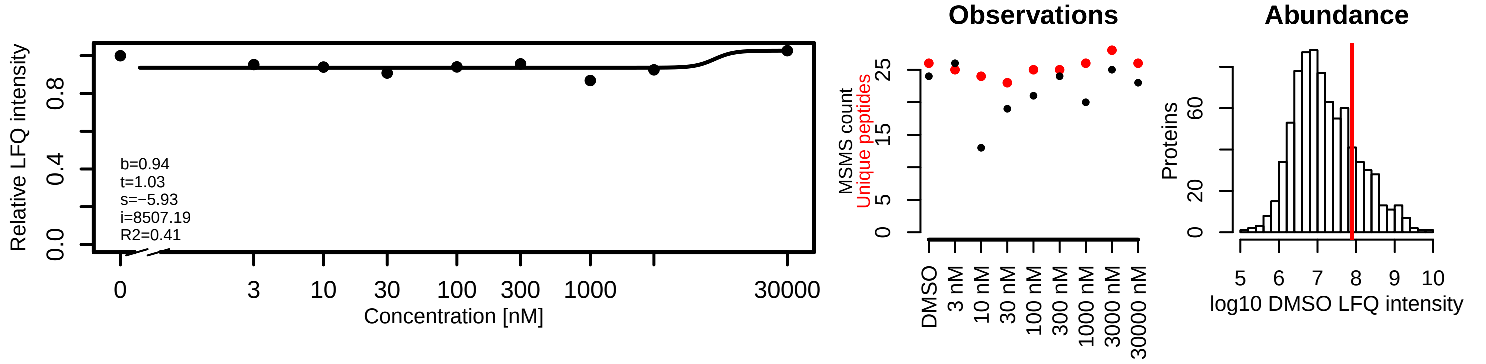


# SRSF7

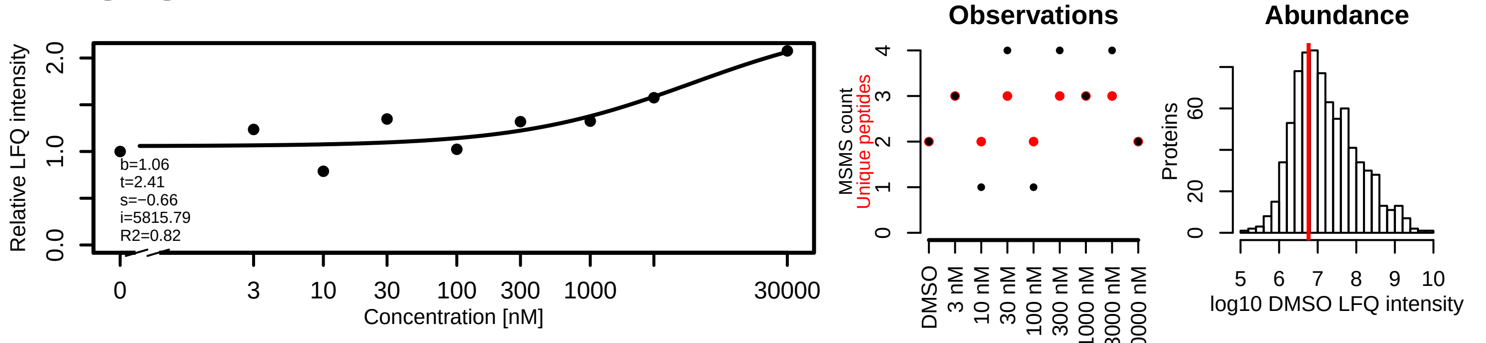

# HMGN2

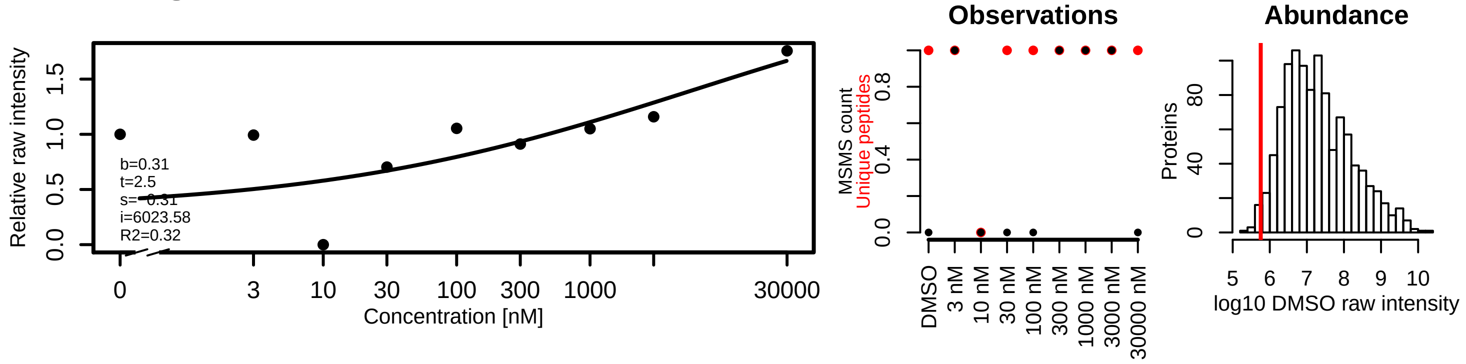


# BET1;DKFZp781C0425

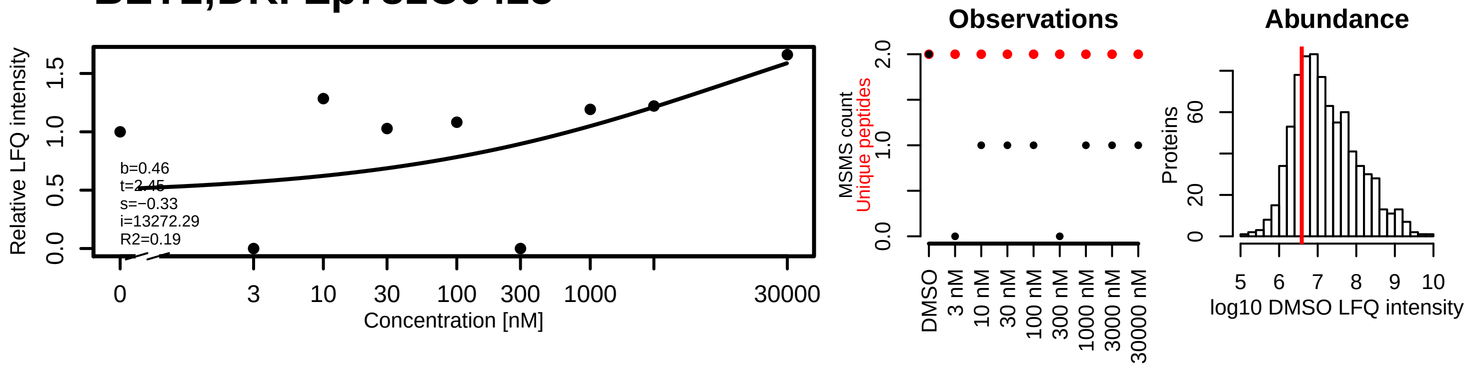


# TBATA

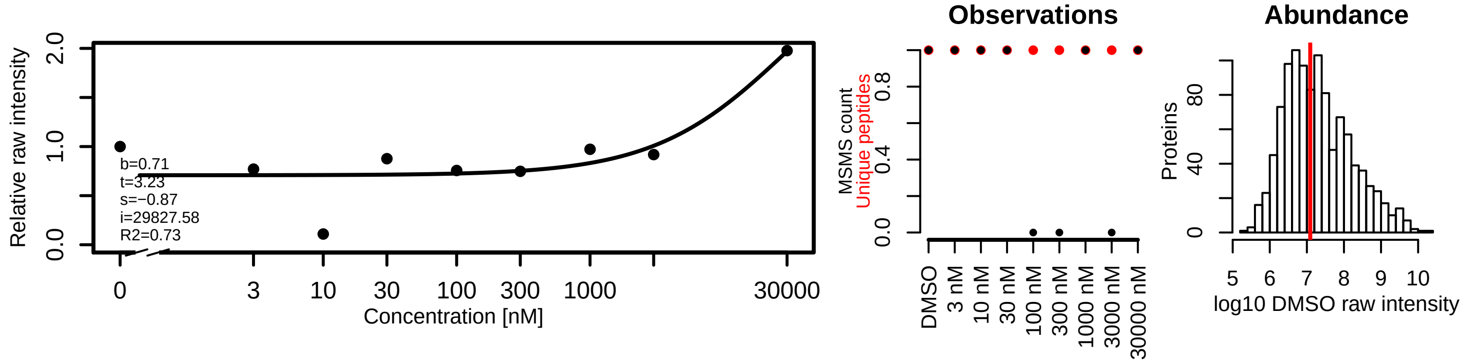


# ICMT

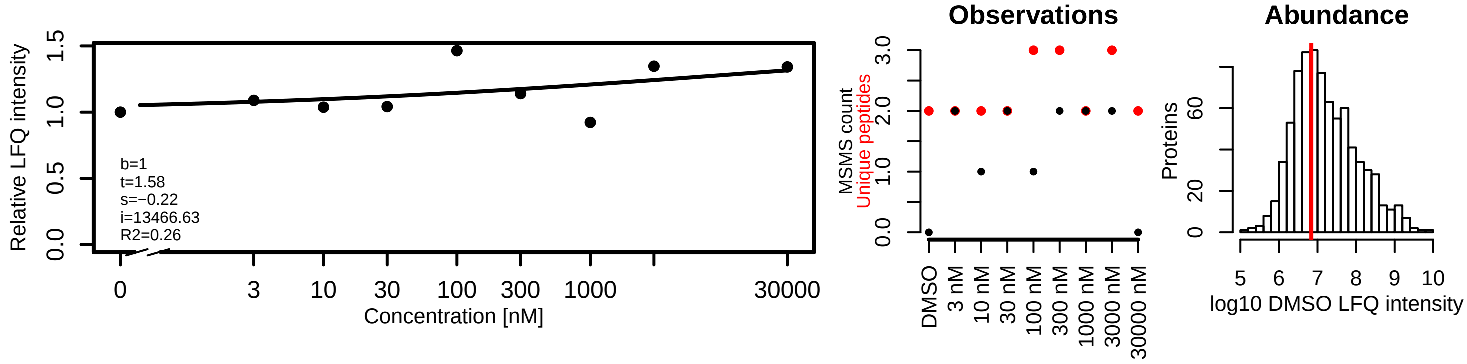


# HLA−C;HLA−B;HLA−A

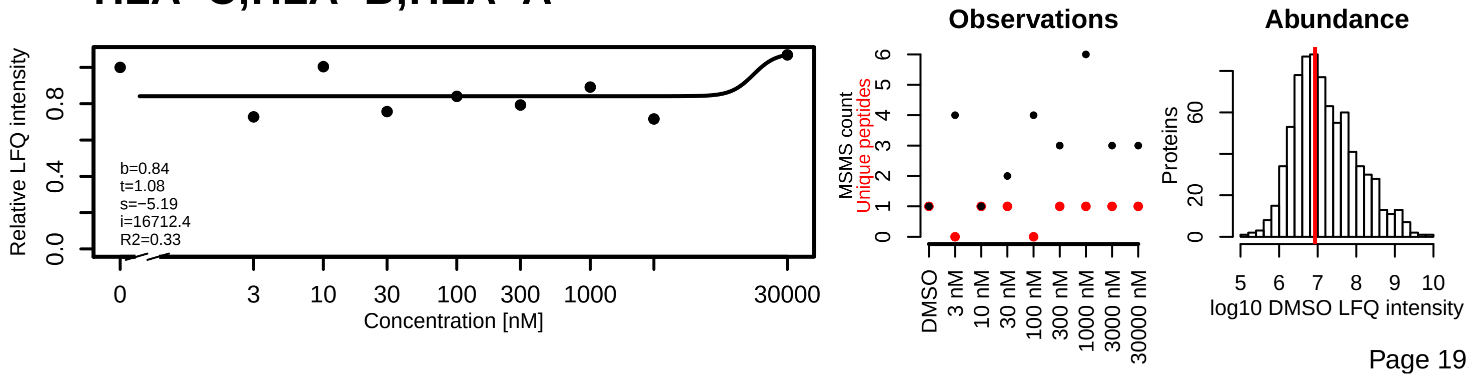

# CCNB1

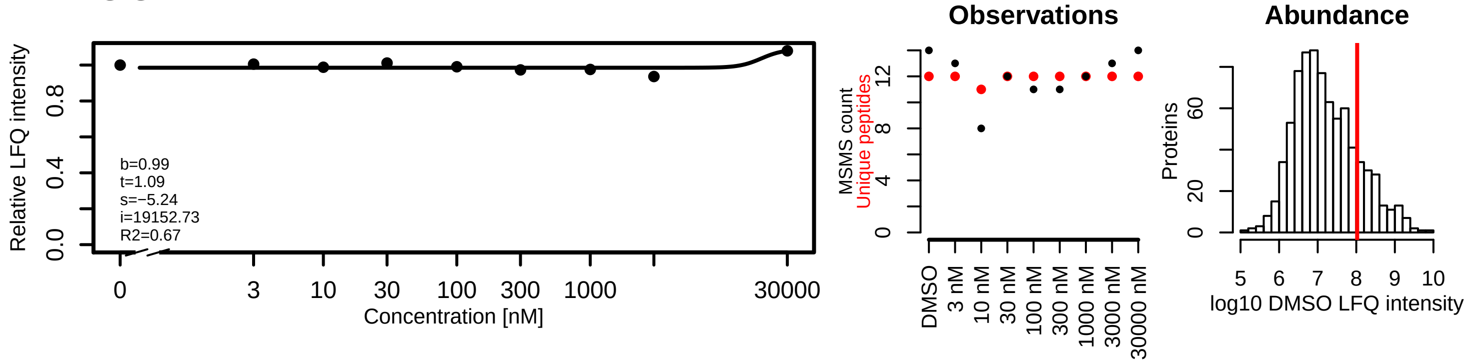


# UBAP2

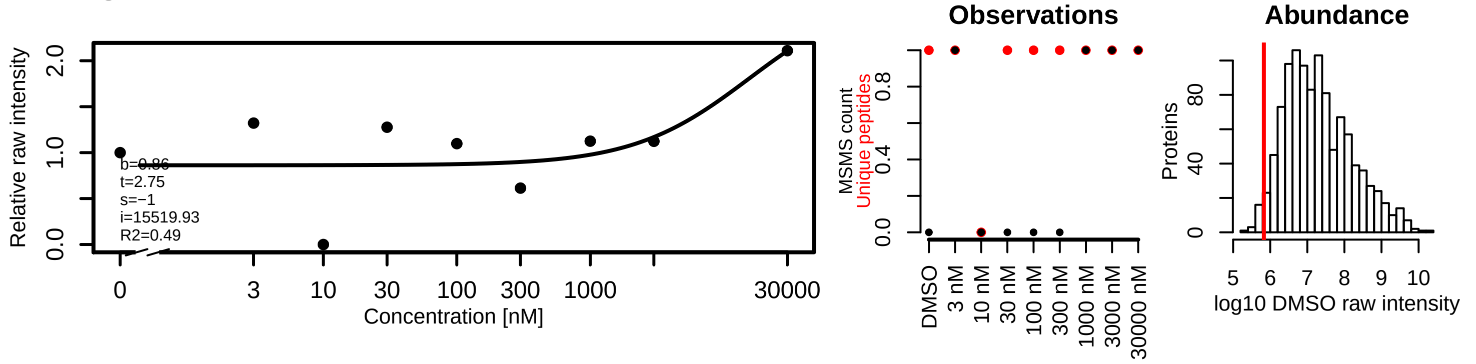


# KIAA1715;LNP

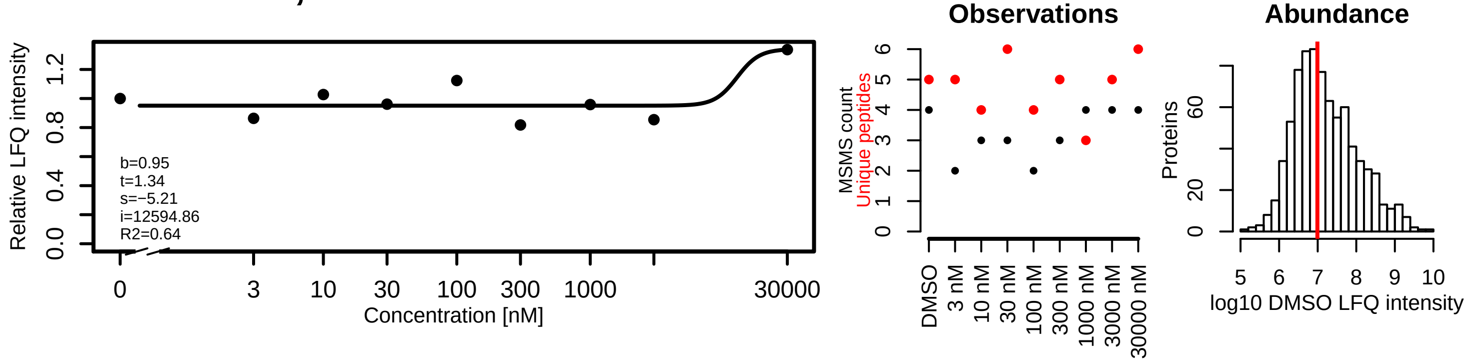


# CALU

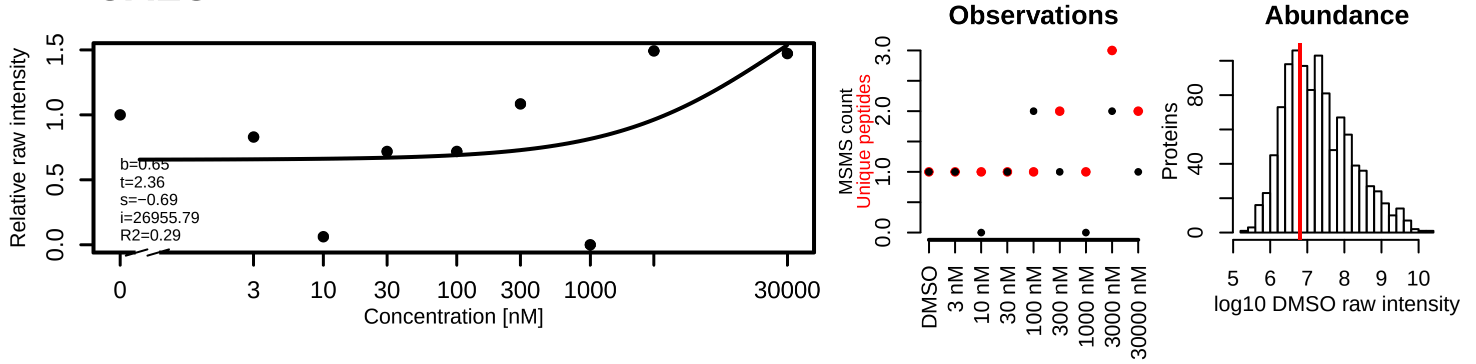


# RPS14

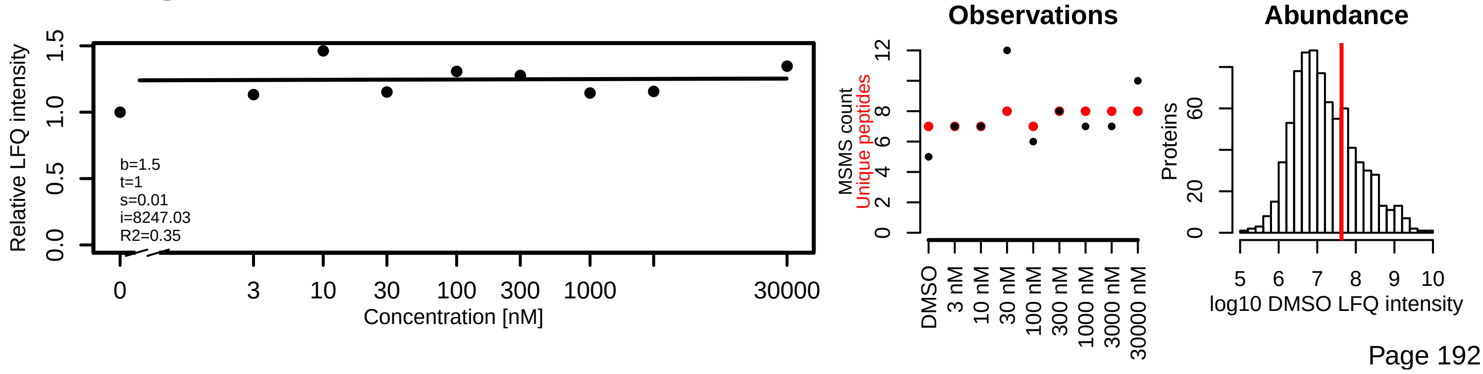

# UPP1

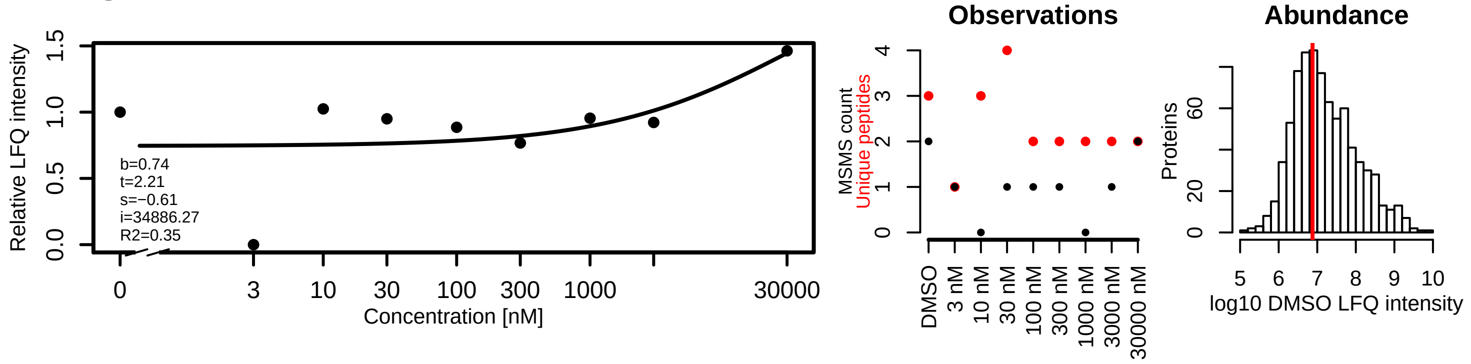


# RPS4X

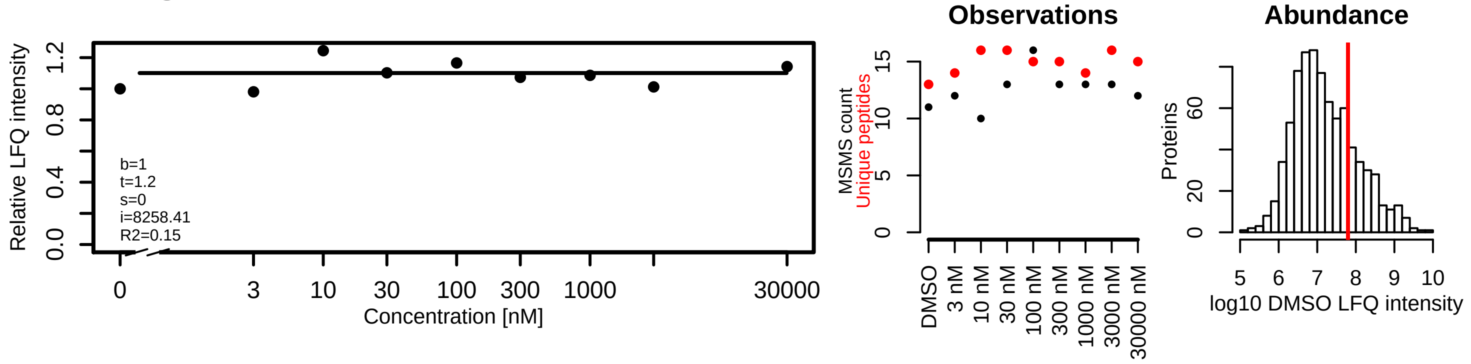


# PSMD8

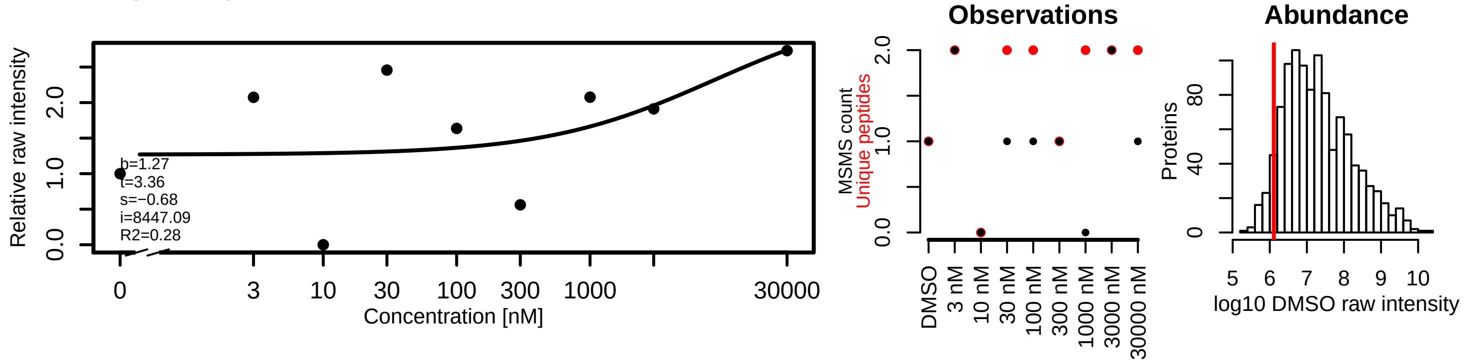


# SDHA

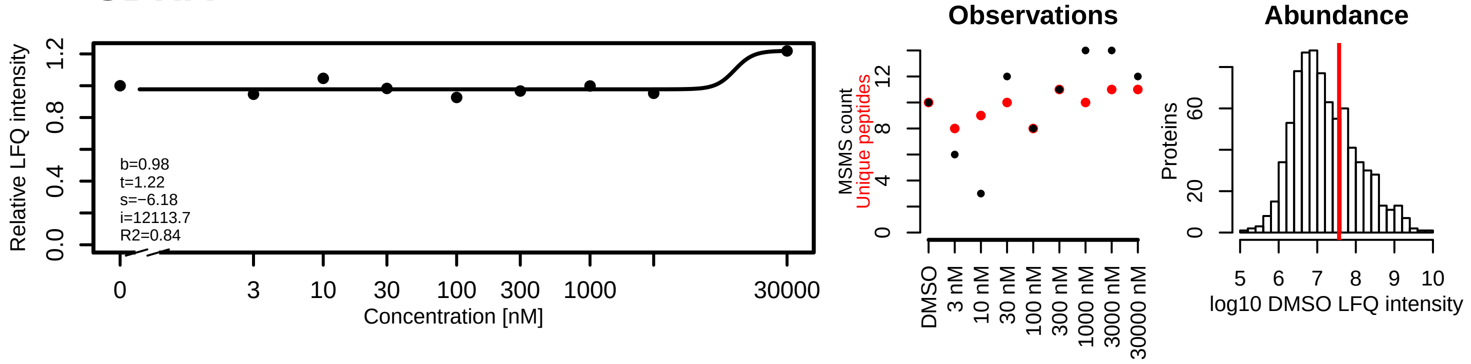


# DLST

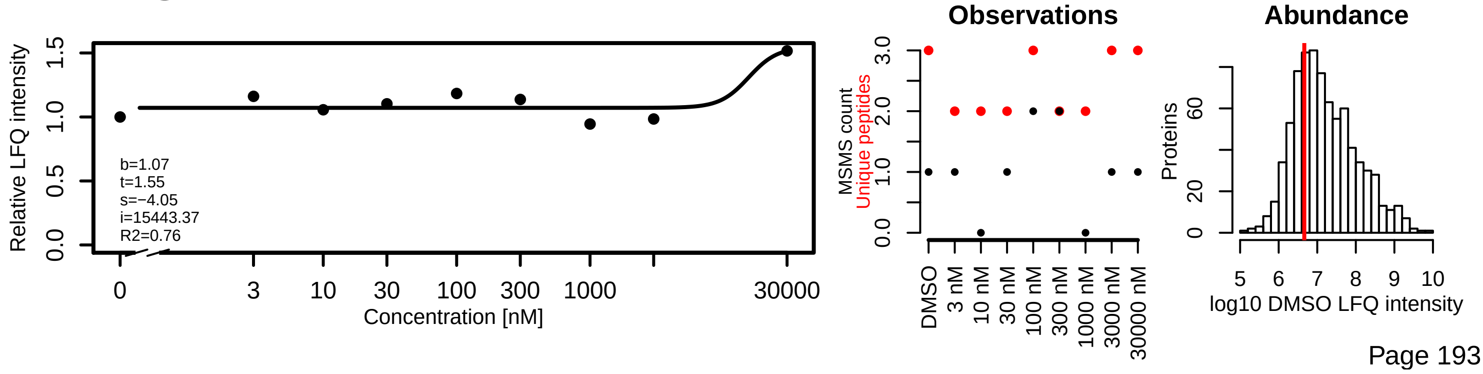

# PGK1

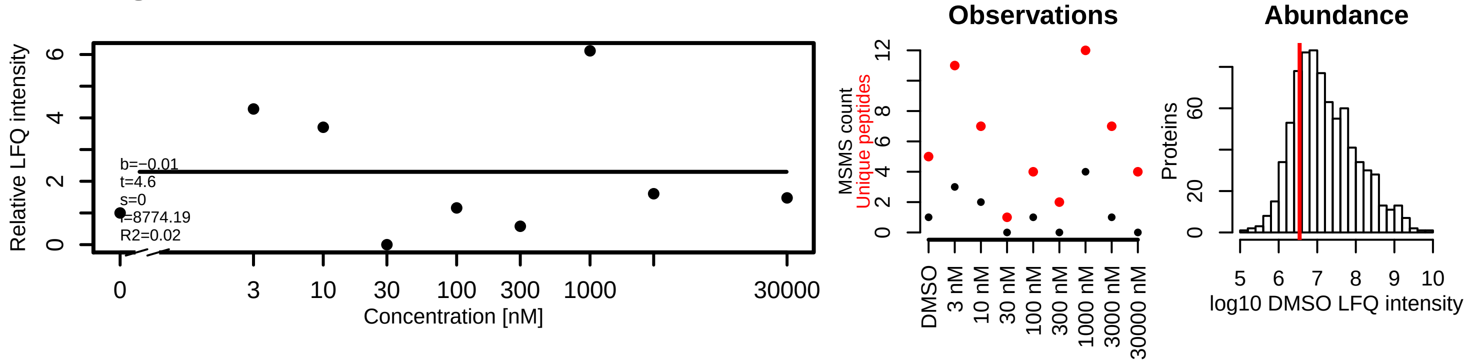


# NDUFB10

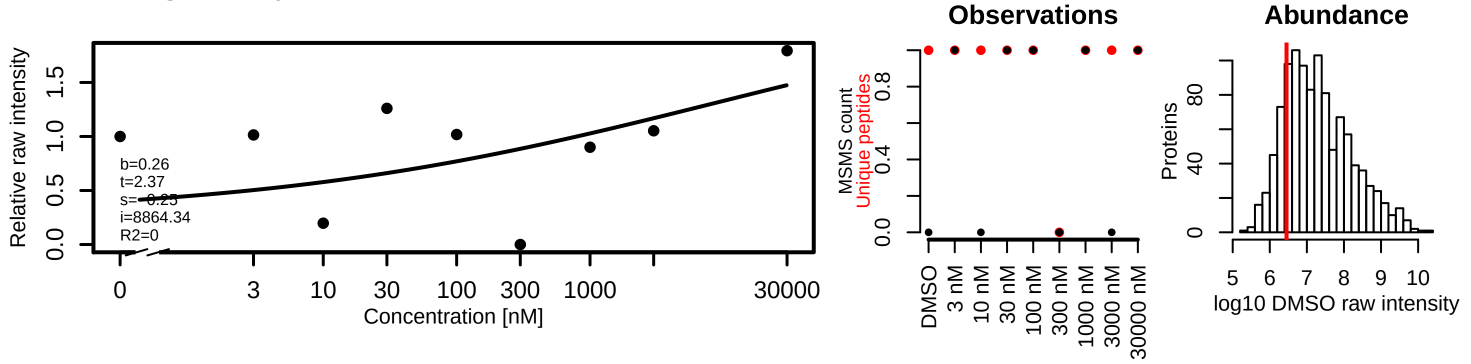


# RPS15A − P62244;I3L3P7;H3BVC7

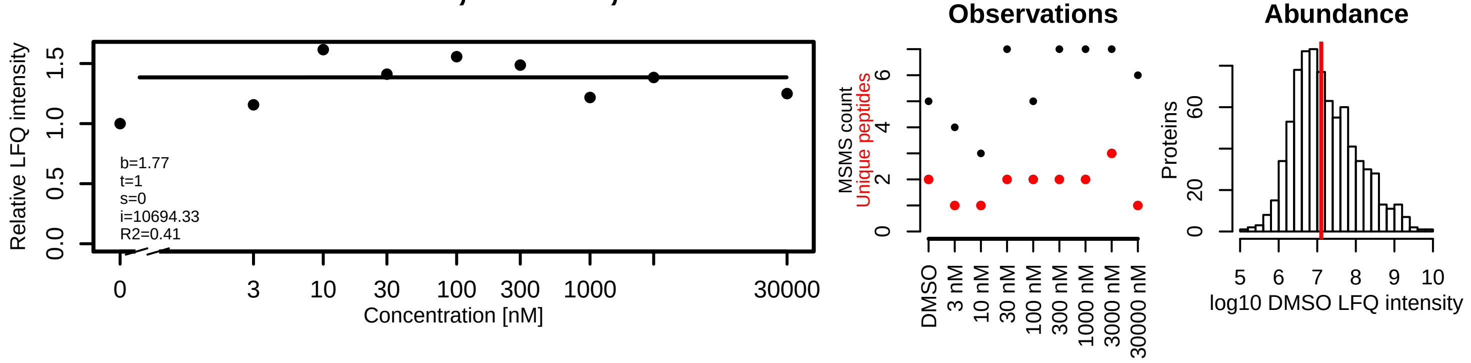


# H1FX

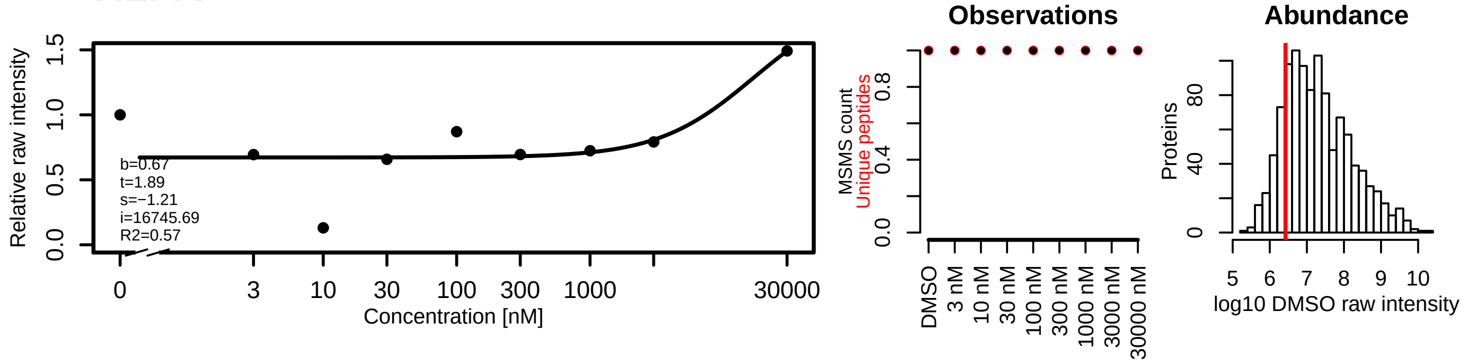


# RAB10

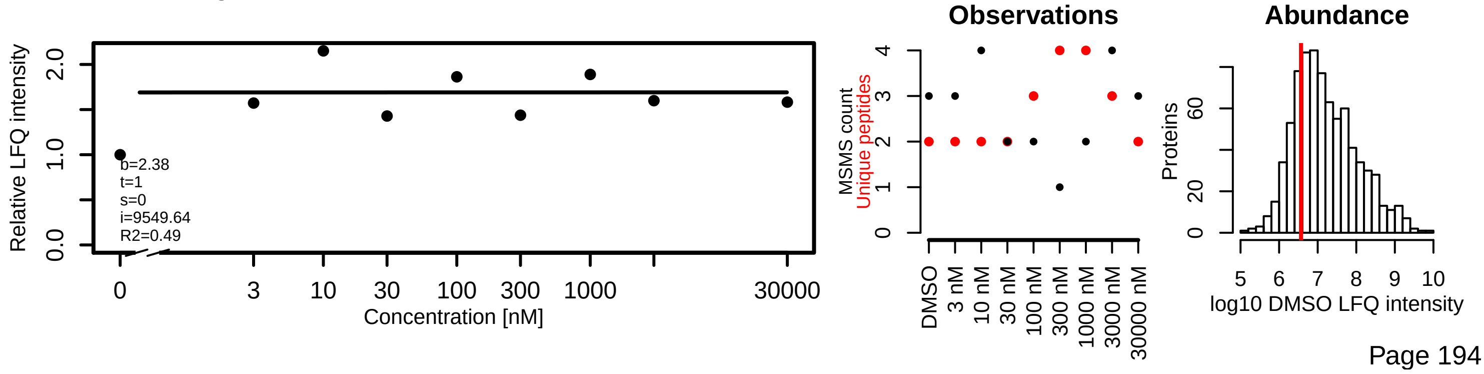

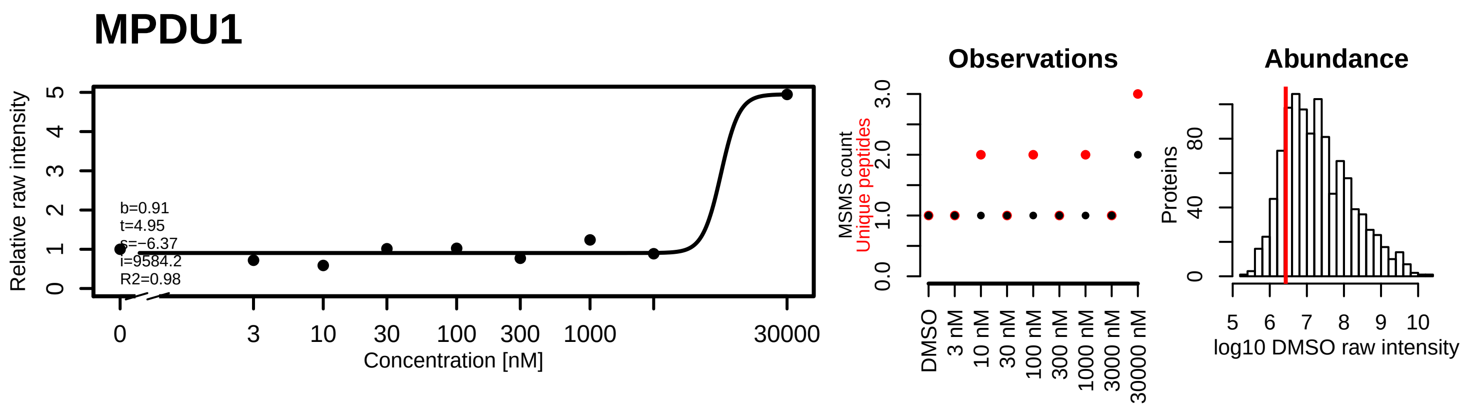
MPDU1
b=0.91
t=4.95
s=−6.37
i=9584.2
R2=0.98
Relative raw intensity
Concentration [nM]
0
3
10
30
100
300
1000
30000
Observations
MSMS count
Unique peptides
DMSO
3 nM
10 nM
30 nM
100 nM
300 nM
1000 nM
3000 nM
30000 nM
Abundance
Proteins
log10 DMSO raw intensity

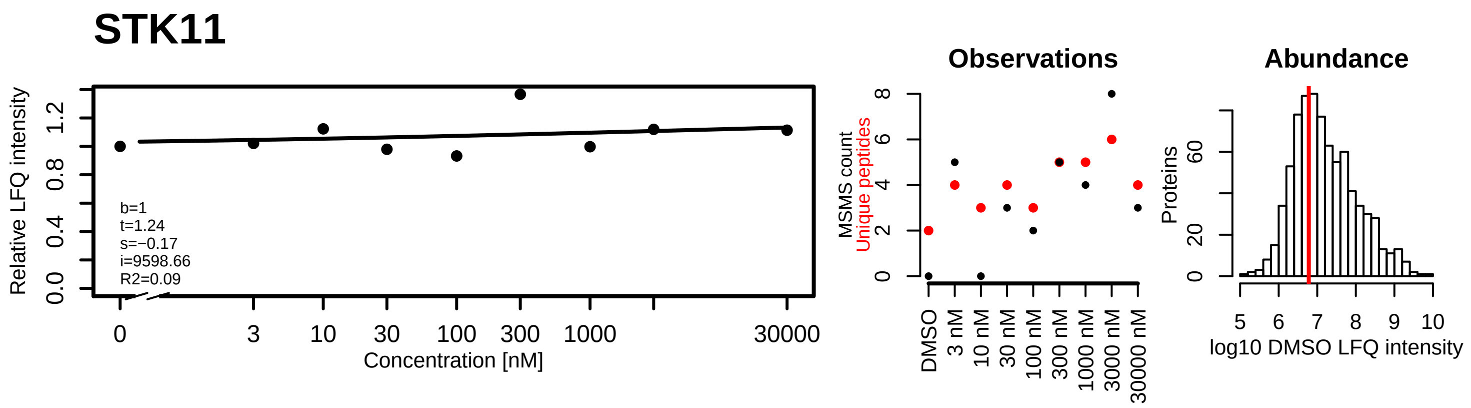
STK11
b=1
t=1.24
s=−0.17
i=9598.66
R2=0.09
Relative LFQ intensity
Concentration [nM]
0
3
10
30
100
300
1000
30000
Observations
MSMS count
Unique peptides
DMSO
3 nM
10 nM
30 nM
100 nM
300 nM
1000 nM
3000 nM
30000 nM
Abundance
Proteins
log10 DMSO LFQ intensity

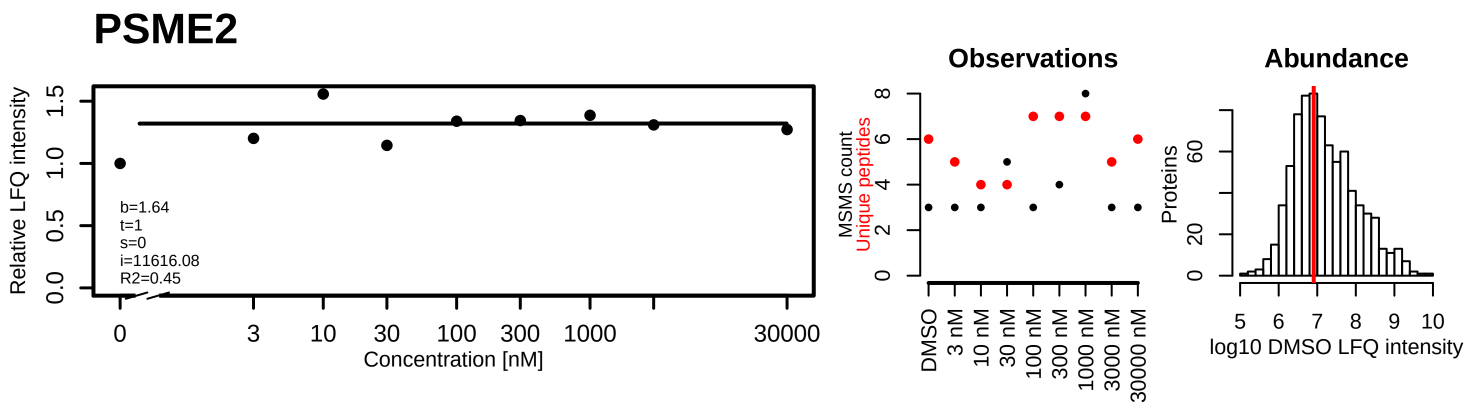
PSME2
b=1.64
t=1
s=0
i=11616.08
R2=0.45
Relative LFQ intensity
Concentration [nM]
0
3
10
30
100
300
1000
30000
Observations
MSMS count
Unique peptides
DMSO
3 nM
10 nM
30 nM
100 nM
300 nM
1000 nM
3000 nM
30000 nM
Abundance
Proteins
log10 DMSO LFQ intensity

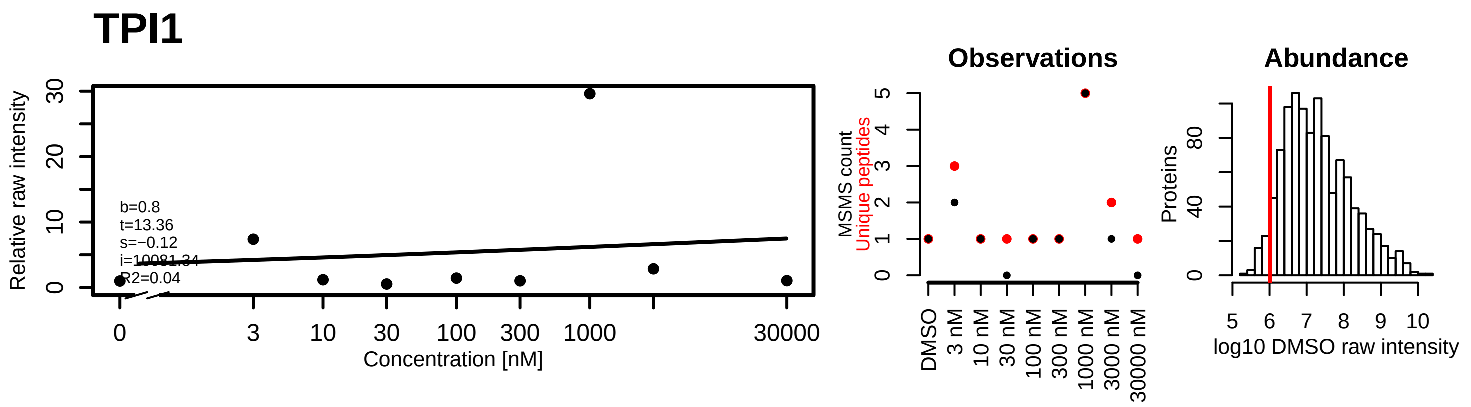
TPI1
b=0.8
t=13.36
s=−0.12
i=10081.24
R2=0.04
Relative raw intensity
Concentration [nM]
0
3
10
30
100
300
1000
30000
Observations
MSMS count
Unique peptides
DMSO
3 nM
10 nM
30 nM
100 nM
300 nM
1000 nM
3000 nM
30000 nM
Abundance
Proteins
log10 DMSO raw intensity

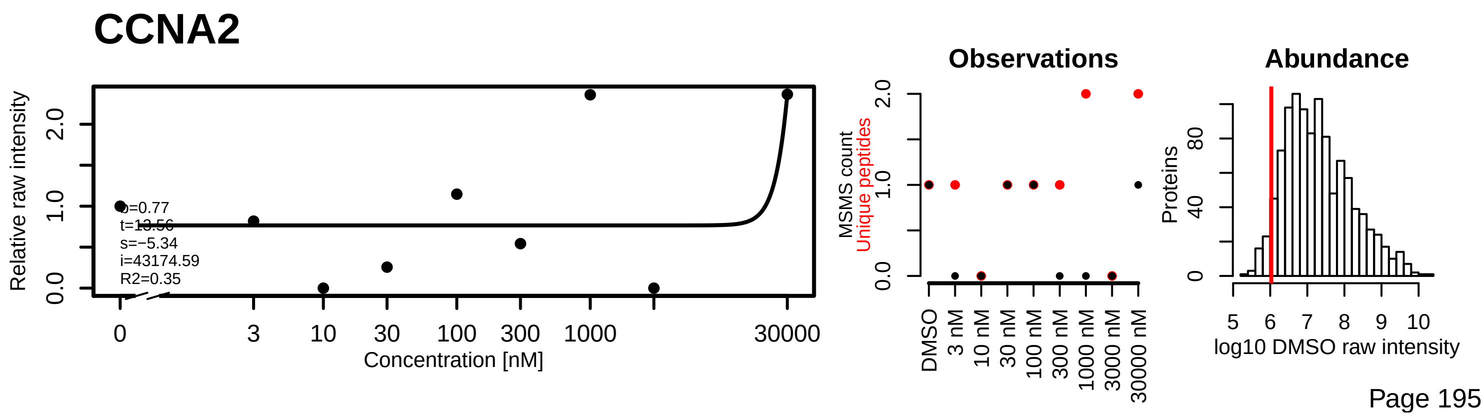
CCNA2
b=0.77
t=13.56
s=−5.34
i=43174.59
R2=0.35
Relative raw intensity
Concentration [nM]
0
3
10
30
100
300
1000
30000
Observations
MSMS count
Unique peptides
DMSO
3 nM
10 nM
30 nM
100 nM
300 nM
1000 nM
3000 nM
30000 nM
Abundance
Proteins
log10 DMSO raw intensity

# CDC73

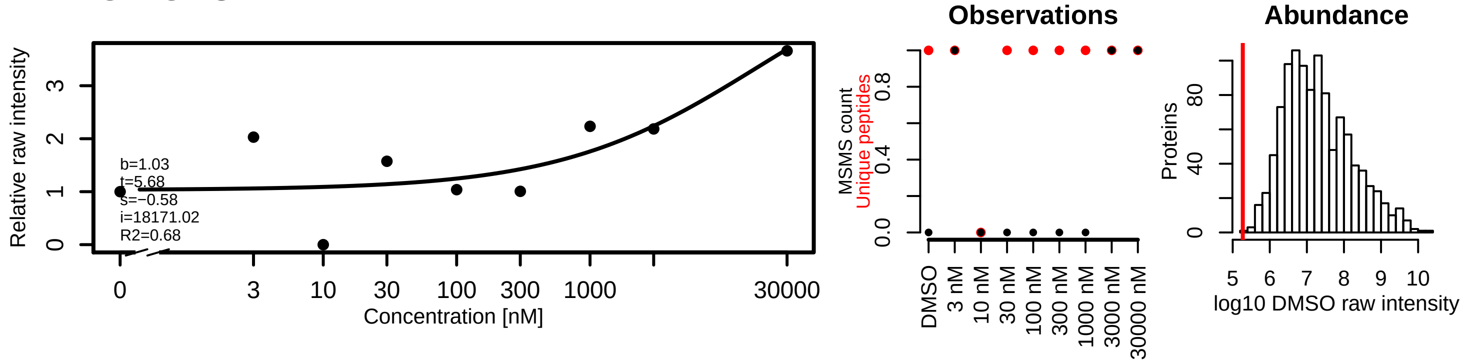


# ALYREF

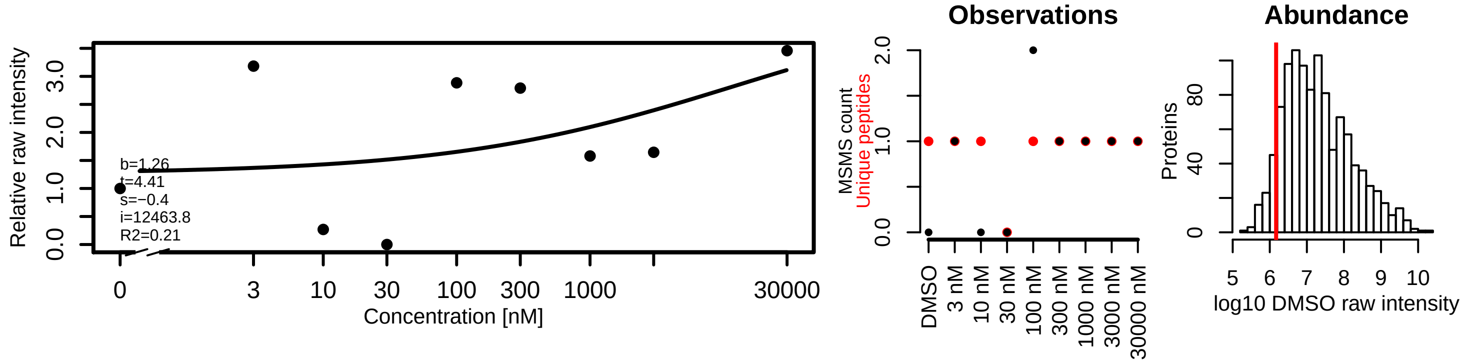


# LACTB

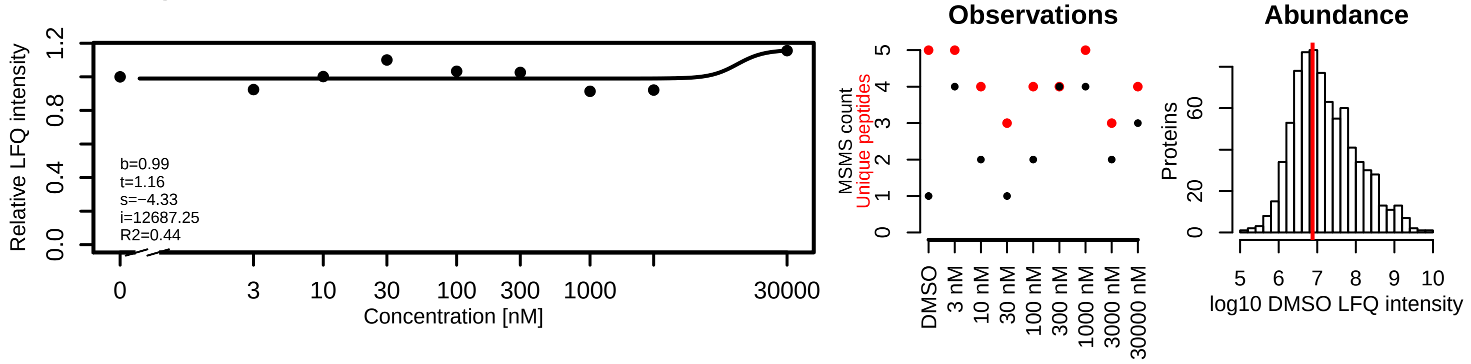


# XRCC6

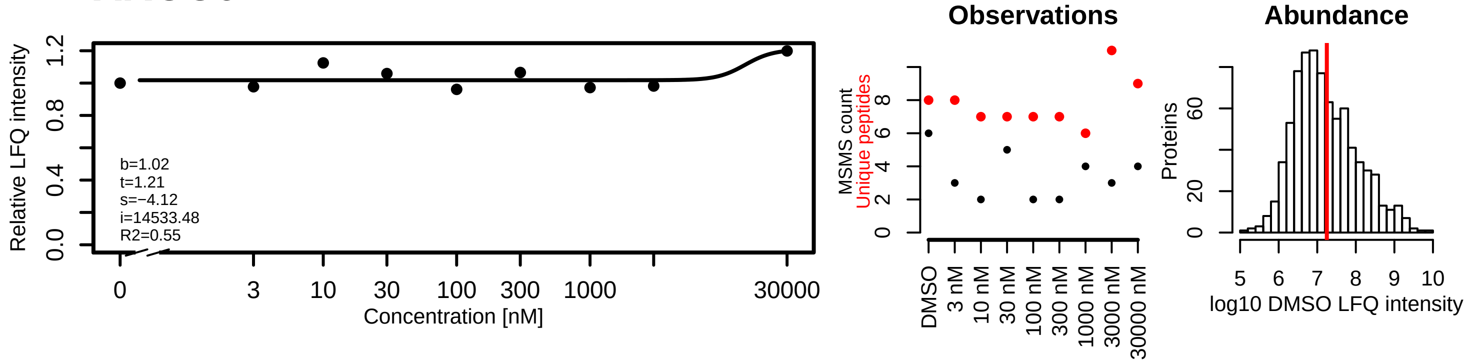


# IGF2BP3

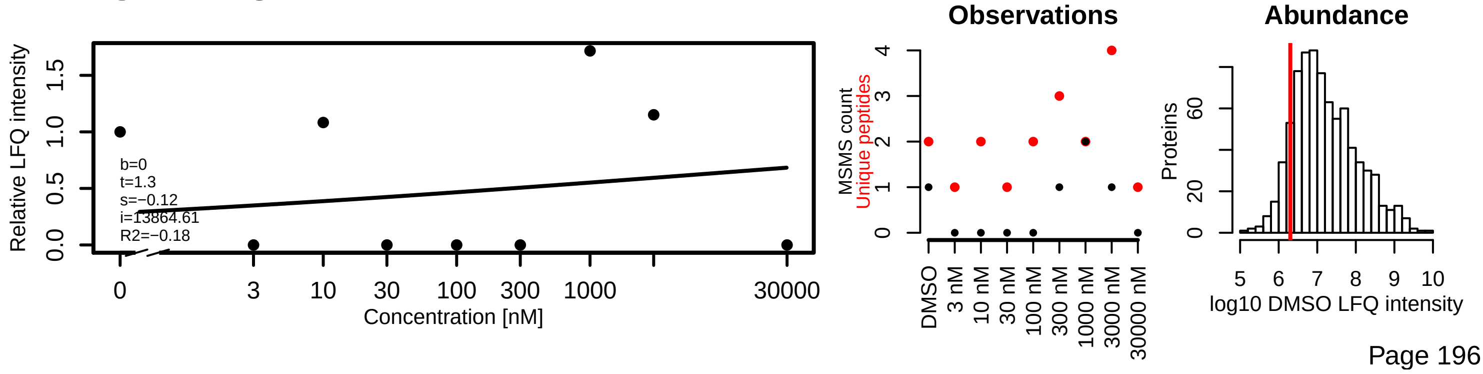

# NOLC1

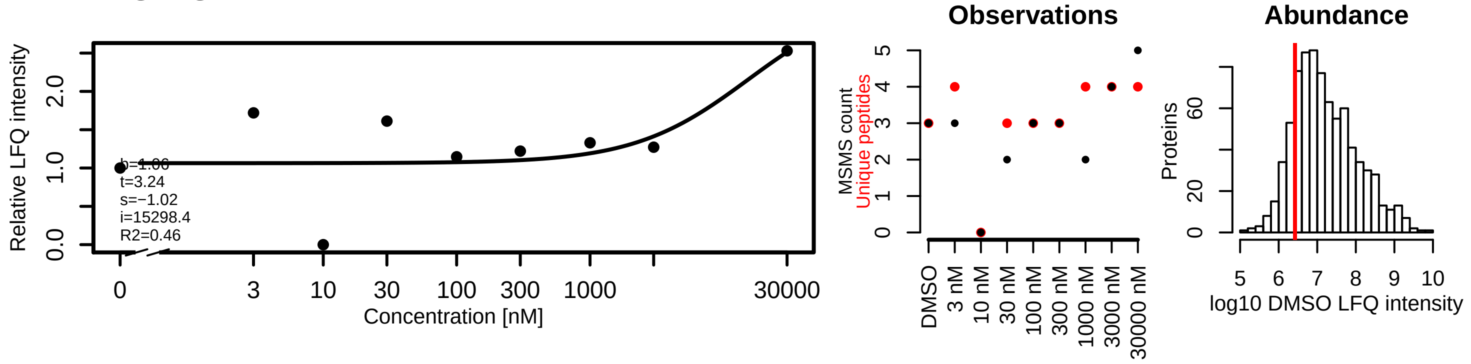


# ACTN1

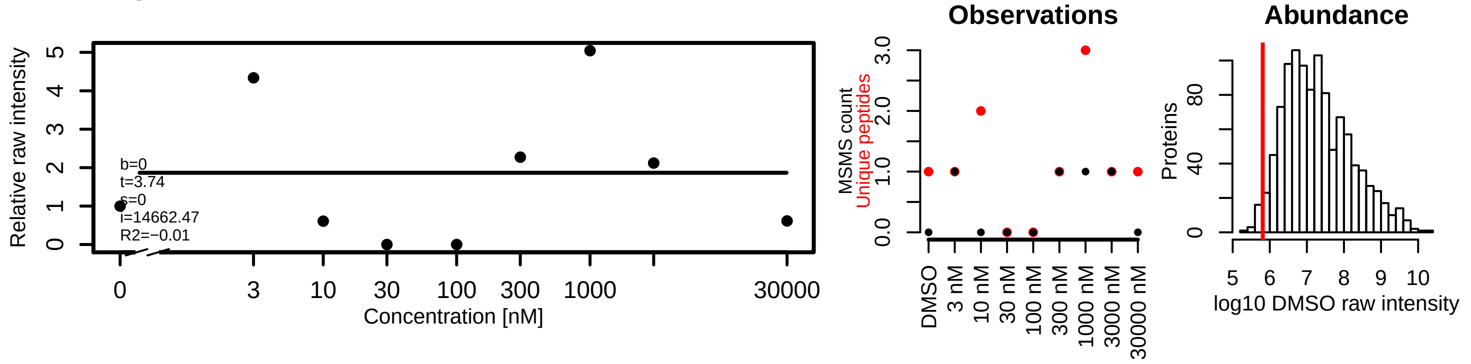


# AIMP2

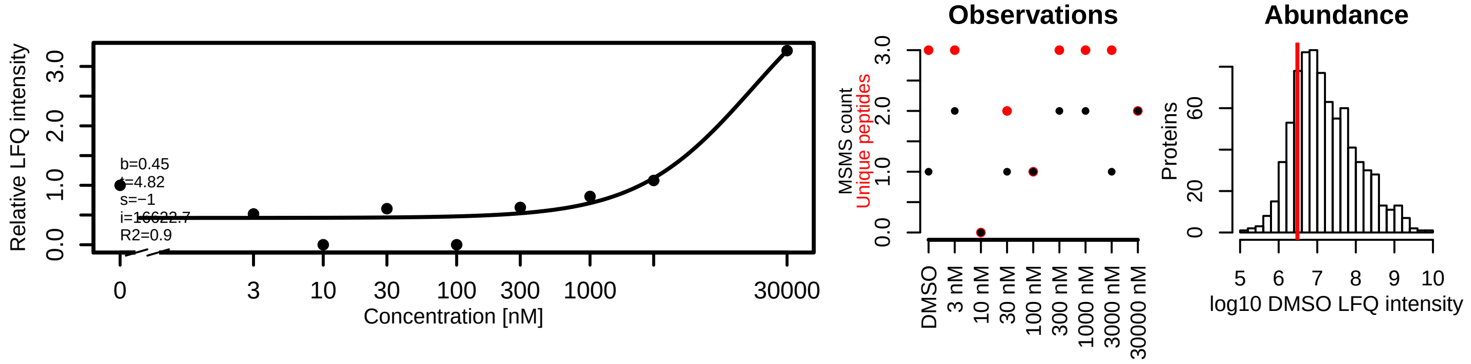


# SUB1

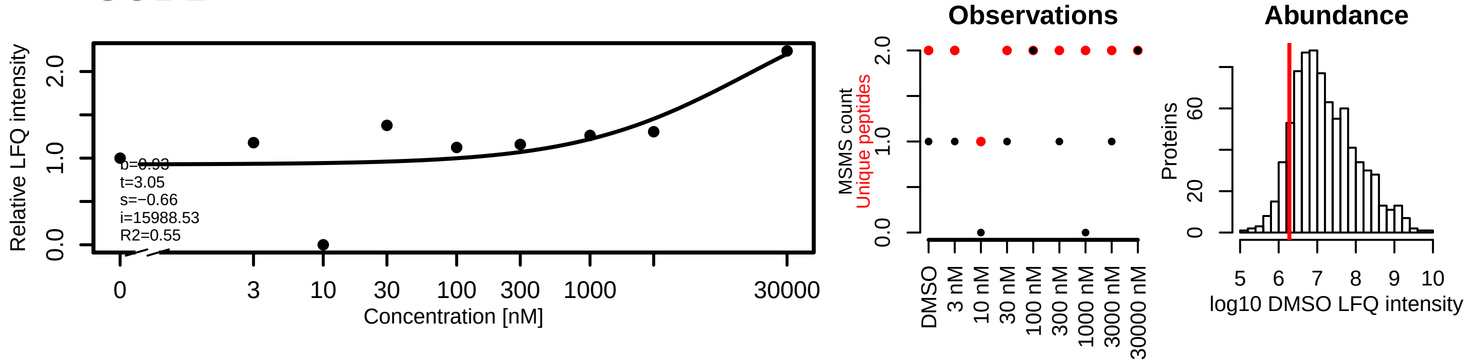


# HSP90AA1

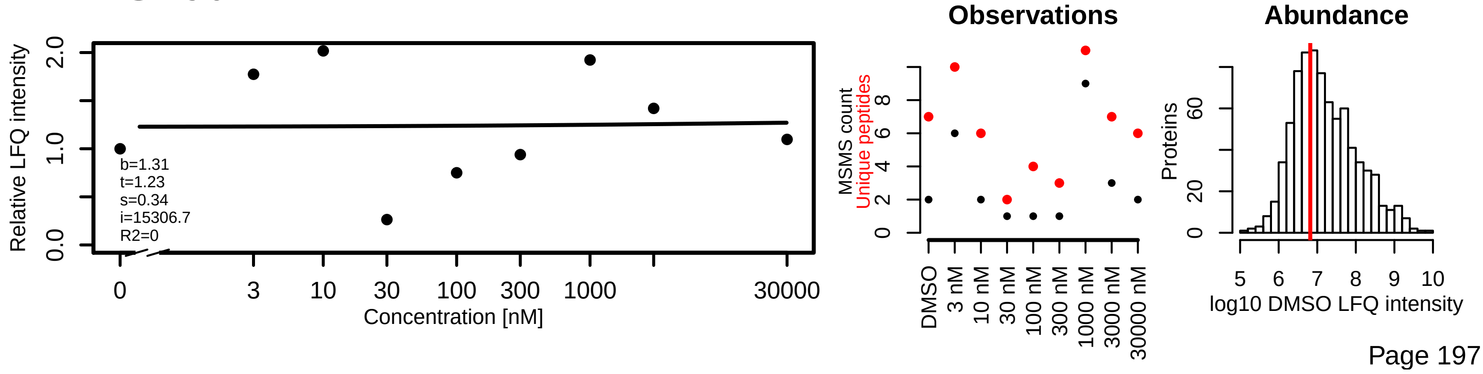

## APP;APLP2

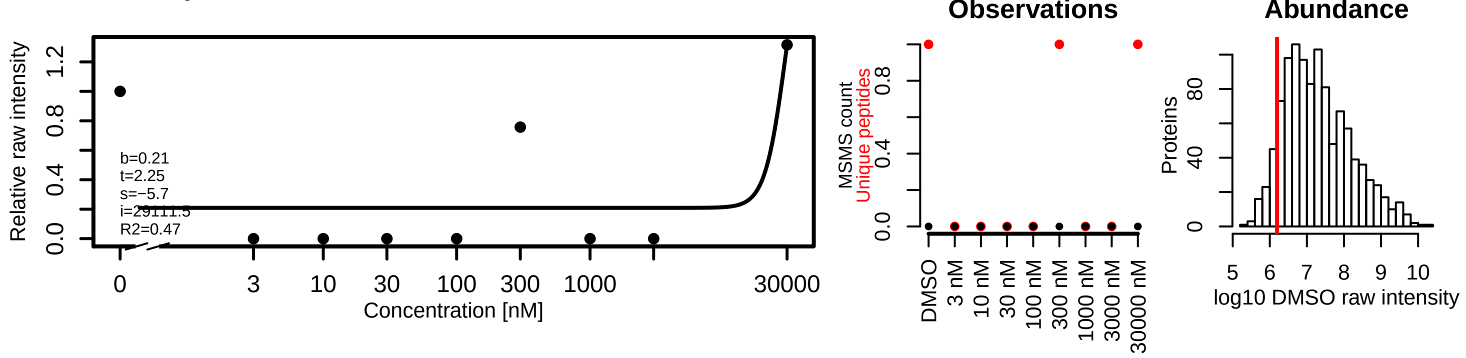


## COPA

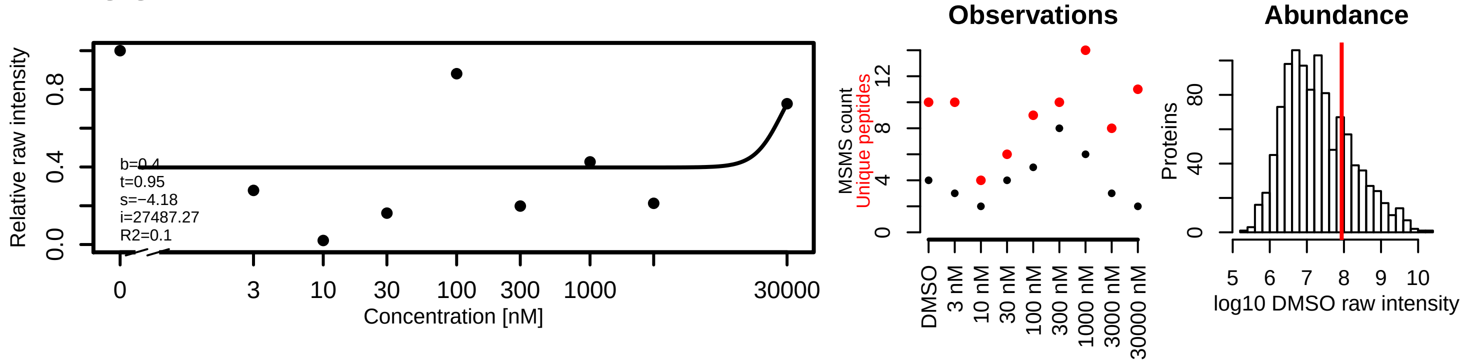


## CTPS1

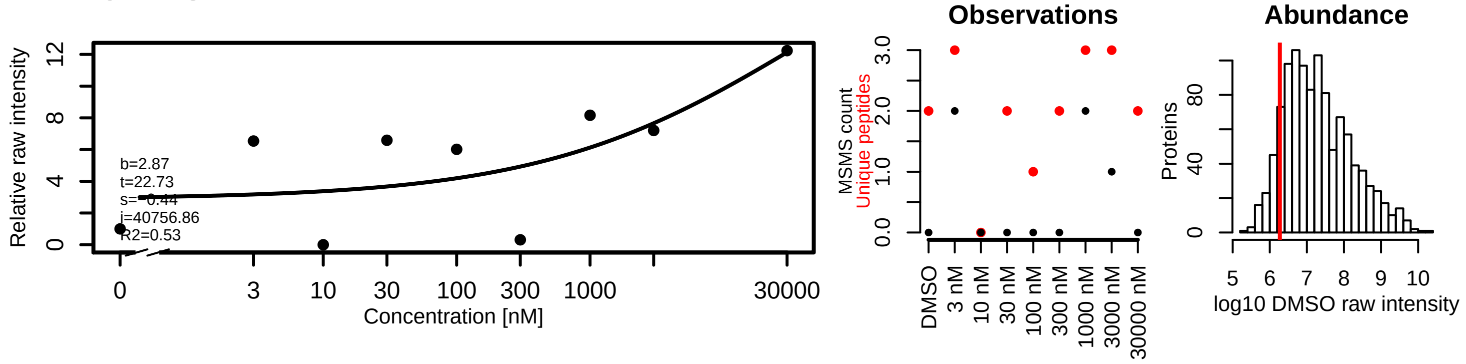


## FARSB

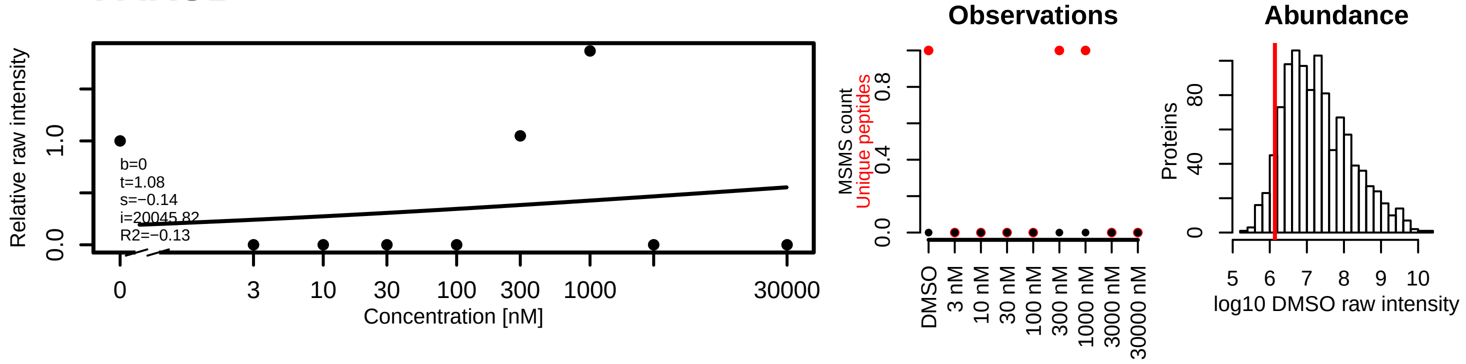


## PSME1

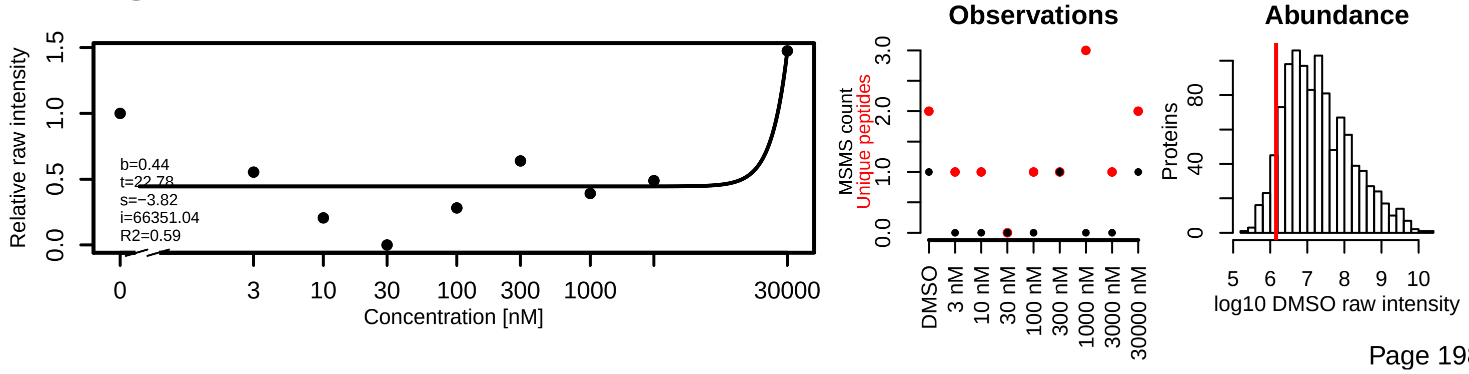

## TUFM

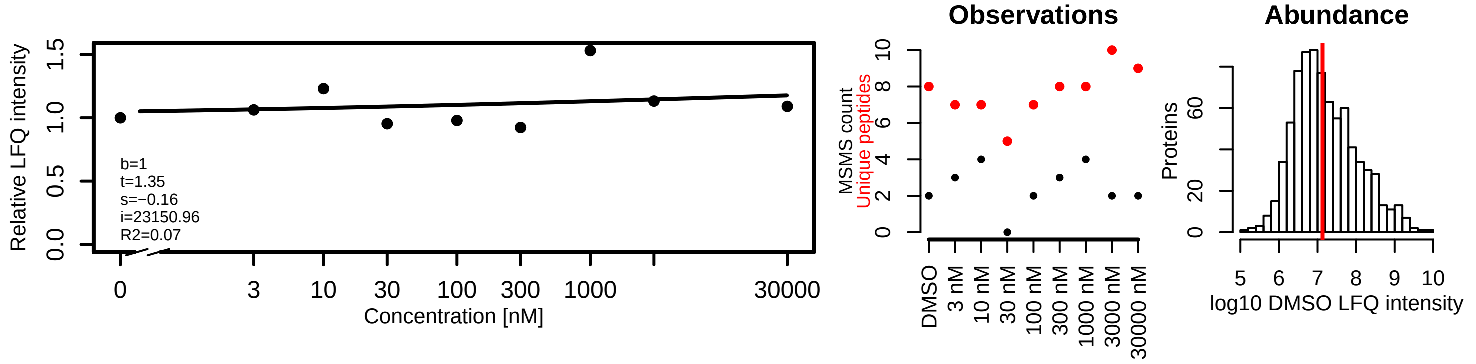


## NUDT14

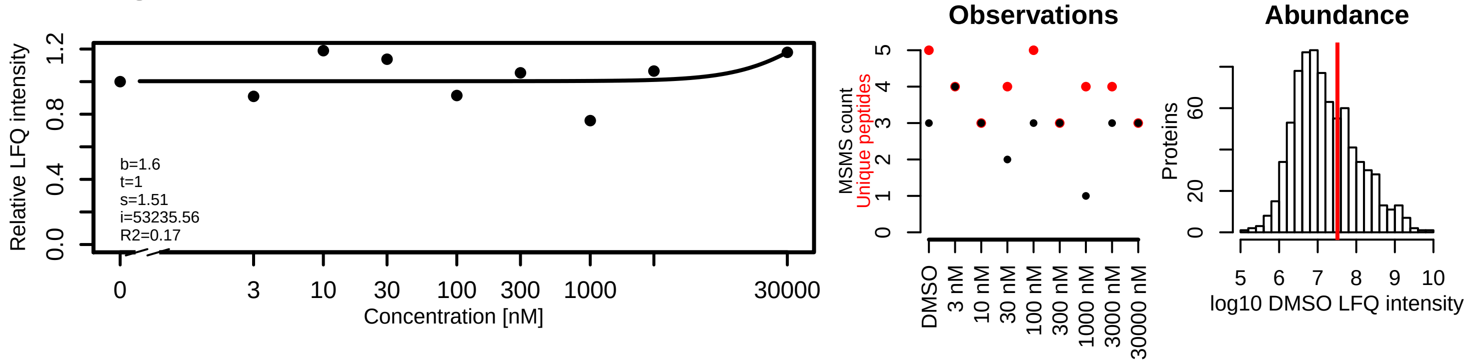


## HLA−A

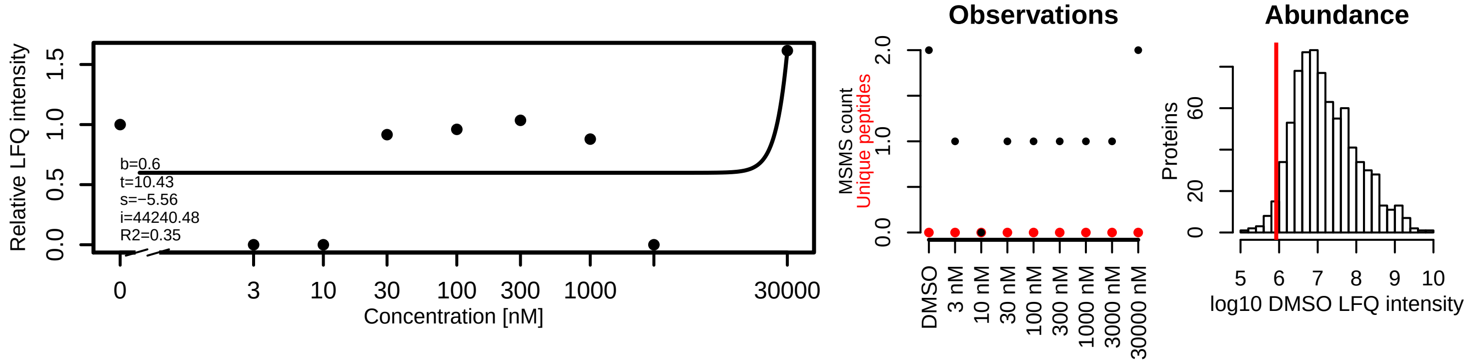


## PABPC4

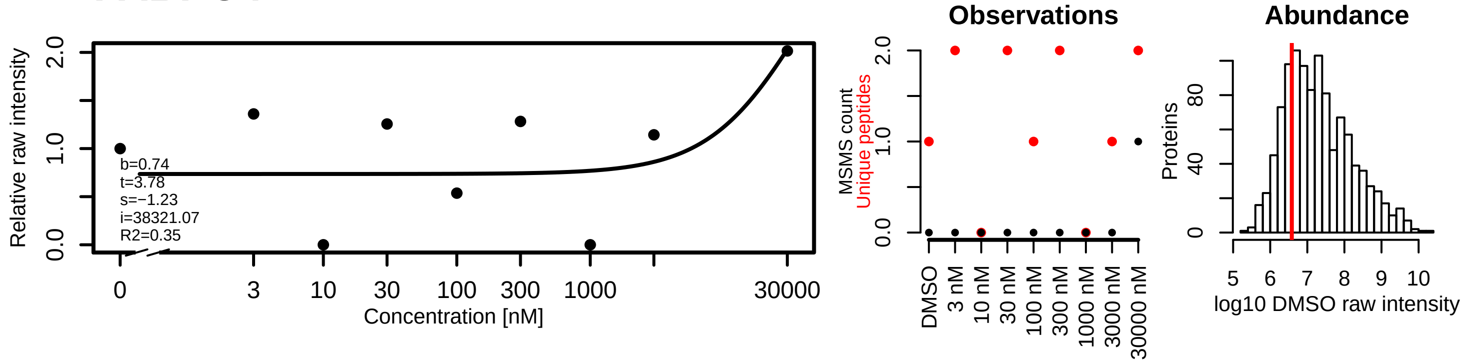


## KRT18

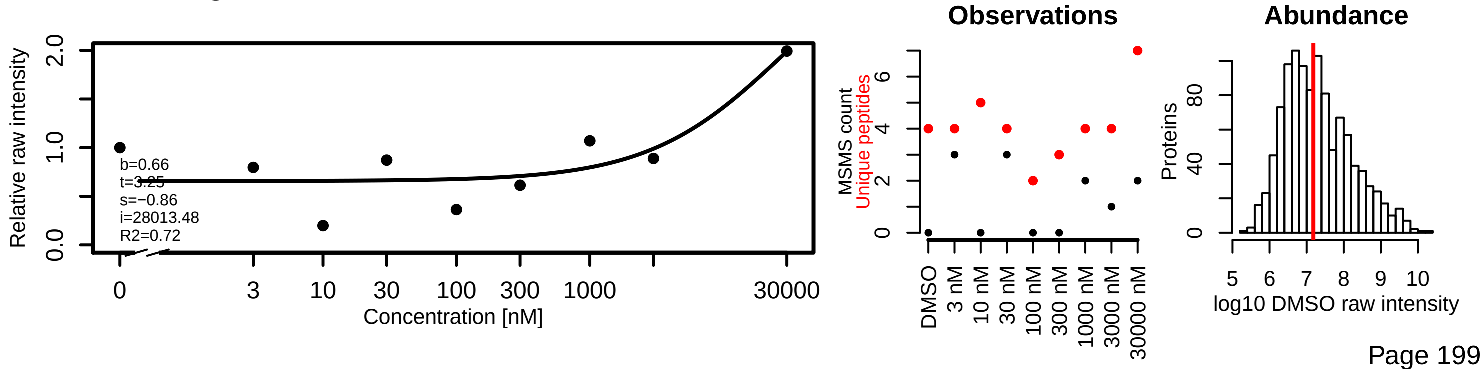

# CDK6

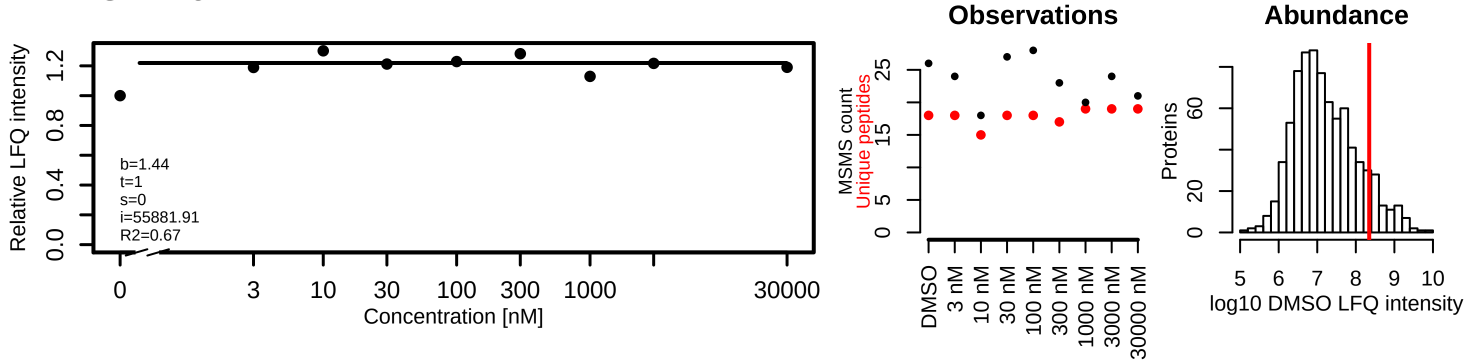


# DNAJC9

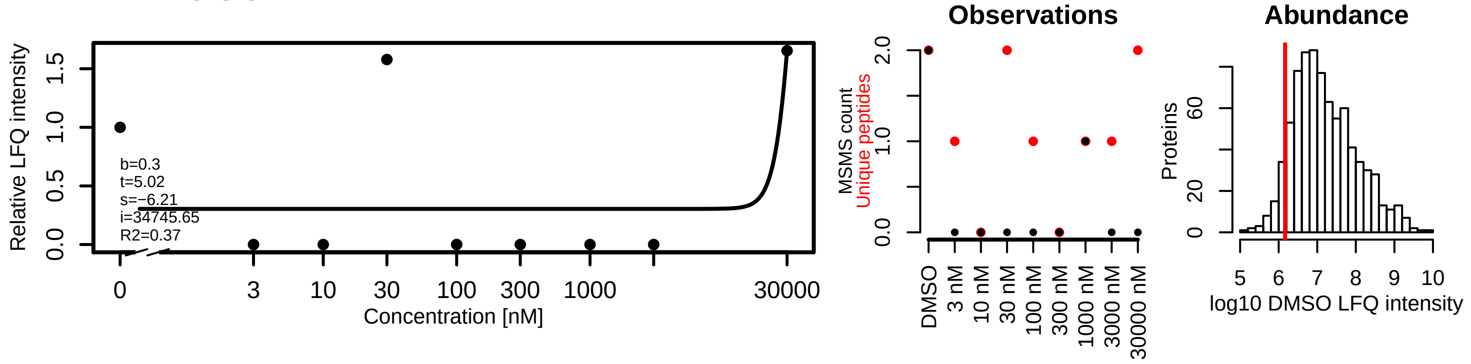


# COPG1

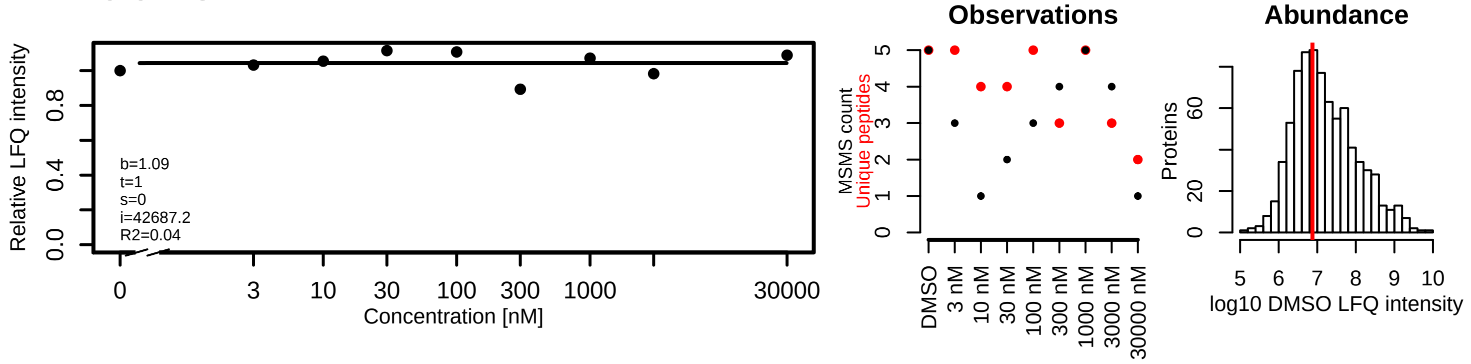


# TARBP1

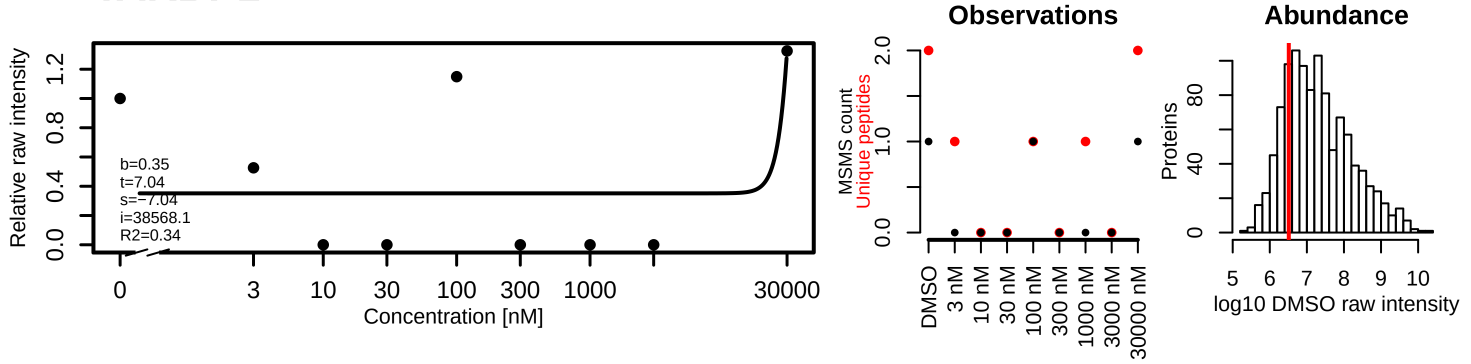


# SMIM7

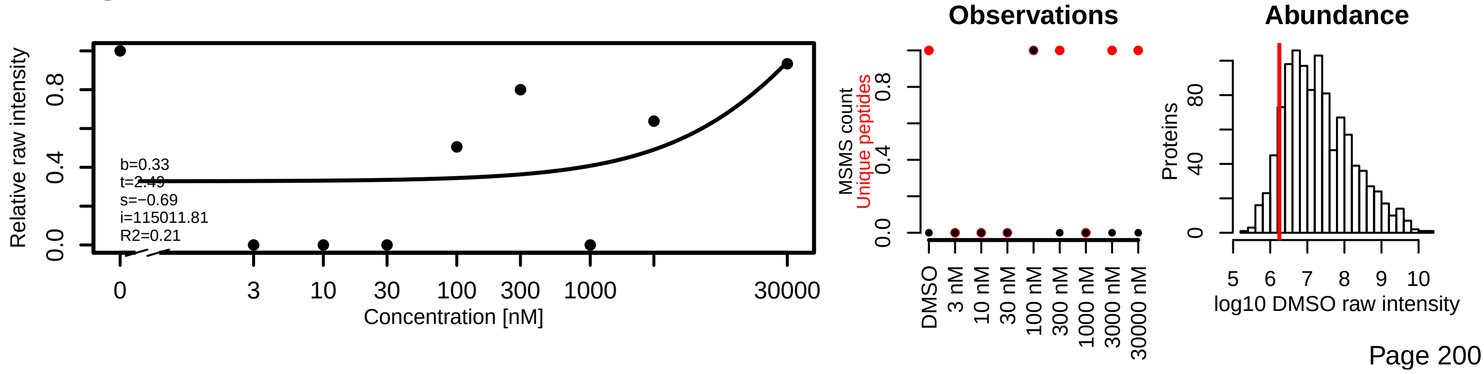

## ACAD9

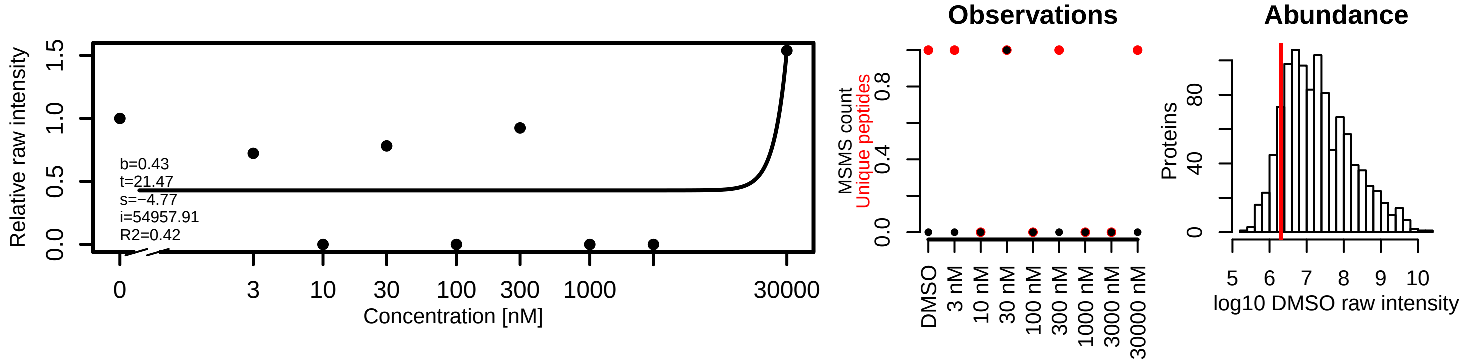


## HEATR1

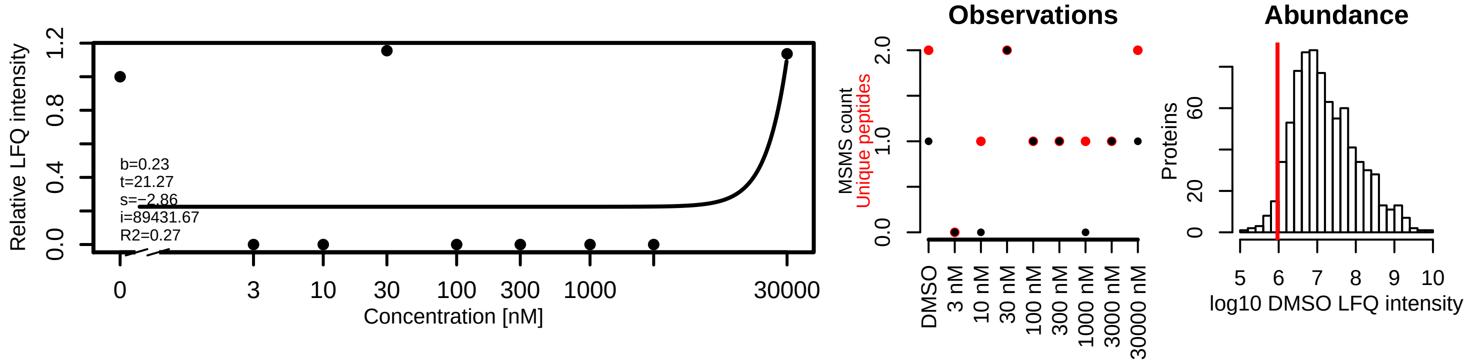


## RPS13

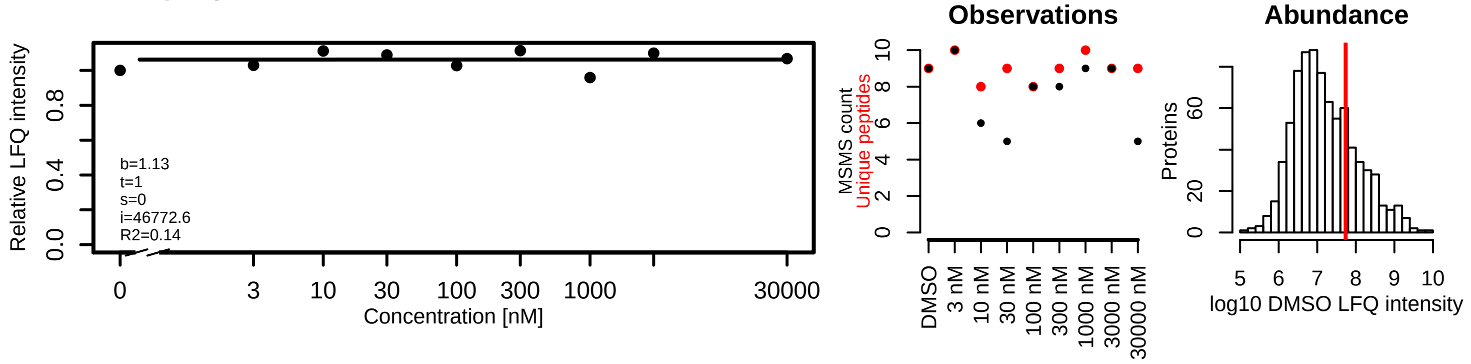


## MRPL13

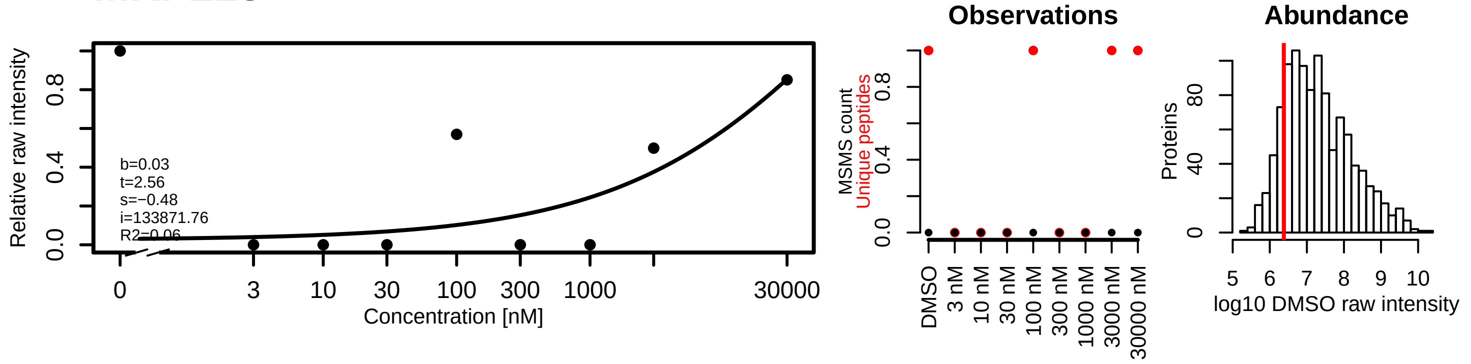


## CAMK2D − H0Y9C2

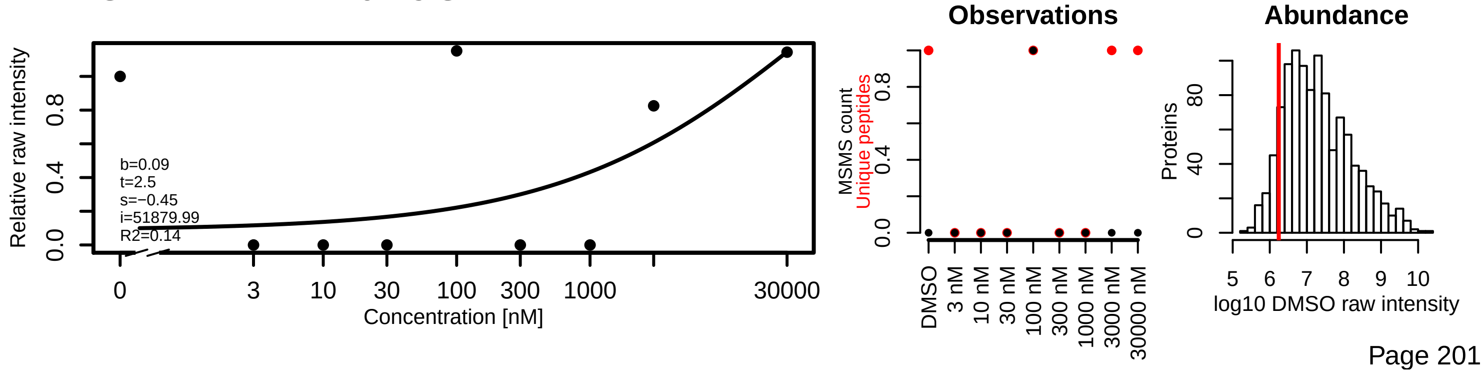

# ZC3HAV1

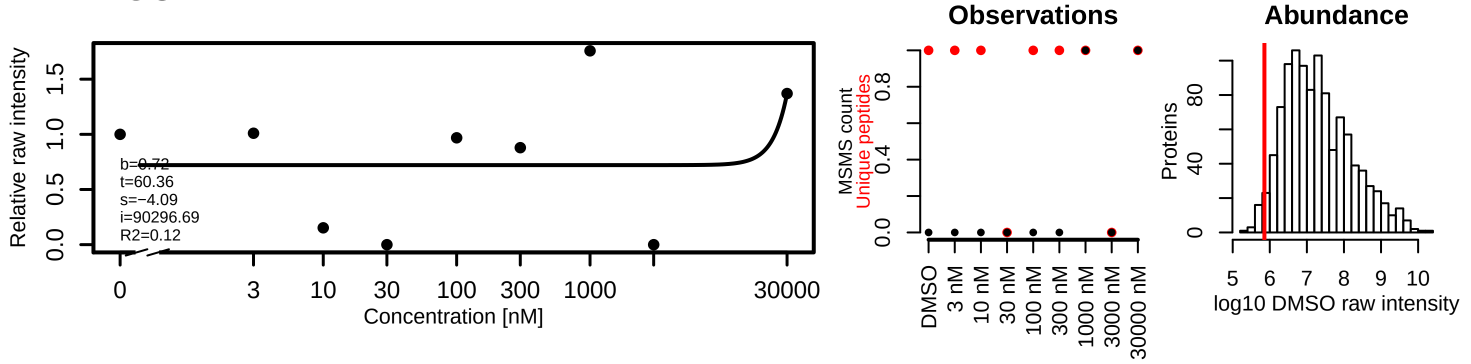


# FARSA

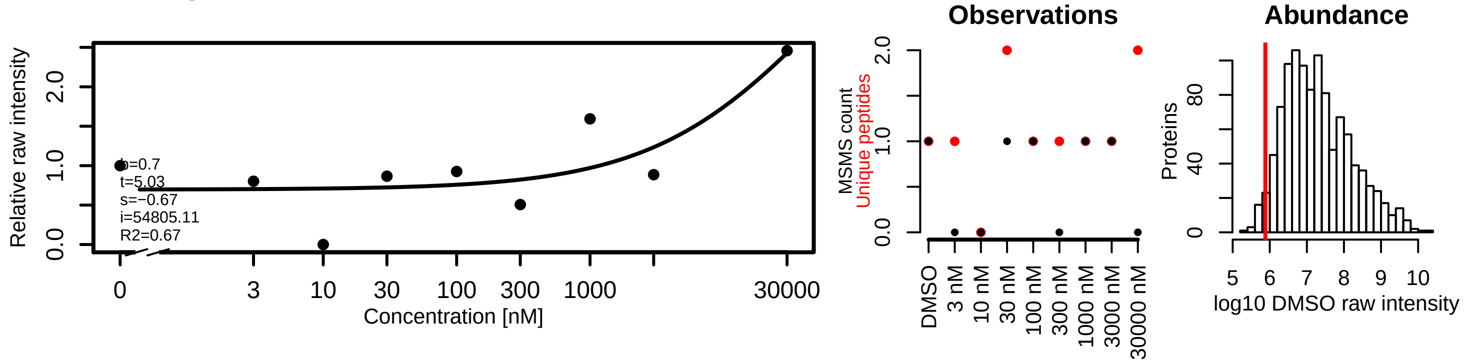


# ATP6AP2

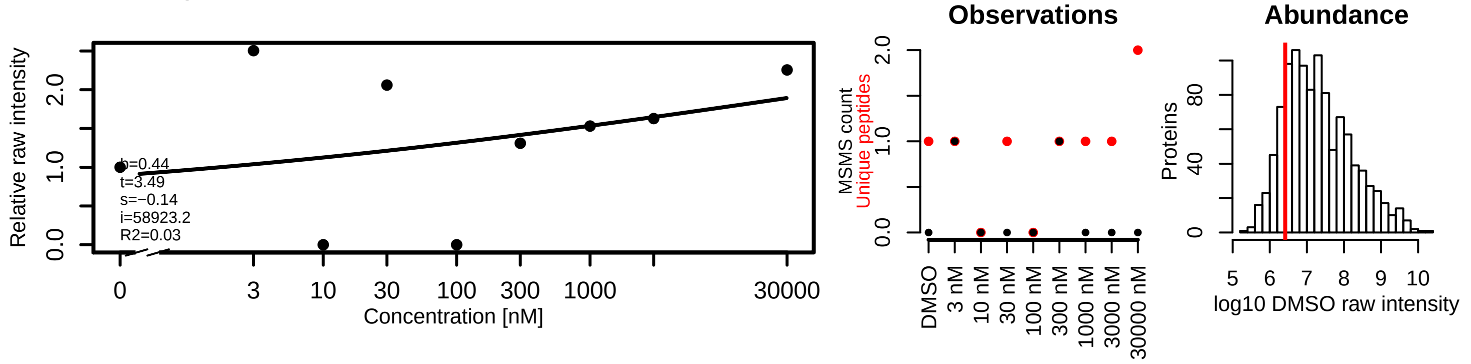


# ERGIC1

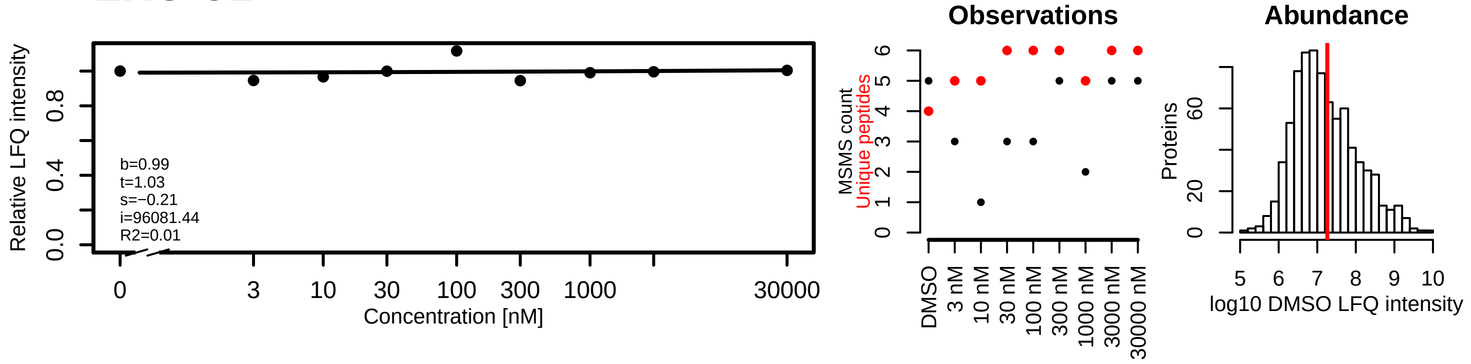


# DSC1

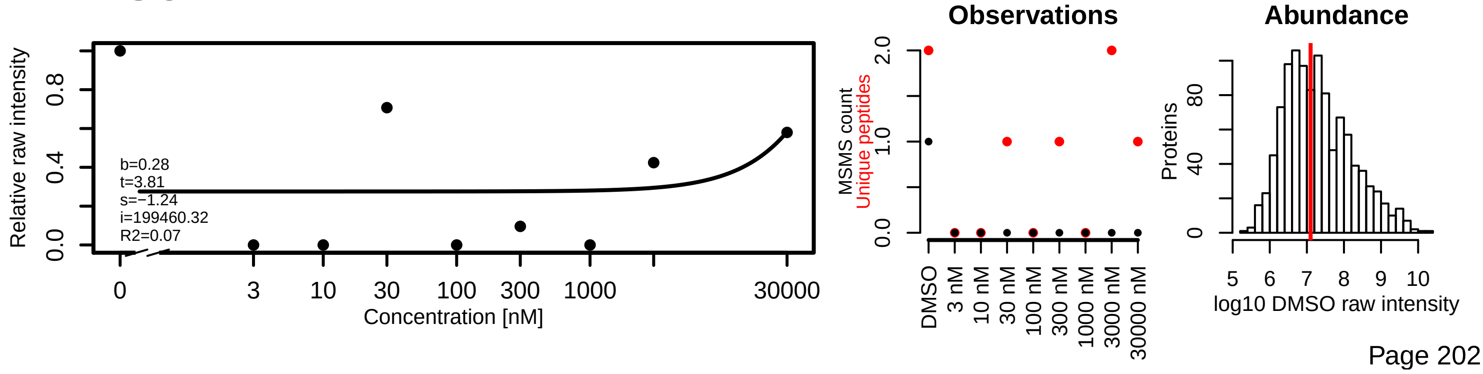

## PYCRL

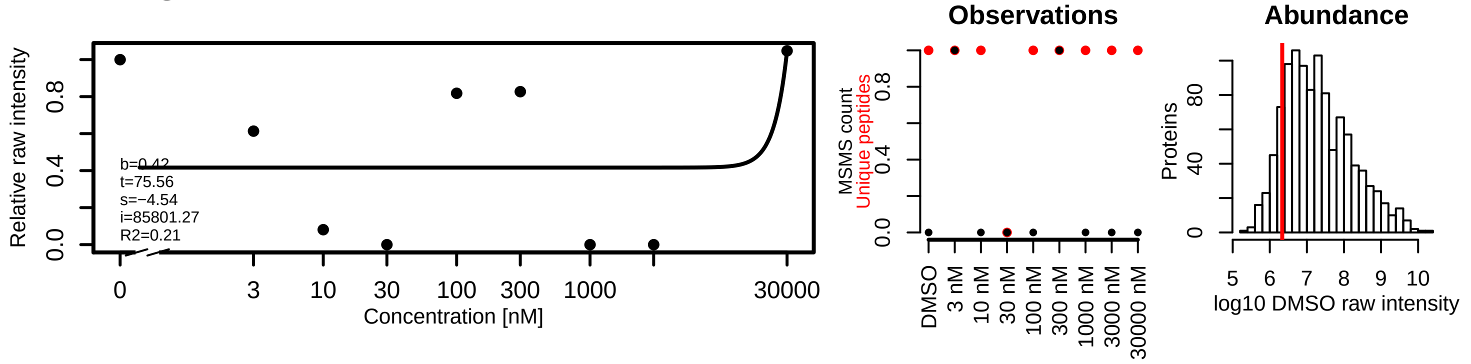


## SRSF1

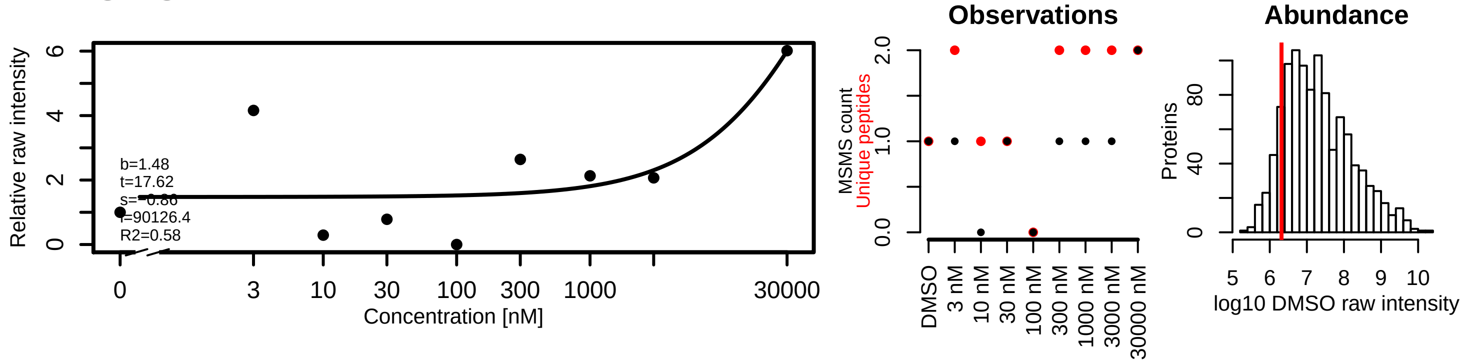


## FUBP1

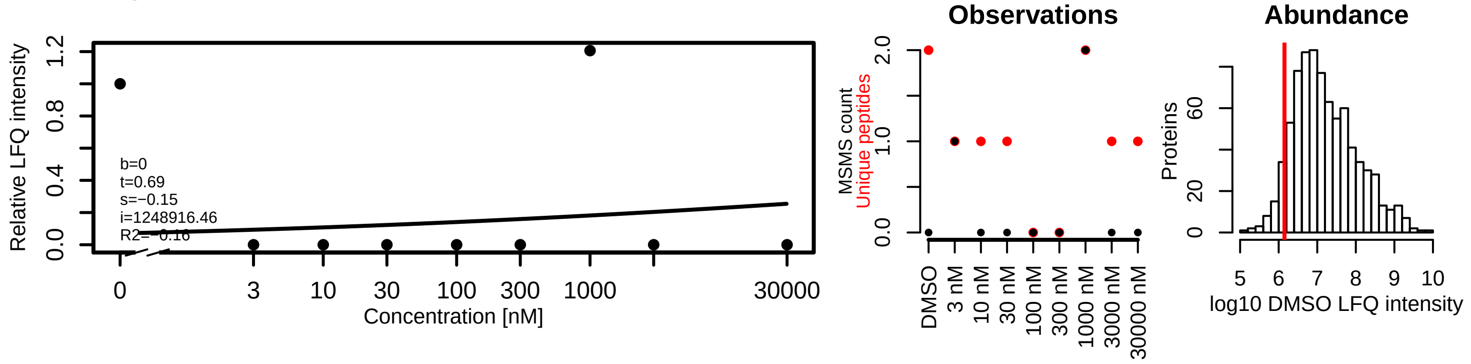


## RAB21

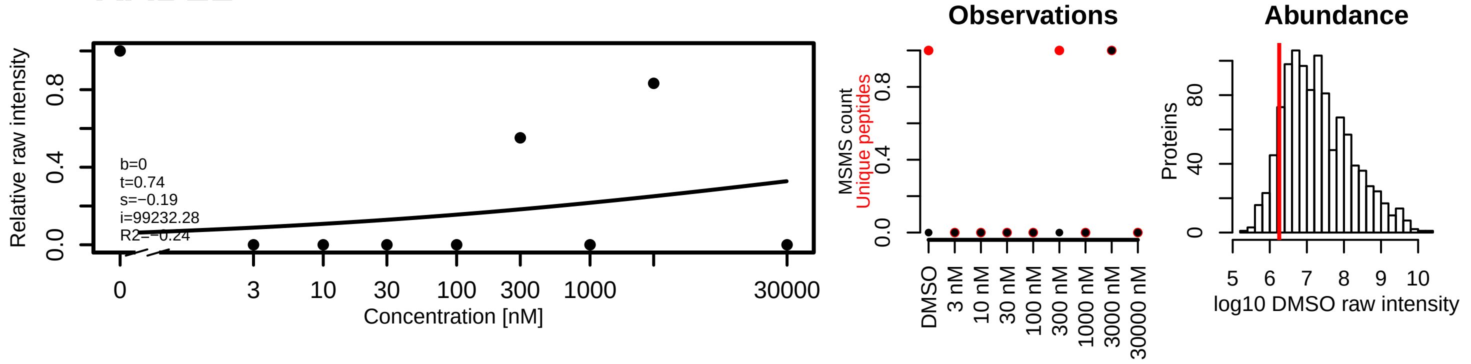


## TMPO

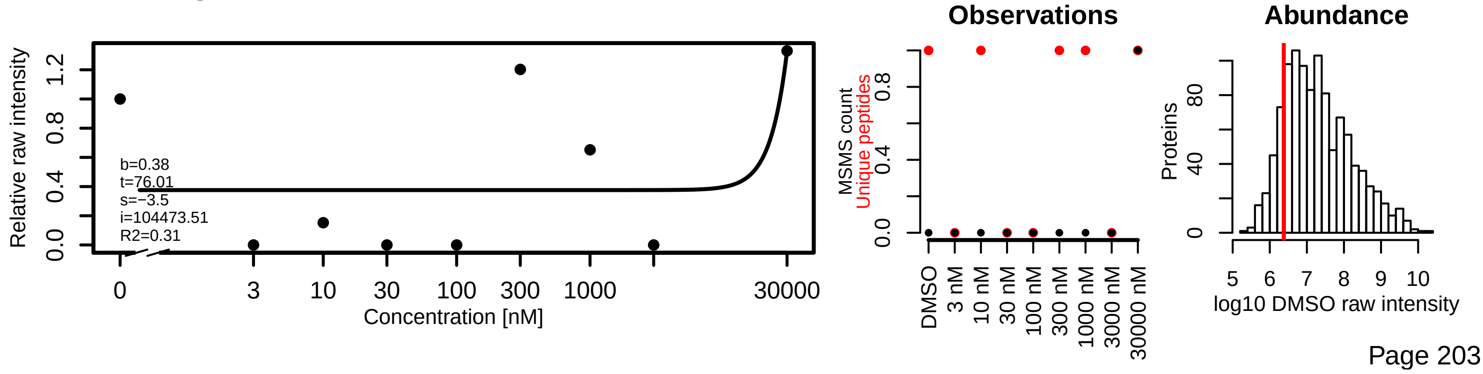

# ERMP1

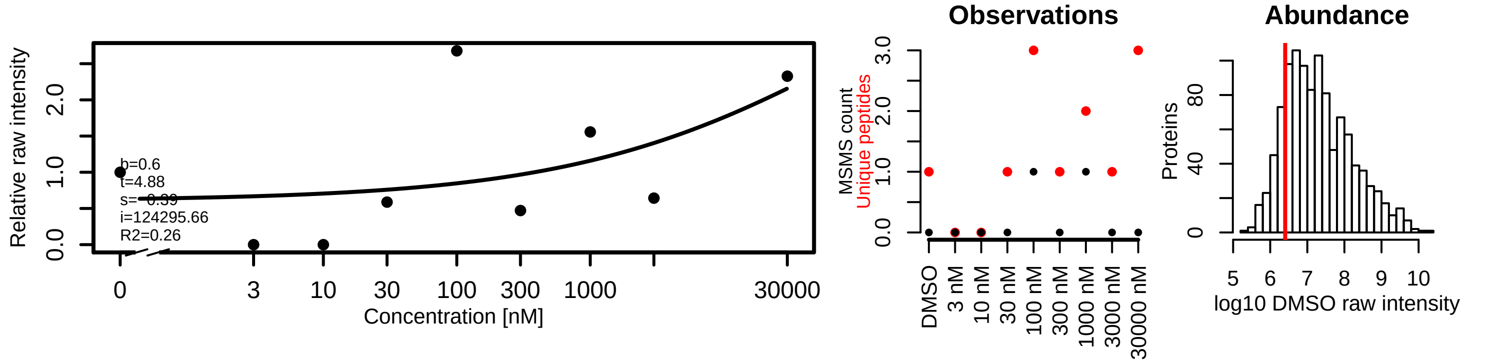


# TIMM50

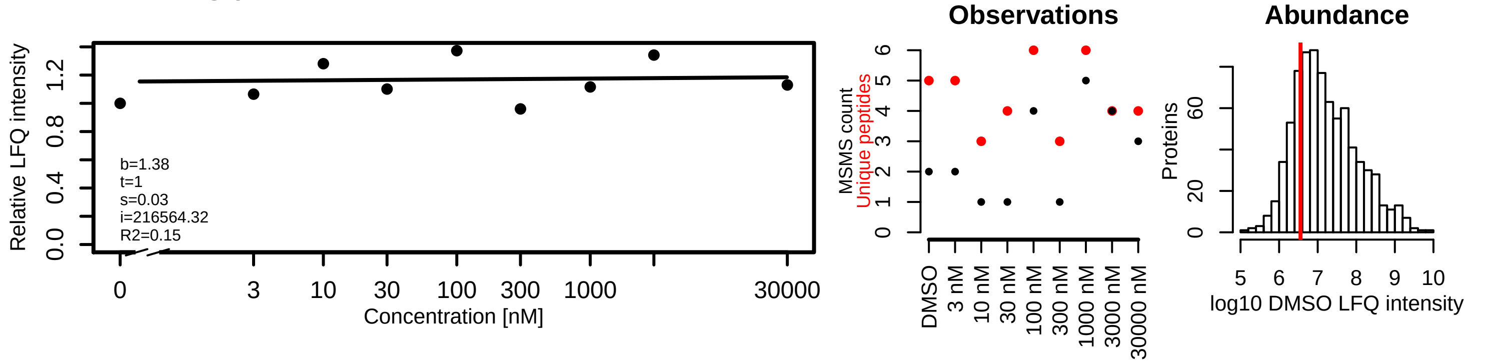


# EIF3B

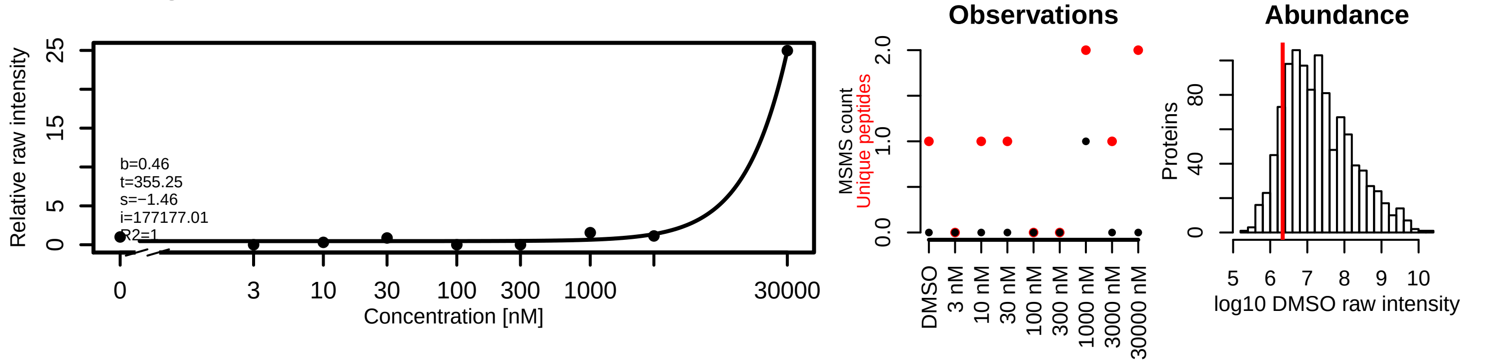


# ITGA3

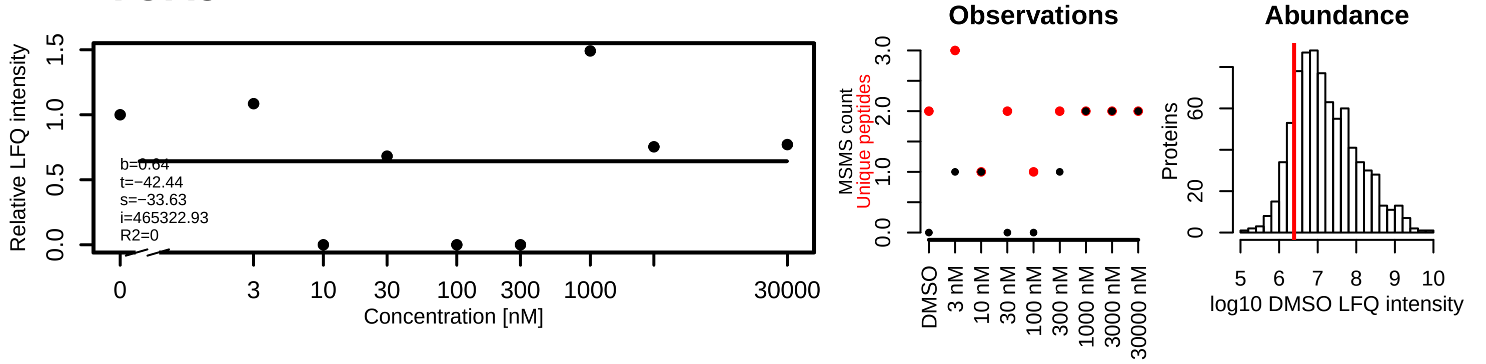


# MAP1B

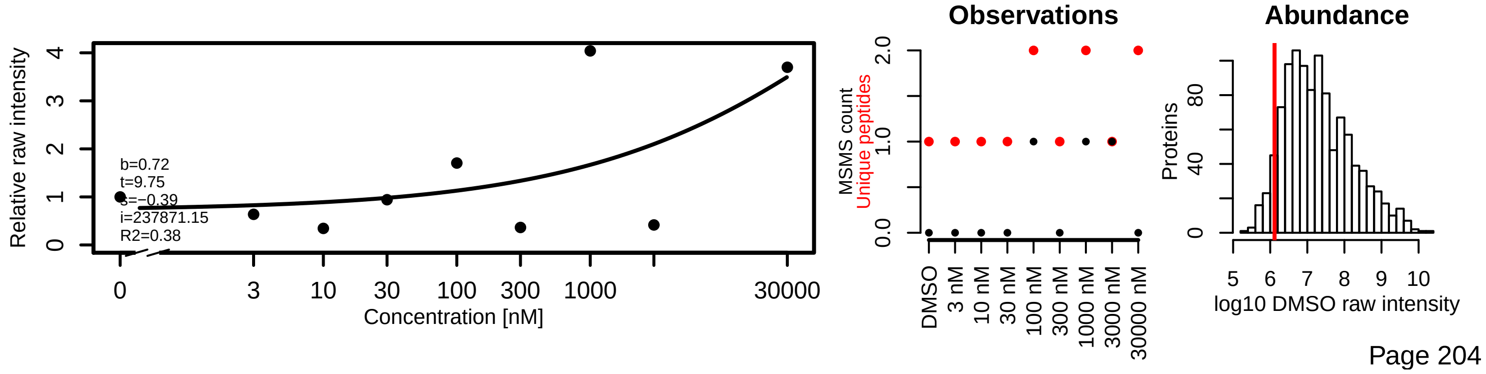

# TCIRG1

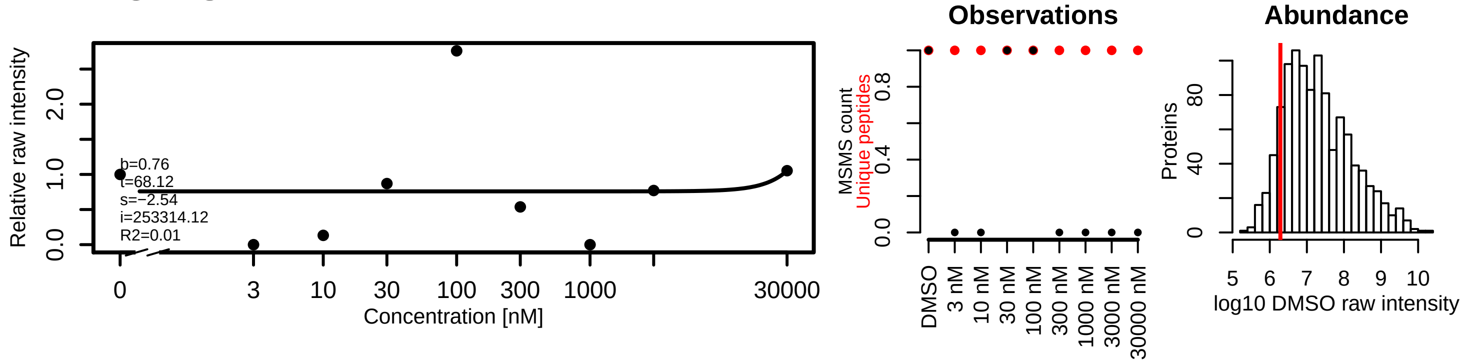


# CEPT1

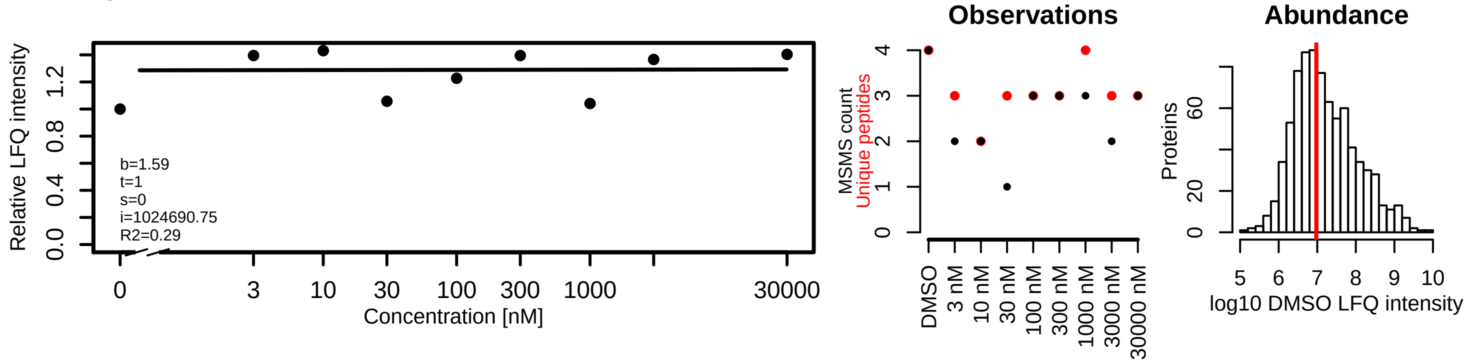


# PPIB

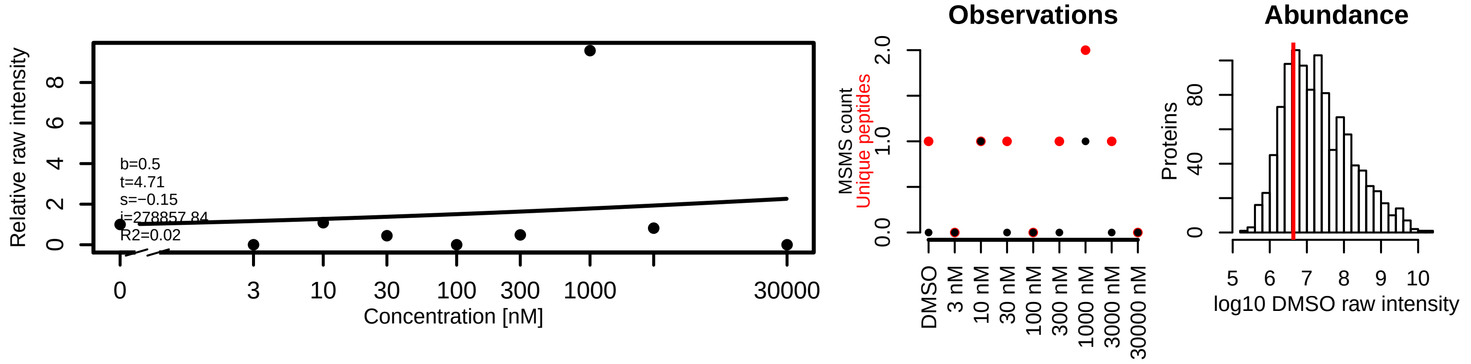


# SRP14

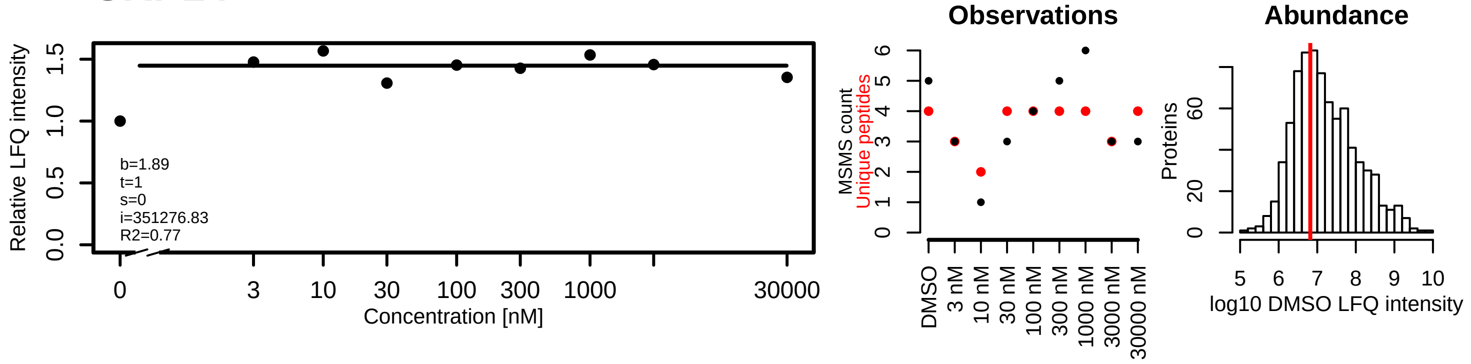


# TAGLN2

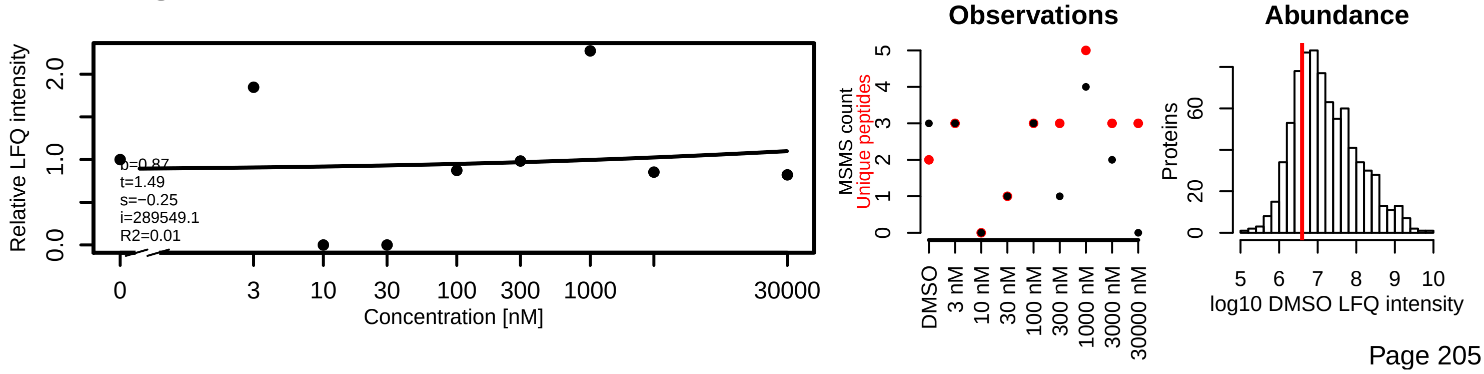

# CYB5D2

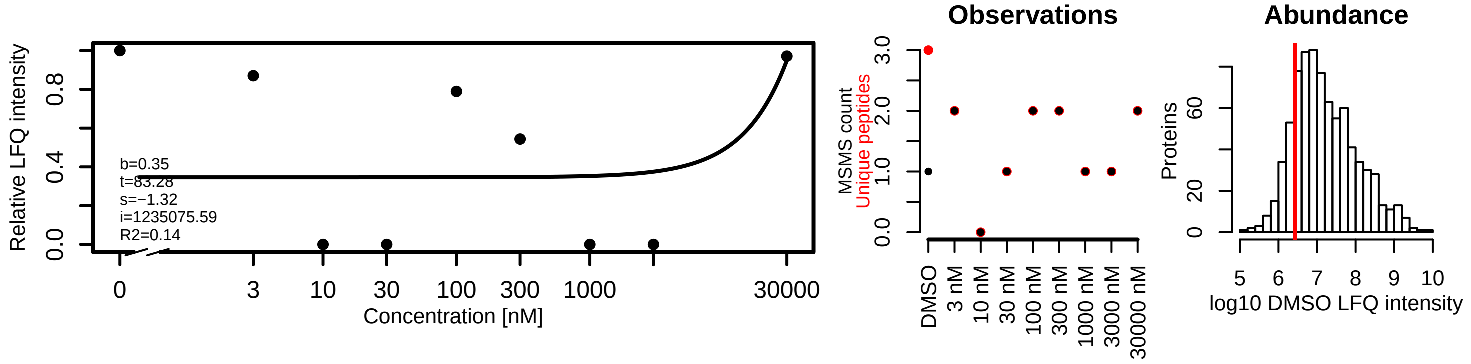


# PKM

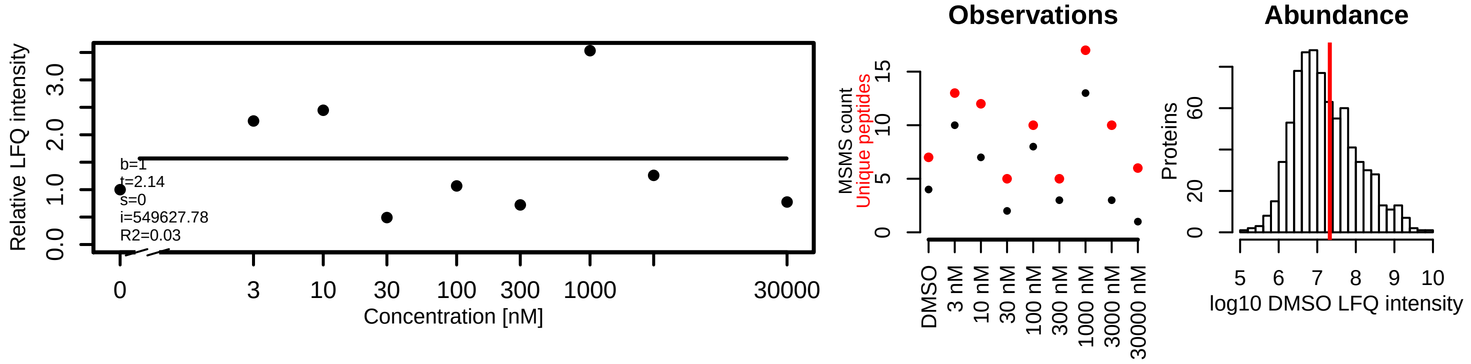


# CNBP

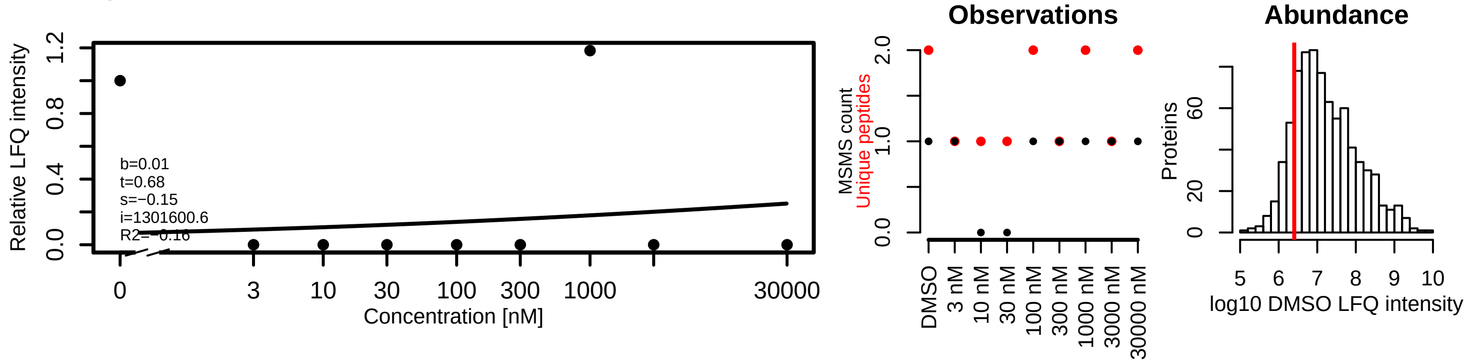


# IGF1R

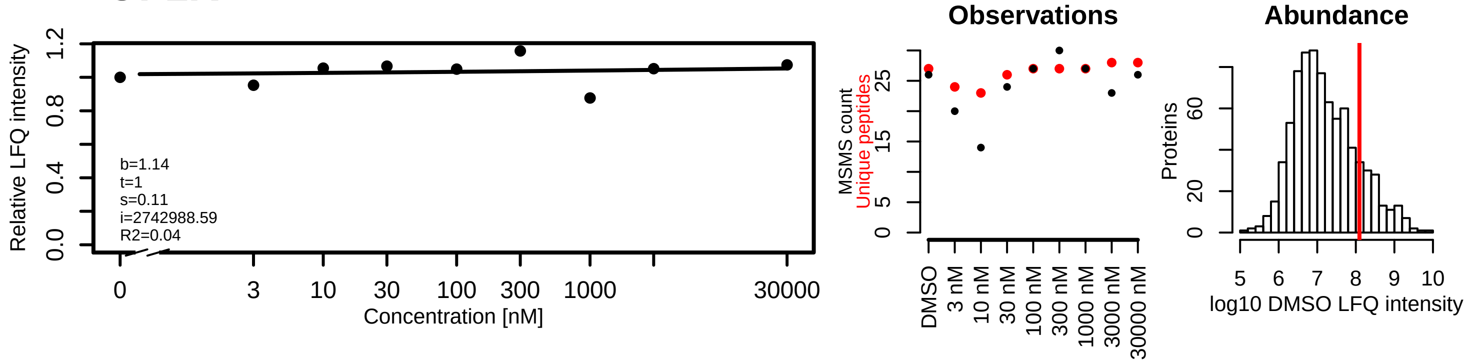


# RPL6

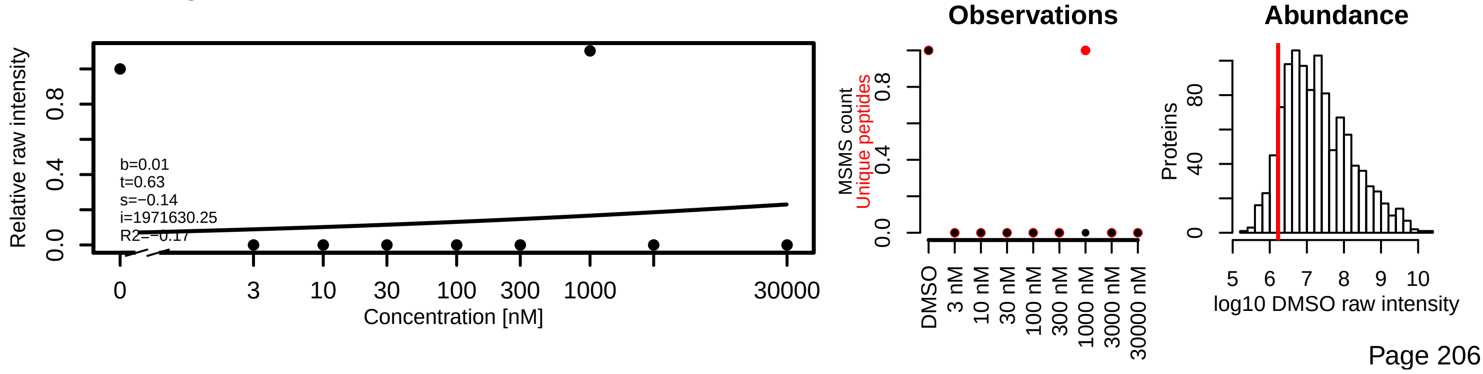

# LMNB2

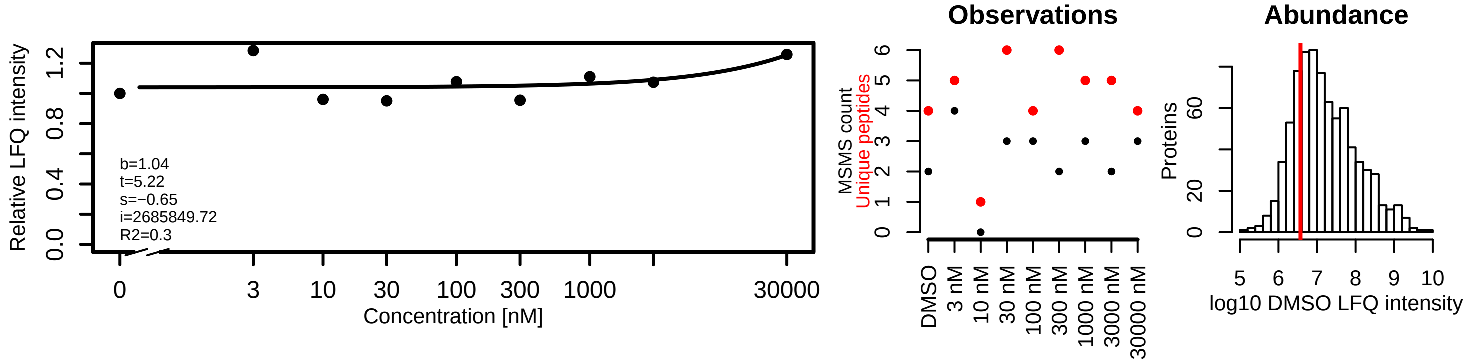


# PTBP1

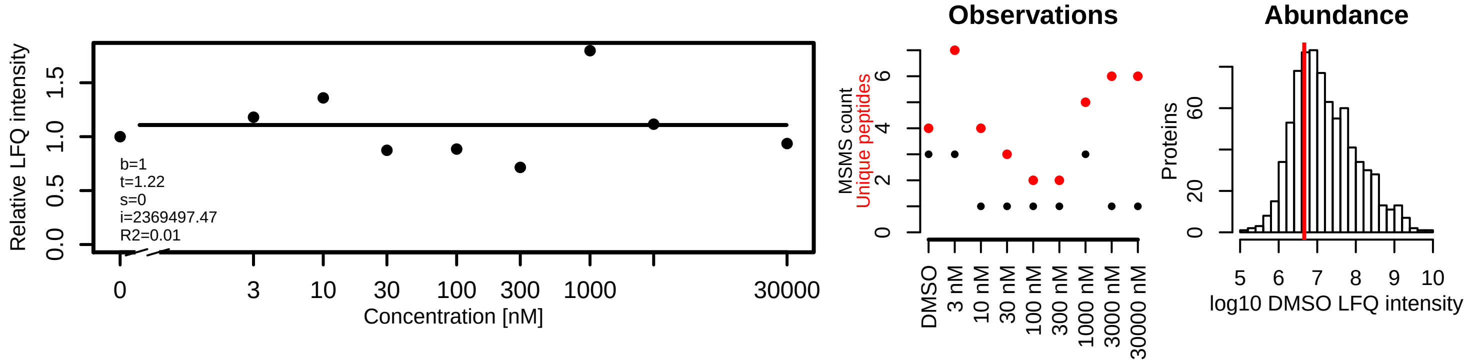


# RPL10

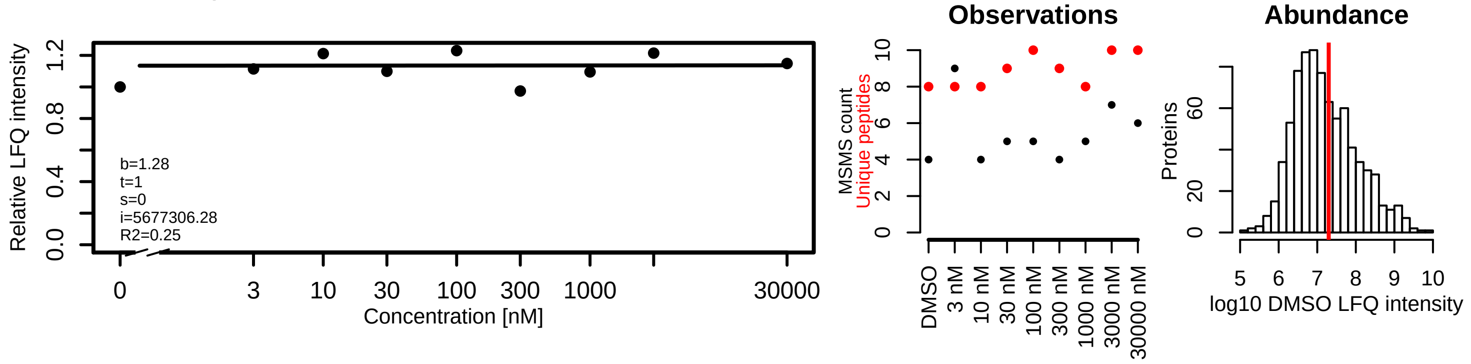


# FSCN1

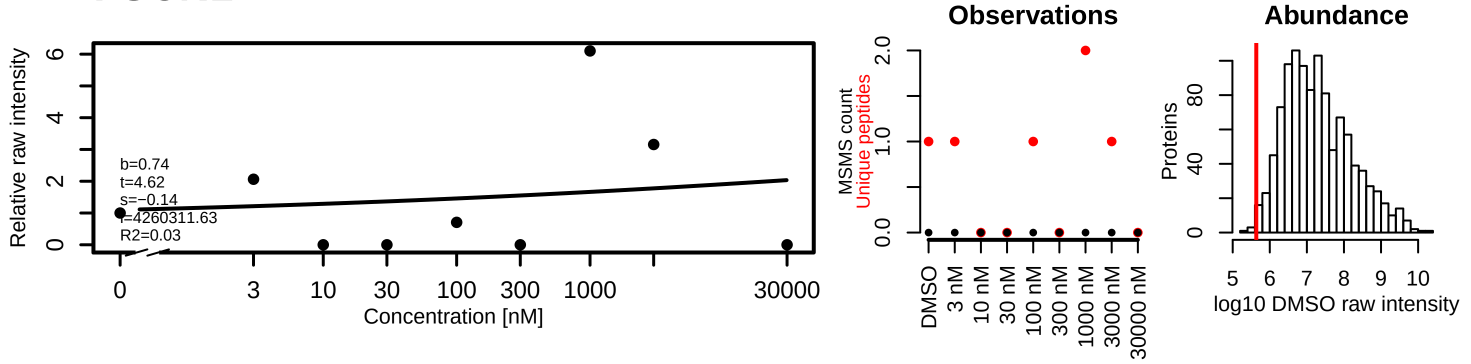


# NME2;NME1−NME2;NME2P1;NME1

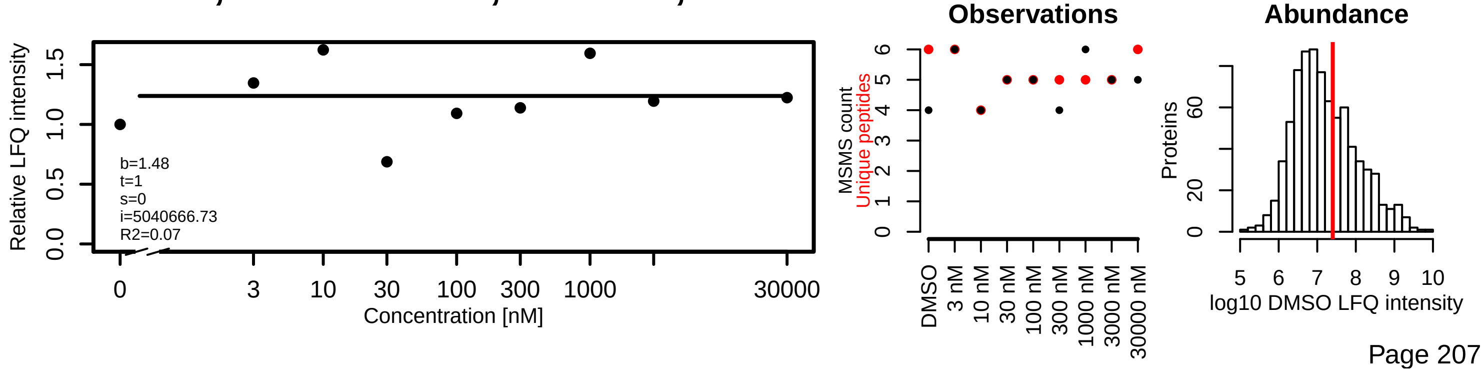

## MRPS9

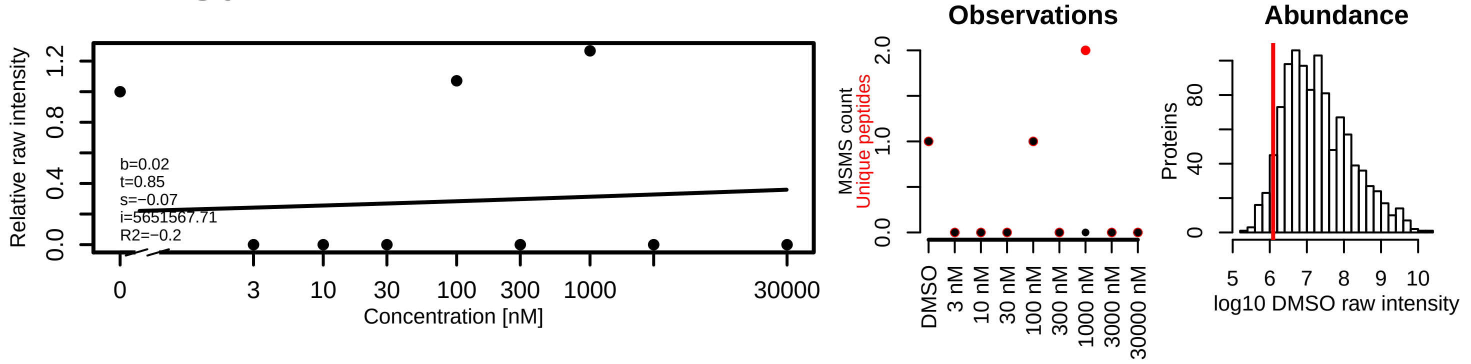


## ENO1

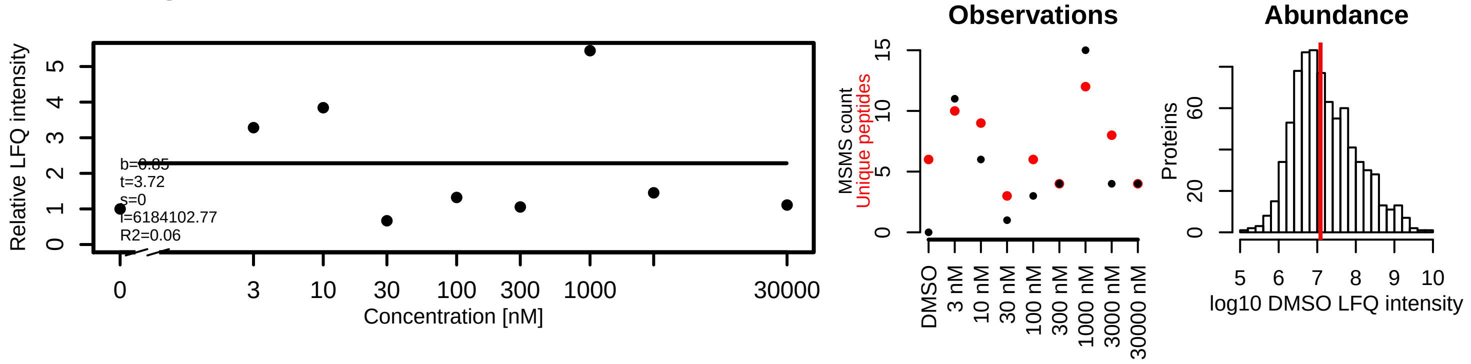


## EEF2

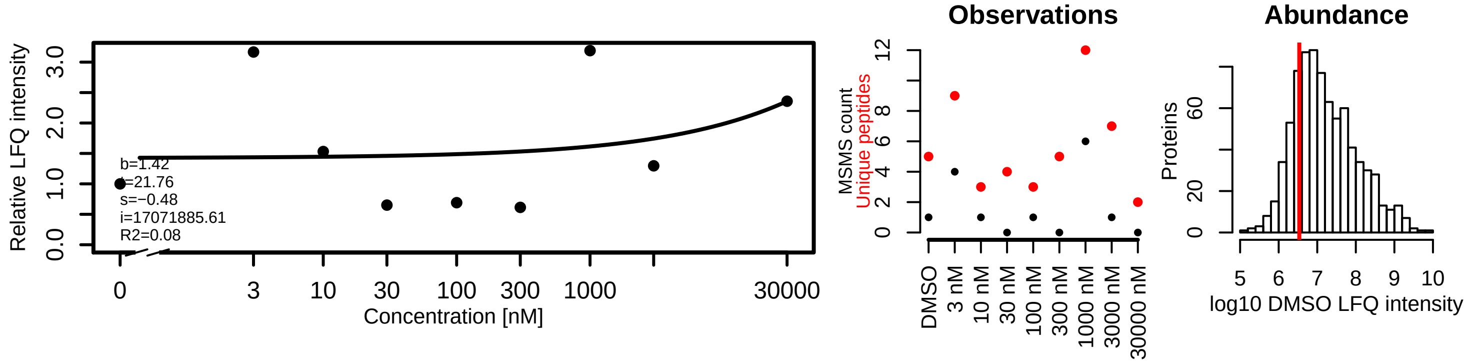


## P4HA1

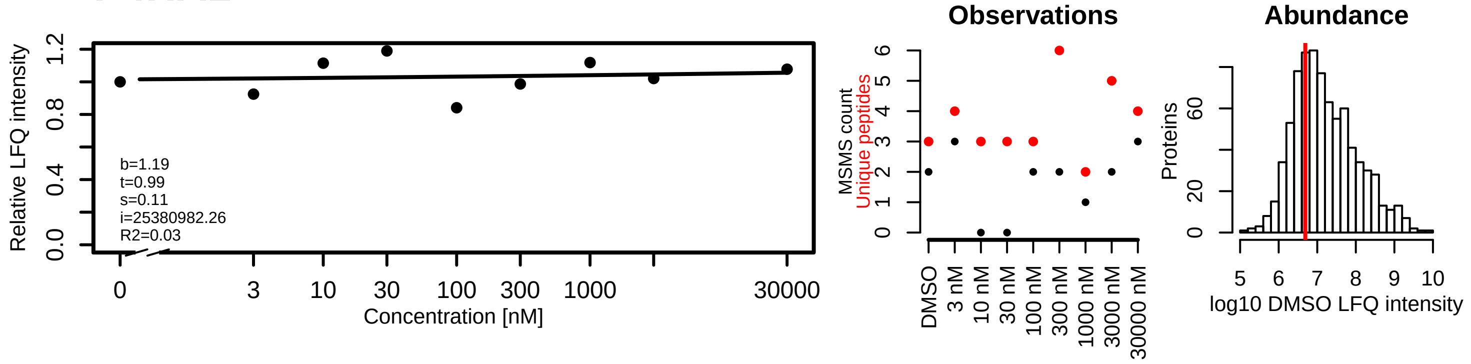


## HIST2H2BE;HIST1H2BB;HIST1H2BO;HIST1H2BJ;HIST3H2BB

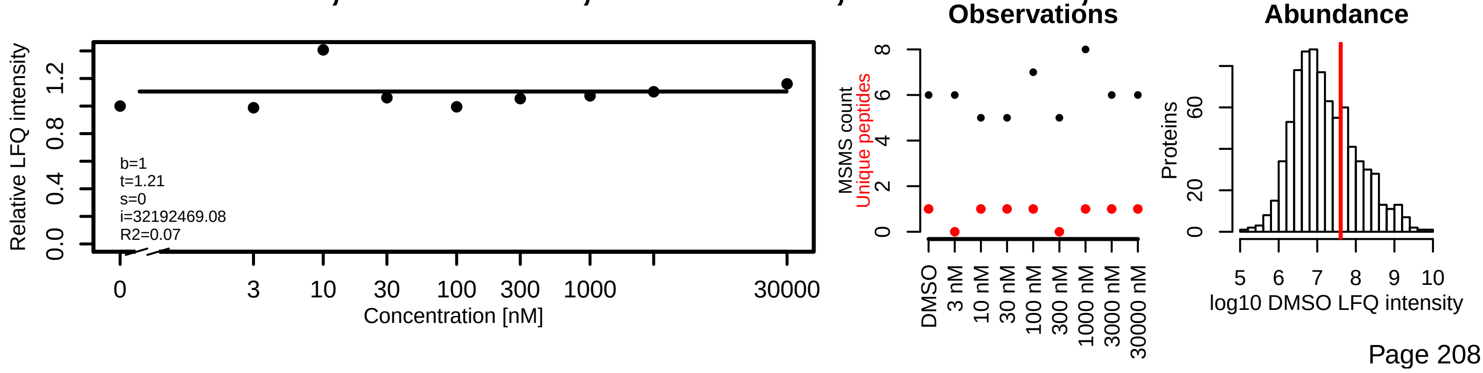

# TRIM59

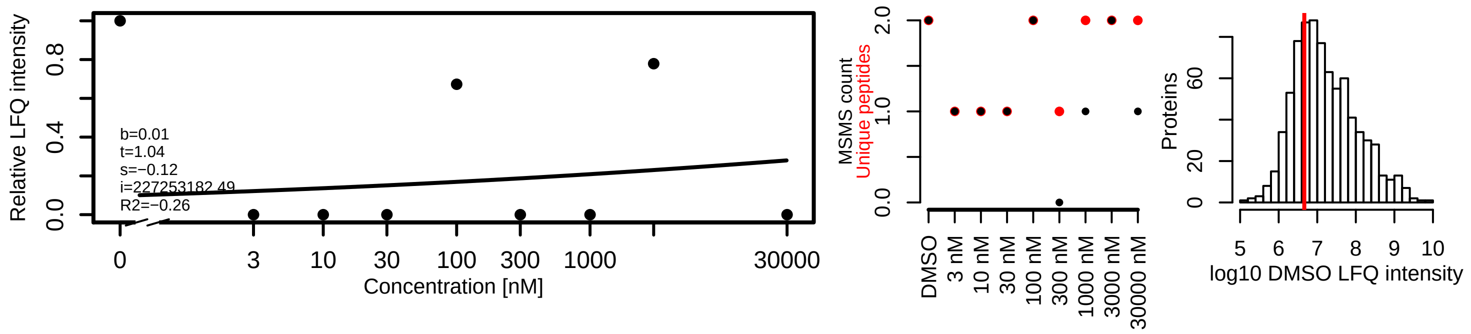


# C1QBP

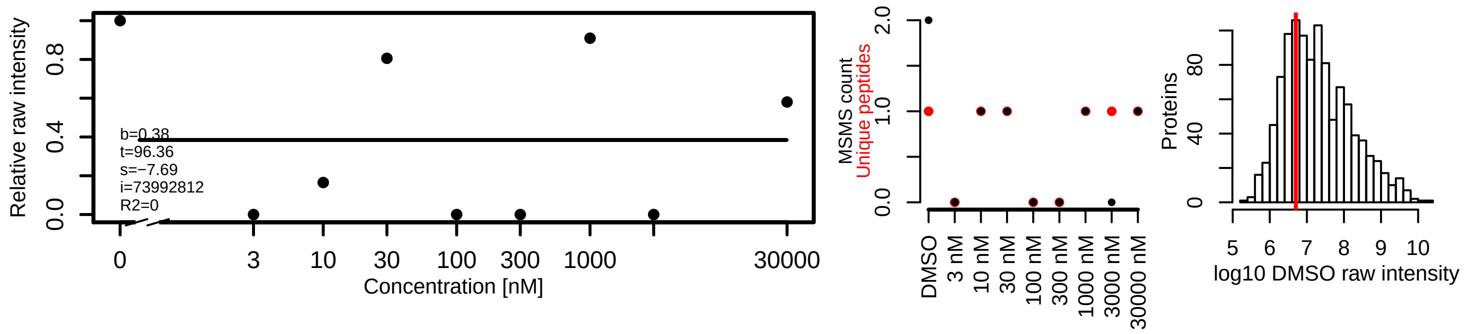


# RPL28

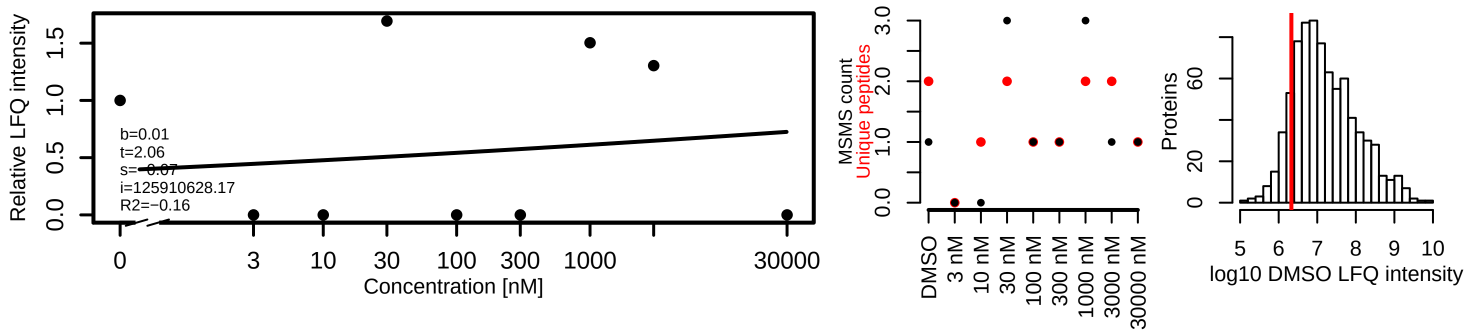


# HIST1H2AJ;HIST1H2AH;H2AFJ;HIST2H2AC;HIST2H2AA3;HIST1H2AI

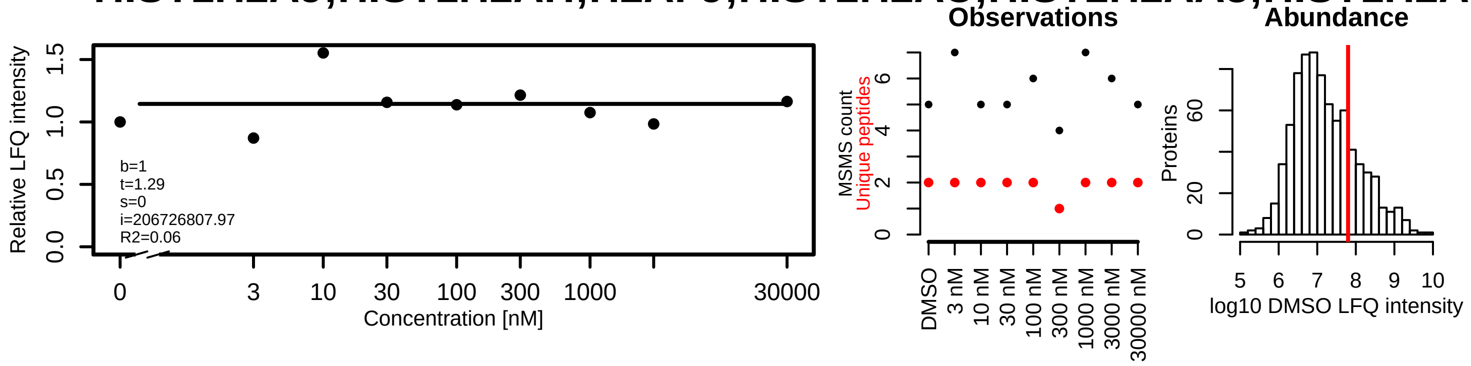


# SPR

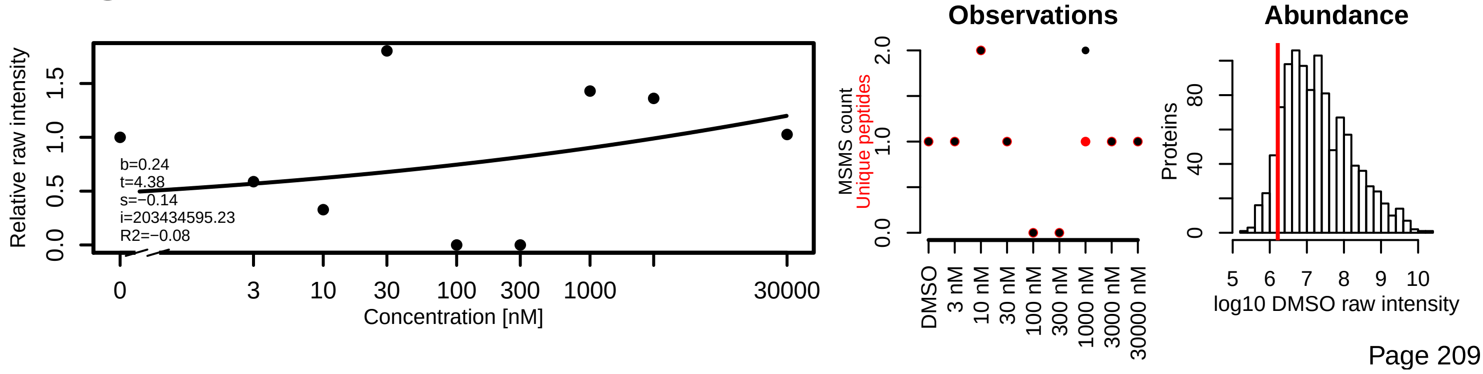

## RPL22

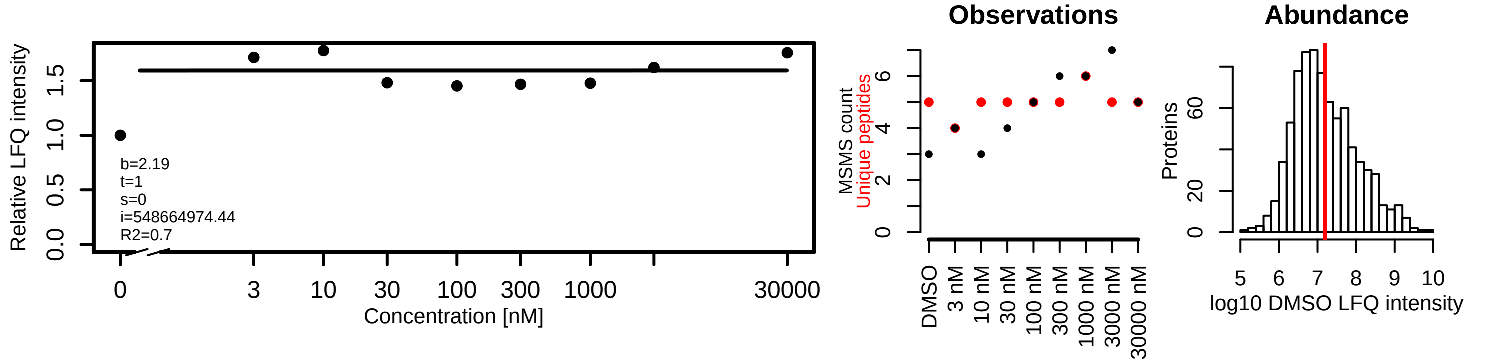


## RPL15

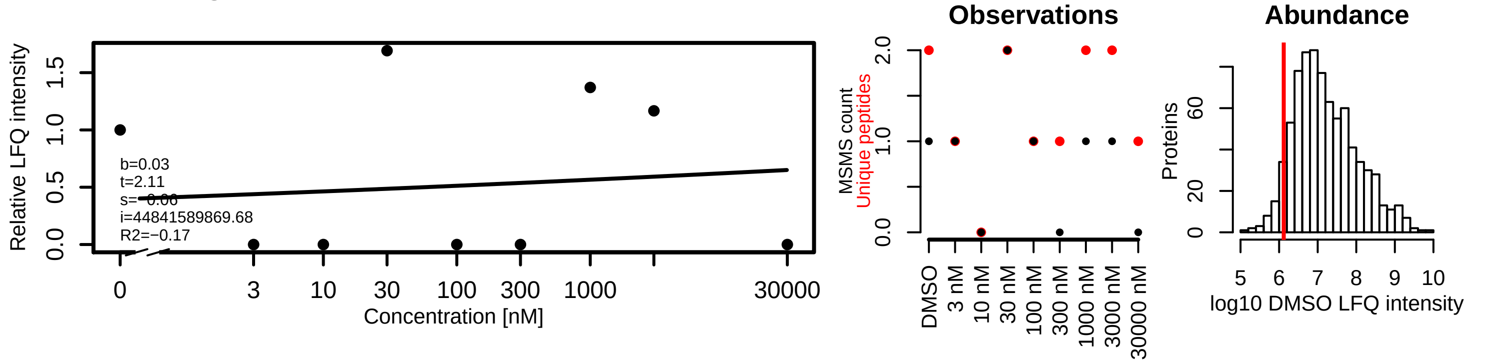


## TLN1

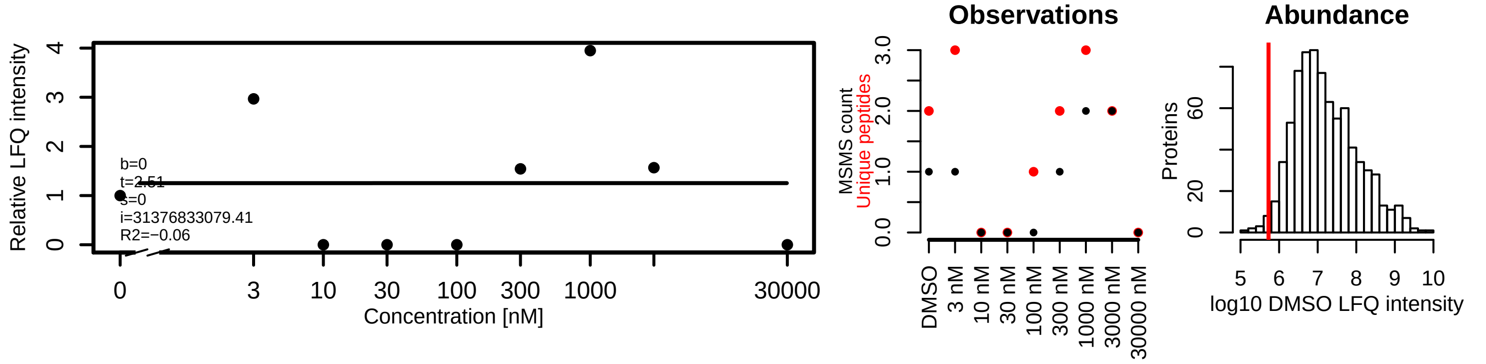


## EZR

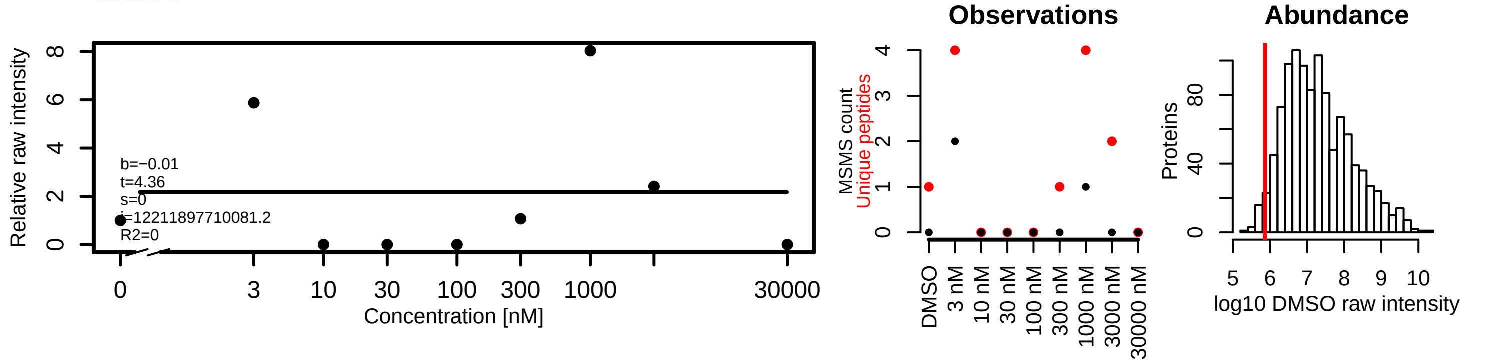


## IGF2BP2

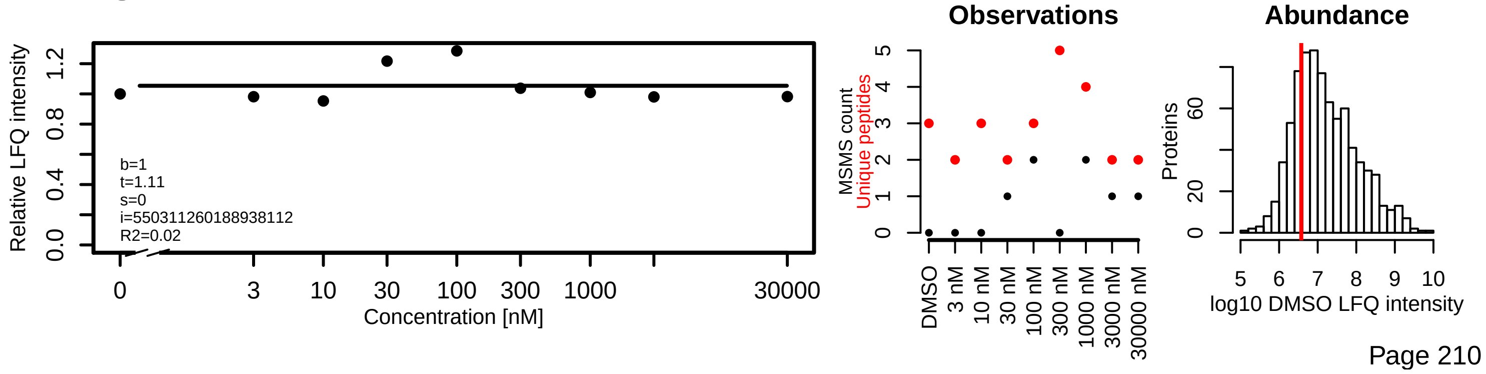

# GPRC5A

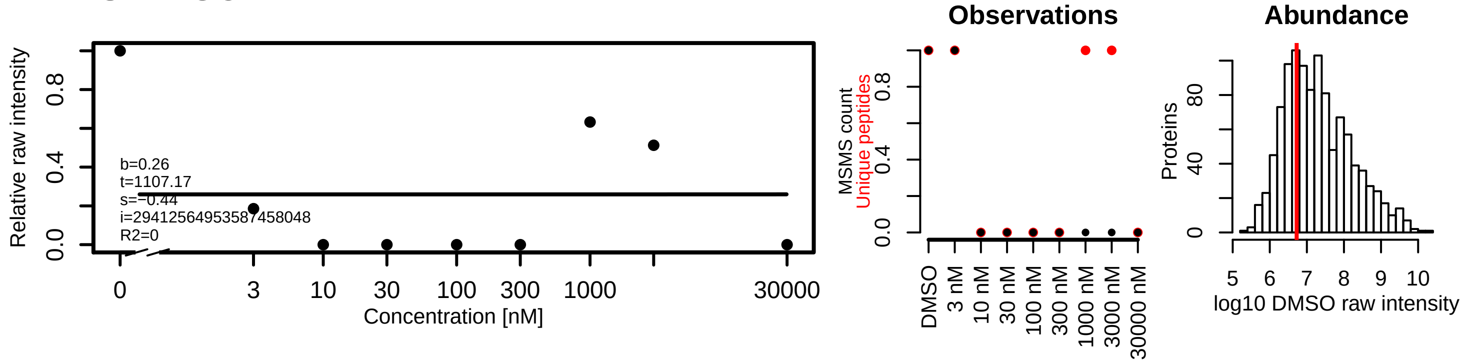


# ACADM

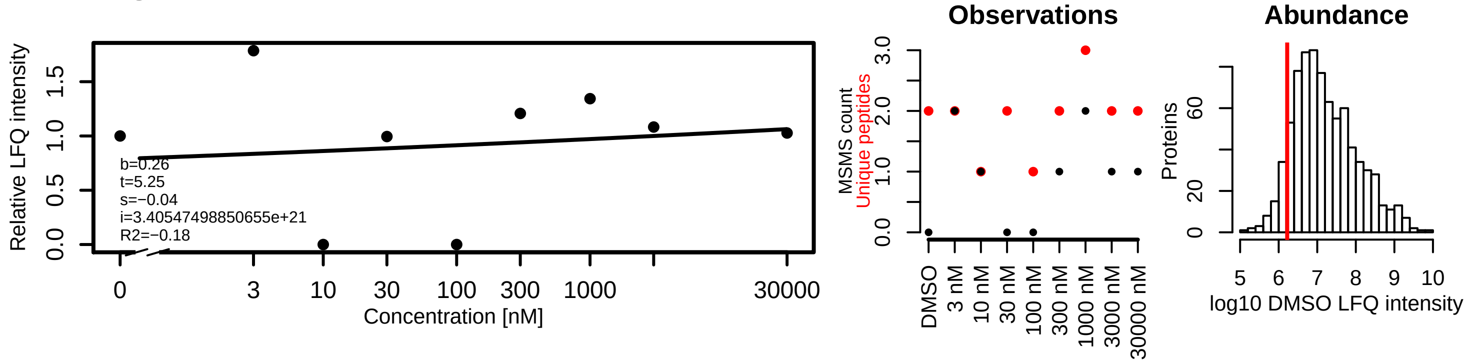


# ANXA1

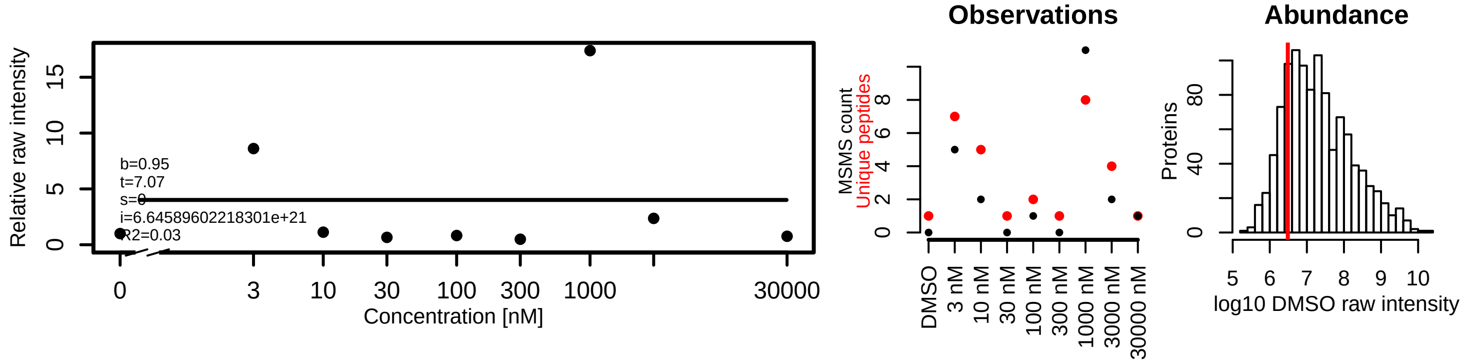


# CCNI

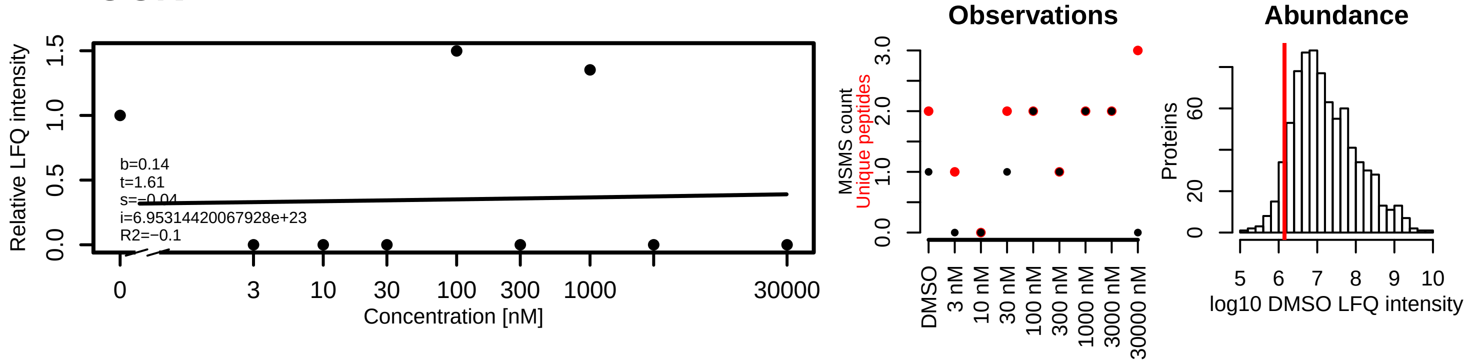


# PPIA

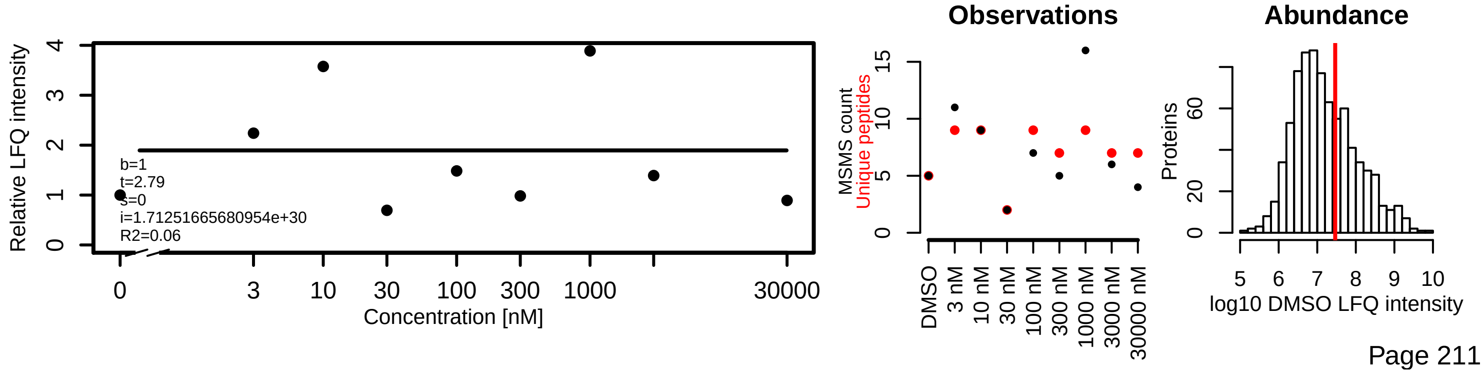

# CAMK2G − Q13555−9

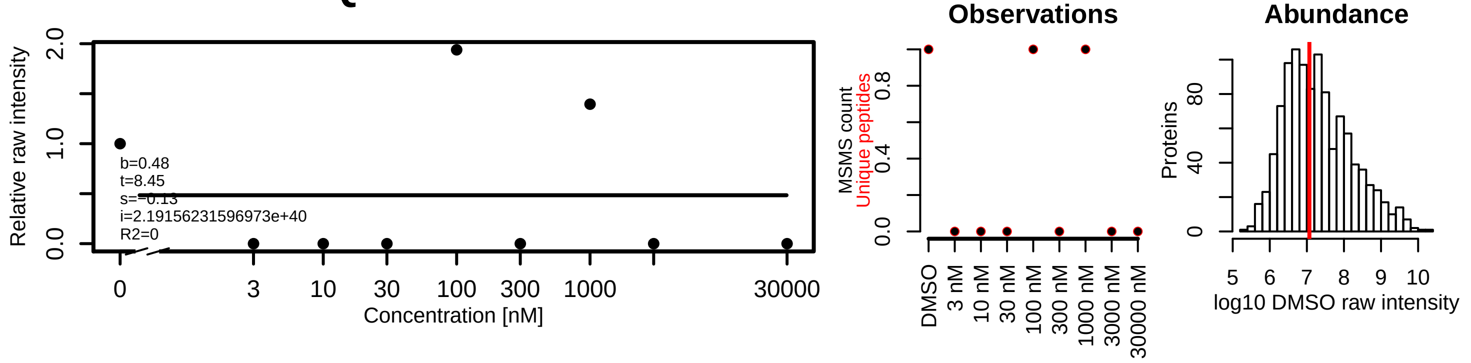


# RAB5C

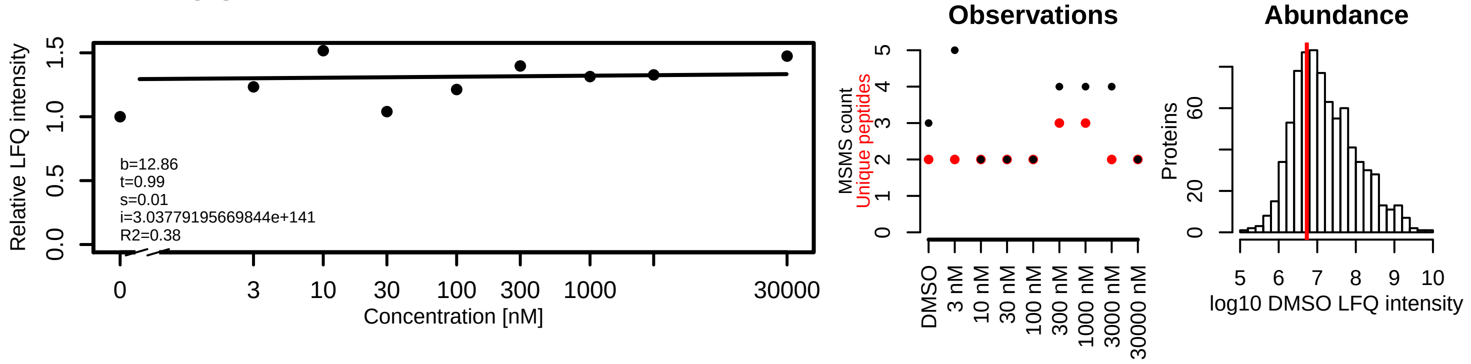


# 2−Sep

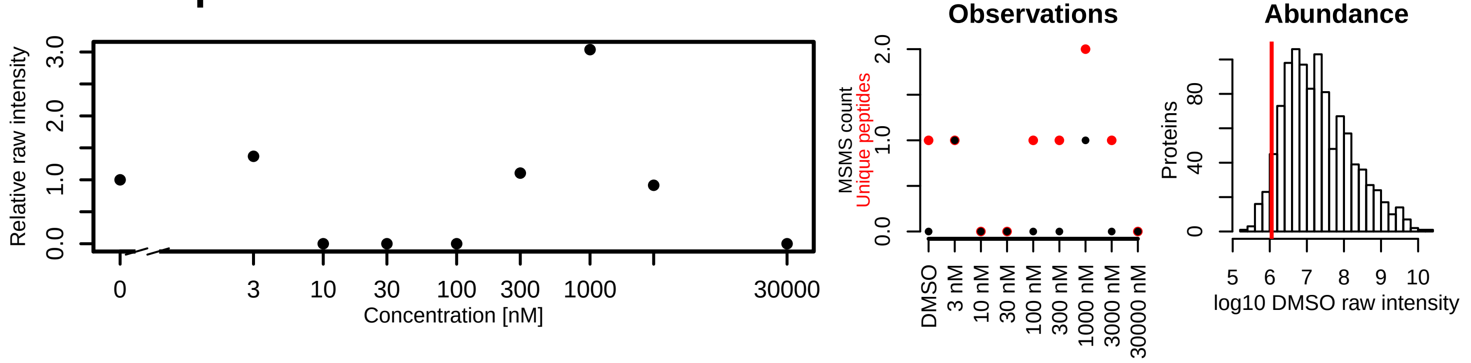


# CCDC113

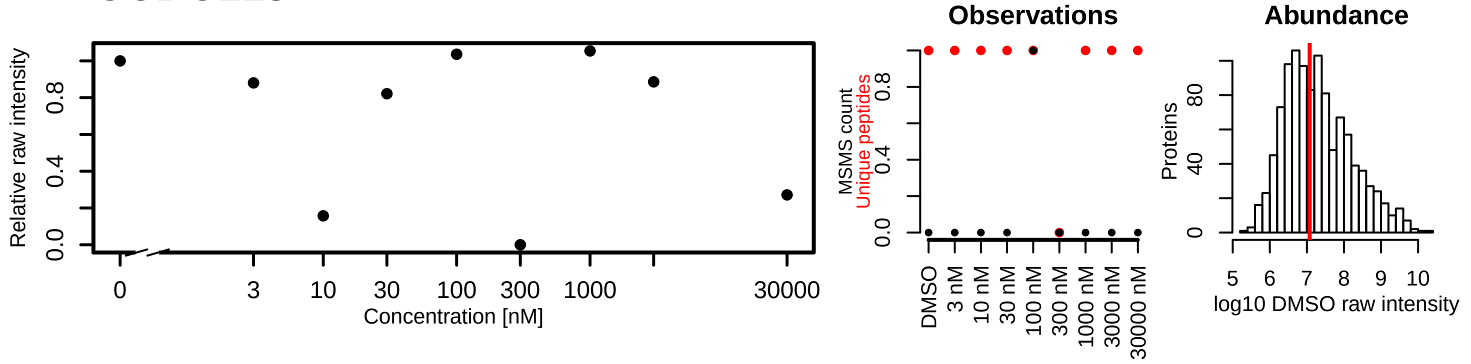


# DSG1

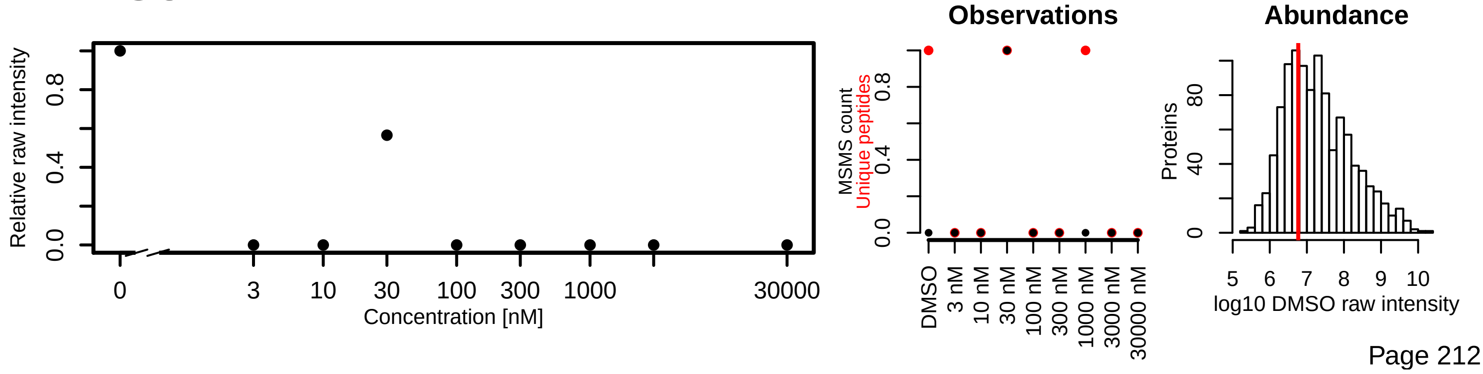

# EI24

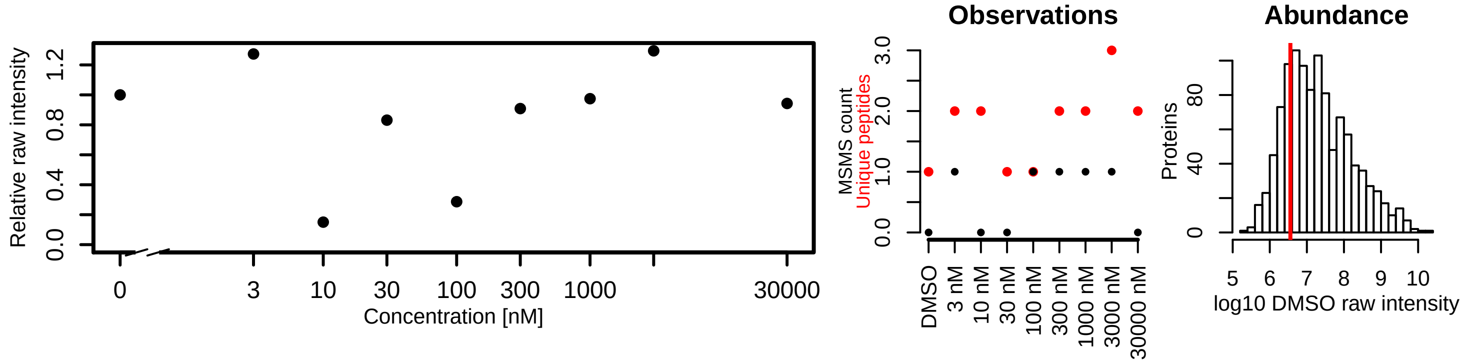


# GARS

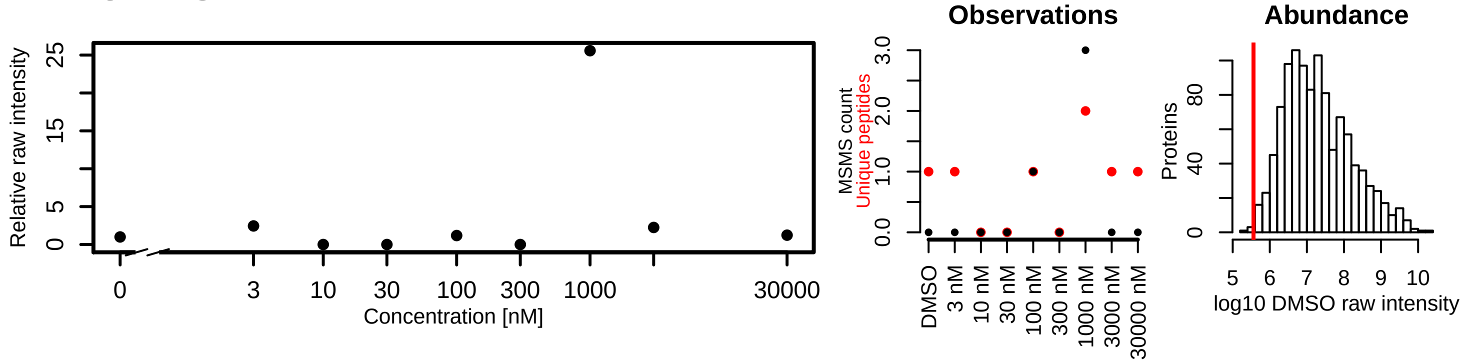


# GPI

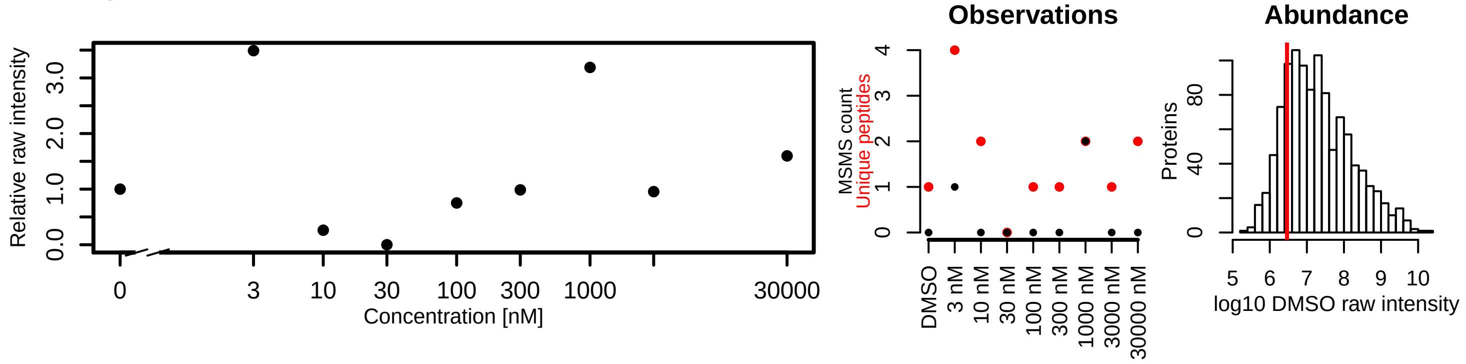


# HSP90AB2P

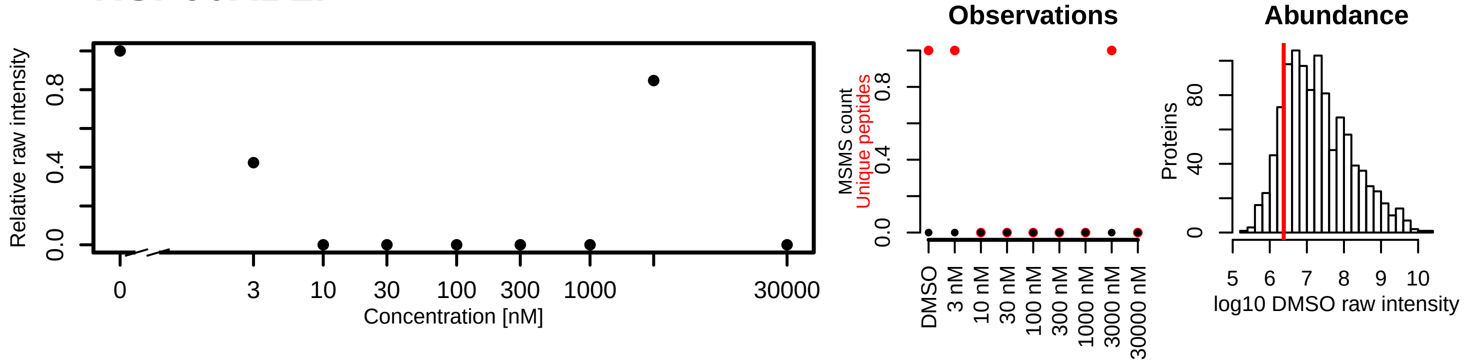


# IGSF10

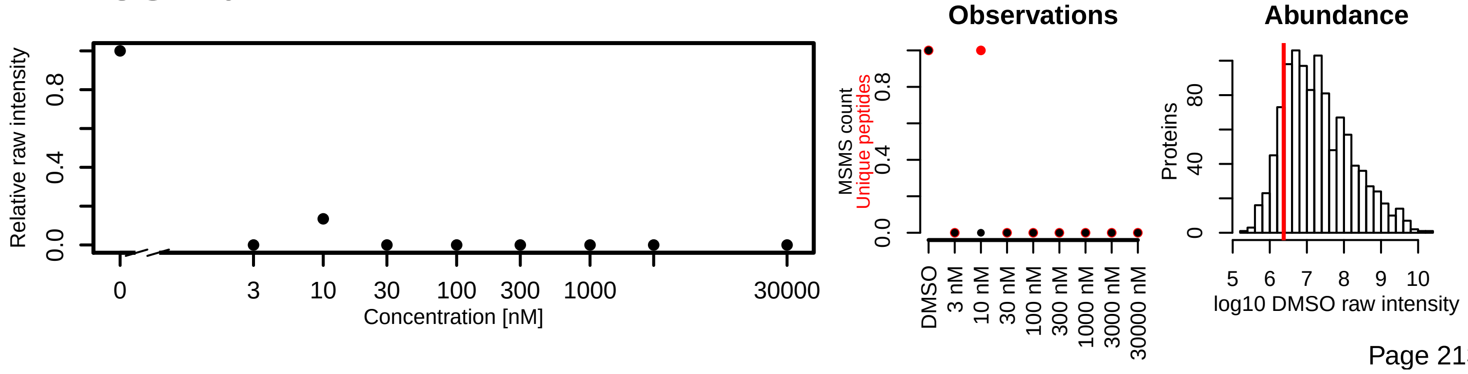

## KIAA0195

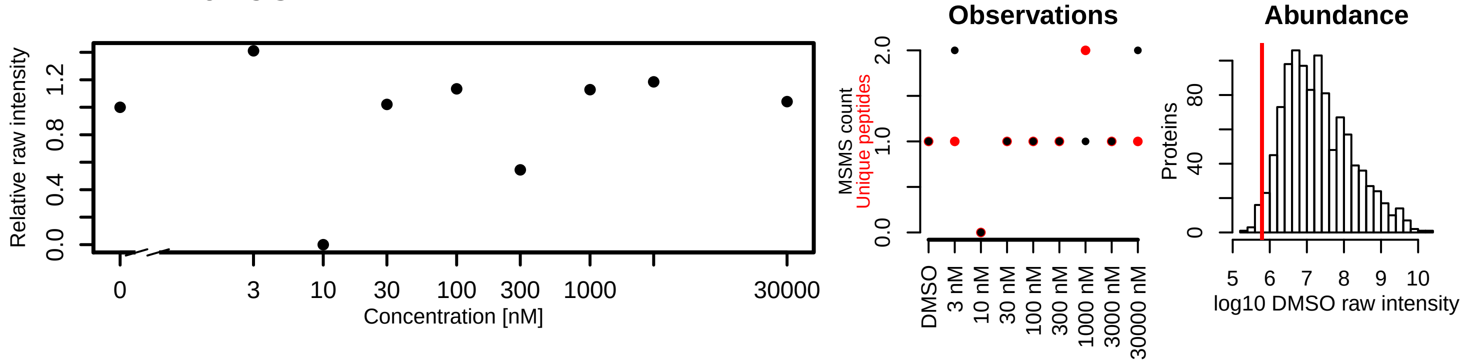


## LMAN2

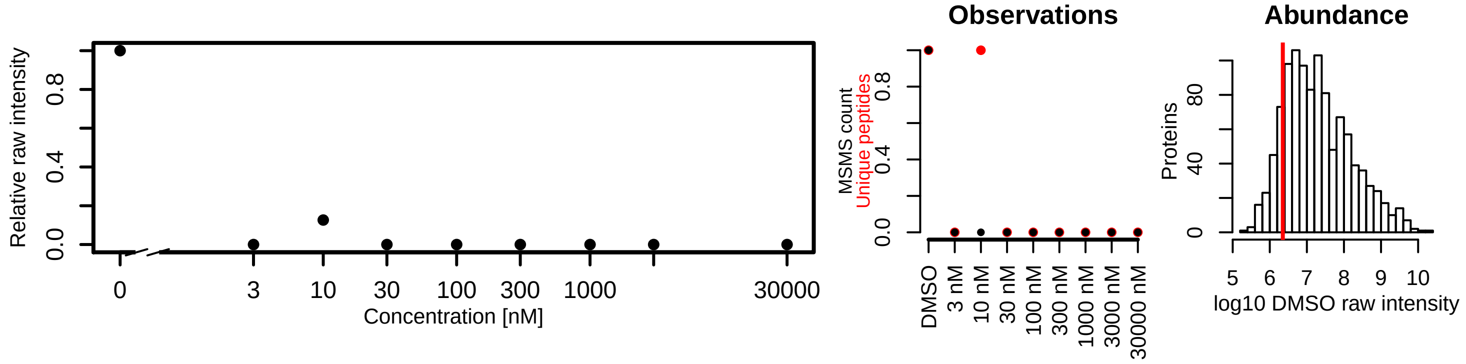


## LYPLA2

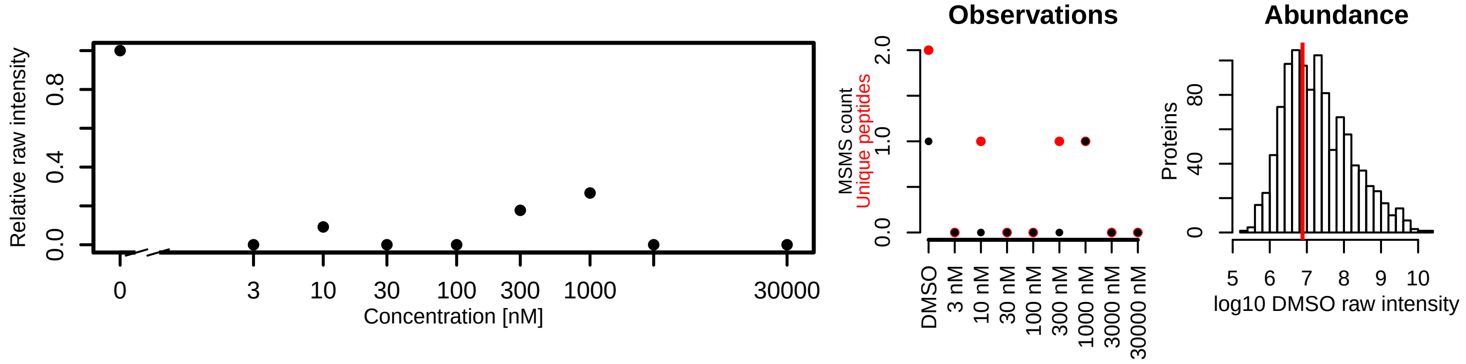


## MDH1

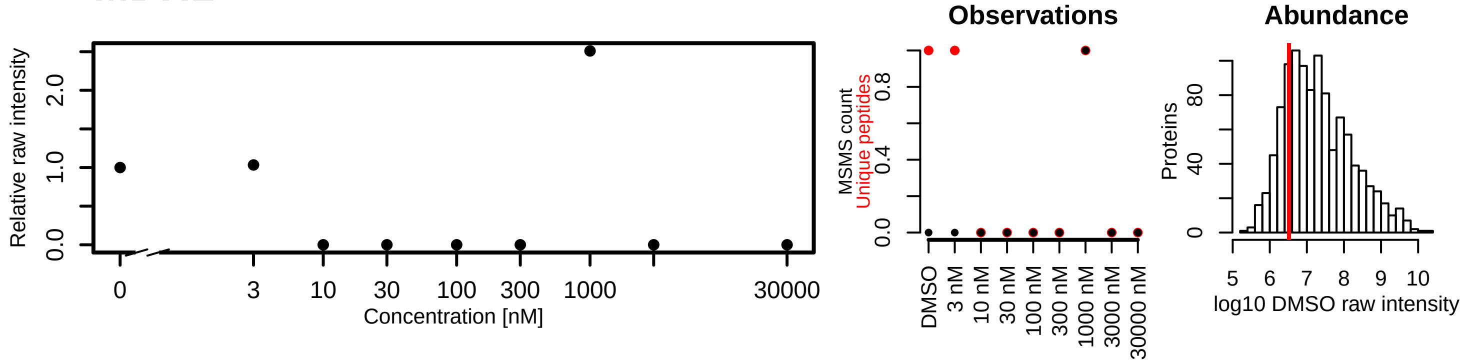


## MYL12A;MYL9;MYL12B

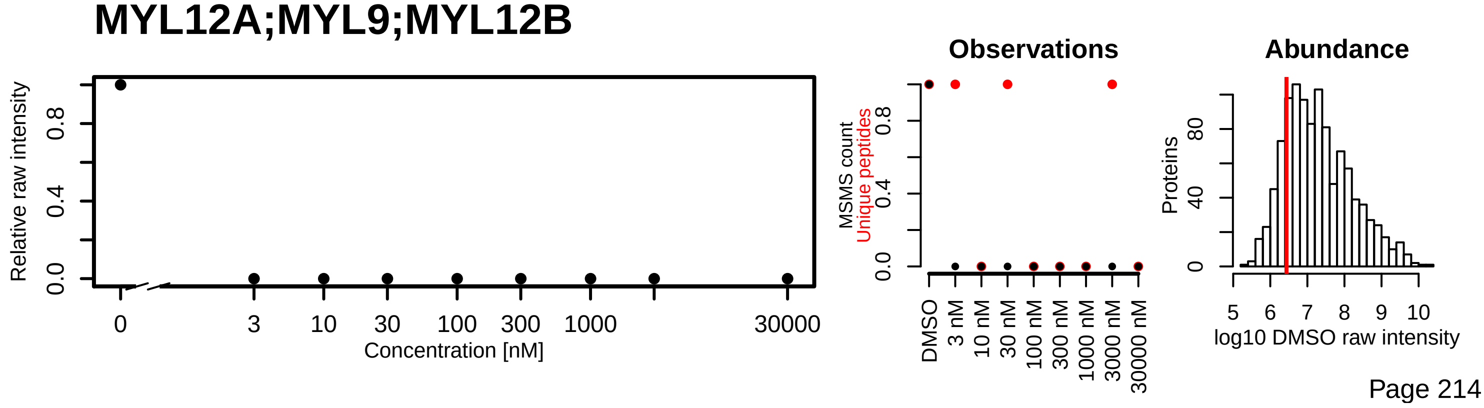

## PPHLN1

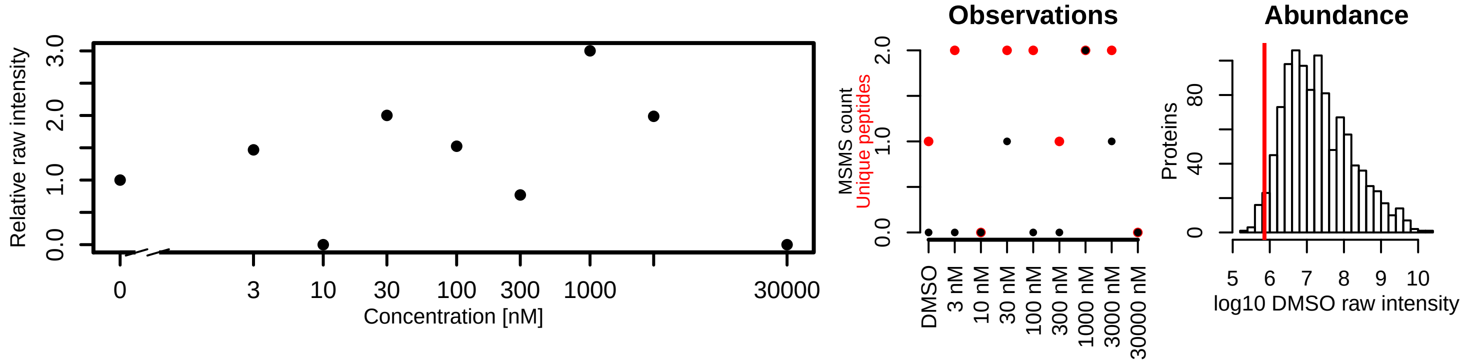


## PSMC2

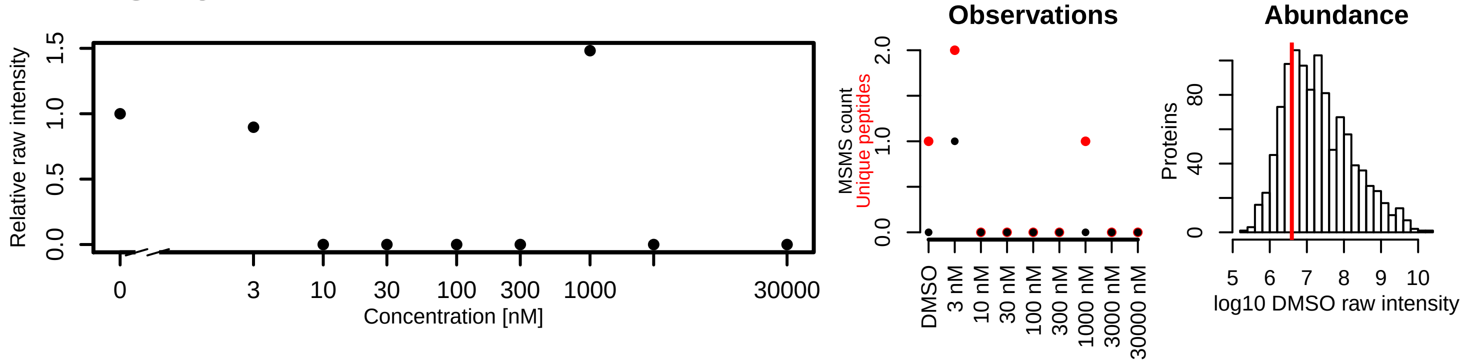


## PSMD14

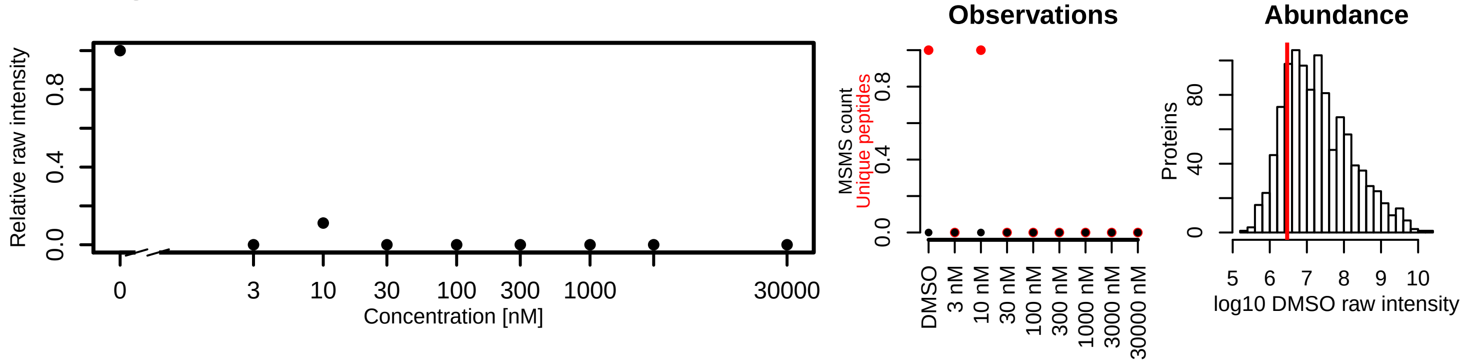


## PTK2 − Q05397−2

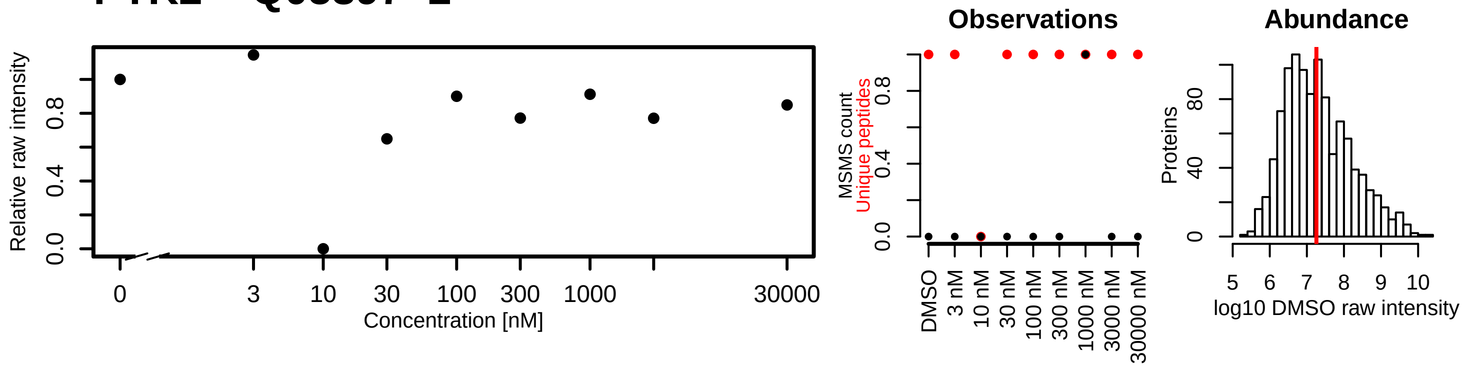


## RELN

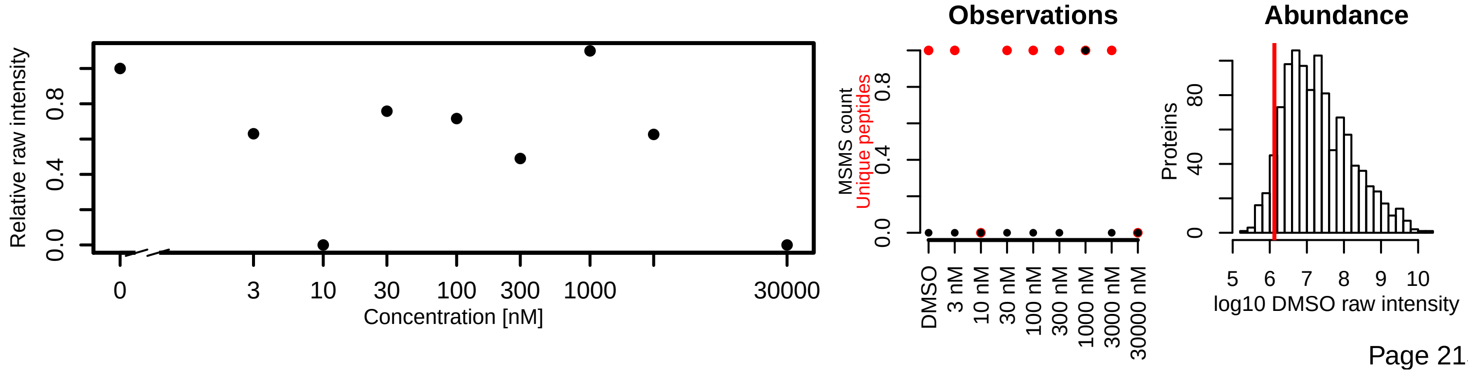

## SAAL1

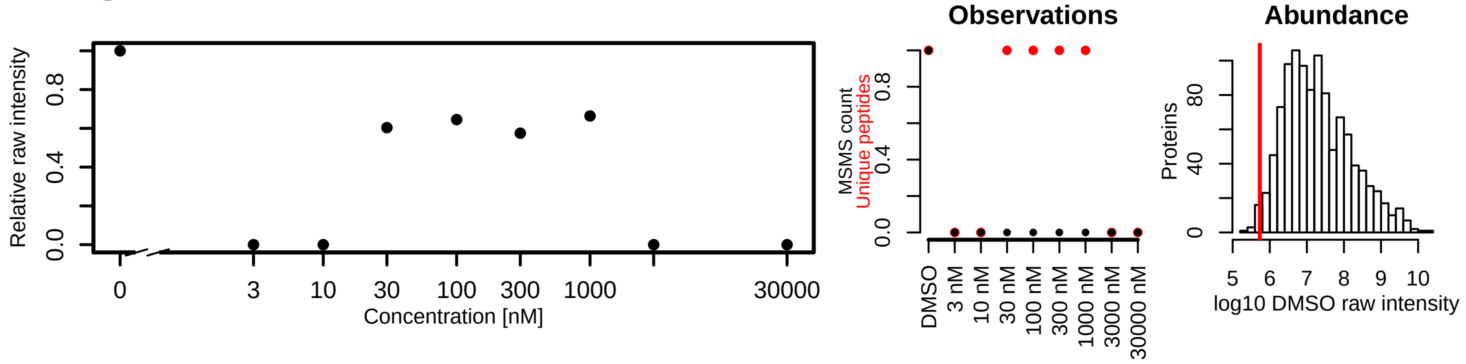


## SPATA1

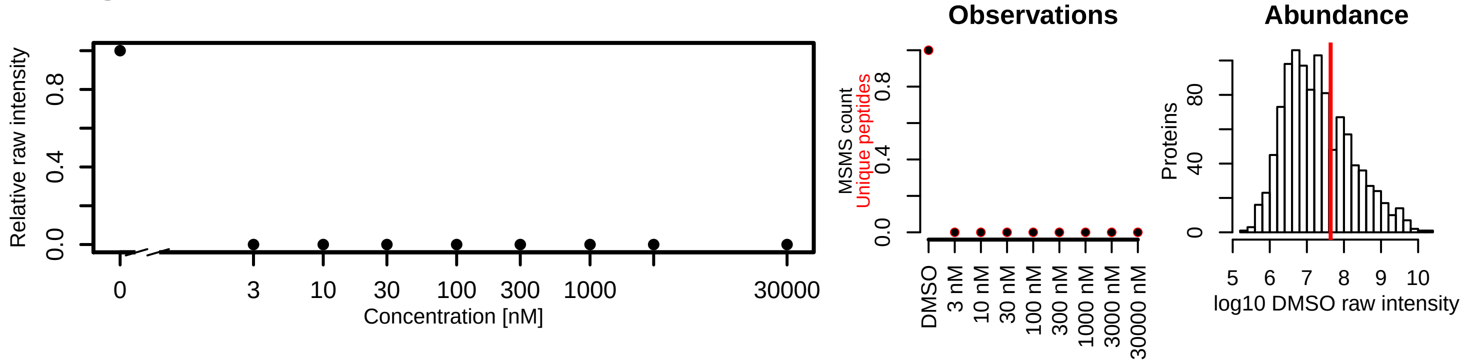


## SRP54

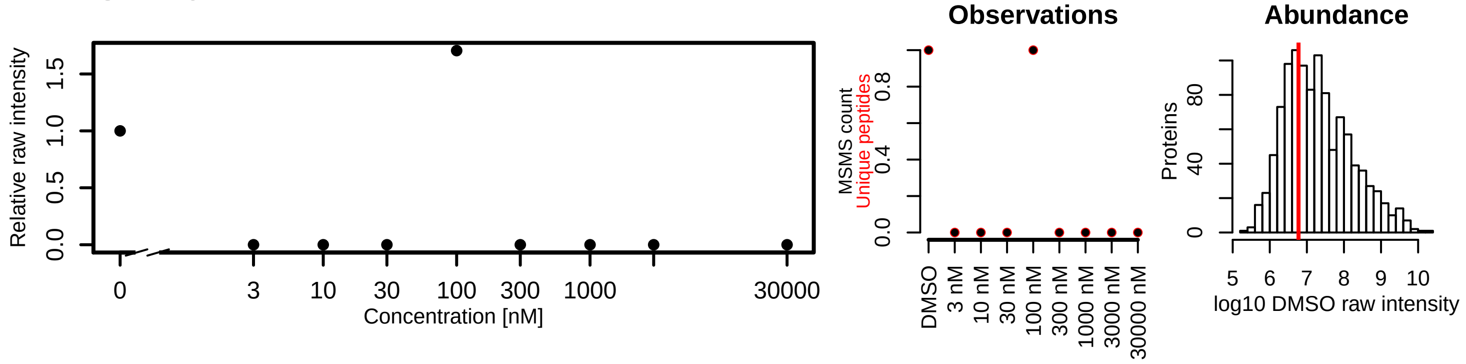


## SYNCRIP

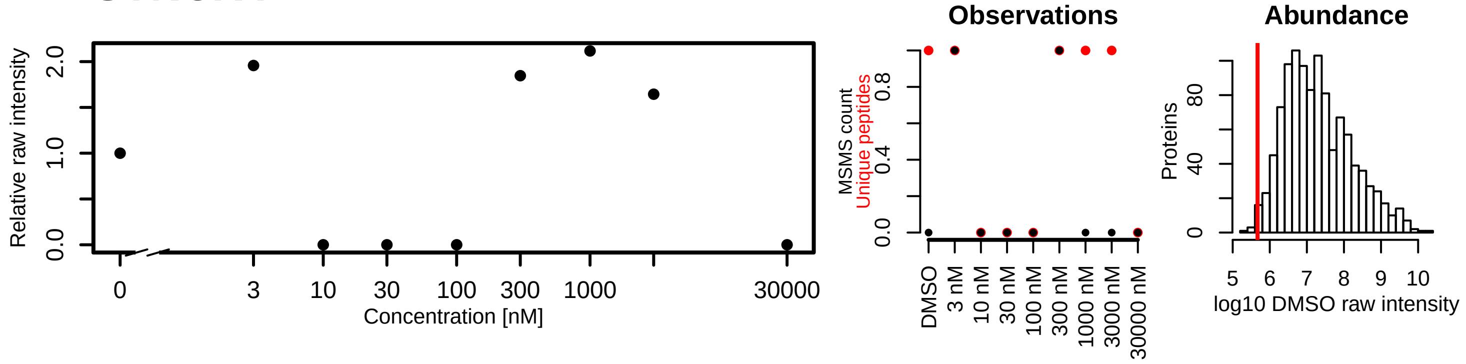


## TIMM9

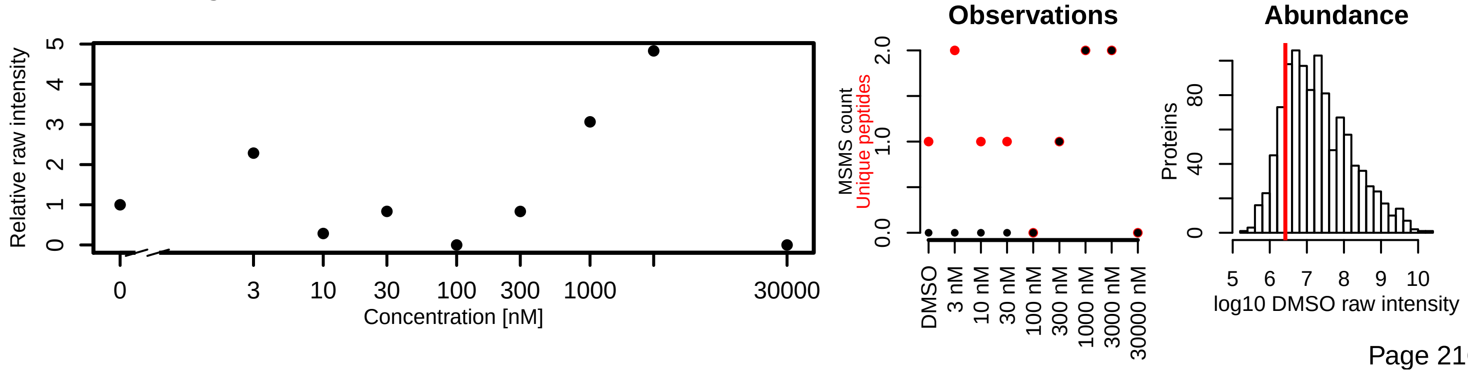

# TM7SF2

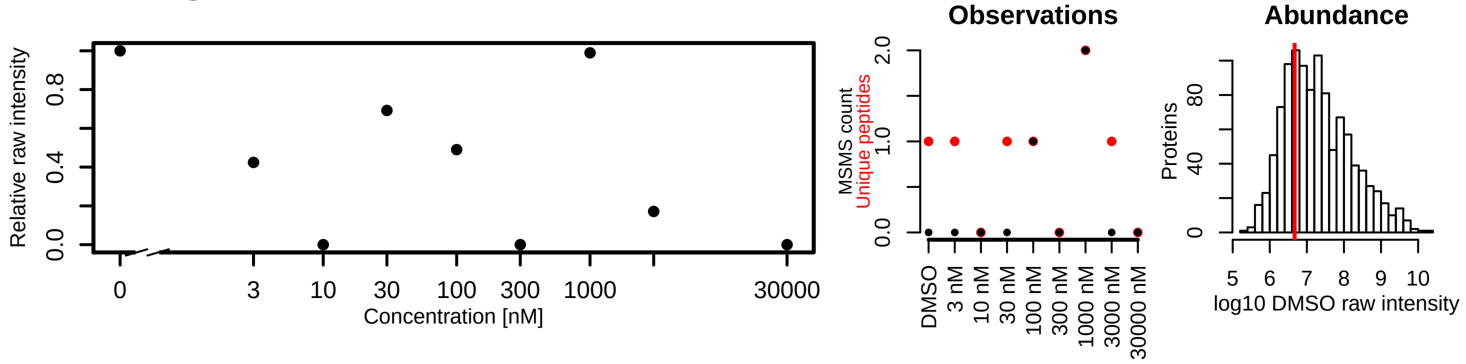


# TTN

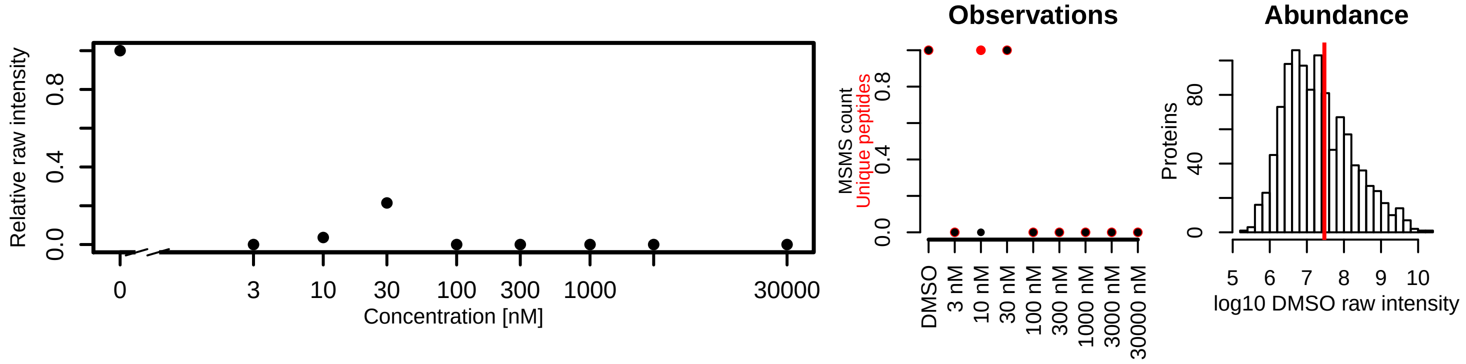


# TUBGCP3

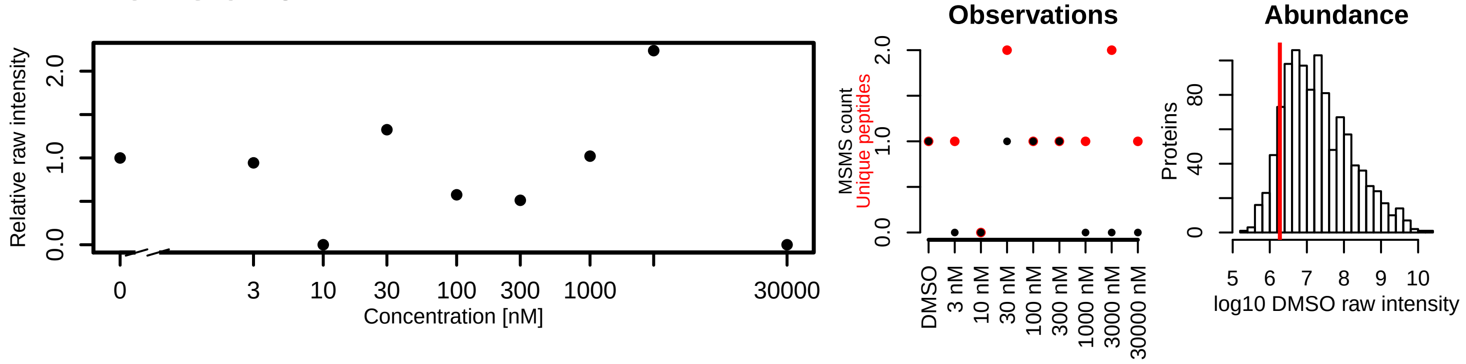


# VMA21

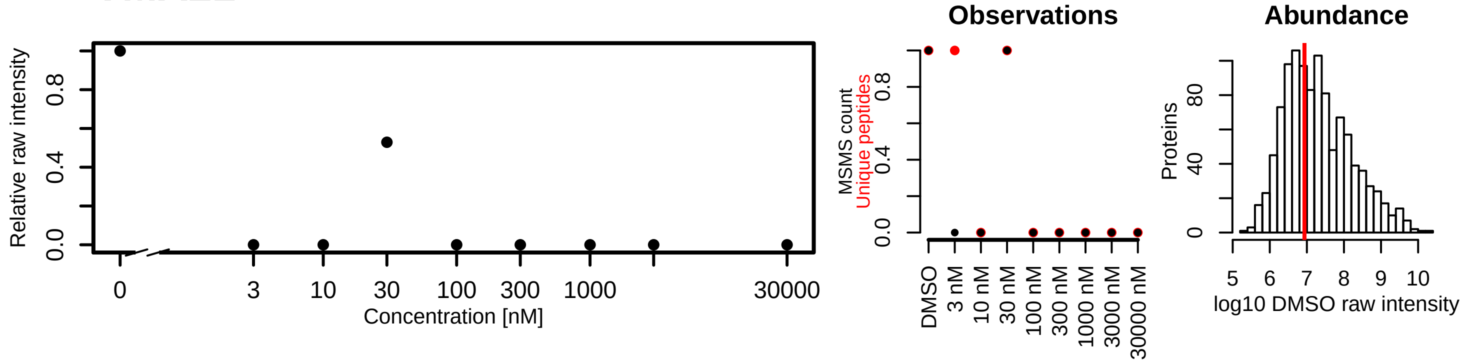

Supplement: Supplementary file 2 [file oncotarget-11-535-s002.pdf]
