# Supplementary material for: Identification of molecular targets for the targeted treatment of gastric cancer using dasatinib
Source: Oncotarget. 2020 Feb 4;11(5):535–49. doi: 10.18632/oncotarget.27462 (PMC7007292; doi:10.18632/oncotarget.27462)
Supplement: Supplementary file 3 [file oncotarget-11-535-s003.pdf]

LYN – P07948;E5RJ37*

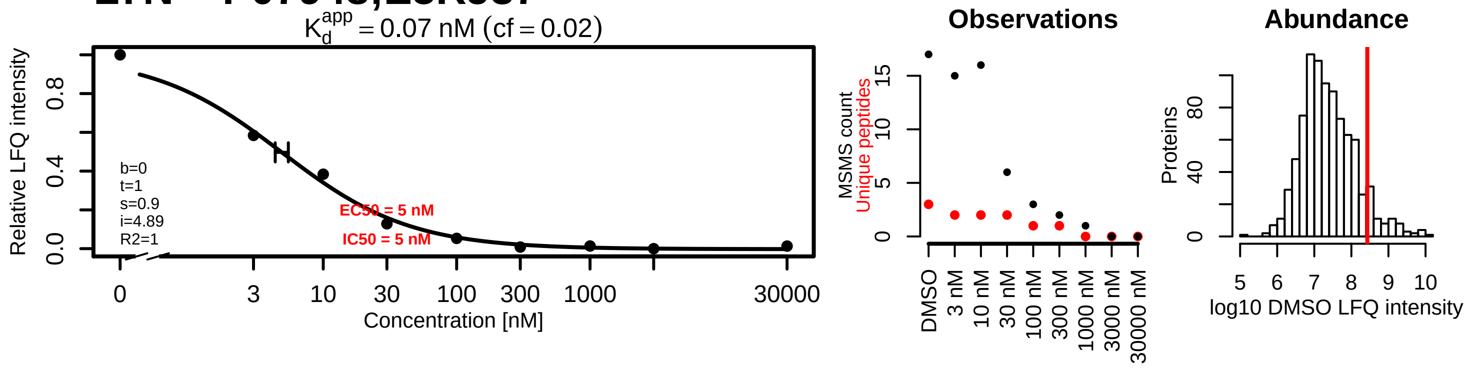

YES1*

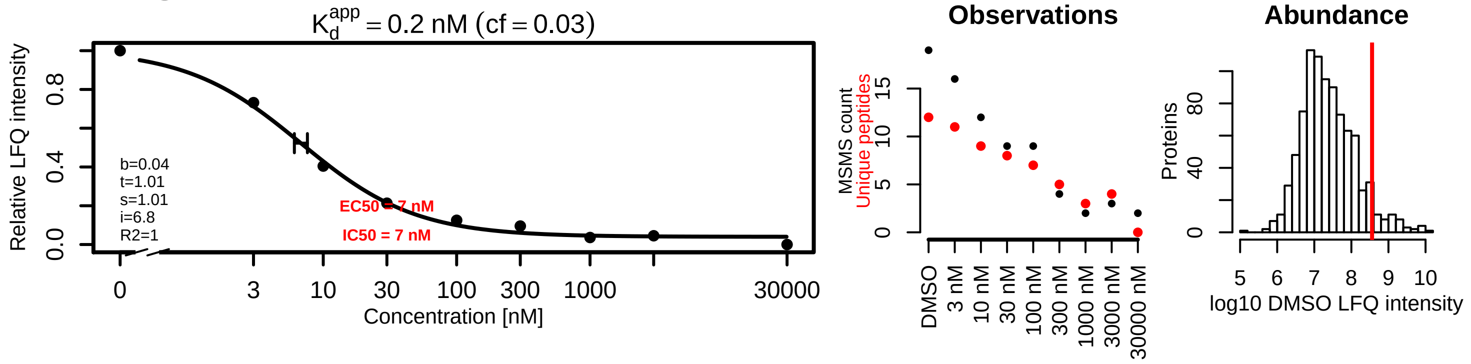

SRC*

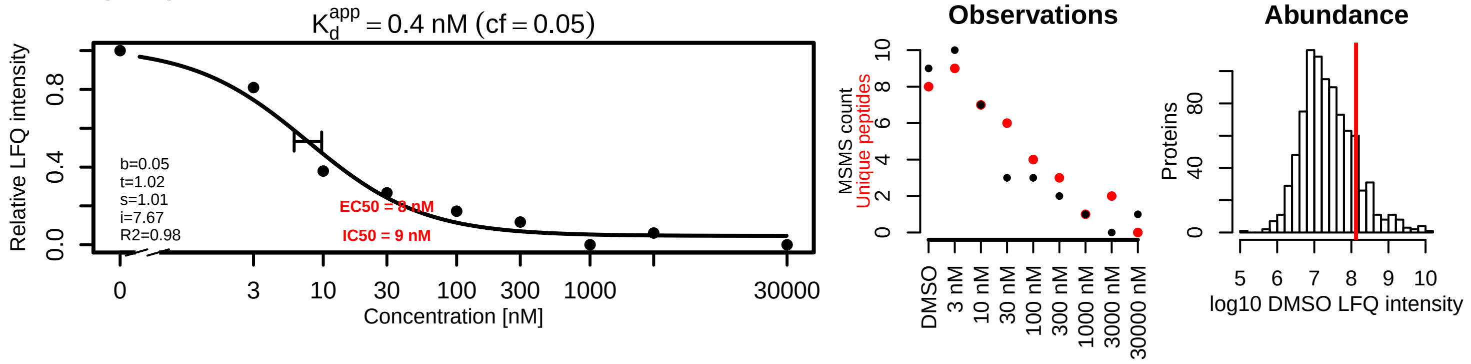

EPHA2*

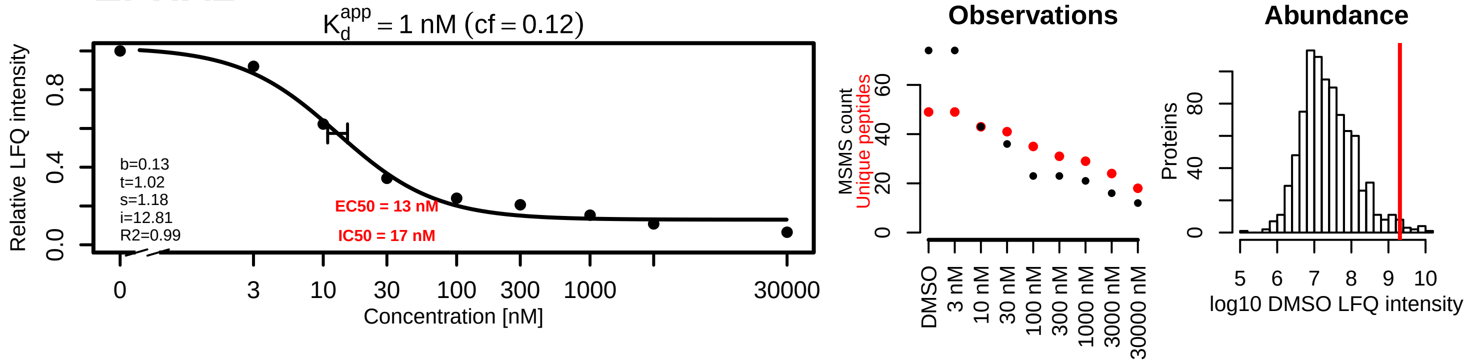

EPHB2*

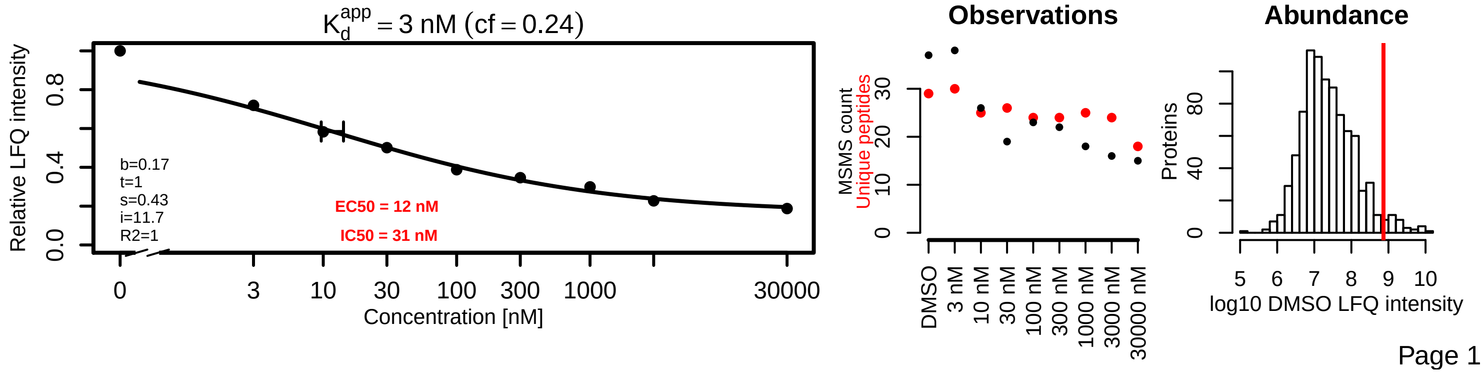

ABL2*

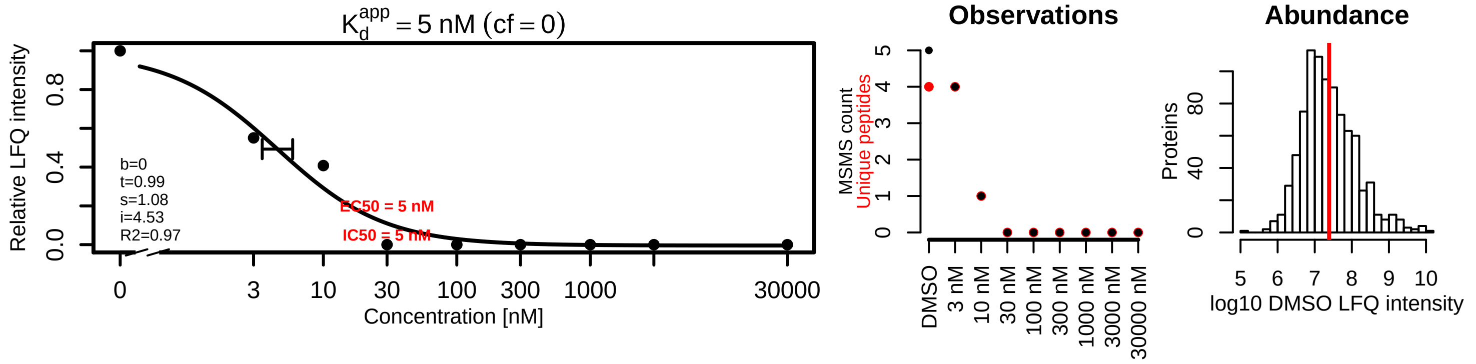

SIK2*

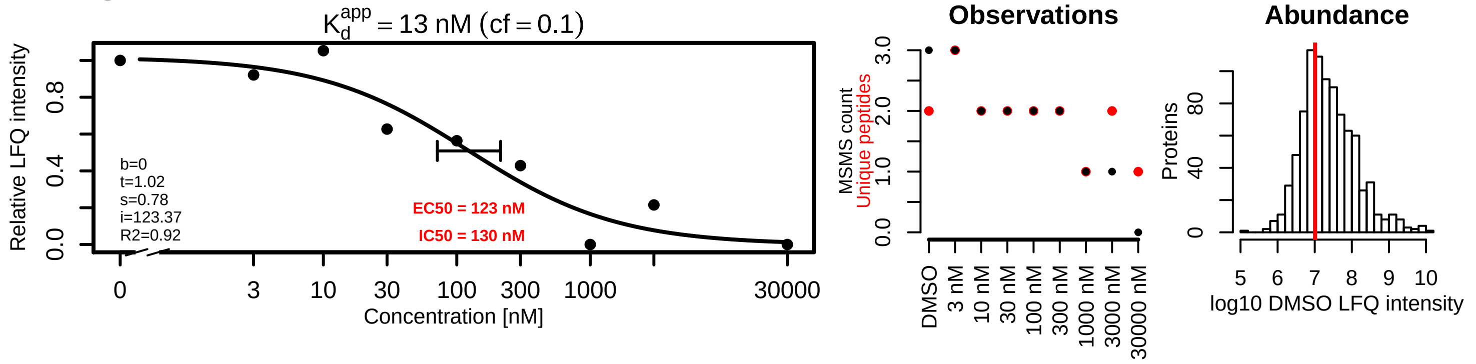

FRK*

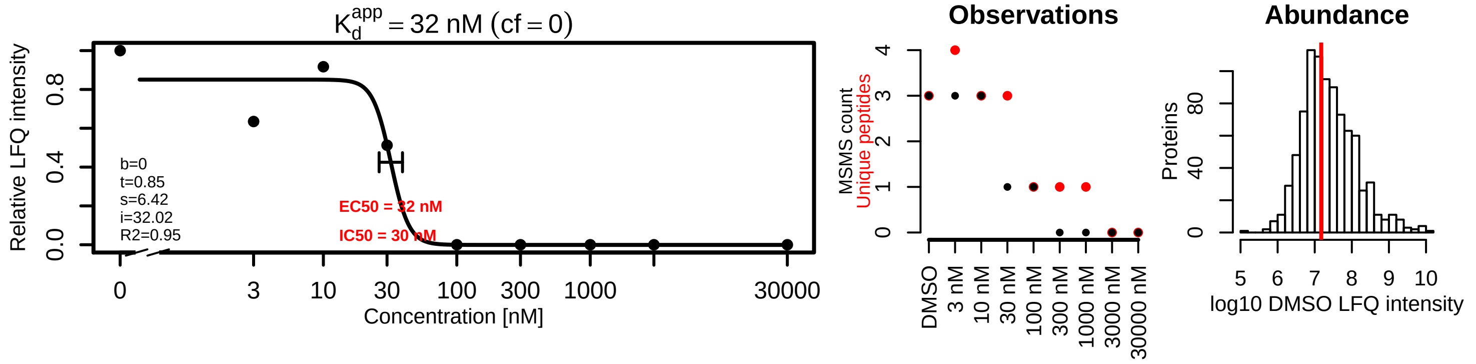

DDR1*

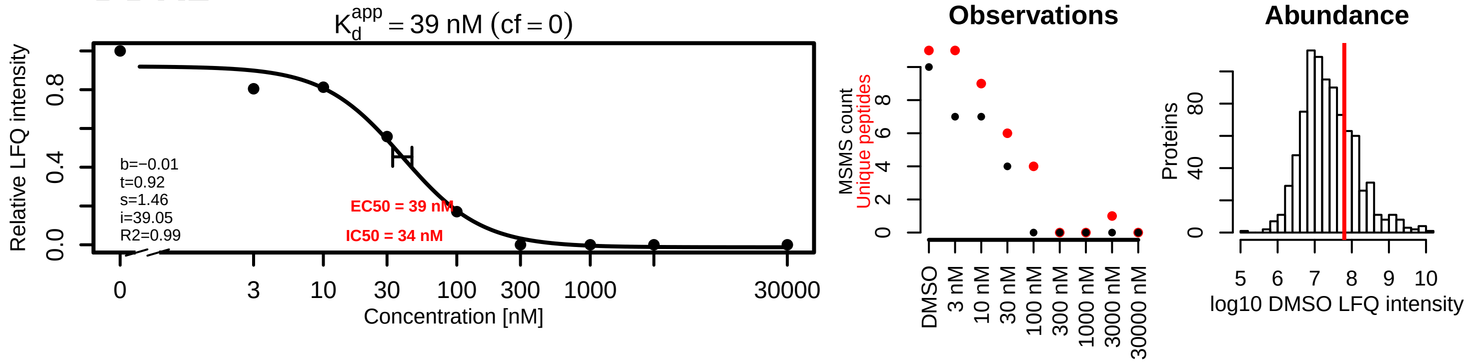

RIPK2*

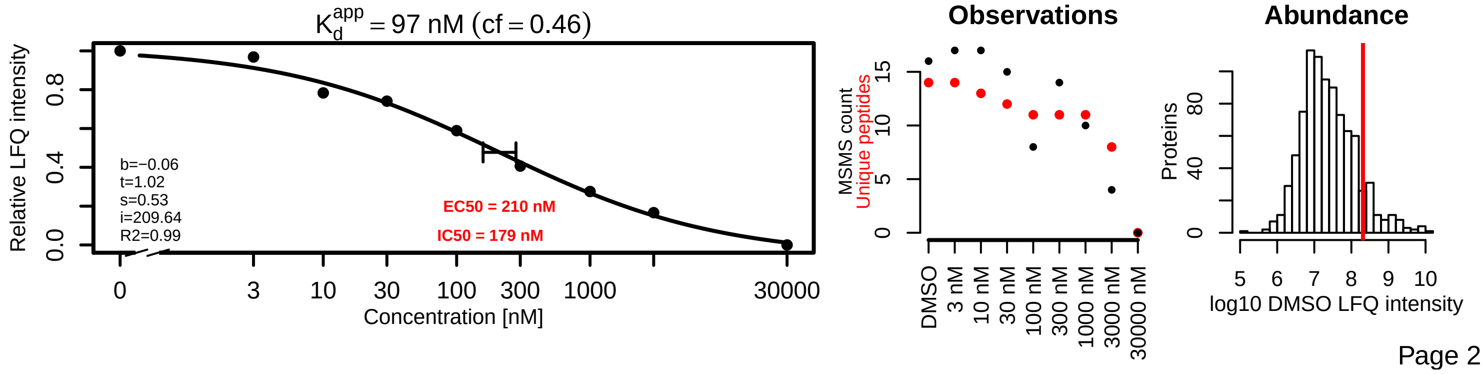

CSK*

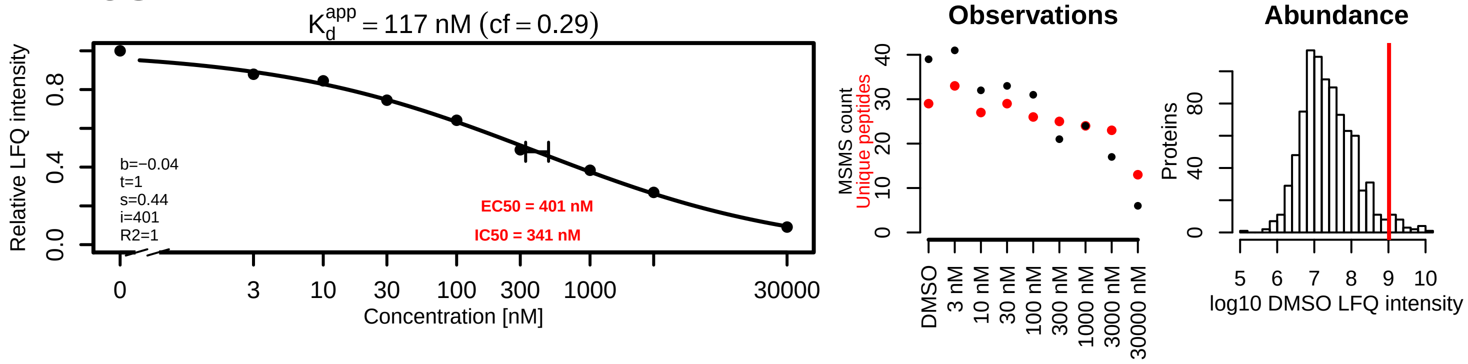

RTL1*

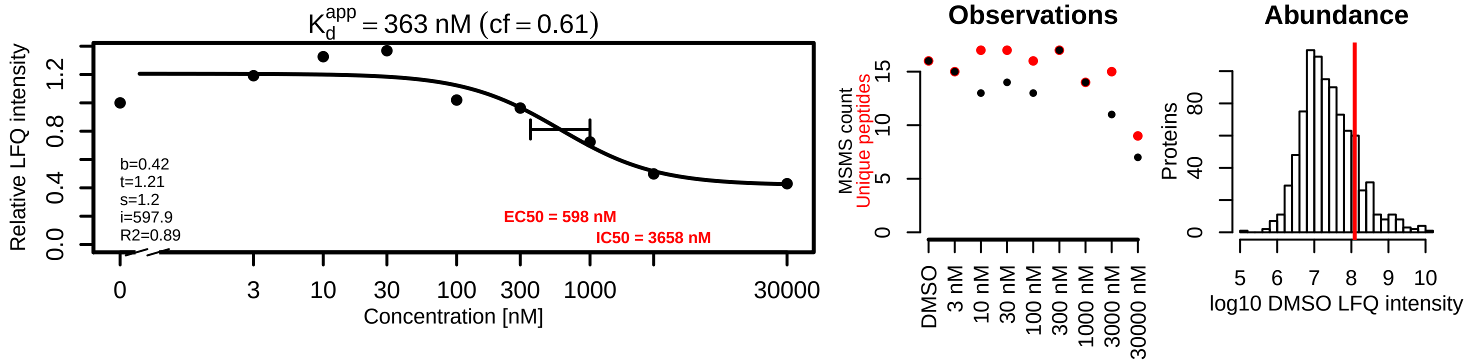

TNK2*

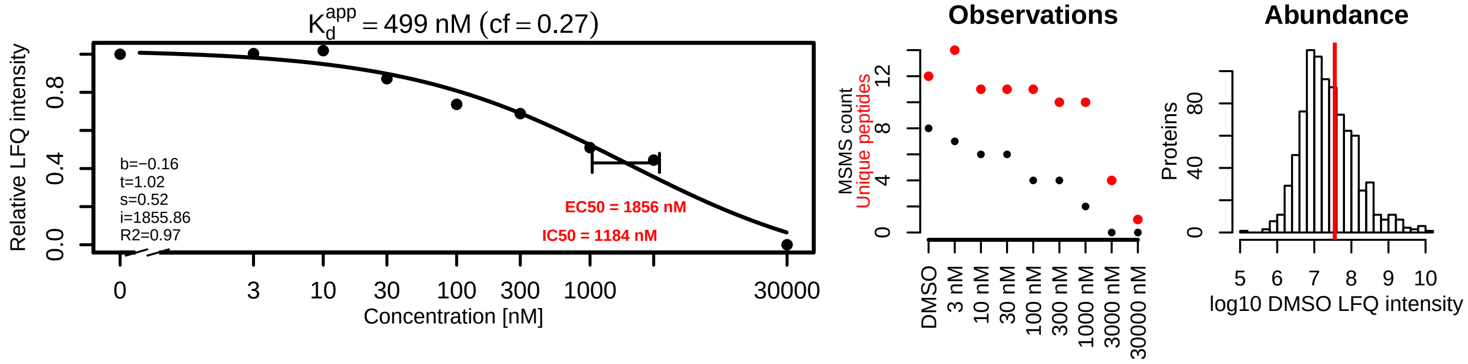

SIK3;KIAA0999*

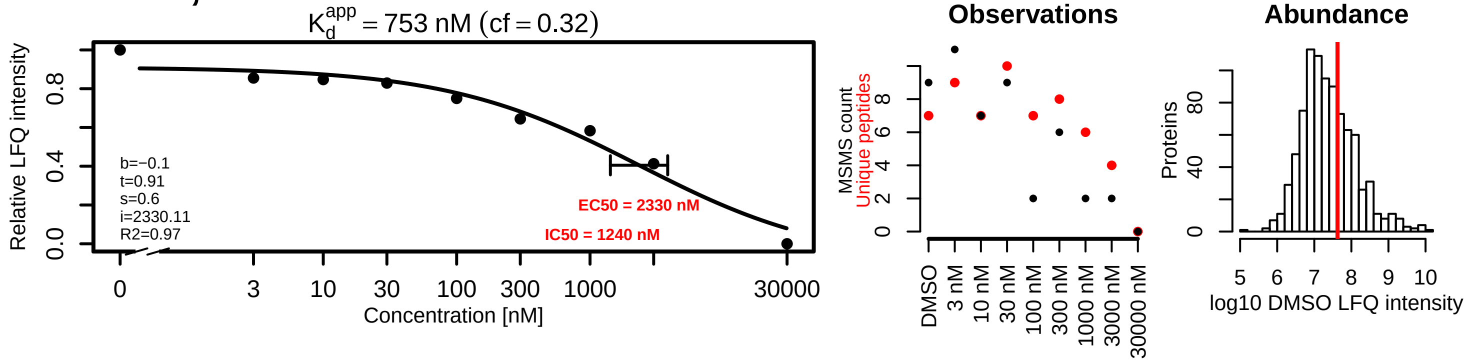

MAP4K5*

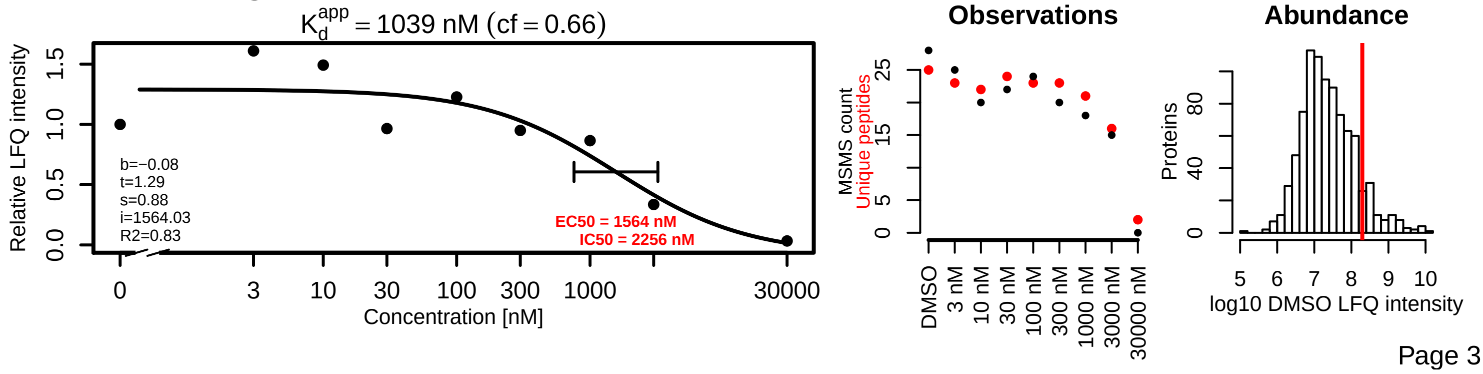

GAK*

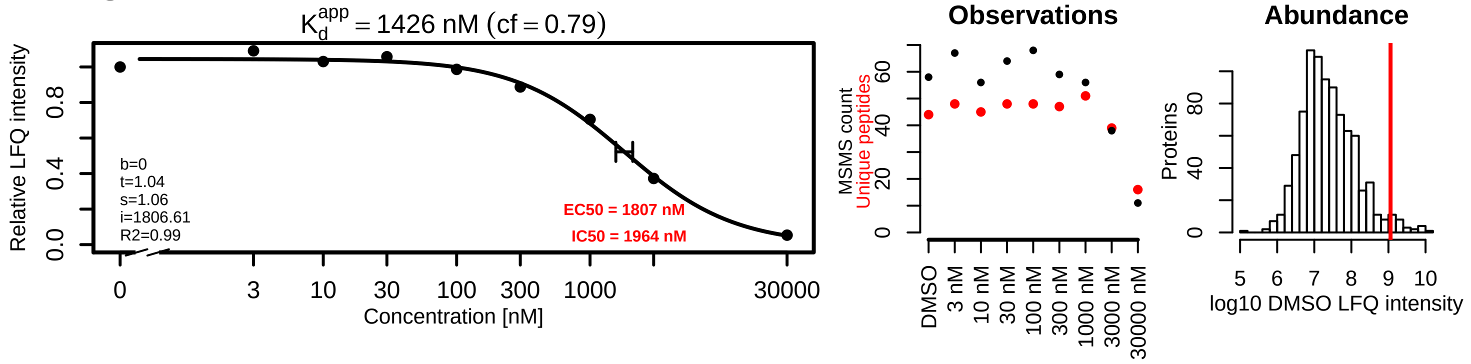

MAP2K5*

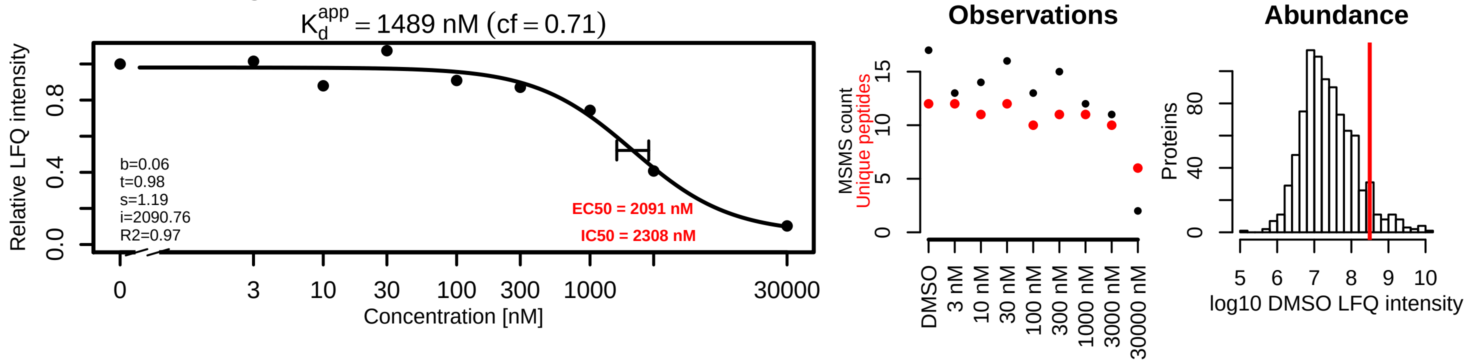

MLTK*

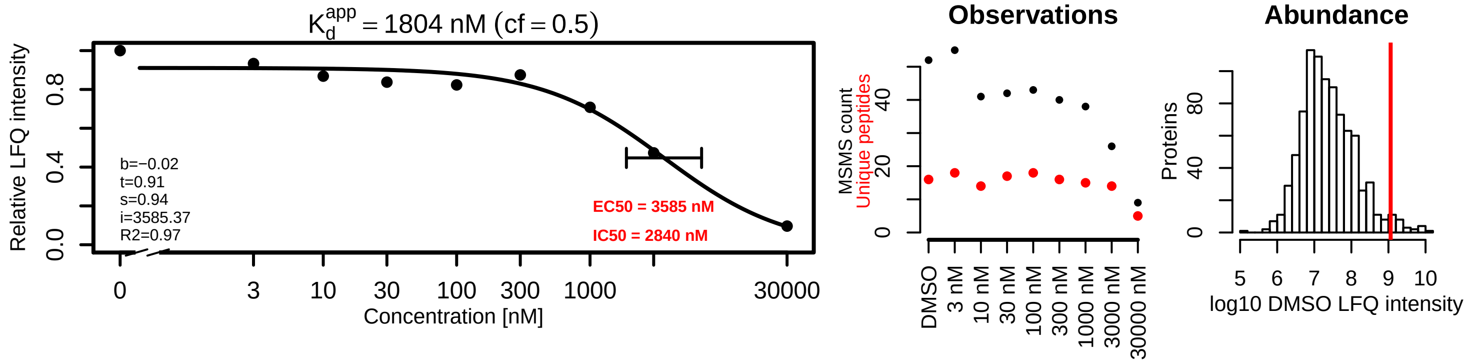

TEC*

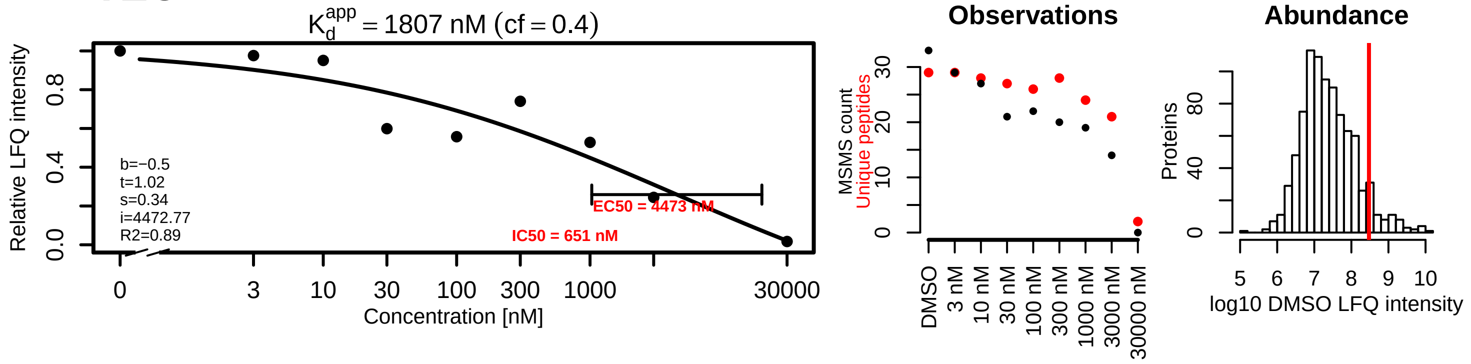

TYK2*

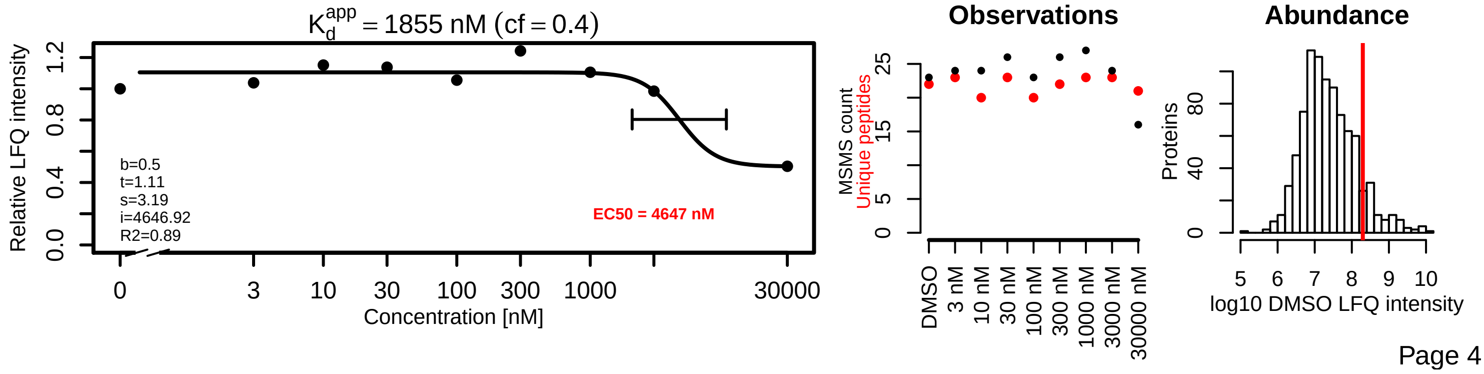

MAP3K2*

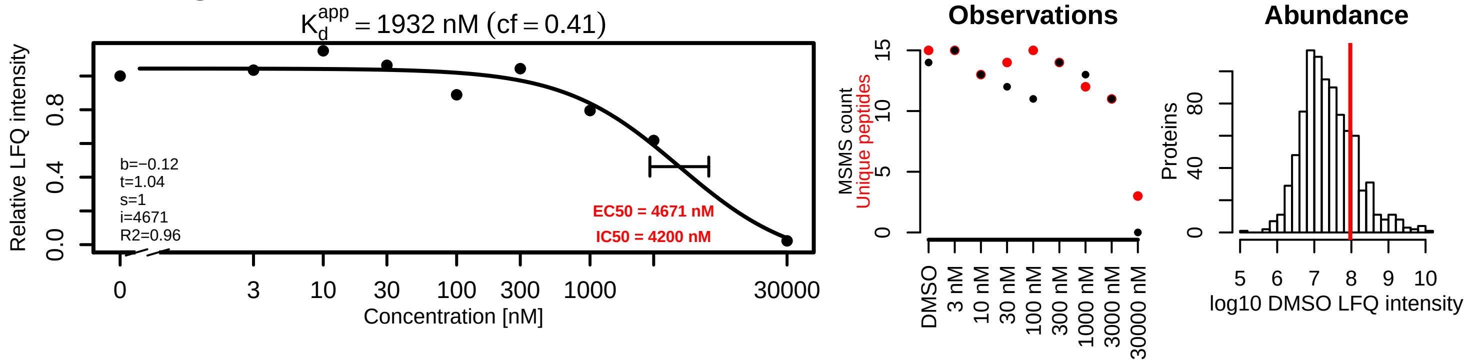

YWHAE*

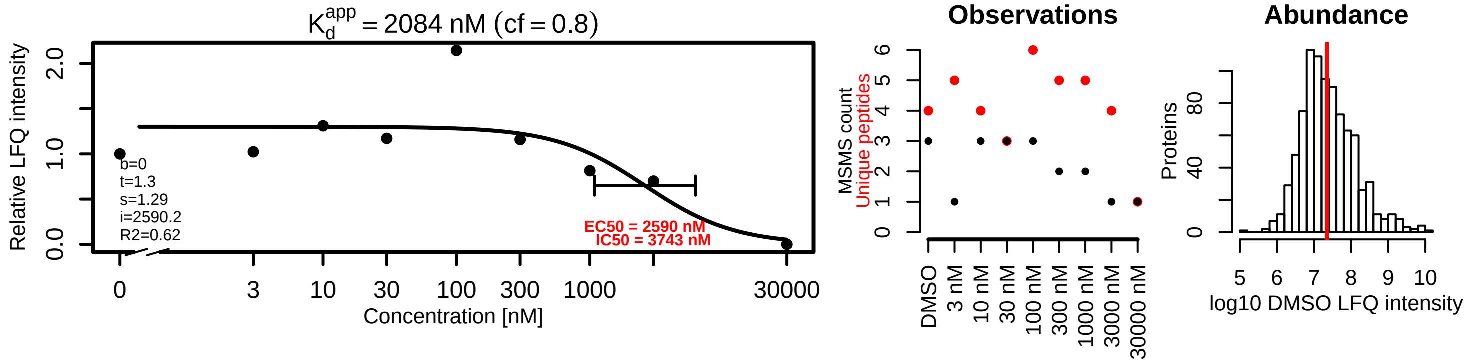

JAK1*

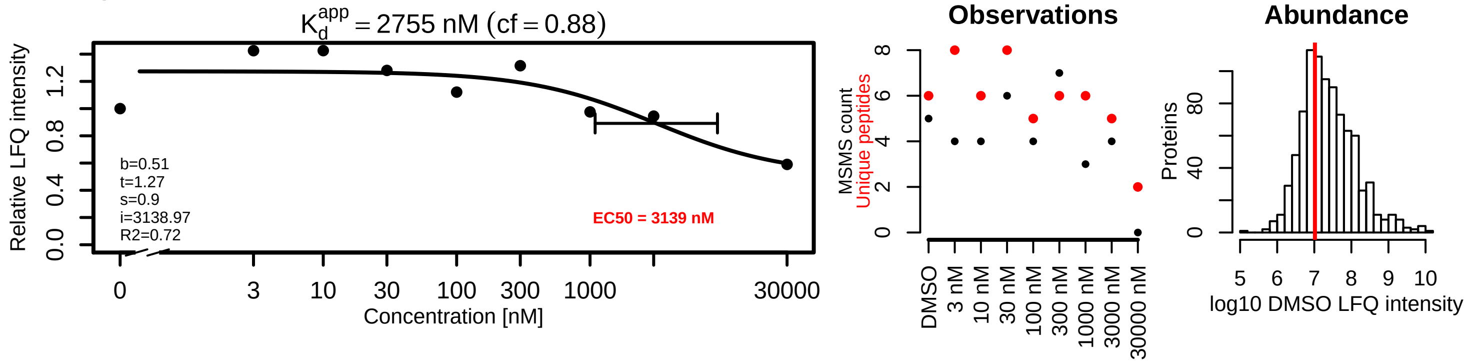

YWHAG*

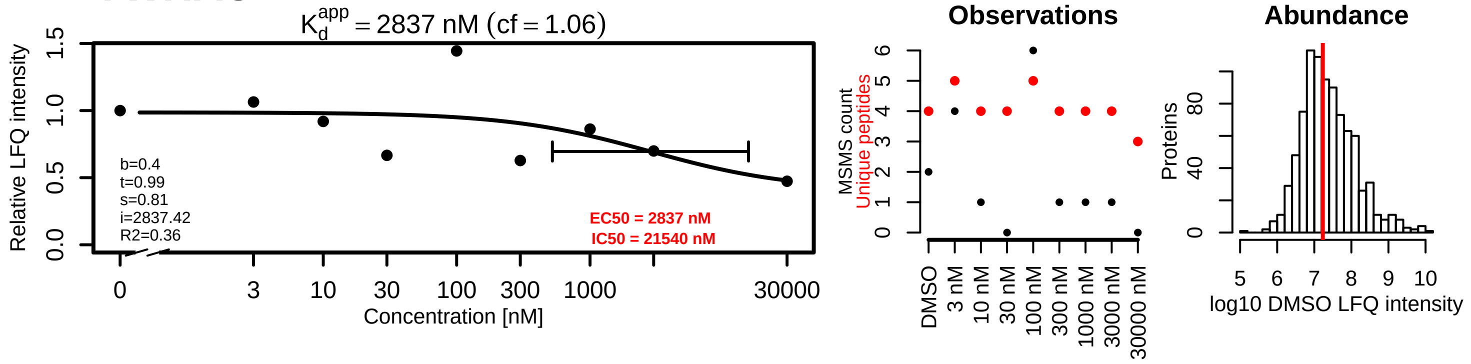

PKMYT1*

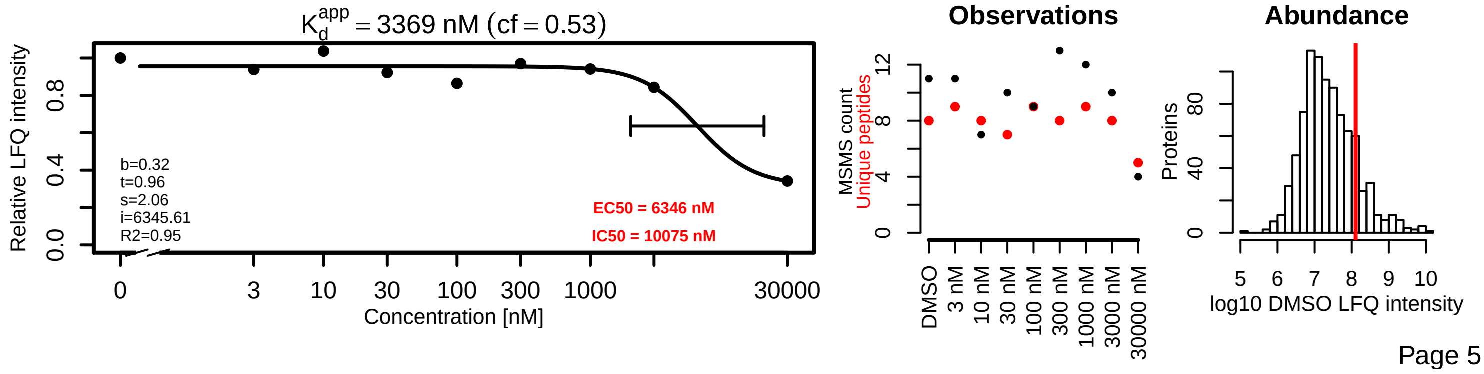

PARVB*

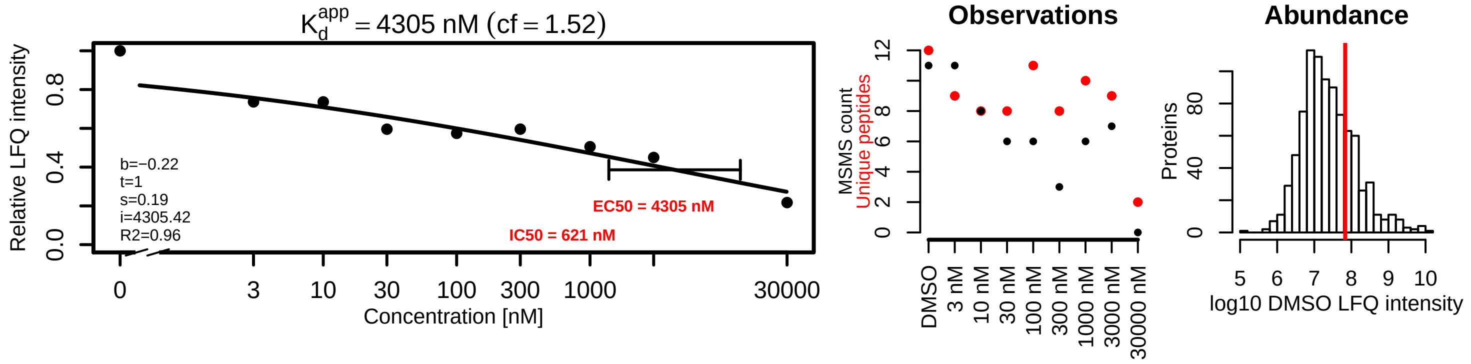

LIMK2*

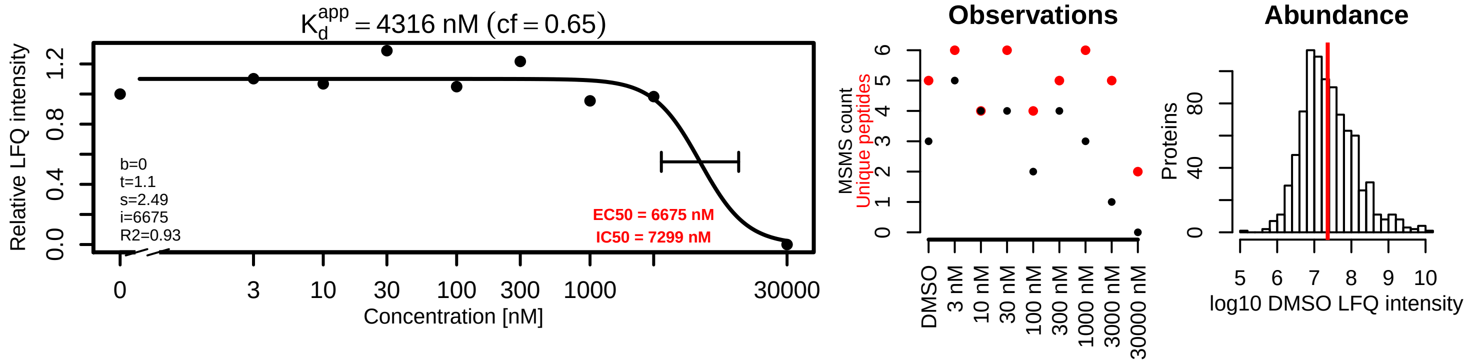

RSU1*

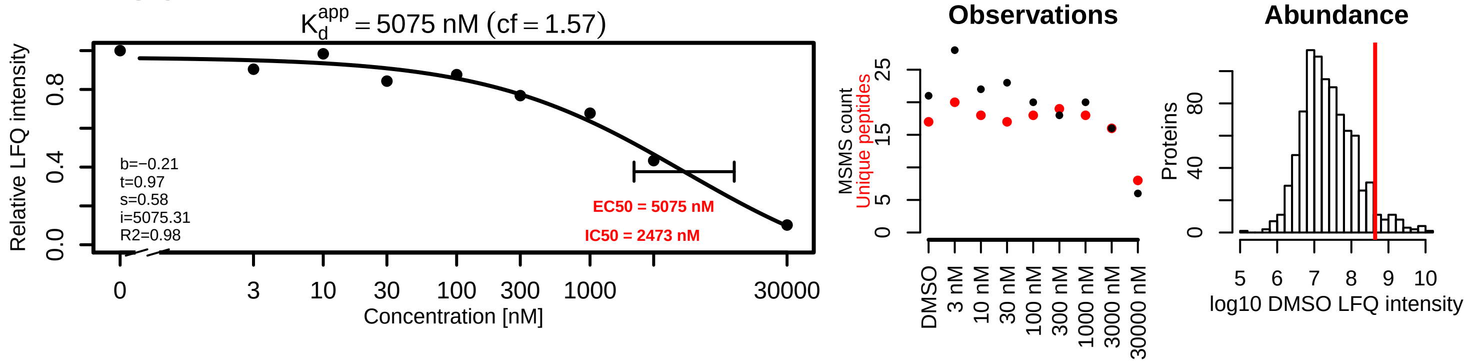

ZAK*

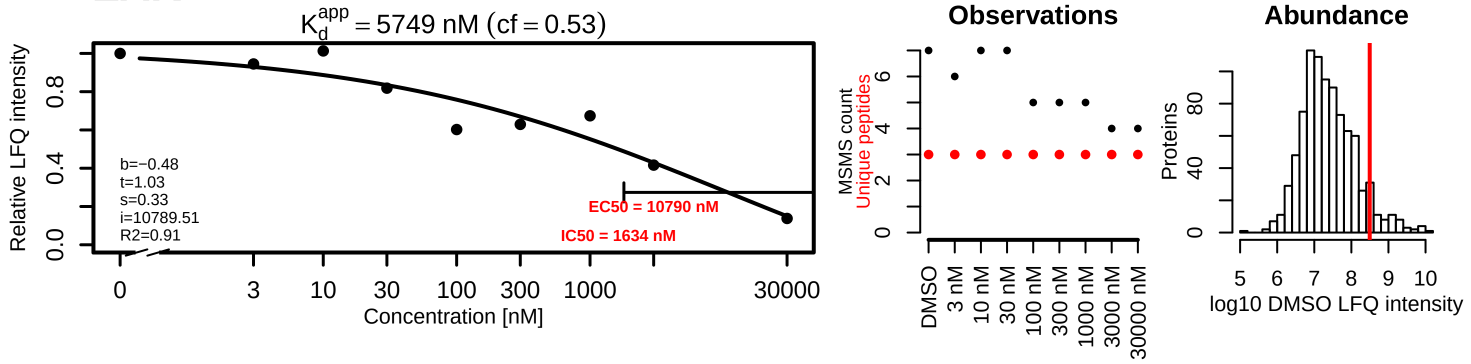

EGFR*

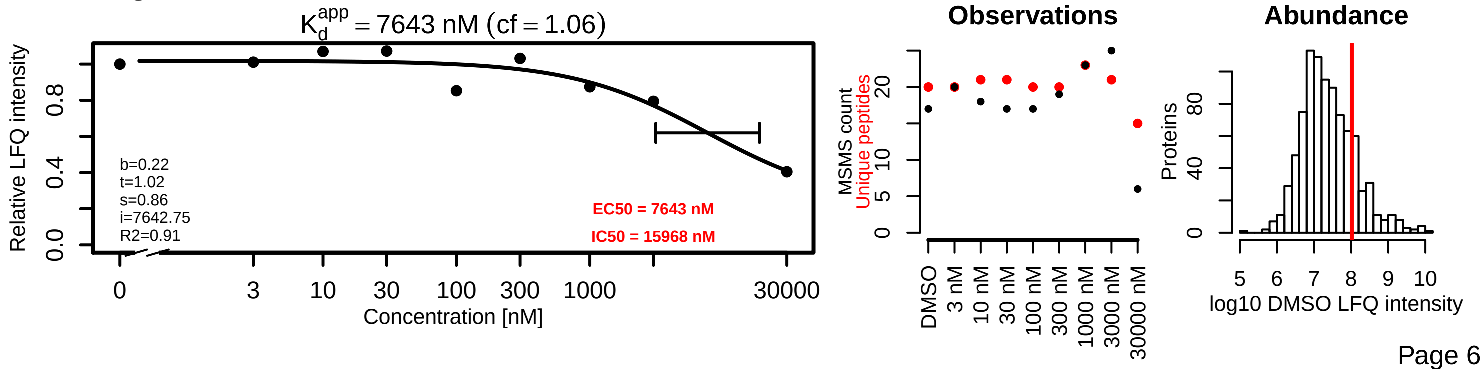

TGFBR1*

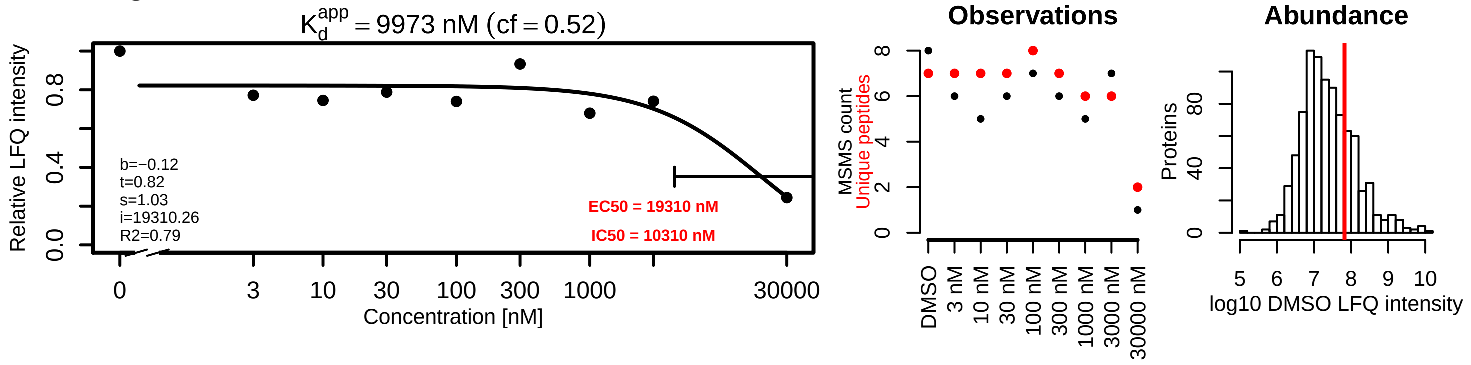

LIMS1*

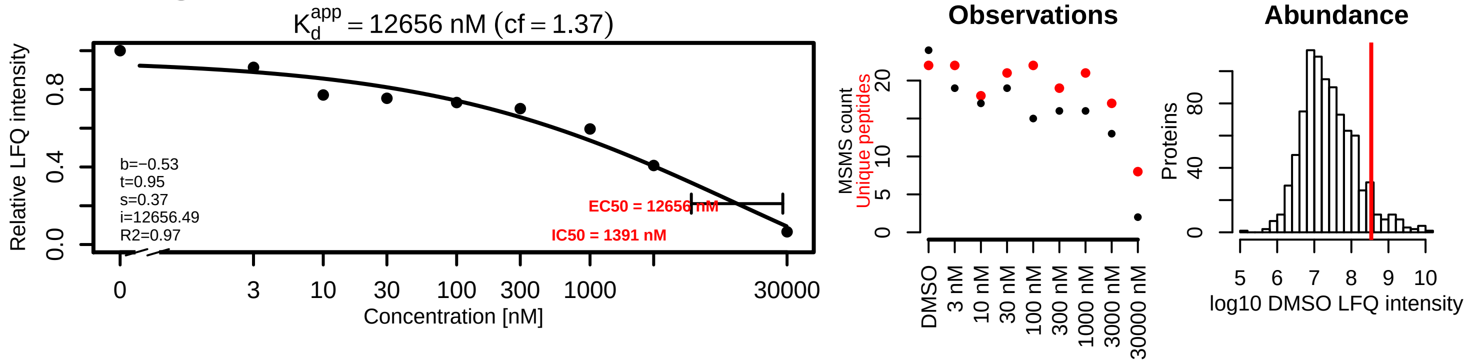

ILK*

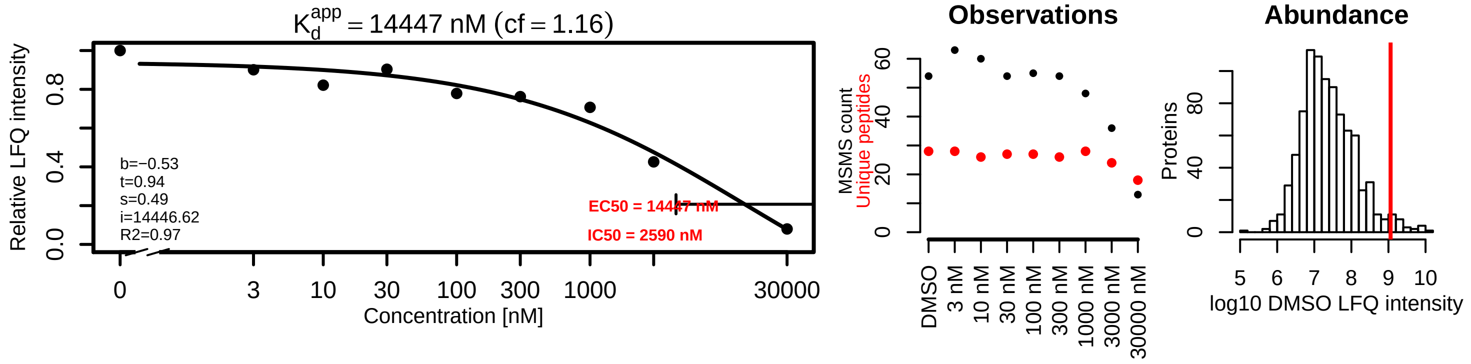

PARVA*

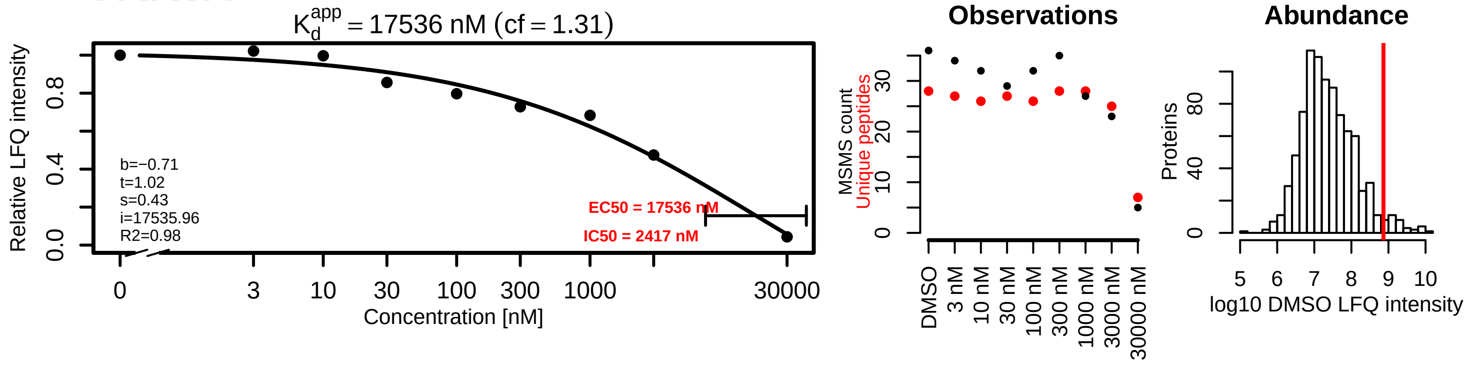

MAPK14 − Q16539;Q16539−3;Q16539−4;B4E0K5;B5TY33;E7EX54;H7

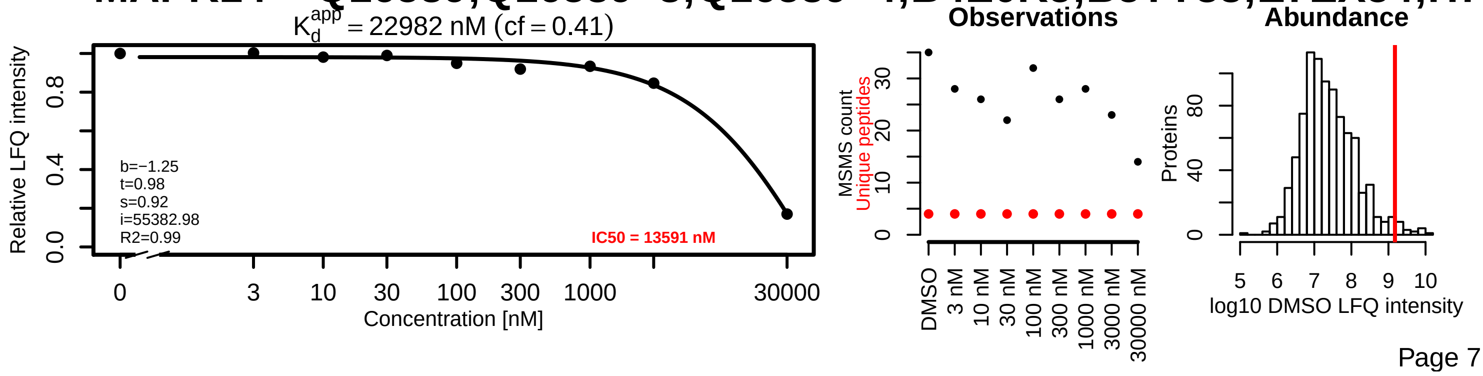

LIMK1*

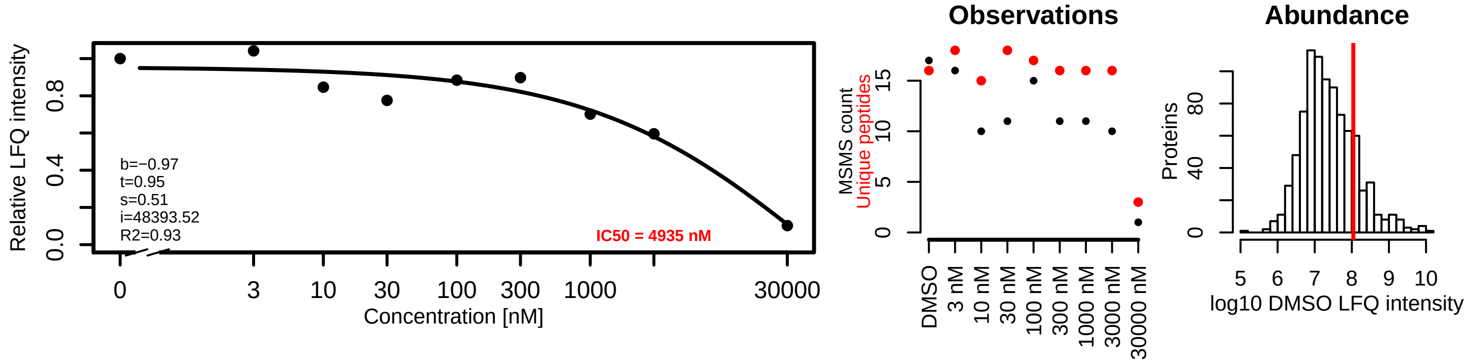

MAP3K1*

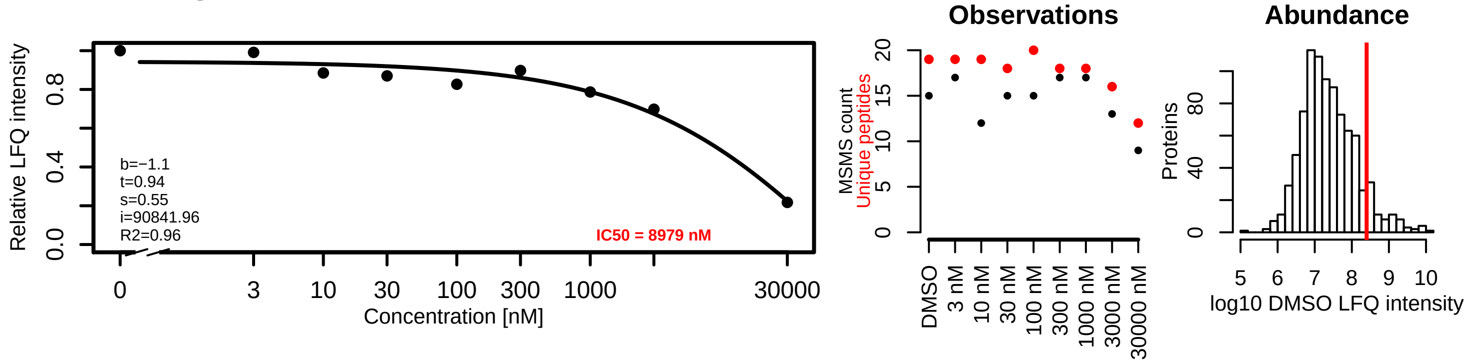

ADCK3*

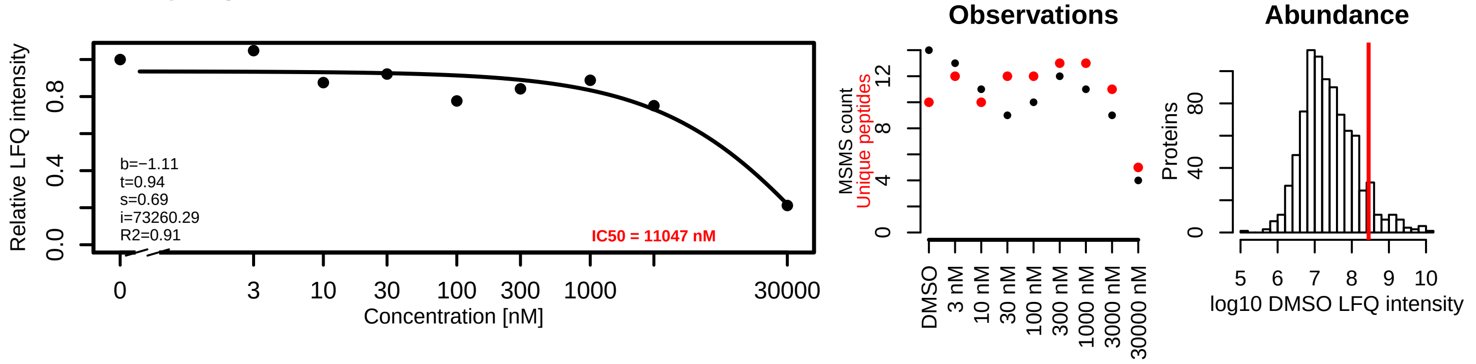

MT−ND1

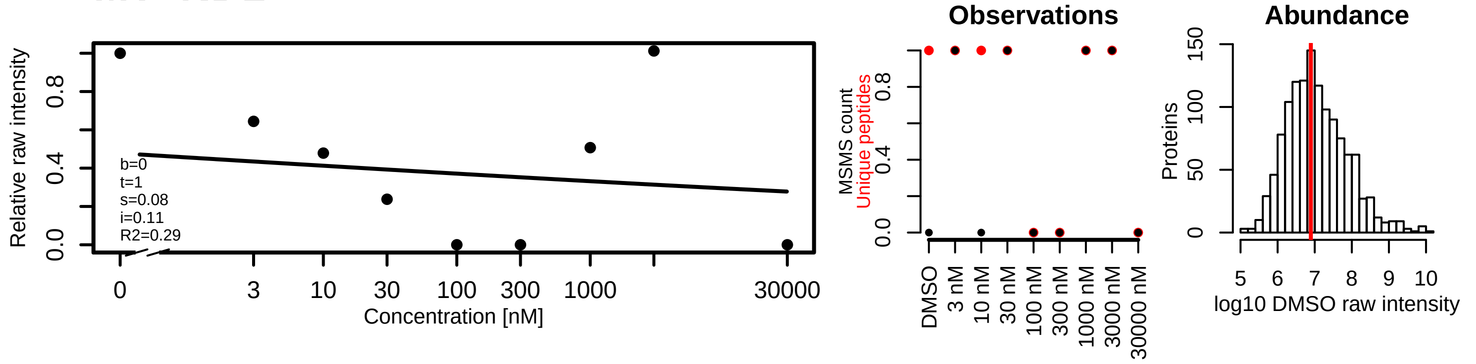

TMED10

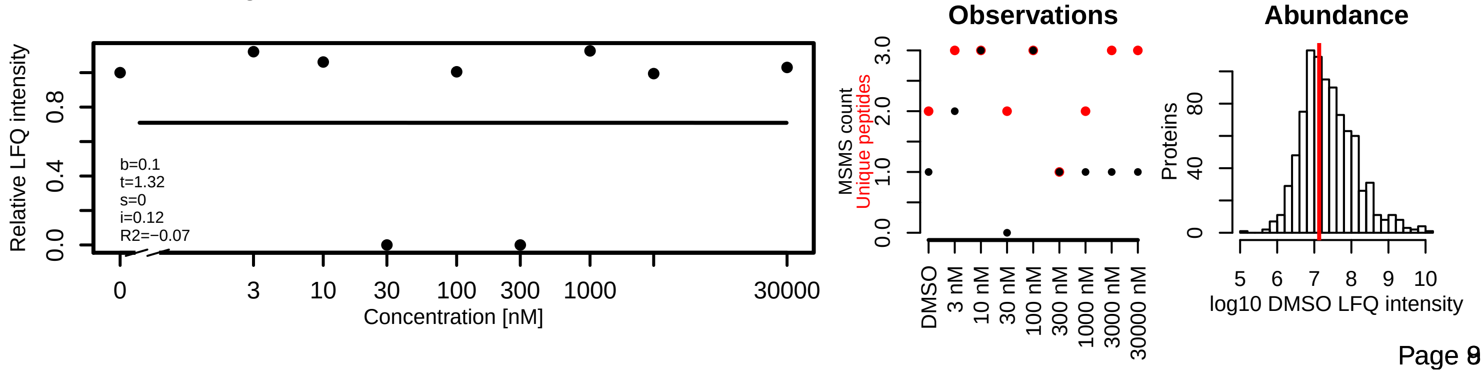

PRAF2

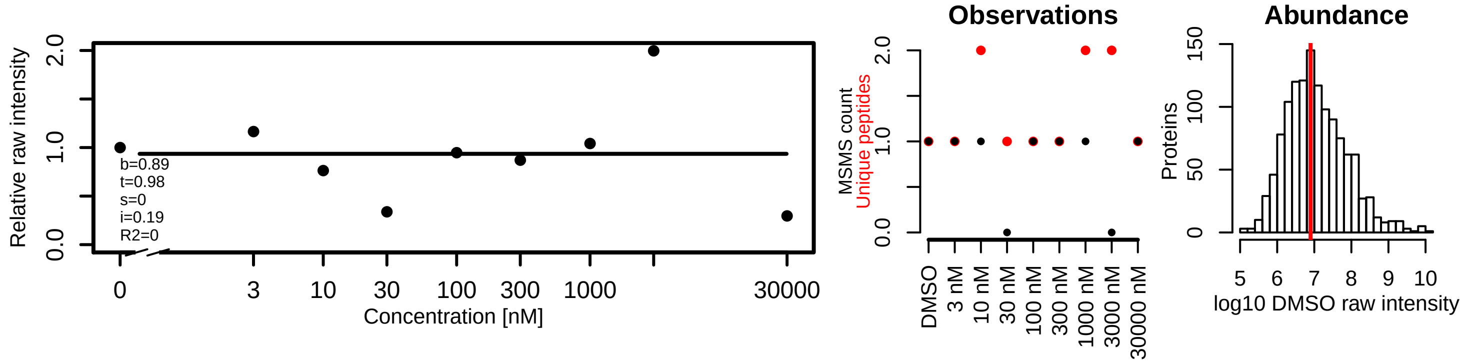

AIFM2

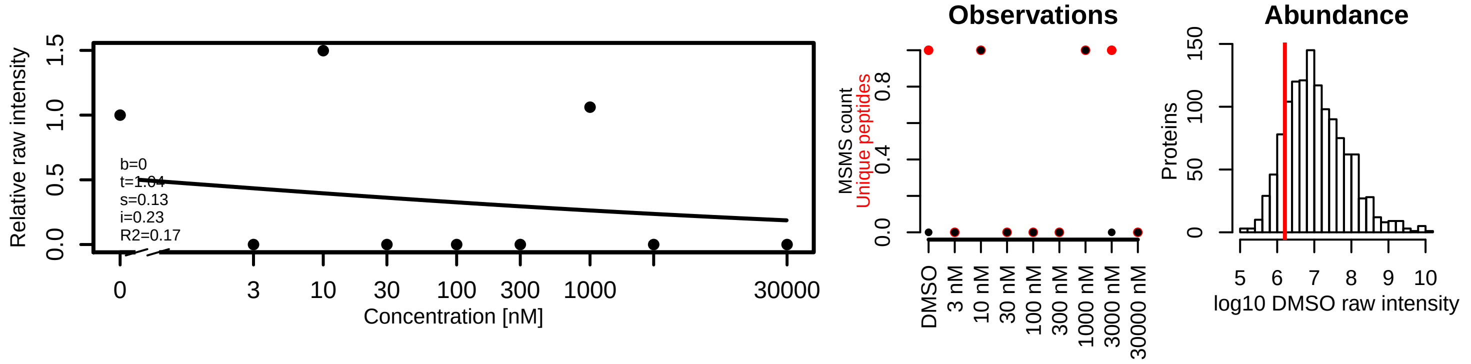

SLC30A9

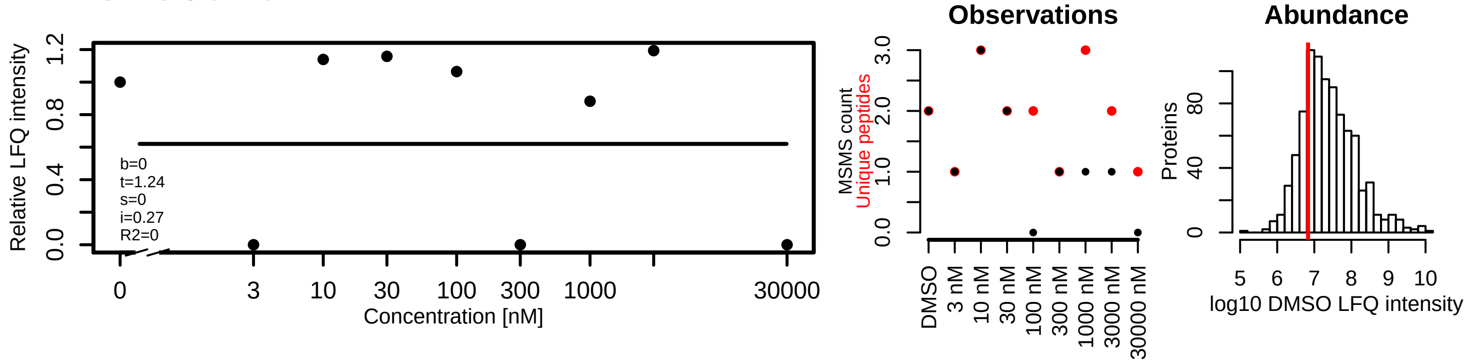

CCDC88A

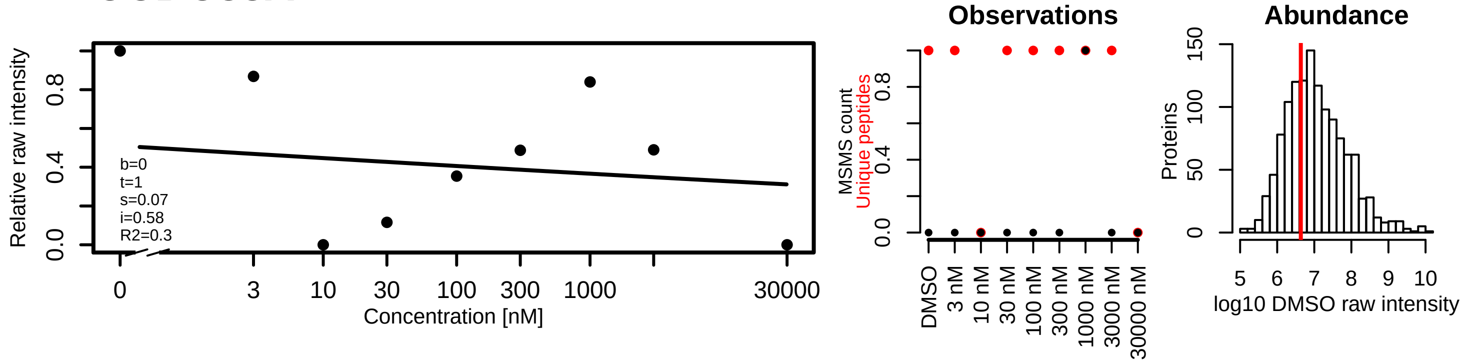

YWHAH

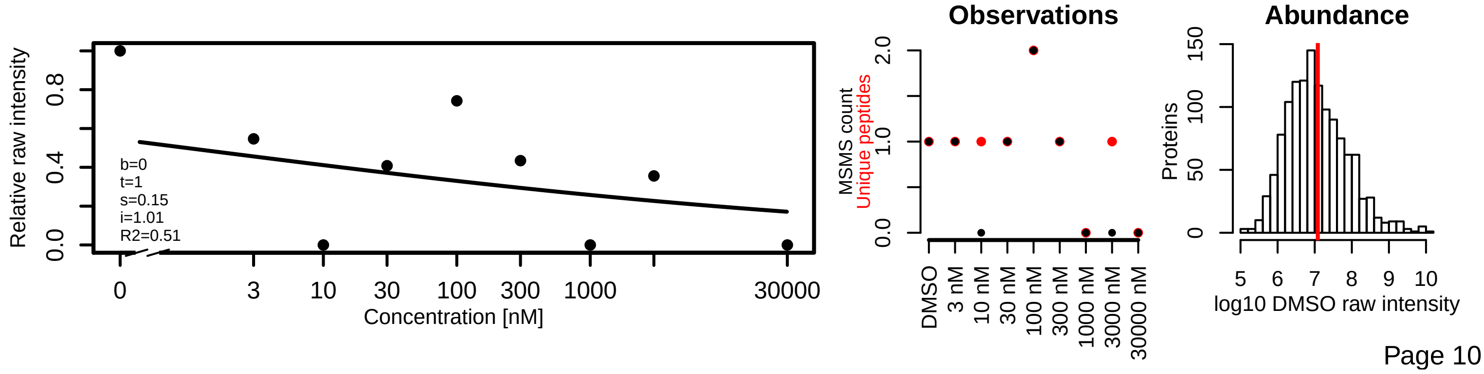

SIK1

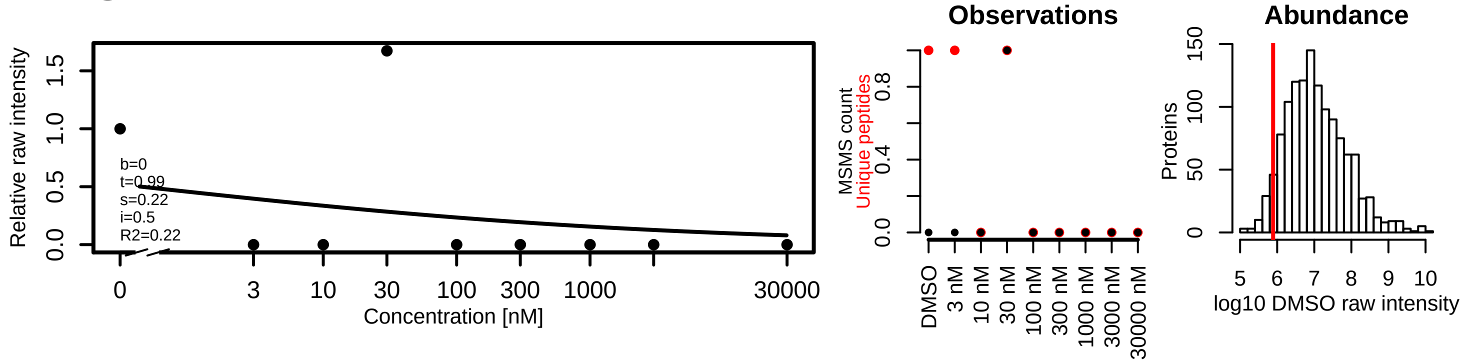

TFRC

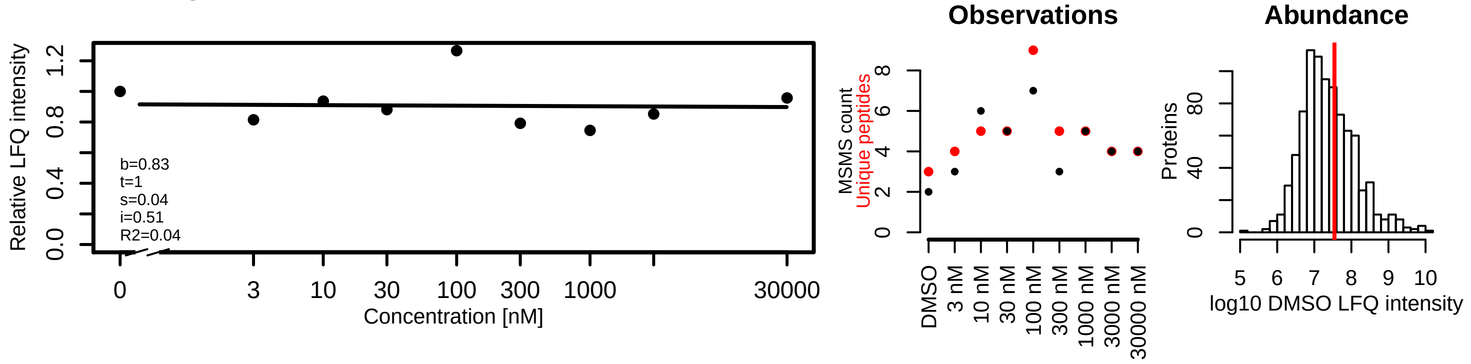

AK2

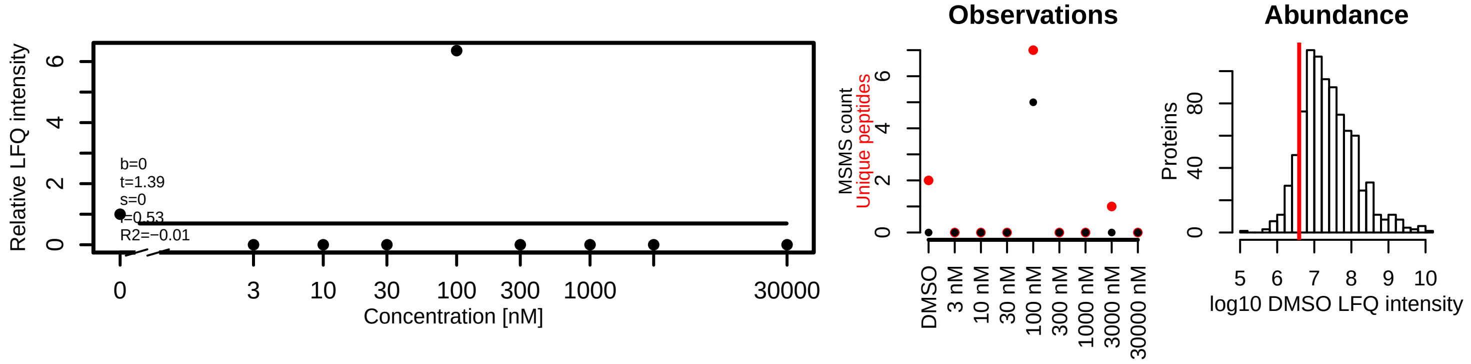

C2orf18;SLC35F6

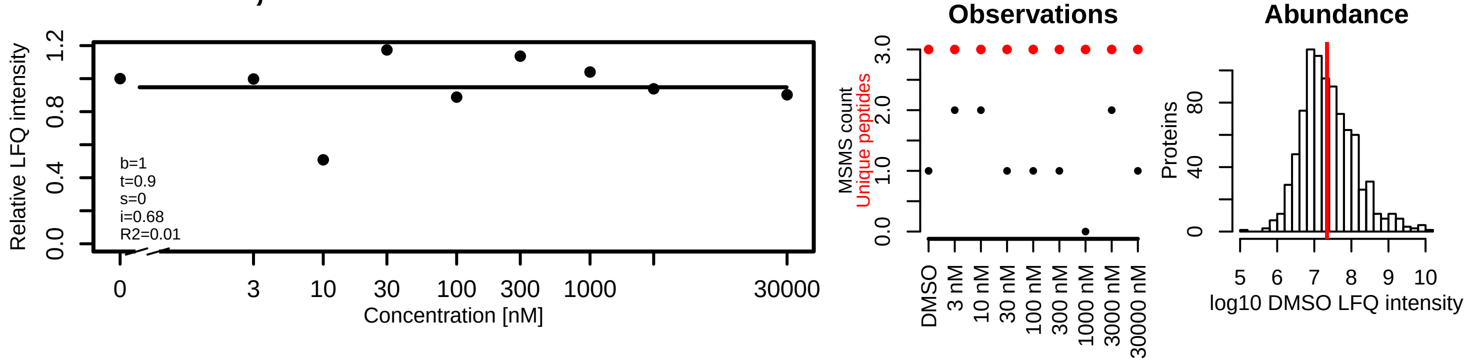

RAB5A;RAB5C;RAB5B

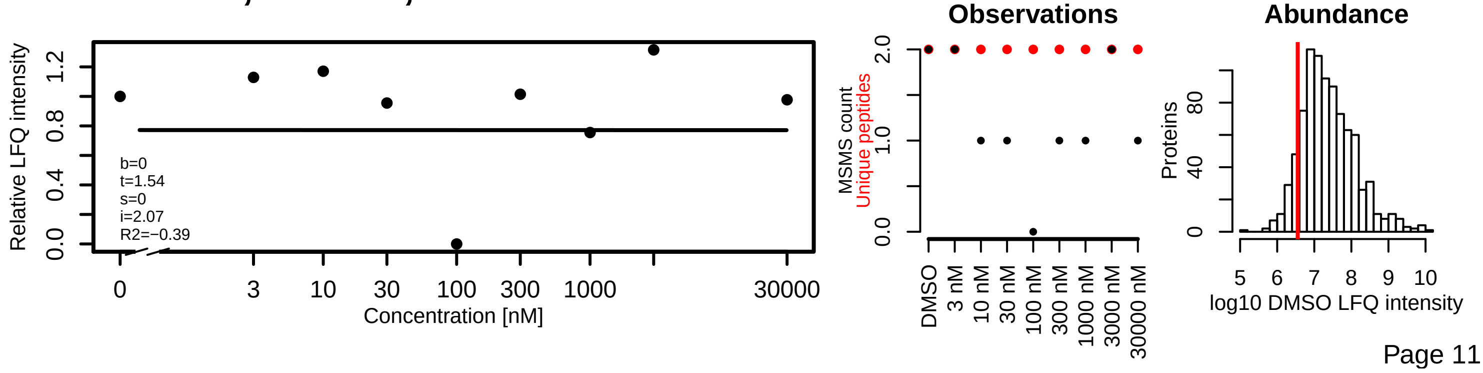

TMEM55B

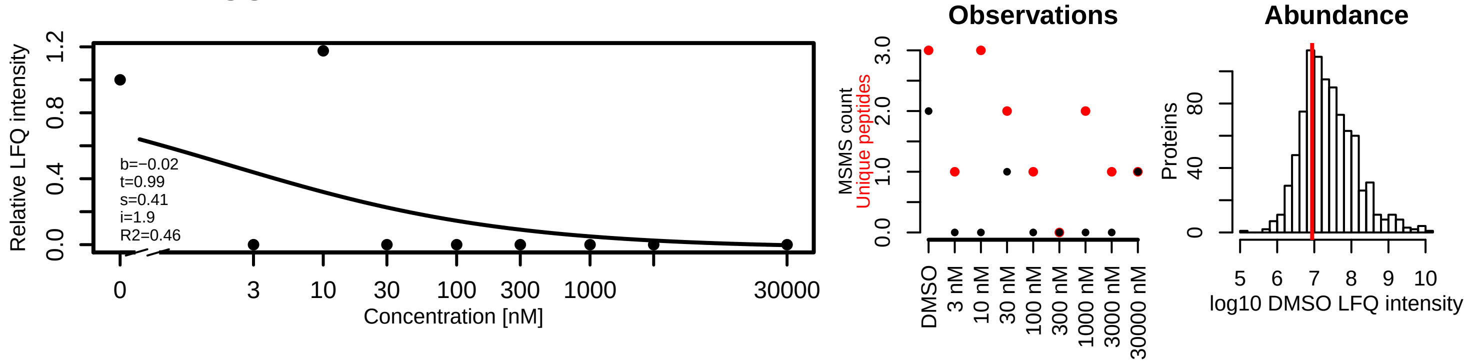

FAM20B

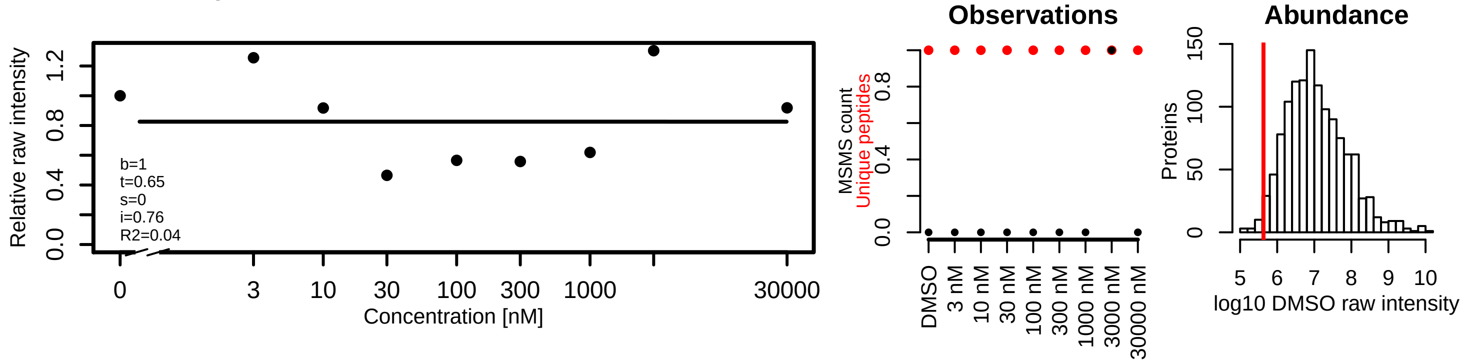

RMND1

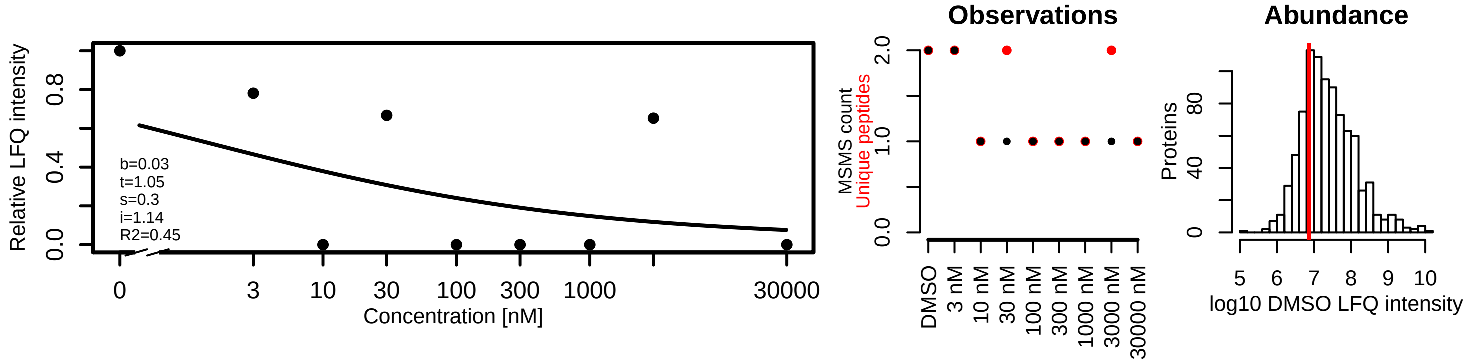

HLA−B;HLA−E

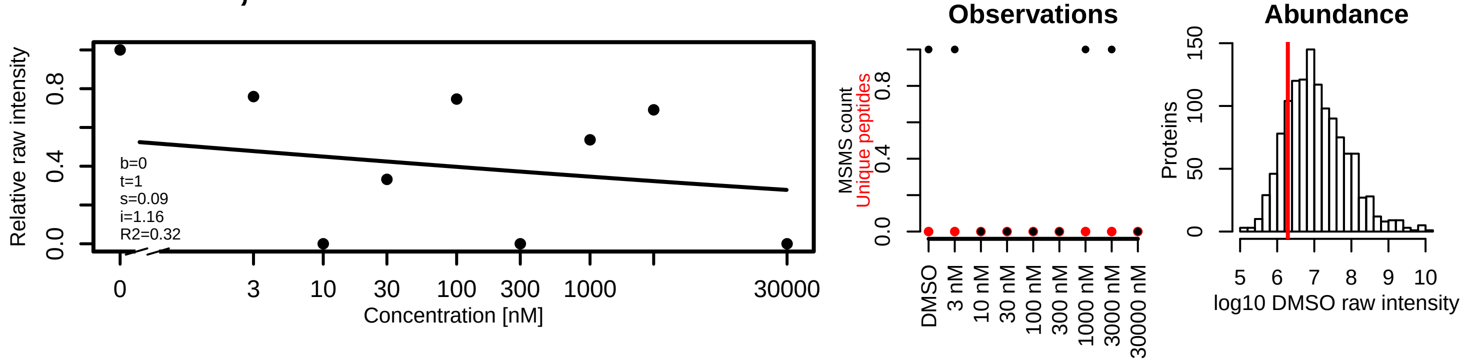

GNB1

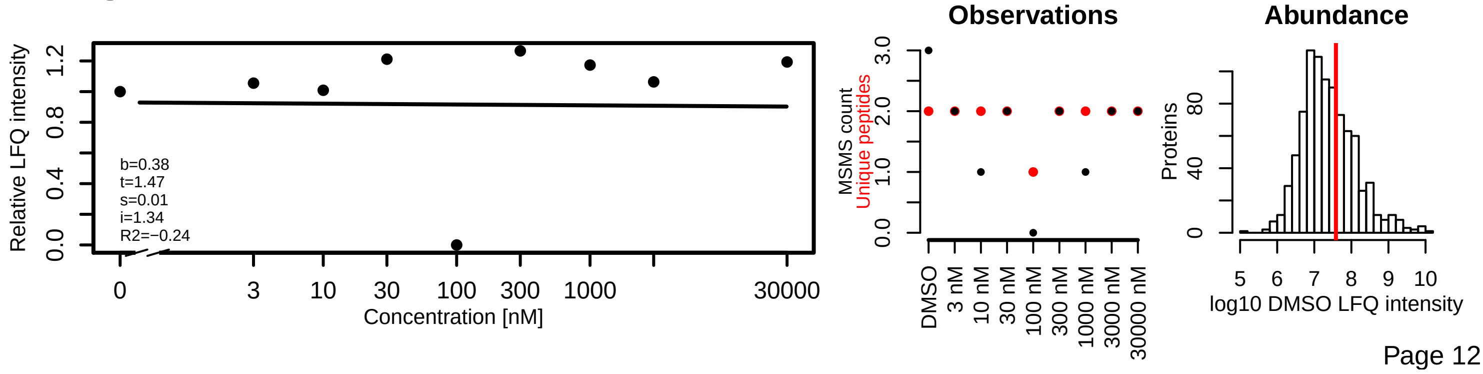

CNOT2

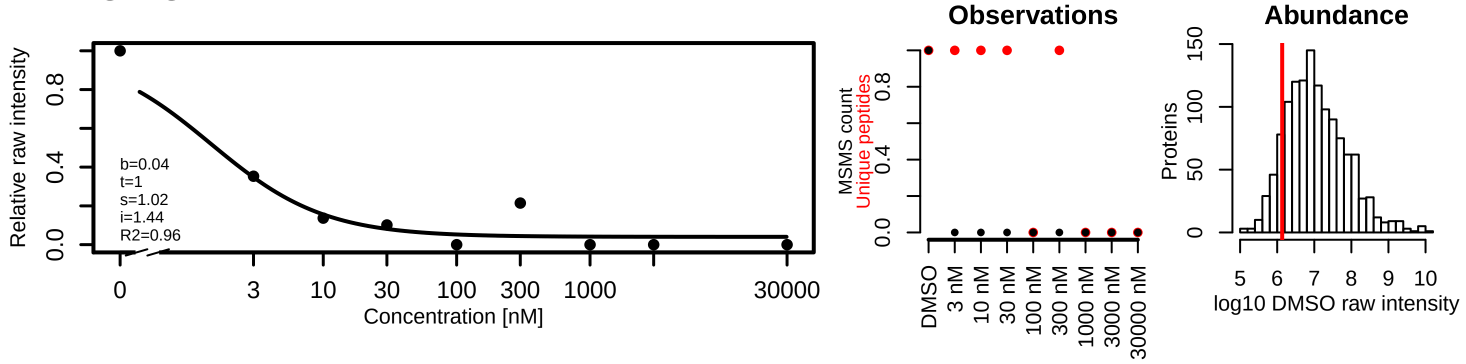

KRT18

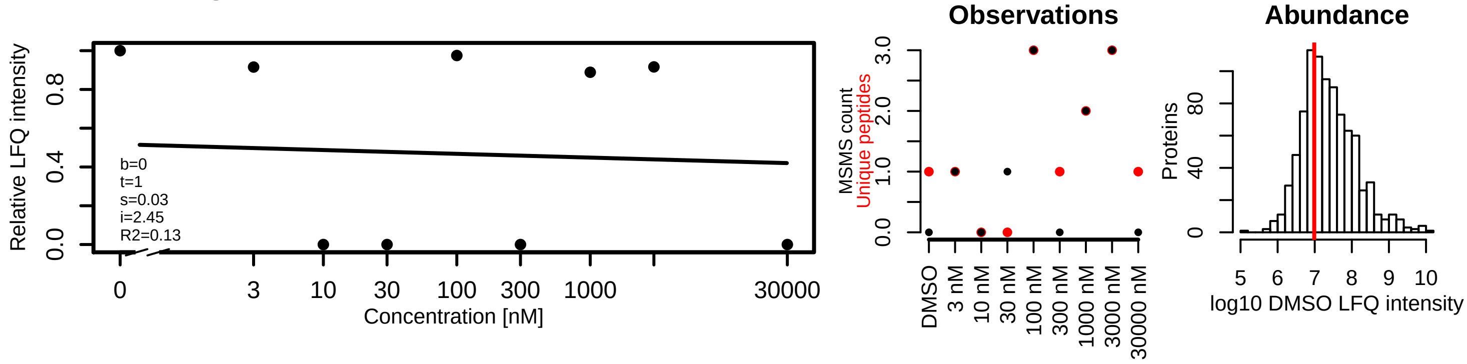

SQRDL

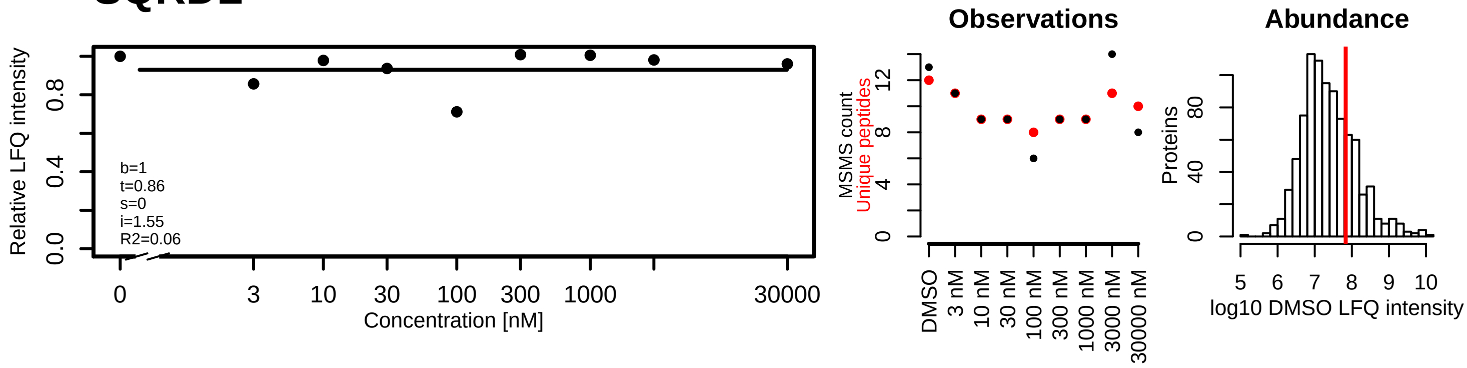

PSME2

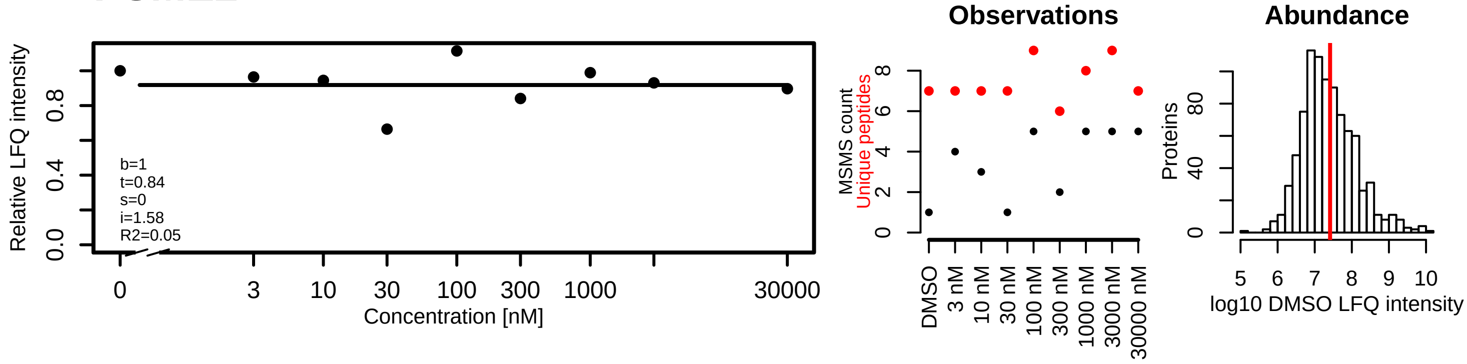

TMEM41A

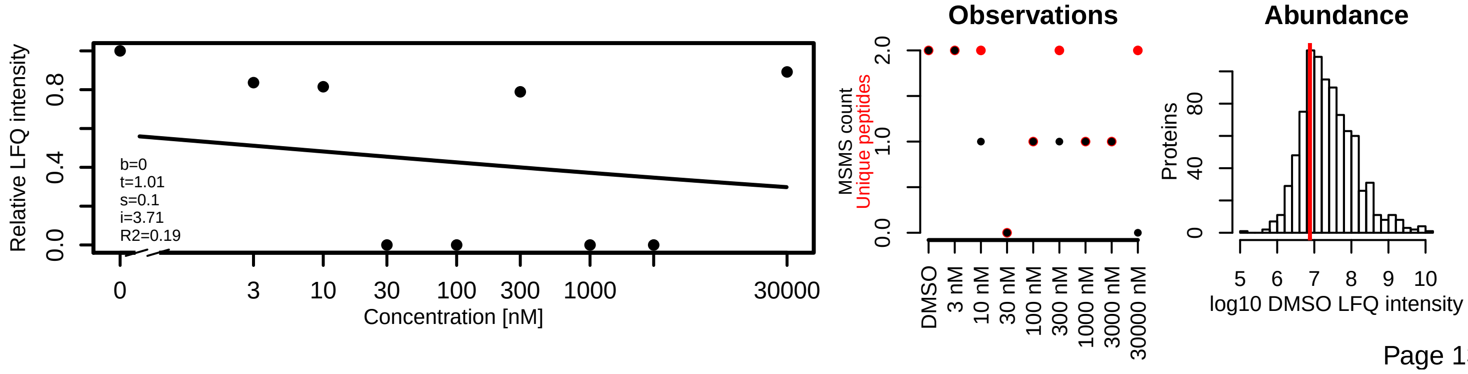

MMS19

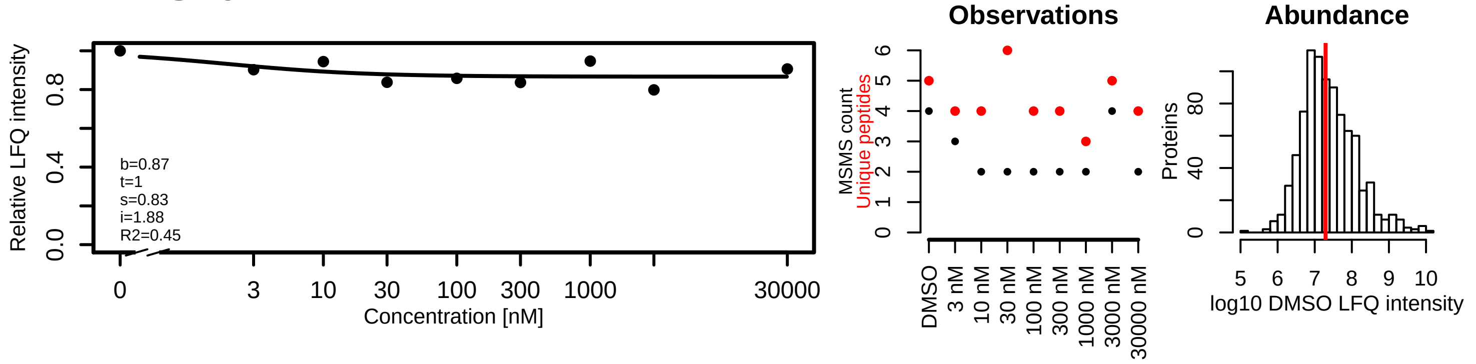

NCBP1

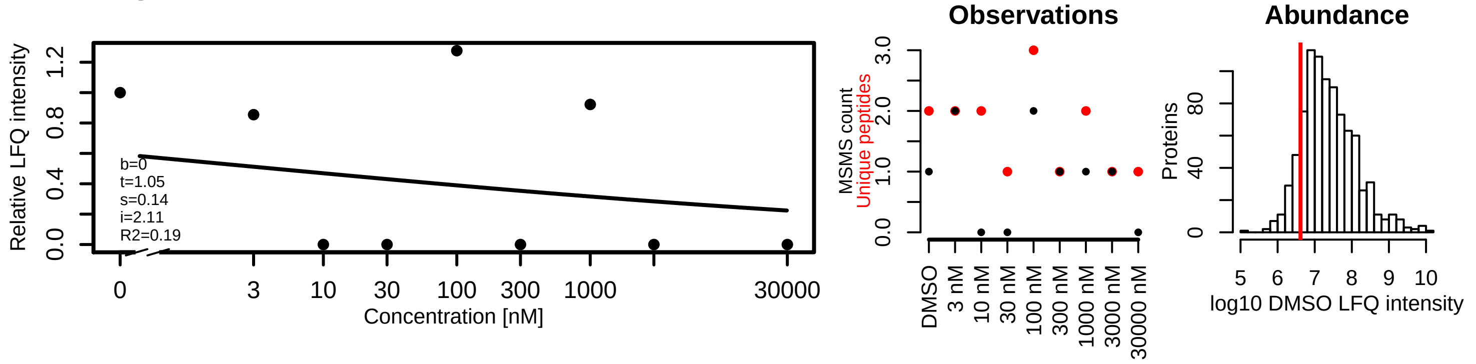

HAX1

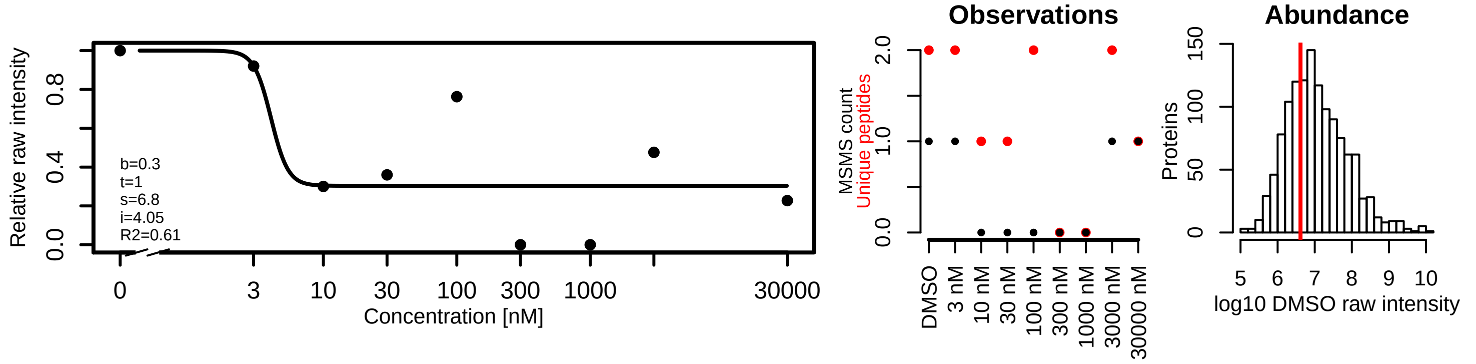

EXOC4

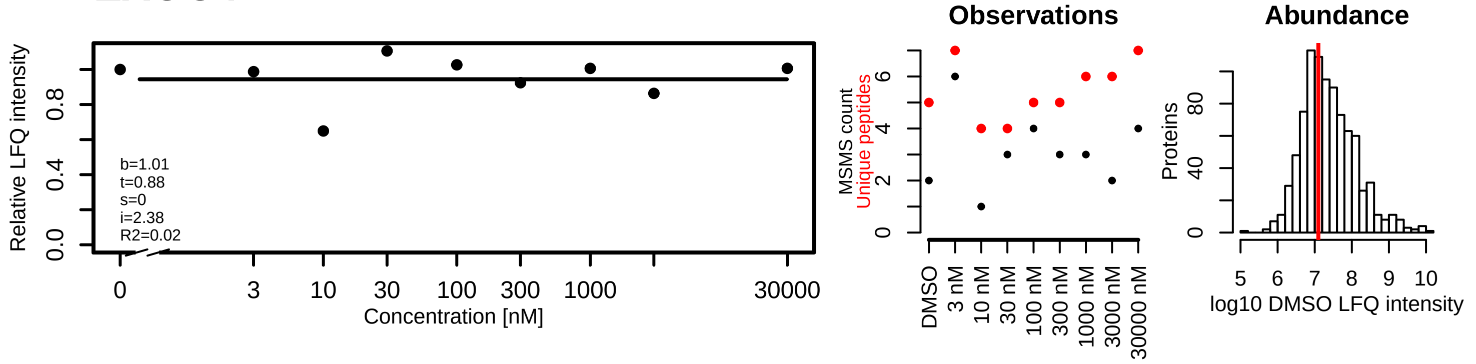

CDK1

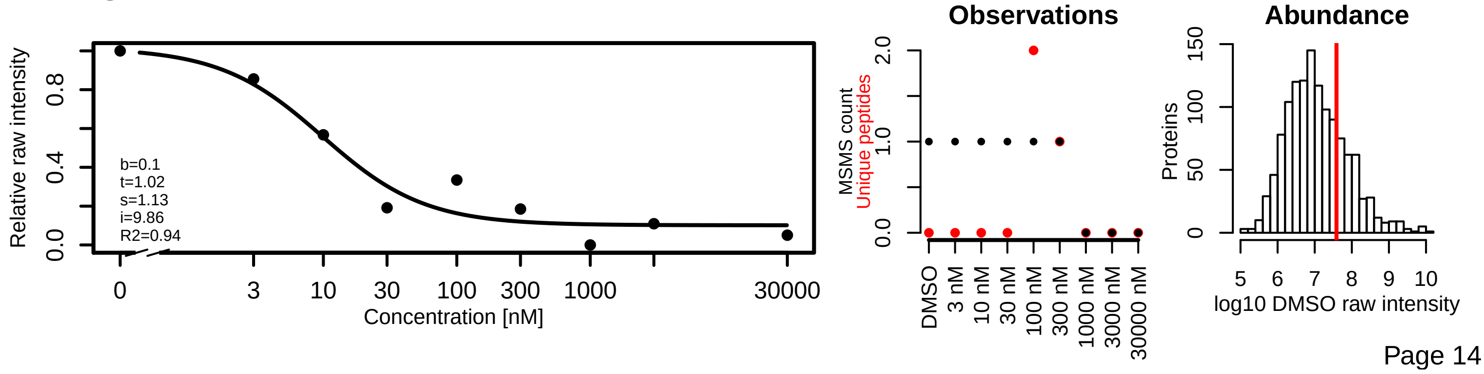

CEPT1

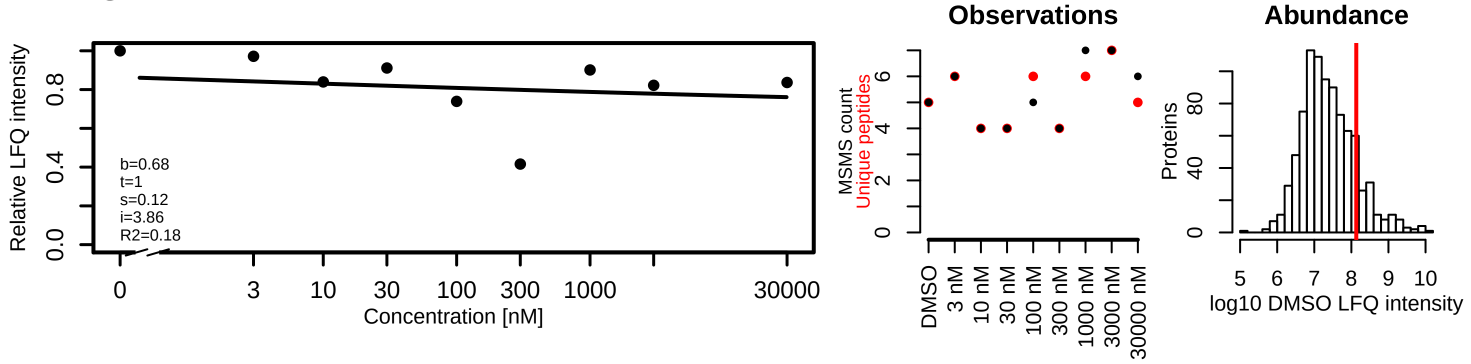

PGAP1

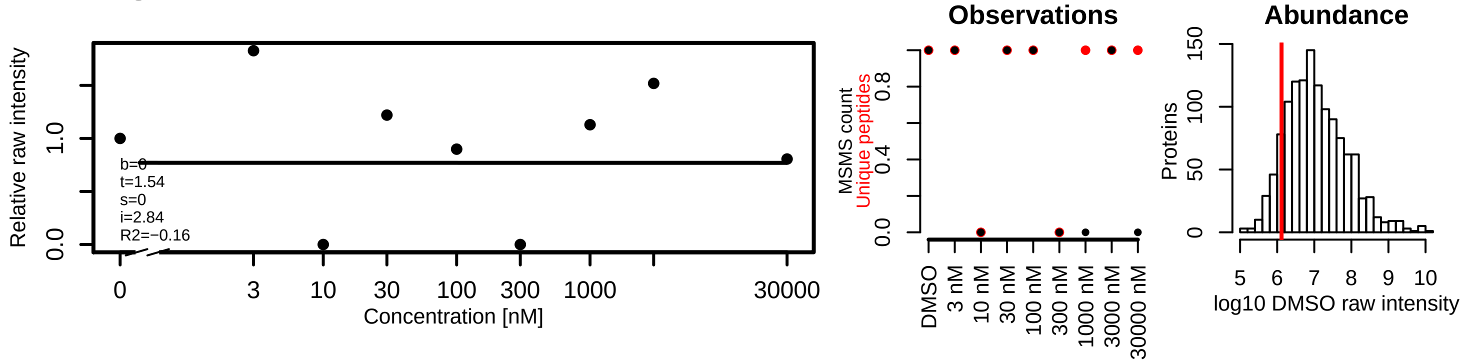

COG8

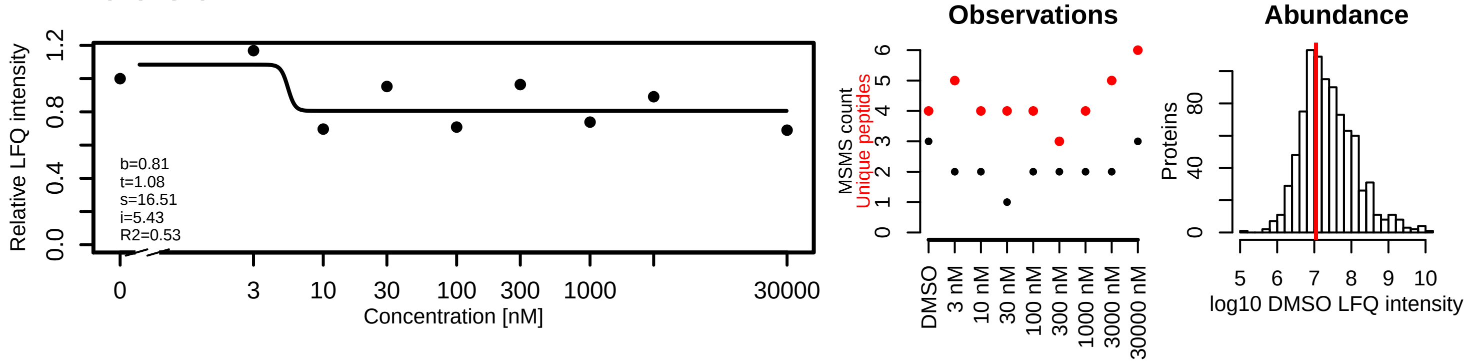

MPC2

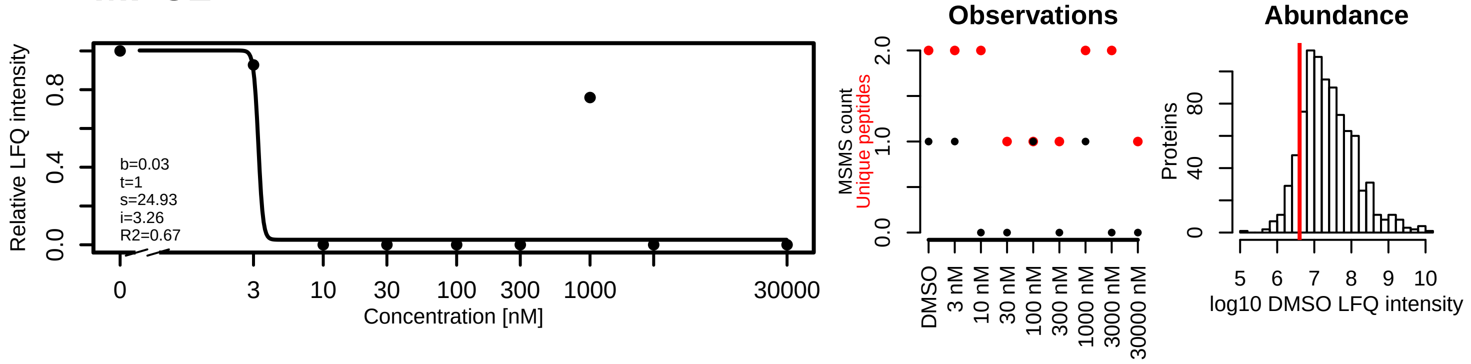

ACSL3

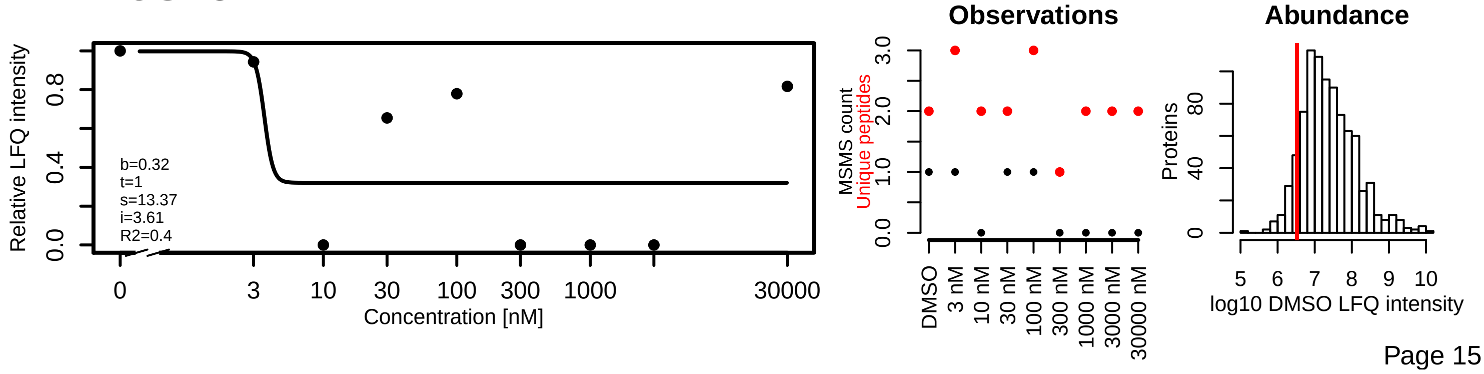

SPNS1

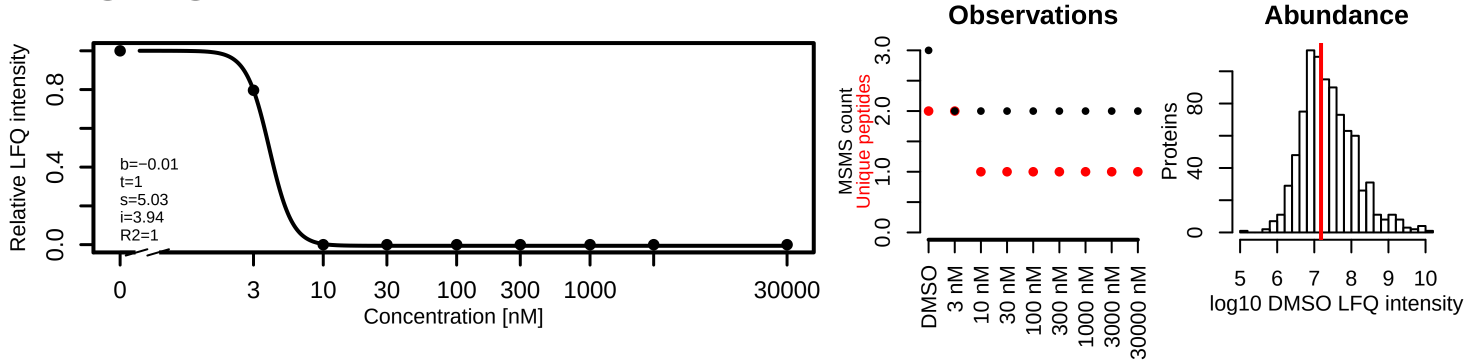

ABTB2

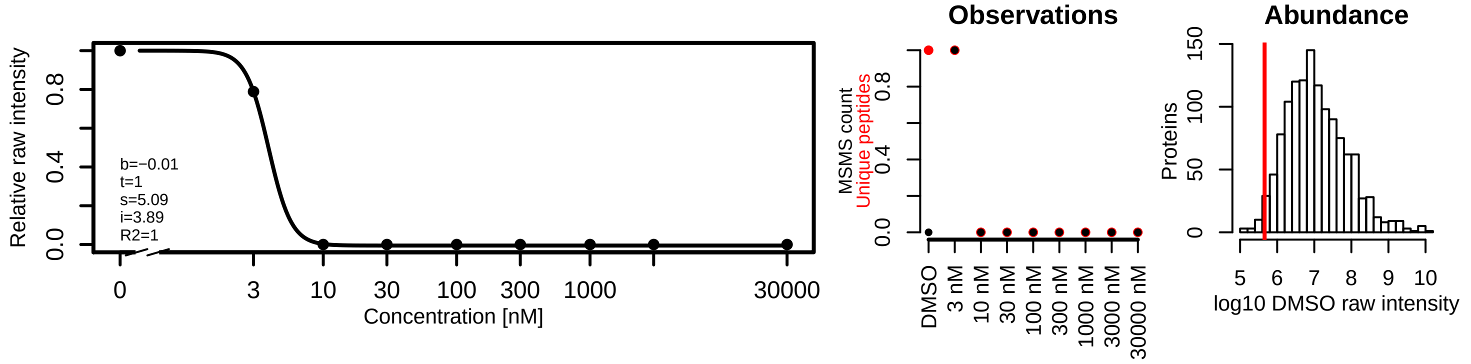

CASD1

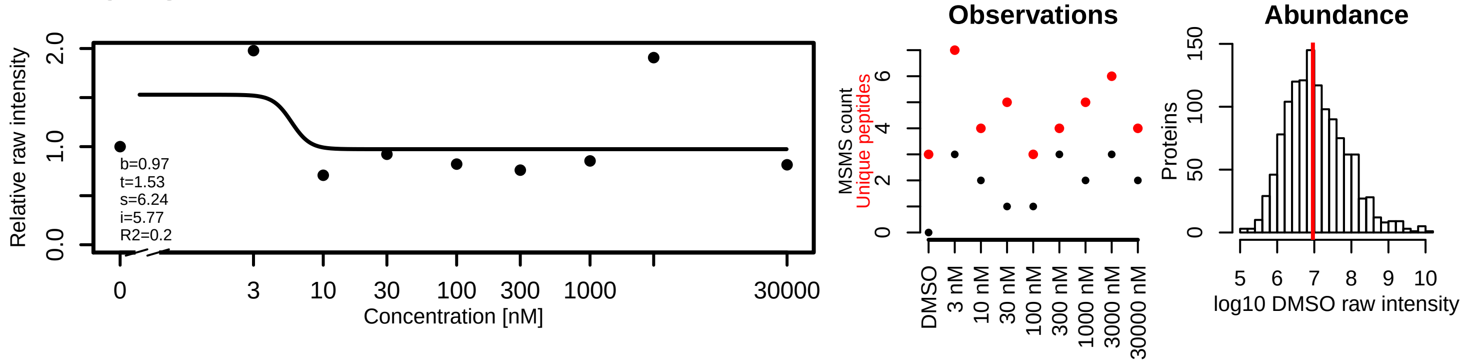

TBL2

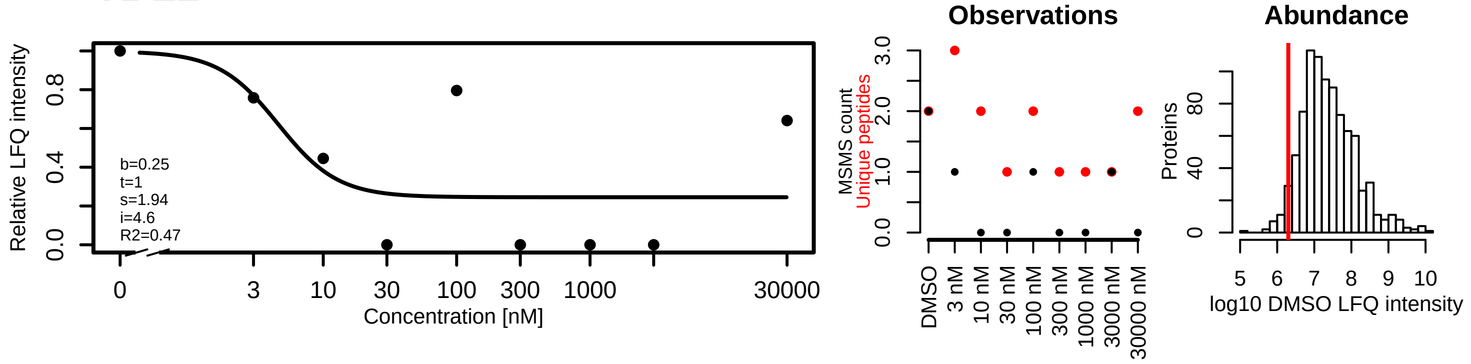

LETMD1

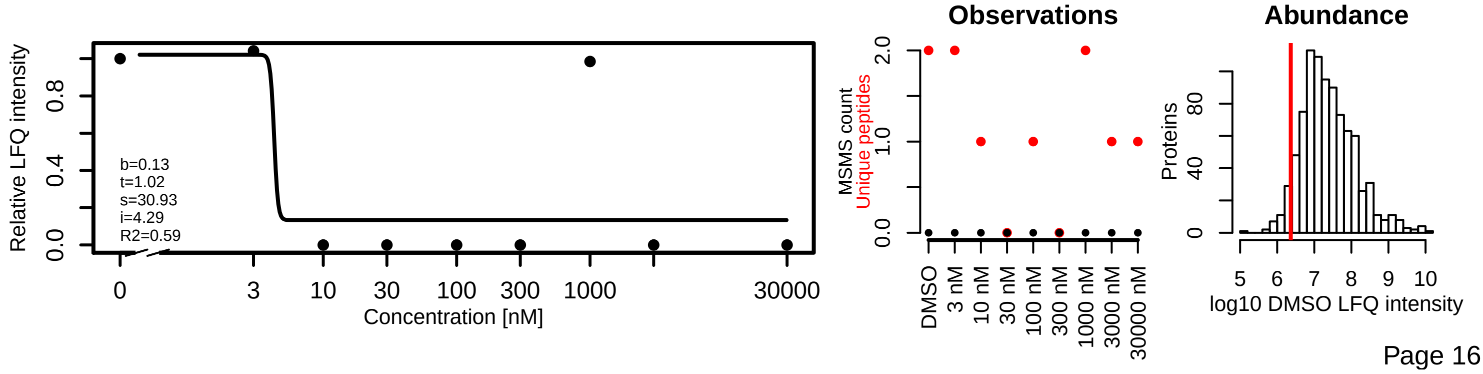

PTDSS1

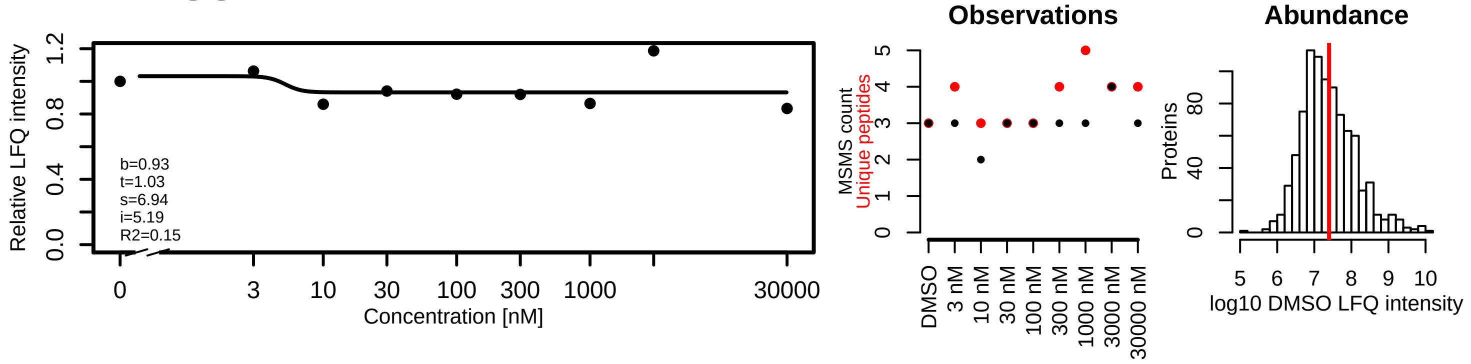

SLC7A11

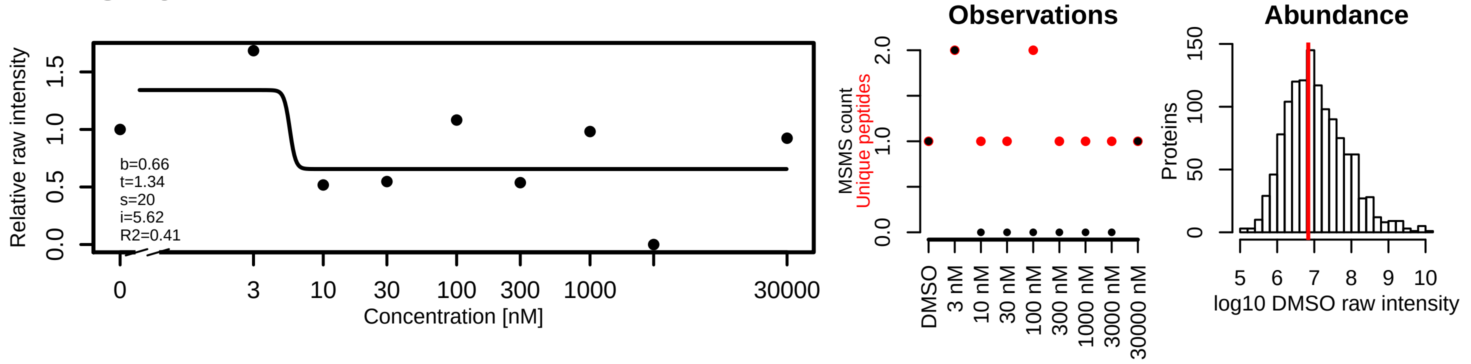

PGAM5

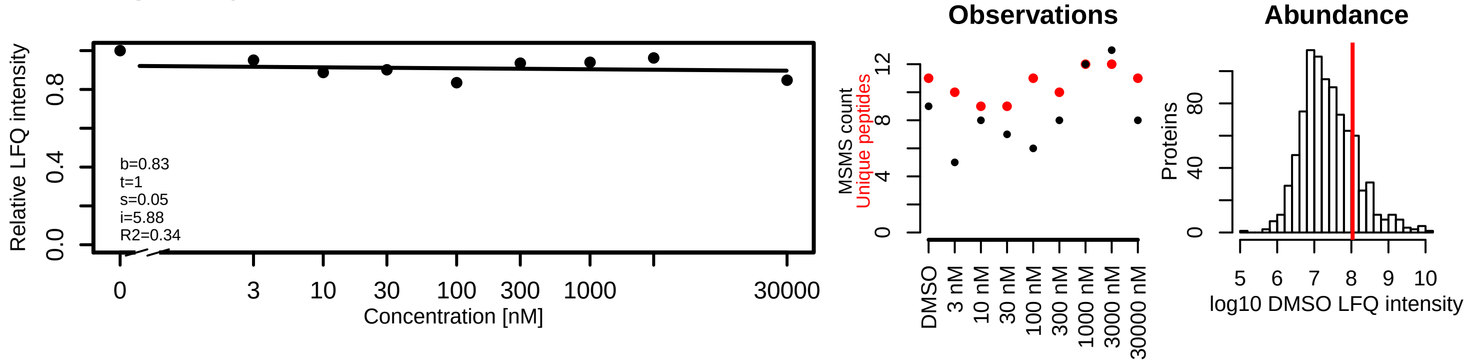

RPN1

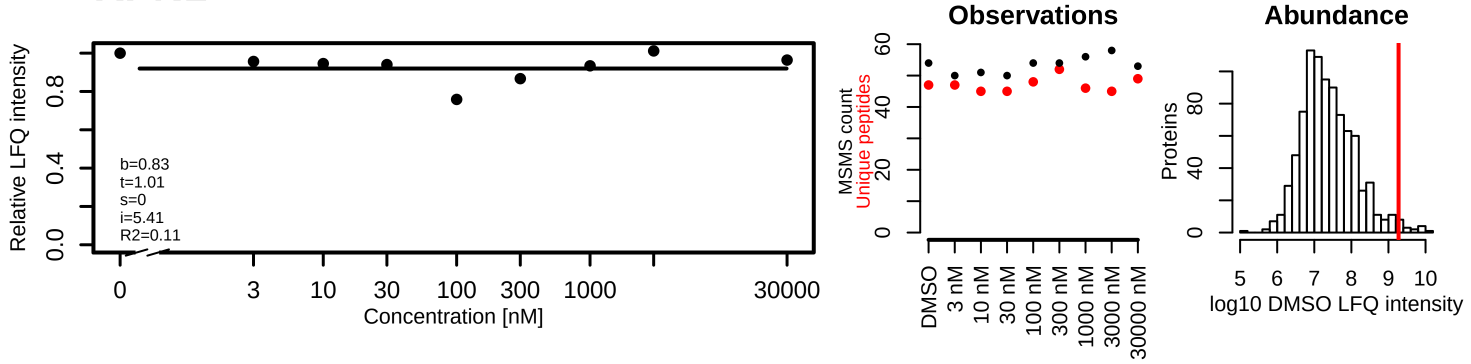

DERL2

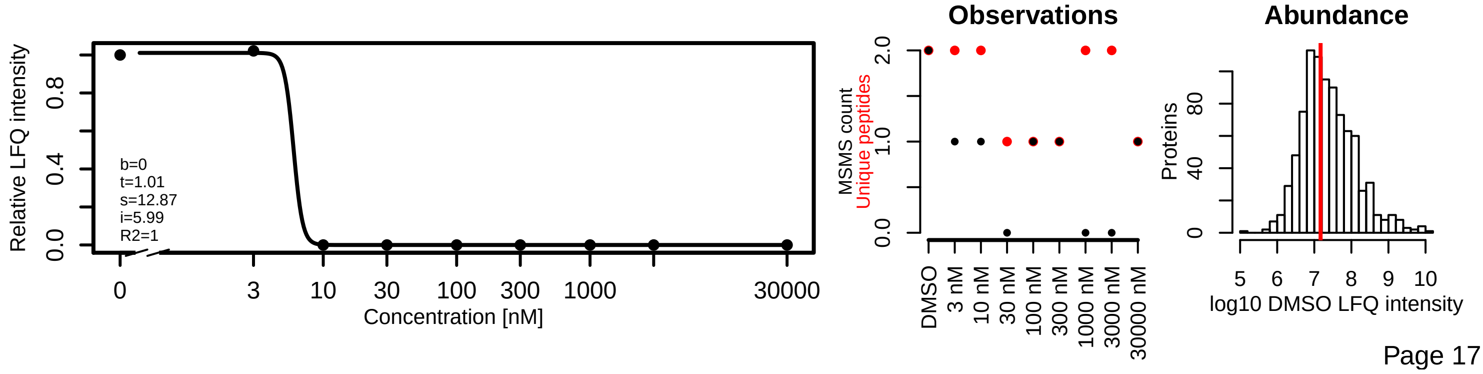

TMED5

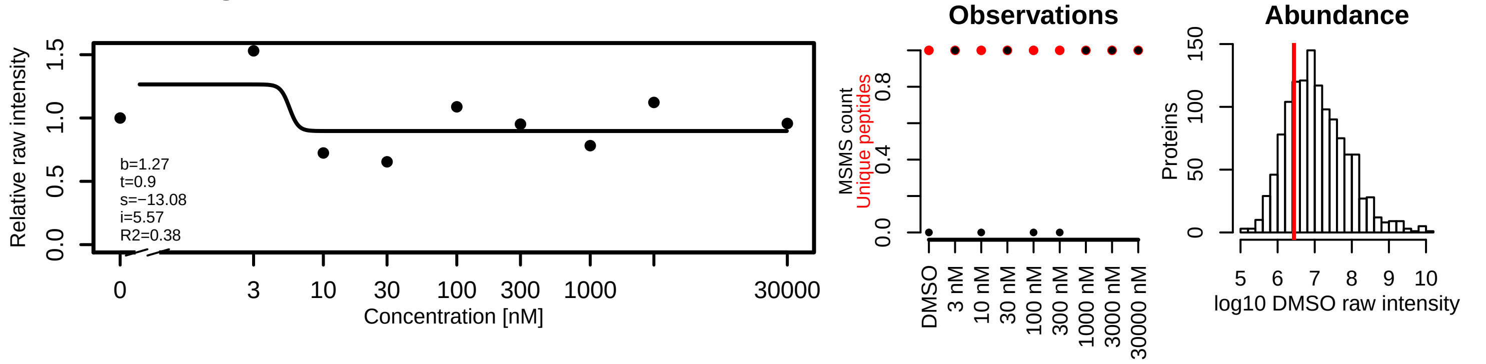

KIAA2013

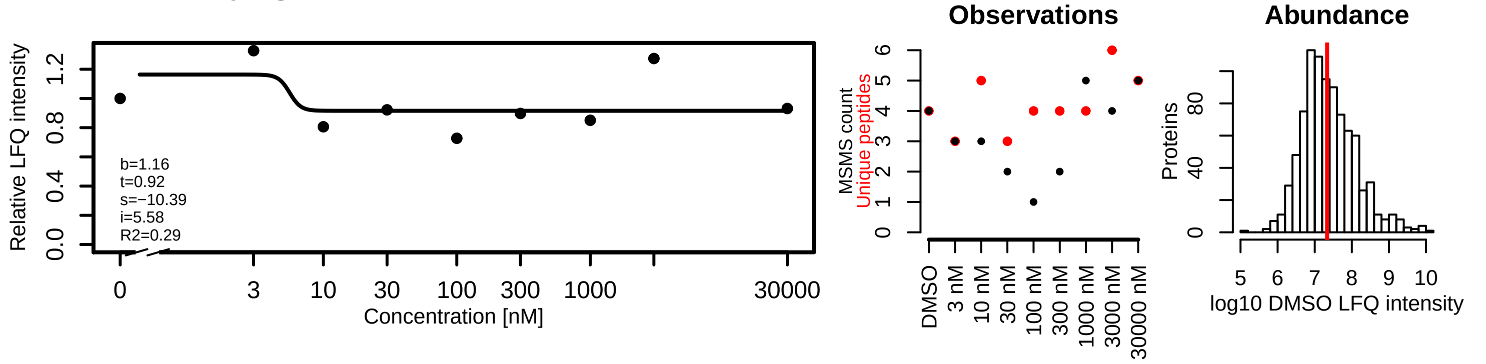

CERS6

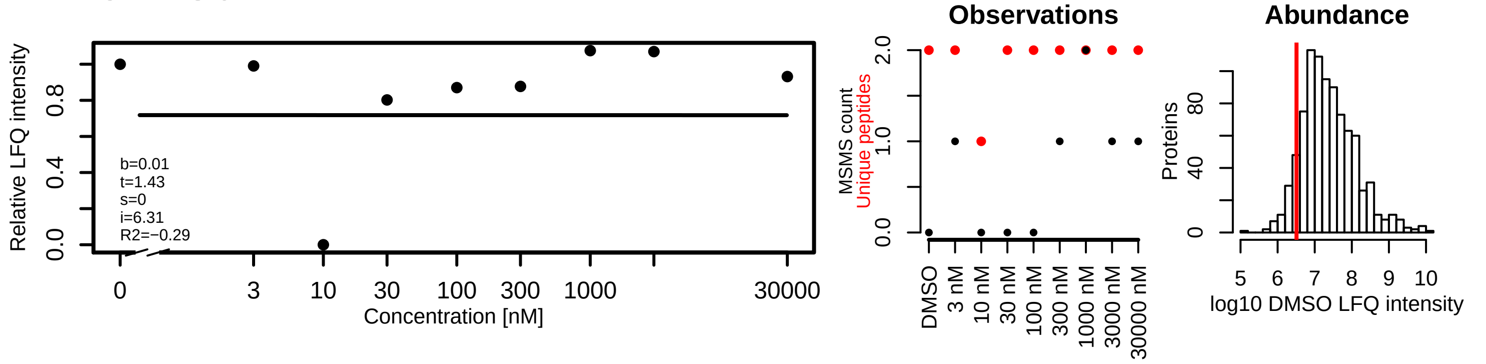

SLC2A1

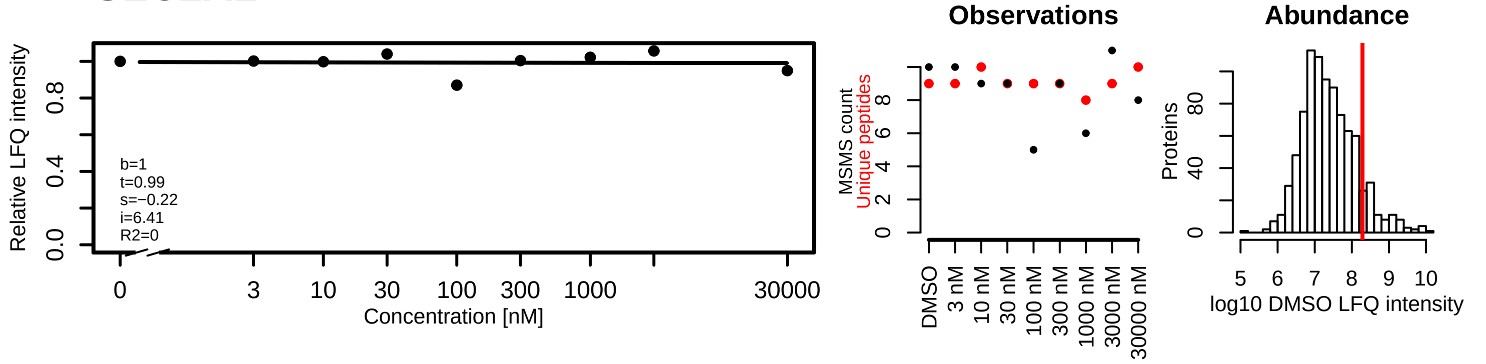

ATP1B1

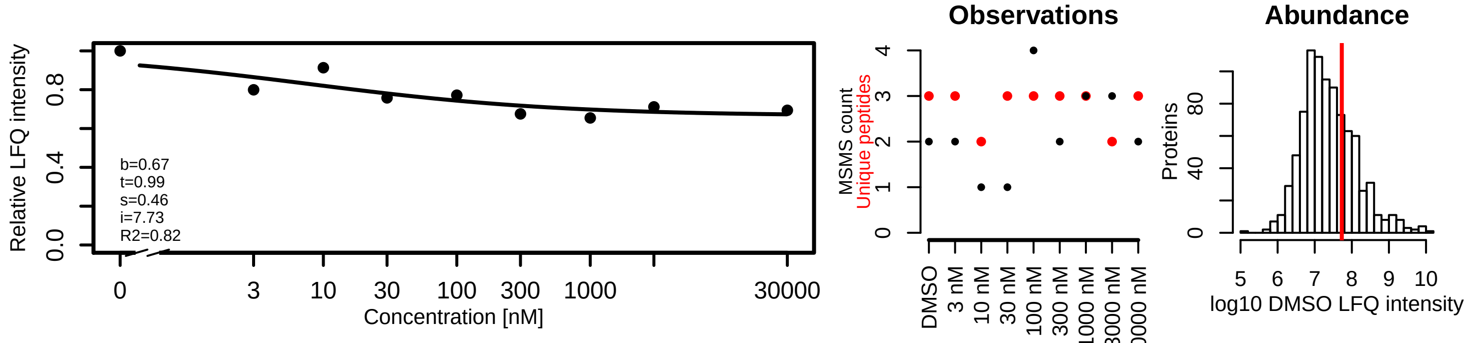

ATP5A1

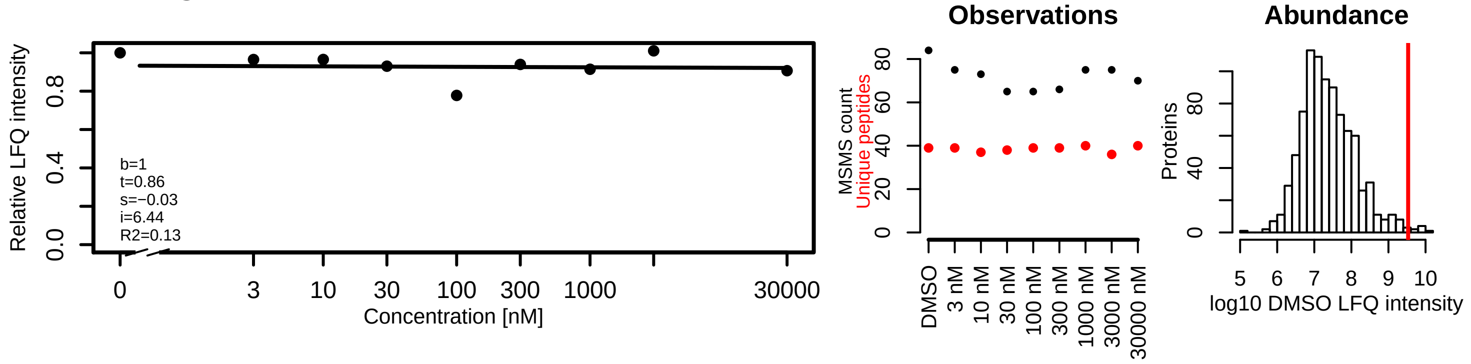

DNAJA3

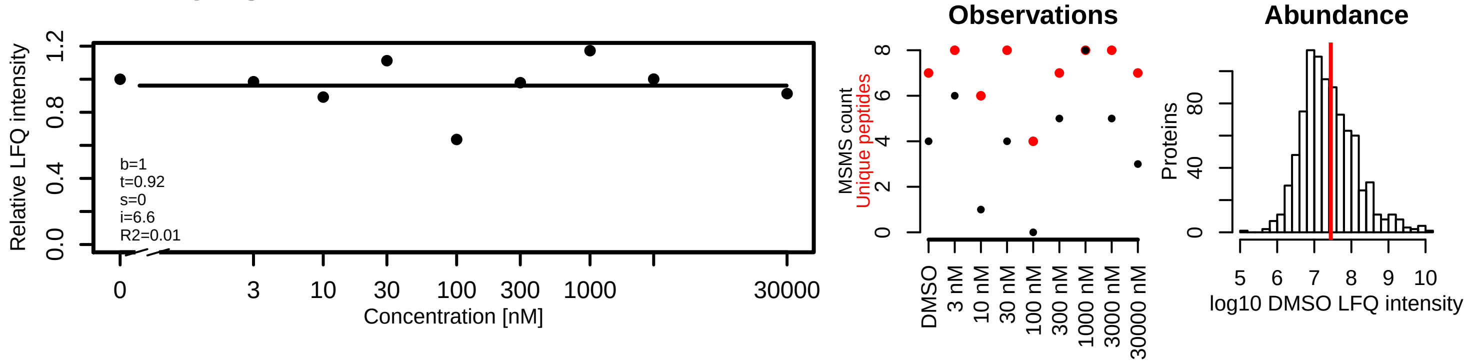

HEATR6

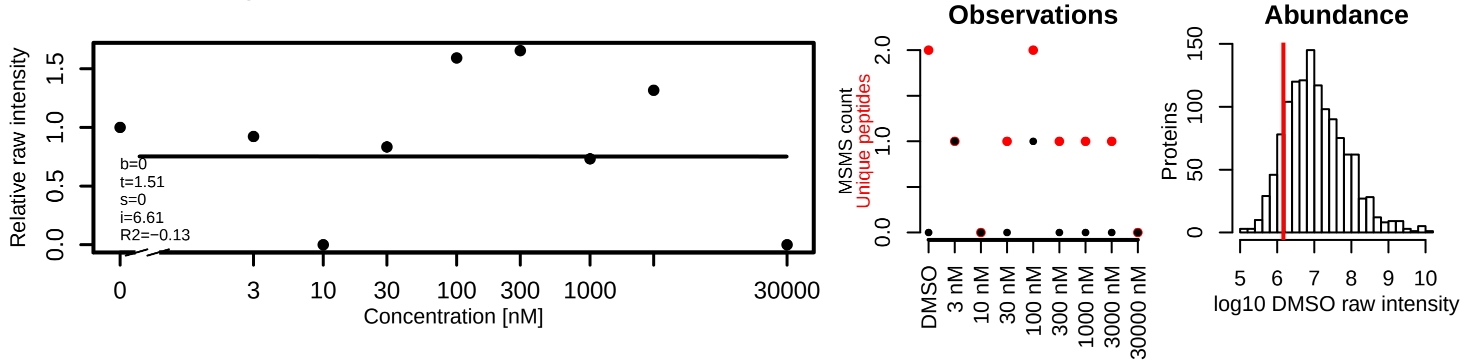

HSDL2

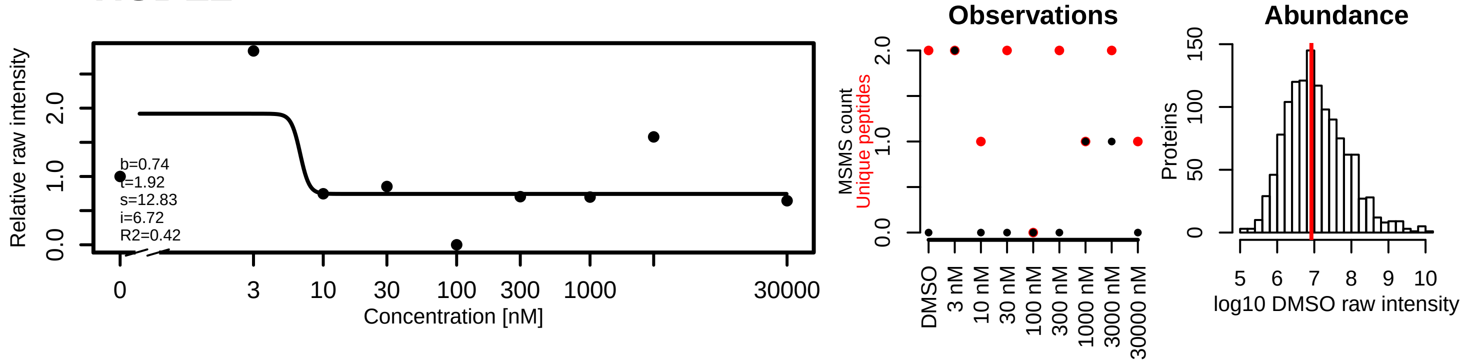

ABL1

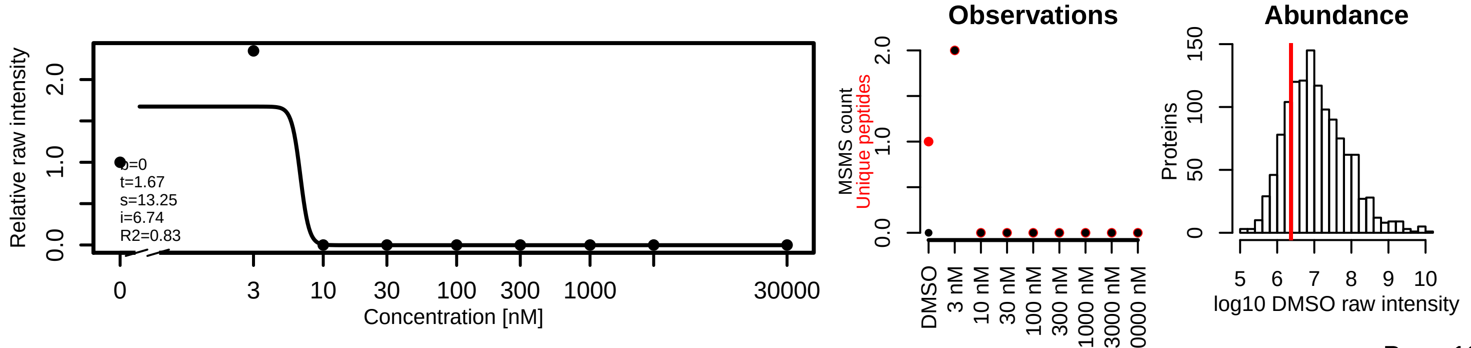

CNIH4

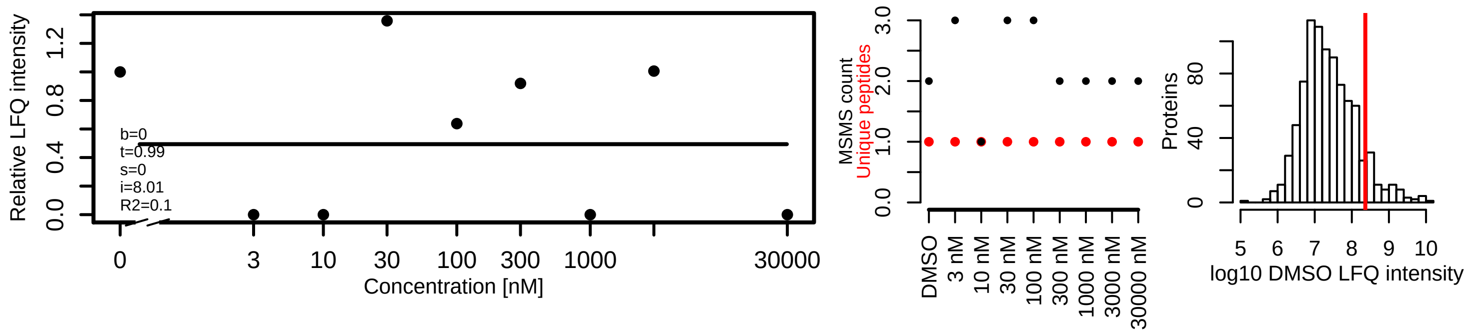

EFR3A

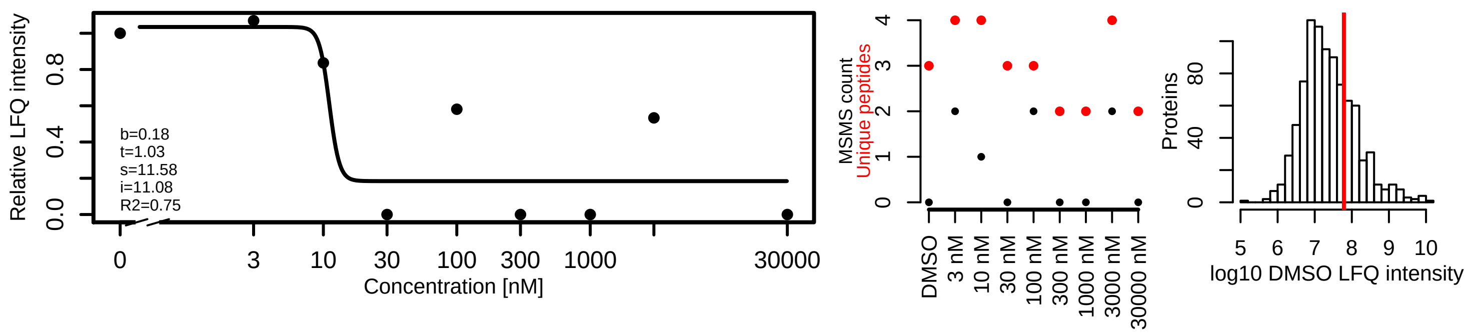

HDAC6

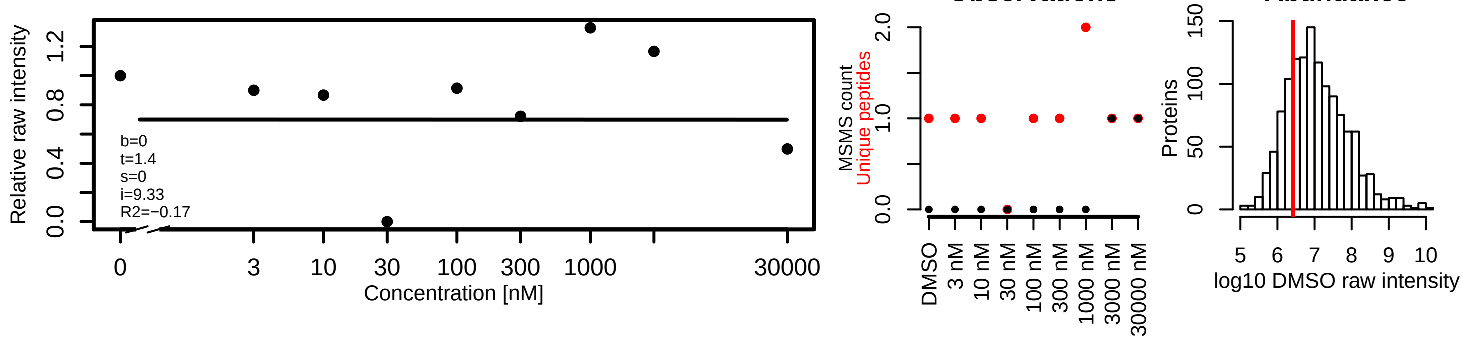

CLDN2

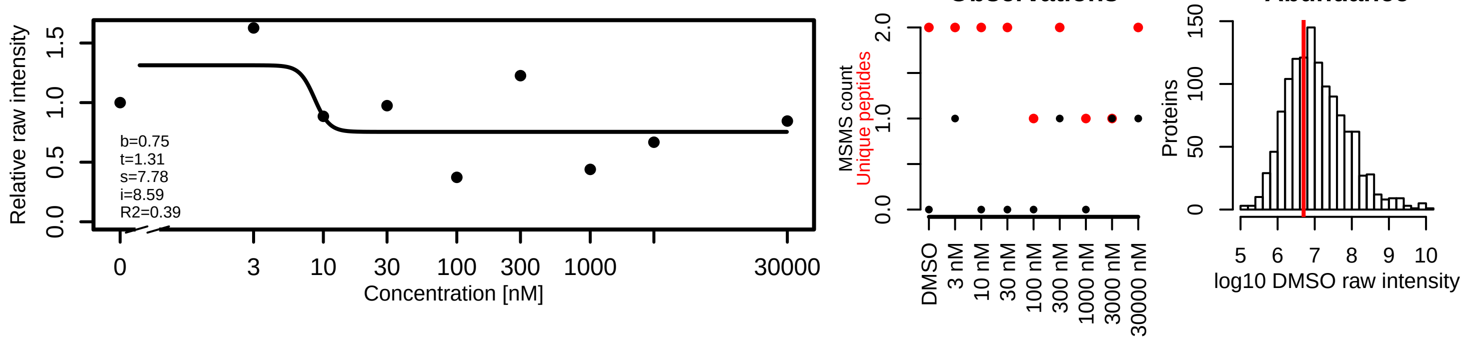

ARAF

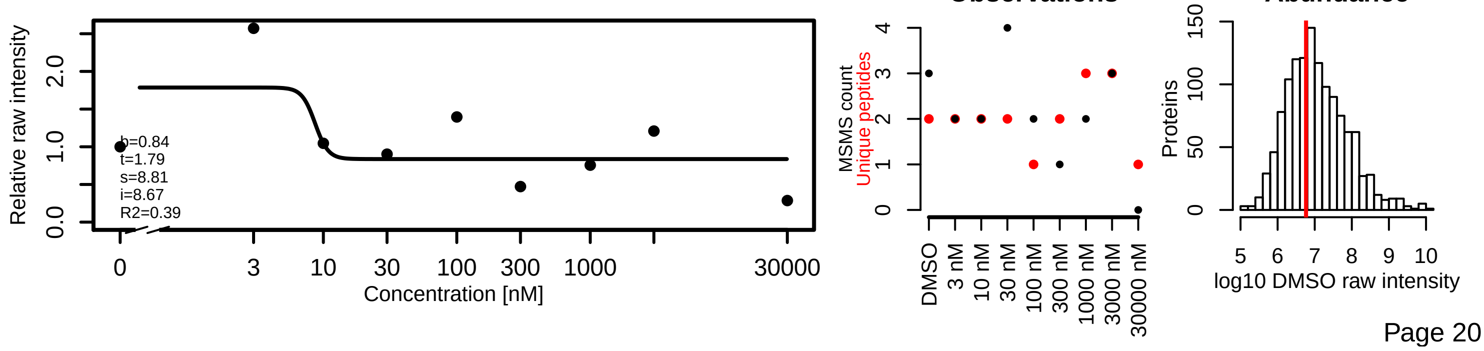

ADTRP

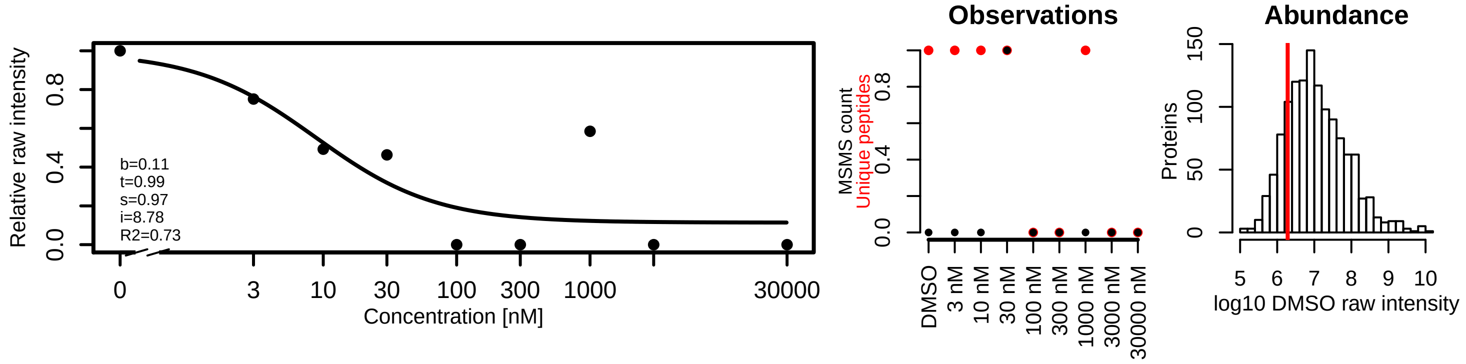

XXYLT1

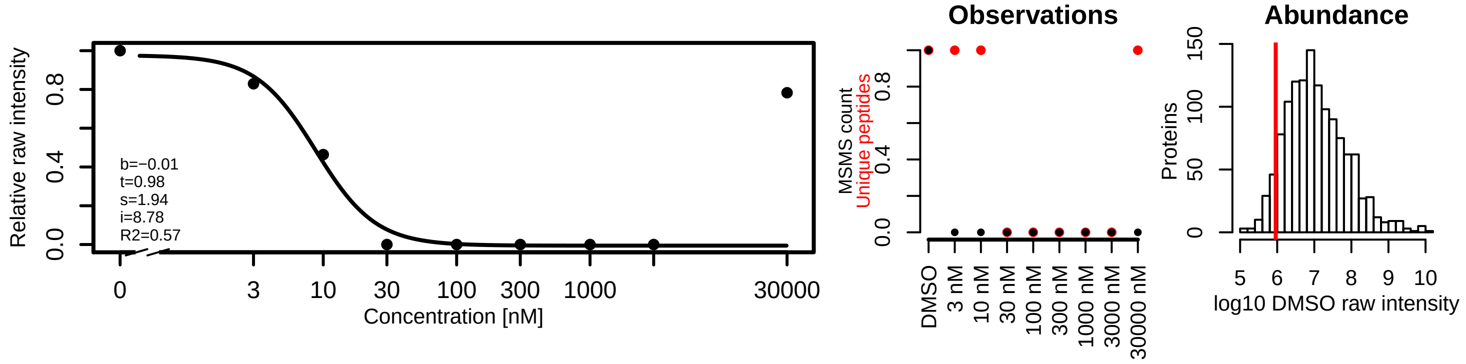

CANX

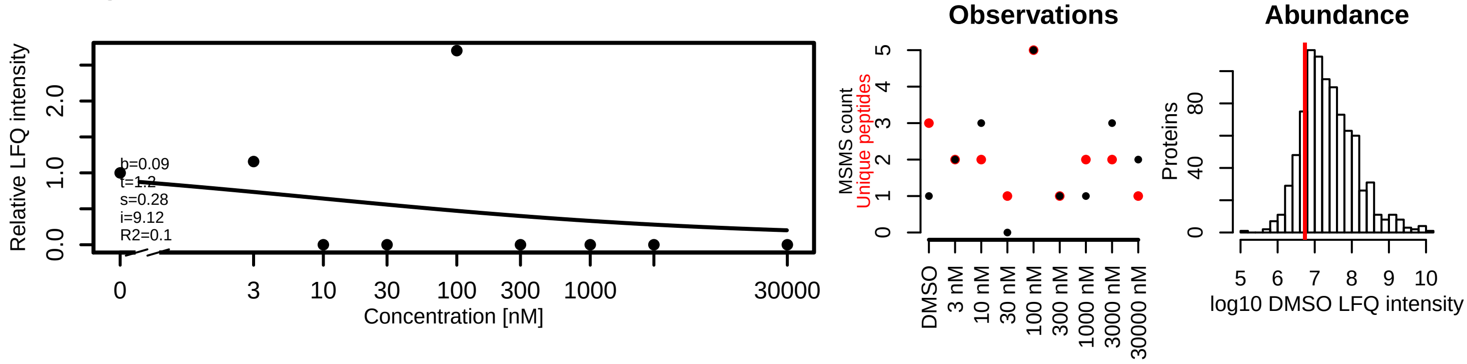

ITGA4

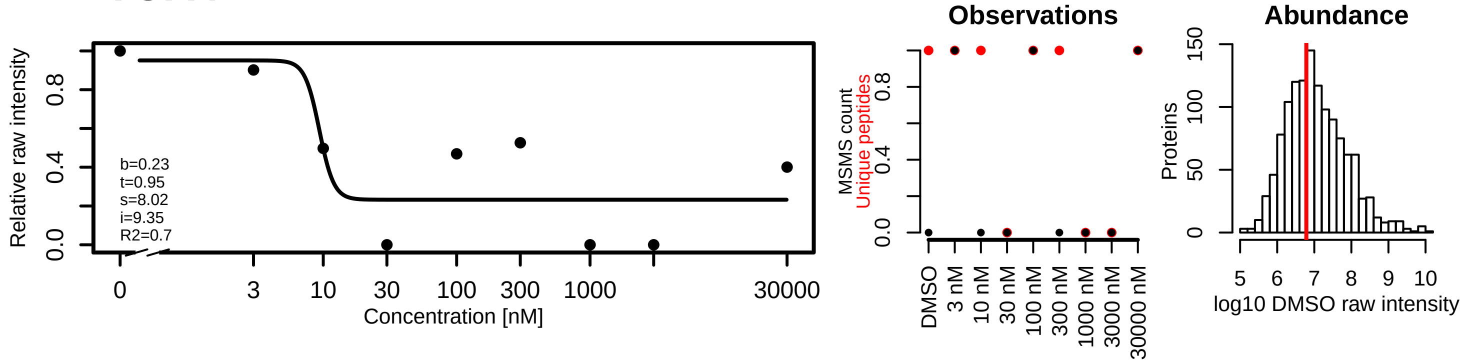

COX7A2

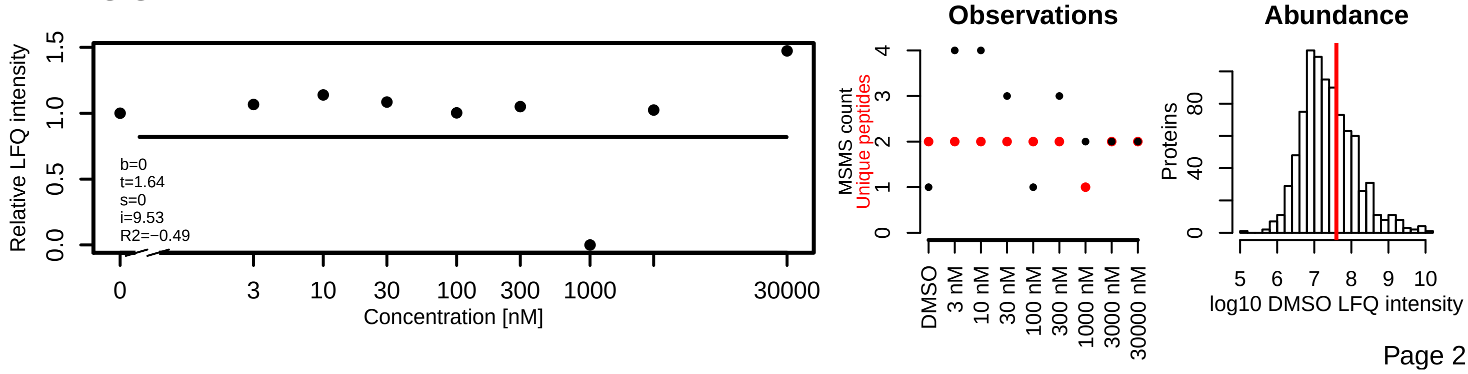

ADCK4

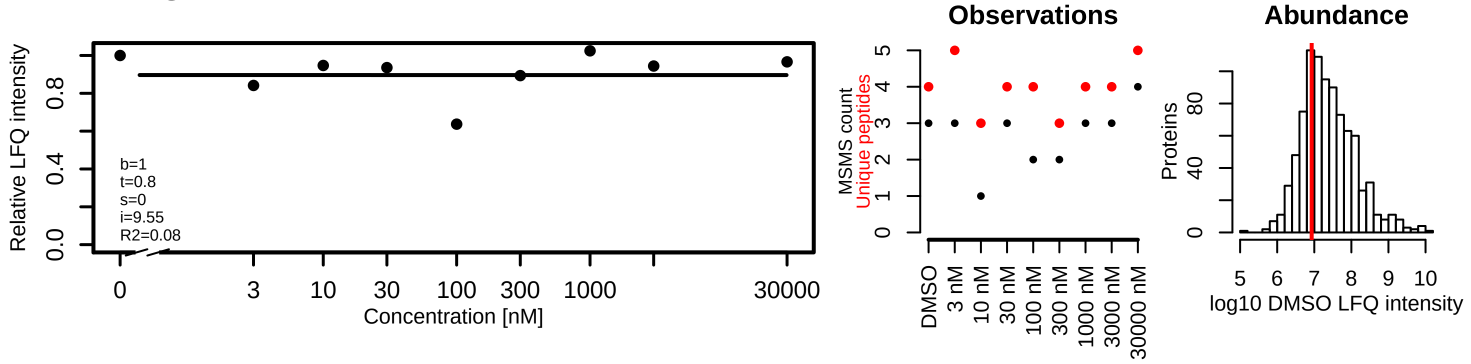

ABCA3

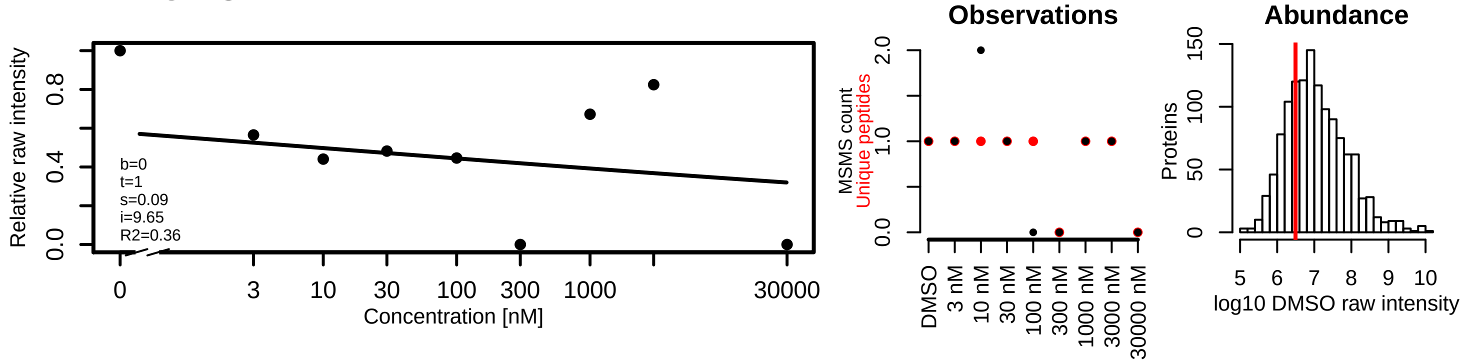

SLC18A3

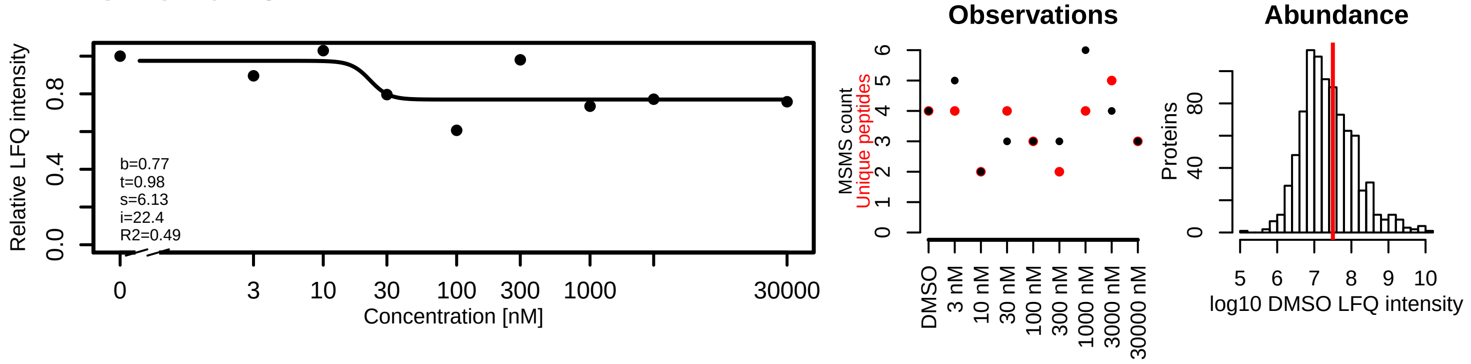

NUP93

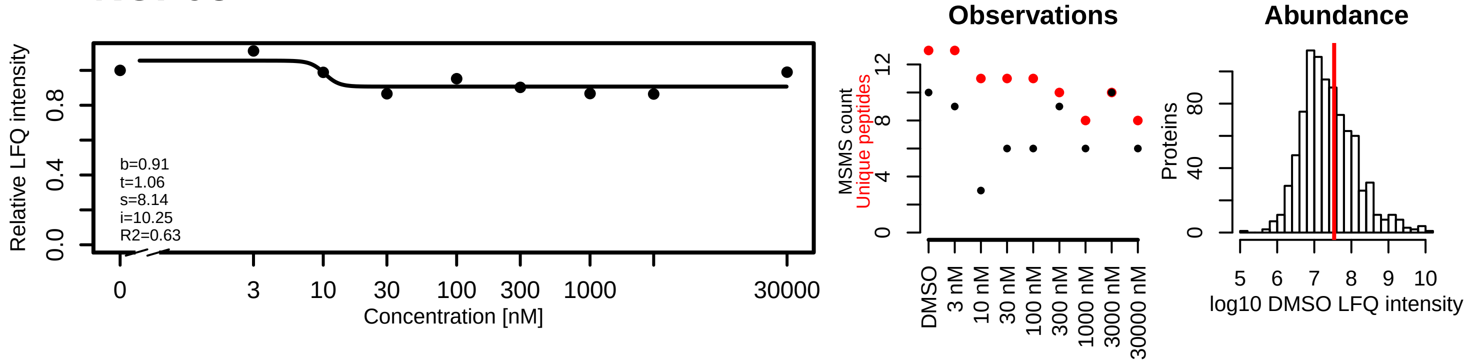

MRPL22

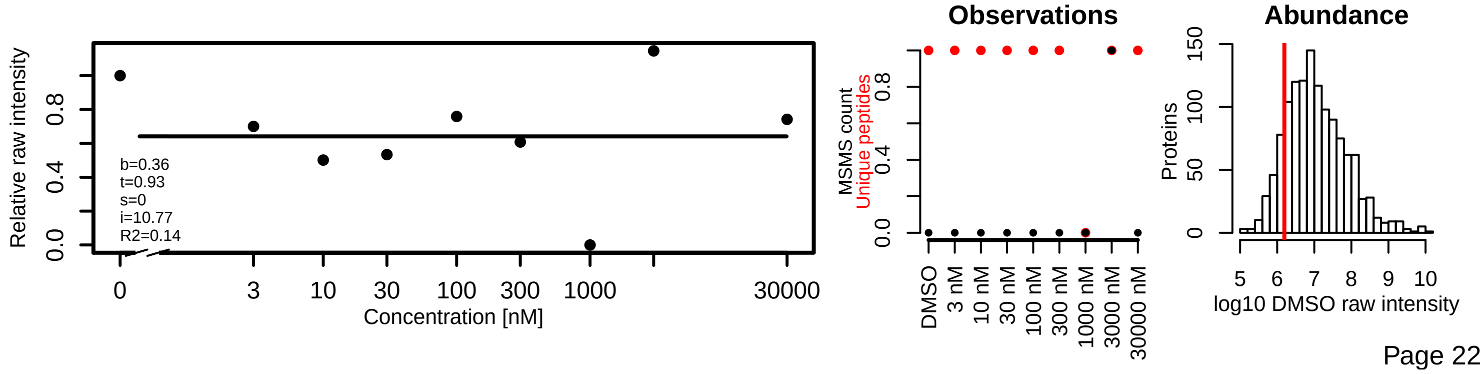

TMEM43

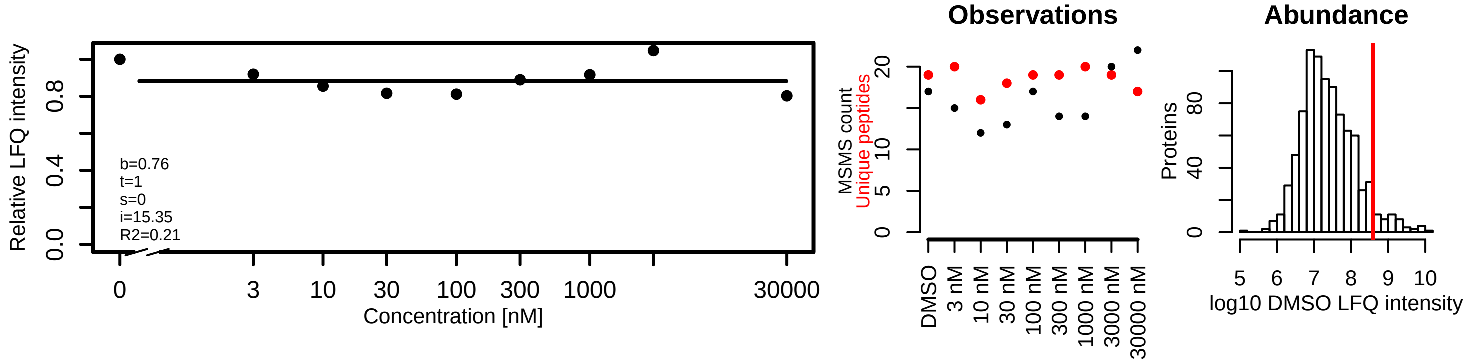

LYN − P07948−2;P08631−3;P08631−2;A8K4G3;P08631−4;H0Y3C5;P08

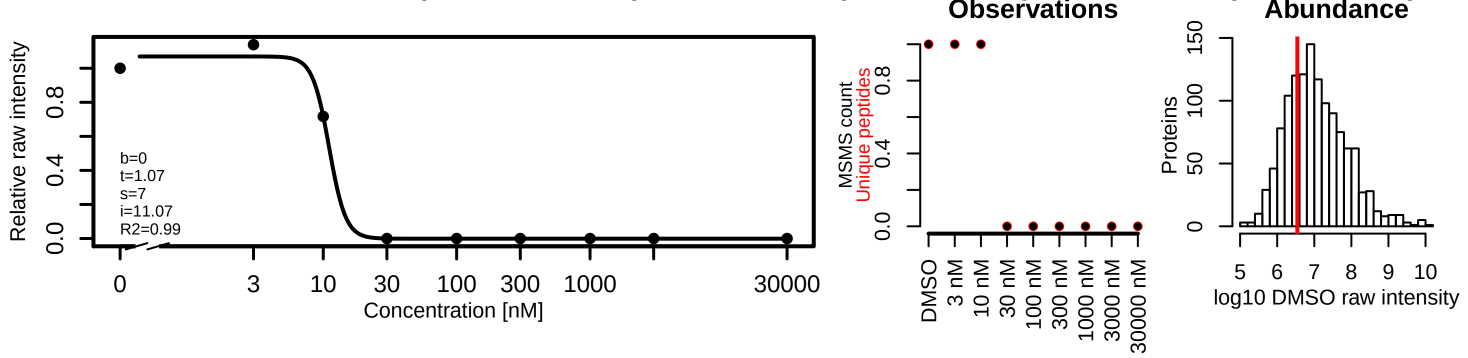

SLC15A4

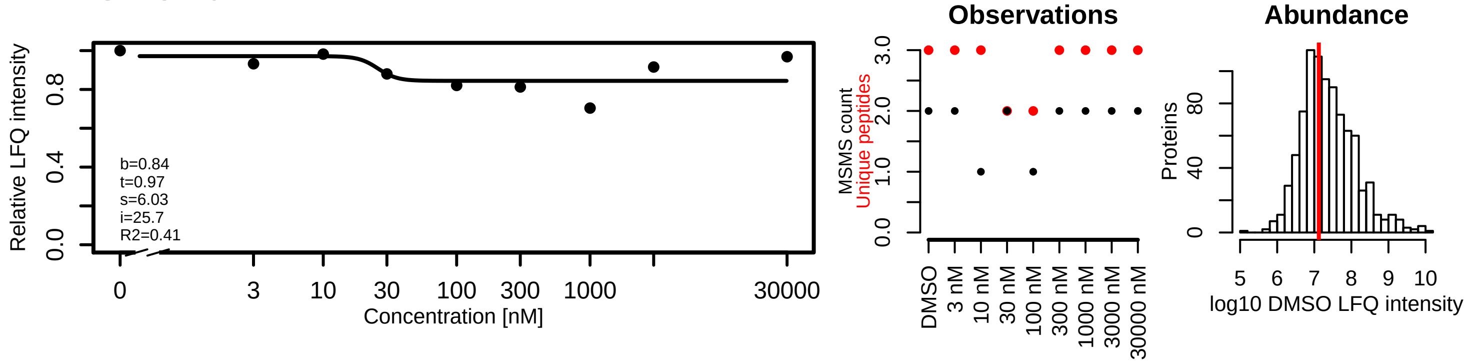

SLC25A12

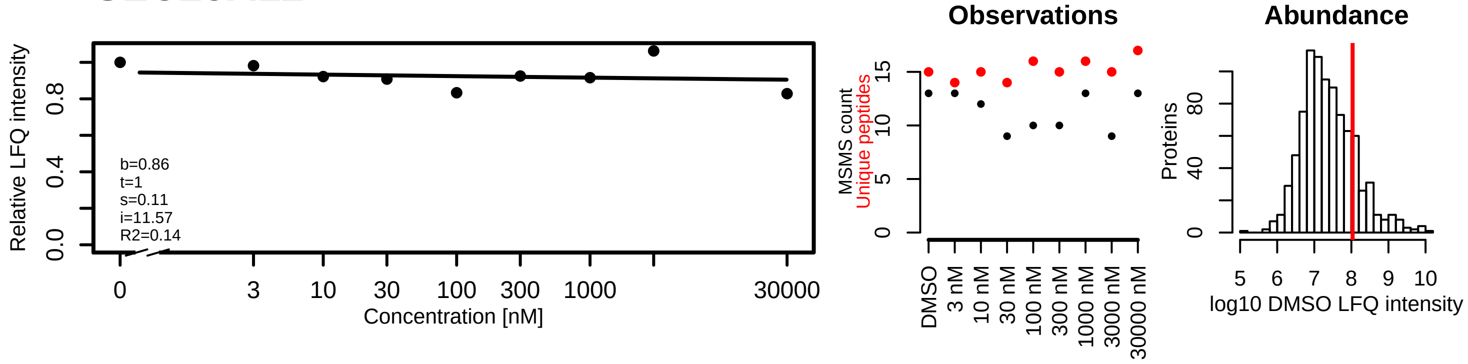

RNF170

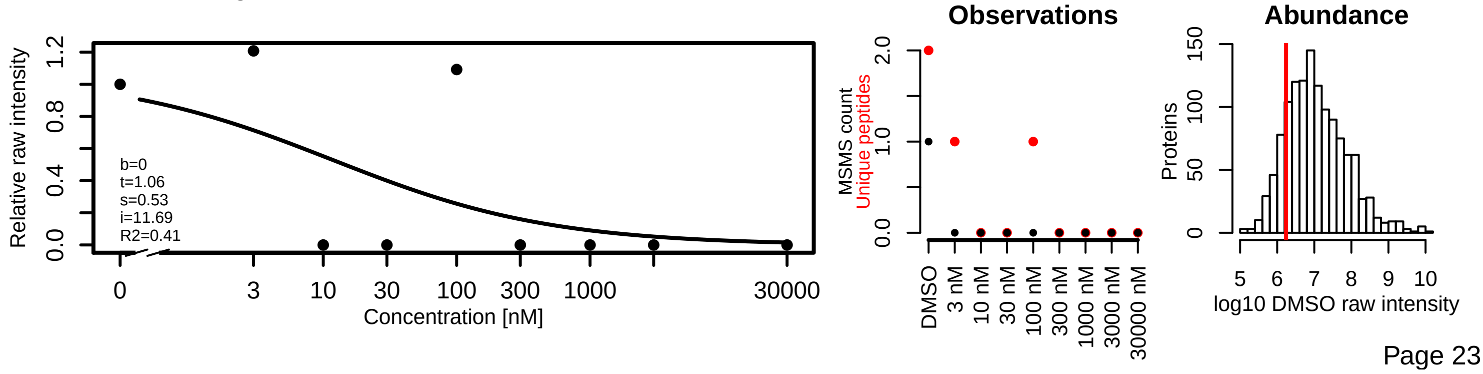

TEX261

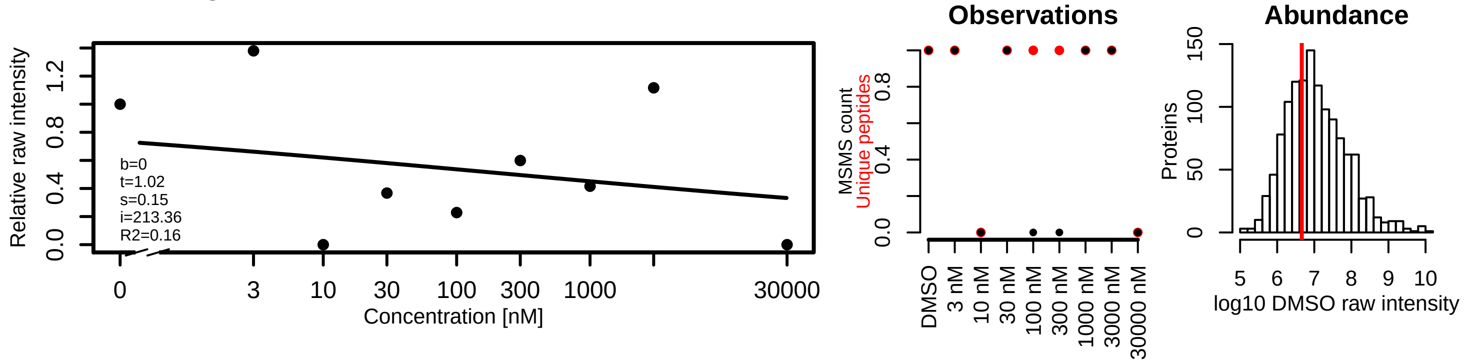

JUP – P14923;C9JTX4;C9J826;C9JK18;C9JKY1

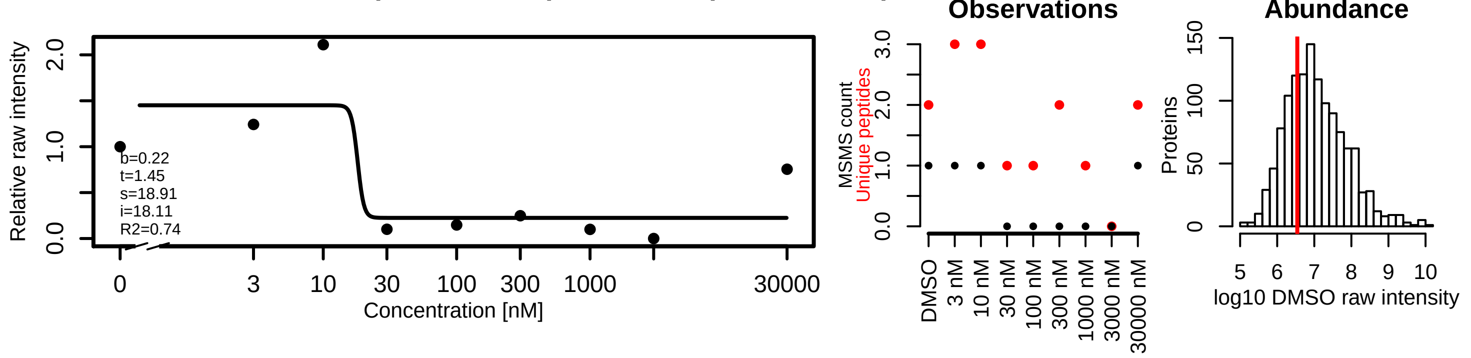

ERMP1

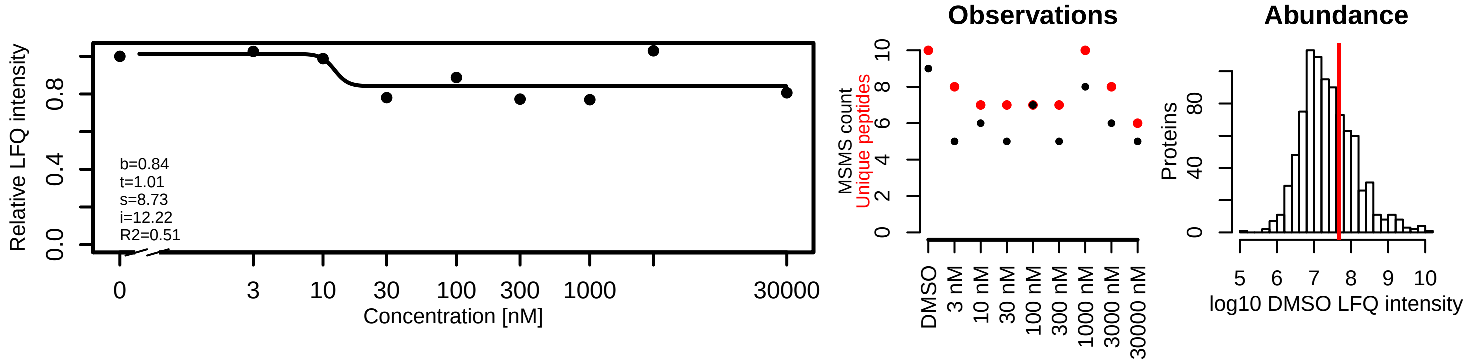

PIGQ

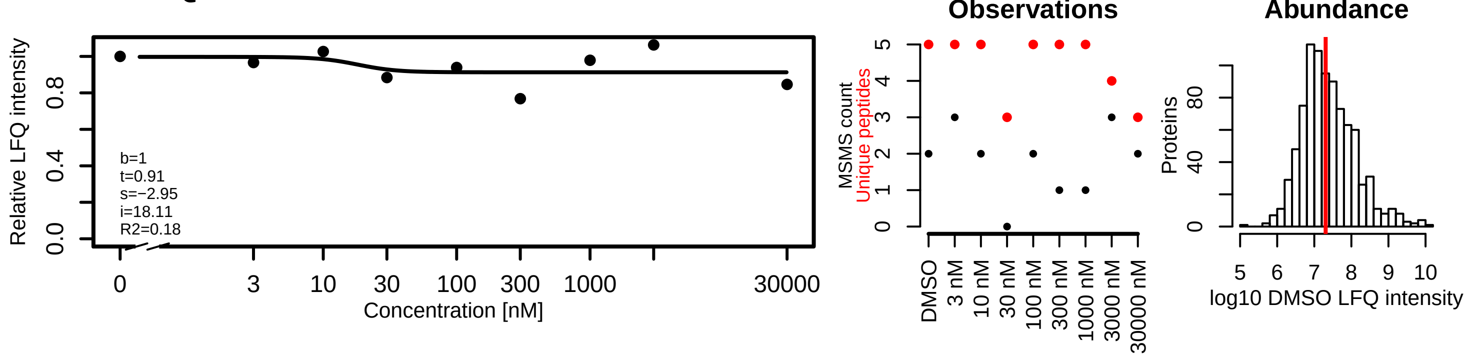

DNAJC11

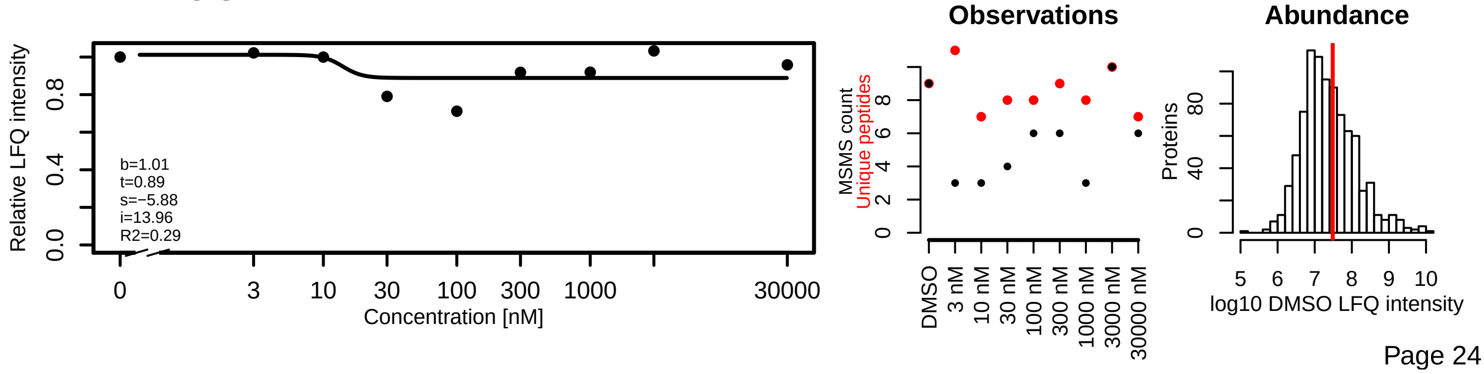

ERAP1

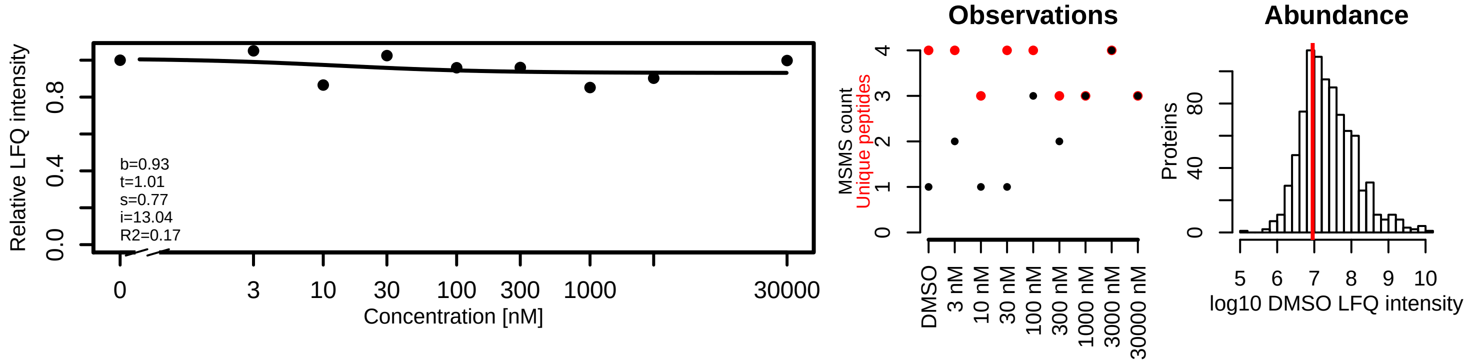

DGAT1

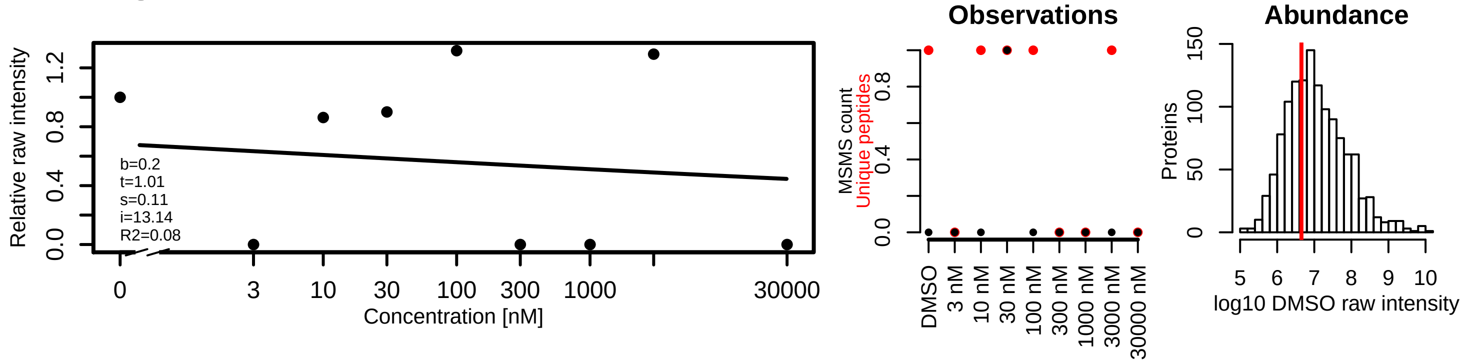

PSMA2

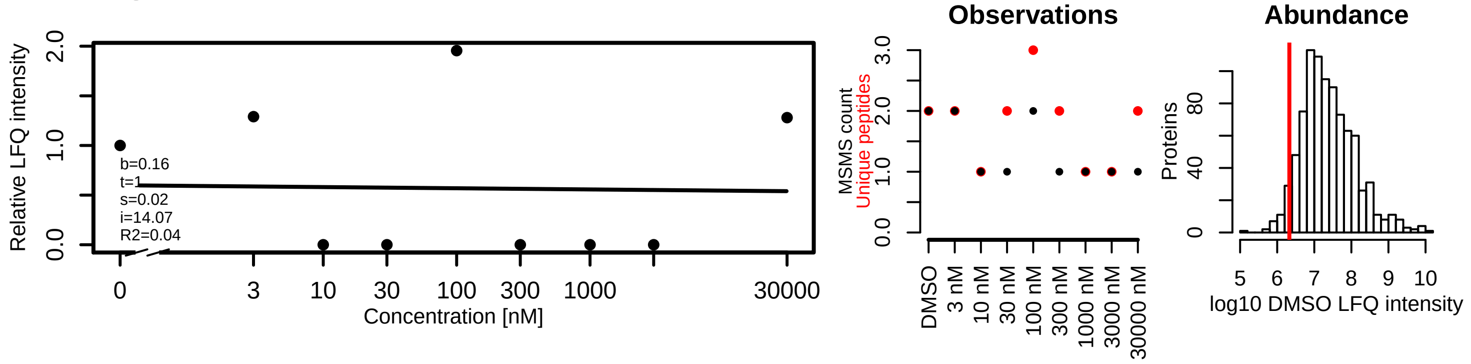

EMC8

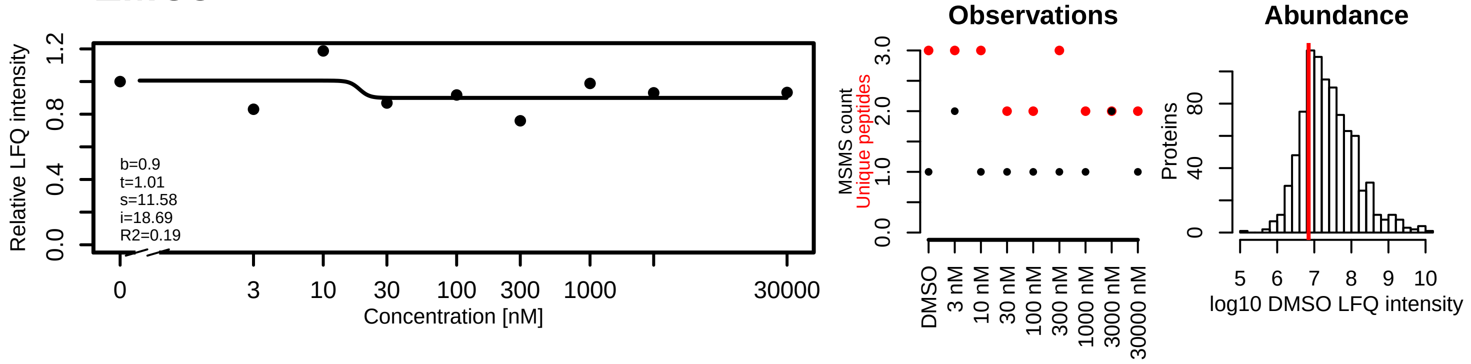

LMAN1

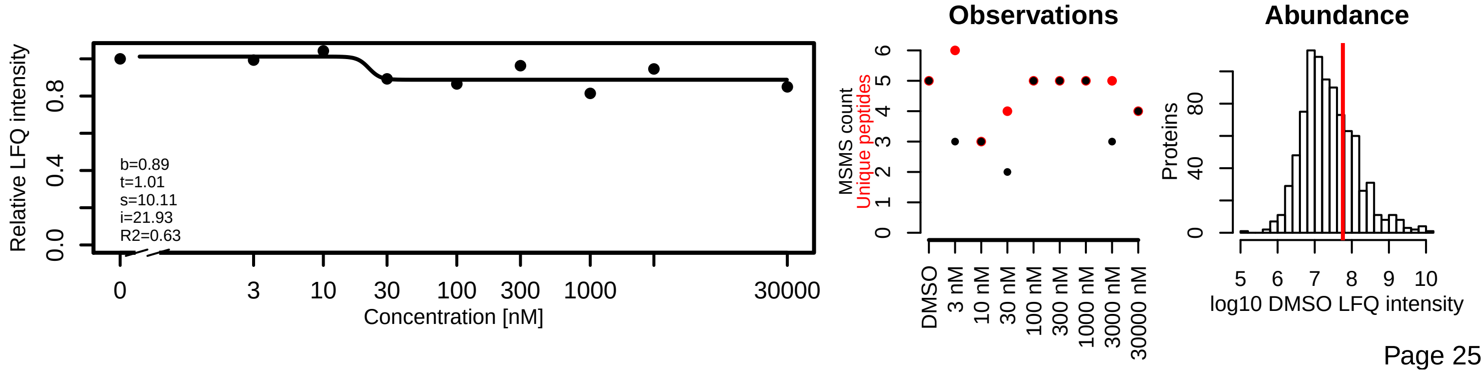

COX5B

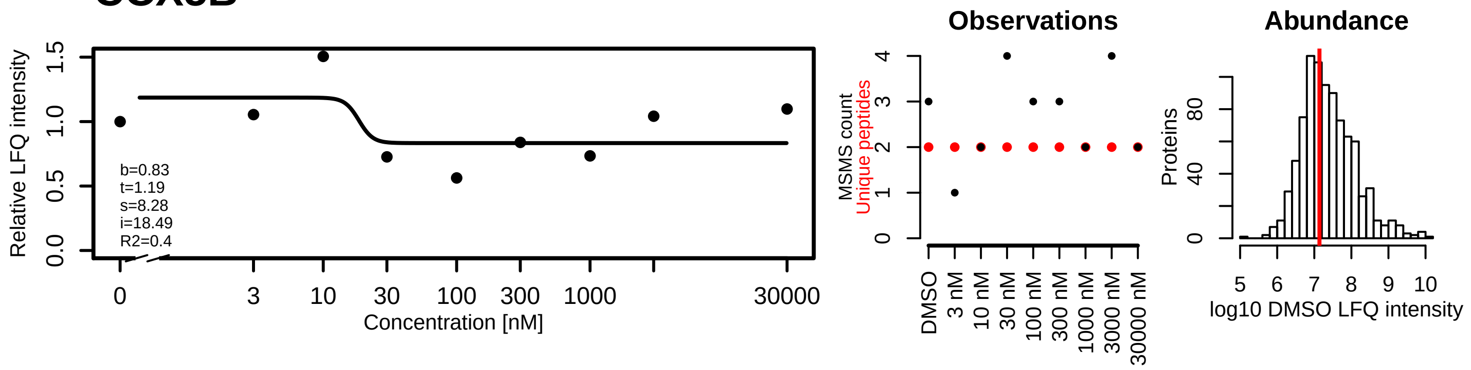

P4HA1

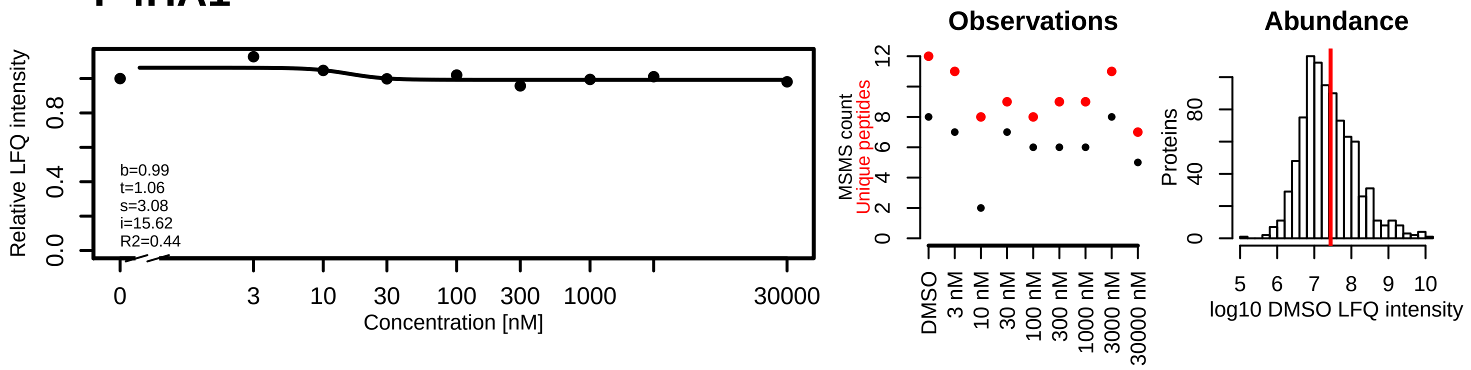

HSD17B2

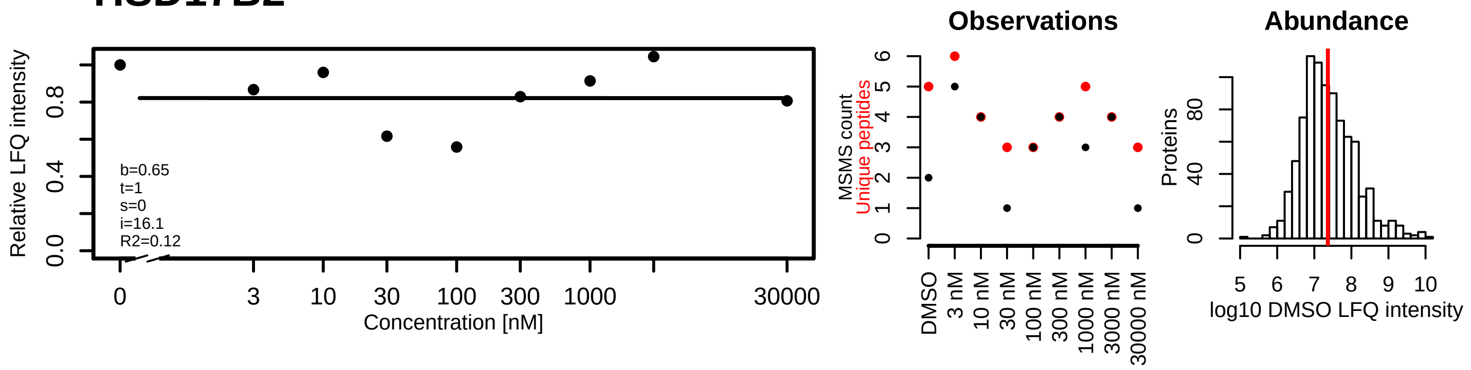

CYP27A1

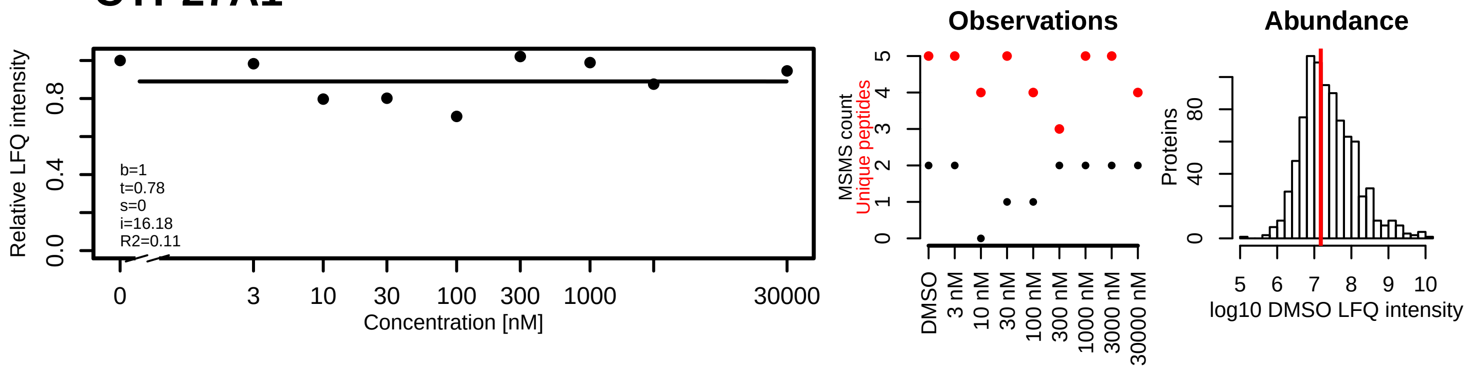

ITGB4

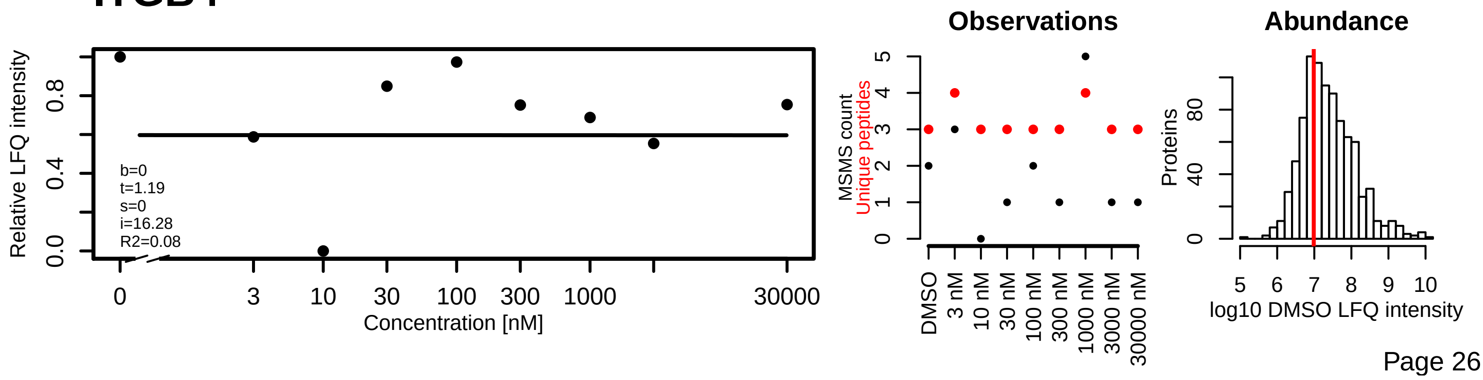

TMEM63B

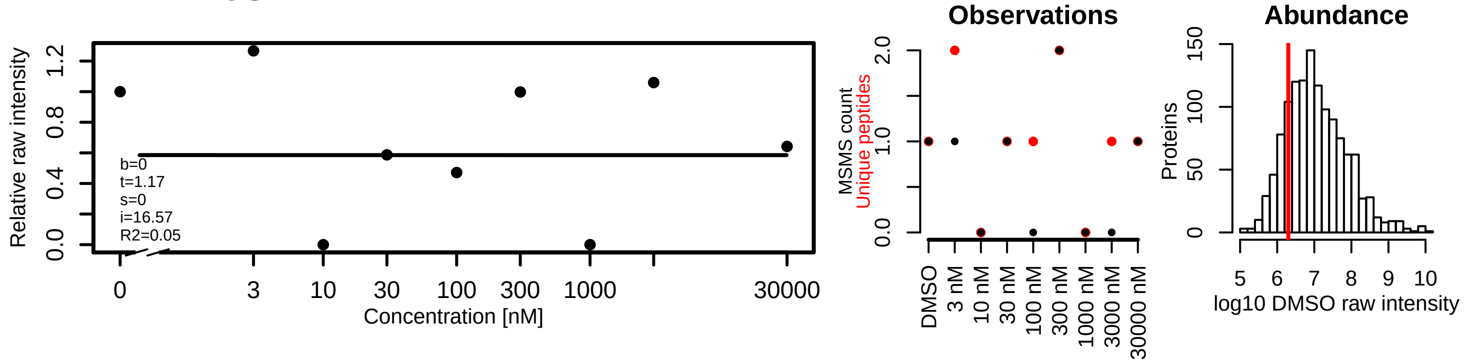

SCAMP2

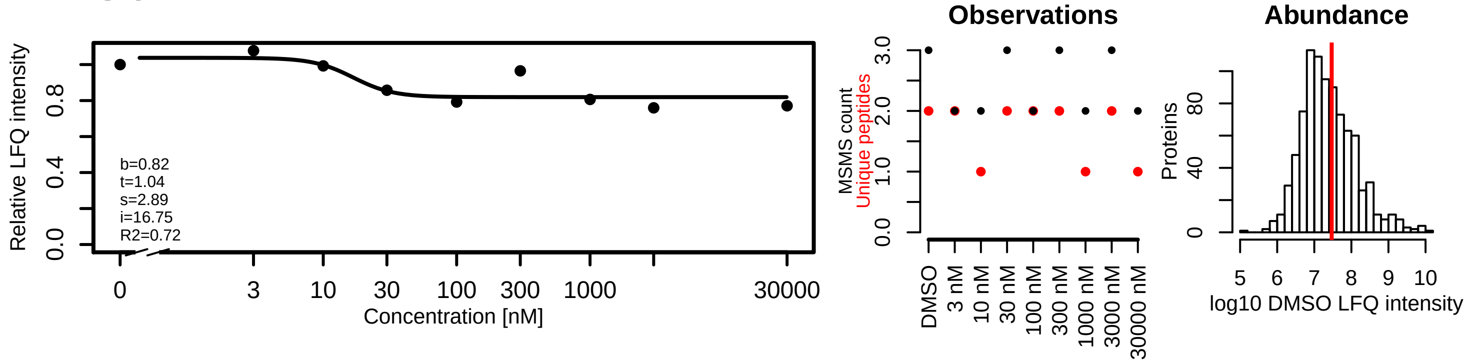

KARS

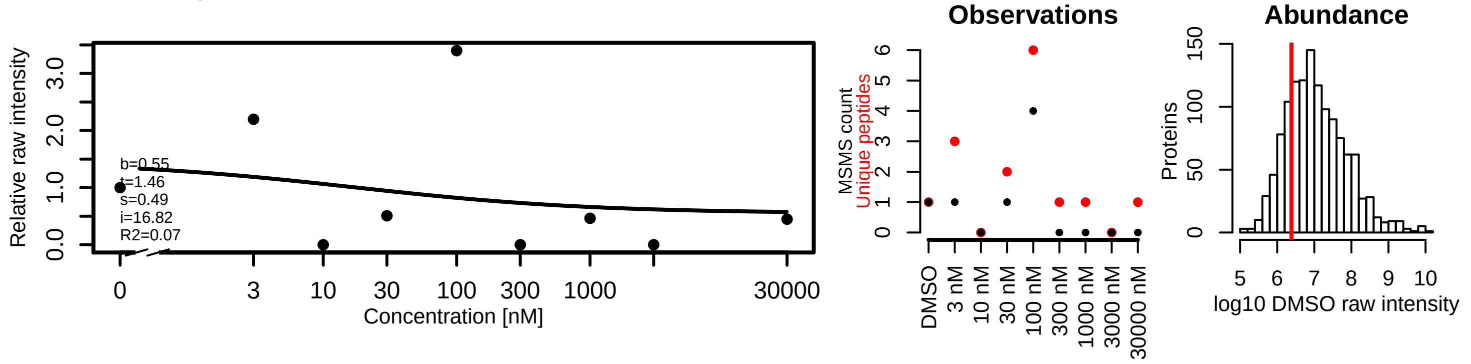

DSG1

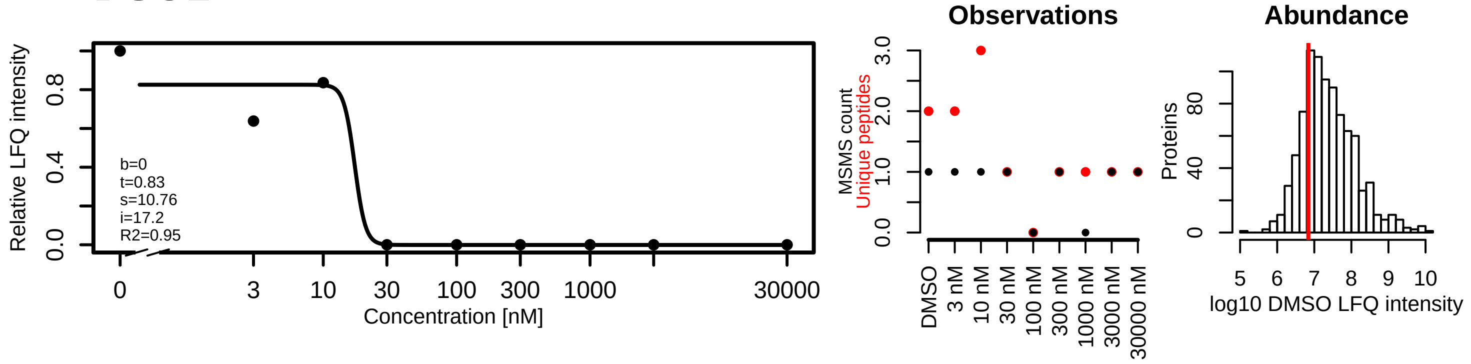

SFXN1

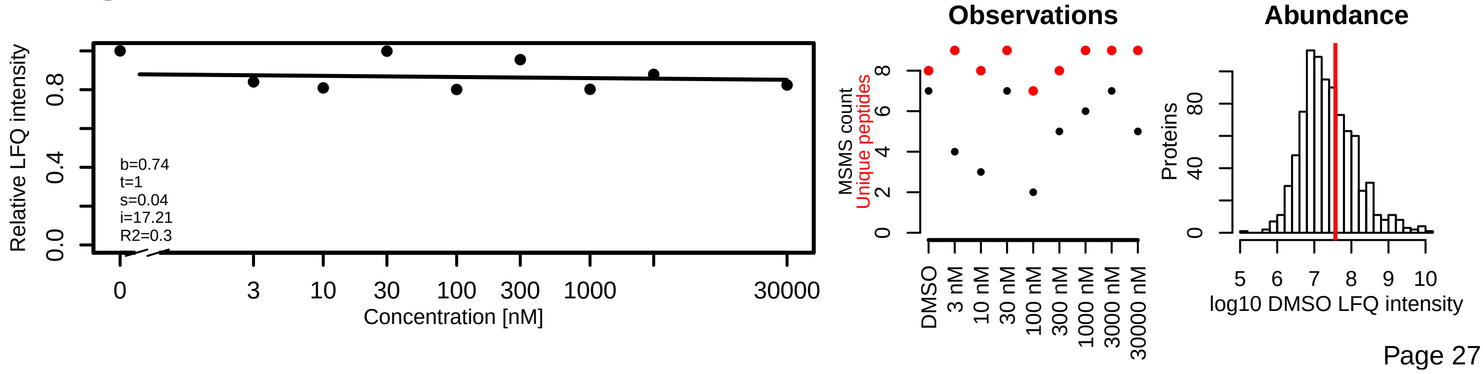

RTN4

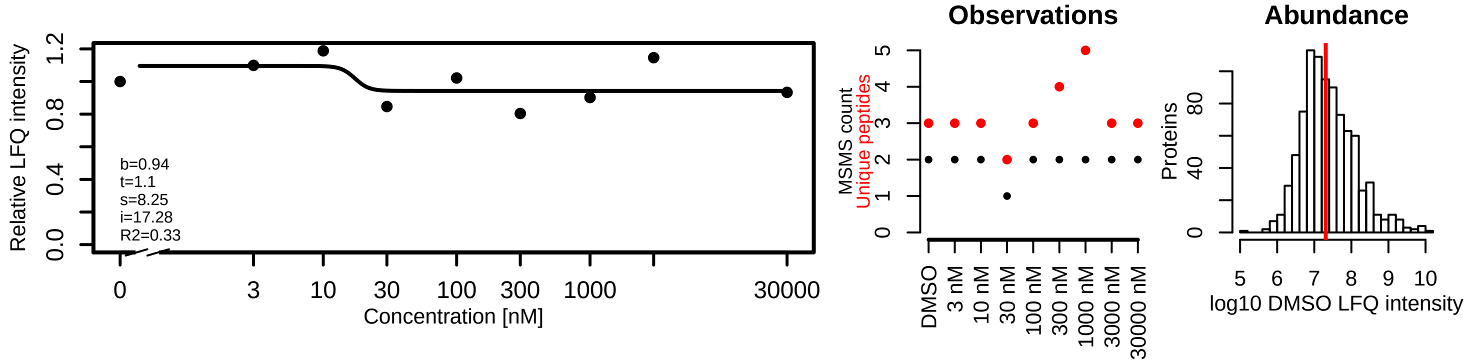

FAF2

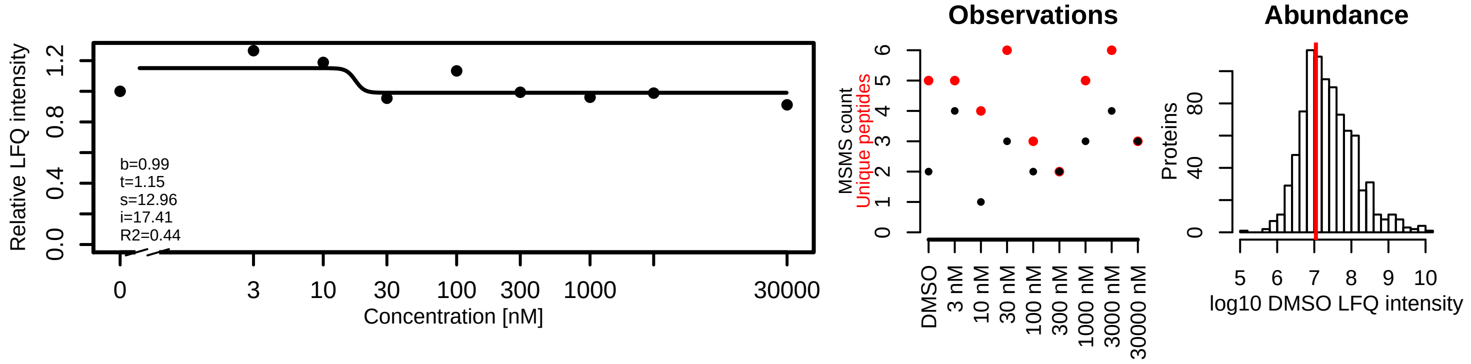

YTHDF2

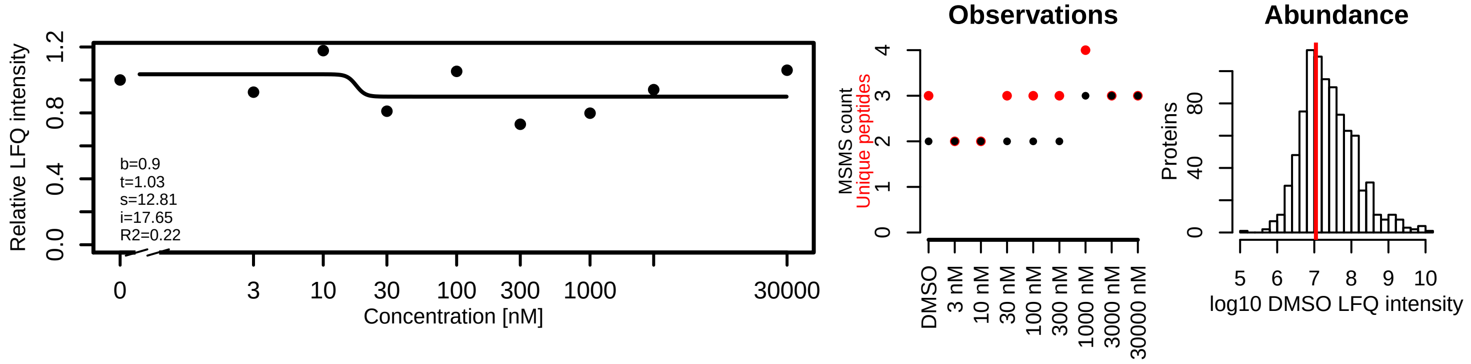

EPHB3

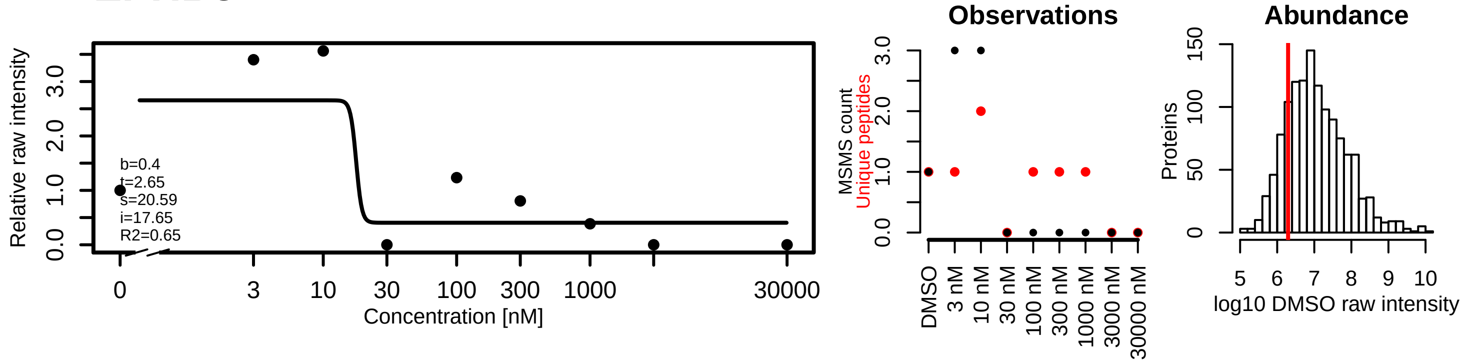

DNAJB6

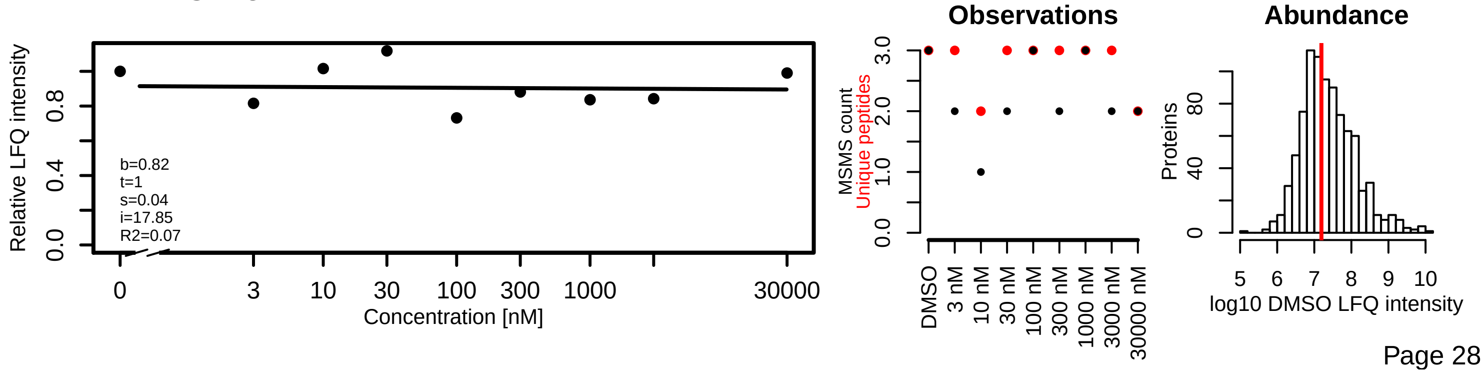

ATAD1

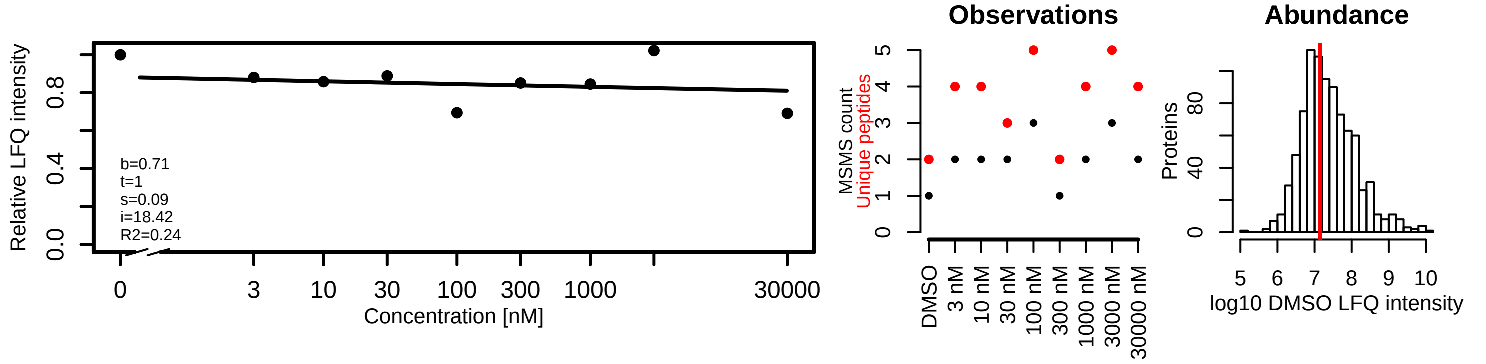

G3BP1

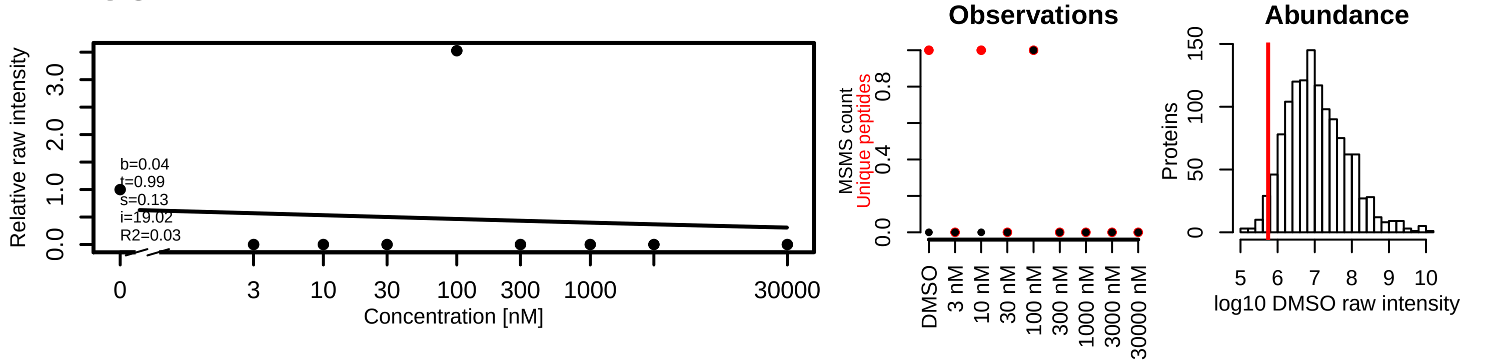

PORCN

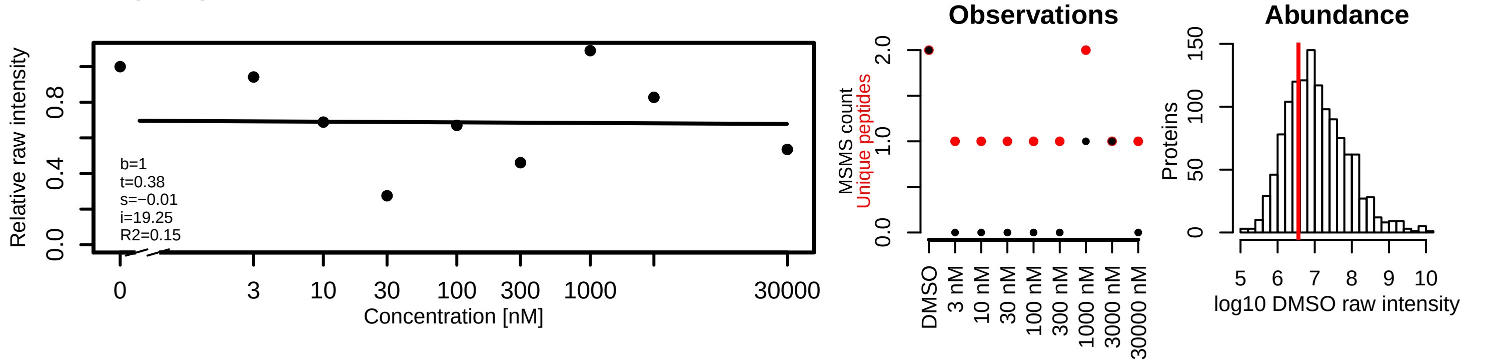

ACVR2A

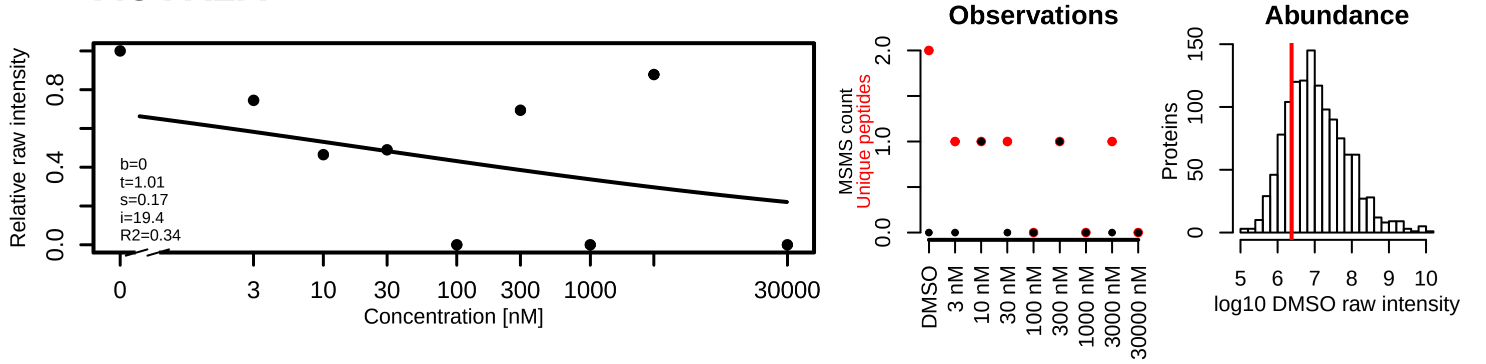

TBRG4

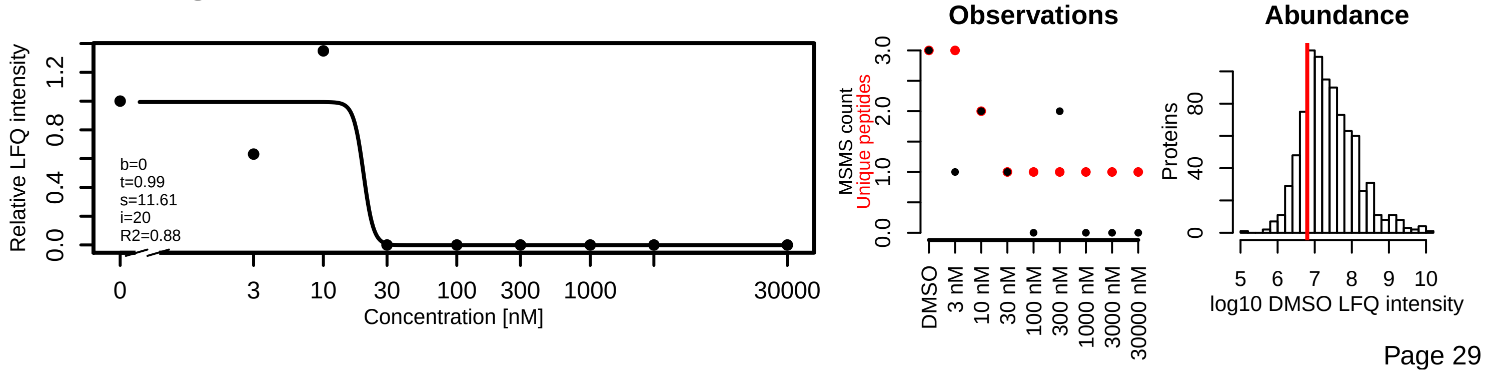

SCAMP3

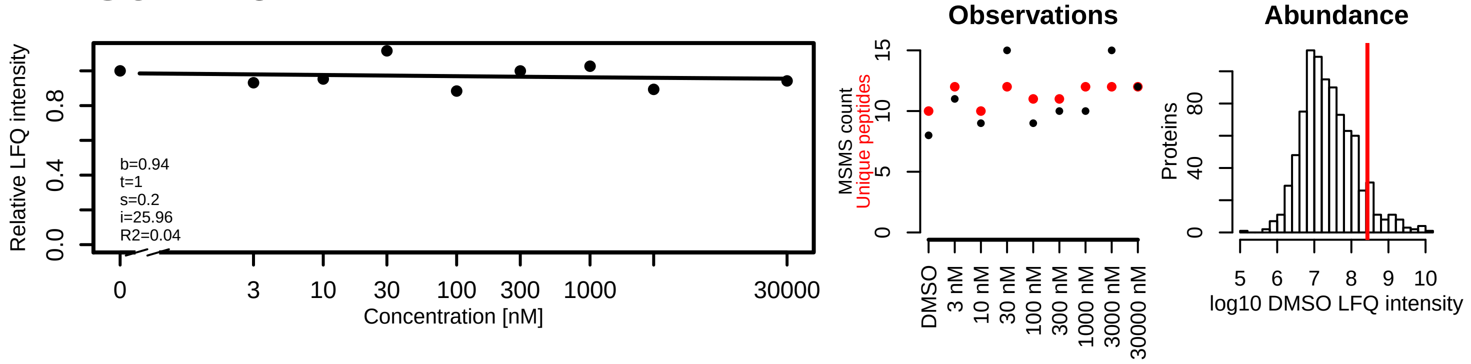

EMC2

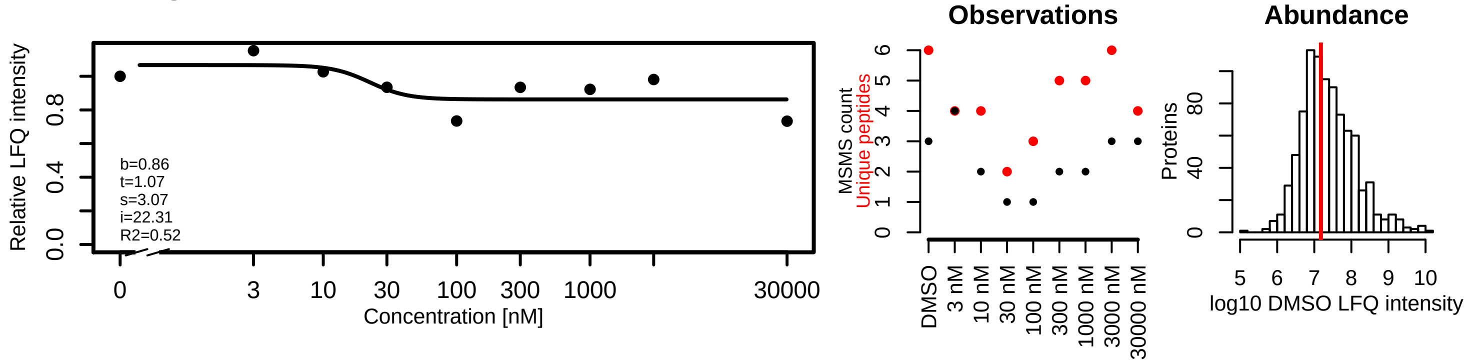

RAF1;BRAF

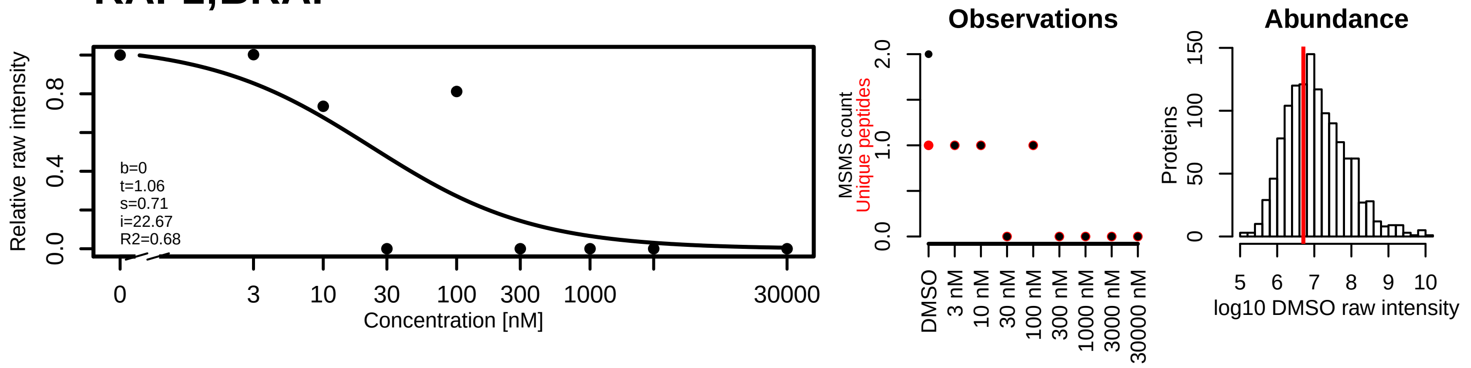

KDELR1

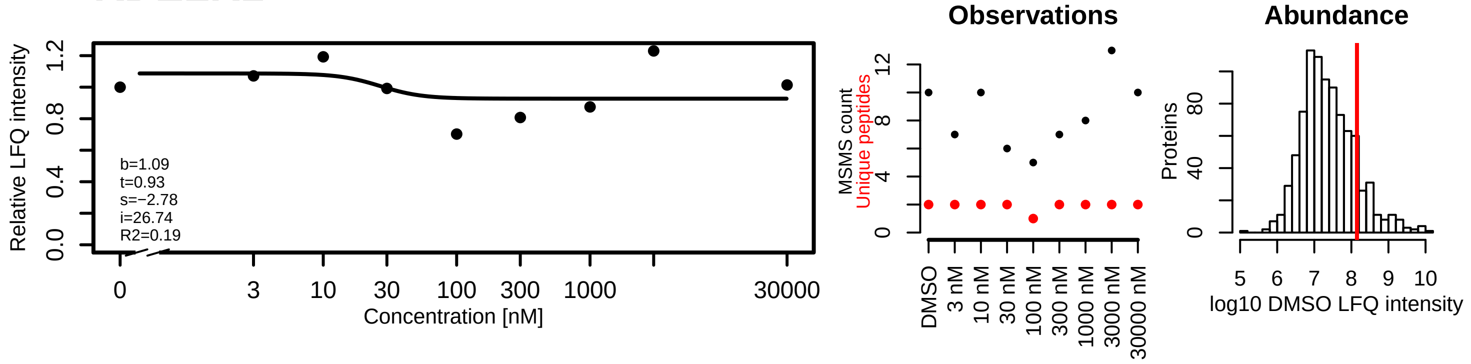

WEE1

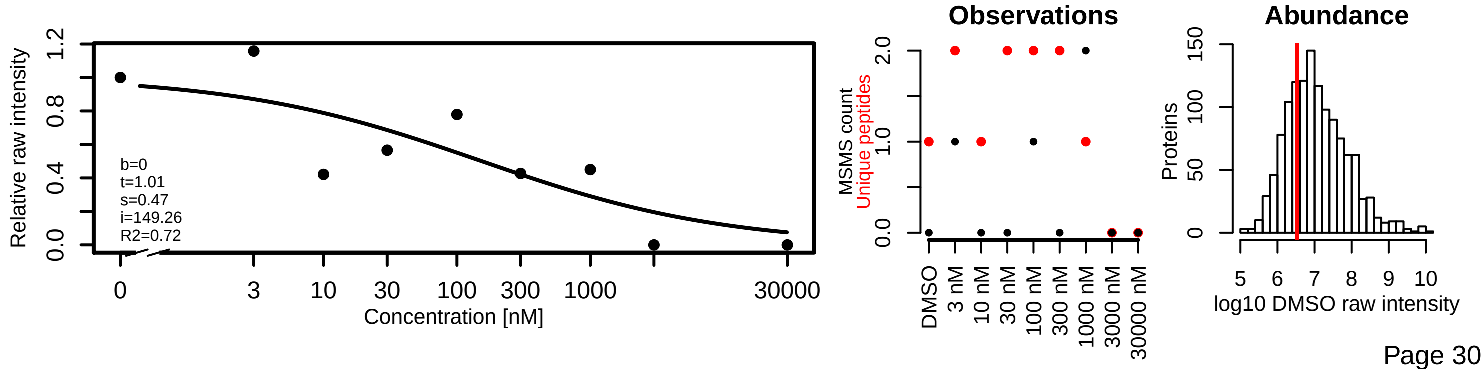

PYCRL

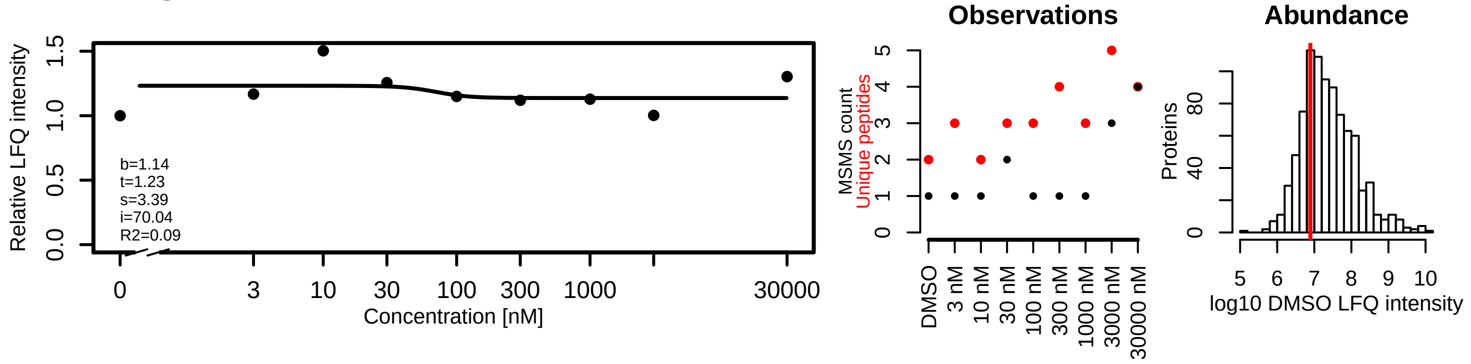

TUBB4A

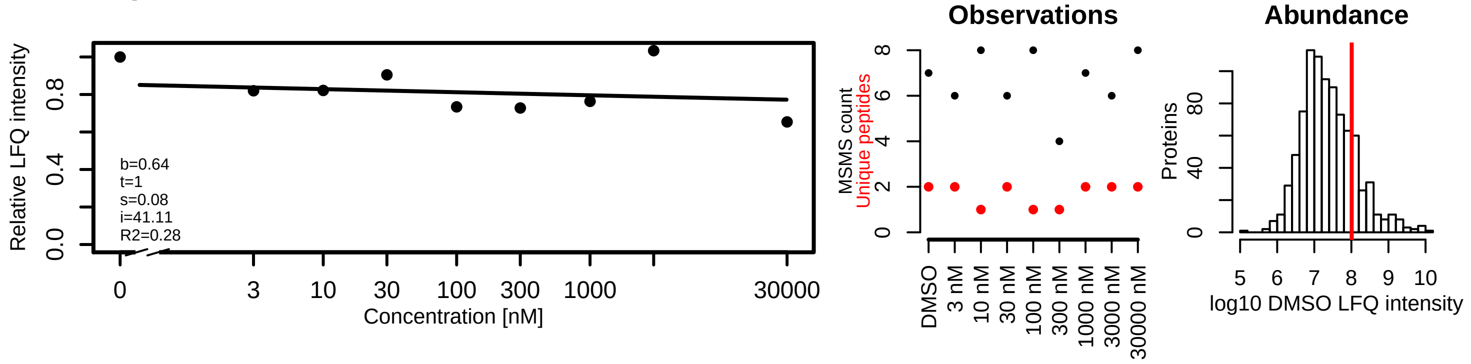

LPHN2

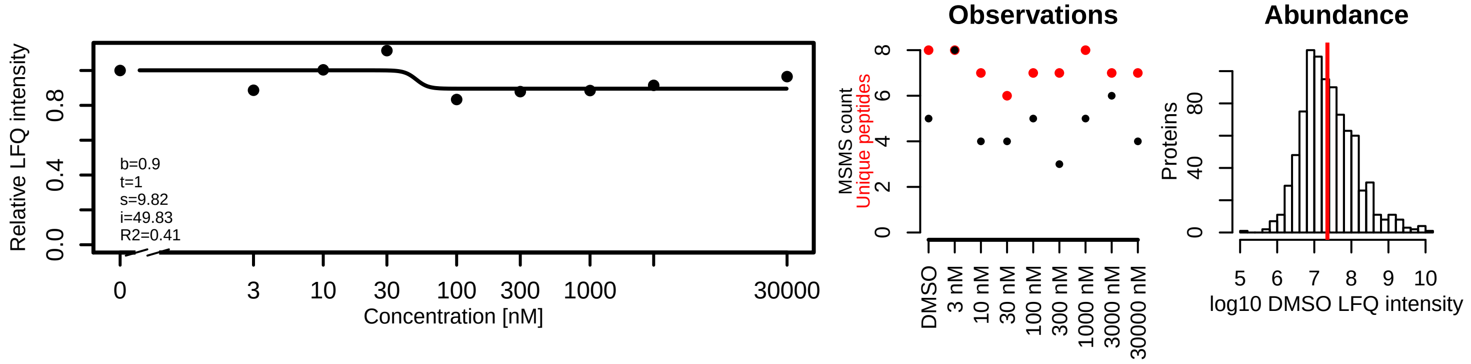

TEX10

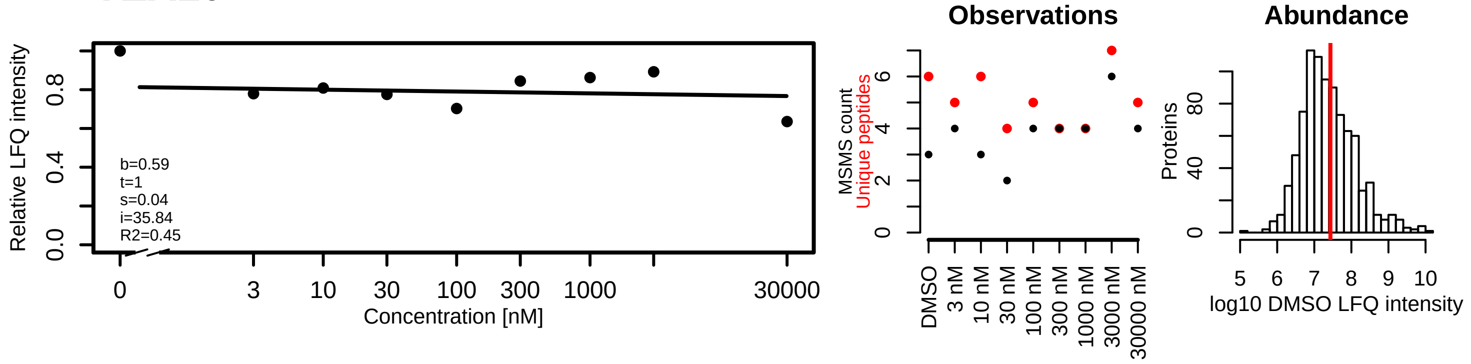

AGK

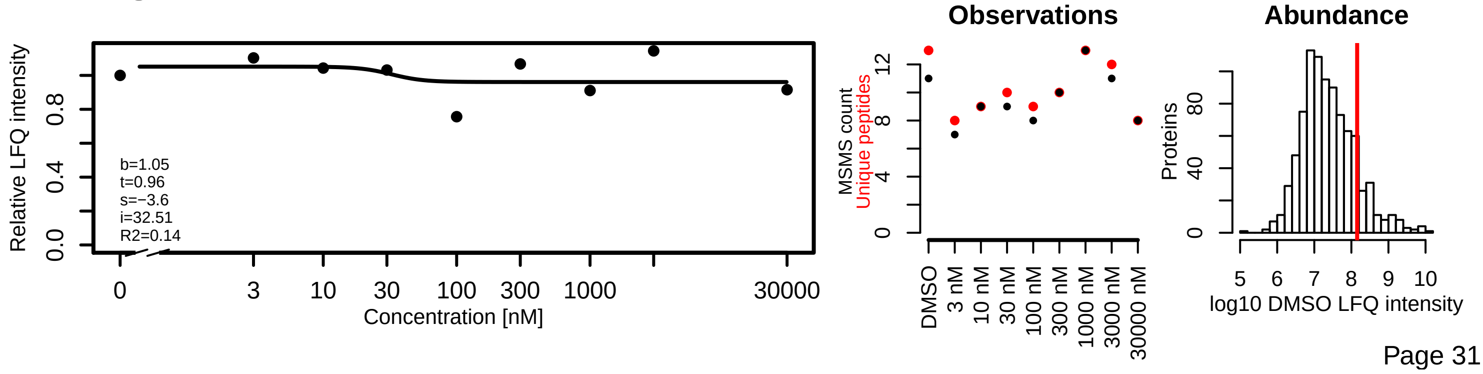

CELSR1

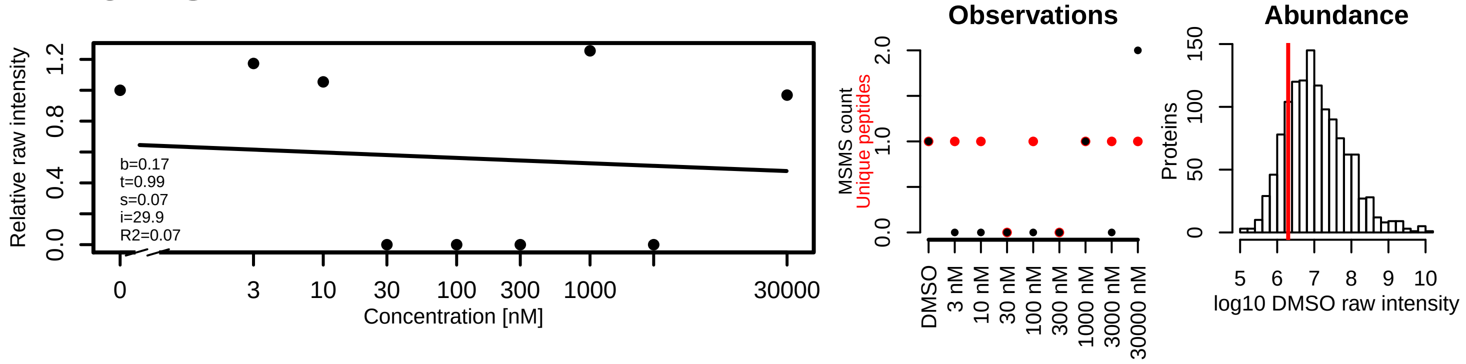

KRT6B

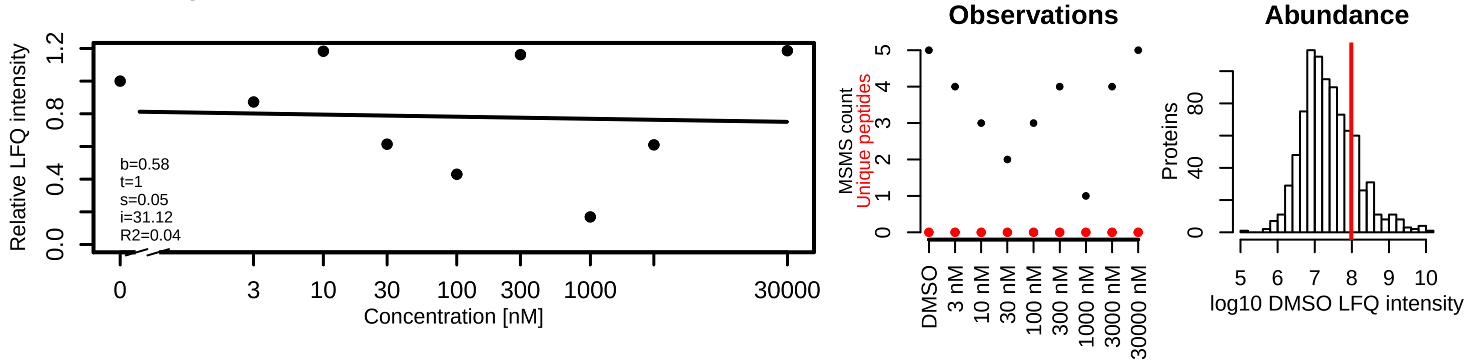

TUBB4B

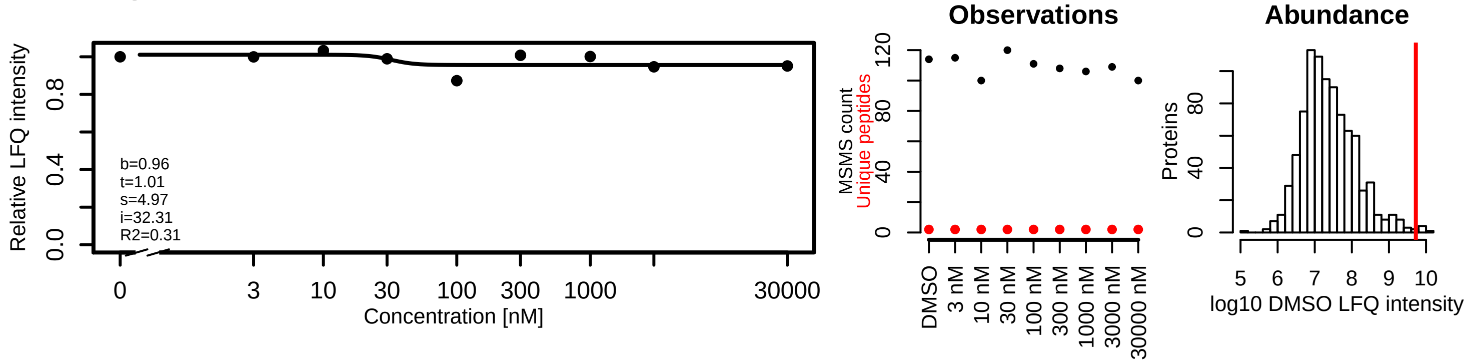

ITGB1

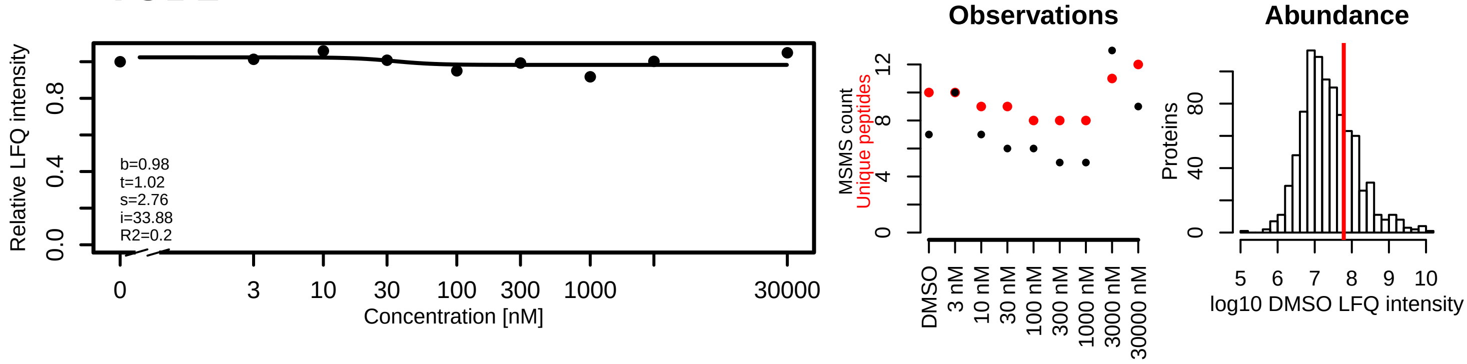

ALG6

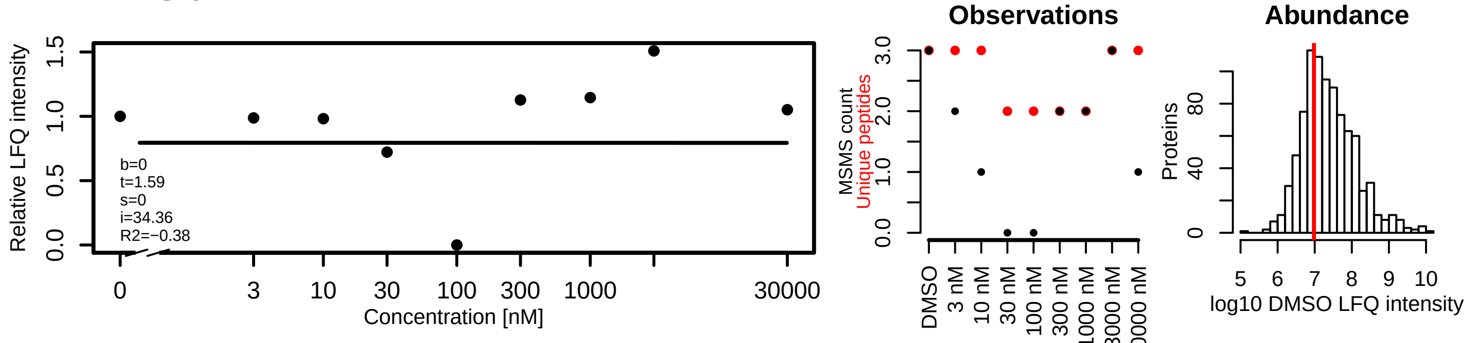

UBAC2

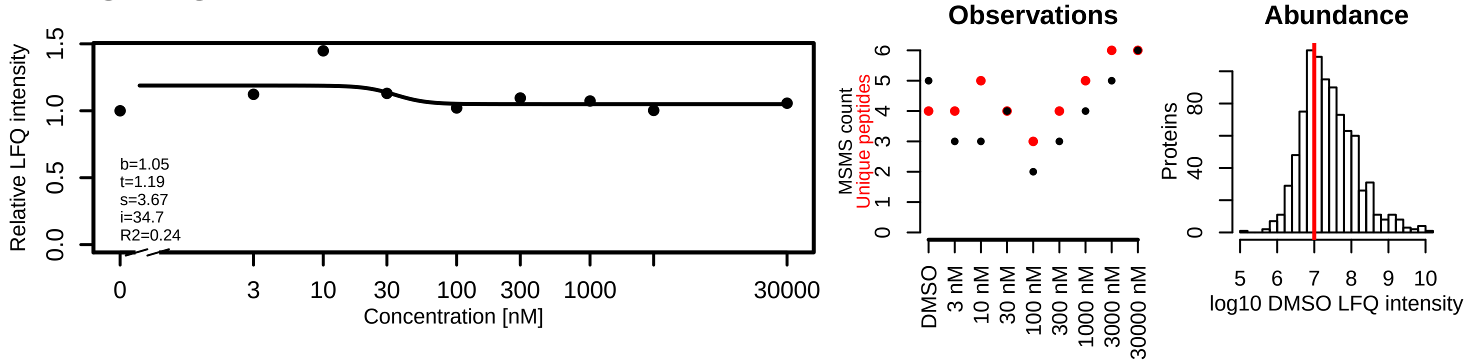

ARF4

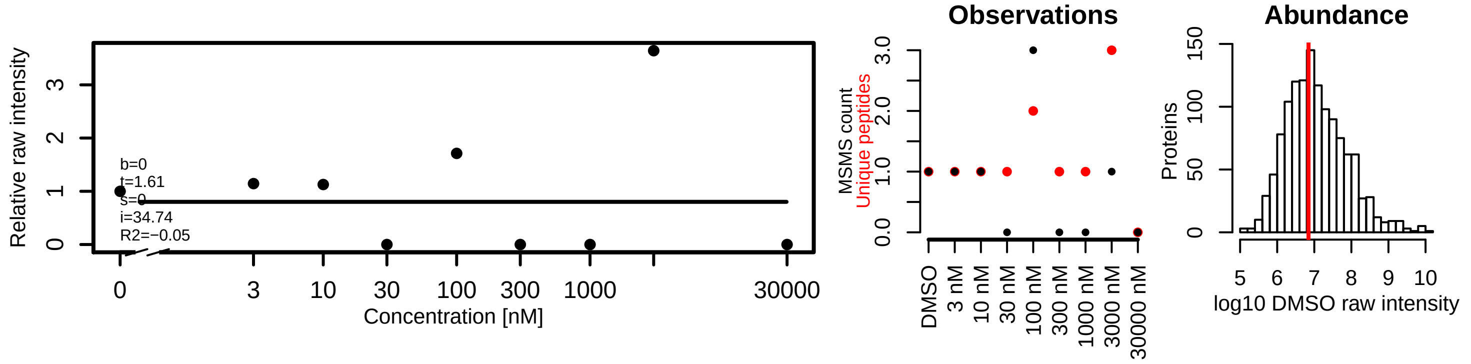

DAD1

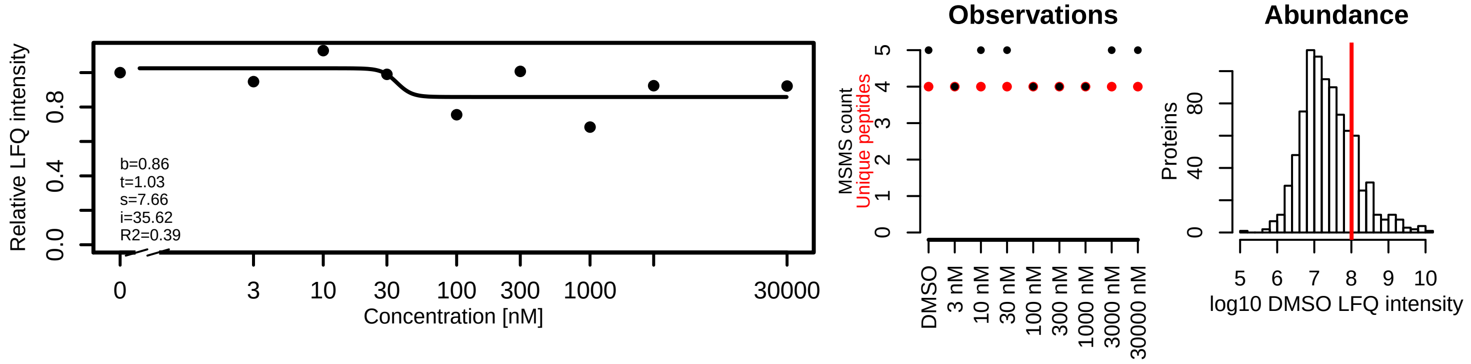

C4orf3

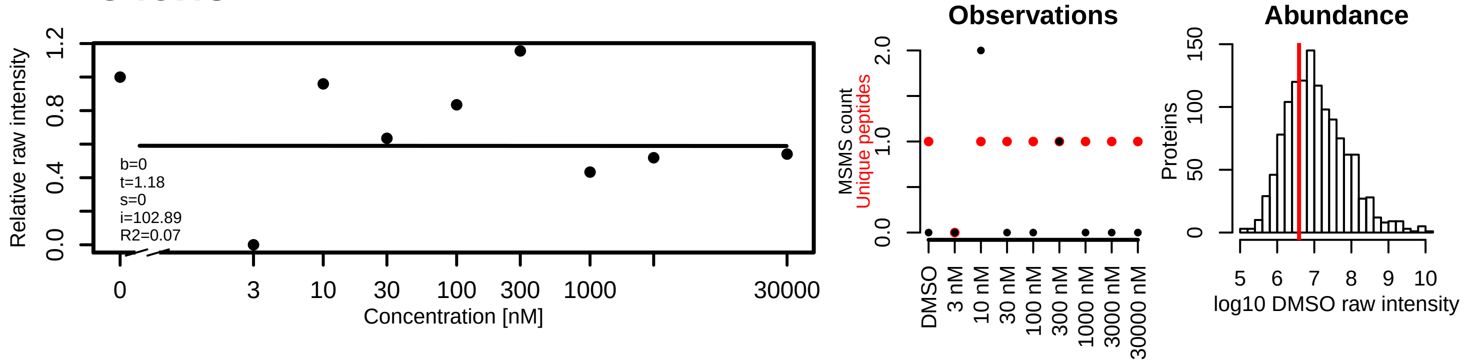

ORMDL3

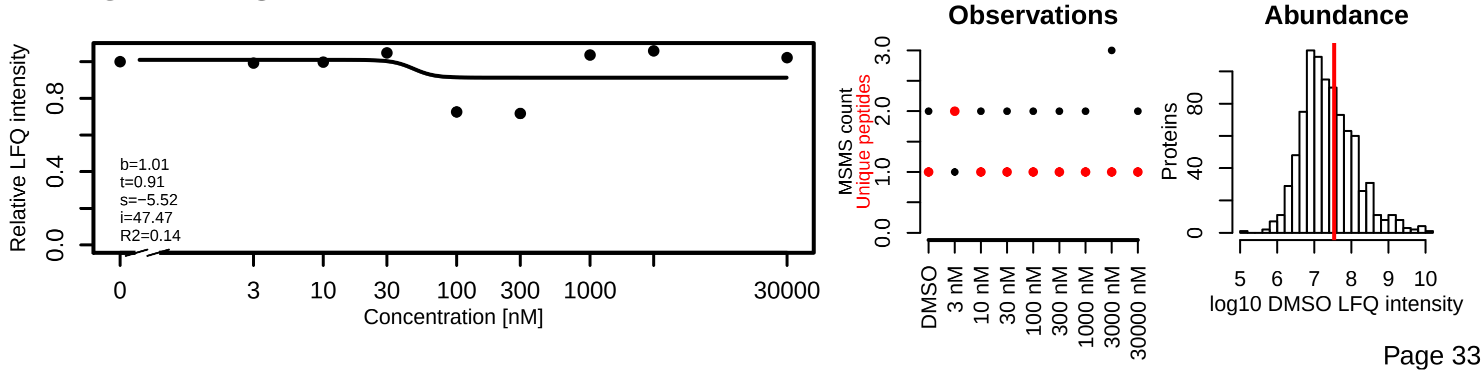

CCDC127

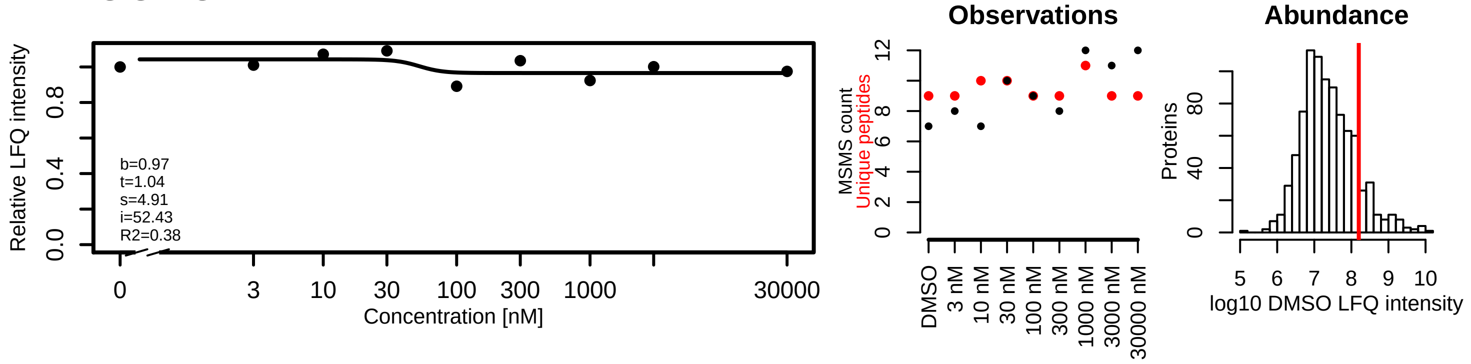

MBOAT1

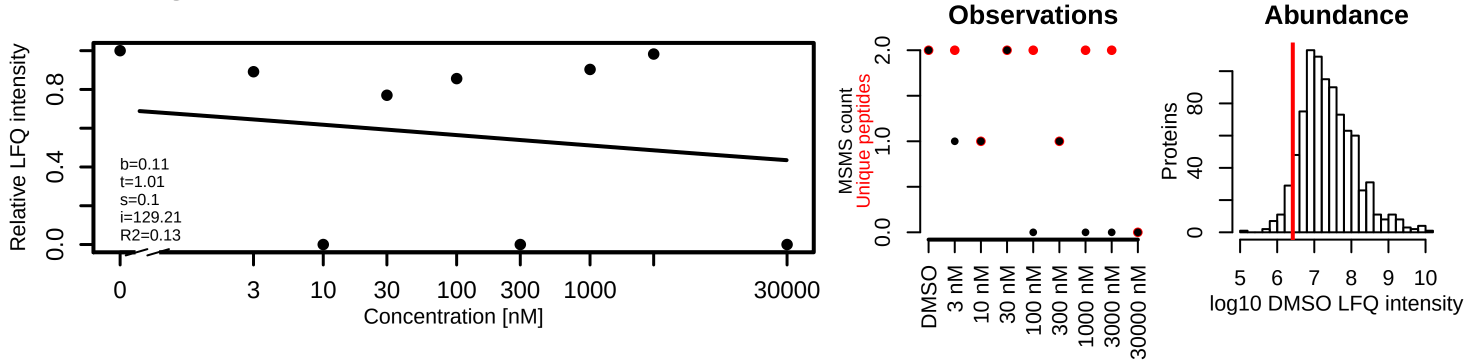

PXMP4

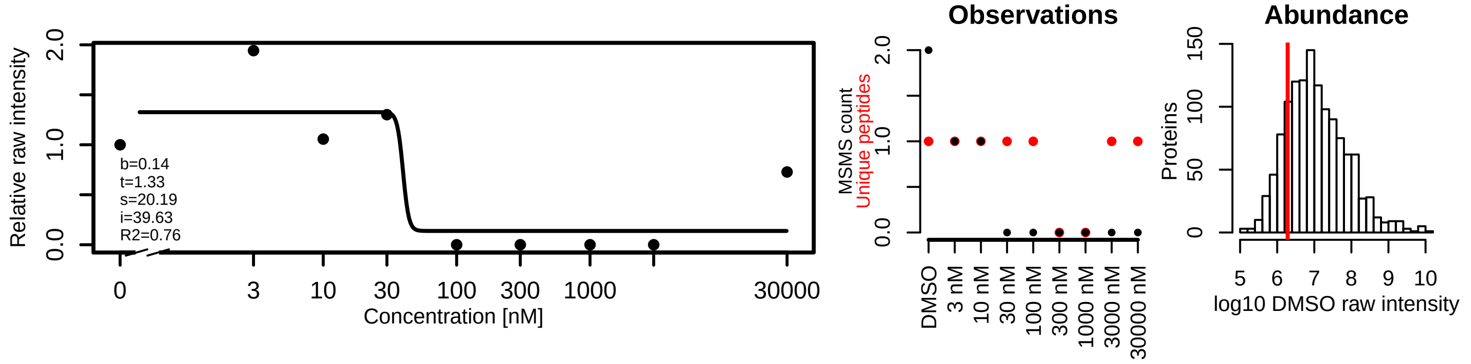

INTS7

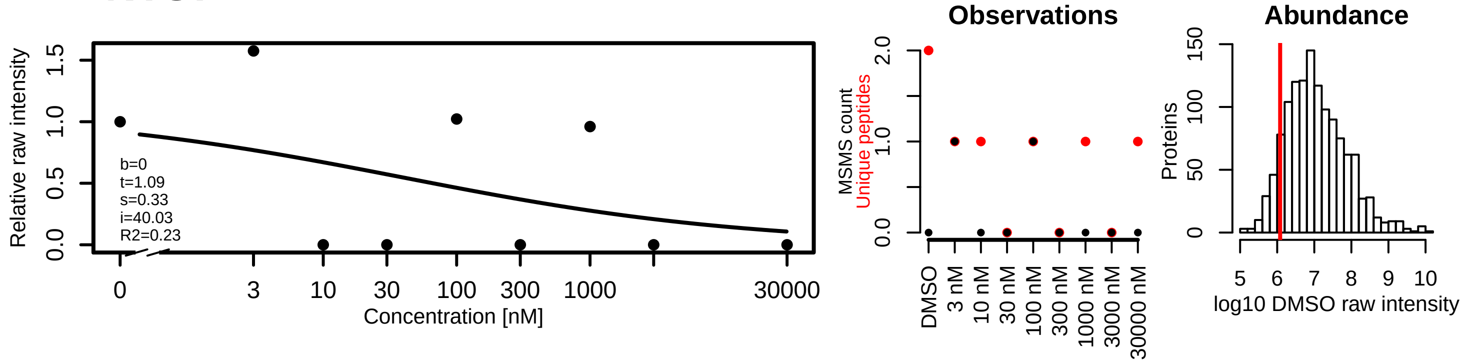

BRI3

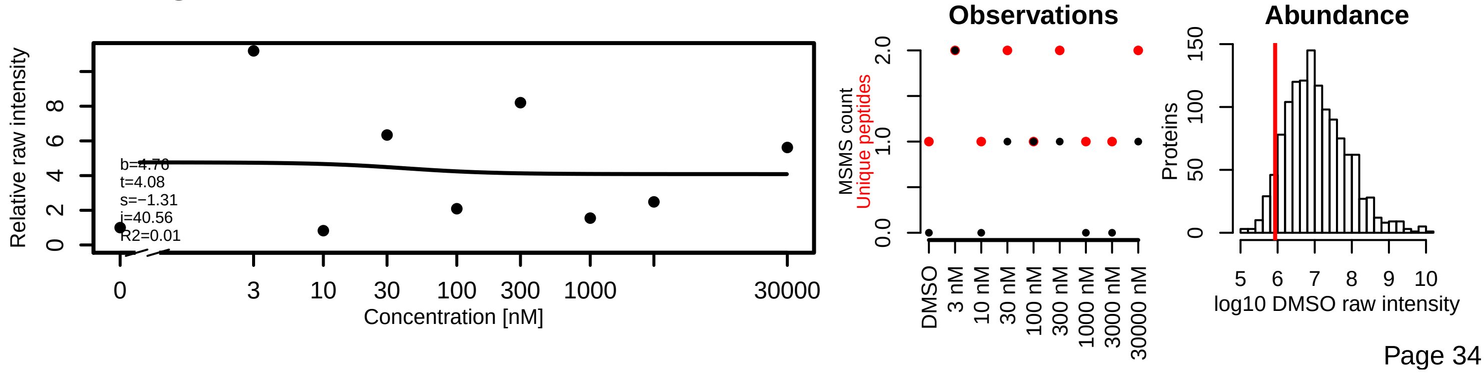

ATP1A1

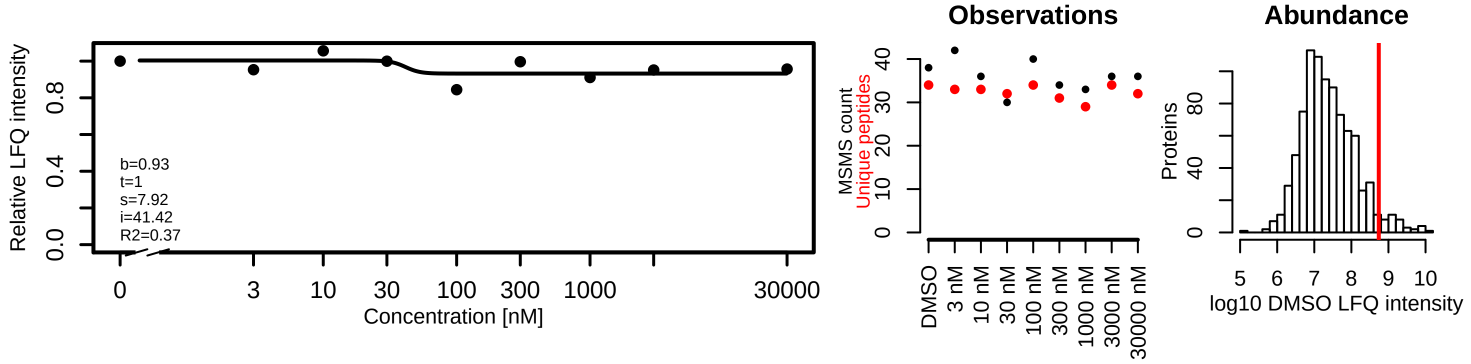

NDUFV1

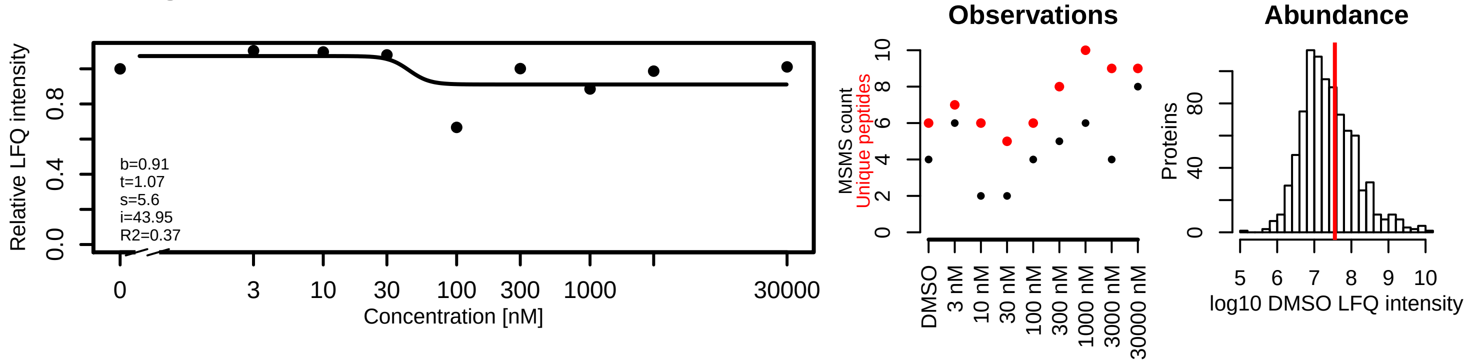

URB1

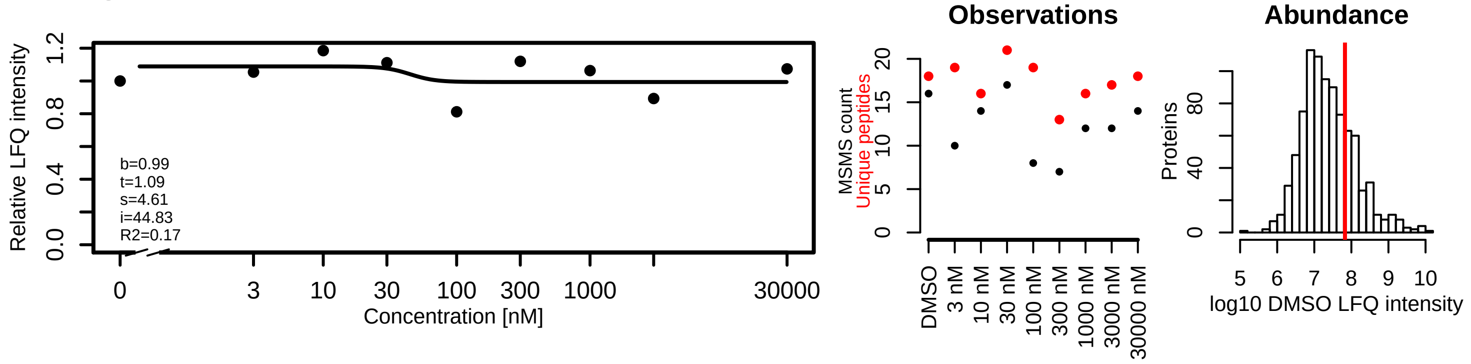

CHCHD6

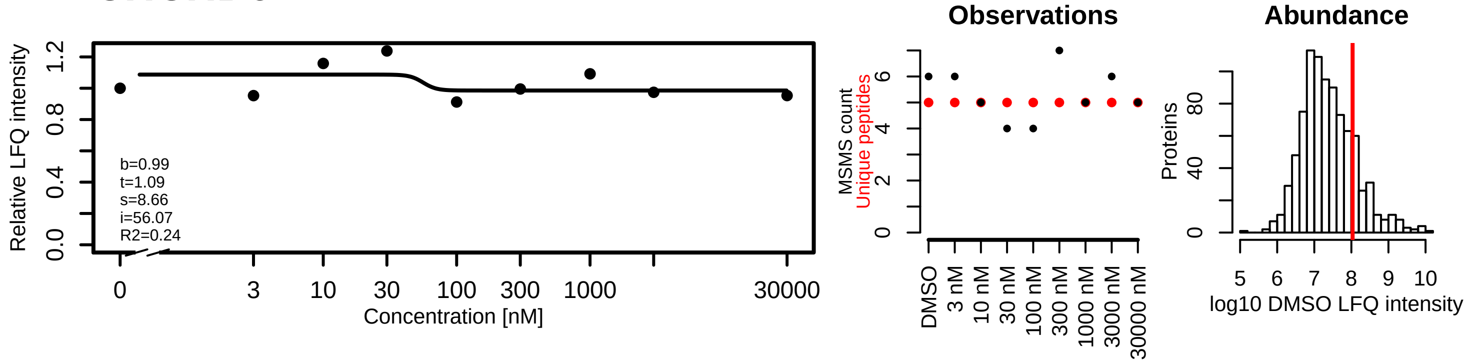

NCLN

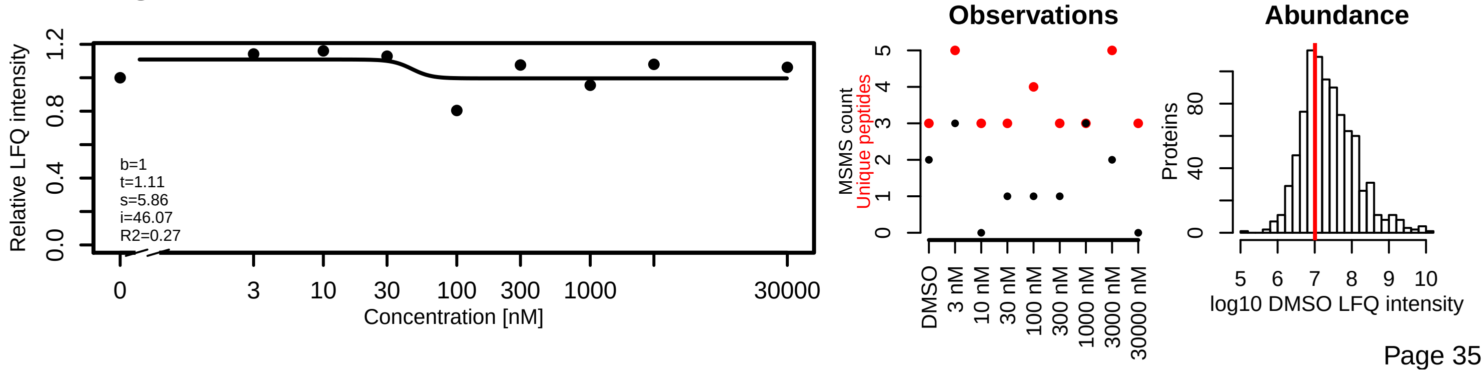

VAPA

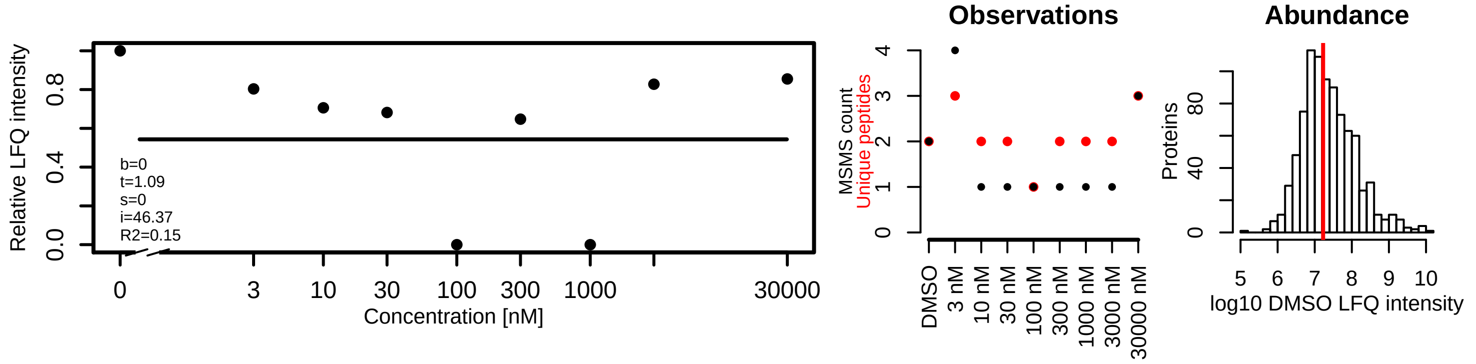

ATP5F1

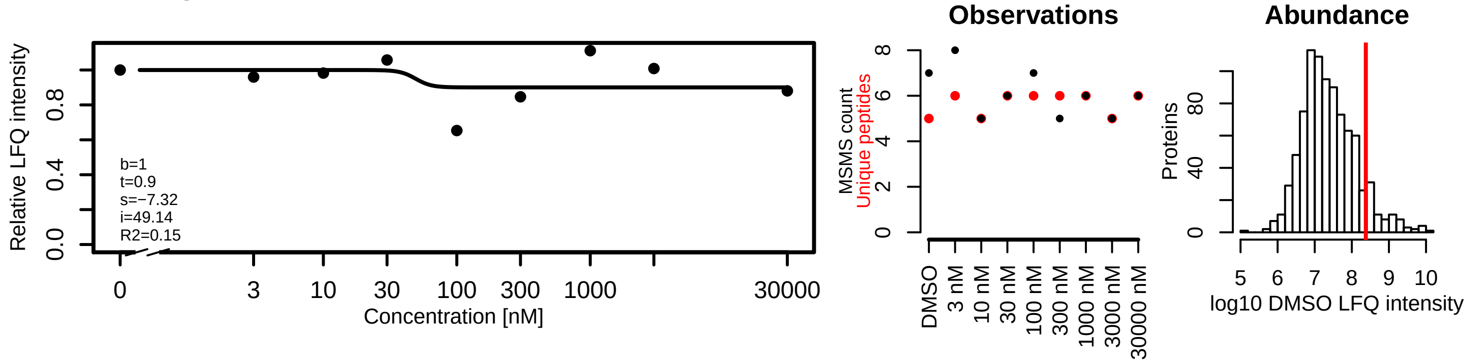

INTS5

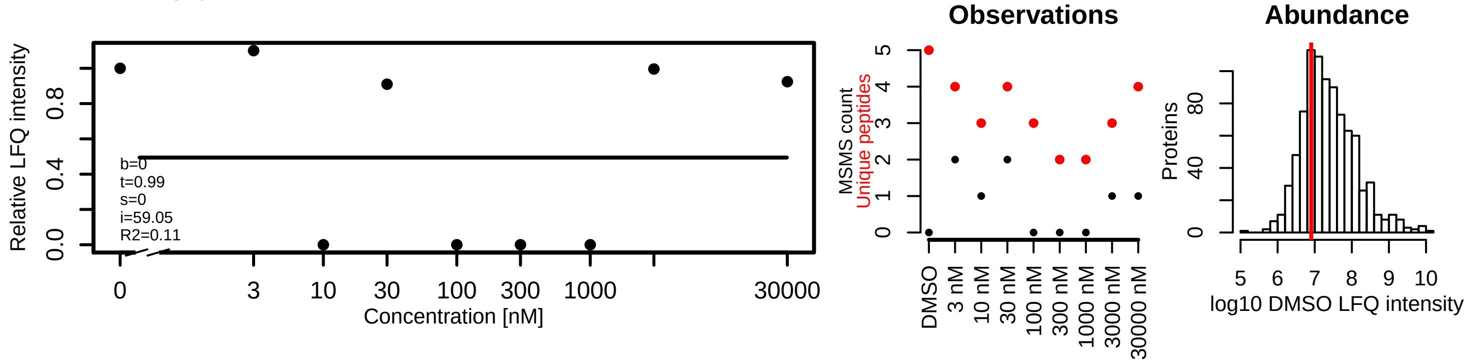

INTS1;DKFZP586J0619

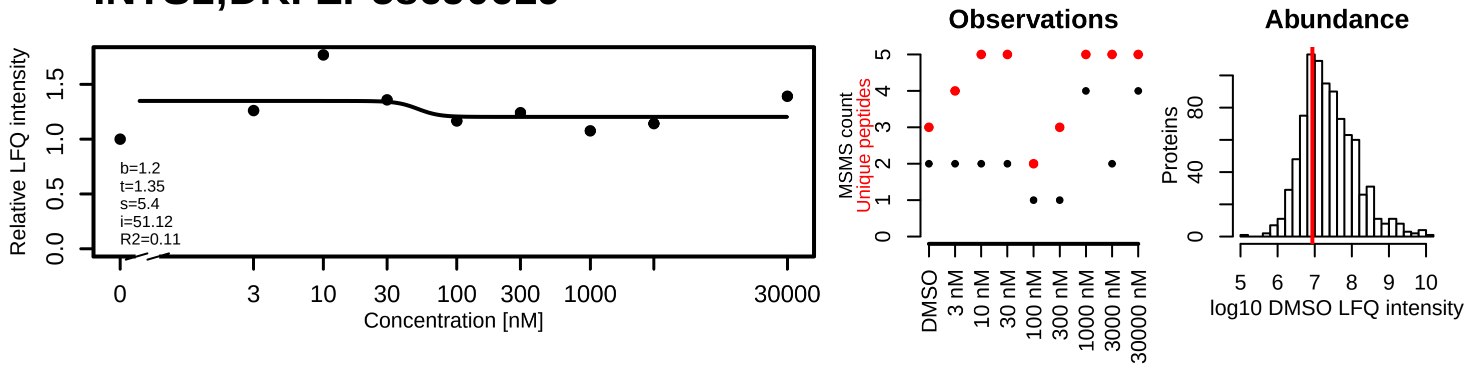

COX6C

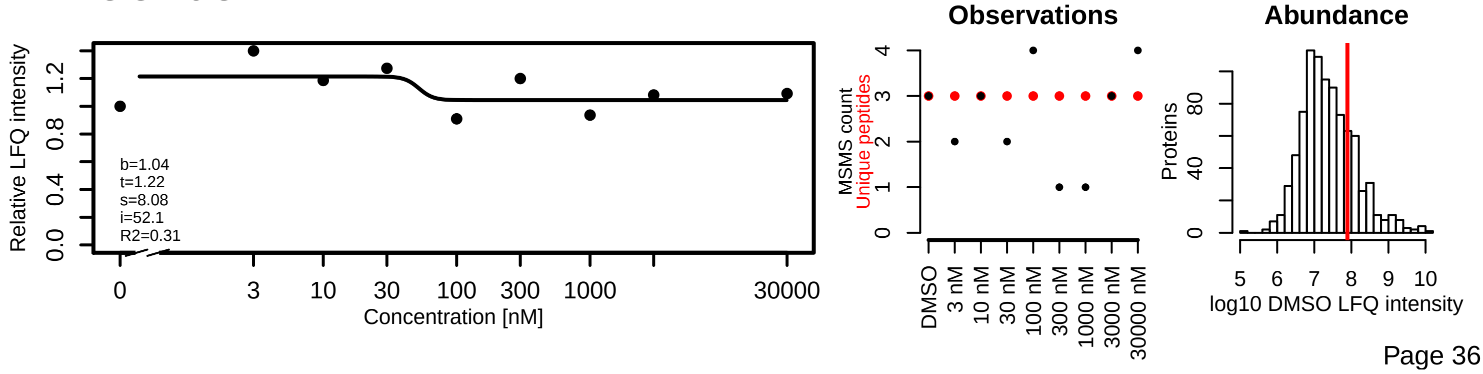

NUP160

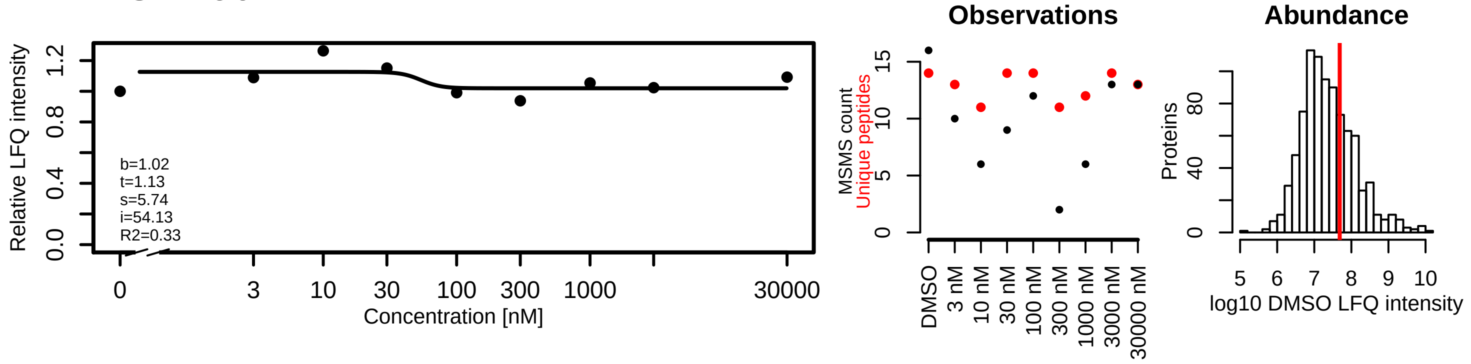

PIEZO1

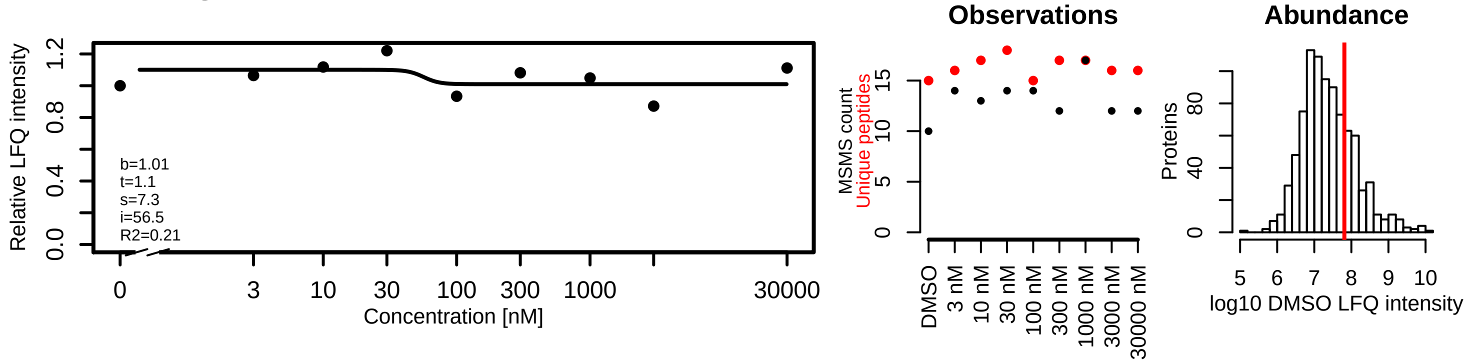

NUP210

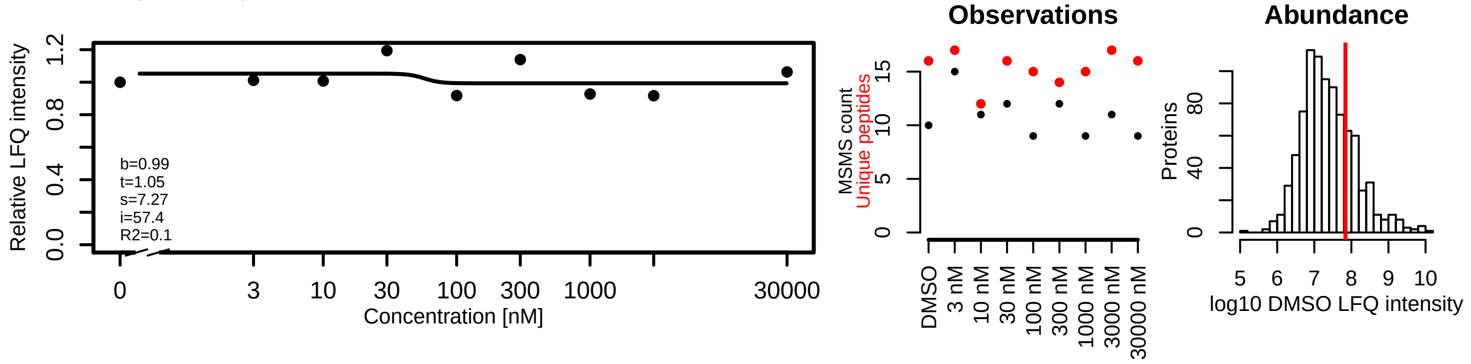

RPL23

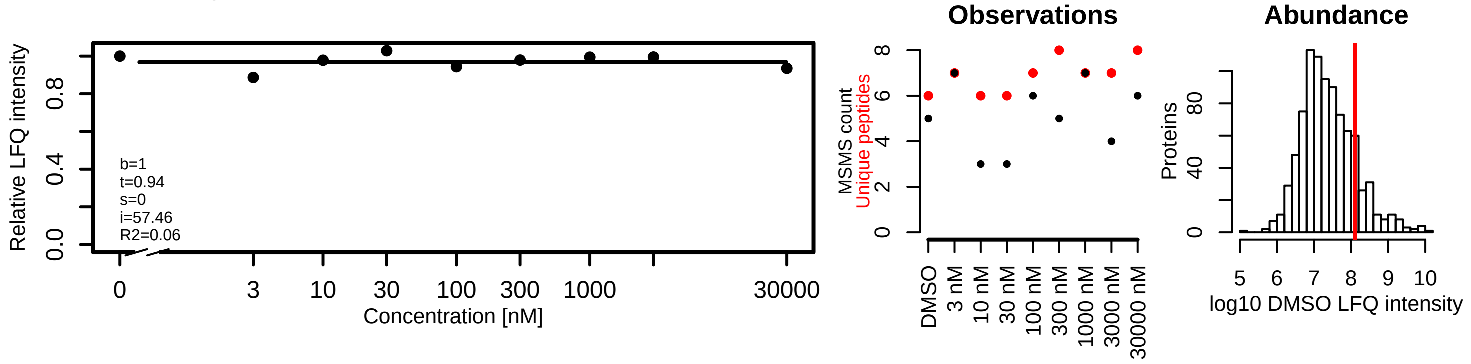

CNOT1

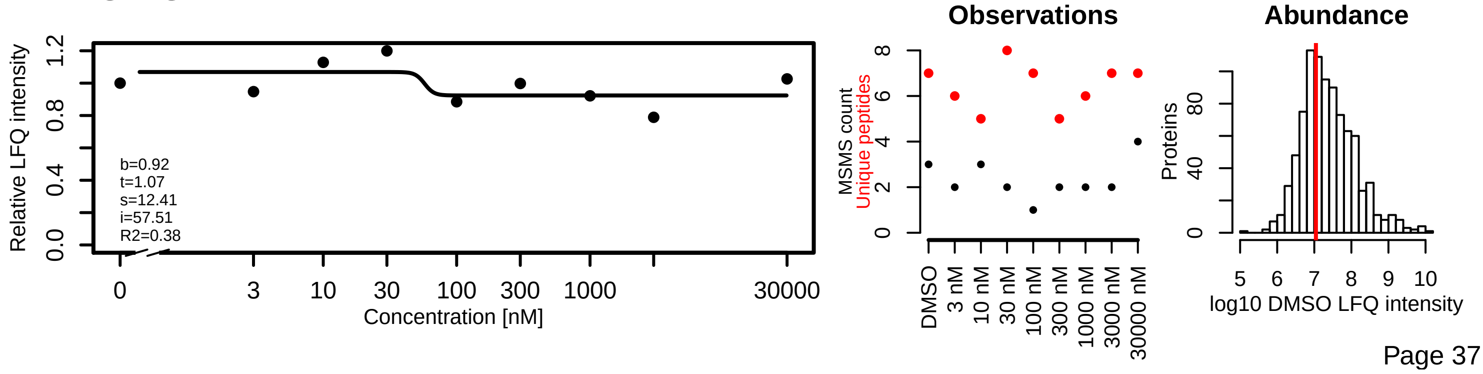

DOPEY2

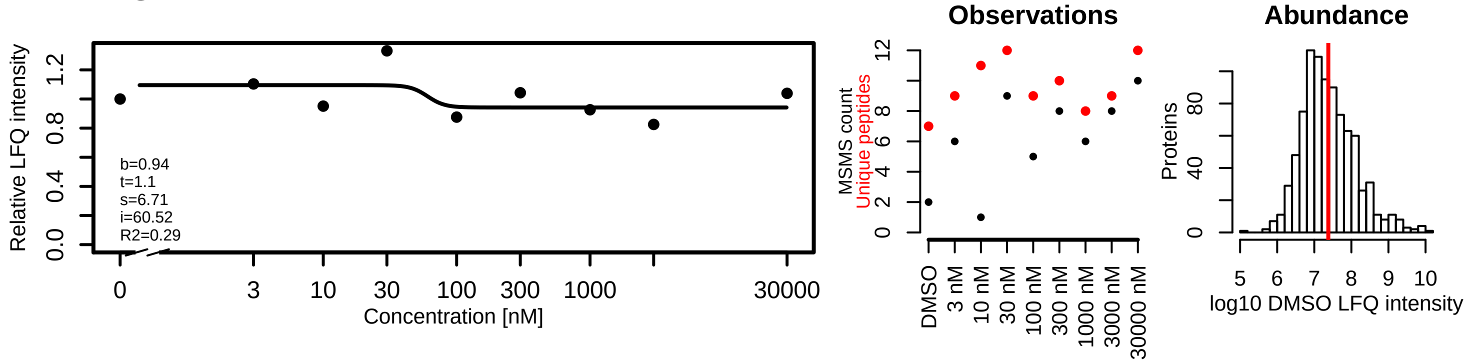

NDUFS1

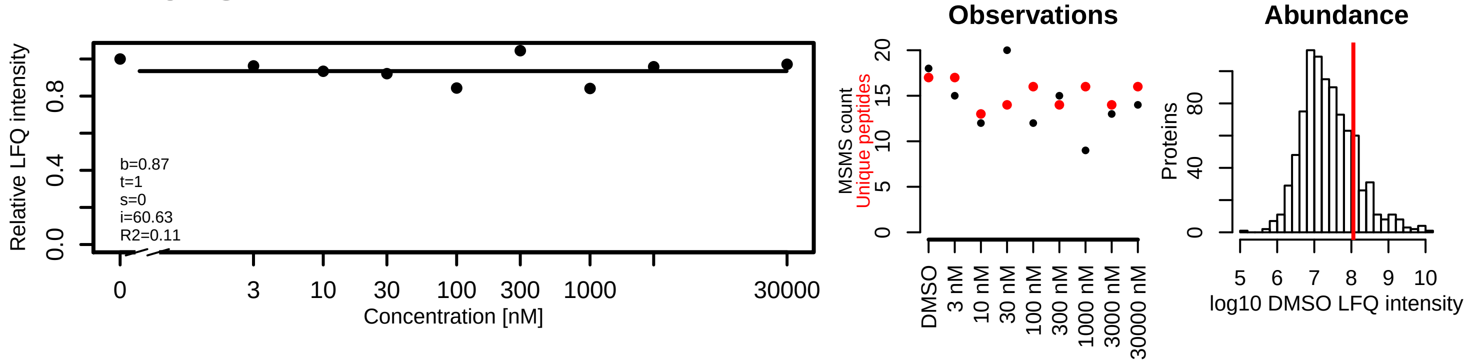

PIGU

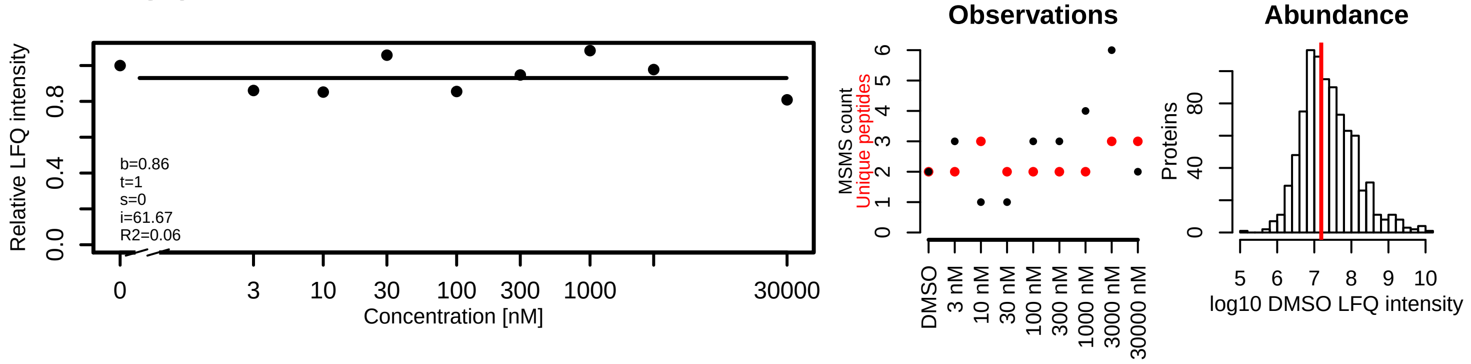

B3GALT6

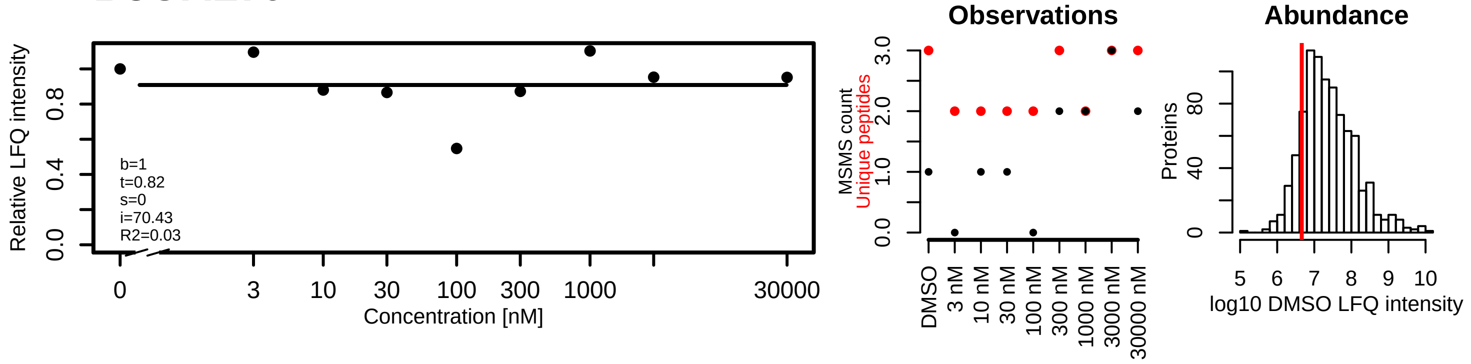

SMPD4

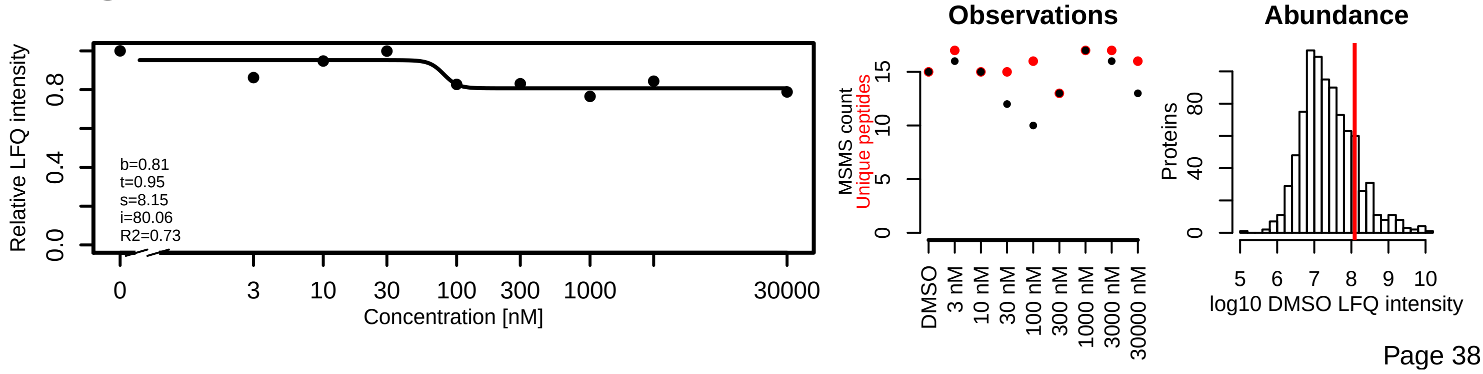

PON2

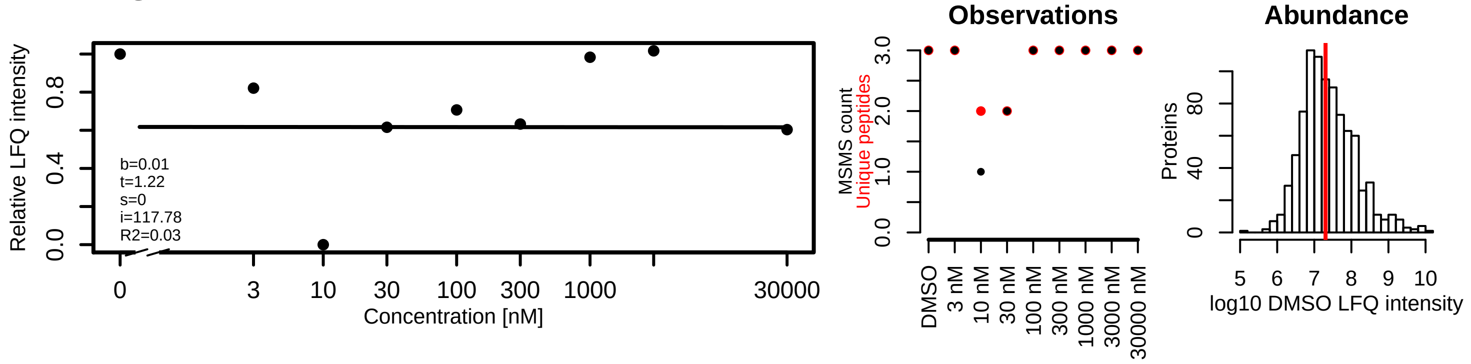

LMBR1;DKFZp547F222

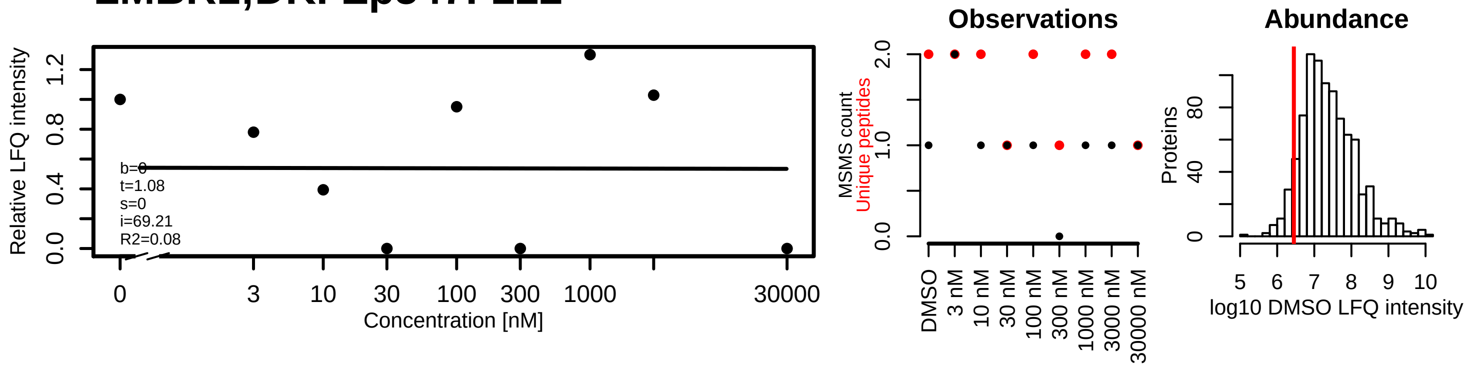

CKAP4

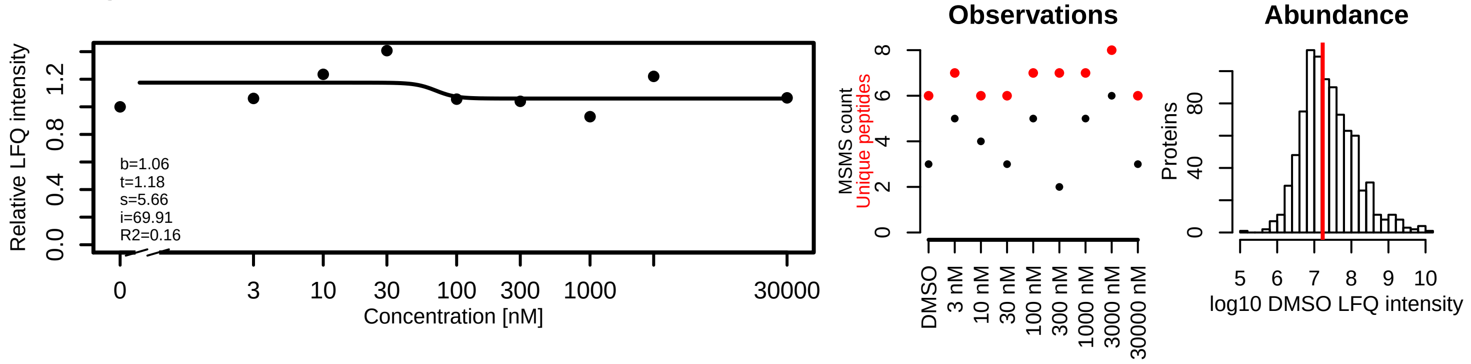

TMEM126A

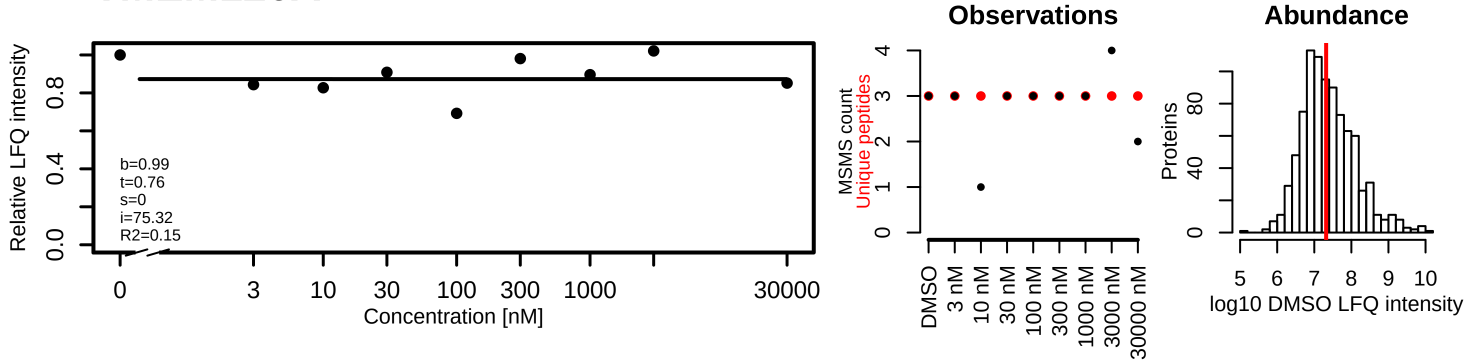

FLOT1

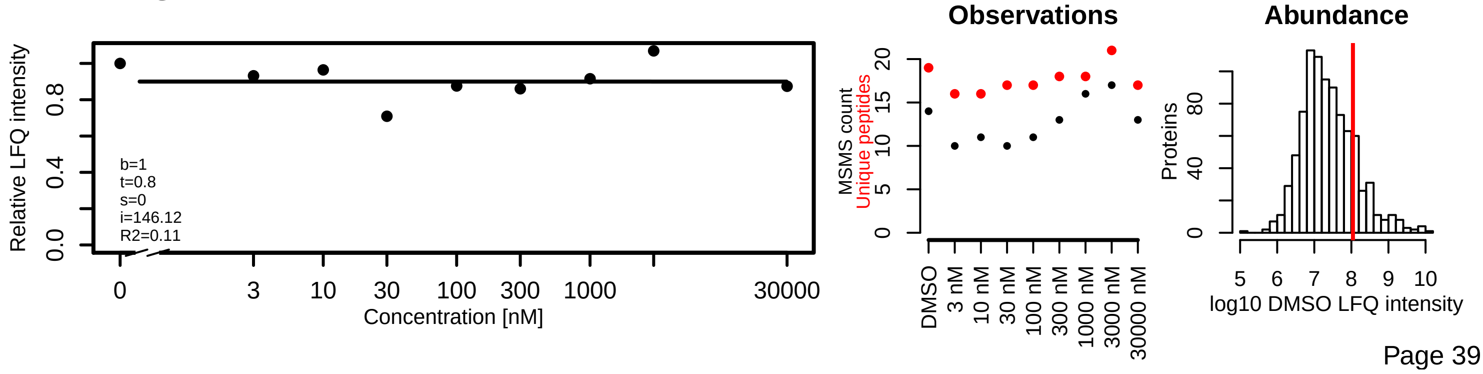

ABCB10

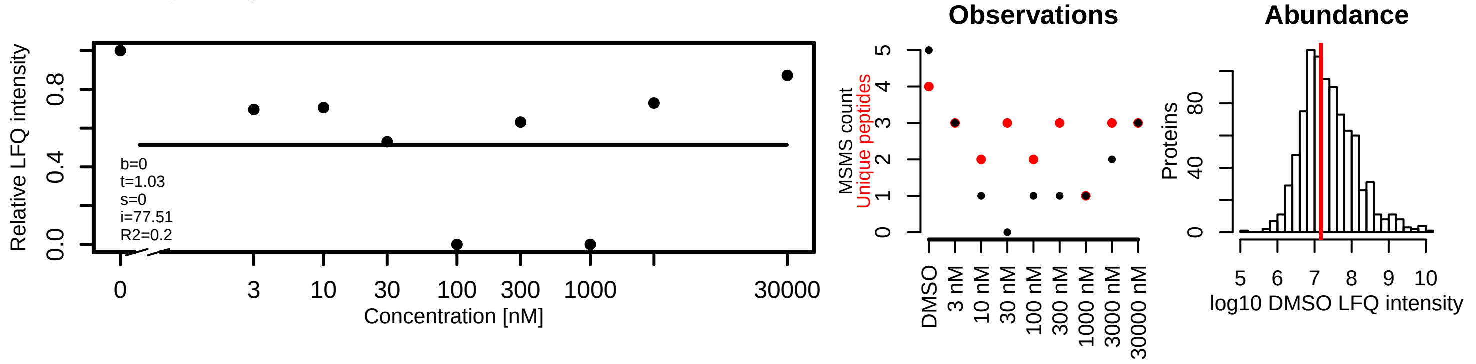

NLK

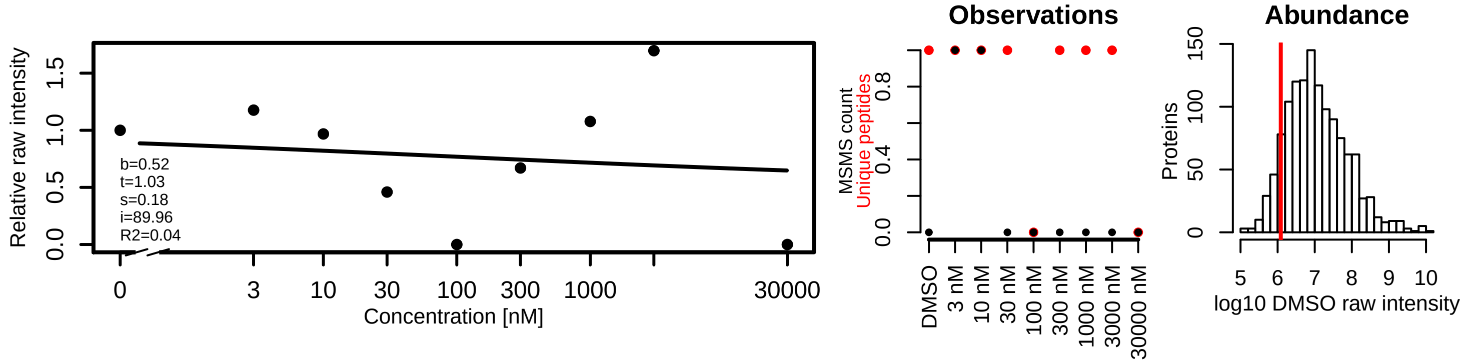

DOLK

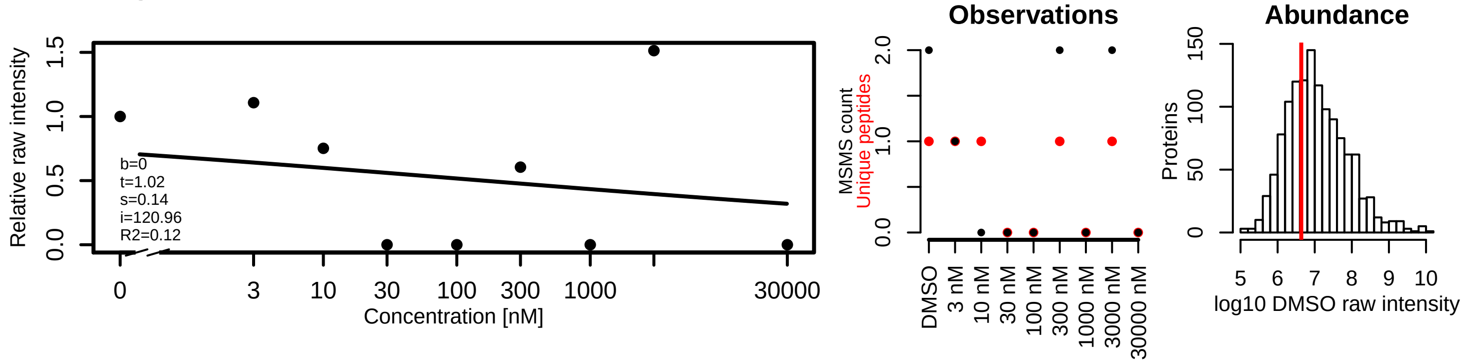

LGMN

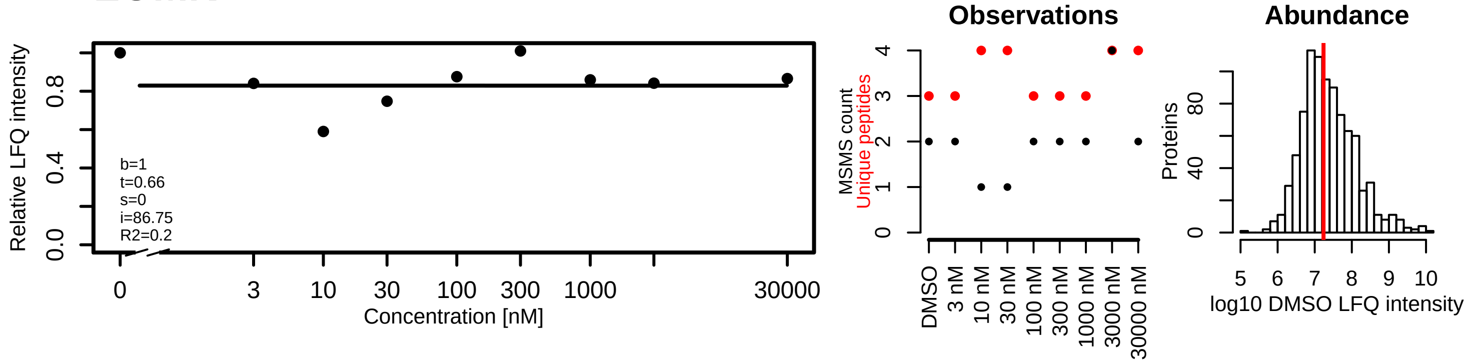

MYL12A;MYL12B

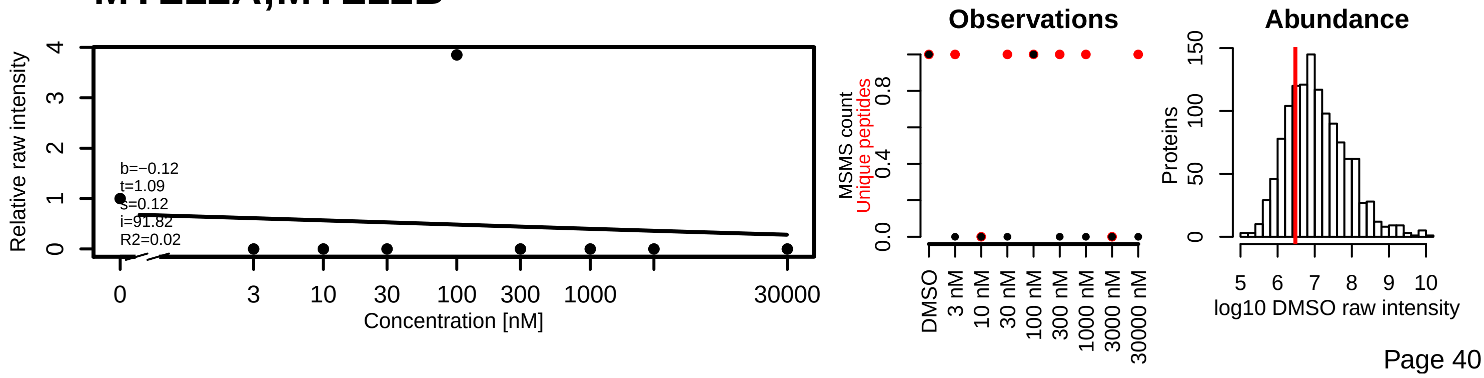

SMC2

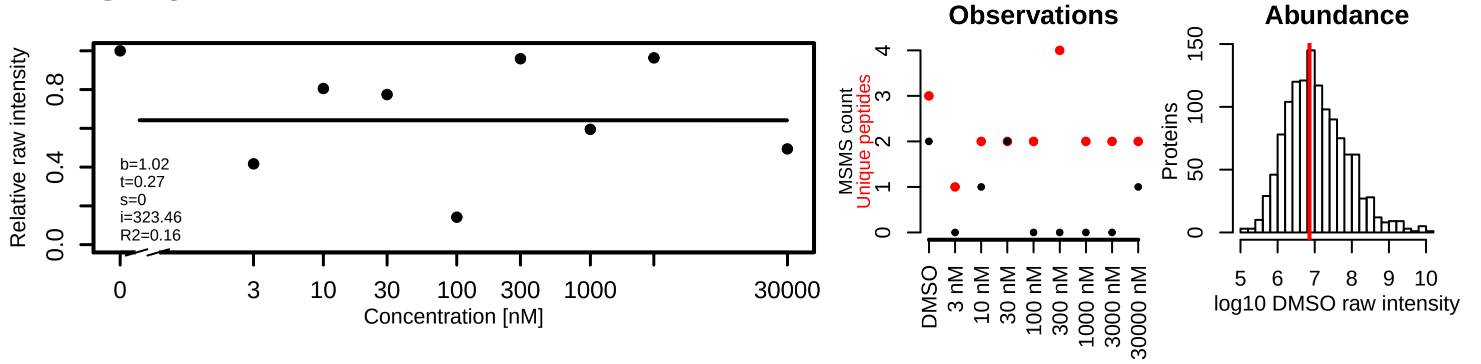

LMNB2

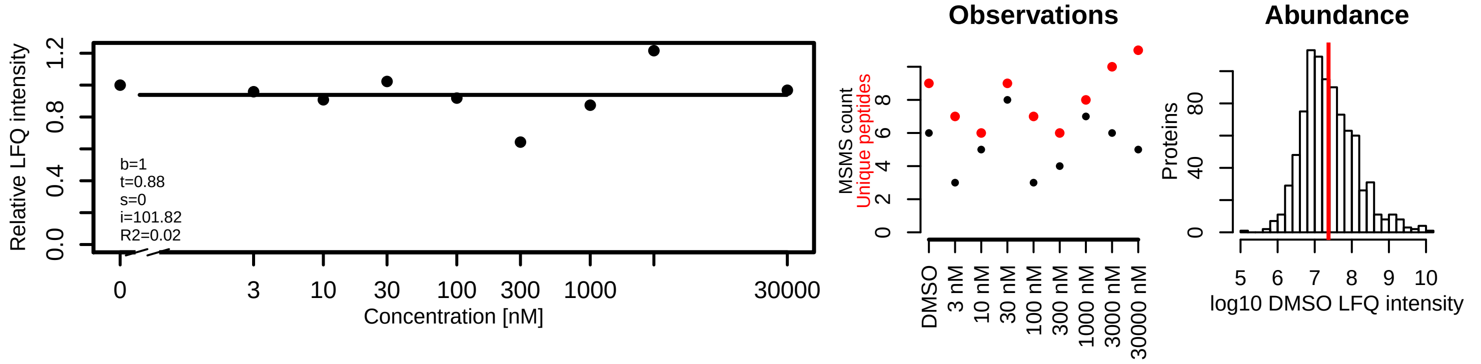

SGPL1

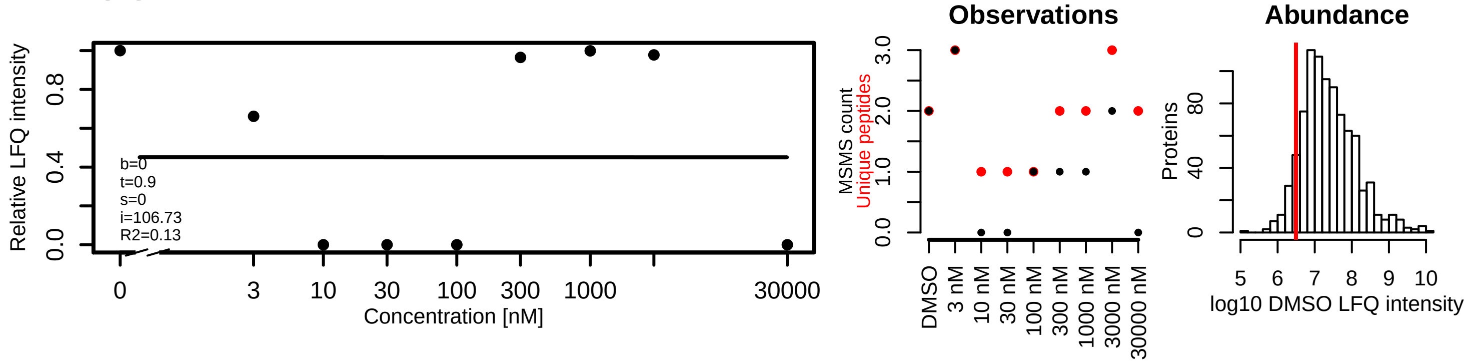

SLC25A10 − B4DLN1;P52815

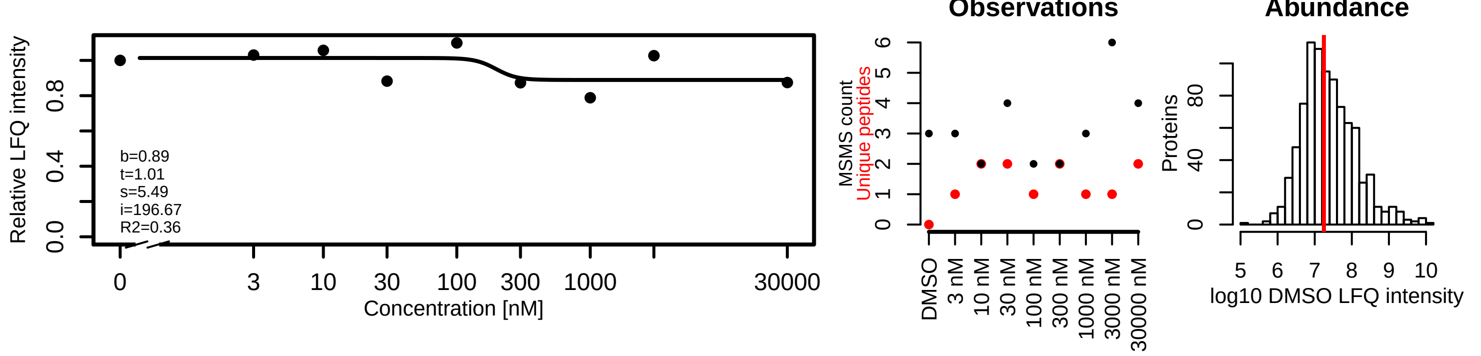

ABCB6

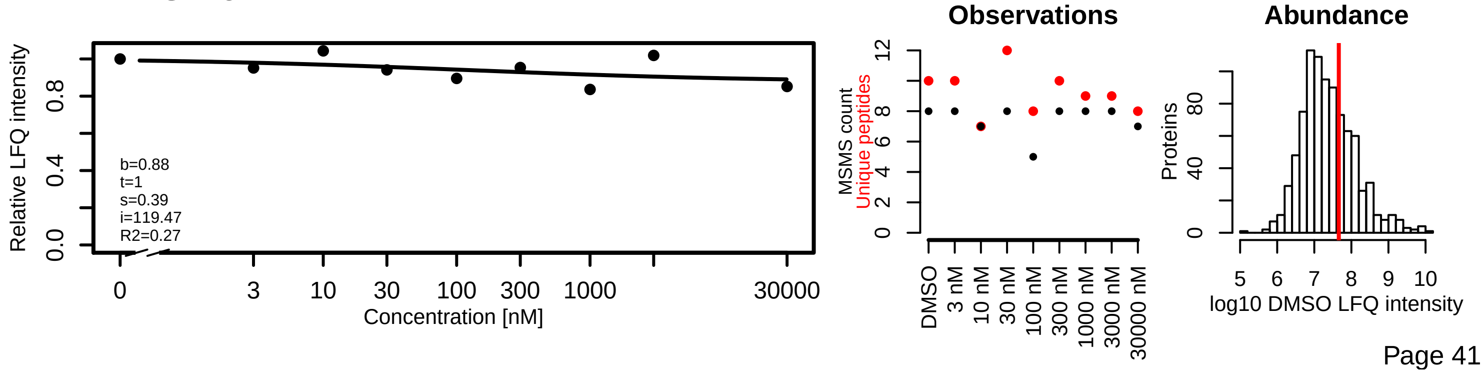

SLC16A3

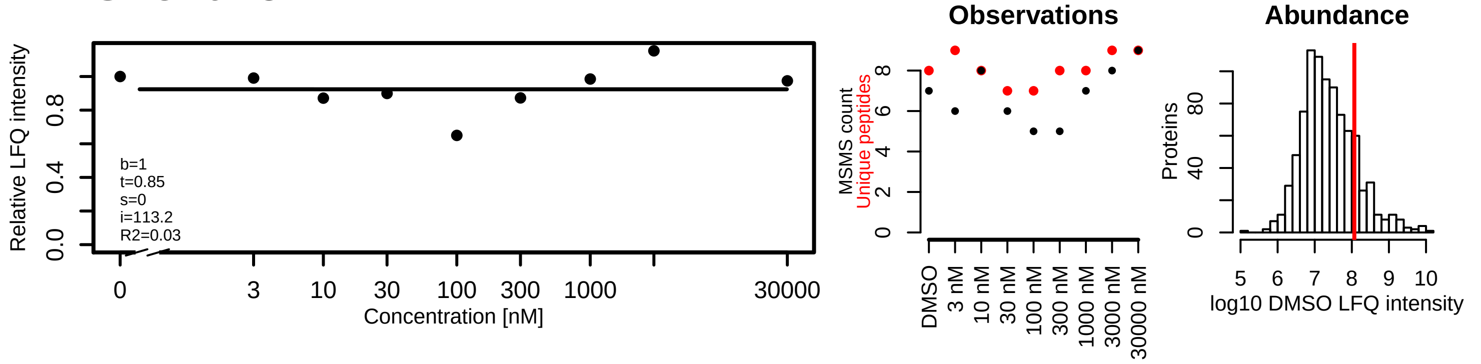

AURKA

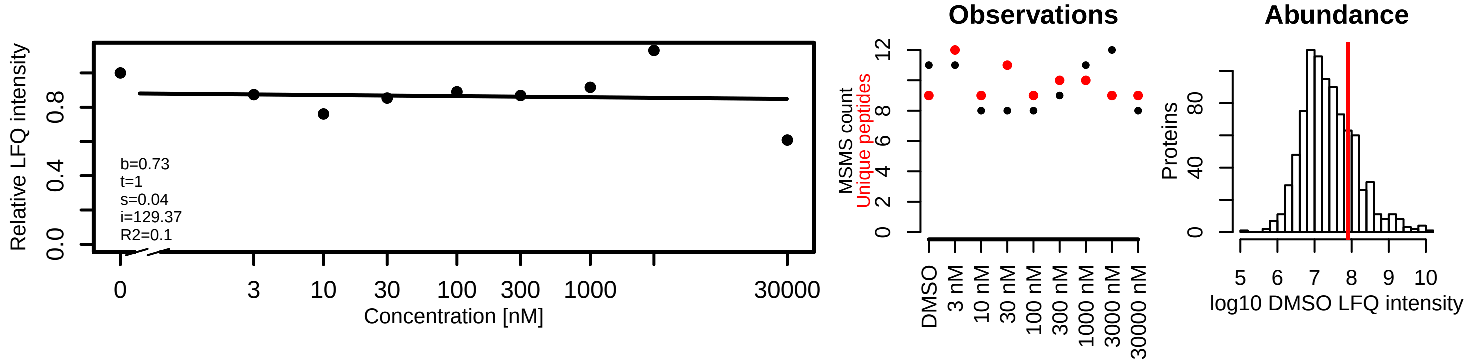

PABPN1L

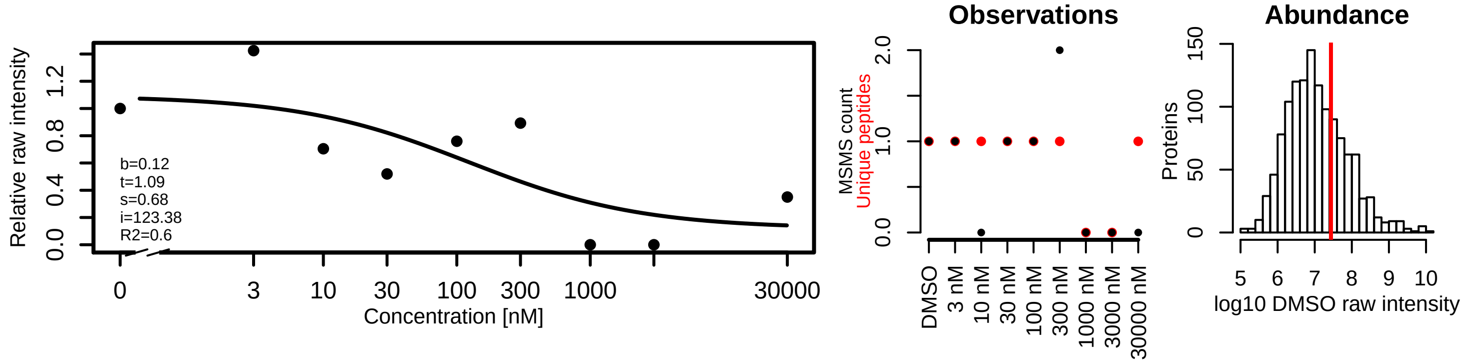

CLDN1

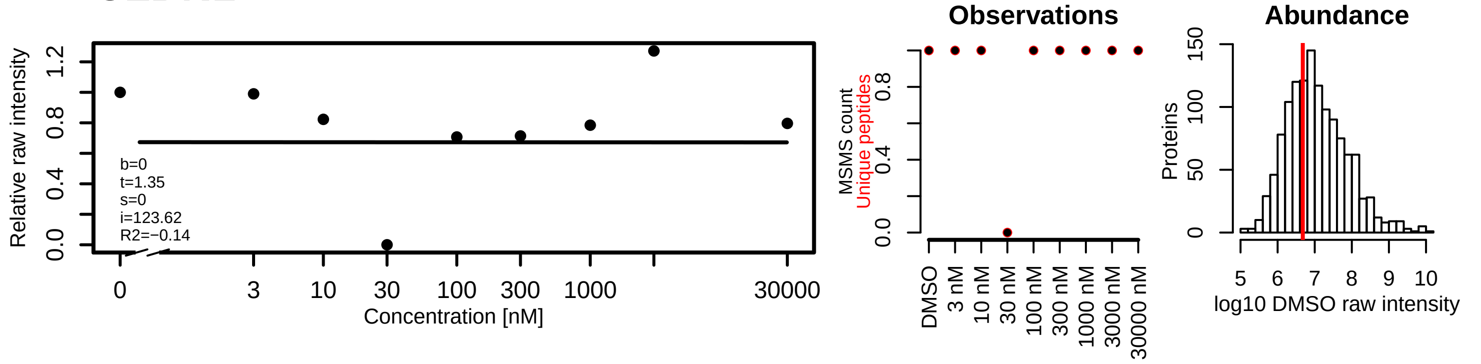

C11orf83

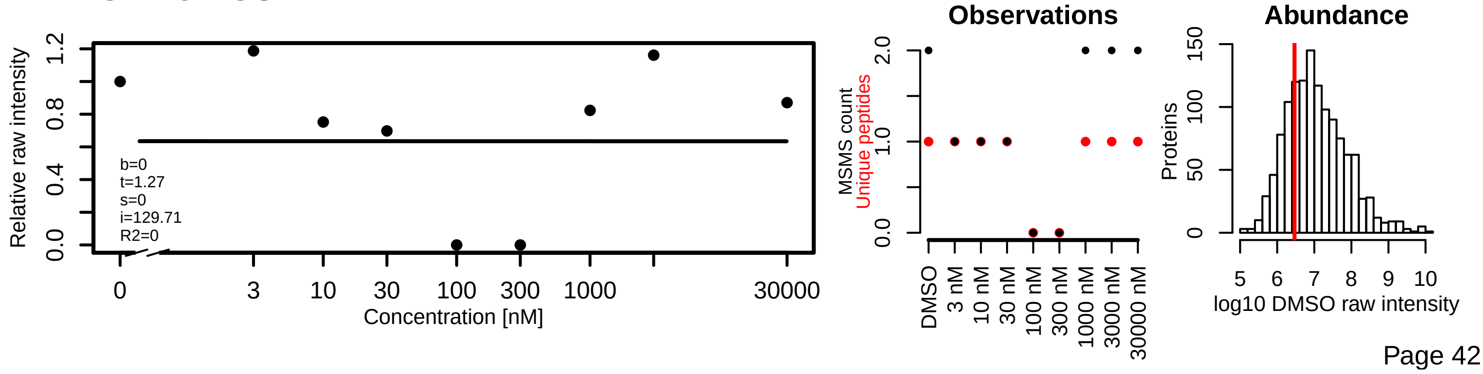

IQGAP1

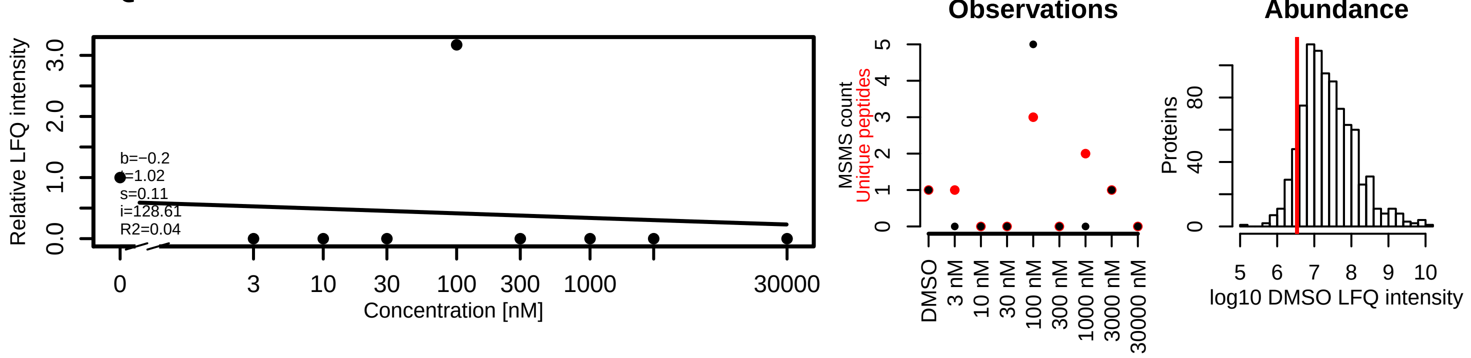

SF3B3

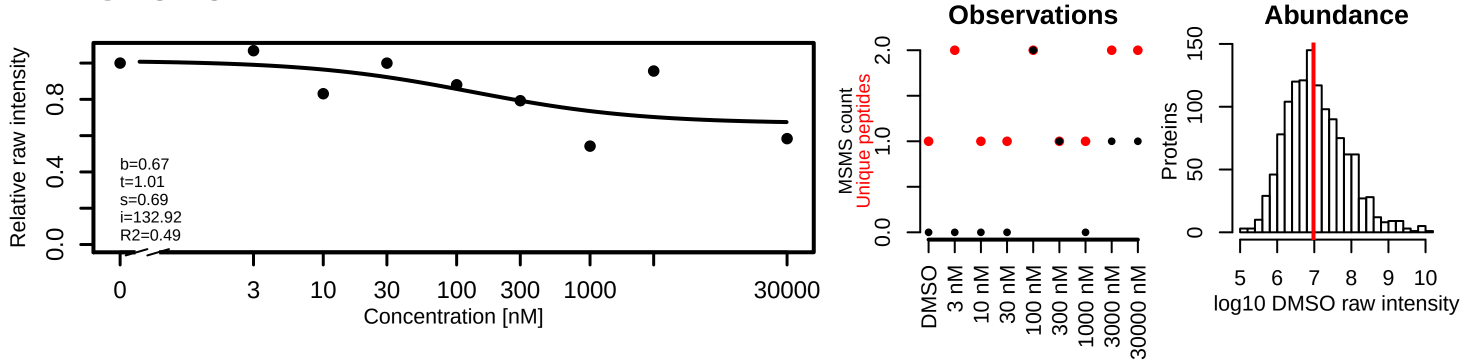

ACADSB

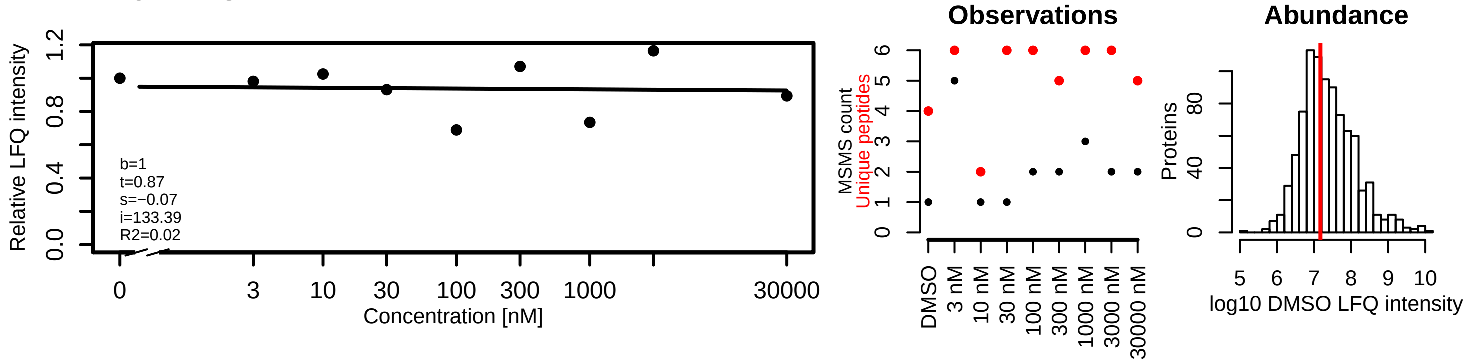

TMCO1

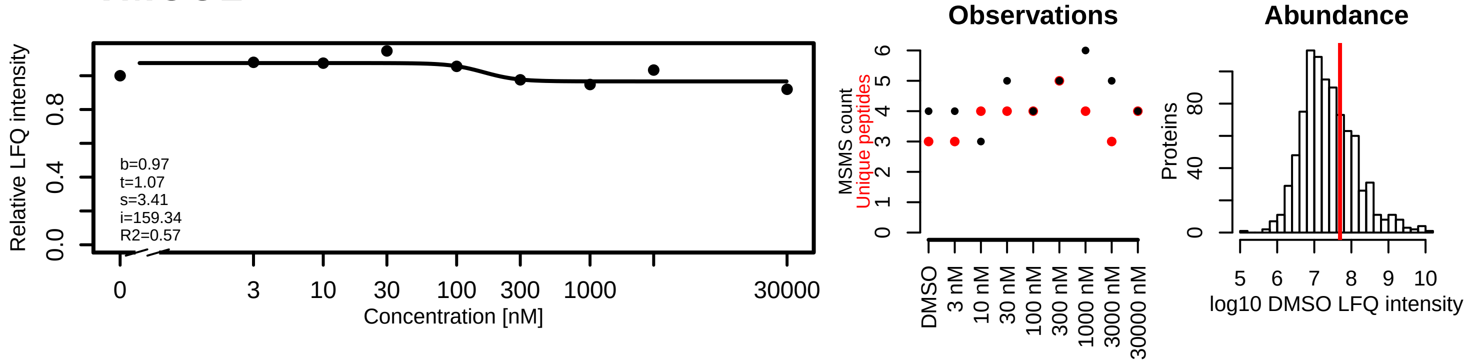

PCBP2;PCBP3

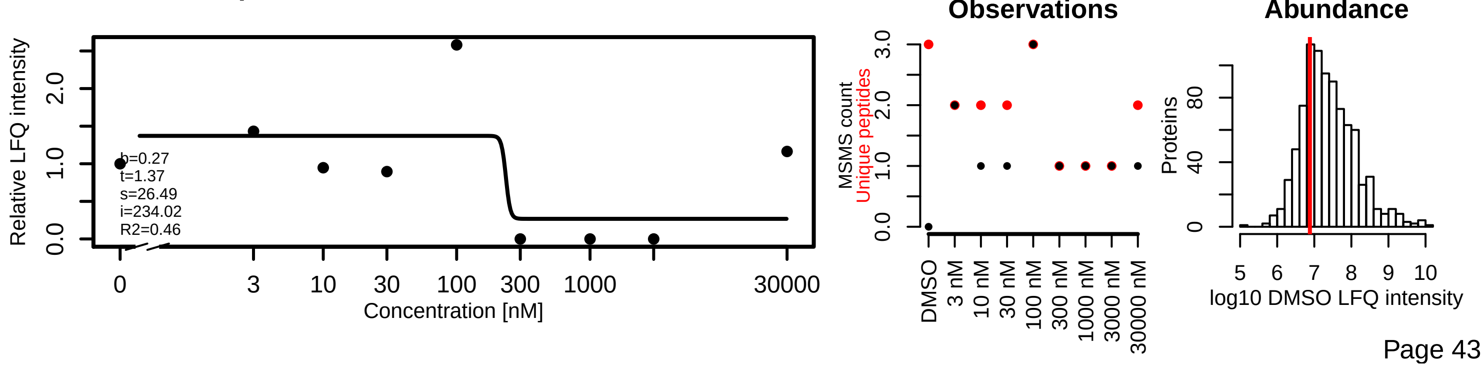

ZDHHC6

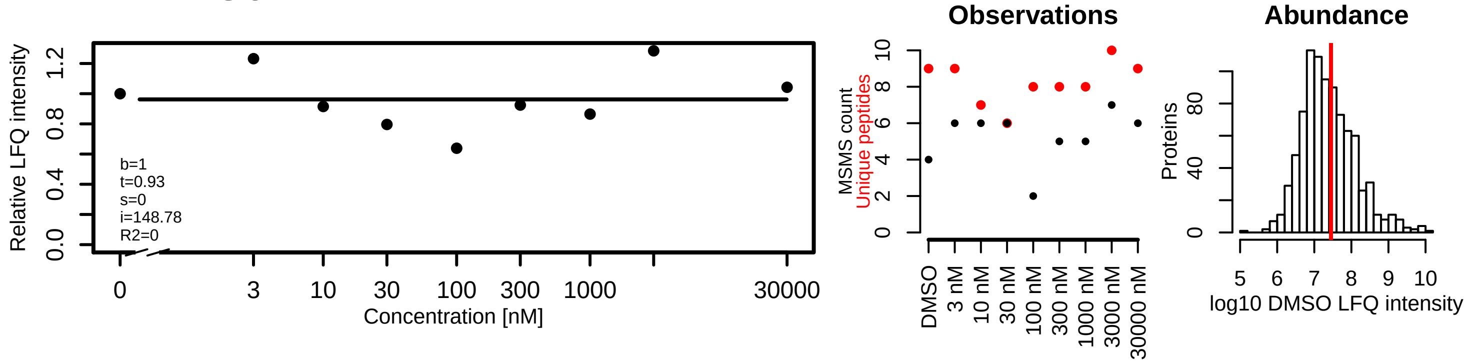

ABCD3

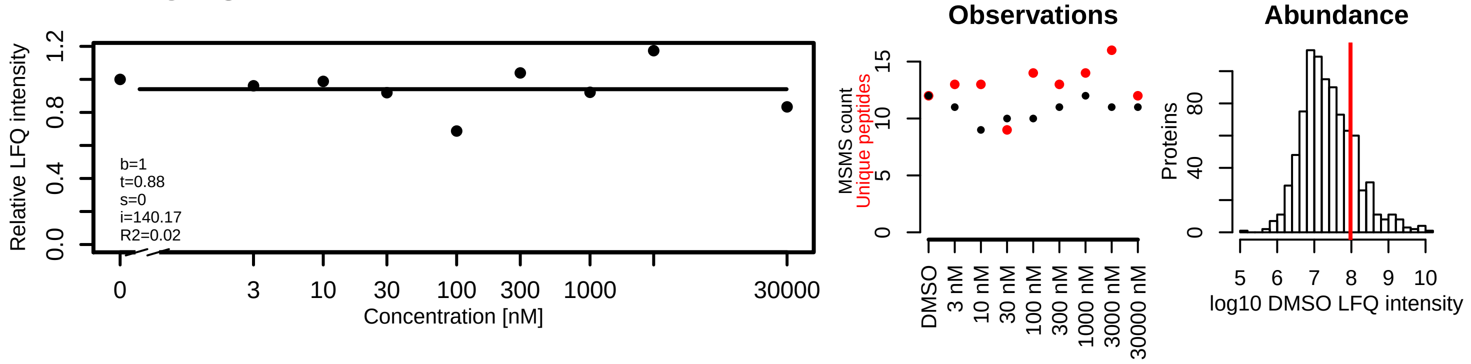

TNPO2

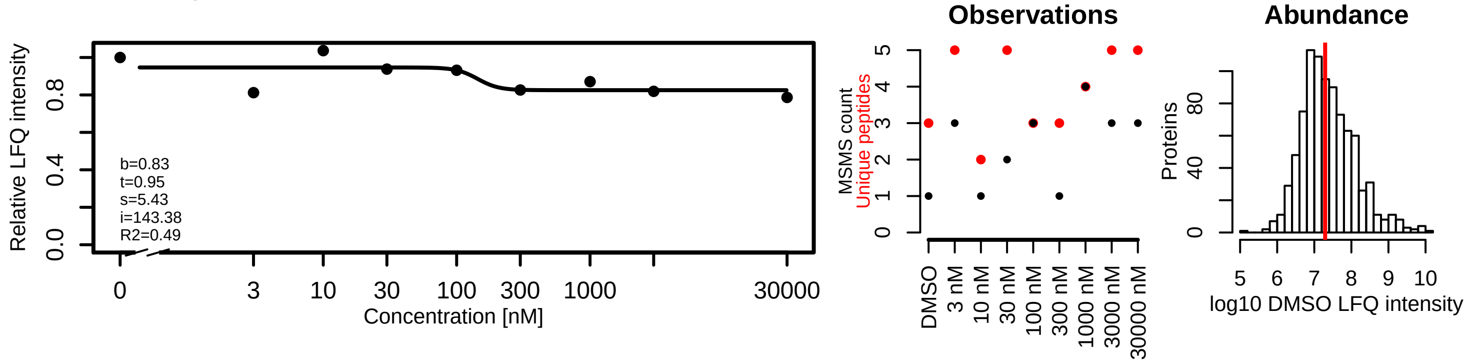

SEC23A

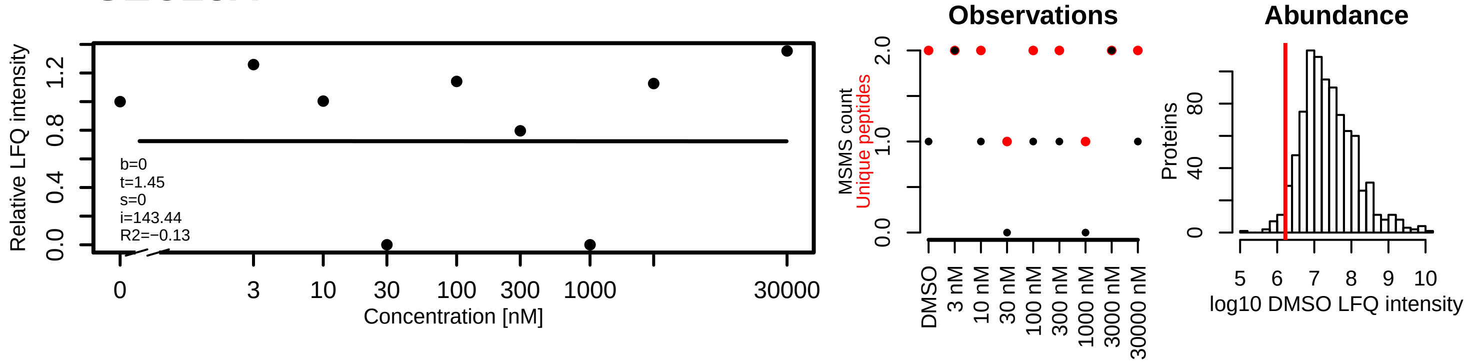

CCDC47

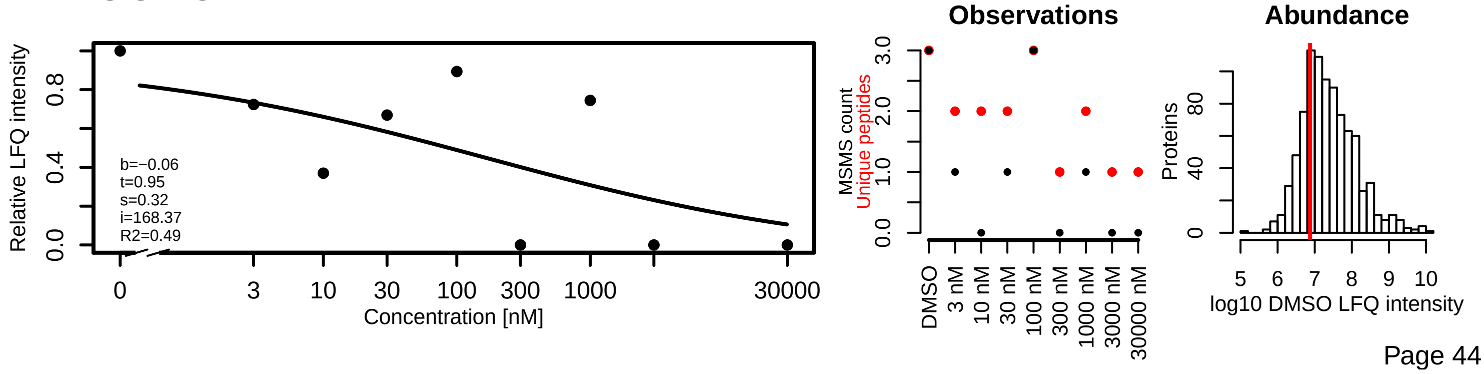

USP9X

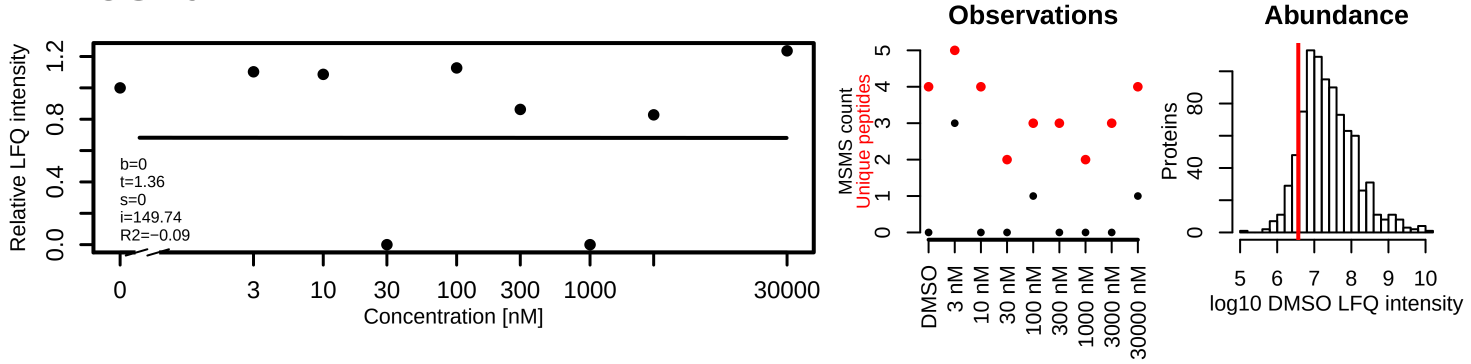

SERPINH1

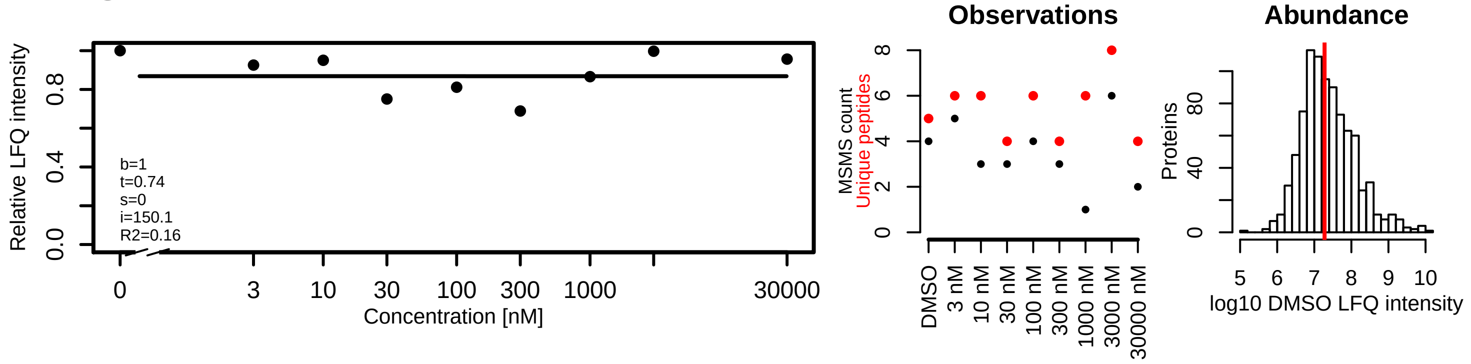

SPG7

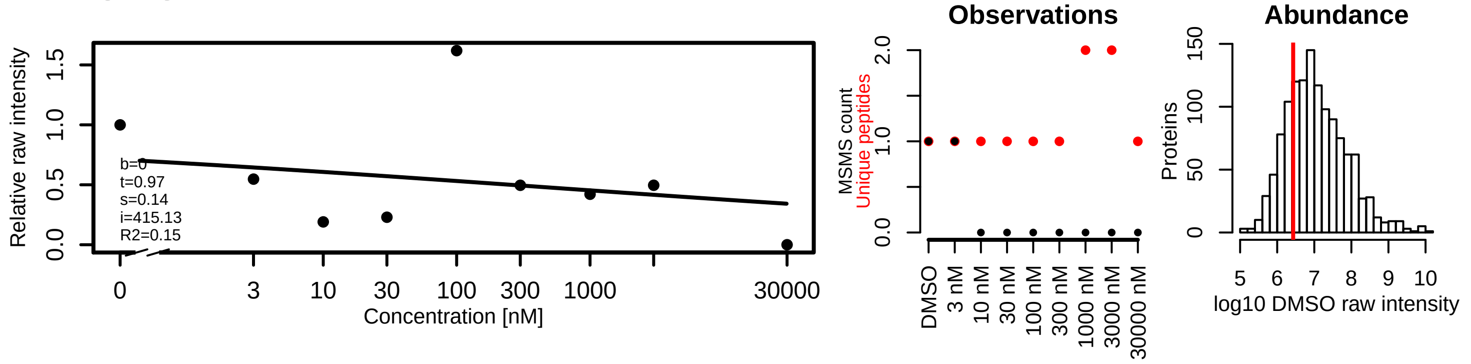

CAD

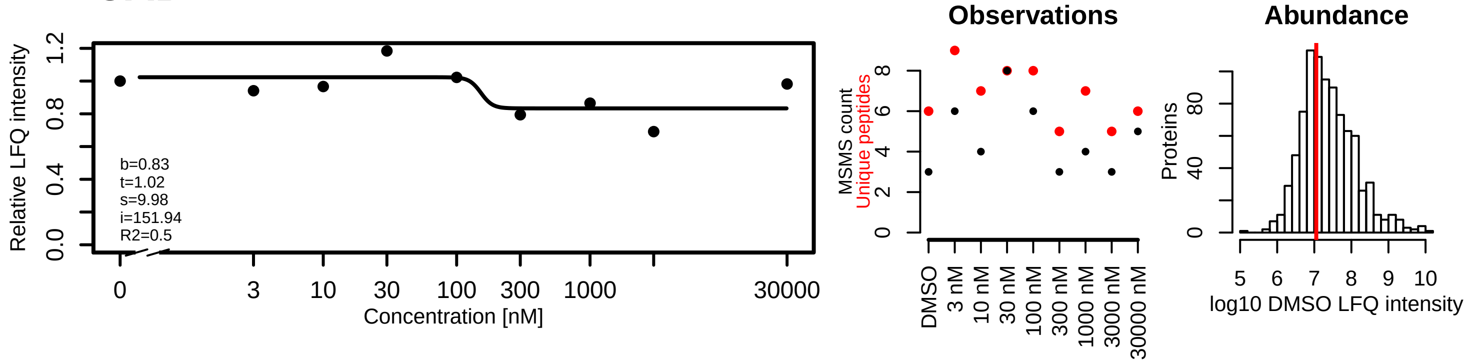

PFKP

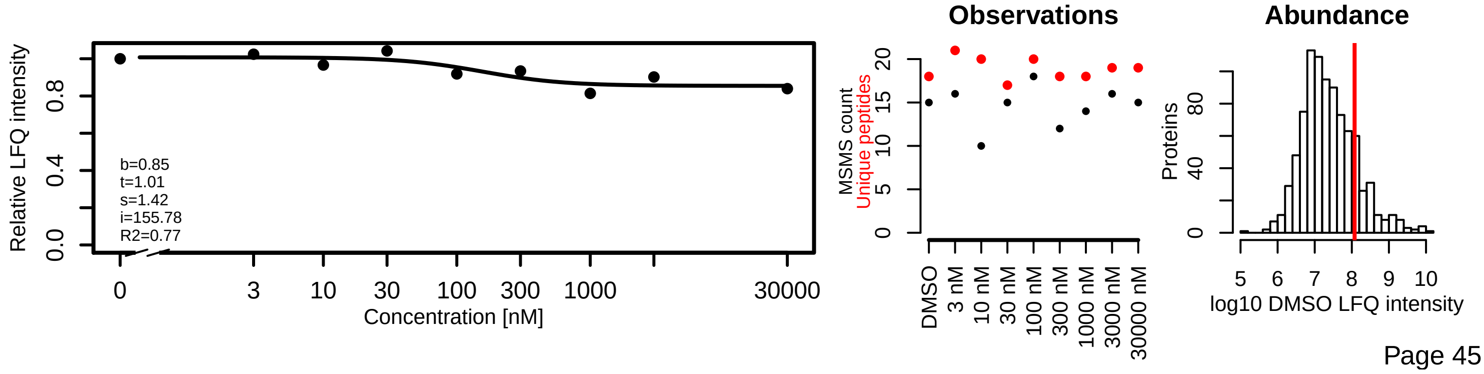

GLUD1;GLUD2

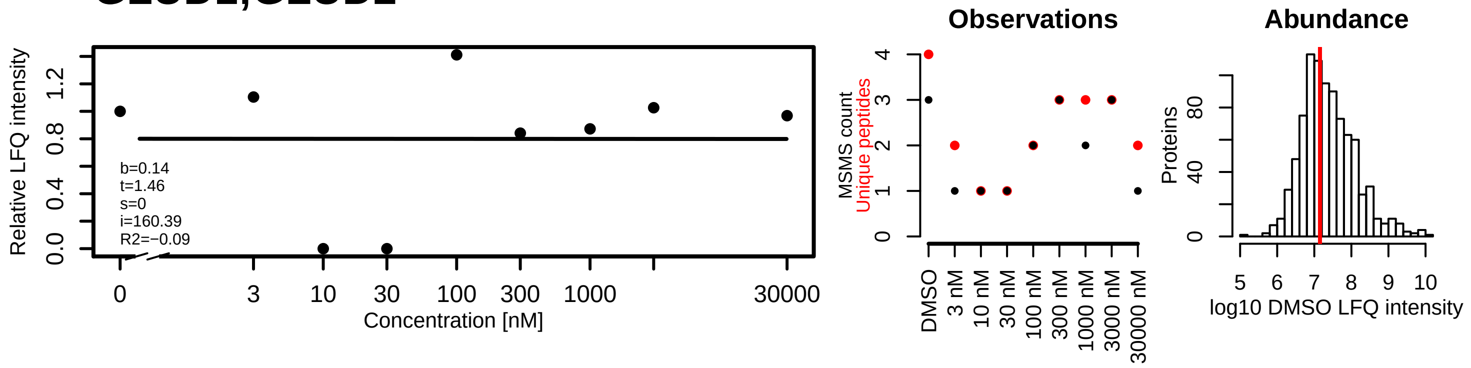

SPTLC2

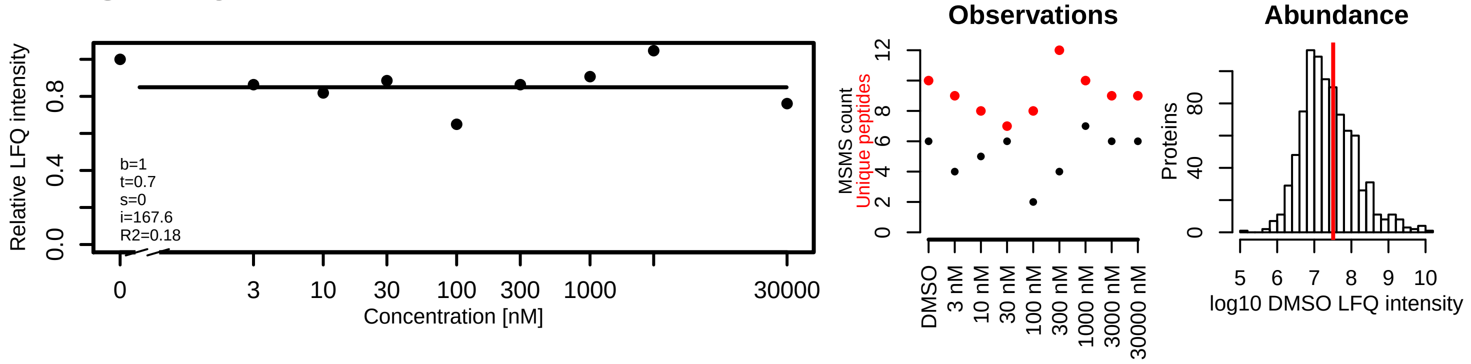

TECR

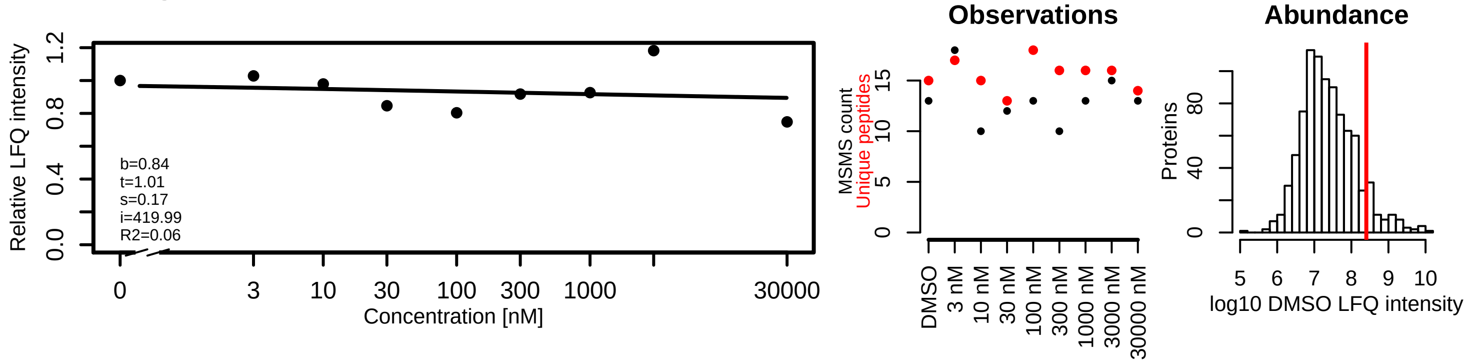

RPS6

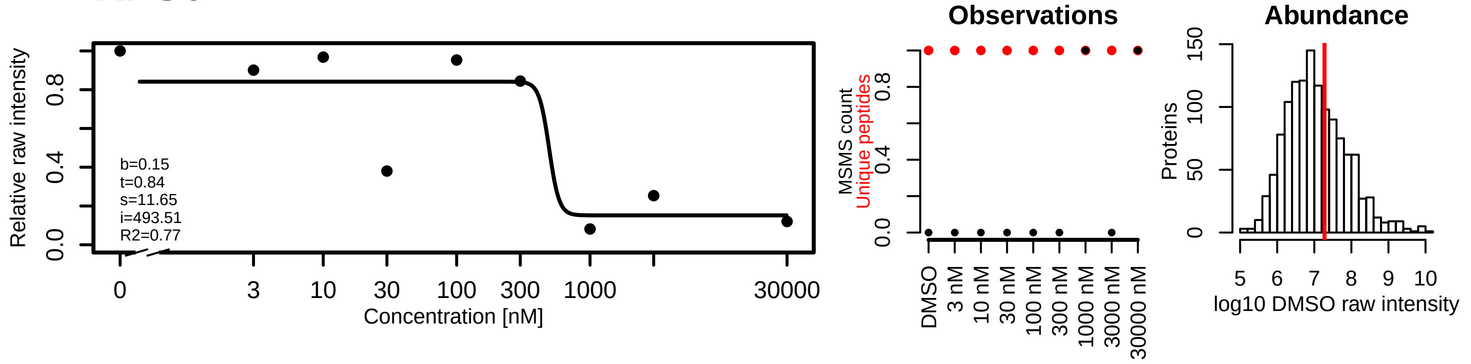

FLNC

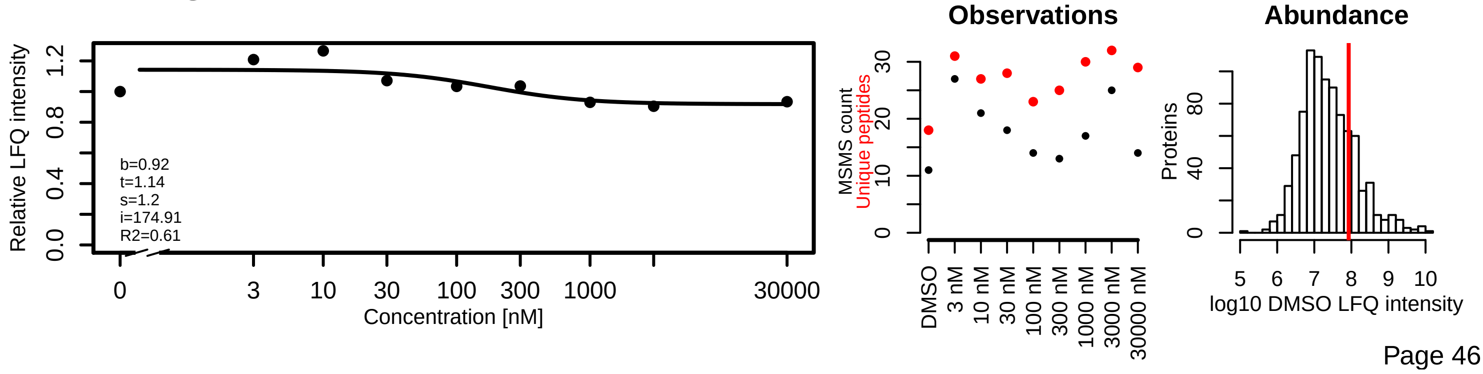

NPTN;DKFZp566H1924

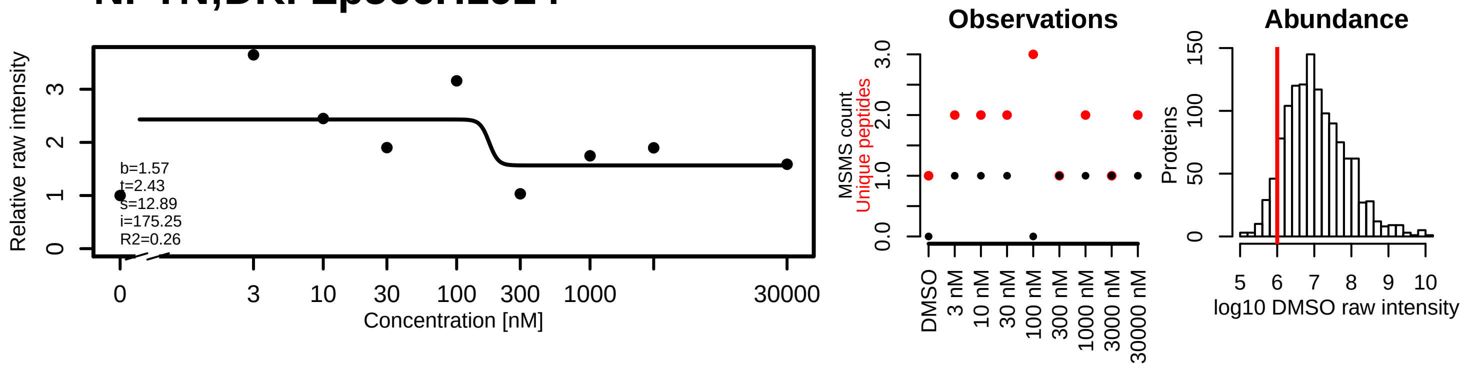

RPS7

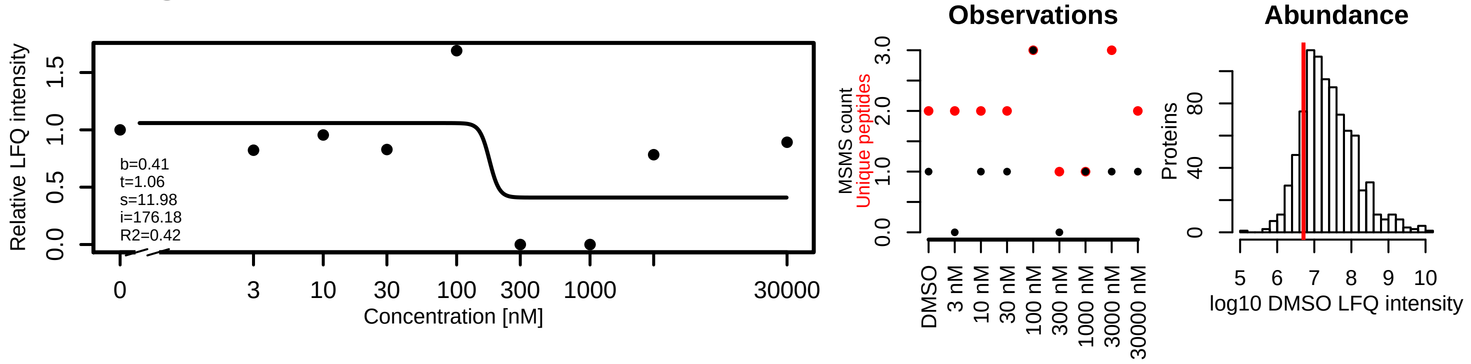

HSPA1A

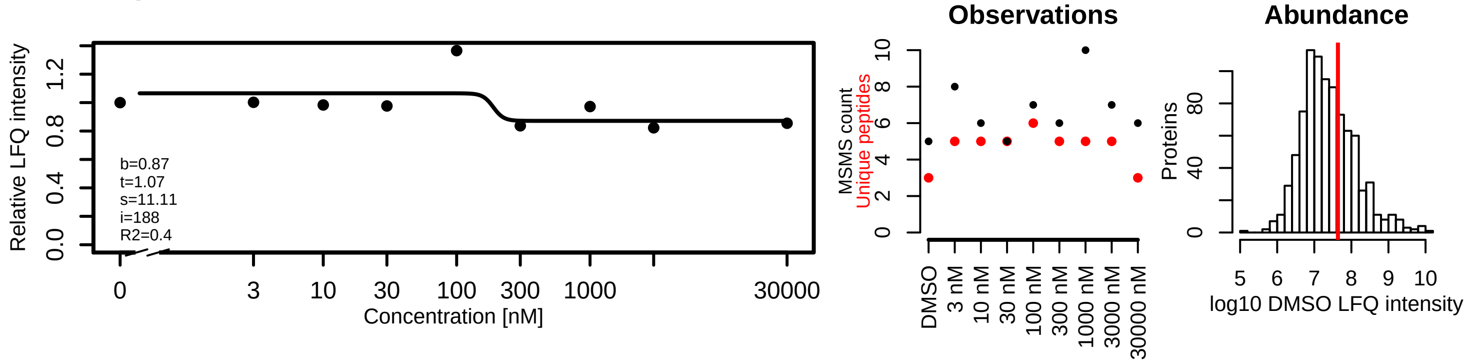

RPS28

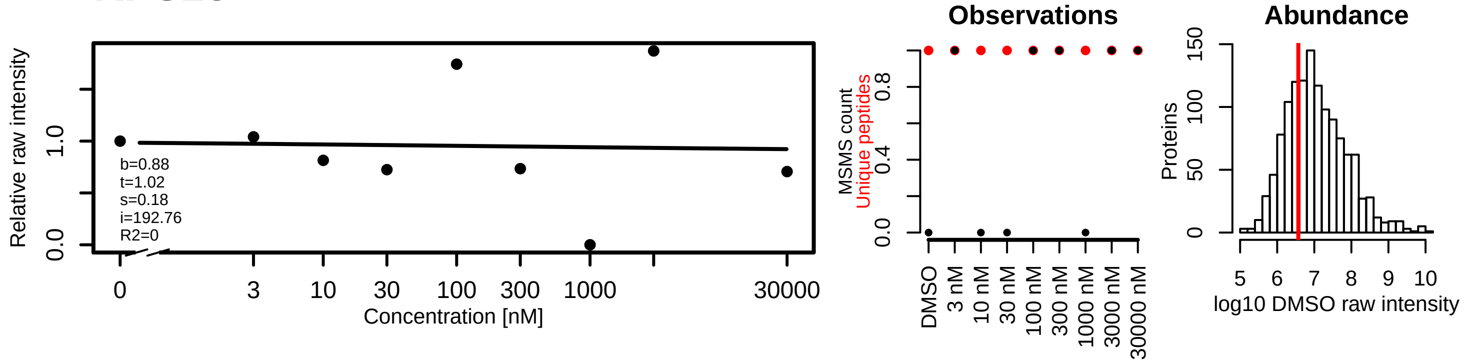

SPTBN1

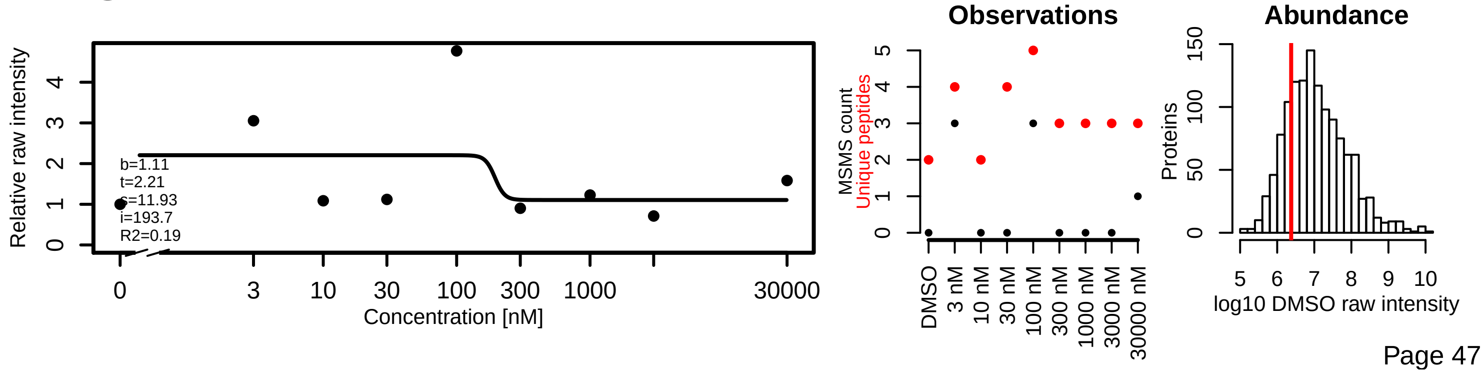

YWHAQ

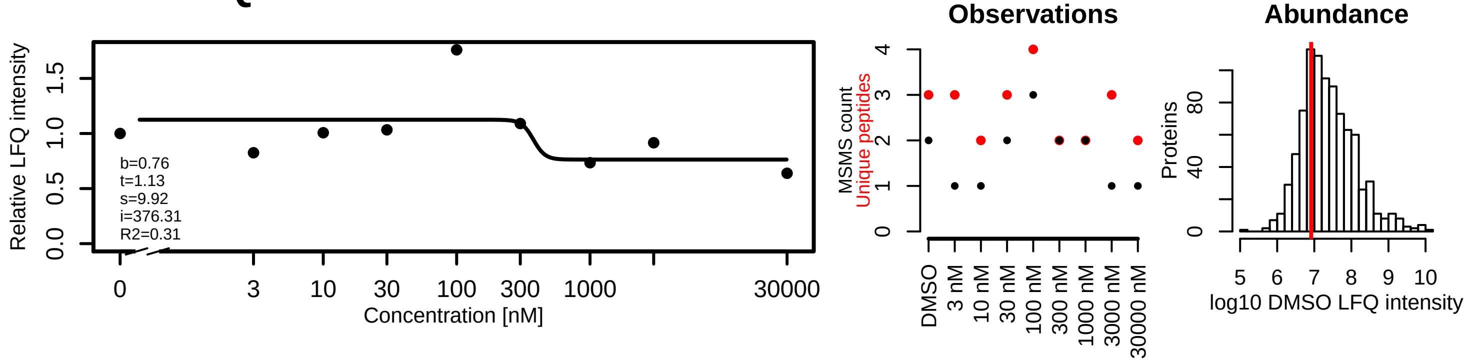

COPG1

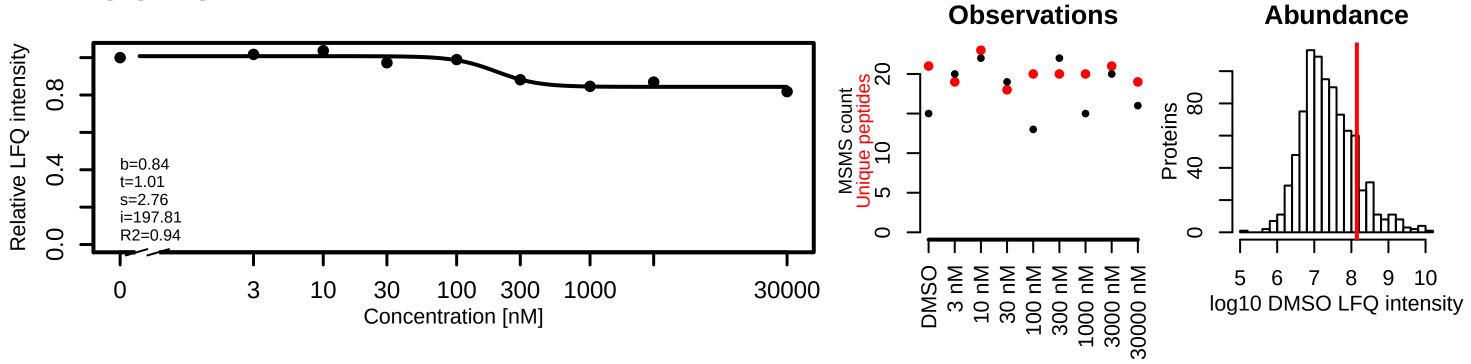

TUBB2A;TUBB2B

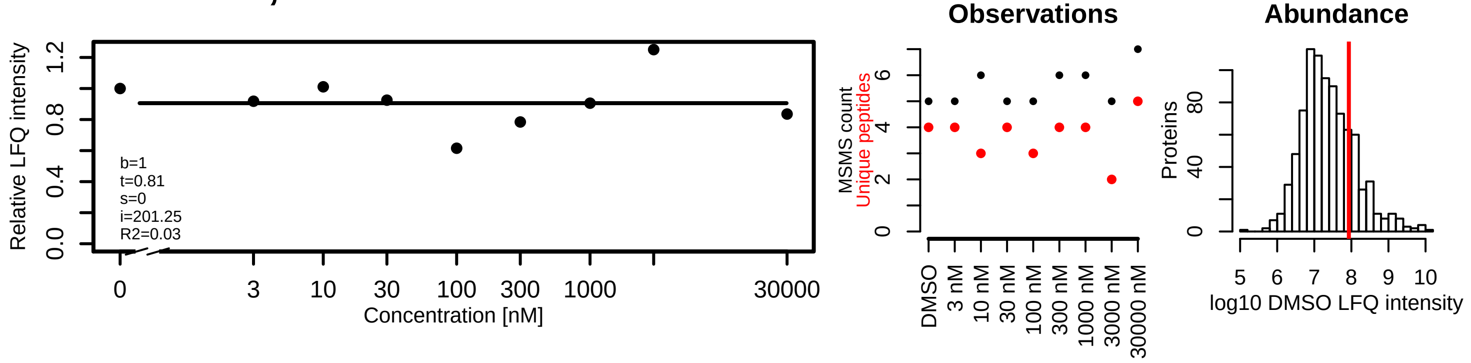

CCT2

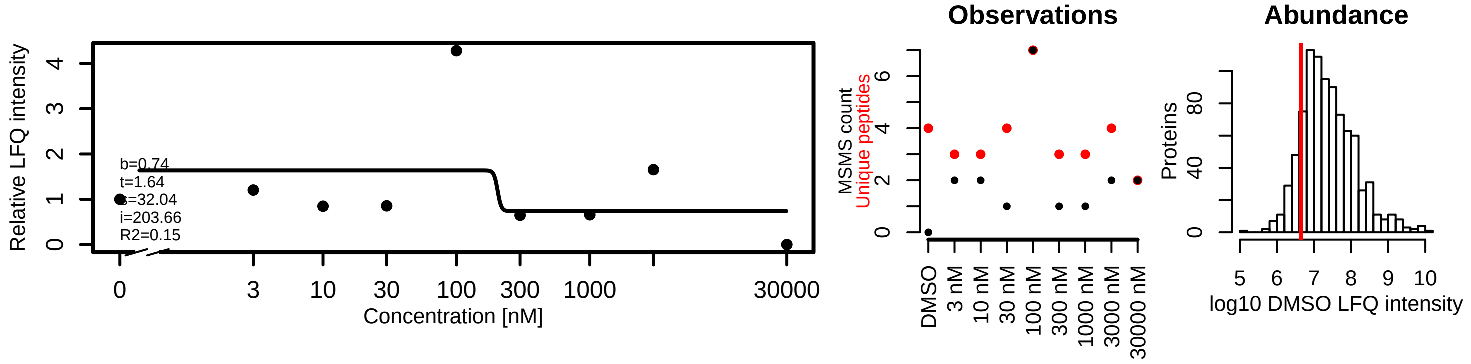

EIF3E

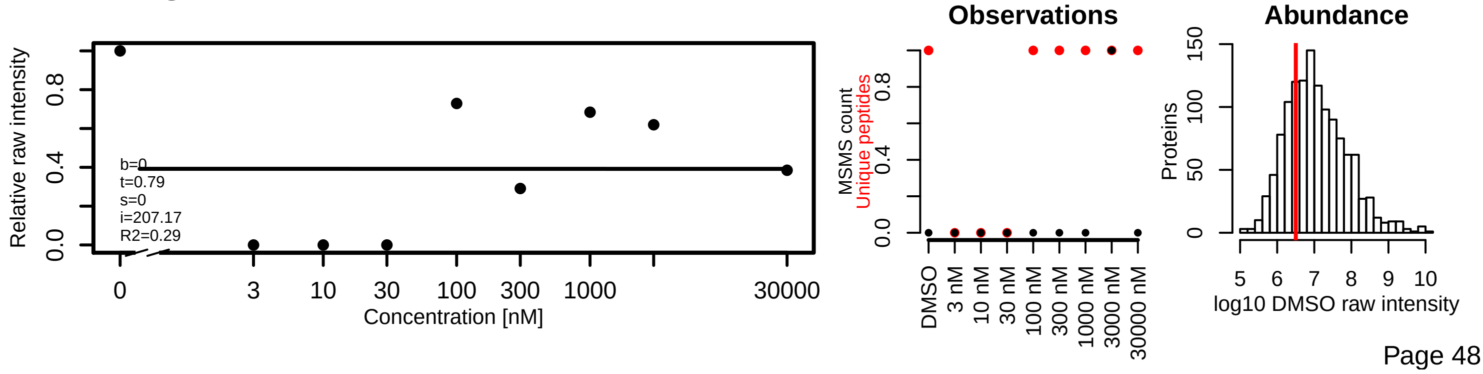

OXA1L

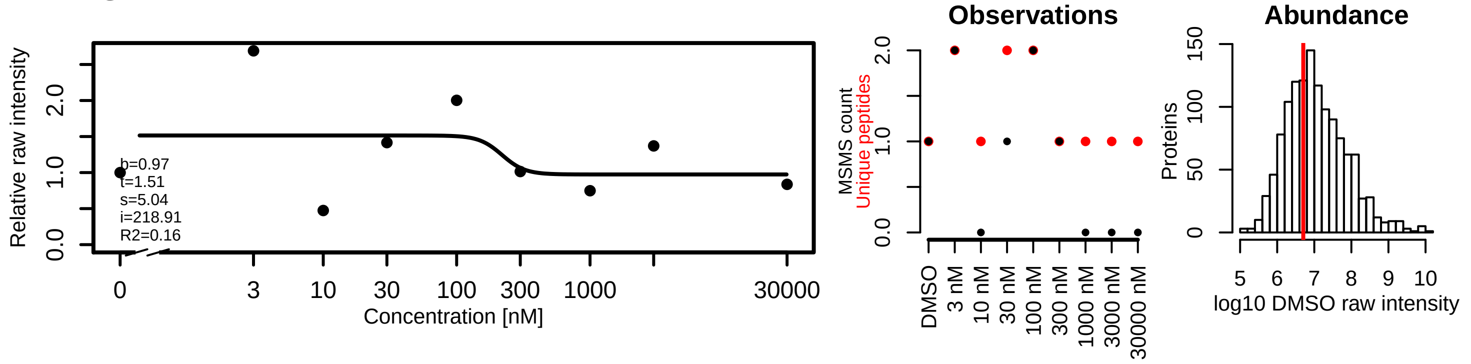

SYNJ2BP

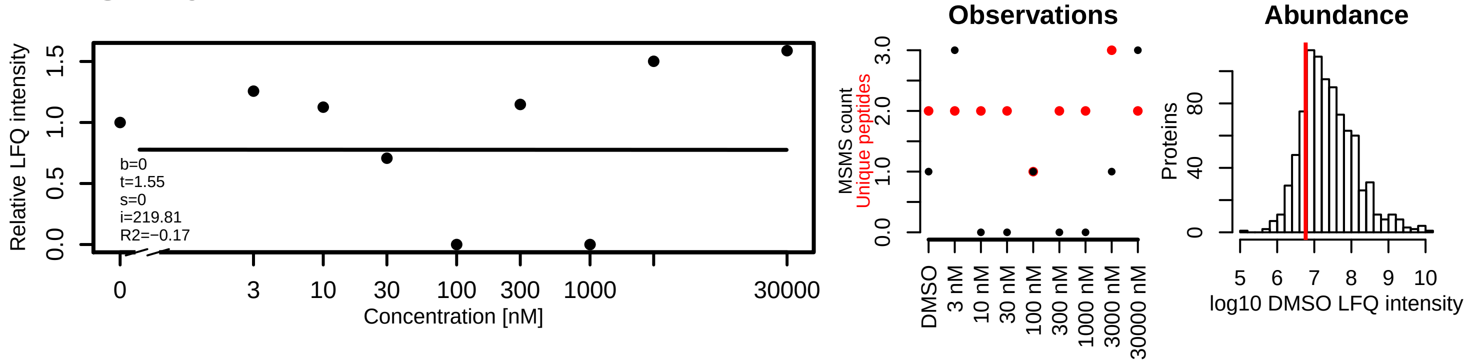

SPTLC1

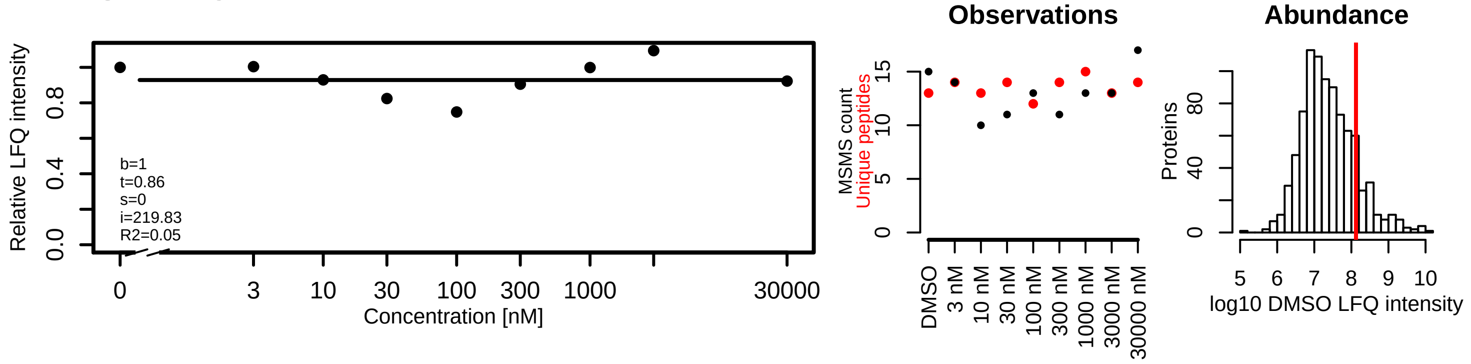

SFN

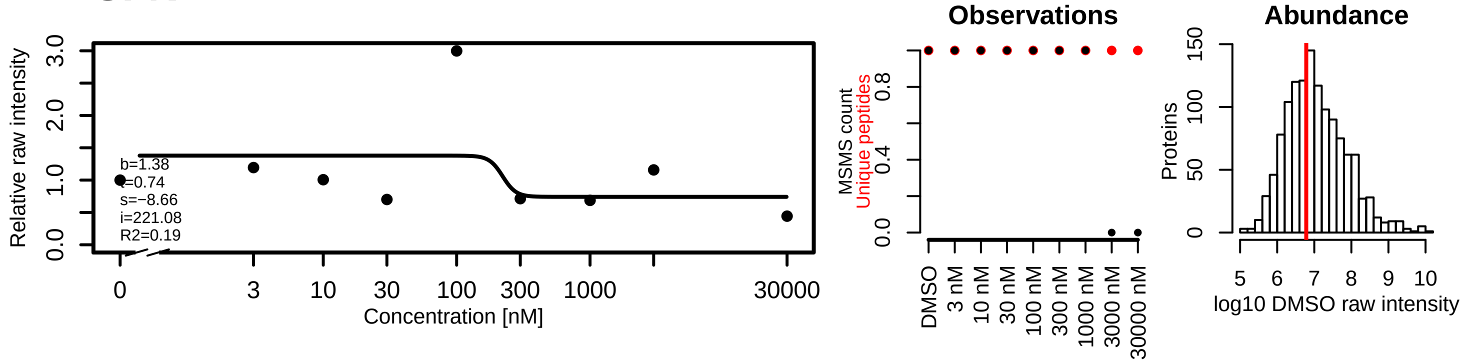

HSPA5

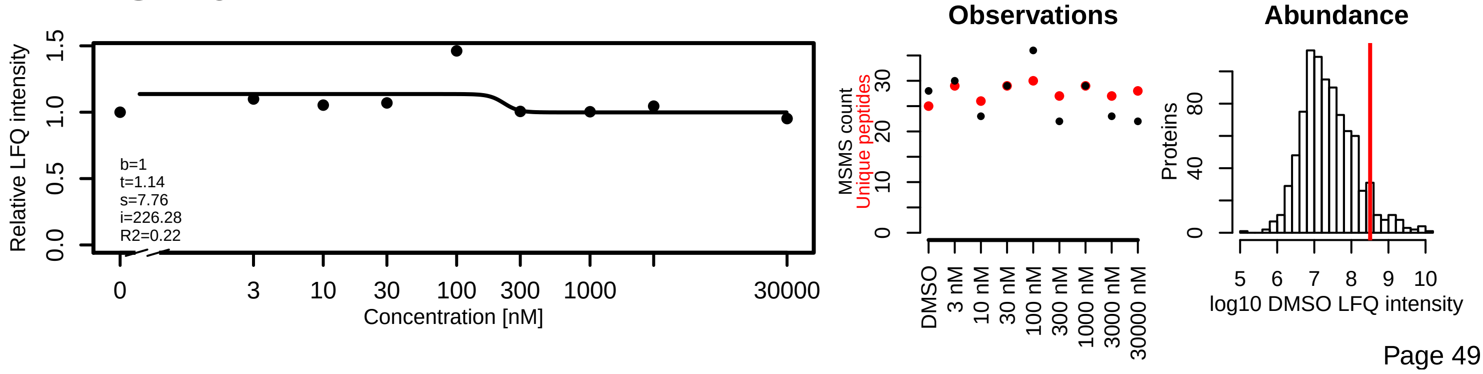

ACVR1B

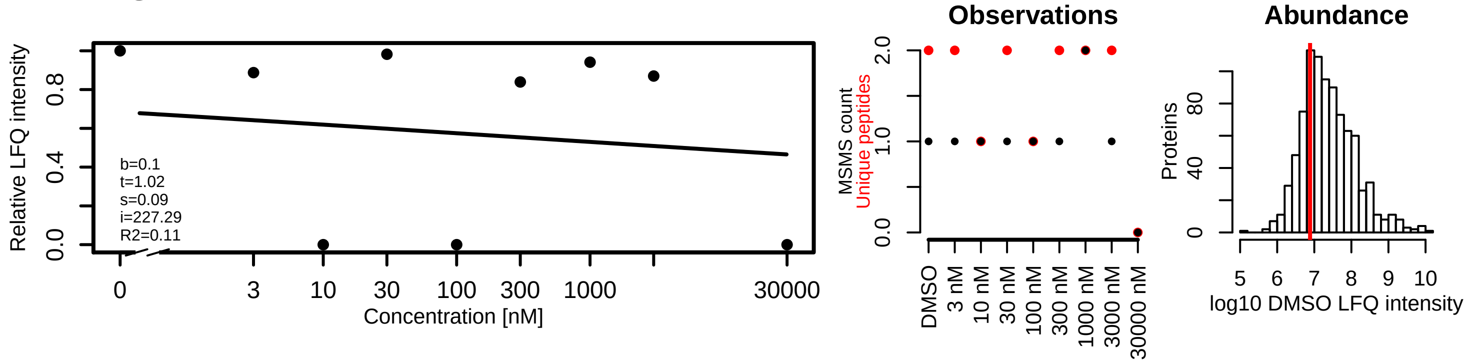

TM9SF3

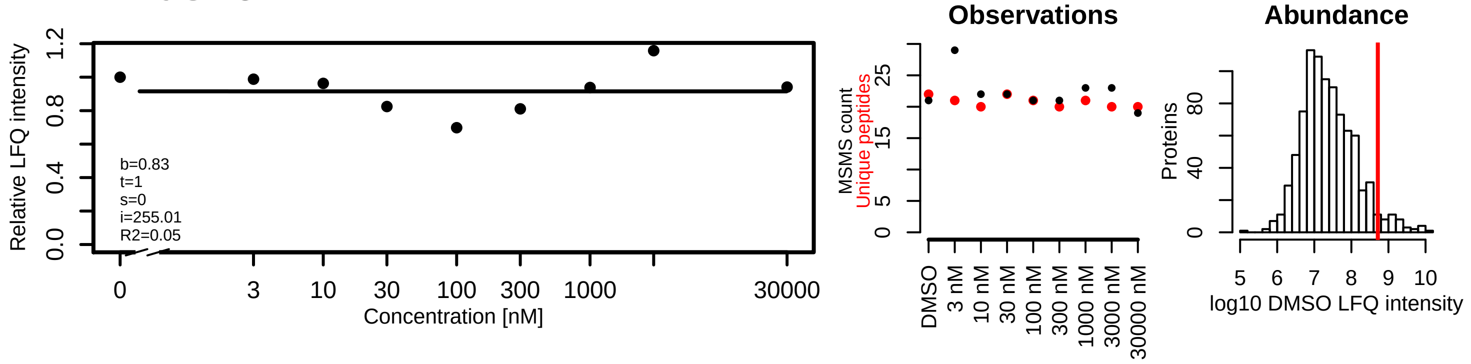

SUN1;UNC84A

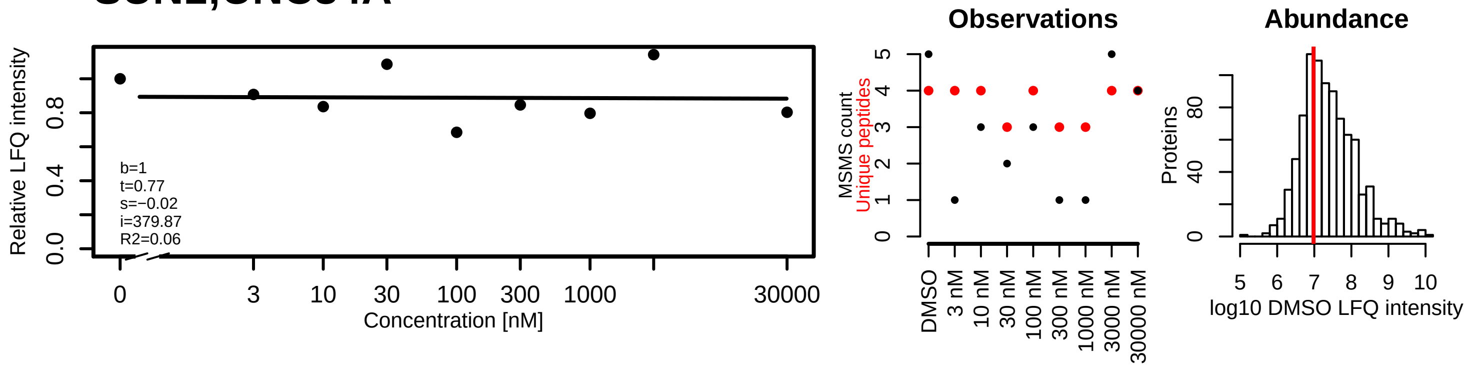

MBOAT7

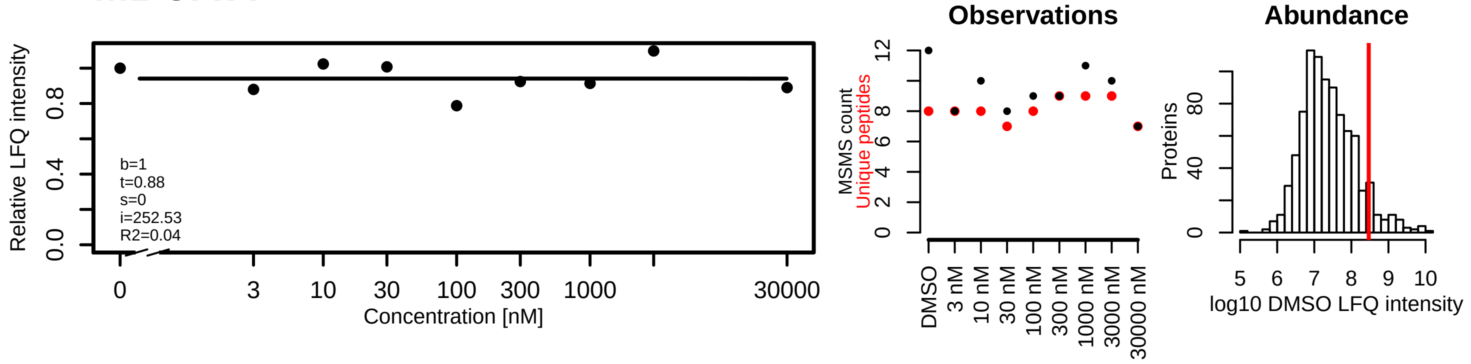

SEC61A2

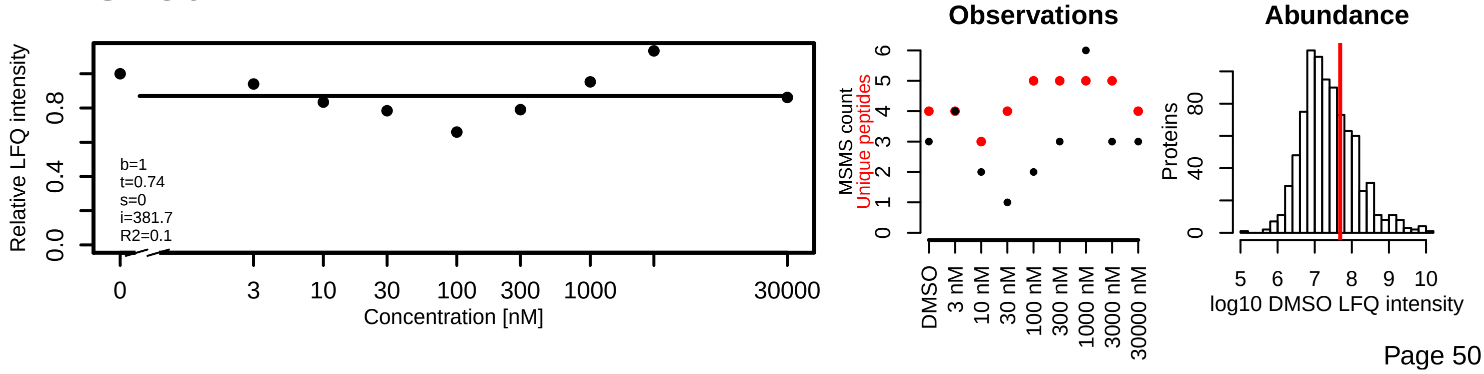

AGPS

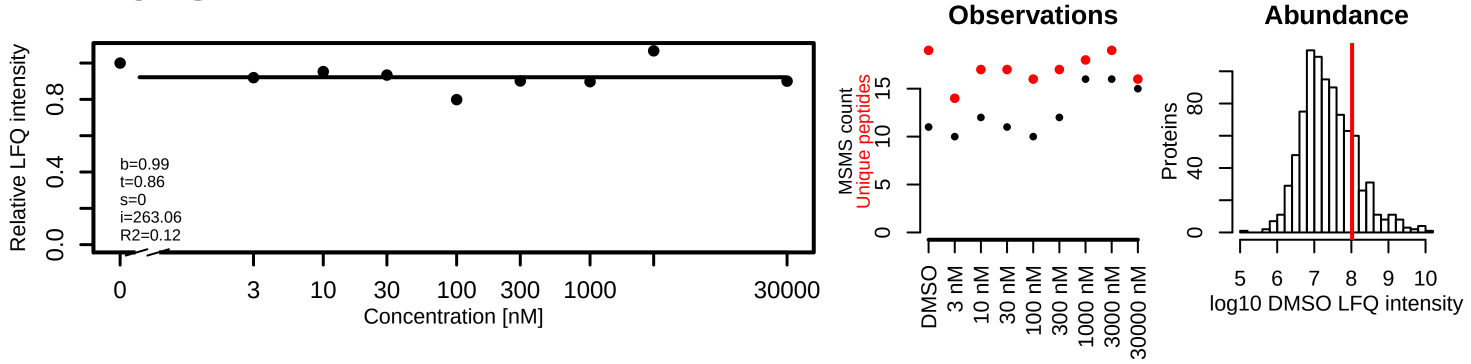

YWHAZ

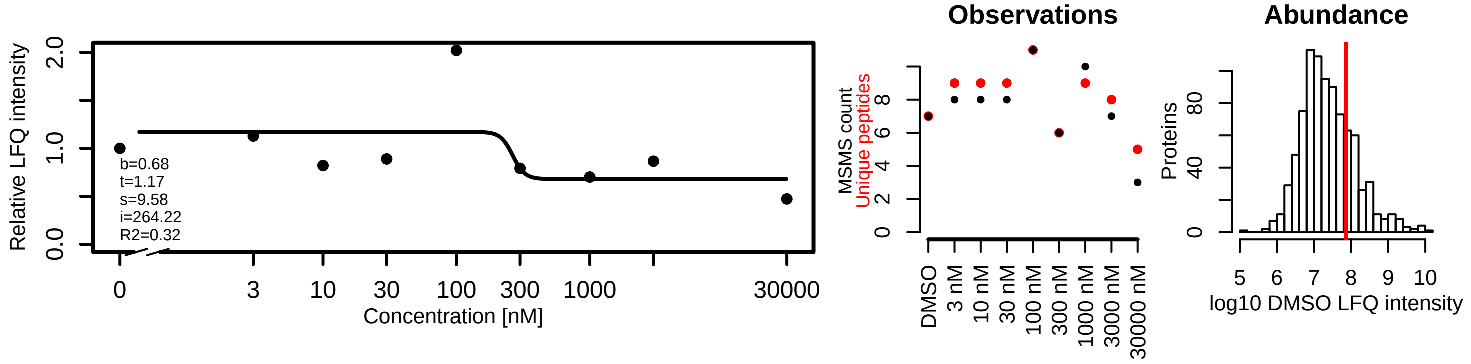

HSPA9

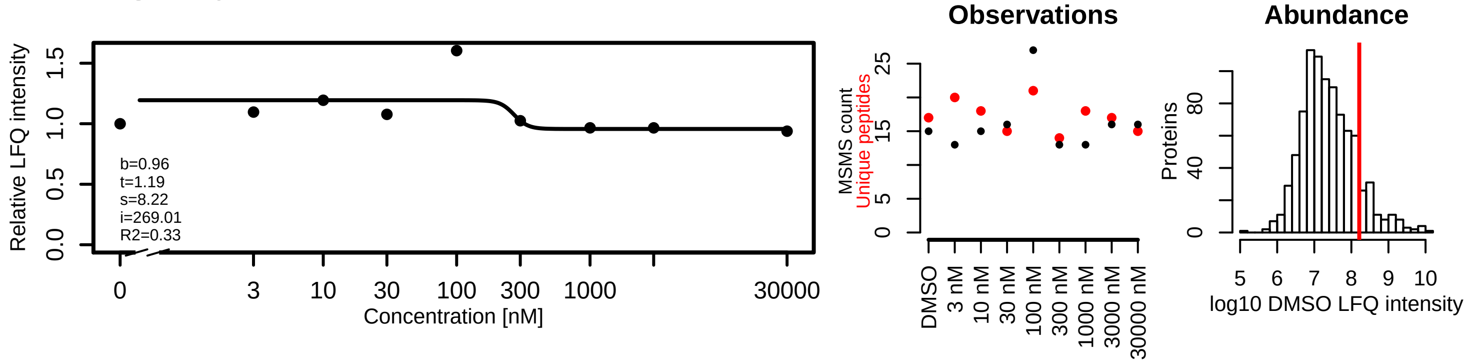

TMEM245

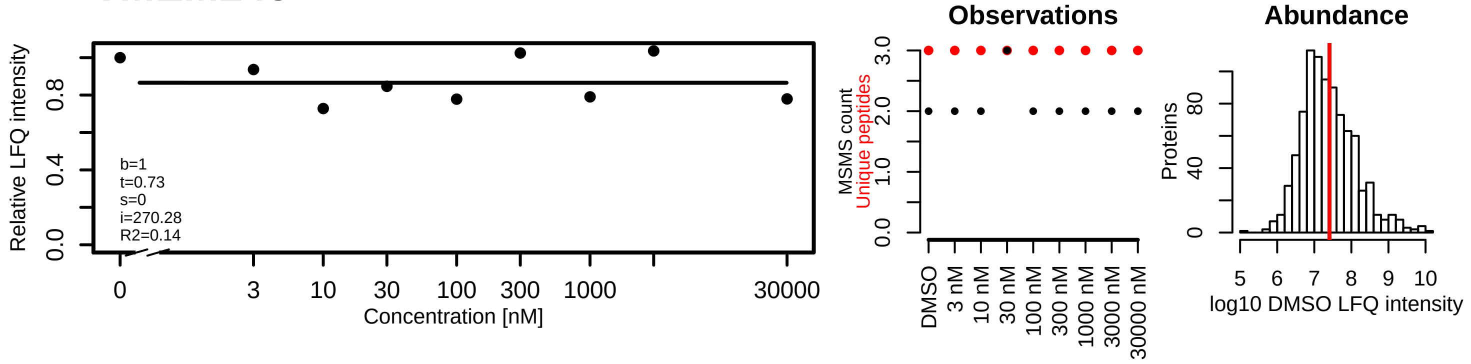

ZDHHC21

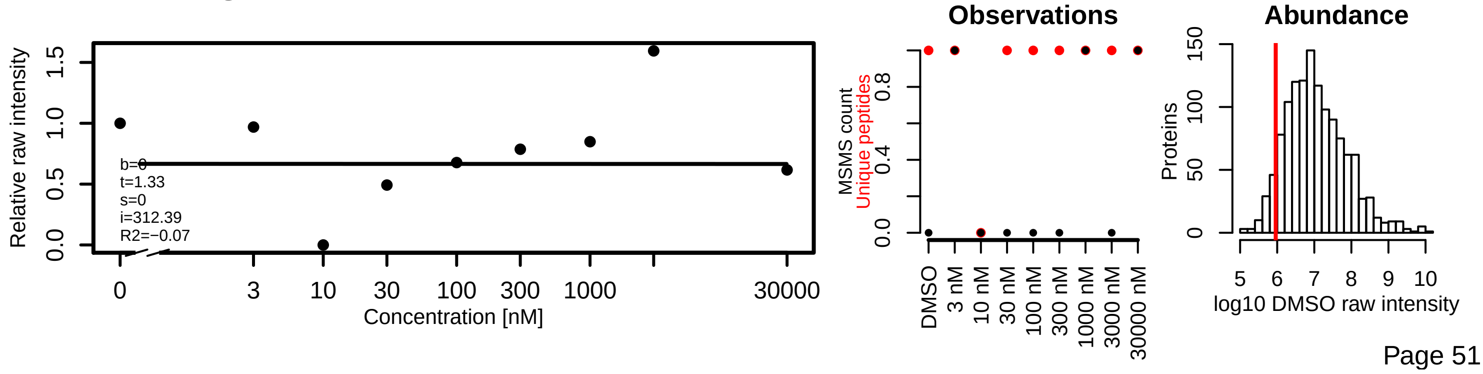

ACTG1

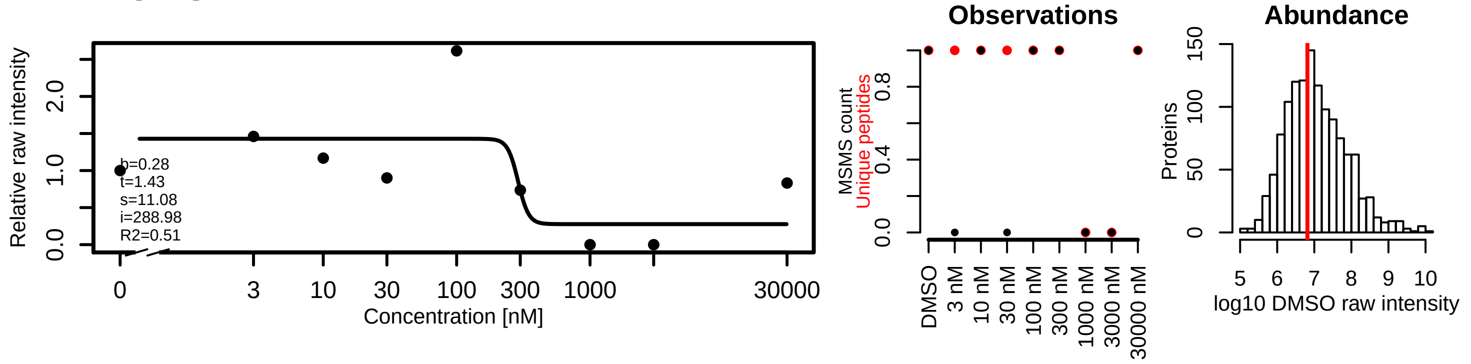

TUFM

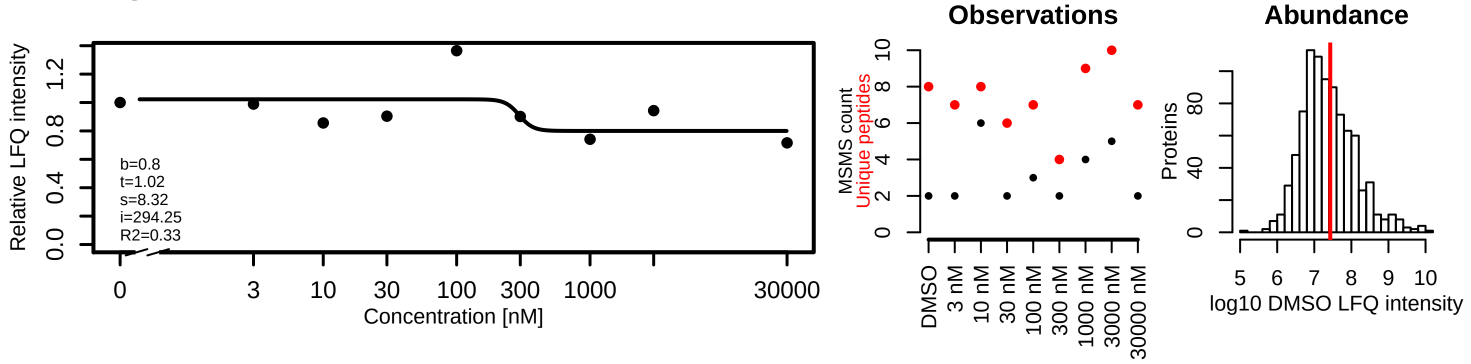

RPAP1

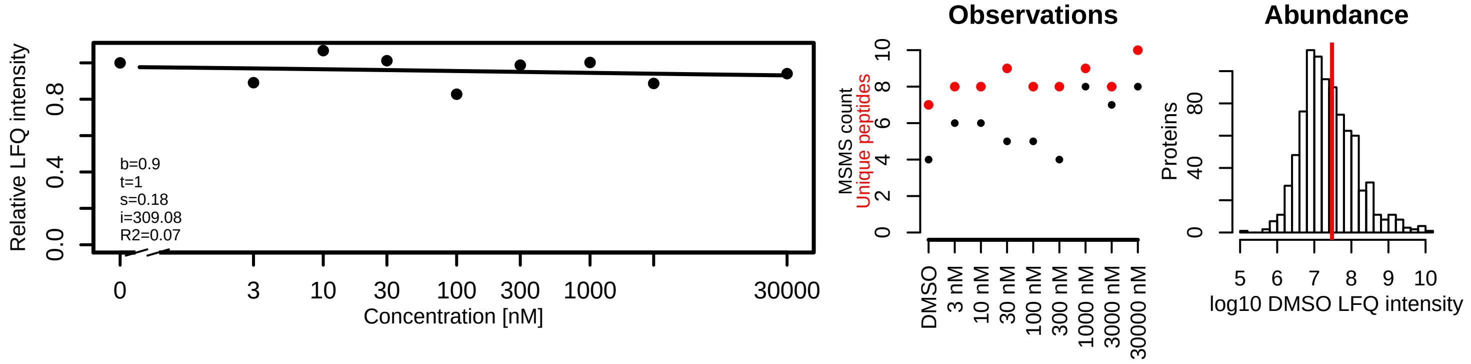

TESK1

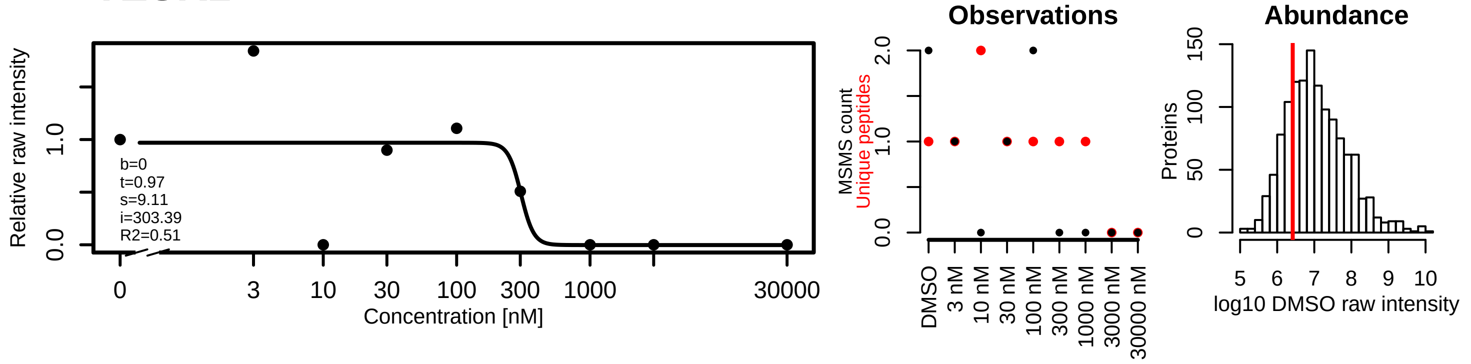

VAPB

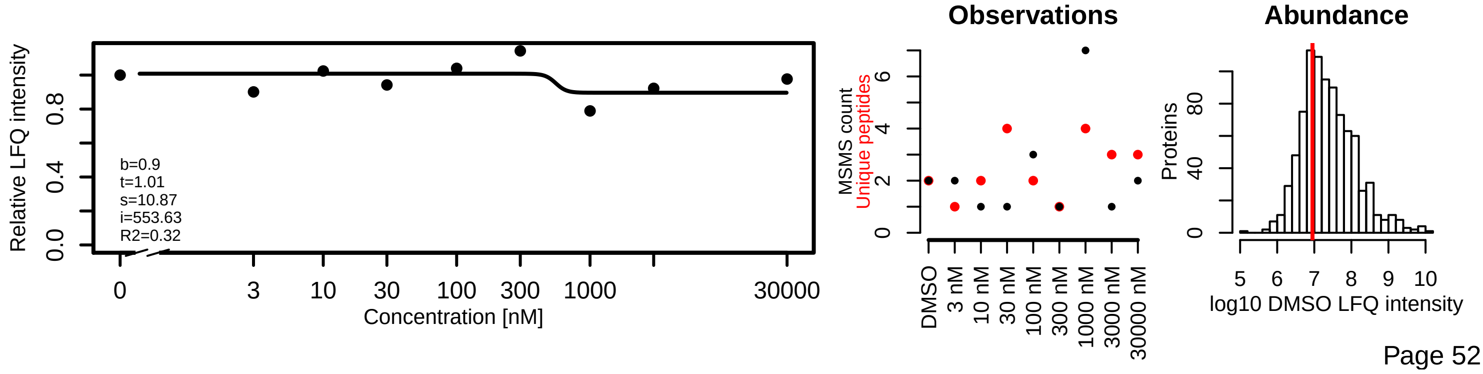

INTS8

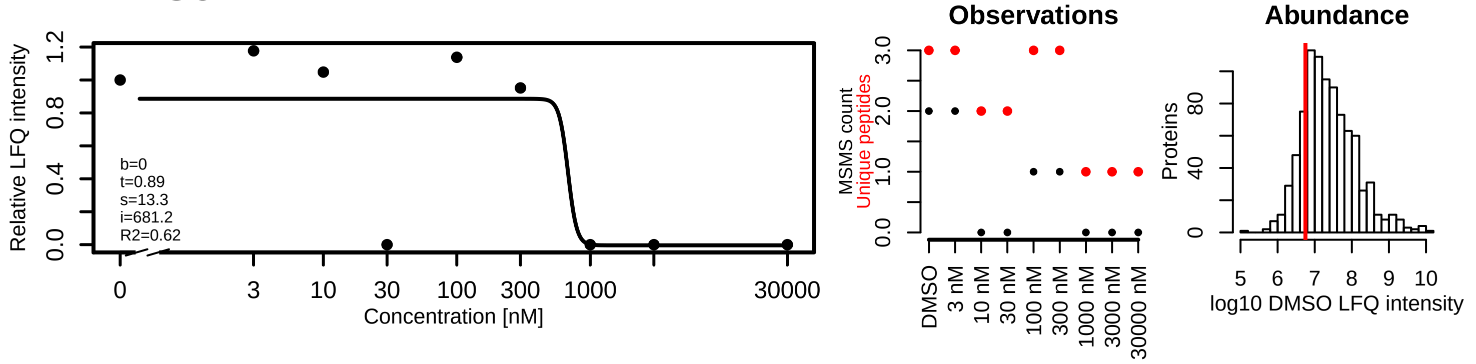

MLLT11

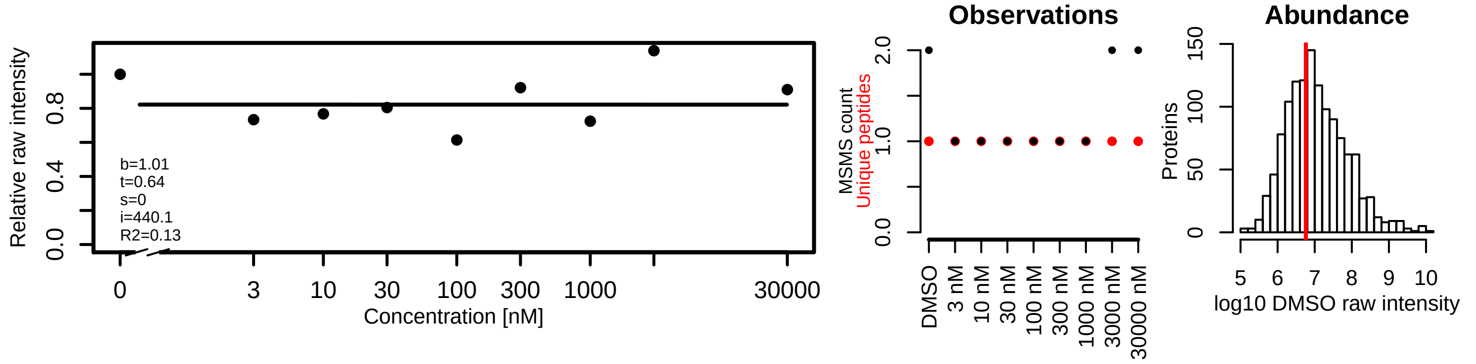

BFAR

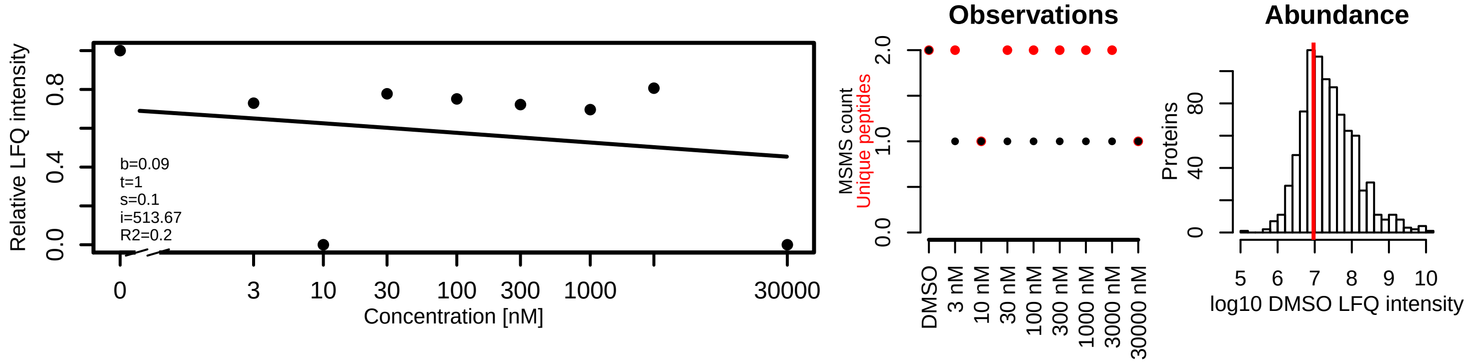

6−Mar

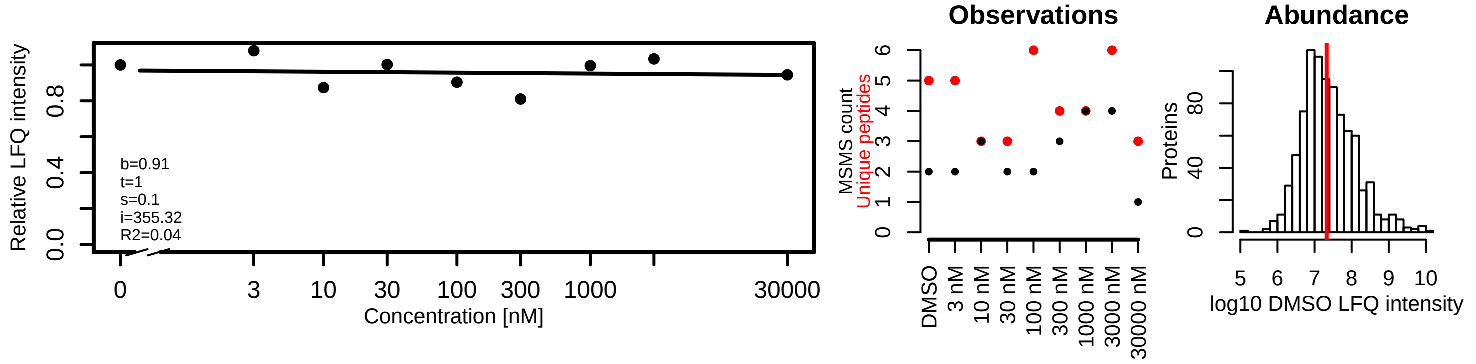

UBE2G2

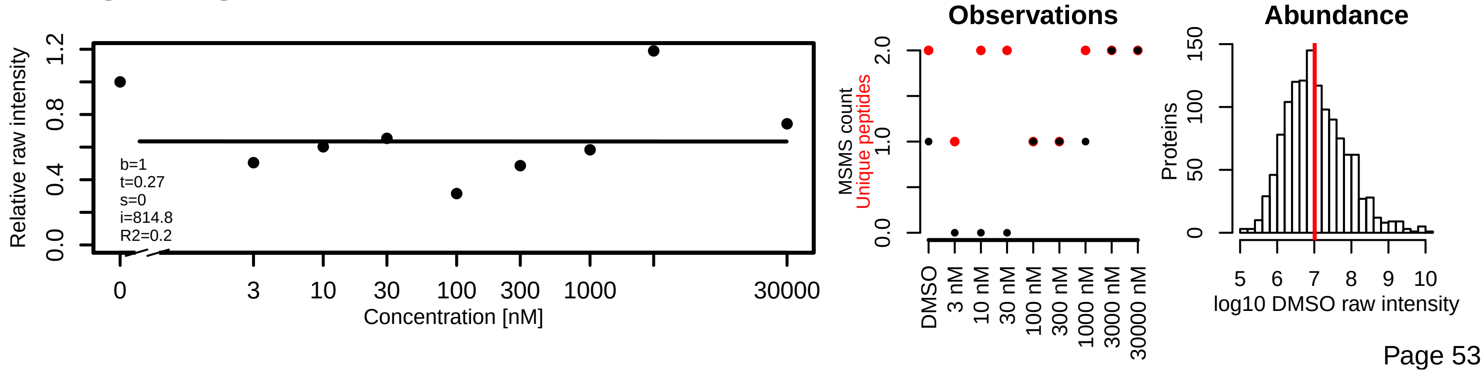

ESYT1

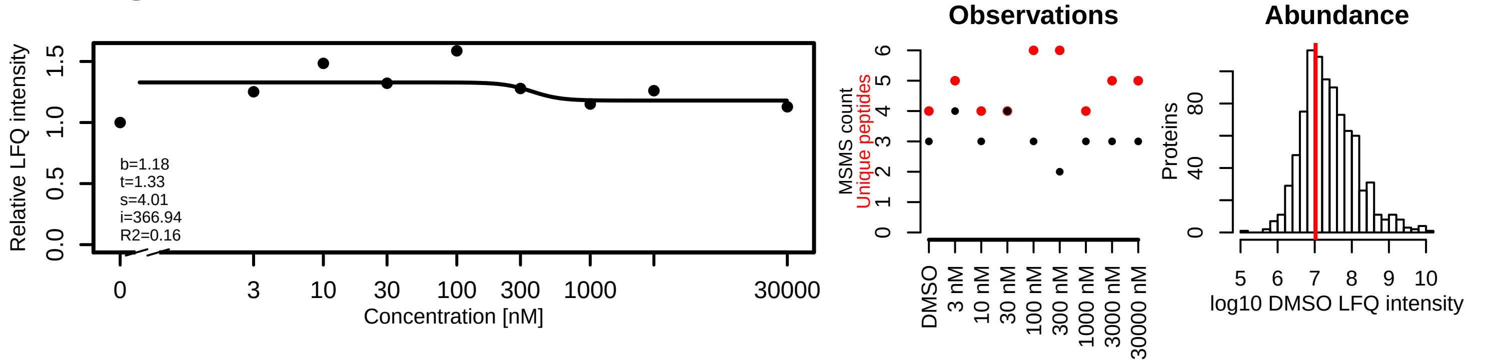

SLC4A2

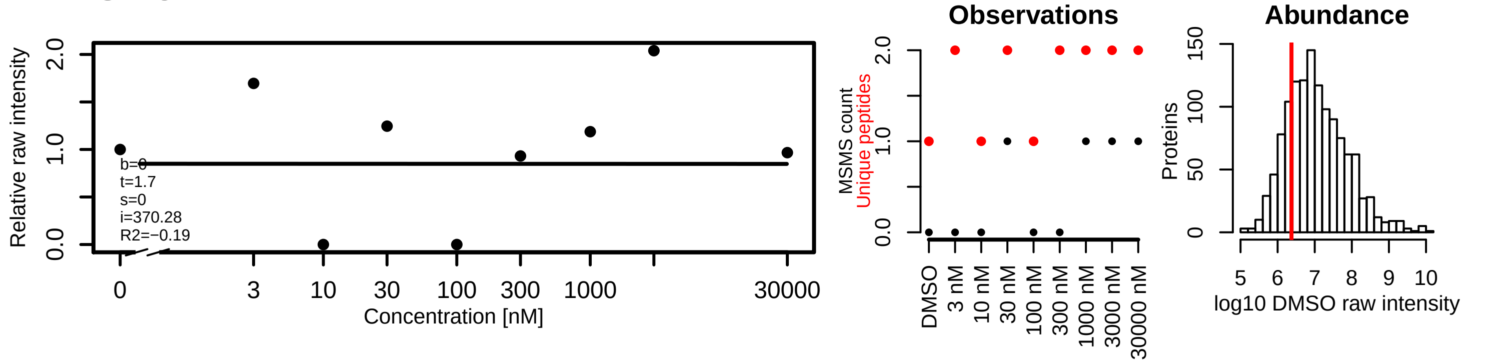

TMEM161A

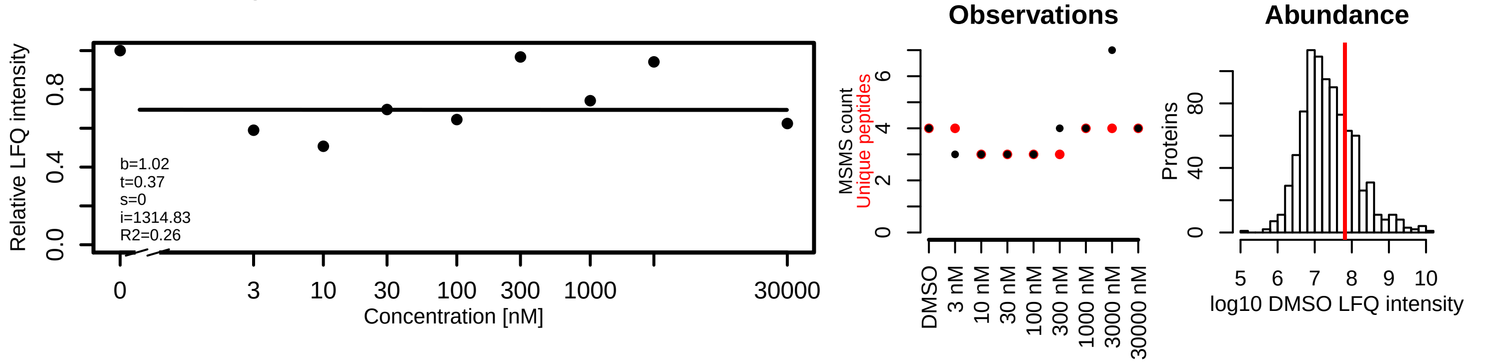

PIGO

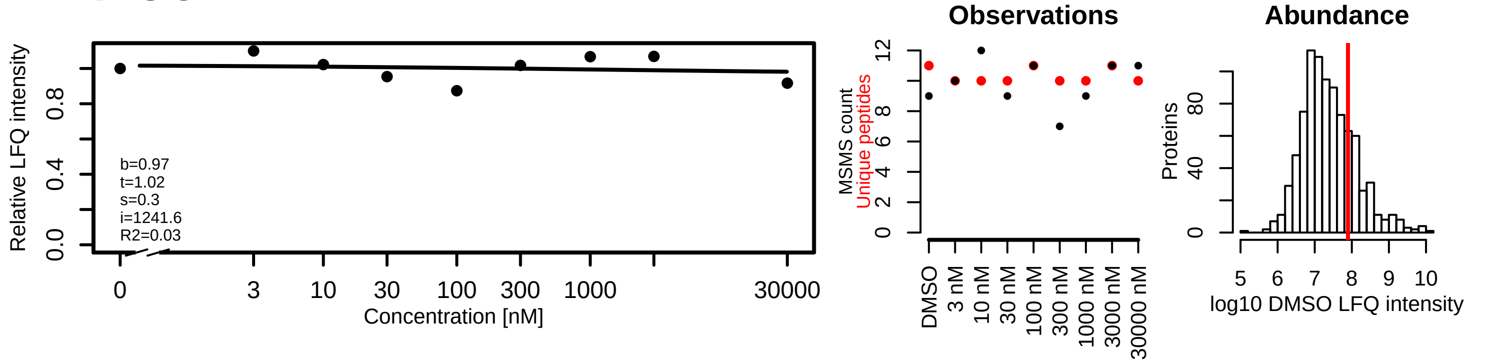

CTTNBP2NL

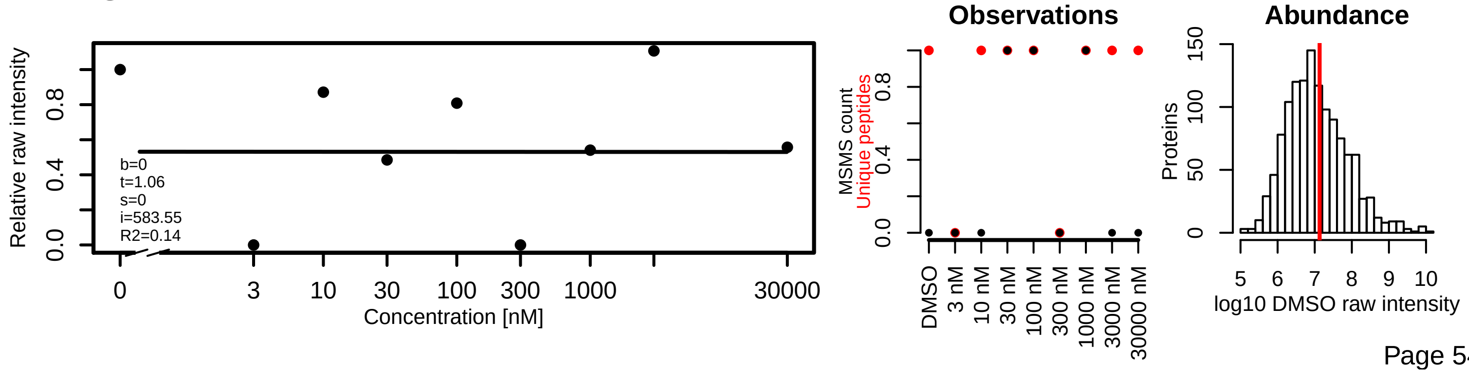

PSENEN

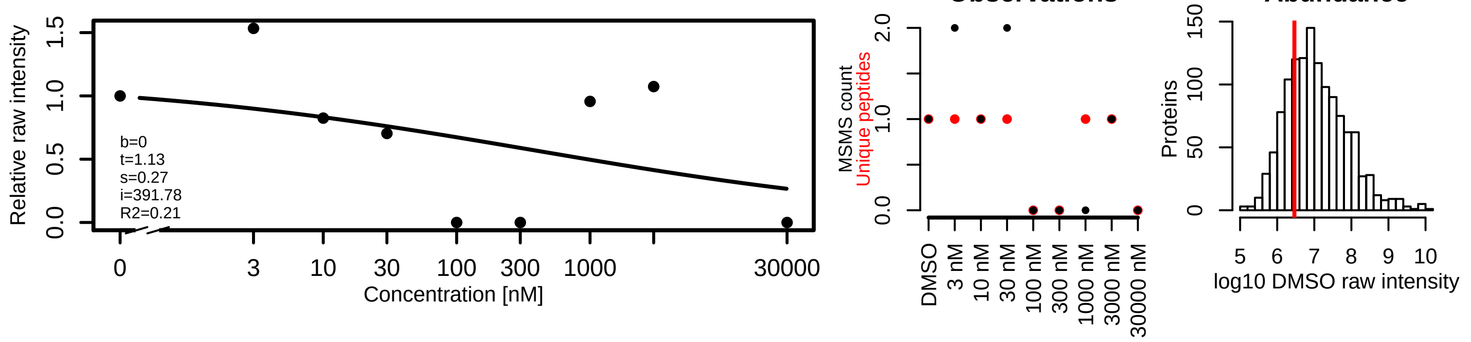

LRRC59

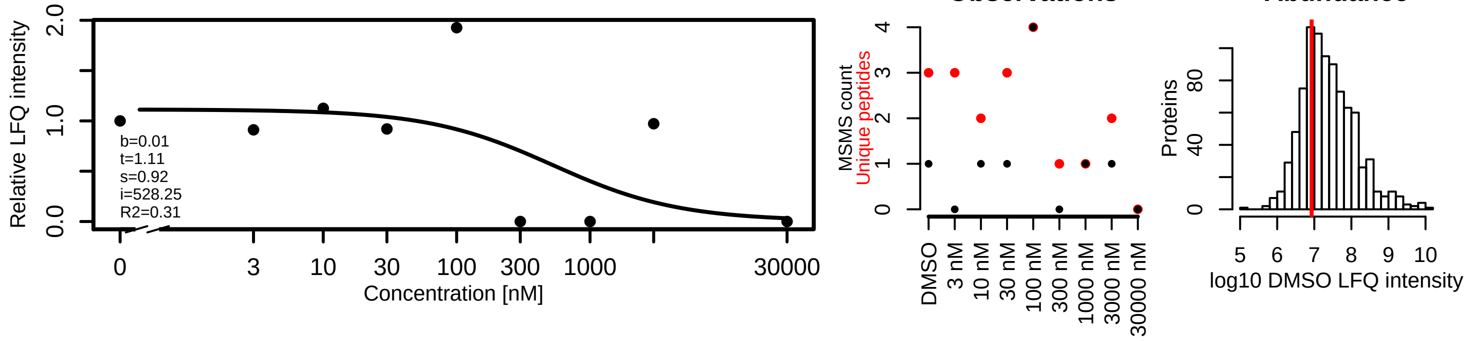

FLNA

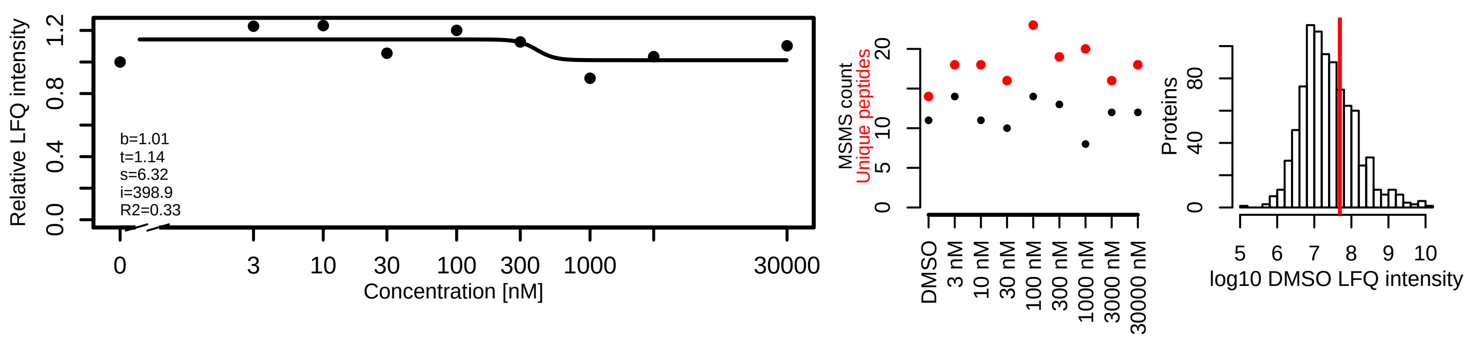

QARS

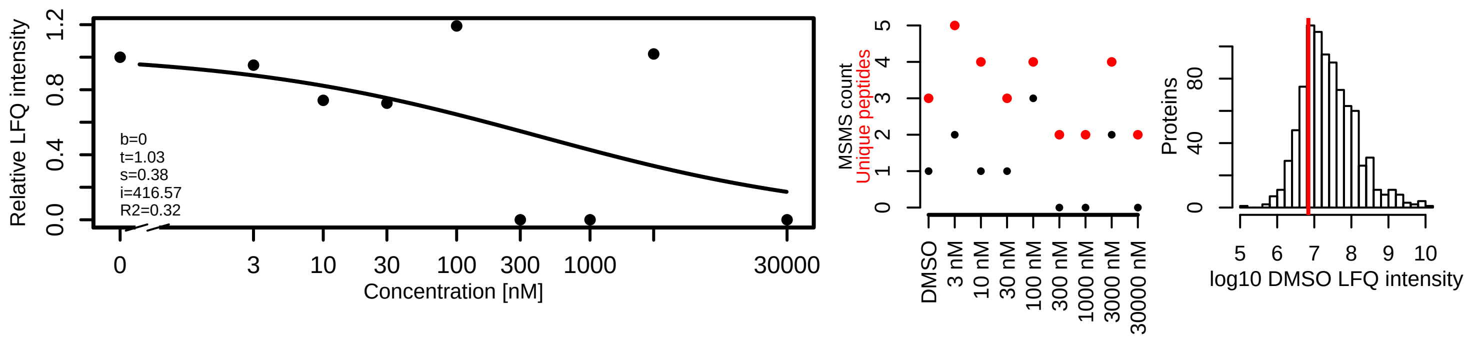

SLC30A6

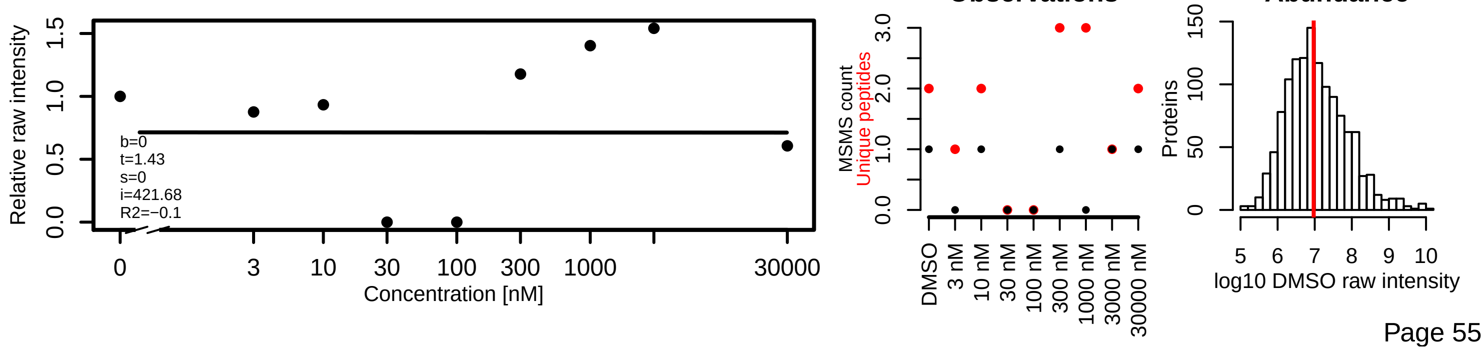

VDAC2

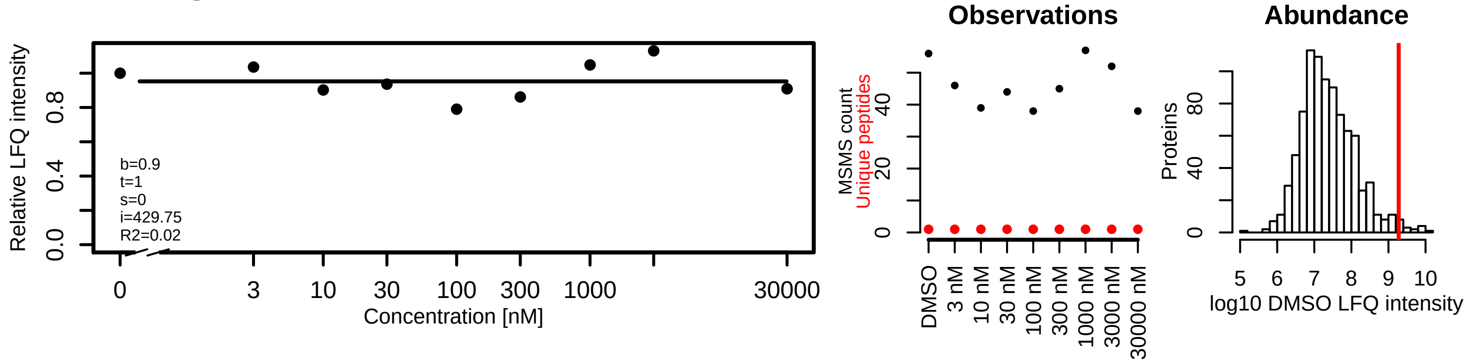

PPM1B;PPM1A

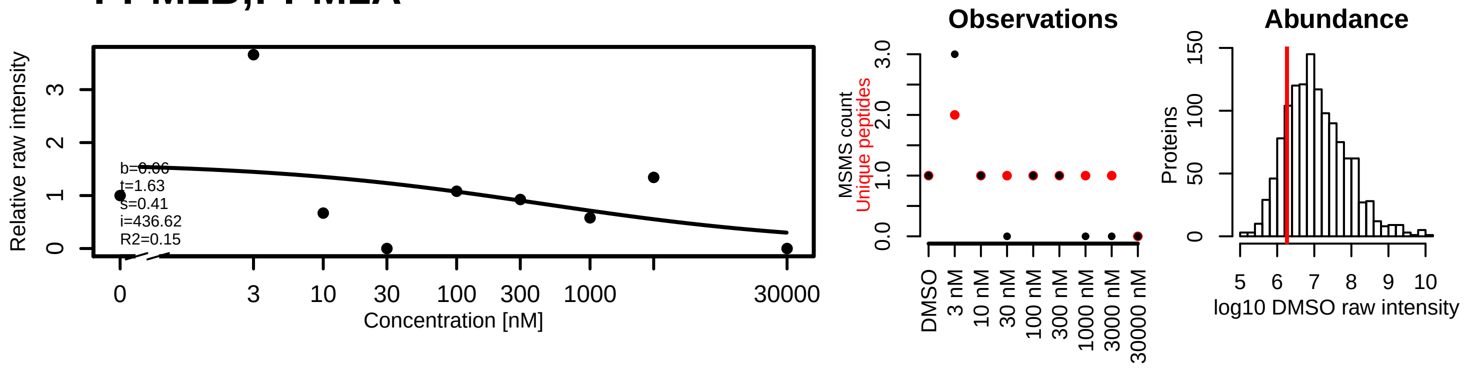

ADCK1

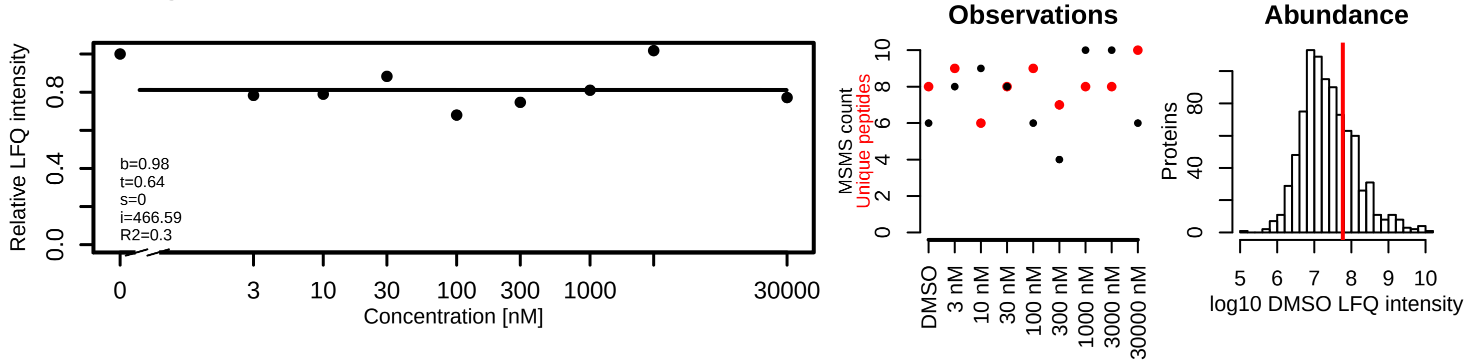

ORMDL1;ORMDL2

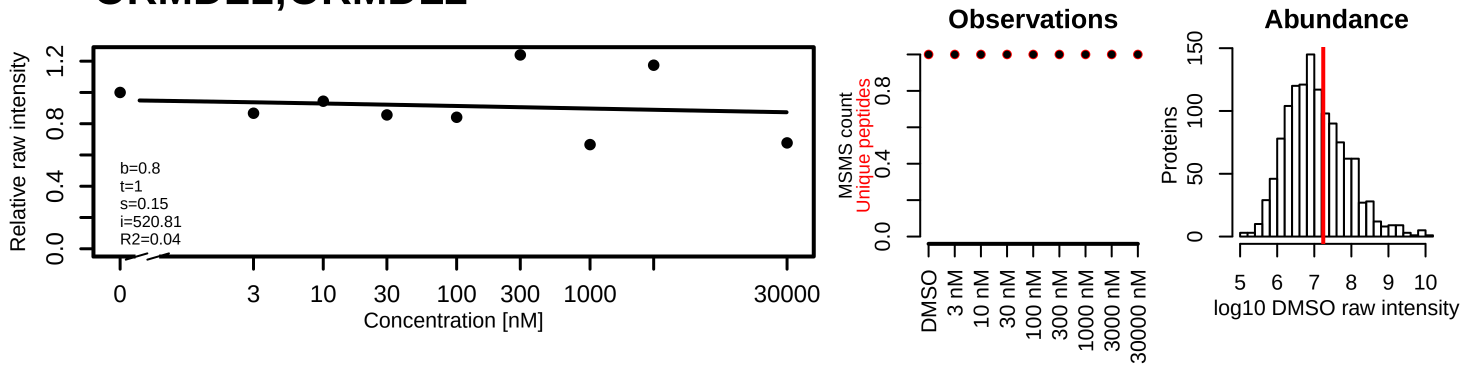

SLC3A2

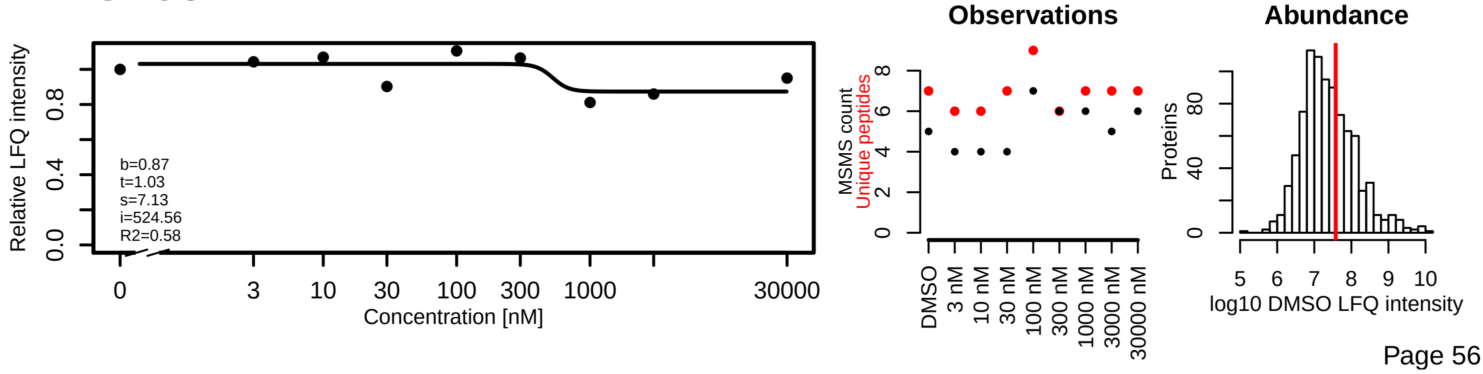

TUBA1A;TUBA3C;TUBA3E

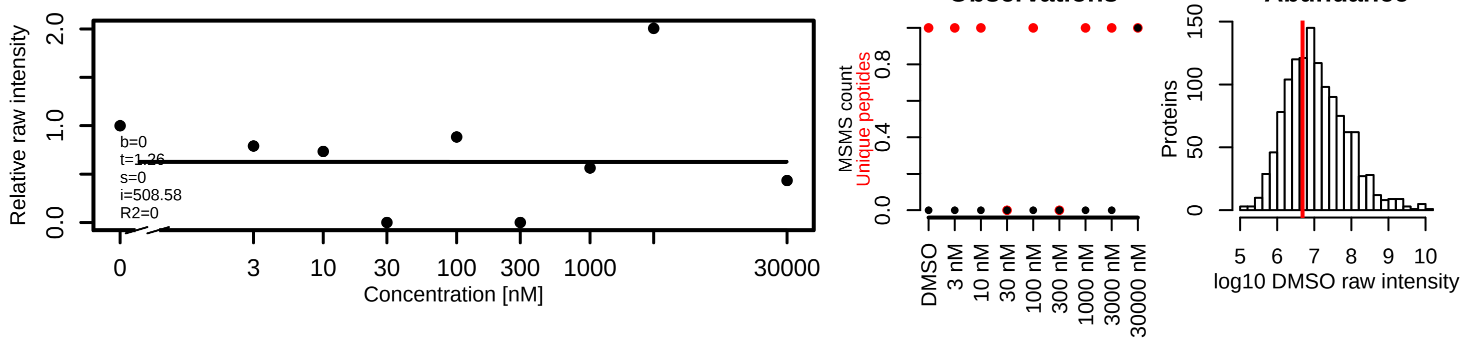

DERL1

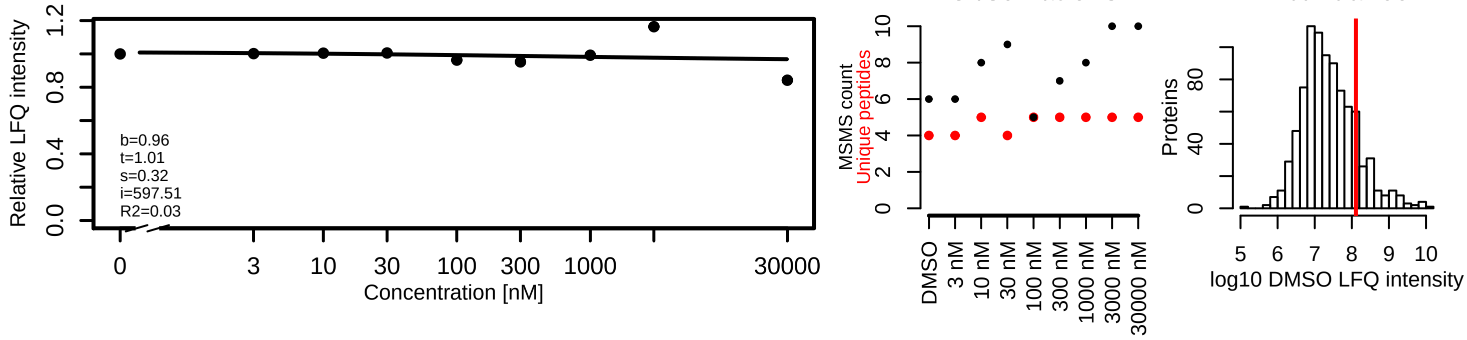

SLC25A13

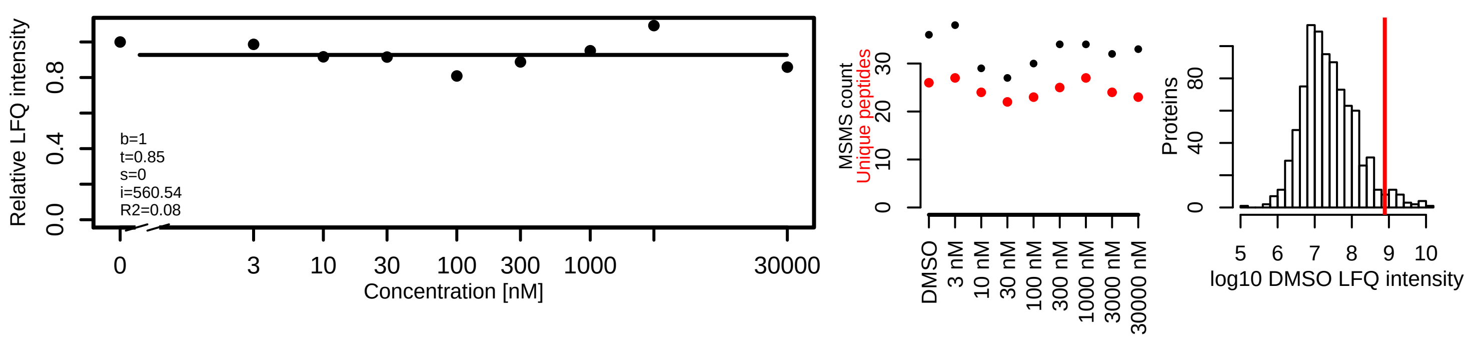

ANXA1

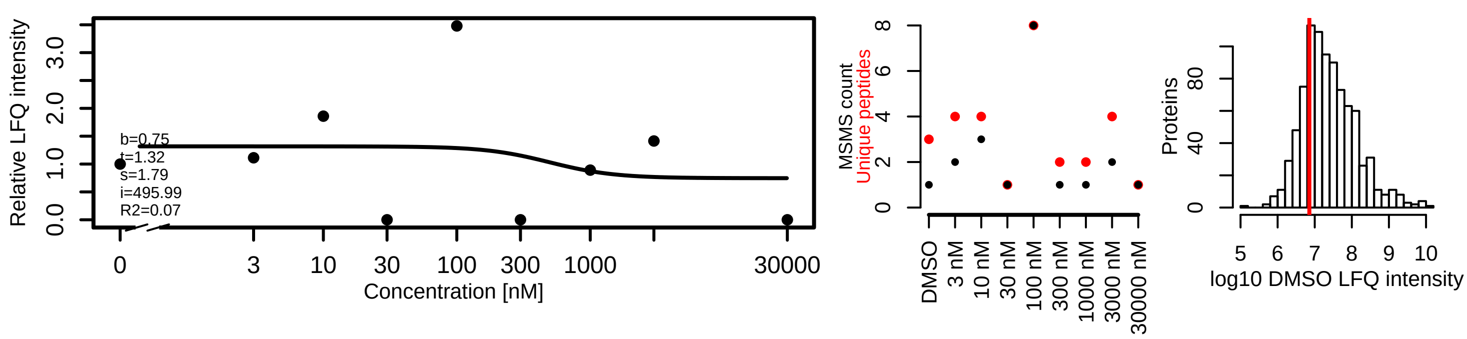

RAC1;RAC2;RAC3

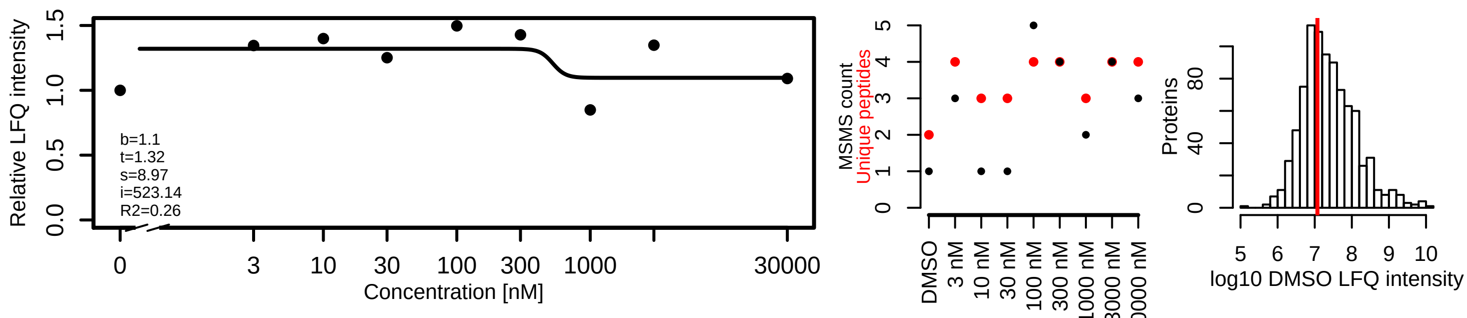

COX6A1P2;COX6A1

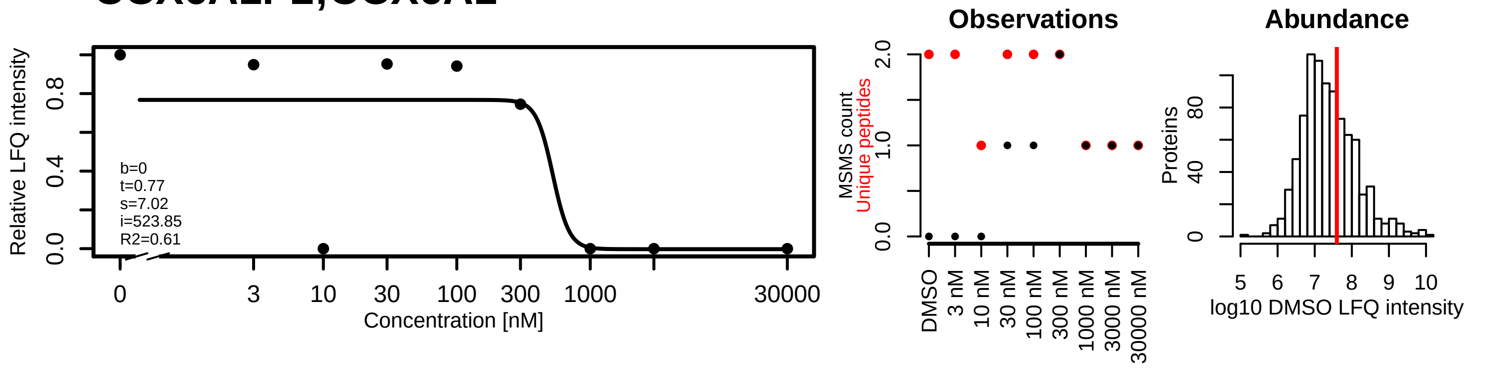

TUBGCP4

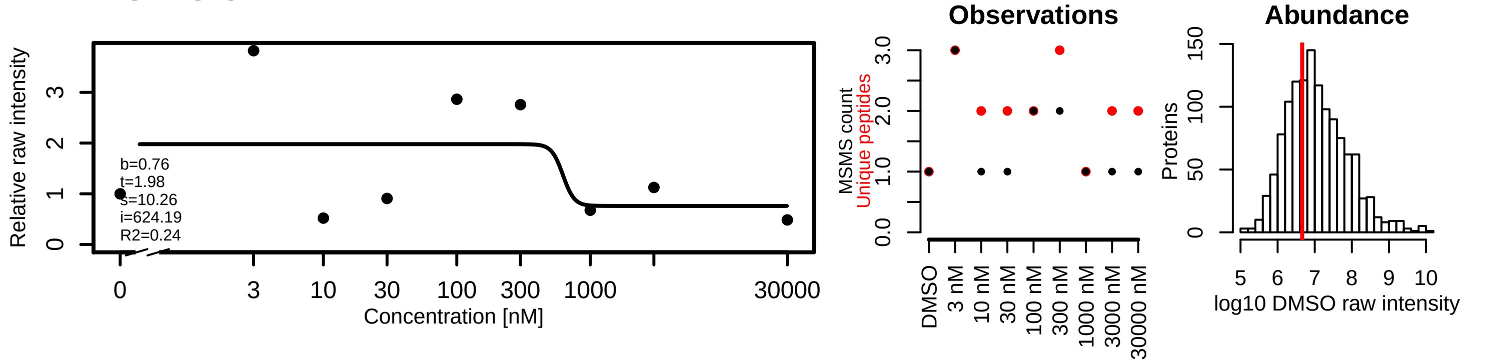

HLA−C − Q07000;P30508;P30501;Q9TNN7;P30505;A2BF24;A2BF25;A

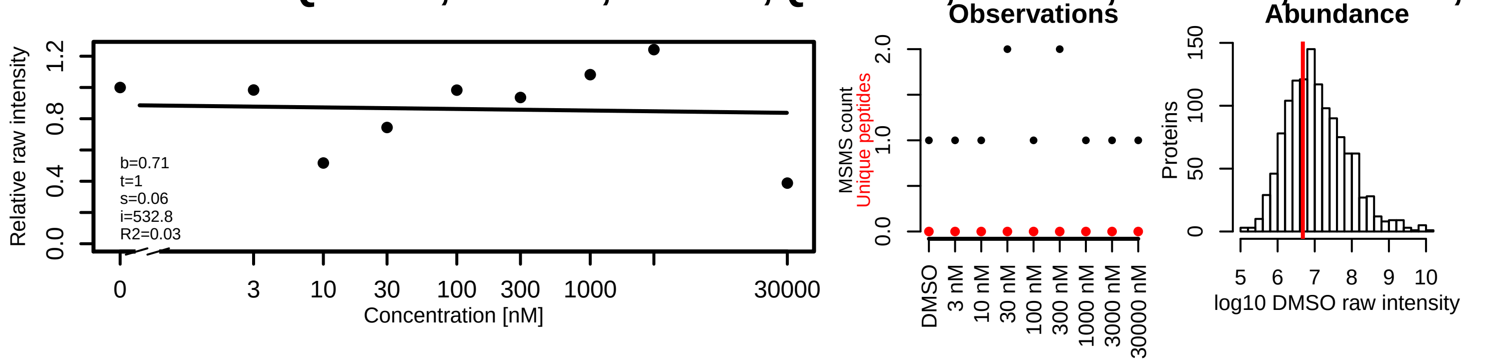

MYH14

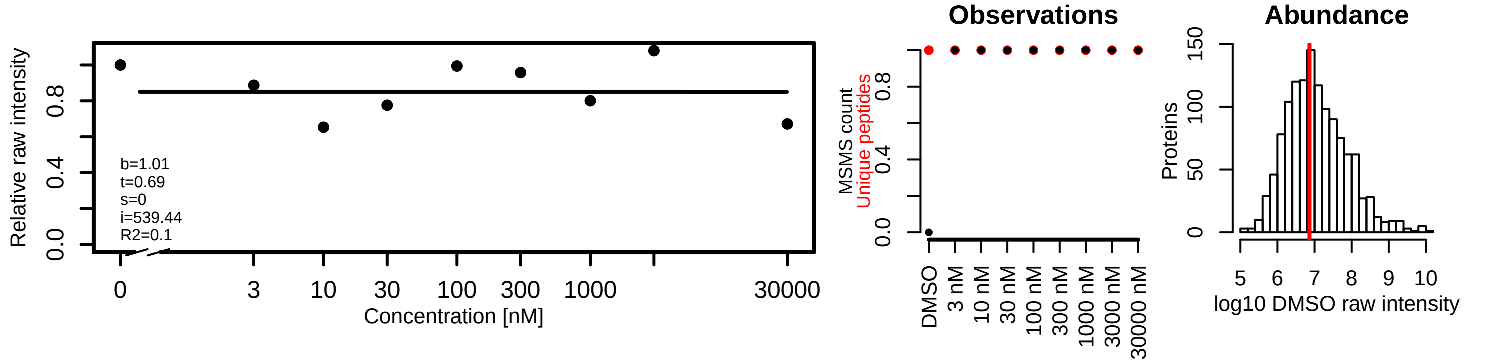

NNT

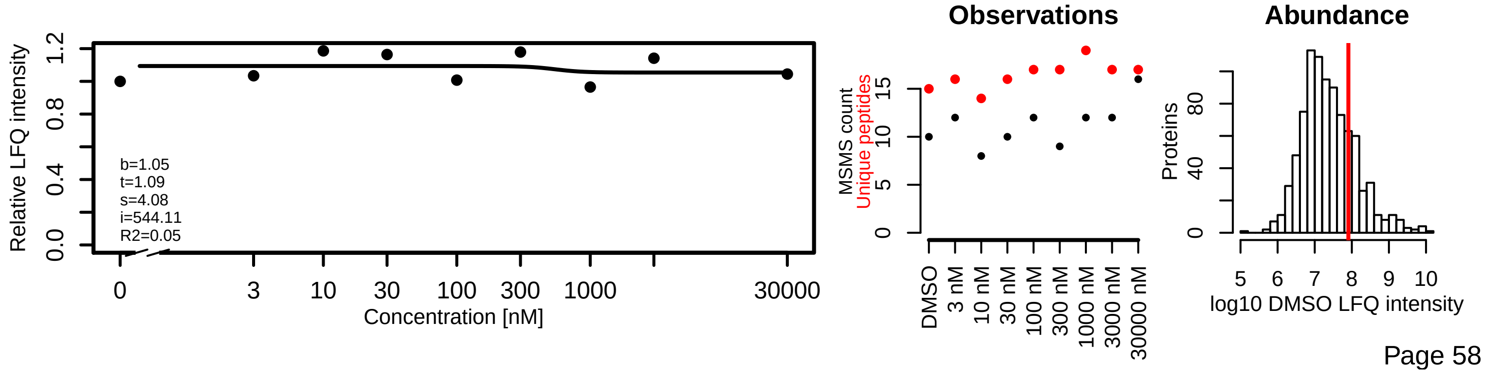

AKAP8L

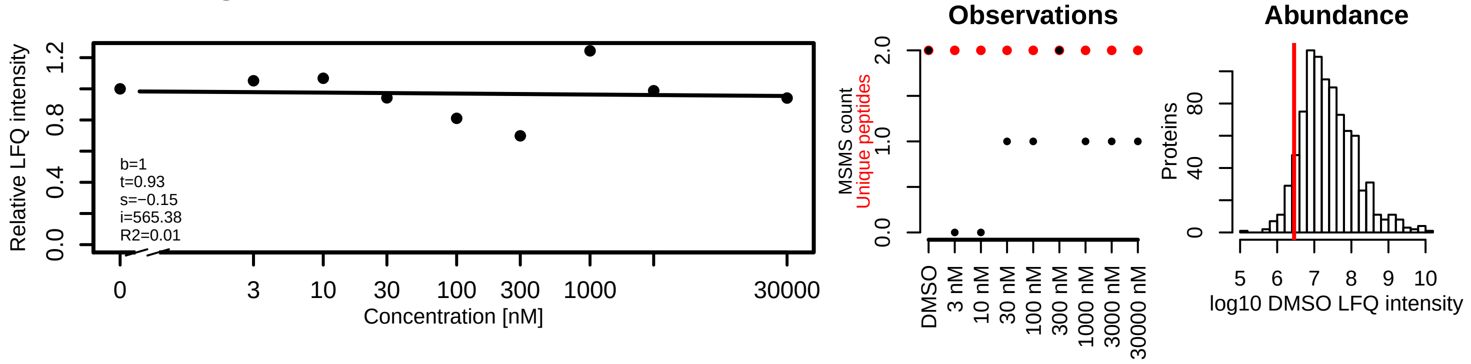

TMEM50A

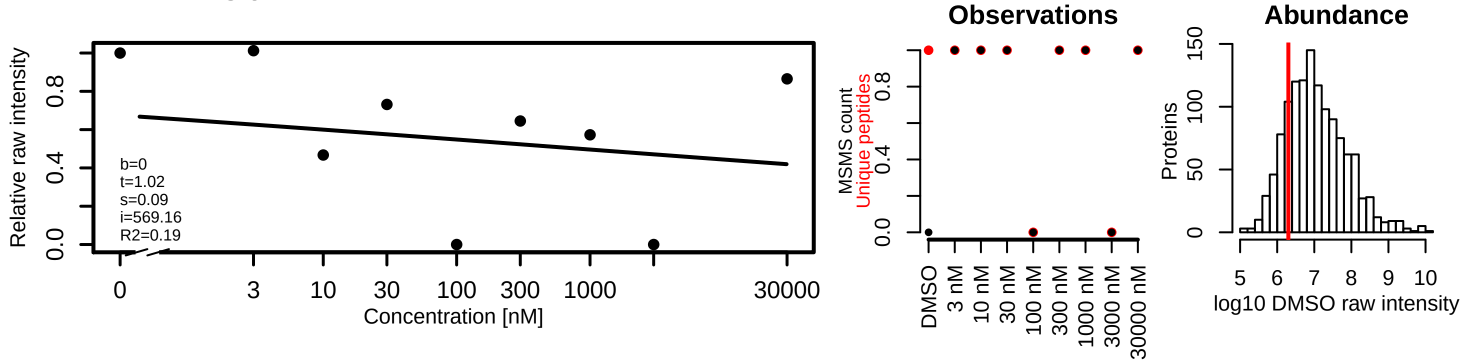

HSPA6

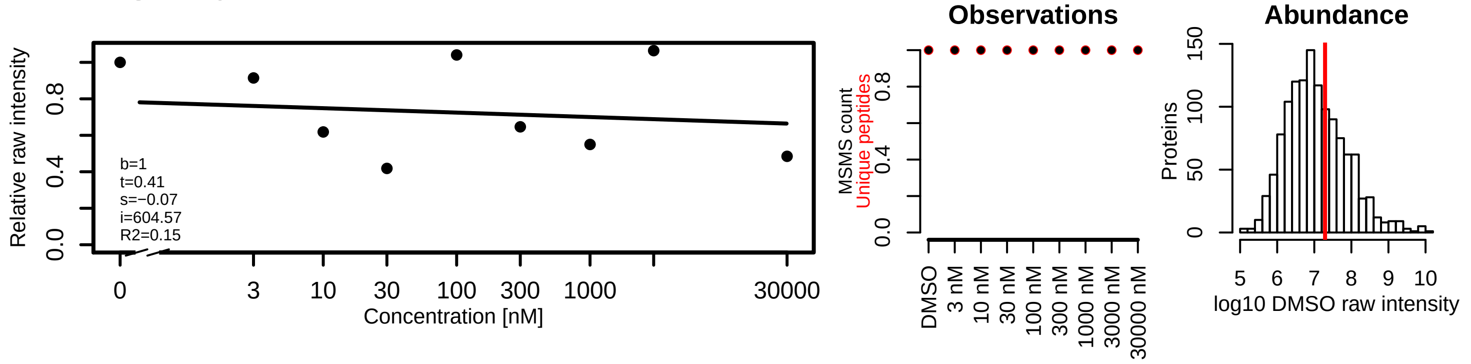

MKRN3

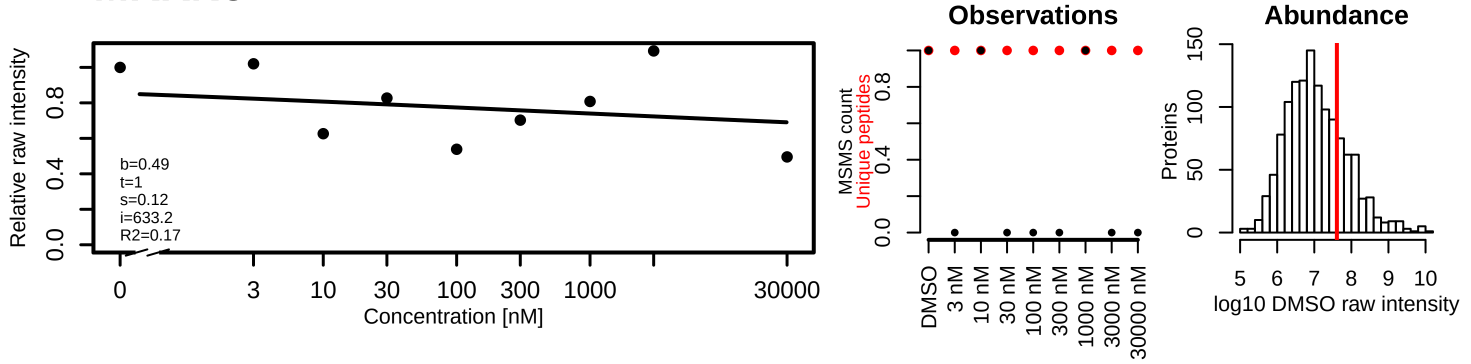

AUP1

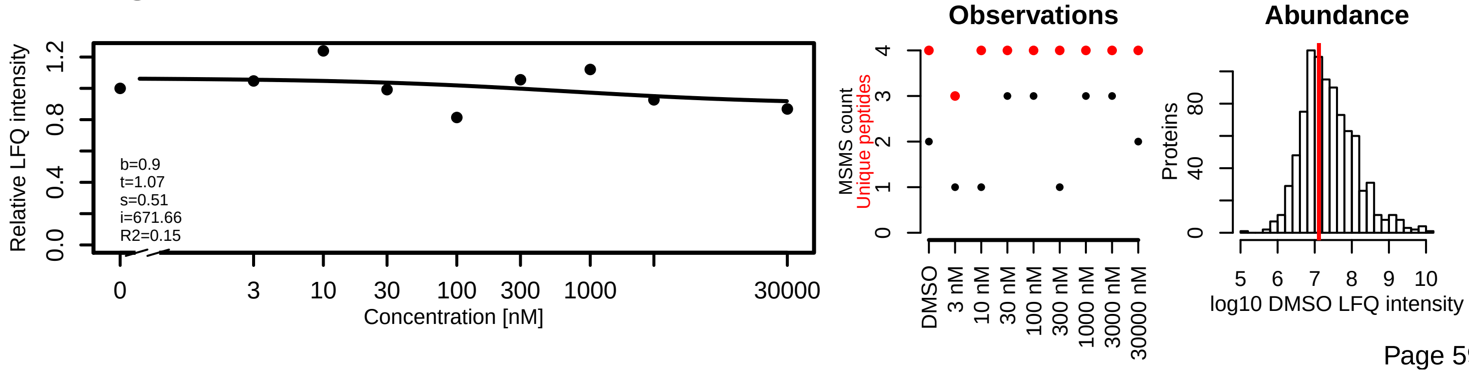

NDUFA9

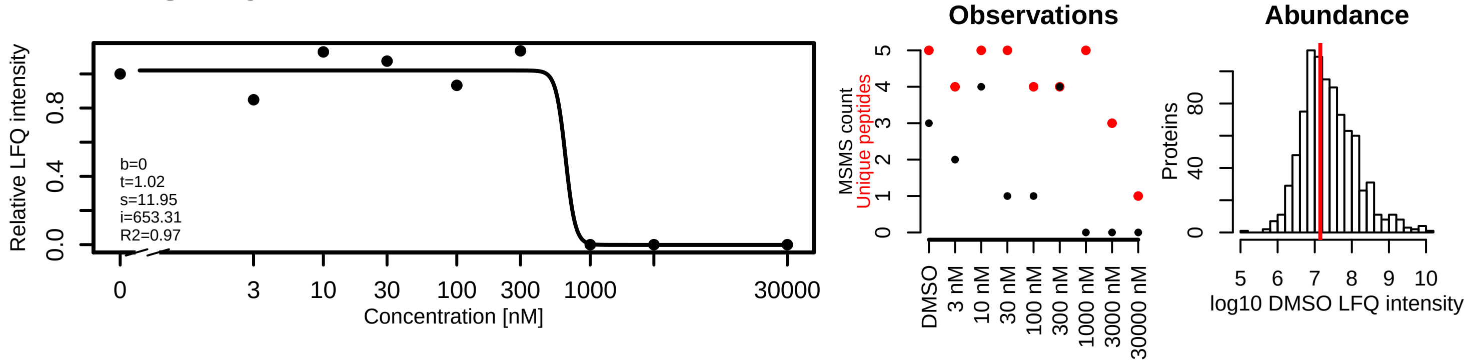

DNAJA1

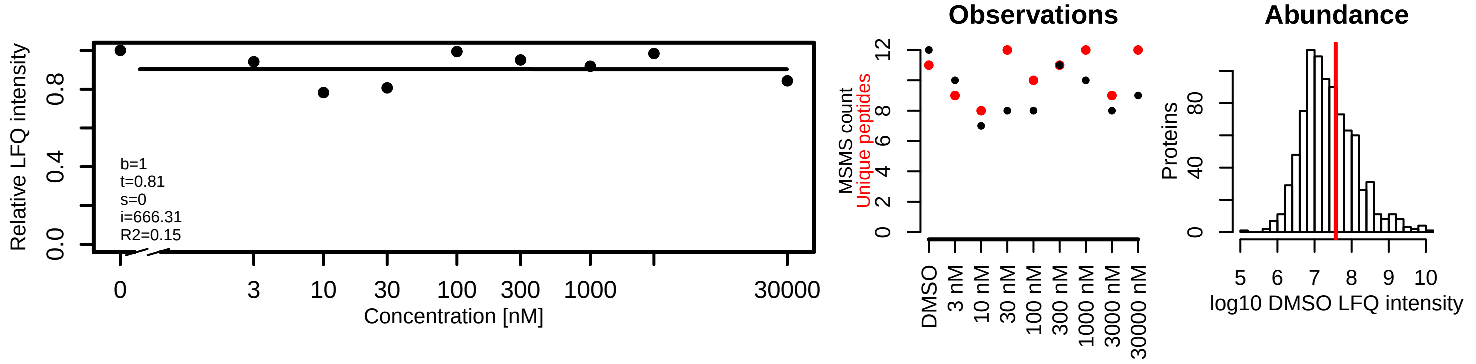

LPGAT1

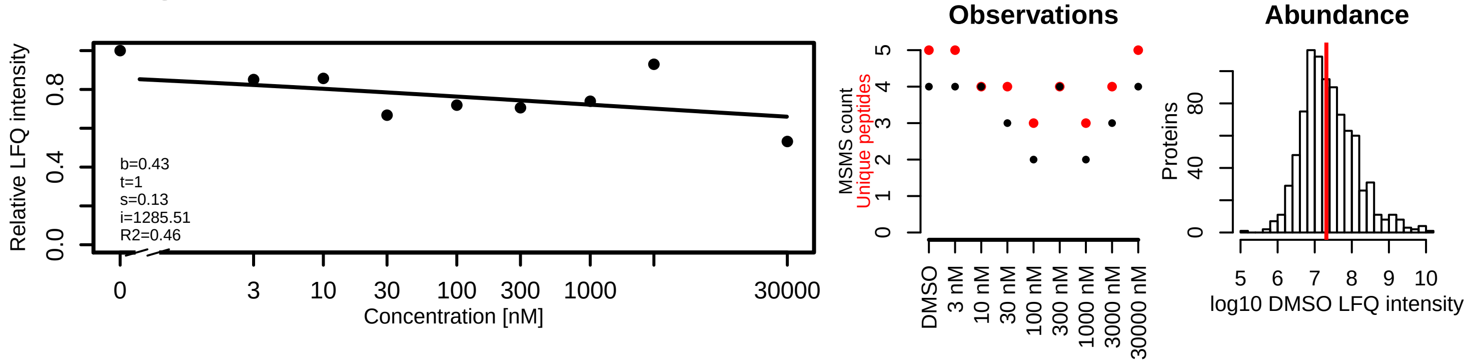

2−Mar

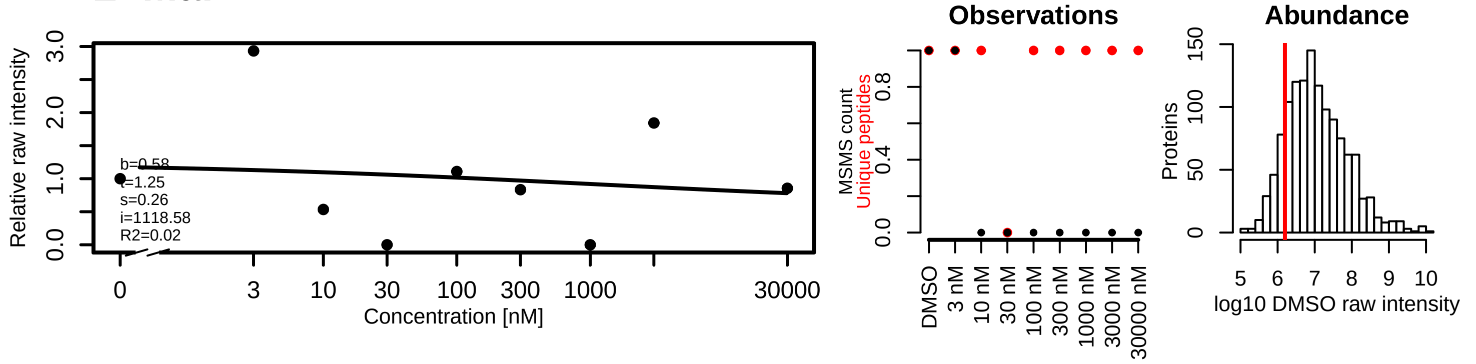

UBE4A

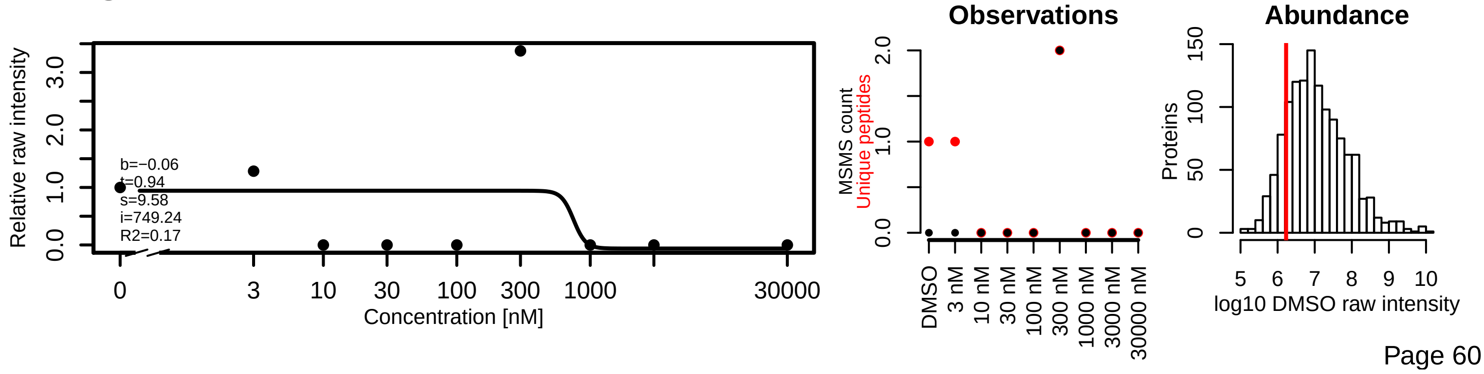

CCDC109B

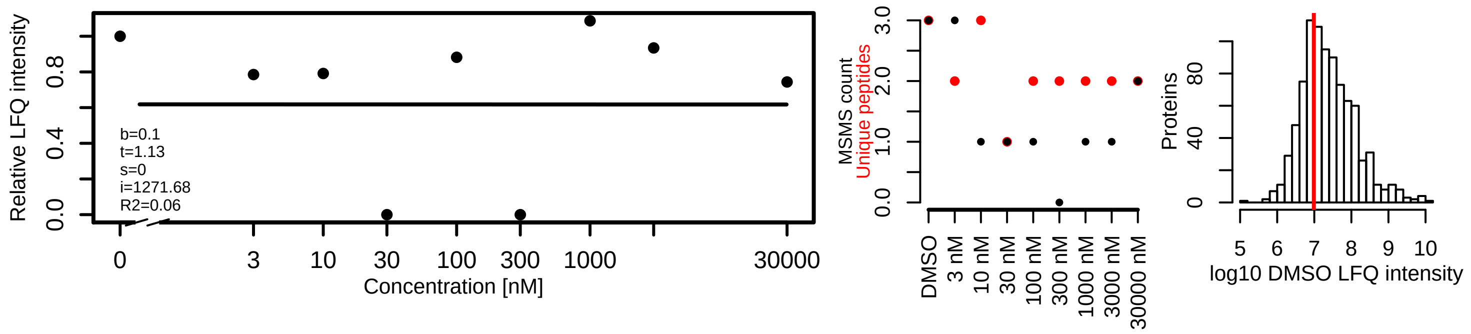

PTGFRN

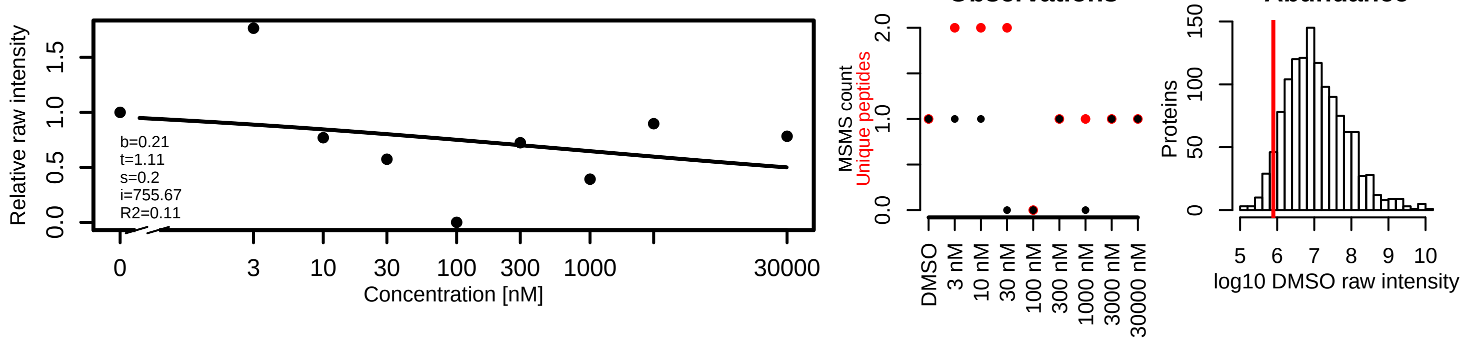

SFXN5

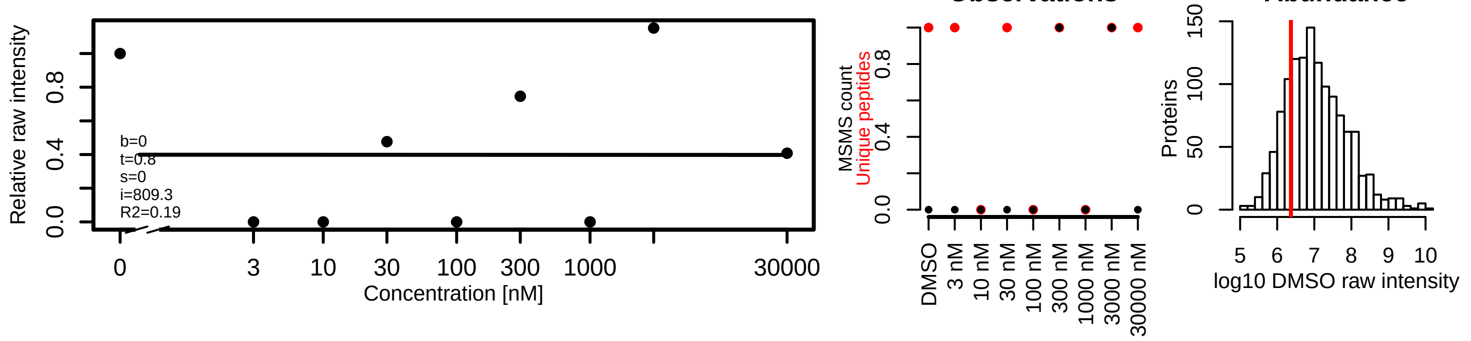

ARCN1

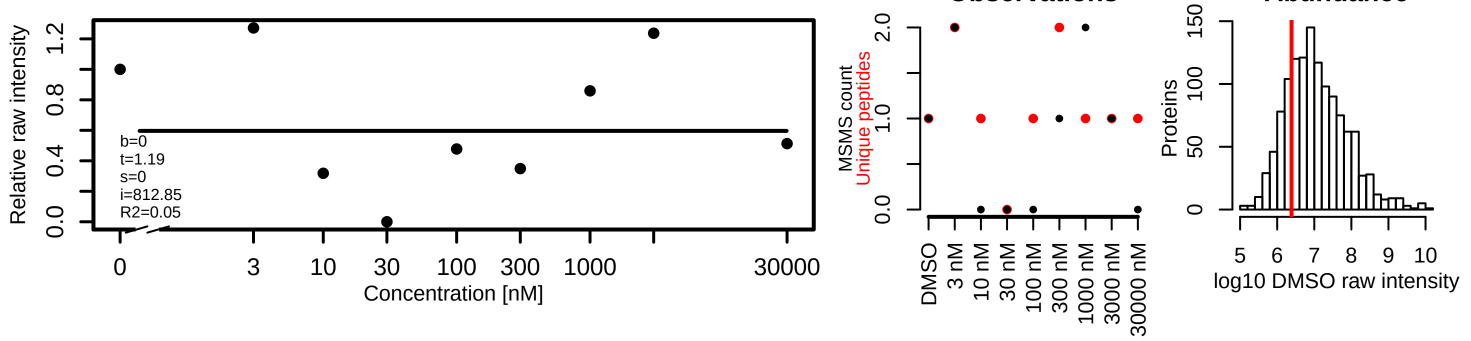

ATP2B4

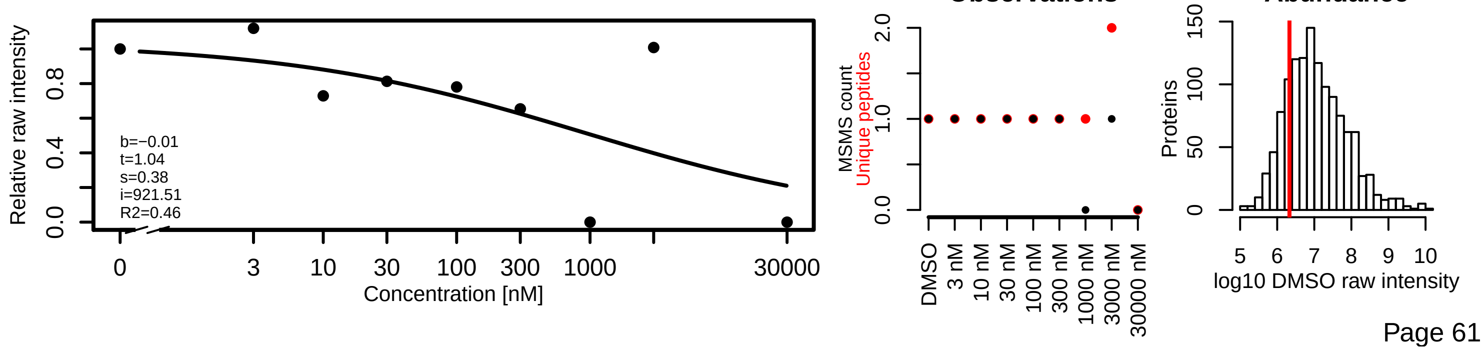

RFT1

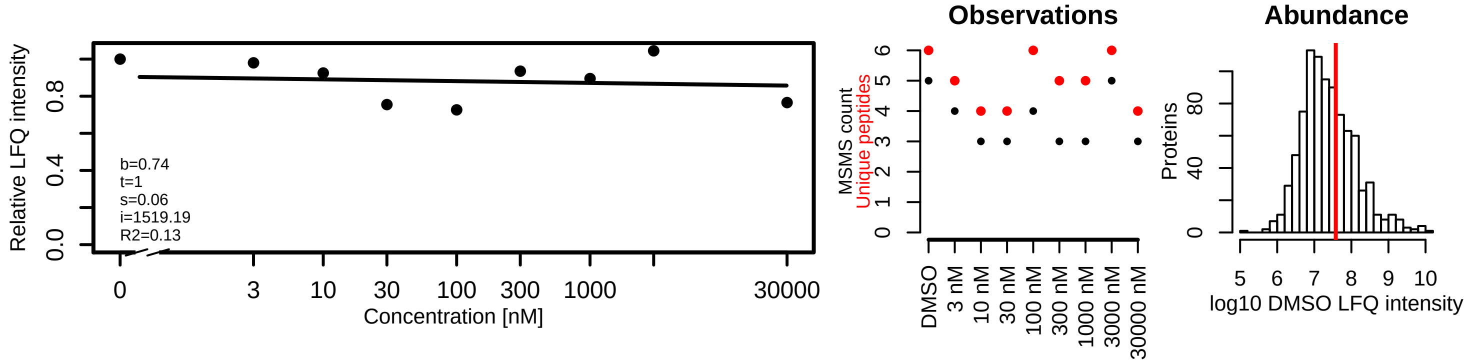

CNBP

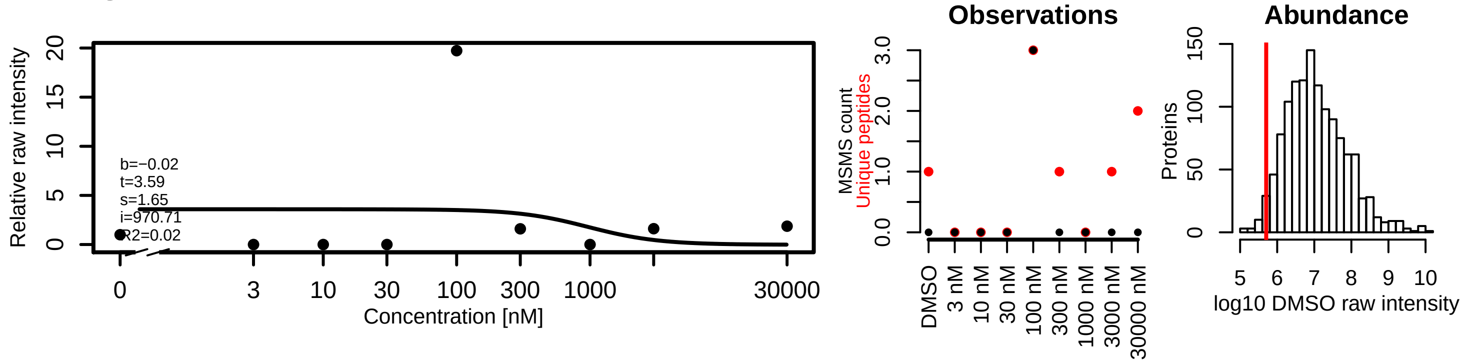

ALDH3A2

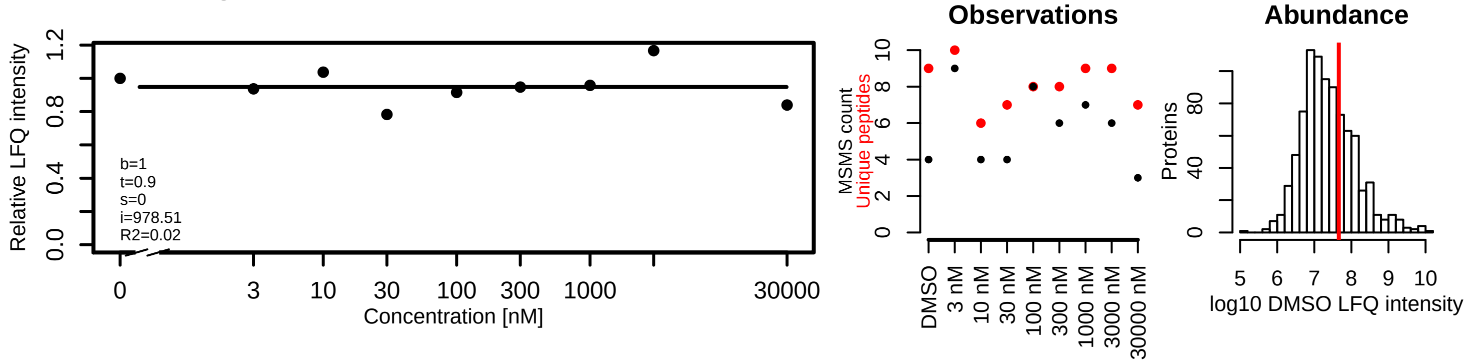

PIGS

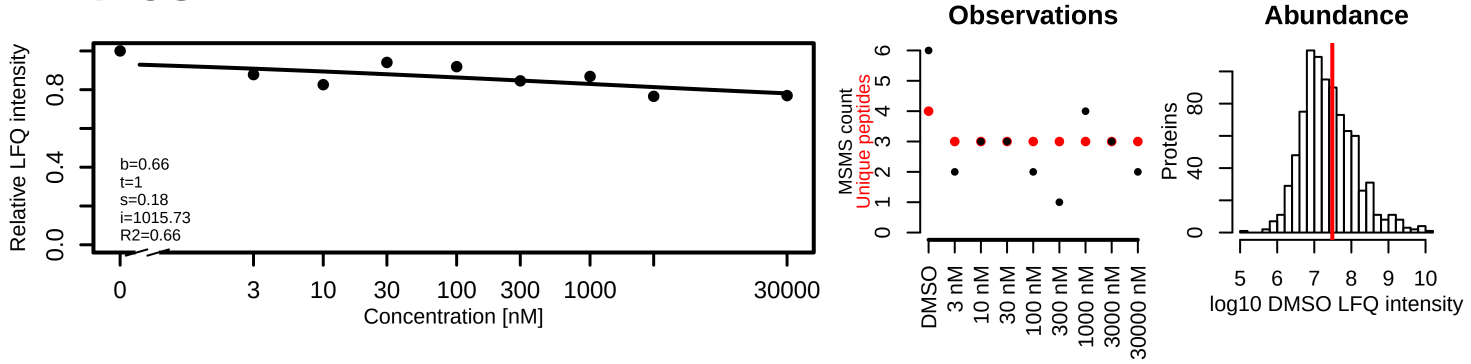

PSMC4

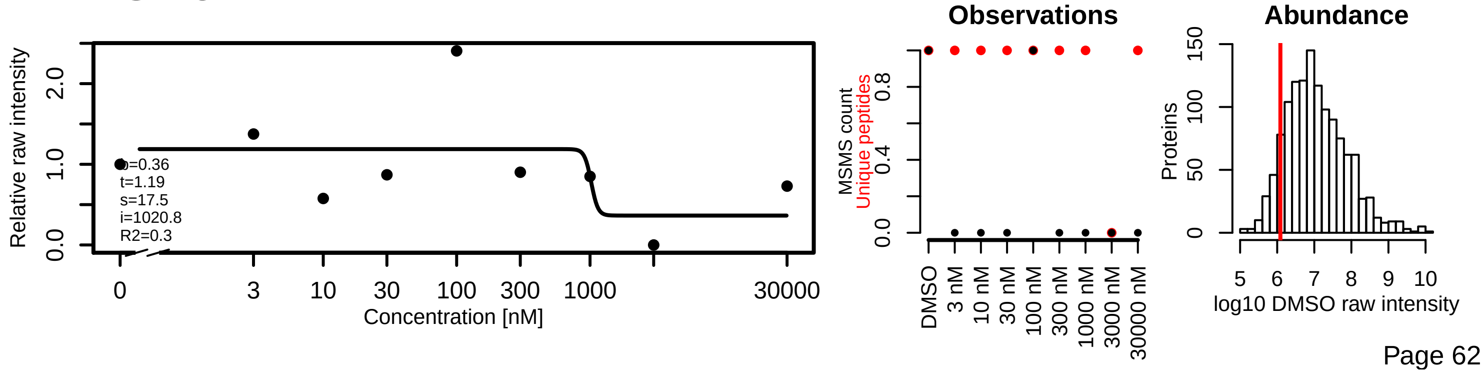

LMF2

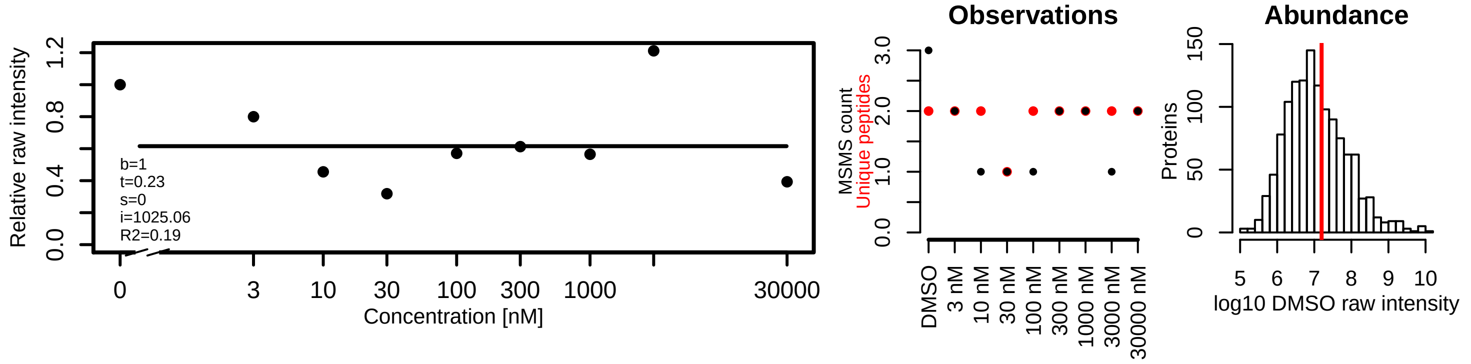

TPP1

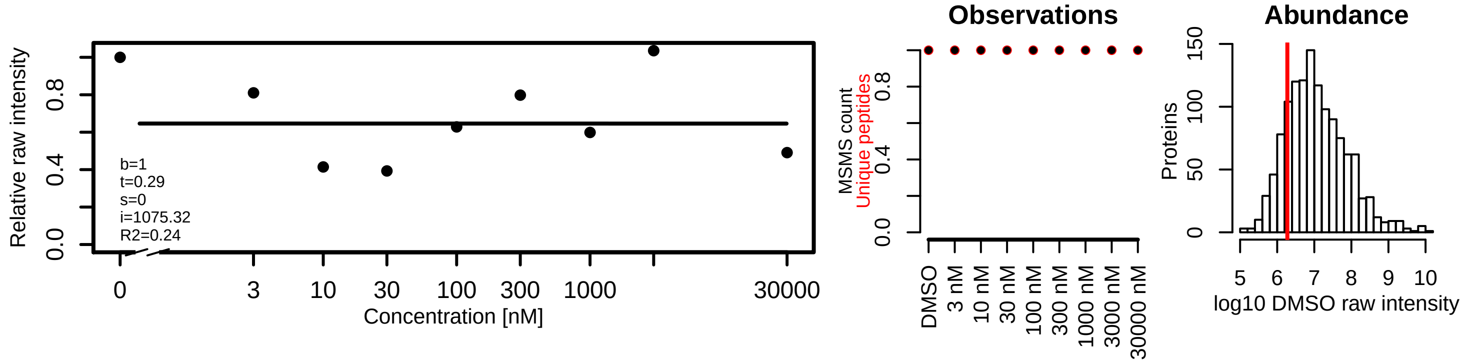

TMED1

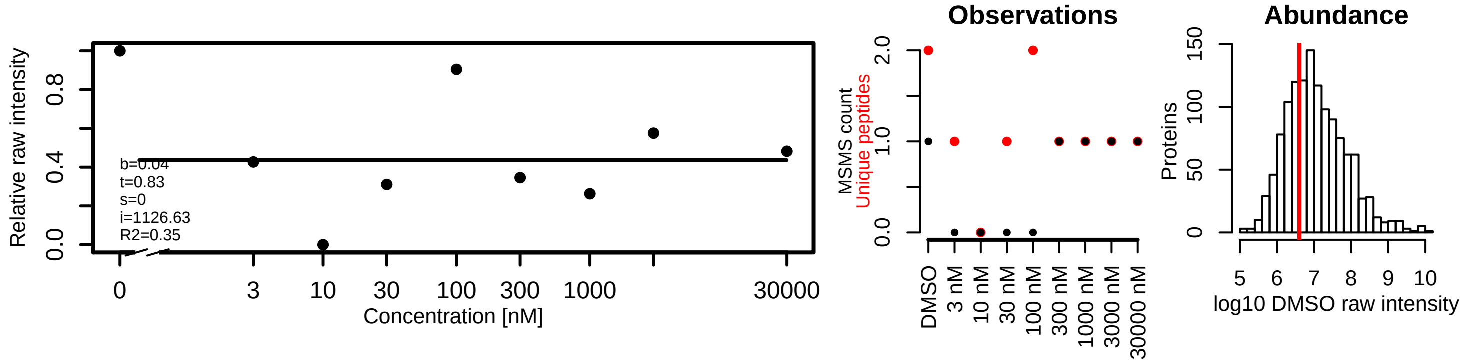

DYNC1H1

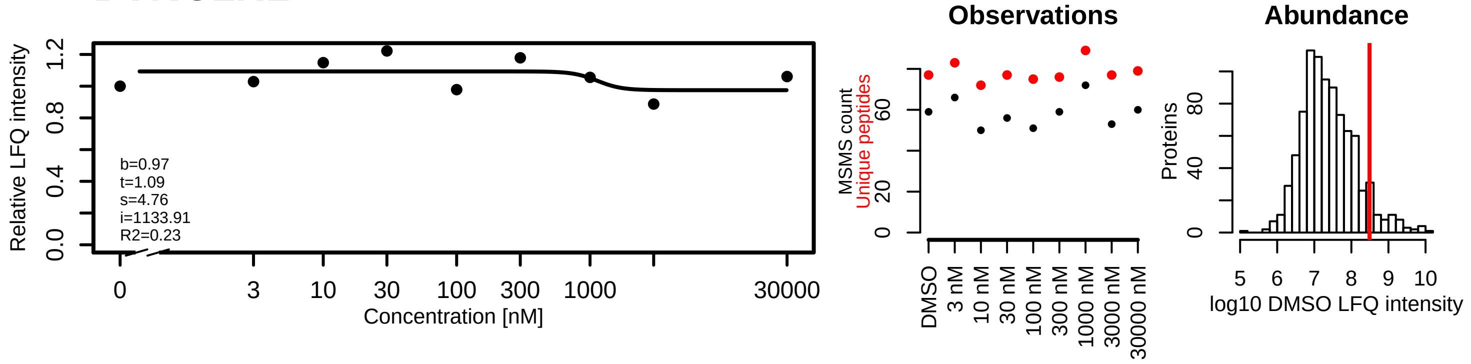

ITGA3

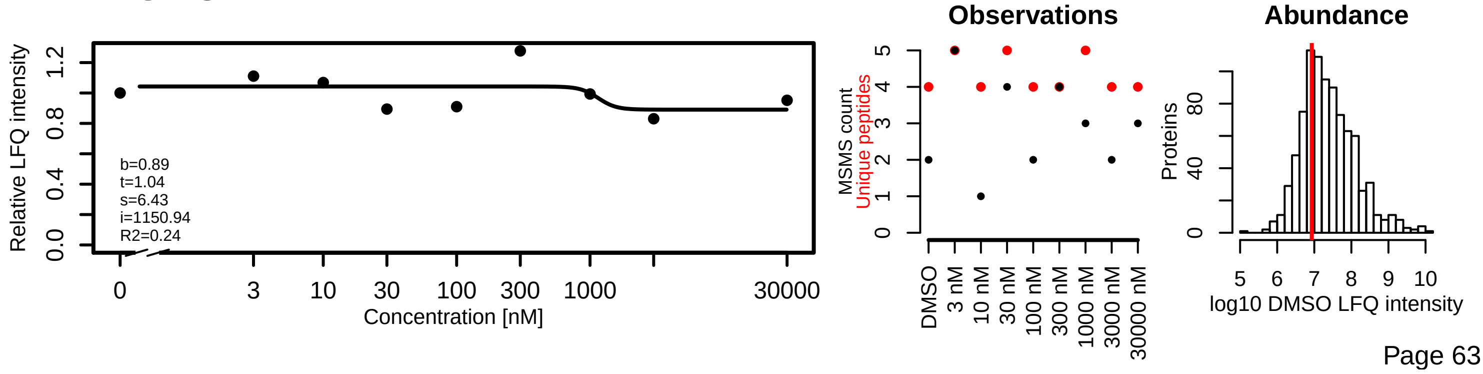

RPL26;KRBA2;RPL26L1

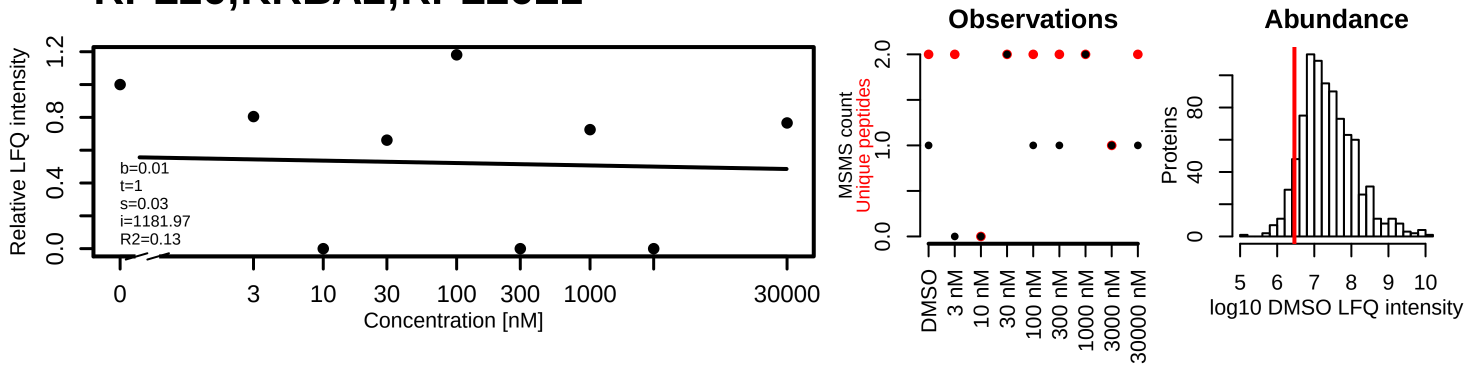

STARD3NL − C9JKL2;O95772;C9JPX5;C9JJ39;C9J3F5

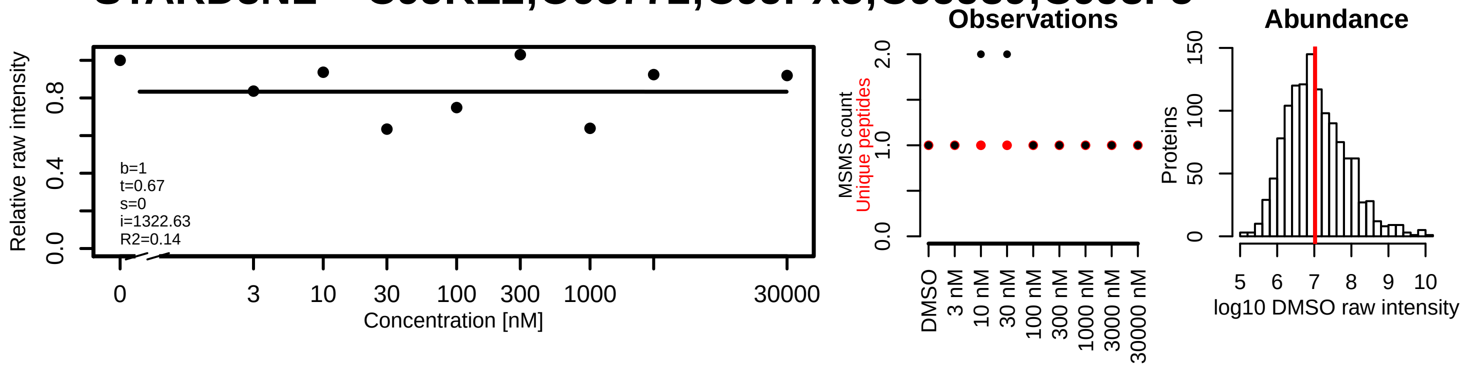

DNAJB12

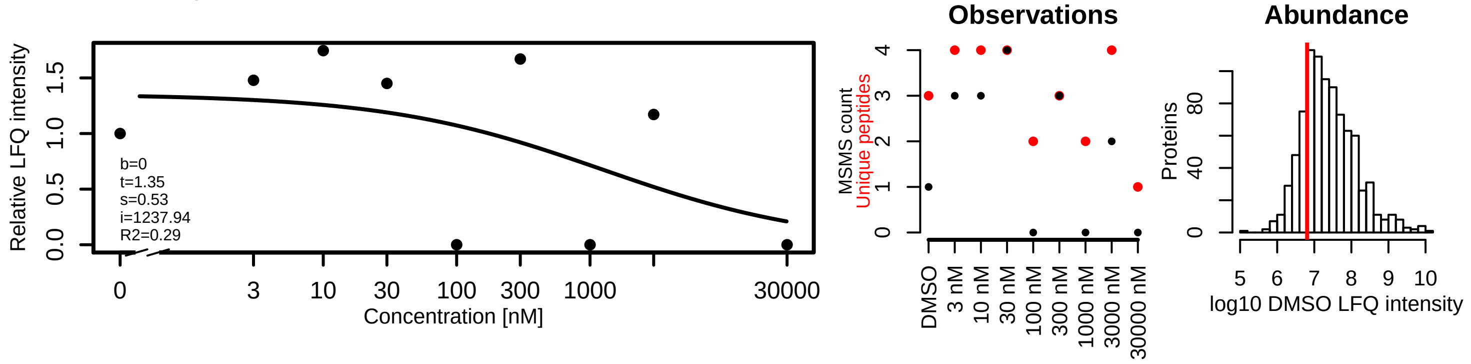

GOLT1B

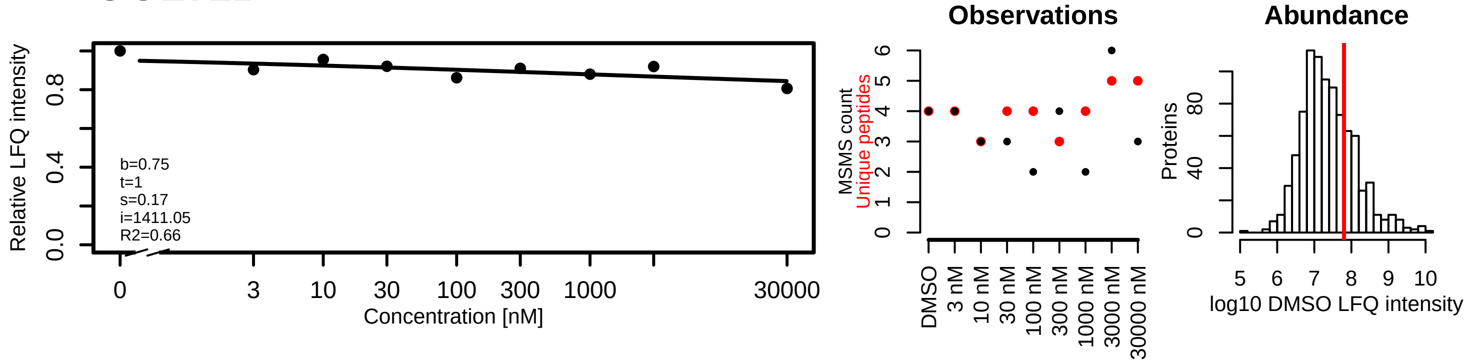

IGF2R

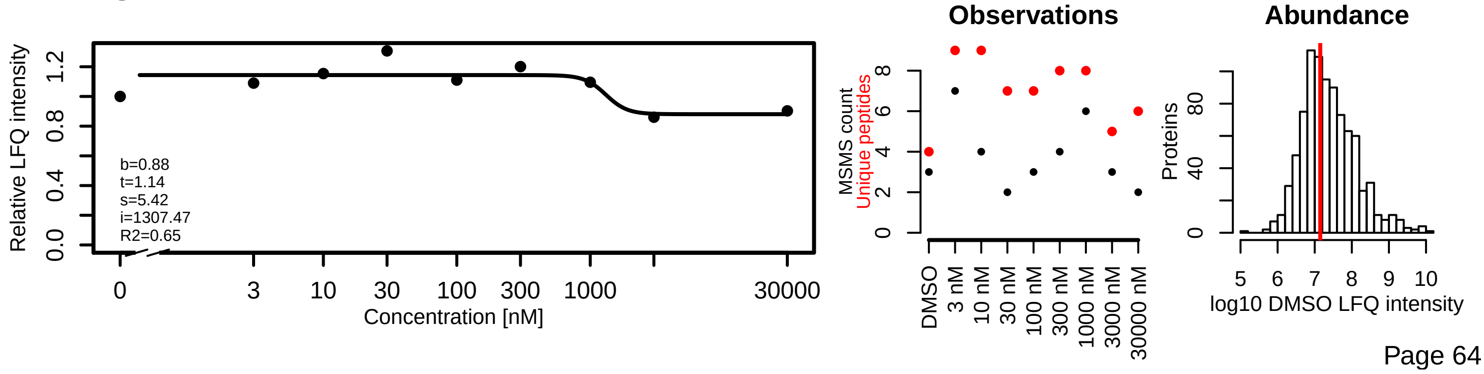

DPY19L1

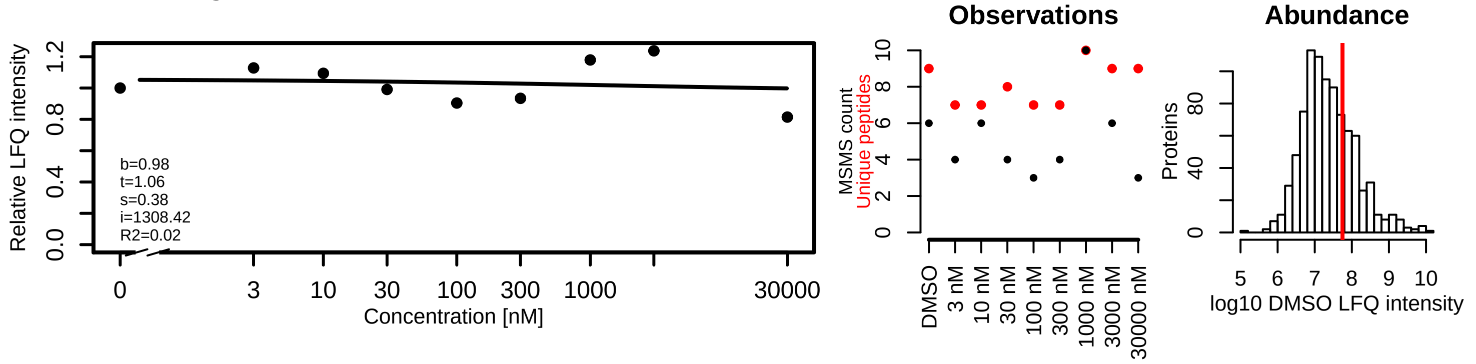

UTP20

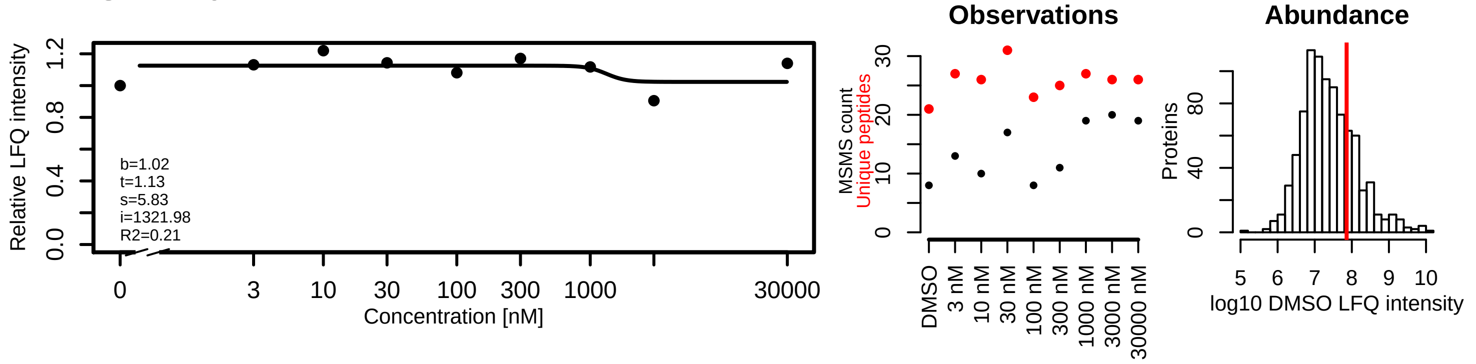

NLRX1

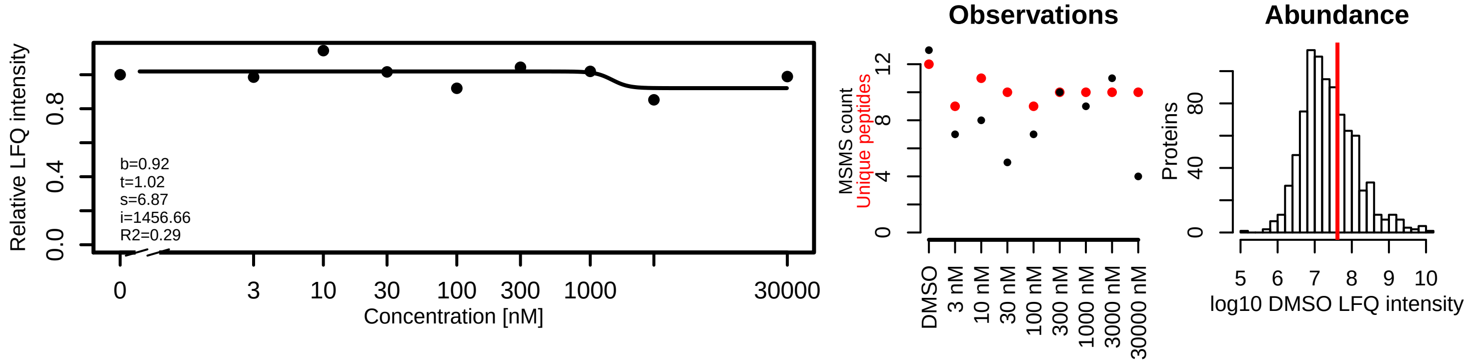

PIGM

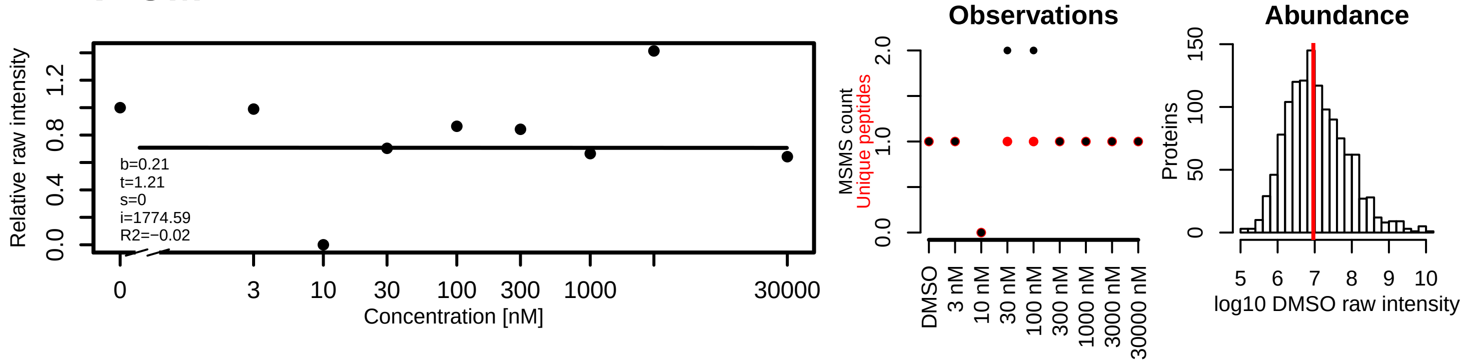

RPN2

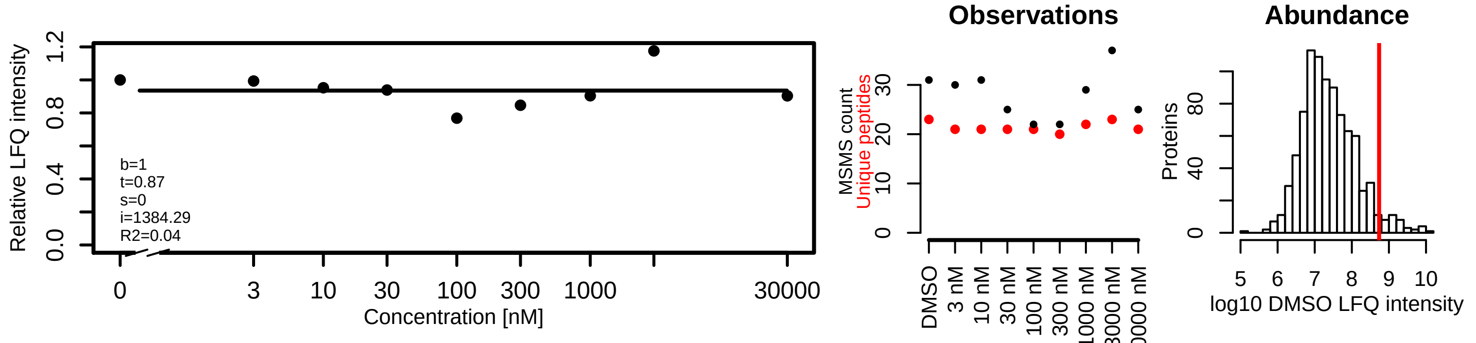

RAB1B;RAB1C

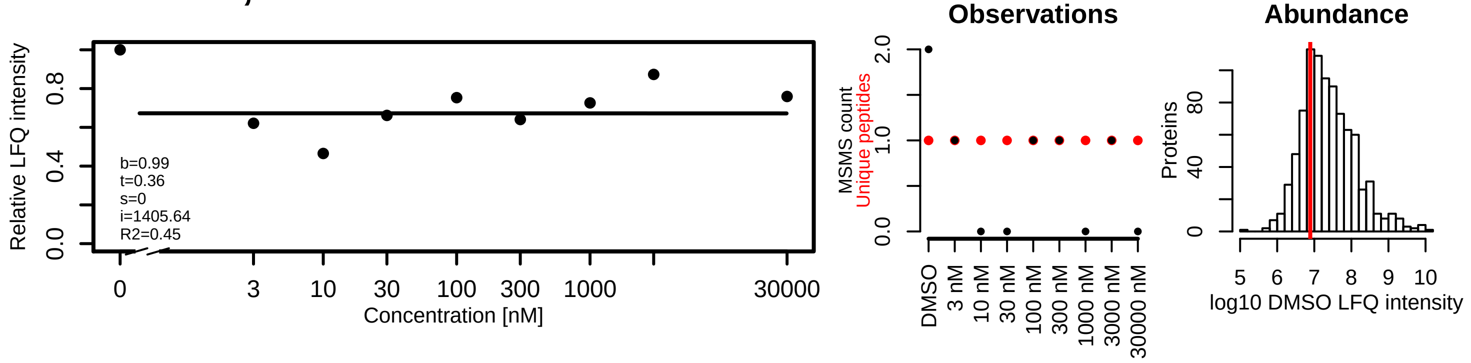

TSPO

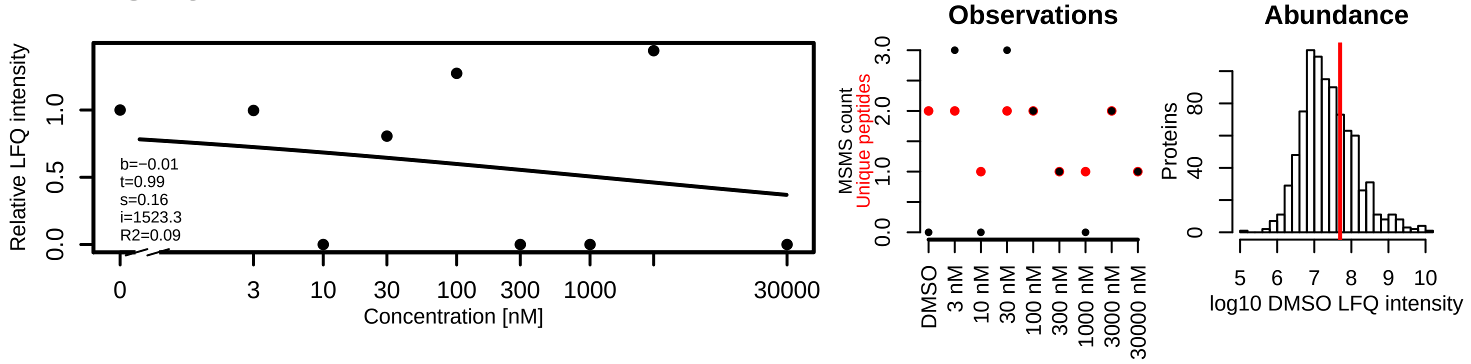

ACVR1

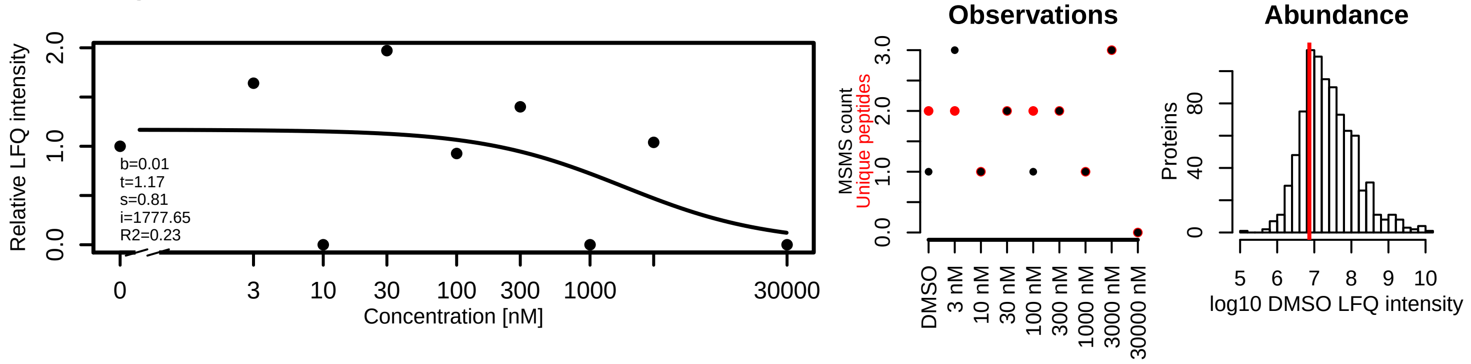

DDB1

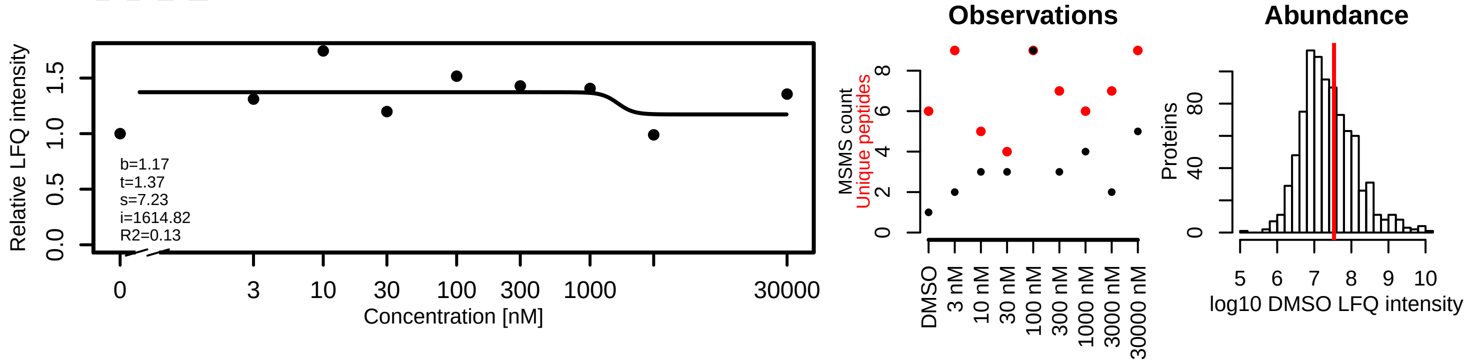

CAPN1

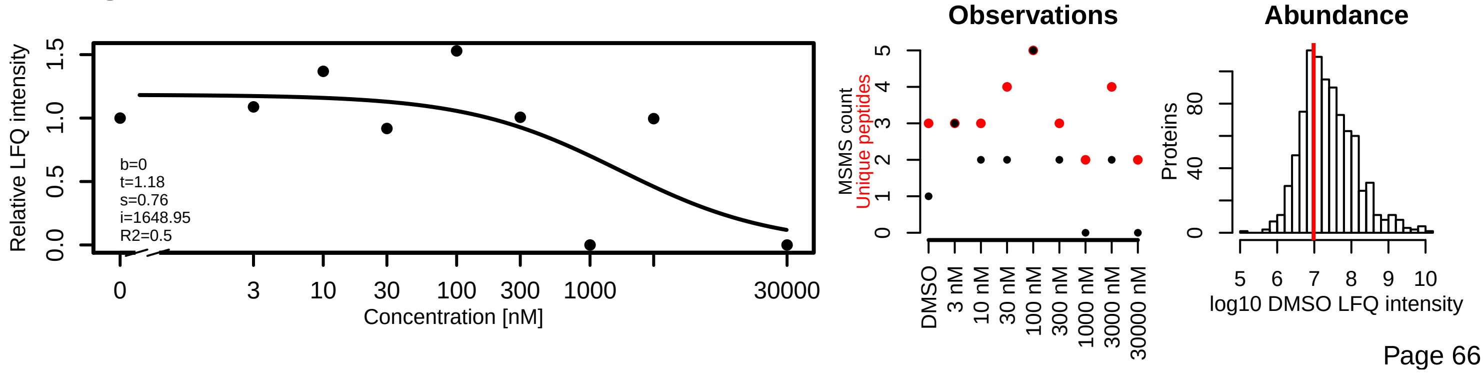

OCLN

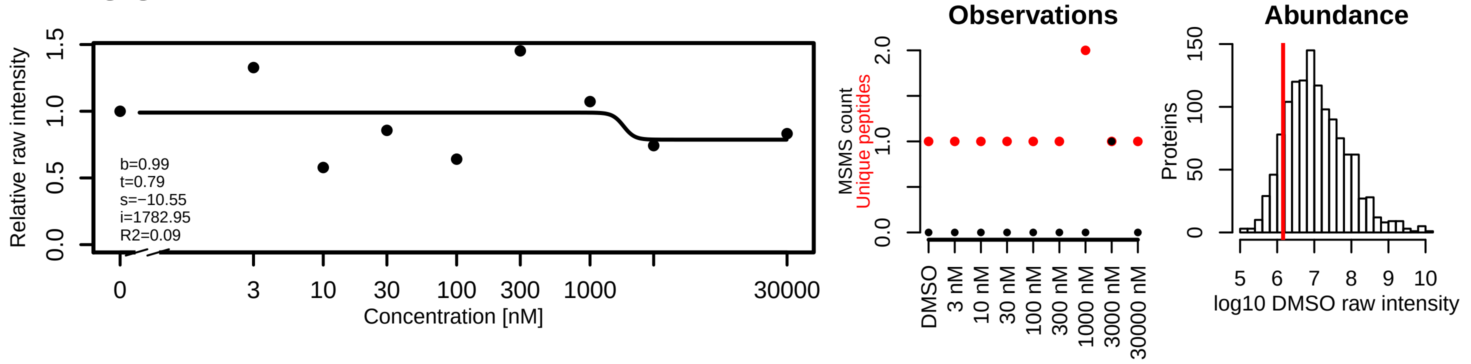

SLC5A6

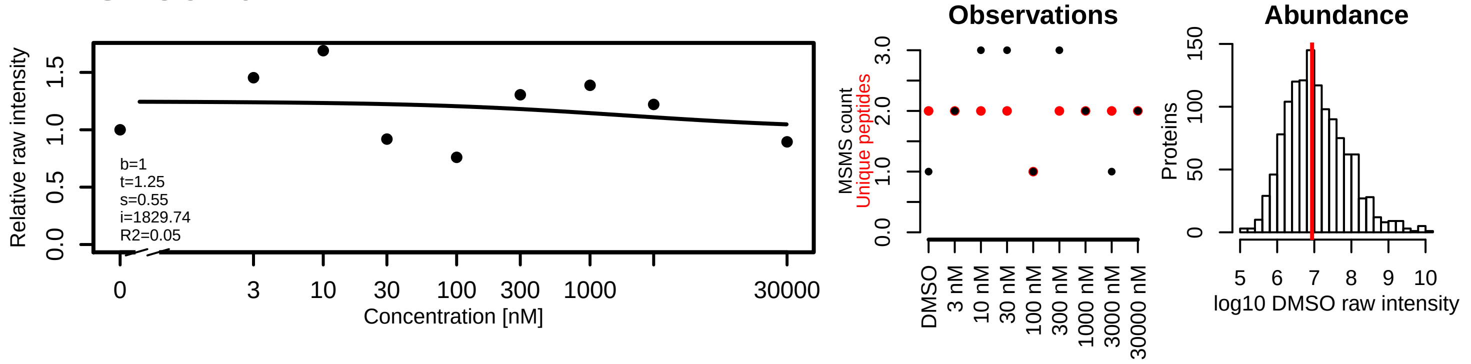

HEATR2

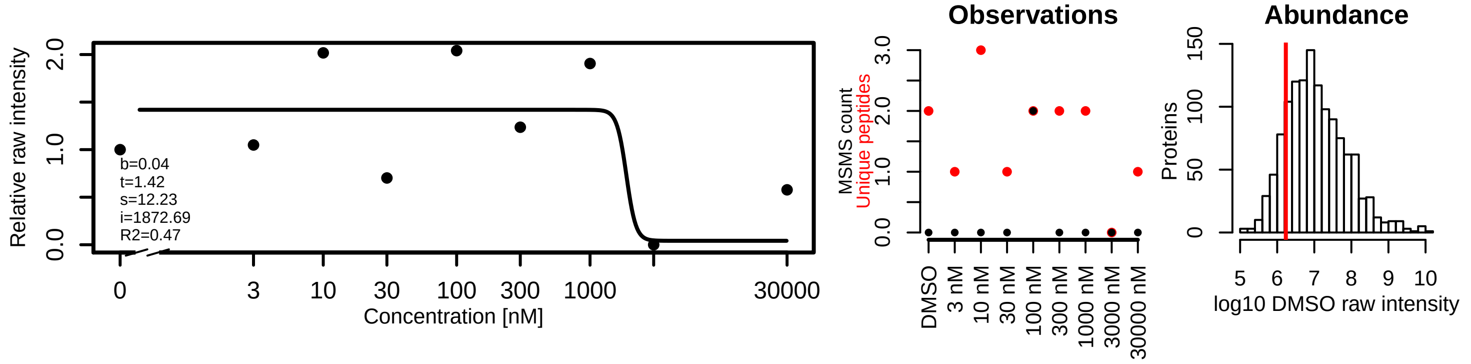

FANCG

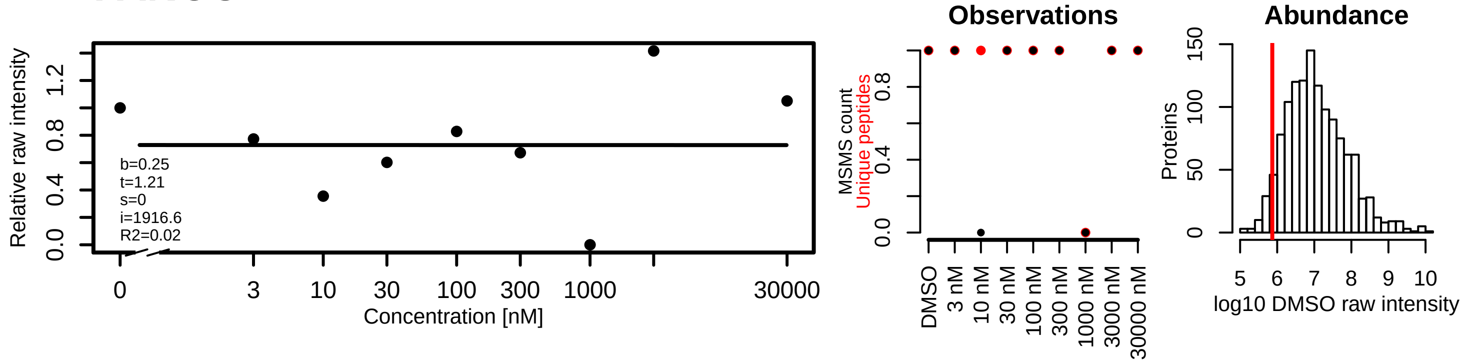

NOP56

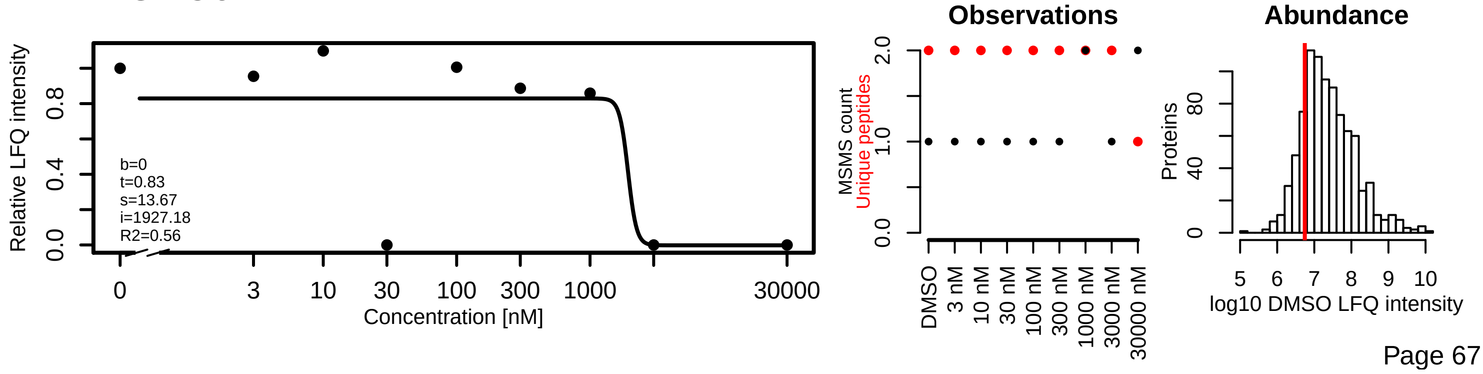

MAPK11

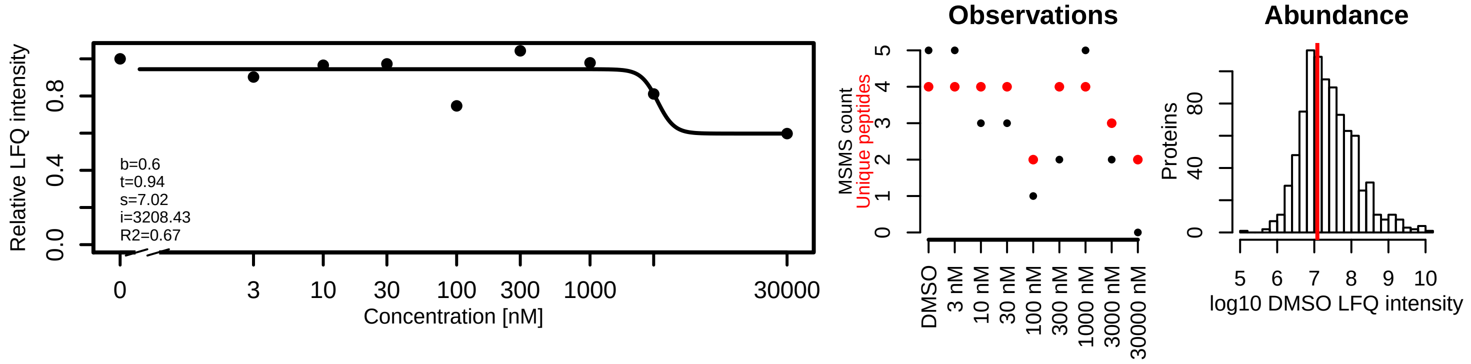

DECR1

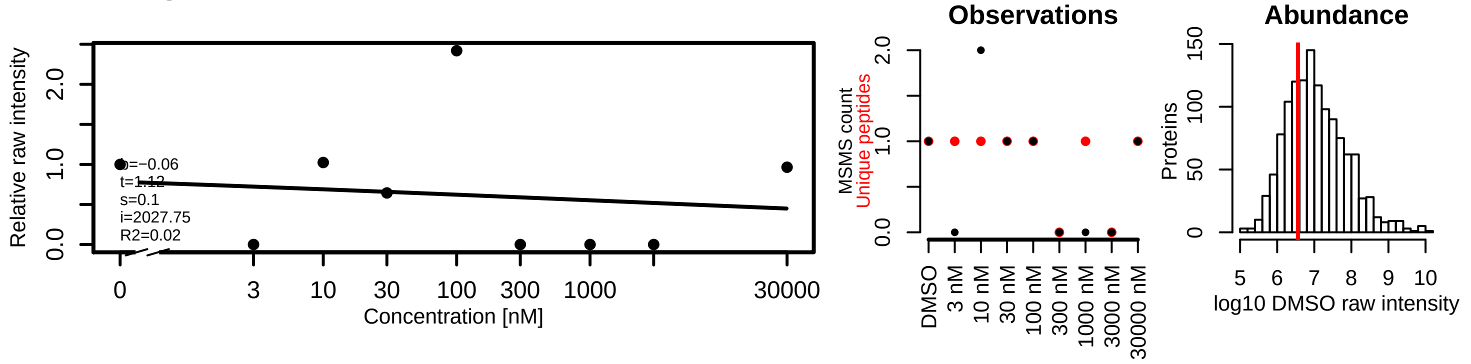

TUBA1C

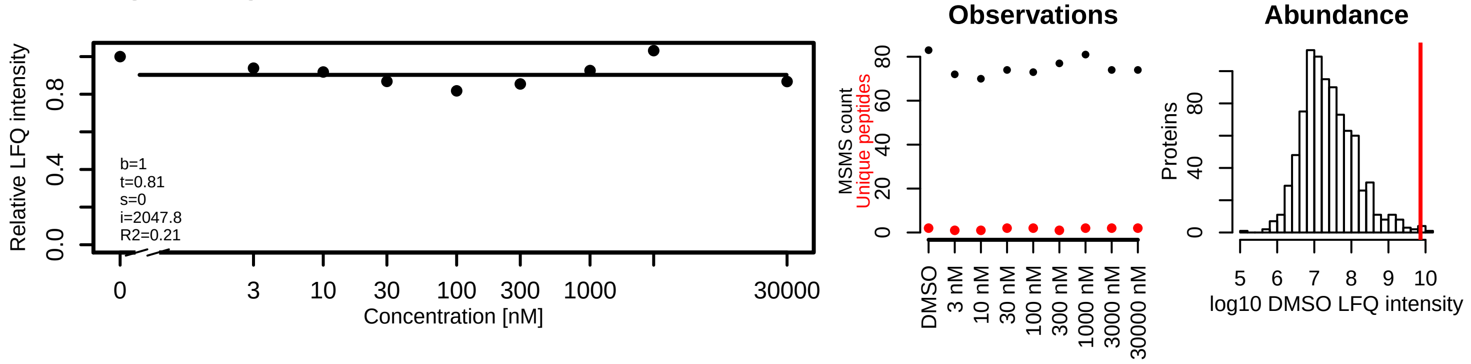

NDUFA10

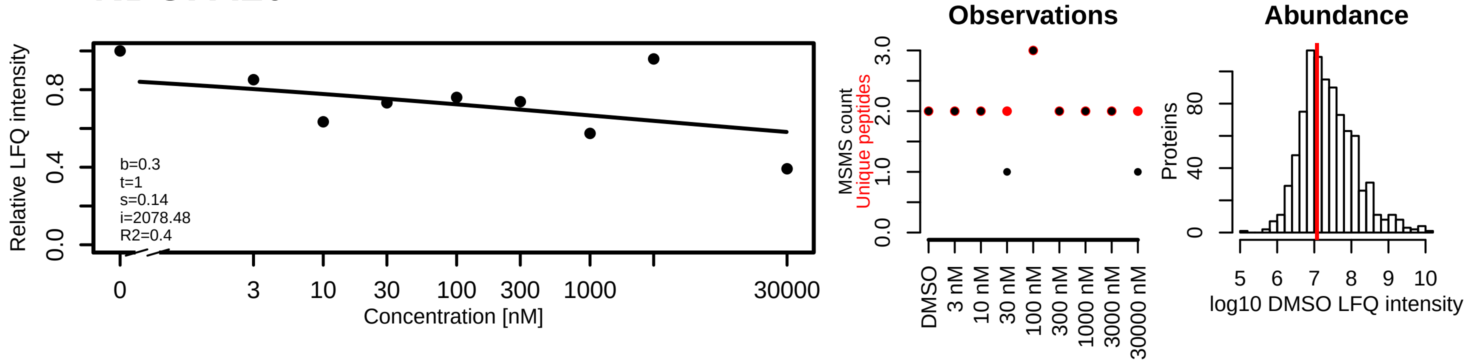

NUMA1

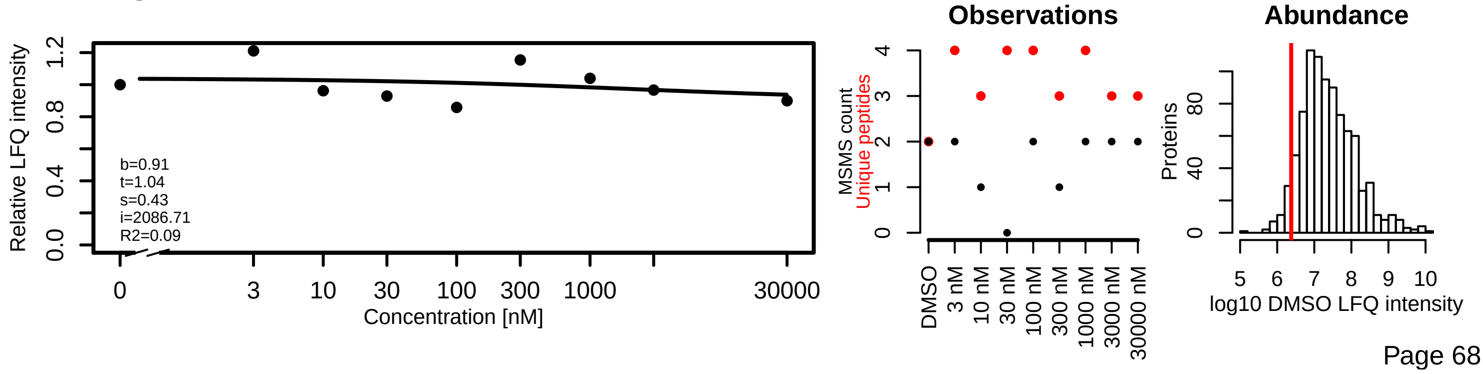

NDUFA13;YJEFN3

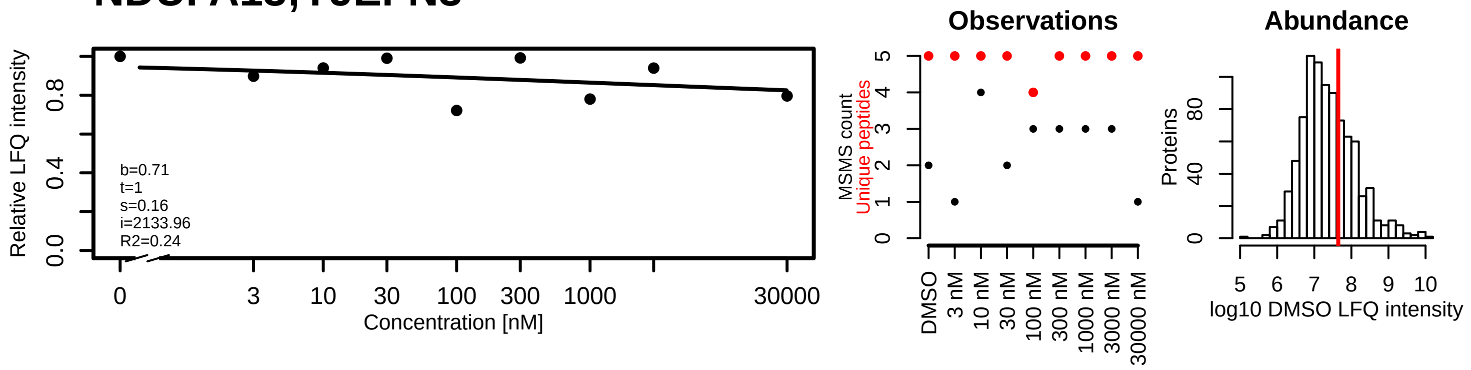

HNRNPL

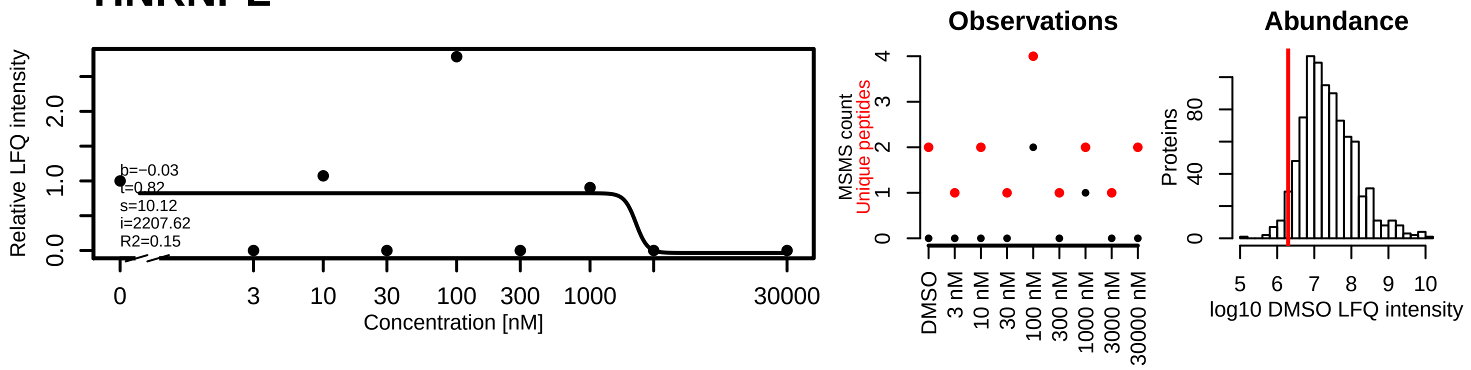

CNN3

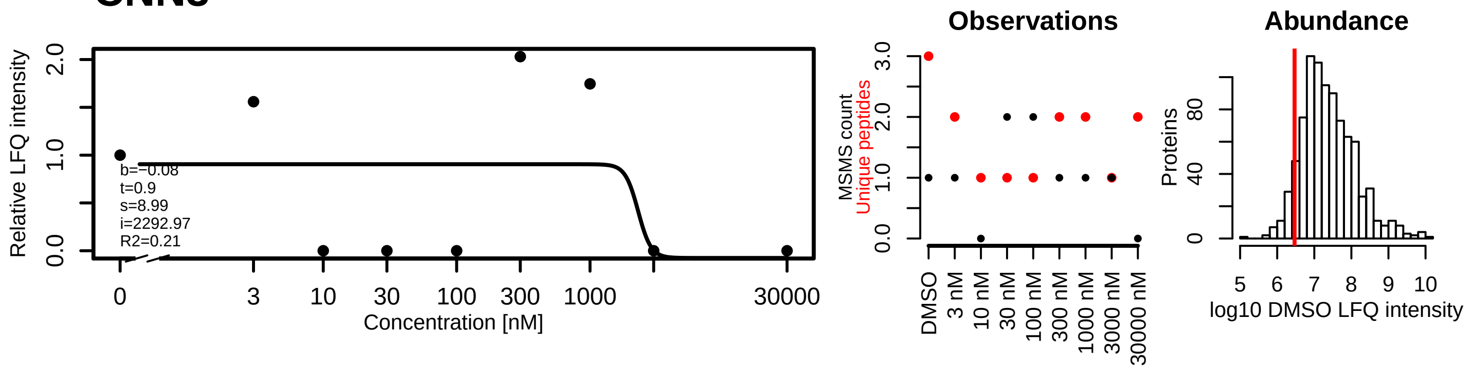

RINT1

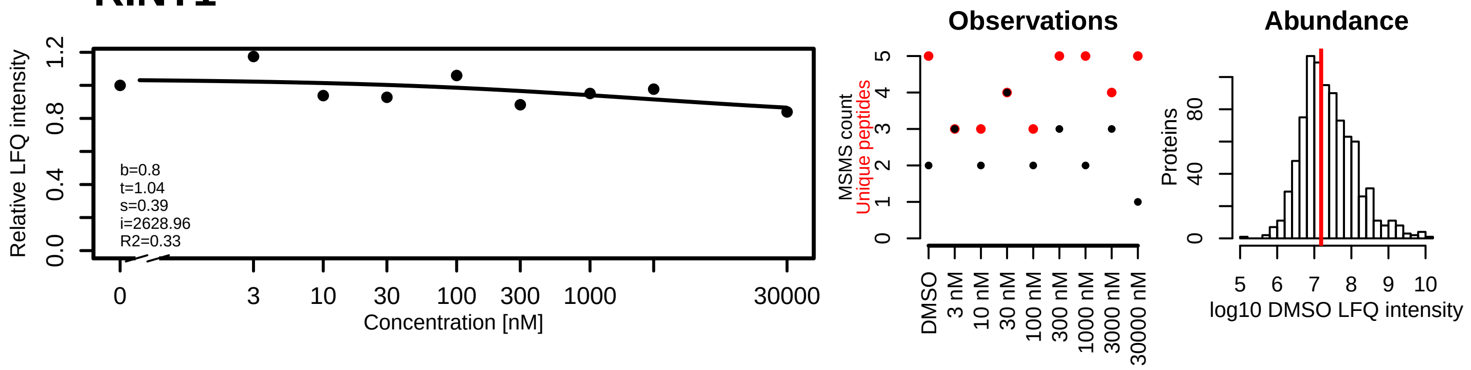

RARS

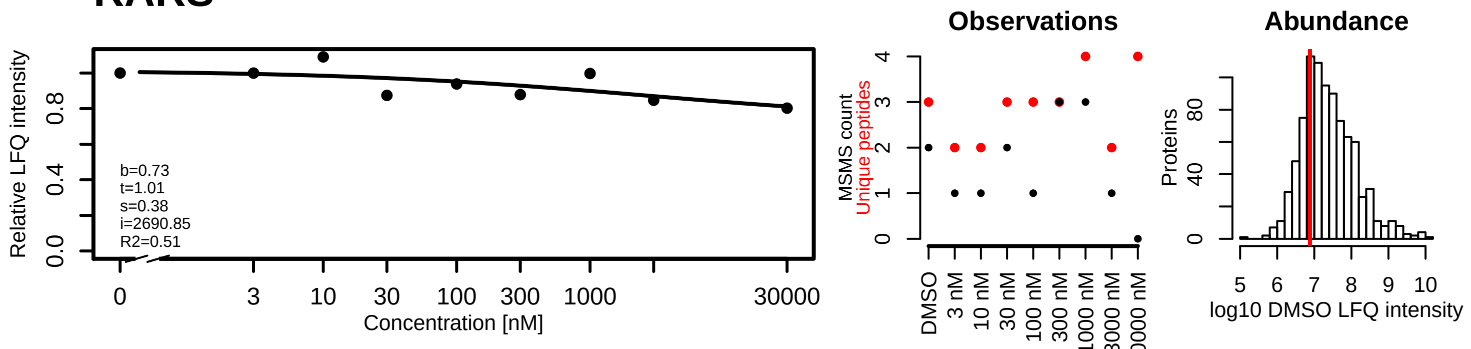

ABCD1

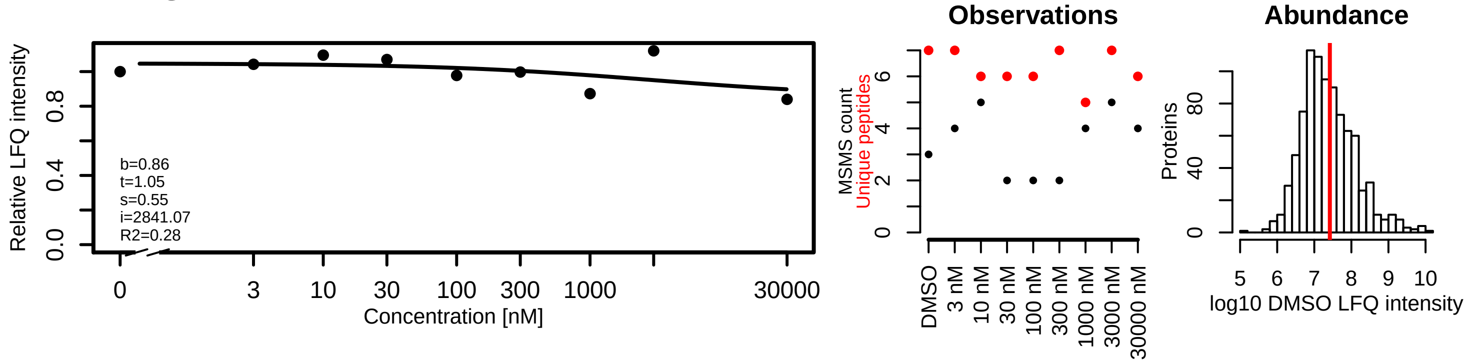

CHPT1

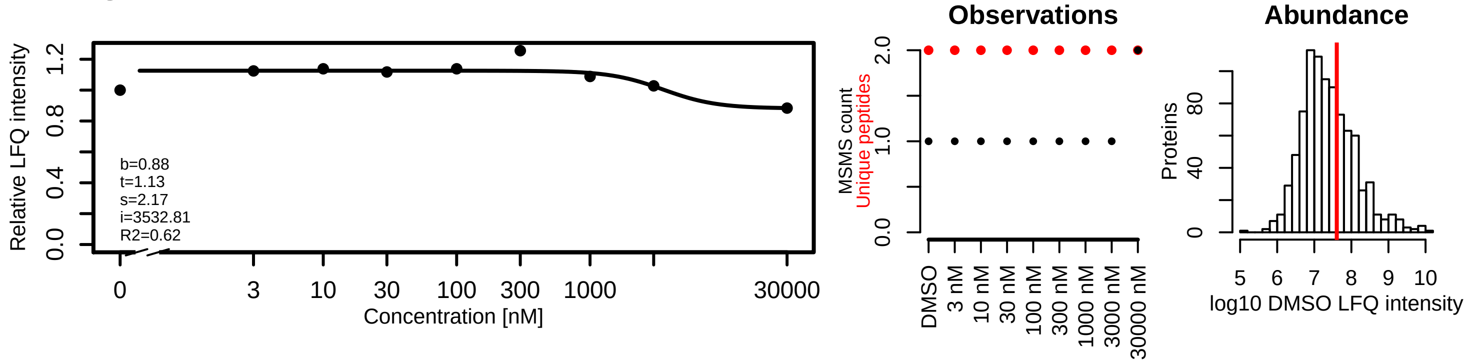

PFKL

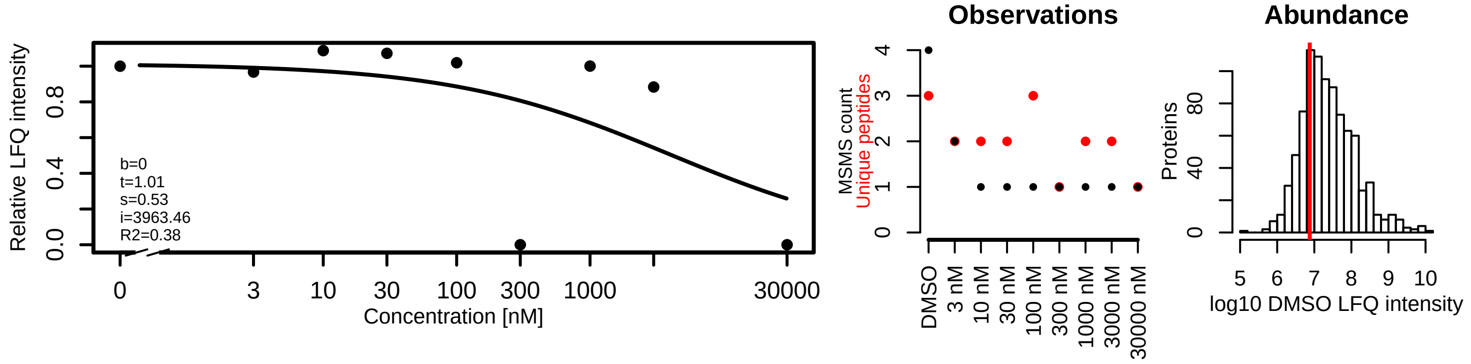

CCDC113

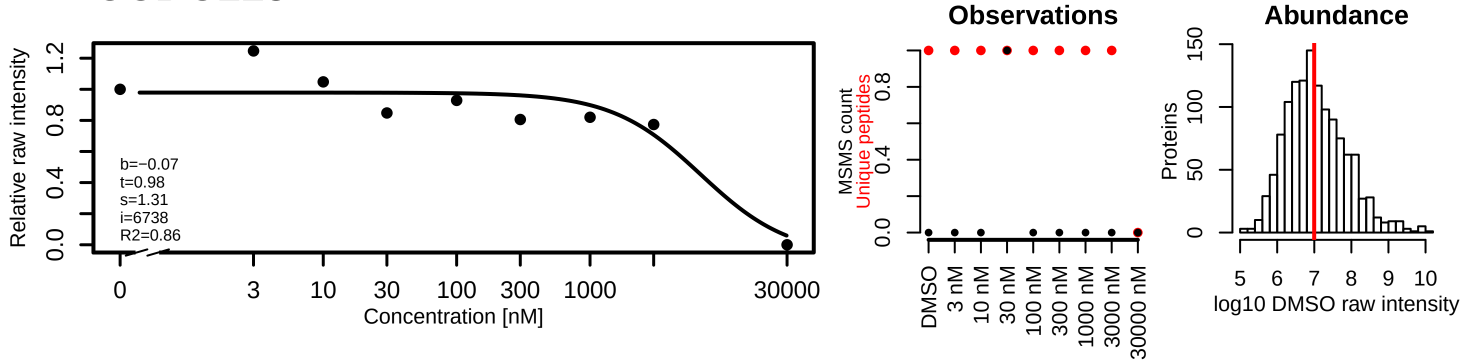

PCNXL3

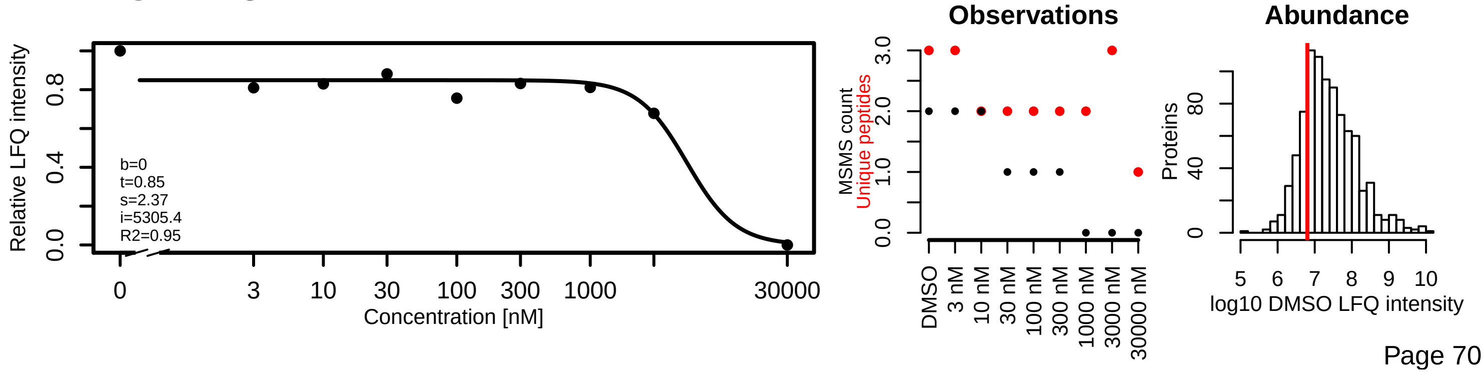

RMDN3

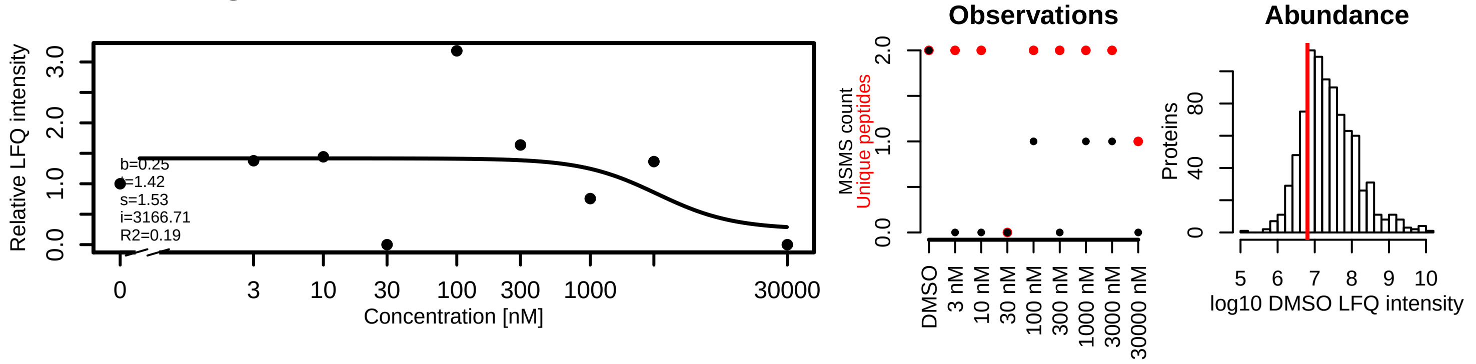

GOLM1

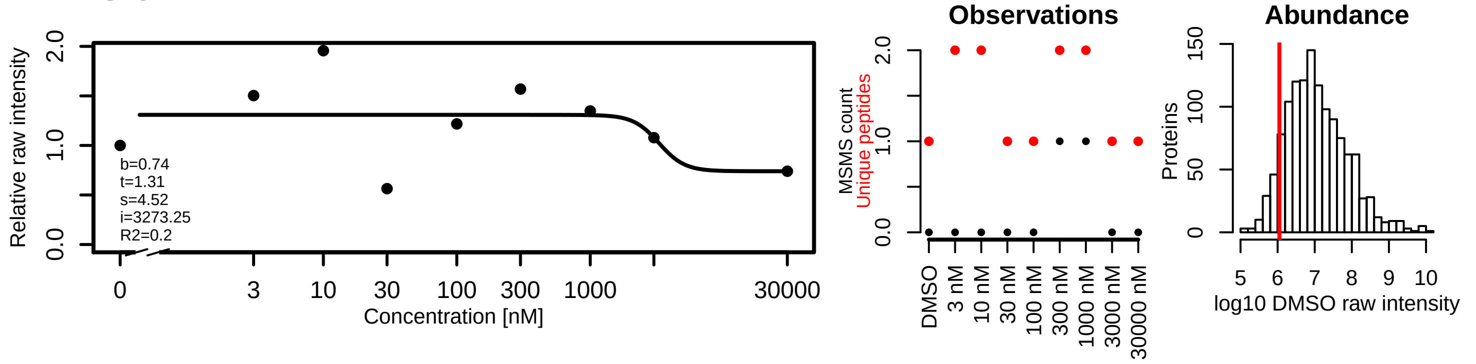

COPG2

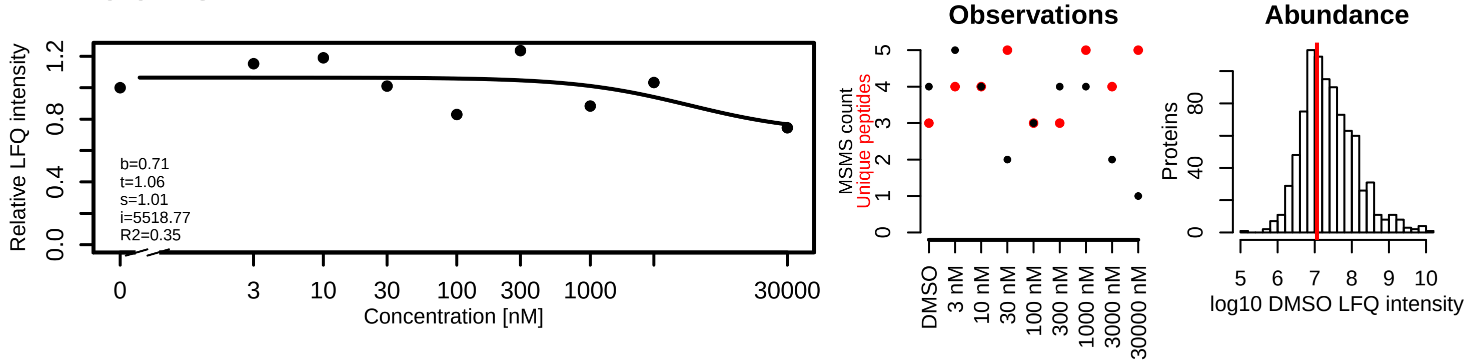

A8MWL6

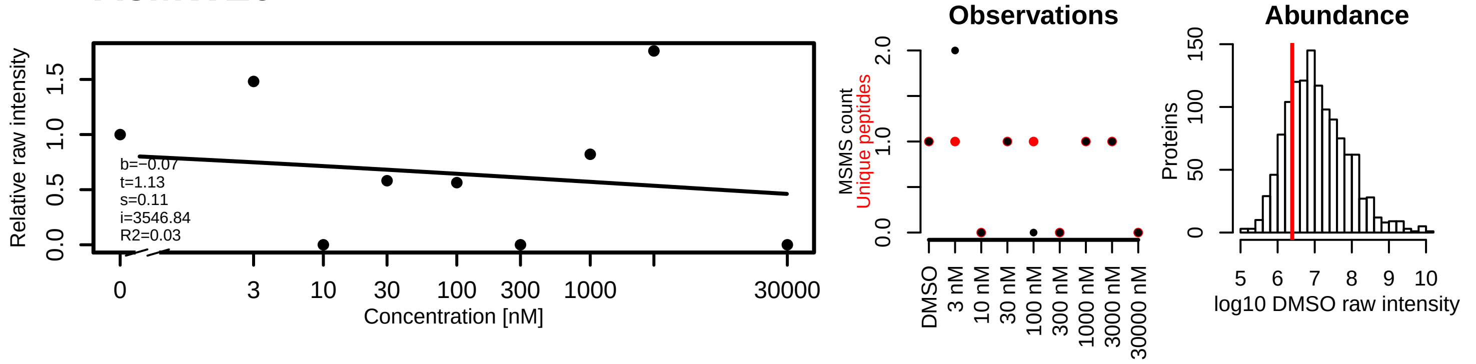

PSEN1

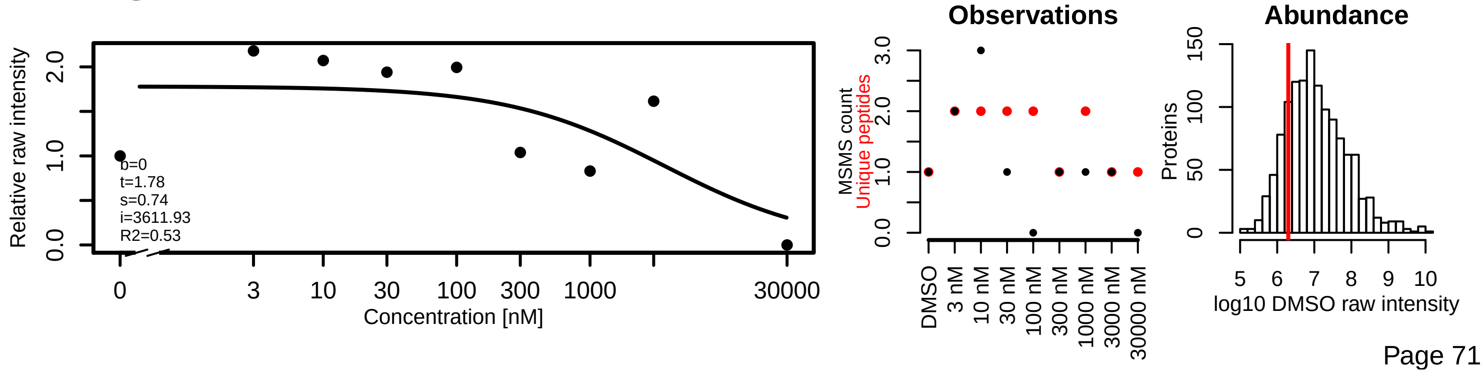

SQSTM1

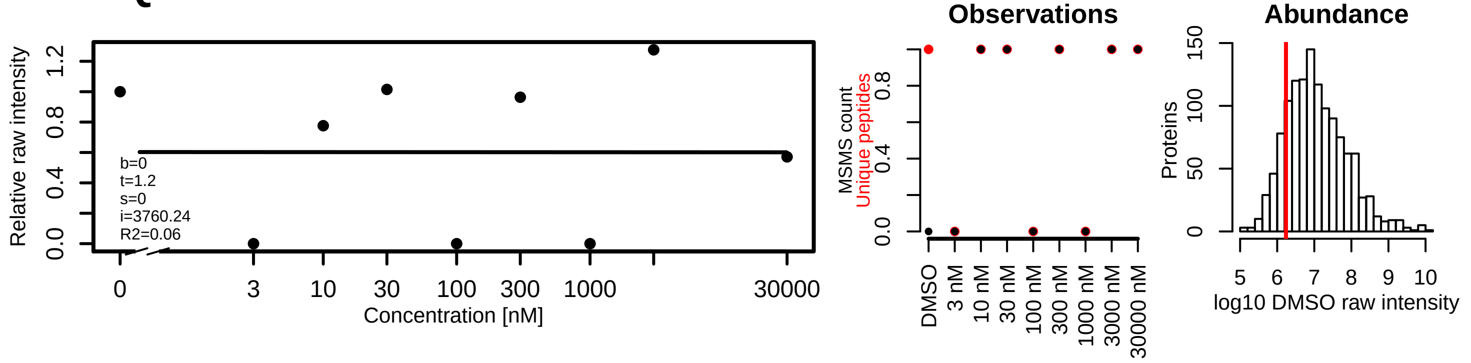

SFXN3

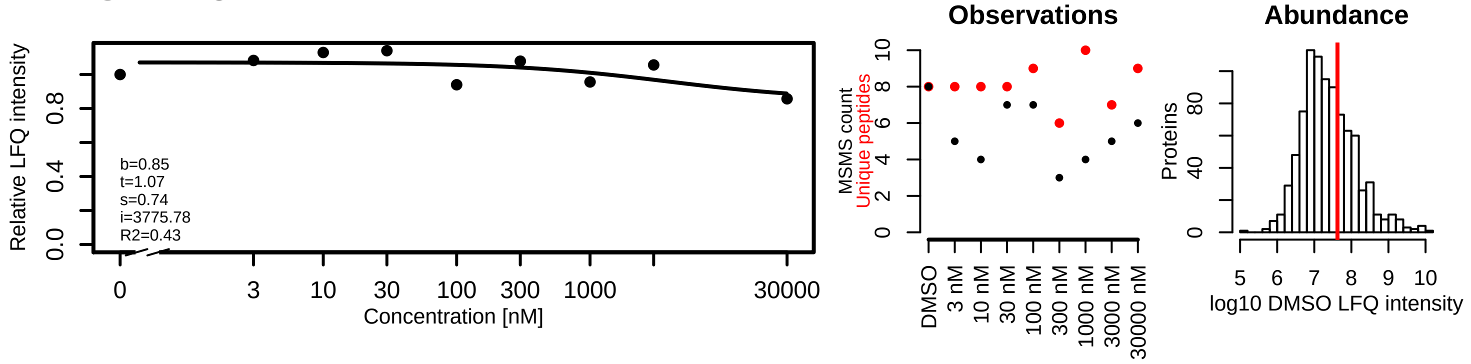

NDUFB10

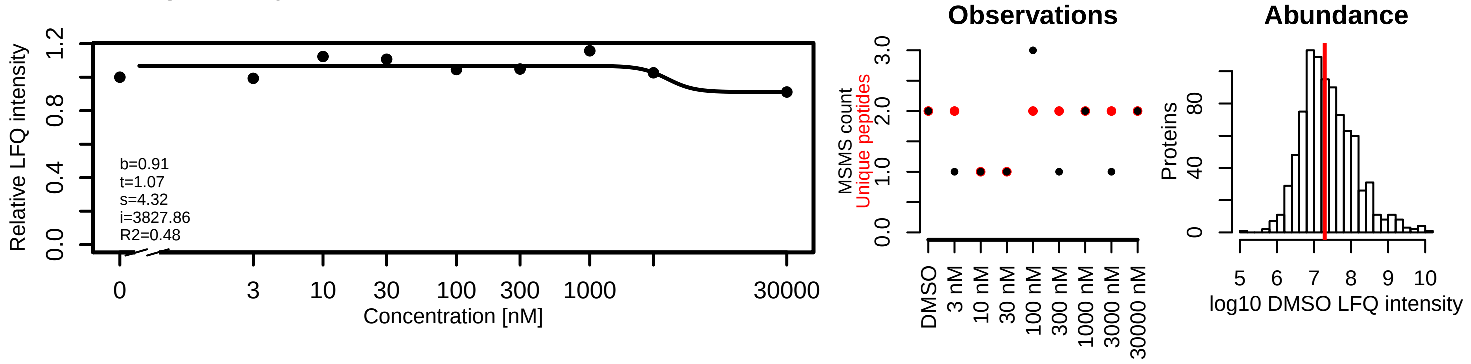

TGM2

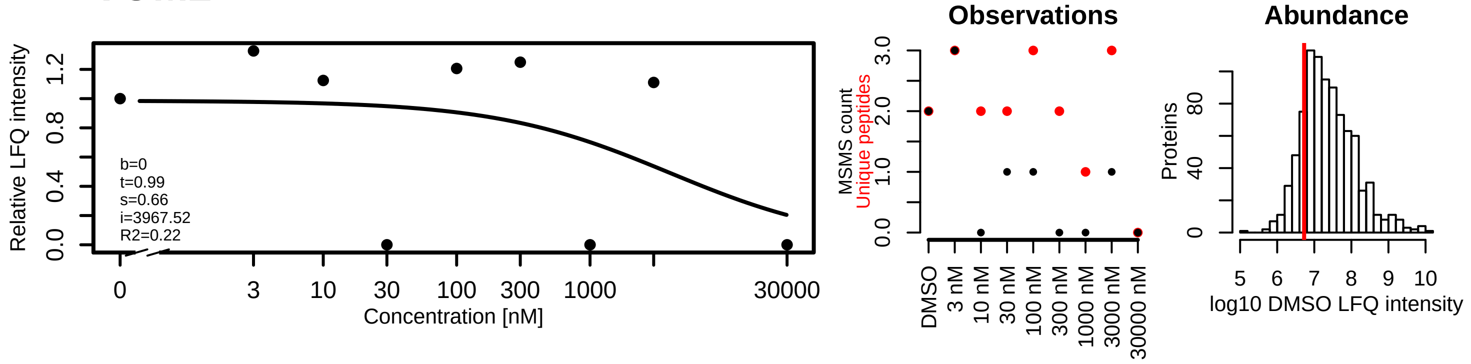

ATP5B

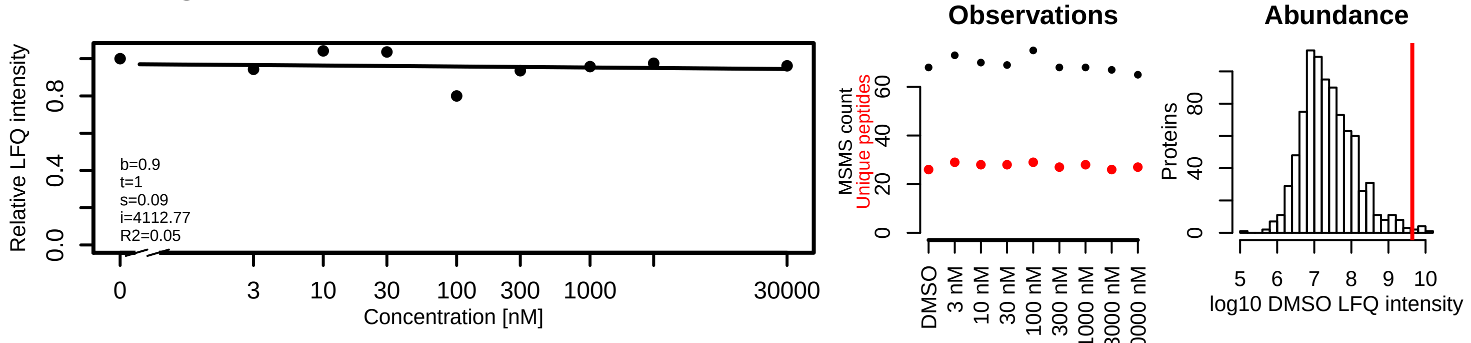

ESYT2

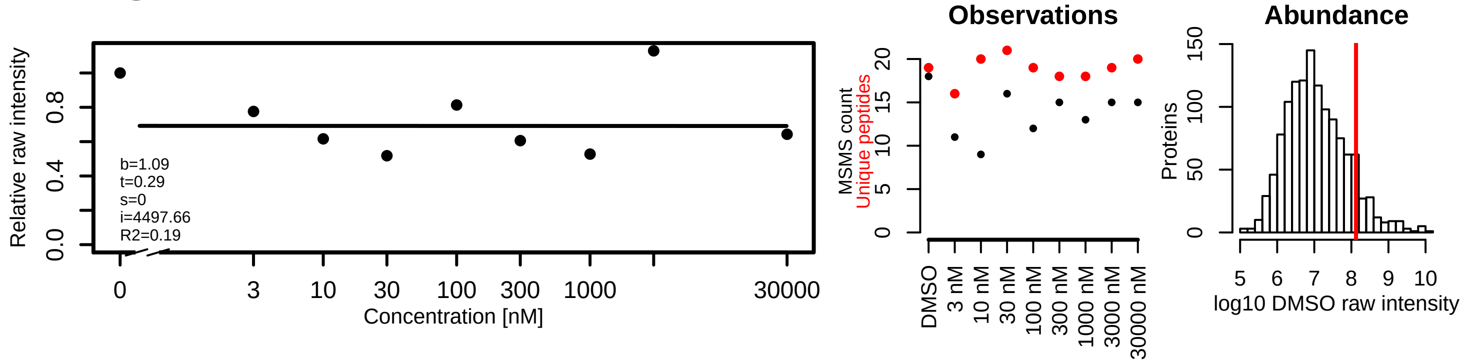

TUBGCP3

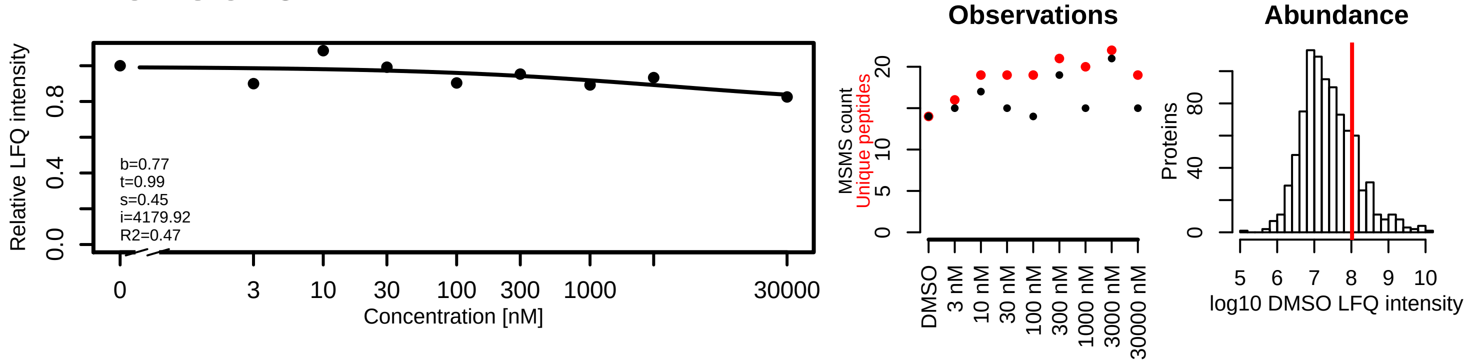

SRP14

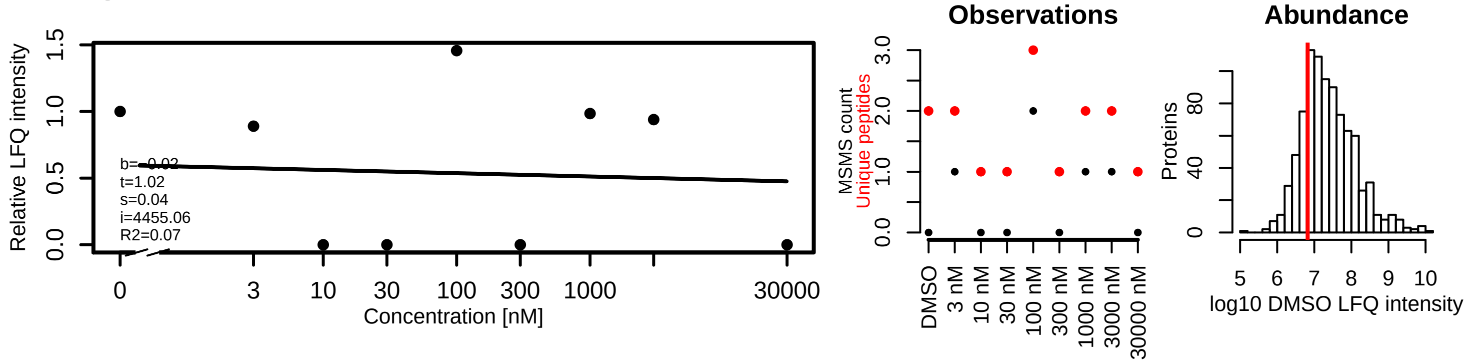

MAPK14 – Q16539–2

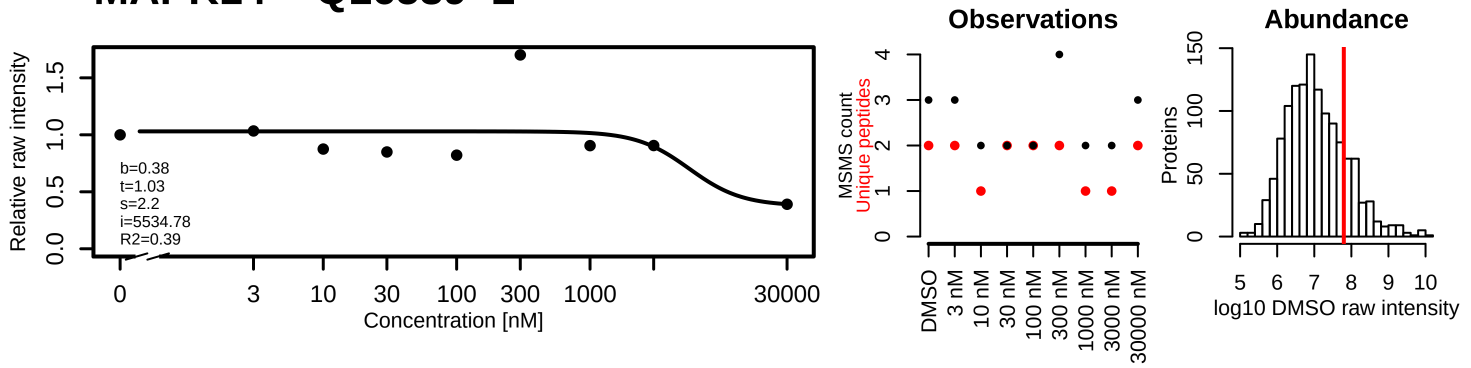

ATP2B3

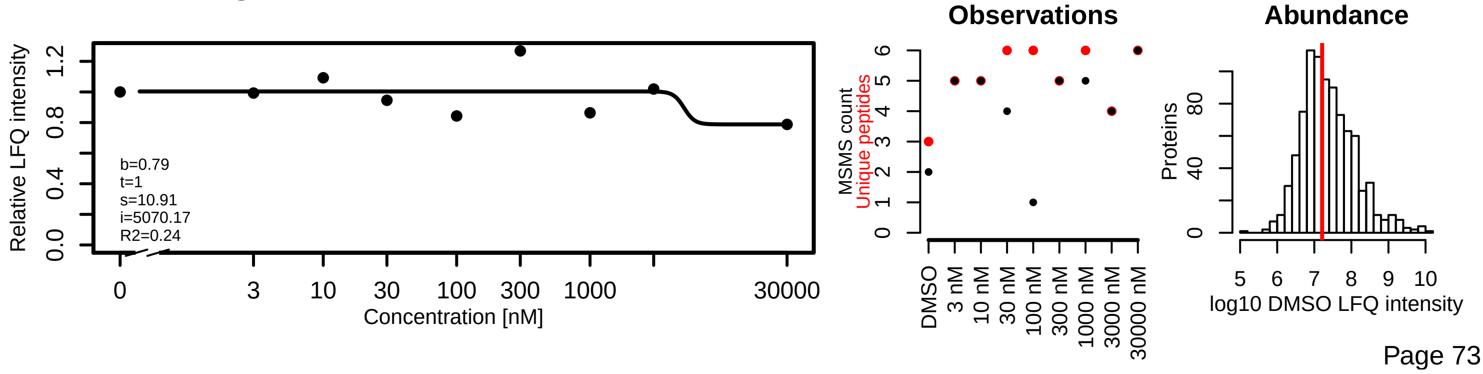

GEMIN5

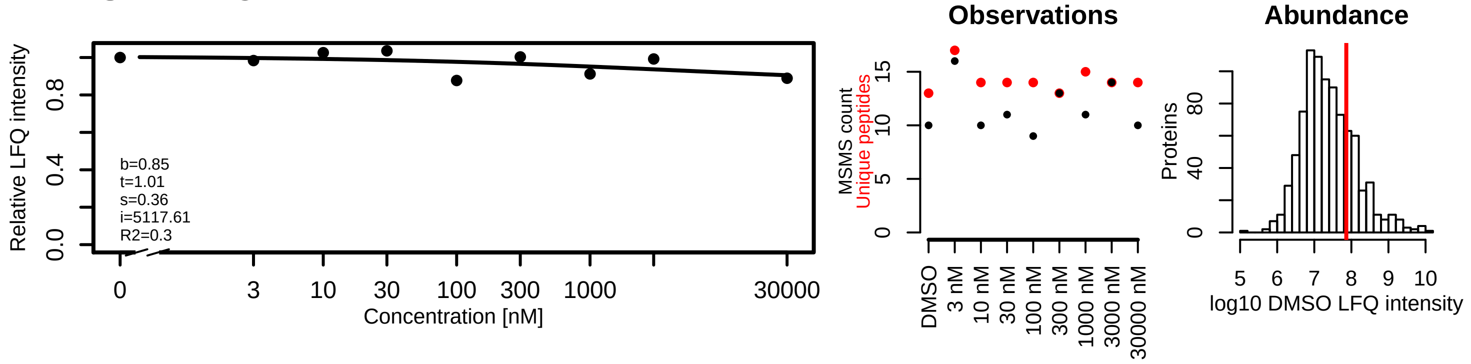

GPR56

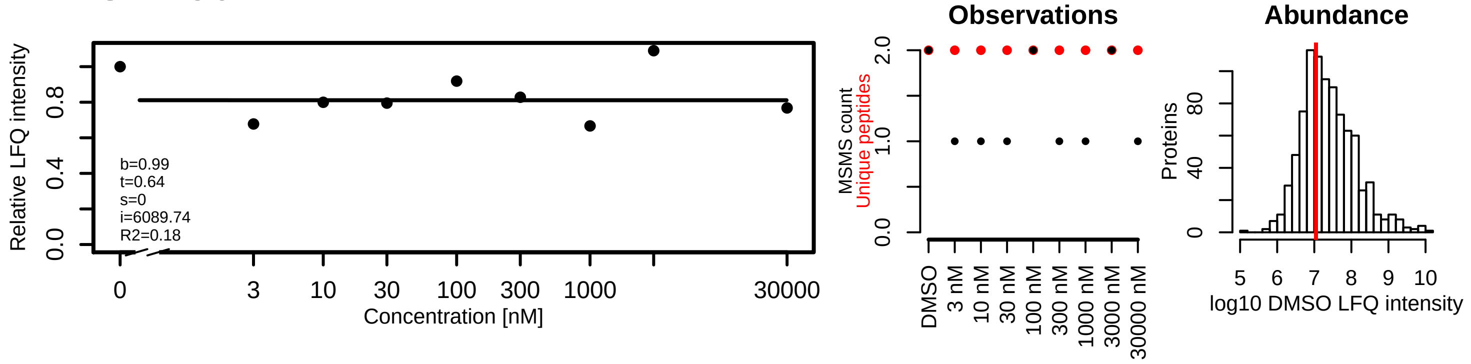

TMEM33

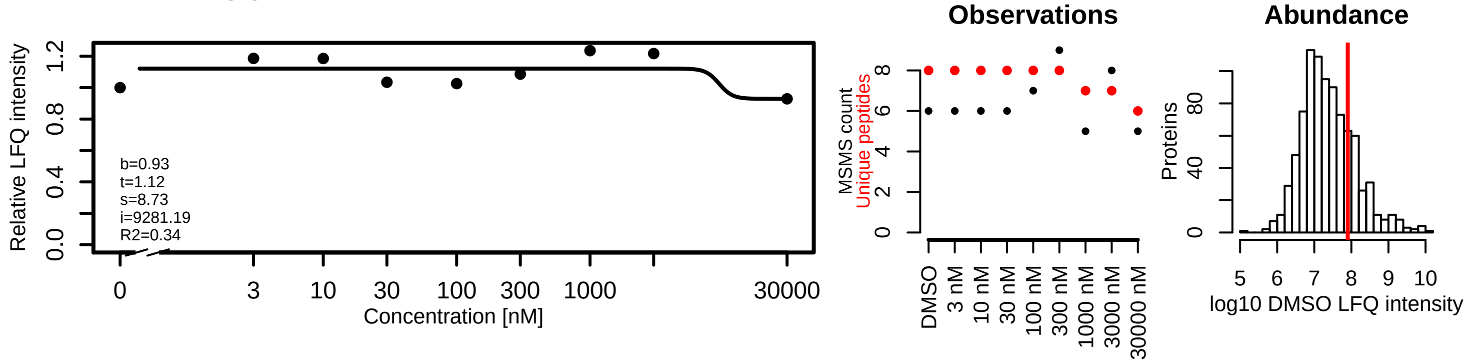

MMGT1

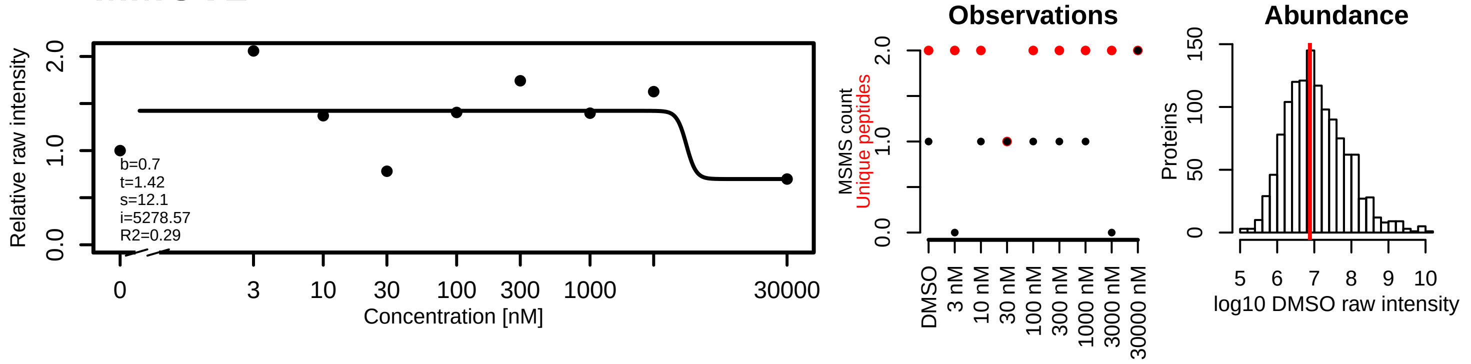

HSPA8

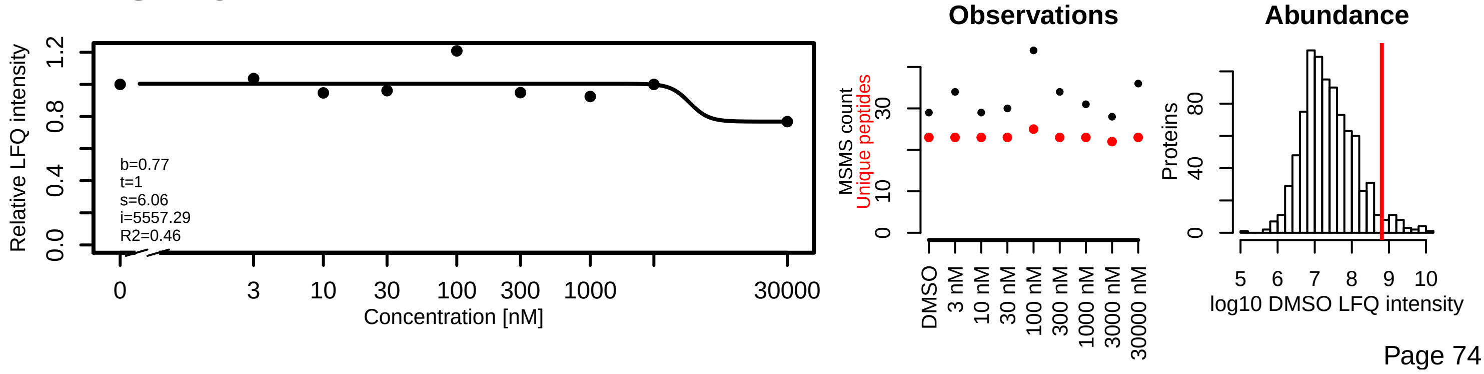

SERPINE1

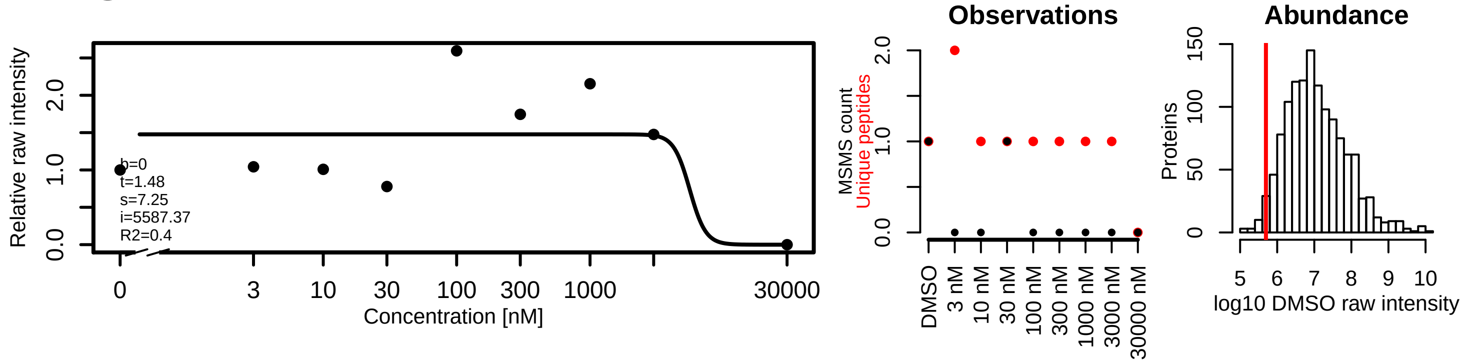

CTTN

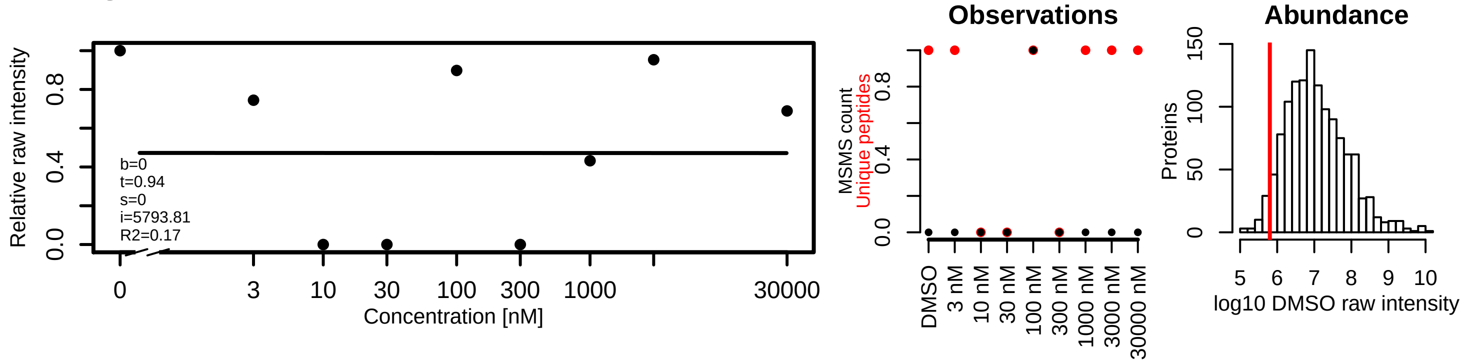

NOM1

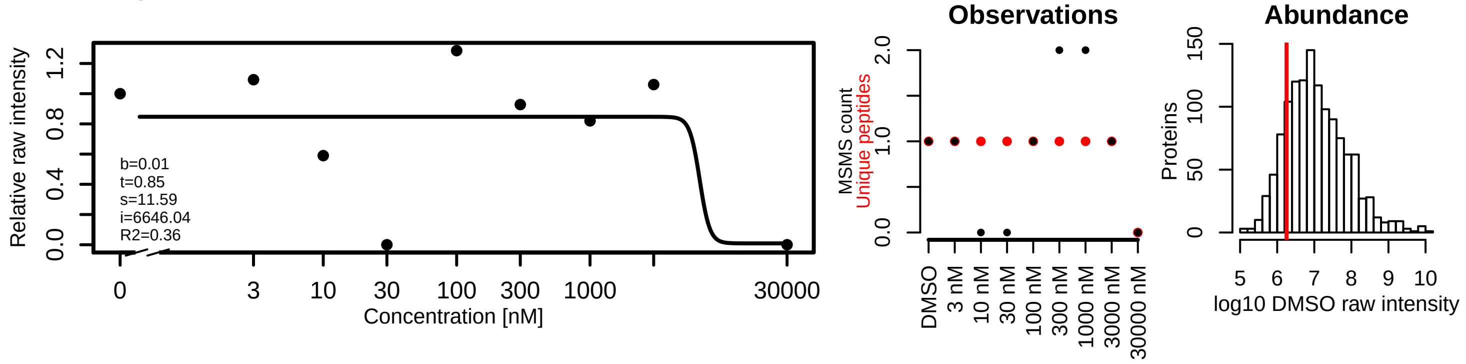

VKORC1

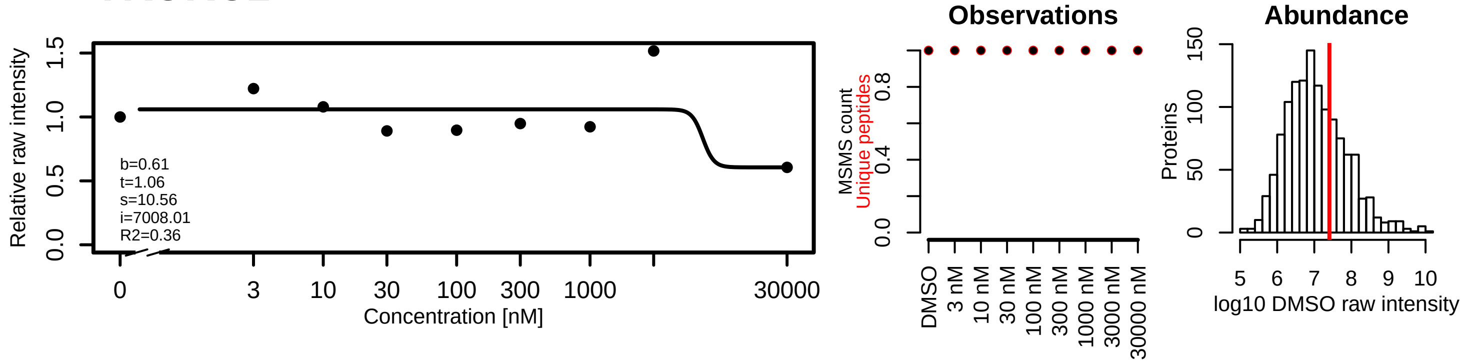

NCOA2

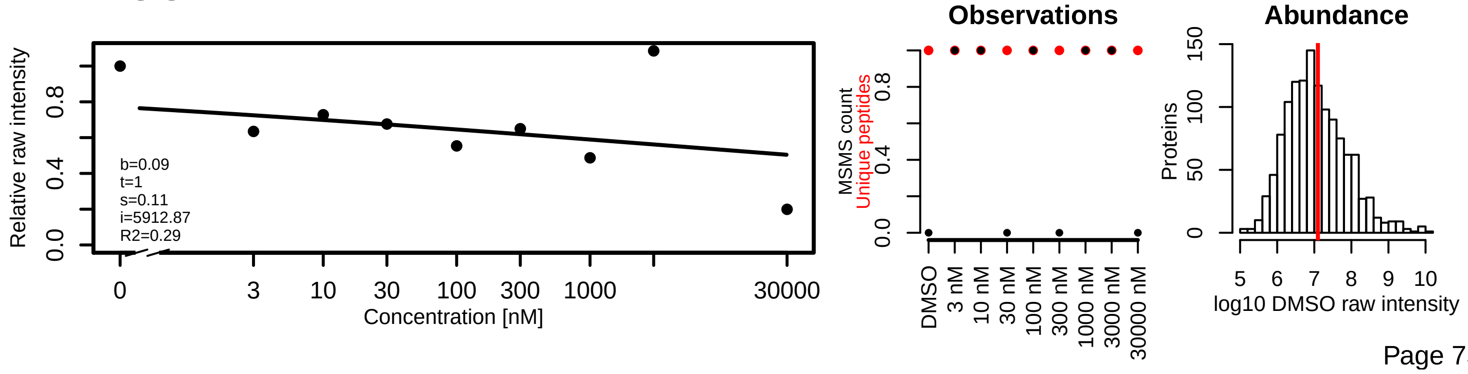

TIMM17B

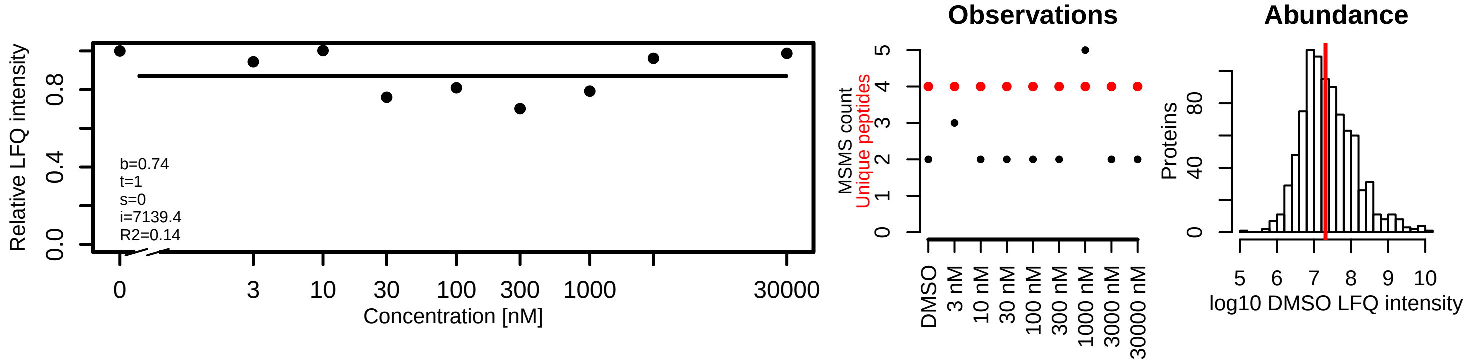

COX18

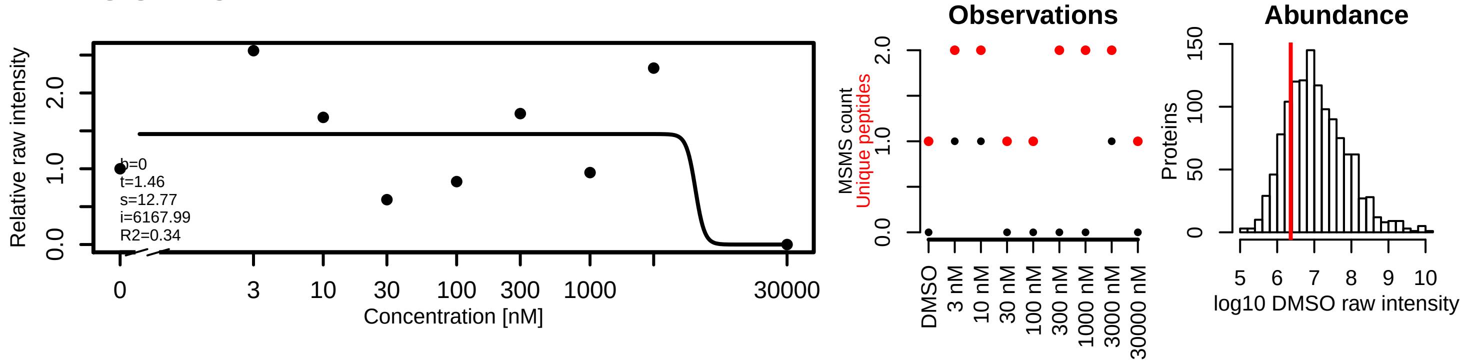

ITPR3

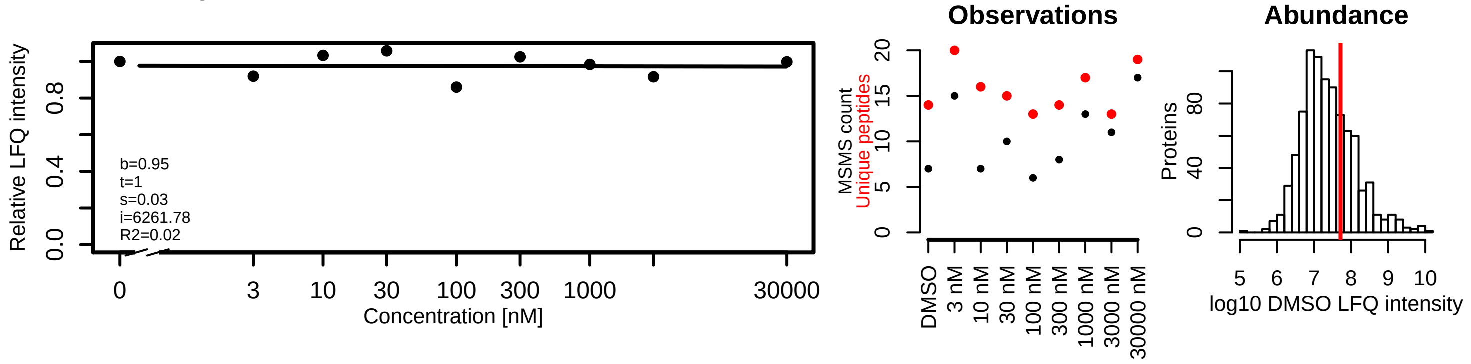

CSNK1E;CSNK1D

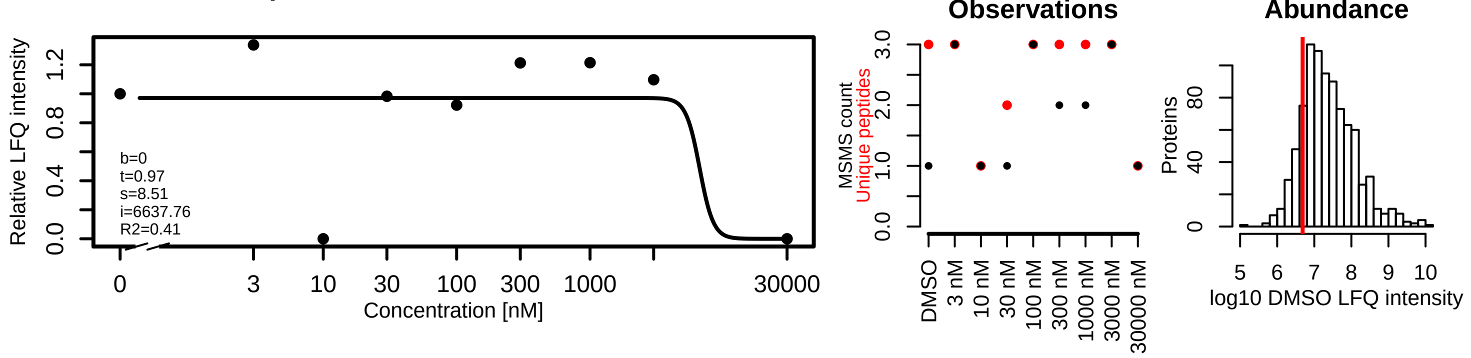

DDOST

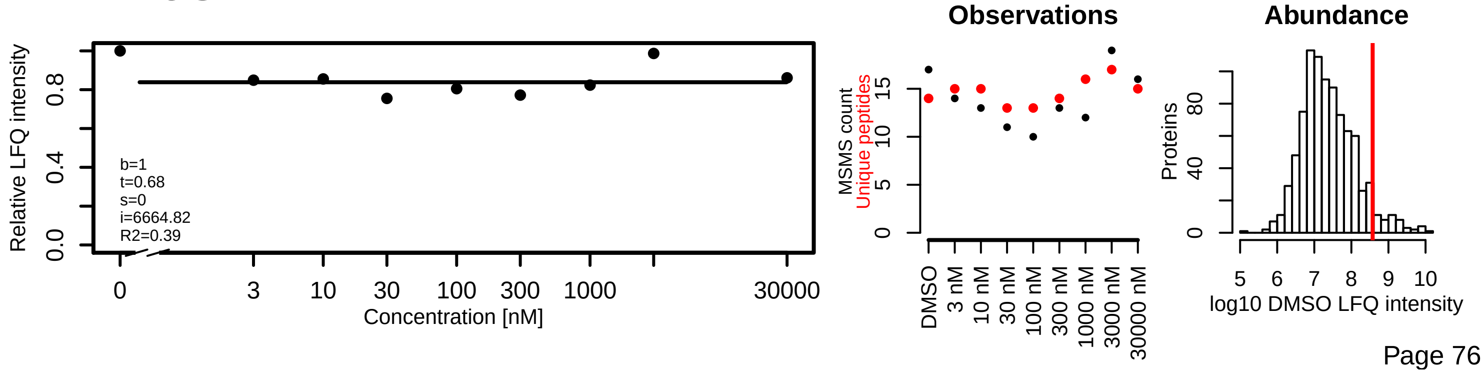

CYB5D2

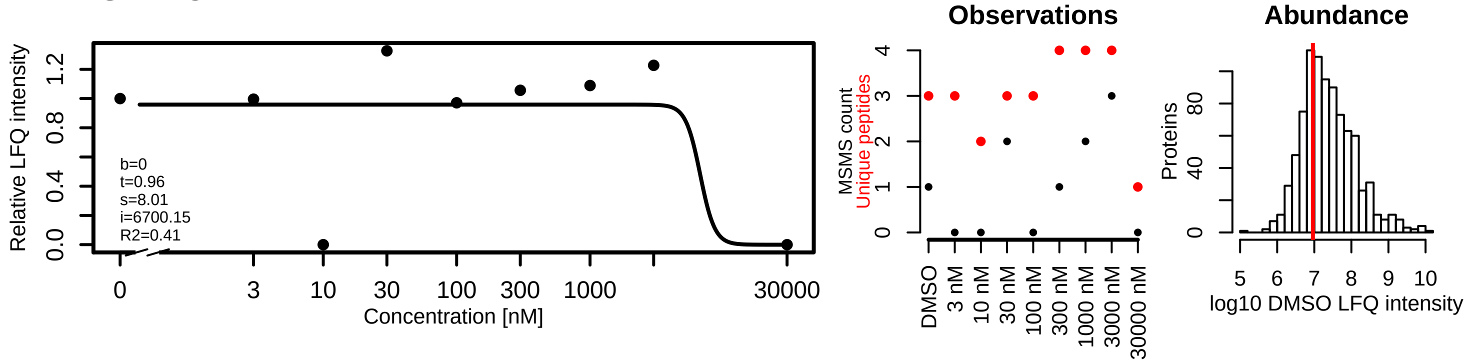

EIF3L

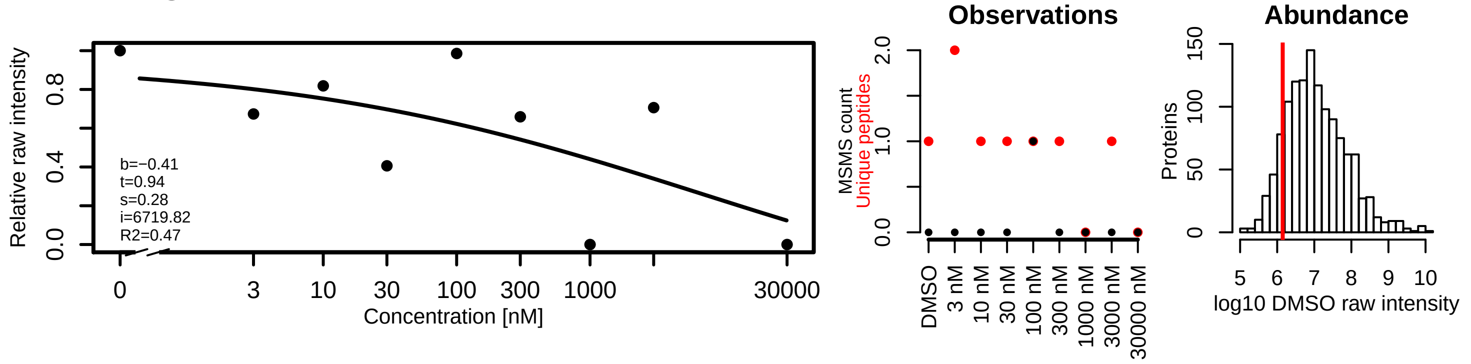

ADCK5

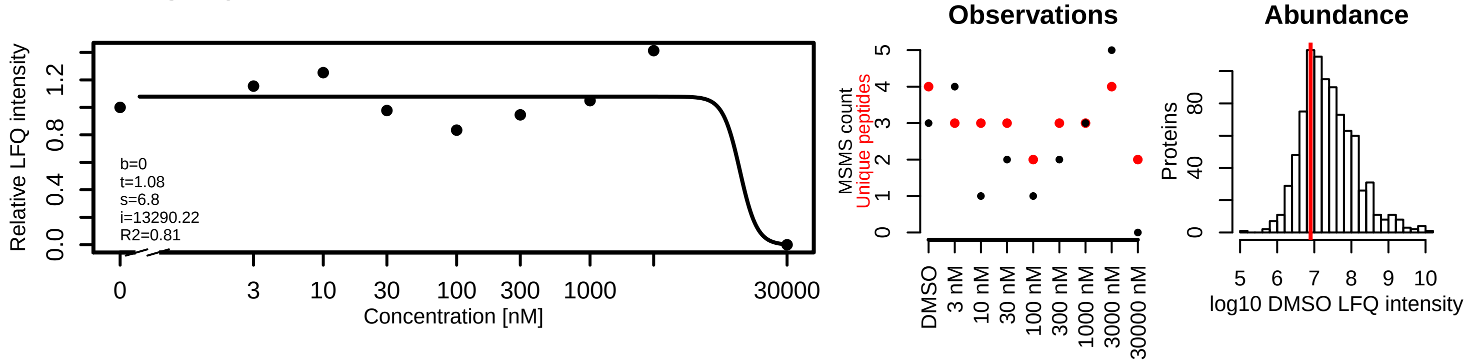

SLC12A9

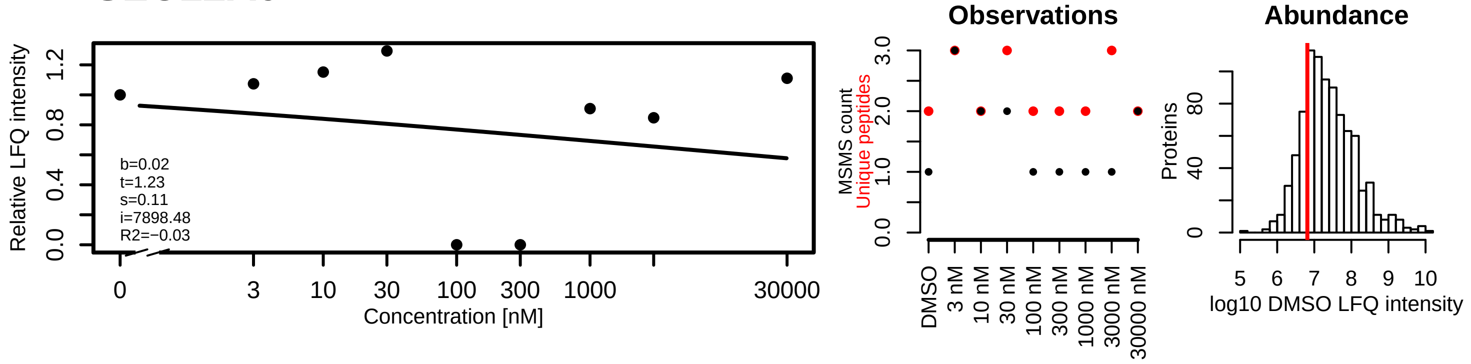

PPAT

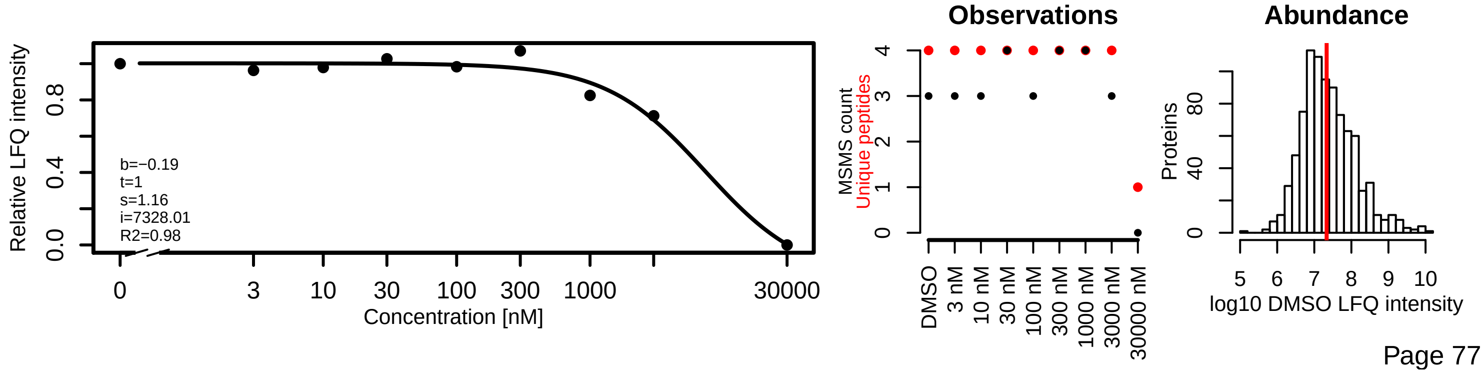

CTSB

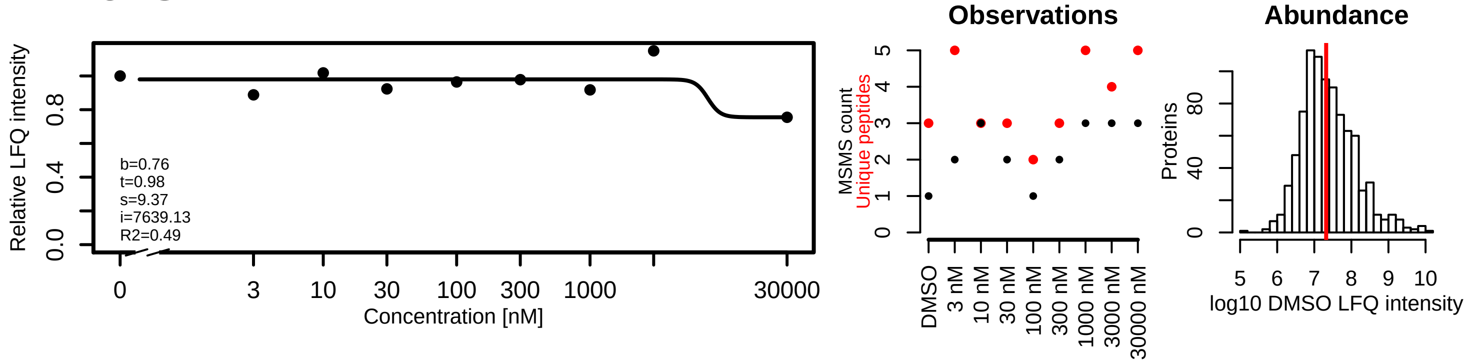

KRTCAP2

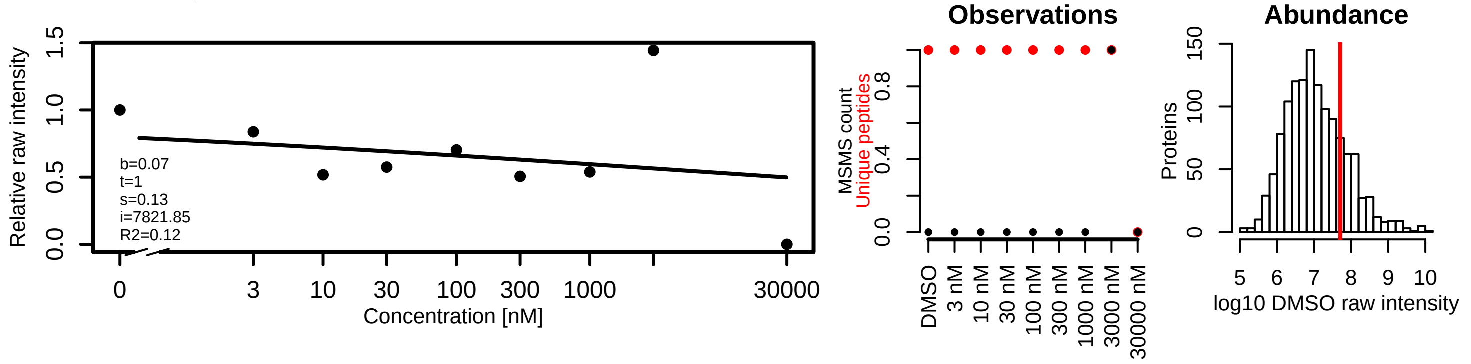

TELO2

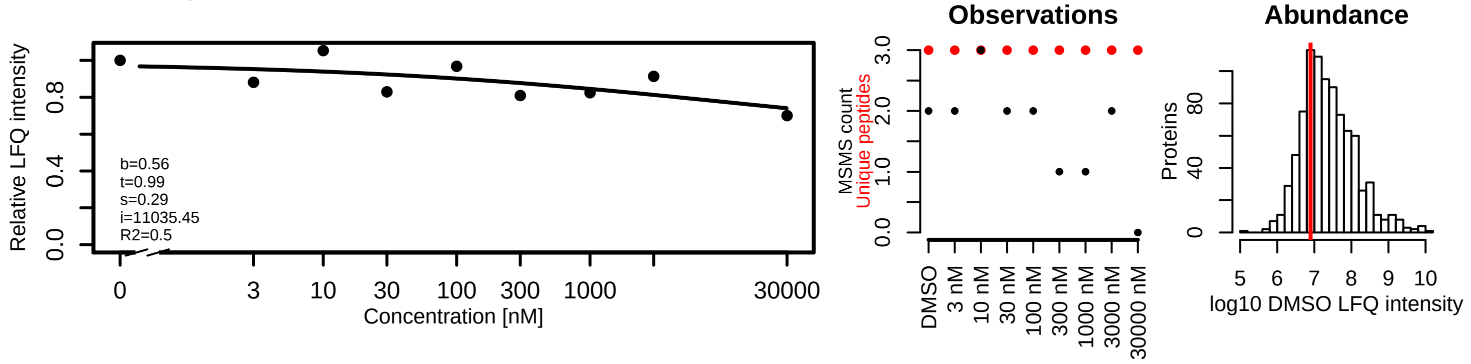

PTPN13

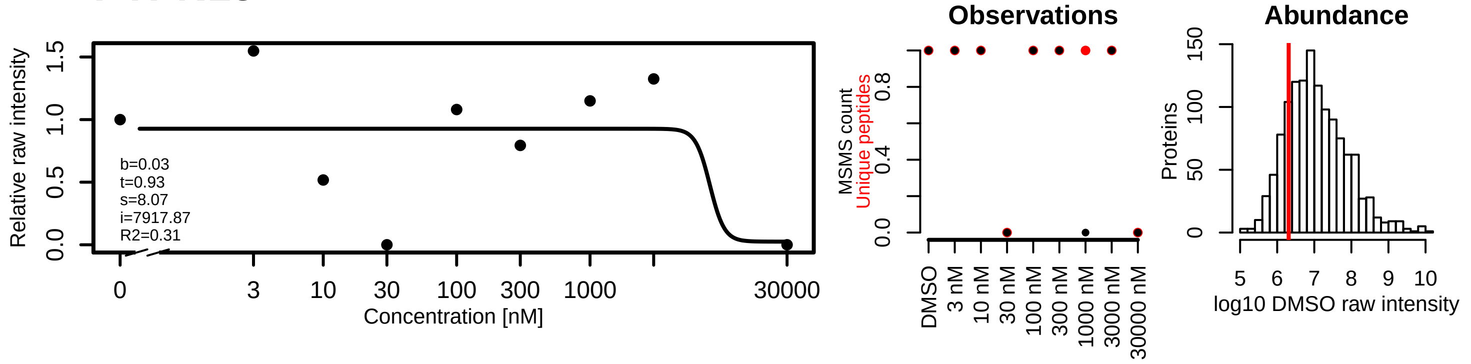

ATP6V0A1

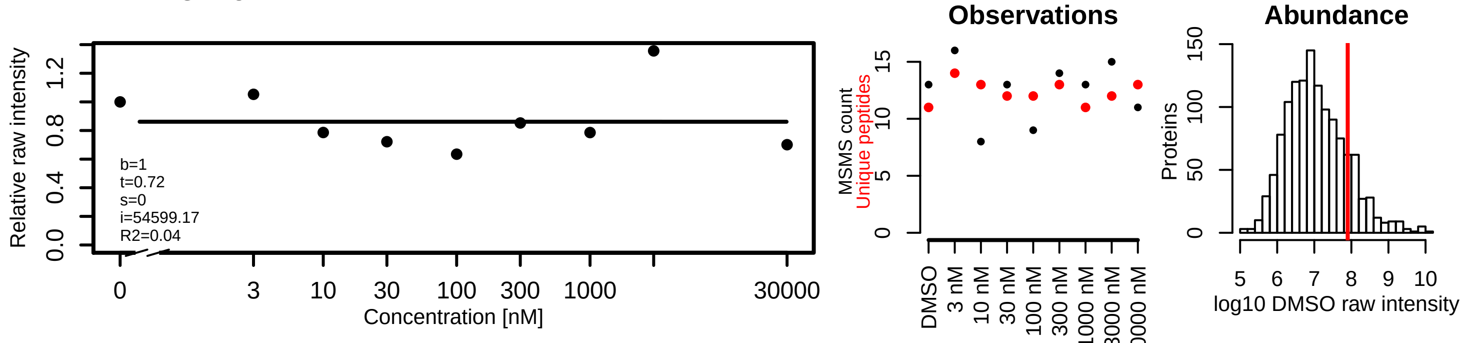

CAPZA1

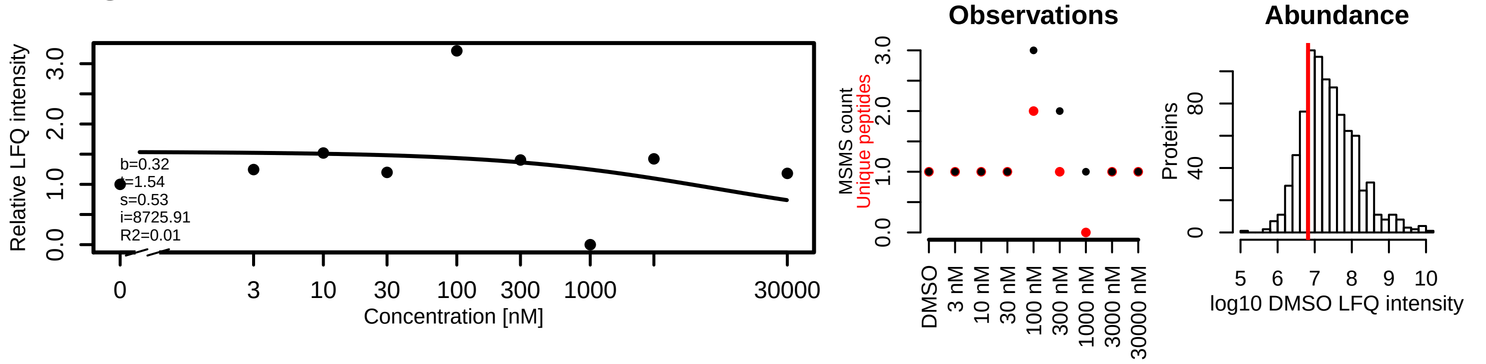

VDAC3

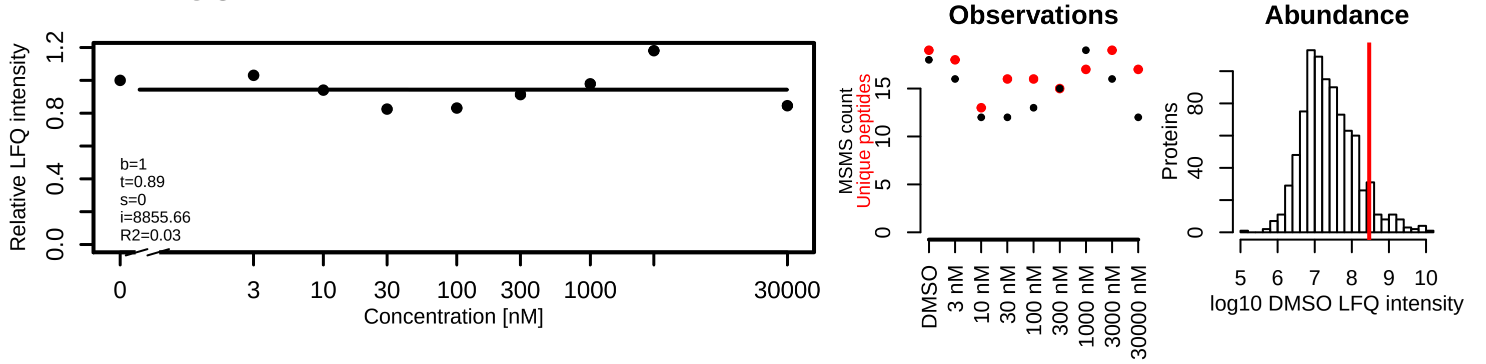

TM9SF4

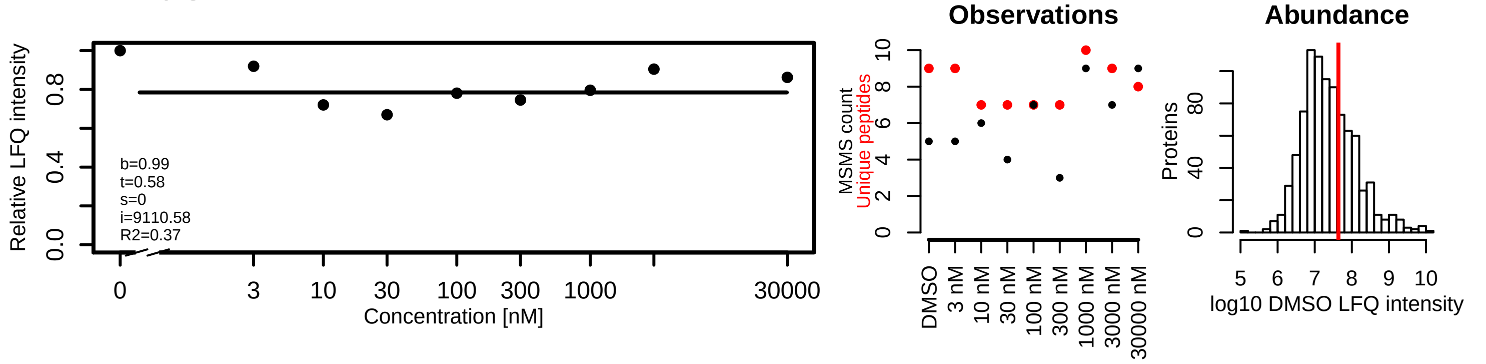

SLC25A11

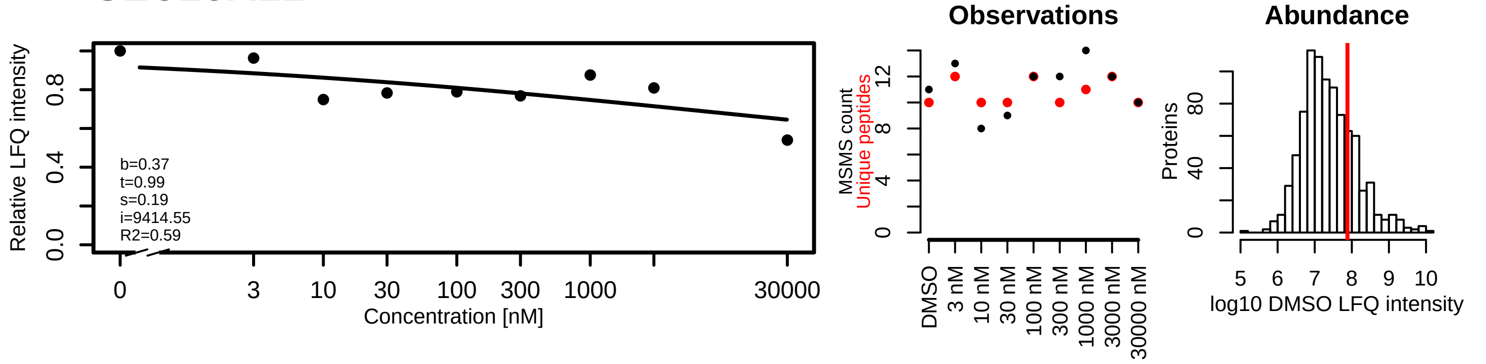

AFG3L2

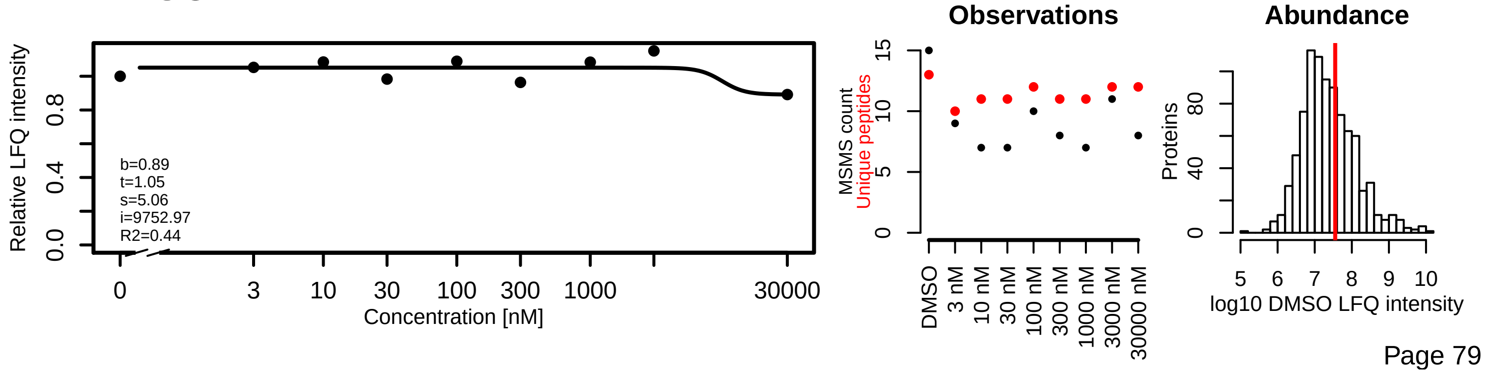

RPS10

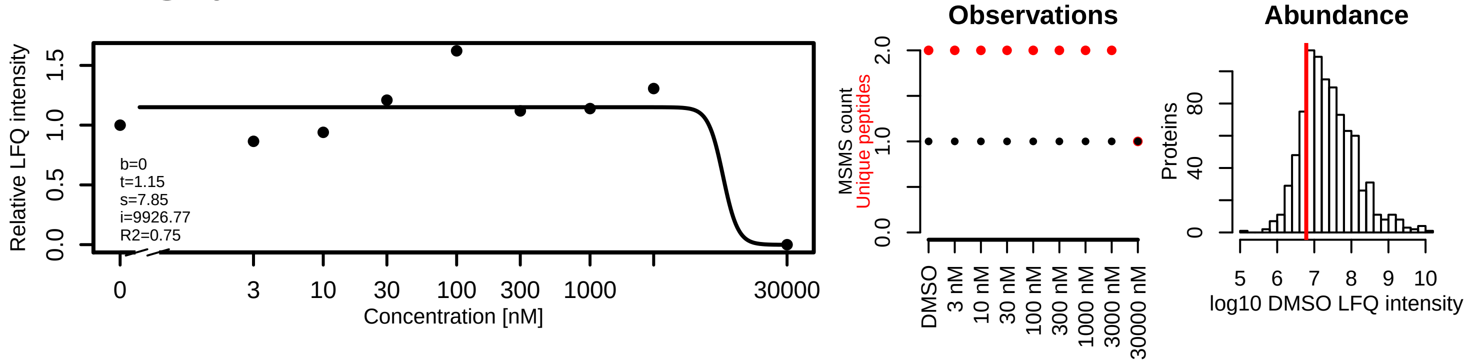

CISD1

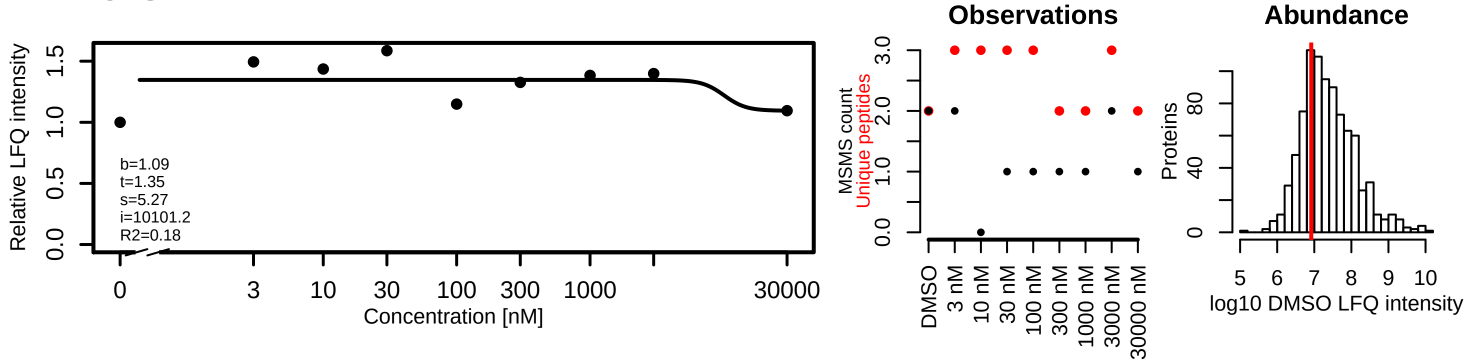

COX20

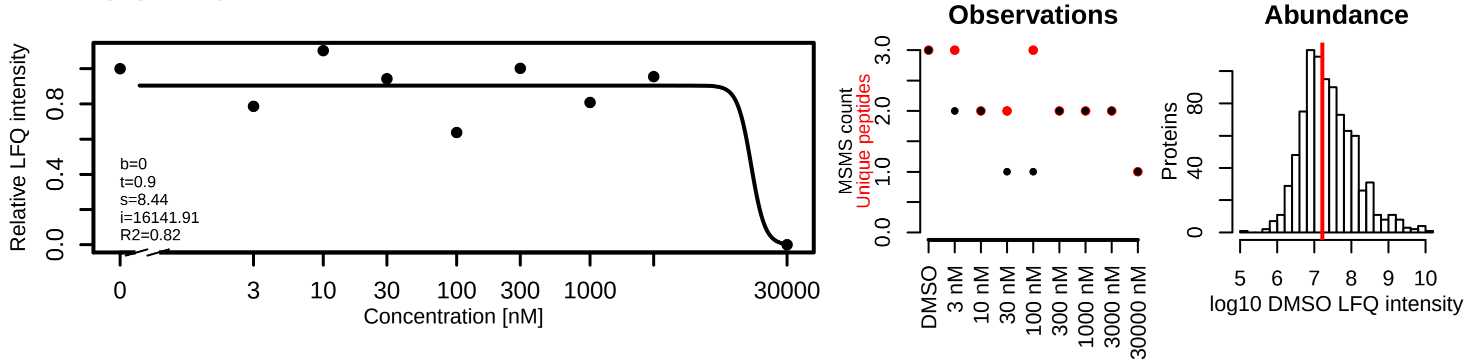

STIM1

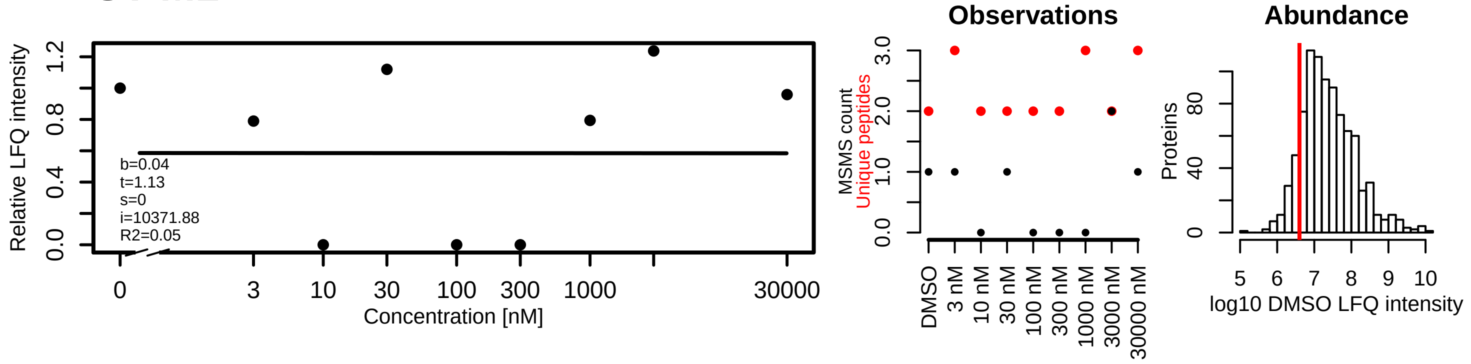

KIAA1468

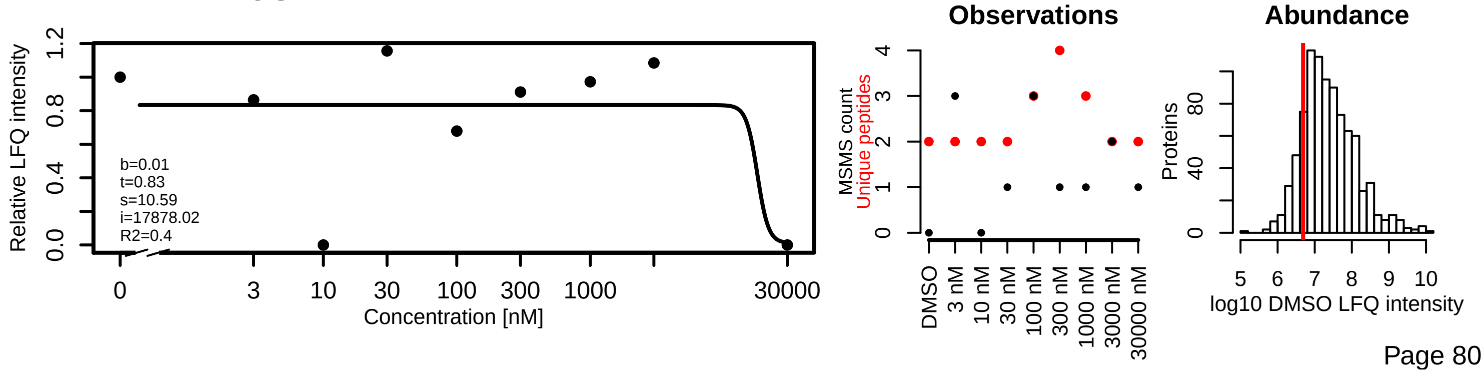

MBTPS2

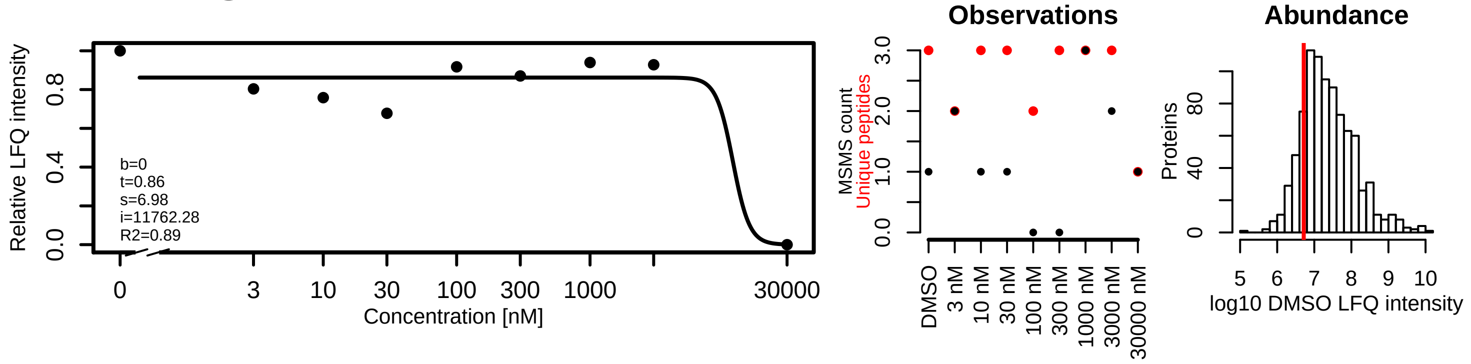

PEX16

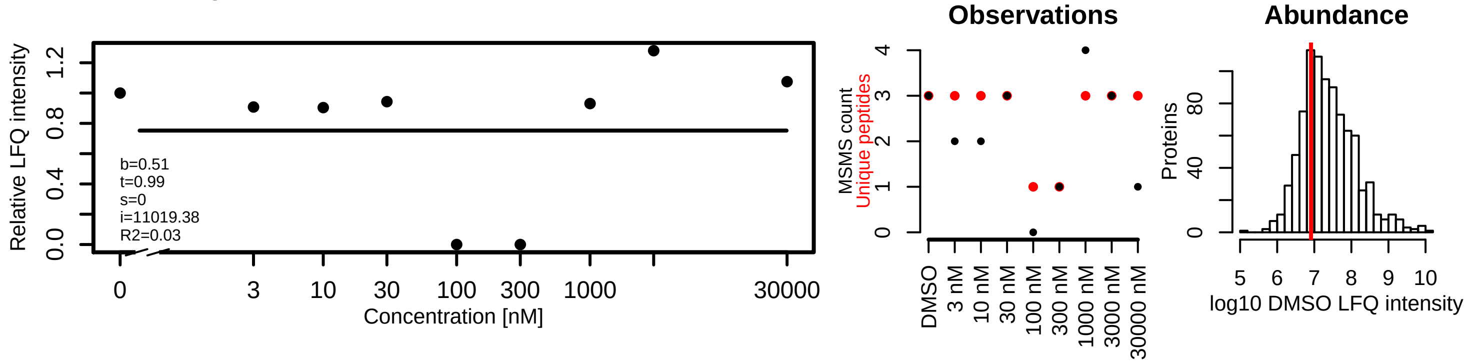

NUP98

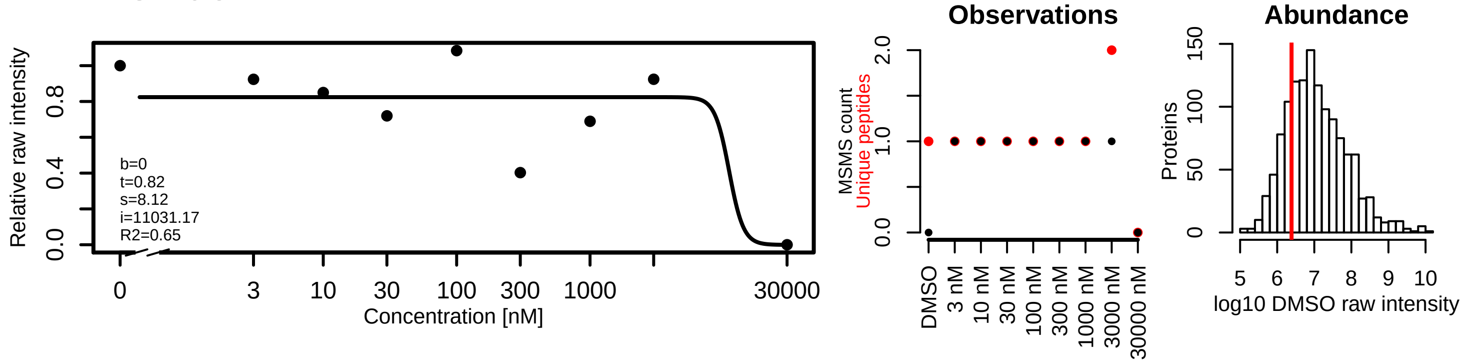

ECSIT

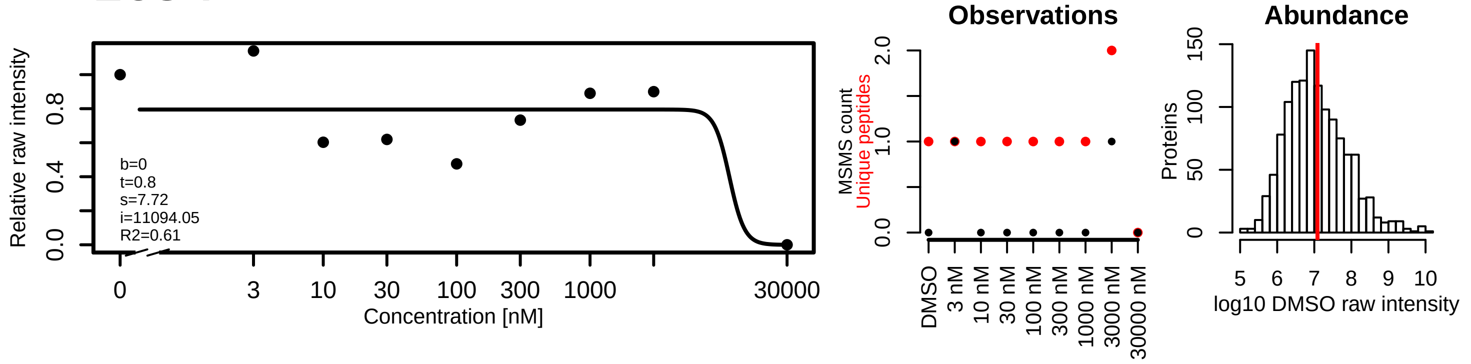

TUBB8

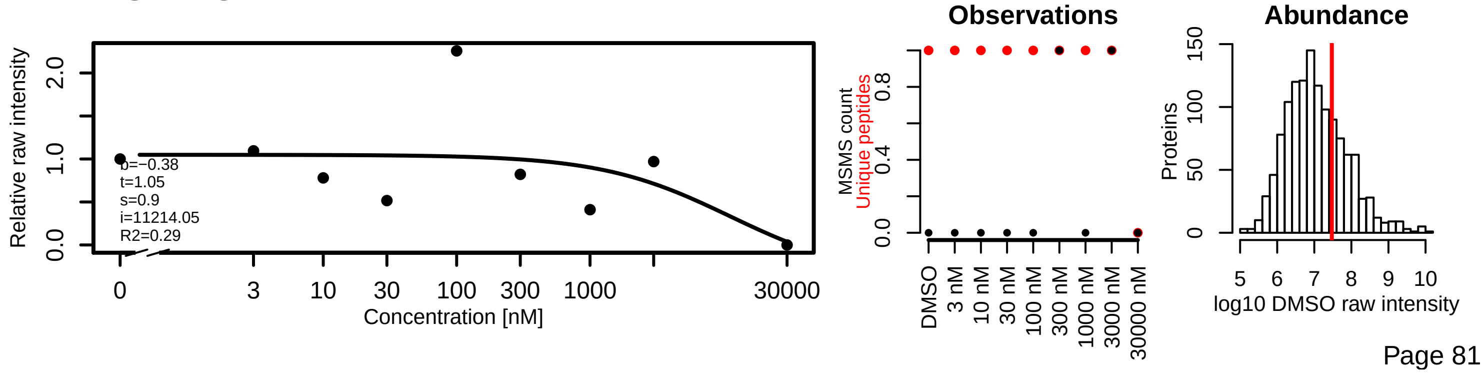

PPT1

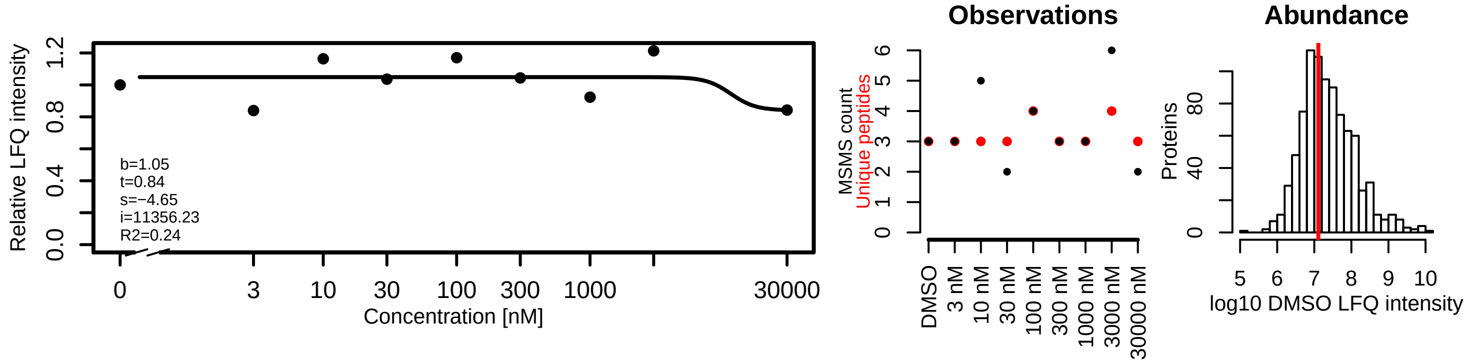

COPB2

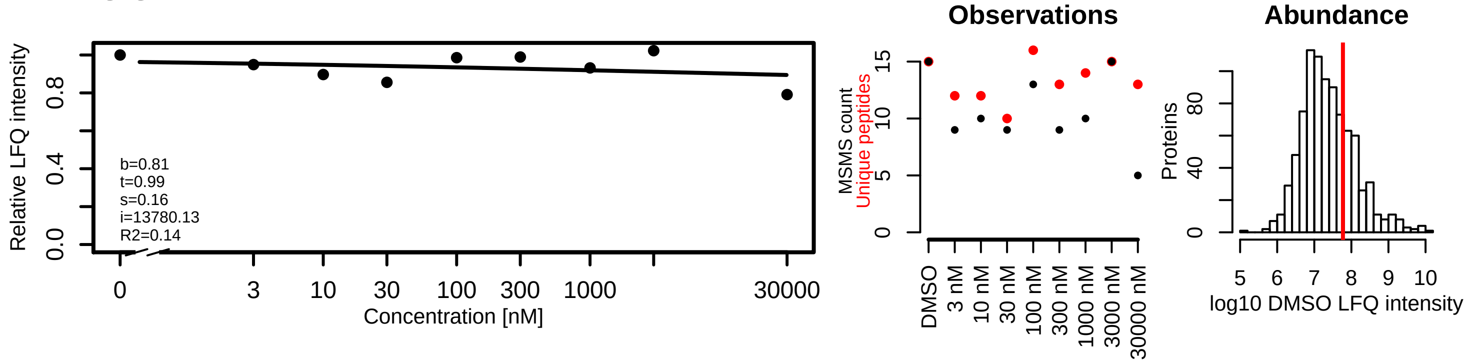

2−Sep

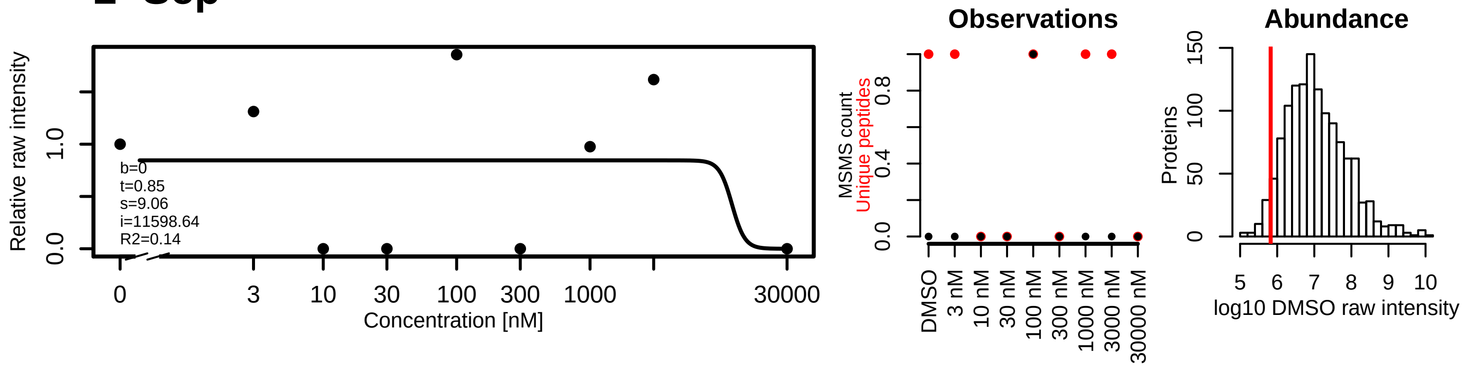

SCEL

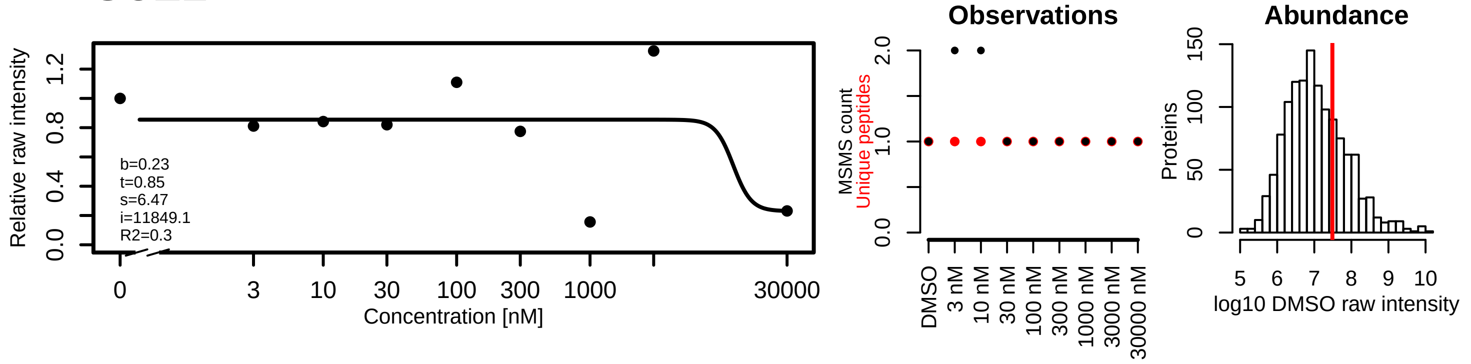

LAMC3

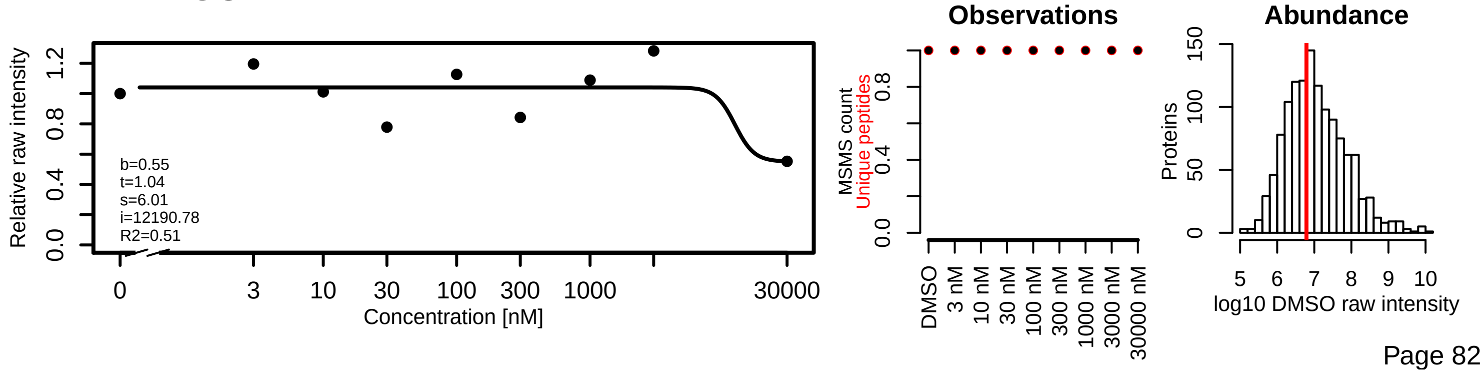

CGN

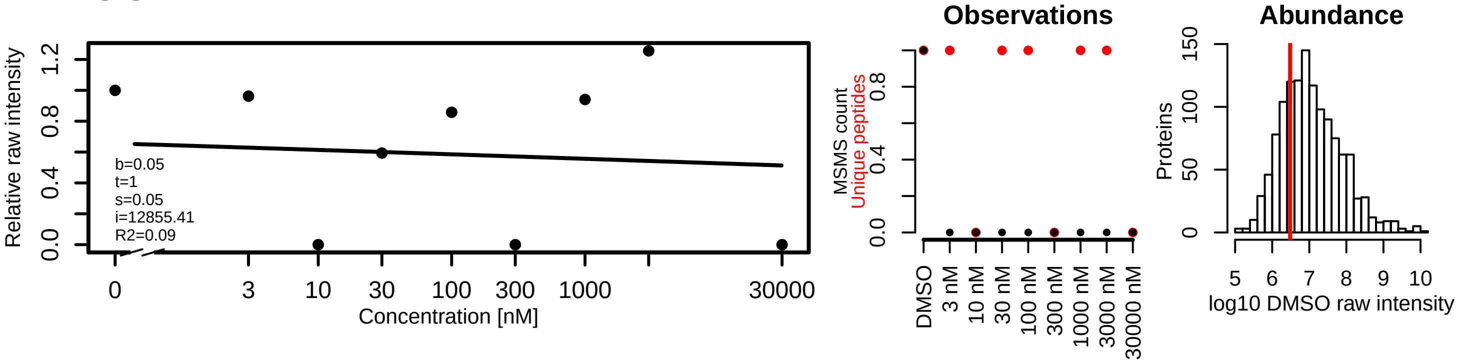

HLA−B − Q31612

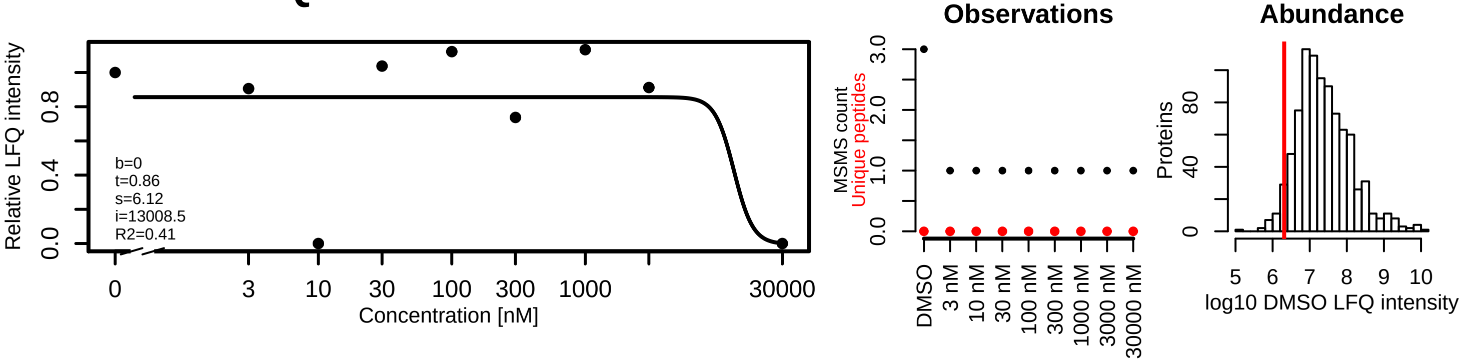

PIGH

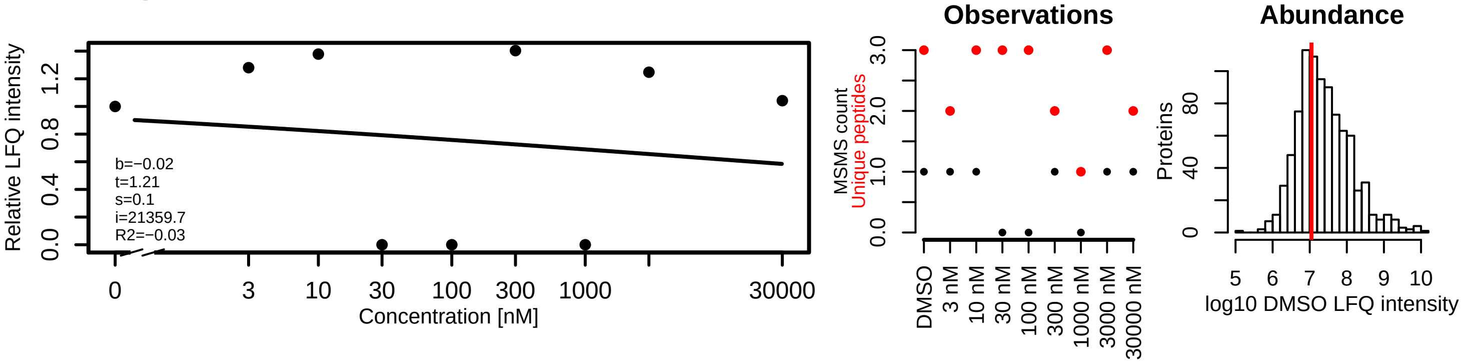

LMAN2

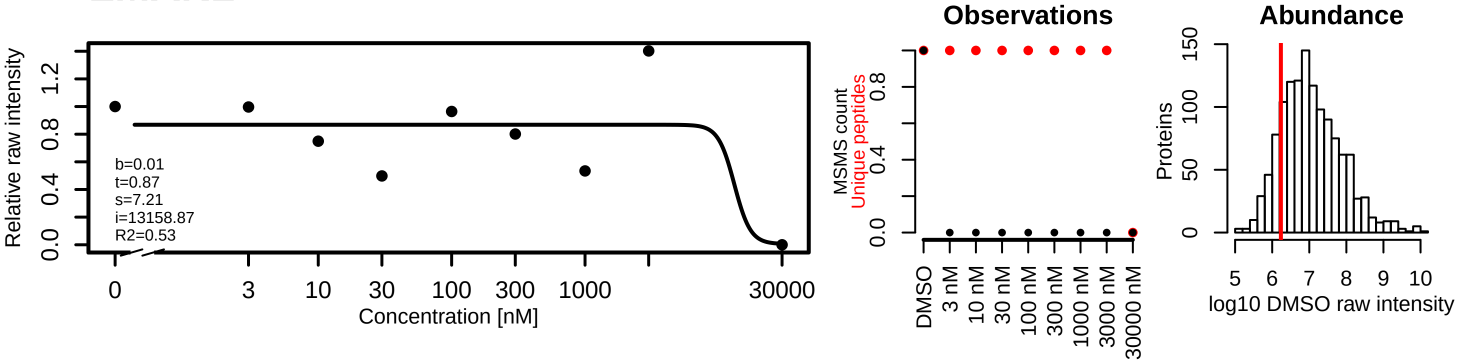

GPAA1

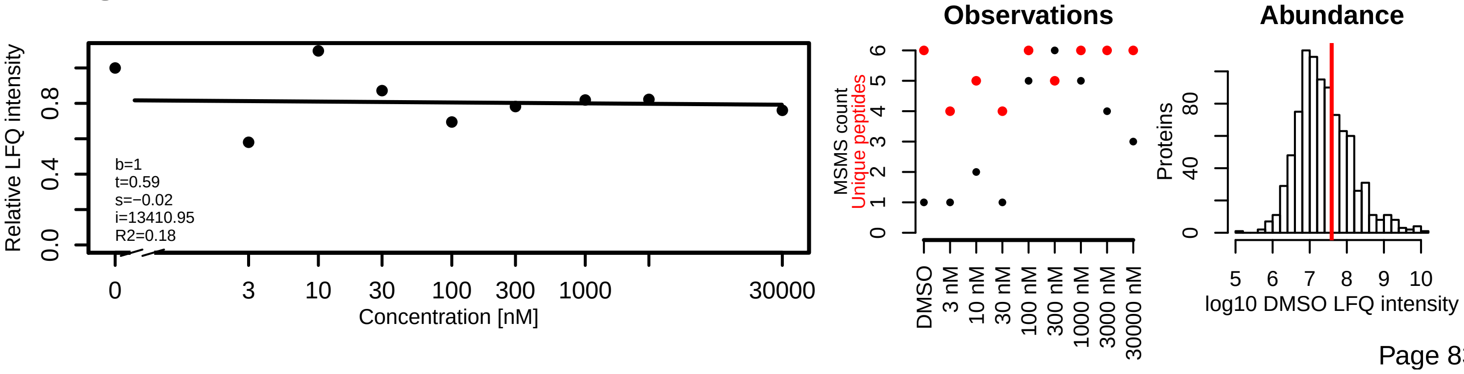

ZHX2

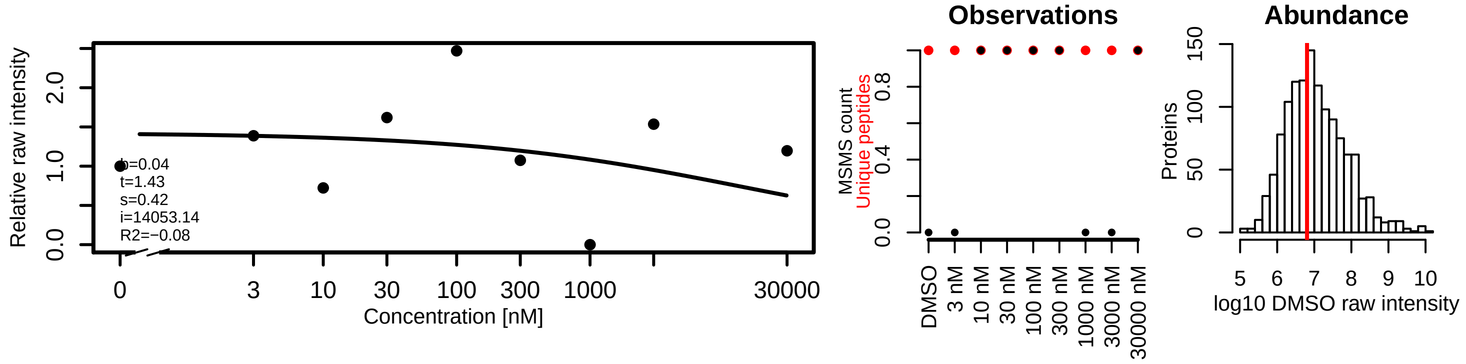

B3GAT3

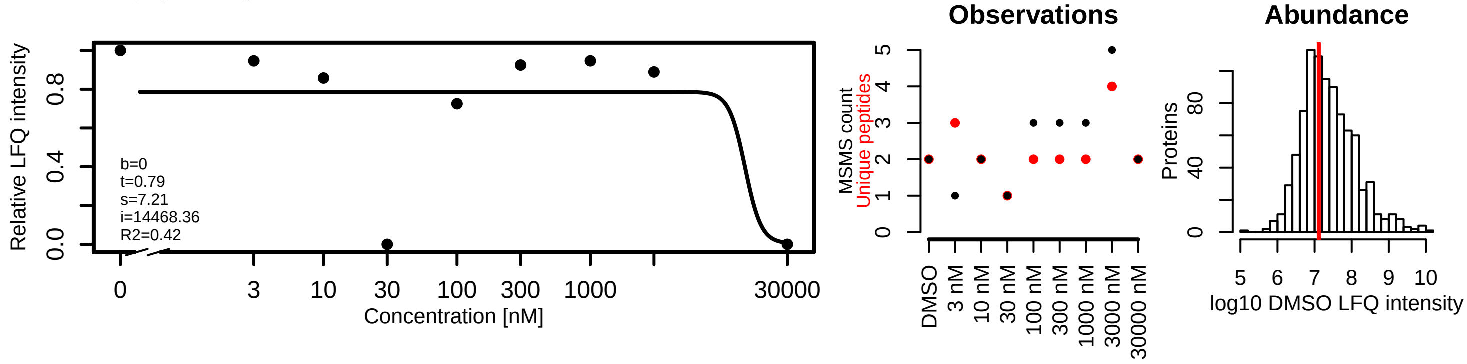

ANO10

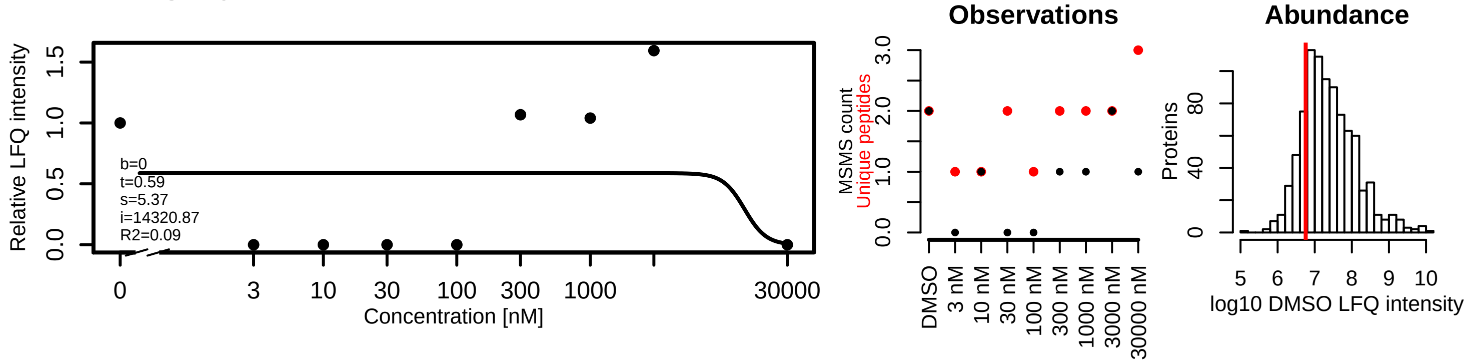

PLXNB2

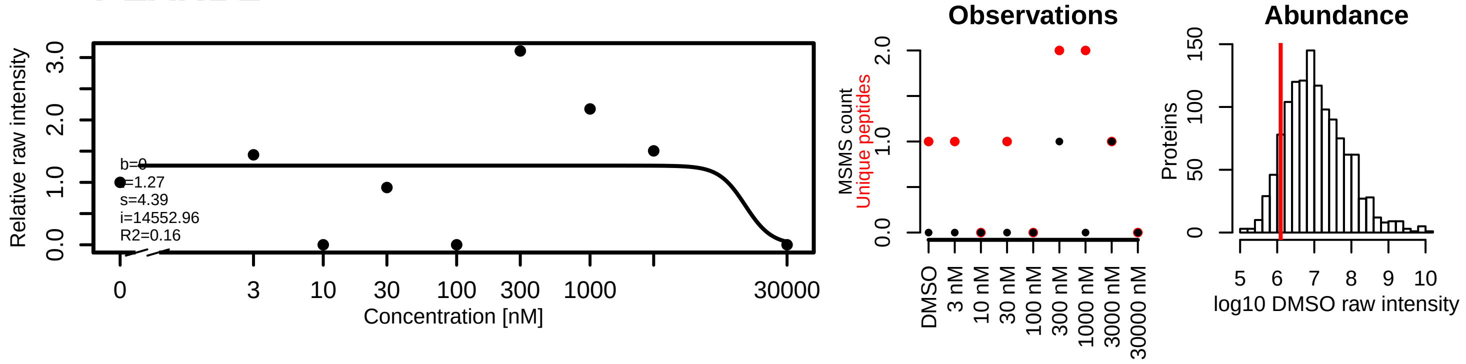

POM121C;POM121

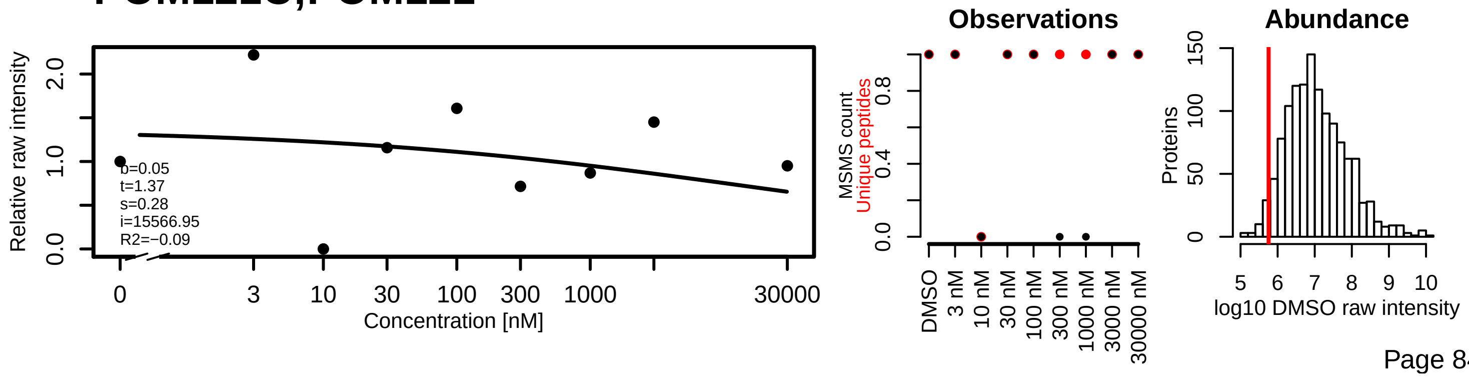

MYLK

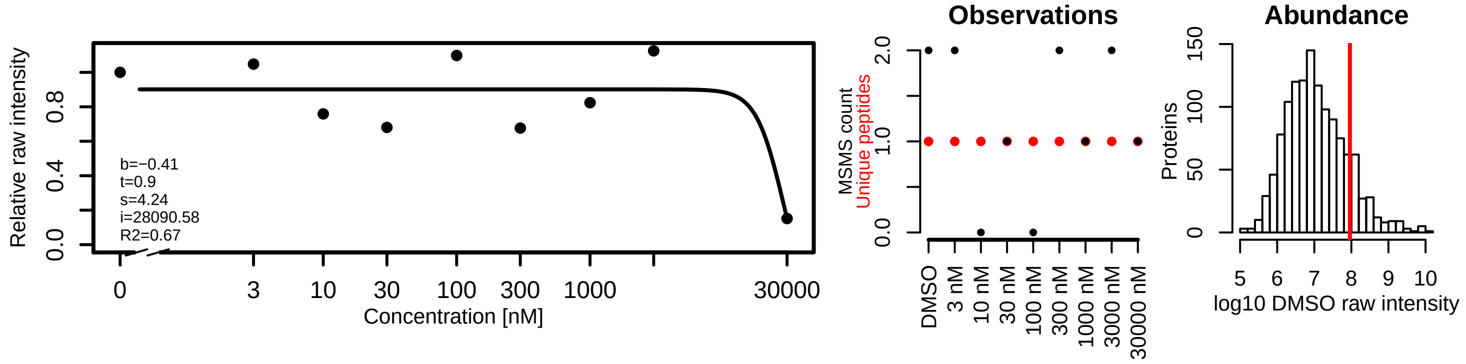

LPCAT4

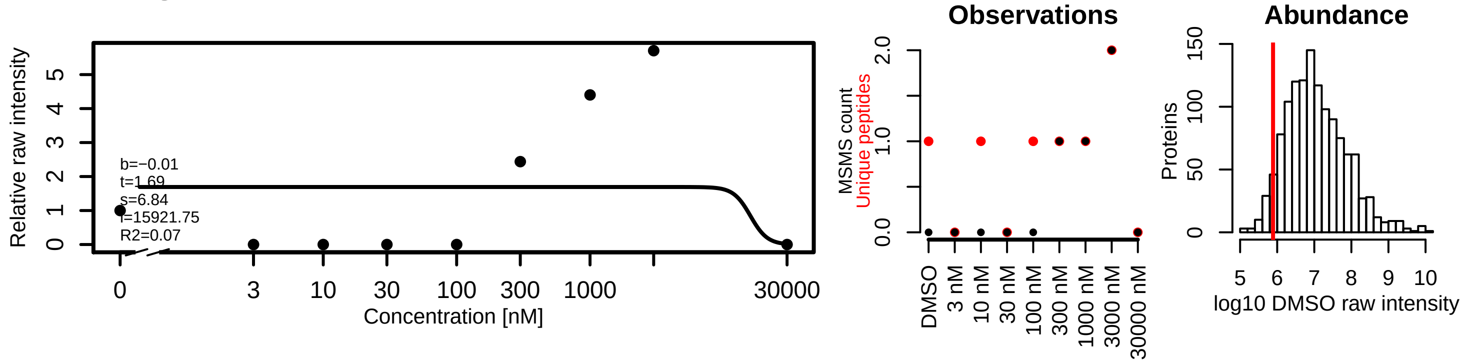

RHOT1;RHOT2

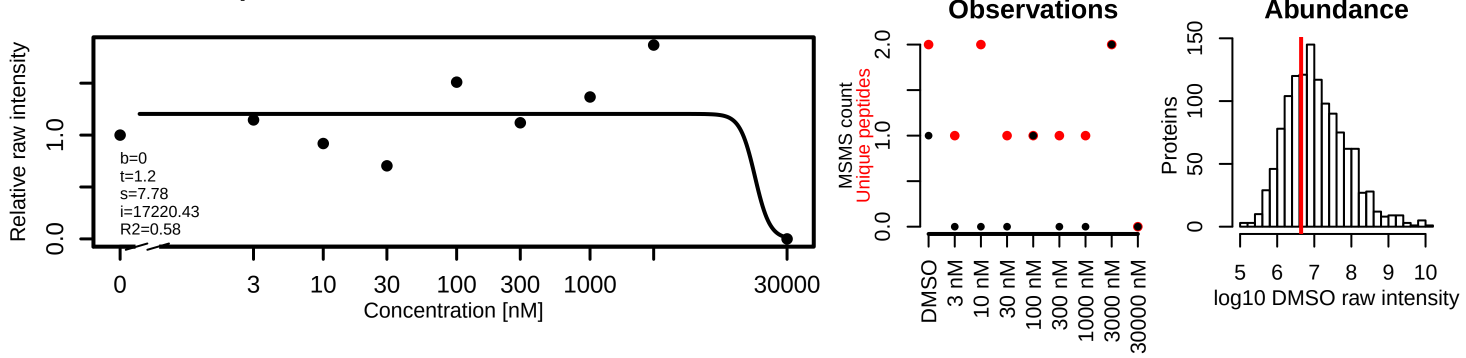

GNAI2;GNAS;GNAT1;GNAI1;GNAT2;GNAO1;GNAI3;GNAT3;GNAL

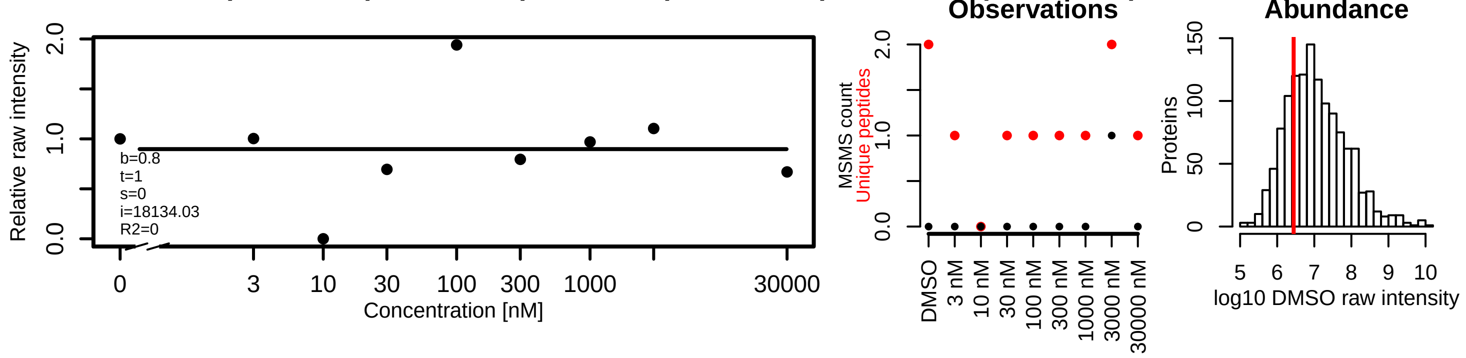

SEC61G

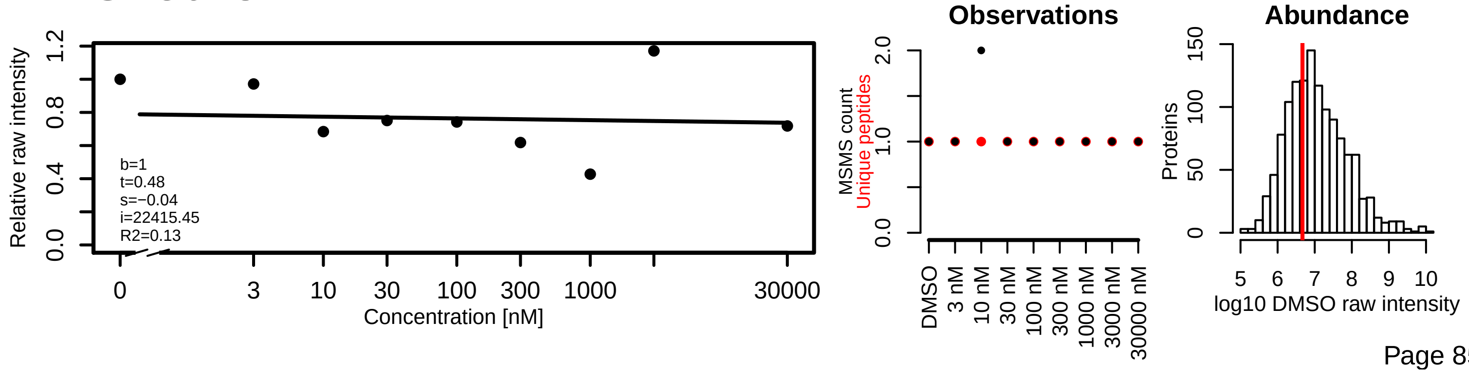

UQCRH

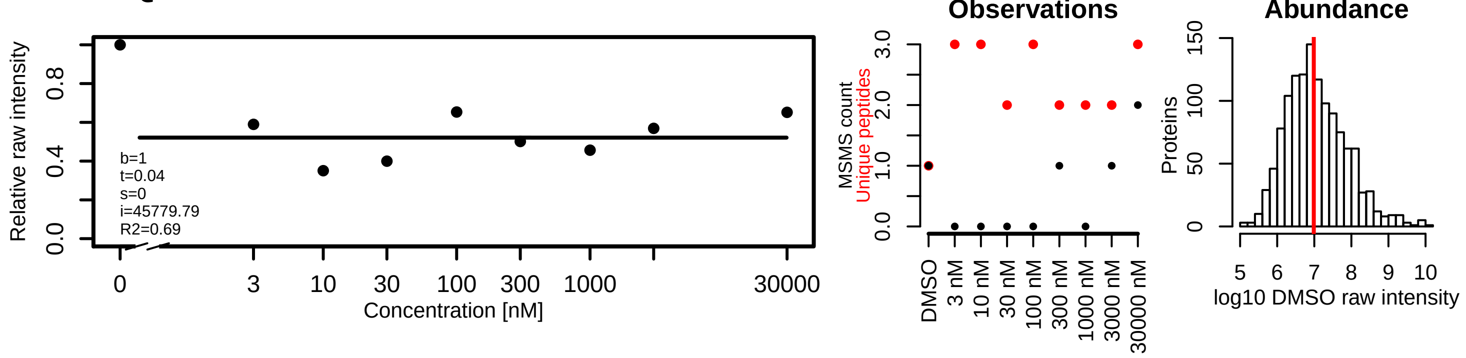

STARD3

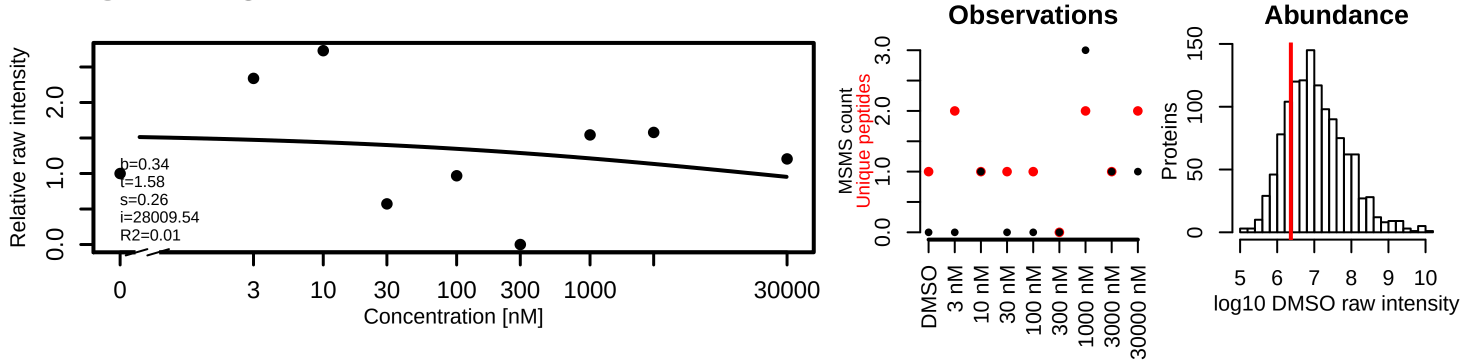

HADHB

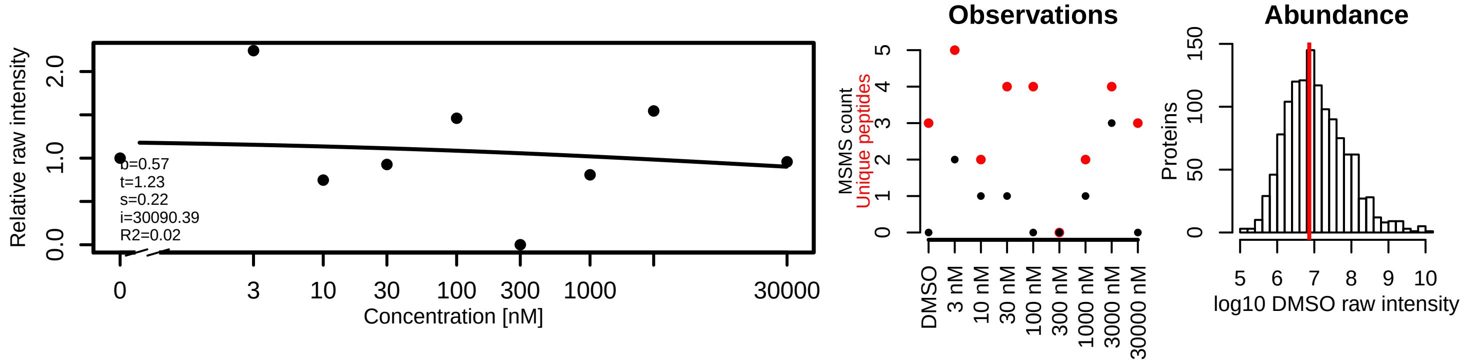

PLSCR1

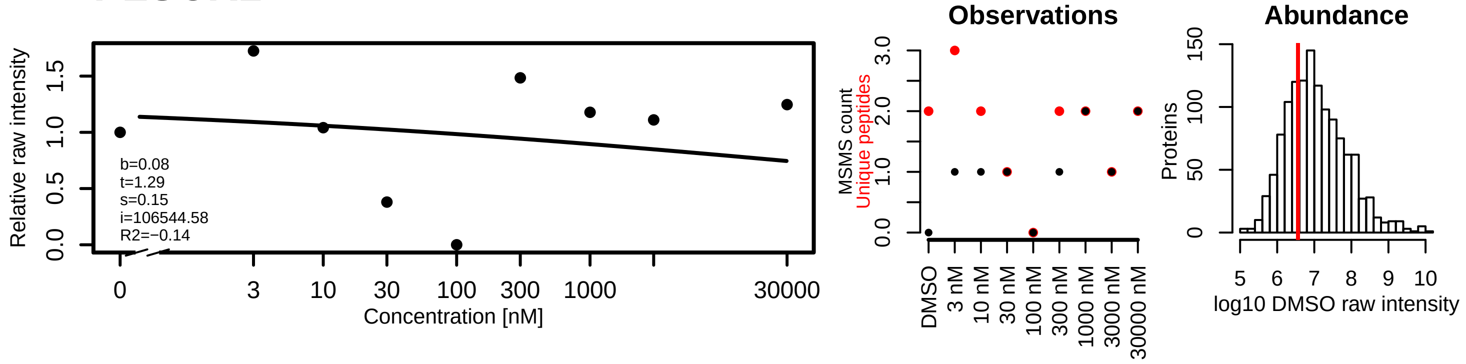

CAMLG

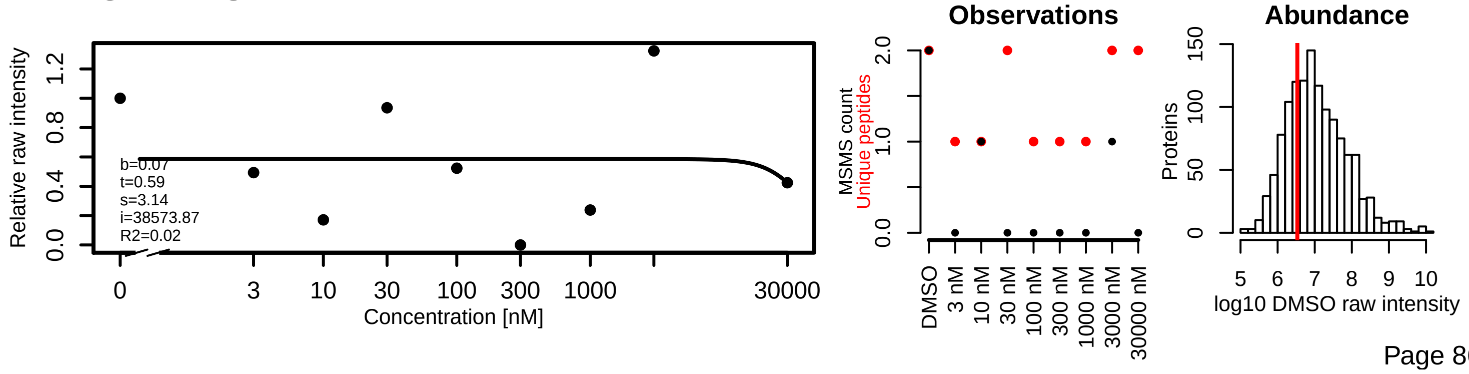

SLC25A17

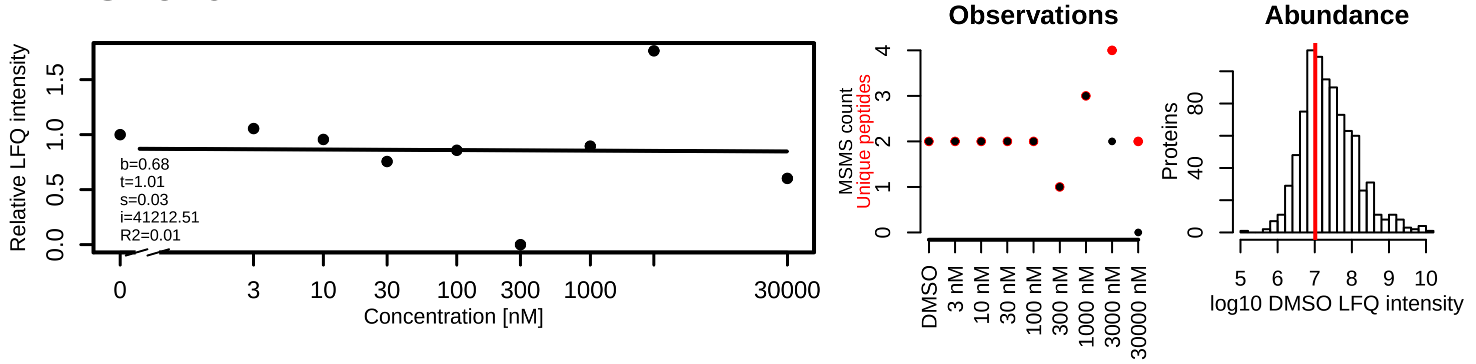

TUBGCP5

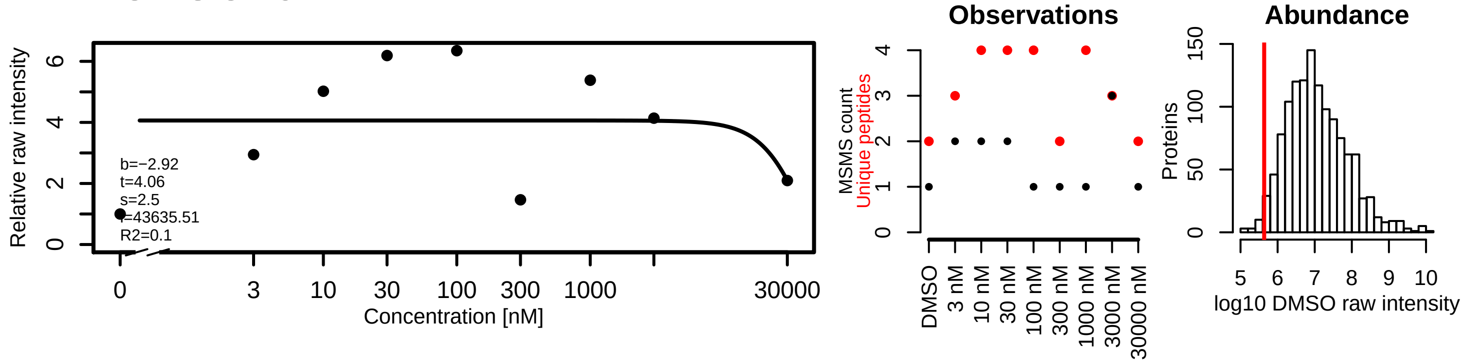

TARDBP

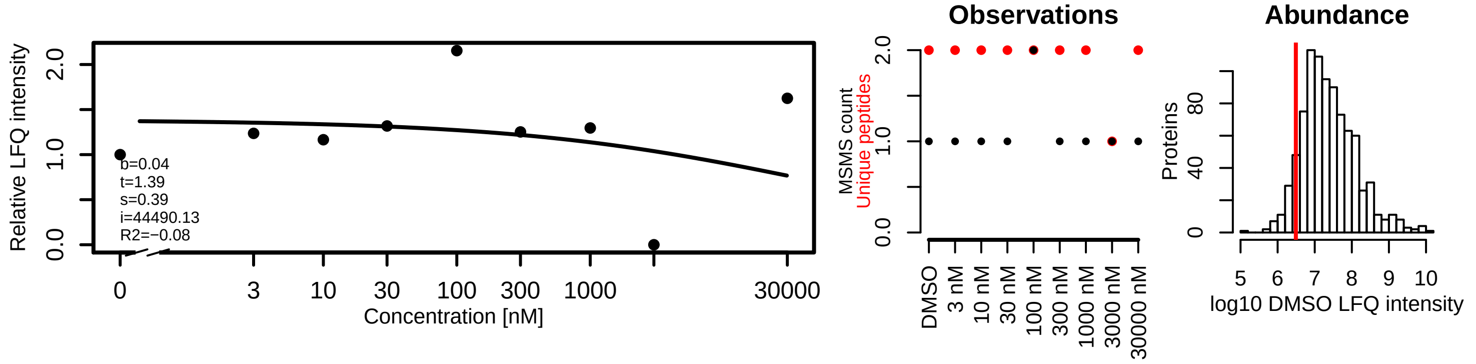

TM2D3

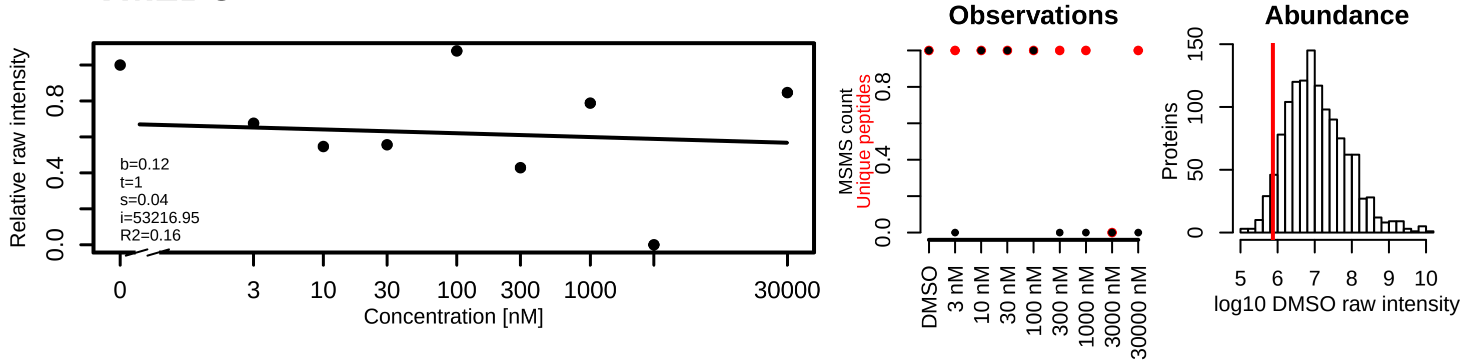

FIS1

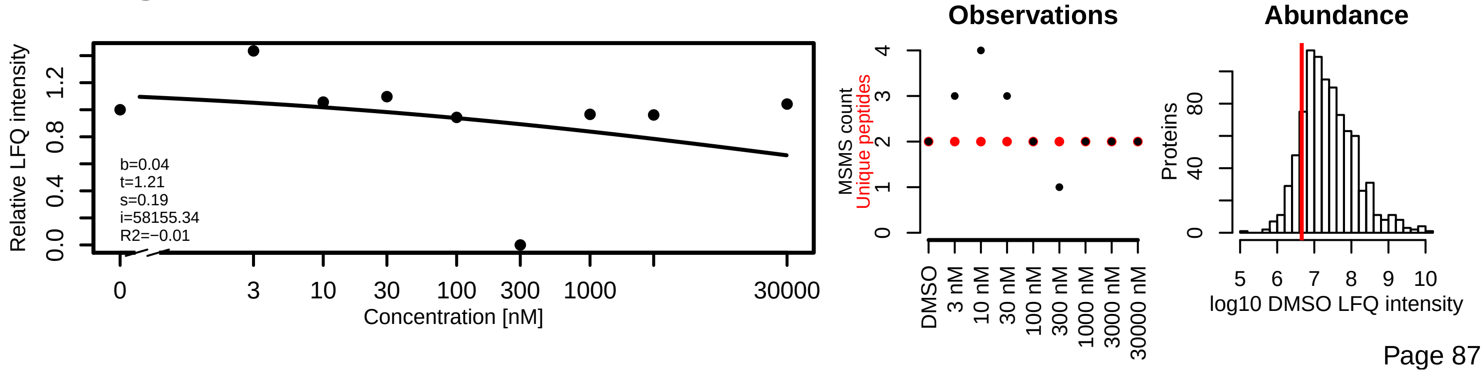

YIPF6

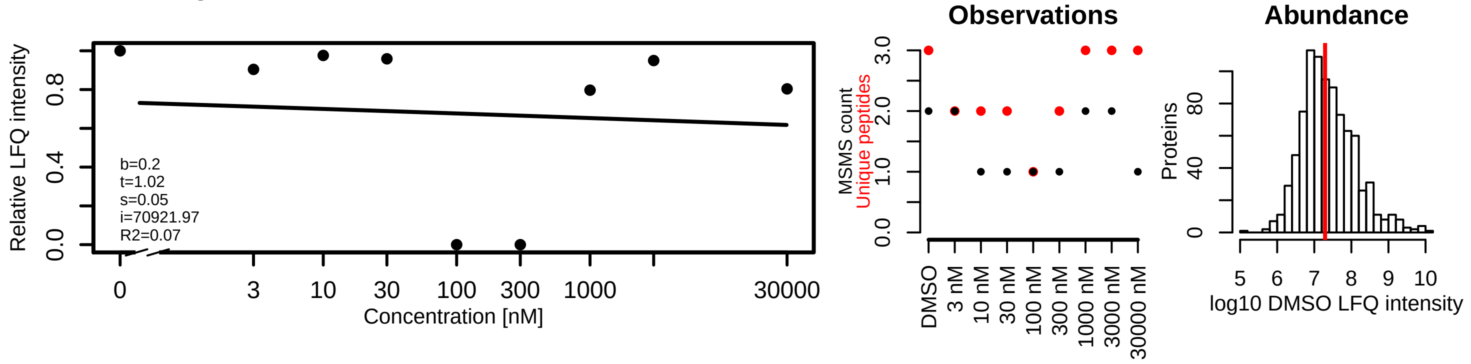

EMC7

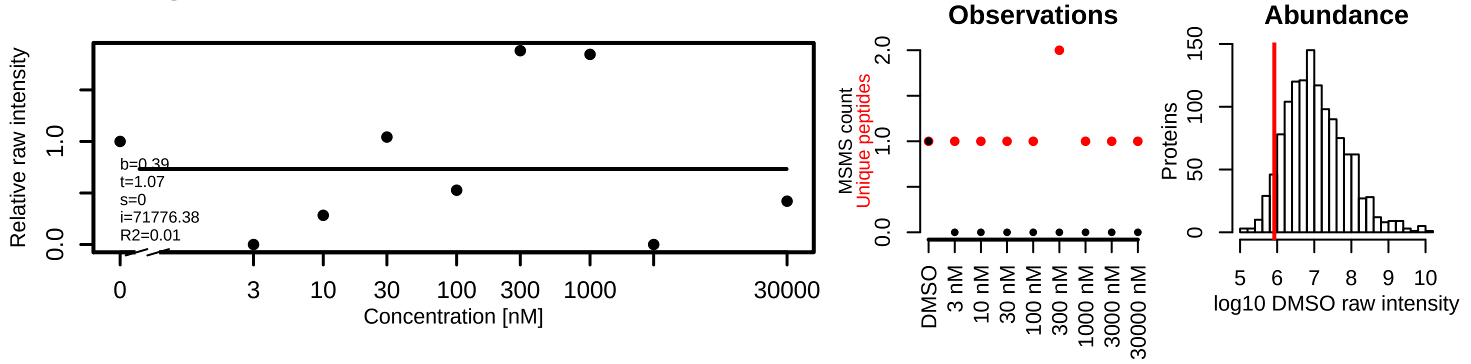

BDH1

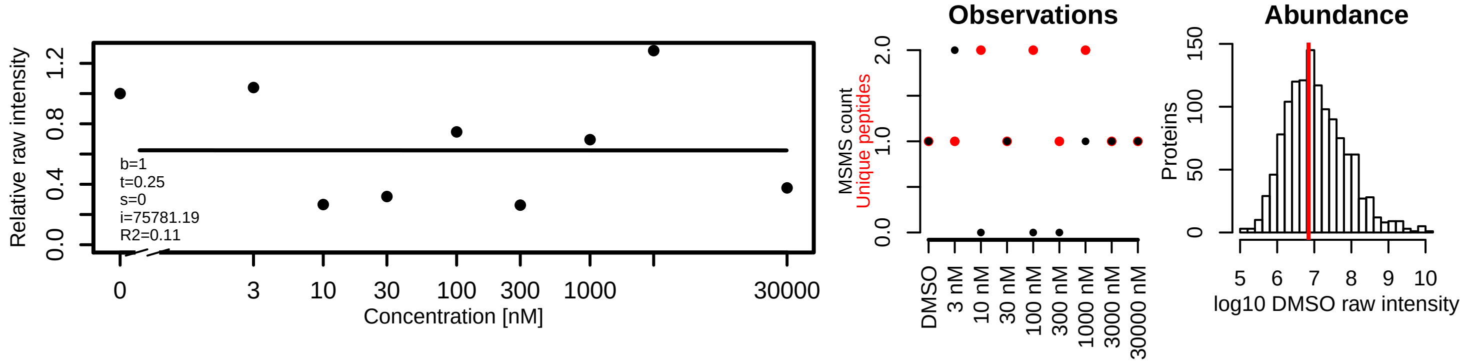

RPS27;RPS27L

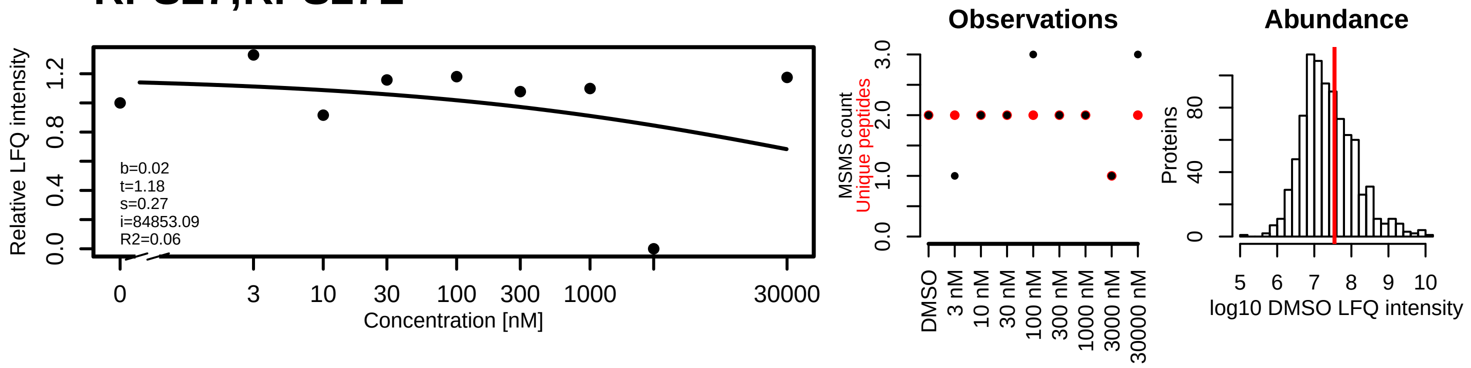

MAN1B1

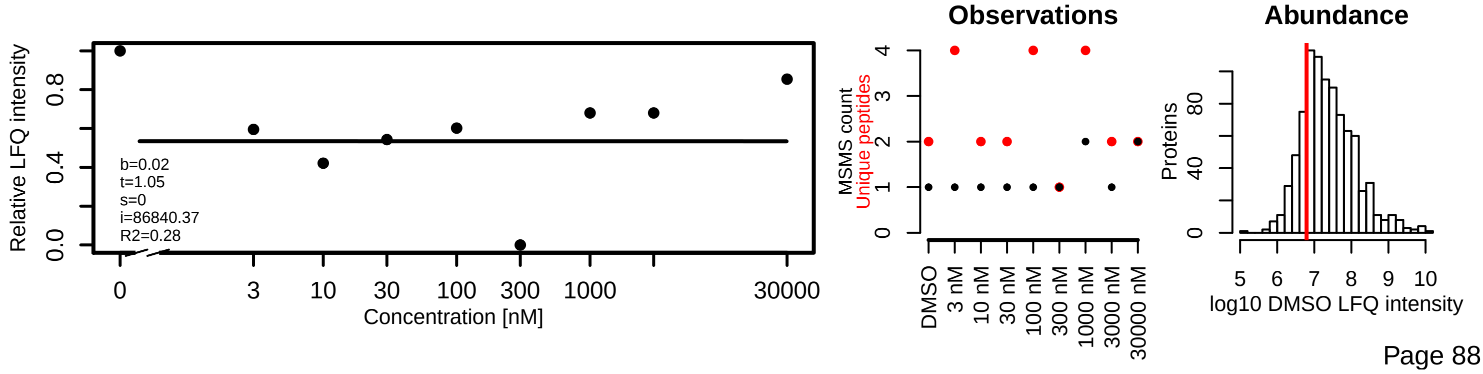

MLEC

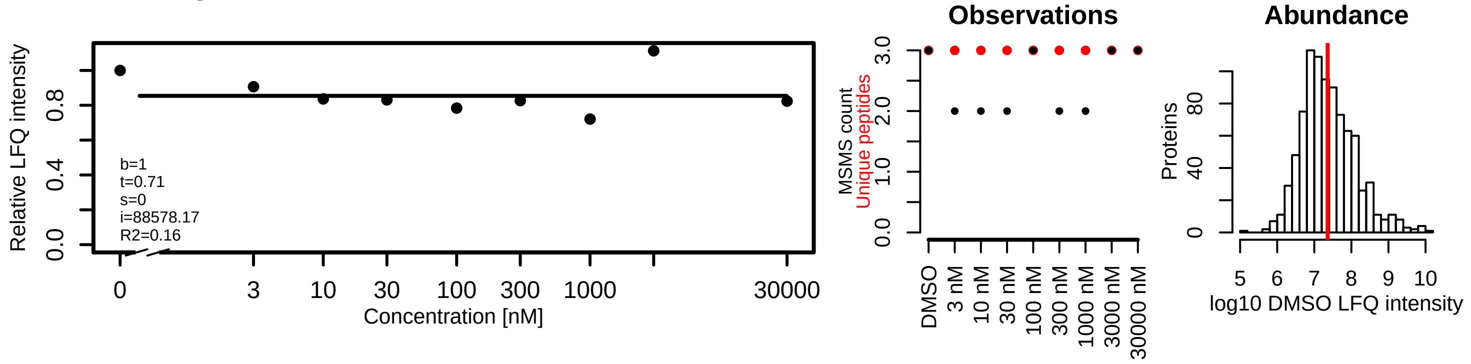

NOL9

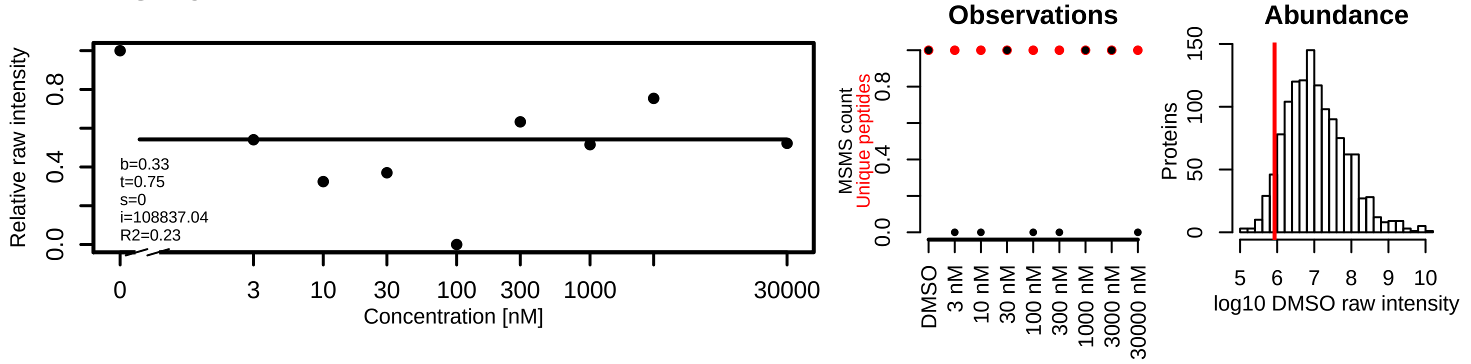

NCAPG2

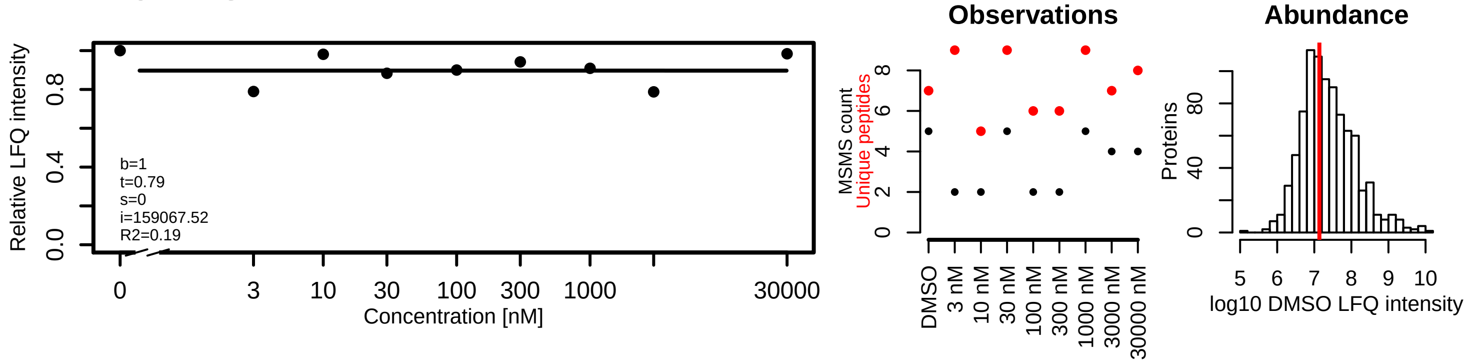

SCAMP1

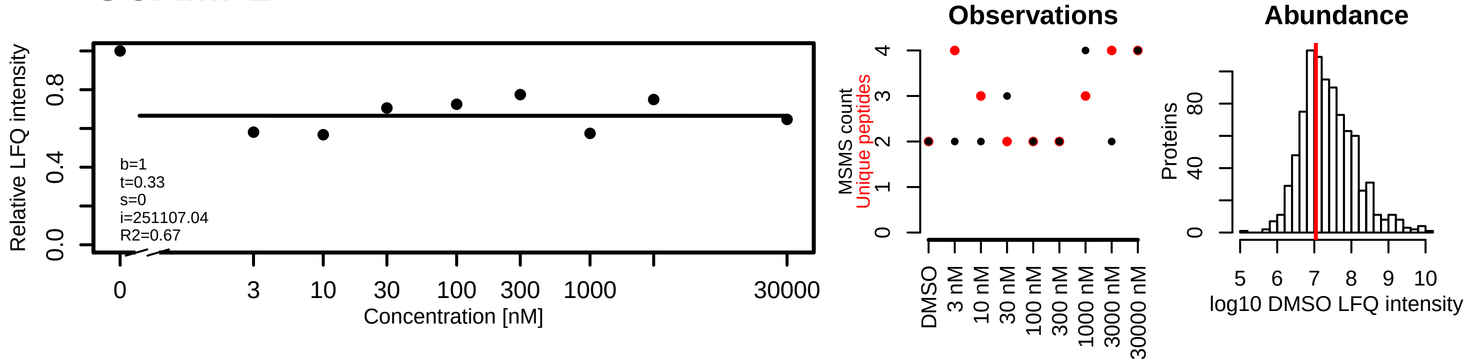

PSMB1

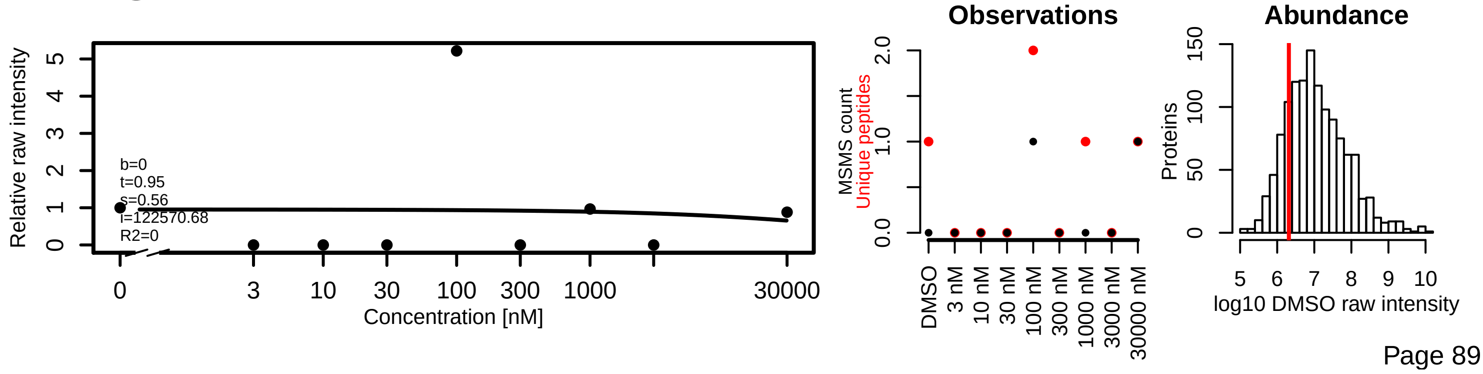

TMTC3

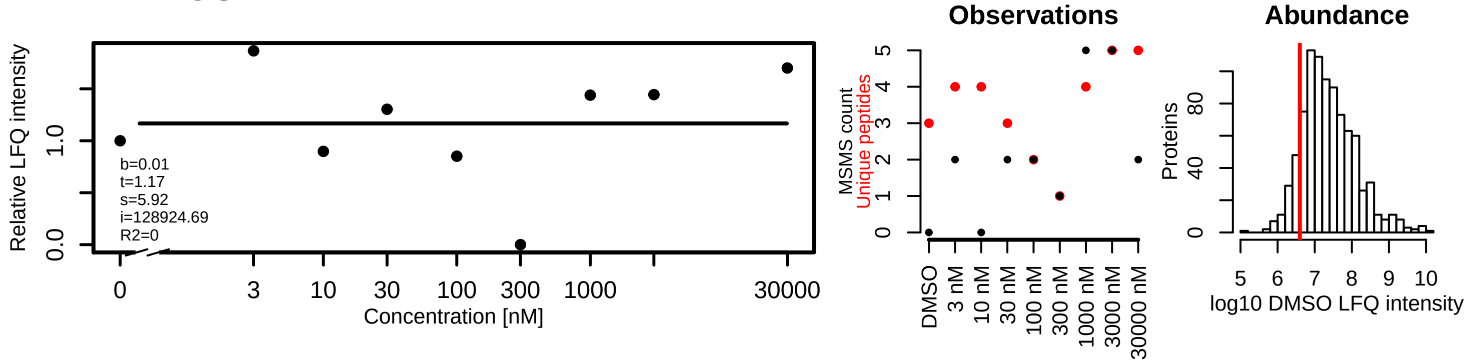

MTFP1

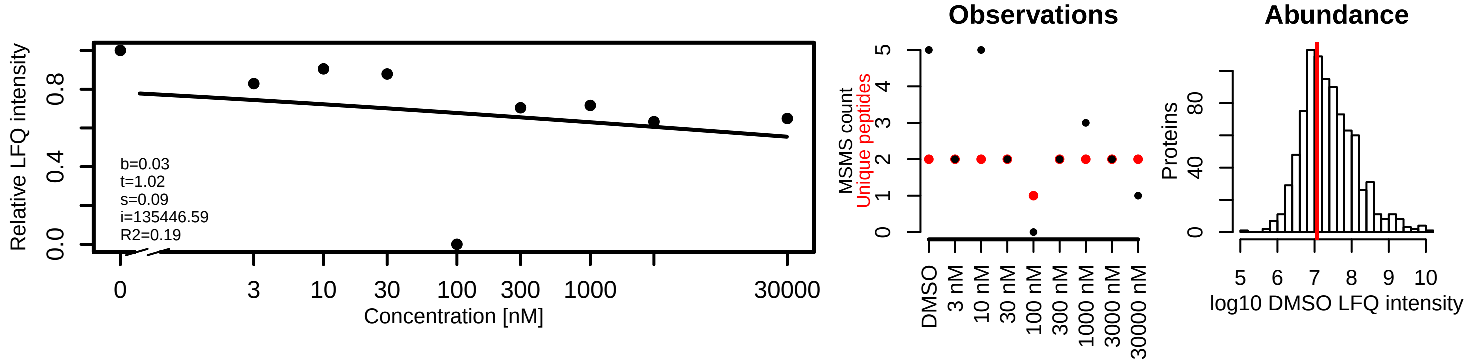

NUP85

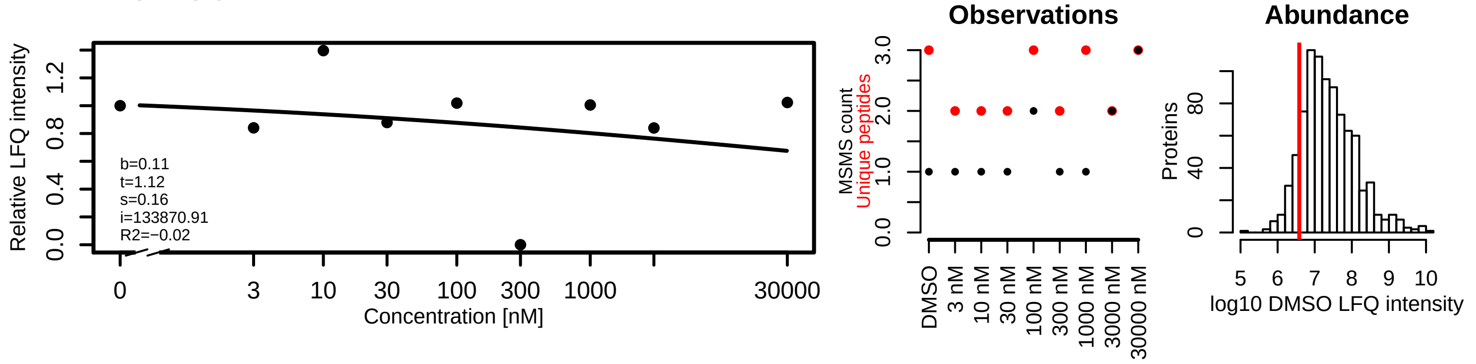

RAN

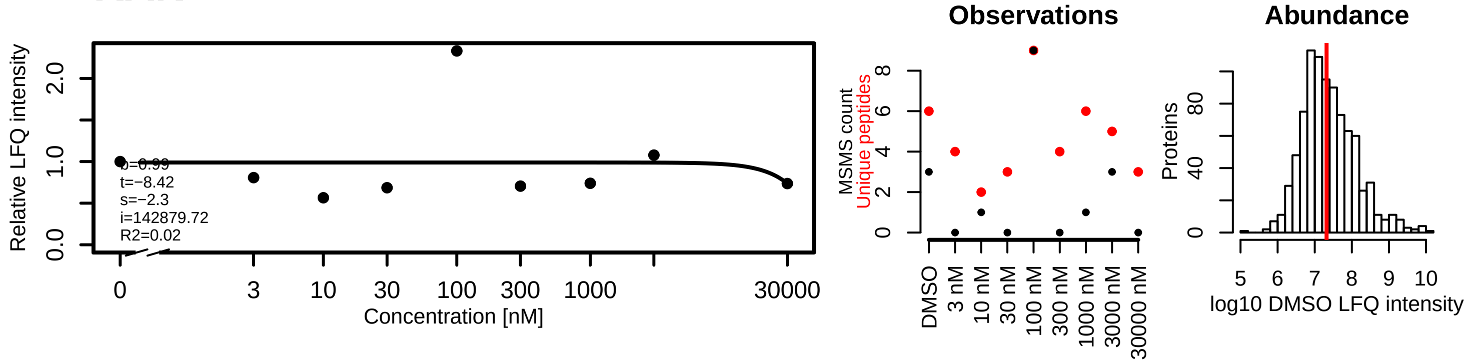

SSR1

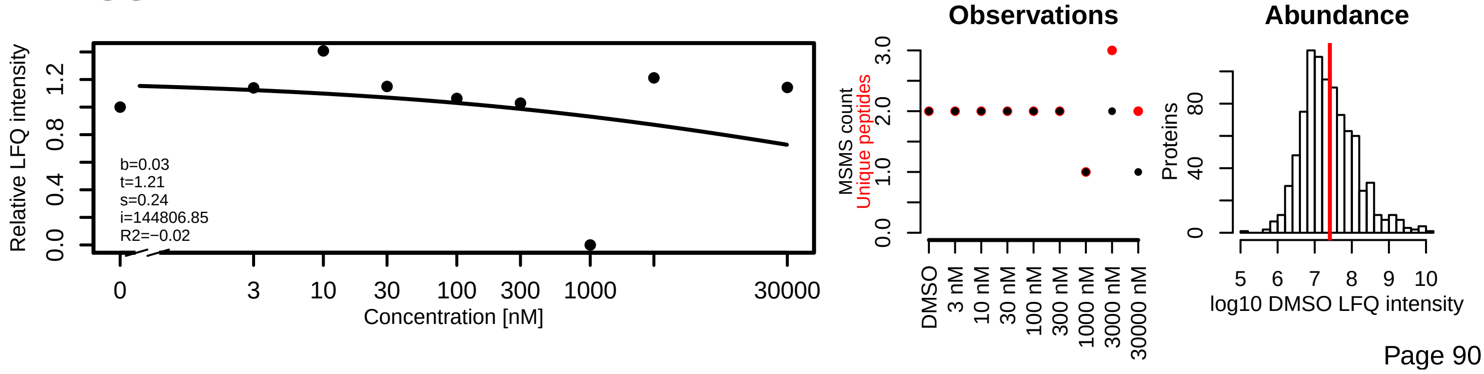

RAB13

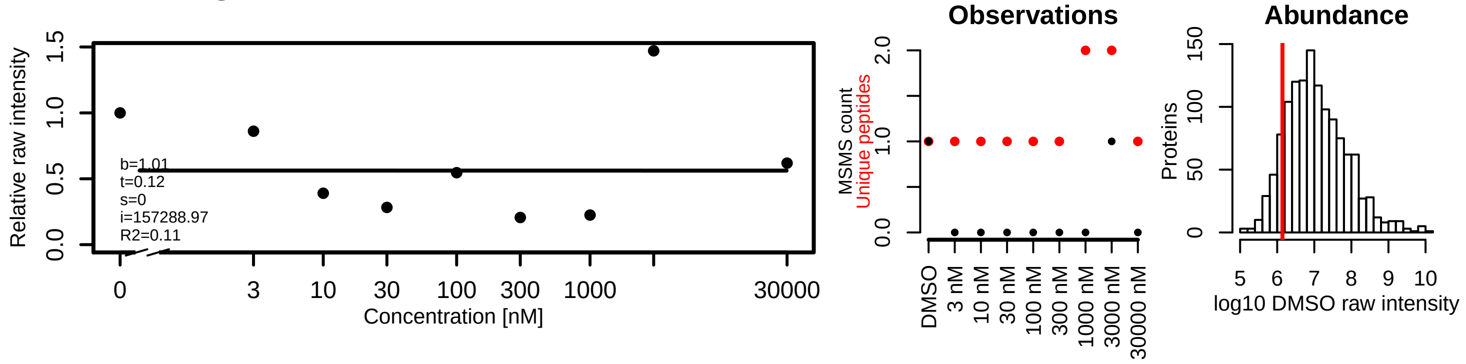

SLC35B4

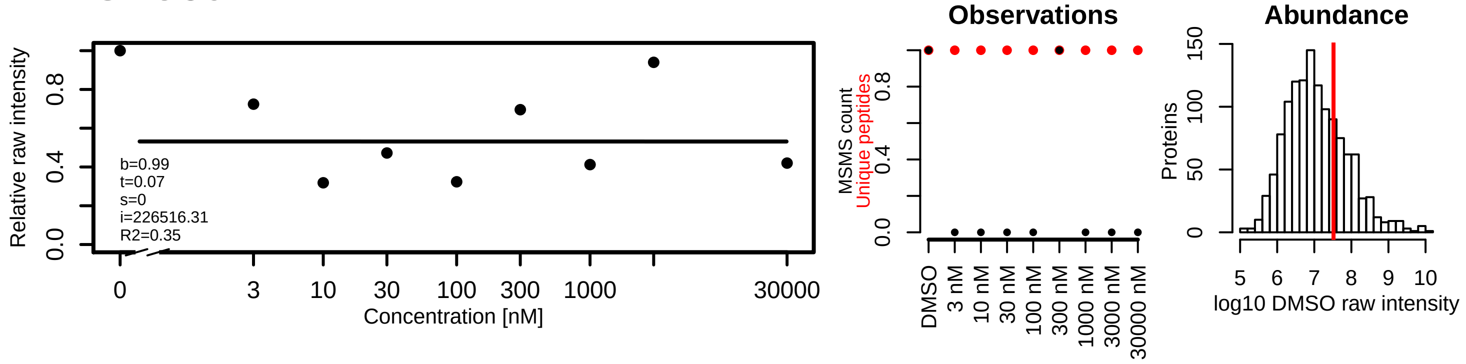

CYBA

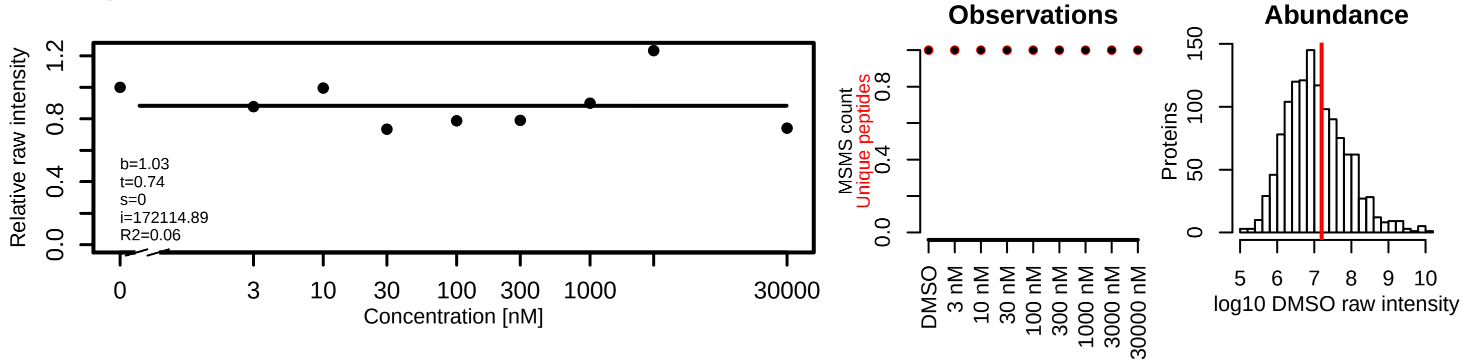

AMFR

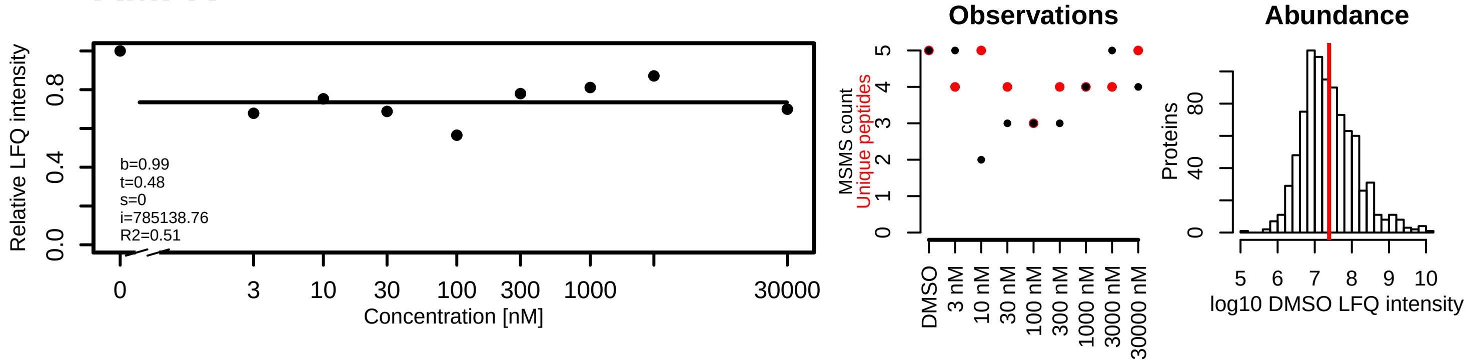

SMN2;SMN1

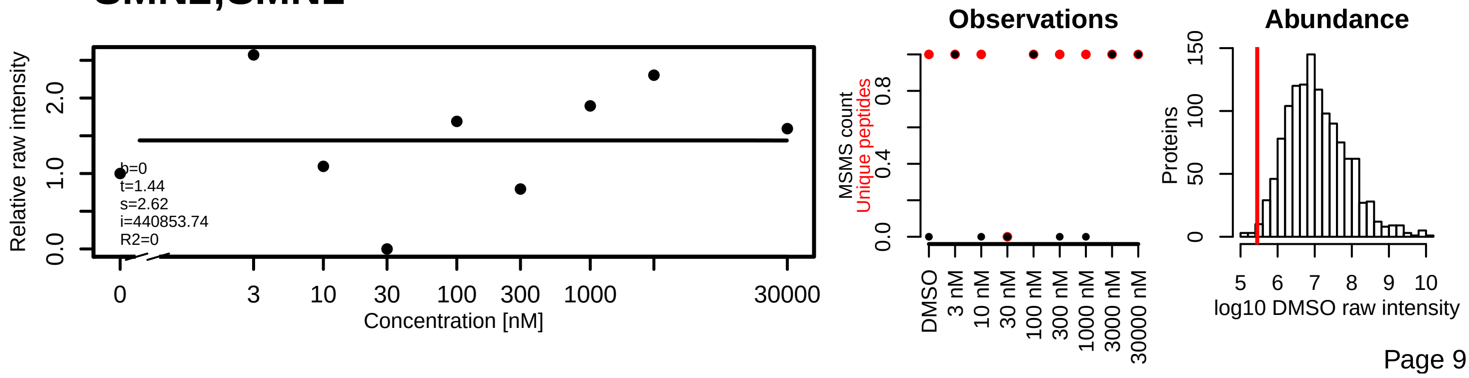

COG6

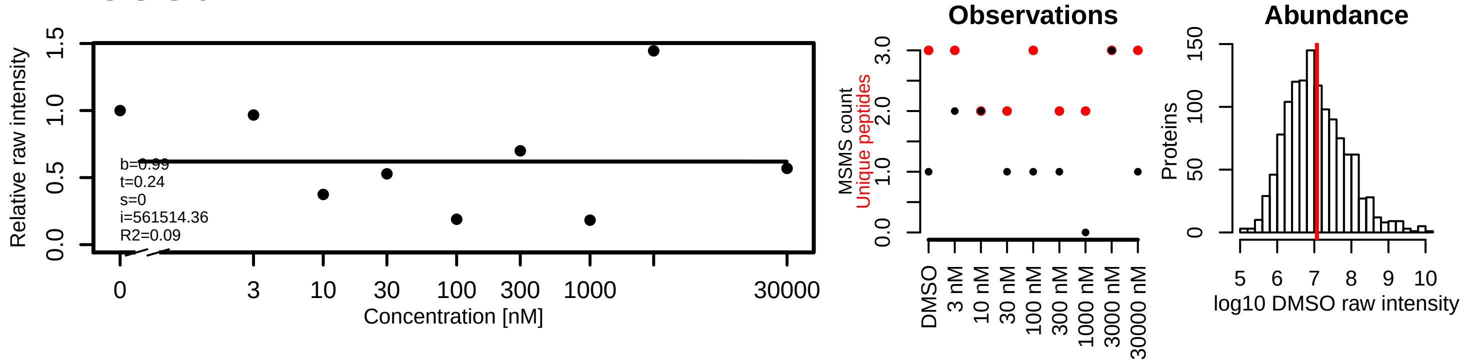

IPO4

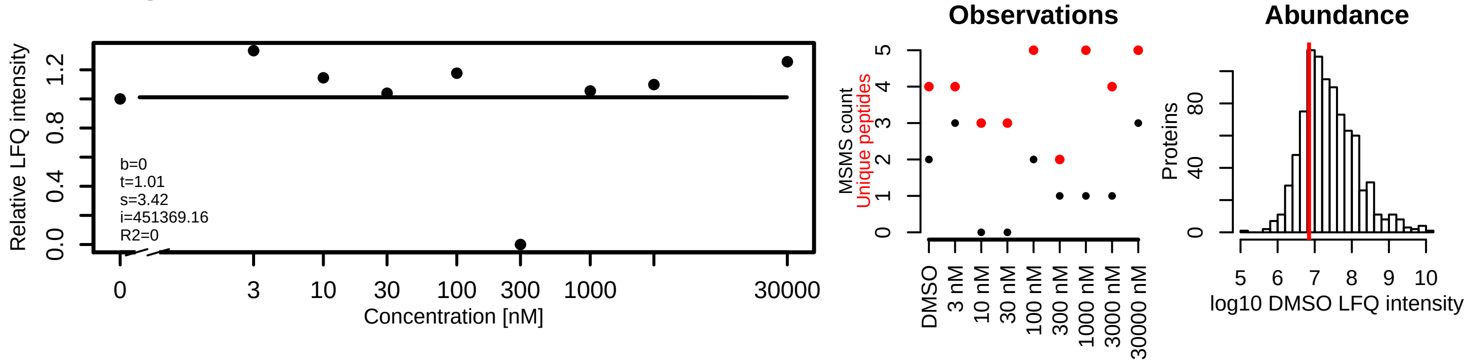

GBF1

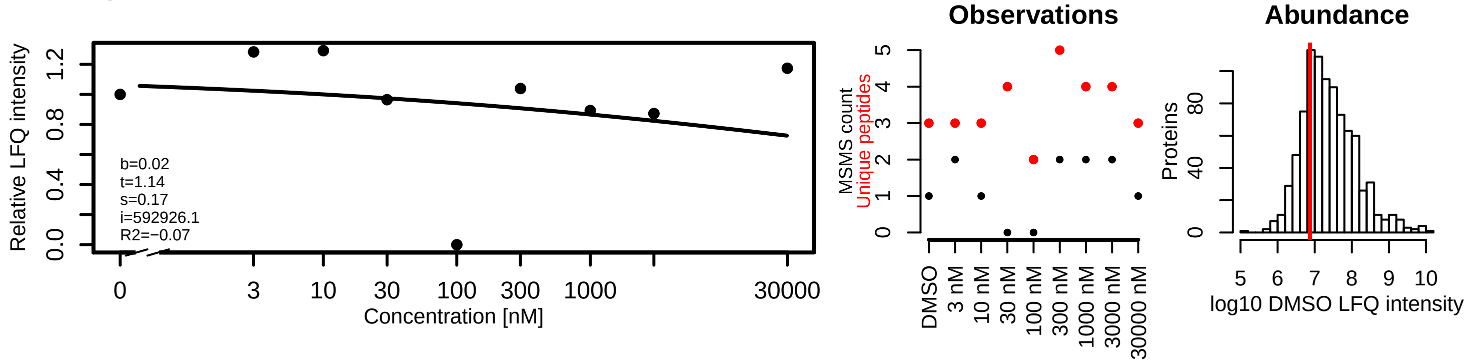

MCU

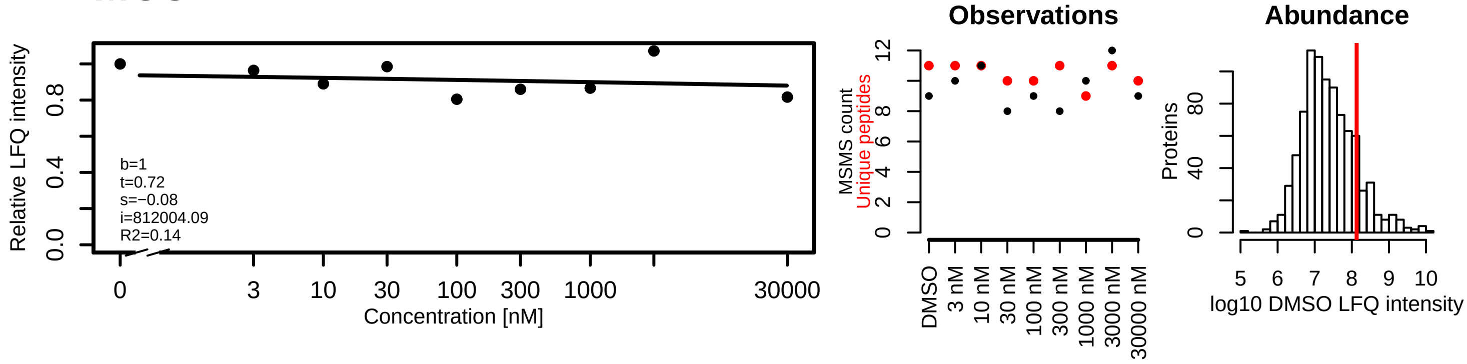

PSMD1

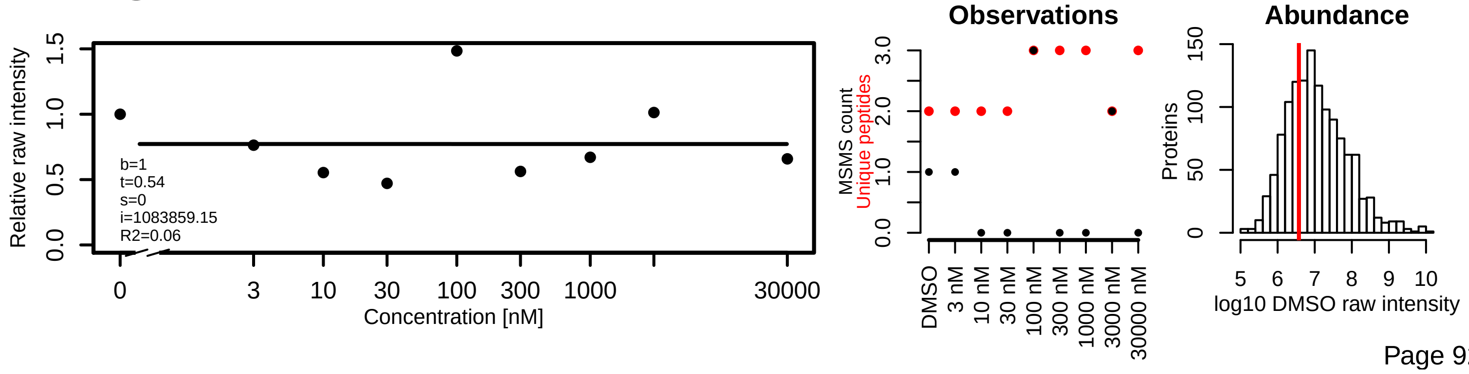

AIMP2

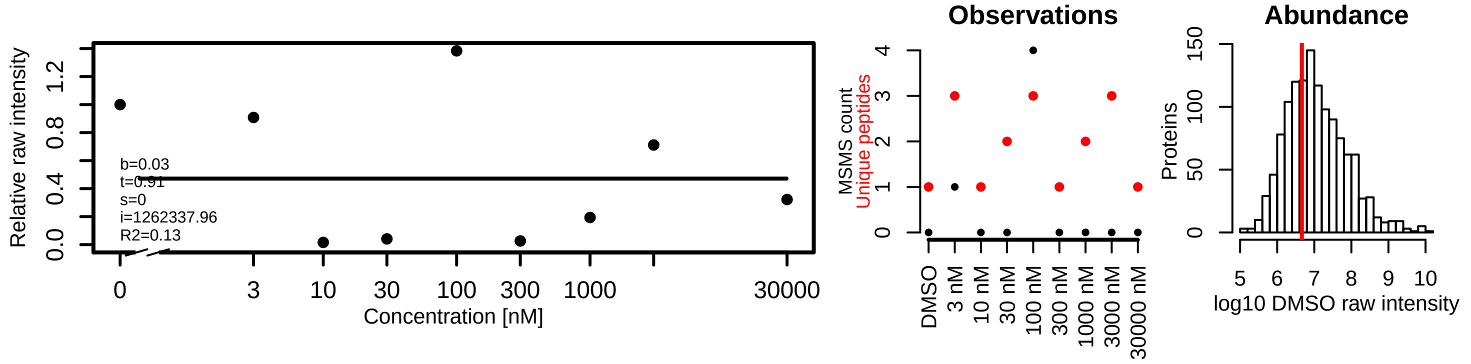

KDSR

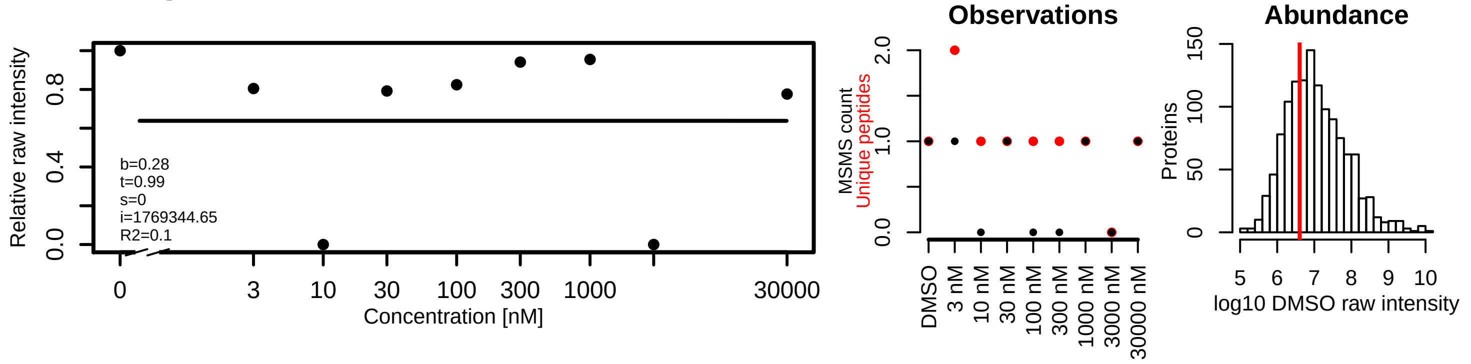

SLC39A7

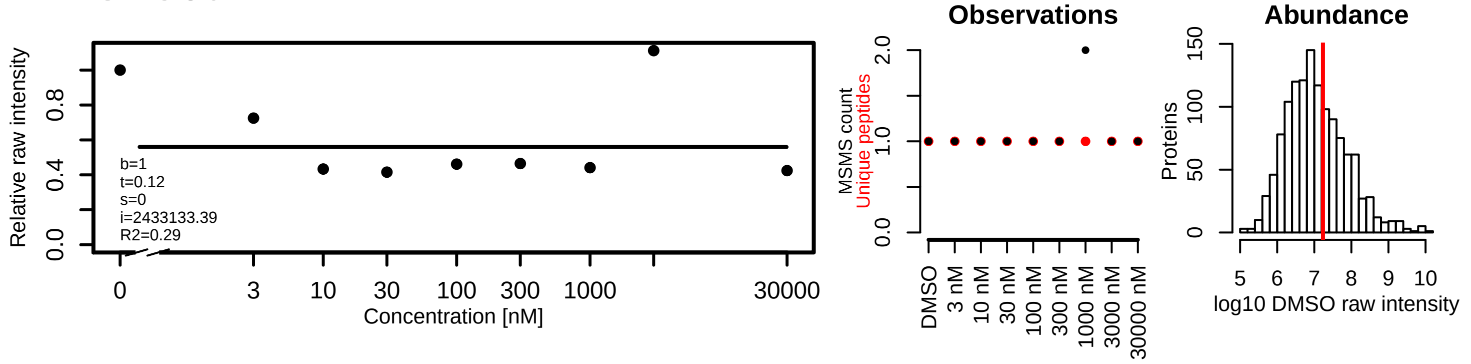

NDUFB8

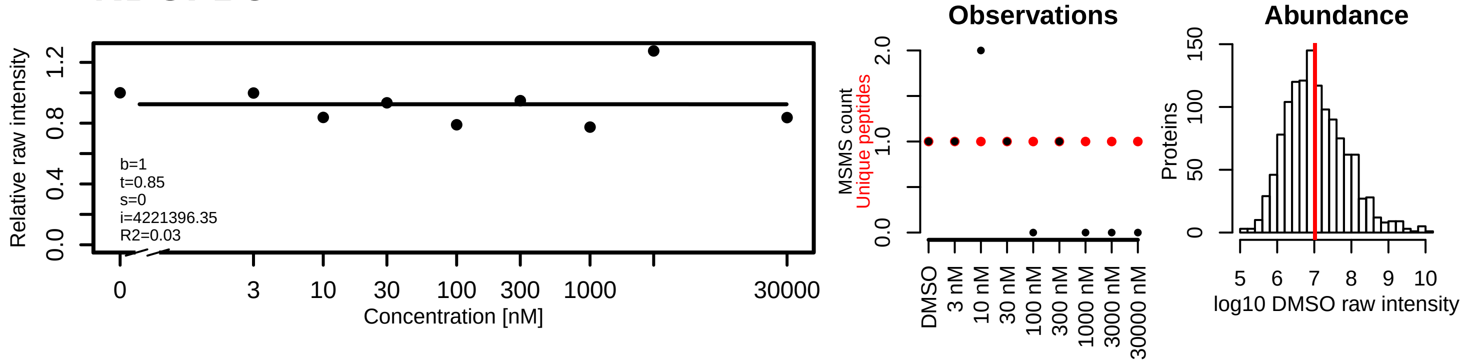

ATP9A

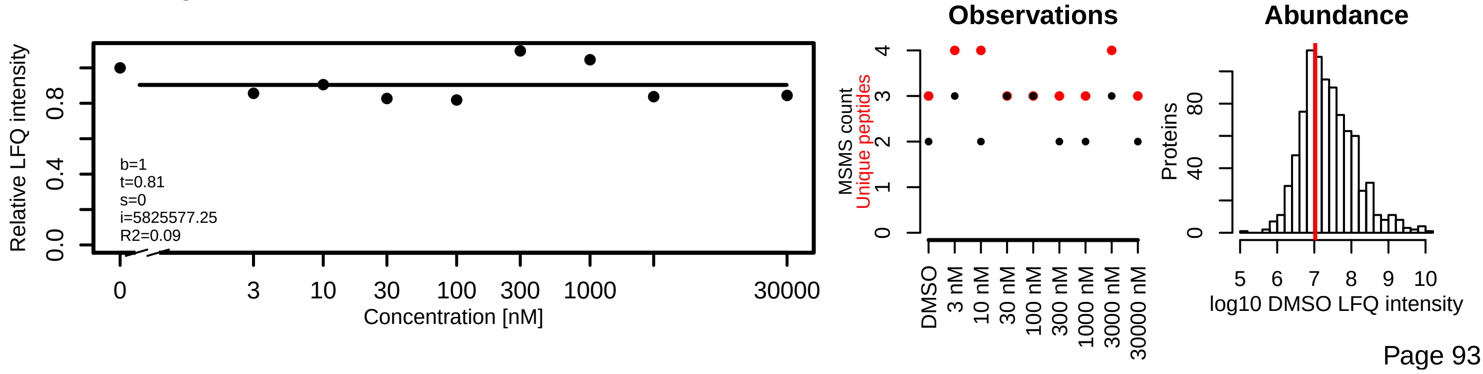

ATP6AP2

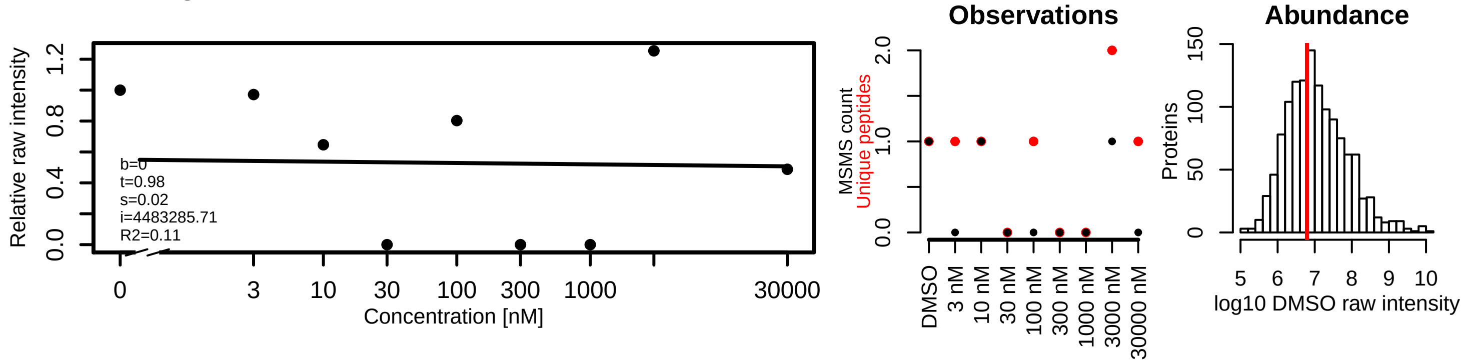

RDH10

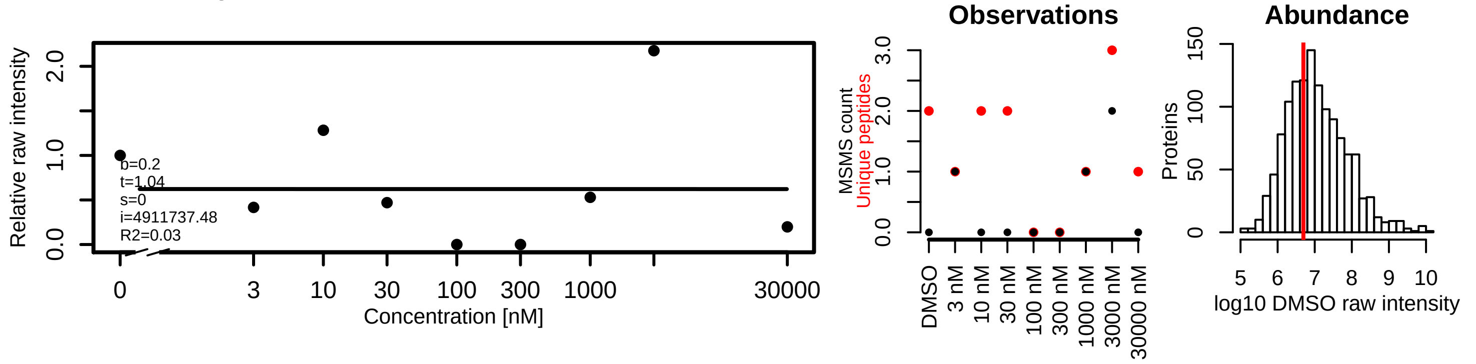

SLC25A1

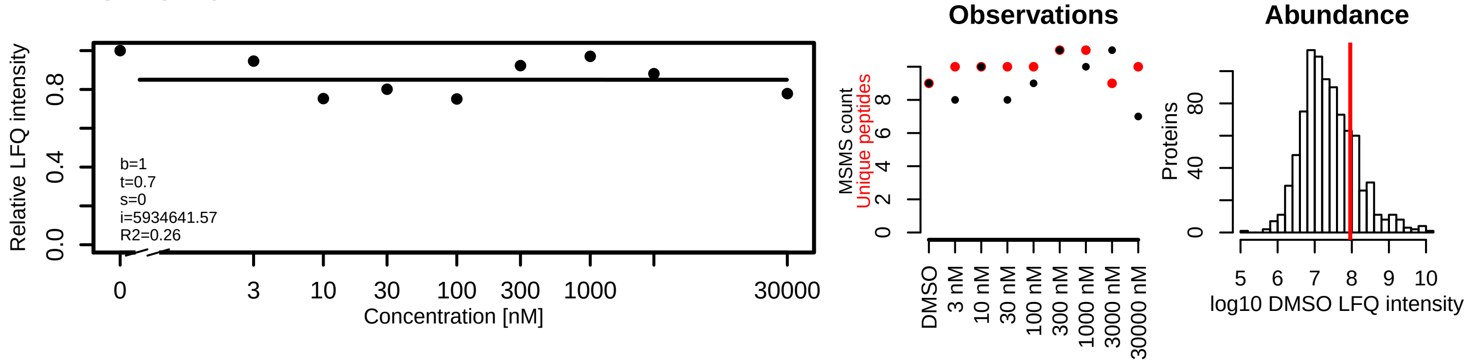

TIMM50

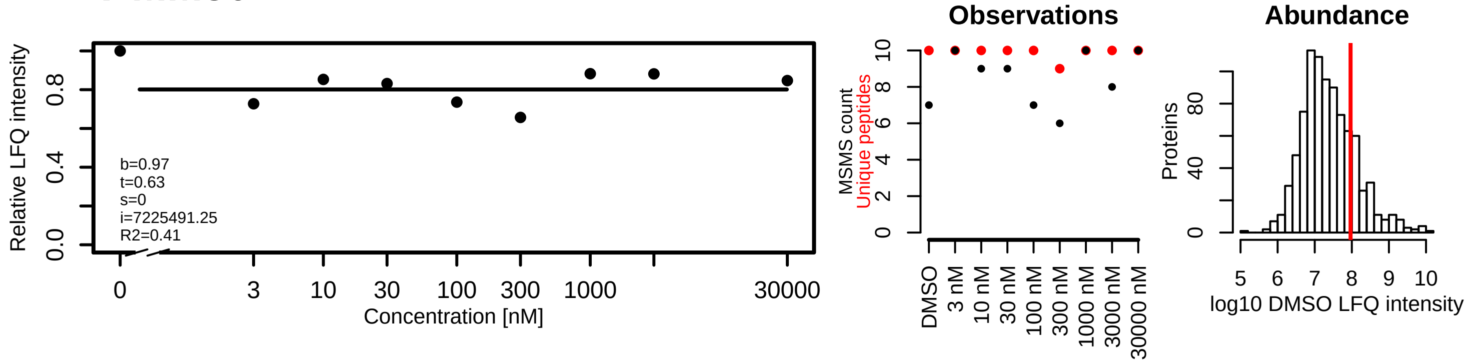

RPS27L

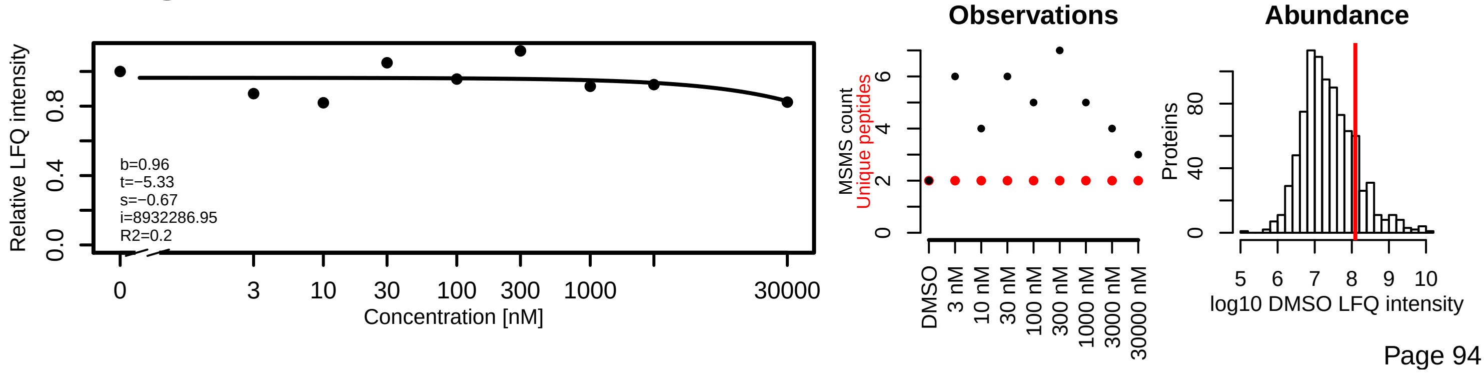

FASTKD5

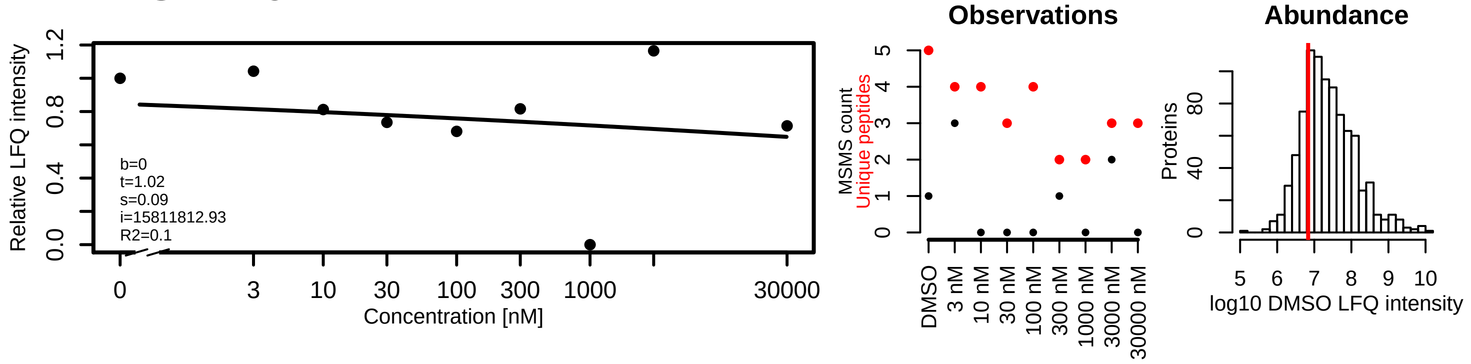

CLPTM1

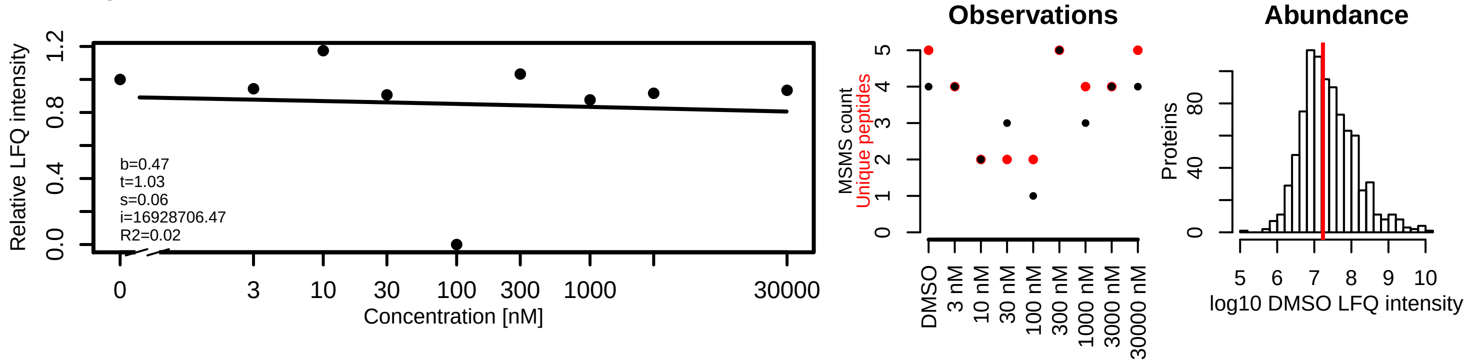

SEL1L

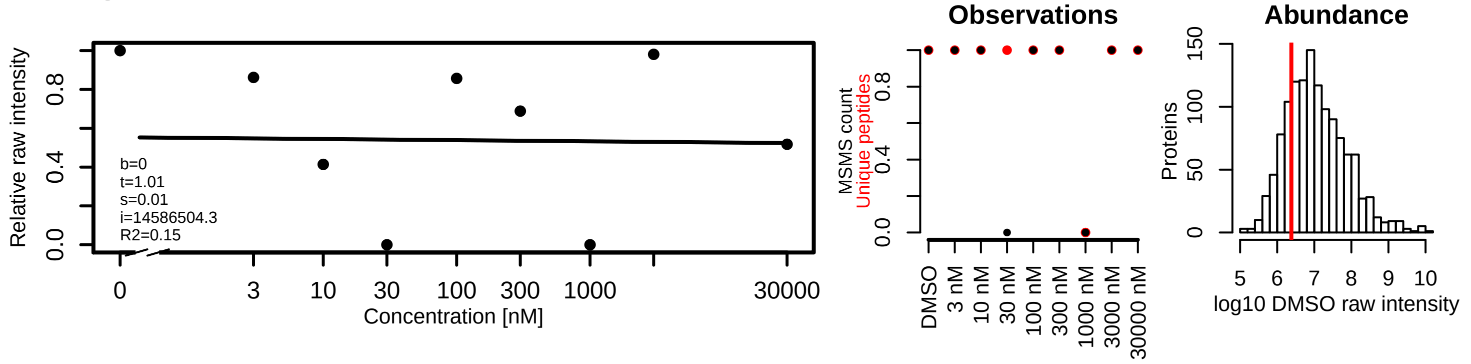

PSMB2

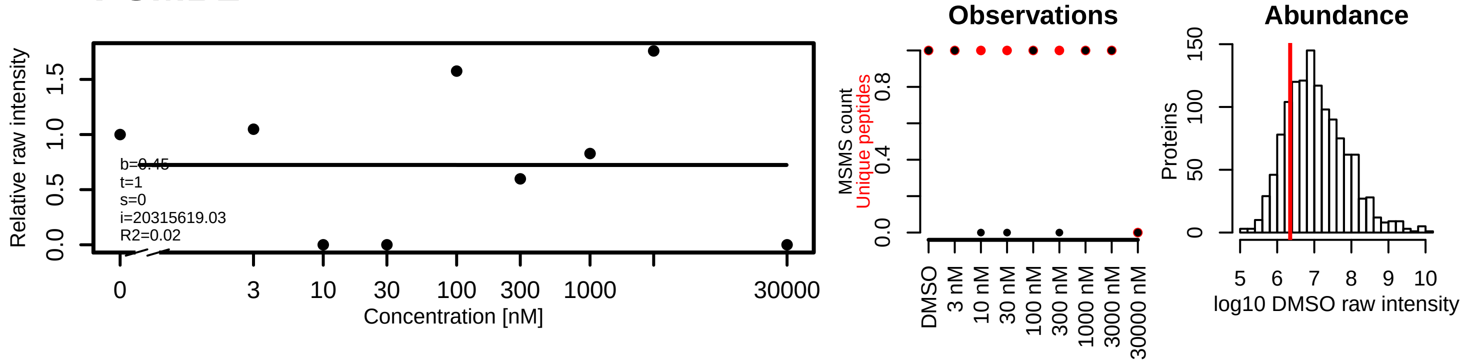

UBIAD1

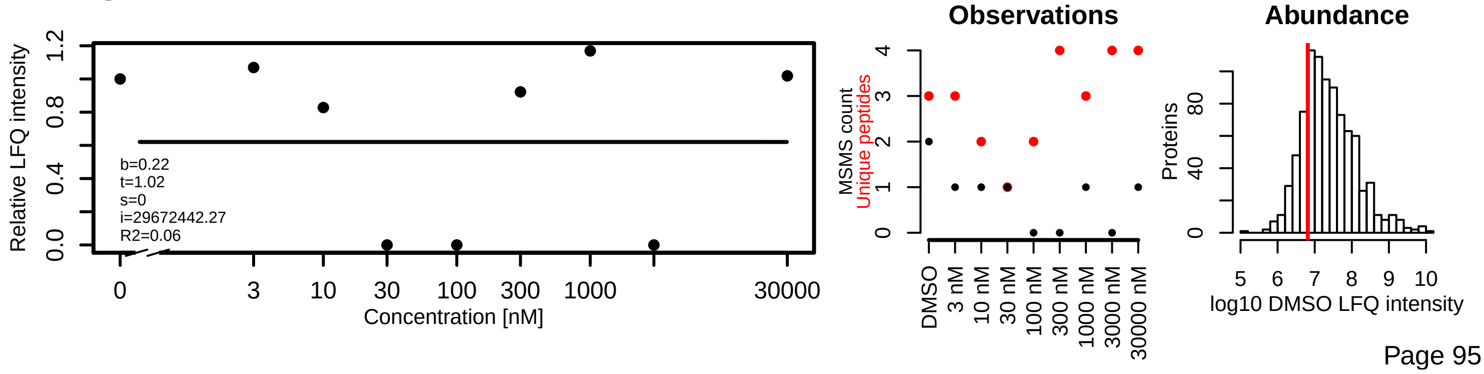

CYB5R3

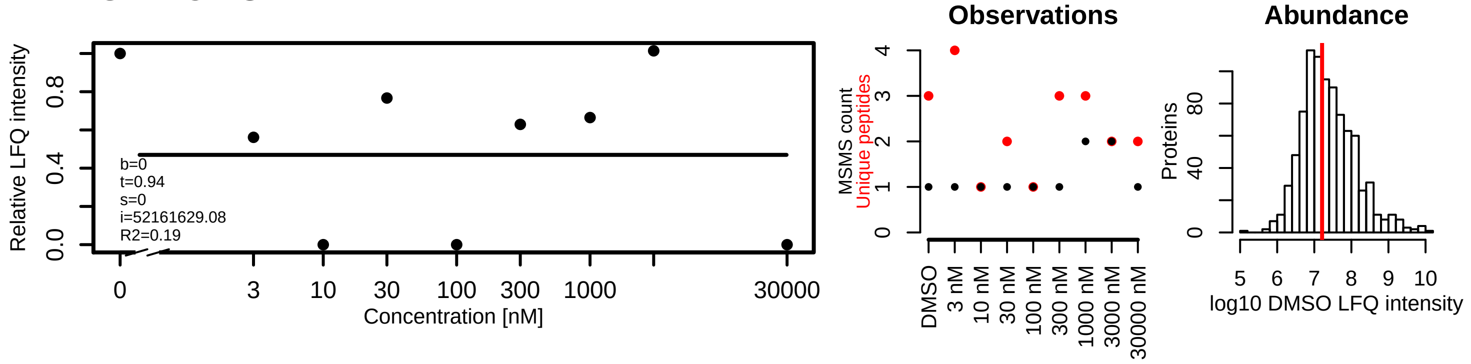

ILVBL

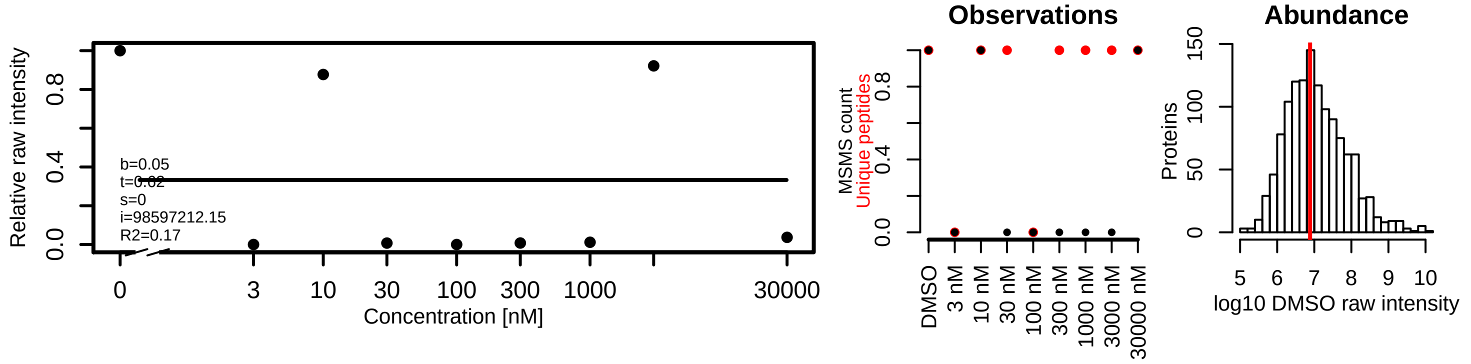

EPDR1;UCC1

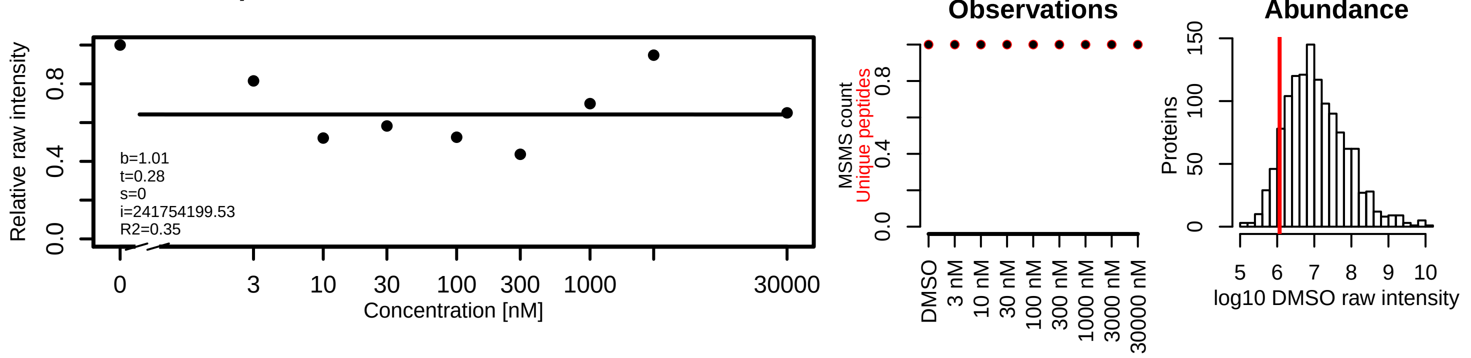

CPT1A

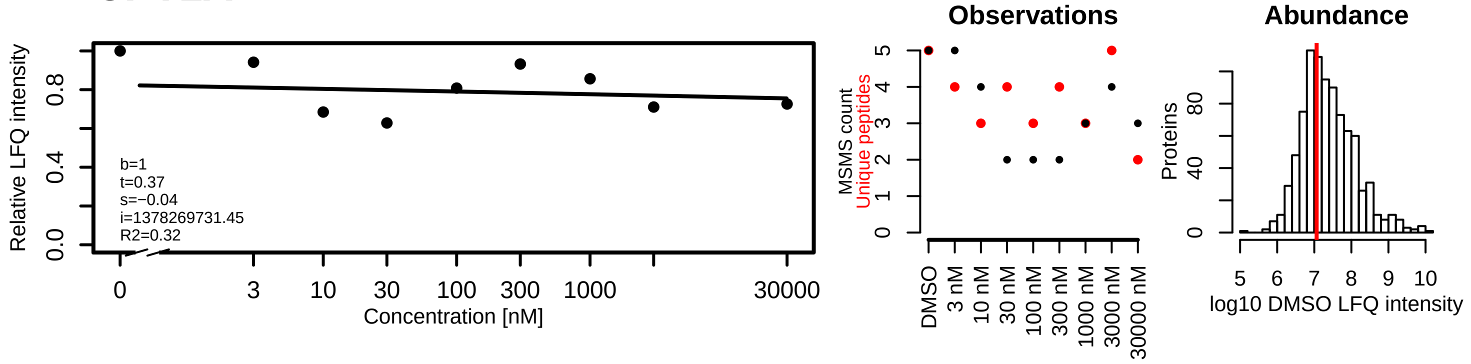

STT3B

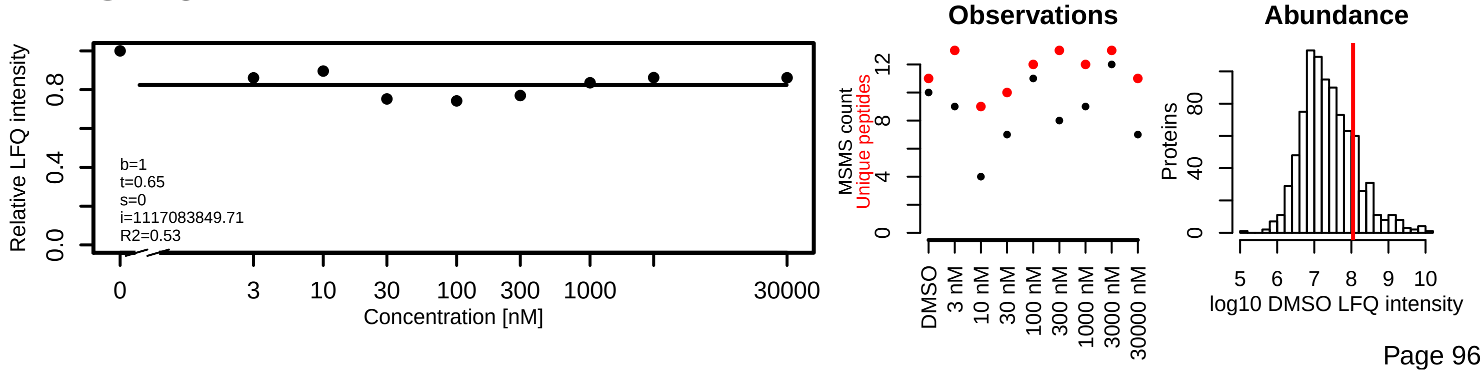

UPP1

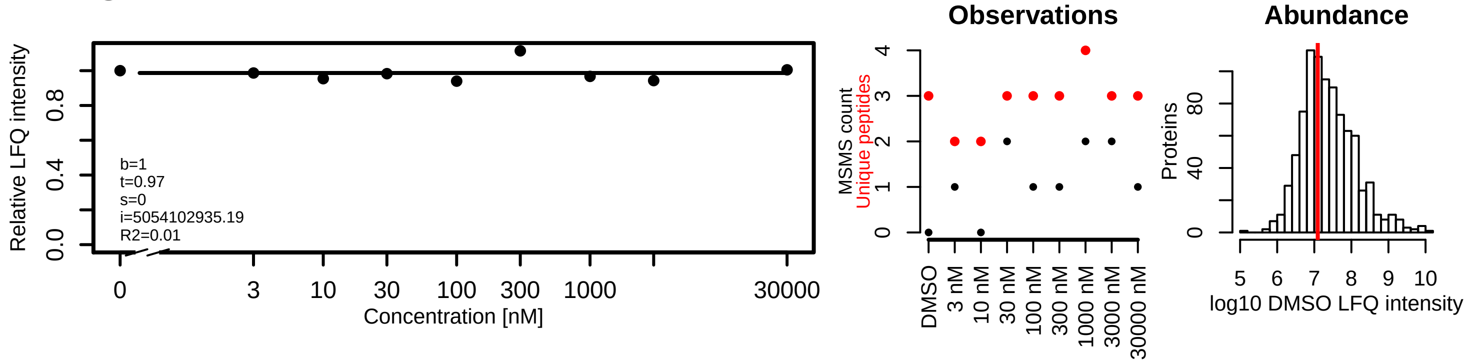

TCIRG1;C17orf96

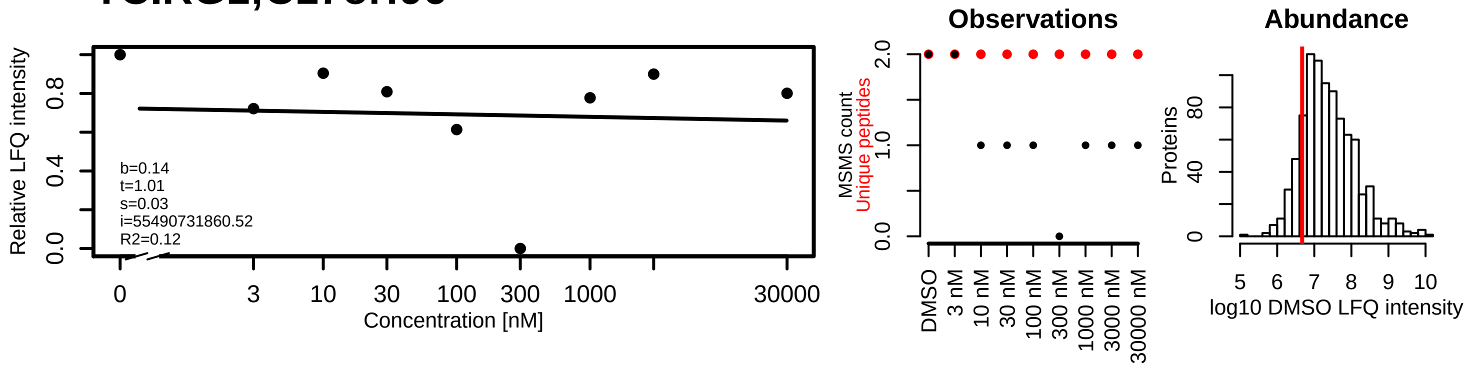

RANBP2

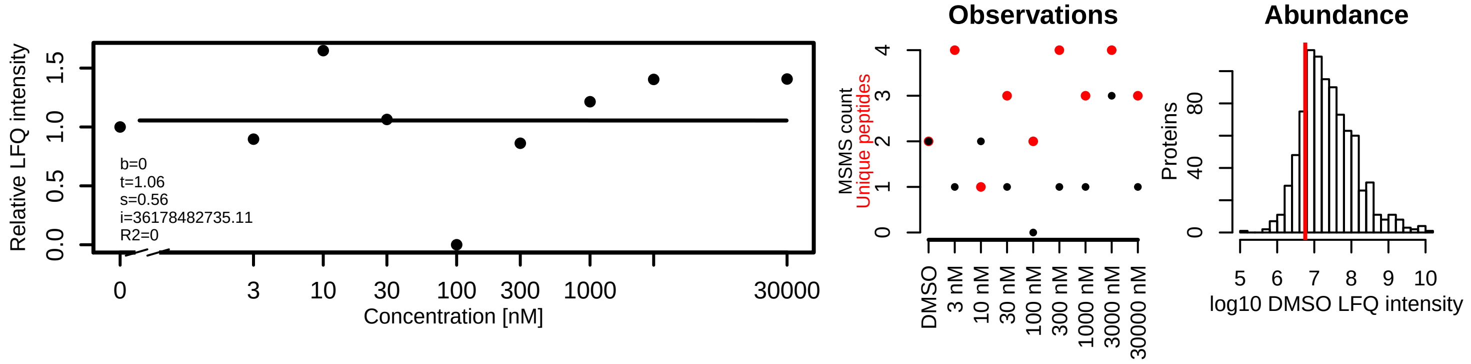

TMEM106B

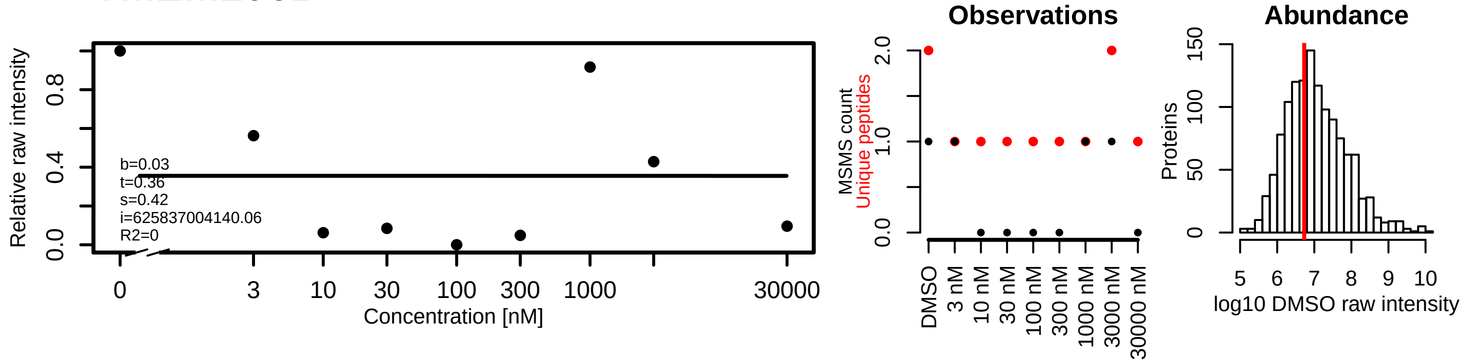

TMEM14C

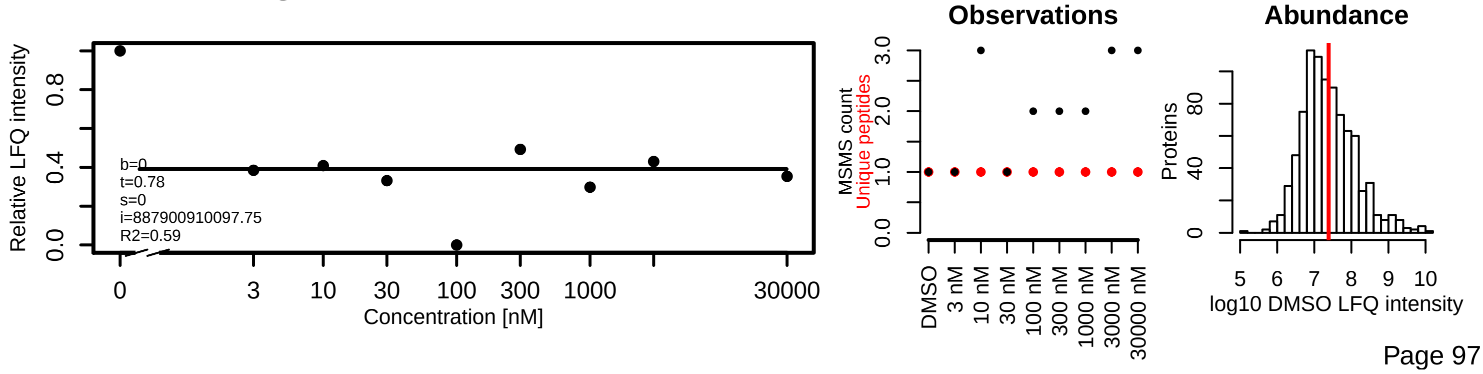

SSBP1

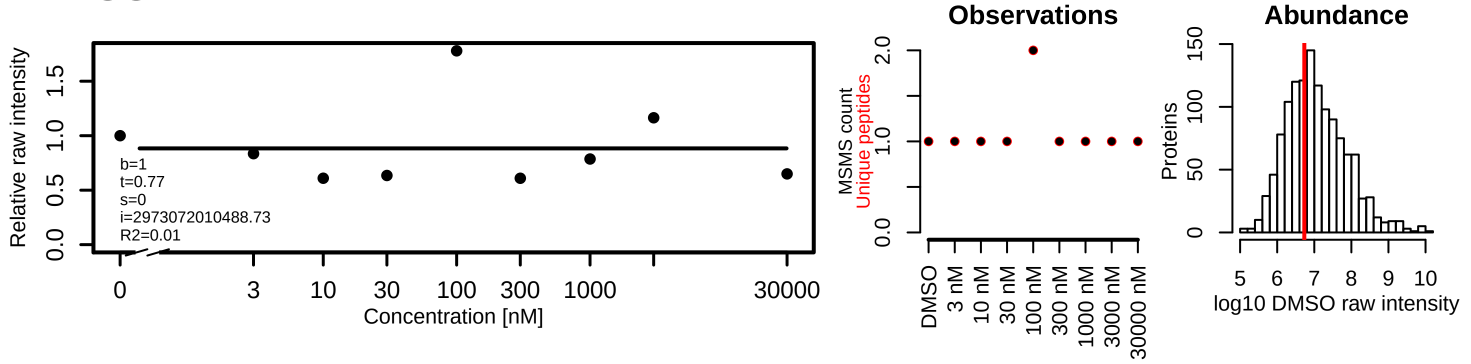

TPCN1

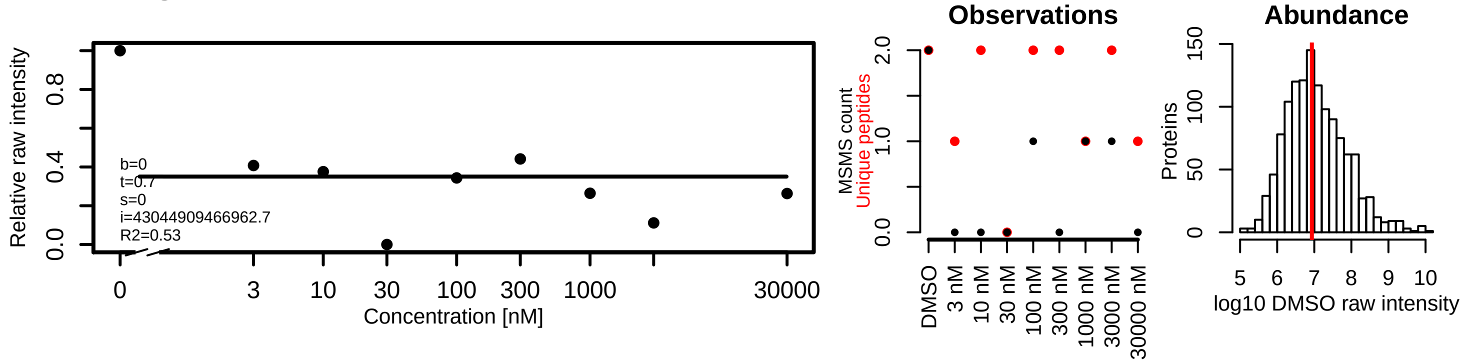

RPS12

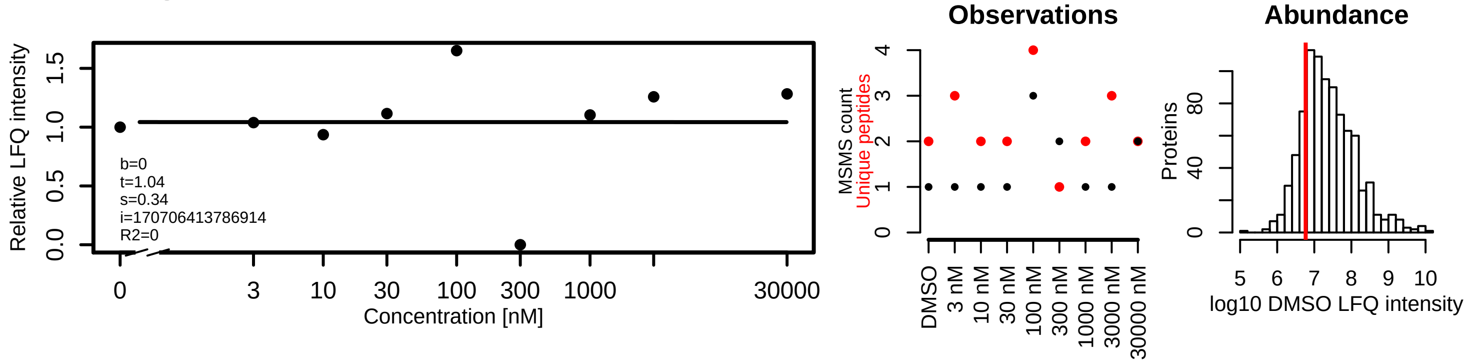

PQLC3

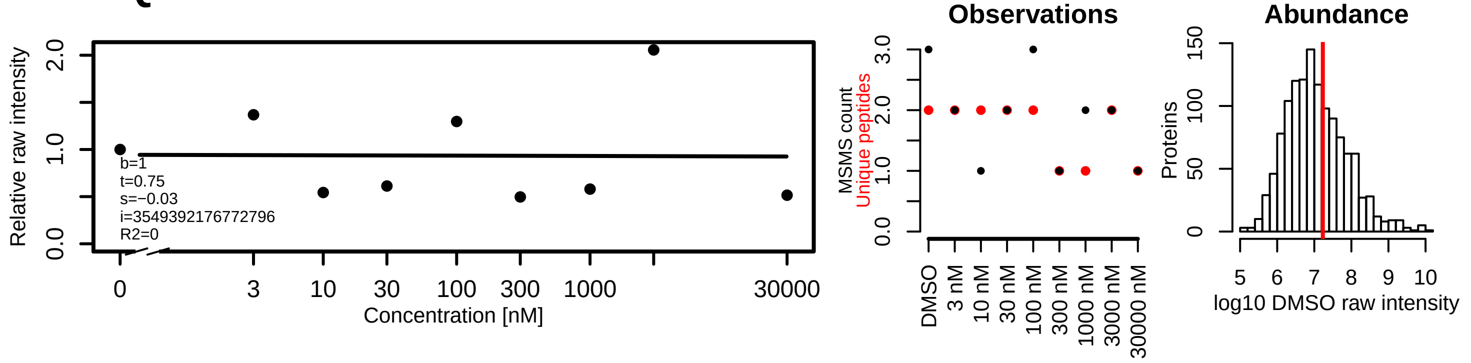

ABCB7

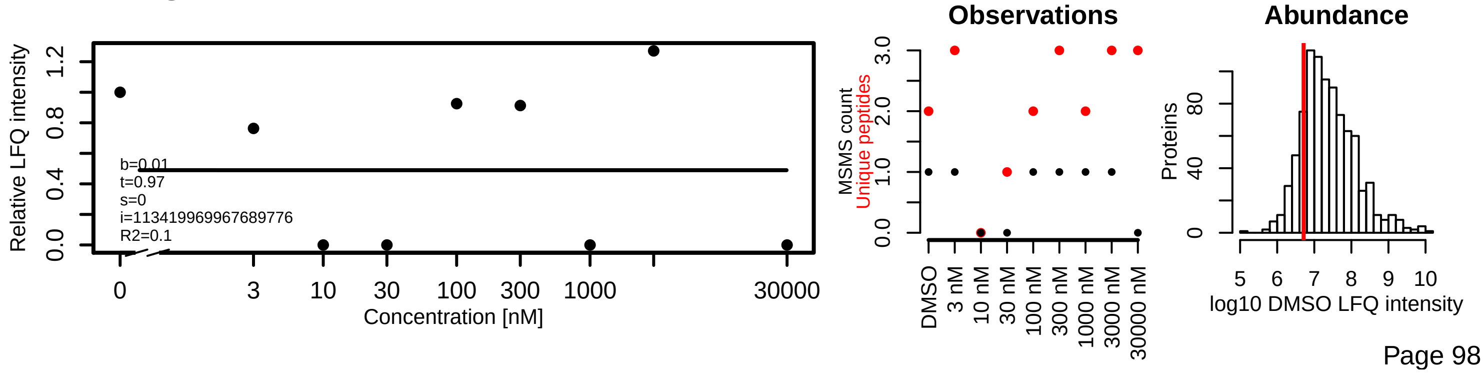

NONO

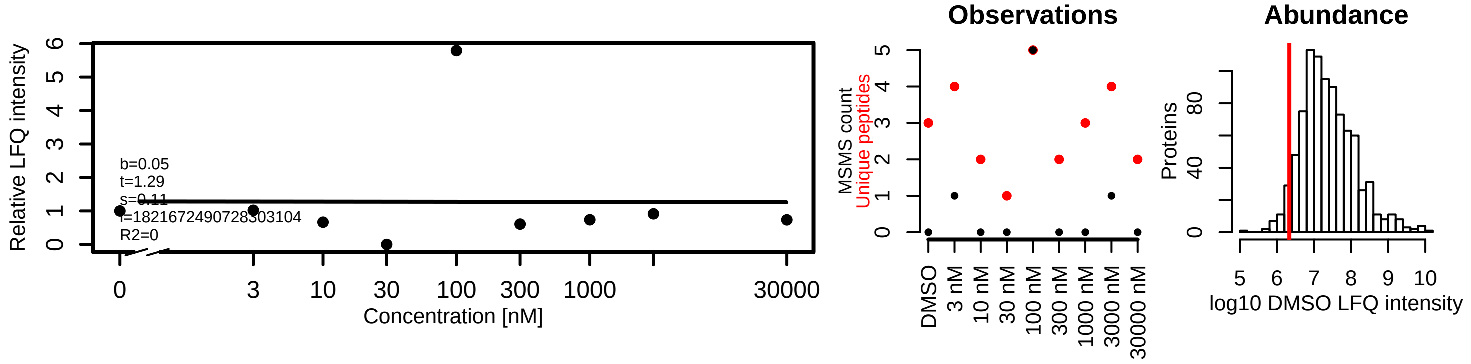

HSD17B7

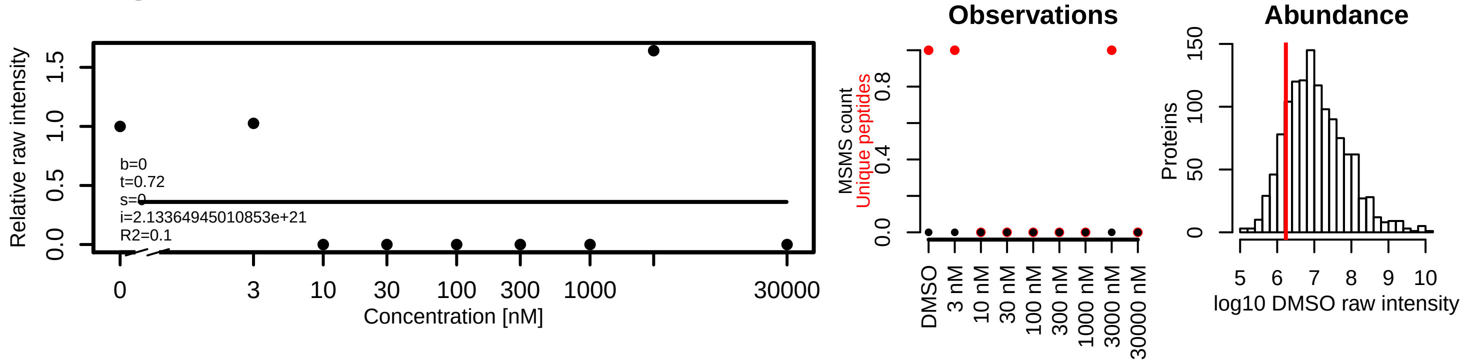

SGPP1

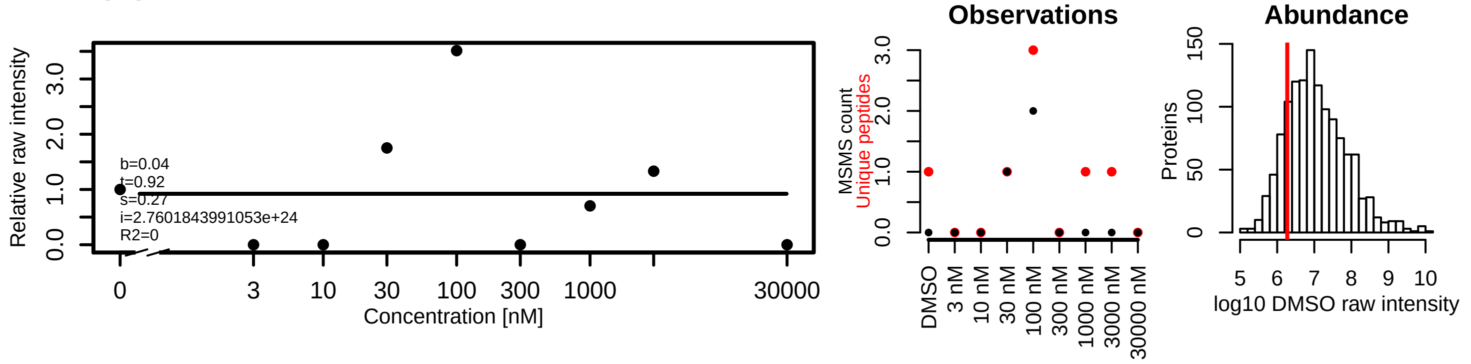

XRCC6

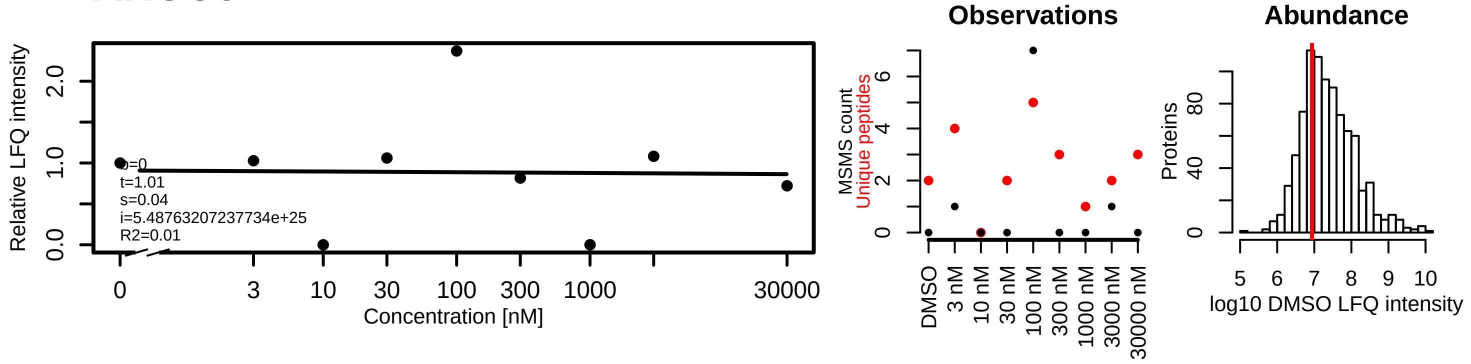

NQO1

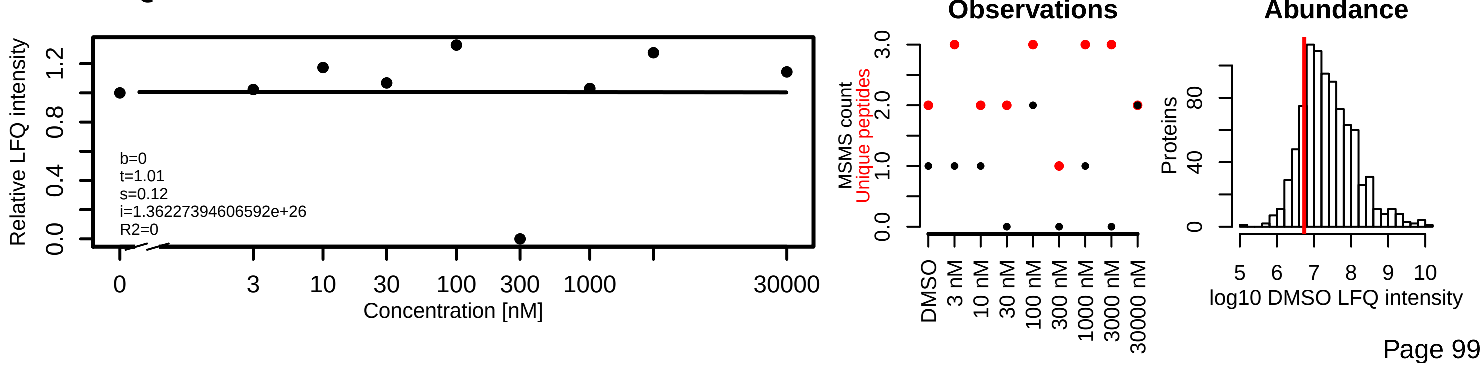

EXOC2

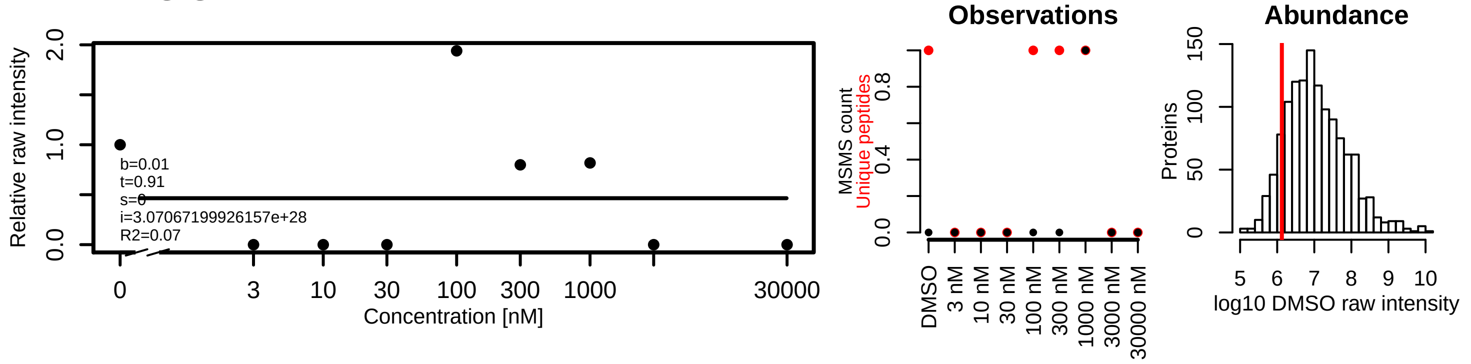

PA2G4

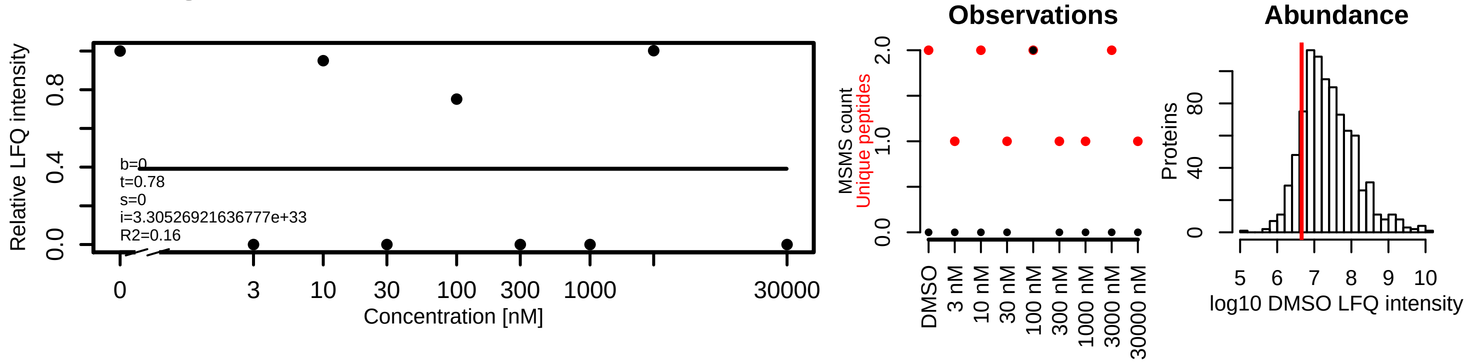

DYM

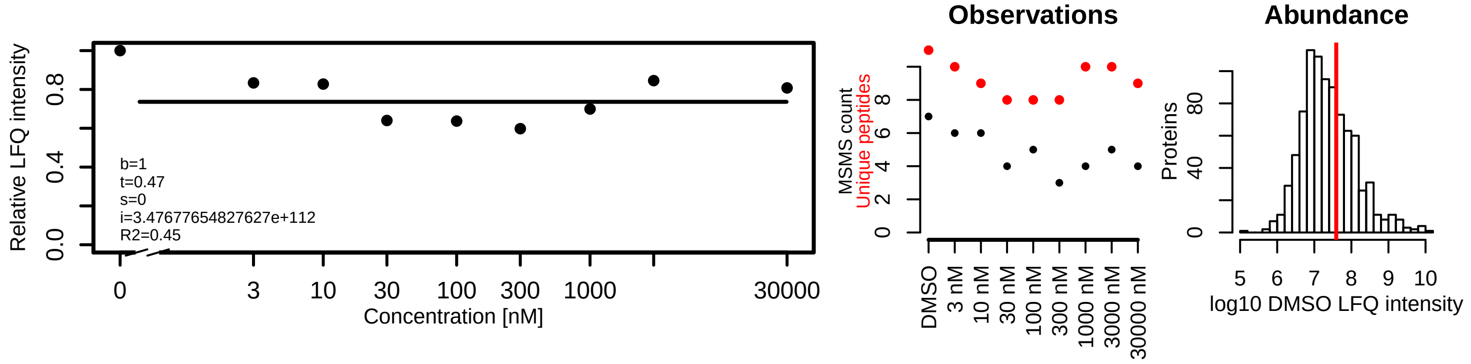

SLC39A6

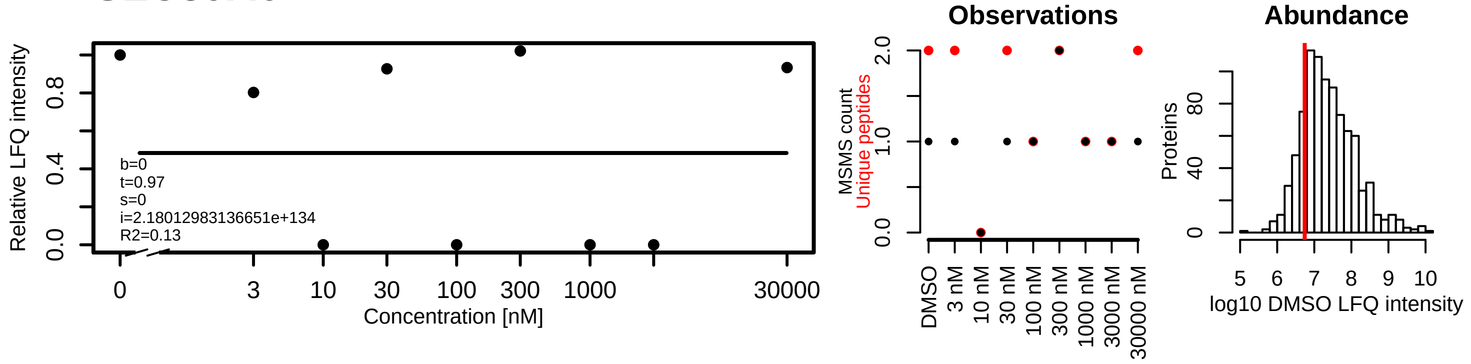

TMEM120B

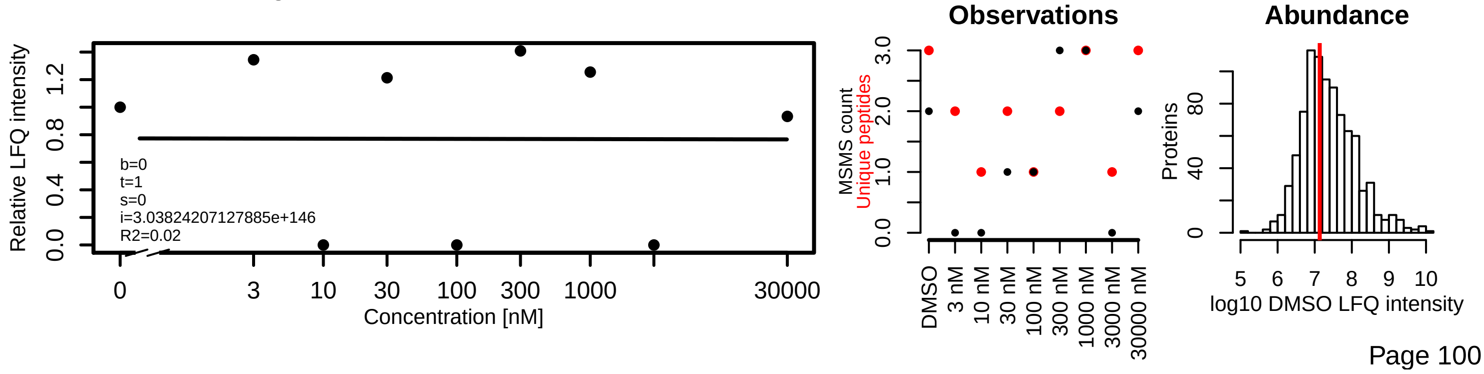

HADHA

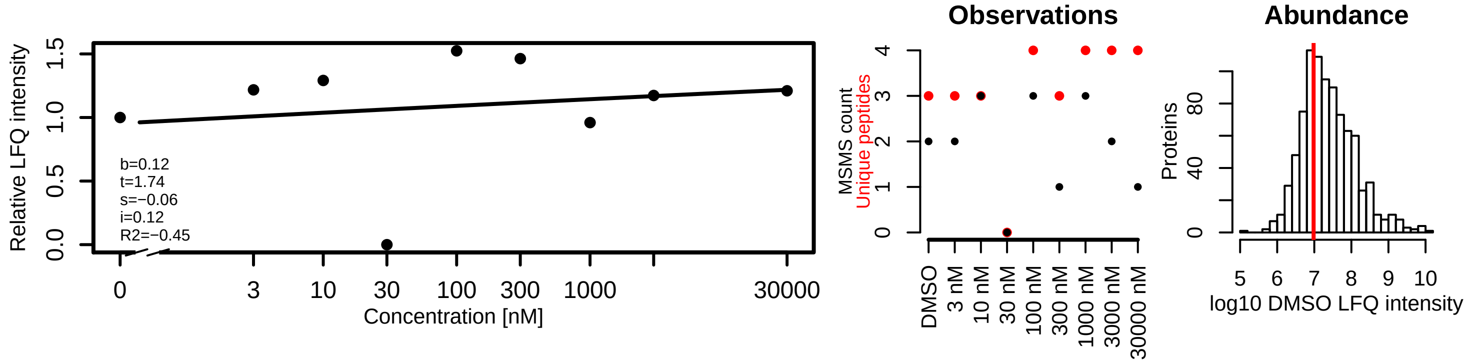

S100A11

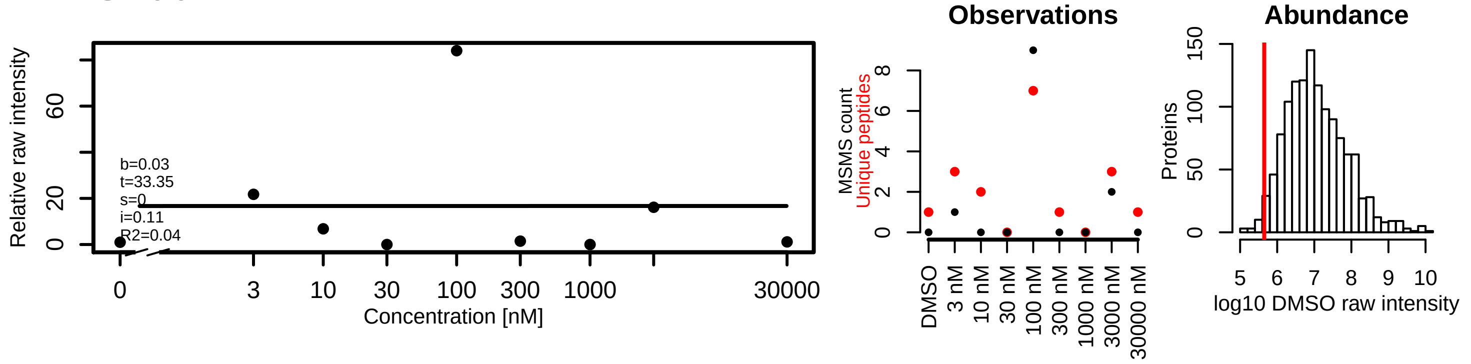

HEATR5A

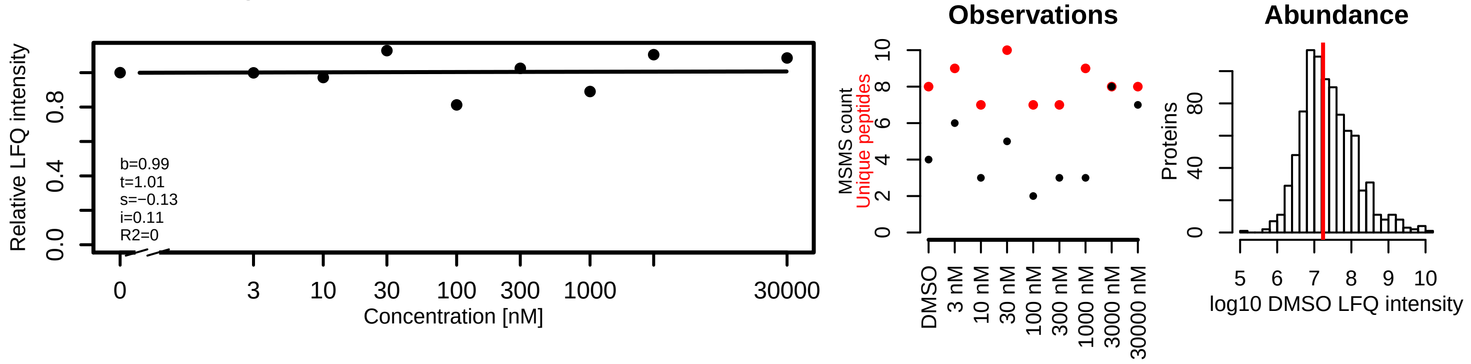

SLC25A22

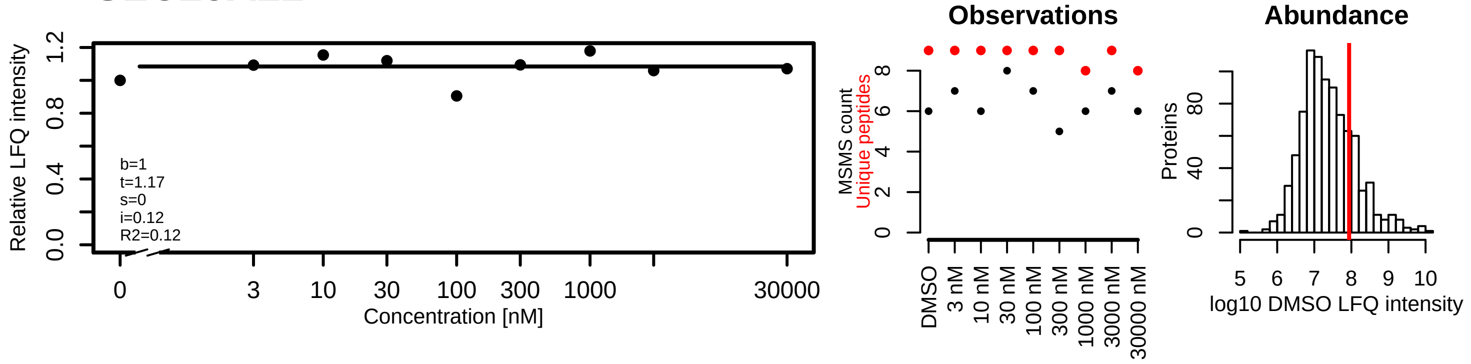

ATP5C1

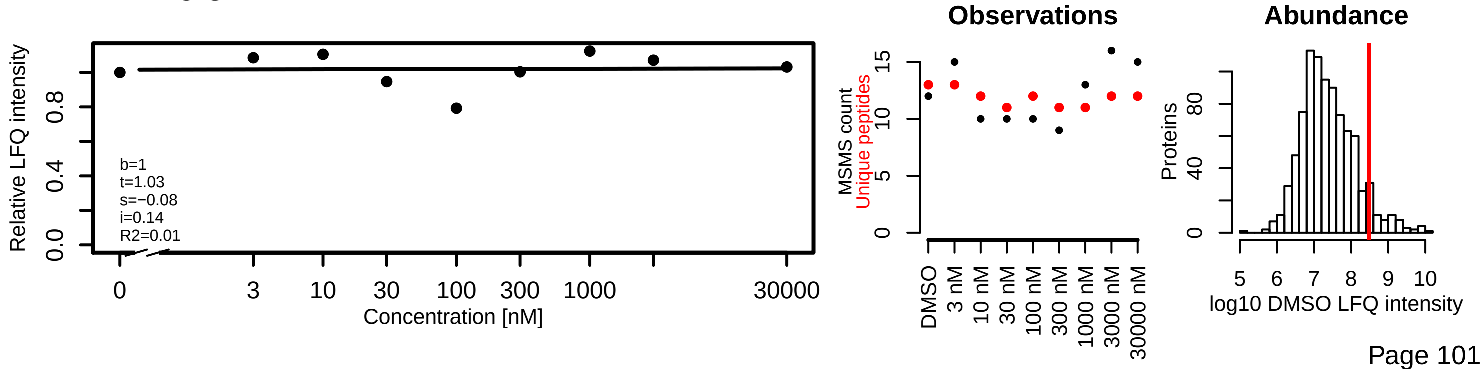

PSMC2

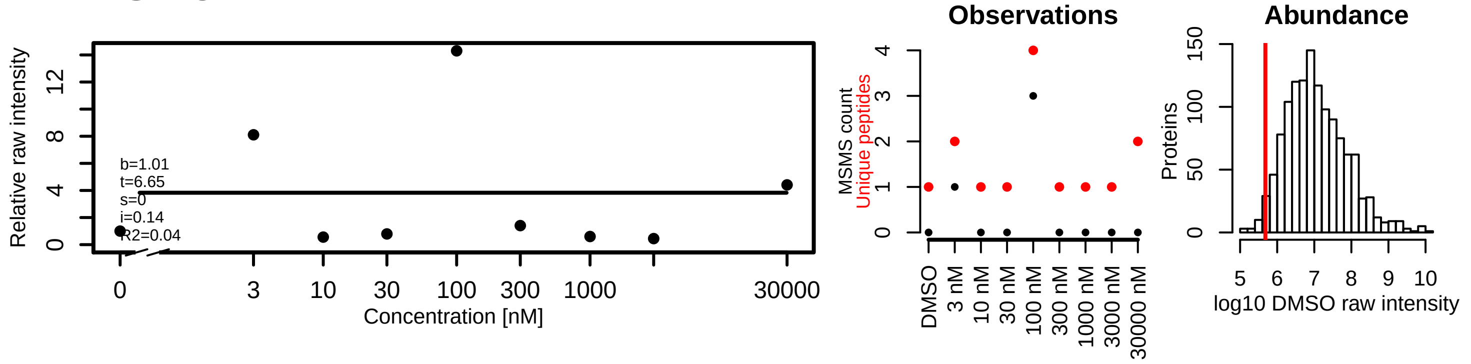

C22orf28

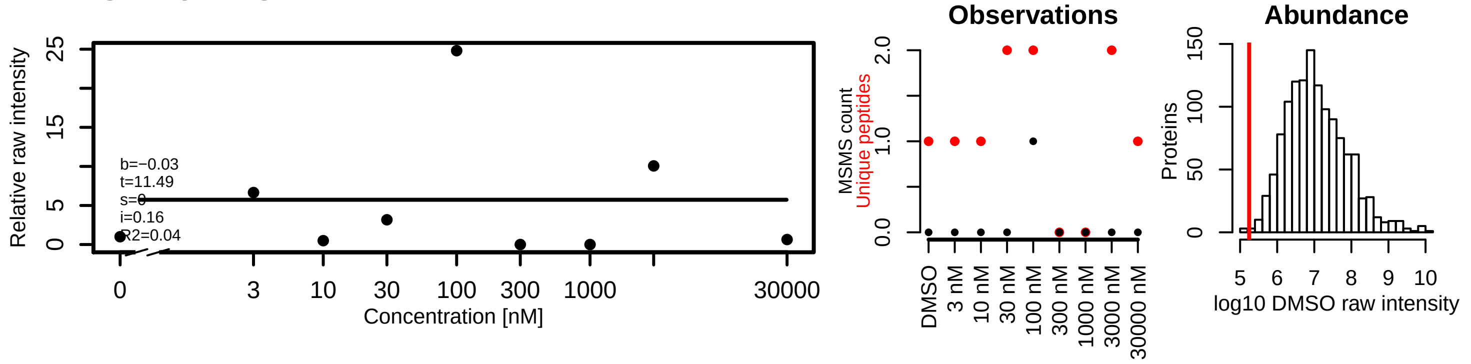

TAGLN2

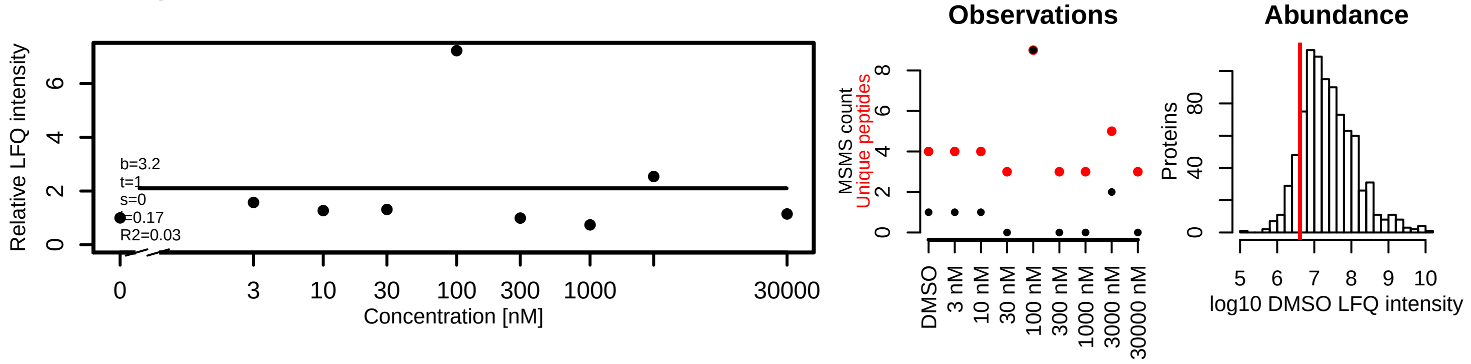

XRCC5

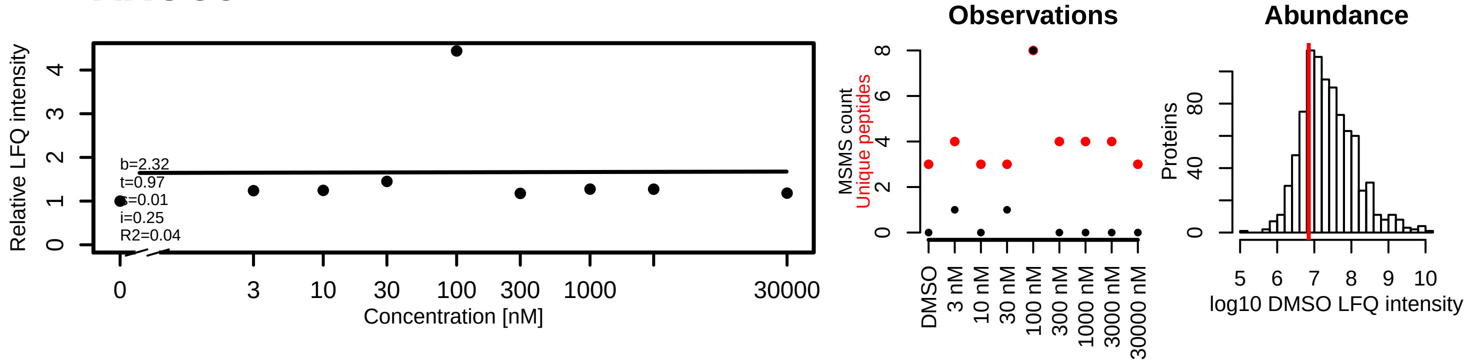

UQCR10

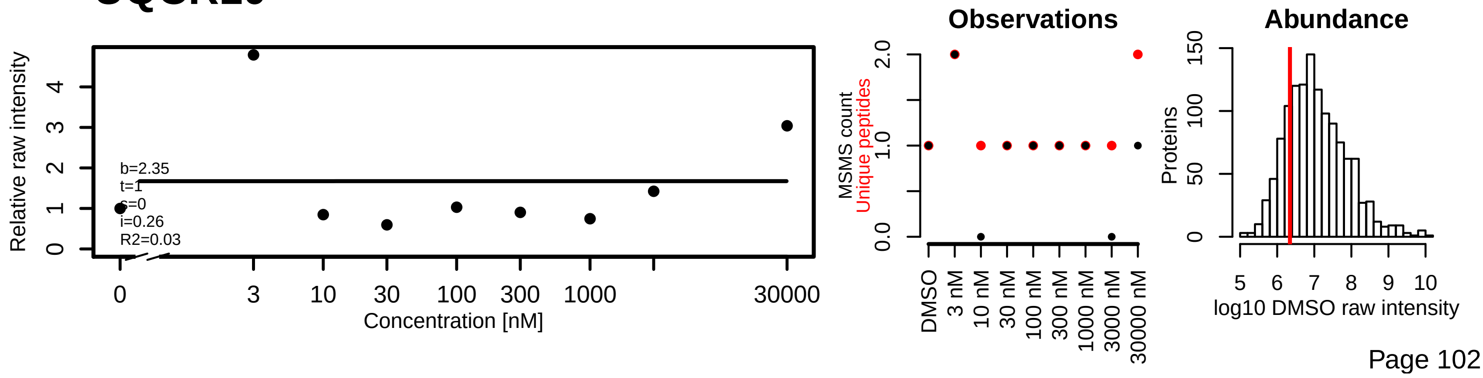

KNTC1

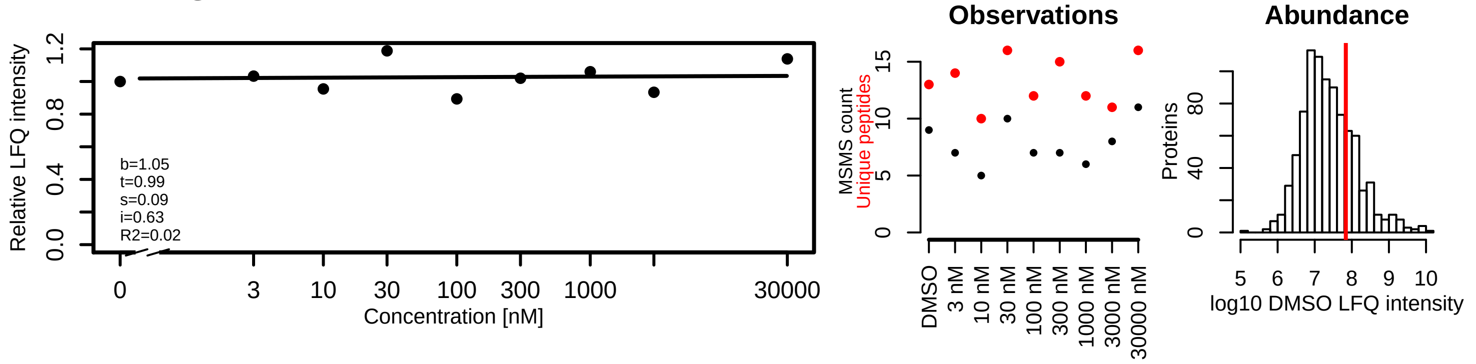

CALM1;CALM2;CALM3;TNNC2;CALML3

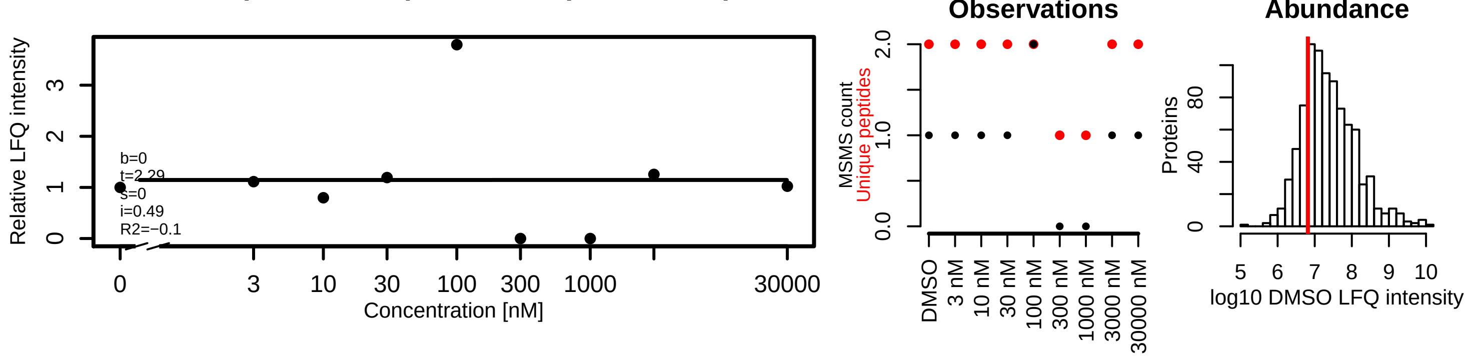

ENO1

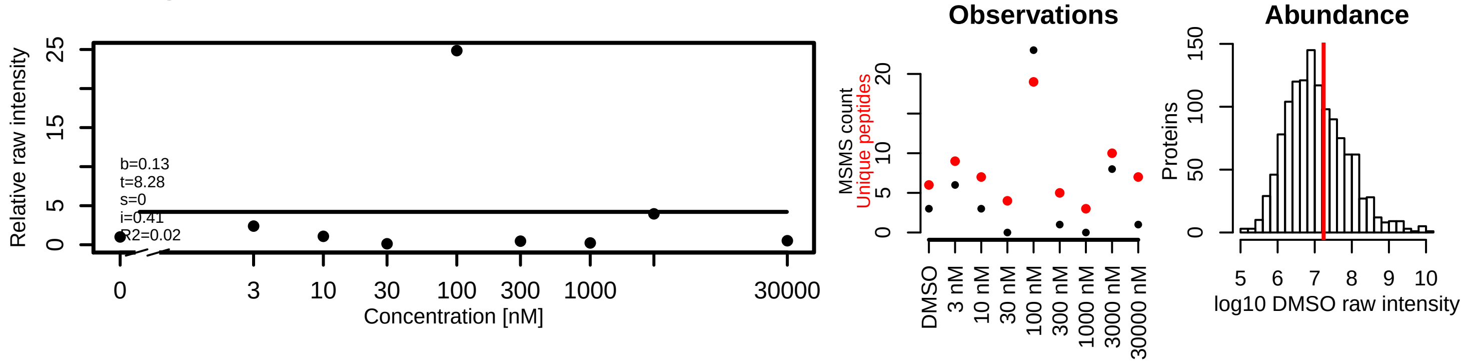

SF3B1

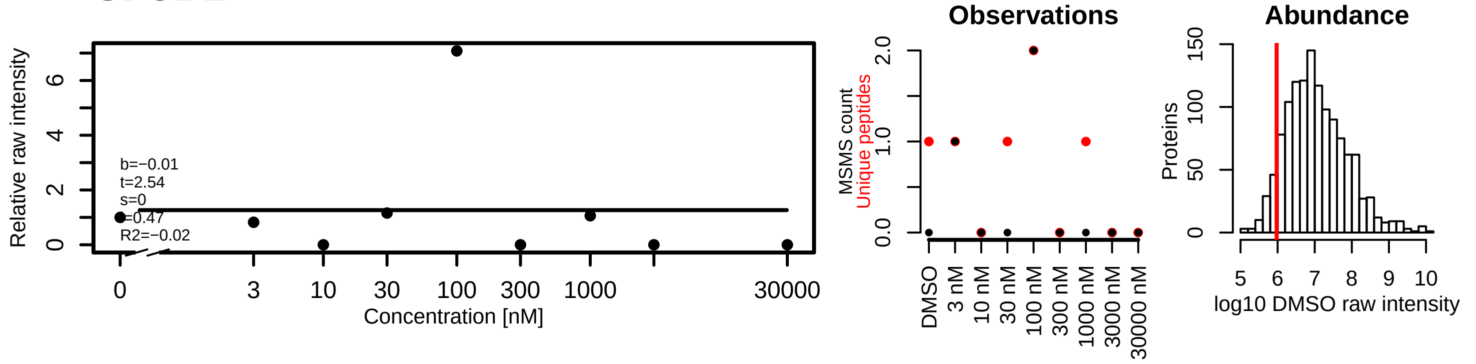

ATP9B

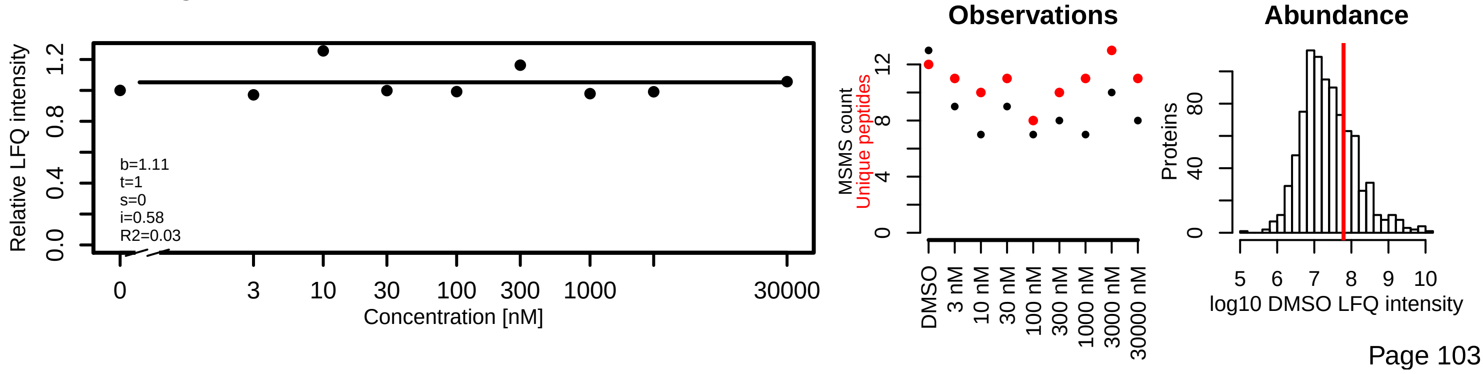

TM9SF1

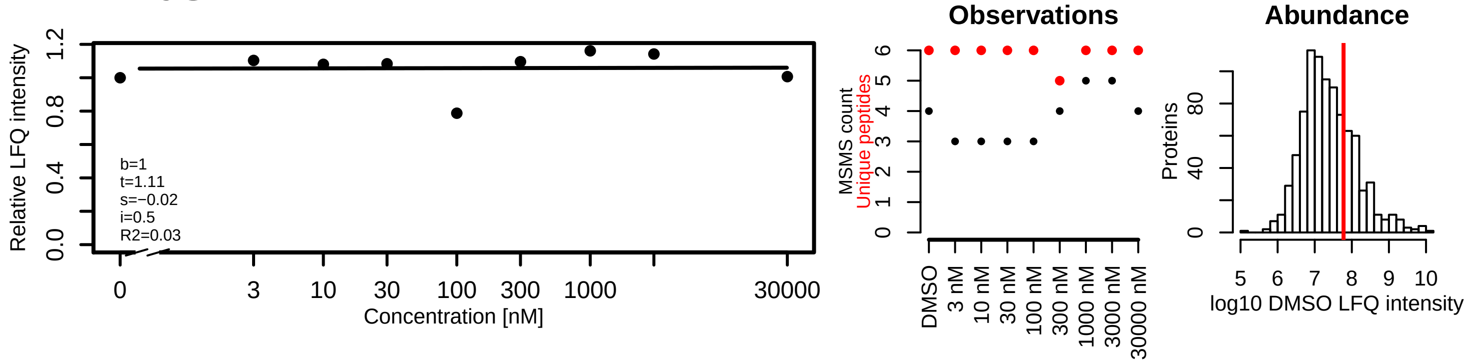

RTTN

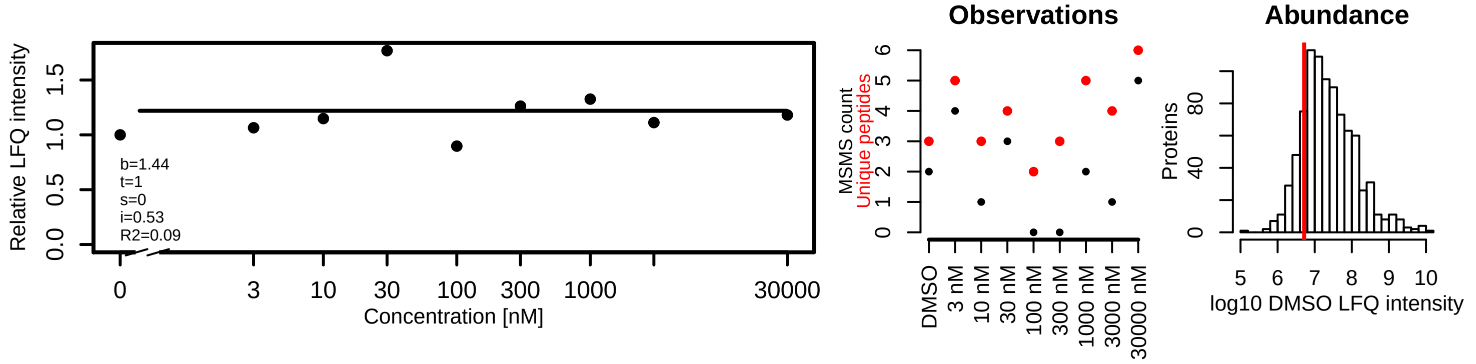

RAB7A

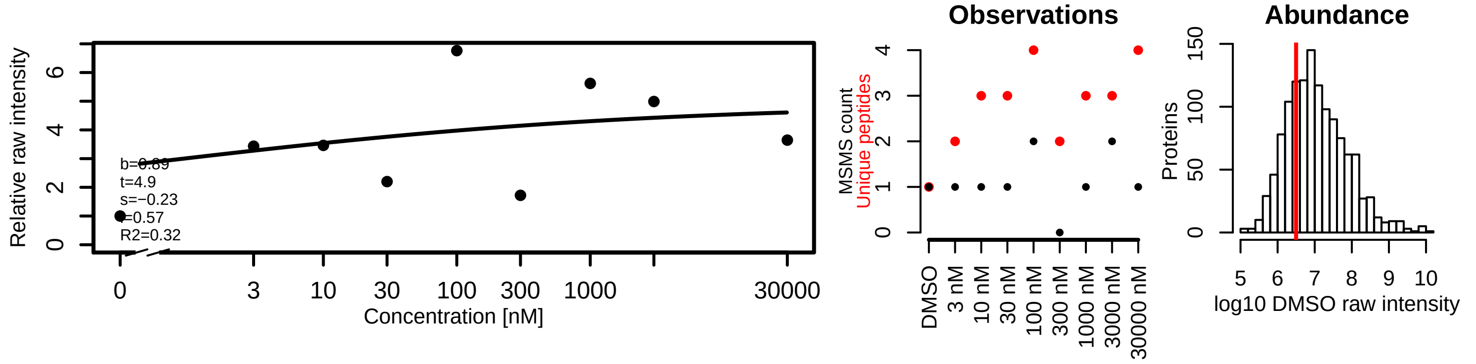

MATR3

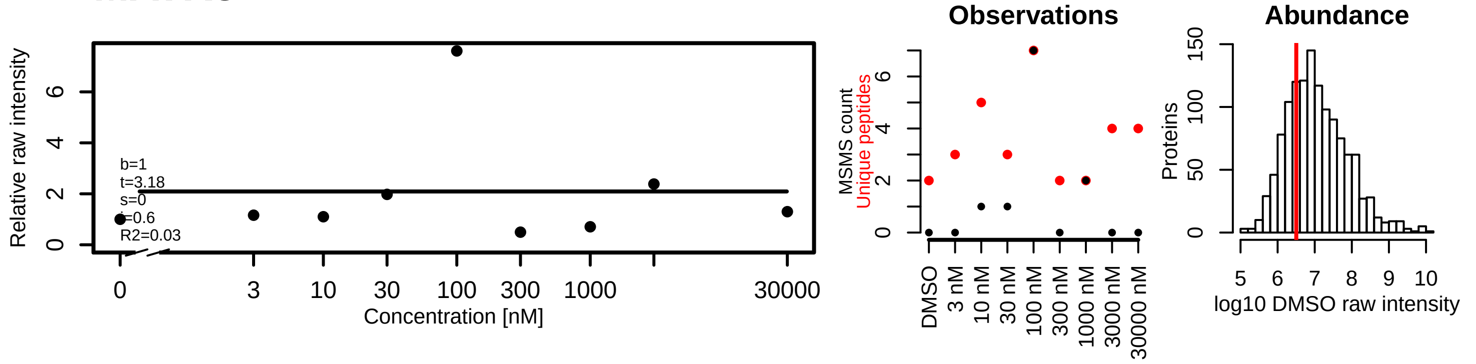

TRIM28

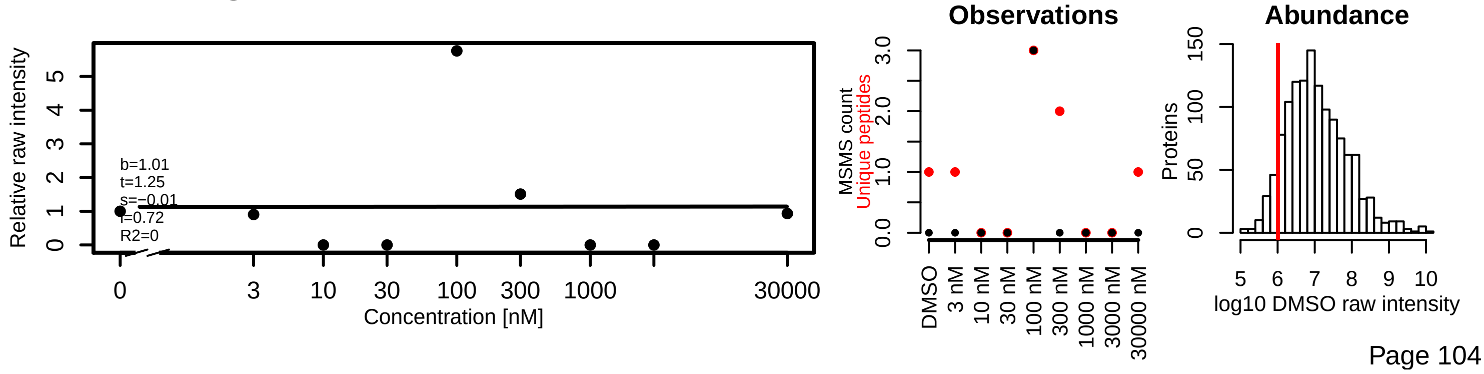

ELOVL5

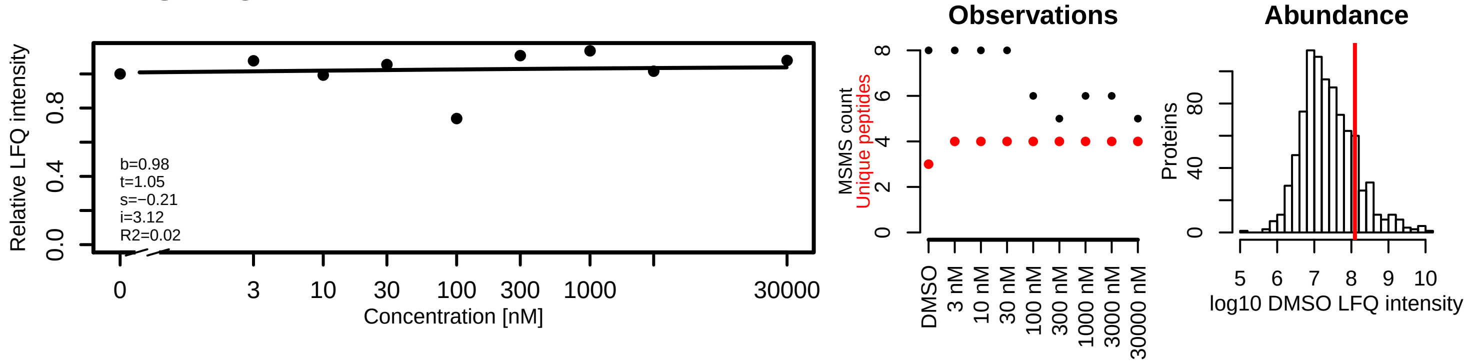

GART

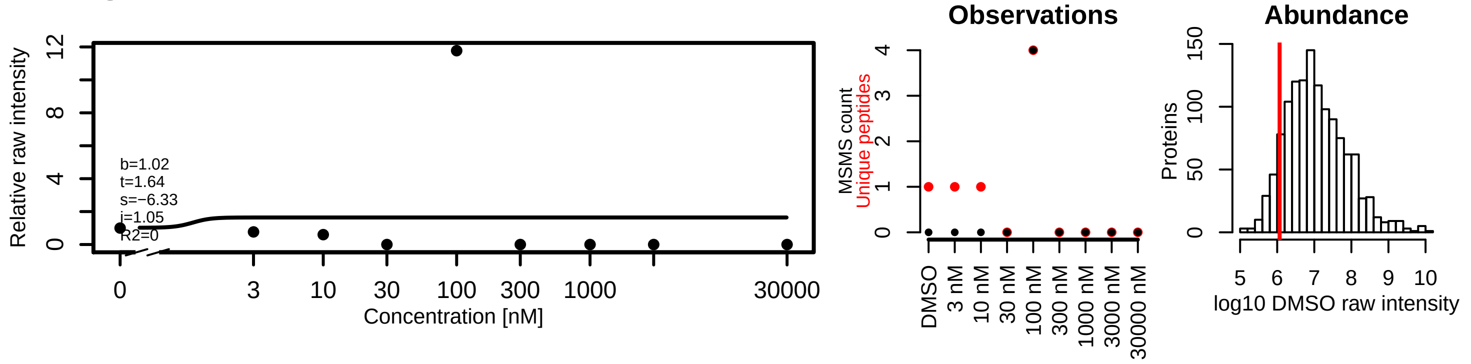

EIF4A1;EIF4A2

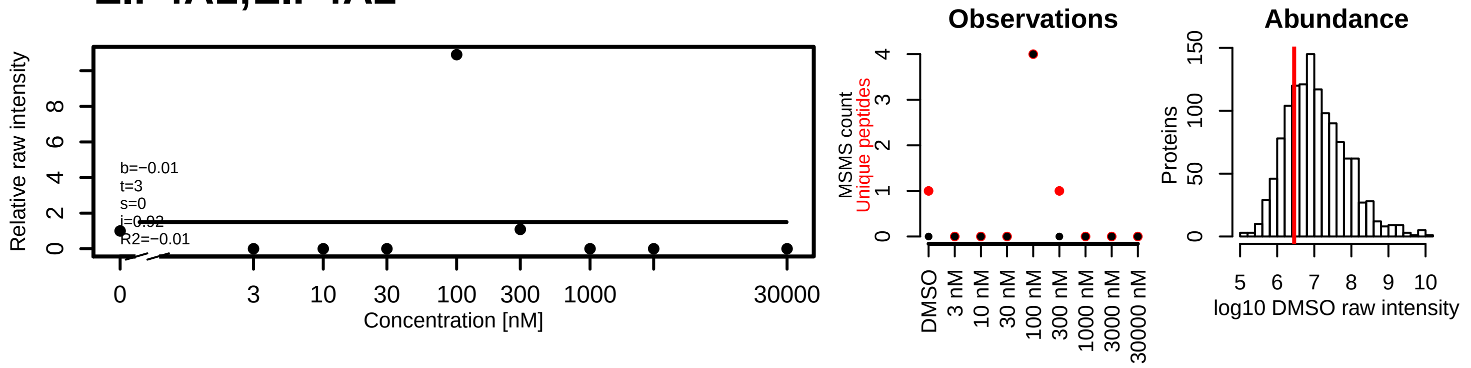

PKM

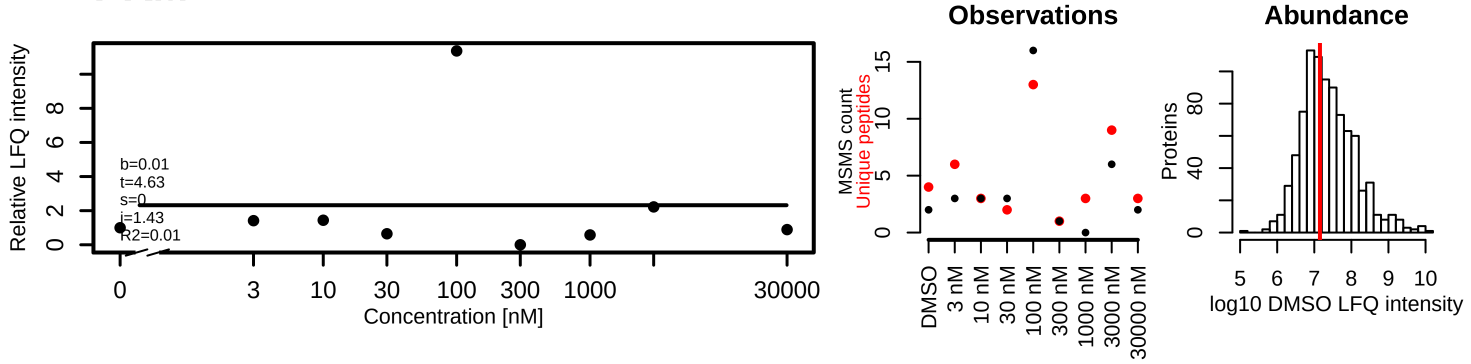

LGALS1

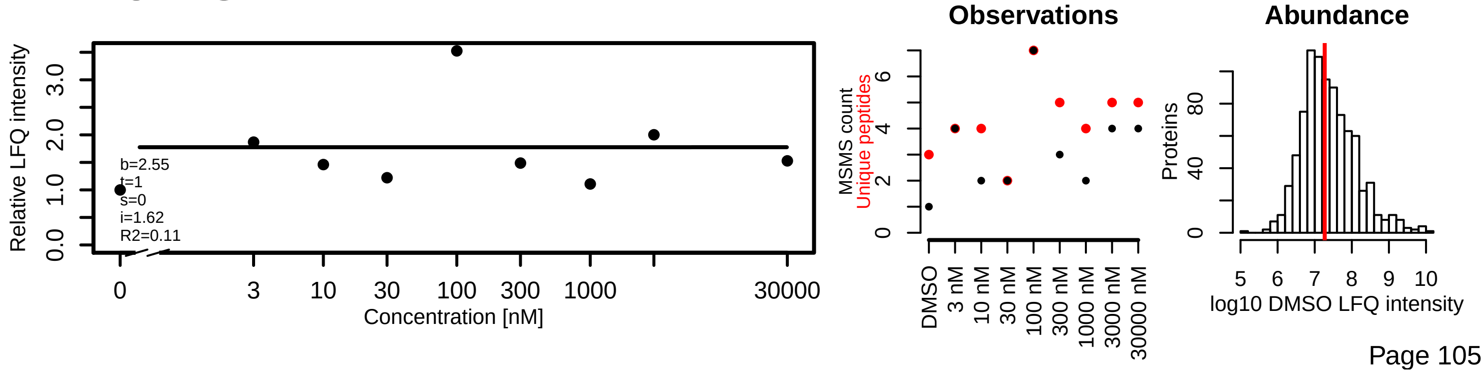

VCP

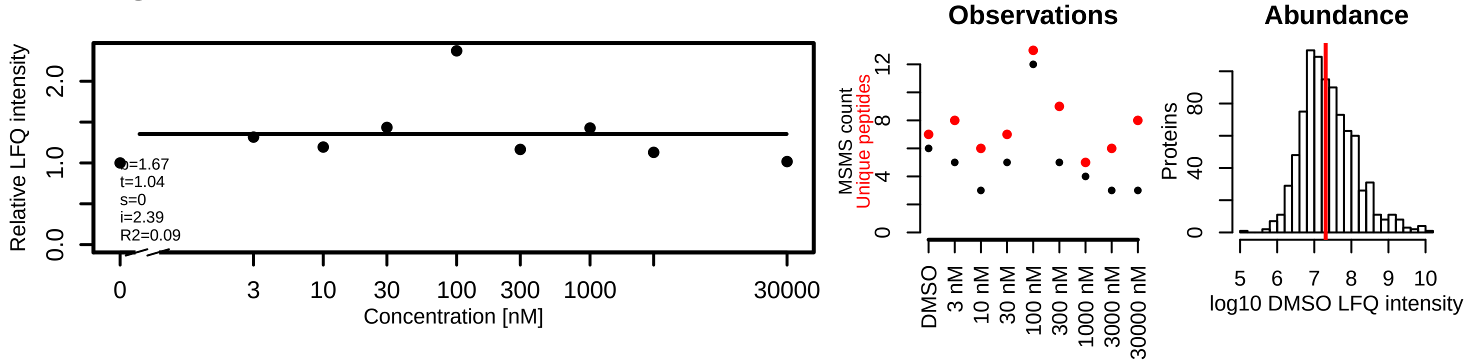

MIF

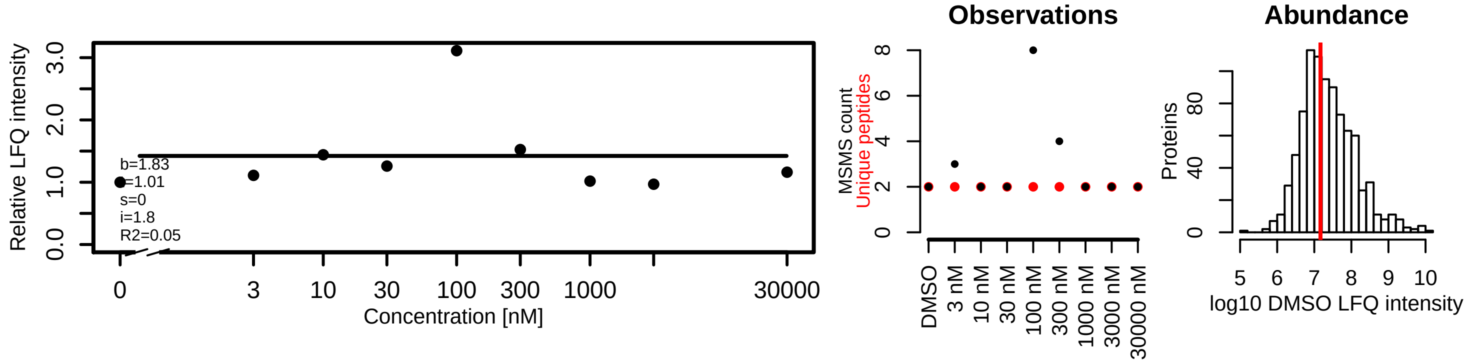

CCT6A

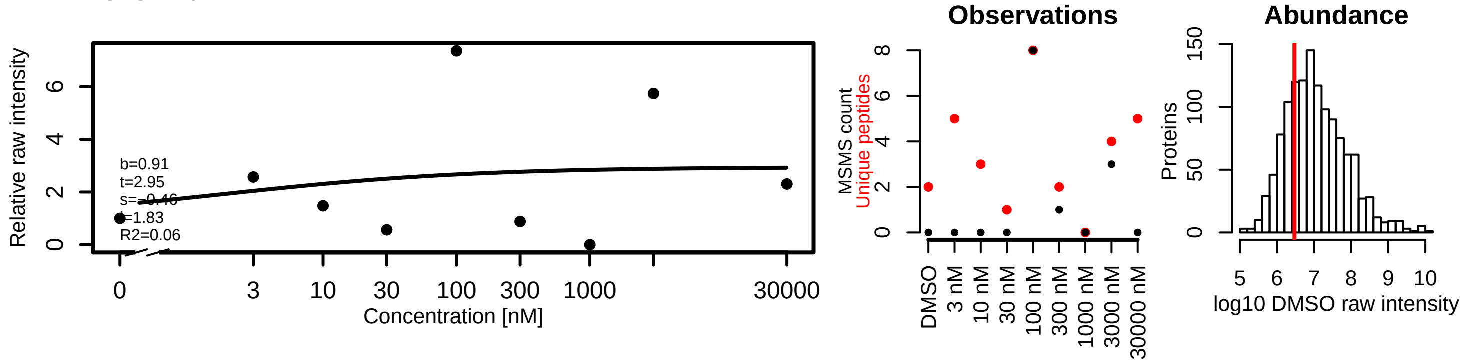

OGDH

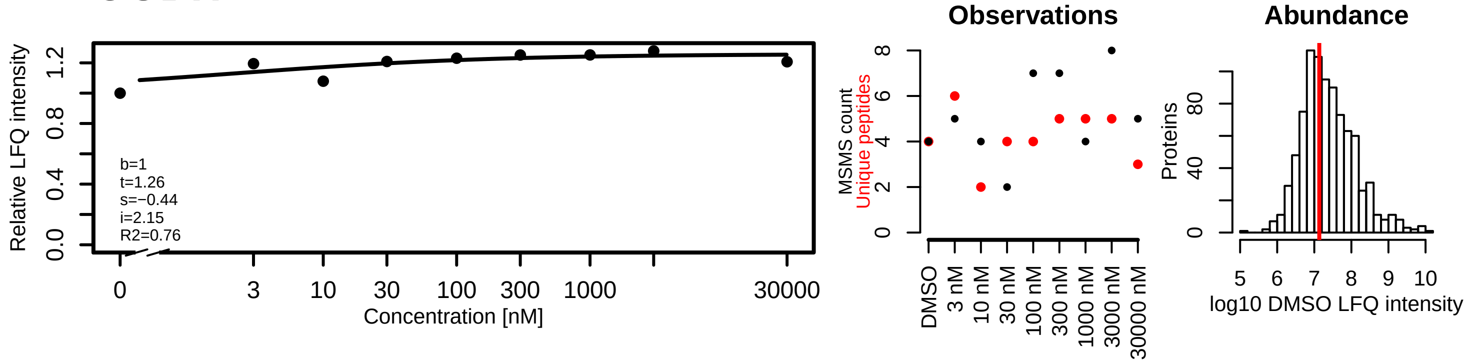

CCT3

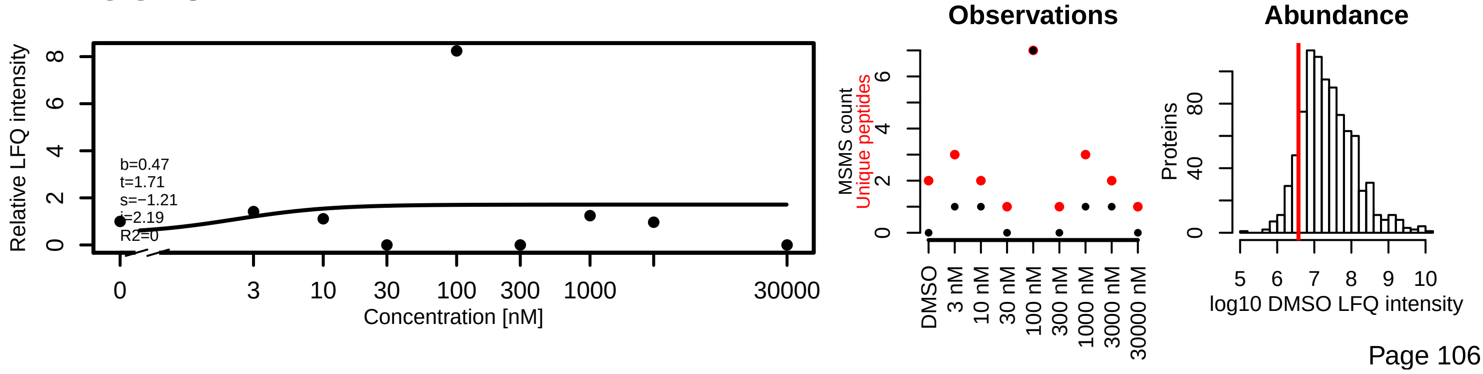

RPL24

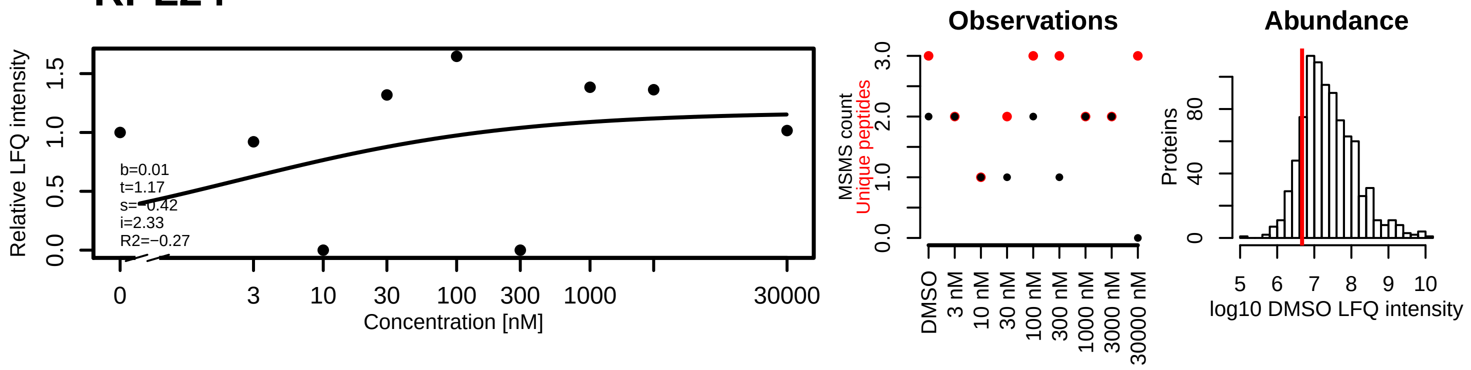

CAV1 − Q03135−2;C9JKI3

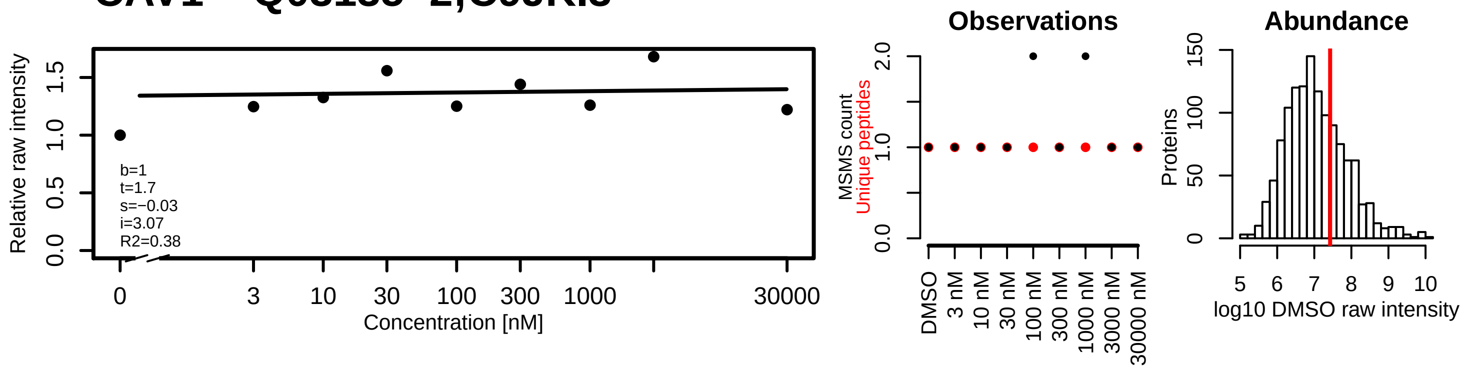

RPL8

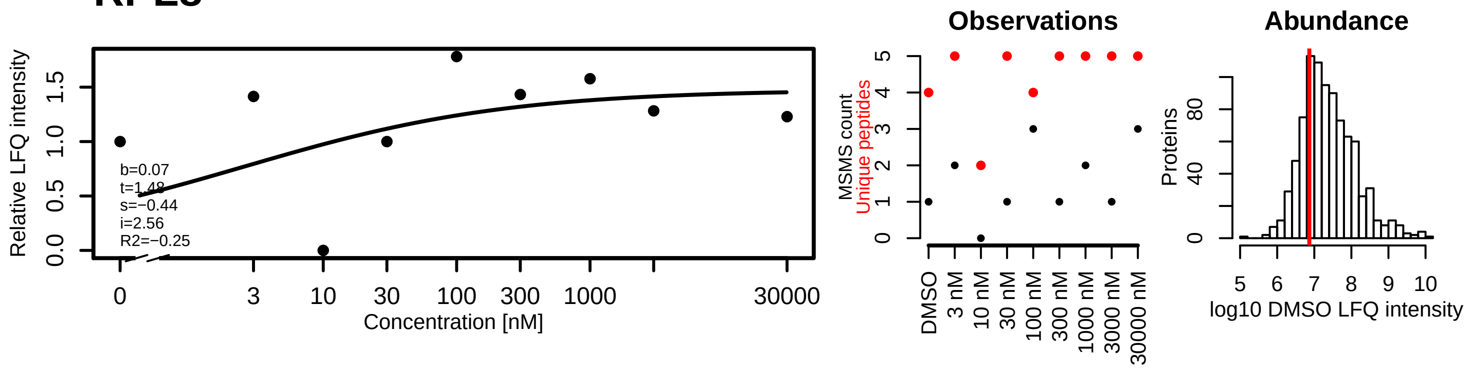

AKR1B1

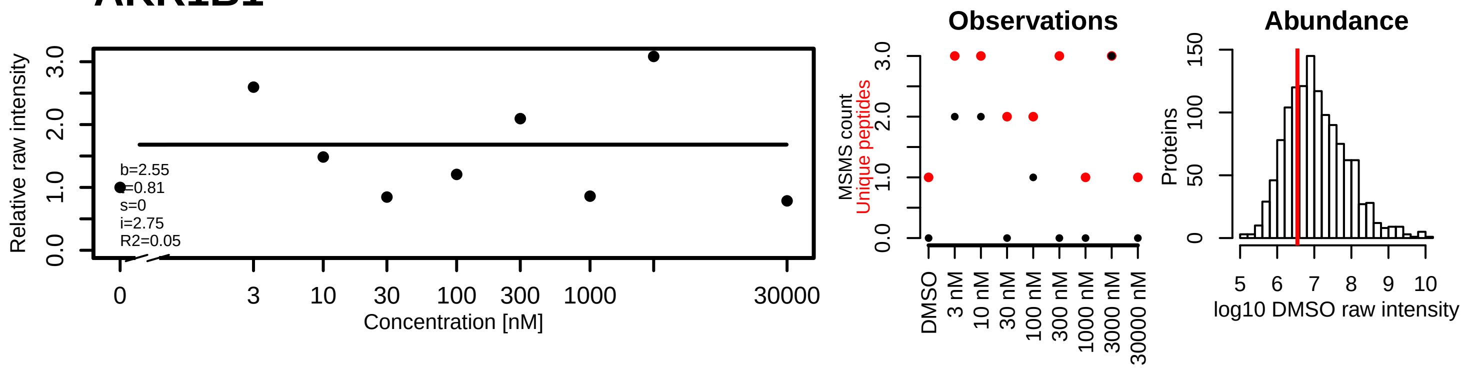

GAPDH

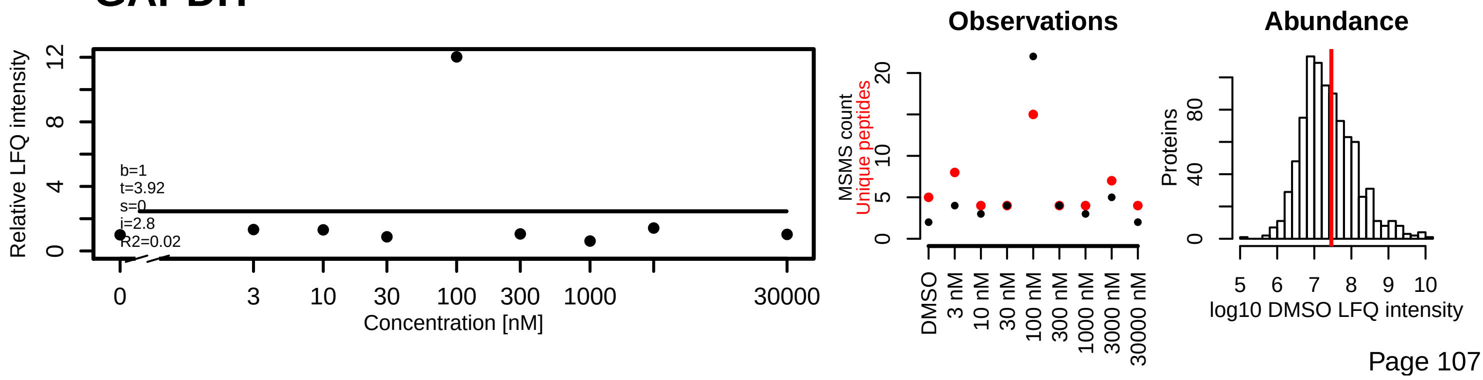

LDHA

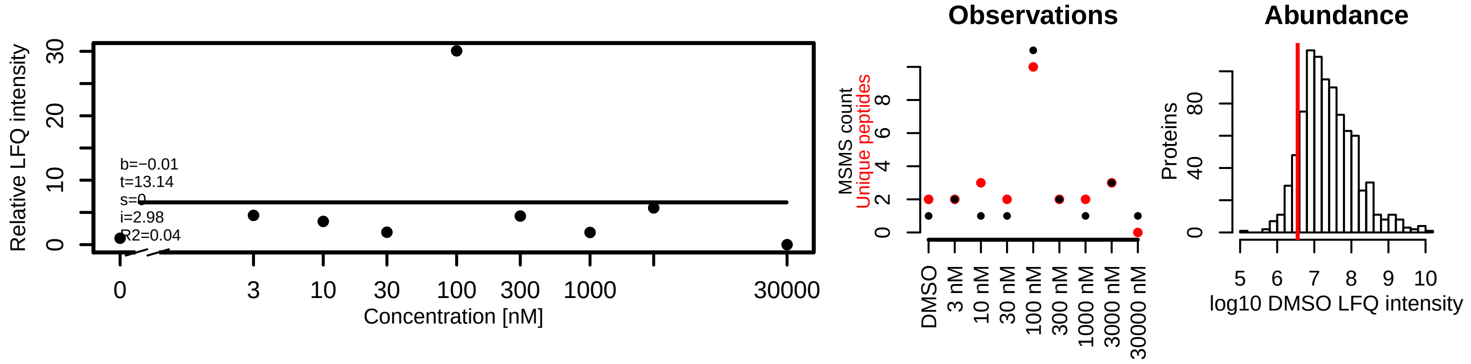

UBA52;UBB;RPS27A;UBC;UBBP4

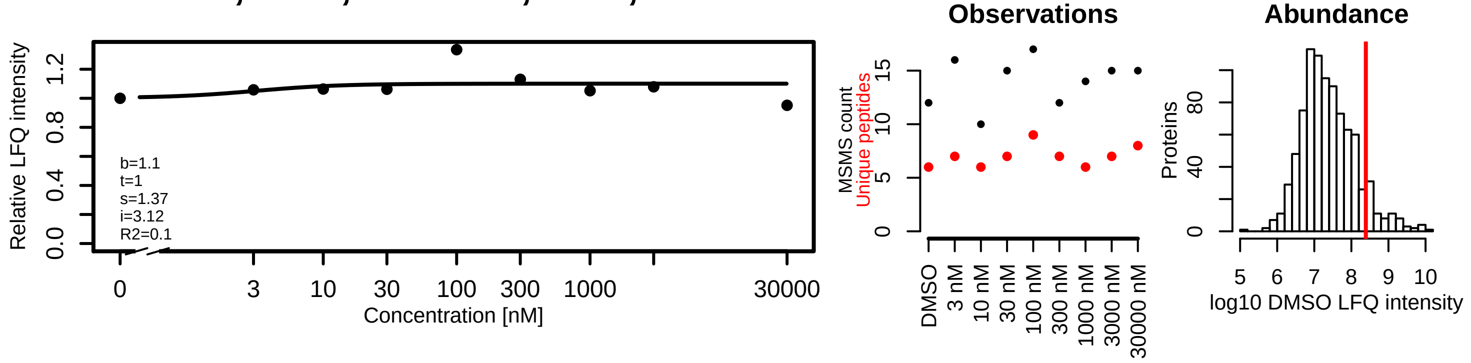

HSP90B1

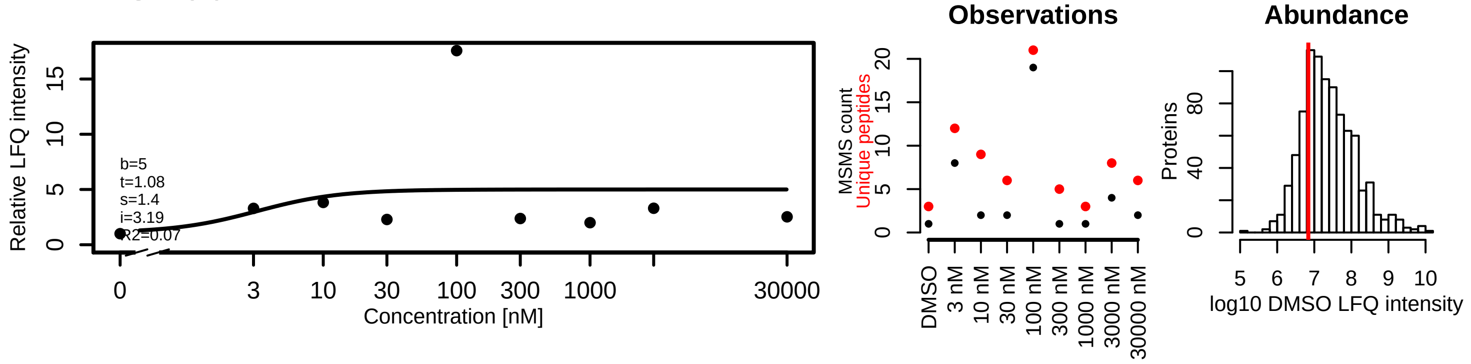

UQCRC2

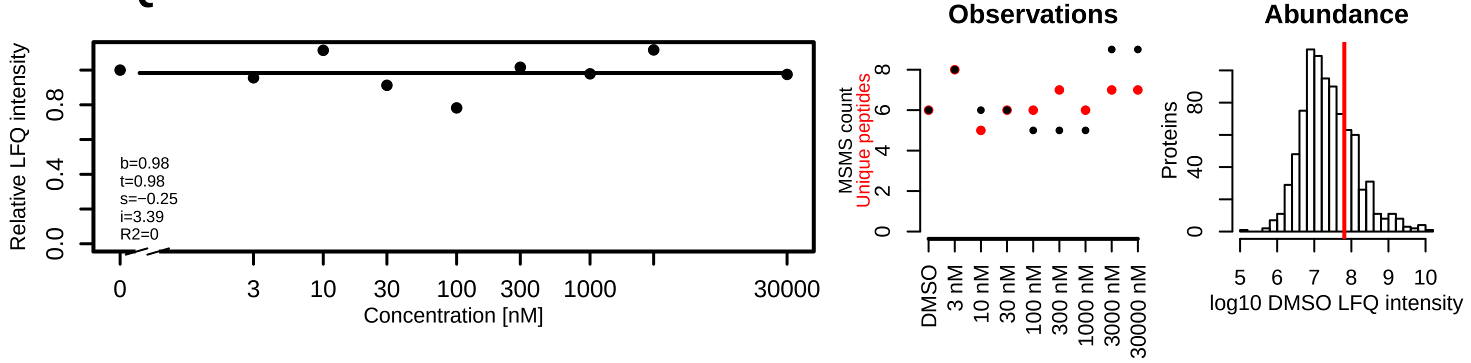

RPS25

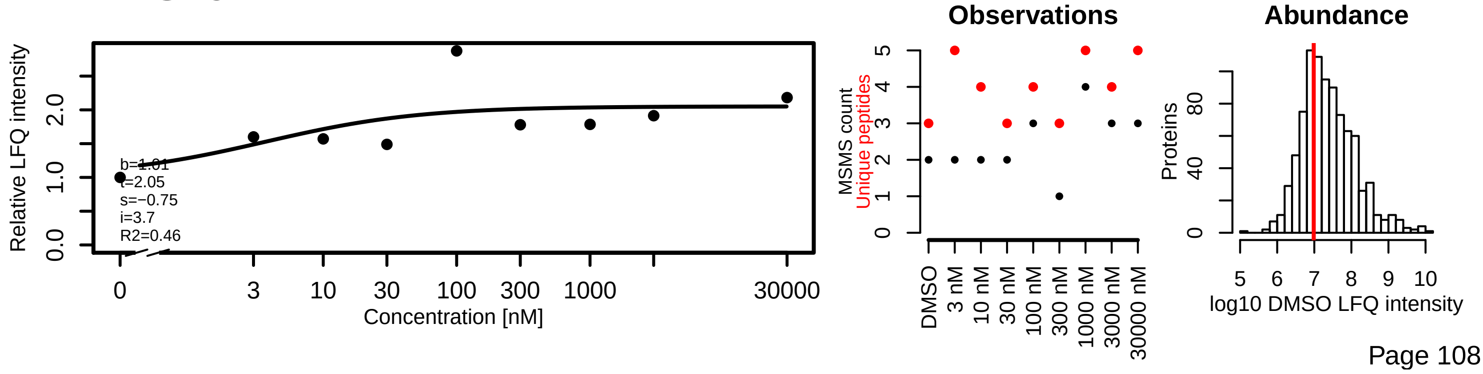

EPRS

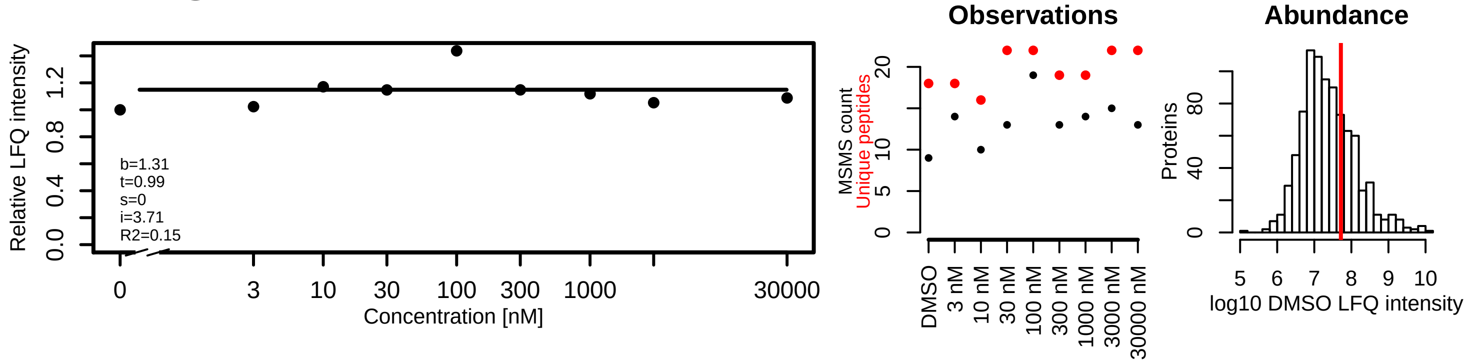

HSP90AB1

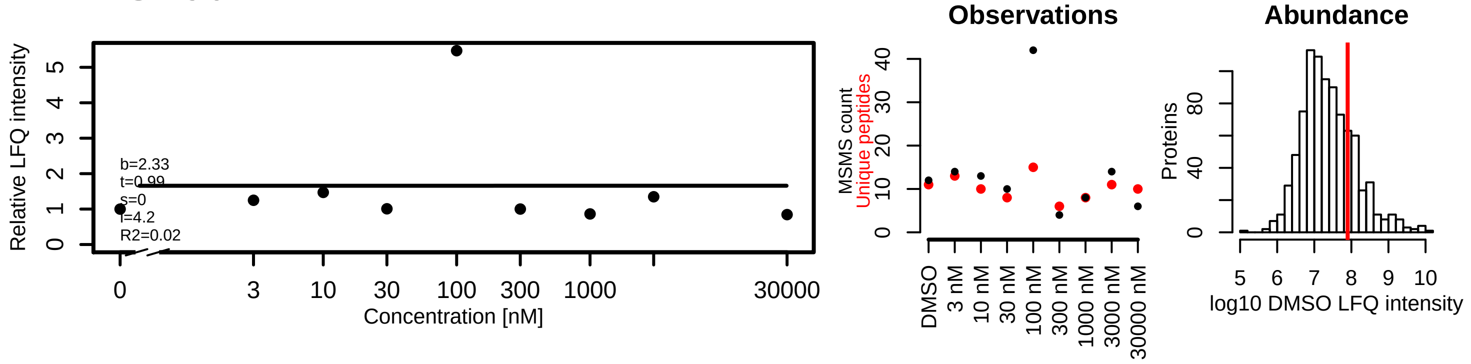

PLEC

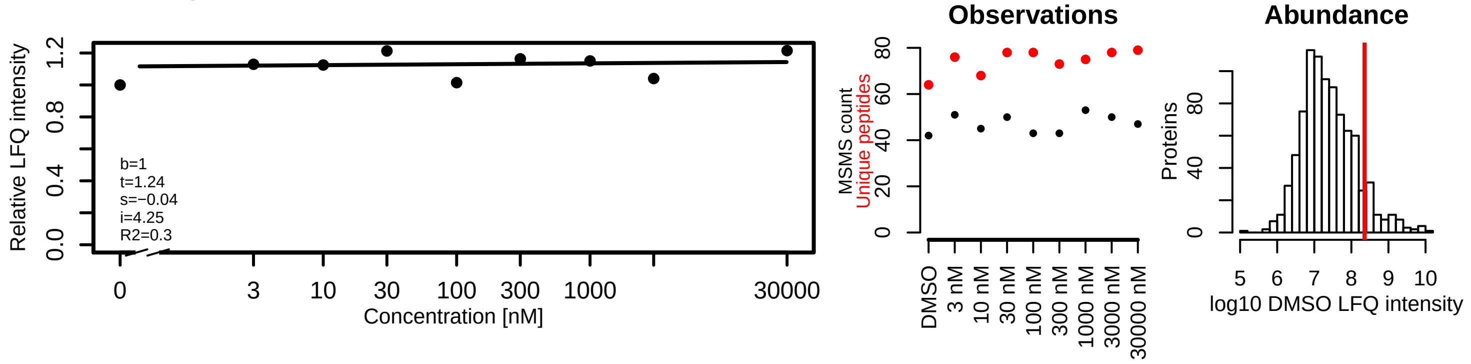

RPS19

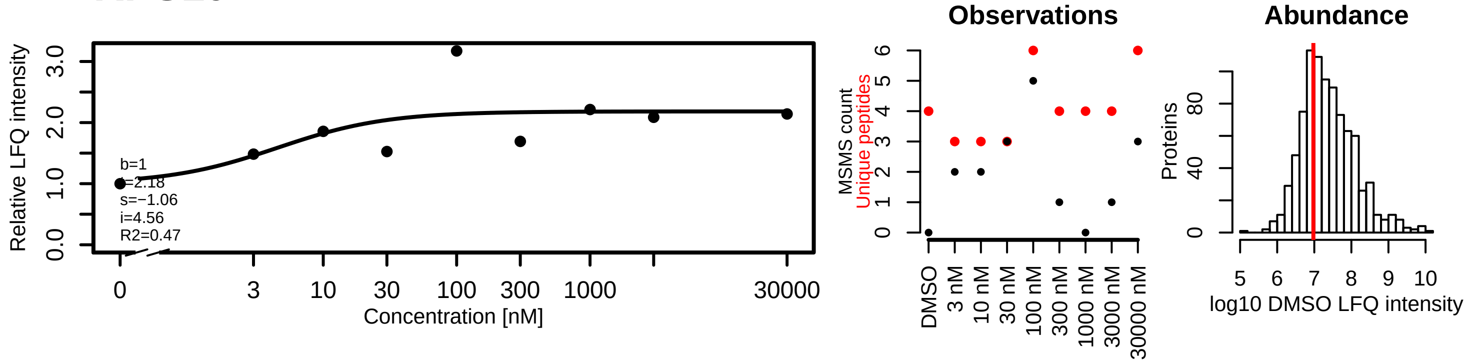

IARS

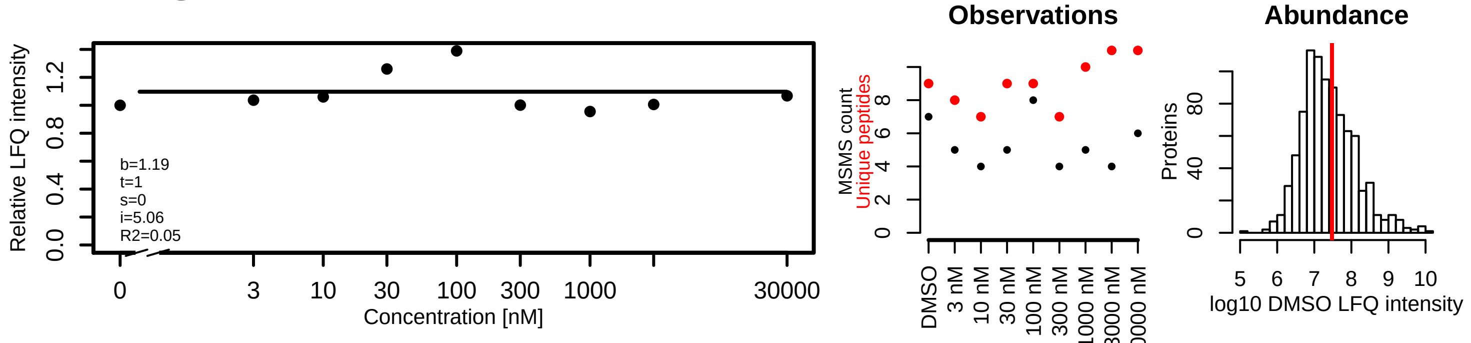

RPL11

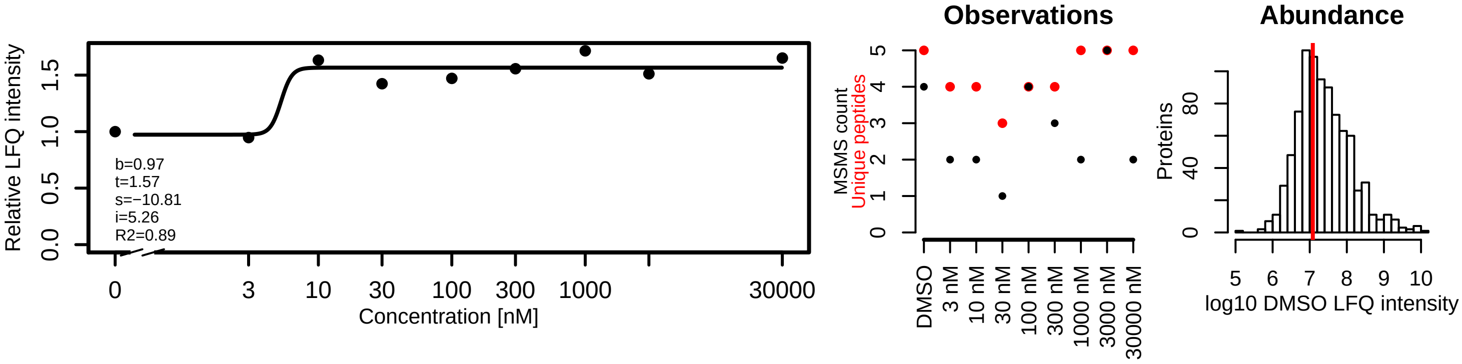

UBE3C

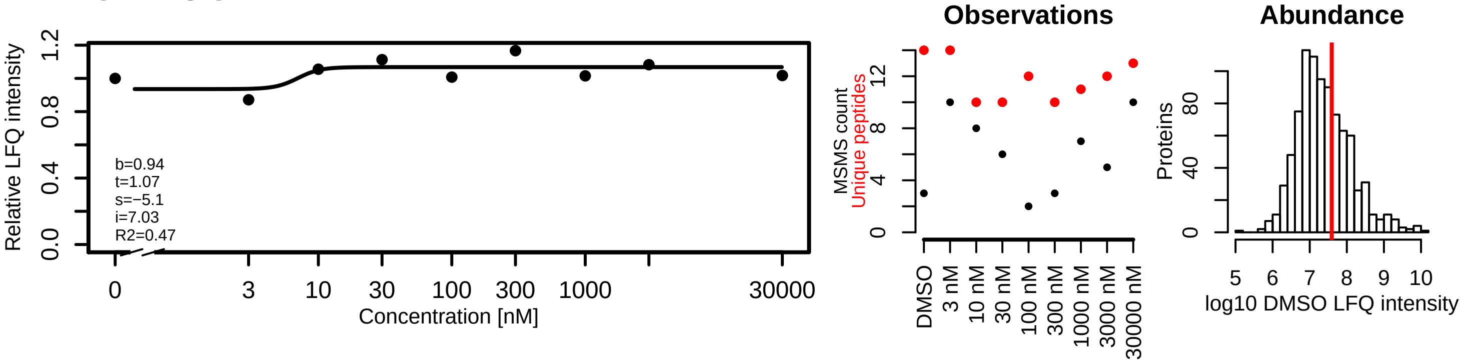

COX5A

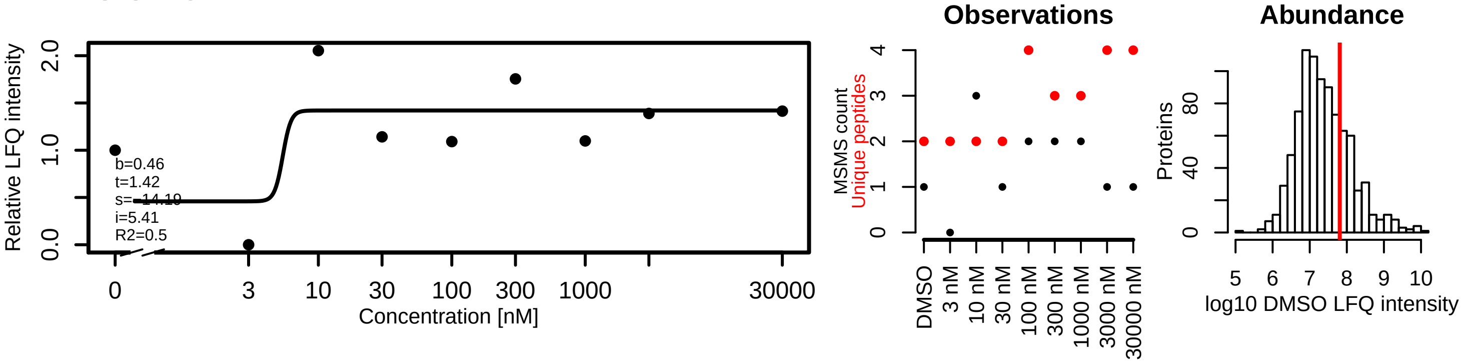

TRIM59

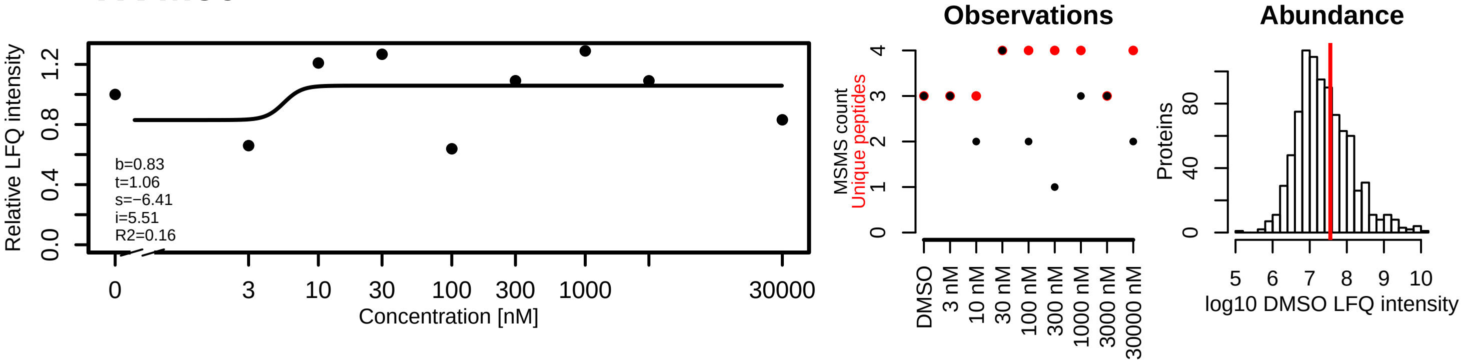

PTCD1;ATP5J2−PTCD1

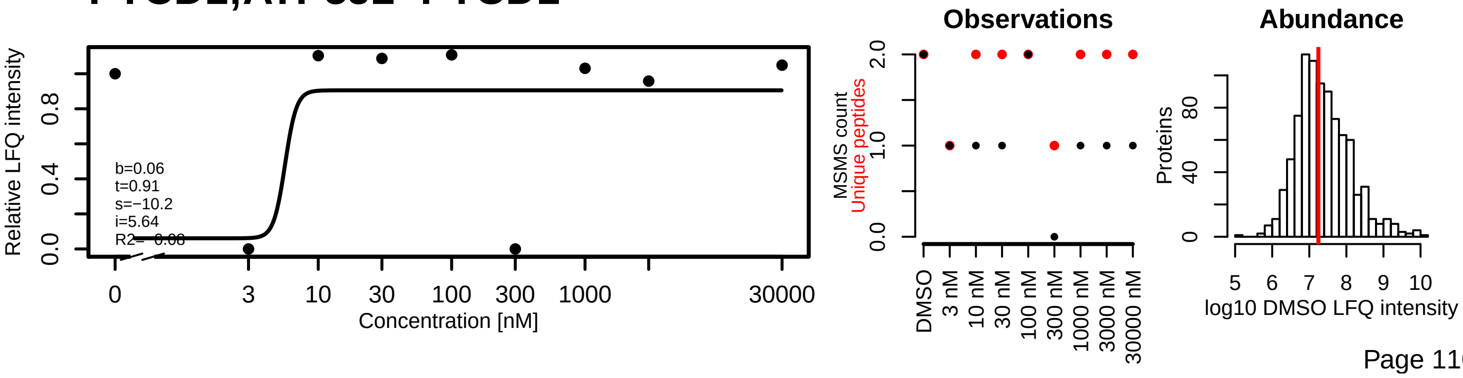

RPS16

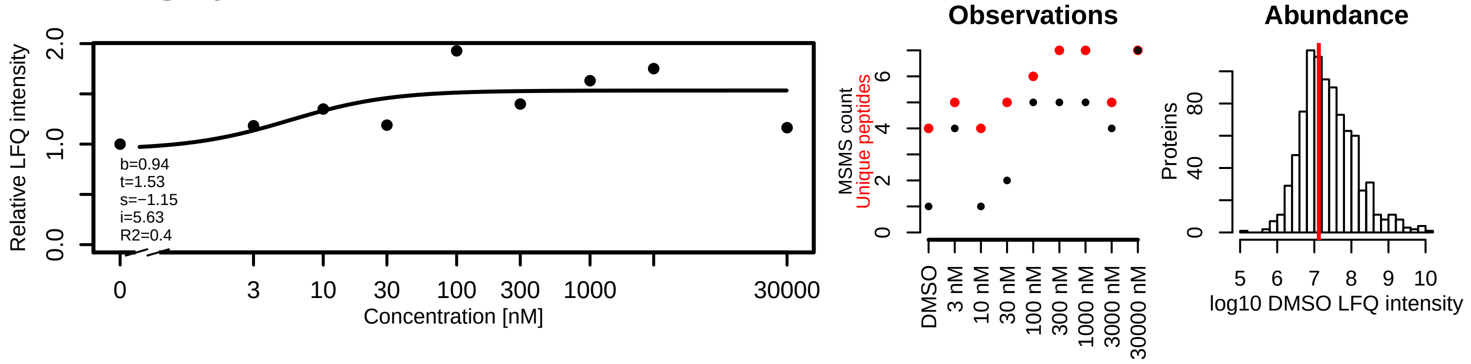

UBR4

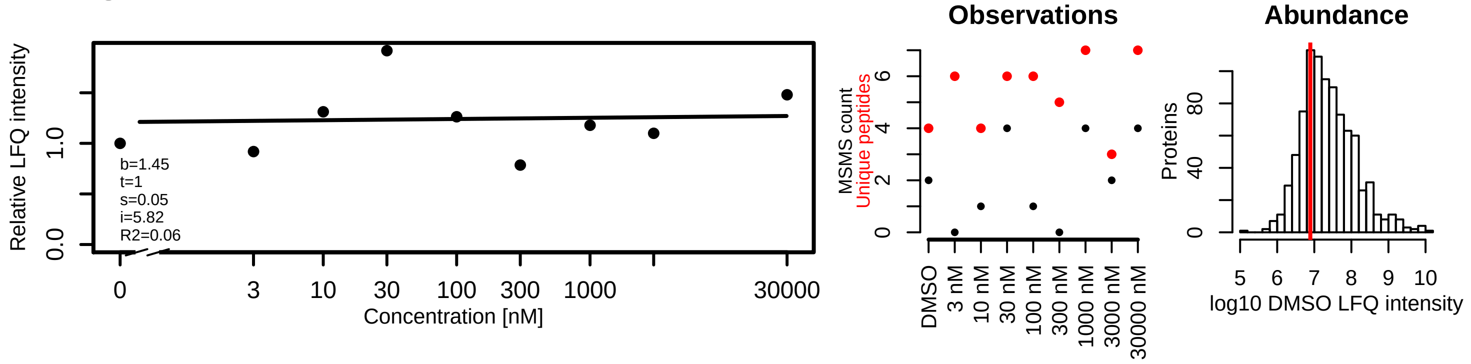

RUVBL2

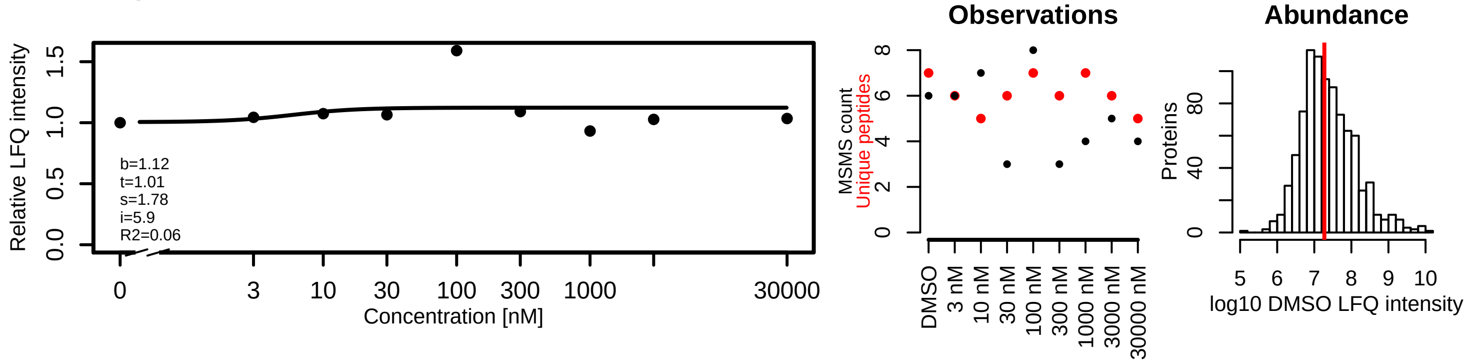

CMTM7

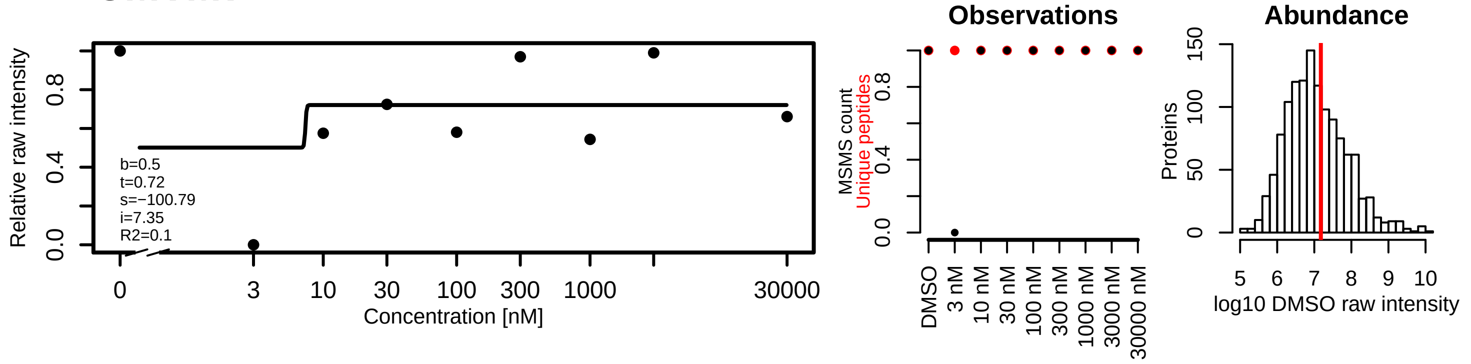

RPLP2

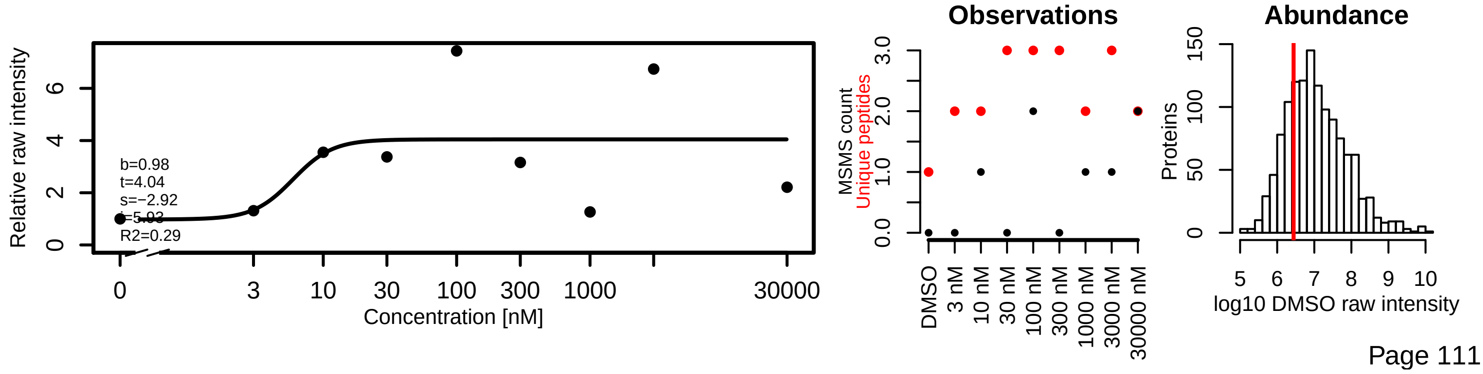

NDUFS6

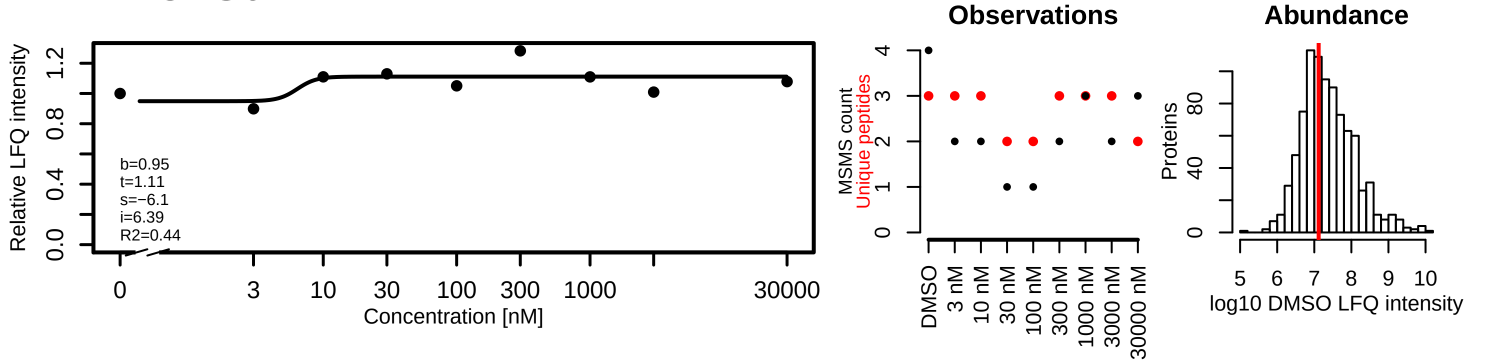

RPL17

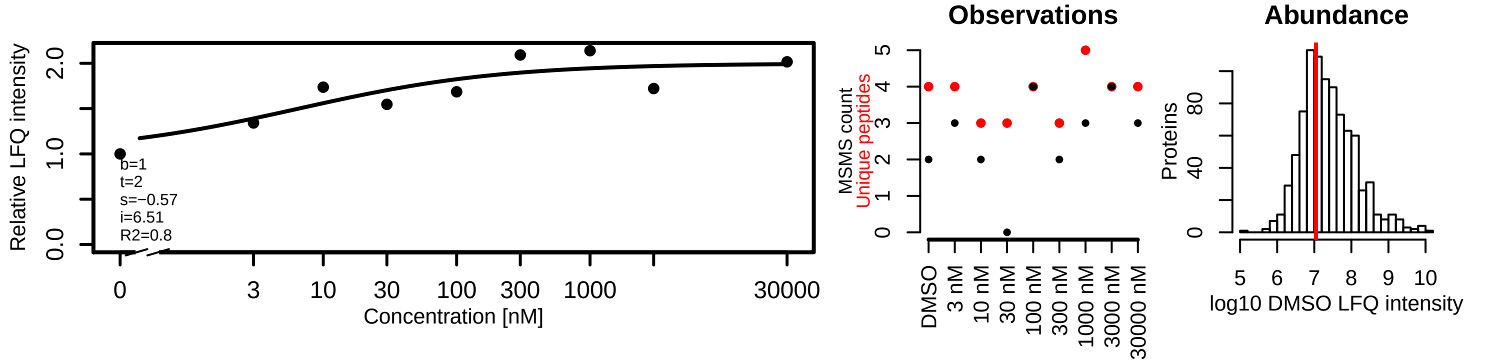

ALDOA

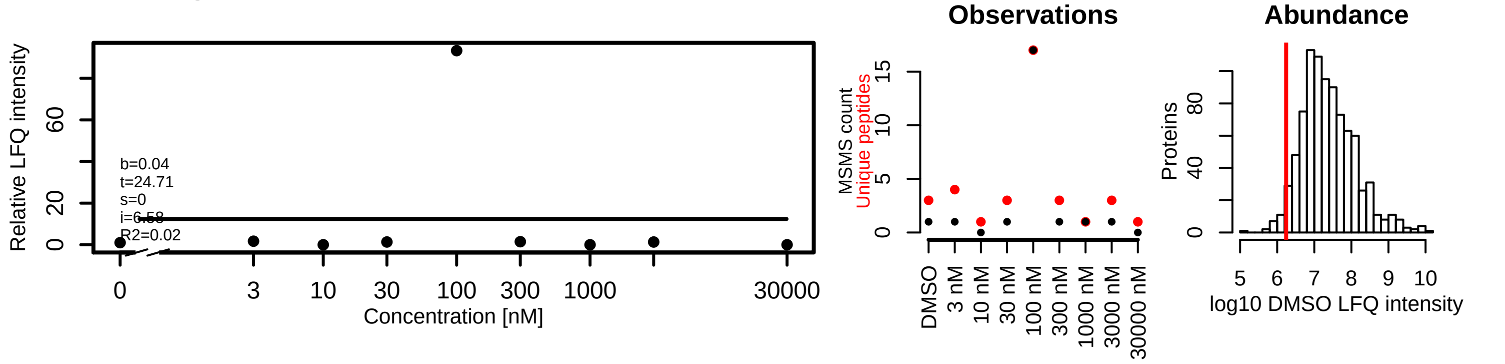

DDX17

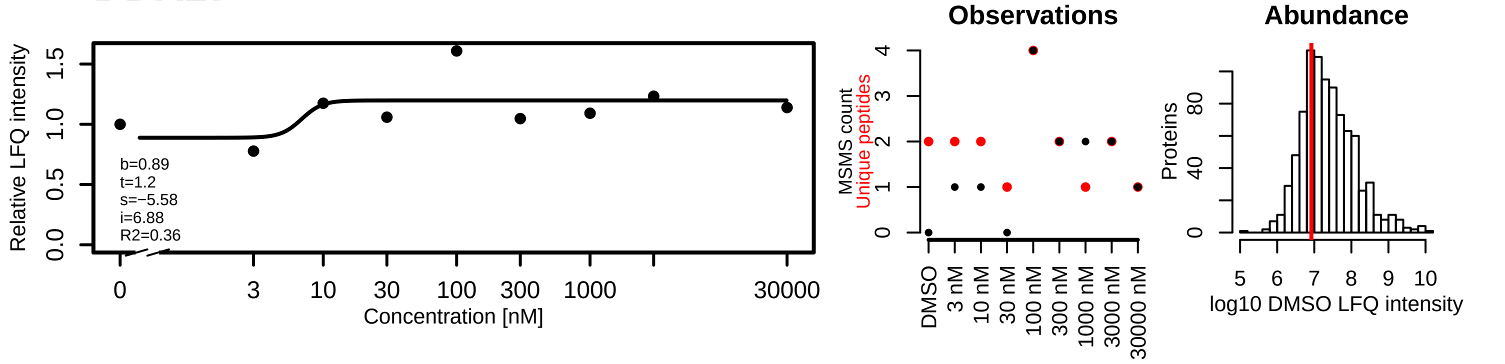

OCIAD1

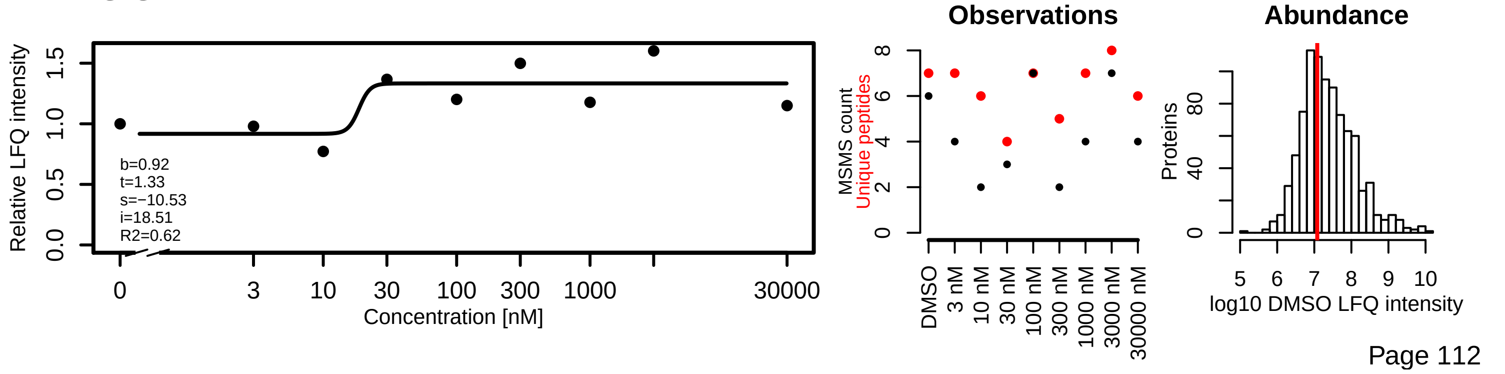

NDUFB9

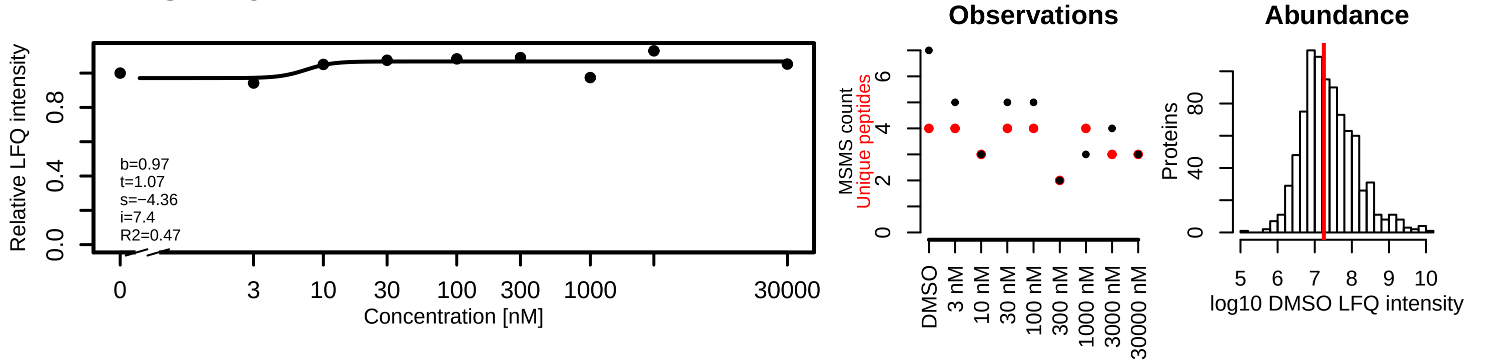

TPI1

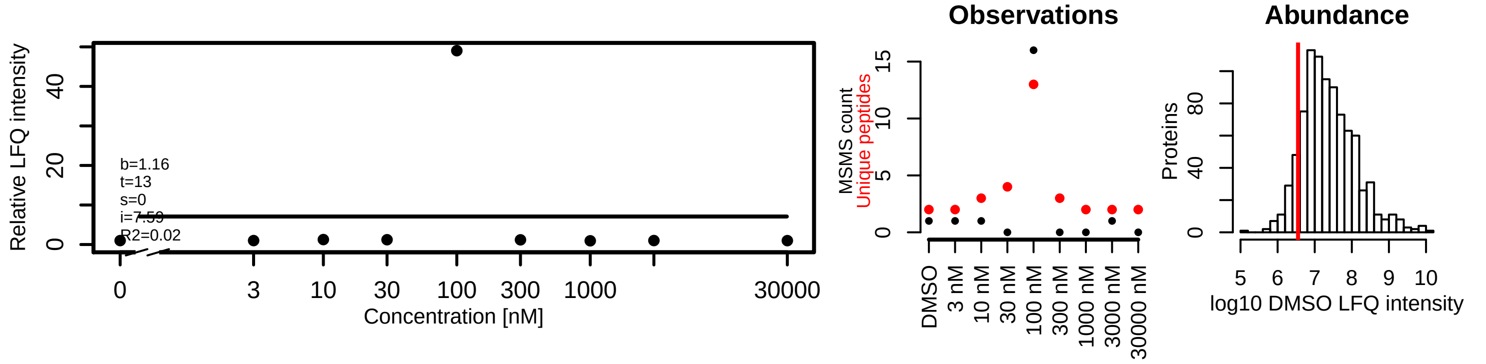

DYNLL1;DYNLL2

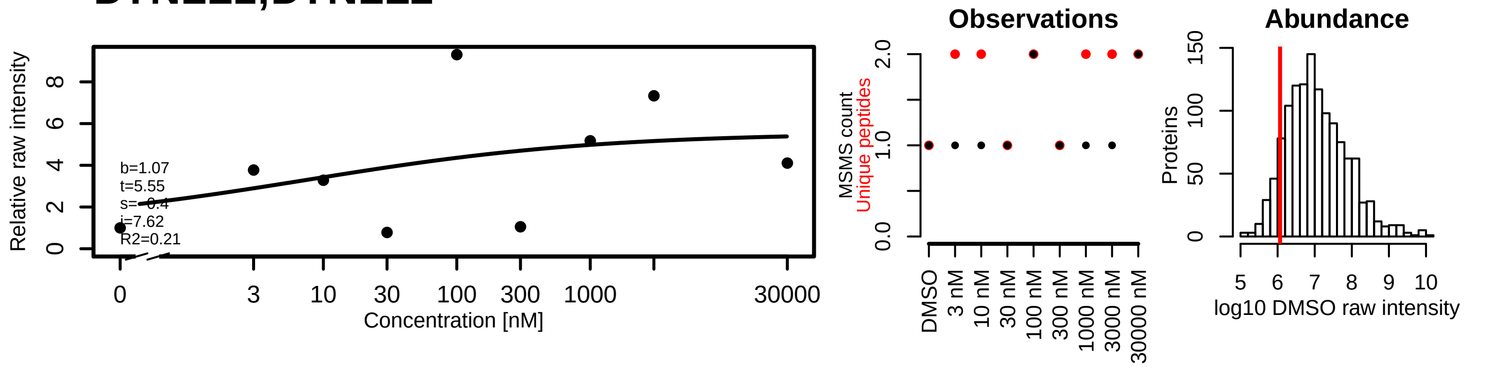

CHCHD2;CHCHD2P9

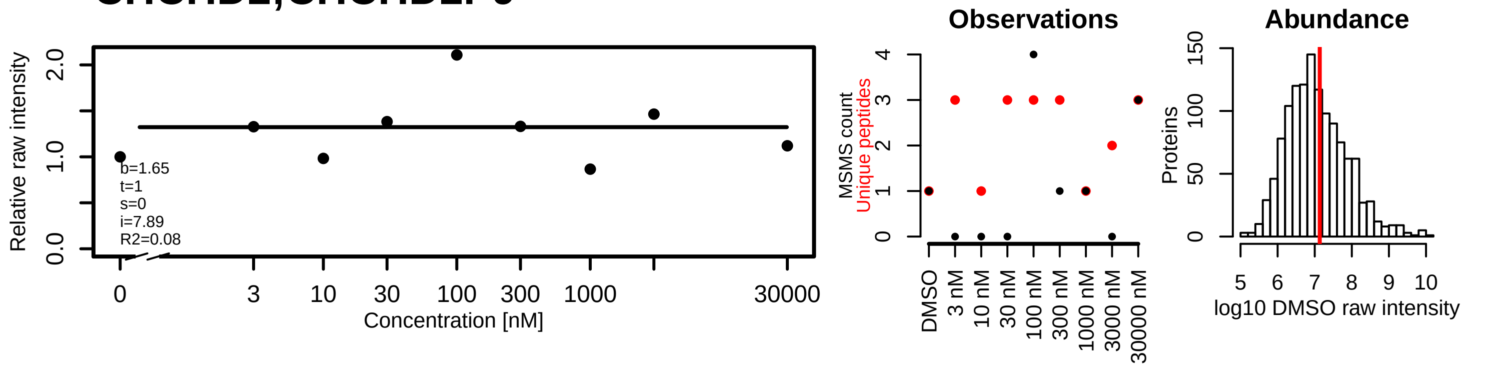

DYNLRB2;DYNLRB1

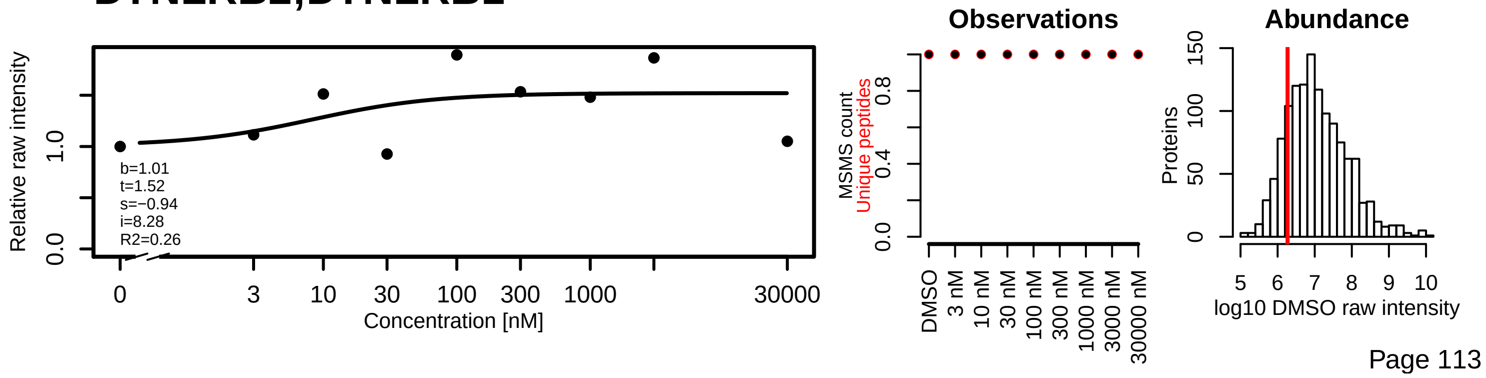

RQCD1

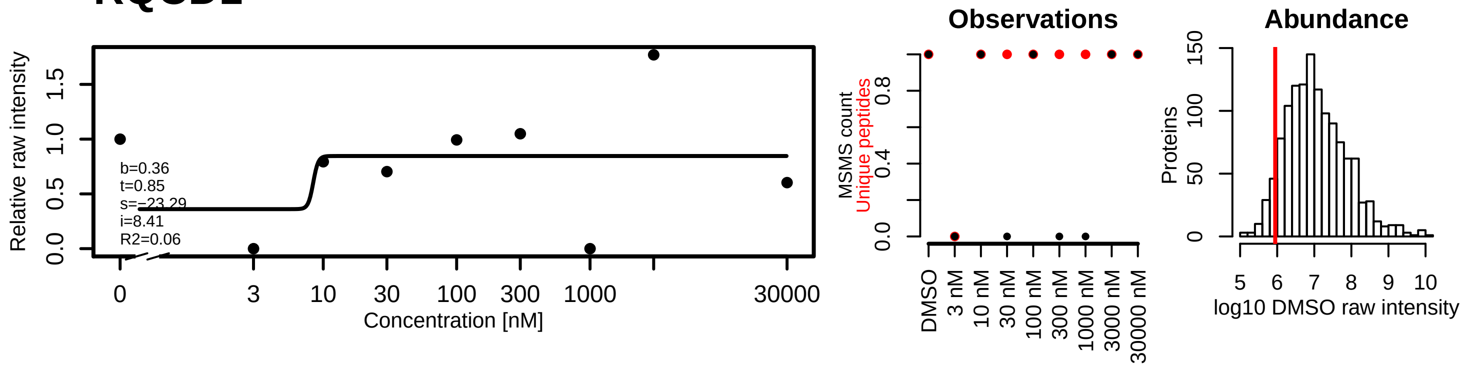

VARS

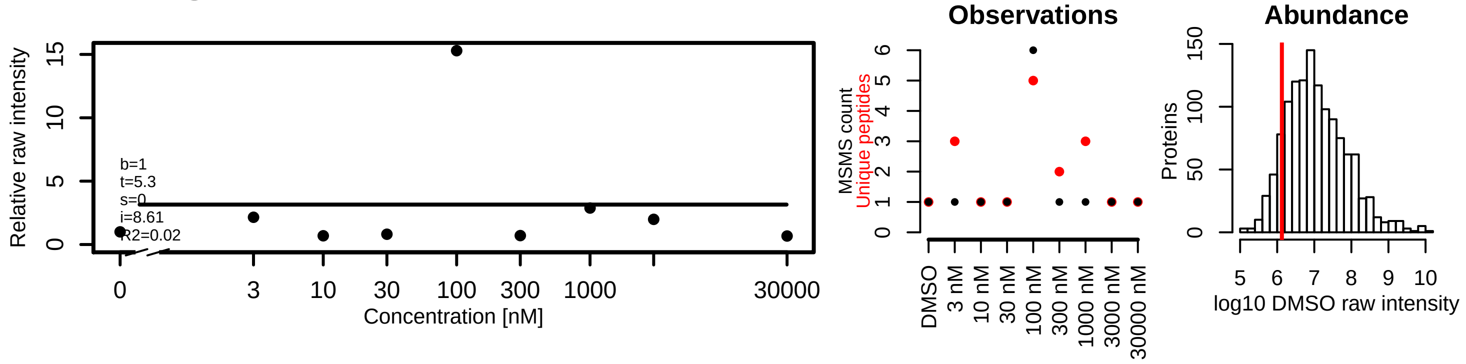

TIMM44

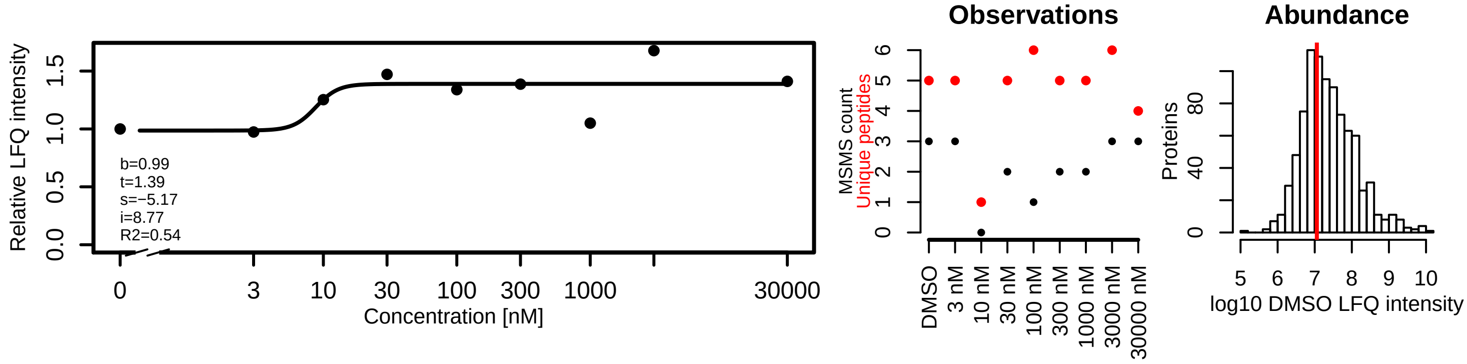

EZR

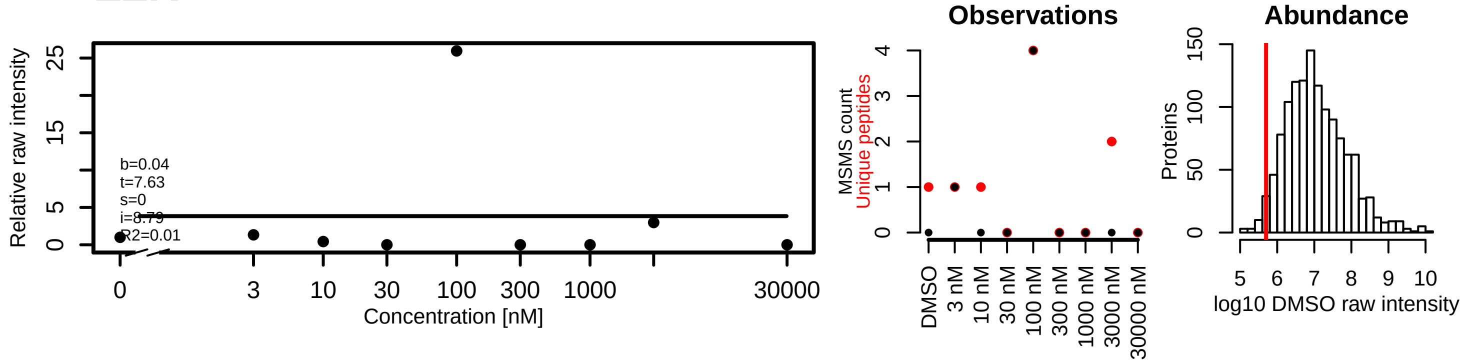

ATG9A

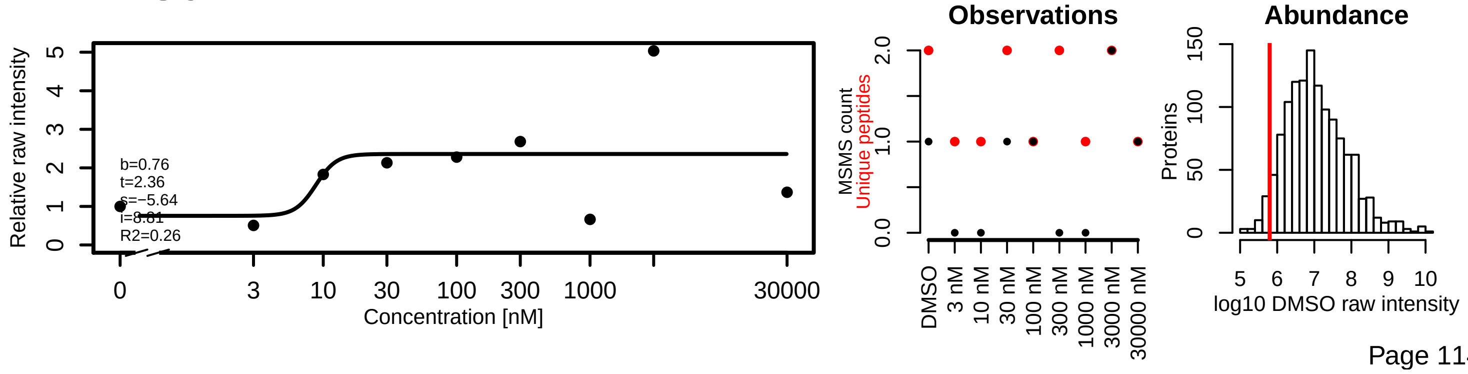

CLN8

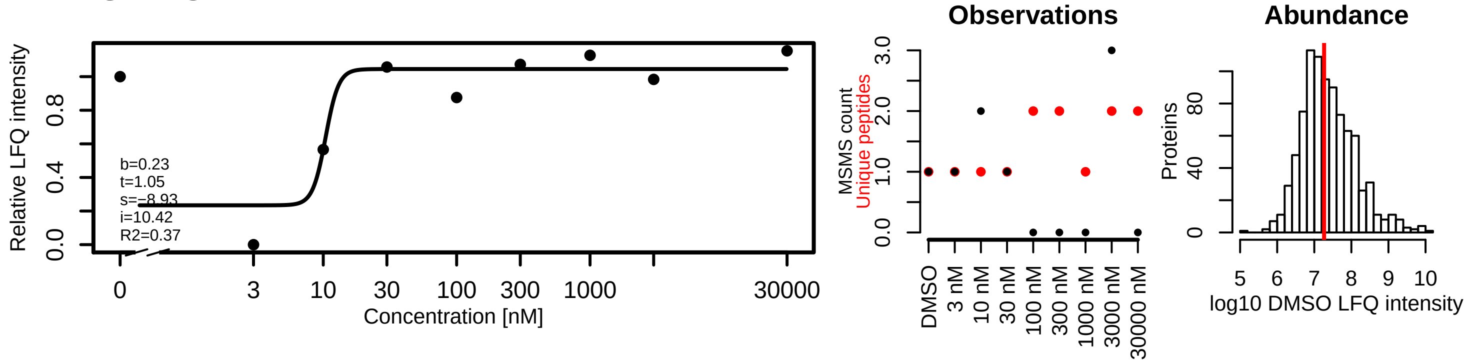

WNT5A

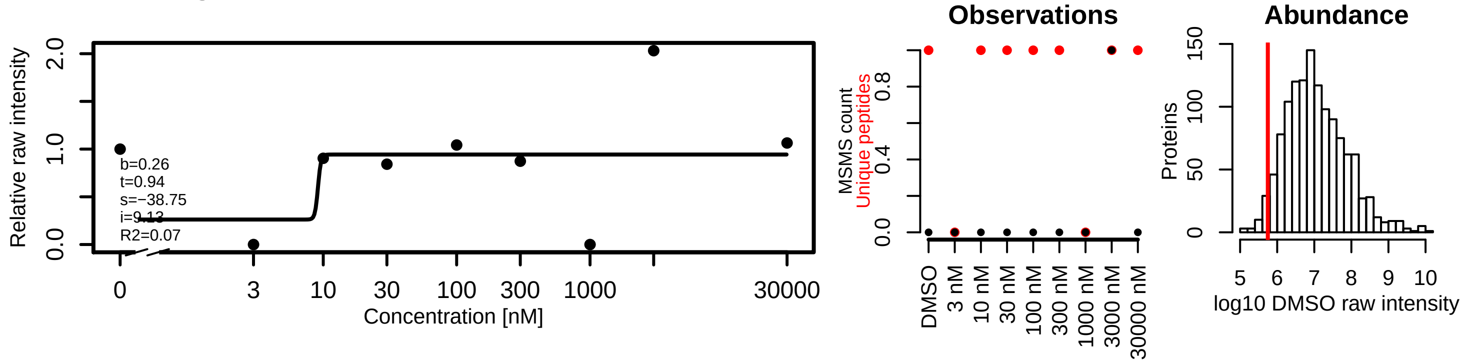

C16orf58

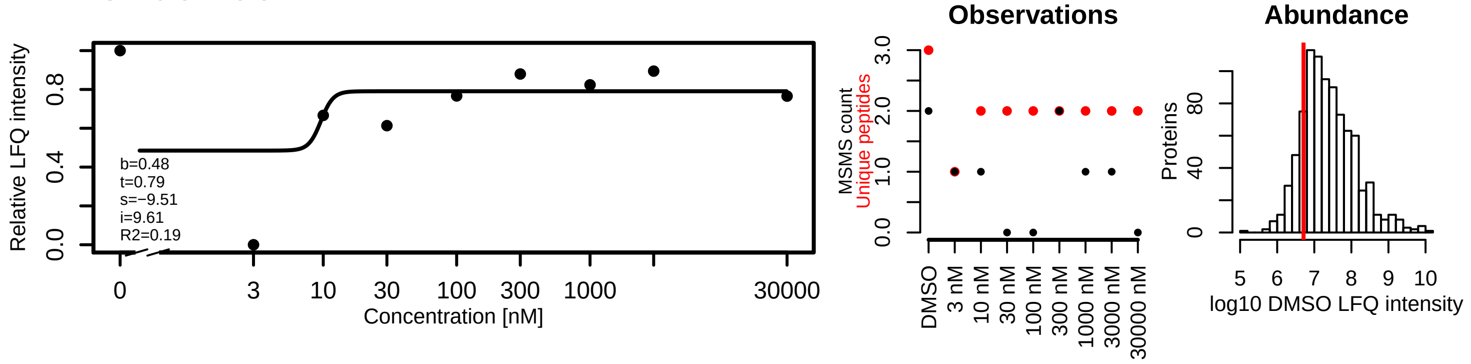

LAMP1

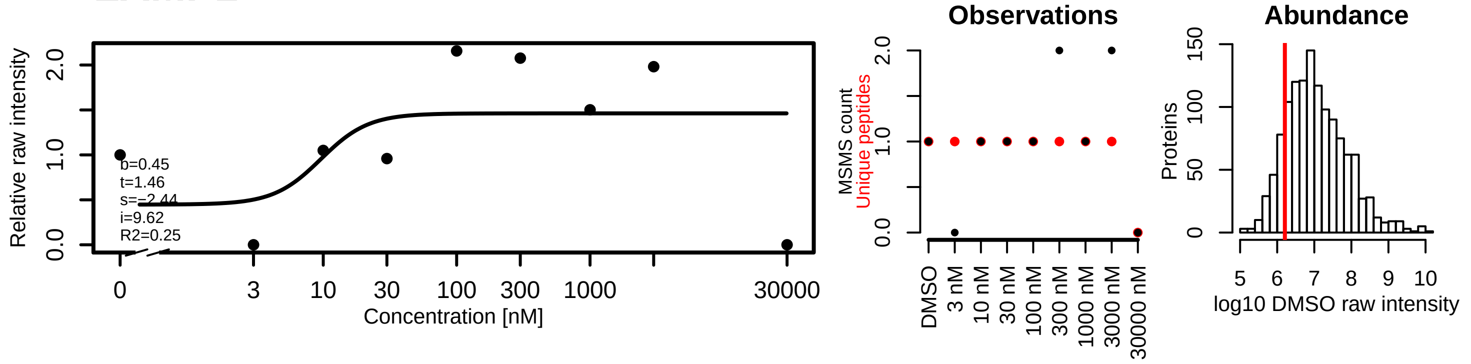

SYNGR2

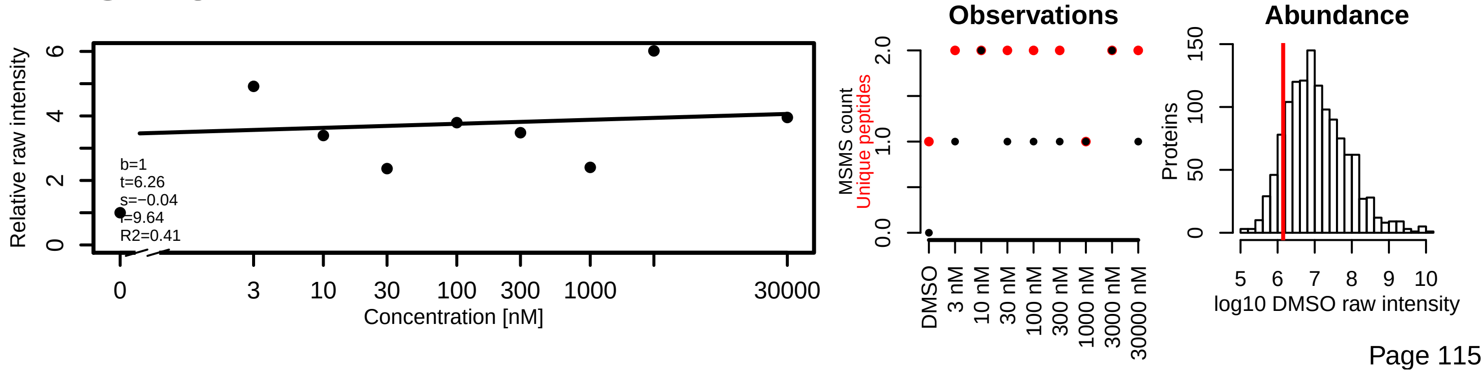

BZW2

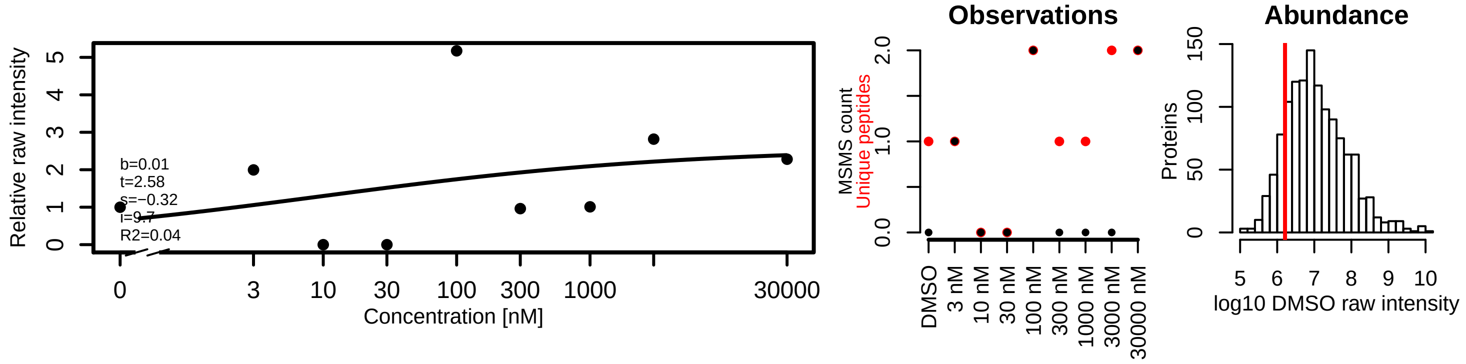

SNRPF

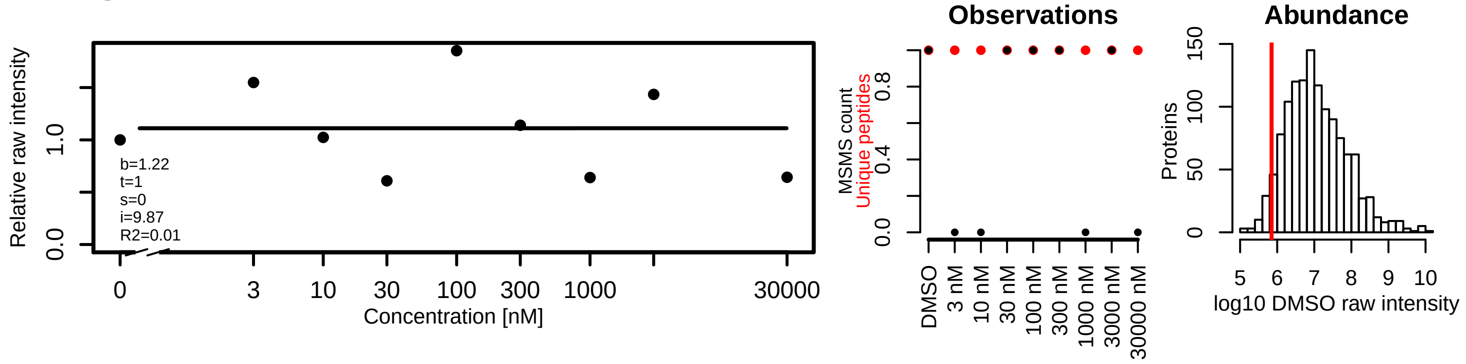

KIAA1033

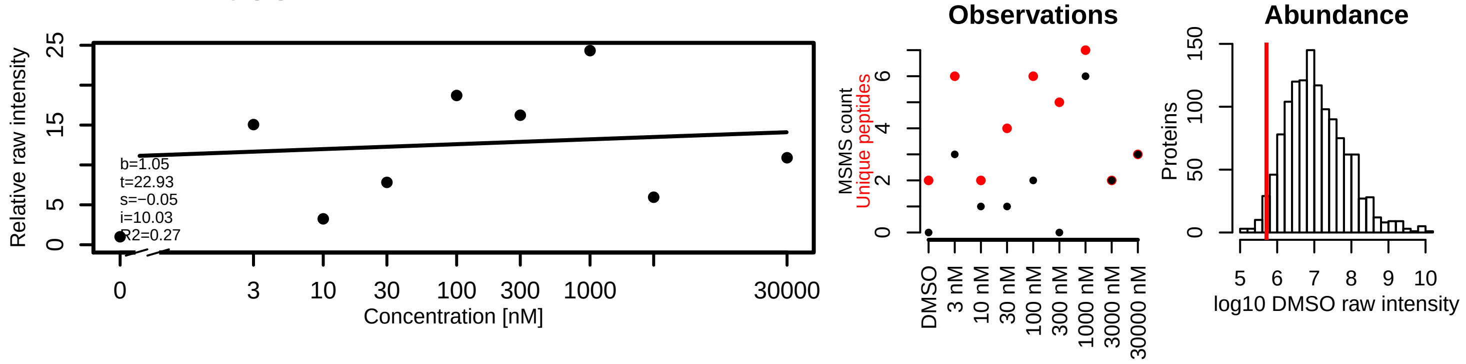

PEX6

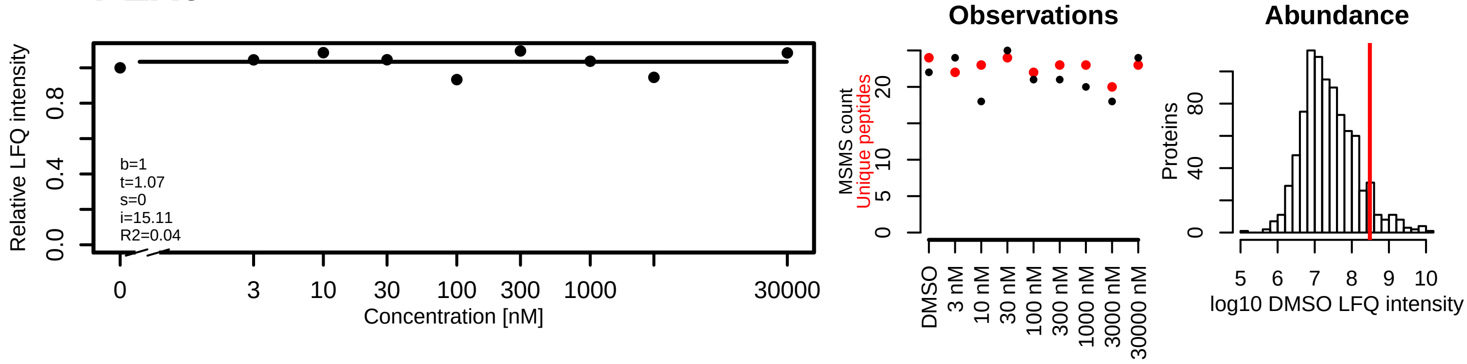

TKT

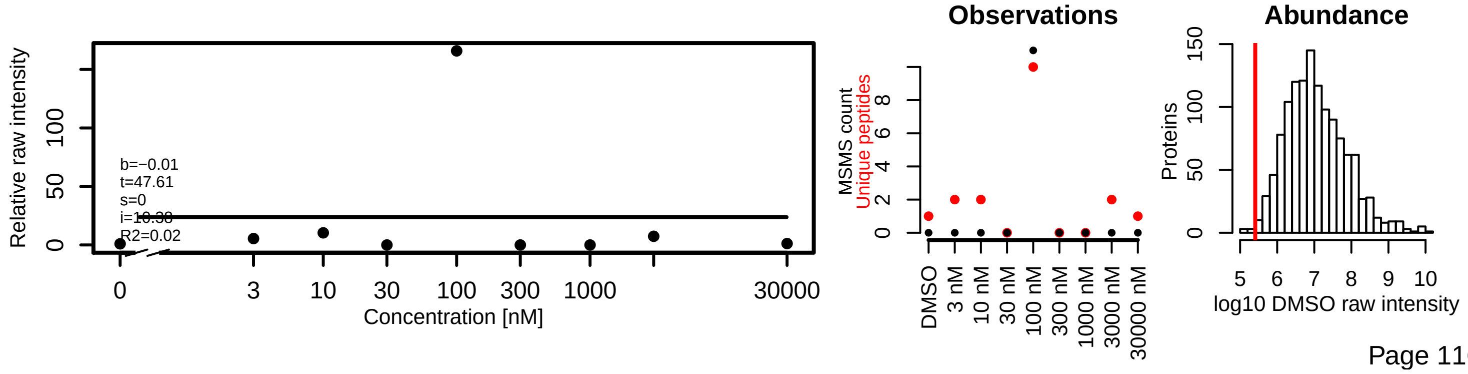

RPS18

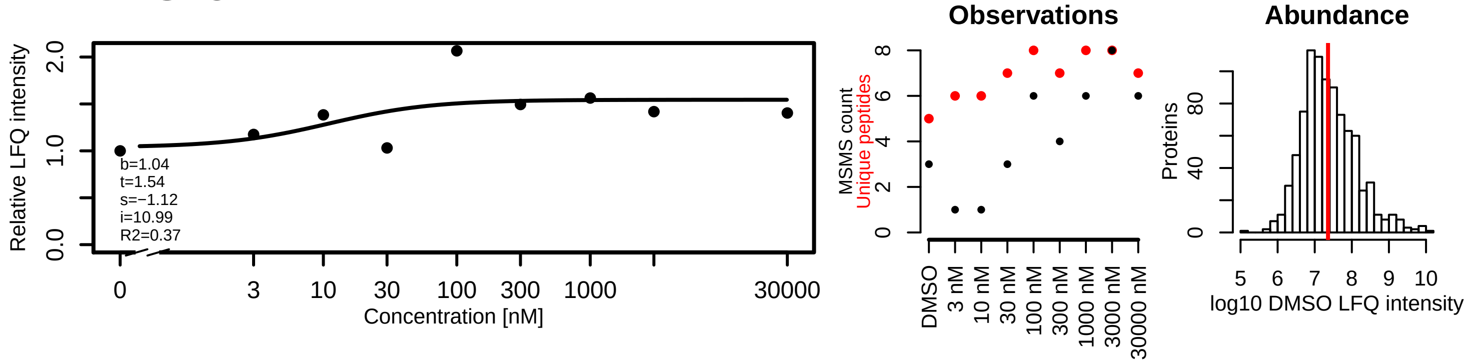

APH1A

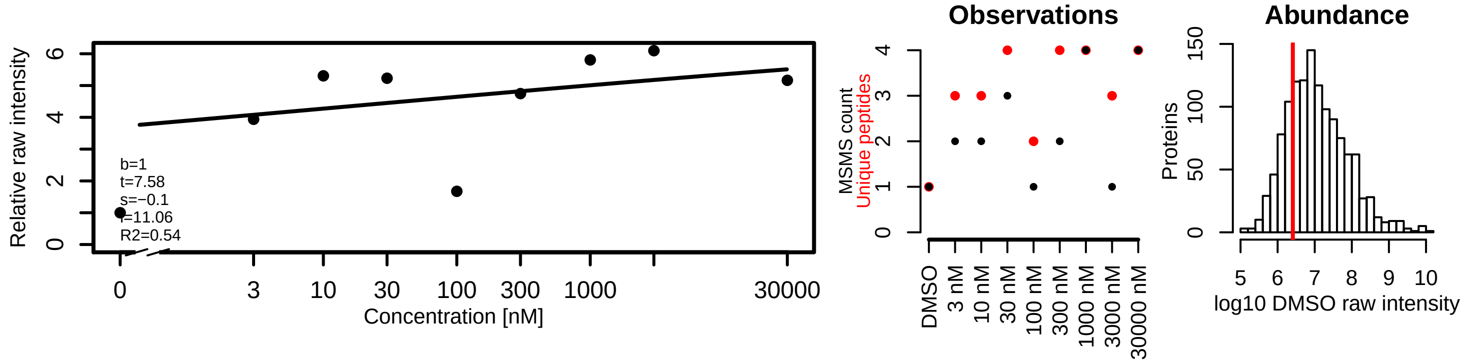

RPL27

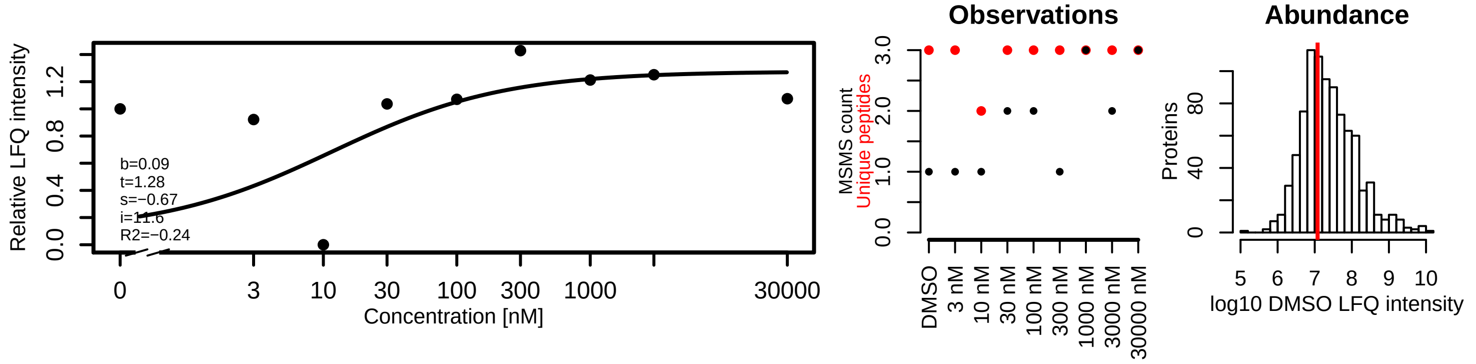

ACADS

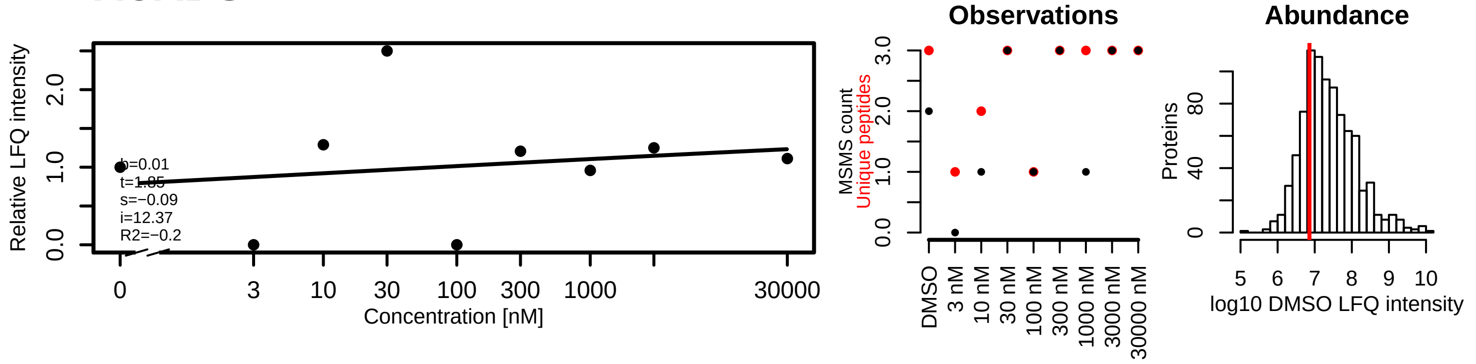

RELN

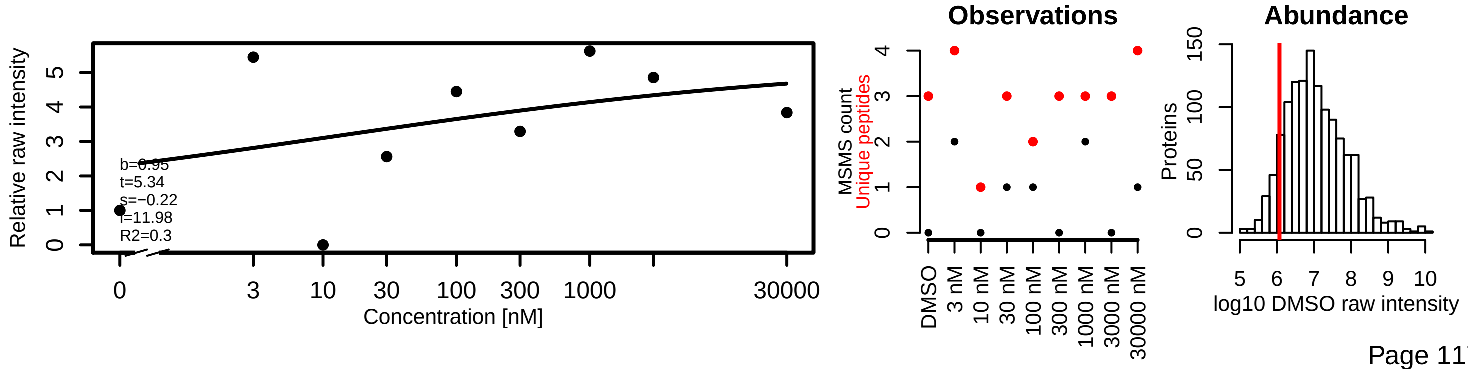

VAC14

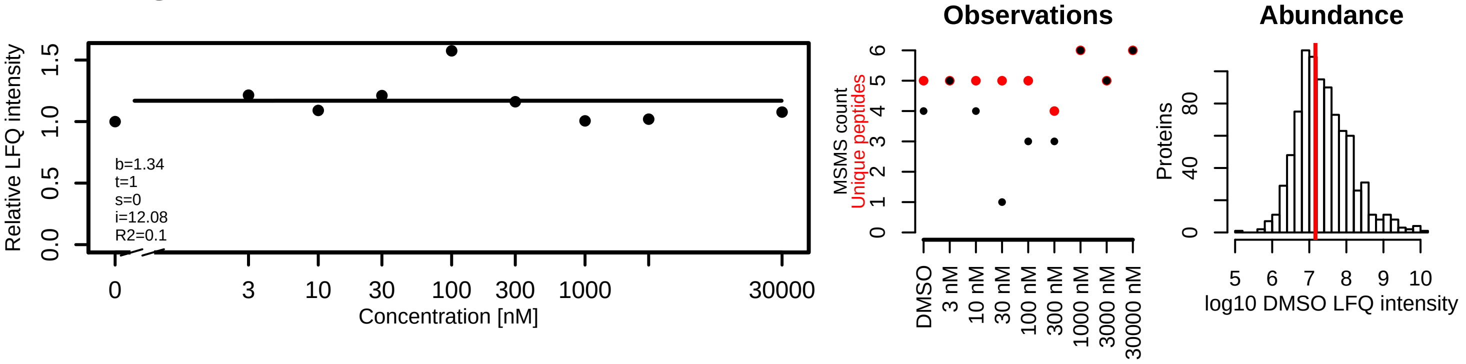

DPM1

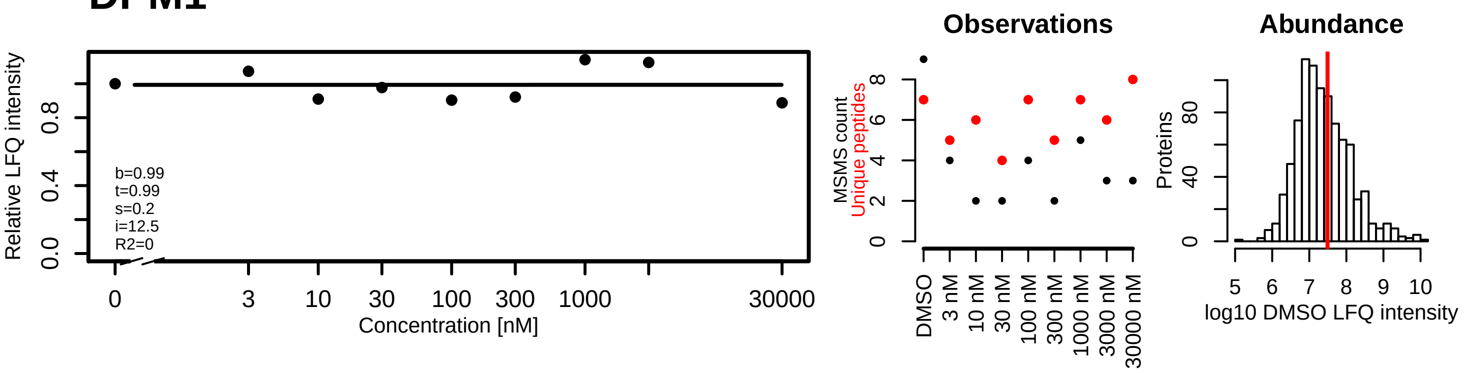

HSP90AA1

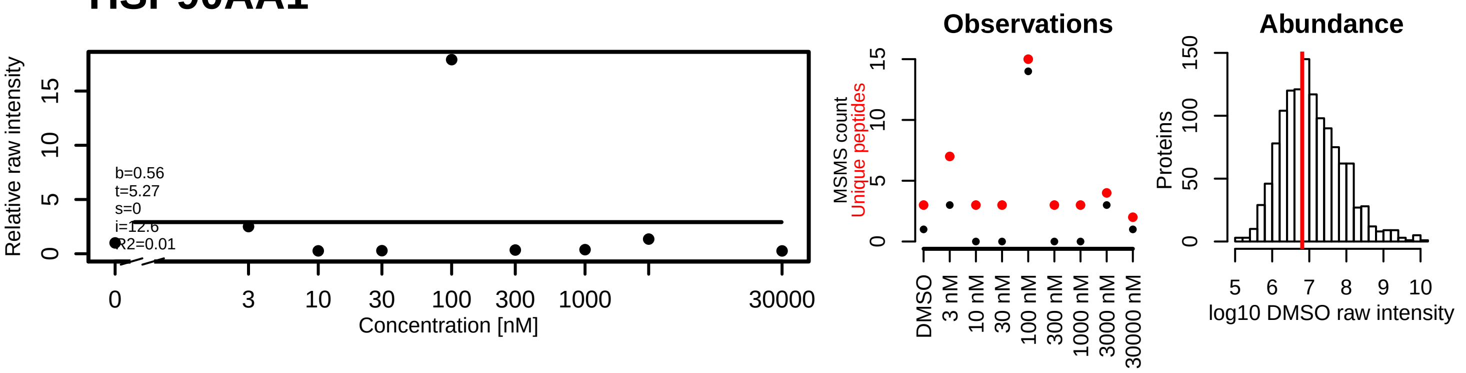

ESD

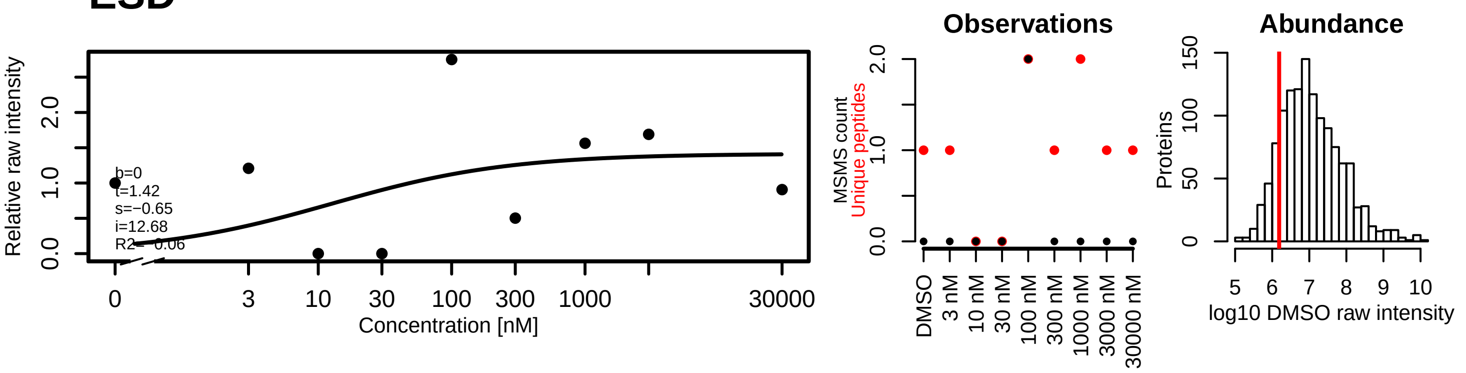

FITM2

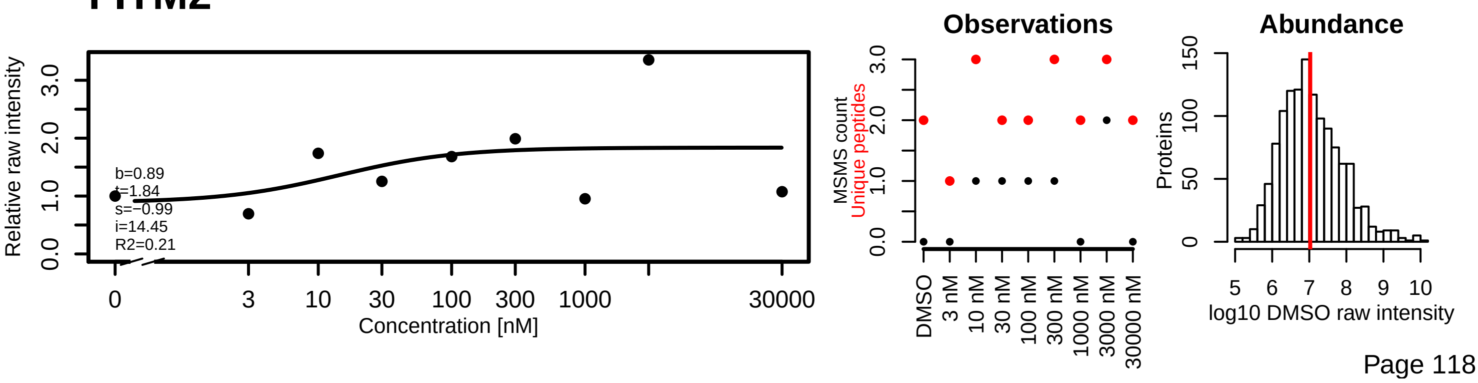

LARS

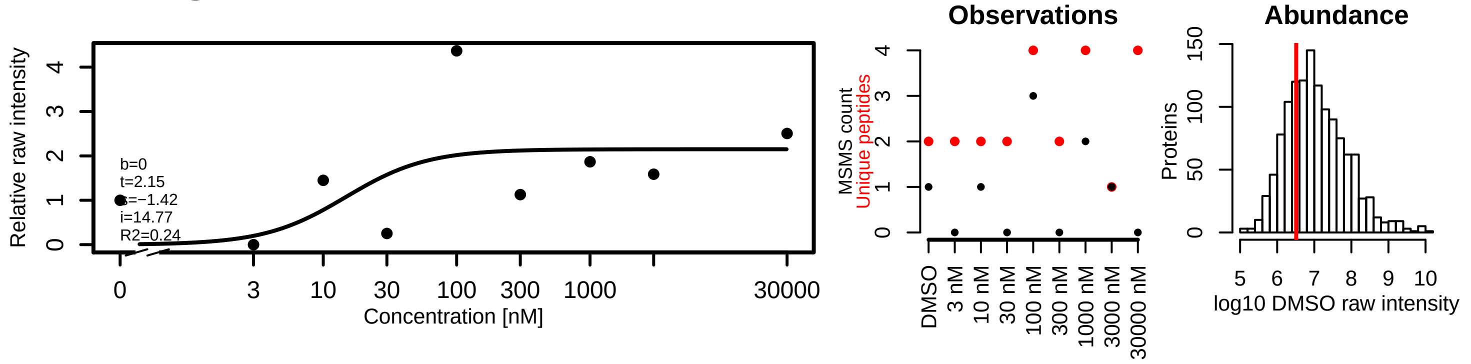

RPL10A

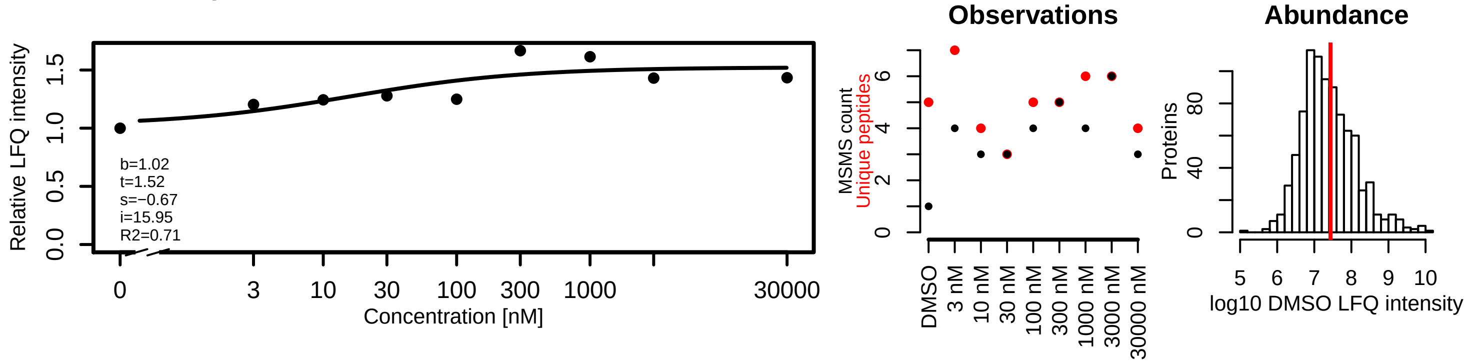

PDIA3 − G5EA52;P30101

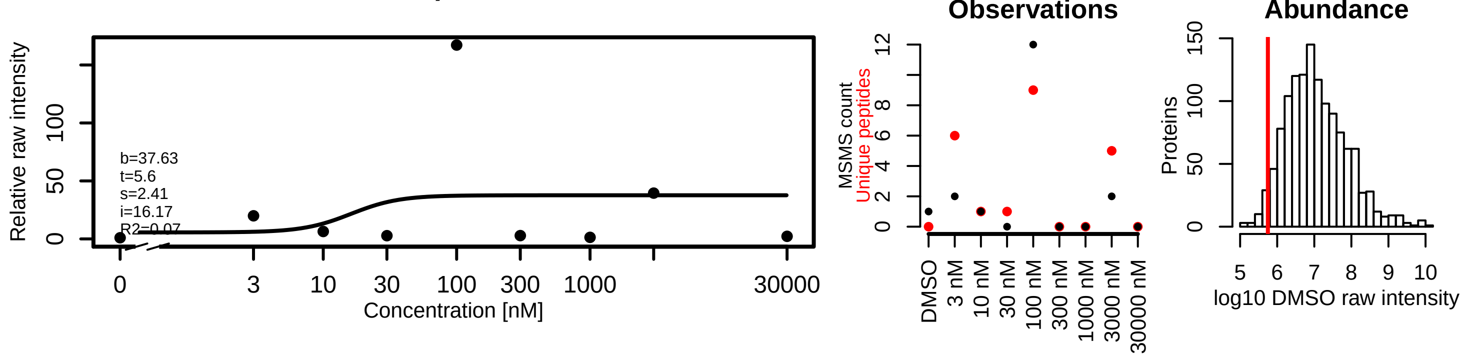

HIST1H2AJ;HIST1H2AH;H2AFJ;HIST2H2AC;HIST2H2AA3;HIST1H2AI

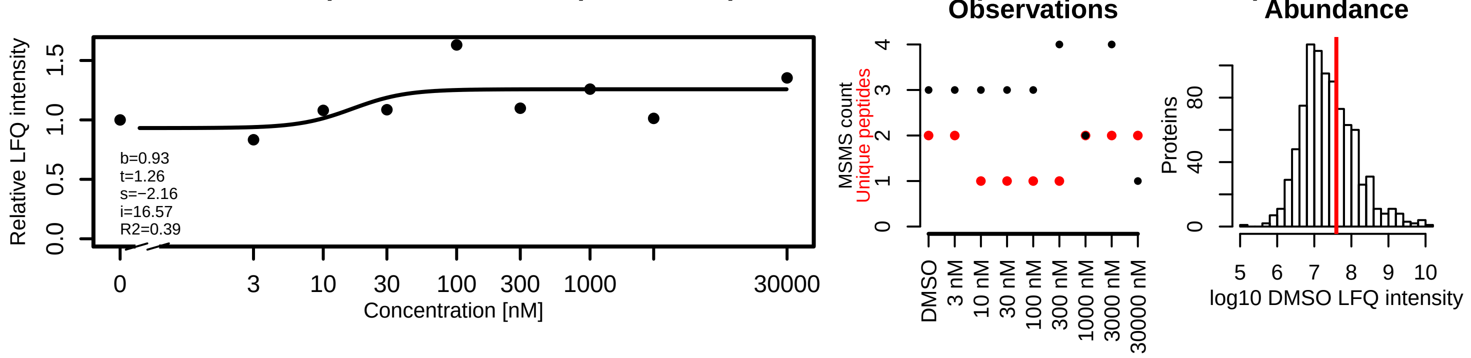

MAP1B

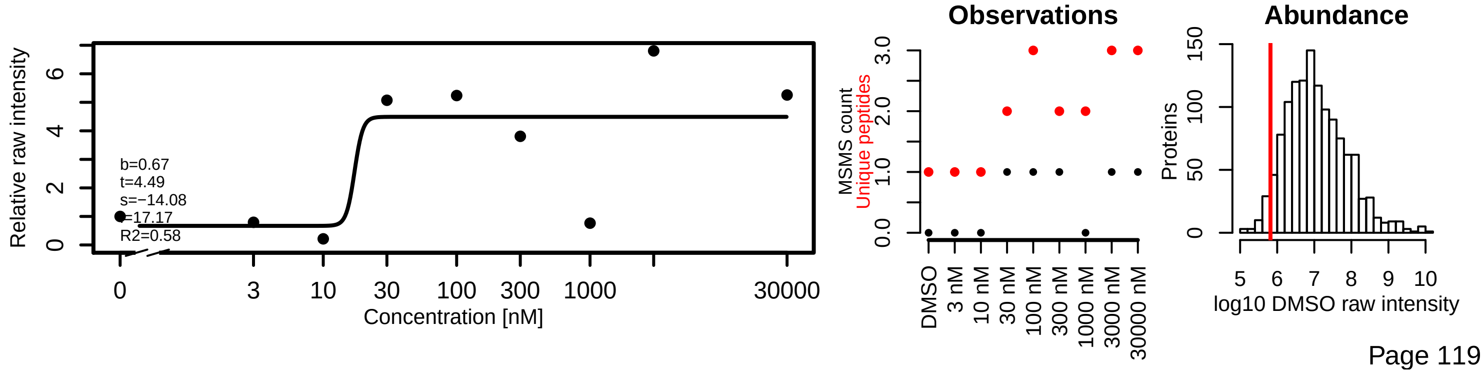

MYH9

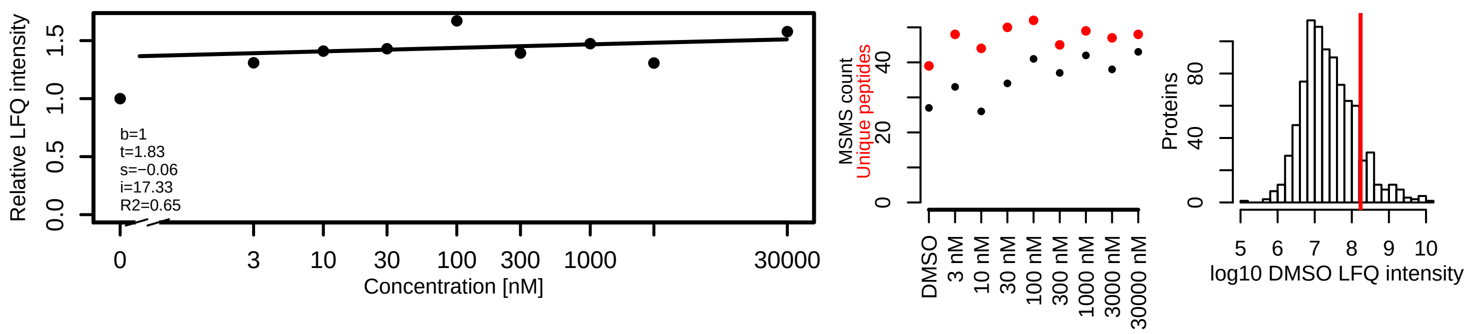

ACSL5

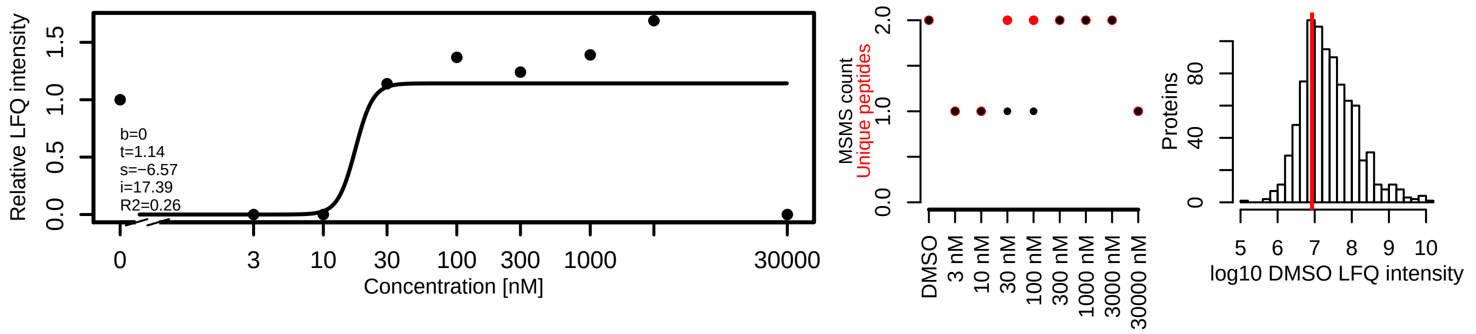

PTRF

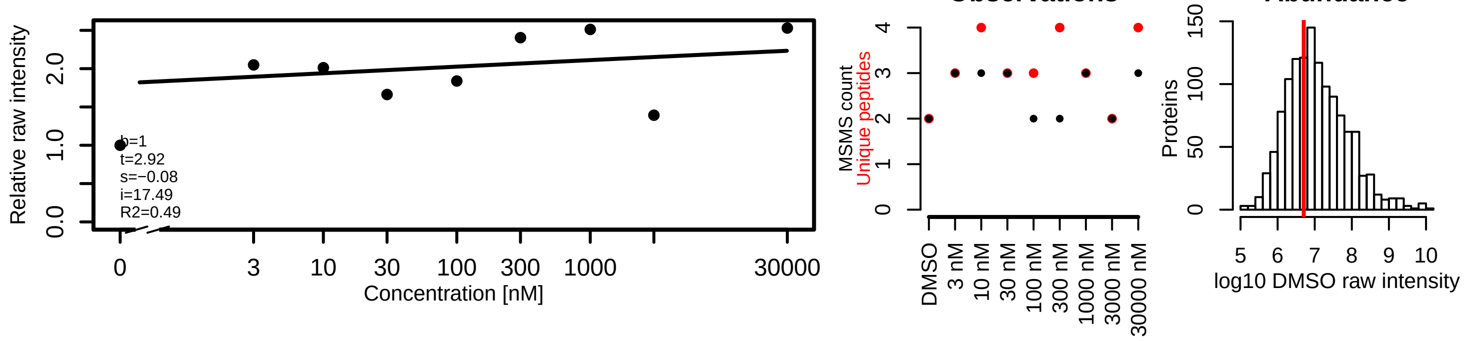

RPS13

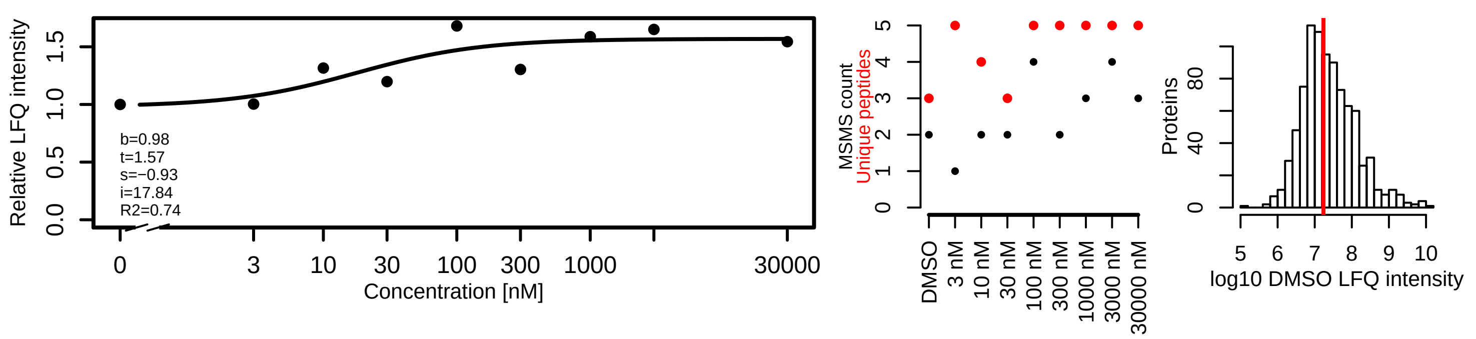

NUDC

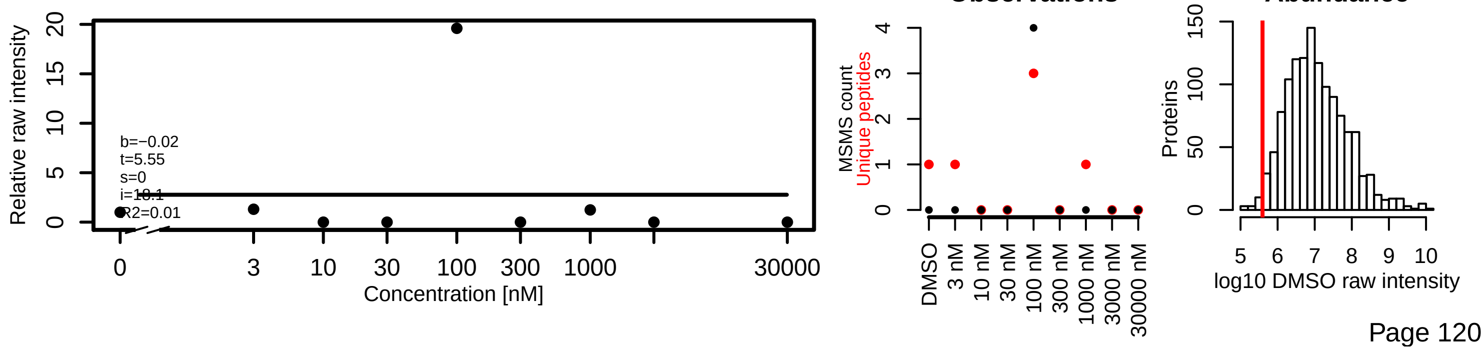

FADS3

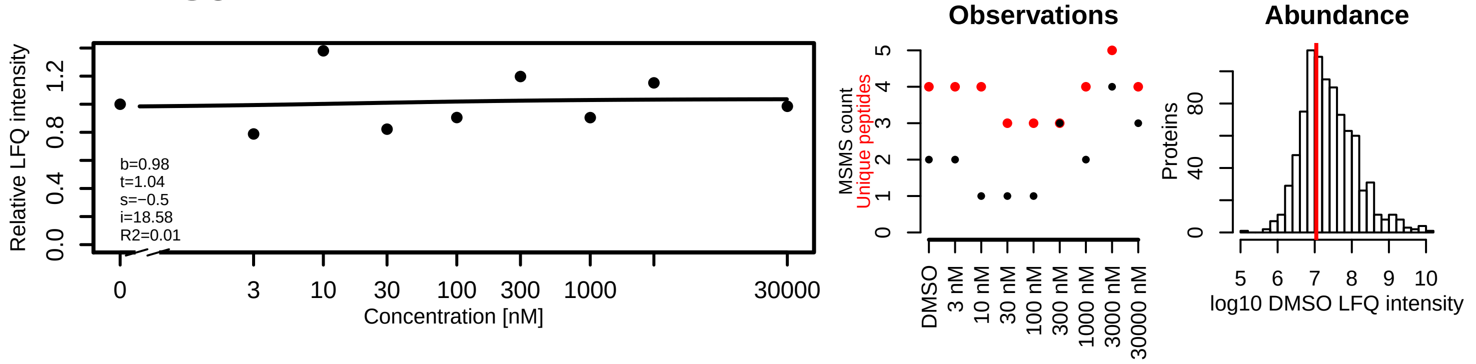

SLC22A1

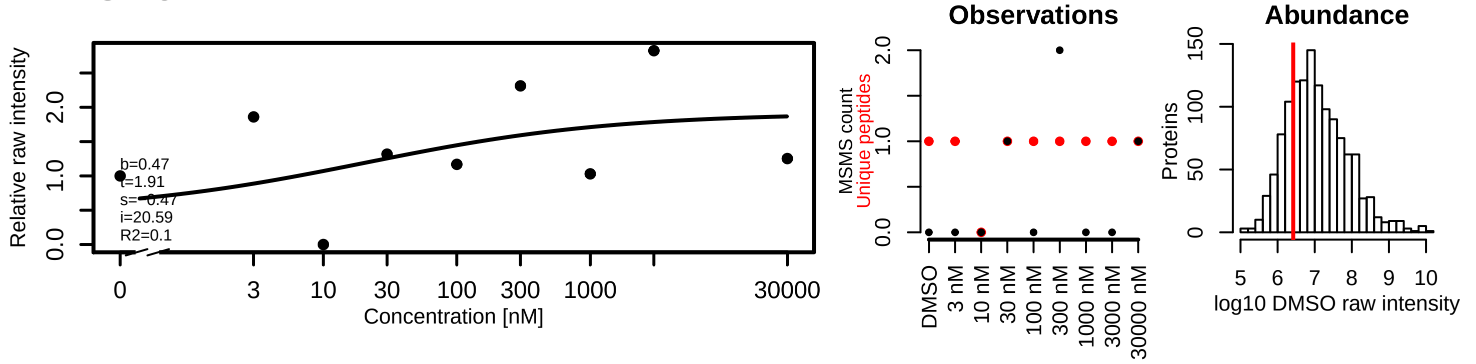

GANAB

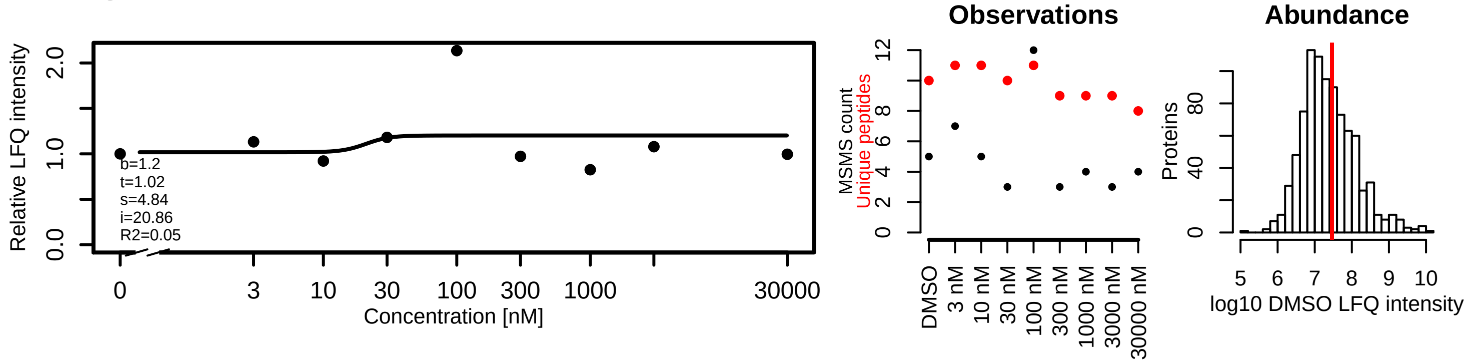

MTOR

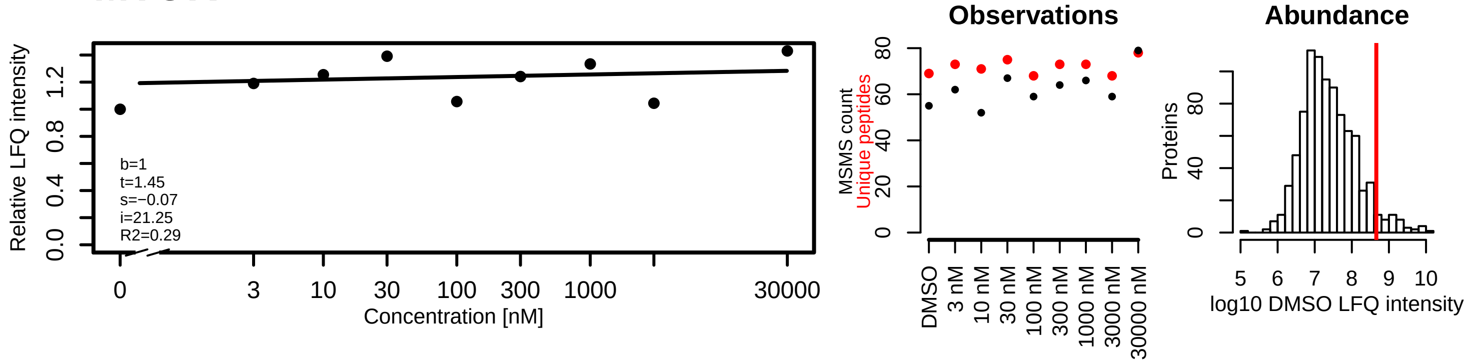

SPCS2

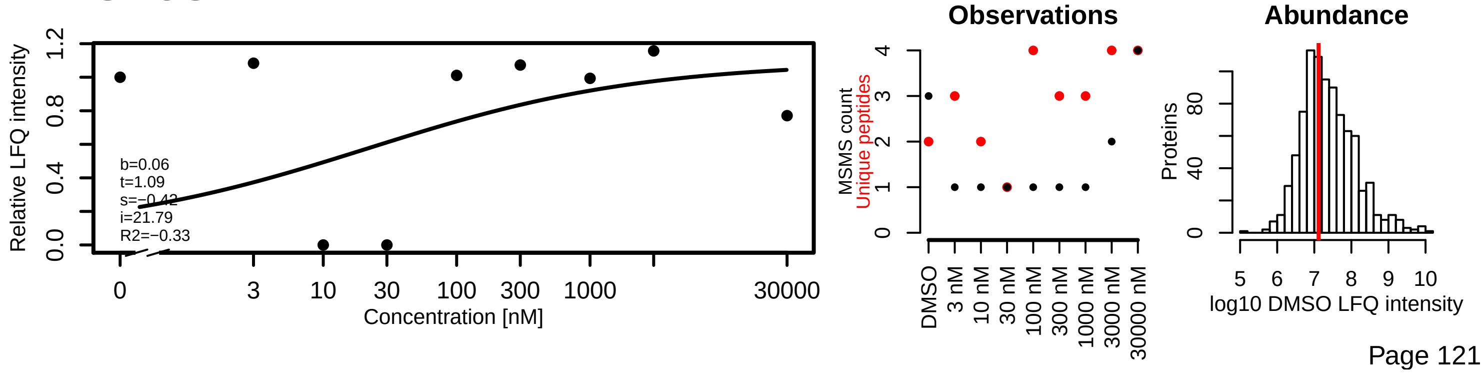

NUP188

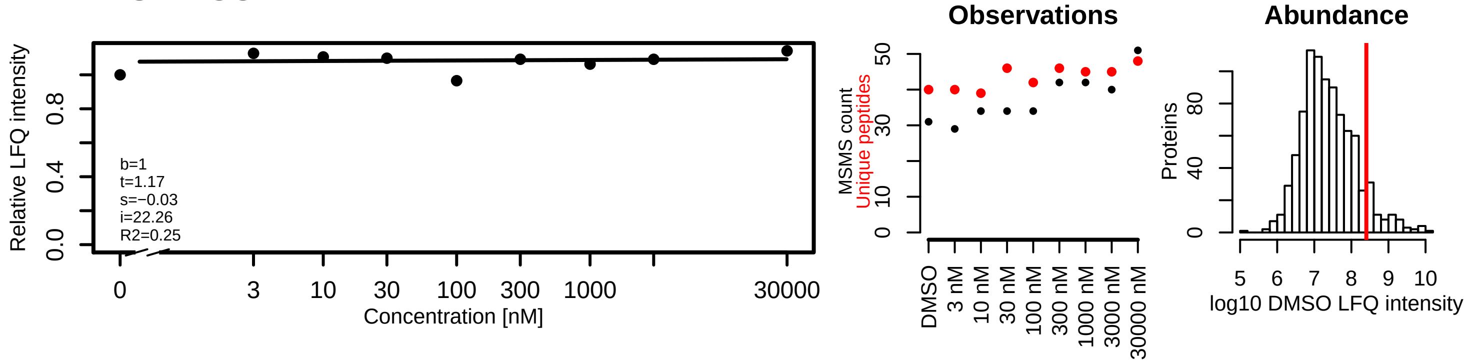

SLC1A4

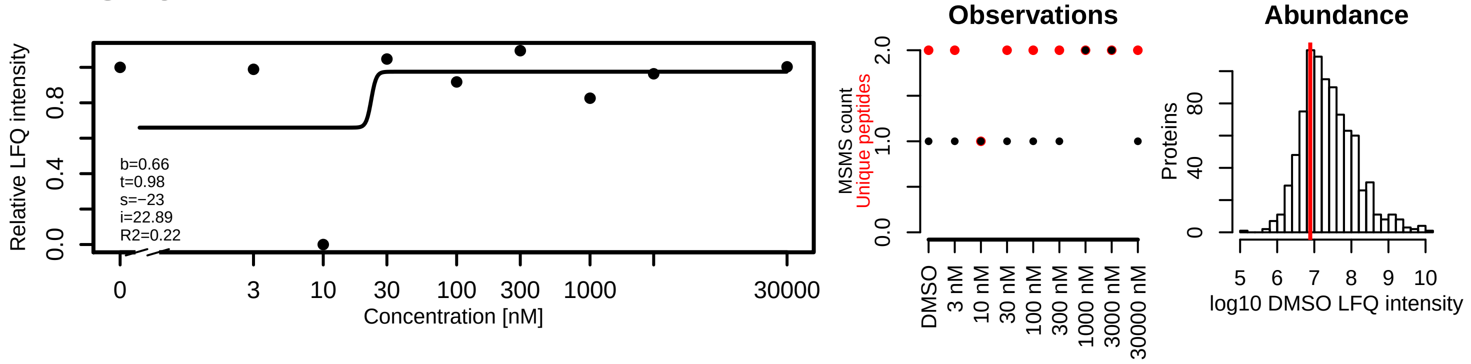

HSPE1

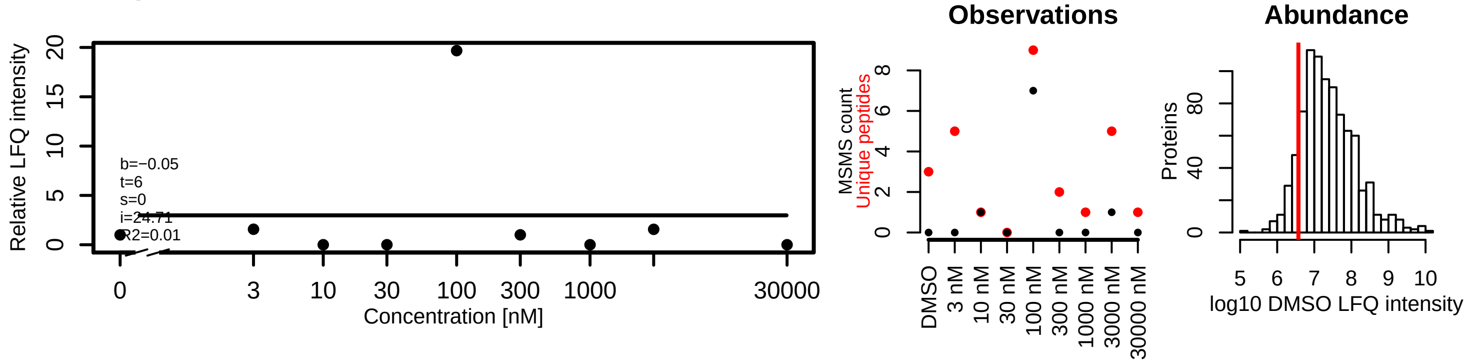

USE1

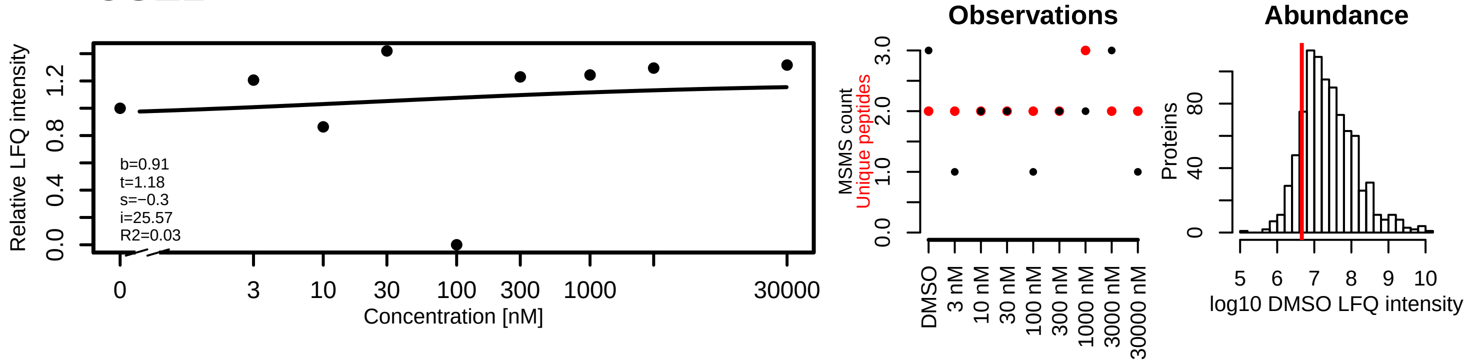

LMNA

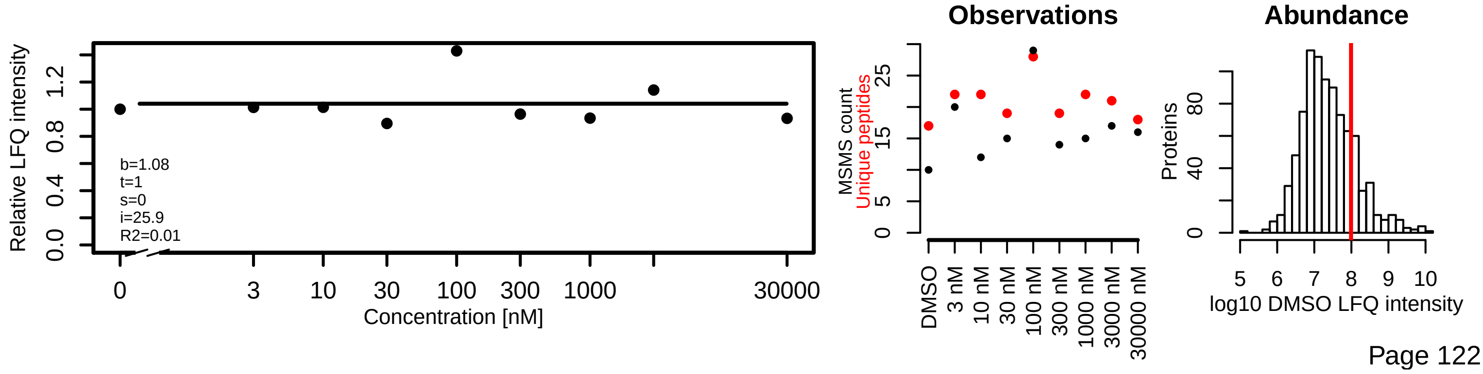

OPA1

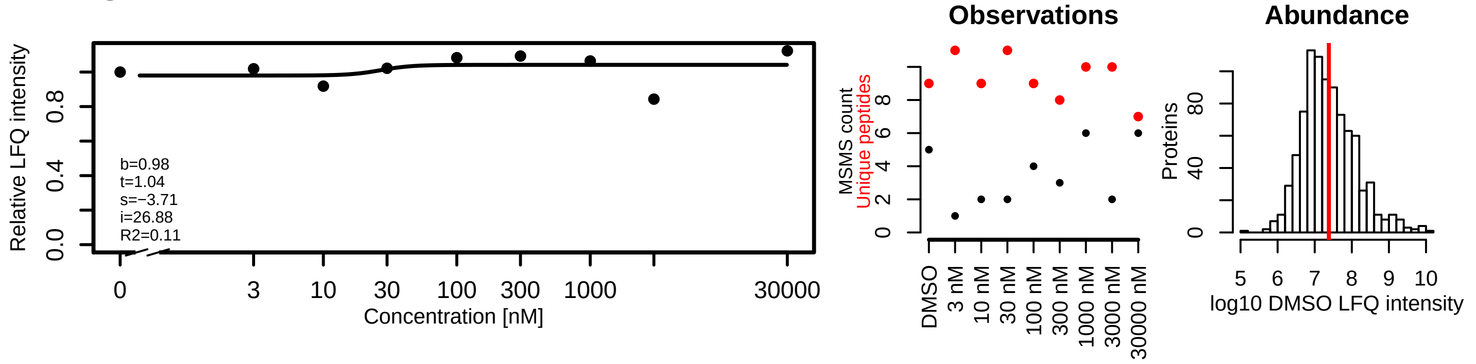

RPL5

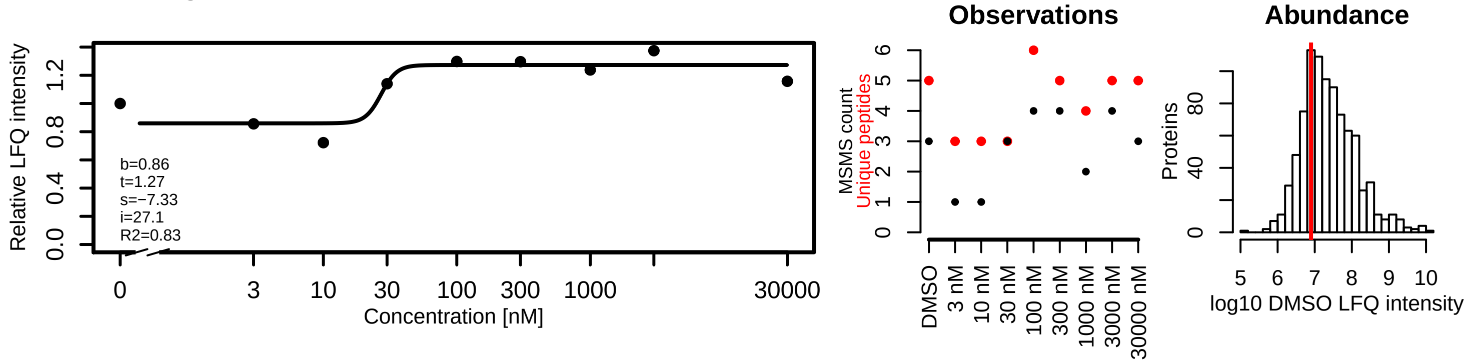

RPL10

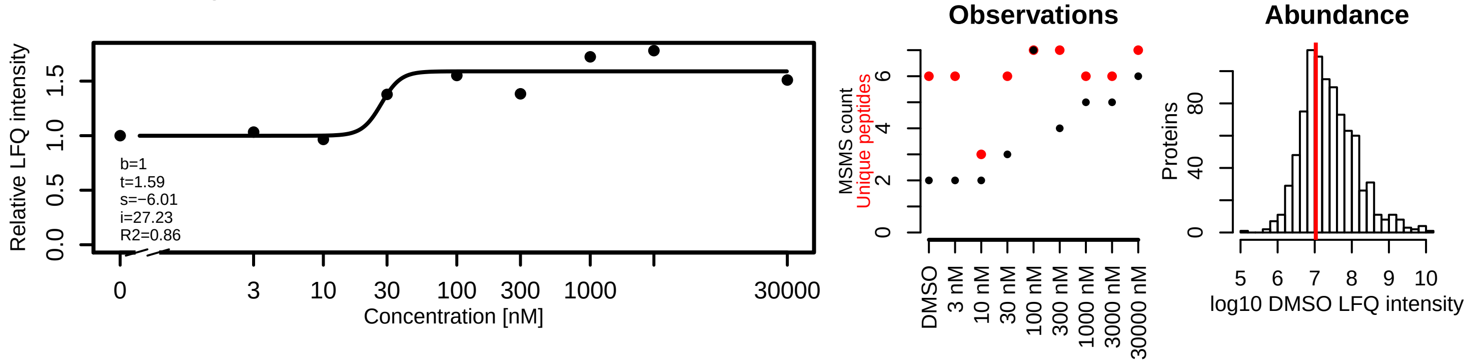

DDX5

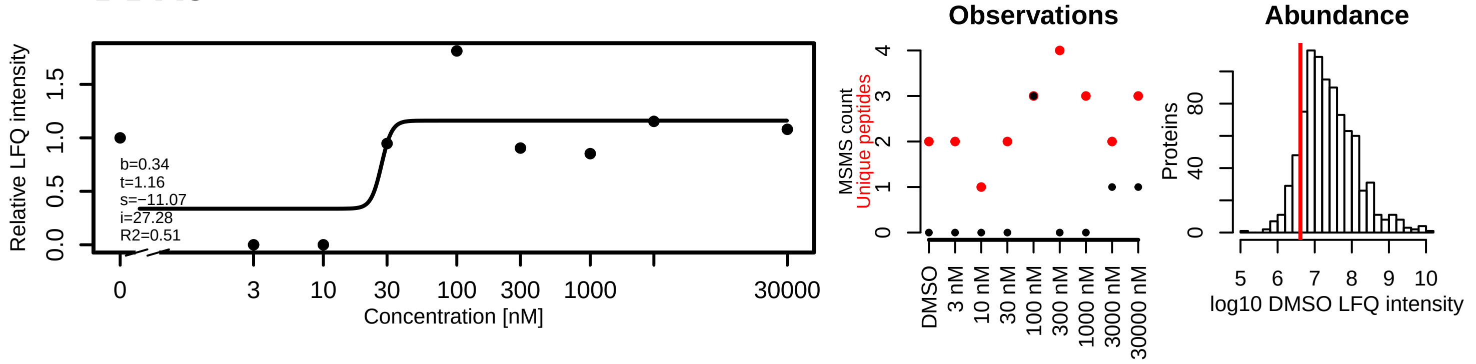

ATAD3A;ATAD3B

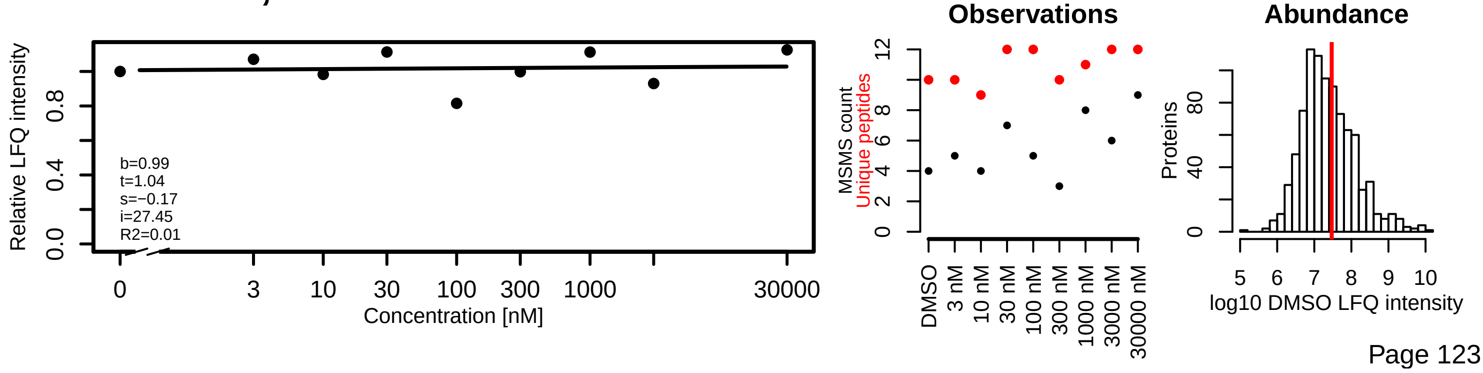

RPL12

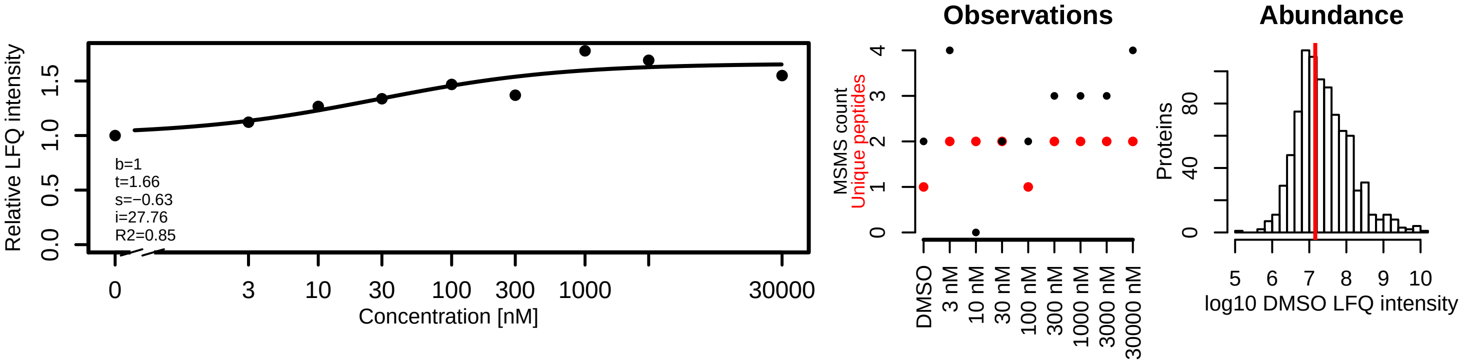

RRM1

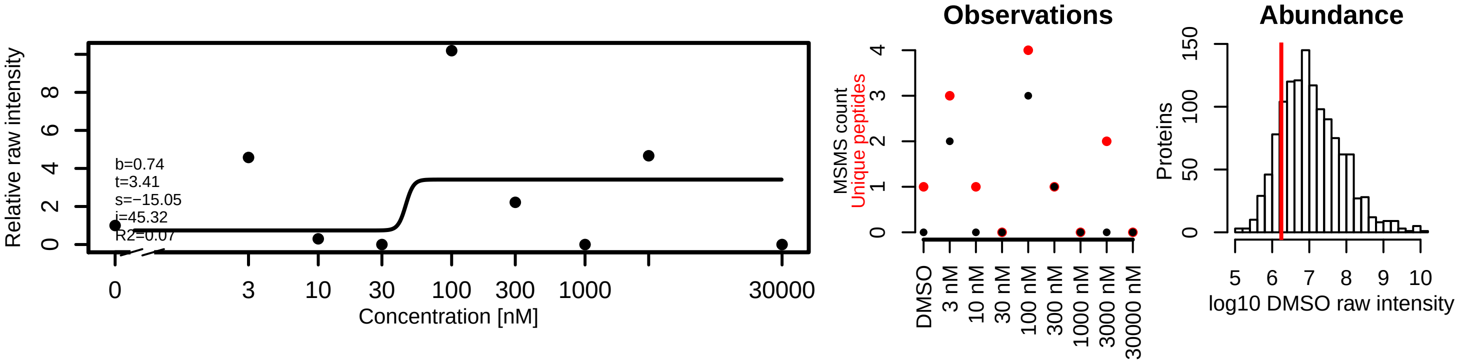

ACADVL

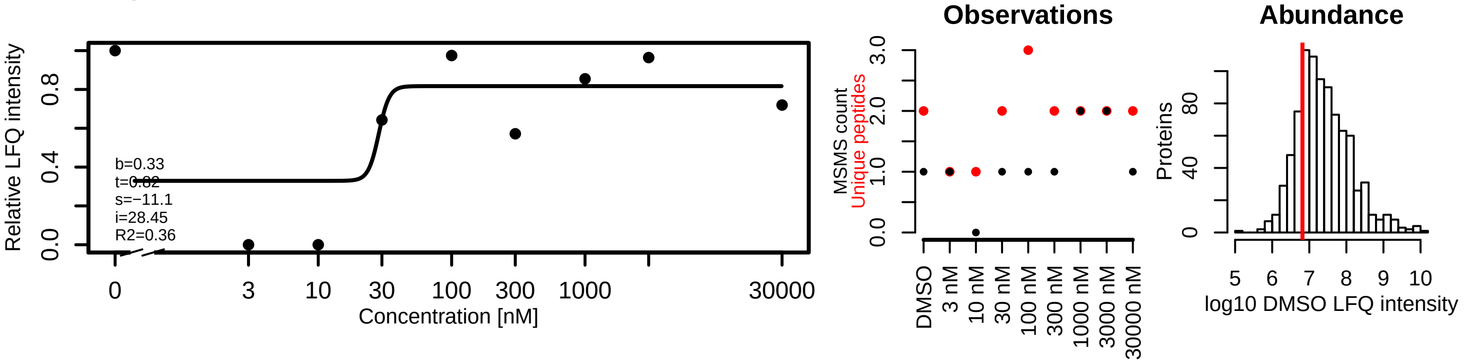

SCLY

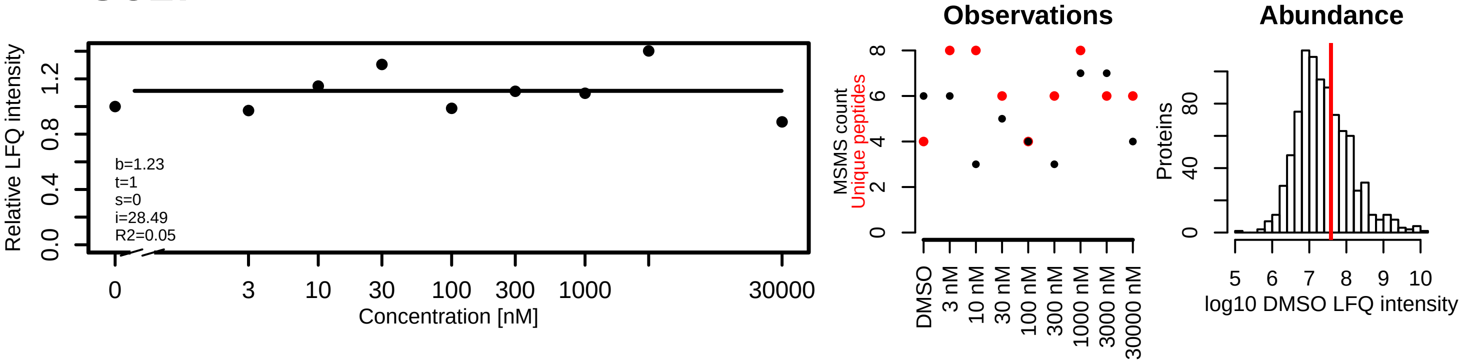

NDUFA4

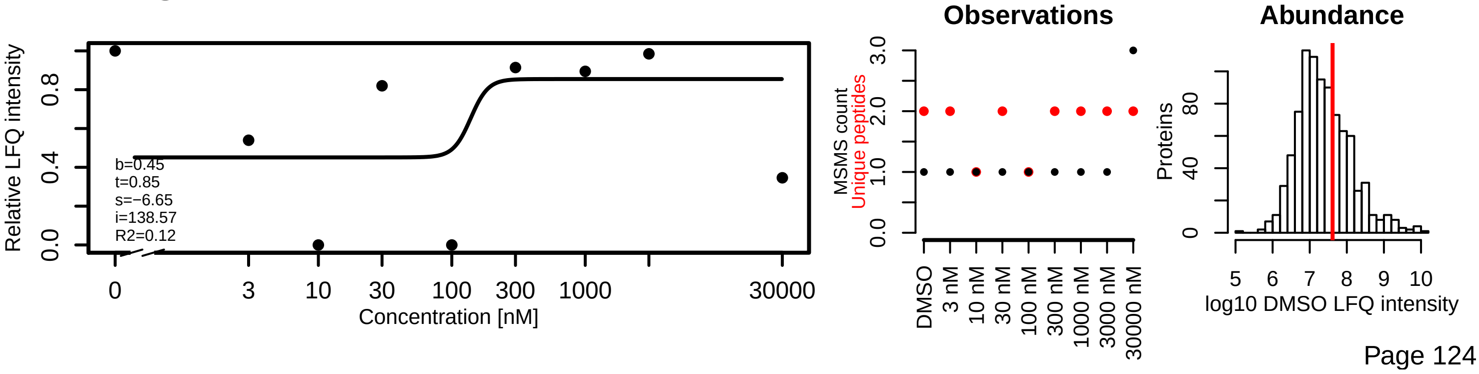

BMPR1A

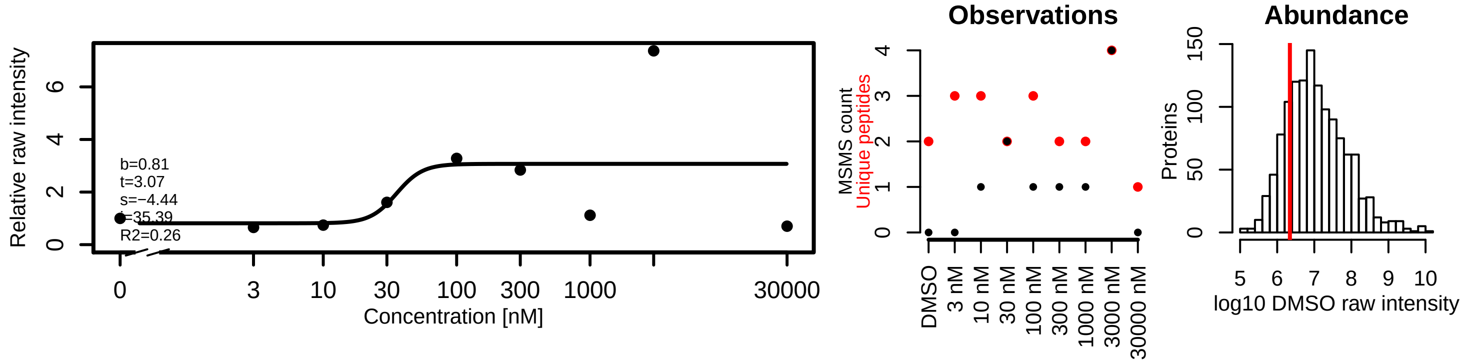

RPS23

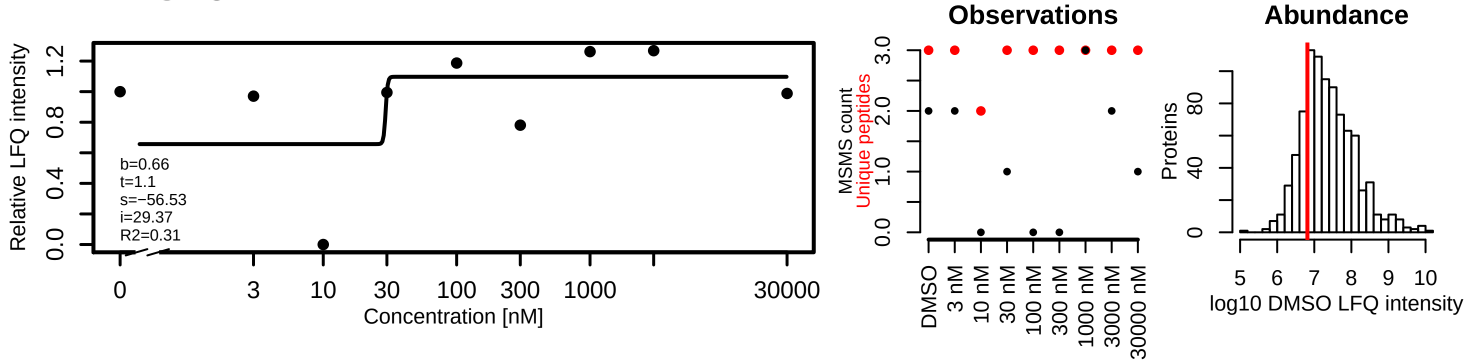

HNRNPC

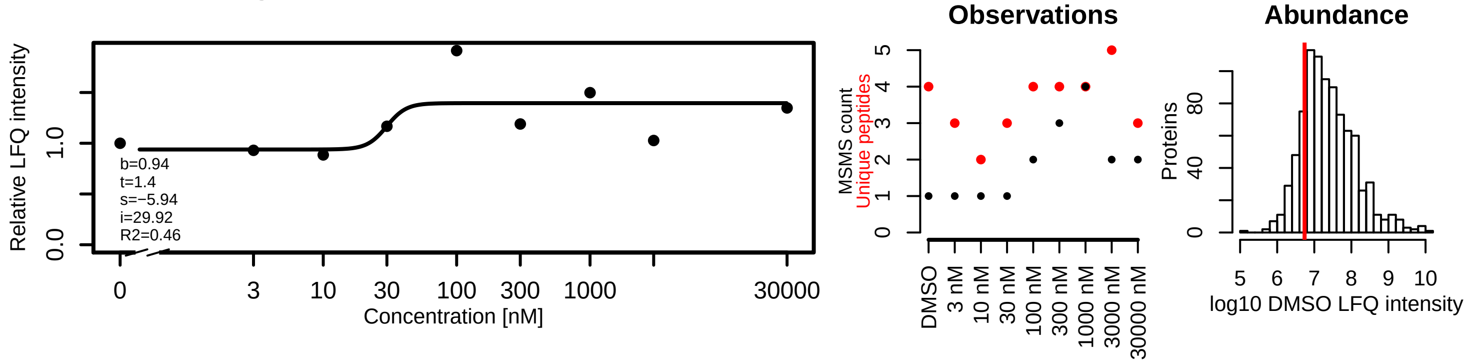

PRKDC

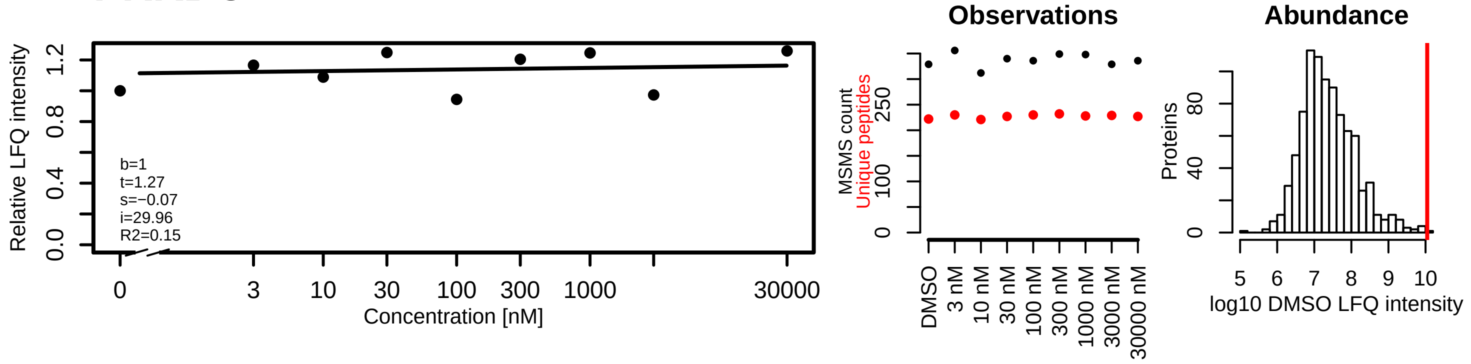

RPLP0;RPLP0P6

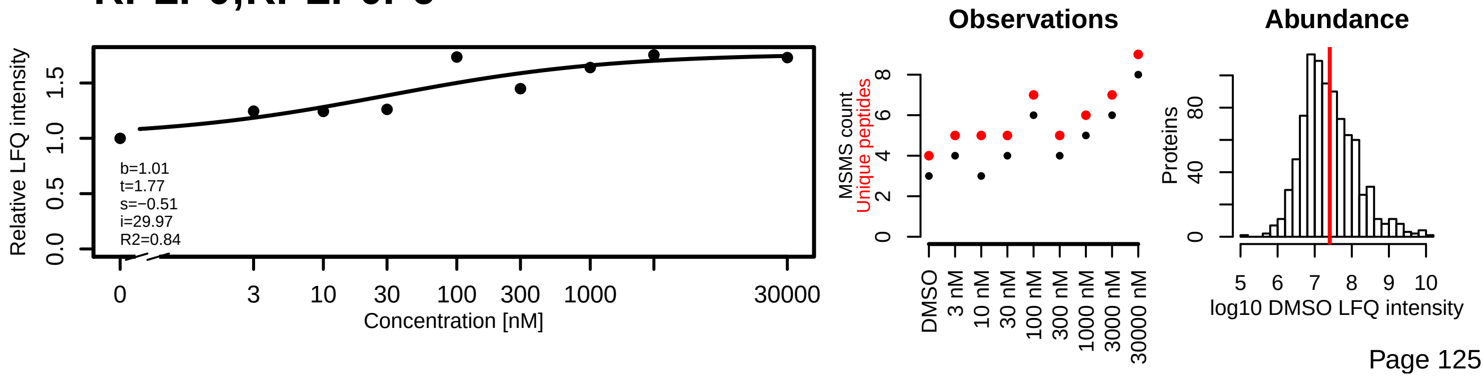

SPTAN1

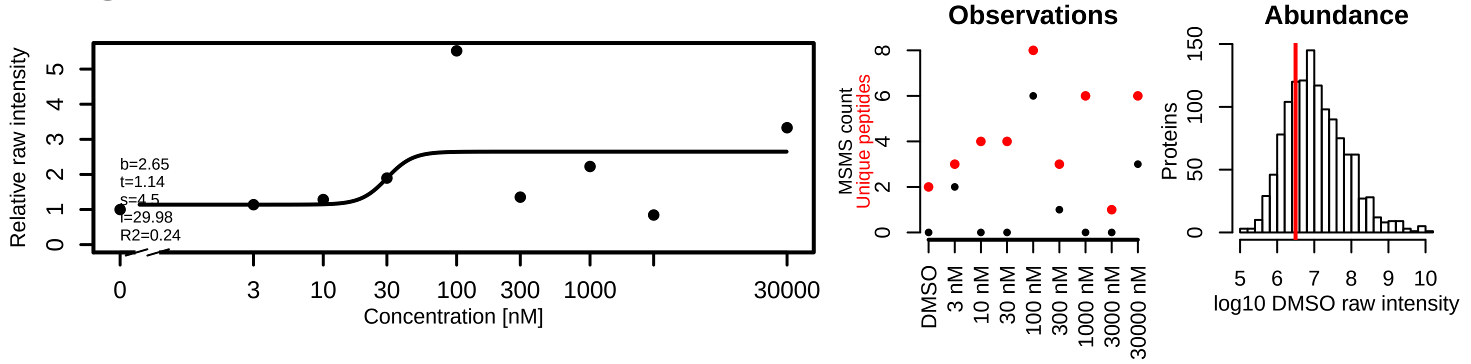

HSPB1

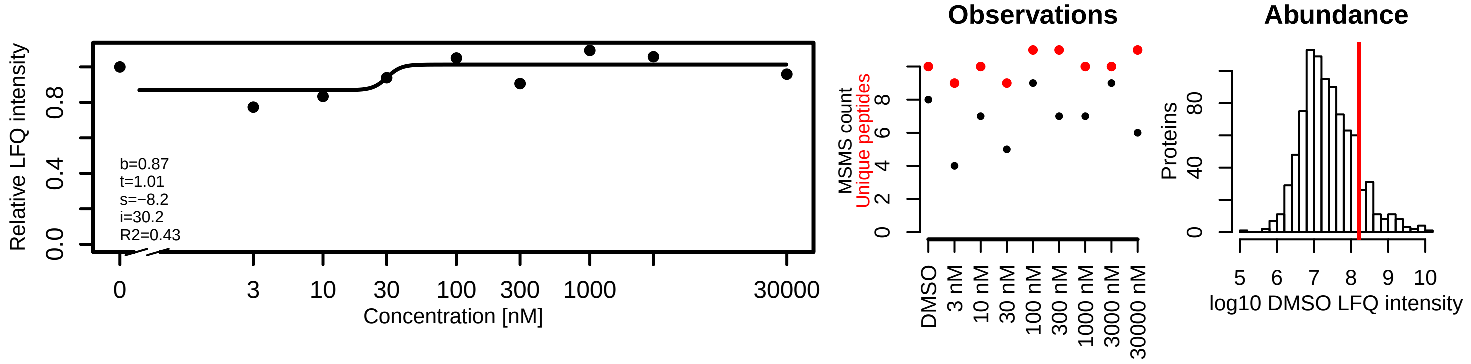

TMEM120A

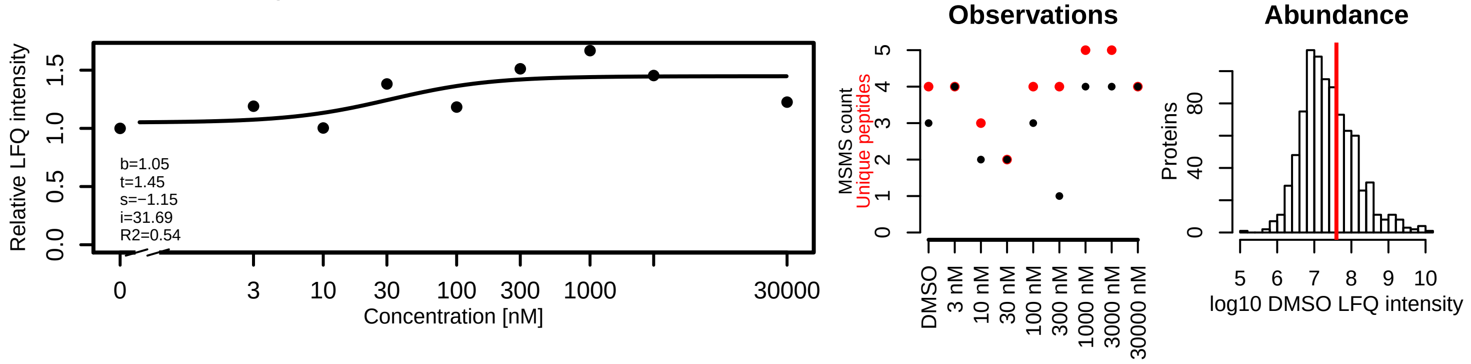

RPL3

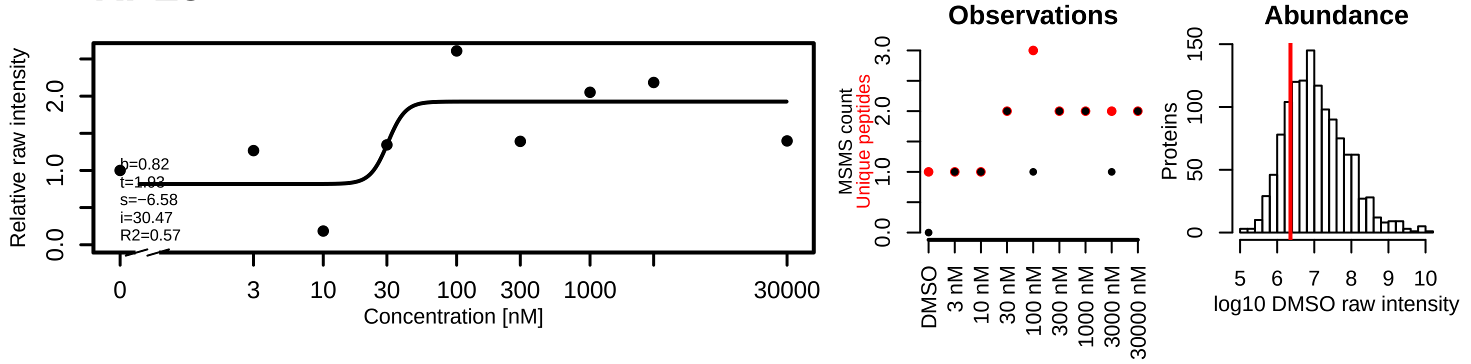

PTPMT1

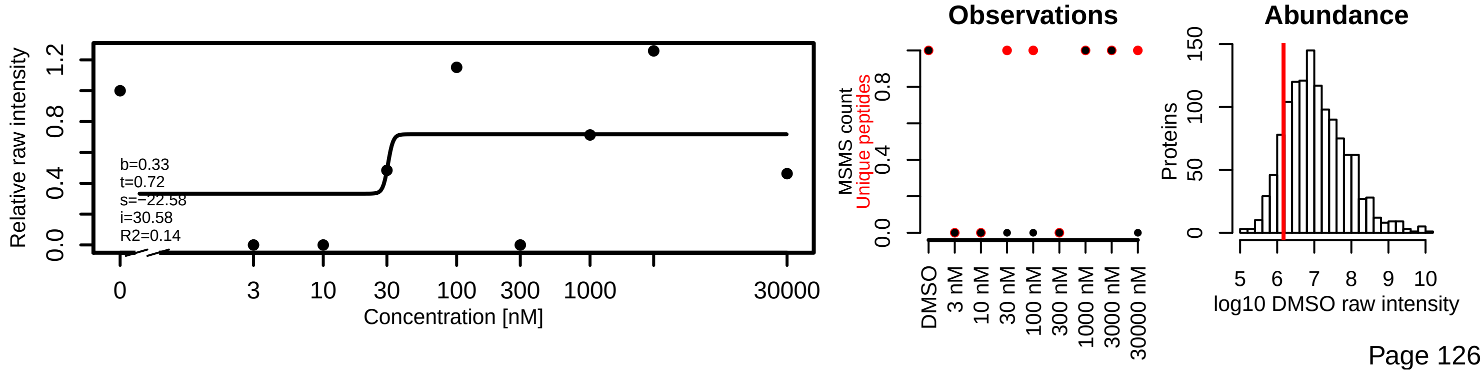

PEX10

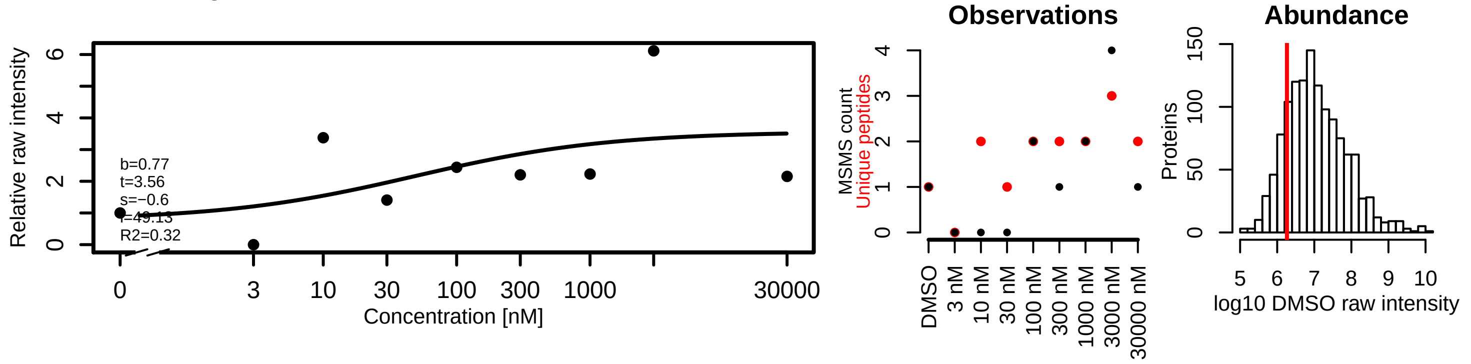

ABHD12

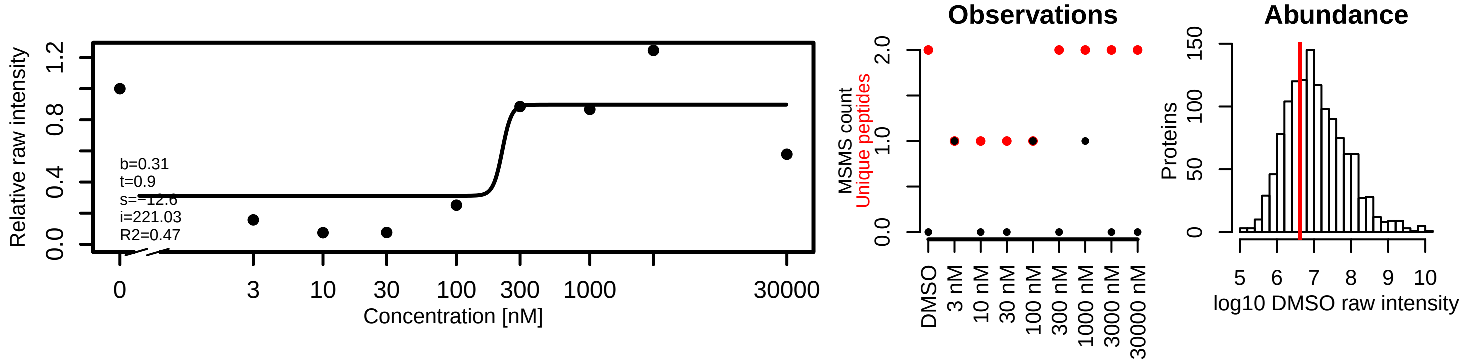

TAP1

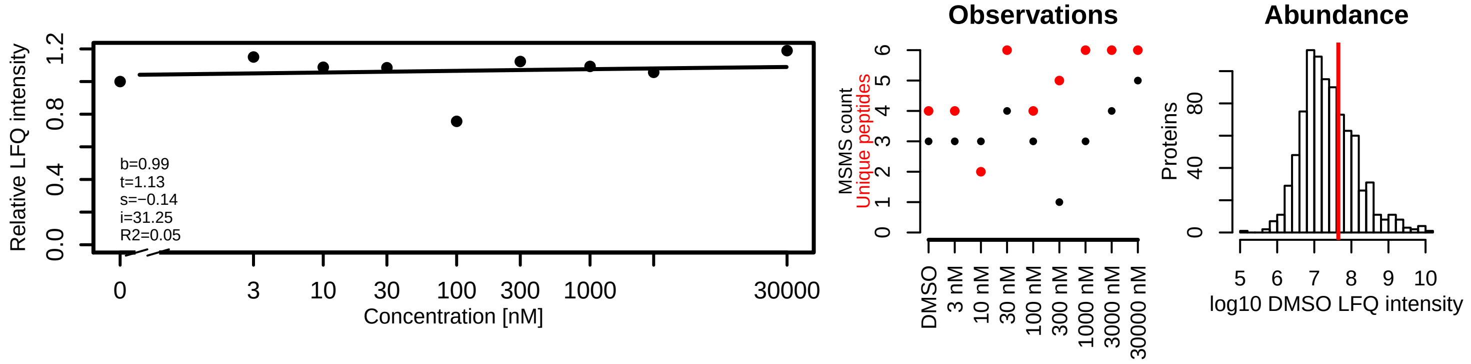

CTSD

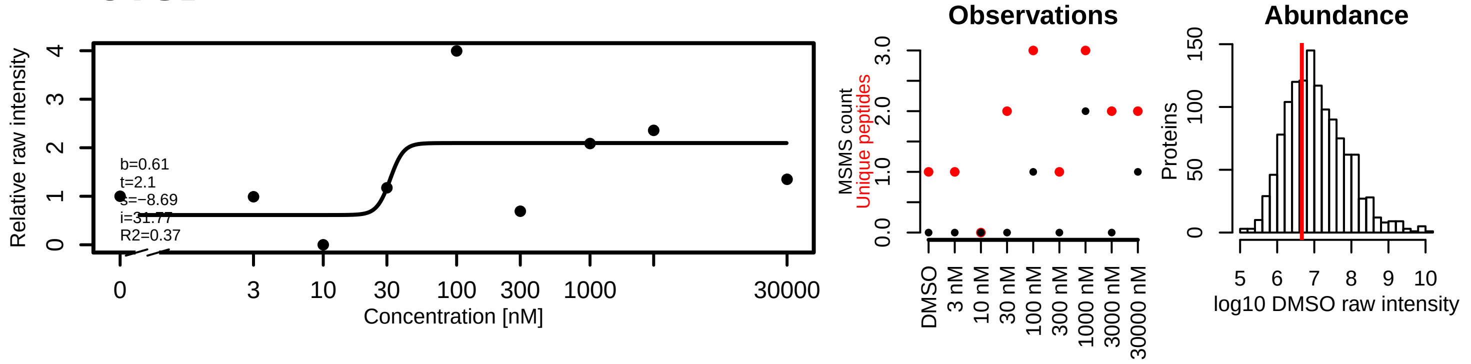

LBR

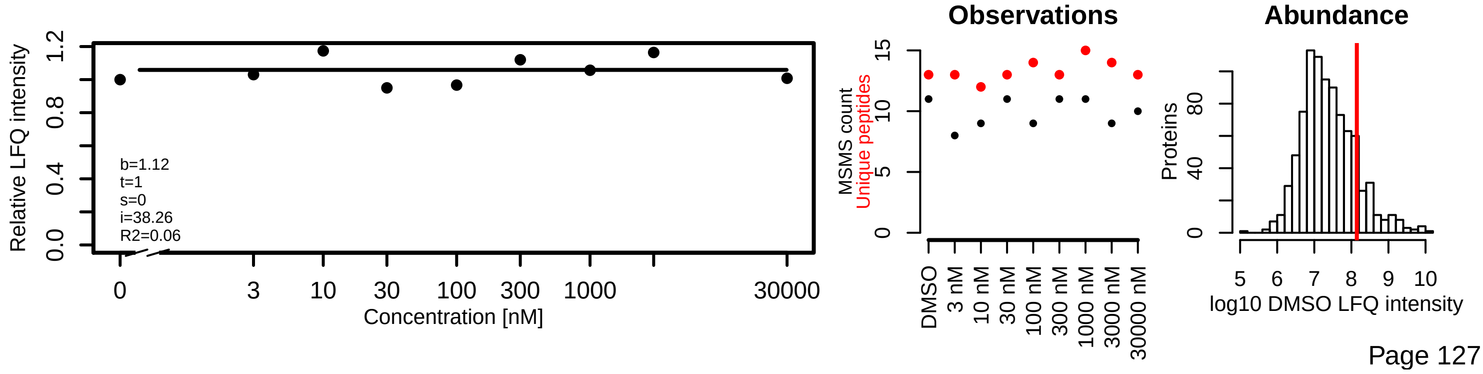

PABPC1

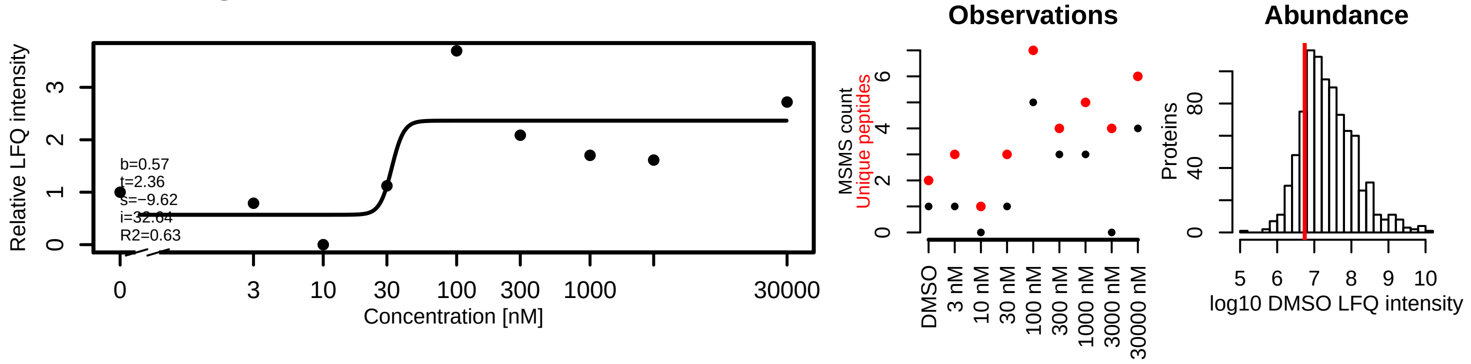

CLN6

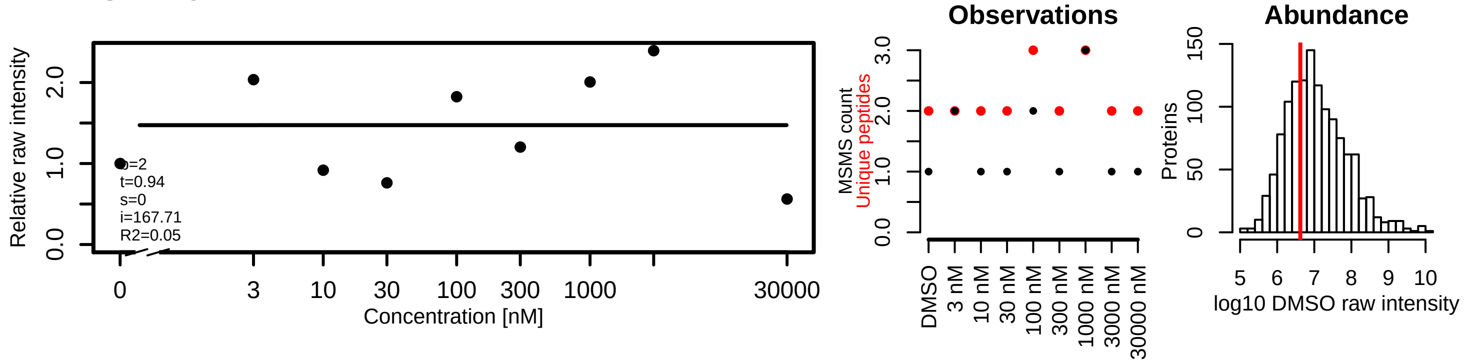

RPL23A

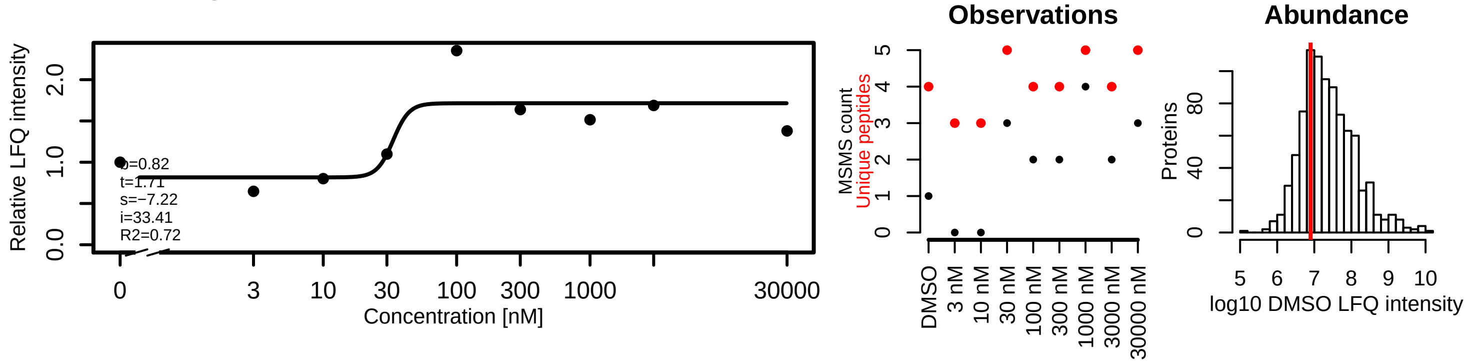

CISD2

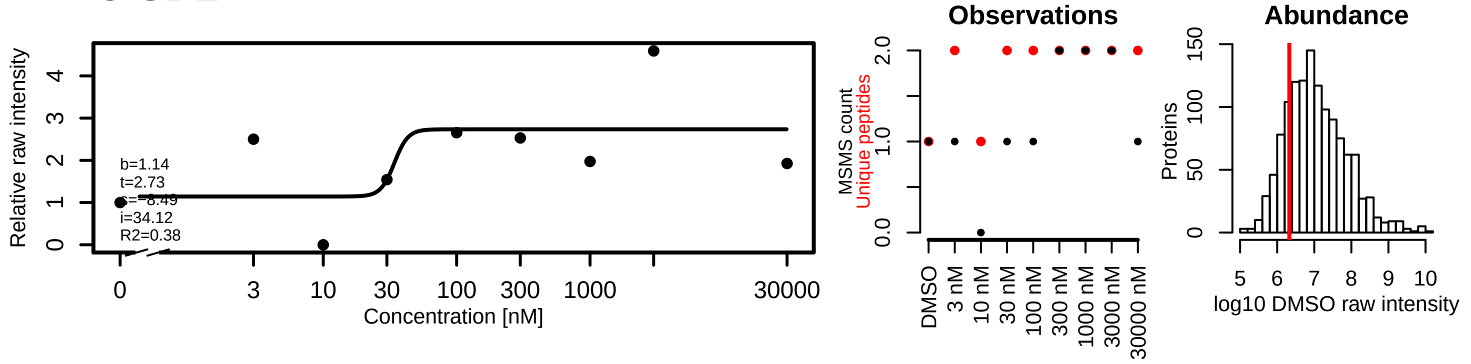

RGPD4;RGPD3

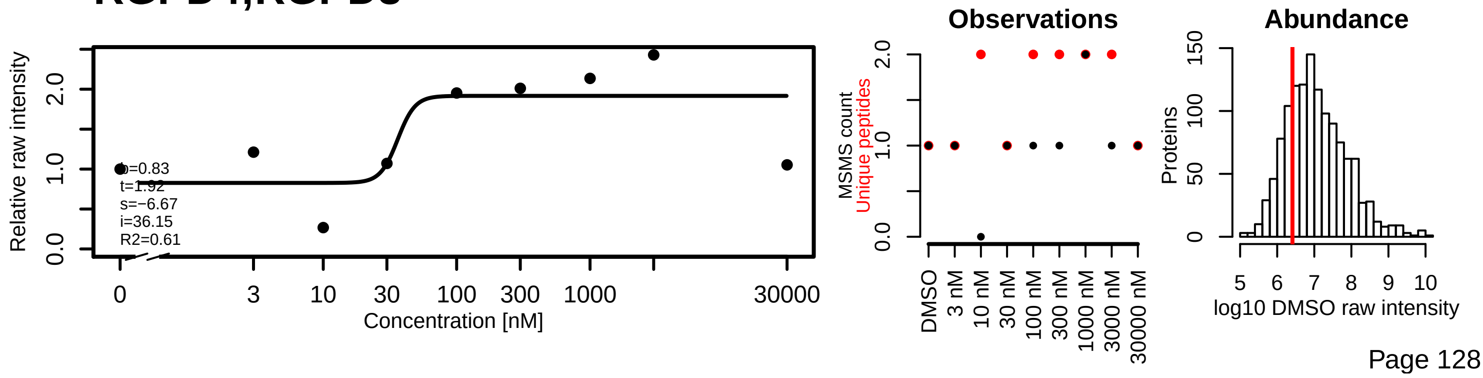

CAPZB

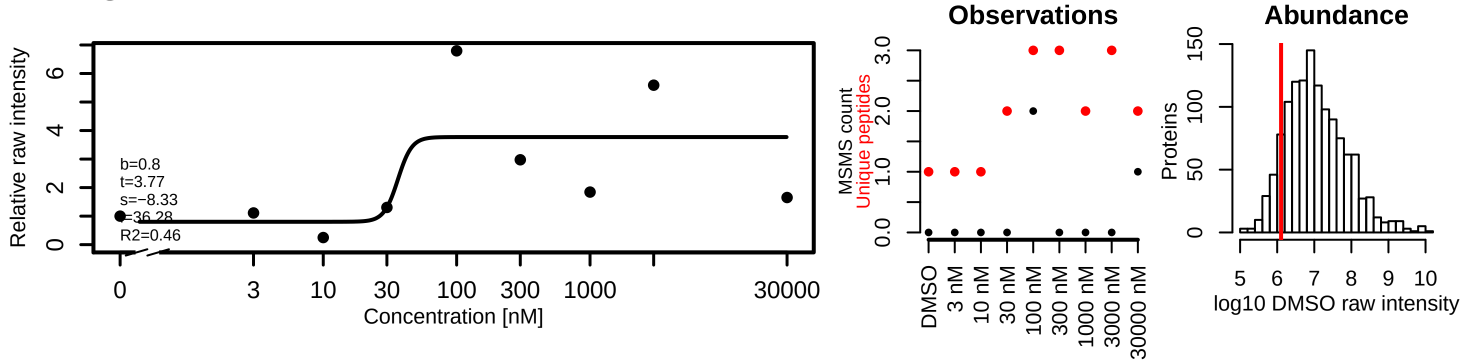

RPL31

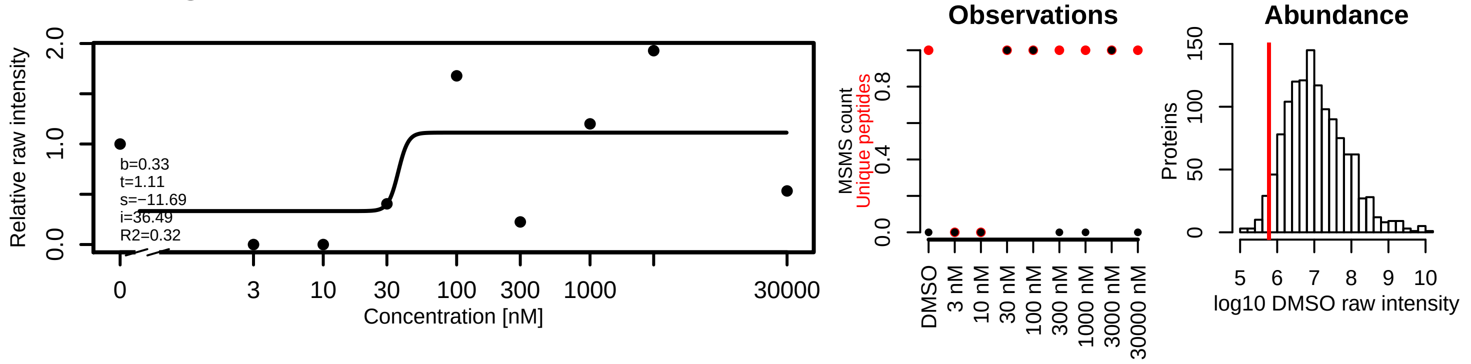

FAM134C;LOC162427

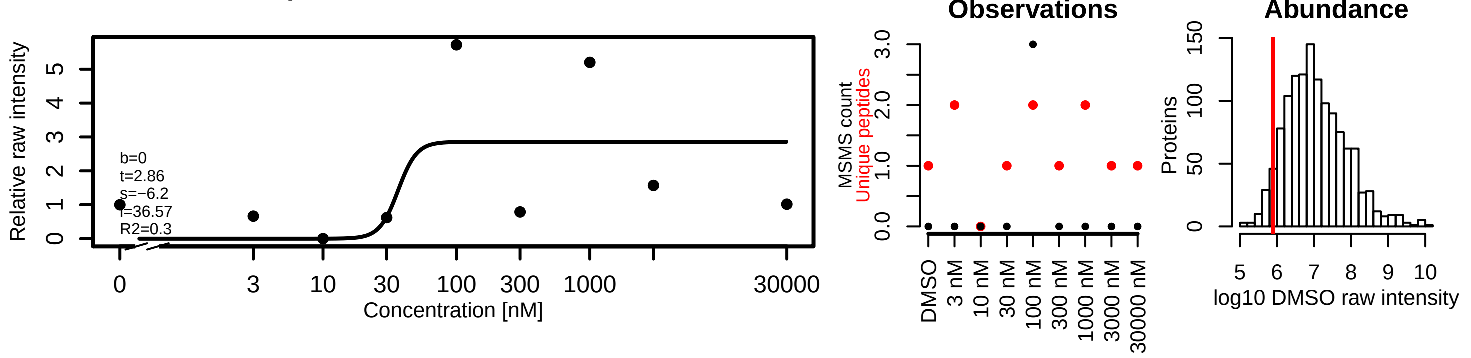

YWHAB

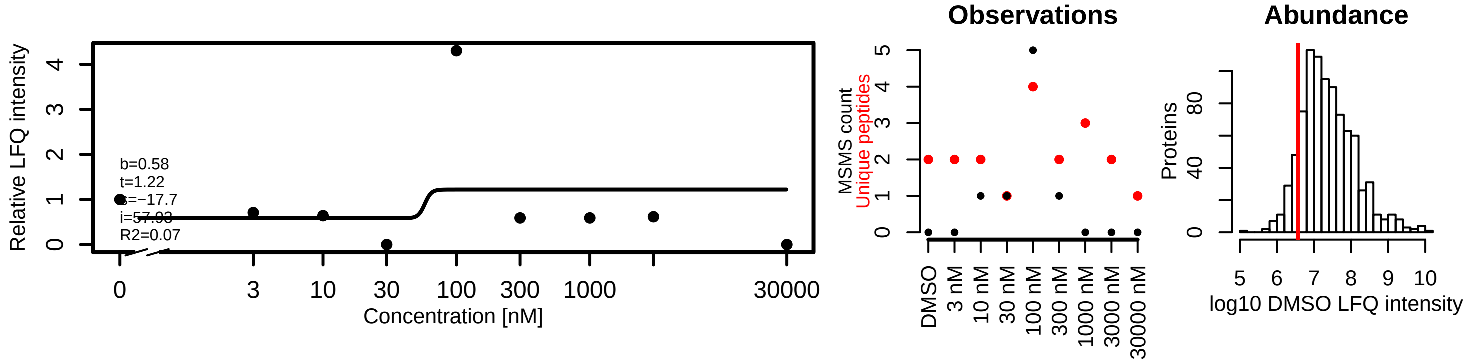

RPL27A

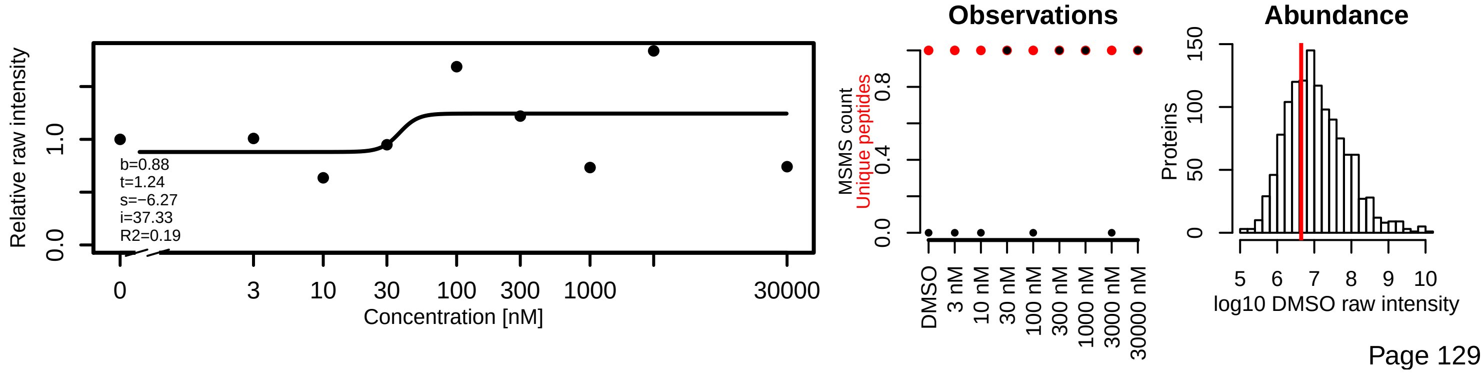

RPS17L;RPS17

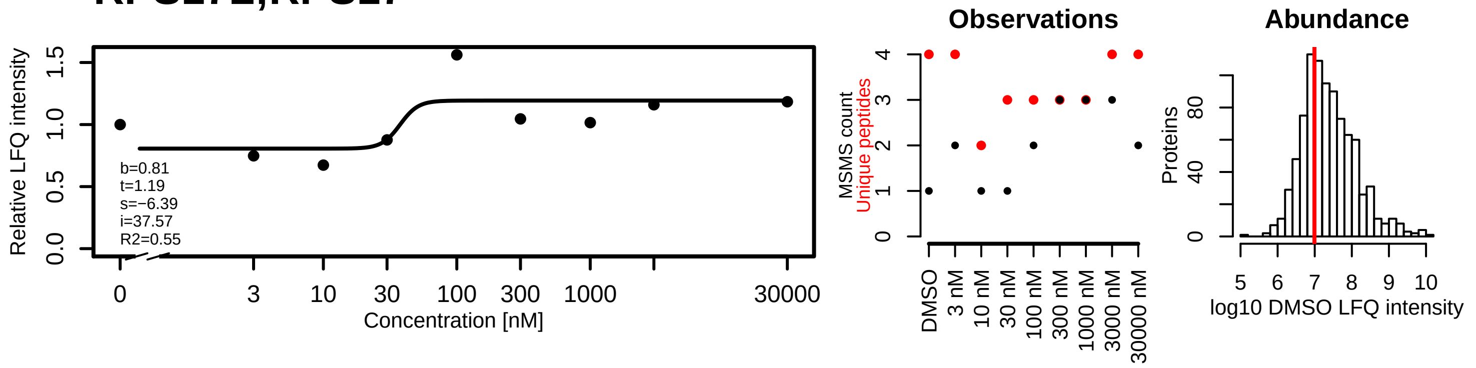

ERH

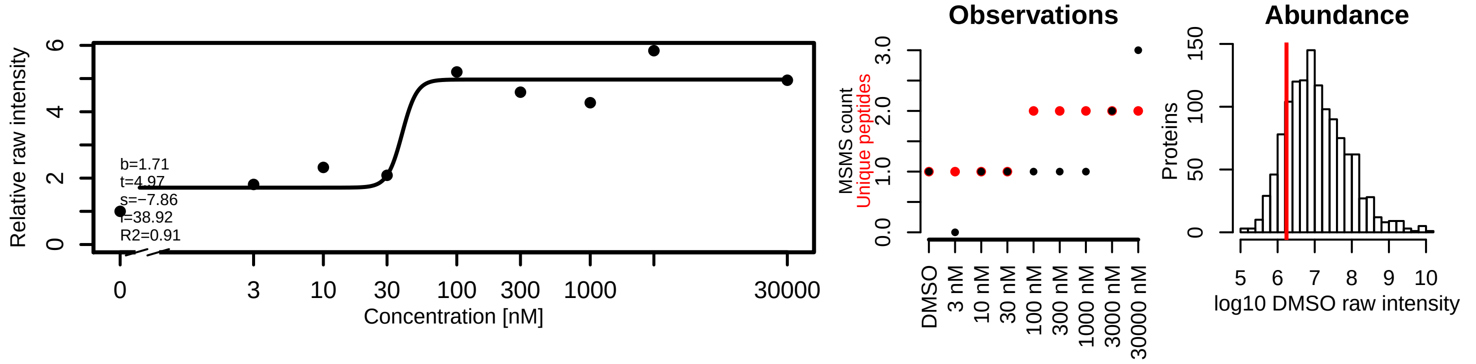

HIST1H2BL;HIST1H2BM;HIST1H2BN;HIST1H2BH;HIST2H2BF;HIST1H

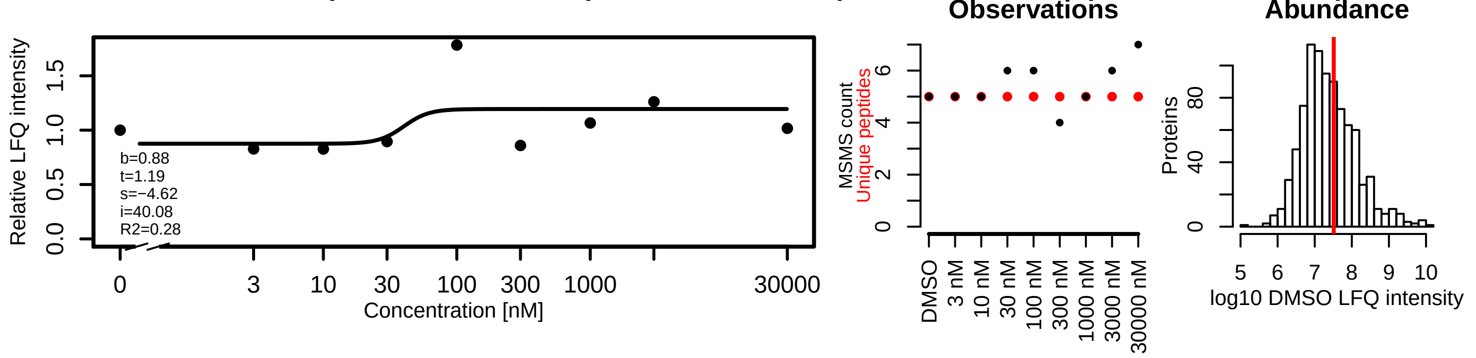

HNRNPA1

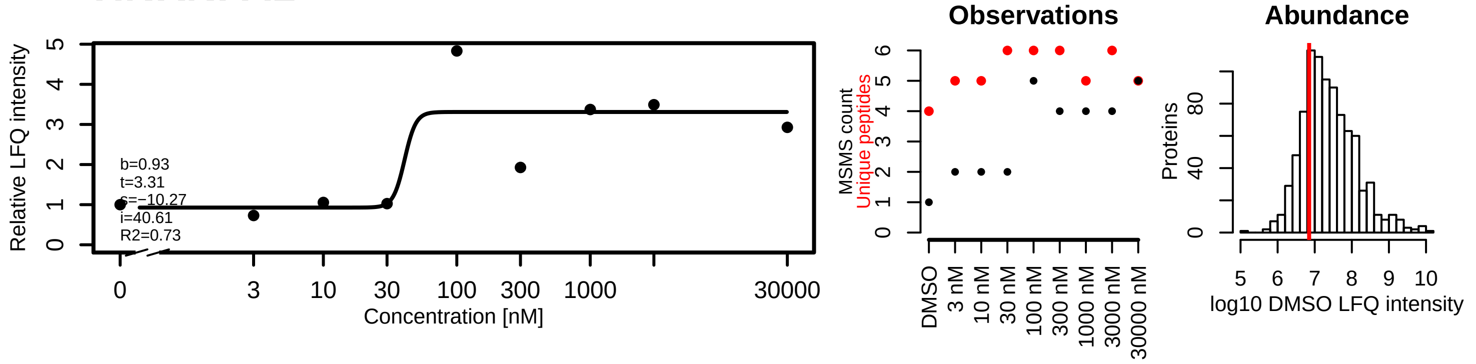

HEATR1

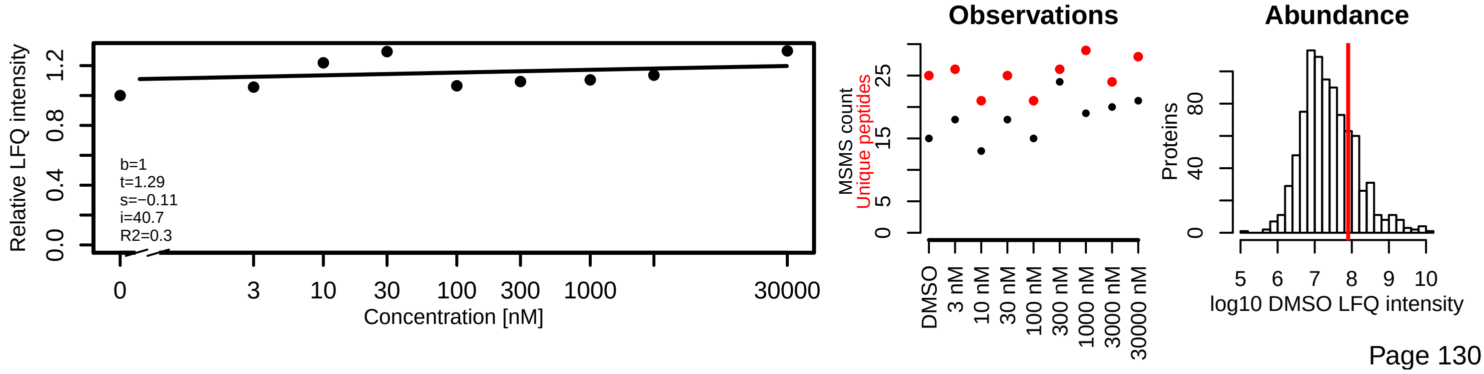

RPS5

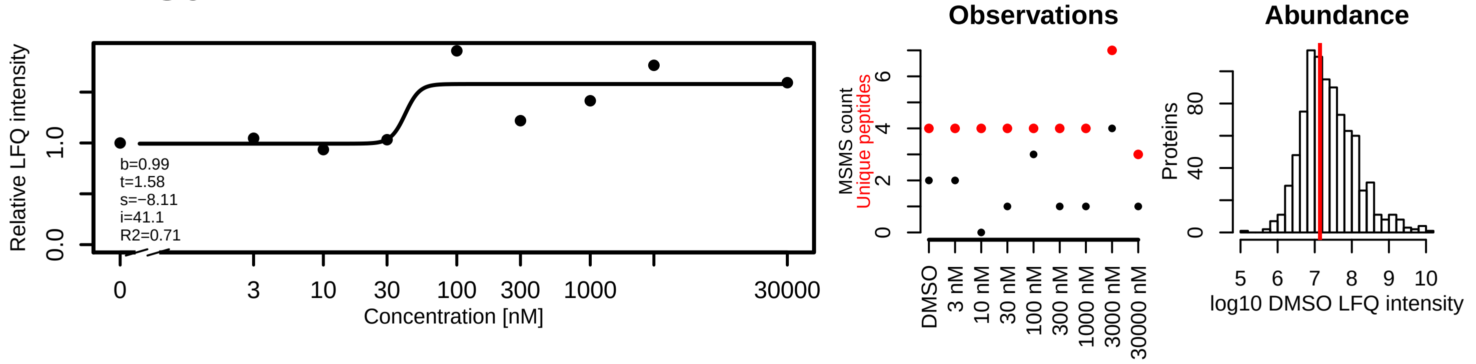

RPL7

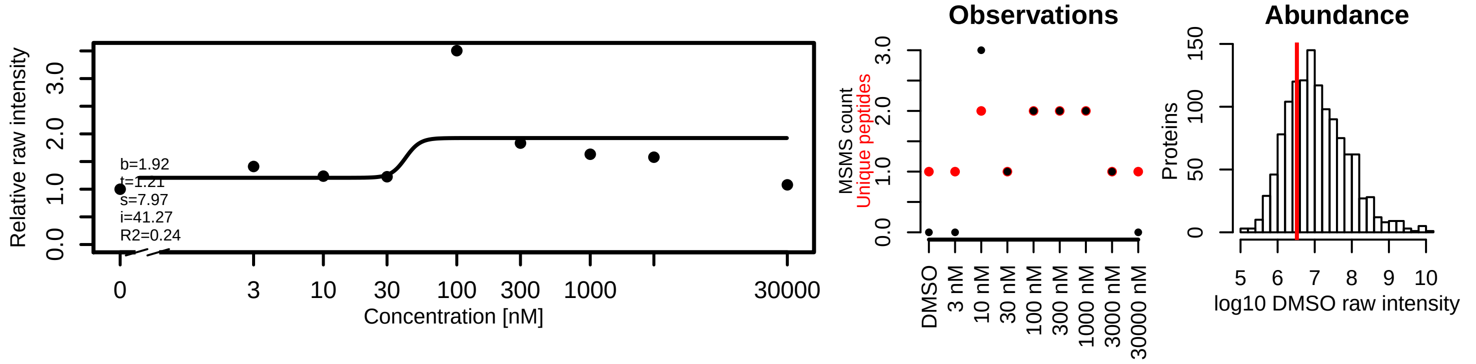

NCL

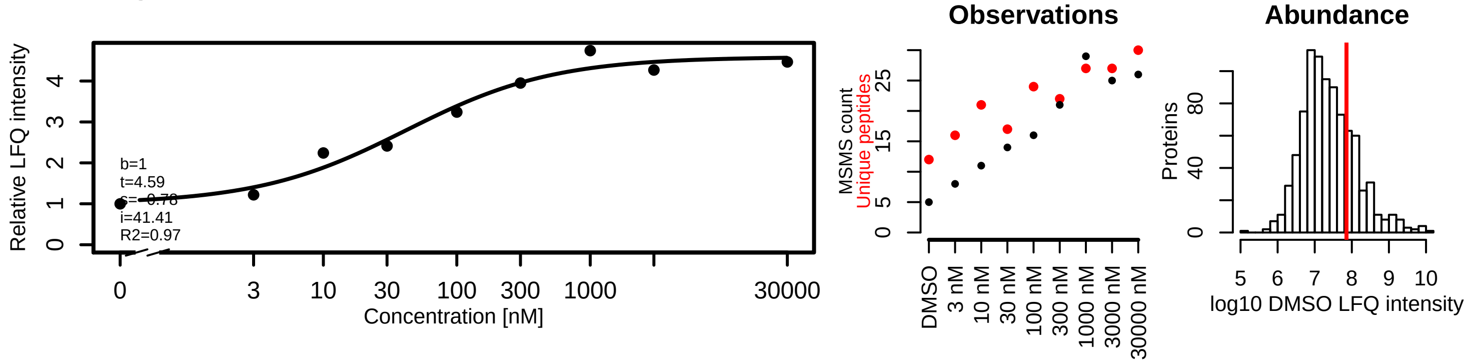

FUBP1

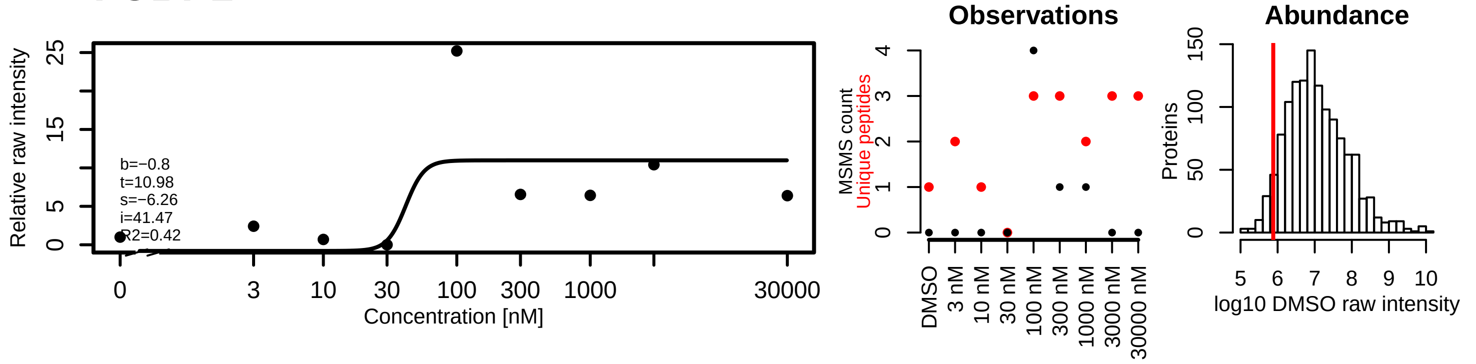

RCE1

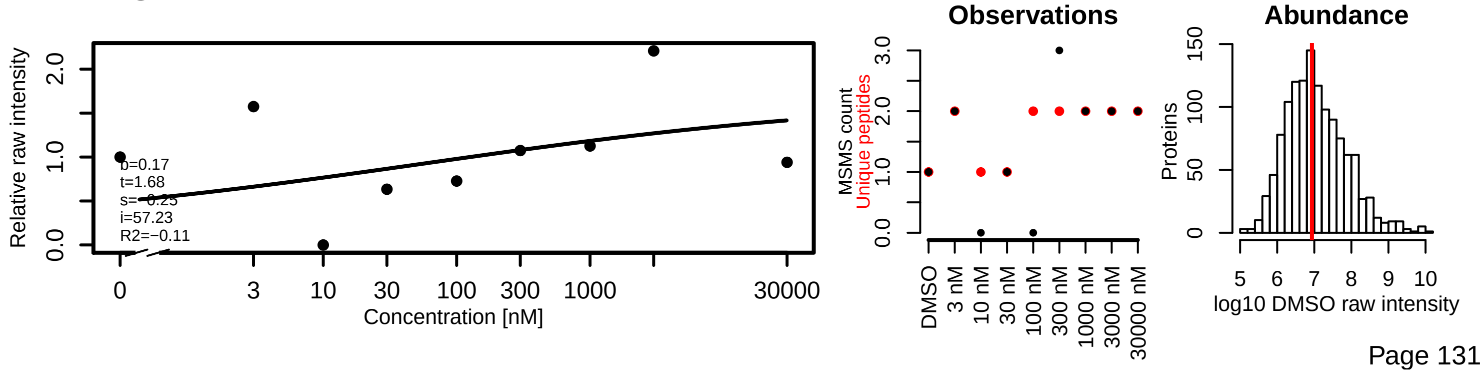

DDX3X;DDX3Y

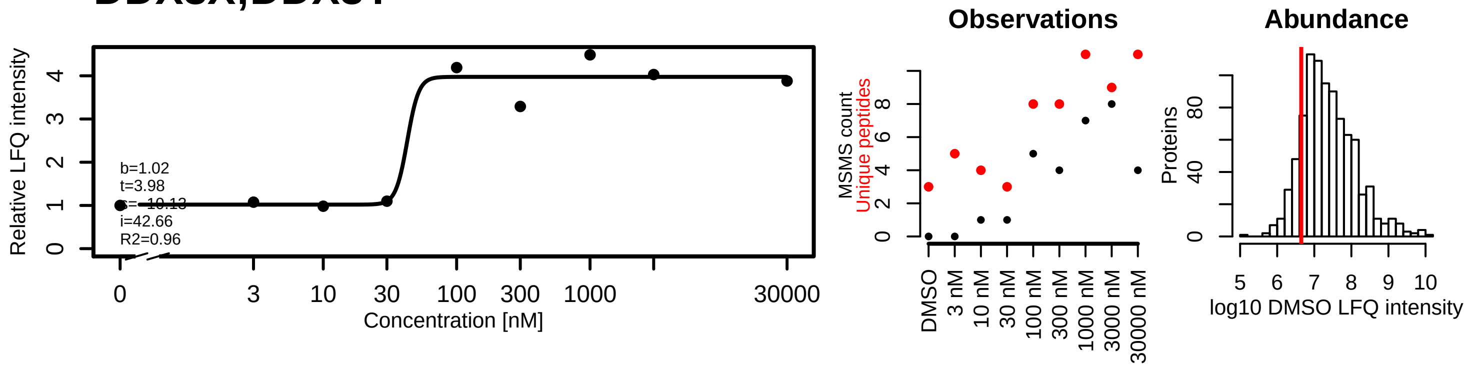

TMPO

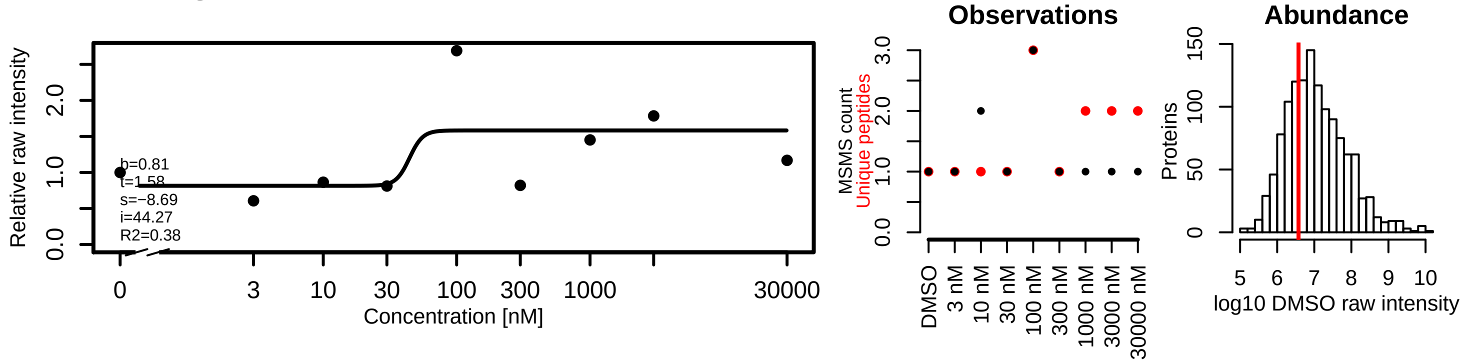

RPS29

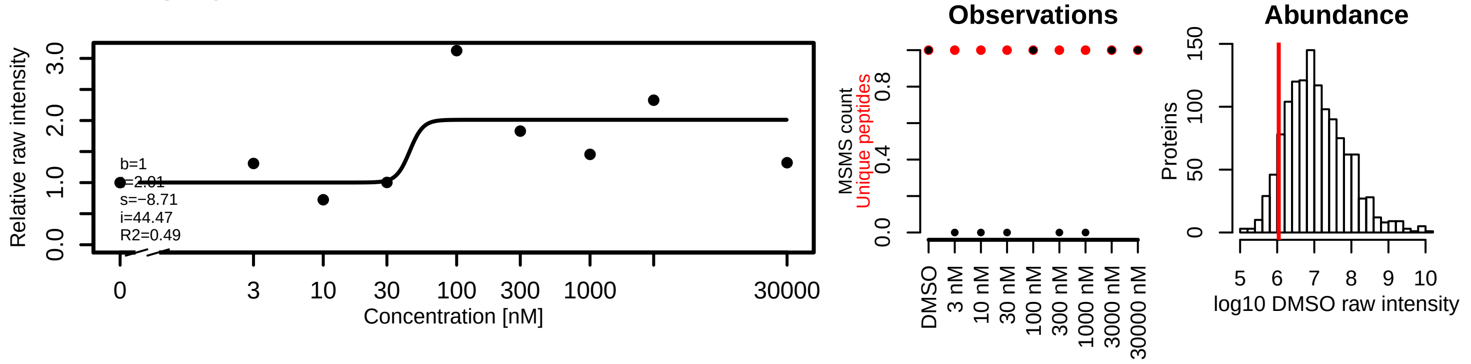

LDHB

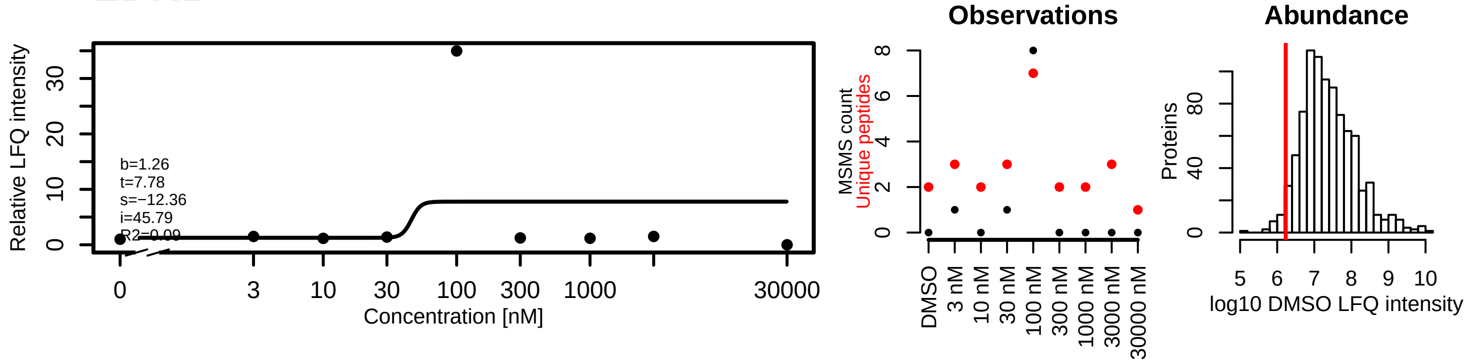

RPS3

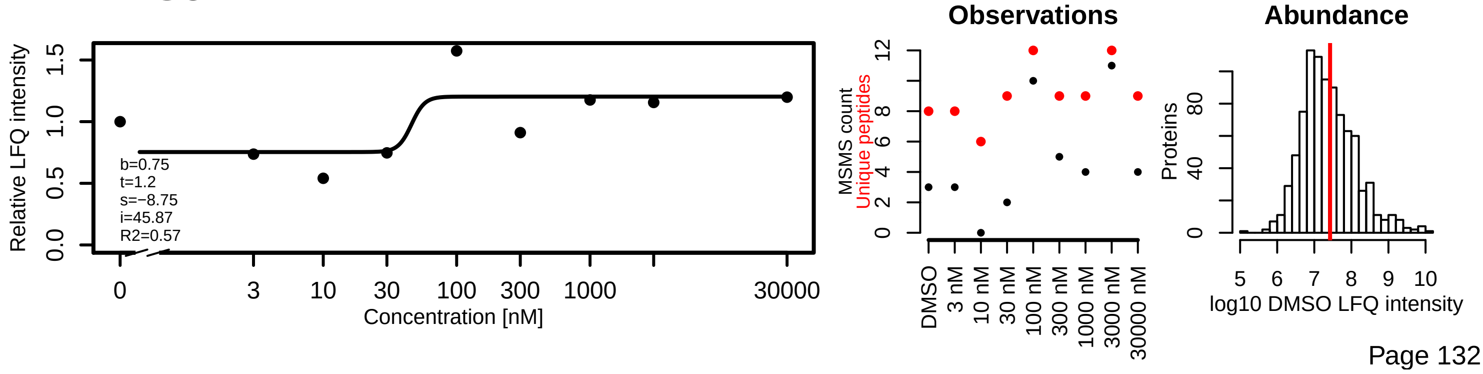

PGK1

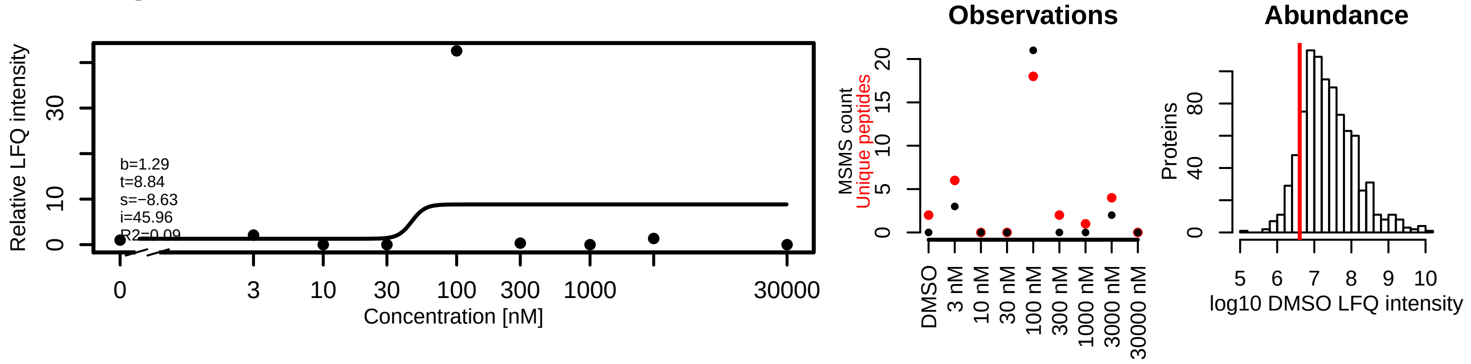

PSMC3

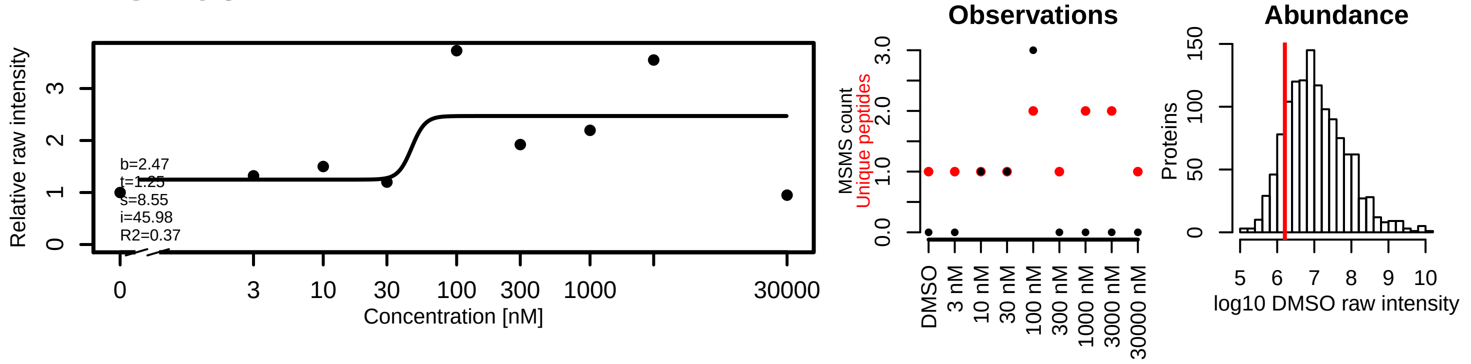

RPS14

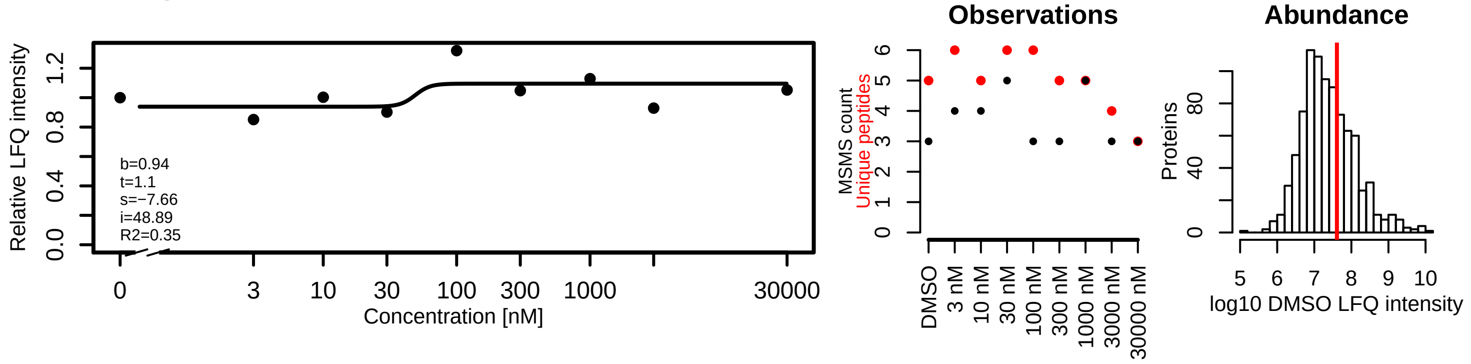

TMEM115

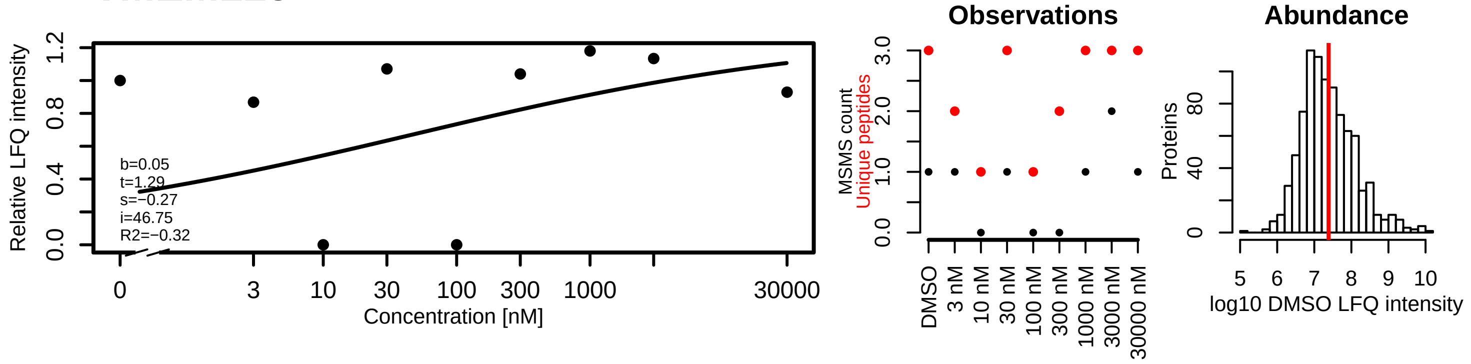

ANXA2;ANXA2P2

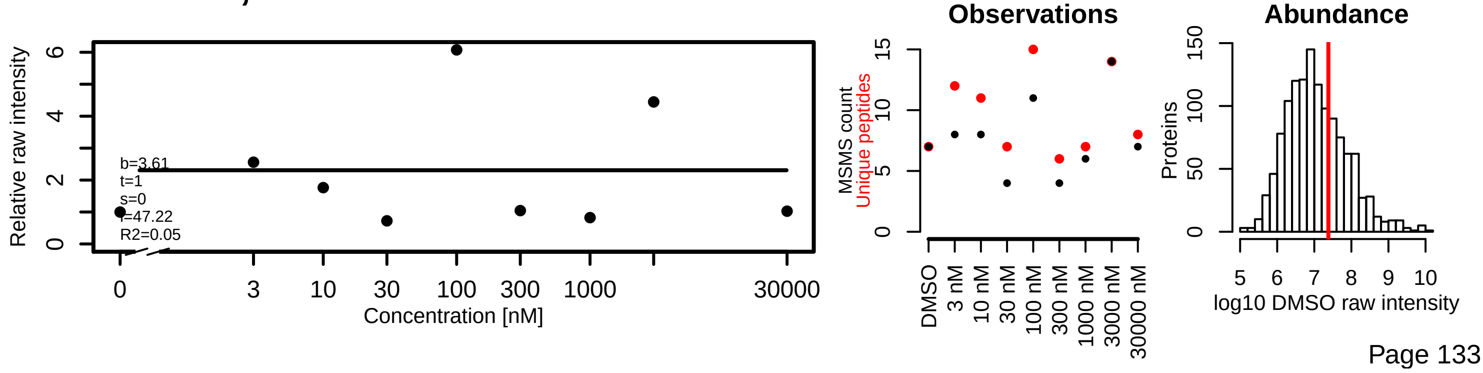

AHCY

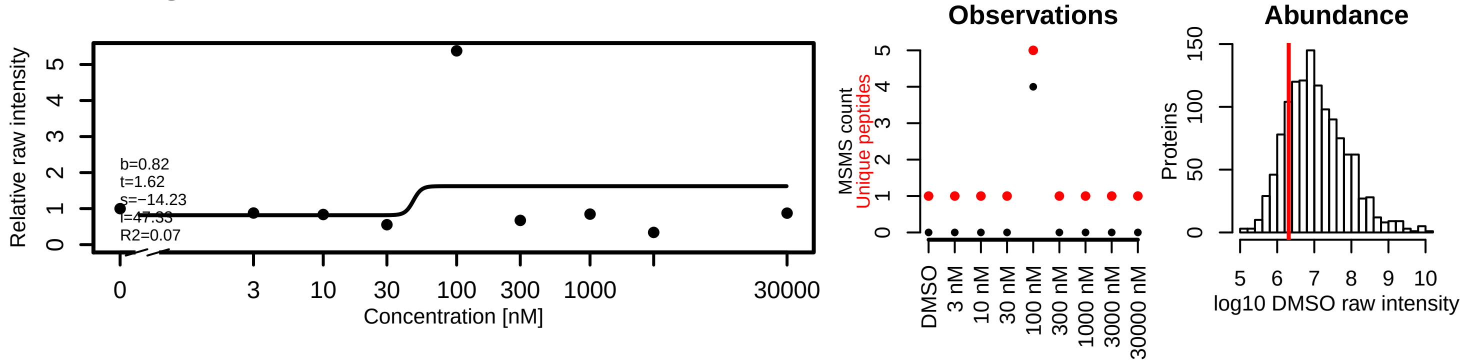

NME1−NME2;NME2;NME1;NME2P1

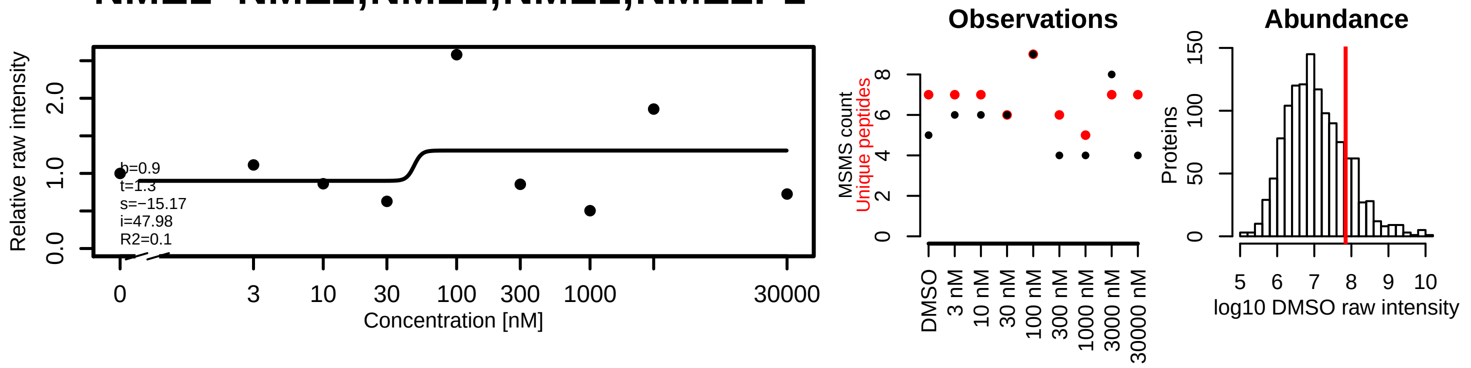

EEF1D

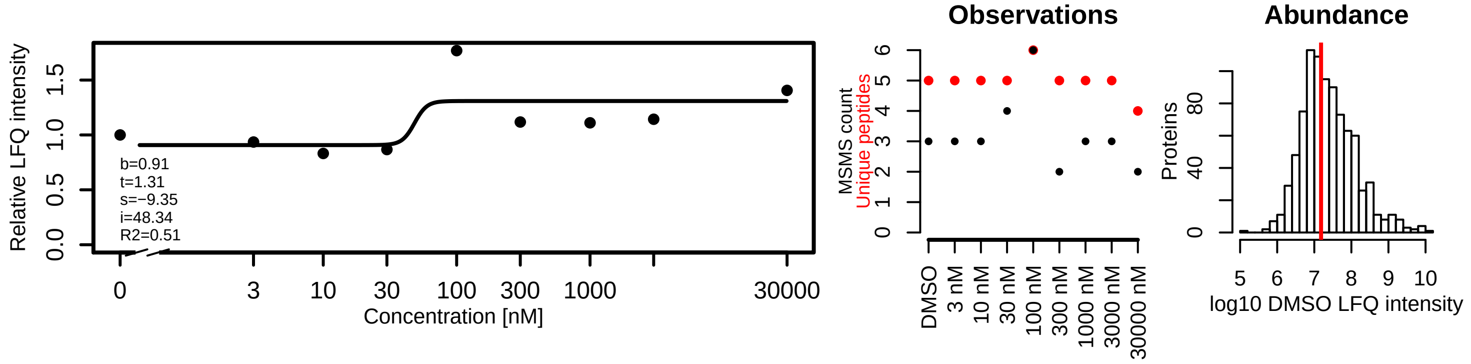

SMC4

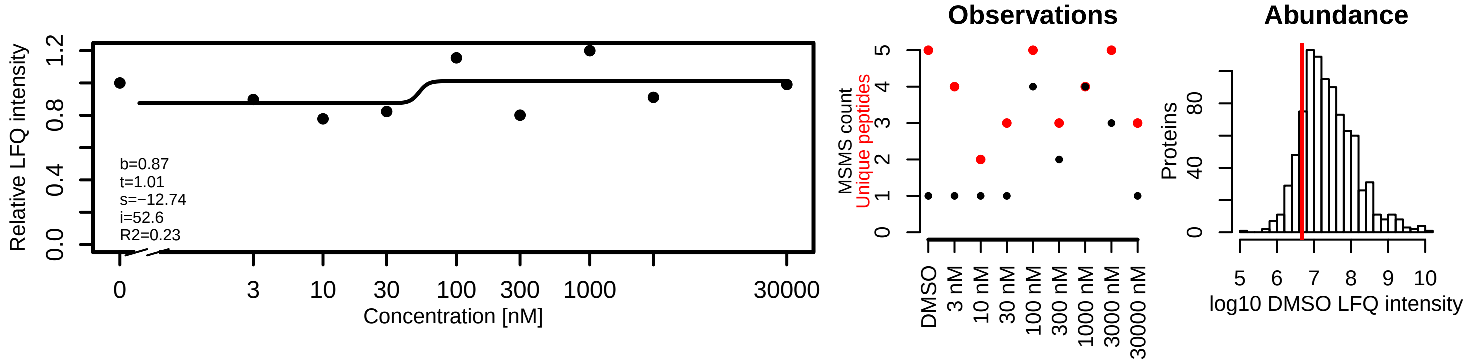

EEF2

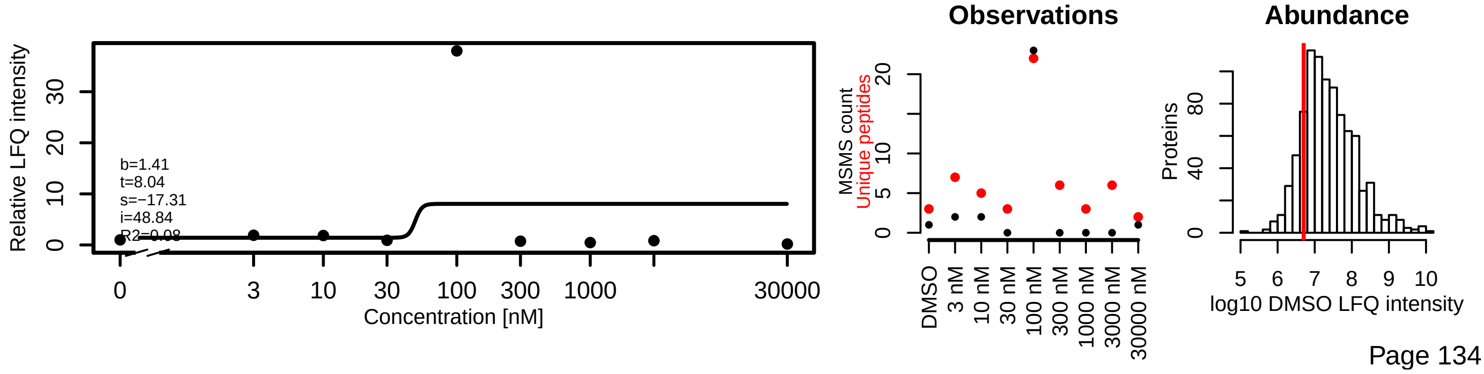

COPE

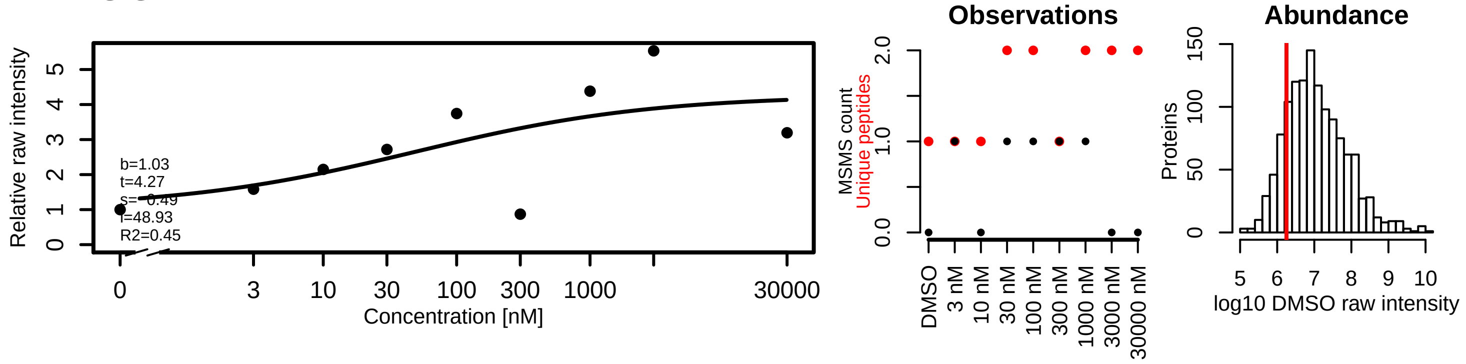

EI24

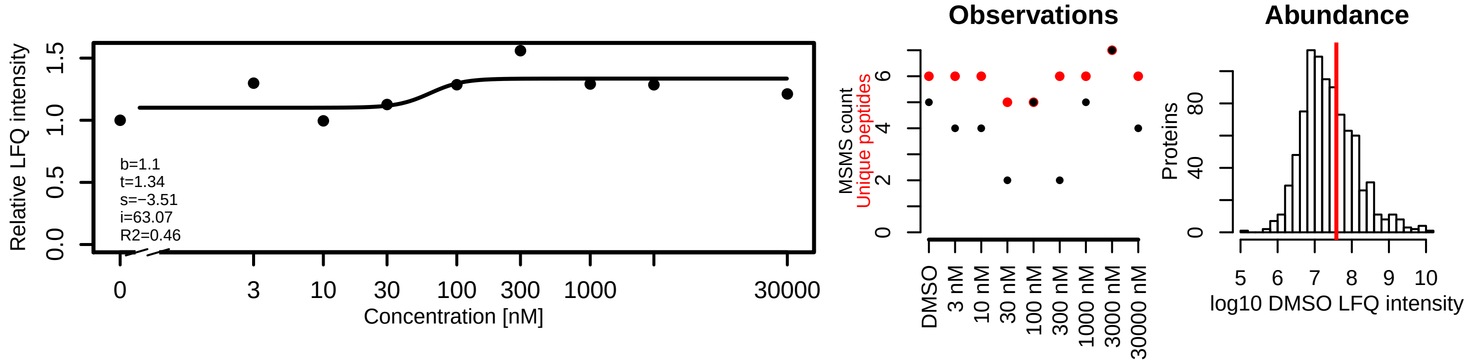

COG1

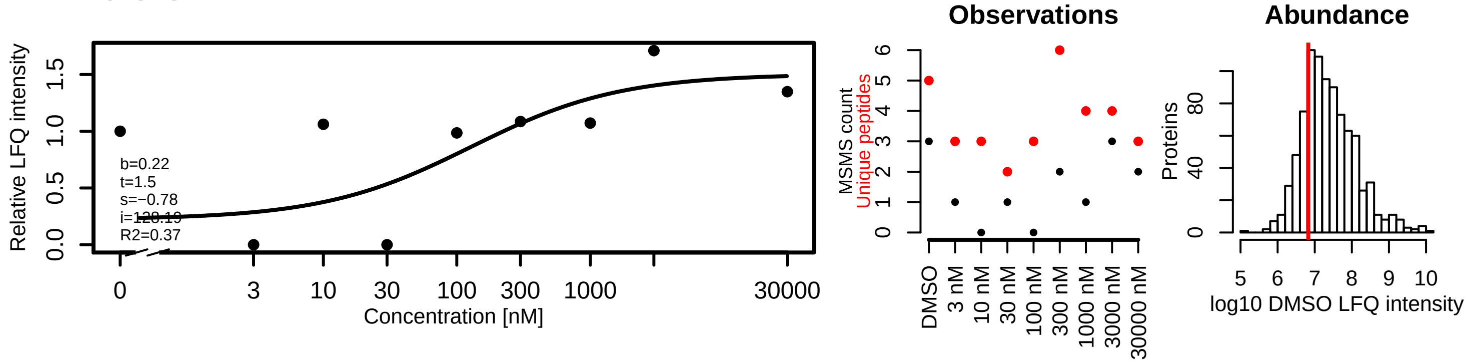

CLTC

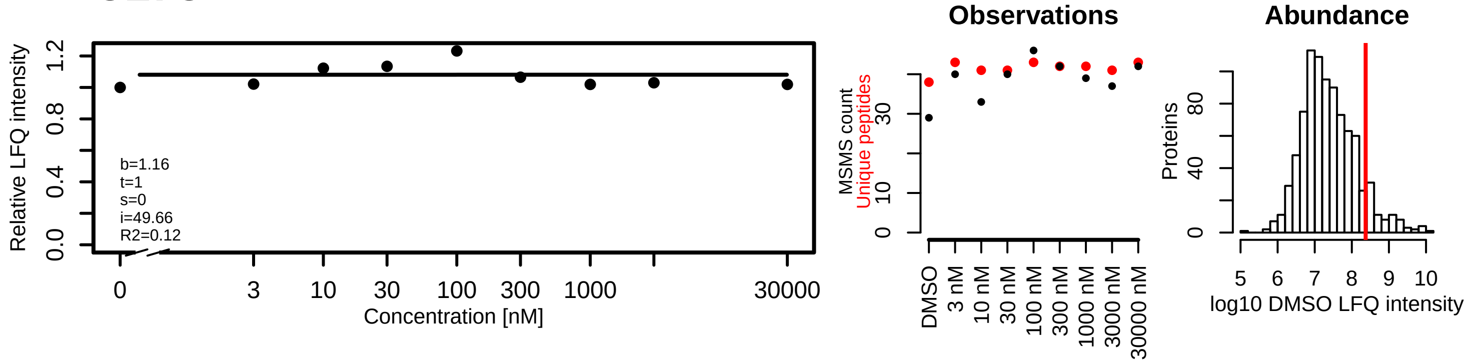

RPS20

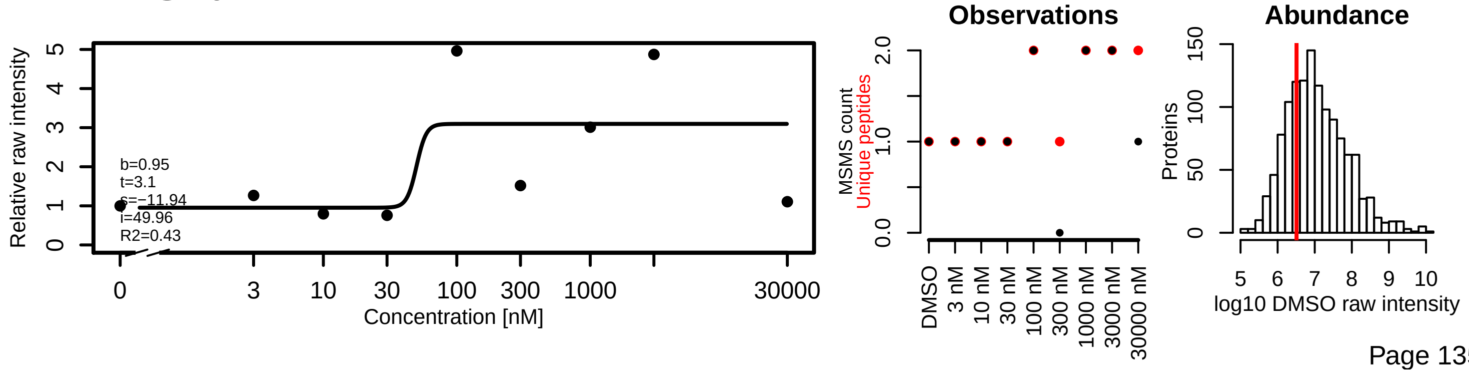

PSMC5

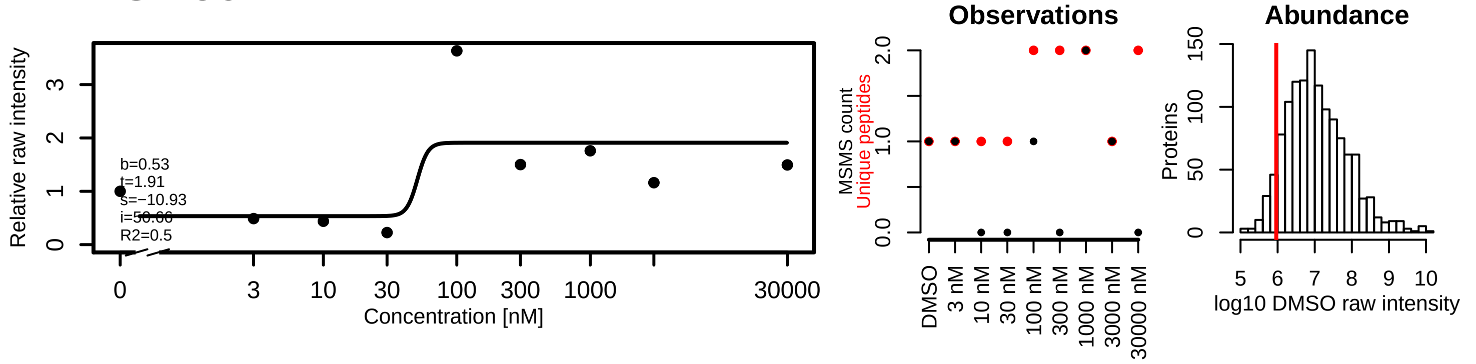

LASP1

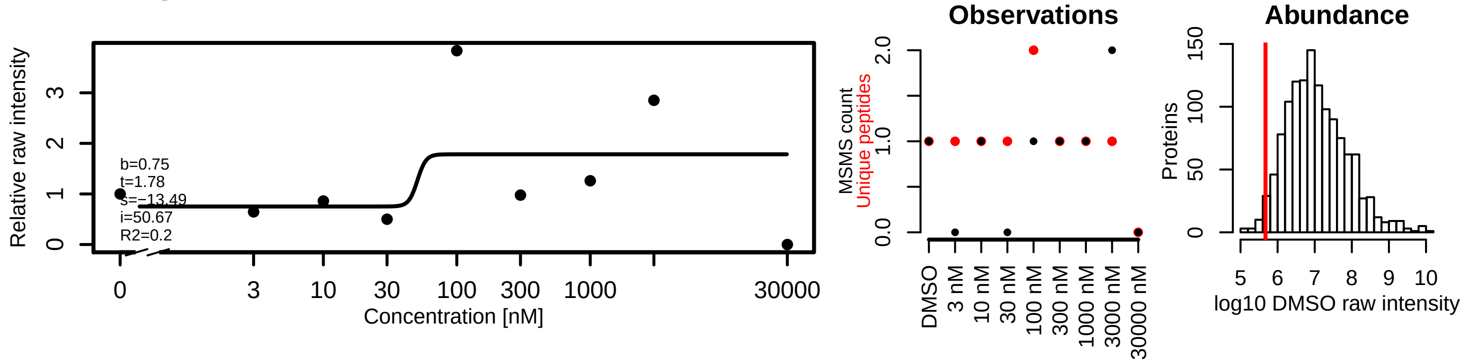

HIST1H1E;HIST1H1C;HIST1H1D

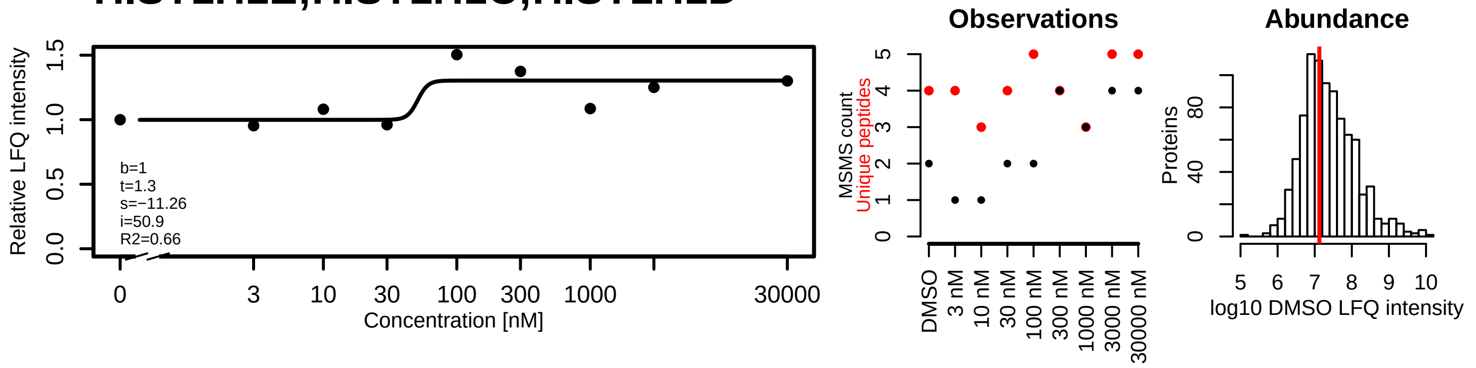

RPL38

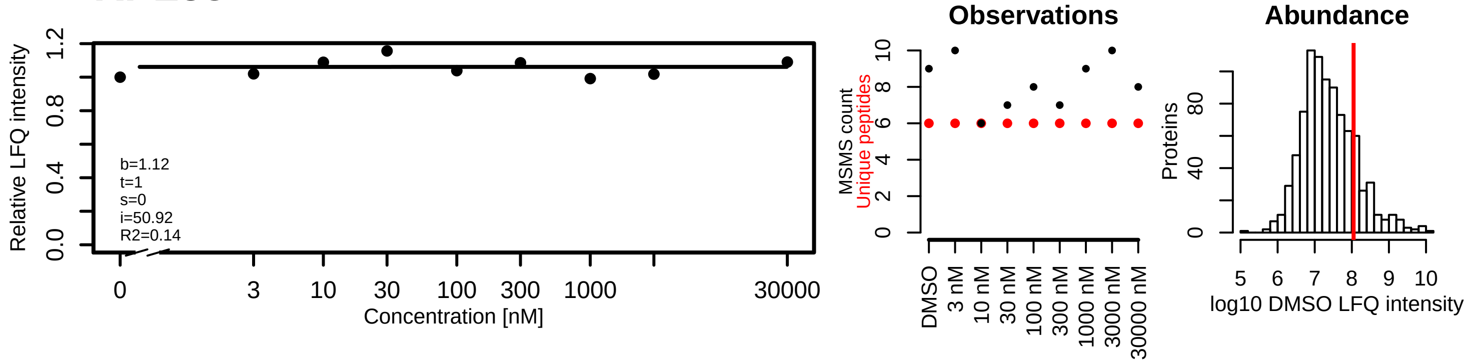

CDC37

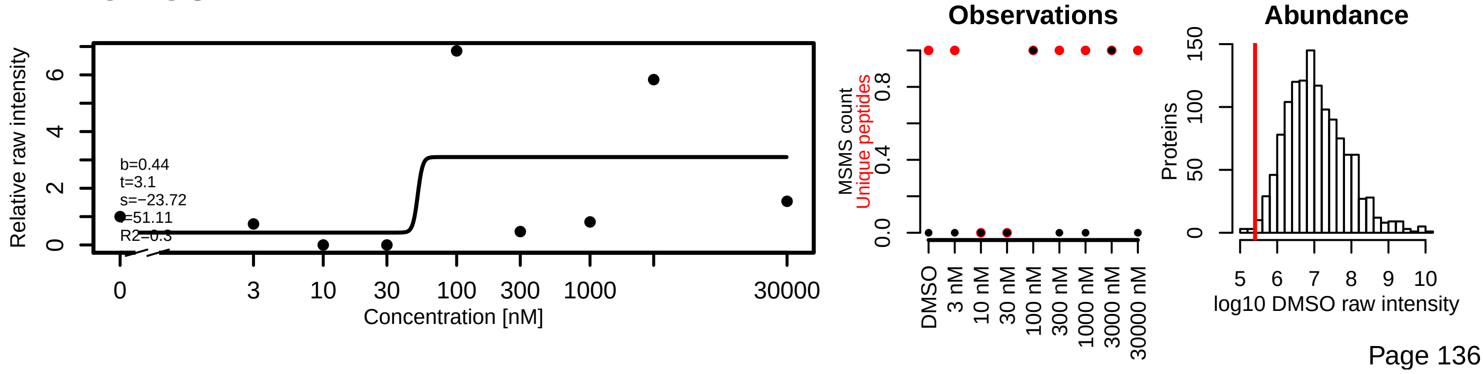

MAPK8IP3;SPAG9

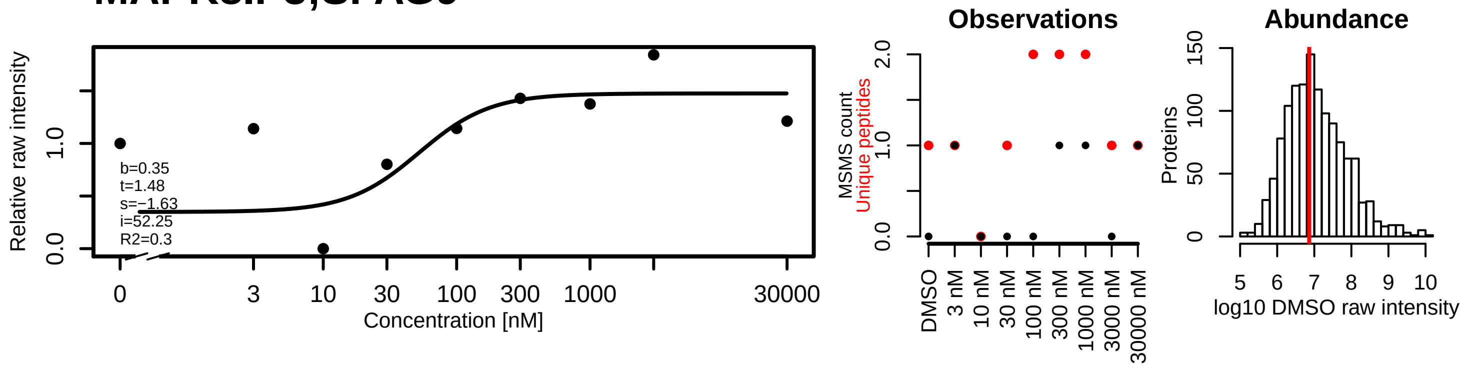

SLC38A10

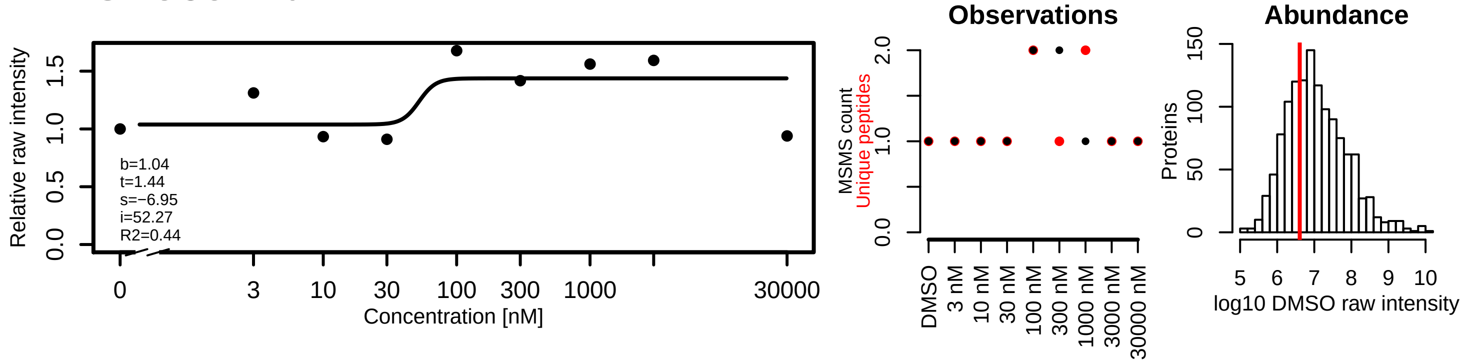

ZDHHC13

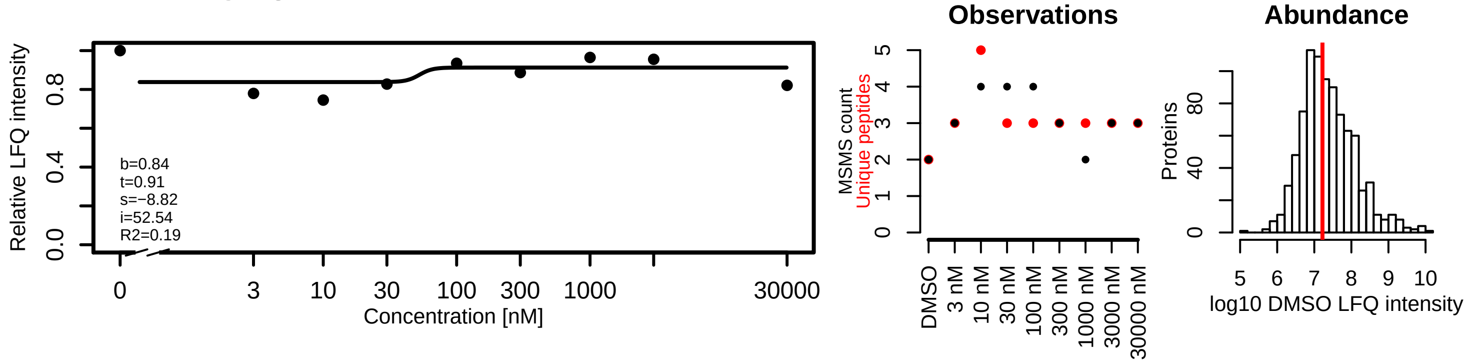

EEF1A1P5;EEF1A1;EEF1A2

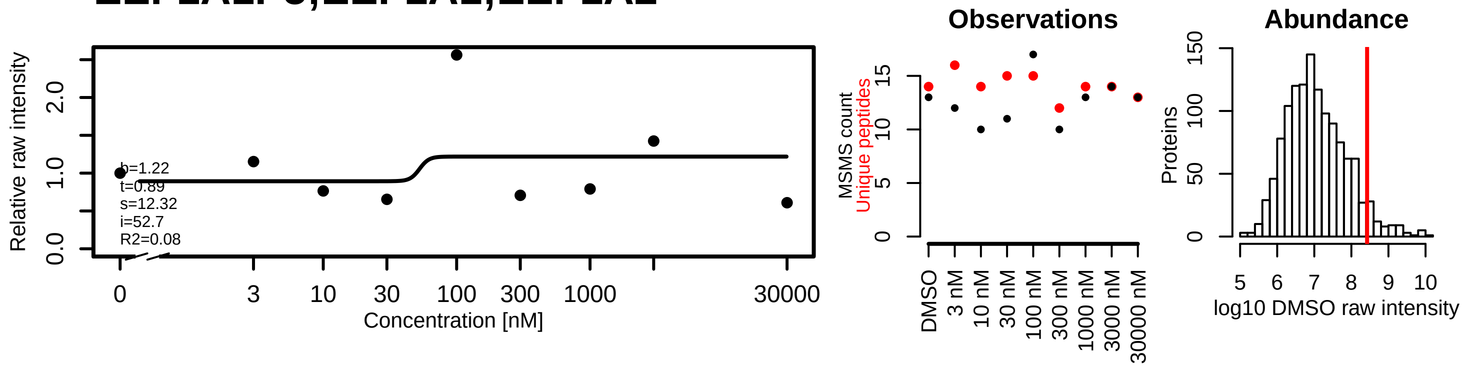

TMED9

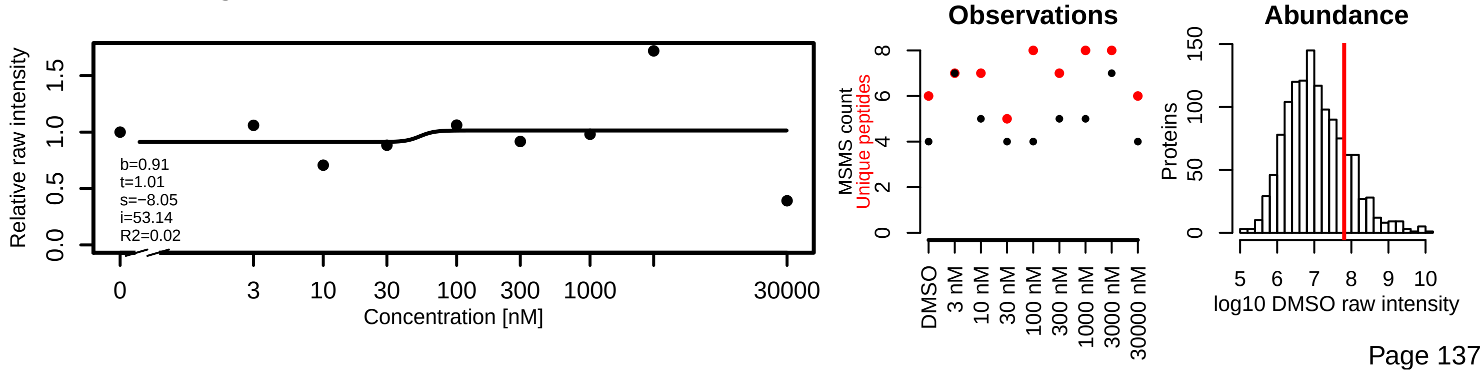

OTUB1

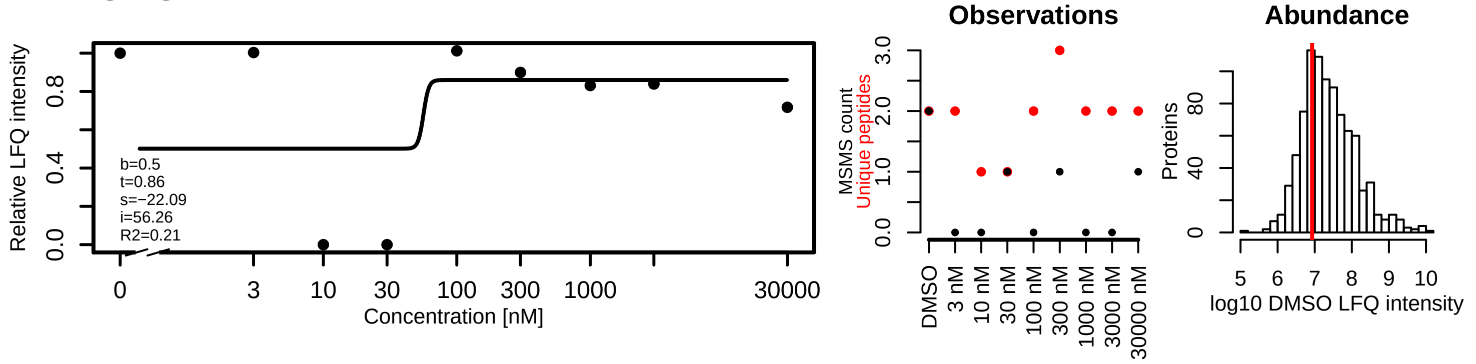

DKFZp586I031;SEPT7

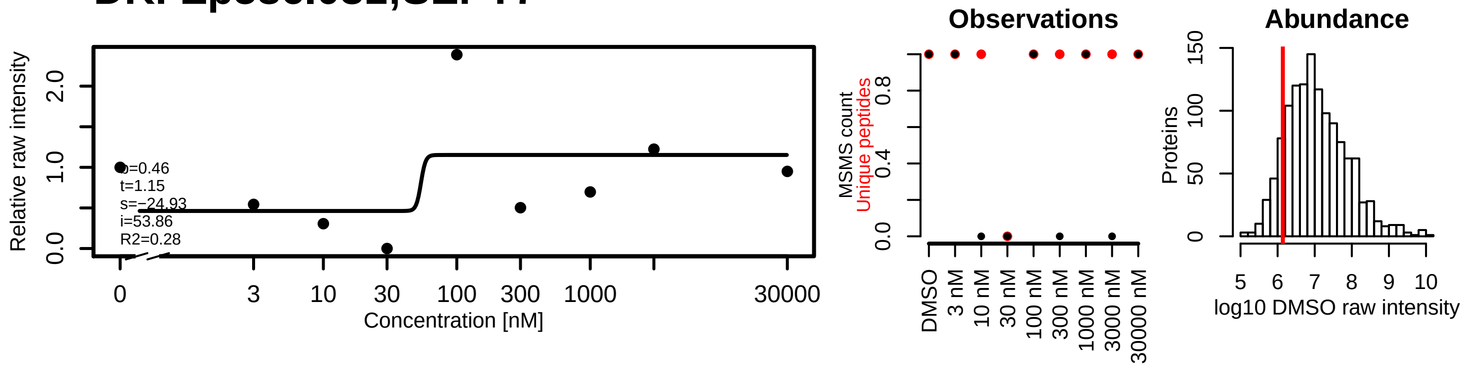

PPIB

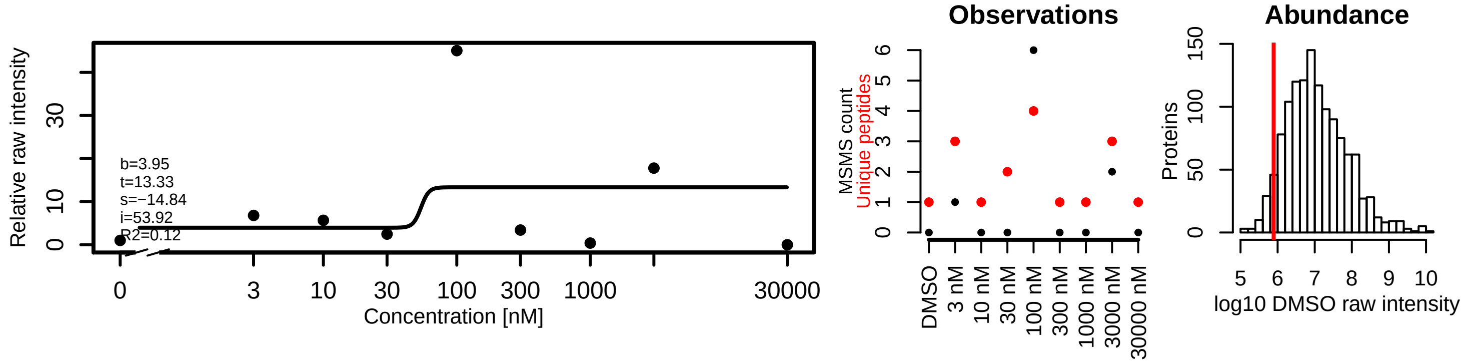

SDHA

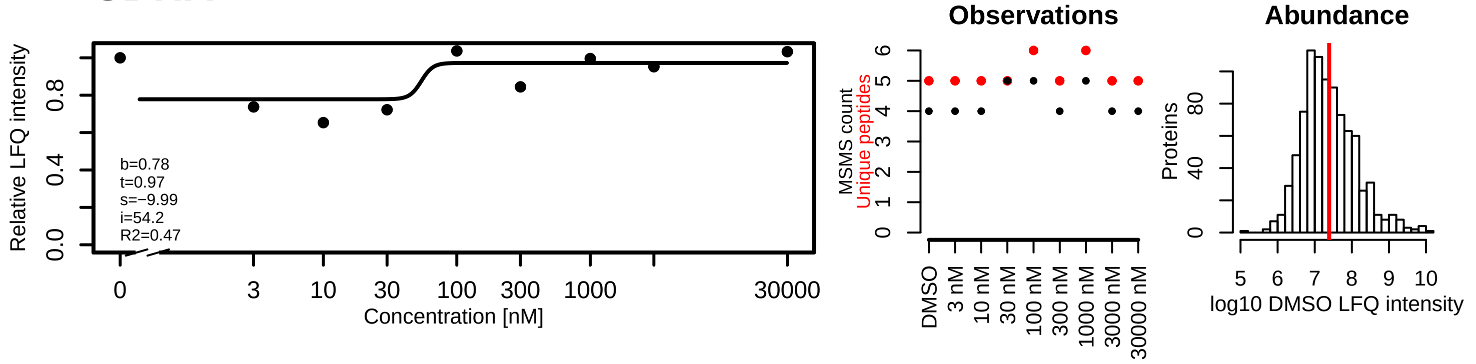

RPS21

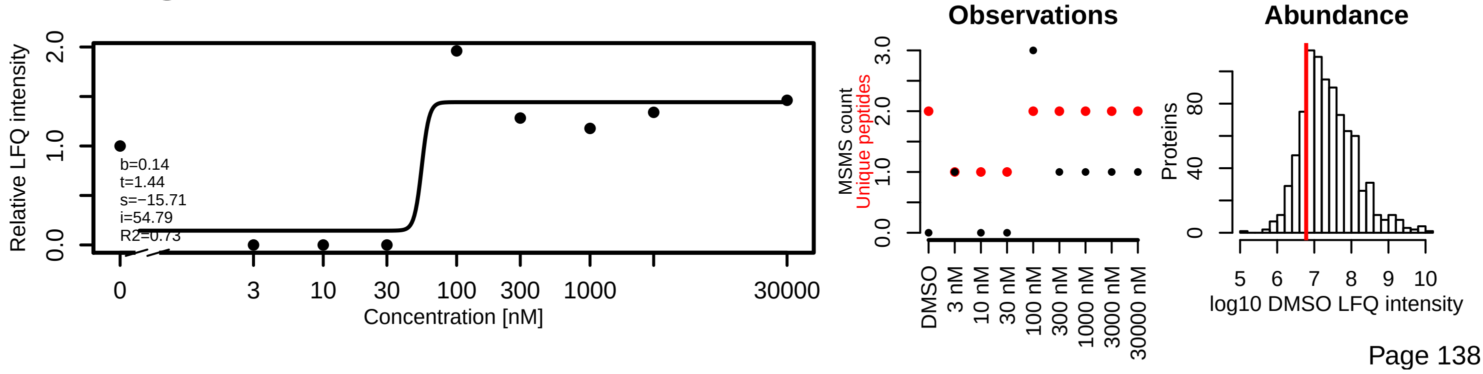

FAR1

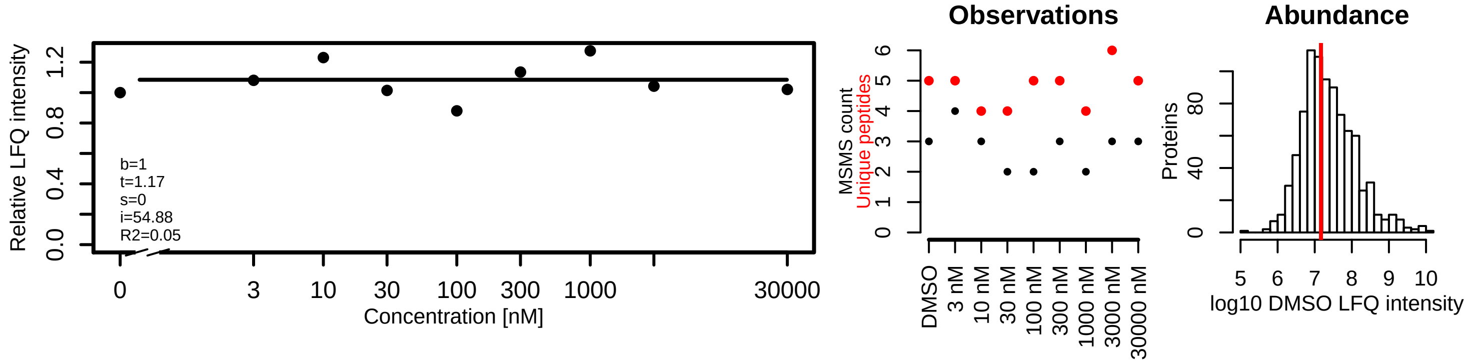

KIDINS220

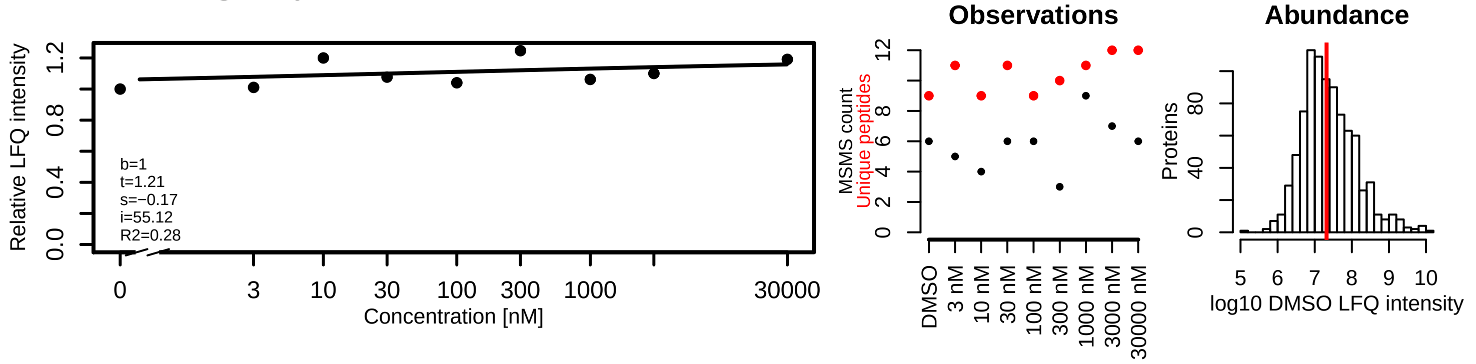

SRSF3

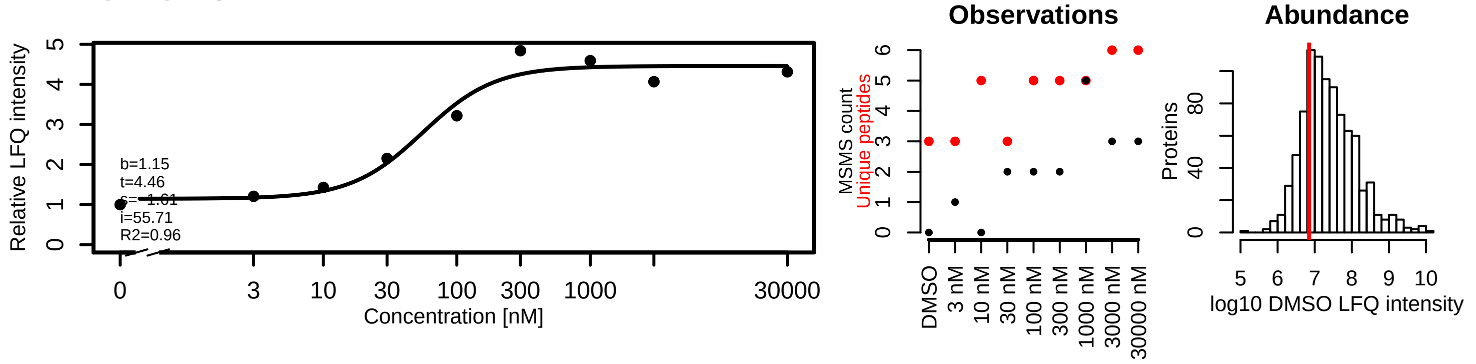

DYNC1LI1

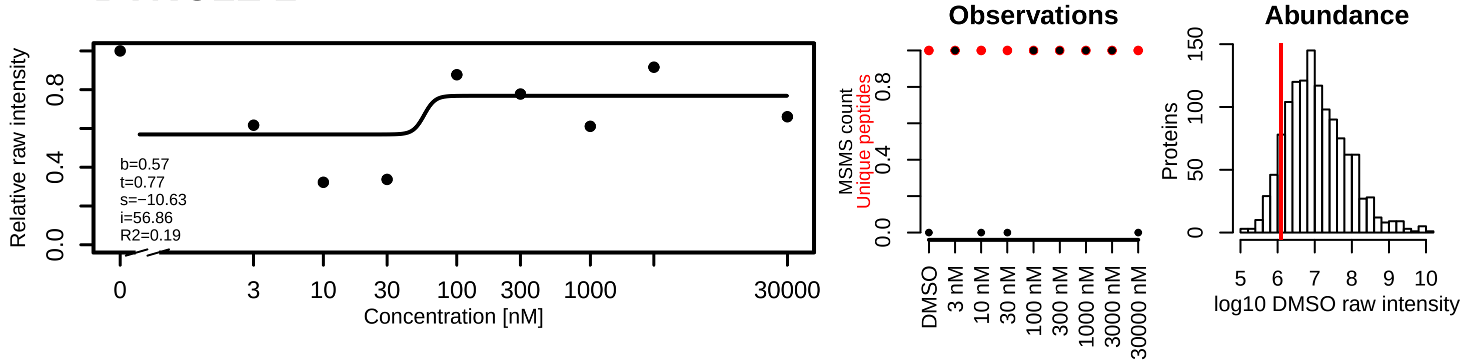

FAM96B

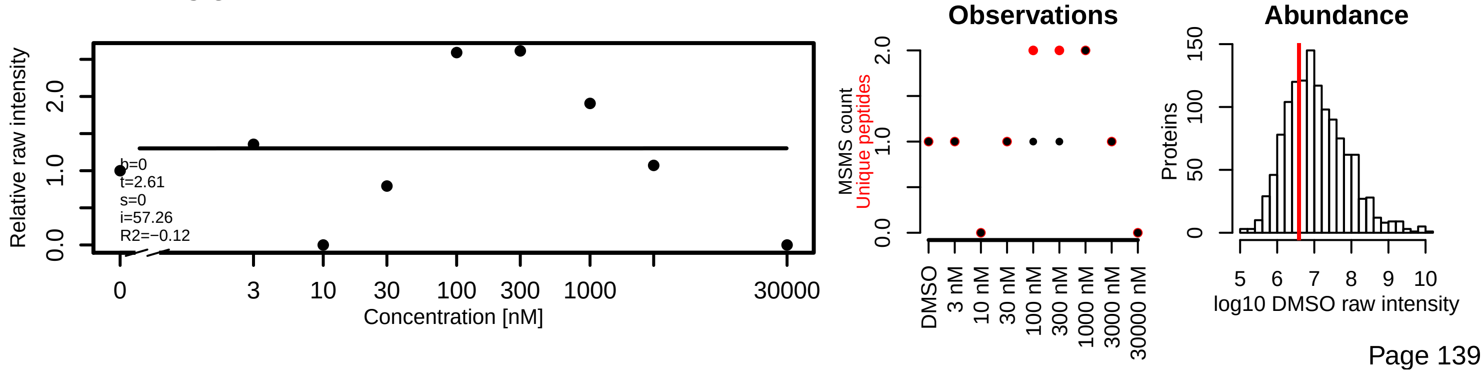

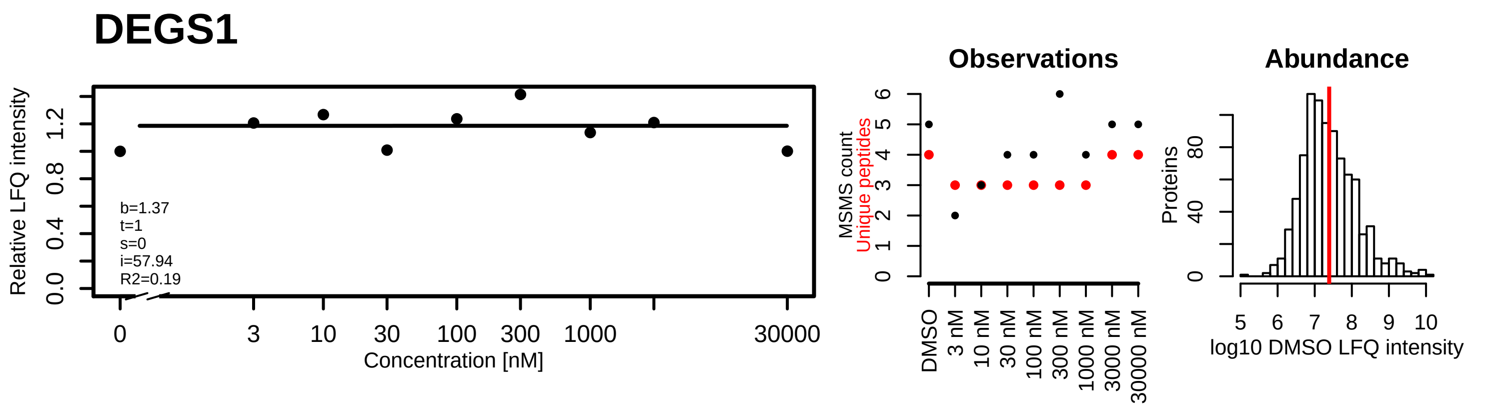

DEGS1
Relative LFQ intensity
b=1.37
t=1
s=0
i=57.94
R2=0.19
Concentration [nM]
Observations
MSMS count
Unique peptides
Abundance
Proteins
log10 DMSO LFQ intensity

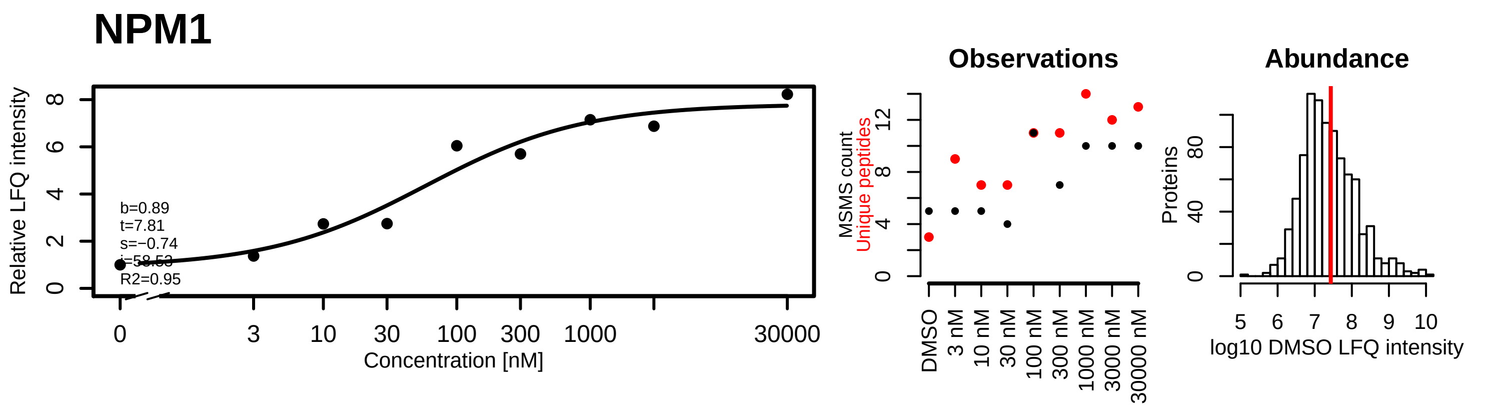

NPM1
Relative LFQ intensity
b=0.89
t=7.81
s=−0.74
i=[illegible]
R2=0.95
Concentration [nM]
Observations
MSMS count
Unique peptides
Abundance
Proteins
log10 DMSO LFQ intensity

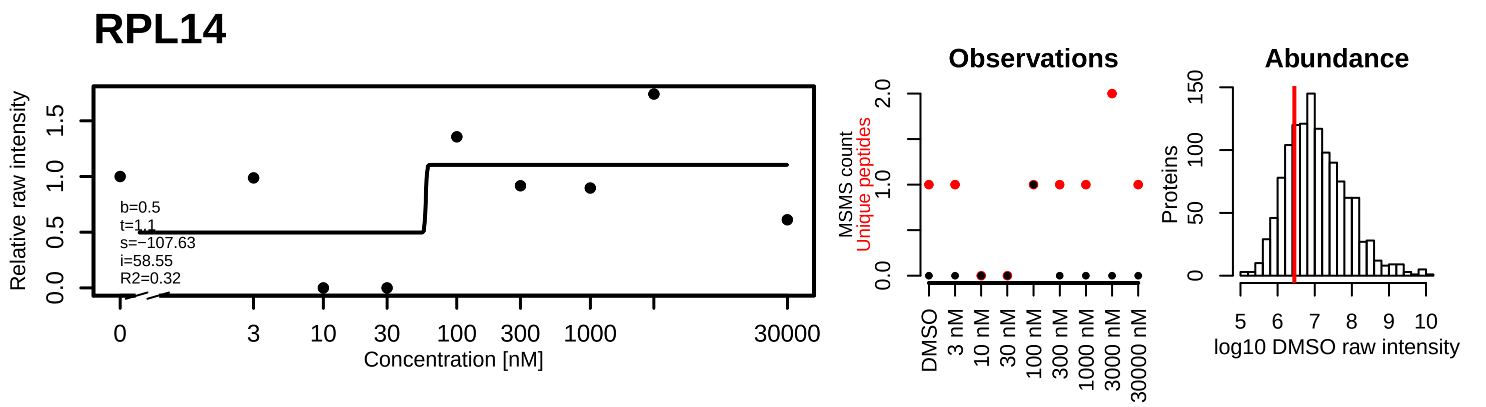

RPL14
Relative raw intensity
b=0.5
t=1.1
s=−107.63
i=58.55
R2=0.32
Concentration [nM]
Observations
MSMS count
Unique peptides
Abundance
Proteins
log10 DMSO raw intensity

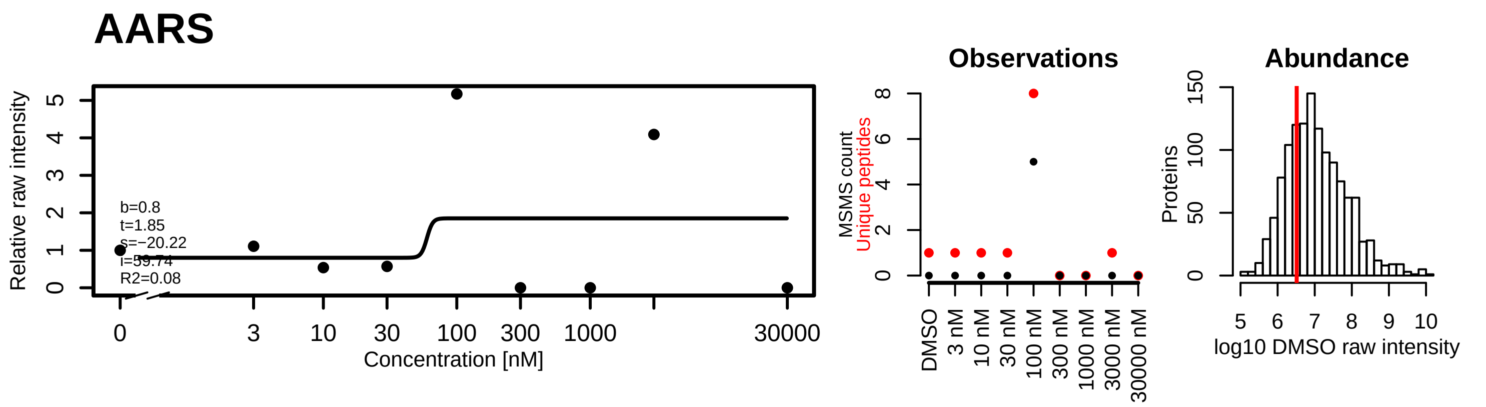

AARS
Relative raw intensity
b=0.8
t=1.85
s=−20.22
i=[illegible]
R2=0.08
Concentration [nM]
Observations
MSMS count
Unique peptides
Abundance
Proteins
log10 DMSO raw intensity

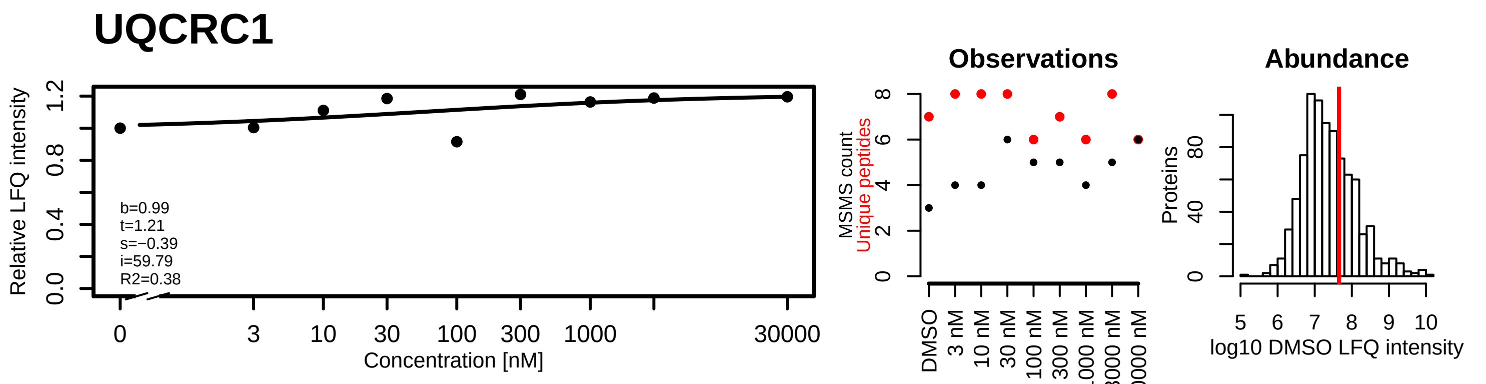

UQCRC1
Relative LFQ intensity
b=0.99
t=1.21
s=−0.39
i=59.79
R2=0.38
Concentration [nM]
Observations
MSMS count
Unique peptides
Abundance
Proteins
log10 DMSO LFQ intensity

TUBB3

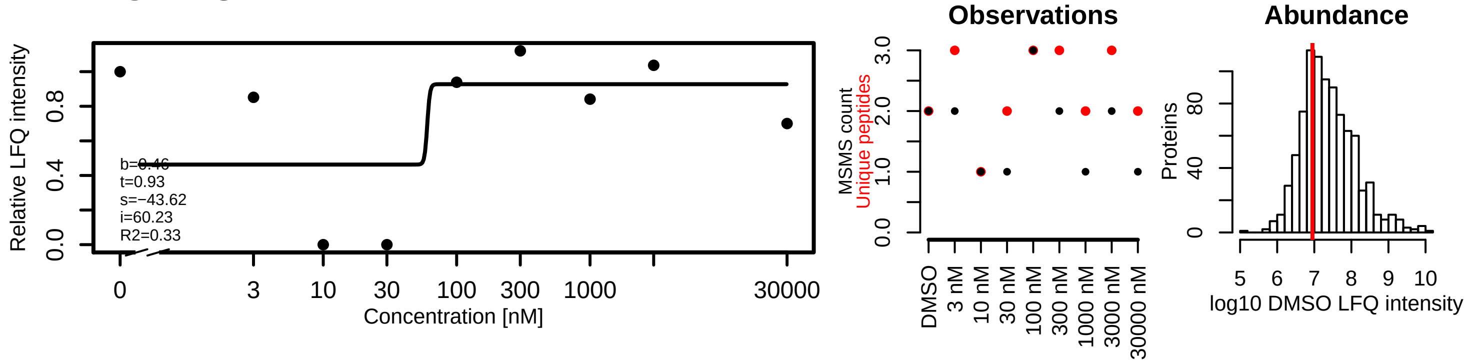

SLC39A10

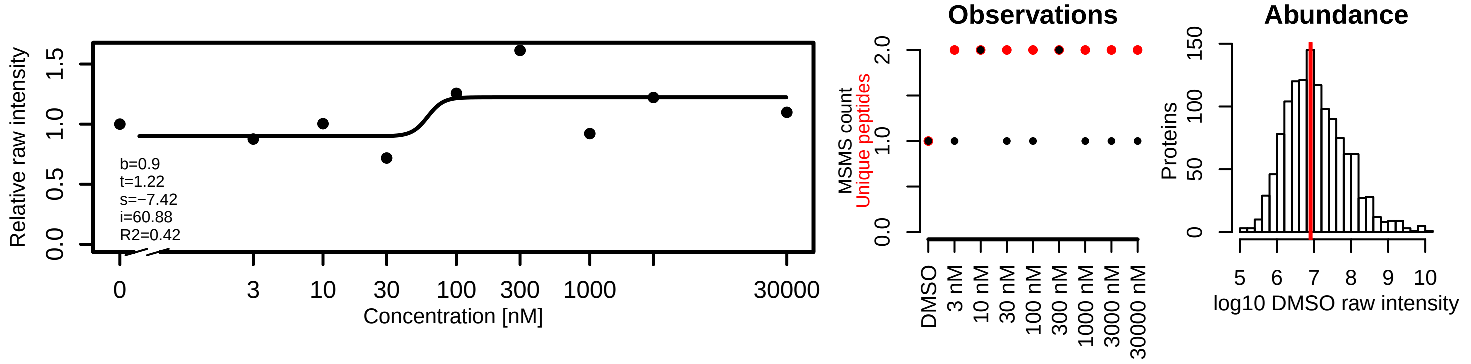

TARBP1

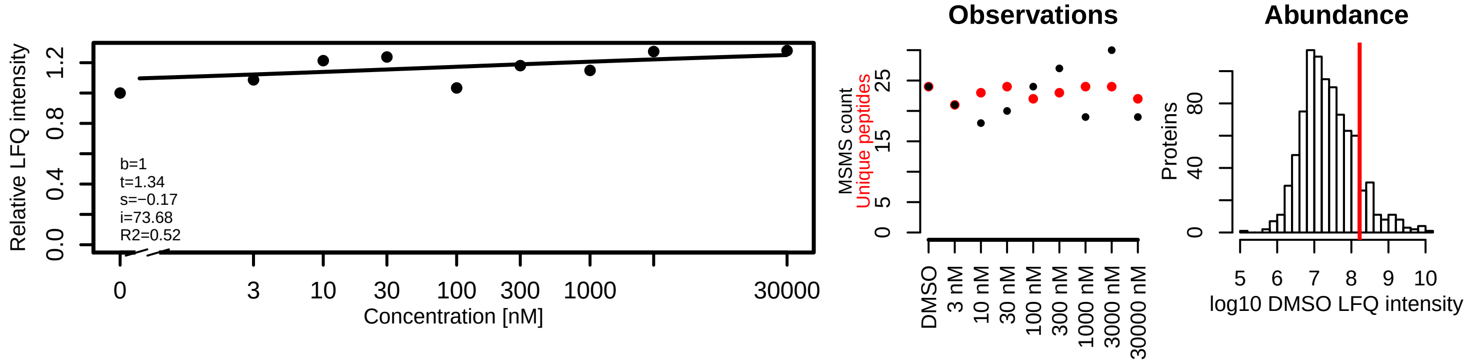

ATP5H

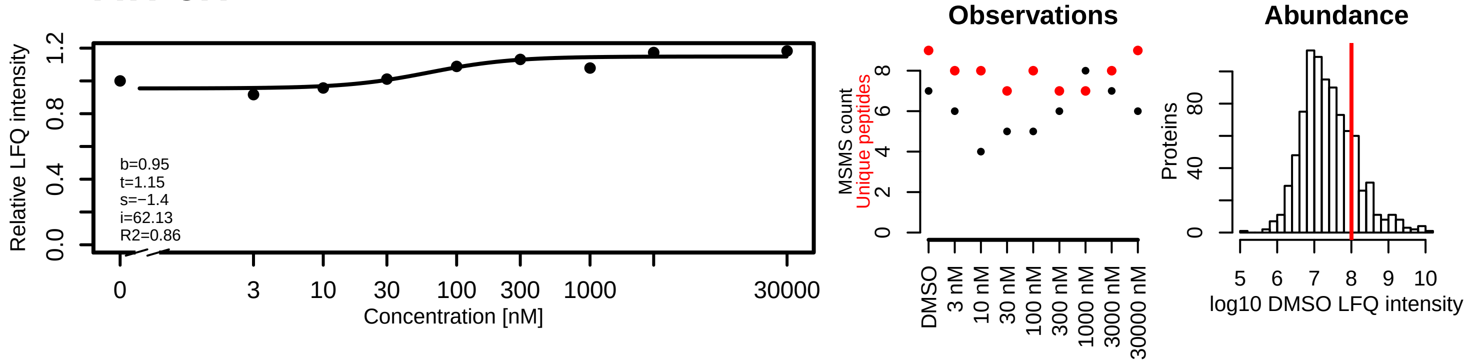

SLC25A6

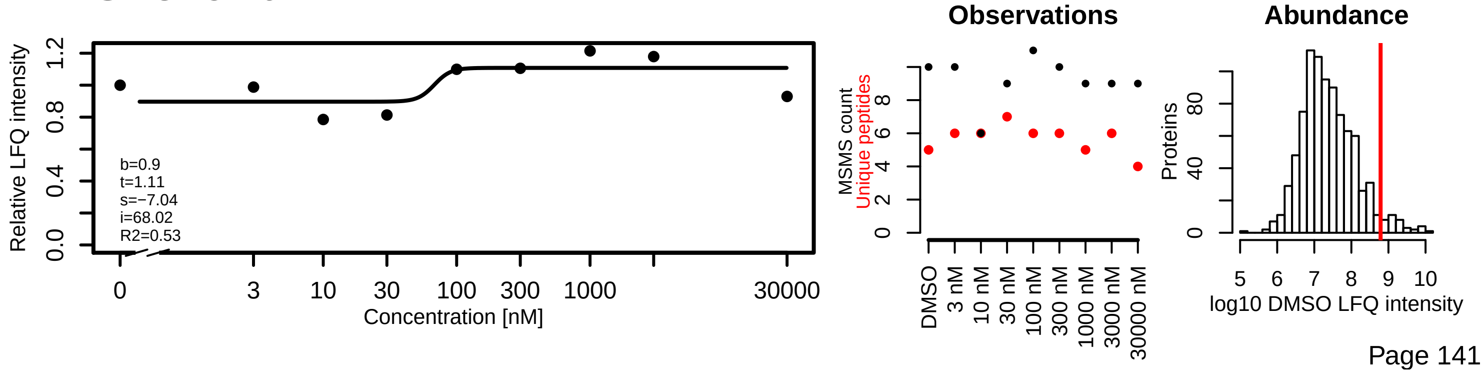

RPLP1

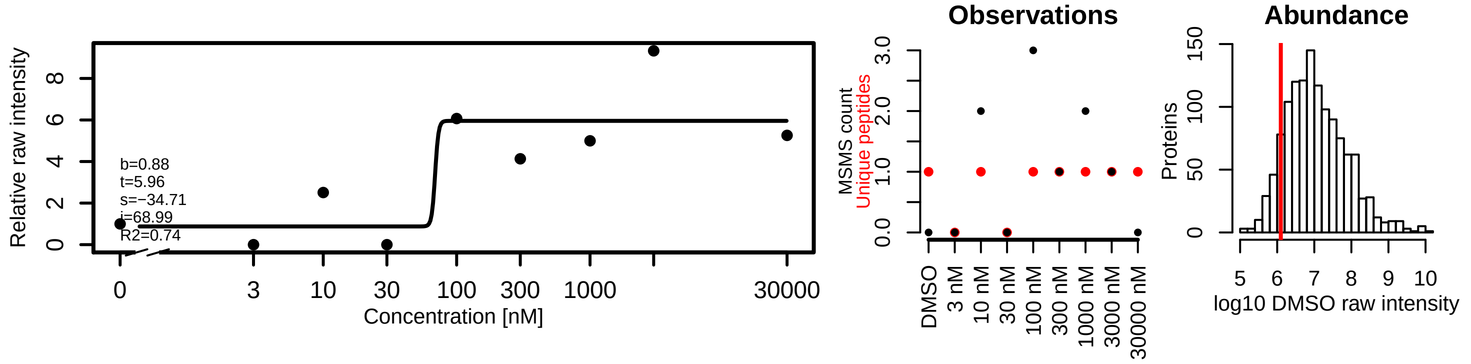

TOMM22

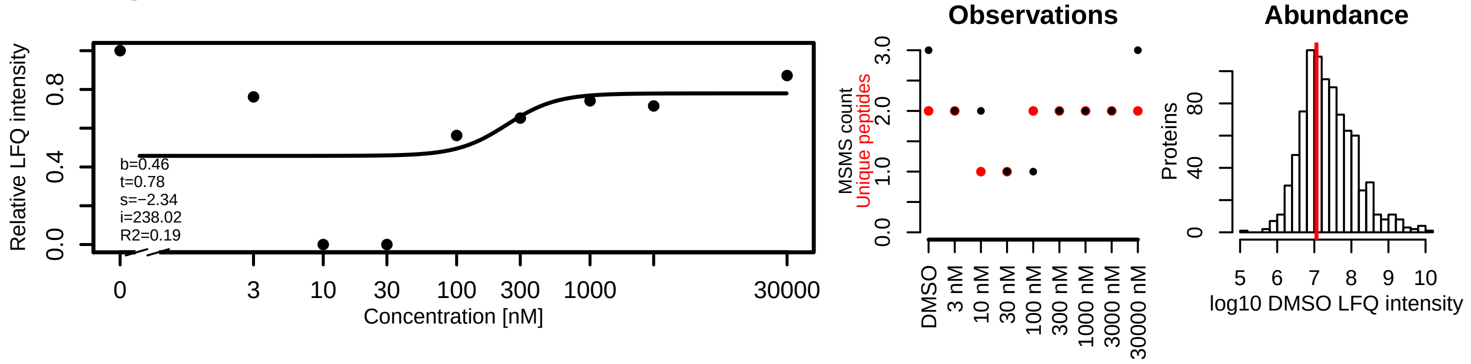

NUP107

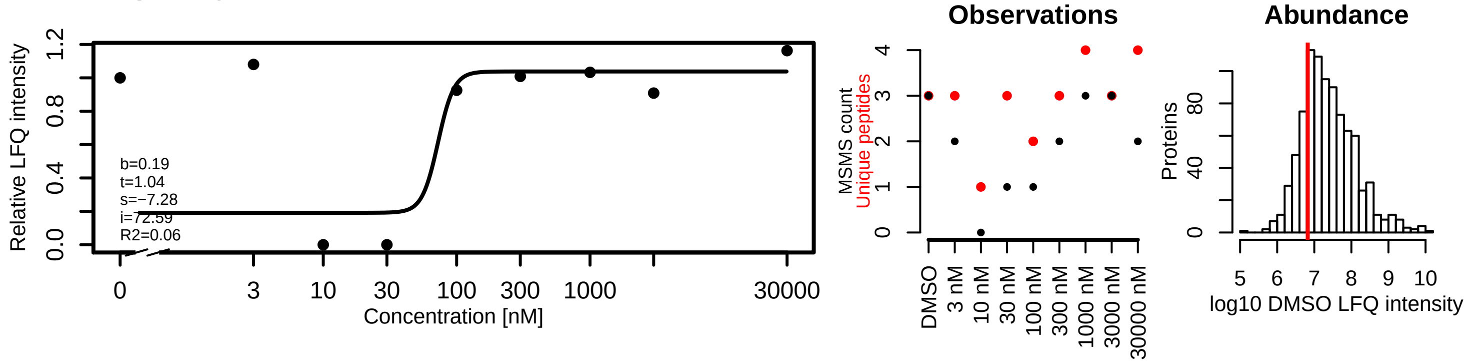

ATP5I

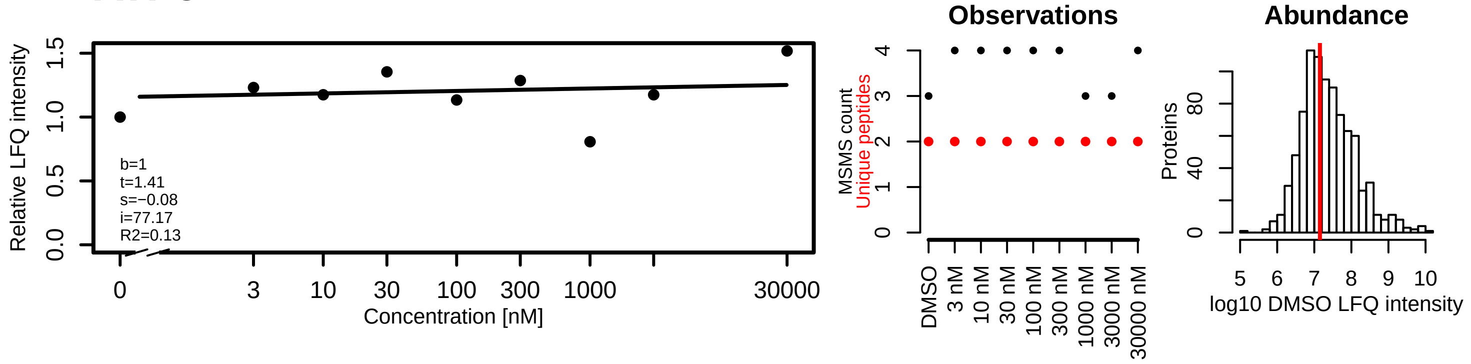

GHITM

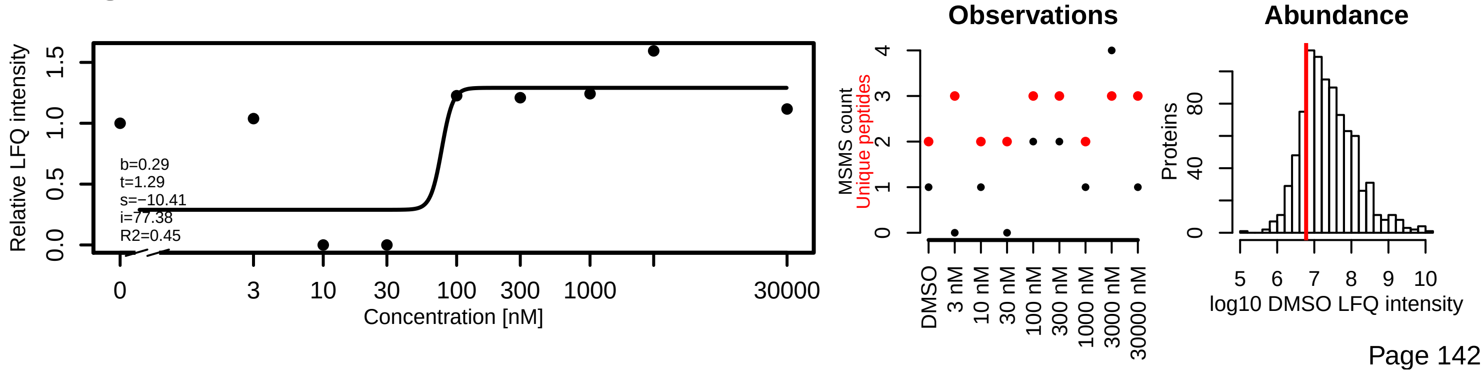

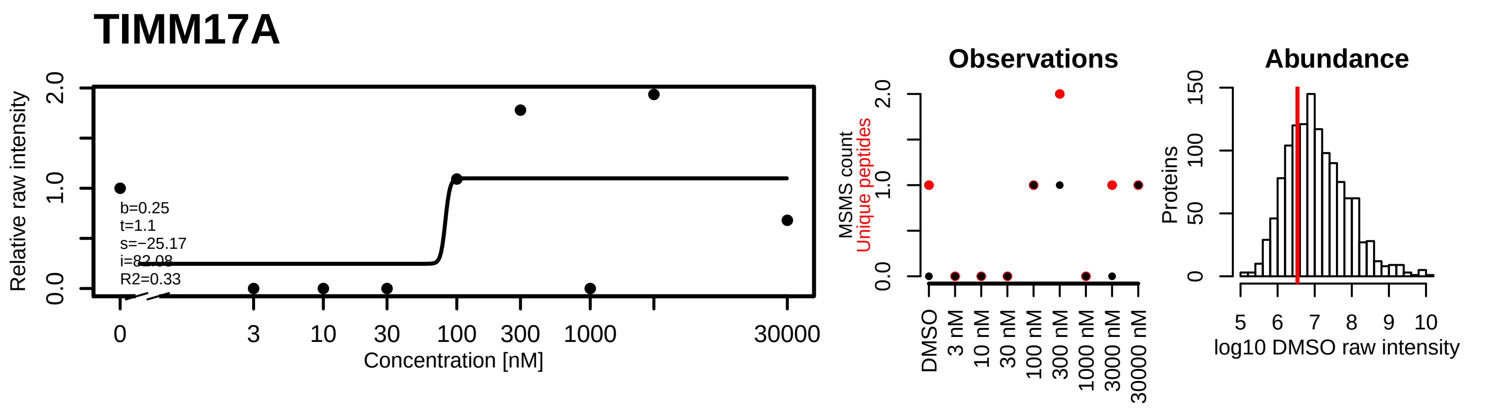
TIMM17A
Relative raw intensity
b=0.25
t=1.1
s=−25.17
i=82.08
R2=0.33
Concentration [nM]
Observations
MSMS count
Unique peptides
Abundance
Proteins
log10 DMSO raw intensity

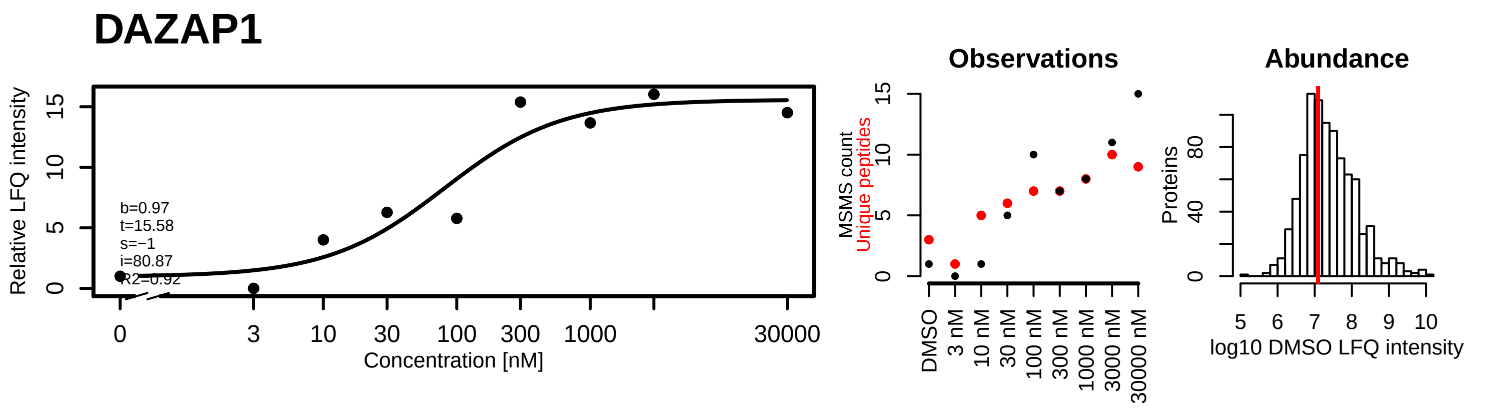
DAZAP1
Relative LFQ intensity
b=0.97
t=15.58
s=−1
i=80.87
R2=0.92
Concentration [nM]
Observations
MSMS count
Unique peptides
Abundance
Proteins
log10 DMSO LFQ intensity

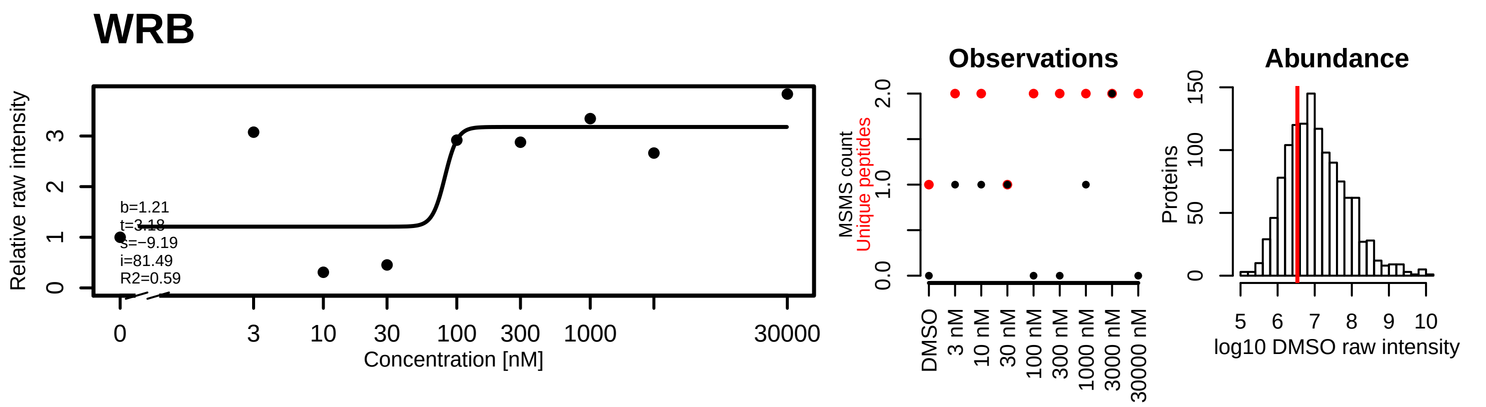
WRB
Relative raw intensity
b=1.21
t=3.18
s=−9.19
i=81.49
R2=0.59
Concentration [nM]
Observations
MSMS count
Unique peptides
Abundance
Proteins
log10 DMSO raw intensity

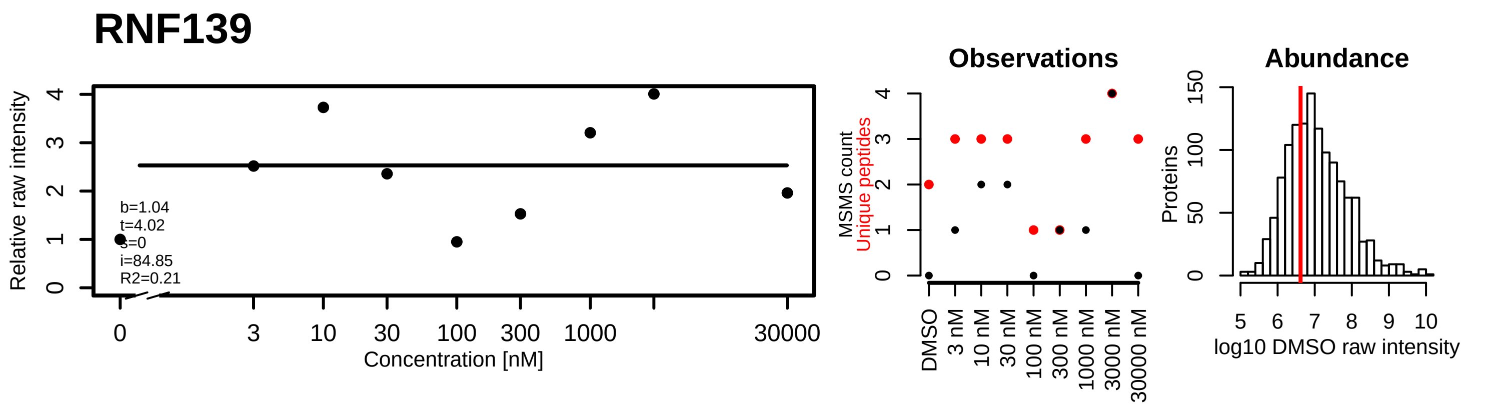
RNF139
Relative raw intensity
b=1.04
t=4.02
s=0
i=84.85
R2=0.21
Concentration [nM]
Observations
MSMS count
Unique peptides
Abundance
Proteins
log10 DMSO raw intensity

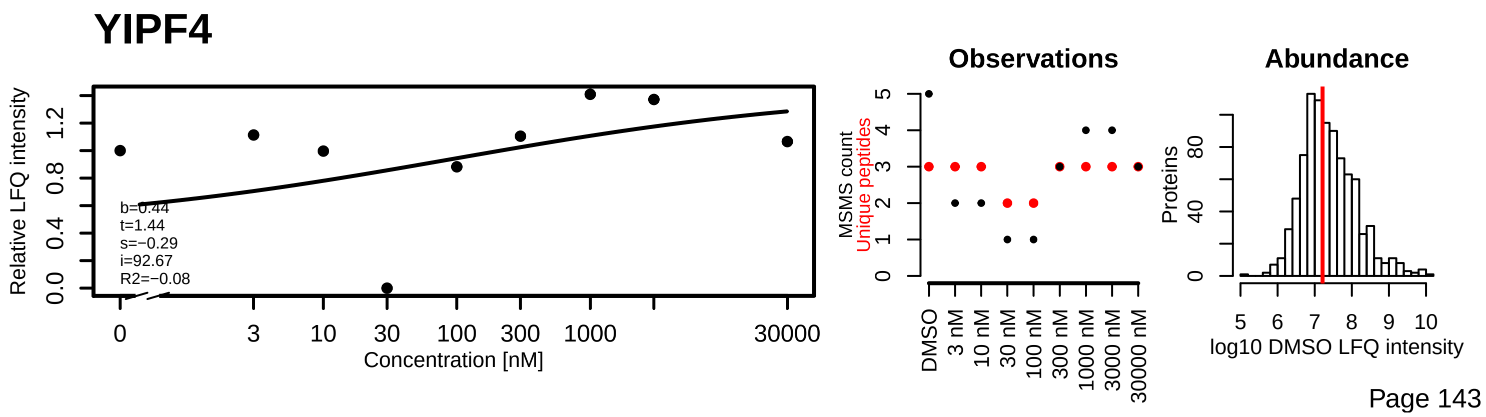
YIPF4
Relative LFQ intensity
b=0.44
t=1.44
s=−0.29
i=92.67
R2=−0.08
Concentration [nM]
Observations
MSMS count
Unique peptides
Abundance
Proteins
log10 DMSO LFQ intensity

TP53I11

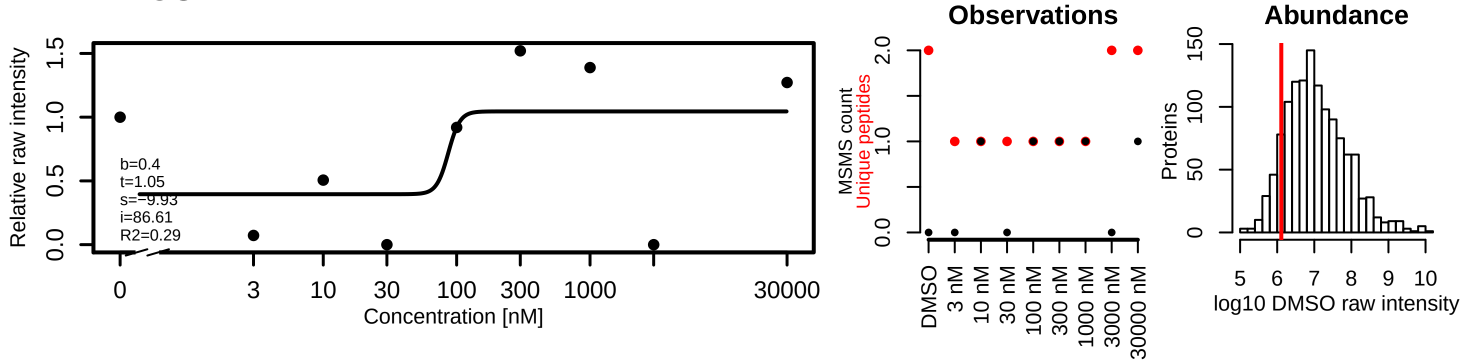

NCSTN

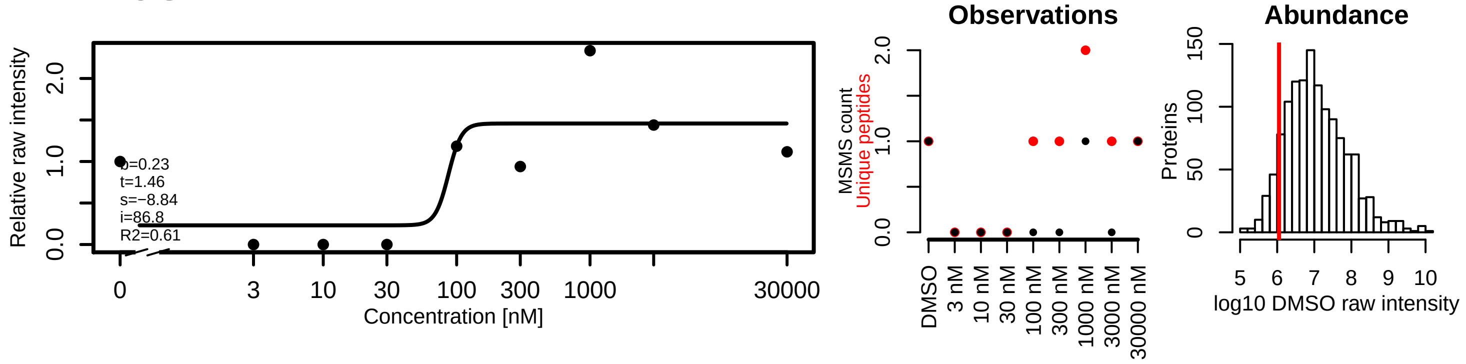

GPR155

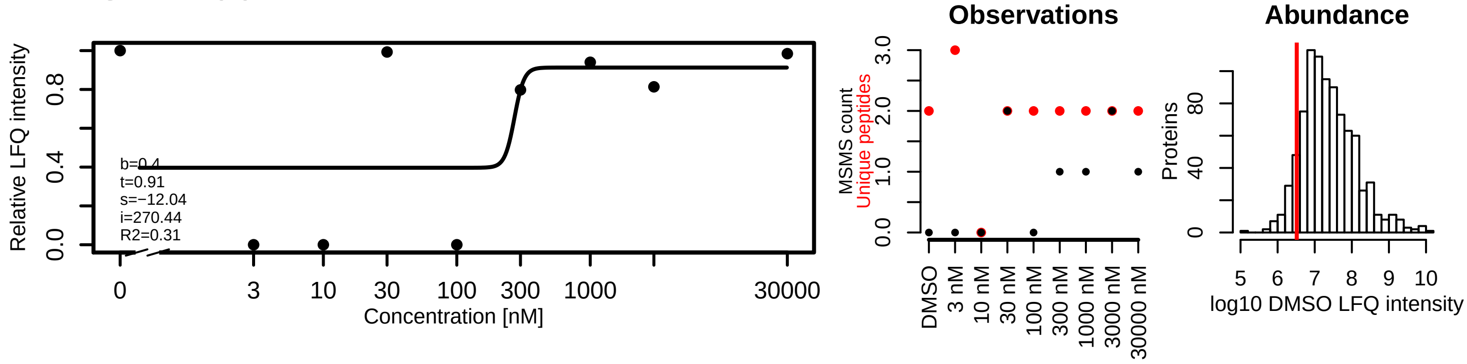

C9orf89

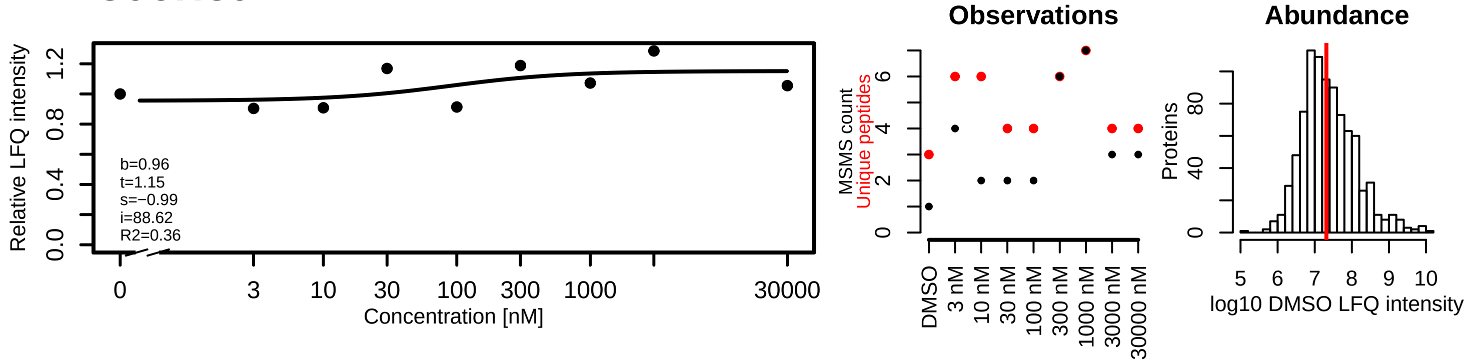

CAND1

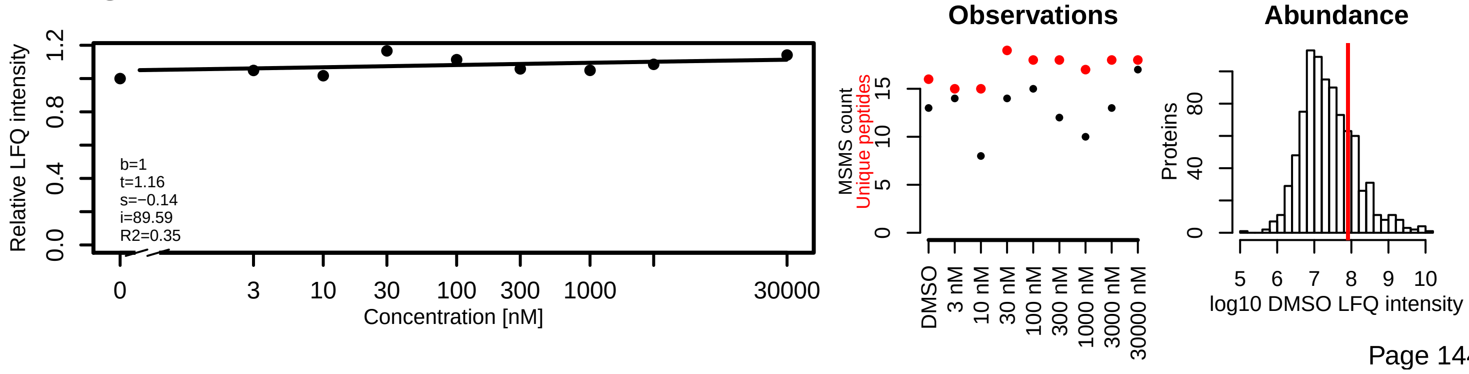

SLC35F2

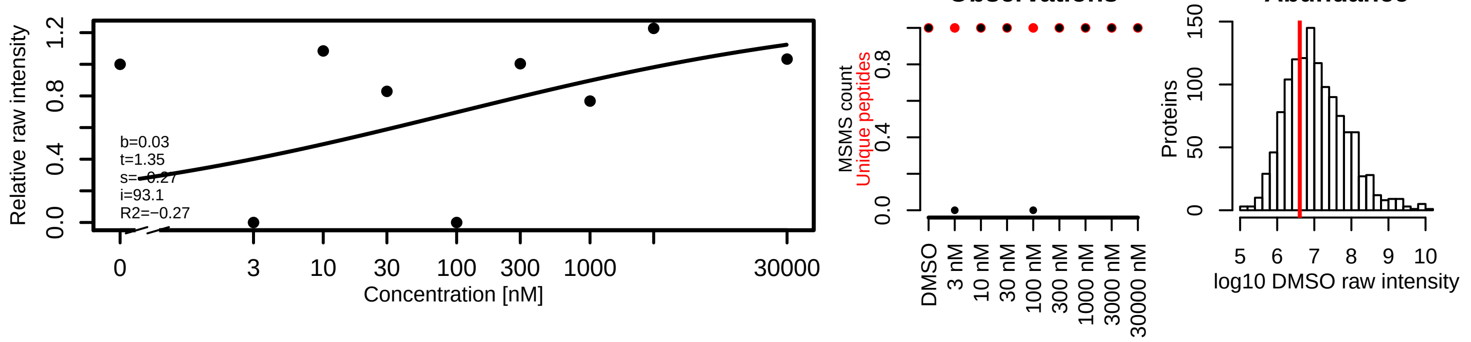

SIGMAR1

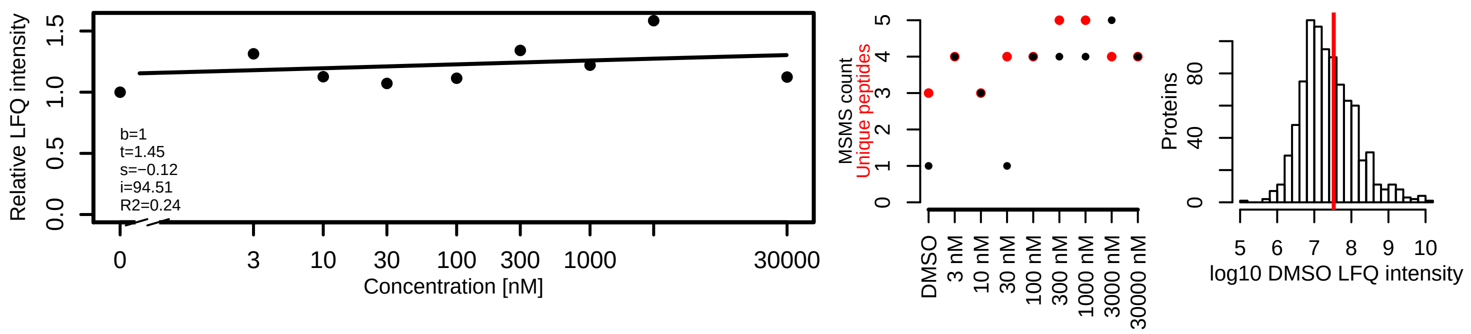

BNIP1

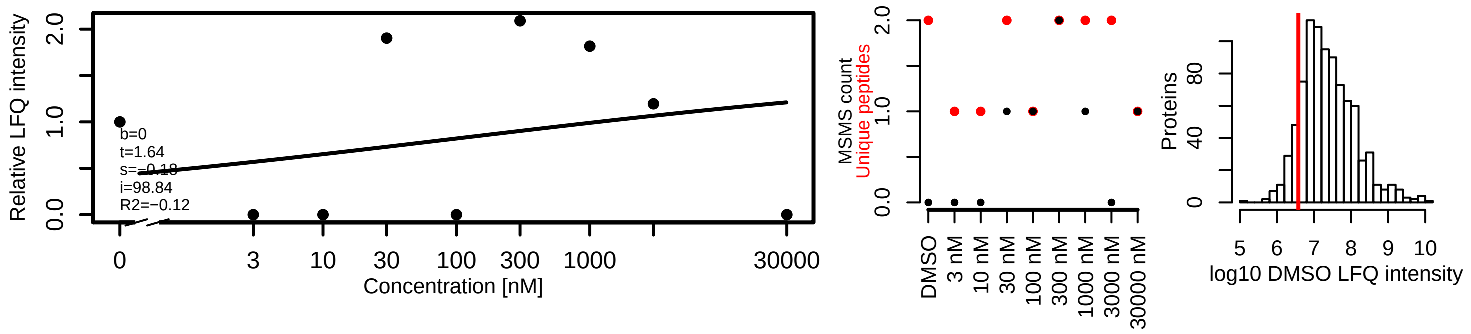

RHBDD1

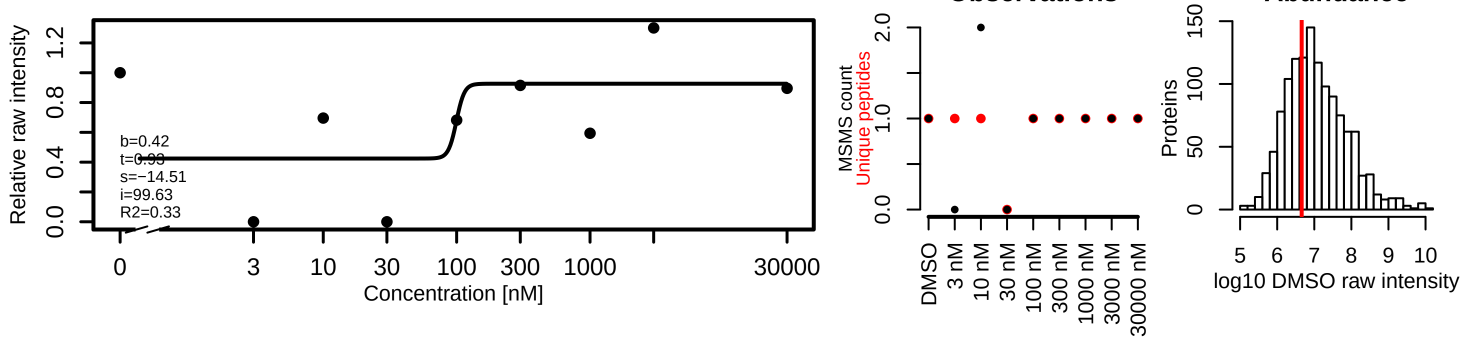

ATP2C1

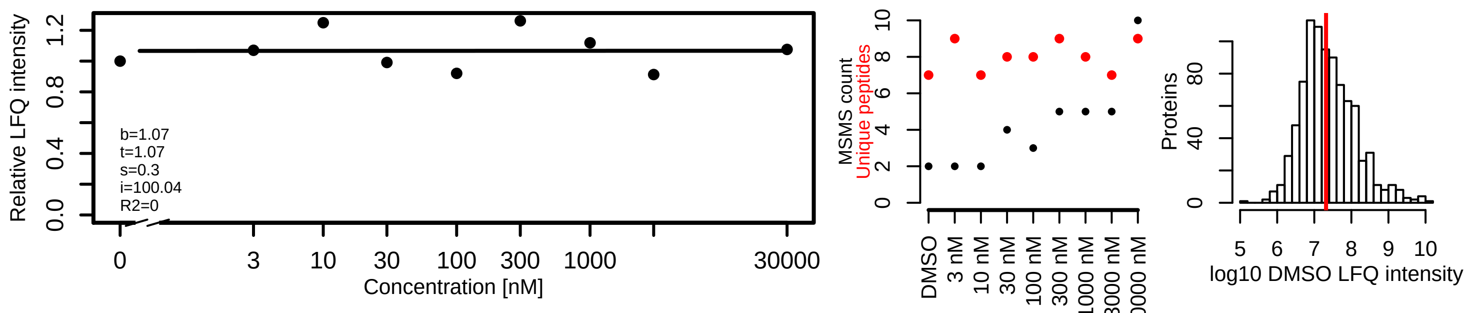

RTN3

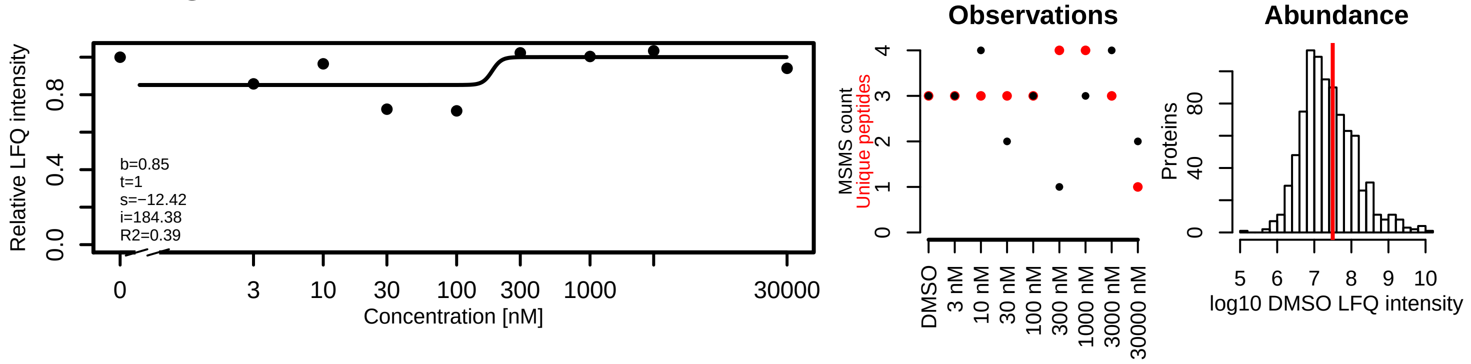

SRSF6

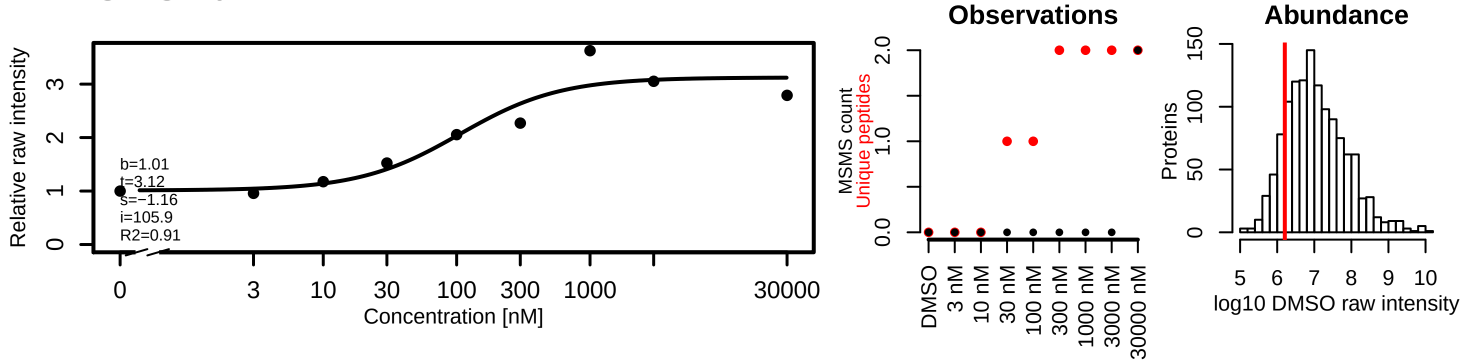

HNRNPM

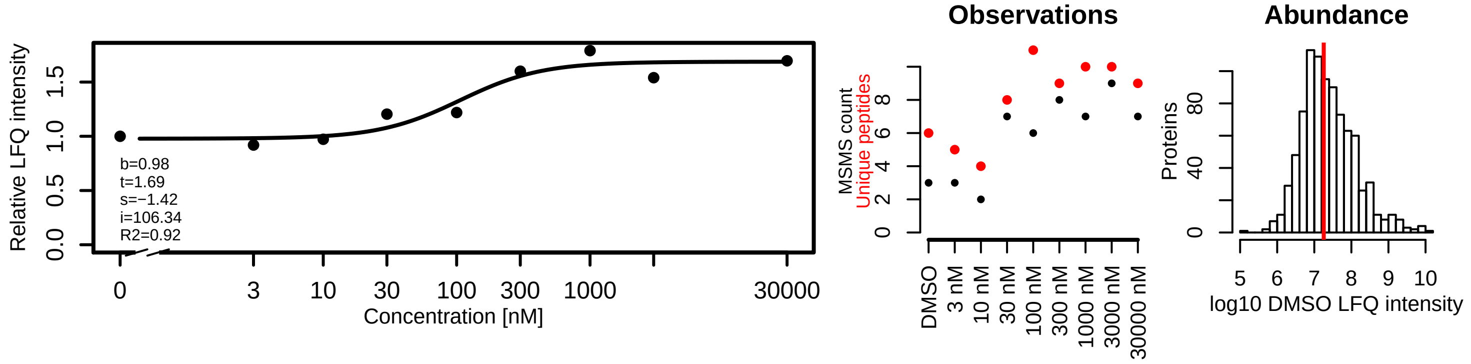

CRLS1

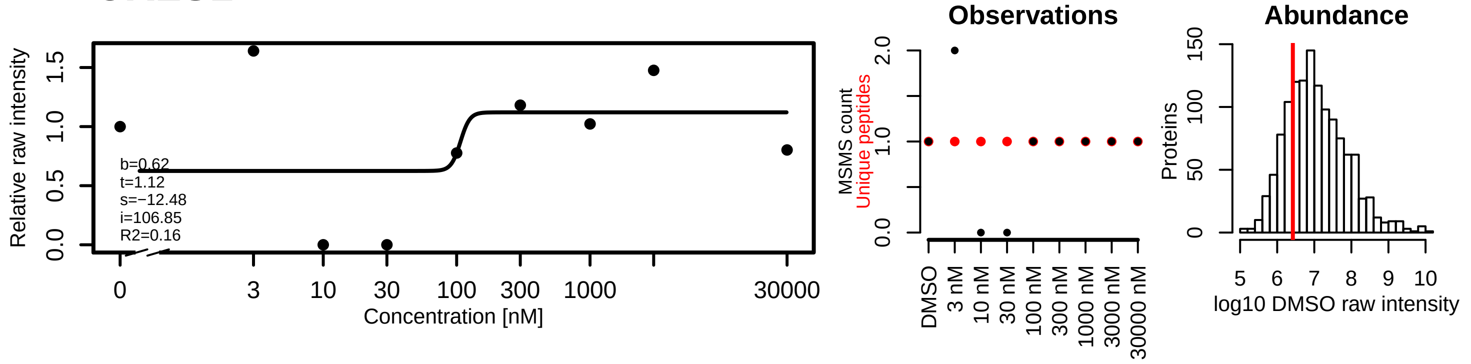

REEP5

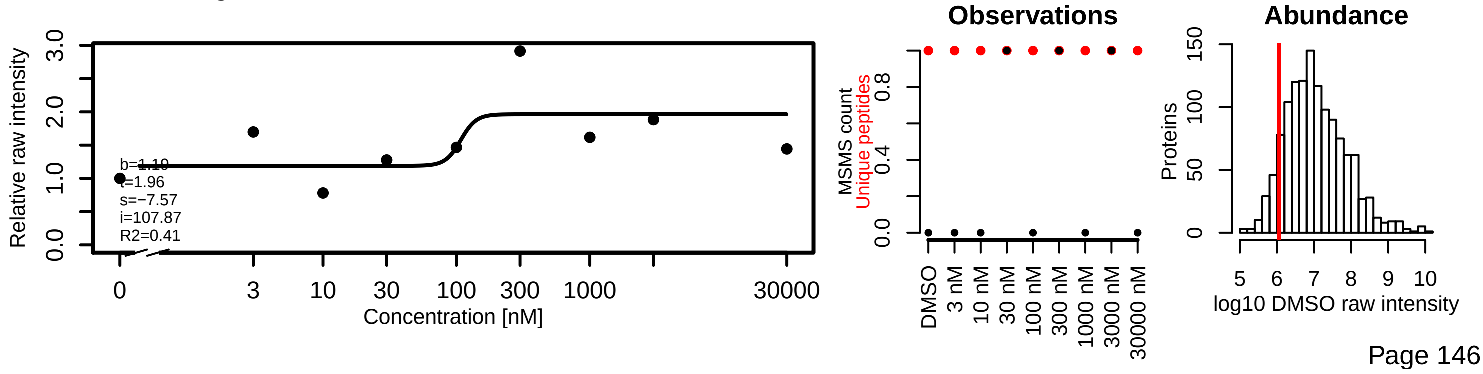

FECH

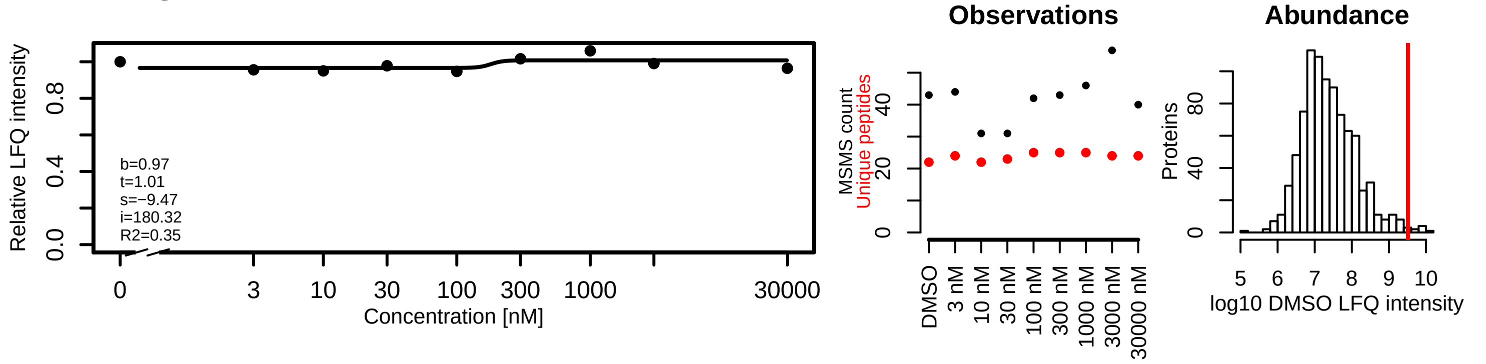

COG2

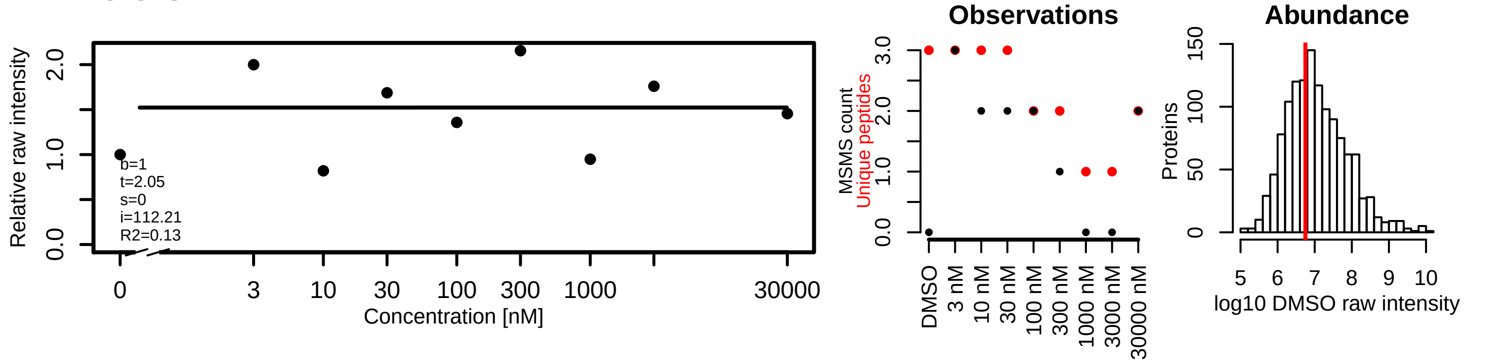

RPS15A − H3BN98;I3L246;H3BV27;H3BT37;I3L303

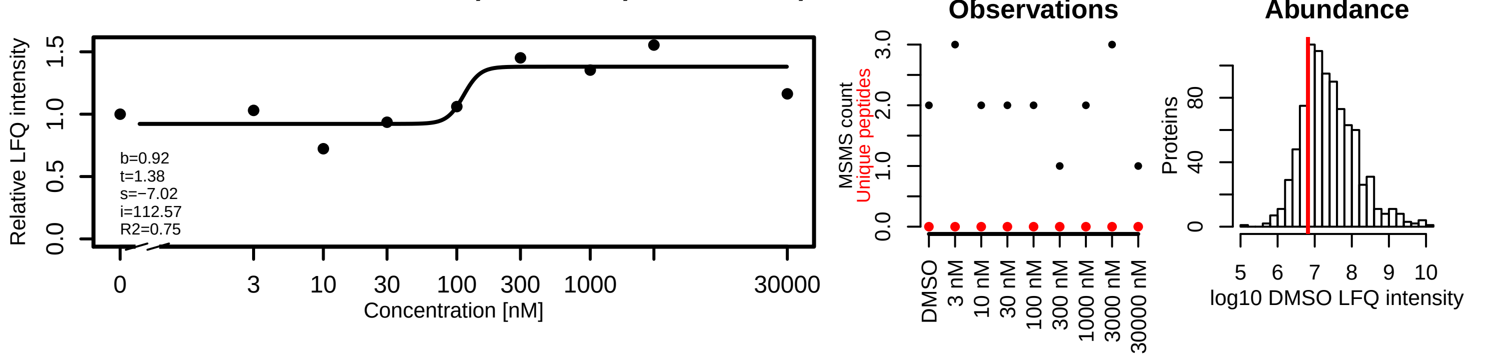

ATP5O

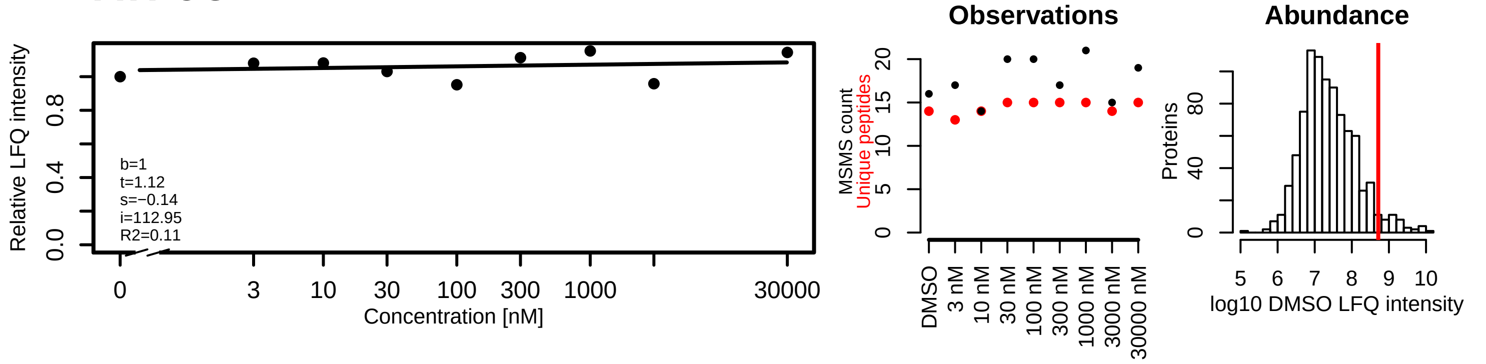

DEK

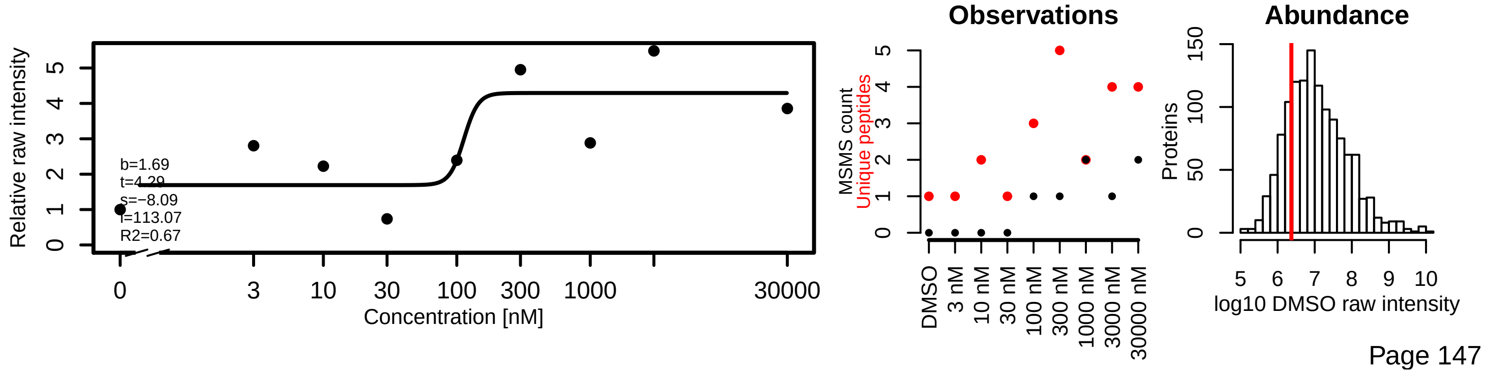

TRA2B

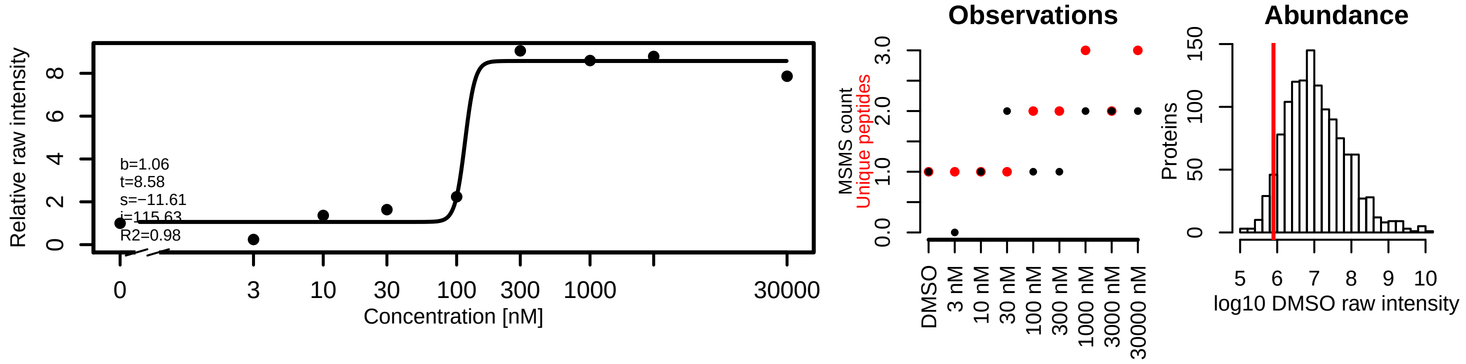

FAM8A1

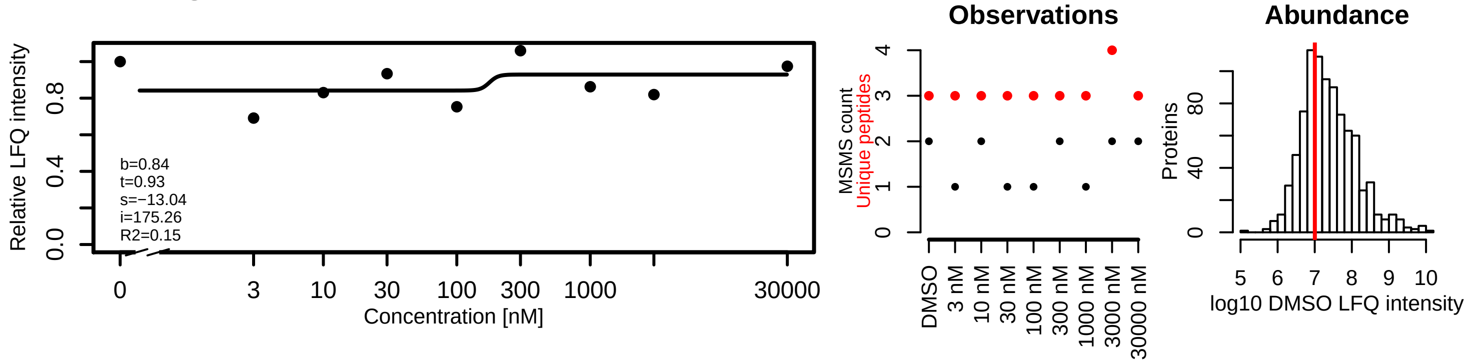

GCN1L1

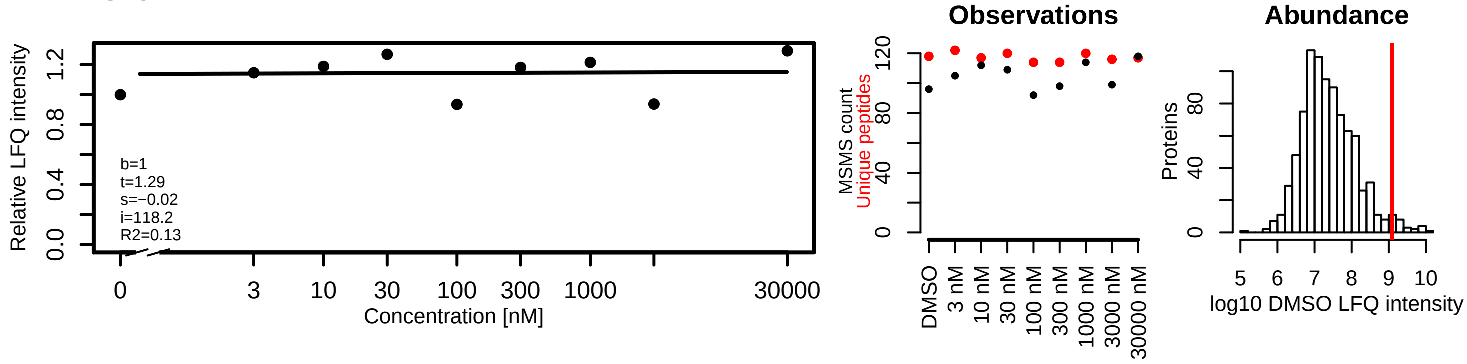

NOC2L

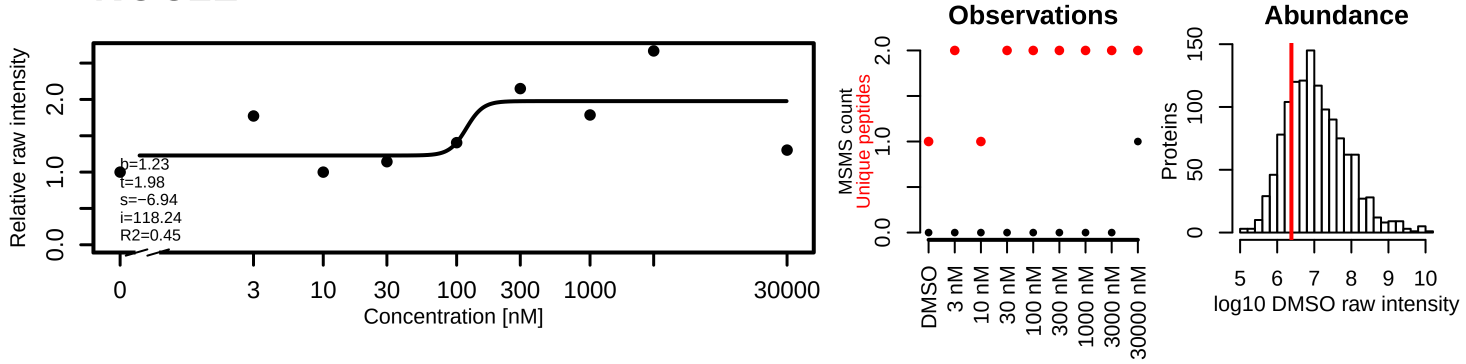

LGR5

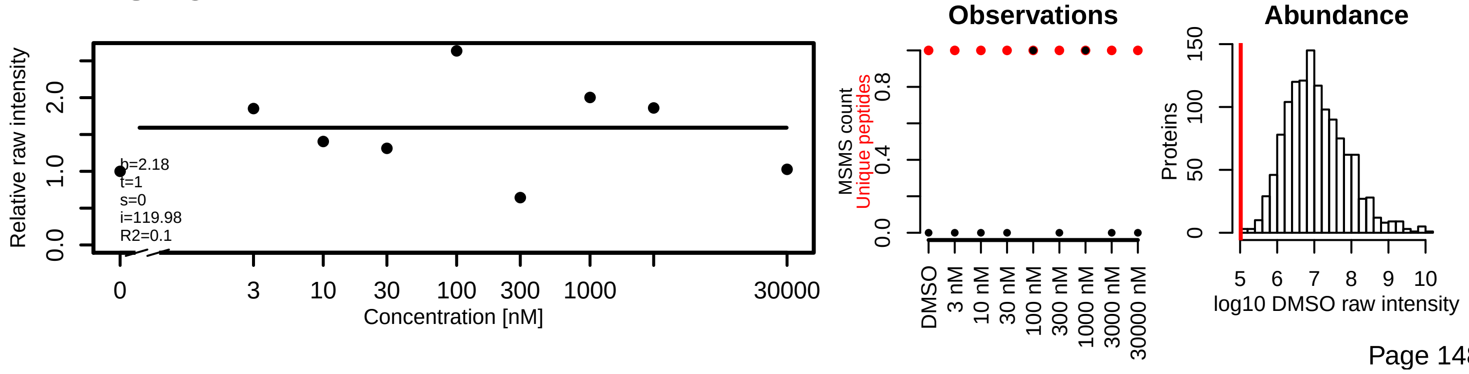

FAM57A

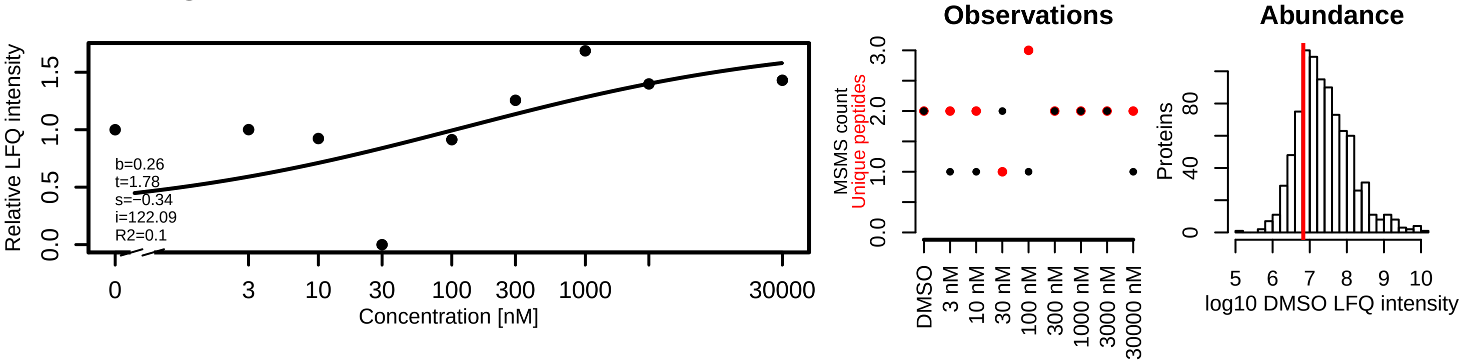

NDUFS8

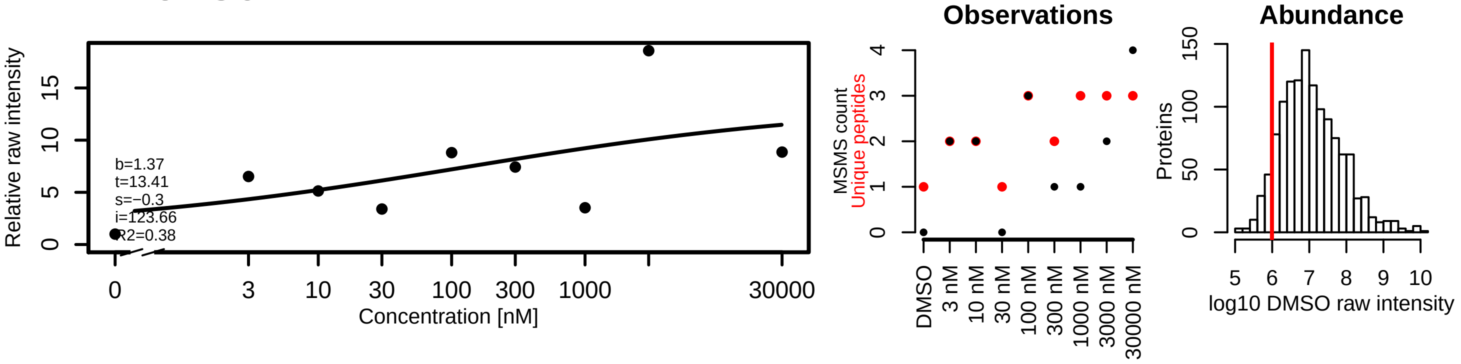

FZD2

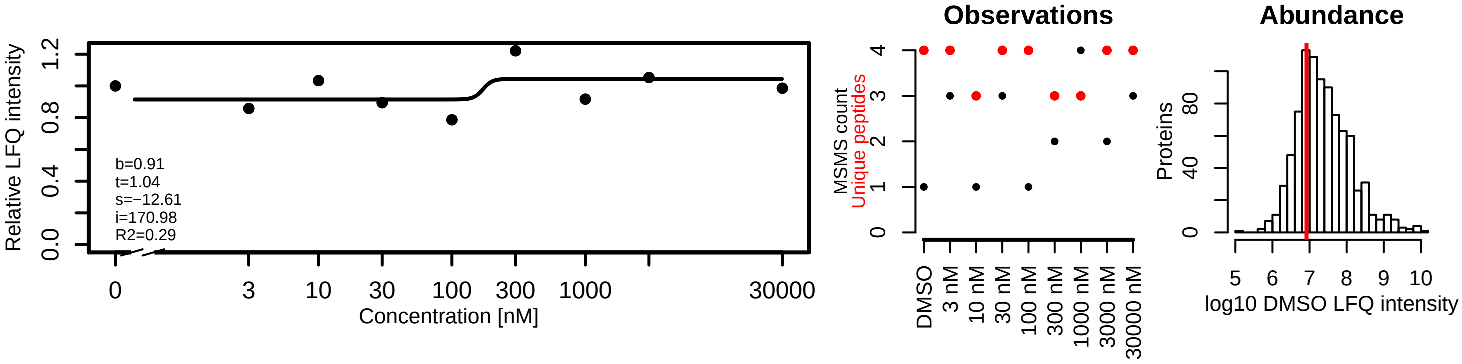

THEM6

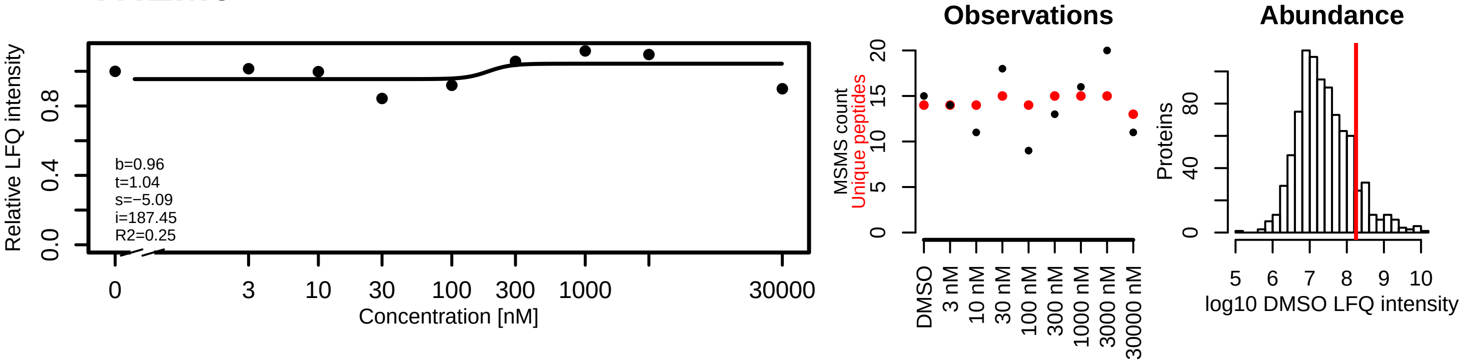

SLC25A4

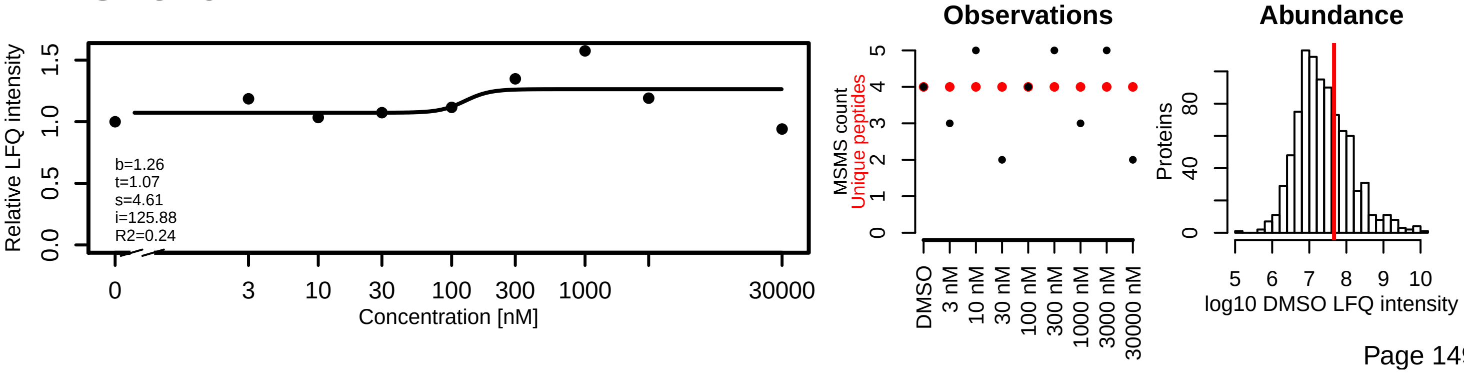

ATP2A2

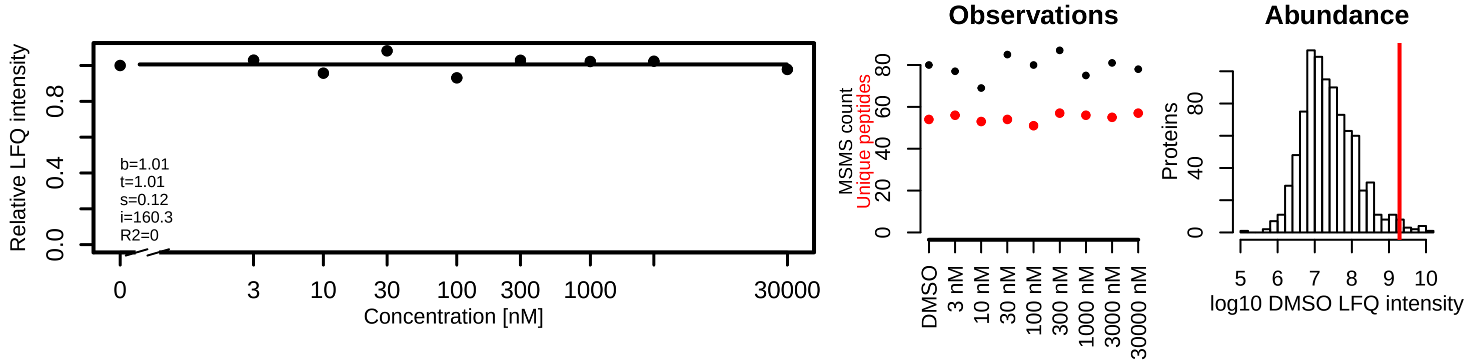

KHSRP

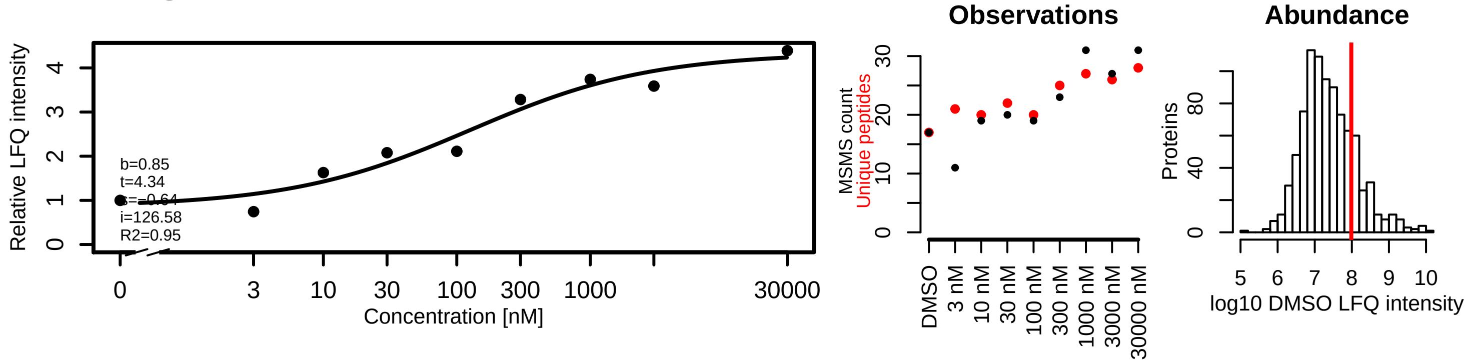

STARD3NL − O95772−2

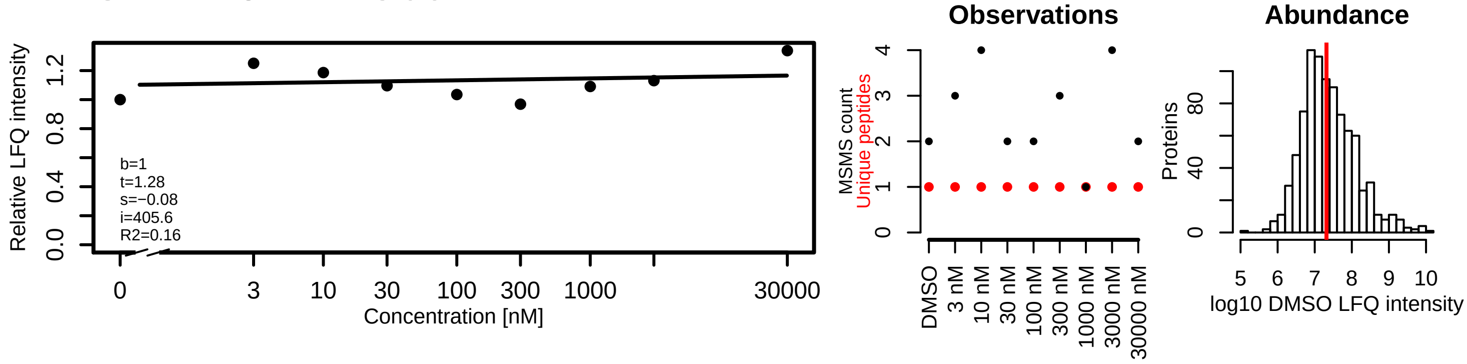

RABL3

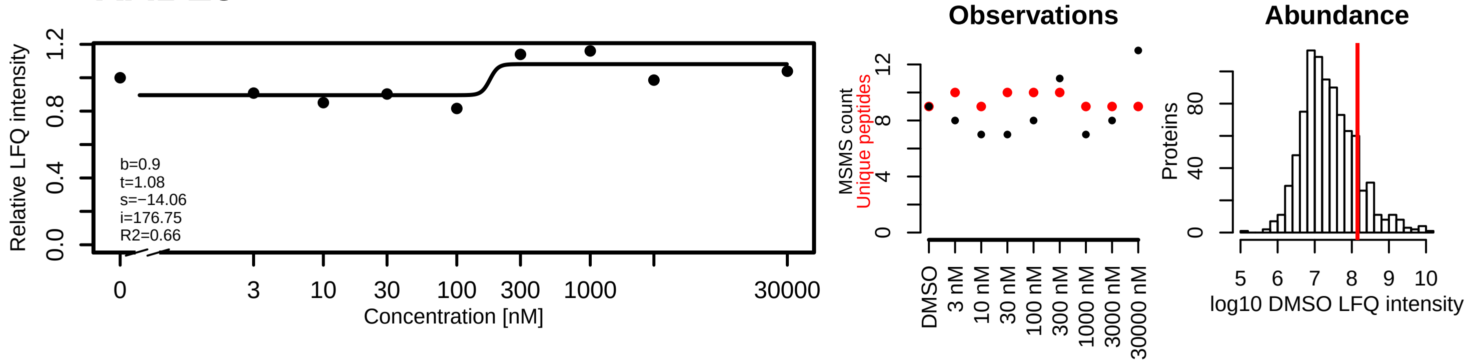

STEAP3

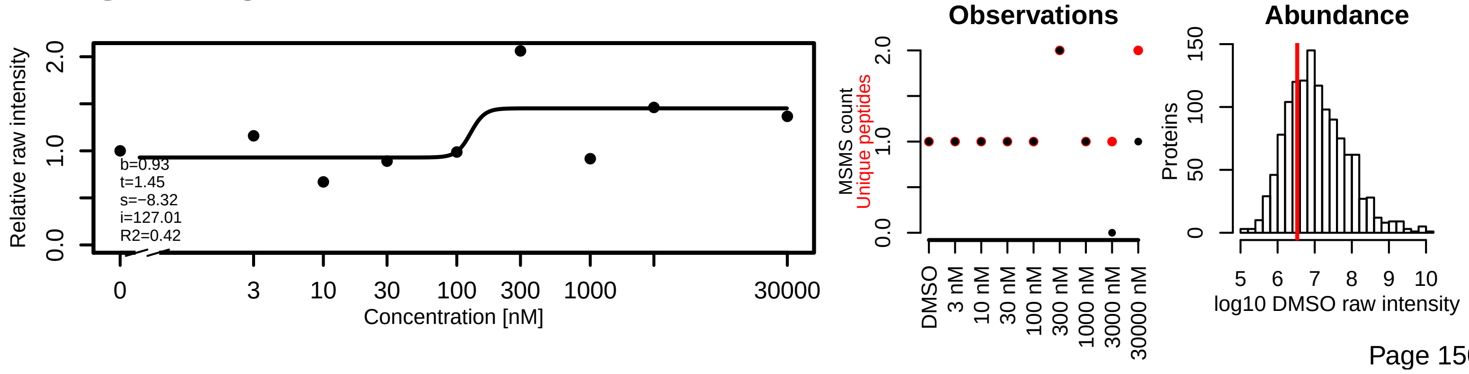

UQCRB

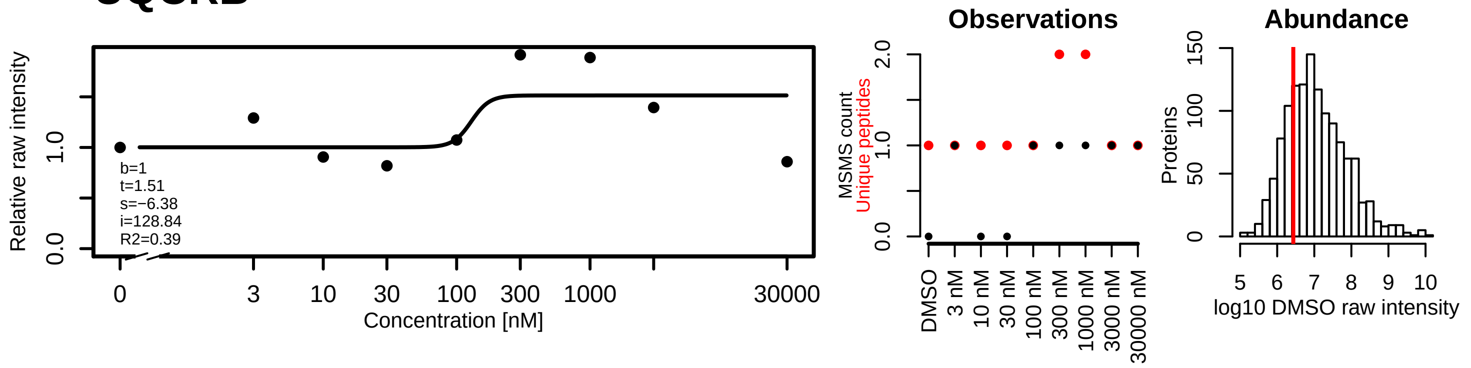

PDCD6

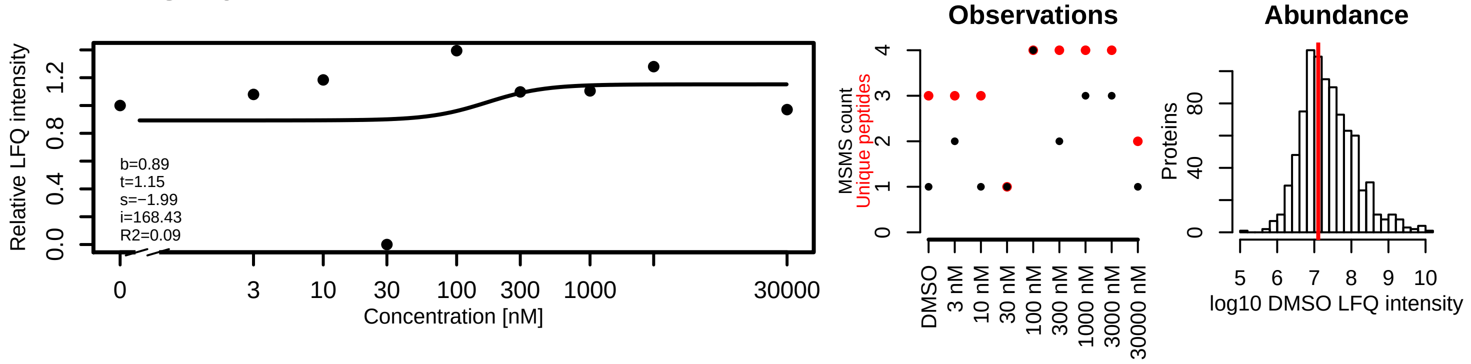

RPS4X

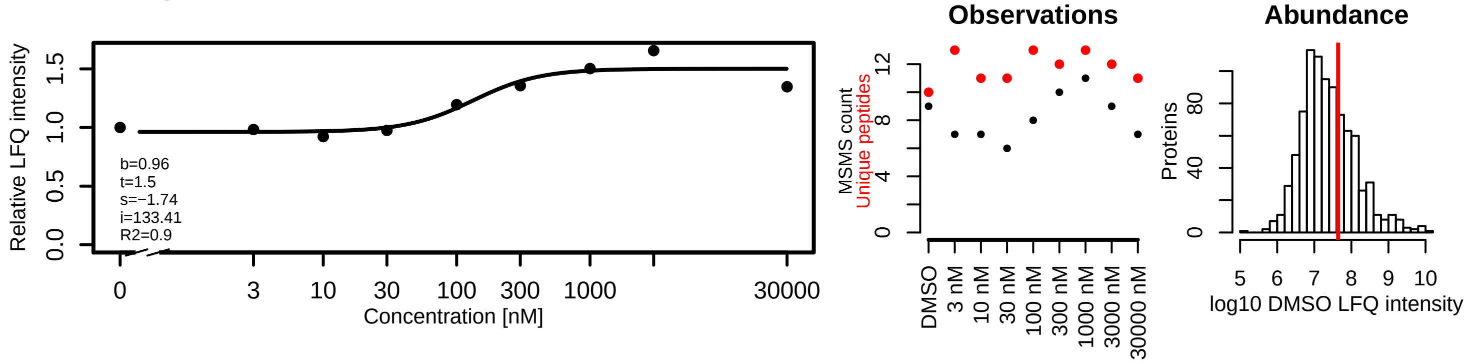

SLC46A1

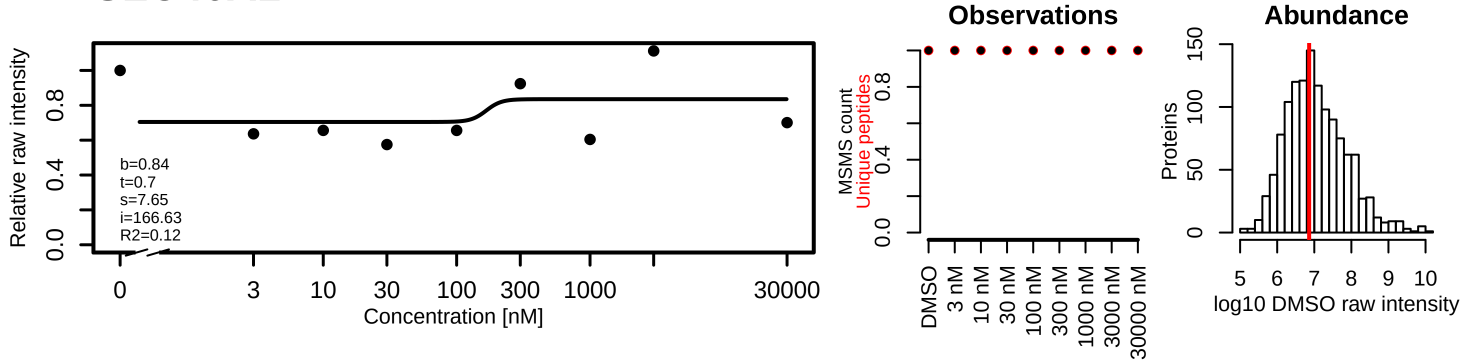

ICMT

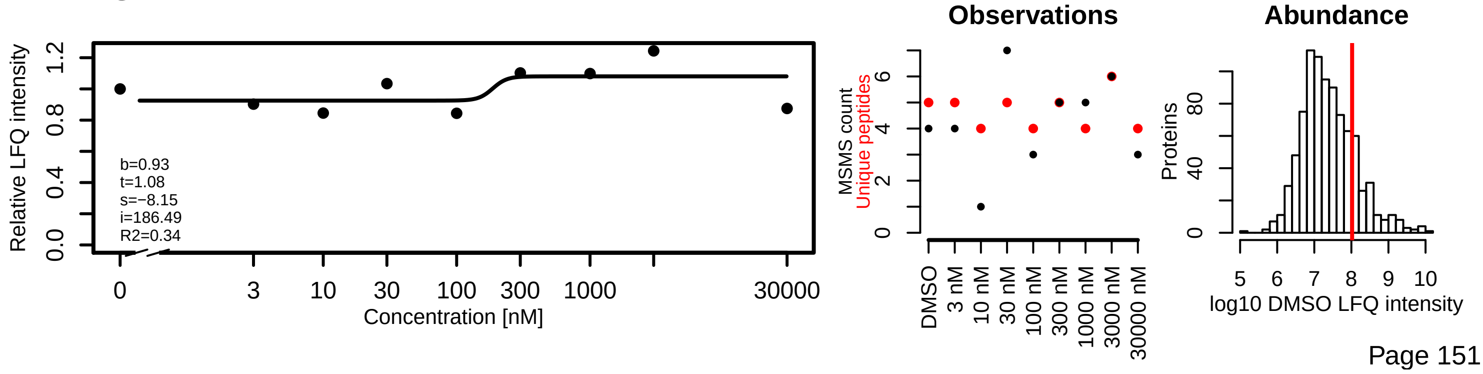

MON2

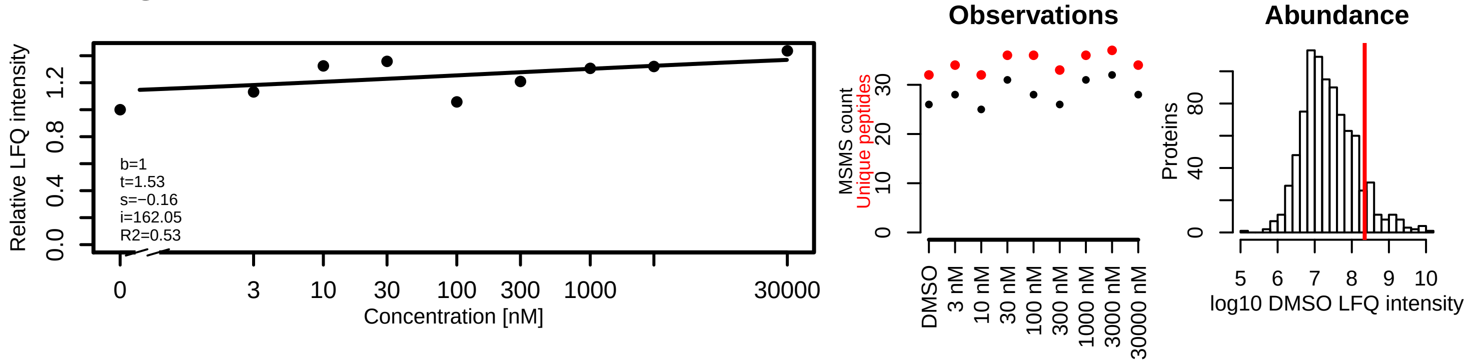

FADS1

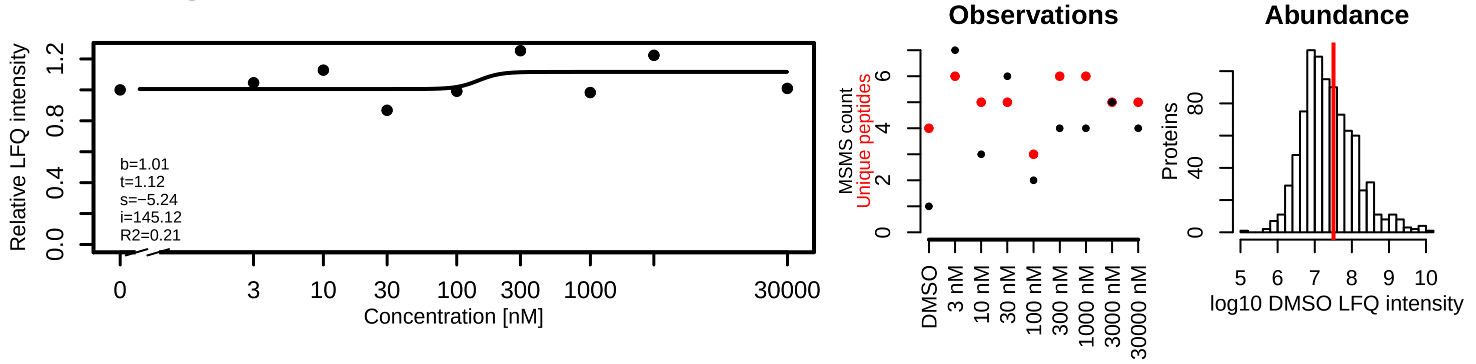

CCT5

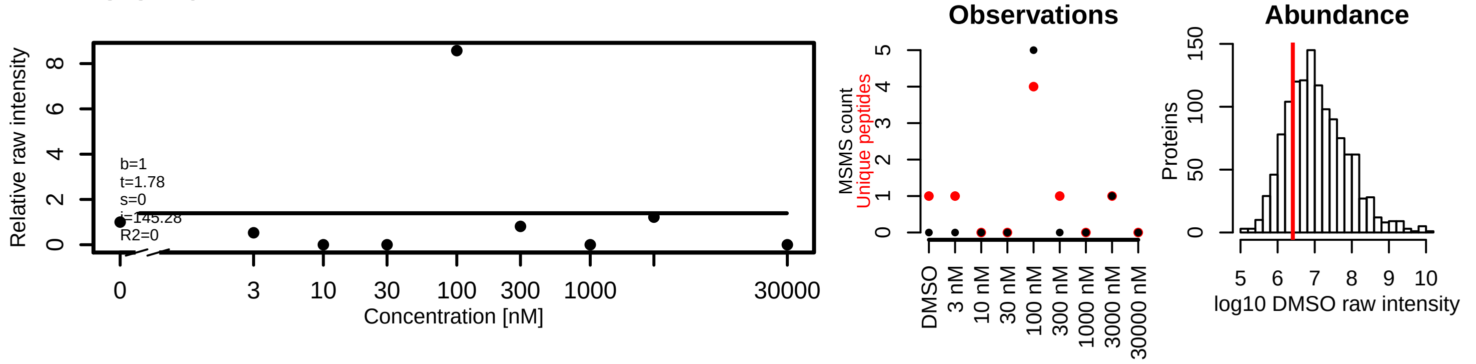

DLST

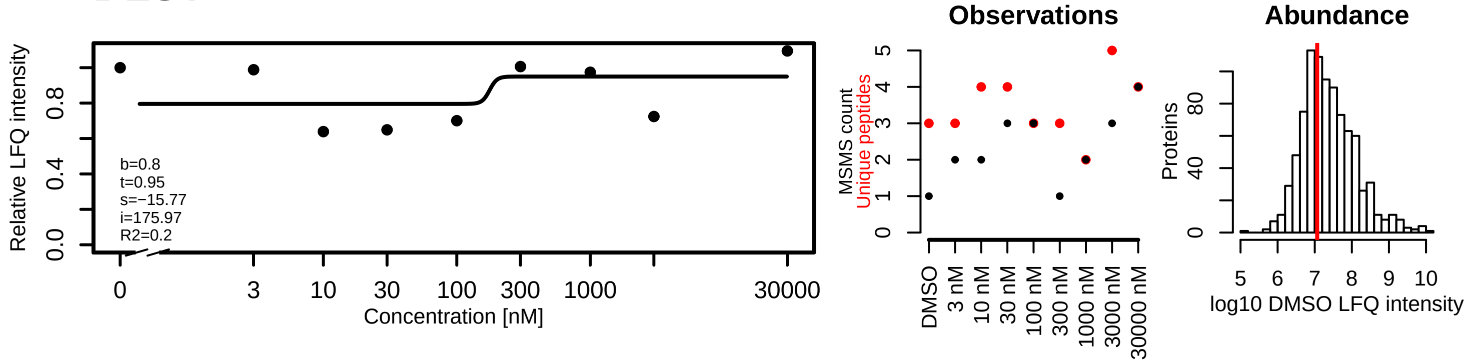

SLC25A5

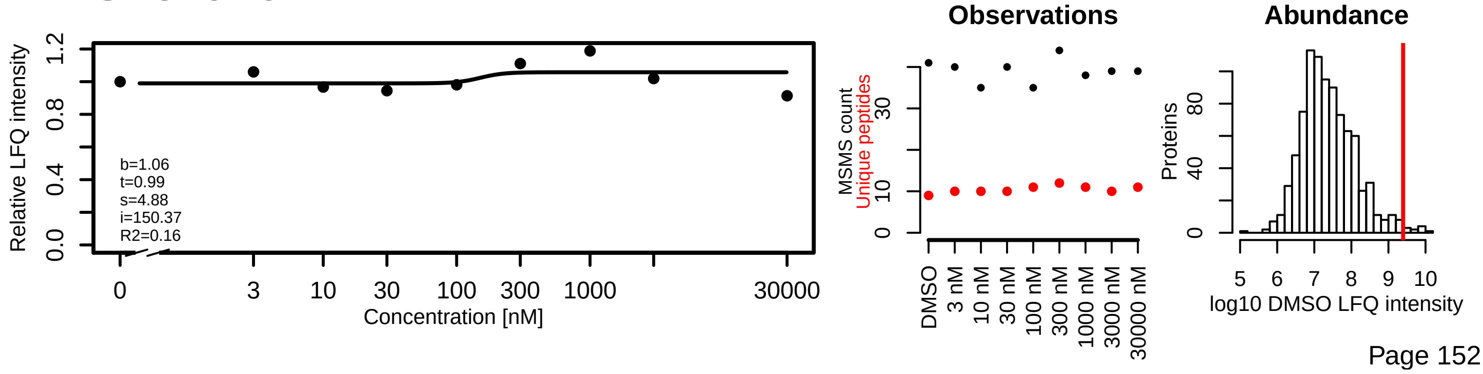

AHCTF1

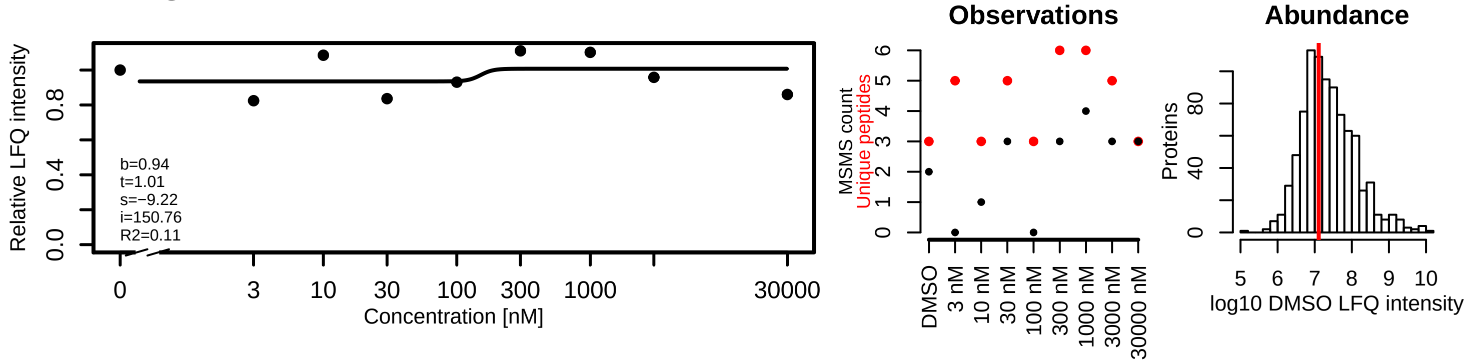

C19orf52

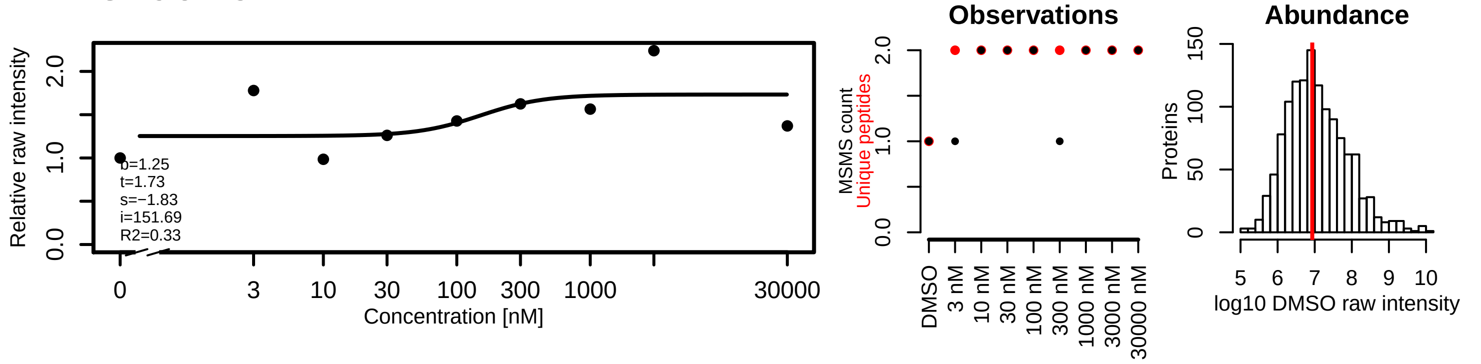

SLC25A35

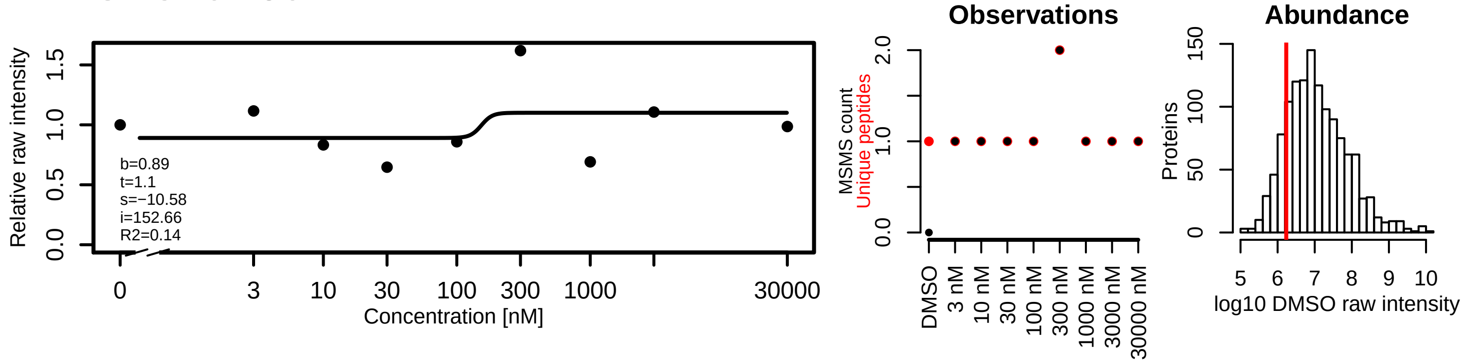

TMEM63A

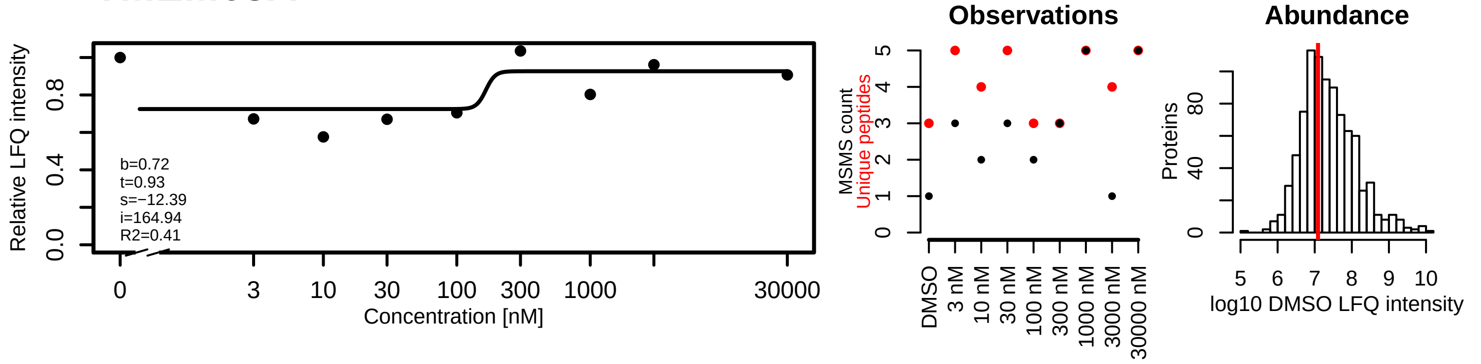

VIM

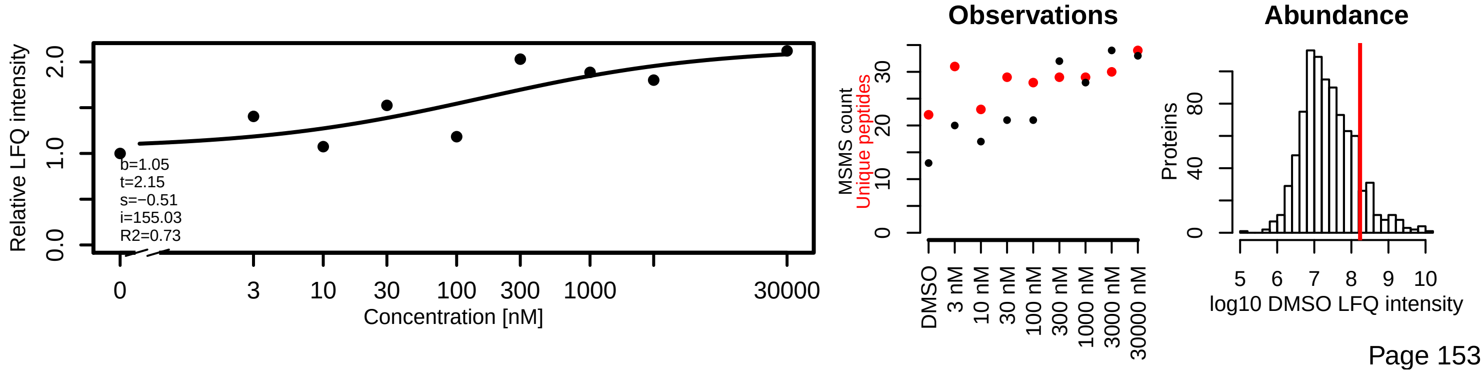

GGCX

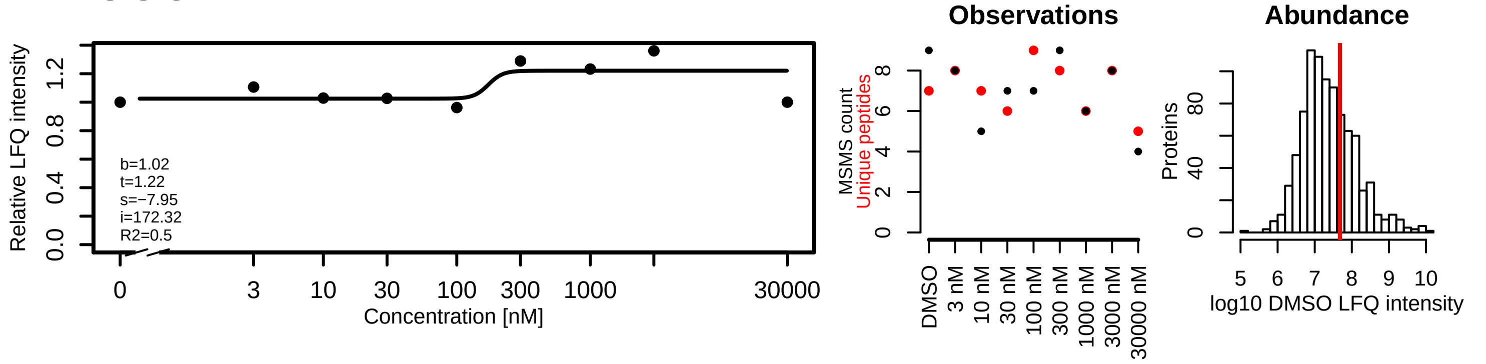

TMEM48

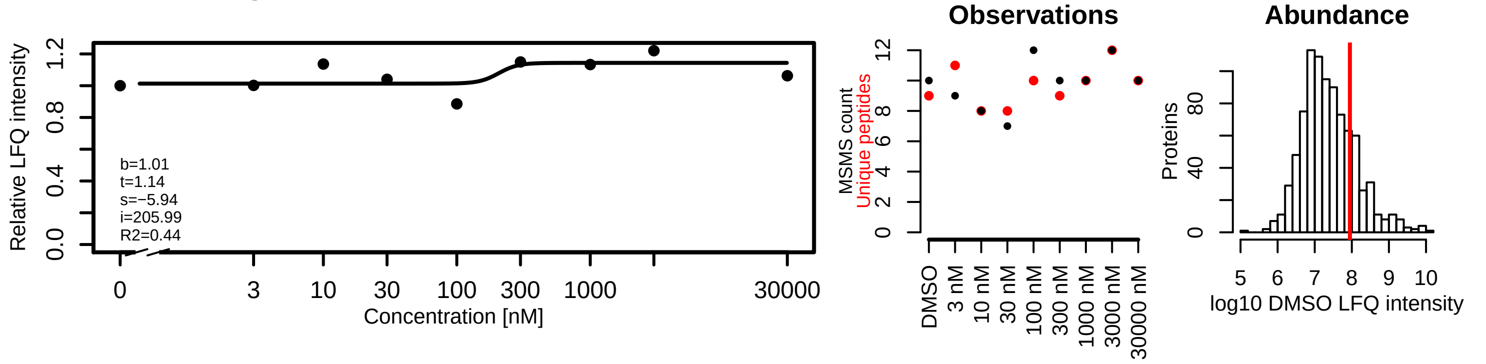

SLC25A10 − Q9UBX3;Q9UBX3−2;B4E1E9;I3L1E8

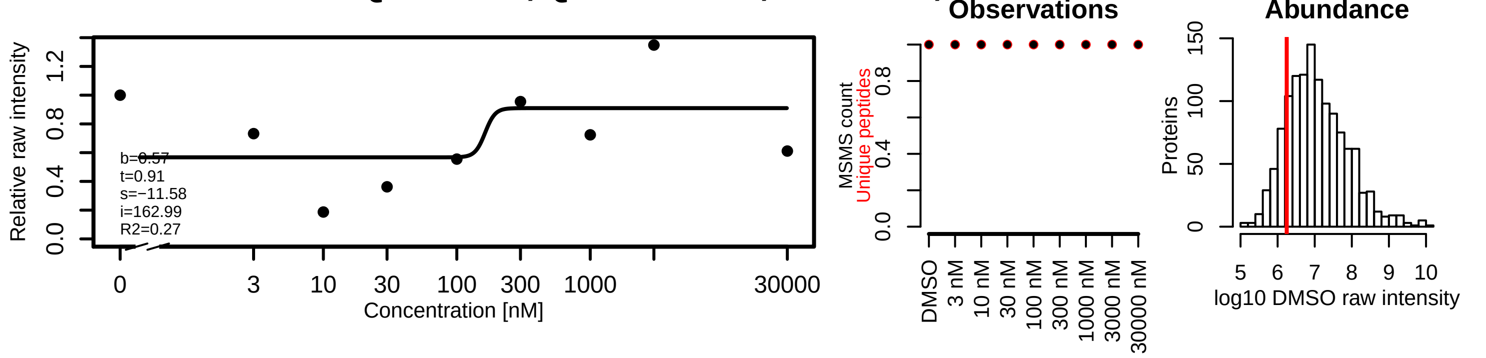

SURF4

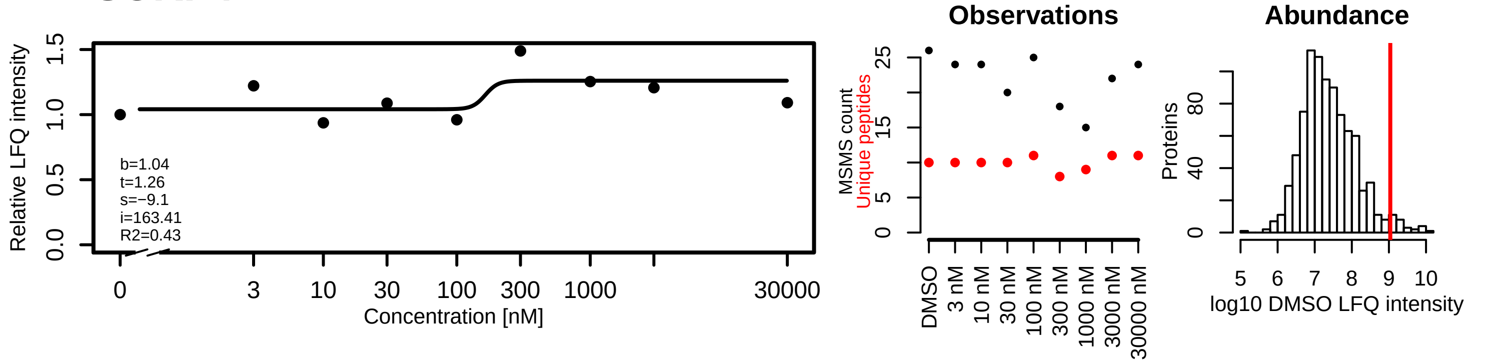

YIPF3

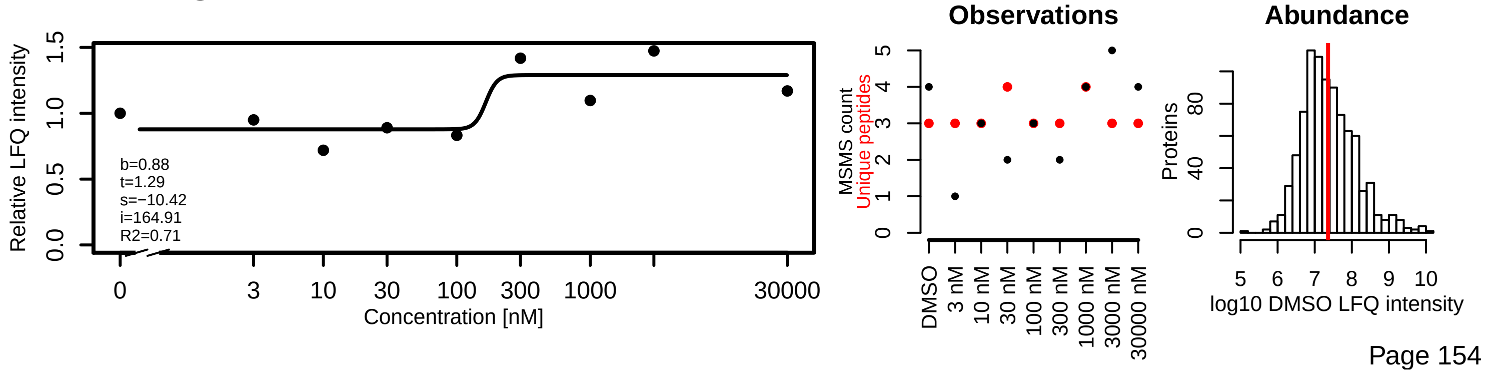

PEX26

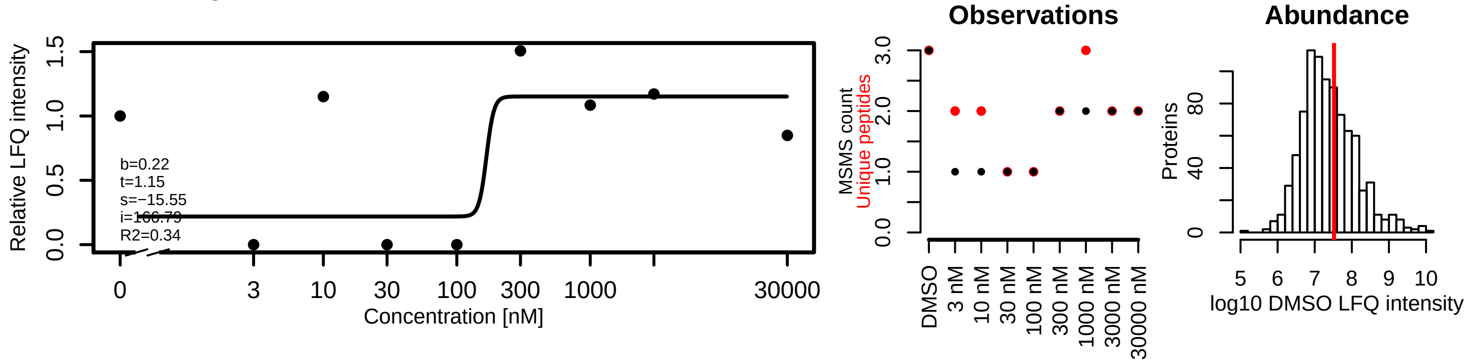

RAB9B;RAB9A

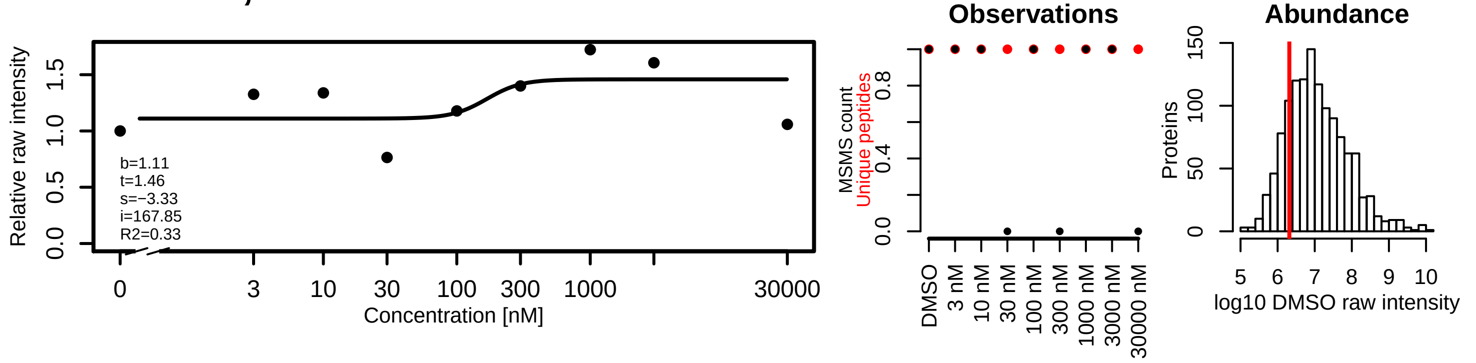

COG7

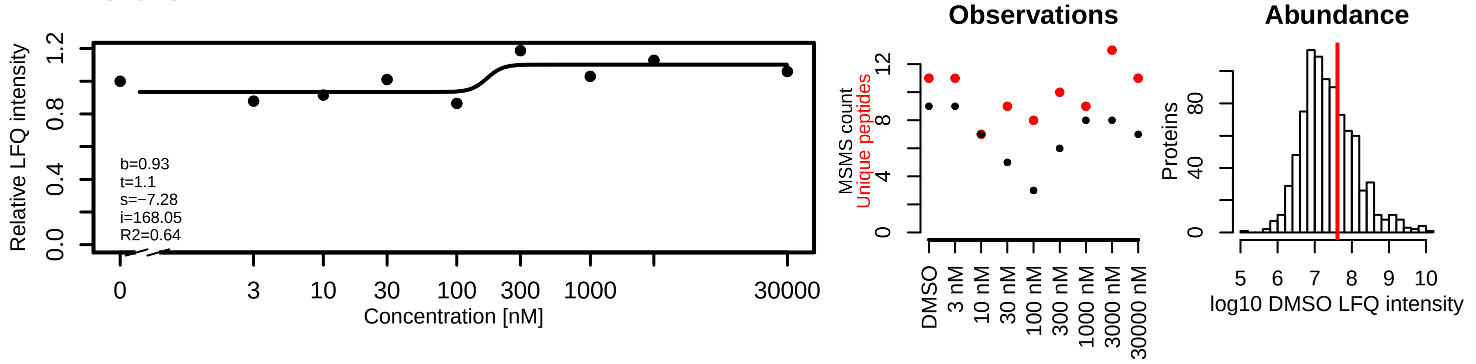

LMBRD2

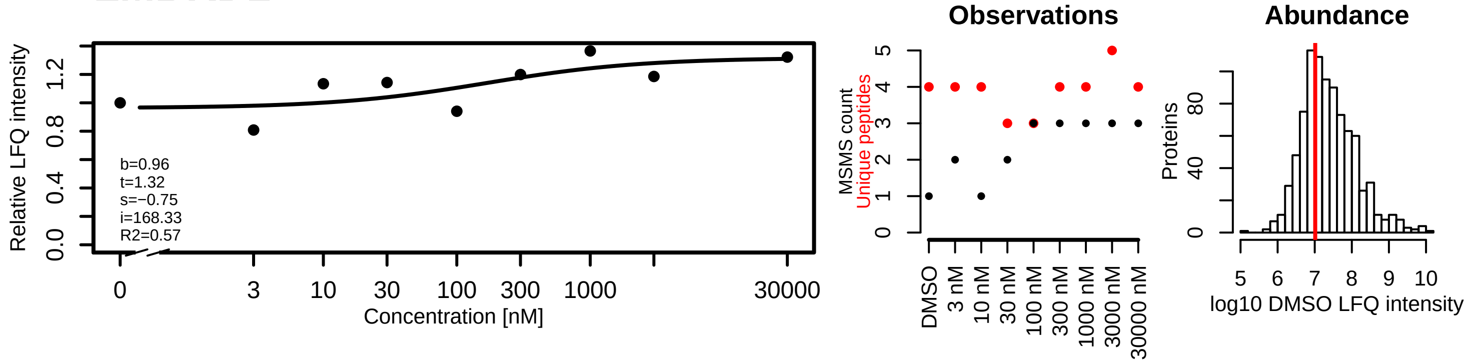

HCCS

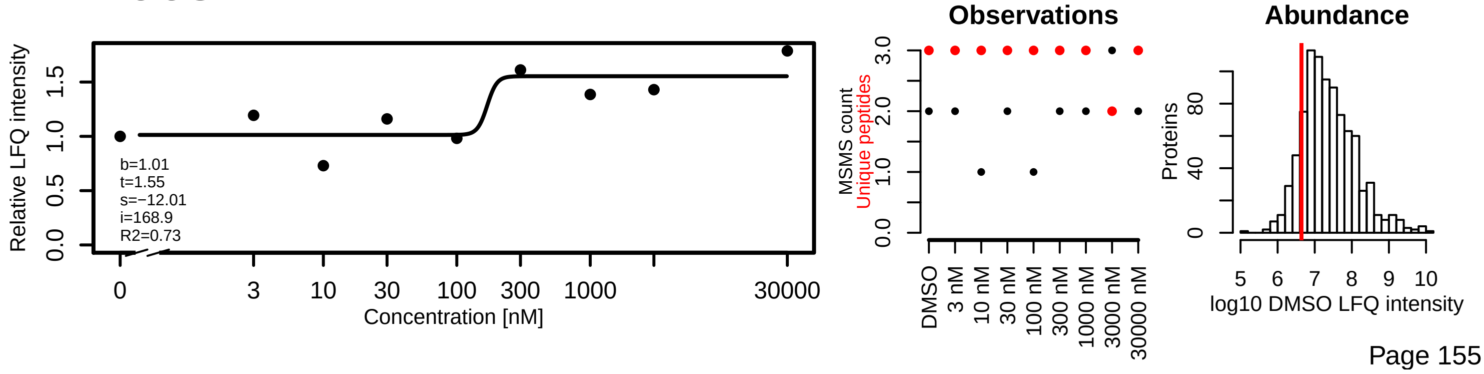

XPO7

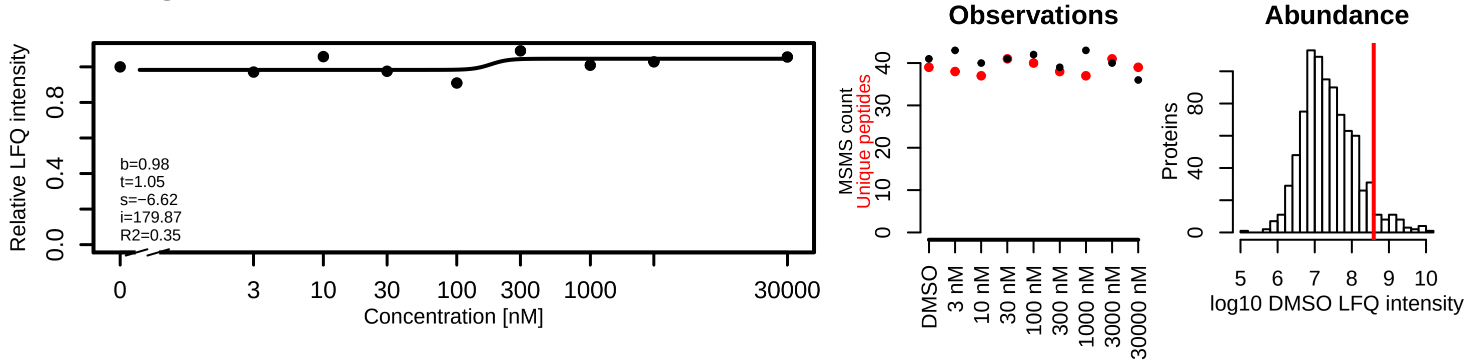

ABCC1

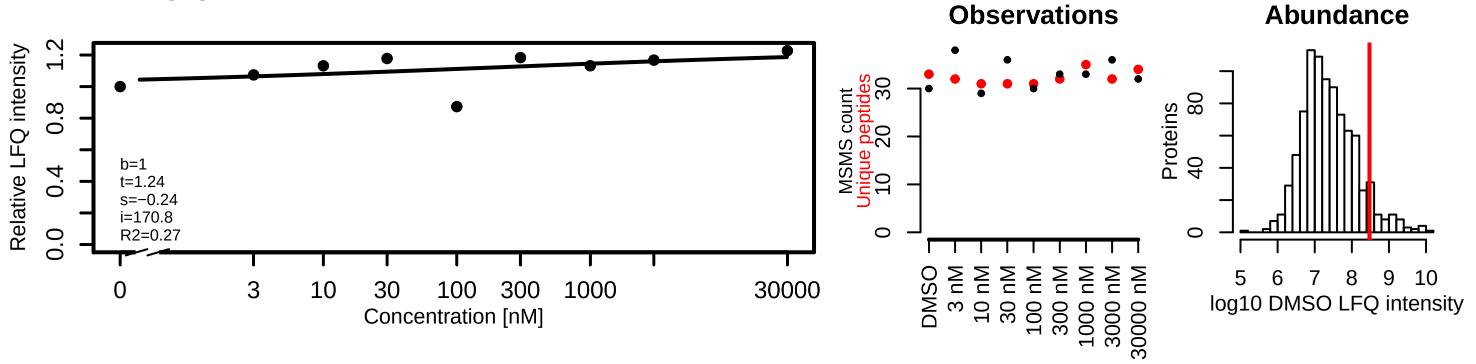

COX11

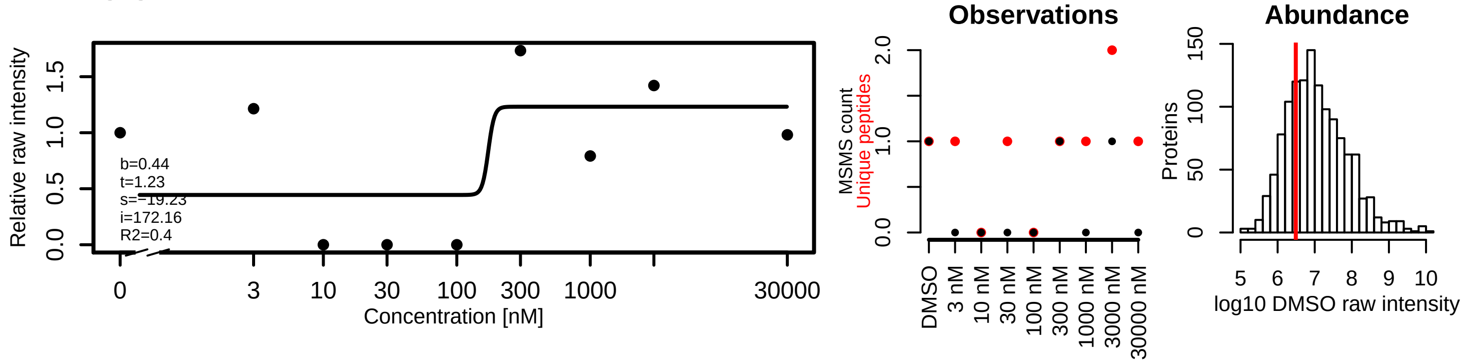

BSG

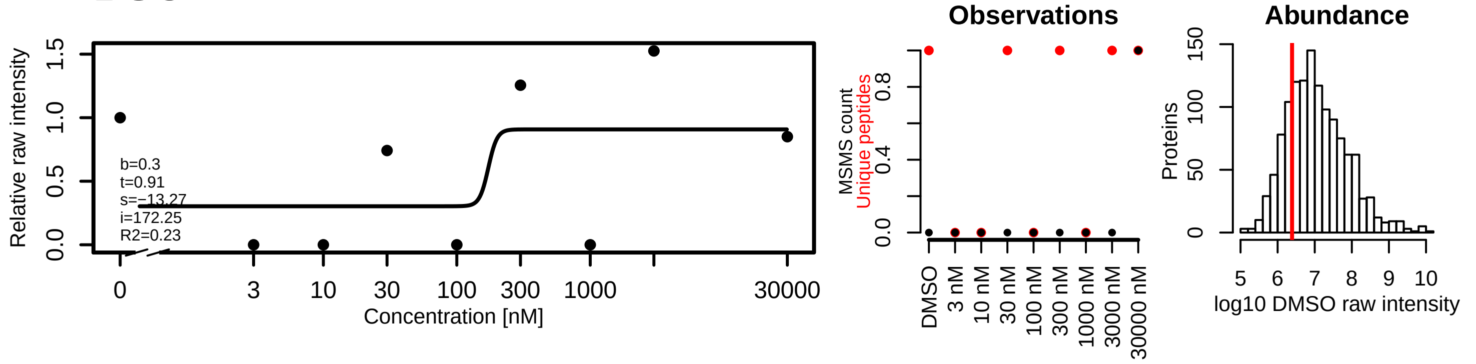

RBM14

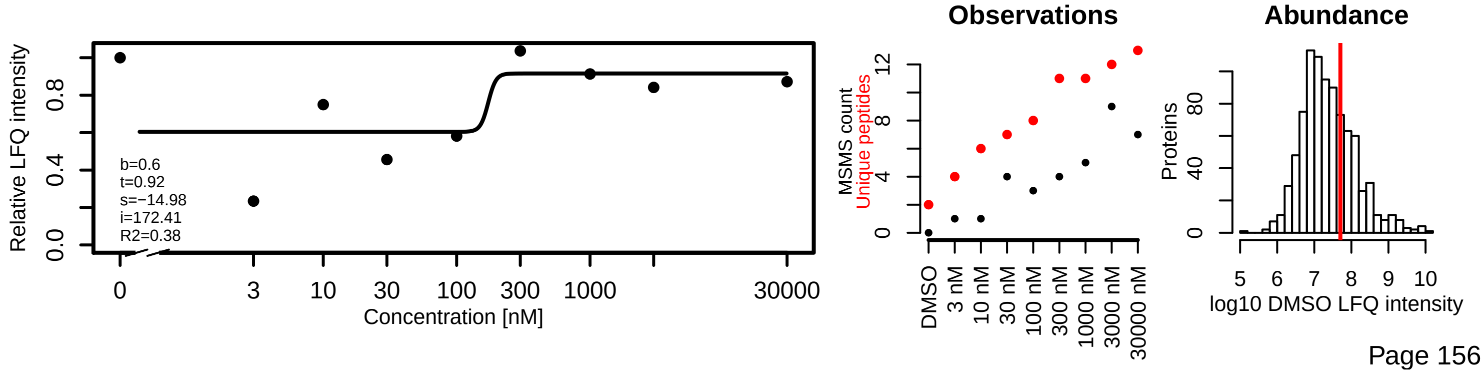

MPDU1

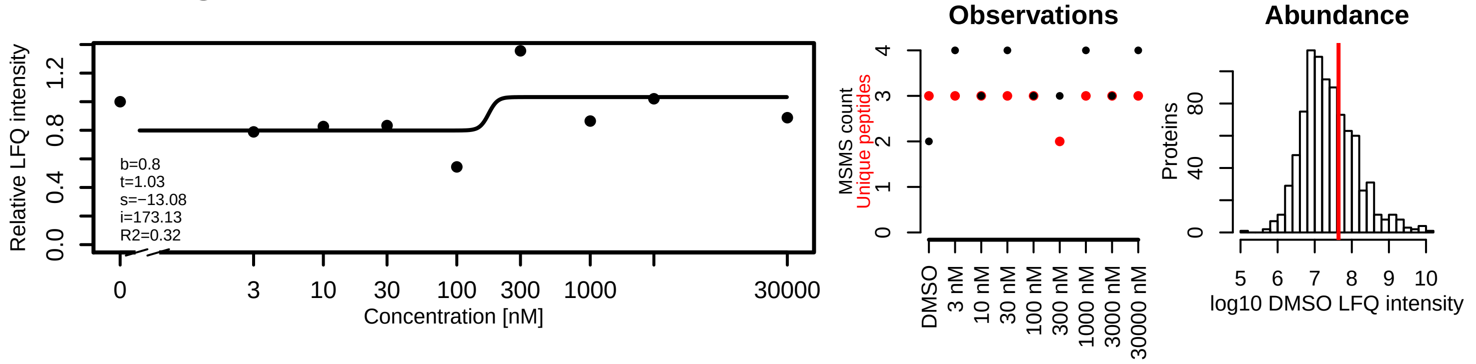

TIMM9

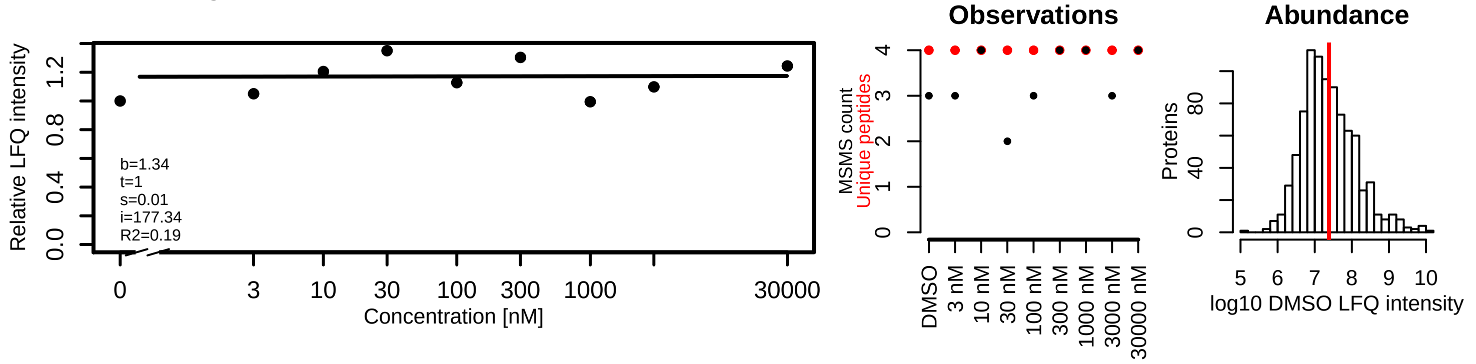

S100A2

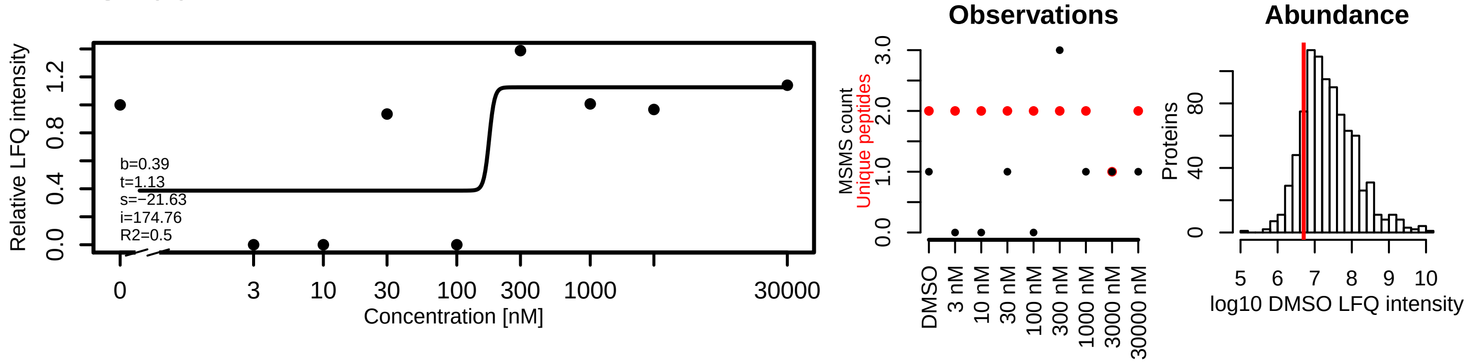

AGPAT9

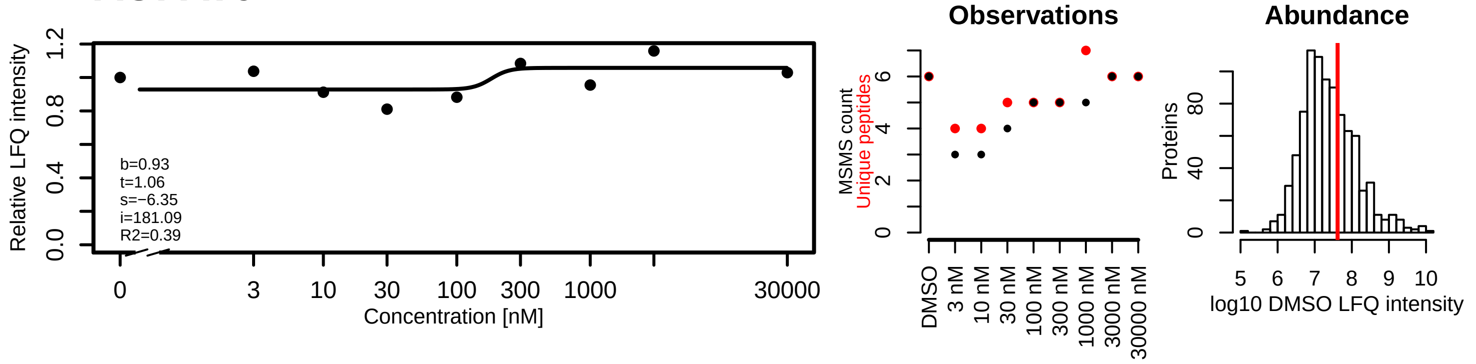

ALG10;ALG10B

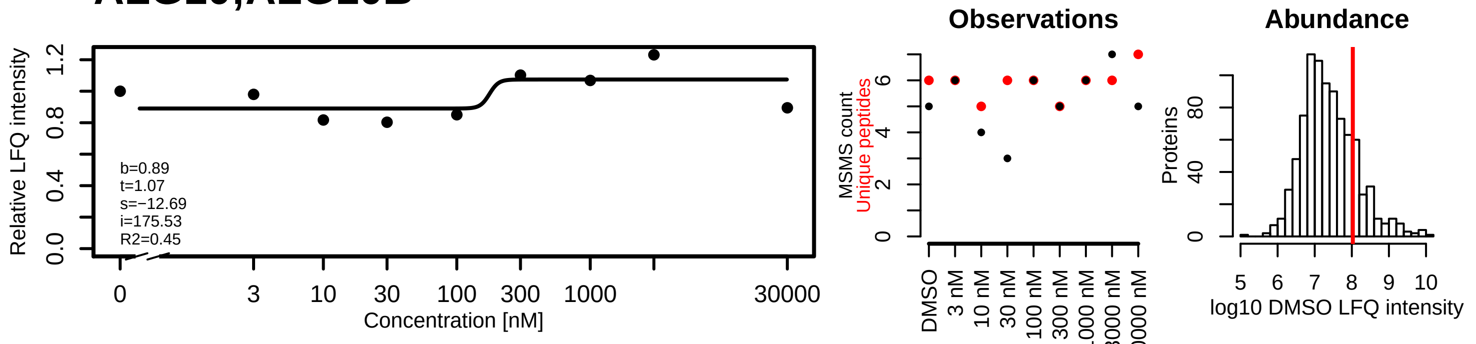

RRP12

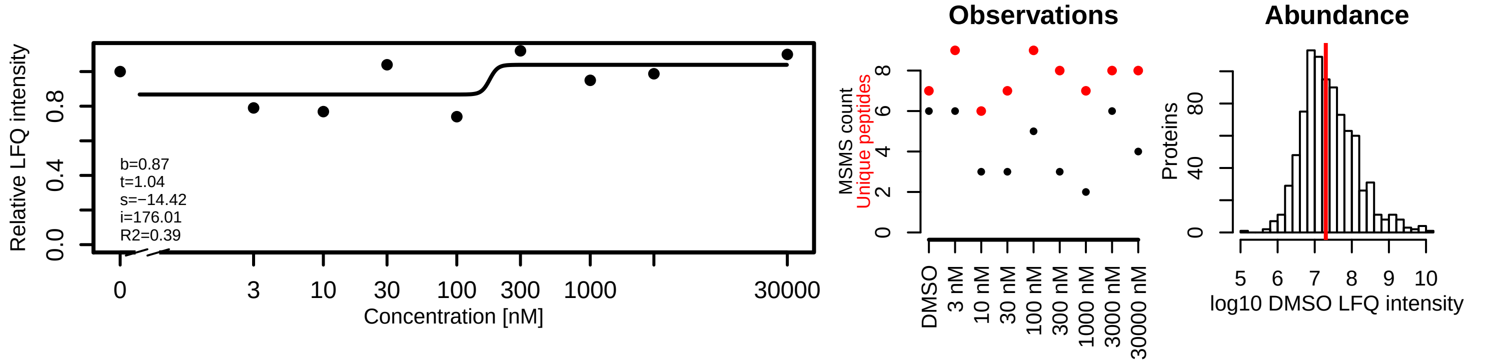

APOOL

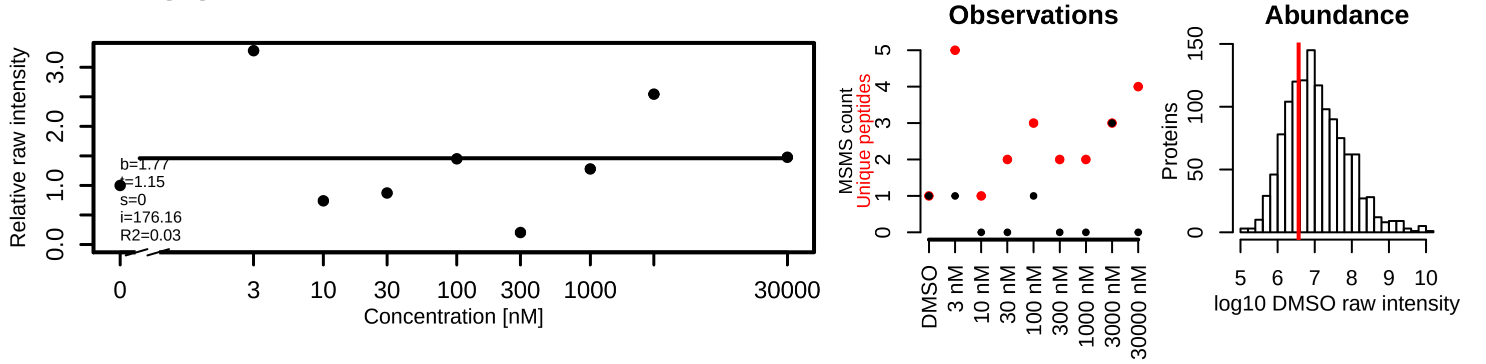

KIAA1524

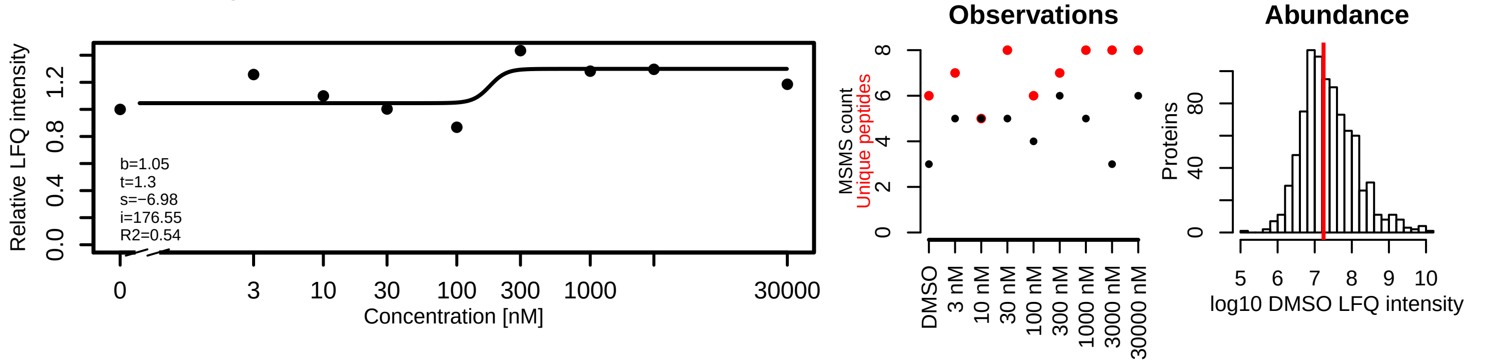

RRAS2

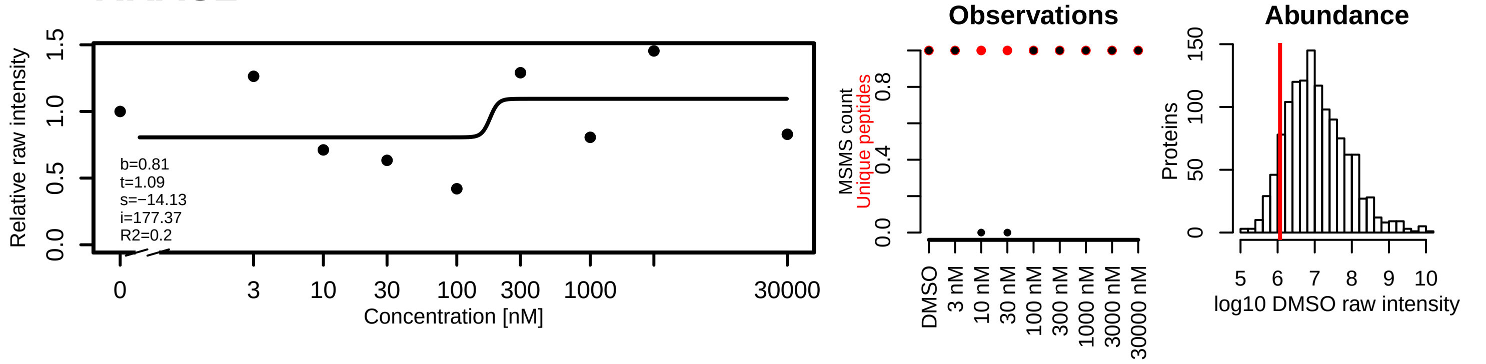

FANCI

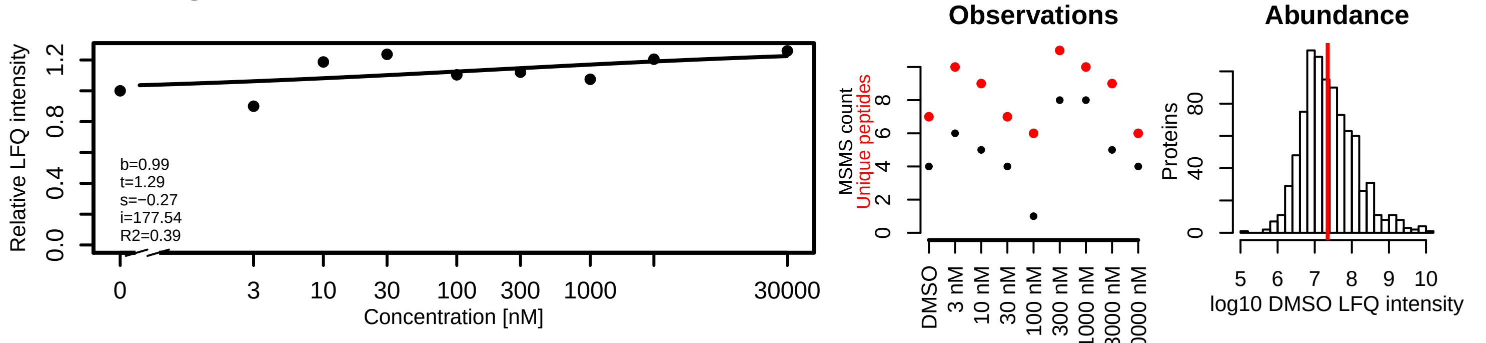

IPO9

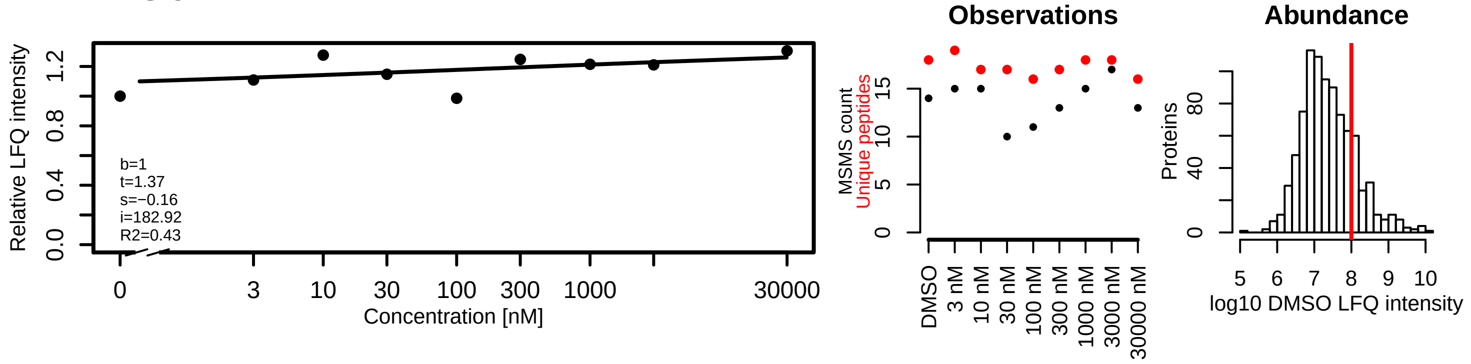

HKDC1

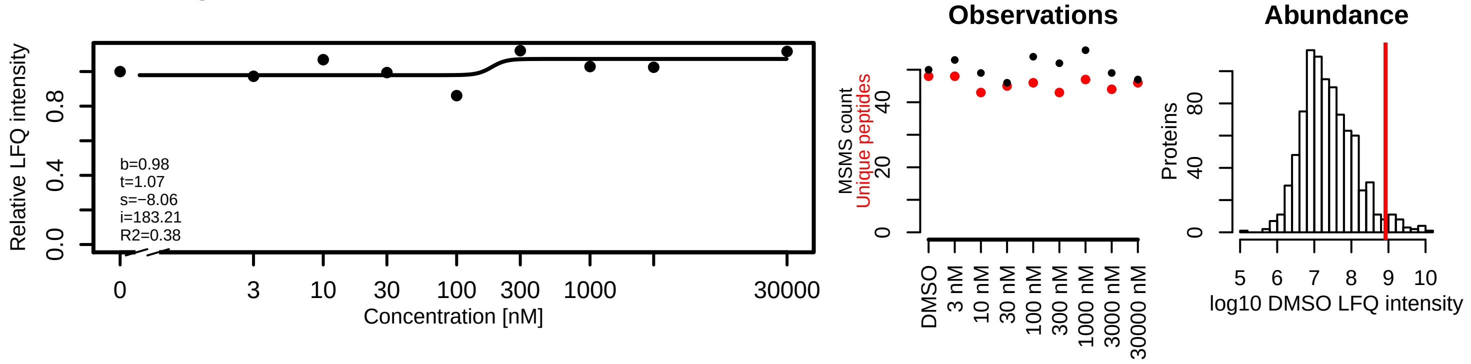

PIGT

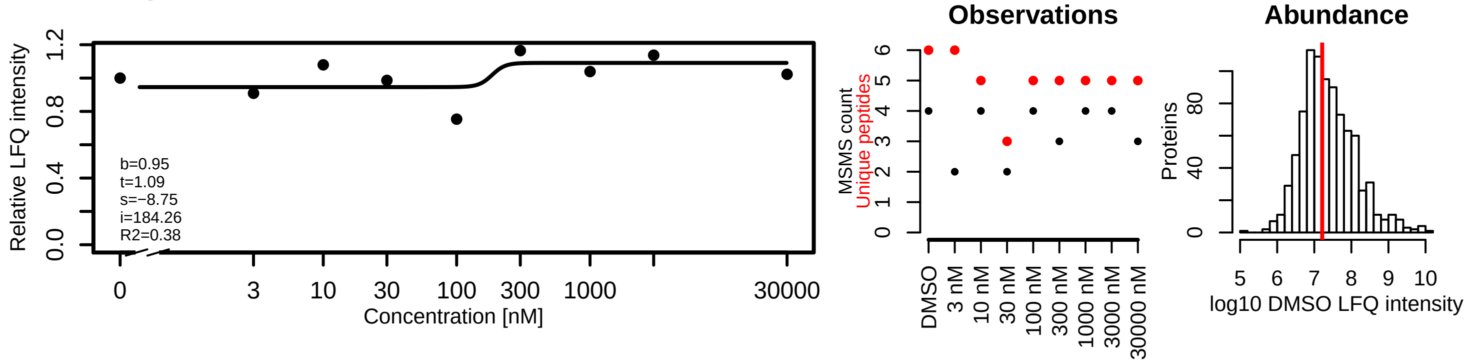

CERS2

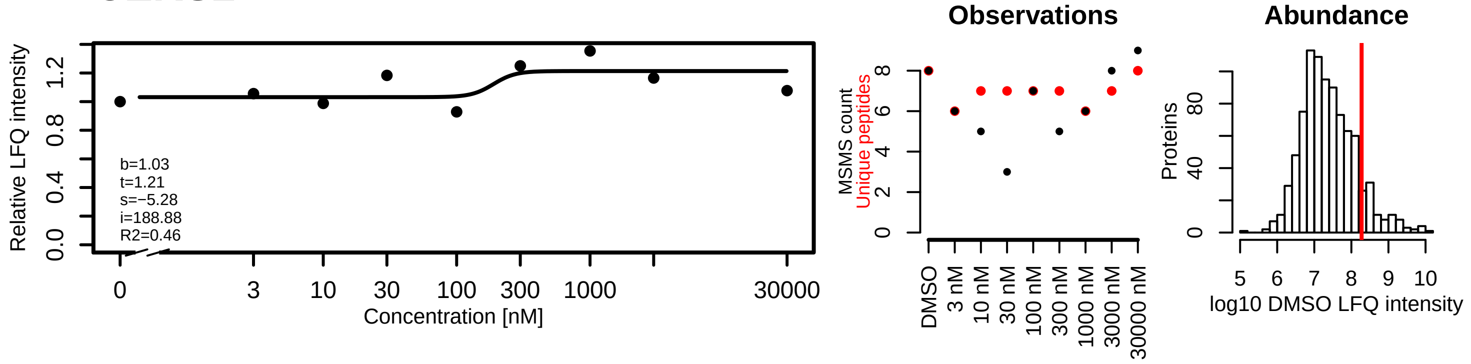

LTN1

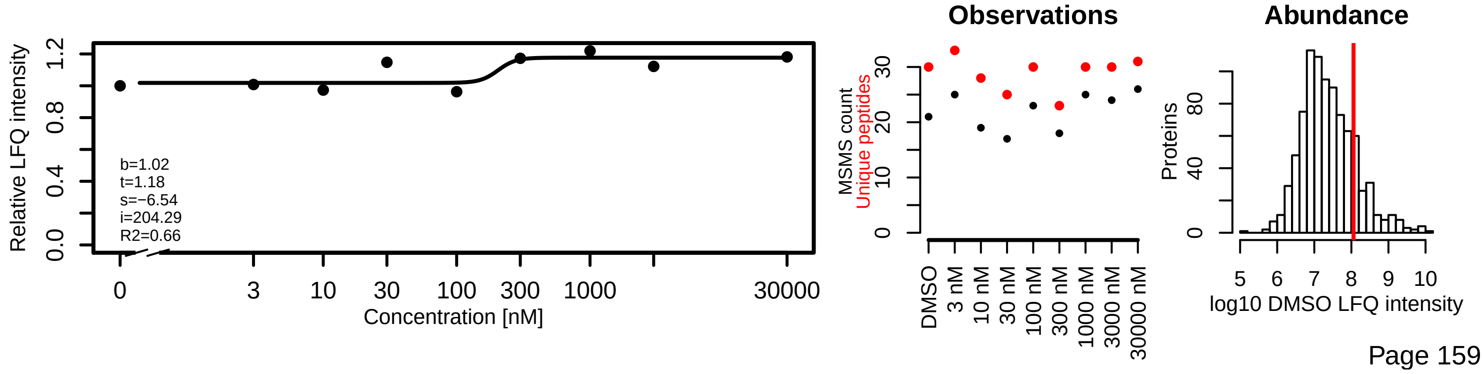

TUBGCP2

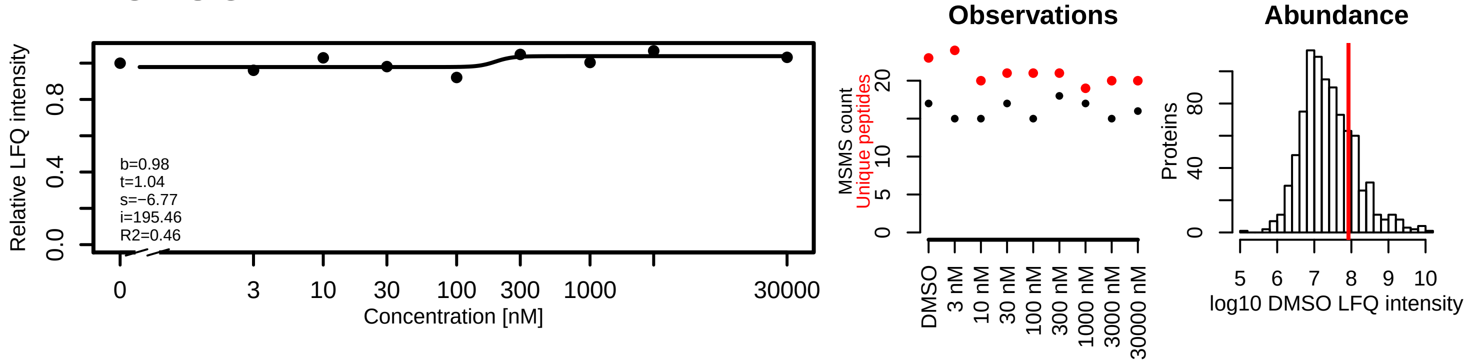

MTCH2 − Q9Y6C9;F5H3Y2

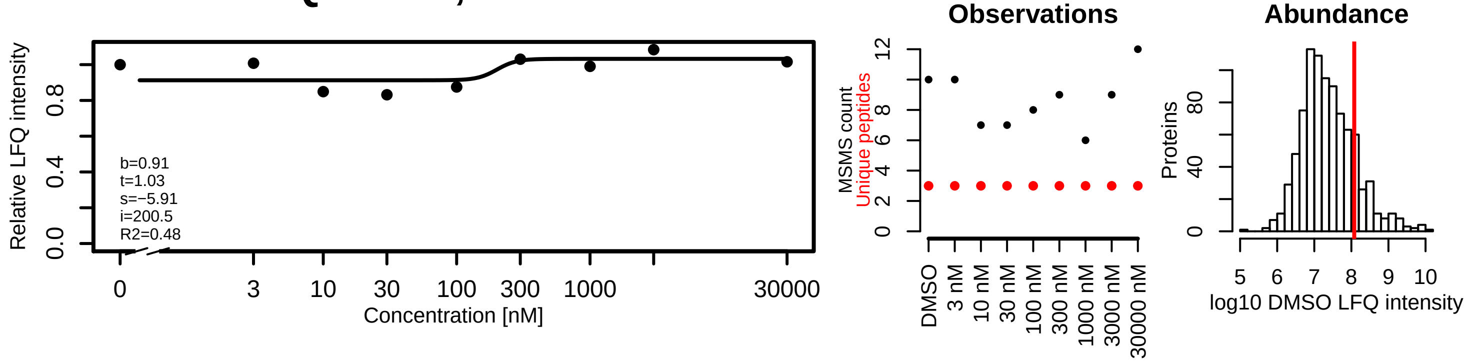

NT5DC2

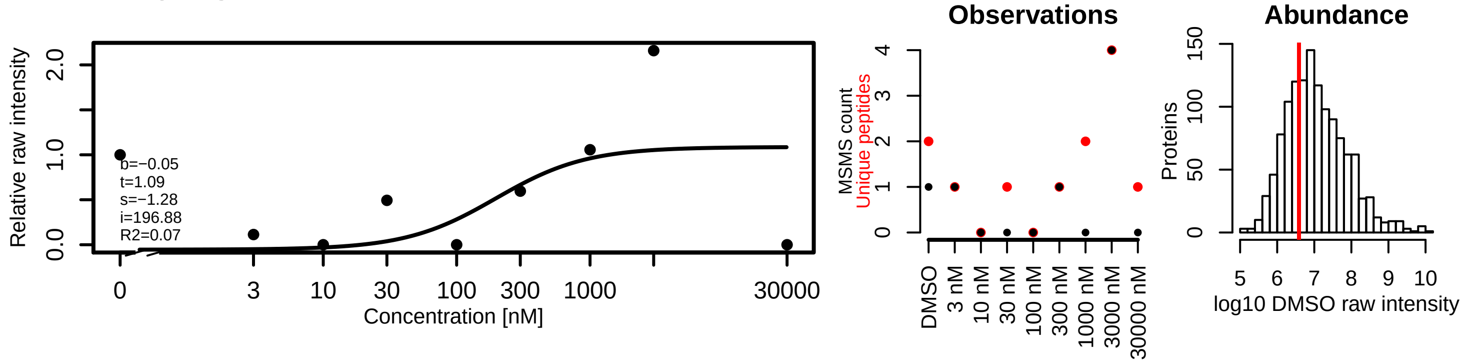

STK36

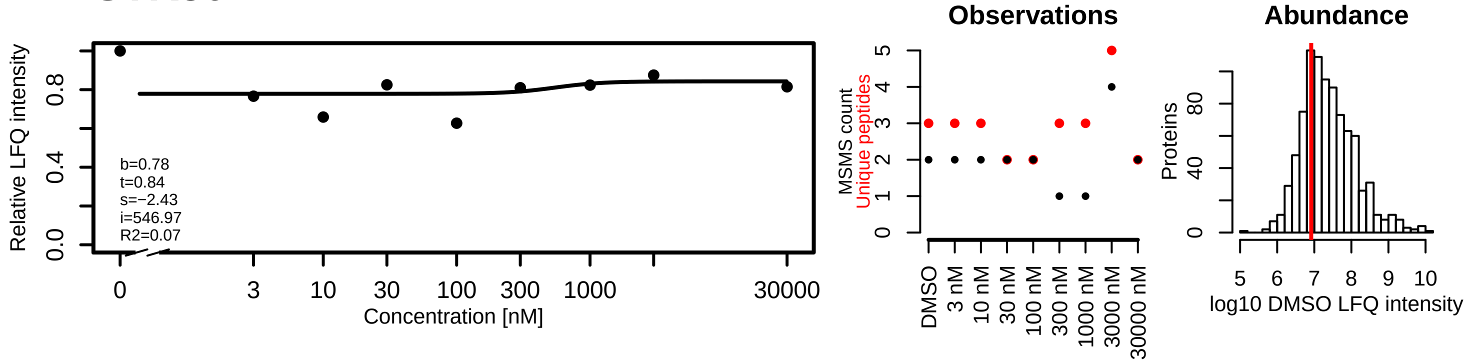

STOML2

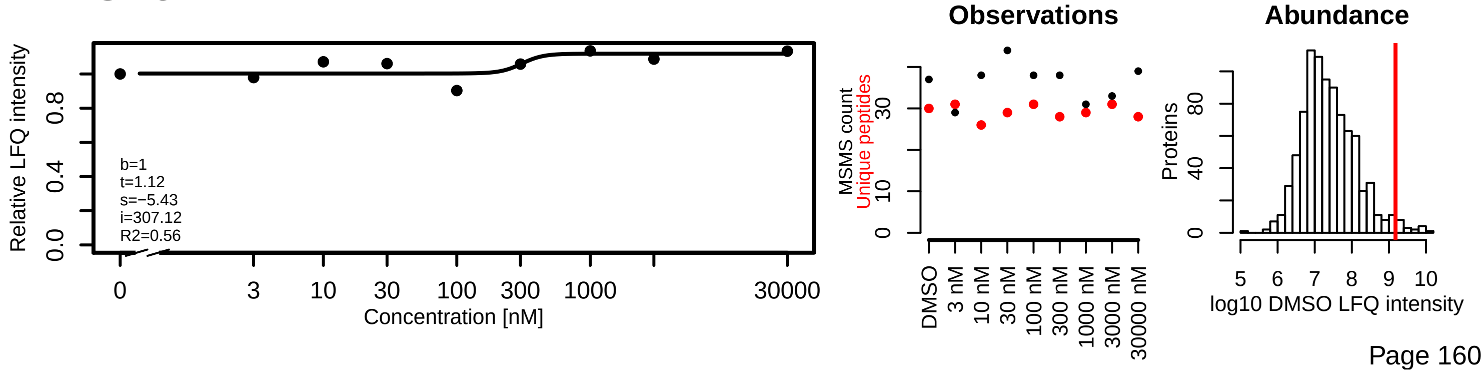

ATP6V1H

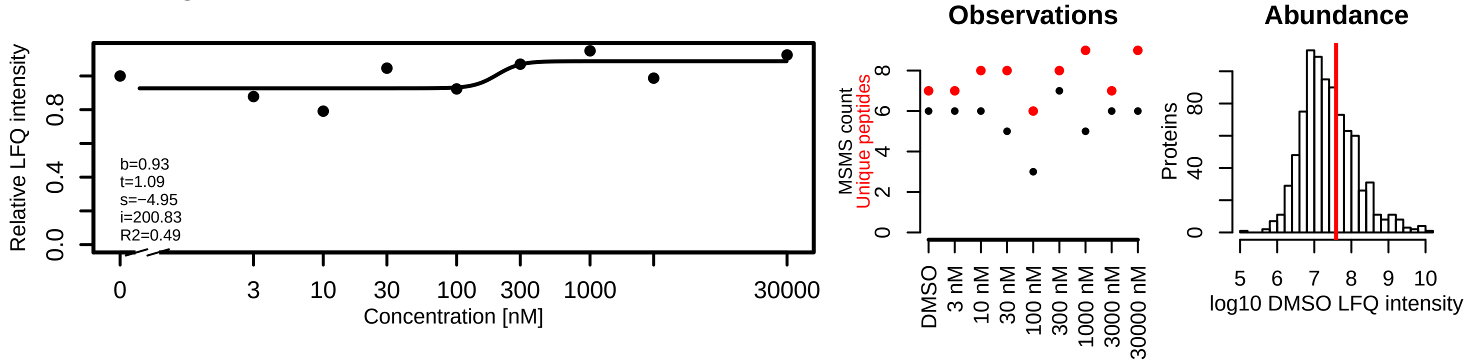

KTN1

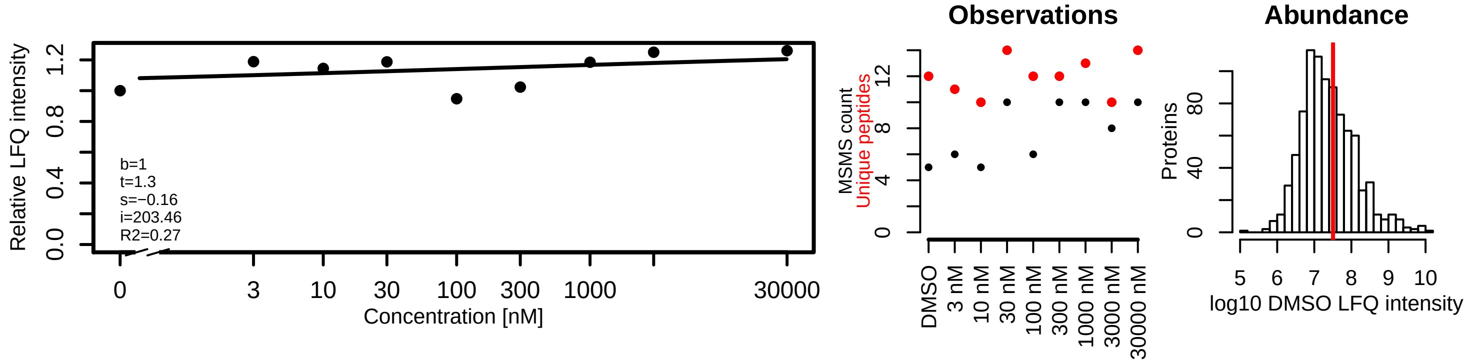

YME1L1

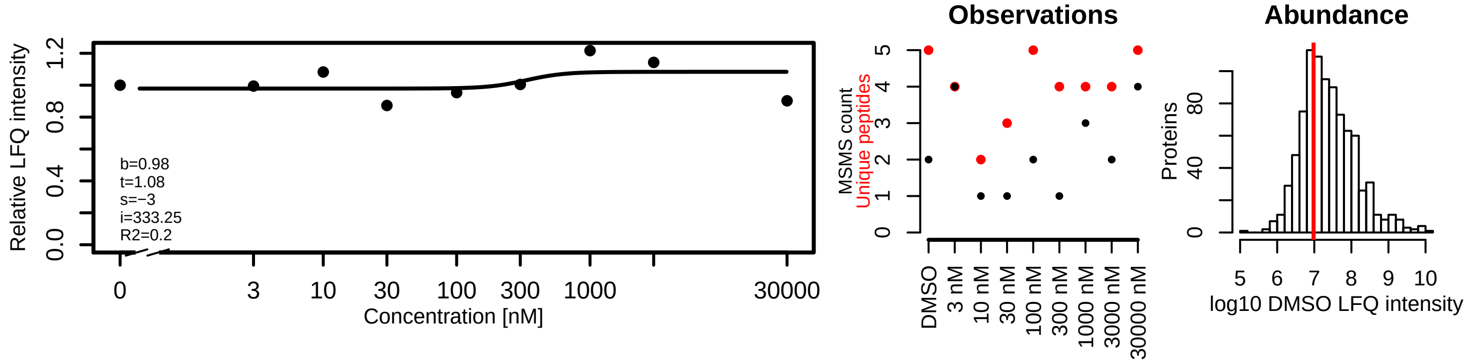

RCN1

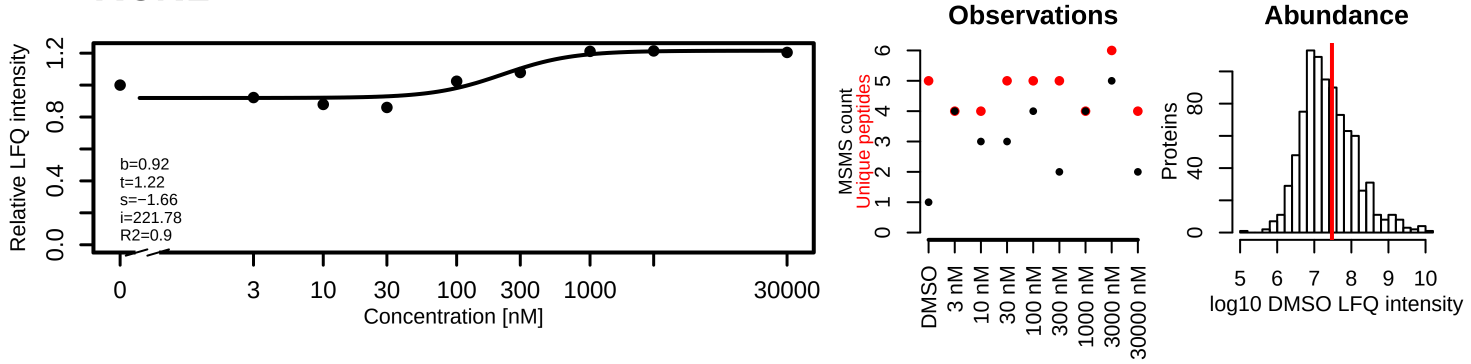

TRAM1

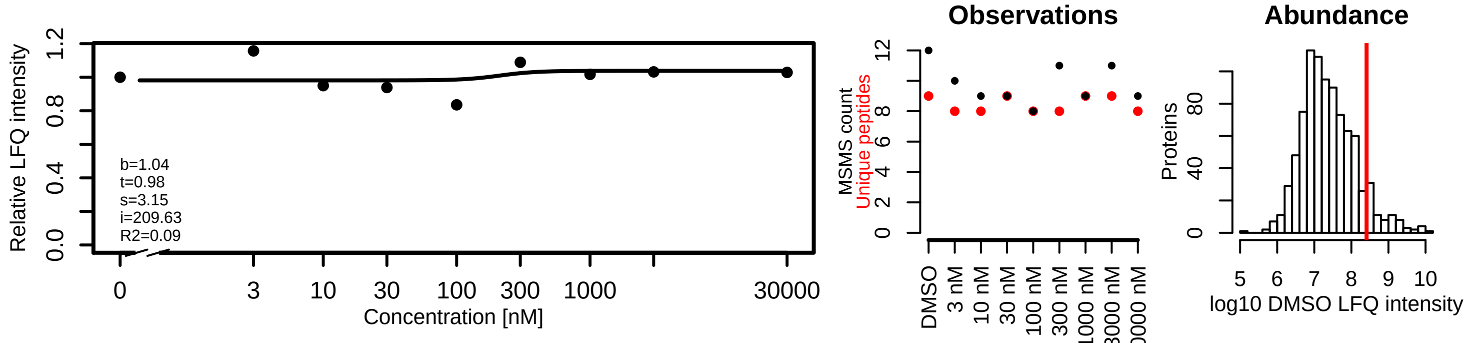

TMEM209

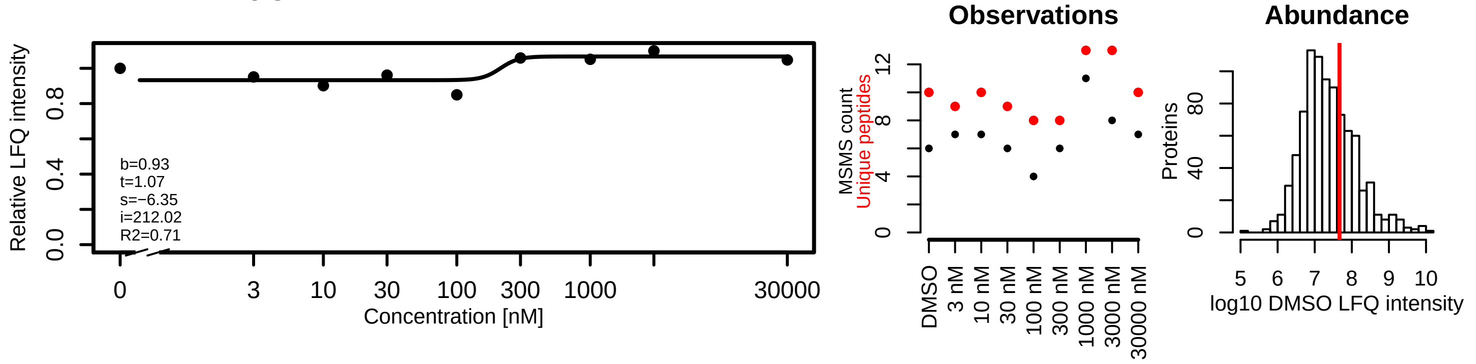

CTSL1;CTSL3P

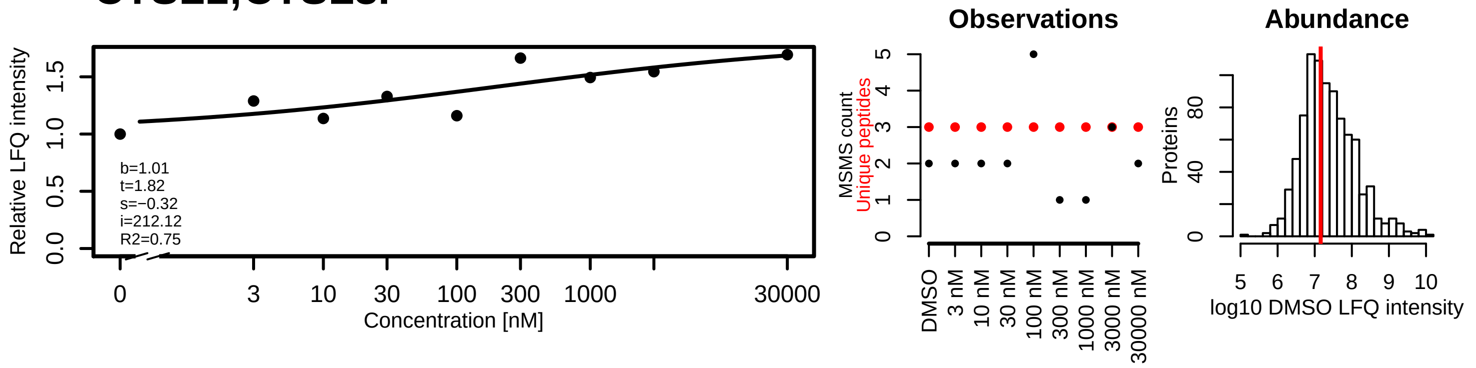

HLA−C;HLA−H;HLA−B;HLA−A

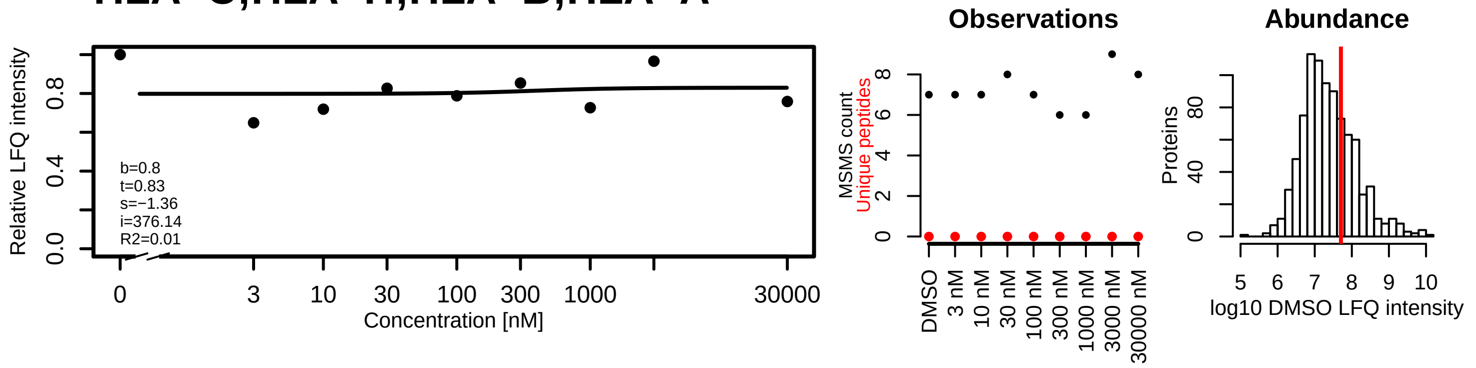

MED24

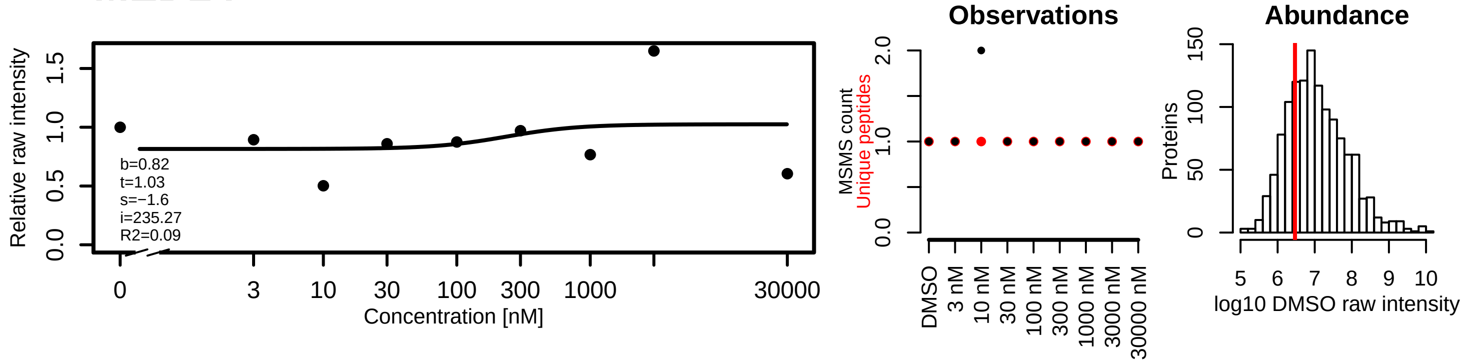

NOLC1

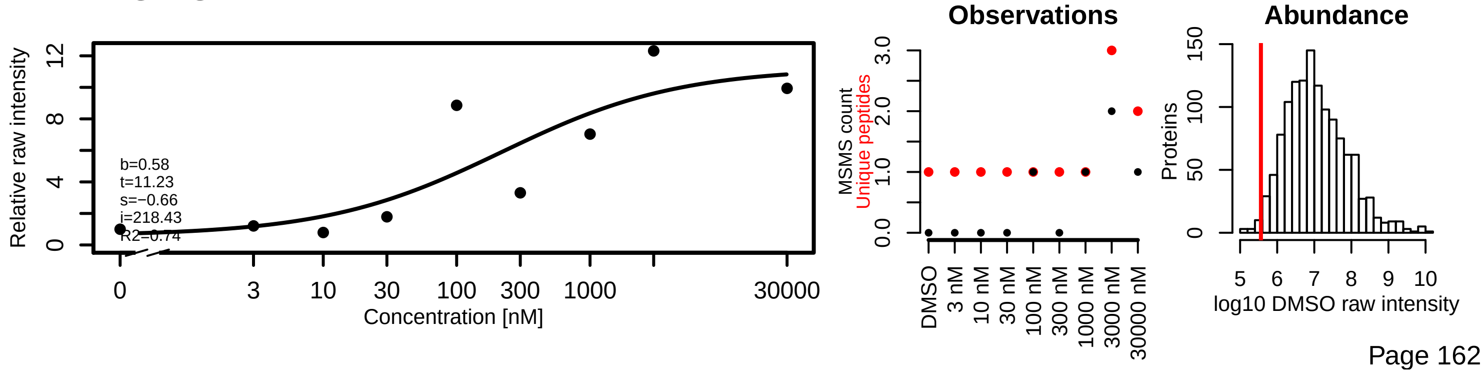

EMD

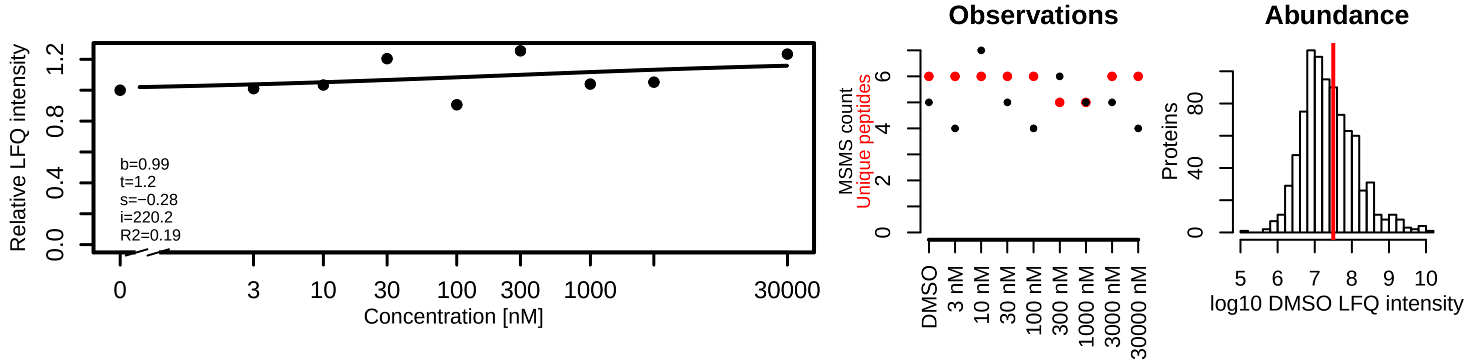

CYP3A5;CYP3A7;CYP3A4

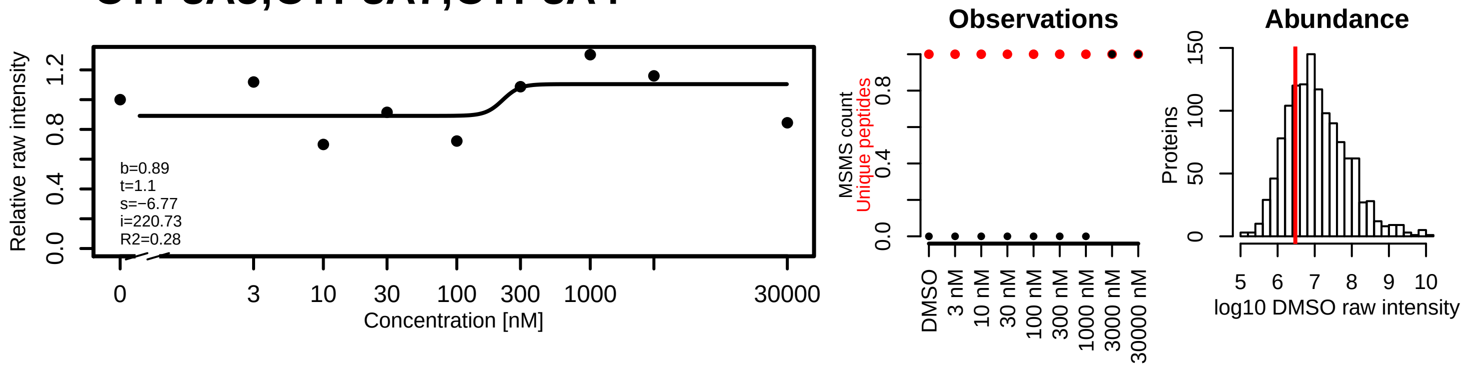

ROMO1

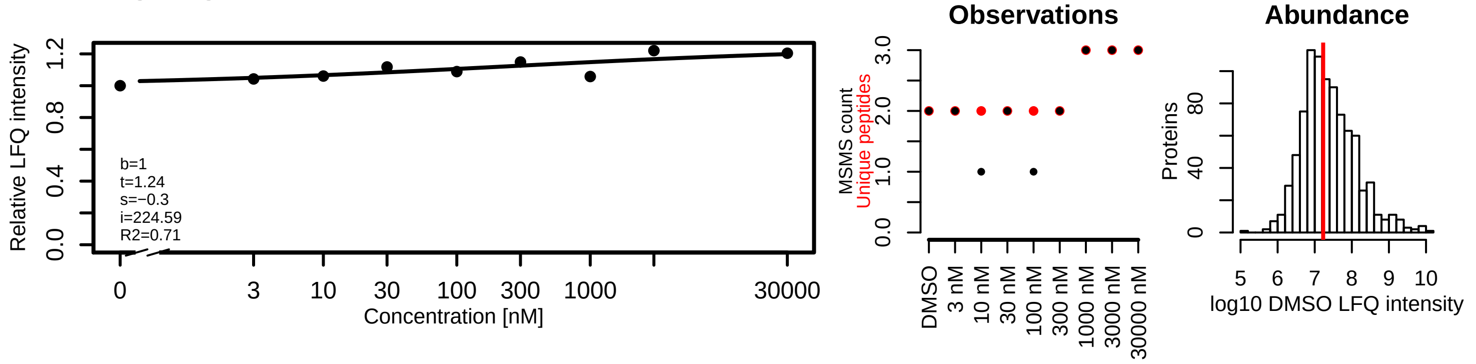

NME3

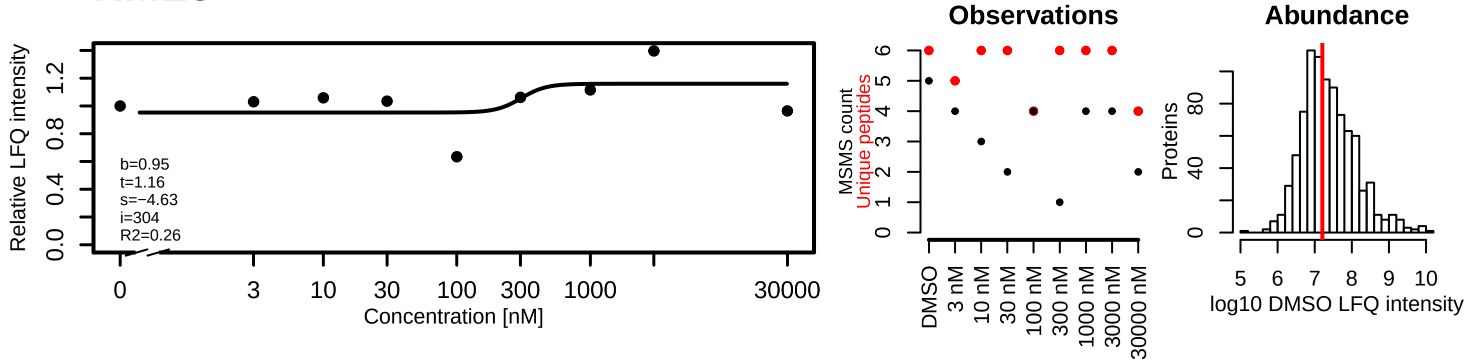

POMT1

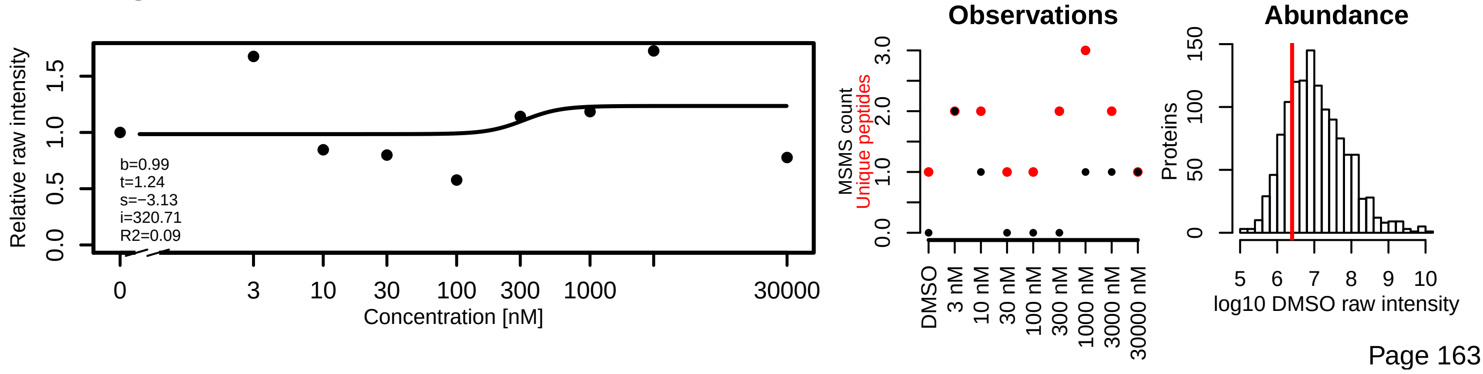

SLC20A1

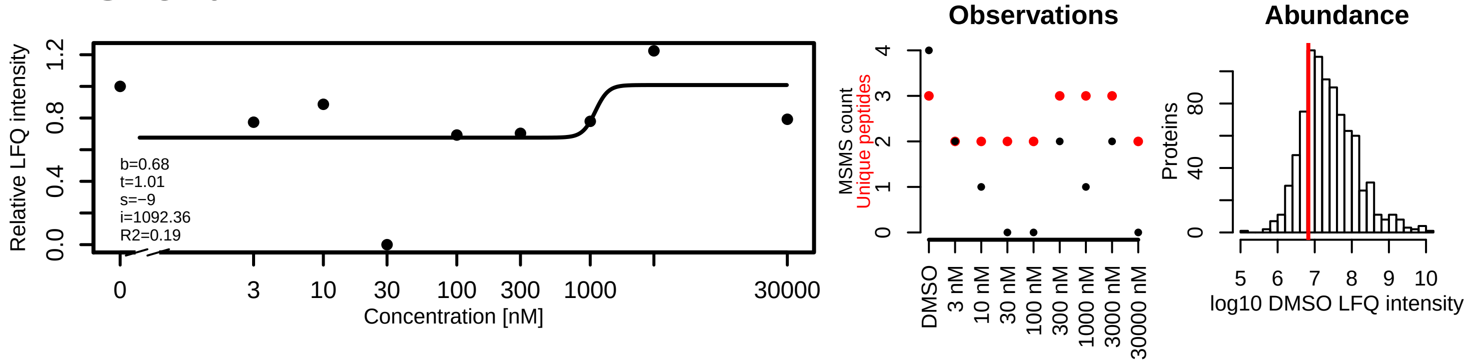

MLL

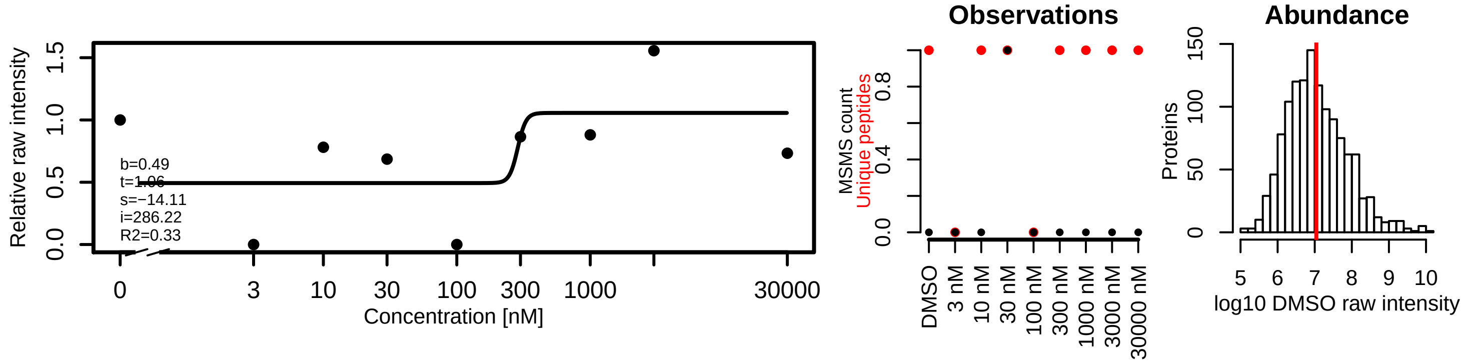

SNRPD2

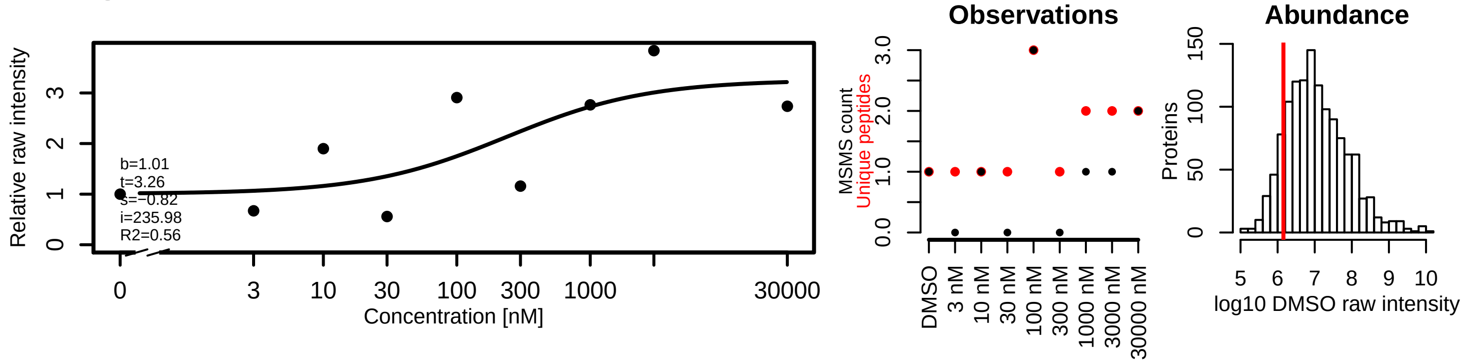

SEC22A

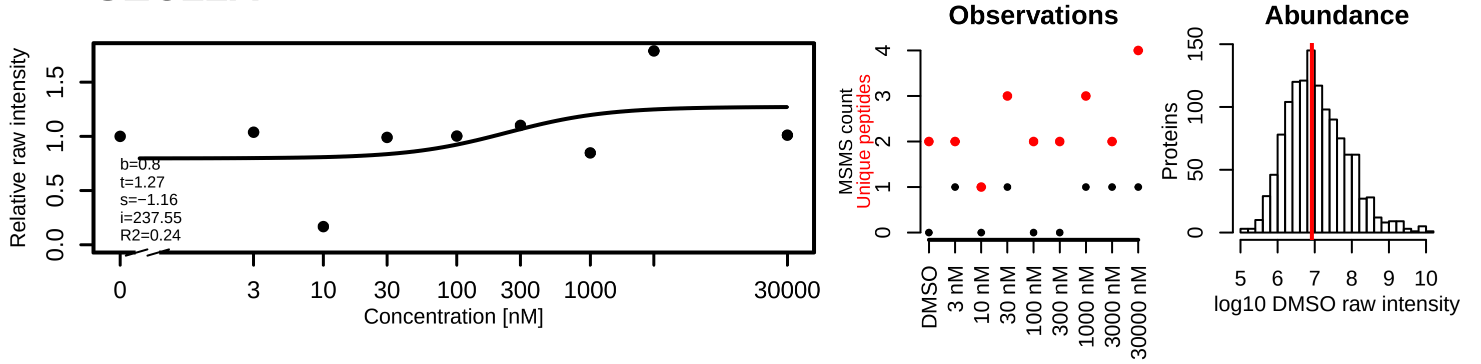

LMNB1

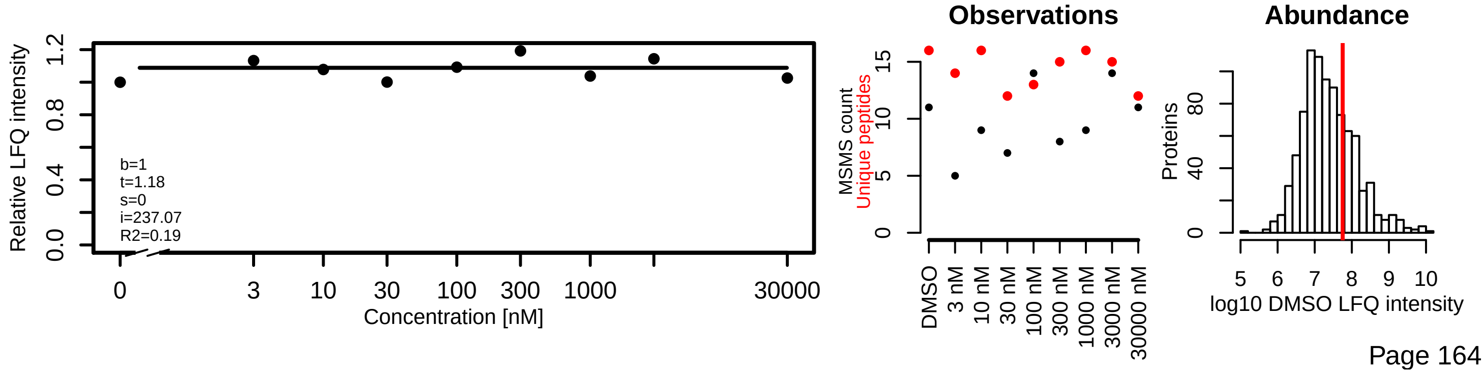

EMC1

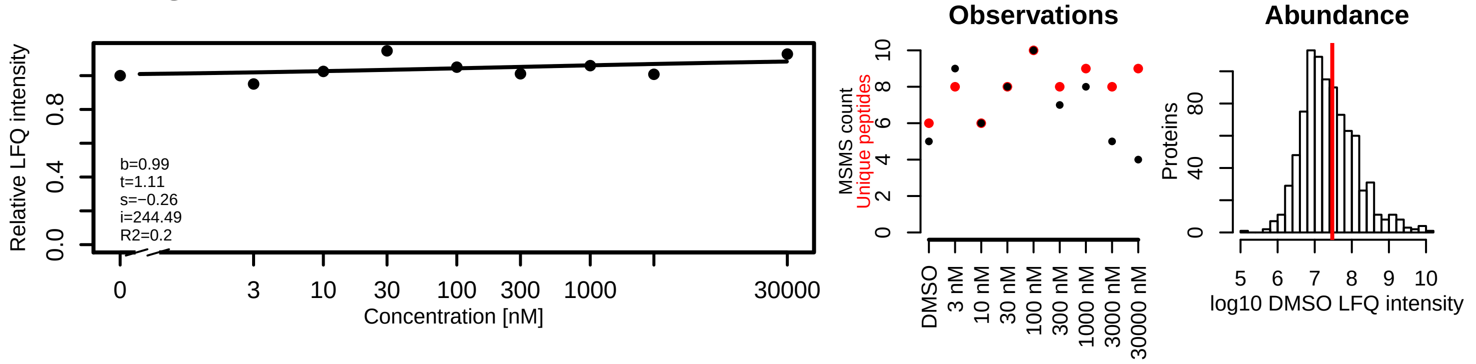

NDUFS2

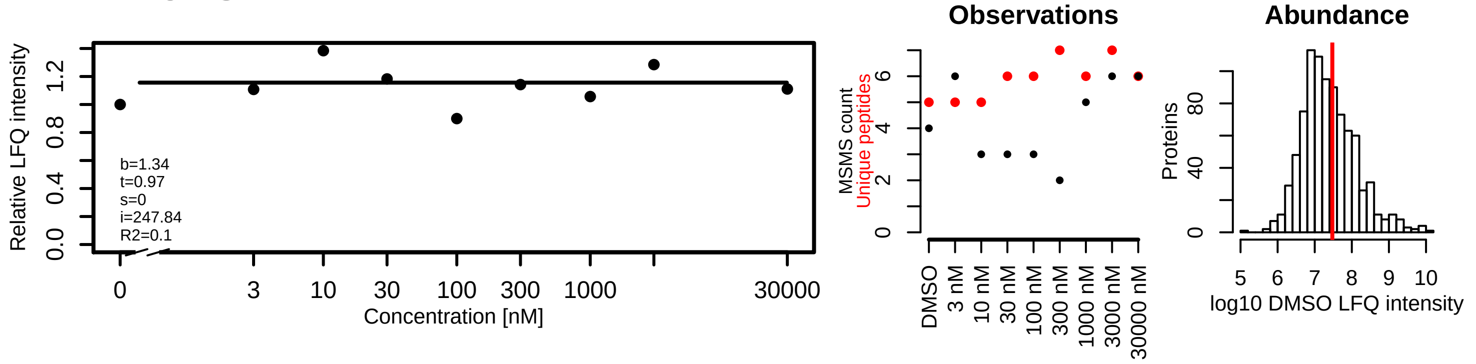

RAP1A;RAP1B

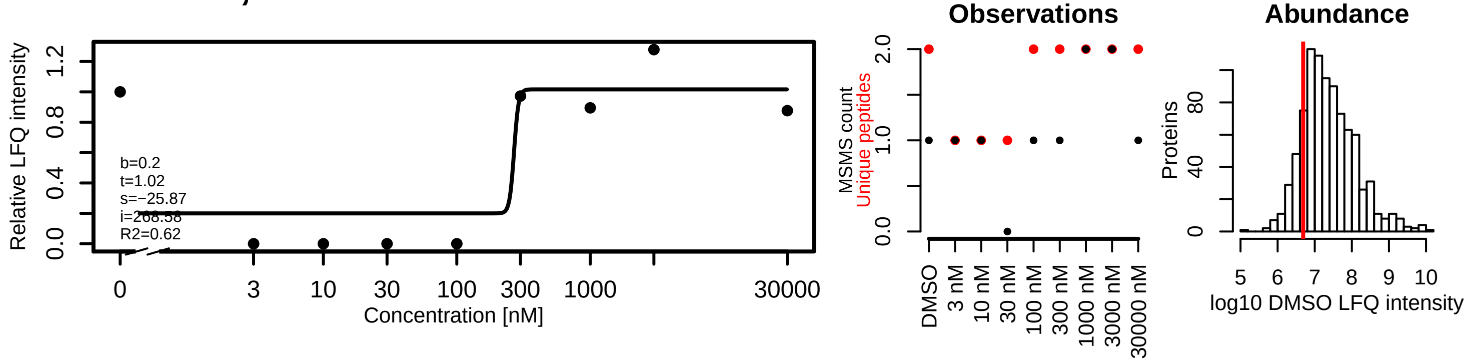

CDC73

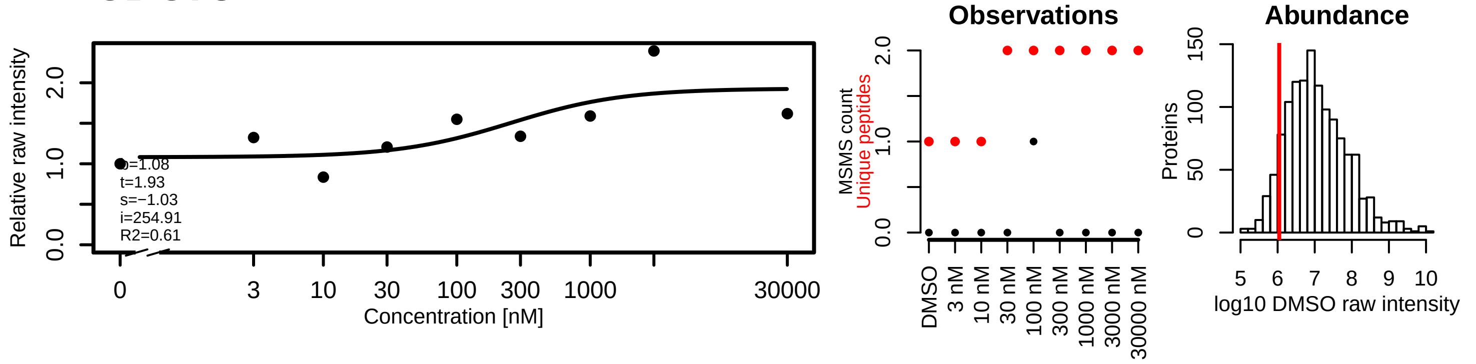

PIGG

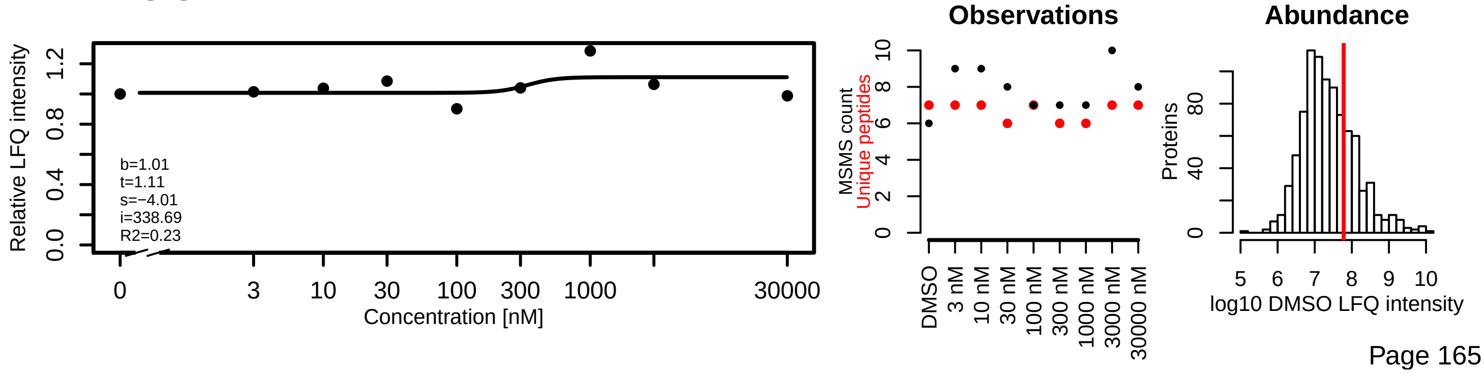

ZDHHC24

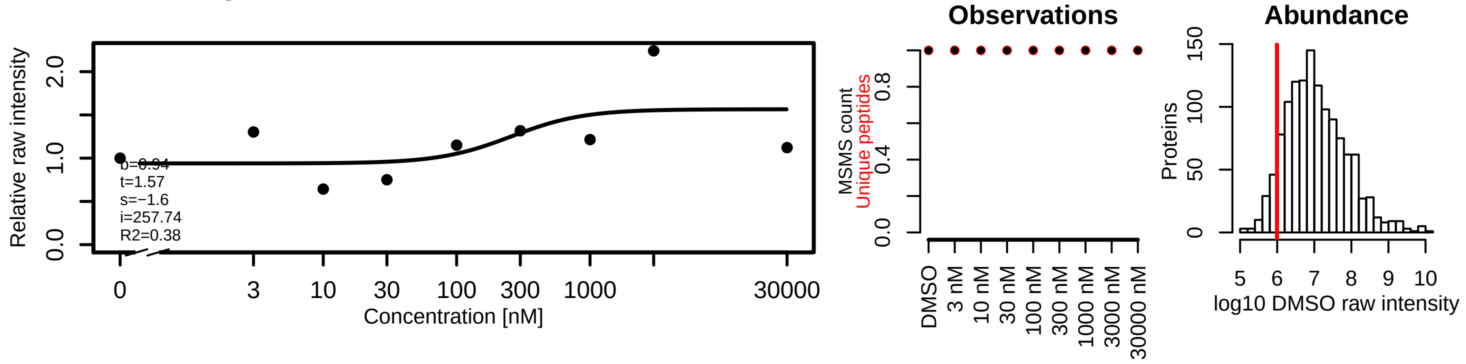

SRPRB

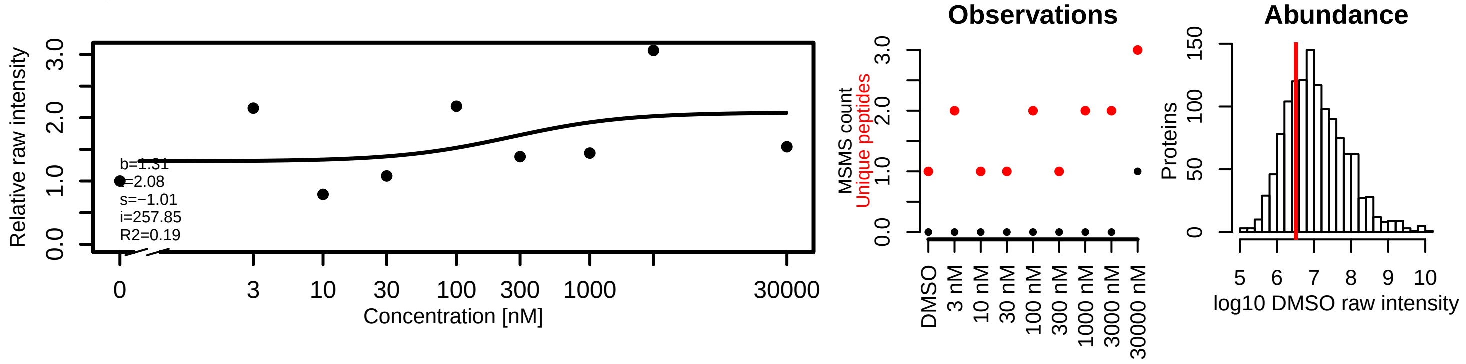

S100A6

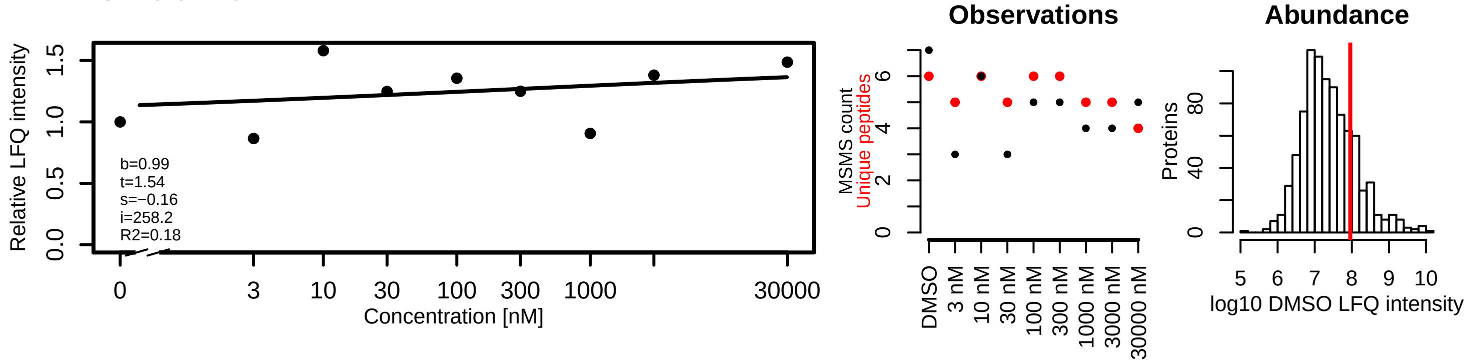

KIAA1715;LNP

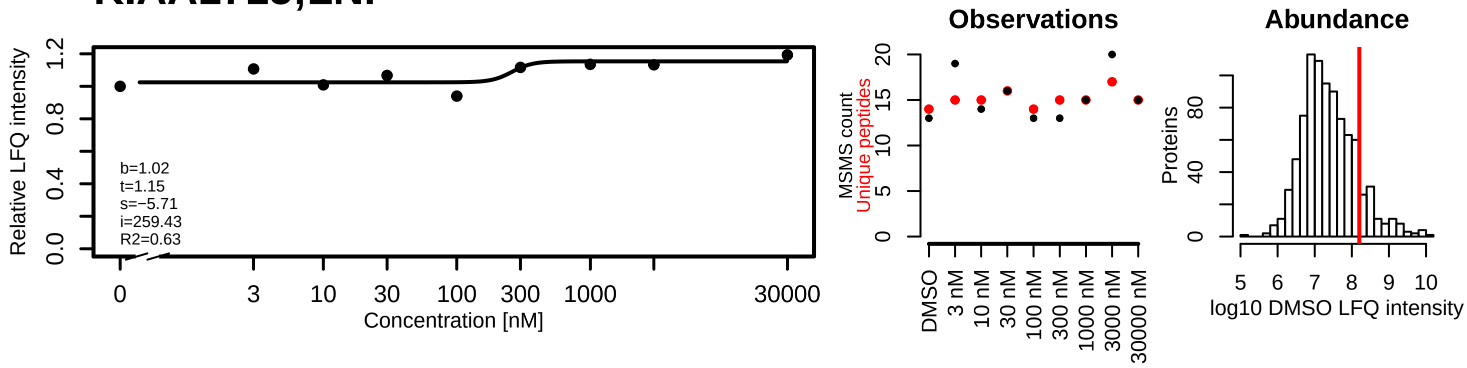

HNRNPH3

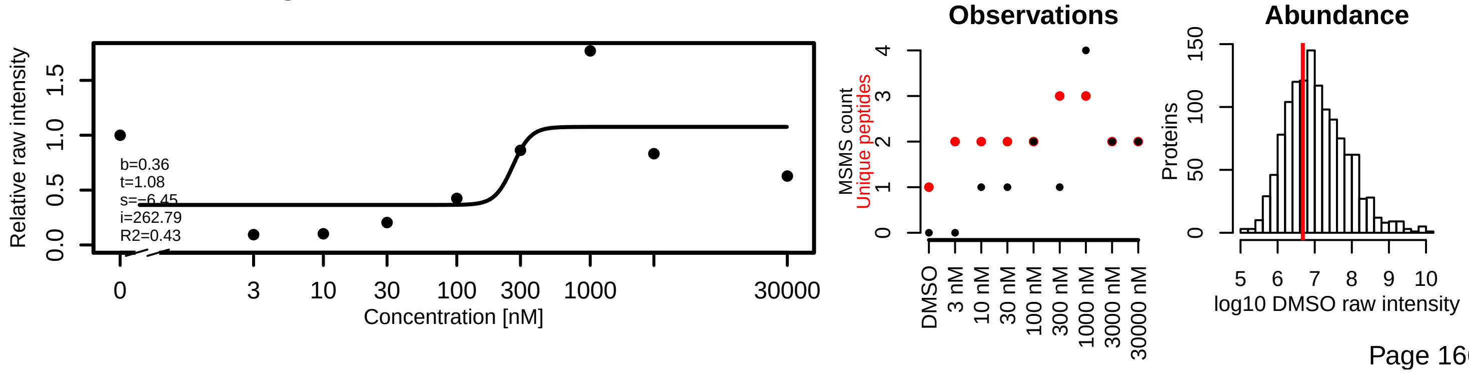

YIPF5

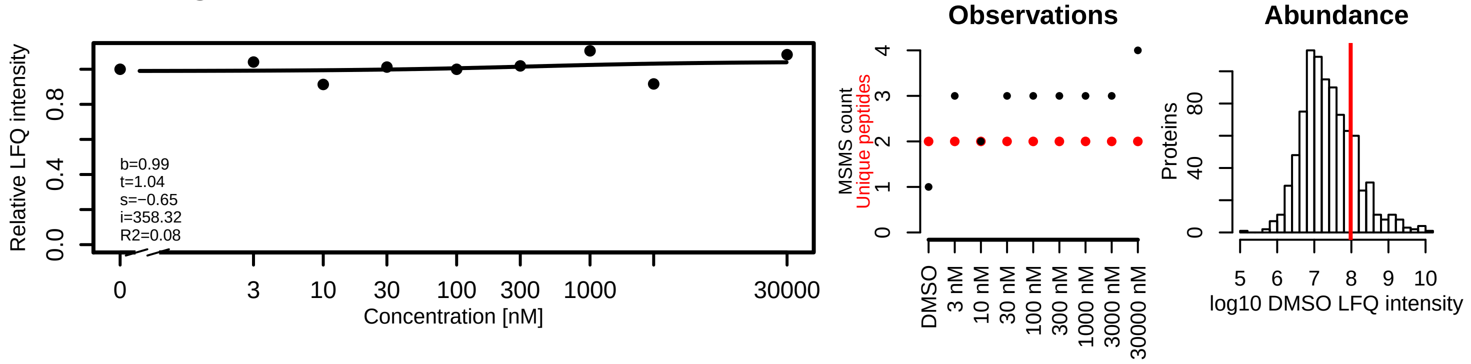

CRELD1

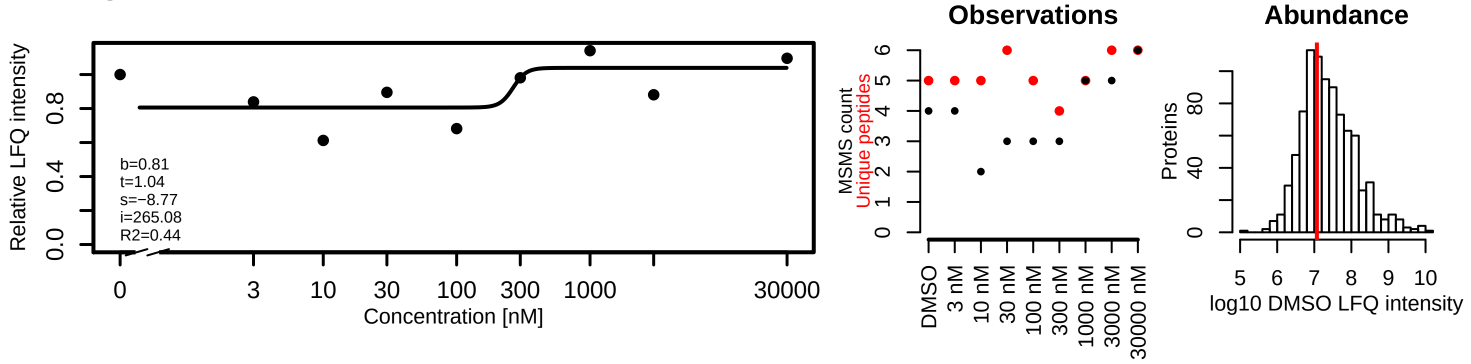

GALNT2

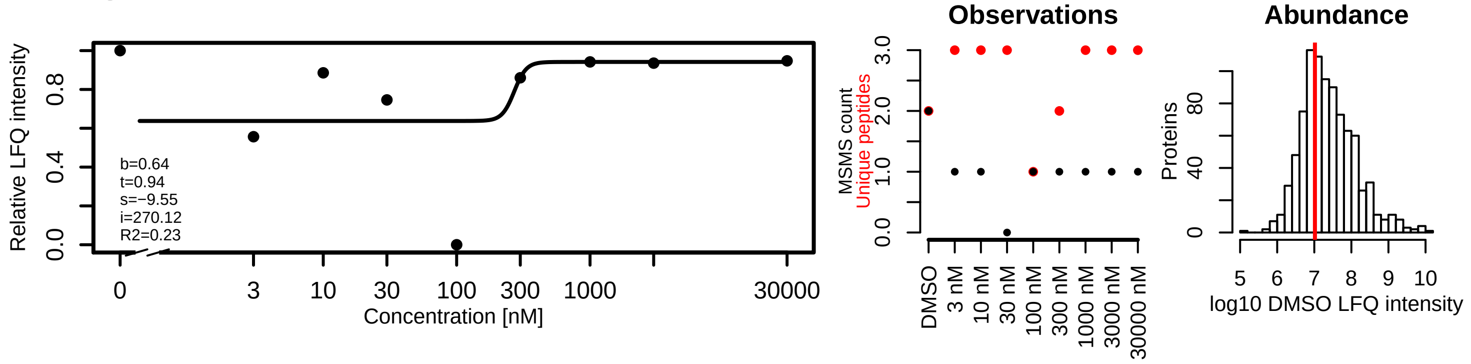

MTCH2 − E9PIE4

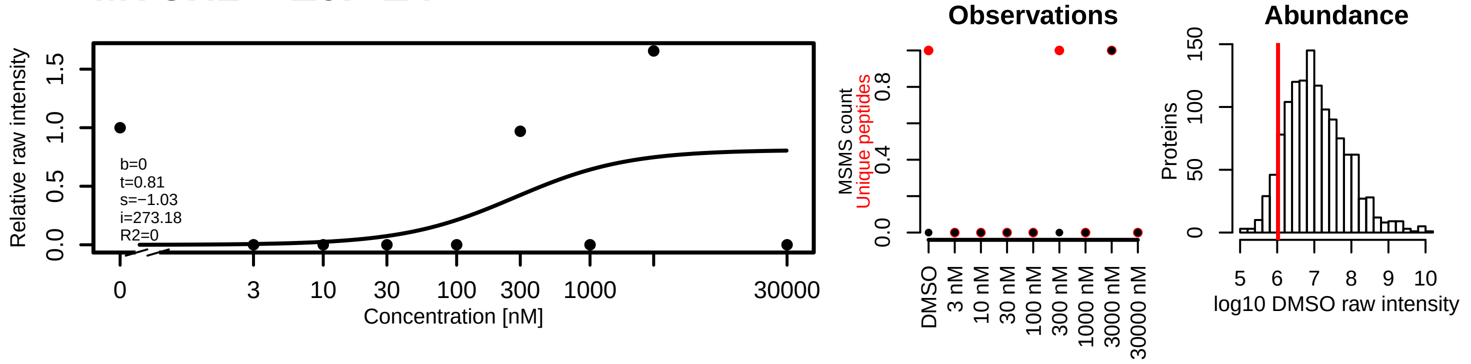

SLC25A40

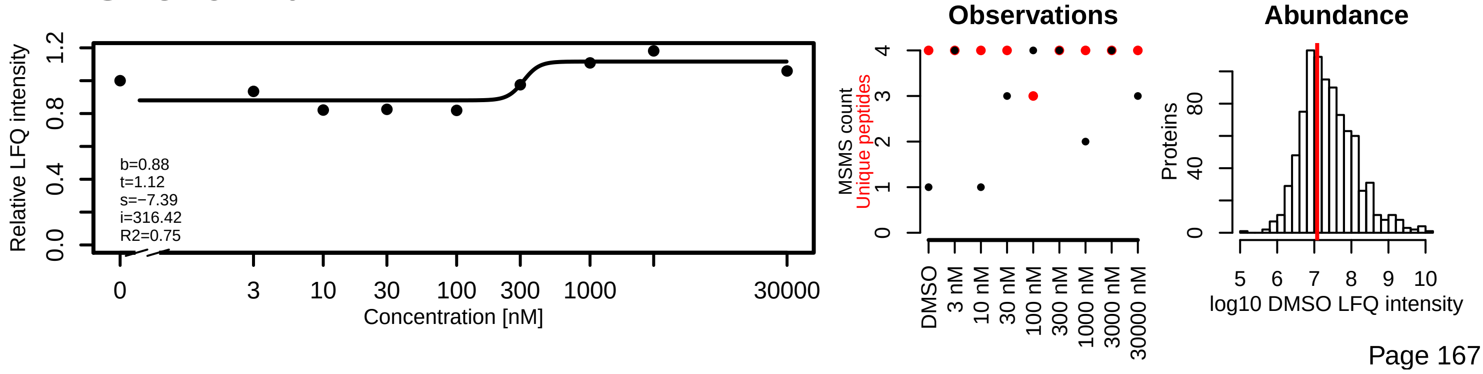

ULK3

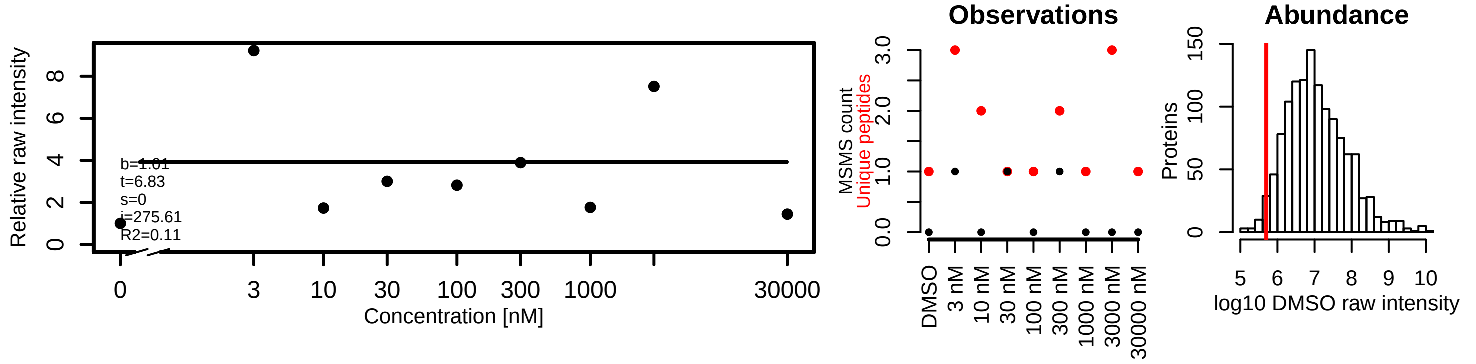

LACTB

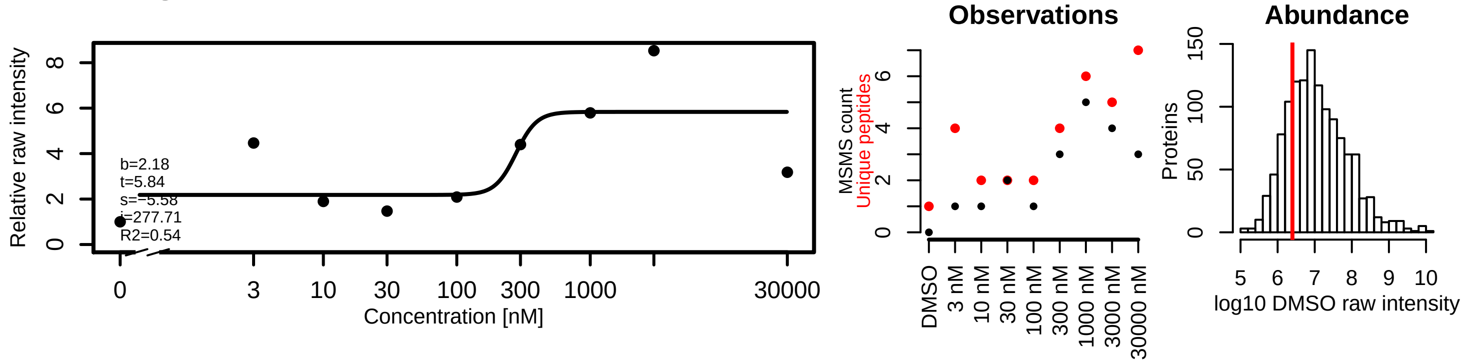

KCNMA1

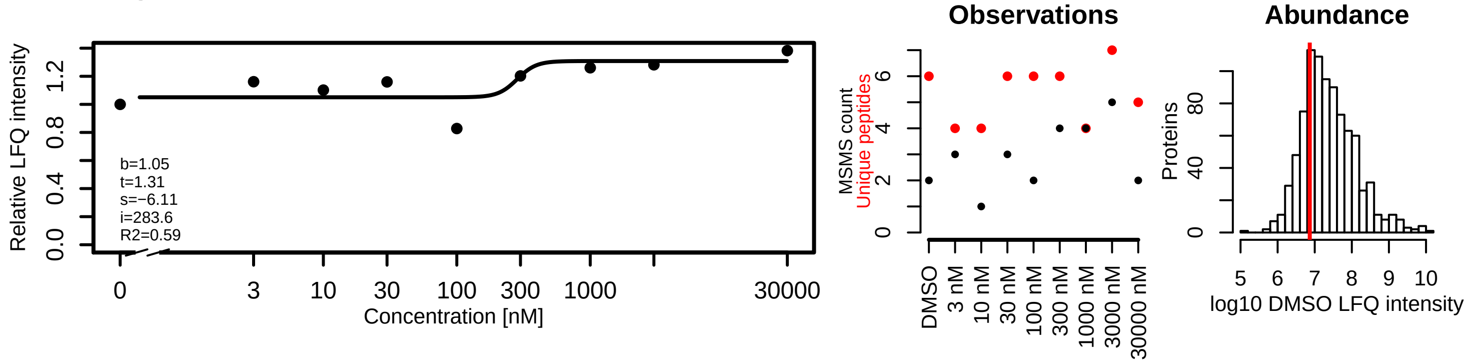

PLGRKT

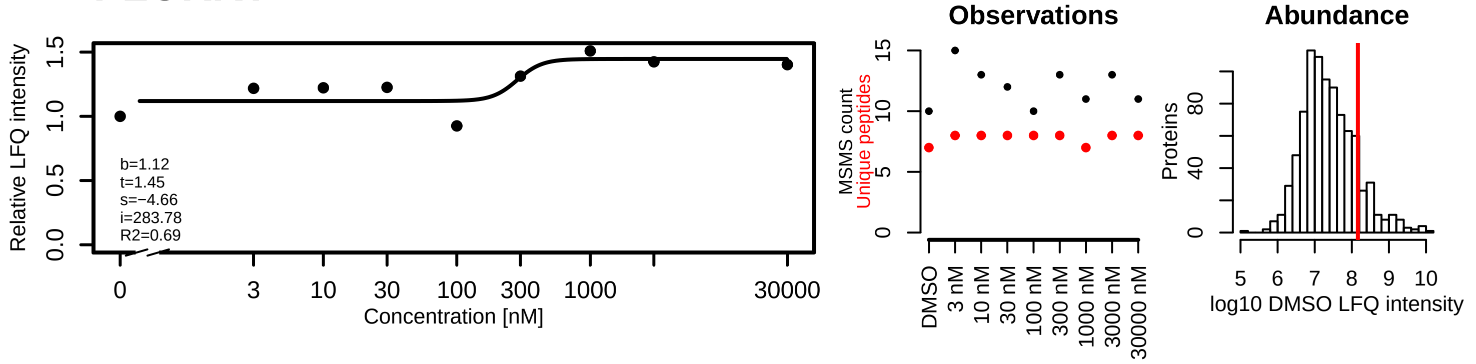

ERLIN1

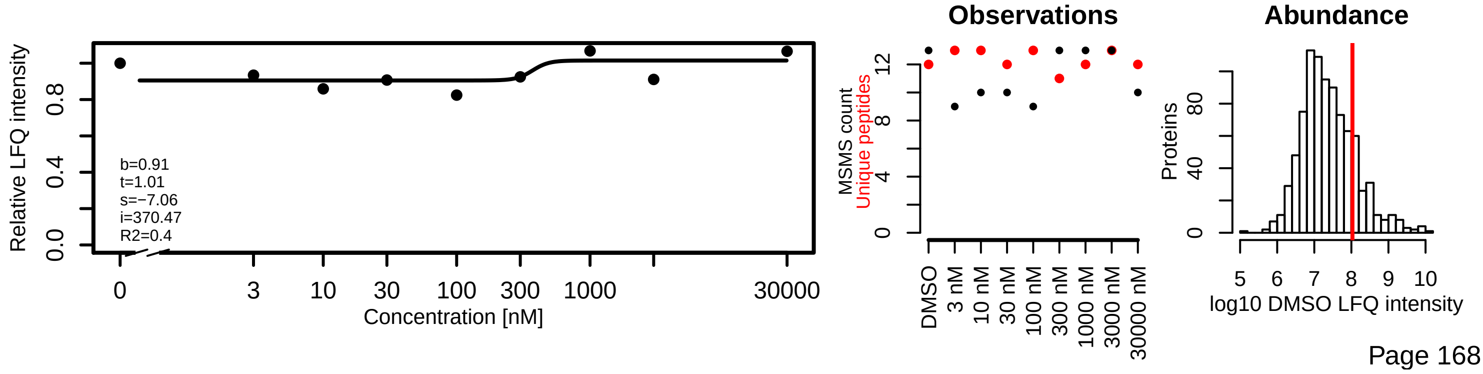

TMEM184C

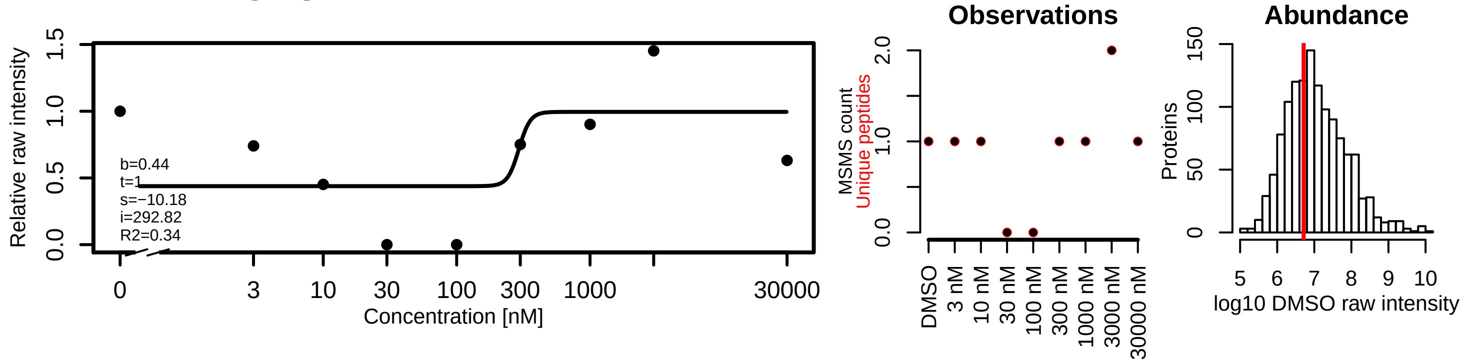

HM13

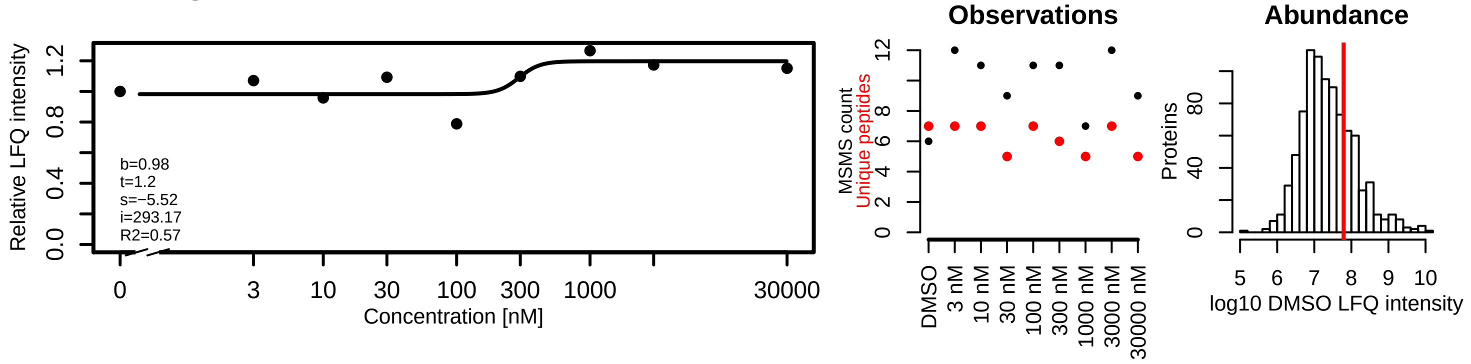

TM7SF2

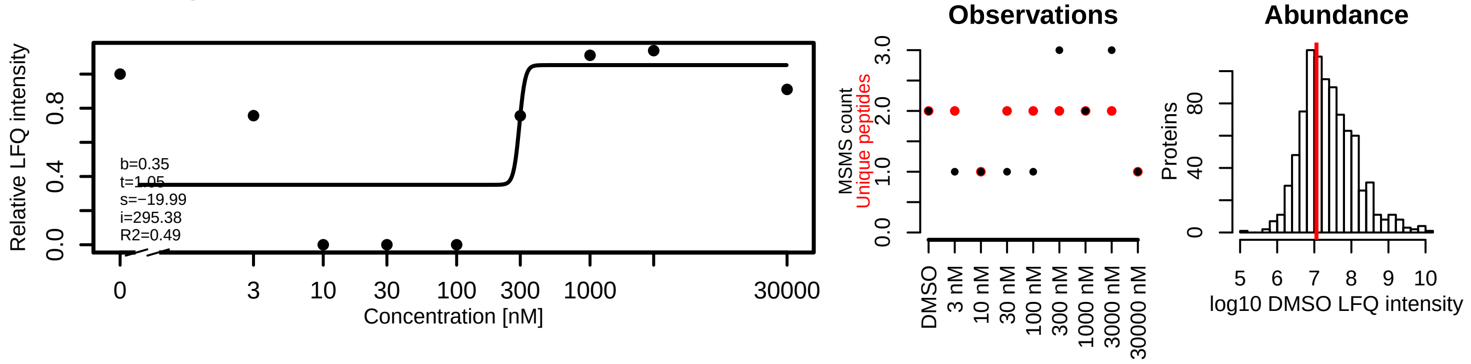

HLA−A

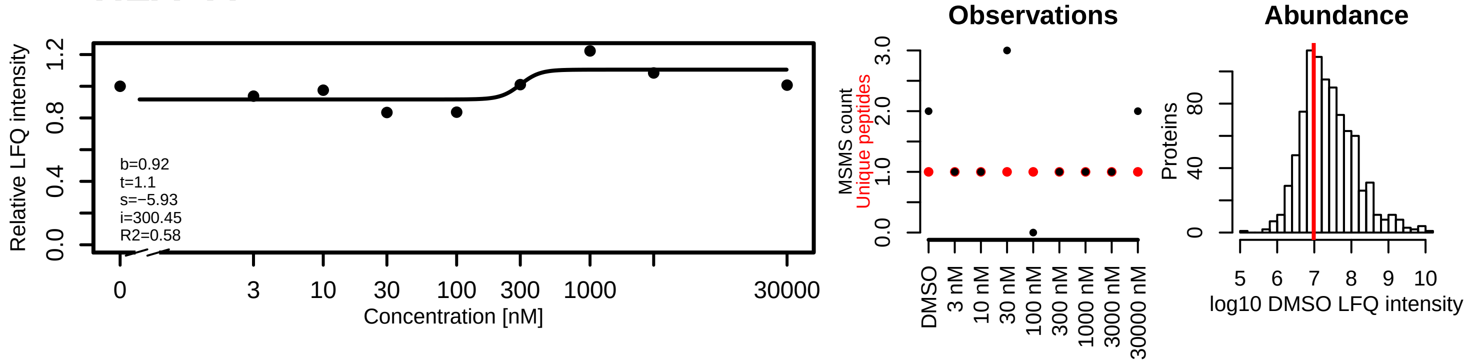

SRSF2;SFRS2

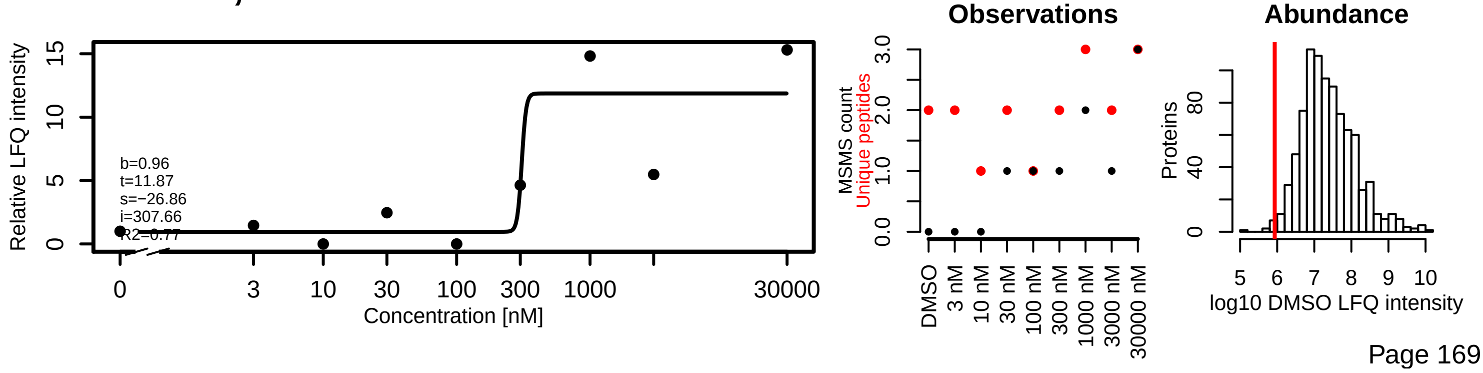

LPCAT1

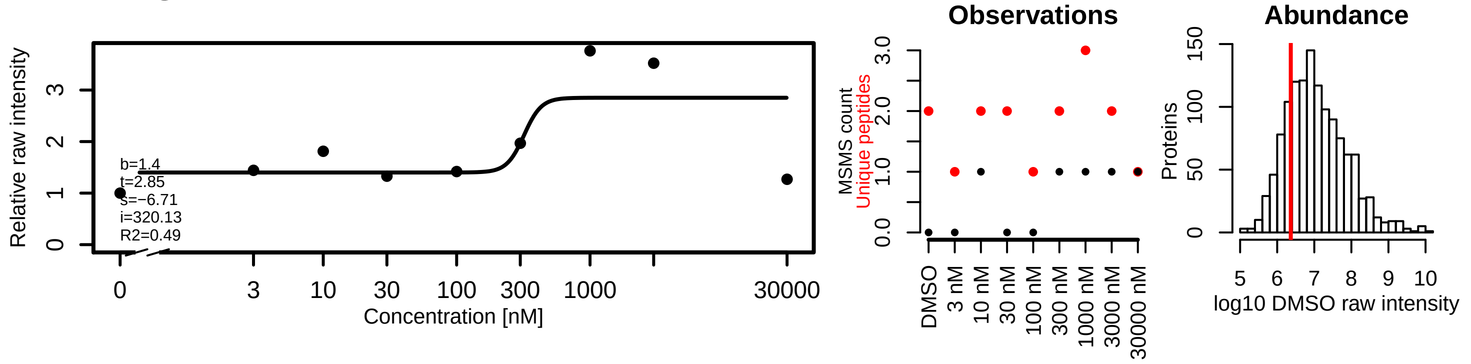

COG5

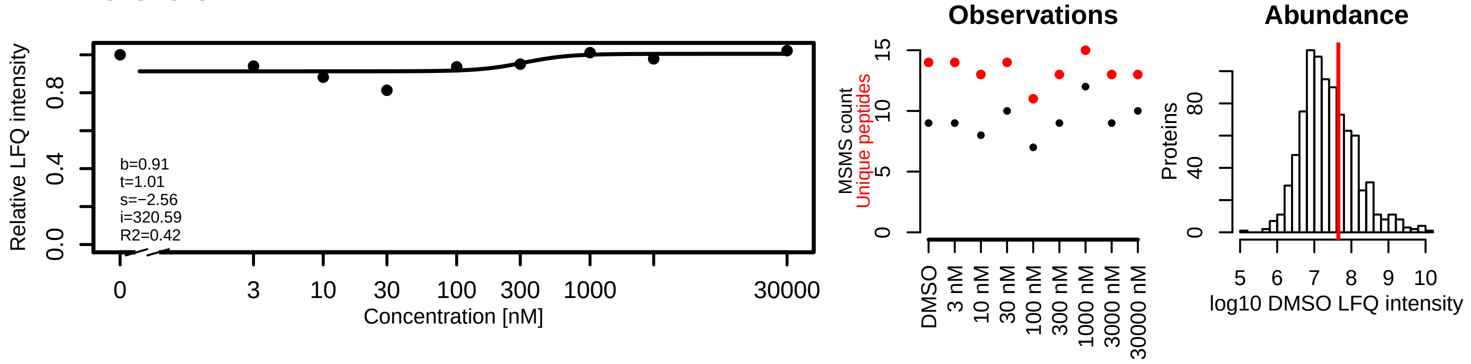

PAM16

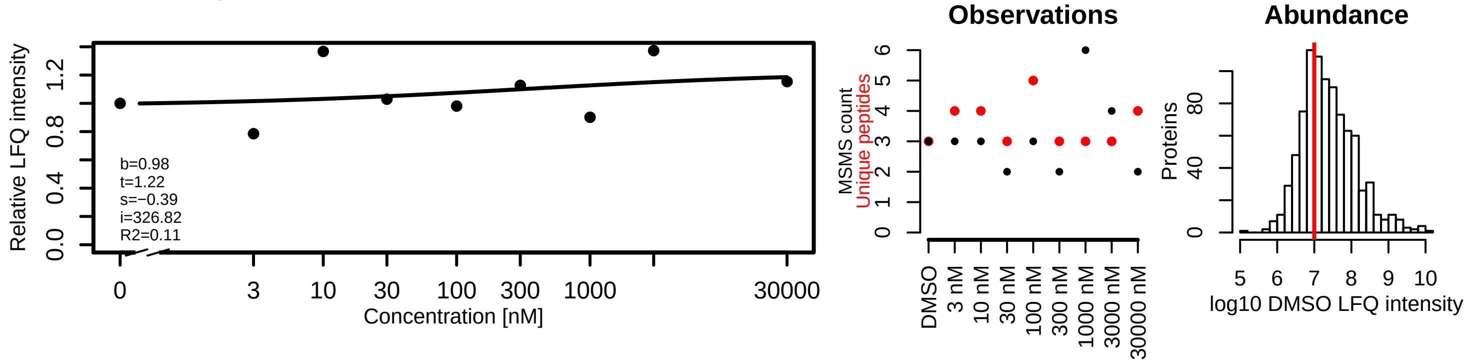

SAMM50

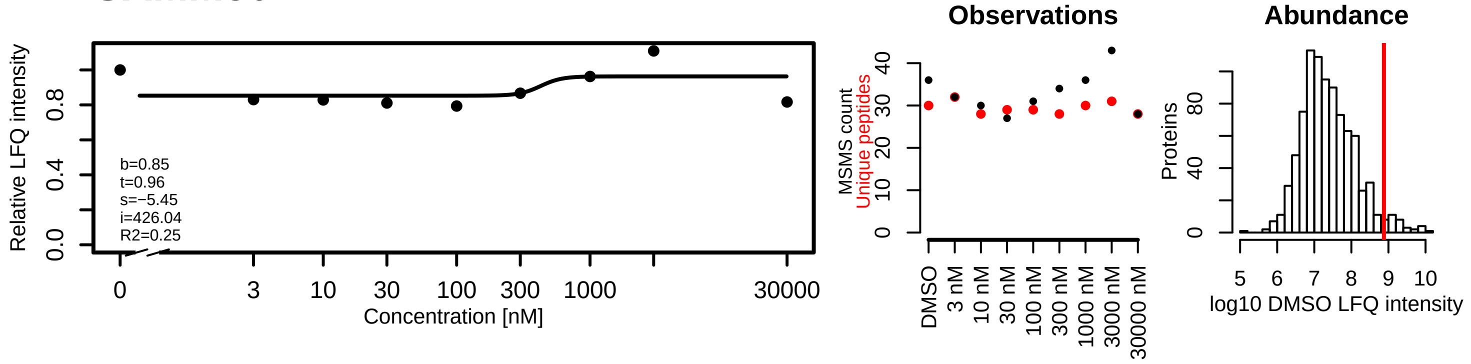

BRAT1

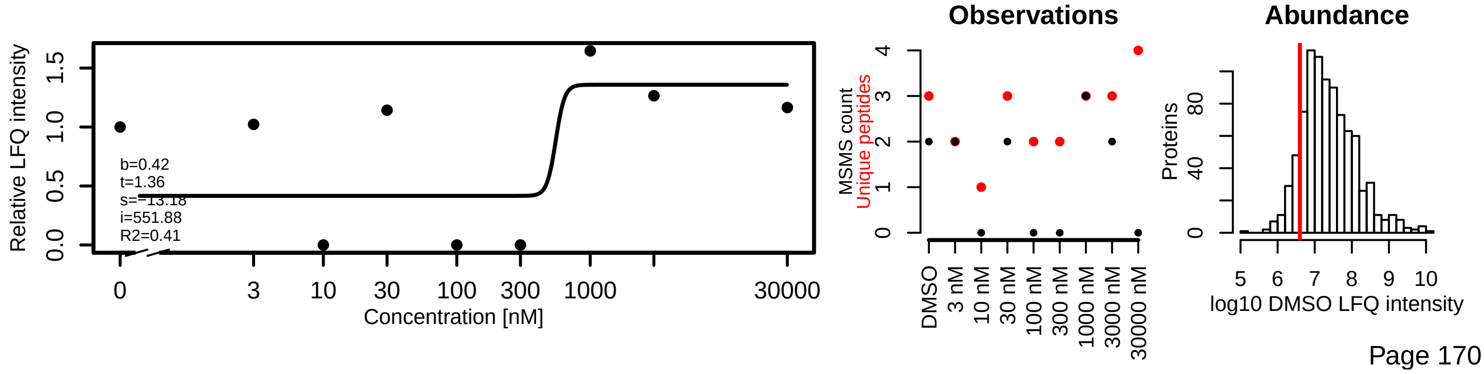

NCKAP1

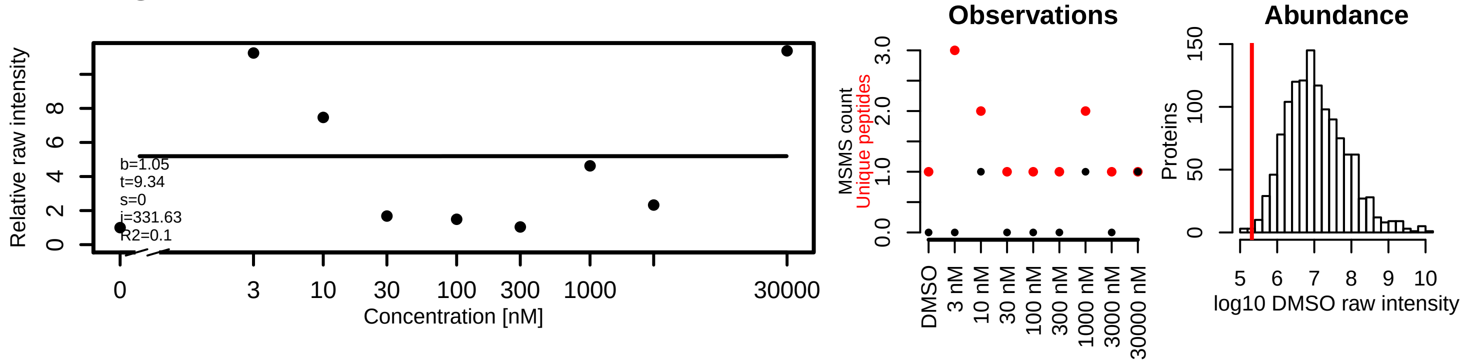

TTI1

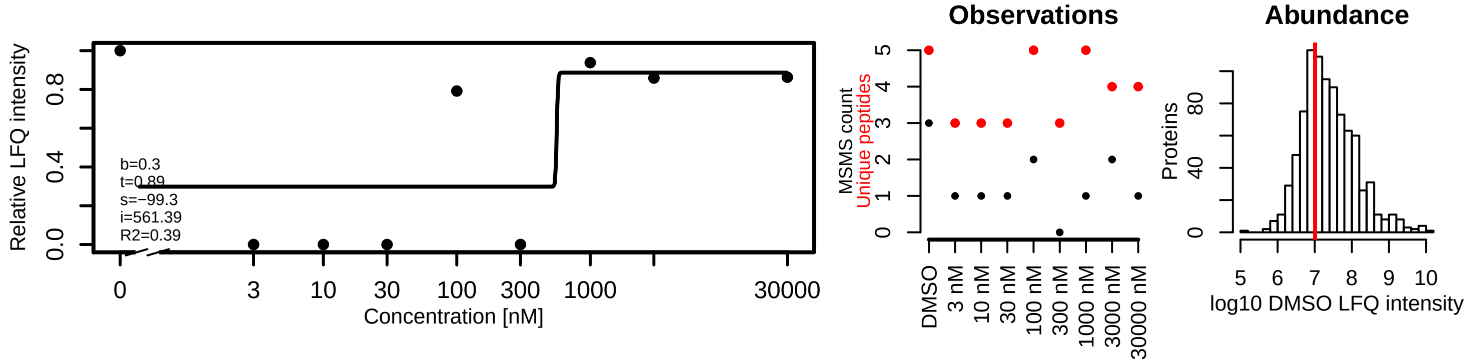

TPR

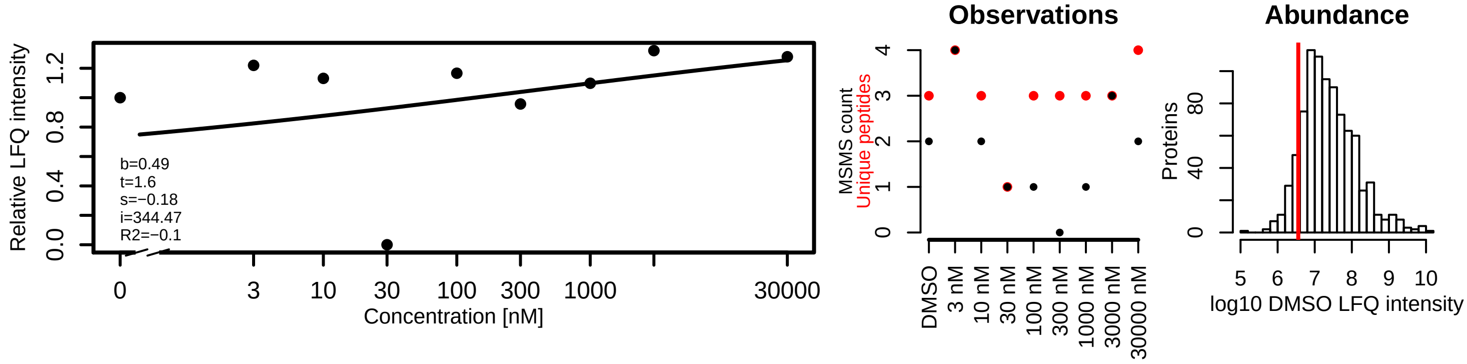

CNNM3

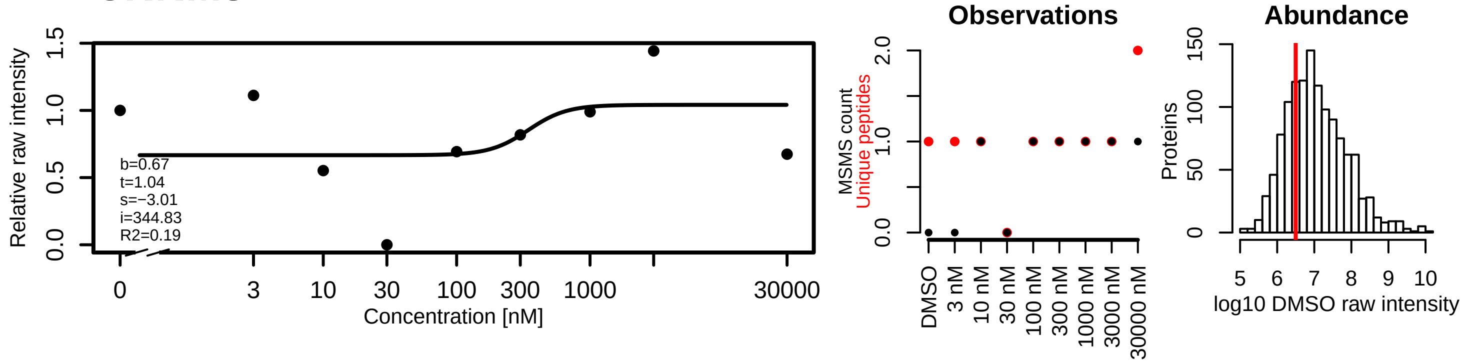

SERINC1

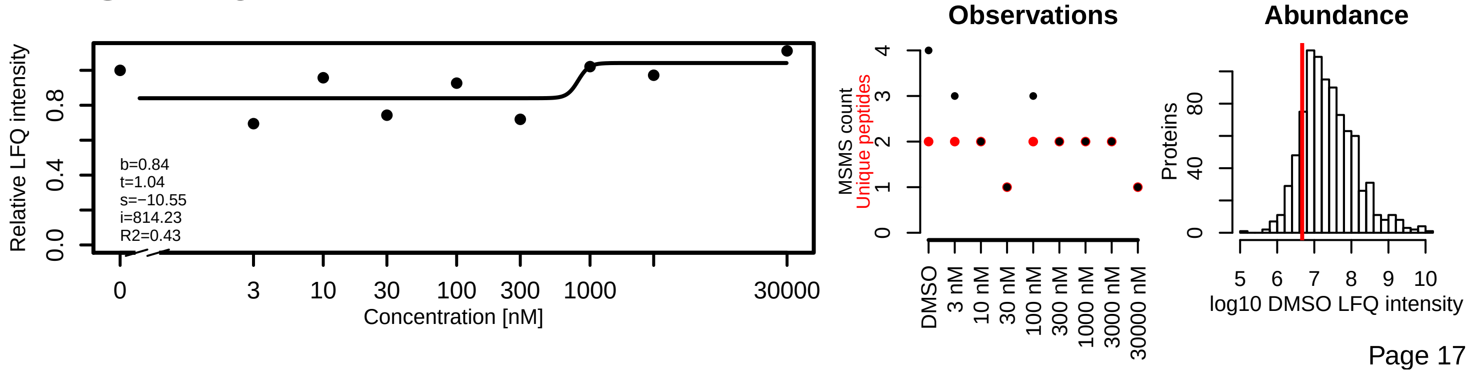

SRSF7

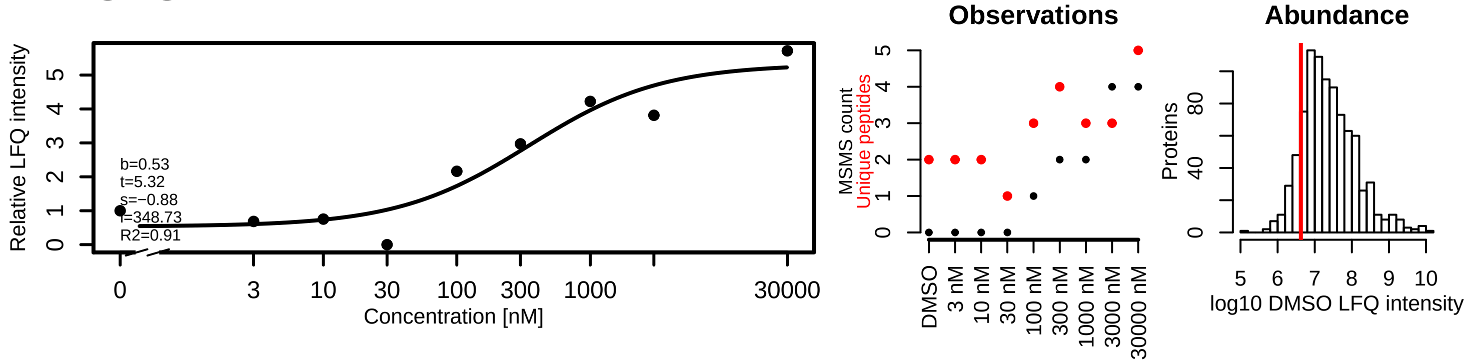

DDX20

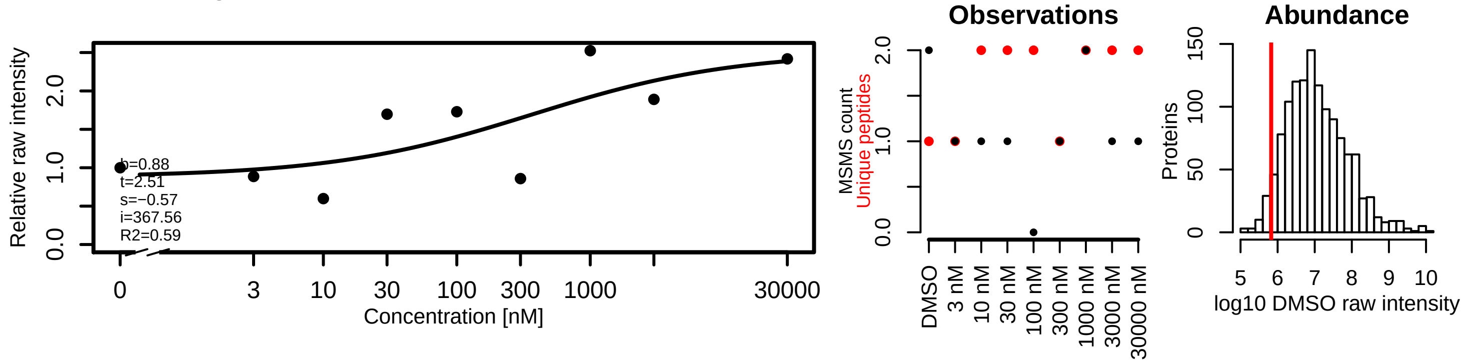

IMMT

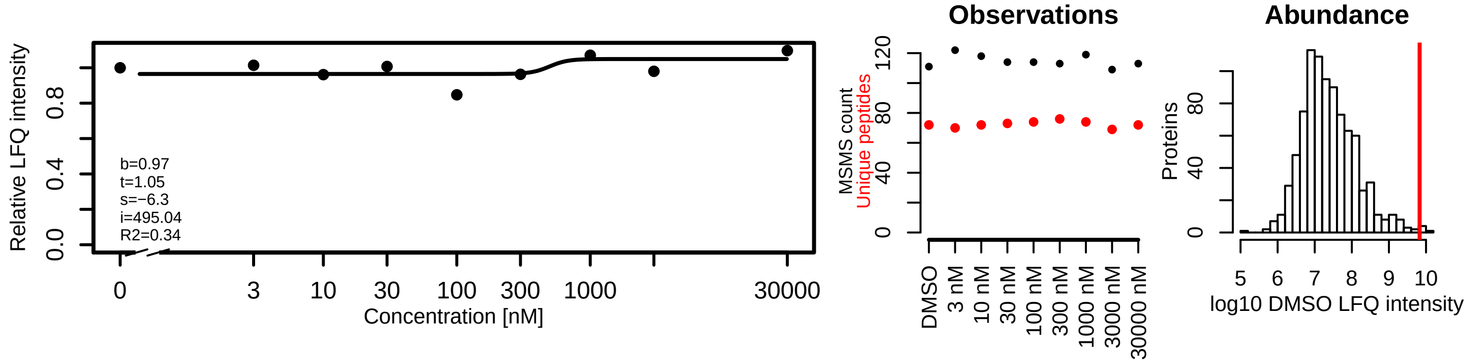

GPC4

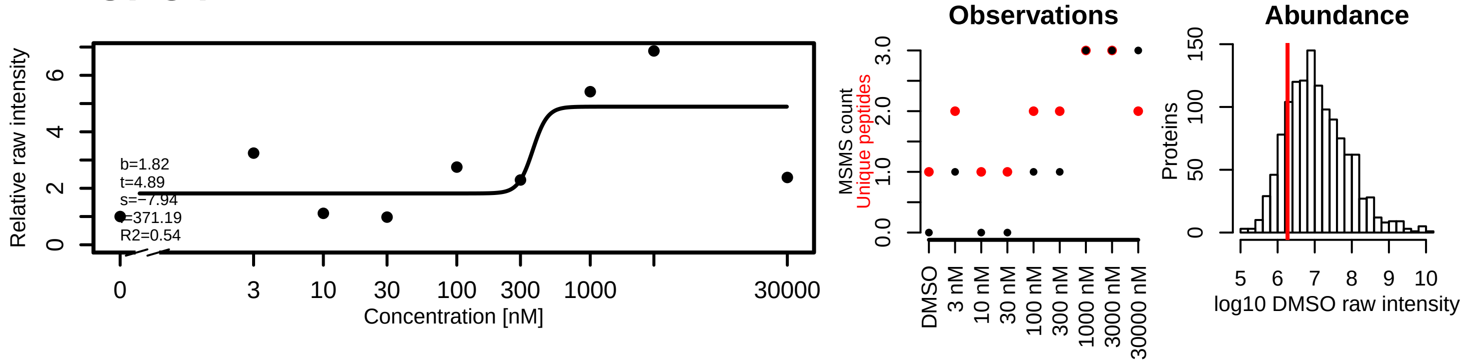

QPCTL

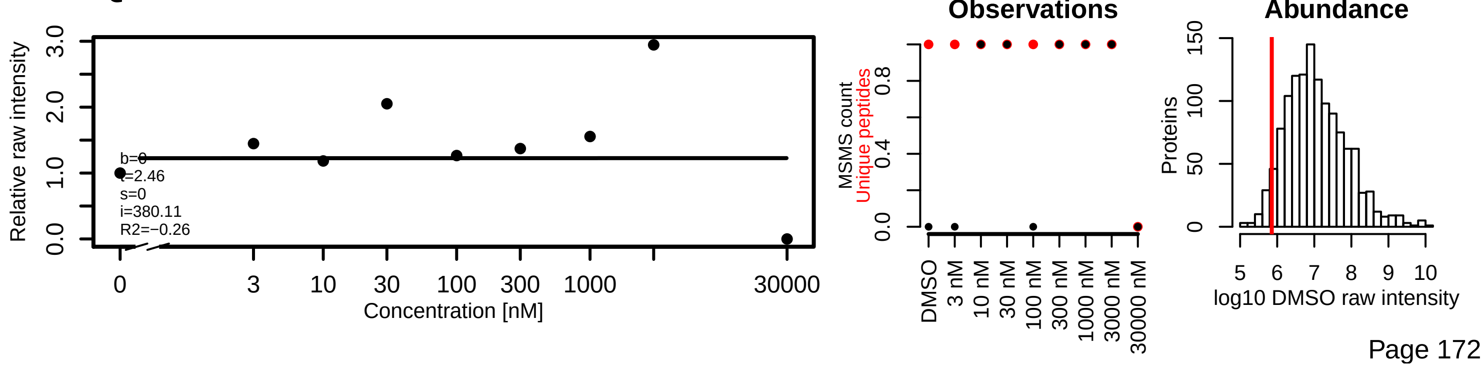

YIF1A

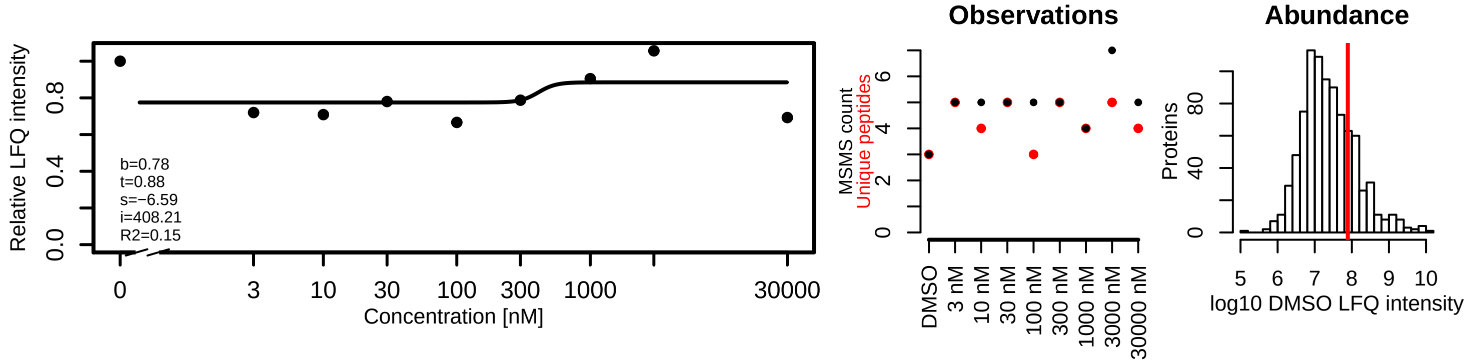

SSR3

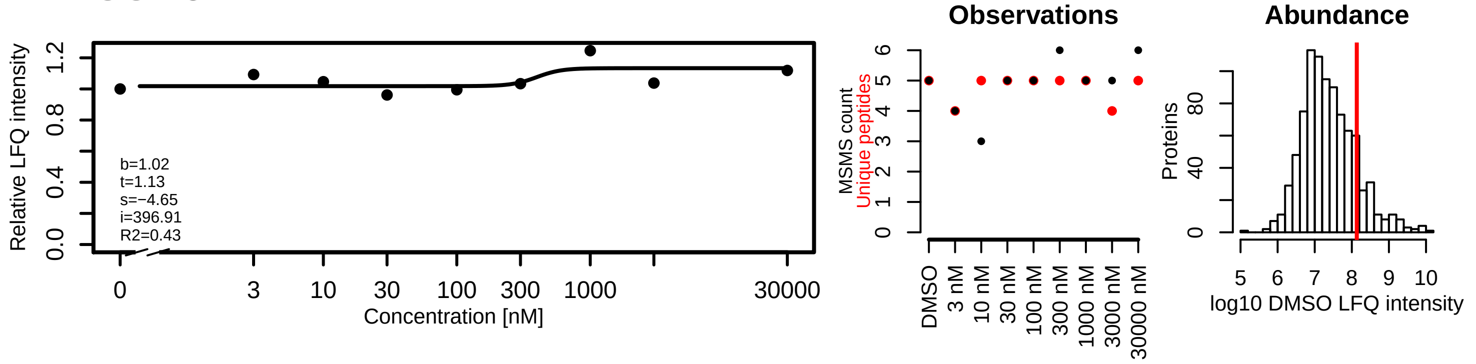

MTX3

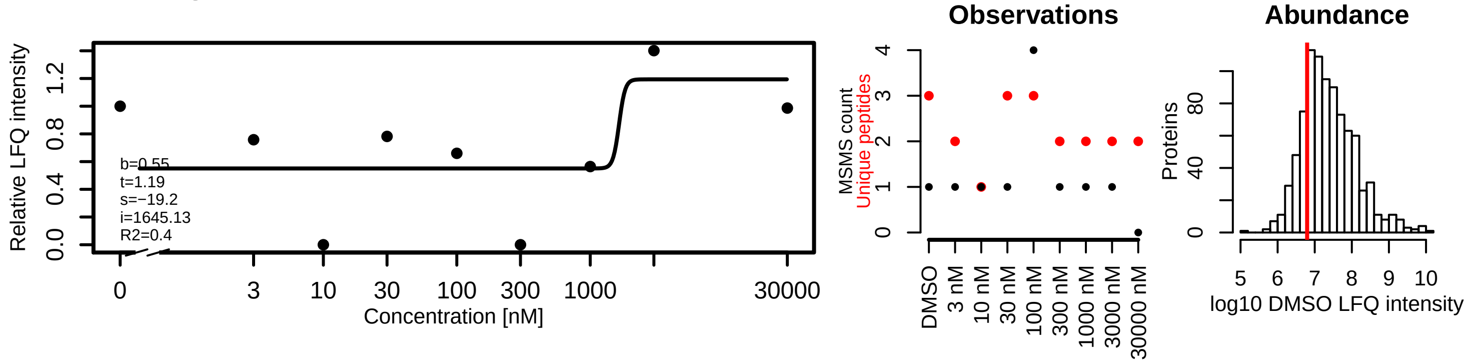

SLC11A2

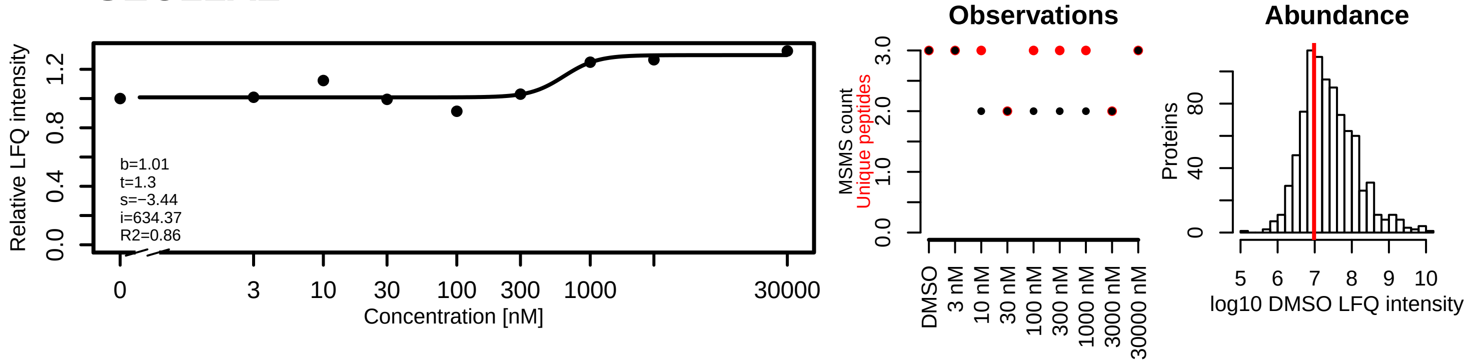

KIAA0196

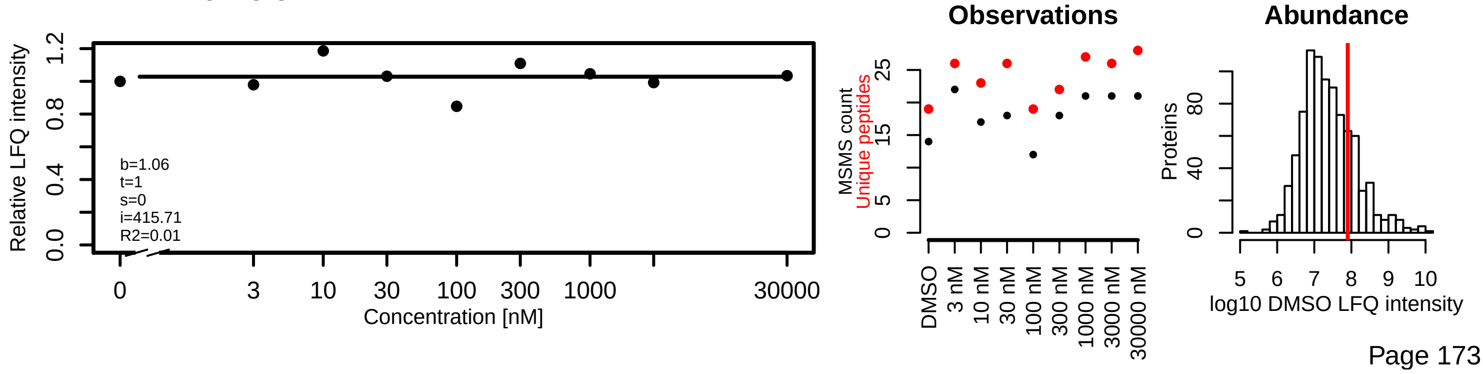

GPR111

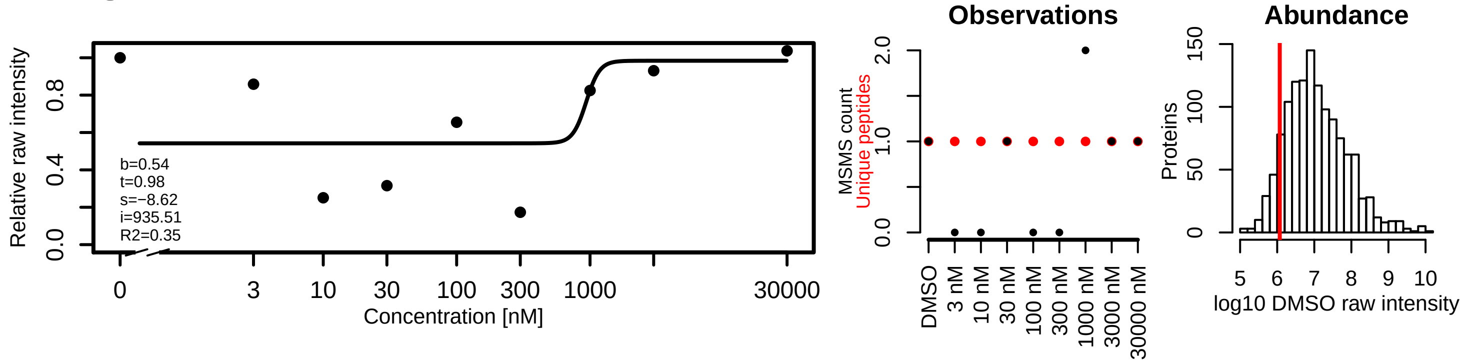

SH3BP4

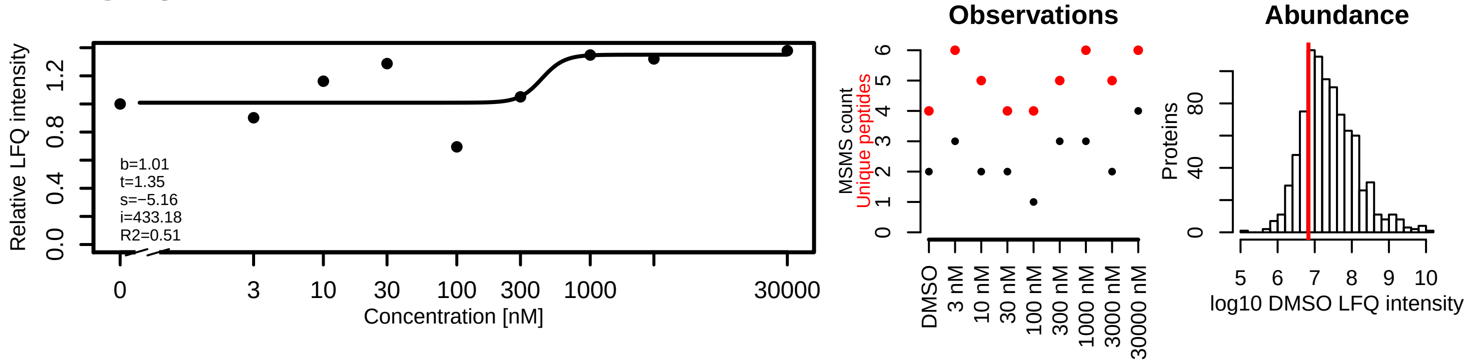

NPC1

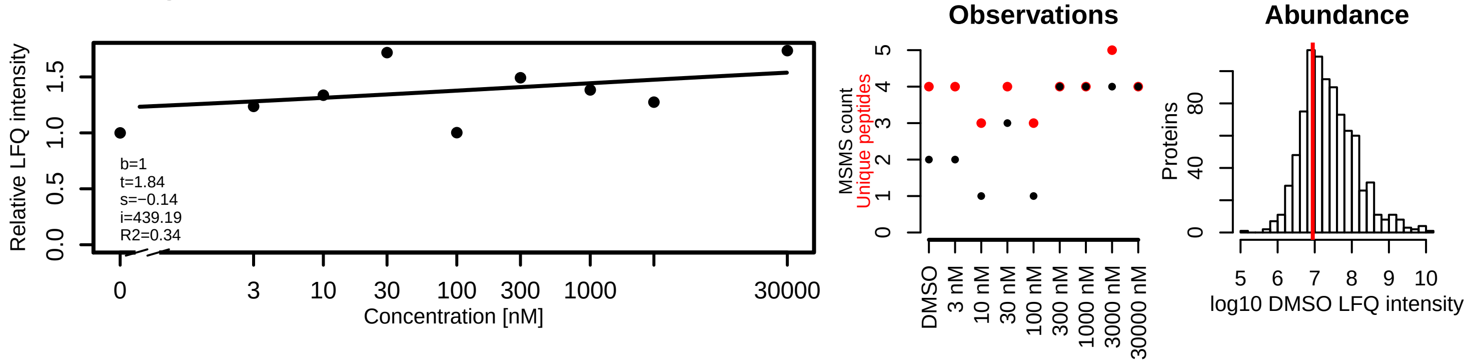

DNAJC10

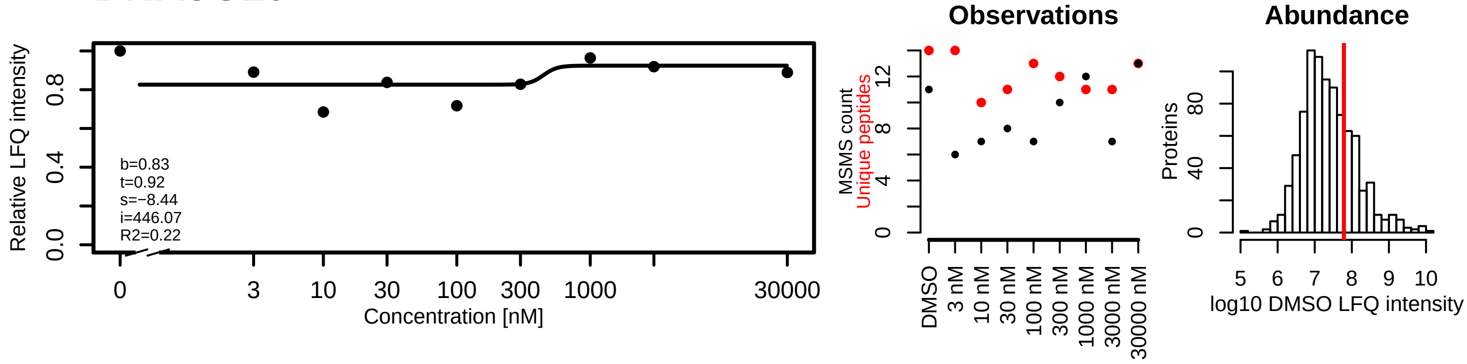

TRPM4

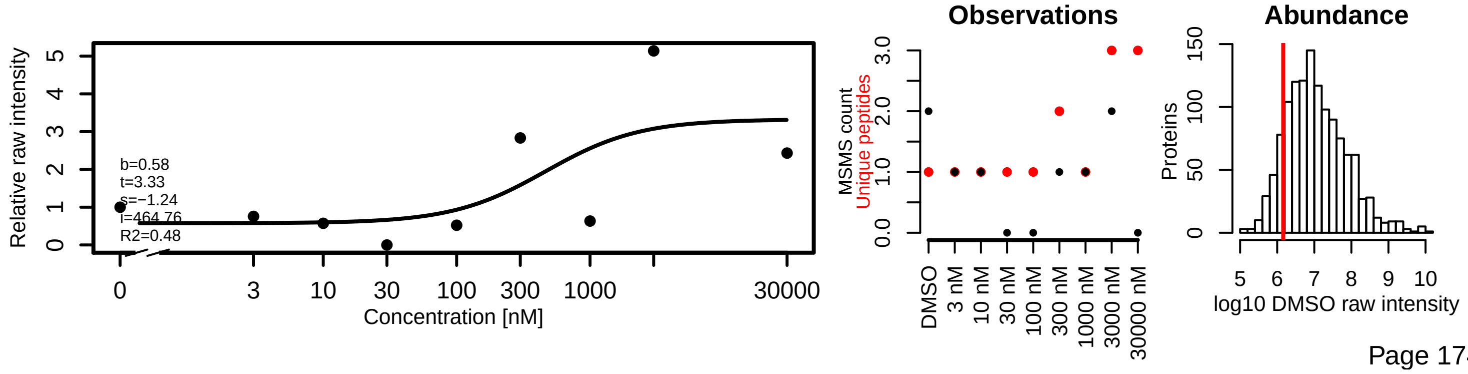

SLC6A8

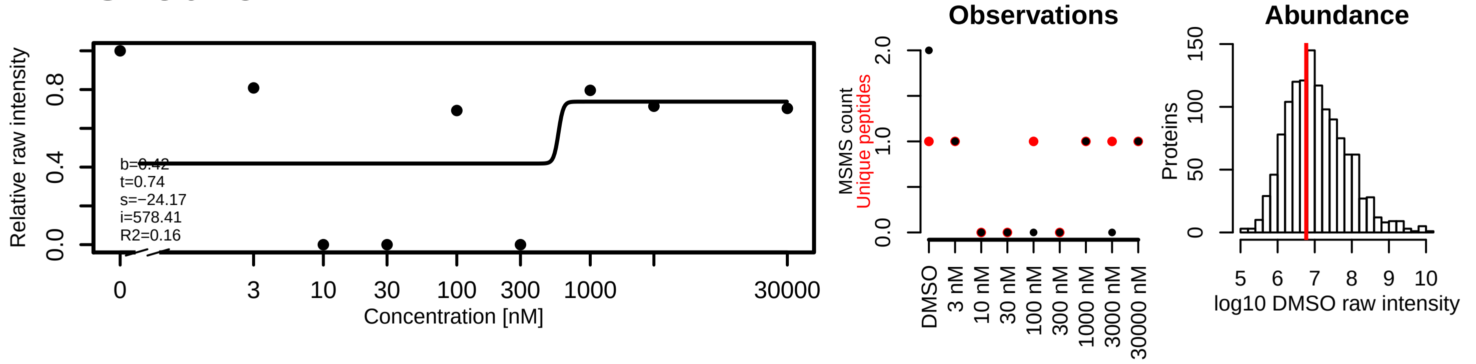

MTX2

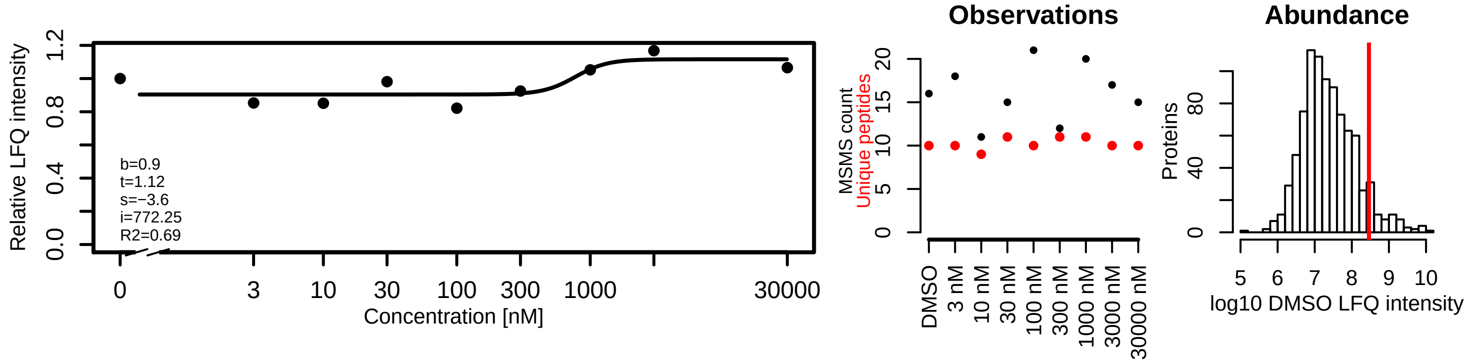

ARL6IP5

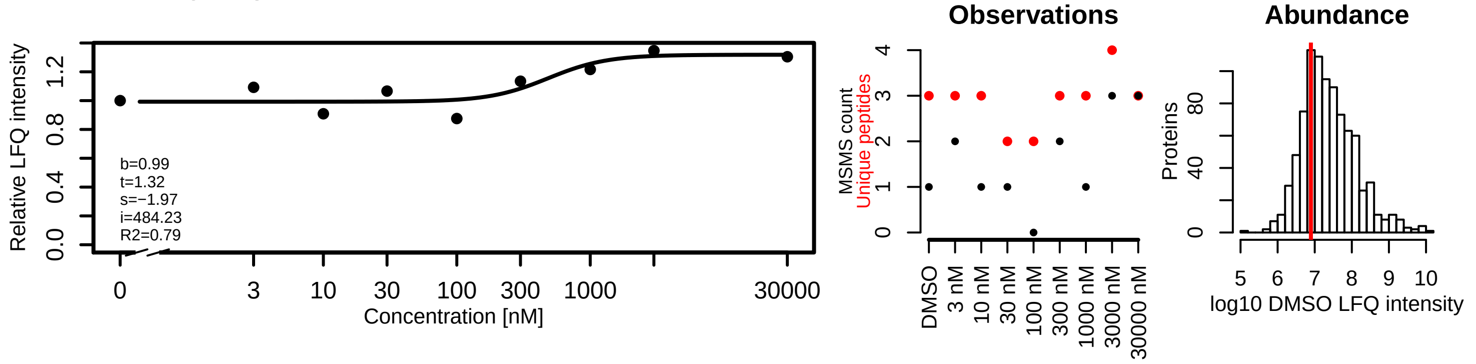

SLC33A1

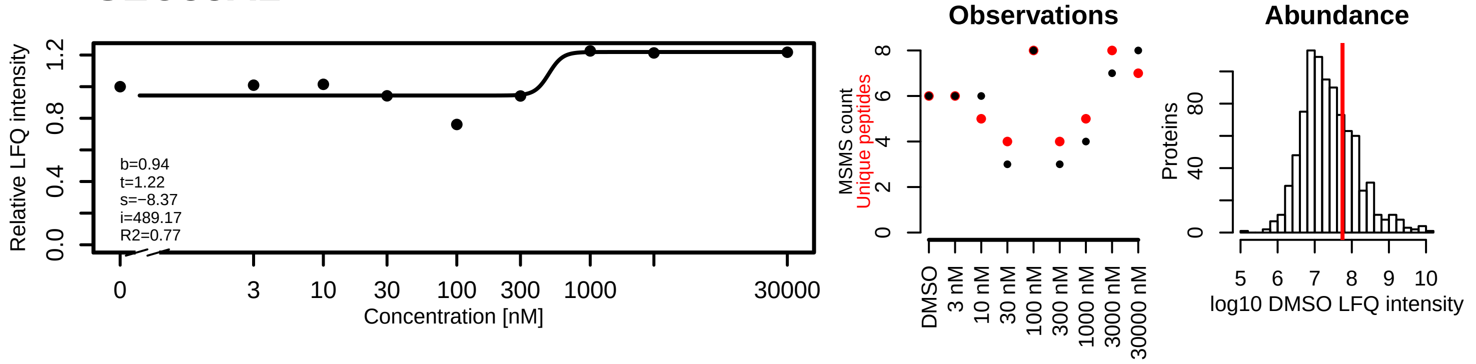

HNRNPF

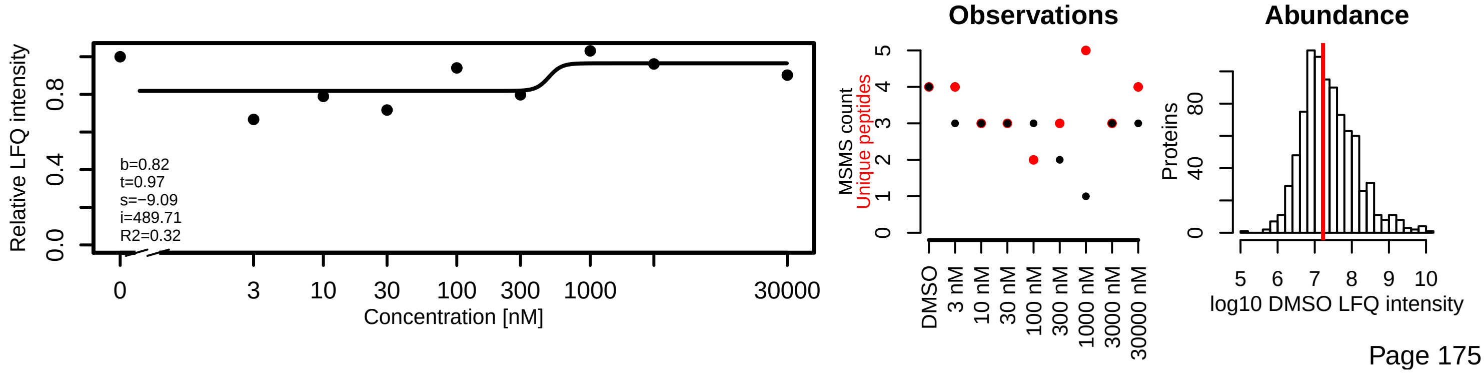

MYADM

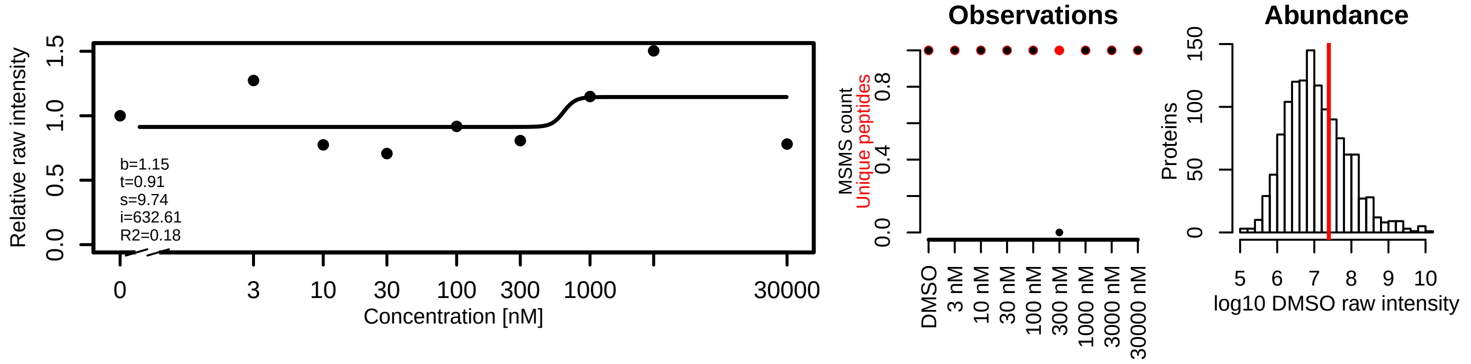

COQ5

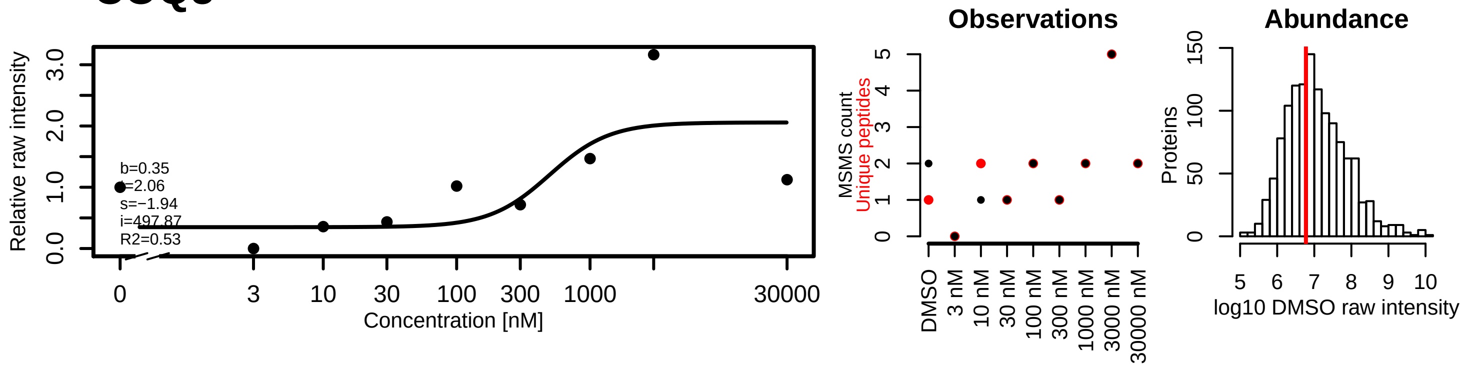

RPS3A

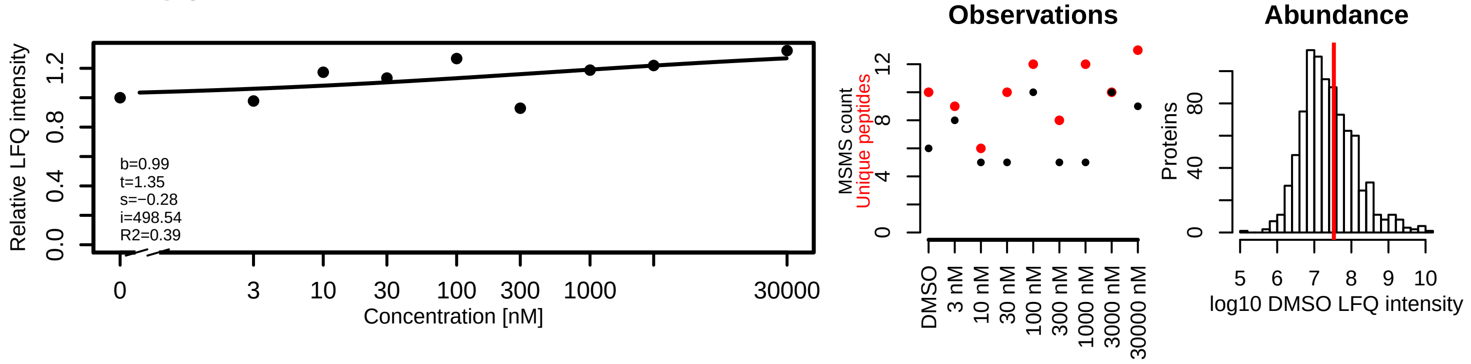

TBCD

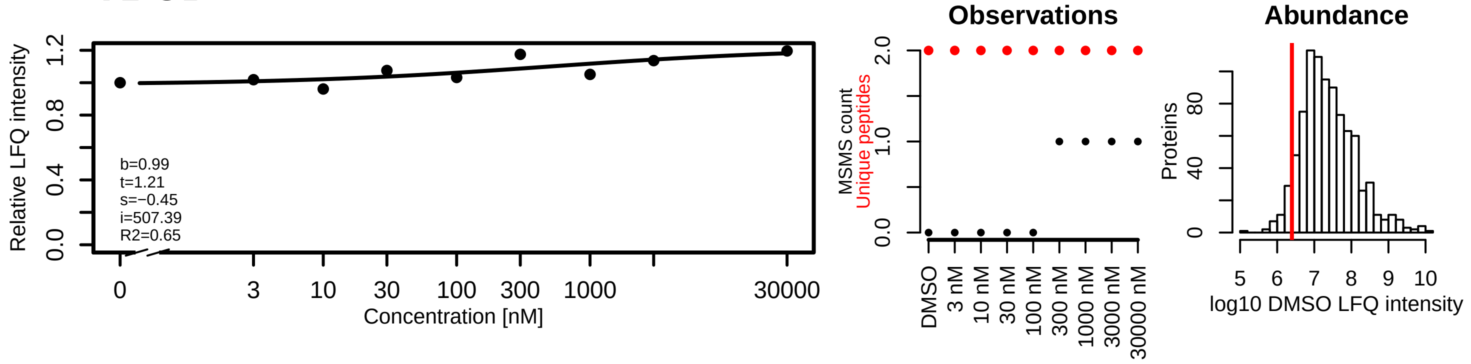

RAB5C

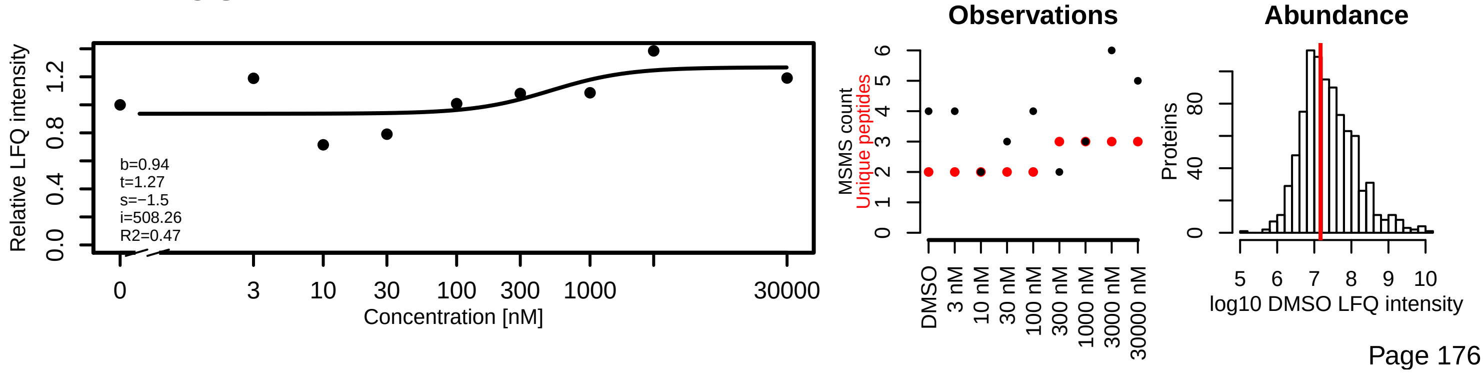

CDC42

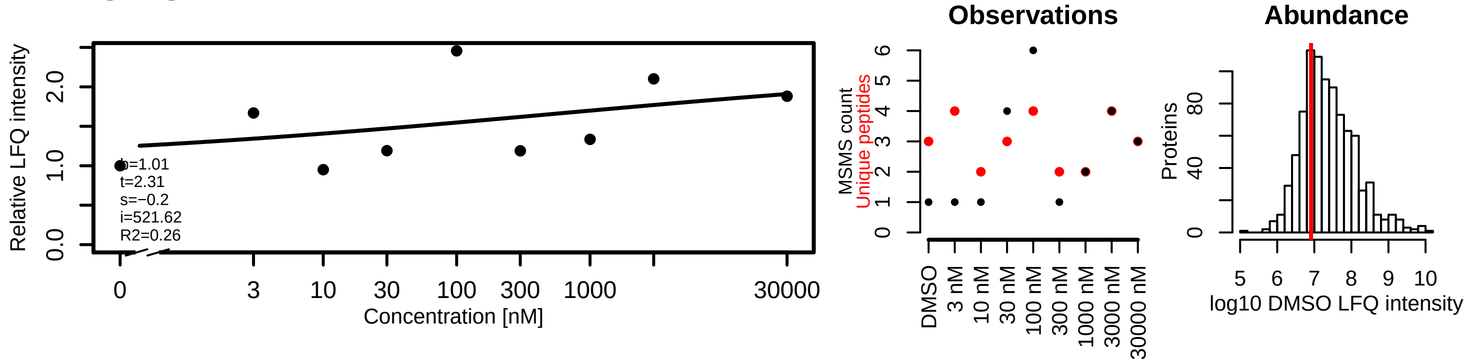

SLC35B3

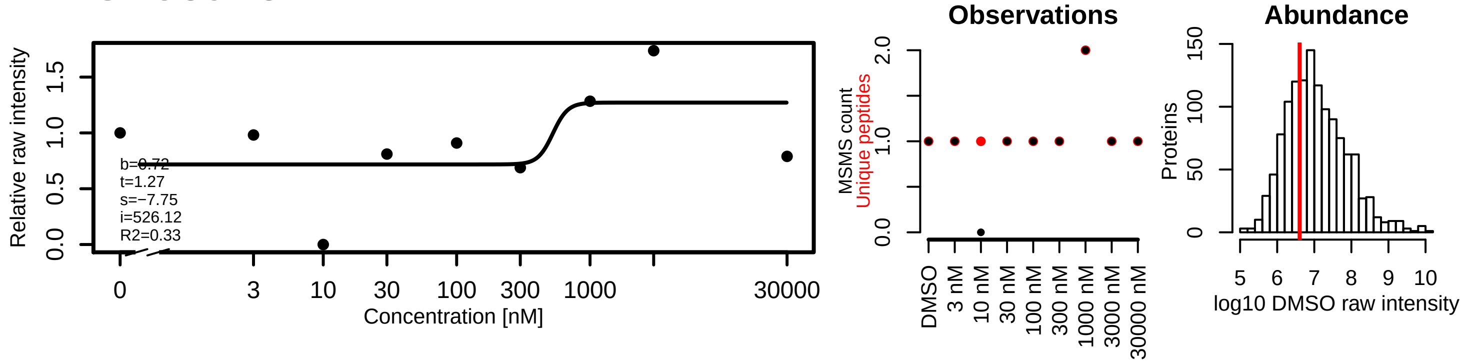

HSD17B12

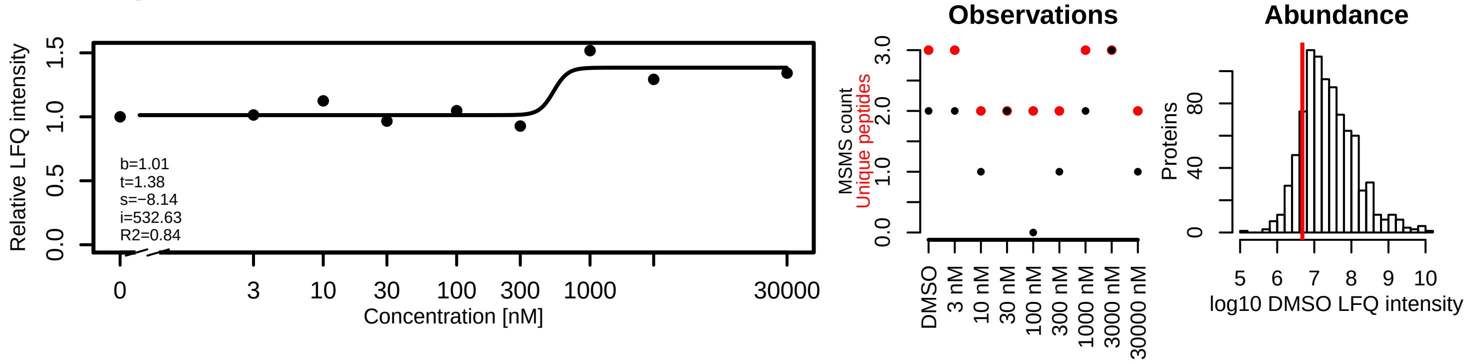

ROPN1B;ROPN1

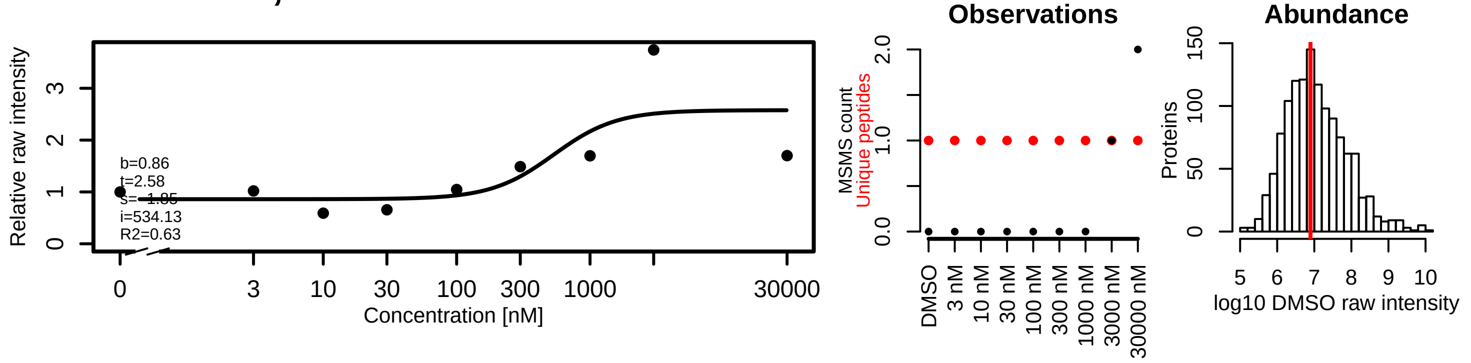

ECM29;KIAA0368

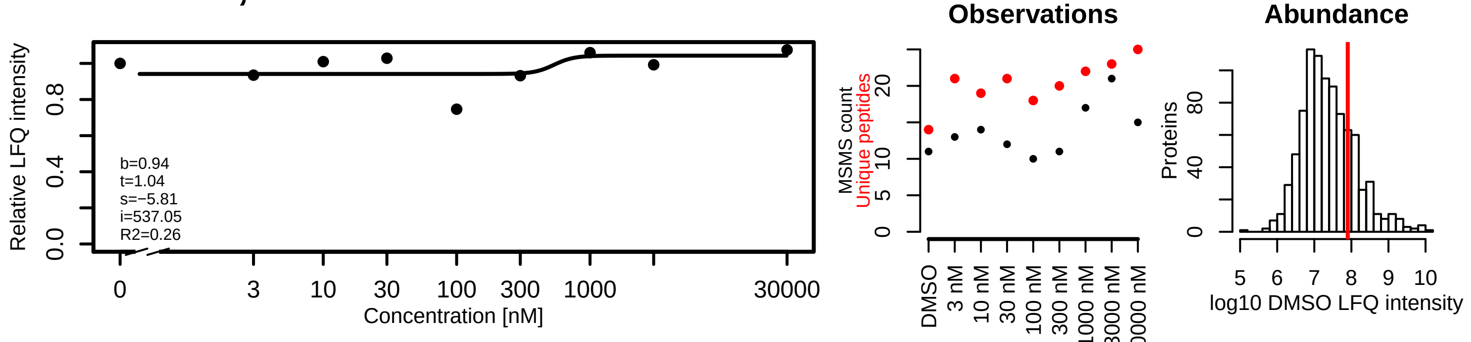

CAV1 – Q03135;E9PCT5;P56539

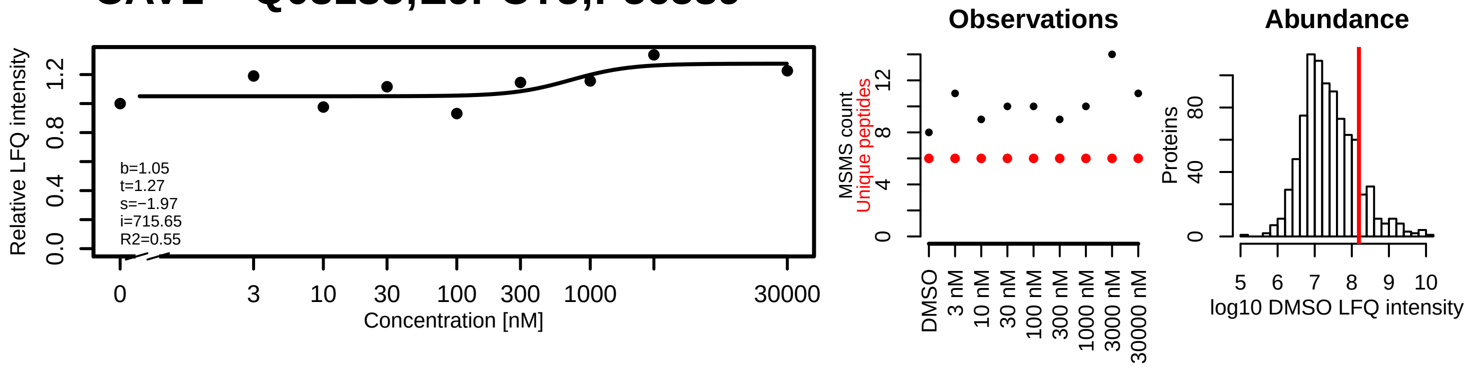

SLC23A2

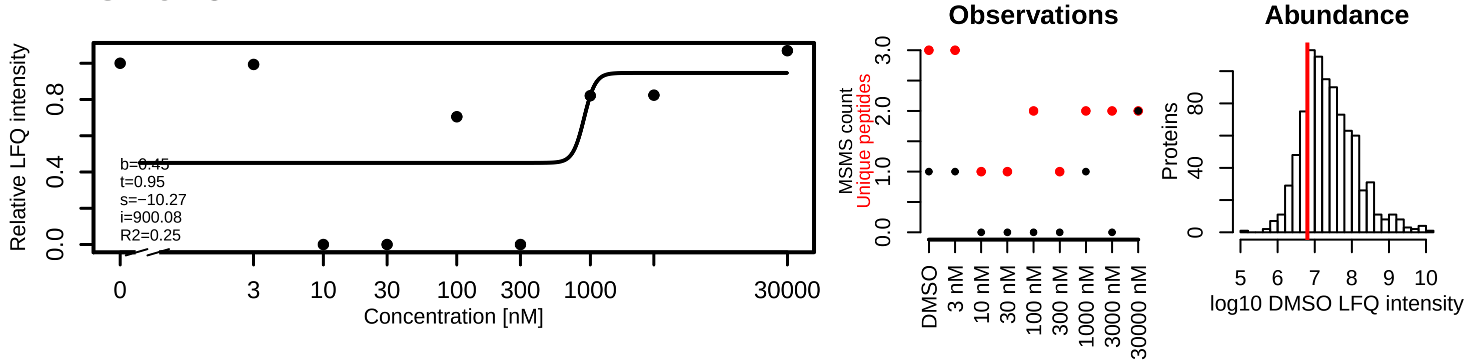

SLC39A14

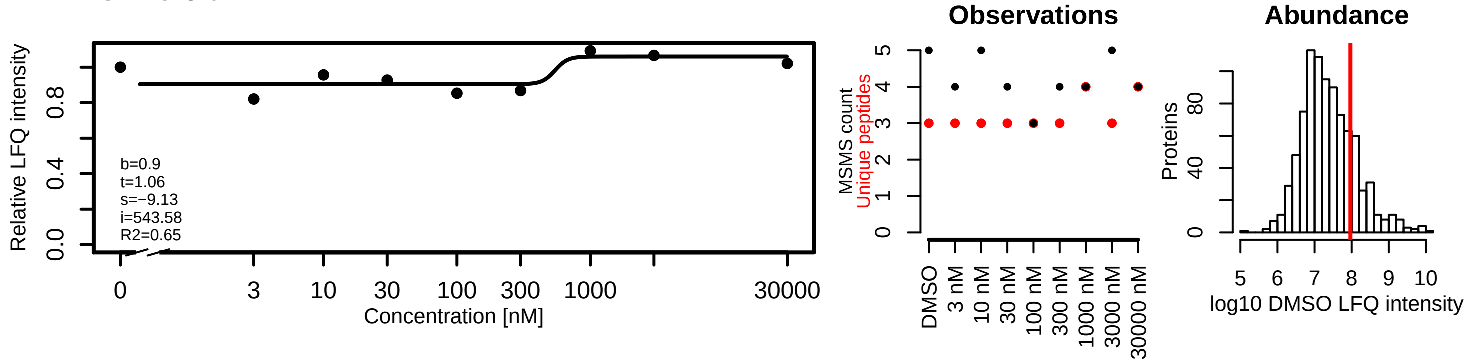

RAB5B

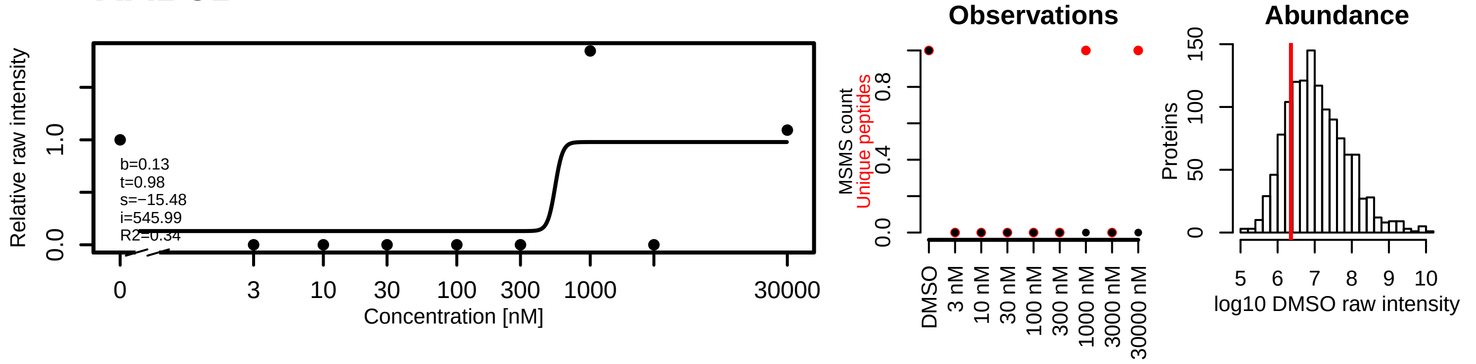

ABCC10

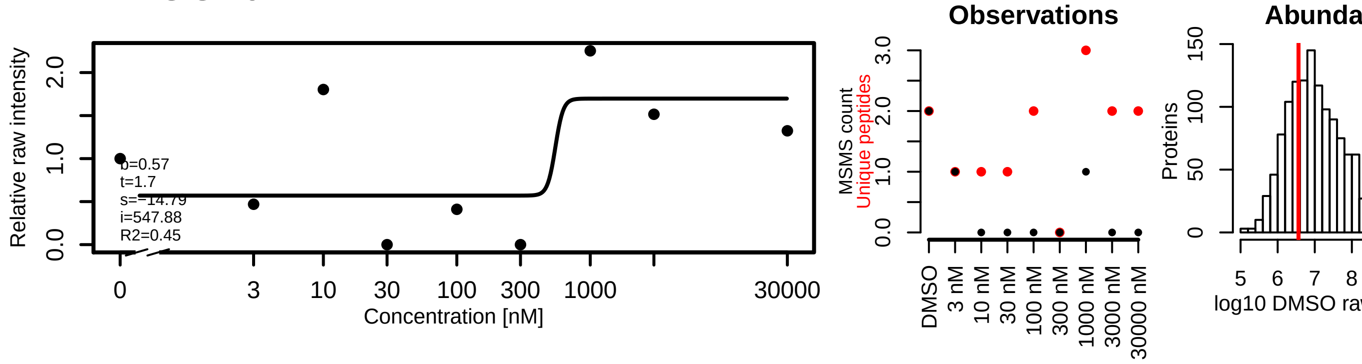

ERGIC1

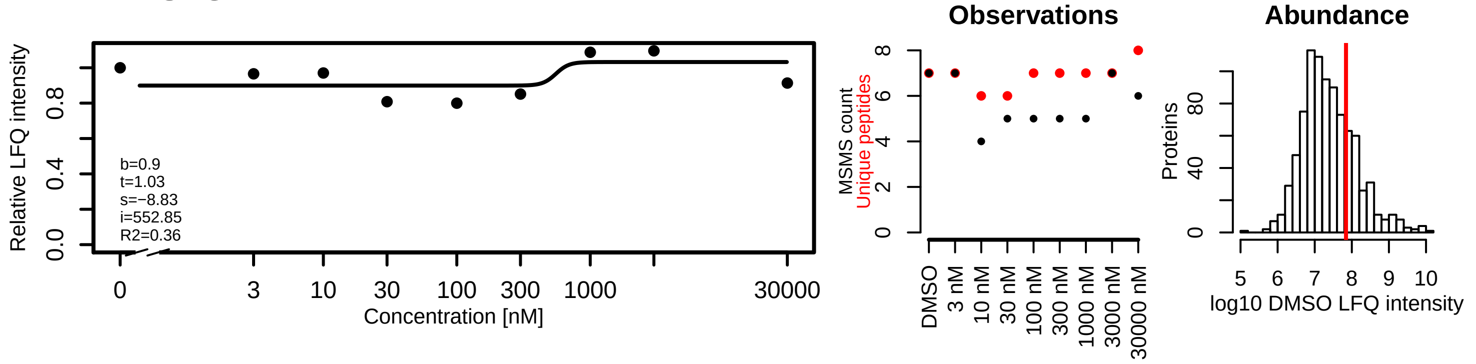

SEC61A1

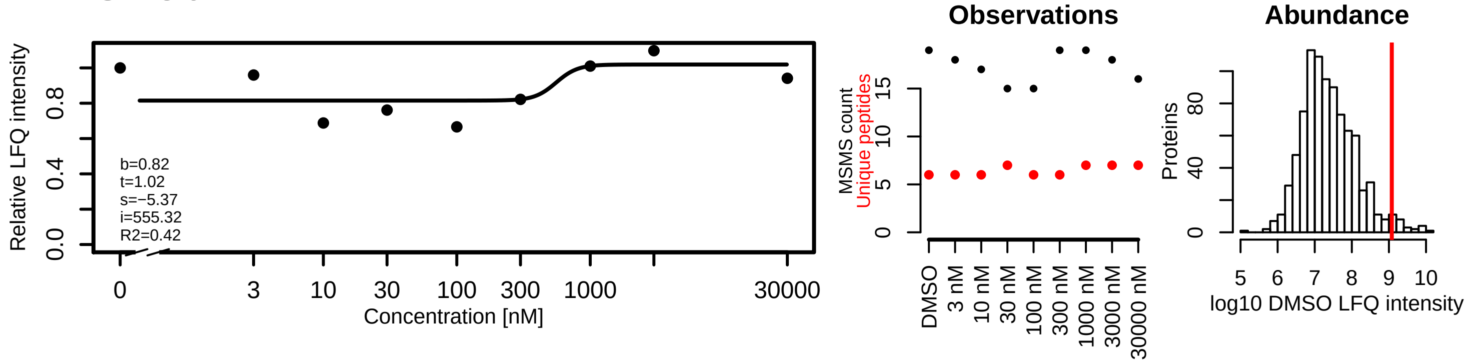

TAP2

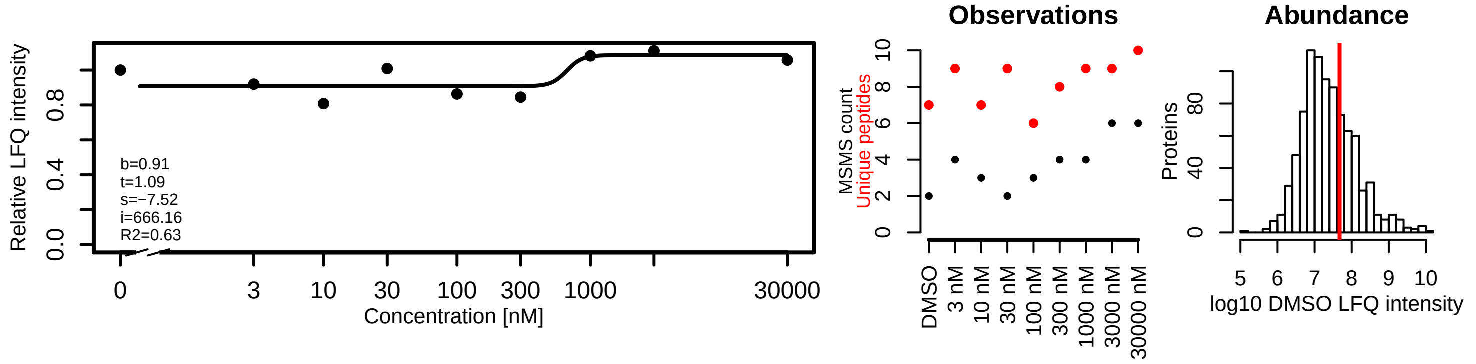

SLC7A5

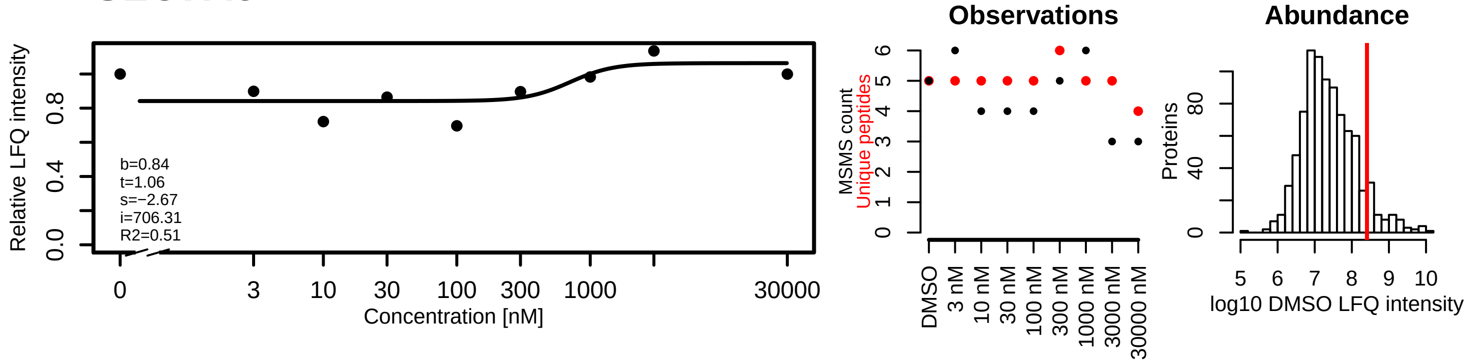

SLC1A3

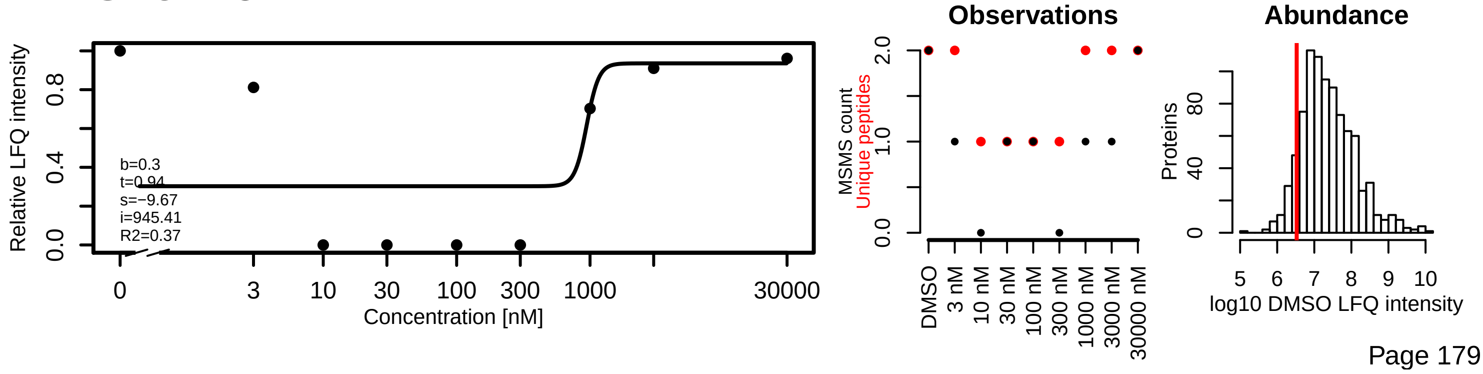

HLA−C − P10321;A2AEA2;F5GXA6

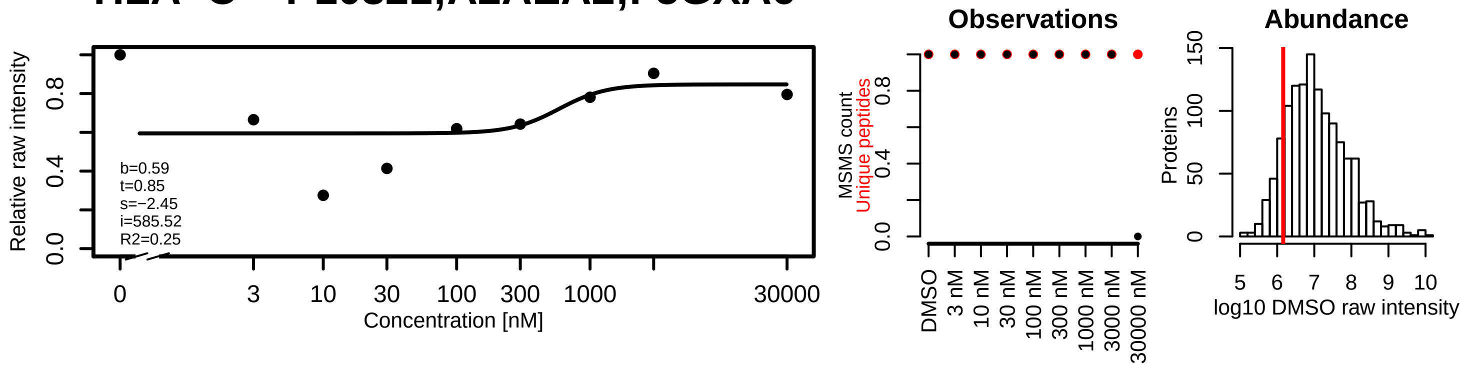

DYNC2H1

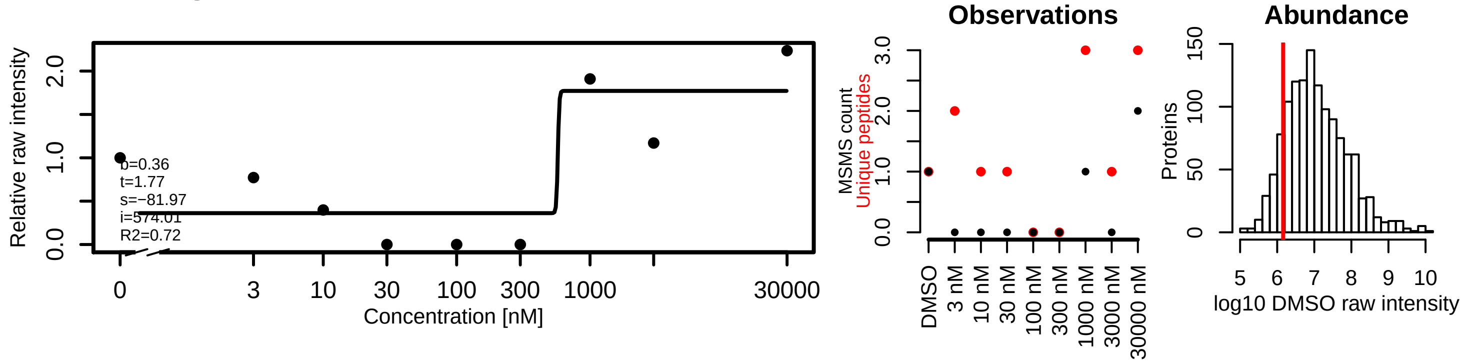

THADA

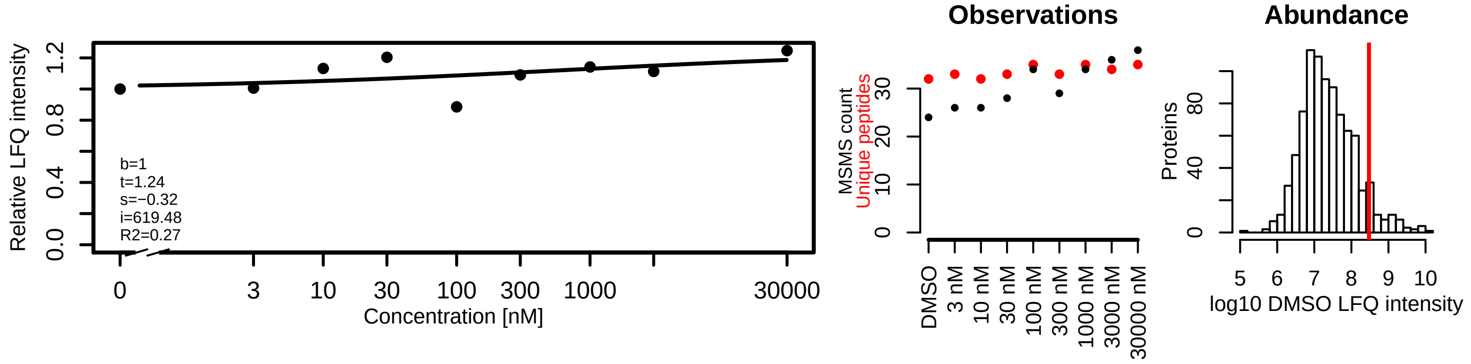

ANO6

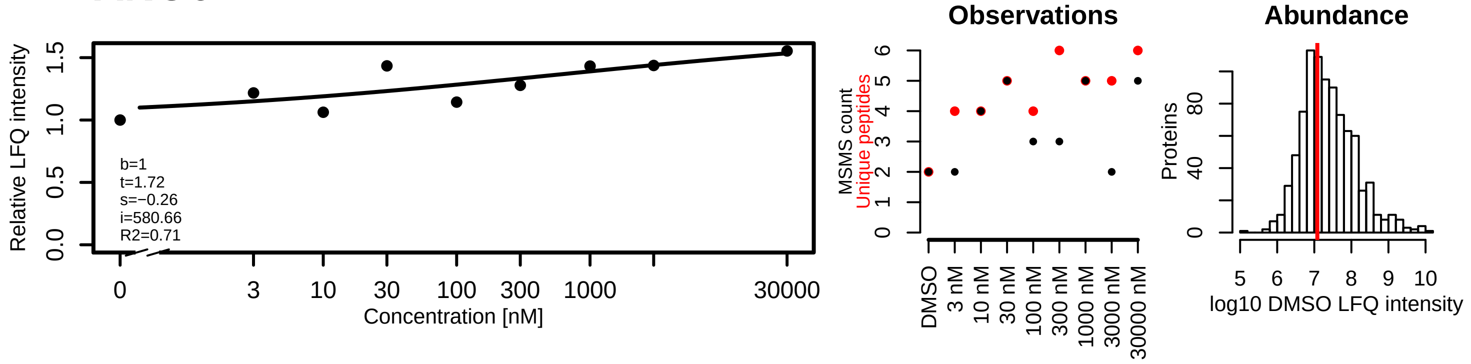

WDFY3

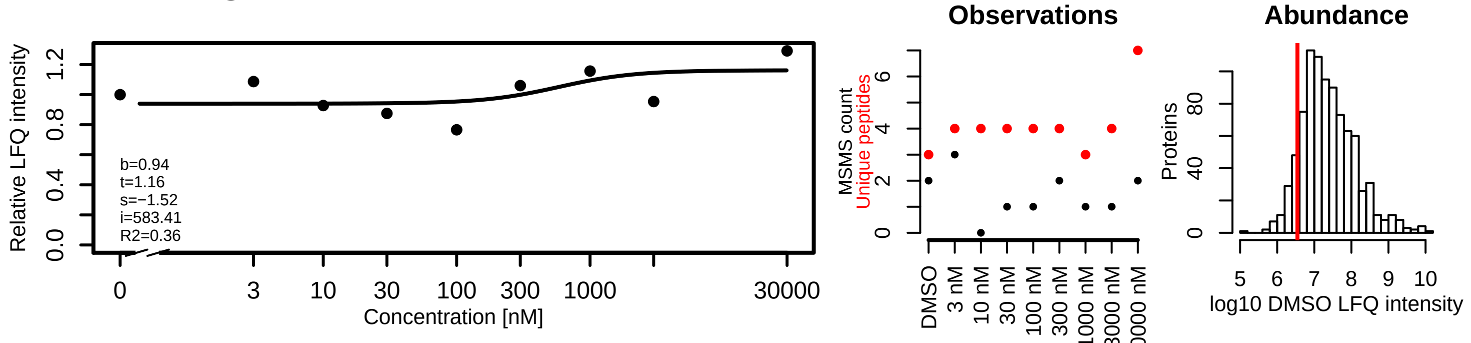

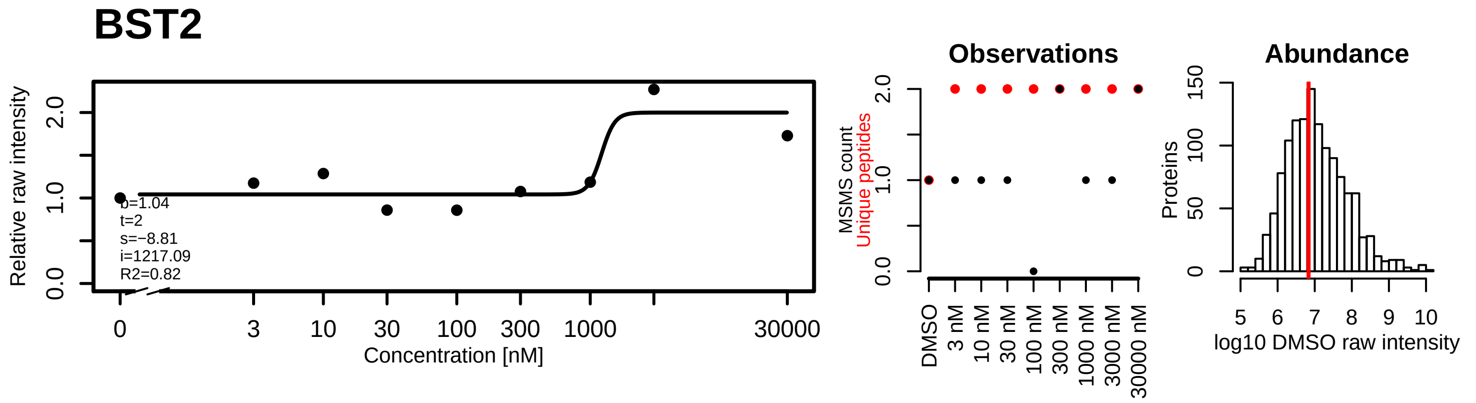
BST2
Relative raw intensity
b=1.04
t=2
s=−8.81
i=1217.09
R2=0.82
Concentration [nM]
Observations
MSMS count
Unique peptides
Abundance
Proteins
log10 DMSO raw intensity

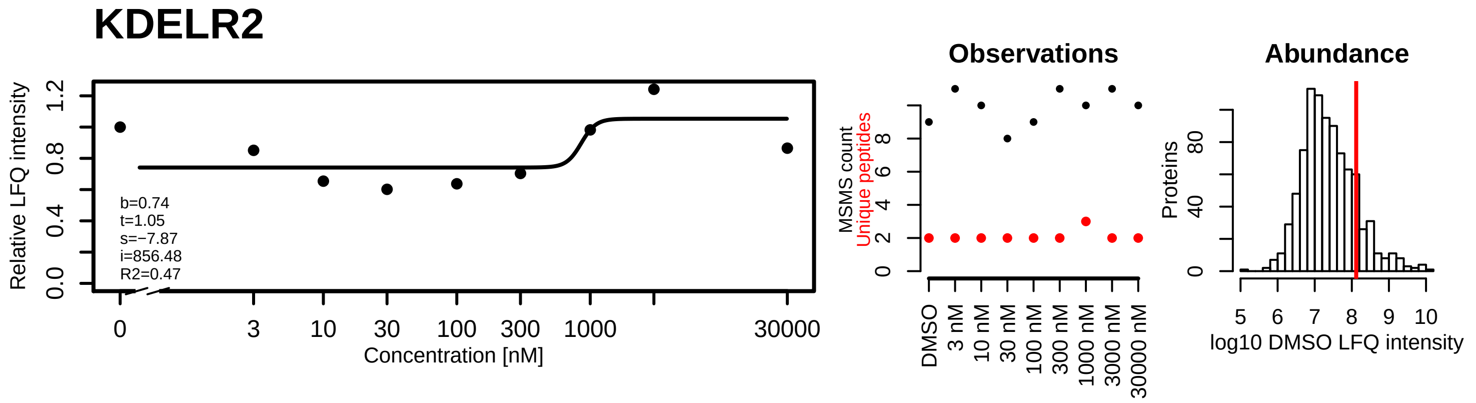
KDELR2
Relative LFQ intensity
b=0.74
t=1.05
s=−7.87
i=856.48
R2=0.47
Concentration [nM]
Observations
MSMS count
Unique peptides
Abundance
Proteins
log10 DMSO LFQ intensity

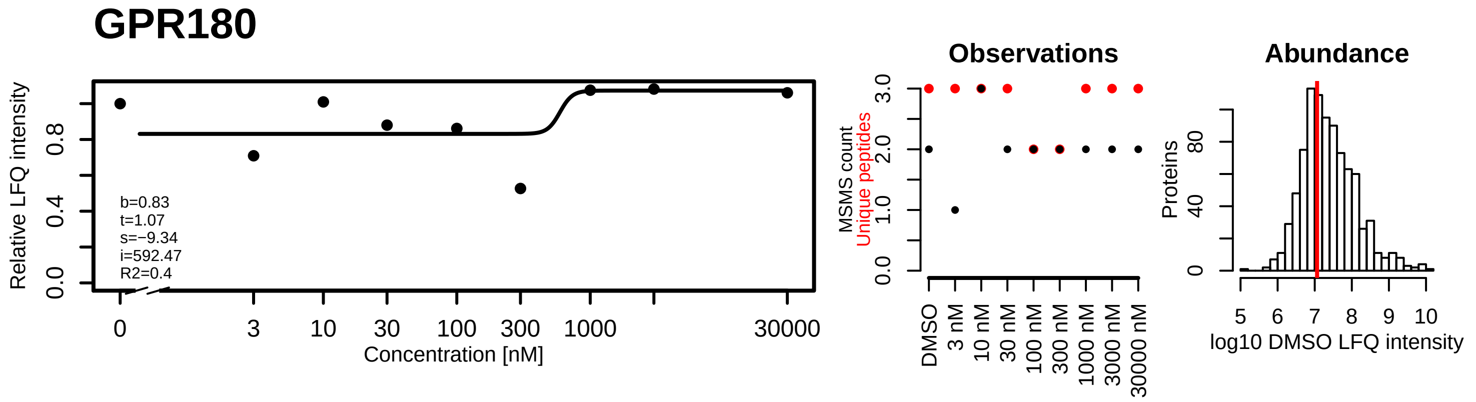
GPR180
Relative LFQ intensity
b=0.83
t=1.07
s=−9.34
i=592.47
R2=0.4
Concentration [nM]
Observations
MSMS count
Unique peptides
Abundance
Proteins
log10 DMSO LFQ intensity

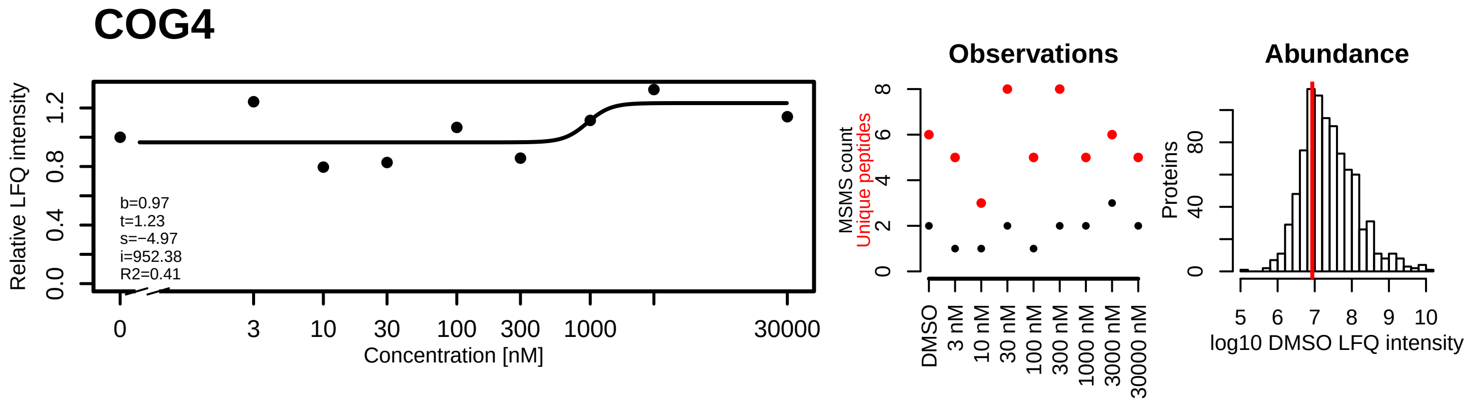
COG4
Relative LFQ intensity
b=0.97
t=1.23
s=−4.97
i=952.38
R2=0.41
Concentration [nM]
Observations
MSMS count
Unique peptides
Abundance
Proteins
log10 DMSO LFQ intensity

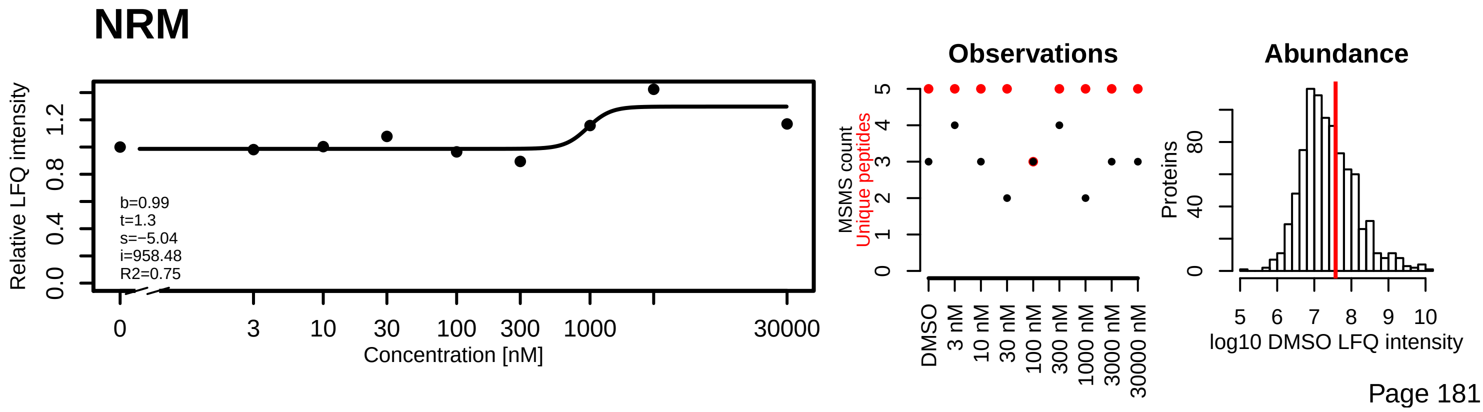
NRM
Relative LFQ intensity
b=0.99
t=1.3
s=−5.04
i=958.48
R2=0.75
Concentration [nM]
Observations
MSMS count
Unique peptides
Abundance
Proteins
log10 DMSO LFQ intensity

TAZ

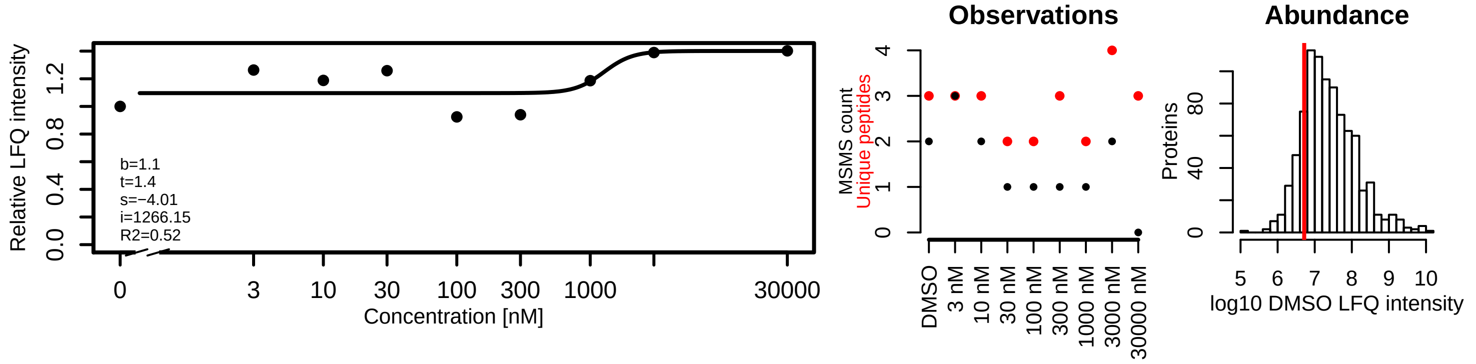

MYBBP1A

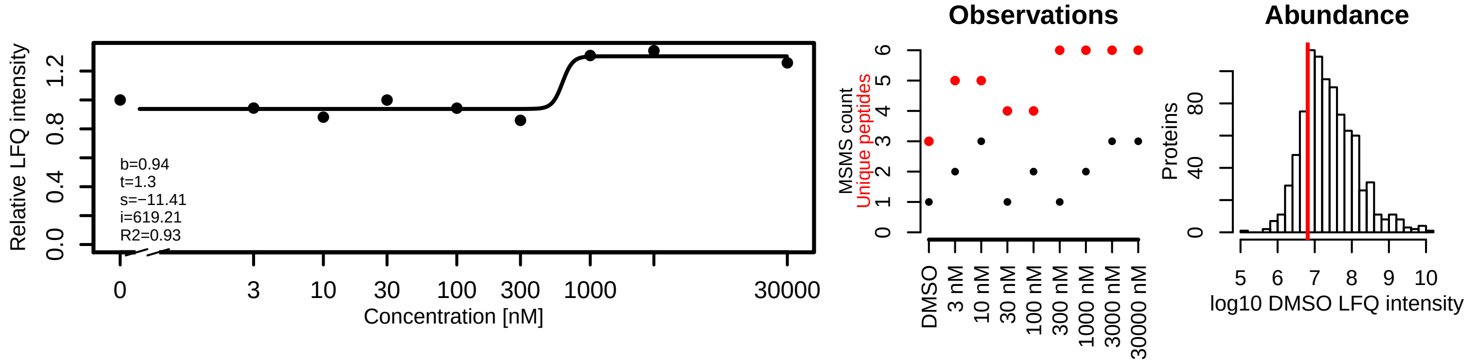

ELOVL4

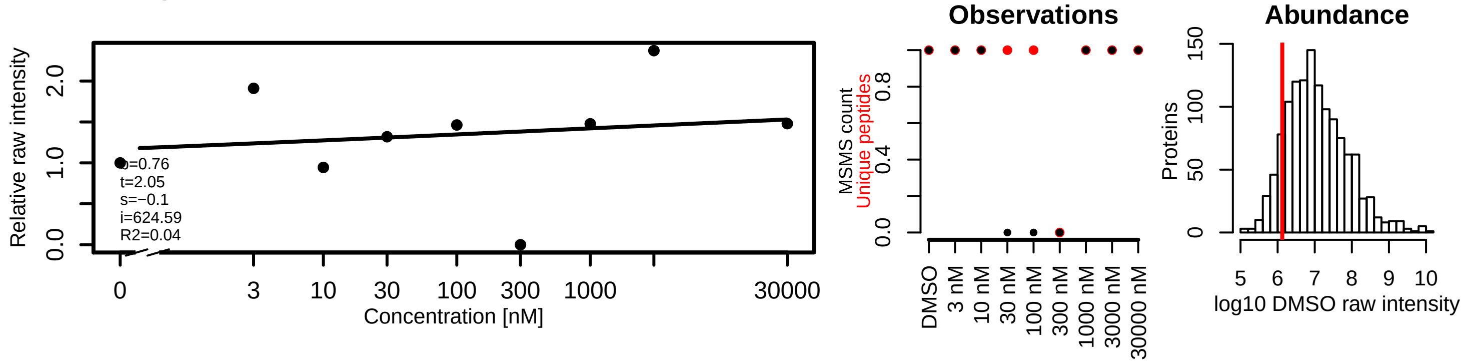

HSD17B10

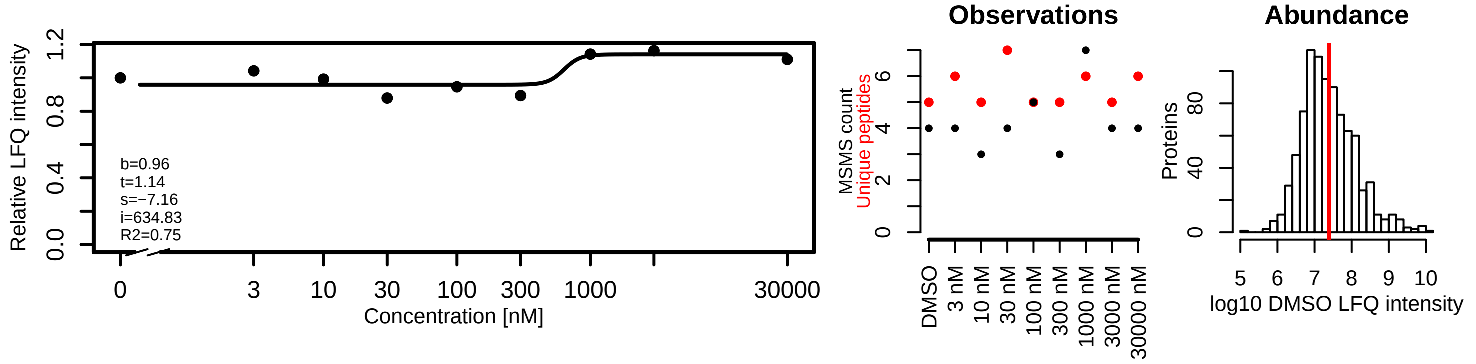

SYVN1

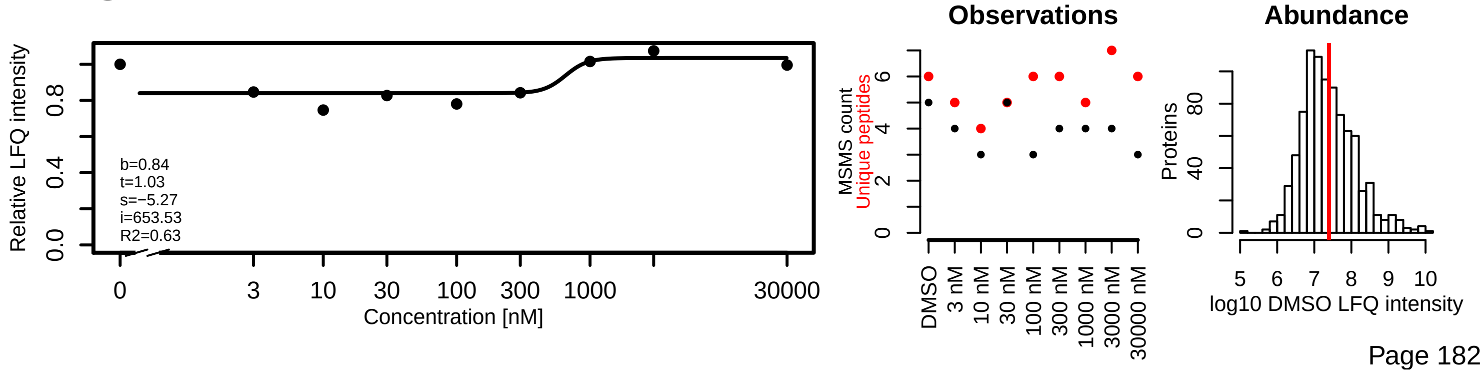

XPO6

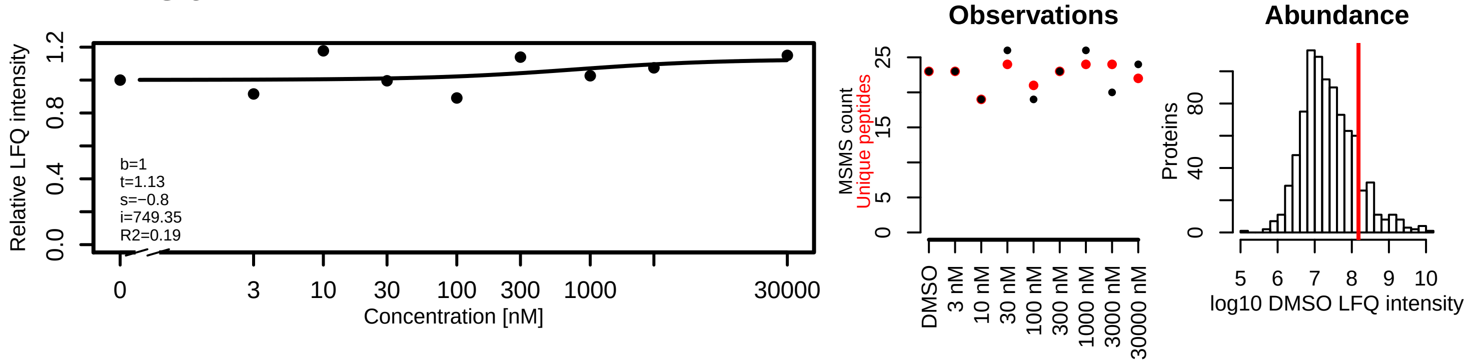

OCIAD2

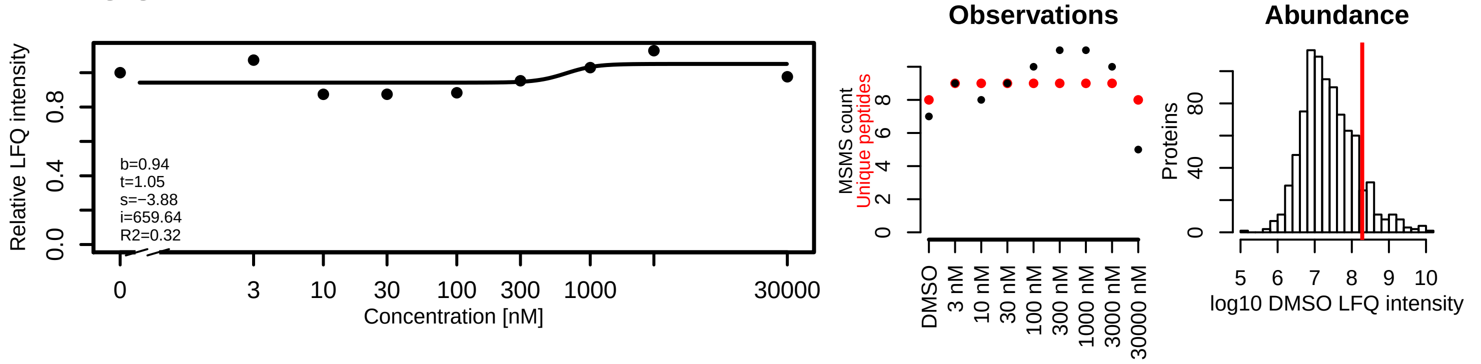

ACAD9

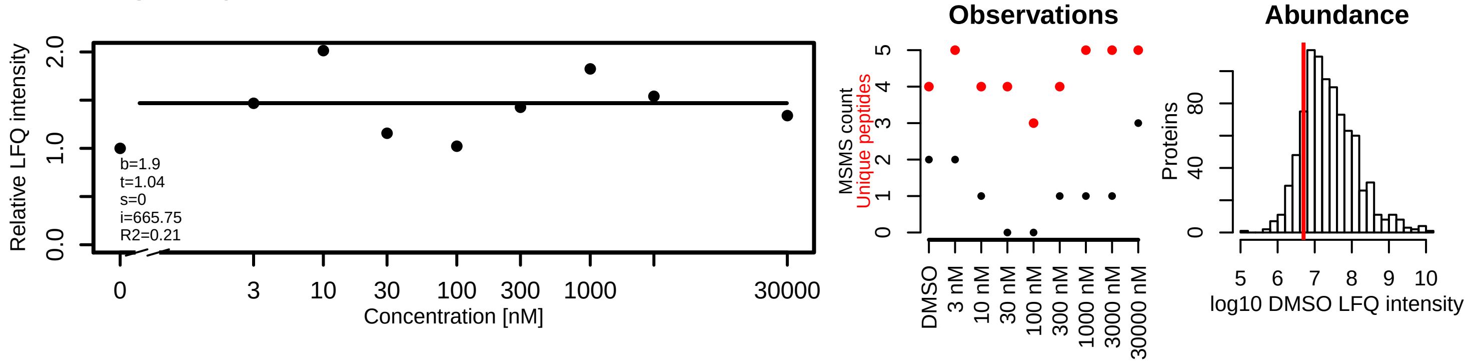

YIF1B

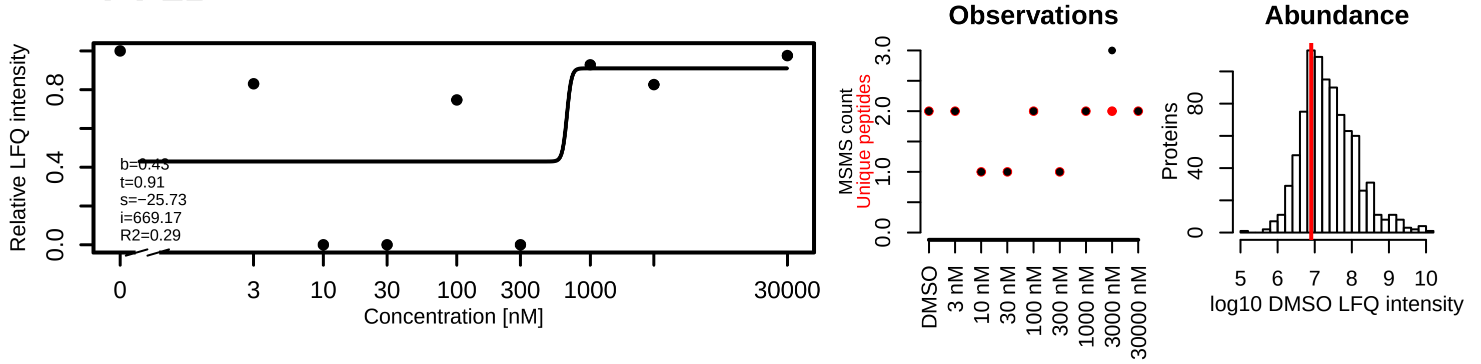

VDAC1

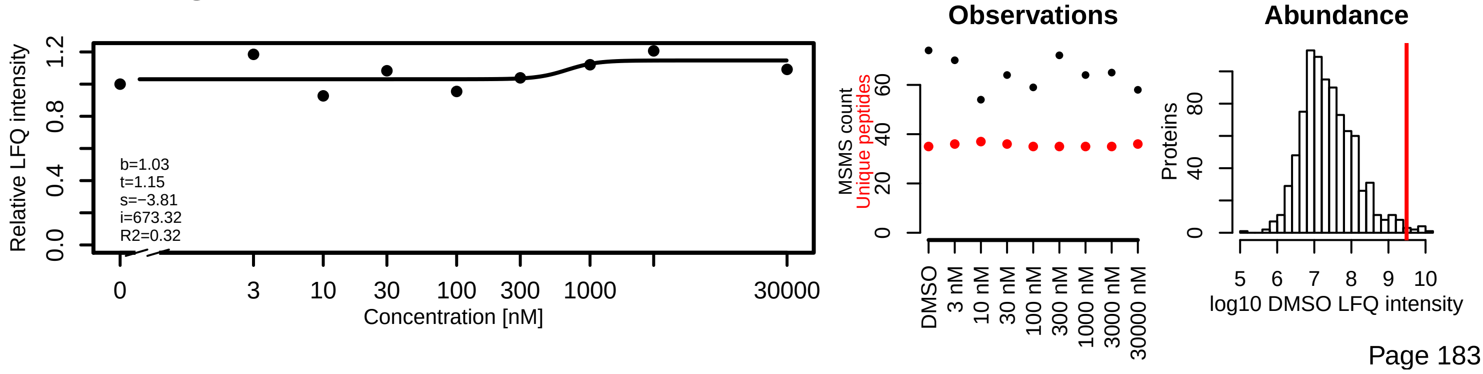

SLC25A3

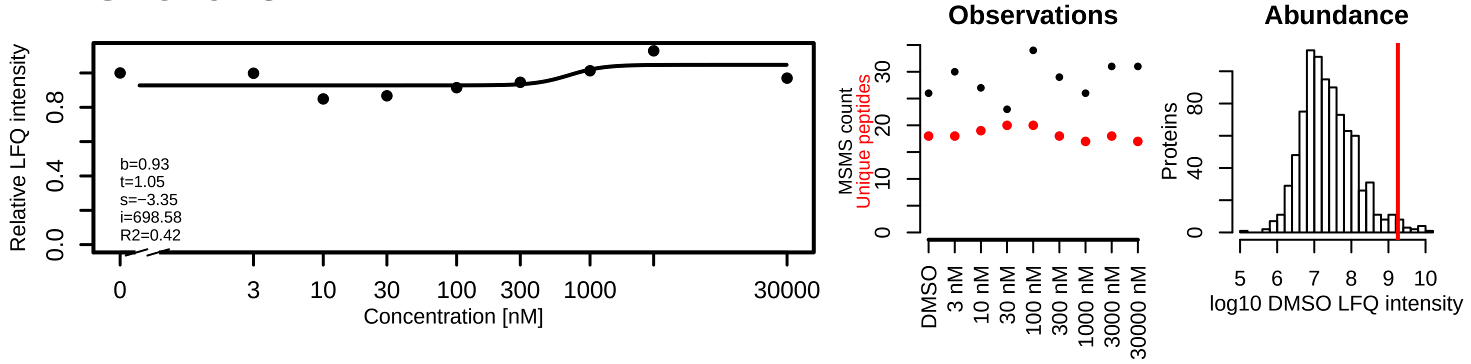

NDUFA8

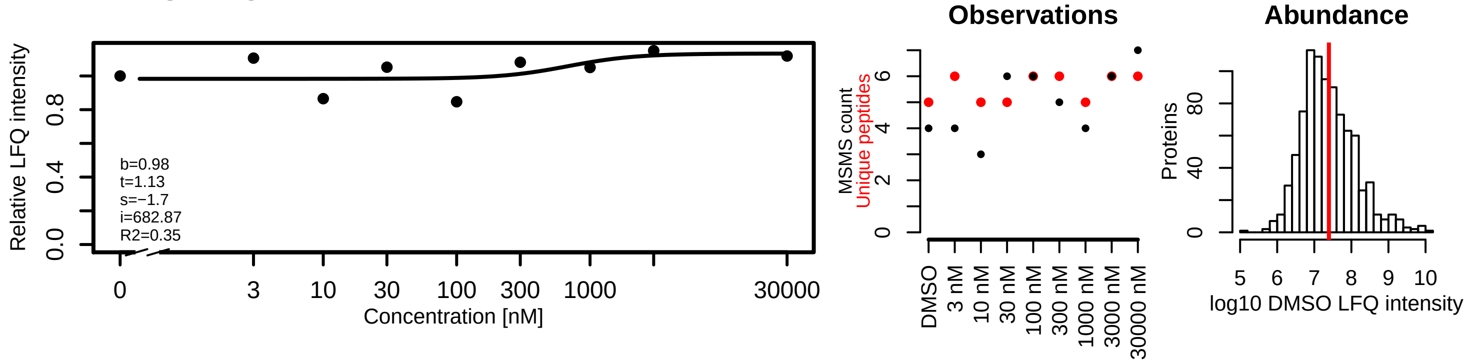

MTX1

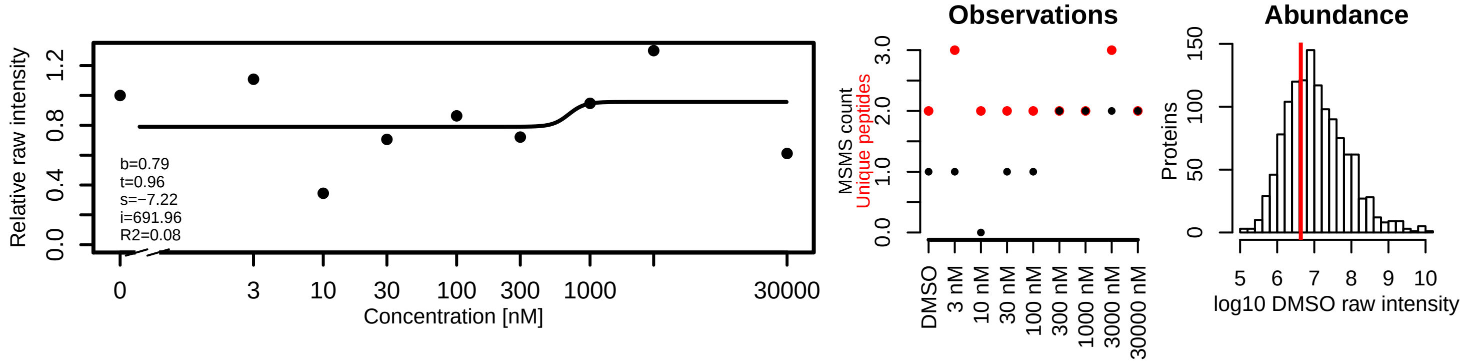

TMEM19

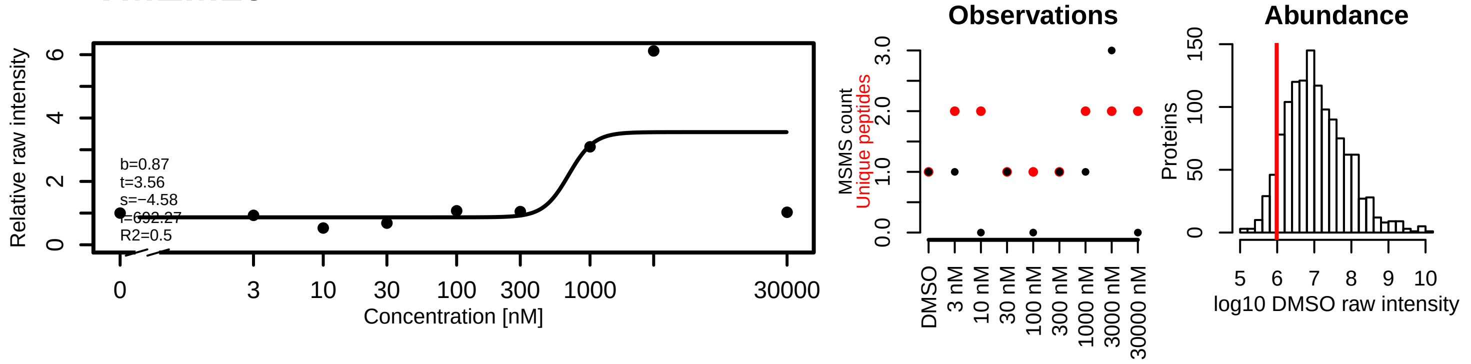

ERLIN2

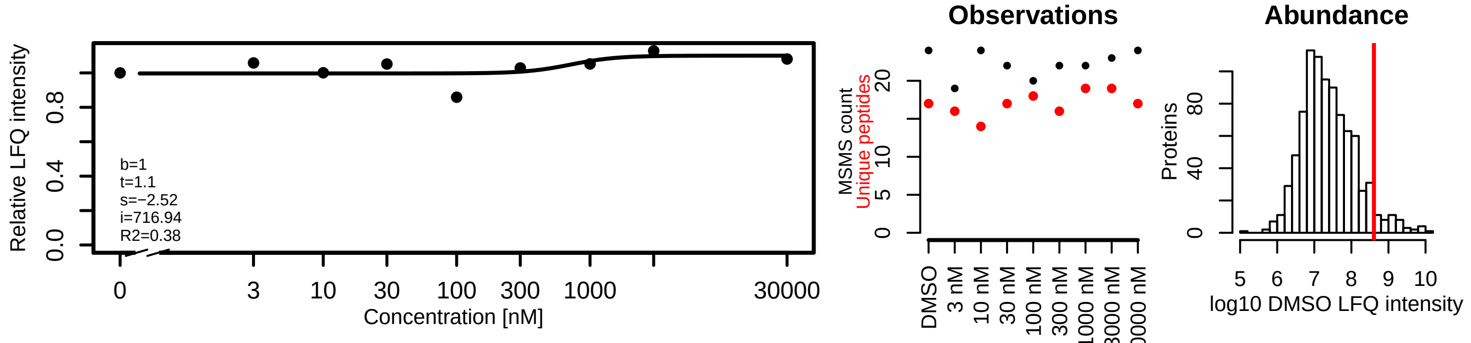

ZW10

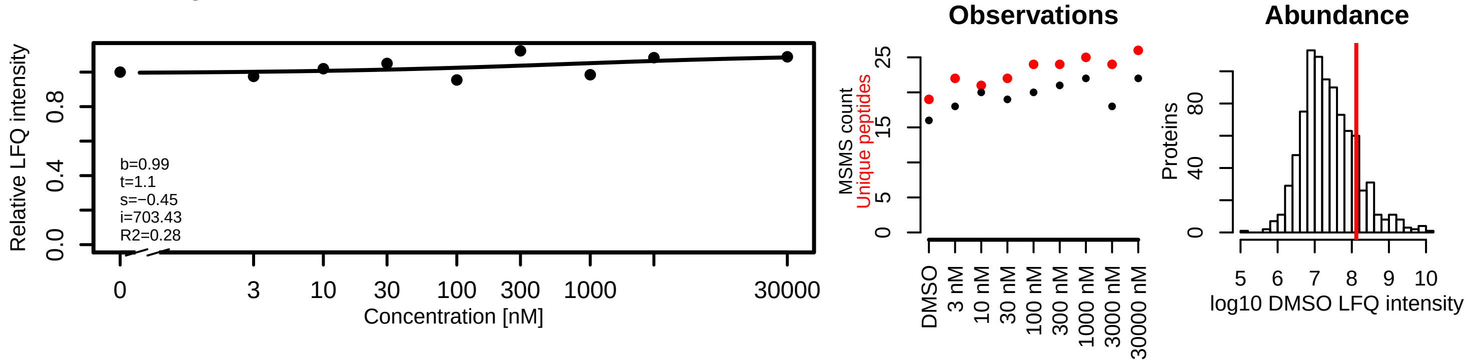

SSR4

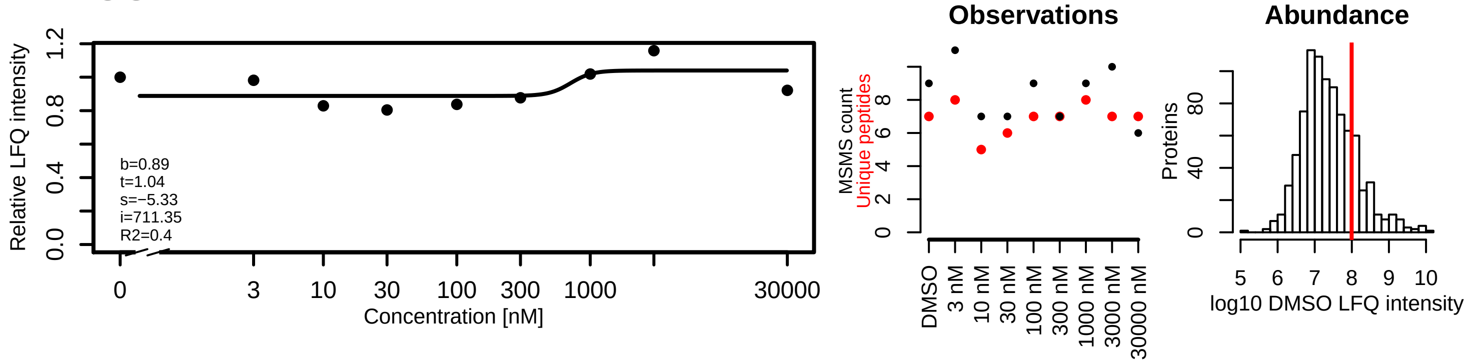

SRBD1

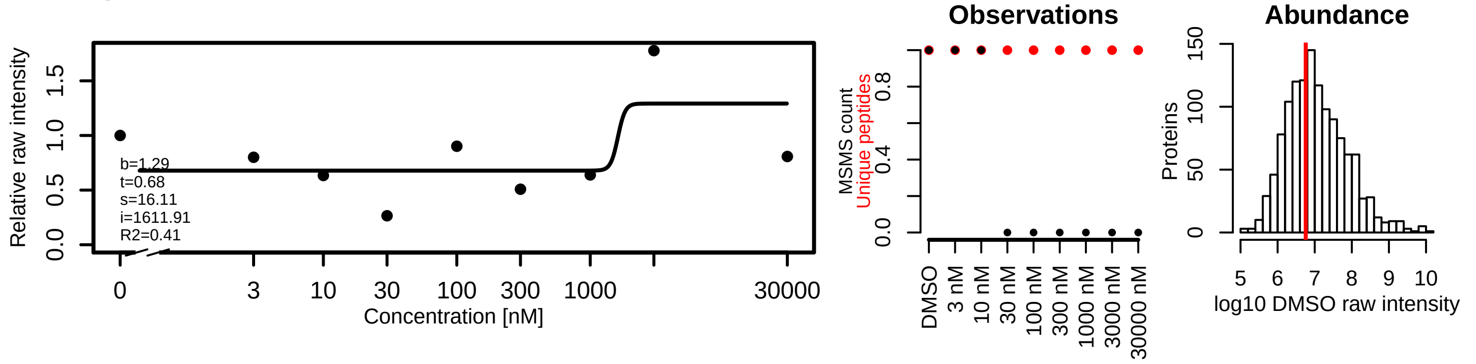

ATR

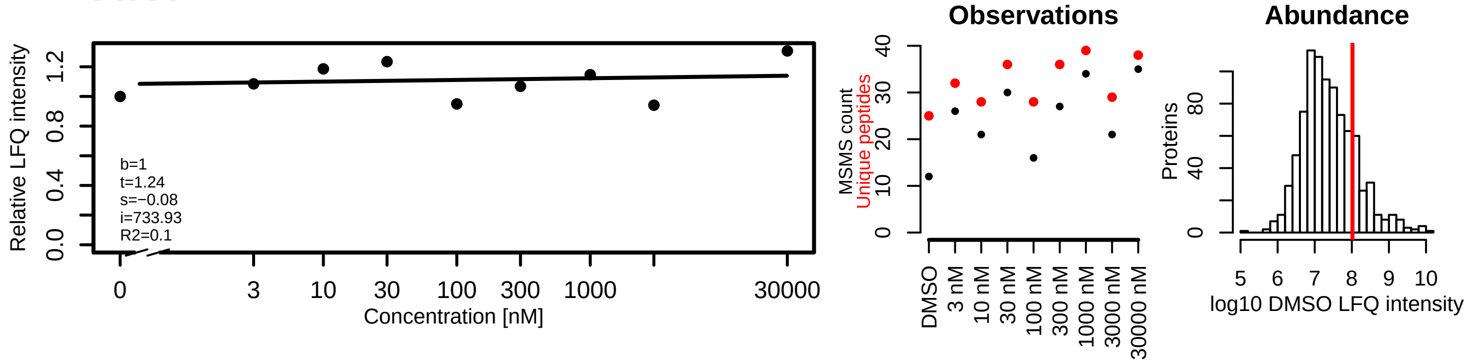

HIST1H4A

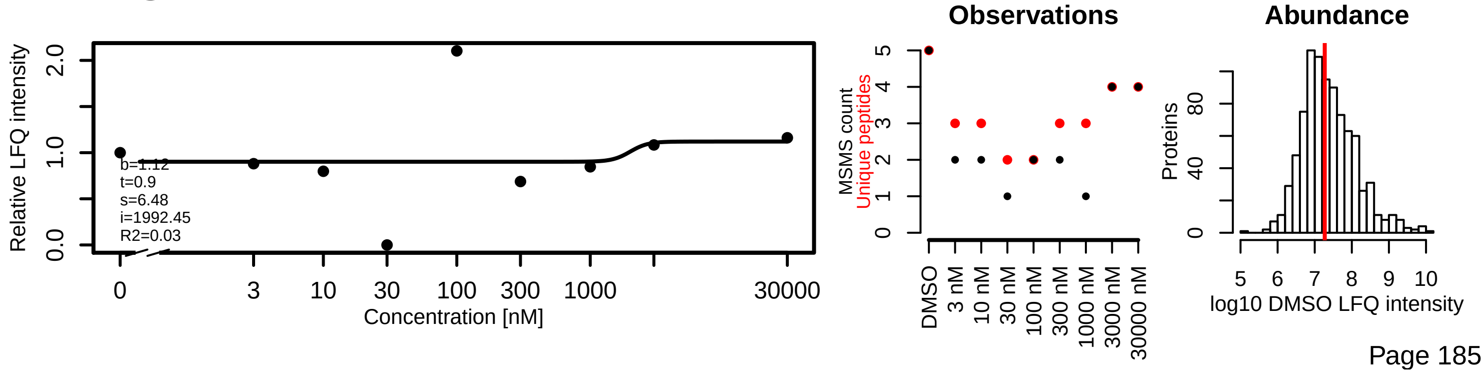

COL6A1

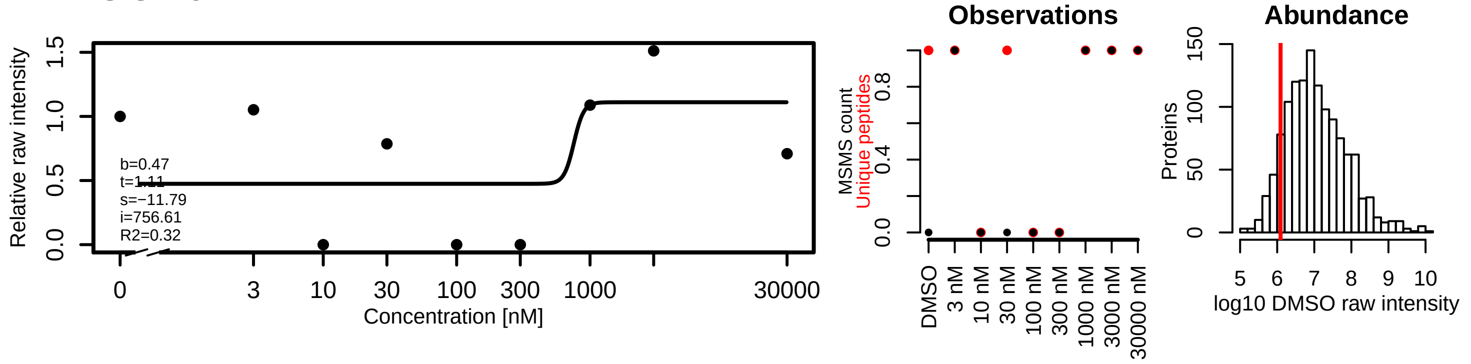

MPV17L2

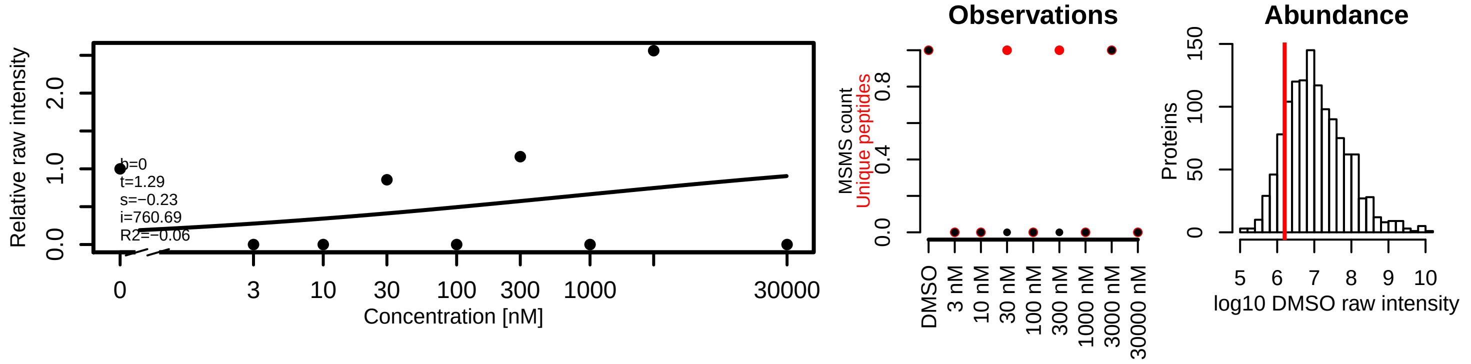

CDIPT

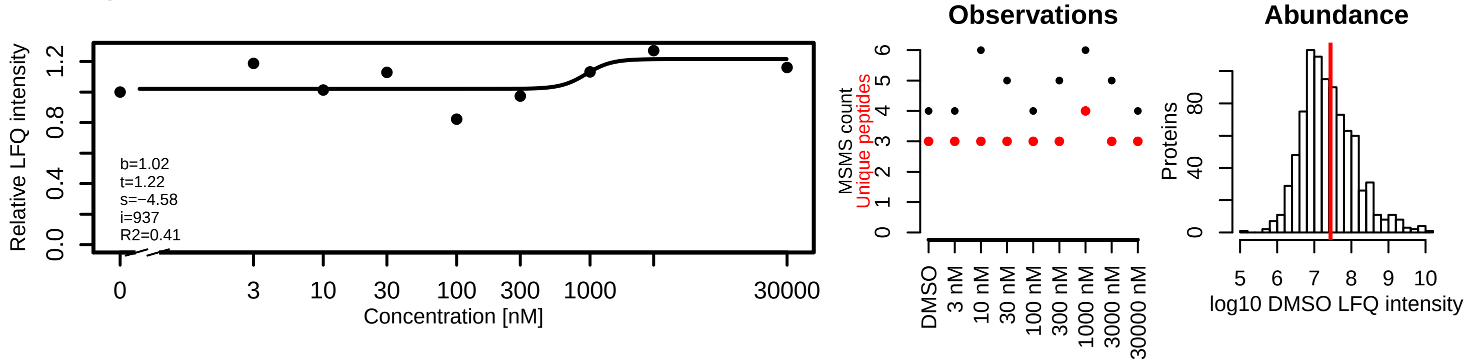

PTPLB

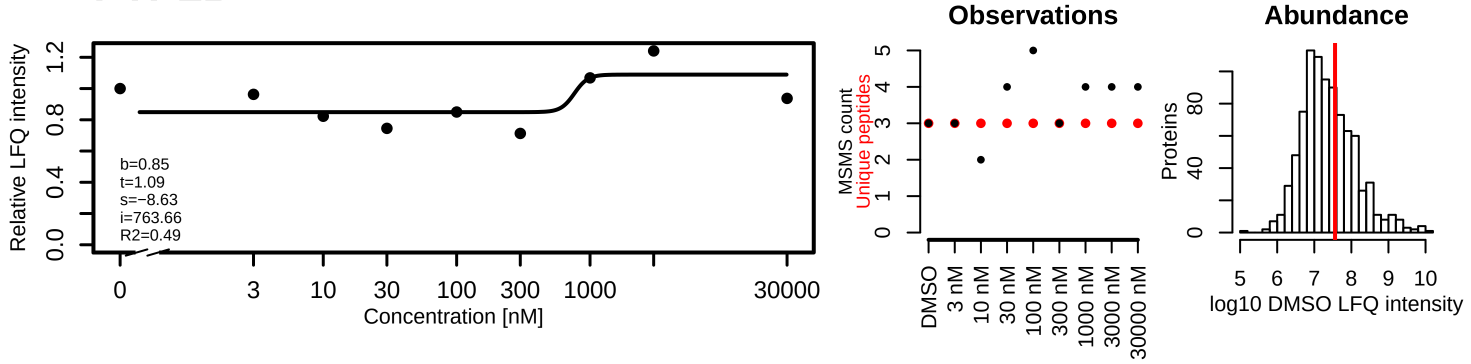

MSH6

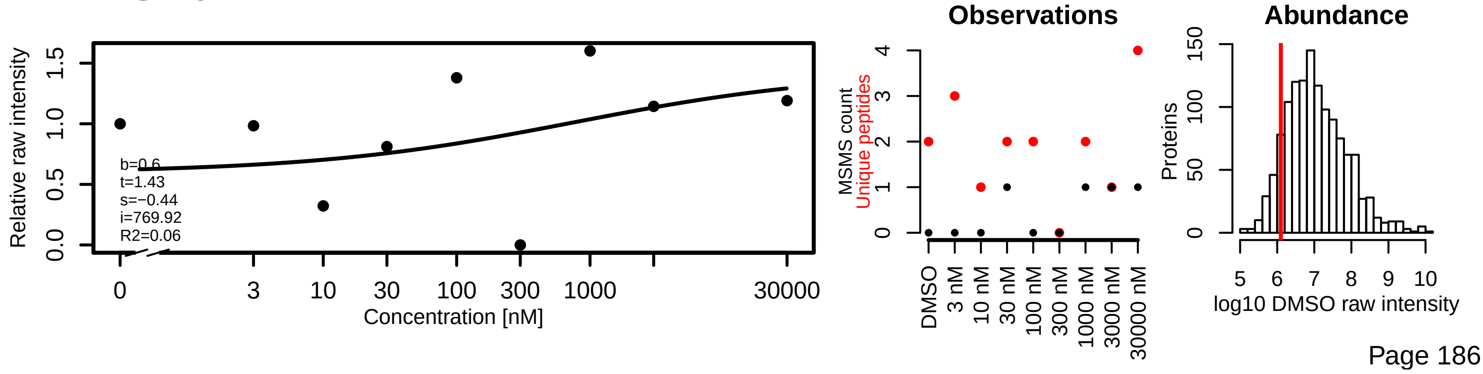

DCK

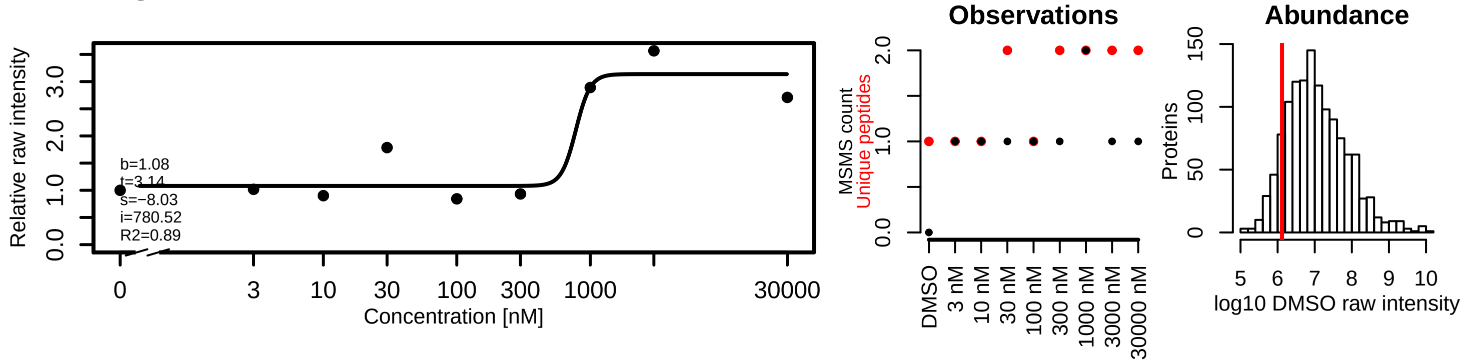

USMG5

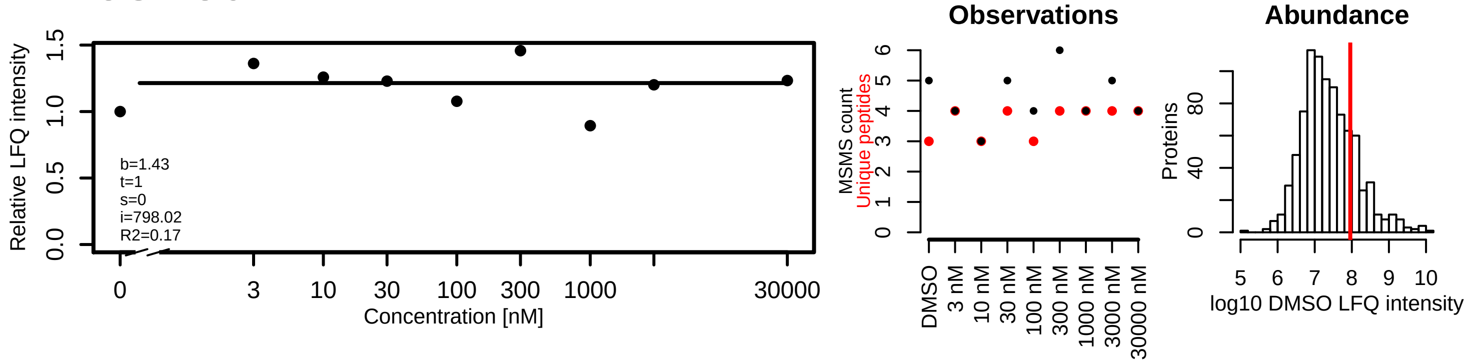

NUP133

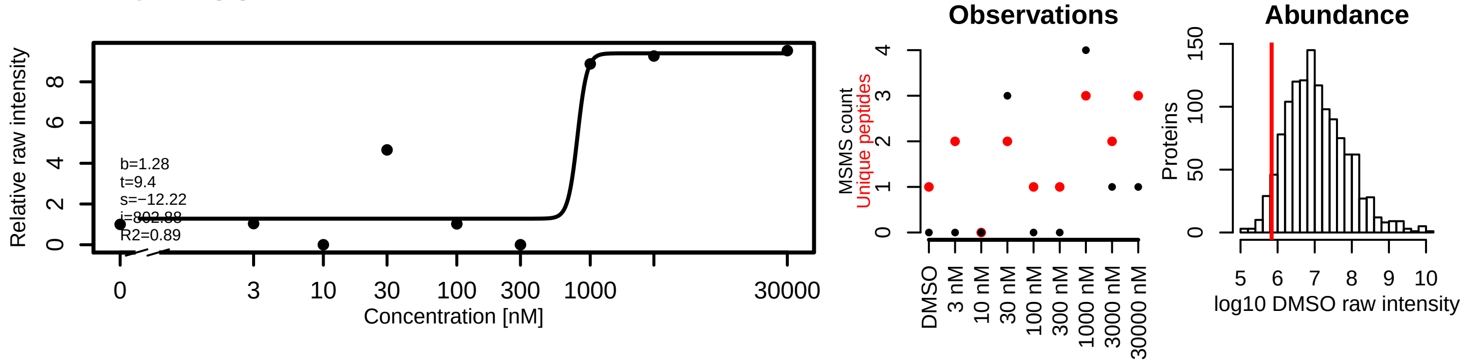

TCEB1

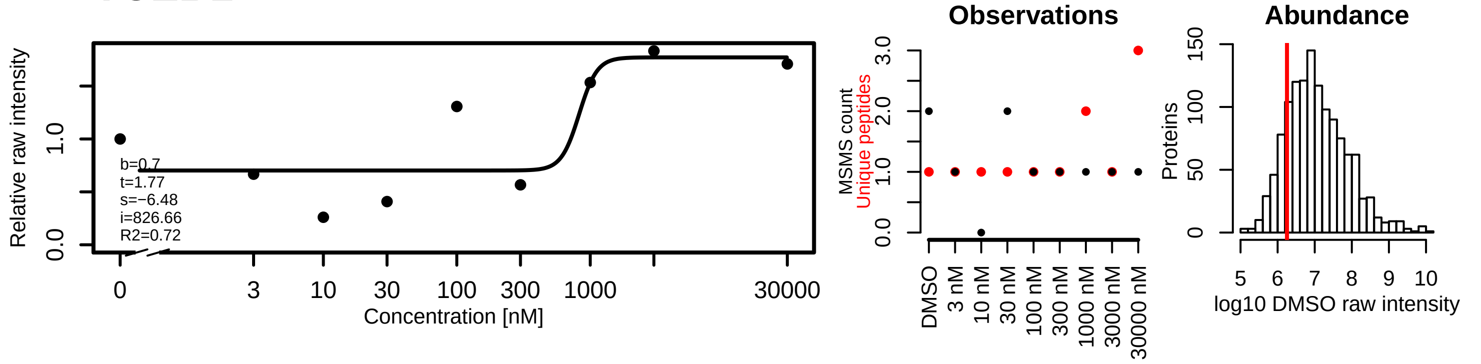

ATP6V0D1

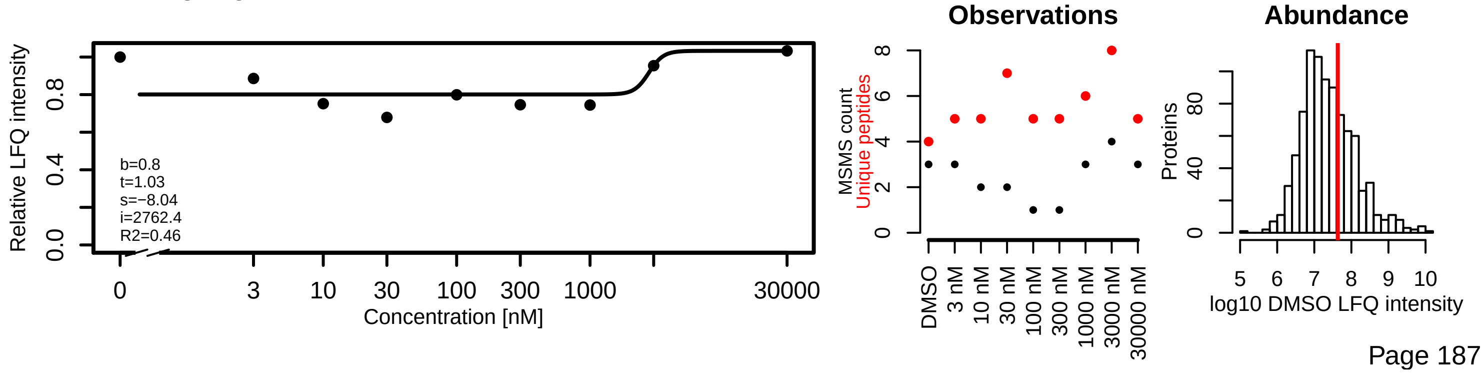

TUBA4A

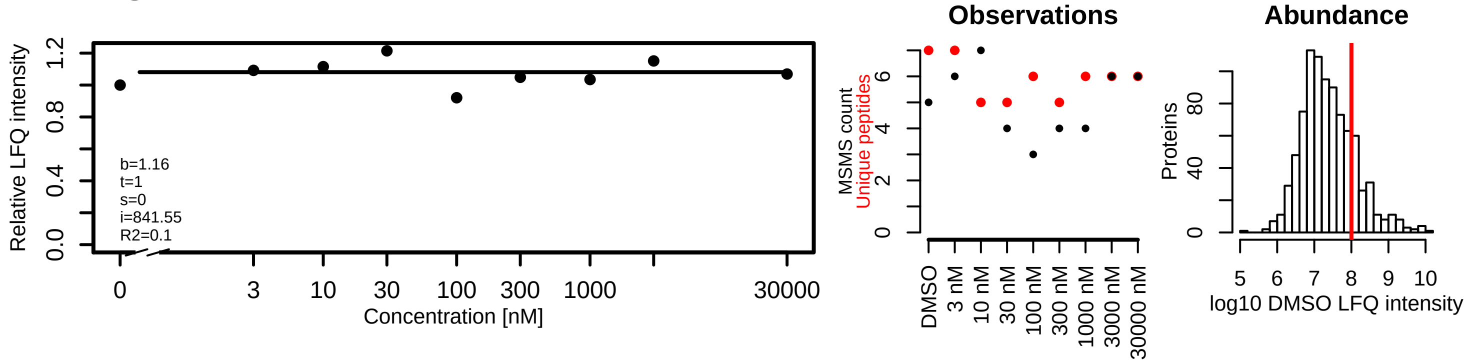

PEX1

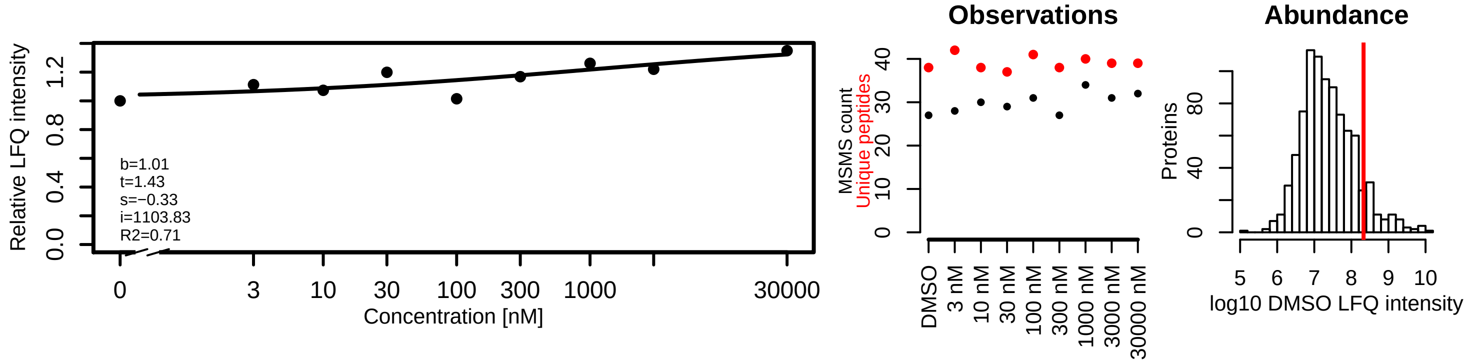

PUM1

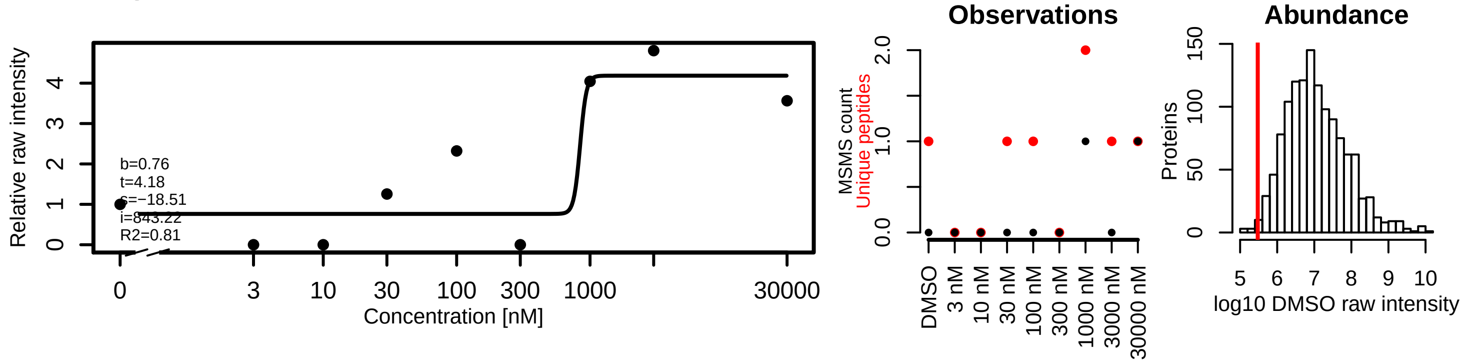

SLC30A7

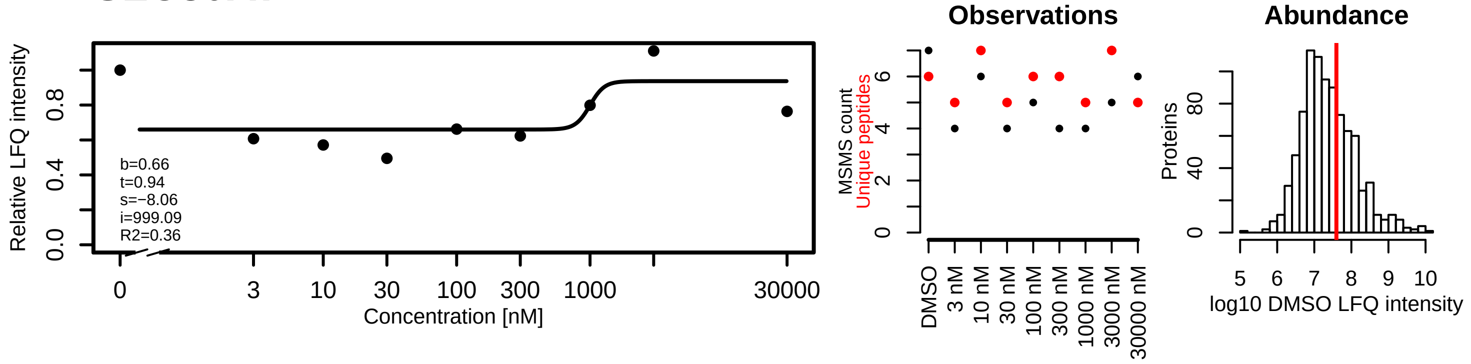

FLOT2

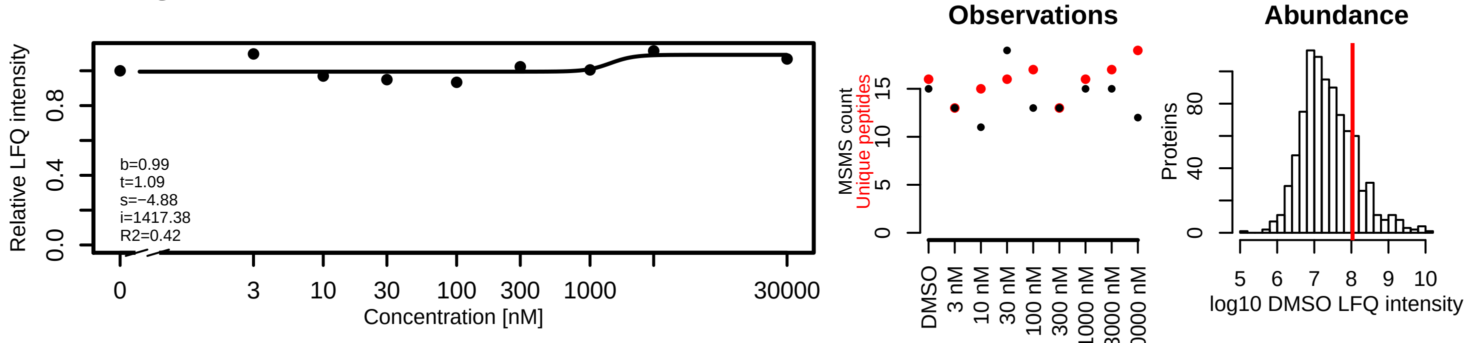

BET1;DKFZp781C0425

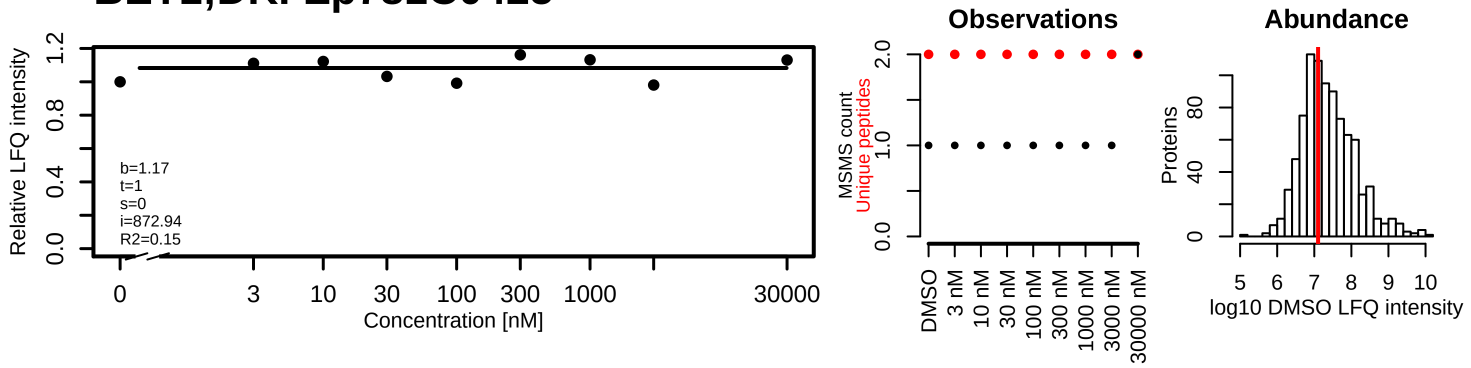

IPO11

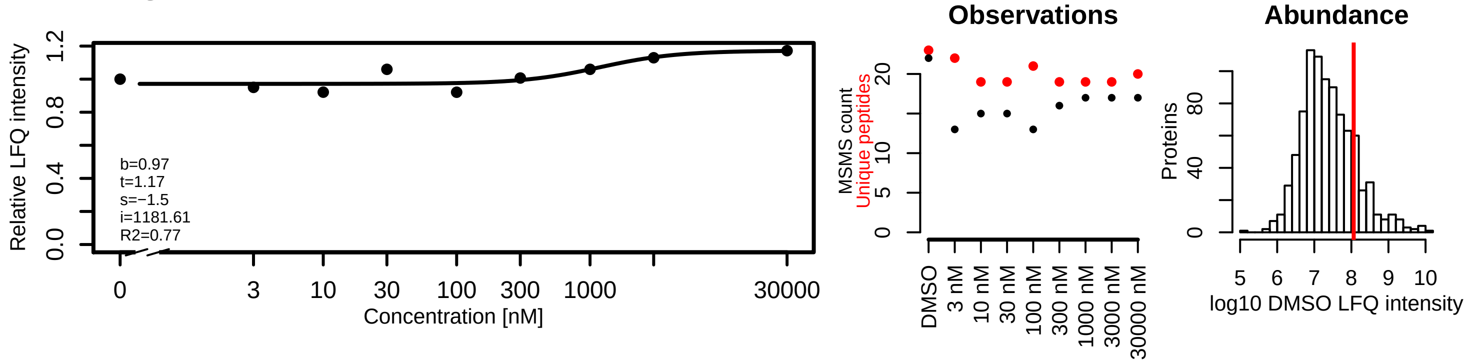

RBFOX1;RBM9;RBFOX2;RBFOX3

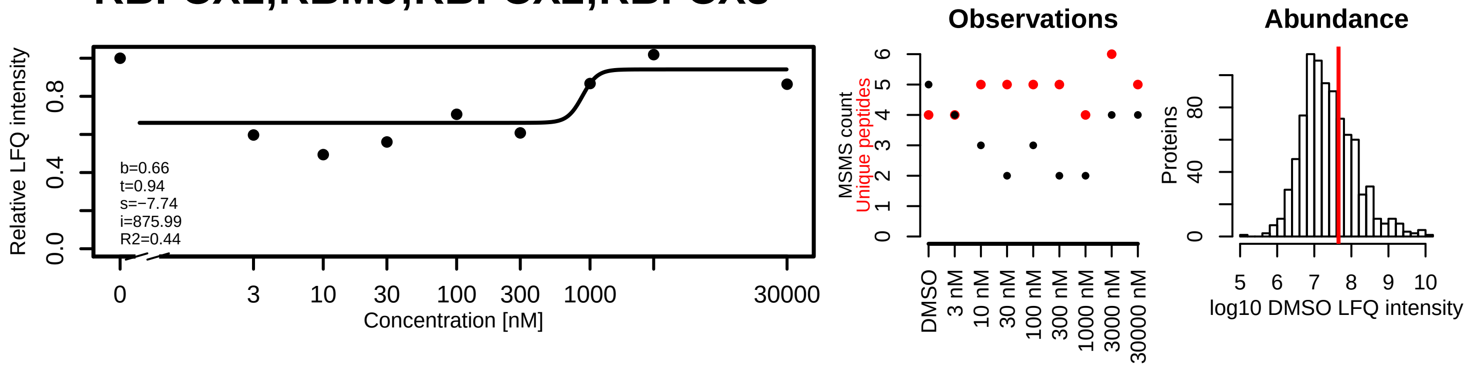

MT−CO1

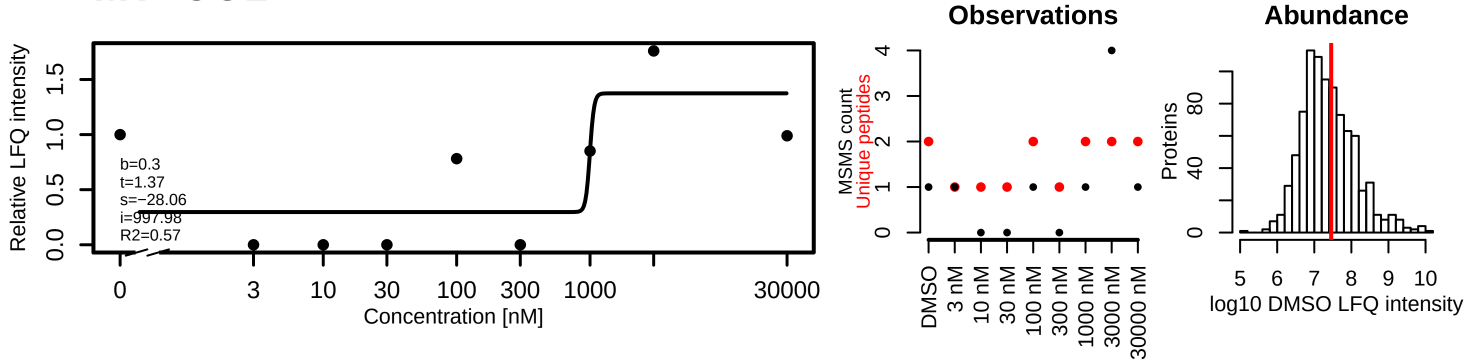

DHCR7

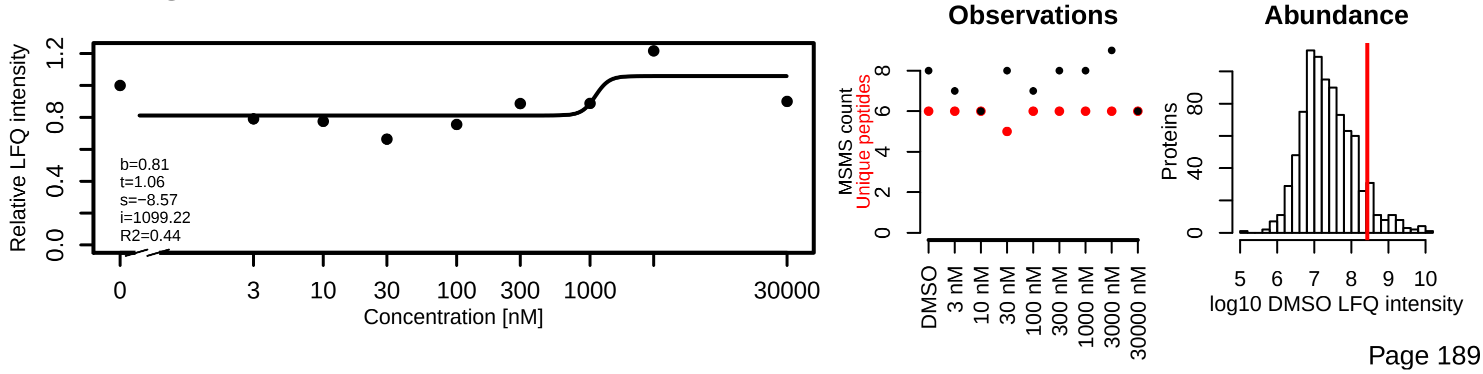

STT3A

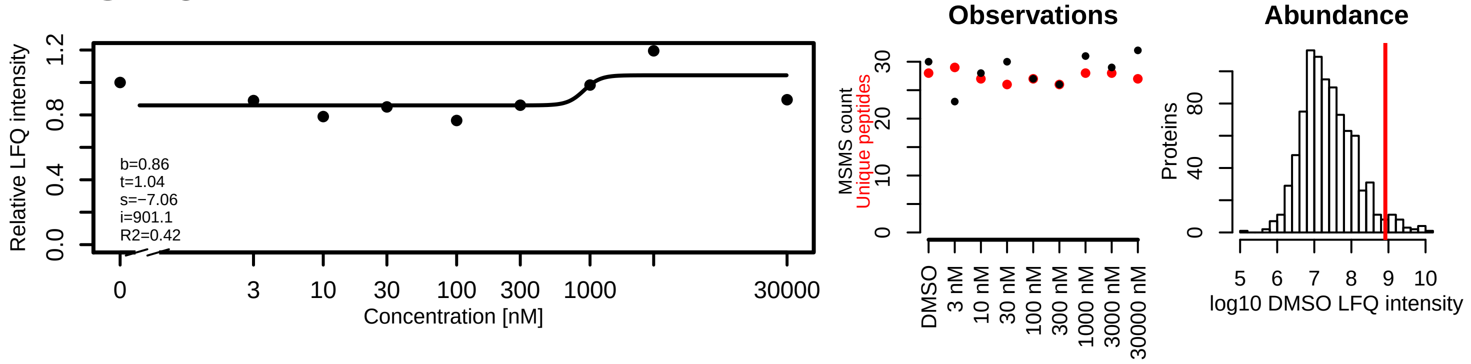

IGSF8

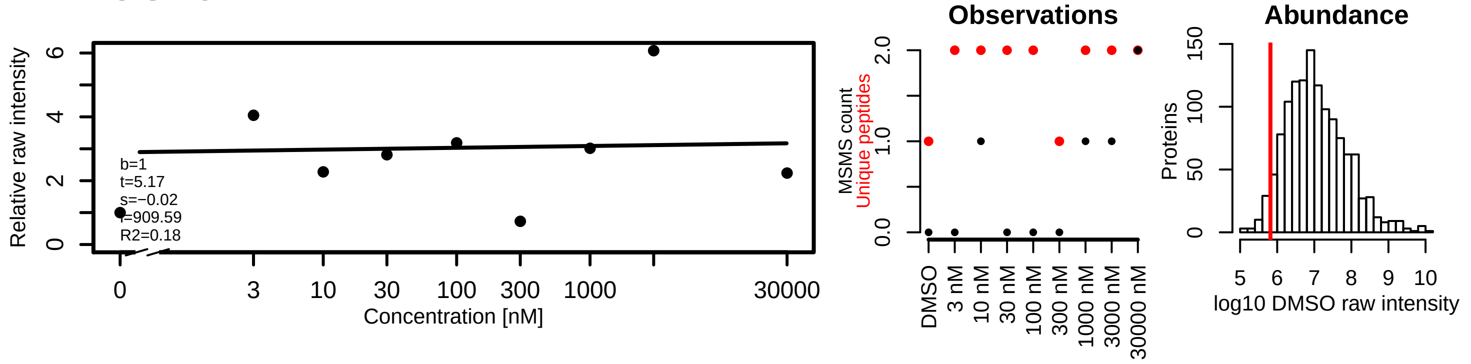

IPO13

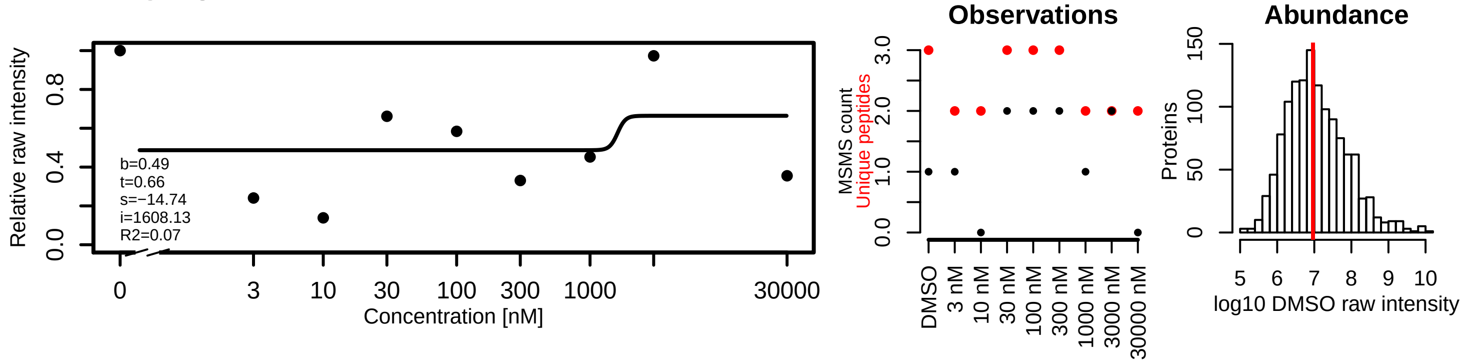

XPOT

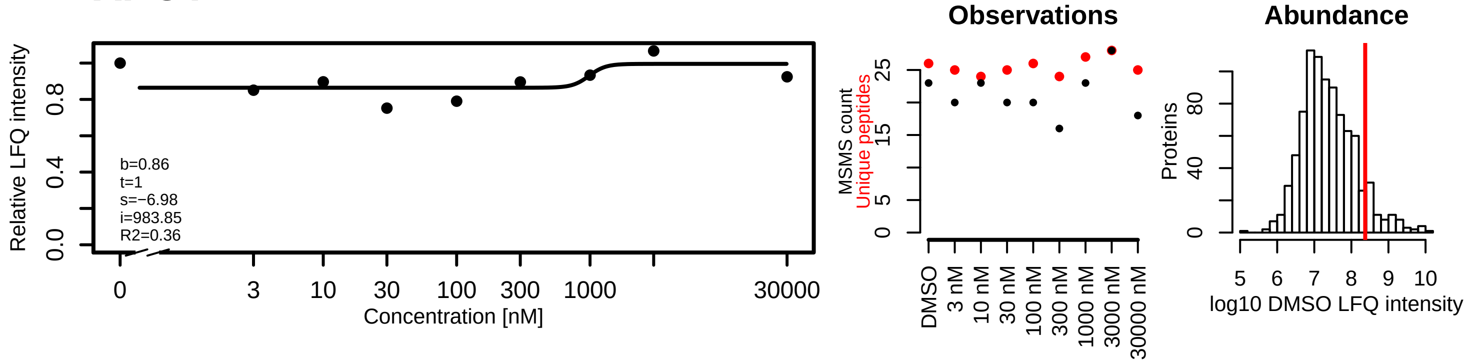

ABCE1

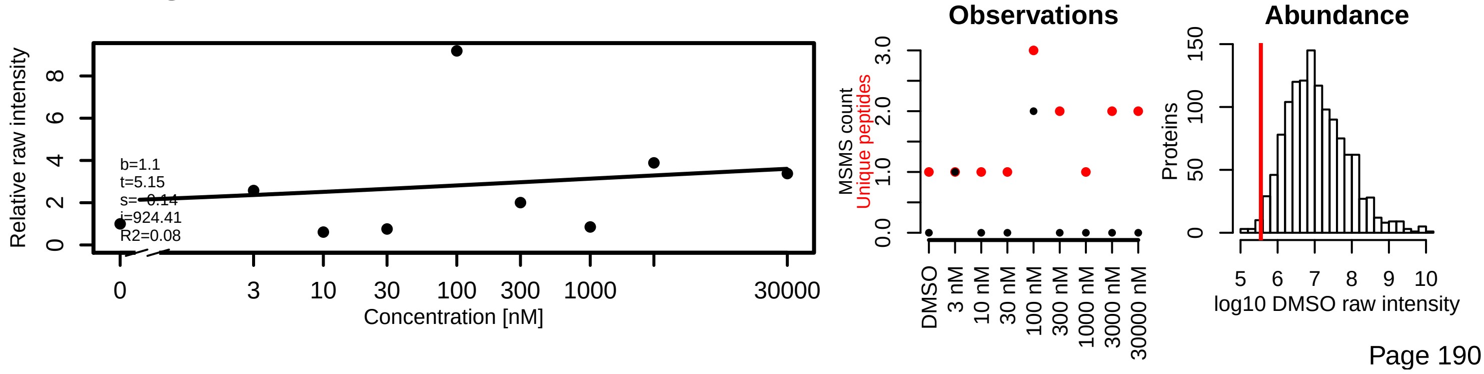

KDELR3

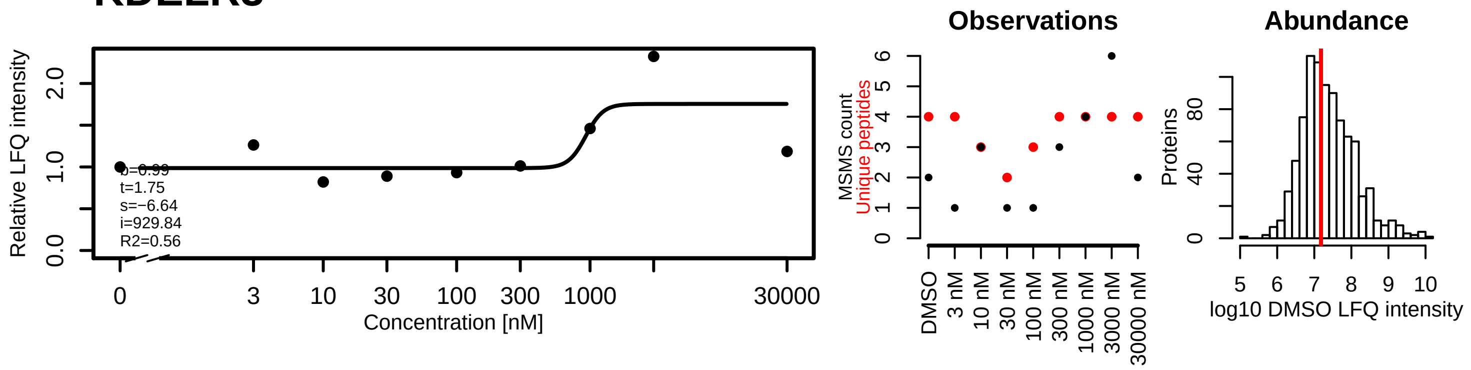

CHP1

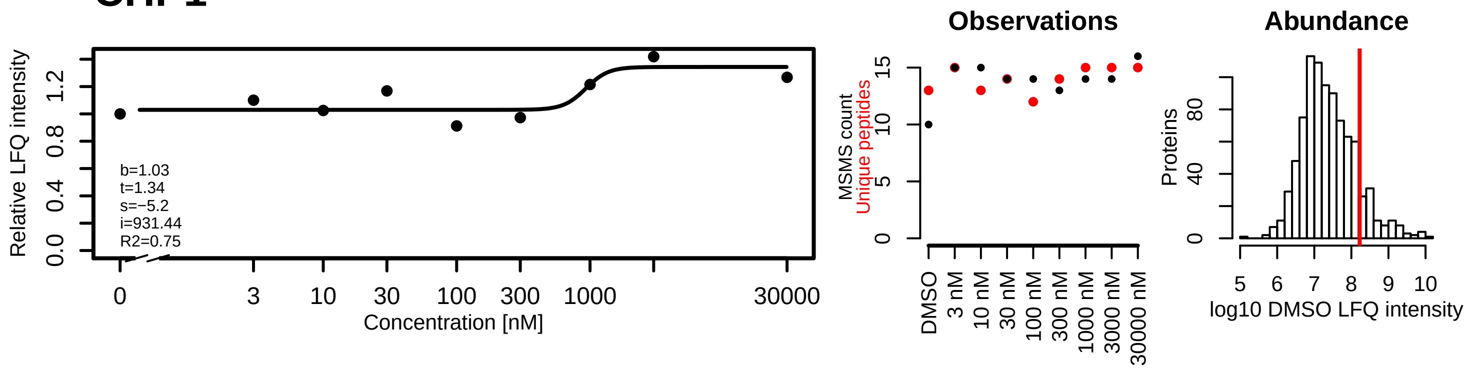

GPR89B;GPR89A;GPR89C

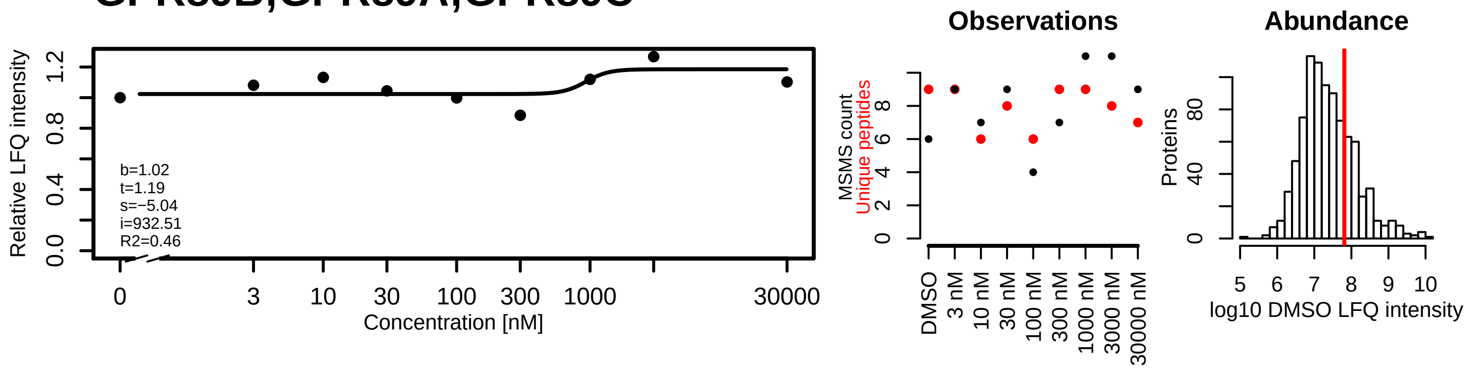

SLC35B2

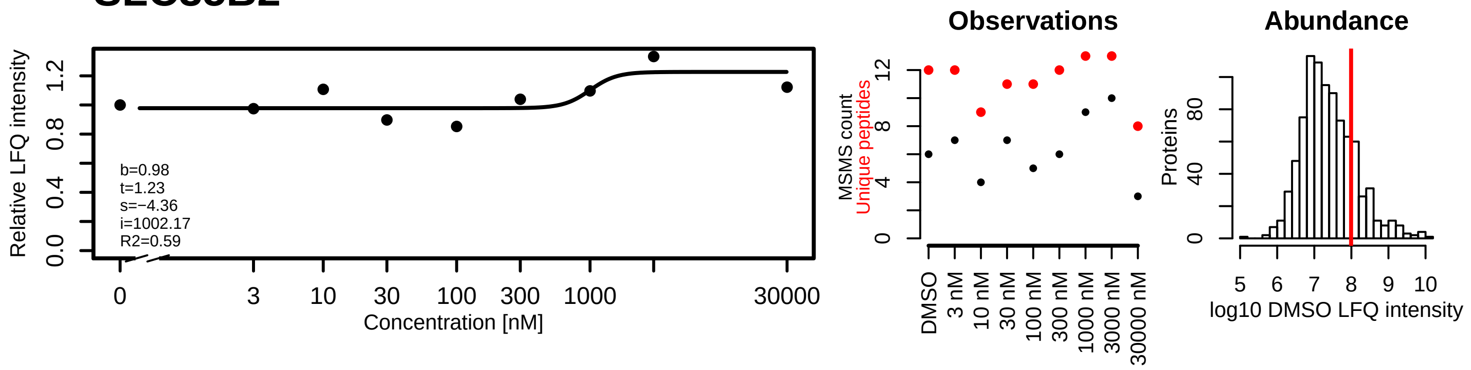

SLC16A13

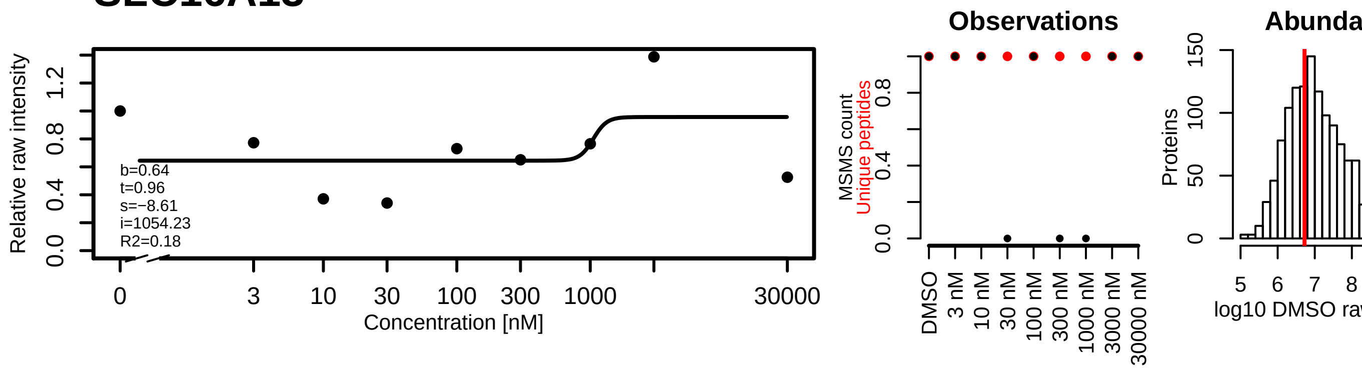

TMEM165

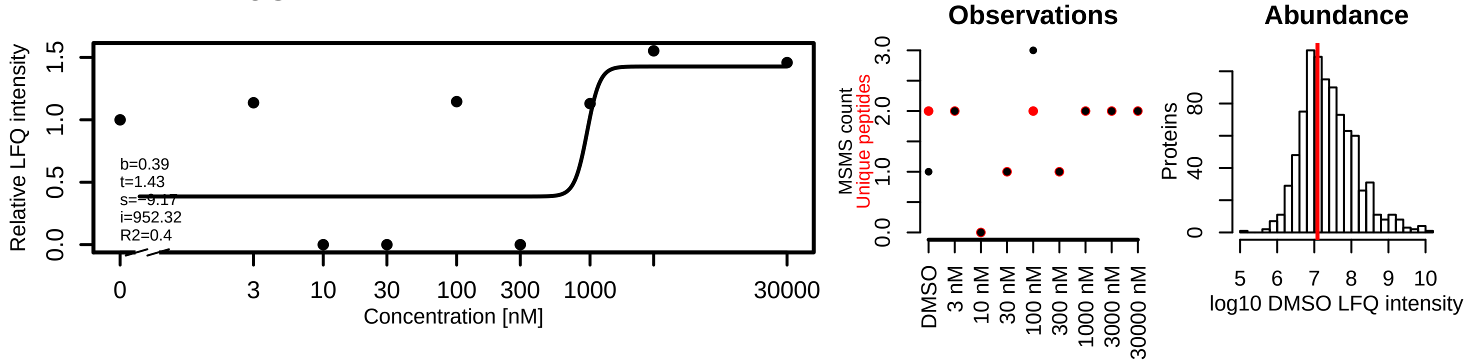

AGPAT6

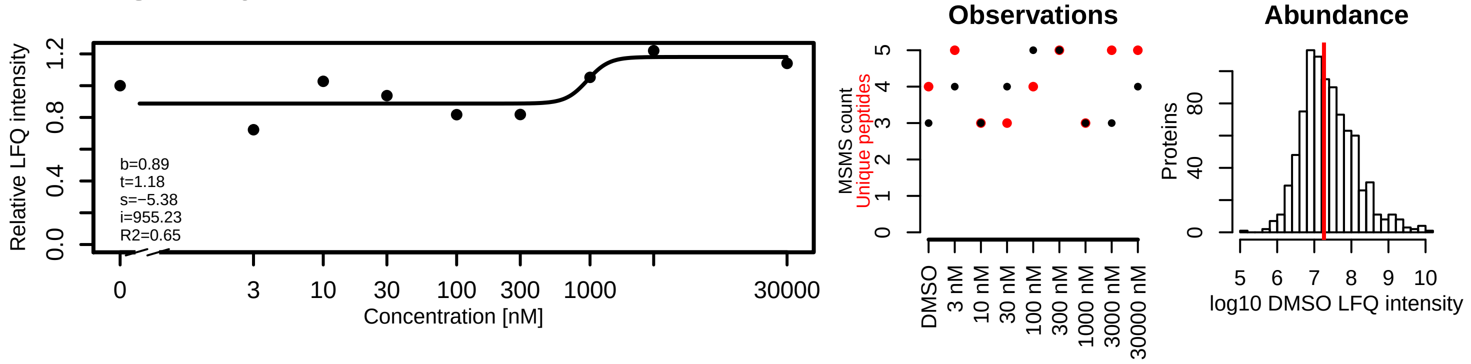

SUMO1

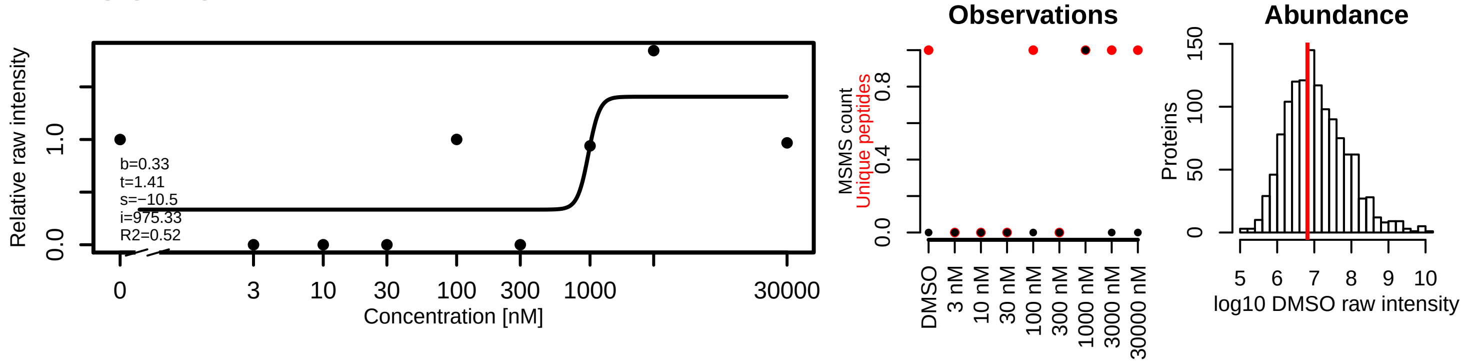

GLG1

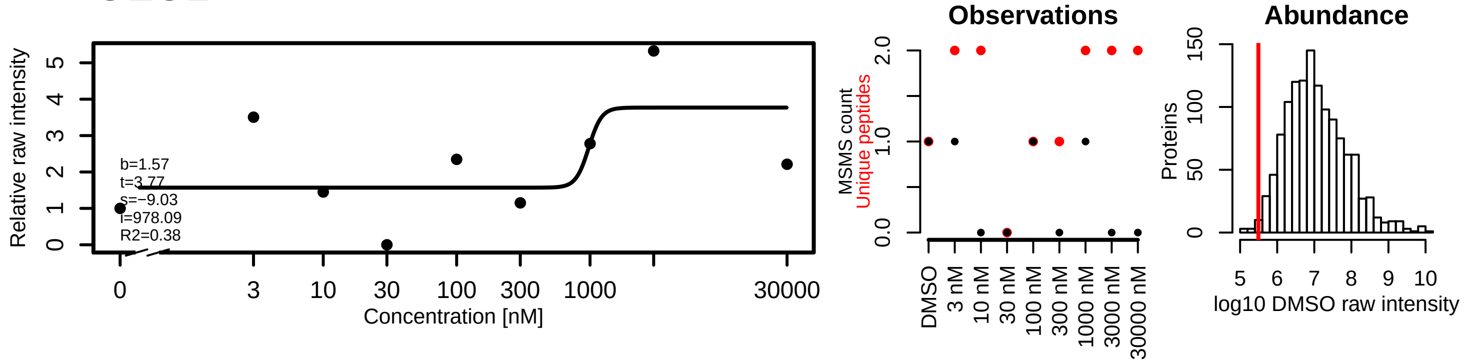

TIMM10

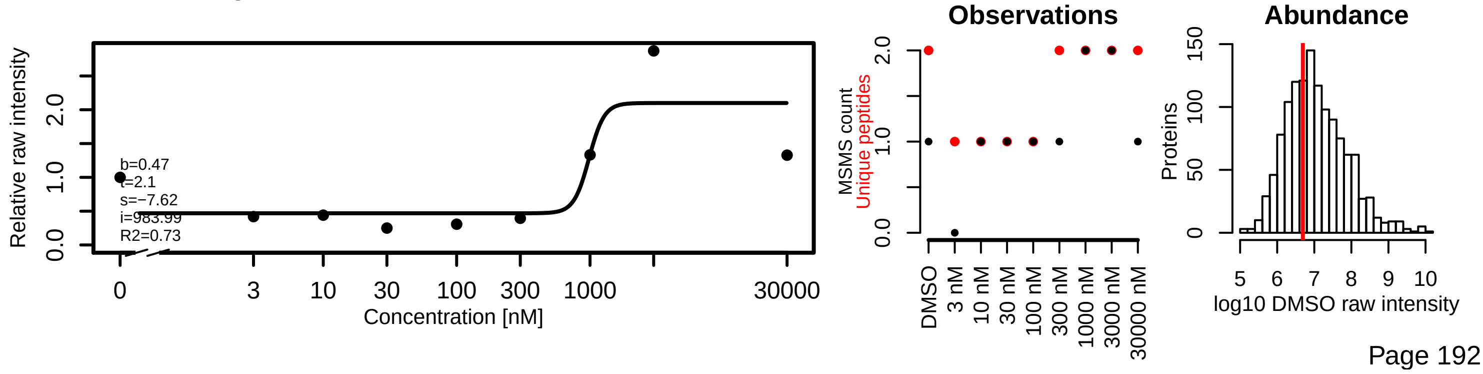

BCAP31

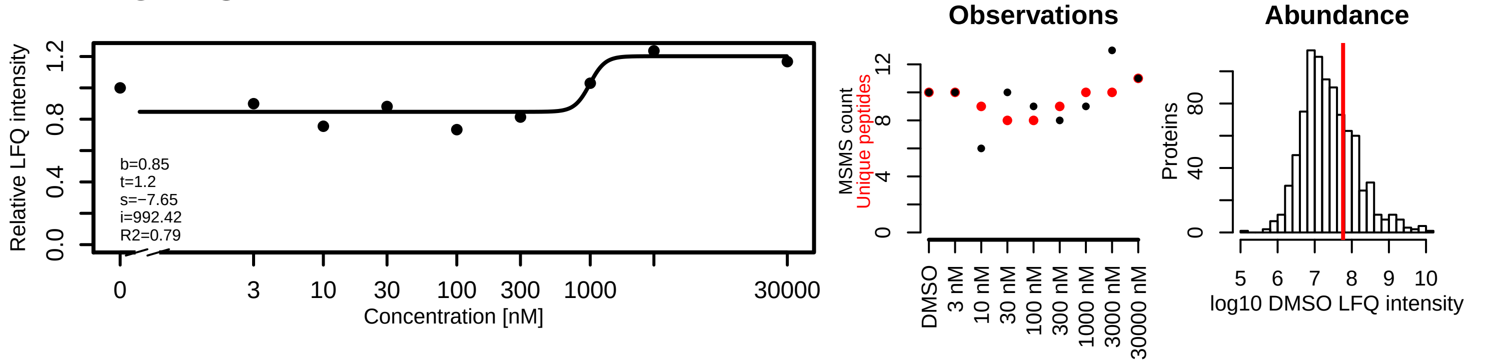

NDUFS7

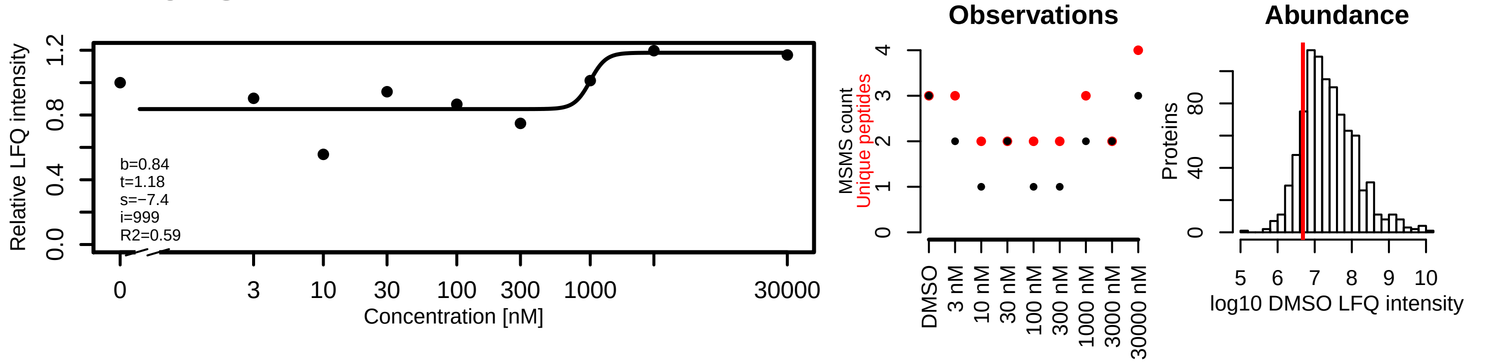

PARL

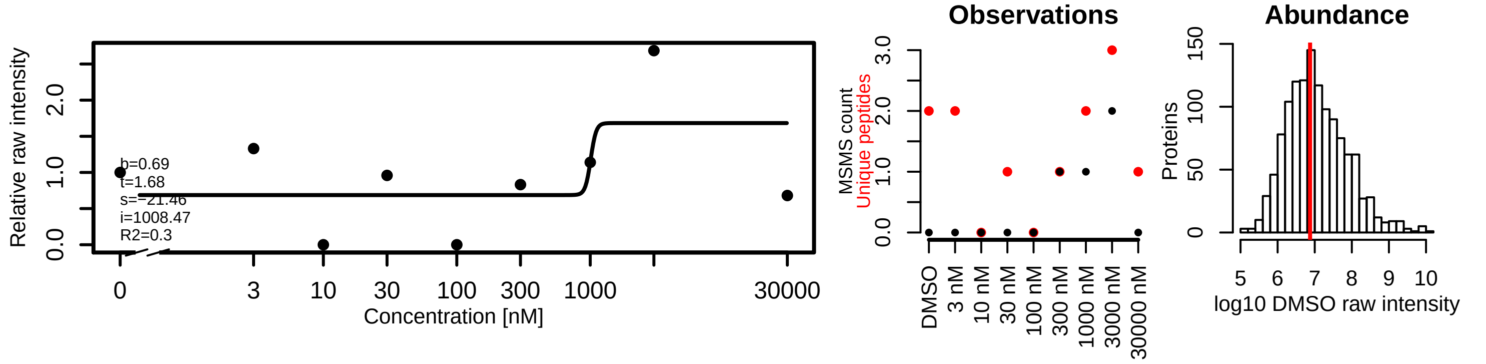

CD97

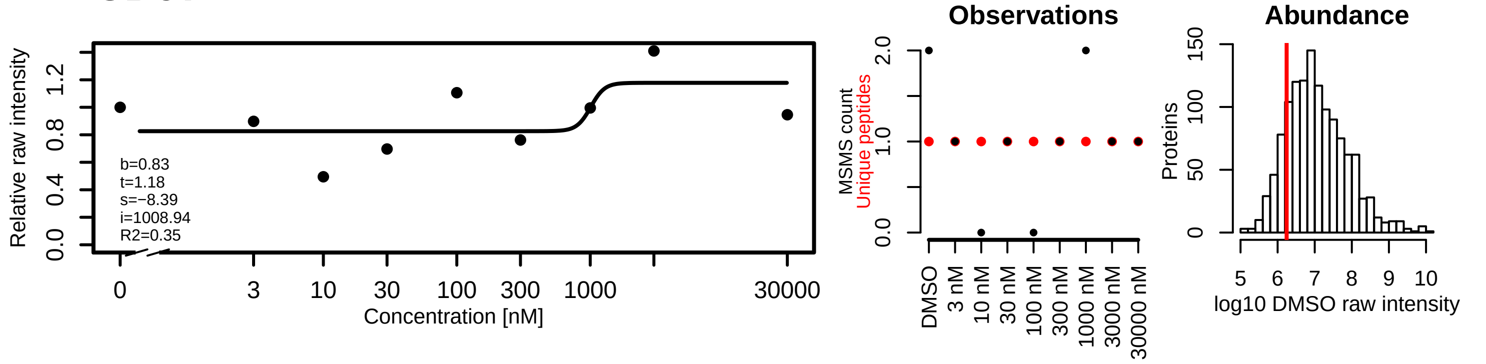

ALG3

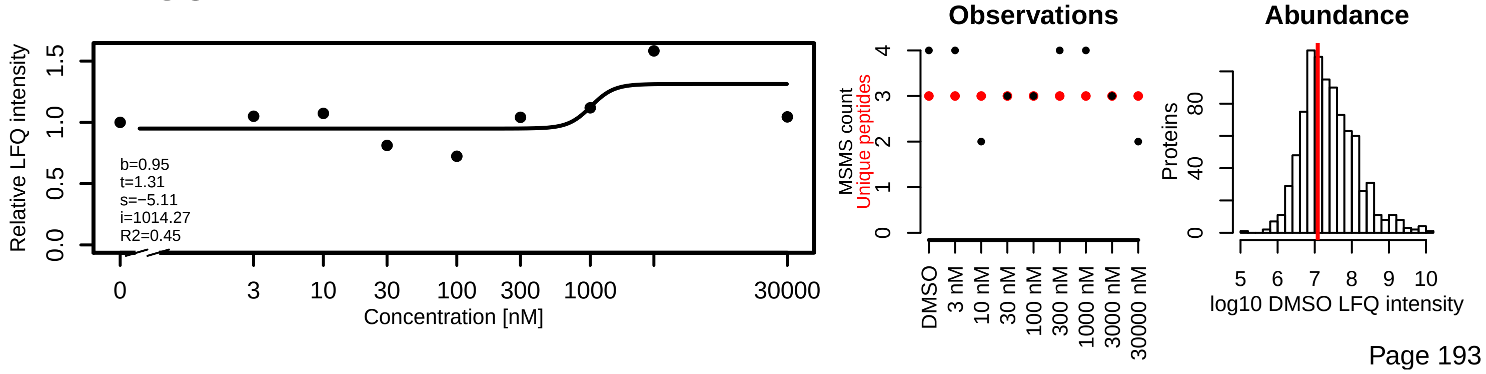

HGSNAT

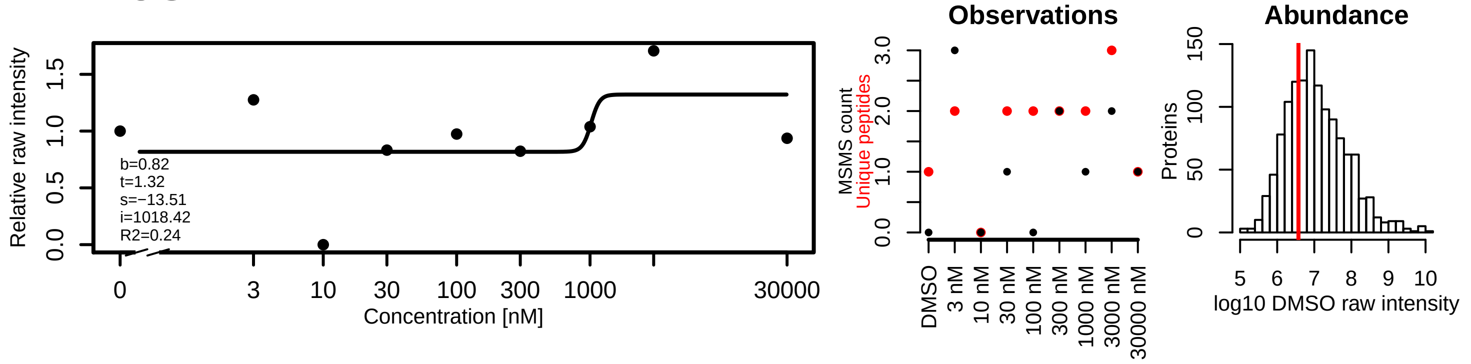

SOAT1

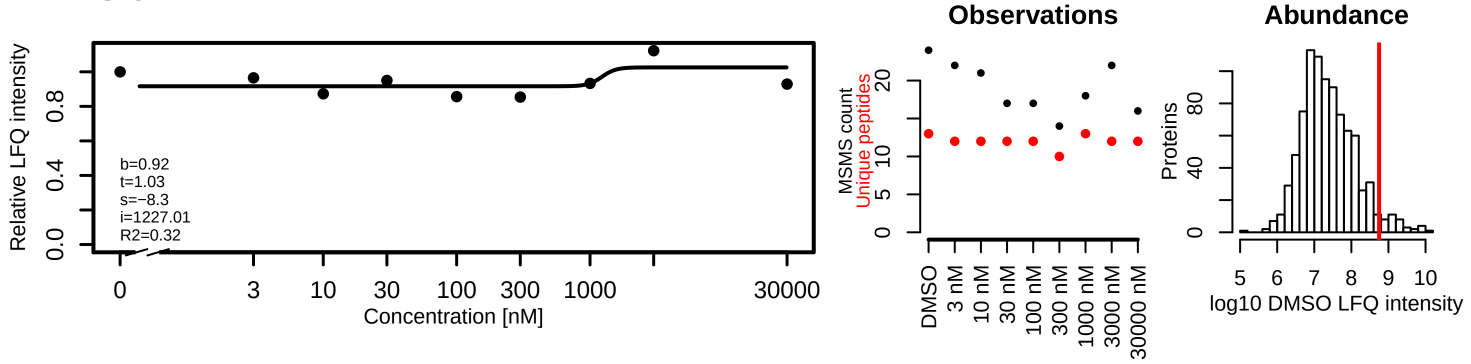

C1GALT1

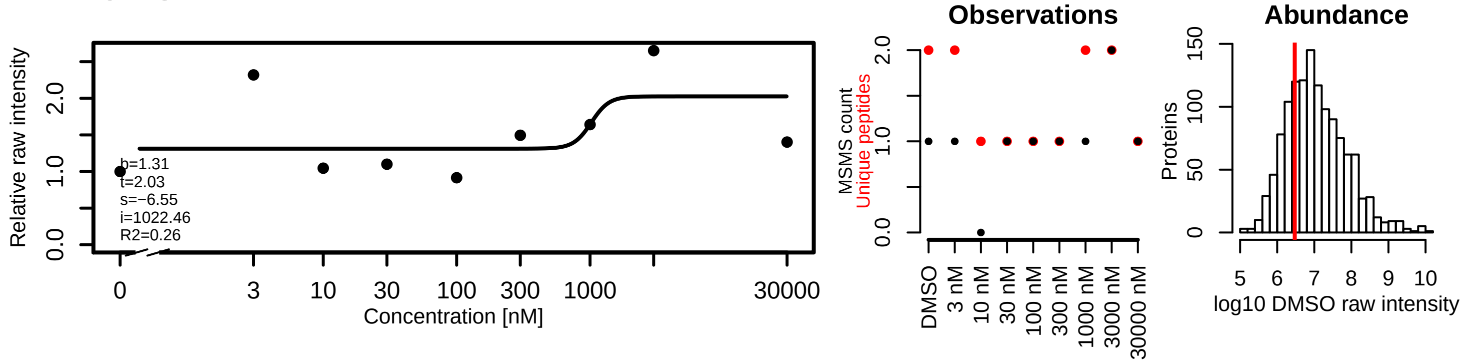

HNRNPH1

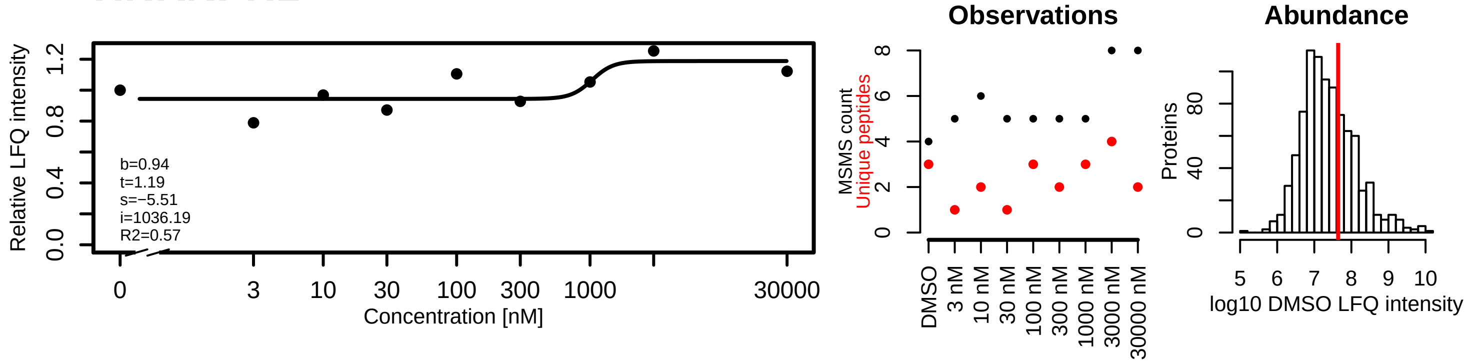

CYC1

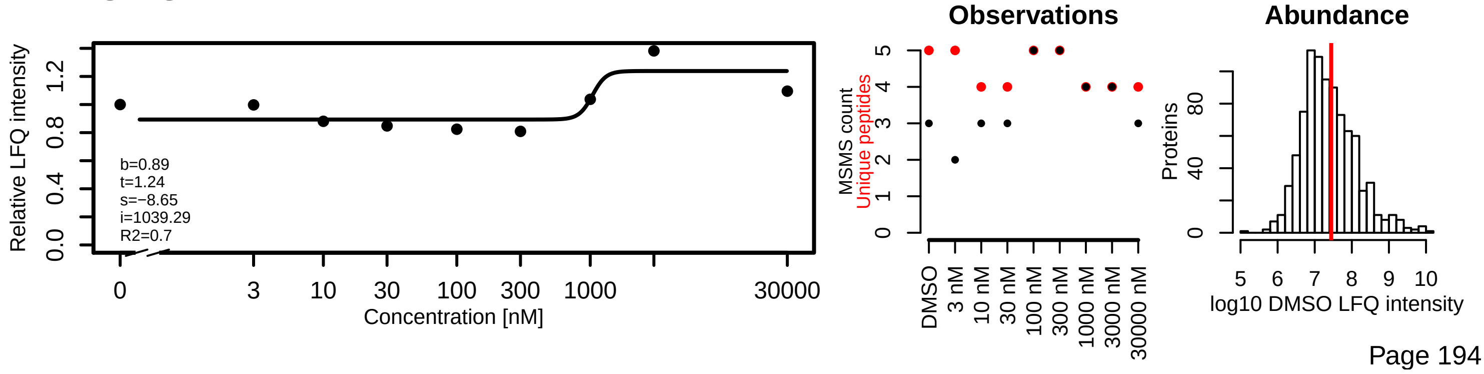

AIFM1

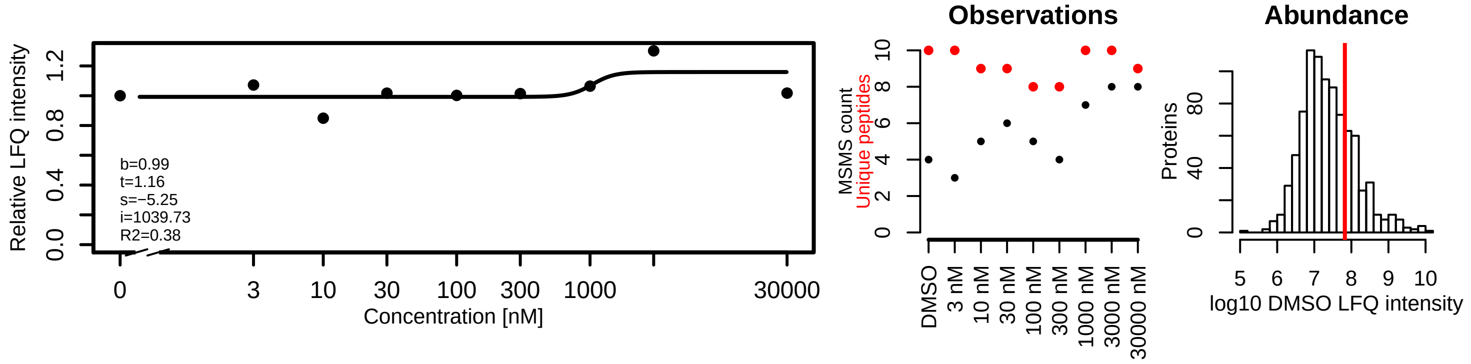

AGPAT3

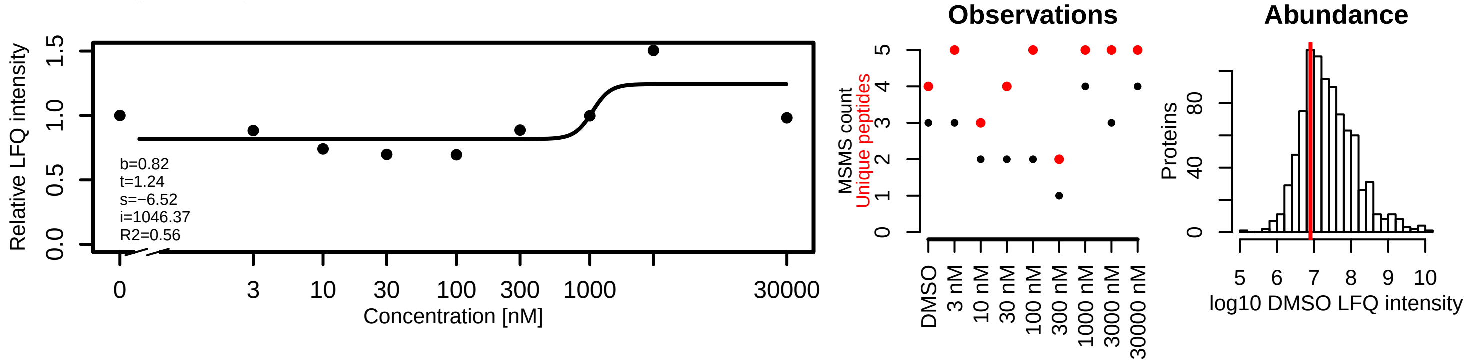

PSMD8

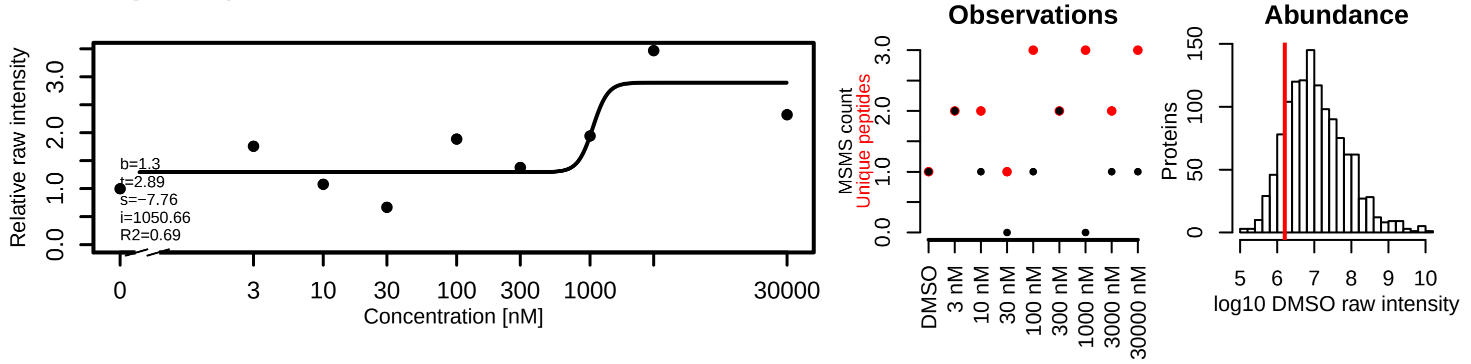

TOMM40

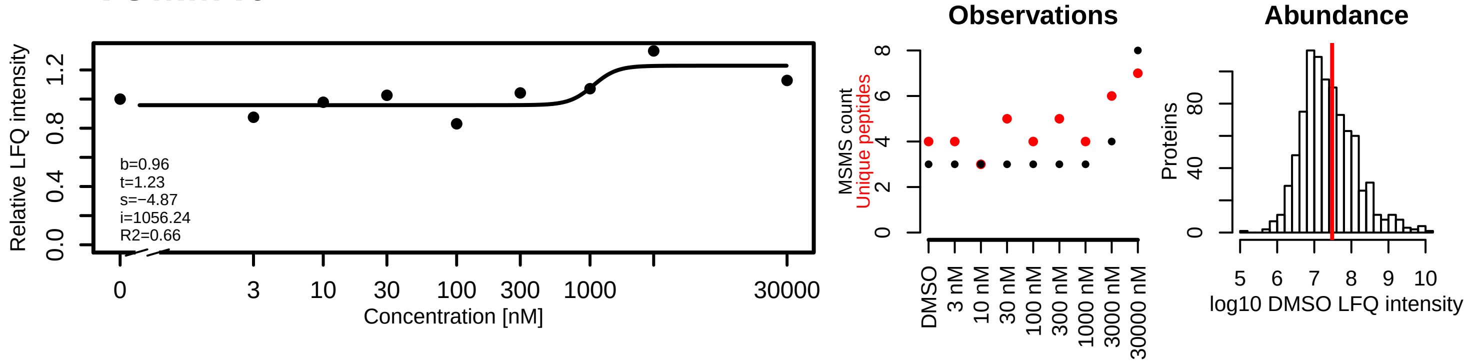

LASS5;CERS5

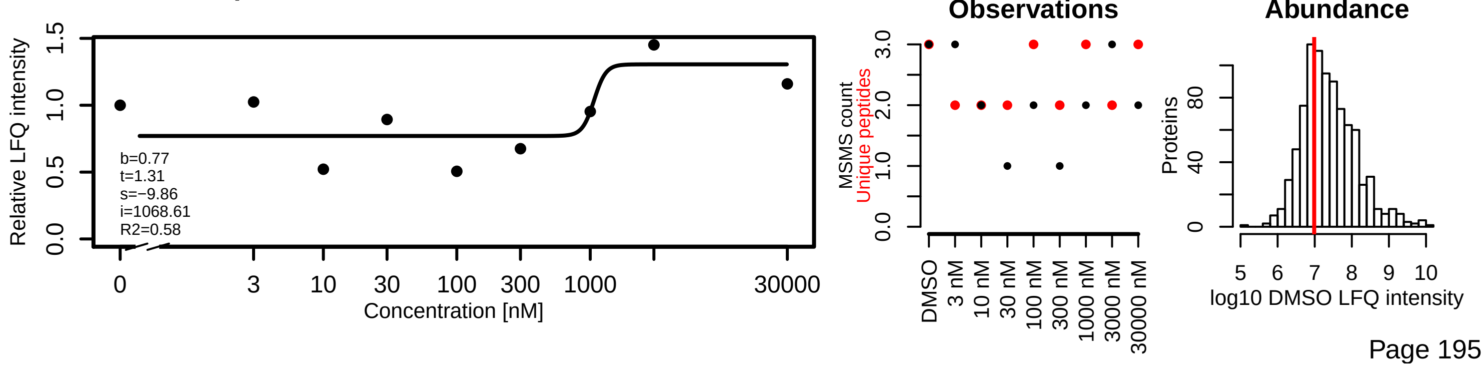

AIG1

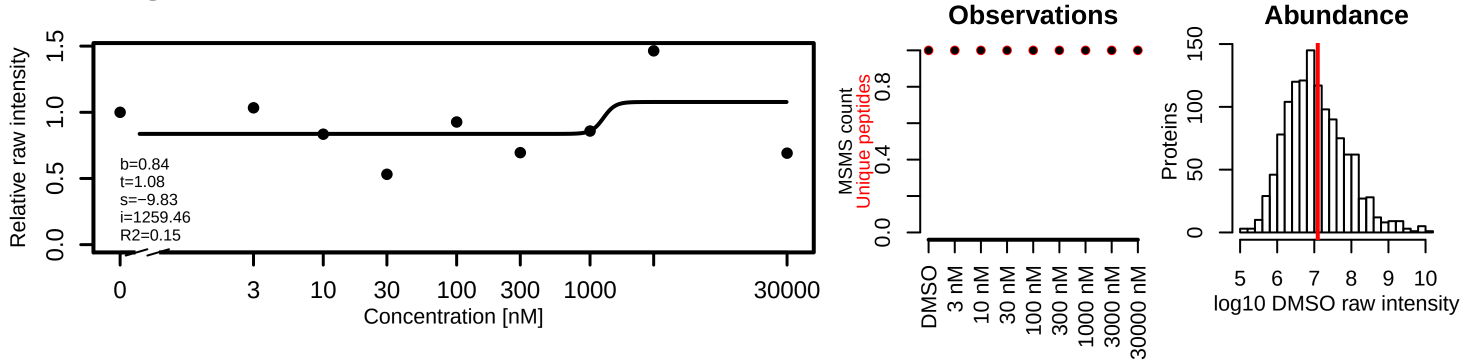

IPO5

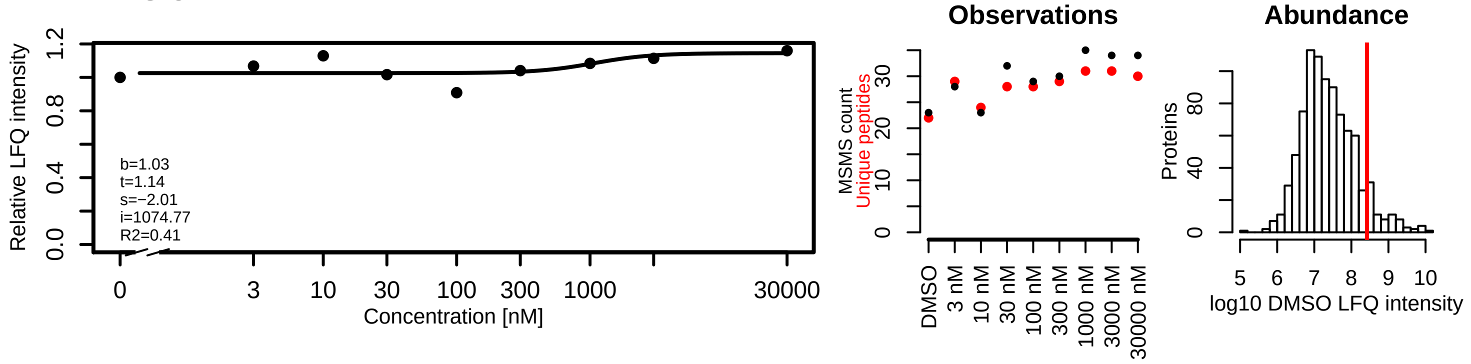

TM9SF2

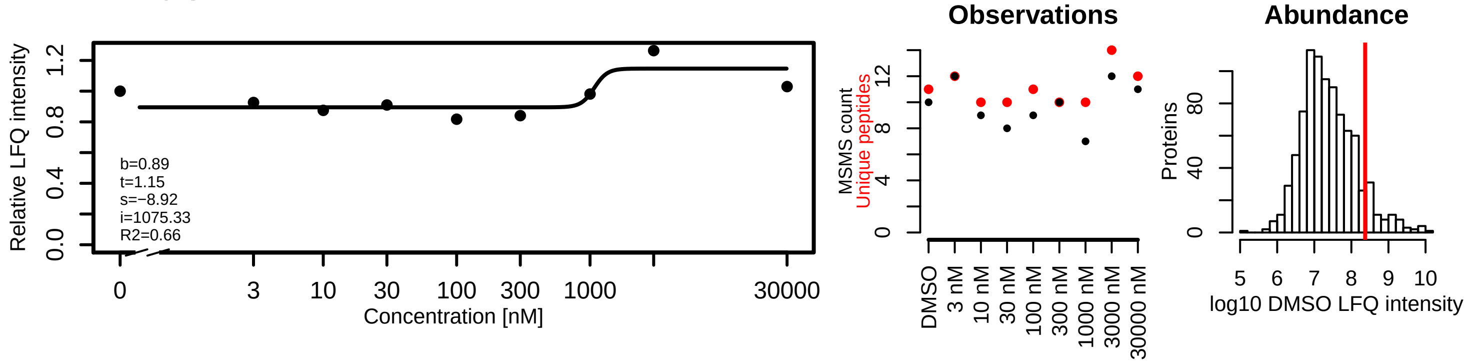

STOM

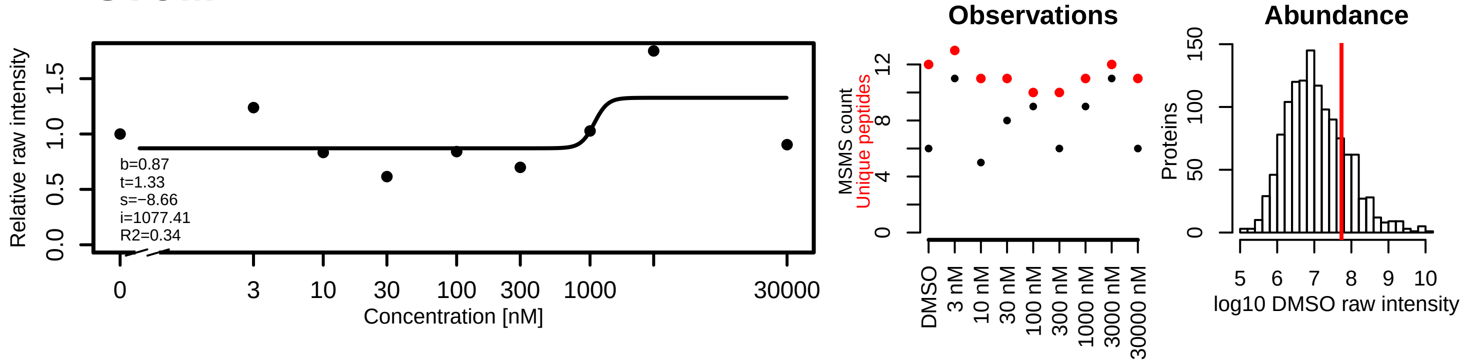

RNF213

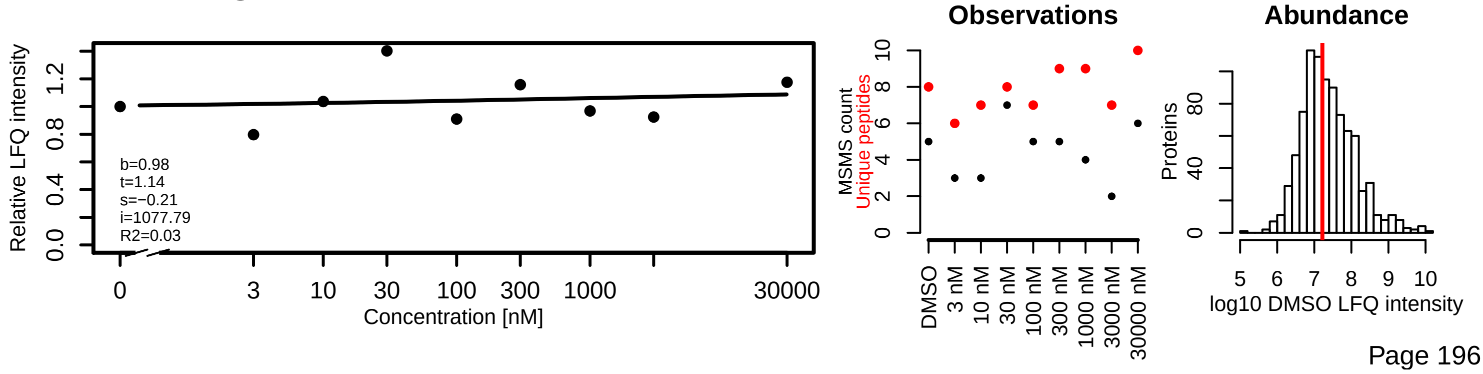

SLC35G1

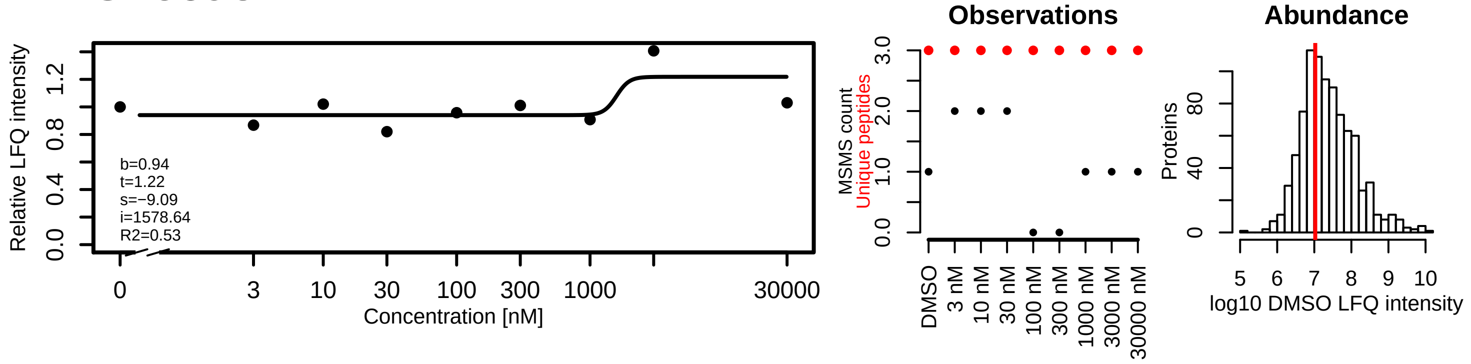

NEIL3

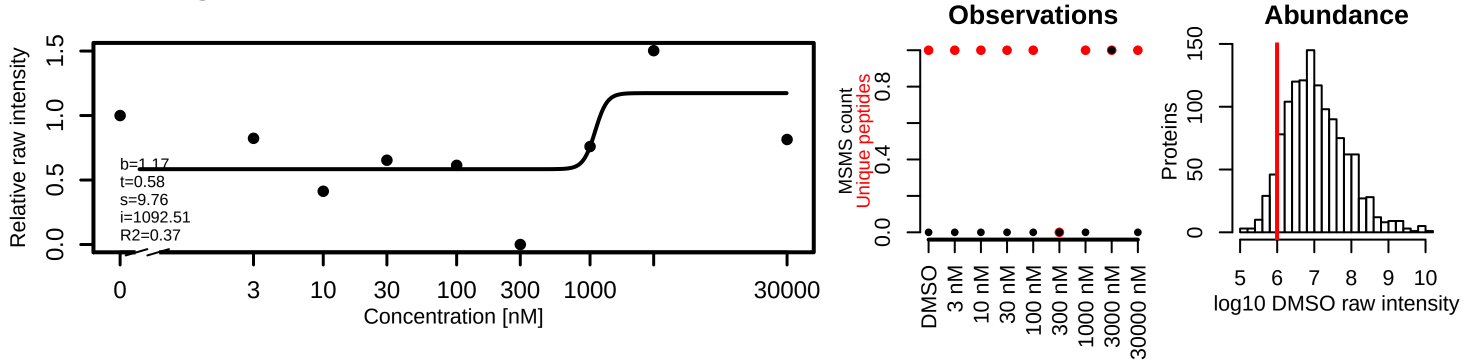

DHRS3

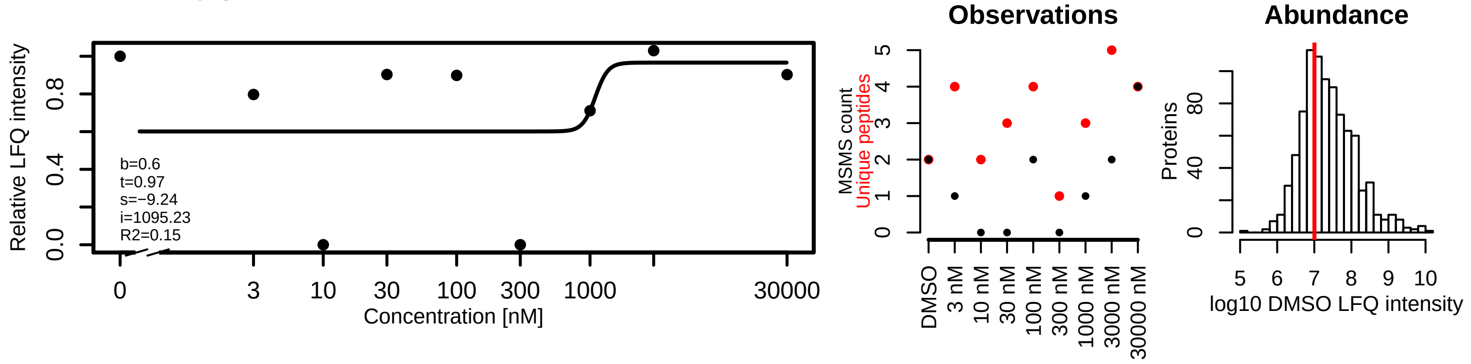

CISD3

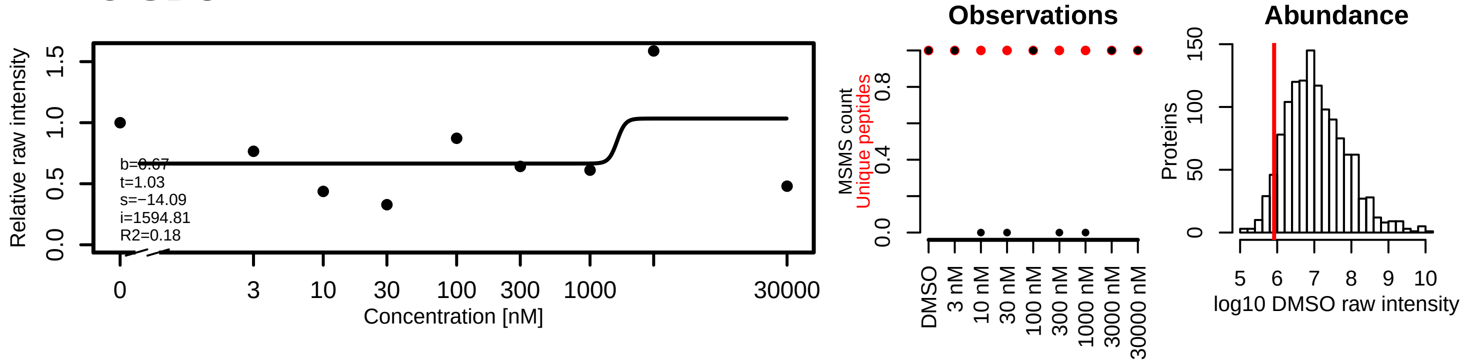

COPB1

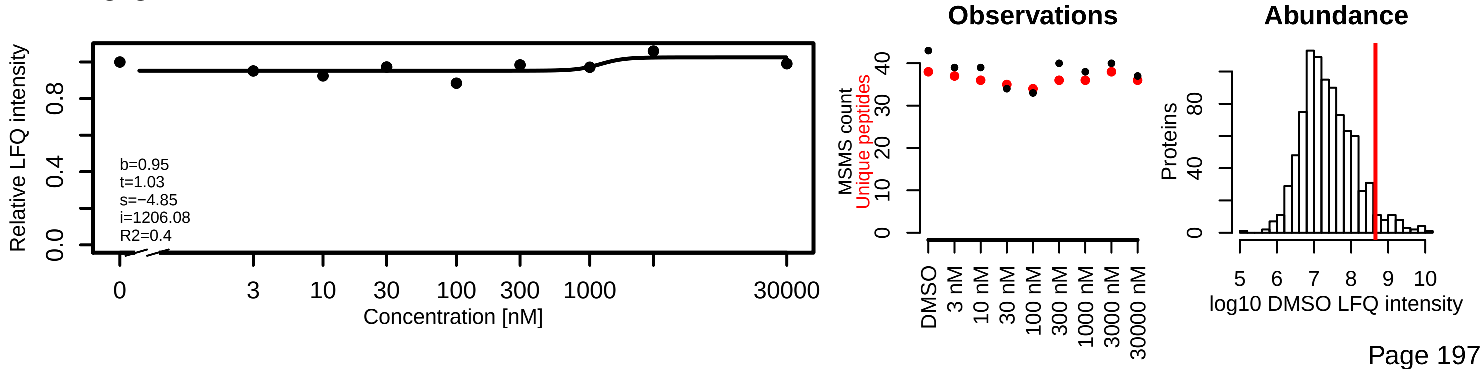

CALU

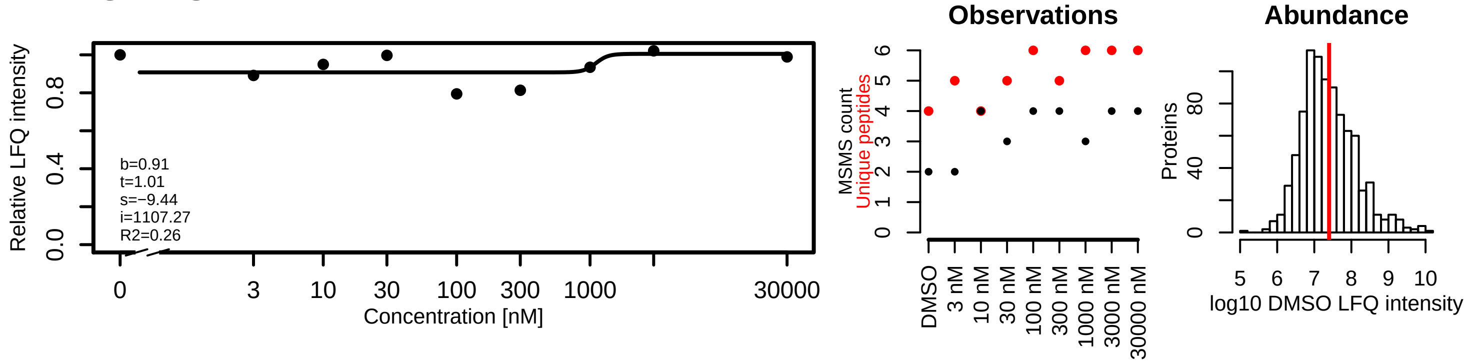

JAGN1

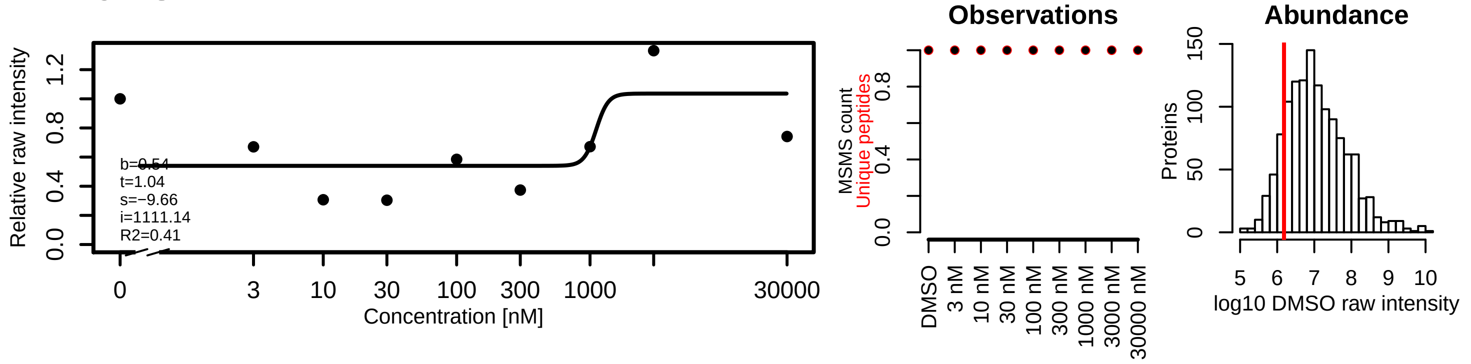

CDS2

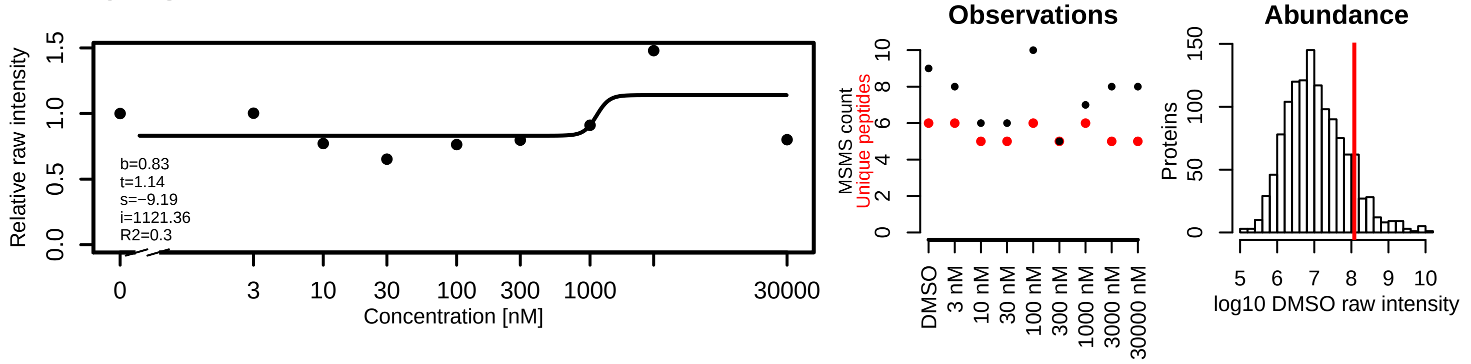

TUBB

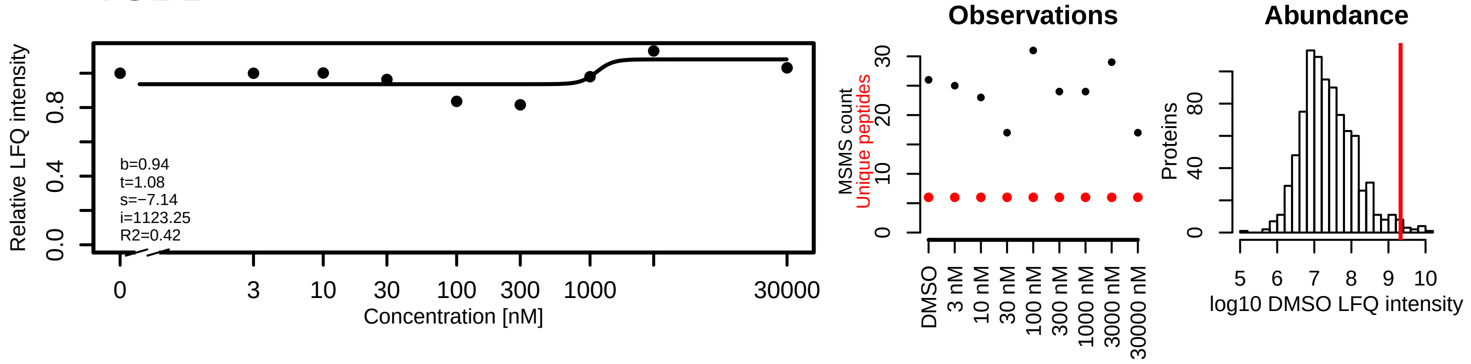

MRPL3

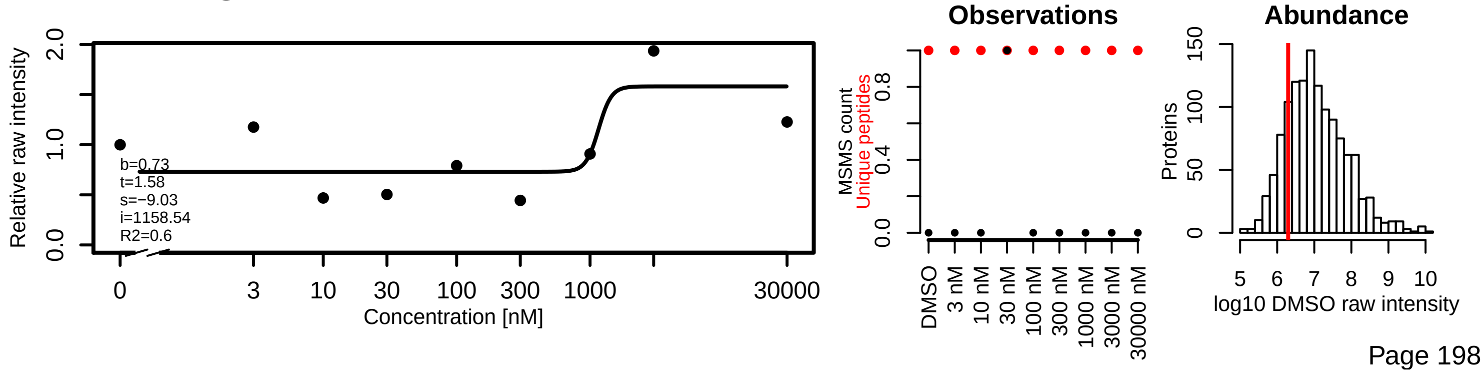

NUP155

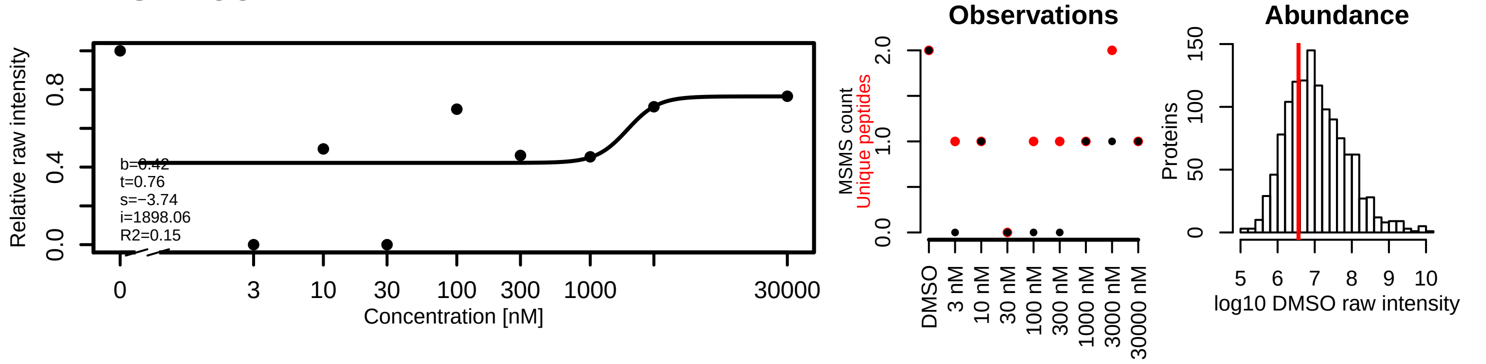

SLC47A1

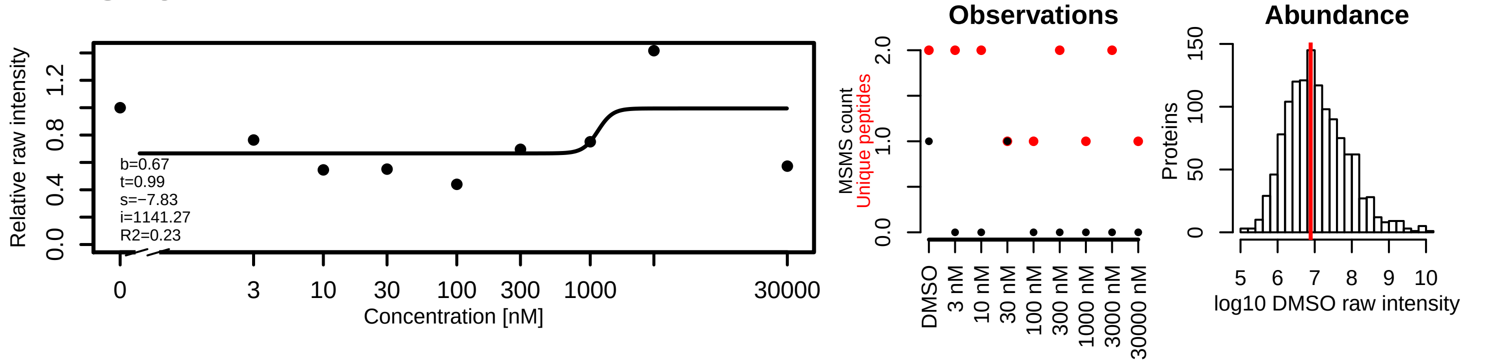

PIGW

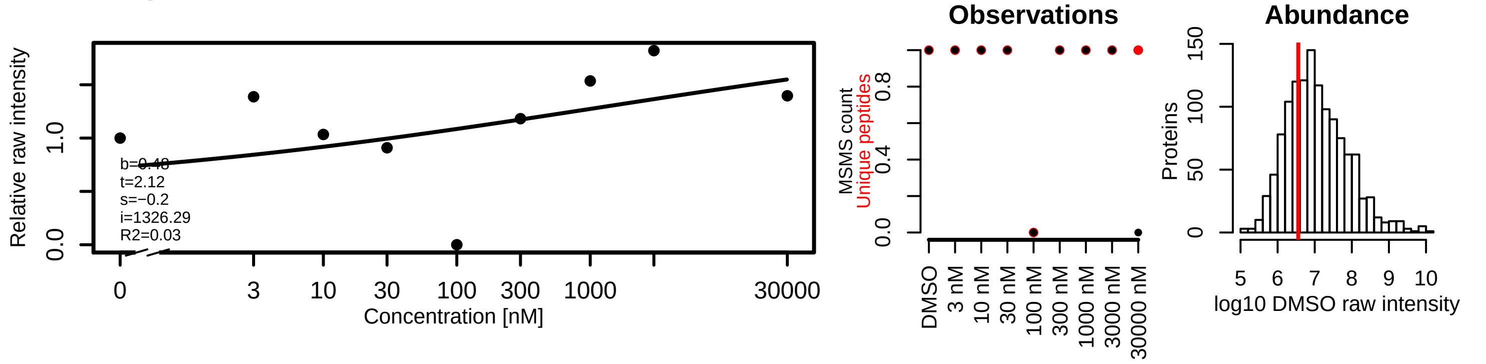

SFXN2

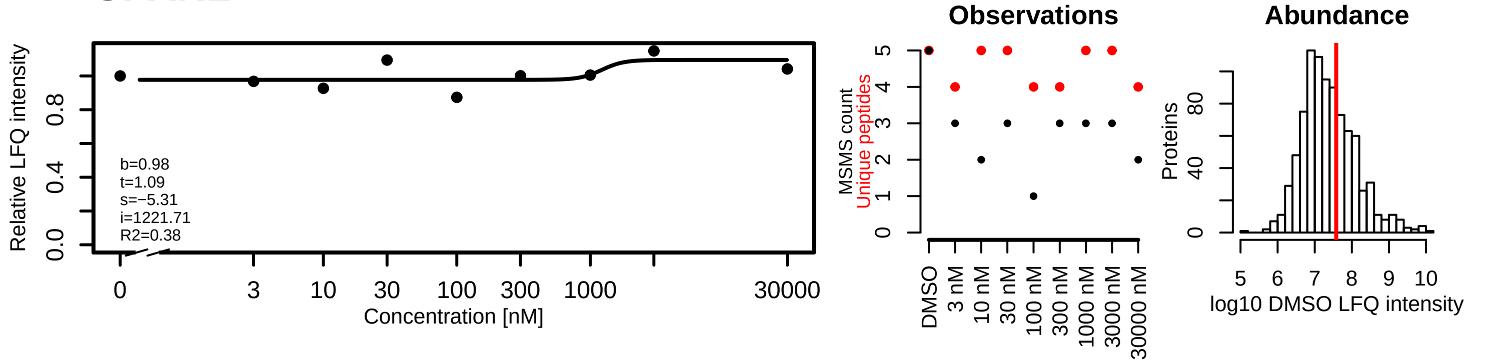

WFS1

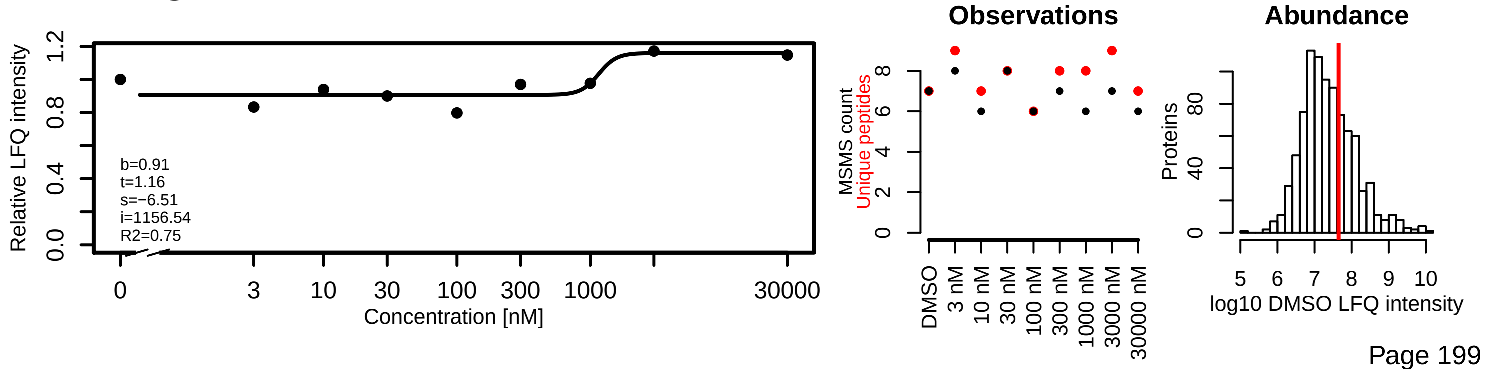

MGST1

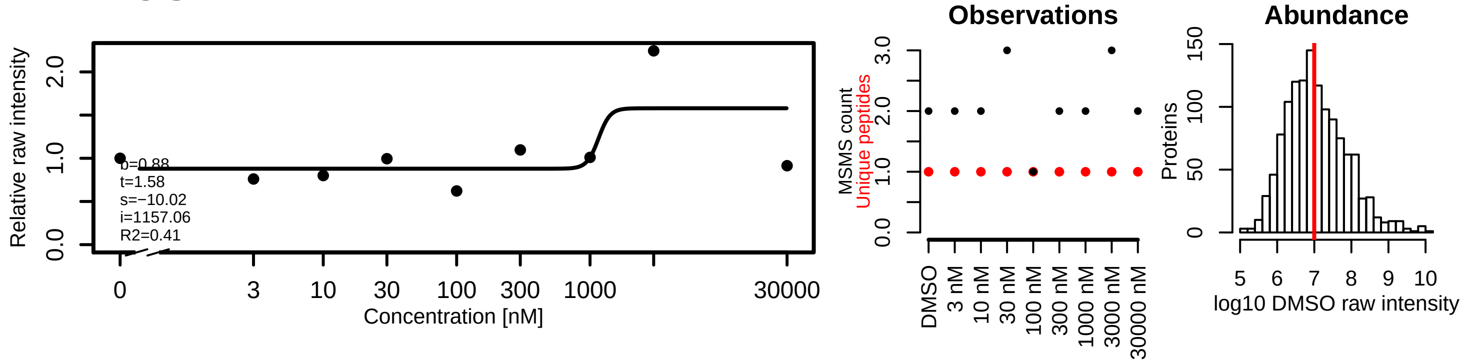

RHOA

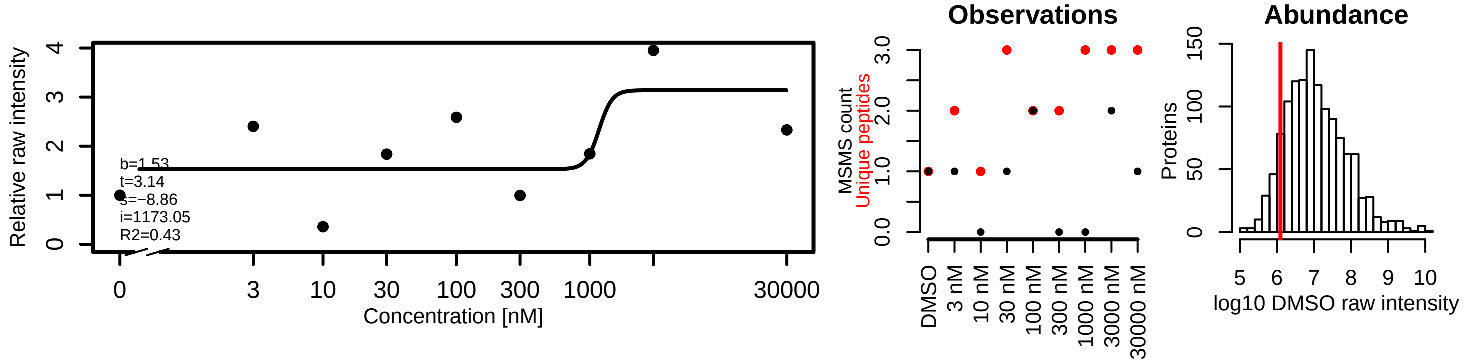

ST7

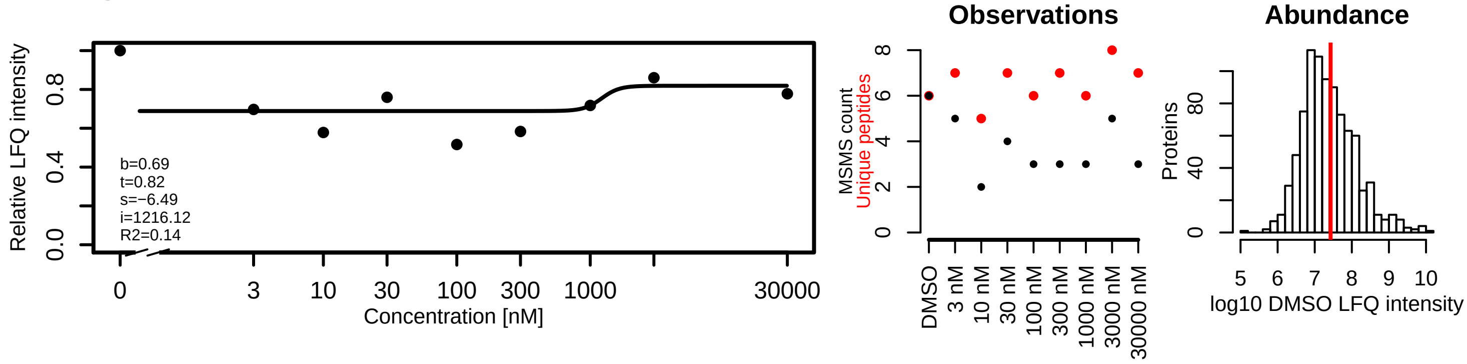

SF1

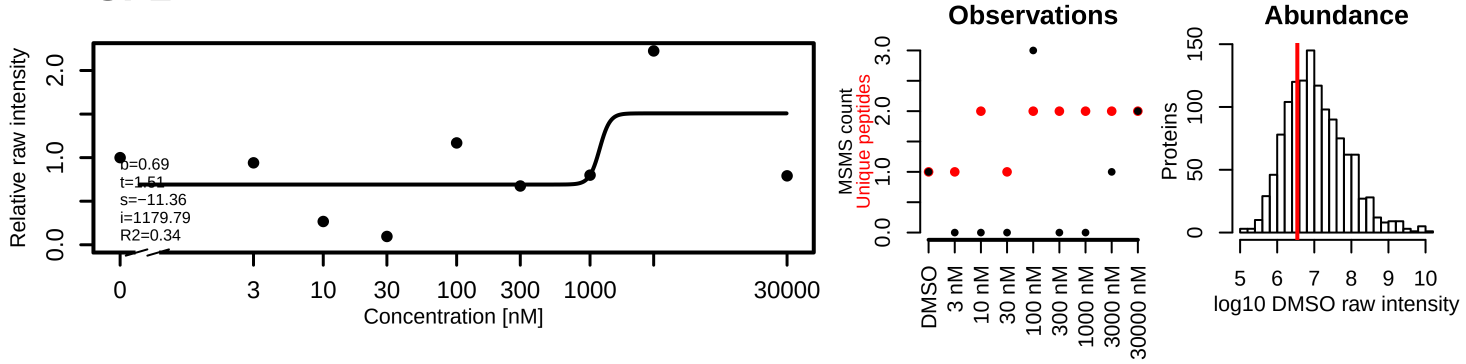

NF1

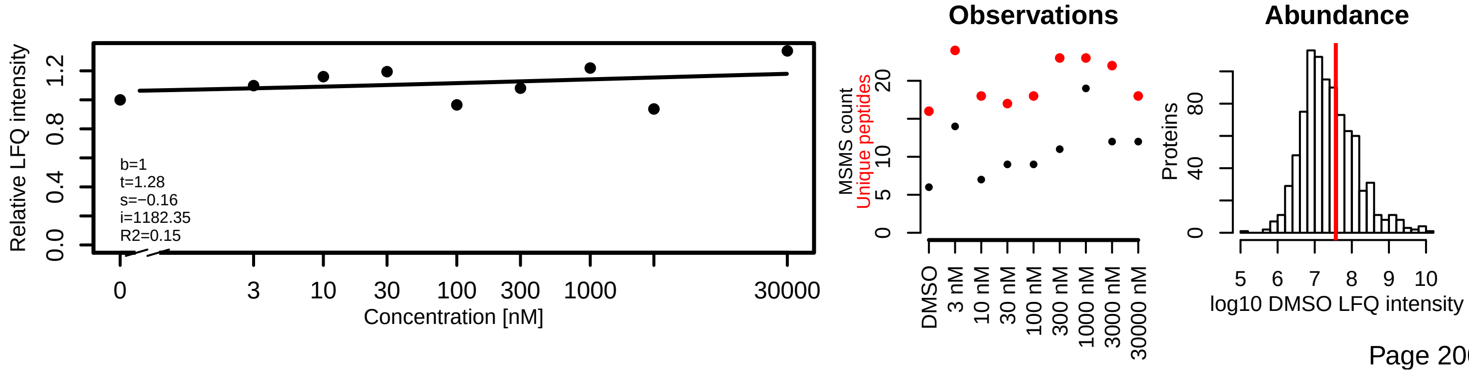

C2orf47

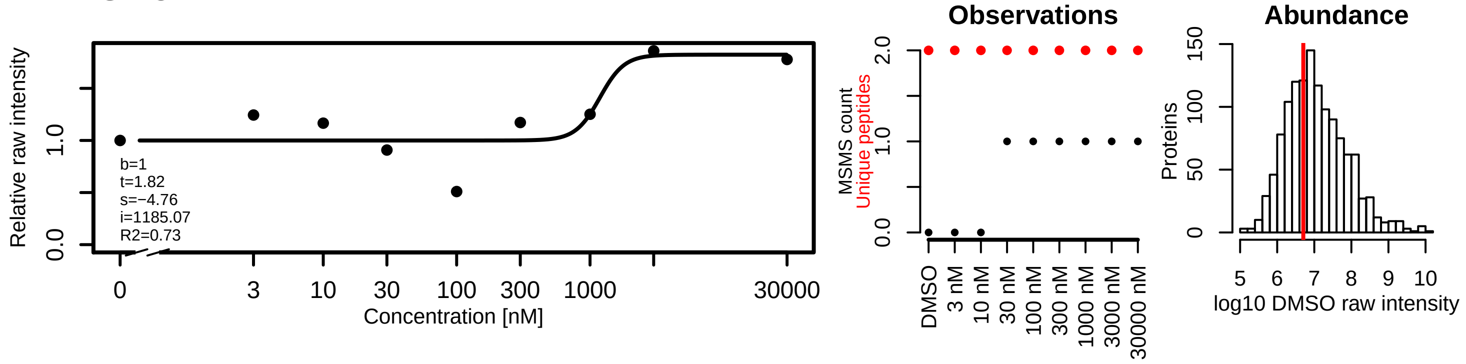

ATP2B1

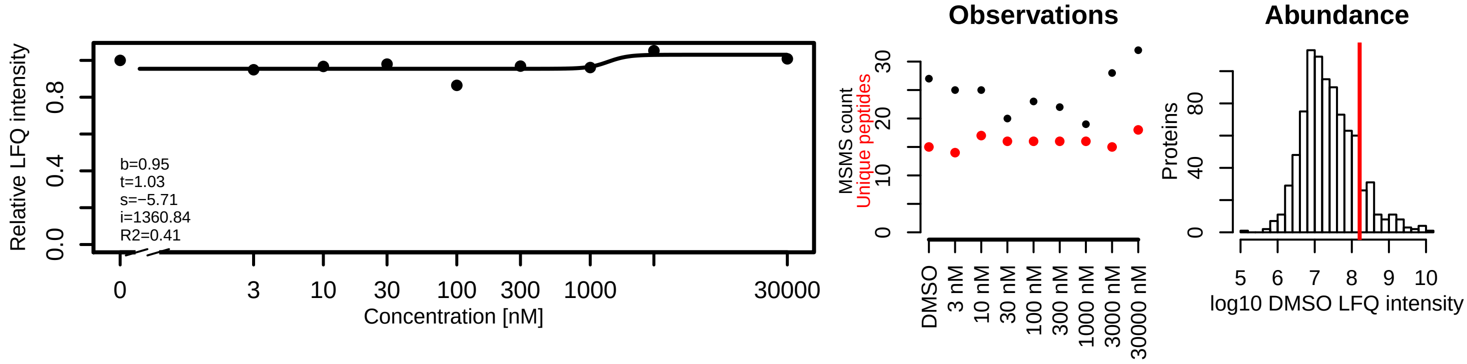

ABCB8

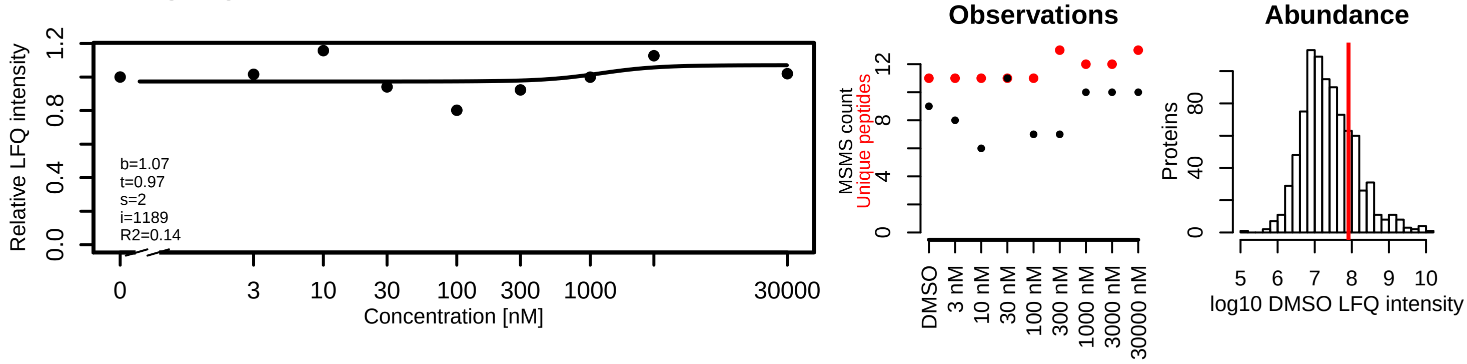

LINC00116

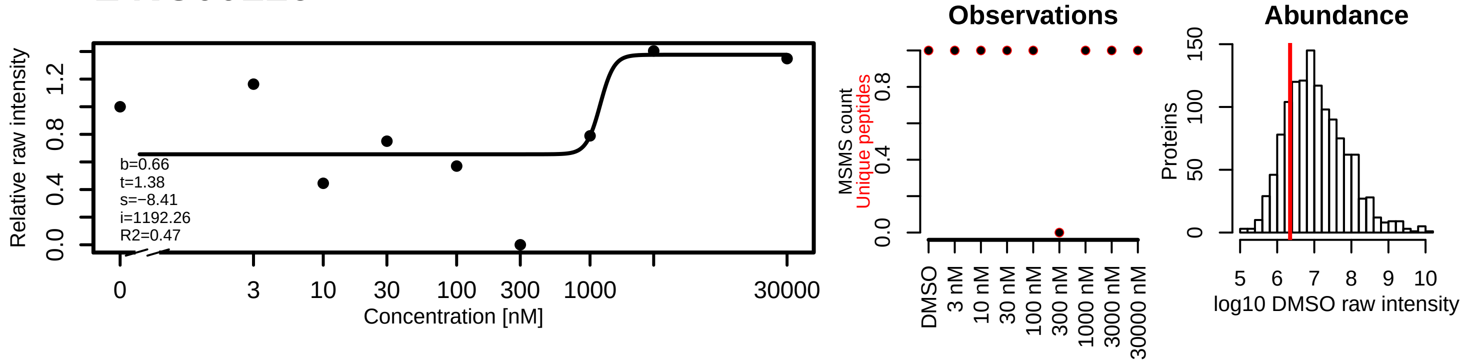

SYNGR1

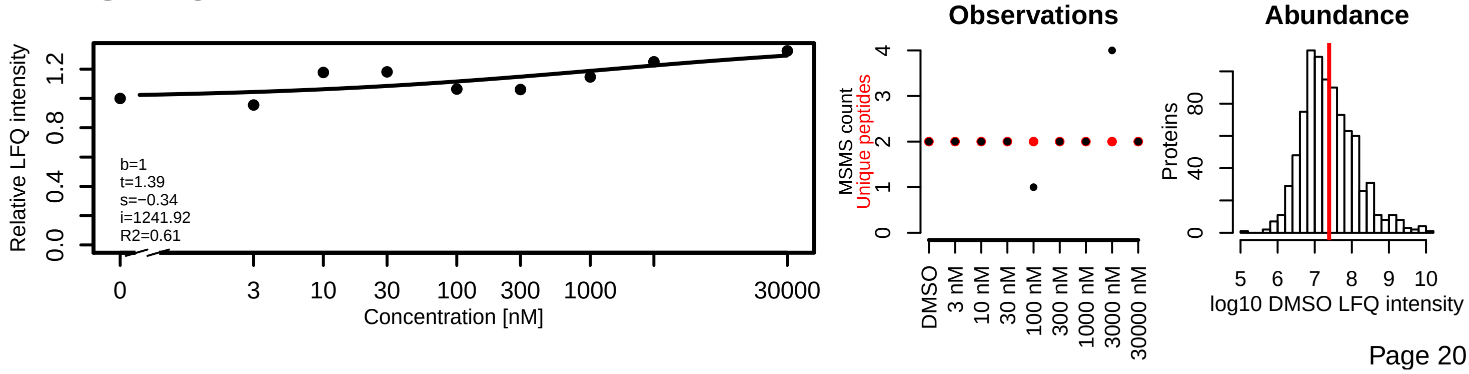

SLC38A1

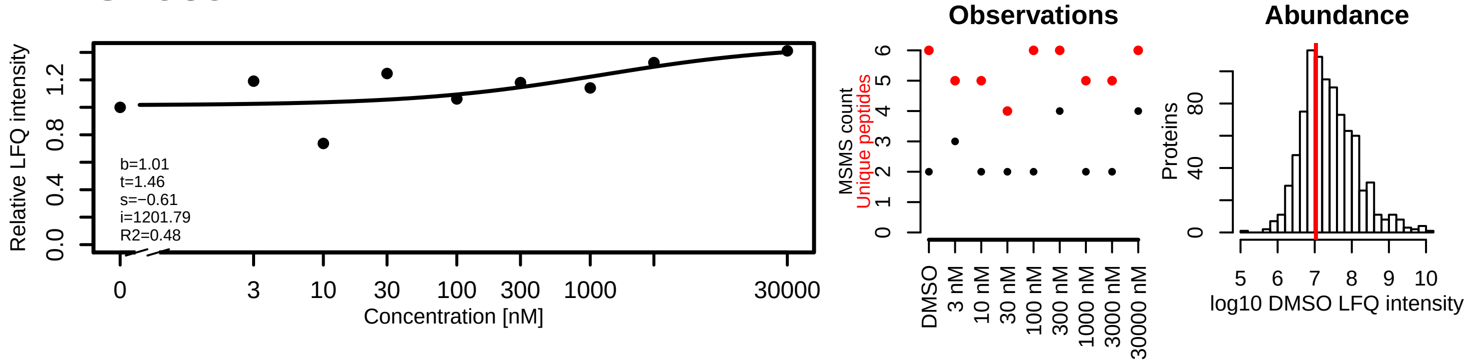

RAB21

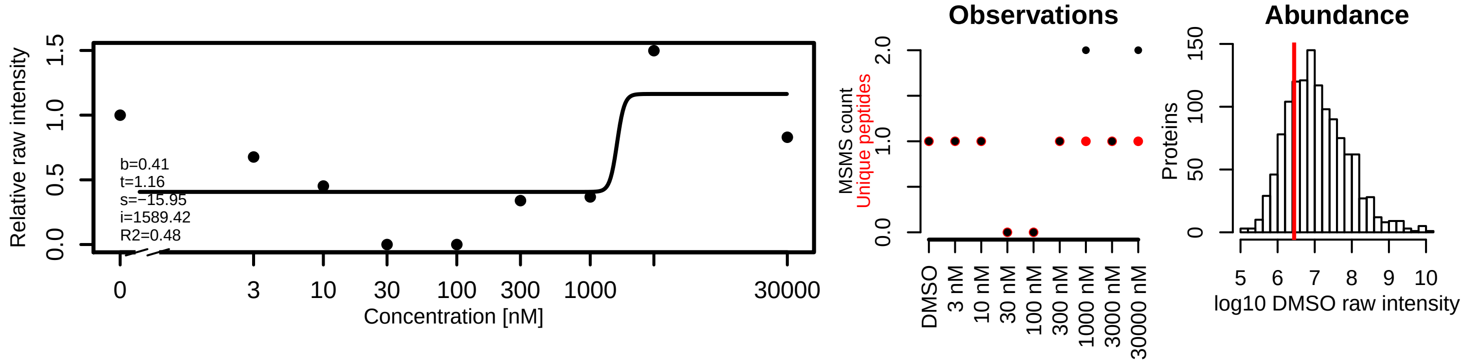

RUVBL1

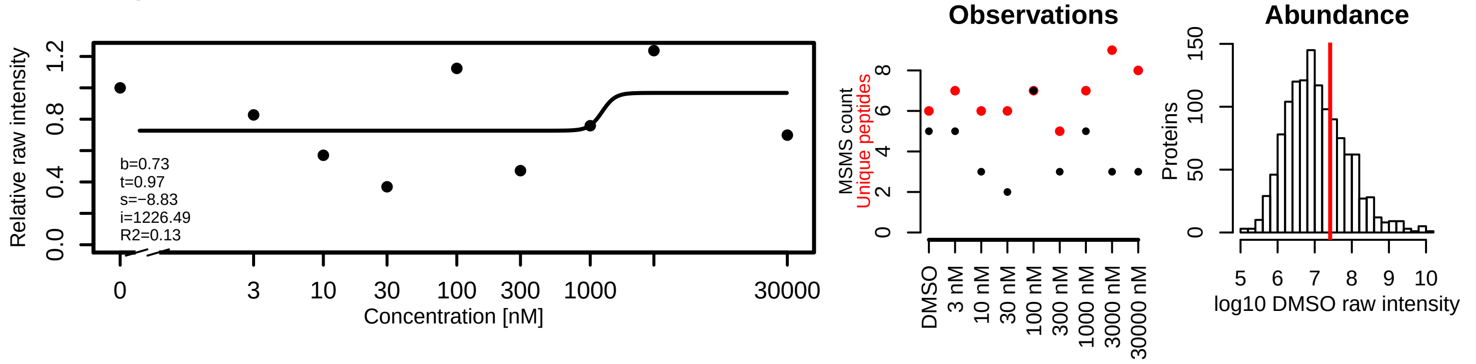

LPCAT3

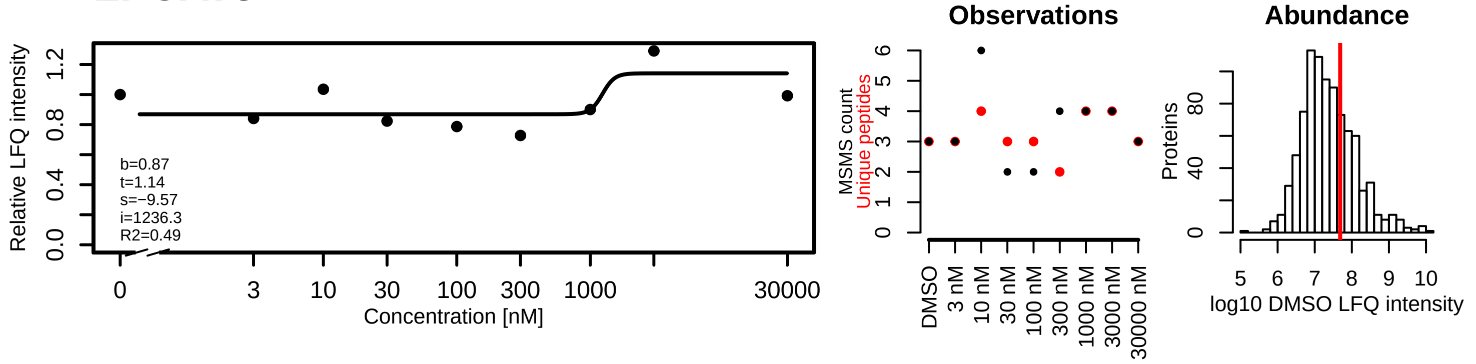

PEX11B

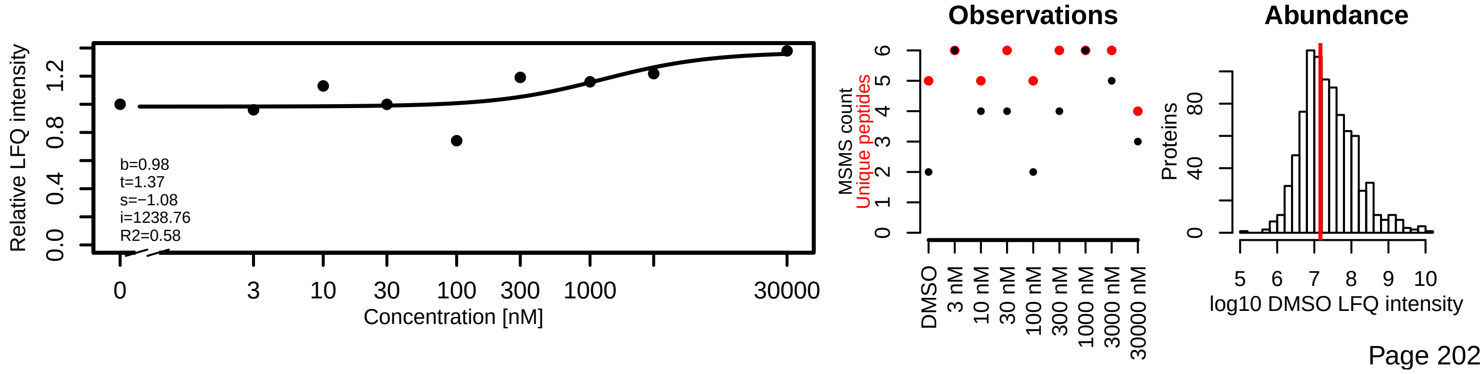

NDUFS3

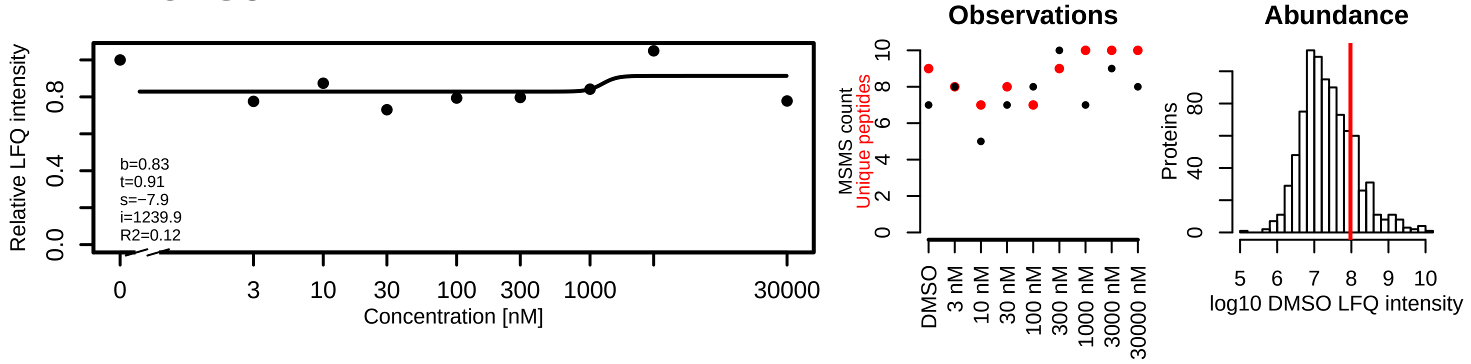

PNKD

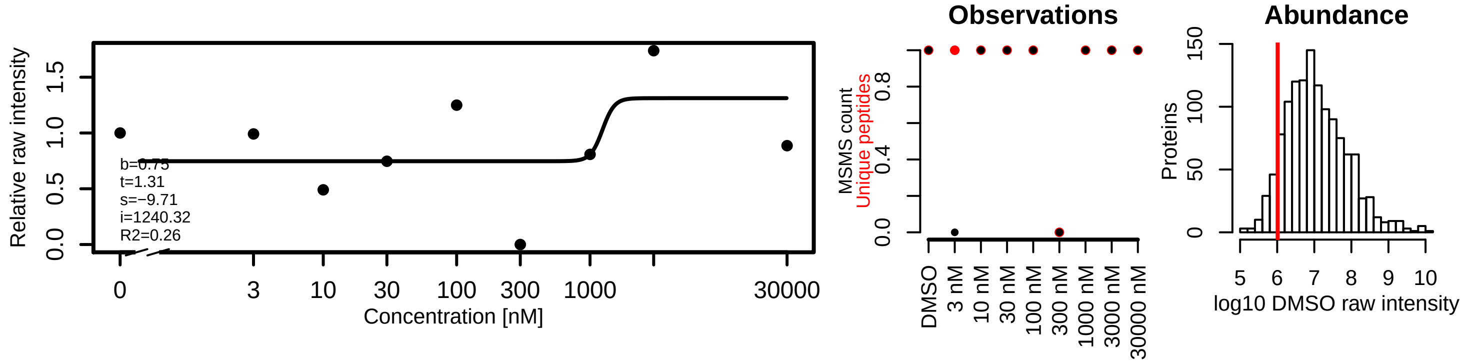

PHB2

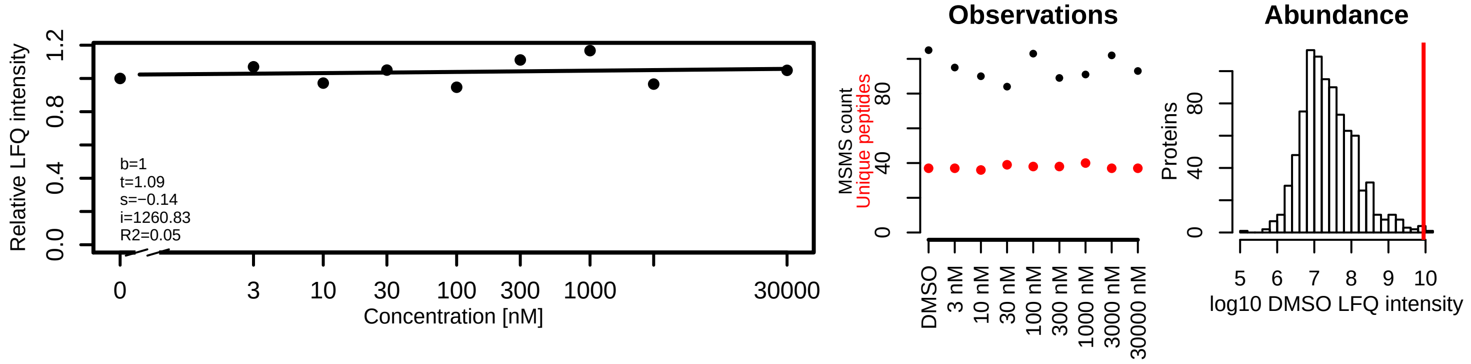

STON2

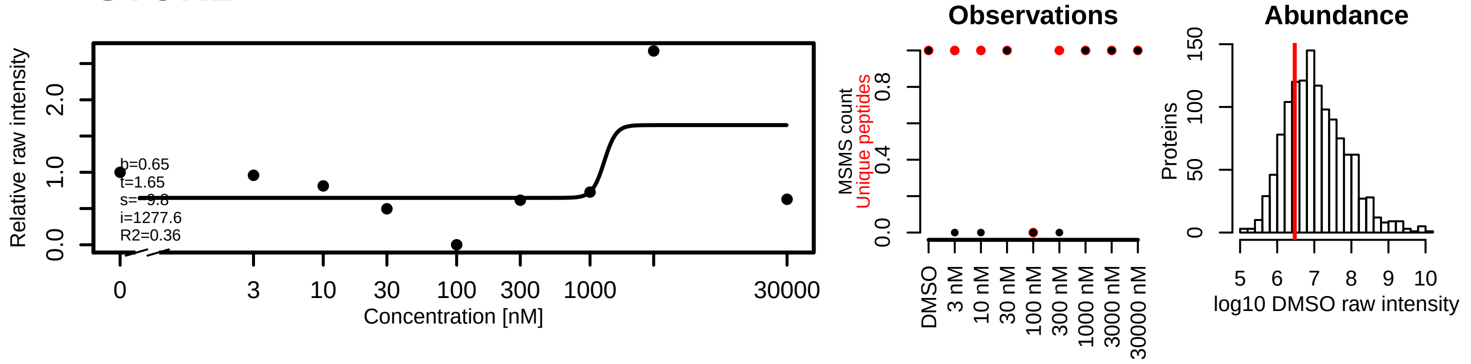

NDUFA6

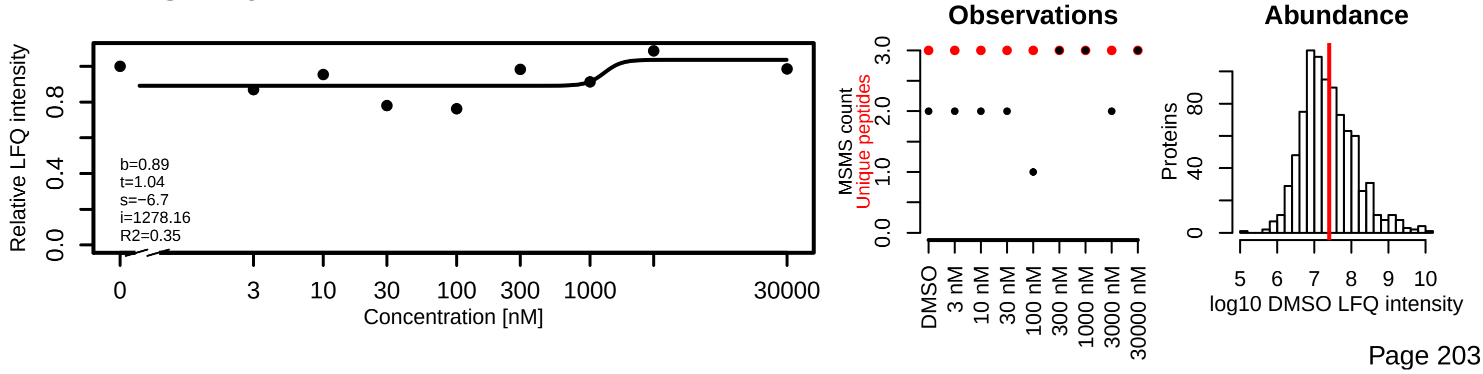

SEC22B

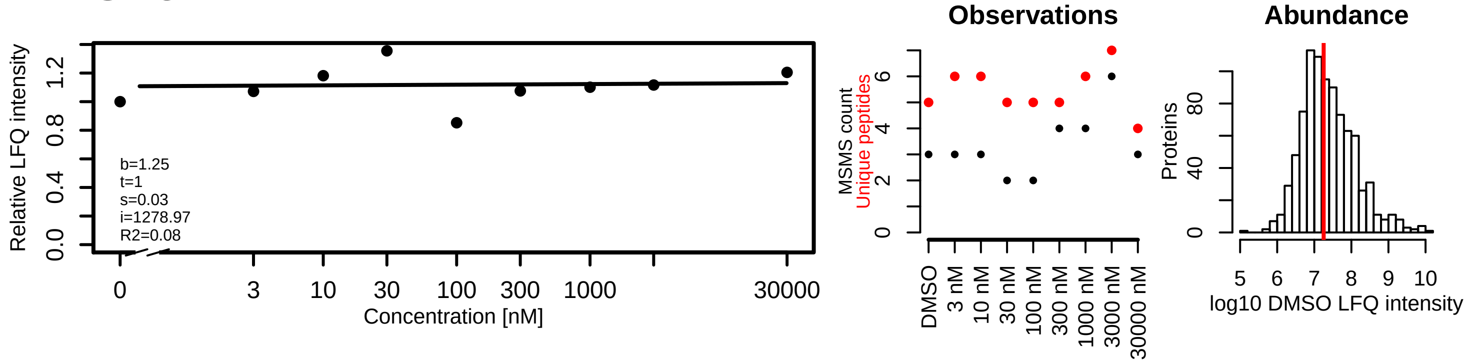

IPO7

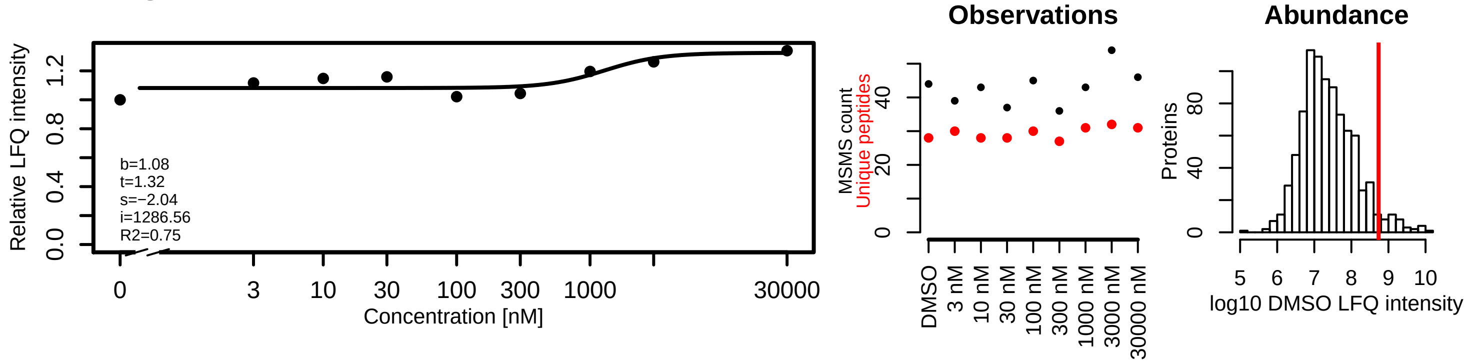

UQCRFS1;UQCRFS1P1

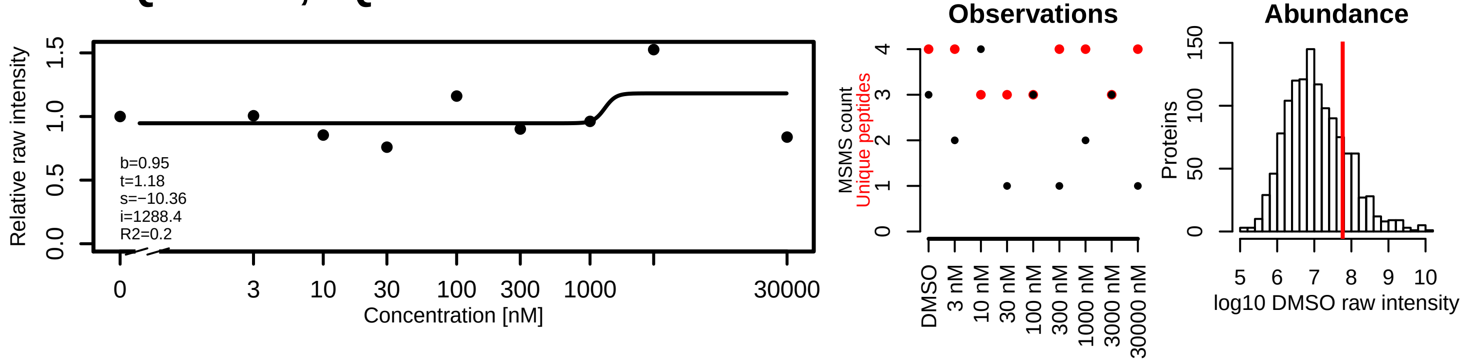

TIMM23;TIMM23B

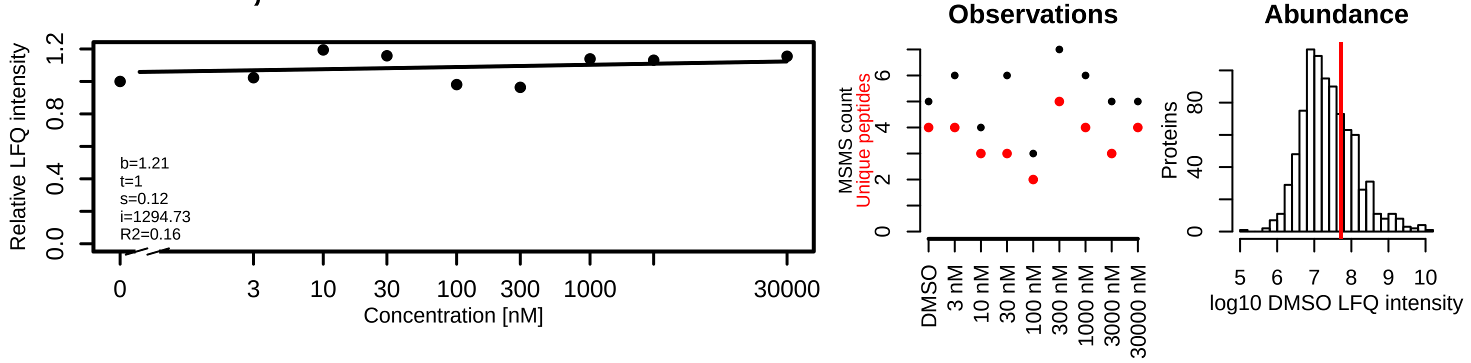

CTNNB1

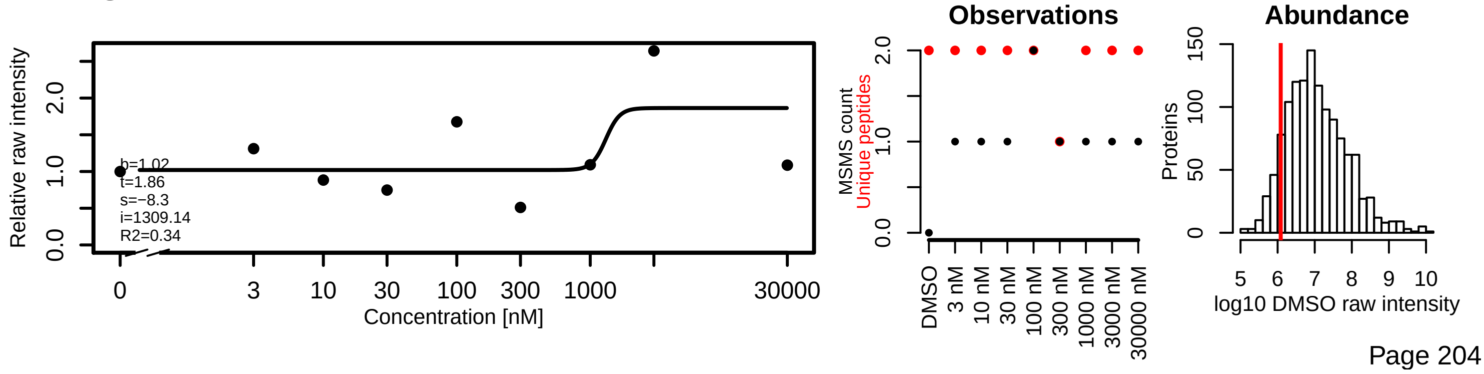

RER1

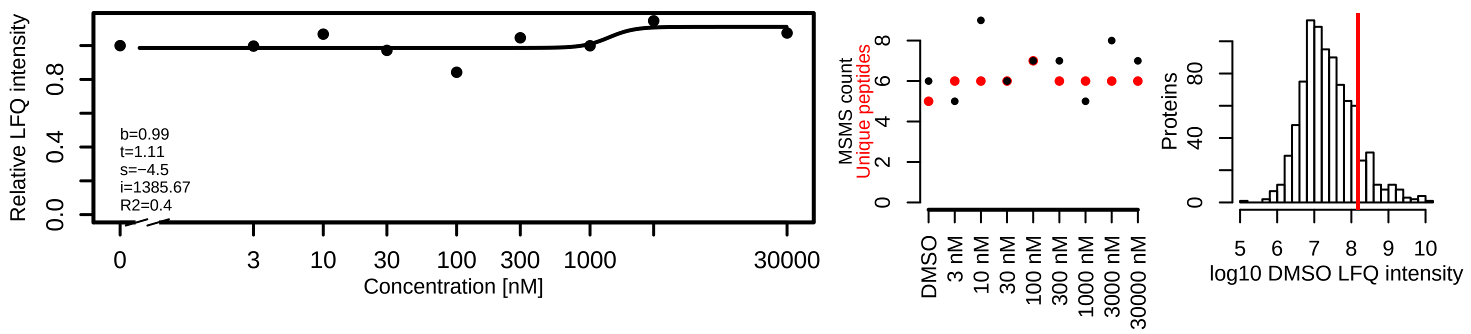

ATP6V1A

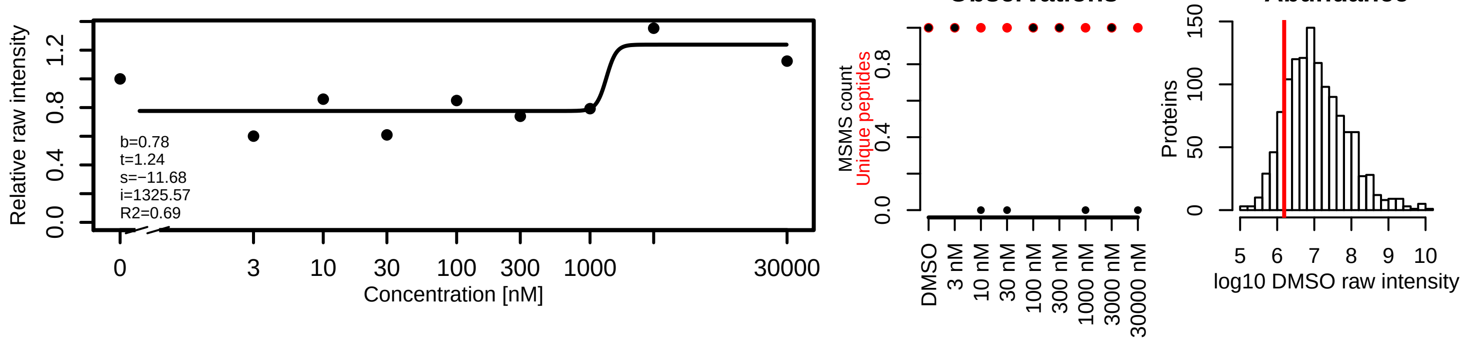

MED23

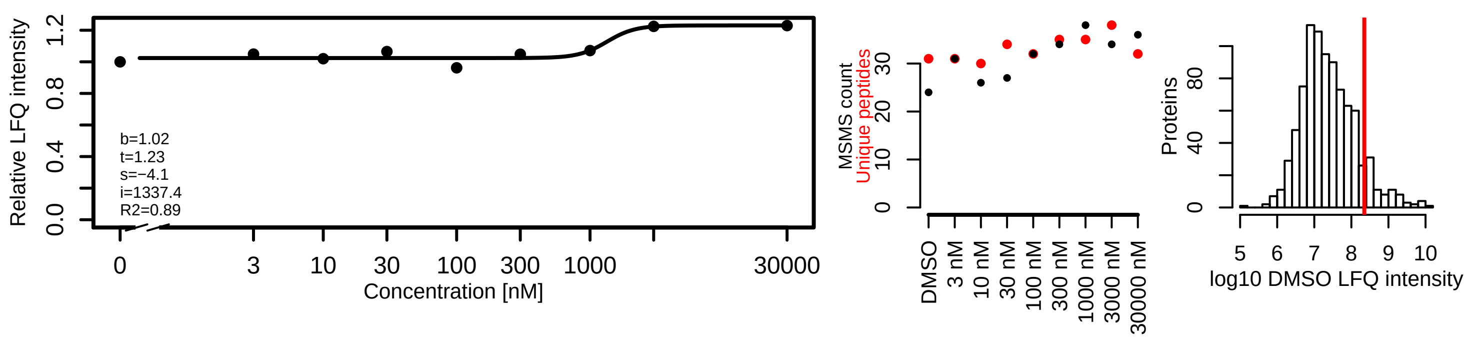

TMEM258

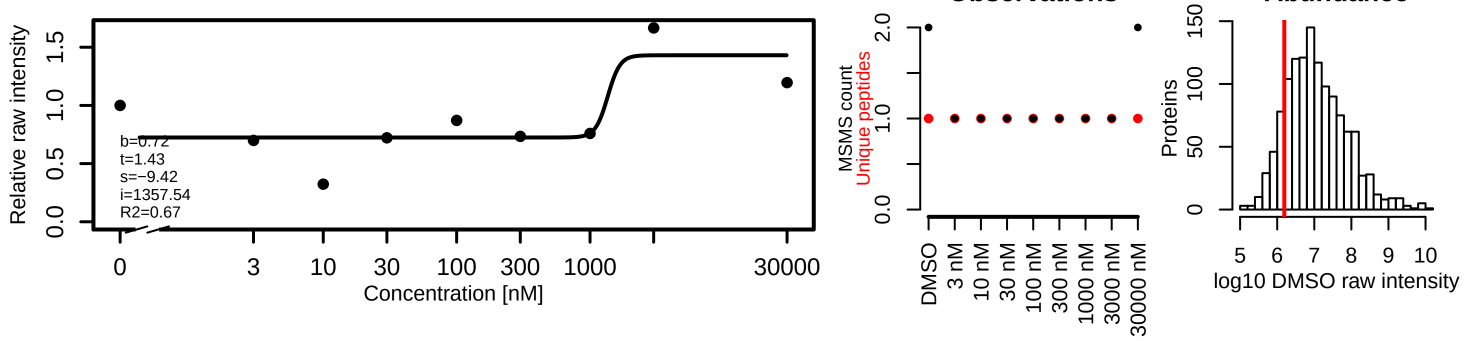

MFSD5

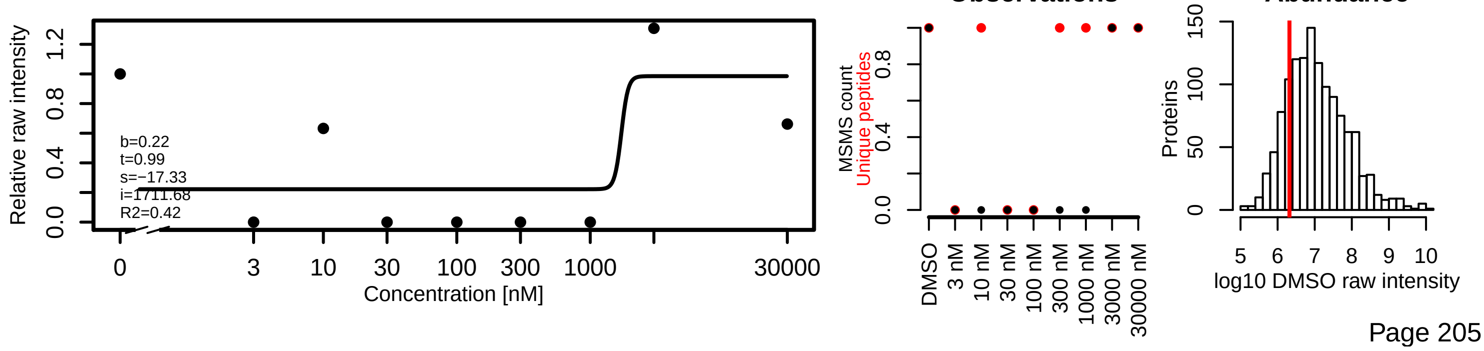

HLA−F

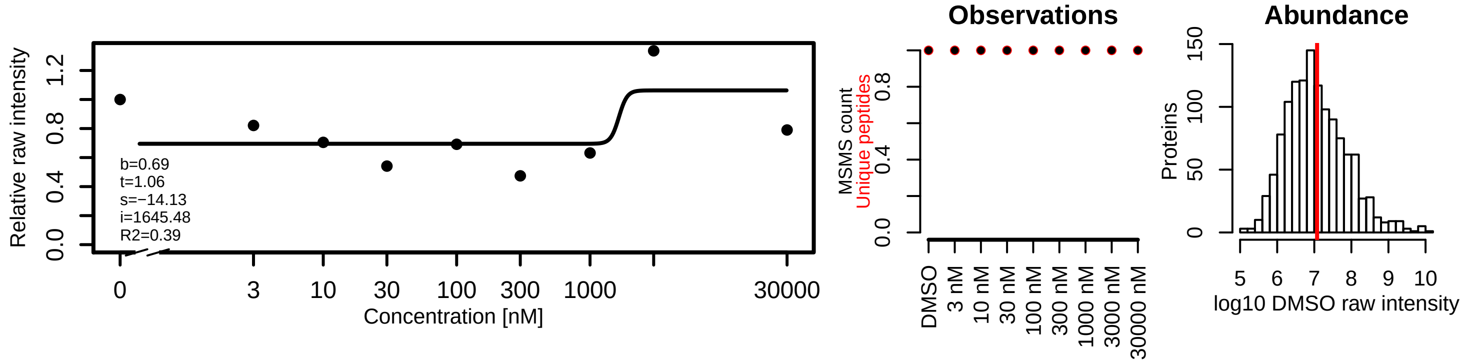

NDUFA5;DKFZp781K1356

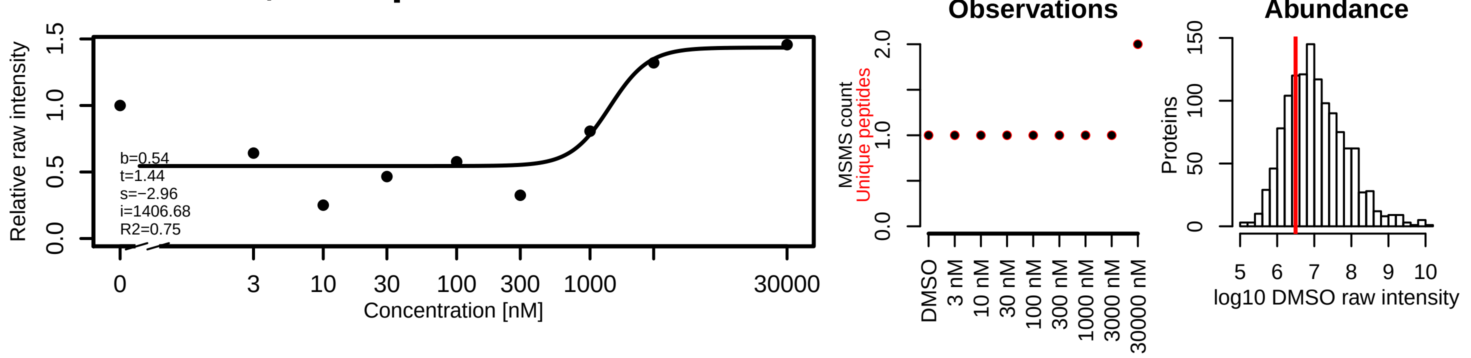

TUBB6

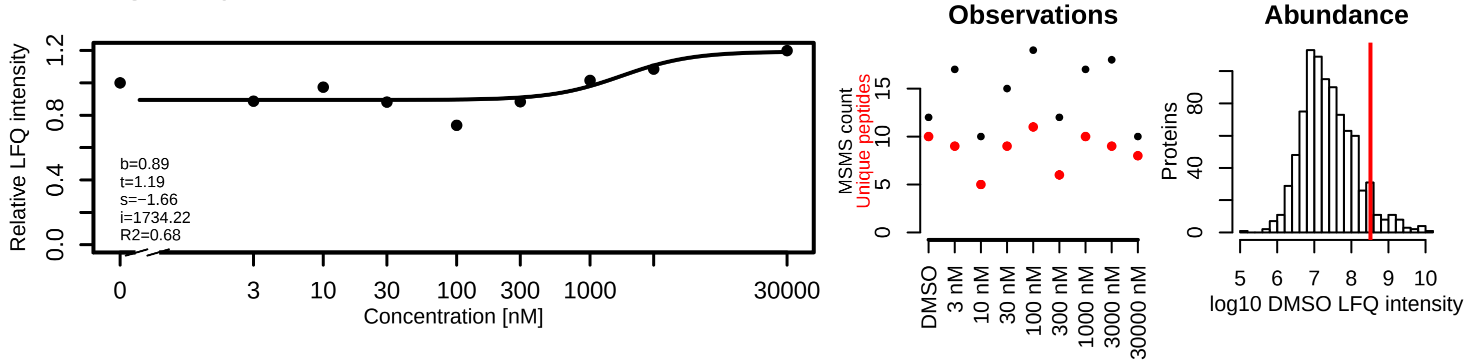

MINOS1

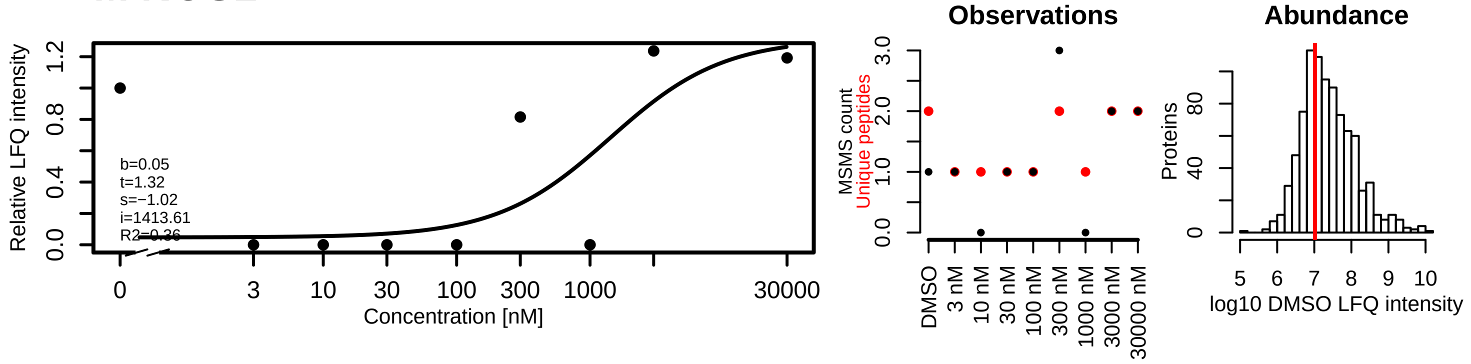

MAGT1

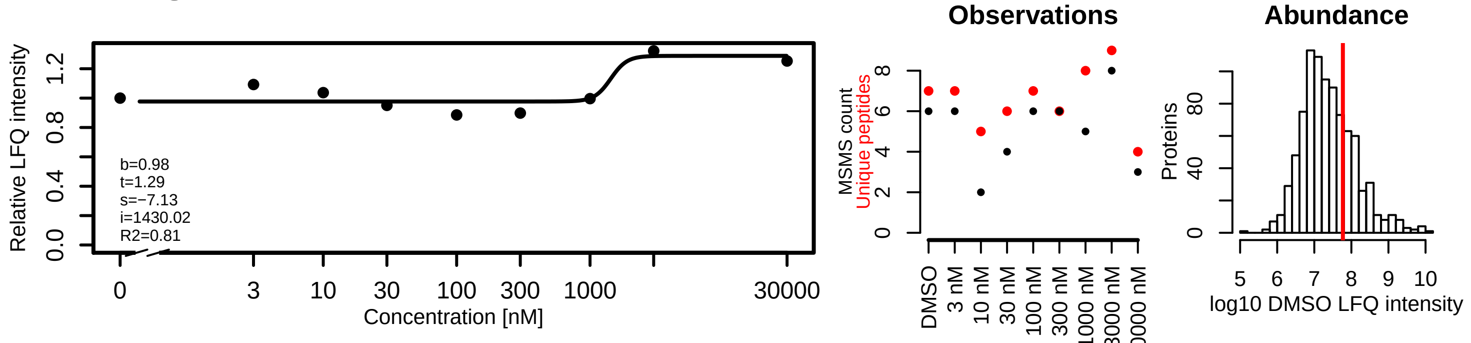

LEPROT

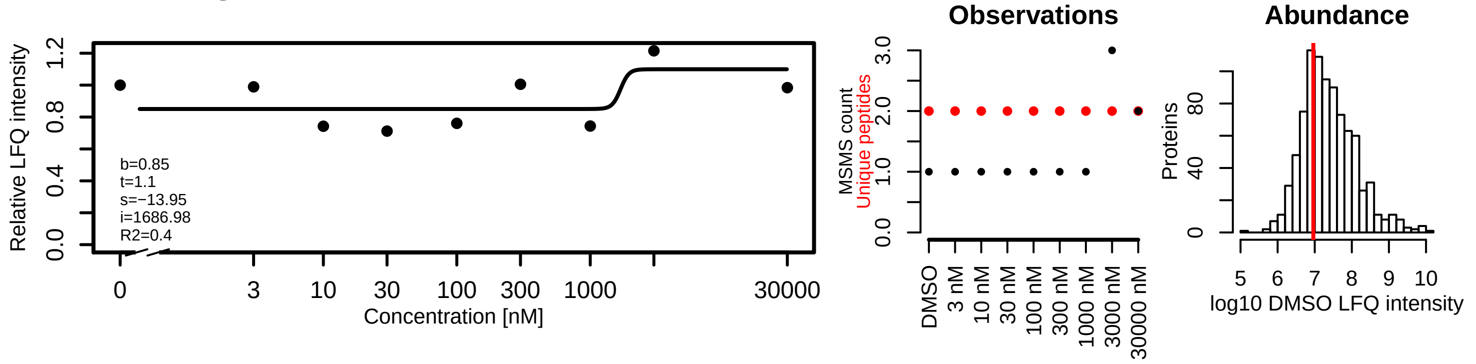

UNC93B1

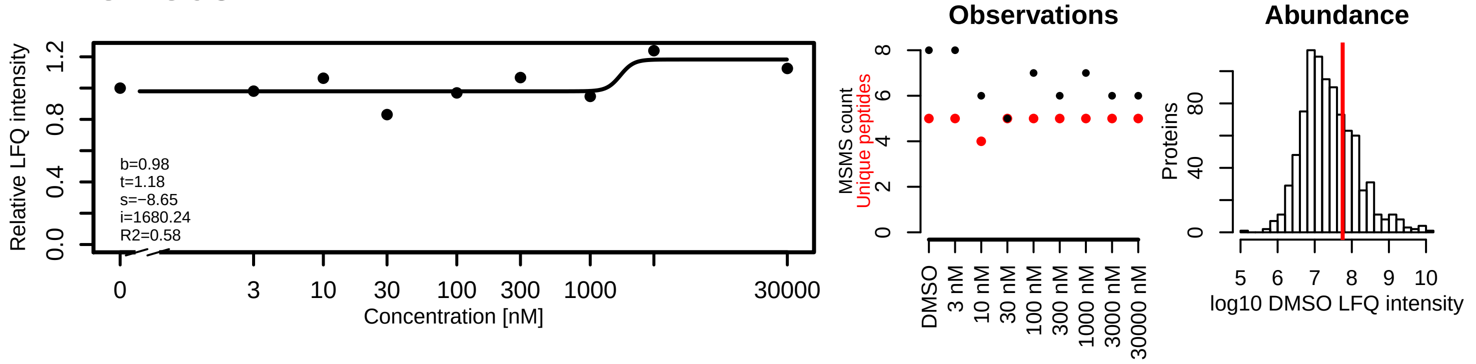

TRAM2

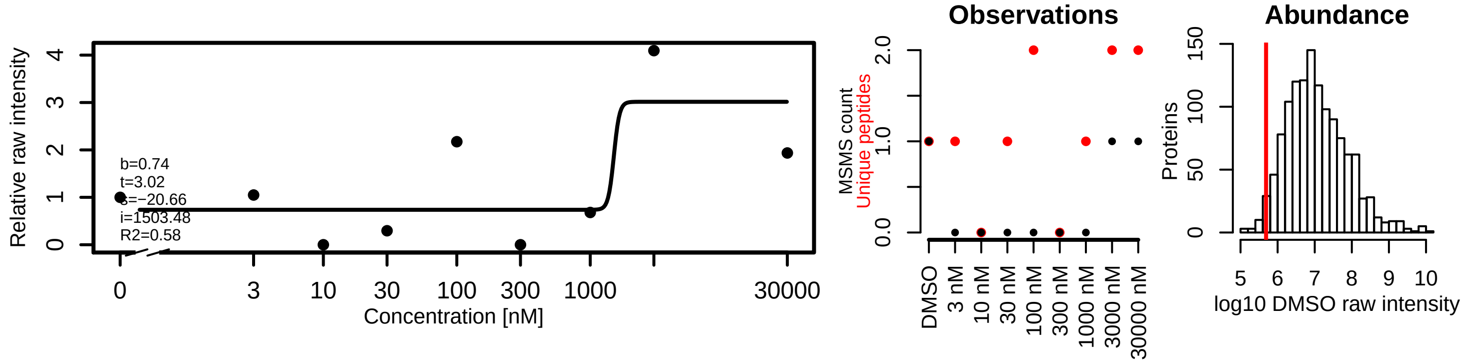

ITGB3

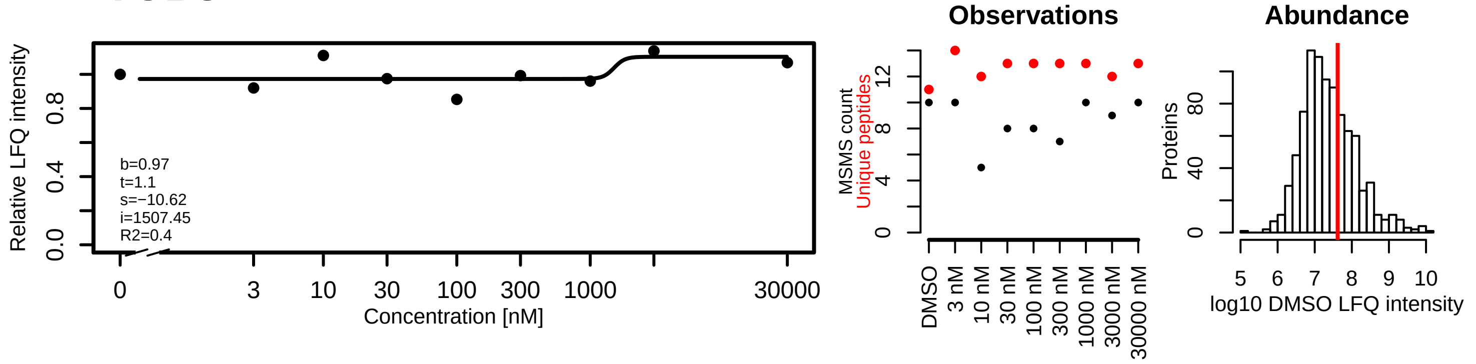

TMEM147

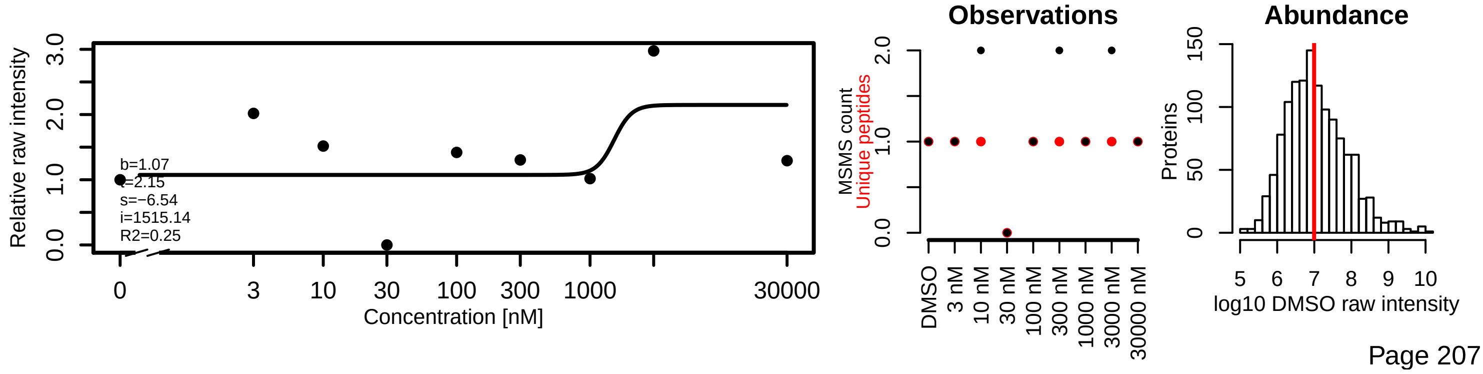

APH1B

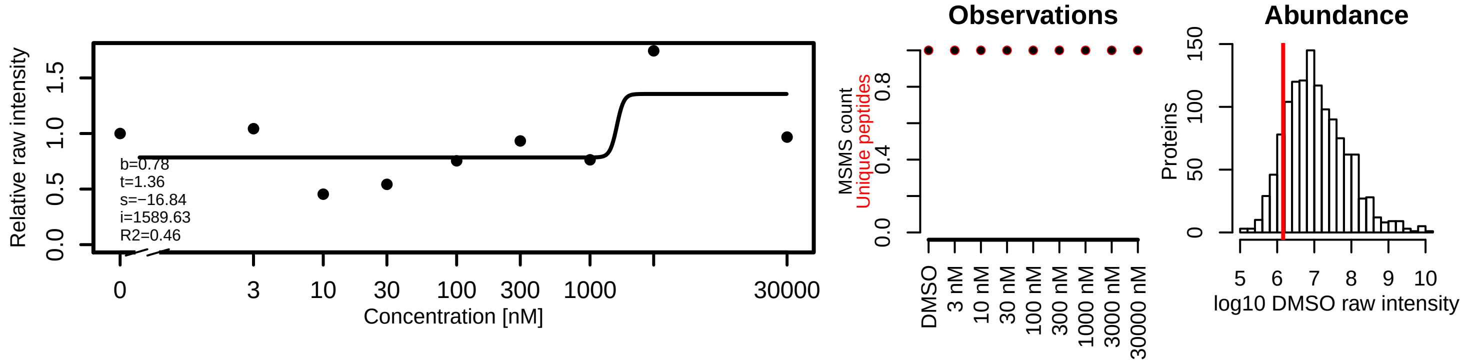

NDUFV3

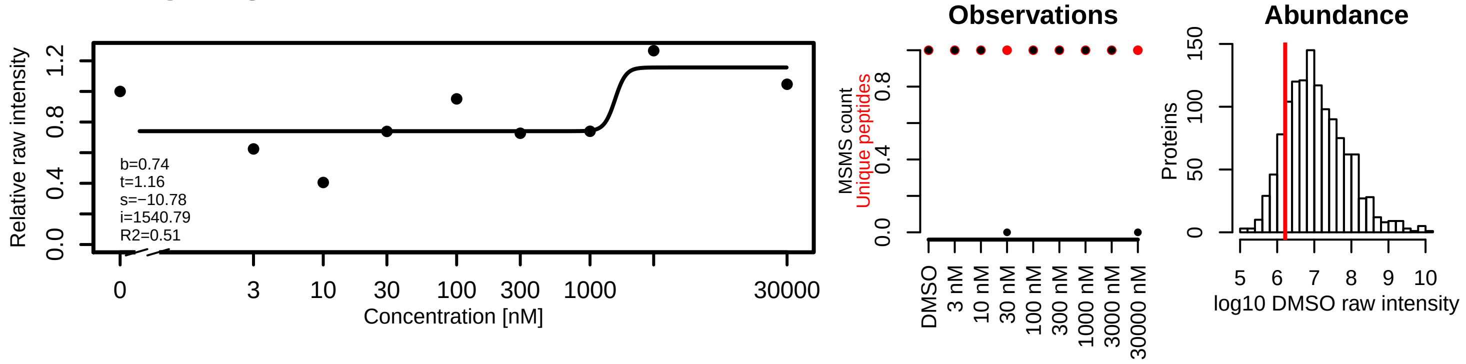

ATP12A

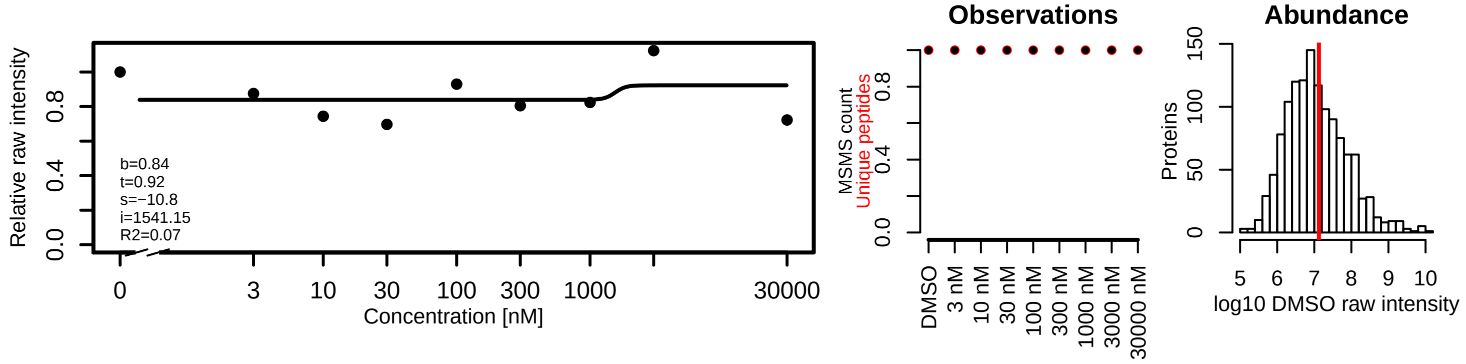

DNAJA2

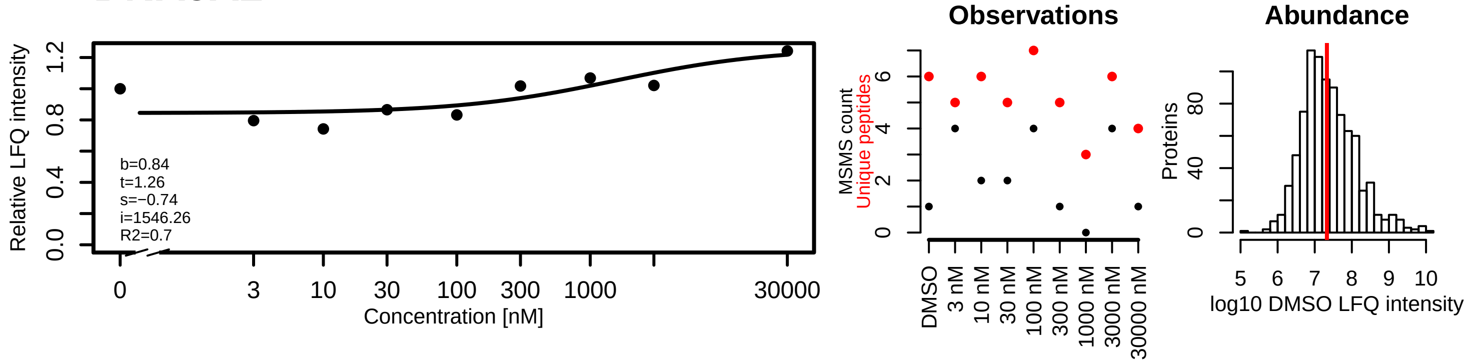

LRP1

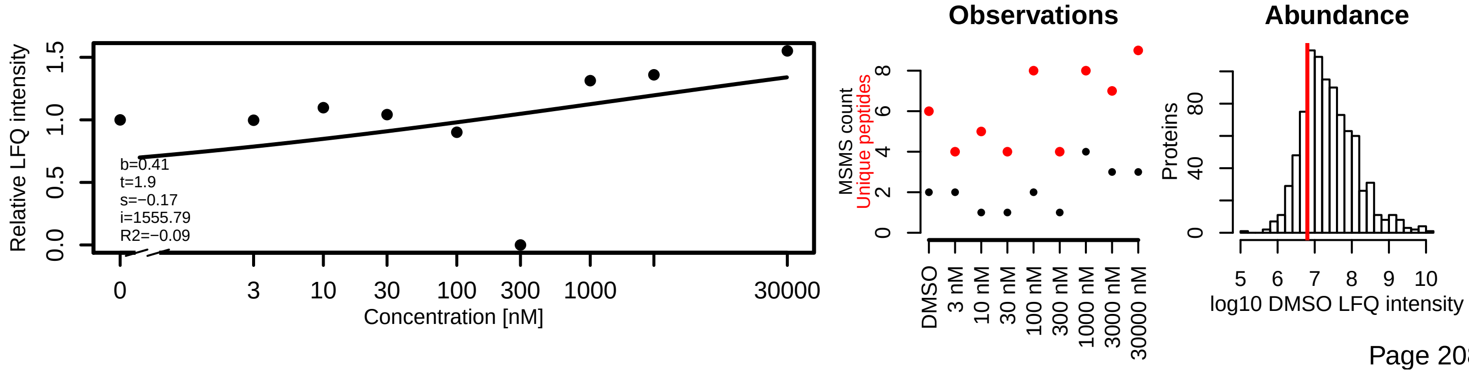

RAB10

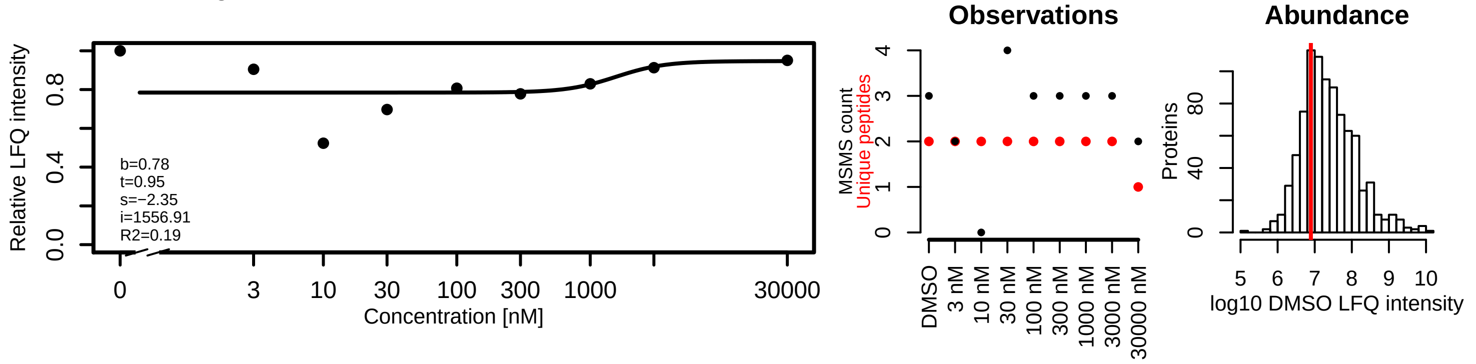

CDKL3

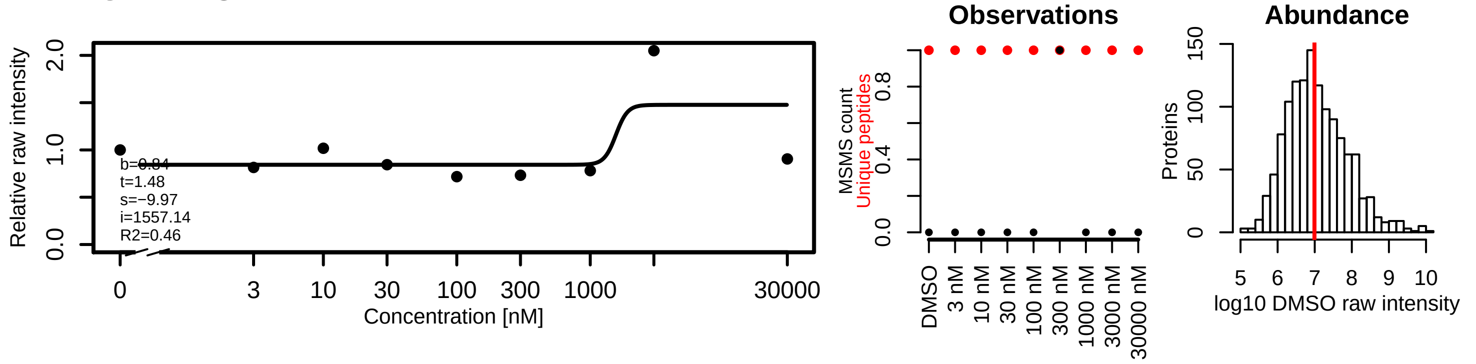

SCO1

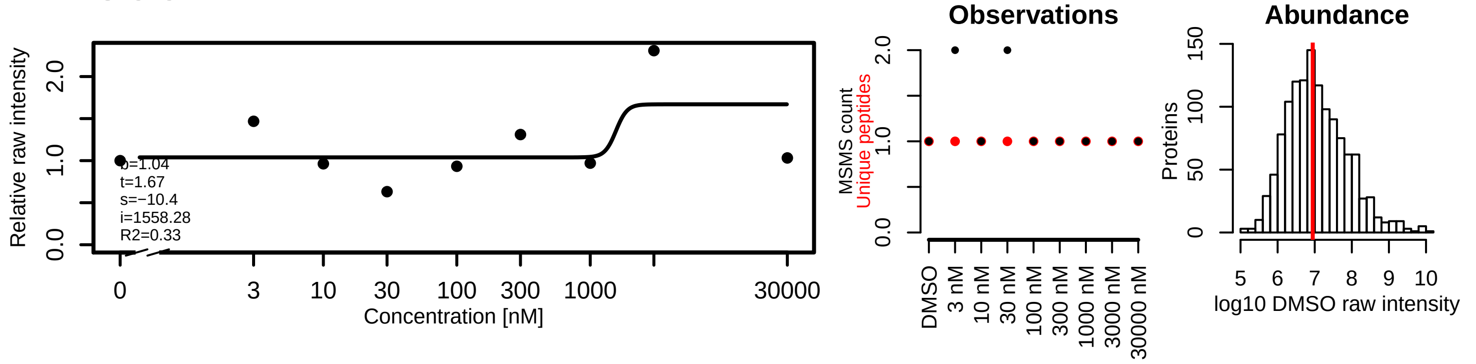

GALNT7

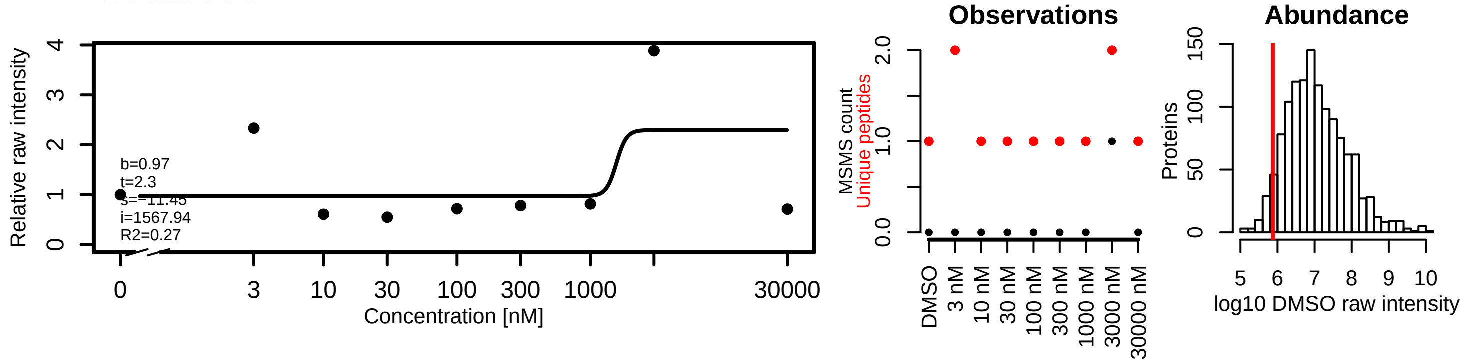

SLC39A1

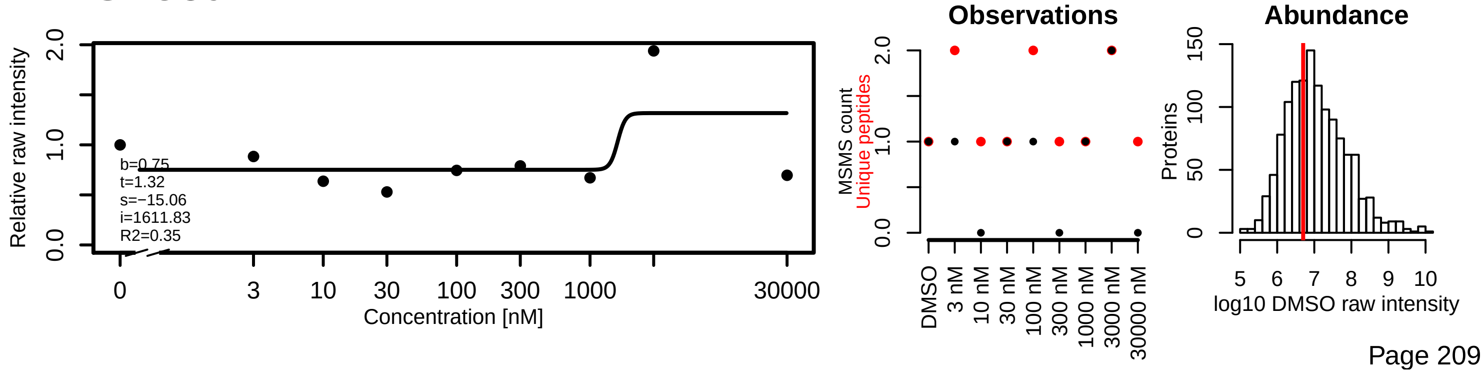

NDUFA11

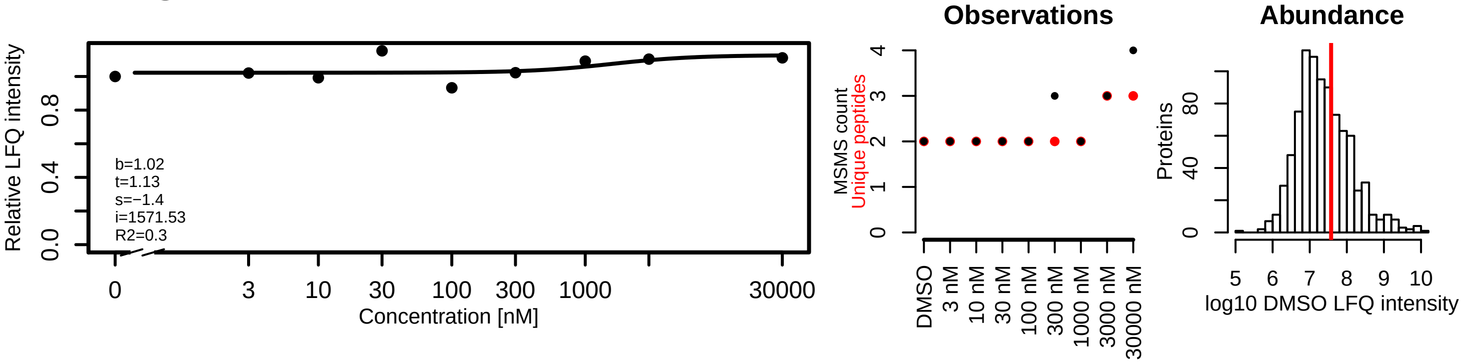

RANBP6

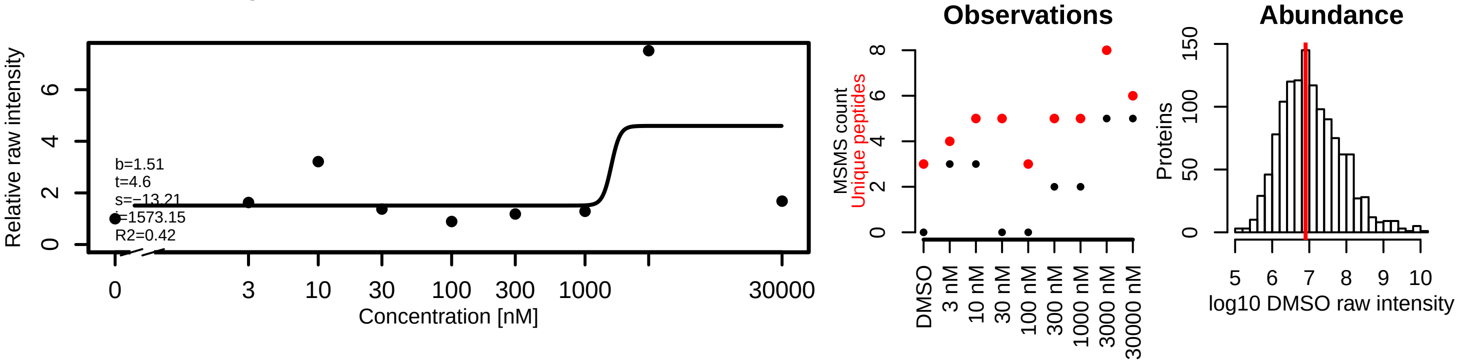

IPO8

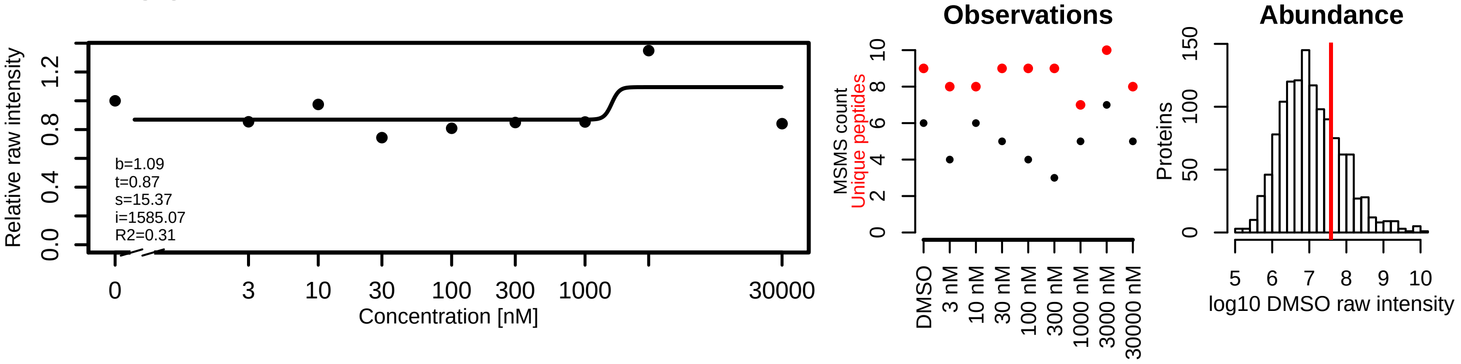

VKORC1L1

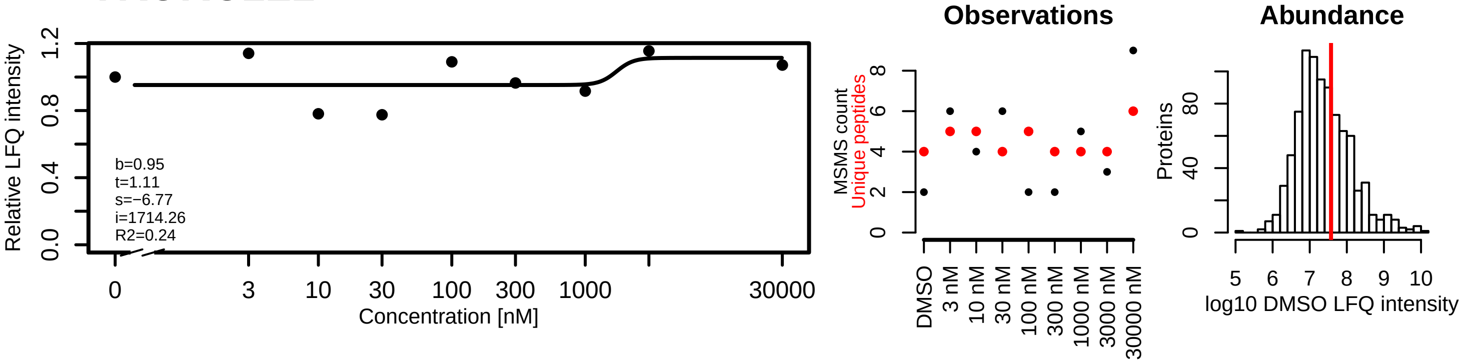

SAAL1

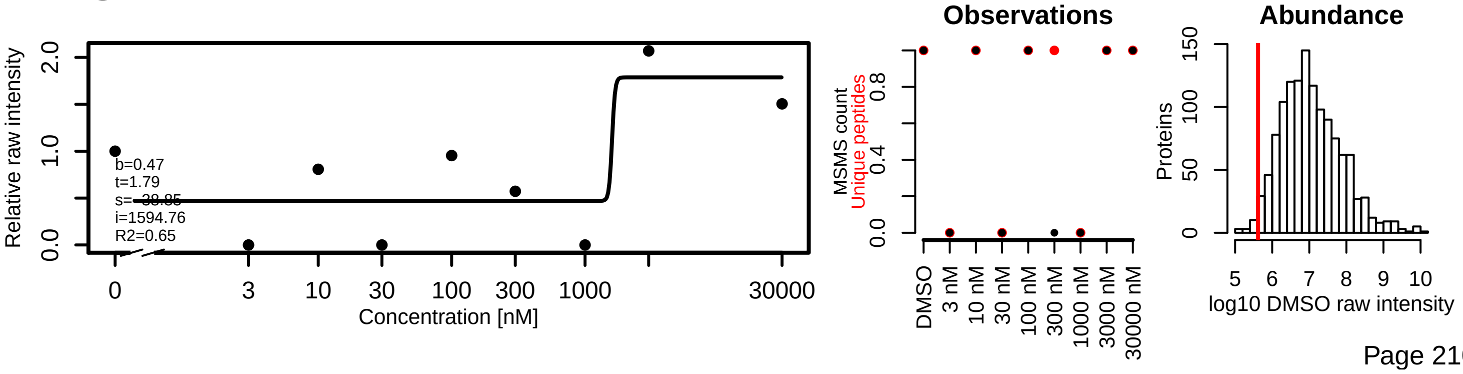

XPO5

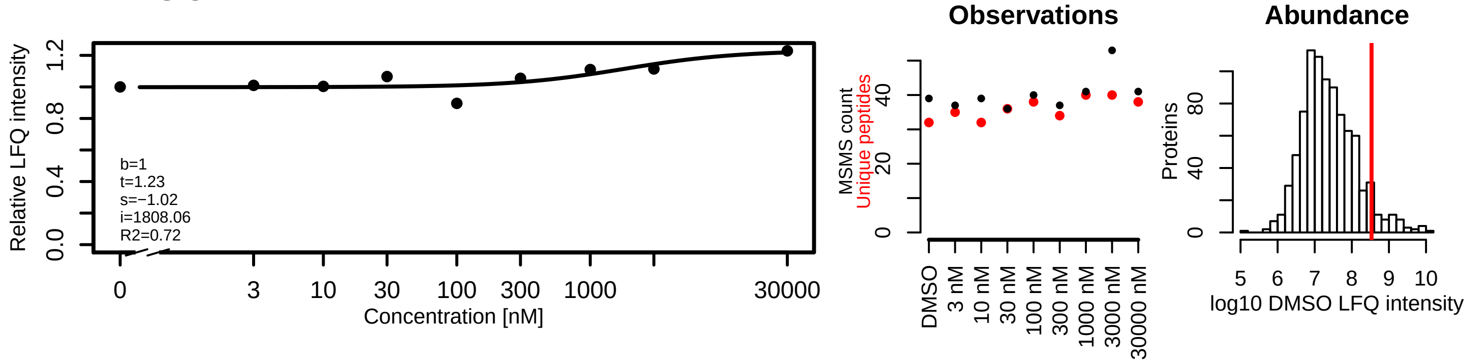

COX15

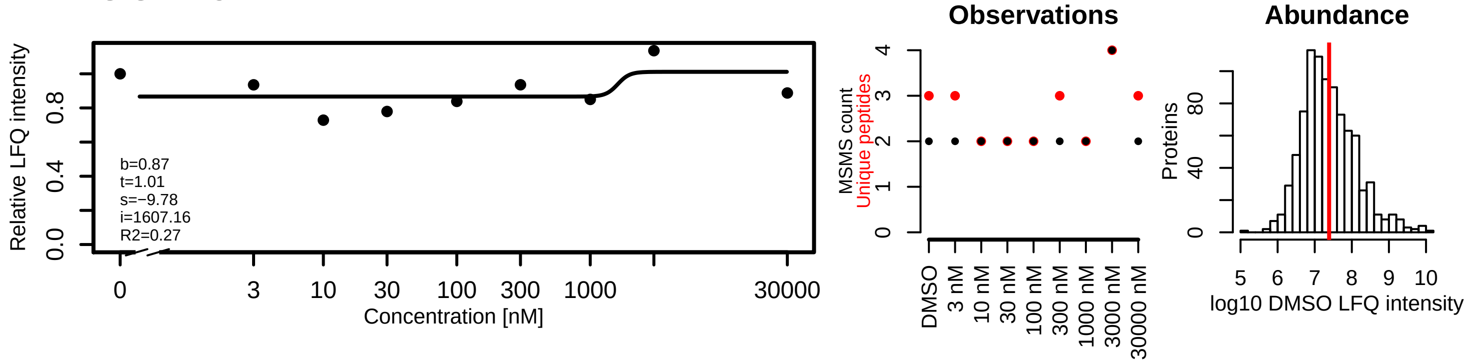

CD9

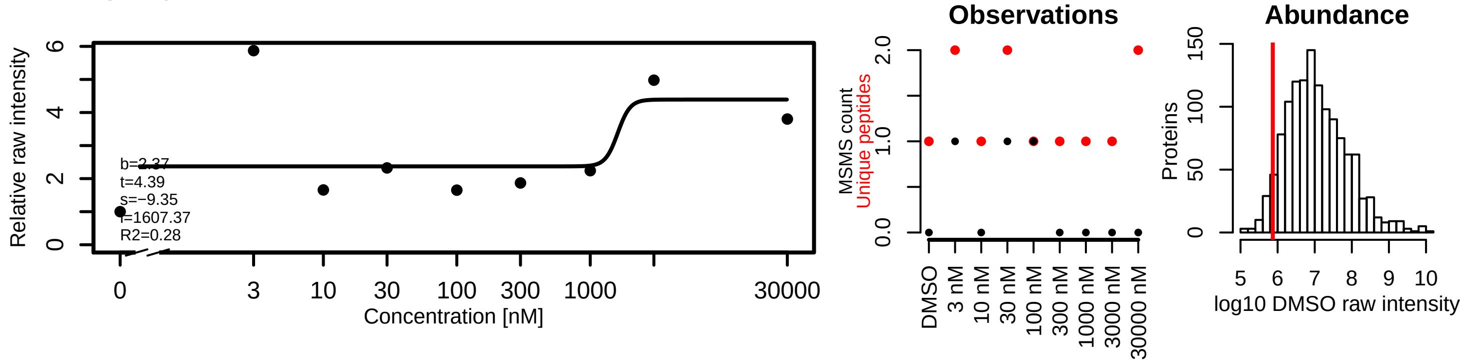

TRIM4

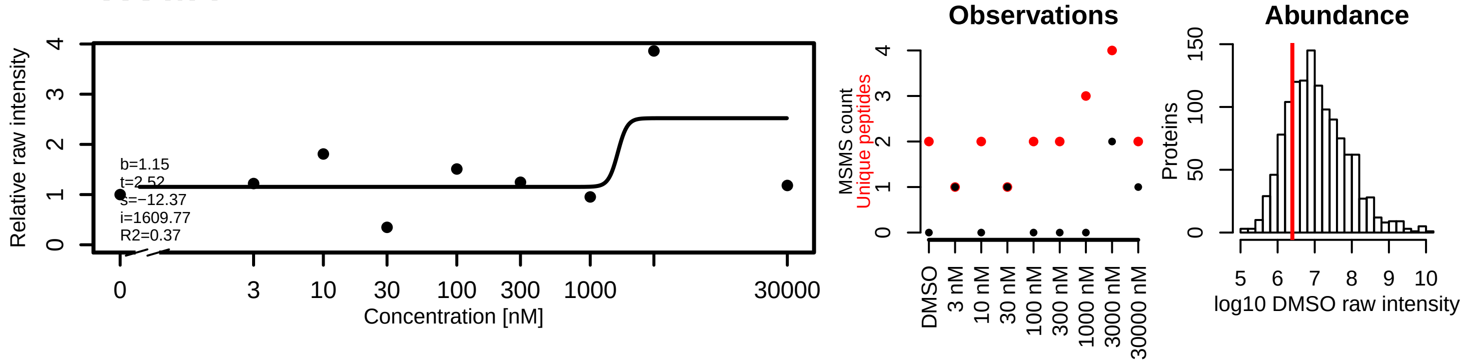

TMEM39A

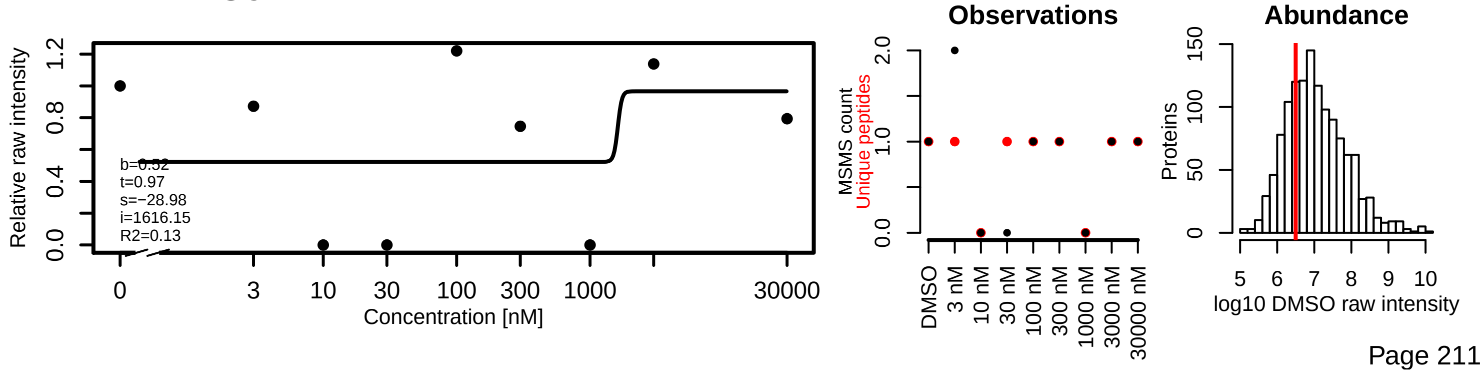

TNPO3

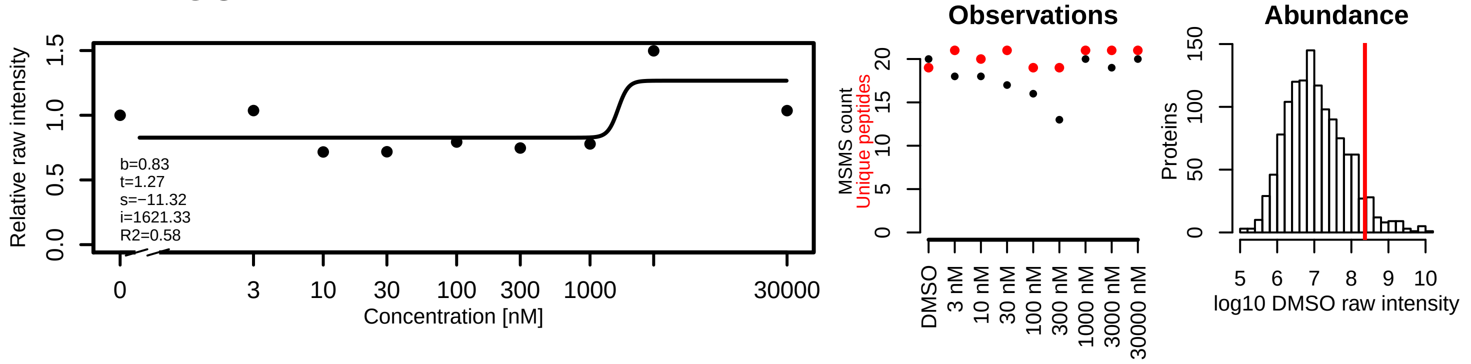

SLC30A5

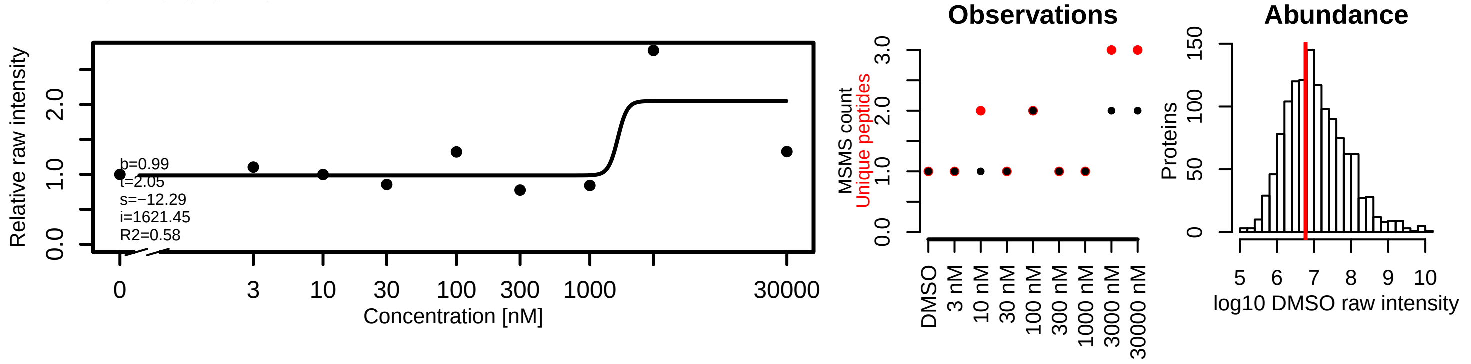

COX4I1

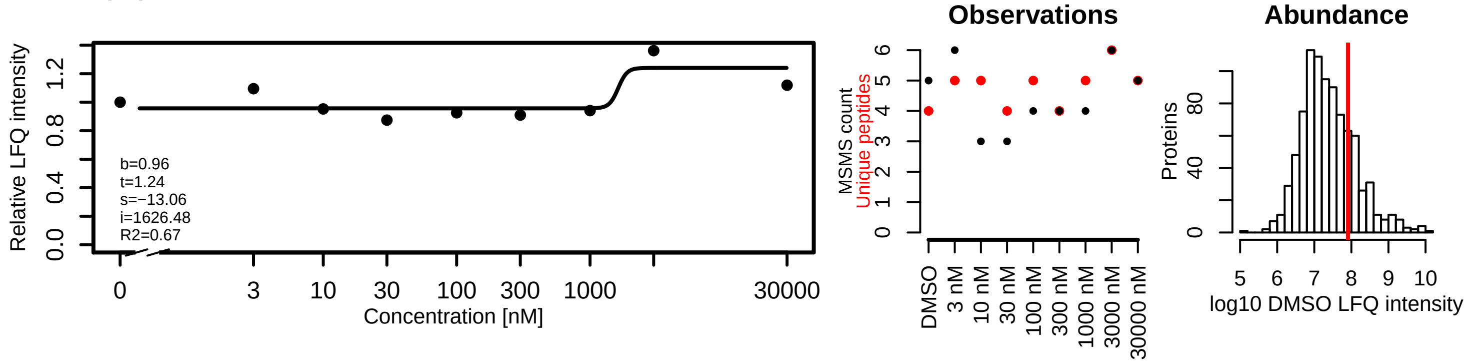

MET

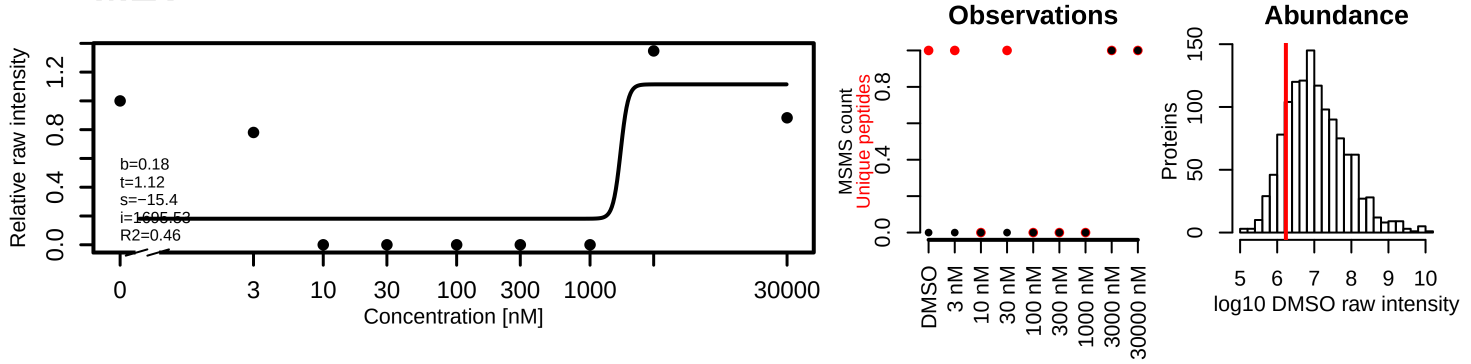

PLP2

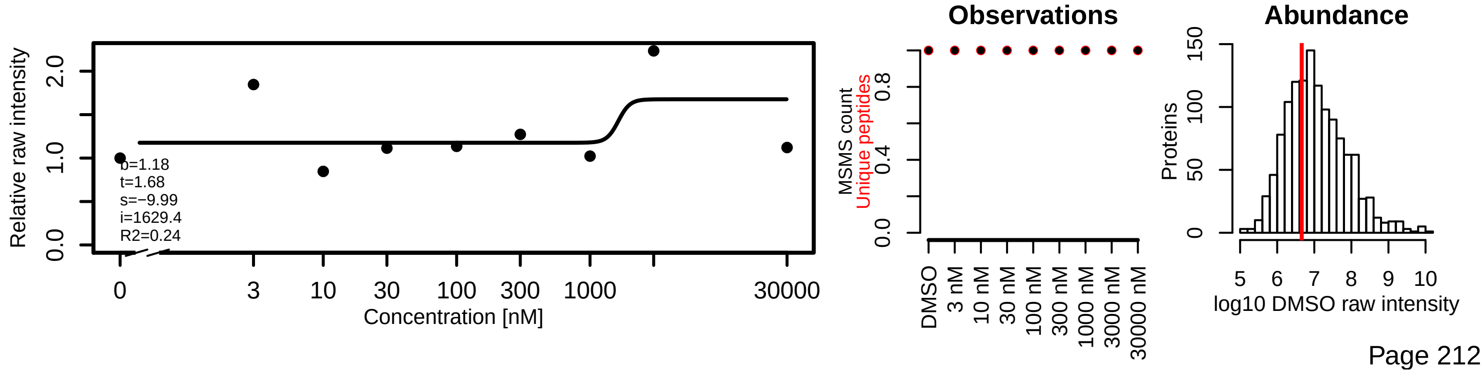

ARL10

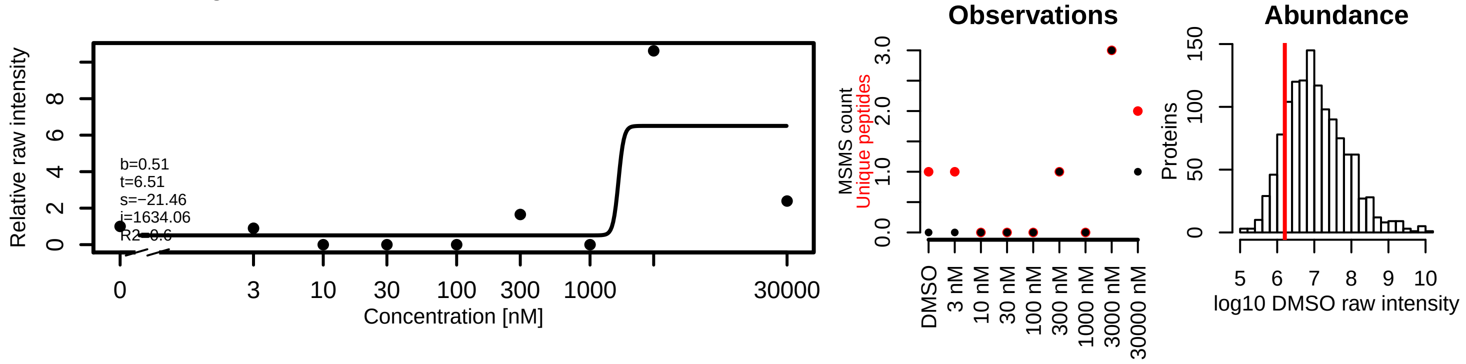

TMEM194A

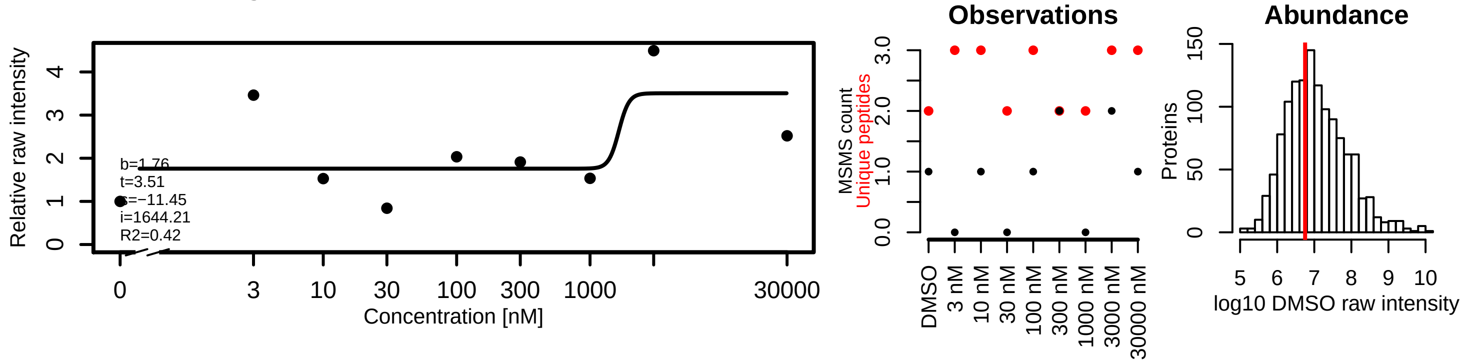

TMEM161B

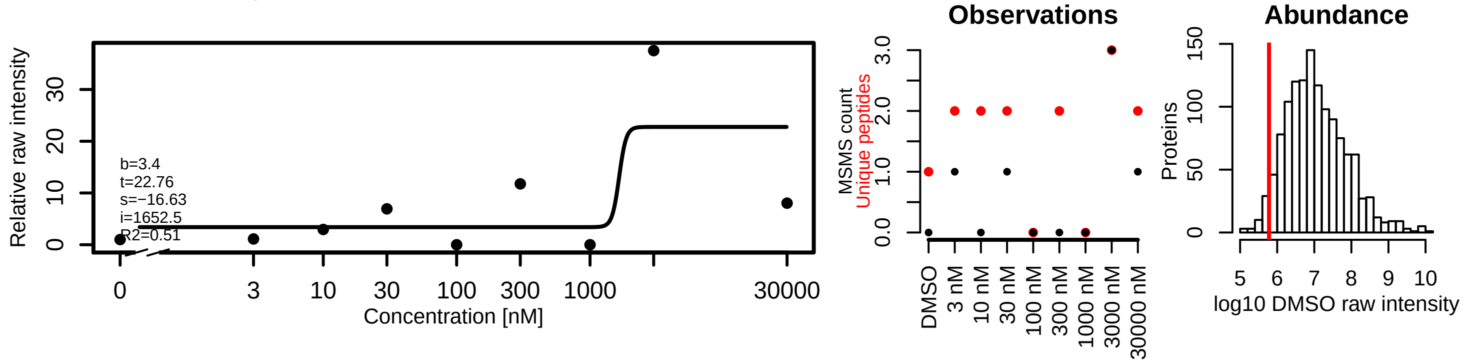

NOL4

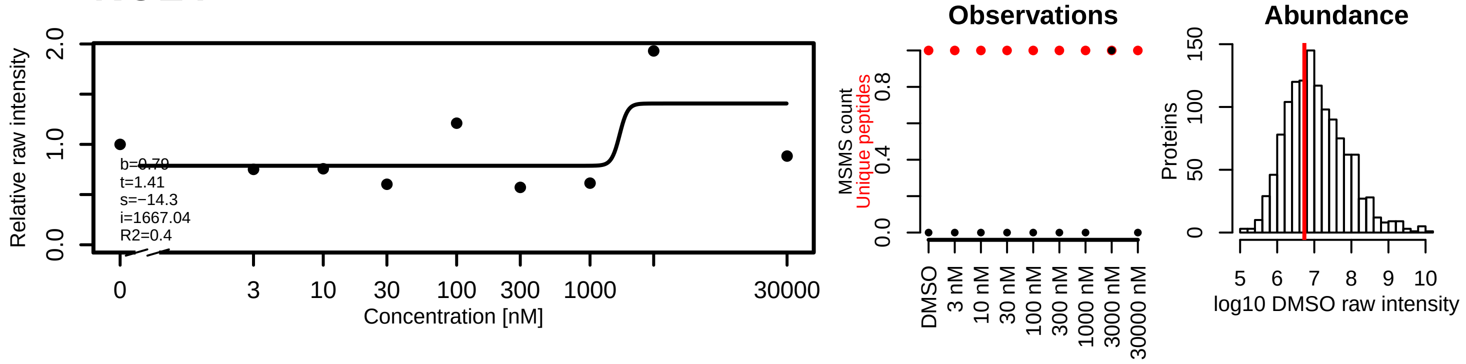

HNRNPH2

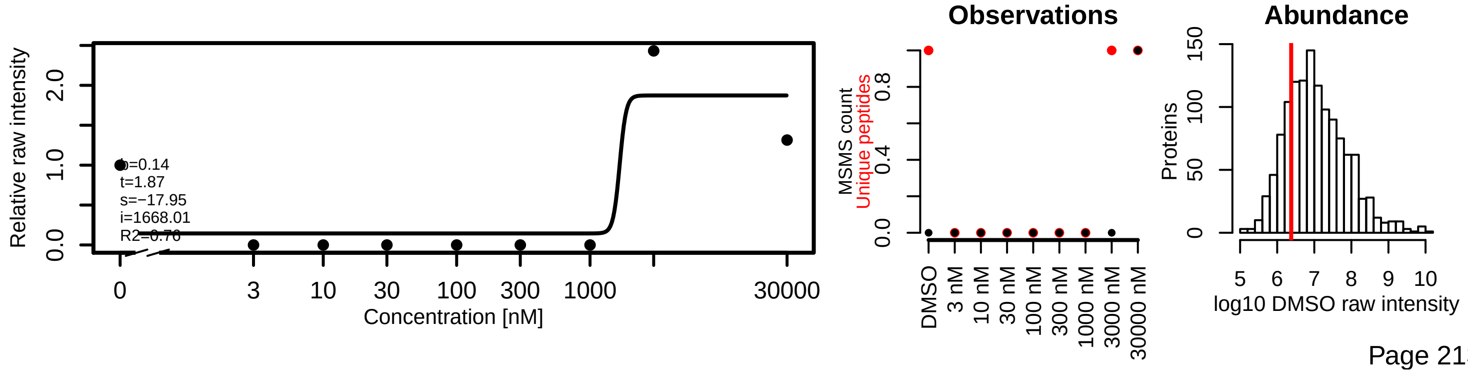

RUFY1

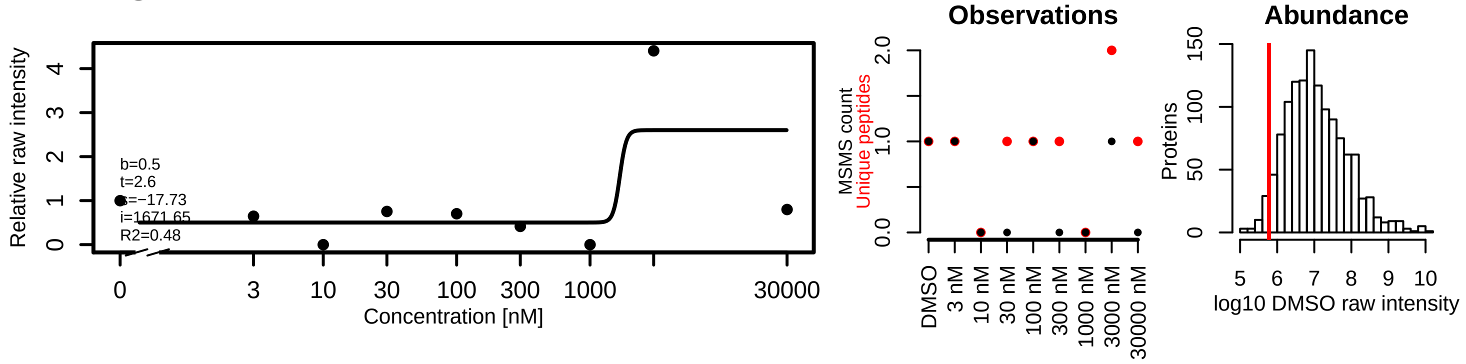

RP13−360B22.2;TMEM164

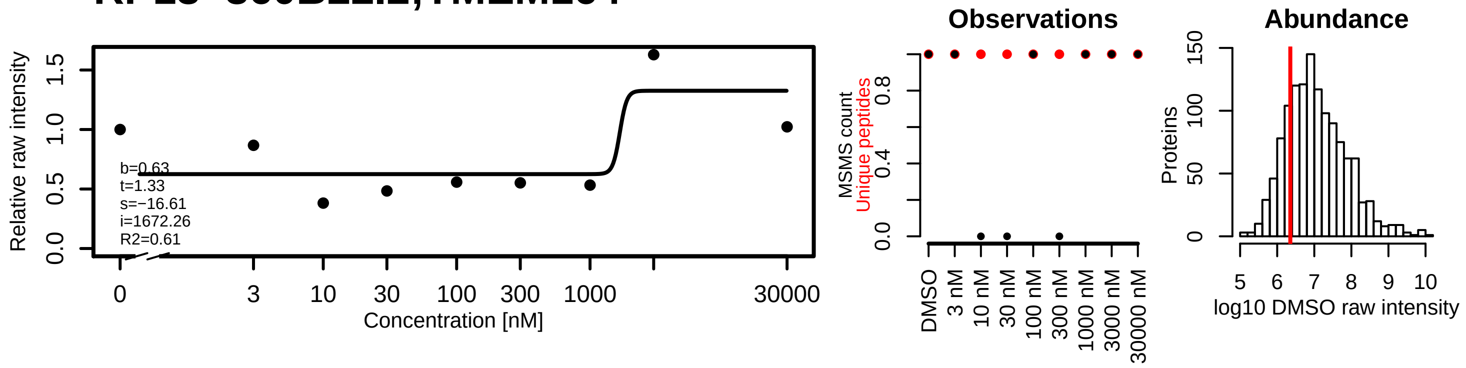

SMIM7

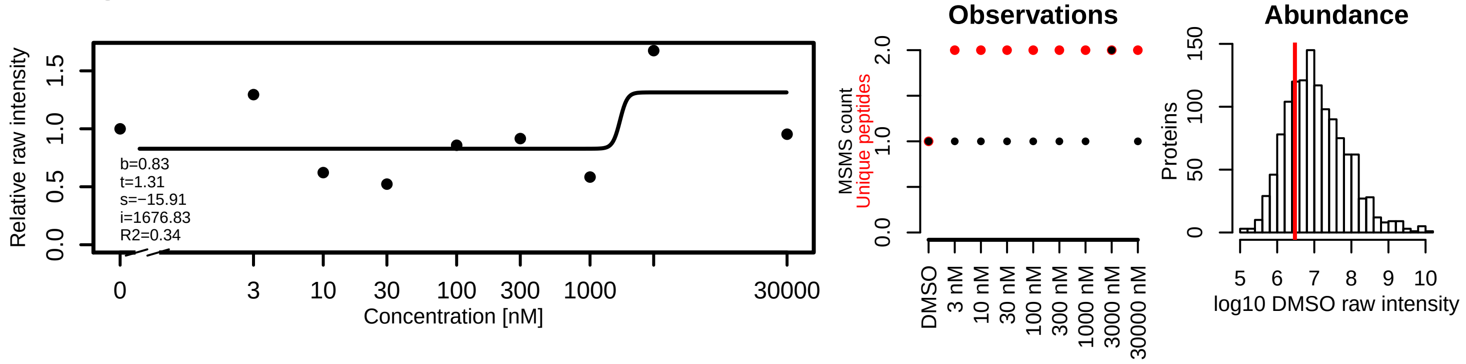

ELOVL1

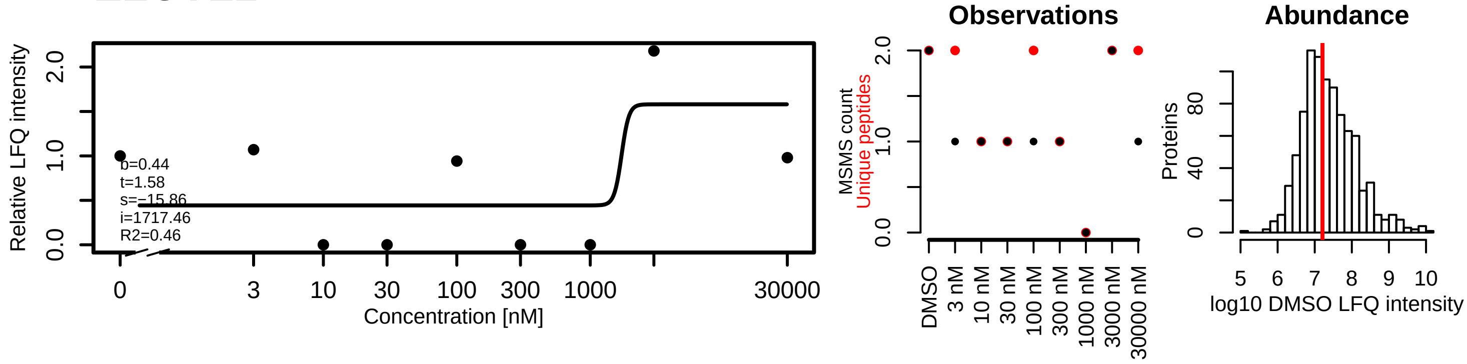

SLC26A6

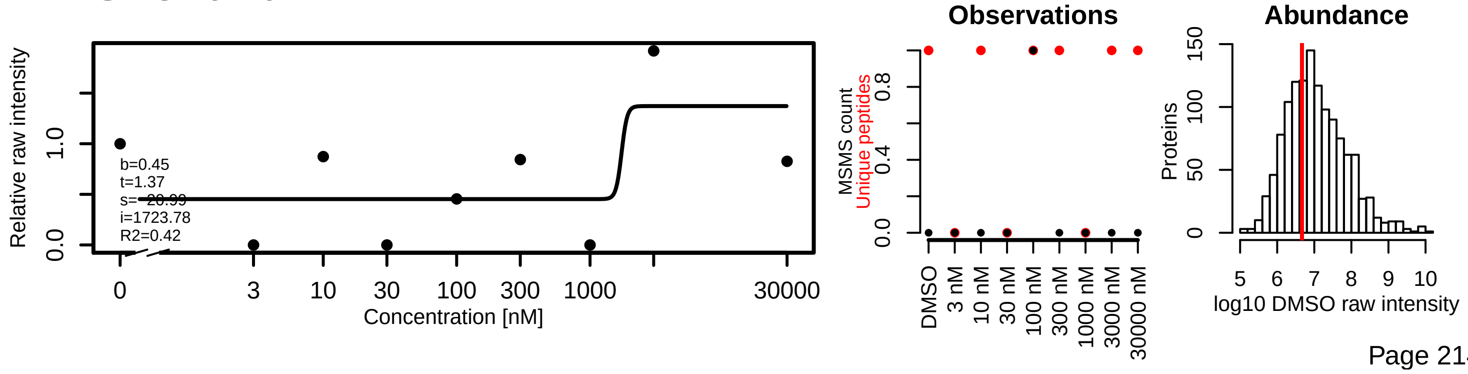

MFSD8

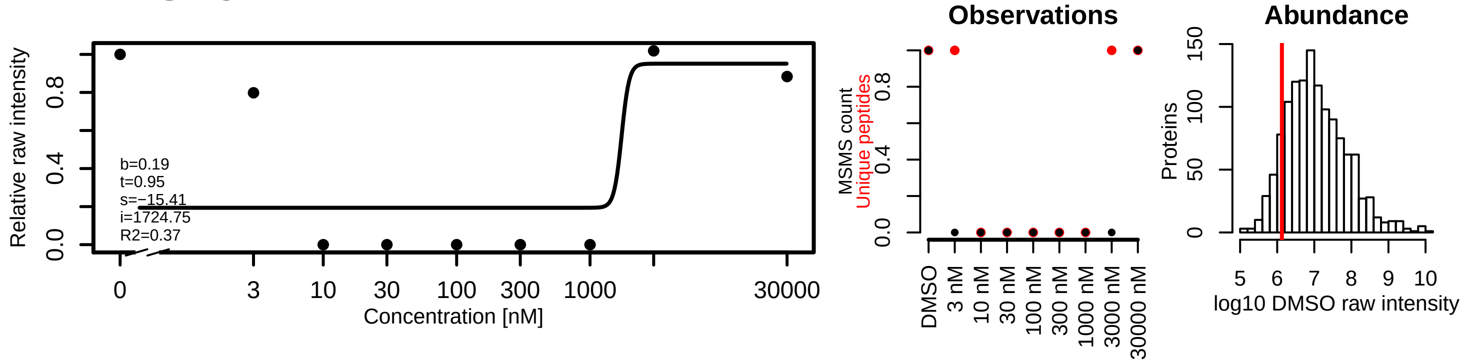

FAAH

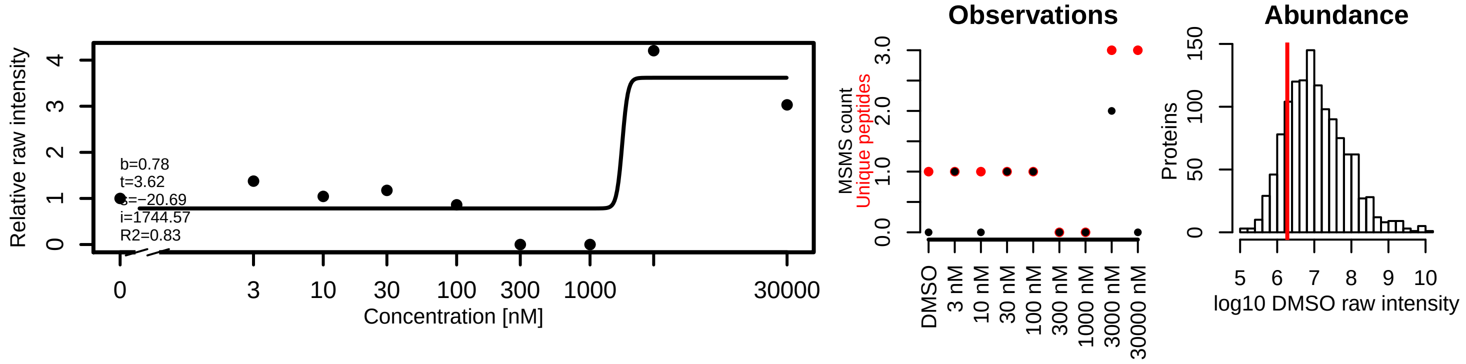

ATP1B3

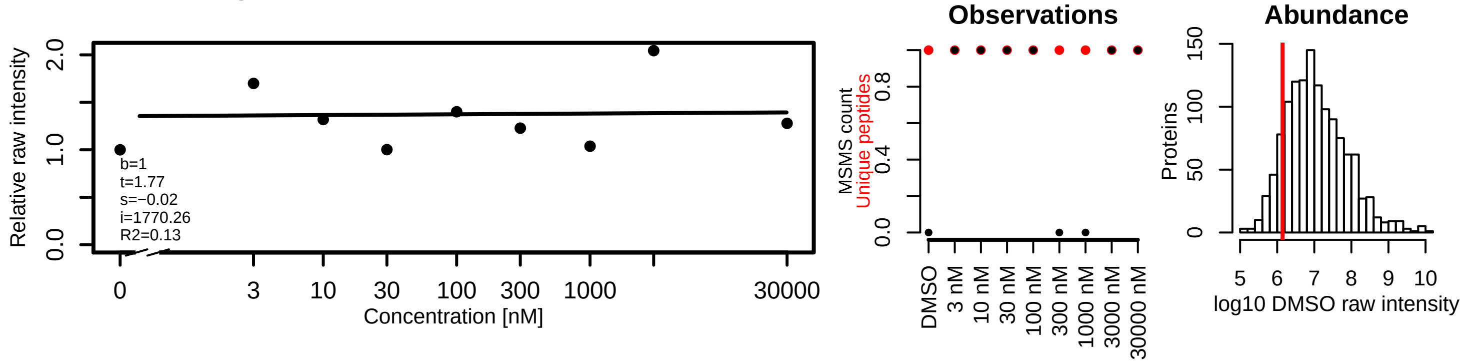

PHB

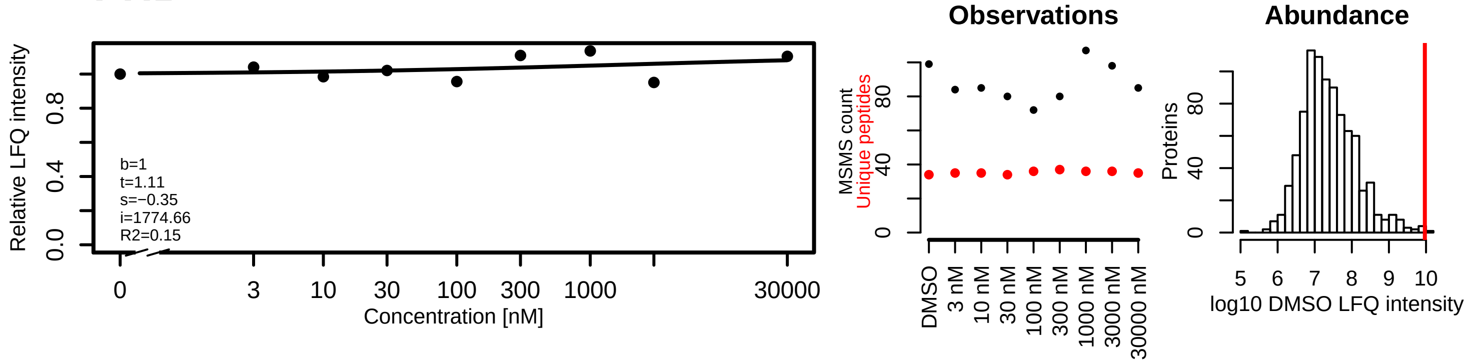

SMDT1

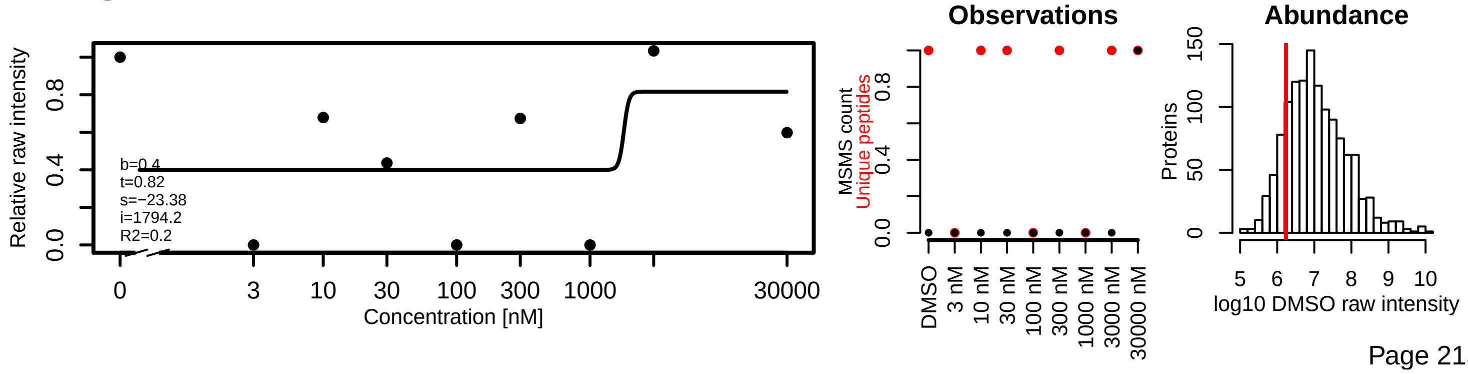

TMEM205

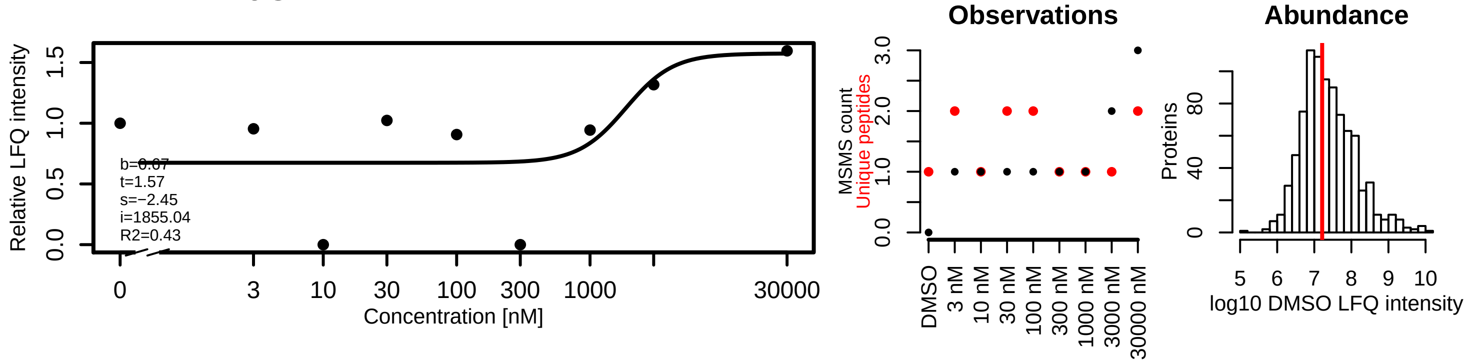

GPNMB

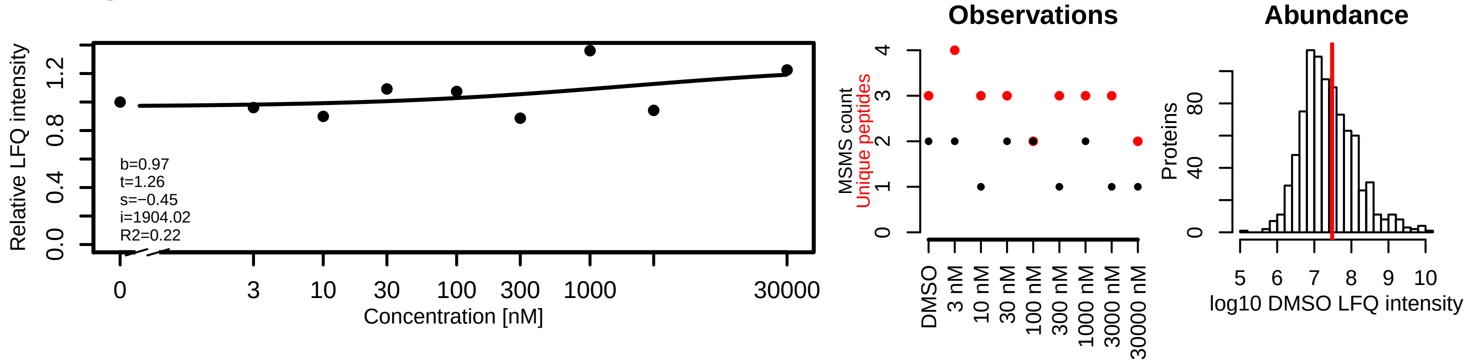

CLDN4

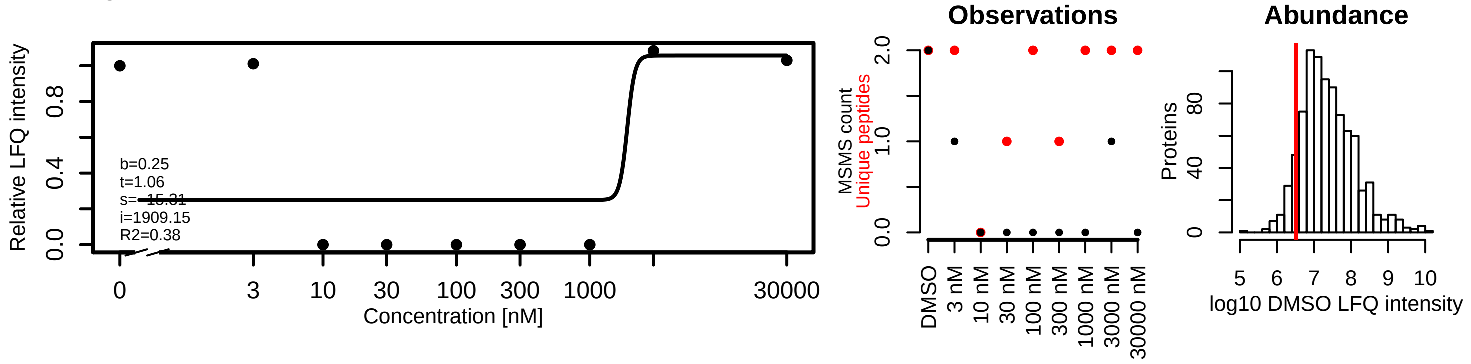

GOLIM4

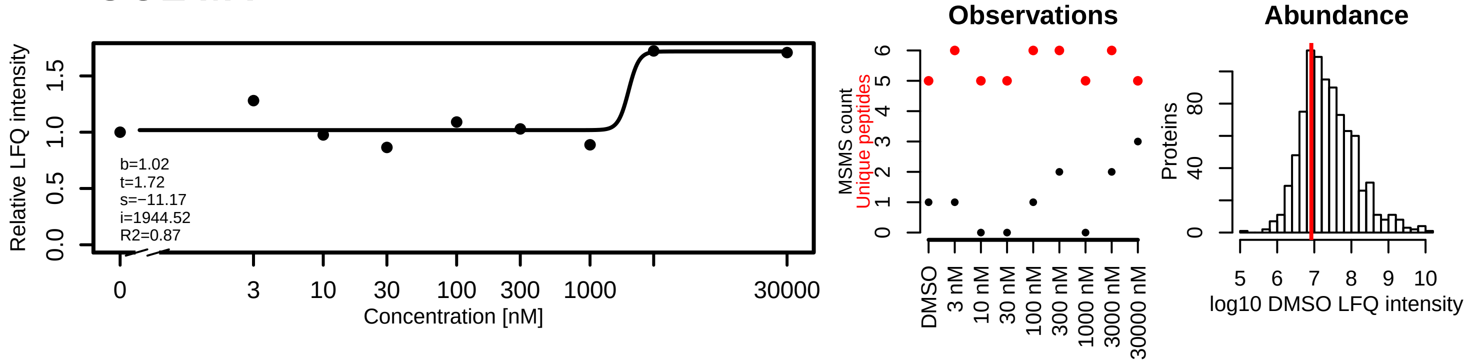

NDUFV2

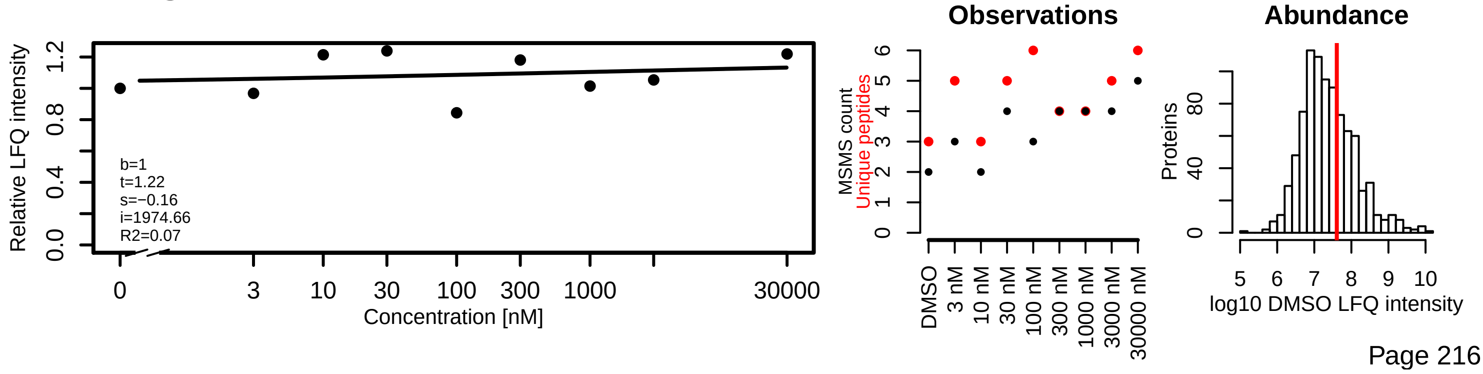

H1F0

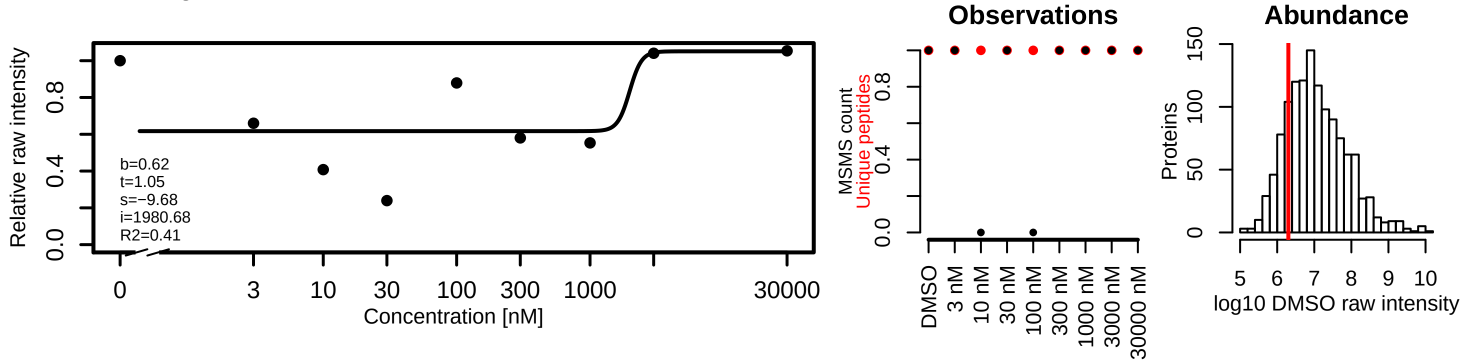

CSE1L

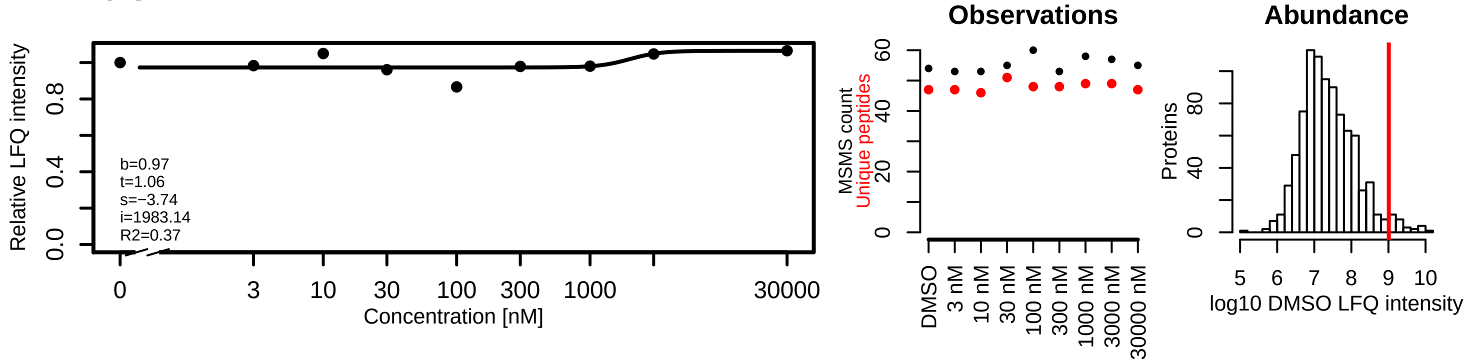

RAB8A

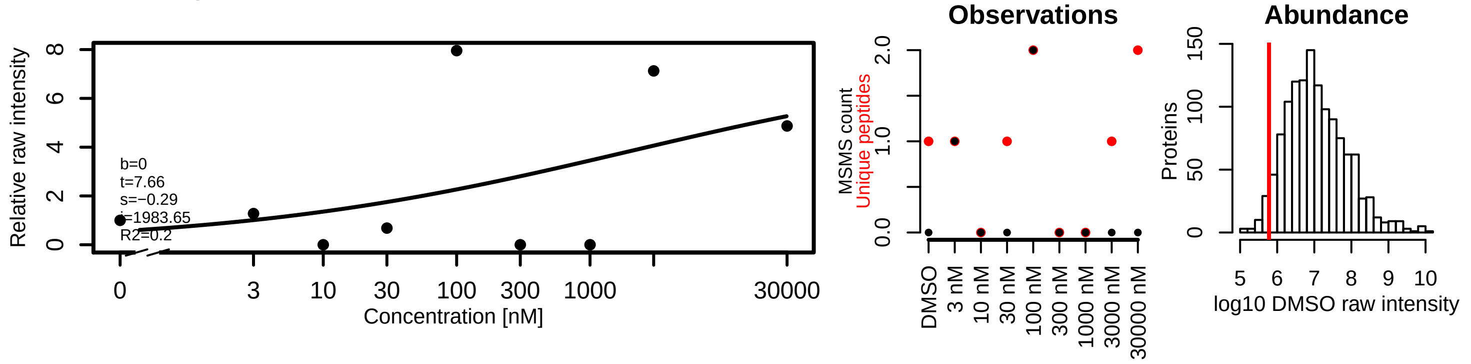

SCFD1

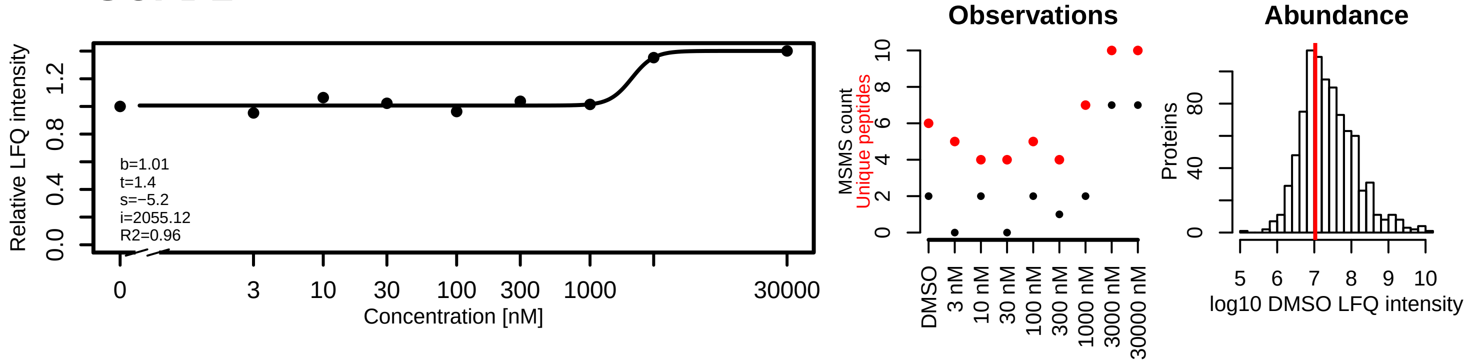

TBC1D8B − J3KN75;Q0IIM8;Q0IIM8−2;D6RFZ2;H0Y552;D6RC82

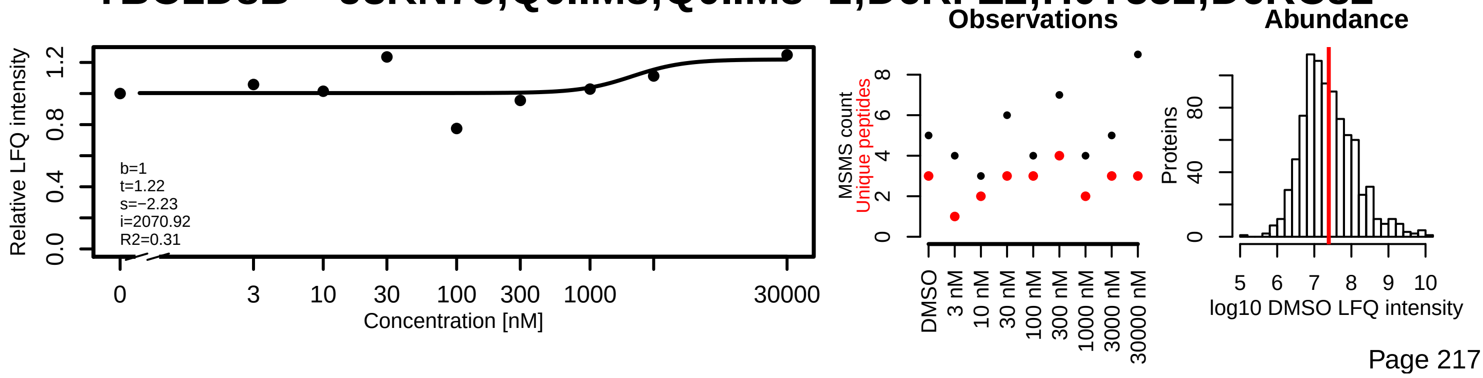

FN1

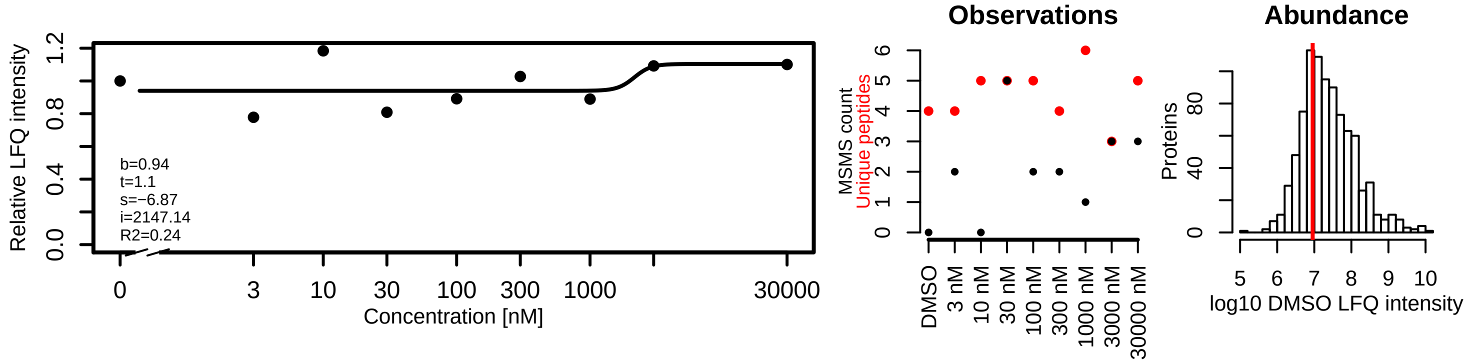

FLVCR1

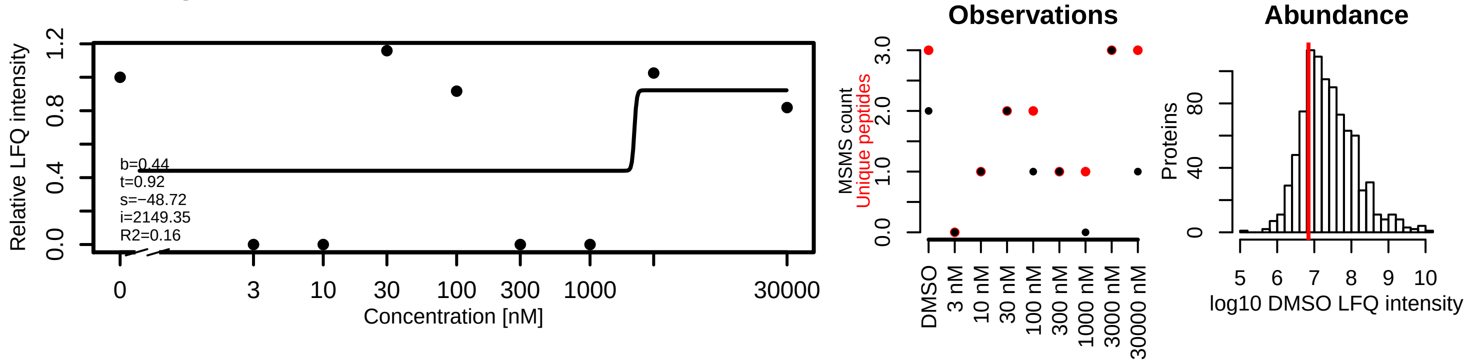

RPSA;RPSAP58

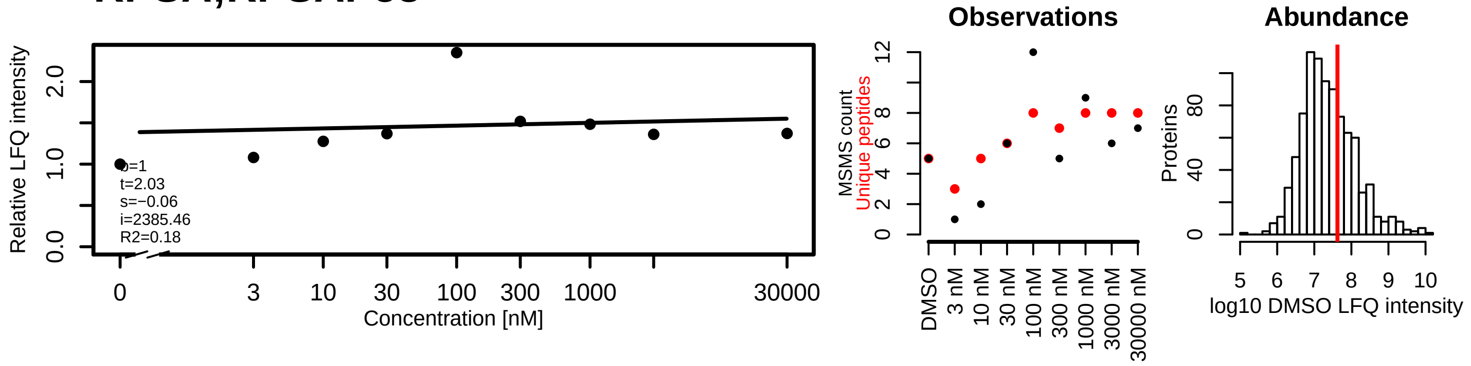

NUDT5

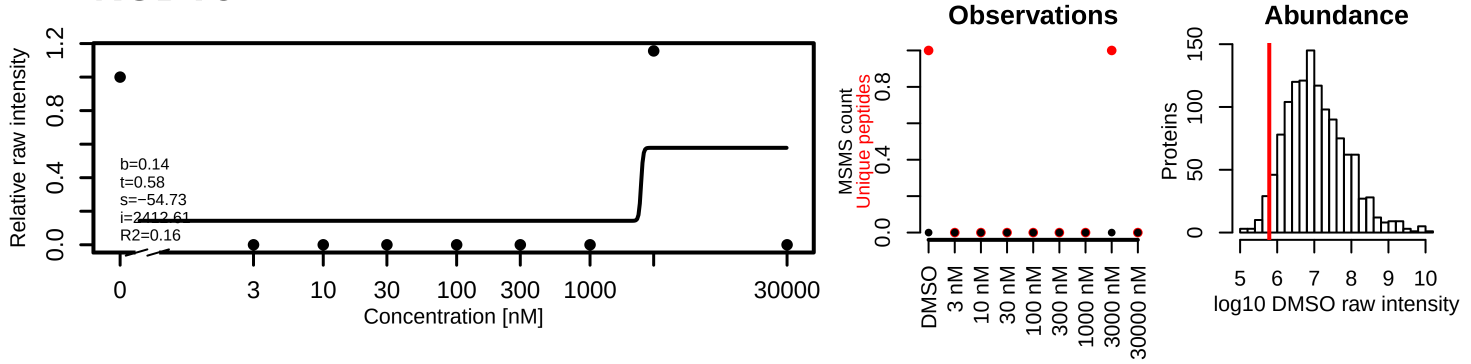

LPAR1

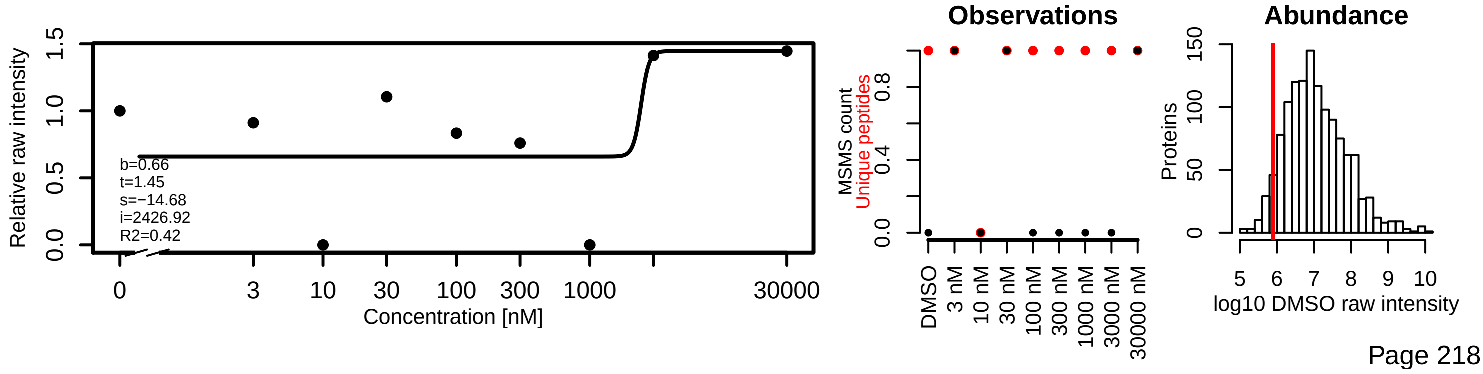

PI4KA

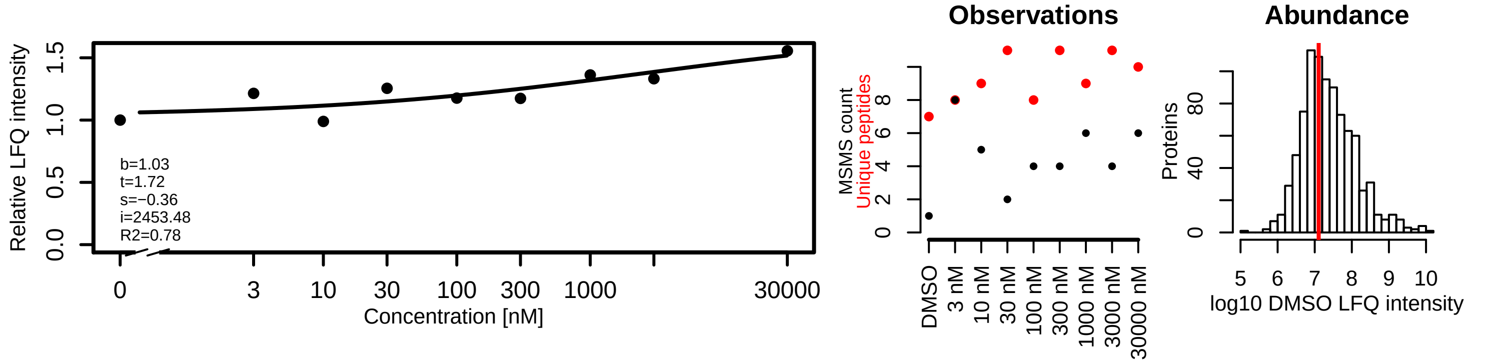

KPNB1

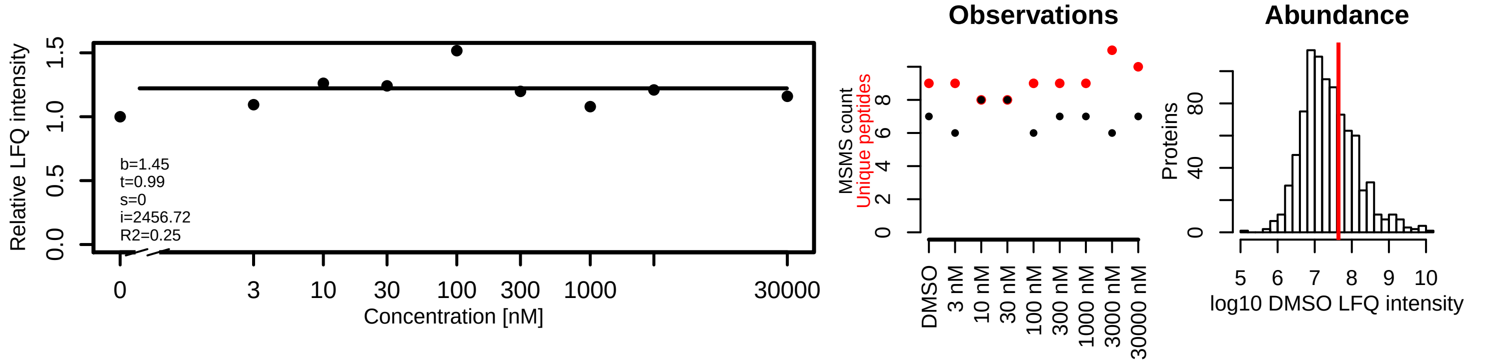

ENDOD1

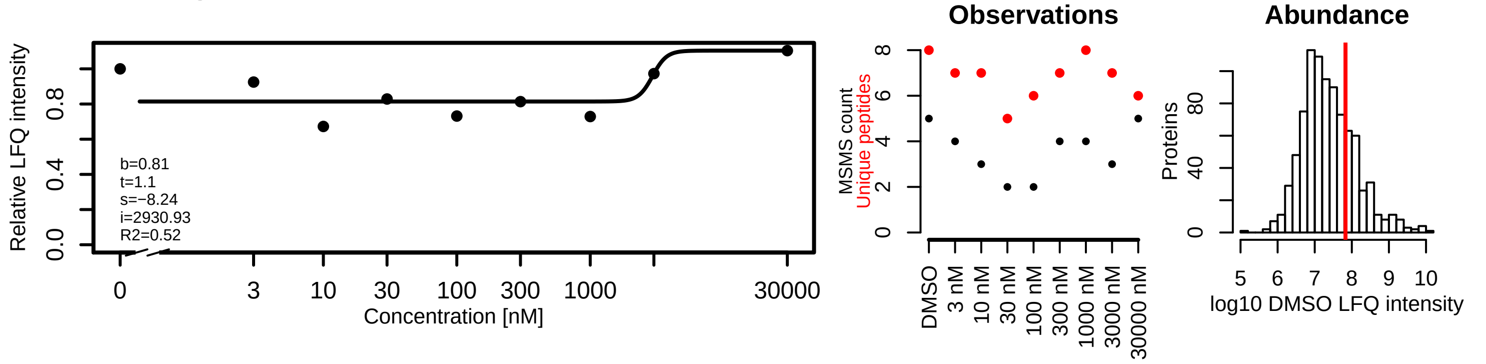

C1QBP

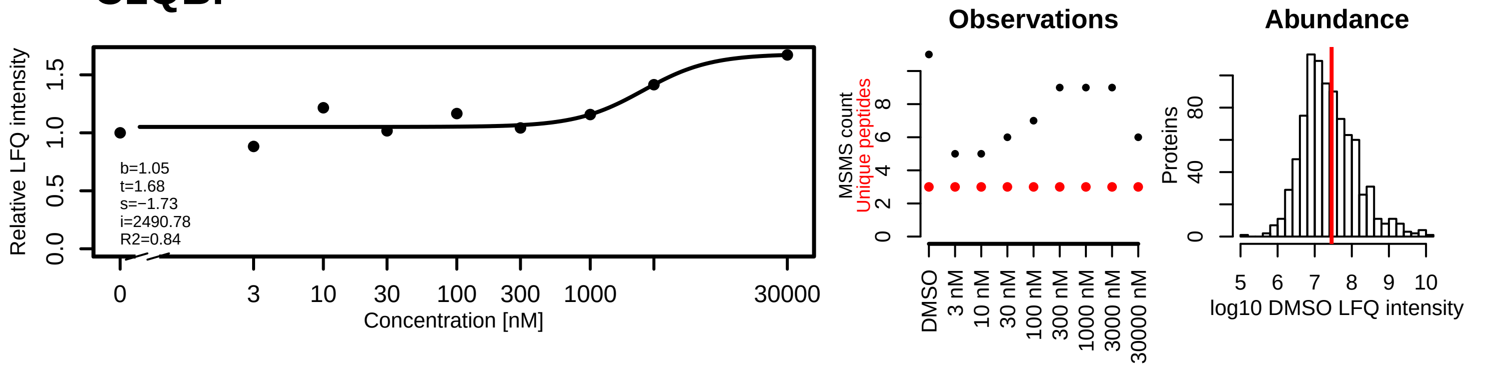

ATP5J

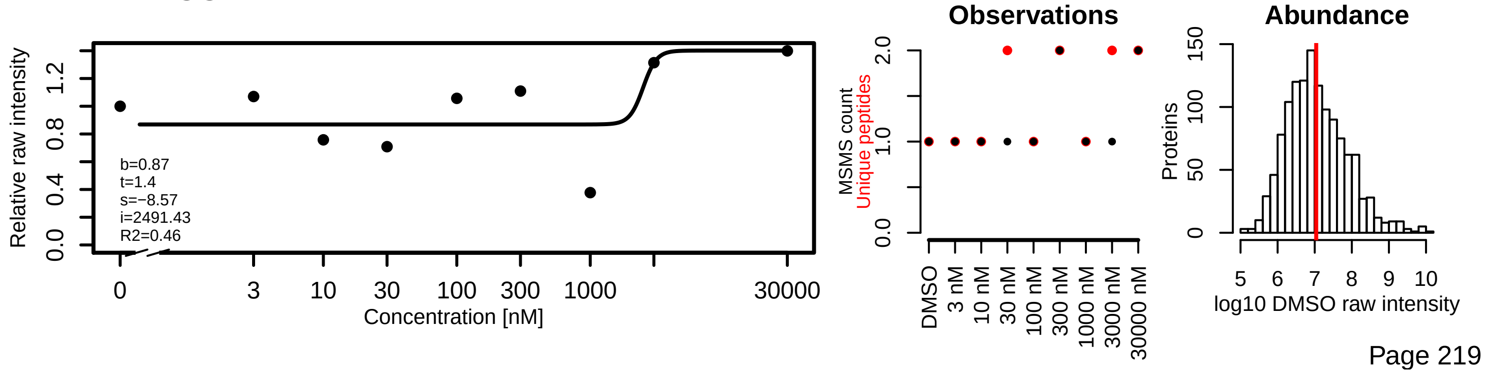

ARFGEF2

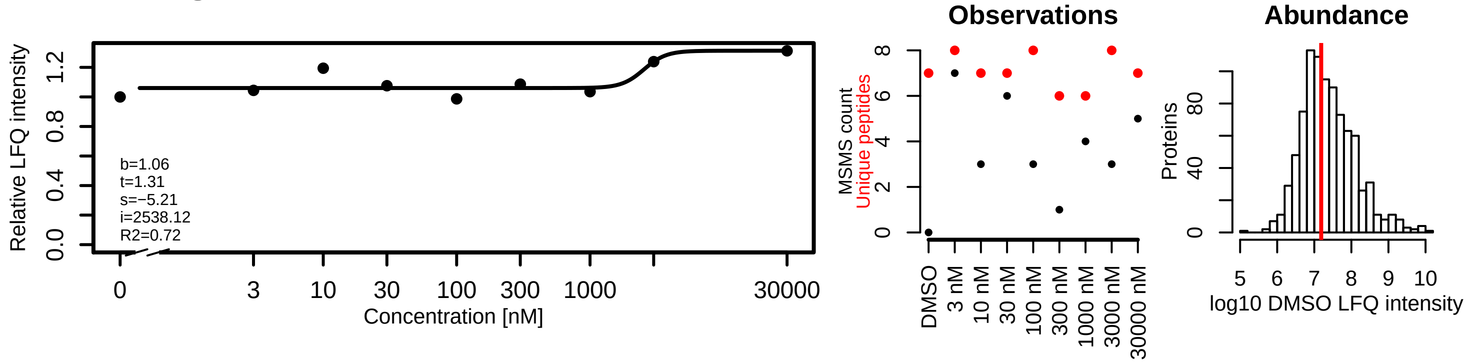

OSTC

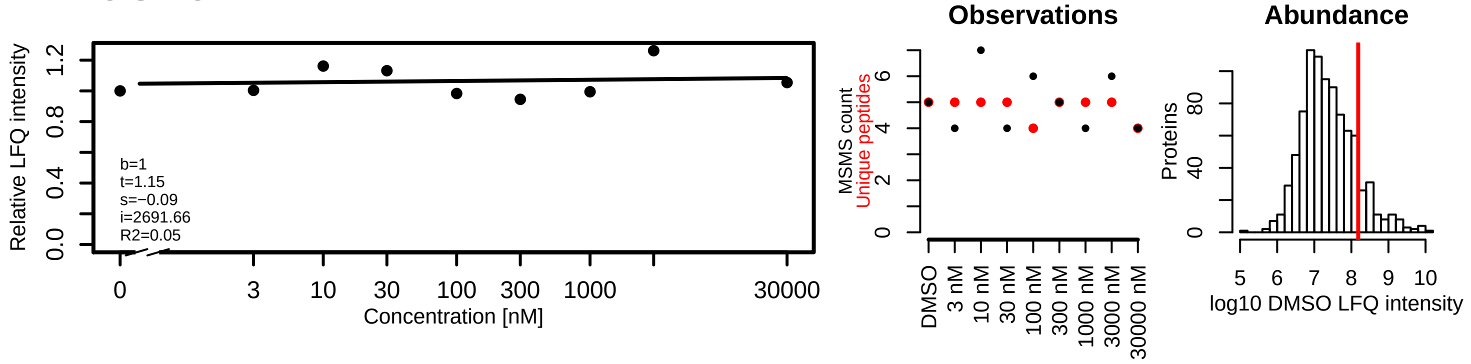

RCN2

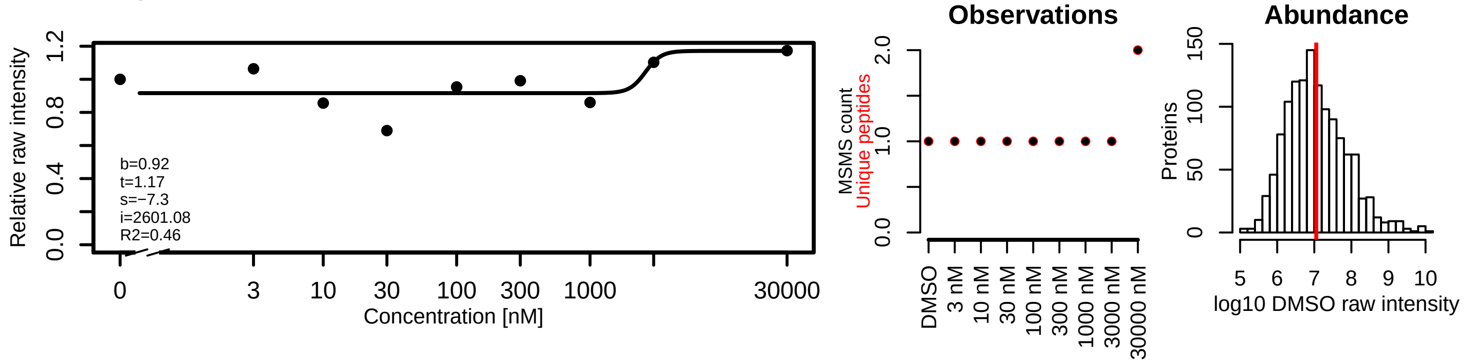

ABCB1

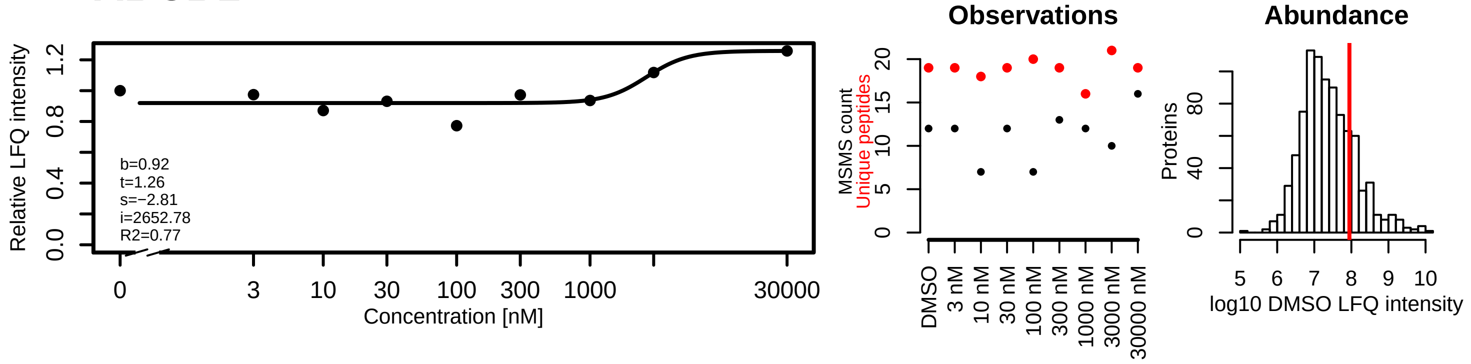

CLPTM1L

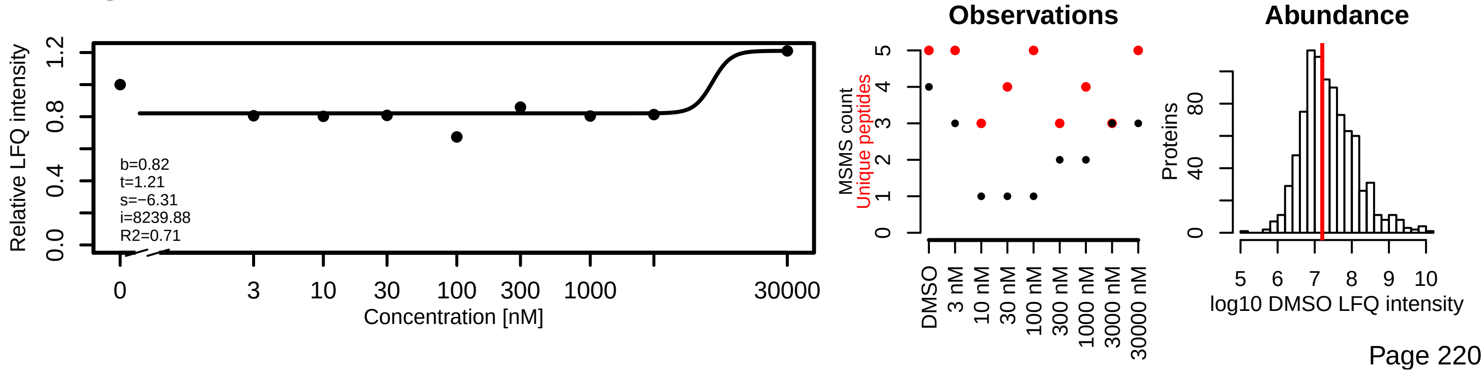

IER3IP1

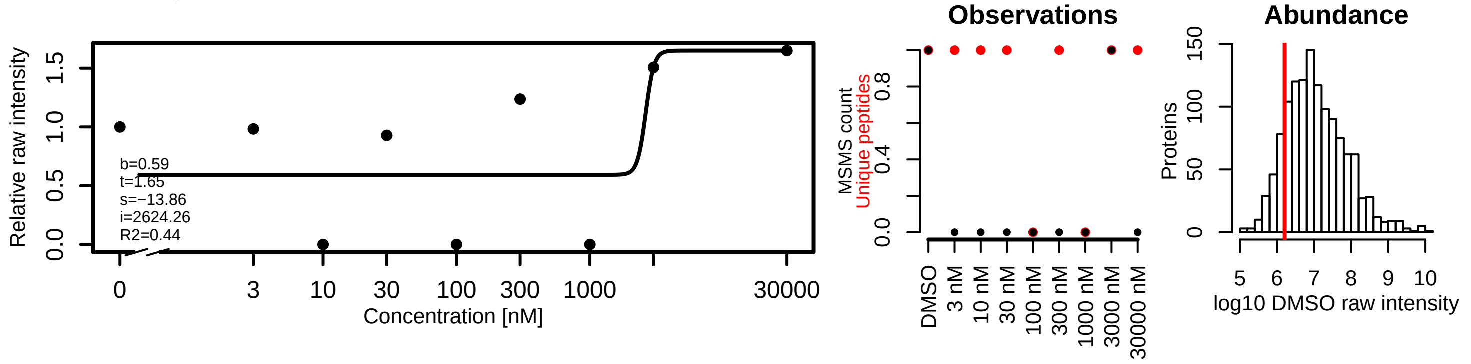

NDUFB3

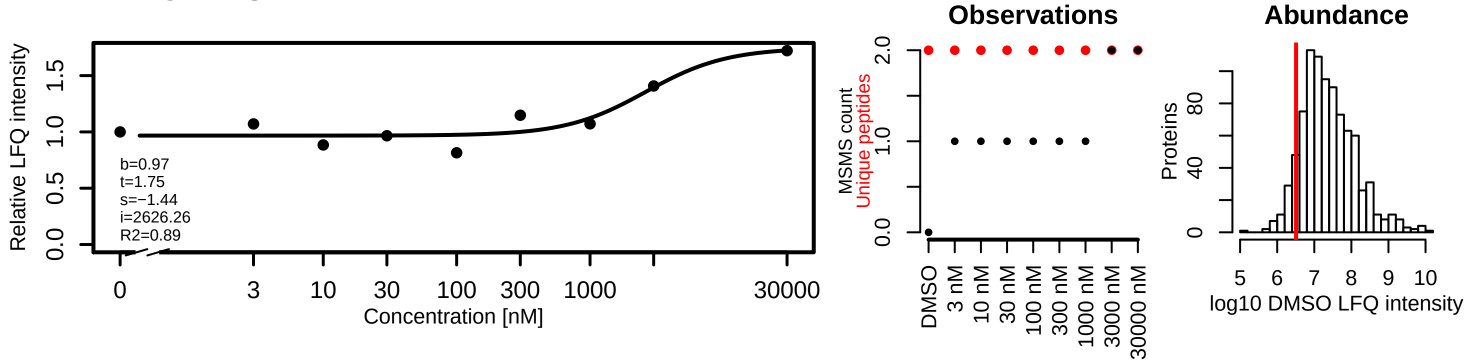

HLTF

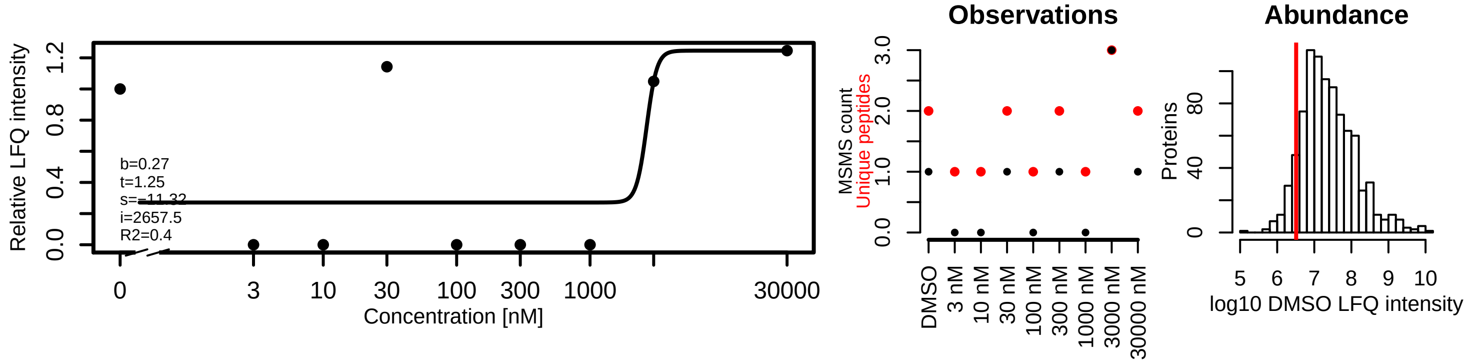

NUP35

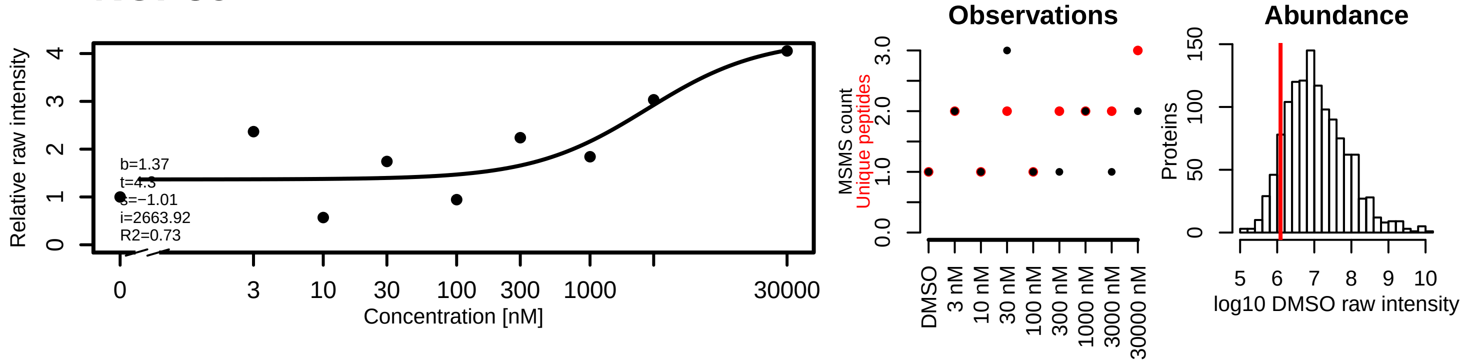

ABCC4

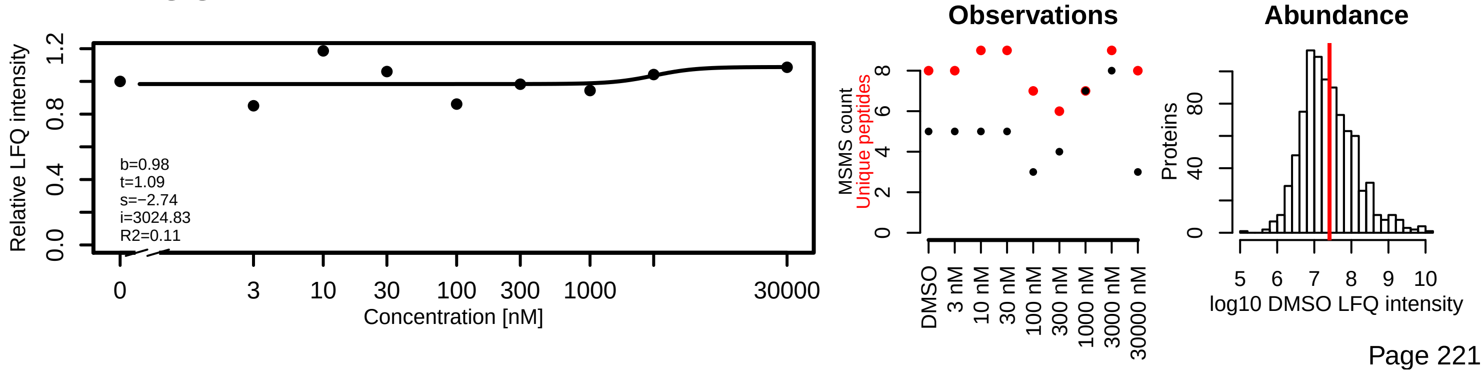

EPT1

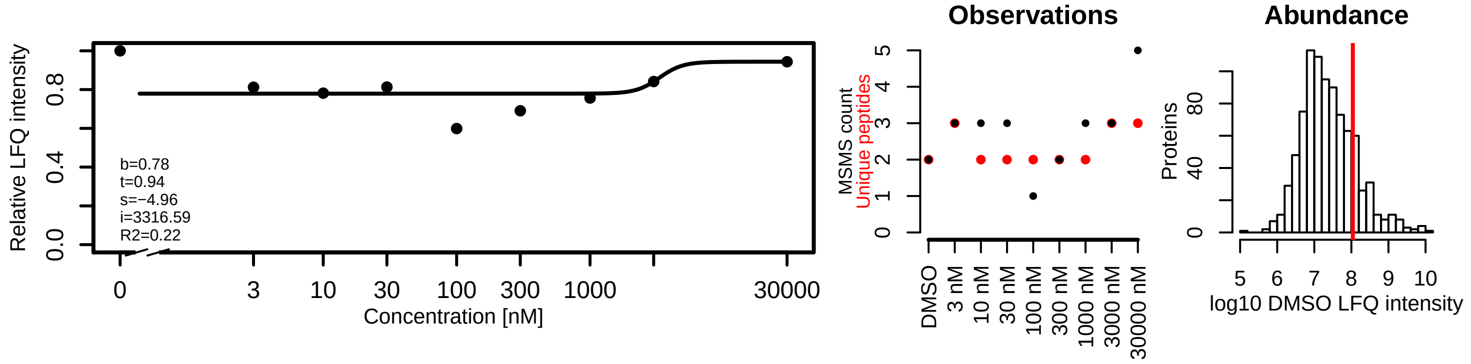

SDF4

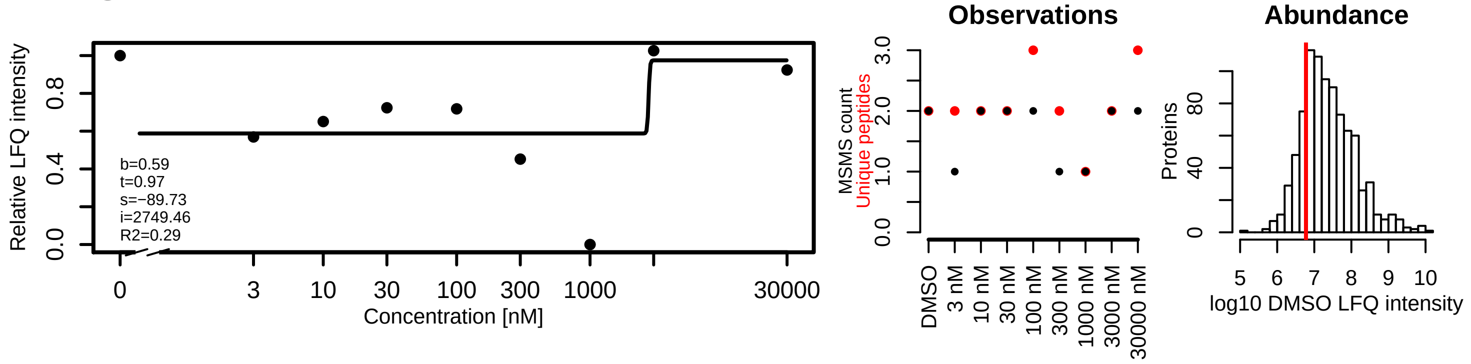

SCAMP4

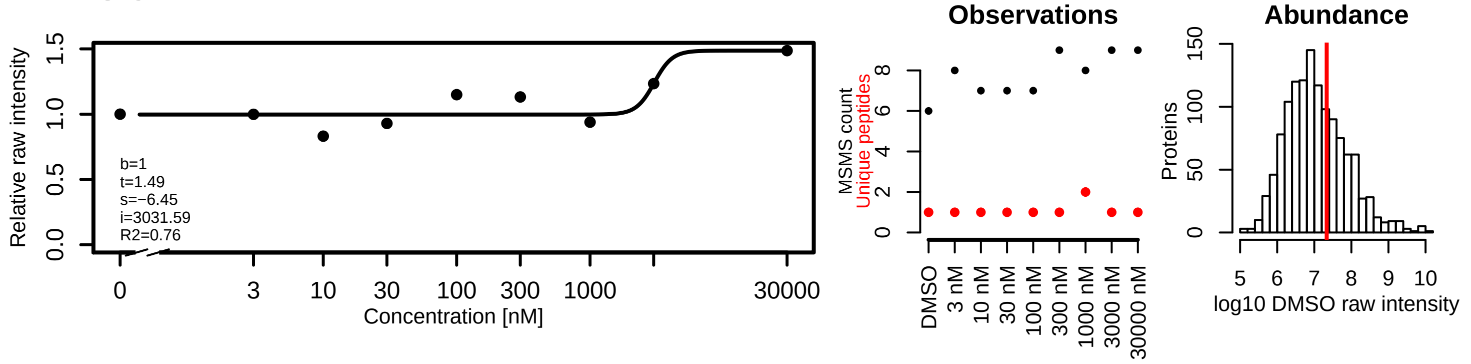

XPO1

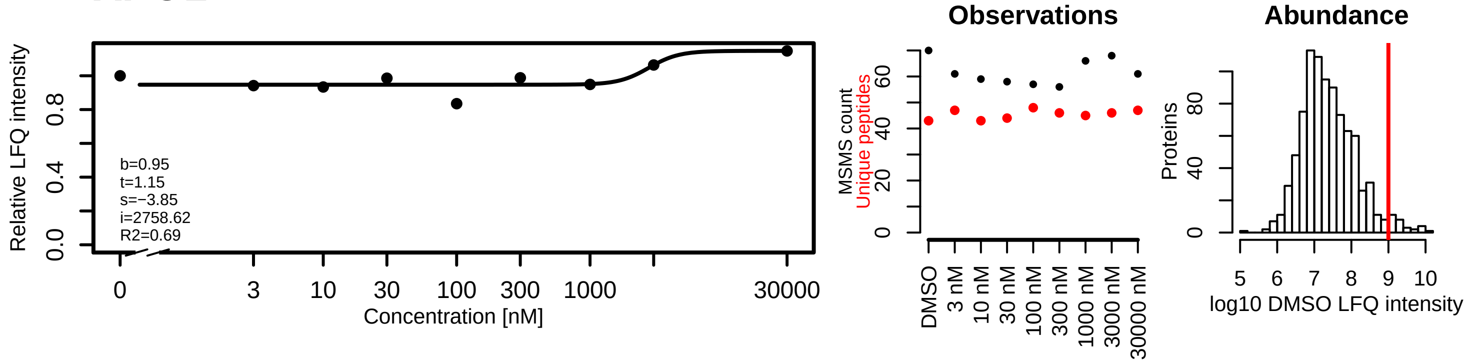

PTPLAD1

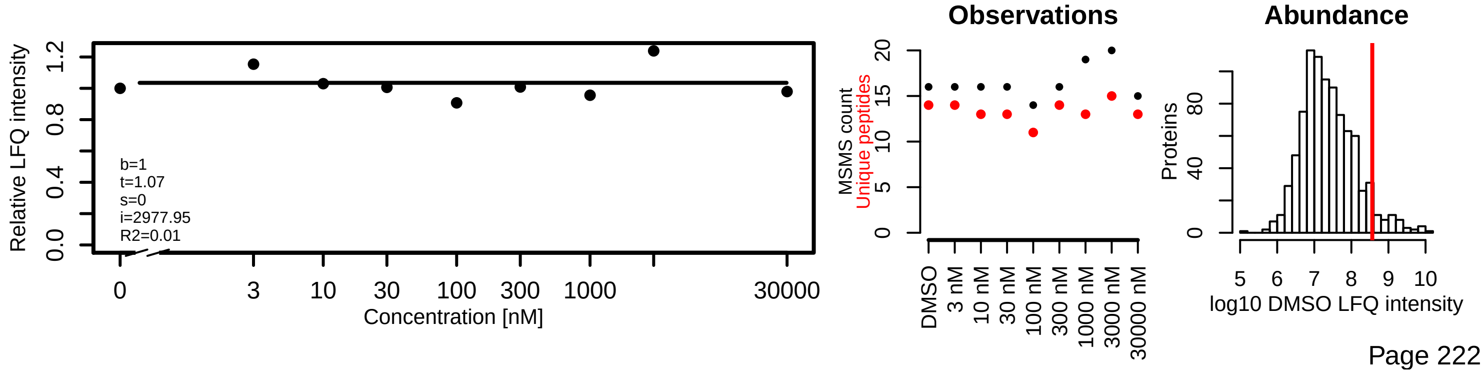

ATP13A1

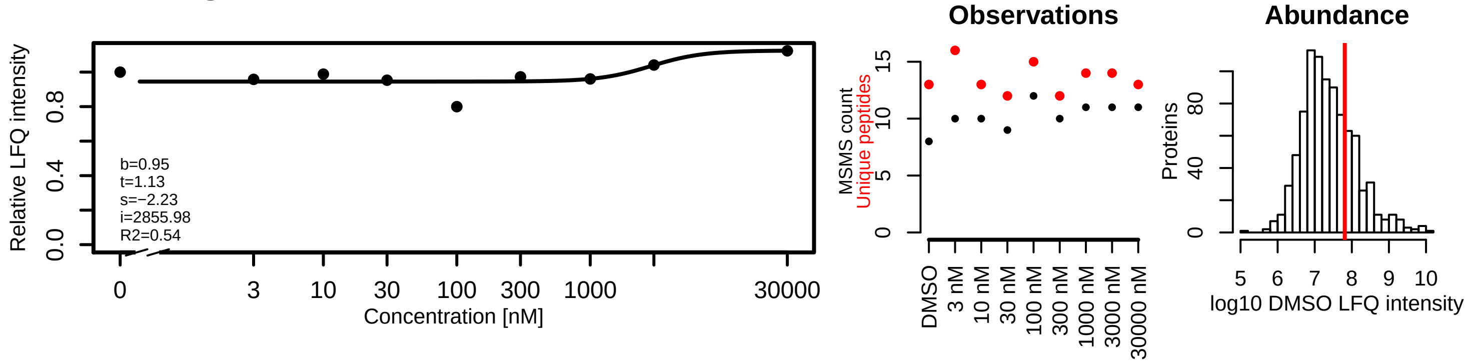

BAX

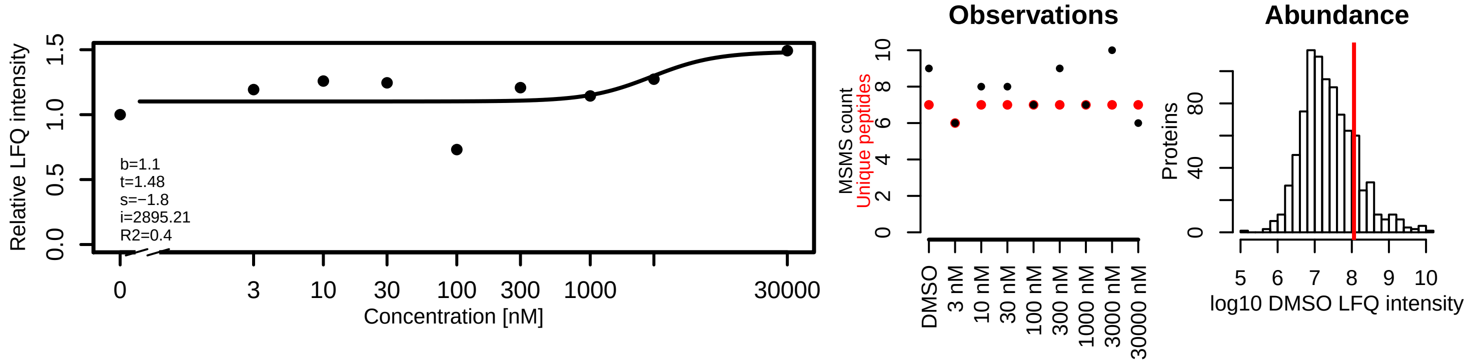

DNAJC19

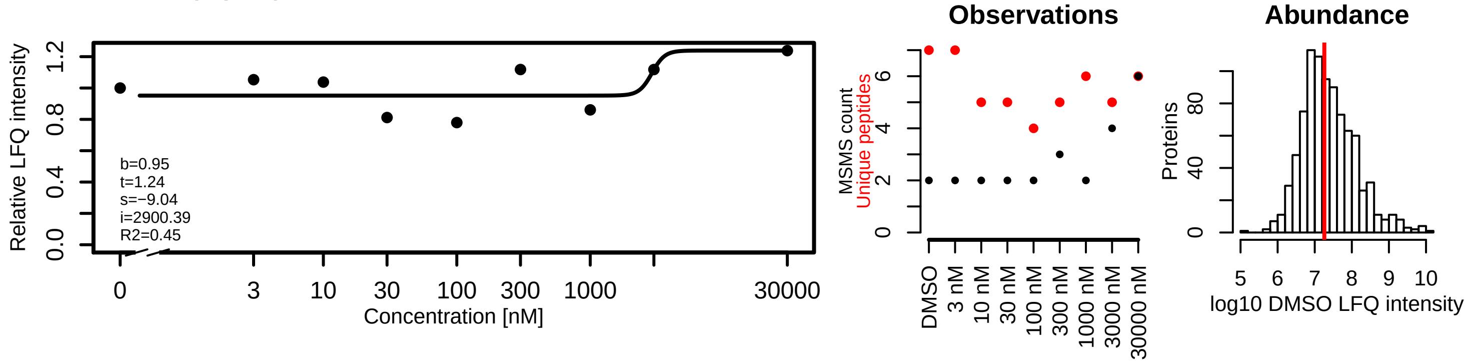

CNN2

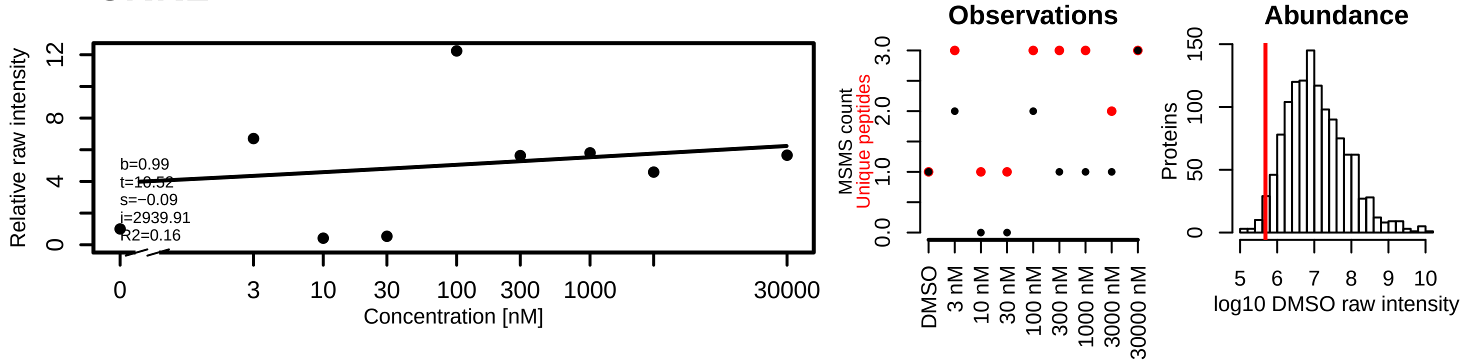

ATP5L

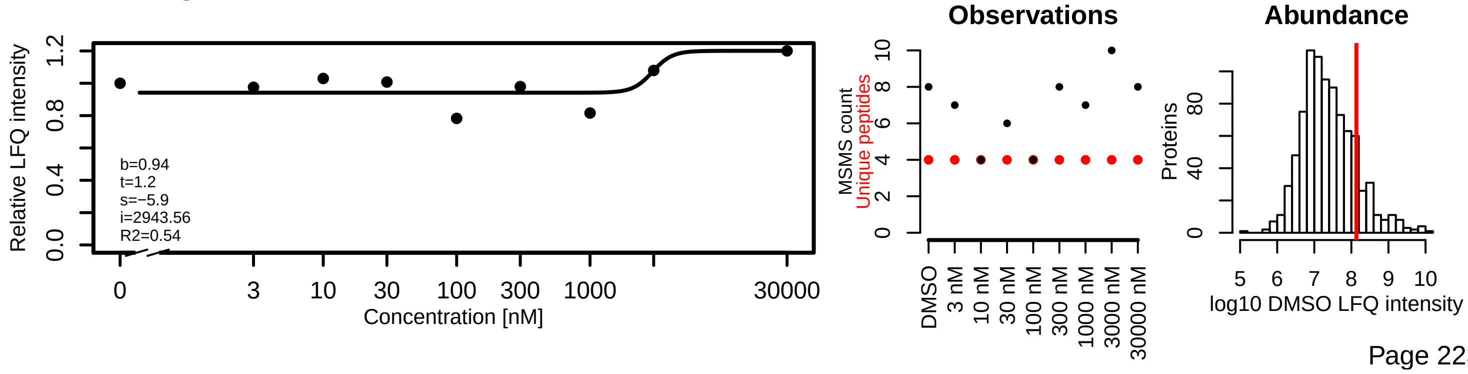

URB2

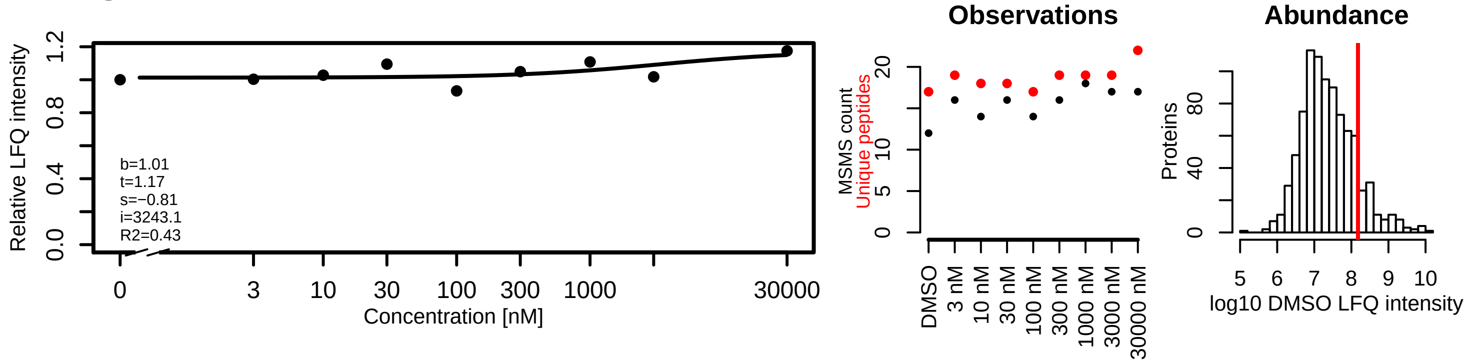

DPM3

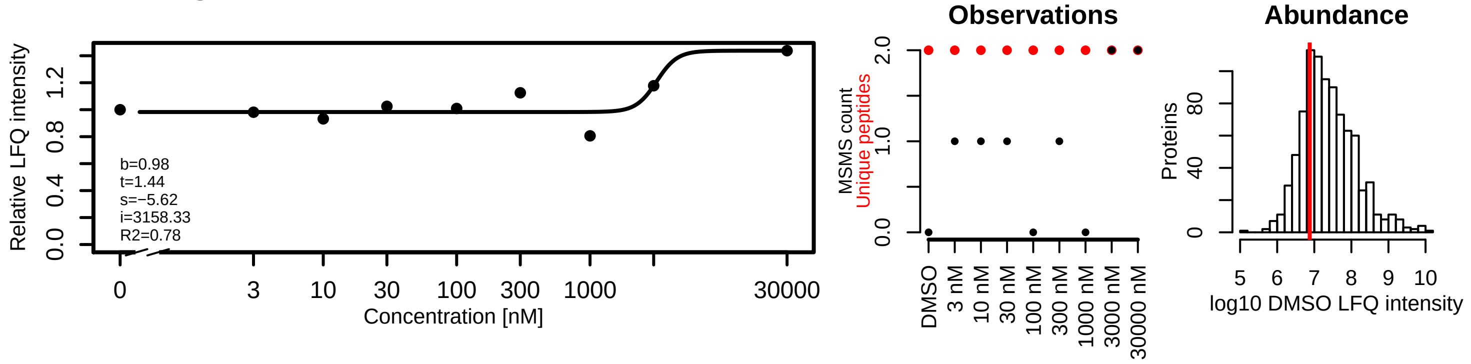

COX6B1

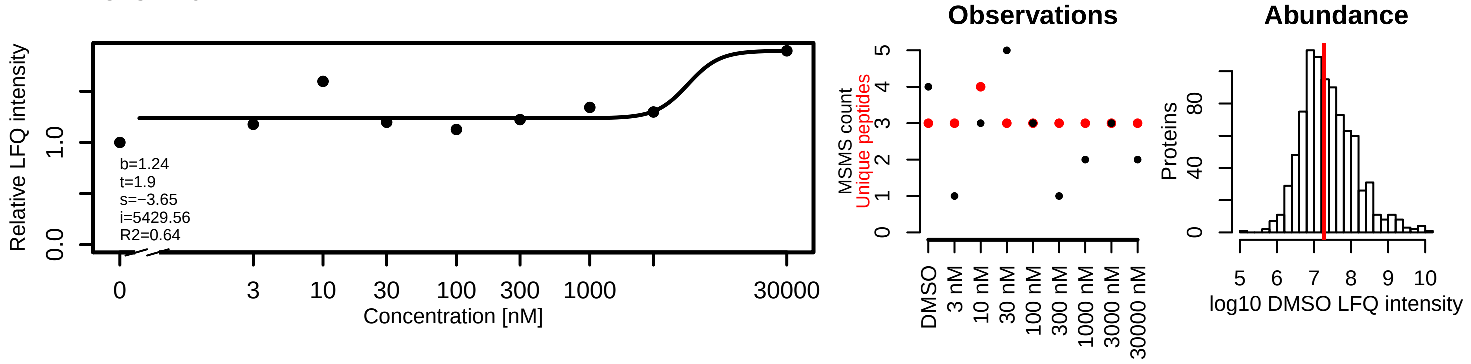

MGST3

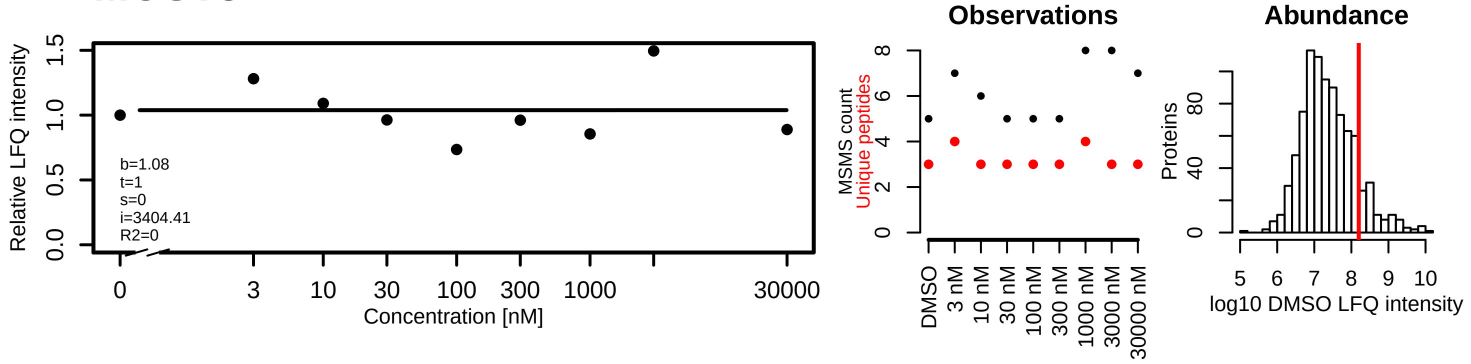

COPA

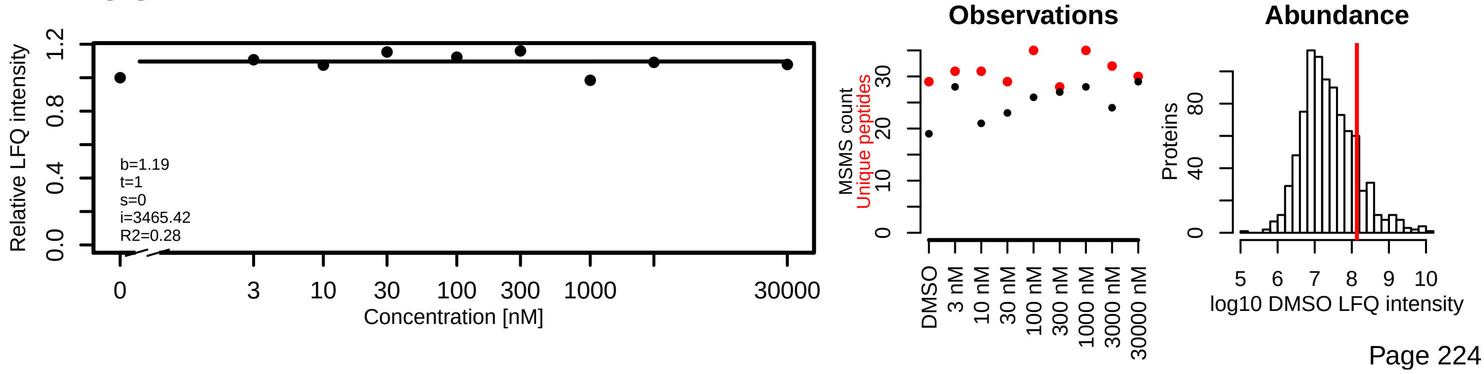

ALG2

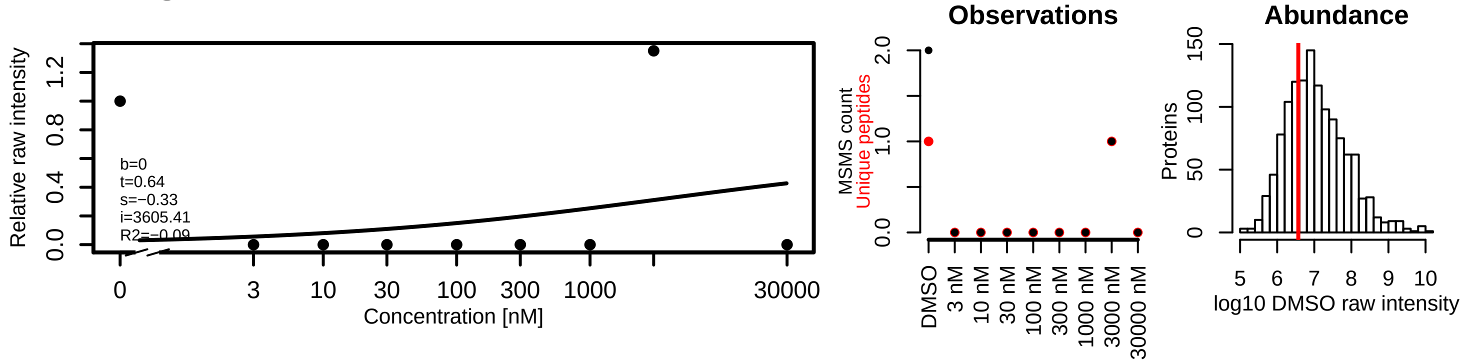

NDUFA7

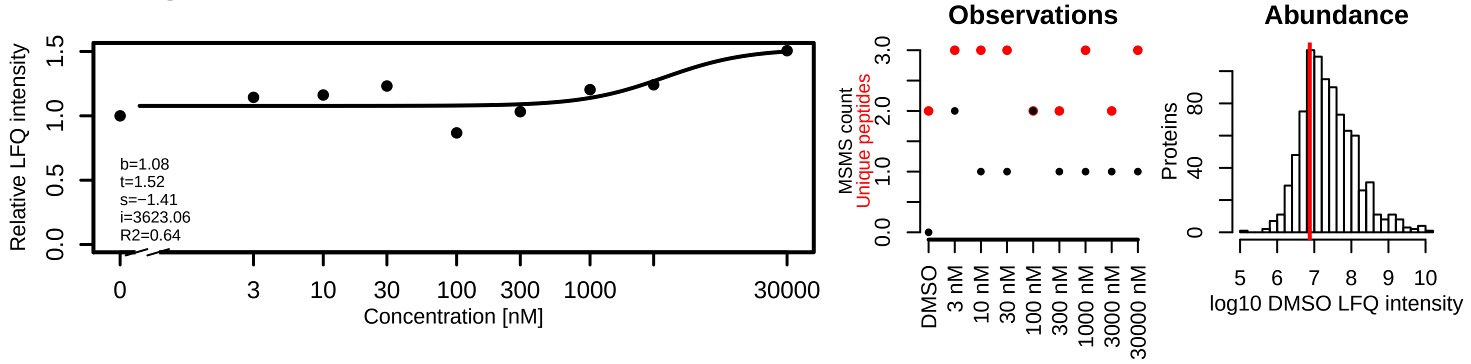

AHNAK

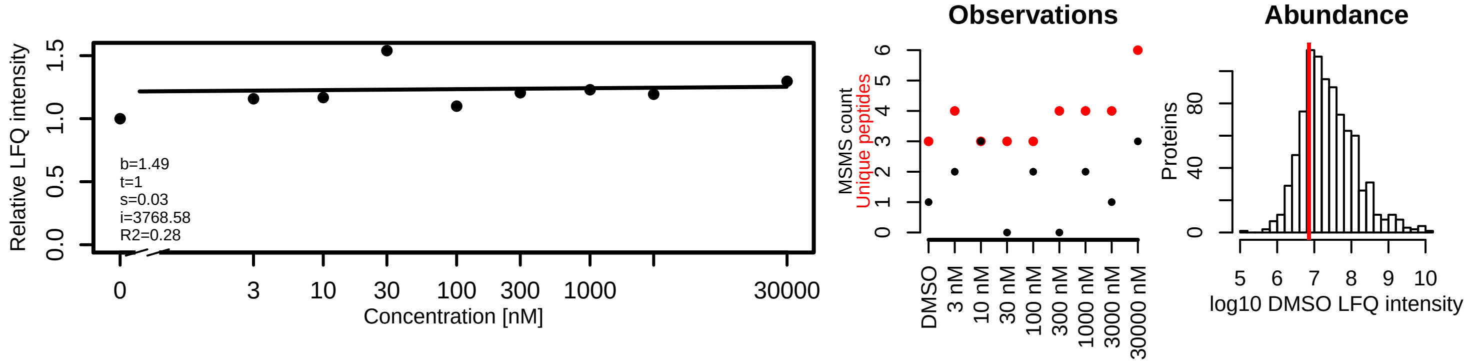

VMP1;TMEM49

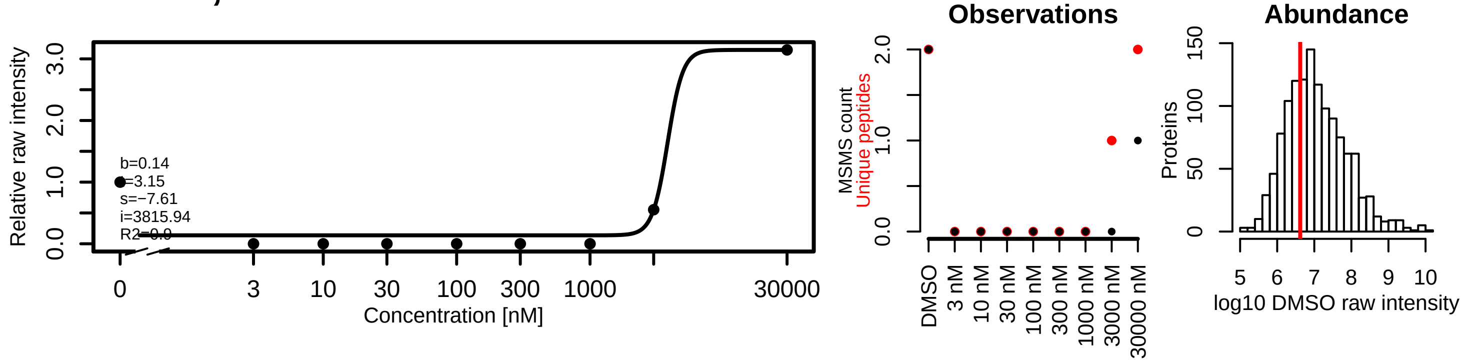

ELOVL2

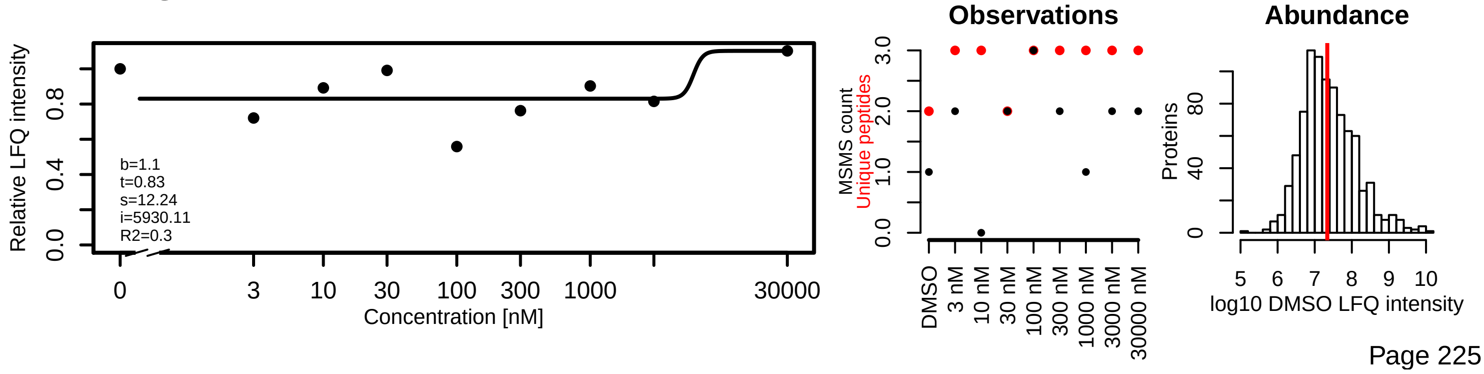

MYOF

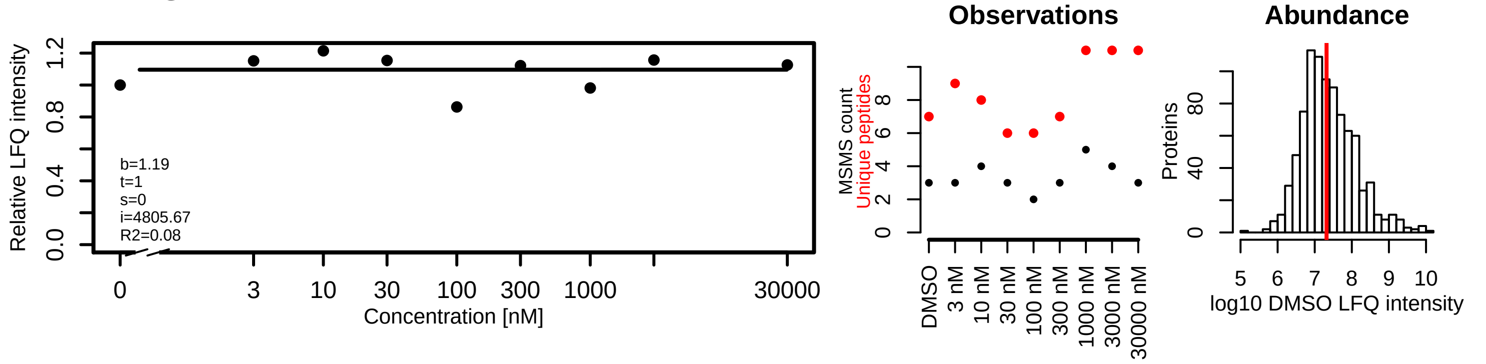

ATM

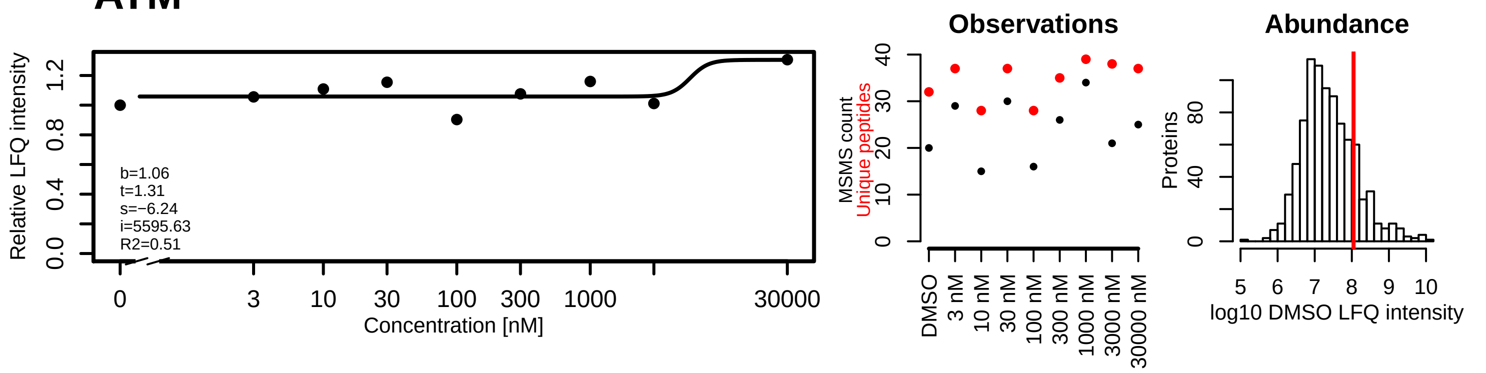

HIGD1A

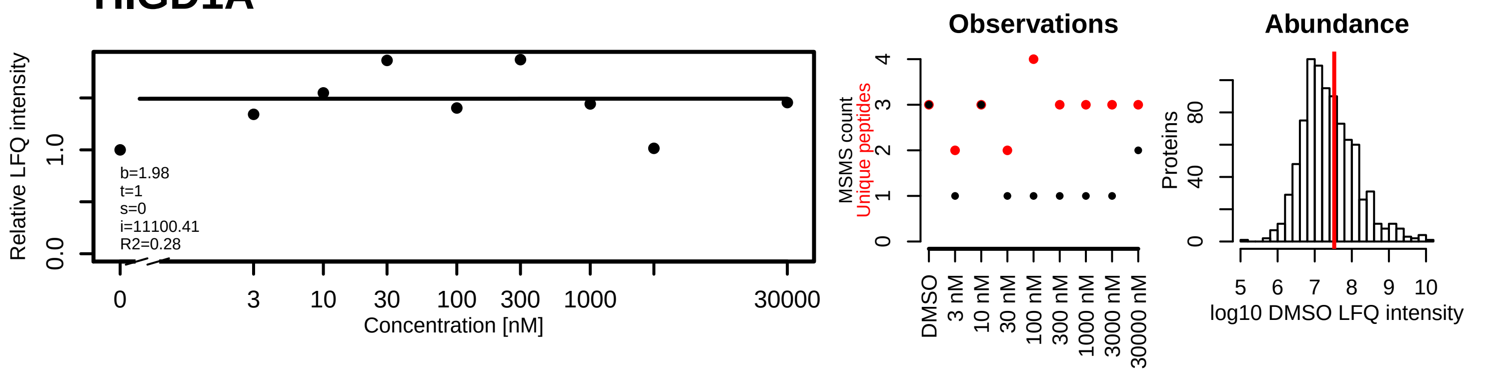

ARFGEF1

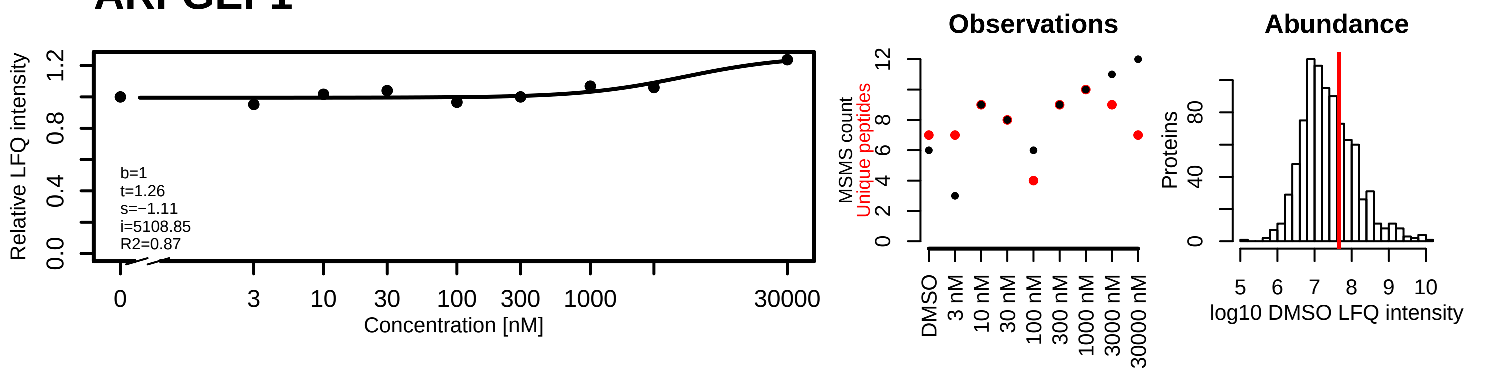

PHKA1

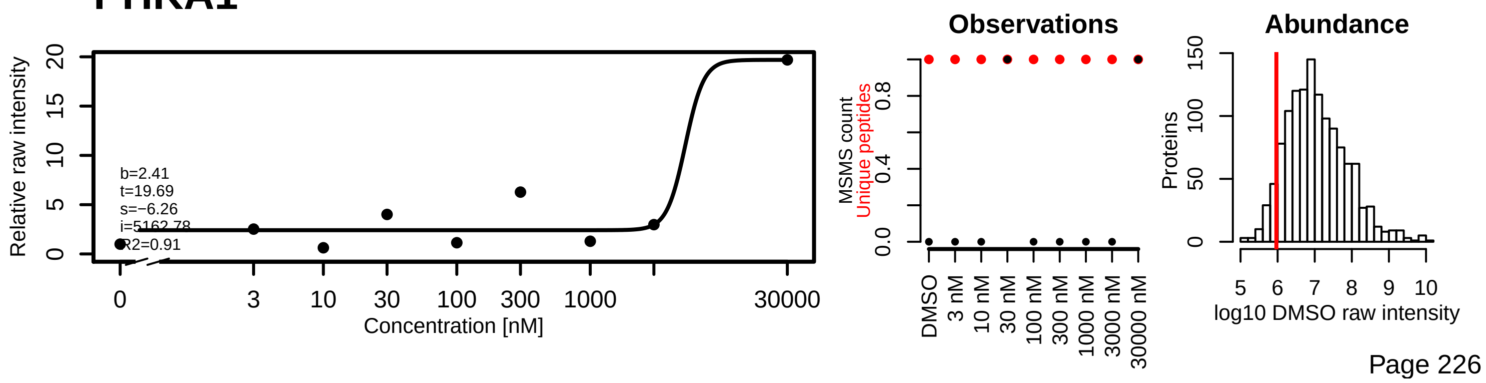

NDUFA2

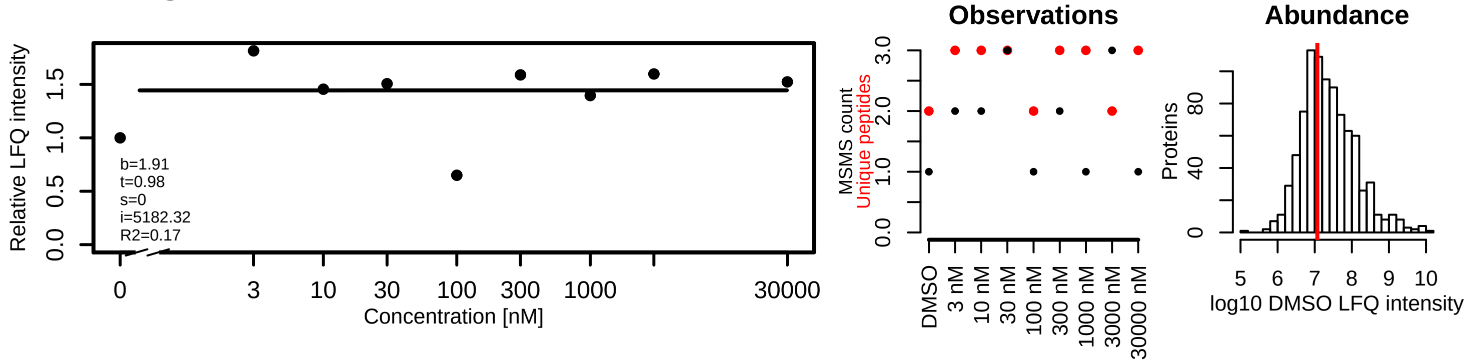

TMED2

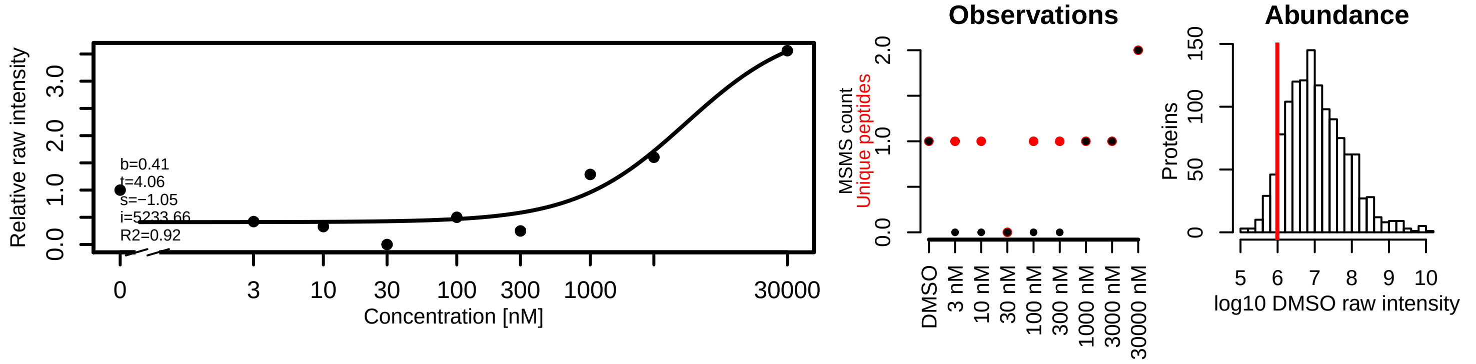

CYFIP1

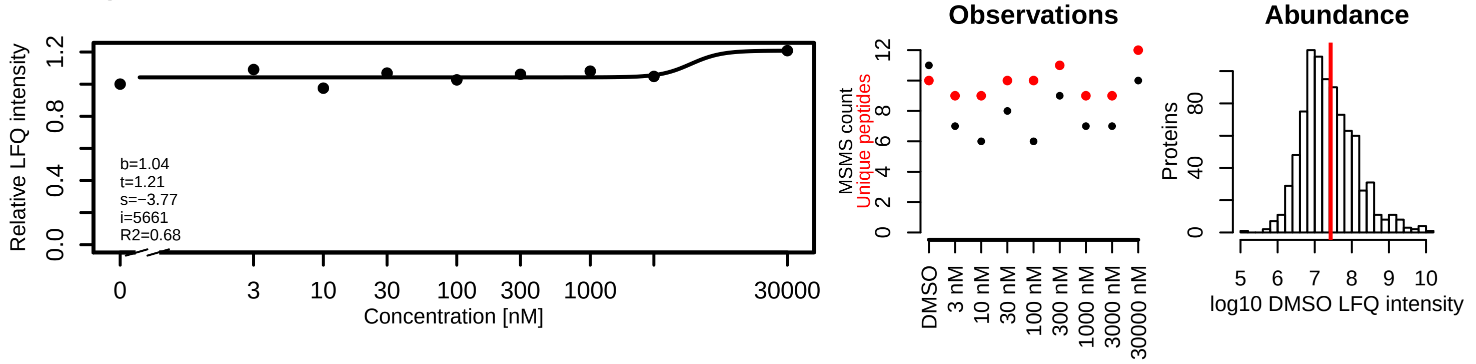

NOMO1;NOMO3;NOMO2

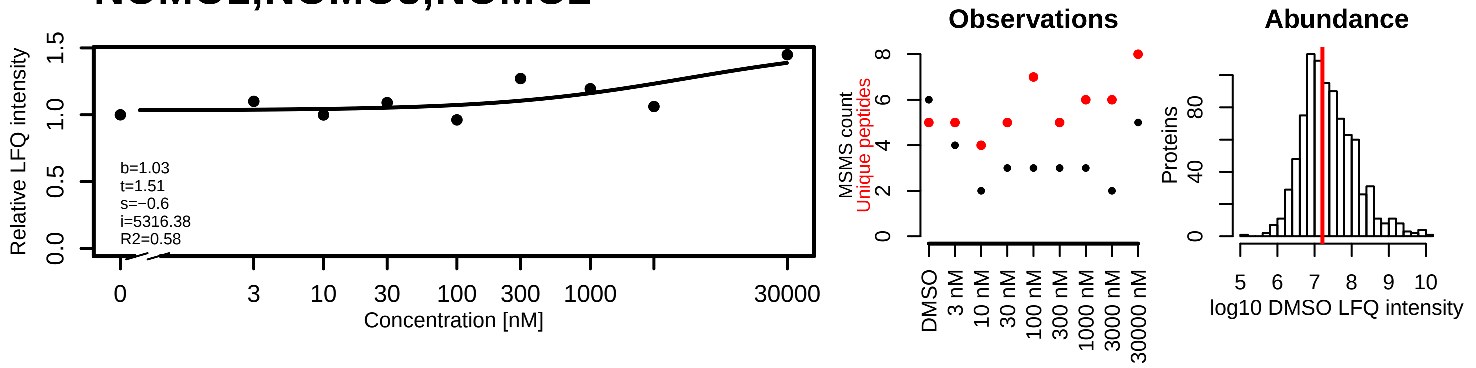

H3F3B;H3F3A;H3F3C;HIST2H3A;HIST3H3;HIST1H3A

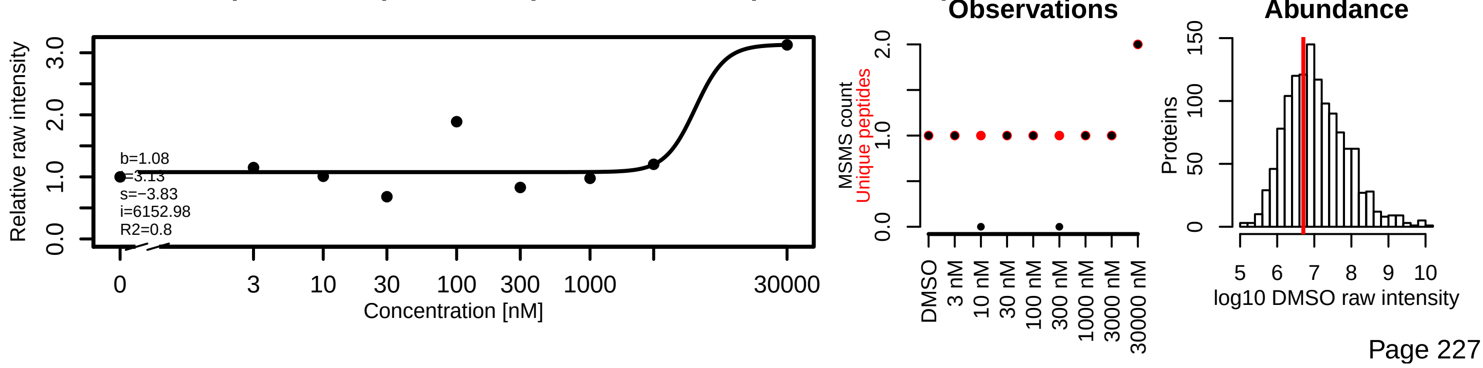

SLC7A1

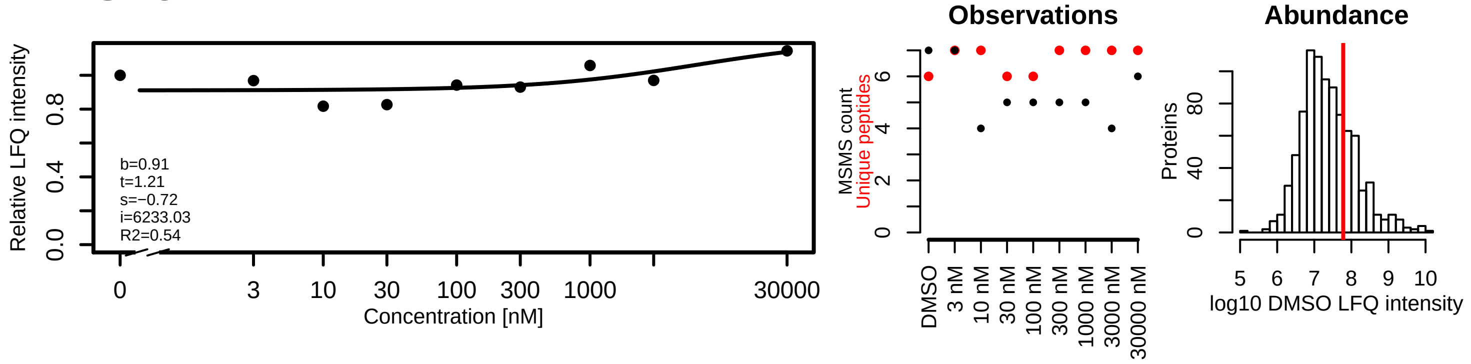

SDHB

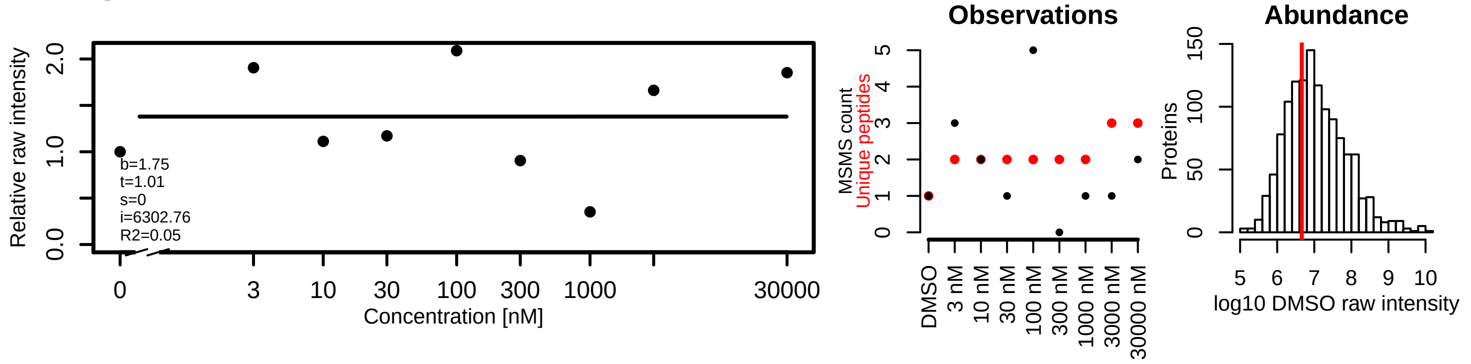

PTK7

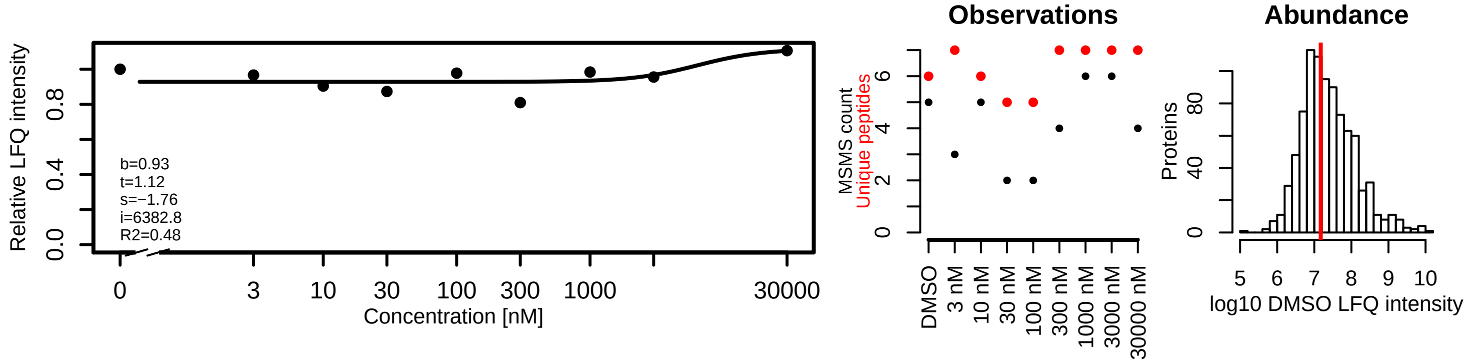

TIMMDC1

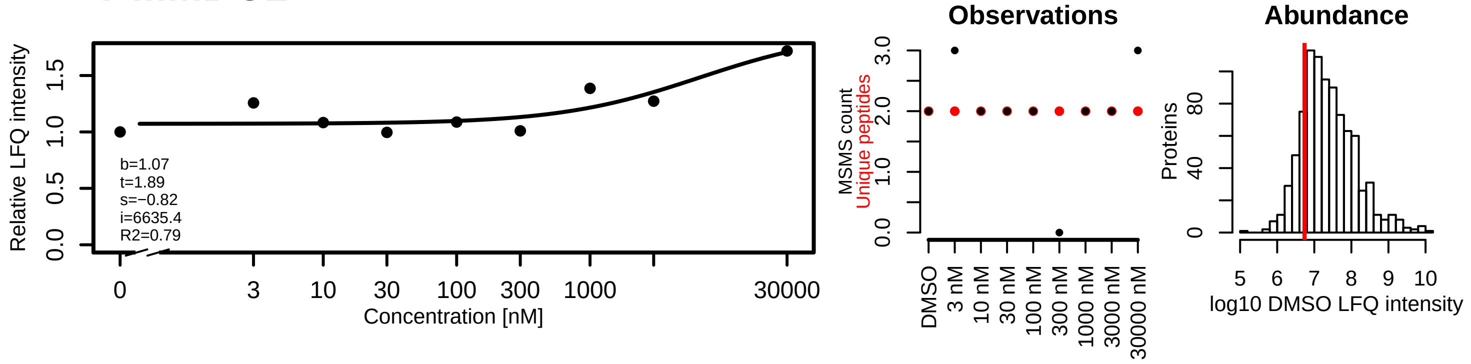

NAP1L1

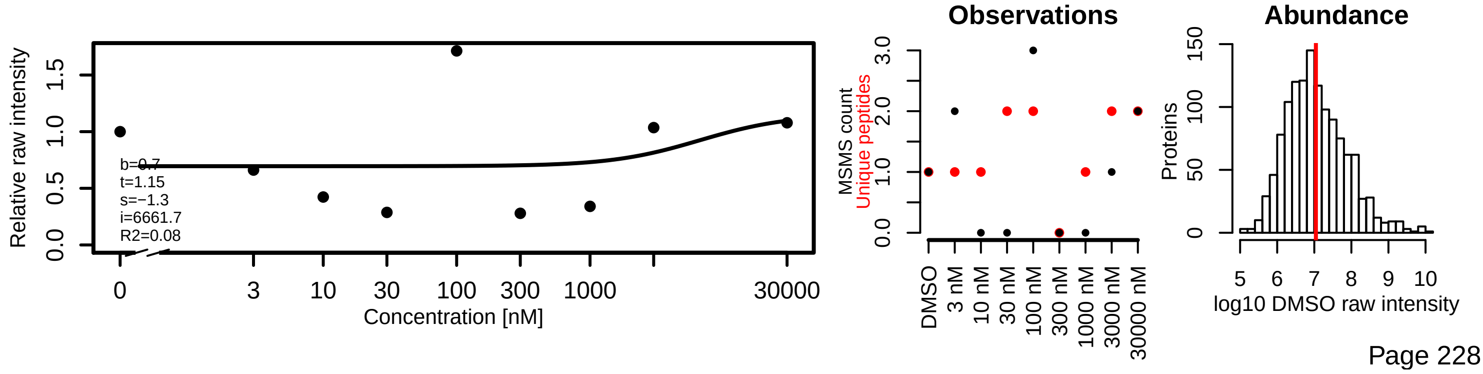

ACADM

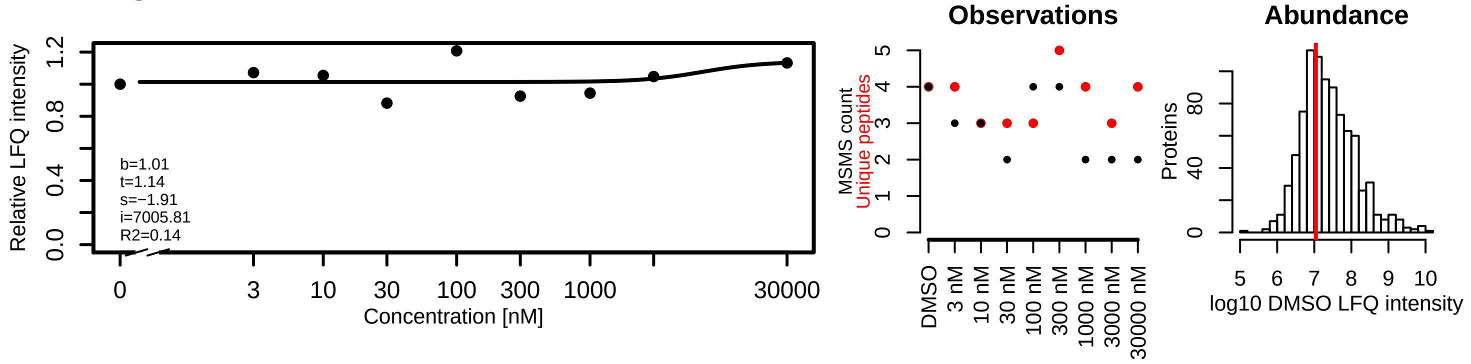

RAB14

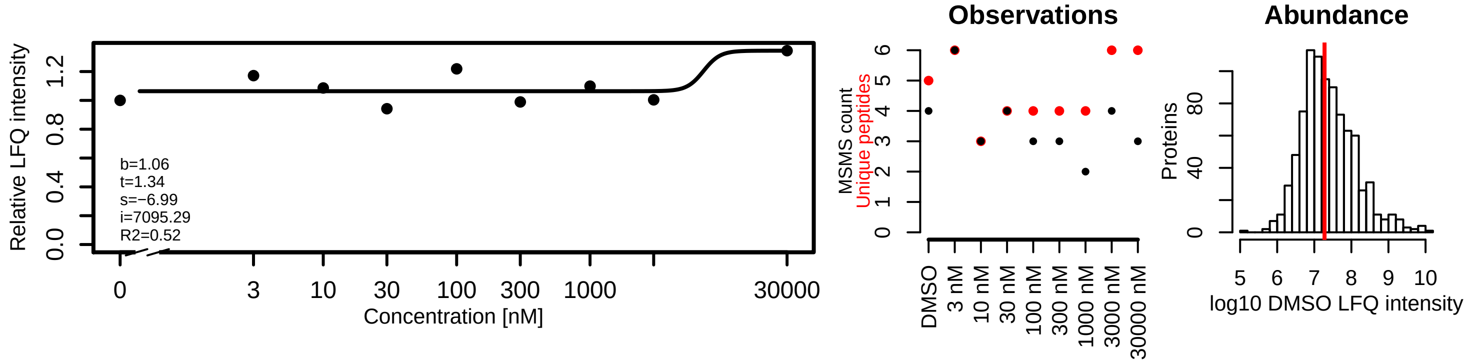

TNPO1

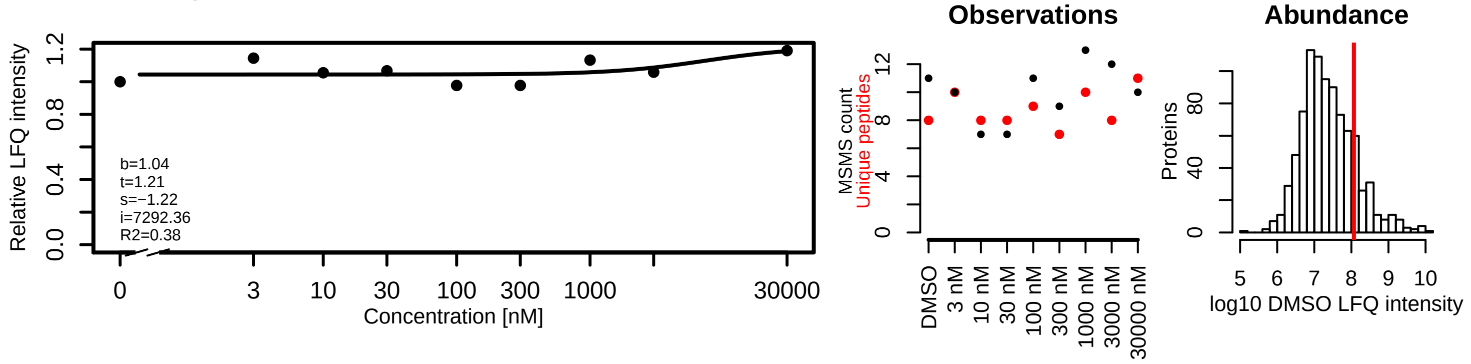

ATPIF1

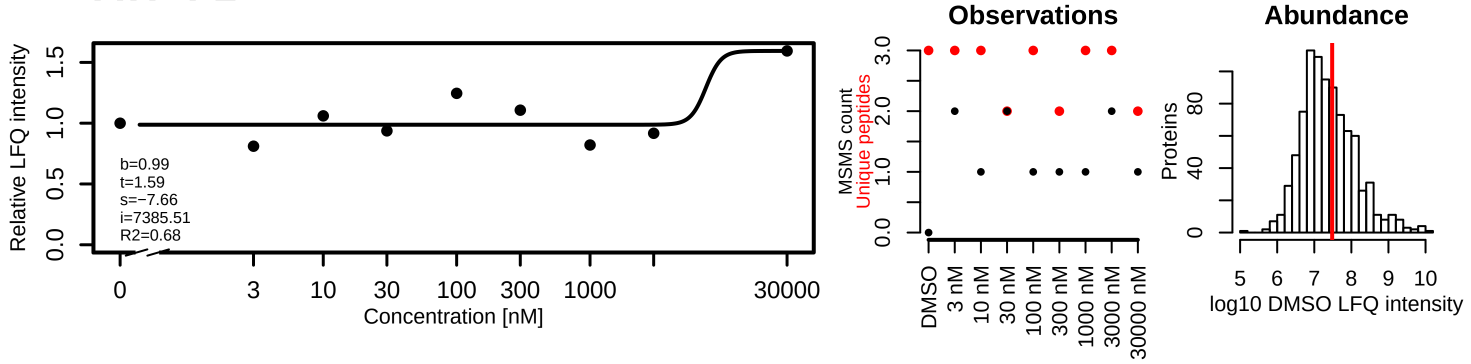

MDN1

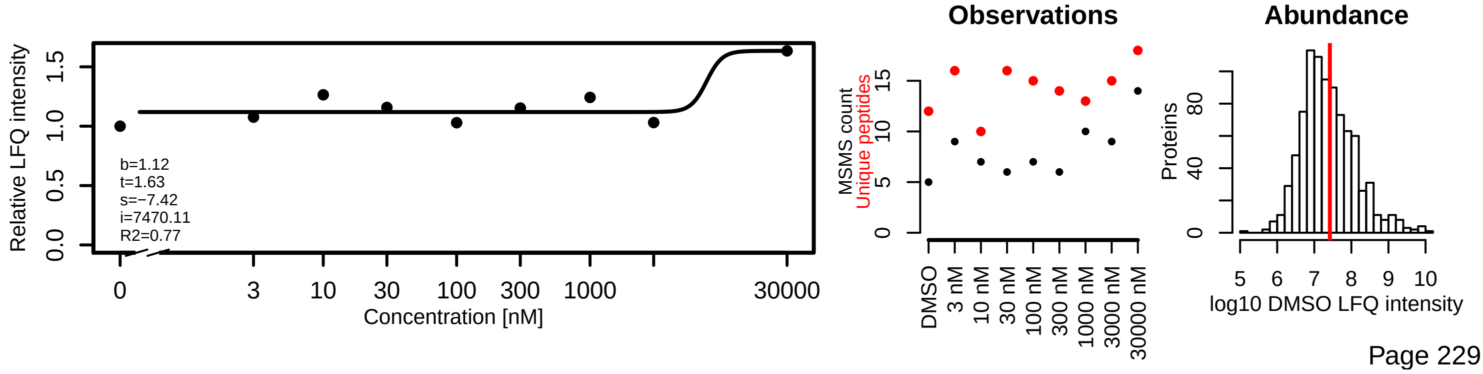

SEC11A;SEC11L1

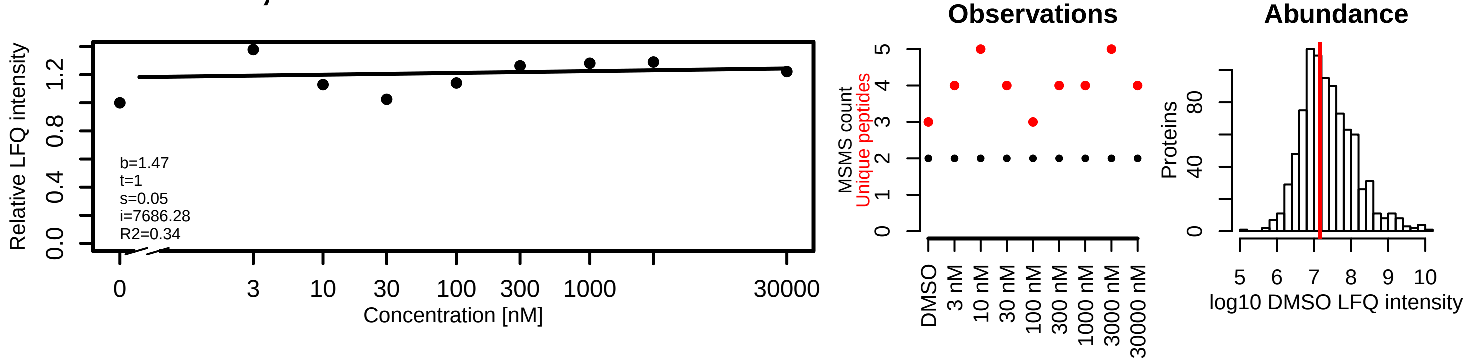

EPG5

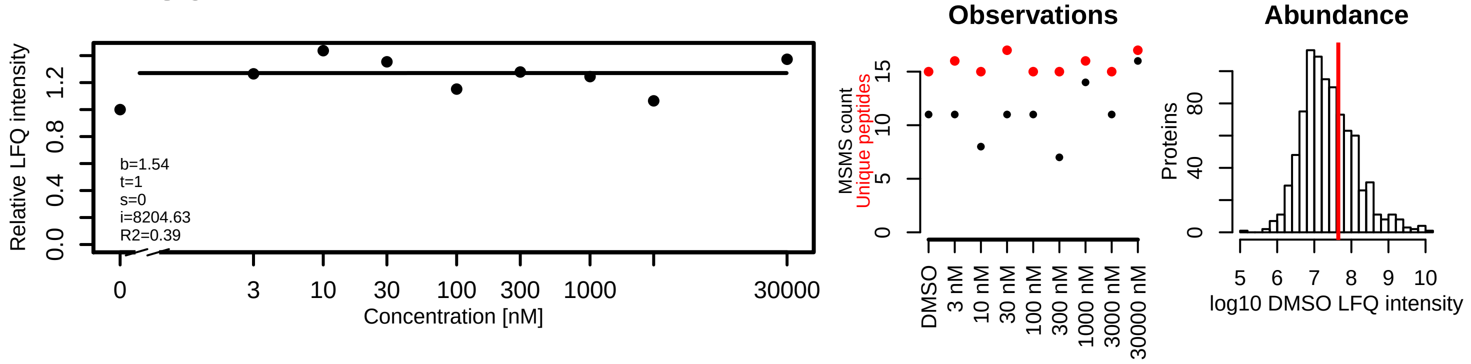

MROH1

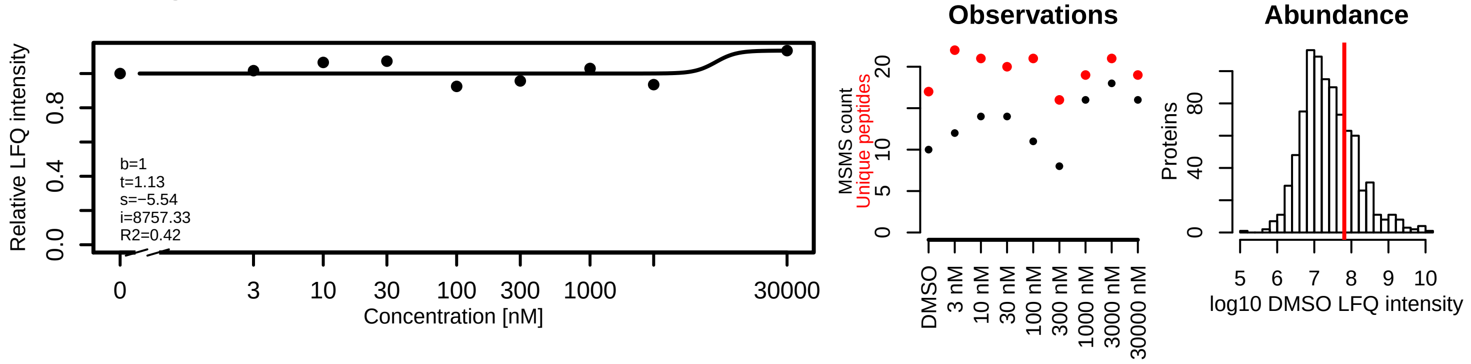

CD44

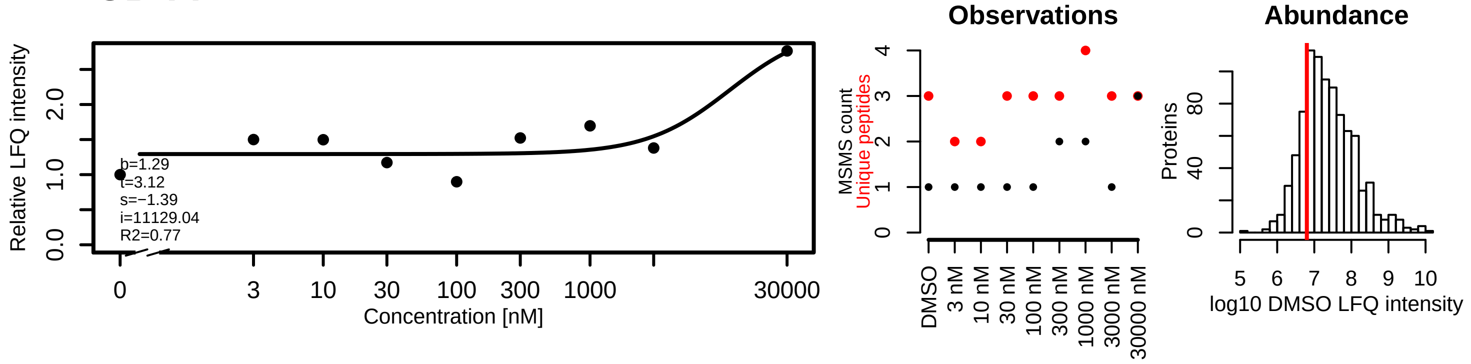

TXN

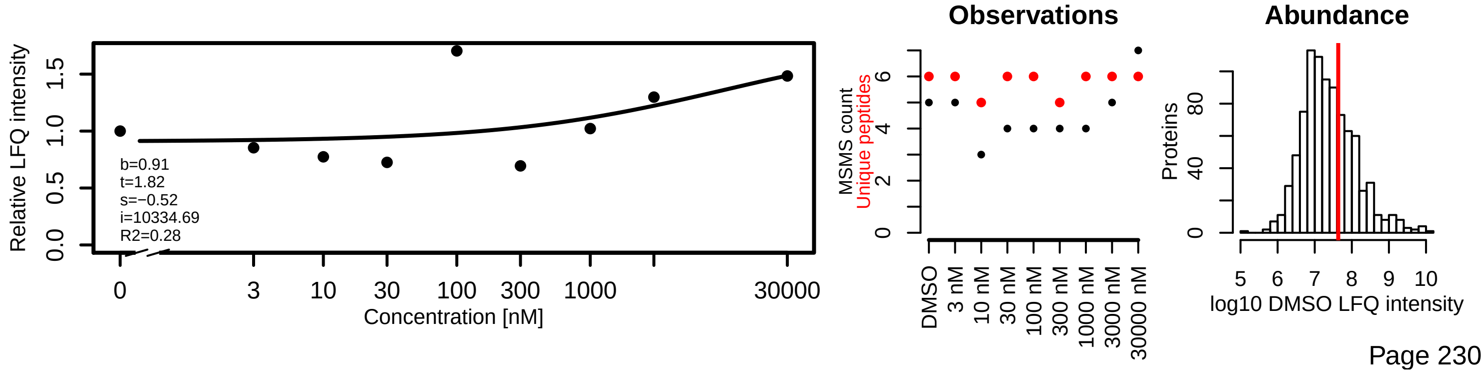

SEC61B

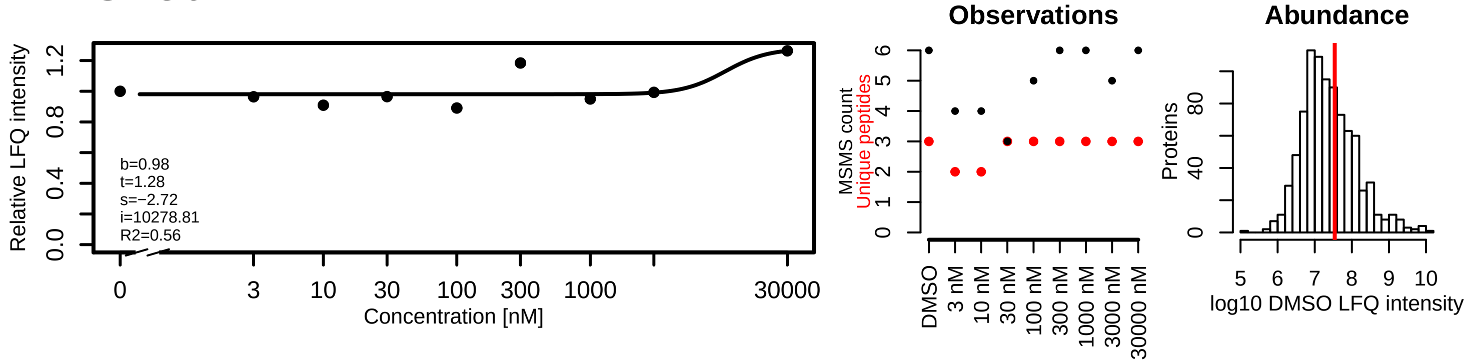

OSBPL3

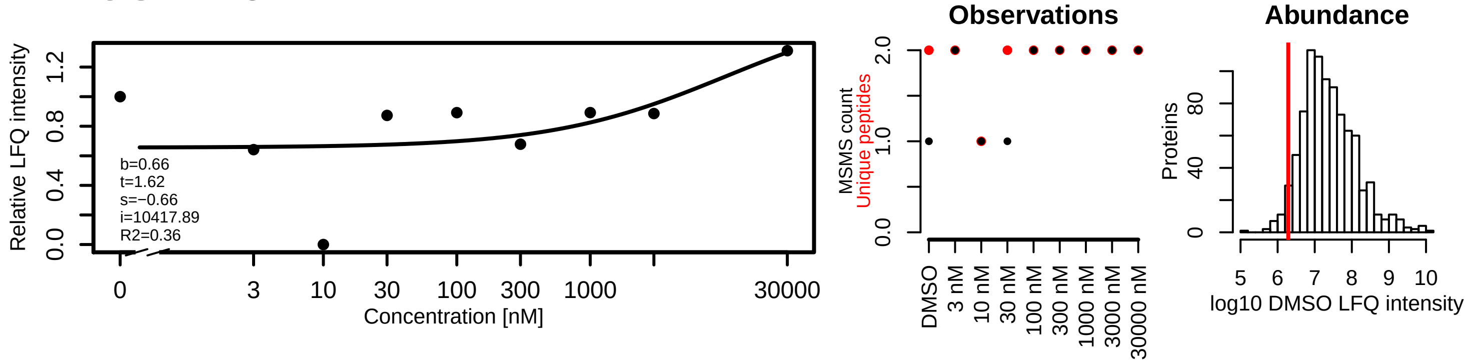

FANCD2

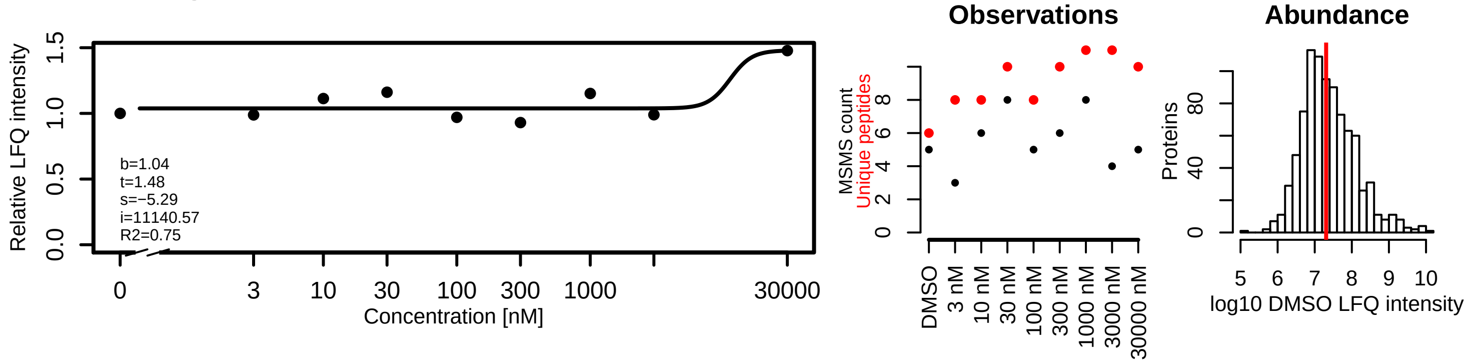

COX7A2L

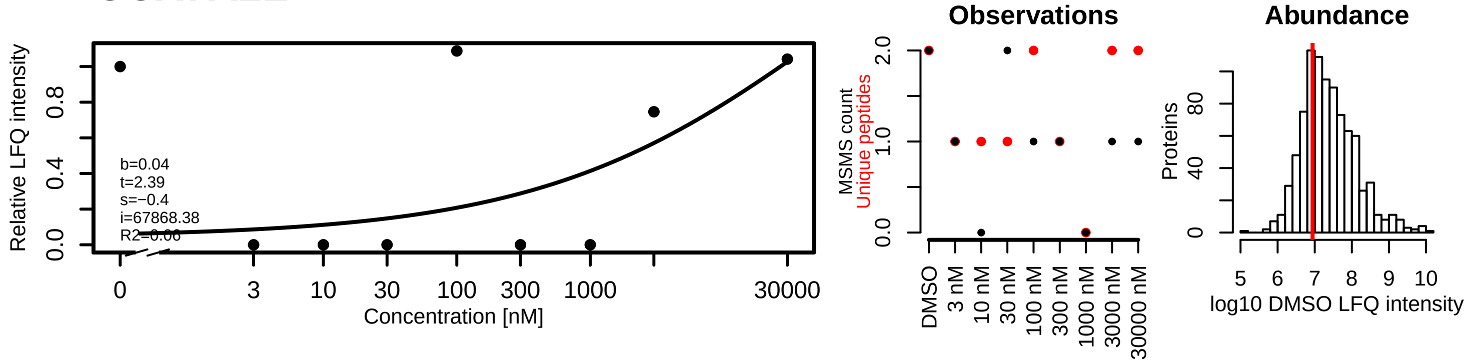

SLC25A24

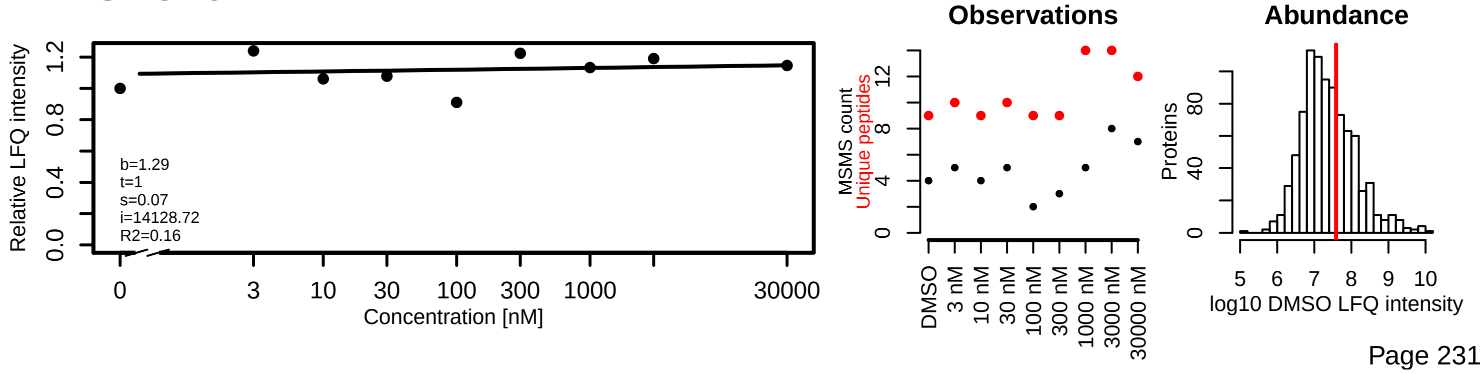

RPS11

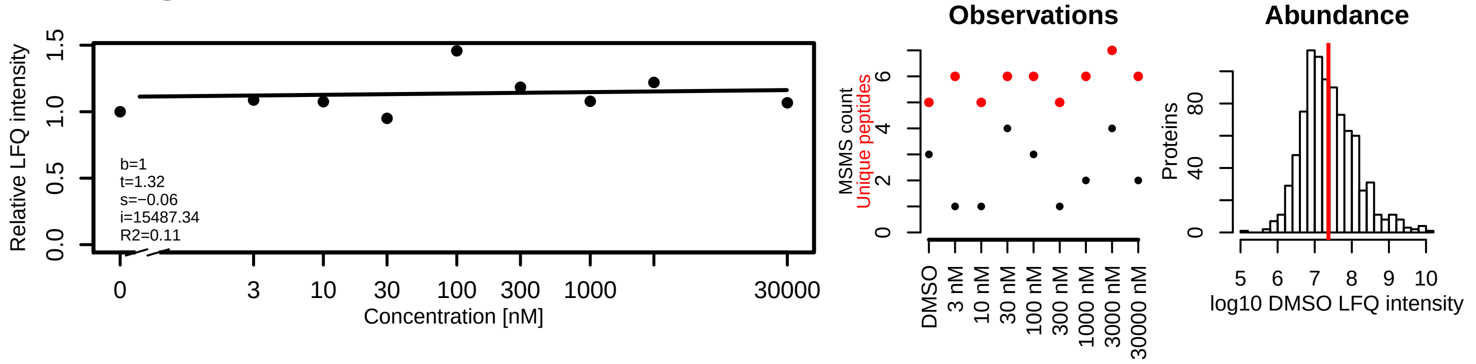

SYPL1

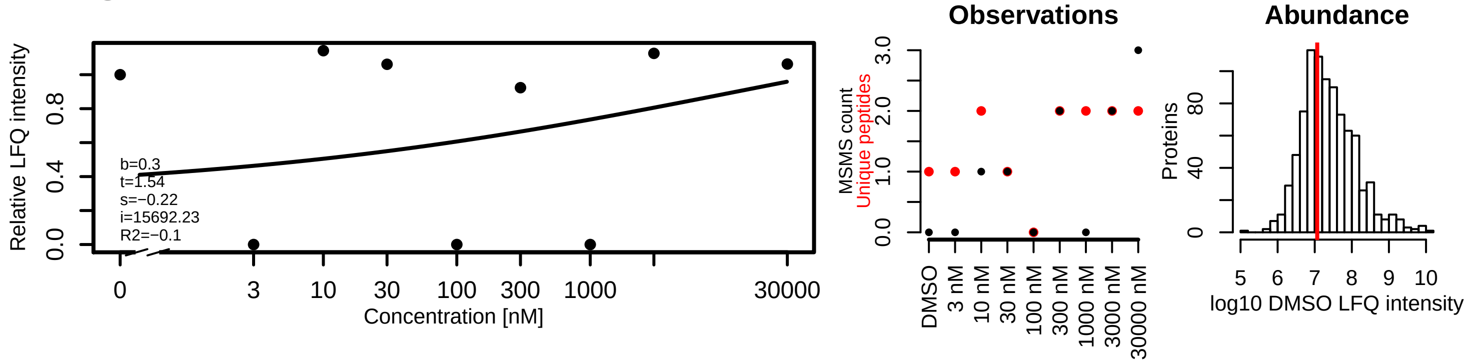

ATP5E;ATP5EP2

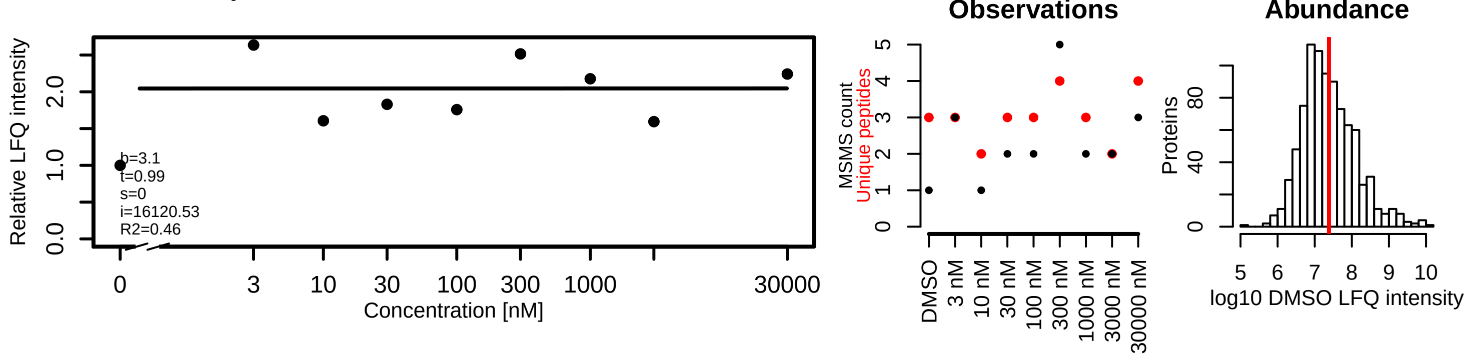

BZW1

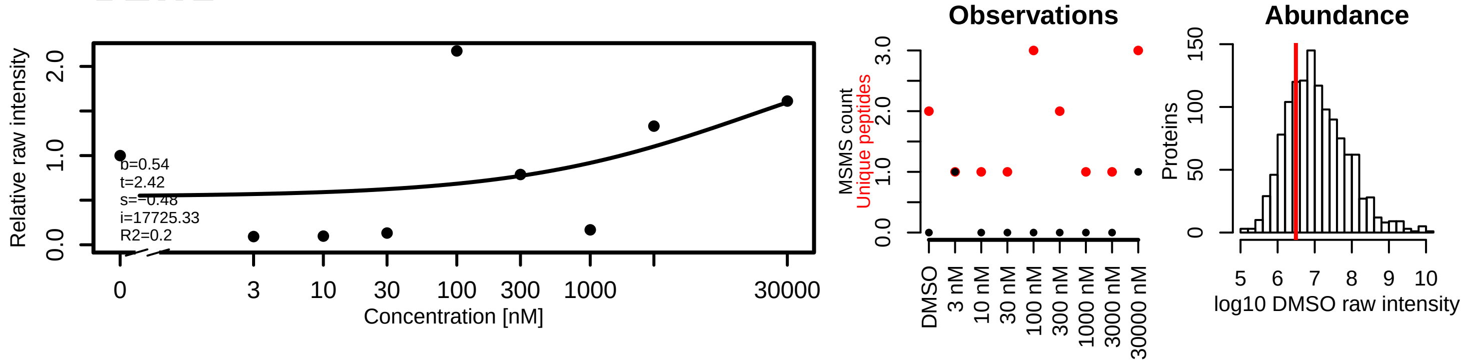

SLMAP

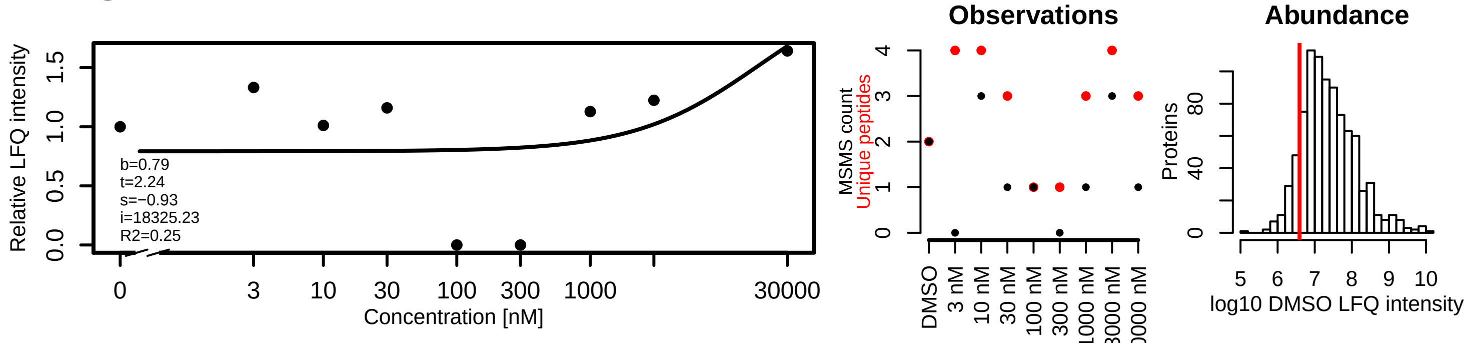

WLS

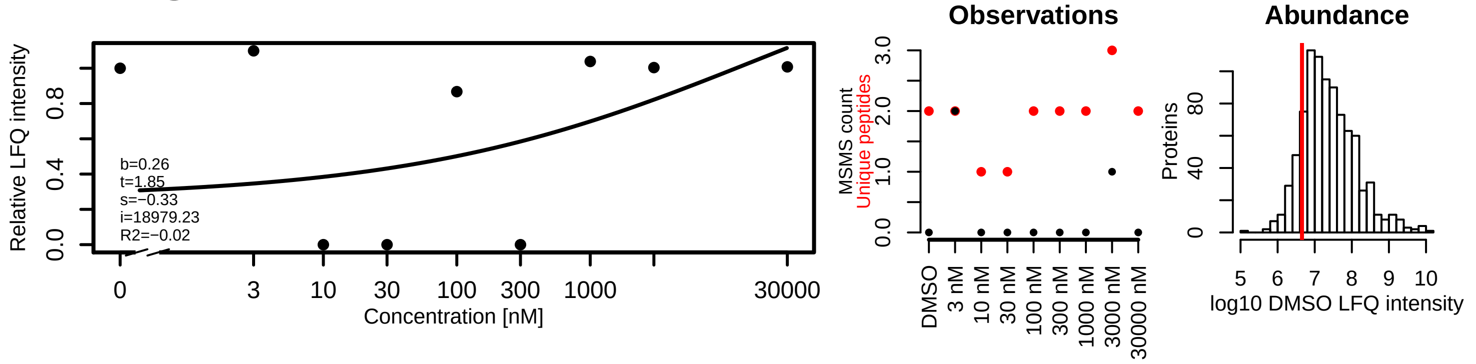

TBC1D9B

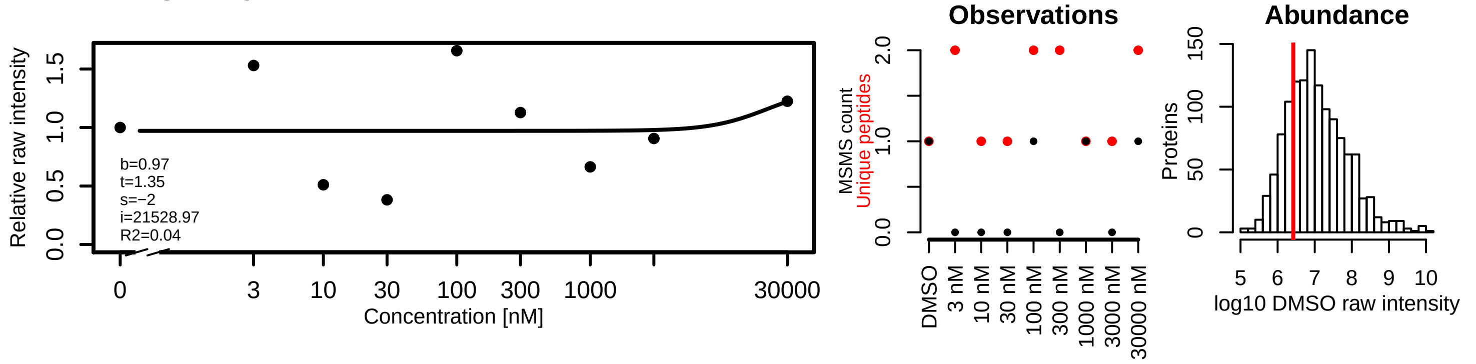

PTDSS2

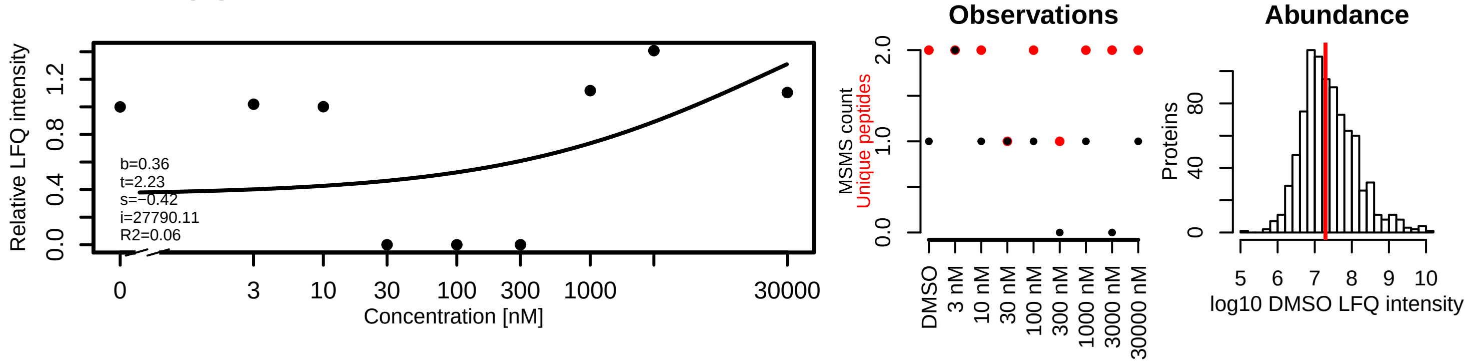

PCBP1

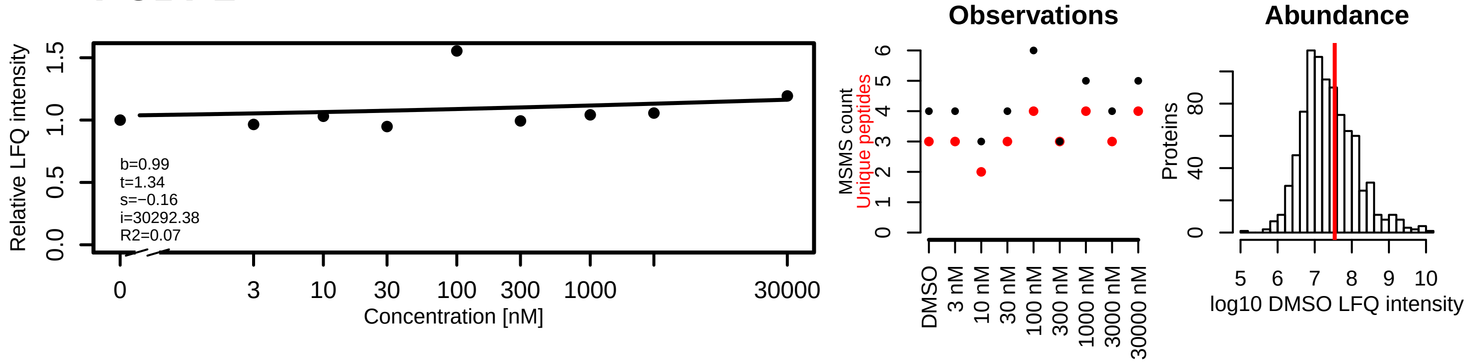

CAPZA2

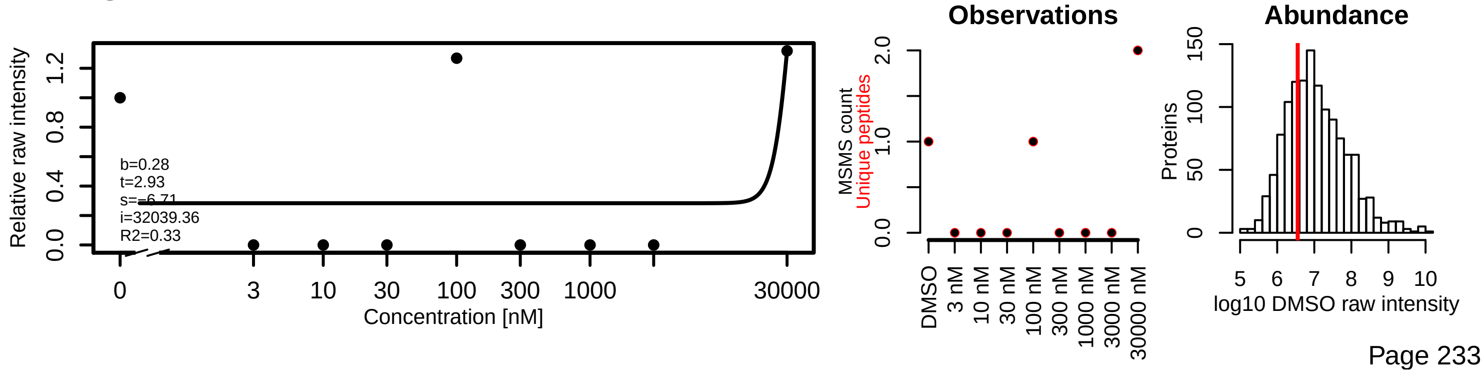

HMOX2

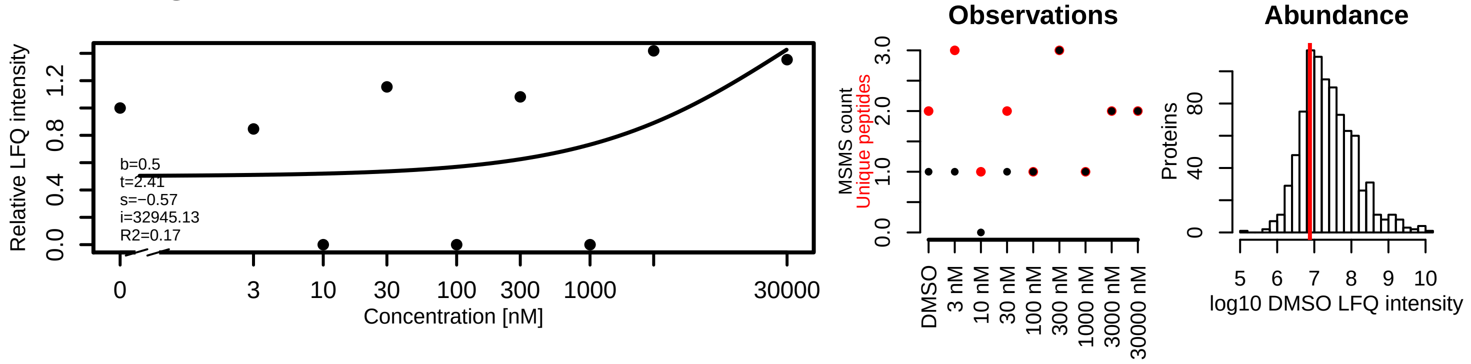

STX18

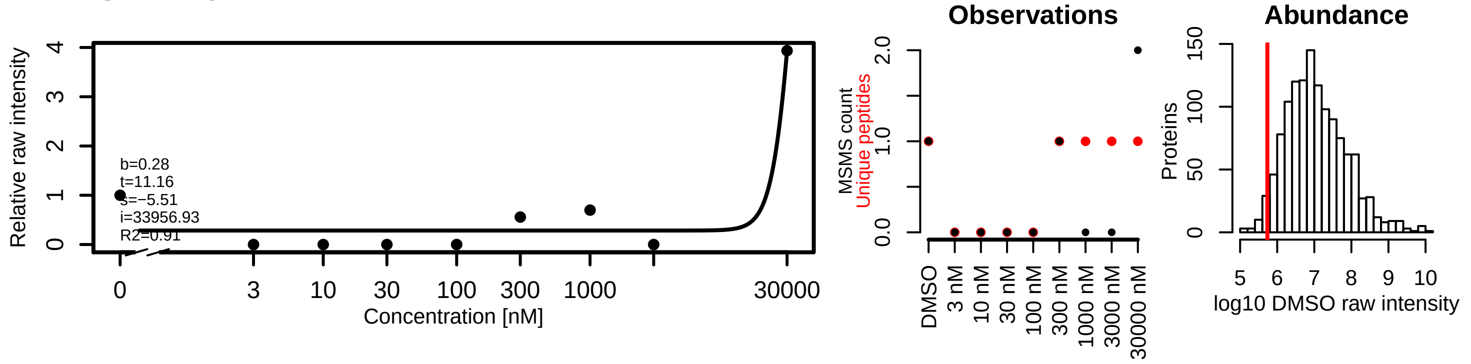

MARS

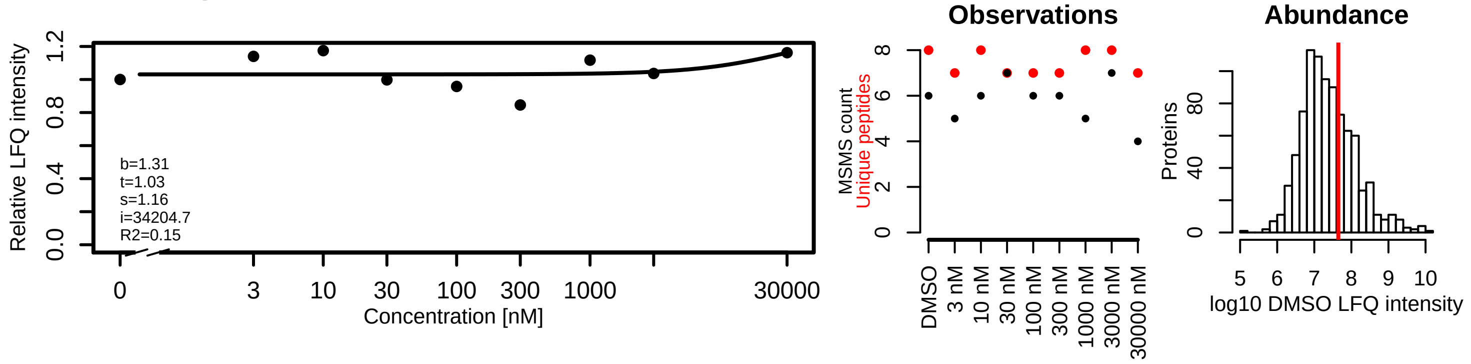

HNRNPD

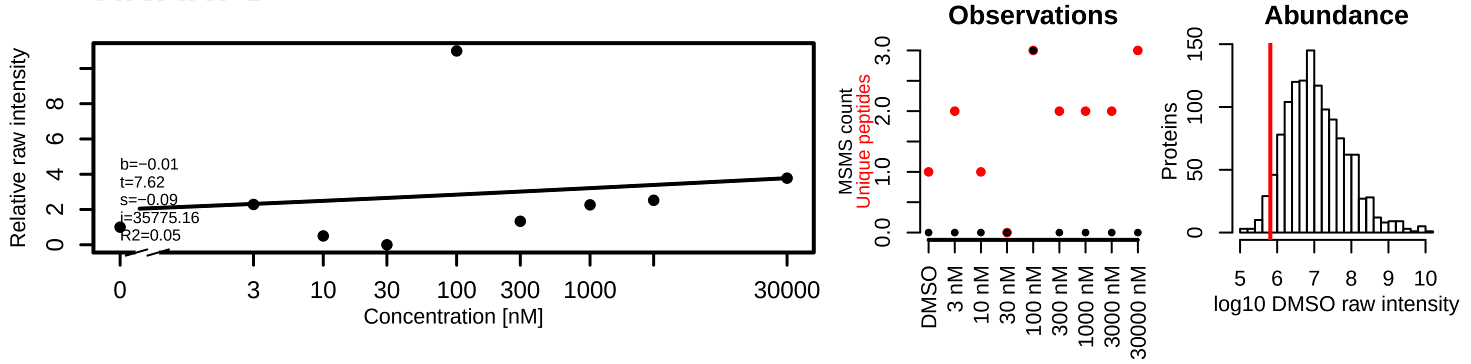

GEMIN4

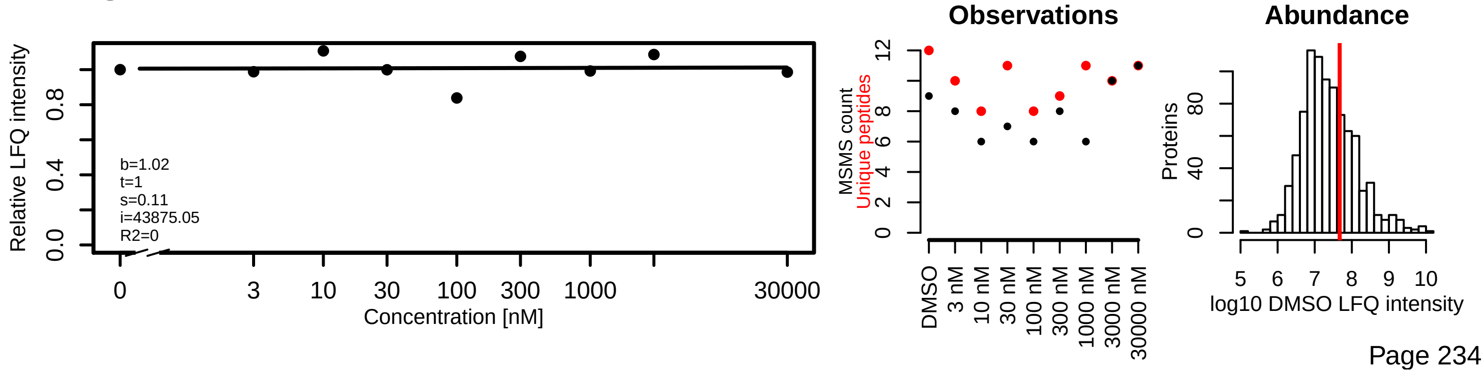

PGRMC2

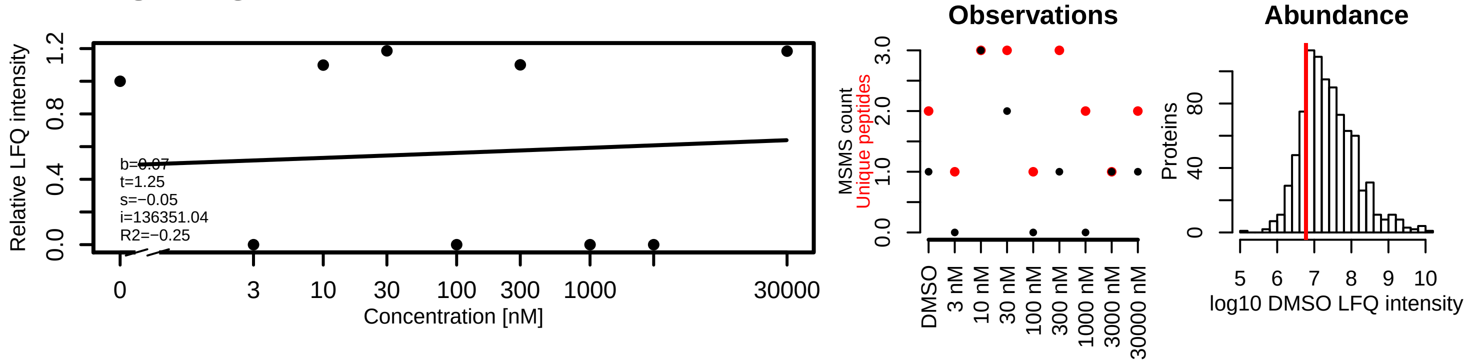

PNPLA6

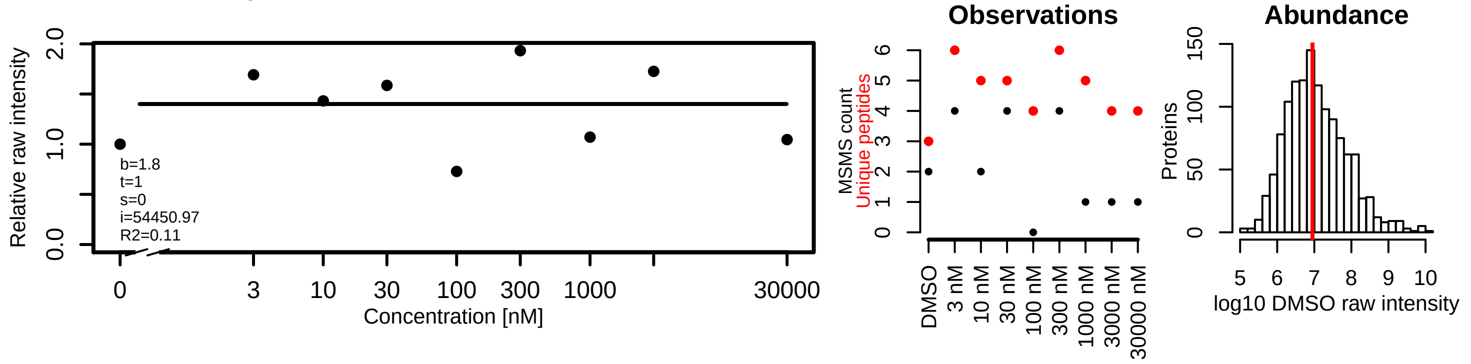

JUP − F5GWP8;K7ESE1;K7ELP7

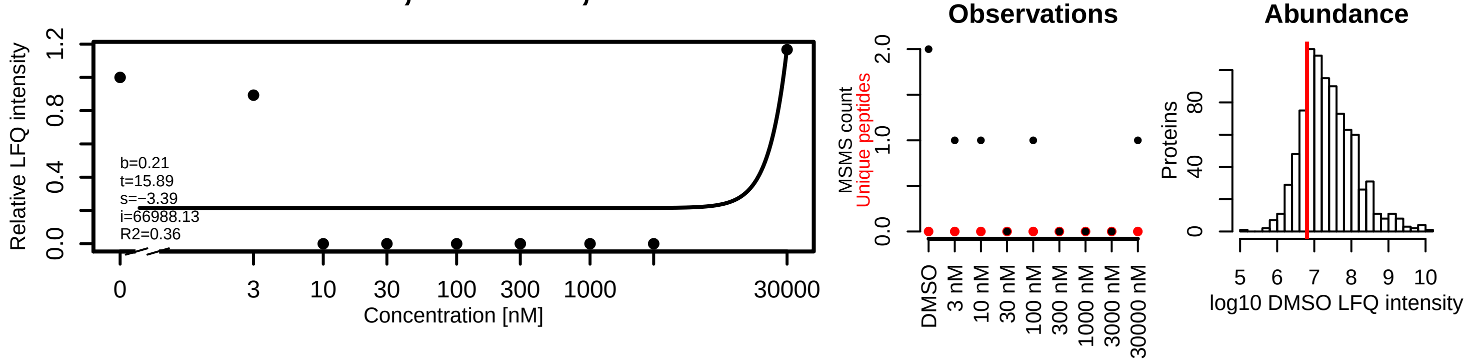

SCARB2

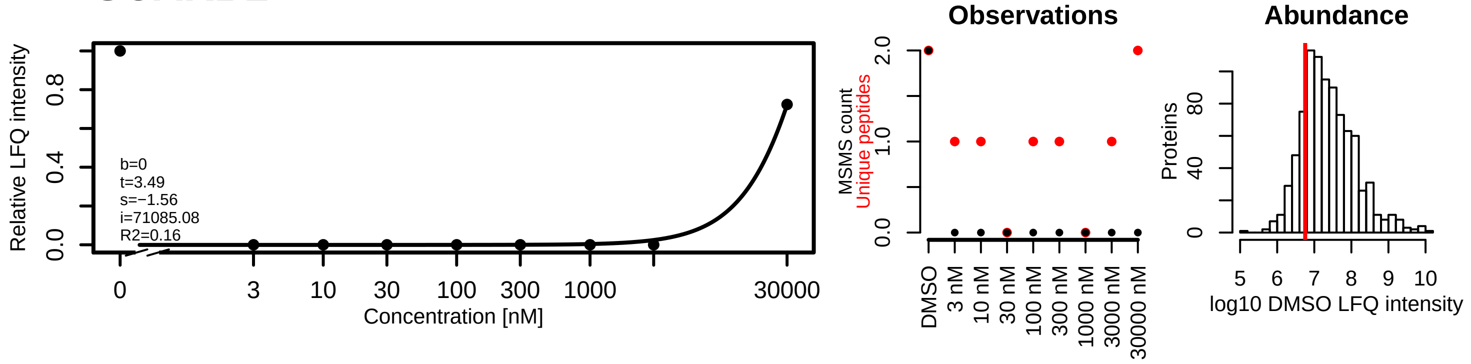

SLCO4A1

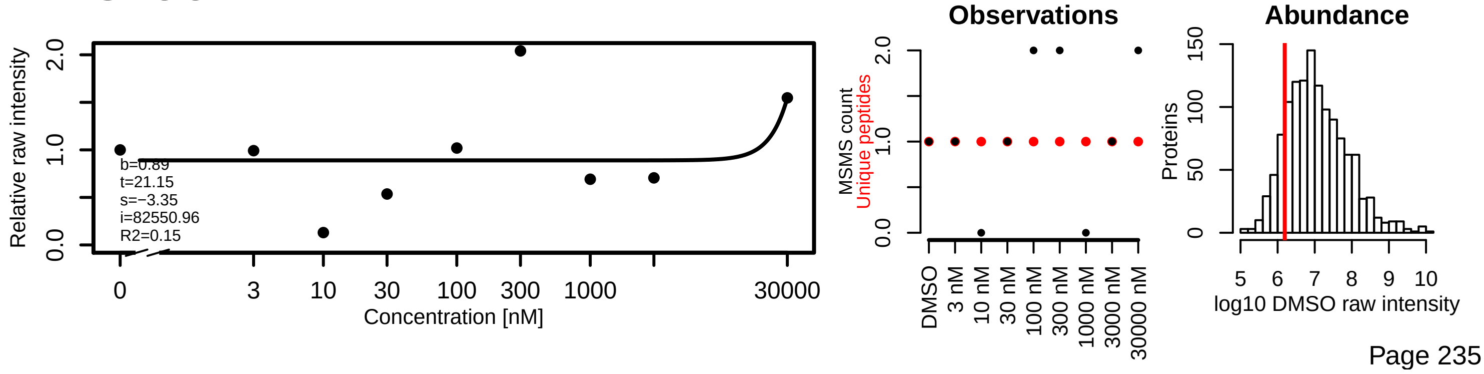

SLC25A46

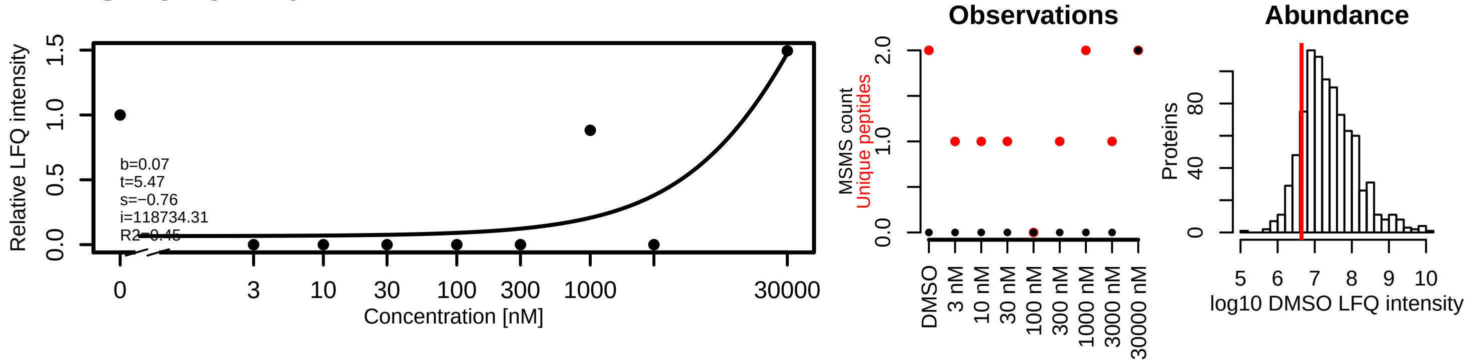

RNF121

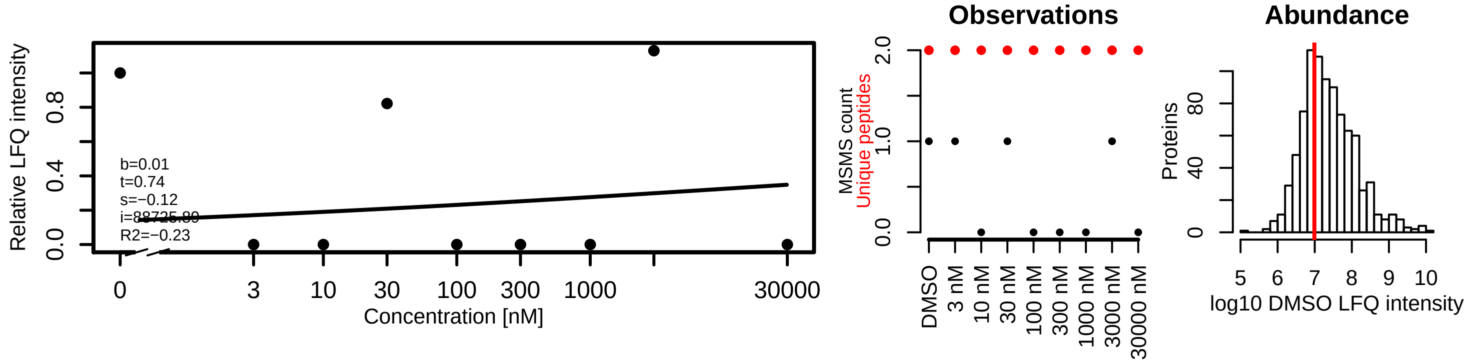

NBAS

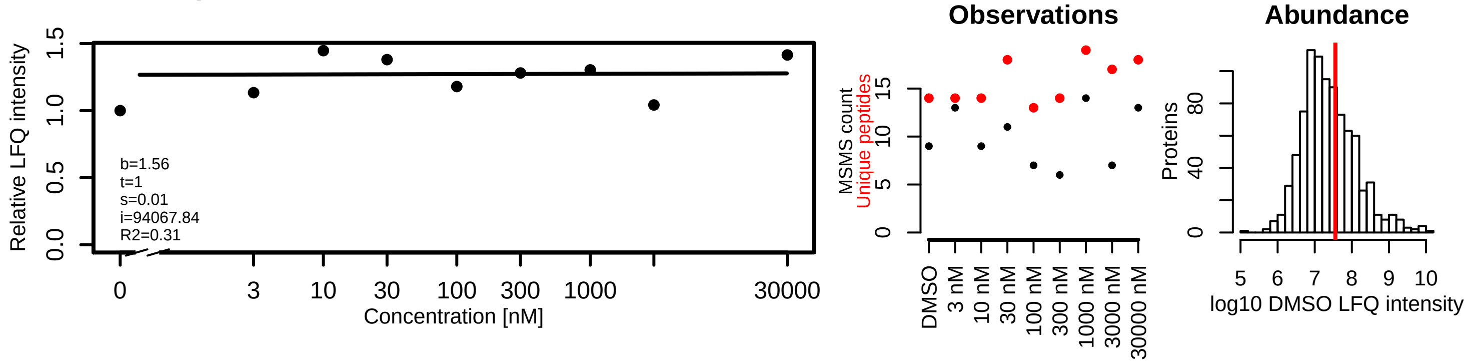

TUBG2;TUBG1

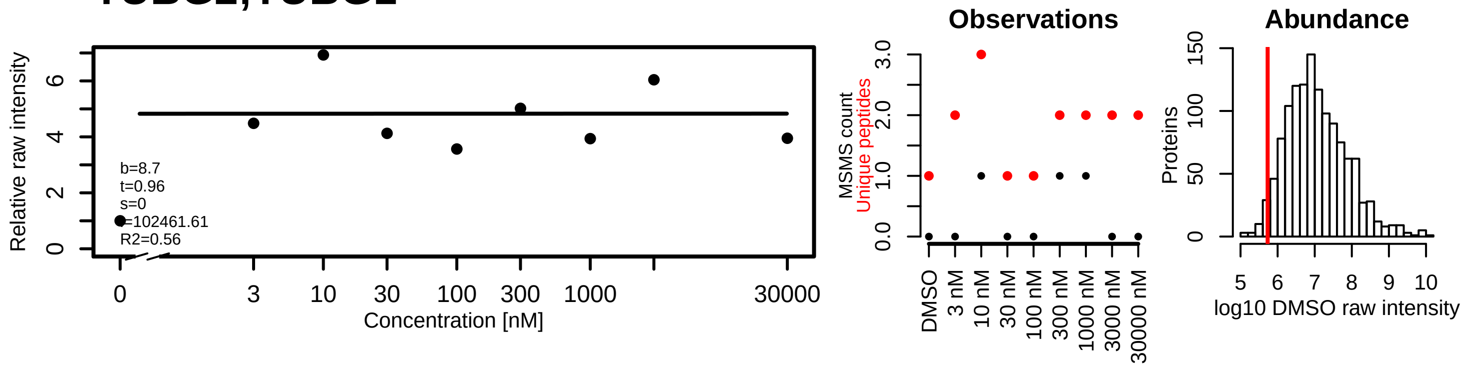

SLC35E1

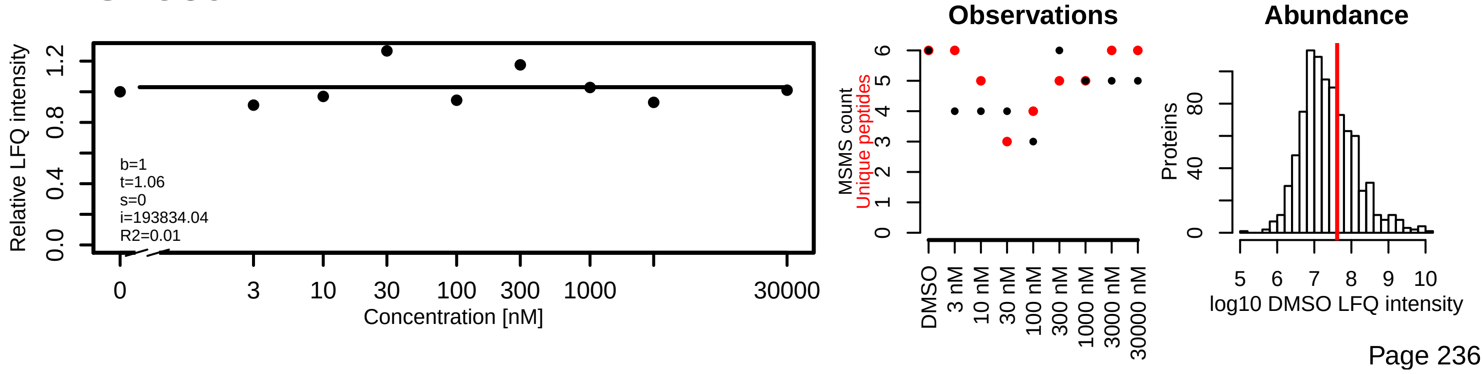

PSMD2

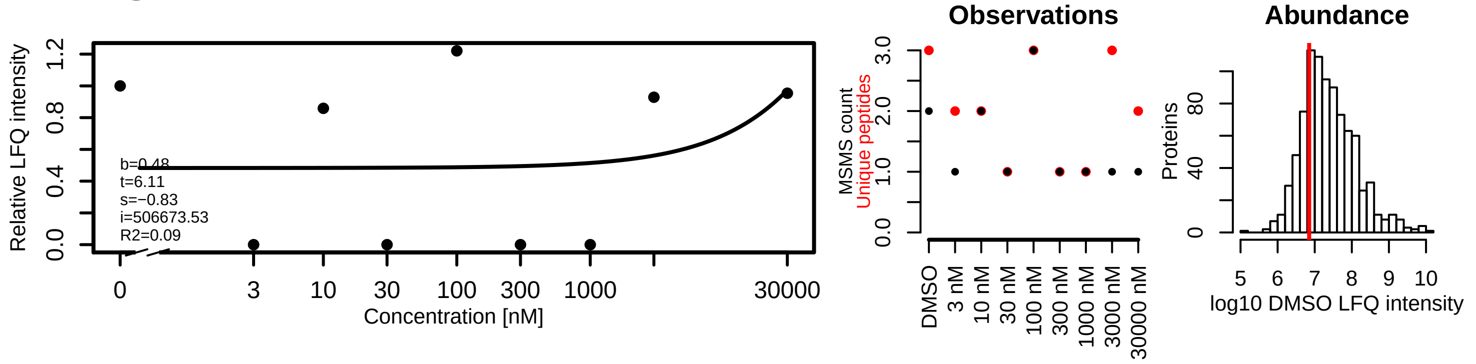

HNRNPK

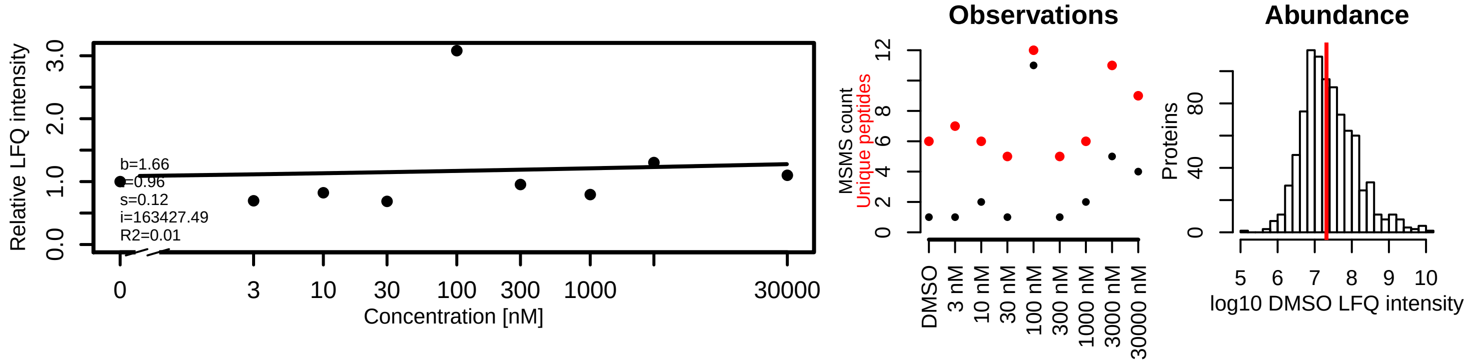

SEMA7A

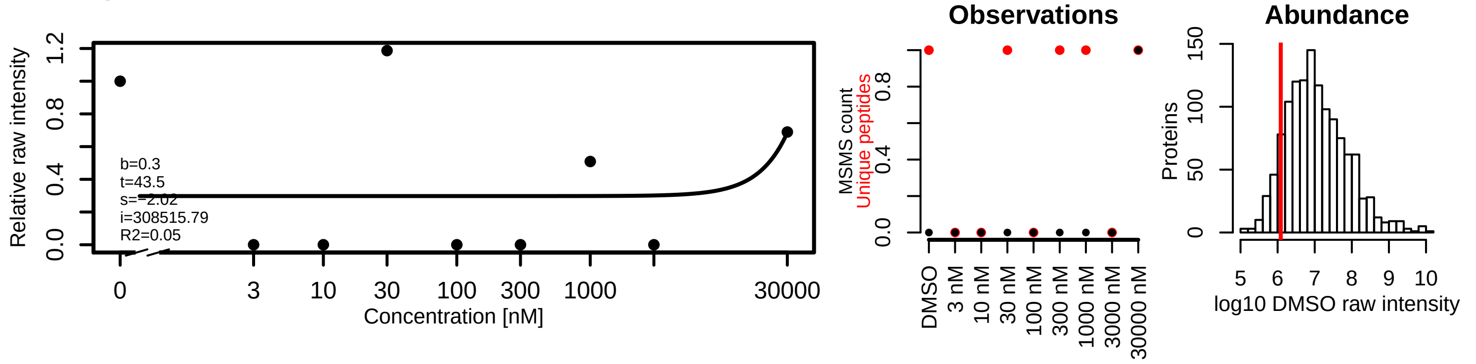

SUN2

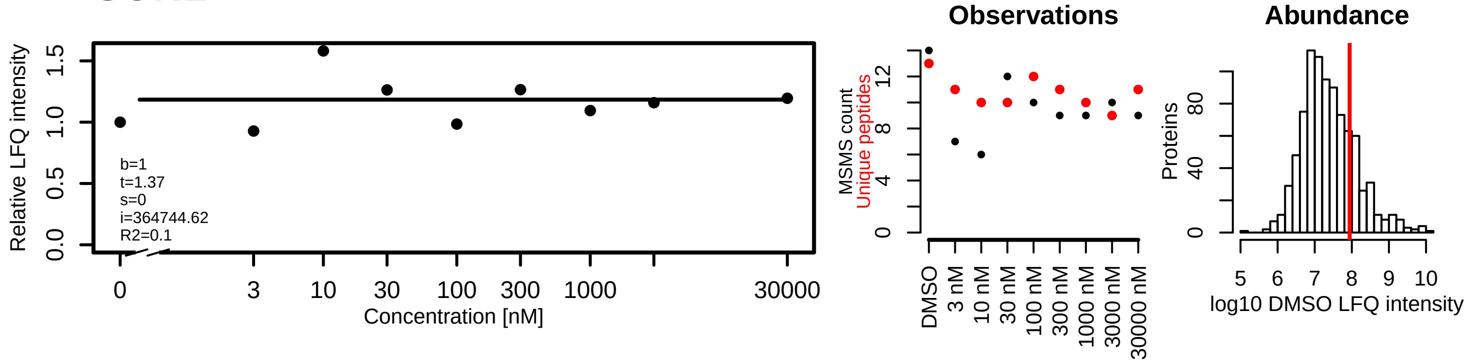

KRT19

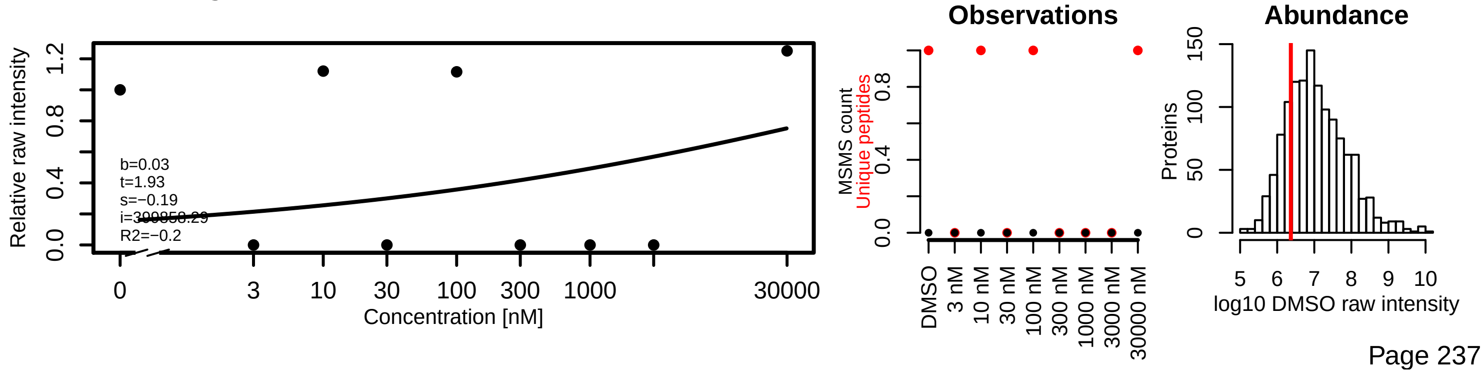

RAB39A;RAB6B;RAB6A

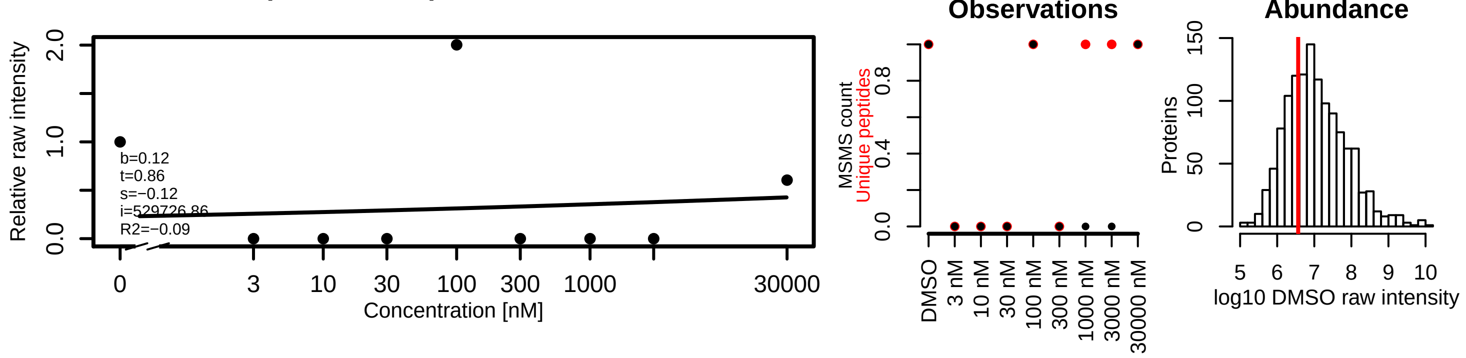

HMGB1

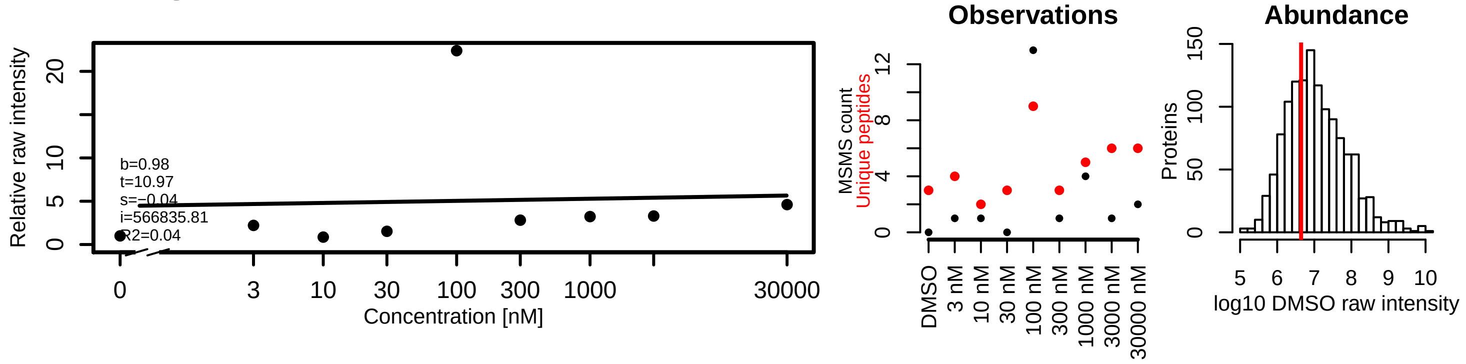

FBL;FBLL1

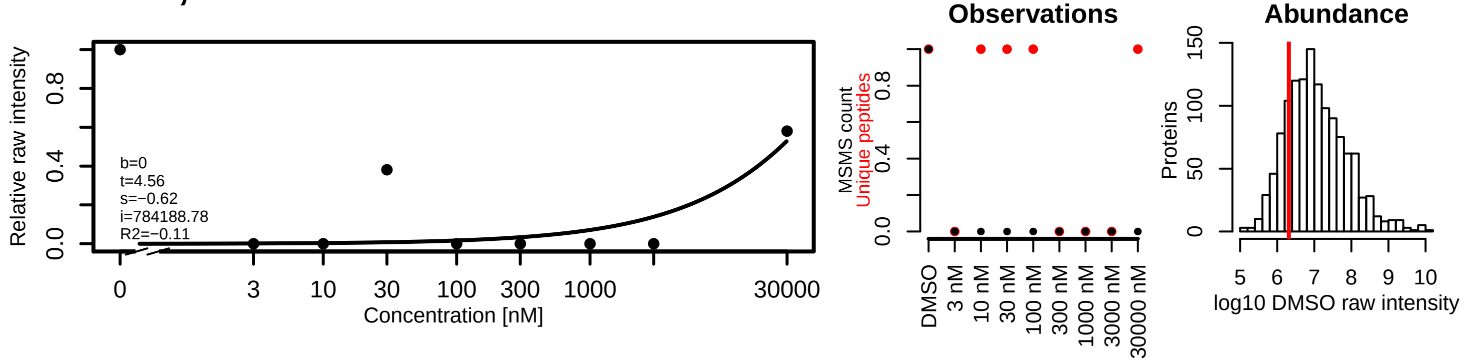

HNRNPR;SYNCRIP

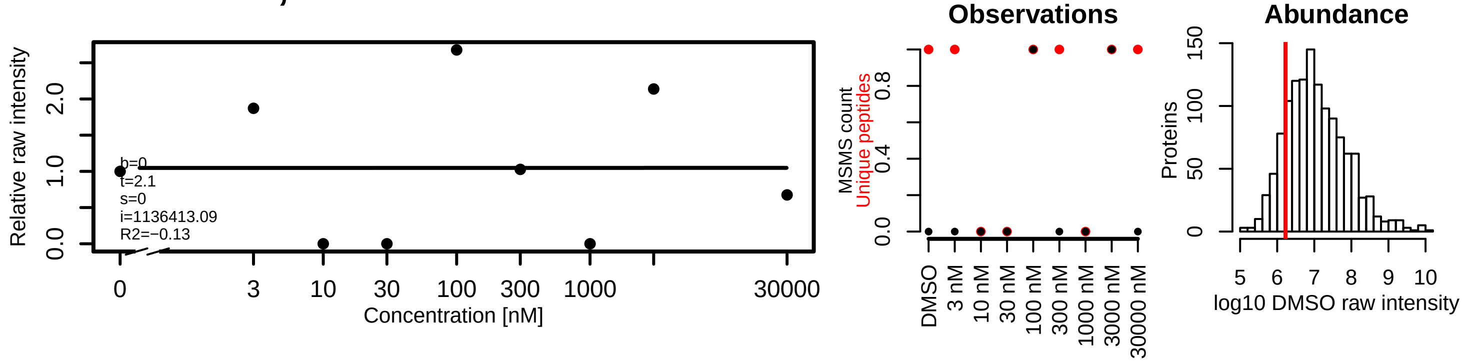

MLF2

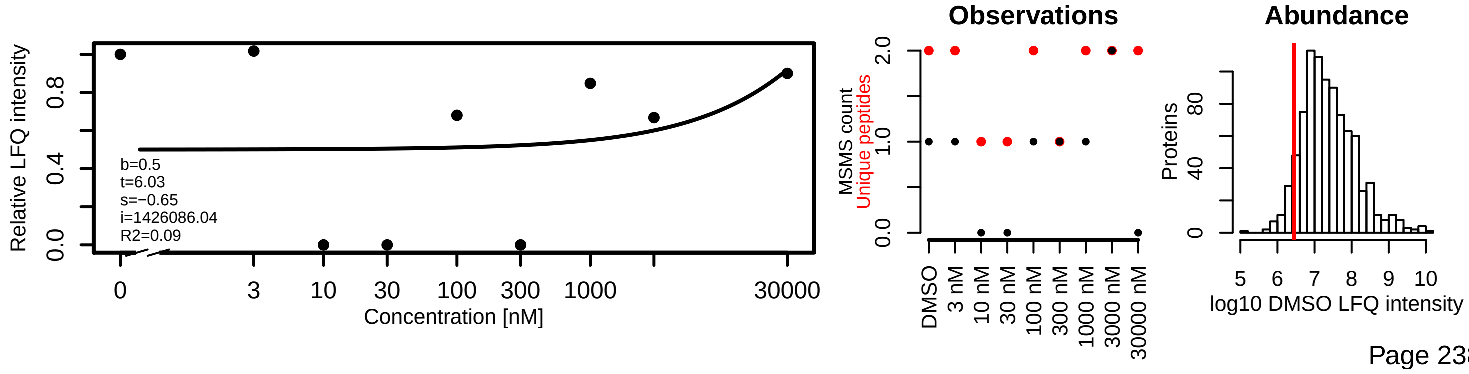

DNAJC13

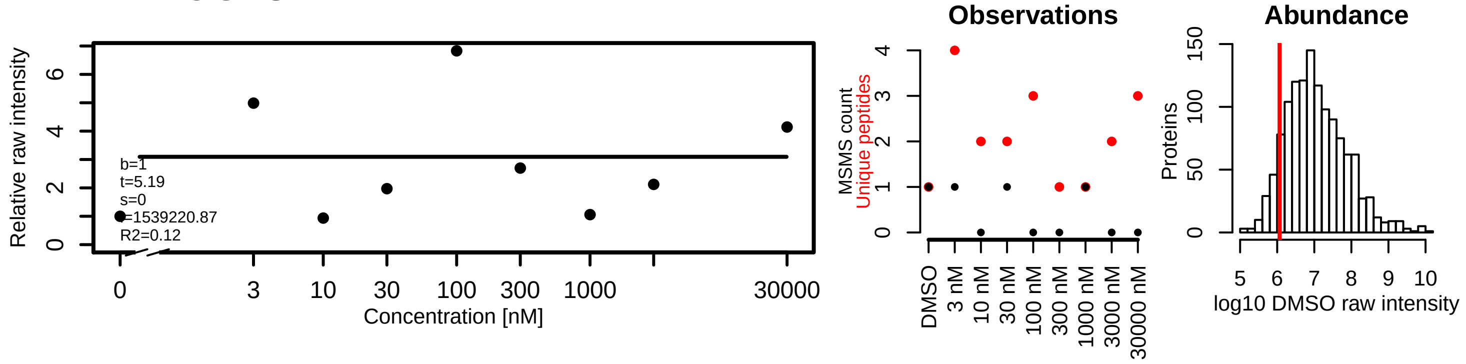

CYCS

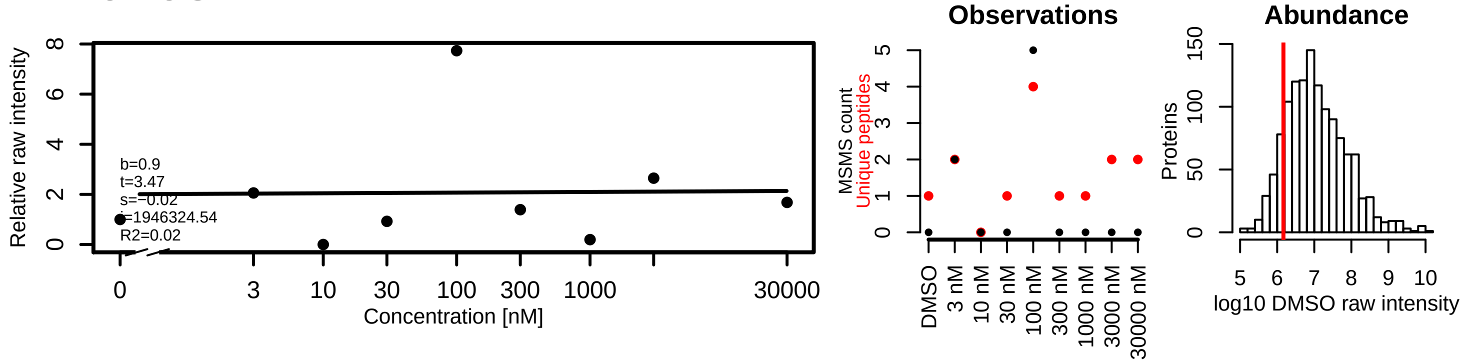

ARL1

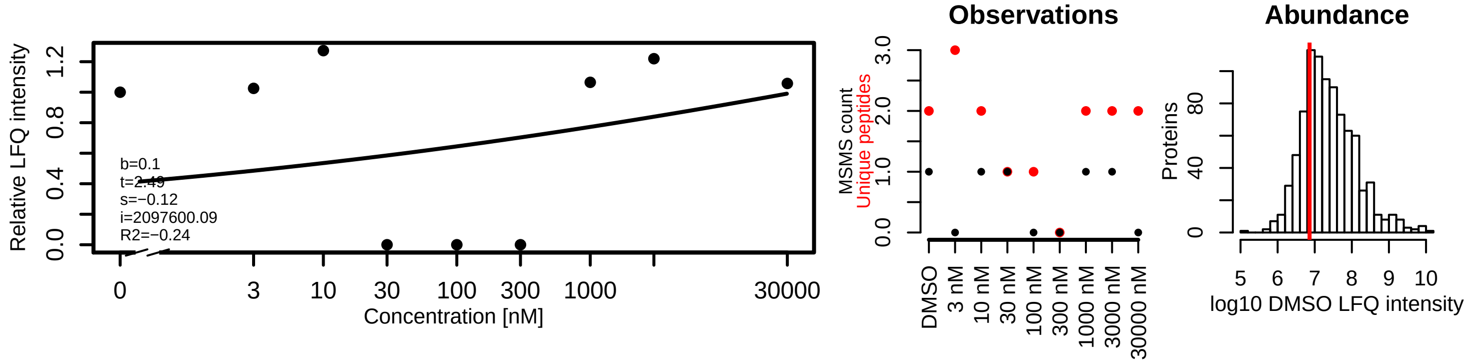

PIGK

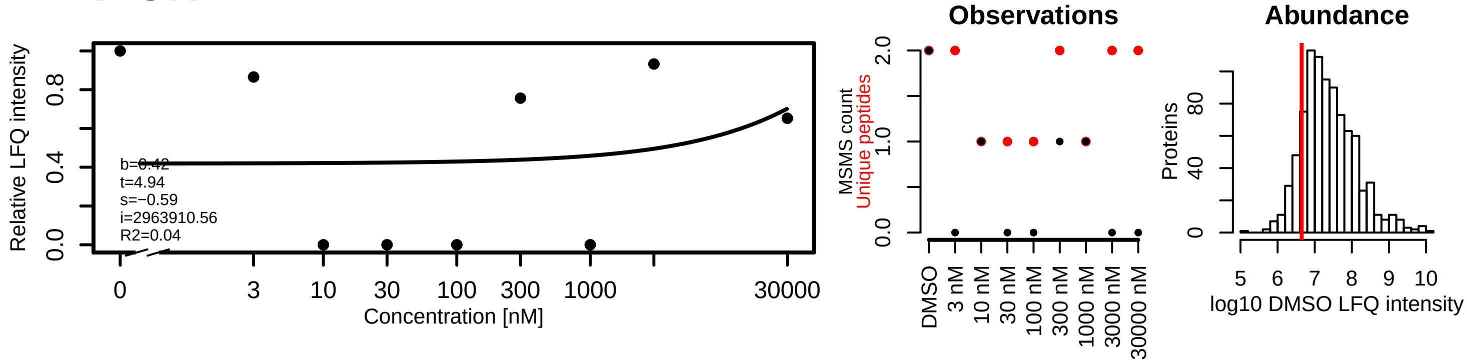

EEF1G

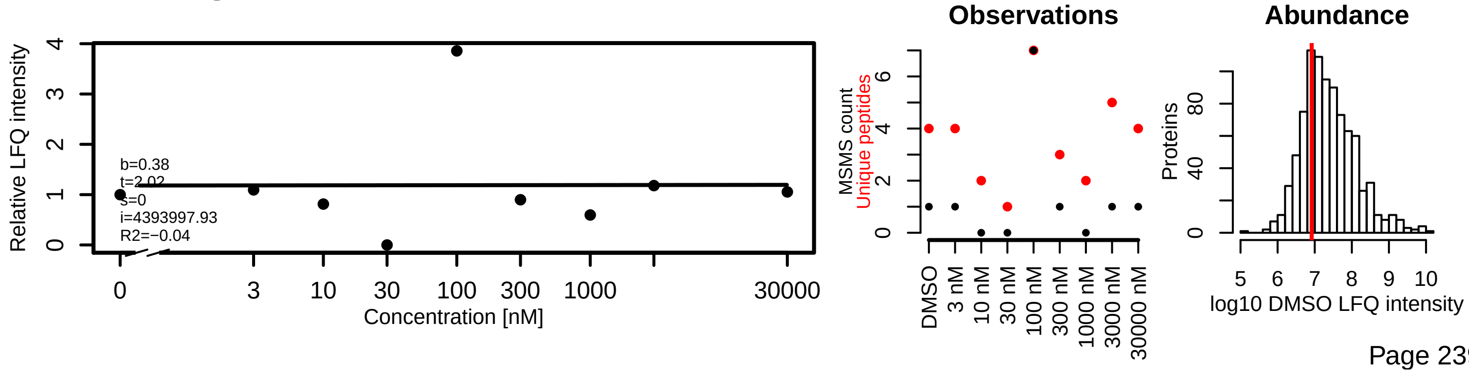

BRI3BP

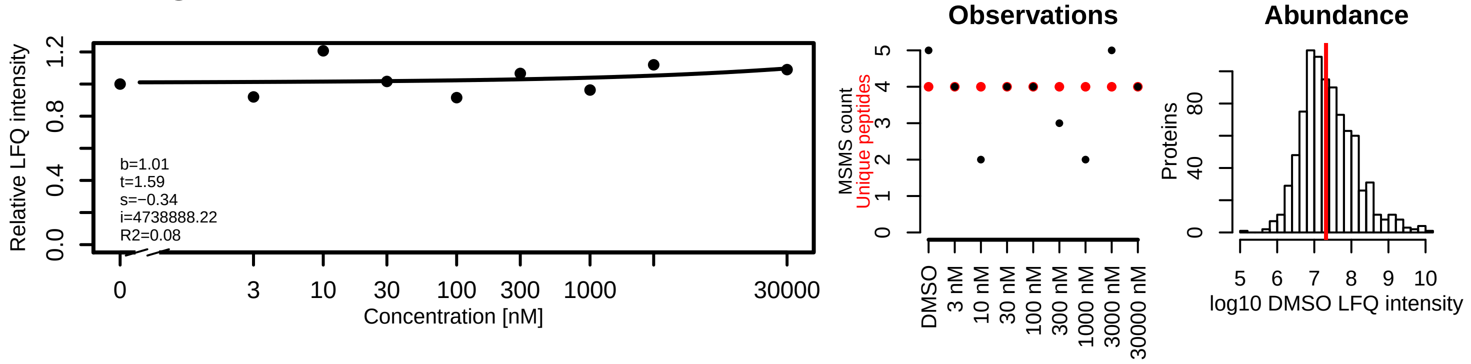

ACTN1

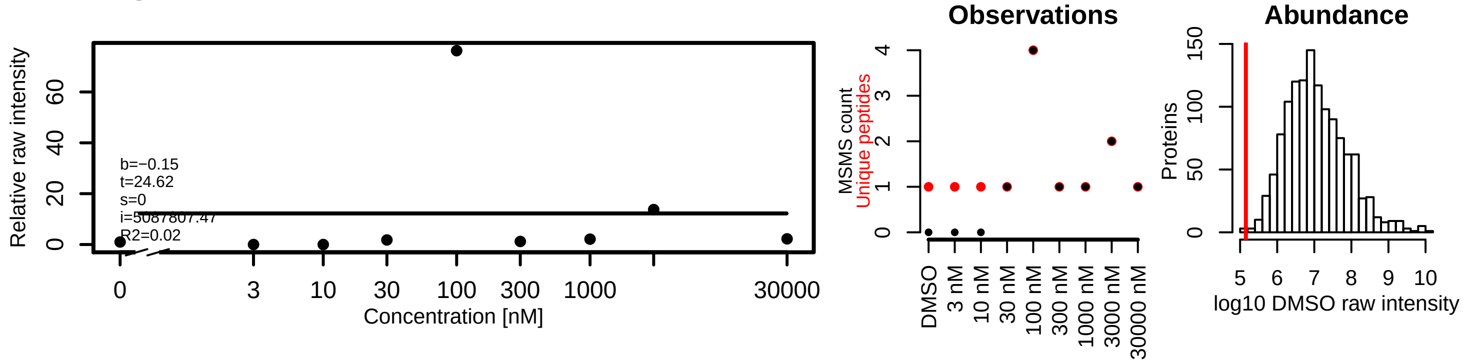

COX16

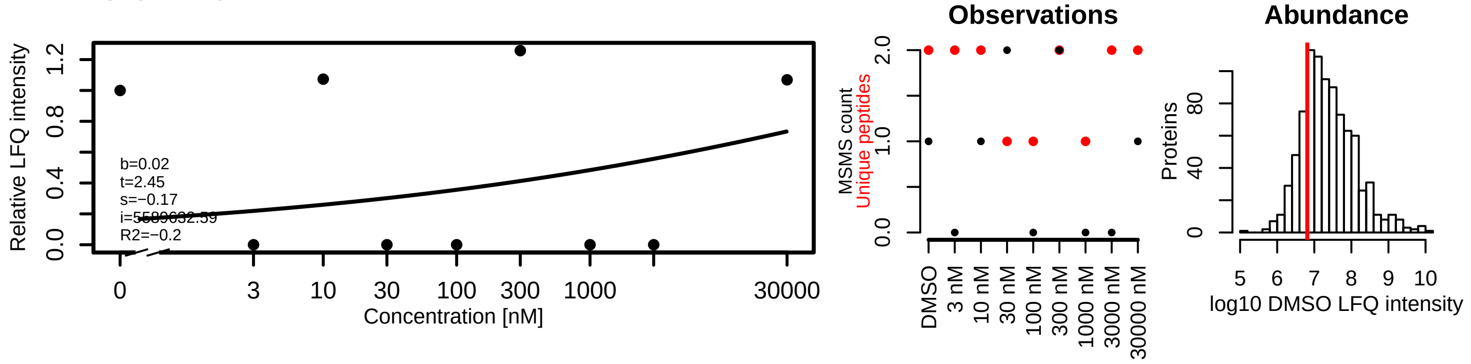

STK17B

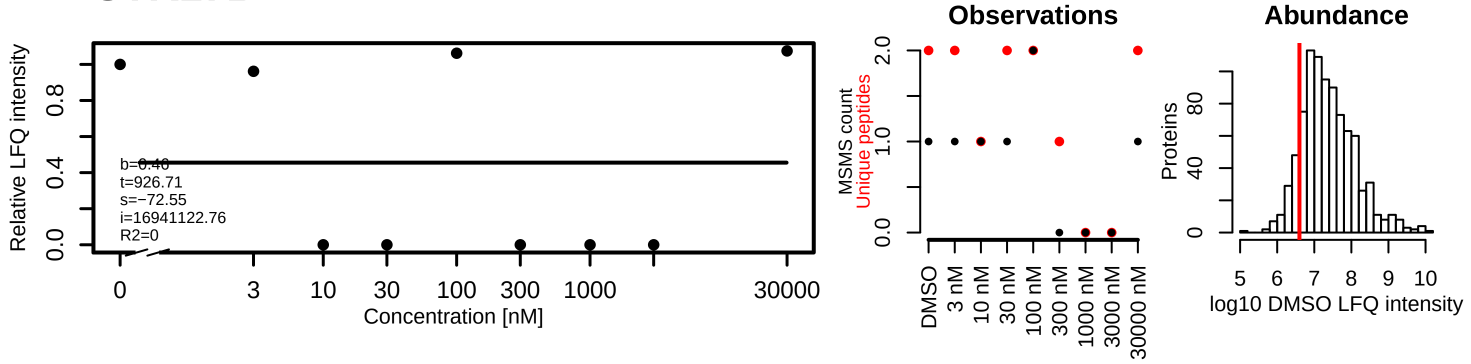

DYNC1I2

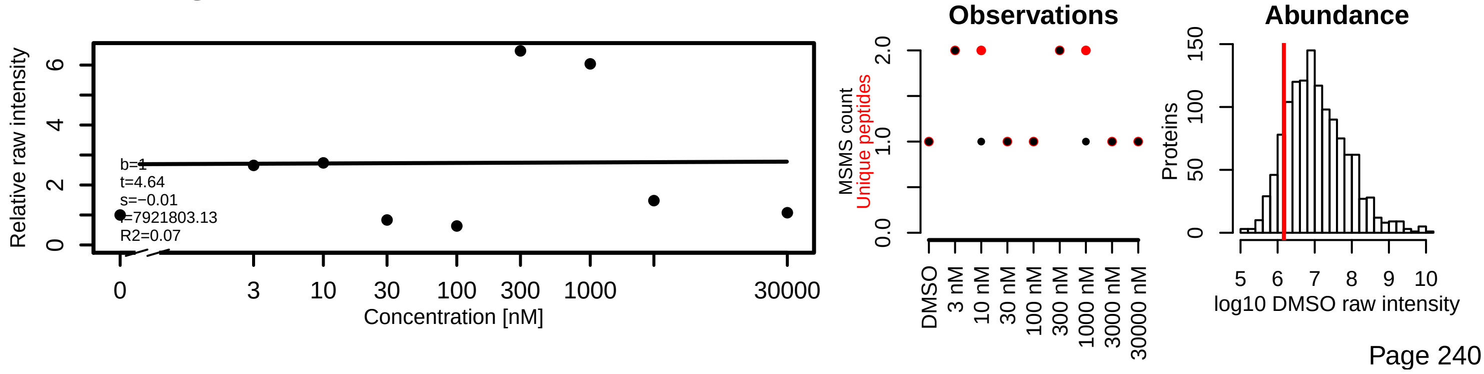

MCUR1

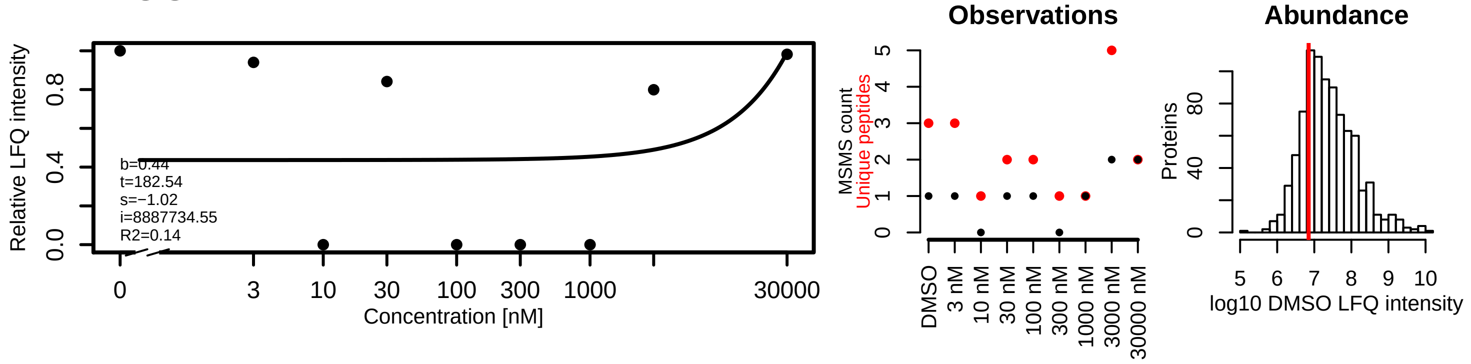

XPO4

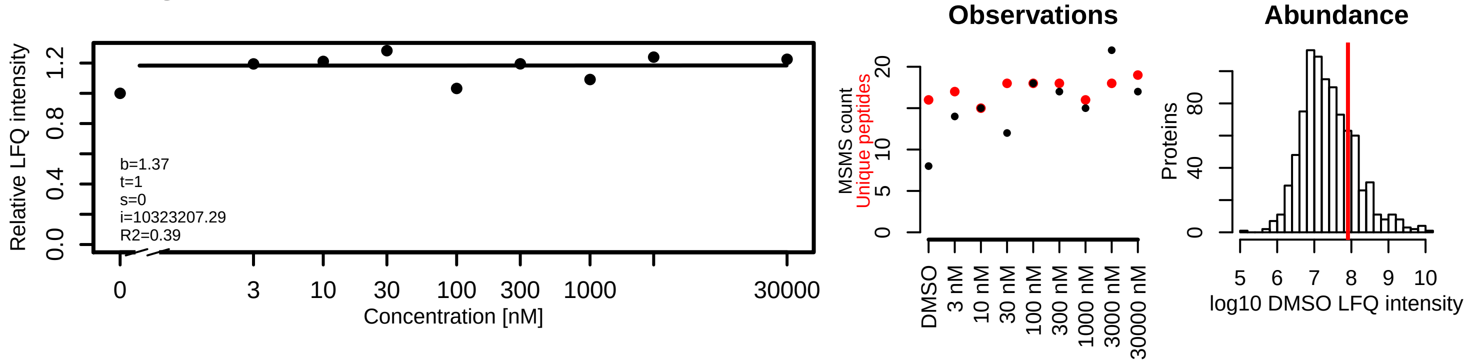

DSP

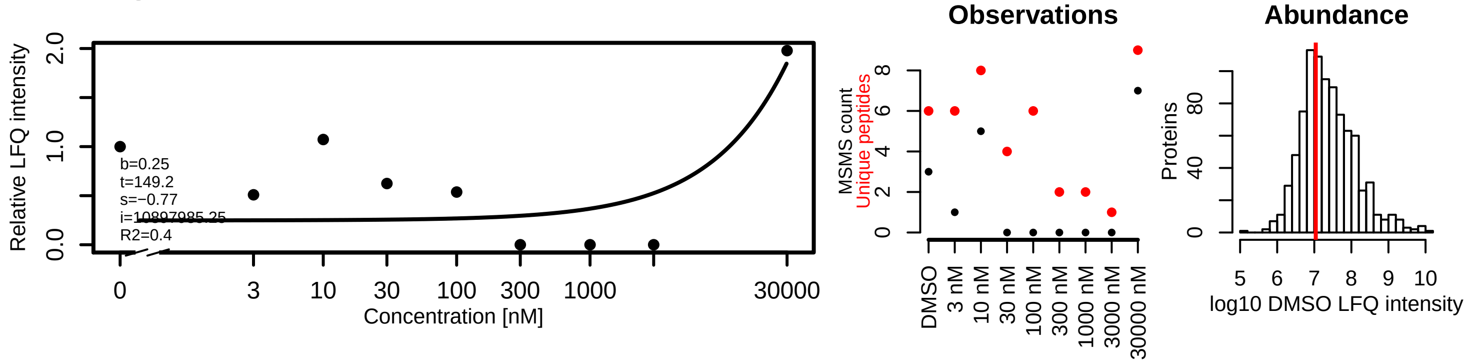

AURKB

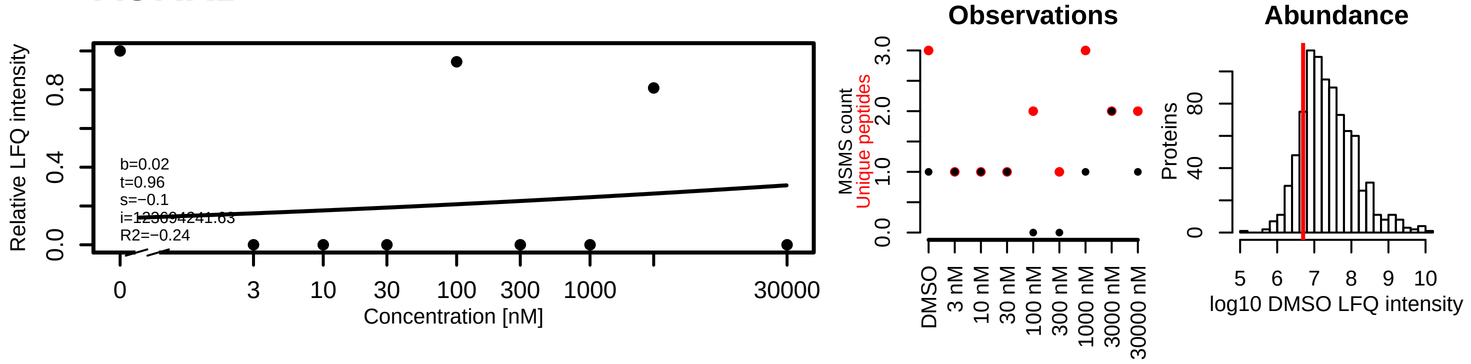

SFPQ

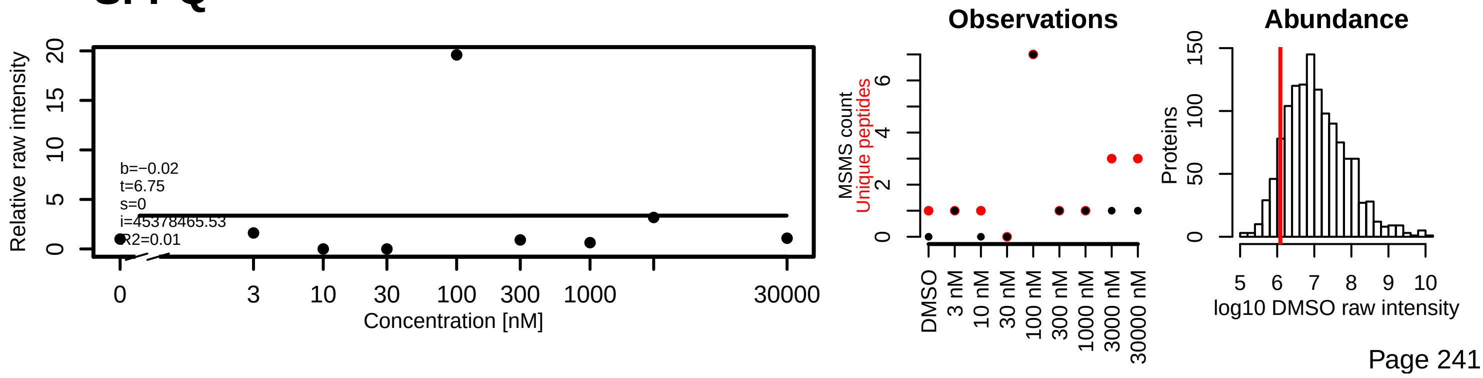

SLC38A9

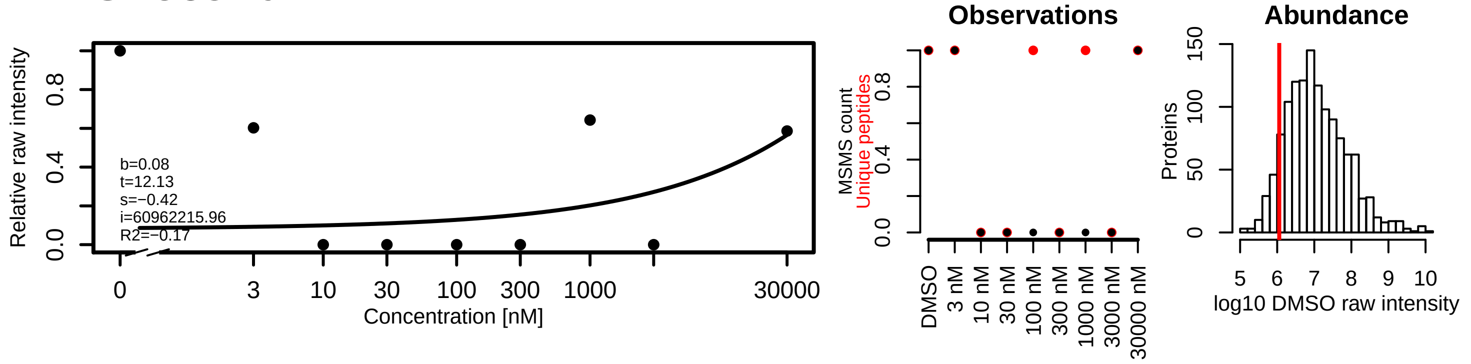

GNB2;GNB4

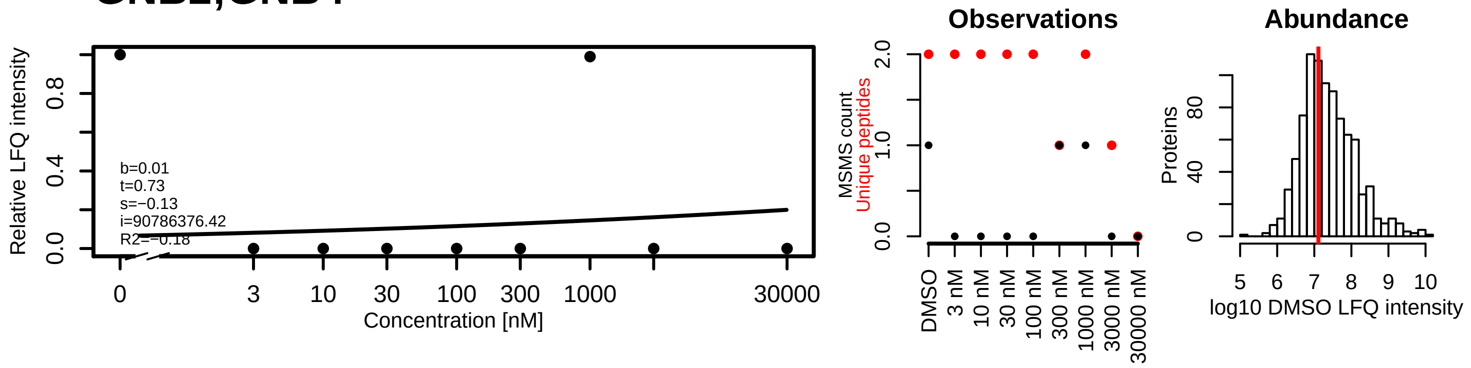

QIL1

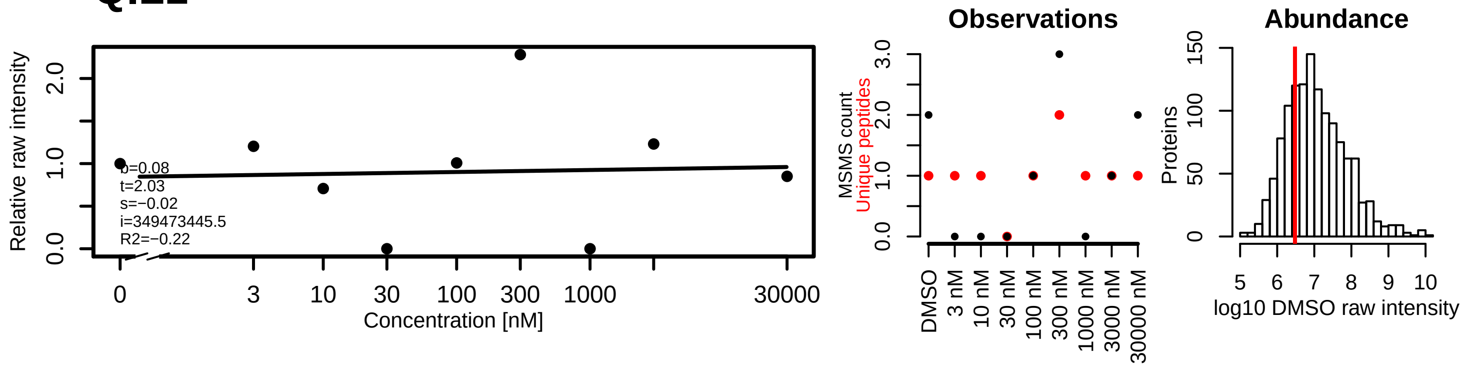

C16orf62

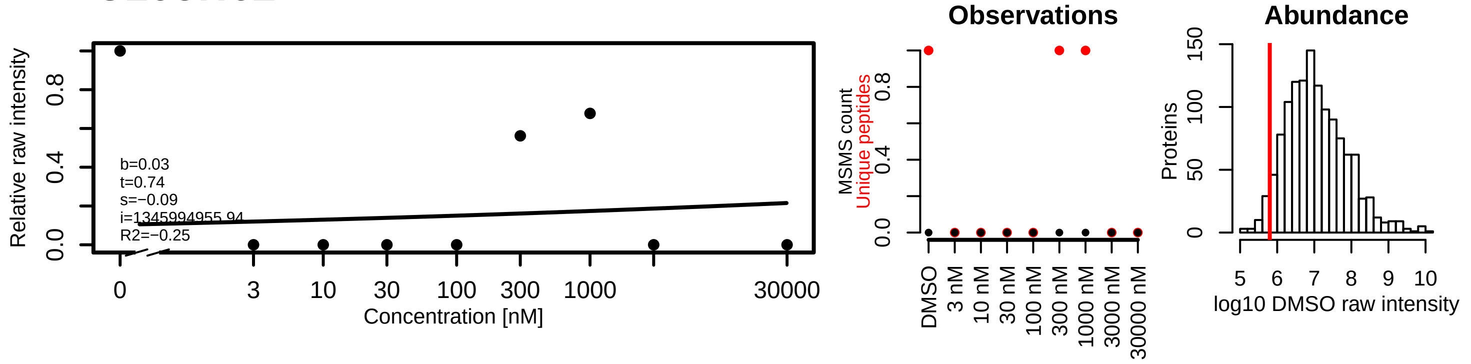

PRDX1

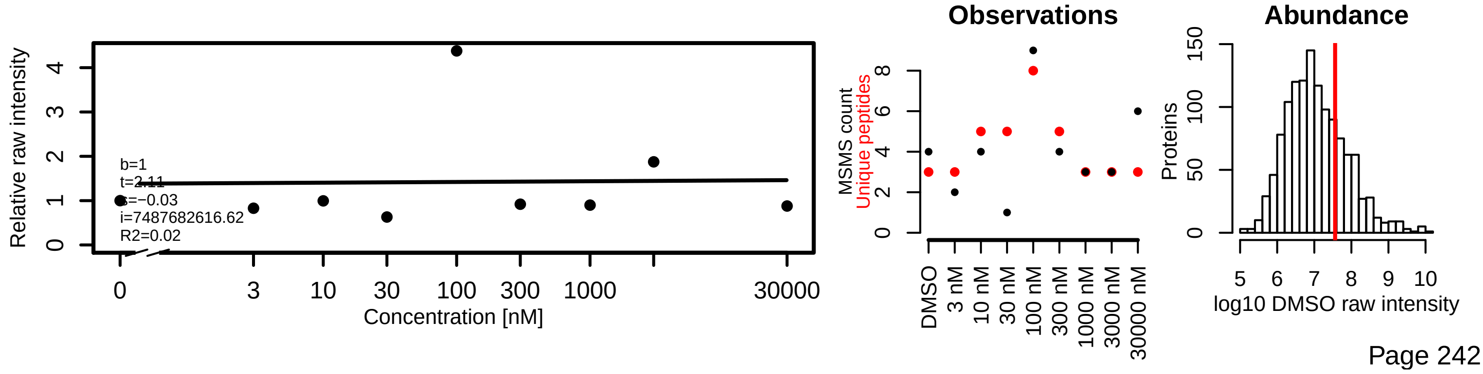

DHX9

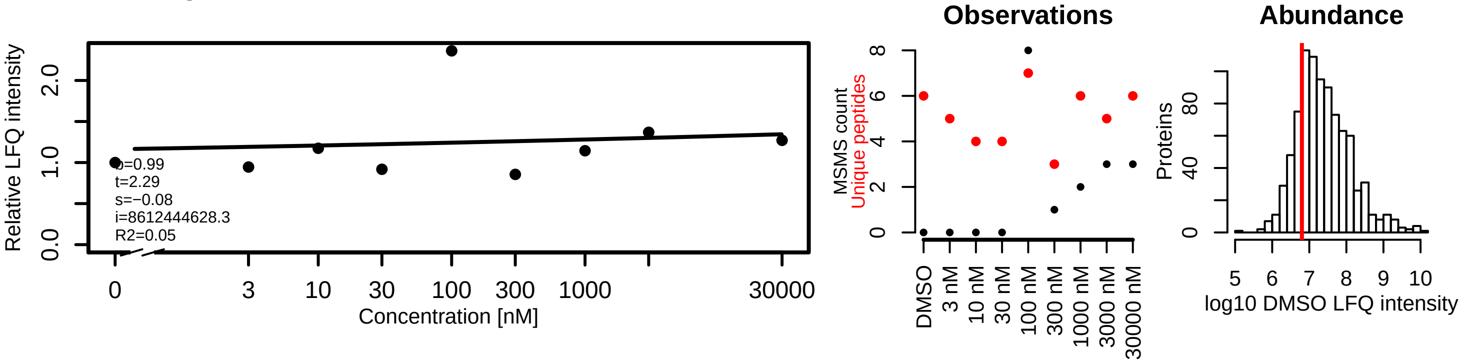

STIP1

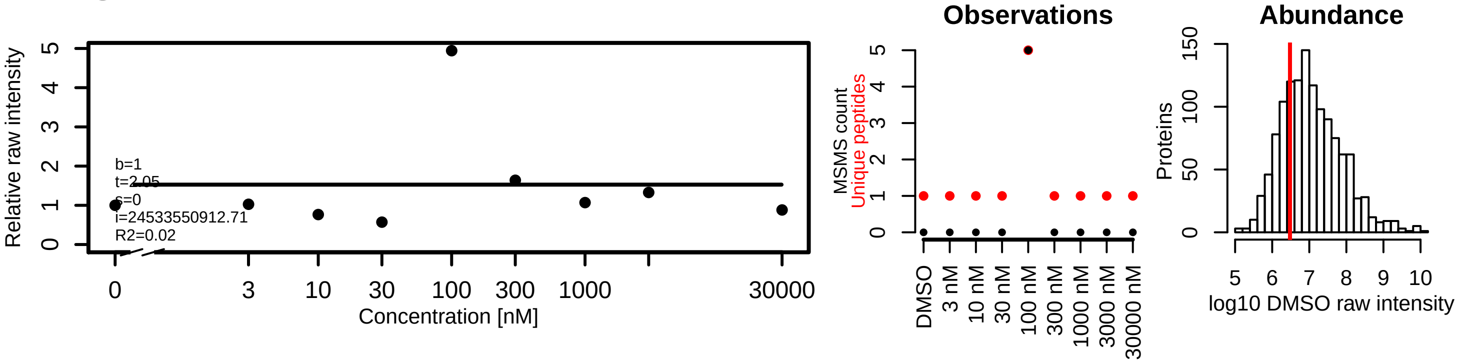

ANXA4

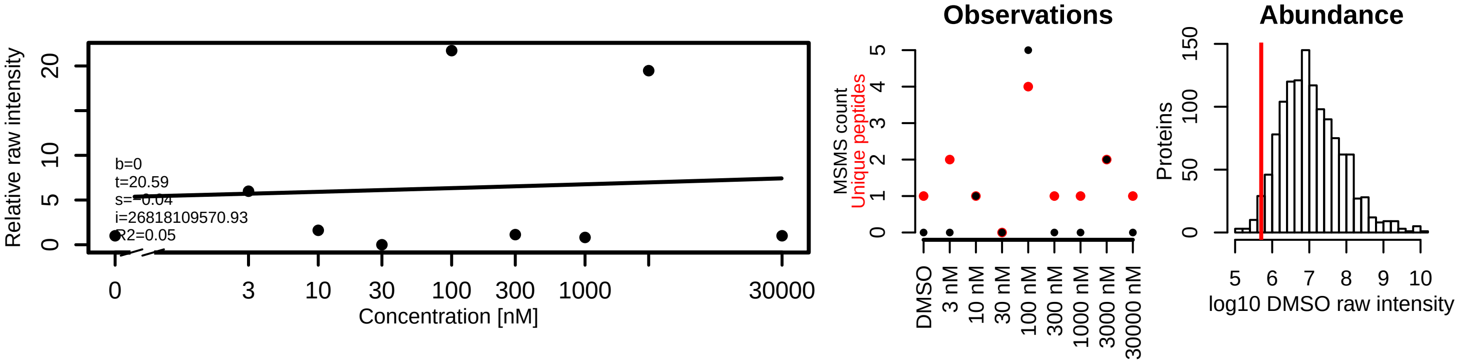

CPM

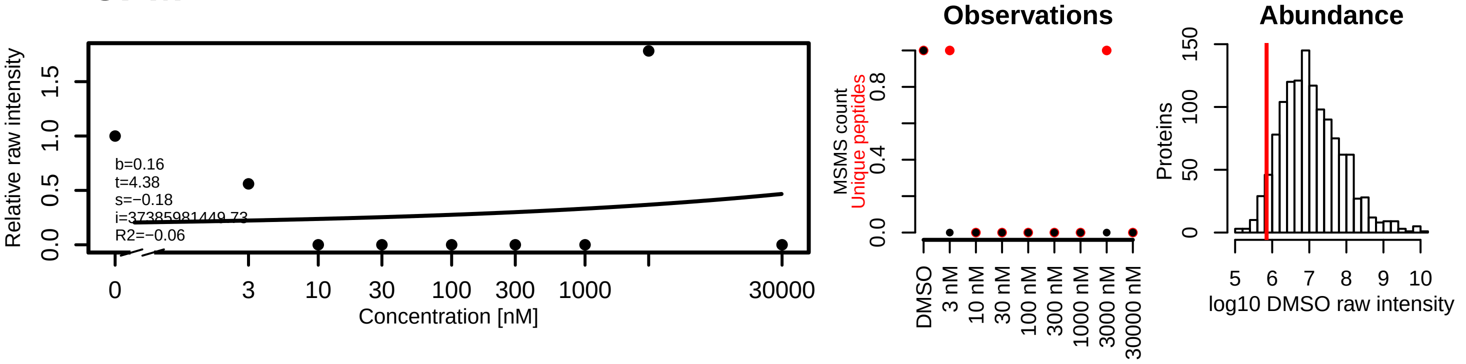

ABCC3

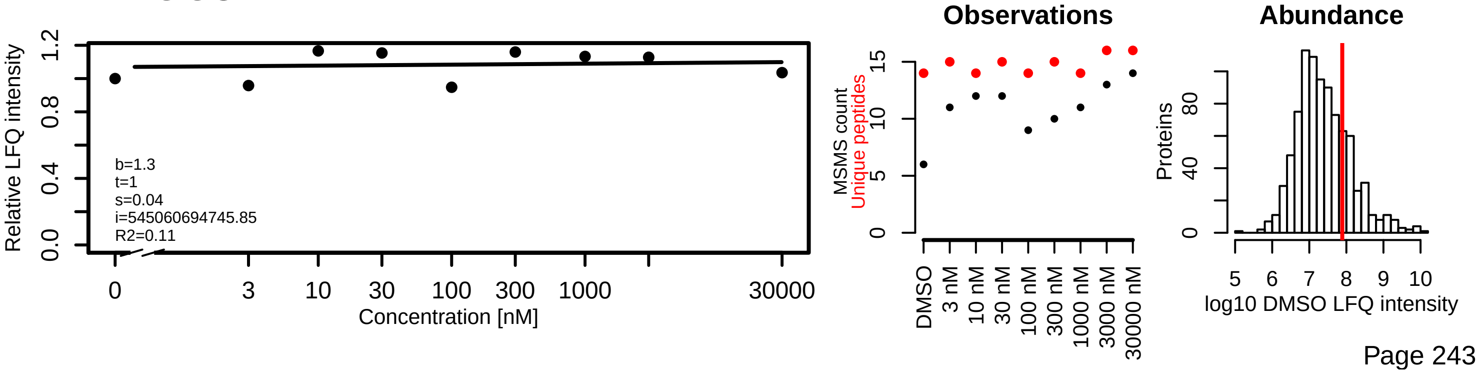

PGRMC1

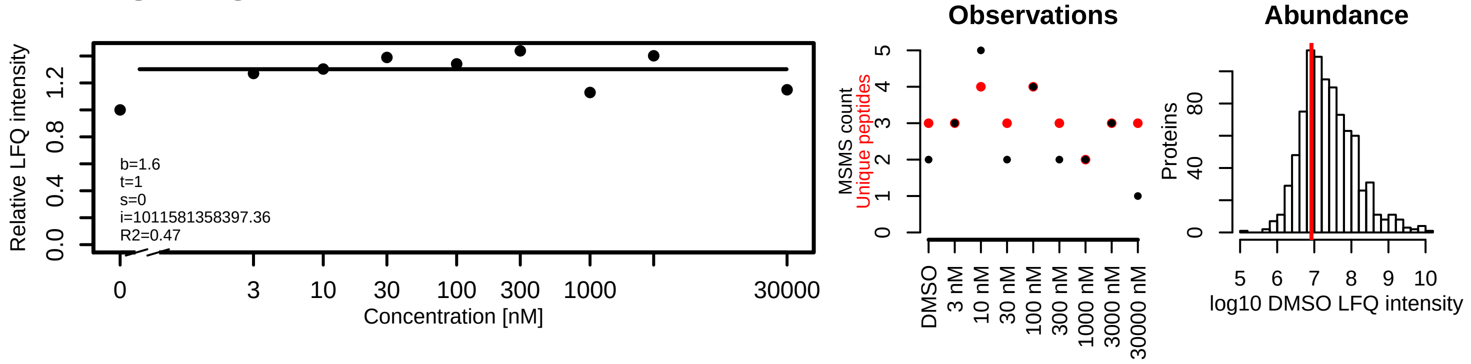

HNRNPU

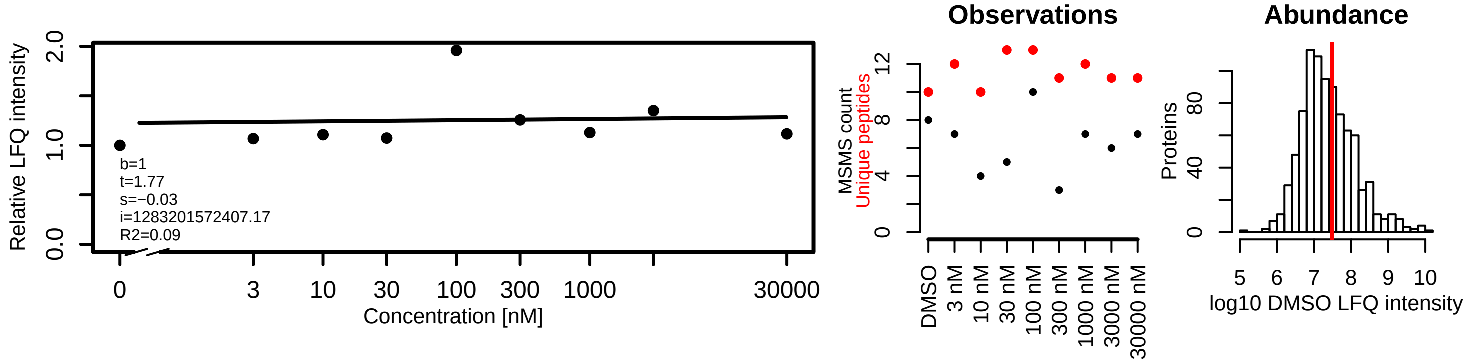

NUP205

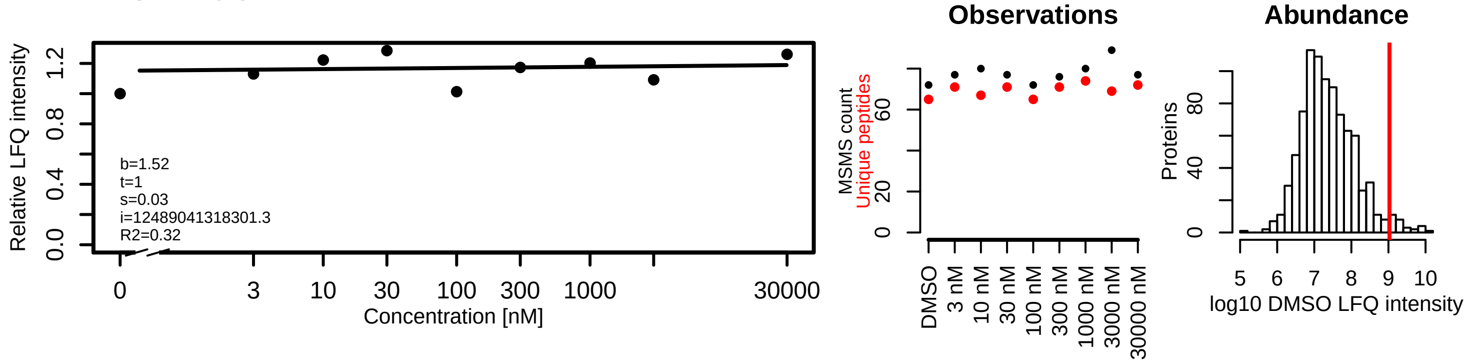

HSPD1

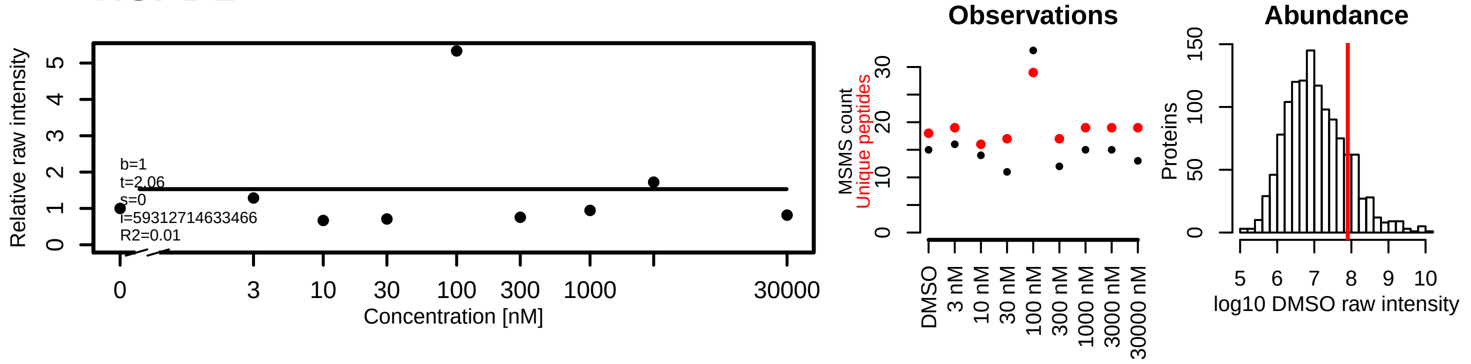

MSN

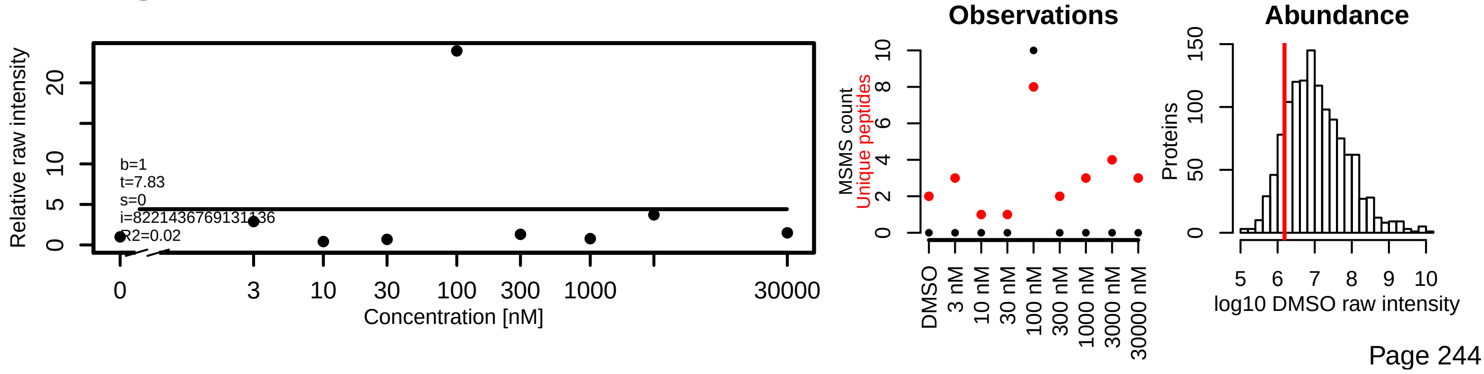

P4HB

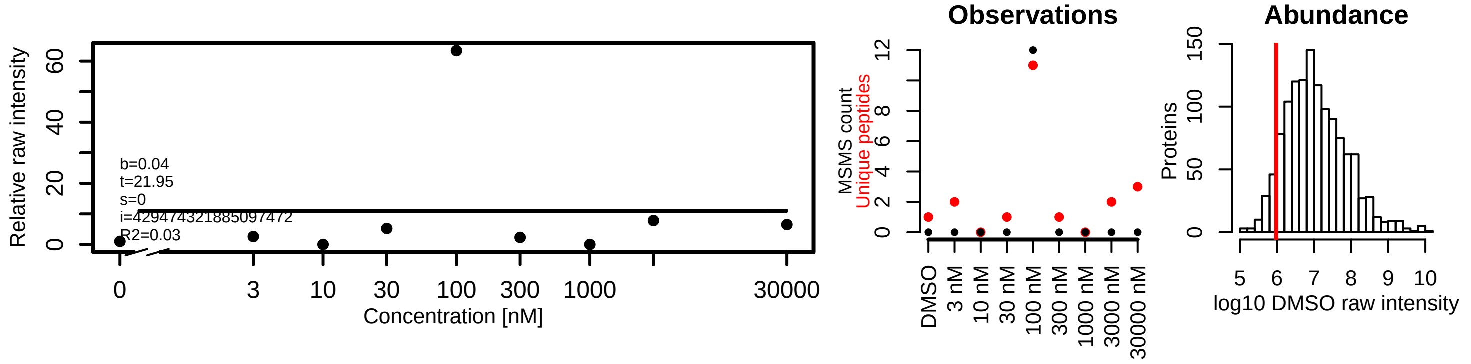

MYL6

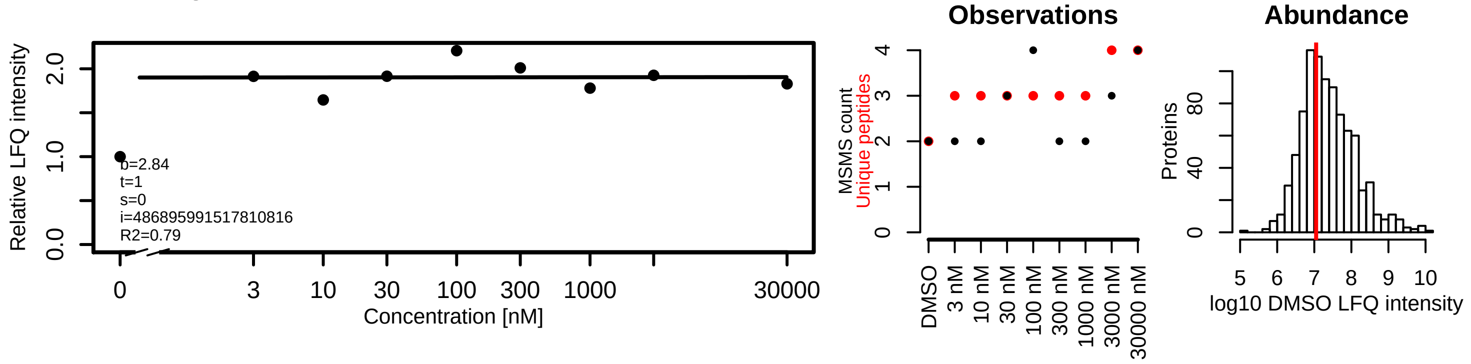

ALG1

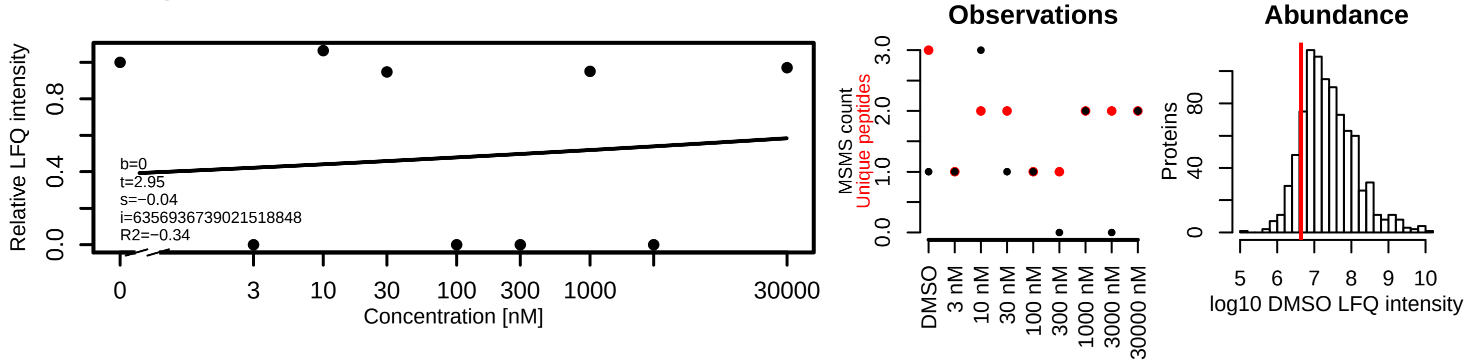

NPEPPS

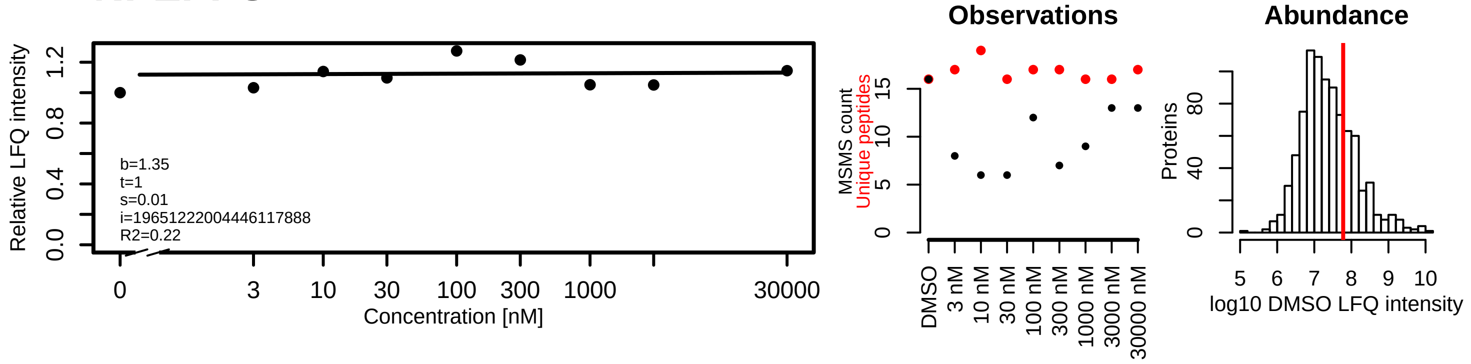

TRRAP

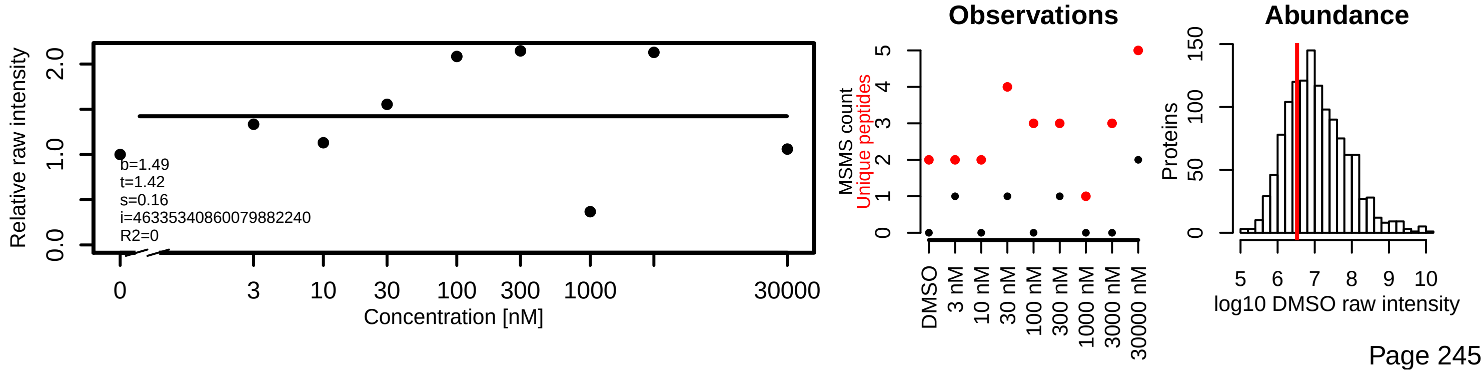

CLIC1

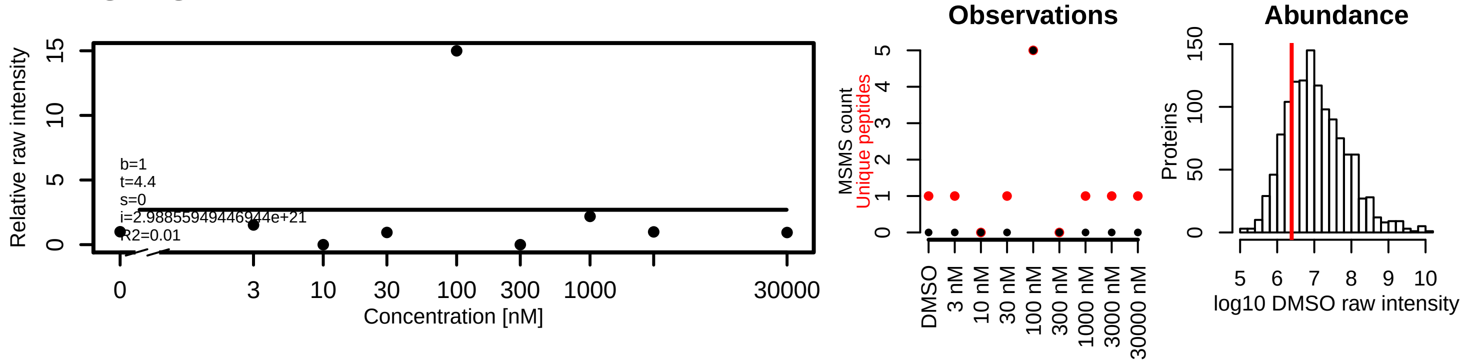

CHCHD3

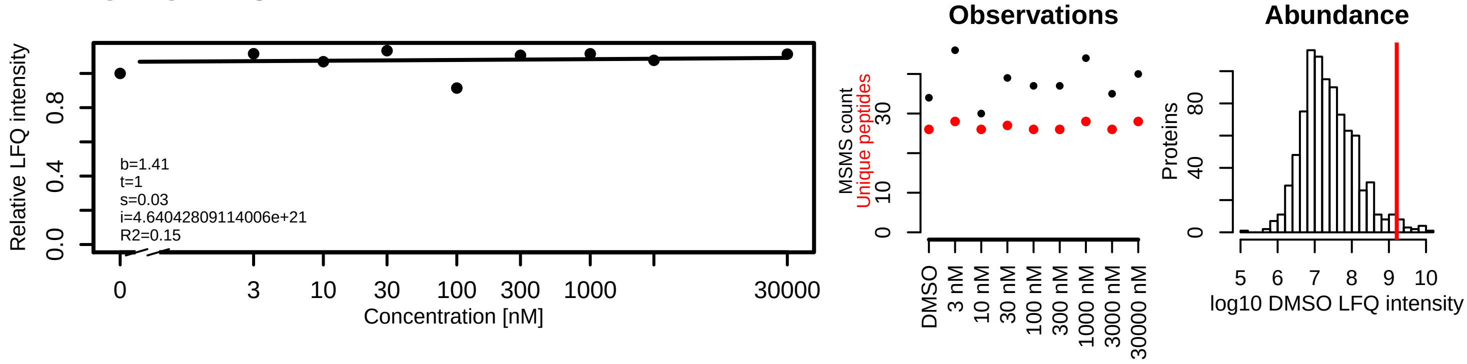

EIF3A

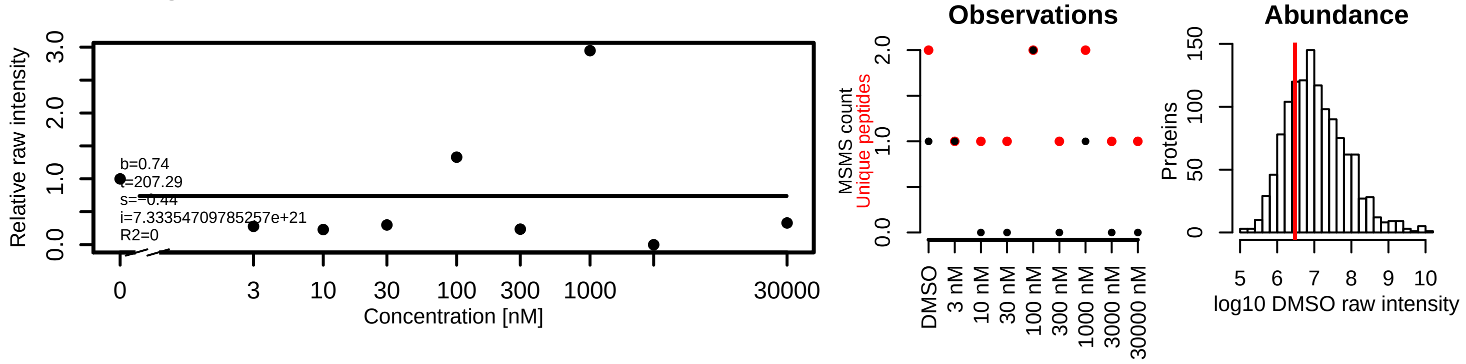

DKFZp686E0752;DCTN1

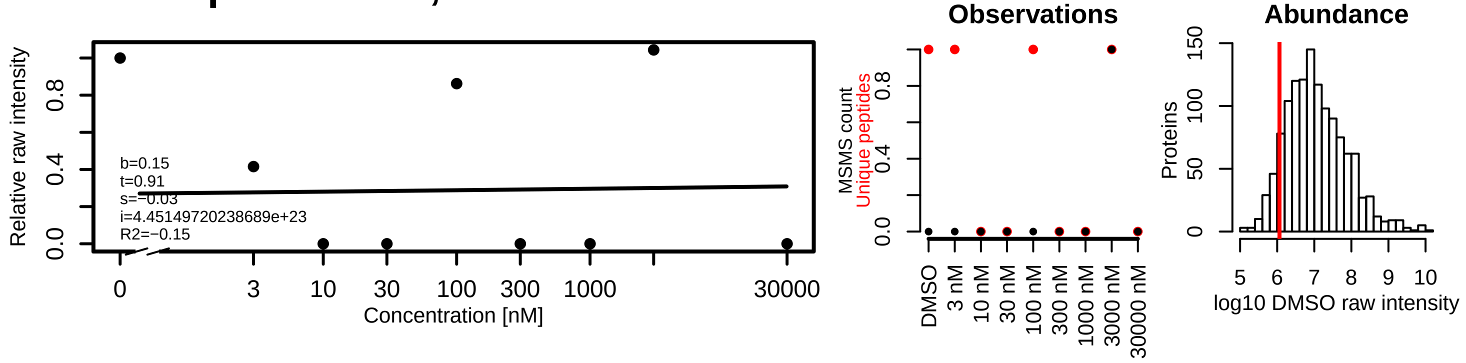

ACLY

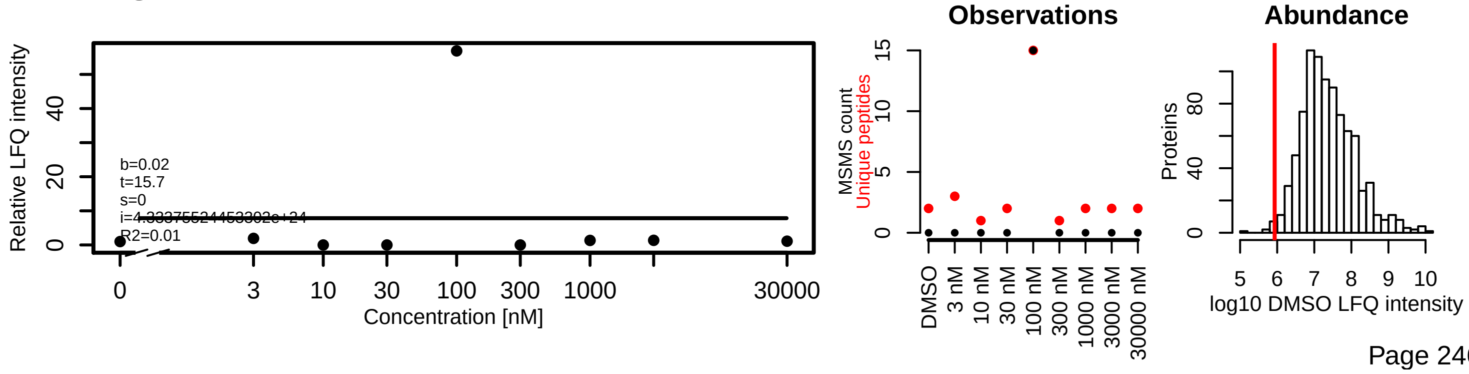

CDK6

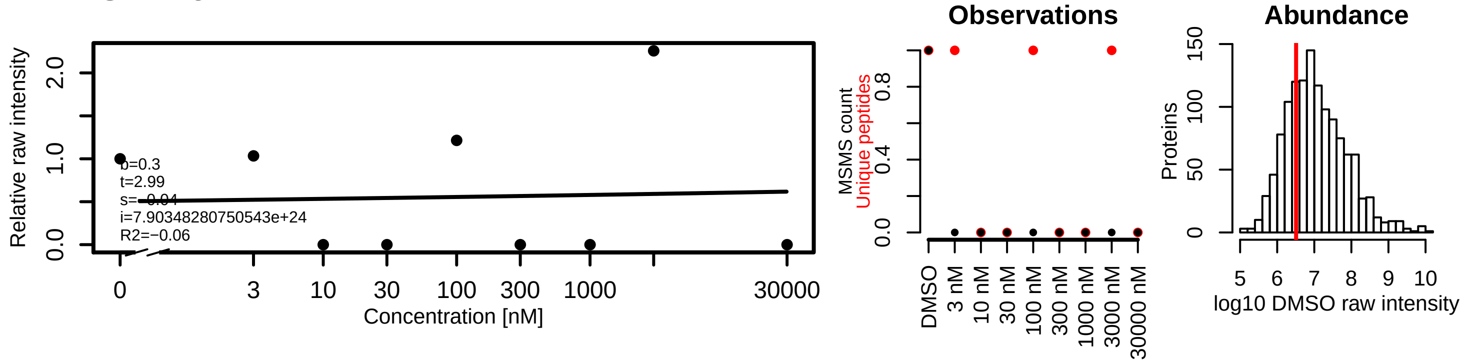

LANCL1

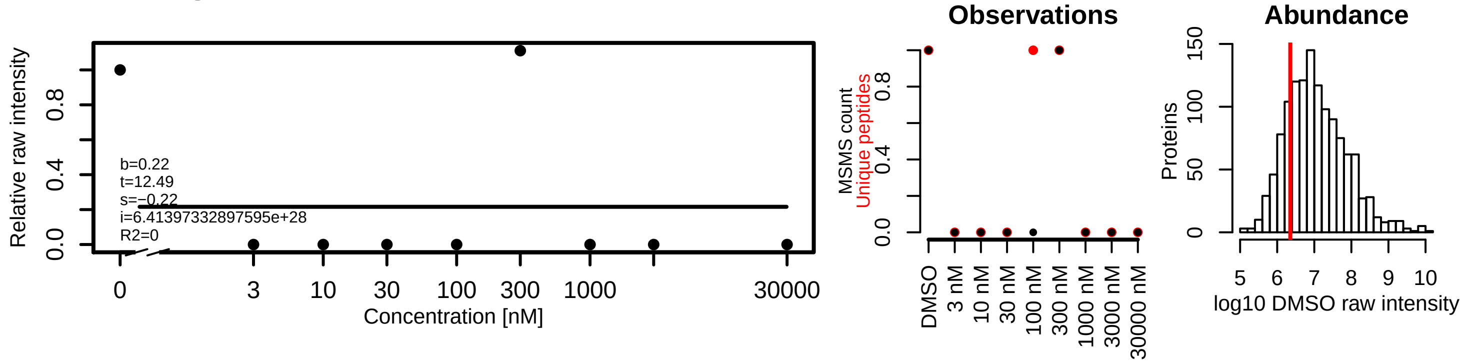

SLC24A2

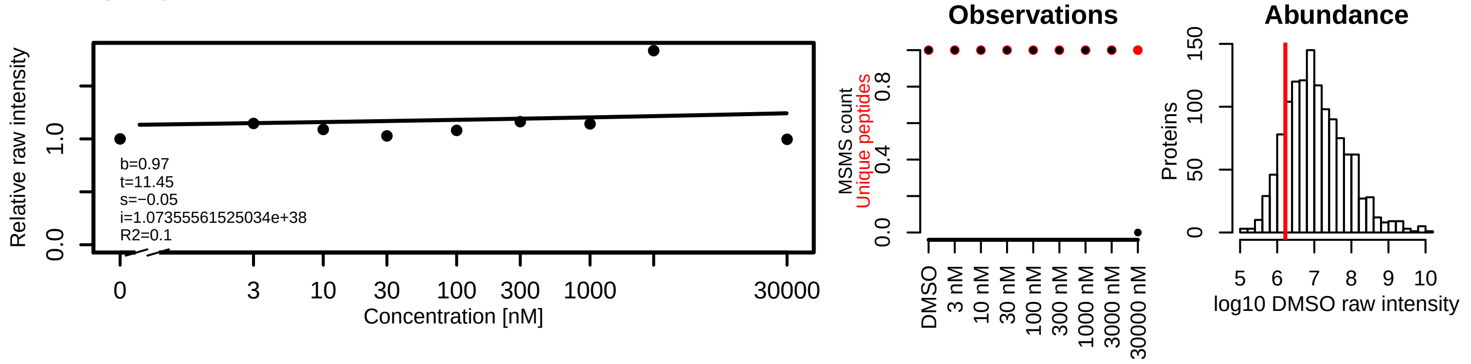

SHMT2

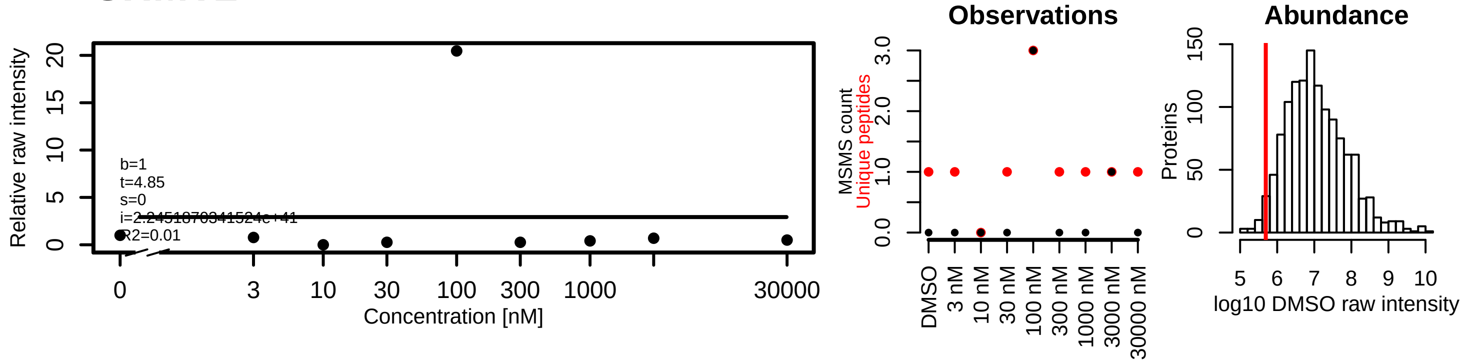

TLN1

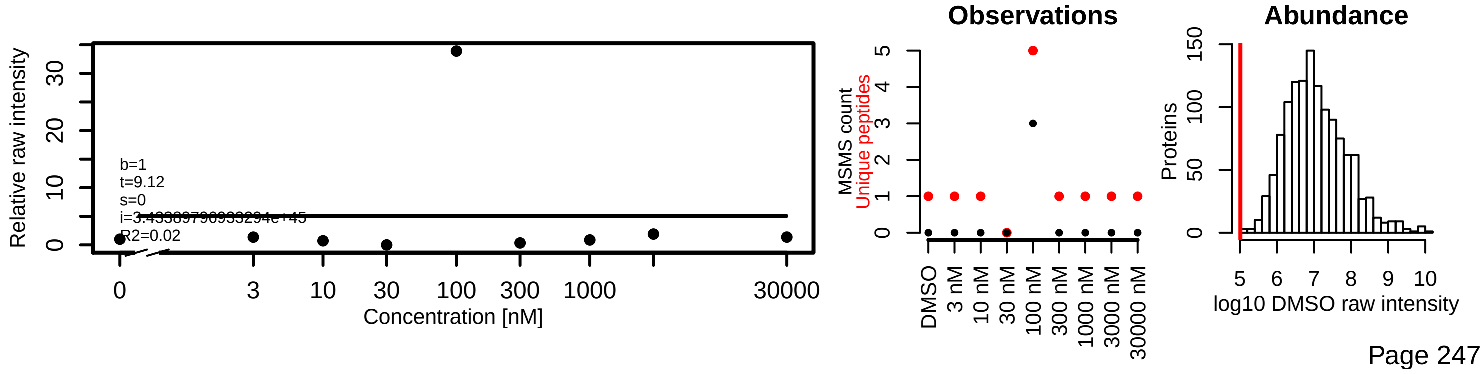

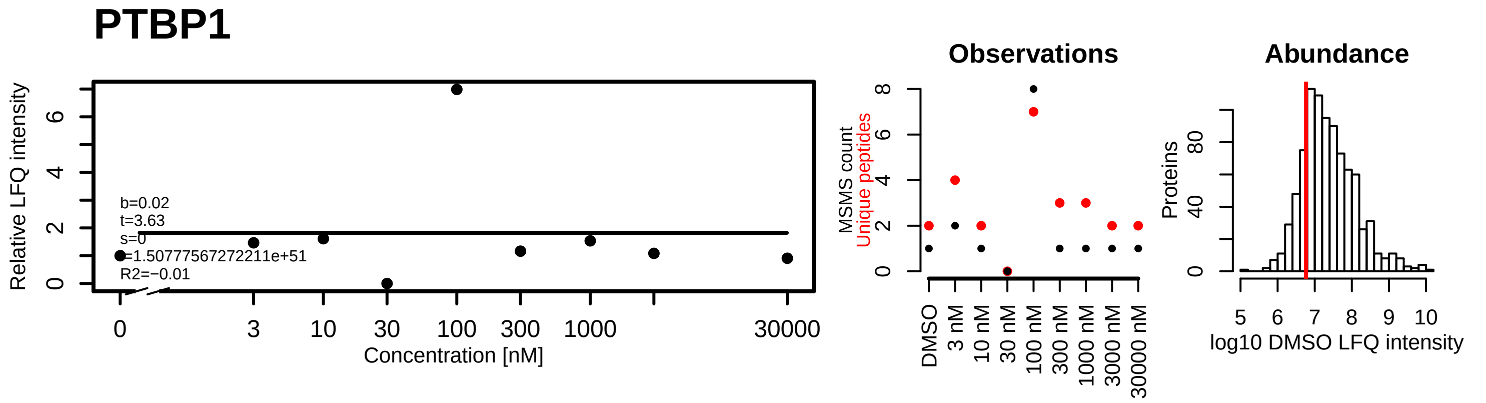
PTBP1
Relative LFQ intensity
b=0.02
t=3.63
s=0
i=1.50777567272211e+51
R2=−0.01
0 3 10 30 100 300 1000 30000
Concentration [nM]
Observations
MSMS count
Unique peptides
DMSO 3 nM 10 nM 30 nM 100 nM 300 nM 1000 nM 3000 nM 30000 nM
Abundance
Proteins
log10 DMSO LFQ intensity

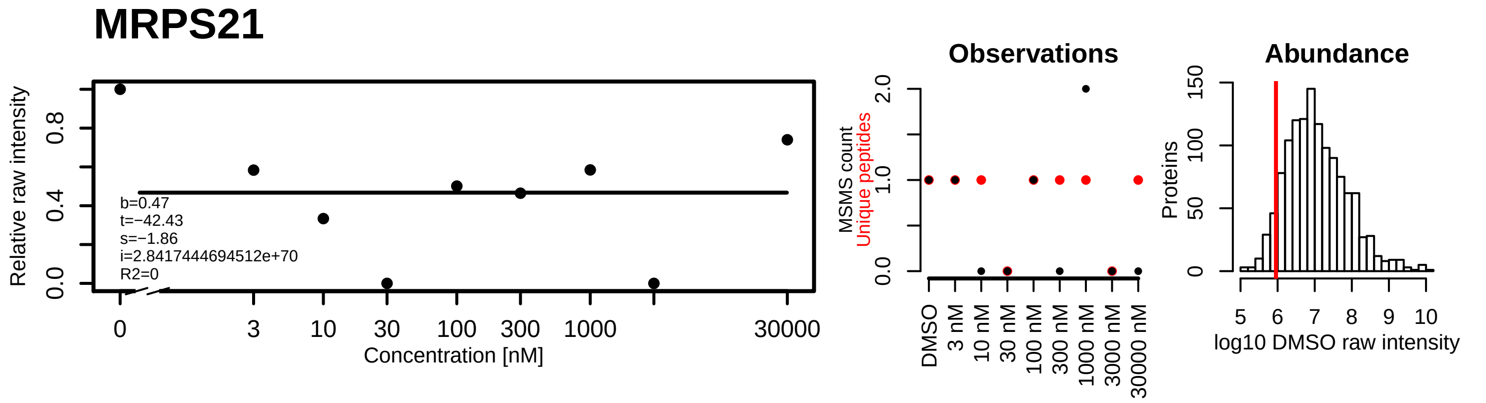
MRPS21
Relative raw intensity
b=0.47
t=−42.43
s=−1.86
i=2.8417444694512e+70
R2=0
0 3 10 30 100 300 1000 30000
Concentration [nM]
Observations
MSMS count
Unique peptides
DMSO 3 nM 10 nM 30 nM 100 nM 300 nM 1000 nM 3000 nM 30000 nM
Abundance
Proteins
log10 DMSO raw intensity

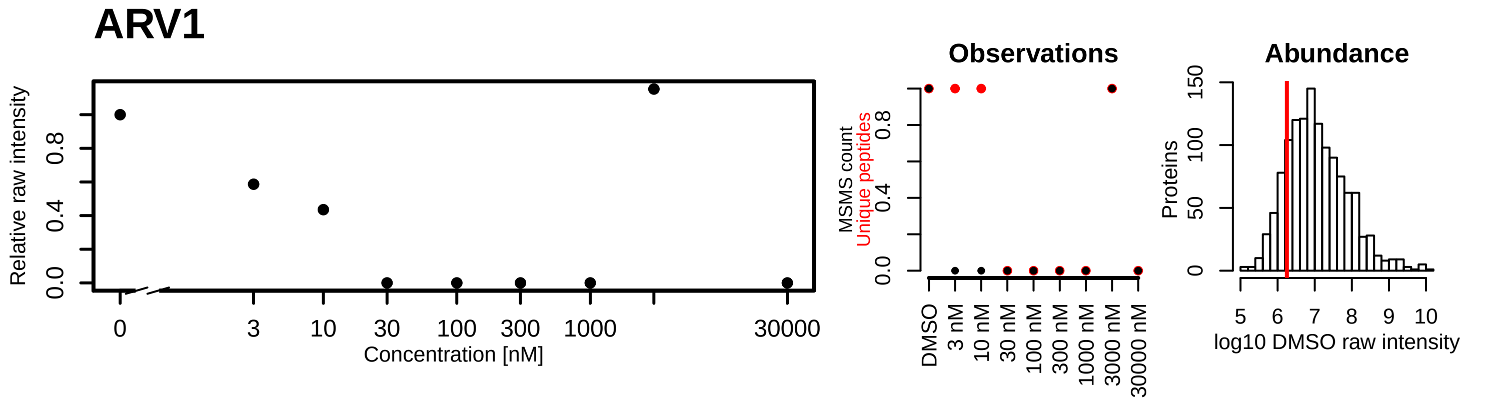
ARV1
Relative raw intensity
0 3 10 30 100 300 1000 30000
Concentration [nM]
Observations
MSMS count
Unique peptides
DMSO 3 nM 10 nM 30 nM 100 nM 300 nM 1000 nM 3000 nM 30000 nM
Abundance
Proteins
log10 DMSO raw intensity

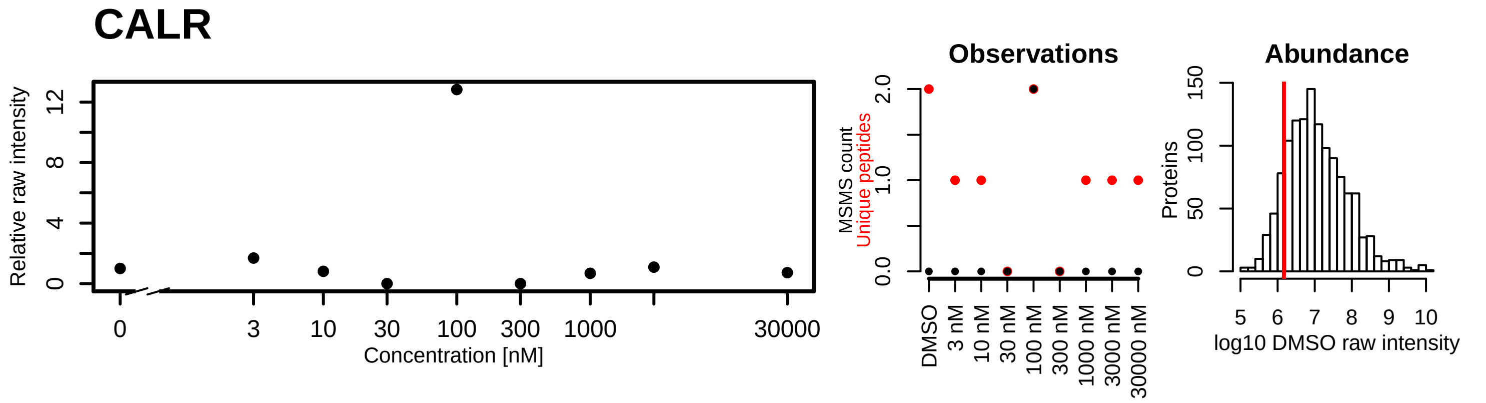
CALR
Relative raw intensity
0 3 10 30 100 300 1000 30000
Concentration [nM]
Observations
MSMS count
Unique peptides
DMSO 3 nM 10 nM 30 nM 100 nM 300 nM 1000 nM 3000 nM 30000 nM
Abundance
Proteins
log10 DMSO raw intensity

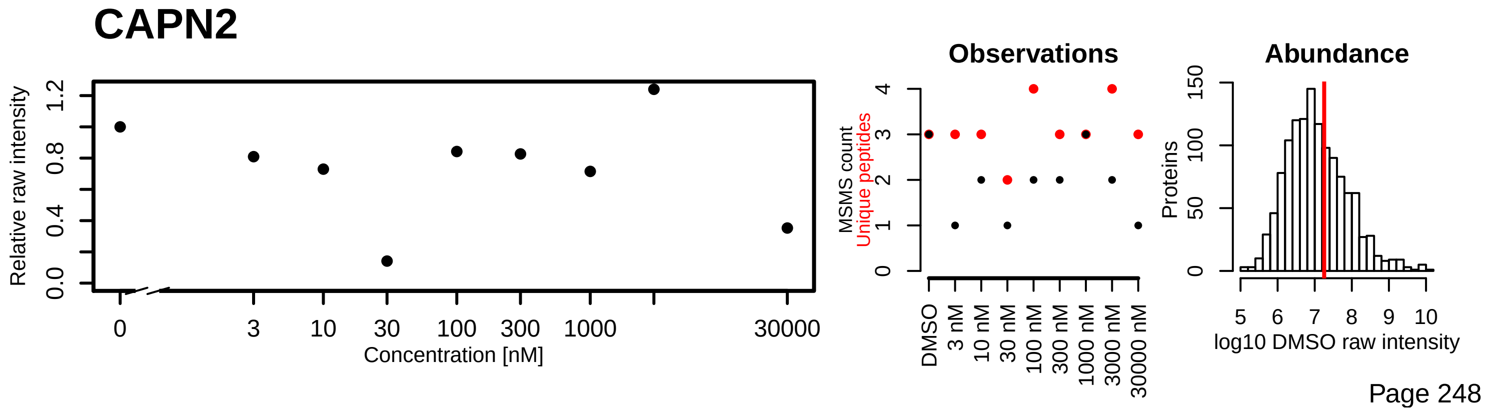
CAPN2
Relative raw intensity
0 3 10 30 100 300 1000 30000
Concentration [nM]
Observations
MSMS count
Unique peptides
DMSO 3 nM 10 nM 30 nM 100 nM 300 nM 1000 nM 3000 nM 30000 nM
Abundance
Proteins
log10 DMSO raw intensity

CCT7

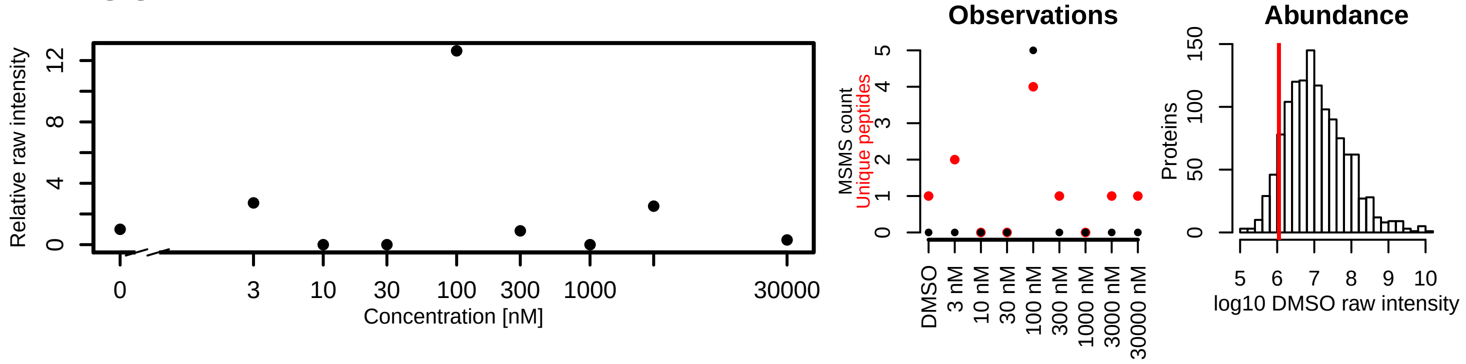

DLAT

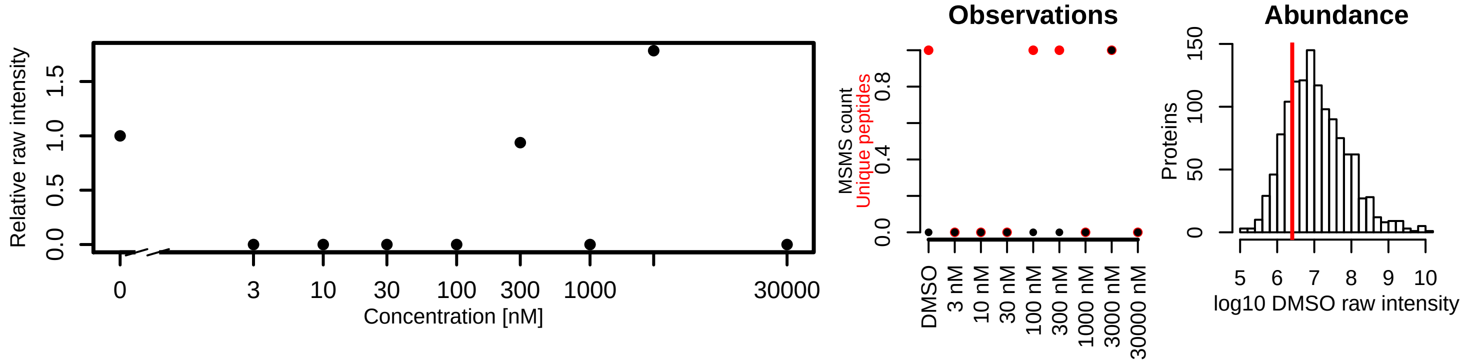

DRAP1

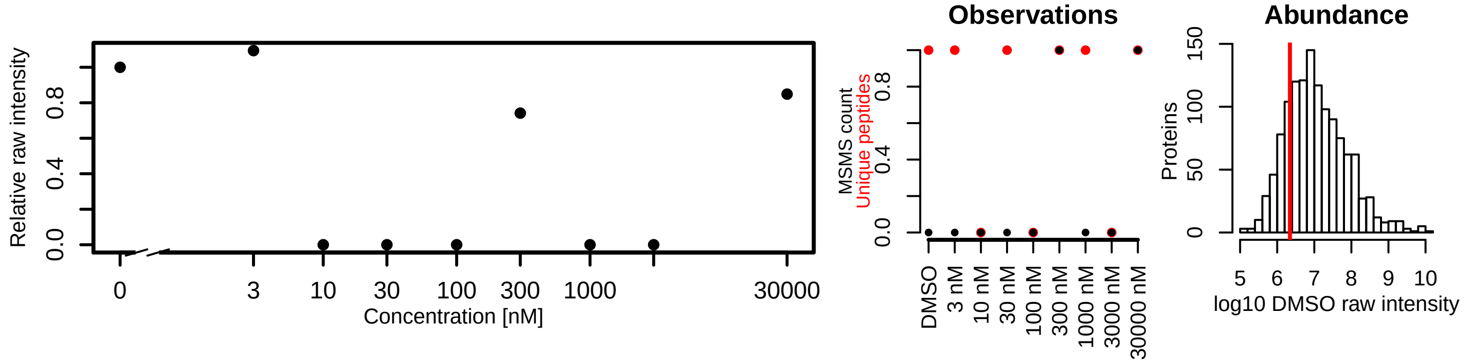

DYNC1LI2

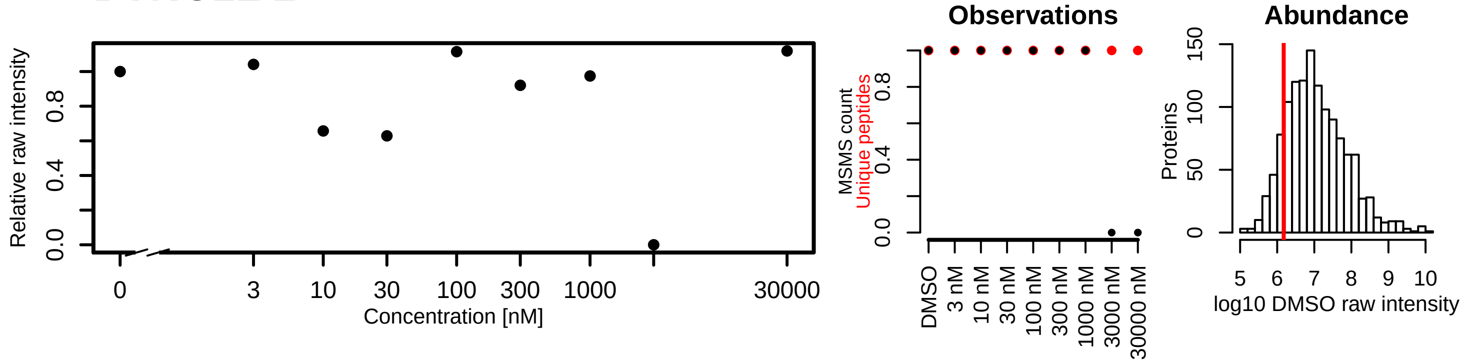

EIF4B

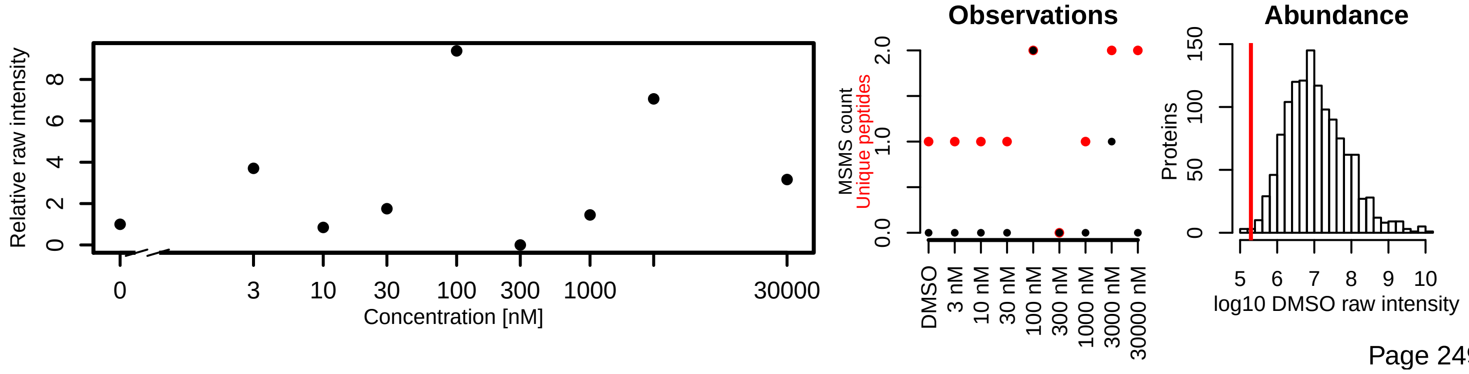

FUS

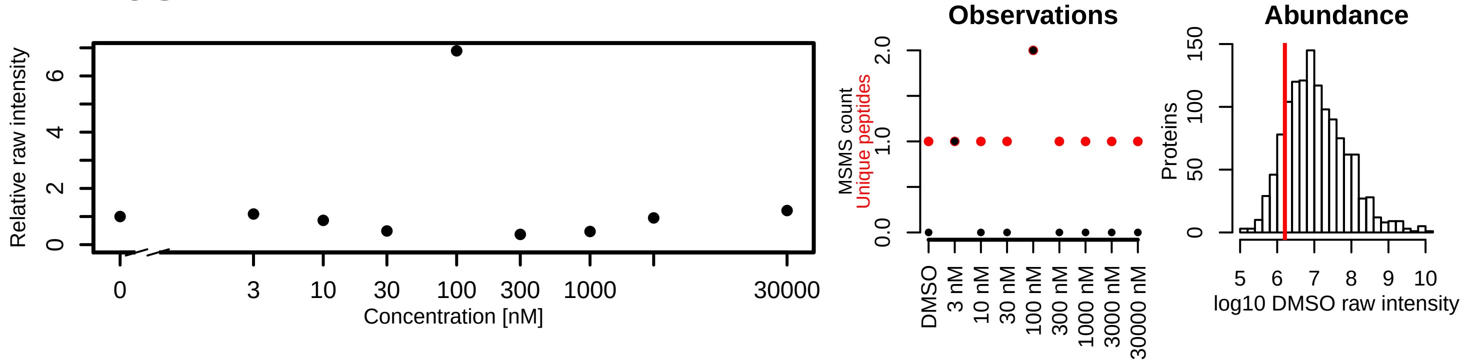

ILF3

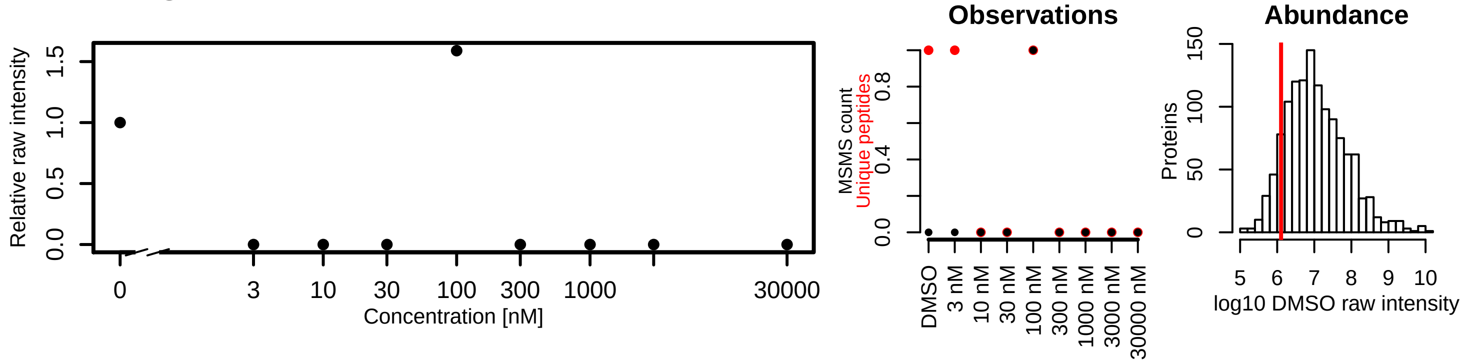

KHDRBS1

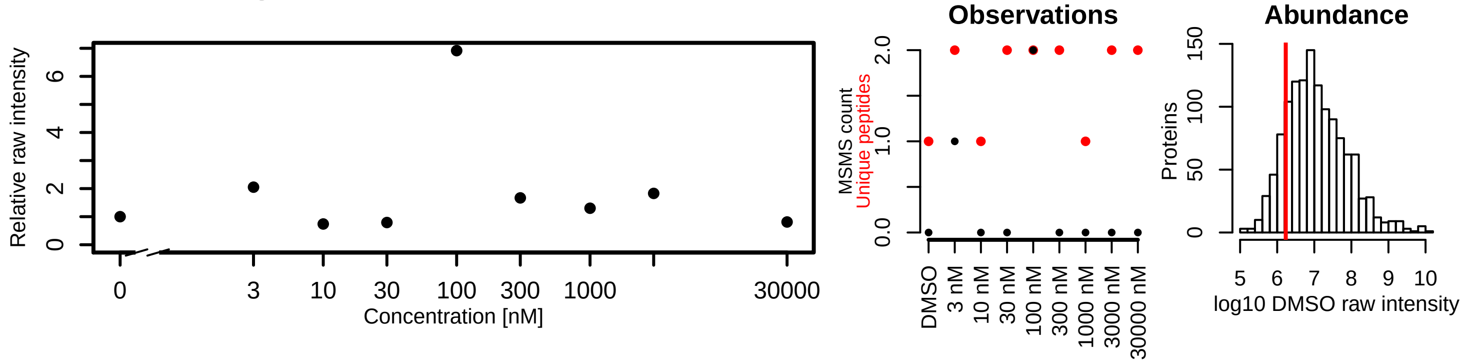

MBD2

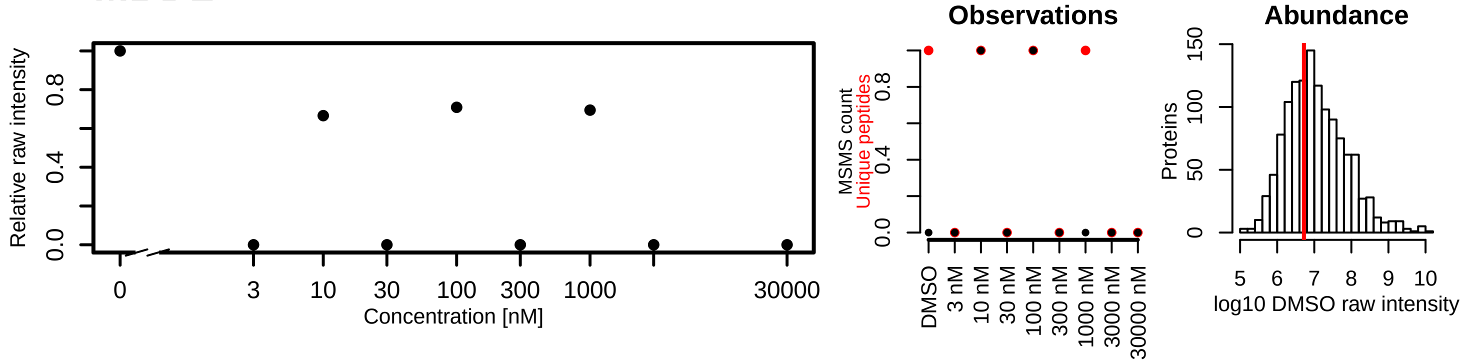

MCM3AP

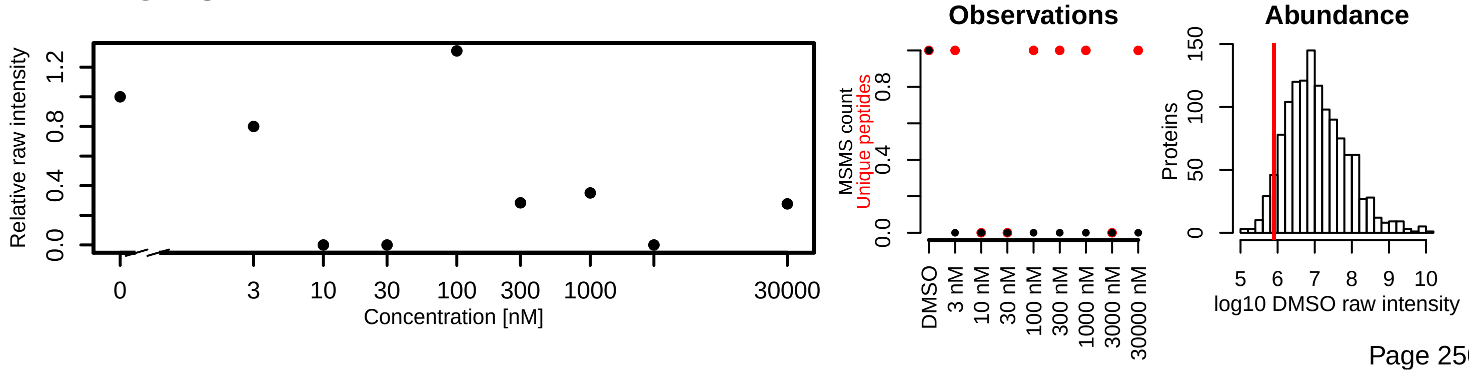

MMS22L

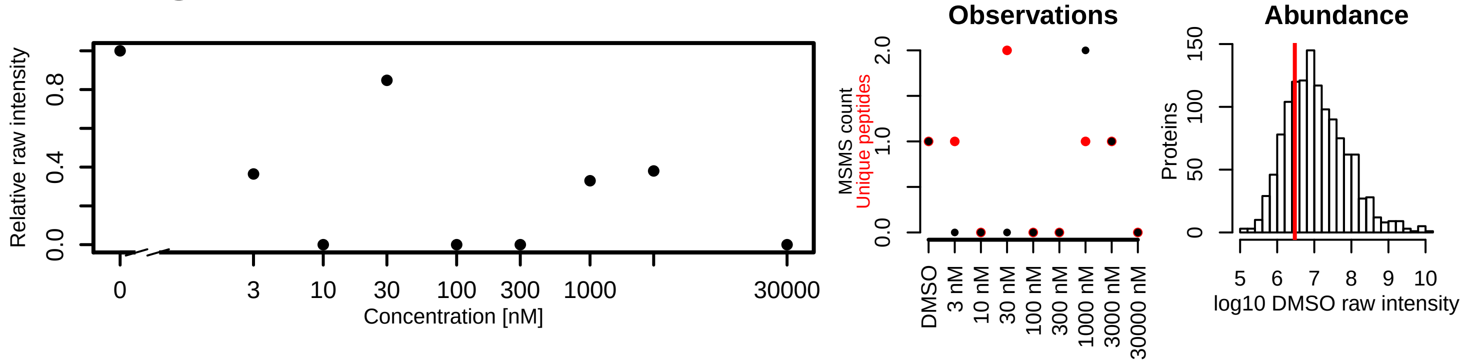

MTDH

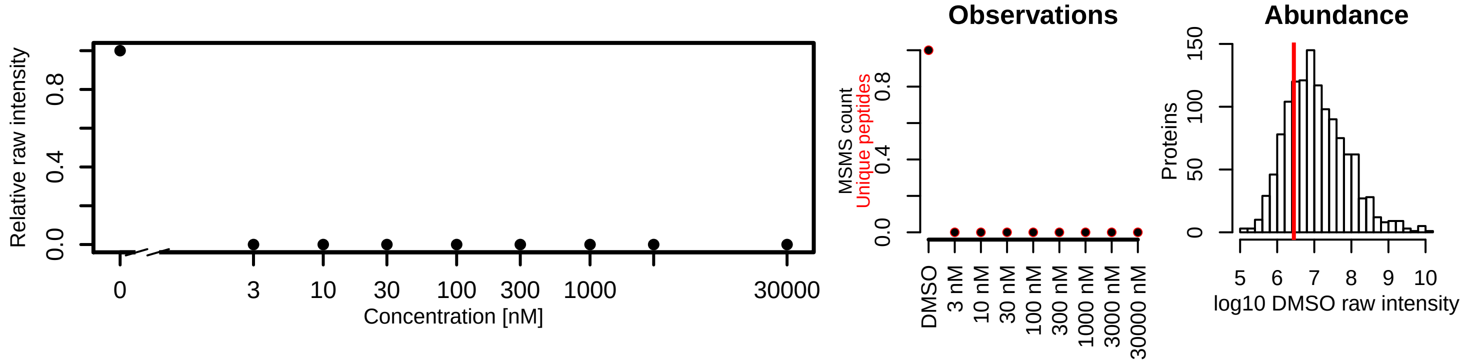

MYO1B

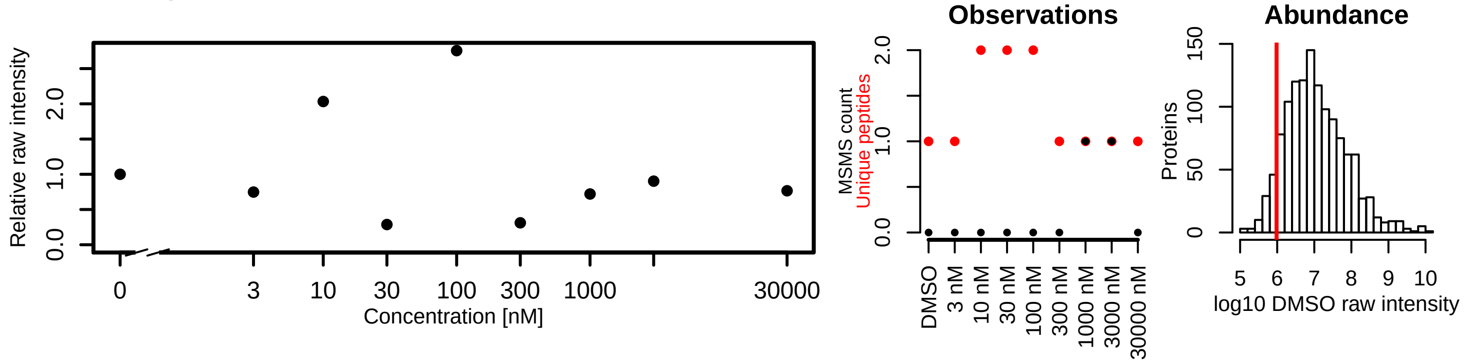

NCDN

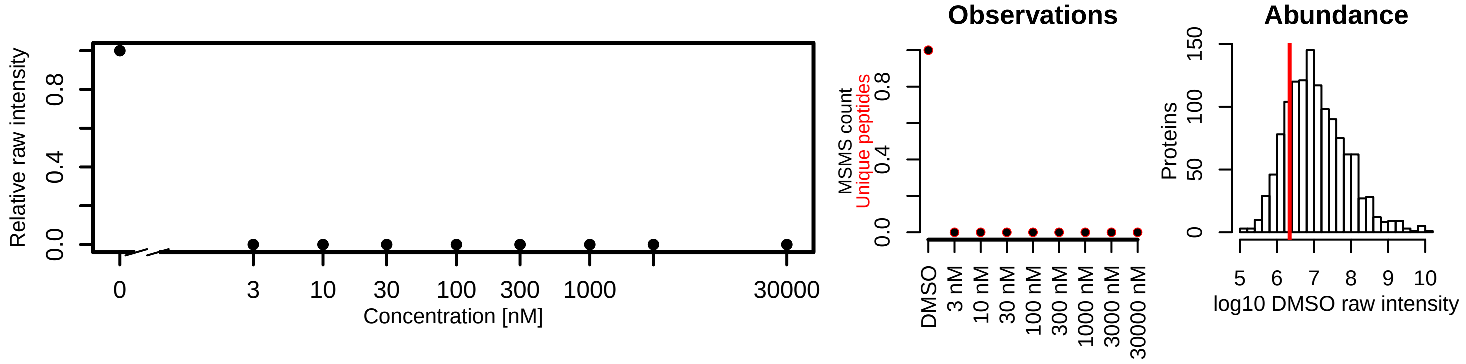

NSDHL

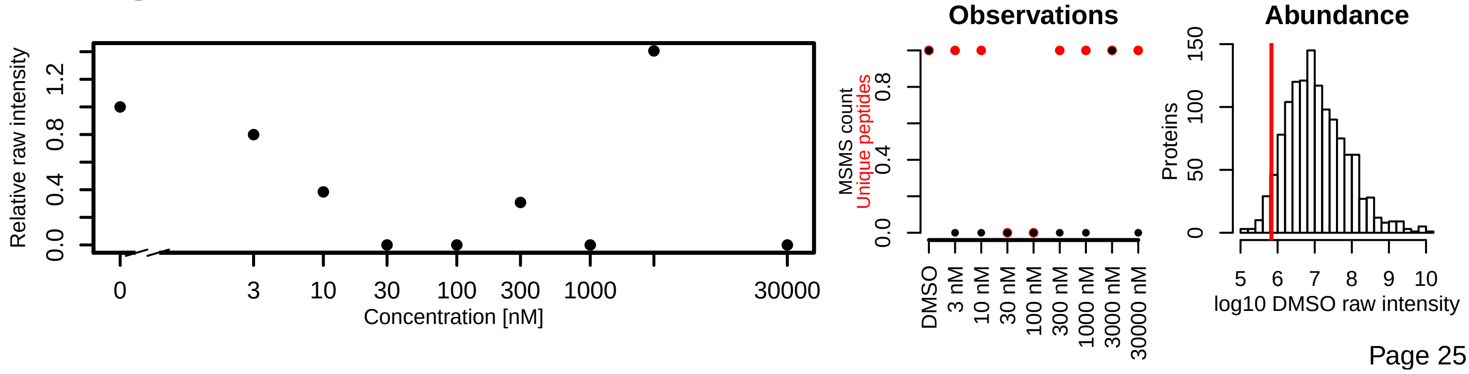

PLD3

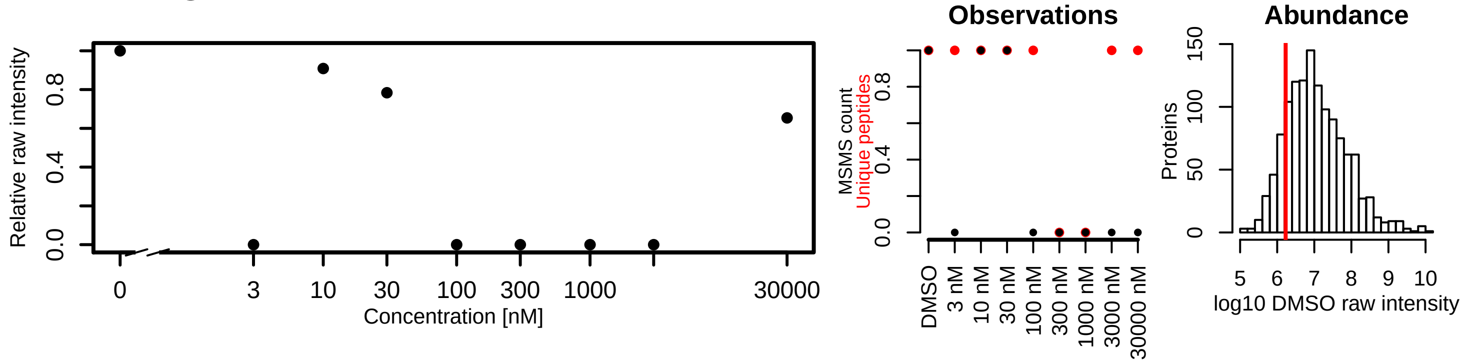

PPIA

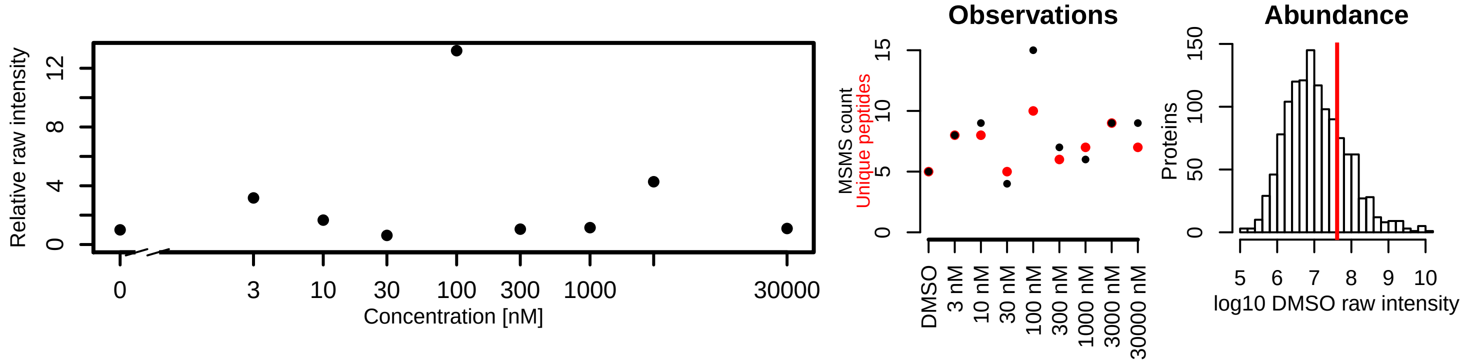

PREB

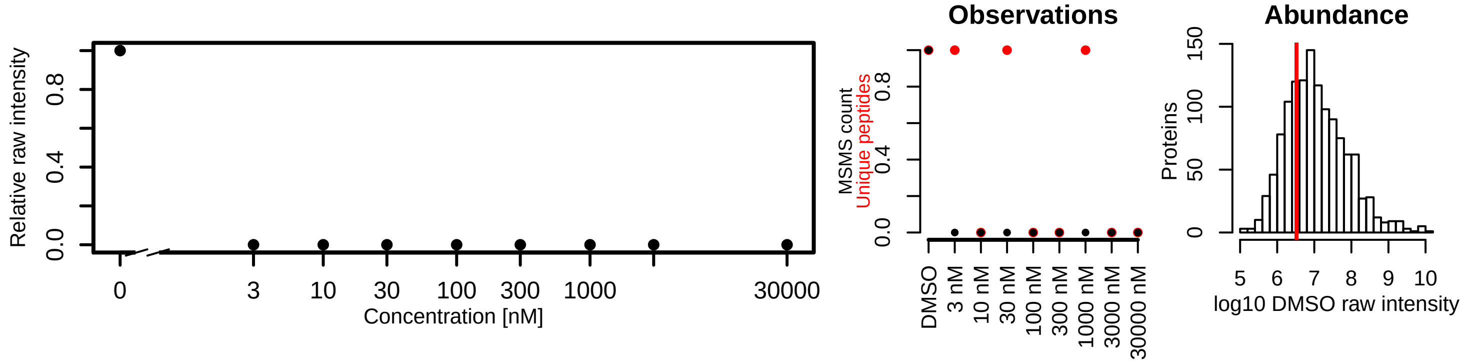

PSMA1

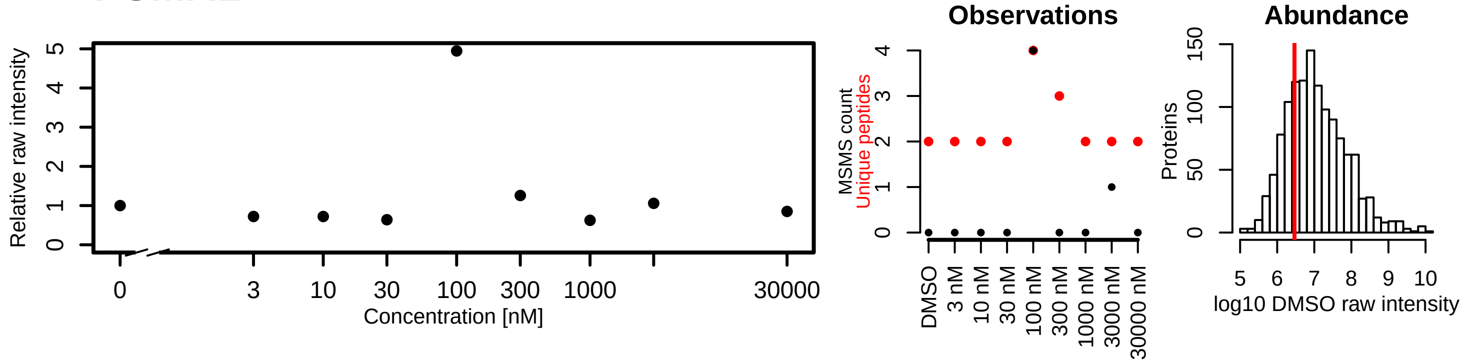

PSMA4

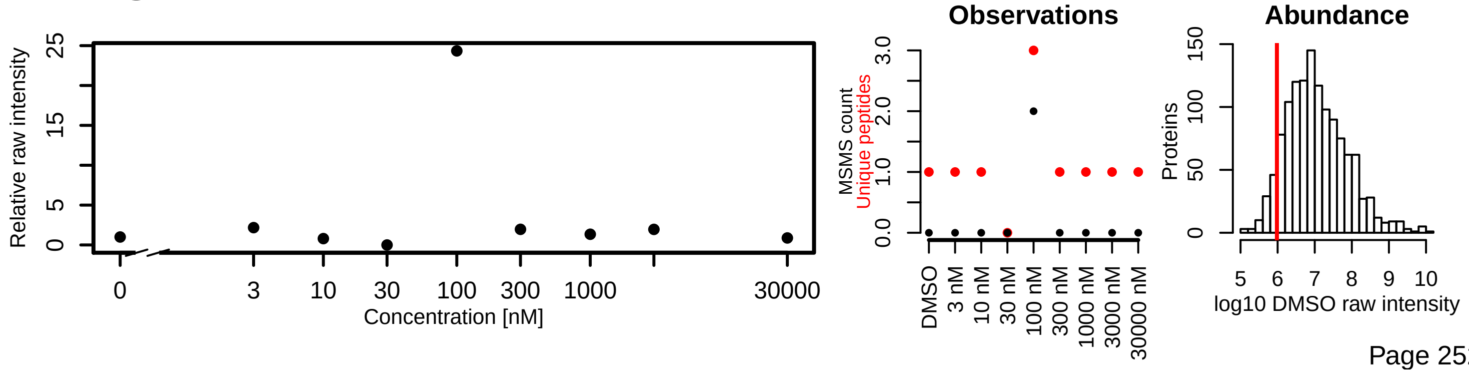

RANBP1

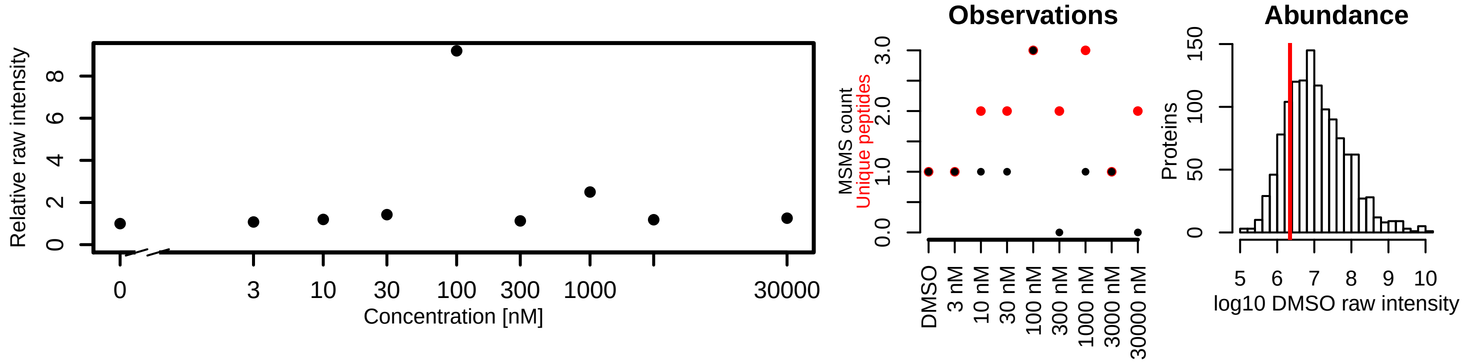

RFC3

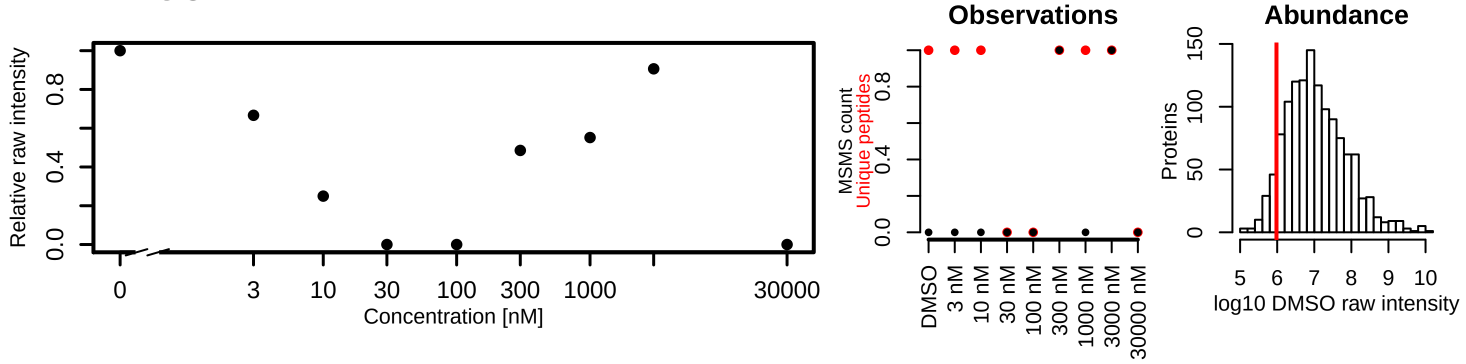

RPS15A – I3L3P7;P62244

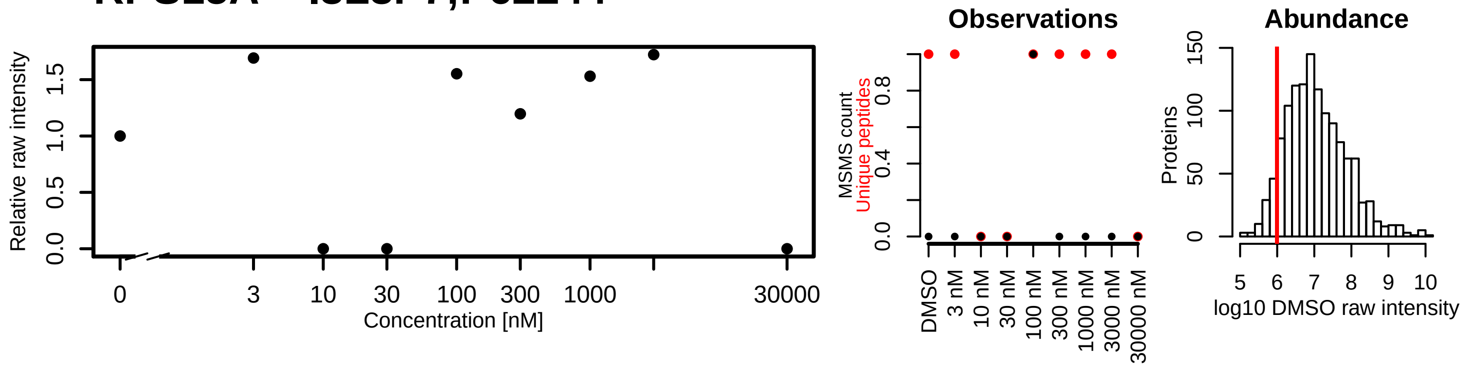

SERBP1

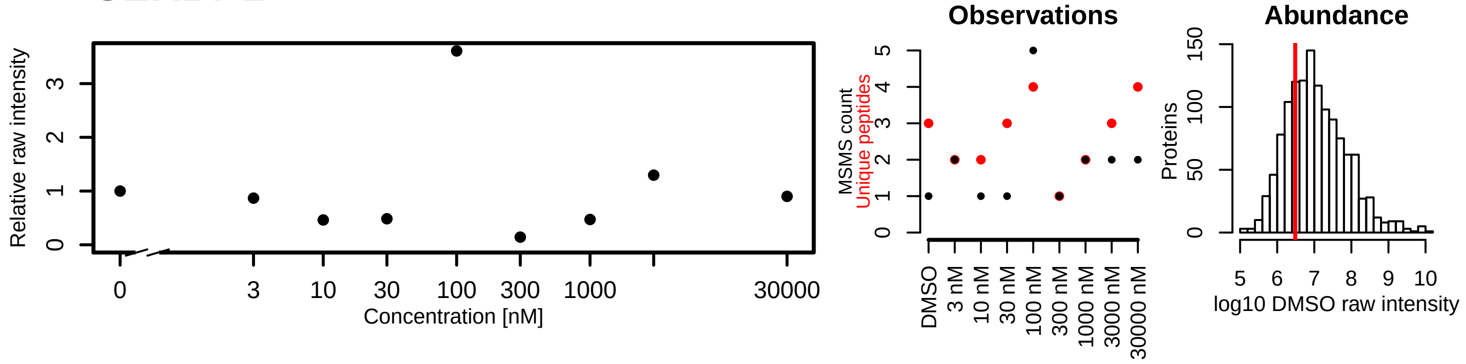

SLC20A2

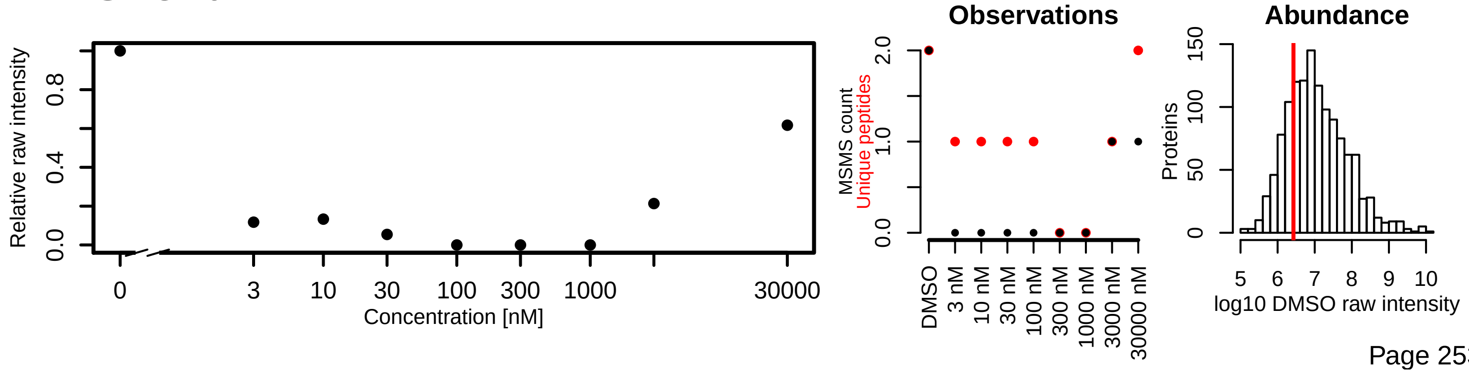

TMED4

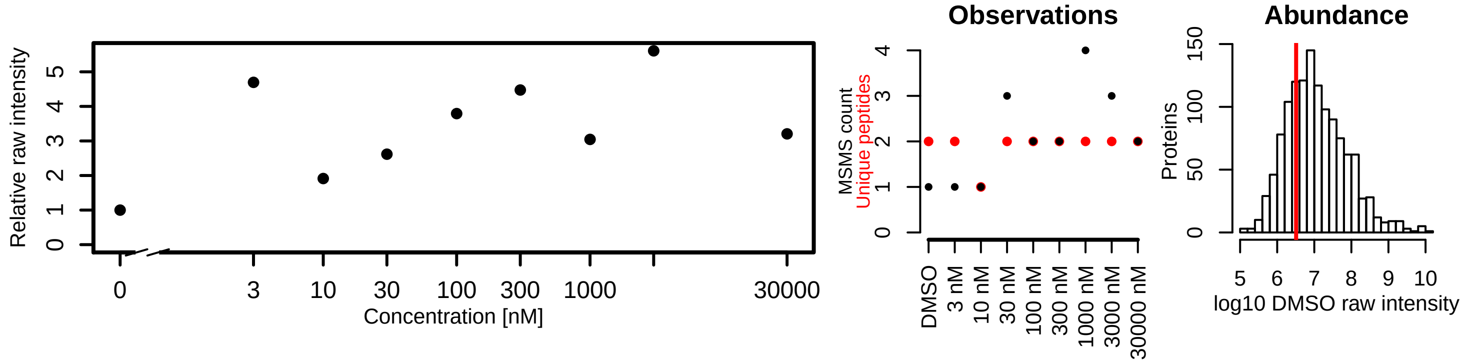

TMEM11

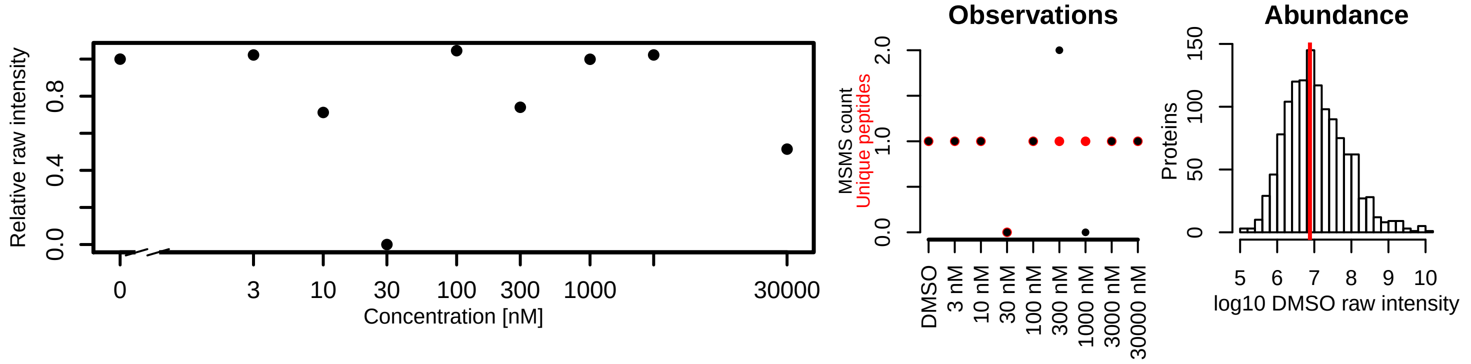

TMEM30A

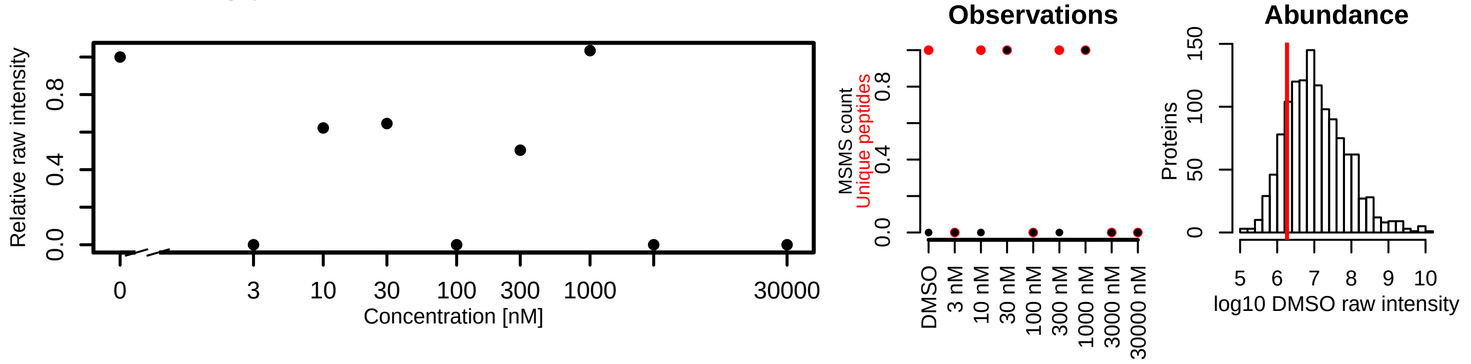

TPT1

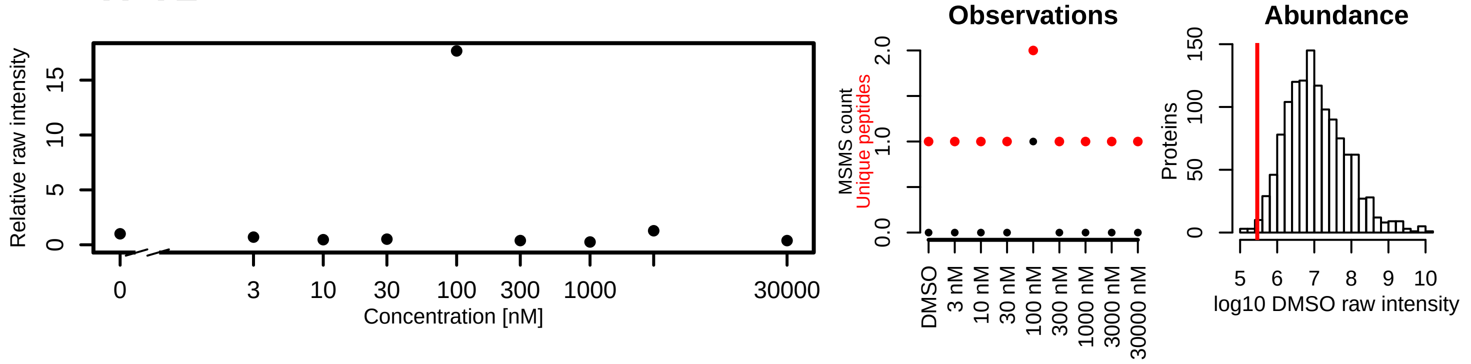

VPS18

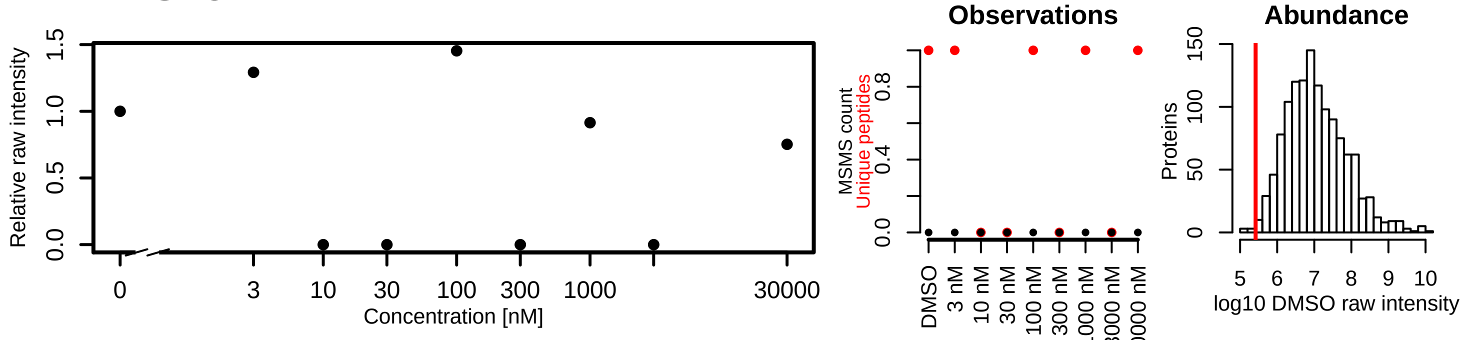

ZMPSTE24

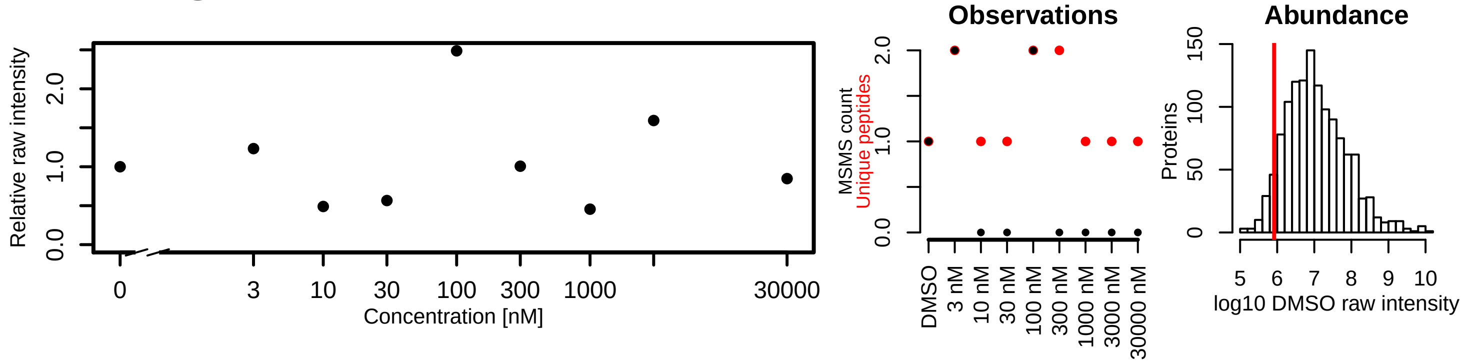
Observations
Abundance
Relative raw intensity
0.0
1.0
2.0
0
3
10
30
100
300
1000
30000
Concentration [nM]
MSMS count
Unique peptides
DMSO
3 nM
10 nM
30 nM
100 nM
300 nM
1000 nM
3000 nM
30000 nM
Proteins
0
50
100
150
5
6
7
8
9
10
log10 DMSO raw intensity
